
JOURNAL INFORMATION
==============================
NLM Title Abbreviation: Intensive Care Med Exp
Journal ID: Intensive Care Med Exp
NLM Journal ID: 101645149

Intensive Care Medicine Experimental

EISSN: 2197-425X
Publisher: Springer

ARTICLE INFORMATION
==============================
PMCID: PMC6763544
PMID: 31559498
DOI: 10.1186/s40635-019-0265-y
Article ID: 265
Article version: 1
Subjects: Meeting Abstracts

ESICM LIVES 2019

Berlin, Germany. 28 September - 2 October 2019

Electronic publication date: 2019 Sep 27
Publication date: 2019 Sep
Volume: 7
Issue: Suppl 3
Issue sponsor: Publication of this supplement has not been supported by sponsorship.
Electronic Location ID: 55
Copyright: © The Author(s). 2019
License: Open AccessThis article is distributed under the terms of the Creative Commons Attribution 4.0 International License (http://creativecommons.org/licenses/by/4.0/), which permits unrestricted use, distribution, and reproduction in any medium, provided you give appropriate credit to the original author(s) and the source, provide a link to the Creative Commons license, and indicate if changes were made.
License URL: https://creativecommons.org/licenses/by/4.0/

Erratum in: Correction to: A case of intracoronary and endovenous levosimendan as a rescue therapy for refractory cardiac arrest 8 21 1 2020 4 Intensive Care Medicine Experimental PMC6973955 31965386
Conference name: 32nd ESICM Annual Congress
Conference Acronym: ESICM LIVES 2019
Conference location: Berlin, Germany
Conference date: 28 September - 2 October 2019
issue-copyright-statement: © The Author(s) 2019

==============================

### AKI / ARF / SIS - Miscellanea of mechanical ventilation, AKI and sepsis

#### 001611

**The outcome of Acute kidney injury in the intensive care unit of A sub Saharan Tertiary Hospital**

M. Oladimeji[1], G. Asiyanbi[2], A. Fadeyi[3], O. Belle[4], S. Olanipekun[3], O. Adekola[5]
[1]Anesthesia & intensive care unit, Lagos University Teaching Hospital, LAGOS, Nigeria, Federal Republic of; [2]Anaesthesia & intensive care, Lagos University Teaching Hospital, LAGOS, Nigeria, Federal Republic of; [3]Intensive care unit, Onelife Hospital, LAGOS, Nigeria, Federal Republic of; [4]Surgery, Lagos University Teaching Hospital, Lagos, Nigeria, Nigeria, Federal Republic of; [5]Intensive care unit, Onelife Hospital, LAGOS, France
**Correspondence:** O. Adekola
*Intensive Care Medicine Experimental* 2019, **7(Suppl 3):**001611

**INTRODUCTION.** Acute kidney injury is characterized by sustained rise in serum creatinine and reduction in urine output. It may also be accompanied by retention of nitrogen products and electrolyte disturbances. The incidence of AKI varies between 36 and 67% among critically ill patients with a mortality rate of 50 to 70%.
**OBJECTIVES.** We determined the incidence and outcome of acute kidney injury in critical care patients
**METHODS.** A total of 177 patients, 18 years and older were studied. Data were collected on admission and daily during hospitalization until discharge or death. AKI was defined as:1-2 1) absolute increase in serum creatinine ≥0.3 mg/dL or ≥1.5 times the baseline level, or 2) requirement for renal replacement therapy, or 3) oliguria defined as urine output <400 ml in 24 hours
**RESULTS.** AKI was observed in 34.3% of our ICU admission, among of whom 4.7% developed AKI during their ICU stay. The mean duration of onset of AKI was 1 (25th to 75th percentile 1-2) days. The overall ICU 30 days mortality was 42.4%, however the 30 days mortality in patients with AKI was 85.5%. Renal replacement therapy was only possible in 36.6% patients. Inotropic support was administered in 59.1% patients with AKI. Factors mitigating against dialysis included proctated hypotension in 63.6%, lack of fund in 18.1%, delayed screening for HIV and Hepatitis B in 18.3%.
**CONCLUSION.** Acute kidney injury is a common problem in the critically ill patient and is associated with a high mortality rate at our institution.

##### REFERENCE(S)

1. Mehta RL, Kellum JA, Shah SV, Molitoris BA, Ronco C, Warnock DG, et al. Acute Kidney Injury Network: report of an initiative to improve outcomes in acute kidney injury. Crit Care 2007; 11: R31.
2. Uchino S, Doig GS, Bellomo R, Morimatsu H, Morgera S, Schetz M, et al. Diuretics and mortality in acute renal failure. Crit Care Med 2004; 32: 1669-1677.

#### 000742

**Acute kidney failure in the post operatory of peripheral vascular surgery, a prospective single- center experience**

GL. Valente, BMN. Lucena, SRS. Fonseca, LMDS. Malbouilsson, MJC. Carmona
Department of anesthesiology, Faculty of Medicine, University of São Paulo, São Paulo, Brazil
**Correspondence:** G.L. Valente
*Intensive Care Medicine Experimental* 2019, **7(Suppl 3):**000742

**INTRODUCTION.** Acute kidney failure (AKF) is identified in 30-40% of cases in post-operative patients. There is limited literature on the importance and the correlation between peripherical vascular surgeries and the development of AKF.
**OBJECTIVES.** Analysis of the connection between factors related to AKF or acute-on-chronic-kidney failure (AOCKF) in the ICU during the first 7 days after peripherical vascular surgeries, for example: arterial bypasses, amputations, angioplasties and embolectomies.
**METHODS.** The definition of AKF is defined by the AKIN: abrupt increase in serum creatinine ≥ 0.3 mg/dL or 50% from the baseline. This study is prospective, observational and non-randomized. Patients on previous dialysis were excluded. Continuous variables were summarized as medians and ranges, and categorical variables as percentages.
**RESULTS.** A database of 65 patients was evaluated. The data was acquired between April 2018 and March 2019. The median age was 70 (range 22-88 y); 73% were male. 25 patients (38%) had AKF.
The average age in both groups is similar, as well the prevalence of comorbidities (hypertension and DM), distribution of sexes and mortality. 46% of the patients with chronic-kidney disease developed an AOCKF (28% of the AKF group). The frequencies of the following values are bigger in AKF group: Re-surgery, emergency surgery, use of vancomycin, gentamicin or amikacin.
CPK values were different in the two groups (4332 AKF; 2937 no AKF), but our sample was inconclusive to demonstrate a real correlation with AKF. When CPK in the first 24h was divided in three categories (<10.000; 10000-20000 and >20000), it was observed in a Kaplan Meier analyses a correlation between these categories and post-operative hemodialysis.
**CONCLUSION.** Despite our small sample, CPK when analyzed as a categorical variable, showed a statistical significance in patients submitted to hemodialysis. Despite differences in both groups, as CPK average as well as further factors related to a more serious condition; like patient urgency surgery, re-surgery and the use of antibiotics; our analysis was inconclusive to stablish those factors as predictive to AKF. The study has several limitations: the number of patients is insufficient for stronger evidences, and it is a single center study. We suggest that a multi-center study can resolve these problems.

**Table 1 (abstract 000742).** See text for description

| Total (65) | AKF (25) | No AKF (40) |
|---|---|---|
| Average CPK in first 24h | 4332 | 2937 |
| Mean basal urea | 46,8 | 47,9 |
| Urgency | 52% | 32% |
| Contrast use | 70% | 80% |
| Nephrotoxic antibiotics | 36% | 20% |
| Re-surgery | 16% | 7% |
| Mortality | 4% | 5% |
| Male | 72% | 78% |
| Fem. | 28% | 22% |
| Average age | 69 | 68 |
| DM | 48% | 42% |
| Chronic renal insufficiency | 28% | 20% |
| Hypertension | 84% | 82% |
| Tabak consume | 35% | 55% |

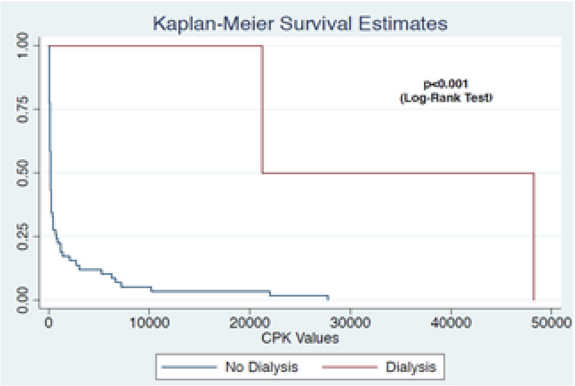

**Fig. 1 (abstract 000742).** See text for description

### 001365

#### Utility of classic Weaning Predictors in decision-making in patients submitted to mechanical ventilation > 48 hours

LE. López, JF. Martínez Carmona, MFA. Hijano, FMÁ. Barbancho, AMJ. Delgado
Intensive care unit, Hospital Carlos Haya, Málaga, Spain
**Correspondence:** J.F. Martínez Carmona
*Intensive Care Medicine Experimental* 2019, **7(Suppl 3):**001365

**INTRODUCTION.** The withdrawal of respiratory support is key in the evolution of the patient, the identification of the optimal moment to advance in the weaning is fundamental, avoiding the problems associated with both an early and delayed withdrawal with increased morbidity and mortality.
The decision based solely on the clinic has proved unreliable. Weaning predictors offer support for decision making. Among the classic predictors is the combination of two of them, MIP / P0.1, which assesses the capacity of the respiratory musculature and the intensity of the stimulus of the respiratory center.
**OBJECTIVES.** To assess the predictive capacity of MIP / P0.1 with respect to the classic predictors in patients undergoing prolonged mechanical ventilation.
**METHODS.** Prospective study including 17 patients submitted to MV due to different causes (TBI, CCV, urgent surgery, respiratory failure). Once criteria for advancing weaning are met, an SBT is made in PSV 5 - 8 cmsH2O over 5 cmsH2O of PEEP, and the following determinations are made: MIP, P0.1, MIP / P0,1, RSBI, FR, Vt. Epidemiological variables are collected, cause of IOT, VM time, weaning failure, mortality.
**RESULTS.** The average age was 56.12 years +/- 13.95. 70.9% were male. Reason for admission: TBI (29.4%), CCV (29.4%), Urgent Surgery (23.5%). The average duration of MV was 12.76 days +/- 8.82. 64.7% presented weaning failure.
We observe a significant relationship between MIP / P0.1 and weaning failure (Test Chi2 p 0.011). ANOVA was performed, we found a relationship between the variables (F 8.08, p 0.012). We performed a COR curve presenting AUC 0.92 (p 0.005, 95% CI 0.78-1). We found the best cut-off at 0.095 (S 90%, E 83%). If we analyze subgroups (Group 1: MV <10 days, Group 2: MV> 10 days) we did not observe a significant relationship between MIP / P0.1 and failure of weaning
**CONCLUSION.** - In our sample, MIP / P0.1 is a good predictor of weaning failure in patients undergoing short-term mechanical ventilation, if we compare it with the rest of the classic parameters.
- However, in patients undergoing prolonged mechanical ventilation, their prognostic capacity is limited

**REFERENCE(S)**

1. Nemer SN, Barbas CS. Predictive parameters for weaning from mechanical ventilation. J Bras Pneumol 2011; Sep-Oct 37(5): 669-79.
2. Sassons CS, Mahutte CK. Airway occlusion pressure and breathing pattern as predictors of weaning outcome. Am Rev Respir Dis 1993; Oct;148 (4 Pt 1): 860-6.
3. Fernandez et al. Extubation failure: diagnostic value of occlusion pressure (P0.1) and P0.1-derived parameters. Intensive Care Med 2004; Feb 30 (2); 234-240.

### 000007

#### Early Predictors of the duration of mechanical ventilation in the critical patient: Oxigenation and age idex

C. Pacheco; D. Petrucci; MG. Sanchez
Medicina critica, University Hospital Of Caracas, Caracas, Venezuela
**Correspondence:** C. Pacheco
*Intensive Care Medicine Experimental* 2019, **7(Suppl 3):**000007

**INTRODUCTION.** EARLY PREDICTORS OF THE DURATION OF MECHANICAL VENTILATION IN THE CRITICAL PATIENT:OXYGENATION AND AGE INDEX
**OBJECTIVES.** To determine the early predictors of MV duration, with emphasis on age and the inspired oxygen/inspired fraction of oxygen (PaO2/FiO2), in critically ill patients.
**METHODS.** Descriptive, retrospective study. Sample consisting of 143 patients, ventilated for more than 24 hours. Demographic, clinical and risk factors were studied in relation to time in MV and mortality.
**RESULTS.** The mean number of days in VM (8,5 +/- 7). (53,4%)remained for less than 5 days in MV and the duration of ventilatory support was shorter in young patients (18-50 years). Patients older than 70 years, with APACHE II>15pts, were associated with higher mortality. There was a linear relationship between age and oxygenation index. PaO2/FiO2 between 101-200, was associated with longer duration in MV and with values of PaO2/FiO2<100, mortality increased. The positive water balance(BH+) was linked to PaO2/FiO2<100. Shock, FiO2>60%, Peep>10cmH20,bronchoaspiration, pneumonia, renal failure, BH+ and use of benzodiazepines were associated with mortality; while the duration of MV was associated with FiO2>60%, shock,bronchoaspiration and hypoalbuminemia. Tracheostomies were performed in (6,9%)patients and general mortality (39,1%)
**CONCLUSION.** PaO2/FiO2 and age can be considered as determining factors in the duration of MV and mortality in critically ill patients.

**REFERENCE(S)**

1. Macintyre NR, Epstein SK, Carson S, Scheinhorn D, Christopher K, Muldoon S; National Association for Medical Direction of Respiratory Care. Management of patients requiring prolonged mechanical ventilation: report of a NAMDRC consensus conference. Chest 2005; 128(6): 3937-54.
2. Clark PA, Lettieri CJ. Clinical model for predicting prolonged mechanical ventilation. J CritCare. 2013; 28(5): 880.

3. Sellares J, Ferrer M, Cano E, Loureiro H, Valencia M, Torres A. Predictors of prolonged weaning and survival during ventilator weaning in a respiratory ICU. Intensive Care Med 2011; 37: 775– 784.
4. Pesau B, Falger S, Berger E, Weimann J, Schuster E, Leithner C, Frass M. Influence of age on outcome of mechanically ventilated patients in an intensive care unit. Crit Care Med. 1992; 20(4): 489-92.
5. Meinders AJ, Hoeren JG, Meinders AE. The outcome of prolonged mechanical ventilation in elderly patients. Age Ageing 1996; 25: 353-356.
6. Funk GC, Anders S, Breyer MK, Burghuber OC, Edelmann G, Heindl W, Hinterholzer G, Kohansal R, Schuster R, Schwarzmaier-D'Assie A, Valentin A, Hartl S. Incidence and outcome of weaning from mechanical ventilation according to new categories. Eur Respir J. 2010; 35(1): 88-94
7. Ely EW, Evans GW, Haponik EF. Mechanical ventilation in a cohort of elderly patients admitted to an intensive care unit. Ann Intern Med. 1999; 131(2): 96-104.
8. Gajic O, Afessa B, Thompson BT, Frutos-Vivar F, Malinchoc M, Rubenfeld GD, et al. for the Second International Study of Mechanical Ventilation and ARDS-net Investigator: Prediction of death and prolonged mechanical ventilation in acute lung injury. Crit Care. 2007;11:R53.
9. Velmahos GC, Belzberg H, Chan L, Avari S, Cornwell EE, Berne TV. Factors predicting prolonged mechanical ventilation in critically injured patients: introducing a simplified quantitative risk score. Am Surg. 1997;63:811-7.
10. Troché G, Moine P. Is the duration of mechanical ventilation predictable? Chest. 1997;112:745-51.
11. Special thanks to the university hospital of Caracas.

### 000182

#### PREVENT OR RESCUE? A Review of the Application of Airway Pressure Release Ventilation (APRV) for Patients with Acute Respiratory Distress Syndrome (ARDS) in one District General Hospital (DGH) Intensive Care Unit (ICU)

T. Sanderson[1], T. Samuels[2], R. Kumar[2], A. Falek[2], R. Phelan[2], J. Burns[2], M. Alice[2]
[1]Critical care, East Surrey Hospital, London, United Kingdom; [2]Critical care, East Surrey Hospital, Redhill , United Kingdom
**Correspondence:** T. Sanderson
*Intensive Care Medicine Experimental* 2019, **7(Suppl 3):**000182

**INTRODUCTION.** Trial evidence suggests that early application of APRV is associated with better oxygenation and shorter length of stay in ICU (1). Animal studies suggest early APRV may improve oxygenation, minimise histological lung damage and even prevent ARDS development entirely (2, 3). However, the use of APRV is inconsistent and not widespread (4).

**OBJECTIVES.** To review the use of APRV within our ICU and describe the course of ARDS and clinical outcomes in those patients.

**METHODS.** A protocol for intital settings, weaning and troubleshooting APRV is in place in our 16 bed mixed medical and surgical ICU. Data were collected between 09/2018-03/2019 on patients receiving APRV for management of ARDS (using the Berlin definition) (5). Nursing charts were reviewed to retrieve PaO2 & FiO2 values. PaO2:FiO2 ratios (PF) were calculated for admission, initiation of APRV, and 6 & 24 hours post initiation. Electronic patient records were reviewed to find mortality rates at 28 and 90 days.

**RESULTS.** 9 patients were initiated on APRV for management of ARDS. On admission to ICU, 5 were classified severe, 2 moderate. Mean time between the point where the patient first met the ARDS criteria on ICU (T0) and initiation of APRV was 12 hours (range: 0-47 hours). PF deteriorated in 5 patients from T0 to APRV initiation by 12-50%; by which point 7 patients were severe, and 2 moderate ARDS. 3 of the 4 patients who died were noted to have the most significant drop in PF (31%, 38% and 50%). In 2 cases APRV was used within 2 hours of T0 and PF was not observed to deteriorate. In the remaining 2 cases, PF improved between T0 and APRV initiation by 13% & 28%. 4 patients were discussed with ECMO, and 2 transferred. 1 was lost to follow up as they were transferred within 4 hours of APRV initiation. PF improved on APRV by 16-342% at 6 hours (with one patient's PF ratio dropping by 41%), 4-364% at 24 hours, and 49-321% at point of APRV termination. 7 of the remaining 8 patients still met the criteria for ARDS on APRV cessation (4 = Moderate, 3 = Mild). Mortality was 44% at 28 days (unchanged at 90 days).

**CONCLUSION.** In this small study, most patients were admitted to ICU with already well-established ARDS, suggesting that it may not have been possible for them to achieve the benefits of early APRV. In addition, further delay before initiation of APRV was associated with deterioration in PF in 5 out of 9 patients. The most significant deterioration in PF was seen in 3 of the 4 patients who died. There was variation in the apparent indication to start APRV (with no obvious PF threshold) implying inconsistency with the practice of APRV. In addition many patients with ARDS will have undergone prone positioning instead of APRV, as there is little evidence to guide which patients will respond optimally to each technique. Further work is needed to clarify optimal timing of APRV use and the role of earlier ICU admission, in order to reduce ARDS progression, and to guide where APRV may be more appropriately used than prone positioning.

**REFERENCE(S)**

1) Zhou, Y. et al (2017). Early application of airway pressure release ventilation may reduce the duration of mechanical ventilation in acute respiratory distress syndrome. Intensive Care Medicine, 43(11), pp.1648-1659.
2) Roy, S. et al (2013). Preemptive Application of Airway Pressure Release Ventilation Prevents Development of Acute Respiratory Distress Syndrome in a Rat Traumatic Hemorrhagic Shock Model. Shock, 40(3), pp.210-216.
3) Roy, S. et al (2012). Early stabilizing alveolar ventilation prevents acute respiratory distress syndrome. Journal of Trauma and Acute Care Surgery, 73(2), pp.391-400.
4) Lakhani, R. et al (2019). Use of prone positioning and airway pressure release ventilation (APRV) for patients with acute respiratory distress syndrome (ARDS) in intensive care units in London and South-East England. Intensive Care Medicine Experimental, 6(2).
5) ARDS Definition Task Force, et al (2012). Acute Respiratory Distress Syndrome: the Berlin Definition. JAMA, 307(23).

### 000230

#### Use of inverse ratio ventilation in prehospital emergency medicine

G. Jansen; N. Kappelhoff, R. Borgstedt; S. Rehberg
Anaesthesiology, Prostestant Hosipital of the Bethel Foundation, Bielefeld, Germany
**Correspondence:** G. Jansen
*Intensive Care Medicine Experimental* 2019, **7(Suppl 3):**000230

**INTRODUCTION.** The increasing availability of modern emergency respirators, allows for differentiated ventilation strategies, such as Inverse ratio ventilation (IRV) also in the pre-hospital emergency care setting. While there are detailed guidelines for preclinical airway management , recommendations for preclinical ventilation are just based on guidelines for in-hospital intensive care. The present survey was designed to determine the use of IRV in the prehospital setting by German emergency physicians (GEP).

**METHODS.** An anonymous web-based questionnaire encompassing 7 questions was sent to GEP from September to December of 2018. They were asked to specify their medical specialty; their education level, their experience in intensive and prehospital emergency care, their average number of assignments per month, their used ventilator model and ventilation mode.

**RESULTS.** 60 % of the questionnaires were completed (157/261). 95 % of participants had preclinical access to respirators with the ability to perform IRV. 9.9% of respondents (male 11, female 4) stated that they were already performing preclinical IRV (I:E 1.5=1; I:E 2:1 = 1). The proportion of consultants in the IRV group was higher than in the non-IRV group. The proportion of experience in intensive care medicine and the number of assignments per month differed significantly between both groups. The corresponding group characteristics are shown in tables 1 and 2.

**CONCLUSION.** Approximately 10% of the surveyed GEP employ IRV in the pre-hospital setting. Compared to their colleagues not using

IRV, IRV using GEP had a longer experience in intensive care and a higher rate of monthly missions. These results suggest, that , training in differentiated ventilation strategies such as IRV should be integrated into the training of GEP.

**Table 1 (abstract 000230).** Specialist discipline of the surveyed emergency physicians

| | Anaesthesiology | Internal Medicine | Surgery | General medicine | Paediatrics | Other |
|---|---|---|---|---|---|---|
| IRV [n(%)] | 11 (73,3 %) | 2 (13,3 %) | 1 (6,7 %) | 0 (0 %) | 1 (6,7%) | - |
| Non-IRV[n(%)] | 102 (70,8%) | 19 (13,2%) | 10 (6,9 %) | 6 (4,2 %) | 2 (1,4 %) | 5 (3,5 %) |

**Table 2 (abstract 000230).** User characteristics IRV vs. Non-IRV

| | IRV | Non-IRV |
|---|---|---|
| Consultants | 14/15 (93,3%) | 105/144 (72,9%) |
| Experience in intensive care (y) | 3,03 # | 1,96 |
| Experience in prehospital emergency medicine (y) | 3,53 | 3,26 |
| Number of monthly missions (n/mon) | 37,6 # | 26,5 |
| # p < 0.05 vs. non-IRV | | |

### 001348

#### Vitamins use in septic shock in a polivalent ICU of a third level hospital

D. Robaglia, A. Vidal, L. Polanco, JJ. Paez Vargas, VA. Hortigüela Martín, JM. Milicua, A. Tejero, CC. Perez
ICU, Hospital Universitario Fundación Jiménez Díaz, Madrid, Spain
**Correspondence:** D. Robaglia
*Intensive Care Medicine Experimental* 2019, **7(Suppl 3):**001348

**INTRODUCTION.** Septic shock is a severe pathology in ICUs, with high cost for the society. There is a recent interest about vitamins therapy in septic shock patients in our unity as much as in the critical medical literature.
**OBJECTIVES.** Describes epidemiological, clinical data and treatments dispended (vitamins and hemodynamic) of all patients admitted with septic shock in our ICU of a third level hospital.
**METHODS.** Retrospectively, all patients diagnosed with septic shock according to SOFA criteria between January 2018 and November 2018 were analyzed. Epidemiological characteristics, gravity scores, admission causes, treatment dispended (vitamins and steroids), complications (acute renal failure, acute liver failure, acute myocardial failure, ARDS and multiorgan failure) and outcome like ICU stay and mortality were collected. We also analyzed hemodynamic data (type of monitoring, need of noradrenalin, use of second vasopressor and use of ionotropic support).
**RESULTS.** A total of 39 patients were analyzed (63.2% ± 2.59% males), the average age was 63.23±2.59 years, the SAPPS II gravity score at admission was 47 ± 2.81, SOFA was 9 ± 1.58 and PCR was 34 ± 14.3. Lactate peak of the first 72 hours was 52.53 ±6.14.
The ICU admission most frequent causes were septic shock (65.8%), post operatory (15.8%) and low conscience level (5.6%).
55.3% of the patients receive surgical treatment, 44.75% emergently. 65.8% had acute renal failure (ARF), 39.5% receive extra renal depuration therapy (ERDT), 15.8% develop an acute liver failure (ALF), 13.2% an ARDS, 26.7% an acute myocardial failure (AMF) and 36.8 % a multiorgan failure (MOF). Use of noradrenalin in 94.7% and in 44.7% of theses case in higher concentration than 0.5 5 µg/kg/min, use of a second vasopressor in 7.9%, dobubutamin was receive in 39.5% and second ionotropic agent in 5.3%.
55.3% of the patients receive thiamine (Vitamin B1), the same percentage steroids and 21.1% vitamin C. The use of vitamin B1 is statistically related with use of steroids (p=0.001) and vitamin C (p=0.018), develop ARDS (p=0.003) and need to ERDT (p=0.037). The use of vitamin C is statistically related with use of steroids (p=0.039) and vitamin B1 (p=0.032), develop ALF (p=0.012), need a second vasopressor (p=0.006) and need to ERDT (p=0.039).
Mortality at hospital discharge was 36.1% in which 46.13% was for multiorgan failure and the same percentage for limitation of effort.
**CONCLUSION.** Vitamin B1 is use more frequently than vitamin C, more than half of the patients with septic shock receive it. Both of them are use in a refractory septic shock with the same indication that we use steroids. Vitamin C is dispended in a few desperate cases with data of high severity like acute liver failure or use of second vasopressor. It is, in our unit a compassionate treatment.

### 001136

#### Usefulness of blood gas analysis as predictor of hospital mortality in patients with septic shock: Cohort study

OE. Palacios Calderon, P. Palacios Moguel, JE. Monter Vigueras, CA. Rojas Gomez, R. Carbajal Serrano, J. Franco Granillo, G. Camarena Alejo, J. Aguirre Sánchez, A. Aisa Alvarez
Intensive care unit, The American British Cowdray Medical Center, Mexico City, Mexico
**Correspondence:** O.E. Palacios Calderon
*Intensive Care Medicine Experimental* 2019, **7(Suppl 3):**001136

**INTRODUCTION.** Blood gas analysis is probably the most common diagnostic tool used in intensive care. A proper understanding and use of arterial and pulmonary/central venous blood gas analysis makes it possible to correctly interpret most of the respiratory, circulatory and metabolic derangements which may occur in septic shock patients1.
**OBJECTIVES.** To assess the effectiveness of arterial and venous blood analysis in predicting mortality of patients with septic shock.
**METHODS.** Prolective cohort study conducted in two general intensive care units, between June 2018–April 2019. Adults >18yo with septic shock diagnosis based on SEPSIS-3 criteria were included. Time 0 when diagnosis was made. All patients monitored from admission to hospital discharge. Simultaneously, blood gas analysis were carried out to obtain: PaO2/FiO2, ScvO2, Qs/Qt, P(v-a)CO2, C(a-v)O2, P(v-a)CO2/C(a-v)O2, O2ER. Primary outcome was usefulness of arterial and pulmonary/central venous blood analysis as mortality predictor. Standard deviation and medians with interquartile ranges for quantitative variables. Frequencies and percentages for qualitative variables. Student´s t-distribution or Mann-Whitney U-test for groups comparison depending on distribution. Binary logistic regression to control for confoundings. Pearson test for correlation analysis between two quantitative variables. Multiple linear regression analysis to generate an equation with the significant variables of the model.
**RESULTS.** 66 patients with mean age of 66.5 ± 17 years, 35 (53%) men, 16 (24%) diabetics and 27 (40%) with hypertension. SAPS II 41.5 (12 – 97) and SOFA 8.4 ± 3.2. 13 (19.7%) patients died. Patients who died had CKD (30 vs 8; *p* = 0.05), higher BMI (29.14 ± 10.53 vs 24.92 ± 5.6; *p* = 0.05) and lower PaO2/FiO2 (139.51 ± 48.48 vs 218.48 ± 98.07; *p* = 0.04). Logistic regression analysis showed that PaO2/FiO2 in time 0 was associated with mortality

[OR 0.95 (IC95% 0.90 to 0.99); $p$ = 0.04]. There is correlation of PaO2FiO2 with SvcO2 (r 0.3; $p$ = 0.013) and Qs/Qt (r 0.6; $p$ < 0.001), by these results we generate the following equation with a multiple linear regression analysis: **PaO2/FiO2 = 84,748 + 3.136 (SvcO2) - 4.411 (Qs/Qt) [r2 0.45;*p*< 0.001].**

**CONCLUSION.** We found association of PaO2/FiO2 with mortality in patients with septic shock. There is correlation of PaO2/FiO2 with SvcO2 and Qs/Qt, so we believe that this should be adjusted to these variables. To increase statistical power, a larger sample size is required.

## AKI - Critical care nephrology 1

### 000210

#### Influence of contrast media on renal function and outcomes in critically ill patients

Y. Goto[1], T. Fujii[2], S. Uchino[3], K. Doi[4], S. Katayama[1]
[1]Department of anesthesiology and intensive care, Jichi Medical University, Tochigi, Japan; [2]Department of epidemiology and preventive medicine, Kyoto University Graduate School of Medicine, Kyoto, Japan; [3]Intensive care unit, department of anaesthesiology, Jikei University School of Medicine, Tokyo, Japan; [4]Department of emergency and critical care medicine, The University of Tokyo, Tokyo, Japan
**Correspondence:** Y. Goto
*Intensive Care Medicine Experimental* 2019, **7(Suppl 3):**000210

**INTRODUCTION.** In recent years, several papers reported that renal dysfunction caused by contrast media is less frequent than previously reported in critically ill patients(1). However, it remains unclear whether the administration of contrast media for critically ill is harmful, as previous studies focused on specific subpopulations in the ICU(2) or were conducted in a single center(3-5).

**OBJECTIVES.** To assess the risk of contrast media administration by examining the renal function and prognosis of patients with or without receiving contrast media in critically ill patients.

**METHODS.** This was a post-hoc analysis of the Japan AKI Database study, which was a prospective cohort study on acute kidney injury (AKI) conducted in the 13 mixed ICUs in Japan from July through December in 2016(6). Adult patients who stayed for >24 hours in the ICU were consecutively registered. Patients were divided into Group C, who received contrast media within 48 hours before the ICU admission and Group NC, who did not receive contrast media. The primary outcome was deterioration of renal function (DRF) defined as elevation of serum creatinine level (>0.3mg/dL or 1.5-fold from baseline) or decreased urine output (<0.5ml/kg/hr for 6 hours). ICU mortality and hospital mortality were also assessed as the secondary outcomes. The effects of contrast media on the outcomes were analyzed using propensity score matched analysis.

**RESULTS.** A total of 2246 patients were eligible to the study. Of which, 606 patients were given contrast media within 48 hours before the ICU admission. After propensity score matching, the proportions of DRF were similar between group C and group NC (35.9 vs 33.8%, *P*= 0.706). As for secondary outcomes, ICU and hospital mortality (4.0 vs 2.8%, *P* =0.623 and 13.8 vs 9.3%, *P* =0.159, respectively) were similar between two groups.

**CONCLUSION.** The effect of contrast media on the deterioration of renal function was not evident in critically ill patients.

**REFERENCE(S)**

1. Aycock RD, Westafer LM, Boxen JL, Majlesi N, Schoenfeld EM, Bannuru RR. Acute Kidney Injury After Computed Tomography: A Meta-analysis. Ann Emerg Med. 2018;71(1):44-53 e4.
2. Ng CS, Shaw AD, Bell CS, Samuels JA. Effect of IV contrast medium on renal function in oncologic patients undergoing CT in ICU. AJR Am J Roentgenol. 2010;195(2):414-22.
3. McDonald JS, McDonald RJ, Williamson EE, Kallmes DF, Kashani K. Post-contrast acute kidney injury in intensive care unit patients: a propensity score-adjusted study. Intensive Care Med. 2017;43(6):774-84.
4. Cely CM, Schein RM, Quartin AA. Risk of contrast induced nephropathy in the critically ill: a prospective, case matched study. Critical care (London, England). 2012;16(2):R67.
5. Haveman JW, Gansevoort RT, Bongaerts AH, Nijsten MW. Low incidence of nephropathy in surgical ICU patients receiving intravenous contrast: a retrospective analysis. Intensive Care Med. 2006;32(8):1199-205.
6. Fujii T, Uchino S, Doi K, Sato T, Kawamura T, group Js. Diagnosis, management, and prognosis of patients with acute kidney injury in Japanese intensive care units: The JAKID study. Journal of critical care. 2018;47:185-91.

**Table 1 (abstract 000210).** See text for description

| | Contrast (n= 248) | No contrast (n= 248) | P value |
|---|---|---|---|
| Renal function deterioration, n(%) | 89 (35.9) | 84 (33.8) | 0.706 |
| - Creatinine >0.3mg/dL increase | 41 (16.5) | 45 (18.2) | 0.722 |
| - 1.5fold increase in creatinine | 23 (9.3) | 24 (9.7) | 1.000 |
| - Urine output < 0.5ml/kg/hr for >6hr | 69 (27.8) | 56 (22.6) | 0.215 |
| ICU death within 3 days | 4 (1.6) | 2 (0.8) | 0.686 |
| RRT during ICU stay, n(%) | 24 (9.7) | 20 (8.1) | 0.636 |
| ICU length of stay, days(IQR) | 3.4 (1.9-6.7) | 3.0 (1.9-6.0) | 0.236 |
| Hospital length of stay, days(IQR) | 26 (16-52) | 30 (17-53) | 0.434 |
| Chronic HD after discharge, n(%) | 6 (2.4) | 2 (0.8) | 0.285 |
| Serum creatinine at discharge | 0.75 (0.59-1.09) | 0.74 (0.58-1.02) | 0.148 |
| ICU mortality | 10 (4.0) | 7 (2.8) | 0.623 |
| Hospital mortality | 34 (13.8) | 23 (9.3) | 0.159 |

### 000232

#### The effect of long-term duration renal replacement therapy on outcomes of critically ill patients with acute kidney injury: A retrospective cohort study

M. Yang[1], K. Hongjun[2]
[1]Departmentof critical care medicine, Department of Critical Care Medicine, Chinese PLA General Hospital, Beijing,China, Beijing, China; [2]Departmentof critical care medicine, Chinese PLA General Hospital, Beijing, China,
**Correspondence:** M. Yang
*Intensive Care Medicine Experimental* 2019, **7(Suppl 3):**000232

**INTRODUCTION.** Renal replacement therapy (RRT), as a cornerstone of supportive treatment, has long been performed in critically ill patients with acute kidney injury (AKI). However, the majority of studies may have neglected the effect of the duration of RRT on the outcome of AKI patients. To explore the effect of the long duration of RRT on the outcome of critically ill patients with AKI

**METHODS.** This retrospective study was conducted by using the Multiparameter Intelligent Monitoring in Intensive Care II (MIMIC-II) database, which is a publicly available clinical database developed by the Massachusetts Institute of Technology (MIT), Phillips Healthcare, and Beth Israel Deaconess Medical Center (BIDMC). This database contains data from more than 32,000 critically ill patients who were treated in the ICUs at BIDMC from 2001 to 2008.The primary outcome measure of this study was the survival rate at 28 days, 60 days and 90 days in the long-duration RRT group and the non-long-duration RRT group. The secondary outcomes assessed the difference in clinical outcome in these two groups. Lastly, the effect of the duration of RRT on mortality in AKI patients was determined as the third outcome.

**RESULTS.** We selected 1,020 patients in total who received RRT treatment according to the MIMIC-IIdatabase. According to the inclusion and exclusion criteria, we finally selected 506 patients with AKI: 286 AKI patients in the non-long-duration RRT group and 220 in the long-duration RRT group. After 28 days, there was a significant difference in all-cause mortality between the long-duration RRT group and the non-long-duration RRT group ($p$=0.001). However, the difference disappeared after 60 days and 90 days ($p$=0.803 and $p$=0.925, respectively). The length of ICU stay, length of hospital stay and duration of mechanical ventilation were significantly longer in the long-duration RRT group than those in the non-long-duration RRT group. Considering 28-day mortality, the longer duration of RRT was shown to be a protective factor (HR=0.995, 95% CI 0.993-0.997, $p$< 0.0001),while 60-day and 90-day mortality were not correlated with improved protection.

**CONCLUSION.** The long duration of RRT can improve the short-term prognosis of AKI patients, but it does not affect the long-term prognosis of these patients. Prognosis is determined by the severity of the illness itself. This suggests that RRT can protect AKI patients through the most critical time, however, the final outcome cannot be altered.

**REFERENCE(S)**

1. Gaudry S, Hajage D, Schortgen F,et al (2016) Initiation Strategies for Renal-Replacement Therapy in the Intensive Care Unit.N Engl J Med 375:122-133.
2. Palevsky PM, Zhang JH, O'Connor TZ,et al (2008) Intensity of renal support in critically ill patients with acute kidney injury.N Engl J Med 359:7-20.
3. Wald R, Shariff SZ, Adhikari NK,et al (2014) The association between renal replacement therapy modality and long-term outcomes among critically ill adults with acute kidney injury: a retrospective cohort study*.Crit Care Med 42:868-877.
4. De Corte W, Dhondt A, Vanholder R,et al (2016) Long-term outcome in ICU patients with acute kidney injury treated with renal replacement therapy: a prospective cohort study.Crit Care 20:256.
5. Kawarazaki H, Uchino S (2016) Validity of low-efficacy continuous renal replacement therapy in critically ill patients.Anaesthesiol Intensive Ther 48:191-196.
6. Akhoundi A, Singh B, Vela M,et al (2015) Incidence of Adverse Events during Continuous Renal Replacement Therapy.Blood Purif 39:333-339.
7. Honore PM, De Waele E, Jacobs R,et al (2013) Nutritional and metabolic alterations during continuous renal replacement therapy.Blood Purif 35:279-284.
8. Economou CJP, Wong G, McWhinney B,et al (2017) Impact of beta-lactam antibiotic therapeutic drug monitoring on dose adjustments in critically ill patients undergoing continuous renal replacement therapy.Int J Antimicrob Agents 49:589-594.
9. Ben-Hamouda N, Charriere M, Voirol P,et al (2017) Massive copper and selenium losses cause life-threatening deficiencies during prolonged continuous renal replacement.Nutrition 34:71-75.
10. Baird JS, Wald EL (2010) Long-duration (>4 weeks) continuous renal replacement therapy in critical illness.Int J Artif Organs 33:716-720.
11. Vats HS, Dart RA, Okon TR,et al (2011) Does early initiation of continuous renal replacement therapy affect outcome: experience in a tertiary care center.Ren Fail 33:698-706.
12. Saeed M, Villarroel M, Reisner AT,et al (2011) Multiparameter Intelligent Monitoring in Intensive Care II: a public-access intensive care unit database.Crit Care Med 39:952-960.
13. Villa G, Neri M, Bellomo R,et al (2016) Nomenclature for renal replacement therapy and blood purification techniques in critically ill patients: practical applications.Crit Care 20:283.
14. Neri M, Villa G, Garzotto F,et al (2016) Nomenclature for renal replacement therapy in acute kidney injury: basic principles.Crit Care 20:318.
15. Clark WR, Neri M, Garzotto F,et al (2017) The future of critical care: renal support in 2027.Crit Care 21:92.
16. Ji Q, Mei Y, Wang X,et al (2011) Timing of continuous veno-venous hemodialysis in the treatment of acute renal failure following cardiac surgery.Heart Vessels 26:183-189.
17. Lin YF, Ko WJ, Chu TS,et al (2009) The 90-day mortality and the subsequent renal recovery in critically ill surgical patients requiring acute renal replacement therapy.Am J Surg 198:325-332.
18. Tatum JM, Barmparas G, Ko A,et al (2017) Analysis of Survival After Initiation of Continuous Renal Replacement Therapy in a Surgical Intensive Care Unit.JAMA Surg 152:938-943.
19. Zhang J, Tian J, Sun H,et al (2018) How Does Continuous Renal Replacement Therapy Affect Septic Acute Kidney Injury?Blood Purif 46:326-331.
20. Lee S, Huen S, Nishio H,et al (2011) Distinct macrophage phenotypes contribute to kidney injury and repair.J Am Soc Nephrol 22:317-326.
21. Castro BA, Flanigan P, Jahangiri A,et al (2017) Macrophage migration inhibitory factor downregulation: a novel mechanism of resistance to anti-angiogenic therapy.Oncogene 36:3749-3759.
22. Wewer Albrechtsen NJ, Kuhre RE, Pedersen J,et al (2016) The biology of glucagon and the consequences of hyperglucagonemia.Biomark Med 10:1141-1151.
23. Ono S, Tsujimoto H, Hiraki S,et al (2018) Mechanisms of sepsis-induced immunosuppression and immunological modification therapies for sepsis.Ann Gastroenterol Surg 2:351-358.
24. Heering P, Morgera S, Schmitz FJ,et al (1997) Cytokine removal and cardiovascular hemodynamics in septic patients with continuous venovenous hemofiltration.Intensive Care Med 23:288-296.
25. Park S, Lee S, Kang MW,et al (2018) Postdischarge Major Adverse Cardiovascular Events of ICU Survivors Who Received Acute Renal Replacement Therapy.Crit Care Med 46:e1047-e1054. 26. Violo L, De Francesco M (2018) Timing of Continuous Renal Replacement Therapy.JAMA Surg 153:289-290.
27. Zarbock A, Kellum JA, Schmidt C,et al (2016) Effect of Early vs Delayed Initiation of Renal Replacement Therapy on Mortality in Critically Ill Patients With Acute Kidney Injury: The ELAIN Randomized Clinical Trial.JAMA 315:2190-2199.
28. Gaudry S, Hajage D, Schortgen F,et al (2018) Timing of Renal Support and Outcome of Septic Shock and Acute Respiratory Distress Syndrome. A Post Hoc Analysis of the AKIKI Randomized Clinical Trial.Am J Respir Crit Care Med 198:58-66.

29. The authors thank all research participants for their assistanc

### 000246

#### Rising GGT during ICU admission protects against acute kidney injury and is associated with lower hospital mortality

G. Jansma, P. Freire Jorge, M. Nijsten
Department of critical care, University Medical Center Groningen, Groningen, Netherlands
**Correspondence:** G. Jansma

*Intensive Care Medicine Experimental* 2019, **7(Suppl 3):**000246

**INTRODUCTION.** It has been demonstrated in various ICU patient populations that an elevated gamma-glutamyl transpeptidase (GGT) between the 6th and 14th ICU day is associated with a better outcome [1-3]. Whether a relatively high GGT also confers a lowered risk for acute kidney injury (AKI) has not been investigated. In this study we examined the dynamics of GGT during the first ICU week and its relation with AKI and outcome.

**METHODS.** A cohort of patients previously studied for the glucose/lactate interaction with liver and renal function was analyzed [4]. Daily creatinine levels were used for the assessment of the development of AKI during the first ICU week. GGT levels were determined frequently. We selected patients with initially normal GGT (i.e. <55 IU/L). GGT levels were compared between AKI and no AKI and hospital survivors and hospital non-survivors. We also analyzed the relation of GGT with AKI in the group of survivors.

**RESULTS.** The cohort existed of 9074 patients (63% male; median age 64 years) and a hospital mortality of 11%. The incidence of AKI in the cohort was 21% (n=1877) and was significantly lower in the group hospital survivors compared to the group hospital nonsurvivors (17 vs 51%, p<0.001). After selecting patients with a normal GGT at ICU day 1 (n = 5399), median [IQR] GGT levels were significantly higher in the group AKI vs no AKI on the first 2 days of ICU admission (26 [17-37] vs 22 [15-32] p=0.02 resp 27 [17-37] vs 24 [16-34] p<0.001). On ICU day 6 GGT was significantly higher in the patients with no AKI vs AKI (80 [45-154] vs 70 [39-119] p<.01). On the first 2 days of ICU admission GGT was also significantly higher in the group non-survivors (29 [19-40] vs 22 [15-33] p<0.001 resp 28 [19-38] vs 24 [16-34] p<0.001) and on day 6 GGT was higher in the group survivors compared to non-survivors (79 [44-148] vs 66 [38-103] p<0.01). Also when only survivors were analyzed, higher GGT at ICU day day 6 was associated with lower risk of AKI (p=0.04).

**CONCLUSION.** In patients with a normal GGT level at day 1 of ICU admission, higher GGT on the first 2 days of ICU admission were associated with a higher incidence of AKI and hospital mortality. However, during ICU admission overall GGT levels progressively increased and higher GGT levels on day 6 of ICU admission were associated with a lower incidence of AKI and hospital mortality. This suggests a protective role of GGT itself or that elevated GGT may be a marker of regeneration and recovery of the liver or kidneys.

**REFERENCE(S)**

[1] Haveman JW, Zeebregts CJ, Verhoeven EL, van den Berg P, van den Dungen JJ, Zwaveling JH, Nijsten MW. Changes in laboratory values and their relationship with time after rupture of an abdominal aortic aneurysm. Surg Today. 2008;38(12):1091-101

[2] Alkozai EM, Lisman T, Porte RJ, Nijsten MW. Early elevated serum gamma glutamyl transpeptidase after liver transplantation is associated with better survival. F1000Res. 2014 Apr 3;3:85

[3] Alkozai EM, Mahmoodi BK, Decruyenaere J, Porte RJ, Oude Lansink-Hartgring A, Lisman T, Nijsten MW. Systemic comparison of routine laboratory measurements with in-hospital mortality: ICU-Labome, a large cohort study of critically ill patients. Clin Chem Lab Med. 2018 Jun 27;56(7):1140-1151

[4] Jorge PF, Wieringa N, de Felice E, van der Horts ICC, Oude Lansink A, Nijsten MW. The association of early combined lactate and glucose levels with subsequent renal and liver dysfunction and hospital mortality in critical ill patients. Critical Care. 2017 21:218

### 000292

#### One-year prognostic value of kidney biomarkers at discharge from the intensive care unit

L. Matthieu,[1], H. Alexa[2], D. François[1], H. Oliver,[3], S. Joachim,[3], F. Marie-Céline,[1], A. Mebazaa[1], G. Etienne,[4]
[1]Department of anaesthesiology, surgical intensive care and burn unit, Saint-Louis Hospital, Paris, France; [2]Department of anesthesia, surgical intensive care, University Hospital of Basel, Basel, Switzerland; [3]Sphingotec gmbh, Sphingotec GmbH, Hennigsdorf, Germany; [4]Department of anaesthesiology, surgical intensive care, Hospital Lariboisière, Paris, France
**Correspondence:** D. François
*Intensive Care Medicine Experimental* 2019, **7(Suppl 3):**000292

**INTRODUCTION.** Serum creatinine (Screat) may overestimate renal function at discharge from the intensive care unit (ICU). The objective was to evaluate the association between biomarkers of kidney function and injury at ICU discharge and one -year survival.

**METHODS.** Ancillary study of the FROG-ICU cohort, a prospective observational cohort in 21 ICUs in France and Belgium, including ICU patients receiving mechanical ventilation and/or hemodynamic support, with one-year follow-up. Kidney biomarkers were measured at ICU discharge among ICU survivors (plasma Cystatin C (pCystc); plasma and urinary Neutrophil gelatinase associated lipocalin (pNGAL and uNGAL); plasma proenkephalin (penKid®)). A Sub-group analyses were performed in the sub-group of patients with discharge serum creatinine < 1.5-fold of baseline.

**RESULTS.** Among 1207 ICU survivors with complete biomarker assessment and follow-up, 231 died during the year following ICU discharge (19.4%). One-year non-survivors had higher discharge biomarkers level (79 (56.5 to 113.5) vs. 63 (50 to 86) mmol/L for Screat, 91.9 (31.6 to 266.9) vs. 47.7 (23.1 to 141.2) ng/mL for uNGAL, 201 (106 to 350.8) vs. 115 (71 to 189.2) ng/mL for pNGAL, 1.6 (1.2 to 2.4) vs. 1.1 (0.9 to 1.6) 1 mg/L for pCystC and 80.7 (53.3 to 132.9) vs. 55 (40.7 to 82.9) ng/mL for penKid, all p<0.0001). Un-adjusted and adjusted (i.e. on prognostic factors previously identified in the FROG cohort: Age, Charlson comorbidity score, vascular disease, severe valvular disease, chronic kidney diseases, cancer, loss of autonomy, systolic blood pressure, body temperature, total protein and platelet counts, and white blood cell count at ICU discharge, red blood cell transfusion and prolonged ICU length) odds ratios showing association between kidney biomarkers and one-year mortality are presented in Table 1.

**CONCLUSION.** Kidney biomarkers measured at ICU discharge are associated with one-year outcome, including patients with low serum creatinine at ICU discharge. These results suggest that patients with sub-clinical acute kidney injury at discharge are identified using these biomarkers.

**Table 1 (abstract 000292).** Adjusted OR and 95% confidence interval for one-year mortality in all ICU survivors, as well as in the subgroup of patients with low serum creatinine at discharge (i.e. <1.5 fold baseline)

| | All patients | Screat <1.5 baseline at discharge |
|---|---|---|
| sCreat | 1.79 [1.17 - 2.73] | 1.68 [1.04 - 2.69] |
| eGFR | 2.06 [1.30 - 3.27] | 1.94 [1.15 - 3.29] |
| uNGAL | 2.08 [1.35 - 3.21] | 1.99 [1.23 - 3.21] |
| pNGAL | 2.61 [1.71 - 3.97] | 2.63 [1.61 - 4.27] |
| pCystC | 3.11 [1.88 - 5.16] | 2.84 [1.67 - 4.83] |
| penKid | 2.20 [1.44 - 3.38] | 2.08 [1.29 - 3.37] |

### 000325

#### Elevated lactate levels were related with an increase in overall 90-day mortality among NCU patients. This association was specifically attributed to neurologic disease-related 90-day mortality

I. Chami[1], TK. Oh[2], S. In-Ae[3], C. Hyunhee[3]
[1]Surgery, Seoul National University Bundang Hospital, Seongnam, Republic of Korea; [2]Anesthesiology, Seoul National University Bundang Hospital, Seongnam, Republic of Korea; [3]Anesthesiology and pain medicine, Seoul National University Bundang Hospital, Seongnam, Republic of Korea
**Correspondence:** T.K. Oh
*Intensive Care Medicine Experimental* 2019, **7(Suppl 3):**000325

**INTRODUCTION.** It is unclear as to whether intensivist coverage has an effect on improving outcomes of patients postoperatively admitted to the surgical ICU.

**OBJECTIVES.** This study aimed to assess the effect of intensivist coverage on the incidence rate of postoperative acute kidney injury (AKI) and postoperative ventilator time among patients postoperatively admitted to the ICU

**METHODS.** Adult patients postoperatively admitted to the ICU between January 2012 and December 2017 were retrospectively enrolled. The incidence rate of AKI within 72 hours after surgery and the postoperative ventilator time were compared between the intensivist coverage group and the non-intensivist coverage group.
**RESULTS.** In total, 5,494 subjects were included in the final analysis (2,747 subjects in each group). The incidence rate of AKI was significantly higher in the non-intensivist coverage group than that in the intensivist coverage group (22.8% vs 20.2%; *P*=.017). Moreover, logistic regression analysis showed that the incidence rate of postoperative AKI in the non-intensivist coverage group has increased by 17% compared to that in the intensivist coverage group (odds ratio: 1.17, 95% confidence interval: 1.03-1.33; *P*=.017). Lastly, the median time of ventilator use in the non-intensivist coverage group was significantly longer than that in the intensivist coverage group [7.8 hours [interquartile range, IQR: 2.7-13.5] vs 5.5 hours [IQR: 2.1-8.5]; *P*<.001).
**CONCLUSION.** High-intensity intensivist coverage is associated with lower risk of postoperative AKI and shorter postoperative ventilator time, indicating the need for full-time intensivist coverage at the surgical ICU.

### 000348

#### Reading Between the "Lines": Improving Renal Replacement Therapy in a District General Hospital

N. French[1], R. Sundaram[2], L. Gemmell[2], J. Hunter[2]
[1]N H S Greater Glasgow & Clyde, Glasgow, United Kingdom; [2]Intensive care, N H S Greater Glasgow & Clyde, Glasgow, United Kingdom
**Correspondence:** N. French
*Intensive Care Medicine Experimental* 2019, **7(Suppl 3):**000348

**INTRODUCTION.** The Kidney Disease Improving Global Outcomes (KDIGO) guidelines recommend using regional citrate anticoagulation for continuous renal replacement therapy (RRT) in critically ill patients with acute kidney injury. We introduced citrate RRT in May 2017 in our intensive care unit.
**OBJECTIVES.** To assess the effect of citrate RRT on filter usage, RRT days, and filter downtime in critically ill patients in a district general hospital.
**METHODS.** We performed a retrospective observational study looking at patients admitted to our 7 bedded Level 3 critical care unit (370 patients per annum) for one year after the introduction of citrate RRT. We included patients over 16 years of age who had received RRT in our unit. Data was collected from WardWatcher, a Scottish Intensive Care Society Audit Group (SICSAG) database, electronic patient records (EPR) and PACS (a radiology information system). 53 patients received RRT on the unit during the year. A total of 8 patients were excluded; five of these patients had no RRT prescription chart on the EPR and three of them had received heparin.
**RESULTS.** There were a total of 53 patients, median age 58 years with 60% males. Of the 45 patients whose data were available, the average time on citrate RRT was 3.1 days vs 4.6 in 2015 giving a p value of 0.157. Average number of set changes was unchanged with 1.8 set changes per patient after introduction of citrate vs 1.8 in 2015, giving a p value of 0.362. We achieved the prescribed blood pump speed 80% of the time with citrate RRT as opposed to only 50% of the time in 2015. In 40% of the patients who received citrate RRT, the filter ran 100% uninterrupted. Filter clotting was still the commonest reason for filter set changes. A subgroup analysis based on line site (i.e. right internal jugular (RIJ), left internal jugular (LIJ), femoral veins (FV), or subclavian veins (SV)) was carried out due to the high proportion of LIJ lines in our unit. This revealed an average of 1.2 set changes for RIJ lines, 1.4 for LIJ, 0.67 for FV and 0 for the single SV line inserted. These included changes due to routine filter set changes at 72 hours.
**CONCLUSION.** It was predicted that the introduction of citrate RRT would reduce number of filter set changes per patient and filter downtime, ultimately reducing cost. The reasons for not being able to demonstrate reduction in filter set usage may include small numbers, line position (LIJ), and not recommencing filter after short planned interruptions such as CT transfers. It has not been possible to do a composite cost analysis at this stage to demonstrate cost reduction. We hope to feedback results of this study to the multidisciplinary team, improve awareness regarding choice of site for dialysis lines, and re-examine our outcomes in a year's time.

### 000441

#### Clinical prediction model for acute kidney injury recovery in the intensive care unit

CY. Huang, F. Guiza Grandas, M. Schetz, J. Gunst, M. Casaer, G. Van den Berghe, G. Meyfroidt
Laboratory of intensive care medicine, academic department of cellular and molecular medicine, KU Leuven, Leuven, Belgium
**Correspondence:** C.Y. Huang
*Intensive Care Medicine Experimental* 2019, **7(Suppl 3):**000441

**INTRODUCTION.** Acute kidney injury (AKI) occurs in up to 50% of patients admitted to the intensive care unit (ICU) [1]. Predicting recovery from AKI could be valuable, for patient and family counseling, prognostication, and post-ICU care planning. Patients with lower predicted probability of AKI recovery could benefit more from a focused health care management. However, early prediction of AKI recovery remains a major challenge.
**OBJECTIVES.** The purpose of this study was to identify predictors for AKI recovery. For this purpose, we developed and validated an AKI recovery prediction model in adult ICU patients, to predict AKI recovery at ICU discharge.
**METHODS.** This study was a retrospective preplanned secondary analysis of the EPaNIC multicenter randomized controlled trial database. AKI recovery was defined as no longer having AKI stage 3 [2], not receiving renal replacement therapy (RRT), and not being deceased at ICU discharge. The development cohort (n= 227) consisted of patients with AKI stage 3 and/or RRT during the ICU stay. Clinical data were retrieved from the EPaNIC research database and from the clinical patient data management system database. The model was developed by using a random forest (RF) machine-learning algorithm. Feature selection was performed with a RF-based selection approach. Performance was evaluated by discrimination, calibration, and decision curve, and internally validated (with 95% confidence intervals (CI)) by bootstrapping with 200 replications.
**RESULTS.** A total of 49 patients (21.59%) recovered from AKI at ICU discharge. Multivariate predictors for AKI recovery included age, baseline serum creatinine (SCr), SCr before onset of AKI stage 3 (mean, 25th percentile, mean of difference above mean, median, and slope), maximum lactate on day1, bilirubin on day1, heart rate before onset of AKI stage 3 (proportion above 75th percentile, and mean of difference above 75th percentile), mean arterial blood pressure before onset of AKI stage 3 (mean of difference below 25th percentile), C-reactive protein (CRP) level on day1, urea level on day1, and urine output volume on day1. The AKI recovery prediction model had an area under the receiver operating characteristic curve (AUROC) of 0.76 (95% C.I. 0.64 – 0.83), calibration slope of 0.98 (95% C.I. 0.43 – 1.73), and calibration in the large of 0.00 (95% C.I. -0.07 – 0.07). At the classification threshold that maximized sensitivity and specificity, sensitivity was 0.76 (95% C.I. 0.63 – 0.89), specificity was 0.74 (95% C.I. 0.61 – 0.86), net benefit with respect to treat-none was 0.09 (95%

C.I. 0.05 – 0.16), and net benefit with respect to treat-all was 0.16 (95% C.I. -0.00 – 0.48).

**CONCLUSION.** Although the determinants of AKI recovery are difficult to define, a prediction model based on routinely collected clinical data was able to fairly predict AKI recovery at ICU discharge.

**REFERENCE(S)**

[1] Chawla LS, Bellomo R, Bihorac A, et al (2017) Acute kidney disease and renal recovery: Consensus report of the Acute Disease Quality Initiative (ADQI) 16 Workgroup. Nat Rev Nephrol. https://doi.org/10.1038/nrneph.2017.2

[2] Khwaja A (2012) KDIGO clinical practice guidelines for acute kidney injury. Nephron - Clin. Pract.

[3] Chao-Yuan Huang receives a Taiwan-KU Leuven scholarship.

### 000442

#### How does admission creatinine affect critical care outcome?: A retrospective cohort database study

A. O'Docherty[1], M. Alice[2], T. Samuels[3], P. Morgan[3]
[1]East Surrey Hospital, Redhill, United Kingdom; [2]Icu, East Surrey Hospital, Redhill, United Kingdom; [3]Critical care, East Surrey Hospital, Redhill , United Kingdom
**Correspondence:** A. O'Docherty
*Intensive Care Medicine Experimental* 2019, **7(Suppl 3):**000442

**INTRODUCTION.** Many patients admitted to the intensive care unit (ICU) have some element of kidney injury, whether acute or on a background of chronic renal failure. Acute kidney injury (AKI) requiring renal replacement therapy (RRT) develops in around 5-10% of patients admitted to ICU[1]. It has previously been found that a low serum creatinine is associated with a poor outcome[2]. It has also been shown that AKI is an independent risk factor for mortality[1]. There are many parameters that can be used to identify and monitor AKI, such as urine output, serum potassium, fluid balance and serum creatinine. Raised serum creatinine is also a marker of chronic kidney disease. However, there is little research into the relationship between serum creatinine and critical care outcome. Our primary aim was to investigate whether there is a correlation between serum creatinine and ICU outcome, and hospital outcome.

**METHODS.** We searched our local ICU database, Ward Watcher, which contains data from 13,927 patients from 1993-April 2019. Emergency and non-emergency admissions were included. Patients with incomplete data regarding outcome were excluded. We assessed whether an association existed between the highest serum creatinine and critical care/hospital outcome.

**RESULTS.**

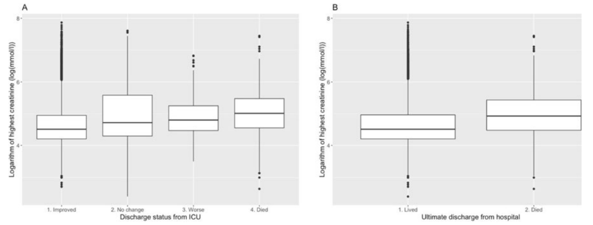

**Fig. 1 (abstract 000442).** A Shows a statistically significant association (p<0.0001 with Kruskal-Wallis rank sum test) between higher levels of creatinine and worse outcomes on discharge from ICU. B Illustrates a statistically significant difference in highest creatinine between the 'Lived' and 'Died' groups (on ultimate discharge from hospital), with p-value <0.0001 (unpaired Wilcoxon rank sum test), further supporting the hypothesis that patients with poor outcomes have worse renal function

**CONCLUSION.** Our data suggest a relationship between raised serum creatinine, and poor critical care and hospital outcome. Creatinine is a readily available test and is usually measured daily in critical care, so could be considered to be a useful aid in considering a patient's prognosis. While serum creatinine may not be the best marker of renal function (GFR)[3], there does appear to be a relationship between creatinine and ICU outcome. In our study we did not isolate emergency admissions, nor did we account for baseline creatinine or any prior chronic kidney disease. Further work is needed to study the effects of these parameters on unit outcome.

**REFERENCE(S)**

1. Delanaye P, Mariat C. Detection of decreased glomerular filtration rate in intensive care units: serum cystatin C versus serum creatinine. BMC Nephrology. 2014;15:9
2. Udy A, Bailey M. The Association Between Low Admission Peak Plasma Creatinine Concentration and In-Hospital Mortality in Patients Admitted to Intensive Care in Australia and New Zealand. Critical Care Medicine. 2016;44(1):73-82
3. Weisbord S, Palevsky P. Acute Renal Failure in the Intensive Care Unit. Seminars in Respiratory and Critical Care Medicine. 2006;27(3):262-73

### 000461

#### Propofol has a protective effect on renal function in patients undergoing thoracoscopic and laparoscopic esophageal surgery

A. Ogawa, H. Koumura, Y. Hara, N. Kuriyama, T. Nakamura, C. Yamashita, J. Shibata, O. Nishida
Anesthesiology and Critical care medicine, Fujita Health University, Toyoake, Japan
**Correspondence:** A. Ogawa
*Intensive Care Medicine Experimental* 2019, **7(Suppl 3):**000461

**INTRODUCTION.** Volatile anesthetic agents have been reported to exert a protective effect on the lungs and heart, whereas intravenous anesthetic agents exert a protective effect on the brain. Acute kidney injury (AKI) is a serious complication associated with morbidity and mortality. The effect of anesthetic agents on renal function remains unclear.

**OBJECTIVES.** We aimed to investigate the incidence and risk factors of AKI in relation to the use of different anesthetic agents in patients undergoing thoracoscopic and laparoscopic esophageal surgery.

**METHODS.** We reviewed the electronic medical records and laboratory results of all patients who underwent thoracoscopic and laparoscopic esophageal surgery at Fujita Health University Hospital between November 2015 and February 2018. We excluded patients undergoing hemodialysis. We analyzed sex, age, weight, height, body mass index, anesthesia time, operative time, thoracoscopic and laparoscopic time, volume of fluid infused, urinary output, intake and output balance, blood urea nitrogen, creatinine(Cr), aspartate transaminase, alanine aminotransferase, white blood cell, C-reactive proteinand hemoglobin.We used Mann-Whitney U-test and chi-squared test for statistical analysis. We used EZR statistical software. Postoperative AKI was defined by the Kidney Disease Improving Global Outcomes guidelines. This study was approved by the FHU Ethics Review Committee(HM17-286).

**RESULTS.** A total of 83 patients [43 inhalation anesthesia (sevoflurane or desflurane), 40 propofol] were included in this review. There were 12 AKI cases in the inhalation anesthesia group (12/43: 28%) but none in the propofol group (0/40: 0%) (p=0.015).Urinary output volume was significantly increased in the propofol group compared with that in the inhalation anesthesia group (p=0.000002) and was significantly decreased in the AKI group compared with that in the non-AKI group (p=0.000006).There were no significant differences between the AKI and non-AKI groups except for height (p=0.036).

**CONCLUSION.** There was a significant decrease in AKI with propofol. Propofol may have a protective effect on renal function in patients undergoing laparoscopic esophageal surgery. Our investigation was a retrospective single center study. Further studies are required.

**REFERENCE(S)**

1. Bang JY, et al. Anesth Analg. 2016 Aug;123(2):363-70
2. None.

**Table 1 (abstract 000461).** See text for description

| | Propofol | Volatile anesthesia | |
|---|---|---|---|
| | all(N=40) | all(N=43) | AKI(N=12) |
| Age, y | 67.5(59-71.5) | 68(63-72) | 65(62.3-73) |
| Sex, male | 33(82.5%) | 36(83.7%) | 11(91.7%) |
| Height, cm | 165(159-169) | 165(158-168) | 168(165-173) |
| Weight, kg | 57(48-64.4) | 55(47.6-62.9) | 62.2(51.1-71) |
| BMI, kg/m2 | 19(18.6-22.8) | 21.3(18.6-22) | 22.2(18-23.5) |
| preoperative Cr, mg/dL | 0.9(0.72-1.05) | 0.8(0.72-0.91) | 0.89(0.8-1.07) |
| operation time, min | 775(692-880) | 715(645-809) | 693(603-758) |
| Intraoperative urinary output, ml/kg/h | 2.0(1.4-2.5) | 0.9(0.7-1.4) | 0.5(0.4-0.7) |

### 000499

#### Aprotinin reduces renal edema, but does not preserve renal perfusion and function following cardiopulmonary bypass in rats

N. Dekker[1], A. Van Leeuwen[1], A. Vonk[2], C. Boer[1], C. Van Den Brom[1]
[1]Anesthesiology, Amsterdam UMC, locatie VUmc, Amsterdam, Netherlands; [2]Cardiothoracic surgery, Amsterdam UMC, locatie VUmc, Amsterdam, Netherlands
**Correspondence:** N. Dekker
*Intensive Care Medicine Experimental* 2019, **7(Suppl 3):**000499

**INTRODUCTION.** Cardiopulmonary bypass (CPB) during cardiac surgery impairs microcirculatory perfusion, which is paralleled by microvascular leakage and associated with acute kidney injury. Thrombin is excessively released during CPB, which leads to endothelial and platelet activation and increased endothelial permeability. Aprotinin (Trasyslol), an anti-fibrinolytic, is also suggested to inhibit thrombin/PAR1-induced endothelial hyperpermeability. Therefore, this study investigated whether aprotinin reduces renal edema formation and whether this is paralleled by preserved renal and cremaster perfusion and reduced renal injury following CPB.

**OBJECTIVES.** To show that reduction of CPB-induced microvascular leakage by targeting the thrombin/PAR1 system using aprotinin preserves renal perfusion and reduces renal injury.

**METHODS.** Male rats were anesthetized and subjected to 75 minutes of CPB after treatment with aprotinin (n=15) or PBS (n=15) as control. Microcirculatory perfusion was measured in the cremaster muscle using intravital microscopy and in the renal cortex using contrast echography before CPB, and 10 and 60 minutes after weaning from CPB (post-CPB). Renal perfusion was calculated by multiplying microvascular filling velocity and microvascular blood volume. Wet/dry weight ratios were determined from harvested kidney tissue. Plasma creatinine, kidney injury molecule-1 (KIM-1) and neutrophil gelatinase-associated lipocalin (NGAL) were measured by ELISA as markers for kidney injury.

**RESULTS.** Onset of CPB decreased hematocrit levels (39±3 to 22±2 %, $P<0.01$) and blood pressure (88±15 to 73±9 mmHg, $P=0.02$). In addition, CPB resulted in a 2-fold reduction in the number of perfused capillaries in the cremaster muscle ($P<0.01$), which did not restore in the first hour post-CPB. One hour post-CPB, renal microvascular filling velocity (1.3±0.4 to 0.9±0.5 /sec, $P<0.01$) and renal perfusion (258±173 to 135±88, $P=0.03$) were reduced, paralleled by increased plasma levels of creatinine (28±3 to 58±13 nmol/ml, $P<0.01$) NGAL (123±37 to 2621±577 ng/ml, $P=0.003$) and KIM-1 (197±39 to 273±81 pg/ml, $P<0.01$).

Aprotinin preserved cremaster perfusion following CPB ($P=0.002$), whereas renal microvascular filling velocity ($P=0.5$) and perfusion ($P>0.9$) were not affected compared to untreated CPB animals. In parallel, no differences were observed in plasma levels of creatinine ($P>0.9$), NGAL ($P=0.7$) or KIM-1 ($P=0.2$). Aprotinin treated animals required less additional fluids (3.9±3.3 vs 7.5±3.0 ml, $P=0.006$) during CPB and reduced kidney wet/dry weight ratios (4.6±0.2 vs 4.4±0.2, $P=0.046$) were found 1 hour post-CPB compared to untreated CPB animals.

**CONCLUSION.** Treatment with aprotinin preserved cremaster microcirculatory perfusion following CPB, but did not prevent renal perfusion disturbances nor renal injury following CPB despite reducing renal edema formation. Future studies should focus on identifying therapeutic strategies to improve renal perfusion and function following CPB.

### 000504

#### Mechanisms of renal-splenic axis involvement in septic AKI process mediated by α7nAChR-NF-κB signaling pathway

Y. Gao[1], JB. Zheng[1], K. Kang[2], HL. Wang[1], KJ. Yu[2]
[1]Department of critical care medicine, the Second Affiliated Hospital of Harbin Medical University, Harbin, China; [2]Department of critical care medicine, the First Affiliated Hospital of Harbin Medical University, Harbin, China
**Correspondence:** K.J. Yu
*Intensive Care Medicine Experimental* 2019, **7(Suppl 3):**000504

**INTRODUCTION.** Spleen plays an important role in the regulation of immune response during sepsis and acute kidney injury (AKI) [1]. α7 nicotinic acetylcholine receptor (α7nAChR) is a key factor involved in the cholinergic anti-inflammatory pathway. Dexmedetomidine (Dex) can exert protective effects against sepsis through α7nAChR signaling, which is mediated by spleen [2].

**OBJECTIVES.** To investigate the role of splenectomy in Dex-activated cholinergic anti-inflammatory pathway in alleviating septic AKI.

**METHODS.** C57BL/6 mice were randomly divided into Control group, LPS group (10mg/kg LPS intraperitoneal injection), Dex+LPS group (Dex 40μg/kg before LPS injection), Splenectomy group, Splenectomy+LPS group and Splenectomy+Dex+LPS group. The mice in splenectomy groups survived for 7days after splenectomy and re-entered the group. Mice were terminated 16 h after LPS challenge.

**RESULTS.** The septic AKI model was established successfully (Figs1-3). The apoptosis in LPS group increased significantly compared to control group. The Dex reduced the apoptosis of kidney cell, however, the apoptosis increased after splenectomy plus Dex (Fig4). Cr and Cys-C were significantly increased after LPS, which were reduced by Dex treatment. However, Cr and Cys-C increased again after splenectomy combined with Dex (Fig5). The levels of IL-6, NF-κB p65, p-STAT3, Caspase-3 and Bax were significantly increased and the levels of α7nAChR and Bcl-2 were significantly decreased in LPS group. Dex treatment resulted in an attenuation of IL-6, NF-κB p65, p-STAT3, Caspase-3 and Bax, and an increase of α7nAChR and Bcl-2. Splenectomy down-regulated the expression of IL-6, NF-κB p65 and p-STAT3 (Fig6).

**CONCLUSION.** Dex effectively alleviated septic AKI, while splenectomy could inhibit anti-inflammatory, anti-apoptotic and renal protective effects of Dex. Splenectomy reduced the production of proinflammatory cytokines and had a protective effect on kidney. The Kidney-spleen axis mediated by α7nAChR signaling pathway was involved in the development of septic AKI.

**REFERENCE(S)**

1. van der Poll T, van de Veerdonk FL, Scicluna BP, et al. The immunopathology of sepsis and potential therapeutic targets. Nat Rev Immunol. 2017;17(7):407-420.
2. Liu Z, Wang Y, Ning Q, et al. The role of spleen in the treatment of experimental lipopolysaccharide-incuced sepsis with demedetomidine. Springerplus. 2015;4:800.
3. This project was funded by the National Natural Science Foundation of China (No. 81571871).

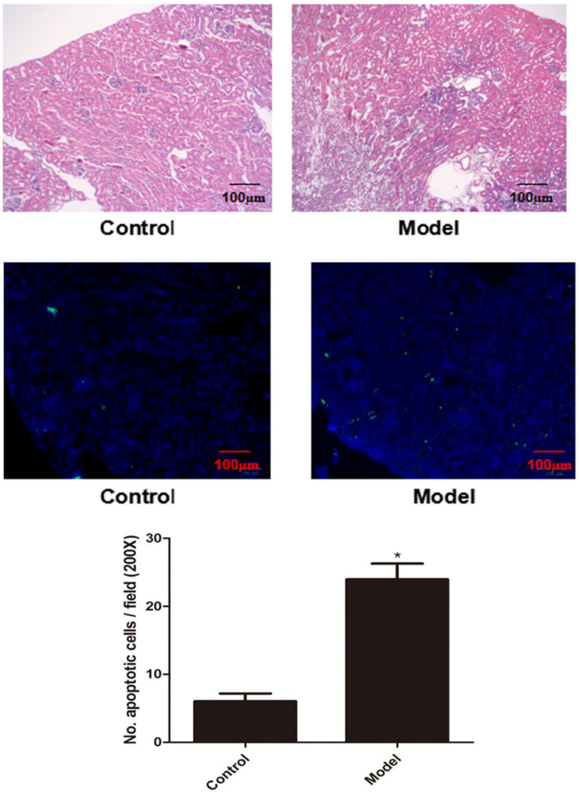

**Fig. 1 (abstract 000504).** HE staining results

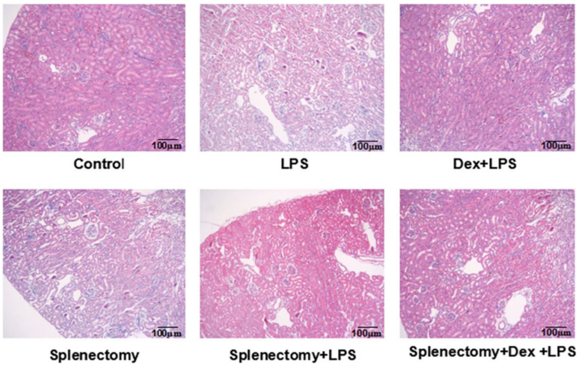

**Fig. 2 (abstract 000504).** Tunel results

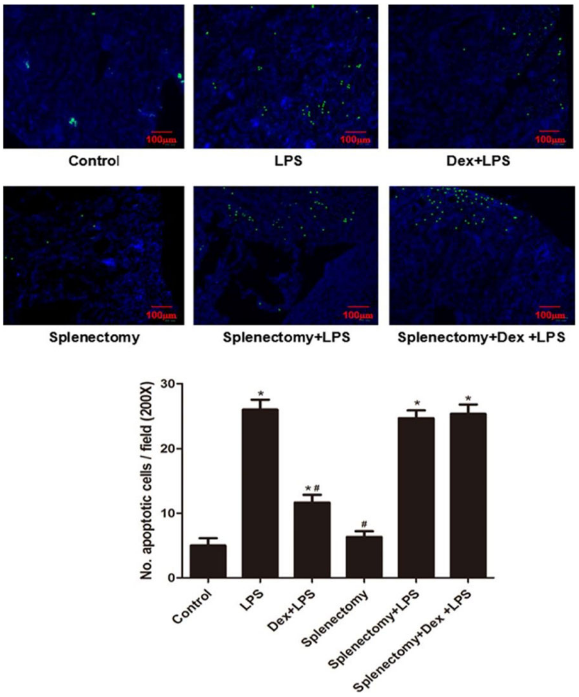

**Fig. 3 (abstract 000504).** HE results of renal pathology in each group

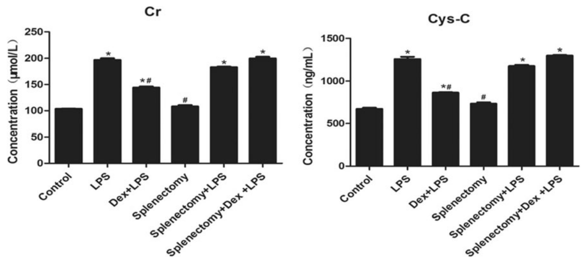

**Fig. 4 (abstract 000504).** Tunel apoptosis results in kidney tissues of each group

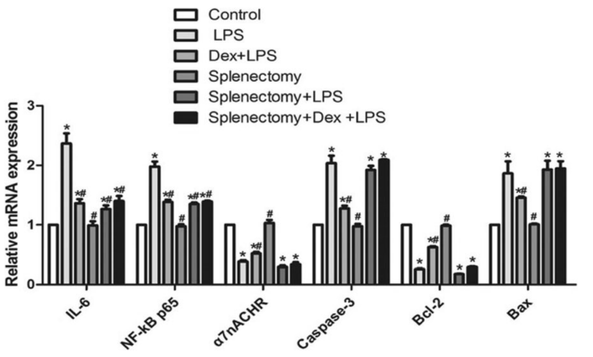

**Fig. 5 (abstract 000504).** The contents of Cr and Cys-C in different groups

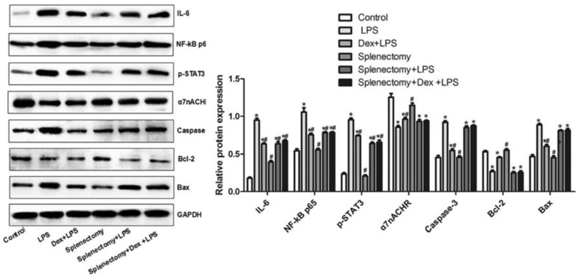

**Fig. 6 (abstract 000504).** IL-6, NF-kB p65, p-STAT3, α7nACHR, Caspase-3, Bcl-2 and Bax expression in different groups (*P<0.05 vs. Control; #P<0.05 vs. LPS)

### 000565

#### Dexmedetomidine Alleviates Kidney Fibrosis via Inhibiting Connexin32-mediated Senescent Bystander Effect after Renal Ischemia/Reperfusion

C. Chen[1], W. Yao[1], H. Fang[1], L. Ye[2], C. Luo[1], Q. Zhang[2], Z. Hei[3]
[1]Department of anesthesiology; laboratory of anesthesiology, The Third Affiliated Hospital, Sun Yat-sen University, Guangzhou, China;
[2]Guangdong provincial key laboratory of liver disease research, The Third Affiliated Hospital, Sun Yat-sen University, Guangzhou, China;
[3]Department of anesthesiology, The Third Affiliated Hospital, Sun Yat-sen University, Guangzhou, China
**Correspondence:** Z. Hei
*Intensive Care Medicine Experimental* 2019, **7(Suppl 3):**000565

**INTRODUCTION.** Renal ischemia/reperfusion (IR) can induce acute kidney injury (AKI), which often progresses to chronic kidney disease (CKD). Our previous study demonstrated that Dexmedetomidine (Dex), a highly selective α2 adrenergic receptor (α2-AR) agonist, improves renal fibrosis to moderate the AKI-to-CKD transition. However, the precise underlying mechanisms were largely unknown.

**OBJECTIVES.** This study focuses on the underlying mechanisms related to gap junction (GJ) composed of connexin32 (Cx32)-induced senescent bystander effect responsible for Dex renal protection.

**METHODS.** Cx32 knockdown mice (Cx32-/-), kidney specific Cx32-overexpressing mice (Cx32-rAAV), and wild-type C57BL/6 mice (Cx32+/+) underwent surgical bilateral renal IR in the absence or presence of treatments with Dex(10μg/kg). Histopathological changes, renal dysfunction, cell senescence and tubular fibrotic markers, reactive oxygen species, inflammatory factors and the expression of NF-κB were studied.

**RESULTS.** Cx32 deficiency alleviated, while Cx32 overexpression enhanced renal IR-induced AKI, renal senescence and chronic tubulointerstitial fibrosis in later stages (Fig1). Cx32 GJ was found to transfer ROS and further activate NF-κB and the senescence-associated secretory phenotype (SASP) (Fig2). Similar to Cx32 knockdown, pretreatment with Dex also decreased the number of senescent tubular cells and tubulointerstitial fibrosis, and weakened the protein expression of senescence and fibrosis-associated markers including p21, p16, α-SMA and TGF-β1 (Fig3). Furthermore, the expression of SASP markers was also decreased in Dex-treated IR mice; and these protective effects of Dex could be abolished by Cx32 overexpression (Fig4).

**CONCLUSION.** Cx32 plays a critical role in Renal IR-induced senescence and chronic tubulointerstitial fibrosis and that inhibition of Cx32 function may represent a new and major mechanism whereby Dex alleviated the senescent bystander effect by inhibiting the Cx32 GJ, which further inhibited ROS/NF-κB-mediated senescent bystander effect.

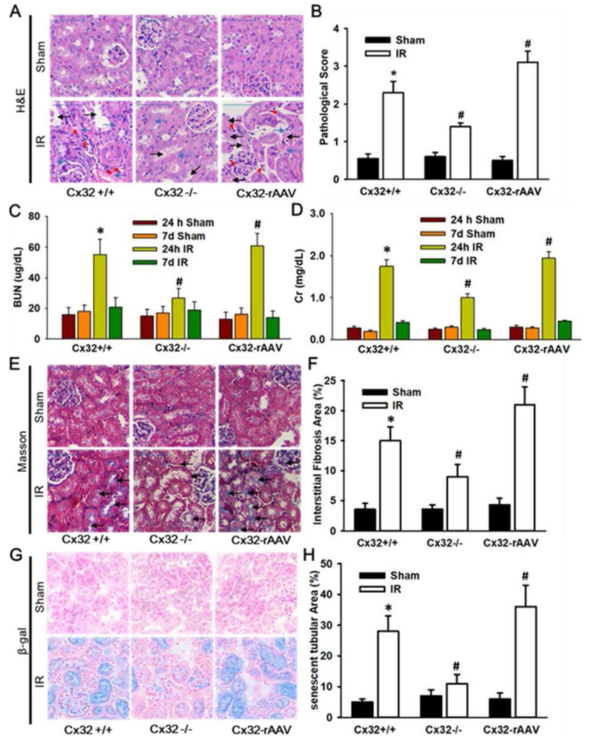

**Fig. 1 (abstract 000565).** Cx32 deficiency Attenuated AKI and renal fibrosis after renal IR with Concomitant Reduction of Post-Renal IR cellular senescence

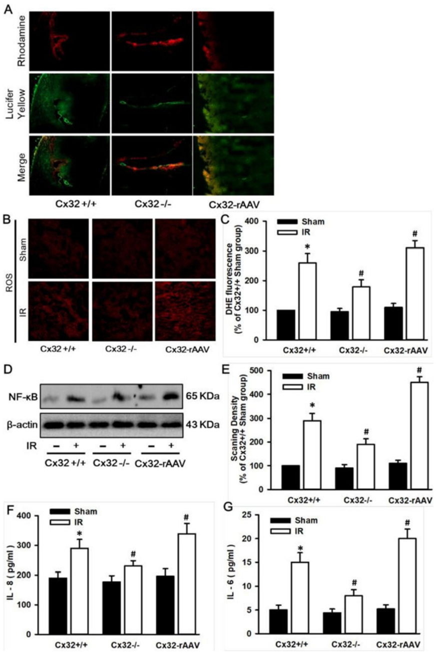

**Fig. 2 (abstract 000565).** Cx32 GJ regulates ROS/ NF-κB axis-mediated senescent bystander effect following renal IR

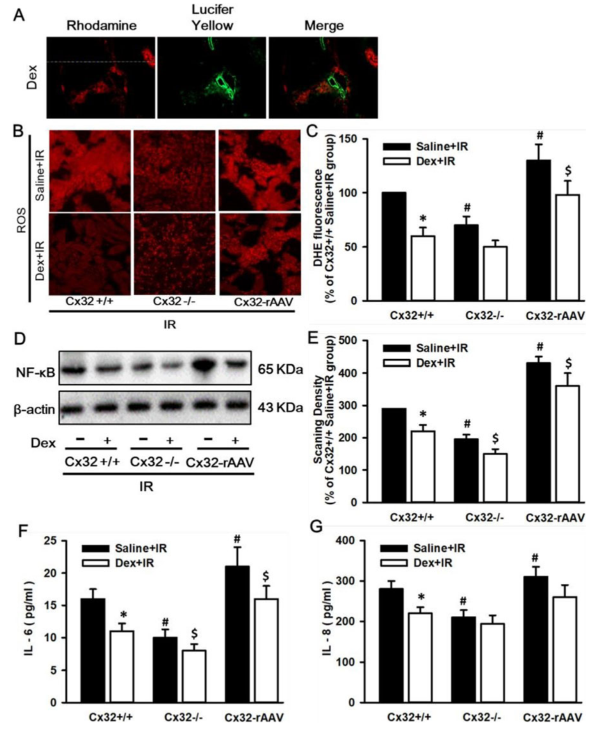

**Fig. 4 (abstract 000565).** Effects of Dexmedetomidine on ROS/ NF-κB axis-mediated senescent bystander effect

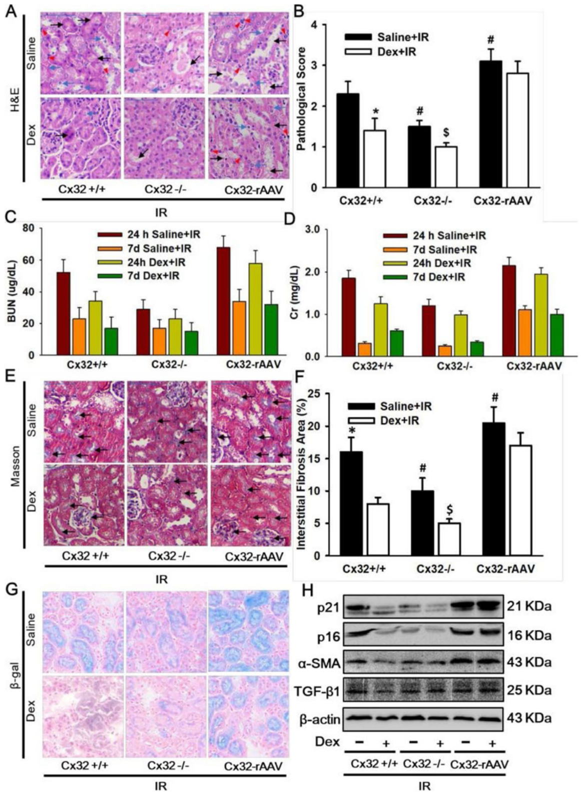

**Fig. 3 (abstract 000565).** Dexmedetomidine Protected against HR-induced senescence-associated fibrosis by Inhibiting Cx32 GJ

### 000570

### Effect of Different Urine Output for the Prognosis of Acute Kidney Injury in Critically Ill Patients

L. Zhang[1], J. Cheng[2], H. Huang[1], X. Xi[3]
[1]Department of critical care medicine, Beijing Tiantan Hospital, Capital Medical University, Beijing, China; [2]Department of critical care medicine, Yantai Affiliated Hospital of Binzhou Medical University, Yantai, China; [3]Department of critical care medicine, Fu Xing Hospital, Capital Medical University, Beijing, China
**Correspondence:** L. Zhang
*Intensive Care Medicine Experimental* 2019, **7(Suppl 3):**000570

**INTRODUCTION.** Acute kidney injury (AKI) is a critically ill syndrome with a high incidence and mortality. It was reported that severity of oliguria and anuria were associated with worse outcomes and a rapid decline in urine output might be the earliest indication of renal dysfunction ahead of other indicators. However, the utility of urine output as a specific indicator for renal dysfunction is somewhat controversial. Therefore, we performed this study to evaluate the prognostic value of different level of urine output for critically ill patients with AKI.

**METHODS.** We conducted a secondary data analysis of the Beijing Acute Kidney Injury Trial (BAKIT) database, which was a multicenter, prospective, observational study involving 9,049 adult patients admitted to 30 ICUs in 28 tertiary hospitals in Beijing [1]. Overall, there were 3,107 patients recruited consecutively into the database. Those who lacked body weight, daily creatinine and hourly urine volume were excluded from the study. All recruited patients were evaluated by the Kidney Disease: Improving Global Outcomes (KDIGO) criteria and four types of urine output [2]. The urine output criteria were as following: UO1, a urine volume of less than 0.5 mL/kg/hour for six hours; UO2, an average urine volume of less than 0.5 mL/kg/hour for six hours; UO3, a urine volume of less than 0.3 mL/kg/hour for six hours; and UO4, an average urine volume of less than 0.3 mL/kg/hour for six hours. Demographic information, underlying disease, diagnostic information, lab and physiological data and patient outcomes were collected. The prognostic indicators included the renal replacement therapy (RRT), ICU mortality, hospital mortality, 28-day mortality, length of ICU stay, and hospitalization costs. Differences in mortality and other prognostic indicators among the KDIGO criteria and the four different urine volume criteria were compared. Logistic regression analysis was used to assess the association of different criteria with prognostic indicators. P<0.05 was considered to be significant.

**RESULTS.** Of the 1,080 patients enrolled in the final analysis, 555 (51.4%) patients met the KDIGO criteria, and 191 (17.7%), 290 (26.9%), 110 (10.2%) and 170 (15.7%) met the UO1, UO2, UO3 and UO4 criterion, respectively. The hospital mortality based on the different criteria was 23.1% (KDIGO), 39.8% (UO1), 33.5% (UO2), 47.3% (UO3) and 43.5%(UO4), and UO3 was significantly higher than other groups (P<0.05) (Figure1). The other prognostic indicators including the RRT rate, ICU mortality, 28-day mortality, length of ICU stay, and hospitalization costs were incompletely equal, with the highest in the UO3 (Table 1).

**CONCLUSION.** Alterations in urine output might be a sensitive prognostic indicator of AKI. A 6-hour average urine volume of less than 0.3 mL/kg/hour might be associated with worse clinical outcomes, such as higher mortality, more RRT, longer ICU stay and higher medical costs.

**REFERENCE(S)**

1. Luo X, Jiang L, Du B, Wen Y, Wang M, Xi X, et al. A comparison of different diagnostic criteria of acute kidney injury in critically ill patients. Crit Care. 2014;18:R144.
2. KDIGO AKI Work Group. KDIGO clinical practice guideline for acute kidney injury. Kidney Int Suppl. 2012;17:124–138.

**Table 1 (abstract 000570).** Comparison of clinical outcomes based on the different criteria

| | KDIGO | UO1 | UO2 | UO3 | UO4 | P value |
|---|---|---|---|---|---|---|
| RRT, n (%) | 92 (16.6) | 75 (39.3) | 85 (29.3) | 63 (57.3) | 75 (39.5) | < 0.05 |
| ICU mortality, n (%) | 104 (20.0) | 64 (33.5) | 80 (27.6) | 42 (38.2) | 62 (36.5) | < 0.05 |
| Hospital mortality, n (%) | 128 (23.1) | 76 (39.8) | 98 (33.8) | 52 (47.3) | 74 (43.5) | < 0.05 |
| 28-day mortality, n (%) | 126 (22.7) | 74 (38.7) | 94 (32.4) | 52 (47.3) | 73 (42.9) | < 0.05 |
| Length of ICU stay, days | 5 (3-10) | 7 (4-13) | 6 (4-12) | 7 (5-12) | 7 (5-13) | < 0.05 |
| Hospitalization costs, CNY×104 | 7 (3-14) | 9 (4-13) | 8 (3-13) | 9 (5-16) | 9 (5-15) | < 0.05 |

Categorical variables are shown as number (%). Continuous data are expressed as median (interquartile range).

RRT, renal replacement therapy; CNY, Chinese Yuan

The KDIGO (the Kidney Disease: Improving Global Outcomes) criteria were as follows: an increase in serum creatinine by at least 26.4 μmol/L within 48 hours or an increase in serum creatinine to 1.5 times baseline, which is known or presumed to have occurred within 7 days before, or a urine volume of less than 0.5 mL/kg per hour for 6 hours.

The urine output criteria were as following: UO1, a urine volume of less than 0.5 mL/kg/hour for 6 hours; UO2, an average urine volume of less than 0.5 mL/kg /hour for 6 hours; UO3, a urine volume of less than 0.3 mL/kg/hour for 6 hours; UO4, an average urine volume of less than 0.3 mL/kg /hour for 6 hours.

P value is the results of comparison based on the KDIGO criteria and different urine outputs.

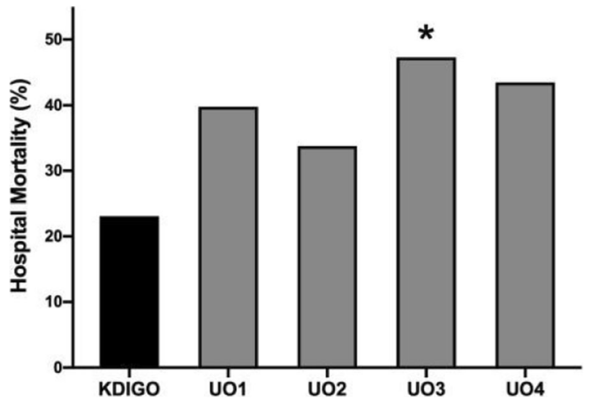

**Fig. 1 (abstract 000570).** The hospital mortality based on the different criteria

### 000595

#### Chronic Kidney Disease Epidemiology Collaboration formula (CKD-EPI) for the assessment of renal function in the critically ill patient

V. Philibert[1], P. Marcos Neira[1], Y. Rovira Vallès[1], L. Bielsa Berrocal[1], S. Triginer Roig[1], A. Campos Gómez[1], JM. Manciño Contreras[1], M. Sánchez Satorra[1], S. Malumbres Serrano[2], TM. Tomasa Irriguible[1]

[1]Intensive care department, Hospital Germans Trias i Pujol, Badalona, Spain; [2]Clinical analysis department, Hospital Germans Trias i Pujol, Badalona, Spain

**Correspondence:** V. Philibert,

*Intensive Care Medicine Experimental* 2019, **7(Suppl 3):**000595

**INTRODUCTION.** Equations (as CKD-EPI) for renal function estimation are usually used in the critical patient despite not having been validated in this population. Utilization of estimation formulas of renal function may lead to incorrect drugs dosage.

**OBJECTIVES.** To analyse the concordance between CKD-EPI and the gold standard glomerular filtration rate (GFR) to verify its validity for the correct estimation of renal function in the critically ill patient.

**METHODS.** Observational study conducted in a polyvalent ICU of a 3rd level hospital. Between March 2017 and August 2018 blood samples and 24h urine samples were collected to determine serum and urine creatinine levels. We graded the sample in stages of CKD for grading severity, and we added a new stage for augmented renal clearance (GFR> 130 mL/min). To evaluate the concordance between CKD-EPI and GFR in the overall sample and in each stage we applied Passing-Bablok and Bland Altman statistic tests.

**RESULTS.** 949 samples of 255 patients (a mean age of 58 years and 66% of males) were collected. The obtained *n* was excessive for an adequate power for the normal GFR stage so that the size of the sample was automatically and randomly reset and thus the global sample was reduced to 643. In the GFR<30 mL/min stage sample group there is a proportional discordance between both tests. In the other subcategories and the overall sample there is a proportional and constant discordance.

**CONCLUSION.** There is no concordance between CKD-EPI and GFR. The obtained results question the validity of the CKD-EPI formula for correct renal function estimation in the critically ill patient. Physicians should take into account that CKD-EPI is not accurate for dosing drugs with renal elimination mechanism.

**REFERENCE(S)**

1. Ruiz S, Minville V, Asehnoune K, Virtos M, Georges B, Fourcade O, Conil JM. Screening of patients with augmented renal clearance in ICU: taking into account the CKD-EPI equation, the age, and the cause of admission. Ann Intensive Care. 2015 Dec;5(1):49. doi: 10.1186/s13613-015-0090-8. Epub 2015 Dec 14.
2. Bragadottir G, Redfors B, Ricksten SE. Assessing glomerular filtration rate (GFR) in critically ill patients with acute kidney injury–true GFR versus urinary creatinine clearance and estimating equations. Crit Care. 2013 Jun 15;17(3):R108. doi: 10.1186/cc12777.
3. Levey AS, Stevens LA, Schmid CH, Zhang YL, Castro AF 3rd, Feldman HI, Kusek JW, Eggers P, Van Lente F, Greene T, Coresh J; A new equation to estimate glomerular filtration rate. CKD-EPI (Chronic Kidney Disease Epidemiology Collaboration). Ann Intern Med. 2009 May 5;150(9):604-12. Erratum in: Ann Intern Med. 2011 Sep 20;155(6):408.

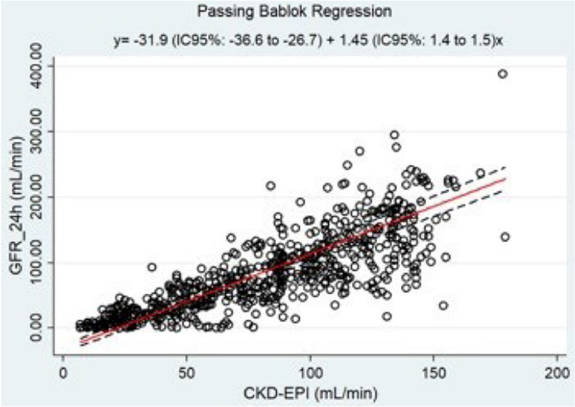

**Fig. 1 (abstract 000595).** See text for description

**Table 1 (abstract 000595).** See text for description

| Sample | n | GFR | CKD-EPI | Passing-Bablok regression | |
|---|---|---|---|---|---|
| | | | | Intercept (95% CI) | Slope (95% CI) |
| Global | 643 | 83.65± 61.60 | 82.16± 40.52 | -31.86 (-36.76 to -26.70) | 1.45 (1.38 to 1.52) |

### 000731

#### Immediate aspiration of the drug infused via central venous catheter through the distally positioned central venous dialysis catheter: an experimental study

J. Stanaitis[1], J. Tutkus[1], V. Vicka[2], D. Ringaitiene[3]
[1]Faculty of medicine, Vilnius University, Vilnius, Lithuania; [2]Clinic of anesthesiology and intesive care, Vilnius university Hospital Santaros Clinic, Vilnius, Lithuania; [3]Clinic of anesthesiology and intensive care, Vilnius University Hospital, Santaros Clinic, Vilnius, Lithuania
**Correspondence:** J. Stanaitis
*Intensive Care Medicine Experimental* 2019, **7(Suppl 3):**000731

**INTRODUCTION.** The incidence of acute kidney injury (AKI) in the intensive care units ranges from 32% to 50%. Only 5% of these patients are treated with renal replacement therapy (RRT), but the mortality is high, often reaching up to 50%. In spite of the modality of the RRT, the access point to the circulation is a central venous dialysis catheter (CVDC). The CVDC and the central venous catheter (CVC) are often placed in the same vessel in order to salvage the veins. As a consequence, the tips of both catheters end up in the superior vena cava (SVC). Therefore, there have been speculations that this might cause immediate aspiration of the drug infused via CVC through the CVDC.

**OBJECTIVES.** Determine the degree of immediate aspiration of the drug infused via central venous catheter (CVC) through the distally positioned central venous dialysis catheter (CVDC).

**METHODS.** A 9.53mm polyvinyl chloride (PVC) tube was used to mimic the SVC. We used water to simulate blood. A centrifugal pump was used to keep a constant flow rate of 1.2 liters per minute. 1mg/mL (0.1%) methylene blue aqueous solution was selected as a tracer and was infused into the tube using a syringe pump via the CVC as a constant infusion (200ml/hour) and as a bolus infusion (800ml/hour). To simulate RRT we performed a manual aspiration through the CVDC using 20ml syringes at the speeds of 100ml/min and 300ml/min. The CVC point was moved in reference to a fixed CVDC point. The most distal point was at +2 cm, and the most proximal point was at -8 cm distance. Four different sets of samples were generated: continuous infusion - slow aspiration (situation – A), continuous infusion - fast aspiration (situation – B), bolus infusion - slow aspiration (situation – C), bolus infusion – fast aspiration (situation – D). The samples were obtained at every of the 6 different distances between the CVC and CVDC tips. 3 samples were taken per each measurement, mixed to one medium sample, amounting to 24 different sample syringes. Spectrophotometry and Beer-Lambert-Bouguer law were used to evaluate the degree of aspiration.

**RESULTS.** When the CVC tip was placed at +2cm distance the aspiration of the tracer was <0.027% in all situations. The levels of aspiration were also minimal at the 0cm distance (<0.27%). Moving the CVC catheter up resulted in increased aspiration of the tracer reaching its peak at -4cm: situation A (1.37%), situation B (0.35%), situation C (5.16%), situation D (2.61%). The levels of the aspirated tracer were higher in bolus infusion setting, reaching an average of 2.07% in situation C and 1.77% in situation D compared to 0.46% in situation A and 0.32% in situation B.

**CONCLUSION.** Our experimental study showed that the degree of immediate drug aspiration is influenced by the position of the adjacent catheters and the speed of infusion. There was no aspiration seen in the +2cm and 0cm positions. These are the safest positions in the setting of adjacent catheters in order to avoid unwanted drug aspiration.

### 000748

#### Can Pneumococcal pneumonia be really associated to acute kidney injury?

MG. Galli, M. Cecchia, M. Giovini, M. Barbera, E. Antonucci
Intermediate care unit, Ospedale "Guglielmo da Saliceto", Piacenza, Italy
**Correspondence:** E. Antonucci
*Intensive Care Medicine Experimental* 2019, **7(Suppl 3):**000748

**INTRODUCTION.** Acute kidney injury (AKI) is a recurrent finding in critically ill patients and is linked to some short- and long-term adverse outcomes. In addition, previous studies(1) showed that Streptococcus pneumoniae (SP) infections could be associated with an increased risk of AKI even if that relationship was not extensively analyzed in critically ill patients.

**OBJECTIVES.** We compared patients with SP pneumonia and similar patients affected by other types of pneumonia (OP) hypothesizing that SP infection could be linked with increased AKI and mortality rates.

**METHODS.** We performed a retrospective analysis, reviewing all patients with pneumonia admitted to our Intermediate Care Unit (IMCU) from December 2015 to December 2018. The inclusion criteria were: age ≥ 18 years old; radiological evidence of pneumonia; positive microbiological samples (urinary antigen for SP or Legionella pneumophila; endotracheal aspirate or bronchoalveolar lavage; blood culture; pleural culture). Exclusion criteria were: patients with bronchitis, aspiration pneumonia and lung tumors. We analyzed two groups of patients (SP and OP) matched for age, SAPS2 score, SOFA score, PaO2/FiO2 ratio at IMCU admission, chronic kidney disease (CKD) and circulatory failure (CF). KDIGO criteria were used to classify AKI. We investigated rates of AKI, IMCU mortality and mortality at 90 days.

**RESULTS.** We reviewed 110 patients and 34 respected the inclusion criteria (17 in SP group and 17 in OP group). The two groups were well matched (age: 78 ± 9 (SP), 74 ± 12 (OP) p= 0.3; CKD 6/17 (SP), 6/17 (OP) p = 1; PaO2/FiO2 149 ± 79 (SP), 134 ± 37 (OP) p = 0.5; SAPS2 42 ± 11 (SP), 40 ± 13 (OP) p = 0.7; SOFA 6 ± 1 (SP), 5 ± 1 (OP) p = 0.3; CF 5/17 (SP), 4/17 (OP) p = 0.7). Furthermore, there was no significant difference in the two groups concerning the use of iodinated contrast media (p= 0.09), aminoglycosides (p= 1), glycopeptides (p= 0.4), colistin (p=1), except for ACEi/sartan use (p= 0.03 in OP). No significant difference was found in AKI rate between the two groups (AKI 9/17 SP; 7/17 OP p = 0.5; KDIGO stage: 1 (5/9 SP; 5/7 OP p= 0.5), 2 (3/9 SP; 1/7 OP p= 0.4), 3/renal replacement therapy (RRT) (1/9 SP; 1/7 OP p = 0.8). As well, IMCU mortality (OR 4.1, 95% CI 0.69-24.24, p= 0.1) and 90-day mortality (OR 1.6, 95% CI 0.41-6.96, p= 0.5) were similar in the two groups.

**CONCLUSION.** In our cohort of patients, SP pneumonia was not associated with increased risk of AKI and mortality when compared with other types of pneumonia in similar adult patients.

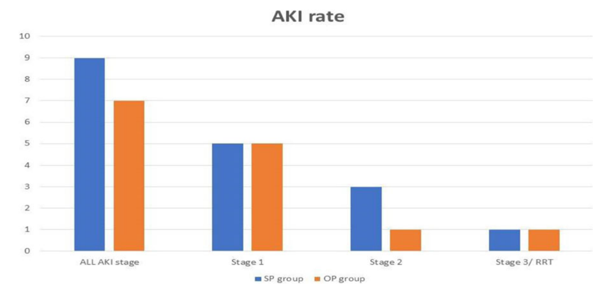

**Fig. 1 (abstract 000748).** See text for description

### 000770

#### Acute Kidney Injury in Patients with Diabetes Mellitus after Major Abdominal Surgery

T. Klancir, T. Goranović, V. Nesek Adam
University department of anaesthesiology, resuscitation and intensiv care medicine, University Hospital Sveti Duh, Sveti Duh 64, Croatia, Zagreb, Croatia
**Correspondence:** T. Klancir
*Intensive Care Medicine Experimental* 2019, **7(Suppl 3):**000770

**INTRODUCTION.** Among many conditions and interventions diabetes mellitus and major abdominal surgery have been recognized as risk factors in acute kidney injury (AKI) (1). However, some research studies denied association between diabetes mellitus and AKI in patients after surgery (2, 3, 4).

**OBJECTIVES.** To determine the association and clinical features of AKI in patients with unrecognized and/or poorly regulated diabetes mellitus after major abdominal surgery.

**METHODS.** This prospective, observational, monocentric study included sixty patients who underwent major abdominal surgery and were admitted to the intensive care unit after the surgery. Patients' follow-up, anamnestic, medical, measured and laboratory data were obtained on the 0, 1st, 2nd, 3rd, 7th, and 14th postoperative days as well as data on patients' condition at hospital discharge. Hemoglobin A1c (HbA1c) was used as a screening tool for previously unrecognized or unregulated diabetes.

**RESULTS.** AKI was recorded in 33.3% of patients. Patients with previously known diabetes mellitus, poorly regulated diabetes mellitus and unrecognized diabetes mellitus did not have a higher incidence of AKI ($P$>0.05). In patients with hypertension ($P$= 0.025) and patients who received furosemide ($P$=0.034, $P$=0.036, $P$=0.033), vasopressor ($P$=0.036, $P$=0.036) and blood derivatives ($P$=0.022) a higher incidence of AKI was observed at one or more days. The appearance of AKI did not affect the length of stay in the intensive care unit ($P$= 0.567), readmission in the intensive care unit ($P$=0.548), total hospitalization time ($P$=0.504) and mortality ($P$=0.595).

**CONCLUSION.** The results of this study did not confirmed association between diabetes mellitus and AKI after major abdominal surgery. Use of HbA1c helped in avoiding inadequate interpretation of results due to poorly controlled and unrecognized diabetic patients.

##### REFERENCE(S)

1. Kdigo.org. (2018). [online] [cited 2018 Jul 29]. Available from: https://kdigo.org/wp-content/uploads/2016/10/KDIGO-2012-AKI-Guideline-English.pdf.
2. Teixeira C, Rosa R, Rodrigues N, et al. Acute Kidney Injury after Major Abdominal Surgery: A Retrospective Cohort Analysis. Crit Care Res Pract. 2014; 2014: 132175.
3. Moschopoulou M, Ampatzidis, FC, Loutradis C, et al. Diabetes mellitus does not affect the incidence of acute kidney injury after cardiac surgery; a nested case-control study. Nephrol. 2016; 29: 835–45.
4. Hertzberg D, Sartipy U, Holzmann MJ. Type 1 and type 2 diabetes mellitus and risk of acute kidney injury after coronary artery bypass grafting. Am Heart J. 2015; 170: 895–902.
5. None

## ARF - Acute respiratory failure 1

### 000013

#### Neurally Adjusted Ventilatory Assist: A cross-sectional staff survey

D. Hadfield
*Intensive Care Medicine Experimental* 2019, **7(Suppl 3):**000013

**INTRODUCTION.** Clinical effectiveness of Neurally Adjusted Ventilatory Assist (NAVA) has not been demonstrated and feasibility data are required. The aim of this survey was to assess NAVA from the perspective of staff users, and inform a feasibility trial prior to a definitive RCT.

**METHODS.** Web (SurveyMonkey) and paper-based, anonymous, self-administered cross-sectional survey, approved as part of a concurrent randomised feasibility trial (NCT01826890). Organised into four domains:

- Training and experience
- Advantages and disadvantages
- Barriers and attitudes
- Views on research

**RESULTS.** Of the 462 distributed surveys, 301 (65.4%) were returned from 236 nurses (78.4%), 56 doctors (18.6%) and 12 physiotherapists (4.0%). Responses were well distributed between levels of staff seniority.

NAVA training had been received by >50% of participants; 227/301 (70.4%) had clinical experience of NAVA and 123 (64.4%) of those had used Edi monitoring. Despite this, NAVA was perceived as infrequently used with staff citing 'low confidence' and 'lack of experience' as key barriers to acceptance.

Most participants agreed or strongly agreed that the NAVA mode was safe (136/177, 76.8%) and clinically effective (99/176, 56.3%), and that Edi monitoring was clinically effective (101/172, 58.7%), citing improved synchrony, comfort and reduced time on ventilation as the main perceived advantages. 'Technical issues' was the most cited clinical disadvantage (129, 68.3%). Mode cross-over was also perceived to occur often, with 'Edi signal problems' the most cited cause (39/168, 23.2%).

In comparison to PSV, respondents perceived NAVA as performing equally well, and to be 'easier' in respect to synchrony, comfort and weaning (all p<0.0001), with no increase in workload. NAVA was, however, perceived as generally harder to use (105/174, 60.3%), particularly in relation to 'set-up and start' and 'reliability' (p<0.01). PSV was preferred to NAVA for ventilator weaning (93/171 [54.4%] versus 29/171 [17.0%]; p=<0.0001), although a large proportion (49/171, 27.1%) were ambivalent.

In relation to research, almost all 161/180 (89.4%) were generally supportive; 110/180 (61.1%) had cared for a patient on the trial; no participants felt that the protocol was unacceptable, with the majority (60/119, 50.4%) finding it very or completely acceptable.

**CONCLUSION.** These results highlight key factors that may interact with parameters in a future trial. The difficulty and lack of confidence perceived by respondents may be modified via training and NAVA usage guidelines. Of more concern are the technical difficulties as reported here and elsewhere 1. Further assessment of these factors may be necessary prior to a definitive RCT.

##### REFERENCE(S)

1. National Institute for Health Research Clinical Doctoral Research Fellowship, Mr Daniel Hadfield (CDRF-2014-05-056). Additional funding from The Moulton Charitable Foundation and Guy's and St Thomas' Biomedical Research Centre.

2. Di Mussi R, et al. Impact of prolonged assisted ventilation on diaphragmatic efficiency: NAVA versus PSV. Critical care. 2016; 20: 1
3. Hadfield D, et al. Neurally Adjusted Ventilatory Assist versus Pressure Support in prolonged mechanical ventilation: A randomised feasibility study. Am J Respir Crit Care Med 2018; 197: A2551-A2551; A2104

### 000022

#### Predicting ARDS in critically ill patients: derivation of a new score

M. Ahmed[1], K. Taema[2], S. Fawzi,[2], G. Hamed,[2]
[1]Intensive care medicine, Al Haram hospital, Cairo, Egypt; [2]Critical care medicine, Cairo University, Faculty Of Medicine, Kasr Al Ainy, Cairo, Egypt
**Correspondence:** K. Taema
*Intensive Care Medicine Experimental* 2019, **7(Suppl 3):**000022

**INTRODUCTION.** Different Lung Injury Prediction Scores (LIPS) had been derived to identify patients at risk for acute respiratory distress syndrome (ARDS).
**OBJECTIVES.** This study was intended to derive a new ARDS prediction score in high risk critically ill patients.
**METHODS.** We recruited 200 patients [63(43-70)years old, 120(60 %)males] admitted to ICU with APACE-II≥15 and at least one ARDS risk factor after excluding patients with ARDS on admission, cardiac patients, and readmission. The presence of risk factors together with the admission and 48-hours CRP (CRP-0&CRP-48) were tested in univariate then multivariate regression models for identifying significant predictors whose weights were assigned according to the β-coefficient of the regression model. Our score was compared with a previously derived score by Trillo-Alvarez et al on 2011 (LIPS-T). The primary and secondary outcomes were the development of ARDS and in-hospital mortality respectively.
**RESULTS.** ARDS developed in 88 patients(44%). Logistic regression revealed that only pneumonia, tachypnea, increased heart rate and increased CRP-48 are significant ARDS predictors. The weight of each predictor was estimated according to its β-coefficient. The new score was 35.5(27-44) and 14(9-24.3) in ARDS and non-ARDS patients respectively(P=0.000). The AUC of the new score was 0.827 compared to 0.74 for the LIPS-T(P=0.014). A score of 20 had a sensitivity and specificity of 82 % and 71 % in predicting ARDS. Our score was significantly lower in survivors compared to non-survivors(P=0.000). Its AUC in predicting in-hospital mortality was 0.761 compared to 0.657 for the LIPS-T(P=0.0045).
**CONCLUSION.** This study derived new simple LIPS score which could be better than the previously derived scores in terms of predicting ARDS and in-hospital mortality in critically ill ICU patients.

### 000024

#### Automated closed-loop versus conventional ventilation mode during daily routine nursing procedures in intensive care unit: the I-NURSING prospective randomized crossover study

J. Chelly, S. Mazerand, S. Jochmans, LV. Vong, CM. Weyer, F. Pourcine, O. Ellrodt, N. Thieulot-Rolin, J. Serbource-Goguel, O. Sy, M. Monchi
Intensive Care Unit, Groupe Hospitalier Sud Ile de France, Melun, France
**Correspondence:** J. Chelly
*Intensive Care Medicine Experimental* 2019, **7(Suppl 3):**000024

**INTRODUCTION.** Blood oxygen desaturation is the most common physiological change in mechanically ventilated patients during nurse cares periods that are routinely performed in the intensive care unit (ICU).
**OBJECTIVES.** To assess the ability of an automated closed-loop ventilation mode (Intellivent-ASV®) to reduce blood oxygen desaturation incidence and depth during daily nurse cares.
**METHODS.** We performed a prospective randomized crossover study in a 22 beds French mixed ICU, including mechanically ventilated patients with FiO2 ≤ 60% and without prone position and/or neuromuscular blocking agent. After inclusion, patients had two consecutive nurse cares periods (each separated by six hours) performed in a randomized order: one using Intellivent-ASV® and another using conventional mode. Ventilation modes were set by the attending physician 30 minutes before the procedure. The primary endpoint was the minimal pulse oxygen saturation level (SpO2min) during the procedure.
**RESULTS.** Among the 265 included patients, 93% were admitted for a medical pathology, and were intubated for pneumonia (50%), coma (32%), sepsis (32%) and acute respiratory distress syndrom (20%). Pressure support was the most common ventilation mode used in the conventional arm (91%). There was no difference between both modes regarding use of sedation, nurse cares duration and ventilator parameters except for spontaneous cycling higher and peak pressure lower in Intellivent-ASV® ($p < 0.001$ respectively). SpO2min ≤ 85% incidence was significantly lower and more patients had a SpO2min in the optimal range (between 90 and 95%) with Intellivent-ASV® ($p = 0.02$ and 0.002 respectively). Nurses had to manually increase FiO2 to 100% less frequently with Intellivent-ASV® ($p = 0.004$). Finally, multivariable logistic regression showed that nurse cares periods performed in Intellivent-ASV® reduced significantly the SpO2min ≤ 85% incidence (OR 0.5; 95%CI [0.3 – 0.9]; $p = 0.01$).
**CONCLUSION.** In this monocentric study, Intellivent-ASV® mode seems to be an interesting tool for nurses to stay focus on patients cares by reducing blood oxygen desaturation incidence and depth, with less manual interventions in comparison with conventional modes.

### 000036

#### The effect of using Airway Pressure release ventilation (APRV) mode on physiological dead space in comparison to Biphasic positive airway pressure (BiPAP) mode. A randomized controlled trial

A. Elkahwagy[1], W. Hamimy[2], M. Elayashy[3], A. Lotfy[4], S. Farouk[4]
[1]anesthesia , surgical ICU and pain management, Kasr El Aini Teaching Hospital, Al Manial, Egypt, Cairo, Egypt; [2]Anesthesia , surgical icu and pain management, Kasr El Aini Teaching Hospital, Cairo, Egypt, Egypt; [3]Anesthesia , surgical icu and pain management, Kasr El Aini Teaching Hospital, Al Manial, Egypt, Cairo, Egypt, Egypt; [4]Anesthesia , surgical icu and pain management, Kasr El Aini Teaching Hospital, Cairo, Egypt
**Correspondence:** A. Elkahwagy
*Intensive Care Medicine Experimental* 2019, **7(Suppl 3):**000036

**INTRODUCTION.** APRV is a relatively new mode of mechanical ventilation which can be used in treatment of patients with impaired oxygenation, but this mode is not widely used by clinicians. Physiological dead space (VDphys.) is an important parameter to assess respiratory function which is equal to the the volume of airway plus ventilated non- perfused alveoli. We conducted this study to compare APRV and BiPAP modes regarding VDphys. and other respiratory variables (pressures and blood gases). Our hypothesis is that the long inspiratory phase in APRV may increase VDphys. (by impairing perfusion) and may affect other respiratory variables.
**OBJECTIVES.** To compare APRV and BiPAP modes regarding VDphys., respiratory pressures and blood gases parameters.
**METHODS.** Mechanically ventilated patients with PO2/FiO2 ratio< 300 were randomized into two groups: Group A (APRV group) :APRV mode was used with high pressure (Phi) 20 cmH2O , low pressure (Plo) 5 cmH2O with I:E ratio ( Phi phase: Plo phase ratio ) 4:1 for 3 hours. Group B(BiPAP group) : BiPAP mode was used with high pressure (Phi) 20 cmH2O, low pressure (Plo) 5 cmH2O with I: E ratio (Phi phase: Plo phase ratio) 1:1 for 3 hours. Then all patients were left for ventilation according to the preference of the physician in charge. VDphys. was measured using the metabolic module of GE ventilators. Respiratory variables were also recorded. All measurements were done at 30 minutes and at the end of the intervention period (3 hours).

**RESULTS.** Sixty patients were available for final analysis (30 in each group). There was no statistically significant difference in age (51.7±15.2 vs 54.3±16.1, p=0.52) and weight (75.80±13.28 vs 76.57±15.93, p=0.840) between the two groups. VDphys/tidal volume (VT) at 30 minutes was (0.47 ±0.09 vs 0.46 ± 0.08, P=0.61) and at 3 hours was (0.44 ± 0.10 vs 0.43 ± 0.11 , p=0.67) in the two groups respectively. there was a reduction in VDphys/VT within each group at 3 hours but it was not statistically significant (p=0.11 for group A and 0.13 for group B). There was improvement of dynamic compliance within each group (36.17 ± 9.1 vs 39.7± 8.33, p=0.002 for group A) and (37.87 ± 7.84 vs 42.37 ± 7.84, p=0.001 for group B). PCO2 was increased in group B (39.17 ± 8.72 vs 42.67 ± 7.73, p=0.013) but it was not clinically significant.

**CONCLUSION.** APRV mode didn't increase physiological dead space compared to BiPAP mode. Both modes improved dynamic compliance.

**REFERENCE(S)**

1. Daoud EG, Chatburn RL. Comparing surrogates of oxygenation and ventilation between airway pressure release ventilation and biphasic airway pressure in a mechanical model of adult respiratory distress syndrome. Respir Investig. 2014 Jul;52(4):236–41.
2. Yu G. The effect of bi-level positive airway pressure mechanical ventilation on gas exchange during general anaesthesia. Br J Anaesth. 2006 Jan 27;96(4):522–32.
3. Anderson CT, Breen PH. Carbon dioxide kinetics and capnography during critical care. Crit Care. 2000;4(4):207–15.
4. Verscheure S, Massion PB, Verschuren F, Damas P, Magder S. Volumetric capnography: lessons from the past and current clinical applications. Crit Care. 2016 Dec 23;20(1):184.
5. Rogers K. Airway Pressure Release Ventilation: Theory and Practice. AACN Clin Issues Adv Pract Acute Crit Care. 2001;12(2):327–9.
6. Baum M, Benzer H, Putensen C, Koller W, Putz G. [Biphasic positive airway pressure (BIPAP)–a new form of augmented ventilation]. Anaesthesist. 1989 Sep;38(9):452–8.
7. Hörmann C, Baum M, Putensen C, Mutz NJ, Benzer H. Biphasic positive airway pressure (BIPAP)–a new mode of ventilatory support. Eur J Anaesthesiol. 1994 Jan;11(1):37–42.
8. Seymour CW, Frazer M, Reilly PM, Fuchs BD. Airway Pressure Release and Biphasic Intermittent Positive Airway Pressure Ventilation: Are They Ready for Prime Time? J Trauma. 2007;62:1298–309.
9. Haitsma JJ, Lachmann RA, Lachmann B. Lung protective ventilation in ARDS: role of mediators, PEEP and surfactant. Monaldi Arch chest Dis = Arch Monaldi per le Mal del torace. 59(2):108–18.
10. Girard TD, Bernard GR. Mechanical Ventilation in ARDS. Chest. 2007 Mar;131(3):921–9.
11. First and foremost, thanks are due to Allah, the most kind and merciful. I gratefully acknowledge the sincere advice and guidance of Prof. Dr. Waleed Ibrahim Hamimy, Professor of Anesthesiology, Faculty of Medicine, Cairo University, for his constructive guidance, encouragement and valuable help in accomplishing this work. I am greatly honored to express my deep appreciation to Dr. Mohamed Elayashy Mohamed, Lecturer of Anesthesiology, Faculty of Medicine, Cairo University, for his continuous support, sincere supervision, direction and meticulous revision of this work. I am really thankful to Dr. Ahmed Mohamed Lotfy, Lecturer of Anesthesiology, Faculty of Medicine, Cairo University, for his great and continuous help, advice, precious time, kindness, and moral support. Words will never be able to express my deepest gratitude to all those who helped me during preparation of this study and to my wife for her non-stoppable support and encouragement. Ahmed Samir Elkahwagy

### 000049

#### Hyperoxia in a General UK Intensive Care Unit (ICU)

R. Charlton[1], M. Carpenter[2]

[1]Newcastle University Medical School, Newcastle upon Tyne, United Kingdom; [2]Intensive care, Sunderland Royal Hospital, Sunderland, United Kingdom

**Correspondence:** R. Charlton

*Intensive Care Medicine Experimental* 2019, **7(Suppl 3):**000049

**INTRODUCTION.** Whilst the dangers of hypoxia are well documented, it has recently been proposed that too much oxygen, hyperoxia, may also be harmful to patients. There is a greater risk of mortality associated with hyperoxia, due in part to increased production of reactive oxidative species and vasoconstriction. Due to the widespread use of supplemental oxygen in ICU patients, this group is at a high risk of hyperoxia.

**OBJECTIVES.**

- To determine the prevalence of hyperoxia in patients receiving supplemental oxygen in ICU.
- To determine whether action is taken to reduce the amount of oxygen delivered to patients based on hyperoxic capillary oxygen saturation ($SpO_2$) and arterial partial pressure of oxygen ($PaO_2$).

**METHODS.** As part of a medical school project, a prospective audit was undertaken in a UK general mixed ICU/HDU of $SpO_2$ and $PaO_2$ of 53 consecutive patients receiving oxygen therapy, over 255 patient bed days, between 19/1/2019 and 6/2/2019.

Data was obtained from patient observation charts, and $SpO_2$ >96% (or >92% in the case of carbon dioxide retention), and $PaO_2$ >14kPa were considered 'hyperoxic'. Any action taken to reduce the fraction of inspired oxygen ($FiO_2$) as a result of hyperoxia was documented.

**RESULTS.** Over 255 bed days on ICU 4338 $SpO_2$ and 609 $PaO_2$ of 53 consecutive patients receiving supplemental oxygen were analysed to identify the prevalence of hyperoxia and the likelihood of action being taken to reduce the $FiO_2$ as a result. 2364 $SpO_2$ (54%) and 135 $PaO_2$ (22%) were found to be 'hyperoxic'.

Action was taken to reduce $FiO_2$ in a total 372 instances of recorded hyperoxia (15%). A $PaO_2$ above target was more likely to prompt action ($FiO_2$ reduced in 33% hyperoxic recordings) than an $SpO_2$ above target ($FiO_2$ reduced in 16%) as illustrated in Figure 1.

**CONCLUSION.** A significant proportion of recorded $SpO_2$/$PaO_2$ in 53 consecutive ICU patients were above target showing that there is a high prevalence of hyperoxia in ICU patients receiving supplemental oxygen. The $FiO_2$ delivered to patients was reduced in a small fraction of those found to be hyperoxic. A $PaO_2$ above target was more likely to prompt action to reduce $FiO_2$ than an $SpO_2$ above target.

Staff education with regards to the importance of recognition and management of hyperoxia and conservative oxygenation targets for patients may reduce the risks associated with hyperoxia.

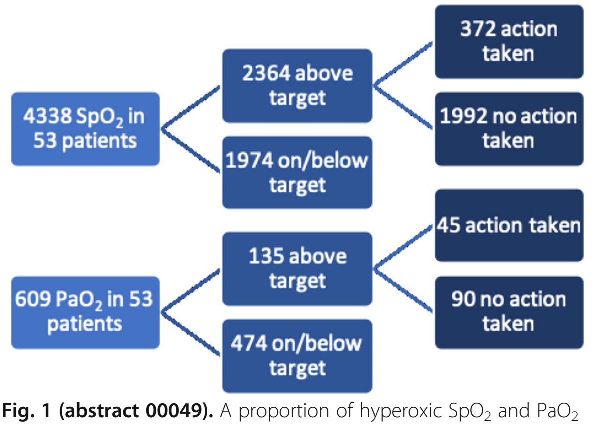

**Fig. 1 (abstract 00049).** A proportion of hyperoxic $SpO_2$ and $PaO_2$ in 53 consecutive ICCU patients resulted in a reduction in $FIO_2$.

### 000058

#### Comparison of various noninvasive respiratory support methods in cardiovascular surgery patients with postextubation respiratory failure

A. Eremenko, P. Polyakova

Intensive care unit, Petrovsky Russian Research Center of Surgery, Moscow, Russia

**Correspondence:** P. Polyakova

*Intensive Care Medicine Experimental* 2019, **7(Suppl 3):**000058

**INTRODUCTION.** Postextubation respiratory failure in the early postoperative period occurs in 5-25% [1,2] of cardiac surgery patients and various methods of noninvasive respiratory support are available for it's treatment.
**OBJECTIVES.** Comparative evaluation of the effect on gas exchange of conventional oxygen therapy (COT), noninvasive positive airway pressure mask ventilation (NIPPV) and high-flow nasal cannula therapy (HFNC) in the patients with postextubation respiratory failure after cardiovascular surgery.
**METHODS.** 52 cardiac surgery patients (age 60±11years) with respiratory failure manifested in the postoperative period after tracheal extubation were included into the study. Respiratory failure criteria: $PaO_2/FiO_2 \leq 300$ mmHg or $SpO_2 \leq 88\%$ on room air. Patients with pleural effusion, pneumothorax and paresis of diaphragm were excluded from the study. In each patient sequentially performed blood gas analysis when breathing room air, COT (mask with pre-volume bag), HFNC and NIPPV. Duration of application of each method before taking of analyses was 1 hour. Respiratory rate (RR) and SpO2 were monitored throughout the study. Optimal parameters for HFNC were: FiO2 47±9%, flow rate - 36,7±9 l/min, and for NIPPV: PIP - 13,3±4,0 mmH2O, PEEP - 6,65±2,04 mmH2O, FiO2 - 50±8%, TV - 7,2±1,27 ml/kg.
**RESULTS.** During COT, mean PaO2/FiO2 ratio was 193±73 mmHg. After change to HFNC it increased to 247±87 mmHg (p=0.0025), and on NIPPV - to 234±67 mmHg (p=0.0097). The same tendency was observed with SpO2: at HFNC and NIPPV it was significantly higher than at COT (97.27±2.29% and 96.37±2.1% versus 94.63±3.51%, p= 0.000028 and p=0.0058, respectively). PaCO2 during COT was higher than on air (42.16±8.33 mmHg and 38.64±6.58 mmHg, respectively, p=0.04). After change to the noninvasive ventilatory support PaCO2 decreased by 9.82% (38.02±7.26 mmHg, p=0.015) on HFNC and by 5.86% (39.69±7.59 mmHg, p=0.16) on NIPPV. Significant differences in values were found in RR during COT (21.02±6.59 bpm) compared to HFNC (17.27±6.5 bpm, p=0.0042) and on NIPPV it was not significant (18.89±5.1 bpm, p= 0.072).
**CONCLUSION.** NIPPV and HFNC in patients with postextubation respiratory failure after cardiac surgery have similar positive effect on gas exchange to compare to the mask with pre-volume bag. HFNC has better effect on RR and CO2 elimination than NIPPV.

**REFERENCE(S)**

1. Filsoufi F, Rahmanian PB, et al. Logistic risk model predicting postoperative respiratory failure in patients undergoing valve surgery. Eur J Cardiothorac Surg.2008;34(5):953-9. DOI:10.1016/j.ejcts.2008.07.061. PMID:1883518
2. Yamagishi, T, Ishikawa S, et al. Postoperative oxygenation following coronary artery bypass grafting. J Cardiovasc. Surg. (Torino).2000Apr;41(2):221–225. PMID:10901525

### 000061

#### Low pressure support level provides respiratory assistance above endotracheal tube resistance compensation during spontaneous breathing trial in patients ready-to-wean from mechanical ventilation

C. Guérin[1], L. Baboi[1], M. Mezidi[1], N. Chebib[1], H. Yonis[1], N. Terzi,[2], B. Louis[3]
[1]Médecine intensive réanimation, Hospital La Croix-Rousse - Hcl, Lyon, France; [2]Médecine intensive réanimation, C.H.U de Grenoble, La Tronche, France; [3]Inserm 955, IMRB, Creteil, France
**Correspondence:** C. Guérin
*Intensive Care Medicine Experimental* 2019, **7(Suppl 3):**000061

**INTRODUCTION.** Spontaneous breathing trial (SBT) during weaning from mechanical ventilation aims at testing ability of respiratory system to work without breathing assistance. SBT can be delivered through T-piece trial or low pressure support (PS) level. Low PS is thought to compensate for endotracheal tube (ETT) resistance (RET), and, hence no further assistance would be provided by the ventilator. We reasoned that this may not be true with the modern ICU ventilators. The real way to compensate for RET only is the automatic tube compensation (ATC) option. Our hypothesis was that low PS was associated with a significant reduction in work of breathing (WOB) as compared to ATC.
**METHODS.** We underwent a prospective, short-term, physiological, cross-over study (NCT02939963) where patients with criteria for SBT after at least 24 hours of mechanical ventilation in our ICU received PS7 cmH2O positive end-expiratory pressure (PEEP) 4cmH2O without ATC (low PS group) and ATC PEEP4cmH2O without PS (ATC group) as SBT trial. Patients with chronic respiratory failure under long term home oxygen therapy and/or noninvasive ventilation before ICU admission, tracheotomy, nasogastric tube contra-indicated, thoracic tube in place, no agreement to participate were not included. Low PS and ATC were randomly applied for 30 minutes each and preceded by a 30-min control period with the PS level before the study. ATC was set at 100% compensation of patient's ETT size. Esophageal and airway pressures and flow were measured at ETT proximal end. At the end of each period resistive and elastic components of WOB done by the patient were obtained from the Campbell diagram over a 2-min recording at the end of each period. Power of breathing (W) and W/liter ventilation were determined as WOB times respiratory rate and W divided by minute ventilation, respectively. Primary end-point was total (resistive + elastic) W. We computed that 16 patients should be required for a 4±4 J/min reduction in W with low PS as compared to ATC be statistically significant at 5% and 20% risks, respectively. Analysis was done using linear mixed model testing effect of treatment (low PS vs ATC), period and interaction between them. Values are median (1st-3rd quartiles).
**RESULTS.** Twenty patients were included over 12 months. Between low PS and ATC, W was 6.4 (4.3-9.3) and 9.8 (5.3-22.0) J/min (P= 0.011) with no significant effect of both period and interaction between treatment and period. Both resistive (3.2 (2.1-4.6) vs. 4.7 (1.5-9.7)J/min P=0.035) and elastic (3.6 (2.3-4.5) vs. 5.2 (3.6-8.8) J/min P= 0.0096) W were significantly lower with low PS than ATC. W/liter minute ventilation was significantly lower in low PS (0.74(0.53-0.93)) than in ATC group (0.97 (0.68-1.54)) J/L (P=0.004). Both resistive and elastic components of W/liter minute-ventilation (0.33 (0.23-0.53) vs. 0.47 (0.22-0.74) (P=0.04) and 0.40 (0.32-0.47) vs. 0.63 (0.45-0.69) J/L) (P=0.00005) were significantly lower with low PS than with ATC, with no significant effect of period and no interaction.
**CONCLUSION.** When SBT is done by using low PS a respiratory support is provided to the patient above the compensation of RET. Low PS does more than just compensating for RET.

### 000098

#### Differences in prognostic factors between direct and indirect ARDS in pediatric patients

DH. Kim, EJ. Ha, WK. Jhang, SJ. Park
Pediatrics, Asan Medical Center, Seoul, Republic of Korea
**Correspondence:** D. Kim
*Intensive Care Medicine Experimental* 2019, **7(Suppl 3):**000098

**INTRODUCTION.** Direct (Pulmonary) and indirect (extrapulmonary) acute respiratory distress syndrome (ARDS) differs responses and therapeutic effects of both groups were heterogenous. However, there were few studies to definite results about it, especially critically ill pediatric ARDS (PARDS) patients.
**OBJECTIVES.** This study aims to compare two ARDS groups in the clinical outcomes and the risk factors for mortality.
**METHODS.** This was a single center, retrospective study. Among the children admitted to the pediatric intensive care unit (PICU) of a tertiary care children's hospital between October 2010 and October 2018, 144 pediatric patients classified as either pulmonary (n=119) or extrapulmonary (n=25) ARDS were included. Patients who had L-R shunt and heart failure were excluded. We used the unpaired student's t-test and Univariate logistic regression for risk factors of mortality in each sutyptes of ARDS.
**RESULTS.** There was significant difference in the PICU mortality rate by PARDS level between two groups. The mortality was significant increased by PARDS level in direct ARDS group but not in indirect ARDS group ($p$=0.002 vs $p$=0.774). The direct ARDS group showed that PaO2/FiO2 (P:F ratio, $p$=0.005), Oxygen index (OI, $p$=0.037), FiO2 ($p$<0.001), peak expiratory end pressure ($p$=0.002), driving pressure ($p$=0.035), peak inspiratory pressure ($p$<0.001) and mean airway pressure ($p$<0.001) were significant risk factor in mortality, while the indirect ARDS group had significant risk factor as lactate ($p$=0.007) and percentage of neutrophil ($p$=0.002) in mortality

**CONCLUSION.** Our results, showing that clinical outcomes and risk factors for mortality rate differ by ARDS types suggest that pulmonary and extrapulmonary ARDS could have different clinical courses and thus should be managed distinctly

**REFERENCE(S)**

1. Keisuke Anan, Kodai Kawamura, Moritaka Suga, Kazuya Ichikado: Clinical differences between pulmonary and extrapulmonary acute respiratory distress syndrome : a retrospective cohort study of prospectively collected data in Japan. J Thorac Dis 2018; 10:5796-5803
2. Gattinoni L, Pelosi P, Suter PM, et al: Acute respiratory distress syndrome caused by pulmonary and extrapulmonary disease: different syndromes? American journal of respiratory and critical care medicine 1998; 158:3-11
3. Ware LB, Matthay MA: The acute respiratory distress syndrome. New England Journal of Medicine 2000; 342:1334-1349
4. Nadir Yehya, Garrett Keim, Neal J.Thomas: Subtypes of pediatric acute respiratory distress syndrome have different predictors of mortality. Int Care Med 2018; 44:1230-1239
5. Luo L, Shaver CM, Zhao Z, et al: Clinical Predictors of Hospital Mortality Differ Between Direct and Indirect ARDS. Chest 2017; 151:755-763
6. Shaver CM, Bastarache JA: Clinical and biological heterogeneity in acute respiratory distress syndrome: direct versus indirect lung injury. Clin Chest Med 2014; 35:639-653

### 000107

#### Plasma Fibrinogen as a Biochemical Marker for Acute Respiratory Distress Syndrome (ARDS) Among Patients with Severe Pneumonia in a Philippine Tertiary Hospital

O. Ubaldo[1], MA. Lazaro,[2], JE. Cinco,[1], E. Aventura[2]
[1]Adult Critical Care, The Medical City Hospital, Ortigas Avenue, Pasig, Metro Manila, Philippines, Pasig, Philippines; [2]Pulmonary medicine, The Medical City Hospital, Ortigas Avenue, Pasig, Metro Manila, Philippines, Pasig, Philippines
**Correspondence:** O. Ubaldo
*Intensive Care Medicine Experimental* 2019, **7(Suppl 3):**000107

**INTRODUCTION.** Severe pneumonia is major risk factor to develop Acute Respiratory Distress Syndrome (ARDS). Early recognition of patients at risk to develop ARDS is not only crucial but pivotal. The Berlin criteria aids in diagnosis of ARDS however, the absence of a standard diagnostic biomarker may lead to under-recognition and delayed evidence-based interventions. To date, most studies on ARDS biomarkers are retrospectively done. Among these, plasma fibrinogen has shown association with ARDS progression in patients with severe pneumonia.

**OBJECTIVES.** At present, the clinical utility of plasma fibrinogen for ARDS is unknown. With this study, we sought to find association between plasma fibrinogen levels and progression to ARDS among patients with severe pneumonia.

**METHODS.** In this prospective cohort study, patients diagnosed with severe pneumonia based on IDSA criteria were enrolled from July 2018 to February 2019. Baseline clinical and laboratory parameters were recorded and disease severity were scored using APACHE II. Two determinations of plasma fibrinogen were done, first within 24 hours of disease recognition and the second after 48 hours. The participants were then followed for 7 days with development of ARDS as the primary outcome. The diagnosis of ARDS was made by the intensivist on-duty and/or the attending pulmonologist.

**RESULTS.** Forty-seven patients were prospectively monitored within 7 days of enrolment then divided into two sub-groups between twelve (25%) patients who developed ARDS and thirty-five (75%) patients who did not. Fibrinogen levels at baseline had sensitivity and specificity of 41.7% and 57.1% respectively (p=0.932), levels after 48 hours had sensitivity and specificity of 55.6% and 65.6% respectively (p=0.729), and delta fibrinogen levels had sensitivity of 55.6% and specificity of 62.5% (p=0.581) (Table 1). Low platelet counts and higher PEEP levels used were found to be statistically significant parameters for ARDS progression.

**CONCLUSION.** Based on this study, plasma fibrinogen is an unreliable biomarker for predicting ARDS development in patients with severe pneumonia. To our knowledge, this is the first attempt to investigate on a biomarker for ARDS progression in the Philippines. A higher-powered study with more robust sample size is recommended. However, further analysis regarding local incidence of ARDS should be made. Hopefully, this study will serve as benchmark for further research as relevant information and local data are still lacking. Other biomarkers that can predict ARDS development amongst patients at risk should be further investigated.

**REFERENCE(S)**

1. Luo J, Yu H, Hu Y-H, Liu D, Wang Y-W. Early identification of patients at risk for acute respiratory distress syndrome among severe pneumonia: a retrospective cohort study. Journal of Thoracic Disease. October 2017:1-17. doi:10.21037/jtd.2017.09.20.
2. Thompson BT, Chambers RC, Liu KD. Acute Respiratory Distress Syndrome. Drazen JM, ed. N Engl J Med. 2017;377(6):562-572. doi:10.1056/NEJMra1608077.
3. Bellani G, Laffey J, Pham T, Fan E, Brochard L. Epidemiology, Patterns of Care, and Mortality for Patients With Acute Respiratory Distress Syndrome in Intensive Care Units in 50 Countries. JAMA. July 2016:1-13. doi:10.1001/jama.2016.0291&utm_campaign=articlePDF%26utm_medium=articlePDFlink%26utm_sour ce=articlePDF%26utm_content=jama.2016.0291.

**Table 1 (abstract 000107).** AUROC Analysis

| Discriminant Variable | Area Under the Curve | Std. Error | P-Value | Sensitivity | Specificity |
|---|---|---|---|---|---|
| Fibrinogen q0h | 0.492 | 0.094 | 0.932ns | 41.7% | 57.1% |
| Fibrinogen q48h | 0.538 | 0.112 | 0.729ns | 55.6% | 65.6% |
| Change in Fibrinogen (delta fibrinogen) | 0.561 | 0.121 | 0.581ns | 55.6% | 62.5% |

### 000112

#### Peak inspiratory muscle pressure estimated by PAV+ during spontaneous breathing trial is a potential predictor of post-extubation respiratory failure

A. Ishizuka[1], J. Kataoka[2], Y. Norisue[1], S. Fujitani[3]
[1]Intensive care medicine, Tokyo Bay Urayasu Ichikawa Medical Center, Urayasu, Japan; [2]Intensive care medicine, Nerima Hikarigaoka Hospital, Nerima City, Japan; [3]Department of emergency and critical care medicine, St. Marianna University School of Medicine, Kawasaki, Japan
**Correspondence:** A. Ishizuka
*Intensive Care Medicine Experimental* 2019, **7(Suppl 3):**000112

**INTRODUCTION.** A spontaneous breathing trial is commonly performed to assess whether a patient can tolerate without ventilator support. However, the reintubation rate is 10-20% even after successful SBT. Although physiologic parameters during SBT, such as the work of breathing (WOB) or the pressure time product (PTP), may inform the risk of reintubation, measurement of such parameters is not technically simple. Proportional assist ventilation with load-adjustable gain factors (PAV+) can easily estimate the WOB and the peak inspireatory muscle pressure (Pmus).

**OBJECTIVES.** We investigated whether the WOB and the Pmus estimated by PAV+ during SBT can predict post-extubation respiratory failure and necessity of reintubation.

**METHODS.** This study was a prospective observational study in a single center in Japan. All patients over 18 years old who were mechanically ventilated and passed SBT using a mode of PAV+ with a support rate 20% were included. We recorded the average of WOB, peak inspiratory pressure (Ppeak), compliance and resistance measured by PAV+ during SBT. And we calculated the peak Pmus from Ppeak. The primary outcome of this study was post-extubation respiratory failure within 72 hours.

**RESULTS.** A total of 50 mechanically ventilated patients were included. Eighteen patients had post-extubation respiratory failure within 72 hours. The patients with post-extubation respiratory failure showed significantly higher peak Pmus than those without post-extubation failure (14.0 vs 12.3 cmH2O, p=0.025). There were no siginificant differences in WOB (3.81 vs 3.21 J/min, p=0.115), compliance (63 vs 61 mL/cmH2O, p=0.846), and resistance (5.3 vs 4.8 cmH2O/L/s, p=0.623) measured by PAV+ between the two groups.

**CONCLUSION.** Peak Pmus estimated by PAV+ during SBT is a possible predictor of post-extubation respiratory failure. Larger sample size is needed to further determine the usefulness of the test.

1. none

### 000142

#### Quantitative correlation of B lines with lung compliance and oxygenation index in critically ill patients on mechanical ventilation: A prospective observational study

V. Saini[1], S. Saini[2], N. Yaddanapudi[3]

[1]Post Graduate Institute of Medical Education & Research, Chandigarh, Chandigarh, India; [2]Anarsthesia, Post Graduate Institute of Medical Education & Research, Chandigarh, Chandigarh, India; [3]Anaesthesia, Post Graduate Institute of Medical Education & Research, Chandigarh, Chandigarh, India

**Correspondence:** V. Saini

*Intensive Care Medicine Experimental* 2019, **7(Suppl 3):**000142

**INTRODUCTION.** EVLW accumulates in the lungs even before the changes in blood gases and chest x-ray become apparent. The lungs which have high compliance tend to expand to a greater extent as compared to the lungs with low compliance.Martelius et al found that a significant decrease in the number of B lines is associated with improved static lung compliance in a prospective observational study of 34 infants. However, till date, no study has been done in adults to correlate EVLW with lung compliance. Therefore, we planned to do this study in adult patients.

**OBJECTIVES.** To assess quantitative correlation of B lines on bedside lung ultrasound with static lung compliance in adult patients on mechanical ventilation. and to assess correlation of B lines with oxygenation index.

**METHODS.** The study commenced after the clearance of the ethics committee of the institute. Informed consent was taken from the patient or the patient's legal guardian to enroll them in the study. Following approval from institutional ethics committee study was conducted from October 2017 to June 2018. (Ref.No.NK/3868/MD/299).During the study a total of 200 lung ultrasound readings were recorded along with static lung compliance and oxygenation index.

Static compliance was recorded along with lung USG. For static compliance, there should be no patient effort. It was calculated with a tool given in eVent ventilator 10 mins after giving short-acting muscle relaxant like atracurium(0.5mg/kg) and sedatives like midazolam or fentanyl.

Oxygenation Index(OI) = FIO2/PaO2×MAP

FIO2 = Fraction of inspired oxygen, Pao2= Partial pressure of arterial oxygen, MAP= Mean airway pressure

**RESULTS.** Sixty-one patients, with 200 USG readings in total, were included in our study. Minimum gap between 2 readings was 24hrs.

All patients had a comparable demographic profile. The B line score and static lung compliance appear to be inversely related which means higher the B line score, lower is the static lung compliance. Correlation showed R2 of 0.43 which explains the 43% changes in static lung compliance values could be explained by changes in the B line scores.

A positive linear correlation was found between B line score and oxygenation index. This suggests as number B lines in lung increases the oxygenation index also increases. Correlation showed R2 value of 0.403 explains 40% of change in B line scores correlated positively with the oxygenation index as shown by R2 value of 0.40.

**CONCLUSION.** The most important finding in our study was a negative correlation of B line score with static lung compliance having a significant R2 value of 0.43. Other findings were a positive correlation of B line score with oxygenation index and a negative correlation of lung compliance with oxygenation index. Through our analysis, we can conclude that increase in number of B lines is associated with concomitant decrease in lung compliance and increase in oxygenation index in adult patients.

**REFERENCE(S)**

1. Martelius L, Süvari L, Janér C, Helve O, Kaskinen A, Kirjavainen T, et al. Lung Ultrasound and Static Lung Compliance during Postnatal Adaptation in Healthy Term Infants. Neonatology. 2015;108(4):287–92.
2. Jozwiak M, Teboul J-L, Monnet X. Extravascular lung water in critical care: recent advances and clinical applications. Ann Intensive Care. 2015;5:38.
3. Papandrinopoulou D, Tzouda V, Tsoukalas G. Lung Compliance and Chronic Obstructive Pulmonary Disease. Pulm Med. 2012;2012:542769.
4. Picano E, Gargani L, Gheorghiade M. Why, when, and how to assess pulmonary congestion in heart failure: pathophysiological, clinical, and methodological implications. Heart Fail Rev. 2010 Jan 1;15(1):63–72.
5. No grant taken for this study

### 000144

#### A systematic bench assessment of automatic tube compensation provided by intensive care unit ventilators

N. Terzi,[1], LM. Galerneau[1], Z. Riad[2], E. Turbil,[3], C. Schwebel[1], C. Guérin[4], B. Louis[5]

[1]Médecine intensive réanimation, C.H.U de Grenoble, La Tronche, France; [2]Anesthesie réanimation, Hospital Cardiovascular And Pneumologique Louis Pradel, Bron, France; [3]Anesthesiology and intensive care, Università degli Studi di Sassari, Sassari, Italy; [4]Service de réanimation médicale, hôpital de la croix rousse, Grande Rue de la Croix Rousse, Lyon, France; [5]Inserm 955, IMRB, Creteil, France

**Correspondence:** C. Guérin

*Intensive Care Medicine Experimental* 2019, **7(Suppl 3):**000144

**INTRODUCTION.** Automatic tube compensation (ATC) is an option available in intensive care unit (ICU) ventilators to compensate for endotracheal tube (ETT) resistance. To achieve this ICU ventilator delivers a certain amount of pressure/flow that compensates for the resistive pressure drop across ETT. It requires notifying size of ETT and percent of compensation. We reasoned that if ATC works properly tidal volume (VT) should be the same without ATC and no ETT as with ATC and ETT. We tested the performance of ICU ventilators on a bench, expecting furthermore differences between them.

**METHODS.** Seven ICU ventilators (Evita XL and V500 infinity (Draeger), C6 and S1 (Nihon-Khoden), Elisa 800 (Lowenstein), 980 (Medtronic), Carescape 860 (GE)) were set in pressure support 0 cmH2O, PEEP 4 cmH2O, FIO2 21% and equipped with the same double limb ventilator circuit (Intersurgical) without any humidification device. ASL 5000 bench model was set with 3 inspiratory/expiratory resistance (R) and compliance (C) combinations: R13/12-C54, R12/14-C39 and R22/18-C59 mimicking normal, ARDS and COPD conditions, respectively (1). Inspiratory effort generated by ASL 5000 consisted of 30 consecutive breaths obtained from the esophageal pressure in a real patient at the time of a spontaneous breathing trial. For each ICU ventilator and RC combination, two steps were performed: in the first, ATC was not activated and ventilator attached to ASL 5000 without ETT (ATC-ETT-); in the second, ATC was set on at 100% compensation for an ETT 8 mm ID and such an ETT (Shiley Hi contour, Covidien) joined ICU ventilator to ASL 5000 (ATC+ETT+). The null hypothesis is that VTATC+ETT+ minus VTATC-ETT- is 0. Primary end point was the breath by breath paired difference betwen ATC+ETT+ and ATC-ETT-. It was tested to zero for each ventilator in each RC condition.

**RESULTS.** Median VT was 213 ml. Table 1 displays mean (±SD) difference in VT (ml) between ATC+ETT+ and ATC-ETT-: a negative value means that ATC under delivers and a positive value that ATC over delivers VT for a given patient's inspiratory effort and RC. In four ventilators (C6, S1, Elisa 800 and 980) ATC almost systematically under delivered VT. In several instances under compensation was greater than 10% median VT. By contrast ATC performed better with the other three ventilators (Evita XL, V500 and Carescape 860).

**CONCLUSION.** ATC tended to under deliver VT. Furthermore, there were marked differences between ICU ventilators the clinician should be aware of when using the ATC option.

**Table 1 (abstract 000144).** See text for description

| | Evita | Carescape 860 | C6 | S1 | Elisa 800 | 980 | V500 |
|---|---|---|---|---|---|---|---|
| ARDS | -5±4** | 3±14 | -21±4↑ | -16±13↑ | -17±3↑ | -39±8↑ | 0±11 |
| COPD | -3±9 | 9±10↑ | -12±4↑ | -12±2↑ | -12±2↑ | -24±6↑ | -8±30** |
| Normal | -8±5** | -9±51* | -33±7↑ | -16±14↑ | -20±4↑ | -51±7↑ | -9±14* |

*P<0.05 **P<0.01 ↑ P<0.001 versus 0

### 000164

#### Estimating oxygen consumption during rehabilitation in mechanically ventilated patients recovering from critical illness

C. Black[1], R. Klapaukh,[2], M. Singer,[3]
[1]Therapies and Rehabilitation, University College London Hospitals NHS Trust, London, United Kingdom; [2]Research software development, University College London, London, United Kingdom; [3]Bloomsbury institute of intensive care medicine, University College London, London, United Kingdom
**Correspondence:** C. Black
*Intensive Care Medicine Experimental* 2019, **7(Suppl 3):**000164

**INTRODUCTION.** Exercise is considered an important intervention to facilitate weaning from mechanical ventilation and limit long-term functional dependence. However, the exercise intensity of individual patients during rehabilitation is not measured. This potentially leads to under training of some and overtraining of others.

Breath-by-breath gas exchange analysis (BBGEA) is the gold standard for measuring oxygen consumption ($VO_2$) to calculate workload. While an invaluable research tool, it is impractical for use in routine clinical practice. Alternative methods of monitoring exercise intensity during rehabilitation are required.

During BBGEA data collection for previous studies, we noted (i) how closely minute ventilation (VE) tracked $VO_2$ and (ii) recovery of VE to the pre-exercise state was indicative of $VO_2$ returning to baseline. While VE could potentially be used as an indicator of recovery between exercise bouts during a rehabilitation session, the complexity of the relationship between VE and $VO_2$ currently negates its use as a direct estimate of absolute exercise intensity.

**OBJECTIVES.** We aimed to establish whether exercise $VO_2$ could be estimated from exercise VE, RR, VT and resting end-tidal $CO_2$ ($PETCO_2$).

**METHODS.** VE, RR, $PETCO_2$, VT and $VO_2$ were recorded using the Medgraphics Ultima device during rehabilitation of 37 patients on 106 occasions.

Principle component analysis (PCA) was used to describe the variation in the data between, VE, RR, VT. The first 2 PCA components accounting for 98% of the variance, were added to a linear mixed effects model along with the explanatory variables; resting $PETCO_2$, age, gender and weight. The fixed effect of the model was patient.

The measured $VO_2$ values were classed as low 3.5 - 4.5 ml/kg/min, medium >4.5 - 5.5 ml/kg/min and high > 5.5ml/kg/min. The performance characteristics of the model were assessed using a confusion matrix.

**RESULTS.** In a small sample size we demonstrate good agreement between actual and predicted $VO_2$ from a model using VE, RR, $PETCO_2$, VT, age, gender and weight. The MROC area under the curve for this model was 88%. The performance classifiers are given in table 1. This model requires future validation in an independent sample to confirm its utility.

**CONCLUSION.** Predicting $VO_2$ from easily obtainable observations in ICU may be possible during rehabilitation.

**REFERENCE(S)**

1. Intensive Care Society New Investigator Award 2017
2. UK National Institute for Health Research (NIHR) Clinical Doctoral Fellowship Award

**Table 1 (abstract 000164).** Model performance characteristics

| Classification | Sensitivity | Specificity | Positive Predictive Value | Negative Predictive Value | Prevalence Rate |
|---|---|---|---|---|---|
| Low | 0.85 | 0.79 | 0.80 | 0.85 | 0.49 |
| Medium | 0.69 | 0.82 | 0.75 | 0.79 | 0.42 |
| High | 0.69 | 0.97 | 0.72 | 0.97 | 0.09 |

### 000170

#### Teaching Mechanical Ventilation for Residents in Intensive Care. A randomized Trial Using Traditional Lectures VS Computer-Based Simulation (SimVA©)

H. Roze[1], E. Rivière[2], R. Dubois[3], A. Ouattara[4]
[1]South department of anesthesiology and intensive care, Bordeaux University Hospital, Pessac, France; [2]Départment de médecine interne, hôpital haut leveque, Bordeaux University Hospital, Bordeaux, France; [3]Ihu liryc, Fondation Bordeaux Université, Bordeaux, France; [4]South department of anesthesiology and intensive care, Bordeaux University Hospital, Bordeaux, France
**Correspondence:** H. Roze
*Intensive Care Medicine Experimental* 2019, **7(Suppl 3):**000170

**INTRODUCTION.** During educational process, trainees apply their knowledge to treat patient in intensive care before achieving full clinical competency. Moreover, advances in knowledge regarding mechanical ventilation in particular lung protective ventilation and asynchronies have been shown to be associated with mortality. For these reasons we developed a simulator of controlled and spontaneous artificial ventilation (Sim-VA) and virtual breathing patients. Mathematical model resolved differential equations of chest and lung movements according to inspiratory effort or not, in order to match with a clinical database.

**OBJECTIVES.** The aim of this study was to compare two teaching modalities on mechanical ventilation: traditional lectures versus virtual simulation.

**METHODS.** This randomized controlled study involved 54 residents. One group of 22 participants attended the same didactic lecture on mechanical ventilation (3 hours) whereas the other 22 were in the simulator group (3 hours). Performance was measured using a pre and post-test evaluation of knowledge on respiratory settings and pressure flow time curves monitoring in ARDS and COPD patients. A retention test was done at 3 months (The same questioner was used for pre, post and retention test). Comparison was individual in each group (*ANOVA, multiple comparison) and between groups (Mann-Whitney), p< 0,05 was considered significant.

**RESULTS.** Baseline knowledge was not different between groups; post-test was significantly improved in both groups (figure) but was significantly higher in the simulator group. Retention test was only significantly different from the pre-test in the simulator group.

**CONCLUSION.** A computer-based simulation with a modelisation of controlled and spontaneous mechanical ventilation has the potential to improve knowledge and skills in ventilator settings in comparison to traditional didactic lectures.

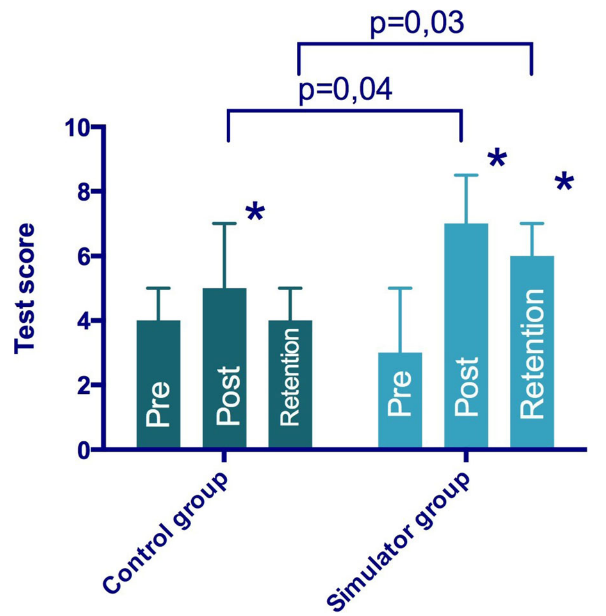

**Fig. 1 (abstract 000170).** See text for description

### 000172

#### Hypercapnic respiratory insufficiency: a retrospective comparison between treatment by high- flow nasal cannula therapy and non-invasive positive pressure ventilation

D. Snijders[1], V. Van Dooren[1], D. Bussmann-Willems[2], R. Van Der Horst[3], M. De Kruif[1]
[1]Department of pulmonary diseases, Atrium MC, Heerlen, Netherlands; [2]Department of emergency medicine, Atrium MC, Heerlen, Netherlands; [3]Department of intensive care, Atrium MC, Heerlen, Netherlands
**Correspondence:** D. Snijders
*Intensive Care Medicine Experimental* 2019, **7(Suppl 3):**000172

**INTRODUCTION.** In numerous studies, high-flow oxygen through nasal cannula (HFNC) has proven beneficial for patients with hypoxic respiratory failure (1-3). In these studies, patients with hypercapnia are generally excluded. Therefore, use of HFNC in hypercapnic patients is controversial.
**OBJECTIVES.** To compare the need for endotracheal intubation and mortality risk in patients with acute respiratory failure with hypoxemia and hypercapnia, admitted to the intensive care unit (ICU) and treated with either HFNC or non-invasive positive pressure ventilation (NIPPV).
**METHODS.** Retrospective cohort study of adult, hypoxic and hypercapnic patients admitted to the ICU, identified by start of treatment with either HFNC or NIPPV at admission. Patients were included between September 2015 and August 2018 at Zuyderland Medical Center, The Netherlands.
**RESULTS.** After screening 676 patients with 1109 episodes, 95 patients fulfilled all inclusion criteria. Out of these patients, 35 received HFNC and 60 NIPPV. In both treatment groups, pH and $paCO_2$ values improved 6 and 24 hours after start therapy. Arterial pH improved in the first 24 hours from 7.27 to 7.40 in the HFNC group and from 7.26 to 7.32 in the NIPPV group (p=0.001 between groups at t=24 hours). The $pCO_2$ improved from 9.3 to 7.1 kPa in the HFNC group and from 9.2 to 8.3 kPa for patients treated with NIPPV (p=0.05 between groups at t=24 hours). Need for intubation did not differ significantly between HFNC and NIPPV (35% and 22% respectively; p=0.4). In contrast, mortality was increased using HFNC instead of NIPPV (41% versus 12% in HFNC; p=0.039). Subgroup analysis revealed that in patients with chronic obstructive pulmonary disease (COPD), both the need for intubation and mortality risk were not different between HFNC and NIPPV (p=0.6 and p=1.0, respectively). Time analysis showed no significant difference between groups in time between admission and death (Log Rank p=0.5).
**CONCLUSION.** This study shows a proof of principle of HFNC in patients with hypercapnic respiratory failure at the ICU in terms of improvement of arterial blood gas (ABG) values. However, when comparing all hypercapnic patients admitted to the ICU in this study, treatment with HFNC seems to be associated with an increased mortality risk. In patients admitted for COPD, there is no increased risk for intubation or mortality when compared to NIPPV. These data suggest that HFNC might be useful for hypercapnic patients with an acute exacerbation of COPD, but may have an adverse outcome in other hypercapnic patient groups.

##### REFERENCE(S)

1. Frat JP, Thille AW, Mercat A, Girault C, Ragot S, Perbet S, et al. High-flow oxygen through nasal cannula in acute hypoxemic respiratory failure. The New England journal of medicine. 2015;372(23):2185-96.
2. Rittayamai N, Tscheikuna J, Praphruetkit N, Kijpinyochai S. Use of High-Flow Nasal Cannula for Acute Dyspnea and Hypoxemia in the Emergency Department. Respiratory care. 2015;60(10):1377-82.
3. Leeies M, Flynn E, Turgeon AF, Paunovic B, Loewen H, Rabbani R, et al. High-flow oxygen via nasal cannulae in patients with acute hypoxemic respiratory failure: a systematic review and meta-analysis. Systematic reviews. 2017;6(1):202.

### 000177

#### Does the adaptive support ventilation have advantages in the postoperative period after uncomplicated cardiac surgery?

R. Komnov[1], A. Eremenko[2]
[1]Cardiac Intencive Care Unit, Petrovsky Russian Research Center of Surgery, Moscow, Russia; [2]Intensive care unit, Russian Research Center of Surgery, Moscow, Russia
**Correspondence:** R. Komnov
*Intensive Care Medicine Experimental* 2019, **7(Suppl 3):**000177

**INTRODUCTION.** Respiratory support plays an important role in patient's recovery after cardiovascular surgery. Adaptive support ventilation (ASV) is an automatic closed-loop ventilation mode where minute volume is controlled by a combination of tidal volume (Vt) and respiratory rate settings based on patient's respiratory mechanics. There is an ability to reduce workload for physicians without compromising patient's safety with using intellectual modes of mandatory ventilation.
**OBJECTIVES.** To compare the effect(s) of ASV and conventional ventilation modes.
**METHODS.** In this randomized controlled trial 40 adult patients were ventilated with ASV and 38 with conventional ventilation modes after uncomplicated cardiac surgery. Hamilton G5 ventilators were used and 8 physicians were involved into the study. All actions of physician's, ventilator settings and changes were monitored and recorded during mechanical ventilation and weaning. Care of both groups was standardized, except modes of postoperative ventilation.
We compared the physician's workload, through accounting number of manual ventilator settings made before tracheal extubation and time, which they spent near the ventilator in every group.
The secondary endpoints were duration of tracheal intubation in ICU, the frequency of undesirable events and the length of ICU and hospital stay.
**RESULTS.** There were no significant differences in the duration of respiratory support in ICU: 267,6±76,6 min (ASV) vs 270,5±80 min (control).
In ASV group the number of manual ventilator settings and time spent near the ventilator before tracheal extubation was lower: 2 (1-4) vs 4 (2-6), (p=0,0000) and 99±35 sec vs 166±70 sec (p=0,0001)

respectively. In the control group the time from changing mandatory to spontaneous mode after restoration of patient's respiratory activity took 36,5(12-76) min. In ASV group spontaneous breathing began immediately after recover of patient's respiratory drive, which meant less risk of patient-ventilator asynchrony.

ASV provided more protective ventilation: the driving pressure was significantly lower - ΔP on mechanical ventilation was 7,2± 1,6 cm H2O vs 9,3±2,1 cm H2O (p=0,000001) and on pressure support - 5,5±1 cm H2O vs 7,9±1,5 cm H2O (p=0,0000). There were no significant differences between the groups in Vt values: 7,0 (6-8,5) ml/kg (ASV) vs 7 (6-10) ml/kg (control) during mechanical ventilation and 7,5 (6,6-9,5) ml/kg in ASV vs 8 (7-10) ml/kg in control group during pressure support ventilation as well as in undesirable events and duration of ICU and hospital stay.

**CONCLUSION.** Application of ASV mode after uncomplicated cardiac surgery provides more protective mechanical ventilation and reduces the physician's workload without compromising the quality of respiratory support and safety of patients.

### 000192

**The effect of airway pressure release ventilation(APRV) compared with low tidal volume ventilation(LTV) on respiratory variables and outcomes in acute respiratory distress syndrome(ARDS) patients: A Systematic Review and Meta-Analysis**

J. Fan[1], J. Ming[2], G. Kaijie[2], X. Mingcheng[2], J. Chen[2]
[1]Henan University of Science and Technology, Xuchang Central Hospital, Xuchang, China; [2]Critical care medicine, Henan University of Science and Technology, Xuchang Central Hospital, Xuchang, China
**Correspondence:** J. Fan
*Intensive Care Medicine Experimental* 2019, **7(Suppl 3):**000192

**INTRODUCTION.** Some recent studies have shown that airway pressure release ventilation(APRV) might have some potential benefits in the patients with acute respiratory distress syndrome(ARDS). However, these results are inconsistent.

**OBJECTIVES.** The aim of our study is to conduct a meta-analysis to analyze the effect of APRV on respiratory variables and outcomes in ARDS patients.

**METHODS.** All eligible articles from MEDLINE, EMBASE, CENTRAL, and Chinese BioMedical Literature Database (January 2000–January 2019) were incorporated into this study. Studies were assessed independently by two reviewers. We extracted the data(Standard Mean Difference (SMD) for continuous variables and Risk Ratio (RR) for dichotomous variables) from the included studies. Heterogeneity was defined as P < 0.10 or I2 > 50%. Fixed-effect model was used to combine effective sizes if homogeneity was fine (P > 0.10,I2 < 50%),otherwise a random-effect model was used. Results are provided with a 95% confidence interval (CI) and associated p-value. STATA 13.0 software was used for the meta-analysis.

**RESULTS.** A total of 446 patients from eight studies were included in this meta-analysis. Compared with the LTV group, the patients in APRV group had a shorter length of hospital stay (SMD -0.37[-0.64, -0.10] p=0.006), shorter length of ICU stay (SMD -0.25[-0.45,-0.05] p=0.013) and higher success rate of extubation (RR =1.40[1.02, 1.96] p=0.048).After 72h ventilation, higher mean airway pressures (SMD 1.08[0.37,1.80] p=0.003) ,higher PaO2/FiO2(SMD 0.56[0.08,1.04] p=0.022), greater respiratory system compliance(SMD 0.68[0.11,1.25] p=0.019), lower PEEP(SMD -1.44[-1.75,-1.14] p<0.001) and lower total minute ventilation(SMD -0.74[-1.27,-0.21] p=0.007) were seen in APRV patients.

**CONCLUSION.** APRV may be a promising ventilation mode for ARDS patients, which can reduce the duration of both hospital and ICU stays, contribute to successful extubation and improve some respiratory indicators in comparison to LTV. However, more prospective and randomized trials are needed to further clarify these issues in the future.

**REFERENCE(S)**

1. Daoud EG, Farag HL, Chatburn RL. Airway pressure release ventilation: what do we know? Respiratory care 2012;57: 282-292
2. Jain SV, Kollisch-Singule M, Sadowitz B, Dombert L, Satalin J, Andrews P, Gatto LA, Nieman GF, Habashi NM. The 30-year evolution of airway pressure release ventilation (APRV). Intensive care medicine experimental 2016;4: 11

## ARF - Acute respiratory failure 2

### 001290

**Hospitalized Adult Patients with 2009 H1N1 Influenza in the city of Laredo (Texas,USA) from September 2018 to April 2019**

RJ. Deliz[1], R. Deliz-Aguirre[2], S. Zaidi[3]
[1]Pulmonary and Critical Care Medicine, Doctors Hospital of Laredo, Laredo, Texas, United States of America; [2]Pulmonary and critical care medicine, Rafael J Deliz, MD PA, Laredo, Texas, United States of America; [3]Internal medicine, Laredo Medical Center, Laredo, Texas, United States of America
**Correspondence:** R.J. Deliz
*Intensive Care Medicine Experimental* 2019, **7(Suppl 3):**001290

**INTRODUCTION.** During the period of September 2018 to April 2019, the number of patients admitted to the intensive care unit with influenza A H1N1 in the city of Laredo was alarming. We decided to investigate the characteristics of our population for future health care interventions.

**OBJECTIVES.** Describe the clinical characteristics of adult patients who were hospitalized with the influenza A 2009 H1N1 virus in the city of Laredo (Texas, USA) from September 2018 to April 2019

**METHODS.** Using medical charts, we collected data on 38 adult patients who were hospitalized for at least 24 hours for influenza bronchopulmonary infection and who tested positive for influenza A 2009 H1N1 virus with the use of a real-time reverse-transcriptase-polymerase-chain-reaction assay.

**RESULTS.** Of the 38 adults of patients we studied, 42% were admitted to the intensive care unit for acute respiratory failure. Ninety-five percent of the patients belonged to the Hispanic ethnic group. Twenty-six percent were on intubated on mechanical ventilation and 16% received non-invasive mechanical ventilation. Only 2 patients were identified to be vaccinated against the influenza virus. None of the patients in the ICU had the influenza virus vaccine. There were 6 cases of ARDS of whom two required ECMO. The average body mass index was 23 ± 2.7 Kg/m2 in ARDS population versus 33 ± 9.9 kg/m2 in the non-ARDS patients. There was a moderately statistical significant correlation between BMI and ARDS patients (n=38,r=-0.44, P=0.005). Twenty-one percent of patients were on vasopressors of whom all were on mechanical ventilation. The most common comorbidities were diabetes mellitus (26%), COPD (24%), obstructive sleep apnea (10%), liver cirrhosis (8%) and chronic renal insufficiency (8%). All the patients received oseltamivir on admission. The average age of patients was 58 years old with 20 males and 18 females. The average age of ARDS patients was 51 years old.

**CONCLUSION.** During the evaluation period, 2009 H1N1 influenza caused a significant number of admissions to the intensive care unit. The most remarkable findings were the moderately significant correlation between a low BMI in the ARDS group vs. the non-ARDS group. Notable, also, was the minimal amount of patients who received the vaccine before admission. The city of Laredo is predominantly of Hispanic ethnicity located at the border with Mexico. This is the first study, in our knowledge, that includes the intensive care unit epidemiological data of influenza A 2009 H1N1 in the city of Laredo, Texas, USA. The impact of higher vaccination rate by patient education and closer surveillance with lower BMI are strategies to be considered for future studies.

REFERENCE
1. Jain,S, Kamimoto,L, Bramley, A et al. Hospitalized patients with 2009 H1N1 Influenza in the United States, April-June 2009. N Engl J Med 2009:1935-44.

### 001296

#### Prevalence of diaphragmatic dysfunction and its impact in patients under non-invasive mechanical ventilation in intensive care unit

C. Rubio Madrigal[1], LE. Varela Sanchez[2], J. Franco Granillo[3], J. Aguirre Sánchez[4], AG. Camarena[5]
[1]Internal medicine, intensive care, The American British Cowdray Medical Center, Ciudad de México, Mexico; [2]Critical care, ABC Medical Center Campus Observatory, Ciudad de México, Mexico; [3]Intensive care unit, The American British Cowdray Medical Center, Mexico City, Mexico; [4]Intensive care unit, The American British Cowdray Medical Center, Ciudad de México, Mexico; [5]Intensive care unit, ABC Medical Center Campus Observatory, Ciudad de México, Mexico
**Correspondence:** C. Rubio Madrigal
*Intensive Care Medicine Experimental* 2019, **7(Suppl 3):**001296

**INTRODUCTION.** More than two thirds of patients who enter in Intensive care unit (ICU) require invasive or non-invasive mechanical ventilation (NIMV), diagnosis of ICU weakness and neuromuscular dysfunction are detected in 25-50% of the patients who require more than five days of mechanical ventilation. However there is lack of data of prevalence of diaphragmatic dysfunction (DD) in patients under non-invasive mechanical ventilation in ICU.

**OBJECTIVES.** The primary objective of this study was to stablish the prevalence of diaphragmatic dysfunction in non-invasive mechanical ventilation.

The secondary objective is to determine if the patients with diaphragmatic dysfunction had a larger duration of NIMV *vs* patients without diaphragmatic dysfunction.

**METHODS.** Cohort Study, Observational, Longitudinal, prospective.

73 patients with NIMV were recruited prospectively at the Department of critical care medicine ABC Medical Center. The thickness of the right diaphragm (RDT) was measured in the area of apposition of the diaphragm to the chest in both hemithorax, using an ultrasound transducer of 4 MHz and the right diaphragmatic fraction index (RDFI) and the change in diaphragm thickness(ΔTdi), was calculated.in patients with NIMV. The presence of DD was defined according to the presence of ΔTdi bilaterally less than 20% or less than 15 mm of diaphragm thickness.

**RESULTS.** The inclusion criteria were met for 73 patients. Overall prevalence of diaphragmatic dysfunction was 20% for a cutoff of 15 mm of thickness of the diaphragm or ΔTdi bilaterally less than 20%. the mean age was 70.4 SD ± 14, with APACHE II mean score of 20 SD±8.2. Charlston comorbidity index mean score of 4.3 SD ±1.6. The variables obtained from USG measurements were as follow. Right diaphragm thickness (RDT) 15.5 mm ± 5 mm, right diaphragmatic fraction index (RDFi) 23.1 ± 10.7% and change in diaphragm thickness(ΔTdi) was 27% ± 10. Media of Length of VMNI was 11.6 days in patients without diaphragmatic dysfunction and 16.6 days in patients in VMNI with diaphragmatic dysfunction. The diagnosis of DD was associated with increment in Length of VMNI with media 5 days OR 5.7 (IC 18.2-7.8).

**CONCLUSION.** A high prevalence of DD was identified in in patients with VMNI. The presence of DD was associated with an increment in days of VMNI.

REFERENCE(S)
1. Alessandro Marchioni1, Ivana Castaniere1, Roberto Tonelli1, Riccardo Fantini1, Matteo Fontana1, Luca Tabbì1, Andrea Viani2, Francesco Giaroni2, Valentina Ruggieri1, Stefania Cerri1 and Enrico Clini1*. Ultrasound-assessed diaphragmatic impairment is a predictor of outcomes in patients with acute exacerbation of chronic obstructive pulmonary disease undergoing noninvasive ventilation.Critical Care (2018) 22:109
2. Fan E, Cheek F, Chlan L et al (2014) An official American Thoracic Society Clinical Practice guideline: the diagnosis of intensive care unitacquired weakness in adults. Am J Respir Crit Care Med 190:1437–1446. doi:10.1164/rccm.201411-2011ST
3. Boussuges A, Gole Y, Blanc P. Diaphragmatic motion studied by m-mode ultrasonography: methods, reproducibility, nd normal values. Chest. 2009;135:391-400.
4. Ferrari G, De Filippi G, Elia F, Panero F, Volpicelli G, Aprà F. Diaphragm ultrasound as a new index of discontinuation from mechanical ventilation. Crit Ultrasound J. 2014;6(1):8.
5. Levine S, Nguyen T, Taylor N, Friscia ME, Budak MT, Rothenberg P, Zhu J, Sachdeva R, Sonnad S, Kaiser LR, Rubinstein NA, Powers SK, Shrager JB (2008) Rapid disuse atrophy of diaphragm fibers in mechanically ventilated humans. N Engl J Med 358:1327–1335.
6. Hussain SN, Mofarrahi M, Sigala I, Kim HC, Vassilakopoulos T, Maltais F, Bellenis I, Chaturvedi R, Gottfried SB, Metrakos P, Danialou G, Matecki S, Jaber S, Petrof BJ, Goldberg P (2010) Mechanical ventilation-induced diaphragm disuse in humans triggers autophagy. Am J Respir Crit Care Med 182:1377–1386
7. Sassoon CS, Caiozzo VJ, Manka A, Sieck GC (2002) Altered diaphragm contractile properties with controlled mechanical ventilation. J Appl Physi 92:2585–2595.
8. Anzueto A, Peters JI, Tobin MJ, de los Santos R, Seidenfeld JJ, Moore G, Cox WJ, Coalson JJ (1997) Effects of prolonged controlled mechanical ventilation on diaphragmatic function in healthy adult baboons. Crit Care Med 25:1187–1190.
9. Esteban A, Frutos-Vivar F, Muriel A, Ferguson ND, Peñuelas O, Abraira V, et al. Evolution of mortality over time in patients receiving mechanical ventilation. Am J Respir Crit Care Med. 2013;188(2):220–30.

### 001321

#### Alkaline Phosphatase Activity Strongly Correlates with Inflammatory Cytokines in the Pulmonary Compartment of Critically-Ill Patients

J. Juschten[1], PR. Tuinman,[1], LDJ. Bos,[2], NP. Juffermans,[3], A. Girbes, T. Van Der Poll[4], MJ. Schultz,[5]
[1]Intensive care, Vrije Universiteit Amsterdam, Amsterdam, Netherlands; [2]Pulmonology, Academic Medical Centre, Amsterdam, Netherlands; [3]Intensive care, Academic Medical Centre, Amsterdam, Netherlands; [4]Center for experimental and molecular medicine, Academic Medical Centre, Amsterdam, Netherlands; [5]Intensive care, Mahidol Oxford Tropical Medicine Research Unit, Bangkok, Thailand
**Correspondence:** J. Juschten
*Intensive Care Medicine Experimental* 2019, **7(Suppl 3):**001321

**INTRODUCTION.** In patients with chronic pulmonary disease, high levels of alkaline phosphatase (ALP) are associated with a neutrophilic inflammatory pattern within the pulmonary compartment. (1) Preclinical studies demonstrate markedly elevated ALP levels in animals with acute lung injury along with increased inflammatory markers. (2, 3) The role of ALP in critically-ill patients with acute pulmonary inflammation remains unknown.

**OBJECTIVES.** This study aimed to investigate if pulmonary ALP activity is associated with pulmonary inflammation in critically-ill patients. We hypothesized that patients with acute respiratory distress syndrome (ARDS) present with higher pulmonary ALP activity levels compared to non–ARDS patients.

**METHODS.** ALP activity levels were measured in bronchoalveolar fluid (BALF) and plasma samples of 125 critically–ill patients with ≥ 2 SIRS criteria from the prospective cohort trial 'Molecular Diagnosis and Risk Stratification for Sepsis' (MARS). Correlations between ALP activity and inflammatory cytokines were assessed by Spearman's correlation coefficient. Comparisons between groups were performed using Mann-Whitney U test according to data distribution.

**RESULTS.** There is a strong positive correlation between pulmonary ALP activity and pulmonary inflammatory cytokines. (r = 0.71 for IL–6 (Figure 1A); r = 0.80 for IL–8 (not shown)). In critically-ill patients, no difference in BALF ALP activity was detected between ARDS and non-ARDS patients (*p = 0.413*, Figure 1B), neither between patients with a direct or indirect cause of ARDS (*p = 0.36*, Figure 1C).

**CONCLUSION.** In this cohort of critically–ill patients, ALP activity strongly correlates with IL–6 and IL–8 levels within the pulmonary compartment. Pulmonary ALP activity is not different in patients with the clinical syndrome ARDS compared to patients without ARDS.

**REFERENCE(S)**

(1) J Toxicol Environ Health A. 1999;57(5):329-43.

(2) Am J Physiol Lung Cell Mol Physiol. 2018;314(1):L83-L92.

(3) Exp Lung Res. 1999;25(2):99-111.

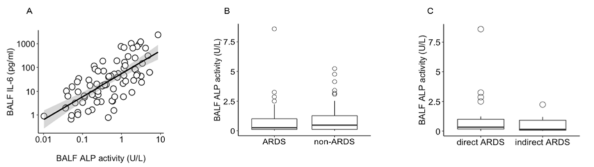

**Fig. 1 (abstract 001321). a** Correlation between IL-6 and ALP activity levels in BALF of all critically-ill patients. **b** ALP activity in ARDS compared to non-ARDS patients. **c** ALP activity in ARDS patients comparing direct vs. inrect causes of ARDS

### 001328

#### The expression and function of Piezo1 in ventilator associated lung injury in ARDS model

J. Guo

West China Hospital, Chengdu, China

*Intensive Care Medicine Experimental* 2019, **7(Suppl 3):**001328

**INTRODUCTION.** Acute respiratory distress syndrome (ARDS) is a common critical syndrome in critical care medicine, and lung protective mechanical ventilation has been recommended in ARDS patients. However, the mechanism is still not clear. Piezo1 protein is a recently discovered, which is a stretch-activated channel (SAC) and closely related to the mechanical signal transduction of eukaryotic cells1-3. But, there is still no evidence about its function in the ventilator associated lung injury in ARDS.

**OBJECTIVES.** To observe the expression of Piezo1 in the ventilator associated lung injury in ARDS in vivo., and study its role in LPS induced type II alveolar epithelial cells (AECII) injury, at last, we explained it`s protective mechanism on AECII injury.

**METHODS.** We carried animal experiments based on ARDS model treated with ventilator, and cell experiment under cyclic mechanical stretch stress(MSS) in vitro. Different tidal volume was given to ARDS animal induced by oil acid, and lung tissue,blood sample were collected to testify the diagnostic of ARDS, and then observe the expression of Piezo1 in ACEII cells. In vitro, we observed Piezo1 in ACEII injury induced by LPS, then we observed the cell apoptosis changes and studied the influence mechanism of Piezo1 on cells injury which induced by different cyclic MSS. At last, we combined with Piezo1-siRNA to study its role in ACEII injury.

**RESULTS.** From the artery blood gas and pulmonary tissue detection results, we had built successful ARDS animal model. After treated with ventilator, the lung injury can be improved, and compared with high tidal volume ventilation group (HV), low tidal volume (LV) group improved better. In cells apoptosis of lung tissue and AECII, we found the apoptosis rate was significantly increased in ARDS, and mechanical ventilation significantly reduced the apoptosis, most importantly, LV had lower apoptosis rate compared with HV ventilation. For Piezo 1, we had evidenced that it was enhanced in ARDS animal model and cyclic stretch stress in ACEII cells by western blot and immunofluorescence. The mechanical ventilation could decrease its expression, compared with HV, the gray value was less in LV group. There was same trend of Piezo1`s expression in cyclic stretch stress ACEII, and the less stretch was associated less expression of Piezo1. Further, we detected the lever of Calcium ion concentration and found intracellular Ca2+ was increased by flow cytometry, Immunofluorescence and Laser scanning confocal microscope from lung tissue and ACEII. The intracellular Ca2+ was influenced by the MSS, and it had lower lever in low cyclic MSS group compared with high cyclic MSS. At last, we combined with Piezo1-siRNA to study the piezo1`s role in cell injury, and found Piezo1-siRNA inhibited the Calcium influx, decreased intracellular Ca2+. In cell apoptosis, we found Piezo1-siRNA significantly decreased cell`s apoptosis in ACEII induced by cyclic MSS.

**CONCLUSION.** Piezo1 was high expression in ARDS animal and cyclic MSS cells, and its different expression was associated with the degree of MSS, the more, the higher. Piezo1-siRNA could decrease intracellular Ca2+ and cell apoptosis induced by cyclic MSS. Piezo1 maybe a new direction in ventilator associated lung injury in ARDS.

**REFERENCE(S)**

1. Coste B, Xiao B, Santos JS, et al. Piezo proteins are pore-forming subunits of mechanically activated channels. Nature. 2012;483(7388):176-181.
2. Tsuchiya M, Hara Y, Okuda M, et al. Cell surface flip-flop of phosphatidylserine is critical for PIEZO1-mediated myotube formation. Nat Commun. 2018;9(1):2049.
3. Lacroix JJ, Botello-Smith WM, Luo Y. Probing the gating mechanism of the mechanosensitive channel Piezo1 with the small molecule Yoda1. Nat Commun. 2018;9(1):2029.

### 001334

#### Is it possible to predict prognosis of Chronic Obstructive Pulmonary Disease exacerbation in Intensive Care Unit with a routine blood test?

A. Bueno-Gonzalez[1], M. Portilla-Botelho[1], M. Sanchez-Casado[2], M. Alvarez-Mon[3], J. Monserrat[4], J. Vejo[5], R. Ortiz-Diazdemiguel[6]

[1]Intensive care unit, Hospital General Universitario de Ciudad Real, Ciudad Real, Spain; [2]Intensive care unit, Hospital Universitario Virgen de la Salud, Toledo, Spain; [3]Immunology department, Hospital Príncipe de Asturias, Alcalá de Henares, Spain; [4]Immunology department, University of Medicine of Alcalá de Hemares, Alcala de Henares, Spain; [5]Intensive care unit, Hospital Universitario La Paz, Madrid, Spain, Spain; [6]Intensive care unit, Hospital General Universitario de Ciudad Real, Ciudad Real, Spain, Spain

**Correspondence:** M. Portilla-Botelho

*Intensive Care Medicine Experimental* 2019, **7(Suppl 3):**001334

**INTRODUCTION.** Chronic Obstructive Pulmonary Disease (COPD) is the third leading cause of death worldwide and it is responsible of important morbidity and healthcare costs. White blood cell populations are involved in the pathophisiology of COPD. Neutrophil to lymphocyte ratio (NLR), a simple, low cost index, has been recently proposed as prognostic marker of several inflammatory and cancer diseases.

**OBJECTIVES.** Neutrophil to lymphocyte ratio (NLR) and eosinophil to basophil ratio (EBR) could be considered as potential markers of systemic inflammation. We hypothesize that diagnosis and prognosis of severe patients admitted to Intensive Care Unit (ICU) due to exacerbated COPD, can be predicted with a routine blood test that include these variables.

**METHODS.** We made a prospective observational cohort study. We analyse consecutive patients who need hospitalization in ICU due to COPD exacerbation that require mechanical ventilation. We exclude patients with other inmunologic disease or antiinflammatory treatment. We analyse NLR and EBR at the moment of ICU admission, and we follow-up them in stable phase in external consultation, during 24 month period. We compare inflammatory markers in both phases and their relation with prognostic factors. Statistical analysis: STATA version 13

**RESULTS.** We include 55 patients. In the comparative study of inflammatory markers in exacerbated patients vs stable COPD patients, we found significant differences between leucocytes (12,3 x vs 5,6 p<0.001); neutrophils (10,5 vs 5,3; p<0.001); lymphocytes (1,02 vs 1,64; p:0.018); and eosinophils (0.039 VS 0.119; p<0.001). The NLR was significantly higher in exacerbated patients (19.310 vs 4.697; p<0.001). EBR was significantly higher in stable patients (1.305 vs 3.322; p:0.009).

The results of our study showed correlation between NLR, EBR and hospital mortality. EBR was significantly lower in exitus than survivors (1.816 vs 1.290; p:0.017); while NLR was significantly higher in exitus than survivors (28,567 vs 18,043; p<0.001) (cut-off point of 5.23 with AUC: 0.90, S: 86,27% E: 84,62%).

**CONCLUSION.** NLR and EBR could be considered as new inflammatory markers for diagnosis of severe exacerbation of COPD and for the prognosis. The results of this study show that higher NLR an lower EBR correlated with poor prognosis.

**REFERENCE(S)**

1. Chen N, Liu S, Huang L, et al. Prognostic significance of neutrophil-to-lymphocyte ratio in patients with malignant pleural mesothelioma: a meta-analysis. Oncotarget. 2017;8:57460-57469
2. Yang Z, Zhang Z, Lin F, et al. Comparisons of neutrophil-monocyte, eosinophil, and basophil-lymphocyte ratios among various systemic autoimmune rheumatic diseases. APMI. 2017;125:863-871
3. Fundacion Sociosanitaria de Castilla-La Mancha. AN-2010/19

### 001346

#### D-RSBI as a Weaning Predictor: Improves the predictive capacity of Rapid Shallow Breathing Index?

AMJ. Delgado, JF. Martínez Carmona, MFA. Hijano, LE. López, FMÁ. Barbancho
Intensive care unit, Hospital Carlos Haya, Málaga, Spain
**Correspondence:** J.F. Martínez Carmona

*Intensive Care Medicine Experimental* 2019, **7(Suppl 3):**001346

**INTRODUCTION.** The decision-making to move towards weaning must be based on objective criteria, not only on the criterion of the responsible physician. In the literature, a large number of predictors have been described to facilitate this decision-making with the lowest possible risk, thus minimizing the complications associated with weaning failure. One of the best known is the Rapid Shallow Breathing Index (RSBI), but it has a limited prognostic capacity. Recently a modification has been proposed (D-RSBI) that takes into account the diaphragm, fundamental in the generation of tidal volume.
**OBJECTIVES.** The aim of the study is to assess the predictive capacity of D-RSBI and compare it with its predecessor in patients subjected to MV> 48 hours
**METHODS.** Prospective study including 17 patients submitted to MV due to different causes (TBI, CCV, urgent surgery, respiratory failure). Once criteria for advancing weaning are met, a SBT is performed in PSV 5 - 8 cmsH2O over 5 cmsH2O of PEEP, and the following determinations are made: RSBI, FR, Vt, Diaphragmatic excursion, D-RSBI. Epidemiological variables are collected, reason for admission, VM time, weaning failure, mortality.
**RESULTS.** The average age was 56.12 years +/- 13.95. 70.6% were male. Reason for admission: TBI (29.4%), CCV (29.4%), Urgent surgery (23.5%). The median duration of MV was 11 days. 64.7% presented weaning failure. We observed a significant relationship between D-RSBI and weaning failure (Test Chi2 p 0.016). ANOVA was performed, showing a good correlation between variables (F 8.6 p 0.01). We performed a COR curve presenting AUC 0.86 (p 0.016, 95% CI 0.688-1), finding a cut-off in 1.17 (S 72%, E 83%). RSBI presents AUC 0.79 (p 0.05, 95% CI 0.57-1). However, in the subgroup analysis (Group 1: MV <10 days, Group 2: MV> 10 days), in group 2 we did not observe a significant relationship in the D-RSBI and weaning failure
**CONCLUSION.** - In our sample, the D-RSBI improves the predictive capacity of the RSBI in patients with short-term respiratory support
- In patients with prolonged mechanical ventilation, the predictive capacity of D-RSBI is limited, similar to the RSBI.

**REFERENCE(S)**

1. Spadaro et al. Can diaphragmatic ultrasonography performed during the T-tube trial predict weaning failure? The role of diaphragmatic rapid shallow breathing index. Crit Care (2016); 20:305.
2. Yang KL, Tobin MJ. A prospective study of indexes predicting the outcome of trials of weaning from mechanical ventilation. N. Engl J Med. 1991; 324: 1445 - 50. - Ferrari et al. Diaphragm ultrasound as a new index of discontinuation from mechanical ventilation. Crit Ultrasound J. 2014; 6:8.
3. Pirompanich P, Romsaiyut S. Use of diaphragm thickening fraction combined with rapid shallow breating index for predicting success of weaning from mechanical ventilator in medical patients. J. Intensive Care (2018) 6:6.

### 001352

#### Ventilatory support with non-invasive mechanical ventilation in patients with severe asthmatic crisis

B. Llorente Ruiz, A. Acha, B. Gracia, M. Daguerre, E. Nevado
Intensive care unit, Hospital Príncipe de Asturias, Alcalá de Henares, Spain
**Correspondence:** A. Acha

*Intensive Care Medicine Experimental* 2019, **7(Suppl 3):**001352

**INTRODUCTION.** Approximately 10% of the asthma-related hospitalizations do include an ICU stay. Around 2–4% require mechanical ventilation. The use of non-invasive mechanical ventilation (NIMV) for acute respiratory failure has increased and the indications for its use have expanded over the past decade. Although it seems to have many advantages and its use has been extended in asthma exacerbation, its benefits are not well established and no recommendations had been made in the guidelines.
**OBJECTIVES.** To describe the use of NIMV in adults presenting severe asthma exacerbation with respiratory insufficiency admitted to our ICU.
**METHODS.** Prospective observational study conducted in a 14-bed intensive care unit of a second level hospital during 18 months. Patients were all adults with a severe asthmatic crisis who failed with first step standard treatment in spite of which they had symptoms of severe respiratory failure with increased in respiratory work with global use of accessory muscles, intercostal retractions and nasal flaring. Demographic data, clinical variables at admission and intensive care unit and hospital length of stay were recorded.
Qualitative variables are described as number and percentages and quantitative variables with normal distribution as mean ± S.D.
**RESULTS.** The total sample comprises 17 patients, having ruled out the patients that required invasive mechanical ventilation upon admission due to encephalopathy or cardiac arrest. 73.7% were women, with an average age of 48.13 ± 14,04 years and a mean APACHE II score of 11.26 ± 6.2. On admission, the mean pH was 7.22 ± 0.139 with mean pCO2 in arterial blood of 60.5 ± 21.05 mmHg. A therapeutic trial with NIMV was carried out in all of them. In 66.7% the ventilatory mode used was BiPAP, with the use of CPAP being a 13.3%. All patients were monitored both clinically and analytically: average heart rate (115.87 ± 19.31 bpm), average respiratory rate (29.8 ± 6 bpm) and mean FiO2 required (0.5 ± 0.18). Control arterial blood gas was taken two hours after the start of respiratory support. The average pH was 7.35 ± 0.614, with a pCO2 of 40.19 ± 8.29 mmHg. The mean stay in the ICU was 4.93± 4.65 days and in hospital was 15.61 ± 10.33 days. In our group only two patients (11,76%) had NIMV failure.
**CONCLUSION.** In our experience, the use of respiratory support with NIMV in patients with severe asthma crisis, performed in a safe environment in which there is no delay in recognizing the failure of the same, may limit both ICU and hospital stay and the rate of orotracheal intubation with the complications derived from it.

**REFERENCE(S)**

1. Official ERS/ATS clinical practice guidelines: noninvasive ventilation for acute respiratory failure Bram Rochwerg et al. Eur Respir J 2017; 50: 160242
2. Non-invasive ventilation use in status asthmaticus: 16 years of experience in a tertiary intensive care Kirsten RL BOND et alEmergency Medicine Australasia (2017)doi: 10.1111/1742-6723.12876

### 001353

#### External expiratory resistances act as expiratory brake and reduce eccentric diaphragmatic contraction during spontaneous breathing ventilation

M. Pellegrini[1], M. Gudmundsson,[2], R. Benzce,[1], M. Segelsjö[3], F. Freden,[1], C. Rylander,[2], G. Hedenstierna,[4], SA. Larsson,[1], G. Perchiazzi,[1]
[1]Hedenstierna laboratory, department of surgical sciences and, Department of Anesthesia and Intensive Care Medicine of Uppsala University Hospital, Uppsala, Sweden; [2]Department of anaesthesiology and intensive care medicine, Institute of Clinical Sciences, Sahlgrenska Academy, University of Gothenburg, Gothenburg, Sweden; [3]Department of radiology, Uppsala University Hospital, Uppsala, Sweden; [4]Department of medical sciences, uppsala university, Hedenstierna Laboratory, Uppsala, Sweden
**Correspondence:** M. Pellegrini

*Intensive Care Medicine Experimental* 2019, **7(Suppl 3):**001353

**INTRODUCTION.** During spontaneous breathing (SB) a post-inspiratory expiratory contraction of the diaphragm, also defined as eccentric diaphragmatic contraction (EDC), has been described [1, 2]. EDC is minimizing the expiratory flow, preserving the end-expiratory lung volume and reducing atelectasis formation. However, EDC has also been described as cause a of ventilator-induced diaphragm dysfunction (VIDD).

**OBJECTIVES.** To describe the effects of an additional expiratory resistance (ExpR) during conditions that promote EDC. We hypothesized that ExpR could reduce EDC, while preserving the positive effects of expiratory brake. If it holds true, we expect throughout the expiration a reduction in: 1. expiratory electrical activity of the diaphragm (EAdi exp), 2. expiratory transdiaphragmatic activity (Pdi exp), 3. expiratory flow (Flow exp).

**METHODS.** Nine anesthetized, tracheostomized, SB pigs underwent lung lavages to achieve mild ARDS (PaO2/FIO2 of 250 mmHg). The animals breathed at three continuous airway pressures (CPAP 12, 5 and 0 cmH2O). For each CPAP, three different conditions were studied, depending on the application of an additional respiratory resistance to the expiratory ventilatory limb: 1) no resistance added (R0), 2) ExpR of 4.8*10-2 cmH2O/mL/sec measured at a flow of 1 L/s (R1), 3) ExpR of 9.6*10-2 cmH2O/mL/sec measured at a flow of 1 L/s (R2). For each breath EAdi exp, Pdi exp and Flow exp were divided in four quartiles. EAdi exp, Pdi exp quartiles were expressed as percent of the end-inspiratory peak. Flow exp quartiles were expressed as absolute values [L/s]. Statistical analysis: Student-T test.

**RESULTS.** During SB, the application of an external expiratory resistance decreased the EDC: both EAdi exp and Pdi exp were significantly reduced by the application of an ExpR. The higher the expR, the lower the EDC (Figure, first and second rows) at all the applied CPAP levels. The use of an ExpR reduced the expiratory flow, at all the applied CPAP levels (Figure, third row). The increase in CPAP reduced the EDC.

**CONCLUSION.** Expiratory modulation by application of an external ExpR preserves the positive effects of the expiratory brake while minimizing diaphragmatic eccentric contraction, thus potentially reducing the VIDD and the consequent lung injury in SB subjects.

**REFERENCE(S)**

1. Pellegrini M et al (2017). Am J Respir Crit Care Med 195:1608–16.
2. Schepens T et al. (2019). Curr Opin Crit Care 25:77–85.
3. European Society of Intensive Care Medicine (NEXT Grant Award 2017).
4. The Swedish Heart Lung Foundation, the Swedish Research Council (K2015-99X-22731-01-4).

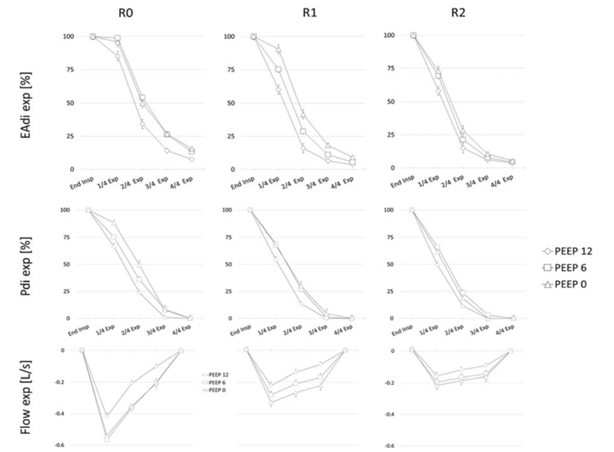

**Fig. 1 (abstract 001353).** See text for description

### 001357

#### No "single" EIT parameter can determine optimal ventilator settings

SJH. Heines, U. Strauch, MC. Van De Poll, PM. Roekaerts, D. Bergmans
Icu, Academic Hospital Maastricht, Maastricht, Netherlands
**Correspondence:** S.J.H. Heines
*Intensive Care Medicine Experimental* 2019, **7(Suppl 3):**001357

**INTRODUCTION.** Electrical impedance tomography (EIT) enables calculation of alveolar collapse (CL) and overdistension (OD) during a PEEP trial[1]. The best balance between OD and CL (ODCL), where the percentage of CL is subtracted from OD is one of the parameters developed to optimize ventilator settings.

**OBJECTIVES.** The aim of this study is to describe the effect of PEEP on ODCL, OD and CL in hypoxic respiratory failure (HRF), and patients with healthy lungs as a reference group (control).

**METHODS.** Twenty control and 43 HRF patients (PaO2/FiO2-ratio <250) were analysed from retrospective data and our prospective EIT-trial data. The ODCL, OD and CL was calculated at each PEEP step by EIT during an incremental and decremental PEEP trial. The analysis is performed in 4 consecutive incremental followed by 4 decremental PEEP steps of 2 cmH2O. An ODCL of 0 would indicate an optimal balance between OD and CL. Characteristics between HRF and control patients were compared using independent *t*-test. Changes in ODCL, OD and CL between HRF and control patients were tested using two-way ANOVA.

**RESULTS.** There was a significant difference between HRF and control patients in APACHE II, PaO2/FiO2-ratio and set PEEP (12 cmH2O (±3) and 8 cmH2O (±0,5) respectively), $p<0.05$. Changes in both OD and CL were directly proportional with changes in PEEP (Fig. 1). The increase in OD is more pronounced by increasing PEEP in HRF then in controls ($p<0.05$). A low ODCL did not exclude OD or CL in HRF nor control patients, mean ODCL 2,5 (±2) with an OD of 7 (±4), CL 7(±3) and ODCL 2,6 (±3) with an OD 8 (±5), CL 7 (±4) respectively.

**CONCLUSION.** Using solely ODCL to titrate PEEP does not exclude the presence of OD and CL. Ventilation distribution varies widely among patients, especially in HRF. No single optimal PEEP value exist for the entire lung. This reinforces the need for personalised titration of PEEP and tidal volume because the latter also contributes to tidal recruitment and OD. Using EIT regional lung mechanics can reliably be assessed at different PEEP levels and tidal volumes. Therefore, EIT can be very helpful for patient tailored lung protective ventilation.

**REFERENCE(S)**

1. E Costa, et al. Intensive Care Med 35(6):1132-7, 2009
2. Not Applicable

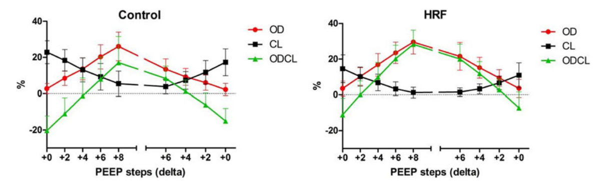

**Fig. 1 (abstract 001357).** Changes in percentages of OD (red), CL (black) and ODCL (green) in patients with HRF and healthy control patients during an incremental and decremental PEEP trial

### 001369

#### External expiratory resistances optimize lung inflation in collapse-prone lungs

M. Pellegrini[1], M. Gudmundsson,[2], R. Benzce,[3], M. Segelsjö[4], F. Freden,[3], C. Rylander[2], G. Hedenstierna,[5], SA. Larsson,[3], G. Perchiazzi,[3]
[1]Department of anesthesia and intensive care medicine of uppsala university hospital, Hedenstierna Laboratory, Department of Surgical Sciences, Uppsala, Sweden; [2]Department of anaesthesiology and intensive care medicine, Institute of Clinical Sciences, Sahlgrenska Academy, University of Gothenburg, Gothenburg, Sweden; [3]Hedenstierna laboratory, department of surgical sciences and, Department of Anesthesia and Intensive Care Medicine of Uppsala University Hospital, Uppsala, Sweden; [4]Department of radiology, Uppsala University Hospital, Uppsala, Sweden; [5]Department of medical sciences, uppsala university, Hedenstierna Laboratory, Uppsala, Sweden
**Correspondence:** M. Pellegrini
*Intensive Care Medicine Experimental* 2019, **7(Suppl 3):**001369

**INTRODUCTION.** Diaphragmatic expiratory brake has been shown to preserve end-expiratory lung volume and avoid/reduce atelectasis formation [1]. However, the eccentric diaphragmatic contraction (EDC), that naturally brakes expiration, is a possible cause of ventilator-induced diaphragm dysfunction (VIDD) [2] during spontaneous breathing (SB).

**OBJECTIVES.** To investigate if an external resistance (ExpR) inserted into the expiratory circuit can mimic the natural EDC and improve lung inflation.

**METHODS.** Nine anesthetized, tracheostomized SB pigs underwent lung lavages to achieve mild ARDS (PaO2/FIO2 of 250 mmHg). The animals were studied with high frequency (20 Hz) dynamic Computed Tomography (CT) at three Continuous Positive Airway Pressures (CPAP 12, 6 and 0 cmH2O) and three ExpR: 1) no resistance added (R0), 2) ExpR of 4.8*10-2 cmH2O/mL/sec measured at a flow of 1 L/s (R1), 3) ExpR of 9.6*10-2 cmH2O/mL/sec measured at a flow of 1 L/s (R2). Image analysis was performed on CT images acquired at end-inspiration and at four quartiles of the expiratory phase (1/4 Exp, 2/4 Exp, 3/4 Exp, 4/4 Exp). Inflation was defined based on voxels radiodensity, according to Gattinoni et al.[3]. Four lung compartments were so defined (atelectatic, poorly-, normally- and hyper- inflated) and reported as percentage of the total lung parenchyma in the same slice. Intrinsic-Positive End Expiratory Pressure (PEEPi) was calculated. Statistical analysis: Student-T test.

**RESULTS.** The application of an additional ExpR (R1 or R2 vs R0) (Figure) significantly reduced the amount of atelectasis at CPAP 0 and 6 cmH2O; and significantly increased normally inflated areas at CPAP 0 and 6 cmH2O. PEEPi (mean ± SE) varied between a minimum of 0.12±0.06 cmH2O at CPAP 12 and R0 and a maximum of 1.32±0.58 cmH2O at CPAP 0 and R2.

**CONCLUSION.** The expiratory brake produced by ExpR improved lung inflation in conditions promoting lung collapse (low CPAP) and was not associated with hyperinflation. If confirmed in human patients, external expiratory brakes might gain clinical relevance in ventilated lungs prone to collapse (e.g. laparoscopic procedures, cardiothoracic surgery, pediatric ventilation, prolonged weaning and critical illness).

**REFERENCE(S)**

1. Pellegrini M et al (2017). Am J Respir Crit Care Med 195:1608–16.
2. Schepens T et al. (2019). Curr Opin Crit Care 25:77–85.
3. Gattinoni L, et al. (1998) Anesthesiology 69:824–832.
4. European Society of Intensive Care Medicine (NEXT Grant Award 2017).
5. The Swedish Heart Lung Foundation, the Swedish Research Council (K2015-99X-22731-01-4)

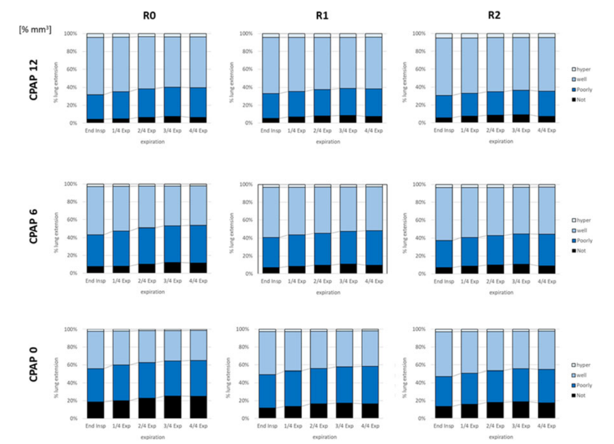

**Fig. 1 (abstract 001379).** See text for description

### 001374

#### Predictors of mortality in ICU-admitted patients with an acute exacerbation of COPD (AECOPD)

H. Kallel[1], I. Ben Saida[2], W. Zarrougui[2], MA. Boujelbèn[1], R. Chelbi[1], W. Ammar[1], S. Rouis[1], K. Meddeb[2], M. Boussarsar[2]

[1]Medical intensive care unit, Farhat hached university hospital, Sousse, Tunisia; [2]Medical intensive care unit, farhat hached university hospital, Université de Sousse, Faculté de Médecine de Sousse, LR N° LR12SP09.Heart Failure, Sousse, Tunisia

*Intensive Care Medicine Experimental* 2019, **7(Suppl 3):**001374

**INTRODUCTION.** COPD is the third leading cause of mortality around the world according to the World Health Organization. It is interesting to identify COPD patients at the time of ICU admission who are likely to have poor short-term outcomes.

**OBJECTIVES.** To identify the characteristics and predictors of mortality of ICU patients admitted for AECOPD.

**METHODS.** A prospective cohort study was performed in a 9-bed Tunisian medical ICU between December 2017 and April 2019. All patients admitted for AECOPD during the study period were included. For all included patients, demographic, clinical characteristics, therapeutic interventions and outcomes were recorded. Univariate and multivariate regression analyses were carried out to identify factors independently associated to poor prognosis.

**RESULTS.** During the study period, 372 patients were hospitalized in the ICU. 99(26%) COPD patients were included. Patients' characteristics were: mean age, 64.0±9.2yrs ; male, 71(91%) ; mMRC≥2, 67(88.2%) ; median SAPSII, 27[22-32] ; invasive mechanical ventilation (IMV) on admission, 10(10.1%). The most common comorbidity was hypertension 31(31.3%), followed by diabetes mellitus 19(19.2%), cardiovascular diseases 19(19.2%) and psychiatric disorders 7(7.1%). The triggering factor of AECOPD was tracheobronchitis 73(73,7%) followed by pneumonia 7(7.1%) and pulmonary edema 3(3%).

During their ICU stay, 46(46.46%) required intubation, 25(25.3%) were put under vasoactive drugs and 12(12.1%) had hospital-acquired infections. The median duration of mechanical ventilation and the length of stay were respectively 11[5.7-15.5]days and 9[6-14.5]days. Mortality rate was 23.3%.

Univariate analysis identified four factors associated with ICU mortality : SAPS II on admission (34[29.5-44] vs 25[20.25-30] ; p=0.001) ; IMV use on admission ( 21.7% vs 6.6% ; p=0.035) ; vasopressors use ( 60.9% vs 14.5% ; p<0.001) and NIV failure (78.9% vs 27.8% ; p<0.001). In multivariate analysis, NIV failure was an independent risk factor associated with mortality in critically ill COPD patients (HR, 9.75 ; 95%CI, [2.9-32.9] ; p<0.001).

**CONCLUSION.** NIV failure was the sole independent risk factor associated with ICU mortality in patients admitted with AECOPD.

### 001382

#### Delta of mechanical power in patients with severe ARDS and prone position

AH. Morales-Morales, JC. Gasca-Aldama, SE. Zamora Gómez, LA. Gorordo-Delsol, KJ. Castillo-Medrano, NI. Medveczky-Ordoñez, ML. Pacheco-Rivera, S. Sosa-Santos, LE. Gaytán-Medina, I. Maldonado-Beltrán, A. Rodríguez-Peredo, D. Sanabria-Cordero, JA. Zepeda-Pérez, GD. Hernández-López

Adult intensive care unit, Hospital Juárez de México, Ciudad de México, Mexico

**Correspondence:** S.E. Zamora Gómez

*Intensive Care Medicine Experimental* 2019, **7(Suppl 3):**001382

**INTRODUCTION.** 10 % of all patients admitted to the Intensive Care Unit and 23 % of mechanically ventilated patients has ARDS (1). The mortality rate is of 46.1 % for patients with severe ARDS (2). The mainstay of treatment is lung protective ventilation with low tidal volumes and optimal PEEP, prone positioning is a validated strategy for patients with moderate and severe ARDS (3). The driving pressure, more than tidal volume, is the best predictor of VALI, as inferred by increased mortality, DP is one of the components of the mechanical power, which also includes respiratory rate, flow and PEEP. Mechanical Power is the energy delivered per unit of time to the respiratory system by the mechanical ventilator. Finding the threshold for mechanical power could simplify assessment and prevention of VALI (4).

**OBJECTIVES.** To analyze the change in Mechanical Power (ΔMP) after prone position and outcome in patients with severe ARDS

**METHODS.** : An analytical, retrospective and cross-sectional study in patients with severe ARDS and prone position strategy in the Intensive Care Unit of Hospital Juarez de México (January 01, 2018 to March 31, 2019).

**RESULTS.** 71 patients with ARDS were admitted, 46 (64.7 %) had severe ARDS and received prone position. Global Mortality was 42.2%, Mortality in severe ARDS group was of 65.2%. Average MP before prone position in survivors was 19.5 J/min in contrast with 24.16 J/min in nonsurvivors (OR 9.6, CI 95 %, 0.97 – 95.67, P: 0.052). Average MP after prone position in survivors was 18.5 J/min in contrast with 28.59 J/min in nonsurvivors (OR 9.6, CI 95 %, 0.97 – 95.67, P: 0.052). ΔMP was -0.504 in survivors, but it was +4.103 in nonsurviors (OR: 0.064, (IC 95 %, 0.109 – 3.72, P: 0.61).

| | Survivors (n) | Non-Survivors (n) | OR | Confidence interval (95%) | P value |
|---|---|---|---|---|---|
| MP before prone (J/min) | 19.50 (16) | 24.16 (30) | 9.66 | 0.97 - 95.67 | 0.0524 |
| MP after prone(J/min) | 18.50 (16) | 28.59 (30) | 9.66 | 0.97 - 95.67 | 0.0524 |
| Δ MP (J/min) | -0.504 (16) | +4.103 (30) | 0.64 | 0.10 - 3.72 | 0.619 |

**CONCLUSION.** In patients with severe ARDS MP was higher in non-survivors before and after prone position in comparison with survivors. ΔMP showed a trend to reduce in survivors and to increase in nonsurvivors after prone position.

**REFERENCE(S)**

1. Thompson B. T., Chambers R. C.and Liu K. D., M. Acute Respiratory Distress Syndrome. New England Journal of Medicine. 2017;377:562-72.
2. Matthay M. A., Zemans R. L., Zimmerman G. A. (et al). Acute respiratory distress syndrome. Nature Reviews. (2019) 5:18
3. Koulouras V., Papathanakos G., Papathanasiou A. (et al). Efficacy of prone position in acute respiratory distress syndrome patients: A pathophysiology-based review. World J Crit Care Med 2016 May 4; 5(2): 121-136
4. Tonetti T., Vasques F., Rapetti F. (et al). Driving pressure and mechanical power: new targets for VILI prevention. Ann Transl Med 2017;5(14):286
5. No financial support or grant were received for this manuscript

### 001385

#### Acute respiratory failure in very old intensive care patients (VIP)

GL. Schwarz, H. Flaatten[1], D. De Lange[2], B. Guidet[3], C. Jung[4], RP. Moreno[5], A. Artigas[6]

[1]Department of anaesthesia and intensive care, Haukeland University Hospital, Bergen, Norway; [2]Department of intensive care medicine, Utrecht University, Utrecht, Netherlands; [3]Réanimation Médicale, Hôpital Saint-Antoine, Paris, France; [4]Department of cardiology, pulmonology and angiology, Heinrich-Heine-University, Düsseldorf, Germany; [5]Unidade de cuidados intensivos neurocríticos, Hospital de São José, Centro Hospitalar de Lisboa Central, Nova Médical School, Lisbon, Portugal; [6]Critical care center, Universitat Autònoma de Barcelona - UAB, Sabadell, Spain

**Correspondence:** G.L. Schwarz

*Intensive Care Medicine Experimental* 2019, **7(Suppl 3):**001385

**INTRODUCTION.** Severe acute respiratory failure (ARF) is a main cause of ICU admission, also for elderly patients (1), frequently warranting respiratory support. The proportion of elderly patients receiving mechanical ventilation is reported to be comparable to younger ICU patients (2). Despite representing a rapidly growing ICU population, outcome data are scarce for ARF with or without respiratory support in elderly ICU patients.

**OBJECTIVES.** The objective is to describe the modality of respiratory support and short-term outcomes in an unselected group of very old intensive care patients (VIP) ≥ 80 years admitted with ARF.

**METHODS.** This is a predefined sub-study of the VIP-1 study; a prospective observational study enrolling 5132 VIP admitted to 311 ICUs in 21 European countries (3). The patients for the present study are selected from the VIP-1 study population by ARF reported as the main admission diagnosis. Patients presenting with both ARF and acute circulatory failure upon ICU admission are excluded.

**RESULTS.** A total of 986 patients are included. 864 patients (87.6%) were given respiratory support. Invasive mechanical ventilation (IMV) compared to non-invasive ventilation (NIV) was associated with a significantly longer ICU stay and a trend towards increased mortality, but patients receiving IMV also had a significantly higher degree of organ failure.

**CONCLUSION.** Acute respiratory failure in very old ICU patients carries a high mortality, ranging from 25% in patients without any form for respiratory support to almost 50% in patients with IMV after a trial of NIV. Patients having received NIV only were less sick and had a shorter ICU stay; however no significant survival benefit of NIV compared to IMV could be shown.

**REFERENCE(S)**

1. Sjoding, M. W., Prescott, H. C., Wunsch, H., Iwashyna, T. J., & Cooke, C. R. (2016). Longitudinal Changes in ICU Admissions Among Elderly Patients in the United States. CCM, 44; 1353–1360.
2. Andersen , Kvåle R. (2012). Do elderly intensive care unit patients receive less intensive care treatment and have higher mortality? Acta Anaesthesiol Scand 56; 1298–1305.
3. Flaatten H, deLange D, Morandi A et al (2017). The impact of frailty on ICU and 30-day mortality and the level of care in very elderly patients (≥ 80 years). ICM; 43: 1820–1828.
4. This study was endorsed by ESICM and received the ESICM research grant 2017.

**Table 1 (abstract 001385).** Baseline and outcome data grouped by the level of respiratory support

| | No Ventilation | NIV only | IMV only | NIV + IMV |
|---|---|---|---|---|
| N= | 122 | 383 | 315 | 166 |
| Age (mean) | 84 | 84 | 83 | 83 |
| SOFA (median+IQR) | 5 (5-7) | 5 (5-6) | 8 (8-9) | 8 (8-9) |
| LOS ICU (days; median+IQR) | 2.0 (1.8-2.7) | 2.8 (2.3-3.0) | 6.1 (4.9-7.0) | 7.6 (6.3-9.6) |
| Vasoactive drugs (%+95%CI) | 15.0% (10.0-22.8) | 17.8% (14.2-21.0) | 64.8% (59.4-69.9) | 77.1% (70.3-83.0) |
| RRT (%+95%CI) | 4.1% (1.6-8.7) | 3.9% (2.3-6.2) | 7.3% (4.8-10.6) | 15.1% (10.2-21.1) |
| ICU-mortality (%+95%CI) | 13.9% (8.7-20.9) | 16.4% (13.0-20.4) | 29.2% (24.4-34.4) | 32.5% (25.8-39.9) |
| 30d-mortality (%+95%CI) | 25.4% (18.3-33.6) | 31.3% (26.6-36.1) | 46.3% (40.9-51.9) | 48.8% (41.3-56.4) |

### 001390

#### Use of the Integrated Pulmonary Index as a predictor of success or failure in the progress of invasive mechanical ventilation (IMV)

JE. Monter Vigueras[1], OE. Palacios Calderon, J. Franco Granillo[1], E. Monares Zepeda[2], CA. Rojas Gomez[3]

[1]Intensive care unit, The American British Cowdray Medical Center, Mexico City, Mexico; [2]Department of critical care medicine, Hospital San Angel Inn Universidad, Mexico City, Mexico; [3]Intensive care unit, American British Cowdray Medical Center Mexico, Mexico City, Mexico

**Correspondence:** J.E. Monter Vigueras

*Intensive Care Medicine Experimental* 2019, **7(Suppl 3):**001390

**INTRODUCTION.** The Integrated Pulmonary Index (IPI) use an algorithm based on the measurement of exhaled carbon dioxide, respiratory rate, heart rate and oxygen saturation to provide an assessment of the patient's ventilatory status. IPI has been maintained as part of respiratory monitoring but not as part of a mechanical ventilation removal protocol.
**OBJECTIVES.** Determine whether the lung integration rate predicts success or failure in the removal of mechanical ventilation.
**METHODS.** In a cohort study, prospective and observational, patients admitted to UTI with support from VMI were analyzed, using the IntelliVue MX800 monitor software to obtain the IPI algorithm. Demographic, hemodynamic and respiratory variables were recorded to perform a multivariable statistical analysis, using regression models to compare patterns in 2 repeated IPI measurements. The extubation protocol kept the patient in support pressure mode (PS) with PS 7 and PEEP 0.
**RESULTS.** IPI was collected in 90 patients, 79 successfully extubated (88%) and 11 with failure (12%), IPI was taken at the time of weaning from mechanical ventilation and 5 minutes before extubating the patient. The value of IPI at the moment of the extubation had an specificity of 97% and sensitivity of 57%, it was observed that an increase of +2 in the basal number of IPI predicts success to the extubation, but a decrease of -2 may or may not determine failure at extubation, with a p of 0.001.
**CONCLUSION.** In this study a variation of the basal IPI at the beginning of the mechanical ventilation removal protocol was observed and compared with the IPI at the time of extubation, with a delta of +2 being found, when the patient had an increase in IPI was observed success at extubation, instead when the variation was in decline (-2), extubation could fail or not.

### 001392

#### Changes of diaphragm function before and after withdrawal of high flow nasal cannula in patients with acute respiratory failure

T. Takashima[1], N. Nobuto[2], U. Yoshitoyo[1], T. Natsuki[1], T. Yumiko[1], I. Taiga[1], O. Jun[2]
[1]Emergency and critical care medicine, Tokushima University Hospital, Tokushima, Japan; [2]Emergency and disaster medicine, Tokushima University, Tokushima, Japan
**Correspondence:** T. Takashima
*Intensive Care Medicine Experimental* 2019, **7(Suppl 3):**001392

**INTRODUCTION.** High flow nasal cannula (HFNC) is increasingly used in managements of acute respiratory failure (ARF). HFNC can contribute to improve diaphragm function due to washout of carbon dioxide in anatomical dead space, increasing lung volume, and decreasing work of breathing. However, there are few clinical data to assess the changes of diaphragm function during weaning from HFNC.
**OBJECTIVES.** To evaluate the changes of diaphragm function before and after withdrawal of HFNC in patients with ARF.
**METHODS.** We included adult patients with ARF who were stable and ready to have HFNC withdrawn. Diaphragm function were assessed by thickening fraction (TF) and diaphragm excursion (DE) using ultrasonography before and after liberation from HFNC. HFNC was set to deliver 30 L/min flow at FIO2 of 0.21 to 0.30. After liberation from HFNC, patients were treated with low flow oxygen of 1 to 3 L/min through nasal cannula. TF was measured at the zone of apposition, 0.5 to 2 cm below the costophrenic sinus. DE was measured in the subcostal area. TF and DE were measured three times, and the mean values were used for evaluation. We compared the values of TF and DE before and after withdrawn of HFNC.
**RESULTS.** Twenty subjects (12 male, 8 female; mean age 68 ± 16 years) were enrolled in this study. APACHE II score was 18 (inter-quartile range: IQR, 13, 20), and duration of HFNC was 2 days (IQR, 2, 3 days). There were no significant difference in TF (13.8 vs. 12.5 %, p=.48) and DE (1.4 vs. 1.5 cm, p=0.37) before and after withdrawn of HFNC. However, four patients presented with paradoxical motion of the diaphragm after withdrawal of HFNC.
**CONCLUSION.** In patients with ARF, HFNC at 30 L/min had no significant effect on diaphragm function before and after withdrawal of HFNC. However, in some cases, paradoxical diaphragmatic movement became noticeable after withdrawal of HFNC. Novel weaning protocol of HFNC to prevent diaphragm dysfunction after liberation from HFNC is warranted.

### 001401

#### A single-breath bedside method to assess lung recruitability in patients with acute respiratory distress syndrome

L. Chen[1], L. Del Sorbo[1], DL. Grieco[1], D. Junhasavasdikul,[1], N. Rittayamai[1], I. Soliman,[1], M. Sklar[1], M. Rauseo[1], N. Ferguson[1], E. Fan[1], JC. Richard[2], L. Brochard[1]
[1]Interdepartmental division of critical care medicine, University of Toronto, Toronto, Canada; [2]Emergency department, General Hospital of Annecy, Annecy, France
**Correspondence:** L. Chen
*Intensive Care Medicine Experimental* 2019, **7(Suppl 3):**001401

**INTRODUCTION.** Positive end-expiratory pressure (PEEP) is an essential treatment for patients with acute respiratory distress syndrome (ARDS) to reopen collapsed or flooded alveoli and closed airways. However, PEEP may also overdistend previously open lung units and/or worsen circulation. The individual response to PEEP depends on lung recruitability but assessing recruitability at the bedside is challenging (1). We propose a simple approach to estimate the recruited volume (ΔVrec) at the bedside, based on the hysteresis-like behavior, which requires only one prolonged expiration maneuver (2). We also propose to standardize the ΔVrec by the real change in pressure for assessing the lung recruitability, taking into account the possible presence of complete airway closure (3). This standardized ΔVrec is defined as the compliance of the recruited lung (Crec) in our study.
**OBJECTIVES.** To validate an experimental method for measuring ΔVrec and calculating Crec; and to test whether Crec differentiates patients with different responses to PEEP in other dimensions.
**METHODS.** Patients with moderate or severe ARDS were passively ventilated at two PEEP levels different by 10 cmH2O when possible (e.g., 15 vs. 5 cmH2O). Respiratory mechanics, absolute lung volumes, and low-flow inflation pressure-volume curves were assessed at each PEEP levels. We used the multiple pressure-volume curves as the reference method (4). A reduction in lung volume between two PEEP levels at a given elastic pressure was measured as ΔVrec (4). The airway opening pressure (AOP) was also measured(3). The "real change in pressure" was not always 10 cmH2O but the difference between high PEEP and AOP in patients with complete airway closure (3). ΔVrec was thus standardized in mL per cmH2O, termed as Crec. In other words, Crec was the ratio of recruited volume to the real change in pressure over which recruitment was assessed. To define lung recruitability, we arbitrarily divided patients into "high recruiters" and "low recruiters" by using the median of Crec. We then tested whether the patients have different response to PEEP in terms of gas exchange, mechanics, and circulation.
**RESULTS.** Forty-five patients were enrolled. In four patients with airway closure, higher PEEP was insufficient to reopen airways and recruitment could not be assessed. In others, the experimental method (single breath) was strongly correlated with the reference method (multiple curves) in measuring ΔVrec and Crec ($P < 0.001$, $R2= 0.798$ and 0.817, respectively). Bias in measuring Crec was -2 mL/cmH2O and limits of agreement were -14 to 10 mL/cmH2O. At PEEP of 15 cmH2O, only high recruiters had better oxygenation compared to lower PEEP 5 ($P = 0.020$), whereas only low recruiters experienced lower mean arterial pressure ($P = 0.009$).
**CONCLUSION.** Our simple method can quantify both the recruited volume and the compliance of the recruited lung at the bedside, allowing clinicians to personalize PEEP based on lung recruitability.

**REFERENCE(S)**

1. Chen L, Brochard L. Lung volume assessment in acute respiratory distress syndrome. Curr Opin Crit Care 2015; 21: 259-264.

2. Chen L, Chen GQ, Shore K, Shklar O, Martins C, Devenyi B, Lindsay P, McPhail H, Lanys A, Soliman I, Tuma M, Kim M, Porretta K, Greco P, Every H, Hayes C, Baker A, Friedrich JO, Brochard L. Implementing a bedside assessment of respiratory mechanics in patients with acute respiratory distress syndrome. Crit Care 2017; 21: 84.
3. Chen L, Del Sorbo L, Grieco DL, Shklar O, Junhasavasdikul D, Telias I, Fan E, Brochard L. Airway Closure in Acute Respiratory Distress Syndrome: An Underestimated and Misinterpreted Phenomenon. Am J Respir Crit Care Med 2018; 197: 132-136.
4. Maggiore SM, Jonson B, Richard JC, Jaber S, Lemaire F, Brochard L. Alveolar derecruitment at decremental positive end-expiratory pressure levels in acute lung injury: comparison with the lower inflection point, oxygenation, and compliance. Am J Respir Crit Care Med 2001; 164: 795-801.

#### 001420

#### Interpleural location of chest drain on ultrasound excludes pneumothorax and can be predicted from low degree of chest drain foreshortening taken from anteroposterior chest X-ray

M. Balik[1], C. Mokotedi[1], M. Maly[1], V. Matousek[1], T. Brozek[1], M. Otahal[1], J. Rulisek[1], M. Porizka[1], L. Lambert[2]

[1]Dept of anaesthesia and intensive care, General University Hospital in Prague, Prague, Czech Republic; [2]Dept of radiology, General University Hospital in Prague, Prague, Czech Republic

**Correspondence:** M. Balik

*Intensive Care Medicine Experimental* 2019, **7(Suppl 3):**001420

**INTRODUCTION.** Chest ultrasound (CUS) is mandatory to confirm a full lung expansion after pleural drainage for pneumothorax in the critically ill. With regards to high rate of chest drain (CD) malposition its subsequent controls by chest X-ray (CXR) during ICU radiology rounds are warranted. Even a small PNO is still important because it may easily enlarge due to positive inspiratory pressure (IPPV). Our pilot paper (1) utilizing CXR and chest CT showed that greater foreshortening of the CD and a steep angle of inclination of the CD above the horizontal at chest entry taken from CXR should raise suspicion of CD migration. They mandate further investigation by CUS to rule out residual pneumothorax occult on CXR. The role of CD location by CUS in the diagnosis of pneumothorax has not been explored yet.

**OBJECTIVES.** CD foreshortening taken from CXR may associate with an absence of CD detection between ventral pleural layers on CUS in a supine patient and presence of an occult pneumothorax confirmed on CUS.

**METHODS.** Patients were prospectively monitored with CUS and CXR after drainage for pneumothorax performed by intensivists using 16-20F CDs and blunt forceps technique in the safe triangle. All drains were connected to a closed suction system providing a pressure of -20 mbar. The foreshortening was estimated as a decrease of chest drain index (CDI=length of CD in chest taken from CXR/depth of insertion on CD scale+5 cm). The angle of inclination of the CD was measured as the angle between the horizontal line and CD at the pleural space entry on CXR. Pneumothorax was diagnosed on CUS according to current standards (2).

**RESULTS.** 85 pneumothoraces were prospectively monitored in 61 patients on IPPV, age 56.2±19.8, APACHE II 22±4, SOFA 9±2.2. CDs were located on CUS in 55 patients, the full interpleural course of a CD until its tip was detected in 43 patients. There were 6 small occult pneumothoraces in this group (13.9%) particularly due to a steep angle of the CD >50°(n=4), the CDI was 0.96±0.13. In 30 patients the CDs could not be located by CUS, 6 of those were excluded for subcutaneous emphysema. There were 13 pneumothoraces in this group (54%), the CDI was 0.76±0.23 (p<0.001). The risk ratio for pneumothorax in a patient with low CDI on CXR and associating absence of the interpleural CD location on CUS is 3.88, CI 1.70-8.89, p=0.001, NNT 2.49.

**CONCLUSION.** A low CDI on CXR calls for a CUS verification of a CD position and exclusion of an occult pneumothorax not detected by the CXR.

##### REFERENCE(S)

1. Mokotedi MC, Lambert L, Simakova L, Lips M, Zakharchenko M, Rulisek J, Balik M. X-ray indices of chest drain malposition after insertion for drainage of pneumothorax in mechanically ventilated critically ill patients. J Thorac Dis. 2018;10:5695-5701
2. Volpicelli G, Elbarbary M, Blaivas M, et al. International Liaison Committee on Lung Ultrasound for International Consensus Conference on Lung Ultrasound. International evidence-based recommendations for point-of-care lung ultrasound. Intensive Care Med 2012;38:577-591.

### INF - Prevention of infection: Ways of improvement

#### 000078

#### Knowledge and Perceptions of the Critical Care Workers about Hand Hygiene

BN. Santana-Lopez[1], YG. Santana-Padilla[1], JD. Martin Santana[2], JL. Santana Cabrera[1], MJ. Molina Cabrillana[3]

[1]Intensive care unit, Maternal and child Hospital, Las Palmas de Gran Canaria, Spain; [2]Economics, Universidad de las Palmas de Gran Canaria, Las Palmas de Gran Canaria, Spain; [3]Preventive medicine unit, Maternal and child Hospital, Las Palmas de Gran Canaria, Spain

**Correspondence:** B.N. Santana-Lopez

*Intensive Care Medicine Experimental* 2019, **7(Suppl 3):**000078

**INTRODUCTION.** There is a great concern to carry out surveillance, prevention and control programs of Healthcare-associated infections (HAIs), with emphasis on hand hygiene (HH). It is important to know the perceptions of the healthcare professionals to try to improve the adherence to these programs.

**OBJECTIVES.** To identify the perceptions and knowledge of the healthcare workers of an Adult Intensive Care Unit (AICU) and a Pediatric Intensive Care Unit (PICU) about HH and correlating them with the adherence of the compliance program to HH.

**METHODS.** Cross-sectional, prospective, descriptive and comparative study, about the knowledge and perceptions of the health professionals with respect to HH, through the realization of a survey published and validated by the World Health Organization. In addition, these perceptions are compared with the evaluation of the adherence to the HH that had been done prior to this study by direct observation of the workers during their daily work.

**RESULTS.** 187 surveys were obtained (142 from AICU and 43 from PICU); >80% of total the population. >90% had received formal training on HH in the last 3 years and regularly use of alcohol-based solution (ABS). Regarding knowledge, there are no significant differences between the units. 35% of the AUCI professionals and almost 50% from the PICU consider that hand washing (HW) is more effective than hand friction (HF) with alcohol-based solutions (ABS) for the elimination of microorganisms. >30% believe that it is advisable to perform the HW and HF sequentially. Only 55.8% know the minimum adequate time to eliminate microorganisms by HF. They overestimate the percentage of occasions in which they perform HH correctly with respect to adherence rates to HH, through direct observation, (PICU 89.32% vs 73,8% and AICU 82.93% vs 51,4%)(p = 0.0001).

**CONCLUSION.** Despite being a sample of professionals with formal training on HH, the results indicate that they have incomplete knowledge and overvalue their perception compared with the adherence rates obtained through observational studies.

##### REFERENCE(S)

1. World Health Organization (WHO). Hand Hygiene Knowledge Questionnaire for Health-Care workers. 2009. Available in: https://www.mscbs.gob.es/organizacion/sns/planCalidadSNS/docs/cuestionario_conocimientos_prof_oms.pdf
2. World Health Organization (WHO). Perception Survey for Health-Care workers. 2009. Available in: http://www.mscbs.gob.es/organizacion/sns/planCalidadSNS/docs/cuestionario_percepcion_prof_oms.pdf
3. Torán Mateos M, Pareja Bezares A. Perceptions and knowledge about hand hygiene in resident doctors of Son Llàtzer Hospital 2011-2017

Medicina Balear 2018; 33; 33-47. Disponible en: http://ibdigital.uib.cat/greenstone/collect/medicinaBalear/index/assoc/Medicina/_Balear_/2018_vol/33_n1p03.dir/Medicina_Balear_2018_vol33_n1p033.pdf
4. Cobo Sánchez JL, Pelayo Alonso R, Menezo Viadero R, Incera Setién E, et al. Hand hygiene perceptions and knowledge of healthcare workers in a Nephrology Unit: a comparative study. Enferm Nefrol 2014: Enero-Marzo; 17: 28/34. Disponible en: http://scielo.isciii.es/scielo.php?script=sci_arttext&pid=S2254-28842014000100005
5. Pérez-Pérez P, Herrera-Usagre M, Bueno-Cavanillas A, Alonso-Humada MS, Buiza-Camacho B, Vázquez-Vázquez M. Hand hygiene: health professionals' knowledge and areas for improvement. Cad. Saúde Pública, Rio de Janeiro, 2015. 31:149-160. Disponible en: http://www.scielo.br/pdf/csp/v31n1/0102-311X-csp-31-01-00149.pdf

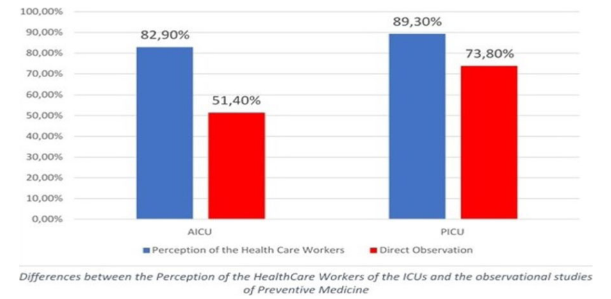

**Fig. 1 (abstract 000078).** See text for description

### 000080

#### Adherence to Hand Hygiene in an Adult and other Pediatric ICU

BN. Santana-Lopez[1], MJ. Molina Cabrillana[2], YG. Santana-Padilla[1], JL. Santana Cabrera[1], JD. Martin Santana[3]
[1]Intensive care unit, Maternal and child Hospital, Las Palmas de Gran Canaria, Spain; [2]Preventive medicine unit, Maternal and child Hospital, Las Palmas de Gran Canaria, Spain; [3]Economics, Universidad de las Palmas de Gran Canaria, Las Palmas de Gran Canaria, Spain
**Correspondence:** B.N. Santana-Lopez
*Intensive Care Medicine Experimental* 2019, **7(Suppl 3):**000080

**INTRODUCTION.** The World Health Organization (WHO) promotes that proper hand hygiene (HH) is the main practice, with the lowest economic cost and the easiest to perform to reduce the incidence and spread of antimicrobial resistant microorganisms, which improves patient safety in all health areas. However, WHO rates the HH compliance index by health professionals is insufficient (less than <40%).

**OBJECTIVES.** To know the adherence rates to the HH, between the Healthcare workers (HCWs) in an adults ICU (AICU) and other pediatric (PICU).

**METHODS.** An observational study was conducted on the compliance of HH for the five WHO moments. HCWs were observed during their work shift. The observers also measured the technique of HH through hand washing or HH with alcohol-based disinfectant. HH opportunities and attempts were designated as appropriate or inappropriate per WHO criteria.

The percentage of adherence was calculated as the number of opportunities of HH (with soap and water or alcohol-based solutions (ABS)) multiplied by 100 and dividing by the total of identified opportunities.

**RESULTS.** 391 opportunities were identified in the AICU and 320 in the PICU, a HH adherence rate in the AICU of 51.40% and 73.80% in the PICU was determined. By professional profile it was observed that the nursing staff is significantly the most adherent (64% in the AICU and 82% in the PICU). It was found that in the AICU the adherence is greater after being in contact with the patient), unlike the PICU where they perform it predominantly before in all professional profiles.

**CONCLUSION.** The adherence to hand hygiene in the AICU is low. The adherence to HH is greater before contacting the patient in the PICU, unlike the AICU where it is predominantly carried out afterwards. So it is necessary to implement effective education programs that improve adherence to hand hygiene compliance.

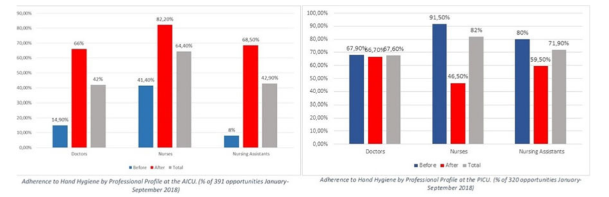

**Fig. 1 (abstract 000080).** See text for description

### 000124

#### Stethoscopes- potential sources of cross infections in ICU

N. Navilehal Rajasab, N. Cemm; S. Patel
Intensive care department, King's College Hospital, London, United Kingdom
**Correspondence:** N. Navilehal Rajasab
*Intensive Care Medicine Experimental* 2019, **7(Suppl 3):**000124

**INTRODUCTION.** –Emergence of antimicrobial resistance and MDROs has resulted in a global health burden, increasing cost of health care, length of hospital/ ICU stay and mortality. Risk of bacterial transmission through equipment transfer is not given as much importance as hand hygiene.

–Studies have proven contamination of stethoscopes with both non-pathogenic and pathogenic (including MDR) organisms. Jones et al showed that out of 150 stethoscopes used by emergency medical staff, 89% grew staphylococci and 19% S aureus[1]. Marinella et al showed that coagulase-negative staphylococcus was present on 100% of stethoscopes and Staphylococcus aureus on 38% of 40 random stethoscopes examined [2].

–CDC guidelines suggest performing low level disinfection for non-critical patient care surfaces and equipment that touch intact skin e.g. bed rails, blood pressure cuffs and stethoscopes

**OBJECTIVES.** - To determine whether stethoscopes can be potential sources of cross-infection/cross contamination in our ICU

**METHODS.** -All 4 adult ICUs (total 73 beds) were surveyed between Nov-Dec 2018 for the number of bedside stethoscopes at random times for a total of 2 weeks. Each bedside stethoscope was also labelled with unique identfier codes, and movement of stethoscopes between bed spaces and patients was tracked on a daily basis.

-A survey questionnaire was also sent to all the ICU medical staff regarding infection control practices with respect to use of bedside stethoscopes in ICU.

**RESULTS.** -Average number of stethoscopes found in ICU's 1,2,3 & 4 were 71.4%, 66.6%, 77.7% and 110% respectively and number of times stethoscopes were found to be misplaced were 24.4%, 31.7%, 34.92% and 8% respectively

-Unit acquired infection rates from April 2018-March 2019 for ICUs 1,2, 3 & 4 were 17.1/1000, 33/1000, 20.3/1000 & 5.8/1000 respectively

-A total of 210 people responded to the survey questionnaire (23% doctors, 74% nurses). 69% said they share stethoscopes between bedspaces, because of non-availability of 1:1 dedicated bedside stethoscopes (5%), poor quality of stethoscopes (17%), or (50%). 100% said they clean stethoscopes after every use. Asked whether there was a checklist to ensure stethoscopes are cleaned, 7% answered "yes", 65% said "no" and 28% were not sure.

**CONCLUSION.** –Our audit revealed that there is evident sharing of stethoscopes between bed spaces mainly because of poor quality and lack of adequate number of dedicated bedside stethoscopes in ICU.

-Units with the highest proprotion of stethoscopes and the lowest frequency of misplaced stethoscopes had the lowest unit-acquired infection rates, suggesting that stethodscopes may play a role in cross infection

–The survey questionnaire showed that there is inadequate education around cleaning stethoscopes and documentation, and importance of having dedicated 1:1 bedside stethoscopes

–CDC recommends monitoring for adherence to recommended environmental cleaning practices as an important determinant for success in controlling transmission of MDROs and other pathogens in the environment[5].

–Based on the above factors, having 1:1 dedicated bed side stethoscopes would be a prudent solution to reduce risk of cross contamination.

**REFERENCE(S)**

1. Jones JS, Hoerle D, Riekse R. Stethoscopes: a potential vector of infection? Ann Emerg Med. 1995;26(3):296-299
2. Marinella MA, Pierson C, Chenoweth C. The stethoscope. A potential source of nosocomial infection? Arch Intern Med. 1997;157(7):786-790
3. Merlin MA, Wong ML, Pryor PW, et al. Prevalence of methicillin-resistant Staphylococcus aureus on the stethoscopes of emergency medical services providers. Prehosp Emerg Care. 2009;13(1):71-74
4. Knecht VR, McGinniss JE, Shankar HM, et al. Molecular analysis of bacterial contamination on stethoscopes in an intensive care unit Infect Control Hosp Epidemiol. doi: 10.1017/ice.2018.319
5. https://www.cdc.gov/infectioncontrol/guidelines/mdro
6. This audit was supported by the Intensive Care Department, King's College Hospital. I thank all my colleagues [doctors and nurses] for sincerely answering the survey questionnaire that helped support the audit results and constant encouragement by asking questions to me when I was collecting data. I thank my junior colleague Nicholas Cemm for being active, sincere and help me with data collection. I like to show my gratitude to my audit supervisor Sam Patel [Consultant, Intensive Care] for his valuable guidance, time and ongoing support.

### 000148

#### Presence of microorganisms in the mobiles phones of professionals in a polivalent ICU

YG. Santana-Padilla[1], JL. Santana Cabrera[1], BN. Santana-Lopez[1], ME. Dorta-Hung[2], MJ. Molina-Cabrillana[2]
[1]Intensive care unit, Maternal and child Hospital, Las Palmas de Gran Canaria, Spain; [2]Preventive medicine, Complejo Hospitalario Universitario Insular-Materno Infantil, Las Palmas de Gran Canaria, Spain, Spain
**Correspondence:** Y.G. Santana-Padilla
*Intensive Care Medicine Experimental* 2019, **7(Suppl 3):**000148

**INTRODUCTION.** Many studies have researched about the contamination of differents surface that there are in the different units of hospitals. In the Intensive Care Units (ICU), several studies have found important microorganism in the personal mobile phones of health workers. Some studies have detected germs such as P. aeruginosa, Acinetobacter, S. aureus and Enterococcus. These findings make its important to consider this surface as an element to monitor in the transmission of nosocomial diseases.

**OBJECTIVES.** Identify the presence of microorganisms in the mobile phones of the profesionals of an Intensive Care Unit (ICU).

**METHODS.** Experimental controlled trial for 5 months among heath and non-health profesionals who had mobile devices during their workday. The collection was performed by a wet swab in Brain Heart Infusion (BHI). A single researcher was in charge of collecting the samples after receiving specific training. Follow up was done in the microbiological findings of the patients in charge of the professionals of which samples were taken. The Medicine Preventive unit collaborates in the microbiological analysis of the samples collected. This study was approved by the ethical and research committee.

**RESULTS.** In our study, 111 samples of mobile devices were collected. In these samples, the 49.5 per cent were contaminated by relevant microorganism. Of this samples 64 microorganisms were detected, with the following distribution by groups: Gram (+) 71.8% predominated, followed by Gram (-) 18.7% and fungi 9.3%. It was relevant for the research to find in the contaminated mobiles, the presence of multi-resistant microorganisms of the type of methicillin-resistant Staphylococcus aureus (10.9%), Pseudomonas aeruginosa (12.5%) and Stenotrophomonas maltophilia. (4.7%)

**CONCLUSION.** Despite not being able to establish causality between the isolations of patients and the findings in mobile phones, if pathogenic microorganisms have been detected, they should alert us that they could serve as a reservoir of transmission to the critical patient. There were only two cases: a nurse and a doctor where a germ of the same type that the patients had, grew in the culture plates. The training in hand hygiene should be carried on. The high adherence in hand hygiene could have an important impact to reduce the contamination of the mobiles.

**REFERENCE(S)**

1. Russotto V, Cortegiani A, Raineri SM, Giarratano A. Bacterial contamination of inanimate surfaces and equipment in the intensive care unit. J Intensive Care. 10 december 2015;3(1):54.
2. Heyba M, Ismaiel M, Alotaibi A, Mahmoud M, Baqer H, Safar A, et al. Microbiological contamination of mobile phones of clinicians in intensive care units and neonatal care units in public hospitals in Kuwait. BMC Infect Dis. 15 october 2015;15(1):434.
3. Ulger F, Dilek A, Esen S, Sunbul M, Leblebicioglu H. Are healthcare workers' mobile phones a potential source of nosocomial infections? Review of the literature. J Infect Dev Ctries. 29 october 2015;9(10):1046
4. Ulger F, Esen S, Dilek A, Yanik K, Gunaydin M, Leblebicioglu H. Are we aware how contaminated our mobile phones with nosocomial pathogens? Ann Clin Microbiol Antimicrob. 6 march 2009;8(1):7.
5. Loyola S, Gutierrez L, Avendaño E, Severino N, Tamariz J. Multidrug-resistant bacteria isolated from cell phones in five intensive care units: Exploratory dispersion analysis. Germs. 4th april 2018;8(2):85-91.

### 001467

#### "Effect of antibiotic oral decontamination therapy compared to oral care with chlorhexidine in intubated ICU patients on prevention of IVAC and VAP"

S. Papoti, E. Setsidou, E. Koletsou, A. Kosmas, E. Lazoudi, E. Siomos, N. Kapravelos, C. Iasonidou
B icu, General Hospital "G. Papanikolaou", Thessaloniki, Greece
**Correspondence:** S. Papoti
*Intensive Care Medicine Experimental* 2019, **7(Suppl 3):**001467

**INTRODUCTION.** Ventilator-associated pneumonia(VAP) occurs in 9-27% among patients on mechanical ventilation. The main risk factor is the endotracheal tube and microaspirations. The bacteriology of mouth flora responsible for that, are Gram-negative bacteria and fungal species. Through the past years, different strategies of decontamination of oropharyngeal cavity have been applied with conflicting evidence on effectiveness and safety.

**OBJECTIVES.** The aim of our study was to compare the effectiveness of antibiotic Selective Oropharyngeal Decontamination(SOD) along with the use of oral antiseptic agent (Chlorhexidine) versus standard care with topical chlorhexidine alone, concerning the prevention of IVAC (Infection-related ventilator-associated complications) and VAP.

**METHODS.** A randomized controlled study of patients in a general ICU. Inclusion criteria contained all intubated patients of age≥18 years old who were mechanically ventilated for≥48h. Patients admitted in the ICU with respiratory infection were excluded from the study. Two groups during a 6month period were studied. The control group was treated with oral application of Chlorhexidine 2% three times a day. To the other group, antibiotic paste with colistine and fluconazole was applied to the oropharynx twice a day additionally to Chlorhexidine for a period of ten days. We recorded the episodes of Ventilator-associated Complications (VACs)/Infection-related (IVACs) and Ventilator-associated pneumonia (VAP) using the Centers for Disease Control and Prevention (CDC) classification, during the first 10 days of ICU stay.

**RESULTS.** Overall 72 patients were studied, 37 assigned to standard care with Chlorhexidine and 35 to additional application of antibiotic paste. The patient's characteristics concerning median age (63 vs 57 years), APACHE II score (17.8 vs 17.7), mean ICU days (16.2 vs 16.4days) and death rate (22.8% vs 21.6%) among the two groups respectively were comparable. Using statistical analysis, we compared the VAC/IVAC occurrence between the two groups (95% confidence interval [CI], 0.332, 13.082; p= 0.472; OR 1.87) and VAP (X-squared = 1.3455, p-value=0.246).

**CONCLUSION.** Applying antibiotic decontamination therapy in addition to standard care with oral Chlorhexidine in our ICU, didn't prove to reduce the development of VAC/IVAC and VAP in intubated patients, in a statistically significant way. Although the comparison between two groups (Figure) showed higher VAP occurrence in the group treated with chlorhexidine alone (21.62% vs 11.43%) this was not statistically significant (p=0.246). Our results are limited by the small size of our sample and more patient recruitment is needed.

**REFERENCE(S)**

1. Klompas M: Oropharyngeal Decontamination with Antiseptics to Prevent Ventilator-Associated Pneumonia: Rethinking the Benefits of Chlorhexidine. Semin Respir Crit Care Med. 2017;38(3):381–90
2. Oostdijk EA, Wittekamp BH, Brun-Buisson C, Bonten MJ. Selective decontamination in European intensive care patients. Intensive Care Med. 2012;38(4):533–538.

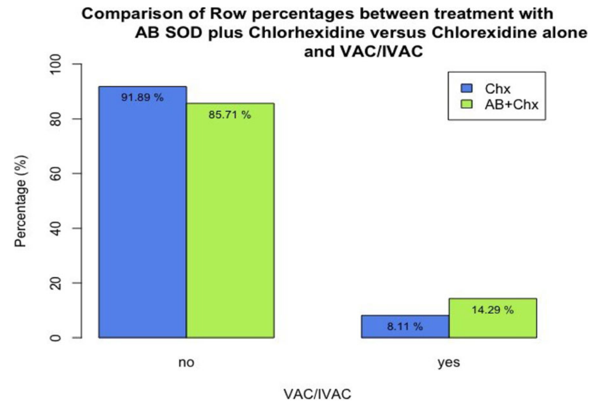

**Fig. 2 (abstract 001467).** See text for description

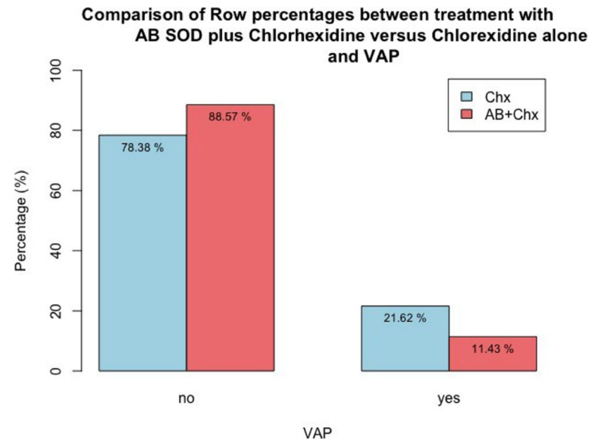

**Fig 1 (abstract 001467).** See text for description

### 001544

#### Perioerative MDR colonisation and surgical prophylaxis

A. Lerose, K. Donadello, D. Cigolini, V. Schweiger, E. Bonora, E. Polati
Anesthesia and intensive care b unit, University of Verona, AOUI-University Hospital Integrated Trust of Verona, Verona, Italy
**Correspondence:** K. Donadello

*Intensive Care Medicine Experimental* 2019, **7(Suppl 3):**001544

**INTRODUCTION.** Multi-drug resistant (MDR) colonization is a major issue worldwide (WHO, 2019), mostly for perioperative and intensive care medicine, frequently resulting in life-threatening infections.

**OBJECTIVES.** Within our ICU admissions, the aim of this study was to evaluate MDR colonized patients undergoing surgery and their post-operative course compared to non colonized patients.

**METHODS.** We retrospectively analyzed all consecutive patients undergoing surgery in our hospital for a period of 12 months (July 2017-July 2018) who required ICU admission on POD 0. According to their surveillance swab, we defined patients as negative, negative for MDR bugs, MDR positive (*Enterobacteriaceae* beta-lactamase producer-ESBL, *Enterobacteriaceae* carbapenemase producer-e.g.KPC, non-lactose fermenting-e.g. *Pseudomonas and Acinetobacter spp, MRSA*). We then compared MDR colonized (rectal and/or pharyngeal swab) patients (MDR+) to non colonized patients (MDR-) for post operative complications, ICU and hospital LOS and mortality.

**RESULTS.** 274 patients were included in the study (186 men), median age 70(61-77) years; they underwent either elective (n=234, 85.4%) or emergency (n=40, 14.6%) surgery; 119 pts (43.4%) were submitted to pancreatic surgery, 67 (24.5%) to hepato-biliary surgery, 51 (18.6%) to intestinal resection and the rest to other abdominal surgery; they were admitted to ICU on POD 0 for scheduled (n =244, 89.1%) or unscheduled intensive PO monitoring. 43 pts were pre operatively MDR+ (15,7%), 13 of whom were either Klebsiella Pneumoniae Carbapenemasis Producer or Enterococcus Faecium VRE colonized and 28 were ESBL colonized. 50 pts resulted colonized early on ICU admission screening. ICU LOS was significantly higher in MDR+ compared to MDR- (6.2±13 vs 2.1±4 days, p <0.05). Hospital mortality occurred in 17 patients, 9 were MDR+, 6 of them died in the ICU. Complication rate and type were significantly different between the two groups. Only 19 MDR+ patients (6.9%) received targeted pre-operative antibiotic therapy compared to standard prophylaxis but, despite a positive trend with regards to infectious complications, LOS and mortality, our results were significant in terms of outcome.

**CONCLUSION.** Pre-operative surveillance swab positivity correlates with both ICU LOS and post-operative mortality. Might these results be confirmed, targeted perioperative antibiotic prophylaxis should be seriously settled so as to be routinely used to improve patients' outcome. More studies are needed to further investigate the possibile therapeutic options in MDR+ patients undergoing surgery.

### 001585

#### Impact of orotracheal intubation and mechanical ventilation on the microbiota of the lung in the development of Ventilator-Associated Pneumonia

D. calabretta[1], D. Piazzai[1], R. Pinciroli[1], A. Vargiolu[2], P. Adriana[2], L. Alagna[3], A. Bandera,[3], GM. Migliorino[4], F. Minardi[5], S. Rossi,[5], E. Picetti[5], M. Ventura[6], A. Gori[3], G. Citerio[2]
[1]Department of anesthesia and critical care, ASST Grande Ospedale Metropolitano Niguarda, Milano, Italy; [2]School of medicine and surgery, University of Milano-Bicocca, Monza, Italy; [3]Infectious diseases unit - department of internal medicine, University of Milan, Milan, Metropolitan City of Milan, Italy, Italy; [4]Infectious diseases unit - department of internal medicine, ASST Monza - san gerardo, Monza, Province of Monza and Brianza, Italy, Italy; [5]Department of anesthesia and intensive care, Parma University Hospital, Parma, Italy; [6]Laboratory of progenomics, department of chemistry, University of Parma, Parma, Italy
**Correspondence:** D. calabretta

*Intensive Care Medicine Experimental* 2019, **7(Suppl 3):**001585

**INTRODUCTION.** Thanks to the development of the most recent culture-independent methods, changes in lung microbiota composition has been observed in pulmonary diseases. Its role among critically ill patients need to be largely investigated because many questions are still unanswered in particular regarding pathogenesis of infective complications. In our study we tried to understand which are the major changes that occur during mechanical ventilation and which are critical for the development of VAP (Ventilation Associated Pneumonia).

**METHODS.** Tracheal aspirates were sampled from 23 patients without pulmonary diseases in progress at the moment of the intubation (T0), after 72 hours (T1) and then every 48 hours later until T7 (day 15) or until the moment of the extubation or the dismission/death of the subject. Analysis of microbiota was performed by 16s rRNA amplifications. Results from QIIME related to taxa classification will be described according diversity measures. Bacterial communities diversity will be analysed applying the Principal Coordinate Analysis (PCoA) based on phylogenetic distance (UNIFRAC) among all samples.

**RESULTS.** The preliminary molecular analysis have shown the presence of 360 different bacterial taxa. In the first part of our study we focused on the description of the microbial community at the moment of the intubation (T0) and after 72 hours (T1). Three out of 23 patients developed VAP during follow up respectively at day 5, 13 and 15 after mechanical intubation. Overall, the comparison of the samples collected at two different times showed that: Bergeyella Veillonella, Leptotrichia, Variibacter, Stenotrophomonas significantly are less present at T1 than T0 while Peptostreptococcus in T0 than T1. Among three patients with VAP genera Eubacterium, Lachnospiraceae, Lachnospira, Lactobacillus, Leptotrichia, Bradyrhizobium and Anaeroplasma were significantly higher in T0 than T1 while commensal strains characteristic of the upper airway such as Porphyromonas, Alloprevotella, Prevotella, Howardella, Solobacterium, Dialister, Veillonella and Fusobacterium increased in T1 than in T0.

**CONCLUSION.** Preliminary results highlighted the impact of orotracheal intubation procedure on the pulmonary microbiota changes, in agreement with the previous data emerged in literature (Kelly, 2016) (Vladimir Lazarevic, 2014). Given the few cases of VAP, comparisons between VAP and no-VAP patients do not allow to identify microbiota characteristics associated to development of pneumonia. Larger studies are needed to compare healthy controls and cases that develop VAP and in order to clear the significance of our findings and to achieve a better understanding of the phenomenon.

**REFERENCE(S)**

1. Vladimir Lazarevic, N. G.-B. (2014). Challenges in the culture-independent analysis of oral and respiratory samples from intubated patients. Frontiers in Cellular and Infection Microbiology.
2. Kelly, B. J. (2016). Composition and dynamics of the respiratory tract microbiome in intubated patients. Microbiome.
3. Supported by Società Italiana di Terapia Antimicrobica (SITA).

### 001695

#### A prospective study on Carbapenem Resistant Enterobacteriaceae (CRE) rectal colonization in Indian living donor liver transplant recipients – Incidence & Outcomes

A. rajakumar[1], P. Velusamy[1], B. Govindarao[2], L. Gopal[2], I. Kaliamoorthy[1], M. Rela[3]

[1]Liver anaesthesia and intensive care, Dr. Rela Institute and Medical Centre, Chennai, India; [2]Laboratory medicine, Gleneagles Global Health City, Chennai, India; [3]Liver transplantation and hpb surgery, Dr. Rela Institute and Medical Centre, Chennai, India

**Correspondence:** A. rajakumar

*Intensive Care Medicine Experimental* 2019, **7(Suppl 3):**001695

**INTRODUCTION.** Immunosuppressive therapy following liver transplantation(LT) makes these patients more susceptible to infections which can lead to poor outcomes following LT.1 Recurrent hospital admissions predispose these patients to colonization with drug resistant organisms and gut is the most common site.2. We therefore decided to study the incidence of Carbapenem Resistant Enterobacteriaceae (CRE) colonization in the gut in our LT recipients, risk factors and the outcome of CRE colonization in those LT recipients.

**METHODS.** This study was conducted between September 2017 and July 2018 at Gleneagles Global Hospital. All adult living donor liver transplant (LDLT) recipients except acute liver failure, re transplantation and combined organ transplants who consented were included. Rectal swab was done in the week preceding the transplant and immediately cultured on Hi chrome KPC Agar. Any growth was subcultured on Blood agar and MacConkey agar, followed by Identification and Antimicrobial Susceptibility Testing in Vitek 2-Compact system. Antibiotics for perioperative period were started as per institution protocol. Preoperative parameters like MELD score, hospital admissions, episodes of infection and antibiotics used, spontaneous bacterial peritonitis (SBP), UGI bleeding, encephalopathy, acute kidney injury (AKI) large volume paracentesis(LVP) and hydrothorax requiring drainage (HTD) were noted. Intra op details like graft versus body weight ratio (GRWR), surgical duration, blood components used and other complications were noted. Postoperative parameters recorded include prolonged ventilator/ vasopressor requirement, AKI requiring continuous renal replacement therapy(CRRT), graft function & other graft related complications, intraabdominal collections & pleural effusions requiring drainage, infections, duration of ICU and hospital stay and mortality. Data collected was analysed using STATA statistical software. Two-sided independent-sample t test to compare means across dichotomous variables & the one-way ANOVA test for comparison of means across multilevel variables were used. A p value $<0.05$ was considered statistically significant.

**RESULTS.** Please see uploaded table. 40 recipients were included. 15 (37.5 %)recipients had CRE colonization - CREpos & 25 (62.5%) were CREnegative. No difference in medical comorbidities were noted except high MELD score in CREpos. Ascitis, LVP,SBP,HTD, malnutrition, AKI, UGI bleeding, encephalopathy, preop ICU were more frequent in CREpos. Requirement of PRBC and other blood components, vasopressors, surgical duration and lactate were higher in the CREpos group. Postoperatively graft function was similar but CREpos had more prolonged requirement of vasopressor/ ventilator and CRRT, reintubations, drainage of intraabdominal collection and pleural effusion, more ICU readmissions, wound infection and bacteremia. The duration of ICU and hospital stay was higher in the CREpos. 2 (13.33%)patients died in CREpos while 1(4 %) died in the CREneg group. Statistically significant difference was noted in the requirement for LVP, preop AKI, PRBC requirement, duration of ICU stay and CRRT requirement. More preop carbapenem exposure was noted in CREpos though it was not statistically significant.

**CONCLUSION.** LT recipients who require more interventions and hospitalizations in the preoperative period seem more susceptible to CRE colonization. These patients are at a higher risk of complications in the intraoperative and postoperative period with higher mortality rates. The most probable cause for CRE colonization is presumed to be gut translocation although the yield of positive cultures is low. Preemptive selective digestive decontamination needs to be vigorously tested in these immunocompromised patients keeping in mind the presumed risk of emergence of more resistant microorganisms.3,4

**REFERENCE(S)**

1. Kashyap R, Jain A, Reyes J, Demetris AJ, Elmagd KA, Dodson SF, et al. Causes of death after liver transplantation in 4000 consecutive patients: 2 to 19 year follow-up. Transplant Proc 2001;33:1482 – 1483.
2. Doycheva I, Leise MD, Watt KD.The Intestinal Microbiome and the Liver Transplant Recipient: What We Know and What We Need to Know. Transplantation. 2016 Jan;100(1):61-8
3. De Smet et al.Decontamination of the Digestive Tract and Oropharynx in ICU Patients .N Engl J Med 2009;360:20-31.
4. Nick Daneman, Syed Sarwar, Robert A Fowler, Brian H Cuthbertson, on behalf of the SuDDICU Canadian Study Group. Effect of selective decontamination on antimicrobial resistance in intensive care units: a systematic review and meta-analysis: Lancet Infect Dis 2013; 13: 328–41
5. No grants were received for this study

### 001723

#### Antibiotic resistant gram-negative bacteria in Intensive Care in a large London Hospital

D. Morley[1], M. Laundy[2], J. Ball[3]
[1]Intensive care unit, St George's Hospital Atkinson Morley Wing, LONDON, United Kingdom; [2]Microbiology, St George's Hospital Atkinson Morley Wing, London, United Kingdom; [3]General intensive care unit, St George's Hospital Atkinson Morley Wing, London, United Kingdom
**Correspondence:** D. Morley
*Intensive Care Medicine Experimental* 2019, **7(Suppl 3):**001723

**INTRODUCTION.** Multidrug resistant infection in Intensive Care is increasingly common and is associated with significant patient mortality and morbidity. St Georges hospital is a Level 1 trauma and tertiary referral centre covering the catchment area of South West London and Surrey. The Adult Critical Care directorate provides 61, level 2 and 3 beds spread across three units; General, Cardiothoracic and Neuro Intensive Care. The aim of this retrospective observational study is to assess the frequency and degree of antimicrobial resistance among common Gram Negative (GN) and Gram Positive (GP) bacteria isolated from patients in a large London Intensive Care Facility.

**METHODS.** Clinically indicated specimens collected from the three intensive care units over a four year period (2013-2016) were included consisting of community and hospital acquired infection, and colonisation specimens. Resistant categories were defined by Minimum Inhibitory Concentration (MIC) using EUCAST breakpoint recommendations. Data was collected and analysed using WHONET software

**RESULTS.** 2911 patient isolates over a 4-year period from January 2013 to December 2016 were included. Of these isolates 59% (1726) were gram negative organisms and 40% (1185) gram positive. Among Enterobactericae, all tested isolates were sensitive to meropenem. 3rd Generation cephalosporin resistance was most prevalent among *Enterobacter clocae* with 22.8% and 24.4% of isolates resistant to ceftazidime and cefotaxime respectively. 10.6% of *Escherichia coli (E. coli)* isolates were resistant to ceftazidime and 13.3% to cefotaxime. Ceftazidime and cefotaxime resistance was present in 8.6% and 9.3% of *Klebsiella pneumonia (K. pneumonia)* isolates. Ciprofloxacin resistance was highest among *E. coli* (15.5%) followed by *K. pneumonia* (8.9%). Meropenem resistance was present in 3.7% of *Pseudomonas aeruginosa* (*P. aeruginosa*) isolates, ciprofloxacin resistance in 7.8% and piperacillin/tazobactam resistance in 6.1%. Vancomycin resistance was present among 14.3% of *Enterococcus faecium* isolates.

**CONCLUSION.** Intensive care is commonly considered to be a reservoir for infection due to its vulnerable immunosuppressed population, frequency of invasive procedures, and use of medications such as muscle relaxants and sedation. A significant percentage of *E. coli* and *K. pneumonia* at our ICU were resistant to 3rd line cephalosporin. This is comparable to national data demonstrating resistance to 3rd generation cephalosporin's to be 10.8-12.4% for E. coli and 10.0-11.5% for K. pneumonia over similar period (2012-2016). Carbapenem resistance among *P. aeruginosa* is a growing concern nationally. London has the highest level of resistance with over 10-14% resistance recorded between 2012-2016. Interestingly the percentage resistant isolates was significantly lower among our critically ill patients (3.7%).This study provides an interesting insight into the ecology of a large London ICU and how it compares to national UK nosocomial data. It reinforces the need for appropriate and diligent infection control practices and antimicrobial stewardship going forward

##### REFERENCE(S)

1. Bassetti M, Poulakou G, Ruppe E, Bouza E, Van Hal SJ, Brink A. Antimicrobial resistance in the next 30 years, humankind, bugs and drugs: a visionary approach. Intensive Care Med. 2017;43(10):1464-75.
2. De Waele JJ, Akova M, Antonelli M, Canton R, Carlet J, De Backer D, et al. Antimicrobial resistance and antibiotic stewardship programs in the ICU: insistence and persistence in the fight against resistance. A position statement from ESICM/ESCMID/WAAAR round table on multi-drug resistance. Intensive Care Med. 2017.
3. English Surveillance Programme for Antimicrobial Utilisation and Resistance (ESPAUR) Public Health England 2017.

### 000643

#### Are surveillance cultures useful for predicting multi-drug resistant bacterial infections?

E. Rodriguez-Ruiz[1], M. Robelo Pardo[1], C. Martin López[1], L. Sayagues Moreira[1], P. Barral Segade[1], MC. Domínguez Antelo[1], A. Virgos Pedreira[1], Al. Suarez Freire[1], G. Barbeito Castiñeiras[2], P. Rascado Sedes[1]
[1]Intensive Care Medicine Department, Complejo Hospitalario Universitario de Santiago de Compostela, Santiago de Compostela, Spain; [2]Microbiology department, Complejo Hospitalario Universitario de Santiago de Compostela, Santiago de Compostela, Spain
**Correspondence:** E. Rodriguez-Ruiz
*Intensive Care Medicine Experimental* 2019, **7(Suppl 3):**000643

**INTRODUCTION.** Early administration of appropriate empirical antibiotic therapy has been shown to reduce the morbidity and mortality in severe infections. Nowadays, in the context of increased antibiotic resistance, clinicians who treat critically ill patients at risk of multidrug resistant bacteria (MDRB) infections initiate broad-spectrum antibiotic therapy with one or more antibiotics, entailing an additional risk of increased resistance. Many studies have shown that surveillance cultures allow us to assess the dimension of the multi-drug resistance problem in an ICU.

**OBJECTIVES.** Our aim was to analyse the impact of surveillance cultures in predicting multi-drug resistant bacterial infections.

**METHODS.** We conducted a prospective observational study in an 8-bed medical ICU between January 2016 and November 2018. All patients included in the ENVIN-HELICS database (ICU-acquired infection surveillance program promoted by the Spanish Society of Intensive and Critical Care Medicine and Coronary Units, SEMICYUC) were analysed. ENVIN-HELICS database collects data from all infections within the ICU. We routinely performed a nasal and rectal swab test to all patients upon admission and on a weekly basis for the detection of MDRB. We describe demographics, severity at admission (APACHE-II), ICU length of stay (LOS), MDRB colonisation on admission and ICU-acquired MDRB infections. We analysed previous MDRB colonisation in patients with ICU-acquired MDRB infections.

**RESULTS.** 735 patients (57% males) were included during the study period. Mean age was 68.5 years (SD 16.37), mean APACHE-II at admission was 21.5 (SD 8.08) and mean ICU LOS was 7.5 days (SD 13.45). Intra-ICU mortality among our cohort of patients was 16.3%. On admission, 16.6% of the patients were colonised by a MDRB. Most frequent microorganisms were: *ESBL-producing Escherichia coli* (32.3%), *carbapenemase-producing Klebsiella spp.* (19.8%), *methicillin-resistant Staphylococcus aureus* (MRSA - 17.6%) and *ESBL-producing Klebsiella spp.* (13.2%). During ICU stay (after 48 hours of admission) a MDRB was isolated in 7% of the patients. *Carbapenemase-producing Klebsiella spp.* (49.1%), *ESBL-producing Klebsiella spp.* (12.3%), *Pseudomonas spp.* (7%) and MRSA (7%) were the most frequent microorganisms. Twenty-one patients (2.9%) had 23 ICU-acquired MDRB infections. Ventilator-associated tracheobronchitis (VAT - 47,8%), ventilator-associated pneumonia (VAP - 13%), urinary tract infection (13%) e skin and soft tissue infection (13%) were the most frequent infections. Most frequent microorganisms were: *carbapenemase-producing Klebsiella spp.* (47,8%), *ESBL-producing Escherichia coli* (13%) and *Acinetobacter baumanni* (13%). Among patients with an ICU-acquired MDRB infection, 69.5% were previously colonised by a MDRB and in 21.7% a MDRB was isolated in a rectal swab and in clinical samples simultaneously. Only 8.7% of the patients were not colonised at the time of infection.

**CONCLUSION.** In the majority of ICU-acquired MDRB infections, infection is preceded by colonization. VAT is the most frequent ICU-acquired MDRB and *carbapenemase-producing Klebsiella spp.* is the most frequently isolated microorganism.

### 000762

#### Monitoring quality of care for Peripheral Intravenous Catheters; feasibility and reliability of the Peripheral Intravenous Catheters mini Questionnaire (PIVC-miniQ)

LH. Høvik[1], KH. Gjeilo,[2], S. Lydersen[3], CM. Rickard[4], B. Røtvold[5], JK. Damås[6], E. Solligård[7], LT. Gustad[8]
[1]Clinic of anaesthesia and intensive care, St. Olav's Hospital, Trondheim, Norway; [2]Department of cardiothoracic surgery, department of cardiology, St Olav's Hospital, Trondheim, Norway; [3]Regional centre for child and youth mental health and child welfare, department of mental health, St. Olav's Hospital, Trondheim, Norway; [4]Alliance for vascular access teaching and research, school of nursing and midwifery, Griffith University, Brisbane City, Australia; [5]Department of anesthesia, Levanger Hospital, Clinic of Surgery, Nord-Trøndelag Hospital Trust, Levanger, Norway; [6]Department of infectious diseases, St. Olavs Hospital, Trondheim, Norway; [7]Clinic of anaesthesia and intensive care, St.Olavs Hospital, Trondheim, Norway; [8]Institute of circulation and medical imaging, Norwegian University of Science and Technolog, Levanger, Norway
**Correspondence:** L.H. Høvik
*Intensive Care Medicine Experimental* 2019, **7(Suppl 3):**000762

**INTRODUCTION.** Peripheral intravenous catheter (PIVCs) is the easiest, quickest and least resource demanding way to administer intravenous fluids, medication and blood transfusion. PIVCs are considered harmless devices, however they account for a mean of 38% of catheter associated bloodstream infections (CABSI) of *Staph.aureus* (1), which is a serious complication often in need of long antibiotic treatment, ICU stay and with high mortality. PIVC related CABSI are preventable complications if PIVC quality is addressed properly. However, there exist no quick validated tool to assess and improve PIVC quality and thereby CABSI reduction (2). Thus, we aimed to develop and test validity for an efficient screening tool regarding overall PIVC quality for systematic measurements of quality improvement to reduce PIVC related CABSI.

**METHODS.** The *PIVC-miniQ* consists of 16 items (yes/no) regarding observation of problems related to the insertion site, condition of dressing and equipment, documentation, and indication for use. Each problem gives one point and all items can be summed up in a total score (0-16). Two hospitals tested the *PIVC-miniQ* for feasibility and inter-rater agreement. Each PIVC was assessed twice, 2-5 minutes apart by two independent raters. We calculated the intraclass correlation coefficient (ICC) for each hospital and overall. For each of the 16 items, we calculated negative agreement, positive agreement, absolute agreement and Scott's pi.

**RESULTS.** Sixty-three raters evaluated 205 PIVCs in 177 patients. ICC between raters was 0.678 for hospital A, 0.577 for hospital B, and 0.604 for the pooled data. Mean time used for each PIVC assessment was 3.02 (SD 1.76) minutes, where most of the time was used to answer the documentation item. The most frequent insertion site symptom was "pain and tenderness" (14.4%), followed by "redness" (12.6%), whereas the most prevalent overall problem was lack of documentation of the PIVC (26.8%). Up to 50% of PIVCs were placed near joints or were inserted under suboptimal conditions, i.e. emergency department or ambulance.

**CONCLUSION.** We found the *PIVC-miniQ* sum score to be a reliable and efficient outcome measure for quality control, taking only 3 minutes on average to complete. The measure of consistency can be described as moderate to high with an ICC of 0.604 for the sum score. The observed PIVC quality were far from optimal and the *PIVC-miniQ* can thus reliably measure development in PIVC quality in point prevalence audits and evaluate interventions to reduce CABSI.

##### REFERENCE(S)

1. Mermel LA. Short-term Peripheral Venous Catheter-Related Bloodstream Infections: A Systematic Review. Clin Infect Dis. 2017;65(10):1757-62.
2. Ray-Barruel G, Polit DF, Murfield JE, Rickard CM. Infusion phlebitis assessment measures: a systematic review. J Eval Clin Pract. 2014;20(2):191-202.
3. The project is funded by The Liaison Committee for education, research and innovation in Central Norway.

### 000763

#### Should CDC's recommendations for promptly removing unnecessary central venous catheters be enhanced?

S. Iordanou,[1], N. Middleton[2], A. Kastoris[1], O. Lambrianidou,[1], C. Timiliotou-Matsendidou,[1], L. Palazis,[3], E. Papathanasoglou[1], V. Raftopoulos,[2]
[1]Intensive care unit, Limassol General Hospital, Kato Polemidia, Cyprus; [2]School of health sciences, Cyprus University of Technology, Limassol, Cyprus; [3]Intensive care unit, Nicosia General Hospital, Nicosia, Cyprus
**Correspondence:** A. Kastoris
*Intensive Care Medicine Experimental* 2019, **7(Suppl 3):**000763

**INTRODUCTION.** Catheter related bloodstream infections (CR-BSIs) not only increase hospital length of stay, affecting the cost of hospitalisation but may, as an independent factor, affect mortality. According to the CDC there is consensus regarding the strong recommendation for the prompt removal of intravascular access that is no longer essential, and the preference of peripheral venous catheters that seem to be rarely associated with bloodstream infection. The implementation of ultrasound guided peripheral venous cannulisation (UGPVC) seems to be helpful according to the relevant literature and clinical practice especially in cases of difficult or failed peripheral cannulisation especially in ICU patients.

**OBJECTIVES.** The main aim of this study is the incorporation of UGPVC and the subsequent examination of the effect of the use of ultrasound guidance for peripheral vein cannulisation on CR-BSIs.

**METHODS.** A retrospective cohort study was performed in the ICU of the Limassol General Hospital, Cyprus from January 2015 until December 2016. During the study data was collected regarding CR-BSIs using ICU protocol (ECDC-NHSN, HAI-ICU Protocol, v1.01 standard edition). Additionally, during 2016 data was collected regarding the use of UGPVC on patients where methods for vein cannulisation such as palpation from experienced practitioners were expected to be difficult or else failed. Patients were included in the study that were admitted in ICU for more than 48 hours. Data was collected regarding demographics, CVC utilization, acute physiology and chronic health evaluation (APACHE II), simplified acute physiology score, days of patient's device exposure, length of stay and outcome on discharge from ICU. During 2016 a UGPVC survey was conducted with additional data being collected regarding attempts of cannulation, number of succeeded or/and failed cannulations, peripheral cannulation site, cannula diameter size, and body mass index.

**RESULTS.** During 2015, surveillance data was collected for 198 (125 males) patients hospitalized in the ICU for a total of 2,269 ICU days. A total of 43 instances of DA-HAIs were detected in 25 of the 198 patients. CR-BSIs was the most commonly encountered type of infection accounting for 21(48.8%) incidents with a rate of 15.93 (9.9-24.3) per 1000 CVC days. During the 2016 period of the study, surveillance data was collected for 184 (113 males) patients hospitalized in the ICU for a total of 2,029 ICU days. A total of 24 instances of DA-HAIs were detected in 16 of the 184 patients. CR-BSIs was the least commonly encountered type of infection accounting for 4 (16.7%) incidents and a rate of 4.16 (1.1-10.6) per 1000 CVC days. CVC utilization ratios was reduced by 10.7% ($p<0.05$) (58% to 47%) and CR-BSI incidence rate was reduced by 11.7 per thousand device–days (15.9 to 4.16/1000 CVC days).

**CONCLUSION.** The reduction of CR-BSIs found in the current study (74% reduction) seems to be more than the reduction described in relevant literature (about 66%). The combination of five evidence-based procedures recommended by the CDC together with the utilisation of UGPVC, may effectively decrease the CR-BSIs, affecting costs, length of stay and even patient outcome.

### 000768

#### When MDRO positive ICU patient isolation and cohorting is not feasible, what comes next? "The Z Concept Approach"

S. Iordanou,[1], C. Timiliotou-Matsendidou,[1], N. Middleton[2], A. Kastoris[1], M. Mendris,[3], L. Palazis,[4], S. Kyratzi[3], V. Raftopoulos,[2]
[1]Intensive care unit, Limassol General Hospital, Kato Polemidia, Cyprus; [2]School of health sciences, Cyprus University of Technology, Limassol, Cyprus; [3]Microbiology department, Limassol General Hospital, Kato Polemidia, Cyprus; [4]Intensive care unit, Nicosia General Hospital, Nicosia, Cyprus
**Correspondence:** A. Kastoris
*Intensive Care Medicine Experimental* 2019, **7(Suppl 3):**000768

**INTRODUCTION.** Increased hospital exposure and transmission of Multi-Drug Resistant Organisms (MDROs), increase costs, length of hospital stay, morbidity and mortality and facilitate the need for increased surveillance and facility level prevention strategies. The CDC recommends in cases of positive MDROs cultures, single room isolation and specific dedicated staff allocation additional to standard contact precaution measures, and other prevention strategies. In the absence of single room facilities, the CDC advises the cohorting of patients with similar culture results. The compliance nevertheless with the recommendations of single room isolation and cohorting of patients may be impossible due to the ICU configuration.
**OBJECTIVES.** The development and implementation in the ICU of a unique MDRO transmission prevention and infection approach was evaluated. The approach was named the "Z Concept Approach".
**METHODS.** The study was conducted in the ICU of the Limassol General Hospital in Cyprus. Since isolation rooms and cohorting MDRO-positive patients was not feasible, the open plan ICU space was divided in theory in two grading zones (red and green zone) with application of behavioural restrictions, together with contact precautions based on the abovementioned zones. The effectiveness of the approach was studied in a prospective cohort study. Data regarding active MDRO screening was collected using a protocol based on the ICU (ECDC HAI-ICU Protocol, v1. 01 standard edition) for a period of four months. All patients admitted in the ICU, were screened for MRSA & MDROs by obtaining nasal and rectal culture swabs during admission, followed by weekly screening and on discharge. In the event of MDRO positive patients, pathogen/s species and resistance pattern were correlated with the rest of the infection positive ICU patients (colonization pressure), in order to locate or decline the patient/infection source.
**RESULTS.** During the 4-month study period, 78 patients were admitted to the ICU for a total of 942 patient days. In total 432 swabs for the detection of MRSA and MDROs were collected during the study period. Patients were monitored for a total of 942 ICU days leading to MDROs incident density of colonisation on admission of 17 per 1000 days [95% CI, 12. 1 – 31. 4]. Furthermore, the overall colonisation pressure during the study period, was 19/78 (24. 4%). A total of 17 (21. 8%) patients were admitted with a pre-existing infection and 2 (2. 5%) patients acquired an infection during their stay. The two microorganisms involved in the two acquired ICU infections were Acinetobacter baumanni and Pseudomonas aeruginosa, with one acquisition respectively. The acquisition rates for each of the MDROs in our ICU were 1.28 instances of A. baumanni and P. aeruginosa per 1000 patient days, while no acquisitions were documented for Klebsiella pneumoniae, Enterococcus faecalis and Staphylococcus aureus.
**CONCLUSION.** The low MDRO acquisition rates documented in the current study, indicated that the development and implementation of unique infection control strategies may effectively prevent pathogen transmission in the ICU. This strategy was successful even in an open plan ICU with high colonization pressure, inadequate space separation, and understaffing by nursing professionals.

### 000792

#### Catheter Associated Urinary Tract Infection Rate in Intensive Care Unit Patients

C. Kaymak[1], CA. Hatipoglu[2], M. Kotanoglu[1], FS. Erdinc[2], A. Ozcan[1], GT. Ertem[2], S. Kinikli[2], H. Basar[1]
[1]Department of anesthesiology and reanimation, University of Health Sciences, Ankara Health Application and Research Center, Ankara, Turkey; [2]Department of clinical microbiology and infectious diseases, University of Health Sciences, Ankara Health Application and Research Center, Ankara, Turkey
**Correspondence:** C. Kaymak
*Intensive Care Medicine Experimental* 2019, **7(Suppl 3):**000792

**INTRODUCTION.** The most important risk factor for the development of nosocomial Urinary Tract Infection (UTI) is the presence of a urinary catheter (UC). The duration of catheterization is also important for CAUTI development in patients with long-term Intensive Care Unit (ICU) requirements.
**OBJECTIVES.** Between the years of 2014-2018, the aim of this study was to evaluate the catheter associated urinary tract infection (CA-UTI) rate, invasive device use rate and causative microorganisms isolated in these infections.
**METHODS.** This study was conducted in an Anesthesiology and Reanimation Intensive Care Unit. Five year's surveillance data was evaluated in this study. CA-UTI rate per 1000 UC-days and device utilization ratios are retrospectively evaluated. Microbiological culture results of invasive device associated urinary tract infection were also evaluated. Standard laboratory methods were used to identify and test the susceptibility of the microorganisms, and standardized US Centers for Disease Control and Prevention (*CDC*) definitions was used for CA-UTI diagnosis (CA-UTI rate = CA-UTI number / Urinary catheter day x 1000).
**RESULTS.** 2944 patients were included in the study**.** Urinary catheter usage ratio was 97.2 % in the ICU for 41.972 catheter days. The mean rate of CA-UTI was 7.56. The total CA-UTI number was 271and the mean was 54.2/year. The CA-UTI rate was 10.8 in 2014. This rate was decreased to 5.3 in 2018, although the urinary catheter use ratio were similar. Most commonly detected causative agents were Escherichia coli (20.9 %), Klebsiella spp. (20.4 %), and Enterococcus spp. (14.8 %) for catheter associated urinary tract infections during the five-year study period. When we analyze by years, the most commonly isolated microorganisms were Candida spp. (20.2 %), Escherichia coli (21.6 %), Klebsiella spp. (18.7 %), Escherichia coli (26.4 %) and Klebsiella spp. (31.1 %), in 2014, 2015, 2016, 2017 and 2018, respectively.
**CONCLUSION.** Patients admitted to ICU are the most appropriate candidates for CA-UTI due to their more frequent necessity of urinary catheterization and longer duration of catheter use. CA-UTI rate in studies involving European countries was 62.2 %. Short-term catheterization rate was 90.8 %. Risk factors leading to CAUTI in ICU and microorganisms should be considered in order to prevent CAUTIs.

### 000828

#### A quality improvement strategy implementation for sternal wound infections after cardiac surgery. The infection control nurse perpective

E. Conoscenti[1], O. Campanella[2], L. Pensato[3], ML. Fazzina[3], C. Spina,[4], S. Caruso[5], M. Barone[3], R. Lombardo[6], SM. Gioe'[7], G. Arena[8], G. Martucci[9], A. Mularoni[10], M. Carvalho Laborne Valle[3], L. Massaud Ribeiro[3], A. Gonçalves Panisset[3]
[1]Rn infection control dept, ISMETT, Palermo, Italy; [2]Rn infection control dept., ISMETT, Palermo, Italy; [3]Quality control dept, ISMETT, Palermo, Italy; [4]Universita' degli studi di milano bicocca, ISMETT, Palermo, Italy; [5]Rn infection control dept., ISMETT, Palermo, Italy; [6]Rn education dept., ISMETT, Palermo, Italy; [7]Jr attending chief medical officer, ISMETT, Palermo, Italy; [8]Chief of nursing, ISMETT, Palermo, Italy; [9]Md dept of anaesthesia of intensive care, ISMETT, Palermo, Italy; [10]Md dept of infection control, ISMETT, Palermo, Italy
**Correspondence:** E. Conoscenti
*Intensive Care Medicine Experimental* 2019, **7(Suppl 3):**000828

**INTRODUCTION.** Sternal wound infections (SWI) following open-heart surgery are a serious complication that have a significant impact on patient morbidity, mortality and length of hospitalization. Despite the implementation of a SWI policy in 2017 in our in center , after one year the incidence of infection is still significant and it needs a proactive action from our team.
**OBJECTIVES.** Identify risks factors for SWI and lack of adherence to our policy. Plan strategies to decrease SWI.
**METHODS.** Review of our SWI prevention policy based on Lazar at al.2016 recommendations.Retrospective review of 684 cardiac surgical procedures performed in 2018. A dedicated Infection Control Nurse (IC) reviewed charts and conducted audits during the pre-op admission, assessing for SWI risks factors and surgeons' performance. Descriptive statistics and plan for quality improvement are presented
**RESULTS.** 684 procedures were performed. Age 64±12.4 , 65% were male. Surgeries: 259 valve replacements, 38%; 116 valvuloplasty, 17%; 116 CABG, 17%; 13 transplants, 2 %; 26 type A aortic dissection 4%; 150 others, 22%.EuroScore 3.39± 4.98,length of stay in ICU 3,56± 6,3,lactate level 4,06 ±4,45,SOFA score4,9±2,48,CEC 111.1±53,38.

SWI were n=24 (3,4%) compared to 3,6% in 2017with a benchmark in literature of 0.5%.

SWI age : 64 ± 12.4, 33% were male. Surgeries: 15 Valve replacements 63%; 6 CABG 25%; 1 transplant,4%; 1 type a Aortic dissection 4%,1 other4%

15 Patients(63%) had pre-op outpatient consultation, 9(37%) were transferred from other hospitals and had no infection prevention screening. No evidence of Pre- op patient education on nutritional status or smoking cessation.

N=9 ;60% had a nasal swab performed pre-op, 4 (44%) were positive to Staph. Aureus, and for them there was no evidence of Mupirocin therapy and Chlorhexidine 4% shower prescription. Showers with chlorhexidine 4% were prescribed for 21 patients (88%), but a lack of accurate documentation makes it impossible to assess this.

Oncall antibiotics were administered to 100% of patients, however, there was no standardized documentation on timing for the second dose. Intraoperative glycemic control was rarely performed. On 86% of the 15 patients, insulin drip was started post-op.

We identified grey areas in which we need to improve.Mupirocin will be prescribed to all patients undergoing cardiac surgeries, whether they are positive or negative;our IT team will make some changes on the electronic medical and nursing notes in order to obtain accurate nursing and medical records, an educational brochure and video on smoking cessation, nutrition, diabetes control, and oral care will be provided to patients pre-op.
**CONCLUSION.** Despite the presence of an up to date policy, the rate of SWI is still high at our center.The audit was able to highlight grey zone in clinical practice that were used to revise the current policy in SWI prevention.The role of an IC nurse dedicated to SWI will be crucial in identifying SWI champions to further train staff and create a multidisciplinary network for SWI prevention.The involvement of leadership will be decisive in order to put into place what we have on policy.

### 001067

#### Assessment of enteral paromomycin to eradicate colistin and carbepenemase resistant microrganisms in rectal colonization to prevent ICU- acquired infections

C. Sánchez Ramírez[1], MA. Hernández Viera[1], RE. Morales Sirgado[1], M. Cabrera Santana[1], S. Hípola Escalada[1], L. Caipe Balcázar[1], SM. Marrero Penichet[2], CF. Lübbe Vázquez[1], F. Artiles Campelo[3], S. Ruiz-Santana[1]
[1]Intensive care medicine, University Hospital of Gran Canaria Dr. Negrin, Las Palmas de Gran Canaria, Spain; [2]Pharmacy department, University Hospital of Gran Canaria Dr. Negrin, Las Palmas de Gran Canaria, Spain; [3]Microbiology department, University Hospital of Gran Canaria Dr. Negrin, Las Palmas de Gran Canaria, Spain
**Correspondence:** C. Sánchez Ramírez
*Intensive Care Medicine Experimental* 2019, **7(Suppl 3):**001067

**INTRODUCTION.** Paromomycin is a aminoglycoside antibiotic. Emergence of bacterial resistance led to many of us to consider the combination of antimicrobial agents. We tested the effect of paromomycin on isolates of multidrug resistant pathogens (MDR) including: *Pseudomonas aeruginosa, Klebsiella pneumoniae, Escherichia coli,* and methicillin-resistant *Staphylococcus aureus*. Paromomycin combined with ceftriaxone, ciprofloxacin, ampicillin/sulbactam, azithromycin, clindamycin or doxycycline showed mostly synergistic effect on these clinically important MDR pathogens. We routinely apply Selective Digestive Decontamination (SDD) with topic colistin. However, some patients had rectal colistin and / or carbepenemase (CPN) resistant microorganisms colonization. The aim of this study was to administrate enteral paromomycin to decontaminate these MDR microorganisms and to prevent the development of ICU nosocomial infections.
**METHODS.** All consecutive patients admitted to the ICU from October 2011 to June 2017, expected to require tracheal intubation for longer than 48 hours were given SDD with a 4-day course of intravenous cefotaxime, plus enteral colistin, tobramycin and nystatin in an oropharyngeal paste and in a digestive solution. Patients with rectal swabs colonized by colistin and / or carbepenemase resistant microorganisms were treated with enteral paromomycin 1 gr every 6 hours a day, in order to become it negative and prevent nosocomial infections. Categorical variables were summarized as frequencies and percentages and the continuous ones as medians and interquartile ranges (IQR) or means and standard deviations. Statistical significance was set at $p \leq 0.05$.
**RESULTS.** We applied paromomycin treatment to 102 colonized patients with rectal colistin resistant microorganisms. All of them but six had colonization by Extended Spectrum Beta-lactamases (ESBLs) producing *Klebsiella pneumoniae.* Two patients was colonized by ESBL producing

Table 1. Patients data

| | |
|---|---|
| **Patients, n** | 102 |
| **Age, years, SD** | 65,1± 14,3 |
| **Male/female, n, %** | 69(67,69/ 31 (30,4) |
| **APACHE II on admission, n(IQR)** | 22(16;7,28) |
| **SOFA on admission, n(IQR)** | 9,5(7;11,2) |
| **APACHE II at paromomycin treatment, n(IQR)** | 17(13;21) |
| **SOFA at paromomycin treatment, n(IQR)** | 5(3;9) |
| **Glasgow Coma Score, n(IQR)** | 14 (6;15) |
| **ICU stay days, n(IQR)** | 78 (42,2;129) |
| **Renal Replacement Therapy, n (IQR)** | 43 |
| **Trauma patients, n** | **9** |
| **Medical patients on admission, n** | **51** |
| **Parenteral nutrition, n** | **23** |
| **Diabetes mellitus, n** | **43** |
| **Neutropenic patients, n** | **2** |
| **Immunosuppression, n** | **11** |
| **Deaths, n** | 47 |
| **Paromomycin treatment days, n(IQR)** | 13.5 (8; 27) |

ICU: Intensive Care Unit; n: number; MV. Mechanical Ventilation; SD: estándar deviation; IQR:interquartile range; n:number

*Enterobacter spp* , other one by ESBL producing *Escherichia coli*., 1 by *Acinetobacter baumannii* and 2 by *Serratia marcescens.* Demographic data and type of admission are shown in Table 1. Seventy-nine out of 102 (80.5%) negativized the rectal exudate after paromomycin. Of those negativized, 26 patients received an appropriate antibiotic during the application of paromomycin. Five out of the seven patients with CPN were decolonized. 47 patients died in the ICU and 24 of them, died without being de-colonized being two of them with CPN resistant microorganisms. Only 3 patients developed infections after decolonization.

**CONCLUSION.** Our data show that enteral paromomycin is effective in treating rectal colistin and / or carbapenemase resistant microorganism colonization to prevent the development of ICU nosocomial infections.

#### 001088

**Selective digestive decontamination in a mixed ICU: seven years impact on colonization, nosocomial multi-resistant infection and antibiotic consumption**

C. Sánchez Ramírez[1], M. Cabrera Santana[1], MA. Hernández Viera[1], S. Hípola Escalada[1], RE. Morales Sirgado[1], L. Caipe Balcázar[1], SM. Marrero Penichet[2], CF. Lübbe Vázquez[1], F. Artiles Campelo[3], JL. Vicente Arranz[1], P. Saavedra-Santana[4], S. Ruiz-Santana[1]
[1]Intensive care medicine, University Hospital of Gran Canaria Dr. Negrin, Las Palmas de Gran Canaria, Spain; [2]Pharmacy department, University Hospital of Gran Canaria Dr. Negrin, Las Palmas de Gran Canaria, Spain; [3]Microbiology department, University Hospital of Gran Canaria Dr. Negrin, Las Palmas de Gran Canaria, Spain; [4]Mathematics and informatics deparment, University of Las Palmas, Las Palmas de Gran Canaria, Spain
**Correspondence:** C. Sánchez Ramírez
*Intensive Care Medicine Experimental* 2019, **7(Suppl 3):**001088

**INTRODUCTION.** Selective digestive decontamination (SDD) have been associated with reduced mortality and lower ICU-acquired rates However, the effect SDD in areas where multidrug-resistant Gram-negative bacteria are endemic is less clear. We want to prospectively.evaluate the impact of SDD application on nosocomial muli-resistant (MR) infections (NI) and colonization rates, after 7 years in a mixed ICU.

**METHODS.** This study was conducted in a 30-bed-medical-surgical ICU. All consecutive patients admitted to the ICU from October 1, 2011 to September 30, 2018 expected to require tracheal intubation > 48 hours were given SDD (SDD study group) with a 4-day course of intravenous cefotaxime, plus enteral colistin, tobramycin, nystatin in an oropharyngeal paste and in a digestive solution. Oropharyngeal and rectal swabs were obtained on admission and once weekly. We used ENVIN NI criteria. We compared all patients admitted to ICU with ICU NI from October 1, 2010 to September 30, 2011 (non-SDD group) to the SDD study group. A univariate and a multivariate logistic regression analysis was performed. For each one of the infections the incidences per 1000 days of exposure in each cohort and the corresponding relative risks were obtained using the Poisson regression. Statistical significance was $p \leq 0.05$.We analyzed colistin- and tobramycin-resistant colonization and also antibiotic consumption as Defined antibiotics Daily Doses (DDD).

| | *Selective decontamination decontamination* No N = 110 | Yes N = 494 | P |
|---|---|---|---|
| Age, years | 59.5 ± 15.8 | 61.6 ± 15.0 | 0.200 |
| APACHE II score | 21.2 ± 7.7 | 22.2 ± 7.5 | 0.211 |
| Sex male | 74 (67.3) | 322 (65.3) | 0.125 |
| Trauma patients | 17 (15.4) | 56 (11.4) | 0.234 |
| Coronary arteria disease patient | 19 (17.3) | 112 (22.7) | 0.387 |
| Emergency surgery | 34 (30.9) | 121 (24.5) | 0.164 |
| Immunosupression | 8 (7.3) | 52 (10.5) | 0.302 |
| Neutropenia | 3 (2.7) | 21 (4.2) | 0.596 |
| Immunodepression | 3 (2.7) | 2 (0.4) | 0.044 |
| Parenteral nutrition | 26 (23.6) | 141 (28.5) | 0.298 |
| RRT | 34 (30.9) | 192 (38.9) | 0.287 |
| Malnutrition | 12 (10.9) | 49 (9.9) | 0.198 |
| Diabetes mellitus | 34 (30.9) | 155 (31.4) | 0.924 |
| COPD | 9 (8.2) | 82 (16.6) | 0.026 |
| Renal failure | 40 (36.4) | 101 (20.4) | < 0.001 |
| Cirrhosis | 6 (5.5) | 27 (5.5) | 0.264 |
| Neoplasm | 10 (9.1) | 52 (10.5) | 0.654 |
| VAP | 59 (53.6) | 168 (34.0) | < 0.001 |
| CRB | 26 (23.6) | 190 (38.5) | 0.003 |
| Secondary bacteremia | 31 (28.2) | 127 (25.7) | 0.699 |
| Urinary infection | 29 (26.4) | 131 (26.5) | 0.973 |
| ATB 48 hours before admission | 28 (25.4) | 135 (27.3) | 0.028 |
| Death | 37 (33.6) | 190 (38.5) | 0.345 |
| *Acinetobacter baumannii* | 13 (11.8) | 5 (1.0) | < 0.001 |
| MRSA | 4 (3.6) | 8 (1.6) | 0.246 |
| ESBL | 38 (34.5) | 104 (21.1) | 0.003 |
| *MR Pseudomonas* | 10 (9.1) | 37 (7.5) | 0.638 |
| MR GNB | 12 (10.9) | 21 (4.2) | 0.005 |
| Admission: | | | 0.165 |
| Medical | 79 (71.8) | 354 (71.8) | |
| Scheduled surgery | 10 (9.1) | 71 (14.4) | |
| Emergency surgery | 21 (19.1) | 68 (13.8) | |
| Non sepsis | 2 (1.8) | 23 (4.7) | |
| Sepsis | 23 (20.9) | 151 (30.6) | |
| Septic shock | 57 (51.8) | 320 (64,7) | |
| ICU days | 28.0 (16.0 - 44.8) | 32.5 (19.0 - 50.0) | 0.052 |

RRT: Renal replacement therapy; VAP: ventilator associated pneumonia;CRB: Catheter related bacteremia; COPD: chronic obstructive pulmonary disease; MRSA: methicillin resistant Sthaphyloccocus aureus; ESBL: extended spectrum belalactamase; MR: multiresistant; GNB: gram negative bacteria

| | P | OR (95% CI) |
|---|---|---|
| ***VAP*** | < 0.001 | 0.473 (0.306 - 0.731) |
| ***Renal failure*** | 0.002 | 0.481 (0.301 - 0.768) |
| ***Acinetobacter infections*** | < 0.001 | 0.094 (0.032 - 0.277) |

SDD: Selective Digestive Decontamination; VAP: ventilator associated pneumonia

| | | *SDD* No | Yes | P | RR (95% CI) |
|---|---|---|---|---|---|
| *VAP /MV* | VAP/1000 days of MV | 10.31 | 4.04 | < 0.001 | 0.392 (0.295 - 0.522) |
| *Urinary infections* | Infections/1000 days of urinary catheter | 3.79 | 2.41 | 0.019 | 0.637 (0.437 - 0.927) |
| *CRB* | CRB/1000 days of CVC | 3.59 | 3.80 | 0.785 | 1.058 (0.704 - 1.590) |
| *Secondary bacteremias* | Bacteremias./1000 ICU days | 4.69 | 2.08 | < 0.001 | 0.444 (0.315 - 0.624) |
| *Multiresistant germs* | Multiresistant germs/1000 ICU days | 9.59 | 2.67 | < 0.001 | 0.279 (0.216 - 0.360) |

VAP: ventilator associated pneumonia; MV: mechanical ventilation; CRB: Catheter related bacteremia; CVC: Central venous catéter.

**RESULTS.** Results are shown in Tables 1, 2 and 3. There were no statistical significant differences between both groups in type of admission or demographic data. Patients with SDD had significantly less Extended Spectrum Betalactamase (ESBL), Gram Negative Bacteria Multirresistant (GNB-MR) and *Acinetobacter spp* infections. We had also a significant reduction in ventilator associated pneumonias ( VAP), urinary infections and other secondary bacteremias and antibiotic resistant bacteria infection rates, in SDD group versus non SDD. There was no infection by *Clostridium difficile*. The exogenous infections were 499 (74%). Colistin resistant colonization was 16,5% and tobramycin resistant colonization was 25,7% of samples. There was a decrease on the DDD/100 ICU stays after SDD.

**CONCLUSION.** After 7 years applying SDD a significant reduction of infections by ESBL, GNB-MR and *Acinetobacter,* was observed. A significant decrease of VAP, secondary bacteremias, urinary and ARB infections rates was also shown. An antibiotic consumption reduction was found after SDD. Low rates of colistin and tobramycin-resistant colonization bacteria were observed

## CD - Managing haemodynamic challenges

#### 000017

**Should I admit this patient with pulmonary embolism to the ICU?**

D. Pérez-Torres, V. Fraile-Gutiérrez, E. Prol-Silva, JA. De Ayala-Fernández, C. Díaz-Rodríguez, GJ. Posadas-Pita, P. Blanco-Schweizer, JJ. Sanz-Hernán, PM. Enríquez-Giraudo
Department of intensive care medicine, Hospital Universitario Río Hortega, Valladolid, Spain
**Correspondence:** D. Pérez-Torres
*Intensive Care Medicine Experimental* 2019, **7(Suppl 3):**000017

**INTRODUCTION.** Risk stratification is essential to decide the best location and resource use for each patient.

**OBJECTIVES.** To describe the risk factors and their association with mortality of the patients with pulmonary embolism (PE) who are admitted to the Intensive Care Unit (ICU).

**METHODS.** We conducted a retrospective observational study in the Intensive Care Unit of a single university hospital. All the patients who were admitted to the ICU with PE as a primary diagnosis were included, over an 8-year period. Demographics, comorbidities and worst values of vital signs and laboratory tests within the first 24 hours of admission were registered. Risk stratification was performed with the simplified Pulmonary Embolism Severity Index (sPESI) and according to the recommendations of the European Society of Cardiology (ESC). Chi-square, Fisher's exact test and Student's T test were applied as appropriate. Bivariate analysis was performed to show the relationship between variables and mortality.

**RESULTS.** A total of 82 patients were included, 43% male, aged 64 (44-77), APACHE-II 12 (8-17), 42% fibrinolytic therapy, 15% hospital mortality. Mortality risk according to sPESI: high 82%, low 18%. Mortality risk according to ESC: high 34%, intermediate 56%, low 10%. Common comorbidities were: arterial hypertension 34%, dyslipidaemia 22%, hospital admission/surgery/trauma (HA/S/T) within the previous 3 months 22%, smoking 18%, previous episode of deep venous thrombosis (DVT) 12%, use of oral contraceptives 12%, diabetes mellitus 11%, neoplasm 10%, autoimmune disorder 8%, thrombophilia 6%, previous episode of PE 6%. Reasons for admission were: recovered cardiopulmonary arrest (CPA) 5%, cardiogenic shock (CS) 11%, acute heart failure 7%, acute respiratory failure (AFR) requiring mechanical ventilation (MV) 17%, ARF with no need for MV 29%, high-risk stratification with clinical stability 30%. Risk factors for mortality were: male sex (OR 3.3, p=0.07), presence of ≥1 cardiovascular risk factor (OR 1.7, p=0.39), HA/S/T (OR 3.1, p=0.07), previous DVT/PE (OR 1.1, p=0.93), presence of ≥2 items in the sPESI (OR 16.5, p<0.01), severe form of presentation (CPA, CS, ARF with MV) (OR 37.1, p<0.01). Vital signs and laboratory test results, survivors vs deceased: MAP 107 vs 81 mmHg, p<0.01; HR 103 vs 122 bpm, p=0.02; SpO2 91 vs 85%, p=0.05; RR 25 vs 30 bpm, p=0.05; haemoglobin 13.2 vs 11.8 g/dl, p=0.03; platelets 232 vs 168×$10^3$/μl, p=0.03; creatinine 0.95 vs 1.59 mg/dl, p<0.01.

**CONCLUSION.** The presence of ≥2 items in the simplified PESI score predicts mortality better than the simply positive result (≥1 item) within patients with PE who are admitted to the ICU. These patients might benefit from a closer and more aggressive management in the ICU.

Severe forms of presentation (CPA, CS or ARF requiring MV) dramatically increased the risk of mortality, and should be admitted to the ICU. However, high-risk stratification with clinical stability was not associated with mortality, so admission in a regular ward might be safe.

Patients who died because of a PE showed more hypotension, tachycardia and kidney failure within the first 24 hours of presentation, so admission to the ICU should be considered when these parameters are abnormal.

### 000054

#### Mortality predictors in patients admitted in Non-cardiac ICU with cardiogenic shock

H. Miranda, I. Milet, I. Militão, N. Barros, H. Leite, F. Esteves
Intensive Medicine Care, Hospital São Pedro de Vila Real (Centro Hospitalar de Trás-os-Montes e Alto Douro, EPE), Vila Real, Portugal
**Correspondence:** H. Miranda
*Intensive Care Medicine Experimental* 2019, **7(Suppl 3):**000054

**INTRODUCTION.** Cardiogenic shock (CS) is a state of critical end-organ hypoperfusion due to primary cardiac dysfunction. Most epidemiological data for CS focus on patients with acute myocardial infarction managed in intensive care units (ICUs) of cardiology departments. Little is known about the most severe forms of CS, with multi-organ failure, managed in general ICUs.

**OBJECTIVES.** Identify main predictors of mortality in CS and evaluate their impact in the outcome. Evaluate usefulness of SOFA, APACHE II and SAPS II scores in predicting the outcome in CS patients (P) and identify the most useful one, if applicable

**METHODS.** Retrospective analysis of patients (P) admitted in our ICU, with confirmed diagnosis of CS, within a period of 5 years (January 2012- December 2016). We analyzed common epidemiological variables, evolution during ICU stay, established therapeutics and outcome.

**RESULTS.** 90 P were included. The mean age of the population was 69,59 ± 12,23 years, with a predominance of males (56,7%). Majority of P coming from the Emergency Room (45,6%) and 26,7% presenting cardiopulmonary arrest at admission. Admission SOFA of 10,39 ± 3,19. The main cause of CS was non-ischemic (66,7%). In 27,8% of P the presence of mixed shock was verified. 68,9% needed mechanical invasive ventilation. Maximum PEEP of 8,22 ± 2,76 and median weaning of 3 days during ICU stay, respectively. PaO2/FiO2 ratio and lactates at admission of 178,5 and 2,85, respectively. All the patients needed aminergic support. 34,4% needed renal replacement therapy. At discharge, the patients presented median ICU stay of 5 days with SOFA, APACHE II and SAPS II of 7.6±5,06, 24,5 and 56,61 ± 19,71, respectively. Hospital mortality of 45,6%.

We found a statistically significant association between outcome and:1) etiology (p=0,024), 2) admission SOFA (p=0,006), 3) APACHE II (p< 0,001), 4) SAPS II (< 0,001) and 5) lactates variation in the first 24h (p=0,027). We also point out that after applying a logistic regression, to compare the impact of the used scores, only APACHE II (OR: 1,13; IC95%: 1,028-1,253) had relevant prediction power.

**CONCLUSION.** CS requires rapid diagnosis and appropriate therapy to have a positive influence on the outcome. In our study, we found that APACHE II was the best score to use in this kind of patients.

### 000093

#### Modifiable risk factors of prolonged dependence on vasopressors after cardiac surgery: retrospective cohort study

L. KONTAR[1], W. Beaubien-Souligny,[2], V. Bouchard-Dechêne[3], A. Rochon[2], S. Levesque[4], Y. Lamarche[5], A. Denault[2]
[1]intensive care , Montreal Heart Institute, Montréal, Canada; [2]Intensive care, Montreal Heart Institute, Montréal, Canada; [3]Intensive care, Basilique Notre Dame, Montréal, Canada; [4]Montreal health innovations coordinating centre, Montreal Heart Institute, Montréal, Canada; [5]Cardiac surgery, Montreal Heart Institute, Montréal, Canada
**Correspondence:** L. KONTAR
*Intensive Care Medicine Experimental* 2019, **7(Suppl 3):**000093

**INTRODUCTION.** Cardiac surgery performed with cardiopulmonary bypass (CPB) is frequently complicated by hemodynamic [1] instability due either to an inflammatory response [2] or to cardiac dysfunction during or immediately after the weaning from CPB [3]. There are limited studies on the incidence and the risk factors of prolonged vasopressor dependence following cardiac surgery [4].

**OBJECTIVES.** The objective of the study was to identify risk factors related to prolonged dependence on vasopressors after weaning from bypass.

**METHODS.** Approval has been obtained by our Research Ethics Committee. In this single-center retrospective cohort study, we analyzed data from consecutive adult patients who underwent cardiac surgery with CPB enrolled in two prospective studies between November 2016 and July 2017 in a specialized cardiac surgery center. The study excluded all cardiac surgery without CPB, heart transplantation and ventricular assist devices. Postoperative prolonged vasopressor dependence was defined as the need for at least one vasopressor agent from the end of CPB for a duration greater than 24 hours. Vasopressor agents included norepinephrine, vasopressin, epinephrine, dopamine and phenylephrine. A follow-up was conducted for all patients until the end of intensive care unit (ICU) day 1. A multivariable logistic regression model using a forward stepwise selection approach was developed to identify independent predictors of vasopressor dependency.

**RESULTS.** A total of 247 out of 263 patients underwent cardiac surgery with CPB between November 2015 and July 2017 using our exclusion criteria. The mean age of the study patients was 65 ± 12 years old and 126 (51%) were female (Table 1). The incidence of prolonged vasopressor dependence was 40% and was associated with more prolonged mechanical ventilation (5h (IQR4-9) vs. 4h (IQR3-5); p<0.001), prolonged ICU stay (3 days (IQR1-2) vs. 1 day (IQR1-2); p< 0.001) as well as hospital stay (7 days (IQR6-10) vs. 5 days (IQR4-7); p<0.001). In multivariable analysis, pre-existing reduced left ventricular ejection fraction (LVEF<30%) (OR: 9.52, 95%CI:1.14-79.24; p=0.03), preoperative pulmonary hypertension (PH) (moderate PH (sPAP≥30 but <55mmHg) OR:2.5, 95%CI:1.14-5.52, severe PH (sPAP>55mmHg) OR:8.12, 95%CI:2.53-26.02; p=0.001) and first 24h cumulative fluid balance (OR:1.78, 95%CI:1.41-2.24; p<0.0001) were independently associated with the development of prolonged vasopressors dependence: with a good ability to predict vasoplegia after cardiac surgery based on ROC analysis (AUC=0.80, 95%CI: 0.73-0.86; p<0.0001).

**CONCLUSION.** Vasopressor dependency remains a frequent complication after CPB surgery. Its associations with PH and large fluid balance is unreported and potentially reversible. Prospective studies and clinical trials should explore the role of these two factors in future studies.

**REFERENCE(S)**

1. J Thorac Cardiovasc Surg 1998;116:973-980.
2. J Card Surg 2000;15:347-353.
3. Curr Opin Anaesthesiol 2013;26:71-81.
4. Anaesthesia 2006;61:938-942.
5. yes

### 000108

#### The use of levosimendan on weaning from venous-arterial extracorporeal mechanical oxygenation support: results of a tertiary centre

F. Caetano[1], V. Templeman[1], C. Remmington[1], M. Passariello[1], R. Trimlett[2], S. Price[1]
[1]Adult intensive care unit, Royal Brompton & Harefield Nhs Foundation Trust, London, United Kingdom; [2]Cardiac surgery, Royal Brompton & Harefield Nhs Foundation Trust, London, United Kingdom
**Correspondence:** F. Caetano
*Intensive Care Medicine Experimental* 2019, **7(Suppl 3):**000108

**INTRODUCTION.** Levosimendan (Lv) is a calcium sensitizing inotrope indicated for short-term treatment of acutely decompensated severe chronic heart failure (1). Venous-arterial extracorporeal membrane oxygenation (VA-ECMO) provides temporary mechanical circulatory support in patients with refractory cardiogenic shock (2). Few data is available on the impact of Lv on VA-ECMO weaning.

**OBJECTIVES.** Evaluate the safety and efficacy of Lv on VA-ECMO weaning in patients admitted to intensive care.

**METHODS.** Retrospective study of 68 patients (57% male; mean age 48.9 years) who received Lv to assist weaning from VA-ECMO.

Successful outcome was described as VA-ECMO decannulation within 48 hours after Lv infusion and patients had also to be alive for, at least, 48 hours after the procedure.

Safety data (hypotension, arrhythmias and hypokalaemia) was recorded.

**RESULTS.** More than half of the patients (54.4%) were established on VA-ECMO after cardiac surgery.

The first Lv infusion was started ± 7 days after admission to intensive care. 2 patients received loading dose. The infusion ran for ± 23 hours and the maximum infusion rate (0.2mcg/kg/min) was achieved in 66.2%.

After the first Lv infusion, 4 patients (5.9%) were successfully bridged to a long term ventricular assist device (VAD) and 26 (40.6%) were successfully weaned off VA-ECMO. On the remaining group of 38 P, 10 received at least one further dose of Lv, 7 or more days after the first dose, enabling successful VA-ECMO decannulation in other 4 patients and bridging to VAD in 1 patient.

Side-effects analysis documented: 53% of clinical significant hypotension and 25% of new atrial fibrillation. One patient had ventricular fibrillation and 3 patients hypokalaemia.

The average ITU length of stay was 25 days and 44.1% of patients survived intensive care admission.

**CONCLUSION.** In this heterogeneous group of patients that required VA-ECMO for cardiogenic shock, the administration of Lv prior to trial of decannulation was associated with successful weaning in 51% of the patients. Nevertheless, the incidence of known side-effects, namely hypotension and arrhythmias, cannot be neglected, highlighting the high level of monitoring that this specific population demands.

The identification of surrogate markers of potential successful decannulation is warranted, enabling a more tailored therapy.

**REFERENCE(S)**

(1) De Luca L, Heringlake M, et al. Evidence-based use of levosimendan in diferente clinical settinggs. Vol 27, European Heart Journal. 2006, p. 1908-20.
(2) Wilson-Smith AR, et al. Outcomes of venoarterial extracorporeal membrane oxygenation for refractory cardiogenic shock: systematic review and meta-analysis. Ann Cardiothorac Surg. 2019 Jan;8(1):1-8.

### 000147

#### The accuracy and trending ability of cardiac index measured by the fourth-generation of FloTrac/Vigileo and transpulmonary thermodilution devices in septic shock patients

B. Khwannimit, J. Rattina
Prince of songkla university, Department of Internal Medicine, Faculty of Medicine, Hat Yai, Thailand
**Correspondence:** B. Khwannimit
*Intensive Care Medicine Experimental* 2019, **7(Suppl 3):**000147

**INTRODUCTION.** Cardiac index (CI) is one of the hemodynamic variables to be assessed and monitored in shock patients. FloTrac/Vigileo is a less-invasive uncalibrated arterial pressure waveform analysis. However, its validation is limited in hemodynamically unstable septic shock patients.

**OBJECTIVES.** The aim of our study was to compare CI measured by 4th generation of FloTrac/Vigileo device with transpulmonary thermodilution obtained by PiCCO in septic shock patients.

**METHODS.** A prospective observational study was performed in the Medical Intensive Care Unit. We simultaneously measured the CI using a Vigileo device (CIv) and compared it with the CI derived from transpulmonary thermodilution (CItp) as well as the pulse contour-derived CI using PiCCO (CIp). The measurement agreements were evaluated using the Bland-Altman analysis, percentage error and concordance analysis.

**RESULTS.** Thirty-one septic shock patients were included. Their mean APACHE II score was 26.5±8.1. All of the patients received norepinephrine with a mean dose of 0.33±0.18 ug/kg/min. In the comparison of CIv with CItp, there were 156 data pairs. The CIv and CItp values ranged from 1.5 to 6.8 and 1.5 to 6.9 L/min/m2, respectively. CIv correlated with CItd as indicated by r = 0.62 (p< 0.001). The Bland-Altman analysis corrected for repeated measurements showed a bias of 0.14, and the limits of agreement were -1.62 to 1.91 L/min/m2 with a percentage error of 48.4%. When comparing CIv with CIp (n=352 paired measurements), CIv ranged from 1 to 6.2 and CIp ranged from 1.4 to 6.9 L/min/m2. Here, the Bland-Altman analysis revealed a bias of -0.163, and the limits of agreement were -1.46 to 1.79 L/min/m2 with a percentage error of 45.7%. The overall correlation coefficient between CIv and CIp was 0.63 (p<0.001). Moreover, a four-quadrant plot analysis was performed to evaluate the trending ability of ΔCI change between the two devices. The concordance rate between CIv *vs.* CItd was 95.6%, and that for CIv *vs.* CIp was 85.4%. There were 56 and 16 pairs of CI measurement performed before and after the increase of norepinephrine dose and fluid bolus, respectively. In terms of the absolute changes in CIv and CIp induced by the increased dose of norepinephrine, the bias value was -0.47, and the limits of agreement were -1.73 to 0.8 L/min/m2. In addition, the coefficient of correlation and concordance rate between the percent changes in CIv and in CIp were 0.8 (p< 0.001) and 95.8%, respectively. However, the bias was 0.05 and the limits of agreement between the absolute changes in CIv and CIp induced by volume expansion were -0.28 to 0.38 L/min/m2. Finally, concerning the percent changes in CIv and in CIp induced by fluid expansion, the coefficient of correlation and concordance rate was 0.49 (p=0.05) and 83%, respectively.

**CONCLUSION.** Our study demonstrated a moderate agreement between the CI derived from a 4th generation FloTrac/Vigileo device with that using the transpulmonary thermodilution method. However, the uncalibrated CI measured using updated FloTrac software exhibited a more reliable trending ability to track changes in CI compare to transpulmonary thermodilution CI obtained by PiCCO.

### 000151

#### Pharmacological scores for monitoring of hemodynamic profile and clinical outcomes in the cardiac surgery

A. Ksendikova, S. Belolipetskiy, A. Radovskiy, L. Karpova, A. Bautin
Anesthesiology and intensive care medicine, Almazov National Medical Research Centre, Saint Petersburg, Russia
**Correspondence:** A. Ksendikova
*Intensive Care Medicine Experimental* 2019, **7(Suppl 3):**000151

**INTRODUCTION.** Pharmacological scores, such as inotrope score (IS) and vasoactive-inotropic score (VIS) were created to improve the adrenergic dose evaluation in infants cardiac surgery. Later these scores have started to use in adult. However, the significance of IS and VIS as an indicator of hemodynamic profile and predictor of the clinical outcomes in adult cardiac surgery remains unclear.

**OBJECTIVES.** To estimate IS and VIS as an approach for monitoring of the hemodynamic profile and clinical outcomes in adult cardiac surgery.

**METHODS.** 144 patients over 18 year had undergone cardiac surgery with CPB were enrolled in prospective observational study. The average age was 63.6 ± 10.4 years. Hemodynamic was estimated by a Swan-Ganz catheter, at the same time points we calculated IS and VIS using standard formulas. For further analysis, we used data that was obtained 6 h after ICU admission. Differences were assessed by the Mann-Whitney and Fisher's exact tests. Data are presented as: median (25th percentile, 75th percentile).

**RESULTS.** At 6 h after ICU admission, VIS ≥10 was observed in 53/144 patients (36.8%), when only 7/144 (4,9%) patients had IS ≥10.

VIS ≥10 did not show high prognostic significance for adverse outcomes. Thus, in the ROC-analysis, the area under the curve (AUC) of the duration of respiratory support (RS) was only 0.58, and AUC of ICU length of stay (LOS) was 0.59. There was no significant difference between VIS ≥10 and VIS <10 in the duration of RS (12 (6; 18) vs 9 (5; 16) h, p = 0,07), LOS (24 (20; 58) vs 22 (19; 24) h, p = 0,06) and mortality (3.8% vs 0, p = 0,13).

IS ≥10 was significantly associated with adverse postoperative outcomes. ROC analysis showed good prognostic models for both the duration of RS (AUC = 0.86) and the LOS (AUC = 0.91). We found significant differences between IS ≥10 and IS <10 in the duration of RS (83 (15; 120) vs 9 (5; 16) h, p = 0,002), LOS (85 (60; 143) vs 22 (19; 26) h, p = 0,001) and mortality (28.6% vs 0, p = 0,02).

VIS ≥10 was not associated with low cardiac output syndrome (LCOS). There were no significant differences between VIS ≥10 and VIS < 10 in stroke volume index (SVI), SvO2 and lactate. 43.4% patients with VIS ≥10 were treated only with vasopressors without any inotropes.

In contrast, patients with IS ≥10 tended to a lower SVI than ones with IS > 10 (31(17; 47) vs 35(30; 41) ml/m2, p = 0,36). Moreover, patients with IS ≥10 had significantly higher lactate level (7,6(6; 10) vs 2,2(1,7; 3) mmol/l, p = 0,001).

**CONCLUSION.** We suggest that VIS ≥10 is less sensitive than IS ≥10 for adverse postoperative outcomes. The results of our study indicate that VIS ≥10 characterizes patients with postoperative vasoplegia, while IS ≥10 is associated with LCOS and organ perfusion disorder.

### 000158

#### An infraclavicular, real-time, ultrasound-guided, approach to the axillary artery for arterial line placement in critically ill patients: a preliminary report of an AxFemArt randomized controlled trial

R. Gawda[1], T. Czarnik[1], R. Kaplon[2], M. Piwoda[1], M. Marszalski[1], K. Filipiak[1], M. Molsa[1], M. Pietka[1]
[1]Department of anesthesiology and intensive care, Opole University Hospital, Opole, Poland; [2]Faculty of computer science and management, Wroclaw University of Science and Technology, Wrocław, Poland
**Correspondence:** R. Gawda
*Intensive Care Medicine Experimental* 2019, **7(Suppl 3):**000158

**INTRODUCTION.** Axillary artery catheterization from the axillary fossa is an alternative approach for arterial line placement in intensive care. However, this access is cumbersome and has many drawbacks. Another technique - an ultrasound-guided approach via an infraclavicular route has been described, but to date, it is unknown whether this method is clinically useful in critically ill patients [1].

**OBJECTIVES.** To examine the efficacy of the infraclavicular, real-time, ultrasound-guided, in-plane percutaneous catheterization of the axillary artery in mechanically-ventilated critically ill patients.

**METHODS.** A prospective randomized controlled study was conducted in a 11-bed mixed ICU. We included mechanically-ventilated adult patients who had an arterial catheter inserted to monitor arterial pressure or to perform hemodynamic monitoring. An arterial catheter for arterial pressure monitoring was inserted only when radial artery catheterization was impossible. Patients were randomly assigned into two groups. In the A-group, an infraclavicular ultrasound-guided, real-time, in-plane catheterization of the axillary artery was performed using an arterial catheter 3.8Fr (Arrow/Teleflex, USA) or PiCCO catheter 4Fr (Pulsion, Germany). In the F-group, an ultrasound-guided, real-time, out-of-plane catheterization of the common femoral artery was performed using an arterial catheter 3.8Fr (Arrow/Teleflex, USA) or PiCCO catheter 5Fr (Pulsion, Germany). In both groups Seldinger's technique was used. The artery puncture success rate, catheterization success rate, and early mechanical complications were assessed.

**RESULTS.** From January 2018 to February 2019, a total of 51 patients were randomized: 25 to the A-group and 26 to the F-group. There was no difference in demographic variables. Out of 51 patients, 88.2% were in shock and treated with norepinephrine. Hemodynamic characteristics were similar in both groups (Table 1). The puncture success rate of the artery was 100% in both groups. The catheterization success rate of the axillary and femoral artery was 96% and 92.3%, respectively (difference – 3.7%, 95%CI: [-9.1%, 16.5%]). Directly after the procedure, a small periarterial hematoma in six patients in each group (11.7%) was identified using an ultrasound examination. The rate of inadvertent puncture of the opposite wall of the artery was 15.4% in the F-group and 4% in the A-group (difference – 11.4%, 95%CI: [-4.5%, 27.2%]). No puncture of the vein in the A-group occurred. There was one inadvertent puncture of the vein in the F-group.

**CONCLUSION.** Preliminary results indicate that the catheterization success rate of the ultrasound-guided axillary artery catheterization via an infraclavicular route is comparable to the femoral approach – we have not found a sufficient evidence against that claim. In terms of early mechanical complications, the axillary approach is not inferior to the femoral approach.

**Table 1 (abstract 000158).** Hemodynamic characteristics. Data are presented as mean (SD)

| | A-group | F-group | p value |
|---|---|---|---|
| Heart rate | 97 (21) | 102 (24) | 0.434 |
| Mean arterial pressure (mmHg) | 71 (11) | 67 (14) | 0.217 |
| Norepinephrine (μg/kg/min) | 0.43 (0.42) | 0.47 (0.50) | 0.746 |

### 000166

#### Effect of early negative fluid balance on the early postoperative recovery in children after surgical repair of complex congenital heart disease with enlarged right ventricle

X. GONG
Cardiac ICU, Shanghai Children's Medical Center, Shanghai, China
*Intensive Care Medicine Experimental* 2019, **7(Suppl 3):**000166

**INTRODUCTION.** To estimate the effect of early fluid removal on the early postoperative recovery in children after surgical repair of complex congenital heart disease with enlarged right ventricle via a randomized trial.

**METHODS.** One hundred and twenty patients with right-sided complex congenital heart defect who underwent cardiac surgery by cardiopulmonary bypass during 2017.1-2017.6 were enrolled and were divided to two groups when the hemodynamics was stable within 3 hours after the surgery. Patients in Group-E were treated with early fluid removal, but Group-C remained conventional therapy. The Data of the hemodynamics and outcomes were collected from the postoperative day to the 2ndday post-operation.

**RESULTS.** Fluid removal therapy was started in Group-E at 4.39±0.85hr postoperatively vs. 10.17±2.77hr(*P*<0.05)in Group-C. Patients in Group-E showed lower extravascular lung water index (ELWI) , lower fluid overload and lower NT-proBNP compared with patients in Group-C on the first day of post-operation(*P*<0.01). Also, the advantages remained in Group-E on the second day but the ELWI showed no remarkable difference. The rate of reintubation(*P*<0.05), the duration of mechanical ventilation and the length of ICU stay (*P*< 0.01)were significantly reduced in Group-E.

**CONCLUSION.** In patients with right ventricle enlarged complex congenital heart defect who underwent cardiac surgery by cardiopulmonary bypass, utilizing early negative fluid balance when the hemodynamics were stable and the right ventricle is obviously enlarged could achieve negative fluid balance, get extubated and discharge from ICU earlier, also present lower extravascular lung water index, lower incidence of weaning-induced pulmonary edema and reintubation.

**REFERENCE(S)**

[1] Vincent JL, De Macker D. Circulatory shock [J]. N Engl J Med, 2013, 369: 1726-1734. DOI: 10.1056/NEJMra1208943.[2] Hazle MA, Gajarski RJ, Yu S, et al. Fluid overload in infants following congenital heart surgery [J]. Pediatr Crit Care Med. 2013; 14(1):44-49. DOI: 10.1097/PCC.0b013e3182712799. [3] Lex DJ, Tóth R, Czobor NR, et al. Fluid overload is associated with higher mortality and morbidity in pediatric patients undergoing cardiac surgery [J]. Pediatr Crit Care Med. 2016; 17(4):307-314. DOI: 10.1097/PCC.0000000000000659. [4] Wilder NS, Yu S, Donohue JE, et al. Fluid overload is associated with late poor outcomes in neonates following cardiac surgery [J]. Pediatr Crit Care Med. 2016; 17(5):420-427. DOI: 10.1097/PCC.0000000000000715. [5] Selewski DT, Askenazi DJ, Bridges BC, et al. The impact of fluid overload on outcomes in children treated with extracorporeal membrane oxygenation: A multicenter retrospective cohort study [J]. Pediatr Crit Care Med. 2017; 18(12):1126-1135. DOI: 10.1097/PCC.0000000000001349. [6] Hoste EA, Maitland K, Brudney CS, et al. Four phases of intravenous fluid therapy: a conceptual model [J]. Br J Anaesth. 2014;113(5):740-7. DOI: 10.1093/bja/aeu300. [7] Cullen S, Shore D, Redington A. Characterization of right [8] ventricular diastolic performance after complete repair of tetralogy [9] of Fallot. Restrictive physiology predicts slow postoperative [10] recovery. Circulation. 1995;91:1782–1789. [11] Cullen S, Shore D, Redington A. Characterization of right [12] ventricular diastolic performance after complete repair of tetralogy [13] of Fallot. Restrictive physiology predicts slow postoperative [14] recovery. Circulation. 1995;91:1782–1789. [15] Cullen S, Shore D, Redington A. Characterization of right [16] ventricular diastolic performance after complete repair of tetralogy [17] of Fallot. Restrictive physiology predicts slow postoperative [18] recovery. Circulation. 1995;91:1782–1789. [7] Cullen S, Shore D, Redington A. Characterization of right ventricular diastolic performance after complete repair of tetralogy of Fallot. Restrictive physiology predicts slow postoperative recovery [J]. Circulation. 1995;91:1782-1789. [8] 王小亭、刘大为、张宏民等。重症右心功能管理专家共识[J]. 中华内科杂志. 2017, 56(12): 962-973. DOI: 10.3760/cma.j.issn.0578-1426.2017.12.019. [9] van der Bruggen CEE, Tedford RJ, Handoko ML, et al. RV pressure overload: from hypertrophy to failure [J]. Cardiovasc Res, 2017, 113(12): 1423-1432. DOI: 10.1093/cvr/cvx145. [10] Dres M, Teboul JL, Anguel N, et al. Extravascular lung water, B-type natriuretic peptide, and blood volume contraction enable diagnosis of weaning-induced pulmonary edema [J]. Crit Care Med. 2014; 42(8):1882-1889. DOI: 10.1097/CCM.0000000000000295. [11] Teboul JL. Weaning-induced cardiac dysfunction: where are we today [J]? Intensive Care Med. 2014; 40(8):1069-1079. DOI: 10.1007/s00134-014-3334-4. [12] Dres M, Teboul JL, Monnet X. Weaning the cardiac patient from mechanical ventilation [J]. Curr Opin Crit Care. 2014; 20(5):493-498. DOI: 10.1097/MCC.0000000000000131. [13] Vieillard-Baron A, Matthay M, Teboul JL et al. Experts' opinion on management of hemodynamics in ARDS patients: focus on the effects of mechanical ventilation [J]. Intensive Care Med. 2016; 42(5):739-749. DOI: 10.1007/s00134-016-4326-3. [14] Cecconi M, Hofer C, Teboul JL et al. Fluid challenges in intensive care: the FENICE study: A global inception cohort study. Intensive Care Med. 2015 Sep;41(9):1529-37. doi: 10.1007/s00134-015-3850-x. [15] Gattinoni L, Carlesso E. Supporting hemodynamics: what should we target? What treatments should we use? Crit Care. 2013;17 Suppl 1:S4. doi: 10.1186/cc11502. [16] Pinsky MR. The right ventricle: interaction with the pulmonary circulation. Crit Care. 2016 Sep 10;20:266. doi: 10.1186/s13054-016-1440-0. [17] Penny DJ, Redington AN. Function of the Left and Right Ventricles and the Interactions Between Them. Pediatr Crit Care Med. 2016 Aug;17(8 Suppl 1):S112-8. doi: 10.1097/PCC.0000000000000794. [18] Liu J Shen F, Teboul JL et al.Cardiac dysfunction induced by weaning from mechanical ventilation: incidence, risk factors, and effects of fluid removal. Crit Care. 2016 Nov 12;20(1):369. [19] Song IK, Kim EH, Lee JH et al. Utility of Perioperative Lung Ultrasound in Pediatric Cardiac Surgery: A Randomized Controlled Trial. Anesthesiology. 2018 Apr;128(4):718-727. doi: 10.1097/ALN.0000000000002069.

### 000168

#### A multi-center survey of current status for postoperative application of vasoactive agents in children undergoing congenital heart surgery with cardiopulmonary bypass

L. ZHU
Cardiac ICU, Shanghai Children's Medical Center, Shanghai, China
*Intensive Care Medicine Experimental* 2019, **7(Suppl 3):**000168

**INTRODUCTION.** The aims of this survey is to know the current status and specialty of postoperative hemodynamic monitoring and perioperative application vasoactive agents in 17 pediatric heart centers in China, and to establish some basics for further investigations.

**METHODS.** A survey was raised with perioperative hemodynamic monitoring, prophylactic using of vasoactive agents during surgery with cardiopulmonary bypass and postoperative application of vasoactive agents according some articles and surveys published recently.

**RESULTS.** Basic hemodynamic monitoring can be 100% implemented, but only 77% and 41% of the centers measured cardiac output and microcirculation. Dopamine was preferred by most centers for preventing low cardiac output syndrome (LCOS) following heart surgeries. Patients suffering from LCOS with high systemic vascular resistance (SVR) , LCOS with low SVR and LCOS with high pulmonary vascular resistance were more likely be treated with Milrinone, Dopamine or Adrenaline and Milrinone+Catecholamines+Pulmonary vasodilators, which was similar to the results in western countries.

**CONCLUSION.** Basic hemodynamic monitoring can be implemented well in Chinese pediatric heart centers, but the application of advanced hemodynamic monitoring still has a great disparity comparing to some centers abroad. The using of vasoactive drugs was similar to foreign centers but differs obviously, lacking symposiums or guidelines. More randomized control tests and clinical guidelines were prospected to instruct intensivists to utilize vasoactive agents in the future.

**REFERENCE(S)**

1. Hoffman TM, Wernovsky G, Atz AM, Kulik TJ, Nelson DP, Chang AC, Bailey JM, Akbary A, Kocsis JF, Kaczmarek R, Spray TL, Wessel DL. Efficacy and safety of milrinone in preventing low cardiac output syndrome in infants and children after corrective surgery for congenital heart disease. Circulation. 2003; 107: 996–1002.
2. Morales DLS, Almond CSD, Jaquiss RDB, Rosenthal DN, Naftel DC, Massicotte MP, Humpl T, Turrentine MW, Tweddell JS, Cohen GA, Kroslowitz R, Devaney EJ, Canter CE, Fynn-Thompson F, Reinhartz O, Imamura M, Ghanayem NS, Buchholz H, Furness S, Mazor R, Gandhi SK, Fraser CD Jr. Bridging children of all sizes to cardiac transplantation: the initial multicenter North American experience with the Berlin Heart EXCOR ventricular assist device. J Heart Lung Transplant. 2011; 30: 1–8. doi: 10.1016/j.healun.2010.08.033.
3. Jones B, Hayden M, Fraser JF, Janes E. Low cardiac output syndrome in children. Curr Anaesth Crit Care. 2005;16(6): 347-358. doi:10.1016/j.cacc.2006.02.011
4. Algarni KD, Maganti M, Yau TM. Predictors of low cardiac out- put syndrome after isolated coronary artery bypass surgery: trends over 20

years. Ann Thorac Surg. 2011;92(5): 1678-1684. doi:10. 1016/ j.athoracsur.2011.06.017.
5. Vogt W, Laer S. Treatment for paediatric low cardiac output syn- drome: results from the European EuLoCOS-Paed survey. Arch Dis Child. 2011;96(12): 1180-1186. doi:10.1136/archdischild- 2011-300370.
6. Pérez Vela JL, Martín Benitez JC, Carrasco Gonzalez M, de la Cal López MA, Hinojosa Pérez R, Sagredo Meneses V, del Nogal Saez F, et al. Summary of the consensus document: "Clinical practice guide for the management of low cardiac output syndrome in the postoperative period of heart surgery". Med Intensiva. 2012;36(4):277-87. doi: 10.1016/ j.medin.2012.01.016.
7. 中国医师协会心脏重症专家委员会。低心排血量综合征中国专家共识。解放军医学杂志，2017年11期933-944。doi: 10.11855/j.issn.0577-7402.2017.11.01.
8. Marino BS, Tabbutt S, MacLaren G, Hazinski MF, Adatia I, Atkins DL, Checchia PA, DeCaen A, Fink EL, Hoffman GM, Jefferies JL, Kleinman M, Krawczeski CD, Licht DJ, Macrae D, Ravishankar C, Samson RA, Thiagarajan RR, Toms R, Tweddell J, Laussen PC; American Heart Association Congenital Cardiac Defects Committee of the Council on Cardiovascular Disease in the Young; Council on Clinical Cardiology; Council on Cardiovascular and Stroke Nursing; Council on Cardiovascular Surgery and Anesthesia; and Emergency Cardiovascular Care Committee. Cardiopulmonary Resuscitation in Infants and Children with Cardiac Disease: A Scientific Statement from the American Heart Association. Circulation. 2018;137(22): e691-e782. doi: 10.1161/CIR.0000000000000524.
9. Brissaud O, Botte A, Cambonie G, Dauger S, de Saint Blanquat L, Durand P, Gournay V, Guillet E, Laux D, Leclerc F, Mauriat P, Boulain T, Kuteifan K. Experts' recommendations for the management of cardiogenic shock in children. Ann Intensive Care. 2016;6(1):14. doi: 10.1186/s13613-016-0111-2.
10. Burkhardt BE, Rücker G, Stiller B. Prophylactic milrinone for the prevention of low cardiac output syndrome and mortality in children undergoing surgery for congenital heart disease. Cochrane Database Syst Rev. 2015;(3):CD009515. doi: 10.1002/14651858.CD009515.pub2
11. Rizza A, Bignami E, Belletti A, Polito A, Ricci Z, Isgrò G, Locatelli A, Cogo P. Vasoactive Drugs and Hemodynamic Monitoring in Pediatric Cardiac Intensive Care: An Italian Survey. World J Pediatr Congenit Heart Surg. 2016;7(1):25-31. doi: 10.1177/2150135115606626.
12. Pei XB, Ma PL, Li JG, Du ZH, Zhou Q, Lu ZH, Yun L, Hu B. Extensive variability in vasoactive agent therapy: a nationwide survey in Chinese intensive care units. Chin Med J (Engl). 2015;128(8):1014-20. doi: 10.4103/ 0366-6999.155064.
13. van Diepen S, Katz JN, Albert NM, Henry TD, Jacobs AK, Kapur NK, Kilic A, Menon V, Ohman EM, Sweitzer NK, Thiele H, Washam JB, Cohen MG; American Heart Association Council on Clinical Cardiology; Council on Cardiovascular and Stroke Nursing; Council on Quality of Care and Outcomes Research; and Mission: Lifeline. Contemporary Management of Cardiogenic Shock: A Scientific Statement from the American Heart Association. Circulation. 2017;136(16): e232-e268. doi: 10.1161/ CIR.0000000000000525.
14. Cecconi M, De Backer D, Antonelli M, Beale R, Bakker J, Hofer C, Jaeschke R, Mebazaa A, Pinsky MR, Teboul JL, Vincent JL, Rhodes A. Consensus on circulatory shock and hemodynamic monitoring. Task force of the European Society of Intensive Care Medicine. Intensive Care Med. 2014 Dec;40(12):1795-815
15. Daniel De Backer. Detailing the cardiovascular profile in shock patients. Critical Care 2017, 21(Suppl 3):311. DOI 10.1186/s13054-017-1908-6
16. Yuerek M, Rossano JW, Mascio CE, Shaddy RE. Postoperative management of heart failure in pediatric patients. Expert Rev Cardiovasc Ther. 2016;14(2):201-15. doi: 10.1586/14779072.2016.1117388.
17. Saugel B, Vincent JL. Cardiac output monitoring: how to choose the optimal method for the individual patient. Curr Opin Crit Care. 2018;24(3):165-172. doi: 10.1097/MCC.0000000000000492.
18. Fuda G, Denault A, Deschamps A, Bouchard D, Fortier A, Lambert J, Couture P. Risk Factors Involved in Central-to-Radial Arterial Pressure Gradient During Cardiac Surgery. Anesth Analg. 2016;122(3):624-32. doi: 10.1213/ANE.0000000000001096.
19. Dubin A, Henriquez E, Hernández G. Monitoring peripheral perfusion and microcirculation. Curr Opin Crit Care. 2018;24(3):173-180. doi: 10.1097/ MCC.0000000000000495
20. Fornier W, Jacquet-Lagrèze M, Collenot T, Teixeira P, Portran P, Schweizer R, Ovize M, Fellahi JL. Microvascular effects of intravenous esmolol in patients with normal cardiac function undergoing postoperative atrial fibrillation: a prospective pilot study in cardiothoracic surgery. Crit Care. 2017;21(1):302. doi: 10.1186/s13054-017-1889-5.
21. Cavigelli-Brunner A, Hug MI, Dave H, Baenziger O, Buerki C, Bettex D, Cannizzaro V, Balmer C. Prevention of Low Cardiac Output Syndrome After Pediatric Cardiac Surgery: A Double-Blind Randomized Clinical Pilot Study Comparing Dobutamine and Milrinone. Pediatr Crit Care Med. 2018;19(7):619-625. doi: 10.1097/PCC.0000000000001533.
22. Vogt W, Läer S. Drug use patterns for the prevention of paediatric low cardiac output syndrome in Europe. Intensive Care Med. 2011; 37: 1390–1391. doi: 10.1007/s00134-011-2266-5.
23. Boehne M, Sasse M, Karch A, Dziuba F, Horke A, Kaussen T, Mikolajczyk R, Beerbaum P, Jack T. Systemic inflammatory response syndrome after pediatric congenital heart surgery: Incidence, risk factors, and clinical outcome. J Card Surg. 2017;32(2):116-125. doi: 10.1111/jocs.12879.
24. Davis AL, Carcillo JA, Aneja RK, Deymann AJ, Lin JC, Nguyen TC, Okhuysen-Cawley RS, Relvas MS, Rozenfeld RA, Skippen PW, Stojadinovic BJ, Williams EA, Yeh TS, Balamuth F, Brierley J, de Caen AR, Cheifetz IM, Choong K, Conway E Jr, Cornell T, Doctor A, Dugas MA, Feldman JD, Fitzgerald JC, Flori HR, Fortenberry JD, Graciano AL, Greenwald BM, Hall MW, Han YY, Hernan LJ, Irazuzta JE, Iselin E, van der Jagt EW, Jeffries HE, Kache S, Katyal C, Kissoon NT, Kon AA, Kutko MC, MacLaren G, Maul T, Mehta R, Odetola F, Parbuoni K, Paul R, Peters MJ, Ranjit S, Reuter-Rice KE, Schnitzler EJ, Scott HF, Torres A Jr, Weingarten-Abrams J, Weiss SL, Zimmerman JJ, Zuckerberg AL. American College of Critical Care Medicine Clinical Practice Parameters for Hemodynamic Support of Pediatric and Neonatal Septic Shock. Crit Care Med. 2017;45(6):1061-1093. doi: 10.1097/ CCM.0000000000002425.
25. Abman SH, Hansmann G, Archer SL, Ivy DD, Adatia I, Chung WK, Hanna BD, Rosenzweig EB, Raj JU, Cornfield D, Stenmark KR, Steinhorn R, Thébaud B, Fineman JR, Kuehne T, Feinstein JA, Friedberg MK, Earing M, Barst RJ, Keller RL, Kinsella JP, Mullen M, Deterding R, Kulik T, Mallory G, Humpl T, Wessel DL; American Heart Association Council on Cardiopulmonary, Critical Care, Perioperative and Resuscitation; Council on Clinical Cardiology; Council on Cardiovascular Disease in the Young; Council on Cardiovascular Radiology and Intervention; Council on Cardiovascular Surgery and Anesthesia; and the American Thoracic Society. Pediatric Pulmonary Hypertension: Guidelines from the American Heart Association and American Thoracic Society. Circulation. 2015 Nov 24;132(21):2037-99. doi: 10.1161/CIR.0000000000000329.

### 000200

#### Prognostic accuracy of qSOFA in patients with Heart Failure

T. Wagner[1], J. Haumann[1], S. Blankenberg[1], H. Grahn[2]
[1]Cardiology, University Heart Center Hamburg GmbH, Hamburg, Germany; [2]University Heart Center Hamburg GmbH, Hamburg, Germany
**Correspondence:** H. Grahn
*Intensive Care Medicine Experimental* 2019, **7(Suppl 3):**000200

**INTRODUCTION.** The Sepsis-3 Task Force proposed the quick Sequential (Sepsis-related) Organ Failure Assessment (qSOFA) to replace SIRS as a new screening tool for the identification of patients with sepsis [1]. Diagnostic and prognostic value for patients outside the ICU is controversial. Further, there are no data regarding the usability in patients with heart failure.

**OBJECTIVES.** To assess the prognostic accuracy of qSOFA score in patients with heart failure admitted to a Heart Failure Unit (HFU).

**METHODS.** Data from patients admitted to HFU were retrospectively analyzed. qSOFA, and sSOFA score as well as SIRS criteria were assessment at admission. In addition, demographic, clinical, laboratory and echocardiographic value were recorded. A follow-up was performed 30 days after discharge. Primary outcome was death or readmission to hospital due do worsening of heart failure symptoms

**RESULTS.** Of 240 patients (73% male, median age 64 years), 25 patients (10%) had a qSOFA score ≥2 points and 126 patients

(53%) fulfilled none of qSOFA criteria (0 points). Within these patients 28 patients (12%) fulfilled the criteria of cardiogenic shock.
Patients with a qSOFA score ≥1 point more frequently had a severe systolic heart failure (EF ≤30%) and had a higher risk to develop multi organ failure during hospital stay (28% vs. 9%, P=0.005).
Within 30 days, the primary endpoint occurred in 46 patients (19%). Seventeen patients (7%) died and 34 patients (14%) were readmitted to hospital due to heart failure. Patients with positive initial qSOFA score reached this endpoint significantly more frequent (48% vs. 19%, p=0.002). Follow-up showed that with positive qSOFA score reported a more often a worsening of heart failure symptoms (p=0.021) representing a higher median NYHA class (p=0.008).
**CONCLUSION.** In our study, qSOFA was useful to identify patients with heart failure with a high risk to have a worse outcome.Therefore, identification of these patients is of vital importance but the diagnosis of sepsis is often not easy made in patients with heart failure because of overlapping symptoms (hypotonia, dyspnoe, tachycardia). qSOFA score is useful to operationalize disease severity in adult in-patients with heart failure.

### 000205

#### Forecasting Hemorrhagic Shock by Analyzing Plethysmograph Response to Preoperative Blood Draws

X. Li[1], M. Pinsky, , G. Clermont[2], A. Dubrawski[1]
[1]Auton lab, Carnegie Mellon University, Pittsburgh, United States of America; [2]School of medicine, University of Pittsburgh, Pittsburgh, United States of America
**Correspondence:** X. Li

*Intensive Care Medicine Experimental* 2019, **7(Suppl 3):**000205

**INTRODUCTION.** Substantial blood loss can induce Irreversible Hemorrhagic Shock (IHS), which can lead to adverse patient outcomes and mortality [2]. We hypothesize that a patient's response to rapid but short blood loss, such as blood draw, can be indicative of cardiovascular reserve and thus predictive of the risk of developing IHS when subject to prolonged bleeding.
**OBJECTIVES.** To develop an approach to predict the likelihood of IHS due to blood loss during surgery by analyzing the physiological patterns of response to pre-operative blood draws.
**METHODS.** 36 healthy pigs were anesthetized and had a 20 mL of blood rapidly drawn while stable, then bled at 20 mL/min until mean arterial pressure (MAP) fell to 30 mmHg. 10 pigs had IHS (defined as MAP < 20 mmHg) despite cessation of bleeding. Plethysmograph (PLE) was recorded at 250 Hz. One-minute segments of data centered at blood draw start were statistically featurized and normalized using pre-draw stable baseline data. Patterns were extracted from data using Graphs of Temporal Constraints [1], and a decision forest (GTC-DF) model was trained to identify patterns most informative of IHS. To mitigate overfitting and for easy interpretation, only a few patterns with the highest information gain were used to assess the subjects (GTC-Top).
**RESULTS.** In a leave-one-subject-out cross-validation, GTC-Top and GTC-DF respectively identify 70% (95% CI: [55.6%, 84.4%]) and 60% (95% CI: [44.8%, 75.2%]) of the subjects who are prone to develop IHS, while giving only 1 false alert in 10,000 predictions on average. They both outperform logistic regression (LR) and random forest (RF) models [Fig 1, Tab 1].
**CONCLUSION.** We can identify, pre-operatively, the majority of patients who are likely to develop IHS due to blood loss during surgery, by using a tractable number of highly interpretable patterns found in plethysmograph waveform changes during pre-operative blood draws.

**REFERENCE(S)**

[1] Guillame-Bert et al. JMLR, 18(121):1–34, 2017.
[2] Gutierrez et al. Critical care. Crit Care, 8(5):373, 2004.

[3] This work is funded by NIH R01GM117622 and DARPA FA8750-17-2-0130.

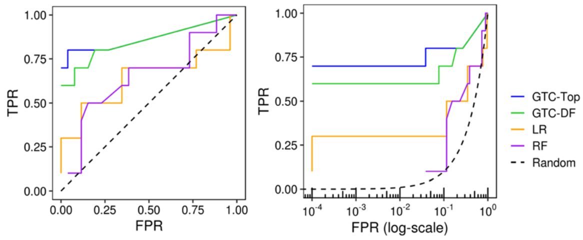

**Fig 2 (abstract 000205).** ROC curves. FPR in (b) is log scaled to emphasize performance at low FPR settings.

**Table 1 (abstract 000205).** Performance of models with 95% CI in square brackets

| Model | TPR at low FPR (FPR=0.0001) | AUC |
|---|---|---|
| **GTC-Top** | **0.7 [0.556, 0.844]** | **0.869 [0.758, 0.980]** |
| GTC-DF | 0.6 [0.448, 0.752] | 0.848 [0.732, 0.964] |
| Logistic regression | 0.3 [0.156, 0.444] | 0.638 [0.488, 0.788] |
| Random forest | 0.0 [0.000, 0.048] | 0.648 [0.499, 0.797] |

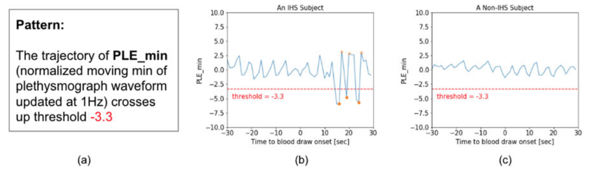

**Fig. 2 (abstract 000205).** An example pattern from the GTC-Top model which manifests in 7/10 IHS subjects and 0/26 non-IHS subjects, and its manifestation on one example IHS subject.

### 000220

#### Effect of early fibrinolysis in the preservation of left ventricular ejection fraction of patients included in a pharmacoinvasive therapy protocol. ARIAM ANDALUCIA

I. Valiente Aleman[1], D. Fortet Cortes[1], MJ. Dominguez Rivas[1], M. Recuerda Nuñez,[1], S. Fernandez Coello[1], R. Jimenez Gomez[1], JP. Benalcazar Arias[1], A. Fregosi[2], X. Romani[3], JC. Rodriguez Yañez[1], I. Diaz-Torres, I[1]
[1]Medicina intensiva, University Hospital of Puerto Real, Puerto Real, Spain; [2]Medicina intensiva, Hospital Universitario Punta de Europa, Algeciras, Spain; [3]Medicina intensiva, Hospital Comarcal La Línea de la Concepción, La Línea de la Concepción, Spain
**Correspondence:** J.C. Rodriguez Yañez

*Intensive Care Medicine Experimental* 2019, **7(Suppl 3):**000220

**INTRODUCTION.** Primary percutaneous coronary intervention (pPCI) is the ideal method of reperfusion for acute STEMI. Although, in some areas it is very difficult to implement pPCI between two hours, and it is well-known that 'Time is myocardium'.

**OBJECTIVES.** To evaluate the preservation of left ventricular ejection fraction (LVEF) in patients undergoing a pharmacoinvasive therapy protocol in a setting without primary percutaneous coronary intervention , Campo de Gibraltar (CG) area.

**METHODS.** Multicentre observational prospective study of consecutive patients with STEMI treated with fibrinolysis in the CG area and transferred to our intensive care unit for coronarography and PCI between January 1, 2012 and March 31, 2018, included in the ARIAM ANDALUCIA registry. We considered as early fibrinolysis those given in the first 3 hours from symptom onset. LVEF was measured by post-fibrinolysis echocardiography and/or by ventriculography during coronarography. Preserved LVEF was defined as >55%, moderately-reduced LVEF 35-50% and severely reduced LVEF <30%.R-comander statistic pack was used for analyses. All continuous variables are described as their mean values ± standard deviation and chi square tests for discrete variables. CI 95%. P< 0.05.

**RESULTS.** 437 patients were included, mean age (sd) 59.79 (11.89), 242 were inferior STEMI and 194 anterior STEMI. Culprit arthery : none 44, ACD 192, ADA 163 and ACX 38. Distribution (n) of Killip grades I, II, III and IV were 383, 10, 13 and 31 respectively. Mean GRACE (sd) was 149.59(33.49).

80.3% of fibrinolysis treatments were given early and 79.4% in hospital, with a median symptoms-to-needle time of 95 minutes. TIMI 3 was achieved in 79.17% of the cases. 264 coronarographies were performed in the first 24 hours (76 rescue-PCI) and 188 in 48-72 horus. PCI were effective in 382 (87.8%) cases. 210 ventriculographies and 230 echocardiographies were performed. 265 had a preserved LVEF (64.6%). Preserved LVEF was significantly associated to early fibrinolysis (p<0.05). We did not find significant differences between PCI performed in 24 hours or more than 48h.

**CONCLUSION.** Fibrinolysis in the first 3 horus favours LVEF preservation in patients undergoing pharmacoinvasive therapy. Early fibrinolysis is an effective alternative to primary PCI for for CG patients unable to achieve a symptoms-to-ballon time lesser than 180 minutes.

**REFERENCE(S)**

1. Armstrong PW, Gershlick A, Goldstein p, et al. for the STREAM Investigative Team. Fibrinolysis or primary PCI in ST-segment elevation myocardial infarction. N Engl J Med 2013;368:1379-1387
2. To memory of Javier Lozano Cid MD who performed numerous coronary interventions with excellence.

### 000248

#### Prediction of Low Cardiac Output Syndrome after Coronary Artery Bypass Surgery in patients with low preoperative Ejection Fraction(<40%)

F. Ampatzidou[1], P. Ntouma[2], R. Ioannidis[2], A. Dimaki[1], G. Kechagioglou[3], N. Mihail[3], T. Asteri[2], G. Drossos[3]
[1]Cardiothoracic icu, G.Papanikolaou Hospital, Thessaloniki, Greece; [2]Cardiothoracic anesthesia, G.Papanikolaou Hospital, Thessaloniki, Greece; [3]Cardiothoracic surgery, G.Papanikolaou Hospital, Thessaloniki, Greece
**Correspondence:** F. Ampatzidou
*Intensive Care Medicine Experimental* 2019, **7(Suppl 3):**000248

**INTRODUCTION.** Low cardiac output syndrome (LCOS) after cardiac surgery,- defined as the need for postoperative intraaortic balloon pump or inotropic support for longer than 30 minutes to maintain the systolic blood pressure greater than 90 mm Hg and the cardiac index greater than 2.2 L/min/m2 -is associated with significant morbidity and mortality

**OBJECTIVES.** Aim of this study is to identify risk factors for LCOS after isolated CABG surgery with the use of cardiopulmonary bypass in patients with low ejection fraction(<40%).

**METHODS.** Patients who underwent isolated elective CABG from June 2012 to March 2019, in our Cardiothoracic Department were retrospectively investigated. The following factors were compared between patients with postoperative LCOS and the rest of the cohort: Age>75years old, gender, smoking habit, Diabetes Mellitus(DM), history of percutaneous coronary intervention(PCI), history of myocardial infarction(MI), preoperative pulmonary hypertension(PHT) defined as systolic pulmonary pressure >30mmHg (ECHO study)and CPB time >120 min. Chi square test was used for statistical analysis.

**RESULTS.** A total of 2024 pts underwent isolated CABG. From 395 pts who had preop EF<40%, LCOS complicated 108 pts (27,3%) Results of our study are shown in table 1

**CONCLUSION.** In our study preoperative pulmonary hypertension and prolonged CPB time found to have statistical significant correlation with postoperative LCOS in patients with low EF.

**Table 1 (abstract 000248).** See text for description

| | LCOS n=108 | Control group n=287 | p |
|---|---|---|---|
| Age >75 | 15(13,9%) | 44(15,3%) | 0,75 |
| Females | 16(14,8%) | 28(9,8%) | 0,2 |
| Smoking | 46(42,6%) | 106(36,9%) | 0,35 |
| DM | 47(43,5%) | 109(38%) | 0,35 |
| PCI | 22(20,4%) | 47(16,4%) | 0,37 |
| MI | 80(74,1%) | 197(68%) | 0,64 |
| PHT | 51(47,2%) | 56(19,5%) | <0,01 |
| CPB >120 | 41(38%) | 67(23,3%) | 0,05 |

### 000254

#### Analyzing Myocardial Infarction with Non-obstructive Coronary Arteries in the ICU of a regional hospital. ARIAM database

R. Torcuato, I. Fernández, A. Alvarez, M. Salgado, P. Cobo, A. Ubeda
Intensive care unit, Hospital Point Europe, Algeciras, Spain
**Correspondence:** A. Ubeda
*Intensive Care Medicine Experimental* 2019, **7(Suppl 3):**000254

**INTRODUCTION.** Acute myocardial infarction (AMI) with coronary arteries without significant obstructions (MINOCA) is a frequent syndrome that accounts for 5% to 25% of all AMI. It is a challenge in clinical practice since there are gaps in their knowledge and therapeutic attitude. It assumes an adjusted 1-year mortality risk significantly higher than patients with non STEMI (*Non-ST-elevation myocardial infarction)* and associated obstructive coronary disease.

**OBJECTIVES.** To analyze the differences observed among patients with AMI with and without angiographically significant coronary lesions (MINOCA) in patients admitted to the Intensive Care Unit (ICU) of Hospital Punta Europe, using the ARIAM database.

**METHODS.** Retrospective descriptive analysis, on a prospective cohort, accomplished in an ICU of 12 beds for 6 years (2013-2018). Demographic variables have been registered, comorbidities, risk factors, previous illnesses, previous medication, diagnosis initial, initial Killip, location of AMI, stratification scales (TIMI, GRACE) and risk intrahospital hemorrhage (CRUSADE), arrhythmias, ICU medication, complications and attention times and procedures. Statistical analysis: variables categorical (frequencies and percentages), numerical (mean and standard deviation or median and interquartile range). Comparisons: chi2 (percentages), Student's T test (means). Statistical significance with p <0.05.

**RESULTS.** 767 patients were included. 564 men (73.53%), 203 women (26.47%). STEMI (ST-segment elevation myocardial infarction) n=362 (47.2%), non-STEMI (*Non-ST-elevation myocardial infarction)* n=405 (52.8%). Non-MINOCA ACS comparison n=722 (94.1%) vs. MINOCA n=45 (5.86%): female (24.3% vs. 62.2%, p =<.001), DM (37.4% vs. 22.2%; p=.038), obesity (14.5% vs. 26.7%, p=.021), previous history of AMI (15.8% vs. 2.2%, p=.045), sinus bradycardia (2.12% vs. 6.8%, p=.047), shock during ICU stay (9.2% vs. 0%; p=.033), recurrent angina (4.9% vs. 13.3%, p=.015).

Worst Killip (p=.026): I (45.2% vs. 68.4%), II (27.7% vs. 21.1%), III (18.7% vs. 10.5%), IV (8.5% vs. 0%). ICU length of stay (LOS) (days): 3.8 ± 4.9 vs. 3.3 ± 2.8, p=.543. Hospital LOS (days): 9.5 ± 8.3 vs. 13.9 ± 16.9, p=.308. Mortality 7.1% vs. 0%, p=.065. Multiple logistic regression: female (OR 7.47, 95% CI [3.80-14.68], p=<.001), age (OR 0.97, 95% CI [0.94-0.99], p=.016) and DM (OR 0.44, 95% CI [0.21-0.94], p=.035). AUROC: 0.765 (95% CI 0.685-0.845).

**CONCLUSION.** In our Unit, the prevalence of MINOCA was 5.86%, similar to the one described in literature. Female sex, a younger age and the absence of diabetes mellitus were independently associated with diagnosis of MINOCA. No differences were observed in mortality among MINOCA and ACS with significant coronary lesions.

1. No grant acknowledgment.

### 000256

#### STEMI vs. NSTEMI in a regional hospital. ARIAM database

I. Fernández, R. TorcuatoA. Alvarez, M. Salgado, P. Cobo, A. Ubeda
Intensive care unit, Hospital Point Europe, Algeciras, Spain
**Correspondence:** A. Ubeda
*Intensive Care Medicine Experimental* 2019, **7(Suppl 3):**000256

**INTRODUCTION.** Eventhough the incidence of STEMI has been declining over the past 20 years, to produce an accurate assessment of risk and mortality individual patients have a combination of clinical features that influence prognosis, and these factors must be appropriately weighted. Time is really important, as nearly half of potentially salvageable myocardium is lost within 1 hour of the coronary artery being occluded, and two-thirds is lost within 3 hours.

**OBJECTIVES.** To analyze the differences between patients diagnosed with ST-segment elevation myocardial infarction (STEMI) and those diagnosed with non ST-segment elevation myocardial infarction (NSTEMI) admitted to the intensive care unit (ICU) of Punta de Europa hospital using ARIAM database.

**METHODS.** Descriptive retrospective analysis on a prospective cohort performed in a 12-bed ICU for 6 years (2013-2018). Two groups were compared, STEMI vs. NSTEMI. Demographic variables, comorbidities, risk factors, initial Killip, extension, TIMI, GRACE, CRUSADE have been registered. Statistical analysis: categorical (frequencies and percentages) and numerical variables (mean and standard deviation). Comparisons: X2 test (percentages), Student's t (means). Statistical significance with p<0.05.

**RESULTS.** 767 patients were included: STEMI 362 (47.19%) vs. NSTEMI 405 (52.80%): male (76.8% vs. 70.6%, p=.053), age (62.14 [±13.16] vs. 67.17 [±12.55], p<.001), arterial hypertension (AH) (39.2% vs. 52.1%, p=.002), smoker (46.1% vs. 32.6%, p=.001), diabetes mellitus (DM) (27.9% vs. 44.2%, p<.001), previous myocardial infarction (MI) (8.3% vs. 21%, p<.001), previous coronary lesions (8.6% vs. 18.8%, p<.001), heart failure (HF) (1.1% vs. 9.6%, p<.001), stroke (3% vs. 8.1%, p=.006), chronic renal failure (CRF) (1.4% vs. 11.4%, p<.001), coronariography (83.2% vs. 49.2%, p<.001), TIMI (3.44 [±2.71] vs. 2.72 [±1.41], p<.001), GRACE (155.86 [± 38.63] vs. 147.86 [±46.03], p=.021), CRUSADE (28.78 [±15.45] vs. 35.97 [± 18.66], p<.001). Responsible artery (p<.001): anterior descending artery (LAD) (35.7% vs. 45.6%), right coronary artery (RCA) (40.9% vs. 18.3). Diagnosis (p<.001): unstable angina (2.5% vs. 44.2%), MI (94.2% vs. 52.3%). Mortality 6.4% vs 6.8% (p=.756). Time (expressed in minutes): first contact-EKG (14.6 [±24.8] vs. 20.9 [±22.7], p=.019), first contact-ICU (149.9 [±284.3] vs. 493.5 [±674.0], p<.001), first contact-percutaneous coronary intervention (PCI) (2316 [±3928.7] vs. 7245.2 [±8647.7], p<.001). Time (expressed in days): first symptoms-ICU discharge (3.5 [±4.2] vs. 4.8 [5.7], p<.001), ICU admission-ICU discharge (3.3 [±3.9] vs. 4.2 [±5.5], p=.006), PCI-ICU discharge (1.7 [±4.7] vs. 0.27 [±6.0], p=.002).

**CONCLUSION.** Patients diagnosed with STEMI were older and had higher prevalence of AH, DM, previous MI, HF, stroke, CRF and smoking. RCA was the most commonly affected in STEMI while LAD was in those diagnosed with NSTEMI. Time indicators were worse for STEMI than NSTEMI except ICU length of stay after PCI.

### 000284

#### Plasma zinc concentration is associated with postoperative vasopressor requirement and hospital length of stay in cardiac surgery patients

A. Jansen, M. Kox, P. Pickkers
Intensive care, Radboud University Medical Center, Nijmegen, Netherlands
**Correspondence:** A. Jansen
*Intensive Care Medicine Experimental* 2019, **7(Suppl 3):**000284

**INTRODUCTION.** Zinc is an essential trace element involved in a plethora of functions, including wound healing, neurological signaling and immune function. Zinc deficiency is associated with the development of atherosclerosis and is prevalent among cardiac surgery patients.

**OBJECTIVES.** To assess the relationship between zinc status and the inflammatory response, endothelial activation and clinical outcomes in cardiac surgery patients.

**METHODS.** One hundred and seventy-nine patients undergoing on-pump cardiac surgery were included. Blood samples were obtained at predefined time points before, during, and after surgery to assess zinc levels (preoperatively) and markers of inflammation (TNF-α, IL-6, IL-8, IL-10, IL-1RA, MCP-1, MIP-1α, MIP-1β) and endothelial activation (VCAM, ICAM) serially. The correlation between zinc concentrations and the area under the curve (AUC) of circulating cytokines, chemokines, and markers of endothelial cell activation was determined. The relationship between zinc status and clinical outcomes was assessed by dividing patients into a low-zinc (below median) and a high-zinc (above median) group.

**RESULTS.** The median zinc level of the enrolled patients was (median [IQR]) 7.0 [5.7-9.2] μmol/L and 84% of the study population displayed below-normal levels (<10 μmol/L). Cardiac surgery induced a substantial increase in postoperative plasma levels of all measured inflammatory mediators, except TNF-α. Zinc status was not related to plasma cytokine levels, but weak negative correlations were observed for the chemokines MIP-1α and MIP-1β (r=-0.16, p=0.04 and r=-0.15, p=0.04, respectively). Furthermore, a weak, but statistically significant, inverse correlation was found between zinc status and VCAM (r=-0.21; p<0.01), and a trend for ICAM (r=-0.14; p=0.06). The proportion of patients requiring vasopressors on the day of surgery and on the following day did not differ between the low and high-zinc groups (84.1% vs. 76.7%, p=0.22 and 53.4% vs. 53.5%, p= 0.99, respectively), but was significantly higher in the low-zinc group on the second postoperative day (20.5% vs. 7.1%, p=0.01). Zinc status was not related to ICU length of stay (21.5 [19-24] vs. 22.0 [19-24] hours in the low and high zinc groups, respectively, p=0.65), but hospital length of stay was significantly prolonged in the low-zinc group (220 [192-297] vs. 195 [171-264] hours, p=0.02).

**CONCLUSION.** A majority of cardiac surgery patients has low zinc levels and this is weakly associated with increased levels of chemokines and endothelial activation markers. Furthermore, low zinc status is related with increased postoperative vasopressor requirement and prolonged hospital stay. Further research is warranted to elucidate the mechanisms behind these findings and to assess a possible role for preoperative zinc supplementation in deficient patients.

### 000302

#### Acute coronary syndrome: predictive factors for the need of invasive mechanical ventilation

I. Fernández, R. Torcuato, A. Alvarez, M. Salgado, P. Cobo; A. Ubeda
Intensive care unit, Hospital Point Europe, Algeciras, Spain
**Correspondence:** A. Ubeda
*Intensive Care Medicine Experimental* 2019, **7(Suppl 3):**000302

**INTRODUCTION.** Patients with acute coronary syndromes (ACS) may develop serious complications and require prolonged intensive care. The need for invasive mechanical ventilation (IMV) is a common reason for prolonged intensive care in patients who are admitted with an ACS.

**OBJECTIVES.** To identify factors associated with the need of IMV in patients diagnosed with ACS admitted to the intensive care unit (ICU) of Hospital Punta de Europa using ARIAM database.

**METHODS.** Retrospective descriptive analysis using a prospective cohort performed in a 12-beds ICU for 6 years (2013-2018). Two groups were compared: patients who suffered ACS and did not required IMV vs.

those diagnosed with ACS and required IMV. Demographic variables, associated comorbidities, risk factors, Killip classification at the beginning, damage extension, TIMI, GRACE, CRUSADE, complications occurred during ICU admission, lesions founds in coronary angiography, diagnosis at ICU discharge and mortality were collected. Statistical analysis was performed: categorical variables (expressed in percentages and frequencies), continuous variables (expressed using mean and standard deviation). Comparison: Chi-squaretest (percentages) and T-student test(mean). Multiple logistic regression. Statistical significance was set at p-value < 0.05.

**RESULTS.** 756 patients were included. 35 patients (4.63%) were under IMV, and 721 patients (95.37%) were not. Those who were under IMV: male (54.38% vs. 74.3%, p=.009), age (69.7 [±11.7] vs. 64.4 [±13.1], p=.019), body mass index (29.01 [±5.64] vs. 27.02 [±3.87], p=.031), diabetes mellitus (DM) (60.0% vs. 35.1%, p=.003), previous ACS (37.1% vs. 13.9%, p<.001), creatinine values (1.91 [±1.13] vs. 1.23 [±0.95], p=.006), heart rate (91.9 [±22.65] vs. 78.94 [±19.67], p=.002), systolic blood pressure (102.14 [±20.66] vs. 124.21 [±26.01], p<.001). Complications occurred during ICU admission: shock (71.4% vs. 5%, p<.001), infectious complications (20% vs. 0.4%, p<.001), renal complications (14.3% vs. 1.5%, p<.001), encephalopathy (5.7% vs. 0.6%, p=.001). Myocardial infarction location (p=.027): anterior(53.6% vs. 43.2%), inferior (28.6% vs. 45.2%). Coronary angiography findings (p=.009): left anterior descending artery (60.9% vs. 39.2%), right coronary artery (26.1% vs. 31%), left main coronary artery (13% vs. 3%), circumflex artery (0% vs. 15.2%). ICU length of stay (LOS) (days): 10.46 ± 12.13 vs. 3.41 ± 3.80; p=.002. Mortality: (48.6% vs. 4.2%, p<.001). Multiple logistic regression: female (OR 2.82, CI 95% [1.12-7.13], p=.028), infectious complication (OR 176,37, CI 95% [30.74-1011.90], p<.001), shock (OR 62.40, CI 95% [23.36-166.70], p<.001), previous MI (OR 2.30, CI 95% [0.86-6.14]. AUROC: 0.92 (CI 95% [0.85-.98]).

**CONCLUSION.** Female sex, shock and infectious complications were predictive factors for the need of IMV in patients diagnosis with ACS. The need of IMV was associated with higher rates or mortality in ICU stay.

## HSRO - Improving treatment in the ICU

### 000026

#### Persistent Inflammation, Immunosuppression and Catabolism Syndrome (PICS) as a marker of the chronic critical illness

MÁ. García-Martínez[1], A. Arrascaeta Llanes[2], F. Ortuño Andériz[3], I. Martínez De Lagrán Zurbano[4], M. Zamora Elson[5], C. Lorencio Cárdenas[6], Á. Jordá Miñana[7], JF. Martínez Carmona[8], S. Chacón Alves[9], F. Martínez Lozano[10], L. Macaya Redín[11], R. Carreño Ponfil[12], C. Serón Arbeloa[13], S. Borras Palle[14], R. Gastaldo Simeón[15], J. Molina Jaime[16], EG. Masdeu[17], JC. Lopez Delgado[18]
[1]Intensive care unit, University Hospital of Torrevieja, Torrevieja, Spain; [2]Internal medicine, Long Island Community Hospital, New York, United States of America; [3]Intensive care unit, Hospital Clinico Universitario San Carlos, Madrid, Spain; [4]Intensive care unit, Hospital Universitari Germans Trias i Pujol, Badalona, Spain; [5]Intensive care unit, University Hospital Arnau de Vilanova, Lleida, Spain; [6]Intensive care unit, Hospital Universitari de Girona Doctor Josep Trueta, Girona, Spain; [7]Intensive care unit, Hospital Clínic Universitari de València, València, Spain; [8]Intensive care unit, Hospital Carlos Haya, Málaga, Spain; [9]Intensive care unit, University Hospital 12 de Octubre, Madrid, Spain; [10]Intensive care unit, Reina Sofia University Hospital, Córdoba, Spain; [11]Intensive care unit, Royal Navarre Hospital, Pamplona, Spain; [12]Intensive care unit, Hospital Universitario de Fuenlabrada, Fuenlabrada, Spain; [13]Intensive care unit, Hospital General San Jorge, Huesca, Spain; [14]Intensive care unit, Hospital Público Universitario Doctor Peset, València, Spain; [15]Intensive care unit, Hospital de Manacor, Manacor, Spain; [16]Intensive care unit, Clínica HLA Vistahermosa, Alacant, Spain; [17]Intensive care unit, Hospital Verge de la Cinta, Tortosa, Spain; [18]Intensive care unit, Hospital Universitari de Bellvitge, L'Hospitalet de Llobregat, Spain
**Correspondence:** M.Á. García-Martínez
*Intensive Care Medicine Experimental* 2019, **7(Suppl 3):**000026

**INTRODUCTION.** Persistent Inflammatory, Immunosuppressed, Catabolic Syndrome (PICS) is a phenotype of critically ill patients that correlates as a potential origin of chronic critical illness (CCI). We defined PICS as a combination of clinical and laboratory criteria to predict the occurrence of CCI.

**OBJECTIVES.** To evaluate the prognostic capacity of our predefined criteria of PICS on the 8th day of admission (PICS-8) for the development of CCI. We analyzed the association between the presence of PICS-8, one event of PICS, or the accumulation of PICS events with intensive care unit (ICU) length of stay (LOS), hospital-LOS, mortality, and functional capacity up to 12 months after hospital discharge.

**METHODS.** We designed a multicenter prospective study enclosing all patients on 8th day of ICU admission over 3 consecutive months. Patients were monitored for PICS-8, every 7 days for PICS status and evaluated 3 times within one year after discharge for functional capacity. When data follows a normal distribution, categorical variables are expressed as frequencies and percentages; and continuous variables as mean and standard deviation (SD), or median and interquartile range as needed. If hospital mortality occurred then ICU-LOS and Hospital-LOS were censored to the right. The distributions of both times were estimated in each group, defined by the status of PICS, by the method of Kaplan-Meier (KM) and compared using the log-rank test. We performed multivariate logistic regression analysis to identify the factors that maintain independent association with PICS (at least once throughout the follow-up period). The variables showing significant association with PICS in univariate analysis were entered into the multivariate analysis. The model was summarized as p-values and OR with confidence intervals at 95% (CI95%). For multivariate Poisson regression model, the days between admission to ICU or Hospital mortality were censored again; these time intervals were summarized using KM. The number of PICS events along the follow-up period was considered as the marker of PICS severity. Statistical significance was set at p<0.05.

**RESULTS.** In the study period, 3398 patients were admitted to 15 hospitals right across Spain. We identified 478 patients that met the inclusion criteria. ICU LOS (p=.001), hospital LOS (p=.018), and ICU mortality (p=.022) showed predictive capacity for PICS-8. The medians of ICU-LOS and Hospital-LOS were increased in PICS-8-patients (25 and 47 days respectively) compared with Non-PICS patients (18 and 36 days respectively). Mortality in the ICU was greater for patients with PICS-8 (OR 1.74 CI95% 1.08;2.81). The factors associated with the number of PICS were: male gender, mechanical ventilation (MV)>16 days, Total parenteral nutrition/complementary nutrition (TPN/C), and Simplified Acute Physiology Score (SAPS3). There was a greater incidence of at least one episode of PICS in those patients who died (43.2% vs 29.7%; p=.004). However, the multivariate logistic regression showed that PICS was not an independent factor of mortality.

**CONCLUSION.** PICS-8 showed higher ICU LOS, hospital LOS, and hospital mortality. PICS severity was greater in males, patients with high SAPS3, MV time>16 days, and recipients of TPN/C.

**REFERENCE(S)**

1. Xiao W, Mindrinos MN, Seok J, Cuschieri J, Cuenca AG, Gao H, et al. A genomic storm in critically injured humans. J Exp Med 2011;208:2581–90. doi:10.1084/jem.20111354.
2. Gentile LF, Cuenca AG, Efron PA, Ang D, Bihorac A, McKinley BA, et al. Persistent inflammation and immunosuppression: a common syndrome and new horizon for surgical intensive care. J Trauma Acute Care Surg 2012;72:1491–501. doi:10.1097/TA.0b013e318256e000.

### 000052

#### Analysis of intensive care unit bed management by an interactive simulation model

L. Esparza[1], D. García De Vicuña[2], F. Mallor[2], C. Azcárate[2], J. Barado[1]
[1]Critical Care Department, Hospital Complex of Navarre, Pamplona, Spain; [2]Department of statistics, computer science and mathematics, UPNA, Pamplona, Spain
**Correspondence:** L. Esparza
*Intensive Care Medicine Experimental* 2019, **7(Suppl 3):**000052

**INTRODUCTION.** The proper bed management of an ICU is crucial for its performance, as well as for the prognosis of patients.

Simulation has a long history in medical education (1), valued for its ability to reproduce some conditions in clinical practice and to allow students to act in a safe environment. More recently, it has been used as a technique to support improvement in health care systems and processes (2,3). Virtual simulation allows analysing decision-making processes in a secure environment and assessing their impact (4).

**OBJECTIVES.** A computer application development that simulates the operation of an ICU and which allows analysing how bed management decisions are made for optimization.

**METHODS.** All information was obtained by Big Data analysis of data collected, and previously anonymized, by MetaVision® system (iMD Sof, Tel Aviv, Israel) implemented in the ICU of the Hospital Complex of Navarre, Spain (a tertiary care center). Simulator was based on a dynamic, discrete and stochastic model that represents the evolution of an ICU over time. The discrete event simulation model was programmed in JAVA language with a visual interface that reproduces the general condition of the ICU, surgical programming for the following days and provides the clinical information of each patient, through screens that mimic those consulted by physicians in their usual practice and enough for decision making about discharge (Figure 1). The simulator will advance in time generating both urgent and scheduled patient arrivals according to real or simulated patterns and events that evolve the health status of each patient admitted (Figure 2), having enough flexibility to be able to define different ICUs and specific scenarios. The decision-making process about the discharge of patients is reproduced, so that, in certain moments (clinical sessions, urgent or programmed arrivals, exitus…) it will interact with the user waiting for indications about possible discharges or admissions, allowing them to modify the occupation of the ICU. The program will record all the events that occurred during the simulation, including all decisions made by users related to bed management. Finally, validation and verification of the simulator was carried out.

**RESULTS.** We have developed an interactive simulation program that, according to ICU staff, reflects the operation of an ICU in a real way and allows the analysis of the bed management decision making. The simulator facilitates the definition of different types of ICU. It allows analyzing the consequences of decisions in a virtual environment and proposing better management strategies. It provides performance measures of the management of the ICU, in relation to the admission (acceptance or rejection), the shortening of the stays and the delay in admission. It analyses the level of dissimilarity in decisions among users in a quantitative way, identifies its causes and quantifies its influence on bed management, detecting situations or patients in which there is a greater discrepancy.

**CONCLUSION.** A valid simulator has been built, which gathers the characteristics to reproduce the real operation of an ICU. The quantitative analysis of decisions concerning bed management is possible in virtual environments that faithfully reproduce the characteristics and evolution of an ICU. The proposed methodology allows for the first time to collect relevant and objective information for the analysis of medical decisions, being able to present scenarios that cause variability. In this way, the best management strategies are identified, quantifying their influence on bed management.

**REFERENCE(S)**

1. This work is integrated into the development of the research carried out by the DECYL research group within the framework of the project MTM 2016-77015-R.
2. Lamé G, Simmons RK. From behavioural simulation to computer models: how simulation can be used to improve healthcare management and policy. BMJ Simulation and Technology Enhanced Learning Published Online First: 20 October 2018. doi:10.1136/bmjstel-2018-000377.
3. Cheng A, Grant V, Auerbach M. Using simulation to improve patient safety: dawn of a new era. JAMA Pediatr. 2015;169:419.
4. Slak ey DP, Simms ER, Rennie KV, et al. Using simulation to improve root cause analysis of adverse surgical outcomes. Int J Qual Health Care. 2014;26:144–50.
5. Cook DA, Hatala R, Brydges R, et al. Technology-enhanced simulation for health professions education: a systematic review and meta-analysis. JAMA. 2011;306:978–88.

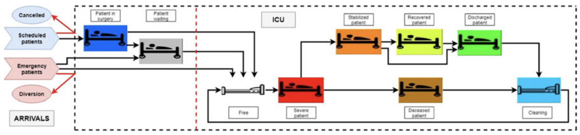

**Fig. 1 (abstract 000052).** Representation of the ICU dynamics through the change of bed's state.

### 000074

### Prospective Clinical Surveillance Using Trigger Tools in Critically Ill Patients

W. Montenegro[1], C. Vasconcelos[2], M. Silva[3], C. Sousa[2], J. Azevedo[4]
[1]Intensive care unit, Hospital São Domingos, São Luis, Brazil; [2]Icu, Hospital São Domingos, São Luis, Brazil; [3]Intensive care unit, Hospital São Domingos, Sao Luis, Brazil; [4]Intensive care unit, Hospital São Domingos, São Luís, Brazil
**Correspondence:** J. Azevedo

*Intensive Care Medicine Experimental* 2019, **7(Suppl 3):**000074

**INTRODUCTION.** In the 1999 landmark report, "To Err is Human: Building a Safe Health System", the Institute of Medicine estimated that avoidable errors in health care contributed to 44 to 98,000 deaths and more than 1 million injuries annually in the United States. Prospective clinical surveillance using triggers as a tool to identify the risk of adverse events, along with prompt implementation of interventions may be the answer to improving patient safety, which remains to be a major public health problem 20 years after the publication of the reference "To err is human".

**OBJECTIVES.** To evaluate the impact of prospective trigger tools and near-real-time interventions on the mortality of critically ill patents, and to determine the impact of these triggers on time taken to stabilize and specific clinical outcomes.

**METHODS.** All adult patients admitted to one of the three intensive care units of a tertiary hospital between November, 2017 to October, 2018 were observed by a multidisciplinary surveillance team composed of physicians, nurses, physiotherapists and pharmacists. If any one of the five triggers was activated, a team member approached the care team responsible for the patient within 24 hours, and the data was presented in the daily multidisciplinary round. This moment was used to establish change in the therapeutic plan in order to achieve the expected goal of each trigger. The triggers used were: **KDIGO**: early identification of AKI to prevent RRT. **Hypoglycemia**: glycemia < 60 mg/dL to prevent recurrence. **Drug interation (D or X)**: early identification to prevent ADR; **Antimicrobial stewardship**:new/modified antimicrobial. Adjustments with MALDI-TOF, MIC or antibiogram to optimize antibiotic therapy. **Delta SOFA**. 3rd >admission SOFA. Expected result 5th day SOFA < 3rd day SOFA.

**RESULTS.** The analysis was conducted with 948 patients, out of which 391 had not identified triggers (group 1) and 557 (group 2) had identified triggers and were subjected to interventions. In relation to the patient population baseline characteristics there was no significant difference between the two groups except for a worse SAPS 3 score observed in group 2. Patients in group 2 had a longer duration of ICU stay; median duration was 5 (IQR, 4 – 10) days for group 1 and 7 (IQR, 4 – 14) days for group 2 (p < 0.001). Stabilization within estimated time was comparable between the two groups and preventable and non-preventable complications were higher in group 2. There was no difference in the mortality rate between the two groups (p= 0.436). Interventions prevented adverse events in the triggers KDIGO (p<0.001); Hypoglycemia (p=0.005); Risk/incompatibility (p<0.001) and antibiotic stewardship (p<0.001) but not in the trigger Delta SOFA. (p=0.433). In the logistic regression analysis of each trigger against time taken to stabilize which was the dependent variable the only trigger intervention that impacted on time taken to stabilize was hypoglycemia.

**CONCLUSION.** This methodology of prospective surveillance using trigger tools and near-real-time interventions was effective in the prevention of adverse events in critically ill patients. Although patients that had identified triggers were more severely ill, their mortality rate was comparable to the patients who did not have identified triggers.

**REFERENCE(S)**

1. Wong BM, Dyal S, Etchells EE, Knowles S, Gerard L, Diamantouros A, Mehta R, Liu B, Baker GR, Shojania KG. Application of a trigger tool in near real time to inform quality improvement activities: a prospective study in a general medicine ward. BMJ Qual Saf. 2015;24(4):272-81
2. Forster AJ, Worthington JR, Hawken S, Bourke M, Rubens F, Shojania K, van Walraven C. Using prospective clinical surveillance to identify adverse events in hospital. BMJ Qual Saf. 2011;20(9):756-63.

### 000087

#### An exploration of the relationships between staff perceptions of safety in adult intensive care (ICU) and patient and staff characteristics

C. Leon-Villapalos[1], M. Wells[2], SJ. Brett[1]

[1]Critical care, Imperial College Healthcare NHS Trust, London, United Kingdom; [2]Directorate of nursing, Imperial College Healthcare NHS Trust, London, United Kingdom

**Correspondence:** C. Leon-Villapalos

*Intensive Care Medicine Experimental* 2019, **7(Suppl 3):**000087

**INTRODUCTION.** Providing a staffing model that meets patients' needs is essential to the delivery of high-quality care. There is incomplete evidence for staffing models in ICU. These are primarily influenced by staff:patient ratios and draw on indices of illness severity (Table 1), which may not be sensitive to patient need. Uni-professional staffing guidance may not reflect the multi-professional nature of ICU. Staff perceptions of safety, of which staffing is a factor, are important organisationally. Perceived safety deficits may contribute to stress and "burnout" in ICU staff, a current field of concern (Seaman 2018).

**OBJECTIVES. Aim** To explore relationships between staff perceptions of shift safety, (how safe patient care felt) and patient and staff characteristics, using a bedside professional reported safety score (BPR). **Objective 1** To explore the relationship between BPR scores and the percentage of patients classified as Level 1, 2 or 3. **Objective 2** To explore the relationship between BPR scores and the percentage of nursing staff with a specialised critical care course (CCC).

**METHODS.** UK Health Research Authority approval was obtained (ID249248). Data were collected for 29

consecutive days at Imperial College Healthcare Trust (comprising 70 general critical care beds on 3 sites). The BPR asked all ICU staff to rate each shift as "safe, unsafe, or very unsafe". These scores were explored with relation to; the percentage of Level 1, 2 and 3 patients per shift and the percentage of nurses per shift who had completed a CCC. Relationships between reported perceptions of safety and these factors were analysed for correlation and regression using PRISM.

**RESULTS.** A total of 2836 BPR scores were recorded. Results were received from 98% of 174 shifts. We found;a significant, positive relationship between perceptions of safety and the percentage of Level 3 patients (p=0.0001, r= 0.32); no significant relationship between perceptions of safety and the percentage of Level 2 patients (p= 0.9729, r= 0.003) and a significant inverse relationship between perceptions of safety and the percentage of Level 1 patients on a shift (p=<0.0001, r= -0.42). We also found a significant relationship between perceptions of safety and the percentage of staff with a specialist CCC (p=0.0001, r= 0.42).

**CONCLUSION.** Staffing models in ICU categorise patients by acuity and allocate staff accordingly. In this study, staff reported feeling less "safe": when there were a larger proportion of less acutely unwell patients in ICU and conversely reported feeling things were safer with higher volumes of more acute patients. This may indicate that staffing models and training are not fully responsive to the needs of intensive care patients, these relationships should be investigated further. The study supports the guidance that 50% of nursing establishment should have a CCC. The mechanism by which this improves perceived safety could be explored.

**REFERENCE(S)**

1. Funding acknowledgment: This study was supported by Imperial Health Charity as part of a Pre-doctoral research grant funded by the National Institute of Health Research (NIHR) Imperial Biomedical Research Centre (BRC)
2. Seaman J, JAMA, 320 (19) 1981-1982, 2018
3. Department of Health, Comprehensive Critical Care; DoH, 2000

**Table 1 (abstract 000087).** Summary of classification of levels of Care (DoH 2000).

| | |
|---|---|
| Level 0 | Acute ward |
| Level 1 | Acute ward with support from critical care |
| Level 2 | At least 1 organ in failure |
| Level 3 | 2 organs in failure / respiratory failure |

### 000103

#### Continuous ambulatory vital sign monitoring – the virtual High Dependency Unit (vHDU) project

C. Areia[1], S. Vollam[1], J. Ede[1], L. Young[1], P. Piper[2], L. Morgan[1], M. D Santos[3], MA. Pimentel,[3], L. Tarassenko[3], P. Watkison[1]

[1]Nuffield Department of Clinical Neurosciences, University of Oxford, Oxford, United Kingdom; [2]Adult intensive care unit, Oxford University Hospitals NHS Foundation Trust, Oxford, United Kingdom; [3]Department of engineering science, Oxford Institute of Biomedical Engineering, Oxford, United Kingdom

**Correspondence:** C. Areia

*Intensive Care Medicine Experimental* 2019, **7(Suppl 3):**000103

**INTRODUCTION.** Timely recognition and escalation of physiological indicators of worsening conditions in acute hospital wards remains challenging. Vital signs monitoring using Early Warning Scoring (EWS) is time consuming and the prescribed observation frequency may be suboptimal or undeliverable. Wearable ambulatory monitoring systems (AMS) may provide an alternative in the form of continuous monitoring, whilst maintaining patient mobility and comfort. AMS can also reduce nursing burden of intermittent vital signs monitoring, improve early detection of abnormal physiological values, facilitate timely escalation and offer an intermediate monitoring option for patients being stepped down from intensive care unit (ICU).

**OBJECTIVES.** This project directly follows previous research and involves a series of linked studies. There are 3 main objectives: test currently available and validated AMS; capture and integrate data into current hospital systems; and evaluate implementation of AMS in clinical practice.

**METHODS.**Phase 1: In-house testing of AMS wearability, to identify the most wearable available devices to move into the next phases. Phase 2: Semi-structured interviews with staff and patients, exploring current monitoring practices within an emergency surgical unit. Phase H-Hypoxia study: Accuracy test of selected AMS in the detection of hypoxia and movement artefacts during clinical use in healthy volunteers. Phase 3: Locational implementation of the AMS to provide continuous data recording in a clinical setting. A human factors approach will direct modifications to both hardware and software and further wearability and user satisfaction assessments will be conducted on clinical staff. Phase 4: Development of a functional user interface (UI) integrating AMS data within current hospital systems. We will introduce the devices to patients, collect wearability data and finalise the locational testing. Phase 5: Implementation of the AMS and UI in clinical practice to test the acceptability and usability of the system with clinical staff. We will also analyse patient specific outcomes such as mortality, length of stay (LOS) and deterioration episodes captured by the AMS.

**RESULTS.** Phase 1 provided wearability data from 15 volunteers and shortlisted half of the AMS to next phases (1 chest patch and 3 wrist-worn) collecting HR, RR and SpO2 data. The selected devices are now being subject to locational (phase 3, n=20) and accuracy testing (hypoxia study, n=30). Fifteen clinical staff have been interviewed and identified challenges with current monitoring practices, such as the impact on nursing workload and patient mobility. Current perceptions of an AMS were also explored. These themes will be considered during user interface development in phase 4 (n=50), alongside continuous user input (Figure 1).

**CONCLUSION.** Technology to monitor patients' vital signs is advancing and clinical vital signs documentation systems need to be synchronous with these changes. Our proposed work aims to develop an electronic system to continuously capture and report vital signs. This will integrate real-time AMS data into current hospital systems via a user interface. The final system has the potential to offer benefits to patient outcomes, nursing workload and facilitate quicker referral and smoother step-down from ICU. We will discuss current outcomes and future plans for this project, the first of its kind outside of a critical care department.

**REFERENCE(S)**

1. This study/project is funded by the NIHR Oxford Biomedical Research Centre.
2. Prgomet, M., Cardona-Morrell, M., Nicholson, M. et al (2016). Vital signs monitoring on general wards: clinical staff perceptions of current practices and the planned introduction of continuous monitoring technology. International Journal for Quality in Health Care, 28(4), 515–521. https://doi.org/10.1093/intqhc/mzw062

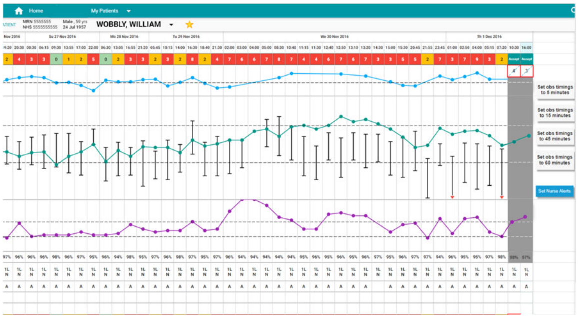

**Fig. 1 (abstract 000103).** See text for description

### 000120

#### Pressure injury prevention programs in adult intensive care patients: a systematic review

F. Lin[1], W. Chaboyer[1], F. Coyer,[2], Z. Wu,[3], B. Song,[3]
[1]School of nursing and midwifery, Griffith University, Gold Coast, Australia; [2]School of nursing, QUT Gardens Point Campus, Brisbane City, Australia; [3]School of nursing, China Medical University Shenbei Campus, Shenyang Shi, China
**Correspondence:** F. Lin
*Intensive Care Medicine Experimental* 2019, **7(Suppl 3):**000120

**INTRODUCTION.** Pressure injuries are costly, potentially preventable but frequently occurring adverse events (Padula et al., 2018). Intensive care patients have major risk factors for pressure injury with a reported pressure injury prevalence of 12-33% (Chaboyer et al., 2018). Multi-faceted interventions, also known as programs or care bundles, are recommended to prevent pressure injuries.

**OBJECTIVES.** This systematic review aimed to: identify pressure injury prevention programs and their components; the way these programs were implemented; and their effectiveness.

**METHODS.** We followed the recommendations from the Preferred Reporting Items for Systematic Reviews and Meta-Analysis. We searched databases including PubMed, EMBASE, MEDLINE, CINAHL, and Cochrane Library databases for papers published in English and Chinese from the year of 2000 to May 2018. Data extraction and quality assessment was conducted by one and checked by a second author. Content analysis was conducted to summarise the findings.

**RESULTS.** Twenty-one peer reviewed papers (12 quality improvement projects, and 9 research papers from 8 studies) were included in this review. Pressure injury prevention care bundles with 2-11 components were commonly implemented. Common components of programs included clarification of staff roles/introducing new roles, repositioning, staff and patient education, support surfaces use, pressure injury risk assessment, skin assessment, nutrition needs assessment, documentation, multidisciplinary team involvement, and mobilisation. Implementation strategies commonly used were education, audit and feedback, and standardising documentation. Five of the research studies reported significant decrease in pressure injury prevalence, significant increase in family satisfaction and participation, and increase in compliance to pressure injury prevention protocols and strategies. Two quality improvement study reported cost savings of $1 million and £2.6 million respectively after the implementation of the programs.

**CONCLUSION.** Most of the included papers reported findings from either qualitive improvement projects or before and after research studies. However, positive outcomes and strong theoretical rationales for the components in the programs suggest they are beneficial. In addition, the success of the programs did not seem to be associated with the number of components included in or complexity of the programs. This calls for future high-quality research such as randomised controlled trials to test the effectiveness of multi-component programs.

**REFERENCE(S)**

1. Padula, W.V., Pronovost, P.J., Makic, M.B.F., Wald, H.L., Moran, D., Mishra, M.K., Meltzer, D.O., 2018. Value of hospital resources for effective pressure injury prevention: a cost-effectiveness analysis. BMJ Quality & Safety 28, 132-141.
2. Chaboyer, W., Thalib, L., Harbeck, E.L., Coyer, F., Blot, S., Bull, C., Nogueira, P., Lin, F., 2018. Incidence and prevalence of pressure injuries in adult intensive care patients: A systematic review and meta-analysis. Critical Care Medicine Online First (P), 1-8.

### 000156

#### Antithrombin deficiency has lower prognostic value in comparison with increased INR in ICU patients with SIRS

E. Neporada, A. Travkov, S. Astrakov
Anaesthesiology and intensive care department, Novosibirsk State University, Novosibirsk, Russia
**Correspondence:** E. Neporada
*Intensive Care Medicine Experimental* 2019, **7(Suppl 3):**000156

**INTRODUCTION.** Low platelet count and increased INR are frequent laboratory findings in patients with systemic inflammatory response syndrome (SIRS) and have prognostic value. Antithrombin (AT) deficiency is reported to be observed early in SIRS, but this test is not used routinely.

**OBJECTIVES.** The aim of the study is to determine if AT deficiency is a useful prognostic tool in ICU patients.

**METHODS.** 135 consecutive ICU patients with 3 or 4 criteria of SIRS were included. During 4 days after SIRS manifestation patients were assessed for thrombocytopenia <120 cells/mcL or severe platelet decline ≥50% during 24 hours in combination with INR>1,2. Also general condition, clinical and laboratory test results and 30-days mortality were evaluated.
**RESULTS.** Significant laboratory haemostasis disorders (thrombocytopenia in combination with increased INR) were observed in 26.6% of patients with SIRS. In patients with severe AT deficiency<50% odds ratio for laboratory haemostasis disorders was 3.5 (95% CI 1.2; 10.4).
ratio for laboratory haemostasis disorders was 3.5 (95% CI 1.2; 10.4).
However AT deficiency did not demonstrate significant prognostic value (p=0.18), while INR was a significant predictor of outcome (p= 0.024)
**CONCLUSION.** AT deficiency is a frequent finding in patients with SIRS the same as low platelet count and increased INR. Yet such common laboratory test as INR in comparison with AT deficiency has higher prognostic value in ICU patients with SIRS.

**Table 1 (abstract 000156).** Variables, predicting laboratory haemostasis disorders (thrombocytopenia in combination with increased INR)

| Independent variables | Regression coefficient | p | Odds Ratio | 95% CI for OR |
|---|---|---|---|---|
| Gender (females/ males) | 1.58 | 0.007 | 4.85 | 1.54; 15.30 |
| AT deficiency <50% | 1.26 | 0.022 | 3.52 | 1.20; 10.40 |
| Fibrinolysis time | -0.02 | 0.049 | 0.98 | 0.96; 1.00 |

**Table 2 (abstract 000156).** Variables, predicting outcome at the 30th day

| Independent variables | Regression coefficient | p | Odds Ratio | 95% CI for OR |
|---|---|---|---|---|
| INR | 1.94 | 0.020 | 6.92* | 1.36; 35.25 |
| APACHE II | 0.12 | 0.014 | 1.13* | 1.02; 1.24 |
| Age (years) | 0.03 | 0.065 | 1.03 | 1.00; 1.07 |
| AT deficiency <50% | -0.85 | 0.180 | 0.43 | 0.12; 1.48 |

### 000157
### Use of a digital checklist and database to improve patient safety in critical care

J. Turner, A. Kuravi
Theatres, anaesthesia and critical care services, Walsall Healthcare NHS Trust, Walsall, United Kingdom
**Correspondence:** J. Turner
*Intensive Care Medicine Experimental* 2019, **7(Suppl 3):**000157

**INTRODUCTION.** Safety huddles and checklists are recognised methods to improve patient safety, and computer-based tools such as automatic early warning score systems and electronic patient records can be used to further augment patient care. In a 13-bed critical care unit within a 550-bed UK district general hospital, a delay in identification and treatment of critically ill patients, and identification of potential patient safety issues was noted. A structured electronic communication tool was developed with the aim of providing accurate, real-time data on patient safety issues, allowing rapid multi-disciplinary response.
**METHODS.** A digital checklist was designed using Microsoft Access, and a morning multi-disciplinary safety huddle was instituted. Data on the number and location of patients at risk of deterioration as well as other safety critical issues were systematically highlighted, recorded electronically, and automatically audited by the software.
**RESULTS.** In two months, 202 patients were identified as requiring urgent review, of whom 68 were outside the critical care unit. Nurse staffing matched patient acuity only 61% of the time, with equipment issues present on 40% of days. Airway equipment had been checked 91% of the time, and high flow nasal oxygen or non-invasive ventilation machines were available to outreach on 73% of days. There were infection control issues on 52% of occasions. When surveyed, staff reported more rapid resolution of patient safety issues, and feeling more engaged with the process of task prioritisation following the introduction of the safety huddle.
**CONCLUSION.** Use of an automatically audited digital checklist to facilitate a daily safety huddle has allowed recognition of potentially significant patient safety issues, and ensured that these are highlighted to everyone including the most senior members of staff, allowing corrective action to be undertaken promptly. The data has led to the ward round being redesigned to facilitate rapid senior review of the most unwell patients. The digital format obviates the need for laborious data collection, in addition to allowing remote monitoring by senior management. Following the success of this, we have increased the huddle to twice daily.

**REFERENCE(S)**
1. https://improvement.nhs.uk/resources/safety-huddles/ Accessed 28/03/2019
2. GlobalSurg Collaborative (2019). "Pooled analysis of WHO Surgical Safety Checklist use and mortality after emergency laparotomy". British Journal of Surgery. 106 (2): 103–112
3. Heller A et al. Detection of Deteriorating Patients on Surgical Wards Outside the ICU by an Automated MEWS-Based Early Warning System With Paging Functionality. Annals of Surgery. Published ahead of print May 16 2018.
4. Sittig D, Singh H. Electronic Health Records and National Patient-Safety Goals. New England Journal of Medicine. 2012; 367:1854-1860

### 000184
### Towards better governance in Focused Intensive Care Echocardiography (FICE)

MSF. Chong, J. Aron
General Intensive Care Unit, St. George's Hospital, London, United Kingdom
**Correspondence:** M.S.F. Chong
*Intensive Care Medicine Experimental* 2019, **7(Suppl 3):**000184

**INTRODUCTION.** With the introduction of Focused Intensive Care Echocardiography (FICE) training programmes in the UK, it is essential that each FICE training unit should have a local echo governance structure.
**OBJECTIVES.** Our quality improvement project involved auditing the process for local image storage and review and quality assurance of echo reporting and referral pathways before and after introduction of a formal echo reporting form.
**METHODS.** A prospective observational study was performed to examine all transthoracic echos performed in our tertiary referral general intensive care unit over a two week period in November 2018 for: (i) local image storage, (ii) network archiving, (iii) echo reporting documentation and (iv) referral for formal echo. After a teaching session and introduction of an echo reporting form on the electronic health record system (Cerner), the data was reaudited two months later in January 2019.
**RESULTS.** 20 scans were performed by FICE trainees on ICU in November 2018 and 22 scans in January 2019 over the two week periods. Image storage on the local ultrasound hard drive occurred 100% in both periods and network archiving increased from 45% to 50%. Echo reporting documentation improved from 30% to 68% after introduction of the echo reporting form. Formal echo referral increased from 20% to 45% with performance of formal echo on ICU occurring 75% and 80% respectively prior to ward discharge.
**CONCLUSION.** Introduction of a formal echo reporting form has led to improved quality assurance of reporting and referral pathways.

**REFERENCE(S)**
1. Fletcher SN, Grounds RM. Critical care echocardiography: cleared for take-up. BJA 2012; 109: 490-2

2. Intensive Care Society. Focused Intensive Care Echocardiography (FICE) accreditation pack. https://www.ics.ac.uk/ICS/Pdfs/FICE_Accreditation_.aspx. Accessed on 5 Feb, 2019

### 000190

#### Is after-hours intensivist staffing required in a surgical intensive care unit?

MK. Kim, IK. Kim
Department of surgery, Yonsei University College of Medicine, Seoul, Republic of Korea
**Correspondence:** M.K. Kim
*Intensive Care Medicine Experimental* 2019, **7(Suppl 3):**000190

**INTRODUCTION.** The need for 24-hour intensivist coverage of the intensive care unit (ICU) presents several controversial issues. Surgical patients are rapidly deteriorated compared with medical patients. In this study, we evaluated the necessity of after-hours intensivist staffing in a surgical intensive care unit (SICU).
**METHODS.** This study was performed in a tertiary referral teaching hospital in Korea from March 2018 until February 2019. A total of 586 cases admitted to SICU. The work-hours was defined as 07:00-19:00, from Monday to Friday. Among them, 571 SICU admissions which had complete medical records were enrolled in this retrospective study. We determined the in-hospital mortality as the primary outcome according to the time of SICU admission.
**RESULTS.** 333 SICU admissions and 238 SICU admissions were developed in work-hours and after-hours respectively. The mean age of the work-hours group was higher than those of the after-hours group (work-hours vs. after-hours = 68.55±12.52 vs. 64.06±13.84, $p<0.001$). APACHE II score of 30 or more of the after-hours group was two times more than those of work-hours group (work-hours vs. after-hours = 6.0% vs. 12.6%, $p$=0.004). Unplanned admission during work-hours was about one and half times more during after-hours (work-hours vs. after-hours = 33.3% vs. 47.1%, $p$<0.001). When compared to the work-hours group, the proportion of patients with ventilator treatment was two times more in the after-hours group (work-hours vs. after-hours = 17.4% vs. 34.0%, $p$<0.001). Among 571 SICU admissions, 27 patients died in hospital. In multivariate analyses for clarifying whether the time of SICU admission affected in-hospital mortality, the time of SICU admission was not the independent factor for in-hospital mortality (OR = 2.385; 95% CI 0.928-6.133, $p=0.071$).
**CONCLUSION.** In this study, the time of SICU admission did not foment greater in-hospital mortality. However, these results revealed our specific situation, high-intensity ICU model. Therefore, in order to generalize these results, related studies should be preceded.

**REFERENCE(S)**

1. Br J Anaesth. 2018 Jun;120(6):1420-1428.
2. N Engl J Med. 2013 Jun 6;368(23):2201-9
3. Crit Care Med. 2016 Aug;44(8):1553-602

### 000219

#### Long-term physical recovery after critical illness is limited

LFE. Beumeler,[1], NA. Bruins,[2], CM. De Jager[2], H. Buter[2], M. Koopmans[2], T. Van Zutphen[1], EC. Boerma[2]
[1]Campus fryslân, University of Groningen, Leeuwarden, Netherlands; [2]Department of intensive care, Medical Center Leeuwarden, Leeuwarden, Netherlands
**Correspondence:** L. Beumeler
*Intensive Care Medicine Experimental* 2019, **7(Suppl 3):**000219

**INTRODUCTION.** Post-ICU sequellae including physical and mental problems interfere with successful rehabilitation [1]. However, data on the incidence of long-term non-recovery after critical illness seem to be lacking.
**OBJECTIVES.** To assess the incidence of and to identify risk factors for non-recovery in long-stay ICU patients.
**METHODS.** In this retrospective, single-centre study, we identified long-stay ICU patients who visited a specialized post-ICU clinic at 3 months after ICU-discharge. During this visit a Dutch 36-item Short Form (SF-36) questionnaire was completed. At 12 months, patients were requested to repeat the SF-36 questionnaire and return by mail.
**RESULTS.** Between 2012 and 2018 591 patients visited the specialized post-ICU clinic. 241 Patients with a mean age of 64±13 and an APACHE III score of 79±32 completed a SF-36 questionnaire at both time points. At 3 months 52 patients (21.5%) recovered to reference values of the physical functioning (PF) SF-36 domain. However, after 12 months only 36 remained within this normal range (14.9%). At 3 months 190 patients (78.5%) were below the reference values of the PF SF-36 domain. At 12 months 152 (62.8%) still remained below this reference values. In total 168 (69.4%) did not reach reference values for the PF SF-36 domain at 12 months. Between 3 and 12 months 126 patients (52.1%) improved over time, whereas in 116 patients (47.1%) the PF SF-36 score decreased or remained equal over time. In a binary multivariate analysis diabetes (OR 0.028, 95% CI 0.001-0.788, p=0.036), days on mechanical ventilation (OR 0.901, 95% CI 0.821-0.988, p=0.026), need for renal replacement therapy (OR 9.249, 95% CI 1.286-66.551, p=0.027) and the Morton Mobility Index score (OR 1.063, 95% CI 1.017-1.111, p=0.007) all remained independent predictors for recovery between 3 and 12 months.
**CONCLUSION.** In the majority of long-stay ICU patients the PF SF-36 score did not fully recover at 12 months after ICU-discharge. Moreover, only half of the patients improved between 3 and 12 months. Determination of risk factors may help to identify patients in danger of non-recovery, and to design individualized rehab strategies.

**REFERENCE(S)**

1. Hofhuis J. et al. Health-related quality of life in critically ill patients: how to score and what is the clinical impact? Curr Opin Crit Care 2009; 15: 425-30.
2. NA

### 000224

#### Influence of income and education on outcomes of intensive care in a healthcare system with full universal health insurance - a nationwide analysis of individual-level data

S. Walther[1], L. Orwelius,[2], M. Kristensson,[3], F. Sjöberg,[4]
[1]Department of cardiothoracic anaesthesia and intensive care, Linkoping University Hospital, Linköping, Sweden; [2]Department of anaesthesia and intensive care, Linköping University, Linköping, Sweden; [3]Department of medical and health sciences, Linköping University, Linköping, Sweden; [4]Department of clinical and experimental medicine, Linköping University, Linköping, Sweden
**Correspondence:** S. Walter
*Intensive Care Medicine Experimental* 2019, **7(Suppl 3):**000224

**INTRODUCTION.** Socioeconomic deprivation is associated with less access to health care services, including intensive care. Choice and quality of treatment during intensive care may also be influenced by socioeconomic factors leading to inferior care and poor outcomes among deprived patients.
**OBJECTIVES.** Our purpose was to examine if low education and low income was associated with worse survival after critical illness.
**METHODS.** We examined records of adult patients in the Swedish Intensive Care Registry with an ICU stay > 48 hrs during 2008-2016. The Charlson comorbidity index (CCI) before critical illness was computed by linking to the Swedish hospital discharge register and SAPS3 was used to describe critical illness severity. Linking with Statistics Sweden provided disposable household income for the year preceding ICU admission, level of education (low, medium, high) and survival. Patients were grouped in quintiles by their income, from low to high. Outcomes were odds ratios for survival on discharge from ICU or hospital and at 90 days after admission. The influence of education and income, separately and combined, was modelled using multi-level logistic regression after adjusting for age, gender, CCI and SAPS3 scores. The study was approved by the regional ethical review board.

**RESULTS.** We analysed 55,091 patients admitted to 74 ICUs (mean age 64 yrs, SAPS3 risk 0.25, CCI score 2.2; median ICU and hospital stay was 4.1 and 12 days, respectively). Low income and low education was not associated with decreased survival to discharge from ICU or hospital but they were associated with decreased 90-day survival (Table).

**CONCLUSION.** Low education and low disposable income was not consistently associated with adjusted lower ICU or hospital survival indicating that ICU and hospital care was not inferior for deprived patients. However, adjusted 90-day survival was significantly lower suggesting that post-discharge care was inferior.

**Table 1 (abstract 000224).** Odds ratios (ORs) for the separate models and the combined model. All models were adjusted for age, gender, CCI and SAPS3 scores ** and *** denotes that ORs were significantly different from 1 (P<.01 and <.001, respectively). NA=not applicable

| | Education model | | | Income model | | | Combined model | | |
|---|---|---|---|---|---|---|---|---|---|
| Outcomes | ICU | Hosp. | 90 d. | ICU | Hosp. | 90 d. | ICU | Hosp. | 90 d. |
| Education, low | Reference | | | NA | NA | NA | Reference | | |
| Education, medium | .99 | 1.02 | 1.09*** | NA | NA | NA | 1.01 | 1.02 | 1.07** |
| Education, high | 1.07 | 1.04 | 1.22*** | NA | NA | NA | 1.12** | 1.06 | 1.15*** |
| Income 1st quint. | NA | NA | NA | Reference | | | Reference | | |
| Income 2nd quint | NA | NA | NA | .94 | .95 | 1.04 | .94 | .95 | 1.04 |
| Income 3rd quint. | NA | NA | NA | .98 | .99 | 1.18*** | .97 | .98 | 1.16*** |
| Income 4th quint. | NA | NA | NA | .87** | .97 | 1.18*** | .85** | .94 | 1.14*** |
| Income 5th quint. | NA | NA | NA | .88** | .96 | 1.27*** | .85** | .94 | 1.21*** |

### 000251

#### Self-perceived discomfort in critically ill patients: comparison between stays in 2 separate units of a single department, ICU vs. intermediate care unit

J. AUDIBERT, P. Bachelier, A. Bensalah, I. Rabaud, A. Conia, O. Gontier, M. Hamrouni, C. Jourdain, G. Badre, L. Lehaie, M. Pépineau, B. Mauchien, P. Kalfon

Intensive care unit, Hospital Louis Pasteur, Le Coudray, France

**Correspondence:** J. AUDIBERT

*Intensive Care Medicine Experimental* 2019, **7(Suppl 3):**000251

**INTRODUCTION.** A tailored multicomponent program for discomfort reduction in critically ill patients decreases self-perceived discomfort in unselected adult critically ill patients (1), with a probable positive impact on the post-traumatic stress disorder assessed at 1 year (2). Patient-centered outcomes and long-term psychological side effects of the critical illness have gained a growing interest and must now be taken into consideration.

**OBJECTIVES.** Should the dissemination of such a program be actively promoted also for patients requiring intermediate care? Which organization of care should be recommended? The aim of our pilot study was to assess and compare discomforts reported by patients after a stay in an intensive care unit (ICU) and after a stay in a dedicated, separate, intermediate care unit, both units belonging to a single department and managed by the same healthcare team.

**METHODS.** Our intensive care department was composed of a 12-bed ICU and a 6-bed dedicated, separate, intermediate care unit. On the day of discharge, the bedside nurse (or the assistant nurse) administered the 18-item version of the French questionnaire on ICU-related self-perceived discomforts (IPREA) to adult patients who survived a stay of 3 calendar days or more (3). IPREA contains the following discomfort items: noise, excess of light, discomfort related to sleeping in a different bed from home, sleep deprivation, thirst, hunger, feeling of cold, feeling of heat, pain, being tied down by perfusion lines, tubes or other connected devices, no respect for privacy, anxiety, isolation, limited visiting hours, absence of phone, lack of information, shortness of breath, and feeling depressed. Each item was scored from 0 to 10 (minima to maximal) leading to an overall discomfort score (from 0 to 100) used as the primary outcome.

**RESULTS.** Between May 1, 2018 and April 20, 2019, 191 and 174 patients were interviewed using IPREA, respectively after an ICU stay and after a stay in the intermediate care unit. Stays in the ICU and in the intermediate care unit were similar for gender (female gender resp. 42% and 36%), age (resp. 62±17 and 62±17 years), and median length of stay, resp. 6 (IQR 4-11) and 6 (IQR 4-8) days. Patients hospitalized in the ICU reported a higher overall discomfort score than patients hospitalized in the intermediate care unit: 23±15 vs. 18±12, *P=0,0008*. The greatest differences in favor of the intermediate care unit were found for feeling of cold (0.9±2.0 vs. 2.3±3.2), shortness of breath (2.1±2.7 vs. 3.3±3.5), lack of phone (0.5±1.7 vs. 1.6±3.0), and thirst (2.4±3.0 vs. 3.4±3.6). In contrast, patients complained more about heat in the intermediate care unit in comparison with the ICU.

**CONCLUSION.** Patients hospitalized in a dedicated, separate, intermediate care unit reported a lower overall discomfort score than patients hospitalized in the ICU. Although patient severity was different depending on the unit, these preliminary results raise the question of the optimal organization of care for patients receiving intermediate care: hospitalization inside the ICU for all the patients or hospitalization in a dedicated, separate, intermediate care unit, before, after, or without the ICU stay, according the course of the critical illness? Further studies are needed to compare perceived discomforts according these two different organisations of care for patients requiring intermediate care.

**REFERENCE(S)**

1. Kalfon, P., Baumstarck, K., Estagnasie, P. et al. Intensive Care Med (2017) 43: 1829
2. Kalfon, P., Alessandrini, M., Boucekine, M. et al. Intensive Care Med (2019) 45: 223
3. Baumstarck et al. Health and Quality of Life Outcomes (2019) 17:29

### 000261

#### Evaluation of quality of life and its different components in patients with traumatic brain injury after one year of ICU admission

M. Guerrero Marin[1], M. Ruiz Garcia[1], M. Delange Van Der Kroff[2], D. Arias-Verdú[3], E. Curiel Balsera[3], A. Muñoz-Lopez[3], JF. Fernandez-Ortega[3], E. Aguilar-Alonso[4], M. Prieto-Palomino[3]

[1]Intensive care, Hospital of Jaen, Jaén, Spain; [2]Intensive care, Hospital Comarcal Axarquia, Vélez-Málaga, Spain; [3]Intensive care, Hospital Carlos Haya, Málaga, Spain; [4]Hospital Cabra, Cabra, Spain

**Correspondence:** E. Aguilar-Alonso

*Intensive Care Medicine Experimental* 2019, **7(Suppl 3):**000261

**INTRODUCTION.** Many instruments have been developed to evaluate hospital mortality, but less attention has been paid to the long-term functional status and quality of life of traumatic brain injury patients
**OBJECTIVES.** To analyze the quality of life (QOL) and its different components one year after ICU admission to traumatic brain injury (TBI).
**METHODS.** Prospective cohort study. TBI in Carlos Haya Hospital (Málaga, Spain) between 2004-2008. Evaluation of QOL with questionnaire PAECC (Project of Epidemiological Analysis of Critical Patients), We analyzed physiological activities (oral communication, urinary and defecation control, food intake), daily life (mobility, work activity, etc.) and emotional state. (0 normal to 29 major deterioration).
**RESULTS.** N=531. Age 40.35±19.75 years, APACHE-II 17.94±6.97, GCS admission 7.53±3.83 points. TAC admission: Type I (10.4%), type II (28.1%), type III (24.5%), type IV (8.3%), mass evacuated (22.6%), not evacuated (6.2%). Hospital mortality: 28.6%. Mortality 1 year: 32.2% (lost:6.6%). Quality of life at one year (n=324) 9.44±8.73 points (significant deterioration).
Almost 80% patients have normalized the basic physiological activities, except oral communication that only maintain 54.8%, another 16.6% communicate with difficulty although with coherent dialogue. Low percentage of work reintegration and to make great efforts. They present normality in a high percentage in tolerance to minimum efforts, movements of precision and social relations. Low percentage presents normality in the items on emotional state or subjective aspects of the patient.
**CONCLUSION.** Patients who enter the ICU due to TBI at one year present significant deterioration in quality of life. Although a high percentage present normality in basic physiological activities, social relationships and physical activities that require little effort. On the contrary, a low percentage present normality in the subjective aspects, make great efforts and work activity.

**Table 1 (abstract 000261).** See text for description

| Normal 1º year (%) | | | |
|---|---|---|---|
| Basic physiological activities | | Normal daily life activities | |
| Oral communication | 54,8 | Movements | 83,1 |
| Urinary control | 79,1 | Minor effort | 71,2 |
| Defecation control | 81,9 | Major effort | 35,3 |
| Food intakes | 81 | Walking | 63,8 |
| Emotional status | | Mobility | 46,6 |
| Subjective well being | 41,1 | Dressing | 63,8 |
| State of mind | 40,2 | Working life activities | 28,5 |
| Vitality | 64,4 | Social relationship | 70,2 |

### 001304
### Nationwide analysis of primary cardiogenic shock epidemiology and mortality in Intensive Care Unit

G. Tavazzi[1], S. Finazzi,[2], A. Bottazzi,[3], G. Bertolini,[2], G. Giviti[2]
[1]Department of clinical, surgical, diagnostic and pediatric sciences; intensive care unit, University of Pavia; Fondazione Policlinico San Matteo IRCCS, Pavia, Italy; [2]Dipartimento di epidemiologia clinica, GiViTI Coordinating Center, IRCCS, Istituto di Ricerche Farmacologiche 'Mario Negri', Ranica, Italy; [3]Anesthesia and intensive care department, Fondazione I.R.C.C.S. Policlinico San Matteo, Pavia, Italy
**Correspondence:** G. Tavazzi
*Intensive Care Medicine Experimental* 2019, **7(Suppl 3):**001304

**INTRODUCTION.** Cardiogenic shock (CS) describes a physiological state of end-organ hypoperfusion characterized by reduced cardiac output. Almost all the data in literature pertains only to CS related to myocardial infarction (AMI-CS), reporting a mortality up to 45%(1).
**OBJECTIVES.** We sought to analyse clinical and epidemiological data on patients admitted to general ICU enrolled in nationwide GiViTi registry (Gruppo italiano per la Valutazione degli interventi in Terapia intensiva) with a diagnosis of primary CS
**METHODS.** Observational prospective study. Out of 485509 admitted in 301 general ICUs from 2011 to 2017, 19250 patients met the enrolment criteria: > 18 years old, diagnosis of CS due to primary cardiocirculatory failure. Only patients with all data from the admission to the outcome in the last discharge hospital have been included in the analysis. CS secondary to other reasons of circulatory shock have been excluded (2). Patients demographic, admission, complications, treatment and mortality data have been analysed
**RESULTS.** Patients mean age admission was 70.9 (±12.7), 61.9 % were male and 92.3% presented cardiovascular comorbidities in the past medical history. 16709 (86.8 %) patients were transferred in ICU from of the same hospital; 12.7% were referred from another hospital and 0.5% from hospices. 52% were transferred from the emergency department while 10.5% were centralized from an another ICU. The main cardiovascular disorder leading to ICU admission were: Cardiac arrest (36.9%); Acute decompensated heart failure (40.1%); myocardial infarction (17.9%); pulmonary embolism (3.9%). Right ventricular heart failure was diagnosed in 6.9% of the population. 96.2% required catecholamine; 90.8% of patients underwent invasive ventilation during the admission, 11.7% renal replacement therapy; 13.8% intra-aortic balloon pump, 2 % received ECMO and 0.5% implanted a ventricular assisting device.ICU mortality was 50.4%, In-hospital mortality was 57.3% and the mortality at discharge from the last hospital further increased to 59.5% (Table 1). The mean ICU admission duration of survived patients was 9.3 days (±11.9) and for those who did not survive was 11 day (± 19.1)
**CONCLUSION.** Primary CS has a unacceptably ICU and intra-hospital mortality.This preliminary descriptive nationwide analysis underlines the urgency of a better delineation of the pathophysiology and treatment of CS. The organization of large registry aiming at the CS clinical phenotypization in the view of therapy titration has effectively reduced the burden of adverse outcome in acute heart failure and myocardial infarction, and it should be considered also for the CS syndrome

**REFERENCE(S)**
1. Reyentovich A., Barghash M.A. and Hochman J. Management of refractory cardiogenic shock. Nat Rev Cardiol. 2016 Aug;13(8):481-92
2. Vincent JL., De Backer D. Circulatory Shock. N Engl J Med 2013;369:1726-34

**Table 1 (abstract 001304).** ICU and in hospital mortality split by main CS aetiology

| Aetiology | ICU mortality | In hospital mortality |
|---|---|---|
| Cardiac Arrest | 57.4% | 62.8% |
| Decompensaed heart failure | 44.7% | 51% |
| Myocardial infarction | 44.4% | 52.8% |
| Pulmonary Embolism | 61% | 65.3% |

### 000735
### Lung Protective Ventilation in the Emergency Department

A. Rasheed
St John of God Hospital Group, Perth, Western Australia, Perth, France
*Intensive Care Medicine Experimental* 2019, **7(Suppl 3):**000735

INTRODUCTION. Mechanical ventilators in the Emergency Department (ED) are becoming more common and more advanced. The benefits of lung protective ventilation versus conventional ventilation have been well established in the ICU setting1. The LOV-ED trial2 is the first trial of its kind that displays the benefits on lung protective ventilation in the ED setting, showing a decrease in patients developing Acute Respiratory Distress Syndrome (ARDS) when ventilated using lung protective strategies.
We carried out this audit in our ED to discover how many patients were receiving lung protective ventilation. Secondary outcomes included adequate oxygenation and time taken to perform the first arterial blood gas (ABG) while receiving mechanical ventilation.
METHODS. We retrospectively recruited all patients who received mechanical ventilation in the first nine months of 2018. Patients with restricted medical files were excluded. Using the ventilator observation sheet in each patient's medical record, the initial ventilator settings were identified. Initial ABG times and measurements were recorded from the ClinicalLabs application and cross referenced with the CIS database.
Patient weight was based on recorded actual weight or estimated weight, there was no recordings of height and therefore no access to predicted body weight.
RESULTS. 49 patients were recruited. 8 patients had to be excluded due to restricted records. Of the 41 patients audited, 17 patients (41%) had lung protective tidal volumes (4- 6mls/kg) initiated in the ED. Mean tidal volume was 6.5ml/kg.
15 patients (37%) of patients had no ABG performed during the duration of their stay in ED while ventilated. Average length of stay (LOS) for these patients with no ABG performed in ED was 3.6 hours – the same as the overall LOS of the whole cohort.
Of the remaining 26 patients, only 12 had an ABG performed in the first 30 minutes of being intubated. Average time to ABG was 72 minutes.
Of the patients who did have an ABG performed post ventilation in the ED, 17 (65%) were hyperoxaemic (paO2 >120mmHg). Mean length of stay in the ED post intubation was 3.6 hours.
CONCLUSION. The lack of predicted body weight had a very negative effect on this audit. Lung protective ventilation settings are all based of lean body weight, rather than actual or estimated weight. This resulted in mostly over-estimating the tidal volumes required for lung protective settings. On initial glance the mean tidal volume (6.5ml/kg) may seem quite close to the recommended lung protective volume (4-6ml/kg), however if the overestimated weights were to be taken into account, the mean tidal volume would be much higher.
A surprising secondary outcome was the time to first ABG. 15 patients not having an ABG performed at all until in a different department is a poor statistic. Furthermore, of the patients who did have a gas done while in the department, most were much too
delayed. A large proportion of patients received high FiO2 resulting in hyperoxaemia,
thus making the initial ABG timing even more crucial to allow adequate titration.

##### REFERENCE(S)

1. Fuller B, Ferguson I, Mohr N, Drewry A, Palmer C, Wessman B et al. Lung-Protective Ventilation Initiated in the Emergency Department (LOV-ED): A Quasi- Experimental, Before-After Trial. Annals of Emergency Medicine. 2017;70(3):406- 418.e4.
2. Acute Respiratory Distress Syndrome Network. Ventilation with Lower Tidal Volumes as Compared with Traditional Tidal Volumes for Acute Lung Injury and the Acute Respiratory Distress Syndrome. New England Journal of Medicine. 2000;342(18):1301-1308.

## TEM - New insights in trauma, toxicology and burn management

#### 000696

#### Strict glycaemic control in the polytrauma patient: A Systematic Review and Meta-Analysis

K. arjan[1], F. Bird[2], G. Grier[3]
[1]Institute of pre-hospital care, Barts and The London School of Medicine and Dentistry, London, United Kingdom; [2]Institute of pre-hospital care, Barts Health NHS Trust, London, United Kingdom; [3]Institute of pre-hospital care, The Royal London Hospital, London, United Kingdom
**Correspondence:** K. arjan

*Intensive Care Medicine Experimental* 2019, **7(Suppl 3):**000696

**INTRODUCTION.** Stress-induced hyperglycaemia in the critically ill trauma patient is associated with increased mortality and morbidity [1]. The benefits and risks of strictly controlling glucose have yet to be determined in this population.
**OBJECTIVES.** To investigate the effects of strict glycaemic control, in polytrauma patients, on adverse outcomes through systematic review and meta-analysis.
**METHODS.** This review was prospectively registered on the Prospero database (CRD42018102948). A systematic literature search was carried out in three electronic databases: Medline, EmBase, and Web of Science from 1980-2018. Both randomised controlled trials (RCTs) and non-randomised controlled trials (nRCT) were included. Studies were included if they compared two groups under either a strict or loose glycaemic regime. Risk of bias was carried out by a single reviewer. The primary outcome was risk of mortality and the secondary outcomes were risk of hypoglycaemia (<3.33mol/L), days in hospital, days on ventilation, and occurrence of septicaemia. Data was analysed using the random-effects model in RevMan 5.3. Dichotomous outcomes are given as risk ratios (RR) and continuous outcomes as mean difference (MD), both with 95% confidence intervals.
**RESULTS.** 2 RCTs (n=957) and 7 nRCTs (n=6104) were identified. Risk of bias was high among the studies. The RCTs revealed: no change in mortality RR 1.07 [0.43, 2.71], increased risk of hypoglycaemia RR 15.66 [3.02, 81.09], and reduced risk of septicaemia RR 0.69 [0.59, 0.80]. The nRCTs revealed: no change in mortality RR 0.83 [0.63, 1.09], increased risk of hypoglycaemia RR 2.14 [1.65, 2.77], no reduction in days on ventilation MD -3.19 [-6.64, 0.27], no reduction in risk of septicaemia RR 0.95 [CI 0.66, 1.35], however a reduction in hospital days MD -6.70 [-13.06, -0.34].
**CONCLUSION.** Strict glycaemic control in the polytrauma patient may not reduce mortality and days on ventilation. There is a significant increase in the risk of hypoglycaemia, however the risk of septicaemia is inconclusive. There is weak evidence suggesting a reduction is hospital days. High risk of bias, especially within the nRCTS, limits strong conclusions. High quality RCTs are needed to further investigate strict glycaemic control in the polytrauma patient.

#### 000771

#### The use of 3% Hypertonic Saline Solution Reduces Multi-Organ Failure in Polytrauma Patients

L. Estupiñan,[1], S. Moreano-Ortiz,[1], G. Montaño,[1], AJ. Arango,[2], DA. Garay,[3], P. Marcela[3], R. Buitrago[3], A. Bastidas,[4], A. Rodríguez,[5], LF. Reyes[6]
[1]Resident, Universidad de La Sabana, Bogotá, Colombia; [2]Student, Universidad de La Sabana, Bogotá, Colombia; [3]Critical care medicine department, Universidad de La Sabana, Bogotá, Colombia; [4]Neumology, Universidad de La Sabana, Bogotá, Colombia; [5]Critical care department, Hospital Universitari de Tarragona Joan XXIII, Tarragona, Spain; [6]Critical Care Medicine - Infectious Disease Department, Universidad de la sabana, Bogotá, Colombia
**Correspondence:** L.F. Reyes

*Intensive Care Medicine Experimental* 2019, **7(Suppl 3):**000771

**INTRODUCTION.** Trauma is one of the ten leading causes of dead in the world. Responsible for about 1.4 million deaths a year. Death secondary to trauma is classified as early (post-injury) due to blood loss followed by hypovolemic shock; and late (post-resuscitation), if dead occurs more than 7 days post-injury, due to development of systemic inflammatory syndrome, tissue damage and multi-organ failure. In order to improve clinical outcomes permissive hypotension and minimizing fluid administration is considered as an effective strategy to reduce coagulopathy. Therefore, Hypertonic saline solutions have emerged as a promising for "low volume" fluid resuscitation in trauma patients. Moreover, some animal models have shown that using HSS in trauma may have immunomodulatory effects and may prevent injury of the glycocalyx. However, these effects have not been studied in polytrauma human patients. Therefore, this study will attempt to resolve this gap in the literature.
**OBJECTIVES.** To determine the impact of 3% hypertonic saline solutions in clinical outcomes in polytrauma patients.
**METHODS.** This an analysis of a retrospective cohort of patients hospitalized due to polytrauma in a reference hospital in Bogotá, Colombia, between 2010 and 2016. Only patients over the age of 18, admitted to the intensive care unit (ICU) due to polytrauma were included. Trauma severity, APACHE, fluid strategy, use of vasopressors, development of MOF at 72 hours after ICU admission and 7-days mortality rate were recorded. Categorical variables were studied by Fisher's test and the continuous variables by Wilconxon and Anova test. Logistic regression (independent association) was used in order to study the association between variables and clinical outcomes.
**RESULTS.** A total of 217 patients were included in the study. Among them, 123/217 (57%) patients were resuscitated with HSS during the first 72 hours of ICU admission. Out of patients treated with HSS, 59/123 (48%) were treated with 3% HSS. A total of 101/217 (47%) patients developed MOF and 42 (19%) died within the 7-days after ICU admission. Demographic characteristics were similar between patients treated with 3% HSS and those who did not. After adjusting for APACHE, trauma severity and type of HSS used in the multivariate analysis; 3% HSS was associated with decreased of MOF (OR: 0.37, 95% CI: 0.17-0.78). However, ICU length of stay, vasopressor requirement and mortality at 7-days among groups remain unchanged in the multivariate analysis.
**CONCLUSION.** Hypotensive reanimation with 3% HSS may decrease multiorgan failure in ICU patients admitted due to polytrauma. Further randomized controlled trials are needed to confirm this beneficial effect and to determine underling mechanisms.

**REFERENCE(S)**

1. Blanchard IE, Ahmad A, Tang KL, et al. The effectiveness of prehospital hypertonic saline for hypotensive trauma patients: a systematic review and meta-analysis. BMC Emerg Med 2017;17:35.
2. Cherkas D. Traumatic hemorrhagic shock: advances in fluid management. Emerg Med Pract 2011;13:1-19; quiz -20.
3. Coimbra R. 3% and 5% hypertonic saline. J Trauma 2011;70:S25-6.
4. Cross JS, Gruber DP, Burchard KW, Singh AK, Moran JM, Gann DS. Hypertonic saline fluid therapy following surgery: a prospective study. J Trauma 1989;29:817-25; discussion 25-6.
5. Galvagno SM, Jr., Mackenzie CF. New and future resuscitation fluids for trauma patients using hemoglobin and hypertonic saline. Anesthesiol Clin 2013;31:1-19.
6. Han J, Ren HQ, Zhao QB, Wu YL, Qiao ZY. Comparison of 3% and 7.5% Hypertonic Saline in Resuscitation After Traumatic Hypovolemic Shock. Shock 2015;43:244-9.
7. Junger WG, Coimbra R, Liu FC, et al. Hypertonic saline resuscitation: a tool to modulate immune function in trauma patients? Shock 1997;8:235-41.
8. Junger WG, Rhind SG, Rizoli SB, et al. Resuscitation of traumatic hemorrhagic shock patients with hypertonic saline-without dextran-inhibits neutrophil and endothelial cell activation. Shock 2012;38:341-50.
9. Kolsen-Petersen JA. Immune effect of hypertonic saline: fact or fiction? Acta Anaesthesiol Scand 2004;48:667-78.
10. Maningas PA, Mattox KL, Pepe PE, Jones RL, Feliciano DV, Burch JM. Hypertonic saline-dextran solutions for the prehospital management of traumatic hypotension. Am J Surg 1989;157:528-33; discussion 33-4.

### 000834

#### Enzimatic escharolysis with NEXOBRID® in critical burn patients at Vall d`Hebrón University Hospital: a retrospective analysis

C. Vizcaino Urresta[1], J. Baena[2], M. S.[2], A. M.[3], L. Lagunes[4], S. J.[5], J. Barret[6], M. Baguena,[2]
[1]Critical care, Vall d'Hebron University Hospital, Barcelona, Spain; [2]Trauma and burns critical care, Vall D Hebron, Barcelona, Spain; [3]Trauma and burns critical care, Vall d'Hebron, Barcelona, Spain; [4]Critical care, Vall D Hebron, Barcelona, Spain; [5]Plastic and reconstructive surgery and burn unit, Vall d'Hebron, Barcelona, Spain; [6]Plastic and reconstructive surgery and burn unit, Vall D Hebron, Barcelona, Spain
**Correspondence:** C. Vizcaino Urresta

*Intensive Care Medicine Experimental* 2019, **7(Suppl 3):**000834

**INTRODUCTION.** Enzymatic escharolysis is a non-surgical treatment method for removing necrotic tissue in severe burn injuries, leaving intact neighboring healthy tissue. In addition it reduces blood loss and the need for autologous skin grafting.
**OBJECTIVES.** To describe our experience in the use of NEXOBRID® in critical burn patients admitted in the Burn Centre at Vall d'Hebron University Hospital; between January 2015 and December 2018, and to assess differences between survivors and non-survivors.
**METHODS.** Retrospective monocentric cohort study of patients of at least 18 years with major burn injury; defined as total body surface area (TBSA) burned over 15% who had NEXOBRID® treatment method for remove total or partial necrotic tissue. Demographic data, comorbidities, mechanism of burn, vasopressor support requirement, length of mechanical ventilation (MV-LOS), length of stay (LOS) and mortality were collected from hospital medical records.
**RESULTS.** One hundred and thirty-three patients were admitted with major burns during the study period, 18 received enzymatic escharolysis (13.5%). When comparing patients treated with either enzymatic or surgical debridement no differences in gender, age, TBSA, Baux score and comorbidities were observed. Seventeen (94.4%) patients in the enzymatic group required mechanical ventilation versus 57 (49.56%) in the surgical group p= < 0.001, without difference in the MV-LOS median days (15, IQR25-75:7-38 versus 14, IQR25-75:5-33). No differences were observed in blood transfusion units (7, IQR25-75:4-19 versus 5.9, IQR25-75: 0-8). A higher proportion of patients in the surgical group required renal replacement therapy 13 (11.3%) versus 3 (16.3%) in the enzymatic group p= 0.06. Four patients (22%) died in the enzymatic debridement group versus 30 in the surgical group (26%) p= 0.003.
**CONCLUSION.** Patients treated with NEXOBRID® required more commonly mechanical ventilation, however no difference in the length of mechanical ventilation was noticed. Less mortality was observed in the enzymatic escharolysis when compared to surgical debridement.

**REFERENCE(S)**

1. Rosenberg L, Krieger Y, Bogdanov-Berezovski A, Silberstein E, Shoham Y, Singer AJ. A novel rapid and selective enzymatic debridement agent for burn wound management: A multi-center RCT. Burns, 2014; 40(3): 466-474.
2. Rosenberg L, Shoham Y, Krieger Y, et al. Minimally invasive burn care: a review of seven clinical studies of rapid and selective debridement using a bromelain-based debriding enzyme (Nexobrid®). Ann Burns Fire Disasters. 2015;28(4):264–274.

### 000872

#### Angioembolization versus Laparotomy in Patients with Isolated Blunt Splenic Injury; cluster-exact propensity score matching

T. Suzuki[1], A. Shiraishi[2], Y. Otomo[3]
[1]Interventional radiology center, Teikyo University Chiba Medical Center, Ichihara, Chiba, Japan; [2]Emergency and trauma center, Kameda Medical Center, Kamogawa, Chiba, Japan; [3]Trauma and acute critical care medical center, Tokyo Medical and Dental University, Bunkyo-ku, Tokyo, Japan
**Correspondence:** T. Suzuki

*Intensive Care Medicine Experimental* 2019, **7(Suppl 3):**000872

**INTRODUCTION.** Angiography with embolization (angioembolization) is an emerging hemostatic procedure for bleeding from splenic injuries. However, its mortality benefit compared with laparotomy remains unclear in patients with blunt splenic injury.
**METHODS.** This retrospective matched cohort study used data from the Japan Trauma Databank (2004 to 2017), a nationwide trauma registry of injuries with the injury severity score ≥9 at 272 institutions in Japan. Trauma patients who underwent angioembolization or laparotomy for isolated blunt splenic injury were studied. Patients were excluded if they needed cardiopulmonary resuscitation before hospital or in the emergency department, or had unsurvivable injury of any region of the body as defined by the Abbreviated Injury Scale. Missing values were handled by multiple imputation. Logistic regression analysis was done to estimate propensity scores for angioembolization from known outcome predictors, including the age, gender, vital signs, resuscitative procedures in the emergency department, severity of injury, and type of splenic injury. Cluster-exact propensity score matching was performed to select pairs of patients from the same center, who undergoing angioembolization or laparotomy. The primary outcome was hospital mortality, while secondary outcomes were the 28 hospital-free days and complications (abdominal complications, organ failure, infection, and central nervous system complications).

**RESULTS.** Of 294,274 trauma patients registered in the JTDB, 1,796 patients had blunt splenic injury treated by angioembolization (N= 1,333) and/or laparotomy (N=564). 101 patients of whom underwent both angioembolization and laparotomy. Propensity score matching selected 240 patients each undergoing angioembolization or laparotomy. Hospital mortality was similar in both groups(8.0% versus 8.9%, odds ratio 0.90, 95%CI [0.46-1.73], P=0.74), but 30 patients receiving primary angioembolization (12.5%) required subsequent laparotomy.

There were no significant differences of secondary outcomes (the 28 hospital-free days (7 versus 7 difference 0 [-1, 1]), abdominal complications (5.4% versus 5.9%, odds ratio 0.91, 95%CI [0.41, 2.04]), organ failure (7.2 versus 7.3 odds ratio 0.99, 95%CI [0.48, 2.03]), infection (5.4 versus 10.1 odds ratio 0.51, 95%CI [0.25, 1.07]), and central nervous system complications(4.1 versus 2.3, odds ratio 1.82, 95%CI [0.60, 5.51])).

**CONCLUSION.** This propensity score-matched observational study found that angioembolization did not significantly reduce hospital mortality compared with laparotomy.

**REFERENCE(S)**

1. Zarzaur BL, Rozycki GS. An update on nonoperative management of the spleen in adults. Trauma Surg Acute Care Open 2017;2:1–7.
2. Aiolfi A et al. Splenic artery embolization versus splenectomy: Analysis for early in-hospital infectious complications and outcomes. J Trauma Acute Care Surg. 2017 Sep;83(3):356-360.

### 000881

#### Inflammatory parameters in trauma patients

R. PRIETO JURADO[1], S. Gonzalez[2], A. Iglesias Santiago[1], A. Lopez[1], A. RUIZ[3], F. Pino[1], F. Guerrero[1]
[1]Critical care department, Hospital Virgen de las Nieves, granada, Spain; [2]Critical care department, Hospital Virgen de las Nieves , granada, Spain; [3]INTENSIVE CARE UNIT, Hospital Universitario Virgen de las Nieves Hospital General Urgencias, Granada, Spain
**Correspondence:** R. PRIETO JURADO
*Intensive Care Medicine Experimental* 2019, **7(Suppl 3):**000881

**INTRODUCTION.** Descriptive study in trauma patients about the correlation betwwen inflammatory parameters and mortality, severity of illness, multiorganic dysfunction (MODS) and nosocomial infection.

**OBJECTIVES.** OBJECTIVES: To analyse the levels of procalcitonine, C-reactive protein and leukocytes in the first 24 hours as well as their correlation with severity scores and association with mortality, MODS and nosocomial infection.

**METHODS.** MATERIAL AND METHODS: Cohort study of trauma patients admitted at intensive care unit from January 2018 to March 2019. Independent variables: age, sex, type of trauma, levels of procalcitonine, C-reactive protein and leukocytes in the first 24 hours. Dependent variables: nosocomial infection, MODS and mortality. Descriptive statitic with mean and standard deviation or median and interquartile range for quantitative variables; absolute and relative frequencies for cualitative variables. Bivariant statitic; Student's t-distribution for independent samples with normal distribution and Chi-squared test for cualitative variables.

**RESULTS.** RESULTS: 157 patients were included during the period under review. Mostly were males (114, 72.6%) and blunt trauma (149, 94.9%) with a mean age of 47.45 ± 19.3. The mean stay at ICU was 5,04 days. ISS score, NISS and APACHE II scores were respectively 23.3 ± 12.4; 31.6 ± 16.8 and 15.2 ± 8.4. The median of leukocytes in the first 24 hours was 15800. C-reactive protein levels were analyzed in 118 patients with a median of 29.55 and procalcitonine was analyzed in 74 patients with a median of 0.74. The level of procalcitonine has a positive and significative correlation with APACHE II, NISS and ISS scores. The level of leukocytes has only a weak correlation with APACHE II score. The level of C-reactive protein has no correlation with severity scores. 21 patients developed MODS, 20 of them during the first three days (12.7%) and the last one later (0.6%). 33 patients (21%) were diagnosed of nosocomial infection during ICU stay with no significative differences in leukocytes and procalcitonine levels in comparison with the non-infected patients. Nevertheless, the levels of C-reactive protein were higher in those with nosocomial infection (p< 0.01). 13.5% of patients died during ICU stay and 16.1% of patients after ICU discharge during hospital stay. There are no statistically significant differences in the levels of leukocytes, C-reactive protein and procalcitonine during the first 24 hours between died and alive.

**CONCLUSION.** CONCLUSIONS: During the first 24 hours the levels of inflammatory parametres are not specific of infection and there is no differences in mortality and nosocomial infection incidence. There is statistically significant association between these parametres and MODS. Procalcitonine could be valuable to predict the development of MODS and nosocomial infection

### 000908

#### D-dimer screening for venous thromboembolism in patients with spinal cord injury

T. Yamamoto[1], Y. Koga[1], T. Yagi[1], M. Todani[1], T. Nakahara[1], Y. Kawamura[2], K. Kaneda[1], M. Fujita[1], R. Tsuruta[2]
[1]Advanced medical emergency and critical care center, Yamaguchi University Hospital, Ube, Yamaguchi, Japan; [2]Department of acute and general medicine, Yamaguchi University Graduate School of Medicine, Ube, Yamaguchi, Japan
**Correspondence:** T. Yamamoto
*Intensive Care Medicine Experimental* 2019, **7(Suppl 3):**000908

**INTRODUCTION.** Patients with spinal cord injury require long-term hospitalization and rest, so they are more likely to develop venous thromboembolism (VTE). However, the screening for VTE in spinal cord injury patients has not yet been established.

**OBJECTIVES.** To clarify the optimal measurement timing and threshold level of the plasma D-dimer for the screening of VTE in patients with spinal cord injury who require long-term hospitalization and rest.

**METHODS.** Of the patients with spinal cord injury who were transported to our center between April 2011 and March 2019, those who required at least 10 days of rest and underwent diagnostic imaging for VTE, with either ultrasonography or enhanced computed tomography of the veins of the lower extremities, were included. We retrospectively accessed their medical records to determine whether they were positive or negative for deep vein thrombosis (DVT), the changes in their plasma D-dimer levels, etc. The subjects were classified into two groups, the VTE group and non-VTE group, based on their VTE status.

**RESULTS.** Of the 87 patients with spinal cord injury, 26 patients were analyzed. The median age was 67 years (61–77) and 16 patients (62%) were male. VTE was detected in 12 patients (46%). Prophylactic anticoagulant administration was given to eight patients (31%). Although the VTE group and non-VTE group showed almost the same changes in their median plasma D-dimer levels until hospital day 10, a sharp upward trend was then observed in the VTE group, which peaked at hospital day 13. The area under a receiver-operator characteristic curve for positive DVT vs D-dimer level at hospital day 13 was

0.857 (p=0.042), and the optimal cutoff point for D-dimer was 19.8 μg/dL because at that level, sensitivity (80%) and specificity (86%) were well balanced.

**CONCLUSION.** The plasma D-dimer levels may be used to screen for VTE in spinal cord injury patients who require long-term hospitalization and rest. The optimal measurement timing is 13 days after injury, and optimal threshold level is 19.8 μg/dL.

1. None

### 001023

#### Development of an ovine model of haemorrhagic shock: Characterisation of systemic and local oxygen supply/demand imbalance

G. Li Bassi[1], W. Dyer[2], JP. Tung,[2], K. Wildi[1], S. Livingstone[1], S. Rozencwajg[1], G. Simonova[2], R. Wellburn[2], S. Chiaretti,[2], F. Temple[2], C. Ainola[1], S. Colombo[3], N. Bartnikowski[1], M. Passmore[1], M. Bouquet[1], T. Shuker[1], L. Pugh[4], D. Irving[2], J. Suen[1], J. Fraser[1]
[1]Critical care research group, The Prince Charles Hospital, Chermside, Australia; [2]R&d, Australian Red Cross, Sydney NSW, Australia, Australia; [3]Critical care medicine, University of Milan, Milan, Italy; [4]Internal medicine, St Andrew's War Memorial Hospital, Spring Hill, Australia
**Correspondence:** G. Li Bassi
*Intensive Care Medicine Experimental* 2019, **7(Suppl 3):**001023

**INTRODUCTION.** The global burden of haemorrhagic shock (HS) is substantial and mostly caused by severe physical trauma. Essential pathophysiologic features of HS are imbalance between oxygen delivery and demand, hyper or hypocoagulatory states and compensatory anti-inflammatory response syndrome, ultimately resulting in multi-organ failure (1). Animal models of HS are essential to study underlying pathobiology and develop new treatments.

**OBJECTIVES.** To develop an ovine model of severe HS caused by blood loss > 30% of the estimated blood volume (EBV) and study dynamics of oxygen supply/demand imbalance systemically and at end-organ tissue level.

**METHODS.** Six adult female Leicester cross breed sheep (46±5 Kg) were anaesthetised, intubated and on mechanical ventilation. We cannulated the femoral artery for blood sampling and mean arterial pressure (MAP) monitoring. Swan-Ganz catheter was inserted through the right jugular vein. Animals were haemorrhaged through consecutive collections of 300 mL of blood over 90 min, up to approximately 30% of the EBV. HS was halted in case of MAP < 50 mmHg, heart rate (HR) >200 beats/min, or venous oxygen saturation (SvO2) <50%. At baseline, and every 15-min thereafter, haemodynamic parameters were recorded, and arterial blood gas analysis performed, oxygen delivery (D02) computed. Microdialysis probes were positioned into various organs to measure, at baseline and end of bleeding period, interstitial lactate and lactate/pyruvate ratio.

**RESULTS.** All sheep survived HS. On average, sheep were haemorrhaged 1055±193 mL of blood (33.6±5.2% of EBV). At baseline, and at the end of HS period, Hb varied from 10.3±2.1 to 7.6±10.8 g/dL (p<0.01), arterial lactate from 2.0±1.5 to 4.3±1.2 mmol/L (p<0.01), HR from 102±14 to 154±30 bpm (p=0.06), MAP from 95.6±8.8 to 51.3±15.2 mmHg (p<0.01), cardiac output from 4.4±1.4 to 2.1±0.8 L/min (p<0.01), SvO2 from 76.3±9.8 to 54.0±24.3% (p=0.192) and D02 from 552±218 to 241±114 ml/min (p<0.01). Table below depicts development of organ-specific oxygen debt, measured by microdialysis.

**CONCLUSION.** We described an ovine model of HS, characterized by significant impairment in cardiac output and oxygen delivery, resulting in multi-organ oxygen supply/demand imbalance. The model will be first used to compare the efficacy of different resuscitation fluids.

**REFERENCE(S)**

1. Cannon J, N Engl J Med 2018; 378:370-379
2. Australian Red Cross Blood Service and ANZSBT

**Table 1 (abstract 001023).** See text for description

| | Brain | Renal cortex | Liver | Skeletal muscle |
|---|---|---|---|---|
| Lactate (mmol) | | | | |
| Baseline | 3.3±1.3 | 0.9±0.6 | 2.4±1.4 | 3.4±0.9 |
| End of haemorrhage | 5.0±1.7 | 11.8±16.9 | 5.4±1.3 | 7.6±2.5 |
| (P-value) | (0.25) | (0.05) | (0.06) | (0.04) |
| Lactate/Piruvate Ratio (%) | | | | |
| Baseline | 50.6±62.1 | 24.9±12.1 | 87.0±49.4 | 43.2±14.1 |
| End of haemorrhage | 60.8±52.2 | 53.6±30.6 | 76.7±22.9 | 145±116 |
| (P-value) | (1.00) | (0.05) | (0.31) | (0.18) |

### 001119

#### Inhaled Argon improves neurological outcome in experimental traumatic brain injury

F. Fossi[1], F. Moro,[2], A. Magliocca,[3], E. Micotti,[2], G. Citerio[4], N. Stocchetti[5], R. Latini,[3], G. Ristagno,[3], E. Zanier[2]
[1]Department of, University of Milano-Bicocca, Milano, Italy; [2]Neuroscience, Mario Negri Institute for Pharmacological Research, Milano, Italy; [3]Cardiovascular research, Mario Negri Institute for Pharmacological Research, Milano, Italy; [4]School of medicine and surgery, University of Milano-Bicocca, Milano, Italy; [5]Neurointensive care unit, Fondazione IRCCS Ca'Granda Ospedale Maggiore Policlinico, Milano, Italy
**Correspondence:** F. Fossi
*Intensive Care Medicine Experimental* 2019, **7(Suppl 3):**001119

**INTRODUCTION.** While supportive treatment in the management of Traumatic Brain Injury (TBI) has progressed over the past 20 years, specific drug treatments are lacking [Maas *et al*]. New strategies are needed. Altering how patients are ventilated, could be an easily modifiable variable in TBI management. Data in *in vitro* and *in vivo* models of ischemic heart [Ristagno *et al*] and brain injury [Loetscher *et al*] show that the gaseous agent Argon is endowed with neuroprotective potential. Whether Inhaled Argon (iAr) is protective in experimental TBI is presently unknown.

**OBJECTIVES.** To test the effects of inhaled Argon administered after experimental TBI in mice on neurological functions and structural outcome by longitudinal behavioural assessments and magnetic resonance imaging (MRI) including T2W and DWI sequences.

**METHODS.** Severe TBI was performed in anesthetized mice (C57BL/6J, 8 weeks old, male) over the left parietotemporal cortex by controlled cortical impact as previously described [Zanier *et al*]. Immediately after TBI, mice were randomized to 24h treatment by iAr 70%-O2 30% (n=10) or air (n=10) from 10 minutes after TBI. Sensorimotor deficits were evaluated at the end of treatment (24h post TBI) and at 1 week by neuroscore and simple neuroassessment of asymmetric impairment (SNAP) tests. MRI (7 Tesla, Bruker) was performed at 3 days post TBI to evaluate contusion volume by T2W. The effect of iAr on acute brain edema, was analysed in a subset of mice (n=3 per group) by DWI-MRI. Maps of the apparent diffusion coefficient generated by DWI-MRI were used to evaluate iper/ipo intense regions as a proxy of vasogenic and cytotoxic edema, respectively. A simple random allocation was applied to assign a subject to an experimental group. Data acquisition and analysis were done blindly. Data were normally distributed. A t-test was used to evaluate differences between iAr and air treated TBI mice.

**RESULTS.** Argon inhalation significantly improved neurological function at 24 hours and 7 days after TBI (Neuroscore 24h post TBI iAr 6.1±0.5 vs. Air 3.7±0.7, p=0.0102). Contusion volume was reduced by 16% in iAr than air breathing TBI mice. Vasogenic brain edema showed a reduction in iAr treated TBI mice close to significance (p= 0.056).

**CONCLUSION.** iAr induces an acute and persistent improvement of sensorimotor function when administered for 24h starting 10 minutes after TBI. This outcome is reinforced by preliminary MRI data showing a trend in toward a decrease in vasogenic edema in iAr treated mice. Our data support future studies to understand the potential of iAr as an accessible treatment in TBI.

**REFERENCE(S)**

1. Zanier ER, et al (2011) Crit Care Med 39:2501–2510.
2. Loetscher PD, et al (2009). Crit Care 13:R206
3. Ristagno G, et al. Shock. (2014) 41:72-78
4. Maas AIR, et al (2017). Lancet Neurol 16:987–1048

### 001243

#### The impact of different intensivist staffing models on drug-drug interactions in adult trauma intensive care units

A. vazin[1], M. Masjedi[2], M. Mirjalili[2]
[1]Clinical pharmacy department, Shiraz University of Medical Sciences, Shiraz, Fars Province, Iran, Islamic Republic of Iran; [2]Critical care department, Shiraz University of Medical Sciences, Shiraz, Fars Province, Iran, Islamic Republic of Iran
**Correspondence:** A. vazin
*Intensive Care Medicine Experimental* 2019, **7(Suppl 3):**001243

**INTRODUCTION.** Drug-drug interactions (DDIs) are considered as concerning issues for public health especially in those admitted to intensive care units (ICUs). There are many studies that show involvement of intensivists in the ICUs improves outcome and limits costs. Different types of intensivist-driven care exist. This study evaluated the effect of academic versus non-academic (therapeutic) intensivist as well as hours of coverage and attendance of intensivist on DDIs in six adult trauma ICUs of a level one trauma center

**OBJECTIVES.** Considering the high incidence of pDDIs in the ICU, and the importance of ADRs caused by DDIs in critically ill patients, we aimed to investigate the effect of presence of an academic versus therapeutic intensivists as well as hour of coverage of intensivist on DDIs in six adult trauma ICUs of a level one trauma center in Shiraz, Iran.

**METHODS.** Two hundred patients were included in this prospective study in a 6-month period. The DDIs were classified into 5 categories, including type A, B, C, D, and X. Type D and X were considered as potential DDIs (pDDIs). Effect of three different types of intensivist staffing models including type A (once daily therapeutic intensivist visit followed by 24 hour on-call), B (twice daily academic intensivist visit, 8 hours attendance in ICU and 16 hours on–call) and C (all criteria just like ICU type B except presence of therapeutic instead of academic intensivist).

**RESULTS.** 3735 drug orders and 3869 drugs (193 different types) were assessed and 1826 potential DDIs were identified including 1107 (60.6%) type C, 648 (35.48%) type D and 12 (0.6%) type X. The mean of DDI per patient was significantly higher (P value <0.001) in ICU type A than ICU type C and type B. The frequency of DDIs was highest in type A. There was a statistically significant relationship between the number of prescribed drugs and ICU length of stay (P value <0.001 and P=0.009, respectively).

**CONCLUSION.** Different types of intensivist models affect DDIs to varying degrees. In this regard, academic versus therapeutic intensivist, twice versus once daily visit and 8 hours attendance with16 hours on-call versus 24 hours on-call is associated with more reduction in DDIs in adult trauma ICUs.

**REFERENCE(S)**

1. Papadopoulos J, Smithburger PL. Common drug interactions leading to adverse drug events in the intensive care unit: management and pharmacokinetic considerations. Critical care medicine. 2010;38:S126-S35.
2. Spriet I, Meersseman W. Relevant CYP450-mediated Drug Interactions in the ICU. Intensive Care Medicine: Springer; 2009. p. 870-7.
3. We would also like to thank staffs of ICUs of Rajaee hospital for support to accomplish this study.

### 001291

#### Airway Management in Spinal Cord Injury: Preventing and Decreasing Tracheostomy Morbidity

R. Onders, M. Elmo
Surgery, University Hospitals Cleveland Medical Center, Cleveland, United States of America
**Correspondence:** R. Onders
*Intensive Care Medicine Experimental* 2019, **7(Suppl 3):**001291

**INTRODUCTION.** High level cervical spinal cord injuries (SCI) often result in respiratory failure and the need for tracheostomy mechanical ventilation. The National Trauma Data Bank reports tracheostomy rate for high cervical injuries to be over 30% and even 20% of patients with injury below C4 require a tracheostomy. The benefits of tracheostomy are documented however they do have risks and in chronic long term use, there is sub optimal management. Early complications include bleeding(2-5%), stomal infection (2-5%) and dislodgement 1%. Late complications approach 65% including tracheomalacia and stenosis.

**OBJECTIVES.** This is a report of tracheostomy use in all traumatic SCI patients who underwent diaphragm pacing (DP).

**METHODS.** This is a retrospective review of prospective IRB and or FDA approved protocols involving SCI and DP. Airway management of traumatic SCI who underwent DP was analyzed pre and post DP implant.

**RESULTS.** Out of over 500 implanted DP patients 99 were traumatic SCI with complete tracheostomy data on 84 patients. Fifty nine or 63% of patients had a cuffed tracheostomy at the time of DP evaluation. Average age at time of injury was 27. 8 years (1 day to 74 years). The average time spent on mechanical ventilation prior to DP was 9.7 years (6 days to 25.6 years). Within this group are 13 pediatric patients age 0 to 17 in which 54% presented with a cuffed tracheostomy tube. Post DP implant, 7 patients were decannulated, 15 patients had tracheostomy converted to cuffless tube and 2 patients went to a stoma stent. One patient required laryngectomy due to tracheal damage. Two patients went directly from Endotracheal tube mechanical ventilation to DP to extubation avoiding tracheostomy altogether. T Median survival was 22.2 years (95% CI 14.0 - not reached) with only 31 deaths. Subgroup analysis showed that earlier DP implantation leads to greater 24 hour use( 72% ) of DP and no need for any MV.

**CONCLUSION.** There continues to be a widely held belief that positive pressure ventilation must be delivered via cuffed and generally inflated cuffed tracheostomy tube. Inflated cuffed tracheostomy tubes has significant morbidity including speech difficulty, aspiration increases from difficulty swallowing, tracheal stenosis and tracheomalacia. The efficacy and benefits of the cuffless tracheotomy was first described in 1990. Publications in SCI and chronic mechanical ventilation describe the use of cuffless tracheotomy but this report confirms cuffed tube usage is dominant. The choice of tracheostomy style needs to be better scrutinized. DP implantation allowed for downsizing and decannulation. It obviated the need for tracheostomy in two patients. Early DP use may significantly alter the morbidity of tracheostomies leading to improved survival.

**REFERENCE(S)**

1. Posluszny JA Jr, Onders R, Kerwin AJ, Weinstein MS, Stein DM, Knight J, Lottenberg L, Cheatham ML, Khansarinia S, Dayal S, Byers PM, Diebel L., Multicenter review of diaphragm pacing in spinal cord injury: successful not only in weaning from ventilators but also in bridging to independent respiration. J Trauma Acute Care Surg. 2014 Feb; 76(2):303-309
2. Kerwin AJ, Yorkgitis BK, Ebler DJ, Madbak FG, Hsu AT, Crandall ML., Use of diaphragm pacing in the management of acute cervical spinal cord injury. J Trauma Acute Care Surg. 2018 Nov;85(5):928-931.
3. Onders RP, Elmo M, Kaplan C, Schilz R, Katirji B, Tinkoff G. Long-term experience with diaphragm pacing for traumatic spinal cord injury: Early implantation should be considered. Surgery. 2018 Oct;164(4):705-711.

### 001302

#### Alcohol and drug-related admissions in a mixed Norwegian ICU-population

KH. Tollisen[1], M. Bjerva[2], C. Lund Hadley[3], GT. Dahl[4], LM. Högvall[2], L. Sandvik[5], F. Heyerdahl[2], D. Jacobsen[3]
[1]Institute of clinical medicine, faculty of medicine, University of Oslo, Oslo, Norway; [2]Department of anesthesiology, Oslo University hospital, Oslo, Norway; [3]Department of acute medicine,, Oslo University Hospital, Norway, Oslo, Norway; [4]Department of anesthesiology, Diakonhjemmet Hospital, Oslo, Norway; [5]Oslo centre for biostatistics and epidemiology, University of Oslo, Oslo, Norway
**Correspondence:** K.H. Tollisen
*Intensive Care Medicine Experimental* 2019, **7(Suppl 3):**001302

**INTRODUCTION.** Alcohol and drug abuse are potentially modifiable risk factors for critical illness. Increased knowledge regarding the impact of substance abuse on cause of admission in different ICU populations is needed.
**OBJECTIVES.** To describe patients with substance abuse-related admissions in a mixed intensive care (ICU) population, and to compare these patients with patients with non-substance abuse-related admissions on selected parameters.
**METHODS.** Cross-sectional prospective study of ICU-patients in Oslo aged ≥18 years included over a one year period from February 3rd 2014 to February 2nd 2015. Data were collected consecutively from a questionnaire, medical records and toxicology results. Substance abuse-related admissions were defined as ICU-admissions due to acute or chronic complications of alcohol or drug use, and included the following subgroups: (A) cause of admission directly associated with acute substance abuse (e.g. acute intoxication) (B) indirect influence of acute substance abuse (e.g. trauma in intoxicated patient) and (C) medical complications of chronic substance abuse (e.g. alcoholic liver disease).
**RESULTS.** Of the 861 patients included, 537 (62%) were medical and 324 (38%) surgical, 632 (73%) received mechanical ventilation and 279 (32%) died during the hospital stay. Overall, 168 (20%) patients had substance abuse-related admissions; 104 (12%) were alcohol-related and 64 (7%) drug-related. Of the 168 patients with substance abuse-related admissions, 59 (35%) patients were in group A, 77 (46%) in group B and 32(19%) in group C. In age groups 18-39 and 40-59 males had a much higher proportion of substance abuse-related admissions than females (47/99, 48% vs 10/40, 25%, p<0.02 and 57/167, 34% vs 11/71, 16%, p<0.01, respectively), while there were no difference between the genders in patients 60 years and older. Among the 191 trauma patients, substance abuse was predisposing factor in 69 (36%). Patients with admissions associated with acute substance abuse (subgroups A and B) were significantly younger (mean age 43 and 46 vs. 63), had lower mean Charlson comorbidity index (1.2 and 0.8 vs. 2.5) and shorter median time of mechanical ventilation (0.5 and 1.0 vs 4 days) than patients with non-substance abuse-related admissions. There were no such differences between patients in group C and patients with non-substance abuse-related admissions. Age-adjusted logistic regression analysis showed lower hospital mortality for patients in group B (OR 0.5, p< 0.04), but not in the other subgroups.
**CONCLUSION.** One in five ICU-admissions was associated with alcohol or drug abuse. Acute substance abuse caused the majority of the substance abuse-related admissions, and was particularly prevalent among young, male patients with otherwise few risk factors for critical illness. Nearly half of the admissions in males aged 18-39 were associated with substance abuse, and more than one third of the trauma patients were influenced by alcohol or drugs at time of injury. Routine screening for alcohol and drugs in Norwegian trauma patients should be considered implemented.

### 001326

#### Resuscitation with albumin using BET formula keeps at bay fluid administration in burned patients. An observational study

P. Blanco-Schweizer[1], J. Sánchez[1], B. Bendito[2], A. Martín[1], L. Fernández[1], JM. Piqueras[2], P. Enríquez[1]
[1]Department of intensive care medicine, Hospital Universitario Río Hortega, Valladolid, Spain; [2]Department of plastic surgery, Hospital Universitario Río Hortega, Valladolid, Spain
**Correspondence:** P. Blanco-Schweizer
*Intensive Care Medicine Experimental* 2019, **7(Suppl 3):**001326

**INTRODUCTION.** The use of albumin to resuscitate burn patients has been a controversial issue for more than 50 years. Currently the most accepted formulas use crystalloids for this purpose and colloids only as a rescue fluid in order to avoid "fluid creep". In contrast, BET formula administers albumin from the beginning of the resuscitation period using it in a progressively decreasing concentration.
**OBJECTIVES.** The purpose of our study was to review and analyze resuscitation related variables of all burned patients treated in our unit using BET formula
**METHODS.** Retrospective, observational study conducted in a 5 bed critical care unit for major burns in a tertiary hospital in Spain. BET formula estimates fluid resuscitation as a function of total burn surface area (TBSA) (ml/h = TBSA (m2) x 220) and administers it through a combination of Lactated Ringer and 20% Albumin starting at a 1:1 relationship. The proportion of albumin is decreased every 8 hours during the first 48 hours (resuscitation period) and the infusion rate is modified according to urinary output. Study period was from January 2013 to January 2018. Inclusion criteria: TBSA > 20% or > 10% plus inhalation injury, admission during the first 12 hours after the injury, who had all documentation regarding fluid resuscitation available. Data are expressed as means, medians, and proportions and analyzed using T-Test, Wilcoxon Rank Sum, Chi-Square and Fishers´ Exact Test as needed. Relationship between variables is expressed as Pearson´s correlation coefficient
**RESULTS.** 131 patients were admitted in the unit during the study period. 40 patients met all inclusion criteria. Patients´ characteristics: age 58 ± 19, 26 (65%), TBSA% 36 ± 17, mechanism fire 28 (70%), inhalation 8 (20%), ABSI 9 ± 2, time injury-admission 4.5h (2.7-6.1), referring hospital 24 (60%). Resuscitation volume during the first 24 hours (VOLBSi24) was 2.58 ml/kg/%BBSA, significantly less than Parkland´s estimation (4 ml/kg/%BBSA; P<0.05). Patients were successfully resuscitated showing a significant base excess and lactate clearance during the resuscitation period (base excess 135%; lactate 35%; P<0.05). A slight but significant correlation was found between time from injury to admission (admission delay) and indexed volume administered in the first 24 h (r 0.32, p<0.05). An inverse correlation was found between indexed volumes and TBSA% during the first 24h (r -0.38, p<0.05). Resuscitation related complications: mechanical ventilation 27 (67.5%), ARDS 12 (30%), renal failure (KDIGO ≥ 1) 21 (53%), continous renal replacement therapy 5 (12.5%), wound deepening 8 (20%), abdominal compartment syndrome 2 (4.5%), limb compartment syndrome 0.
**CONCLUSION.** BET formula is capable of resuscitating burn patients successfully, limiting fluid administration, and being able to keep burn related complications low.

### 001379

#### Biomarkers for multiple organ dysfunction syndrome in severely injured trauma patients

D. Kleinveld[1], A. Tuip-De Boer[1], M. Hollmann[2], N. Juffermans[1]
[1]Intensive care and laboratory of experimental intensive care and anesthesiology, Amsterdam UMC - Location AMC, Amsterdam, Netherlands; [2]Anesthesiology and laboratory of experimental intensive care and anesthesiology, Amsterdam UMC - Location AMC, Amsterdam, Netherlands
**Correspondence:** D. Kleinveld
*Intensive Care Medicine Experimental* 2019, **7(Suppl 3):**001379

**INTRODUCTION.** Improvements in the early resuscitation phase of trauma comes at a cost of increased morbidity and mortality in later phases, including the occurrence of multiple organ dysfunction syndrome (MODS). If patients at risk for MODS could be identified early using different cytokine production profiles, preventative treatment measures could be taken.

**OBJECTIVES.** The aim of this study is to investigate whether specific pro- and/or anti-inflammatory biomarkers are associated with development of MODS.

**METHODS.** Adult (18 years or older) multiple trauma patients with an injury severity score of 16 or higher presenting to a level 1 trauma center in the Netherlands between 2008 and 2018 were included. Blood was drawn at presentation to the hospital and analyzed for 19 markers of systemic and pulmonary inflammation, endothelial activation and coagulation using a Luminex assay. MODS was defined as a SOFA score of three or more in two organ systems. Comparisons were made between patients with and without MODS. Univariate and multivariate logistic regression analysis was used to determine association between specific biomarkers and MODS. Data are presented as median with interquartile range. A p-value of less than 0.05 was considered to be statistically significant.

**RESULTS.** In total, 147 multiple trauma patients were included, of which, 32 patients developed MODS. Compared to patients without MODS, patients with MODS had higher injury severity scores (25 [22 – 30] vs 20 [17 – 27], p=0.010), more often had traumatic brain injury (75 vs 56 %, p=0.048), were deeper in shock with lower base excess (-2.7 [-6.1 – -0.6] vs -1.2 [-3.1 – 0.8], p=0.005) and were more coagulopathic as determined by higher prothrombin time (12.7 [11.7 – 14.7] vs 11.7 [11.3 – 12.4] seconds, p=0.001). Overall, baseline levels of both pro- as well as anti-inflammatory biomarkers were higher in MODS patients, indicative of a severe host immune reaction. In the multivariate analysis, the combination of interleukin-1 receptor antagonist (OR 1.27 [1.07 – 1.51], p=0.002) and clara cell protein 16 (1.06 [1.01 – 1.05], p=0.031) was most strongly associated with the development MODS. Receiver operating curves showed an AUC of 0.73 (IL-1RA) and 0.70 (CC-16), respectively.

**CONCLUSION.** In trauma, biomarkers IL-1RA and CC-16 have the potential to identify patients at risk for development of MODS. Further research is warranted to prospectively validate these results.

### 001410

#### Identifying poisoning patients in need of intensive care in EMS: study of factors associated with intensive care admission in patients in contact with the EMS

L. Koskela[1], L. Raatiniemi[2], A. Ehrola,[3], J. Liisanantti,[1]
[1]Study group of surgery, anesthesiology and intensive care, Oulu University, Medical Research Center, Oulu, Finland, Finland; [2]Centre for pre-hospital emergency care, Oulu University Hospital, Oulu, Finland; [3]Emergency medical services, Oulu-Koillismaa Rescue Department, Oulu, Finland, Finland
**Correspondence:** L. Koskela
*Intensive Care Medicine Experimental* 2019, **7(Suppl 3):**001410

**INTRODUCTION.** Acute poisoned patients are a common occurrence in intensive care units (ICU). It is estimated that three to six percent of patients admitted to ICU are due to acute intoxication.(1) ICU admission rate of patients admitted to hospital in Nordic countries varies largely: from 7.3 % in Finland up to 49 % in Norway, mostly due to different poisoning treatment policies. (1–3) Majority of poisonings are presented to hospitals by ambulance operated by emergency medical services (EMS). (3)

**OBJECTIVES.** We set out to map out factors associated with hospitalization and intensive care admission to better recognize patient population in risk of death or other serious sequelae.

**METHODS.** A retrospective study was conducted in all EMS missions of poisoning patients in Oulu University Hospital (OUS) catchment area for three years, from 2015 to 2017. We combined data from Oulu-Koillismaa Emergency Medical Services database with OUS hospital discharge registry.

**RESULTS.** From a total of 1,336 EMS missions labeled as poisoning-related, 682 patients were included in the study after excluding patients under 16 years, those who were discharged with other diagnosis and those who were never transported to hospital. 4.8% of poisoning patients were admitted into ICU. Data for 682 patients are presented in Table 1.

**CONCLUSION.** Significant factors associated with intensive care admission in poisoning patients were pre-hospital intubation, negative alcohol finding and intravenous fluid resuscitation of >500 ml. Risk factors should be taken into account in further poisoning missions, and results of this study may provide support when a poisoning patient's need for intensive care is in question.

**REFERENCE(S)**

1. Lindqvist E, Edman G, Hollenberg J, Nordberg P, Ösby U, Forsberg S. Intensive care admissions due to poisoning. Acta Anaesthesiol Scand. 2017;61(10):1296–304.
2. Koskela L, Raatiniemi L, Liisanantti J. How does socioeconomic status affect the incidence of hospital-treated poisonings? A retrospective study. Unpubl Manuscr. 2019.
3. Lund C, Drottning P, Stiksrud B, Vahabi J, Lyngra M, Ekeberg I, et al. A one-year observational study of all hospitalized acute poisonings in Oslo: complications, treatment and sequelae. Scand J Trauma Resusc Emerg Med. 2012;20(1):49
4. Finnish Medical Foundation Eka Grant

**Table 1 (abstract 001410).** Factors associated with admission to Intensive Care Unit in 682 poisoning patients transported by EMS to Oulu University Hospital in 2015-2017. Odds ratio values result from logistic regression analysis

| | ICU admission N=33 | No ICU admission N=649 | P value | OR (95% CI) | P value for OR |
|---|---|---|---|---|---|
| Age, median | 40.2 [31.3-55.0] | 39.1 [26.7-51.6] | 0.357 | | |
| Male gender | 18 (54.5) | 371 (57.5) | 0.736 | | |
| Priority dispatch | 30 (90.9) | 414 (63.8) | 0.001 | | |
| Priority transport | 33 (100.0) | 277 (42.7) | < 0.001 | | |
| SAP lowest, mmHg | 102 [78-123] | 123 [106-137] | < 0.001 | | |
| SpO2 lowest <94% | 11 (37.9) | 169 (32.5) | 0.544 | | |
| NEWS ≥7 | 11 (33.3) | 73 (11.2) | 0.001 | | |
| GCS <9 | 13 (46.4) | 59 (11.4) | < 0.001 | 2.3 (0.9-6.1) | 0.085 |
| Intravenous fluid >500ml | 23 (69.7) | 250 (38.5) | < 0.001 | 3.7 (1.3-10.4) | 0.012 |
| Pre-hospital intubation | 8 (24.2) | 17 (2.6) | < 0.001 | 4.4 (1.4-14.0) | 0.013 |
| Negative for alcohol | 24 (72.7) | 235 (36.2) | < 0.001 | 4.1 (1.6-10.8) | 0.004 |
| Suicidal intention | 4 (12.1) | 70 (10.8) | 0.81 | | |

### 001602

#### The role of computed tomography in patients admitted to the intensive care unit following overdose

J. mccarthy, C. Ward, R. Sundaram
Intensive care department, Royal Alexandra Hospital, Paisley, UK, United Kingdom
**Correspondence:** J. mccarthy
*Intensive Care Medicine Experimental* 2019, **7(Suppl 3):**001602

**INTRODUCTION.** Self-inflicted overdose remains the third most common reason for admission to intensive care units in Scotland. Computed tomography (CT) scanning is often performed, however its value in this patient group has been debated. CT requires intrahospital transfer of the critically ill patient, which has been associated with increased risk of respiratory and cardiovascular instability.
**OBJECTIVES.** We wished to ascertain the diagnostic yield of CT head in patients admitted to the intensive care unit following overdose. We hoped to identify any differences in length of stay or mortality between those patients who received a CT head and those who did not. We wished to identify clinical features which were associated with positive scan results, and to determine if a positive CT head had any impact on decision making.
**METHODS.** We performed a retrospective analysis of patients admitted to a single Scottish intensive care unit over a 13 year period (2005-2018). Patients presenting with "self-poisoning" were identified using the ward watcher national audit database. Descriptive statistics were used to analyse patient demographics, pre-intubation GCS, length of stay, and predicted and actual mortality. Differences between groups were analysed using unpaired t tests and p values of < 0.05 were considered statistically significant. Diagnostic yield was calculated using percentages. Where a CT head was reported as abnormal, we determined if this affected the treatment plan by reviewing all associated electronic patient records.
**RESULTS.** 179 patients admitted to intensive care following overdose were identified, with a median age of 39. 55% were male. 38% had a CT scan performed. 15% of these scans were reported as "abnormal" but only 6% represented an acute pathology. None required intervention or resulted in a change in management. Pre-intubation GCS was lower in those patients who had CT scans than those who did not (p = 0.0013). Patients with a recent history, or clinical evidence of, head injury, were more likely to receive a CT head. There was no increase in the rate of positive scan in this group. In the small group of patients with a positive CT scan, there was no history of head injury, seizures, localising neurology or anticoagulation.
**CONCLUSION.** The yield of CT head scanning following overdose was low. Only 6% revealed an acute intracranial pathology and non required intervention or resulted in a change in management. Mortality rates in this patient population are low compared to the general ICU population and having a CT head had no demonstrable effect on mortality. CT scans were more likely to be performed in those patients with a lower pre-intubation GCS and those with a head injury, but we did not find any increase in positive findings. We could not identify any clinical characteristics associated with increased likelihood of a positive scan. We cannot make any recommendations regarding which patients may benefit from early scanning. Any decision to proceed to intrahospital transfer to facilitate diagnostic imaging must be balanced against the very real risks of instability during transfer. Larger studies are required to provide appropriate statistical power to identify patient subgroups who may benefit from early imaging following overdose.

**REFERENCE(S)**

1. ED utilization of computed tomography in a poisoned population. Patel, M et al. American journal of emergency medicine 2002;20:212-217
2. Brain computed tomographic scan findings in acute opium overdose patients. American Journal of Emergency Medicine, 31(1),50-53
3. Complications during intrahospital transport of critically ill patients: focus on risk identification and prevention. Knight et al. International journal of critical illness and injury science. 2015;5(4):256-64
4. Audit of Critical Care in Scotland 2018 Reporting on 2017. Scottish intensive care society audit group. https://www.sicsag.scot.nhs.uk/publications/_docs/2018/2018-08-14-SICSAG-report.pdf

### 001659

#### Risk factors of delirium in ICU patients with acute poisoning

HY. Kim, KM. Cha, BH. So
Department of emergency medicine, St. Vincent's Hospital, College of Medicine, The Catholic University of Korea, Seoul, Republic of Korea
**Correspondence:** B.H. So

*Intensive Care Medicine Experimental* 2019, **7(Suppl 3):**001659

**INTRODUCTION.** Delirium is a common clinical syndrome in intensive care unit, correlated with various adverse clinical outcomes.
**OBJECTIVES.** This study aims at estimating the incidence of delirium and investigating the associated risk factors and outcomes in ICU patients with acute poisoning.
**METHODS.** The data was collected from the ICU patients admitted via the emergency center over 18 years old presenting with poisoning from 2010 to 2015. Delirium was assessed retrospectively using Intensive Care Delirium Screening Checklist (ICDSC). Risk factors were evaluated with univariate and multivariate analysis.
**RESULTS.** 199 patients participated in this study and a total of 68 (34.2%) of the patients were diagnosed with delirium based on ICDSC score. The delirium group showed statistically significantly higher association with prolonged length of stay in the hospital and ICU in comparison with non-delirium group. The use of physical restraints and altered mental status were identified as significant risk factors for delirium with an odd ratio of 1.92 and 3.90. Pharmaceutics poisoning group was observed to develop delirium faster than non-pharmaceutics poisoning group. There was no significant difference between the two groups with respect to age, sex, past history, GCS score, vital sign, and application of ventilator care and renal replacement therapy.
**CONCLUSION.** In ICU patients with acute poisoning, the development of delirium was found to be common and associated with the use of physical restraints and altered mental status.

### 001713

#### Clinical prediction score to predict massive blood transfusion in trauma patients

O. Akaraborworn[1], B. Siribumrungwong,[2], B. Sangthong[1], K. Thongkhao[1]
[1]Division of trauma and critical care, department of surgery, Prince of Songkla University, Hat Yai, Songkhla, Thailand; [2]Division of vascular and endovascular surgery, Department of Surgery, Faculty of Medicine, Thammasat University, Bangkok, Thailand
**Correspondence:** O. Akaraborworn

*Intensive Care Medicine Experimental* 2019, **7(Suppl 3):**001713

**INTRODUCTION.** Exsanguination is the most common cause of early trauma death. Rapid blood transfusion is crucial in trauma resuscitation. Well-performed scoring system to predict massive blood transfusion(MBT) aids resource utilization with promptly blood preparation. Previous scoring systems take long time to obtain after admission and inaccurate in Thailand with a different trauma system.
**OBJECTIVES.** To create a scoring system to predict MBT in trauma patients.
**METHODS.** All patients who met one of trauma activated criteria which were 1) Systolic blood pressure < 90mmHg 2) Gun shot wound(GSW) at the chest, abdomen or back 3) Stab wound at the chest or abdomen 4) Respiratory rate <12/min or >30/min 5) Pulse rate > 120/min 6) Glasgow coma scale score ≤ 8 7)Present of free fluid from focused assessment with sonography for trauma(FAST) from January 2012 to December 2018 were included. MBT was defined as receiving packed red cell(PRC) > 10 units in 24 hours or > 4 units in the first hour. Univariable analysis was conducted on all possible predictors which included patients characteristic, mechanism of injury, and laboratory results. Factor with p value < 0.2 were included in the stepwise backward multivariable logistic regression. The score was assigned by coefficient. Sensitivity, specificity and positive likelihood ratio was estimated. Cutoff point was selected based on Receiver Operator Characteristic curve analysis then internal validation was performed with bootstrap replications.
**RESULTS.** Among of 878 patients, 102(11.6%) received MBT. Nine factors were significantly associated with MBT with the score of 2.5 for age ≥ 60 years, 1.5 for female, 2 for GSW, 3 for pelvic fracture from physical examination, 2 for femur fracture from physical examination, 1 for PR ≥ 105/min, 2 for base excess ≤ -10mEq/L, 2.5 for lactate > 4mmol/l, and 2 for present of free fluid on FAST. The area under the curve(AUC) was 0.824 (95% CI 0.778 - 0.871). At the cutoff point ≥ 7, this score has a positive likelihood ratio of 10.22. Internal validation demonstrated AUC 0.805 (95%CI 0.754 -0.856).

**CONCLUSION.** The new score has an excellence discrimination to prediction of MBT. The parameters were simple and easy to obtain a few minutes after arrival thus should be applied in clinical practice. Further, cost-effectiveness and implemental studies should be done.

**REFERENCE(S)**
1. Chaochankit W, Akaraborworn O, Sangthong B, Thongkhao K. Combination of blood lactate level with assessment of blood consumption (ABC) scoring system: A more accurate predictor of massive transfusion requirement. Chin J Traumatol. 2018 Apr;21(2):96–9.
2. This study was funded by "Targeted Research Grants, Faculty of Medicine, Prince of Songkla University."

## SIS - Sepsis phenotypes

### 000008

#### Epidemiology of septic patients admitted to the Intensive Care Unit. Analysis of incidence and outcomes

AB. López Pérez, HG. Khandji Aslan, JB. López Messa
Intensive care unit, Hospital Río Carrión, Palencia, Spain
**Correspondence:** A.B. López Pérez
*Intensive Care Medicine Experimental* 2019, **7(Suppl 3):**000008

**INTRODUCTION.** Sepsis incidence is increasing compared to incidence for other leading causes of mortality such as acute myocardial or stroke. Estimates of sepsis incidence and trends are also essential to estimate the resources needed to care for these patients. We would like to emphasize the importance of using local data to monitor trends in results over time. This information has significant implications for health-care service planning and may be useful to estimate future care requirements.
**OBJECTIVES.** To examine the most recent epidemiological characterestics of sepsis and the temporal changes in its incidence and outcome.
**METHODS.** A retrospective study was carried out during a consecutive six years period in a polyvalent ICU of a secondary hospital from January 1, 2010 to December 31, 2016. To identify episodes of sepsis we applied the International Classification of Diseases, Ninth Revision, Clinical Modification. The statistical analysis was perfomance with IBM-SPSS-22. The hospitalization rate was defined as the yearly number of admissions per 100.000 population. Qualitative variables are expressed as frecuency and percentage, and quantitative variables as mean and standard deviation. The Student t test was used to compare means of the quantitative variables and when the hypothesis of normality was no accepted, the nonparametric Mann-Whitney U test was used. And Chi-square test for categorical variables. We performed a logistic regression analysis to identify factors associated with in-hospital mortality. Odds ratios (ORs) with 95% confidence intervals were computed.
**RESULTS.** Seven hundred and twenty four patients were included at ICU admission. Sepsis was present in 180 patients (24.9%) and septic shock in 544 (75.1%). The mean age was 69.2 years and 67% were male. 66.9% of all episodes of sepsis were among people aged 65 years or more. Hypertension (53%), non-metastatic cancer (30.7%) and diabetes (26.8%) were the most common categories of comorbility. Gram-negative bacteria were the most frequently involved microorganisms (42.7%). The observed mortality was 38.7%. Elderly patients (≥65 yrs) were more likely to have 3 or more organ failures (73.1% vs 61.7%). Between 2010 and 2016 there was an annualized increase in the incidence of sepsis from 35.4 per 100.000 population to 53.3 per 100.000 population. The total in-hospital mortality rate fell from 52.9% in 2010 to 34.6% in 2016. The risk of death was significantly associated with the respiratory failure, a low pH, SAPS 3 and mechanical ventilatory support.
**CONCLUSION.** Our analysis shows that cases of sepsis occur in older people and develop a greater number of organ failures. Sepsis incidence has risen in recent years in our region while mortality has fallen despite increase in age and severity of sepsis.

**REFERENCE(S)**
1. Yébenes JC, Ruiz-Rodriguez JC, Ferrer R, et al. Epidemiology os sepsis in Catalonia: analysis of incidence and outcomes in a European setting. Annals of Intensive Care.2017;7:19.
2. Azkárate I, Choperena G, Salas E, Sebastián R, Lara G, Elósegui I et al. Epidemiology and prognostic factors in severe sepsis/septic shock. Evolution over six years. Med Intensiva.2016;40:18-25.
3. Bouza C, López-Cuadrado T, Saz-Parkinson Z, Amate-Blanco JM. Epidemiology and recent trends of severe sepsis in Spain. BMC Infect Dis.2014;14:3863.

### 000014

#### Presepsin as a predictor of sepsis outcome in comparison to procalcitonin and CRP

A. Mahmoud,[1], K. Taema[2], H. Saber,[1], H. Sherif,[2]
[1]Critical care medicine, Benisuef University Hospital, Beni Suef, Egypt; [2]Critical care medicine, Cairo University, Faculty Of Medicine, Kasr Al Ainy, Cairo, Egypt
**Correspondence:** K. Taema
*Intensive Care Medicine Experimental* 2019, **7(Suppl 3):**000014

**INTRODUCTION.** Identification of predicted sepsis related mortality is important for patient stratification.
**OBJECTIVES.** We evaluated the significance of Presepsin in predicting sepsis mortality.
**METHODS.** We enrolled 83 sepsis patients according to the SCCM/ESICM/ACCP/ATS/SIS International Sepsis Definitions Conference in a prospective observational study. After excluding 28 patients due to different exclusion criteria, 55 continued the study. Their age was 58(47-65) years-old with 33 (60%) males.
We measured serum Presepsin, Procalcitonin, and CRP on admission, 24 and 72 hours later. APACHE-II score and Capillary leak index (CLI) were estimated. The primary outcome was the in-hospital mortality.
**RESULTS.** The median(Q1-Q3) Presepsin24 and Presepsin72 levels were 1127.5(835.75-2137.5) and 883(429-1214.5)pg/ml in survivors compared to 2321(1264-3456) and 3421(1900-5432)pg/ml in non-survivors (P=0.01 and 0.000 respectively). The serum CRP24 and CRP72 were 123(76-154) and 94(42.5-127)mg/L in survivors compared to 156(101-201) and 187(139-233)mg/L in non-survivors (P= 0.02 and 0.000). PCT72 was 111.5(66-186.25)pg/ml in survivors compared to 231(187-324)pg/ml in non-survivors (P=0.000). Presepsin0, CRP0, PCT0 and PCT24 were not significantly different between survivors and non-survivors (P=0.4, 0.7, 0.5, and 0.2 respectively). The APACHE-II score was 18(15-20.8) in survivors compared to 21 (19-24) in non-survivors (P = 0.02) while the CLI was 42(27.6-57.7) and 42.4(33.3-62.3) in survivors and non-survivors respectively (P = 0.8). The AUC was the highest for Presepsin72 (0.918). Presepsin72 of 1262pg/ml was seen to be 92.3% sensitive and 81.3% specific for mortality prediction.
**CONCLUSION.** This study showed that the serum Presepsin could be a valuable biomarker for predicting in-hospital mortality in sepsis.

### 000025

#### Immunomodulatory effects of antibiotics during sepsis: the endothelial cell's role

S. Pons[1], M. Loiselle[1], E. Arrii[1], M. Nouacer[1], J. Lion[1], A. Cross[1], E. Azoulay[2], N. Mooney[1], L. Zafrani[2]
[1]U976 "human immunology, pathophysiology, immunotherapy", INSERM, Paris, France; [2]Medical icu, Hôpital Saint-Louis, Paris, France
**Correspondence:** S. Pons
*Intensive Care Medicine Experimental* 2019, **7(Suppl 3):**000025

**INTRODUCTION.** Sepsis is defined as the host's inflammatory response to a life-threatening infection potentially leading to a septic shock and the failure of organs initially not infected [1]. Currently, the most efficient weapon to fight against bacterial infections is antibiotics. New elements suggest that some antibiotics could have an intrinsic immunomodulatory effect in addition to their antimicrobial effect [2]. Endothelial cells are able to detect danger signals from pathogens and to initiate the innate immune response, and in some specific conditions, the adaptive immunity [3].

**OBJECTIVES.** The aim of this study was to highlight the effects of two antibiotic classes on endothelial cells' immune properties during sepsis.

**METHODS.** Human Microvascular Endothelial Cells (HMEC) were stimulated by Lipopolysaccharide, Interferon γ and Tumor Necrosis Factor α (Cytomix) during 24 hours before the administration of different antibiotics. The surface expression of Inter-Cellular Adhesion Molecule 1 (CD54), Program-Death Ligand 1 (CD274), Human Leucocyte Antigen (HLA) class 1 and HLA-DR were determined using flow cytometry 24 hours after antibiotics' administration. The production of cytokines as Interkeukin (IL)-6 or IL-8 by HMEC was determined by Enzyme-Linked Immunoabsorbent Assay (ELISA). Quantitative polymerase chain reaction (qPCR) was used to analyze GAPDH (control), IL-6, HLA-A and HLA-DR gene expression. To study the effect of antibiotics on lymphocytes, the activated HMEC exposed to antibiotics were co-cultured with peripheral blood mononuclear cells obtained (PBMC) from healthy donors.

**RESULTS.** Compared to Cytomix alone, Clarithromycin (1 μg/mL) associated to Cytomix significantly decreased the expression of HLA-1 (fold change HLA-1 expression to Cytomix 0,67 to 1 p<0,01) and HLA-DR (0,65 to 1 p<0,01) on HMEC surface. Rovamycin and Erythromycin also significantly decreased both expressions of HLA-1 and HLA-DR. In qPCR, Clarithromycin (10 μg/mL) significantly decreased HLA-DR gene expression (1 versus 0,78 HLA-DR fold expression to GAPDH p<0,01) and IL-6 gene expression (1 versus 0,67 IL-6 fold expression to GAPDH p=0,03) compared to Cytomix. Compared to Cytomix, Amoxicillin (1 μg/mL) significantly decreased the expression of HLA-1 on HMEC surface (0,55 to 1 p<0,01) but did not affect HLA-DR expression. IL-6 and IL-8 production were not significantly different between Cytomix stimulation alone and Cytomix associated with antibiotics treatment. Co-cultures of HMEC with Rovamycin and PBMC did not change lymphocytes polarization nor regulatory or memory T cells proliferation.

**CONCLUSION.** Macrolides and Penicillin modulate the expression of HLA molecules on microvascular endothelial cells' surface. The use of a second endothelial cell line, co-cultures of endothelial cells with sorted CD8+ lymphocytes and *in vivo* experiments could better enlighten the potential pathophysiological implications of those results.

**REFERENCE(S)**

1. Singer M, Deutschman CS, Seymour CW, et al (2016) The Third International Consensus Definitions for Sepsis and Septic Shock (Sepsis-3). JAMA 315:801–810. https://doi.org/10.1001/jama.2016.0287
2. Yoshioka D, Kajiwara C, Ishii Y, et al (2016) Efficacy of β-Lactam-plus-Macrolide Combination Therapy in a Mouse Model of Lethal Pneumococcal Pneumonia. Antimicrob Agents Chemother 60:6146–6154. https://doi.org/10.1128/AAC.01024-16
3. Taflin C, Favier B, Baudhuin J, et al (2011) Human endothelial cells generate Th17 and regulatory T cells under inflammatory conditions. Proc Natl Acad Sci U S A 108:2891–2896. https://doi.org/10.1073/pnas.1011811108

4. SRLF/ MSD grant
5. ESICM Basic Science Award

### 000063

#### Right ventricular function and long-term outcome in sepsis: a retrospective cohort study

JC. Winkelhorst, SN. Voorrips, IT. Bootsma, PM. Koetsier, F. De Lange, EC. Boerma[1]
Department of intensive care, Medical Centre Leeuwarden, Leeuwarden, Netherlands
**Correspondence:** J.C. Winkelhorst
*Intensive Care Medicine Experimental* 2019, **7(Suppl 3):**000063

**INTRODUCTION.** Sepsis-related myocardial dysfunction is associated with impaired outcome. Traditionally, in this setting the main focus has been on left ventricular performance. As of now, specific knowledge on the prognostic importance of right ventricular dysfunction is scarce. The aim of this study was to determine whether right ventricular ejection fraction (RVEF) is predictive of long-term mortality in sepsis.

**METHODS.** Single-centre retrospective cohort study in adult patients admitted to the ICU with severe sepsis and septic shock, and equipped with a pulmonary artery catheter within 24 hours following admission. RVEF was recorded as an average over the first 24 hours (sample rate of 1 per minute). Patients were a priori separated into subgroups according to their RVEF: RVEF<20% (A), RVEF 20-30% (B), and RVEF>30% (C). The primary endpoint was one-year all-cause mortality; secondary endpoints included haemodynamic variables and use of norepinephrine in relation to RVEF.

**RESULTS.** In a 7-year period 101 patients fulfilled all entry criteria and 98 were included in the study. Demographic data, medical history, reason for admission, and source of infection did not statistically differ between groups. However, patients in group A had a significantly higher APACHE II score and signs of shock were more profound, as indicated by a lower cardiac index, SvO2, and pH, a higher lactate, and more use of norepinephrine in comparison to other groups. One-year all-cause mortality was significantly different between groups: 57.1% in group A (n=21), 18.2% in group B (n=55), and 22.7% in group C (n=22); p=0.003. Kaplan-Meier survival analysis revealed a clear separation between group A and B/C (chi square= 14,00; p=0.001). In a multivariate logistic regression analysis, RVEF, both as a categorical variable (RVEF<20%) and as a continuous variable, remained independently associated with the primary endpoint (OR 4.1; 95% CI 1.3-13.4; p=0.018 and OR 0.92; 95% CI 0.85-0.99; p= 0.018, respectively).

**CONCLUSION.** RVEF is independently associated with one-year all-cause mortality in a highly selected group of patients with severe sepsis and septic shock. In addition, a low RVEF was associated with an increase in signs of circulatory failure, suggesting maladaptation.

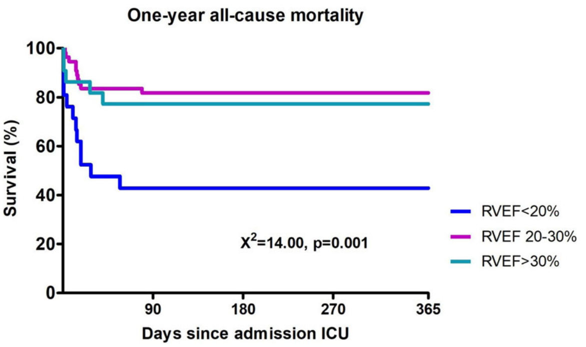

**Fig. 1 (abstract 000063).** See text for description

### 000123

#### The forgotten hemodynamic [ PCO2 gap] in patients with sepsis

Z. Bitar[1], O. Maadarani[2], A. El-Shably[3]
[1]Critical care unit, New Ahmadi hospital KOC -, Ahmadi, Kuwait; [2]Cardiology, Ahmadi hospital , Ahmadi, Kuwait; [3]Critical care unit, New Ahmadi hospital KOC, Ahmadi, Kuwait
**Correspondence:** Z. Bitar
*Intensive Care Medicine Experimental* 2019, **7(Suppl 3):**000123

**INTRODUCTION.** The central venous-arterial carbon dioxide differences (pCO2 gap) is a marker of the adequacy of cardiac output with the global metabolic conditions that are less affected by an impairment of oxygen extraction capacities. (PCO2 gap =K. VCO2(CO2 production)/COP)(1)

**OBJECTIVES.** To investigate the relation between the pCO2 gap, serum lactate and cardiac index (CI) and prognostic value on admission. We also investigated the pattern of Ultrasound chest (US).

**METHODS.** We performed a prospective observational study and recruited 28 patients, over 6 months, with severe sepsis and septic shock in mixed ICU. We determined central venous PO2, PCO2, PCO2 gap, lactate, CI at 0 and 6 hours after critical care unit (CCU) admission. The cardiac index was measured using echocardiography (non-invasive) in a blinded fashion. The population was divided into two groups based on pCO2 gap (cut off value 0.8 kPa).

**RESULTS.** CI was significantly lower in the high gap group (p = 0.001). The high gap CO2 group, on admission, required more bolus fluid administration and vasopressors (P= 0.01 and 0.009 respectively) table1. The hospital mortality rate for all patients was 24.5 % (7/28). The in-hospital mortality rate was 20 % (2/12) for the low gap group and 30 % (5/16) for the high gap group; the odds ratio was 1.6 (95 % CI 0.5–5.5), p = 0.53. Patients with a persistent or rising pCO2 gap larger than 0.8 kPa at T = 6 and 12, , had a higher mortality change (n = 6; in-hospital mortality 21.4 %) compared to patients with a pCO2 gap less than 0.8 kPa at T = 6 (n = 1; in-hospital mortality 3%); this odds ratio was 5.3 (95 % CI 0.9–30.7); p = 0.08. PCO2 gap has no relation with chest ultrasound pattern.

**CONCLUSION.** PCO2 gap is an important hemodynamic variable in the management of sepsis-induced circulatory failure. PCO2 gap can be a marker of the adequacy of the cardiac output status in sepsis. A high-value PCO2 gap (>0.8 kPa) can identify situations where increasing cardiac output can be attempted with fluid resuscitation in severe sepsis. PCO2 gap carries an important prognostic value in severe sepsis

**REFERENCE(S)**

1. Monnet and Teboul . My patient has received fluid. How to assess its efficacy and side effects? Ann Intensive Care (2018) 8:54
2. Kuwait Oil Company

**Table 1 (abstract 000123).** See text for description

| | Total population (n = 28) | Low gap (n = 12) | High gap (n = 16) | P value# |
|---|---|---|---|---|
| Age (year) | 71 ± 14 | 69± 17 | 73.62 ± 13 | 0.7 |
| SOFA | 10.39± 3.9 | 8.5±3.4 | 11.75± 3.8 | 0.03* |
| Lab & Therapy | | | | |
| Lactate (mmol/L) | 2.7 ± 2.3 | 1.3 ± 0.9 | 3.76 ± 2.6 | 0.003* |
| CI (L/min/m2) | 2.6 ± 0.41 | 3.2 ± 0.37 | 2.1 ± 0.46 | 0.001* |
| Ultrasound chest A/B profiles | | 8/4 | 8/8 | 0.8 |
| vasopressor | 18 | 5 | 13 | 0.009* |
| Fluid boluses | 28 | 10 | 18 | 0.01* |
| Mean BP | 60 ± 10 | 65 ± 9 | 55 ± 11 | 0.01* |
| SvO2 (%) | 71.9 ± 10.7 | 73.2 ± 9.1 | 70.3 ± 6.6 | 0.01* |

### 000132

#### C-reactive protein and albumin kinetics before community-acquired bloodstream infections – a Danish population-based cohort study

OS. Garvik,[1], P. Povoa[2], B. Magnussen,[1], PJ. Vinholt,[3], C. Pedersen,[4], TG. Jensen,[5], HJ. Kolmos,[5], AT. Lassen,[6], KO. Gradel,[1]
[1]Research unit of clinical epidemiology, institute of clinical research, usd, Center for clinical epidemiology, Odense University Hospital, Odense, Denmark; [2]Polyvalent intensive care unit, São Francisco Xavier Hospital, CHLO, Lisbon, Portugal; [3]Department of clinical biochemistry and pharmacology, Odense University Hospital, Odense, Denmark; [4]Department of infectious diseases, Odense University Hospital, Odense, Denmark; [5]Department of clinical microbiology, Odense University Hospital, Odense, Denmark; [6]Department of emergency medicine, Odense University Hospital, Odense, Denmark
**Correspondence:** P. Povoa
*Intensive Care Medicine Experimental* 2019, **7(Suppl 3):**000132

**INTRODUCTION.** Early changes in biomarker levels probably occur sometime before bloodstream infection (BSI) is diagnosed. It has clinical relevance to understand when the host inflammatory response begins. So far, this issue has not been fully addressed.

**OBJECTIVES.** We aimed at evaluating the kinetics of C-reactive protein (CRP) and plasma albumin (PA) in the 30 days before the community-acquired (CA) BSI.

**METHODS.** From a population-based BSI database we identified 658 patients with at least one measurement of CRP or PA from day −30 (D −30) through day −1 (D−1) before the day of CA-BSI (D0) and a measurement of the same biomarker at D0 or D1. Amongst these, 502 had both CRP and PA measurements which fit the above defined criteria.

**RESULTS.** CRP and PA concentrations began to change inversely some days before the diagnosis of CA-BSI, CRP increasing by day −3.1 and PA decreasing by day −1.3. From D−30 to D−4, CRP kinetics (expressed as slopes – rate of concentration change per day) was −1.5 mg/L/day. From D−3 to D1, CRP slope increased to 36.3 mg/L/day. For albumin, the slope between D−30 to D−2 was 0.1 g/L/day and changed to −1.8 g/L/day between D−1 and D1.

**CONCLUSION.** We showed that biomarker levels, used as surrogates of host inflammatory response, begin to change some days before the CA-BSI diagnosis, CRP 3.1 days and PA 1.3 days before.

### 000133

#### Real-life data patterns of C-reactive protein and albumin level trajectories around Community-Acquired Bloodstream Infections

KO. Gradel,[1], P. Povoa[2], PJ. Vinholt,[3], B. Magnussen,[1], C. Pedersen,[4], TG. Jensen,[5], HJ. Kolmos,[5], AT. Lassen,[6]
[1]Research unit of clinical epidemiology, institute of clinical research, usd, Center for clinical epidemiology, Odense University Hospital, Odense, Denmark; [2]Polyvalent intensive care unit, São Francisco Xavier Hospital, CHLO, Lisbon, Portugal; [3]Department of clinical biochemistry and pharmacology, Odense University Hospital, Odense, Denmark; [4]Department of infectious diseases, Odense University Hospital, Odense, Denmark; [5]Department of clinical microbiology, Odense University Hospital, Odense, Denmark; [6]Department of emergency medicine, Odense University Hospital, Odense, Denmark
**Correspondence:** P. Povoa
*Intensive Care Medicine Experimental* 2019, **7(Suppl 3):**000133

**INTRODUCTION.** The ideal study cohort should have the same number of biochemical specimens with equal time intervals between all specimens (as in well-designed prospective studies). This is a limitation of population-based studies with different numbers of samples per patient and highly variable time intervals between specimens. To date no study has assessed trajectories of C-reactive protein (CRP) and plasma albumin (PA) levels in real-life data around Community-Acquired Bloodstream Infections (CA-BSI).

**OBJECTIVES.** To assess trajectory patterns of CRP and PA levels before and after CA-BSI.

**METHODS.** Population-based study, with 2418 CA-BSI patients, CRP and PA specimens from 30 days before through 30 days after CA-BSI episode (day 0). Subgroups were established based on whether specimens were occurring or not in days -30/-1, 0, 1/7 or 8/30. Mean daily CRP and PA levels on day -30/30 were computed for each subgroup.

**RESULTS.** Mean CRP rose on day -5 and reached its peak on day 1. Mean steady PA on day −30/0 declined abruptly on day 1 and increased slowly thereafter. Trajectories did not differ between subgroups, in other words the course of CRP and PA around the BSI episode was independent of the number of specimens. 30-day mortality odds ratios for age, gender, co-morbidity, bacteria group, sepsis status, and albumin level did not change materially when amending 0 versus ≥1 specimen in day -30/0 to the multivariate model, though the subgroup with ≥1 specimen had 50% higher mortality.

**CONCLUSION.** Trajectories of CRP and PA levels around CA-BSI did not differ in relation to number of specimens from each patient. This enables longitudinal analyses of real-life data in population-based studies.

### 000149

#### Revising clinical phenotypes of sepsis using microbiology

H. Zhao[1], J. Kennedy[1], S. Wang[2], G. Bernard[3], K. Demerle[1], CCH. Chang,[2], D. Angus[1], CW. Seymour[1]
[1]Department of critical care medicine, University of Pittsburgh, Pittsburgh, United States of America; [2]Department of biostatistics, University of Pittsburgh, Pittsburgh, United States of America; [3]Division of allergy, pulmonary & critical care medicine, Vanderbilt University, Nashville, United States of America
**Correspondence:** H. Zhao
*Intensive Care Medicine Experimental* 2019, **7(Suppl 3):**000149

**INTRODUCTION.** There is wide heterogeneity in sepsis. The identification of sepsis phenotypes may lead to more precise therapy. The contribution of pathogen data to sepsis phenotypes is unknown.

**OBJECTIVES.** We aim to assess the effects of adding microbiological variables on clinical phenotypes determined using clinical data alone.

**METHODS.** We conducted a secondary analysis of data from the Recombinant Human Activated Protein C Worldwide Evaluation in Severe Sepsis (PROWESS) Study. We used latent class analysis (LCA) to identify phenotypes using 24 clinical-only variables (*C*) (including demographic variables, vital signs, markers of inflammation and organ dysfunction) versus 24 clinical and 3 microbiological variables (e.g. sites of infection, type of pathogen, and drug resistance) (*CM*) using measurements collected prior to randomization. We assessed model fit using Bayesian Information Criteria (BIC), entropy, and the probability of cluster membership. We describe clinical characteristics and outcomes of phenotypes before and after adding microbiology variables. We tested for heterogeneity of treatment effect of activated protein C (APC) by phenotype using logistic regression.

**RESULTS.** Among 1,690 septic patients enrolled in the PROWESS study, we identified an optimal 4-class model for both *C* and *CM* variables. In clinical-only phenotypes, the α-type (N=327, 19.4%) was younger and had more pulmonary dysfunction; the β-type (N=518, 30.7%) was old and had greater comorbidity; the γ-type (N=532, 31.5%) had more pulmonary dysfunction; the δ-type (N=313, 18.5%) had more liver, renal, and hematologic dysfunction and shock. Microbiological variables rearranged 772 of 1690 (45.7%) patients (**Figure 1A**) and increased the median probability of the clustering across 4 groups from 0.952 to 0.971. When microbiology was added to clinical variables, the β-type had more abdominal infection (from 19.7 to 40.1%), while γ-type had increased lung infection (from 50.4 to 77.8%). The 28-day mortality was significantly different across phenotypes (both chi2 $P<0.001$), but was similar between *C* and *CM* models (**Figure 1B**). No heterogeneity of treatment effect by phenotype was present with either *C* (*P* value for interaction=0.25) or *CM* models ($P=0.40$). However, the treatment effect of APC in γ-type significantly changed from benefit ($P=0.04$) to neutral ($P=0.73$) when microbiological variables were added.

**CONCLUSION.** Compared to sepsis phenotypes determined using clinical data alone, the addition of microbiological variables such as site of infection or type of pathogen revised phenotype membership, increased the probability of cluster members, and contributed to clinically meaningful rearrangement of sepsis patients. Microbiology data are an important domain for the investigation of sepsis phenotypes.

**REFERENCE(S)**

1. Seymour CW, Gomez H, Chang CH, et al: Precision medicine for all? Challenges and opportunities for a precision medicine approach to critical illness. Crit Care 2017; 21:257.
2. Vincent JL: The coming era of precision medicine for intensive care. Crit Care 2017; 21:314.
3. This study was supported in part by Dr. Seymour's grant from the National Institutes Health (R35GM119519).

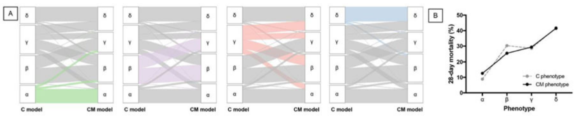

**Fig. 1 (abstract 000149).** Alluvial plot showing the change of membership between *C* and *CM* phenotypes (panel *A*); 28-day mortality by phenotype (panel *B*)

### 000269

#### Outcomes in patients with ventilated nosocomial pneumonia (NP) and organ failure treated with ceftolozane/tazobactam (C/T) vs meropenem (MER) – analysis of the ASPECT-NP randomized, controlled trial

I. Martin-Loeches[1], CJ. Bruno,[2], N. Shime[3], RG. Wunderink[4], M. Kollef[5], Ü. Kivistik[6], M. Nováček,[7], A. Réa-Neto,[8], JF. Timsit, B. Yu,[9], JA. Huntington[9], E. Jensen,[10], JR. Butterton,[2], EG. Rhee[2], AF. Shorr,[11]
[1]Intensive care medicine, St. James's Hospital, Dublin, Ireland; [2]Clinical research, Merck & Co., Inc., Kenilworth, United States of America; [3]Department of emergency and critical care medicine, Hiroshima University, Hiroshima, Japan; [4]Pulmonary and critical care division, Northwestern University Feinberg School of Medicine, Chicago, United States of America; [5]Division of pulmonary and critical care medicine, Washington University School of Medicine, St. Louis, United States of America; [6]Anaesthesiology clinic, North Estonia Medical Centre, Tallinn, Estonia; [7]Department of anaesthesia and intensive care, General Hospital of Kolin, Kolin, Czech Republic; [8]Department of clinical medicine, Universidade Federal do Paraná, Curitiba, Brazil; [9]Clinical operations, Merck & Co., Inc., Kenilworth, United States of America; [10]Biostatistics, Merck & Co., Inc., Kenilworth, United States of America; [11]Pulmonary and critical care medicine, Georgetown University, Washington D.C., United States of America
**Correspondence:** I. Martin-Loeches
*Intensive Care Medicine Experimental* 2019, **7(Suppl 3):**000269

**INTRODUCTION.** NP is a frequently occurring healthcare-acquired infection often caused by gram-negative pathogens and associated with high mortality rates. Rapid initiation of appropriate antibacterial therapy is key to improving survival, especially in patients (pts) with sepsis. In the recently completed, randomized double-blind, multicenter, phase 3 ASPECT-NP trial, C/T (at double the currently approved dose) was noninferior to MEM for ventilated NP in both primary and key secondary endpoints.

**OBJECTIVES.** Retrospectively evaluate outcomes in pts from ASPECT-NP who had greater severity of illness and organ failure, by SOFA component scores.

**METHODS.** Mechanically ventilated pts with NP were randomized 1:1, stratified by NP type (ventilator-associated pneumonia [VAP] vs ventilated hospital-acquired pneumonia [vHAP]) and age (<65 y vs ≥65 y), to receive 3 g C/T or 1 g MEM, by 1-h IV infusions every 8 h for 8-14 d. The primary endpoint was 28-d all-cause mortality in the intent-to-treat (ITT) population, and the key secondary endpoint was clinical response at test-of-cure (TOC; 7-14 d after end-of-therapy in the ITT population). SOFA scores were collected at baseline. We conducted a post-hoc analysis comparing outcomes by treatment arm in pts with a SOFA respiratory component score ≥2 (RS2) and in pts with a cardiovascular component score ≥2 (CVS2), regardless of their other component scores.

**RESULTS.** The majority of ITT pts in this trial met one or both SOFA score criteria, with distributions balanced across arms: in the C/T arm, 312/362 (86.2%) had RS2 and 84/362 (23.2%) had CVS2; in the MEM arm, 321/364 (88.2%) had RS2 and 99/364 (27.2%) had CVS2. In the CVS2 subgroup, 69 (82.1%) C/T and 91 (91.9%) MEM pts also had RS2. Baseline characteristics, including causative pneumonia pathogens (mostly Enterobacteriaceae and *Pseudomonas aeruginosa*), were generally similar between both subgroups and across treatments. Clinical outcomes in both the RS2 and CVS2 subgroups were comparable between C/T and MEM (Table). In RS2, outcomes were consistent with the previously presented overall ITT results (i.e., 28-d mortality ~25% and TOC clinical cure ~55% in both arms). As expected, mortality rates in pts with shock (i.e., CVS2) were slightly higher than in the overall ITT population.

**CONCLUSION.** These data confirm that ASPECT-NP enrolled a critically-ill patient population, reflected in the prevalence of acute lung injury (RS2) and shock (CVS2). In these retrospective analyses, 28-d mortality and TOC clinical cure rates were generally not affected by severity of illness. Furthermore, greater severity of illness did not affect the relative efficacy of C/T vs MEM in these high-risk patients with gram-negative ventilated NP.

1. Funding for this research was provided by Merck Sharp & Dohme Corp., a subsidiary of Merck & Co., Inc., Kenilworth, NJ, USA.

| Endpoint | C/T n/N (%) | MEM n/N (%) | % Difference (95% CI)[a, b] |
|---|---|---|---|
| **SOFA Respiratory Component Score ≥2** | | | |
| 28-day all-cause mortality (ITT) | 74/312 (23.7%) | 77/321 (24.0%) | 0.3 (-6.37, 6.89) |
| Clinical cure at TOC (ITT) | 174/312 (55.8%) | 174/321 (54.2%) | 1.6 (-6.16, 9.25) |
| **SOFA Cardiovascular Component Score ≥2** | | | |
| 28-day all-cause mortality (ITT) | 28/84 (33.3%) | 30/99 (30.3%) | -3.0 (-16.43, 10.27) |
| Clinical cure at TOC (ITT) | 45/84 (53.6%) | 55/99 (55.6%) | -2.0 (-16.14, 12.22) |

CI, confidence interval. C/T, ceftolozane/tazobactam. ITT, intent-to-treat. MEM, meropenem. SOFA, sequential organ failure assessment. TOC, test-of-cure.

[a]Positive differences are in favor of C/T, negative differences are in favor of MEM. [b]Unstratified Newcombe CIs.

### 000181

#### Evaluation of the Diagnostic Effect of Sequential Organ Failure Assessment (SOFA) on Septic Shock: a Retrospective Analysis of Clinical Database MIMIC-III and Multicenter Data

K. Lui, C. Cai
Department of critical care medicine, The First Affiliated Hospital Of Sun Yat-sen University, Guangzhou, China
**Correspondence:** C. Cai
*Intensive Care Medicine Experimental* 2019, **7(Suppl 3):**000181

**INTRODUCTION.** In the Sepsis 3.0 definition, sepsis is only divided into 2 groups: sepsis and septic shock. Septic shock is still one of the most major causes of intensive care unit (ICU) admission and death in the world[1,2]. However, in Sepsis 3.0, it didn't tell us what the accurate mean of adequate fluid resuscitation and provide an objective and detectable indicator in septic patients.

**OBJECTIVES.** Our aim in the present study was to evaluate the diagnostic effect of SOFA on septic shock in septic patients.

**METHODS.** Firstly, we conducted a retrospective study of patients with sepsis diagnosis admitted to the ICU using the MIMIC-III database. They were divided into septic shock and non-shock based on ICD-9 and SOFA≥2. The multivariate logistic regression analysis was used to find out the independent risk factors affecting the incidence of septic shock. ROC curve was used to analyze those risk factors, SOFA, qSOFA, SAPS and SAPSII. What's more, we collected clinical data of sepsis and septic shock patients from three different hospitals within 24 hours after admission. SOFA, qSOFA, SAPS II and APACHE II were also compared with ROC curve analysis.

**RESULTS.** A total of 5724 patients were enrolled in MIMIC-III, with 2811 in septic shock group and 2913 in non-septic shock. The result of multivariate logistic regression analysis indicated that lactate and SOFA were the independent risk factors ($p$<0.05). The result of ROC analysis showed that the AUC of pH-value, lactate and BUN were 0.587, 0.553 and 0.539. SOFA had the best predictive effect with the statistical results of AUC (0.643) (95%CI 0.550–0.723, $p$=0.001), sensitivity (0.620), specificity (0.600); and also shown that SOFA ≥7 meant the patients have supposed to be septic shock. Secondly, SOFA, qSOFA, SAPS II and APACHE II were also compared with ROC by our multicenter data. A total 216 patients were enrolled in our data, with 80 septic shock group and 136 in sepsis. It also shown that SOFA have a higher diagnostic efficiency (AUC=0.687, 95%CI 0.550–0.723, sensitivity=0.727, specificity=0.601, $p$=0.002) than qSOFA (AUC=0.666, 95%CI 0.582–0.750, $p$<0.001), SAPS II (AUC=0.642, 95%CI 0.559–0.724, $p$=0.001) and APACHE II (AUC=0.642, 95%CI 0.558–0.727, $p$=0.001) within 24 hours after admission. It also indicated that SOFA ≥8 meant the patients have supposed to be septic shock。

**CONCLUSION.** SOFA score is the best choice for diagnosing the possibility of septic shock. Our findings suggest that the SOFA≥8 within 24 hours after admission in ICU provides better diagnostic effect for septic shock in septic patients.

**REFERENCE(S)**

1. Mira JC, Gentile LF, Mathias BJ, Efron PA, Brakenridge SC, Mohr AM, Moore FA, Moldawer LL: Sepsis Pathophysiology, Chronic Critical Illness, and Persistent InflammationImmunosuppression and Catabolism Syndrome. Crit Care Med. Feb; 45(2):253-262, 2017.
2. Singer M, Deutschman CS, Seymour CW, et al. The Third International Consensus Definitions for Sepsis and Septic Shock (Sepsis-3). JAMA, 2016;315:801–10.
3. This study was supported by two Guangzhou Science and Technology Programs (Grant No. 201607010186, 201704020153). The funders had no role in study design, data collection and analysis, decision to publish, or preparation of the manuscript.

### 000196

#### Evaluation of Empiric Antimicrobial Therapy in ICU Patients With Severe Sepsis and Septic Shock

M. HASHHOUSH[1], K. Olsen,[2], M. Al Khamis[3], A. Zikri,[4], N. Al Ashi[4], S. Humaid,[4]
[1]Pharmaceutical care services administration, King fahad Specialist Hospital Dammam, Dammam, Saudi Arabia; [2]College of pharmacy, University of Nebraska Medical Center, Omaha, United States of America; [3]Adult critical care department, King Fahad Specialist Hospital - Dammam., Dammam, Saudi Arabia; [4]Pharmaceutical care services administration, King Fahad Specialist Hospital - Dammam., Dammam, Saudi Arabia
**Correspondence:** M. HASHHOUSH
*Intensive Care Medicine Experimental* 2019, **7(Suppl 3):**000196

**INTRODUCTION.** Prompt initiation of appropriate empiric antimicrobial therapy decreases mortality and improve clinical outcomes. However, the excessive use of antibiotics has been associated with antibiotic resistance that has been linked to increased morbidity and mortality. This study evaluated the appropriateness of empiric antimicrobial therapy in patients with severe sepsis and septic shock

**OBJECTIVES.** The objective of the study is to evaluate the appropriateness of empiric antimicrobial therapy in patients with severe sepsis and septic shock admitted to intensive care

**METHODS.** This retrospective study included adult patients admitted with severe sepsis and septic shock to a 16-bed intensive care unit (ICU) at a tertiary care facility in the eastern province, Saudi Arabia. The ICU database was utilized to identify all patients who had an admission diagnosis of severe sepsis or septic shock in 2015, which was defined based on the 2012 Surviving Sepsis Campaign guidelines. Demographic data, empiric antimicrobials, source of infection, and results of antimicrobial sensitivity testing were recorded. The primary goal was the appropriateness of empiric antimicrobial therapy in severe sepsis and septic shock patients initiated within 24 hours of ICU admission against the isolated organisms

**RESULTS.** A total of 98 ICU admissions were included, among whom 70 (71%) had septic shock while the remaining had severe sepsis. Among the patients, 57 were males, 53 (54%) had malignancies and the mean age was 55 years. The most common sources of infection were pneumonia (35, 36%), intra-abdominal (24, 24%), bacteremia (14, 14%), urinary tract (8, 8%), and skin/soft tissue (7, 7%). Cultures were positive in 47 episodes (48%). Empiric antimicrobial therapy was appropriate in 48 out of 55 (87%) the total isolates causing infections. Only 13% of the isolated organisms were resistant to initial empiric therapy, of which three isolates were carbapenem-resistant Pseudomonas aeuroginosa. There was a single isolate of carbapenemresistant Klebsiella pneumoniae, carbapenem-resistant Acineotbacter baummannii, methicillin-resistant Staphylococcus aureus and candida glabrata. The most frequently prescribed antibiotics were vancomycin prescribed in 66 (67%) cases, carbapenems in 59 (60%) cases followed by piperacillin/tazobactam (n=37, 38%), amikacin (n=21, 21%), and levofloxacin (n=13, 13%). There was a variation as regard to patients type, inappropriateness of empirical antimicrobial was relatively highest among oncology patients (p= 0.025)

**CONCLUSION.** The majority of the patients in this study received empiric antimicrobial therapy for which the pathogens identified were susceptible to. Institutional antibiotic susceptibility profile should be considered in optimizing empirical antibiotics therapy in settings with a high rate of resistance.

**REFERENCE(S)**

1 Brusselaers N, Vogelaers D, Blot S. The rising problem of antimicrobial resistance in the intensive care unit. Ann Intensive Care 2011; 1: 47

2 Vincent JL, Sakr Y, Sprung CL, et al. Sepsis in European intensive care units: results of the SOAP study. Crit Care Med 2006; 34: 344-53

3 Vincent JL, Rello J, Marshall J, et al. International study of the prevalence and outcomes of infection in intensive care units. JAMA 2009; 302: 2323-29

4 Kumar A, Roberts D, Wood KE, et al. Duration of hypotension before initiation of effective antimicrobial therapy is the critical determinant of survival in human septic shock. Crit Care Med 2006; 34: 1589-96

5 Kavar KS, Jha RK, Gaikwad NR, et al. Antibiotic use density in medicine ICU in a tertiary care rural hospital of central India. Res J Pharm Biol Chem Sci.2012;3:133–42

6 Vincent JL, Marshall JC, Amendys-Silva SA, et al. Assessment of the worldwide burden of critical illness: the Intensive Care Over Nations (ICON) audit. Lancet Respir Med. 2014; 2:380–6.

7 Heddini A, Cars O, Qiang S, et al. Antibiotic resistance in China— a major future challenge. Lancet. 2009;373(9657):30.

8 Habibzadeh F. Lancet (London, England). 2013. http://www.thelancet.com/pb/assets/raw/Lancet/global-health/middle-east/Nov13_MiddleEastEd.pdf?elsca1=220713.

9 Bin Abdulhak AA, Altannir MA, Almansor MA, et al. Non prescribed sale of antibiotics in Riyadh, Saudi Arabia: a cross sectional study. BMC Public Health. 2011;11:538.

10 World Health Organization. Antimicrobial resistance global report on surveillance: 2014 summary. 2014. Available from: URL:http://www.who.int/iris/bitstream/10665/112642/1/9789241564748_eng.pdf

11 Mahin Jamshidi, et al. Antimicrobial resistance pattern among intensive care unit patients. African Journal of Microbiology Research. 2009; (10): 590-4.

12 Kaushal V Sheth, et al. Antibiotic Sensitivity Pattern of Bacterial Isolates from the Intensive Care Unit of a Tertiary Care Hospital in India. Tropical Journal of Pharmaceutical Research December 2012; 11 (6): 991-9.

13 Radjia M, Fauziaha S, Aribinuko N. Antibiotic sensitivity pattern of bacterial pathogens in the Intensive Care Unit of Fatmawati Hospital, Indonesia. Asian Pac J Trop Biomed 2011; 1: 39-42.

14 Saravu K, et al. Clinico-microbiological profile and outcomes of nosocomial sepsis in an Indian tertiary care hospital–a prospective cohort study. Pathog Glob Health. 2015 Jul;109(5):228-35.

15 Hanssens Y, Ismaeil BB, Kamha AA, et al. Antibiotic prescribing pattern in a medical intensive care unit in Qatar. Saudi Med J. 2005 Aug;26(8):1269-76.

16 Surviving Sepsis Campaign: International Guidelines for Management of Severe Sepsis and Septic Shock: 2012. Critical Care Medicine. 2013;41(2).

17 Ivan Surya Pradipta, Dian Chairunnisa Sodik, Keri Lestari, et at. Antibiotic Resistance in Sepsis Patients: Evaluation and Recommendation of Antibiotic Use. N Am J Med Sci. 2013 Jun; 5(6): 344–352.

18 Orsini J, Mainardi C, Muzylo E, Karki N, Cohen N, Sakoulas G. Microbiological profile of organisms causing bloodstream infection in critically ill patients. J Clin Med Res. 2012;4:371–7.

19 Kumar A, Ellis P, Arabi Y, Roberts D, Light B, Parrillo JE, et al. Initiation of inappropriate antimicrobial therapy results in a five-fold reduction of survival in human septic shock. Chest. 2009; 136:1237–48.

20. Kumar A, Safdar N, Kethireddy S, Chateau D. A survival benefit of combination antibiotic therapy for serious infections associated with sepsis and septic shock is contingent only on the risk of death: a meta-analytic/meta-regression study. Crit Care Med. 2010; 38:1651–64. A useful study reconciling divergent findings regarding the potential utility of combination therapy in septic shock.

21 Kumar A, Zarychanski R, Light B, Parrillo J, Maki D, Simon D, et al. Early combination antibiotic therapy yields improved survival compared with monotherapy in septic shock: a propensity-matched analysis. Crit Care Med. 2010; 38:1773–85.

22 Micek ST, Welch EC, Khan J, Pervez M, Doherty JA, Reichley RM, et al. Empiric combination antibiotic therapy is associated with improved outcome against sepsis due to Gram-negative bacteria: a retrospective analysis. Antimicrob Agents Chemother. 2010; 54:1742–8.

23 Martinez JA, Cobos-Trigueros N, Soriano A, Almela M, Ortega M, Marco F, et al. Influence of empiric therapy with a beta-lactam alone or combined with an aminoglycoside on prognosis of bacteremia due to gram-negative microorganisms. Antimicrob Agents Chemother. 2010; 54:3590–6. [PubMed: 20585123]

24 Delannoy PY, Boussekey N, Devos P, Alfandari S, Turbelin C, Chiche A, et al. Impact of combination therapy with aminoglycosides on the outcome of ICU-acquired bacteraemias. Eur J Clin Microbiol Infect Dis. 2012; 31:2293–9. [PubMed: 22350387]

25 Diaz-Martin A, Martinez-Gonzalez ML, Ferrer R, Ortiz-Leyba C, Piacentini E, Lopez-Pueyo MJ, et al. Antibiotic prescription patterns in the empiric therapy of severe sepsis: combination of anti-microbials with different mechanisms of action reduces mortality. Crit Care. 2012; 16:R223.

26 Esteban A, Frutos-Vivar F, Ferguson ND, Peñuelas O, Lorente JA, Gordo F, et al. Sepsis incidence and outcome: Contrasting the intensive care unit with the hospital ward. Crit Care Med. 2007; 35:1284–9.

27 Wang HE, Shapiro NI, Angus DC, Yealy DM. National estimates of severe sepsis in United States emergency departments. Crit Care Med. 2007;35:1928–36.

28 John I. J, Devi P, John J, et al. Drug utilization study of antimicrobial agents in medical intensive care unit of a tertiary care hospital. Asian J Pharm

Clin Res, Vol 4, Issue 2, 2011, 8184 29 Severe sepsis screening tool version 7.2.13

### 000236

#### Prognostic accuracy of Sepsis-induced coagulopathy score in critically ill patients with sepsis: a post hoc analysis of nationwide multicentre registry in Japan

C. Tanaka[1], T. Takashi[2], S. Shin[1], T. Akiko[1], K. Junya[1], N. Fumihiko[1], F. Reo[1], K. Saori[1], K. Masamune[1], U. Kyoko,[1]
[1]Emergency and critical care medicine, Nippon Medical School Tama Nagayama Hospital, Tama, Japan; [2]Emergency and critical care medicine, Nippon Medical School Musashi Kosugi Hospital, Kawasaki, Japan
**Correspondence:** C. Tanaka
*Intensive Care Medicine Experimental* 2019, **7(Suppl 3):**000236

**INTRODUCTION.** Disseminated intravascular coagulation (DIC) is one of the major causes of organ dysfunction and death in sepsis; however, there are no standard criteria for DIC. Recently, sepsis-induced coagulopathy (SIC) was developed as a new criterion for coagulopathy of sepsis. It includes the sequential organ failure assessment (SOFA), similarly the new definition of sepsis revised in 2016. Previous studies suggested that SIC score might be a prognostic value for patients with sepsis and coagulopathy.
**OBJECTIVES.** To evaluate the generalizability of SIC score as a prognostic value generally for patients with sepsis.
**METHODS.** Data from a multicenter observational study (Japan Septic Disseminated Intravascular Coagulation [JSEPTIC-DIC] study), which investigated patients with sepsis in 42 intensive care units from 2011 to 2013, were analyzed. This contained information about patients' backgrounds, examination results, treatments, and mortality. Primary outcome measure was in-hospital mortality. SIC score, Japanese Association for Acute Medicine (JAAM) DIC score, and the International Society of Thrombosis and Haemostasis (ISTH) DIC score were calculated. Then, patient characteristics and other variables for survivor and non-survivor groups were compared, using $\chi 2$ test or Fisher's test. Finally, receiver operating characteristic (ROC) analysis was performed by estimating the corresponding areas under the curve (AUC).
**RESULTS.** This study included 2283 patients (mean age, 68.7 years; standard deviations [SD] 14.6); 1364 were males (59.7%). All-cause mortality rate was 32.1%. The mean APACHE II score was 23.4 (SD 8.8) and the mean SOFA score was 9.7 (SD 4.1). A total of 1647 patients (72.1%) were diagnosed with SIC – 1291 patients (56.5%) were positive JAAM DIC and 727 patients (31.8%) met the ISTH DIC criteria. The proportion of the JAAM DIC and ISTH DIC were significantly higher in non-survivor group (66.5% vs 51.8% p<0.01, 43.7% vs 26.2% p<0.01, respectively); however, there was no statistically significant difference in SIC score between non-survivor and survivor groups (74.2% vs 71.2%, p=0.07). ROC curve showed that AUC for SIC, JAAM DIC, ISTH DIC, APACHE II, and SOFA scores were 0.52 (95% confidential interval [CI], 0.49-0.54), 0.57 (95%CI, 0.55-0.60), 0.59 (95%CI, 0.56-0.61), 0.72 (95%CI, 0.70-0.74), and 0.71 (95%CI, 0.69-0.74), respectively.
**CONCLUSION.** Generally, SIC score might not be a good prognostic value for mortality among sepsis patients.

##### REFERENCE(S)

1. Toshiaki Iba, Marcello Di Nisio, Jerrold H Levy, Naoya Kitamura, Jecko Thachil: New criteria for sepsis-induced coagulopathy (SIC) following the revised sepsis definition: a retrospective analysis of a nationwide survey. BMJ Open 2017; 7: e017046.
2. Hayakawa M, Yamakawa K, Saito S, Uchino S, Kudo D, Iizuka Y, Sanui M, Ta kimoto K, Mayumi T: Nationwide registry of sepsis patients in Japan focu sed on disseminated intravascular coagulation 2011-2013. Sci Data 2018, 5:180243.

### 000245

#### Systematic review on epidemiology of health care-associated sepsis

H. Saito[1], T. Harder[2], R. Markwart[2], S. Tomczyk[2], T. Eckmanns[2]
[1]Department of emergency and critical care medicine, St. Marianna University School of Medicine Yokohama City Seibu Hospital, Yokohama, Japan; [2]Infectious disease epidemiology department, Robert Koch Institute, Berlin, Germany, Germany
**Correspondence:** H. Saito
*Intensive Care Medicine Experimental* 2019, **7(Suppl 3):**000245

**INTRODUCTION.** Sepsis is reported to affect more than 30 million people every year and is a priority on the global health agenda.[1,2] It is estimated that 70% of sepsis is community-onset while 30% is health care-associated (HA).[2] However, there has been only one study conducted to estimate the global sepsis burden, and specifically, the global burden of HA sepsis as compared to community-onset is still not well understood. A systematic review was conducted to assess the burden of HA sepsis and its epidemiology.
**METHODS.** The following electronic bibliographic databases were searched from 1 January 2000 to 31 December 2017: MEDLINE, EMBASE, Global Index Medicus, and PubMed. Epidemiological studies that described the incidence or prevalence of HA sepsis were included. Languages were restricted to English, French, Spanish, Arabic, Russian, German, Italian, Japanese, and Portuguese. Title/abstract screening, full-text screening, and data extraction were conducted by at least two reviewers. The study was registered to PROSPERO (CRD42018089554).
**RESULTS.** Title/abstract screening and full-text screening was performed for 8922 and 1772 studies, respectively. Among these, 4 population-based studies and 12 hospital-based studies with clear definitions of HA sepsis were identified. Studies came from 13 countries, including 38.5% from low- and middle-income settings, and 4 geographic regions. Two population-based studies focused on a pediatric population; incidence of HA sepsis ranged from 1.28-6.88 cases per 100,000 population-years. Two studies focused on an adult population including 1 study which defined only severe sepsis and septic shock; incidence of HA sepsis ranged from 12.28-13.92 cases per 100,000 population-years. The proportion of HA sepsis out of all sepsis cases ranged from 22.8% to 49.1%.
**CONCLUSION.** Our systematic review highlighted the important epidemiological burden of HA sepsis. HA infections leading to sepsis are preventable, and infection prevention and control measures remain important to mitigate the sepsis burden in the healthcare setting.

##### REFERENCE(S)

[1] Fleischmann C, Scherag A, Adhikari NKJ, et al. Assessment of Global Incidence and Mortality of Hospital-treated Sepsis. Current Estimates and Limitations. American Journal of Respiratory and Critical Care Medicine. 2016;193(3):259-272.
[2] Reinhart K, Daniels R, Kissoon N, Machado FR, Schachter RD, Finfer S. Recognizing Sepsis as a Global Health Priority — A WHO Resolution. New England Journal of Medicine. 2017;377(5):414-417.

### 000249

#### Immunoglobulins levels in septic shock

M. Tosi, E. Munari, S. Venturelli, E. Roat, I. Coloretti, M. Girardis
Intensive care unit, Policlinico of Modena University Hospital of Modena, Modena, Italy
**Correspondence:** M. Tosi
*Intensive Care Medicine Experimental* 2019, **7(Suppl 3):**000249

**INTRODUCTION.** Septic shock has growing incidence and mortality rates above 50%. Impairment of immune defences may be common among this patients and underpin the development of unfavourable outcomes1. Immunoglobulins deficit is related to higher mortality rates in septic patient2 3. Hence immunoglobulin supplement is used as adjunctive treatment with immune-adjuvant purposes. Nonetheless it was not clarified yet if Ig baseline titers can affect the outcome in patients treated with IgGAM adjunctive treatment.

**METHODS.** We performed an observational retrospective study analysing a registry specifically conceived to collect information on immune system's functionality in patients undergoing septic shock. Patients admitted to polyvalent intensive care unit of the University Hospital of Modena from January 2014 to February 2019 with diagnosis of septic shock and who received adjunctive treatment with polyclonal immunoglobulins enriched in IgM and IgA (IgGAM) were included in the analysis. Patients <18 years, with an end-of-life decision or too sick to benefit and patients with no Ig titers measured at baseline were excluded. The population was splitted in tertiles of basal IgM titers. Survival ad day 30 from shock occurrence was compared among the three groups.

**RESULTS.** 84 patients were included. Mean age was 65, median SAPS II score at admission was 62 (IQR 48-76) and baseline SOFA score was in median 11 (IQR 8-13), more common comorbidities were neoplasm, cirrhosis and diabetes. 12 patients had a previous state of immune impairment due to haematological disease, HIV, immunosuppressant or chemotherapic drugs. Overall 30-days mortality was 47.6% (40 patients). Median length of stay in ICU was 9 days (IQR 6-19) and in hospital 22 days (IQR 11-38). IgM titers at T0 were in median 49.5 (IQR 28.5-94). Tertiles division according to IgM basal titers splitted the population using threshold values of IgM of 37 and 71 mg/dL. The three groups did not differ for age, sex, basal SAPS II and SOFA score, immune impairment state, rate of nosocomial and MDR infections and comorbidities except for cirrhosis, that was more common in 3° tertile and hematologic diseases, more frequent in 1° tertile. 30 days mortality in 1st, 2nd and 3rd tertile was 53%, 37% and 51.9% respectively (P-value 0.407).

**CONCLUSION.** Patients with septic shock who received adjunctive treatment with IgGAM had higher survival rates when IgM at admission had intermediate concentrations (from 37 to 71 mg/dL) compared to lower or higher IgM titers. Further studies are needed to clarify whether patients with normal or high levels of IgM at diagnosis do take advantage from Ig supplementation therapy.

**REFERENCE(S)**

1. Hotchkiss, R. S., Monneret, G. & Payen, D. Immunosuppression in sepsis: a novel understanding of the disorder and a new therapeutic approach. Lancet Infect. Dis. 13, 260–268 (2013).
2. Bermejo-Martín, J. F. et al. Immunoglobulins IgG1, IgM and IgA: a synergistic team influencing survival in sepsis. J. Intern. Med. 276, 404–412 (2014).
3. Giamarellos-Bourboulis, E. J. et al. Kinetics of circulating immunoglobulin M in sepsis: relationship with final outcome. Crit. Care 17, R247 (2013).

### 000250

#### Epidemiology and characteristics of ICU acquired infections using ENVIn database

I. Fernández, R. Torcuato, A. Alvarez, M. Salgado, A. Ubeda
Intensive care unit, Hospital Point Europe, Algeciras, Spain
**Correspondence:** A. Ubeda
*Intensive Care Medicine Experimental* 2019, **7(Suppl 3):**000250

**INTRODUCTION.** Patients admitted in an intensive care unit (ICU) are at the greatest risk of acquiring nosocomial infections. Infectious diseases are associated with higher rates of morbi-mortality and hospital and ICU length of stay (LOS).

**OBJECTIVES.** To identify the clinical and epidemiological features of the infections registered in the ICU of Hospital Punta de Europa using ENVIN database.

**METHODS.** Retrospective descriptive analysis was performed using a prospective cohort of 330 patients obtained from a 12-bedsICUcollected during the year 2018. Demographic variables, ICU admission diagnoses and their origin (community, ward or another ICU), risk factors, prognosis scores, ICU LOS, mortality, microorganism isolated and antimicrobial therapy used were collected. Statistical analysis was performed: continuous variables (mean and standard deviation), categorical variables (percentages and frequencies).

**RESULTS.** 330 patients were included: male (63.03%), age: 64 [± 15.22], SAPS II 35.78 [±16.23], APACHE II 15.73 [±7.94]. ICU LOS (days) 6.93 [±6.02]. ICU mortality: 88 patients (26.67%).Community-acquired infections (64 patients, 19.39%): 18 non ventilator-associated pneumonias, 8 surgical infections, 8 soft tissue infections. Microbiological isolation: *E. coli*(19.51%), *H. influenzae*(7.32%) and *S. aureus*(7.32%). Antibiotic therapy: ceftriaxone (17.89%), piperacillin/tazobactam (PTZ) (12.11%). Hospital-acquired infections outside the ICU (30 patients, 9.09%): 13 surgical related infections and 3 non ventilator-associated pneumonia. Microbiological isolation: *E. faecium*(16.67%), *E. coli* (13.33%) and *Candida albicans*(13.33%). Antibiotic therapy: metronidazole(12.12%),ceftriaxone(11.11%).ICU-acquired infections (20 patients, 6.06%): 11 ventilator-associated tracheobronchitis, 5 ventilator-associated pneumonia (VAP), 5 bacteremia due to intra-abdominal infections and 3 catheter-related bloodstream infections (CRBSIs):*E. coli*(12.90%), *P. aeruginosa* (12.90%) and *S. aureus*(12.90%). Antibiotic therapy: levofloxacin (10.61%), PTZ (10.61%), linezolid (10.61%). CRBSI: 1.36 per 1000 central venous catheter days. Isolated:*E. faecalis*(33.33%), *K. penumoniae*(33.33%) and *S. aureus*(33.33%). VAP: 168 patients under invasive mechanical ventilation (50.91%), 5.17 VAP per 1000 ventilator-days. 80% of the cases had not microbiological isolation.

**CONCLUSION.** Most of the infections registered were community-acquired, being *E. coli*the most frequently isolated.*E. coli*and *P. aeruginosa*were the most frequently detected pathogens in ICU-acquired infections. About antibiotic therapy, ceftriaxone, PTZ, levofloxacin and meropenem were more frequently used.

### 000268

#### Aspiration pneumonia and drug overdose requiring invasive ventilation : impact of a care protocol on the antibiotic prescription

G. LE BOUAR[1], PL. Declercq[2], M. Bousta[3], JB. Michot[4], JP. Rigaud[2], L. Lagache[3], O. Delastre[4], D. Boyer[5], S. Boubeche[5], D. Carpentier[5], T. Clavier[5], E. Demarest[1], C. Girault[5], J. Glenisson[5], E. Godeau[5], S. Grangé[5], D. Cerasuolo[6], F. Tamion[5], G. Beduneau[7]
[1]Medical-surgical intensive care unit, District Hospital Center, Évreux, France; [2]Medical-surgical intensive care unit, District Hospital Center, Dieppe, France; [3]Medical-surgical intensive care unit, District Hospital Center, Le Havre, France; [4]Medical-surgical intensive care unit, District Hospital Center, Elbeuf, France; [5]Medical intensive care unit, Rouen University Hospital, Rouen, France; [6]Unit of biostatistics, Rouen University Hospital, Rouen, France; [7]Medical intensive care unit, Rouen University Hospital; Normandie Univ, UNIROUEN, EA 3830, Rouen, France
**Correspondence:** G. LE BOUAR
*Intensive Care Medicine Experimental* 2019, **7(Suppl 3):**000268

**INTRODUCTION.** Aspiration pneumonia (AP) is a frequently suspected complication of drug overdose requiring mechanical ventilation (MV) and admitted to intensive care unit (ICU) (1). In the absence of reliable biomarkers for distinguishing between aspiration pneumonia and aspiration pneumonia, antibiotic therapy is frequently prescribed (2). Latest studies suggest that a care protocol could better select patients requiring antibiotic therapy (3).

**OBJECTIVES.** To determine the impact of a care protocol on the antibiotic prescription among patient admitted to ICU for toxic coma with MV.

**METHODS.** We conducted a prospective observational cohort study in four ICU. We included all patients admitted for toxic coma with MV. In the University-affiliated ICU, a care protocol was applied. In the three others ICU, physicians declared that they did not follow formalized conduct within the service and did as usual.

**RESULTS.** We included 43 patients in care protocol group and 42 in control group. The mean SAPS II was 43.3 (±15.3) with a mean Glasgow Coma Scale score at 4.9 (±2.1) before intubation. Within the total population, 40 patients (47%) had a pulmonary bacteriologic sample (PBS), mostly because purulent tracheobronchial aspirate and new infiltrates on the chest X-ray (respectively 36.4% and 29.4% of the population with a bacteriological sample). Among the patients with a bacteriological sample, 34 (85%) were culture positive. The incidence of probabilistic antibiotherapy did not differ between the care protocol group (n=16) and the control group (n=16). There was no difference for the incidence of PBS (20 in each group). The others secondary outcomes did not differ either (Table 1).

**CONCLUSION.** Our study does not show that a care protocol allows a reduction of antibiotic prescription among patient admitted to ICU for toxic coma with MV. Our incidence of antibiotic prescription is lower than the previous studies (4). The absence of difference can be explain by two reasons: some of physicians of control group had been trained in the university-affiliated ICU in the last years and may follow a management approach similar to that of the control group; despite our precautions, the existence of the study could have modify the practices in the control group.

**REFERENCE(S)**

1. Christ A, Arranto CA, Schindler C, Klima T, Hunziker PR, Siegemund M, et al. Incidence, risk factors, and outcome of aspiration pneumonitis in ICU overdose patients. Intensive Care Med. sept 2006;32(9):1423-7.
2. Mandell LA, Niederman MS. Aspiration Pneumonia. Longo DL, éditeur. N Engl J Med. 14 févr 2019;380(7):651-63.
3. Lascarrou JB, Lissonde F, Le Thuaut A, Bachoumas K, Colin G, Henry Lagarrigue M, et al. Antibiotic Therapy in Comatose Mechanically Ventilated Patients Following Aspiration: Differentiating Pneumonia From Pneumonitis. Crit Care Med. août 2017;45(8):1268-75.
4. Rebuck JA, Rasmussen JR, Olsen KM. Clinical aspiration-related practice patterns in the intensive care unit: A physician survey: Crit Care Med. déc 2001;29(12):2239-44.

**Table 1 (abstract 000268).** Outcome in patients included

| Results | "Care protocol" group (n=43) | Control group (n=42) | p |
|---|---|---|---|
| Incidence of probabilistic antibiotherapy, n (%) | 16 (38.1) | 16 (39) | 0.93 |
| Incidence of antibiotherapy within 12 hours after intubation, n (%) | 3 (7.7) | 7 (16.6) | 0.16 |
| Incidence of PBS, n (%) | 20 (46.5) | 20 (47.6) | 0.91 |
| MV-free days by day 28 (d), mean ± SD | 25.3 (4.5) | 25.6 (2.4) | 0.73 |
| Antibiotherapy-free days by day 28 (d), mean ± SD | 25.3 (3.8) | 25.4 (3.2) | 0.87 |
| Length of stay in ICU (d), mean ± SD | 4.6 (8.5) | 3.8 (3) | 0.57 |
| Mortality in ICU, n (%) | 0 | 2 (4.7) | 0.49 |

### 000309

#### Monocyte Function in Sepsis: Their Role in Explaining Sepsis-Response-Signatures

P. Hickland[1], DB. Antcliffe[1], JK. Ward[1], C. Bradley[1], KP. O'Dea[1], AC. Gordon[1], DF. Mc Auley[2], KC. Tatham[1]
[1]Section of anaesthetics, pain medicine, and intensive care, Imperial College London, London, United Kingdom; [2]Centre for experimental medicine, Queen's University Belfast, Belfast, United Kingdom
**Correspondence:** P. Hickland

*Intensive Care Medicine Experimental* 2019, **7(Suppl 3):**000309

**INTRODUCTION.** There remains no specific effective therapy for sepsis, thought to be due to heterogeneity within the syndrome. However, recent transcriptomic work has identified two distinct sepsis-response-signature (SRS) profiles; with SRS1 being associated with increased mortality compared to SRS2 [1, 2]. The genes that are differentially expressed between these two groups are involved in a range of immune processes, the functional implications of which are not yet understood, although SRS1 appears to be the relatively immunosuppressed endotype compared to SRS2. Monocytes are implicated in the pathogenesis of sepsis through direct involvement in the innate and modulation of the adaptive immune responses.

**OBJECTIVES.** The same SRS endotypes have been identified using transcriptomic methodology applied to samples collected from patients with septic shock from the VANISH trial [3, 4]. This aim of this study was to condition healthy monocytes in plasma taken from these patients, to model septic monocytes, and assess their functional status via surface phenotype and phagocytic activity, which may explain the some of the differences between SRS endotypes. Cell surface markers of interest included those involved with defining monocyte subsets (CD14, CD16), antigen presentation (HLA-DR), apoptosis (MerTK) and costimulation (CD86, PD-1, PDL-1).

**METHODS.** Healthy monocytes were obtained from fresh whole blood using a two step process of density-gradient centrifugation to isolate peripheral blood mononuclear cells, and then magnetic cell sorting to isolate CD14+ monocytes. These were conditioned overnight in complete media and either SRS-profiled or healthy volunteer plasma (previously frozen).

Fluorochrome associated antibodies were used to assess surface phenotype and fluorescent E. coli bioparticles for phagocytic activity. Results were acquired by flow cytometry, and analysed with FlowJo V10. Fluorescent activity was reported as percentage of cells positive (PP) or geometric mean fluorescence intensity (GMFI). Statistical analyses were performed using Graphpad Prism 8.0.2.

**RESULTS.** CD14+ monocytes were conditioned using plasma samples from 28 SRS1 and 28 SRS2 patients with septic shock recruited to the VANISH trial, and 7 healthy volunteers. There were no significant differences in baseline characteristics between the two groups. The most marked difference in monocyte function was increased expression of CD14 in SRS1 (PP 39.3% vs 23.4%, $p < 0.01$), and increased expression of PD-1 in SRS2 (PP 18.2% vs 11.7%, $p < 0.05$). No difference in phagocytic activity was seen between the two SRS groups. Serum cytokine levels were compared between the two profiles (from the same plasma samples), with increased levels of IL-6, IL-10 and MCP-1 in SRS1 ($p < 0.01$).

**CONCLUSION.** These results indicate that the transcriptomic differences that distinguish SRS1 and SRS2 translate to the cellular level, with the main finding being increased CD14 expression on healthy monocytes conditioned in SRS1 plasma. In this *in vitro* model, it could be assumed that this is an effect of the increased cytokine levels found in SRS1 plasma. However, *in vivo* this may instead represent a greater proportion of 'classical' monocytes, producing more cytokines. This should be validated by performing similar immunophenotyping on monocytes taken directly from patients who have been SRS-profiled.

**REFERENCE(S)**

1. Davenport EE. et al. Genomic landscape of the individual host response and outcomes in sepsis: A prospective cohort study. Lancet Respir Med 2016; 4: 259-271
2. Burnham KL. et al. Shared and distinct aspects of the sepsis transcriptomic response to fecal peritonitis and pneumonia. Am J Respir Crit Care Med 2017; 196: 328-339
3. Gordon AC, et al. Effect of Early Vasopressin vs Norepinephrine on Kidney Failure in Patients With Septic Shock: The VANISH Randomized Clinical Trial. JAMA 2016; 316: 509-518
4. Antcliffe DB, et al. Transcriptomic Signatures in Sepsis and a Differential Response to Steroids: From the VANISH Randomised Trial. Am J Respir Crit Care Med 2018; [epub ahead of print]

5. This work was supported by the Wellcome Trust, Grant Number 204835/Z/16/Z.

## MEN - Assessment, macro- and micro-nutrients

### 000042

#### Pharmacokinetics of meropenem in critically ill patients with and without acute-on-chronic liver failure undergoing continuous venovenous hemodialysis

J. Grensemann[1], D. Busse[2], C. König[3], W. Jäger[4], D. Jarczak[1], S. Iwersen-Bergmann[5], C. Kloft[2], S. Kluge[1], V. Fuhrmann[1]
[1]Department of Intensive Care Medicine, University Medical Center Hamburg-Eppendorf, Hamburg, Germany; [2]Department of clinical pharmacy and biochemistry, institute of pharmacy, Freie Universitaet Berlin, Berlin, Germany; [3]Hospital pharmacy, University Medical Center Hamburg-Eppendorf, Hamburg, Germany; [4]Department of pharmaceutical chemistry, University of Vienna, Vienna, Austria; [5]Legal medicine, University Medical Center Hamburg-Eppendorf, Hamburg, Germany
**Correspondence:** J. Grensemann
*Intensive Care Medicine Experimental* 2019, **7(Suppl 3):**000042

**INTRODUCTION.** Organ dysfunction during sepsis or septic shock may alter antibiotic drug concentrations via changes of drug distribution and elimination. We therefore studied the pharmacokinetics of meropenem in critically ill patients with acute-on-chronic liver failure (ACLF) and without concomitant liver failure (NLF) during continuous venovenous hemodialysis (CVVHD).

**METHODS.** In this prospective cohort study, all patients received meropenem 1g tid. Meropenem pre- and postfilter serum and ultradialysate samples were obtained at the following time points: T0, T1, T2, T4, T8, T24, T25, T48, T49 and analyzed by high pressure liquid chromatography with diode array detection (HPLC-DAD). Nonlinear mixed-effects modelling was performed in NONMEM® 7.3.

**RESULTS.** 19 patients were studied. Of these, 8 patients suffered from ACLF. A two-compartment model with linear clearance (CL) from the central compartment adequately described meropenem pharmacokinetics in both patient populations. CL was 5.2 L/h with a coefficient of variation of 28%. The population estimate of the peripheral volume of distribution (Vd) was 19.7 L for critically ill patients without liver failure and 38.6 L for the ACLF-population (p = 0.05). Deterministic simulations of meropenem concentration over 24 hours using typical parameter values for NLF and ACLF showed differences in trough levels that decreased with consecutive doses.

**CONCLUSION.** Patients with ACLF receiving CVVHD have a higher Vd and may require a higher initial dose of meropenem to reach microbiological defined targets (i.e. time above minimal inhibitory concentration) compared to critically ill patients without ACLF undergoing CVVHD.

### 000076

#### Electrical muscle stimulation on upper and lower limb muscle in critically ill patients

N. Nakanishi[1], J. Oto[2], R. Tsutsumi[3], T. Yamamoto[3], Y. Ueno[1], E. Nakataki[4], T. Itagaki[1], M. Nishimura[4]
[1]Emergency and critical care medicine, Tokushima University Hospital, Tokushima, Japan; [2]Emergency and disaster medicine, Tokushima University Hospital, Tokushima, Japan; [3]Department of nutrition and metabolism, Tokushima University Graduate School of Biomedical Sciences, Tokushima, Japan; [4]Critical care medicine, Tokushima Prefectural Central Hospital, Tokushima, Japan
**Correspondence:** N. Nakanishi
*Intensive Care Medicine Experimental* 2019, **7(Suppl 3):**000076

**INTRODUCTION.** Early mobilization prevents muscle atrophy and functional disability in critically ill patients, while its implementation is challenging due to the severity of illness and limited resources. Electrical muscle stimulation (EMS) evokes muscle contraction without input from the central nervous system, and it can be applied to critically ill patients. However there has been much debate if EMS improves functional ability of the patients.

**OBJECTIVES.** To investigate if early EMS on upper and lower limb muscles prevents muscle atrophy and improve physical function in critically ill patients.

**METHODS.** We included consecutive adult patients admitted to ICU and expected to be mechanically ventilated longer than 48 hours and to stay more than 5 days. The patients were randomly assigned to EMS or control group. EMS was applied with a low-frequency therapy equipment (Solius, Minato Medical Science Co., Ltd, Osaka, Japan). EMS group of patients received EMS sessions of bilateral upper and lower extremities daily for 30 min from day 1 to 5 in addition to the usual rehabilitation. Patients in control group received only the usual rehabilitation. The primary outcome was the change of upper and lower limb muscle thickness and cross-sectional area, measured by ultrasound from day 1 to 5. The secondary outcomes were Medical Research Council score and the incidence of ICU-acquired weakness at day 5, and ICU Mobility scale at the discharge from the ICU.

**RESULTS.** Thirty-seven patients were enrolled, and 4 patients were excluded (death, pain, and insufficient muscle contraction due to edema or obesity). Fourteen patients in EMS group and 19 in the control group were evaluated. Mean age was 73±3 vs. 66±3 years, (p=0.16), and median APACHE II score was 23(18-28) vs. 22(19-30) (p=0.94) in EMS and in control, respectively. The change of thickness in upper limb muscle was -1.0±2.5% vs. -11.2±2.1% (p<0.01), and in the lower limb was 3.2±3.7% vs. -14.7±3.2% (p<0.01), the change of cross-sectional area in upper limb muscle was -5.0±3.6% vs. -10.0±2.4% (p=0.18), and in the lower limb was -1.0±3.4% vs. -10.4±2.9% (p=0.046) in EMS and in control, respectively. There was no significant difference in Medical Research Council score (54±4 vs. 46±4, p=0.18), ICU-acquired weakness (13% vs. 40%, p=0.20), and ICU Mobility scale (2.2±0.5 vs. 1.5 ±0.5, p=0.31) between EMS group and the control group.

**CONCLUSION.** In critically ill patients, EMS on upper and lower limb muscles preserved muscle mass. Further research should be done to clarify if EMS improves physical function and long-term outcomes.

### 000084

#### Association of glycemic parameters at admission in intensive care unit with outcomes in critically ill patients: a prospective study

T. Rech[1], P. Bellaver[1], A. Schaeffer[2], M. Viana[1], D. Dullius[3], C. Leitao[4]
[1]Intensive care division, Hospital de Clínicas de Porto Alegre, Porto Alegre, Brazil; [2]Medical school, Universidade Federal do Rio Grande do Sul, Porto Alegre, Brazil; [3]Department of plastic surgery, Hospital de Clínicas de Porto Alegre, Porto Alegre, Brazil; [4]Endocrine division, Universidade Federal do Rio Grande do Sul, Porto Alegre, Brazil
**Correspondence:** T. Rech
*Intensive Care Medicine Experimental* 2019, **7(Suppl 3):**000084

**INTRODUCTION.***Introduction*: Hyperglycemia is a compensatory metabolic response to acute stress. It reflects the development of insulin resistance to preserve life during life-threatening conditions, but is associated with worse prognosis during critical illness (1, 2).

**OBJECTIVES.***Objectives*: The aim of the present study is to investigate the association of glycemic parameters at intensive care unit (ICU) admission with outcomes in critically ill patients.

**METHODS.***Methods:* From September 2017 to February 2018, adult critically ill patients admitted to ICU were prospectively included in the study. Blood samples were collected at study entry for random blood glucose and glycated hemoglobin (HbA1c) and were used to calculate glycemic gap and stress hyperglycemia ratio (SHR). All patients were assessed for presence of hyperglycemia (>140 mg/dL at admission), hypoglycemia (<70 mg/dL in 24h) and glycemic variability (higher - lower capillary glucose in 24h).

**RESULTS.***Results:* A total of 542 patients were enrolled (mean age 59; 30% with previous diabetes) and were followed until hospital discharge or death for 180 days. Hypoglycemia was associated with increased mortality (54.8% vs. 35.8%, p=0.004), need for renal replacement therapy (RRT; 45.1% vs. 22.3%, p <0.001) mechanical ventilation (MV; 72.6% vs. 57.5%, p=0.024) and higher shock incidence (62.9% vs. 35.8%, p<0.001). Hyperglycemia was associated with higher mortality (44.3% vs.34.9%, p=0.031) and increased need for MV (66.1% vs. 55.7%, p=0.018). Patients with glycemic gap >80 mg/dL had increased need for RRT (37.7% vs. 23.7%, p=0.025) and higher shock incidence (54.7% vs. 37.4%, p=0.014), but no difference in mortality. SHR >1.1 was associated with increased need for MV (65.8% vs. 2.8%, p=0.001). Glycemic variability was associated with an increased need for RRT (28.3% vs. 14.4%, p=0.002, for variability >40 mg/dL; 31.6% vs. 16.6%, p<0.001, for variability > 60 mg/dL and 34% vs. 18%, p<0.001, for variability > 80mg/dL) and higher shock incidence (41.4% vs.31.2%, p=0.039, for variability >40 mg/dL; 42.9% vs. 34%, p=0.034, for variability >60 mg/dL; and 64.2% vs. 55.9%, p= 0.010, for variability >80 mg/dL). The associations of hypoglycemia and hyperglycemia with mortality remained after adjustments for disease severity.

**CONCLUSION.***Conclusions:* In this sample of medical-surgical critically ill subjects, including patients with and without previous diagnosis of diabetes, hypoglycemia and hyperglycemia were independently associated with increased mortality, while glycemic variability, glycemic gap and SHR were associated with worse outcomes, such as shock incidence, need for RRT and need for MV, but not with mortality.

**REFERENCE(S)**

1. Van den Berghe G (2002) Beyond diabetes: saving lives with insulin in the ICU. International Journal of Obesity 26, Suppl 3, S3–S8
2. Chao H-Y, Liu P-H, Lin S-C, Chen C-K, Chen J-C, Chan Y-L, et al (2017) Association of InHospital Mortality and Dysglycemia in Septic Patients. PLoS ONE 12(1): e0170408. doi:10.1371/ journal.pone.0170408
3. This work was supported by Fundo de Incentivo à Pesquisa e Ensino (FIPE) of Hospital de Clínicas de Porto Alegre (project number 17-0386) and Conselho Nacional de Desenvolvimento Científico e Tecnológico (CNPq – process number 420323/2018-0).

### 000235

#### The Determination of Serum Micronutrient Levels in Patients Transferred From the ICU to the Service and Investigation of the Relationship Micronutrient Levels with 90-Day Mortality and Readmission to the ICU

K. Gundogan[1], Y. Gunay[2], M. Nilgun[3], R. Coskun[3], GG. Sahın[4], S. Temel[3], M. Güven[3], M. Sungur[3]
[1]Department of internal medicine, division of intensive care, Erciyes University, School of medicine, Kayseri, Turkey; [2]Department of internal medicine, Erciyes University, School of medicine,, Kayseri, Turkey; [3]Department of internal medicine, division of intensive care, Erciyes University, School of Medicine, Kayseri, Turkey; [4]Division of clinical nutrition, Erciyes University Health Sciences Institute, Kayseri, Turkey
**Correspondence:** K. Gundogan
*Intensive Care Medicine Experimental* 2019, **7(Suppl 3):**000235

**INTRODUCTION.** Micronutrients included in nutritional support have a central role in maintaining human physiological functions.

**OBJECTIVES.** The aim of this study is to identify serum micronutrient levels in patients who are transferred from intensive care unit (ICU) towards and their relation with 90-day mortality rate and readmission to ICU.

**METHODS.** This study was conducted prospectively in Erciyes University Medical ICU. Patients who are above 18 years old and stayed in the ICU more than 48 hours and then transferred to a ward were included into the study. Blood samples for micronutrient levels were taken at the time of transfer.

**RESULTS.** We enrolled 100 patients. Mean APACHE II score was 15.4± 7.8. Median SOFA score at the time of discharge from ICU was 3 (range 0-7). The route for nutrition was oral in 41%, enteral in 21%, oral plus parenteral in 18%, parenteral in 17% and oral plus enteral in 3% of the patients in last day ICU. Low levels rate of thiamine (98%), vitamin B6 (98%), vitamin B12 (11%), copper (21%), zinc (90%), selenium (36%), chromium (98%), and cobalt (35%) were identified in the patients. Low levels of vitamin B6 was an independent risk factor for 90 day mortality and re-admission to the ICU in multivariate analysis (OR: 0.283, CI 95%:0.099-0.812, p: 0.019 OR: 0.231 CI 95%:0.071-0.745, p: 0.014 respectively). Median duration of ICU stay was 5.5(range 3-32) days. Re-admission rate to the ICU within 90 days was 24% and 90 day mortality rate was 29%.

**CONCLUSION.** Vitamin B1, B6, zinc and chromium levels were very low rate in these patients. Low levels of vitamin B6 identified as an independent risk factor for 90 day mortality rate and re-admission to the ICU.

**REFERENCE(S)**

1. Li, Z., et al., Nutrition support in hospitalized cancer patients with malnutrition in China. Asia Pac J Clin Nutr, 2018. 27(6): p. 1216-1224.
2. Borrelli, E., et al., Plasma concentrations of cytokines, their soluble receptors, and antioxidant vitamins can predict the development of multiple organ failure in patients at risk. Crit Care Med, 1996. 24(3): p. 392-7.
3. This work was supported Erciyes University Scientific Research Committee (TTU-2014-5461)

### 000260

#### Putting the PN in PatieNts: Why are ICU patients missing their parenteral nutrition?

R. Carrington[1], J. Zekavica[2], M. Bossy[1]
[1]Intensive care, Royal Surrey County Hospital, Guildford, United Kingdom; [2]Nutrition and dietetics in critical care, Royal Surrey County Hospital, Guildford, United Kingdom
**Correspondence:** R. Carrington
*Intensive Care Medicine Experimental* 2019, **7(Suppl 3):**000260

**INTRODUCTION.** Critically ill patients are at risk of significant nutritional deficits leading to delayed recovery. Nutritional support has been shown to improve outcomes in these patients(1).

Parenteral nutrition (PN), intravenously administered artificial nutrition, is often thought to ensure secure delivery of the prescribed nutrition. However, studies have demonstrated a discrepancy between the amount of PN prescribed and the amount received(2). In critical illness, immobilisation and increased inflammatory and stress responses mean that the catabolic response to an energy deficit is much greater than in a healthy fasting person. If these patients miss periods of their PN prescription, they will accumulate a caloric deficit which can rapidly reach levels that contribute to skeletal muscle loss and impaired healing. This in turn leads to prolonged intensive care unit (ICU) admission and rehabilitation(3).

**OBJECTIVES.**

1. To identify whether the amount of PN received by patients in the Royal Surrey County Hospital (RSCH) ICU matches the number of hours prescribed
2. To identify the causal factor in instances where 100% of prescribed PN is not received
3. To identity the type of vascular access by which the PN is being administered, and whether this affects the amount of PN received

**METHODS.** Retrospective data collection from all patients admitted to RSCH ICU who received PN during the 6 month period between 1st April and 31st September 2018. The number of hours of PN prescribed each day and the number of hours received each day were recorded. The reason for any deficit was documented where 100% administration was not achieved. First bag prescription start time was taken as 19.00 (2 hours after routine delivery from pharmacy).

**RESULTS.** 637 patients were admitted to RSCH ICU during this time. 77 (12%) were prescribed PN. 9395 hours of PN were prescribed: 9129 hours (97%) were received, and 266 hours (3%) were missed. The reasons for the missed hours are displayed in Figure 1. PN was administered via central venous catheter (CVC) in 62 patients (81%), peripherally inserted central catheter (PICC) in 10 patients (13%) and peripheral cannula (PC) in 5 patients (6%). The mean percentage delivery for each access type is shown in Table 1.

**CONCLUSION.** The largest reason for PN deficit was the first PN bag being started late. This was due to a combination of factors: pharmacy delivery time, time required for PN to return to room temperature, and nursing handover time. This has identified an area for an improvement initiative that we are implementing to enable the PN bags to be started earlier. Also, our results suggest PN may be received more consistently via a PICC vs other types of access, but a larger study with greater statistical power would be required to investigate this further.

**REFERENCE(S)**

1. Barr J, Hecht M, Flavin KE, Khorana A, Gould MK. Outcomes in critically ill patients before and after the implementation of an evidence-based nutritional management protocol. Chest 2004;125:1446-57
2. Harvey SE, Parrott F, Harrison DA, et al.; CALORIES Trial Investigators. Trial of the route of early nutritional support in critically ill adults. N Engl J Med 2014;371:1673–84.
3. Casaer MP, Van den Berghe G (2014) Nutrition in the acute phase of critical illness. N Engl J Med 370:1227–1236

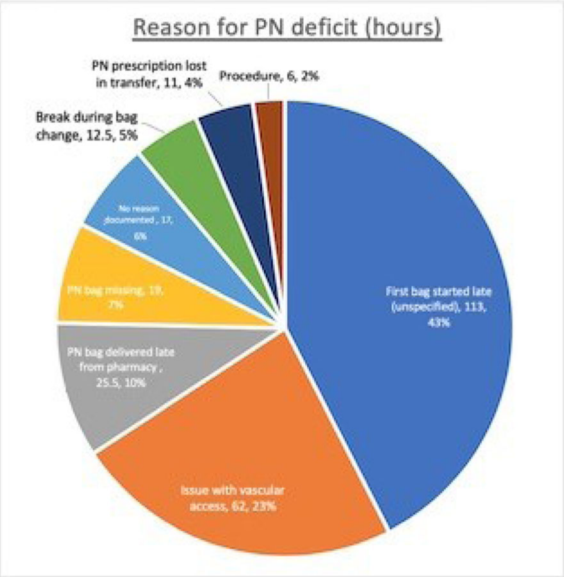

**Fig. 1 (abstract 000260).** See text for description

**Table 1 (abstract 000260).** See text for description

| Type of Vascular Access | Number of patients | Mean PN delivery |
|---|---|---|
| Central Venous Catheter (CVC) | 62 | 95% |
| Peripherally Inserted Central Catheter (PICC) | 10 | 99% |
| Peripheral Cannula (PC) | 5 | 92% |
| Total (all access types) | 77 | 97% |

### 000274

### Enhance caloric intake from enteral nutrition rather than parenteral nutrition decreased hospital mortality in ARDS patients with the prone position

CY. Wang[1], CH. Wang[2]

[1]Department of critical care medicine, Taichung Veterans General Hospital, Taichung, Taiwan; [2]Graduate institute of education, National Changhua University of Education Jin De Campus, Changhua City, Taiwan

**Correspondence:** C.Y. Wang

*Intensive Care Medicine Experimental* 2019, **7(Suppl 3):**000274

**INTRODUCTION.** Feeding intolerance is common in acute respiratory distress syndrome (ARDS) patients with the prone position. Prone position has been proved to improve mortality in ARDS patients but only a few small sample sized studies talked about the feeding adequacy in these patients. In literatures, there was no outcome difference regarding feeding amount of ARDS patients. However, the optimal amount of caloric intake for ARDS patients with the prone position is still unknown. We hypothesize adequate caloric delivery influence clinical outcomes in ARDS patients received prone position therapy.

**OBJECTIVES.** We retrospectively collected patients' data from Sep 2015 to Nov 2018 in medical intensive care units at a tertiary medical center. The inclusion criteria were age older than 20 years old, ARDS defined by Berlin criteria, ICU stay more than 48 hours, received mechanical ventilation and prone position therapy. Exclusion criteria were any non per os order by physicians.

**METHODS.** Data collection including demographic data, BMI, calories of parenteral nutrition(PN), enteral nutrition(EN) and hospital mortality, etc. Nutritional risk was evaluated by modified NUTRIC score. In addition to descriptive analysis and univarate analysis, we used cox regression modeling to control multiple variables and to calculate 95% confidence intervals.

**RESULTS.** There were 110 patients enrolled in the study. The hospital mortality rate was 58% (64/110). The average age was 61±17 years old and APACHE II score was 31±7. The first 7 days average EN calories intake were higher and PN calories intake were lower in survival group. (EN: 672 kcal/day vs 547 kcal/day, p=0.03; PN: 103 kcal/day vs 179 kcal/day, p=0.001). The cutoff value of average EN calories intake higher than 564 kcal/day and PN calories intake less than 79 kcal/day have the lowest mortality by receiver operating characteristic(ROC) curve analysis. (p<0.05) In cox regression model, EN calories supplement more than 564 kcal/day (HR:1.85, 95% CI:1.10-3.13, p<0.021) and PN calories supplement less than 79 kcal/day (HR:0.52, 95% CI:0.29-0.94, p<0.03) decreased hospital mortality after adjust potential confounders.

**CONCLUSION.** In order to decrease hospital mortality in ARDS patients with the prone position, enhance caloric intake from enteral nutrition rather than parenteral nutrition should be considered.

**REFERENCE(S)**

1. Guerin C, Reignier J, Richard JC, et al. Prone positioning in severe acute respiratory distress syndrome. N Engl J Med. 2013;368(23):2159-2168.
2. Rice, T.W.; Wheeler, A.P.; Thompson, B.T.; Steingrub, J.; Hite, R.D.; Moss, M.; Morris, A.; Dong, N.; Rock, P. Initial trophic vs full enteral feeding in patients with acute lung injury: the EDEN randomized trial. Jama 2012, 307, 795-803
3. Reignier J, Dimet J,Martin-Lefevre L, et al. Before-after study of the standardized ICU protocol for early enteral feeding in patients turned in the prone position. Clin Nutr. 2010;29(2):210-216.
4. Reignier J, Thenoz-Jost N, Fiancette M, et al. Early enteral nutrition in mechanically ventilated patients in the prone position. Crit Care Med. 2004;32(1):64-99.
5. Saez de la Fuente I, Saez de la Funte J, Estelles MD, et al.Enteral nutrition in patients receiving mechanical ventilation in a prone position. JPEN J Parenter Enteral Nutr. 2016;40(2):250-255.
6. Rahman, A.; Hasan, R.M.; Agarwala, R.; Martin, C.; Day, A.G.; Heyland, D.K. Identifying critically-ill patients who will benefit most from nutritional therapy: Further validation of the "modified NUTRIC" nutritional risk assessment tool. Clinical nutrition (Edinburgh, Scotland) 2016, 35, 158-162,

### 000304

#### Impact of vitamin deficiency on septic patient's outcome

L. Bielsa Berrocal, V. Philibert, Y. Rovira, S. Triginer, A. Herraiz, E. Valls, D. Salat, L. Bordejé, P. Marcos, T. Tomasa
Intensive Care Unit, Hospital Germans Trias i Pujol, Badalona, Spain
*Intensive Care Medicine Experimental* 2019, **7(Suppl 3):**000304

**INTRODUCTION.** Our aim was to analyze plasma levels of vitamins A, B6, C, E and zinc in septic patients, and to determine their impact on patient's outcome.

**METHODS.** Observational and prospective study. Inclusion criteria: patients admitted to the ICU due to sepsis (January-July 2018). We analysed vitamins A, B6, C, E and zinc serum levels at ICU admission. Statistical analysis: quantitative variables in means (SD) and medians (min-max) according to their normality and qualitative variables in proportions and 95% CI. Bivariate analysis: Kruskal-Wallis and khi-square.

**RESULTS.** 37 patients (67.6% male). Median age was 62 years old (45-69). SOFA at admission was 7 (3.5) and the APACHE II was 27 (9.9). They remained in the ICU for 8 days (1-53) and 22 days (1-132) in the hospital. 5.4% of patients were re-admitted to the ICU. ICU mortality was 10.8% (95% CI 3.9-26.5) and in-hospital mortality was 21.6% (95% CI 10.8-38.5).

**CONCLUSION.** 1) We found a remarkable deficit of all micronutrients analysed, except for Vit E.

2) Septic patients with zinc deficiency had longer stays and higher mortality in the ICU.

3) Vitamin deficiency was not associated with an increased mortality, AKI or stay, in this small sample of septic patients.

**Table 1 (abstract 000304).** See text for description

| Normal Levels | | VIT A (0.3-0.6 mg/L) | VIT B (3.6-18 ng/mL) | VIT C (0.4-2 mg/dL) | VIT E (5-18 mg/L) | ZINC (80-120 mcg/dL) |
|---|---|---|---|---|---|---|
| Sample | | 30 | 31 | 37 | 32 | 32 |
| Levels | Low | 0.14 (0.07-0.3) | 1.61 (0.4-3.4) | 0.19 (0.1-0.4) | 3.98 | 56.6 (30.9-78.5) |
| | Normal | 0.37 (0.3-0.6) | 5.65 (3.9-14.9) | 0.53 (0.4-1.3) | 9.57 (6-15.2) | 112.6 (84.3-155.5) |
| Occurrence | Low | 20 (66.7%) | 23 (74.2%) | 26 (70.3%) | 1 (3%) | 27 (84.4%) |
| | Normal | 10 (33.3%) | 8 (25.8%) | 11 (29.7%) | 29 (90.6%) | 4 (12.5%) |
| ICU Stay | Low | 7.5 (1-53) | 5 (1-36) | 11 (3-53) | 12 | **8 (3-53)*** |
| | Normal | 10 (3-21) | 28 (3-53) | 5 (1-45) | 7 (1-53) | 9 (4-13) |
| Hosp. Stay | Low | 25 (1-132) | 21 (1-69) | 27 (3-132) | 16 | 29 (3-132) |
| | Normal | 33 (14-69) | 33.5 (5-132) | 22 (1-62) | 22 (1-132) | 20.5 (10-69) |
| ICU Mort. | LOW | 4 (20%) | 2 (8.7%) | 2 (7.7%) | 0 | **3 (11.1%)**** |
| | Normal | 0 | 2 (25%) | 2 (18%) | 4 (13.8%) | 0 |
| Hosp. Mort. | Low | 6 (30%) | 5 (21.7%) | 5 (19%) | 0 | 6 (22%) |
| | Normal | 1 (10%) | 2 (25%) | 3 (27%) | 6 (20.7%) | 0 |
| AKI | Low | 12 (60%) | 14 (61%) | 18 (69%) | 1 (100%) | 17 (63%) |
| | Normal | 5 (50%) | 3 (37.5%) | 4 (36%) | 16 (55%) | 1 (25%) |

* p 0.04 ** p 0.02

### 000326

#### Augmented Renal Clearance and Drugs Dosage. An Epidemiological Multicentre Study

TM. Tomasa-Irriguible[1], J. Sabater,[2], M. Pérez,[3], P. Ortiz,[4], M. Torrens,[5], A. Navas,[6], Y. Díaz,[7], C. Rovira,[8], I. Oliva,[9], M. Ibarz,[10], J. Xirgu,[11], RM. Catalán,[12], S. Cano,[13], A. Olmo,[14], M. Rodríguez,[15], E. Vendrell,[16], J. González De Molina[17], M. Miralbés,[18], P. Marcos,[19]
[1]Intensive Care Department, Hospital Germans Trias i Pujol, Badalona, Spain; [2]Intensive care, Bellvitge, L'Hospitalet de Llobregat, Spain; [3]Intensive care, Vall d'Hebron University Hospital, Barcelona, Spain; [4]Intensive care, Josep Trueta Hospital, Girona, Spain; [5]Intensive care, Hospital de la Santa Creu i Sant Pau, Barcelona, Spain; [6]Intensive care, Corporació Sanitària i Universitària Parc Taulí, Sabadell, Spain; [7]Intensive care, Hospital del Mar, Barcelona, Spain; [8]Intensive care, Hospital Universitari Sant Joan de Reus, Reus, Spain; [9]Intensive care, Hospital Universitari de Tarragona Joan XXIII, Tarragona, Spain; [10]Intensive care, Hospital Universitari Sagrat Cor - Grup Quirónsalut, Barcelona, Spain; [11]Intensive care, Hospital General de Granollers, Granollers, Spain; [12]Intensive care, Hospital Universitari de Vic, Vic, Spain; [13]Intensive care, ALTHAIA Centre Hospitalari, Manresa, Spain; [14]Intensive care, Hospital General de Catalunya, Sant Cugat del Vallès, Spain; [15]Intensive care, Hospital de Sant Joan Despí Moisès Broggi, Sant Joan Despí, Spain; [16]Intensive care, Hospital de Mataró, Mataró, Spain; [17]Intensive care, Mútua Terrassa University Hospital, Terrassa, Spain; [18]Intensive care, University Hospital Arnau de Vilanova, Lleida, Spain; [19]Intensive care, Hospital Germans Trias i Pujol, Badalona, Spain
**Correspondence:** T.M. Tomasa-Irriguible
*Intensive Care Medicine Experimental* 2019, **7(Suppl 3):**000326

**INTRODUCTION.** Augmented Renal Clearance (ARC) is currently defined as a Glomerular Flitration Rate (GFR) greater than 130 mL/min/1.73 m2. ARC is a phenomenon that can lead to therapeutic failure in critically ill patients. Inadequate antibiotic dose may worsen sepsis outcome and may increase resistance generation.

**OBJECTIVES.** To analyze clinical characteristics of patients with ARC in comparison to patients with normal GFR (nonARC, 90-130 mL/min/1.73 m2) admitted to the Intensive Care Unit (ICU), and to compare the prescribed dose of antibiotics (AB), antiepileptic (AE) and diuretic (DI) drugs between both groups.

**METHODS.** We set up 3 points of prevalence according to different seasonal times, November'17, February'18 and May'18, with a follow-up period of 2 months, measuring the GFR (mGFR) in 4-hour urine collection twice a week. Inclusion criteria: Adult patients admitted to the ICU with a bladder catheterization. Exclusion criteria: renal replacement therapy, bladder washings and absence of bladder catheterization. Variables: patients' features and type and dose of prescribed drugs were recorded. Statistical analysis: Pearson Chi-square Test.

**RESULTS.** 18 hospitals were enrolled and 561 patients were included (61% male) with a median age of 65.5 years old. At ICU admission 10.7% had already ARC. Overall, we found 31% ARC and 22% non-ARC patients. Prescribed drugs: 67% AB, 17.5% AE and 41% DI. Mechanical ventilation was required in 64% and vasopressors agents in 51%. Median SOFA was 6. ICU mortality was 14% and in-hospital mortality was 18%. Median ICU and hospital length of stay (LOS) were 15 and 29 days, respectively. Median mGFR was 176 in ARC and 106 mL/min/1.73 m2 in nonARC. ARC remained with GFR>130 for several days during their LOS. Bivariate analysis: ARC patients were younger, 56.5 vs. 66 years (P<0.001). ICU mortality was 7% in ARC vs. 14.5% in nonARC group (P=0.003) and in-hospital mortality was 10% vs. 16% (P=0.001), respectively. ICU-LOS was 13 vs. 19 days (P=0.028) and hospital-LOS was 27 vs. 33 days (P=0.022), respectively. Regarding the use of drugs, neither the number nor the delivered dose were different between ARC and nonARC groups.

**CONCLUSION.** 1) Up to 30% of ICU patients had ARC during ICU-LOS and 10% had already ARC when admitted.

2) ARC patients received the same dosage of AB, AE and DI, as non-ARC patients.
3) ARC patients were youger, had shorter LOS and lower mortality than nonARC patients.

**REFERENCE(S)**

1. Cook AM, Hatton-Kolpek J. Augmented Renal Clearance. Pharmacotherapy. 2019 Feb 5. doi:10.1002/phar.2231.
2. Roberts JA, Paul SK, Akova M, Bassetti M, De Waele JJ, Dimopoulos G, et al. DALI: defining antibiotic levels in intensive care unit patients: are current beta-lactam antibiotic doses sufficient for critically ill patients? Clin Infect Dis.2014; 58(8): 1072-83.

### 000361

#### Uptake of dietary amino acids into arterial blood during continuous enteral feeding in critically ill patients and healthy subjects

F. Liebau[1], E. Király[1], D. Olsson[2], O. Rooyackers[3]
[1]Perioperative Medicine and Intensive Care, Karolinska University Hospital Huddinge, Stockholm, Sweden; [2]Unit for Medical Statistics at Department of Learning, Informatics, Management and Ethics, Karolinska Institute, Stockholm, Sweden; [3]Anaesthesia and Intensive Care at Department of Clinical Science, Intervention and Technology, Karolinska Institutet, Stockholm, Sweden
**Correspondence:** F. Liebau

*Intensive Care Medicine Experimental* 2019, **7(Suppl 3):**000361

**INTRODUCTION.** Sufficient nutrition is necessary to counteract catabolism in critical illness. Protein anabolism requires amino acid availability, one source of which is uptake from enteral nutrition. Continuous enteral feeding is a common but unphysiological approach, and little is known about its effects on amino acid uptake.
**OBJECTIVES.** To characterize the time course of dietary amino acid uptake into arterial plasma during continuous enteral feeding in ICU patients and healthy subjects, using a stable-isotope-labeled phenylalanine tracer as a specific indicator for dietary amino acids.
**METHODS.** 10 healthy subjects and 10 ICU patients were studied. Patients had invasive ventilatory support and ongoing enteral feeding at >80% of energy expenditure. All subjects were given enteral nutrition by constant continuous infusion via nasogastric tube or jejunostomy at ≥80% (patients) or 100% (healthy subjects) of energy expenditure.
After a 12-hr equilibration period, a primed constant infusion of 13C-phenylalanine (13C-Phe) was added to the enteral infusion. Arterial blood samples were then drawn every 30 minutes for 12 hrs. For isotopic analysis, plasma samples were analyzed by gas chromatography-mass spectrometry. Plasma aminograms were determined by HPLC.
**RESULTS.** Data are expressed as median (range). Patients' age was 64 (44-74) yrs, SAPS 3 score was 60.5 (57-85), primary diagnoses were n=3 surgical, n=3 neurological and n=4 medical, and studies were performed on ICU day 20 (5-41).
Plasma essential amino acid (EAA) concentration and 13C-Phe enrichment were largely stable in aggregate, but highly variable individually. Variability over time of 13C-Phe, expressed as coefficient of variation, was 25.8% (17.5-30.3) in healthy subjects and 20.9% (12.3-46.3) in ICU patients. Variability of EAA was 10.5% (8.5-18.3) and 10.2% (7.8-13.2), respectively. Significant co-variation between timepoint-to-timepoint changes of 13C-Phe enrichment and EAA was found in 9/10 healthy subjects and 3/10 ICU patients.
**CONCLUSION.** Plasma EAA concentrations and 13C-Phe enrichment reached a tentative steady state on average, but there was large intra-individual variability during 12 hours of feeding. The magnitude of variation was similar to that seen in the only previous study reporting comparable data (de Betue et al., 2017). Variation in aminoacidemia may be physiologically relevant for anabolic stimulation (Bouillanne et al., 2013), but its significance in critical illness remains unclear.
The physiological mechanism underlying the variability in aminoacidemia during continuous enteral feeding is unknown. Co-variation of changes in EAA concentrations and 13C-Phe enrichment (here a specific indicator of dietary uptake) suggests a common underlying process such as gastric emptying, but this appears less obvious in the ICU patients.

**REFERENCE(S)**

1. Supported by the Regional Agreement on Medical Training and Clinical Research between Stockholm County Council and Karolinska Institutet.
2. Bouillanne, O., et al., Impact of protein pulse feeding on lean mass in malnourished and at-risk hospitalized elderly patients: a randomized controlled trial. Clin Nutr, 2013. 32(2): p. 186-92.
3. de Betue, C.T.I., et al., 24-Hour protein, arginine and citrulline metabolism in fed critically ill children - A stable isotope tracer study. Clin Nutr, 2017. 36(3): p. 876-887.

### 000423

#### Does infection in acute liver failure influence patients outcome?

T. Isidoro Duarte[1], J. Avila[2], J. Estevão[1], N. Germano[1]
[1]Intensive Care Medicine Department, Curry Cabral Hospital, Central Lisbon Hospital Center, Lisboa, Portugal; [2]Intensive care medicine department, Espirito Santo de Evora Hospital, EPE, Evora, Portugal
**Correspondence:** T. Isidoro Duarte

*Intensive Care Medicine Experimental* 2019, **7(Suppl 3):**000423

**INTRODUCTION.** Acute liver failure (ALF) accounts for up to 6% of liver related deaths and is characterized by the acute onset of jaundice, coagulopathy and hepatic encephalopathy in patients without pre-existing hepatic disease. Due to multiple immunological dysfunctions, systemic inflammation and bacterial translocation, patients with ALF are at higher risk of developing sepsis. Bacterial infections have been documented in 60-80% of these patients, most commonly pneumonia (50%), urinary tract infections (22%) and spontaneous bacteraemia (15%) and are a significant cause of mortality.
**METHODS.** We assessed a retrospective cohort of 39 patients with ALF admitted to an Intensive Care Unit (ICU) of a Liver Transplant Center from January 2015 until December 2018. Baseline characteristics, admission respiratory, kidney and hemodynamic support and culture samples were recorded.
**RESULTS.** The median age (IQR) was 40 (28-52) years; 17 were male (43,6%). Paracetamol etiology was found in 7 patients (17,9%). On ICU admission, median SAPS II score was 49 (30,8-59,5) with an associated risk of mortality of 43,85% (12-71). Paracetamol overdose or others drugs-related toxicity and unknown causes represented more than 50% of the ALF aetiologies. Invasive mechanical ventilation, vasopressor support and renal replacement therapy on admission were required in 10 (27%), 11 (28%) and 8 (21%), respectively. Admission hepatic encephalopathy higher than grade 3 was present in 10 patients (27%) with a median arterial ammonia of 248 (161-316) mg/dL. Median INR, total bilirubin and lactate levels were 2,7 (2,3-3,4), 9,90 (2,52-19,57) mg/dL and 2,6 (1,6-6,0) mmol/L, respectively. King's College (KCC) and Clichy's Criterias were fulfilled by 14 (36%) and 5 (13%) patients. Eleven patients (28%) received liver transplant. Infection was present in 17 patients (43,6%), predominantly gram-negative organisms. Median day of infection diagnostic was 3 (2-5). Time-to-event analysis (Cox regression) with Kaplan-Meyer curves adapted for the presence of infection revealed a worst 30 day survival for those patients who developed infection (67% *versus*50%, $p$ = 0,08). Hospital mortality rate was 36%.
**CONCLUSION.** In a Portuguese cohort, bacterial infection was present in almost half of the patients admitted with ALF. As the diagnosis is difficult, a high level of clinical suspicion, particularly in patients who have signs of SIRS, refractory hypotension or unexplained progression to higher grades of hepatic encephalopathy may indicate the presence of infection. So, careful assessment for ongoing infections should be performed and treated in order to optimize patient's outcome.

REFERENCE(S)
1. Nanchal R, Ahmad S. Infectious in Liver Disease. Crit Care Clin. 2016; 32, 411-424;
2. Cheng A, Tanna S et al. Infectious Complications in Critically ill Liver Failure Patients. Seminars in Respiratory and Critical Care Medicine. 2018; vol.39, 578-587;
3. EASL Clinical Practical Guidelines on the management of acute (fulminant) liver failure. European Association for the Study of the Liver. Journal of Hepatology. 2017; vol.66, 1047-1081;

### 000453

### Intrahospital infections and glycemic variability among critically ill patients

S. Mulet Mascarell[1], MM. Juan Díaz[2], B. Furquet López[2], A. González Díaz[2], M. Rodríguez Gimillo[2], C. Sanchis Piqueras[2], R. Huerta Bravo[1], A. Serrano Lazaro[1], M. García Simón[1], N. Carbonell Monleón[2], J. Ferreres Franco[1], ML. Blasco Cortes[1]
[1]Intensive care unit, Hospital Clínic Universitari de València, València, Spain; [2]Intensive care, University Hospital, València, Spain
**Correspondence:** S. Mulet Mascarell
*Intensive Care Medicine Experimental* 2019, **7(Suppl 3):**000453

**INTRODUCTION.** Previous research has demonstrated that high Glycemic Variability (GV) is associated with increased mortality among critically ill patients, but its relationship with other factors such as nosocomial infections has not yet been determined.

**METHODS.** Observational prospective study of critically ill patients receiving artificial nutrition (first 48h). Our exclusive criteria were diabetic ketoacidosis or those not receiving artificial nutrition. The study was carried out at a Medical ICU, from November 2015 until December 2018.

Admission data: demographic data, clinical characteristics,APACHEII,-SOFA, nutritional parameters during the 1st&7th day, GV during first 7 days, infectious complications, days of mechanical ventilation (MV), ICU length of stay (LOS) and mortality.

We calculated GV as VC= SD/Mx100. We performed univariate statistical analysis considering the significance $p<0.05$, T-test [LP1] for quantitative independent group variables and $\chi 2$ for qualitative variables. We also employed linear regression for continuous dependent variables and binary logistic regression for dichotomous dependent variables.

**RESULTS.** The sample consists of 118 patients, 67% men with a mean age of 62±14 years, BMI 29±16, diabetics (DM) 24%, dyslipidemics 38%, Arterial hypertension 54% and 38% were smokers. From the sample 86% presented stress hyperglycemia, 70% needed vasoactive drugs, 90.5% enteral nutrition (EN) and 20.5% parenteral nutrition (PN). Mechanical ventilation median 8 days (min 0 – max60), ICU length of stay (LOS) 10 days (min 2 - max 69), hospital LOS 23 days (min 3 – max 123) and 32% of mortality.

APACHE II 19.9±8 and SOFA on admission 8±3.

35% suffered nosocomial infections: ventilated-associated pneumonia (VAP) 26%, tracheobronchitis48%, bacteraemia 1% and catheter related bacteraemia 4.7%, urinary tract infection7%, ventriculitis 2.3% and others 4.7%). We divided our sample into quartiles depending on the level of GV: (1) 8.7–15.49; (2) 15.5–20.37; (3) 20.38–26.48; (4) 26.49–43.9. Mean GV 21.8±8. Table 1 with Results:

**CONCLUSION.**

- Patients with higher illness severity scores (APACHE II) show higher GV.
- We observe higher GV among patients receiving PN.
- It is observed that patients with DM present higher rates of GV.

REFERENCE(S)
1. Diabetes-specific enteral nutrition formula in hyperglycemic, mechanically ventilated, critically ill patients: a prospective, open-label, blind-randomized, multicenter study
2. Intensive versus Conventional Glucose Control in Critically Ill Patients

**Table 1 (abstract 000453).** See text for description

| | Quartile1(n29) | Quartile2(n30) | Quartile3(n29) | Quartile4(n29) | p |
|---|---|---|---|---|---|
| DM(n%) | 25% | 21% | **39%** | 15 | **<0.05*** |
| APACHEII(mean) | 18±8 | 17±7 | **21±8** | **23.3±7** | **<0.05*** |
| PN(n) | 9% | 10% | 17% | **38%** | **<0.05*** |
| EN(n) | 96.5% | 90% | 88% | 88% | NS |
| Vasoactivedrugs(n) | 59% | 60% | 72% | 90% | NS |
| PCT7thDay | 1.08±2 | 1.18±2 | 3.7±8 | 4.08±8 | NS |
| MV (days) | 9.2±8 | 10±13 | 12±7 | 14±16 | NS |
| ICU LOS | 12.5±100 | 13±14 | 16±10 | 17±15 | NS |
| Hospital LOS | 33±31 | 34±27 | 23±19 | 40±34 | NS |
| Infectiouscomplications(n) | 30% | 43% | 31% | 31% | NS |
| Mortality(coef.ß) | -0.18 | 0.18 | 1.18 | 1.2 | NS |

### 000489

### Simplifying the NUTRIC score

R. Lozano Zúñiga[1], MCA. Galindo[2], RL. Carrera Duarte[3], A. Garza de la Maza[4], EA. Ojeda Izquierdo[5], JO. Guamán Crespo[6], ZE. Monares[5]
[1]Critical Care, Hospital San Angel Inn Universidad, Mexico City, Mexico; [2]Chief nutrition department, Hospital San Angel Inn Universidad, Mexico City, Mexico; [3]Nutritiont student, Hospital San Angel Inn Universidad, Mexico City, Mexico; [4]Critical care unit, Hospital San Angel Inn Universidad, Mexico City, Mexico; [5]Intensive critical care unit., Hospital San Angel Inn Universidad, Mexico City, Mexico; [6]Intensive Care Unit, Hospital San Ángel Inn Universidad, Ciudad de México, France
**Correspondence:** R. Lozano Zúñiga
*Intensive Care Medicine Experimental* 2019, **7(Suppl 3):**000489

**INTRODUCTION.** Recent guidelines suggest using the Nutritional Risk in Critically ill (NUTRIC) score to detect those patients who would benefit the most of nutritional therapy by giving them a category of high (≥5 points) or low (<5 points) nutritional risk.(1) To obtain this score the clinician most calculate the Acute Physiology and Chronic Health Evaluation score II (APACHE II) and the Sepsis Related Organ Failure Assessment score (SOFA) to assign them specific points, finally adding the obtained points from the age, number of comorbidities and days of hospitalization before admission to the intensive care unit (ICU).(2,3)To our consideration some collinearity is given by using two severity scores in the same calculation, implying a high probability of obtaining similar results if either one of the severity scores is omitted from the original model.(4)

**OBJECTIVES.** To assess the practical validity of using the NUTRIC score omitting the calculation of either the APACHE II or SOFA scores given their collinearity.

**METHODS.** Retrospective observational study including every adult patient with nutritional assessment (NUTRIC score included) admitted to the ICU excluding obstetric patients, in a period of 9 months. The population was divided in two groups: 1) generation sample (first 50% of the population) 2) validation sample (second 50% of the population). In the generation group two receiver operating characteristics (ROC) curves were generated: 1) NUTRIC score without APACHE II points (model 1) 2) NUTRIC score without SOFA score points (model 2) and a cutoff point for every curve was assigned to detect high nutritional risk according to the original NUTRIC score. In the validation group, 2x2 tables were generated and sensitivity (Se), specificity (Sp), positive predictive value (PPV) and negative predicted value (NPV) were calculated for the detection of high nutritional risk according to the original NUTRIC score using the new cutoff points previously generated (model 1 and model 2). Comparisons between groups were also made.

**RESULTS.** 174 and 173 patients were included in the generation and validation groups respectively. The generation model had higher

SOFA score and higher proportion of patients with 2 or more comorbidities: 3 (2-6) vs 2 (1-4) points (p<0.05) and 70.7% vs 52.6% (p< 0.05). In the generation phase the model 1 obtained an area under the curve (ABC) of 0.929 (p<0.05, confidence interval at 95% (CI95%) 0.892-0.966) selecting a cutoff value of ≥3 points (Se 93.7% and Sp 78.4%), the model 2 obtained an ABC of 0.973 (p<0.05, CI9%% 0.953-0.993) selecting a cutoff value of ≥4 points (Se 98.4% and Sp 77.5%). In the validation phase, the model 1 obtained a Se 98.18%, Sp 81.36%, PPV 71.05% and NPV 98.97% and the model 2 obtained a Se 100%, Sp 70.34%, PPV 61.11% and NPV 100%.

**CONCLUSION.** Using the NUTRIC score omitting the calculation of either SOFA or APACHE II scores could be a practical way to detect patients with low nutritional risk; this does not imply that both obtained scores detect high nutritional risk according to the original NUTRIC in a reliable manner. This could translate to a faster assessment that excludes only low risk patients leaving more time for complete calculation for patients with a high score.

**REFERENCE(S)**

1. Tolles J, Meurer WJ. Logistic Regression Relating Patient Characteristics to Outcomes JAMA Guide to Statistics and Methods. JAMA August [Internet]. 2016;2(5):533. Available from: http://jama.jamanetwork.com/
2. Rahman A, Hasan RM, Agarwala R, Martin C, Day AG, Heyland DK. Identifying critically-ill patients who will benefit most from nutritional therapy: Further validation of the "modified NUTRIC" nutritional risk assessment tool. Clin Nutr [Internet]. 2016;35(1):158–62. Available from: http://dx.doi.org/10.1016/j.clnu.2015.01.015
3. Heyland DK, Dhaliwal R, Jiang X, Day AG. Identifying critically ill patients who benefit the most from nutrition therapy: the development and initial validation of a novel risk assessment tool. Crit Care [Internet]. 2011;15(6):R268. Available from: http://ccforum.biomedcentral.com/articles/10.1186/cc10546
4. McClave SA, Taylor BE, Martindale RG, Warren MM, Johnson DR, Braunschweig C, et al. Guidelines for the Provision and Assessment of Nutrition Support Therapy in the Adult Critically Ill Patient: Society of Critical Care Medicine (SCCM) and American Society for Parenteral and Enteral Nutrition (A.S.P.E.N.). J Parenter Enter Nutr. 2016;40(2):159–211.
5. The authors declare not having conflict of interest to the present study

### 000532

#### Patients with Acute ST-Segment Elevation Myocardial Infarction and multivessel disease: is there association with glycosilated hemoglobin ?

AM. García-Bellón, AM. González González, M. Cano-García, M. De Mora-Martin
Cardiology, Regional Hospital of Malaga, Málaga, Spain
**Correspondence:** A.M. González González
*Intensive Care Medicine Experimental* 2019, **7(Suppl 3):**000532

**INTRODUCTION.** Glycosylated hemoglobin (HbA1c), is currently the best existing parameter of metabolic control in patients with diabetes mellitus. Cardiovascular disease is the main cause of morbidity and mortality in this group of patients

**OBJECTIVES.** To determinate the association between Hb1Ac and multivessel coronary disease in patient with ST elevated myocardial infarction (STEMI)

**METHODS.** We included 246 patients with STEMI admitted in our center. Patients were classified according to their Hb1Ac levels at admission (elevated ≥6.5%; normal < 6.5%).Multivessel coronary disease was assessed by invasive coronary angiography. The association between Hb1Ac and multivessel coronary disease was evaluated using logistic regression.

**RESULTS.** 36% of patients had elevated Hb1Ac. 91,6% of patients with known diabetes had Hb1Ac ≥6.5%. Those with Hb1Ac ≥ 6.5% had increased odds of having multivessel disease as compared with those with hb1Ac < 6.5% (OR: 2.15, 95% CI: 1.01, 4.55, p 0.02)

**CONCLUSION.** Hb1Ac was positively associated with multivessel coronary disease in patients with STEMI

**Table 1 (abstract 000532).** Baseline Characteristics

| | N | % |
|---|---|---|
| Men | 208 | 84,5 |
| Women | 38 | 15,5 |
| Age (years) | 62,86 +/-9,2 | |
| Diabetes Mellitus | | 32% |
| Hypertension | | 48% |
| Dyslipidemia | | 16,1% |
| Current smokers | | 42% |

### 000574

#### Endogenous production of glutamine in ICU patients related to plasma glutamine concentration

M. Smedberg, O. Rooyackers, Å. Norberg, I. Tjäder, J. Wernerman
Department of clinical science, intervention and technology, Karolinska Institute, Stockholm, Sweden
**Correspondence:** M. Smedberg
*Intensive Care Medicine Experimental* 2019, **7(Suppl 3):**000574

**INTRODUCTION.** According to several studies one third (31-38%) of ICU patients have a low plasma glutamine concentration (<420 μmol/L) at admission. Moreover, this hypoglutaminemia has been shown to be associated with an increased morbidity and mortality. It has been hypothesized that an increased utilization of glutamine cannot be met by the production in spite of an increased net efflux from skeletal muscle to plasma resulting in the low concentrations. However, glutamine kinetics in ICU patients has only been studied scarcely and data to relate endogenous production to glutamine levels has not been available. It remains important to understand the mechanisms behind the low glutamine concentrations in order to determine when, how and if glutamine should be supplemented in the ICU setting.

**OBJECTIVES.** To elucidate the relationship between plasma glutamine levels and the endogenous glutamine production in ICU patients.

**METHODS.** Glutamine kinetics was studied in ICU patients. A tracer bolus injection method employing 13C-glutamine was used. It has been validated to give minimum effect on plasma glutamine and insulin concentrations. After bolus injection 18 plasma samples during 90 minutes were taken and the area under the curve for C13-glutamine calculated.

**RESULTS.** There was a weak statistical correlation between de novo endogenous production of glutamine (not emanating directly from protein breakdown) and plasma glutamine concentration in stabilized ICU patients (n=11), with R2 = 0,308. Two sided P=0,0764 with a power of 41%, which was not statistically significant.

**CONCLUSION.** Plasma concentration of glutamine did not directly reflect the de novo production of glutamine, although a statistical correlation was at hand. A larger cohort of ICU patients representing a wider concentration interval will be needed to fully explore the relationship.

**REFERENCE(S)**

1. Tsujimoto T, Shimizu K, Hata N, Takagi T, Uejima E, Ogura H, et al. Both high and low plasma glutamine levels predict mortality in critically ill patients. Surg Today. 2017;47(11):1331-8.
2. Oudemans-van Straaten HM, Bosman RJ, Treskes M, van der Spoel HJ, Zandstra DF. Plasma glutamine depletion and patient outcome in acute ICU admissions. Intensive Care Med. 2001;27(1):84-90.
3. Nienaber A, Dolman RC, van Graan AE, Blaauw R. Prevalence of glutamine deficiency in ICU patients: a cross-sectional analytical study. Nutr J. 2016;15(1):73
4. The study is supported by a grant from the local County Council.

### 000609

#### Nutritional Goals and time of Pressure Injury development in Intensive Care Unit patients

F. Wenzel, I. Whitaker
Escola paulista de enfermagem, UNIFESP, São Paulo, Brazil
**Correspondence:** F. Wenzel
*Intensive Care Medicine Experimental* 2019, **7(Suppl 3):**000609

**INTRODUCTION.** Pressure Injuries (PI) are characterized by damage to the skin and either soft tissues resulting from compression of the bony prominence against a hard surface or related to the use of some medical device. The time until PI development can be related to several factors present in critically ill patients, such as their clinical condition and nutritional status.

Nutritional support is essential for wound healing and it is important for patients at risk of developing PI. Inadequate nutrition and negative energy balance combined with catabolic stress and inflammation caused by severe disease increase the risk of complications, including PI and bad wound healing.

**OBJECTIVES.** To compare the time until PI development between groups of critical care patients with enteral nutrition which achieved or not the nutritional goals and verify the risks associated with the PI development over time.

**METHODS.** Observational, prospective study conducted at the Intensive Care Center of a University Hospital in São Paulo (Brazil) during 2016. Patients admitted to ICU without PI and which received exclusive Enteral Nutrition were selected for the sample. The PI development and time to PI were considered dependent variables. The statistical analysis was performed by applying the survival curve with the Kaplan-Meier method and the Cox regression model was used to verify the risks associated with the PI development over time, observing a significance level of < 5% and confidence interval of 95%.

**RESULTS.** The sample included 181 patients, 56.4% was male, the mean age was 55.1 years, the mainly location before ICU admission was operating room (84.0%) and the main reason for hospitalization was the neurological causes (44.8%). The mean of ICU stay was 17.5 days and mortality was 30.4%. The mean of the SAPS3 was 59.9 and the SOFA was 6.7. The incidence of PI was 31.5%. The survival curves did not show difference in time until PI development, considering the patients who achieved caloric goal (p = 0.532) or protein goal (p = 0.694) or caloric + protein goals (p = 0.648). The nutritional goals were not observed among the risks associated with the time of PI development. The identified risks were location before ICU admission (emergency department - HR = 2.05 and ward - HR = 2.40), length of ICU stay (HR = 1.01), Braden scores (HR = 0.63) and EVARUCI scores (HR = 1.33).

**CONCLUSION.** The time of PI development in critically ill patients with enteral nutrition was not influenced by the achievement of nutritional goals. Moreover, the nutritional goals were not included among the risks associated with the development of PI over time. Considering that the occurrence of PI is multifactorial, the early identification of patients at risk is essential for preventive measures to be adopted immediately.

**REFERENCE(S)**

1. National Pressure Ulcer Advisory Panel. National Pressure Ulcer Advisory Panel Announces a Change in Terminology From Pressure Ulcer to Pressure Injury and Updates the Stages of Pressure Injury. Available at: www.npuap.org/national-pressure-ulcer-advisory-panel-npuap-announces-a-c.... Acessed april 26, 2018.
2. Cox J, Rasmussen L. Enteral Nutrition in the Prevention and Treatment of Pressure Ulcers in Adult Critical Care Patients. Critical Care Nurse. 2014; 34(6). DOI: 10.4037/ccn2014950
3. McClave AS, Taylor BE, Martindale RG, et al; Society of Critical Care Medicine; American Society for Parenteral and Enteral Nutrition. Guidelines for the Provision and Assessment of Nutritional Support Therapy in the Adult Critically Ill Patient: Society of Critical Care Madicine (SCCM) and American Society for Parenteral and Enteral Nutrition (ASPEN). Journal of Parenteral and Enteral Nutrition. 2016; 40(2): 159-211. DOI: 10.1177/0148607109335234
4. O'Leary-Kelley C, Bawel-Brinkley K. Nutrition Support Protocols: Enhancing Delivery of Enteral Nutrition. Critical Care Nurse. 2017; 37 (2):15-23. https://doi.org/10.4037/ccn2017650
5. Cox, J. Pressure Injury Risk Factors in Adult Critical Care Patients: A Review of the Literature. Ostomy Wound Management. 2017; 63 (11). https://doi.org/10.1186/s13643-017-0451-5

### 000690

#### Lactate kinetics in ICU patients using a bolus of 13C-labeled lactate

J. Grip[1], T. Falkenström[2], P. Promsin[3], J. Wernerman[1], Å. Norberg[1], O. Rooyackers[1]
[1]Clintec, dept. of anesthesiology and intensive care, Karolinska Institute and University Hospital, Huddinge, Stockholm, Sweden; [2]Dept of perioperative medicine and intensive care, Karolinska University Hospital, Huddinge, Stockholm, Sweden; [3]Division of critical care, department of medicine, Faculty of Medicine Siriraj Hospital, Mahidol University, Bangkok, Thailand
**Correspondence:** J. Grip
*Intensive Care Medicine Experimental* 2019, **7(Suppl 3):**000690

**INTRODUCTION.** The correlation between elevated plasma lactate concentration and increased mortality has been firmly established in ICU patients. Although plasma lactate is heavily relied upon for clinical decision making, the kinetics of lactate in critically ill patients is still sparsely studied.

**OBJECTIVES.** To establish and validate a feasible method to study whole body lactate kinetics in critically ill patients.

**METHODS.** Healthy volunteers (n=6) received a bolus dose of 13C-labeled lactate (20µmol/kg body weight) and 43 blood samples were drawn over two hours to determine the decay in labeled lactate. Data was analyzed using non-compartmental modeling, calculating rates of appearance (Ra) as an estimate of lactate production, and clearance of lactate. The area under the curve (AUC) was calculated using a linear-up log-down trapezoidal approach with extrapolation beyond 120 minutes using the terminal slope to obtain the whole AUC. After evaluation, the same protocol was used in an unselected group of critically ill patients (n=10).

**RESULTS.** Ra for healthy volunteers and ICU patients were 12.8 ± 3.9 vs 22.7 ± 11.2 µmol/kg/min (p=0.026) and clearance 1.57 ± 0.39 vs 1.10 ± 0.43 L/min (p=0.047), respectively. ICU patients (n=6) with normal lactate concentrations showed kinetics very similar to healthy volunteers (Ra 14.5 (range 12.3 – 18.3) µmol/kg/min, lactate clearance 1.3 (range 0.77 – 1.77) L/min). For ICU patients, there were statistically significant correlations between plasma concentration and Ra of lactate, $r2=0.85$ ($p<0.0002$) and lactate clearance, $r2= 0.50$ ($p=0.023$). Simulations showed that reducing the number of samples from 43 to 14 gave the same results. Our protocol yielded results on lactate kinetics very similar to previously published data.

**CONCLUSION.** This minimally invasive protocol using an isotopically labeled bolus dose of lactate was accurate and feasible for studying lactate kinetics in critically ill ICU patients.

## INF - Novelties in prevention and diagnosis of ICU-acquired infections

### 000263

#### Subglottic Secretion Drainage for Preventing Ventilator-associated Pneumonia in Intensive Care Units, a Clustered, Randomized, Cross-over Multicenter Medico-economic Trial: the « DEMETER Study»

JC. Lacherade[1], F. Meziani[2], O. Martinet[3], C. Cléophax[4], JE. Herbrecht[5], SM. Au[6], JP. Quenot[7], P. Courouble[8], I. Runge[9], A. Veinstein[10], J. Reignier[11], D. Couret[12], G. Mourembles[13], D. Garot[14], M. Piagnerelli[15], O. Pajot[16], D. Thévenin[17], D. Schnell[18], E. Tavernier[19], J. Frenkiel[20]
[1]Medical-surgical intensive care unit, Centre Hospitalier Départemental - site de La Roche-sur-Yon, La Roche-sur-Yon, France; [2]Medical intensive care unit, CHU de Strasbourg Nouvel Hôpital Civil, Strasbourg, France; [3]Medical-surgical intensive care unit, CHU Felix-Guyon, Saint-Denis, La Réunion, France; [4]Medical-surgical intensive care unit, CH Pontoise, Pontoise, France; [5]Medical intensive care unit, CHU de Strasbourg Hôpital Hautepierre, Strasbourg, France; [6]Medical intensive care unit, Hospital Center Intercommunal Poissy/Saint-Germain-En-Laye, Poissy, France; [7]Medical intensive care unit, Chu Dijon, Dijon, France; [8]Medical-surgical intensive care unit, Hospital De Saint-Nazaire, Saint-Nazaire, France; [9]Medicine intensive reanimation, The Regional Hospital of Orleans, Orléans, France; [10]Medical intensive care unit, Poitiers University Hospital, Poitiers, France; [11]Médecine intensive réanimation, Nantes University Hospital Hotel-Dieu, Nantes, France; [12]Neurological intensive care unit, CHU de la Réunion, Saint-Pierre, France; [13]Medical-surgical intensive care unit, CHU de la Réunion, Saint-Pierre, France; [14]Medical intensive care unit, Chru Hôpitaux De Tours, Hospital Bretonneau, Tours, France; [15]Medical-surgical intensive care unit, C.H.U. Charleroi, Charleroi, Belgium; [16]Medical-surgical intensive care unit, C.H. Victor Dupouy, Argenteuil, France; [17]Medical-surgical intensive care unit, Hospital Center De Lens, Lens, France; [18]Medical-surgical intensive care unit, C.H. d'Angoulême, Angoulême, France; [19]Centre d'investigation clinique inserm cic 1415, Chru Hôpitaux de Tours, Tours, France; [20]Urc-eco ile de france, AP-HP, Hôpital Hôtel Dieu, Paris, France
**Correspondence:** J.C. Lacherade
*Intensive Care Medicine Experimental* 2019, **7(Suppl 3):**000263

**INTRODUCTION.** Subglottic secretion drainage (SSD) is one of the recommended strategies to prevent ventilator-associated pneumonia (VAP) with a high level of evidence, especially regarding early-onset pneumonia. Despite published recommendations, SSD has not been widely implemented in ICUs and remains underused. Several factors could account for this: the uncertainty regarding the safety of SSD, the lack of evidence of benefits in other clinical outcomes than prevention of VAP and the extra cost of the specific endotracheal tubes (ETs) allowing SSD compared with usual endotracheal tube. To assess the medico-economic impact of SSD implementation, we performed a multicenter, pragmatic trial: the DEMETER study (ClinicalTrials.gov, identifier NCT02515617).
**OBJECTIVES.** To assess the cost utility of the implementation of SSD in a VAP prevention bundle with a time horizon of one year.
**METHODS.** It is a multicenter cluster-randomized and cross-over study. 26 ICUs were involved (25 located in France and 1 in Belgium). Two periods of 10 months (period A and B) were planned to include patients. During the period A, the ICU's team used endotracheal tubes not allowing SSD and during the period B, specific endotracheal tubes allowing SSD were used. A 2-month interval between the two periods was planned to allow the switch of ET type. Both inclusion periods correspond to the same seasonal period. Moreover, during period B, specific endotracheal tubes allowing SSD were also available in the emergency rooms and the prehospital emergency units of hospitals located around the ICUs involved in the study. Regardless of the period (A or B), mechanically ventilated patients benefited from the regular VAP prevention policy which included: measures to reduce the duration on mechanical ventilation (MV) as well as the exposure to the medical device (sedation algorithm, standardization procedure for weaning of the MV) and specific measures to prevent VAP (maintaining a cuff pressure between 25 and 30 cm H20, semi-recumbent positioning of the patient, oropharyngeal care). During period B, in addition, SSD was performed intermittently every 2 hours by using a 10ml syringe.
The economic evaluation was performed in accordance with the CHEERS statement. The prospective analysis was conducted from the health care perspective to determine the cost per quality-adjusted life year (QALY) gained with the SSD implementation over a 1-year period. Hospital resources were considered as well as the cost of the medical device and the cost of the nursing time required to perform the SSD procedure during the mechanical ventilation period. Health-related quality of life was assessed using the EuroQol 5 Dimensions at baseline, 3-month, 6-month, and 1-year. The utility values are based on French tariffs for the corresponding EQ5D scores.
**RESULTS.** During the periods of inclusion (nov 2015-aug 2016 and nov 2016-aug 2017), 2600 patients were included. Patient's follow-up ended in August 2018. The analyses are currently ongoing. The results will be available and presented in exclusivity at LIVES 2019.
**CONCLUSION.** The DEMETER study is the first prospective and large study assessing the medico-economic impact on the SSD use and could afford new insights on this VAP prevention strategy.

**REFERENCE(S)**

1. Caroff DA1, Li L, Muscedere J, Klompas M. Subglottic Secretion Drainage and Objective Outcomes: A Systematic Review and Meta-Analysis. Crit Care Med. 2016 Apr;44(4):830-40.
2. Husereau D, Drummond M, Petrou S, et al. Consolidated Health Economic Evaluation Reporting Standards (CHEERS)—explanation and elaboration. Value Health. 2013;16(2):231-250.
3. The DEMETER study was supported by a grant from the French Ministry of Health (Programme de Recherche Médico-Economique 2014).

### 000478

#### Efficacy and Safety Results of the SAATELLITE Phase 2 Study of Suvratoxumab, a Staphylococcus aureus Alpha Toxin-Neutralizing Human Monoclonal Antibody in Mechanically Ventilated Patients in the Intensive Care Units

B. Francois[1], M. Sánchez García[2], P. Eggimann[3], PF. Dequin[4], PF. Laterre[5], V. Huberlant[6], D. Escudero[7], T. Boulain[8], C. Bretonnière[9], J. Pugin[10], SJ. Trenado[11], AC. Hernandez Padilla[1], SO. Ali[12], K. Shoemaker[13], A. Ruzin[12], V. Pierre[14], J. Vignaud[1], T. Bellamy[12], F. Dubovsky[15], H. Jafri[15]
[1]Inserm cic 1435, Centre Hospitalier Universitaire de Limoges, Limoges, France; [2]Medicina intensiva, Hospital Clínico San Carlos - Medicina Intensiva, Madrid, Spain; [3]Department of locomotor system, Lausanne University Hospital, Lausanne, Switzerland; [4]Medecine intensive reanimation, Chru Hôpitaux De Tours, Hospital Bretonneau, Tours, France; [5]Intensive care unit, Cliniques Universitaires Saint-Luc, Brussels, Belgium; [6]Soins intensifs, C. H. Jolimont-Lobbes Site, Lobbes, Belgium; [7]Intensive care unit, Hospital Universitario Central de Asturias, Oviedo, Spain; [8]Médecine intensive et réanimation, The Regional Hospital of Orleans, Orléans, France; [9]Soins intensifs – pneumologie, Hôpital Guillaume et René Laennec, Nantes, France; [10]Intensive care division, department of acute medicine, University Hospitals of Geneva, Geneva, Switzerland; [11]Department of critical care medicine, Mútua Terrassa University Hospital, Terrassa, Spain; [12]Microbial sciences, AstraZeneca, Gaithersburg, United States of America; [13]Clinical biostatistics and data management, AstraZeneca, Gaithersburg, United States of America; [14]Clinical pharmacology and safety sciences, AstraZeneca, Gaithersburg, United States of America; [15]Clinical biologics, AstraZeneca, Gaithersburg, United States of America
**Correspondence:** H. Jafri
*Intensive Care Medicine Experimental* 2019, **7(Suppl 3):**000478

**INTRODUCTION.** Morbidity and mortality of *Staphylococcus aureus* (SA) pneumonia remain high in mechanically ventilated, intensive care unit (MV ICU) patients despite best clinical care. Suvratoxumab (MEDI4893) is an extended half-life, SA alpha toxin-neutralizing human monoclonal antibody that may reduce incidence of SA pneumonia in MV ICU patients.

**OBJECTIVES.** To assess efficacy, safety, pharmacokinetics (PK), population PK modeling, anti-drug antibody (ADA) responses, and healthcare resource utilization (HRU) benefits of suvratoxumab in MV ICU patients at risk for SA pneumonia.

**METHODS.** MV ICU subjects with PCR-confirmed SA colonization of the lower respiratory tract were randomized to either a single intravenous infusion of 5000 mg suvratoxumab (n=96) or placebo (n=100) and followed for 190 days post dose. Efficacy endpoints included relative risk reduction (RRR) vs. placebo within 30 days post dose in: incidence of SA pneumonia (primary endpoint; tested at 2-sided α=0.1), all-cause pneumonia, and all-cause pneumonia or death (all events determined by the Endpoint Adjudication Committee). HRUs, PK and ADA were measured through 90 days post dose and TEAEs and serious TEAEs through 190 days.

**RESULTS.** Baseline characteristics were similar between groups. Suvratoxumab provided 31.9% RRR in incidence of SA pneumonia vs. placebo (17.7% vs 26%; *P*=0.166), 30% RRR (*P*=0.146) in incidence of all-cause pneumonia, and 23% RRR (*P*=0.164) in incidence of all-cause pneumonia or death. Suvratoxumab provided 38% RRR in incidence of SA pneumonia vs. placebo, when adjusted for duration of initial MV. Suvratoxumab provided greater prevention of SA pneumonia in subjects aged ≤65 years (RRR=47.4%), low SA colonization status (RRR=66.7%), and 5 VAP bundles (RRR=46.3%) vs. placebo. Suvratoxumab was associated with 3.0 fewer hospital days and 2.4 fewer ICU days for every 90 days patient follow-up and greater HRU reductions in subjects aged ≤65 years (10.1 fewer hospital days, 3.7 fewer ICU days). Over 190 days, proportions of subjects with TEAEs and serious TEAEs were similar between suvratoxumab and placebo groups: ≥1 TEAE (90.6% vs 90.0%); ≥1 serious and/or ≥grade 3 severity TEAE (55.2% vs 52.0%). Mean serum suvratoxumab concentrations were above the target serum level of 211 μg/mL through day 31 (mean±SD concentration 296±131 μg/mL). Population PK model estimated mean±SD terminal half-life of 72±33 days. No ADA responses were detected post-dose through 90 days.

**CONCLUSION.** A single intravenous dose of suvratoxumab produced a trend toward reduced incidence of SA pneumonia and reduced HRU in high-risk ICU patients compared with placebo. Greater clinical benefits were observed in certain subgroups. Suvratoxumab demonstrated acceptable safety, PK and ADA profiles. These results support continued development of suvratoxumab in MV ICU patients.

**REFERENCE(S)**

1. on behalf of The SAATELLITE Study Group
2. This research project receives support from the Innovative Medicines Initiative Joint Undertaking under grant agreement number 115523, resources of which are composed of financial contribution from the European Union Seventh Framework Programme (FP7/2007-2013) and EFPIA companies in kind contribution.

### 001006

#### Significance of mHLA-DR expression on circulating monocytes in ICU patients: results of a large survey

C. de ROQUETAILLADE[1], C. Dupuis,[2], V. Faivre[3], AC. Lukaszewicz[4], D. Payen,[5]

[1]Anesthesiology and Intensive Care Unit, Hospital Lariboisière, Paris, France; [2]Intensive care unit, Bichat-Claude Bernard Hospital, Paris, France; [3]Anesthesiology laboratory, Hospital Lariboisière, Paris, France; [4]Anesthesia & critical care, Hospital Édouard Herriot, Lyon, France; [5]Intensive care, Paris Diderot University, Paris, France

**Correspondence:** C. de ROQUETAILLADE

*Intensive Care Medicine Experimental* 2019, **7(Suppl 3):**001006

**INTRODUCTION.** To test the interest to monitor the expression of human monocytic Human Leukocyte Antigen-D Related (mHLA-DR) to predict mortality and the occurrence of secondary infections in a large single-center Intensive Care Unit (ICU) population.

**METHODS.** We conducted a single-center, prospective, observational study in a tertiary hospital in France. All patients admitted from 2013 to 2015 in our ICU were included from our data base if mHLA-DR measurement was performed within the first days (< 4 days) post admission. Collected parameters were severity score on admission (SAPS II and SOFA), sex, age, mortality and secondary infections. The association between mHLA-DR expression and outcomes was tested by the adjusted Fine and Gray sub-distribution competing risk models.

**RESULTS.** 1053 patients having an early mHLA-DR measurement were included and analyzed: 151 patients (14.3%) died in the ICU; based on the logarithm cutoff value for median (mHLA-DR < 9.2 log), a low mHLA-DR was independently associated with mortality (HR = 0.71 [0.57; 0.95], p < 0.01). The performance of mHLA-DR to predict ICU death was inferior to the gravity scores (mHLA-DR: AUC = 0.65 [0.6-0.69], SAPS II: 0.8 [0.77-0.84], p<0.01) and the performance of the combination of mHLA-DR and SAPS II was similar to SAPS II alone (p = 0.81). Two successive mHLA-DR measurements within the first week were performed in 543 patients allowing to test the 2nd value or the slope between the 2 values (positive, negative or stable). Among these patients, 223 patients
(37.7%) had a secondary infection with a sustained lower mHLA-DR than other patients (mHLA-DR = 9.0 log vs. 9.3 log, p < 0.01). The second mHLA-DR value was independently associated with the development of subsequent secondary infection, regardless of initial severity (HR = 0.66 [0.51 ; 0.84], p = 0.001). A negative slope between mHLA-DR 1 and 2 was strongly associated with later occurrence of a secondary infection (HR = 0.62 [0.43; 0.89], p = 0.009).

**CONCLUSION.** mHLA-DR expression is early decreased in ICU patients and is associated with ICU
mortality (the higher mortality, the lower mHLA-DR), but with no superiority to severity scores even in combination. For a given severity, persisting low mHLA-DR expression or decreasing mHLA-DR, are independent and reliable predictors of secondary infection occurrence. This large survey may then be used to design clinical trials testing immune-stimulating drugs based on mHLA-DR monitoring to prevent or cure secondary infections.

**REFERENCE(S)**

1. Paul A. ROCHE et Kazuyuki FURUTA. The Ins and Outs of MHC Class II-Mediated Antigen Processing and Presentation. Nat. Rev. Immunol. 15.4 (avr. 2015), 203–216.
2. Carmen Andrea PFORTMUELLER, Christian MEISEL, Michaela FUX et Joerg C. SCHEFOLD. Assessment of Immune Organ Dysfunction in Critical Illness : Utility of Innate Immune Response Markers. Intensive Care Medicine Experimental 5.1 (23 oct. 2017), 49.
3. Fabienne VENET et Guillaume MONNERET. Advances in the Understanding and Treatment of Sepsis-Induced Immunosuppression. Nat. Rev. Nephrol. 14.2 (fév. 2018), 121– 137.
4. Wolf-Dietrich DÖCKE, Felix RANDOW, Uta SYRBE, Dietmar KRAUSCH, Khusru ASADULLAH, Petre REINKE et al. Monocyte Deactivation in Septic Patients : Restoration by IFN- Treatment. Nat. Med. 3.6 (juin 1997), 678–681.
5. Andrew CONWAY MORRIS, Deepankar DATTA, Manu SHANKAR-HARI, Jacqueline STEPHEN, Christopher J. WEIR, Jillian RENNIE et al. Cell-Surface Signatures of Immune Dysfunction Risk-Stratify Critically Ill Patients : INFECT Study. Intensive Care Med 44.5 (1er mai 2018), 627–635.
6. Anne-Claire LUKASZEWICZ, Marion GRIENAY, Matthieu RESCHE-RIGON, Romain PIRRACCHIO, Valérie FAIVRE, Bernadette BOVAL et al. Monocytic HLA-DR Expression in Intensive Care Patients : Interest for Prognosis and Secondary Infection Prediction* : Crit. Care Med. 37.10 (oct. 2009), 2746–2752.
7. Richard S. HOTCHKISS, Guillaume MONNERET et Didier PAYEN. Sepsis-Induced Immunosuppression : From Cellular Dysfunctions to Immunotherapy. Nat. Rev. Immunol. 13.12 (déc. 2013), 862–874.

### 000582

#### BreathDx – Molecular Analysis of Exhaled Breath as a Diagnostic Test for Ventilator–Associated Lower Respiratory Tract Infections: a European multicentre study

PMP. van Oort[1], T. Nijsen[2], I. White[3], H. Knobel[4], T. Felton,[5], N. Rattray,[6], O. Lawal,[7], M. Bulut,[2], W. Ahmed[7], A. Artigas[8], P. Povoa[9], I. Martin-Loeches[10], P. Sterk[1], M. Schultz[1], P. Dark[7], S. Fowler[7], LDJ. Bos,[1]
[1]Intensive Care, Academic Medical Centre, Amsterdam, Netherlands; [2]Research, Philips Research, Global Headquarters, Eindhoven, Netherlands; [3]Analytics, Manchester Institute of Biotechnology, Manchester, UK, United Kingdom; [4]Analytics, Eurofins Materials Science Netherlands B.V., Eindhoven, Netherlands; [5]Intensive care, Wythenshawe Hospital, Wythenshawe, United Kingdom; [6]Pharmacy, University of Strathclyde, Glasgow, United Kingdom; [7]Division of infection, immunity and respiratory medicine, The University of Manchester, Manchester, United Kingdom; [8]Critical care center, Universitat Autònoma de Barcelona - UAB, Sabadell, Spain; [9]Polyvalent intensive care unit, São Francisco Xavier Hospital, CHLO, Lisbon, Portugal; [10]School of medicine, Trinity College Dublin, Dublin, Ireland
**Correspondence:** P.M.P. van Oort
*Intensive Care Medicine Experimental* 2019, **7(Suppl 3):**000582

**INTRODUCTION.** Patients suspected of ventilator-associated lower respiratory tract infections (VA-LRTI) receive antimicrobial therapy, even though subsequent bronchoalveolar lavage (BAL) microbiological culture results deem this inappropriate in almost half of these patients [1]. Breath contains volatile organic compounds (VOCs) produced by host response or pathogen metabolism, which may be used to diagnose pneumonia [2].

**OBJECTIVES.** We hypothesize that exhaled breath analysis can discriminate between patients suspected of VA-LRTI with positive respiratory tract cultures (confirmed) and those who are negative (nonconfirmed).

**METHODS.** This was an international European multicentre observational cohort study in intubated and ventilated ICU patients who received antibiotics for suspected VA-LRTI [3]. Gas-chromatography mass-spectrometry was used to detect and identify VOCs. A (mini-)BAL semi-quantitative culture ≥10^4 CFU was considered positive for VA-LRTI. Comparison of all measured VOCs was performed using the "limma" function in *R statistics*, resulting in *p*-values adjusted for multiple testing and fold changes.

**RESULTS.** 108 patients were included consecutively: 52 (48%) had positive cultures, most commonly *Pseudomonas aeruginosa* (N=9, 17%) and *Staphylococcus aureus* (N=15, 29%). The clinical pulmonary infection score (CPIS) was higher in confirmed VA-LRTI patients ($p<$ 0.001). 5 out of 184

VOCs were significantly higher in culture positive patients ($p<$ 0.05). The AUROCC for the 5 VOCs combined was 0.82 and increased to 0.85 when added to the CPIS.

**CONCLUSION.** Exhaled breath analysis can be used to discriminate between patients with suspected VA-LRTI who had confirmed positive or negative respiratory cultures, with the potential to reduce delays in diagnosis. Further analysis will result in the identification of the molecules and further validation of the diagnostic algorithm.

##### REFERENCE(S)

1. Fowler SJ, Basanta-Sanchez M, Xu Y, et al (2015) Surveillance for lower airway pathogens in mechanically ventilated patients by metabolomic analysis of exhaled breath: a case-control study. Thorax 320–325. https://doi.org/10.1136/thoraxjnl-2014-206273
2. van Oort PMP, Bruin S De, Weda H, et al (2017) Exhaled Breath Metabolomics for the Diagnosis of Pneumonia in Intubated and Mechanically-Ventilated Intensive Care Unit ( ICU ) -Patients. Int J Mol Sci 18:1–14. https://doi.org/10.3390/ijms18020449
3. van Oort PMP, Nijsen T, Weda H, et al (2017) BreathDx – molecular analysis of exhaled breath as a diagnostic test for ventilator–associated pneumonia: protocol for a European multicentre observational study. BMC Pulm Med 17:1. https://doi.org/10.1186/s12890-016-0353-7
4. The project was funded by the European Union FP7 Marie Curie Actions (MC-IAPP BreathDx 611951)
5. NIHR's Manchester Biomedical Research Centre (UK)

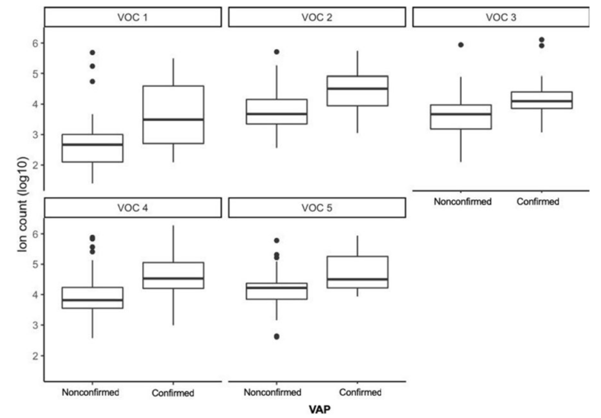

**Fig. 1 (abstract 000582).** See text for description

### 000760

#### Effects of topical antibiotics on eradication and acquisition of 3rd-generation cephalosporin and carbapenem resistant gram-negative bacteria in ICU patients

BH. Wittekamp[1], N. Plantinga[2], C. Brun-Buisson[3], MJM. Bonten[4], T. R-Gnosis Icu Study Group[5]
[1]Intensive care center, University Medical Center Utrecht, Utrecht, Netherlands; [2]Medical microbiology, University Medical Center Utrecht, Utrecht, Netherlands; [3]Université paris-est créteil, Medical ICU & Infection control Unit, Hôpital Henri-Mondor Ap-Hp, Créteil, France; [4]Medical microbiology, julius center for health sciences, University Medical Center Utrecht, Utrecht, Netherlands; [5]., ., ., Netherlands
**Correspondence:** B.H. Wittekamp
*Intensive Care Medicine Experimental* 2019, **7(Suppl 3):**000760

**INTRODUCTION.** The effectiveness of decontamination strategies for eradication of antibiotic resistant GNB in ICU patients remains controversial and has not been studied extensively in ICUs with higher levels of antibiotic resistance than Dutch ICUs (1). We, therefore, aimed to determine the efficacy SDD, SOD and CHX 1-2% mouthwash on eradication of 3GCR-E and CR-GNB from the rectum and respiratory tract and on acquisition of these bacteria during ICU stay.

**OBJECTIVES.** To quantify the effects of selective digestive tract decontamination (SDD), selective oropharyngeal decontamination (SOD) and 1-2% chlorhexidine (CHX) mouthwash on eradication and acquisition of carriage of third generation cephalosporin resistant Enterobacteriaceae (3GCR-E) and carbapenem resistant Gram-negative bacteria (CR-GNB) in ICU patients.

**METHODS.** Nested cohorts study within a European cluster-randomized cross-over trial (2) with 8,665 patients. Eradication and acquisition of 3GCR-E and CR-GNB were investigated separately in the rectum and respiratory tract for the three interventions and compared to standard care (SC) using Cox-regression competing events analyses.

**RESULTS.** Adjusted cause specific hazard ratios (CSHR) for eradication of rectal carriage for SDD were 1.76 (95%CI 1.31-2.36) for 3GCR-E and

3.17 (95% CI 1.60-6.29) for CR-GNB, compared to SC. Adjusted CSHR for eradication of 3GCR-E from the respiratory tract were, compared to SC, 1.47 (95% CI 0.98-2.20) for SDD and 1.38 (95%CI 0.92-2.06) for SOD. Adjusted CSHRs for acquisition of rectal carriage during SDD (compared to SC) were 0.51 (95%CI 0.40-0.64) for 3GCR-E and of 0.56 (95%CI 0.40-0.78) for CR-GNB. Adjusted CSHRs for acquiring respiratory tract carriage with 3GCR-E, compared to SC, were 0.55 (95%CI 0.42-0.71) for SOD and 0.38 (95%CI 0.28-0.50) for SDD, and 0.60 (95%CI 0.44-0.81) and 0.46 (95%CI 0.33 - 0.64) for CR-GNB during SOD and SDD, respectively.

**CONCLUSION.** SDD was associated with faster eradication of 3GCR-E and CR-GNB from the rectum than SC, and SDD and SOD were associated with less acquisition of both 3GCR-E and CR-GNB, compared to SC, in the respiratory tract.

**REFERENCE(S)**

1. de Smet AM, Kluytmans JA, Blok HE, Mascini EM, Benus RF, Bernards AT, et al. Selective digestive tract decontamination and selective oropharyngeal decontamination and antibiotic resistance in patients in intensive-care units: an open-label, clustered group-randomised, crossover study. The Lancet Infectious diseases 2011;11(5):372-80.
2. Wittekamp BH, Plantinga NL, Cooper BS, Lopez-Contreras J, Coll P, Mancebo J, et al. Decontamination Strategies and Bloodstream Infections With Antibiotic-Resistant Microorganisms in Ventilated Patients: A Randomized Clinical Trial. JAMA 2018;320(20):2087-98.
3. The R-GNOSIS ICU study was funded European Commission under the Seventh Framework Programme (FP7-HEALTH-2011-single-stage, grant agreement number 282512).

## Abstract Award Winning Session

### 000023

#### Early use of Norepinephrine improves survival in septic shock: earlier than early

M. Elbouhy[1], M. Soliman[2], K. Taema[2], A. Abdelaziz[2]
[1]Intensive care medicine, Elaraby Hospital, Cairo, Egypt; [2]Critical care medicine, Cairo University, Faculty Of Medicine, Kasr Al Ainy, Cairo, Egypt
**Correspondence:** K. Taema

*Intensive Care Medicine Experimental* 2019, **7(Suppl 3):**000023

**INTRODUCTION.** The timing of initiation of Norepinephrine (NEP) in septic shock is still controversial. The most recent guidelines recommend its use after failed trial of fluid resuscitation.

**OBJECTIVES.** We intended in this study to evaluate the impact of early starting NEP simultaneously with fluids resuscitation immediately after diagnosis of septic shock.

**METHODS.** We recruited 101 patients admitted to the emergency department (ED) with septic shock who were randomized in 1:1 ratio to early initiation of NEP simultaneously with IV fluids (early NEP group) and starting NEP after failed fluid resuscitation trial (late NEP group). Our primary outcome was the in-hospital survival while the secondary outcomes were the time of restoring a mean arterial pressure (MAP) of 65 mmHg, lactate clearance and volume of fluids used in resuscitation.

**RESULTS.** There was no statistically significant difference between the two groups regarding the baseline hemodynamics, laboratory findings and the source of infection. NEP infusion started after 25 (20-30) minutes from ED admission in the early group compared to 120 (120-180) minutes in the late group [P=0.000,95% CI:-100(-140,-100)]. Target MAP of 65mmHg was achieved faster in the early group [2 (1-3.5)hours Vs 3(2-4.75)hours in the late group][P=0.003,95% CI:-1(-1,0)]. The post resuscitation serum lactate was decreased by 37.8(24-49)% and 22.2(3.3-38)% in the early and late groups respectively [P=0.005,95% CI:13.96(4,24.2)]. Serum lactate less than 2mmol/L after resuscitation was achieved in 29 patients (50.9%) in the early group compared to 12 (27.3%) in the late group (P=0.017). Patients with early NEP were resuscitated by significantly lower volume of fluids [25(18.8-28.7)mL/Kg Vs 32.5(24.4-34.6)mL/Kg in the early&late groups] [P=0.000,95% CI:-5.5(-8.2,-2.8)]. The early group had survival rate of 71.9 % (41 patients) compared to 45.5 % (20 patients) in the late group (P = 0.007). The survivors had NEP started after 30(20-120) minutes Vs 120(30-165) minutes in the non-survivors [P= 0.013,95%CI:-15(-60,0)].

**CONCLUSION.** We concluded that the early use of Norepinephrine at the onset of diagnosis of septic shock might cause earlier restoration of blood pressure, better lactate clearance and consequently better in-hospital survival

### 000660

#### Frailty is an independent predictor of mortality in very old patients (VIPs) admitted to intensive care unit after acute and elective intervention

B. Wernly[1], C. Jung[2], D. De Lange[3], B. Guidet[4], H. Flaaten[5]
[1]Department of cardiology, Paracelsus Medical University, Salzburg, Austria; [2]Department of cardiology, pulmonology and angiology, University Hospital of Düsseldorf, Düsseldorf, Germany; [3]Department of intensive care medicine, Utrecht University, Utrecht, Netherlands; [4]Réanimation Médicale, Hôpital Saint-Antoine, Paris, France; [5]Department of anaestesia and intensive care, University of Bergen, Bergen, Norway
**Correspondence:** B. Wernly

*Intensive Care Medicine Experimental* 2019, **7(Suppl 3):**000660

**INTRODUCTION.** Mortality of old intensive Patients (80 years and older; VIP) admitted to an intensive care unit (ICU) for various admission diagnoses is high, and morbidity causes significant health costs and leads to human suffering in survivors. Risk stratification is crucial in VIPs to optimize outcomes. Established clinical scores including Sequential Organ Failure Assessment score (SOFA) score are available to stratify risk. However, these scores were not developed for VIPs. Assessing frailty through clinical assessment might help, and the Clinical Frailty Scale (CFS) has been successfully used to risk-stratify elderly patients. However, patients after elective intervention versus after acute intervention might differ significantly with regards to characteristics, outcomes, and predictors of outcomes.

**OBJECTIVES.** We evaluated differences between patients admitted to intensive care unit (ICU) after elective versus acute intervention in a multinational cohort of very old patients (≥80 years; VIP; NCT03134807). Differences in baseline characteristics, outcomes, and predictors of mortality, with particular emphasis on frailty, were assessed.

**METHODS.** In total, 5063 VIPs were included in this analysis, 922 were admitted after elective surgery or intervention, 4141 acutely, with 402 after acute surgery. Differences were assessed using Mann-Whitney-U test and Wilcoxon test in continuous variables. Associations with mortality were determined by univariate and multivariable logistic regression. We further matched 296 patients admitted after acute intervention for age, sex, frailty and SOFA score to 296 patients admitted after elective intervention.

**RESULTS.** Compared to patients admitted after acute intervention, patients admitted after elective intervention suffered less often from frailty as defined as CFS (28% vs. 46%; p<0.001), evidenced lower SOFA scores (4±5 vs. 7±7; p<0.001). Presence of frailty (CFS >4) was associated with significantly increased mortality both in elective surgery patients (7% vs 12%; p=0.01), in acute surgery (7% vs 12%; p= 0.02).

In the matched cohorts both ICU mortality (mean difference 5.07%; 9.37-0.76%; p=0.03) and 30-day mortality (mean difference 10.12%; 3.58-16.65%; p<0.01) was lower in patients admitted after elective internvention.

**CONCLUSION.** In VIPs, patients admitted after acute intervention were compared to patients admitted after elective intervention clinically less sick, younger and less frail as assessed by CFS. Admission after acute intervention was an independent predictor of mortality even after correction for relevant confounders and in a matched-pair analysis. Frailty assessed by CFS was useful for risk prediction and independently associated with 30-day mortality both in VIPs admitted after elective and acute intervention.

1. Submitted on behalf of the VIP1 study group

**001121**

**Low versus high blood pressure targets after acute myocardial infarction and cardiac arrest**

P. Jakkula[1], K. Ameloot[2], C. De Deyne[3], J. Dens[2], S. Janssens[4], M. Reinikainen[5], V. Pettilä[1], J. Hästbacka[1], M. Skrifvars[1]
[1]Perioperative, intensive care and pain medicine, University of Helsinki and Helsinki University Hospital, Helsinki, Finland; [2]Department of cardiology, Ziekenhuis Oost-Limburg, Genk, Belgium; [3]Department of anaesthesiology and critical care medicine, Ziekenhuis Oost-Limburg, Genk, Belgium; [4]Department of cardiology, University Hospitals Leuven, Leuven, Belgium; [5]Department of anaesthesiology and intensive care, Kuopio University Hospital, Kuopio, Finland
**Correspondence:** P. Jakkula
*Intensive Care Medicine Experimental* 2019, **7(Suppl 3):**001121

**INTRODUCTION.** In patients resuscitated from cardiac arrest (CA) with acute myocardial infarction (AMI), the optimal level of inotropic and vasopressor support is unknown. While overuse may increase myocardial oxygen consumption and induce arrhythmias, diastolic hypotension may reduce coronary perfusion and increase infarct size.

**OBJECTIVES.** We aimed to determine the optimal mean arterial pressure (MAP) in AMI patients after CA.

**METHODS.** We performed a pooled analysis of post-CA AMI patients randomised in the Neuroprotect (NCT02541591) [1] and COMACARE (NCT02698917) [2] trials to either MAP 65 mmHg or MAP 80/85-100 mmHg targets for the first 36 hours after ICU admission. AMI was defined as either ST-elevations in the electrocardiogram at hospital admission or identification of a clear culprit lesion (a coronary lesion with at least 70% stenosis and the presence of characteristics of plaque disruption) in coronary angiography performed within 2 hours after admission.

**RESULTS.** Out of 235 patients originally included in both trials, 120 patients qualified for our definition of AMI. Of these, 58 patients were randomised to the MAP 80/85-100 mmHg group and 62 patients to the MAP 65 mmHg group. Patients assigned to the higher MAP target received significantly higher doses of norepinephrine ($p$=0.004) and dobutamine ($p$=0.01) and had significantly higher MAP ($p$<0.001) and diastolic blood pressure ($p$<0.001) levels. Myocardial infarct size, as quantified by the area under the 72-hour high-sensitivity troponin T (HS-TnT) curve, was significantly lower in patients assigned to the higher MAP target (median [IQR] 73 μg [24-136 μg] vs 109 μg [46-317 μg], $p$=0.01). Additional inotropic and vasopressor support did not increase the risk of a new CA (8/58 [14%] in the higher MAP group vs. 9/61 [15%] in the lower MAP group, OR 0.92 [95% CI 0.33-2.58], $p$=0.88) or new onset atrial fibrillation (4/58 [7%] in the higher MAP group vs. 4/61 [7%] in the lower MAP group, OR 1.05 [95% CI 0.25-4.43], $p$=0.94).

**CONCLUSION.** Myocardial infarct size, as measured with HS-TnT, remained significantly smaller in the group targeting MAP between 80/85-100mmHg. The risk for re-arrest or arrhythmia was not increased.

**REFERENCE(S)**

1. Ameloot K, De Deyne C, Eertmans W, et al (2019) Early goal-directed haemodynamic optimization of cerebral oxygenation in comatose survivors after cardiac arrest: the Neuroprotect post-cardiac arrest trial. European Heart Journal 369:2197–11. doi: 10.1093/eurheartj/ehz120
2. Jakkula P, Pettilä V, Skrifvars MB, et al (2018) Targeting low-normal or high-normal mean arterial pressure after cardiac arrest and resuscitation: a randomised pilot trial. Intensive Care Med 44:2091–2101. doi: 10.1007/s00134-018-5446-8

**000436**

**Effect of hyperbaric oxygen therapy in preventing delayed neurological sequelae in patients with carbon monoxide poisoning: a multicenter, prospective, observational study in Japan**

M. Fujita[1], K. Kaneda[2], S. Suzuki[3], S. Wakai[4], S. Kikuta[5], S. Sasaki[6], N. Hattori[7], K. Yagishita[8], K. Kuwata[9], R. Tsuruta[10]
[1]Advanced medical emergency and critical care center, Yamaguchi University Hospital, Ube, Yamaguchi, Japan; [2]Advanced medical emergency and critical care center, Yamaguchi University Hospital, Ube, Japan; [3]Department of emergency medicine, Kameda Medical Center, Kamogawa, Japan; [4]Department of emergency and critical care medicine, Tokai University School of Medicine, Isehara, Japan; [5]Department of emergency and critical care medicine, Hyogo Emergency Medical Center, Kobe, Japan; [6]Advanced medical emergency department and critical care center, Japan Red Cross Maebashi Hospital, Maebashi, Japan; [7]Department of emergency and critical care medicine, Chiba University Graduate School of Medicine, Chiba, Japan; [8]Hyperbaric medical center, Tokyo Medical and Dental University, Bunkyo City, Japan; [9]Division of medicine, Japan Self Defense Forces Hospital Yokosuka, Yokosuka, Japan; [10]Acute and general medicine, Yamaguchi University Graduate School of Medicine, Ube, Japan
**Correspondence:** M. Fujita
*Intensive Care Medicine Experimental* 2019, **7(Suppl 3):**000436

**INTRODUCTION.** Hyperbaric oxygen (HBO2) therapy is thought to be essential for preventing delayed neurological sequelae (DNS) in patients with carbon monoxide (CO) poisoning, based on the results of a randomized controlled trial (RCT) reported by Weaver et al. [1]. However, the beneficial effect of HBO2 therapy remains debated because subsequent reports have been contradictory [2].

**OBJECTIVES.** To identify the appropriate clinical treatment for acute CO poisoning in Japan and to clarify the effects of HBO2 therapy in the acute phase of CO poisoning.

**METHODS.** We conducted a multicenter, prospective, observational study of acute CO poisoning in Japan, the "COP-J Study". Patients with acute CO poisoning were enrolled in the study. The treatments, including HBO2 therapy, were performed according with each institution's policy, and about 30% of institutions did not administer HBO2 therapy [3]. The primary endpoint was the onset of DNS within 2 months of CO exposure. DNS was defined as cognitive dysfunction after an improvement in disturbed consciousness.

**RESULTS.** A total of 311 patients were registered form 57 institutions and 255 were analyzed in this study. Of these patients, 171 received HBO2 therapy (HBO2 group) and 84 did not (NBO2 group). The mean ages were 49 ± 19 and 54 ± 22 years in the HBO2 and NBO2 groups, respectively (P = 0.063). There were no significant differences between the HBO2 and NBO2 groups in the estimated exposure time (202 ± 256 and 181 ± 376 min, respectively; P = 0.605) or the time between exposure and arrival at hospital (279 ± 350 and 240 ± 382 min, respectively, P = 0.420). The carboxyl hemoglobin concentration upon arrival did not differ between the HBO2 and NBO2 groups (18.6 ± 11.3% and 18.9 ± 10.1%, respectively; P = 0.831). HBO2 therapy was performed once, twice, or three times within the first 24 h in 11.1%, 30.4%, and 56.1% of patients in the HBO2 group, respectively. The treatment pressure in the first HBO2 session was 2.8

ATA (51.6% of patients), 2.0 ATA (37.3%), 2.5 ATA (8.5%), or other (2.6%). The number of patients with DNS was 13 (7.6%) in the HBO2 group and three (3.6%) in the NBO2 group (P = 0.212).

**CONCLUSION.** In this study, the total incidence of DNS was 6.3% in patients with acute CO poisoning, which is lower than in previous reports [1,2]. The superiority of HBO2 therapy over NBO2 therapy in terms of the prevention of DNS was not demonstrated. Furthernore, the clinical practice of HBO2 therapy varied in this study. Further investigations are required to clarify the effect of HBO2 therapy on the prevention of DNS after CO poisoning.

**REFERENCE(S)**

[1] Weaver LK, Hopkins RO, Chan KJ, et al. Hyperbaric oxygen for acute carbon monoxide poisoning. N Engl J Med. 2002; 347: 1057-1067.
[2] Annane D, Chadda K, Gajdos P, et al. Hyperbaric oxygen therapy for acute domestic carbon monoxide poisoning: two randomized controlled trials. Intensive Care Med. 2011; 3: 486–492.
[3] Fujita M, Oda Y, Kaneda K, et al. Variability in Treatment for Carbon Monoxide Poisoning in Japan- A multicenter retrospective survey. Emerg Med Int. 2018; 2018: 2159147.

### 001246

#### SAPS 3 performance in cancer patients admitted to an intensive care unit in Brazil

L. Taniguchi, E. Siqueira
Intensive care unit, Hospital Sirio Libanes, Sao Paulo, Brazil
**Correspondence:** L. Taniguchi
*Intensive Care Medicine Experimental* 2019, **7(Suppl 3):**001246

**INTRODUCTION.** Simplified Acute Physiology Score 3 (SAPS 3) is the most accurate prognostic model for Brazil [1]. However, recent evaluation of its performance in solid cancer patients has not been performed in our country.

**OBJECTIVES.** To compare the performance of SAPS 3 in patients with and without solid cancer admitted to the intensive care unit (ICU) of a comprehensive oncological hospital in Brazil.

**METHODS.** Retrospective cohort analysis of Epimed® administrative database of adult patients (>18 years) first admission to the intensive care unit (ICU) of Hospital Sírio Libanês (São Paulo, Brazil) from 2012 to 2016. Patients were categorized according to the presence of solid cancer at ICU admission. Hematological patients were excluded. We evaluated discrimination using the area under the receiver operating characteristic curve (AUROC) and the agreement between observed and expected mortality rates (calibration) using the calibration belt.

**RESULTS.** We studied 7,390 patients (age 66.1±18.4 years, 54.1% male, SAPS 3 score 41.6± 13.3, 41.6% had cancer, 12.1% died during hospitalization). Cancer patients were younger compared to non-cancer patients (62.6±16.5 years vs 68.7±19.3 years respectively, p<0.001), had lower SAPS 3 (40.9±14 vs 42.1±12.8 respectively, p<0.001), were admitted more frequently after elective surgery (60.6% vs 24.9%, p<0.001), but had higher hospital mortality (14.1% vs 10.6%, p<0.001). SAPS 3 discrimination was better for cancer patients (AUROC = 0.85) than for non-cancer patients (AUROC = 0.79) (p < 0.001). After we applied the calibration belt, we observed that in cancer patients the SAPS 3 match the average observed rates for the confidence level of 95%. In non-cancer patients the SAPS 3 overestimated mortality in those with low-middle risk (figure).

**CONCLUSION.** SAPS 3 has different performances in cancer and non-cancer patients in our single-center cohort.

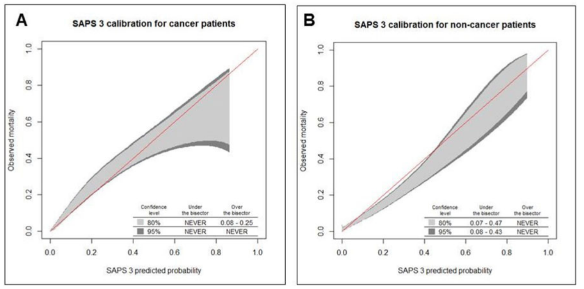

**Fig. 1 (abstract 001246).** See text for description

## TEM - New insights in cardiac arrest management and emergency medicine

### 000439

#### Effect of standard therapy plus plasmapheresis or only standard therapy on serum pseudo-cholinesterase and plasma erythrocyte acetyl cholinesterase levels in critically ill patients with organophosphate poisoning: Randomized controlled, open-label, clinic

GC. Sezgin[1], H. Sipahioglu[2], R. Coskun[2], K. Gundogan[3], S. Temel[2], M. Sungur[2], M. Güven[2]
[1]Department of internal medicine, division of gastroeneterology, Erciyes University, School of Medicine, Kayseri, Turkey; [2]Department of internal medicine, division of intensive care, Erciyes University, School of Medicine, Kayseri, Turkey; [3]Department of internal medicine, division of intensive care, Erciyes University, School of medicine,, Kayseri, Turkey
**Correspondence:** K. Gundogan
*Intensive Care Medicine Experimental* 2019, **7(Suppl 3):**000439

**INTRODUCTION.** Organophosphates (OP) have been used commonly worldwide as insecticides. OP intoxication is almost a public health problem in some countries leading to significant morbidity and mortality. Diagnosis and treatment have many difficulties.

**OBJECTIVES.** The aim of the study was to determine the effect of standard therapy plus plasmapheresis or only standard therapy on serum pseudo-cholinesterase (PChE) and plasma erythrocyte cholinesterase (AChE) levels in critically ill patients with OP poisoning.

**METHODS.** This study was performed prospectively Medical Intensive Care Unit (ICU). OP poisoning patients above 18 years of age were included into the study. Patients were randomized into two groups; Study (intervention) group received standard therapy plus plasmapheresis and control (standard) group received only standard therapy.

**RESULTS.** We enrolled 40 patients (Intervention group: 21, standard group: 19 patients). The mean age was 37±16 years. The mean APACHE II score was 9±5. The most common cause OP intoxication compound was dichlorvos. Serum PChE values of all patients included in the study were 482.5 (177-3883) u / L at baseline, 3723 (227-7078) u / L after plasmapheresis, 1529 (174-7078) u / L on 2nd day, 1956 (181-10918) u / L on 3rd day, 2206 (161-9993) u / L on 4th day, 1258 (178-8464) u / L on 5th day, and 2590 (121-10597.00) u / L at the time of ICU discharge.Serum PChE values of the intervention group and standard group patients were compared in the first 5 days and the last day of intensive care hospitalization. No statistically significant difference was found between the two groups (p> 0.05). Plasma erythrocyte AChE values of all patients included in the study were 1.9050 (0.12- 4.85) u / mL on admission, 2.5300 (0.06-4.47) u / mL after plasmapheresis, 2.18 (0.01-4.27) u / mL on day 2, 2.13 (0.07-4.30) u / mL on day 3, 2.015 (0.07-4.24) u / mL on day 4, 2.18 (0.12-4.19) u / mL on day 5 and 1.90 (0.09-4.37) u / mL at the time of ICU discharge. Plasma erythrocyte AChE values of the intervention group and standard group patients were compared on the daily from the admission to intensive care of patients, the first 5 days and the last day of the intensive care unit stay. No statistically significant difference was found between the two groups (p > 0.05).

**CONCLUSION.** As a result of this study, in addition to standard treatment, plasmapheresis therapy did not show any additional effect on serum PChE and erythrocyte AChE levels compared to standard treatment.

1. This work was supported Erciyes University Scientific Research Committee by TT-07-27.

### 001191

#### EEG reactivity testing for prediction of good outcome in patients after cardiac arrest

M. Admiraal[1], J. Horn[2], C. Hoedemaekers[3], J. Hofmeijer[4], M. Van Putten[4], M. Schultz[5], A. Van Rootselaar[6]

[1]Intensive care unit, Academic Medical Centre, Amsterdam, Netherlands; [2]Intensive care, Academic Medical Centre, Amsterdam, Netherlands; [3]Intensive care, Radboud University Medical Center, Nijmegen, Netherlands; [4]Myra, University of Twente / Universiteit Twente, Enschede, Netherlands; [5]Intensive care, Amsterdam University Medical Centers, University of Amsterdam, Amsterdam, Netherlands; [6]Neurology and clinical neurophysiology, Academic Medical Centre, Amsterdam, Netherlands

**Correspondence:** J. Horn

*Intensive Care Medicine Experimental* 2019, **7(Suppl 3):**001191

**INTRODUCTION.** Prognostication after cardiac arrest (CA) remains challenging (Rossetti 2016). Traditionally, most emphasis was put on prediction of poor outcome, but with the introduction of advanced supportive therapies, such as extra corporeal membrane oxygenation, it becomes increasingly important to identify patients with a chance of good neurological recovery. For prediction of good outcome after CA, presence of EEG reactivity (EEG-R) has been proposed as a valuable marker, but the relation to EEG background pattern has not been described. The EEG background pattern itself between 12 and 24h post CA is already a reliable predictor of good outcome (Hofmeijer 2015).

**OBJECTIVES.** In this study, the primary aim was to define the *additional* prognostic value of EEG-R per EEG background pattern. Secondary aims were to explore whether time after CA and sedative medication influence the prognostic value of EEG-R and to determine optimal methods of EEG-R testing

**METHODS.** A post-hoc analysis of a prospective cohort study. EEG-R was tested twice a day, using a strict protocol of different stimuli. Good outcome was defined as a Cerebral Performance Category of 1-2 within 6 months. The additional value of EEG-R per EEG background pattern was evaluated using the diagnostic odds ratio (DOR). Furthermore, prognostic value (sensitivity and specificity) of EEG-R was investigated in relation to the timing after CA, sedative medication, different stimuli, and repeated testing.

**RESULTS.** Patients with a discontinuous (< 50% suppression) normal voltage (> 20 uV) background pattern and reactivity were eight times more likely to have a good outcome (DOR 8.0, 95%-CI 1-64, p-value < 0.05). In continuous normal voltage background patterns, DOR of EEG-R was 3.4 (95%-CI 1-12, p-value 0.06). (108 patients, with and without sedation, 12-24h after CA). EEG-R was not observed in other background patterns. In 119 patients with (dis)continuous normal voltage background, prognostic value was highest in sedated patients (sensitivity 81.3% (95%-CI 70.7-89.4), specificity 59.5% (95%-CI 42.1-75.2)), irrespective of time after CA. EEG-R induced by handclapping and sternal rubbing, especially when combined, had highest prognostic value for good outcome. Repeated EEG-R testing increased sensitivity from 65% to 89% with a decrease in specificity from 71% to 46%.

**CONCLUSION.** These results suggest that EEG-R testing is of additional value for prediction of good outcome in patients with a (dis)-continuous normal voltage background pattern. The best stimuli were clapping and sternal rubbing.

REFERENCE(S)

1. Rossetti AO, Rabinstein AA, Oddo M. Neurological prognostication of outcome in patients in coma after cardiac arrest. Lancet Neurol. 2016;15:597–609
2. Hofmeijer J, Beernink TMJ, Bosch FH, Beishuizen A, Tjepkema-Cloostermans MC, van Putten MJAM. Early EEG contributes to multimodal outcome prediction of postanoxic coma. Neurology. 2015;85:137–143.

### 000580

#### A relationship between serum osmolality, electrolyte and neurologic outcome in patients with post-cardiac arrest syndrome

M. Lee[1], GJ. Suh[2], WY. Kwon[3], YH. Jo[4], J. Shin[5], KS. Kim[3], YS. Jung[1], T. Kim[3], SM. Shin[3]

[1]Emergency medicine, critical care medicine, Seoul National University Hospital, Seoul, Republic of Korea; [2]Emergency medicine, Seoul National University, Seoul, Republic of Korea; [3]Emergency medicine, Seoul National University Hospital, Seoul, Republic of Korea; [4]Emergency medicine, Seoul National University Bundang Hospital, Seongnam-si, Republic of Korea; [5]Emergency medicine, Seoul Metropolitan Government-Seoul National University Boramae Medical Center, Seoul, Republic of Korea

**Correspondence:** M. Lee

*Intensive Care Medicine Experimental* 2019, **7(Suppl 3):**000580

**INTRODUCTION.** Normal saline is routinely used in post-cardiac arrest patients based on the belief that management in sepsis setting will be similarly beneficial in cardiac arrest setting. (1) However, there is no established evidence supporting such management in cardiac arrest patients. It is known that brain edema is a key prognostic factor in post-cardiac arrest syndrome (PCAS). (2-5, 7, 8) A clinical study have shown that hypertonic saline use during cardiopulmonary resuscitation might be beneficial to patients with PCAS. (6) But hypertonic fluid could be harmful to those patients after vasogenic edema occurs. (2, 3)

**OBJECTIVES.** Therefore, the aim of this study is to evaluate the correlation between electrolyte or osmolality alteration and neurologic outcome of PCAS patients, thereby providing evidence to the optimal fluid management in post-cardiac arrest care.

**METHODS.** This is a retrospective observational study based on a multicenter prospective cohort registry of out-of-hospital cardiac arrest patients from December 2013 to February 2018. 653 patients were finally included in this study, excluding those with poor baseline cerebral performance category (CPC 3, 4, or 5) and those with incomplete data. Outcome variables were defined as 28-day CPC, 6-month CPC, and 1-year CPC after discharge. We conducted univariate and multivariate logistic regression analyses to find the relationship between serum electrolyte at 0, 24 hours after return of spontaneous circulation (ROSC), calculated osmolality, and main outcomes. The final multivariate logistic regression model included osmolality at 24 hours after ROSC, total low-flow time, total no-flow time, witnessed arrest, bystander CPR, ROSC achieved by prehospital emergency medical service, sequential organ failure assessment (SOFA) score, initial ECG rhythm after ROSC and initial light reflex after ROSC.

**RESULTS.** 139 [35%] patients had good CPC (1 or 2) at 28-day after discharge otherwise, 514 [65%] patients had poor CPC (3, 4 or 5). Multivariate logistic regression analysis showed that high serum osmolality at 24 hours after ROSC had significant correlation with poor neurologic outcome at 28-day after discharge (adjusted odds ratio [OR], 1.04; 95% confidence intervals [CI], 1.00-1.07; p value, 0.03). Similar results were obtained in the multivariate model using neurologic outcome at 6 months and 1 year.

**CONCLUSION.** High serum osmolality at 24 hours after ROSC was independently associated with short-term and long-term poor neurologic outcome in patients with post-cardiac arrest syndrome.

REFERENCE(S)

1. Web-based Integrated 2015 & 2018 American Heart Association Guidelines for CPR and ECC; Part 8: Post-Cardiac Arrest Care
2. Brain oedema in focal ischaemia: molecular pathophysiology and theoretical implications. Lancet Neurol. 2007 Mar;6(3):258-68.
3. Cerebral Edema After Cardiopulmonary Resuscitation: A Therapeutic Target Following Cardiac Arrest? Neurocrit Care. 2018 Jun;28(3):276-287.
4. Cardiac arrest-induced regional blood-brain barrier breakdown, edema formation and brain pathology: a light and electron microscopic study on a new model for neurodegeneration and neuroprotection in porcine brain. J Neural Transm (Vienna). 2011 Jan;118(1):87-114.
5. Abnormalities of the blood-brain barrier in global cerebral ischemia in rats due to experimental cardiac arrest. Acta Neurochir Suppl (Wien). 1994;60:274-6.

6. Hypertonic saline infusion during resuscitation from out-of-hospital cardiac arrest: a matched-pair study from the German Resuscitation Registry. Resuscitation. 2014 May;85(5):628-36.
7. Outcome prediction in patients after cardiac arrest: a simplified method for determination of gray-white matter ratio in cranial computed tomography. Clin Neuroradiol. 2015 Mar;25(1):49-54.
8. Prognostic value of reduced discrimination and oedema on cerebral computed tomography in a daily clinical cohort of out-of-hospital cardiac arrest patients. Resuscitation. 2015 Jul;92:141-7.

### 001125

#### Neurological Pupil Index correlates with other prognostic markers after cardiac arrest

E. Di Bernardini[1], M. Oddo[2], C. Sandroni[3], G. Citerio[4], JF. Payen[5], J. Horn[6], M. Rundgren[7], A. Cariou[8], C. Storm[9], P. Stammet[10], J. Creteur[1], FS. Taccone[1]
[1]Department of intensive care, Erasme Hospital, Université Libre de Bruxelles, Brussels, Belgium; [2]Department of intensive care medicine, Lausanne University Hospital, Lausanne, Switzerland; [3]Department of anesthesiology and intensive care, Catholic University School of Medicine, Rome, Italy; [4]School of medicine and surgery, University of Milano-Bicocca, Monza, Italy; [5]Department of anesthesia and critical care, Grenoble Alpes University Hospital, Grenoble, France; [6]Intensive care, Academic Medical Centre, Amsterdam, Netherlands; [7]Department of clinical sciences, anesthesiology and intensive care medicine, Skåne University Hospital, Lund University, Lund, Sweden; [8]Medecine intensive reanimation, Hospital Cochin, Paris, France; [9]Department of internal medicine, nephrology and intensive care, Charité-University, Berlin, Germany; [10]Medical and health department, National Fire and Rescue Corps, Luxembourg, Luxembourg
**Correspondence:** E. Di Bernardini
*Intensive Care Medicine Experimental* 2019, **7(Suppl 3):**001125

**INTRODUCTION.** Neurological prognostication after cardiac arrest (CA) is a complex challenge for physicians and requires a multimodal approach. Neurological Pupil Index (NPI) derived from automated pupillometry and assessed at 24 hours after the event has been recently shown a high sensitivity to predict unfavorable neurological outcome after CA. Nevertheless, it is unclear how NPI correlates with other predictors of brain injury in this setting.
**OBJECTIVES.** To assess the correlation of NPI with electroencephalography (EEG) background, serum neuron-specific enolase (NSE) and cortical response to somatosensory evoked potentials (SSEPs).
**METHODS.** Post hoc analysis of an international multicenter (n=10; n=456 patients) prognostic study on automated pupillometry in comatose post-CA patients. The primary study endpoint was the accuracy of NPI in predicting 3-month unfavorable neurological outcome, defined as Cerebral Performance Category (CPC) of 3-5 (severe disability, unresponsive wakefulness or death). The worst finding over the first 3 days after CA among EEG background (dichotomized as "continuous" vs. "'discontinuous"), cortical response to SSEPs (dichotomized as bilaterally absent (N20ABS) vs. "other"), and the highest NSE value were evaluated.
**RESULTS.** On a total of 456 included patients, 398 (87%) had at least one other prognostic tool assessed along with NPI. NPI were inversely correlated with NSE levels (n=228; r2=0.20; p<0.001). Furthermore, NSE values were significantly higher in patients with NPI ≤ 2 vs. those with NPI ≥ 4.0 (152 [113-415] vs. 31 [18-46] mg/L, respectively; p<0.001). NPI values were significantly lower in patients with a discontinuous EEG (n=137) (n=234 - 3.5 [1.4-4.0] vs. 3.8 [3.3-4.2]; p< 0.001) and in patients with N20ABS (n=63) than others (n=123 - 3.3 [1.0-3.7] vs. 3.6 [3.3-4.1]; p<0.001).
**CONCLUSION.** Our data corroborate previous findings indicating that impaired NPI is strongly associated with severe hypoxic-ischemic brain injury and unfavorable neurological outcome after CA.

### 001288

#### Active intrathoracic pressure regulation following hemorrhagic shock reduces vasopressor demand and improves systemic and cerebral hemodynamics

A. Metzger[1], P. Berger,[1], M. Lick,[2], K. Lurie,[3]
[1]Scientific affairs, Zoll Medical, St. Paul, United States of America; [2]Emergency, Hennepin Healthcare Institute, Minneapolis, United States of America; [3]Medicine, University of Minnesota, Minneapolis, United States of America
**Correspondence:** A. Metzger
*Intensive Care Medicine Experimental* 2019, **7(Suppl 3):**001288

**INTRODUCTION.** Following severe hemorrhage, proper response to circulatory dysfunction is essential to immediate survival and long-term outcome. Standard approach is replacement fluids and vasopressors to treat hypotension. No firm consensus as to type and volume of fluids exists, and hemodilution, clotting disruption, and increased bleeding may result. Vasopressor use also varies clinically with some reports documenting increased risk of mortality with use. The CirQPOD (Zoll Medical) is an active intrathoracic pressure regulation (a-IPR) device placed between the endotracheal tube and a ventilation source that generates negative intrathoracic pressure. It has previously been shown to improve systemic and cerebral hemodynamics.
**OBJECTIVES.** This study tested the hypothesis that post-hemorrhage a-IPR use would enhance hemodynamics while reducing vasopressor demand.
**METHODS.** 14 female farm pigs (38.8±1.3 kg) were bled 40% of circulating blood volume from a central arterial line over 15 minutes, followed by a 15-minute stabilization period. Animals were randomized to one of two treatment groups, one with continuous a-IPR therapy, the other without a-IPR therapy. In the a-IPR group, the CirQPOD was started at -5cmH2O and stepwise increased to a maintenance level of -10cmH2O over a 30-minute ramp-up period. At this point, a target mean arterial pressure (MAP) of 65 mmHg was achieved in both groups through controlled infusion of norepinephrine (NE), delivered to effect as needed, to the end of the 3-hour study. After 2 hours of device use, the device was ramped down for 30 minutes. Vasopressor demand and hemodynamic data were recorded throughout the study. All animals were anesthetized with 1.0% isofluorane throughout the study and ventilated with an FiO2 of 0.4 and a tidal volume of 8 ml/kg. Student's t-tests were used for statistical comparisons. Data are expressed as mean±SD.
**RESULTS.** MAP during the study was matched between groups (64.8±6.3 for NE only vs 67.8±4.5 mmHg for a-IPR) through careful dosing of vasopressor. Mean NE demand during the post-bleed period was significantly lower in the a-IPR group compared to NE only group (0.17±0.1 vs 0.04±0.04 ug/kg/min, p<0.05). Pulse pressure variation (PPV) was matched between groups at the end of the post-bleed stabilization period (27.6±6.0 vs 27.7±6.8%) but was lower in the a-IPR group throughout the study, significantly during a-IPR ramp-up from 0-30 minutes (30.1±8.2 vs 17.5±4.9%, p<0.001) and from 60-120 minutes (25.7±2.7 vs 17.7±2.7%, p<0.001). Cerebral perfusion pressure was also consistently higher in the a-IPR group throughout the study (49.4±6.8 vs 54.3±6.3mmHg), significantly during ramp-up from 0-30 minutes (41.2±4.9 vs 50.3±7.2mmHg, p<0.01).
**CONCLUSION.** Addition of a-IPR therapy post-hemorrhage reduced vasopressor demand, decreased PPV, and improved cerebral hemodynamics. Use of a-IPR has the potential to treat the hypotension and systemic circulatory disturbances associated with hemorrhagic shock without risk of hemodilution associated with fluid therapy and the complications associated with high dose norepinephrine use.

## CD - Haemodynamic monitoring

### 000118

#### Multi-centre clinical validation of a novel point of care microcirculation assessment tool - the POEM score

J. Watchorn[1], U. Goebel[2], J. Wollborn[2], M. Mccurdy[3], H. Fargaly[3], M. Gilani[3], J. Assadi[3], AR. Deitchman[3], DN. Naumann[4], SD. Hutchings[1]
[1]Critical care, King's College Hospital, Denmark Hill, London, UK, London, UK, United Kingdom; [2]Critical care, Uniklinik Freiburg, Freiberg, Germany; [3]Critical care, University of Maryland Medical Center, Baltimore, United States of America; [4]Surgery, Royal Centre For Defence Medicine, Birmingham, United Kingdom
**Correspondence:** J. Watchorn
*Intensive Care Medicine Experimental* 2019, **7(Suppl 3):**000118

**INTRODUCTION.** Microcirculatory impairment has an impact on outcome in a variety of disease states (1,2)and has been proposed as a therapeutic target, however interventional studies are hindered by time consuming off-line analysis. We recently described a 5-point ordinal scoring system, the Point Of carEassessment of the Microcirculation (POEM) score (3).

**OBJECTIVES.** To determine the reliability of the Point of Care Microcirculation tool when used at the bedside in critically ill patients.

**METHODS.** All adult intubated patients in critical care were eligible for inclusion using an emergency waiver of consent. A minimum of four standardized video clips were taken by an investigator using an incident dark field (IDF) video-microscope (Cytocam, Braedius Medical, NL) who subsequently calculated a POEM score and recorded the time taken. On completion a second investigator blinded to the results of the first repeated this process. At a later stage (>1 month) investigators re-scored their own and their co-investigators images. Scores were analysed using Cohens weighted Kappa score to provide intra-user, inter-user and test-retest reliability. A kappa statistic was also calculated for test-retest reliability for POEM score 1-3 against POEM score 4-5 which is the threshold for fluid administration used in an ongoing interventional study (Clinical Trials.gov ID).

**RESULTS.** 80 patients were recruited at 3 sites in 3 countries. Poor quality video assessments (cumulative Massey score >10) were excluded resulting in a loss of 15 (18%) of patients. Weighted kappa analysis showed good agreement ( (0.4-0.6 moderate, 0.61-0.8 substantial, 0.8-1 excellent(4)) in all circumstances: Intra-User 0.801 (95% CI 0.722-0.879), Inter-User 0.792(95% CI 0.719-0.861) and Test-Retest reliability 0.701(95% CI 0.589-0.812). Mean time to record videos and assess POEM scores was 7 min. (std deviation 03:38) Reliability of the POEM score used to potentially dichotomise a decision on fluid administration (POEM 1-3 vs 4,5) was also good, 0.726 (95% CI 0.637-0.815)

**CONCLUSION.** Point-of-care assessment of the microcirculation using IDF video microscopy and POEM scoring appears to be both quick and have good agreement within and between investigators in a real life clinical setting. This opens the possibility of using the score as a resuscitation end-point.

##### REFERENCE(S)

1. Hutchings SD, Naumann DN, Hopkins P, Mellis C, Riozzi P, Sartini S, et al. Microcirculatory Impairment Is Associated With Multiple Organ Dysfunction Following Traumatic Hemorrhagic Shock: The MICROSHOCK Study. Critical Care Medicine. Critical Care Medicine; 2018 Sep;46(9):e889–96.
2. Trzeciak S, McCoy JV, Phillip Dellinger R, Arnold RC, Rizzuto M, Abate NL, et al. Early increases in microcirculatory perfusion during protocol-directed resuscitation are associated with reduced multi-organ failure at 24 h in patients with sepsis. Intensive Care Med. Springer-Verlag; 2008 Dec;34(12):2210–7.
3. Naumann DN, Mellis C, Husheer SLG, Hopkins P, Bishop J, Midwinter MJ, et al. Real-time point of care microcirculatory assessment of shock: design, rationale and application of the point of care microcirculation (POEM) tool. Critical Care. Critical Care; 2016 Sep 29;:1–9.
4. McHugh ML. Interrater reliability: the kappa statistic. Biochem Med (Zagreb). Croatian Society for Medical Biochemistry and Laboratory Medicine; 2012;22(3):276–82.
5. Royal Centre For Defence Medicine, UK

### 000829

#### The clinical course and prognostic impact of sustained new-onset atrial fibrillation in critically ill patients: a multicenter prospective cohort study

T. Yoshida[1], S. Uchino[2]
[1]Intensive care unit, department of anesthesiology, Jikei University School of Medicine, Tokyo, Japan; [2]Intensive care unit, department of anaesthesiology, Jikei University School of Medicine, Tokyo, Japan
**Correspondence:** T. Yoshida
*Intensive Care Medicine Experimental* 2019, **7(Suppl 3):**000829

**INTRODUCTION.** Atrial fibrillation (AF) is the most common arrythmia in intensive care unit (ICU). Previous studies reported that development of new-onset AF was associated with poor outcomes. However, information of the clinical course after the AF onset is scarce. Moreover, it is unknown whether sustained new-onset AF contributes to poor outcomes.

**OBJECTIVES.** The primary goal is to describe the clinical course after the AF onset. The secondary goal is to determine whether the duration of sustained AF contributes to poor outcome.

**METHODS.** In a prospective cohort study in 32 ICUs in Japan, we enrolled patients who were admitted to the participating ICUs from 2017 to 2018 and developed new-onset AF during their ICU stay. Following information was collected for the study patients: demographics, medical history, physiological variables before and after the AF onset, AF duration, treatment for AF, mortality and the incidence of ischemic stroke.

**RESULTS.** Of the 14,348 adult ICU patients excluding post-cardiac surgery, 423 patients (2.9%) with new onset AF were enrolled. Medication for rhythm control, rate control and anticoagulation were administered to 30%, 61% and 41% of all the new-onset AF patients, respectively, during the initial episode of AF. Eighty-six patients (20%) died within 30 days after the AF onset and 19 patients (4.5%) experienced in-hospital ischemic stroke. Patients with initial sustained AF longer than 48 hours (long AF) had increased 30-day mortality compared with those with initial sustained AF shorter than 48 hours (15% vs. 28%, p=0.009). Long AF was independently associated with 30-day mortality (adjusted OR 2.13; 95% CI, 1.17–3.85; p = 0.013) in multivariable regression analysis. Repeating multivariable regression analysis for different duration of the new on-set AF (6,12, 18, 24, 30, 36, 42, 48, 72, 96, 120, 144, 168 hours), the longer initial AF duration had greater odds ratios for 30-day mortality. Restricted cubic spline function analysis also showed a dose-response relationship between the initial AF duration and 30-day mortality.

**CONCLUSION.** The duration of initial sustained AF was associated with 30-day mortality by various statistical analyses. Our findings provide strong rationale for further research assessing treatment for new-onset AF in critically ill patients.

### 000851

#### Hemodynamic monitoring parsimony: minimal information for rapid hemorrhage detection

A. Wertz[1], G. Clermont[2], A. Dubrawski[1], M. Pinsky
[1]Robotics institute, auton lab, Carnegie Mellon University, Pittsburgh, United States of America; [2]School of medicine, University of Pittsburgh, Pittsburgh, United States of America
**Correspondence:** A. Wertz
*Intensive Care Medicine Experimental* 2019, **7(Suppl 3):**000851

**INTRODUCTION.** Early detection of occult hemorrhage is important for timely treatment. Yet, availability of sensing modalities may be minimal and knowledge of physiologic baseline often unknown.

**OBJECTIVES.** To characterize the differences in bleeding detection performance of predictive models trained with vital sign waveform data derived from different monitoring modalities, referenced with a personalized baseline or not.

**METHODS.** Waveform data (250Hz) from non-invasive monitoring (NIM), arterial (ART), central venous (CVC) and pulmonary artery (PAC) catheters were collected from 38 Yorkshire pigs during a stable 30-min period followed by bleeding at 20 mL/min until a mean arterial pressure of 30 mmHg. Waveform data as well as beat-to-beat (B2B) parameters were used to compute various secondary measures (features) in a rolling window including basic statistics, entropy, power spectral density, and others. Time series data were either referenced to a personalized stable baseline or not (universal baseline). Classification models were trained to discriminate between stable and bleeding states, and the Activity Monitoring Operating Characteristic (AMOC) curve was used to characterize the tradeoff between time to detection (TTD) of bleeding and false positive rate (FPR). Nested models were trained to evaluate an increasingly rich sensor data, starting from NIM, then adding ART, CVC, PAC, ART+CVC and ART+PAC. The models were optimized and validated in a leave-one-out cross validation framework.

**RESULTS.** Increasing availability of sensing modalities and referencing to personalized baseline decreased TTD of bleeding at lower FPR (Fig. 1). Without personalized baseline all but NIM and CVC based models detected bleed <10min at a 1 in 100 FPR. With a personalized baseline, all models detect bleed <10min. PAC and ART+-PAC detect bleed at 3min.

**CONCLUSION.** The ability of hemodynamic monitoring to detect bleed onset improves with the availability of a personalized baseline and more intense monitoring. Excellent bleed detection can be achieved by all invasive monitors if referenced to personalized baselines whereas NIM performs less well.

1. NIH GM126811

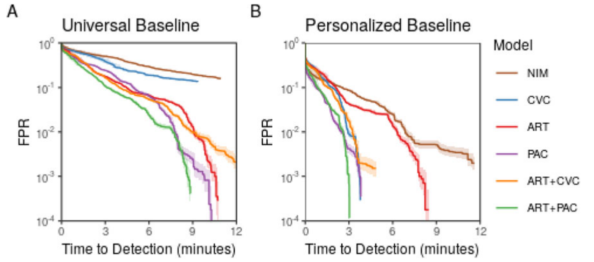

**Fig. 1 (abstract 000851).** Figure 1 - AMOC curves showing tradeoff between false positive rate (FPR) and detection latency (time to detection) for the the models trained using either a universal (A) or personalized baseline (B).

### 000880

#### Atrial Fibrillation after Cardiac Surgery; Impact of Dexmedetomidine

A. Petroulaki, G. Lazopoulos, P. Kalogerakos, S. Palioudakis, M. Kiparakis, D. Pavlopoulos, C. Ioannou
Cardio-thoracic surgery division, University Hospital of Heraklion, Iraklio, Greece
**Correspondence:** A. Petroulaki
*Intensive Care Medicine Experimental* 2019, **7(Suppl 3):**000880

**INTRODUCTION.** Atrial fibrillation is the most common arrhythmia following cardiac surgery, with a percentage up to 50%. It is associated with increased morbidity and mortality. Dexmedetomidine is a highly selective a2 adrenergic agonist. Considering its sympathoinhibitory action, dexmedetomidine could be associated with postoperative atrial fibrillation reduction.

**OBJECTIVES.** The present study aims to evaluate the effect of post-operative dexmedetomidine on the incidence of atrial fibrillation after cardiac surgery.

**METHODS.** This prospective, randomized, controlled, clinical trial was carried out at the cardiac surgery unit of a tertiary care university hospital. 164 patients, undergoing on pump cardiac surgery, were evaluated. 21 were excluded due to preoperative atrial fibrillation or flutter. 143 patients were enrolled. After arrival to the cardiac intensive care unit, patients were randomized in two groups, dexmedetomidine and control group. Patients in dexmedetomidine group received an infusion of dexmedetomidine (0.7 mg/kg/h) during the early postoperative period. Primary endpoint was the incidence of postoperative atrial fibrillation within the first three postoperative days.

**RESULTS.** There were no differences in atrial fibrillation risk factors between the two groups. There were also no differences in postoperative parameters except for catecholamine (44.4% vs 55.6%, p=0.675) and beta-blocker use (45.2% vs 54.8%, p=0.967) in dexmedetomidine and non-dexmedetomidine group respectively. Our results revealed that the dexmedetomidine group was associated with an increased incidence of atrial fibrillation compared to the control. Prevalence of atrial fibrillation in dexmedetomidine group (n=70) was 26 patients (37.1%) and non-dexmedetomidine group (n=73) 16 patients (21.9%). Subgroup analysis indicated that postoperative augmented diuresis and low potassium levels (p<0.001) have an increased influence on occurrence of atrial fibrillation in patients after cardiac surgery.

**CONCLUSION.** In our study, dexmedetomidine use after cardiac surgery was associated with a higher incidence of postoperative atrial fibrillation. More studies are required to determine the dexmedetomidine effect on atrial fibrillation after cardiac surgery.

### 000895

#### Non-invasive blood pressure measurements versus invasive blood pressure measurements in critically ill patients with shock

L. Tijsma[1], T. Kaufmann[1], R. Wiersema[2], B. Hiemstra[1], F. Keus[2], ICC. Van Der Horst[2], Sics Study Group[2]
[1]Anaesthesiology, University Medical Center Groningen, Groningen, Netherlands; [2]Critical care, University Medical Center Groningen, Groningen, Netherlands
**Correspondence:** R. Wiersema
*Intensive Care Medicine Experimental* 2019, **7(Suppl 3):**000895

**INTRODUCTION.** Invasive blood pressure monitoring is recommended in patients admitted to the ICU with shock (1). Non-invasive blood pressure measurements are still often used but may be inaccurate and may lead to erroneous interpretations in patients receiving vasoactive medication in the ICU (2).

**OBJECTIVES.** We aimed to compare mean arterial pressure (MAP) measurements of blood pressure using non-invasive (MAPNi) and invasive (MAPi) methods in a prospective cohort of critically ill patients, both with and without vasoactive medication.

**METHODS.** This was a post-hoc analysis of the SICS-I study, in which all patients acutely admitted to the ICU were included (3). The invasive blood pressure, preferably via an indwelling radial artery catheter, and non-invasive blood pressure, via oscillometric brachial cuff, was measured within 24 hours of ICU admission. Linear correlation was assessed by Pearson coefficient and precision and agreement by Bland-Altman analysis. A difference of more than 10 mmHg was considered clinically inaccurate.

**RESULTS.** Of 1075 patients, 734 patients (68%) had both invasive and non-invasive blood pressure measurements. Overall, MAPi and MAPNi had strong positive linear correlation (r2=0.62, $p$ < 0.001) (fig 1) which decreased if patients were on vasoactive medication (n = 361, r2= 0.44, $p$ < 0.001) compared to patients not on vasoactive medication (n = 373, r2=0.64, $p$ <0.001). Bias was 1.0 (95% CI 0.2–1.7) and limits of agreement of all measurements ranged from -19.5 (95% CI -20.8– -18.2) to 21.4 (95%CI 20.1–22.7) (fig 2). The difference in MAP was acceptable in 69.1% of all measurements, in 71.7% of measurements in patients on vasopressors, and in 66.5% of measurements in patients not on vasopressors (table 1).

**CONCLUSION.** Non-invasive blood pressure measurements are not a reliable alternative for invasive intra-arterial measurements in critically ill patients, regardless of receiving vasoactive medication.

**REFERENCE(S)**

1) Cecconi M, De Backer D, Antonelli M, Beale R, Bakker J, Hofer C, et al. Consensus on circulatory shock and hemodynamic monitoring. Task force of the European Society of Intensive Care Medicine. Intensive Care Med. 2014 Dec 13;40(12):1795–815.
2) Kaur B, Kaur S, Yaddanapudi LN, Singh N V. Comparison between invasive and noninvasive blood pressure measurements in critically ill patients receiving inotropes. Blood Press Monit. 2019 Feb;24(1):24–9.
3) Hiemstra B, Koster G, Wiersema R, Hummel YM, van der Harst P, Snieder H, et al. The diagnostic accuracy of clinical examination for estimating cardiac index in critically ill patients: the Simple Intensive Care Studies-I. Intensive Care Med. 2019 Feb;45(2):190–200.

4) This research received no specific grant from any funding agency in any sector. Currently more data is being analyzed to elucidate and refine the outcomes of the SICS-I. In case of admission to the ESICM congress, revised results will be presented.

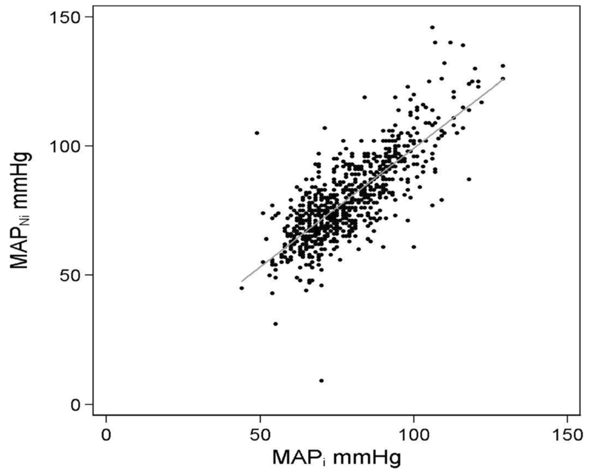

**Fig. 2 (abstract 000895).** See text for description

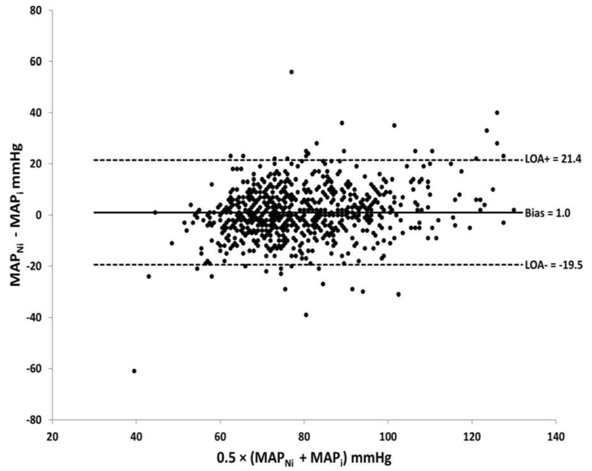

**Fig. 1 (abstract 000895).** See text for description

**Table 1 (abstract 000895).** See text for description

| | Bias (95% CI) | LOA + (95% CI) | LOA – (95% CI) | measurements within limits of agreement [n, (%)] | measurements with bias of ≤ 10 mmHg [n, (%)] |
|---|---|---|---|---|---|
| All patients (n = 734) | 1.0 (0.2 – 1.7) | 21.4 (20.1 – 22.7) | -19.5 (-20.8 - -18.2) | 95.8% | 507 (69.1%) |
| Vasopressors (n = 361) | 1.0 (-0.1 – 2.0) | 21.4 (19.5 – 23.2) | -19.4 (-21.3 – -17.6) | 94.5% | 259 (71.7%) |
| No vasopressors (n = 373) | 1.0 (-0.1 – 2.0) | 21.5 (19.7 – 23.4) | -19.6 (-21.5 – -17.8) | 95.4% | 248 (66.5%) |

## MEN - Metabolic modulation over time

### 000298

#### Plasma citrulline dynamics in intensive care patients

M. Padar[1], J. Starkopf[1], A. Forbes[2], J. Wernerman[3], O. Rooyackers[3], SM. Jakob[4], M. Hiesmayr[5], T. Gold[5], M. Poeze[6], D. Meesters[6], A. Reintam Blaser[7]

[1]Department of Anaesthesiology and Intensive Care, Tartu University Hospital, Tartu, Estonia; [2]Norwich medical school, University of East Anglia, Norwich, United Kingdom; [3]Department of clinical science, intervention and technology, Karolinska Institute, Stockholm, Sweden; [4]University clinic for intensive care medicine, Bern University Hospital, Bern, Switzerland; [5]Division of cardiac thoracic vascular anaesthesia and intensive care, Medical University of Vienna, Wien, Austria; [6]Department of trauma surgery, Maastricht University Medical Centre, Maastricht, Netherlands; [7]Department of anaesthesiology and intensive care, University of Tartu, Tartu, Estonia

**Correspondence:** M. Padar

*Intensive Care Medicine Experimental* 2019, **7(Suppl 3):**000298

**INTRODUCTION.** The amino acid citrulline (CIT) is of interest in various conditions characterized by reduced enterocyte mass or function. Decreased CIT levels have been shown in critical illness, sepsis and shock [1,2]. Less is known about daily dynamics of CIT values in a general ICU patient population.

**OBJECTIVES.** To describe CIT in ICU patients during their first week of stay.

**METHODS.** Consecutive adult patients in 5 ICUs were studied. Plasma was collected immediately after ICU admission and thereafter daily. Following storage at -80 °C, samples were deproteinized and analyzed for CIT, using liquid chromatography-mass spectrometry. Citrulline values between groups were compared using the SPSS Median Test for each day separately.

**RESULTS.** CIT was measured in 224 patients (median age 66.5, range 29-94 years; median APACHE II 18, IQR 11-23; 28 d mortality 10.3%). Median CIT value of all measurements was 19.5 (IQR 14.0-25.8) µmol/L, being higher in 28 d survivors [19.9 (IQR 14.7-26.2)] *vs* nonsurvivors [14.4 (IQR 11.8-22.9) µmol/L] independent of renal dysfunction. Median of the lowest individual CIT value was 16.3 (12.6-22.4) µmol/L and occurred on day 2 in 54% of patients. CIT values were lower in patients with sepsis (significant on days 1-4), emergency surgical patients (days 1 and 2) and those admitted after gastrointestinal surgery (days 1-4). CIT dynamics in all patients, in comparison of 28 d survivors *vs* nonsurvivors and related to early enteral/oral nutrition (within 48 h) and its success are shown in Figures 1-4.

**CONCLUSION.** A majority of patients had normal CIT values on admission to ICU, rapidly decreasing to marginally low normal levels that persisted over the first week. Lowest individual CIT value was reached most often on day 2. CIT increased with early and successful enteral or oral nutrition.

**REFERENCE(S)**

1. This study is a part of the iSOFA study (NCT02613000) endorsed by ESICM and funded by Fresenius Kabi and institutional grant from the Ministry of Education and Research of Estonia (IUT34-24).

2. Piton G, Capellier G. Biomarkers of gut barrier failure in the ICU. Curr Opin Crit Care. 2016. 22:152-160.
3. Wijnands KAP, Castermans TMR, Hommen MPJ, Meesters DM, Poeze M. Arginine and Citrulline and the Immune Response in Sepsis. Nutrients. 2015 Mar; 7(3): 1426–1463.

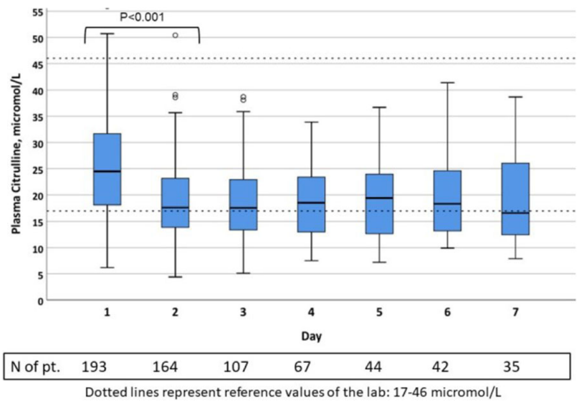

**Fig. 1 (abstract 000298).** Plasma citrulline dynamics in all patients

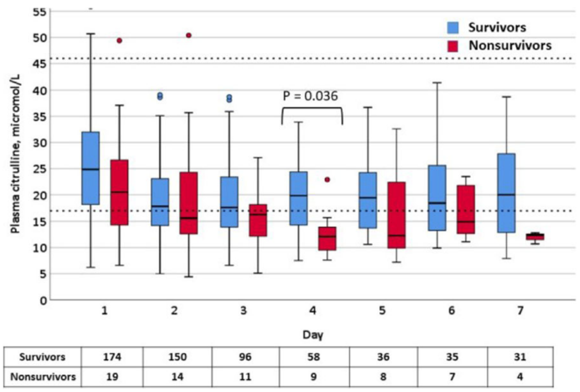

**Fig. 2 (abstract 000298).** Plasma citrulline dynamics in 28 day survivors vs nonsurvivors

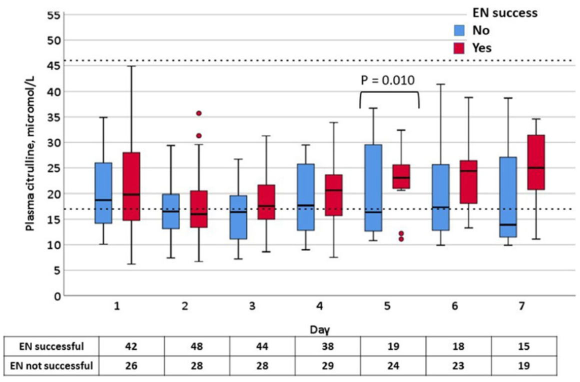

**Fig. 3 (abstract 000298).** Plasma citrulline in early (within 48h) enternal nutrition/oral diet

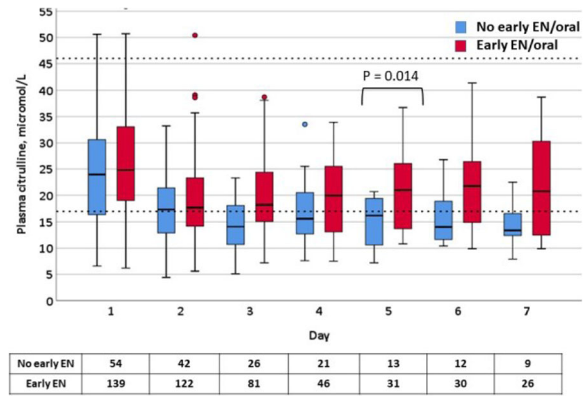

**Fig. 4 (abstract 000298).** Plasma citrulline in patients with successful (80% of caloric goal reached by day 4) *vs* unsuccessful early enteral nutritionn/oral diet

### 000600

#### Omega-3 fatty-acid enriched parenteral nutrition (PN) in the ICU: systematic review with meta-analysis and trial sequential analysis

L. PRADELLI[1], K. Mayer[2], S. Klek[3], AJ. Omaralsaleh[4], MD. Rosenthal[5], AR. Heller[6], M. Muscaritoli,[7]

[1]AdRes HE&OR, Torino, Italy; [2]Department of internal medicine, University Hospital of Giessen and Marburg GmbH, Marburg, Germany; [3]Stanley dudrick's memorial hospital, Stanley dudrick's memorial hospital, Skawina, Poland; [4]He&or, AdRes, Torino, Italy; [5]Department of surgery, division of trauma and acute care surgery, University of Florida College of Medicine, Miami, United States of America; [6]Department of anaesthesiology and intensive care medicine, University of Augsburg, Augsburg, Germany; [7]Department of translational and precision medicine, Sapienza University of Rome, Rome, Italy

**Correspondence:** L. PRADELLI

*Intensive Care Medicine Experimental* 2019, **7(Suppl 3):**000600

**INTRODUCTION.** Fish oil-containing lipid emulsions (LE), a type of LE that is high in omega-3 (ω-3) polyunsaturated fatty acids, have been used over the last years as a long-chain triglycerides source. Evidence on their effectiveness is accumulating, but an updated pooled estimate is currently unavailable.

**OBJECTIVES.** We conducted a meta-analysis (MA), asking if ω-3 fatty-acid enriched PN improves clinical and laboratory outcomes in patients treated in the ICU, and in a subgroup of critically ill patients, with trial sequential analysis (TSA) to evaluate the robustness of the estimates. TSA assesses the conclusiveness of pooled estimates by controlling for type I errors given heterogeneity of the single estimates.

**METHODS.** We searched public databases using keywords "PN", "fish oil", "lipids", "emulsion" and "randomised controlled trial (RCT)". Publications eligible for inclusion were RCTs in hospitalised adults given PN (at least 70% of total caloric delivery) in ICU settings. Intervention and control groups received ω-3 fatty acids-containing and ω-3 fatty acids-free LEs, respectively, both as part of PN. A critically ill (defined by the condition or by ICU stay >48h) subgroup analysis was pre-specified.

**RESULTS.** 24 publications were included. Pooled treatment effect estimates for clinical outcomes are shown in Table 1.

TSA results indicate statistically significant robustness of the estimates on the effect of ω-3-enriched PN regimen compared to standard PN regimen in reducing infection rate, hospital stay and ICU stay, which can be considered conclusive.

**CONCLUSION.** This is the largest MA conducted on this topic. Both in the ICU and in the critically ill subgroup, risk of infection and lengths of stay in hospital and ICU were impressively reduced with ω-3 fatty-acid enriched PN; all effects were confirmed to be conclusive by TSA.

1. The presented research was funded with a grant by Fresenius Kabi

**Table 1 (abstract 000600).** Results for clinical outcomes

| Outcome | Population, N/n | RR/MD (95% CI; p-value) |
|---|---|---|
| Infection rate | Critically ill, 5/659<br>Total, 8/795 | RR 0.65 (0.46, 0.94; p=0.02)<br>RR 0.62 (0.45, 0.86; p<0.01)fe |
| Sepsis rate* | Total, 3/336 | RR 0.56 (0.26, 1.19; p=0.13)fe |
| 30-day mortality | Critically ill, 10/769<br>Total, 12/925 | RR 0.90 (0.69, 1.16; p=0.41)<br>RR 0.90 (0.69, 1.16; p=0.41)fe |
| Hospital stay, days | Critically ill, 8/742<br>Total, 11/872 | MD −3.89 (−6.90, −1.06; p<0.01)<br>MD −3.05 (−5.03, −1.07;p<0.01)re |
| ICU stay, days | Critically ill, 9/826<br>Total, 11/890 | MD −2.14 (−3.89, −0.40; p=0.02)<br>MD −1.89 (−3.33, −0.45; p=0.01)re |
| Mech-ventilation, days* | Total, 6/528 | MD −0.02 (−0.10, 0.05; p=0.60)fe |

*CI* confidence interval, *ICU* intensive care unit, *MD* mean difference, *N* number of studies, *n* number of patients, *RR* relative risk, *fe* fixed effects, *re* random effects

* No subgroup analysis performed due to insufficient number of trials

### 000663

#### New risk classification of developing refeeding syndrome and associated mortality in critically ill patients: A cohort study in Japan

M. Yoshida[1], J. Izawa[2], H. Wakatabe[1], C. Kawabata[3], S. Matsushima[4], M. Kaneko[3], A. Suzuki[5], H. Saito[1], T. Yoshida[1], Y. Masui[1], Y. Taira[1], S. Fujitani[1]

[1]Department of emergency and critical care medicine, St. Marianna University School of Medicine, Kawasaki, Japan; [2]Center for critical care nephrology, department of critical care medicine, University of Pittsburgh School of Medicine, Pittsburgh, United States of America; [3]Department of nursing, St. Marianna University School of Medicine Yokohama City Seibu Hospital, Yokohama, Japan; [4]Department of rehabilitation, St. Marianna University School of Medicine Yokohama City Seibu Hospital, Yokohama, Japan; [5]Department of nutrition, St. Marianna University School of Medicine Yokohama City Seibu Hospital, Yokohama, Japan

**Correspondence:** M. Yoshida

*Intensive Care Medicine Experimental* 2019, **7(Suppl 3):**000663

**INTRODUCTION.** The importance of early recognition, prevention and treatment of refeeding syndrome (RFS) has been emphasized recently[1]. Based on the criteria for high risk of developing RFS proposed by National Institute for Health and Clinical Excellence (NICE)[2], the new risk classification criteria of developing RFS were recently published in 2018[3]. However, the frequency of each applied risk category and the association between the risks and patients' prognosis have not yet been investigated.

**OBJECTIVES.** To describe the frequency of new classification groups of RFS risk in the ICU and to investigate the association between the risks and 30-day mortality.

**METHODS.** This cohort study was conducted at the tertiary-care university hospital located in the metropolitan area in Japan from December 2016 to December 2018. We included critically ill patients who were aged 18 or older, who were admitted to the ICU via the emergency department, and who stayed in the ICU for 24 hours or longer. The registered nurses and dieticians well-trained for this study prospectively evaluated the NICE RFS risk factors at ICU admission for all critically ill patients. NICE RFS risk factors consisted of weight loss, the period of little or no nutritional intake prior to the ICU admission, and so on. The main exposure was risk classification of RFS: no risk, low risk, high risk or very high risk, according to the recently published criteria. The primary outcome was in-hospital mortality censored at 30 day after ICU admission. We performed multivariable analysis using Cox proportional hazard regression. As the primary analysis, the hazard ratio was adjusted for age, sex and presence of chronic illness.

**RESULTS.** We analyzed 541 patients who met the eligibility criteria. The prevalence of four risk classification groups of RFS was 24.4% in no risk, 24.6% in low risk, 48.8% in high risk, and 2.2% in very high risk. The 30-day mortality was 5.3%, 6.8%, 14.8%, and 25.0%, respectively (log-rank trend test: p<0.001) (Figure). In multivariable Cox regression, adjusted hazard ratios as compared to no risk group were 1.19 (95% CI 0.44–3.23) with low risk group, 2.94 (95% CI 1.31–6.60) with high risk group, 5.33 (95% CI 1.35–21.1) with very high-risk group.

**CONCLUSION.** More than half the critically ill patients had high or very high risk of developing RFS according to the recently published risk classification criteria. Patients with high or very high risk had higher 30-day mortality. Early recognition, prevention and treatment might be required for these patients.

**REFERENCE(S)**

[1] Singer P, et al. ESPEN guideline on clinical nutrition in the intensive care unit. Clin Nutr. 2018.
[2] Nutrition Support for Adults: Oral Nutrition Support, Enteral Tube Feeding and Parenteral Nutrition. Nutrition Support for Adults: Oral Nutrition Support, Enteral Tube Feeding and Parenteral Nutrition. London2006.
[3] Friedli N et al. Management and prevention of refeeding syndrome in medical inpatients: An evidence-based and consensus-supported algorithm. Nutrition. 2018;47:13-20.

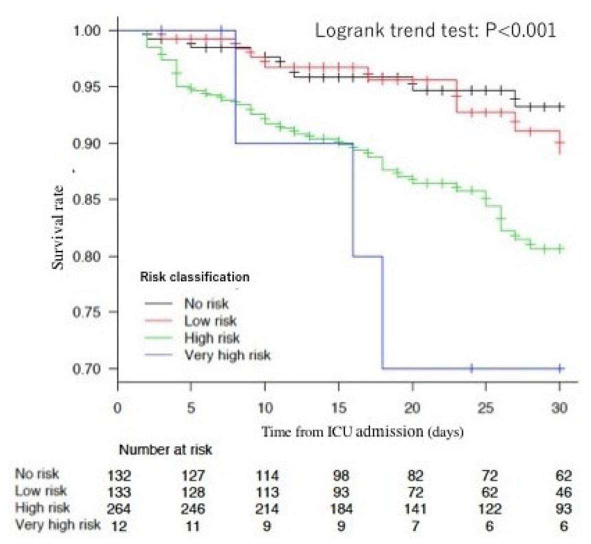

**Fig. 1 (abstract 000663).** See text for description

### 000807

#### Efficacy and tolerance of a semi-elemental solution versus a polymeric solution for enteral nutrition of neurocritically ill patients: a randomized trial

C. Gilli[1], L. Carteron[1], AS. Balon[2], G. Blasco[3], E. Samain[1], S. Pili-Floury[1], G. Besch[1]

[1]Anesthesiology and intensive care medicine, CHRU de Besançon, Besançon, France; [2]Anesthesiology, Clinique Saint Vincent, Besançon, France; [3]Druq, CHRU de Besançon, Besançon, France

**Correspondence:** L. Carteron

*Intensive Care Medicine Experimental* 2019, **7(Suppl 3):**000807

**INTRODUCTION.** A polymeric solution is recommended in first intention for enteral nutrition in critically ill patients. The efficacy to provide optimal amount of calories is often limited by digestive tolerance. Gastroparesis is frequent in neurocritically ill patients, explaining the difficulties to achieve optimal enteral nutrition in the early days after acute brain injury. A semi-elemental solution with hydrolyzed nutrients could facilitate digestive absorption and tolerance. Efficacy has never been evaluated in this specific condition.

**OBJECTIVES.** To evaluate efficacy and tolerance of a semi-elemental versus a polymeric solution for enteral nutrition in neurocritically ill patients.

**METHODS.** Randomized open-label trial (NCT01833624) including adult (age > 18), comatose (Glasgow Coma Scale (GCS) ≤ 8) patients, admitted in our ICU after severe acute brain injury. Next-of-kin's consent was obtained, or inclusion was done accordingly to an emergency procedure, as allowed by local ethic committee. Patients in the SE group received a semi-elemental solution (Peptamen AF®, Nestlé®, 1.5 kcal/ml, 9.4 g of proteins per 100 ml), patients in the PM group received a polymeric solution (Sondalis HP®, Nestlé, 1.5 kcal/ml, 7.5 g of proteins per 100 ml). Protocol considered twice daily residual gastric volume (RGV) to increase enteral nutrition delivering until objective was achieved, before 36th hour in case of good tolerance[1]. Primary judgement criterion was the daily amount of delivered calories. Intervention period lasted 10 days maximum. Secondary judgement criteria were incidence of gastroparesis (defined as RGV > 500 ml/12h) and diarrhea, the need for prokinetic drugs use, daily amount of delivered proteins, 10th day plasma levels of albumin and prealbumin, and 28-day mortality. Results are expressed as mean ± standard deviation, or as number(percent).

**RESULTS.** 205 patients (128 men, age 53 ± 17, admitted for traumatic brain injury (34%), subarachnoid hemorrhage (24%) or polytrauma (15%) – GCS 5 ± 2, IGS2 49 ± 12), respectively 107 and 98 in the SE and PM groups. Mean daily amount of delivered calories did not differ between groups (1501 ± 496 vs. 1539 ± 463 kcal/d, p=0.60). Secondary criteria are presented in table. Mean daily amount of delivered proteins was significantly higher in the SE group (1.24 ± 0.04 vs. 1.05 ± 0.04 g/kg/d, p=0.0006).

**CONCLUSION.** The use of a semi-elemental solution did not increase the daily amount of delivered calories in the first 10 days in neurocritically ill patients, compared to a polymeric solution. The daily amount of delivered proteins was increased, its interest could be evaluated in further studies.

**REFERENCE(S)**

1. SFAR 2012 Abstract 1844

2. The authors thank warmfully Gaëlle Amiotte for her unvaluable assistance.

**Table 1 (abstract 000807).** See text for description

| | SE group | PM group | p |
|---|---|---|---|
| Gastroparesis | 22 (21%) | 16 (16%) | 0.43 |
| Diarrhea | 16 (15%) | 9 (9%) | 0.20 |
| Prokinetic drugs | 20 (19%) | 18 (18%) | 0.95 |
| Albumin D10 (g/L) | 23 ± 4 | 23 ± 4 | 0.73 |
| Prealbumin D10 (g/L) | 0.21 ± 0.08 | 0.18±0.06 | 0.06 |
| Mortality D28 | 23 (22%) | 21 (21%) | 0.99 |

#### 000947

#### Long-stayer patients in the ICU: the nutrition and blood glucose pattern change through the stay

M. Viana, G. Bagnoud, A. Martinez, O. Pantet, E. Favre, P. Eckert, M. Berger
Service of adult intensive care & burns, Lausanne University Hospital, Lausanne, Switzerland
**Correspondence:** M. Berger
*Intensive Care Medicine Experimental* 2019, **7(Suppl 3):**000947

**INTRODUCTION.** The intensive care (ICU) patient population has increasing number of very long ICU stays. Long-stayers are usually defined as requiring more than 1 weeks of mechanical ventilation and of ICU therapy. Little is known about their nutritional and metabolic characteristics. In an attempt to better coordinate their treatment and reduce their length of stay (LICU), the service instituted a dedicated program enrolling patients requiring more than 2 weeks of ICU treatment.

**OBJECTIVES.** The study aimed at investigating the demographic, nutritional and metabolic aspects of their stay, including in the factors associated with Hypoglycemia (<4.1mmol/l) or Hyperglycemia (>10mmol/l) during the different phases of their ICU stay

**METHODS.** Retrospective analysis of 150 consecutive patients admitted to the ICU long-stayer program. Data extracted from the PDMS: age, SAPSII, weight, NRS-2002 score, daily energy, protein, and glucose intakes, blood glucose (Glu) and lactate, 24hr insulin needs to achieve Glu 6-8 mmol/l, 24hr-insulin, length of stay (LICU), and outcome. Data median [IQR 25-75], univariate analysis.

**RESULTS.** Cohort characteristics were: age 62 years [52-71], NRS 5 [3-7], SAPSII score 51[39-66]. LICU was 28 days [22-42] with mortality 18.7%: NRS was significantly higher in those dying (p=0.007). The nutrition intakes were extremely variable during the first week with multiple interruptions, stabilizing thereafter at values below the protocol recommendation. Median protein intakes and energy balances from D1-D7 were 0.92g/kg [0.79-1.15] and -380kcal/d [-1170/19] respectively, and from D8-D30 were 0.97g/kg [0.82-1.11], and -7 kcal [-259/78] respectively. During D1-D5, blood glucose was correlated 24hr-insulin (r2 =0.41, p< 0.001), but only weakly with glucose intake, and not with C-Reactive-Protein, without difference in septic and non-septic patients. Early HyperGlu was associated with higher severity (p=0.040) and lactate (p<0.001), but not with Glu intake, and resulted in higher Insulin (p<0.001) needs; late HyperGlu was observed in younger (p=0.013), heavier patients(p< 0.001), and resulted in higher insulin(p<0.001)needs. Early HypoGlu was observed in younger(p=0.013) and leaner patients(p<0.001), and late HypoGlu, in younger patients (p=0.003) with low Glu intake (p<0.001).

**CONCLUSION.** Long-stayer patients were characterized by a higher NRS-score, highest in those dying. Variability of nutritional intakes compared to the protocol was a striking characteristic. Energy and protein intakes were especially variable during the first week, and below the ICU recommendation. Factors associated with Hypo- and HyperGlu differed over time. Early HyperGlu was associated with higher severity. Late HypoGlu was associated with low Glu intake. Leaner younger patients were at highest risk of HypoGlu, while modestly overweight elder patients were most exposed to HyperGlu. Blood glucose had no clear association with glucose intake nor with inflammatory status.

1. None

### ARF - Novel diagnostics in acute lung injury

#### 000048

#### Role of the chest wall in airway closure and expiratory flow limitation in patients with acute respiratory distress syndrome

N. Terzi[1], LM. Galerneau[1], M. Mezidi[2], H. Yonis[2], L. Baboi[2], B. Louis[3], C. Schwebel[1], C. Guérin[1]
[1]Médecine intensive réanimation, C.H.U de Grenoble, La Tronche, France; [2]Médecine intensive réanimation, Hospital La Croix-Rousse - Hcl, Lyon, France; [3]Inserm 955, IMRB, Creteil, France
**Correspondence:** C. Guérin
*Intensive Care Medicine Experimental* 2019, **7(Suppl 3):**000048

**INTRODUCTION.** When flow does not go up after increasing expiratory driving pressure during tidal breathing expiratory flow limitation (EFL) occurs heralding airway closure (AC). We were wondering whether chest wall can contribute to small airways closure and hence to either EFL or AC occurrence in acute respiratory distress syndrome (ARDS) patients.

**OBJECTIVES.** To investigate the role of chest wall elastance (EcW) relative to lung elastance (EL) as a mechanism by which airways would close in ARDS patients. Our hypothesis was that the lower the EcW/EL ratio the higher the likelihood of EFL and AC.

**METHODS.** Moderate to severe ARDS patients intubated, sedated and paralyzed were prospectively included in two centers. Mechanical ventilation was delivered in volume-controlled mode (Evita XL or V500 ICU ventilators, Draeger) with tidal volume 6 ml/kg predicted body weight at positive end expiratory pressure 5 cmH2O in semirecumbent position. Airway (Paw) and esophageal (Pes) pressures and flow were continuously recorded during 2 minutes by a data logger (Biopac 150). Then, end-expiratory and end-inspiratory occlusions were performed for 5 seconds each, then respiratory system was slowly inflated at constant flow from the ventilator. Finally, patient was allowed to breathe out freely to atmosphere by using a three-way stop lock by-passing expiratory valve. AC and airway opening pressure (AOP) were determined according to Chen et al. (1) from the pressure-volume curve. EFL was assessed by the atmospheric method (2). Dynamic elastances of chest wall (Edyn,cW) and lung (Edyn,L) were obtained from least square linear regression method over 10 consecutive breaths. Static elastances (Est,cW and Est,L) were determined by classic formulas and also by taking into account AOP (Est,cW_aop and Est,L_aop, respectively). The performance of EcW/EL ratio to predict EFL and AC was assessed by area under receiver operating characteristic (AUCROC) curve.

**RESULTS.** Among 21 included ARDS patients EFL was observed in 7 (33%) and AC in 15 (71%). Median AOP was 7.8 (95%CI 7.6-11.7) cmH2O. The AUCROCs for EcW/EL ratios to detect EFL and AC are shown in table 1. Edyn,cw/Edyn,L ratio has the best performance to detect EFL(Edyn,cw/Edyn,L≤0.16 100% sensitivity and 85% specificity). Correction for AOP worsened the performance of Est,cW/Est,L ratio. AC was poorly predicted but prediction was improved with AOP correction. AOP values had an AUCROC of 1 (0.84-1) (P<0.0001) to predict EFL at the threshold of 8.3 cmH2O.

Table 1. Area under ROC curve (95% confidence intervals) for EcW/EL ratio for EFL and AC prediction

**CONCLUSION.** Low Edyn,cW/Edyn,L ratio and AOP predicted EFL occurrence. However, EFL and AC are two distinct phenomena in ARDS patients and should be assessed together to identify specific ARDS subphenotypes in terms of small airway contribution in ARDS.

**Table 1 (abstract 000048).** Area under ROC curve (95% confidence intervals) for EcW/EL ratio for EFL and AC prediction

| | EFL | P value | AC | P value |
|---|---|---|---|---|
| Edyn,cW/Edyn,L | 0.90 (0.84-0.94) | <0.0001 | 0.53 (0.45-0.61) | 0.51 |
| Est,cW/Est,L | 0.79 (0.56-0.93) | 0.0099 | 0.56 (0.33-0.77) | 0.69 |
| Est,cW/Est,L_aop | 0.57 (0.34-0.78) | 0.71 | 0.79 (0.56-0.94) | 0.004 |

### 000247

#### Arterial oxygen tensions in mechanically ventilated patients in the intensive care unit: a descriptive study of hyperoxaemia and associations with mortality

OL. Schjørring[1], AKG. Jensen,[2], CG. Nielsen,[3], A. Ciubotariu,[4], A. Perner,[5], J. Wetterslev[6], T. Lange[7], BS. Rasmussen,[8]
[1]Department of anesthesia and intensive care medicine, Aalborg University Hospital, Aalborg, Denmark; [2]Division of epidemiology and biostatistics, School of Public Health, University of California, Berkeley, United States of America; [3]Department of clinical biochemistry, Aalborg University Hospital, Aalborg, Denmark; [4]Department of anaesthesia and intensive care medicine, North Denmark Regional Hospital, Hjørring, Denmark; [5]Department of intensive care, Copenhagen University Hospital, Rigshospitalet, Copenhagen, Denmark; [6]Copenhagen trial unit, centre for clinical intervention research, department 7812, Copenhagen University Hospital, Rigshospitalet, Copenhagen, Denmark; [7]Center for statistical science, Peking University, Beijing, China; [8]Department of anaesthesia and intensive care medicine, Aalborg University Hospital, Aalborg, Denmark
**Correspondence:** O.L. Schjørring
*Intensive Care Medicine Experimental* 2019, **7(Suppl 3):**000247

**INTRODUCTION.** Oxygen therapy is part of the invasive mechanical ventilation strategy in the intensive care unit (ICU). Hyperoxaemia may however increase mortality.

**OBJECTIVES.** To assess degrees of hyperoxaemia, adjustments of fraction of inspired oxygen ($FiO_2$) in response to hyperoxaemia, and associations between hyperoxaemia and mortality in invasively mechanically ventilated ICU patients.

**METHODS.** We conducted a retrospective study of all invasively mechanically ventilated patients in 5 ICUs admitted from January 2011 to June 2016, retrieving all oxygen tension ($PaO_2$) and $FiO_2$ data. Time between arterial blood gas (ABG) samplings, proportions of hyperoxaemia ($PaO_2$ > 16 kPa), $FiO_2$ adjustments upon hyperoxaemia, and associations between mechanically ventilated exposure-time-divided area-under-the-curve (AUC) $PaO_2$ and ICU mortality was assessed. Primary ICU admissions were included in the mortality analyses, which used a multi-state illness-death model with transition intensities estimated by Cox proportional hazards models. The exposure-time-divided AUC measure represents the average $PaO_2$ level during invasive mechanical ventilation. Permission to use data without patient consent was obtained from the Danish Patient Safety Authority.

**RESULTS.** The study included 4,998 patients and 177,769 ABGs. Median time between ABGs was 3 hours (inter-quartile range: 2-4 hours), $PaO_2$ was 11.3 kPa (9.8-13.6 kPa), and $FiO_2$ was 0.40 (0.35-0.50). Hyperoxaemia was present in 11.9% of the ABGs. In 15.4% of the hyperoxaemic ABGs with $FiO_2 \geq 0.50$, the subsequent ABG remained hyperoxaemic with uncorrected $FiO_2$, whereas this was the case in 64.6% of hyperoxaemic ABGs with $FiO_2 < 0.40$. Exposure-time-divided AUC $PaO_2$ > 16.0 kPa was associated with increased ICU mortality (Table 1). This association was accentuated when censoring at the first $PaO_2$ < 8 kPa to diminish the impact of hypoxaemia (adjusted HR: 1.90 (95% CI: 1.27-2.86)).

**CONCLUSION.** ABG samplings were frequent and hyperoxaemia was common in invasively mechanically ventilated ICU patients. While $FiO_2$ in general was adjusted upon hyperoxaemia, several patients remained hyperoxaemic, especially at $FiO_2 < 0.40$. This may not be optimal as hyperoxaemia was associated with increased ICU mortality in the cohort.

1. Olav L. Schjørring's PhD study was funded by a grant from Innovation Fund Denmark

**Table 1 (abstract 000247).** Hazard ratio (HR) estimates for death in the ICU

| Exposure-time-divided AUC $PaO_2$ | Deaths per 100 person-days (deaths/person-days) | Adjusted HR** (95% CI) |
|---|---|---|
| ≥ 8.0 kPa to < 12.0 kPa* | 2.1 (256/11,930) | 1.0 |
| < 8.0 kPa | 16.2 (9/55) | 6.24 (3.17–12.25) |
| ≥ 12.0 kPa to ≤ 16.0 kPa | 2.0 (164/8,010) | 1.00 (0.82–1.23) |
| > 16.0 kPa | 3.4 (54/1,571) | 1.66 (1.20–2.29) |

Among 3,021 patients alive and on ICU 24 hours after primary ICU admission. *Reference **Adjusted for gender, age, admission type, Simplified Acute Physiology Score II, being currently ventilated, renal replacement therapy within the first 24 hours of ICU admission, and inotropes or vasopressors within the first 24 hours of ICU admission

### 000875

#### Positive end-expiratory pressure affects the position and contractile efficiency of the human diaphragm: MRI and functional analysis

AH. Jonkman[1], D. Jansen[2], HJ. De Vries[1], J. Elshof[3], M. van den Berg[4], MA. Hoofs[1], A. Girbes, JT. Marcus[5], C. Ottenheijm[4], L. Heunks[1]
[1]Intensive care medicine, Amsterdam UMC, locatie VUmc, Amsterdam, Netherlands; [2]Anesthesiology, Radboud UMC, Nijmegen, Netherlands; [3]Pulmonology, UMCG, Groningen, Netherlands; [4]Physiology, Amsterdam UMC, location VUmc, Amsterdam, Netherlands; [5]Radiology and nuclear medicine, Amsterdam UMC, location VUmc, Amsterdam, Netherlands
**Correspondence:** A.H. Jonkman
*Intensive Care Medicine Experimental* 2019, **7(Suppl 3):**000875

**INTRODUCTION.** Mechanical ventilation with positive end-expiratory pressure (PEEP) limits alveolar collapse, thereby improving oxygenation. However, PEEP-induced increase of end-expiratory lung volume may flatten the diaphragm dome. Experimentally, we showed that PEEP induced caudal diaphragm displacement, leading to an acute reduction in diaphragm fiber length [1]. As the muscle is forced to act at this shorter length, PEEP might impair diaphragm function.

**OBJECTIVES.** To test the hypothesis that PEEP acutely induces a caudal displacement and reduction in contractile efficiency of the human diaphragm.

**METHODS.** 18 healthy subjects participated. Non-invasive ventilation (NIV) was used to apply PEEP at 2, 5, 10 and 15 cmH2O, for 15 min. per level. For the first part, two nasogastric catheters were inserted to measure diaphragmatic pressure (Pdi) and diaphragm electromyography (EMGdi). Neuromechanical efficiency (NME) was defined as Pdi/EMGdi during tidal breathing with PEEP. Second, a MRI scan was made to measure diaphragm position at each PEEP level.

**RESULTS.** *Preliminary results:* Fig.1 shows a MRI scan of the diaphragm at end-expiration. A gradual PEEP-induced caudal displacement of the diaphragm dome was found for all subjects (mean±SD 20.2±9.5 mm after increasing PEEP from 2 to 15 cmH2O, p<0.001). In 9 of the subjects, NME could be obtained at all PEEP levels and decreased gradually with higher PEEP. Median NME reduction was 20% (p= 0.31), 27% (p<0.05) and 41% (p<0.05) after increasing PEEP from 2 to 5, 10 and 15 cmH2O, respectively.

**CONCLUSION.** In healthy subjects, acute application of PEEP results in caudal diaphragm displacement and reduction in diaphragm contractile efficiency. If similar effects occur in the critically ill, acute *reduction* in PEEP (i.e. during a spontaneous breathing trial) may result in an overstretched diaphragm and consequently impair diaphragm performance.

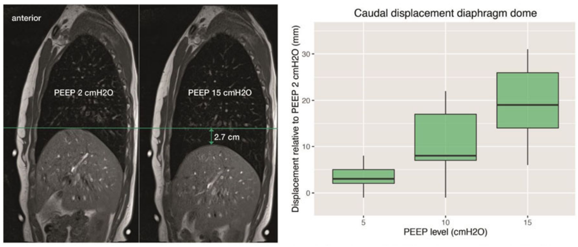

**Fig. 1 (abstract 000875).** Left: Static MRI image of the diaphragm (sagittal right view) at end-expiration. An increase in PEEP from 2 to 15 cmH2O resulted in 2.7 cm displacement of the diaphragm dome (green line). Right: PEEP-induced caudal displacement of the diaphragm dome (p<0.001, for all PEEP levels).

### 001591

#### EtCO2 doesn't always predict paCO2 during mechanical ventilation and negative paCO2-EtCO2 gradient can result because of the development of a new hyperinflated compartment in the lung

M. Bonifazi, F. Vassalli, F. Romitti, M. Busana, I. Pasticci, L. Giosa, MM. Macrì, M. Quintel, L. Gattinoni
Department of anesthesiology, emergency and intensive care medicine, University Hospital Göttingen - University Medical Center Göttingen, Göttingen, Germany
**Correspondence:** M. Bonifazi
*Intensive Care Medicine Experimental* 2019, **7(Suppl 3):**001591

**INTRODUCTION.** End-tidal carbon dioxide pressure is used routinely as an indicator of arterial partial pressure of carbon dioxide, giving an estimation of adequate ventilation. Normally paCO2-etCO2 gradient is positive, representing normal ventilation-perfusion mismatch. This can vary during mechanical ventilation.

**METHODS.** 42 healthy piglets, 24,2±2 Kg, were mechanically ventilated for 48 hours in prone position with a similar mechanical power, delivered by primarily increasing tidal volume (TV), respiratory rate (RR) or PEEP. The ventilation was set as follow:

1) High TV group (14 pigs): TV 803±122 ml/min, RR 11±4,5, PEEP 5,3± 0,8 cmH2O, mechanical power 20,8±7,9 J/min
2) High RR group (14 pigs): TV 299±61 ml/min, RR 40±1, PEEP 8,3±1,5 cmH2O, mechanical power 22,2±8,5 J/min
3) High PEEP group (14 pigs): TV 319±21 ml/min, RR 16,3±5,5, PEEP 24±1,1 cmH2O, mechanical power 21,3±8,5 J/min.

**RESULTS.** We observed after the set of our ventilatory strategy an inversion of the paCO2 and etCO2 values with a negative gradient for the entire experiment and in all groups of animals (EtCO2 30±18 paCO2 29±17 p=0,29)(fig1). The degree of hyperinflation was computed as PEEP volume plus FRC and the median value (511,5 ml) was taken as cut off above which the hyperinflation group was defined. Hyperinflation significantly affected the inversion of EtCO2 and paCO2: in fact the hyperinflation group (EtCO2 38±19,9 paCO2 37± 18 pCO2mixed 43±19) compared to the hypoinflation group (EtCO2 22±9, paCO2 21±10,6 and pCO2mixed 26,38±11,12) was significantly different (p < 0,0001)(fig 2).

**CONCLUSION.** A possible reason for the inverse relationship between etCO2 and paCO2 is indeed the development of hyperinflation and for this reason of areas of the lungs open but without ventilation. These areas will have longer time constants, behaving like a new compartment or reservoir of CO2 for the expired air. This will make the etCO2 more similar to the mixed venous pCO2, having more time to reach equilibrium, while paCO2 will increase less the etCO2. The overall phenomenon is the result of an impaired ventilation/perfusion mismatch.

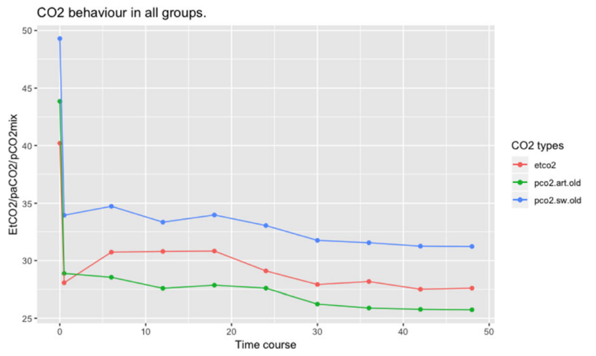

**Fig. 1 (abstract 001591).** behaviour in all groups.

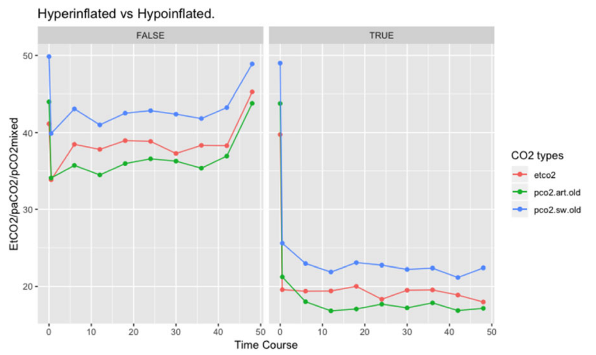

**Fig. 2 (abstract 001591).** Hyperinflated vs Hypoinflated;

#### 001604

**Cytomix Preactivated UC-MSCs as a Treatment of Acute Lung Injury in a New Model of Rodent Staphylococcus Aureus–Induced Pulmonary Acute Respiratory Distress Syndrome**

E. Rezoagli, D. O'toole, EJ. Murphy, JG. Laffey
Anaesthesia, National University of Ireland, Galway, Galway, Ireland
**Correspondence:** E. Rezoagli
*Intensive Care Medicine Experimental* 2019, **7(Suppl 3):**001604

**INTRODUCTION.** Models of gram negative bacteria induced acute respiratory distress syndrome (ARDS) have been well established in rats. The therapeutic role of mesenchymal stromal/stem cells (MSCs) looks promising in such a model (1). Contrarily, no information is available on the role of MSCs in the treatment of pulmonary ARDS induced by gram positive bacteria. *Staphylococcus Aureus* is a clinically relevant gram positive bacteria, which is associated with >40% health care pneumonia cases and with mortality rates of >50% (2).

**OBJECTIVES.** Objectives of our investigation were: 1. to establish a model of gram positive bacterial pneumonia using a clinically relevant strain of *S. Aureus* from a human isolate (Newman) (3); 2. To evaluate the potential therapeutic role of naïve and preactivated umbilical cord (UC) MSCs freshly harvested from culture in the treatment of acute lung injury in a new model of Rodent *S. Aureus*–induced ARDS.

**METHODS.** Adult male Sprague Dawley rats underwent intratracheal instillation of *S. Aureus* Newman to induce pulmonary ARDS. Animals were randomized, within 2 hours post infection, to intravenous administration of: (1) vehicle (phosphate buffered saline (PBS)); (2) 1x10^7/kg fresh UC-MSCs; and (3) 1x10^7/kg fresh UC-MSCs preactivated for 24 hours with cytomix (TNF-α; IL-1β; and IFN- γ [50 ng/mL each]). Comparisons among the groups were tested for differences in bacterial load and white blood cell count in the bronchoalveolar lavage (BAL), and arterial oxygenation after 48 hours.

**RESULTS.** Endotracheal instillation of *S. Aureus* Newman induced a model of moderate ARDS in rats. Fresh naïve UC-MSCs did not treat the lung injury at 48 hour post infection. In contrast, the preactivation of fresh UC-MSCs with cytomix for 24 hours allowed to significantly increase the pulmonary bacterial clearance – as shown by the lower bacterial load in the BAL (**Figure 1A**), to reduce the lung cell infiltrates – as shown by the lower white blood cell count in the BAL (**Figure 1B**), and to improve oxygenation with an average PaO2/FiO2 ratio above 300 at an FiO2 of 1.0 (data not shown).

Data are expressed as mean (SEM). n=6-8 per group. p-UC-MSCs=preactivated UC-MSCs.

**CONCLUSION.** Fresh preactivated UC-MSCs therapy decreased the severity of *S. Aureus* induced ARDS by the reduction of bacterial load and white blood cell infiltrates into the lungs, and by the increase of arterial oxygenation. The use of preactivated UC-MSCs could represent a potential clinically relevant treatment of acute lung injury in patients with gram positive induced ARDS.

**REFERENCE(S)**

1. Devaney J, Horie S, Masterson C, Elliman S, Barry F, O'Brien T, Curley GF, O'Toole D, Laffey JG. Human mesenchymal stromal cells decrease the severity of acute lung injury induced by E. coli in the rat. Thorax. 2015 Jul;70(7):625-35.
2. Tong SY, Davis JS, Eichenberger E, Holland TL, Fowler VG Jr. Staphylococcus aureus infections: epidemiology, pathophysiology, clinical manifestations, and management. Clin Microbiol Rev. 2015 Jul;28(3):603-61.
3. Bubeck Wardenburg J, Patel RJ, Schneewind O. Surface proteins and exotoxins are required for the pathogenesis of Staphylococcus aureus pneumonia. Infect Immun. 2007 Feb;75(2):1040-4.

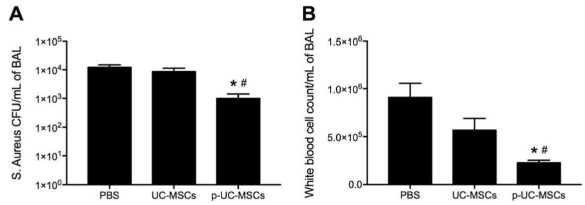

**Fig. 1 (abstract 001604). Bacterial load (A) and white blood cell count (B) in BAL.** Differences among the groups were tested using an ordinary one-way ANOVA. Post-hoc comparisons were performed using the Newman-Keuls test. *p<0.05 vs PBS; # p<0.05 vs UC-MSCs.

### SIS - Septic shock and respiratory infection

#### 000258

**Genetic Association of Body Mass Index With Risk of Dying from a Bloodstream Infection: A Study of 56,000 Subjects from the HUNT Study With 23 Years Follow-Up**

T. Rogne[1], E. Solligård,[1], S. Burgess,[2], BM. Brumpton[3], J. Paulsen[4], HC. Prescott[5], RM. Mohus[1], LT. Gustad[1], A. Mehl[6], BO. ÅSvold[7], AT. Dewan[8], JK. Damås[1]
[1]Department of circulation and medical imaging, Norwegian University of Science and Technology, NTNU, Trondheim, Norway; [2]Mrc biostatistics unit, University of Cambridge, Cambridge, United Kingdom; [3]K.g. jebsen center for genetic epidemiology, Norwegian University of Science and Technology, NTNU, Trondheim, Norway; [4]Department of medical genetics, Norwegian University of Science and Technology, NTNU, Trondheim, Norway; [5]Department of medicine, University of Michigan, Ann Arbor, United States of America; [6]Department of medicine, Levanger Hospital, Levanger, Norway; [7]Department of endocrinology, St Olavs Hospital, Trondheim, Norway; [8]Department of chronic disease epidemiology, Yale University School of Public Health, New Haven, United States of America
**Correspondence:** T. Rogne
*Intensive Care Medicine Experimental* 2019, **7(Suppl 3):**000258

**INTRODUCTION.** In observational studies, higher body mass index (BMI) has been associated with increased incidence and mortality of bloodstream infection (BSI) and sepsis in the general population (1,2), but with reduced mortality among patients with BSI or sepsis (3). However, traditional observational studies are subject to bias and confounding (4). Mendelian randomization studies utilizes the fact that genotypes are randomly assigned at conception, which make these studies less susceptible to reverse causation and confounding (5).

**OBJECTIVES.** To evaluate the causal association of BMI with risk and mortality of BSI in the general population, and with mortality among patients with BSI.

**METHODS.** Non-linear Mendelian randomization study with a 23-year prospective follow-up between 1995 and 2017 using a population-based cohort in Norway (The HUNT Study). Of 93,865 invited subjects, 65,236 (70%) participated, and 55,908 (60%) had complete data on genotype and anthropometric measures. Genetically-predicted BMI was calculated based on 939 single nucleotide polymorphisms identified in an independent genome-wide association study. To account for selection bias, inverse-probability weighting was used in the sub-analysis restricted to patients with BSI.

**RESULTS.** The mean age at enrollment was 48.3 years, 26,324 (47.1%) were men, and mean BMI was 26.3 kg/m2. During median 21 years follow-up, 2,547 (4.6%) subjects experienced a BSI and 451 (0.8%) died from BSI. Compared with a genetically-predicted BMI of 25 kg/m2, a genetically-predicted BMI of 30 kg/m2was associated with a hazard ratio (95% confidence interval) for BSI incidence of 1.78 (1.40 – 2.27) and for BSI mortality of 2.56 (1.31 – 4.99) in the general population, and for mortality of 2.34 (1.11 – 4.94) in the sub-analysis of patients with BSI.

**CONCLUSION.** Supportive of a causal relationship, we found that genetically-predicted BMI was associated with BSI incidence and mortality, even within the normal BMI range. Our findings contradict the "obesity paradox" where previous traditional epidemiological studies have found increasing BMI to be protective in terms of mortality for patients with BSI or sepsis.

**REFERENCE(S)**

1) Twig G, Geva N, Levine H, et al. Body mass index and infectious disease mortality in midlife in a cohort of 2.3 million adolescents. Int J Obes. 2018;42(4):801-807.
2) Paulsen J, Askim Å, Mohus RM, et al. Associations of obesity and lifestyle with the risk and mortality of bloodstream infection in a general population: a 15-year follow-up of 64 027 individuals in the HUNT Study. Int J Epidemiol. 2017;46(5):1573-1581.
3) Wang S, Liu X, Chen Q, Liu C, Huang C, Fang X. The role of increased body mass index in outcomes of sepsis: A systematic review and meta-analysis. BMC Anesthesiol. 2017;17(1):118.
4) Banack HR, Kaufman JS. The "obesity paradox" explained. Epidemiology. 2013;24(3):461-462.
5) Haycock PC, Burgess S, Wade KH, Bowden J, Relton C, Smith GD. Best (but oft-forgotten) practices: the design, analysis, and interpretation of Mendelian randomization studies. Am J Clin Nutr. 2016;103(4):965-978.

6) The first author was funded in part by a Fulbright Scholarship by the U.S-Norway Fulbright Foundation. Ben Michael Brumpton and Bjørn Olav Åsvold work in a research unit funded by Stiftelsen Kristian Gerhard Jebsen; Faculty of Medicine and Health Sciences, NTNU; The Liaison Committee for education, research and innovation in Central Norway; the Joint Research Committee between St. Olavs Hospital and the Faculty of Medicine and Health Sciences, NTNU; and the Medical Research Council Integrative Epidemiology Unit at the University of Bristol which is supported by the Medical Research Council and the University of Bristol [MC_UU_12013/1]. Hallie Christine Prescott is funded in part by K08 GM115859 from the US National Institutes of Health.

### 000305

#### Biological markers of tissue hypoxia to assess lactate kinetics in septic shock: Preliminary Report of the Andromeda – Shock Physiology study

N. Pavez[1], G. Ferri[2], R. Castro[1], L. Alegria[1], ED. Valenzuela Espinoza[1], D. Soto[1], V. Oviedo[1], R. Pairumani[2], C. Santis[2], B. Astudillo[2], S. Bravo[1], G. Ospina-Tascon[3], J. Bakker[1], G. Hernandez[1]

[1]Departamento de medicina intensiva, Pontificia Universidad Católica de Chile, Santiago, Chile; [2]Hospital barros luco trudeau, Unidad de Cuidados Intensivos, Santiago, Chile; [3]Department of intensive care medicine, Fundación Valle del Lili, Universidad ICESI, Cali, Colombia

**Correspondence:** N. Pavez

*Intensive Care Medicine Experimental* 2019, **7(Suppl 3):**000305

**INTRODUCTION.** Targeting normal macro hemodynamics in septic shock (SS) does not ensure adequate tissue perfusion. Thus, assessing biological markers of tissue hypoxia may help to titrate therapeutics. The lactate/pyruvate ratio (L/P) and the venoarterial CO2 gap / arteriovenous O2 content difference (PcvaCO2/CavO2) have been proposed as markers of anaerobic metabolism and as a surrogate of the respiratory quotient, respectively.

**OBJECTIVES.** We attempted to determine if L/P ratio and PcvaCO2/CavO2 correlates adequately as tissue hypoxia markers and if they may predict lactate outcome in SS patients.

**METHODS.** Data from an ongoing prospective clinical randomized control trial performed in 2 ICUs were analyzed. Patients were divided into 2 groups: Lactate improvers (LI), defined as a decrease ≥10% at 6 hours or normalization (<2.0 mmol/L), and no-LI (decrease <10%). L/P ratio and PcvaCO2/CavO2 samples were collected and compared at time 0 (basal), 2 and 6 h.

**RESULTS.** Twenty-seven septic shock patients have been studied until now. Basal demographic and hemodynamic characteristics are described in Table 1. No clinical differences were observed. No correlation was found between L/P and PcvaCO2/CavO2 (r=0,11 p=0,34) (figure 1). L/P ratio did not discriminate between LI and no LI, whereas PcvaCO2/CavO2 did as a whole (1.63±0.31 vs 2.26±0.5; p=0,024) and by 2 and 6h (1.26 vs. 2.07; p=0.006 and 1.79 vs 2.84; p=0.006, respectively). No other perfusion parameter showed a significant difference. These and other perfusion parameters are shown in Fig 2.

**CONCLUSION.** PcvaCO2/CavO2 was accurate to identify lactate improvers and thus might be a reliable surrogate of tissue hypoxia, whereas L/P did not. L/P and PcvaCO2/CavO2 values did not correlate in septic shock patients and might represent different biological mechanisms.

**REFERENCE(S)**

1. Ospina-Tascón, G.A., Hernández, G. & Cecconi, M. Understanding the venous–arterial CO2 to arterial–venous O2 content difference ratio. Intensive Care Med (2016) 42: 1801.

2. Funded by FONDECYT 1170043, ClinicalTrials.gov NCT03762005.

**Table 1 (abstract 000305).** Basal demographic and hemodynamic characteristics at inclusion

| | Total | LI | nLI |
|---|---|---|---|
| **Patients (n)** | 27 | 17 | 10 |
| **Sex (w)** | 16 | 9 | 7 |
| **Age** | 58±19,6 | 62±20,4 | 51,7±17,2 |
| **SOFA** | 9,3±3,9 | 8,3±2,8 | 10,9±3,9 |
| **APACHE II** | 23±10,9 | 20,1±9,6 | 27,2±11,9 |
| **Lactate (mmol/L)** | 4,6±3 | 4,1±2,1 | 5,5±4 |
| **Norepinephrine (mcg/kg/min)** | 0,28±0,18 | 0,28±0,21 | 0,31±0,22 |
| **MAP (mmHg)** | 72,6±10 | 72,3±8,34 | 66,1±11,4 |
| **CVP (cmH2O)** | 8,3±2,7 | 7,9±2,6 | 9,5±3,2 |
| **pCO2 gap (mmHg)** | 7,8±3,5 | 7,45±4,4 | 7,5±3,8 |
| **ScvO2 (mmHg)** | 70,8±9,1 | 70±10,9 | 68,2±17,1 |
| **CRT (s)** | 5 [3 - 6] | 5 [3 - 5,3] | 5 [3 - 7] |

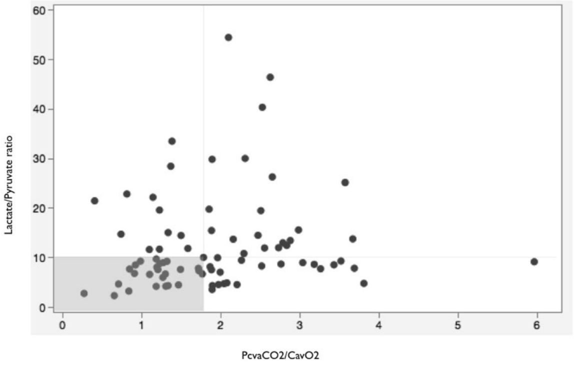

**Fig. 1 (abstract 000305).** Relationship between Lactate/Pyruvate and PcvaCO2/CavO2. Dotted lines: normal limits (L/P<10, PcvaCO2/CavO2<1.8), grey shaded area: normal range. No statistical correlation was found (*r*=0,11; *p*=0,34)

### 000764

#### High Serum Concentration of Neutrophil Extracellular Traps Associates with the Pro-inflammatory State and Mortality in Patients with Community-acquired Pneumonia

LF. Reyes[1], N. Gonzalez-Juarbe[2], E. Gamboa[3], L. Claverias[4], S. Trefler[5], M. Bodí[5], J. Marin-Corral[6], A. García-España[7], A. Rodríguez[5]
[1]Critical Care Medicine - Infectious Disease Department, Universidad de la sabana, Bogotá, Colombia; [2]Infectious diseases department, J. Craig Venter Institute, Rockville, United States of America;
[3]Student, Universidad de La Sabana, Bogotá, Colombia; [4]Critical care department, Hospital Verge de la Cinta, Tortosa, Spain; [5]Critical care department, Hospital Universitari de Tarragona Joan XXIII, Tarragona, Spain; [6]Critical care department, IMIM, Barcelona, Spain; [7]Cell biology department, Instituto de Investigación Sanitaria Pere Virgili, Tarragona, Spain
**Correspondence:** L.F. Reyes
*Intensive Care Medicine Experimental* 2019, **7(Suppl 3):**000764

**INTRODUCTION.** CAP is the most important cause of infectious death worldwide. A major hallmark of CAP physiopathology is the high systemic proinflammatory state that generates in part, when pathogens induce mechanisms of host necrotic cell death, exacerbating inflammation. It is well known that CAP patients experiencing uncontrolled systemic inflammation, have worse clinical outcomes. However, the underlying mechanisms of these proinflammatory reactions are not well understood. NETosis, a recently described pro-inflammatory pathway of neutrophil death and the driver of NET formation, has been shown to be important in patients with pulmonary diseases such as bronchiectasis or COPD, and directly associated with lung inflammation and damage. However, the role of serum NETs has not been investigated as the cause of the proinflammatory state and mortality observed in patients with CAP. Therefore, this study attempted to bridge this knowledge gap.

**OBJECTIVES.** To determine the role of circulating neutrophil extracellular traps (NETs) in the systemic proinflammatory state and mortality observed in patients admitted with community-acquired pneumonia (CAP).

**METHODS.** Here, we report a multicenter cohort study designed to better understand the role of baseline circulating NETs in the systemic inflammation and mortality of CAP patients. Thirty-seven CAP patients and twenty-five controls (patients with stroke and healthy individuals) were prospectively enrolled in 2 university hospitals in Spain. Baseline serum cfDNA was quantified using the Quant-iT PicoGreen® dsDNA Kit. To detect NETs in human serum samples we used a modification of the MPPO-DNA ELISA method. Interleukins were tested by ELISA. Descriptive statistics and logistical regressions were used to assess the results.

**RESULTS.** We show that patients with CAP developed a systemic proinflammatory profile characterized by increased serum concentrations of IL-6 and IL-10 (1,025pcg/mL, 172.5pcg/mL respectively). Patients diagnosed with CAP had higher levels of free-DNA in serum (Median [IQR]; 0.273 [0.216, 0.391] Vs 0.480 [0.357, 0.720], p=<0.001) and NETs (Median [IQR]; 32 [25, 36] Vs 76 [47, 125], p=<0.001). A linear regression among free-DNA and NETs was confirmed with a r2=0.5788. The relation among IL-6 (r2=0.11), IL-10 (r2=0.09) and NETs was not significant. Regarding ICU mortality, 7 (19%) CAP patients died and had lower levels of circulating NETs compared with patients who survive (Median [IQR]; 34.00 [24.00, 70.00] Vs 50.75 [32.25, 94.50], p=0.12).

**CONCLUSION.** Our results provide a mechanistic link to the proinflammatory state of patients with CAP. Serum concentration of NETs associated to up-regulation of NETosis and IL-6/IL-10. However, serum NETs are not associated with mortality due to CAP. Finally, there is no relation among interleukin levels and NETs concentration. Other pro-inflammatory cell death pathways should be investigated to better understand inflammation and mortality in CAP patients.

##### REFERENCE(S)

1. Storisteanu, D. M. L., Pocock, J. M., Cowburn, A. S., Juss, J. K., Nadesalingam, A., Nizet, V. & Chilvers, E. R. (2017) Evasion of neutrophil extracellular traps by respiratory pathogens
2. Gray, R. D. (2018) Nets in pneumonia: Is just enough the right amount? Eur Respir J, 51(4).
3. Ebrahimi, F., Giaglis, S., Hahn, S., Blum, C. A., Baumgartner, C., Kutz, A., Van Breda, S. V., Mueller, B., Schuetz, P., Christ-Crain, M. & Hasler, P. (2018) Markers of neutrophil extracellular traps predict adverse outcome in community-acquired pneumonia: Secondary analysis of a randomised controlled trial. Eur Respir J, 51(4).
4. Dicker, A. J., Crichton, M. L., Pumphrey, E. G., Cassidy, A. J., Suarez-Cuartin, G., Sibila, O., Furrie, E., Fong, C. J., Ibrahim, W., Brady, G., Einarsson, G. G., Elborn, J. S., Schembri, S., Marshall, S. E., Palmer, C. N. & Chalmers, J. D. (2018) Neutrophil extracellular traps are associated with disease severity and microbiota diversity in patients with chronic obstructive pulmonary diseaseJ allergy clin immunol. (vol. 141), 117-27.
5. Brinkmann, V., Reichard, U., Goosmann, C., Fauler, B., Uhlemann, Y., Weiss, D. S., Weinrauch, Y. & Zychlinsky, A. (2004) Neutrophil extracellular traps kill bacteria.

### 001187

#### Effect of cholesterol administration on catecholamine hyporesponsiveness in a septic rat model

C. Gaupp[1], A. Kleyman[1], A. Press[2], M. Bauer[3], M. Singer[4]
[1]University college london, Bloomsbury Institute of Intensive Care Medicine, London, United Kingdom; [2]Nanophysiology group department of anesthesiology and intensive care medicine, Jena University Hospital, Jena, Germany; [3]Department of anesthesiology and intensive care medicine, Jena University Hospital, Jena, Germany;
[4]University College London, Bloomsbury Institute of Intensive Care Medicine, London, United Kingdom
**Correspondence:** A. Kleyman
*Intensive Care Medicine Experimental* 2019, **7(Suppl 3):**001187

**INTRODUCTION.** Hypocholesterolaemia and catecholamine hyporesponsiveness are hallmarks of sepsis, with a greater degree of abnormality associated with worse prognosis [1,2]. Cholesterol is a crucial component of the cell plasma membrane, regulating membrane permeability and responsiveness to hormones though expression and activity of G-protein coupled receptors such as the β-adrenergic receptors. We have previously shown in our rat model of sepsis that membrane cholesterol levels fall in heart and liver, with the extent correlating with mortality.

**OBJECTIVES.** To determine whether restoration of membrane cholesterol concentration levels in a rat model of sepsis would rescue adrenergic signalling and improve catecholamine responsiveness.

**METHODS.** Awake, instrumented male Wistar rats (300±50g) received an i.p. injection of faecal slurry with i.v. fluid resuscitation commenced from 2h. Sham operated rats were treated identically but did not receive slurry. At 6 h post-induction of sepsis, the intervention group received an i.v. infusion of cholesterol containing liposomes (10 mg cholesterol/300g bw) over 15 hours. Catecholamine responsiveness was then tested by consecutively administering dobutamine (10 μg/kg/min for 10 min, and norepinephrine (0.5 μg/kg/min for 10 min) with a 30 min washout period in between. BP (invasive), HR and SV (by echocardiography) were measured before and after each drug was administered. Results are presented as mean ± SE, analysed using Student's t test and considered statistically significant when p <0.05.

**RESULTS.** Animals treated with cholesterol-containing liposomes demonstrated a more pronounced cardiac response after dobutamine (DB) administration, with a significant increase in stroke volume over untreated septic animals (Table 1). No improvement was seen in the blood pressure response to norepinephrine (NE).

**CONCLUSION.** Administration of cholesterol-containing liposomes improved myocardial responsiveness to dobutamine, but not the pressor response to norepinephrine

##### REFERENCE(S)

1. Journal of Critical Care 2010 25, 361.e7 2. Journal of International Medical Research 2018 v46 (4), 1303
2. Therapeutic Acceleration Support (TAS) Fund UCL

**Table 1 (abstract 001187).** See text for description

| % change of | Sham | Septic | Liposome treated |
|---|---|---|---|
| Stroke volume | | | |
| Dobutamine | 21 ± 3 | 5 ± 5 | 32 ± 9 |
| Norepinephrine | 15 ± 18 | 5 ± 5 | 22 ± 13 |
| Heart rate | | | |
| Dobutamine | 11 ± 11 | 13 ± 6 | 7 ± 3 |
| Norepinephrine | 7 ± 9 | 5 ± 4 | 2 ± 3 |
| Cardiac index | | | |
| Dobutamine | 34 ± 16 | 18 ± 11 | 41 ± 12(b) |
| Norepinephrine | 23 ± 14 | 10 ± 9(a) | 24 ± 14(b) |
| Blood pressure | | | |
| Dobutamine | 2 ± 8 | -9 ± 2(a) | -6 ± 3 |
| Norepinephrine | 20 ± 12 | 10 ± 4 | 4 ± 4 |

*a p< 0.05 vs sham, b p< 0.05 vs septic (untreated)*

#### 000615

#### Efficacy of inhalative phage therapy against lethal methicillin resistant Staphylococcus aureus ventilator associated pneumonia in rats - AEROPHAGE

J. Prazak[1], D. Cameron[1], M. Iten[1], L. Valente[2], L. Federer[3], D. Baechler[3], D. Grandgirard[2], S. Jakob[4], J. Takala[1], S. Leib[2], G. Resch[5], M. Haenggi[1], YA. Que[1]
[1]Department of intensive care, Inselspital, Bern University Hospital and University of Bern, Bern, Switzerland; [2]Institute of infectious diseases, University of Bern, Bern, Switzerland; [3]Faculty of medicine, University of Bern, Bern, Switzerland; [4]University clinic for intensive care medicine, Bern University Hospital, Bern, Switzerland; [5]Deptartment of fundamental microbiology, University of Lausanne, Lausanne, Switzerland
**Correspondence:** J. Prazak
*Intensive Care Medicine Experimental* 2019, **7(Suppl 3):**000615

**INTRODUCTION.** Ventilator associated pneumonia (VAP) is common in critically ill patients and associated with high morbidity and mortality, especially when caused by antibiotic resistant bacteria. Recently, phage therapy has emerged as a promising non-antibiotic based treatment of antibiotic resistant bacterial infections. However, proof-of-concept experimental and clinical studies are missing before its wider use in clinical medicine. In our previous study, we have shown, that intravenous phage therapy was as effective as antibiotics (teicoplanin) in improving outcome of rats with VAP caused by methicillin-resistant *S. aureus* (MRSA).

**OBJECTIVES.** The goal of our current study was to evaluate the efficacy of inhalative administration of phage cocktails (aerophage) for the treatment of lethal MRSA VAP in a rat model.

**METHODS.** Four hours after intubation and protective ventilation, rats were inoculated via the endotracheal tube with 1 x 1010 CFU (LD100) of the MRSA clinical isolate AW7. Two hours after bacterial challenge, rats were randomised and received either inhalative (n=10), intravenous (n=10) or combination of both (n=11) administration of a cocktail of four phages (1 x 1010 plaque forming units/ml of 2003, 2002, 3A and K). Therapy was repeated after 12 and 24 hours and then once daily for four days in a blinded manner. The primary outcome was survival at day four. Secondary outcomes were bacterial and phage densities in the lungs and spleen.

**RESULTS.** Application of the multiphage cocktail using a combination of inhalative (aerophage) and intravenous (IV) route significantly improved survival compared to IV or aerophage monotherapies in this lethal pneumonia model (91 % vs. 50 % and 50%, respectively, p< 0.04, log-rank test). Mortality correlated with high bacterial load within the lungs and spleens. Animals that succumbed to infection had over 19,000-times more MRSA in the lung when compared to those that survived until 96 hours (8.8 x 108 CFU/g vs. 4.6 x 104 CFU/g, respectively, p < 0.0001, t test). Similarly, spleen in dying animals had over 150-times more MRSA when compared to those that survived until 96 hours (4.1 x 104 CFU/g vs. 2.6 x 102 CFU/g, respectively, p < 0.0001, t test). Despite improved survival for rats treated with the combination of aerophage and IV, the treatment groups did not differ in the bacterial burden in the lung or the spleen (p=0.20, two-way ANOVA). High phage loads were detected in the lungs of all treatment groups. In contrast to IV administered phages, inhalative phages did not readily accumulate in the spleen.

**CONCLUSION.** The combination of aerosol and intravenous phage therapy significantly improved outcome compared to inhalative or intravenous phage therapy alone.

### HSRO - What should we do better to improve (long term) outcome?

#### 000212

#### A Phase 2 Randomised Clinical Trial to Investigate the Effect of Intermittent versus Continuous Enteral Nutrition on Muscle Wasting in Critical Illness: Adverse Events, Gastric Intolerance, Glucose Variation

A. McNelly[1], DE. Bear[2], B. Connolly[3], G. Arbane[3], L. Allum[3], A. Tarbhai,[4], J. Cooper[5], P. Hopkins[6], M. Wise[7], D. Brealey[8], K. Rooney[9], J. Cupitt[10], B. Carr[11], N. Hart[3], H. Montgomery[4], ZA. Puthucheary[1]
[1]William harvey research institute, Barts and The London School of Medicine and Dentistry, London, United Kingdom; [2]Critical care unit, Guys & St Thomas Nhs Foundation Trust, London, United Kingdom; [3]Lane fox unit, Guys & St Thomas Nhs Foundation Trust, London, United Kingdom; [4]Centre for human health & performance, University College London, London, United Kingdom; [5]Cardiovascular genetics, University College London, London, United Kingdom; [6]Critical care, King's College Hospital, London, United Kingdom; [7]Critical care, University Hospital of Wales, Cardiff, United Kingdom; [8]Critical care, UCL Hospitals NHS Foundation Trust , London, United Kingdom; [9]Critical care, Bristol Royal Infirmary, Bristol, United Kingdom; [10]Critical care, Blackpool Victoria Hospital, Blackpool, United Kingdom; [11]Critical care, University Hospitals of North Midlands, Stoke-on-Trent, United Kingdom
**Correspondence:** A. McNelly
*Intensive Care Medicine Experimental* 2019, **7(Suppl 3):**000212

**INTRODUCTION.** Early rapid loss of skeletal muscle is common in critical illness and is associated with negative outcomes[1-4]. Continuous feeding, routine in most intensive care units (ICUs), suppresses myofibrillar protein synthesis[5], whereas intermittent feeding may augment it[6]. We report safety data from a study comparing muscle mass after intermittent versus continuous enteral feeding during early ICU care.

**METHODS.** Adults (mechanically ventilated ≥48hours, on ICU ≥7days, with multi-organ failure) recruited within 24 hours of admission to one of 8 UK ICUs were randomised to receive four-hourly intermittent feed (intervention) or continuous (control) enteral feed for 10 days (NCT02358512). We recorded events associated with significant morbidity or death (Adverse Events, AEs; Serious Adverse Events, SAEs), including pulmonary aspiration; gastric intolerance (vomiting, diarrhoea [Bristol Stool Score ≥5], need for prokinetics use, gastric residual volume (GRV)>300mls); adverse blood glucose levels (≥10.1mmol/l hyperglycaemia, ≤3.9mmol/l hypoglycaemia); Coefficient of Glucose Variation [CGV]). Daily insulin use was also documented. Data are reported descriptively and analysed by two sample t-tests or Mann-Whitney U-tests as appropriate.

**RESULTS.** Participants (mean [95% Confidence Intervals] age: 57.7 [54.7-60.6] years; 67% male) received intermittent (n=62) or continuous (n=59) enteral feed. No cases of pulmonary aspiration were reported. Groups did not differ in proportion of days in which prokinetics were used, GRVs were >300ml, or in which diarrhoea, vomiting, hypoglycaemia or hyperglycaemia occurred. Daily insulin use was also similar. Glucose variation was greater in those intermittently fed (p<0.001, Table 1).

Ten SAEs and AEs were reported (3, Continuous vs. 7, Intermittent): two of latter AEs were probably/possibly considered to be intervention-related (both due to abnormal glucose levels). One patient transferred from intermittent to continuous feed (consultant decision).

**CONCLUSION.** Intermittent enteral feeding in early critical illness is safe and associated with similar levels of gastric intolerance, but greater glucose variation than continuous feeding; however, daily insulin use was similar between patient groups.

**REFERENCE(S)**

1. Puthucheary, Z.A., Acute skeletal muscle wasting in critical illness. JAMA, 2013. 310(15): p. 1591.
2. Herridge, M.S., One-year outcomes in survivors of the acute respiratory distress syndrome. N Engl J Med, 2003. 348(8): p. 683.
3. Ali, N.A., Acquired Weakness, Handgrip Strength, and Mortality in Critically Ill Patients. Am J Resp Crit Care Med, 2008. 178(3): p. 261.
4. Gamrin-Gripenberg, L., An attenuated rate of leg muscle protein depletion and leg free amino acid efflux over time is seen in ICU long-stayers. Crit Care, 2018. 22(1): p. 13.
5. Atherton, P.J., Muscle full effect after oral protein: time-dependent concordance and discordance between human muscle protein synthesis and mTORC1 signaling. Am J Clin Nutr, 2010. 92(5): p. 1080.
6. Phillips, S.M., Alterations of protein turnover underlying disuse atrophy in human skeletal muscle. J Appl Physiol, 2009. 107(3): p. 645.

• American Society of Parenteral and Enteral Nutrition
• LRCN (London South & North Thames)
• Intensive Care Foundation (New Investigator Award)
•NIHR UCL/UCLH BRC Cardiometabolic research grant
• Moulton Charitable Foundation

**Table 1 (abstract 000212).** Mean percentage of days of specific events occurring out of total possible days (%), 95% Confidence Intervals (CI); INT: Intermittent; CONT: Continuous; GRV: Gastric residual volume; CGV: Coefficient Glucose Variation; *p<0.001

| Mean percent daily events,%; 95%CI | INT | INT | CONT | CONT |
|---|---|---|---|---|
| Diarrhoea | 35.9 | 27.9-43.9 | 28.1 | 20.9-35.3 |
| Vomiting | 0.83 | (-0.2)-1.8 | 3.7 | 0.8-6.6 |
| Prokinetics use | 13.8 | 6.3-21.3 | 20.8 | 13.0-28.7 |
| GRV (>300mls) | 16.1 | 10.0-22.2 | 21.3 | 14.6-28.0 |
| Glucose: <3.9mmol/l | 2.1 | (-0.3)-4.5 | 1.5 | (-0.3)-3.2 |
| Glucose: >10.1mmol/l | 52.0 | 42.7-61.3 | 39.7 | 30.6-48.9 |
| CGV* | 17.8 | 16.9-18.7 | 13.0 | 12.2-13.9 |
| Insulin use | 34.0 | 23.1-45.0 | 34.7 | 23.8-45.6 |

### 001457

#### Associations between anesthesia type and transfer to higher level of care in patients with rapid response team activation within 24 hours after undergoing anesthesia

C. Wu[1], A. Gallo De Moraes[2], Y. Dong[3], RD. Frank[4], JB. Jensen[5]
[1]Anesthesiology, West China Hospital, Sichuan University, Chengdu, China; [2]Pulmonary and critical care medicine, Mayo Clinic, Rochester, United States of America; [3]Anesthesiology and perioperative medicine, Mayo Clinic, Rochester, United States of America; [4]Biomedical statistics and informatics, Mayo Clinic, Rochester, United States of America; [5]Anesthesiology and critical care medicine, Mayo Clinic, Rochester, United States of America
**Correspondence:** C. Wu
*Intensive Care Medicine Experimental* 2019, **7(Suppl 3):**001457

**INTRODUCTION.** Rapid response teams (RRT) provide critical care services for deteriorating patients outside of intensive care units to stabilize them, and if necessary, facilitate transfer to a higher level of care within the hospital [1]. There is a paucity of literature looking into characteristics of RRT activations in the post-operative period. There is no data looking into the correlation of RRT activations and outcomes and the types of anesthesia used for adult patients.
**OBJECTIVES.** Our objective was to explore if specific anesthesia types had any impact in the disposition of patients who required RRT activation during the first 24 hours postoperative period.
**METHODS.** Patients with RRT activations that occurred within 24 hours after receiving anesthesia at Mayo Clinic in Rochester from July 1, 2012 to November 1, 2017 were included. Only the first RRT activation was included in those patients who had multiple RRT activations. Anesthesia types were extracted from established Operation Room DataMart and divided into 6 groups: general anesthesia (GA), regional block (RB), local anesthesia (LA), sedation (SE), monitored anesthesia care (MAC) and combined anesthesia with general anesthesia and regional block (CO).
**RESULTS.** A total of 2,046 (54% men) unique patients aged >=18 years (median 65 years) with RRT activations within 24 hours after procedures were identified and their anesthesia types were: 825 (40 %) GAs, 91 (4 %) RBs, 165 (8 %) LAs, 445 (22%) SEs, 329 (16 %) MACs and 191 (9 %) COs. 62% of those patients were ASA 3 or higher. The median time to the RRT calls after anesthesia was 8 hours (interquartile range: 4, 16). The most common reason for RRT activations in each group was due to cardiovascular issue, accounting 61% for 2046 RRT activations and including bradycardia (5%), tachycardia (24%), hypotension (30%), hypertension (6%), chest pain (6%) and other symptoms (1%). 897 patients (44%) were transferred to a higher level of care unit immediately after RRT activations. Multivariable logistic regression analysis revealed the following factors to be associated with increased transfers to higher levels of care: anesthesia type (p=0.004), the procedure type (p<0.001), preoperative history of hypertension (p<0.001), intraoperative use of vasoactive drugs (p=0.02). Compared with the GA group, patients who underwent LA (OR=2.39; p<0.05) and SE (OR=1.83; p<0.01) had significant more transfers to a higher level of care.
**CONCLUSION.** Patients who had RRT activations during the first 24 hours after local anesthesia or sedation required transferring to the higher level of care more often, when compared to general anesthesia. We hypothesize that this occurs because the above patients who underwent local anesthesia or sedation had a higher ASA status and more serious disease so that they might not be able to undergo the complicated procedures, and the anesthesia type might not be a direct factor. Maintaining vigilance in non-GA patients is critical to ensuring postoperative safety in these patients.

### 000128

#### The correlation between length of ward stay prior to icu admission and mortality

A. Taylor[1], D. Leith[2], T. Samuels[2], P. Morgan[2]
[1]Critical care, East Surrey Hospital, Redhill, United Kingdom; [2]Critical care, East Surrey Hospital, Redhill , United Kingdom
**Correspondence:** A. Taylor
*Intensive Care Medicine Experimental* 2019, **7(Suppl 3):**000128

**INTRODUCTION.** Correlation between mortality and the length of hospital stay (LOS) prior to ICU admission is relatively understudied. Some studies have previously shown that admission to ICU from the ward vs from ED, and LOS prior to ICU admissions, is associated with higher mortality[1-4]. This study aimed to determine if LOS prior to admission to the ICU significantly affects mortality rate in emergency admissions in an undifferentiated patient population at a district general hospital. We looked for correlation between LOS and likelihood of requiring invasive ventilation and renal replacement therapy (RRT).
**METHODS.** This study is a retrospective analysis of 13,270 patients from the WardWatcher Database at the ICU at East Surrey Hospital. Analysis included all unplanned ICU admissions (n=8,623). Patient data on LOS prior to ICU admission was compared against hospital and unit mortality, and requirement for invasive ventilation and RRT. The mean APACHE II score and the absolute number of patients admitted with respect to LOS prior to ICU admission was also recorded. We used regression analysis and bootstrapping to assess for significant correlations, producing an R-square value for mortality, the need for invasive ventilation and RRT.
**RESULTS.**
Both the hospital mortality and the ICU mortality increased as LOS on the ward prior to ITU admission increased (fi.g1). Using bootstrapping (1000 samples), we estimated the R-squared value and 95% confidence interval for mortality in the groups with 14 and 28 day stays on the ward as 0.94 [0.88 - 0.97] and 0.26 [0.01 - 0.66] respectively. There was also a weak positive correlation between the mean APACHE II score (days 0-14) and LOS prior to ICU admission (R= 0.4634). However, there was almost no correlation between LOS prior to admission and the need for ventilation/RRT (fig.4).

**CONCLUSION.** In conclusion, this study found no correlation between duration of stay in hospital prior to ICU admission, and requirement for invasive ventilation and RRT. Interestingly, however, there was a strong positive correlation between length of ward stay and hospital and unit mortality up to 14 days. A similar correlation, albeit not as strong, was seen with APACHE II scores and LOS prior to ICU admission. Patients admitted to ICU from the ward have been previously shown to have higher illness severity scores compared to those admitted from ED[4]. More work looking into ward patients' antecedent events prior to ICU referral is needed to establish whether there are predictors of a requirement for critical care that could prompt earlier referral, and whether earlier critical care input actually improves outcomes in this cohort.

**REFERENCE(S)**

1. Moztkus et al (2017) ICU Admission Source as a Predictor of Mortality for Patients With Sepsis, J Intensive Care Med vol.33 (9) 510-516
2. Molina et al. (2014) Outcomes of direct and indirect medical intensive care unit admissions from the emergency department of an acute care hospital: a retrospective cohort study . BMJ Open 2014;4:e005553. doi:10.1136/bmjopen-2014-005553
3. Hillman et al. (2002) Duration of life-threatening antecedents prior to intensive care admission Intensive Care Med (2002) 28:1629–1634 DOI 10.1007/s00134-002-1496-y
4. Goldhill et al. (2004) The longer patients are in hospital before Intensive Care admission the higher their mortality. Intensive Care Med. 2004 Oct;30(10):1908-13. Epub 2004 Jul 23.

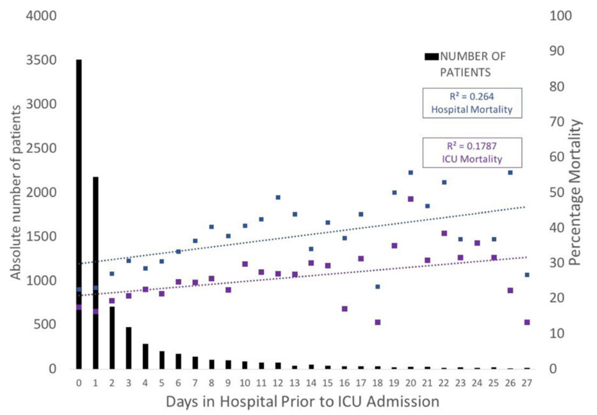

**Fig. 1 (abstract 000128).** Graph showing number of patients admitted to the ICU (days 0-28) on primary y-axis. Graph showing percentage hospital mortality and percentage ICU mortality with respect to number of days in hospital prior to ICU admission secondary y-axis

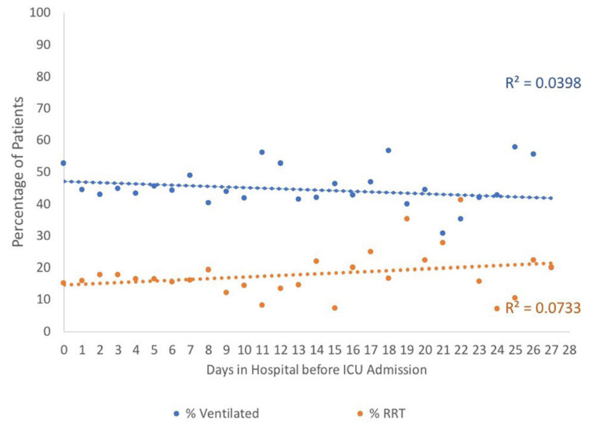

**Fig. 4 (abstract 000128).** Percentage of patients requiring invasiveventilation and RRT with respect to lenght of hospital stay prior toICU admission

### 000331

#### Predictive performance of SAPS 3, SOFA, ISS and New ISS in trauma patients admitted to a specialized trauma ICU

RML. Roepke, RM. Guazzelli, RDD. Oliveira, E. Bassi, EM. Utiyama, LMS. Malbouisson

Trauma and emergency surgery intensive care unit, Hospital das Clínicas Faculdade de Medicina USP, São Paulo, Brazil

**Correspondence:** R. Roepke

*Intensive Care Medicine Experimental* 2019, **7(Suppl 3):**000331

**INTRODUCTION.** Several specific predictive models were developed to predict outcomes in victims of trauma. Among the most widely used are those based on anatomical injury patterns, derived from the Abbreviated Injury Scale (AIS): the Injury Severity Score (ISS) and its later modification New Injury Severity Score (New ISS). However, it's not known whether these scores perform better than general critical care scores for trauma patients admitted to the intensive care unit (ICU).

**OBJECTIVES.** To compare the predictive ability for hospital mortality of a general critical care score (SAPS 3), an organ dysfunction score (SOFA) and anatomical injury based scores (ISS and New ISS).

**METHODS.** Retrospective cohort study of patients admitted to a specialized ICU from a tertiary university hospital in São Paulo, Brazil between March, 2012 and January, 2016. Admission data were prospectively collected in the ICU clinical database. We retrieved SAPS 3 and SOFA within 24 hours of ICU admission from the database. Both ISS and New ISS were retrospectively calculated based on the analysis of whole-body CT results and medical records by three independent and trained evaluators. We compared the different models overall accuracy with area under the receiving operator characteristic (AUROC) curves using the DeLong method.

**RESULTS.**

The study cohort included 1110 victims of trauma admitted to the ICU. Males were 84.1% of the patients and mean age was 40 (±18) years. Main injury mechanism was blunt trauma (89.1%). Traumatic brain injury was present in 733 patients (66%). Whole-body CT scan was performed in 689 patients (62%). At the time of ICU admission, 861 patients (77.6%) were on mechanical ventilation and 549 (49.5%) on vasoactive drugs. Hospital mortality was 26.7% (296 patients), ICU length of stay (LOS) was 7 [4,15] days and hospital LOS was 15 [7,33] days. Mean SAPS 3 was 48.6 (±16.5) and mean SOFA was 6.4 ± 4.2. Mean ISS was 30 (±12) and New ISS 41 (±15). In a complete case analysis, 918 patients were included. AUROCs (95% CI) were as follows: SAPS 3 = 0.79 (0.76; 0.82); SOFA = 0.78 (0.75; 0.81); ISS = 0.60 (0.56; 0.64); 0.67 (0.63; 0.71). In pairwise comparisons, SAPS 3 and SOFA did not differ (p = 0.53), while both outperformed the anatomical scores (p <0.001); New ISS was a better predictor than ISS (p <0.001).
**CONCLUSION.** Critical care predictive scores outperform anatomical based injury scores in predicting hospital mortality in trauma patients admitted to the intensive care unit.

**REFERENCE(S)**

1. Vincent JL, Moreno R, Takala J, et al. The SOFA (Sepsis-related Organ Failure Assessment) score to describe organ dysfunction/failure. Intensive Care Med. 1996;22:707–710.
2. Moreno RP, Metnitz PG, Almeida E, et al. SAPS 3–From evaluation of the patient to evaluation of the intensive care unit. Part 2: Development of a prognostic model for hospital mortality at ICU admission [published correction appears in Intensive Care Med. 2006 May;32(5):796]. Intensive Care Med. 2005;31(10):1345–1355. doi:10.1007/s00134-005-2763-5
3. No funding.
4. Osler T, Baker SP, Long W. A modification of the Injury Severity Score that both improves accuracy and simplifies scoring. J Trauma. 1997;43(6):922-5.
5. Baker SP, O'Neill B, Haddon W, et al. The Injury Severity Score: a method for describing patients with multiple injuries and evaluating emergency care. J Trauma. 1974;14:187–196

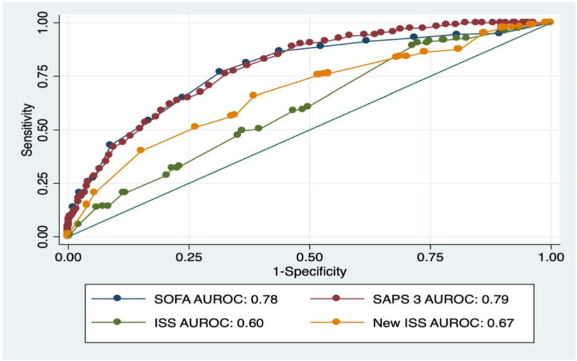

**Fig. 1 (abstract 000331).** See text for description

### 000606

#### Effects of different visiting policies on ICU syndrome: A Systematic review and Meta-analysis

W. Yuchen[1], J. Biantong[2], D. Nannan[2], Z. Zhigang[1]
[1]Department of icu, The First Hospital of Lanzhou University, Lanzhou, China; [2]School of nursing, lanzhou university, Lanzhou University, Lanzhou, China
**Correspondence:** W. Yuchen

*Intensive Care Medicine Experimental* 2019, **7(Suppl 3):**000606

**INTRODUCTION.** At present, there are so many ICUS implement restrictive visiting system at home and abroad, which could reduce ICU acquired infection by limiting visiting time, number of visitors and other ways. The concentrated visiting method enables family members and patients to have certain communication and contact, and that maybe alleviates the anxiety of family members and patients [1-2]. One study found that the restricting relative visits is the main cause of patients' pain [3], and another study also found that ICU patients who lack visitation have a more than threefold increased risk of delirium[4], but unrestricted relative visitation may increase infection, affect medical treatment work, and increase job burnout of ICU medical staff, etc. [2,5]. However, at present, there is no relevant guidance or consensus on the visiting of relatives of ICU patients at home and abroad [1,2], which visiting mode is more beneficial to ICU patients, and there is no clear concept on the visiting time and number of the two visiting modes.
**OBJECTIVES.** To compare the effects of un-restrictive visiting policy(UVP) and restrictive visiting policy(RVP) on ICU patients' delirium and ICU acquired infection, to explore the best visiting policy in ICU.
**METHODS.** Randomized controlled trails (RCTs) and quasi-experiment about UVP and RVP in ICU were retrieved in CBM, CNKI, Wanfang Data, PubMed, Cochrane Library and Web of Science from their foundation to Decmber 31 2018, and other sources as supplement was also retrieved. Data were extracted after strict evaluation of literature quality by two researchers, and Meta-analysis was conducted on literatures that met the inclusion criteria.
**RESULTS.** A total of 18 studies and 4647 patients were included in this study. There are 8 RCT studies and 10 quasi-experimental studies. The included studies were divided into subgroups according to study design and length of visitation. The results of the forest plot showed that UVP can not only effectively decrease the incidence of ICU patients delirium [RR = 0.19, 95% CI (0.10, 0.18), P < 0.00001), reduce the ICU patients anxiety score [RR = 1.60, 95% CI (1.67, 1.54), P = < 0.00001] and depression score [RR = 1.63, 95% CI (2.76, 0.49), P = 0.005), as well as effectively improve the family members' satisfaction [RR=3.56, 95%CI(2.32,5.48),P=<0.00001]. Meanwhile, the UVP did not increase the incidence of ICU acquired infection [RR=0.82, 95%CI(0.53,1.36),P= 0.31], ICU mortality [RR=0.61, 95%CI (0.21,1.79), P=0.37], and length of ICU stay [RR=-0.40, 95%CI(-1.29, 0.50),P=0.39]. In addition, without considering the study design, we found that the UVP may reduce the incidence of ICU acquired infection [OR=0.65, 95%CI (0.51,0.816), P= 0.002], and the longer the visit, the lower the incidence of ICU acquired infection [OR=0.75, 95%CI (0.61,0.91), P=0.004].
**CONCLUSION.** The UVP does not increase the incidence of ICU acquired infection , the length of ICU stay and the ICU mortality. On the other hand, the UVP can effectively decrease the incidence of ICU patients' delirium, reduce the ICU patients' anxiety score and depression score and improve the ICU patients family' satisfaction.

**REFERENCE(S)**

[1] Cappellini E, Bambi S, Lucchini A, et al. Open intensive care units: a global challenge for patients, relatives, and critical care teams[J]. Dimensions of critical care nursing : DCCN. 2014,33(4):181-193
[2] Nassar Junior A P, Besen B, Robinson C C, et al. Flexible Versus Restrictive Visiting Policies in ICUs: A Systematic Review and Meta-Analysis[J]. Crit Care Med. 2018,46(7):1175-1180
[3] Nelson JE, Meier DE, Oei EJ, et al. Self-reported symptom experience of critically ill cancer patients receiving intensive care. Crit Care Med. 2001;29:277-282
[4] Van Rompaey B, Elseviers MM, Schuurmans MJ, et al: Risk factors for delirium in intensive care patients: A prospective cohort study. Crit Care 2009; 13:R77
[5] Mitchell M Laitken L M. Flexible visiting positively impacted on patients, families and staff in an Australian Intensive Care Unit: A before-after mixed method study[J]. Australian critical care: official journal of the Confederation of Australian Critical Care Nurses. 2017,30(2):91-97

## NIC - Neurocritical care 1

### 000110

#### Characteristics and outcomes of elderly traumatic brain injured patients admitted to ICU. Data from Center-TBI

F. Fossi[1], C. Robba[2], F. Graziano[1], C. Iaquaniello[1], P. Rebora[1], E. Banzato[1], E. Wiegers[3], N. Stocchetti[4], M. Carbonara[5], A. Vargiolu[6], G. Citerio[1]
[1]School of medicine and surgery, University of Milano-Bicocca, Milano, Italy; [2]Department of anaesthesia and intensive care, IRCCS AOU San Martino, Genova, Italy; [3]Dept. of public health, Erasmus University Medical Center, Rotterdam, Netherlands; [4]Neurointensive care unit, Fondazione IRCCS Ca'Granda Ospedale Maggiore Policlinico, Milano, Italy; [5]Neurointensive Care, Fondazione IRCCS Ca' Granda Ospedale Maggiore Policlinico, Milano, Italy; [6]Neurointensive care, department of emergency and intensive care, Ospedale San Gerardo di Monza, Monza, Italy
**Correspondence:** F. Fossi
*Intensive Care Medicine Experimental* 2019, **7(Suppl 3):**000110

**INTRODUCTION.** The epidemiology of traumatic brain injury (TBI) is rapidly changing, with an increasing number of old and fragile patients. The aim of this study is to describe the characteristics of elderly TBI and the effect of age on patients' outcome.
**METHODS.** The CENTER-TBI (clinicaltrials.gov registration NCT02210221) is a prospective observational longitudinal cohort study including patients with TBI from centers across Europe. Data were extracted from the CENTER-TBI database v1.1 with Neurobot v2.6. Inclusion criteria were:

- diagnosis of TBI, with indication for Computed Tomography (CT),
- presentation to study center within 24 hours of injury.
- ICU stay >72h.

We excluded patients discharged alive from the ICU in < 72 hrs. Patients aged ≥65 were classified as elderly.
**RESULTS.** A total of 4509 patients were included in the CENTER-TBI study, 2138 were admitted in the ICU and we focused on the 1488 fulfilling the inclusion criteria. Overall, mean age was 48.3 years (sd = 20.37) and 395 patients (26.5%) were considered elderly. Elderly patients presented significant differences (p<0.001) with respect to not elderly both in the admission characteristics and in the discharge status:

- higher incidental fall rate (59% vs 32.9%), mainly at home (43.3% vs 17.3%)
- higher morbidities, as cardiovascular history (70% vs 13.4%), or pulmonary disease (13.8% vs 8.6%)
- higher use of anticoagulants (20.6% vs 1.3%) and antiplatelet (27.6 % vs 3.9%)
- higher Glasgow Coma Score at admission (GCS higher than 13: 24.8% vs 15.5%)
- higher non-evacuated mass lesion (Marshal score 6 in 54.3% vs 31.1%)
- more frequent subdural hemorrhage (73.3% vs 51.8%)
- less Intracranial pressure monitoring (ICP 46.7% vs 63.9%)
- less days in ICU (8 days vs 12 days, in median)
- higher mortality in ICU (32.7% vs 13.2%) and at 6 months (52.7% vs 18.6%)
- more unfavorable status (GOSE ≤4: 79.2 % vs 46.2%) but similar severe disability (lower or upper) at 6 months (26.5% vs 27.6% p= 0.728)

No statistically significant differences according to age were shown in pre-hospital hypotension (14.7% vs 17.3%, p=0.285), hypoxia (13.2% vs 17.7%, p=0.052) and also in the discharge location (in both cases, general ward was the most common 50.7% vs 48.8%, p=0.599).
**CONCLUSION.** Elderly patients have more comorbidities, different mechanism of injury, more severe CT scan findings, and have a higher mortality when compared to the younger population.

### 000588

#### Acute kidney injury in traumatic brain injured patients: results from the CENTER TBI study

C. Iaquaniello[1], C. Robba[2], E. Banzato[1], E. Wiegers[3], F. Fossi[1], F. Cipulli[1], F. Graziano[1], A. Vargiolu[4], P. Rebora[1], G. Citerio[1]
[1]School of medicine and surgery, University of Milano-Bicocca, Milano, Italy; [2]Department of anaesthesia and intensive care, IRCCS AOU San Martino, Genova, Italy; [3]Department of public health, Erasmus University Medical Center, Rotterdam, Netherlands; [4]Neurointensive care, department of emergency and intensive care, Ospedale San Gerardo di Monza, Monza, Italy
**Correspondence:** C. Iaquaniello
*Intensive Care Medicine Experimental* 2019, **7(Suppl 3):**000588

**INTRODUCTION.** Acute kidney injury (AKI) is frequent in polytrauma patients and it is associated with increased mortality, hospital stay, and morbidity. However, the real prevalence and impact on the outcome of AKI in isolated traumatic brain injury (TBI) are unclear. Our aims were

1. to assess the incidence and timing of AKI in a large cohort of prospectively enrolled TBI,
2. to assess in-hospital factors related to AKI development;
3. to assess the association between AKI at different stages and patients' outcome.

**METHODS.** The CENTER-TBI is a prospective observational longitudinal cohort study including patients with TBI from centers across Europe. Data were extracted from the CENTER-TBI database v1.1 with Neurobot v2.6. Inclusion criteria for our study were ICU length of stay >72 h and > two creatinine values or at least one urine output (UO) available measures during the ICU stay.
AKI was defined by applying the RIFLE criteria.
**RESULTS.** 4509 patients were included in the CENTER-TBI study, of which 2138 were admitted to the ICU. 1317 fulfilled the inclusion criteria. Patients were grouped in three categories: normal renal function (N), risk or injury (R/I) and failure (F). The median age in the 3 groups was similar (around 50 y/o). The majority of AKI occurred within the first 10 days from admission, with a median of 2 days (IQR: 1-4 days) for the R/I category, and a median of 4 days (IQR: 1-6 days) for the F group. Patients with history of cardiovascular (CV) disease had a higher occurrence of both R/I and F (F= 45%, R/I = 31% vs N= 25.5%, p = 0.003). During the ICU stay, patients in both the R/I and in the F group suffered more from: episodes of neuroworsening (F= 45%, R/I= 43% vs N= 30%, p < 0.001), raised ICP (F= 34%, R/I= 39% vs N= 27%, p=0.003), respiratory failure (F= 40%, R/I= 44% vs N= 29%, p < 0.001)and pulmonary edema (F= 2%, R/I= 6% vs N= 1%, p < 0.001). Unsurprisingly, patients in the F group underwent more renal replacement therapy (F= 27% vs R/I= 3.5%, N= 2%, p < 0.001).

Median length of stay in ICU for R/I (14 days, IQR 7.5–28.5) and F (15 days, IQR 8 –26) is longer than that of N patients (11 days, IQR 6–19.5). Patients in the R/I and in the F groups showed higher ICU mortality (F 25.5%, R/I 27% vs N 9%, p < 0.001) as well as higher 6-month mortality (F 40%, R/I 28% vs N 17.5%, p < 0.001) and higher incidence of 6-month unfavorable outcome (GOSE≤4 in F 62%, R/I 66% vs N 47.5%, p < 0.001).

**CONCLUSION.** The development of AKI seems to be an early phenomenon, associated with the presence of previous CV history. It is associated with increase LOS, ICU mortality and 6-month unfavorable outcome.

### 000838

#### Burden of intracranial hypertension in subarachnoid hemorrhage in relationship with the cerebrovascular autoregulatory status

G. Carra[1], V. Rass[2], F. Elli[3], I. Piper[4], B. Depreitere[5], BA. Ianosi[2], R. Helbok[2], G. Citerio[3], F. Guiza Grandas[1], G. Meyfroidt[1]

[1]Department and laboratory of intensive care medicine, Katholieke Universiteit Leuven, Leuven, Belgium; [2]Department of neurology, Medizinische Universität Innsbruck, Innsbruck, Austria; [3]School of medicine and surgery, University of Milano-Bicocca, Monza, Italy; [4]Department of clinical physics, University of Glasgow, Glasgow, United Kingdom; [5]Department of experimental neurosurgery and neuroanatomy, Katholieke Universiteit Leuven, Leuven, Belgium

**Correspondence:** G. Carra

*Intensive Care Medicine Experimental* 2019, **7(Suppl 3):**000838

**INTRODUCTION.** In patients with Traumatic Brain Injury (TBI), impaired cerebrovascular autoregulation (CAR), decreases the ability of the brain to tolerate the burden of elevated ICP [1]. This burden is defined as the combination of the intensity and duration of events of elevated ICP. The association with the 6-month Glasgow Outcome Score (GOS) can be visualized with the color-coded plots proposed by Güiza [1]. In these plots, impaired CAR shifted the transition curve, which divides good ad poor neurological outcome, towards lower ICP values. This methodology can be applied to other neuro-monitored patients. The proposed study aimed to investigate whether CAR status plays a role in determining the ability of the brain to sustain elevated dose of ICP after subarachnoid hemorrhage (SAH).

**METHODS.** Retrospective analysis of ICP and mean arterial blood pressure (MABP) time series of 98 patients with severe-grade SAH, prospectively collected in two large European centres, (Innsbruck University Hospital (Austria); San Gerardo University Hospital, Monza (Italy)). The methodology proposed by Güiza [1] was used to visualize the association of the dose of ICP with outcome, for active and passive CAR.

**RESULTS.** The transition curves resulting from the evaluation of ICP dose events during passive and active CAR presented a negligible difference with the "all-events" curve, Fig 1. This may be due to the low prevalence of prolonged elevated ICP, the median [IQR] percentage of ICP monitoring time above 20mmHg was equal to 6.2 % [0.4 - 6.5], where CAR status may have a stronger impact on outcomes.

**CONCLUSION.** No difference in the association between the ICP dose burden and outcome could be demonstrated for active and passive CAR. In absence of prolonged intracranial hypertension other factors, apart from CAR status, may have a more important role in determining the outcomes.

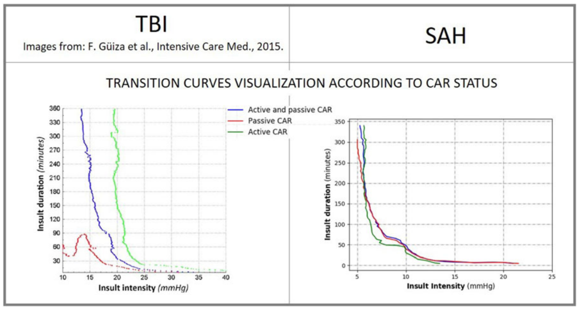

**Fig. 1 (abstract 000838).** See text for description

### 000934

#### Continuous monitoring of frontal slow wave activity in early prediction of neurological outcome after cardiac arrest

J. Kortelainen[1], T. Ala-Kokko[2], M. Tiainen[3], D. Strbian[3], K. Rantanen[3], J. Laurila[2], J. Koskenkari[2], M. Kallio[4], J. Toppila[5], E. Väyrynen[6], M. Skrifvars[7], J. Hästbacka[7]

[1]Physiological signal analysis team, center for machine vision and signal analysis, University of Oulu, Oulu, Finland; [2]Division of intensive care medicine, University of Oulu and Oulu University Hospital, Oulu, Finland; [3]Department of neurology, University of Helsinki and Helsinki University Hospital, Helsinki, Finland; [4]Department of clinical neurophysiology, University of Oulu and Oulu University Hospital, Oulu, Finland; [5]Hus medical imaging center, clinical neurophysiology, Helsinki University Hospital and University of Helsinki, Helsinki, Finland; [6]Cerenion oy, {street_address}, Oulu, Finland; [7]Department of emergency care and services, University of Helsinki and Helsinki University Hospital, Helsinki, Finland

**Correspondence:** J. Kortelainen

*Intensive Care Medicine Experimental* 2019, **7(Suppl 3):**000934

**INTRODUCTION.** Estimation of patients' neurological prognosis after cardiac arrest is challenging and reliable prediction of the outcome can usually be made only after few days. Lack of slow waves in electroencephalogram (EEG) has been associated with hypoxic ischemic encephalopathy [1]. Quantification of slow wave activity offers a potential tool for early prediction of neurological outcome in comatose cardiac arrest survivors.

**OBJECTIVES.** To investigate the association between continuously measured frontal slow wave activity and neurological outcome at six months after cardiac arrest.

**METHODS.** In this study (NCT03485781), data from 66 comatose cardiac arrest survivors treated in the intensive care units (ICUs) of Helsinki University Central Hospital and Oulu University Hospital was used. EEG was recorded from the ICU admission until 48 h from return of spontaneous circulation (ROSC) using self-adhesive disposable forehead electrode and wireless measurement device. The slow wave activity was determined by calculating the low-frequency (< 1 Hz) power of EEG. The low-frequency activity at different time points after ROSC was statistically compared between patients with good and poor neurological outcome using Wilcoxon rank sum test. The outcome was defined by evaluating the patients' neurological recovery at six months using Cerebral Performance Category (1-2 = good outcome; 3-5 = poor outcome).

**RESULTS.** The patients with poor outcome (N = 26) had less frontal slow wave activity in the early phase of ICU admission compared to the patients with good outcome (N = 40). The median low-frequency power at 6-15 h after ROSC in patients with poor outcome was 48.4 ± 9.7 % (mean ± std) compared to the median low-frequency power in patients with good outcome. The difference between groups was statistically significant until approximately 15 h from ROSC.

**CONCLUSION.** Frontal EEG slow wave activity may be a suitable objective measure for the early prediction of neurological outcome in comatose cardiac arrest survivors.

**REFERENCE(S)**

1. Kortelainen J et al. Anesthesiology 126:94-103, 2017
2. Grant 308935 from Academy of Finland

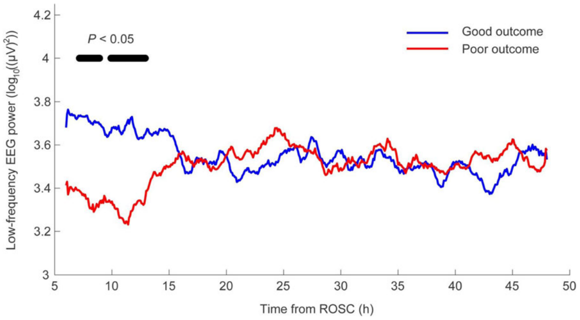

**Fig. 1 (abstract 000934).** Low-frequency EEG power in poor andgood outcome patients. The lines represent medians of groups afterreturn of spontaneous circulation (ROSC)

### 001454

#### Intracranial pressure and cerebral oxygenation at different levels of PaCO2 in critically ill patients with acute cerebral aneurysm rupture and high risk of cerebral vasospasm

A. Solodov[1], ED. Mekhia Mekhia[1], S. Petrikov[1], V. Krylov[2]
[1]Neurosurgical icu, Sklifosovsky Research Institute for Emergency Medicine, Moscow, Russia; [2]Neurosurgery, Sklifosovsky Research Institute for Emergency Medicine, Moscow, Russia
**Correspondence:** A. Solodov

*Intensive Care Medicine Experimental* 2019, **7(Suppl 3):**001454

**INTRODUCTION.** To prevent of secondary ischemic brain damage in critically ill patients with acute cerebral aneurysm rupture, a level of PaCO2 should be adjusted to achieve the best cerebral oxygenation with stable intracranial pressure (ICP). Presently the maximum safety level of hypocapnia has not been determined.

**OBJECTIVES.** To determine intracranial pressure and cerebral oxygenation in different levels of PaCO2 in patients with acute cerebral aneurysm rupture and high risk of cerebral vasospasm.

**METHODS.** We analyzed the acid-base and gas composition of arterial blood, intracranial pressure, mean arterial blood pressure (ABPmean) and cerebral perfusion pressure (CPP), jugular bulb oxygenation (SvjO2) at different levels of PaCO2 in 30 critically ill patients with acute cerebral aneurysm rupture and high risk of cerebral vasospasm (Fisher III-IV, Hunt-Hess III-IV). In this patients were recorded - 84 cases a decrease PaCO2 ≤ 30 mm Hg. and less (group 1), in 50 cases - PaCO2 30.1-33 mm Hg (group 2), in 36 cases - PaCO2 33.1-36 mm Hg (group 3), in 57 cases - PaCO2 ≥ 36.1 mm Hg (group 4).

**RESULTS.** Different levels of PaCO2 were due to different minute ventilations (MV) (Table 1). The medians of SvjO2 at different PaCO2 levels were within normal limits, but the severe hypocapnia (≤ 30.1 mmHg) was accompanied by episodes of a decrease SvjO2 ˂ 60%. The back side of the PaCO2 selection for prevention of cerebral blood flow disturbance is the increase of ICP during the elimination of hypocapnia. We identified highest ICP levels with an increase PaCO2 ≥ 33.1 mm Hg and no negative changes in cerebral oxygenation, despite a decrease in CPP. The minimum levels of cerebral perfusion pressure were noted in group 3 (Table 1).

**CONCLUSION.** Moderate hyperventilation with a decrease PaCO2 to 33.1 mm Hg. can be a safe method for the ICP correction in critically ill patients with acute cerebral aneurysm rupture and high risk of cerebral vasospasm. PaCO2 ˂ 30.1 mm Hg increased risk of cerebral desaturation and cerebral metabolism disorders.

**Table 1 (abstract 001454).** Dynamics of intracranial pressure, mean arterial blood pressure, cerebral perfusion pressure and cerebral oxygenation in different levels of PaCO2

| Parameters | Group 1 | Group 2 | Group 3 | Group 4 |
|---|---|---|---|---|
| PaCO2, mmHg | 27,9 (25;28,9) | 31,9 (31;32,3)* | 34,5 (34,1;35,3)* ≠ | 38,6 (37,3;41,7)* ≠ ¥ |
| MV, l/min | 8,7 (6,8;11,7) | 7,2 (6,6;9,3)* | 7,0 (6,8;7,3)* | 6,8 (6,4;7,1)* |
| SvjO2, % | 70 (56,6;75,5) | 73,0 (70;77,8)* | 75,5 (66;85,8)* | 78,4 (73,2;88,8)* ≠ |
| ABPmean, mmHg | 107 (99,5;114) | 106 (96;114) | 95 (83;101)* ≠ | 100 (92;110) ¥ |
| ICP, mmHg | 12 (7,9;16) | 10 (7;12) | 15 (8,3;19)* ≠ | 15 (12,8;22,8)* ≠ |
| CPP, mmHg | 95 (88;106) | 92,5 (87;103) | 84 (73;87)* ≠ | 87 (74;106) )* ¥ |

(* - p ˂ 0.05 compared 1st group, ≠ - p 0.05 compared 2nd group, ¥ - p ˂ 0.05 compared 3rd group)

## TEM - Emergency medicine: Trauma, burns and cardiac arrest

### 000528

#### Inflammatory serum cytokines in the first 6 hours of severe trauma are inversely related to hospital mortality

PA. Duarte[1], AC. Jorge,[1], TT. Chung,[1], G. Trilo Silva[2], B. Trevizan Padua[2], E. Padilha[2], RA. Menolli[3]
[1]General icu, Hospital Universitario do Oeste do Parana, Cascavel, Brazil; [2]College of biomedicine, UNIPAR – Universidade Paranaense, Cascavel, Brazil; [3]College of pharmacy and biochemistry, UNIOESTE – Universidade Estadual do Oeste do Paraná, Cascavel, Brazil
**Correspondence:** P.A. Duarte

*Intensive Care Medicine Experimental* 2019, **7(Suppl 3):**000528

**INTRODUCTION.** Objectives: To analyze the relationship between serum inflammatory cytokines in adult patients in the first hours of severe trauma, and hospital mortality.

**METHODS.** Prospective cohort study. Consecutive adult patients with trauma admitted to a teaching hospital - reference in trauma (traffic accidents or violence) care. In all patients the serum cytokines were collected within the first 6 hours of trauma. Descriptive statistics and univariate analysis were performed.

**RESULTS.** It were studied 40 patients (mean age 33.8y, 77.5% male, APACHE 21.0); 77.5% traffic accidents, 15% violence, 7.5%

falls/ work accidents, 32.5% had AKI (7% needed dyalisis), 15% ARDS. Mean ICU and hospital LOT of 11.8 and 25.2 days, respectively. Hospital mortality of 15%. Higher serum cytokines IL-1, IL-6 and TNF where inversely related to hospital mortality (table 1 and figure 1).
Figure 1

**CONCLUSION.** In adult patients with severe trauma (most of them with brain trauma), the serum level of inflammatory cytokines (mainly IL-1, IL-6 and TNF-α) in the first 6 hours of trauma is inversely related to hospital mortality.

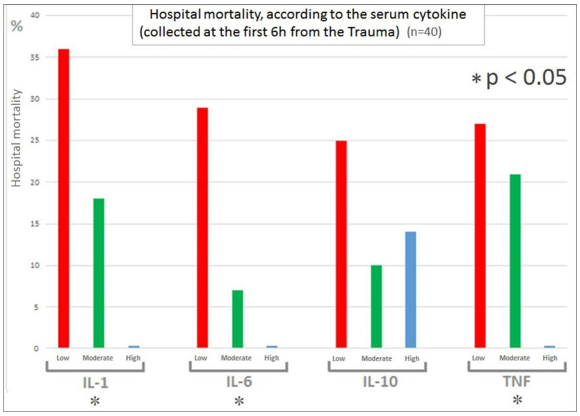

**Fig. 1 (abstract 000528).** See text for description

### 001724

#### Epidemiological patterns for early onset and late onset bacteremia after trauma

TY. Kim, L. Jeong Woo, L. Hak-Jae, H. Suk-Kyung
Dept of surgery, division of critical care and acute care surgery, Asan Medical Center, Seoul, Republic of Korea
**Correspondence:** T.Y. Kim
*Intensive Care Medicine Experimental* 2019, **7(Suppl 3):**001724

**INTRODUCTION.** Bacteremia is a major nosocomial infection problem in view of its high mortality.
The aim of this study was to identify risk factors and to describe epidemiological patterns for early onset (EOB) and late onset (LOB) bacteremia after trauma

**METHODS.** All trauma patients admitted to our surgical intensive care unit (SICU) and general ward (GW) from January 2011 to December 2015 were retrospectively enrolled. The following information was collected for each patient and recorded in a computer database: demography, severity of trauma according to the Injury Severity Score (ISS) and each abbreviated injury score (AIS), severity of coma according to the Glasgow Coma Scale (GCS), presence of shock and transfusion. Early onset bacteremia was defined as EOB when onset occurred within 7 days after trauma, and late onset bacteremia was defined as LOB when appearing after 7 days from trauma.

**RESULTS.** Thirty-seven patients developed bacteremia during their hospital stay: 9 (24.3%) EOB, 28 (75.6%) LOB, and 4 (10.8%) patients developed both of them. Sixty-eight events of bacteremia happened to these patients: 10 (14.7%) EOB and 58 (85.3%) LOB. Gram-positive cocci were isolated more frequently than Gram-negative bacilli in both groups. Gram-positive cocci were more frequently isolated in EOB than in LOB: otherwise, there was no statistical significance (80% versus 67.2%, $P = 0.702$). Central line associated blood stream infection (CLABSI) and surgical site infection (SSI) were the most common identified source for LOB. The risk of EOB was significantly increased by the presence of pelvic and liver injury on arrival in emergency department (ED), transfusion within the first 24 hours after trauma. Presence of liver and pancreas injury, gastrointestinal tract perforation and shock represented risk factors for LOB. LOB had a different pattern and their risk was significantly increased by ISS ($P = 0.002$) and abdominal injury score ($P = 0.003$).

**CONCLUSION.** Presence of pelvic and liver injury on arrival in ED and transfusion within the first 24 hours after trauma appear a significant risk factor for EOB. Scoring with the ISS and intra-abdominal injury at admission to ED appears a useful tool for identifying trauma patients at increased risk of LOB.

**REFERENCE(S)**

1. Hensler, T., et al., Distinct mechanisms of immunosuppression as a consequence of major surgery. Infect Immun, 1997. 65(6): p. 2283-91.
2. Mandragos, E., et al., Survival after multiple traumas is associated with improved outcomes from gram-negative sepsis: Clinical and experimental evidence. J Infect, 2017. 74(2): p. 163-171.
3. Menges, P., et al., Surgical trauma and postoperative immune dysfunction. Eur Surg Res, 2012. 48(4): p. 180-6.
4. Tschoeke, S.K. and W. Ertel, Immunoparalysis after multiple trauma. Injury, 2007. 38(12): p. 1346-57.
5. Sadjadi, J., et al., Transfusion, not just injury severity, leads to posttrauma infection: a matched cohort study. Am Surg, 2009. 75(4): p. 307-12.
6. Laupland, K.B., et al., Cost of intensive care unit-acquired bloodstream infections. J Hosp Infect, 2006. 63(2): p. 124-32.
7. Niven, D.J., et al., Cost and outcomes of nosocomial bloodstream infections complicating major traumatic injury. J Hosp Infect, 2010. 76(4): p. 296-9.
8. Rello, J., et al., Polymicrobial bacteremia in critically ill patients. Intensive Care Med, 1993. 19(1): p. 22-5.
9. Antonelli, M., et al., Early and late onset bacteremia have different risk factors in trauma patients. Intensive Care Med, 1996. 22(8): p. 735-41.
10. Rello, J., et al., Nosocomial bacteremia in a medical-surgical intensive care unit: epidemiologic characteristics and factors influencing mortality in 111 episodes. Intensive Care Med, 1994. 20(2): p. 94-8.

### 001435

#### Arrhythmias in Critically Burn Patients with Inhalation Injury

A. Garcia Muñoz, C. Gutiérrez Mavarez, C. Arevalo Martin, M. Sanchez, B. Civantos Martin, E. Flores, L. Cachafeiro, M. Hernandez, J. Manzanares, A. Agrifoglio, A. García De Lorenzo
Intensive care unit, Hospital La Paz, Madrid, Spain, Spain
**Correspondence:** C. Gutierrez
*Intensive Care Medicine Experimental* 2019, **7(Suppl 3):**001435

**INTRODUCTION.** The burned patient is one of the most complex cases and Intensivist can face, with a high mortality rate and multiples connotations on the hemodynamics of the patient. Those with Inhalation Injury have a worst prognosis, typically associated with respiratory complications. However, the possible effects of inhalation in the cardiac conduction and therefore in the development of critical arrhythmias have been less studied.

**OBJECTIVES.** To Study the possible association of toxic gas inhalation with the development of critical arrhythmias in burned patients with Inhalation Injury.

**METHODS.** Retrospective observational study performed in the Critical Burn Unit (January 2017 to February 2019) including 16 patients over 18 years old with a high suspicion or confirmed Inhalation Injury. Mean and standard deviation were used for normal quantitative variables and median and interquartile range in the opposite case. Qualitative variables were presented by absolute and relative frequencies.

**RESULTS.** We studied 152 patients admitted in our Critical Burn Unit of the La Paz Hospital in Madrid Spain from January 2017 until February 2019. 16 patients (10.5%) with high suspicion or compatible criteria with Inhalation Injury, with initial carboxyhemoglobin over 4% at admission. Mean age was 59 (± 17.1), Median burned TBSA was 17% (RI 6-36%), Median APACHE II was 13 (RI 10-19), Median ABSI score was 7 (RI 5-9). The initial Carboxyhemoglobin at the burn site was not registered in the majority of the patients, so we couldn't study it's correlation with the evolution of the patients. 65% of the patients received treatment with 5grs of hydroxocobalamin before

Hospital Admission. The median initial levels of Carboxyhemoglobin at admission in the ICU was 13 (RI 5-22), mean Lactate at admission was 4.2. 31% of patients had corrected QT interval prolongation. Troponin I was measured only in 8 patients, 75% had elevated levels in the first 24hours. Mortality was 25% (4 patients), 2 patients developed Ventricular tachycardia followed by cardiac arrest.

**CONCLUSION.** There might be correlation between toxic gas mediated Inhalation Injury and electrocardiographic disorders as suggested by the prolongation of the Corrected QT interval, which represents a good indicator of myocardial repolarization, elevation of Troponin I levels and the development of cardiac arrhythmias. Further studies must be performed.

**REFERENCE(S)**

1. Dallas, Mark L et al. "Carbon monoxide induces cardiac arrhythmia via induction of the late Na+ current." American journal of respiratory and critical care medicine vol. 186,7 (2012): 648-56. doi:10.1164/rccm.201204-0688OC
2. Kaita, Yasuhiko et al. Cyanide poisoning is a possible cause of cardiac arrest among fire victims, and empiric antidote treatment may improve outcomes. The American Journal of Emergency Medicine , Volume 36 , Issue 5 , 851 - 853
3. Peers C, Steele DS. Carbon monoxide: a vital signalling molecule and potent toxin in the myocardium. J Mol Cell Cardiol 2012;52:359–365
4. Walker PF, Buehner MF, Wood LA, et al. Diagnosis and management of inhalation injury: an updated review. Crit Care. 2015;19:351. Published 2015 Oct 28.

### 000161

#### Pre-empting In-Hospital Cardiac Arrests

A. Doolan, J. Clarke, S. Clarke, C. Broe, M. Power, G. Curley
Anaesthesia and critical care, Beaumont Hospital, Dublin, Ireland
**Correspondence:** A. Doolan
*Intensive Care Medicine Experimental* 2019, **7(Suppl 3):**000161

**INTRODUCTION.** There continues to be a significant mortality burden associated with cardiac arrests. The percentage discharged home remains low. (5 to 27%) [1,2] A failure to recognise clinical antecedents and involve intensivists has been found in previous studies. [2, 3]

**OBJECTIVES.** 1. Identify the frequency and outcomes of in-hospital cardiac arrests.

2. Asses were the cardiac arrests preceded by clinical antecedents in the 24 hours before arrest and were they recognised and acted upon

**METHODS.** An audit was performed of all in-hospital cardiorespiratory arrests in 2017. Cardiac arrests in ED, endoscopy, theatre and ITU were excluded. In the 24 hours before arrest, all clinical antecedents to suggest deterioration were noted.

**RESULTS.** 37 of the 23,398 inpatients had cardiorespiratory arrests. The median age-adjusted Charlson's comorbidity index (CCI) was 6. The median age was 72. (IQR 81-63) In the 24 hours before arrest, the 4 most common symptoms and signs were dyspnoea, tachypnoea, agitation and arrhythmias. 42% of patients had an EWS of 7 or higher. 45% had RR >/= 22. 42% had HR >/=100. 32% had SBP </= 90mmHg. 33% had 02 saturations </= 92%. **In the previous 24 hours, 32% of patients did not have a registrar or consultant review. (Table 1.)** Only 11% had an ICU review. Only 22% had ABGs performed. 67% of arrests occurred after hours. **(Table 2.)** 65% had ROSC and 30% were discharged home.

**CONCLUSION.** Patients who proceed to cardiorespiratory arrest during their admission have a high pre-existing morbidity burden and one third are expected to die within the year. The rates discharged home after cardiorespiratory arrest remain low. Similar to previous studies, failure to recognise clinical antecedents and involve senior doctors and intensivists still occurs. A critical care outreach team is being introduced to help address this concern.

**REFERENCE(S)**

1. Vetro J, Natarajan DK, Mercer I, Buckmaster JN, Heland M, Hart GK, Bellomo R, Jones DA: Antecedents to cardiac arrests in a hospital equipped with a medical emergency team. Crit Care Resusc 2011, 13(3):162-166.
2. Berlot G, Pangher A, Petrucci L, Bussani R, Lucangelo U: Anticipating events of in-hospital cardiac arrest. Eur J Emerg Med 2004, 11(1):24-28.
3. Frankin , Mathew J: Developing strategies to prevent inhospital cardiac arrest: analysing response of pysicians and nurses in the hours before the event. Crit Care Med 1994,22(2):244-247

**Table 1 (abstract 000161).** Max grade of doctor review in the 24 hours before arrest, No. (%)

| | |
|---|---|
| No review | 8(22) |
| Intern | 3(8) |
| Sho | 1(3) |
| Registrar | 15(41) |
| Team | 3(8) |
| Consultant | 7(19) |

**Table 2 (abstract 000161).** Cardiac Arrest Data (initial rhythm unknown in 23%), No. (%)

| | |
|---|---|
| Out-of-hours (16:00 to 08:00) | 25(68) |
| Weekend/Bankholiday | 14(38) |
| PEA/Asystole | 22(59) |
| Vtach/Vfib | 6(16) |
| Advanced Airway | 27 (73) |
| ROSC | 24(65) |
| Discharged from Hospital | 11(30) |

### 000407

#### The clinical application of Neurone Specific Enolase to prognosticate neurological recovery in cardiac arrests patients

C. Maher[1], J. Worthy[2], R. Grey[2], O. Boyd[2]
[1]Brighton & Sussex Medical School, Falmer, United Kingdom; [2]Intensive caure unit, Brighton & Sussex University Hospitals Nhs Trust, Brighton, United Kingdom
**Correspondence:** C. Maher
*Intensive Care Medicine Experimental* 2019, **7(Suppl 3):**000407

**INTRODUCTION.** Hypoxic-ischaemic brain injury (HIBI) is a common complication following out of hospital cardiac arrest (OOHCA), and is the cause of death in 68% of patients surviving to ICU admission(1). Prediction of neurological outcome is a multimodal approach, with no singular method giving 0% false positive rate(FPR)(2). Neurone specific enolase (NSE) is a biomarker that positively correlates with poor neurological outcome, with European Resuscitation Council (ERC) guidelines having an NSE cut off of 33μg/L for poor neurological outcome(2). Controversially, recent studies have shown higher thresholds. With new techniques such as targeted temperature management (TTM), previous work gives little indication of the utility of NSE in clinical practice. This study looked retrospectively at the use of NSE in a single centre and describes the predictive value for NSE in prognostication for HIBI post OOHCA.

**OBJECTIVES.** The Primary outcome was to find an NSE threshold value with 0% FPR for poor neurological outcome at ICU discharge (GCS <7/15, or death due to neurological causes).

**METHODS.** A retrospective study was carried out on all adults with OOHCA admitted to the Royal County Sussex Hospital ICU between

April 2017-November 2018. NSE levels, TTM, brain imaging, comorbidities, survival and GCS at ICU discharge were recorded. NSE was measured using Chemiluminescent-immunoassay. NSE data was not used for clinical decision making by the ICU team.

**RESULTS.** 89 patients were admitted with an OOHCA, 59 patients had an NSE measured. NSE was sampled 48-72 hours post arrest. 61% had poor neurological outcome, median NSE was significantly higher in this group than those with good neurological outcome (103μg/L vs 20μg/L, p= 0.001). ROC analysis showed AUC of 0.901, sensitivity 77.8%, specificity 100%, and Yougen's Statistic showed an NSE cut off value of 64.5μg/L. A significant negative correlation was found between NSE and discharge GCS.

**CONCLUSION.** Our results show that NSE can reliably and safely be used to prognosticate for HIBI in clinical practice after an OOHCA, an NSE over 64.5μg/L indicates poor neurological outcome with 0% FPR. Furthermore we suggest that ERC guidelines need updating.

**REFERENCE(S)**

1. Laver S, Farrow C et al. Mode of death after admission to an intensive care unit following cardiac arrest. (2004) Intensive Care Med. 30(11):2126–8. doi: 10.1007/s00134-004-2425-z
2. Nolan JP, Deakin C et al (2015) Post-resuscitation care. UK resuscitation council https://www.resus.org.uk/resuscitation-guidelines/post-resuscitation-care

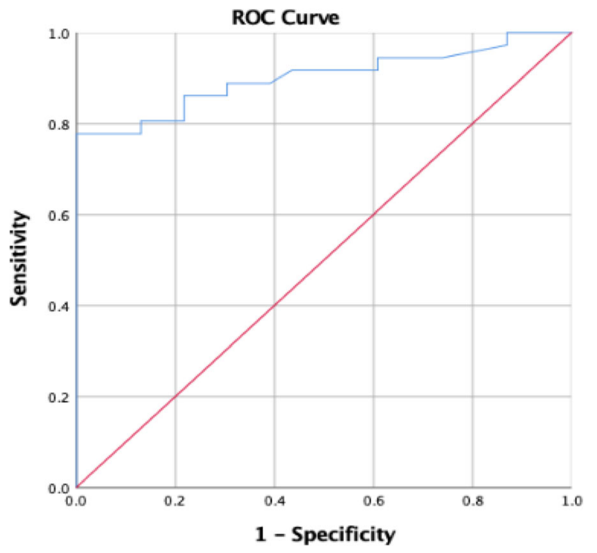

**Fig. 1 (abstract 000407).** Receiver-operating characteristic curves for prediction of poor outcome based on NSE values

### 000713

#### Implementation of an early warning system (EWS) for detection of patients at clinical risk of deterioration in a medical ward (pilot study)

J. Ruiz Izquierdo[1], J. Llabata,[1], ML. Urendes,[1], C. Palencia,[1], B. Sánchez González[1], A. Bertran[2], G. Muñoz Gamito[3], J. Trenado Alvarez[1]
[1]Intensive care medicine, Hospital Universitari MutuaTerrassa, Terrassa, Spain; [2]Coordinadora formació contínua infermeria, Hospital Universitari MutuaTerrassa, Terrassa, Spain; [3]Emergency department, Hospital Universitari MutuaTerrassa, Terrassa, Spain
**Correspondence:** J. Ruiz Izquierdo
*Intensive Care Medicine Experimental* 2019, **7(Suppl 3):**000713

**INTRODUCTION.** EWS allows detection of patients at clinical risk of deterioration in order to establish an early and adequate response.

**OBJECTIVES.** To implement a system for early detection of patients at clinical risk in conventional ward based on the NEWS-2 score, using the SBAR tool as a communication system.

**METHODS.** A unicentric and descriptive pilot study. Period: Weekends ( Friday 4 pm to Monday 8 am) and bank holidays from 13th December 2018 to 14th January 2019. Inclusion criteria: Patients allocated in a medical ward (pilot ward) that deteriorate their condition during the period of study. Exclusion criteria: Palliative patients with indication of no reanimation orders. The score used NEWS-2 (Figure 2). Response protocol to alerts is shown in figure 1.

**RESULTS.** 20 patients included: 4 with NEWS-2 ≥ 7; 2 patients with NEWS-2 = 5-6 and 14 paties with NEWS-2 ≤ 4. 3 patients recorded a determination of 3 points in at least one parameter. NEWS-2 score was correctly performed in 100% of cases.9 patients met the alert criteria. 78% of the cases being high risk (NEWS ≥ 7 and/or value 3 in any individual parameter).Medical team activation was adequate in 67% of cases; in the other 33%, despite register a score of 3 points in only one parameter, the alert protocol was not activated.The time until medical evaluation was adequate in 67% of the casesA single determination of 3 points in one variable was more common in respiratory system variables: 67% of the cases of arterial oxygen saturation and in 45% of the cases the respiratory rate.No patient required admission to ICU, and clinical deterioration was solved with measures implemented in the ward. We indicated no reanimation orders In 2 patients (both with NEWS2 ≥7) after evaluation. These 2 patients died despite treatment implemented.

**CONCLUSION.** Our EWS system could be a very useful tool to detect patients at risk of deterioration in a high percentage.Protocol deviations were related to no medical team activation and delayed response times.Measures to improve training in the ward teams could improve these results.

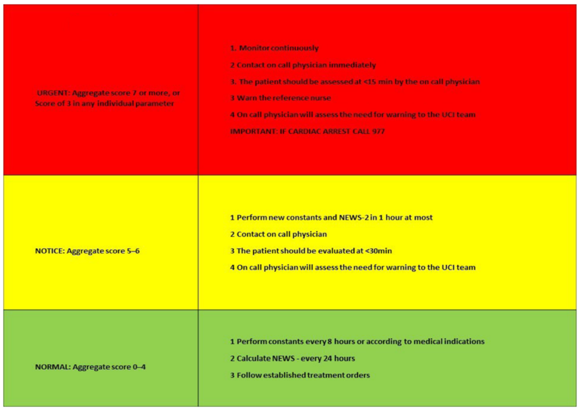

| | |
|---|---|
| URGENT: Aggregate score 7 or more, or Score of 3 in any individual parameter | 1. Monitor continuously<br>2 Contact on call physician immediately<br>3. The patient should be assessed at <15 min by the on call physician<br>3 Warn the reference nurse<br>4 On call physician will assess the need for warning to the UCI team<br>IMPORTANT: IF CARDIAC ARREST CALL 977 |
| NOTICE: Aggregate score 5–6 | 1 Perform new constants and NEWS-2 in 1 hour at most<br>2 Contact on call physician<br>3 The patient should be evaluated at <30min<br>4 On call physician will assess the need for warning to the UCI team |
| NORMAL: Aggregate score 0–4 | 1 Perform constants every 8 hours or according to medical indications<br>2 Calculate NEWS - every 24 hours<br>3 Follow established treatment orders |

**Fig. 1 (abstract 000713).** See text for description

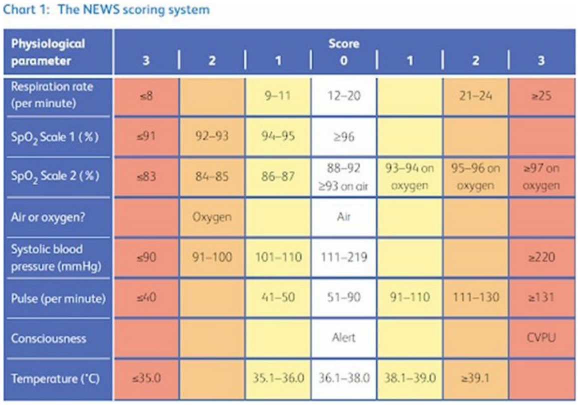

Chart 1: The NEWS scoring system

| Physiological parameter | Score 3 | 2 | 1 | 0 | 1 | 2 | 3 |
|---|---|---|---|---|---|---|---|
| Respiration rate (per minute) | ≤8 | | 9–11 | 12–20 | | 21–24 | ≥25 |
| $SpO_2$ Scale 1 (%) | ≤91 | 92–93 | 94–95 | ≥96 | | | |
| $SpO_2$ Scale 2 (%) | ≤83 | 84–85 | 86–87 | 88–92 ≥93 on air | 93–94 on oxygen | 95–96 on oxygen | ≥97 on oxygen |
| Air or oxygen? | | Oxygen | | Air | | | |
| Systolic blood pressure (mmHg) | ≤90 | 91–100 | 101–110 | 111–219 | | | ≥220 |
| Pulse (per minute) | ≤40 | | 41–50 | 51–90 | 91–110 | 111–130 | ≥131 |
| Consciousness | | | | Alert | | | CVPU |
| Temperature (°C) | ≤35.0 | | 35.1–36.0 | 36.1–38.0 | 38.1–39.0 | ≥39.1 | |

**Fig. 2 (abstract 000713).** See text for description

#### 000736

**Impact of ECG after Out of Hospital Cardiac Arrest (OHCA)**

J. Karasek[1], K. Bouskova[2], R. Pospisil[2], J. Seiner[1], M. Strycek[1], R. Polasek[1], P. Ostadal[3]
[1]Cardiology, Hospital Liberec, Liberec, Czech Republic; [2]3. medical faculty, Charles University, Prague, Czech Republic; [3]Cardiology, Na Homolce Hospital, Prague, Czech Republic
**Correspondence:** J. Karasek
*Intensive Care Medicine Experimental* 2019, **7(Suppl 3):**000736

**INTRODUCTION.** ECG is simply method accesible in prehospital care and is commonly used in management of OHCA. Previous studies was focused mainly on ST elevation. Value of ECG after OHCA could be influenced by haemodynamic instability, acido-basis changes and hyposaturation after resuscitation.

**METHODS.** Observation retrospective study from prospective OHCA registry of Cardiac Arrest Center (CAC). It was described different pathologies and its frequency immediately after ROSC and after Hospital Admission and their relation to coronarography findings and finaly diagnosis.

**RESULTS.** It was included 146 patients after OHCA with Restitution of Spontaneous Circulation (ROSC). Their ECG was provided after OHCA and than after Admission to Hospital. ST elevation was presented by 52% of patients after ROSC and STEMI diagnosis was confirmed by 65,8% of patients ( senzitivity 66%, specificity 96% for STEMI). ACS was confirmed by 68,4% of this patients and signifficant Coronary Artery Disease (CAD) by 91,7% and percutaneous coronary intervention (PCI) by 73,3% ( patients underwent coronarography).

ST elevation were presented by 36% of patients after Admission, diagnosis of STEMI confirmed by 75,5% (senzitivity 75%, specificity 89% for STEMI), ACS was confirmed by 75,5%, significant CAD by by 93,2% and PCI was provided by 77,3% of patients. Between ROSC and Admision ECG is significant difference in STE elevation incidence (p=0,009) and QRS latitude (p=0,003). We observe no significant diferences between both groups in incidence of ACS, of significant CAD, PCI and systolic blood pressure. Time between both curves was median 60 min,( IQR 25-75 ) 45-90 min.

Left bundle branch block (LBBB) was presetend by 9,6% of patients after ROSC and 11,6% after Admission and has low sensitivity and specificity for STEMI and ACS. Incidence of STEMI is 7,14% after ROSC and 11,8% after Admisssion. ACS is present by 21,4% after ROSC and 17,6% after Admission, significant CAD by 62,5% and 75%.

ST depression are by 24,8% of patients after ROSC and 27,8% after Admisssion, sensitivity and specificity for ACS is low (ACS by 36,1% after ROSC and 45,7% after Admission, significant CAD by 79,2% after ROSC and 80,6% after Admission and PCI was provided by 52,4% and 51,6%).

Normal ECG has low incidency after ROSC ( 5,5% after ROSC and 6,85% after Admisssion). ACS was confirmed by 50% of patients after ROSC and 0% after Admission. (sensitivity for ACS exclusion 100%, secificity 56% after Admisssion). Significant CAD was by 100% after ROSC (if coronarography was provided) and 12,5% after Admission. PCI was provided by 100% and 20%.

**CONCLUSION.** ST elevation has for STEMI diagnosis no significant higher sensitivity, if they remain after Admisssion and both ST elevation groups have high incidency of significant CAD and PCI. ST elevation after ROSC has high specificity for STEMI. Normal ECG after ROSC has are not well for ACS exclusion and normal ECG after Admission is very high sensitive for ACS exclusion. LBBB and ST depression has low sensitivity and specificity for ACS and CAD.

#### 000739

**CPR related trauma from autopsy reports, their incidency and seriousness**

J. Karasek[1], B. Blankova[2], T. Bartes[3], D. Nahalka[3], J. Seiner[1], M. Strycek[1], R. Polasek[1], T. Adamek[2], J. Hladik[4], P. Ostadal[5]
[1]Cardiology, Hospital Liberec, Liberec, Czech Republic; [2]Forensic medicine, Hospital Liberec, Liberec, Czech Republic; [3]3. medical faculty, Charles University, Prague, Czech Republic; [4]Forensic medicine, Faculty Hospital Kralovske Vinohrady, Prague, Czech Republic; [5]Cardiology, Na Homolce Hospital, Prague, Czech Republic
**Correspondence:** J. Karasek
*Intensive Care Medicine Experimental* 2019, **7(Suppl 3):**000739

**INTRODUCTION.** CPR related injuries were not properly observed since were established new guidlines for resuscitation (CPR) 2015 with stronger recomandation for bystader and topless CPR. Data were not objectivised by any scale in effort to establish seriousness of injury.

**OBJECTIVES.** To describe incidency and seriousness of injuries related to CPR and compare it by gender, bystander CPR, out vs. in-hospital CPR and try identify factors for seriousness of injury.

**METHODS.** Multicentric study, retrospective analysis of autopsy reports of patients after CPR, cardiac arrests caused by trauma were excluded. We describe damage of particular organs and we objectivised the most serious injury with Abbreviated injury scale (AIS)and summary of all injuries with New injury severity score (NISS).

**RESULTS.** We have enroled 701 autopsy reports , traumatic cardiac arrests were excluded. We have analyzed 628 autopsies: 80,4% men, age median 67 years, out of hospital cardiac arrests 89,2%, bystander CPR 56,8% and cardiac ethiology 78,2%. Ribs injury were founded by 94,6%, injury of lung by 9,9%, sternal injury by 62,4%, liver by 2,5% and spleen by 1,8%. Mechanical CPR was provided by 11,5%. Median of the most serious injury was 3 (serious by Abbreviated injury scale) and median of summry of injuries was 13 by NISS-low risk of fatal injury. By out of hospital cardiac arrest was hifgher incidency of pleural injury and thorax vessles injuries without influence on total seriousness of injury compared to hospital cardiac arrests. Bystanders provided CPR had similar incidency and seriousness of injury like CPR provided only by professional emergency stuff, also by mechanical chest deviced CPR we have observed no differences comapred to manually. Women are significant older (p=0,0001), frequency of their injuries are similar to men, but seriousness of their injuries by NISS is significant higher (p= 0,01). Patients with life threatening injury (AIS 4 and more) has similar baseline profil to their without injury (AIS 0), exept of significant higher cardiac etiology of cardiac arrest by AIS 4+.

**CONCLUSION.** Incidency of CPR related injuries from autopsy reports is very high, but life threatening injuries create only 3%. The highest incidency have injuries of thoreax sceleton, especially ribs. There is no difereneces if patients were resuscitated by bystander or by mechanical chest devices compared to those by professional stuff or manually. Women has similar frequency of injuries like men, but significant more serious by NISS

### MEN - Organ consequences of endocrine and metabolic pathologies

#### 000769

**Cardiac Manifestations of Pheochromocytoma: A review of the literature**

A. Kastoris, F. Georgiou, C. Timiliotou-Matsendidou
Intensive care unit, Limassol General Hospital, Kato Polemidia, Cyprus
**Correspondence:** A. Kastoris
*Intensive Care Medicine Experimental* 2019, **7(Suppl 3):**000769

**INTRODUCTION.** Pheochromocytoma represents a rare tumour that originates in chromaffin tissue, producing distant effects by secretion of catecholamines. Usual clinical manifestations include headaches, palpitations, hyperhidrosis and hypertension. Cardiac manifestations of the disease are rare and throughout the literature sporadic cases of phaechromocytoma mimicking acute coronary or Takotsubo syndrome have been reported. Acute cardiac manifestations in these patients, may be dramatic and even life threatening.

**OBJECTIVES.** A review of the literature regarding cardiac manifestations in patients with pheochromocytoma mimicking acute coronary syndrome, cardiogenic shock or Takotsubo syndrome. In the review of the literature we included a case reported in our ICU.

**METHODS.** A review of the literature was conducted using the key words 'pheochromocytoma' and 'cardiac manifestations', 'acute coronary syndrome', 'Takotsubo syndrome', 'pulmonary oedema' in PubMed, Scopus, and Google Scholar databases. Published case reports and series written in English were included. Abstracts were not included in the review. Data was extracted regarding the presentation of symptoms such as pulmonary oedema, Takotsubo syndrome, ECG changes consistent with acute coronary syndrome, elevated cardiac enzymes and cardiogenic shock in patients with pheochromocytoma. The second part of the study includes a case reported in the ICU of our hospital of a 45-year-old patient, with cardiogenic shock due to pheochromocytoma.

**RESULTS.** A total of 91 studies were included in the review of the literature, 2 case series and 89 case reports, with a total of 128 patients. The mean age of the patients was 46 years of age with ages ranging between 17 and 89 years of age with 1 case report not reporting the age. Seventy nine of the 128 patients were female (62%). Forty nine of the 128 patients (38%) presented symptoms of pulmonary oedema, 38 of the 128 patients (30%) Takotsubo syndrome, 38 of the 128 patients (30%) cardiogenic shock, 101 of the 128 patients (80%) ECG changes consistent with ACS, and 45 of the 128 patients (35%) presented cardiac enzyme elevations. Only 5 of the 128 (4%) patients presented all the above symptoms. A 45-year-old patient admitted in our ICU with chest pain, emesis, and nausea, and a history of smoking, presented all the above symptoms of pulmonary oedema, cardiogenic shock, Takotsubo syndrome, ECG changes consistent with acute coronary syndrome and cardiac enzyme elevation. After percutaneous coronary angioplasty and exclusion of acute coronary syndrome, abdominal CT revealed pheochromocytoma.
**CONCLUSION.** It is rare for pheochromocytoma to present all cardiac manifestations of pulmonary oedema, cardiogenic shock, Takotsubo syndrome, ECG changes consistent with ACS, and elevated cardiac markers. Although reviews of the literature exist regarding the presentation of Takotsubo Syndrome in pheochromocytoma, no other review has presented all possible cardiac manifestations of pheochromocytoma. Every clinician should be aware of the possible presence of pheochromocytoma especially in middle aged, female patients, with negative angiography results, however rare the occurrence.

### 001143

#### Severe acute liver failure: Epidemiology and prognosis

D. Arias-Verdú, G. Seller-Pérez, J. Barrueco-Francioni, C. Aragón-González, M. Lebrón-Gallardo, A. Muñoz-López, G. Quesada-García, M. Herrera-Gutiérrez
Intensive care, Regional Hospital of Malaga, Málaga, Spain
*Intensive Care Medicine Experimental* 2019, **7(Suppl 3):**001143

**INTRODUCTION.** .
**OBJECTIVES.** To analyse the epidemiology and prognosis of acute liver failure in patients admitted to the intensive care unit (ICU).
**METHODS.** Third level ICU covering the provinces of Malaga and Almeria (2,350,000 inhabitants) for liver transplantation. This retrospective cohort study comprised all patients admitted to our unit with acute liver failure between 2010-2016, excluding acute-over-chronic failure and subacute decompensated failure after a first admission. All patients or their relatives signed the consent form for use of their data. The results are described as mean (standard deviation), median (quartiles) or %. Given the low number of patients we applied non-parametric tests and no multivariate analysis could be performed.
**RESULTS.** There were 27 cases, 66.7% women, with an average age of 34.1±13.1 years. The failure was hyperacute in 33% of cases, acute in 37% and subacute in 29.6%. The cause was related to use of medicines in 22.2%, toxic in 14.8%, virus in 7.4%, pregnancy in 7.4%, Wilson in 3.7%, other in 29.6% and cryptogenic in 14.4%. Biopsy was performed before the OLT in 29.6% and in the extracted organ in 51.9%. The results confirmed the suspected diagnosis in 15.4%, did not agree with the initial diagnosis in 22.2%, and suggested the diagnosis although not definitively in 57.7%. Hepatic clearance was used in 96.3% of the cases and 66.7% received a transplant during their admission to the ICU.
The ICU stay was 10 (4-16) days and the hospital stay was 25 (18-35). ICU mortality was 18.5% (5 cases) and hospital mortality 22.2%. None of the variables analysed was related to mortality but the low number of cases detracts from this analysis. However, mortality for transplanted patients was 11.1% vs. 33.3% in non-transplanted patients (ns).
**CONCLUSION.** : Severe liver failure is a condition with a low incidence but nevertheless a very high mortality, especially among patients who do not receive a transplant, which can reach one third of cases. These results emphasize the convenience of treating these patients in reference centres with the possibility of carrying out an emergency transplant. The aetiology of the liver failure is still difficult to determine in many cases and biopsy does not prove conclusive in a high percentage of cases.

### 001574

#### Reaching nutritional goals and negative fluid balance have positive impact on mortality

E. Senturk[1], D. Egeli[2], Z. Cukurova[3], S. Asar[3], GO. Hergünsel[3], S. Erus[4], S. Ugur[1], B. Ozserezli[1], F. Yurdakul[1], SY. Tekdos[3], N. Cakar[1]
[1]Anesthesiology and reanimation, Koc University, Istanbul, Turkey; [2]Anesthesiology and reanimation, American Hospital , Istanbul, Turkey; [3]Anesthesiology and reanimation, Sadi Konuk Research Hospital, Istanbul, Turkey; [4]Thorasic surgery, Koc University , Istanbul, Turkey
**Correspondence:** E. Senturk
*Intensive Care Medicine Experimental* 2019, **7(Suppl 3):**001574

**INTRODUCTION.** The effects of reaching nutritional caloric goals and negative fluid balance on outcomes have not been clearly demonstrated in critically ill patients. Electronic recording systems (ERS) are widely used and may help to answer this question.
**OBJECTIVES.** Our hypothesis was that achieving the nutritional caloric goals along with a negative fluid balance would have a positive effect on survival.
**METHODS.** In our two centered retrospective study, data were reviewed from the ERS (Metavision) between 2015 and 2018. 2814 critically ill patients ≥ 18 years of age and who had been hospitalized ≥ 72 hours were included. Predicted body weight on admission day and data on fluid balance, duration of mechanical ventilation, length of ICU and hospital stay and mortality were recorded. Patients target calorie were calculated from the average of Harris-Benedict and Schofield formula. Last day NUTRIC scores were also noted. Caloric balance on third day was recorded as positive if the target value was achieved and as negative if not. Fluid balance was recorded as positive if balance was ≥ 500 and negative if < 500 according to the total balance on third day; hence, balance-negative, balance-positive, calorie-negative, and calorie-positive groups were formed.
**RESULTS.** There were no differences in demographic data between the groups.
Calorie-positive and negative-groups did not differ significantly in terms of mortality.
**CONCLUSION.** Caloric intake and fluid balance on the first three days does not appear to have an impact on mortality.

**Table 1 (abstract 001574).** See text for description

| Groups and Mortality | |
|---|---|
| | Mortality (%) (number) |
| Calorie + Balance - | 39.2 (249/636) |
| Calorie - Balance + | 34.1 (307/901) |
| Calorie - Balance - | 36.2 (199/550) |
| Calorie + Balance + | 38.5 (276/717) |

**Table 2 (abstract 001574).** See text for description

| Demographic data | | |
|---|---|---|
| | Mean | Standart Deviation |
| Gender (Male %) | 57.7 | |
| Age | 61.4 | 20.1 |
| Predicted Body weight | 62.0 | 9.5 |
| Ventilation Hour | 223.3 | 276.7 |
| Weight (admission day) | 76.4 | 19.1 |
| Weight (last day) | 77.3 | 18.9 |
| Height (cm) | 167 | 8.7 |

#### 001599

**Managing critically ill cirrhotic patients admitted in the ICU. The burden of ACLF**

E. Marques Mendes, R. Corga Da Silva, P. Moura, J. Caldeiro, S. Oliveira, A. Bal
Departamento de medicina crítica, Unidade Local de Saude do Alto Minho, Viana do Castelo, Portugal
*Intensive Care Medicine Experimental* 2019, **7(Suppl 3):**001599

**Correspondence:** E. Marques Mendes

**INTRODUCTION.** It is a common known fact, that cirrhotic patients have worse prognosis, with high mortality associated with their decompensations. Admitting these cases in the intensive care unit (ICU) has been longed discussed, weighting the cost-effectiveness, and the adequacy of resources. Until the canonic study, medical community didn't understand which patients would progress worse, and for what reasons. The definition of the Acute on Chronic Liver Failure (AoCLF) as an individual syndrome, its diagnostic CLIF SOFA and the prognostic CLIF-C ACLF scores where an important improvement. Still, to this day, there is no predicting wich AoCLF will die irrespective of medical intervention, and witch will benefit from monitoring, early identification and early organ support. Studies also suggest that AoCLF development and evolution is independent of the identification of a precipitating event. In our study, we decided to evaluate every cirrhotic patient admitted to the ICU, evaluating if the decompensating event was identified, and the mortality rate (comparing it with the prognostic mortality rate calculated).

**OBJECTIVES.** To prospectively assess and characterize all cirrhotic patients admitted in the Intensive Care Unit of a secondary hospital and evaluate decompensation factors and mortality rates.

**METHODS.** All cirrhotic patients over 18 years, were prospectively enrolled between 01/01/2017 to 31/03/2019. For each patient, grade of ACLF and specific ICU prognostic scores were collected at the time of ICU admission. All cirrhotic patients over 18 years, were prospectively enrolled between 01/01/2017 to 31/03/2019. For each patient, grade of ACLF and specific ICU prognostic scores were collected at the time of ICU admission.

**RESULTS.** A total of 32 cirrhotic patients were admitted in intensive care unit, of which 71.9%(n=23) were male and the median age was 55.38 (±8.85) years. The majority (53.1%; n=17) of these patients died during ICU hospitalization. One-year mortality rate was 33% (n=5). Mean APACHE score was 26.8 and SAPSII score was 59.8. The CHILD score at admission was elevated, and 68.8%(n=22) had a C stage. Mean CLIF-SOFA was 11.4. A total of 81.3% had criteria of AoCLF, with 7.7% grade 1, 30.8% grade 2 and 61.5% grade 3 criteria. Mean CLIF-C-SOFA was 54.1. In only 2 patients (6.3%) there was no trigger identified. The trigger factor mostly identified was infection (56.3%; n=18), particularly spontaneous bacterial peritonitis and nosocomial pneumonia.

**CONCLUSION.** This is a population of adult patients, with high severity scores, that were predicted to have high risk of short-term mortality. Their survival is dependent of diagnosis, intensive monitoring and early organ support, with the difficult decision of admitting and treating them. The mortality rate observed proves that this syndrome, even in CHILD C score patients, doesn't necessarily mean short term fatality, since the mortality observed was not as high as it would be expected. 46.9% of patients survived the ICU stay, even though the majority of patients (61.5%) where grade 3 AoCLF (with correspondent 31.3% ICU survival).

##### REFERENCE(S)

1. R. Moreau, R Jalan, V. Arroyo et al. Acute-on-chronic liver failure: Recent Concepts. Journal of Clinical and Experimental Hepatology. March 2015; vol 15, No1:81-85.
2. Moreau R, Jalan R, Gines P, et al. Acute-on-chronic liver failure is a distinct syndrome that develps in patients with aute decompensation of cirrhosis. Gastreoenterology. 2013;144:1426-2437.
3. Validation of CLIF-C ACLF score to define a threshold for futility of intensive care support for patients with acute-on-chronic liver failure.Engelmann C, Thomsen KL, Zakeri N, Sheikh M, Agarwal B, Jalan R, Mookerjee RP. Crit Care. 2018 Oct 10;22(1):254. doi: 10.1186/s13054-018-2156-0.

## NIC - Acute care of the injured brain

#### 001250

**The Parameters Affecting the Outcome in Drowning Cases in Intensive Care; Eight Years of Retrospective Patient Data**

M. Pehlivanlar[1], AO. Küçük[2], ÇE. ÖZtürk[3], Ö. Kömürcü[4], IK. Bayrak[5], F. ÜLger[6]
[1]Departmen of chest disease, division of intensive care medicine, KTU Faculty of Medicine, Trabzon, Turkey; [2]Department of anesthesiology and reanimation, division of intensive care medicine, KTU Faculty of Medicine, Trabzon, Turkey; [3]Division of intensive care medicine, Samsun Training and Research Hospital, Samsun, Turkey; [4]Department of anesthesiology and reanimation, division of intensive care medicine, Ondokuz Mayıs Universiry, School of Medicine, Samsun, Turkey; [5]Department of radiology, Ondokus Mayıs University, School of Medicine, Samsun, Turkey; [6]Department of anesthesiology and reanimation, division of intensive care medicine, Ondokuz Mayıs Üniversitesi Tıp Fakültesi , Samsun, Turkey
**Correspondence:** A.O. Küçük,
*Intensive Care Medicine Experimental* 2019, **7(Suppl 3):**001250

**INTRODUCTION.** Drowning is a common cause of accidental deaths. Complication rate and mortality of patients requiring intensive care support are high. The aim of our study was to reveal the clinical course and treatment efficiencies of a limited number of patients in the intensive care unit due to the drowning.

**METHODS.** Patients who were admitted to the intensive care unit for >24 hours and who met the inclusioncriterias between January 2010 and August 2018 were examined retrospectively.

**RESULTS.** Thirteen of the 103 patients needed intensive care who admitted emergency service. Five (38.4%) of the patients died. Steroids were used in 6 patients (46.2%) but no statistically significant difference was found in terms of mortality (p=0.592). The presence of diffuse cerebral edema 5(38.5%) was associated with a statistically higher mortality rate compared to 6 (46.2%) patients with normal findings (p=0.003). Mechanical ventilator supports were provided with SIMV mode in 9(69.2%) patients and APRV mode was used in 4 (31.8%) patients. According to our clinical observations, the radiological improvement was observed in patients with APRV mode (Table 1).

**CONCLUSION.** The effect of steroid and prophylactic antibiotherapy on mortality was not determined. APRV can provide the recruitment of consolidated lung areas and prevent the decruitment of alveoli. These effects should be kept as a mode of choice in cases of severe alveolar and interstitial edema in drowning cases. Mechanical ventilation strategies, the degree of metabolic acidosis at admission and the neurological status of the patient may be considered as variable clinical parameters that may affect patient outcome.

##### REFERENCE(S)

1. Orlowski JP, Abulleil MM, Phillips JM. The hemodynamic and cardiovascular effects of near-drowning in hypotonic, isotonic, or hypertonic solutions. Ann Emerg Med. 1989;18(10):1044-9.
2. Grmec S, Strnad M, Podgorsek D. Comparison of the characteristics and outcome among patients suffering from out-of-hospital primary cardiac arrest and drowning victims in cardiac arrest. Int J Emerg Med. 2009;2(1):7-12.
3. Orlowski JP. Drowning, near-drowning, and ice-water drowning. Jama. 1988;260(3):390-1.
4. Bierens JJ, Knape JT, Gelissen HP. Drowning. Curr Opin Crit Care. 2002;8(6):578-86.

**Table 1 (abstract 001250).** Comparison of groups according to general specifies

| Parameter | | Alive n (%) | Death n (%) | Total n | p |
|---|---|---|---|---|---|
| Drowning water type | Freshwater | 0 (0) | 2 (100) | 2 | |
| | Saltwater | 8 (72.7) | 3 (27.3) | 11 | 0.128 |
| CT Findings | Normal | 5 (100) | 0 (0) | 5 | |
| | Minimal Odema | 2 (100) | 0 (0) | 2 | |
| | Diffuse Odema | 1 (16.7) | 5 (83.3) | 6 | 0.003 |
| Inotrope / Vazopressor | No | 6 (100) | 0 (0) | 6 | |
| | Yes | 2 (28.6) | 5 (71.4) | 7 | 0.039 |

### 001123

#### Mortality associated risk factors in patients with subarachnoid hemorrhage in a neurotraumatic ICU

C. Sánchez Ramírez[1], CF. Lübbe Vázquez[1], JL. Vicente Arranz[1], J. López Pérez[1], C. Vázquez Pineda[1], J. Garriga Segarra[1], JM. Ríos Bort[1], A. Sánchez Del Rio[1], P. Saavedra-Santana[2], S. Ruiz-Santana[1]
[1]Intensive care medicine, University Hospital of Gran Canaria Dr. Negrin, Las Palmas de Gran Canaria, Spain; [2]Mathematics and informatics deparment, University of Las Palmas, Las Palmas de Gran Canaria, Spain
**Correspondence:** C. Sánchez Ramírez
*Intensive Care Medicine Experimental* 2019, **7(Suppl 3):**001123

**INTRODUCTION.** Aneurysmal subarachnoid hemorrhage (SAH ) is a worldwide health burden with fatality as permanent disability.To know risk mortality factors is important,with particular regard to the identification of potentially avoidable factors, in order to reduce SAH mortality.

**OBJECTIVES.** To assess the mortality risk factors in patients with subarachnoid hemorrhage (SAH), admitted in a neurotraumatic ICU

**METHODS.** Prospectively collected data of patients admitted from October, 2013 to December 2017 to a 10-bed Neurotraumatic ICU. We analyzed: main diagnosis at admission; demographic data including sex and race; neurological data (clinical examination, pupils reactivity and size, and Glasgow Coma Score: GCS; localization and aneurysm size; presence of intracranial hematoma (ICH); presence and volume of intraventricular bleeding; days to develop vasoespam; development of DCI; Fisher scale, Modified Fisher scale, Hunt and Hess scale, Word Federation of Neurosurgeons (WFNS) scale; presence of vasoespasm in doppler or arteriography; delayed of admission in ICU; treatment of the aneurysm; complications, including infections; Glasgow Outcome Scale (GOS) at ICU discharge and 6 months after ICU discharge and several other risk factors. In order to identify factors that maintain independent association with mortality, a multivariate logistic regression analysis were performed. It was considered significant if $p \leq .05$.

**RESULTS.** Among 83 SHA admitted patients, 18 (36.44%) out of 64 patients died. In a univariate analysis, age, APACHE II, SOFA and GCS at ICU admission, GCS at site and at emergency room, to be oriented and alert, isochoric pupils, posterior cerebral artery aneurysm, decompressive craniectomy, dead after treatment, hidrocephalus, mechanical ventilation > 7 days, ICH, frontal ICH, internal carotid artery aneurysm ICH, rebleeding within 72 hours, McCabe, GOS at ICU discharge and 6 months after ICU discharge, Fisher scale, Hunt and Hess scale and WFNS scale were statistically significant. Among the patients who died versus survivals, ICU stay was not significantly different (Table 1a y 1b). The independent risk factors associated with mortality were: isochoric pupils OR (95% CI): 0.118 (0.022 ; 0.644), frontal ICH OR (95% CI): 7.121 (1.577 ; 32.159), GCS at ICU admission OR (95% CI): 0.824 (0.719 ; 0.944) (Table 2).

From the logistic model also summarized inTable 2, a prediction score was obtained (require further validation) (Figure 1):*SHA mortality Score=-2.143 X Isochoric pupils + 1.963 X Frontal ICH-0.194 X GCS at ICU admission*

**CONCLUSION.** In SHA admitted to a neurotraumatic ICU, risk factors that were independently associated with mortality were: isochoric pupils , frontal ICH and GCS at ICU admission. A mortality prediction score was estimated.

**Table 1a (abstract 001123).** Characteristics of the patients according survival/death

| | Alive N = 64 | Death N = 18 | P |
|---|---|---|---|
| Age, years | 55.6 ± 11.3 | 64.4 ± 16.1 | 0.010 |
| Age > 55 | 33 (51.6) | 11 (61.1) | 0.473 |
| Sex female | 41 (64.1) | 13 (72.2) | 0.519 |
| Apache II at admission | 12.6 ± 7.5 | 18.8 ± 5.4 | 0.002 |
| SOFA at admission | 1 (0 - 4) | 5 (3 - 8) | < .001 |
| GCS on site | 15 (14 - 15) | 13 (6 - 15) | 0.002 |
| GCS in emergency room | 15 (13 - 15) | 12 (6 - 15) | 0.042 |
| GCS at ICU admission | 14 (7 - 15) | 6 (3 - 6) | 0.001 |
| ICU re-admission by vasospasm | 6 (9.5) | 1 (5.6) | 1 |
| Anti-Vitamin K drugs | 2 (3.2) | 1 (5.9) | 0.517 |
| Diabetes | 11 (17.5) | 2 (11.1) | 0.722 |
| Dyslipidemia | 23 (36.5) | 6 (33.3) | 0.804 |
| Chronic renal failure | 3 (4.8) | 1 (5.6) | 1 |
| Neoplasm | 2 (3.2) | 0 | 1 |
| Malnutrition | 0 | 1 (5.6) | 0.222 |
| Smoker | 24 (38.1) | 7 (38.9) | 0.951 |
| Alcoholic | 6 (9.5) | 2 (11.1) | 1 |
| Cocaine use | 2 (3.2) | 2 (11.1) | 0.212 |
| Stroke family history | 0 | 1 (7.1) | 0.203 |
| Caucasian race | 56 (94.9) | 14 (100.0) | 1 |
| Platelet inhibitor | 6 (9.5) | 3 (17.6) | 0.392 |
| Emergency surgery at admission | 5 (7.8) | 2 (11.1) | 0.645 |
| Previous surgery | 1 (1.6) | 1 (5.6) | 0.393 |
| SDD | 15 (25.0) | 6 (40.0) | 0.335 |
| Oriented | 41 (64.1) | 4 (22.2) | 0.002 |
| Alert | 45 (70.3) | 8 (44.4) | 0.043 |
| Confused | 8 (12.5) | 5 (27.8) | 0.146 |
| Stuporous | 3 (4.8) | 5 (27.8) | 0.012 |
| Bilateral mydriasis | 1 (1.6) | 2 (11.1) | 0.125 |
| Anisochoric pupils | 4 (6.5) | 4 (22.2) | 0.071 |
| Isochoric pupils | 58 (93.5) | 12 (66.7) | 0.007 |
| One reactive pupil | 2 (3.2) | 3 (16.7) | 0.072 |
| Both reactive pupils | 54 (87.1) | 12 (66.7) | 0.073 |
| None-reactive pupils | 4 (8.9) | 2 (13.3) | 0.634 |
| Right aneurysm | 12 (19.1) | 7 (46.7) | 0.042 |
| Left aneurysm | 23 (36.5) | 4 (26.7) | 0.472 |
| Bilateral aneurysm | 5 (7.9) | 0 | 0.579 |
| Aneurysm in the midline | 15 (23.8) | 1 (6.7) | 0.175 |
| Anterior cerebral artery aneurysm | 4 (6.5) | 2 (12.5) | 0.597 |
| Anterior communicating artery aneurysm | 21 (32.8) | 5 (29.4) | 0.789 |
| Posterior communicating artery aneurysm | 14 (22.2) | 4 (26.7) | 0.739 |
| Anterior cerebral artery aneurysm | 5 (7.9) | 1 (6.7) | 1 |
| Ophthalmic artery aneurysm | 1 (1.6) | 0 | 1 |
| Middle Cerebral Artery aneurysm | 5 (7.9) | 2 (13.3) | 0.614 |
| Posterior Cerebral Artery aneurysm | 0 | 2 (13.3) | 0.035 |
| Basilar Artery aneurysm | 6 (9.5) | 1 (6.7) | 1 |

Data are means SD (standard deviation), frequencies (%) and medians (IQR: Interquartile range). SDD: selective digestive decontamination

Data are means SD (standard deviation), frequencies (%) and medians (IQR: Interquatile range). SDD selective digestive decontamination

**Table 1b (abstract 001123).** Characteristics of the patients according survival/death

| | Alive N = 64 | Death N = 18 | P |
|---|---|---|---|
| Posterior inferior cerebellar artery anurysm | 3 (4.8) | 1 (6.7) | 1 |
| Internal carotid artery aneurysm | 4 (6.3) | 2 (13.3) | 0.325 |
| Multiple aneurysm | 11 (17.7) | 4 (25.0) | 0.495 |
| Aneurysm clipping | 12 (18.8) | 5 (27.8) | 0.511 |
| Lumbar drainage | 1 (1.6) | 0 | 1 |
| Embolization of the aneurysm | 40 (62.5) | 10 (55.6) | 0.594 |
| Embolization and surgery treatment | 1 (1.6) | 1 (5.6) | 0.393 |
| Conservative treatment | 8 (12.5) | 3 (16.7) | 0.699 |
| Decompressive craniectomy | 0 | 3 (16.7) | 0.010 |
| Intraoperative aneurysm rupture | 3 (4.7) | 3 (16.7) | 0.116 |
| Died after treatment | 0 | 2 (11.1) | 0.046 |
| External ventricular device | 27 (42.9) | 11 (64.7) | 0.109 |
| Cerebrospinal fluid fistula | 0 | 1 (5.6) | 0.220 |
| Hidrocephalus | 24 (38.1) | 12 (66.7) | 0.031 |
| MV > 7 days | 13 (20.3) | 11 (61.1) | < .001 |
| ICH | 8 (12.7) | 7 (38.9) | 0.034 |
| Frontal ICH | 6 (9.7) | 7 (38.9) | 0.007 |
| Perisylvian ICH | 10 (16.1) | 4 (22.2) | 0.506 |
| Temporal ICH | 4 (6.5) | 4 (22.2) | 0.071 |
| Subdural hematoma | 5 (8.1) | 1 (5.6) | 1 |
| Vasoespasm_doppler | 21 (35.0) | 7 (41.2) | 0.640 |
| Vasoespasm_Arteriography | 15 (26.3) | 2 (11.8) | 0.327 |
| Internal carotid artery aneurysm | 4 (6.2) | 5 (27.8) | 0.021 |
| DCI | 18 (28.1) | 6 (33.3) | 0.668 |
| Rebleeding 72 hours | 1 (1.6) | 3 (17.6) | 0.028 |
| Ventriculitis | 6 (9.8) | 3 (16.7) | 0.418 |
| McCabe | | | 0.008 |
| 1 | 9 (14.8) | 7 (50.0) | |
| 2 | 41 (67.2) | 4 (28.6) | |
| 3 | 11 (18.0) | 3 (21.4) | |
| Delayed admission after bleeding | 12 (4 - 24) | 9 (3 - 24) | 0.796 |
| GOS_ICU discharge | 5 (4 - 5) | 1 (1 - 1) | < .001 |
| GOS_6 months after discharge | 5 (5 - 5) | 1 (1 - 1) | < .001 |
| Fisher scale | 3 (2 - 4) | 4 (3 - 4) | 0.013 |
| Fisher modified scale | 4 (3 - 4) | 4 (3 - 4) | 0.099 |
| Hunt and Hess scale | 1 (1 - 2) | 3 (2 - 5) | 0.001 |
| WFNS scale | 1 (1 - 4) | 4 (2 - 5) | 0.002 |

Data are means SD (standard deviation), frequencies (%) and medians (IQR: Interquartile range, MV: mechanical ventilation, ICH: intracerebral hematoma, DCI: delayed cerebral ischemia, GOS: Glasgow outcome score, WFNS: World Federation Neurosurgical Societies

Data are means SD (standard deviation), frequencies (%) and medians (IQR: Interquatile range). SDD selective digestive decontamination

**Table 2 (abstract 001123).** Multivariate logistic regression for death

| | P | OR (95% CI) |
|---|---|---|
| Isochoric pupils | 0.014 | 0.118 (0.022 ; 0.644) |
| Frontal ICH | 0.011 | 7.121 (1.577 ; 32.159) |
| GCS at ICU admission | 0.005 | 0.824 (0.719 ; 0.944) |

ICH: intracerebral hematoma

*ICH* intracerebral hematoma

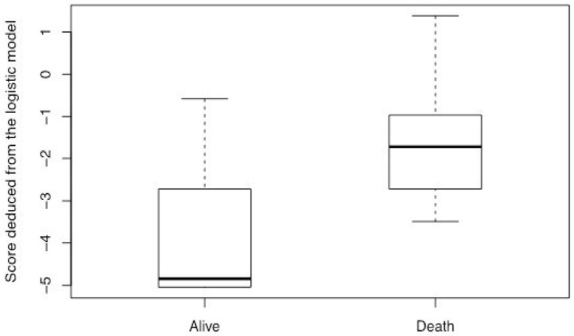

**Fig. 1 (abstract 001123).** See text for description

### 001430

#### External ventricular drainage - related infection in neurocritical patients

M. Segura Pensado[1], B. Porral Sanchez[2], EM. Menor Fernandez[2], S. Vara Adrio[2], A. Garcia Sagastume[2], C. Garcia Redruello[2], A. Touceda[2]
[1]Hospital Álvaro Cunqueiro, Estrada de Clara Campoamor, Vigo, Spain, Vigo, Spain; [2]Intensive medicine, Hospital Álvaro Cunqueiro, Estrada de Clara Campoamor, Vigo, Spain, Vigo, Spain
**Correspondence:** M. Segura Pensado
*Intensive Care Medicine Experimental* 2019, **7(Suppl 3):**001430

**INTRODUCTION.** External ventricular drainage (EVD) is frequently used in different groups of patients in neurocritical care. EVD related infection (ERI) is a common complication in these patients, and it can worsen their condition.

**OBJECTIVES.** Our aim in this study is to analyze which factors are associated with the development of ERI and its effects in the prognosis of these patients.

**METHODS.** A retrospective, observational study including fifty one patients with EVD was performed during 2017 and 2018. Data collected were analyzed using SPSS Statistics v.24.

**RESULTS.** Fifty one patients (51% men) with a mean age of fifty eight ( 58 ± 13 years) and a APACHE II mean score of thirteen (13 ± 6). Seventeen patients were diagnosed of ERI (34%) The mean length of stay was 50 +- 25 days in those with infection and 25 +- 14 p< 0,05 (IC 95% 8-41) in those without it. Total days with EVD were 15 +- 10 in patients without ERI and 28 +- 14 days in patients without ERI (IC 95% 4-20), with significant association (p=0,03). 23.5% were tunnel EVD and were placed in the operating room (OR) (ERI in 50%) and the rest (78.5%) were intraventricular catheters placed bedside in the ICU (ERI in 28,2%), without significant association. Regarding the relationship between infection and the number of handlings (opening for treat Intracranial hypertension (IH) or to provide antibiotics). In 29.4% of patients we used antibiotic through the EVD and in this group we diagnosed ERI in 100% of patients. Mortality was 35,5% in the group diagnosed with ERI and 29.4% in the other group, with no significant association between the two groups.

**CONCLUSION.** ERI is a common complication in patients with EVD. In our study this was associated with more days needing a EVD and increased length of hospital stay. However mortality was not different between groups. Despite no significant differences, it seems that tunnel EVD and handling of these drainage system are associated with a higher infection rate. Further studies with larger sample size are necessary to identify risk factors that have impact in the prevalence and development of EVD related infections.

### 001456

#### External ventricular drainage related infections in a neurocritical ICU

B. Porral Sanchez[1], M. Segura Pensado[2], EM. Menor Fernandez[1], S. Vara Adrio[1], A. Garcia Sagastume[1], C. Garcia Redruello[1], A. Touceda[1]
[1]Intensive medicine, Hospital Álvaro Cunqueiro, Estrada de Clara Campoamor, Vigo, Spain, Vigo, Spain; [2]Hospital Álvaro Cunqueiro, Estrada de Clara Campoamor, Vigo, Spain, Vigo, Spain
**Correspondence:** M. Segura Pensado
*Intensive Care Medicine Experimental* 2019, **7(Suppl 3):**001456

**INTRODUCTION.** External ventricular drainage (EVD) is frequently used in different groups of patients in neurocritical care. EVD related infection (ERI) is a common complication in these patients.

**OBJECTIVES.** In this study, we try to determine the prevalence of external ventricular drainage related infections in our ICU, just as the demographic characteristics of the patients.

**METHODS.** A retrospective and observstional study in which we included 51 patients during 2017 and 2018 with a EVD insertion as a part of the neurological monitoring and treatment in different neurocritical conditions. Data collected was analyze using SPSS Statistics v.24.

**RESULTS.** 51 patients were included in the study, 49% were women and 51% were men. The mean age was fifty eight ( 58 ± 13 years) and the APACHE II mean score was thirteen (13 ± 6). Patients were admitted in the ICU with different conditions. 28 patients (54.9%) had a subarachnoid hemorrhage (SAH), 14 had an hemorrhagic stroke (27,5%), 1 had ischemic stroke (2%), 7 patients (13,7%) had traumatic brain injury (TBI) and 1 had acute hydrocephalus (2%). 23.5% were tunnel EVD and were placed in the operating room (OR) (ERI in 50%) and the rest (78.5%) were intraventricular catheters placed bedside in the ICU (ERI in 28,2%). ERI confirmed with microbiological studies was diagnosed in 24% of patients en in 10% ERI was highly suspected regarding clinical and laboratory data, but without microbiological confirmation. We identified gram positive cocci (GPC) in 70.6% patients with ERI (Staphilococcus epidermidis in 10 patients and Enterococcus faecalis in 2) followed by gram negative bacteria (GNB) in 11.8% (acnetobacter Pitt and pantoea aglomerans). Empiric antibiotic was used in most of cases (94.4% of patients diagnosed with ERI). Mortality was 35,5% in the group diagnosed with ERI and 29.4% in the other group, with no significant association between the two groups

**CONCLUSION.** External ventricular drainage (EVD) is frequently used in different groups of patients in neurocritical care and its been proved as an useful monitoring tool. However ERI is a common complication in these patients, and can worsen their condition. In this study we had analyze the characteristics of our patients during two years. We believe that ERI rate is high in our ICU comparing with the existing publications. Further studies are necessary to identify the cause or the risk factors of this high rate of these infections.

### 001524

#### Aseptic meningitis after carotid artery stenting

I. Luneva[1], R. Yulia[1], P. M.[2]
[1]Icu, Research Center of Neurology, Moscow, Russia; [2]The head of research center of neurology, Research Center of Neurology, Moscow, Russia
**Correspondence:** I. Luneva
*Intensive Care Medicine Experimental* 2019, **7(Suppl 3):**001524

**INTRODUCTION.** Increasing bloodstream after angioreconstructive surgery is usually called as cerebral reperperfusion syndrome. It can manifested by headache, seizures, neurologic symptoms and in severe cases with cortical edema.

**OBJECTIVES.** Hyrperfusion injury after carotid artery stenting (CAS) may engage disruption of the blood-brain barrier (BBB) by sharp rise of cerebral blood fluid. Recent studies have shown that extravasation of gadolinium visible on magnetic-resonance imaging (MRI) in the subarachnoid space are a marker for early BBB damage.

**METHODS.** We collected all clinical data of 3 patients with severe and uncommon complications associated with cerebral hyperperfusion syndrome (CPS) after stenting of the left internal carotid artery. Each patient was performed MRI head near after CAS and repeat MRI with gadolinium on a next day, lumbal puncture was made also.

**RESULTS.** All patients have had aphasia, meningeal syndrome and temperature rising. They were initially with mild aphasia after the CAS and then developed severe aphasia and meningeal syndrome, with a normal blood pressure. MRI head did not show any acute changes. Ultrasound ICA showed non-occluded stent. Their body temperature increased to 39.2°C. Repeat MRI head performed 20 hours post-CAS showed cortical edema of left hemisphere in all patients and leptomeningeal gadolinium-enhanced MRI has been in 2 patients. Cerebrospinal fluid (CSF) revealed a white blood cell count of 96x10^6/L in 2 patients and 1856x10^6/L in 1 patient, that was 90% neutrophils, increase proteins of 1.5g/L and normal glucose. Bacterial culture was negative. These cases with clearly inflammatory CSF and the development of meningeal syndrome in the absence of infection were regarded as aseptic meningitis. MRI with intravenous gadolinium was repeated 48 hours after operating and it shown no increase MR-signal from the subarachnoid space. We repeated the lumbar puncture on the 3st day after surgery to the patient in whose CSF there were 185610^6/L cells and the number of cells in the CFS decreased from 1856 to 4310^6/L. 2 days after CAS their neurologic symptoms were mild and in 3 days it was resolved, patients were discharged home.

**CONCLUSION.** Thus, our presented clinical observations are very rare complications of the hyperfusion syndrome after CAS. Despite a lot of publications on the reperfusion syndrome, observatios of aseptic meningitis after carotid artery stenting are few; in the worldwide literature we have met one clinical case. These clinical manifestations require further research and develop conception of pathogenesis.

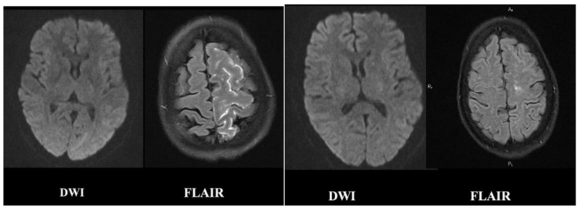

**Fig. 1 (abstract 001524).** See text for description

### 001567

#### Initial results of noninvasive monitoring of intracranial pressure in the postoperative period of cardiovascular surgery

P. TRAVASSOS, CL. Agnes, MS. Maiko, H. Cintya, F. Gustavo, LDSB. Thaís, S. Januário, CV. Viviane, SOR. Salomón
NEUROCRITICAL CARE UNIT, HOSPITAL BP - A BENEFICÊNCIA PORTUGUESA DE SÃO PAULO, SÃO PAULO, Brazil
**Correspondence:** P. TRAVASSOS
*Intensive Care Medicine Experimental* 2019, **7(Suppl 3):**001567

**INTRODUCTION.** Patients with cardiovascular diseases requiring surgical treatment, especially those that compromise the ascending aorta and crust, often require extracorporeal circulation (ECC), aortic clamping and hypothermia. Such procedures are related to poor perfusion. One of the main concerns of the intensive care team is to avoid secondary neurological damage after a long time of blood hypoflow, which can lead to cerebral edema and seizures. The most common parameters for neurological monitoring would be intracranial pressure and electroencephalogram and cerebral oximetry, however, for non-neurological patients, this information is uncommon and impedes optimal management.

**OBJECTIVES.** Our objective was to evaluate the cerebral compliance and the neurological condition of ICU patients in the immediate postoperative period of cardiovascular surgery using a new noninvasive intracranial pressure device (NIICP).

**METHODS.** This new method of noninvasive ICP monitoring consists of a strain gauge attached to a mechanical device that touches the surface of the scalp in the frontoparietal region laterally to the sagittal suture. The device can detect small changes in skull size resulting from changes in ICP, without the need for surgical procedures.

**RESULTS.** Twenty-two patients were included in this study; 20 required CPB. The mean ECC time was 123 minutes. Of those who required ECC, 8 presented alterations in the ICP curves with low cerebral compliance (P2 / P1 ratio> 1.0) evaluated by the morphological analysis of the curve and 9 were submitted to cerebral hypoflow, 4 of which presented cerebral complacency . Volume optimization and neuroprotection measures, such as MAP between 90 and 100 mmHg, temperature control, target pCO2 between 35 and 40 mmHg, sodium between 140 and 150 meq / dL, glycemic control between 140 and 180 mg / dL, and analgesic with RASS target -3 to -5 were initiated based on this NIICP information for the management of these cases. Among those patients with altered PICNI curves, 7 were discharged from ICU with good clinical condition and Glasgow Coma Scale of 15.

**CONCLUSION.** The cerebral monitoring of cardiovascular postoperative patients is important to prevent complications and may be a useful tool for acute neuroprotective treatment in the ICU.

### 001572

#### Correlation of comorbidities and intercurrences in the prognosis of patients with spinal cord trauma in the intensive care unit

J. Alves[1], C. Silva[1], JF. Martins[1], A. Catarino,[1], A. Salgueiro,[1], P. Martins,[2]
[1]Intensive care unit, Hospitalar and University Center of Coimbra, Coimbra, Portugal; [2]Intensive care unit director, Hospitalar and University Center of Coimbra, Coimbra, Portugal
**Correspondence:** J. Alves
*Intensive Care Medicine Experimental* 2019, **7(Suppl 3):**001572

**INTRODUCTION.** Cervical spine injury often occurs after major trauma, impairing motor and sensory impairment and causing complete or incomplete paraplegia or quadriplegia.

Characterization of the patient according to their comorbidities and the presence of complications during hospitalization in relation to the prognosis. Follow-up after hospital discharge with retrospective evaluation of the patient, their evolution as well as their current health status.

**METHODS.** Statistical analysis were performed with SPSS (version 24). Descriptive statistics were presented as means (M) and standard deviations (SD) for symmetrical continuous variables and medians (Mdn) and percentiles P25 and P75 for skewed continuous variables.

Chi-square tests or Fisher exact tests were used to associate prognostic with independent variables. The association of hospital length of stay (days) and the prognosis, as favorable or unfavorable, with the independent variables, was performed with Mann-Whitney test. Statistical significance was considered for p<.05 and marginal significance for p<.10.

Telephone interviews were carried out in 20 of the 24 patients and a questionnaire was applied.

**RESULTS.** A total of 24 patients, mostly males (23; 87.5%), aged from 19 to 89 years old (*M*=48.88; *SD*=19.08) with spinal-cord injury (SCI) were enrolled during 2017 and 2019. Cervical was the most frequent lesion (13; 54.2%). Surgical fixation was performed in 19 (79.2%) patients; 4 (16.7%) patients had non invasive ventilation and 11 (45.8%) tracheostomy. TCE was present in 14 (58.3%) patients, infection in 14 (58.3%) and other complications in 19 (79.2%).

Infection was associated with unfavorable prognostic (p=.005); 73.3% of the unfavorable prognostics had infection against 33.3% of the favorable prognostics. Noninvasive ventilation was associated with hospital length of stay (p=.068) (Mdn=20.5; P25=19.0, P75=25.5). Tracheostomized patients were hospitalized during longer periods (Mdn=21.0; P25=15.0, P75=27.0) (p=.006). The follow-up performed in 20 of the 24 patients involved in the study, 37.5% did not present any memory of hospitalization. Post-discharge clinical progression is positive in about 90% of patients.

**CONCLUSION.** The presence of traumatic brain injury is associated with a worse prognosis. Surgical fixation did not show statistically significant values (p=. 367).

There is association between infection and unfavorable prognosis (p=.006), with significant statistical value. Complications during hospitalization presented slight variations in prognosis, as well as duration of hospitalization.

Tracheostomy showed a statistically significant relationship (p = .006) with duration of hospitalization, but no relation to prognosis (p = .341). These results may have been affected by the size of the sample, by polytrauma or hospitalization complications in ICU.

## ARF - Acute respiratory failure 3

### 000515

#### Altered Airway Ward Round Review: How are we doing three years on?

J. Longo[1], A. Chan-Dominy[2]
[1]Rehabilitation & therapies, Royal Brompton Hospital, London, United Kingdom; [2]Intensive care, Royal Brompton Hospital, London, United Kingdom
**Correspondence:** J. Longo
*Intensive Care Medicine Experimental* 2019, **7(Suppl 3):**000515

**INTRODUCTION.** Tracheostomy insertion is known to be associated with significant morbidity and mortality (Cipriano et al., 2015). An Altered Airway Ward Round (AAWR) was introduced to the Brompton Hospital in 2015 as per the NCEPOD (2014) report 'On the Right Trach' in tracheostomy care recommendations. An initial audit in 2015 highlighted that the presence of the ward round had a positive impact on patient safety 3 months post introduction.

**OBJECTIVES.** The aim of this study was to assess for ongoing improvement in safety, and to measure the number and types of adverse incidents in altered airway patients since the initial audit of the AAWR.

**METHODS.** A retrospective review was carried out of all adult inpatients with altered airways (tracheostomy, laryngectomy, airway stent) for the period of January 2017-September 2018 inclusive using electronically recorded AAWRs, tracheostomy insertion data and all reported incidents. These incidents were collected and analyzed in terms of categories. A 3 month segment of this data (from January-March 2018) was also directly compared to the initial audit performed January-March 2015.

**RESULTS.** A total of 319 patients were seen during the AAWRs from January 2017-September 2018. 45 incidents were identified (45/319; 13%); Lack of insertion form (n=17; 48%), missing or incorrect equipment (n=4; 9%), emergency airway plan related (n=22; 38%), other (n=2; 4%).

A direct comparison of a 3 month snapshot is reported below in the table:

**CONCLUSION.** Three years on from the commencement of an AAWR, we have seen a considerable reduction in reported clinical incidents (down from 30% to 13%), reflecting the ongoing efficacy of a dedicated ward round.

##### REFERENCE(S)

1. NCEPOD. On the Right Trach. A review of the care received by patients who underwent a tracheostomy. https://www.ncepod.org.uk/2014report1/downloads/OnTheRightTrach_Summary.pdf (accessed April 2019)
2. Cipriano, A., Mao, M.L., Hon, H.H., Vazquez, D., Stawicki, S.P., Sharpe, R.P. & Evans, D.C. 2015, "An overview of complications associated with open and percutaneous tracheostomy procedures", International journal of critical illness and injury science, vol. 5, no. 3, pp. 179.

**Table 1 (abstract 000515).** See text for description

| Incident type | Jan-Mar 2015 (n=64) | Jan -Mar 2018 (n=81) |
|---|---|---|
| Total recorded incidences | 19 (30%) | 6 (8%) |
| Lack of insertion form/data | N/A | 4 (5%) |
| Equipment missing/incorrect | 8 (12.5%) | 1 (1%) |
| Bedside emergency airway plan/details | 11 (17%) | 1 (1%) |

### 000519

#### Association of the estimated dead space fraction and the ventilatory ratio with mortality in patients with acute respiratory distress syndrome

L. Morales-Quinteros[1], A. Artigas[2], M. Camprubí-Rimblas[3], J. Bringue[2], M. Schultz[4], O. Cremer[5], J. Horn[6], LDJ. Bos,[6]
[1]Intensive care, Hospital Universitari Sagrat Cor - Grup Quirónsalut, Barcelona, Spain; [2]Critical care center, Universitat Autònoma de Barcelona - UAB, Sabadell, Spain; [3]Institut d'investigació i innovació parc tauli (i3pt), Universitat Autònoma de Barcelona, Cerdanyola del Vallès, Spain; [4]Intensive care, Amsterdam University Medical Centers, University of Amsterdam, Amsterdam, Netherlands; [5]Intensive care, University Medical Center Utrecht, Utrecht, Netherlands; [6]Intensive care, Academic Medical Centre, Amsterdam, Netherlands
**Correspondence:** L. Morales-Quinteros
*Intensive Care Medicine Experimental* 2019, **7(Suppl 3):**000519

**INTRODUCTION.** The prognostic value of oxygenation indexes for mortality prediction in ARDS is limited. Increased dead space fraction (VD/VT) determined during its first week is a predictor of survival independent from oxygenation.

Estimated methods for impaired ventilation such as the estimated dead space fraction calculation and the ventilatory ratio (VR) are feasible to do at the bedside and correlate well with direct dead space fraction measurements.

**OBJECTIVES.** The primary objective was the predictive accuracy of the estimated dead space fraction and ventilatory ratio on mortality at 30 days after ARDS diagnosis measured by area under the receiver operating characteristics curve (AUROCC), net reclassification improvement and integrated discrimination index on top of a model with other predictors of outcome and calibration

**METHODS.** Secondary analysis of 940 patients with ARDS from the "Molecular Diagnosis and Risk Stratification of Sepsis" (MARS) project in mixed ICUs of two teaching hospitals. Different formulas were used to calculate dead space fraction: 1) Unadjusted Harris-Benedict (VD/VTHB); 2) Penn State (VD/VTPS); and 3) Direct estimate (VD/VTdirect). VR was calculated as per the following formula: VEmeasured X PaCO2measured/VEpredicted x PaCO2predicted. where VEpredicted is 100 ml/kg/min on the basis of predicted body weight and PaCO2predicted is 37.5 mm Hg.

**RESULTS.** VD/VT estimations and VR were significantly higher among non-survivors for all estimating equations at both days 1 and 2. VD/VTdirect and VR were statistically independent predictors of death at 30 days on day 2. The addition of each one of the predictors improved the predictive accuracy of the baseline model on day 2.

**CONCLUSION.** Estimated methods for impaired ventilation were associated with mortality in the early course of ARDS patients and added predictive value to indexes of oxygenation and respiratory system mechanics. These indexes offer clinicians information about ventilatory efficiency at the bedside and may provide useful prognostic information. Measurement of the dead-space fraction could help clinical investigators to identify the patients who may benefit most from a particular therapeutic intervention.

REFERENCE(S)
1. Dr. Morales-Quinteros was partially funded by a grant from Fisher & Paykel Healthcare Limited (FPH).
2. Sinha P, et al. Physiological Analysis and Clinical Performance of the Ventilatory Ratio in Acute Respiratory Distress Syndrome. Am J Respir Crit Care Med. 2019; 199(3): 333-341.
3. Beitler JR, et al. Estimating dead-space fraction for secondary analyses of acute respiratory distress syndrome clinical trials. Crit Care Med. 2015;43(5):1026-1035.

### 000534

#### The multi-disciplinary collaboration team to improve the outcome of patients with respiratory failure

CM. Chen
{street_address}, Tainan, Taiwan
*Intensive Care Medicine Experimental* 2019, **7(Suppl 3):**000534

**INTRODUCTION.** Early extubation for the critically ill patients on mechanical ventilation (MV) due to acute respiratory failure (ARF) is the primary treatment aim in intensive care unit (ICU). Overt sedation of ICU patients is associated with extended MV, increased ICU length of stay (LOS) and long-term cognitive impairment.

**OBJECTIVES.** By using the different ways of quality control, team resource management (TRM) and also a bundle-care approach with evidence-based medicine, we hope to improve the quality of care in critically ill patients with MV due to ARF.

**METHODS.** Since 2014, we had established a multi-disciplinary team (physical therapist, nurse, the medical staff and the patient's family) for MV quality improvement in a 19-bed medical ICU at Chi-Mei medical center. We performed early mobilization program (within 72 hours after MV use) with a four-phase rehabilitation protocol, which range from passive movement to active mobilization, and they were named as different Chinese kungfus to help the teams to remember. Although the implementation of this rehabilitation program had shortened the duration of MV from 2014 to 2015, the outbreak of influenza in 2016 with the increased sedation use for patient-ventilator synchrony made the prolonged use of MV. Thus, the performance of early mobilization might not be easily achieved. In order to improve the outcome of patients with MV, we represented the use of an evidence-based, interdisciplinary team approach, patient-centered and integrated protocol, known as ABCDE care bundle (Awake trial, Breathing Trial, Coordination of drugs, Delirium management and Early Mobilization).

**RESULTS.** Before the quality improvement of care (Jan 1 to March 30, 2014) , the average duration of MV was 6.6 days. After the intervention of early mobilization program, the duration of MV was down to 4.4 days (July 1 to September 30, 2014), 4.1 days (November 1to December 31, 2014) and an average of 4.5 days in the majority of 2015. Unfortunately, MV duration raised up to 8.8 days (Dec 1, 2015 to March 31, 2016). After the performance of ABCDE bundle, the average duration of MV was gradual reduced to 4.8 days (September 1to December 31, 2016) and 4.5 days (April 1 to August 31, 2017). Also the percentage of delirium assessment increased from 0% to 100% after ABCDE bundle. The ICU LOS (from 2014 to 2017) decreased gradually each year, as 10.0, 7.5, 7.3 and 6.8 days separately.The hospital cost and delirium rate were also decreased respectively.

**CONCLUSION.** To improve the outcomes of ARF patients with MV, we have performed different way of quality control, TRM and ABCDE Bundle Care. They could effectively decrease the duration of MV, ICU LOS and hospital cost. Moreover, we hope to share, expand and promote this effective strategy, ABCDE bundle care to all hospitals in Taiwan in order to achieve a favorable quality of care in MV patients.

REFERENCE(S)
1. Balas MC, Vasilevskis EE, Olsen KM, et al. Effectiveness and safety of the awakening and breathing coordination, delirium monitoring/management, and early exercise/mobility bundle. Critical Care Medicine. 2014;42(5):1024–1036.
2. Lai CC, ChouW, Chan KS, et al. Early mobilization reduces duration of mechanical ventilation and intensive care unit stay in patients with acute respiratory failure. Arch Phys Med Rehabil 2017;98:931–9.
3. No grant acknowledgment

### 000537

#### Transdiaphragmatic Inspiratory Pressure and Ultrasound Study of Diaphragm for Mechanical Ventilation Weaning Prediction

AA. Menis[1], V. Tsolaki[2], E. Zakynthinos[1], D. Makris[1]
[1]Department of intensive care medicine, University of Thessaly, Medical School, Larissa, Greece; [2]Department of intensive care medicine, University Hospital of Larissa, Larisa, Greece
**Correspondence:** A.A. Menis
*Intensive Care Medicine Experimental* 2019, **7(Suppl 3):**000537

**INTRODUCTION.** Difficult weaning causes a prolongation of the patient's hospitalization and of his dependence from mechanical ventilation (MV).

**OBJECTIVES.** The main objective of this study was to identify whether indices of diaphragmatic function obtained by diaphragmatic pressure measurement or ultrasound imaging could predict successful weaning from MV.

**METHODS.** The study took place in the Intensive Care Unit of a tertiary Greek hospital. Patients who fulfilled criteria for weaning from MV were included in the study and underwent a spontaneous breathing trial (SBT). Successful weaning was defined when a patient remained 48 hours without mechanical ventilation following a successful SBT. Esophageal and gastric pressures were measured with esophageal balloon-tipped-catheters. Diaphragmatic movement was assessed by ultrasound using a Linear 3-11 MHz head. The ultrasound probe was placed in the mid axillary line perpendicular to the zone of apposition between the 8th-10th intercostal spaces.

**RESULTS.** Twenty-five patients were evaluated. Peak esophageal inspiratory pressure, peak transdiaphragmatic inspiratory pressure, Peak Inspiratory (Tdpi) and End Expiratory (Tdee) diaphragmatic thickness, Diaphragmatic Thickening Fraction (Tfdi), Diaphragmatic Thickness (Tdth) and Excursion (Tdex) during SBT were significantly different ($p<0.05$) between those who succeeded and those who failed weaning. The receiver operating characteristics curves to identify those who succeeded weaning were: for Esophageal inspiratory pressure 0.872 ($p<0{,}01$)(C.I.95% 0.733-1), for Transdiaphragmatic inspiratory pressure 0.792 ($p<0.05$) (C.I. 95% 0.616-0.907), for Tdpi 0.868 ($p<0.01$) (C.I. 95% 0.682-1) for Tdee 0.819 ($p<0.01$) (C.I. 95% 0.644 – 0.995), for Tfdi 0.764 ($p<0{,}05$) (C.I. 95% 0.571 – 0.957), for Tdth 0.837 ($p<0.01$) (C.I.95% 0.653-1) and finally for the Tdex 0.893 ($p<0.01$) (C.I. 95% 0.722-1) having a sensitivity of 93.8% and specificity of 85.7%.

**CONCLUSION.** Ultrasound imaging may be useful in identifying patients who are ready for weaning from MV and might be useful in reducing MV duration.

REFERENCE(S)
1. Matamis D. et al. Intensive Care Med. 39, 801–810 (2013).
2. Akoumianaki E. et al. Am. J. Respir. Crit. Care Med. 189, 520–531 (2014).

### 000542

#### Maximal Inspiratory Pressure and Resistive Breathing and Diaphragm Ultrasound for Mechanical Ventilation Weaning Prediction

AA. Menis[1], V. Tsolaki[2], E. Zakynthinos[1], D. Makris[1]
[1]Department of intensive care medicine, University of Thessaly, Medical School, Larissa, Greece; [2]Intensive care, University Hospital of Larissa, Larisa, Greece
**Correspondence:** A.A. Menis
*Intensive Care Medicine Experimental* 2019, **7(Suppl 3):**000542

**INTRODUCTION.** Maximal Inspiratory Pressure trial (MIP) is used as a predictive index for weaning from mechanical ventilation (MV). Resistive Breathing (RB) follows the same physiological rationale as MIP without fully obstructing the airway.

**OBJECTIVES.** The main objective of this study was to identify whether indices of diaphragmatic function obtained by diaphragmatic imaging or relevant pressures during MIP or RB may be useful in predicting successful weaning from MV.

**METHODS.** The study took place in the Intensive Care Unit of a tertiary Greek hospital. Patients who fulfilled criteria for weaning from MV were included in the study and underwent a spontaneous breathing trial (SBT), with an airway of reduced diameter (RB) and a MIP trial. Successful weaning was defined when a patient remained 48 hours without mechanical ventilation following a successful SBT. Esophageal and gastric pressures were measured with esophageal balloon-tipped-catheters. Diaphragmatic movement was assessed by US using a Linear 3-11 MHz head. The US probe was placed in the mid axillary line perpendicular to the zone of apposition between the 8th-10th intercostal spaces.

**RESULTS.** 16 out of 25 (64%) patients succeeded and 9 (36%) failed weaning. Esophageal inspiratory pressure, Diaphragmatic Thicknening (Tdth) and Excursion (Tdex) during RB and Peak Inspiratory (Tdpi) and End Expiratory diaphragmatic thickness (Tdee) during MIP were significantly different (p<0.05) between those who succeeded and those who failed. The receiver operating characteristics curves to identify successful weaning were: for Esophageal pressure during RB 0.748 (p<0.05) (C.I. 95% 0.550-0.946), for Tdth-RB 0.759 (p<0.05) (C.I. 95% 0.540-0.979), for Tdex–RB 0.875 (p<0.01) (C.I. 95% 0.716-1) with a sensitivity (sn) of 93% and specificity (sp) of 75%, for Tdpi-MIP 0.850 (p<0.05) (C.I. 0.575-1) and Tdee-MIP 0.857 (p< 0.05) (C.I. 95% 0.638-1) both having a sn of 100% and sp of 80%.

**CONCLUSION.** Ultrasound imaging during RB and MIP might be useful in predicting patients who are ready for weaning from MV.

**REFERENCE(S)**

1. Matamis D. et al. Intensive Care Med. 39, 801–810 (2013).
2. Akoumianaki E. et al. Am. J. Respir. Crit. Care Med. 189, 520–531 (2014).

### 000543

#### Severe cardiovascular collapse following endotracheal intubation and its association with prolonged ICU length of stay and ICU mortality

A. elsharkawy, S. Al-Awady, T. Helmy

Critical care medicine, Alexandria Faculty of Medicine, Alexandria, Egypt

**Correspondence:** A. elsharkawy

*Intensive Care Medicine Experimental* 2019, **7(Suppl 3):**000543

**INTRODUCTION.** Cardiovascular collapse (CVC) is a serious complication of endotracheal intubation (ETI). From a physiological point of view, it could be attributed to the changes that occur in venous return, intrathoracic pressure, PaCO2 and catecholamine level. Many studies have evaluated risk factors for postintubation hypotension. However, there is no universally agreed definition of postintubation cardiovascular collapse. In this study, we considered severe (CVC) as systolic blood pressure ≤65 mmHg recorded at least once and/or ≤90 mmHg for ≥30 minutes or the requirement of vasopressors and/or inotropes.

**OBJECTIVES.** The aim of this study is to assess the incidence and potential risk factor for severe CVC after ETI and its impact on ICU length of stay and mortality.

**METHODS.** This is a prospective cohort study that was conducted on 300 patients. Subjects were classified into two categories: those who were haemodynamically stable after intubation and those who collapsed. Different variables were documented in both groups and they were compared with the incidence of postintubation CVC. ICU length of stay and 28-day mortality were assessed in both groups.

**RESULTS.** 26.3% of subjects have developed CVC after ETI. Factors that were associated with increased incidence of CVC include: old age, heart failure, CKD, COPD, higher heart rate before and during intubation and propofol usage for intubation. The mean value for ICU length of stay for collapsed patients was 15.08 ± 8.51 days whereas it was 10.97 ± 9.60 days for non-collapsed patients (P < 0.001). 67.2% of collapsed patients have died within 28 days of intubation while only 27.1% of non-collapsed patients have died within the same period (P < 0.001).

**CONCLUSION.** Postintubation CVC is associated with poorer outcomes as regards ICU length of stay and ICU mortality. There are potential risk factors that need further evaluation to predict and/or decrease the incidence of CVC after ETI.

**REFERENCE(S)**

1. Heffner AC, Swords DS, Nussbaum ML, Kline JA, Jones AE. Predictors of the complication of postintubation hypotension during emergency airway management. J Crit Care. 2012 Dec;27(6):587–93.
2. Perbet S, De Jong A, Delmas J, Futier E, Pereira B, Jaber S, et al. Incidence of and risk factors for severe cardiovascular collapse after endotracheal intubation in the ICU: a multicenter observational study. Crit Care Lond Engl. 2015 Jun 18;19:257.
3. Franklin C, Samuel J, Hu T-C. Life-threatening hypotension associated with emergency intubation and the initiation of mechanical ventilation. Am J Emerg Med. 1994 Jul 1 ;12 (4):425–8.
4. I would like to express my gratitude to the medical student Nourhan A.Youssef whose efforts were essential for completion of this project.

### 000562

#### Predictors of early readmission in patients with home mechanical ventilation

EY. Kim[1], HJ. SUH[1], GJ. Seo[1], SB. Hong[2], Y. Koh[2], CM. Lim[2], JW. Huh[2]

[1]Respiratory care services, Asan Medical Center, Seoul, Republic of Korea; [2]Department of pulmonary and critical care medicine, Asan Medical Center, Seoul, Republic of Korea

**Correspondence:** E.Y. Kim

*Intensive Care Medicine Experimental* 2019, **7(Suppl 3):**000562

**INTRODUCTION.** With advanced respiratory care, the number of critically ill patients receiving home mechanical ventilation (HMV) therapy has increased. Readmission due to disease progression and medical emergencies in these fragile patients is also increasing.

**OBJECTIVES.** The purpose of this study was to investigate the factors affecting early readmission of patients who received HMV for the first time.

**METHODS.** This is a retrospective study of adult patients who were admitted to Asan Medical Center from March 2014 to February 2018. The inclusion criterion was readmission of patients within a year of initiating HMV. We compared the clinical characteristics of the patients at the time of discharge between those who had readmission within 30 days (early readmission group) and those who had readmission within a year from the 31st day (late readmission group). Additionally, we investigated the clinical outcomes at the time of readmission.

**RESULTS.** In total, 78 patients had unplanned readmission within a year from discharge, and 27 (34.6%) had early readmission. Among the causes of readmission, aspiration event was significantly higher in the early readmission group than in the late readmission group (29.6% vs 7.8%; $p$ = 0.011). Among the underlying diseases, neoplasm (55.6% vs 21.6%; $p$ = 0.002) and sequelae of acute respiratory failure (63.0% vs 33.3%; $p$ = 0.012) were significantly higher in the early readmission group. In contrast, chronic respiratory failure was higher in the late readmission group (44.4% vs 74.5%, $p$ = 0.008). Among the types of HMV, use of the tracheostomy tube was more common with early readmission than non-invasive mask ventilation (66.7% vs 19.6%; $p$ <0.001), and all-day support was also more common with early readmission (44.4% vs 17.6%; $p$ = 0.011). In a multivariate logistic regression analysis, neoplasm and tube-feeding methods, such as nasogastric and gastrostomy tubes, had a correlation with early readmission.

**CONCLUSION.** Underlying diseases and feeding methods were significantly associated with unplanned early readmission. The medical staff should educate the patients, such as on aspiration prevention, for home mechanical ventilation.

**REFERENCE(S)**

1. Kun SS, Edwards JD, Davidson Ward SL, Keens TG (2012) Hospital readmissions for newly discharged pediatric home mechanical ventilation patients. Pediatr Pulmonol, 47:409-414.

2. None. No funding to declare.

### 000564

#### The use of high flow nasal cannula oxygen (HFNC 02) and its impact on patient outcomes in a tertiary hospital in Singapore

R. Gokhale[1], KGF. Jean[2], CMJ. Ni[3], A. Leylane[3], C. Siau[4]
[1]Intensive care, Changi General Hospital, Singapore, Singapore; [2]Respiratory therapy, Changi General, Singapore, Singapore; [3]Respiratory therapy, Changi General Hospital, Singapore, Singapore; [4]Respiratory and critical care medicine, Changi General Hospital, Singapore, Singapore
**Correspondence:** R. Gokhale
*Intensive Care Medicine Experimental* 2019, **7(Suppl 3):**000564

**INTRODUCTION.** High flow nasal cannula oxygen (HFNC 02) was introduced in December 2017. Changi General hospital is a 1000 bedded hospital which caters to the population in the east of Singapore.We have an 18 bedded Medical Intennsive Care Unit and we recieve around 1000 admissions over a year

**OBJECTIVES.** To identify indications for the use of HFNC 02 in our hospital and to look at the impact on need for Invasive Mechanical ventilation (IMV) and ICU mortality in these patients.

**METHODS.** All patients who required HFNC 02 from February to November 2018 were included and data was collected with regards to their diagnosis, indication for HFNC O2, PF ratio, length of time on HFNC 02, final outcome on discharge from critical care.

**RESULTS.** 60 patients received HFNC 02 during the stated time period. The mean age was 63.5 years, and there were 43 males. The main diagnoses were Community acquired and hospital acquired pneumonia. 47 patients received therapy for Type 1 Respiratory failure, 9 patients were post extubation, 2 for Type 2 respiratory failure and 2 received therapy as palliation.

The mean Pf ratio was 130.5 .The average length of time spent on HFNC 02 was 42 hours. At the end of HFNC 02 therapy, 35 patients were weaned off to face mask oxygen (58.3 %), 2 palliative patients died and 23 patients (38.3%) needed intubation. The most common reason for intubation was rapidly progressive respiratory failure. Of the 23 patients 8 patients died in critical care. Overall mortality at discharge from critical care was 16% including the 2 palliated patients.

**CONCLUSION.** Despite having a cohort of patients with moderate to severe hypoxia, a majority of patients (58.3%) avoided IMV and the average length of therapy was under 2 days. The ability to identify the right patients, who will benefit from HFNC 02 and avoid IMV, continues to pose the biggest challenge. Rapidly progressive respiratory failure was the main reason for the need for IMV.

##### REFERENCE(S)

1. Frat JP, et al. "High-flow oxygen through nasal cannula in acute hypoxemic respiratory failure". The New England Journal of Medicine. 2015. 372(23):2185-2196.
2. Helviz Y, Einav S. A Systematic Review of the High-flow Nasal Cannula for Adult Patients. Crit Care. 2018 Mar 20;22(1):71. doi: 10.1186/s13054-018-1990-4. PubMed PMID: 29558988; PubMed Central PMCID: PMC5861611.
3. None

### 000569

#### The use of airway pressure release ventilation for severe respiratory failure; a retrospective review of the clinical effectiveness and patient safety in an adult critical care unit in the south east of England

S. Bahlool, G. Millen
Critical care, William Harvey Hospital, Willesborough, United Kingdom
**Correspondence:** S. Bahlool
*Intensive Care Medicine Experimental* 2019, **7(Suppl 3):**000569

**INTRODUCTION.** Airway pressure release ventilation (APRV) has been recently gaining more popularity in many adult critical care units in the south east of England. Although it is now widely used in the region as part of the management of severe respiratory failure, it failed to find its way into the national guidelines for the management of ARDS (1). I guess the paucity of clinical trials plus some conflicting results from already published studies could be the reason (2, 3). Since the publication of Guidelines for the Provision of Intensive Care Services (GPICS) by the Faculty of Intensive Care Medicine and the Intensive Care Society in April 2015(4), all critical care units were encouraged to develop a pathway for refractory hypoxaemia. At the William Harvey Hospital in south east of England, we introduced our local pathway for severe respiratory failure that includes APRV as one option.

**OBJECTIVES.** One year following the introduction of the APRV into the local practice, we decided to retrospectively review all the patient who had received APRV from two points of view; clinical effectiveness and safety profile. Effectiveness was measured by the improvement in oxygenation whilst safety was judged by the incidence of serious adverse events like barotrauma.

**METHODS.** We retrospectively reviewed all patients admitted to our critical care unit over a period of a year and had received APRV for at least six hours or more. Data collection was anonymous and only non-identifiable information was used. There were no patients with head injury in our audit. Data collected for each patient included: age, sex, ventilation days and total Intensive care days. Physiological data collected for each patient were the worst daily value for PO2, PCO2, peak airway pressure and the P/F ratio. We also recorded the incidence of barotrauma and patients' final outcome.

**RESULTS.** Seventy six patients received APRV over the period of the review. The average patients' age was 55 (16 – 85) years with male to female ratio of 1.5. The average length for mechanical ventilation was 6 (2 – 38) days and the length of total ITU stay before final destination was 8 (2- 28) days. The average P/F ratio significantly improved over the first three days following the application of APRV. This coincided well with a reduction in the peak airway pressure and no significant hypercapnoea. Pneumothorax occurred in only three patients (incidence of 4%); all those patients had a chest drain sited promptly and two of them were eventually transferred to a tertiary centre. One patient developed Pneumo-mediastinum and was managed conservatively.Regarding the final outcome, 59 (77%) patients were weaned successfully from mechanical ventilation, 14 (18%) patients died and 4 (5%) patients retrieved by a tertiary centre (three of which received ECMO).

**CONCLUSION.** The results showed that application of APRV was associated with dramatic improvement in oxygenation over a very short period of time. There was no significant hypercapnoea associated with the application of APRV and the incidence of barotrauma was 4% which is less than described in the literature for such cohort of patients(5). Although our patients sample is small, this review showed that APRV is very effective tool in the management of severe respiratory failure with no evidence of increased harm to our local patients.

##### REFERENCE(S)

1) https://www.ficm.ac.uk/sites/default/files/ficm_ics_ards_guideline_-_july_2018.pdf.
2) Zhou Y, Jin X, Lv Y, et al. Early application of airway pressure release ventilation may reduce the duration of mechanical ventilation in acute respiratory distress syndrome. Intensive Care Med 2017; 43:1648.
3) Hirshberg EL, Lanspa MJ, Peterson J, et al. Randomized Feasibility Trial of a Low Tidal Volume-Airway Pressure Release Ventilation Protocol Compared With Traditional Airway Pressure Release Ventilation and Volume Control Ventilation Protocols. Crit Care Med 2018; 46:1943
4) https://www.ficm.ac.uk/sites/default/files/GPICS%20-%20Ed.1%20%282015%29_0.pdf.
5) AUAnzueto A, Frutos-Vivar F, et al. Incidence, risk factors and outcome of barotrauma in mechanically ventilated patients. Care Med. 2004;30(4):612.

### 000576

#### ADVOS removes CO2 and corrects acidosis: Mechanism of action following a H+ and HCO3- gradient (physiological approach) or pCO2 and SID variations (quantitative approach)

A. Perez[1], B. Kreymann[2]
[1]Scientific & Clinical Affairs, Hepa Wash GmbH, München, Germany; [2]Chief executive officer, Hepa Wash GmbH, München, Germany
**Correspondence:** A. Perez
*Intensive Care Medicine Experimental* 2019, **7(Suppl 3):**000576

**INTRODUCTION.** Acid-base balance is a complex continuous process regulated by several organs, including the lung, the kidney and the liver. The ADVOS therapy is based on albumin dialysis and can support all three organs by, among others, removing CO2, counterbalancing electrolyte disturbances and correcting acidosis. This is achieved with a recirculating dialysate formed by mixing an acidic and an alkaline concentrate at a modifiable ratio to reach a desired pH.
**OBJECTIVES.** In this work we explain the mechanism of ADVOS to achieve CO2 removal and acidosis correction in an ex vivo blood model with a carbonate-free dialysate using the classical (i.e. Henderson-Hasselbalch) and modern (i.e. Stewart) approach for acid-base balance.
**METHODS.** Briefly, 5 liters of swine blood were dialyzed with the ADVOS system using a high pH carbonate-free dialysate. For experimental purposes a pH of 10.0 was set. Combinations of blood flows (QB = 100, 200 or 400 ml/min) and concentrate flows (QC = 160 or 320 ml/min) were tested for 1 hour each. A pH-probe controlled blood pH continuously. CO2 was supplied to reach a constant blood pH of 7.35-7.45. Blood gas analysis were performed every 15 minutes in the inlet and the outlet of the dialyzer.
**RESULTS.** With a Qc of 160 ml/min a maximum continuous CO2 supply of 114 ml/min was achieved with QB of 400 ml/min. Blood pH was maintained between 7.35-7.45, while pCO2 and HCO3- were elevated. With a QC of 320 ml/min, HCO3- levels were lower. Moreover, every blood gas value remains physiological with a 70 ml/min CO2 supply and a QB of 400 ml/min.
The acid-base balance can be explained through the physiological Henderson-Hasselbalch or the quantitative Stewart approach, which use the relationship between pCO2 and HCO3-, or variations in pCO2, strong ion difference (SID) and total weak acids, respectively. Anyhow for ADVOS, the recirculation of the dialysate, to adjust pH to a desired level, is determinant.
According to dialyzer inlet and outlet values in Table 1, first, the high pH carbonate-free dialysate induces a concentration gradient between blood and dialysate for H+ and HCO3-, which triggers CO2 removal and pH increase. Second, a reduction in pCO2 and SID occurs, which can predict the increased pH level [1].
**CONCLUSION.** ADVOS therapy can remove CO2 and correct acid-base balance using a high pH dialysate. The mechanism of action can be explained through a H+ and HCO3- gradient and by pCO2 and SID variations.

**Table 1 (abstract 000576).** Results from blood gas analysis from the inlet and outlet of the dialyzers during ADVOS treatments. Mean ± S.D.

| Qb (ml/min) | Qc (ml/min) | CO2 supply (ml/min) | Blood pH | | Blood HCO3- | | pCO2 (mmHg) | | SID (mEq/l) | |
|---|---|---|---|---|---|---|---|---|---|---|
| | | | inlet | outlet | inlet | outlet | inlet | outlet | inlet | outlet |
| 100 | 160 | 72 ± 1 | 7.43 ± 0.01 | n.a. | 44 ± 5 | n.a. | 66 ± 10 | n.a. | 43 ± 3 | 27 ± 2 |
| 200 | | 102 ± 1 | 7.42 ± 0.02 | 7.93 ± 0.03 | 54 ± 4 | 38 ± 1 | 97 ± 4 | 12 ± 5 | 50 ± 1 | 39 ± 2 |
| 400 | | 114 ± 3 | 7.34 ± 0.01 | 7.84 ± 0.04 | 63 ± 3 | 50 ± 2 | 117 ± 5 | 29 ± 2 | 54 ± 2 | 47 ± 1 |
| | 320 | 110 ± 3 | 7.33 ± 0.04 | 7.77 ± 0.10 | 35 ± 3 | 28 ± 2 | 66 ± 9 | 19 ± 7 | 38 ± 1 | 39 ± 2 |
| | | 70 ± 1 | 7.35 ± 0.06 | 7.69 ± 0.01 | 21 ± 2 | 14 ± 2 | 36 ± 3 | 12 ± 1 | 34 ± 1 | 29 ± 1 |

### 000583

#### SpO2/FIO2 accurately predicts PaO2/FIO2 value in patients with acute hypoxic respiratory failure supported by nasal high-flow

M. Santafe[1], J. Messika[2], M. Samper[3], B. Sztrymf[4], G. Hernandez[5], M. García-De-Acilu[1], JP. Frat[6], A. Thille[6], T. Mauri[7]; E. Spinelli[7], JR. Masclans[3], JD. Ricard[4], O. Roca[1]
[1]Critical care department, Vall d'Hebron University Hospital, Barcelona, Spain; [2]Ap-hp, Louis-Mourier Hospital (AP-HP), Colombes, France; [3]Critical care department, Hospital del Mar, Barcelona, Spain; [4]Ap-hp, Hôpital Antoine-Béclère Ap-Hp, Clamart, France; [5]Critical care department, Virgen de la Salud University Hospital, Toledo, Spain; [6]Medecine intensive reanimation, Poitiers University Hospital, Poitiers, France; [7]Department of anesthesiology, Fondazione IRCCS Ca'Granda Ospedale Maggiore Policlinico, Milano, Italy
**Correspondence:** M. Santafe
*Intensive Care Medicine Experimental* 2019, **7(Suppl 3):**000583

**INTRODUCTION.** The PaO2/fraction of inspired oxygen (FIO2) (P/F) ratio is correlated with the ratio of the pulse oximetry saturation (SpO2)/FIO2 (S/F) in intubated hypoxic patients. Whether this correlation exists in acute hypoxic respiratory failure (AHRF) patients treated by nasal high flow (NHF) remains to be determined.
**OBJECTIVES.** To describe the correlation between P/F and S/F values in patients with AHRF supported by NHF.
**METHODS.** Measurements of P/F and S/F of the patients enrolled in the ROX index studies (1, 2) were compared to assess the relationship between them (derivation cohort). SpO2 values ≥98% were excluded from the analysis. Scatterplot of S/F and P/F ratios were used to determine the linear correlation. Generalized estimating equations were used to estimate the best regression line. S/F threshold values correlating with P/F ratios of 200 and 100 were determined. The area under receiver operator characteristic (AUROC) curves was calculated to assess the diagnostic ability to discriminate between patients with P/F ratio ≤200 and ≤100. Patients from the FLORALI trial (3) and 3 other physiologic studies (4-6) were used as validation cohort.
**RESULTS.** Using the 638 measurements of the derivation cohort, the relationship between S/F and P/F was described by the equation S/F= 45 + P/F * 0.78, R2=0.68; p<0.0001) (Figure 1). A S/F of 201 corresponds to a P/F of 200, and a S/F of 123 to a P/F of 100. S/F ratios demonstrated high diagnostic accuracy to discriminate between patients with P/F ≤100 and ≤200 (AUROC 0.91 [0.88-0.95] and 0.86 [0.77-0.94], respectively). Sensbility, specificity and positive and negative predictive values are presented in Table 1. Similar results were observed in the validation cohort (280 measurements).
**CONCLUSION.** S/F ratio correlates with P/F ratio in AHRF treated with NHF. S/F values of 201 and 123 correlate with P/F of 200 and 100, respectively. S/F thresholds could be a non-invasive but accurate measure to stratify severity of hypoxic patients treated with NHF.

##### REFERENCE(S)

1. Roca O, et al. Am J Respir Crit Care Med 2018. doi: 10.1164/rccm.201803-0589OC.
2. Roca O, et al. Journal of critical care 2016; 35: 200-205.
3. Frat JP, et al. N Engl J Med 2015; 372: 2185-2196
4. Mauri T, et al. Intensive Care Med 2017; 43: 1453-1463
5. Mauri T, et al. Am J Respir Crit Care Med 2017; 195: 1207-1215
6. Mauri T, et al. Crit Care 2018; 22: 120.

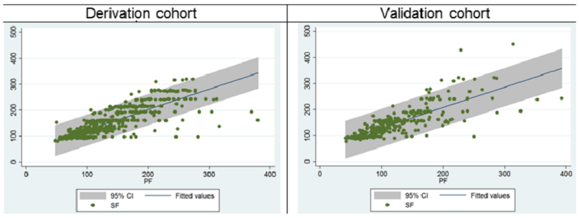

**Fig. 1 (abstract 000583).** Scatterplot of S/F and P/F ratios in the derivation and validation chort.

**Table 1 (abstract 000583).** See text for description

| | Cohort | Se | Sp | PPV | NPV | LR+ | LR- |
|---|---|---|---|---|---|---|---|
| S/F≤201 | Derivation | 89.8% | 71.4% | 95.8% | 49.1% | 3.14 | 0.14 |
| | Validation | 92.8% | 66.7% | 95.1% | 57.1% | 2.78 | 0.11 |
| S/F≤123 | Derivation | 89.4% | 82.8% | 74.0% | 93.4% | 5.19 | 0.13 |
| | Validation | 77.7% | 87.3% | 81.7% | 84.3% | 6.14 | 0.26 |

### 000591

#### Outcomes and prognostication of patients with ARDS given immunosuppression as rescue on a Specialist Respiratory Critical Care Unit

C. Remmington[1], S. Ledot[1], B. Patel[1], C. Morgan[1], M. Passariello[1], S. Price[1], T. Xu[1], J. Doyle[1], S. Desai[2], A. Wells[3], S. Singh[1]
[1]Department of critical care, Royal Brompton & Harefield NHS Foundation Trust, London, United Kingdom; [2]Department of radiology and imaging, Royal Brompton & Harefield NHS Foundation Trust, London, United Kingdom; [3]Department of interstitial lung disease, Royal Brompton & Harefield NHS Foundation Trust, London, United Kingdom
**Correspondence:** C. Remmington
*Intensive Care Medicine Experimental* 2019, **7(Suppl 3):**000591

**INTRODUCTION.** High dose Steroids for ARDS have not shown survival value, and possible harm beyond 14d (1,2). However, dosing, timing and variation in study design influence published outcomes (3). Extended lower doses (1-2mg/kg/d) may improve hospital outcomes (4). High dose immunosuppression (IS) benefits some patients(pts) with severe ILD on mechanical ventilation (MV)(5). Furthermore, steroids in severe pneumonia can improve survival (6). Thus, high dose steroids continue to be given in ARDS when, clinicopathoradiographic features suggest a potentially steroid responsive behavior pattern, or as a last resort, if faced with an inability to wean off MV. The outcomes from this strategy are unclear.

**OBJECTIVES.** We reviewed outcomes of pts with ARDS treated with immunosuppression.

**METHODS.** A retrospective case matched study from a single tertiary respiratory critical care unit(2009-17), identified sequential pts who received intravenous Methylprednisolone (MePred), +/- Rituximab, +/- cyclophosphamide, for ARDS. ARDS pts, (AECC/Berlin criteria - 10% of all ARDS patients), 81 given IS, were case matched with 81 pts without IS (by P/F ratio, gender, age, SOFA-all in 34 cases). Outcomes of survival (30d, 6mo, 12mo), Covariates such as SOFA score, MV days, length of stay (LOS), presence of infection, antibiotic usage were analysed. Cox multiple comparisons regression identified prognosticators.

**RESULTS.** 162 pts (81 ARDS-IS and 81 ARDS). Mean age 51y±4.9 and 44y±12.9. Male 48(59%) and 45(56%). For ARDS-IS 70(86%) had MePred and 6(7%) received MePred+cyclophosphamide. Percentage of pts given IS at <7d 34(42%), 7-14d 24(30%), >14d 23(28%). Veno-venous ECMO in 49(60%) ARDS-IS and 62(77%) ARDS pts. Mean SOFA scores 10.8±3.71 and 13.5±3.47. Neuromuscular blockade in 65(80%) and 68(84%). Mean mechanical ventilator days were 34 and 16 days(p<0.05). Mean length of stay 40 and 19 days(p<0.05). Mean total antibiotic (AB) days 97(±101) and 61(± 20); AB/day 2.42 and 3.25 (p>0.05). AB days pre and post ARDS-IS were 34(±34) and 63(±88.5). In ARDS-IS, respiratory was the highest positive microbiology site. There was a 1.5x increase in positive respiratory and 2x urine/line infection post IS, but not causing worse outcome. Fungal infections were the most frequently encountered organism followed by gram-ve. Overall, survival was 29% v 71.4% for ARDS-IS v ARDS (p<0.0001). Survival at 30d, 6mo and 1 yr was 75, 48 and 46% for ARDS-IS, and 79, 75 and 74% for ARDS. Overall mortality by timing of IS was 70% 24/34(<7d), 58% 14/24(7-14d), 65% 15/23 (>14d). Immunosuppression, diabetes, age, length of stay were univariately associated with a worse outcome. Hazards ratio (HR) for death in ARDS given IS was 2.73 (p<0.0001). Only age (HR 1.03, p=0.002), length of stay (HR 0.47, p=0.0001) and diabetes (HR 2.34, p=0.021) were independently associated with mortality.

**CONCLUSION.** After high dose IS, post discharge survival for ARDS-IS was significantly worse than case matched ARDS pts. Age, length of stay and diabetes were independent factor for increasing risk of death. No survival difference based on early or late timing of IS. High dose IS is associated with significantly worse post discharge survival. Investigation of causes of post 30d deaths and predictors of steroid responsiveness in ARDS is necessary.

**REFERENCE(S)**

1. Steinberg KP, et al. N Engl J Med. 2006; 354:1671–84.
2. Takaki M, et al. Crit Care.2017;21(1):135.
3. Fielding-Singh V, et al. Crit Care Med. 2018; 46(11):1820-1831.
4. Meduri GU, et al. Intensive Care Med. 2016;42(5):829-840.
5. Keir GJ, et al. Eur Respir J. 2012;40(3):641-8.
6. Torres, A, et al. Intensive Care Med 2018; 44: 478.

### 000592

#### Active humidification system used in mechanical ventilation not associated to infectious respiratory complications

R. Muñoz-Bermúdez[1], L. Picazo,[1], X. Durán[2], F. ÁLvarez-Lerma[1], MP. Gracia[1], JR. Masclans[1]
[1]Critical care department, Hospital del Mar, IMIM-GREPAC, Barcelona, Spain; [2]Statistics department, IMIM, Barcelona, Spain
**Correspondence:** R. Muñoz-Bermúdez
*Intensive Care Medicine Experimental* 2019, **7(Suppl 3):**000592

**INTRODUCTION.** The importance of gases administered during invasive mechanical ventilation (IMV) reaching the patient in adequate conditions of heat and humidity through the respirator's circuits is indisputable. Controversy remains about which is the most appropriate humidification system and its influence on the incidence of mechanical ventilation-related respiratory infection.

**OBJECTIVES.** To evaluate differences in the rate of incidence of pneumonia and tracheobronchitis associated with mechanical ventilation (VAP and AVT respectively) with a hygroscopic heat-moisture exchanger with antibacterial filter versus active humidification with heating of inspiratory and expiratory tubulars.

**METHODS.** Retrospective descriptive study in a polyvalent ICU. Included were all patients undergoing IMV for more than 48 hours during 2014, when passive humidification was used (EdithFlex of the brand AirlifeTM®), and 2016, when active humidifiers (Fisher & Paykel MR850®) were used. In both groups, identical measures were established to prevent VAP (Pneumonia Zero project). European Centre for Disease Prevention and Control (ECDC) diagnostic criteria were used to define VAP. Centers for Disease Control (CDC) diagnostic criteria were used to define TAV. Any NAV developing in ≤7 days from the beginning of the IMV was considered early NAV. Incidence rates of VAP and TAV were estimated for 1000 days of IMV in both groups. Late and early VAP were determined as well. Statistically significant differences between both groups were assessed between by Poisson regression. Clinical Research Ethics Committee authorization was obtained.

**RESULTS.** 287 patients were included (116 ventilated with passive humidification and 171 ventilated with active humidification). The mean age of the sample was 62.3 ± 16.2 years, 61.2% males, average APACHE II of 23.5 ± 9.5 points and ICU mortality of 31.1%. Demographic characteristics were not significantly different between both groups. The incidence density of VAP for 1000 days of IMV was 5.68 for the passive humidification group and 5.80 for the active humidification group (p= NS). The incidence density of TAV was 3.41 and 3.26 cases per 1000

days of IMV with passive and active humidification, respectively (p=NS). The duration of IMV was identified as a risk factor for NAV. The incidence density of early VAP per 1000 days of IMV was 3.03 with passive humidification and 2.53 with active humidification (p=NS).
**CONCLUSION.** In our study, active humidification in patients with IMV for more than 48 hours was not associated with an increase of infectious complications.

### 000601

#### The effect of driving pressures during the initial 24 hours of ventilation on 28-day crude mortality rate

A. Rochester, N. Clayden, M. Alice, T. Samuels
Intensive care unit, East Surrey Hospital, Redhill, United Kingdom
**Correspondence:** A. Rochester
*Intensive Care Medicine Experimental* 2019, **7(Suppl 3):**000601

**INTRODUCTION.** It is well documented that lung-protective ventilation strategies are associated with favourable outcomes in patients with ARDS.[1-3] In 2015, Amato et al. showed that increased driving pressure (ΔP) was associated with increased 60-day mortality in patients with ARDS, especially during the first 24 hours of ventilation.[4]
**OBJECTIVES.** To ascertain if there was any association with a median ΔP greater than or equal to 14 cmH2O during the initial 24 hours of ventilation and 28-day crude mortality rate.
**METHODS.** Data were collected at regular intervals during a one-month period. ΔP (cm H2O) was calculated as the difference between the peak pressure and positive end-expiratory pressure. Data were further divided into a survivor and deceased group according to the 28-day crude mortality rate. Patients ventilated via APRV or NIV were excluded from the study, as were patients with incomplete data. Statistical analysis was carried out using R version 3.5.3 (R Foundation).
**RESULTS.** 32 patients met inclusion criteria. Overall, 22 patients survived and 10 patients were deceased at 28 days. The mean ages (SD) for the survivor and deceased groups were 60.8( 18.3) years and 68.5 (16.1) years respectively (Fig A, p=0.20). During the first 24 hours of intubation, 90% (9 patients) of the deceased group and 68% (15 patients) of the survivor group had a median ΔP ≥ 14cmH20 (Fig B, p=0.30).
**CONCLUSION.** On Day 1 of intubation 71.8% of our patients had a median ΔP ≥ 14cmH2O, yet, when data from the initial 24 hours were split into two groups (ΔP < 14cmH2O or ≥ 14cmH2O), a Fisher's exact test showed no significant association with outcome at 28 days (p=0.20; Fig. B). Amato et al. evidenced that 60-day mortality risk was significantly increased by both age and median ΔP when compared to multiple variables.[4] Our data demonstrated neither variable proved significant on crude mortality at 28 days. Nonetheless, our data set is small and looked at all-cause intubation, not just patients with ARDS, as in the Amato study. In fact, patients ventilated using APRV (often used for those with severe ARDS) were excluded from this study, which may have removed a key patient population from the mortality analysis. However, Schmidt et al. found no association with day 1 ΔP and mortality (ICU and at 6-months) in patients without ARDS, suggesting it is the severity of respiratory compromise which has the greatest impact on mortality, not ΔP, which may, therefore, reflect the degree of morbidity rather than mortality.[5] Further investigation is therefore required in a larger patient cohort throughout the full ventilation period to compare ΔP in patients with and without ARDS if an association between mortality and ΔP is to be proven.

##### REFERENCE(S)

1. The Acute Respiratory Distress Syndrome Network. Ventilation with lower tidal volumes as compared with traditional tidal volumes for acute lung injury and the acute respiratory distress syndrome. N Engl J Med2000;342:1301-1308
2. Villar J et al. A high positive end-expiratory pressure, low tidal volume ventilatory strategy improves outcome in persistent acute respiratory distress syndrome: a randomized, controlled trial. Crit Care Med 2006;34:1311-1318
3. Amato MBP et al. Effect of a protective-ventilation strategy on mortality in the acute respiratory distress syndrome. N Engl J Med 1998;338:347-354
4. Amato MBP et al. Driving Pressure and Survival in the Acute Respiratory Distress Syndrome. N Engl J Med 2015; 372:747-755
5. Schmidt MFS et al. Driving Pressure and Hospital Mortality in Patients Without ARDS. Chest 2018; 153: 46-54

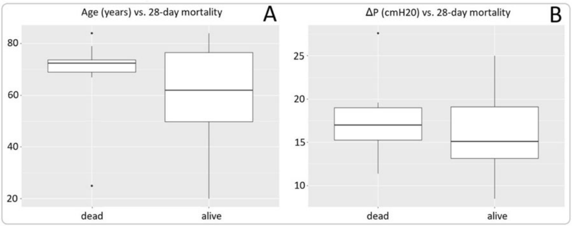

**Fig. 2 (abstract 000601).** See text for description

### 000608

#### Outcome of 153 lung transplant recipients requiring readmission to the intensive care unit: A multicenter prospective study of 5 ICUs

CA. Mazo Torre[1], T. Pont[1], A. Sandiumenge[1], A. Gomez[1], MA. Ballesteros[2], E. López[3], L. Rellán[4], J. Rello[5]
[1]Transplant Coordination, Vall d'Hebron University Hospital, Barcelona, Spain; [2]Intensive care, Marqués de Valdecilla University Hospital, Santander, Spain; [3]Intensive care, 12 de Octubre University Hospital, Madrid, Spain; [4]Intensive care, A Coruña University Hospital, A Coruña, Spain; [5]Vall d'hebron research institute, Vall d'Hebron Barcelona Hospital Campus, Barcelona, Spain
**Correspondence:** C.A. Mazo Torre
*Intensive Care Medicine Experimental* 2019, **7(Suppl 3):**000608

**INTRODUCTION.** Lung transplant (LT) has improved survival (although average is only 5.8 years) [1], whereas the number of LTs has been progressively increasing. Consequently, the interest for intensive care specialists, even those not directly involved in LT, has gone beyond the post-operative period, due to the increase of ICU readmissions (ICUr) of LT subjects [2, 3], also occurring in centres without a LT programme. Acute respiratory failure (ARF) and sepsis have been described as the most frequent conditions determining ICUr in LT recipients [4–9]. However, factors influencing the ICUr of this unique subgroup of immunocompromised patients after the post-operative period have been reported by few, mainly retrospective and single-centre studies. Thus, it is of paramount importance to have an updated snapshot of this emerging condition.
**OBJECTIVES.** We aimed to assess the main causes of ICUr in lung transplant adults and identify potential predictors of ICU-mortality.
**METHODS.** This 5-centre prospective cohort study enrolled all LT adults readmitted to ICU after post-transplantation ICU discharge (2012-2016). Patients were followed until hospital discharge or death. Primary end-point was to identify independent predictors of ICU-mortality (by multivariate logistic regression analysis).
**RESULTS.** We followed 153 patients (with 174 ICUr), readmitted a median of 6 months [6-25] (33, 21.6% within 1 month) post-

LT. Thirty-five (22.9%) had chronic allograft dysfunction (CLAD). ARF (110, 71.9%) was the main condition requiring ICUr. Pneumonia (56, 36.6%) was the main cause (50 admitted due to ARF and 6 for shock), with *P. aeruginosa* (50% MDR) being the predominant pathogen. Graft rejection (6, 5% acute) caused 12 (11%) admissions. The ICU and hospital mortality were 55 (35.9%) and 69 (45.1%) subjects, respectively. CLAD (adjusted-OR: 3 and 3.5) and pneumonia at ICUr (aOR: 3 and 2.4) were identified as independent predictors of ICU and hospital mortality (p<0.05).

**CONCLUSION.** Acute respiratory failure was the main condition requiring ICU readmission in lung trasnplant recipients and it was associated with high mortality. Pneumonia was the main cause and an independent predictor of death.

**REFERENCE(S)**

1. Yusen RD, Edwards LB, Dipchand AI, et al. The registry of the international society for heart and lung transplantation: thirty-third adult lung and heart–lung transplant report 2016; focus theme: primary diagnostic indications for transplant. J Hear Lung Transplant 2016; 35: 1170–1184.
2. Rello J. Lung transplant: an emerging challenge in the ICU. Med Intensiva 2012; 36: 504–505.
3. Fuehner T, Greer M, Welte T, et al. The lung transplant patient in the ICU. Curr Opin Crit Care 2012; 18 (5): 472-478.
4. Pietrantoni C, Minai OA, Yu NC, et al. Respiratory failure and sepsis are the major causes of ICU admissions and mortality in survivors of lung transplants. Chest 2003; 123: 504–9.
5. González-Castro A, Suberviola B, Llorca J, et al. Prognosis factors in lung transplant recipients readmitted to the intensive care unit. Transplant Proc 2007; 39: 2420–2421.
6. Cohen J, Singer P, Raviv Y, et al. Outcome of lung transplant recipients requiring readmission to the intensive care unit. J Hear Lung Transplant 2011; 30: 54–58.
7. Hadjiliadis D, Steele MP, Govert JA, et al. Outcome of lung transplant patients admitted to the medical ICU. Chest 2004; 125: 1040–5.
8. Banga A, Sahoo D, Lane CR, et al. Characteristics and outcomes of patients with lung transplantation requiring admission to the medical ICU. Chest 2014; 146: 590–599.
9. Maurer JR, Tullis E, Grossman RF, et al. Infections complications following isolated lung transplantation. Chest 1992; 101: 1056-59.
10. This work was funded in part by the Instituto Salud Carlos III, Madrid, (ES) and CIBERES, (Fondos FEDER), [Grant number: PI14/1296].

### 000611

#### A Meta-analysis of the Effect of Threshold Inspiratory Muscle Training on Respiratory Muscle Function

W. Yuchen[1], D. Nannan[2], J. Biantong[2], Z. Zhigang[1]
[1]Department of icu, The First Hospital of Lanzhou University, Lanzhou, China; [2]School of nursing, lanzhou university, Lanzhou University, Lanzhou, China
**Correspondence:** W. Yuchen

*Intensive Care Medicine Experimental* 2019, **7(Suppl 3):**000611

**INTRODUCTION.** Respiratory muscle weakness leads to prolonged mechanical ventilation, difficulty in machine withdrawal, tracheal intubation again and tracheotomy caused by respiratory weakness, increased mortality and reduced the quality of life of survivors[1-3]. There are some studies found that withdrawal time accounts for about 40% of patients' mechanical ventilation time [4]. If withdrawal is delayed, significant weakness of respiratory muscles means that 5%-15% patients cannot successfully withdraw[5]. Inspiratory Muscle Training (IMT) has been shown to improve the respiratory muscle strength in patients with chronic obstructive pulmonary disease (COPD) [6], chronic heart failure[7]and asthma [8]. The IMT also could improve patients' mobility and the quality of life [9-10]. However, there is a major controversy whether the threshold inspiratory muscle training(TIMT) could promote weaning mechanical ventilation, and benefical to the respiratory muscle recovery and clinical outcomes.

**OBJECTIVES.** To study whether the Threshold Inspiratory Muscle Training(TIMT) can improve respiratory muscle strength and clinical results for machinery ventilates patients.

**METHODS.** To retrieve the related randomized controlled studies by following databases: the Cochrane Library, PubMed, Embase, Web Of Science, The Chinese Biomedical Literature Database, Wan Fang Database, China National Knowledge Infrastructure (CNKI) and VIP Database, retrieval ends on July 1, 2018. Two researchers strictly evaluated literature quality and extracted information, and then meta-analysis the including literatures which met the inclusion criteria.

**RESULTS.** There are 1 Chinese and 14 English literatures including the study. The total sample size was 657.TIMP can effectively improve the maximal inspiratory pressure(MIP) [*RR*=6.64, 95%*CI*(5.10, 8.17), *P*] and respiratory muscle strength [*RR*=7.64, 95%*CI* (2.99,12.29)], reduce weaning time [RR=-2.24,95%CI (-4.33,-0.15), shorten mechanical ventilation time[*RR*=-1.04, 95%*CI* (-1.66,-0.42), *P*=0.001], P=0.04],the ICU length of stay [*RR*=-2.95, 95%*CI* (-5.38,-0.52), *P*=0.02], and reduce the rate of weaning failure [*RR*=0.57, 95%*CI*(0.36,0.89),*P*], in mechanical ventilation patients compared with conventional treatment. However, TIMP compared with traditional treatment, there was no statistically significant difference in improving the maximum expiratory pressure(MEP) [*RR*=1.22, 95%*CI* (-6.55,9.00), *P*=0.76], reducing the rate of reintubation/tracheotomy [*RR*=0.99, 95%*CI* (0.56,1.73), *P*=0.96] and mortality [*RR*=1.05, 95%*CI* (0.53,2.06) in mechanical ventilation patients.

**CONCLUSION.** The TIMP can significantly improve the mechanical ventilation patients' MIP and respiratory muscle strength, shorten the weaning time, the mechanical ventilation time and the ICU length of stay, reduce the incidence of weaning failure.

**REFERENCE(S)**

[1] Magalhaes P A F, Camillo C A, Langer D, et al. Weaning failure and respiratory muscle function: What has been done and what can be improved?[J]. Respir Med. 2018,134:54-61.DOI.10.1016/j.rmed.2017.11.023
[2] Vorona S, Sabatini U, Al-Maqbali S, et al. Inspiratory Muscle Rehabilitation in Critically Ill Adults A Systematic Review and Meta-Analysis.pdf[J]. Annals ATS Journals. 2018,15(6) : 735-744. DOI.org/10.1513/AnnalsATS
[3] Nepomuceno BRV, Barreto MS, Almeida NC, et al. Safety and efficacy of inspiratory muscle training for preventing adverse outcomes in patients at risk of prolonged hospitalisation[J]. Trials. 2017,18: 56-65. DOI.10.1186/s13063-017-2372-y
[4] Esteban A, Anzueto A, Frutos F, et al. Characteristics and outcomes in adult patients receiving mechanical ventilation: a 28-day international study[J]. JAMA. 2002;287(3):345-355.DOI. 10.1001/jama.287.3.345
[5] Brochard L, Thille AW. What is the proper approach to liberating the weak from mechanical ventilation [J]. Crit Care Med. 2009;37(10 Suppl):S410-415.DOI. 10.1097/CCM.0b013e3181b6e28b
[6] Elbouhy MS, AbdelHalim HA, Hashem AMA. Effect of respiratory muscles training in weaning of mechanically ventilated COPD patients[J]. Egypt J Chest Dis Tuberc. 2014,63(3):679-687.DOI. doi.org/10.1016/j.ejcdt.2014.03.008
[7] Montemezzo D, Fregonezi G A, Pereira D A, et al. Influence of inspiratory muscle weakness on inspiratory muscle training responses in chronic heart failure patients: a systematic review and meta-analysis[J]. Arch Phys Med Rehabil. 2014;95:1398–1407.DOI. 10.1016/j.apmr.2014.02.022.
[8] SHI Hui, LIU Ling, Zufeiya TURDI.Threshold Training of Respiratory Muscles after Stroke: A Meta-analysis [J]. Chin J Rehabil Theory Pract, 2016,03; 22(9):1069-1074.DOI. 10.3969/j.issn.1006-9771.2016.09.019
[9] Doorduin J, van der Hoeven JG, Heunks LM. The differential diagnosis for failure to wean from mechanical ventilation[J]. Curr Opin Anesthesiol. 2016,29(2):150-157.DOI. 10.1097/ACO.0000000000000297
[10] Bissett BM, Leditschke IA, Neeman T, et al. Inspiratory muscle training to enhance recovery from mechanical ventilation: a randomised trial[J]. Thorax. 2016,71(9):812-819.DOI. 10.1136/thoraxjnl-2016-208279

#### 000617

**Determination of device-related dead spaces in controlled ventilation**

M. Klutzny, D. Drees, Al. Georgevici, K. Brückl, T. Weber, M. Bellgardt
Anesthesiology and intensive care medicine, St. Josef-Hospital, Ruhr-University of Bochum, Bochum, Germany
**Correspondence:** M. Klutzny
*Intensive Care Medicine Experimental* 2019, **7(Suppl 3):**000617

**INTRODUCTION.** The clinical practice and the weaning of patients from the ventilator is a highly time dynamic process. The ventilation parameters must be permanently adjusted individually, as the patient can unexpectedly become exhausted at any time. However, a target-oriented weaning therapy can only be realized as soon as all technical dead space (DS) increasing influences are identified. On the ICU, commonly used devices influencing the dead space volume are either an HME (35ml) or anesthetic conserving devices (ACD, AnaConDa, Sedana Medical, Danderyd, Sweden) - ACD (50 ml and 100 ml).

**OBJECTIVES.** The objective was to determine the influence of devices that are widely used in intensive care medicine on ventilation parameters during Controlled Mechanical Ventilation. The elimination of CO2 and the increase in the tidal volume needed to compensate for those devices were our main endpoints. In addition to that, we wanted to quantify the impact of CO2 reflection in anesthetic gas reflection devices.

**METHODS.** After written informed consent, a total of 34 patients undergoing orthopedic surgery under general anesthesia have been included in our trial. Patients were randomly assigned into two groups (16 to 18 each). Anesthesia was induced as a TIVA and maintained as such for the first phase of measurements. In the second phase, anesthesia has been maintained using sevoflurane as the anesthetic agent. Patients were ventilated with the ICU respirator S1, Hamilton Medical (Bonaduz, Switzerland), which is equipped with a flow-through capnometry. After a baseline measurement without any additional dead space (no-DS), the devices have been installed sequentially and we waited for a steady state in etCO2 for at least 5 minutes until proceeding to the next device. Our study has been designed as a case-control study, randomizing the order in which the two different AnaConDa-devices have been measured.

**RESULTS.** As both groups were comparable in age, weight, ASA, sex and ventilation parameters the groups have been analyzed together. While maintaining a steady breathing rate and inspiratory pressure, in TIVA phase the etCO2 (mmHg) changed as following: no-DS-1 30.0±0.0, HME 33.9±2.5, ACD50 40.2±5.3 and ACD100 44.2±5.8 (ANOVA: p< 0.001). Compensating the devices by an increase of inspiratory pressure and maintaining the etCO2 measured in the baseline measurement, the tidal volume (ml) acted as follows: no-DS-1 454.1±94.7, HME 534.1± 100.4, ACD50 603.9±101.8 and ACD100 740.2±113.8 (ANOVA: p< 0.001). After a second baseline measurement with an etCO2 of 30.0± 0.0mmHg, in sevoflurane phase, etCO2 for the ACD50 increased to 35.5±2.4mmHg and 37.4±2.6mmHg for the ACD100 (ANOVA: p< 0.001). For the ACD50 a tidal volume (ml) of 571.3±92.3 was needed and for the ACD100 one of 636.7±119.8 in comparison to a no-DS-2 tidal volume of 446.8±99.9 (ANOVA: p<0.001).

**CONCLUSION.** This study showed, that the usage of devices influencing the dead space volume can drastically change the elimination of CO2. The ventilation parameters have to be altered significantly to level out the increased dead space ventilation. Using an anesthetic gas reflector, the addition of the anesthetic gas sevoflurane reduces CO2 reflection.

### TEM - Clinical studies in trauma and acute poisoning

#### 000604

**Factors affecting hospital survival in comatose out-of-hospital cardiac arrest patients**

J. Mackey, M. O'brien, P. May, L. Eveson, S. Shrestha, M. Peck
ICU, Frimley Park Hospital, Frimley, United Kingdom
**Correspondence:** J. Mackey
*Intensive Care Medicine Experimental* 2019, **7(Suppl 3):**000604

**INTRODUCTION.** Patient selection and optimal timing for coronary angiography (CA) and percutaneous coronary intervention (PCI) following out-of-hospital cardiac arrest (OHCA) have been contentious. PCI is resource-intensive, and whilst large observational studies have concluded that early PCI improves survival in patients with non-ST elevation myocardial infarction, a recent Dutch randomised controlled trial - COACT - demonstrated no survival benefit of immediate over delayed PCI in patients without evidence of STEMI. Our 700-bedded UK DGH has a 24-7 Cardiac Cath Lab service that has routinely investigated OHCA patients with indications pre-ICU admission.

**OBJECTIVES.** To identify factors affecting hospital mortality in OHCA patients admitted to our ICU; in particular, disease severity, CA pre and post ICU admission, and adoption of 36°C targeted temperature management (TTM) target on 01/01/15.

**METHODS.** We analysed prospectively collected data from all comatose OHCA patients admitted to our ICU between 01/01/12 and 31/12/17 using Trust electronic databases. Data were statistically analysed using a key driver analysis to identify factors that correlate most with survival.

**RESULTS.** Of 283 patients identified, summary values [presented as median (range) or percentages] were: age 66 (16-93); APACHE II score 19 (4-44); ICNARC score 28 (9-83); male 70%; presented with VF 61.8%, PEA 24.7%, asystole 8.8%, VT 2.1%, unknown primary arrest rhythm 2.5%; CA pre-ICU 62%; PCI pre-ICU 35%, CA post-ICU 6%; completed 24 hours of TTM 75%; survived to hospital discharge 42%. Of survivors: ICU length-of-stay (LOS) 4.4 (0.5-34) days, hospital LOS 12 (0.5-101). Of non-survivors: ICU LOS 2.5 (0.1-16) days, hospital LOS 3 (0.1-70) days. On univariate analysis, survival was increased by male sex, VF as primary rhythm, completing 24 hours of TTM, CA and PCI before ICU, CA after ICU. On multivariate analysis, drivers for survival were APACHE II score (p=0.000), VF as primary rhythm (p=0.001), having completed 24 hours TTM (p=0.004); undergoing CA after ICU admission (0.01) and ICNARC score (p=0.03).

**CONCLUSION.** Changing TTM target to 36°C did not affect ICU or hospital survival. Neither pre-ICU CA or PCI were found to be significant drivers on multivariate analysis. Strongest independent predictors of survival in our study were APACHE II mortality score and VF rhythm, followed by completing TTM, undergoing CA after ICU admission and ICNARC score, which were all linked by selection criteria. Our results, in keeping with the recent COACT study, demonstrate that performing CA before ICU admission does not improve mortality and we conclude that this should be considered in comatose OHCA patients only on a case-by-case basis.

1. Statistical analysis by D Chilvers, funded by Bard Medical.

#### 000668

**The Value of Arterial Blood Gas Parameters for Prediction of Mortality in Survivors of In and Out-of-hospital Cardiac Arrest**

K. MOHEE[1], M. Protty[2], H. Haboubi[3], S. Pillai[1]
[1]Ed major critical care unit, Morriston Hospital, Morriston, United Kingdom; [2]Systems immunity university research institute, Cardiff University, Cardiff, United Kingdom; [3]Medical school, Swansea University, Swansea, United Kingdom
**Correspondence:** K. MOHEE
*Intensive Care Medicine Experimental* 2019, **7(Suppl 3):**000668

**INTRODUCTION.** Cardiac arrest is one of the leading causes of mortality in Europe, and early prognostication remains challenging. There is a paucity of valid parameters for the prediction of survival after both in hospital (IHCA) and out of hospital cardiac arrest (OHCA). Arterial blood gas (ABG) monitoring evaluates partial pressures of gas and acid-base content.

**OBJECTIVES.** This study aims to investigate if arterial blood gas parameters correlate with mortality of patients after in and out-of-hospital cardiac arrest following admission to intensive care unit (ICU).

**METHODS.** This is a single centre retrospective observational review of the database WardWatcher. Cardiac arrest patients who were admitted between January 2007 to July 2018 to a tertiary ICU were included. The patient's survival 30 days after resuscitation defined the study end-point. For the statistical analysis, the mean, standard deviation, Mann Whitney-U test, Chi-square test, and logistic regression analyses were used (level of significance $p < 0.05$).

**RESULTS.** ABG samples were taken from 1034 patients during the study period. When compared with OHCA cases, patients admitted with IHCA were older 68 vs 62 years, (p<0.0001), had higher GCS score 5.4 vs 4.5, (p<0.0001), higher APACHE II scores 18.9 vs 17.1 (p<0.0001). 30-day mortality was higher in the OHCA cohort but not statistically significant.

In both cohorts (together and independently), lactate ≥ 4.0 mmol/L showed strong and independent correlations with mortality within the first 30 days after admission to ICU [All: OR 2.37 (95% CI 1.77-3.19), IHCA: OR 1.81 (95% CI: 1.19 – 2.76), OHCA: OR 3.26 (95%CI: 2.12-5.07)], whereas a GCS score > 8 showed strong and independent correlations with survival within the first 30 days after admission to ICU [All: OR 0.17 (95% CI:0.11-0.26), IHCA: OR 0.31 (95% CI:0.19-0.52), OHCA: OR 0.06 (95% CI: 0.02-0.13)].

**CONCLUSION.** Our study results indicate that blood gas parameters correlate with mortality of patients after cardiopulmonary resuscitation. In both IHCA and OOHCA, the most relevant parameter is a lactate ≥ 4.0 mmol/L which was observed to be a strong and independent predictor associated with mortality within the first 30 days after resuscitation. In contrast, having a GCS>8 on admission to ICU is associated with good outcome at 30 days. Despite these findings, it is likely that early prognostication relies on multiple factors beyond individual covariates. Nevertheless, these parameters can form a part of a multimodal approach to assessing the patients' prognosis.

### 000670

#### Epidemiology and chronobiology of cardiac arrest in a subpopulation of South Wales admitted to the Intensive Care Unit from 2007-2017- A retrospective study

K. MOHEE[1], H. Haboubi[2], S. Pillai[3]
[1]Ed major critical care unit, Morriston Hospital, Treforys, United Kingdom; [2]Medical school, Swansea University, Swansea, United Kingdom; [3]Ed major critical care unit, Morriston Hospital, Morriston, United Kingdom
**Correspondence:** K. MOHEE
*Intensive Care Medicine Experimental* 2019, **7(Suppl 3):**000670

**INTRODUCTION.** Recent studies have shown temporal variations in the incidence of out of hospital cardiac arrest (OHCA), however, there is a dearth of studies investigating similar variations in in-hospital cardiac arrest (IHCA) especially in the Welsh population.

**OBJECTIVES.** This study aims at investigating the epidemiology, circadian, weekly and seasonal variations of patients with IHCA and OHCA admitted to a tertiary intensive care unit (ICU) in South Wales.

**METHODS.** This is a single centre retrospective observational review of Wardwatcher database. All post cardiac arrest patients admitted to a tertiary referral ICU from January 2007 until December 2017 were included. A variety of data including demographics and comorbidieties were collected.

**RESULTS.** Of the 475 IHCA, 53% were females with mean age of group 68 ± 14.0 years. Conversely, the majority of the 490 OHCA cases were males with 53% with mean age of group 62 ± 16.5 years. From 2007 until 2017, admissions of patients with cardiac arrest increased steadily with an exponential increase within the IHCA group. During that 11-year period, May and June were the months with the lowest number of admissions for both IHCA and OHCA.

The incidence of OHCA admitted to ICU in the studied population was 44.5 patients per year. Admission of patient with OHCA to ICU stayed stable low levels between 22:00 and 4:59 and started to increase at 5:00, with a modal peak: 13:00–14:59. The lowest number of OHCA admitted to ITU occurred from 07:00 to 8:59, the highest from 13:00 to 14:59 (1.2% vs. 8.2%, p < 0.001). The day with the lowest cases of OHCA admitted to ITU was Sunday, the highest Monday (12.7% vs. 16.3%, p = 0.01). Summer was the season of the lowest cases of OHCA admitted to ITU, while winter — the highest (22.4% vs. 29%, p = 0.001).

The incidence of IHCA admitted to ICU in the studied population was 43.2 patients per year. IHCA admitted to ICU increased with age reaching a peak of 95 years. Patients with IHCA admitted to ICU stayed high levels between 07:00 and 1:59 and started to decrease at 02:00, with bi-modal peaks: 00:00–01:59 and 18:00-18:59. The day with the lowest admission of IHCA to ICU was Sunday, the highest Thursday (8.8%% vs. 17.1%, p <0.001). Spring was the season of the lowest number of patients with IHCA admitted to ICU, while winter — the highest (21.7% vs. 27.3%, p = 0.001)

**CONCLUSION.** This is the first study of a large Welsh subpopulation over an 11-year period, which confirmed that times of admission, weekly variability and seasonal differences exists in both IHCA and OHCA patient who are admitted to ICU.

### 000719

#### The effect of veno-arterial extracorporeal membrane oxygenation (VA-ECMO) for patients with out-of-hospital cardiac arrest: A propensity score matched analysis

T. Tsuchida[1], T. Wada[1], S. Gando[2]
[1]Department of acute and critical care medicine, Hokkaido University Graduate School of Medicine, Sapporo, Hokkaido, Japan; [2]Acute and critical care center, Sapporo Higashi Tokushukai Hospital, Sapporo, Hokkaido, Japan
**Correspondence:** T. Tsuchida
*Intensive Care Medicine Experimental* 2019, **7(Suppl 3):**000719

**INTRODUCTION.** Some studies both support and refute the use of veno-arterial extracorporeal membrane oxygenation (VA-ECMO) for out-of-hospital cardiac arrest (OHCA) patients, and there is no sufficient evidence on the characteristics of patients who should receive VA-ECMO.

**OBJECTIVES.** We evaluated the relationship between the use of VA-ECMO, the outcome and some physiological parameters in OHCA patients.

**METHODS.** We extracted 239 OHCA patients from computer-based medical records from February 2000 to December 2017. The 239 patients were all hospitalized after a return of spontaneous circulation (ROSC) from cardiac arrest of cardiac origin at 16 years of age or older. We divided the patients into two groups: patients who underwent VA-ECMO (n=84) and those did not (n=155) and established propensity score (PS) matched groups with patients matched according to pre-hospital factors (gender, age, time from the detection of cardiac arrest to ROSC, and the initial rhythm at cardiac arrest). We compared the following factors in two groups of 24 PS-matched patients: platelet count, coagulation and fibrinolysis marker and lactate levels, International Society on Thrombosis and Haemostasis (ISTH) DIC score, sequential organ failure assessment (SOFA) score, and acute physiology and chronic health evaluation (APACHE) II score. The outcome was measured by all-cause hospital mortality after 28 days of hospitalization.

Furthermore, a sub-group analysis was performed after dividing patients who received VA-ECMO into two groups according to the outcome.

**RESULTS.** The VA-ECMO group had more severe coagulo-fibrinolytic changes and organ dysfunction than the non-VA-ECMO group. In addition, the VA-ECMO group showed poorer outcomes. A subgroup analysis showed that the patients in the VA-ECMO group with a poor outcome had significantly higher ISTH DIC scores and lower PT% on day 2, while these parameters were not significantly different on day 1.

**CONCLUSION.** This study showed no beneficial effect of VA-ECMO on the OHCA patients. Furthermore, among the patients who received VA-ECMO, poor outcomes were observed in patients whose ISTH DIC scores and PT% showed deterioration.

### 000722

#### Early increased citrullinated histone H3 levels are associated with poor neurologic recovery in cardiac arrest survivors

M. Schwameis[1], LM. Mauracher[2], N. Buchtele[3], C. Schörgenhofer[3], H. Herkner[1], B. Jilma[3]
[1]Department of emergency medicine, Medical University of Vienna, Vienna, Austria; [2]Department of medicine i, Medical University of Vienna, Vienna, Austria; [3]Department of clinical pharmacology, Medical University of Vienna, Vienna, Austria
**Correspondence:** N. Buchtele
*Intensive Care Medicine Experimental* 2019, **7(Suppl 3):**000722

**INTRODUCTION.** Mechanisms by which neutrophils contribute to post-resuscitative brain damage are unknown. In recent years, neutrophil extracellular traps (NETs) have emerged as a central player in inflammation, thrombogenesis and cardiovascular disease. NETs are chromatin fibers consisting of histones, cell free DNA (cfDNA) and granular proteins and are released within minutes (1) to hours (2) following activation by various stimuli including ischemia and reperfusion (3). While NETs have primarily been recognized as mediators of antimicrobial host defense, they may exert detrimental inflammatory and procoagulant effects causing endothelial damage, platelet activation, microvessel occlusions and ultimately tissue malperfusion (4). In particular neutrophil histones and DNA are considered cytotoxic and procoagulant components of NETs (5) and have been implicated in organ damage in various medical conditions (6). Despite this, the role of NETs in cardiac arrest has not yet been investigated. Pro-inflammatory and pro-thrombotic properties, however, render them possible mediators of neutrophil-borne brain injury after successful resuscitation.

**OBJECTIVES.** We hypothesized that the formation of NETs in the early phase after successful resuscitation may be associated with neurologic impairment in cardiac arrest survivors.

**METHODS.** This prospective cohort study included adult patients with out-of-hospital cardiac arrest of cardiac etiology who received targeted temperature management. Plasma levels of specific (citrullinated histone H3, H3Cit) and putative (cell free DNA and nucleosomes) biomarkers of NET formation were assessed at 0 and 12 hours after admission and categorized into quintiles prior to analysis. The primary outcome variable of interest was the effect of H3Cit levels at 12 hours on 30-day neurologic function, assessed by logistic regression. Results are given as odds ratio (95% confidence interval).

**RESULTS.** Between January 2014 and January 2017 62 patients (79% male, median age: 57 years) were enrolled at the Emergency Department at the Medical University of Vienna. In total, 52% of patients (n=32) had a poor 30-day neurologic function. The odds of neurologic impairment linearly increased with 12-hour levels of H3Cit (1.6, 1.1-2.3; p=0.029) and were highest in patients in the fifth quintile of the histone level distribution (9, 1.3-63; p=0.027). The effect remained unchanged after bivariate adjustment for covariables.

**CONCLUSION.** Increased plasma levels of H3Cit 12 hours after successful resuscitation are associated with poor 30-day neurologic recovery in adult out-of-hospital cardiac arrest survivors with cardiac etiology.

##### REFERENCE(S)

1. Yipp BG, Petri B, Salina D, et al. Infection-induced NETosis is a dynamic process involving neutrophil multitasking in vivo. Nature medicine 2012;18:1386-93.
2. Fuchs TA, Abed U, Goosmann C, et al. Novel cell death program leads to neutrophil extracellular traps. The Journal of cell biology 2007;176:231-41.
3. Ge L, Zhou X, Ji WJ, et al. Neutrophil extracellular traps in ischemia-reperfusion injury-induced myocardial no-reflow: therapeutic potential of DNase-based reperfusion strategy. American journal of physiology Heart and circulatory physiology 2015;308:H500-9.
4. Gould TJ, Lysov Z, Liaw PC. Extracellular DNA and histones: double-edged swords in immunothrombosis. Journal of thrombosis and haemostasis : JTH 2015;13 Suppl 1:S82-91.
5. Noubouossie DF, Whelihan MF, Yu YB, et al. In vitro activation of coagulation by human neutrophil DNA and histone proteins but not neutrophil extracellular traps. Blood 2017;129:1021-9.
6. Jorch SK, Kubes P. An emerging role for neutrophil extracellular traps in noninfectious disease. Nature medicine 2017;23:279-87.
7. This study was kindly supported by the Austrian Science Fund FWF grant SFB 54/APF05404FW (Special Research Program-Cellular Mediators Linking Inflammation and Thrombosis, Medical University of Vienna).

### 000738

#### Comparing of CPR related trauma: mechanical chest devices vs. manual CPR

J. Karasek[1], B. Blankova[2], T. Pitasova[3], A. Doubkova[3], J. Seiner[1], M. Strycek[1], R. Polasek[1], T. Adamek[2], J. Hladik[4], P. Ostadal[5]
[1]Cardiology, Hospital Liberec, Liberec, Czech Republic; [2]Forensic medicine, Hospital Liberec, Liberec, Czech Republic; [3]3. medical faculty, Charles University, Prague, Czech Republic; [4]Forensic medicine, Faculty Hospital Kralovske Vinohrady, Prague, Czech Republic; [5]Cardiology, Na Homolce Hospital, Prague, Czech Republic
**Correspondence:** J. Karasek
*Intensive Care Medicine Experimental* 2019, **7(Suppl 3):**000738

**INTRODUCTION.** Resuscitation (CPR) with mechanical chest devices are not recomanded for routine CPR according to randomised trials. One of possible explanation could be CPR related trauma caused with mechanical chest devices. Current data are based on subanalysis from randomised trial, but autopsies are limited by law and autopsy results are not objectivised.

**OBJECTIVES.** To compare injuries after CPR in autopsy results by manually resuscitated and mechanical (LUCAS 2, AutoPulse, CORpulse) resuscitated patients and establish possible proportion of CPR related injuries on death without respect to cause of cardiac arrest.

**METHODS.** Retrospective multicentric study based on autopsy reports by patients died after CPR, patients with traumatic cause of cardiac arrest were excluded. Patients were devided in two groups: mechanical and manually CPR. For objective evaluation of injury seriousness we used Abbreviated injury scale scoring for the most serious injury and New Injury Scale Score for summary of all injuries.

**RESULTS.** We have enroled 704 patients, after trauma exclusion we have analyzed 630 autopsies. Manually CPR were provided by 559 patients and mechanical by 64 (11,4%) patients. Both groups are no diferent in age, gender, bystander CPR and cardiac etiology of Arrest. Mechanical CRP was significantly longer (p=0,0005). Both groups have no diferences in incidency of injuries of thoracic vessels, lungs, heart, pericard, pleura, stomach, liver and spleen. We have observed injuries by 80% of manual and 87,5% of mechanical CPR (p=0,18). The most frequent was thorax sceleton injury 85,5% vs. 87,5%. Median of the most seriuos injury was 3 ( serious by Abbreviated injury scale scoring) without statistical difference, median of summary of injuries (New Injury Severity Score) was 13 in both groups ( low probability of fatal injury). If we analysed CPR by LUCAS 2 compared to manual, results are similar, only pericard injuries are higher with LUCAS 2.

**CONCLUSION.** Incidency a seriousness of CPR related injuries according to autopsy reports are no diferent in comapring of manually and mechanical CPR. Mechanical CPR is significant longer a LUCAS 2 leads to significant more pericard injuries without influence to total seriousness of injury

### 000758

#### Post Resuscitation Care: Are Intensive Care Units Adhering To Resuscitation Council (UK) Guidelines?

S. Chaudhry, K. Ooi, M. Ranganathan
Anaesthetics, Warwick Hospital, Warwick, United Kingdom
**Correspondence:** K. Ooi
*Intensive Care Medicine Experimental* 2019, **7(Suppl 3):**000758

**INTRODUCTION.** The pathophysiology of the 'post cardiac arrest syndrome' is complex and often requires multiorgan support, which is best managed on the intensive care unit. Good quality post-resuscitation care can greatly influence overall outcome, particularly neurological recovery. The Resuscitation Council (UK) published guidelines for post-resuscitation care in 2015, in line with those set by the European Resuscitation Council. There are key interventions that should be followed to optimise outcomes and to facilitate accurate prognostication of patients after cardiac arrest.

**OBJECTIVES.** To determine whether intensive care units in England and Wales are following the Resuscitation Council UK Guidelines for post-resuscitation care.

**METHODS.** An online survey (generated using SurveyMonkey) was disseminated via e-mail to all Critical Care Network Leads in England and Wales. A reminder was sent two weeks later. The survey was also advertised in the Faculty of Intensive Care Medicine website and Twitter account.

**RESULTS.** We received 49 responses from across England and Wales. 95% of respondents were aware of the post-resuscitation care guidelines, but only 56% had an established protocol on their ICU. Most physiological targets used correlated with guidelines; however, 49% of respondents used a different target temperature to that recommended in the guideline. With regards to prognostication, the majority (72%) waited 72 hours post-ROSC. Many ICUs have access to EEG (89%) and CT/MRI (78%); however, only 21% of institutions can measure neuronal specific enolase (NSE) levels.

**CONCLUSION.** Despite clear guidance from the Resuscitation Council, there is heterogeneity in the management of the post-cardiac arrest patient. In particular, access to prognostication facilities varies greatly; EEG is not universally available and NSE level measurement is not possible in most centres. The guideline may therefore not be fit for purpose at all institutions and may require revision. At present, we recommend that each institution establishes their own post-resuscitation protocol, tailored to the facilities available locally. This will ultimately improve the quality of care provided to survivors of cardiac arrest.

**REFERENCE(S)**

1. Post resuscitation guidelines, Resuscitation Council 2015: https://www.resus.org.uk/resuscitation-guidelines/post-resuscitation-care/ (Last accessed 13.4.19)
2. None declared

### 000783

#### Targeted Temperature Management Guided by the Severity of Hyperlactatemia for Out-of-Hospital Cardiac Arrest Patients: A Post Hoc Analysis of a nationwide, multicenter prospective registry

T. Okazaki[1], T. Hifumi[2], K. Kenya[3], K. Yasuhiro[3]
[1]Emergency Medical Center, Kagawa University Hospital, Miki, Japan; [2]Department of emergency and critical care medicine, St. Luke's International Hospital, Chuo City, Japan; [3]Emergency medical center, Kagawa University Hospital, Miki, Japan
**Correspondence:** T. Okazaki
*Intensive Care Medicine Experimental* 2019, **7(Suppl 3):**000783

**INTRODUCTION.** The International Liaison Committee on Resuscitation guidelines recommend target temperature management between 32° C and 36° C for patients after out-of-hospital cardiac arrest, but did not indicate patient-specific temperatures. The association of serum lactate concentration and neurological outcome in out-of-hospital cardiac arrest patient has been reported.

**OBJECTIVES.** The study aim was to investigate the benefit of mild therapeutic hypothermia (32ºC–34ºC) in patients with various degrees of hyperlactatemia compared to normothermia (35ºC or 36ºC) .

**METHODS.** This study was a post hoc analysis of the Japanese Association for Acute Medicine out-of-hospital cardiac arrest registry between June 2014 and December 2015. Patients with complete targeted temperature management and lactate data were eligible. Patients were stratified to mild (< 7 mmol/l), moderate (< 12 mmol/l), or severe (≥ 12 mmol/l) hyperlactatemia group based on lactate concentration after return of spontaneous circulation. They were subdivided into mild therapeutic hypothermia or normothermia groups. The primary endpoint was an adjusted predicted probability of 30-day favorable neurological outcome, defined as a cerebral performance category score of 1 or 2.

**RESULTS.** Of 435 patients, 139 had mild, 182 had moderate, and 114 had severe hyperlactatemia. One hundred-eight (78%) with mild, 128 with moderate (70%), and 83 with severe hyperlactatemia (73%) received mild therapeutic hypothermia. The adjusted predicted probability of a 30-day favorable neurological outcome following severe hyperlactatemia was significantly greater with mild therapeutic hypothermia (27.8%, 95% confidence interval: 22.6%–33.1%) than normothermia (15.3%, 95% CI: 6.7%–23.9%; $p$ = 0.015). The differences in outcomes in those with mild and moderate hyperlactatemia were not significant.

**CONCLUSION.** Mild therapeutic hypothermia independently predicted a greater probability of a favorable 30-day neurological outcome in patients with severe hyperlactatemia compared with normothermia.

**REFERENCE(S)**

1. Prause G, Ratzenhofer-Comenda B, Smolle-Juttner F, Heydar-Fadai J, Wildner G, Spernbauer P et al: Comparison of lactate or BE during out-of-hospital cardiac arrest to determine metabolic acidosis. Resuscitation. 2001; 51(3):297-300.
2. Callaway CW, Donnino MW, Fink EL, Geocadin RG, Golan E, Kern KB et al: Part 8: Post-Cardiac Arrest Care: 2015 American Heart Association Guidelines Update for Cardiopulmonary Resuscitation and Emergency Cardiovascular Care. Circulation. 2015; 132(18 Suppl 2):S465-82.

3. The Japanese Association for Acute Medicine out-of-hospital cardiac arrest registry was supported by research funding from the Japanese Association for Acute Medicine and scientific research grants from the Ministry of Education, Culture, Sports, Science and Technology of Japan (16K09034 and 15H05006) and the Ministry of Health, Labor, and Welfare of Japan (grant no. 25112601).

### 000833

#### Correlation between optic nerve sheath diameter, measured by magnetic resonance imaging, and intracranial pressure in cardiac arrest survivors who underwent targeted temperature management

C. Kang[1], JS. Park[2]
[1]Emergency medicine, Chungnam National University Hospital, Daejeon, Republic of Korea; [2]Department of emergency medicine, College of medicine, Chungnam National University, Daejeon, Republic of Korea
**Correspondence:** C. Kang
*Intensive Care Medicine Experimental* 2019, **7(Suppl 3):**000833

**INTRODUCTION.** Optic nerve sheath diameter (ONSD) is considered as one of the prognostic factors in cardiac arrest (CA) survivors because of a parameter reflecting intracranial pressure (ICP) indirectly. We aimed to investigate the correlation between ONSD measured by magnetic resonance imaging (MRI) and ICP in CA survivors who underwent target temperature management (TTM).

**METHODS.** In this retrospective cohort study conducted in 2018, ONSD and cerebrospinal fluid (CSF) pressure were measured before (Day 0 group) and after (Day 3 group) TTM. ICP and ONSD were respectively measured with CSF pressure and an axis perpendicular to the optic nerve 3 mm behind the eyeball in MRI. Increased ICP was defined as an opening pressure over 20 mmHg. The primary outcome was the correlation between ONSD and ICP.

**RESULTS.** A total of 37 patients who underwent ONSD measurement, with simultaneous lumbar drainage catheter placement, were enrolled in this study. The median and interquartile range [IQR] of ICP was significantly higher in the Day 3 group than in the Day 0 group (14.9 mmHg [12.4-19.1] versus 10.5 mmHg [8.4-11.9], $P$ < 0.001). However, ONSD showed no difference between both groups (5.21 mm [4.80-5.58] versus 5.20 mm [4.79-5.70], $P$ = 0.939). Ten cases (26.3%) were found to have an increased ICP. Day 3 showed higher incidence of increased ICP than in the Day 0 group (8% versus 19%, $p$=0.178). The median and interquartile range of ONSD in the group with increased ICP (6.17 mm [5.11-6.45]) was significantly higher than those without increased ICP (5.19 mm [4.80-5.59]) ($P$ = 0.015). The group with increased ICP showed a statistically significant strong linear correlation with ONSD (r = 0.89, P < 0.001) compared to the other (r = 0.14, $P$ = 0.347). ONSD > 5.99 mm indicated a sensitivity of 60.0% and a specificity of 98.0% for identifying increased ICP.

**CONCLUSION.** ONSD strongly correlated with ICP, in the case of increased ICP. Therefore, ONSD measurement at an early stage, without increased ICP from return of spontaneous circulation, may not be useful for predicting neurological outcomes in CA survivors, due to poor correlation with ICP. Prospective studies should be conducted to confirm these results.

**REFERENCE(S)**

1. Naito H, Isotani E, Callaway CW, Hagioka S, Morimoto N. Intracranial Pressure Increases During Rewarming Period After Mild Therapeutic Hypothermia in Postcardiac Arrest Patients. Therapeutic hypothermia and temperature management. 2016;6(4):189-193.
2. Mohamed Kilji AS, Kesavamurthy Bhanu, Balasubramanian Samivel, Lakshmi Ranganathan. Comparison of the Measurement of Optic Nerve Sheath Diameter(ONSD) with B Scan and MRI as a Surrogate for Patients with Idiopathic Intracranial Hypertension. american academy of neurology. 2016;86:6.
3. Nolan JP, Neumar RW, Adrie C, et al. Post-cardiac arrest syndrome: epidemiology, pathophysiology, treatment, and prognostication. A Scientific Statement from the International Liaison Committee on Resuscitation; the American Heart Association Emergency Cardiovascular Care Committee; the Council on Cardiovascular Surgery and Anesthesia; the Council on Cardiopulmonary, Perioperative, and Critical Care; the Council on Clinical Cardiology; the Council on Stroke. Resuscitation. 2008;79(3):350-379.
4. Grasner JT, Lefering R, Koster RW, et al. Corrigendum to "EuReCa ONE-27 Nations, ONE Europe, ONE Registry A prospective one month analysis of out-of-hospital cardiac arrest outcomes in 27 countries in Europe" [Resuscitation 105 (2016) 188-195]. Resuscitation. 2016;109:145-146.
5. Marcella Balestreri MC, Peter Hutchinson, Luzius A. Steiner, Magda Hiler, Piotr Smielewski and John D. Pickard. Impact of Intracranial Pressure and Cerebral Perfusion Pressure on Severe Disability and Mortality After Head Injury. Neurocrit Care. 2006;04:6.
6. Sombat Muengtaweepongsa WS. Targeted temperature management in neurological intensive care unit. world journal of methodology. 2017;7:13.
7. Bernard SA, Gray TW, Buist MD, et al. Treatment of comatose survivors of out-of-hospital cardiac arrest with induced hypothermia. The New England journal of medicine. 2002;346(8):557-563.
8. Cardim D, Griesdale DE, Ainslie PN, et al. A comparison of non-invasive versus invasive measures of intracranial pressure in hypoxic ischaemic brain injury after cardiac arrest. Resuscitation. 2019;137:221-228.
9. Geeraerts T, Newcombe VF, Coles JP, et al. Use of T2-weighted magnetic resonance imaging of the optic nerve sheath to detect raised intracranial pressure. Crit Care. 2008;12(5):R114.
10. Lee DH, Lee SH, Oh JH, et al. Optic nerve sheath diameter measured using early unenhanced brain computed tomography shows no correlation with neurological outcomes in patients undergoing targeted temperature management after cardiac arrest. Resuscitation. 2018;128:144-150.
11. intracranial pressure
12. optic nerve sheath diameter
13. target temperature management
14. cardiac arrest

### 000845

#### MEWS vs CART Scores for Prediction of Cardiopulmonary Arrest At the Philippine Heart Center

AD. Tan, M. Dolor-Torres, C. Permejo
Department of adult cardiology, Philippine Heart Center, Quezon City, Philippines
**Correspondence:** A.D. Tan
*Intensive Care Medicine Experimental* 2019, **7(Suppl 3):**000845

**INTRODUCTION.** Several clinical scores have been developed to facilitate a well-timed transfer to the intensive care unit (ICU) that could potentially prevent a cardiac arrest (CA). The Modified Early Warning Score (MEWS) is the most studied of these scores and is utilized in many countries to activate rapid response teams. The newer Cardiac Arrest Risk Triage (CART) score was shown to have greater accuracy than the MEWS. However, validation studies are lacking. Several clinical scores have been developed to facilitate a well-timed transfer to the intensive care unit (ICU) that could potentially prevent a cardiac arrest (CA). The Modified Early Warning Score (MEWS) is the most studied of these scores and is utilized in many countries to activate rapid response teams. The newer Cardiac Arrest Risk Triage (CART) score was shown to have greater accuracy than the MEWS. However, validation studies are lacking.

**OBJECTIVES.** The aim of this study was to validate the CART score and MEWS for predicting cardiac arrest and ICU transfers among patients admitted at the Philippine Heart Center. To our knowledge, no study has validated similar CA predictor scores in our country.

**METHODS.** This is a case-control study including 82 adult patients (36 cases, 46 controls) admitted at the Philippine Heart Center. Cases included patients who had CA at the wards (18 cases) and those who were transferred to the ICU for any reason (18 cases). Personal profiles and demographic data were collected. Differences in factors were determined between cases and controls. Vital signs and "alert-verbal-pain-unresponsive" (AVPU) scales from 48 hours prior and up to the time of recruitment (CA or ICU transfer for cases) were noted. The MEWS and CART scores for each time point were computed and compared using measures of validity.

**RESULTS.** There was no significant difference between the case and control groups in terms of age, sex, height, weight, diagnosis, disease classification, and prior ICU admissions. A CART score of 12 was found to have a higher accuracy than a MEWS of 4 across all time points prior to CA or ICU transfer. The highest accuracy of the CART score (cut off $\geq$ 12) was 74.39%, noted 8 hours prior to an event. This had a corresponding specificity of 80.43% and sensitivity of 66.67%. At this hour, the MEWS (cut off $\geq$ 4) had a lower accuracy of 68.29% with a higher specificity of 97.83% but a lower sensitivity of 30.56%. However, area under the curve analysis revealed that the differences in scores between the CART and MEWS were not statistically significant. Among CA patients detected by both tools, the CART was able to detect CA earlier at a median of 30 hours prior versus 24 hours prior for the MEWS.

**CONCLUSION.** The accuracy of the CART score is comparable to the MEWS which is currently the internationally recognized tool for detecting CA at the wards. In our opinion, the CART is simpler than the MEWS as: 1) it has fewer variables, and 2) it does not contain a factor requiring a higher level of proficiency (such as the AVPU scale of the MEWS). Thus, we recommend the use of the CART score.

### 000854

#### Bedside microdialysis for detection of early brain injury after out-of-hospital cardiac arrest: A pilot proof-of-concept study

S. Mölström[1], TH. Nielsen[2], CH. Nordström[2], S. Möller[3], S. Venö[1], T. Tencer[1], H. Schmidt[1], P. Toft[1]
[1]Department of anesthesiology and intensive care, Odense University Hospital, J. B. Winsløws Vej, Odense Municipality, Denmark, Odense, Denmark; [2]Neurosurgery, Odense University Hospital, J. B. Winsløws Vej, Odense Municipality, Denmark, Odense, Denmark; [3]Open – odense patient data explorative network, odense university hospital and department of, Odense University Hospital, J. B. Winsløws Vej, Odense Municipality, Denmark, Odense, Denmark
**Correspondence:** S. Mölström
*Intensive Care Medicine Experimental* 2019, **7(Suppl 3):**000854

**INTRODUCTION.** Brain injury remains the leading cause of death in comatose patients resuscitated from out of hospital cardiac arrest (OHCA). Markers measuring global cerebral ischemia, and reflecting the metabolic variations after resuscitation are needed for a more individualized care. The lactate-pyruvate (LP) ratio is an important indicator of compromised cerebral oxidative metabolism. The study aims to investigate whether the LP ratio obtained by microdialysis (MD) of the cerebral venous outflow reflects brain injury after OHCA.

**METHODS.** A feasibility study designed to determine the yield of bedside monitoring of cerebral energy metabolism after OHCA utilizing intravenous MD. 15 unconscious patients were admitted to the intensive care unit following OHCA with presumed cardiac cause. MD catheters were inserted into the jugular bulb and radial artery (reference). The primary end-point was a difference in time-weighted mean MD parameters and LP ratio measured in venous and arterial blood.

**RESULTS.** The median time from OHCA to MD analysis was 369 min (IQR 255-444) and patients underwent a median of 76 h (IQR 65-82) of monitoring. The LP ratio of cerebral venous blood increased (LP ratio > 25) after OHCA indicating compromised cerebral oxidative metabolism during the first 20 hours. The difference between time-weighted mean of lactate, pyruvate and glycerol (in intervals of 12 hours) of the jugular venous and the arterial blood was significant during post-resuscitation care (p < 0.02) when using mixed effects models. In patients with unfavorable outcome (87 %), cerebral venous lactate remained high with mean and peak venous lactate level > 2.2 and 5.5, respectively.

**CONCLUSION.** Isolated neurochemical changes indicating brain injury were found after OHCA and consecutive resuscitation. Jugular bulb MD may provide a reliable global estimate of cerebral metabolic state and can be implemented as a new diagnostic tool for ICU patients after OHCA, with implications for early prognosis and treatment.

### 000927

#### The association of coagulofibrinolyticmarkers with brain death after out-of-hospital cardiac arrest

S. Tahara[1], K. Maekawa[1], M. Hayakawa[1], M. Takahashi[1], K. Moriki[1], M. Hayamizu[1], Y. TAKAHASHI[1], T. Tsuchida[1], Y. Sadamoto[1], Y. Homma[1], T. Oyasu[1], A. Mizugaki[1], T. Saito[1], T. Yoshida[1], K. Katabami[1], T. Wada[1]
[1]Department of emergency medicine, Hokkaido University Hospital, Sapporo, Japan
**Correspondence:** S. Tahara
*Intensive Care Medicine Experimental* 2019, **7(Suppl 3):**000927

**INTRODUCTION.** Despite recent improvements, some patients after out-of-hospital cardiac arrest (OHCA) suffer total loss of brain function, i.e. brain death. Coagulofibrinolyticmarkers and disseminated intravascular coagulation (DIC) score were known to be associated with neurological outcome in patients after OHCA, but the associations between these variables and brain death were not examined.

**OBJECTIVES.** To evaluate the associations of coagulofibrinolyticmarkers and DIC score with brain death after OHCA.

**METHODS.** We performed a retrospective analysis using data from the regional UtsteinRegistry and medical records between 2006 and 2012. We enrolled patients who experienced OHCA with successful return of spontaneous circulation and were admitted to Hokkaido University Hospital. Outcome measure was brain death during hospitalization. Logistic regression model was used to evaluate the associations of coagulofibrinolyticmarkers including platelet count, prothrombin time, plasma levels of fibrinogen, fibrin/fibrinogen degradation products, and calculated DIC score on admission with brain death. Classification and Regression Tree (CART) analysis was used to identify specific thresholds of significant variables.

**RESULTS.** The overall rate of DIC (defined by DIC score >= 5) and brain death were 13.0% (n = 41/315) and 9.5% (n = 30/315), respectively. Compared with non-brain death group, brain death group gained significant lower level of fibrinogen on admission (1.97 vs. 2.45 g/L, p=0.005), but there were no differences about other variables. CART analysis identified fibrinogen level of 2.26 g/L as an optimal threshold. In multivariable logistic regression analysis, fibrinogen level =< 2.26 g/L was associated with increased risk of brain death (adjusted odds ratio 2.87, 95%CI 1.16-7.08, p=0.02).

**CONCLUSION.** Decreased fibrinogen level at admission was an independent prognostic factor for brain death after OHCA.

### 000938

#### Automated pupillometry to predict outcome in cardiac arrest patients undergoing ECMO

M. Menozzi[1], M. Oddo[2], C. Sandroni[3], G. Citerio[4], JF. Payen[5], J. Creteur[1], FS. Taccone[1]
[1]Department of Intensive Care, Erasme Hospital, Université Libre de Bruxelles, Brussels, Belgium; [2]Department of intensive care medicine, Centre Hospitalier Universitaire Vaudois (CHUV), University of Lausanne, Lausanne, Switzerland; [3]Department of anesthesiology and intensive care, Catholic University School of Medicine, Rome, Italy; [4]School of medicine and surgery, University of Milano-Bicocca, Milan, Italy; [5]Department of anesthesia and critical care, Grenoble Alpes University Hospital, Grenoble, France
**Correspondence:** M. Menozzi
*Intensive Care Medicine Experimental* 2019, **7(Suppl 3):**000938

**INTRODUCTION.** Hypoxic-ischemic brain injury (HIBI) is the main cause of death or disability in patients resuscitated from cardiac arrest (CA). Extracorporeal membrane oxygenation (ECMO) is used to treat refractory CA or post-CA shock. However, the potential benefit of this resource-intensive treatment should be balanced against the risk of futility. Automated pupillometry (AP) recently shown to accurately predict HIBI outcome within 24h from CA and may therefore be useful in this setting.

**OBJECTIVES.** To assess the accuracy of early AP for outcome prediction in CA patients undergoing ECMO.

**METHODS.** *Post hoc* analysis of an international multicenter prognostication study. The primary study endpoint was the accuracy of a neurological pupil index (NPI) ≤ 2 to predict 3-month poor neurological outcome defined as severe disability, unresponsive wakefulness or death (Cerebral Performance Category [CPC] 3-5).

**RESULTS.** On a total of 456 included patients, 66 (14%) were treated with ECMO during resuscitation or immediately after the return of spontaneous circulation. Unfavorable outcome was observed in 43 (65%) patients. On admission, 15 (23%) patients had NPI of ≤ 2; 13/15 had CPC 3-5. The two patients with favorable outcome had a continuous EEG background and recovered a normal NPI (> 3.5) within 24 hours. Sensitivity, specificity, and positive predictive value of NPI ≤ 2 on admission for unfavorable outcome was 91%, 30%, and 87%, respectively. On day 1 and 3, NPI ≤ 2 was observed in 11/66 (17%) and 4/42 (10%), respectively; all of them had unfavorable outcome.

**CONCLUSION.** NPI measured with AP in CA patients undergoing ECMO immediately after hospital admission was a sensitive but not specific predictor of poor neurological outcome. Altered pupillary response should not be used as a tool for deciding on ECMO treatment in this phase.

### 001092

#### Early lactate and glucose kinetics after return to spontaneous circulation after out-of-hospital cardiac arrest

P. Freire Jorge[1], R. Boer,[1], KC. Harms,[2], BWJ. Bens[2], M. Nijsten[1]
[1]Department of critical care, University Medical Center Groningen, Groningen, Netherlands; [2]Department of emergency medicine, University Medical Center Groningen, Groningen, Netherlands
**Correspondence:** P. Freire Jorge
*Intensive Care Medicine Experimental* 2019, **7(Suppl 3):**001092

**INTRODUCTION.** Hyperlactatemia(1) and hyperglycemia(2) are frequently observed as part of the stress reaction during critical illness. Shorter times to normalization or higher rates of decrease of lactate and glucose have been associated with better outcomes.(3,4) After return to spontaneous circulation (ROSC) after out-of-hospital cardiac arrest (OHCA), blood gas analyses usually demonstrate a marked hyperlactatemia and hyperglycemia. We hypothesize that after ROSC recovery from hyperlactatemia and hyperglycemia can be faster than described for several other critical conditions.

**OBJECTIVES.** To determine the early kinetics of lactate and glucose in the first 3 hours after ROSC after OHCA in patients presenting with marked hyperlactatemia.

**METHODS.** We retrospectively analyzed patients that presented between 2006 and 2016 after OHCA in whom initial ROSC was achieved at departure of the ED. Arterial lactate and blood glucose levels were determined by point-of-care blood gas analyzers. We only included patients with an initial lactate immediately after ROSC of ≥8 mmol/L and with ≥2 measurements in the first 3 hours after ROSC. With linear regression we estimated the absolute rate of decrease (mmol/L/h) and relative rate of decrease (% of initial/h) per individual patient. We compared the early kinetics between hospital survivors and hospital non-survivors.

**RESULTS.** We studied 602 blood samples taken from 152 patients, 82.2% male, with a mean age of 59 years. The mean (±SD) initial lactate and glucose levels were 12.1±3.7 and 18.6±5.2 mmol/L, respectively. The mean relative lactate and glucose decreases were 30% and 14% of initial/h over the first 3 hours. The mean absolute lactate and glucose decrease was 3.6 mmol/L/h and 2.7 mmol/L/h, respectively. Lactate relative kinetics differed between survivors and non-survivors (33%/h vs 27%/h p=0.007). Glucose relative kinetics did not differ significantly between survivors and non-survivors (16 %/h vs 13 %/h p= 0.27).

**CONCLUSION.** Lactate is rapidly consumed through oxidation and gluconeogenesis early after ROSC. Hospital survivors have relatively higher initial decreases of lactate compared to hospital non-survivors, while there was no difference in the decrease of glucose between the two groups in the first 3 hours after ROSC.

**REFERENCE(S)**

1. Woo JH et al. The Relationship Between the Decreased Rate of Initial Blood Glucose and Neurologic Outcomes in Survivors of Out-of-Hospital Cardiac Arrest Receiving Therapeutic Hypothermia. Neurocrit Care 2017;26:402-410.
2. Donnino MW et al. Initial lactate and lactate change in post-cardiac arrest: a multicenter validation study. Crit Care Med 2014 ;42:1804-11.
3. Marik PE, Bellomo R. Stress hyperglycemia: an essential survival response! Crit Care. 2013 ;17:305.
4. Bakker J, Nijsten MW, Jansen TC. Clinical use of lactate monitoring in critically ill patients. Ann Intensive Care 2013;3:12.

### 001165

#### Efficacy of the cooling method for targeted temperature management in post-cardiac arrest patients: a systematic review and meta-analysis

JG. Kim[1], C. Ahn[2], H. Shin[3], W. Kim[1], TH. Lim[4], BH. Jang[5], Y. Cho[6], KS. Choi[7], J. Lee[8], MK. Na[9]

[1]Department of emergency medicine, College of Medicine, Hallym University, Chuncheon, Republic of Korea; [2]Department of emergency medicine, Armed Forces Yangju Hospital, Yangju, Republic of Korea; [3]Department of emergency medicine, Hanyang University Guri Hospital, Guri, Republic of Korea; [4]Department of emergency medicine, Hanyang University, Seoul, Republic of Korea; [5]Department of preventive medicine, Kyung Hee University, Seoul, Republic of Korea; [6]Department of emergency medicine, Hallym University, Chuncheon, Republic of Korea; [7]Department of neurosurgery, Hanyang University, Seoul, Republic of Korea; [8]Department of emergency medicine, Armed Forces Capital Hospital, Seongnam, Republic of Korea; [9]Department of neurosurgery, Severance Hospital, Yonsei University Health System, Seoul, Republic of Korea

**Correspondence:** J.G. Kim

*Intensive Care Medicine Experimental* 2019, **7(Suppl 3):**001165

**INTRODUCTION.** This study aimed to compare the efficacy of endovascular cooling device (ECD) like Thermogard® compared to surface cooling device (SCD) such as Arctic Sun® in outcomes of adult post-cardiac arrest patients treated undergoing targeted temperature management (TTM).

**METHODS.** We systematically searched MEDLINE, EMBASE, and the Cochrane Library (search date: 14 February 2019) to identify studies on TTM for post cardiac arrest patients. Randomized controlled studies (RCT) and observational studies (OS) comparing in-hospital morality and neurological outcomes between ECD and SCD were selected. We additionally performed subgroup analyses based on confounding factors that could affect patient outcomes. To assess the methodological quality of the included studies, Cochrane Risk of Bias Tool for randomized trials and Risk of Bias Assessment Tool for Non-randomized Studies was used independently by two authors.

**RESULTS.** A total of 4311 patients from nine studies were finally included. Two RCT and seven OS were included in the meta-analysis. Overall pooled analysis showed no significant difference on in-hospital mortality between ECD and SCD (nine studies; RR, 0.93; 95% CI 0.86–1.00; I2 = 0%). Likewise, there was no statistical difference in RCT (RR, 0.80; 95% CI 0.56–1.14; I2 = 0%) and OS (RR, 0.94; 95% CI 0.86–1.01; I2 = 2%)

In the pooled analysis for neurologic outcome, overall pooled data analysis revealed that neurologic outcome in ECD recipients was better than in SCD recipients (RR, 0.91; 95% CI 0.86–0.96; I2 = 0%), However, ECD showed significantly better neurological outcome compared to SCD only in OS (RR, 0.90; 95% CI 0.85–0.95; I2 = 0%). There was no statistical difference in RCT (RR, 0.95; 95% CI 0.75–1.21; I2 = 0%).

**CONCLUSION.** This study suggested that ECD could be associated with better neurologic outcomes compared with SCD. However, further studies would be needed to confirm the obvious benefit of ECD in TTM recipients because that most included studies were observational studies.

### 001460

#### Major trauma: epidemiology and ICU-mortality risk factors from RETRAUCI database

E. Machado[1], G. Moreno[2], A. Rodriguez[3], R. Carbonell[2], J. Leache Irigoyen[2], M. Bodí[3]

[1]Intensive Care, Hospital Joan XXIII, Tarragona, Spain; [2]Intensive care, Hospital Universitari de Tarragona Joan XXIII, Tarragona, Spain; [3]Uci, Hospital Universitari de Tarragona Joan XXIII, Tarragona, Spain

**Correspondence:** E. Machado

*Intensive Care Medicine Experimental* 2019, **7(Suppl 3):**001460

**INTRODUCTION.** According to the World Health Organization, trauma is one of the leading causes of death worldwide. Trauma-related injuries are responsible up to 30% of admissions in the Intensive Care Unit (ICU), mainly in young people and it has been recognized as a serious social and economic issue. As expected, traumatic brain injury (TBI) is the main cause of the majority of trauma-related deaths, followed by haemorrhage-induced hypovolemic shock and multiple organ failure syndrome (MODS).

**OBJECTIVES.** To describe the epidemiology of major trauma and analyse the prognostic factors of ICU-mortality.

**METHODS.** Prospective, observational and single centre study. All admitted trauma patients and registered in RETRAUCI (National database of trauma in ICU) during 18 months, were consecutively included. Demographic data, severity scores, pre- and intra-hospital care variables and outcomes were recorded. Mann-Whitney U test (continuous variables) and Chi-squared test (categorical variables) were used; Mortality risk factors were evaluated by a multivariable analysis with logistic regression (95% CI) and survival analysis with Cox hazard regression analysis.

**RESULTS.** Total population comprises 140 patients admitted on ICU due to major trauma. 73.6% (n = 103) were male. The median age was 55 years old (IQR 39-70), and 32.1% were older than 65 years old. The median ISS (Injury Severity Score) was 21 (IQR 13-26); 72.8% (n = 102) had ISS ≥ 16. Blunt trauma was predominant (95%). The most common causes of trauma were traffic accidents (47.1%) followed by falls (37.4%). Alcohol and drug consumption was frequent (40.7% and 63%, respectively of available data). 62.9% required mechanical ventilation (MV), 27.1% developed shock and intracranial hypertension occurred in 25.7%. Only four patients required massive transfusion, and the mean of blood transfusion in the first 24 hours was 3.45 (± 2.5) packed red blood cells. Neurotrauma was frequent (n = 80, 57.1%); among them, 40 patients had severe TBI. 72.5% of these had intracranial pressure catheter (ICPc) monitoring and 30% needed neurosurgery (17.5% of them required decompressive craniectomy). Overall mortality was 16.4% (n = 23); the mean ICU-length of stay was 13.2 days, and the mean of MV duration was 7 days. The most frequent cause of early mortality (<48 hours) was intracranial hypertension (n=7). The factors independently associated with ICU-mortality were ISS (OR 1.06, CI 1.00-1.12, p=0.034), severe TBI (OR 4.48, CI 1.37-16.61, p=0.014) and shock (4.02, CI 1.21-13.3, p= 0.023). The adjusted survival analysis showed higher ICU-mortality over time in severe TBI patients (HR 2.9, CI 1.18-7.17, p=0.02).

**CONCLUSION.** The factors associated with ICU-mortality were ISS, severe TBI and shock. It emphasizes the importance of neurotrauma for its frequency as well as its implication as a prognosis in mortality.

**REFERENCE(S)**

1. Emmanuel Lilitsis, Sofia Xenaki, Elias Athanasakis, Eleftherios Papadakis, Pavlina Syrogianni, George Chalkiadakis and Emmanuel Chrysos. Guiding Management in Severe Trauma: Reviewing Factors Predicting Outcome in Vastly Injured Patients. J Emerg Trauma Shock. 2018; 11: 80–87.
2. RETRAUCI

#### 001465

**Impairment in endocrine axes due to acute cholinesterase inhibition – case series**

C. Cobilinschi[1], R. Tincu[2], A. Baetu[3], A. Totan[4], I. Grintescu[1]
[1]Anesthesiology and intensive care, Clinical Emergency Hospital, Bucharest, Romania; [2]Toxicology, Clinical Emergency Hospital , Bucharest, Romania; [3]Anesthesiology and intensive care, Clinical Emergency Hospital , Bucharest, Romania; [4]Biochemistry, Carol Davila University of Medicine and Pharmacy, București, Romania
**Correspondence:** C. Cobilinschi

*Intensive Care Medicine Experimental* 2019, **7(Suppl 3):**001465

**INTRODUCTION.** Pesticide self-poisoning accounts as a high percentage in suicidal attempts, especially in agricultural countries. Although many of the systemic effects of organophosphate exposure have been extensively studied, endocrine consequences are still unclear.

**METHODS.** In order to assess the effects of acute organophosphate poisoning we performed a prospective, observational and cross-sectional study on 13 patients admitted in the Intensive Care Department after self-ingestion of pesticides. Patients with documented pre-existing endocrine dysfunction or in treatment with acetylcholinesterase inhibitors were excluded. The final study group included seven patients in whom we determined levels of acetylcholinesterase, cortisol, free triiodothyronine (fT3), free thyroxin (fT4), thyroid-stimulating hormone (TSH) and prolactin on admission and after 24 hours. Results were statistically analyzed using t-test and correlations, setting a standardized significant P value of 0.05.

**RESULTS.** All patients in the study group survived after adequate treatment was administered. Acetyl-cholinesterase level was significantly lower on admission indicating an acute organophosphate intoxication status (mean difference between determinations 1312 U/L, p = 0.0034). Cortisol level was significantly lower on the second measurement with a mean difference of 25 ng/ml (p = 0.011). Levels of fT3, fT4 and TSH were also significantly lower at 24 hours post-exposure (p=0.001). Increase of fT3 correlated with increase of fT4 on admission with a p = 0.053. Moreover, increase of fT4 on admission was proportional with the increase of cortisol levels (p= 0.04). Prolactin levels registered no statistically significant changes.

**CONCLUSION.** The present study demonstrates that acute organophosphate poisoning can induce an endocrine dysfunction. High levels of cortisol can be induced by the accumulation of acetylcholine as well as by the direct effects of organophosphate compound. Therefore, normalizing acetyl-cholinesterase levels can solve the adrenal dysfunction. This study identified changes in thyroid hormone levels, suggesting the possibility of a non-thyroidal illness in these patients.

**REFERENCE(S)**

1. Angelis, S. De et al. Developmental Exposure to Chlorpyrifos Induces Alterations in Thyroid and Thyroid Hormone Levels Without Other Toxicity Signs in Cd1 Mice. 108, 311–319 (2009).
2. Bertolote, J. M., Fleischmann, A., Eddleston, M. & Gunnell, D. Deaths from pesticide poisoning : a global response. (2005).

### INF - Diagnosis and treatment of infection: Strategies for a better use

#### 000090

**Combining Procalcitonin and Rapid Multiplex Respiratory Virus Testing for Antibiotic Stewardship in Elderly Patients with Severe Acute Respiratory Infection**

CC. Lee[1], C. Chia-Yu[2], M. Xiao-Wei[3], H. Wan-Ting[4], C. Shey-Ying[1], C. Yee-Chun[5], H. Chung-Kwang[2]
[1]Department of Emergency, National Taiwan University Hospital, Taipei, Taiwan; [2]Department of emergency, Taiwan Veteran General Hospital, Taipei, Taiwan; [3]Department of medical technology, National Taiwan University Hospital, Taipei, Taiwan; [4]Epidemiology, Harvard T.H. Chan School of Public Health, Boston, United States of America; [5]Division of infectious disease, department of internal medicine, National Taiwan University Hospital, Taipei, Taiwan
**Correspondence:** C.C. Lee

*Intensive Care Medicine Experimental* 2019, **7(Suppl 3):**000090

**INTRODUCTION.** Antibiotics are overused in elderly patients with acute respiratory tract infection because virus infection is under-evaluated. We aimed to evaluate the effectiveness of combining point-of-care multiplex PCR test (FilmArray Respiratory Panel, BioFire Inc.) and serum procalcitonin (PCT) level for antibiotic stewardship in the emergency department (ED).

**OBJECTIVES.** To test whether combining procalcitonin test and rapid molecular test for respiratory virus would change physician's antibiotic prescription and patients' outcome

**METHODS.** We conducted a prospective bi-center cohort study that enrolled elderly (aged ≥ 65 years) patients who presented to the ED with severe acute respiratory illness (SARI) between January 2017 and March 2018. Eligible patients received a rapid molecular test with 17 respiratory viruses and a PCT test. Physicians were recommended to stop or de-escalate antibiotics if a patient had a PCT level < 0.25 ng/mL and a positive test for respiratory viruses. To evaluate the clinical impact, we compared the outcomes of SARI patients between the experimental cohort and a propensity score matched historical cohort. The primary outcome was the proportion of antibiotics discontinuation or de-escalation in the ED. The secondary outcomes included duration of intravenous antibiotics, length of hospital stay, and mortality.

**RESULTS.** Between January 1, 2017, and March 30, 2018, we enrolled 169 eligible patients, of which 36 (27.9%) were tested positive for virus. Influenza A or B (7.7%) and respiratory syncytial virus (5.3%) were the major two viruses identified. By propensity score matching, we selected 507 SARI patients with similar demographic and comorbidity characteristics in 2016 as the control group. Compared with controls, the experimental group had significantly higher proportion of antibiotics discontinuation or de-escalation in the ED (26.0% vs.16.1%, p = 0.007), neuraminidase inhibitor use in the ED (8.9% vs. 0.6%, p < 0.001), shorter duration of intravenous antibiotics (12.5 ± 12.0 vs. 16.8 ± 13.6, p < 0.001), shorter length of stay (17.0 ± 14.5 vs. 19.7 ± 17. 2, p = 0.042). The 30-day mortality rates were not different in the two groups.

**CONCLUSION.** Viral infection is under-evaluated in elderly patients with SARI. Combining FilmArray RP and PCT testing may be a useful strategy for antibiotic stewardship in this group of patients.

**REFERENCE(S)**

1. Nicholson KG, Abrams KR, Batham S, et al. Randomised controlled trial and health economic evaluation of the impact of diagnostic testing for influenza, respiratory syncytial virus and Streptococcus pneumoniae infection on the management of acute admissions in the elderly and high-risk 18- to 64-year-olds. Health technology assessment (Winchester, England). 2014;18(36):1-274, vii-viii.
2. Mengelle C, Mansuy JM, Pierre A, et al. The use of a multiplex real-time PCR assay for diagnosing acute respiratory viral infections in children attending an emergency unit. Journal of clinical virology : the official publication of the Pan American Society for Clinical Virology. 2014;61(3):411-417.

3. Keske S, Ergonul O, Tutucu F, Karaaslan D, Palaoglu E, Can F. The rapid diagnosis of viral respiratory tract infections and its impact on antimicrobial stewardship programs. European journal of clinical microbiology & infectious diseases : official publication of the European Society of Clinical Microbiology. 2018;37(4):779-783.
4. Jeong HW, Heo JY, Park JS, Kim WJ. Effect of the influenza virus rapid antigen test on a physician's decision to prescribe antibiotics and on patient length of stay in the emergency department. PloS one. 2014;9(11):e110978.
5. Brendish NJ, Malachira AK, Armstrong L, et al. Routine molecular point-of-care testing for respiratory viruses in adults presenting to hospital with acute respiratory illness (ResPOC): a pragmatic, open-label, randomised controlled trial. The Lancet Respiratory medicine. 2017;5(5):401-411.
6. Bhavnani D, Phatinawin L, Chantra S, Olsen SJ, Simmerman JM. The influence of rapid influenza diagnostic testing on antibiotic prescribing patterns in rural Thailand. International journal of infectious diseases : IJID : official publication of the International Society for Infectious Diseases. 2007;11(4):355-359.
7. Leber AL, Everhart K, Daly JA, et al. Multicenter Evaluation of BioFire FilmArray Respiratory Panel 2 for Detection of Viruses and Bacteria in Nasopharyngeal Swab Samples. Journal of clinical microbiology. 2018;56(6).
8. Huang HS, Tsai CL, Chang J, Hsu TC, Lin S, Lee CC. Multiplex PCR system for the rapid diagnosis of respiratory virus infection: systematic review and meta-analysis. Clinical microbiology and infection : the official publication of the European Society of Clinical Microbiology and Infectious Diseases. 2018;24(10):1055-1063.
9. Chen H, Weng H, Lin M, et al. The Clinical Significance of FilmArray Respiratory Panel in Diagnosing Community-Acquired Pneumonia. BioMed research international. 2017;2017:7320859.
10. Babady NE. The FilmArray(R) respiratory panel: an automated, broadly multiplexed molecular test for the rapid and accurate detection of respiratory pathogens. Expert review of molecular diagnostics. 2013;13(8):779-788.
11. The study is sponsored by the joint research grant between National Taiwan University Hospital and Taipei Veteran General Hospital (VN106-16). The FilmArray mutiplex PCR test kits were sponsored by the bioFire Inc. (Salt Lake City, UT, USA).

### 000270

#### Ceftolozane/tazobactam (C/T) vs meropenem (MEM) in patients (pts) with ventilated hospital-acquired pneumonia (vHAP) – subset analysis of the ASPECT-NP randomized, controlled, phase 3 trial

JF. Timsit[1], JA. Huntington[2], RG. Wunderink[3], N. Shime[4], M. Kollef[5], Ü. Kivistik[6], M. Nováček[7], A. Réa-Neto[8], I. Martin-Loeches[9], B. Yu[2], CJ. Bruno[10], E. Jensen[11], JR. Butterton[10], DJ. Wolf[12], EG. Rhee[10]
[1]Infectious diseases, Université Paris Diderot/Hôpital Bichat, Paris, France; [2]Clinical operations, Merck & Co., Inc., Kenilworth, United States of America; [3]Pulmonary and critical care division, Northwestern University Feinberg School of Medicine, Chicago, United States of America; [4]Department of emergency and critical care medicine, Hiroshima University, Hiroshima, Japan; [5]Division of pulmonary and critical care medicine, Washington University School of Medicine, St. Louis, United States of America; [6]Anaesthesiology clinic, North Estonia Medical Centre, Tallinn, Estonia; [7]Department of anaesthesia and intensive care, General Hospital of Kolin, Kolin, Czech Republic; [8]Department of clinical medicine, Universidade Federal do Paraná, Curitiba, Brazil; [9]School of medicine, St. James's Hospital, Dublin, Ireland; [10]Clinical research, Merck & Co., Inc., Kenilworth, United States of America; [11]Biostatistics, Merck & Co., Inc., Kenilworth, United States of America; [12]Global scientific & medical publications, Merck & Co., Inc., Kenilworth, United States of America
**Correspondence:** J.F. Timsit

*Intensive Care Medicine Experimental* 2019, **7(Suppl 3):**000270

**INTRODUCTION.** The randomized, double-blind, multi-center, phase 3, noninferiority ASPECT-NP trial evaluated C/T (at double the currently approved dose) vs MEM for treating ventilated nosocomial pneumonia (NP). As part of randomization, pts were stratified by NP type, i.e., ventilator-associated pneumonia (VAP) vs vHAP, the latter of which is associated with worse prognosis. The expected higher 28-day mortality rate in the vHAP subgroup was only seen in the MEM arm (37.0% in vHAP vs 20.3% in VAP) but not the C/T arm (24.2% in vHAP vs 24.0% in VAP).

**OBJECTIVES.** To retrospectively explore potential reasons behind the between-treatment mortality difference observed in the vHAP subgroup of ASPECT-NP.

**METHODS.** Mechanically ventilated pts with NP were randomized 1:1, stratified by NP type (VAP vs vHAP) and age (<65 y vs ≥65 y), to receive 3 g C/T or 1 g MEM, by 1-h IV infusions every 8 h for 8-14 d. Lower respiratory tract (LRT) cultures were obtained from all pts at baseline. Pathogen identification and susceptibility were confirmed at a central laboratory. The primary endpoint was 28-d all-cause mortality in the intent-to-treat (ITT) population, with a 10% noninferiority margin. Pathogens and antibacterial susceptibility are presented for the microbiologic ITT (mITT) population (i.e., pts with ≥1 dose of study drug and with baseline LRT pathogens susceptible to C/T and/or MEM).

**RESULTS.** 362/362 C/T (27.3%) and 108/364 (29.7%) MEM pts had vHAP. Baseline characteristics and causative pathogens (mostly *Klebsiella pneumoniae, Pseudomonas aeruginosa,* and *Escherichia coli*) in pts with vHAP were generally similar between treatment arms (Table). In the MEM arm of the mITT population (MEM MIC50/90 for gram-negative pathogens: <0.064/8 μg/mL), there were 8 MEM non-susceptible baseline isolates in the 71 mITT pts with vHAP; all Enterobacteriaceae were MEM susceptible (MIC ≤1 μg/mL). In the C/T arm of the mITT population (n=55), the C/T MIC50/90 for gram-negative pathogens was 0.5/128 μg/mL. In the ITT population, 28-day mortality was 24.2% (24/99) with C/T and 37.0% (40/108) with MEM (difference: 12.8%; 95% confidence interval: 0.18, 24.75). As assessed by the investigator, the proportion of vHAP patients who died by Day 28 due to fatal AEs was 9/99 (9.1%) in the C/T arm vs 17/108 (15.7%) in the MEM arm; the proportion of pts who died due to underlying conditions was 10/99 (10.1%) vs 16/108 (14.8%), respectively; and the proportion of pts who died due to NP was 5/99 (5.1%) vs 5/108 (4.6%), respectively.

**CONCLUSION.** These exploratory analyses in the vHAP subgroup of the ASPECT-NP trial are limited by their retrospective nature and the smaller sample size. Fewer vHAP patients in the C/T arm died due to fatal AEs/underlying conditions compared with MEM. The difference in 28-day mortality observed in the MEM- vs C/T-treated vHAP pts does not appear to be due to clinically meaningful differences in baseline clinical characteristics, causative pathogens, or antibacterial susceptibility.

1. Funding for this research was provided by Merck Sharp & Dohme Corp., a subsidiary of Merck & Co., Inc., Kenilworth, NJ, USA.

**Table 1 (abstract 000270).** See text for description

| ITT baseline characteristic, n (%) | C/T (N=99) | Meropenem (N=108) |
|---|---|---|
| **Age ≥65 y** | 47 (47.5) | 61 (56.5) |
| **Moderate/severe renal impairment** | 29 (29.3) | 23 (21.3) |
| **Augmented renal clearance** | 9 (9.1) | 5 (4.6) |
| **APACHE II score ≥20** | 31 (31.3) | 40 (37.0) |
| **SOFA score >7** | 42 (42.4) | 41 (38.0) |
| **CPIS >8** | 65 (65.7) | 80 (74.1) |
| **Prior antibacterial use** | 94 (94.9) | 97 (89.8) |
| **Failed prior pneumonia therapy** | 20 (20.2) | 17 (15.7) |
| **Prior hospitalization ≥5 days** | 71 (71.7) | 72 (66.7) |
| **Prior mechanical ventilation ≥5 days** | 11 (11.1) | 12 (11.1) |
| **Bacteremia (gram-negative respiratory pathogen)** | 6 (6.1) | 7 (6.5) |
| **mITT baseline pathogen, n (%)[a]** | **C/T (N=55)** | **Meropenem (N=71)** |
| **Gram-negative pathogen** | 53 (96.4%) | 66 (93.0%) |
| *Pseudomonas aeruginosa* | 12 (21.8%) | 15 (21.1%) |
| Enterobacteriaceae | 37 (67.3%) | 54 (76.1%) |
| ESBL+ Enterobacteriaceae | 17 (30.9%) | 23 (32.4%) |

[a]Patients could have had multiple baseline pathogens.

### 000208

#### Rapid Detection of Multidrug-resistant Acinetobacter baumannii and Methicillin-resistant Staphylococcus aureus using the Novel Loop-mediated Isothermal Amplification (LAMP) in Patients With Ventilator-Associated Pneumonia

SJ. kwon[1], MH. Lee[2], J. Kim[3], IB. Jung[4], JW. Son[5], SR. Yun[6], HS. Jeon[6]
[1]Konyang University Hospital, Daejeon, Republic of Korea; [2]Internal medicine, Konyang University Hospital, Daejeon, Republic of Korea; [3]Allergy, Konyang University Hospital, Daejeon, Republic of Korea; [4]Respiratory and critical care, Konyang University Hospital, Daejeon, Republic of Korea; [5]Respiratory medicine, Konyang University Hospital, Daejeon, Republic of Korea; [6]Research, M monitor, Daegu, Republic of Korea
**Correspondence:** S.J. kwon
*Intensive Care Medicine Experimental* 2019, **7(Suppl 3):**000208

**INTRODUCTION.** Methicillin-resistant *Staphylococcus aureus* (MRSA) and multidrug-resistant *Acinetobacter baumannii* (MRAB) are rising microorganisms in ICUs

**OBJECTIVES.** We evaluate the utility of the novel loop-mediated isothermal amplification (LAMP) to rapidly detect MRAB and MRSA in bronchoalveolar lavage (BAL) fluids for surveillance of colonization and etiologic diagnosis

**METHODS.** Two conditions of LAMP reactions that have different limit of detection have been developed using reference strain that target *mecA*/*femB* gene (Genbank No. AB505630.1/CP010300.1) of MRSA and *bla*OXA-23/*bla*OXA51-like gene (Genbank No. KF305669.1/ DQ385606.1) of MRAB by adjusting composition of primers and reaction time. these two conditions of LAMP reaction were applied to 123 of BAL samples from clinically suspected VAP patients and compared it with quantitative BAL fluid culture method

**RESULTS.** Compared to quantitative BAL fluid culture method, the sensitivity, and specificity of the M-/C- condition of LAMP reaction was 81.8%/65.9% and 84.8%/91.1% in MRSA and 90.2%/97.5% and 71.9%/56.1% in MRAB. And The diagnostic accuracy of the M-/C- condition of LAMP reaction in was 83.73%/82.11% in MRSA and 65.91%/ 78.04% in MRAB

**CONCLUSION.** The LAMP assay is promising method to surveillance and etiologic diagnosis of MDR pathogen in VAP patients

##### REFERENCE(S)

1. Yang HY, Lee HJ, Suh JT, Lee KM. Outbreaks of imipenem resistant Acinetobacter baumannii producing OXA-23 β-lactamase in a tertiary care hospital in Korea. Yonsei medical journal. 2009;50(6):764-70
2. Kobayashi N, Wu H, Kojima K, Taniguchi K, Urasawa S, Uehara N, et al. Detection of mecA, femA, and femB genes in clinical strains of staphylococci using polymerase chain reaction. Epidemiology & Infection. 1994;113(2):259-66.
3. Lin Q, Xu P, Li J, Chen Y, Feng J. Direct bacterial loop-mediated isothermal amplification detection on the pathogenic features of the nosocomial pathogen–Methicillin resistant Staphylococcus aureus strains with respiratory origins. Microbial pathogenesis. 2017;109:183-8.

### 000338

#### Tuberculosis in Critical Care. Phenotypic and genotypic characteristics, and possible molecular biomarkers of severity

FJ. Hurtado[1], N. Nin[2], G. Greif[3], JI. Hurtado,[3], M. Buroni[2], A. Giordano[2], C. Coitinho[4], C. Robello[5]
[1]Pathophysiology dept. school of medicine. universidad de la república., ICU. Hospital Español - ASSE, Montevideo, Uruguay; [2]Intensive care unit, Hospital Español, Montevideo, Uruguay; [3]Molecular biology laboratory, Institut Pasteur de Montevideo, Montevideo, Uruguay; [4]Microbiology laboratory, Antituberculosis and Prevalent Disease Honorary Comission, Montevideo, Uruguay; [5]Depto de bioquímica. facultad de medicina. universidad de la república., Molecular Biology Laboratory. Instituto Pasteur Montevideo., Montevideo, Uruguay
**Correspondence:** J. Hurtado
*Intensive Care Medicine Experimental* 2019, **7(Suppl 3):**000338

**INTRODUCTION.** The global incidence and mortality of Tuberculosis (TB) progressively decreased along the past years around the world. However, some patients coursing advanced stages of the disease may require vital support in Intensive Care Units (ICU). Comorbidities, HIV infection, and other community or nosocomial infections are frequently associated and may contribute to decreasing survival. The clinical course could evolve with respiratory failure, circulatory shock, and sepsis with variable organ dysfunction. Patients who need mechanical ventilation show mortality rates higher than 50%. Since ICU admission occurs in a low percentage of the patients, few communications are describing the clinical phenotype and outcome in the critical care setting.

**OBJECTIVES.** To describe the phenotype and genotype characteristics of critically ill TB patients, and to investigate possible molecular biomarkers of virulence.

**METHODS.** One hundred and four TB patients were admitted in a general ICU. *Mycobacterium tuberculosis* was suspected based on compatible clinical, radiologic, and epidemiological factors. The microbiologic diagnosis was confirmed by a) Positive cultures; b) Acid-Alcohol Resistant Bacilli in sputum or bronchoalveolar lavage; c) Positive GeneXpert for *Mycobacterium tuberculosis*; d) Histopathological findings in tissue autopsy/biopsy when corresponding.

Molecular genotyping of *Mycobacterium tuberculosis* strains were made by MIRU-VNTR and Spoligotyping techniques. miRNA profiles in serum were studied as potential biomarkers of severity. Clinical, radiologic, laboratory and physiological variables were correlated with ICU mortality.

**RESULTS.** During the study period, 104 among 6.353 critically ill patients (1.6%) were admitted to the ICU with active TB disease. The patient population was relatively young with male predominance and low HIV-positive incidence (20%). Genotype investigation identified Ghana, Haarlem, Lam10, Lam9, and Lam3 lineages. Haarlem (49.2%) followed by Lam3 (28%) strains predominated in these ICU patients.

The miRNA profiles from 16 patients were compared with normal serum from a public database. Bioinformatic analysis showed that some specific miRNAs significantly enriched in plasma from TB individuals making them as possible biomarkers for diagnosis, prognosis, and virulence.

The global ICU mortality was 50%, but it was higher in patients under mechanical ventilation (64.5%). After multivariate analysis, APACHE II score and cardiovascular dysfunction ($p<0.05$) were independently associated with death.

**CONCLUSION.** A better phenotype and genotype characterization and the identification of possible miRNA biomarkers of severity could contribute to an earlier and more effective therapeutic intervention in these patients.

##### REFERENCE(S)

1. Funded by the Investigation and Innovation National Agency (ANII, Health Sectorial Grant 2016 (FSS_X_2016_1_127692)
2. World Health Organization. Global Tuberculosis Report 2018. http://www.who.int/tb/publications/global_report/en/ (access March 2019).
3. Otu A, Hashmi M, Mukhtar AM, Kwizera A, Tiberi S, Macrae B, Zumla A, Dünser MW, Mer M. The critically ill patient with tuberculosis in intensive care. Clinical presentation, management, and infection control. J Crit Care 2018; 45:184-196.
4. Ryu YJ, Koh WJ, Kang EH, Suh GY, Chung MP, Kim H, Kwon OJ. Prognostic factors in pulmonary tuberculosis requiring mechanical ventilation for acute respiratory failure. Respirology 2007; 12:406–411
5. Pacheco Duro R, Figueredo Dias P, Azevedo Ferreira A, Margarida Xerinda S, Lima Alves C, Carlos Sarmento A, Campos dos Santos L. Severe Tuberculosis requiring intensive care; a descriptive analysis. Crit Care Research Practice 2017: Article ID 9535463

### 001515

#### Assessment of pleural effusions associated with pneumonia in the ICU

J. Hornsby[1], S. Daly[2], G. Murphy[2], K. Puxty[1]
[1]Intensive care medicine, Glasgow Royal Infirmary, Glasgow, United Kingdom; [2]Respiratory medicine, Glasgow Royal Infirmary, Glasgow, United Kingdom
**Correspondence:** J. Hornsby
*Intensive Care Medicine Experimental* 2019, **7(Suppl 3):**001515

**INTRODUCTION.** The incidence of parapneumonic effusion and empyema in the intensive care unit is not well studied. Pleural effusions in ICU are common with some studies suggesting an incidence of 40-60% of which parapneumonic effusions may account for approximately 40% of these effusions. Pleural infections result in a longer ICU stay compared with those without pleural infections. We aimed to review the assessment of pleural effusions associated with pneumonia and diagnosis of parapneumonic effusions in a mixed level 2/3 ICU in a large teaching hospital (Glasgow Royal Infirmary).

**METHODS.** The Electronic Patient Record (EPR) was interrogated to identify any patient admitted between 01/01/2017 and 01/07/2017 that had a diagnosis of "pneumonia" in the problem list. The radiology reports for these patients were reviewed (Chest X-Ray and CT Thorax) and those that commented on pleural effusion were included for assessment. The radiology was reviewed by two senior respiratory registrars who made a recommendation regarding pleural ultrasound to further assess or facilitate diagnostic aspiration of the effusion. Those patients identified by the electronic search also underwent a case review by a senior intensive care medicine registrar to confirm the diagnosis of pneumonia.

**RESULTS.** Six hundred patients were admitted during the audit period. Sixty patients (10%) were identified using the electronic search parameters. Of these, 16 (27%) met the inclusion criteria – a diagnosis of pneumonia and radiologically confirmed pleural effusion. Nine patients had a diagnosis of Community Acquired Pneumonia, 5 Hospital Acquired Pneumonia, 1 Aspiration Pneumonia and 1 ongoing pleural infection following a community acquired pneumonia. Two patients were excluded as the imaging reviewed was outside the audit period.

Assessment of effusions: Of the 16 patients; 6 (37.5%) had the presence of the effusion recorded in the notes and 2 (12.5%) effusions were assessed by ultrasound. Pleural fluid samples were sent in 2 (12.5%) patients: one who was assessed by ultrasound and underwent pleural aspiration. One of which had fluid sent from a pre-existing drain. Of the patients with potential parapneumonic effusions in ICU, 10 (62.5%), did not have any assessment or investigation performed.

Complications: Of the 16 patients with an effusion associated with pneumonia, 3 (18.75%), developed a complication from pleural infection. This included systemic sepsis, a multi-loculated effusion requiring a prolonged course of antibiotics and a hospital readmission with recurrence of pleural infection.

Review of imaging by Respiratory Registrars: Twenty-four chest x-ray and 12 CT studies were assessed. Ultrasound assessment was suggested by both respiratory clinicians for 12 patients (75%). Only 2 (12.5%) of these patients underwent ultrasound examination.

**CONCLUSION.** Pleural effusions occur commonly in ICU patients with potential parapneumonic effusions seen in 27% of the study group. Complications were significant and developed in over one sixth of patients (18.7%, n=3). We believe that most of these patients should have had an ultrasound evaluation and an aspiration with analysis of pleural fluid considered. This occurred in less than 19% of patients in our study. Education on assessment of potential parapneumonic effusions and thoracic ultrasound is ongoing.

**REFERENCE(S)**

1. Walden, A., Jones, Q., Matsa, R., Wise, M. Pleural effusions on the intensive care unit: hidden morbidity with therapeutic potential. Respirology. 2013;18:246–254
2. Azoulay, E. Pleural effusions in the intensive care unit. Curr Opin Pulm Med. 2003;9:291–297
3. Fysh, E., Yogendran, A., Rosenstengel, A, Roberts, B., Palerma, A., Kay I.,Litton, E. Ho, K. Lee, Y. Pleural Infections in Intensive Care. Chest. 2016;150(6):1419-1420
4. None to declare
5. Vetrugo, L., Bignami, E., Orso, D., Vagas, M., Guadfagnin, G., Saglietti, F., Servillo, G., Volpicelli, G., Navalesi, P., Bove T. Utility of pleural effusion drainage in the ICU: An updated systemic review and META-analysis. Journal of Critical Care 52 (2019) 22-32

### 001592

#### Association between plasma midregional pro-adrenomedullin and sublingual microcirculation in critically ill patients admitted with infection

P. Giaccaglia, R. Domizi, C. Scorcella, J. Montomoli, S. Zuccari, S. Bolognini, E. Damiani, S. Vannicola, A. Carsetti, E. Casarotta, C. D'angelo, L. Lombardi, A. Donati
Marche politechnic university, UNIVPM, Ancona, Italy
**Correspondence:** P. Giaccaglia
*Intensive Care Medicine Experimental* 2019, **7(Suppl 3):**001592

**INTRODUCTION.** Recent studies propose plasma midregional pro-adrenomedullin (MR-proADM) as early marker of organ dysfunction in critically ill patients with infections and sepsis.

**OBJECTIVES.** As severe microcirculatory derangement has been demonstrated in patients with sepsis-related organ failure admitted to critical care unit (ICU), the primary objective of this study is to assess if a correlation existsbetween plasma MR-proADM and Microcirculatory Flow Index (MFI) in 20 adult patients admitted to ICU with suspected infection. It is also purpose of the study to evaluate the relationship between MR-proADM and Total Vessel Density (TVD), Perfused Vessel Density (PVD), Proportion of Perfused Vessels (PPV) and with organ failure in this group of patients.

**METHODS.** Prospective observational study articulated in five days of monitoring (T1-T5). Plasmatic MRproADM was dosed for all timepoints. At T1, T2 and T5 the sublingual microcirculation was assessed using Incident Dark Field technology. Laboratory and clinical data were collected to calculate the Simplified Acute Physiology Score (SAPS) II and Acute Physiology and Chronic Health Disease

Classification System II (APACHE) scores at admission in ICU and daily Sequential Organ Failure Assessment (SOFA) score. Exclusion Criteria: Age<18 years old, ICU stay>24 hours before inclusion, refusal to consent, conditions that do not allow clear monitoring of sublingual microcirculation.

**RESULTS.** This is a half-sample preliminary analysis. MRproADM> 1.5 nmol/l showed good discrimination capacity toward SAPS II and APACHE II scores at admission in ICU, Area Under the Curve (AUC) respectively 0.90[CI 0,69-1] p=0.04 and 0.96[CI 0.84-1] p= 0.06 [Fig. 1]. Persistence of MRproADM>1.5 nmol/l at T5 was able to discriminate worsening microcirculation in term of MFI of either small, AUC 0.92[CI 0.73-1] p=0.03 and total vessels, AUC 0.92[CI 0.73-1]p=0.03 and of PPV, AUC 0.83 [CI 0.57-1] p=0.09 [Fig. 2]

**CONCLUSION.** The preliminary results of the study confirm the association between MR-proADM and severity scores at admission in ICU and they show that a correlation exists between moderate-to-high levels of this marker and microcirculatory dysfunction in critically ill patients with infection.

**REFERENCE(S)**

1. Angeletti et al. Disease Marker, 2015
2. Onal et al. Healthcare, 2018

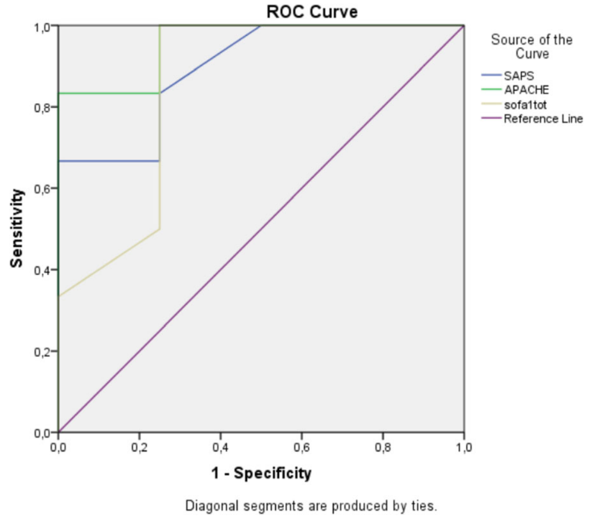

**Fig. 1 (abstract 001592).** Correlation between MR-proADM in patient with level > 1.5nmol/l and SAPS II, APACHE II and SOFA score at the ICU admission (T1). AUC SAPS II, 0.90[CI 0,69-1] p=0.04, AUC APAcHE II, 0.96 [CI 0.84-1] p=0.02, AUC SOFA 0.85 [CI 0.56-1] p= 0.07. MR-proADM: *midregional proadrenomedullin*, SAPS II: *Simplified Acute Physiology Score II*, APACHE II: *Acute Physiology, Age, Chronic Health Evaluation II, SOFA: sequential organ failure* of patients. *assessment, ICU: intensive care unit, unit, CI: confidence interval, AUC: area under the curve.*

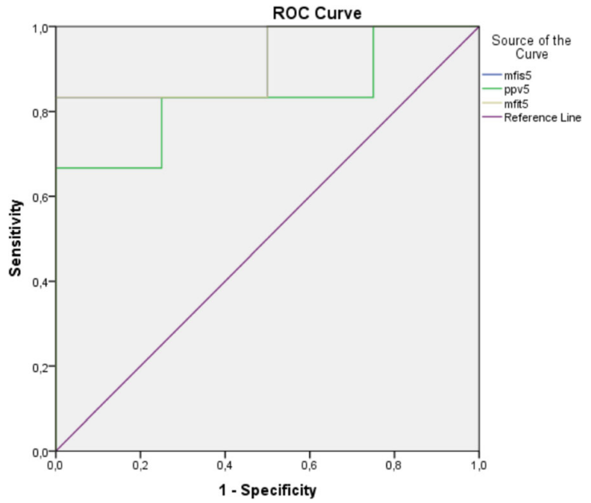

**Fig. 2 (abstract 001592).** Correlation between MR-proADM in patient with level > 1.5nmol/l and MFIs, PPV and MFIt at T5. AUC MFIs, 0.9 [CI 0,7-1] p=0.03, AUC PPV, 0.8 [CI 0.57-1] p=0.09, AUC MFIt 0.9 [CI 0.73-1] p=0.03. MR-proADM: midregional proadrenomedullin, MFIs5: Microvascular Flow Index for small vessels at T5, PPV: percentage of total perfused vessels at T5, MFIt5: Microvascular Flow Index for total vessels at T5, ICU: intensive care unit, CI: confidence interval, AUC: are under the curve.

### 001057

#### Is procalcitonin level is a valuable biomarker for diagnosis of infection in critically ill patients with renal failure?

M. Türkoğlu[1], BB. Aydoğan[2], N. Boyacı,[1], Z. Güllü,[3], H. Aksu,[4], G. Aygencel[1], M. Dizbay[5]
[1]Division of critical care medicine, department of internal medicine, Gazi University School of Medicine, Ankara, Turkey, Ankara, Turkey; [2]Department of critical care medicine, Denizli State Hospital, Denizli, Turkey, Ankara, Turkey; [3]Department of critical care medicine, Yıldırım Beyazıt University, Yenimahalle Hospital, Ankara, Turkey, Ankara, Turkey; [4]Department of critical care medicine, Hakkari State Hospital,Hakkari, Turkey, Ankara, Turkey; [5]Department of infectious disease and clinical microbiology, Gazi University School of Medicine, Ankara, Turkey, Ankara, Turkey
**Correspondence:** M. Türkoğlu
*Intensive Care Medicine Experimental* 2019, **7(Suppl 3):**001057

**INTRODUCTION.** Serum procalcitonin level is a valuble biomarker for diagnosing infection and sepsis in critically ill patients (1). Recent studies suggest that procalcitonin levels can be increased in non critically ill patients with chronic kidney disease (CKD) without any infection (2). The aim of this study was to investigate the effect of renal failure on serum procalcitonin levels and the value of serum procalcitonin for diagnosis of infection in patients with renal failure

**METHODS.** Patients who were admitted to our intensive care unit between January 2012 and November 2015 and whom procalcitonin levels were obtained during their hospitalization were examined retrospectively. The diagnosis of renal failure were made according to RIFLE score.

**RESULTS.** Out of 907 patients included in to the study, 304 patients had normal renal function, 379 patients had acute renal failure (ARF), 133 patients had CKD and 91 patients had ARF on CKD. Serum procalcitonin levels were higher in patients with CKD and in patients with ARF than in ones with normal renal function (p <0.05) (Table 1). According to ROC analysis procalcitonin level was a good predictor for diagnosing infection and sepsis in both patients with and without normal renal function. However higher treshold values were obtained for diagnosing infection and sepsis in patients with renal failure (Table 1).

**CONCLUSION.** Serum procalcitonin level is a good predictor marker for diagnosing infection and sepsis in patients with and without renal failure. However, higher threshold values should be used in patients with renal failure compared to patients without any failure.

**REFERENCE(S)**

1. Wirz Y, Meier MA, Bouadma L et al. Effect of procalcitonin-guided antibiotic treatment on clinical outcomes in intensive care unit patients with infection and sepsis patients: a patient-level meta-analysis of randomized trials. Critical Care 2018;22:191
2. Lee WS, Kang DW, Back Jh et al Cutoff value of serum procalcitonin as a diagnostic biomarker of infection in end-stage renal disease patients. Korean J Intern Med.2015;30:198

**Table 1 (abstract 001057).** Procalcitonin values based on renal function and infection status

| | Patients with CKD n=133 | Patients with ARF n=379 | Patients with normal renal function n=304 |
|---|---|---|---|
| Procalcitonin (ng/mL) | 7.51 (1.80-25.12)* | 4.45 (0.99-23.68)* | 0.57 (0.21-3.05) |
| Procalciton in patients with infection (ng/mL) | 7.94 (2.12-28.54)* | 7.32 (1.90-30.13)* | 1.63 (0.54-8.25) |
| Procalciton in patients without infection (ng/mL) | 0.46 (0.25-9.83)** | 0.39 (0.23-0.99)* | 0.22 (0.10-0.41) |
| AUCinfeksiyon | 0.813 (p=0.00) | 0.871 (p=0.00) | 0.853 (p=0.00) |
| AUCsepsis | 0.760 (p=0.000) | 0.805 (p=0.00) | 0.813 (p=0.00) |
| Procalcitonin treshold for infection (ng/mL) | 0.675 93% sensitivity, 75% spesifity | 0.695 90% sensitivity, 70% spesifity | 0.410 82% sensitivity, 76% spesifity |
| Procalcitonin treshold for sepsis (ng/mL) | 2.105 83% sensitivity, 64% spesifity | 1.565 %83 sensitivity, %62 spesifity | 0.648 %80 sensitivity, %72 spesifity |

*When comparing with patients with normal renal function, p<0.001
**When comparing with patients with normal renal function, p=0.006

### 001076

#### Predicting in-ICU acquired infections in patients over 65 years old. Data from the ENVIN-HELICS registry 2013-16

N. Mas[1], J. Molinuevo[1], E. Gamboa[1], U. Aguirre[2], F. Alvarez-Lerma[3], M. Palomar[4], PM. Olaechea[1], Envin Study Group[5]
[1]Intensive care unit, Hospital Universitario Galdakao-Usansolo, Galdakao, Spain; [2]Research unit, Hospital Universitario Galdakao-Usansolo, Galdakao, Spain; [3]Intensive care unit, Hospital del Mar, Barcelona, Spain, Spain; [4]Intensive care unit, Hospital Arnau de Villanova, Lleida, Spain; [5]., ., ., Spain
**Correspondence:** N. Mas
*Intensive Care Medicine Experimental* 2019, **7(Suppl 3):**001076

**INTRODUCTION.** Elder patients are becoming a noteworthy population in-ICU. Tools aimed to predict their performance in-ICU could have a main role to help the clinicians manage ICU resources.

**OBJECTIVES.** To design predictive models to estimate the risk to acquire different nosocomial infections in-ICU for patients older than 64 years old.

**METHODS.** Data was obtained from the 2013-2016 ENVIN-HELICS registry, prospective multicentric study of in-ICU acquired infections. Data regarding demographics, comorbidities, used techniques and outcome was gathered. The studied infections were: ventilator associated pneumonia (VAP), catheter related urinary infection (CUI) and primary and secondary bloodstream infections (BSI). The population was geographically divided in two groups, for the model to be developed in the first one and validated in the second one. The factors related to the development of in-ICU infections were determined by multivariate analysis, each of them related to its logical population; its predictive value was evaluated by the AUC and goodness of fit by the Hosmer Lemeshow test (HS).

**RESULTS.** A total of 37172 patients older than 64 years old were registered in the study period, most of them for medical causes (65.5%). Mean age was 75,1 years (SD 6,5 years). 41.8% needed mechanical ventilation and 76.0% urinary catheter. 165 VAP, 409 CUI, 472 primary BSI and 404 secondary BSI were diagnosed. Overall in-ICU mortality was 11.5%, higher in patients with any in-ICU acquired infection but mostly remarkable in those who suffered VAP (41.9%) or secondary VSI (40.1%). Regarding factors associated to VAP, gender (male vs. female OR 1.7; CI 95%1.4-2.0), malnutrition (OR 1.70; CI 95% 1.3-2.2), devices such as renal replacement therapies (OR 3.0; CI95% 2.8-3.7), tracheostomy (OR 7.4; CI95% 6.2-8.8) or central venous catheter (OR 3,7; CI95% 2,1-6,6), and the acquirement of a MRSA were related to its diagnosis. In the CUI predicting model similar variables were used, and in those regarding both kinds of BSI most of the variables included in the model regarded used devices. The AUC in all of them was higher than 0,78, showing excellent fit according to the HS test (p value over 0.1 in all of them).

**CONCLUSION.** In-ICU acquired infections are associated to several factors regarding demographics, comorbidities and used devices. The predictive models developed for VAP, CUI and primary and secondary BSI out of the sum of prognosis factors had a good discriminatory power and excellent fit.

### 000015

#### Implementation of an Antimicrobial Stewardship Program in Intensive Care: A Pilot Experience

D. Pérez-Torres[1], JA. De Ayala-Fernández[1], M. Domínguez-Gil González[2], MA. Sacristán-Salgado[3], R. Almendros-Muñoz[4], ME. González-González[3], L. Fuentes-Alvarez De Eulate[5], MC. Ramos-Sánchez[2], JM. Eiros-Bouza[2], LM. Tamayo-Lomas[1]
[1]Department of intensive care medicine, Hospital Universitario Río Hortega, Valladolid, Spain; [2]Department of microbiology, Hospital Universitario Río Hortega, Valladolid, Spain; [3]Department of preventive medicine and public health, Hospital Universitario Río Hortega, Valladolid, Spain; [4]Department of hospital pharmacy, Hospital Universitario Río Hortega, Valladolid, Spain; [5]Faculty of medicine and health sciences, University of Valladolid, Valladolid, Spain
**Correspondence:** D. Pérez-Torres
*Intensive Care Medicine Experimental* 2019, **7(Suppl 3):**000015

**INTRODUCTION.** Patients admitted to the intensive care unit (ICU) are frequently treated with antimicrobial agents. Optimizing antimicrobial use is imperative to reduce infection-related morbimortality and to limit the emergence of multidrug-resistant microorganisms (MDRMs).

**OBJECTIVES.** To describe the process of implementation of an antimicrobial stewardship program (ASP) in a 22-bed mixed ICU of a third-level university hospital in Spain (608 beds) and the results of a 6-month pilot experience.

**METHODS.** A multidisciplinary ASP group was created (intensivist, internist, microbiologist, pharmacist, preventivist, nurse and intensive care resident). Theorical need for antimicrobial agents use was discussed in daily meetings of the group, whenever a result of a microbiological test was positive, taking into account the clinical setting, infectious syndrome, isolated microorganism and antimicrobial pharmacology. Non-mandatory recommendations concerning better use of antimicrobials were proposed to the intensive care physician after each meeting, including de-escalation (switch to narrower spectrum or discontinuation of treatment), adequacy (change to same spectrum or start a new treatment), optimization (duration, dose or route of administration) and escalation (switch to broader spectrum). We report antimicrobial utilization and expense, expressed as daily defined dose (DDD) per 100 bed-days (BD) and euros (€), respectively, in the ASP period and in the same period of the previous year (baseline period), as an exploratory endpoint.

**RESULTS.** A total of 120 meetings were held, where 92 recommendations were proposed over 54 patients (54.3% male, age 61±15 years old), with a 90.2% of adherence (77.8% of the rejected interventions were due to severity of the condition). The most common microbiological samples to trigger an intervention were blood cultures (52.2%) and respiratory samples (26.1%), and the most common microorganisms were Gram-negative bacteria (46.7%), Gram-positive bacteria (37.8%) and fungi (17.4%). Most infections were community-acquired (55.4%), with 9.8% of MDRMs, and the most common sites of infection were the lungs (40.2%), abdominal organs (14.1%) and urinary tract (13%). Antimicrobial stewardship was essentially proposed as de-escalation (70.7%), but also as adequacy (18.5%), escalation (7.6%) and optimization (3.2%). Most interventions were advised over carbapenems (imipenem and meropenem) (20.5%), other antibacterials (linezolid and daptomycin) (20.5%) and penicillins (ampicillin, amoxicillin/clavulanic, cloxacillin and piperacillin/tazobactam) (14.5%). Antibiotic and antifungal use increased from 135.8 to 162.4 and from 40.7 to 72.4 DDD/100 BD, respectively, during the ASP period, with a decrease in expense (33,642 to 32,777 € and 152,590 to 99,547 €). A higher incidence of *Aspergillus* during the influenza season and a change of price of echinocandins during the ASP period may limit the interpretation of antifungal utilization results. Differences in terms of severity and reason for admission may limit the comparison of baseline and ASP periods.

**CONCLUSION.** Implementation of an antimicrobial stewardship program in intensive care is feasible, cost-effective and safe. The most common intervention was de-escalation, with a high adherence rate to the proposed recommendations.

**REFERENCE(S)**

1. Pickens CI and Wunderink RG (2019) Principles and Practice of Antibiotic Stewardship in the ICU. Chest PII:S0012-3692(19)30064-9 [Epub ahead of print]
2. Garnacho-Montero J, Escoresca-Ortega A and Fernández-Delgado E (2015) Antibiotic de-escalation in the ICU: how is it best done? Curr Opin Infect Dis 28(2):193-8
3. Rodríguez-Baño J, Paño-Pardo JR, Alvarez Rocha L et al (2012) Programs for optimizing the use of antibiotics (PROA) in Spanish hospitals: GEIH-SEIMC, SEFH and SEMPSPH consensus document. Enferm Infecc Microbiol Clin 30(1):22.e1–22.e23

### 000038

#### Effect of macrolide treatment in mortality in patients with severe pneumonia due to influenza virus infection

L. claverias[1], E. Papiol[2], L. Vidaur[3], E. Díaz[4], J. Marín-Corral[5], M. Bodí,[6], L. Reyes[7], I. Martín-Loeches[8], M. Restrepo[9], A. Rodriguez[6]

[1]UCI, Hospital Verge de la Cinta, Tortosa, Spain; [2]Uci, Hospital Universitari Vall d'Hebron, Barcelona, Spain; [3]Uci, Donostia Unibertsitate Ospitalea, Donostia, Spain; [4]Uci, Hospital Parc Taulí, Sabadell, Spain; [5]Uci, Hospital del Mar, Barcelona, Spain; [6]Uci, Hospital Universitari de Tarragona Joan XXIII, Tarragona, Spain; [7]Medicine, Universidad de La Sabana, Chía, Colombia; [8]Uci, St. James's Hospital, Dublin, Ireland; [9]Uci, South Texas Veterans Health Care System, San Antonio, United States of America

**Correspondence:** L. claverias

*Intensive Care Medicine Experimental* 2019, **7(Suppl 3):**000038

**INTRODUCTION.** despite macrolides are widely used in chronic respiratory diseases such as cystic fibrosis and chronic obstructive pulmonary disease because of its anti-inflammatory and immunomodulator effects, its use in acute pulmonary infection remains controversial.

**OBJECTIVES.** the main objective of our study was to evaluate the impact in prognosis of macrolide treatment in critical care patients with severe viral pneumonia.

**METHODS.** prospective, cohorts and multicenter study in critically ill patients with confirmed viral pneumonia. Study period went from June 2009 to April 2018, and 181 Intensive Care Units participated. Patients admitted to an ICU due to confirmed influenza pneumonia were included. Demographic, clinic and analytic variables, comorbidities, level of severity according to Acute Physiology and Chronic Health Evaluation II (APACHE II) score and antibiotic treatment received were registered, among other variables. We only considered patients who received combined empiric antibiotic therapy and compared those who received combined therapy with macrolides and those who received other combinations. Differences between groups were assessed using Chi-square and T-Student test as appropriate. We performed a multivariate analysis by binary logistic regression to determine which variables were independently associated with mortality. Significant p<0,05.

**RESULTS.** Of the 4175 patients who presented confirmed viral pneumonia, 2962 (70,9%) received combined empiric antibiotic treatment. Mean age was 54 years with a mean APACHE II score of 17. Global mortality was 22,5%, and it was significantly lower in the group of patients who received macrolides (17,5%) comparing to the ones that received other combinations (25,1%; p<0.05). Receiving macrolides was independently associated with a lower risk of mortality after adjusting the regression logistic model according to age, APACHE II score, need of mechanical ventilation, presence of shock, comorbidities and corticosteroid treatment (OR= OR 0,75, IC 95% 0,60-0,93; p=0,008). Bacterial coinfection was present in 702 patients. Mortality in this group was 29,3% and having received macrolides was not associated with lower risk of mortality (OR 0,92, IC 95% 0,61-1,38, p=0,68). On the other hand, the group of 2260 patients who presented primary viral infection showed lower risk of mortality when receiving combined antibiotic treatment with macrolides (OR= 0,69, IC 95% 0,54-0,89; p=0,005).

**CONCLUSION.** our results suggest there is a beneficial effect in macrolides treatment in patients with influenza virus infection. This effect does not seem to be related with its antibacterial activity. Its possible immunomodulatory effect in this specific population should be addressed in future studies.

### 001409

#### Right Dose, Right Now - Developing a clinical dosing algorithm for AutoKinetics, a clinical decision support system for personalised antibiotic dosing

L. Roggeveen[1], T. Guo,[1], L. Fleuren[1], PJ. Thoral[1], A. Girbes, PHJ. Van Der Voort[2], RJ. Bosman[2], PWG. Elbers[1]

[1]Intensive care medicine, Amsterdam UMC, locatie VUmc, Amsterdam, Netherlands; [2]Intensive care medicine, OLVG location East, Amsterdam, Netherlands

**Correspondence:** L. Roggeveen

*Intensive Care Medicine Experimental* 2019, **7(Suppl 3):**001409

**INTRODUCTION.** Antibiotic dosing in critically ill patients is difficult because of altered pharmacokinetics (PK) [1,2]. Population PK model based dose adaptation plays an important role in optimizing antibiotic dosing due to its ability to include covariate effects and PK variability. Population PK models are able to describe the time course of drug concentration after administration of a dose. Further, PK models can predict concentrations following the dosage regimens under consideration. These techniques are increasingly applied in clinical care though typically require specialized expertise and software [3]. Moreover, the use of software based dose adaptation has to date not been widely investigated and trials are eagerly anticipated [4]. We developed a clinical decision support system for realtime bedside personalised dosing, which is being evaluated in an ongoing randomised controlled trial. For this, we required an automated clinical dosing algorithm to determine the recommend dosage regimen.

**OBJECTIVES.** To develop a clinical dosing algorithm based on PK models to generate real-time antibiotic dosing advices that can be implemented in a software package for personalised antibiotic dosing in critically ill patients.

**METHODS.** Dose recommendations are based on pharmacokinetic principles. The goal is to inversely find a dose which is able to maintain a needed PK exposure of the target given a dosing time schedule. An important presumption that is made in dose calculation is the linearity of PK. Accordingly, PK exposure is proportional to the dose. Thus, if one measures the area under the concentration-time curve (AUC) for a 500 mg dose, one can estimate the AUC for a 750 mg dose in the same patient as being 50% greater. The Target maintenance dose (MD) can be calculated using the following equation:

$$MD_{\text{target}} = \frac{AUC_{\text{target}}}{AUC_{test}} \cdot MD_{test} \quad (1)$$

Particularly, when the clearance (CL) of a patient is expected consistent over the time period of interest, the target maintenance dose can also be calculated through the following equation:

$$MD_{\text{target}} = AUC_{\text{target}} \cdot CL \quad (2)$$

For adaptive dosing advice it is also required to calculate a loading dose. The design of loading dose (LD) calculation is to derive a dose that enables the concentration right after the administration to achieve the target maximum concentration (Cmax) of MD-maintained steady state. Lastly, a dosing interval needs to be chosen.

**RESULTS.** Based on these pharmacokinetic principles we devised an iterative method for deriving a clinical dosage regimen. The final advice consists of a start time, if needed a loading dose, a maintenance dose and a dose interval. They are incorporated in a closed looped algorithm while taking safety precautions into account.

**CONCLUSION.** We successfully developed a clinical dosing algorithm. It is incorporated in AutoKinetics, a clinical decision support system for personalised antibiotic dosing. It alllows for automated adaptive antibiotic dosing regimes. if proven effective in improving clinical outcomes, it could pave the road forward towards true personalised antibiotic dosing.

**REFERENCE(S)**

1. Elbers PWG et al. Right dose, right now: using big data to optimize antibiotic dosing in the critically ill. Anaesthesiol Intensive Ther 2015;47(5):457-463
2. Roberts JA, et al. DALI: Defining antibiotic levels in intensive care unit patients: Are current ß-lactam antibiotic doses sufficient for critically ill patients? Clin Infect Dis. 2014;58(8):1072-1083.
3. Fuchs A, Csajka C, Thoma Y, Buclin T, Widmer N. Benchmarking Therapeutic Drug Monitoring Software: A Review of Available Computer Tools. Clin Pharmacokinet. 2013;52(1):9-22. doi:10.1007/s40262-012-0020-y
4. Roberts JA, Roger C, De Waele JJ. Personalized antibiotic dosing for the critically ill. Intensive Care Med. January 2019:1-4. doi:10.1007/s00134-019-05522-3

5. This work has been partially funded by the ZonMw Rational Pharmacotherapy program

### 001763

#### PCR of MRSA: a tool to improve antibitotics stewardship in our hospital

R. Carmona[1], M. Rodriguez[2], S. Cárcel,[2], M. Bueno,[2], I. Ben,[2], F. Onieva,[2], J. Vilches,[2], RM. Perez,[2], M. Dealba,[2], C. De La Fuente Martos[2]

[1]Medicina Intensiva , Reina Sofia University Hospital, Córdoba, Spain; [2]Medicina intensiva, Reina Sofia University Hospital, Córdoba, Spain

**Correspondence:** R. Carmona

*Intensive Care Medicine Experimental* 2019, **7(Suppl 3):**001763

**INTRODUCTION.** Ventilator-associated pneumonia due to methicillin-resistant Staphylococcus aureus (MRSA) is associated with excess mortality and costs. Molecular biology test allows rapid identification of MRSA. Our hospital reaches an incidence of 28% of infection by MRSA and is responsible for a high cost in the intensive care unit. Clinical suspicion of VAP is the most frequent cause to use Linezolid as a first antibiotics treatment, in spite of Guidelines recommend considering methicillin-resistant Staphylococcus aureus (MRSA) just in case of hight suspicion of infections, according to the bacterial ecology of the unit. Initially designed to identify MRSA in nasal secretions, its use has been then validated in bronchial aspirations. We hypothesized that use of a rapid diag- nostic test was associated with a decreased use of anti-MRSA antibiotics prescribed for patients with suspected VAP. Using this diagnostic test, we checked how we saved on the cost of antibiotics. This is important not only in what implies a lower cost, since linezolid resistant microorganisms appear every time, posing a challenge for the clinical doctor and serious infections in ICU.

**METHODS.** We carry out this observational, descriptive and retrospective study. For this, we chose a total of 50 patients admitted to the ICU for any pathology during January 2016 and December 2017 in our intensive care unit. (Reina Sofía University Hospital, Córdoba, Spain) that had developed NAVM and which had been requested PCR in respiratory samples for etiological diagnosis. Age, mortality in the ICU, the date on which the CRP was requested, the APACHE II scale, SOFA, the days of ICU stay, the days of MV and the antibiotic therapy before and after the PCR outcome of the study were collected as variables.

**RESULTS.** 50 patients admitted during 2016 and 2017 in our ICU, the median age was 56 years and 34 were men (68%) and 16 women (32%). The median length of stay in the ICU was 16 (2-117). Mortality during admission was 48%. We use the APACHE II and SOFA scales as prognostic scales, being 20 and 5 the mean, respectively. The average of the days of mechanical ventilation were 19. With respect to the result of the diagnostic tests by PCR, in 46 of them no bacterial growth was obtained, a sample of MRSA and 3 of MSSA were isolated. The initial antibiotic regimen that included Linezolid was administered in 13 patients prior to the PCR result, of which, it was substituted after the result of the test in 7 patients.

**CONCLUSION.** The use of PCR as a rapid diagnosis of VAP caused by MRSA resulted in a total cessation of the administration of broad-spectrum antibiotic therapy in a patient whose result was negative. The rapidity with which the microbiological diagnosis is obtained with this type of technique, led us to diagnose in a short period of time that the cause of the pneumonia associated with the ventilator was not MRSA, and therefore, de-escalate antibiotic therapy, with the consequent cost savings and improving the usual clinical practice before the remarkable increase of new resistance against these antibiotics at present.

1. Rapid diagnostic test and use of antibiotic against methicillin-resistant Staphylococcus aureus in adult intensive care unit A.-F. Dureau 1 & G. Duclos 1 & F. Antonini 1 & D. Boumaza 1 & N. Cassir 2 & J. Alingrin 1 & C. Vigne 1 & E. Hammad 1 & L. Zieleskiewicz 1 & M. Leone

### 001406

#### Changes in antibiotic consumption structure and antibiotic resistance level in ICU as a result of 5 year-long antimicrobial stewardship implementation

M. Dementienko[1], V. Gusarov[1], D. Kamyshova[2], E. Gritsenko[1], M. Zamyatin[1]

[1]Icu, National Medical and Surgical Center named after N.I.Pirogov, Moscow, Russia, Russia; [2]Clinical pharmaology, National Medical and Surgical Center named after N.I.Pirogov, Moscow, Russia, Russia

**Correspondence:** M. Dementienko

*Intensive Care Medicine Experimental* 2019, **7(Suppl 3):**001406

**INTRODUCTION.** The results of numerous studies on the implementation of antibiotic stewardship programs (ASP), including intensive care units (ICUs), provide conflicting data on their effect on antimicrobial resistance, antibiotic consumption and secondary outcomes [1-3].

**OBJECTIVES.** To assess the effect of ASP on antibiotic consumption, antimicrobial resistance and clinical outcomes in patients with infection in the ICU.

**METHODS.** A prospective interventional single-center study was conducted in the period from January 2011 to December 2017. The intervention: ASP, including multidisciplinary team building, antimicrobial therapy and prophylaxis protocols, infection control measures, personnel training and internal audit. The data from 2012 (pre-intervention) and 2017 (intervention) reports were compared. We assessed consumption of antibiotics in the ICU, antibiotic therapy duration, drug resistance index (DRI) of gram-negative ESKAPE pathogens in the ICU, the share of ESKAPE-bacteremia and candidemia in the ICU, length of hospital stay (LOS) and mortality of the patients with infection, complicated by bacteremia in the ICU.

**RESULTS.** Total consumption of antibiotics has not significantly changed: 227.3 from 238.6 DDD/100 days. Meanwhile, the structure of consumption significantly changed with the decrease in antibiotics for the treatment of the following infections (DDD/100 days): carbapenems from 46.4 to 34.4, aminoglycosides from 10.0 to 2.2, fluoroquinolones from 29.8 to 21.3. The consumption of 1st generation cephalosporins for perioperative antibiotic prophylaxis increased from 4.2 to 98.1. The average duration of therapy in patients with infection in the ICU decreased from 15.7 to 10.4 days, p<0.01, with bacteremia from 22.7 to 12.8, p<0.001. The dynamics of changes in the DRI of gram-negative ESKAPE pathogens in the ICU and the share of ESKAPE-bacteremia and candidaemia in the ICU are shown in Figures 1 and 2. LOS for patients with an infection complicated by bacteremia decreased from 48.8 days to 35.4 days, p = 0.039, Hospital mortality of ICU patients decreased from 38,8% to 31,5%, p<0.536.

**CONCLUSION.** The introduction of ASP into the ICU leads to a significant change in the structure of antibiotic consumption shortening the average duration of antibiotic therapy for infections including those complicated by bacteremia. This leads to the reduction of prevalence and antibiotic resistance of nosocomial pathogens, but does not lead to a significant improvement in secondary outcomes.

**REFERENCE(S)**

1. Bouadma L, et al. Use of procalcitonin to reduce patients' exposure to antibiotics in intensive care units (PRORATA trial): a multicentre randomised controlled trial. Lancet 2010; 375: 463-474 [PMID: 20097417 DOI: 10.1016/S0140-6736(09)61879-1]
2. Jensen JU, et al. Procalcitonin-guided interventions against infections to increase early appropriate antibiotics and improve survival in the intensive care unit: a randomized trial. Crit Care Med 2011; 39: 2048-2058 [PMID: 21572328 DOI: 10.1097/CCM.0b013e31821e8791]
3. Ramsamy Y, et al. Microbiological surveillance and antimicrobial stewardship minimise the need for ultrabroad-spectrum combination therapy for treatment of nosocomial infections in a trauma intensive care unit: an audit of an evidence-based empiric antimicrobial policy. S Afr Med J 2013; 103: 371-376 [PMID: 23725954 DOI: 10.7196/samj.6459]

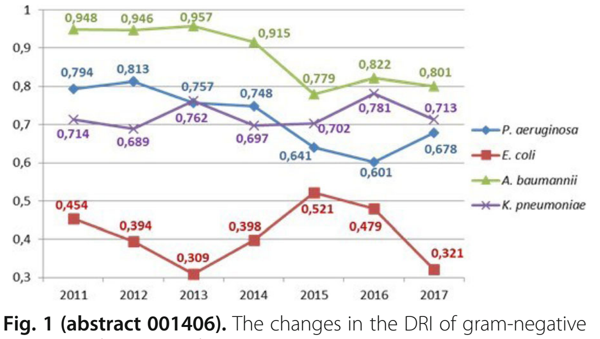

**Fig. 1 (abstract 001406).** The changes in the DRI of gram-negative ESKAPE pathogens in the ICU.

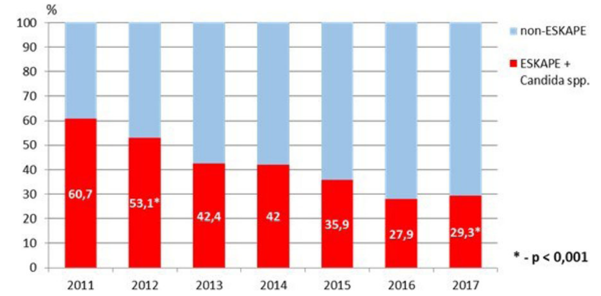

**Fig. 2 (abstract 001406).** The changes in the share of ESKAPE-bacteremia and candidaemia in the ICU.

### 000942

#### Effect of the Antimicrobial Stewardship Program on the pediatric patients of ENVIN-HELICS data base

G. Armero[1], E. Fresan[1], S. Bobillo[1], A. Bustinza[2], P. Garcia[3], Y. Peña[4], I. Jordan Garcia[1]

[1]Picu, H Sant Joan de Déu, Barcelona, Spain; [2]Picu, H Gregorio Marañon, Madrid, Spain; [3]Picu, Carlos Haya Malaga, Málaga, Spain; [4]Picu, H Valle Hebron, Barcelona, Spain

**Correspondence:** I. Jordan

*Intensive Care Medicine Experimental* 2019, **7(Suppl 3):**000942

**INTRODUCTION.** Health care associated infections (HAI) suspicious require immediately empiric antibiotic treatment, most of times with broad spectrum antibiotics. The implementation of Antimicrobial Stewardship Program (ASP) seems to be a useful way to improve antibiotic management, also in HAI.

**OBJECTIVES.** To compare the evolution of antibiotics used for HAI in Paediatric Intensive Care Units (PICU) from the Spanish registry Paediatric-ENVIN-HELICS, after a progressive ASP implementation.

**METHODS.** Multicentre, prospective and observational study. HAI diagnosed in 24 Spanish PICU, from April to June of 2013–2017, were included. The ENVIN diagnostic criteria adapted to paediatrics were used, based on CDC recommendations. ASP was consensuated and recommended, with progressive implementation along 2014-2017. SPSS®21 programme was used.

**RESULTS.** The total number of patients included in 5 years was 8717. Eight PICU (32%) had an Antimicrobial Stewardship Program (ASP) in 2017 compared with none in 2013 (p=0.000). The rate of antibiotics use progressively decreased (4%, p=0.0179).

In 2017 the number of antibiotics indicated per patient was 1.34 and per patient with antibiotics was 1.81. Whereas in 2013 that number was greater: 1.6 and 2.06, respectively.

Regarding the antibiotic indicated for HAI previous PICU admission, there was an increase on meropenem use of 1.64% (not statistically significant), while the use of piperacillin-tazobactam significantly decreased (4.97%, p=0.0123). However, meropenem indication for HAI in PICU patients dropped down (4.62%, p=0.05).

During 2017, the practice of antibiotic stewardship was 13.93% higher (p=0.0048). There was also an increasing tendency for early suspension antibiotic rate (6.78%, not statistically significant). The results also showed a decrease of antibiotic modification due to adverse event (1.19%, p=0,0021), compared with 2013.

**CONCLUSION.**

1. The rate of antibiotics use was high, with almost 2 antibiotics indicated per patient, but results showed a decreasing trend during 2017.
2. The implementation of ASP in PICU probably has led to a better use of carbapenems, and to an increase of antibiotic de-escalation and early suspension rate.
3. Modifications of the antibiotic regime due to adverse event have decreased.

REFERENCE
1. Spanish Society of Pediatric Intensive Care Medicine

### 001062

#### Antimicrobial stewardship from the ICU to the ICU. Impact on antifungal use in critically ill patients

A. Cercos[1], S. Sancho[2], V. Ramirez[2], J. Camarena[3], R. González[3], D. Illa[2], E. Lopez[2], R. Zaragoza[2]
[1]Pharmacy, Hospital Universitario Doctor Peset, València, Spain; [2]Intensive care unit, Hospital Universitario Doctor Peset, Valencia, Spain; [3]Microbiology, Hospital Universitario Doctor Peset, Valencia, Spain, Spain
**Correspondence:** R. Zaragoza
*Intensive Care Medicine Experimental* 2019, **7(Suppl 3):**001062

**INTRODUCTION.** Although antifungal de-escalation therapy has been previously studied in critically ill patients, few data has been provided about its consequences on antifungal use in this setting

**OBJECTIVES.** The aim of this study was to analyze the impact of an Antimicrobial Stewardship Program (ASP) on antifungal consume from ICU to ICU.

**METHODS.** Retrospective study during 24 months from April 2016 to June 2018 with a pre-post intervention design in two ICU in an unique universitary hospital. Medical (16 beds) and Surgical (6 beds) ICUs were included. Preintervention period (Pre ASP implementation) lasted from April 2016 to March 2017 and post implantation period from July 2017 to June 2018. All patients treated with Liposomal amphotericine B, anidulafungin, caspofungin, micafungin, voriconazole and posaconazole were analyzed.in both periods. Clinical and microbiological variables were recorded. Daily doses DDD/100 stays and economic impact (expressed in euros) of the intervention were also studied. Wilcoxon Rank-sum test was performed to compare the pre and post consume of prescribed antifungal drugs.

**RESULTS.** In Medical ICU there were a clearly decrease of DDD/ 100 stays of all the antifungals studied : posaconazole (100%; 0,16 vs. 0,00), liposomal amphotericine B (93,6%; 14,81 vs. 0,95), micafungine (73%; 0,10 vs. 0,03), anidulafungin (54%; 0,98 vs. 0,45), voriconazole (28%; 1,64 vs. 1,18) and caspofungin (11%; 3,63 vs. 3,25). In Surgical ICU, a clearly reduction of DDD/100 stays was also observed in liposomal amphootericine B (100%; 2,18 vs. 0,00), micafungine (61%; 5,87 vs. 2,31), caspofungin (55%; 12,73 vs. 5,77) and anidulafungine (41%; 11,07 vs. 6,51), whereas voriconazole experimented an increased use (18%; 0,89 vs. 1,94). Posaconazole was not used in SICU in the study period

In economical terms , median of cost was reduced with statistical significance 70% in Medical ICU (10.859,4 (5.083,30-19.785,35) vs. 3.154,15 (1.745,45-6.380,28); p=0,042) and 58% in Surgical ICU (16.041,85 (14.601,75-19.762,75) vs. 6.789,45 (5.382,35-9.630,00); p= 0,0005). As a consequence a cost saving of 67,4% in the antifugal consume in Medical ICU (associated saving cost of 94.500 euros) and 58% (associated saving cost of 115.803 euros) in surgical ICU. A global reduction of cost of 210.303 euros in a year was obtained in critically ill patients setting.

**CONCLUSION.** ASP from ICU to ICU had an exceptional impact on antifungal use and was associated with a real, significant and important cost saving.

REFERENCE(S)
1. Garnacho-Montero J, Díaz-Martín A, Cantón-Bulnes L, Ramírez P, Sierra R, Arias-Verdú D, Rodríguez-Delgado M, Loza-Vázquez A, Rodriguez-Gomez J, Gordón M, Estella Á, García-Garmendia JL. Initial Antifungal Strategy Reduces Mortality in Critically Ill Patients With Candidemia: A Propensity Score-Adjusted Analysis of a Multicenter Study. Crit Care Med. 2018 Mar;46(3):384-393.
2. None

### 000086

#### Sepsis and septic shock in onco-haematological patients: a Groupe de Recherche en Réanimation Respiratoire en Onco-Hématologie (GRRR-OH) study

S. Pons[1], V. Lemiale[2], E. Azoulay[2], D. Mokart[3], JP. Mira[4], A. Kouatchet[5], J. Mayaux[6], M. Nyunga[7], F. Bruneel[8], P. Perez[9], AP. Meert[10], E. Borcoman[2], Y. Hourmant[2], M. Bisbal[11], M. Adda[2], M. Legrand[12], D. Benoit[13], M. Darmon[2], L. Zafrani[2]
[1]U976, INSERM, Paris, France; [2]Medical icu, Hôpital Saint-Louis, Paris, France; [3]Medical icu, Institute Paoli-Calmettes, Marseille, France; [4]Medical icu, Hospital Cochin, Paris, France; [5]Medical icu, C.H.U d'Angers, Angers, France; [6]Respiratory and medical icu, University Hospitals Pitié Salpêtrière - Charles Foix, Paris, France; [7]Icu, CH Roubaix, Roubaix, France; [8]Icu, C.H. de Versailles, Le Chesnay, France; [9]Medical icu, CHRU de Nancy - Hôpitaux de Brabois, Vandœuvre-lès-Nancy, France; [10]Icu, Institut Jules Bordet, Bruxelles, Belgium; [11]Anesthesiology and critical care, Institute Paoli-Calmettes, Marseille, France; [12]Anesthesiology and critical care and burn unit, Saint-Louis Hospital, Paris, France; [13]Icu, Ghent University Hospital, Gent, Belgium
**Correspondence:** S. Pons
*Intensive Care Medicine Experimental* 2019, **7(Suppl 3):**000086

**INTRODUCTION.** Cancer affects up to 20% of critically ill patients, and sepsis is one of the leading reasons for ICU admission in this setting [1, 2]. No recent formal estimation of outcome changes has however been performed. Studies to appraise outcomes in critically ill cancer patients with sepsis are warranted.

**OBJECTIVES.** To assess trends in survival rates in cancer patients admitted to the intensive care unit (ICU) for sepsis or septic shock over the last two decades.

**METHODS.** Data from onco-haematological patients admitted to the ICU for sepsis or septic shock were extracted from the GRRR-OH database (1994-2015). A hierarchical model taking into account the year of inclusion and the source dataset as random variables were used to identify risk factors for day 30 (D-30) mortality. A sensitivity analysis within patients with bacterial pneumonia at ICU admission was performed.

**RESULTS.** Overall, 2062 patients were included in the study. 1225 (42%) patients were male and the median age was 59 [48-67] years. Underlying malignancies included solid tumors (n= 638; 31%), acute leukemia (n=591; 29%), non-Hodgkin lymphoma (n= 461; 22%) and myeloma (n=244; 12%). Two hundred and fifty patients (12%) underwent allogenic stem cell transplantation and 640 (31%) were neutropenic at ICU admission. Death at day 30 occurred in 812 (39%) patients. After adjustment on SOFA score at ICU admission, the year of ICU admission was significantly associated with mortality, decreasing over time (OR 0.96; 95%CI 0.94-0.98) (**Figure 1**). Mechanical ventilation (OR 3.43; 95%CI 2.73-4.32), vasopressor use (OR 1.51; 95%CI 1.20-1.90) and renal replacement therapy (OR 1.40; 95%CI 1.10-1.78) were independently associated with D-30 mortality, whereas the type of underlying malignancy, allogenic stem cell transplantation and neutropenia were not. Sensitivity analysis in the subgroup of patients with bacterial pneumonia (n=858) found similar results.

**CONCLUSION.** Survival in critically ill oncology and hematology patients with sepsis improves significantly over time. Studies are needed to identify targets to further improve survival in this growing subgroup of ICU patients.

REFERENCE(S)
1. Vincent J-L, Rello J, Marshall J, et al (2009) International study of the prevalence and outcomes of infection in intensive care units. JAMA 302:2323–2329. https://doi.org/10.1001/jama.2009.17542. Rosolem MM, Rabello LSCF, Lisboa T, et al (2012) Critically ill patients with cancer and sepsis: clinical course and prognostic factors. J Crit Care 27:301–307. https://doi.org/10.1016/j.jcrc.2011.06.014

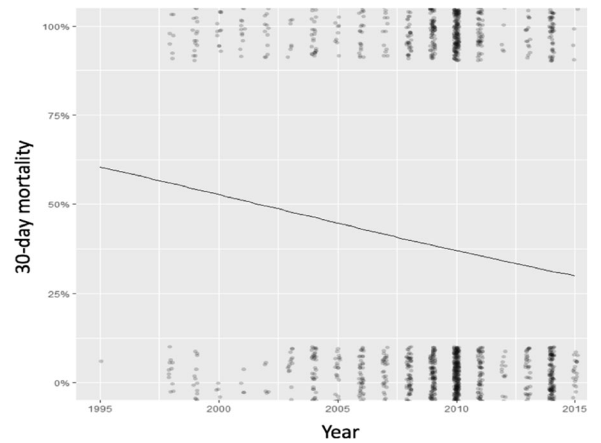

**Fig. 1 (abstract 000086).** See text for description

#### 000275

**Use of intrapulmonary administration of thrombin in hematological malignancy patients with alveolar hemorrhage**

N. JEONG, J. Lee, C. Rhee
Division of pulmonary, allergy and critical care, seoul st. mary's hospital, college of medicine, The Catholic University of Korea Songeui Campus, Seoul, Republic of Korea
**Correspondence:** N. JEONG
*Intensive Care Medicine Experimental* 2019, **7(Suppl 3):**000275

**INTRODUCTION.** Alveolar hemorrhage (AH) is characterized by the acute onset of alveolar bleeding and hypoxemia and can be fatal. Thrombin has been widely used to achieve coagulation and hemostasis. However, the efficacy of thrombin in patients with AH is unclear. Thus, this study aimed to evaluate the efficacy of thrombin administration in patients with hematological malignancy and AH.

**METHODS.** This retrospective study included 15 hematological malignancy patients (eight men and seven women; mean age 47.7 ± 17.3 years) with AH who were administered intrapulmonary thrombin between March 2013 and July 2018. All patients received bovine-origin thrombin (1,000 IU/ml, Reyon Pharmaceutical Co., Ltd., Seoul, Korea) via a fiberoptic bronchoscope. A maximum of 15 ml of thrombin was injected via the working channel to control bleeding. The ability of thrombin to control bleeding was assessed. Additionally, the change in the PaO2/FiO2 (PF) ratio after intrapulmonary thrombin administration was evaluated.

**RESULTS.** Intrapulmonary thrombin was administered a minimum of 3 days after starting mechanical ventilation in all patients, and it immediately controlled the active bleeding in 13 of 15 patients (86.7%). However, AH relapse was noted in 3 of the 13 patients (23.1%). The PF ratio improved in 10 of 15 patients (66.6%), and the mean PF ratio was significantly higher after thrombin administration than before administration (p = .03). No adverse thromboembolic complications or systemic adverse events were observed.

**CONCLUSION.** Thrombin administration was effective in controlling bleeding in hematological malignancy patients with AH. Intrapulmonary thrombin administration might be a good therapeutic option for treating AH.

**REFERENCE(S)**

1. Kinoshita M SR, Wagai F, Watanabe H, Kitamura S. Thrombin instillation therapy through the fiberoptic bronchoscope in cases of hemoptysis. Japan J Thorac Dis. 1982;20(3):251-254.
2. Heslet L, Nielsen JD, Levi M, Sengelov H, Johansson PI. Successful pulmonary administration of activated recombinant factor VII in diffuse alveolar hemorrhage. Crit Care. 2006;10(6):R177.
3. Sharma SK, Kumar S, Singh AK, et al. Diffuse alveolar hemorrhage following allogeneic peripheral blood stem cell transplantation: a case report and a short review. Indian J Hematol Blood Transfus. 2014;30(1):41-44.
4. Escuissato DL, Warszawiak D, Marchiori E. Differential diagnosis of diffuse alveolar haemorrhage in immunocompromised patients. Curr Opin Infect Dis. 2015;28(4):337-342.

### CD - Cardiac problems and extracorporeal management

#### 000316

**Surgical reoperation as a complication after cardiac surgery**

A. RUIZ, R. Prieto, M. Sevilla, S. Ramiro, M. Garcia
INTENSIVE CARE UNIT, Hospital Universitario Virgen de las Nieves Hospital General Urgencias, Granada, Spain
**Correspondence:** A. RUIZ
*Intensive Care Medicine Experimental* 2019, **7(Suppl 3):**000316

**INTRODUCTION.** Surgical reoperation as a complication after cardiac surgery. ARIAM-Andalusia Registry.

**OBJECTIVES.** To analyze the clinical profile and evolution of reoperated patients after cardiac surgery, as wellas the main causes for reoperation.

**METHODS.** Retrospective study of patients undergoing cardiac surgery in our hospital during5 years (January-2014 to December-2018), and included in the ARIAM-Andalusia registry. We excludedpatients undergoing TAVI. The demographic and surgical characteristics, causes of reoperation and themoment of it, complications and mortality are collected. The quantitative variables are expressed as x +sd and the qualitative ones as number and percentage. T student was made for comparing v.quantitativeand X2 for v.qualitative, and a multivariate analysis of risk factors for reoperation.

**RESULTS.** 2152 patients were included during the period of the study. 116 of them (5.4%) were reoperatedat the same hospital admission. The re-operated patients had longer surgical times (ECC: 136 + 60 minvs 115 + 50 min, p <0.05) and higher severity scores (Euroscore I: 13.8 + 13.7 vs 9.7 + 11.3, p <0.05)than the non-reoperated ones. When initial emergent surgery happened, reoperation was more frequent(11.2%). Valve or aortic surgery was a risk factor for reoperation (OR 2.7, 95% CI 1.6-4.6). Mortality wassignificantly higher in reoperated patients (28.4% vs 9.9%, p <0.001), and similarly there were higherrates of renal failure (75.2% vs 48.9%, p <0.05) and prolonged times for mechanical ventilation (62.1 vs15.2). %, p <0.005). Early reoperation (<48 hours) was performed in 62 patients (53.4%), beingtamponade (64.5%) and mediastinal bleeding (30.6%) the most frequent causes. In patients whounderwent a delayed reintervention (> 48 hours), tamponade (46.3%), sternal dehiscence (24.1%) andvalvular problems (14.8%) were the most important causes. There were no differences in age, sex, timeor mortality between the two groups; but early reoperation was more frequent after an emergent surgery(18.3% vs 5.6%, p = 0.05).

**CONCLUSION.** Reoperation after cardiac surgery associates high mortality and more complications, beingtamponade and mediastinal bleeding the most frequent causes.

1. ARIAM- Andalusia Registry

#### 000352

**Alveolar Dead space fraction and arterial saturation predict postoperative course in Fontan patients**

E. Shostak[1], O. Schiller[1], G. Amir[2], G. Frenkel[2], T. Shochat[3], O. Dagan[1]
[1]Pediatric Cardiac Intensive care unit, Schneider Children's Medical Center of Israel, Petah Tikva, Israel; [2]Pediatric cardiothoracic surgery division, Schneider Children's Medical Center of Israel, Petah Tikva, Israel; [3]Epidemiology, Rabin Medical Center, Petah Tikva, Israel
**Correspondence:** E. Shostak
*Intensive Care Medicine Experimental* 2019, **7(Suppl 3):**000352

**INTRODUCTION.** The Fontan surgery, the 3rd procedure in single-ventricle palliation, redirects systemic venous blood into the pulmonary circulation for gas exchange [1]. A decrease in pulmonary blood flow can lead to major complications and grave outcome. Even though data shows that these patients benefit from negative pressure ventilation [2, 3], a delicate balance between sedation & analgesia, pharmacological cardiac support and early extubation may be difficult to achieve. To date there are neither validated protocols nor auxiliary tests to predict tolerability of early extubation and guide clinicians with early postoperative management. Alveolar dead-space fraction (AVDSf) represents the portion of inhaled air that does not participate in gas exchange and quantifies ventilation-perfusion abnormalities in the lung[4]. Increased AVDSf has been associated with prolonged mechanical ventilation and worse outcome after congenital heart surgery [5, 6]. The association of AVDSf with clinical outcomes in patients undergoing Fontan operation has not been reported.

**OBJECTIVES.** Our goal was to find AVDSf and arterial saturation cut-off values which predicts bad post-operative outcome

**METHODS.** A retrospective charts review of all pediatric Fontan patients from 6/2010-11/2018 in a tertiary-care pediatric hospital was performed. Associations between AVDSf and SaO2 to a composite outcome (need for surgical\catheter-based intervention, ECMO use, prolonged ventilation, prolonged hospital LOS or death) were explored. Secondary endpoints were parameters of severity of illness, chest drainage duration, and LOS

**RESULTS.** Of 128 patients undergoing Fontan operation, 34 patients met criteria for composite outcome. AVDSf was significantly higher in the composite groups (0.33±0.14) versus control group (0.25±0.26; p=0.016). AVDSf ≥0.29 indicated a 37% increase in the risk to meet composite criteria. Admission SaO2 was significantly lower in composite versus the control group (93.4% Vs 97.1%, p=0.005). AVDSf was significantly associated with increased durations of mechanical ventilation, ICU LOS, duration of thoracic drainage, and parameters of severity of illness

**CONCLUSION.** We found that high AVDSf and low SaO2 are associated with more complicated postoperative course in children undergoing the Fontan operation. AVDSf value≥0.29 or arterial saturation≤86% should raise concern that the patient may have complex postoperative course

**REFERENCE(S)**

1. Fontan, F. and E. Baudet, Surgical repair of tricuspid atresia. Thorax, 1971. 26(3): p. 240-8.
2. Mac, L., et al., Cardiopulmonary interactions after fontan operations. Circulation, 1999. 100(2): p. 211-4.
3. Shekerdemian, L.S., et al., Cardiopulmonary interactions after Fontan operations: augmentation of cardiac output using negative pressure ventilation. Circulation, 1997. 96(11): p. 3934-42.

4.Frankenfield,
D.C., et al., Predicting dead space ventilation in critically ill patients using clinically available data. Crit Care Med, 2010. 38(1): p. 288-91.

5. Bhalla, A.K., et al., Higher Dead Space Is Associated With Increased Mortality in Critically Ill Children. Crit Care Med, 2015. 43(11): p. 2439-45.
6. Shakti, D., et al., Pulmonary deadspace and postoperative outcomes in neonates undergoing stage 1 palliation operation for single ventricle heart disease. Pediatr Crit Care Med, 2014. 15(8): p. 728-34.

### 000355

#### Calculating pulmonary blood flow from CO2 elimination. An in vitro model

KF. Bachmann[1], R. Vasireddy[1], A. Vogt[1], D. Berger[2]

[1]Department of anesthesiology & pain medicine, inselspital, bern university hospital, university, Insel Gruppe, Bern, Switzerland; [2]Department of intensive care medicine, inselspital, bern university hospital, university of bern, Insel Gruppe, Bern, Switzerland

**Correspondence:** K.F. Bachmann

*Intensive Care Medicine Experimental* 2019, **7(Suppl 3):**000355

**INTRODUCTION.** ECMO is an emerging rescue therapy for severe cardiopulmonary failure. ECMO treatment is technically demanding and assessment of native cardiac output is difficult.

**OBJECTIVES.** We performed an in vitro simulation on an ECMO Model to assess whether it is possible to calculate pulmonary blood flow (QLung) from ECMO Blood flow (QECMO) and carbon dioxide elimination (VCO2) by a modified Fick-principle.

**METHODS.** The in-vitro simulator consisted of two perfusate pumps, simulating QECMO and QLung. Two oxygenators (OxyECMO and OxyLUNG) were used to eliminate CO2 and oxygenate the blood. QLung could be diverted into a shunt, bypassing OxyLUNG. CO2 production and O2 consumption were simulated by two oxygenators in series, rinsed with a mixture of CO2 and N2. The system was filled with discarded human blood and heated to 36.5 °C. The experimental protocol consisted of weaning steps with differing shunt values. Exhaust CO2 was measured with a capnometer. VCO2 was calculated as the exhausted fraction of CO2 times gas flow. The modification of the Fick principle Q_total* Δ_(v-ao) CO2 = Q_LUNG* Δ_(v-LA) CO2+ Q_ECMO* Δ_(v-pm) CO2 can be transferred to 〚ΔQ〛_LUNG=〚ΔQ〛_ECMO*(-Δ〚VCO2〛_LUNG)/〚ΔVCO2〛_ECMO. (Δ_(v-ao): veno-aortal, Δ_(v-LA): veno-left atrium, Δ_(v-pm): veno-post membrane). In cases of V/Q ≠1, a normalization factor f was applied to correct VCO2 towards a V/Q of 1: f(V,Q)= (Q*(V/Q+c))/(V*(1+c)). Constant c was calculated from a blood gas sample as a function of temperature T, pH, CO2 solubility and the gas constant R.q

**RESULTS.**

We normalized both VCO2Lung and VCO2ECMO and calculated QLung. Our QLung calculations showed a small bias with wide limits of agreement and good correlation (Figure 1A, r2=0.6372, p=0.0003). Shunt leads to an underestimation of blood flow and decreases correlation (Figure 1B). Normalizing only QECMO increases bias and reduces correlation (Figure 1C, r2=-0.1576, p=0.4230).

**CONCLUSION.** Our simulation shows that simulated pulmonary blood flow calculations are possible from VCO2 measurements within clinically acceptable limits. V/Q mismatch and shunt will decrease the precision of the method. Measurements are readily available and non-invasive. We will perform animal and human trials to further investigate this method.

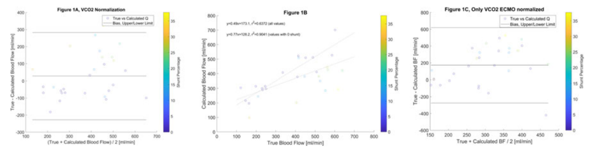

**Fig. 1 (abstract 000355)** See text for description

### 000366

#### Defining vasoplegia following durable, continuous flow left ventricular assist device (CF-LVAD) implantation

J. Swan[1], T. Iso[2], E. Rizk[2], B. Trachtenberg[3], J. Krisl[2], S. Varnado[2], W. Suki[4], A. Frost[4], E. Suarez[5], F. Uddin[6], M. Kassi[3], N. Giesecke[7], A. Bhimaraj[3], F. Masud[6]

[1]Pharmacy; Surgery, Houston Methodist Hospital, Houston, United States of America; [2]Pharmacy, Houston Methodist Hospital, Houston, United States of America; [3]Cardiology, Houston Methodist Hospital, Houston, United States of America; [4]Medicine, Houston Methodist Hospital, Houston, United States of America; [5]Surgery, Houston Methodist Hospital, Houston, United States of America; [6]Critical care, Houston Methodist Hospital, Houston, United States of America; [7]Anesthesiology and critical care, Houston Methodist Hospital, Houston, United States of America

**Correspondence:** J. Swan

*Intensive Care Medicine Experimental* 2019, **7(Suppl 3):**000366

**INTRODUCTION.** Early identification and treatment of vasoplegia following CF-LVAD implantation is imperative to prepare patients for post-surgery recovery. In the absence of a consensus definition of vasoplegia following CF-LVAD, interventions and outcomes can't be reliably assessed.[1-4]

**OBJECTIVES.** This study aimed to construct criteria that would be reproducible, standardized, and associated with clinical outcomes. This study reports incidences and associations with non-favorable discharge disposition (death, hospice, long term acute care, and rehab) for 8 permutations of vasoplegia criteria.

**METHODS.** This retrospective, cohort study included patients who underwent initial CF-LVAD implantation on cardiopulmonary bypass (CPB) at an academic medical center from 08/2016 through 08/2018. Investigators identified 6 considerations for constructing vasoplegia criteria: (1) **Period of observation:** Observation started at CPB termination and lasted for 72 hours; (2) **Unit of time for measurement:** Analysis was conducted at each 15-minute interval; (3) **Clinical constructs:** Three clinical constructs were chosen: low blood pressure using mean arterial pressure (MAP; mmHg) or systemic vascular resistance (SVR; dynesseccm-5), normal cardiac index (CI; L/min/m2), and vasopressor support using norepinephrine equivalents (NEE; mcg/kg/min); (4) **Threshold logic strategies:** Multiple hemodynamic measurements documented within an interval were categorized as having "ever" or "always" crossed the threshold for detection; (5) **Duration**: The number of consecutive intervals where criteria were sustained were analyzed; (6) **Missing data**: The last documented MAP and SVR were carried forward for 3 intervals, and CI for 288 intervals. We evaluated 8 permutations of criteria: two versions, two threshold logic strategies, and two duration cut points. Version 1 used MAP <60 or SVR <800 combined with CI >2.2 and NEE ≥0.1. Version 2 used MAP <60 or SVR <700 combined with CI >2.5 and NEE ≥0.2.

**RESULTS.** This study included 98 CF-LVAD recipients, accounting for 28,224 intervals. Version 1 had a larger incidence and risk for non-favorable discharge compared to version 2. Meeting criteria for ≥2 consecutive intervals decreased incidence but increased risk for non-favorable discharge.

**CONCLUSION.** Version 1 "always" for ≥1 interval and version 1 "ever" for ≥2 consecutive intervals occurred frequently (32% to 40%) and increased risk for non-favorable discharge by 19% to 27%.

**REFERENCE(S)**

1. de Waal EEC, van Zaane B, van der Schoot MM, Huisman A, Ramjankhan F, van Klei WA, Marczin N, (2018) Vasoplegia after implantation of a continuous flow left ventricular assist device: incidence, outcomes and predictors. BMC anesthesiology 18: 185
2. Tecson KM, Lima B, Lee AY, Raza FS, Ching G, Lee CH, Felius J, Baxter RD, Still S, Collier JDG, Hall SA, Joseph SM, (2018) Determinants and Outcomes of Vasoplegia Following Left Ventricular Assist Device Implantation. J Am Heart Assoc 7: 1-9
3. Argenziano M, Choudhri AF, Oz MC, Rose EA, Smith CR, Landry DW, (1997) A prospective randomized trial of arginine vasopressin in the treatment of vasodilatory shock after left ventricular assist device placement. Circulation 96: II-286-290
4. Levin RL, Degrange MA, Bruno GF, Del Mazo CD, Taborda DJ, Griotti JJ, Boullon FJ, (2004) Methylene blue reduces mortality and morbidity in vasoplegic patients after cardiac surgery. Ann Thorac Surg 77: 496-499

5. Dr. Swan received research funding from La Jolla Pharmaceutical Company to support this study.

**Table 1 (abstract 000366).** See text for description

| Version | Threshold logic strategy | Incidence, n (%) (N=98) | Absolute risk difference for non-favorable discharge, % (95% CI) |
|---|---|---|---|
| Criteria met for ≥1 interval | | | |
| 1 | Ever | 60 (61%) | 16% (-4% to 36%) |
| 1 | Always | 31 (32%) | 27% (9% to 44%) |
| 2 | Ever | 27 (28%) | 1% (-21% to 22%) |
| 2 | Always | 14 (14%) | 23% (1% to 45%) |
| Criteria met for ≥2 consecutive intervals | | | |
| 1 | Ever | 39 (40%) | 19% (0% to 37%) |
| 1 | Always | 20 (20%) | 32% (14% to 49%) |
| 2 | Ever | 15 (15%) | 8% (-18% to 33%) |
| 2 | Always | 8 (8%) | 25% (0% to 50%) |

### 000403

### Predictive value for survival in pre-ECMO lactate level of VA ECMO in adult cardiogenic shock

DW. Kim, IS. Jeong,
Thoracic and cardiovascular surgery, Chonnam National University Medical School, Gwangju, Republic of Korea
**Correspondence:** D.W. Kim

*Intensive Care Medicine Experimental* 2019, **7(Suppl 3):**000403

**INTRODUCTION.** The effectiveness of extracorporeal membrane oxygenation for patients with cardiogenic shock is well-established, and lactate is well known as the biochemical biomarker of end organ perfusion. However there were no sufficient data of correlation between pre-ECMO lactate level and survival. We evaluate the efficacy of pre-ECMO lactate level for prediction of survival in patients with cardiogenic shock.

**METHODS.** We respectively reviewed the medical records of patients who underwent ECMO for cardiogenic shock between January 2015 and December 2017. Of these, 79 adult patients underwent the venoarterial ECMO for cardiogenic shock. These patients were divided into survivor and nonsurvivor groups, based on survival to hospital discharge. The patient characteristics in pre-ECMO condition were compared between 2 groups.

**RESULTS.** Mean age was 60.9±14.8 years and the overall survival rate to hospital discharge was 46.8%(n=37). In multivariate analysis, independent predictors of mortality were the pre-ECMO lactate level (OR,1.1703; 95% CI,1.0521-1.3017;P=0.0038). The optimal cut-off value for pre-ECMO lactate was 9.2(AUC 0.696, p=0.0015). Kaplan-Meier survival curves showed that the cumulative survival rate at hospital discharge was significantly higher among patients with pre-ECMO lactate of 9.2 or less compared with patients greater than 9.2 (65.1% versus 28.1%;p=0.0007).

**CONCLUSION.** We conclude that pre-ECMO lactate of 9.2 or less may be an indicator of favorable outcomes with the use of ECMO in adult cardiogenic shock. However it is still difficult to predict survival with only pre-ECMO lactate levels and to determine whether to apply the ECMO for adult cardiogenic shock. Further research on how to predict reversibility more accurately is essential.

**REFERENCE(S)**

1. The journal of Heart and Lung trasplantation. 2017 Apr;36;4. Lactic Acid Is the Most Important Factor Predicting Survival on VA ECMO. A. Kolodziej1, A. Burchett1, T. Tribble1, A.Y. Grigorian2, M. Guglin1
2. J Crit Care. 2017 Dec;42:35-41. Lactate and lactate clearance as valuable tool to evaluate ECMO therapy in cardiogenic shock. Slottosch I1, Liakopoulos O2, Kuhn E2, Scherner M3, Deppe AC2, Sabashnikov A2, Mader N2, Choi YH2, Wippermann J3, Wahlers T2.

### 000406

### Safety and Efficacy of the Gastric Reactance (XL) in Patients Post-operated of Elective Cardiac Surgery: preliminary results

R. Alvarez[1], MDM. Godinez Garcia[2], Y. Guillen[3], MC. Lespron[3], M. Ricaño[1], A. Chavez[1], E. Garcia[1], F. Baranda[1], F. Molina[3], A. Martinez[2], E. Chavez[2], C. Sanchez[4], R. Gaitan[4]
[1]Terapia intensiva cardiovascular, Instituto Nacional de Cardiologia Ignacio Chavez, Mexico City, Mexico; [2]Clinical research department, Alandra Medical, Mexico City, Mexico; [3]Anestesiologia, Instituto Nacional de Cardiologia Ignacio Chavez, Mexico City, Mexico; [4]Biomedical engineering department, Alandra Medical, Mexico City, Mexico
**Correspondence:** M.D.M. Godinez Garcia

*Intensive Care Medicine Experimental* 2019, **7(Suppl 3):**000406

**INTRODUCTION.** The goals of resuscitation in critically ill patients are to ensure adequate tissue perfusion and cellular metabolism [1]. The gut has been hypothesized as the "motor" of critical illness [2,3]. A mucosal injury surveillance system with a special orogastric tube with four electrodes in the distal tip, Florence (Critical Perfusion Inc., California) [4], has been proposed for surveying the integrity of the mucosal tissue of the stomach, in critically ill patients through the reactance biomarker (XL).

**OBJECTIVES.** Evaluate the safety and effectiveness of the XL trend measured by Florence in the prediction of morbimortality (cardiogenic shock, excessive bleeding, vasoplegic syndrome, and death) and with hemodynamic variables (CI, MAP, MPAP, CVP, PCWP), lactate, SVO2, and the risk predictors (EUROSCORE II, STS, APACHE II, and SOFA) of patients post-operated of elective cardiovascular surgery.

**METHODS.** The study was approved by the local ethics committee and regulatory entity (193300912X0085/2019)[5]. Patients with an elective surgery for a coronary artery bypass (CABG) and/or valvular replacement, and complies with the following STS $^{3}$6% or EuroScore II $^{3}$ 6% or LVEF< 45% or TAPSE < 17mm or TSVI < 0.1 m/s, pulmonary artery catheter (PAC) and an orogastric tube were eligible for the study. Subjects with recent gastrointestinal bleeding, paraplegic or hemiplegic, congenital background, maxillofacial malformation, woman in breastfeeding period, were excluded from the study. Following general induction anesthesia and endotracheal intubation, the orogastric tube was positioned in the stomach to measure XL; positioning was confirmed during surgery by aspiration of gastric content and chest radiography after ICU admission. All patients were under cardiopulmonary bypass (CPB), aortic cross-clamp, mechanically ventilated and were monitored during surgery and the first seventy-two postoperative hours or until removal of the orogastric probe during this period. For statistical analysis, we used T-Student and Kruskal-Wallis tests as appropriate considering the significance p< 0.05.

**RESULTS.** 10 patients were included, with mean age 49.30 ± 13.38 years, 6(60 %) male,4 (40%) female, had 7(70%) undergone valvular surgery, 1(10%) CABG surgery and 2(20%) valvular surgery and CABG, ICU length of stay (LOS): 3.97± 3.82 days, Hospital LOS: 8.86 ±6.8 days, ICU mortality: 8.4%. Seven 7(70%) patients developed complications: 4(57%) required vasopressor (more than 48h), 5(71%) had prolonged mechanical ventilation (more than 24h), 2(29%) underwent re-operation due to excessive bleeding (at the first 72h), 3(43%) cardiogenic shock, 4(57%) excessive bleeding, 1(14%) vasoplegic syndrome, and overall mortality was 71%. No significant differences between complications and without complications in age, sex, ICU LOS, CPB time, LVEF @ICU, EuroScore II, STS, APACHE and SOFA was founded. Significant differences between XL, lactate was founded in Post-CPB and in the first 24h at ICU. Lactate and XL trend shows the same behavior in both groups

**CONCLUSION.** The results suggest that XL trend is a relevant biomarker during surgery and the first 24h at ICU and has a similar behavior as lactate. The sample size must be completed to obtain the final results.

**REFERENCE(S)**

1. Ekbal NJ, Dyson A, Black C, Singer M. Monitoring tissue perfusion, oxygenation, and metabolism in critically ill patients. Chest. 2013 Jun;143(6):1799-1808. doi: 10.1378/chest.12-1849. Review. PubMed PMID: 23732592.
2. Mittal R, Coopersmith CM. Redefining the gut as the motor of critical illness. Trends in molecular medicine. 2014 Apr;20:214–223.
3. Clark JA, Coopersmith CM. Intestinal crosstalk: a new paradigm for understanding the gut as the "motor" of critical illness. Shock (Augusta, Ga). 2007 Oct;28:384–393.
4. www.icuflorence.com
5. NCT03799133 https://clinicaltrials.gov/ct2/show/NCT03799133
6. This study was supported by grant CONACYT (FIT) ECO-2016-C01-275223

### 000410

#### Pre-shock state: a forgotten condition at immediate cardiac surgery patients

NA. Chávez Ponce, E. Bucio, F. Baranda, M. Ayala
Terapia intensiva cardiovascular, Instituto Nacional de Cardiología "Ignacio Chávez", México City, Mexico
**Correspondence:** N.A. Chávez Ponce
*Intensive Care Medicine Experimental* 2019, **7(Suppl 3):**000410

**INTRODUCTION.** The shock index (SI) is calculated dividing the heart rate by the systolic blood pressure, it is a bedside assessment tool that has been used to predict outcomes in different states of circulatory failure as trauma, sepsis, and hypovolemia[1]. Different studies regarding acute coronary syndromes, have proposed distinct cutoffs for SI to predict the development of cardiac shock or mortality (0.7, 0.9, 0.93)[2,3].

**OBJECTIVES.** To determine whether SI is a reliable tool to identify pre-shock state in the postoperative period, to anticipate the development of cardiogenic shock.

**METHODS.** A retrospective, observational study was conducted, in which medical information of patients who underwent aortic valve replacement surgery, from January 2017 to December 2018, was collected. The exclusion criteria encompass those patients who had another surgical procedure besides aortic valve replacement in the same surgical time. Additionally, patients in whom an intra-aortic balloon pump (IABP) was placed prior or during surgery, patients with vasopressors in the intensive care unit (ICU), and those who had systolic blood pressure <90 mmHg when the surgery finished were excluded. The data regarding patients with SI ≥0.7 and SI ≥0.9 was analyzed using STATA 12.1, a p value of <0.05 was considered significant.

**RESULTS.** Total 136 patients were included. Male patients 64.7% and female 35.3%, the mean age was 55 years SD 14.4, mean left ventricular ejection fraction (LVEF) 58.2% SD 8.7. SI: 0.72 SD 0.16, SI ≥0.7 52.9%, SI ≥0.9 13.2%. Pearson correlation between SI and LVEF, mortality, cardiogenic shock, cardiac tamponade, and length of stay after surgery did not show correlation but it correlates with low cardiac output syndrome (LCOS) (R=0.23, p=0.006), bleeding during surgery (R=0.17, p=0.044), and the need for a second surgery during the stay at the ICU (R=0.20, p=0.016). The ≥0.7 cutoff did not demonstrate correlation with any variable, albeit the ≥0.9 cutoff showed correlation with LCOS (R=0.22, p=0.009), the need for second surgery (R= 0.22, p=0.008), bleeding during surgery (R=0.20, p=0.019), and length of stay after surgery (R=0.21, p=0.011). In the multivariate analysis, the SI ≥0.9 only demonstrated significance for the need for a second surgery (OR=3.88, p=0.047, CI=1.02-14.87).

**CONCLUSION.** Pre-shock state defined by SI ≥0.7 is present in more than a half of our population but this cutoff showed no correlation with mortality or any other factors related to shock state, nevertheless SI ≥0.9 showed correlation with the performing of a second surgery due to major bleeding or cardiac tamponade, and LCOS. This suggests that SI ≥0.9 could be an assessment tool in the ICU to recognize patients at possible risk of development of circulatory failure before arterial pressure reaches shock values. The SI warrants future investigation in cardiac surgery.

**REFERENCE(S)**

1. Spyridopoulos I, Noman A, Ahmed JM, et al. Shock-index as a novel predictor of long-term outcome following primary percutaneous coronary intervention. Eur Hear J Acute Cardiovasc Care. 2014. doi:10.1177/2048872614561480
2. Wei Z, Bai J, Dai Q, et al. The value of shock index in prediction of cardiogenic shock developed during primary percutaneous coronary intervention. 2018:1-9.
3. Hemradj V V., Ottervanger JP, Boer MJ De, Suryapranata H. Shock Index More Sensitive Than Cardiogenic Shock in ST-Elevation Myocardial Infarction Treated by Primary Percutaneous Coronary Intervention. Circulation. 2017;81(February):199- 205. doi:10.1253/circj.CJ-16-0616

### 000424

#### Influence of central venous pressure in acute renal failure in the early postoperative period after cardiac surgery

A. Gordillo Brenes, S. Alvarez-Ossorio Cisneros, J. Salas Martin, B. Gomez Garcia, B. Marcote Denis, B. Hernandez Alonso, A. Sanchez Rodriguez
Intensive Care Unit, Hospital Universitario Puerta del Mar, Cádiz, Spain
**Correspondence:** A. Gordillo Brenes
*Intensive Care Medicine Experimental* 2019, **7(Suppl 3):**000424

**INTRODUCTION.** The cardiorenal syndrome comprises a spectrum of disorders affecting both the heart and the kidneys, in which acute or chronic organ dysfunction can induce dysfunction in the other, and central venous pressure (CVP) is of paramount importance in its pathophysiology.

**OBJECTIVES.** To analyze the relation of PVC with acute renal failure in the first 48 hours (ARF-48) and at 15 days (ARF-15) in the postoperative period of cardiac surgery.

**METHODS.** Patients in the immediate postoperative period of cardiac surgery. Variables analyzed: history of diabetes mellitus (DM), arterial hypertension (HT), stroke (ST) and previous arrhythmias (ARR). CVP in the first hours (h) postoperatively (1, 3, 6, 12, 24 y 36). Presence of ARF-48 and ARF-15 according to AKIN criteria. The results are shown as means and 95% confidence interval in the case of quantitative variables and as a percentage in qualitative variables.

**RESULTS.** 1016 patients. 65% men and 35% women. Age of 65.3 (64.7-66). Mortality of 6.5%. IRA-48: 31%. AKIN-48 (1: 67,1%; 2: 27,2% y 3: 5,7%). IRA-15: 3.6%. There was a significant relationship of IRA-48 with HT (40,2% vs 22,4%, p= 0.00), DM (45,1% vs 28,5%, p= 0.00), ARR (46,4% vs 29,5%, p= 0.000) and stroke (48,2% vs 33,2%, p= 0.02). CVP was significantly higher in valvular and aortic surgery (p= 0.000). The CVP was higher in the patients who developed IRA-48 (p= 0.00), and with a proportional relation to the AKIN grade, so the greater dysfunction the higher CVP (Table 1). The CVP of the first hours were associated with ARF-15, so that a CVP-12h > 10 mmHg has a risk of up to 10 times higher for ARF-15 (OR: 10.78, 5.08-22,88).

**CONCLUSION.** The elevation of CVP in the first hours of postoperative cardiac surgery are associated with IRA-48. The CVP at 12 hours postoperatively could predict the persistence of renal failure at 15 days.

**Table 1 (abstract 000424).** See text for description

| AKIN-48 | CVP | | | | | |
|---|---|---|---|---|---|---|
| Stage | 1 h | 3 h | 6h | 12 h | 24 h | 36 h |
| *0* | 6,16 | 5,80 | 5,11 | 5,09 | 6,68 | 7,71 |
| *1* | 7,33 | 6,89 | 6,42 | 6,34 | 7,88 | 8,37 |
| *2* | 8,11 | 7,31 | 6,90 | 6,72 | 8,24 | 8,83 |
| *3* | 8,75 | 7,82 | 8,53 | 7,06 | 8,11 | 9,67 |
| Value of p | **0,012** | **0,000** | **0,000** | **0,000** | **0,000** | 0,104 |

### 000467

#### Cardiogenic shock in the intensive care unit developing in patients with ST-elevated myocardial infarction

A. Sánchez, M. Celaya, B. Hernandez, J. Salas, B. Marcote, A. Gordillo
Medicina intensiva, Hospital Universitario Puerta del Mar, Cádiz, Spain
**Correspondence:** A. Sánchez
*Intensive Care Medicine Experimental* 2019, **7(Suppl 3):**000467

**INTRODUCTION.** Cardiogenic shock is defined as an acute haemodynamic disorder due to a critical reduction of heart's pumping capacity, caused by a systolic or diastolic dysfunction leading to a reduced ejection fraction or impaired ventricular filling. A significant proportion of patients suffering a ST-segment elevated myocardial infarction may develop cardiogenic shock, and the early recognition of these would be very important in order to improve their outcome.

**OBJECTIVES.** The aim of the current study was to identify the clinical predictors that may lead to this complication, and describe the outcome of these patients with ST-segment elevated myocardial infarction (STEMI) who have developed cardiogenic shock (CS).

**METHODS.** Data for this study is collected from a prospective cohort of patients admitted to a terciary level ICU after an STEMI from the 1st January 2009 to 31th December 2018. There patients are all included in the ARIAM-ANDALUCIA registry. Statistical analysis was performed using the Statistical Package for the Social Sciences (SPSS), version 20.0 (IBM Corp., Armonk, NY).

**RESULTS.** From January 1, 2009 to December 31, 2018, a total of 1306 patients with STEMI were admitted to the ICU. Upon admission or lately during their stay, 97 of these presented CS.

**CONCLUSION.** The present study shows that a relevant proportion of patients with STEMI develop CS after presentation (7,43%). This complication occurred more frequently in patients with advanced Killip class, anterior IM, low systolic blood pressure (BP) and higher heart rate (HR) at presentation, history of diabetes or female gender. Unsuccessful and delay reperfusión (PCI/Lysis) also increased the risk of developing CS.

**REFERENCE(S)**

1. Awad HH, Anderson FA Jr, Gore JM, Goodman SG, Goldberg RJ (2012) Cardiogenic shock complicating acute coronary syndromes: insights from the Global Registry of Acute Coronary Events. Am Heart J 163:963–971.
2. Kunadian V, Qiu W, Ludman P, Redwood S, Curzen N, Stables R, Gunn J, Gershlick A, National Institute for Cardiovascular Outcomes Research (2014) Outcomes in patients with cardiogenic shock following percutaneous coronary intervention in the contemporary era: an analysis from the BCIS database (British Cardiovascular Intervention Society). JACC Cardiovasc Interv 7:1374–1385.
3. Poss J, Köster J, Fuernau G, Eitel I, de Waha S, Ouarrak T, Lassus J, Harjola VP, Zeymer U, Thiele H, Desch S (2017) Risk stratification for patients in cardiogenic shock after acute myocardial infarction. J Am Coll Cardiol 69:1913–1920.

**Table 1 (abstract 000467).** See text for description

| | Non shock n=1209 | Shock n= 97 |
|---|---|---|
| Age(mean±SD) years | 61,77 ±14 | 66,39 ± 11 |
| Female gender(%)* | 24,25 | 36,36 |
| Diabetes mellitus (%)* | 22,23 | 36,84 |
| Killilp class at presentation > 1(%)* | 17 | 76 |
| Anterior IM(%)* | 39 | 51 |
| Nonanterior IM(%)* | 51 | 34 |
| HR (mean ±SD) bpm* | 132 ±23 | 102 ±27 |
| SBP (mean ±SD) mmHg* | 71 ± 18 | 88 ±25 |
| Lysis reperfusión (%) | 17,5 | 12,3 |
| Primary PCI (%) | 72,6 | 76,7 |
| No reperfusión (%) | 9,9 | 11 |
| Time pain-reperfusion < 120 min (%) | 21,26 | 13,11 |
| Time pain-reperfusion < 180 min (%)* | 51,28 | 37,7 |
| Successful reperfusión (%)* | 81,2 | 59,5 |
| Unsuccessful reperfusión (%)* | 8,9 | 29,5 |
| In-hospital mortality (%)* | 3,33 | 61,11 |

*p<0,005

### 000469

#### Prevalence and Incidence of Deep Vein Thrombosis in Mixed ICU

J. Benes, R. Skulec, J. Jobanek, V. Cerny
Department of anaesthesiology, perioperative medicine and intensive care, Masaryk Hospital, University J.E. Purkyne, Usti nad Labem, Czech Republic
**Correspondence:** J. Benes
*Intensive Care Medicine Experimental* 2019, **7(Suppl 3):**000469

**INTRODUCTION.** Despite prophylactic measures, the reported incidence of deep vein thrombosis (DVT) during ICU stay remains significant and varies between 2 and 15 %[1].

**OBJECTIVES.** To evaluate the incidence of DVT in the mixed ICU, to assess compliance with the local DVT prophylaxis protocol and to measure the course of anti-Xa activity levels in patients receiving enoxaparin in a prophylactic regimen.

**METHODS.** We conducted a prospective observational cohort study in mixed ICU from March to December 2017. Patients with expected ICU stay longer than 72 hours were included and screened for the presence of proximal DVT by compression ultrasound test (CUS). Baseline CUS was performed within the first 24 hours of admission to the ICU, patients with positive finding were excluded. Patients with negative baseline CUS were included. CUS was performed twice a week until ICU discharge or until new DVT or pulmonary embolism occurred. Prophylaxis with enoxaparin was initiated on admission (40 mg of enoxaparin QD in body mass index ≤35, 60 mg QD in body mass index >35, half dose in the presence of renal failure requiring hemodialysis) unless a risk of severe bleeding was present. Mechanical prophylaxis with graduated compression stockings were used until enoxaparin could be administered. Compliance with the protocol was assessed by comparing expected and delivered prophylactic regimen on each day of CUS evaluation. Peak anti-Xa activity levels were measured twice a week 4 hours after enoxaparin administration.

**RESULTS.** A total of 225 patients were enrolled. Two of them were excluded after positive baseline CUS for the presence of DVT (both clinically suspected) and four were not included in the analysis due to failure to obtain baseline CUS. In 219 analyzed patients, mean±SD Simplified Acute Physiology Score II was 60±20. The largest proportion of the patients was medical (38%), 29% were trauma patients, 28% surgical and 8% post-cardiac arrest patients. Median length of ICU stay was 5 days (IQR 3-11). During ICU stay, 6 patients developed deep vein thrombosis, for an incidence of 2,7%. None of the diagnosed DVT was clinically suspected. No new pulmonary embolism developed. The median peak level of anti-Xa activity in patients treated with enoxaparin was 0.2 IU/mL (IQR 0.1-0.3). Up to 29% of the patients were receiving enoxaparin during the first 24h of ICU stay, 68% between the day 2-4 and 85% after day 4. The agreement between the expected and delivered DVT prophylaxis was 94% for pharmacological and 95% for mechanical prophylaxis.

**CONCLUSION.** We observed a low incidence of deep vein thrombosis in our cohort of mixed ICU patients despite very low levels of anti-Xa activity. Compliance with the DVT prophylaxis protocol was excellent. Therefore, enoxaparin dose adjustment based on regular monitoring of anti-Xa activity is unlikely to result in more effective DVT prophylaxis in a mixed ICU population.

### 000473

#### The central venous to arterial CO2 difference (ΔPCO2) is a good marker of tissue hypoperfusion after cardiac surgery

À. Castillo Niell, JM. Morales Pedrosa, S. Foradada Ubach, A. Tache Sala, P. Pujol Valverde, J. Gonzalez Londoño, M. Morales Moli, P. Ortiz Ballujera, JM. Sirvent Calvera
Intensive Care Medicine, Hospital Universitari de Girona Dr Josep Trueta, Girona, Spain
**Correspondence:** À. Castillo Niell
*Intensive Care Medicine Experimental* 2019, **7(Suppl 3):**000473

**INTRODUCTION.** Venous to arterial CO2 difference is a valid marker to evaluate tissue hypoperfusion after cardiac surgery. A pathological gap (>6) correlates with a reduced cardiac index (CI) and tissue hypoperfusion.

**OBJECTIVES.** This observational, prospective study aimed whether the CO2 gap was a faster parameter than serum lactate and central venous saturation (ScvO2) in diagnosing tissue hypoperfusion and reduced cardiac index (CI).

**METHODS.** 62 patients who had undergone cardiac surgery in our centre during 2018 and were admitted to our general Intensive Care Unit (ICU) during the immediate postoperative period and were monitored with VIGILEO or LiDCO. Patients with arrhythmias, extubation <6 hours or death <24 hours were excluded. Demographic variables, type of surgery (valve surgery, CABG), time of extracorporeal circulation (ECC), aortic cross-clamping, SOFA and EuroScore II were collected. The cohort was divided in two groups: initial ΔPCO2≤6 (n=20) and ΔPCO2>6 (n=42). From each we analysed: serum lactate, ScvO2 and CI at the moment of admission (T0) and in six hours (T6); differences in days of admission in the ICU, hospitalization, need of vasopressors, days of invasive mechanical ventilation (IMV) and morbimortality.

**RESULTS.** From our 62 patients, the CI was remarkedly lower in the ΔPCO2>6 group at T0 and T6 and we observed a tendency of the serum lactate and ScvO2 to worsen during the first 6 hours versus the ΔPCO2≤6 group, although none of it was significantly associated. Days of hospital admission were significantly longer in the ΔPCO2>6 group.

**CONCLUSION.** Given the results, we concluded that venous to arterial CO2 difference might be faster than serum lactate and ScvO2 in diagnosing tissue hypoperfusion and reduced cardiac index; that patients with ΔPCO2>6mmHg at the moment of admission have a tendency of needing more vasopressors, IVM days, higher SOFA and EuroSCore II punctuation, and longer ICU stay although the p value is not significant. And finally, hospitalization days were significantly longer in the ΔPCO2>6 group.

**Table 1 (abstract 000473).** See text for description

| | ΔPCO2≤6mmHg | ΔPCO2>6mmHg | p |
|---|---|---|---|
| Dobutamine (hours) | 2,9±13,1 | 2,7±10,1 | N.S. |
| Norepinephrine (hours) | 35,2±74,2 | 51,5±82,1 | N.S. |
| ICU admission days | 4,9±8,9 | 6,9±11,9 | N.S. |
| Hospitalization days | 13,4±7,8 | 21±23,1 | <0,05 |
| IMV days | 2,9±8,5 | 3,1±3,3 | N.S. |
| Euroscore II (%) | 1,1±0,1 | 2,6±0,1 | N.S. |
| SOFA score t0 | 4,2±2,1 | 5,2±2,6 | N.S. |
| Deaths | 5% | 7,14% | N.S. |

### 000480

#### Left ventricular dysfunction in critically ill patients

O. Cavefors[1], J. Holmqvist[1], S. Lundin[1], SE. Ricksten[1], B. Redfors[2], J. Oras[1]
[1]Anesthesiology and intensive care medicine, Sahlgrenska University Hospital, Gothenburg, Sweden; [2]Department of cardiology, Sahlgrenska University Hospital, Gothenburg, Sweden
**Correspondence:** J. Oras
*Intensive Care Medicine Experimental* 2019, **7(Suppl 3):**000480

**INTRODUCTION.** Left ventricular (LV) dysfunction is potentially harmful in the critically ill patient as it might compromise circulatory status. LV dysfunction can be triggered by many conditions in the critically ill e.g. myocardial infarction, toxic cardiomyopathy, hypoxia or severe stress (Takotsubo syndrome). Previous studies of LV dysfunction have focused on specific patient categories but the impact of LV dysfunction in the mixed ICU population has not been sufficiently studied. ClinicalTrials identifier: NCT03787810.

**OBJECTIVES.** To evaluate the incidence, pattern, risk-factors and impact on mortality of LV dysfunction in a mixed ICU population.

**METHODS.** This is a single center, prospective observational study performed at the Sahlgrenska University Hospital, Gothenburg, Sweden. The study was performed during an eight month period, mainly on weekdays. On study days, all patients admitted to the general or neuro ICU within 24 hours were eligible for inclusion. Included patients were examined using a standard echocardiography protocol [1]. Left ventricular dysfunction was defined as having a global hypokinesia with an ejection fraction <50% or detected regional wall motion abnormalities (RWMA). Patients with LV dysfunction were evaluated for myocardial ischemia, or not, based on findings on coronary angiography, troponin levels, ECG-recordings and distribution of RWMA.

**RESULTS.** A total of 426 patients were included and fulfilled the study protocol. Mean age was 61 ± 17 years, mean SAPS 3 score was 59 ± 16 and 60% were male. A total of 113 patients (26%) had LV dysfunction of whom 86 (20%) patients had RWMA and 27 (6%) had global hypokinesia. Of the 86 patients with RMWA, 17 (20%) had myocardial infarction and 68 (80%) were regarded as non-infarction hypokinesia. Twelve of these patients (3%) demonstrated a circumferential pattern of hypokinesia in concordance with the Takotsubo syndrome. In a multivariable model, independent SAPS 3 risk factors of LV dysfunction were cardiac arrest, circulatory chock of any cause or respiratory failure as cause of admission (pseudo R-square 0.117). Neither total SAPS 3 score, any of the physiological SAPS 3 variables or surgical status were independently associated with LV dysfunction. Ninety-day mortality was higher in patients with LV dysfunction (41%) vs patients with normal LV function (21%, p<0.001), also when adjusting for SAPS 3 score (p=0.005). Mortality was higher in patients with regional hypokinesia (44%) vs patients with global hypokinesia (33%), but this was not statistically significant (p=0.379). There were no differences in mortality if having RWMA due to myocardial infarction or not (p=0.790).

**CONCLUSION.** This preliminary analysis suggest that left ventricular dysfunction is common in the critically ill but not easily predicted by the patient SAPS 3 admission status. Regional hypokinesia is more common than global hypokinesia and RWMA is not obviously caused by myocardial ischemia. LV dysfunction is independently associated with an increased risk of death. Future studies need to address the potential causality between LV dysfunction and an increased risk of death to adapt treatment and improve prognosis.

**REFERENCE(S)**

1. Grants: The Swedish Heart-Lung Foundation, The Swedish State Support for Clinical Research
2. Reference: 1. Evangelista, A et al, Eur J Echocardiogr 9(4): 438-448 (2008).

### 000526

#### Inactivation of the endothelial barrier regulator Tie2 induces microvascular leakage and edema in mice

AL. Van Leeuwen[1], N. Dekker[1], M. Van Meurs[2], G. Molema[2], C. Van Den Brom[1]
[1]Anesthesiology, Amsterdam UMC, locatie VUmc, Amsterdam, Netherlands; [2]Pathology and medical biology, University Medical Center Groningen, Groningen, Netherlands
**Correspondence:** A.L. Van Leeuwen
*Intensive Care Medicine Experimental* 2019, **7(Suppl 3):**000526

**INTRODUCTION.** Critical illness is associated with multiple organ failure and increased mortality, potentially due to impairment of the endothelial barrier, resulting in microvascular leakage, edema and disturbances in microcirculatory perfusion. Angiopoietin-2 is a known predictor for organ failure and mortality in critically ill patients. During stress and inflammation, angiopoietin-2 impairs endothelial barrier function via inactivation of the endothelial Tie2 receptor. We previously showed that activation of Tie2 reduced microvascular leakage and restored microcirculatory perfusion in a rat model of critical illness, proposing the angiopoietin/Tie2 system as a promising target in critically ill patients.

**OBJECTIVES.** To further explore the angiopoietin-2/Tie2 system, we studied the direct effect of angiopoietin-2 administration to WT or heterozygous Tie2+/- mice, expressing 50% of normal Tie2 gene and protein levels, on microvascular leakage and edema formation in mice.

**METHODS.** Male mice with a 50% reduction of Tie2 expression (heterozygous exon 9 deletion, Tie2+/-) and wild type controls (WT) received either angiopoietin-2 (WT+Ang-2 n=8, Tie2+/-+Ang-2 n=8) or PBS as control (WT+PBS n=7, Tie2+/-+PBS n=7) and were monitored for one hour. Microvascular leakage and edema formation were determined by extravasation of Evans Blue dye (EBD) or wet/dry ratio, respectively, in kidney and lung tissue.

**RESULTS.** In healthy WT mice, angiopoietin-2 administration did not affect pulmonary or renal EBD extravasation (lung 0.08±0.03 vs 0.06±0.02 μg/g, p=0.15; kidney 0.07±0.02 vs 0.07±0.02 μg/g, p=0.66), but induced pulmonary edema formation (lung 7.0±1.5 vs 4.5±1.4, p< 0.05; kidney 4.8±0.4 vs 4.5±0.5, p=0.20).

Genetically reduced expression of Tie2 did not affect pulmonary and renal EBD extravasation (lung 0.08±0.02 vs 0.06±0.02 μg/g, p=0.10; kidney 0.07±0.02 vs 0.07±0.02 μg/g, p=0.70) compared to WT controls, but increased pulmonary edema formation (lung 8.0±1.8 vs 4.5±1.4, p<0.01; kidney 4.8±0.1 vs 4.5±0.5, p=0.18).

Administration of angiopoietin-2 in mice with genetically reduced Tie2 expression tended to increase pulmonary (0.12±0.05 vs 0.08±0.02 μg/g, p=0.07) and renal EBD extravasation (0.10±0.03 vs 0.07±0.02 μg/g, p=0.07) compared to control mice with genetically reduced Tie2 expression, but edema formation remained unaltered (lung 6.9±1.9 vs 8.0±1.8, p=0.30; kidney 4.8±0.2 vs 4.8±0.1, p=0.88).

**CONCLUSION.** Angiopoietin-2 mediated Tie2 inactivation, as observed during critical illness, leads to microvascular leakage and edema formation in mice. Future studies should reveal whether therapeutically targeting the angiopoietin/Tie2 system reduces microvascular leakage and improves organ function in critically ill patients.

**REFERENCE(S)**

1. Dutch Heart Foundation (Dr. E. Dekker programme 2016)
2. Dutch Society of Anesthesiology (Young Investigator Grant 2017)
3. European Society of Anaesthesiology (Research project grant 2016)
4. European Society of Intensive Care Medicine (Levi-Montalcini Award 2017)

### 000530

#### Acute Coronary Syndrome in young patients: predictors of mortality in long-term follow-up

AM. González González, AM. García-Bellón, JA. Cano-Nieto, M. De Mora-Martin
Cardiology, Regional Hospital of Malaga, Málaga, Spain
**Correspondence:** A.M. González González
*Intensive Care Medicine Experimental* 2019, **7(Suppl 3):**000530

**INTRODUCTION.** Cardiovascular diseases are the main cause of morbidity and mortality in the developing world, currently occurring at younger ages**.** Acute coronary syndrome (ACS) has a high prevalence in mortality in long-term in young adults

**OBJECTIVES.** The purpose of this study was to determine the factors that are associated with poor prognosis in long-term in this population

**METHODS.** We performed a retrospective analysis with consecutive inclusion of 253 patients admitted with a diagnosis of ACS between January 2011 and January 2013, aged equal or less than 60 years old. We classsified these patients into 2 groups: those who have died (n: 32 (12,7%), 77,8% male), and those who have survived (n: 221 (87,3%); 66,4% male) during the follow- up of 5 years. First, we tested for factors that were associated with mortality during the follow-up. Then, we used a multivariable logistic regression to identify the predictors of mortality in a long-term period.

**RESULTS.** During the folow-up of 5 years, age-independent predictors factors of mortality were: chronic kidney diseas (OR 30,199; p = 0,02); myocardial reinfarction (OR 14,022; p = 0,041); cardiac arrhytmias (OR 13,922; p = 0,035); heart failure following myocardial infarction (OR: 7,994; p = 0,001); hemorrhagie complications (OR 5,273; p = 0,17); Cardiac shock (OR 4,62; p = 0,001); and diabetes (OR 2,856; p = 0,035). Regarding continous variables isolated were serum creatinine (OR 2,311; p = 0,011); and heart rate (OR 1,027; p = 0,03).

The patients registered higher mortality rate were the ones that were less frequently submitted to angioplasty (OR 0,309; p = 0,003), that were not as promptly selected and treated in the emergency department (OR 0,298; p0 =0,14) and that suspended dual anti-platelet therapy before 1 year (OR 0,276; p = 0,007). Continous variables isolated were hemoglobin (OR 0,717; p = 0,003); and hematocrit values (OR 0,906; p = 0,012).

Then, we analyzed those variables using Logistic regression analysis, and we observed that Killip class at admission ≥ 2, diabetes mellitus, heart rate; myocardial reinfarction, , serum creatinine were the strongest predictors mortality and standard dual anti-platelet therapy a predictor of survival.
**CONCLUSION.** Acute cardiac ischemia in < 60 years old, the strongest predictors of mortality during the follow-up of 5 years were admission Killip ≥ 2, diabetes mellitus, myocardial reinfarction, heart rate a serum creatinine. The standard dual antiplatelet therapy during the follow-up of 1 year was an independent survival predictor.

### 000533

#### Prognostic impact of major bleeding in patients hospitalized with Non-ST elevation myocardial infarction

AM. García-Bellón, AM. González González, C. Lara-García, M. De Mora-Martin
Cardiology, Regional Hospital of Malaga, Málaga, Spain
**Correspondence:** A.M. González González
*Intensive Care Medicine Experimental* 2019, **7(Suppl 3):**000533

**INTRODUCTION. Major bleeding (MB) is a feared complication in patients with Non-ST elevation acute myocardial infarction (NSTEMI) and may have impact in the therapeutic strategy and prognosis of these patients.The aim was to characterize the population of these patients (defined MB by Gusto criteria) and to assess their impact on therapeutic approach and in-hospital complications and mortality**
**METHODS. We studied 3216 patients with NSTEMI included in ARIAM(Analysis of delay in myocardial acute infarction )We considered 2 groups:Group 1–Patients with MB(n=67p,2,1%)Group 2–Patients without MB(n=3149p,97,9%)We collected: age,gender,Cardiovascular and non-cardiovascular co-morbidities,laboratory parameters,coronary angiography performed,coronary anatomy and type of implanted stent,in-hospital therapy,ejection fraction(EF)In-hospital complications considered:Heart Failure(HF)and cardiogenic shock(CS);cardiac mechanical complications;stroke;need for mechanical ventilation and need of blood transfusion**
**RESULTS. Regarding clinical profile of patients: With MB and without MB respectively : Age: 70 +/- 9 vs 65+/-12(p < 0,001); Hypertension 84,2% vs 75,3% (p=0,03); Previous HF 19,2% vs 9,2% (p < 0,001); Chronic Renal Failure 15,2% vs 7,9% (p= 0,03); Peripheral artery disease 14,6% vs 6,8% (p=0,02); Previous bleeding 11,5% vs 2% (p < 0,001).Table 2. In-Hospital therapy and coronary angiography**
**CONCLUSION. The presence of MB is present in 2,1% of patients with NSTEMI and is associated with an increased in-hospital mortality and heart failure.**

**Table 1 (abstract 000533).** See text for description

| | With MB | Without MB | p |
|---|---|---|---|
| Aspirin | 93,9% | 98,2% | 0,036 |
| UF Heparin | 27,7% | 15% | 0,004 |
| Other antiplatelets or anticoagulants | - | - | ns |
| Loop diuretics | 55,4% | 29,9% | p < 0,001 |
| Aldosterone Antagonists | 18,5% | 9,8% | 0,02 |
| Inotropes | 6,3% | 1,9% | 0,03 |
| Coronary angiographt | 78,8% | 84% | 0,249 |
| Radial acess | 68,1% | 75,7% | 0,228 |
| Multi-vessel disease | 76,1% | 55,7% | 0,006 |

**Table 2 (abstract 000533).** In-hospital complications and mortality

| Complication | Group 1 | Group 2 | p-value |
|---|---|---|---|
| Heart failure | 33,8% | 14,7% | 0,006 |
| Need for non-invasive ventilation | 7,6% | 1,8% | 0,007 |
| Need for invasive ventilation | 6,1% | 1% | 0,006 |
| Need for blood transfusión | 43,9% | 1,5% | p < 0,001 |
| CS, stroke or mechanical complications | - | - | ns |

### 000535

#### Predictors of major bleeding in patients hospitalized with Non-st elevation myocardial infarction

AM. García-Bellón, AM. González González, D. Gaitan-Roman, M. De Mora-Martin
Cardiology, Regional Hospital of Malaga, Málaga, Spain
**Correspondence:** A.M. González González
*Intensive Care Medicine Experimental* 2019, **7(Suppl 3):**000535

**INTRODUCTION. Major bleeding (MB) is a feared complication and a daily reality in patients with Non-ST elevation acute myocardial infarction (NSTEMI) as a result of pharmaological management. This may have impact in the therapeutic strategy and prognosis of these patients**
**OBJECTIVES. To characterize the population of patients with NSTEMI and MB ( defined by Gusto criteria) and to evaluate possible predictors of the onset of MB**
**METHODS. We studied 3216 patients with NSTEMI included in ARIAM (Analysis of delay in myocardial acute infarction)We considered 2 groups:Group 1:Patients with MB(n=67p,2,1%).Group 2:Patients without MB(n=3149p,97,9%)We collected :age,gender,Cardiovascular and non-cardiovascular co-morbidities,laboratory parameters,coronary angiography performed, coronary anatomy and type of implanted stent,in-hospital therapy,ejection fraction(EF)We compared the in-hospital mortality and multivariate analysis was performed to identify the predictors of MB**
**RESULTS.**
**By multivariate analysis, were identified as predictors of MB : age , previous history of bleeding and unfractionated heparin**
**CONCLUSION. The presence of major bleeding is present in 2,1% of patients with NSTEMI .**
**There were identified as major bleeding predictors : age, previous history of bleeding and therapy with unfractioned heparin.**

**Table 1 (abstract 000535).** Clinical profile

| | With MB | Without MB | p |
|---|---|---|---|
| Age | 70 +/- 9 | 65+/-12 | p < 0,001 |
| Hypertension | 84,2% | 75,3% | 0,03 |
| Previous HF | 19,2% | 9,2% | p < 0,001 |
| Chronic Renal Failure | 15,2% | 7,9% | 0,03 |
| Peripheral artery disease | 14,6% | 6,8% | 0,02 |
| Previous bleeding | 11,5% | 2% | p < 0,001 |

**Table 2 (abstract 000535).** In-hospital complications and mortality

| Complication | Group 1 | Group 2 | p-value |
|---|---|---|---|
| Heart failure | 33,8% | 14,7% | 0,006 |
| Need for non-invasive ventilation | 7,6% | 1,8% | 0,007 |
| Need for invasive ventilation | 6,1% | 1% | 0,006 |
| Need for blood transfusión | 43,9% | 1,5% | p < 0,001 |
| CS, stroke or mechanical complications | - | - | ns |

#### 000548

**Low cardiac-output syndrome after heart surgery: are there any echocardiographic patterns with prognostic implications?**

A. Marcos-Morales, Z. Molina-Collado, N. Quílez-Trasobares, R. Ashbaugh-Lavesiera, E. Renes Carreño, JC. Montejo Gonzales
Intensive Care, University Hospital 12 de Octubre, Madrid, Spain
**Correspondence:** A. Marcos-Morales
*Intensive Care Medicine Experimental* 2019, **7(Suppl 3):**000548

**INTRODUCTION.** Low cardiac-output syndrome (LCOS) is a common complication during the first days after heart surgery, increasing the need of circulatory support, length of stay and mortality in the intensive care unit (ICU).(1) This syndrome lacks one precise definition (2), and the role of cardiac ultrasound in its diagnosis and management is also unclear. The purpose of this study is to investigate if any echographic patterns arise in patients with low cardiac-output syndrome after cardiac surgery, and if these findings could have any prognostic implications.

**OBJECTIVES.** The aim of this study is to evaluate echocardiographic findings in patients who develop LCOS after heart surgery, defined as the sustained need of inotropic support during the first 48 hours of admission. A secondary objective is to study the implications of these findings until discharge from the ICU.

**METHODS.** This is a unicentric, descriptive, retrospective study, set in an adult surgical intensive care unit specialized in heart surgery, at the Hospital Doce de Octubre in Madrid, Spain. Patients were included if they showed a sustained cardiac index less than 2.2 L/mn/m2 or were in need of sustained inotropic support for at least 48 hours after surgery in the intensive care unit. To be included these patients had to receive a cardiac ultrasound evaluation during the first 3 days of admission, performed by a trained practitioner.

**RESULTS.** Seventy patients with LCOS were included, 58% were women. Patients were admitted after heart valve surgery (41%), heart transplant surgery (17%), combined procedure of valve repair and coronary artery bypass grafting (CABG) (13%) and isolated CABG (11%). Median length of stay was 10 days (IQR 5-19), and 8 patients died (11%). Median cardiopulmonary bypass time was of 137 minutes (105-168), median aortic cross-clamp time was of 124 minutes (82-147). Median time of inotropic use was 72 hours (48-118). Before surgery, 54% patients had a normal left ventricular ejection fraction, or LVEF ≥50%. After surgery, 25% of this subgroup presented low LVEF <50%, but 74% still had a normal LVEF. Among the latter, impaired right ventricle ejection fraction (RVEF) was found in 68%, and diastolic dysfunction (DD) in 50%. Among patients with low LVEF before surgery, excluding transplants, most of them still had an altered LVEF after surgery. Having a LVEF <30% after surgery was associated with higher mortality.

**CONCLUSION.** LCOS remains a fearful complication during the first days after heart surgery. Cardiac ultrasound is an important tool in its diagnosis and management. In our findings, reduced RVEF and DD were more common in patients with LCOS who had a normal LVEF after surgery. A higher mortality was associated with a LVEF<30%.

**REFERENCE(S)**

1. Lomivorotov V V et al. Low-Cardiac-Output Syndrome After Cardiac Surgery. JCVA. 2017 Feb;31(1):291–308.
2. Perez Vela JL et al. Clinical practice guide for the management of low cardiac output syndrome in the postoperative period of heart surgery. Med intensiva. 2012 May;36(4):e1-44.

## HSRO - Follow up of ICU patients

#### 000262

**Long term evolution of quality life and its different components, in patients with traumatic brain injury after icu admission**

M. Guerrero Marin[1], R. Rivera Fernandez[1], E. Aguilar-Alonso[2], M. Delange Van Der Kroff[3], E. Curiel Balsera[4], A. Muñoz-Lopez[4], JF. Fernandez-Ortega[4], G. Quesada Garcia[4], M. Prieto-Palomino[4]
[1]Intensive care, Hospital of Jaen, Jaén, Spain; [2]Hospital Cabra, Cabra, Spain; [3]Intensive care, Hospital Comarcal Axarquia, Vélez-Málaga, Spain; [4]Intensive care, Hospital Carlos Haya, Málaga, Spain
**Correspondence:** E. Aguilar-Alonso
*Intensive Care Medicine Experimental* 2019, **7(Suppl 3):**000262

**INTRODUCTION.** Many instruments have been developed to evaluate hospital mortality, but less attention has been paid to the long-term functional status and quality of life of traumatic brain injury patients

**OBJECTIVES.** To analyze the evolution of quality of life (QOL) and its different components between the 1st year and 3-4 years of admission to the ICU due to traumatic brain injury (TBI).

**METHODS.** Prospective cohort study. TBI in Carlos Haya Hospital (Málaga) between 2004-2008. Evaluation of QOL with questionnaire PAECC (Project of Epidemiological Analysis of Critical Patients), We analyzed physiological activities (oral communication, urinary and defecation control, food intake), daily life (mobility, work activity, etc.) and emotional state. (0 normal to 29 major deterioration).

**RESULTS.** N = 531. Age 40.35 ± 19.75 years, APACHE-II 17.94 ± 6.97, GCS admission 7.53 ± 3.83 points. Hospital mortality: 28.6%. Mortality 1 year: 32.2% (lost: 6.6%). 3-4 years 33% (lost 16.2%) QOL 1st year: 9.44 ± 8.73 points (significant deterioration) and at 3-4 years 6.77 ± 7.70 points, (p <0.001).

At 3-4 years, almost 90% have normalized physiological activities, except oral communication (64.3%), and another 17.2% with difficulty but with coherent dialogue. First year low percentage in work reintegration and capacity for great efforts , improve to 3-4 years without reaching 50% normality. High percentage in tolerance to minimum efforts, precision movements and social relations at year and 3-4 years. Emotional state presents high percentage of abnormality.

**CONCLUSION.** TCE, a year show significant deterioration of QOL improving to 3-4 years. At one year they present high normality of basic physiological activities, physical ones of small effort, social relation and movements of precision and that even improves something at 3-4 years. First year, normality in ability to make great efforts, work activity and subjective aspects a low percentage that improves to 3-4 years, although it does not reach 50%.

**Table 1 (abstract 000262).** See text for description

| Normal 1º and 3-4 year (%) | | | | | |
|---|---|---|---|---|---|
| Basic physiological activities | | | Normal daily life activities | | |
| Oral communication | 54,8 | 64,3 | Movements | 83,1 | 89,9 |
| Urinary control | 79,1 | 84 | Minor effort | 71,2 | 82,4 |
| Defecation control | 81,9 | 88,2 | Major effort | 35,3 | 47,9 |
| Food intakes | 81 | 88,7 | Walking | 63,8 | 75,6 |
| Emotional status | | | Mobility | 46,6 | 68,1 |
| Subjective well being | 41,1 | 49,2 | Dressing | 63,8 | 78,2 |
| State of mind | 40,2 | 50 | Working life activities | 28,5 | 41,2 |
| Vitality | 64,4 | 73,1 | Social relationship | 70,2 | 80,6 |

### 000281

#### Identification of the sublingual triangle as the optimal sublingual location for microcirculatory assessment using hand-held vital microscopy imaging

Z. Uz[1], O. Dilken[2], M. Hilty[2], D. De Haan[1], L. Shen[1], J. Houtzager[3], LC. Franken[3], TM. Van Gulik[3], C. Ince[1]

[1]Translational physiology, Amsterdam UMC, Amsterdam, Netherlands; [2]Department of intensive care, Erasmus MC, Rotterdam, Netherlands; [3]Experimental surgery, Amsterdam UMC, Amsterdam, Netherlands

**Correspondence:** O. Dilken

*Intensive Care Medicine Experimental* 2019, **7(Suppl 3):**000281

**INTRODUCTION.** Identification of microcirculatory alterations in critically ill patients using hand-held vital microscopes (HVMs) is commonly applied sublingually. The sublingual area however, contains different types of vessel morphology related to capillaries, salivary gland structures and large veins. Although several studies have shown the importance of sublingual microcirculatory assessment during health and disease, no attention has been paid to the heterogeneity of the microcirculatory morphology in the sublingual cave. This study introduces the anatomy guided area in the sublingual cave, which is called the sublingual triangle. The sublingual triangle is most suited to perform sublingual measurements due to its rich vasculature with content conform the international consensus ESICM guidelines on microcirculatory monitoring. (1).

**OBJECTIVES.** The objective of this study is to identify the optimal sublingual location for measurement of the microcirculation and to validate that with suitable training this optimal location can be readily identified.

**METHODS.** A trained observer performed sublingual microcirculation assessments with HVM using incident dark field (IDF) imaging. Measurements were done before and after suitable training aimed at identifying its location(2) , with an interval of 3 months. The instructions consisted of introducing a specific region of interest, guided by the anatomical structure which can be seen adjacent on either side of the frenulum of the sublingual area. Small vessel density (SVD) (vessels <20μm in diameter), Large vessel density (LVD) (vessels between 20 – 100 μm in diameter) and Total vessel density (TVD), of non-instructed (NIM) and instructed (IM) measurements were analyzed and compared. Also the vessel structures were morphologically analyzed, and the appearance of salivary gland structures related microcirculation identified.

**RESULTS.** A total of 9 healthy volunteers were included. During each time point (T0:NIM and T1:IM) 4 video clips of the sublingual microcirculation were captured. Poor quality videos were excluded according to the microcirculation quality assessment (1) before offline analysis. A total of 53 videos were analyzed with MicroTools(3). TVD, but not LVD and SVD, was higher in IM compared to NIM, indicating a bigger contribution to TVD from capillaries, which are associated with oxygen transport to tissue. (TVD NIM vs IM: 24.76 ± 1.74 vs 27.29 ± 3.1 mm/mm2 p=0.044 , LVD NIM vs IM: 6.6 ± 1 vs 7.6 ± 1.3 p=0.092 , SVD NIM vs IM: 18.19 ± 1.65 vs 19.88 ± 3.07 p=0.103). In the NIM 6 video clips were identified containing salivary gland structure related microcirculation, whereas in the IM videos only small and large vessels were detected.

**CONCLUSION.** The sublingual triangle identified in this study showed a higher vessel density and avoided salivary gland structure related microcirculation. These findings indicate that the sublingual triangle is the optimal location for measurement since it can be identified by its morphology and contains the optimal vessel content conform the international guidelines. We propose the sublingual triangle to become the target of microcirculatory assessment using HVM to reduce heterogeneity and increase reproducibility of the studies.

**REFERENCE(S)**

1. Ince C, Boerma EC, et al. (2018) Second consensus on the assessment of sublingual microcirculation in critically ill patients. : Results from a task force of the European Society of Intensive CareMedicine. Intensive Care Med. 2018;44(3):281-299. 15.
2. Uz, Zühre et al. "Intraoperative Incident Dark Field Imaging of the Human Peritoneal Microcirculation." Journal of vascular research vol. 55,3 (2018): 136-143. doi:10.1159/000488392
3. Hilty MP, Arend S, Van Assen M, Toraman F, Ince C. A software tool to quantify capillary blood volume and absolute red blood cell velocity in sublingual incident dark field microscopy video clips. Intensive Care Med Exp 2018; 6: 172–173.

### 000297

#### The impact of antithrombin III level as a prognostic predictor for patients admitted to the ICU

K. Yarimizu[1], Y. Onodera[2], M. Yoshioka[1], M. Kudo[1], M. Nakane[3], K. Kawamae[2]

[1]Department of anesthesia, Nihonkai General Hospital, Sakata, Japan; [2]Department of anesthesiology, Yamagata University Hospital, Yamagata, Japan; [3]Department of emergency and critical care medicine, Yamagata University Hospital, Yamagata, Japan

**Correspondence:** K. Yarimizu

*Intensive Care Medicine Experimental* 2019, **7(Suppl 3):**000297

**INTRODUCTION.** Reduced antithrombin-III (AT-III) is an important component in the latest diagnostic criteria for disseminated intravascular coagulation (DIC) established by the Japanese Society of Thrombosis and Hemostasis. However, some severe cases show reduced AT-III, but do not meet the diagnostic criteria for DIC. In our experience, patients with low AT-III levels show very poor outcomes, regardless of their disease or DIC status. However, few reports have examined the prognostic ability of AT-III in all patients admitted to the ICU.

**OBJECTIVES.** To investigate the relationship between the AT-III level and the outcomes of patients admitted to the ICU and evaluate the prognostic value of the AT-III level.

**METHODS.** We conducted a retrospective observational study of 672 patients who were admitted to our hospital ICU from January to December in 2018. Cases involving patients of <18 years of age, APACHE II scores of <10 points, cases without AT-III measurement, and cases involving patients who died within 1 day of admission to the ICU were excluded. We divided the included cases into 4 groups based on the AT-III values measured at the early stage of hospitalization: AT-III ≥71% (H group), 70-51% (M group), 50-31% (L group), and ≤30% or less (VL group). We also checked the rates of death within 7 days, 28 days, and at hospital discharge. The accuracy of the predicted prognosis was then compared with the APACHE II score using an ROC curve analysis. The JMP software program was used to perform the multivariate and ROC curve analyses.

**RESULTS.** The 56 subjects in this study were classified into the following groups: H group, n=13; M group, n=25; L group, n=12; and VL group, n=6. In most target cases, the basic disorders were postoperative patients, sepsis patients, or acute myocardial infarction patients. The APACHE II scores were 20, 24.5, 21 and 30 (groups H, M, L, and VL, respectively). The rates of death within 7 days were 0%, 4%, 8.3% and 50%; the rates of death within 28 days were 0%, 28%, 25% and 67%, and the rates of death at hospital discharge were 25%, 32%, 50% and 100%, respectively. The multivariate analysis revealed that the AT-III value and APACHE II scores were independent predictors of death at hospital discharge. In comparison to the APACHE II

score, the AT-III value appeared an equally or more powerful prognostic predictor for any outcome (Fig. 1). The cut-off values of the for AT-III level and APACHEII score for predicting 7-day mortality were 47% and 26; those for 28-day mortality were 57% and 26, death and those for death at discharge were 56% and 26, respectively.
**CONCLUSION.** Early morbidly low AT-III values may be a prognostic predictor of hospital mortality in patients admitted to the ICU.

**REFERENCE(S)**

1. Hidesaku Asakura, et al. Diagnostic criteria for DIC by the Japanese Society on Thrombosis and Hemostasis-2017 edition-, Japanese Journal of Thrombosis and Hemostasis, 2017, 28(3), 369-392.
2. None

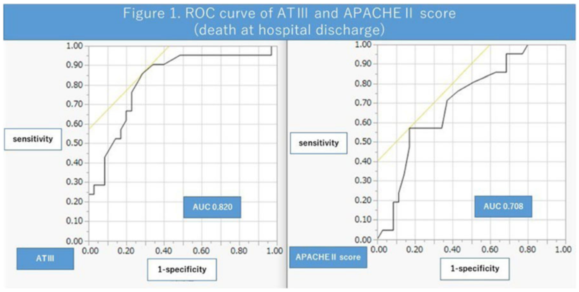

**Fig. 1 (abstract 000297).** See text for description

### 000336

#### Determining Unmet Need for Critical Care as Part of a Business Case for Expansion of Capacity in a Mid-Sized Acute UK Hospital

L. Sharifi, A. Ackerman, J. Brinsley, R. Maeda, B. Boira, D. Higgins
Critical Care, Southend University Hospital NHS Foundation Trust, Southend-on-Sea, United Kingdom
**Correspondence:** L. Sharifi

*Intensive Care Medicine Experimental* 2019, **7(Suppl 3):**000336

**INTRODUCTION.**

- Our medium sized 544 bed acute hospital lacked a dedicated HDU for many years. Accordingly patients were admitted to ITU with higher APACHE scores, and the hospital had an outlying mortality rate. This data led to the construction of a new 8 bedded HDU facility, opened at 50% capacity in May 2018.
- Six months after opening, and encouraged by a fall in admission APACHE to ITU, we audited the degree of unmet need throughout acute and elective care. We present this audit so others can use our methodology to assist in their cases for expansion of Critical Care (CC) capacity within their institutions.

**OBJECTIVES.**

- To identify: Adult patients who would benefit from admission to Critical Care (HDU and ITU) but are not actually admitted, where CC beds are occupied by patients who have recovered but cannot leave due to a shortage of hospital beds
- To determine if additional HDU capacity would help mitigate this problem

**METHODS.**

- 14 day 24 hour data collection of CC bed state, record of all referrals / escalations to CC, record of all theatre / recovery patients potentially requiring CC

**RESULTS.**

- CC services were in high demand, ITU being at 100% capacity for 43% time
- HDU capacity was the biggest cause of delayed discharge from ITU
- 25% of all HDU bed hours were filled with patients fit for step down to the ward
- Elective surgery was cancelled more often than usual due to CC capacity constraints
- Surgical patients were less likely to get a bed on CC than medical patients, with patients being nursed in areas lacking CC resources and training

**CONCLUSION.**

- Surgical patients are being cancelled or nursed in innapropriate areas. Seperately, national data shows below average admission of high risk emergency laparotomies to CC. Thus both elective and emergency patients are experiencing suboptimal care.
- Capacity pressures on wards block HDU discharges, in turn blocking ITU
- This audit informed the business case for additional HDU capacity, and our materials made it repeatable for the audit cycle. We hope this model may be of use to others.

### 000358

#### Physical, mental and cognitive outcomes one year after ICU admission: a prospective cohort study

W. Geense[1], M. Zegers[1], H. Vermeulen[2], H. Van Der Hoeven[1], M. Van Den Boogaard
Department of intensive care medicine, Radboud University Medical Center, Nijmegen, Netherlands; [2]Department of iq healthcare, Radboud University Medical Center, Nijmegen, Netherlands
**Correspondence:** W. Geense

*Intensive Care Medicine Experimental* 2019, **7(Suppl 3):**000358

**INTRODUCTION.** There are numerous studies describing the adverse long-term physical, mental and cognitive outcomes among ICU survivors. However, their pre-ICU health status is often not taken into account.
**OBJECTIVES.** To assess patients' physical, mental and cognitive health status before and one year after ICU admission.
**METHODS.** Adult patients admitted for ≥12 hours to a university hospital ICU between July 2016 and December 2017 were included (MONITOR-IC study, clinicaltrials.gov NCT03246334). Moribund patients were excluded. Patients (or proxies) rated their health status before and one year after ICU admission, by completing the Short Form-36 Physical and Mental Component Summary (PCS and MCS) (range 0-100; higher scores indicate better physical or mental function), Hospital Anxiety and Depression Scale (HADS) (range 0-21; higher scores indicate more symptoms of anxiety and depression) and abbreviated Cognitive Failure Questionnaire (CFQ-14) (range 0-100; higher scores indicating more cognitive failure). Moreover, new physical symptoms within the first year were noted.
**RESULTS.** A total of 1300 patients was included: mean age 61.5 (± 14.8 years), 65% male. Median ICU and hospital stay were 1 [IQR 1-2]

and 9 days [IQR 6-15] respectively, with mainly elective surgery admissions (65%).
One year after ICU admission, mean SF-36 PCS score was significantly higher compared to baseline; 42.6(±11.4) to 45.2(±10.8); p <.001, with a mean difference score of 2.60 (95%CI, 1.79 to 3.41), while 41% of the patients experienced a physical decline. Reported new physical symptoms within the first year were impaired physical function (70%), joint stiffness, dizziness, muscle weakness and/or shortness of breath (40%). Sexual problems were experienced by 25% of the ICU survivors.
Mean SF-36 MCS score increased significantly from 48.5 (±11.3) to 50.0 (±10.3); p<.001, mean difference score 1.55 (95% CI, 0.75 to 2.35), while 46% of the patients experienced a mental decline. Depression scores decreased from median 4 [IQR 1-7] to median 3 [IQR 1-7]; p=.057, and anxiety scores decreased significantly from median 4 [IQR 2-7] to 3 [IQR 1-7]; p<.001.
Contrary to the improvements in physical and mental health, the cognitive status decreased significantly from median 21 [IQR 11.3-29.5]) to median 23.7 [IQR 13.6-33.2]); p<.001, and 60% of the patients reported a cognitive decline.
**CONCLUSION.** Although statistically significant, improvement in physical and mental outcomes and decline in cognitive outcomes were small after one year. Nevertheless, the vast majority of patients experienced new physical problems, such as impaired physical function, muscle weakness and/or shortness of breath. Identifying patients who are at risk of these adverse long-term outcomes may lead to better and tailored care for ICU patients and survivors.

### 000369

#### In-hospital mortality in the older old patients admitted at Intensive Care Units. Predictive factors

D. Díaz Díaz, M. Villanova, L. Lopez, B. Bueno Garcia, G. Andrade, E. Palencia
Intensive care units, Hospital Universitario Infanta Leonor, Madrid, Spain
**Correspondence:** D. Díaz Díaz
*Intensive Care Medicine Experimental* 2019, **7(Suppl 3):**000369

**INTRODUCTION.** Ageing of the world's population is influencing in an increase in the number of elderly patients admitted to intensive care units (ICU). However, invasive treatments tend to be avoided in elderly patients. The identification of predictive mortality factors for older old patients (older than 80 years) admitted to the ICU could assist the medical team, patients and families in planning advanced care and decision-making.
**OBJECTIVES.** Our main objective is to identify predictive factors for hospital mortality in patients older than 80 admitted at an ICU in a community hospital
**METHODS.** Retrospective Study in a cohort of consecutive patients over 80 years admitted to the ICU during the year 2018. We collected the demographic data, comorbidity, Barthel Index prior hospitalisation, reason for ICU admission, severity (SOFA, APACHE II, SAPS III), therapy received in ICU, in-hospital mortality, and in the group of survivors we also collected data on destiny at ICU discharge
**RESULTS.** A total of 71 patients older than 80 years were admitted at the ICU, representing 15.56% of all ICU admissions. The average age was 83.5 years (SD 2.3), 52.1% were women. In hospital mortality was 24 patients (33.65%), being mortality at ICU of 16 patients(22.5 %), the 75% of them due to limitation of therapeutic effort.Patients with in-hospital mortality had non-significant higher frequencies of diabetes , chronic obstructive pulmonary disease, chronic renal disease , cognitive impairment, previous oncological history, as well as higher body mass index than survivors.Up to 70% of survivors came from the emergency department as compared to 50% of non-survivors. The most common reason of ICU admission in the non-surviving group was septic shock (50% vs10, 6%, P = 0,000), followed by cardiogenic shock (20.8% vs 8.5%, P = 0.015). The Severity Indexes at ICU admission (SAPS III, SOFA and APACHE II) were significantly higher in the non-surviving group compared to survivors (P = 0.000). The use of vasoactive drugs was significantly more frequent in non survivors (95.8%vs 61.7%,P= 0.000), with a trend to higher need for invasive/non-invasive mechanical ventilation and for renal replacement purification therapy. For survivors, the Barthel index at discharge remained above 85 (mild disability).
**CONCLUSION.** Older old patients represent up to 15.56% of ICU admissions. The main predictive factor for in-hospital mortality is severity at ICU admission by SAPS III, APACHE II or SOFA,as well as the need for vasoactive drugs. However, survivors had low rates of dependence.

### 000371

#### ICE Cold

C. Baker[1], C. Achary[2], C. Tai[1], B. Post[2], D. Melia[1], V. Parekh[1]
[1]Department of critical care, Whipps Cross University Hospital, London, United Kingdom; [2]Department of anaesthesia, The Royal London Hospital, London, United Kingdom
**Correspondence:** D. Melia
*Intensive Care Medicine Experimental* 2019, **7(Suppl 3):**000371

**INTRODUCTION.** Starting work as a junior doctor in critical care can be intimidating and overwhelming1. Junior doctors starting a clinical placement in intensive care medicine (ICM), can come from a variety of specialties2, with differing levels of knowledge and practical skills. We were unable to find any institution that offers specific training to address these issues.
**METHODS.** We developed a standardised one-day course for novices, timed before their first clinical shift, free of cost to them, and integrated into their normal working hours. We constructed a programme of lectures and practical workshops, covering core knowledge and themes in; mechanical ventilation, non-invasive ventilation, cardiac output monitoring, inotropes and vasopressors, sedation and delirium, renal replacement therapy and airway management - "Intensive Care Essentials (I.C.E)". Allowing comprehensive, protected teaching, to new starters in intensive care, we aimed to improve key knowledge and skills, and, allay anxiety amongst doctors new to ICM. Candidates were asked to rank how strongly they agreed or disagreed with pre-defined questions and statements along a numerical scale (0 (strongly disagree) - 5 (strongly agree)), before the course (n=26), and then after course completion(n=24). Data were compared using independent t-tests.
**RESULTS.** Candidates indicated that they felt significantly more prepared for their first shift in ICU after attending the course (mean pre-course score 2.4 (SD=0.76) vs mean post-course score 3.74 (SD= 0.45), *p*=<0.0001). In addition, they felt their knowledge was more sufficient to start on the critical care unit (mean pre-course 2.28 (SD= 0.68) vs mean-post course 3.7 (SD=0.56), *p*=<0.0001), and were more confident in tackling problems encountered in each of the clinical scenarios.
**CONCLUSION.** It is both feasible and beneficial, to deliver a targeted teaching programme covering basic knowledge and practical skills, required for safe practice in the critical care environment. Furthermore, we have demonstrated that this can be delivered within the framework of the UK Junior Doctors Contract, as protected, mandatory education, built into rostered working hours.

##### REFERENCE(S)

1. Kellett J, Papageorgiou A, Cavenagh P, Salter C, Miles S, Leinster S. "The preparedness of newly qualified doctors – Views of Foundation doctors and supervisors." Medical Teacher 2015;37(10): 949-954

2. "Person Specification 2019: Intensive Care Medicine – ST3" [Internet]. UK; Health Education England; 2019 [cited 2019 Apr 9]. Available from: https://specialtytraining.hee.nhs.uk/portals/1/Content/Person%20Specifications/Intensive%20Care%20Medicine/INTENSIVE%20CARE%20MEDICINE%20–%20ST3.pdf
3. None

**Table 1 (abstract 000371).** See text for description

| Confidence in tackling problems with… | Pre-Course (mean(SD)) | Post-Course (mean(SD)) |
|---|---|---|
| Non-inavsive Ventilation | 2.5 (0.95) | 3.83 (0.89) |
| Mechanical Ventilation | 1.92 (0.98) | 3.57 (0.95) |
| Cardiac output monitoring | 2.12 (0.77) | 3.35 (0.78) |
| Inotropes | 2.00 (0.96) | 3.35 (0.78) |
| Sedation | 2.27 (0.96) | 3.78 (0.80) |
| Renal replacement therapy | 1.69 (0.88) | 2.95 (0.84) |
| Airway management | 2.73 (0.92) | 3.78 (0.67) |

All $p$=<0.0001

### 000438

#### The nature and adverse effects of registration burden on ICU nurses and physicians: a mix-methods study

M. Zegers[1], G. Veenstra[2], R. Verhage[1], J. Hoeven Van Der[1]
[1]Intensive care, Radboud University Medical Center, Nijmegen, Netherlands; [2]Centre of expertise on quality and safety, University Medical Center Groningen, Groningen, Netherlands
**Correspondence:** M. Zegers
*Intensive Care Medicine Experimental* 2019, **7(Suppl 3):**000438

**INTRODUCTION.** Registration of quality indicators is crucial for accountability and quality improvement. However, nurses and physicians suffer from the amount of administrative tasks. Clerical burden is a cause of physician burnout.

**OBJECTIVES.** To analyse the nature and consequences of registration burden experienced by nurses and physicians working in the ICU.

**METHODS.** A mix-methods observational study in an academic hospital in 2017, including a questionnaire survey (N=158), individual semi-structured interviews (N=5), and participative observations (3 days).

**RESULTS.** On average, physicians and nurses spend 54.9 (SD±43.1) minutes per day on quality registrations; nurses more than physicians, median 45 (min-max 0-241) and median 29 (min-max 0-119) respectively. They register 102 quality indicators and 1438 underlying variables for several stakeholders, including government bodies (Healthcare Inspectorate and National Health Care Institute), accreditation institutes, professional societies and registries, and internal demands from the board of directors. In total, 20% is registered for accountability purposes, 25% for institutional governance, and 54% for quality improvement. Almost half (48%) of the quality indicators were requested by multiple stakeholders, but the timing and content (operationalisation of definitions) of the demands were not aligned. Of all 102 quality indicators, 34% was perceived as useful for quality improvement in daily practice.

In the interviews, eight types of registration burden were identified, such as excessive amount of quality registrations, low efficiency of quality registrations to achieve quality improvements, feelings of distrust by mandatory registrations, registrations are not relevant for clinicians and nurses, and ICT-problems. The interviewees mentioned that the amount of registrations and other types of registration burden diverts time and focus from patient care and improving quality, with, paradoxically, lower quality as a consequence.

**CONCLUSION.** In sum, registration burden perceived by physicians and nurses can be classified into four main categories: 1) time spend on quality registrations; 2) amount of quality indicators; 3) usefulness for quality improvement, and 4) inefficiencies in the registration process. Registering less quality indicators, but more what matters (usefulness) is paramount to increase the efficiency of quality registrations, and to mitigate the registration burden and its consequences on nurses, physicians and their patients.

### 000440

#### Quick-Link Learning: introduction of rapid-access educational resources on the intensive care unit

AM. Amphlett, S. Heaton, N. Freeman-Fielding, C. Bourdeaux
Department of intensive care, University Hospitals Bristol NHS Foundation Trust, Bristol, United Kingdom
**Correspondence:** A.M. Amphlett
*Intensive Care Medicine Experimental* 2019, **7(Suppl 3):**000440

**INTRODUCTION.** UK Intensive care units (ICUs) have a high turnover of junior staff across the multidisciplinary team (MDT)1. Our general ICU has a regular intake of new staff who are inexperienced in critical care and in this hospital's specialties. They enter a high intensity, high risk environment and need rapid access to supporting information and resources2. Current information systems within our hospital are not streamlined enough for effective point of care use.

**OBJECTIVES.** Our purpose was to improve access to educational resources and support for junior staff across the MDT. The use of mobile devices to support learning is recognised as a strong emerging educational technology3. Quick Response (QR) codes offer a shortcut to provision of information and we aimed to site them appropriately on the ICU, seeking improvements in assessed confidence and knowledge among medical and nursing staff.

**METHODS.** We took a two-part approach, siting QR codes on posters and on equipment. The posters took a question/answer format, each QR code linking to the appropriate educational source (protocol, guideline etc). We assessed new groups of junior medical staff in their knowledge and confidence about each poster topic at the beginning of their rotations and again after the introduction of the posters. Multiple choice and multiple answer questions were used in the quiz and a seven point Likert-type scale was used to assess confidence.

A QR code was sited on the ABG machine: this linked to an independent website which tests understanding of blood gas results in a quiz format. We assessed nursing staff confidence in blood gas interpretation before and after introducing the code.

**RESULTS.** Both quiz performance and confidence improved among junior doctors after the introduction of the educational posters. Across eight quiz questions, the average number of respondents answering correctly improved from 48% to 75%. Confidence among the same respondents also improved across all topics assessed: there was an average improvement of 1.6 points on the seven point Likert-type scale.

The initial survey of nursing staff showed a wide range of confidence in blood gas interpretation. The QR code on the ABG machine received more than 60 hits in the study month and the post-intervention survey showed an increase in confidence among nursing staff of an average 2.6 points on the Likert-type scale.

**CONCLUSION.** The ICU is challenging for new staff, who are faced with a steep learning curve in a specialised and complex environment. Many aspects of ICU care are highly protocolised, and therefore well suited to independent learning if the appropriate resources can be easily accessed. QR codes facilitate this learning using readily available mobile technology. Introducing QR codes to make resources immediately available at the point of care has shown improvements in both knowledge and confidence, for all tested topics, among staff across the MDT.

**REFERENCE(S)**

1. The Faculty of Intensive Care Medicine. Workforce data bank for adult critical care, May 2018; available from: https://www.ficm.ac.uk/sites/default/files/workforce_data_bank_2018_updated_for_website.pdf. Accessed 10/04/2019.
2. Joyce MF, Berg S, Bittner EA. Practical strategies for increasing efficiency and effectiveness in critical care education. World J Crit Care Med. 2017 Feb 4; 6(1): 1–12. doi: 10.5492/wjccm.v6.i1.1
3. Spector JM. Emerging Educational Technologies and Research Directions. Educ Technol Soc. 2013;16:21–30.
4. With thanks to UHBristol Above and Beyond charity for QR generator subscription funding grant

### 000448

#### Three-year trajectory of patients discharged from ICU: Quality of life and healthcare resource utilization

R. Hauwaert[1], S. Castro[2], L. Melão[2], A. Binnie[1], C. Granja[2]
[1]Departamento de ciências biomédicas e medicina, Universidade do Algarve, Faro, Portugal; [2]Serviço de medicina intensiva 1, Centro Hospitalar Universitário do Algarve, Faro, Portugal
**Correspondence:** S. Castro
*Intensive Care Medicine Experimental* 2019, **7(Suppl 3):**000448

**INTRODUCTION.** Critical care is associated with significant consumption of health resources, both in the intensive care unit (ICU) and after ICU discharge. The number of critically ill patients is anticipated to rise over the coming years, and healthcare systems will need to adjust to meet increasing demands.

**OBJECTIVES.** The aim of this study was to assess the impact of critical illness on patients and the healthcare system during the three years following ICU discharge, including determination of long-term mortality, analysis of patients' healthcare utilization, and assessments of HR-QOL (Health-Related Quality of Life) at six months and three years after ICU discharge.

**METHODS.** The study was a mixed retrospective/prospective observational study conducted at the Centro Hospitalar Universitário do Algarve, a tertiary-level university hospital and regional trauma and neurosurgical centre in Faro, Portugal. All patients admitted during the year of 2015 were eligible (n = 415). Patients were subdivided by prior health status (healthy, chronic non-disabling disease, chronic disabling disease). HR-QOL was assessed using the Euro Quality of Life – Five Dimensions (EQ-5D) questionnaire. Six month EQ-5D results were extracted retrospectively from 2015 follow-up records while three-year EQ-5D telephone assessments were performed prospectively by the same investigators. Patients' trajectories during ICU stay and after ICU discharge were evaluated through hospital and national records and summarized in schematic form.

**RESULTS.** Our results reveal that out of 271 patients who survived to hospital discharge, nearly 20% were discharged to post-acute facilities and 10,7% remained institutionalized at three years (Figure 1). Patients admitted to the ICU with septic shock, emergent neurosurgery and neurologic emergencies were more likely to remain institutionalized. Hospital readmissions during the three years following the critical event were frequent (46,5% of patients) as were emergency department visits (73,4% of patients). At 3 years, cumulative survival was 60% for patients with a "healthy" premorbid status and <20% for patients with pre-ICU chronic disabling illness. Results of the EQ-5D questionnaire applied three-years after hospital discharge revealed a deterioration in HR-QOL relative to the evaluation completed at six months.

**CONCLUSION.** Survivors of critical illness show persistently high healthcare resource utilization during the 3 years after hospital discharge along with a deterioration in HR-QOL at 3 years relative to 6 months post-discharge. Further research in this field is required to better support healthcare systems, improve care for ICU survivors, and contribute to gains in HR-QOL.

**REFERENCE(S)**

1. Griffiths J, Hatch RA, Bishop J, Morgan K, Jenkinson C, Cuthbertson BH, et al. An exploration of social and economic outcome and associated health-related quality of life after critical illness in general intensive care unit survivors : a 12-month follow-up study. Crit Care. 2013;17:R100
2. Cuthbertson B, Wunch H. Long-Term Outcomes after Critical Illness. Am J Respir Crit Med. 2016;194(2):2014–6.
3. Algarve Biomedical Center

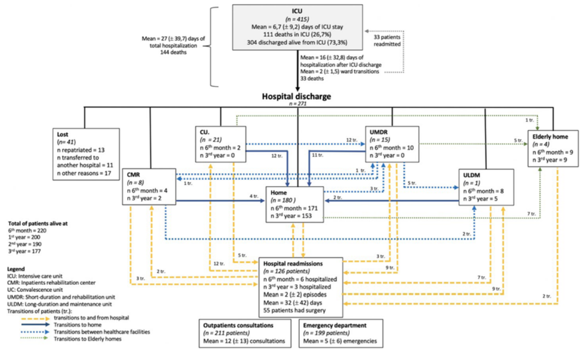

**Fig. 1 (abstract 000448).** See text for description

### 000452

#### Humanizing care in the intensive care unit: Perspectives of clinicians on using the Get to Know Me board

S. Ahmad, G. Ognjen, L. Karnatovskaia
Division of Pulmonary and Critical Care, Mayo Clinic, Rochester, United States of America
**Correspondence:** S. Ahmad
*Intensive Care Medicine Experimental* 2019, **7(Suppl 3):**000452

**INTRODUCTION.** Over a third of critical illness survivors manifest significant psychological impairments following discharge from the intensive care unit (ICU). Memories of frightening delusional experiences and in-ICU psychological distress are the strongest potentially modifiable risk factors. Our prior surveys and interviews of physicians, nurses, patients and their families revealed that inadequate communication was universally viewed as the key aspect contributing to patient distress and fear. The Get to Know Me Board is one of the tools adapted from palliative medicine that can be used to overcome anonymity of the critically ill patient.

**OBJECTIVES.** Given known challenges in communication with critically ill patients and their families, we explored providers' knowledge of the 'Get to Know Me board' as a tool to help facilitate such communication.

**METHODS.** We administered a web-based survey consisting of multiple choice and open ended questions to critical care physicians and nurses in medical and surgical ICUs at the Mayo Clinic.

**RESULTS.** 170 clinicians responded to the survey (23% response rate); of those 118 were nurses (69%) and 52 physicians (30%). All used the Get to Know Me board, although a third were not aware of where the blank forms were located. 152 (89%) found the board helpful in communication, such as in seeing the human side of the patient, being able to apply things patient may like in their interactions, and creating bridges with families. Most common information clinicians found helpful was the knowledge of patient's favorite movie, sport or food, hobbies, and achievements as well as their stressors and de- stressors. Ninety four (55.4%) respondents expressed no difficulty in incorporating the board as a part of routine clinical care, whereas the rest reported some level of difficulty. When asked about barriers to incorporating the board in clinical practice, the most common response was getting too busy with clinical duties (85 or 50.6%) followed by 'whether it gets filled out depends on who is on staff' (73 or 43%); 40 (23.8%) encountered no barriers. Most participants 102 (61%) expressed that the best time to fill out the board was anytime during the stay, particularly when the family arrives or upon admission. About 7% of providers cautioned that patients should be stabilized first. See Figure 1 for an example of the filled out Get to Know Me board.

**CONCLUSION.** The Get to Know Me board is not a consistently utilized instrument in our critical care unit environment. However, when applied, it can be helpful to providers to get to know their patient as a person and not a disease, develop therapeutic relationship with them and their families, thereby improving communication and potentially outcomes. Further studies will be needed to measure effects on patient specific outcomes and ICU experiences of patients as well as providers.

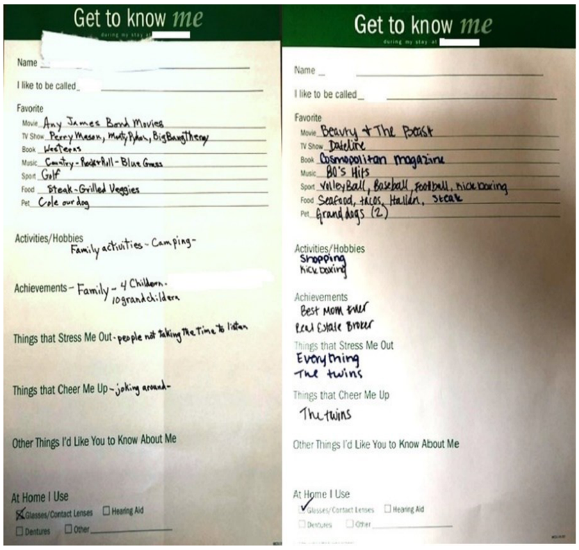

**Fig. 1 (abstract 000452).** See text for description

### 000483

#### Clinical characteristics of patients with very prolonged ICU-stay (≥ 90 days)

K. Roedl[1], D. Amann[1], L. Eichler[1], V. Fuhrmann[1], S. Kluge[1], J. Müller[2]
[1]Department of intensive care medicine, University Medical Center Hamburg-Eppendorf, Hamburg, Germany; [2]Department of anesthesiology, Tabea Hospital Hamburg, Hamburg, Germany
**Correspondence:** K. Roedl

*Intensive Care Medicine Experimental* 2019, **7(Suppl 3):**000483

**INTRODUCTION.** An increasing number of patients are surviving the acute critical illness, however prolonged critical care treatment may be necessary. A prolonged ICU-stay from weeks to months is associated with increased mortality of up to 50%. Data on outcome and functional status of patients with an ICU-stay ≥90 days are scarce. The aim of this study was to investigate mortality and functional outcome of patients with a very prolonged ICU stay.

**METHODS.** Single-center retrospective study at the University Medical Center Hamburg-Eppendorf, Germany including all critically ill adult patients with an ICU stay ≥90 days treated at our institution between January 1st 2008 and December 31st 2016.

**RESULTS.** 65,249 patients were treated during the study period, of these we identified 96 (0.1%) patients with a very prolonged ICU stay. Median age was 61 (49.8 – 67) years, 30 (31%) patients were female. Reason of ICU admission were abdominal/gastroenterological (28%, n=27), followed by sepsis (23%, n=22) and transplantation (15%, n=14). All patients needed mechanical ventilation (MV), with a median duration of 74.1 (55 – 95.1) days. 63 (66%) patients survived the ICU-stay, of these 95% (n=60) had unfavourable overall outcome (Overall performance category III/IV) at ICU discharge. One-year survival rate was 28% (n=27), 44% (n=12) had overall performance category III/IV. Severity of illness (SOFA, SAPS II) on admission were comparable in ICU-survivors and non-survivors. Length of MV, use of renal replacement therapy (both $p < 0.01$) and maximum lactate (5.3 vs 11.5 mmol/l; $p < 0.001$) were significantly higher in ICU non-survivors. ICU-stay was significantly longer in ICU non-survivors (137 vs 107 days; $p < 0.05$)

**CONCLUSION.** Only a small proportion of critically ill patients requires ICU-therapy ≥90 days. Although two-third of these patients survive the ICU stay, almost all of these patients have an unfavourable overall outcome.

### 000491

#### Discordance between online process and patient care as an important source of patient safety events in the intensive care setting: Exploration from the institutional incident reporting system

JS. Jerng[1], LC. Chen[2], YT. Li[2], SF. Huang[3], JS. Sun[2]
[1]Department of internal medicine, National Taiwan University Hospital, No. 7 Zhongshan South Road, Taipei, Taiwan; [2]Center for quality management, National Taiwan University Hospital, Taipei, Taiwan; [3]Department of nursing, National Taiwan University Hospital, Taipei, Taiwan
**Correspondence:** J.S. Jerng

*Intensive Care Medicine Experimental* 2019, **7(Suppl 3):**000491

**INTRODUCTION.** Patient safety events (PSEs) are commonly reported in the ICU setting, despite the advent of information technology with the proliferation of corresponding online processes to be connected to the real-world patient care tasks. Increasing work burden, complex care environment, and high variety of the patients' condition might render the probability of discordance between the corresponding online and real-world processes, threatening safety.

**METHODS.** We retrieve the case records of PSEs from the institutional incident reporting system of the intranet at an academic medical center in Taiwan. Cases related to the care of ICU patients were selected, and the investigators reviewed the reporting contents including the descriptions on the occurrence of PSE, provisional causes for the occurrences, and recommendations and suggestions on the improvement of the processes and further prevention of event occurrence. Online processes related to discordance with the real-world patient are were identified and classified by investigators, who also provided the possible causes related to discordance.

**RESULTS.** From 2011 to 2018, a total of 20,032 PSEs were reported to the institutional incident reporting system. Of these reports, 2,300 were related to the patient care of the ICU setting, resulting in patient harm of varying severity in 723 (31.4%) cases. We identified 832 cases to have corresponding online processes; 559 (67.2%) of them had at least one online process harboring discordance with the real-world patient care tasks, including 412 (74%) errors of the real-world tasks, 80 (14%) wrong timing in performing tasks, including delays and too early provision of care, and 67 (12%) omissions of tasks. The most common related patients care process for the discordance included medication administration (n=312, 56%), transfusion (n=92, 16%), laboratory tests (n=53, 9%), care of tubes (n=33, 6%), and transportation (n=21, 4%). For cases with discordance, errors were found in 90% of events of medication administration, 48% of transfusion events, and 70% of the laboratory test events. Analysis showed that for all 2,300 reports ICU cases, those with a corresponding online process had fewer patient harm than those without a corresponding online process (14.5% vs. 41.0%, p<0.001). However, for the 832 reported ICU cases with corresponding online processes, the cases with discordance with real-world tasks had significantly more patient harm than those without discordance (21.1% vs. 2.6%, p<0.001).

**CONCLUSION.** Although the provision of corresponding online processes for real-world patient care tasks might generally enhance efficiency and safety in the healthcare setting, the discordance of the online process and real-world patient care is an important source of patient harm due to the occurrence of patient safety events at the ICUs. Timely cross-validation between the online and offline process during patient care should be strongly considered in future designing and modification of the implementation of information technology in the intensive care setting.

### 000494

#### Shared Decision Making Impact on Early Tracheostomy in Prolonged Intubated Critical Patient

SH. Kuo, TH. Yang, KC. Lin, HC. Chen, MC. Wu, CP. Yang, HN. Chen, SC. Chang, SY. Chang, HL. Liang, WC. Huang
Department of Critical Care Medicine, Kaohsiung Veterans General Hospital, Kaohsiung City, Taiwan
**Correspondence:** S.H. Kuo
*Intensive Care Medicine Experimental* 2019, **7(Suppl 3):**000494

**INTRODUCTION.** The idea of shared decision making(SDM) was first proposed in 1972, with gradually increasing its importance as the upsurging interest in patient centredness and autonomy in health care interactions since the 1970s. Tracheostomy creation has long been poorly accepted, though with superiorly odds to pros, owing to cultural mis-understanding.
**OBJECTIVES.** Retrospective cohort study from single tertiary medical center adult ICU revealed introducing shared decision making on early Tracehostomy in prolonged intubated critical patient might improve total Tracheostomy rate, reduce ventilator days and length of hospital stay, and shorten the Tracheostomy decision time delay.
**METHODS.** Medical record of tertiary medical center adult ICU admitted respiratory failure patient from Jan. 1st, 2016 to Dec. 31th, 2017 was retrospectively reviewed, and further subdivided into pre-SDM and post-SDM(after Jan. 1st , 2017) intervention period for analysis. Prolonged intubation was defined as using mechanical ventilation support more than 14 days and sub-acute respiratory care center transferral needed. Early and late Tracheostomy were defined as created within 14 days and beyond the 14th day since being intubated and ventilated individually. Patient who was successful weaning, chronic ventilator dependent before admission, post Tracheostomy creation surgery, or expired was excluded. Data including total Tracheostomy rate, early and late Tracheostomy rate, ventilator weaning rate and ventilator days, in-hospital mortality, and length of hospital stay were collected. SPSS was applied for statistically analysis, and a p value less than 0.05 was considered significant difference.
**RESULTS.** Medical records from total 1253 patient from Jan. 1st , 2016 to Dec. 31th , 2017 were reviewed, and total 7137 patient ventilator days were recorded. Among the total 1253 patients, the pre-SDM(2016) total Tracheostomy rate was found 8.8%, in compared to post-SDM(2017) total Tracheostomy rate 9.6%, and 9.1% improvement impressed. By inclusion and exclusion, 128 patients from year 2016 and 112 patients from year 2017 were considered prolonged intubated left for further analysis. Pre-SDM early and late Tracheostomy rate were 42% and 58% individually, with ventilator weaning rate(43(79.6%) v.s 46(62.2%), p: 0.034), ventilator days(35.3+/-18.1 v.s 47.2+/-16.1, p< 0.001), in-hospital mortality(1(1.9%) v.s 6(8.1%), p: 0.237), and length of hospital stay(59.7+/-35.1 v.s 69.2+/-24.6, p: 0.091) of prolonged intubated patient. Post-SDM early and late Tracehostomy rate were 39% and 73% individually, with ventilator weaning rate(27(69.2%) v.s 56(76.7%), p: 0.389), ventilator days(34.6+/-17.6 v.s 47.5+/- 28.4, p: 0.004), in-hospital mortality(4(10.3%) v.s 6(8.2%), p: 0.737), and length of hospital stay(57.2+/-21.8 v.s 68.4+/-26.8, p: 0.028) of prolonged intubated patient. After SDM was introduced, ventilator days and length of hospital stay of prolonged intubated patient was found reduced 13 days and 11 days individually in compared in between early and late groups. The Tracheostomy decision making time delay was found 5 days less after SDM introduced.
**CONCLUSION.** Our retrospective cohort study from a single tertiary medical center adult ICU revealed introducing shared decision making on early Tracheostomy in prolonged intubated critical patient might improve total Tracheostomy rate, reduce ventilator days and length of hospital stay, and shorten the Tracheostomy decision time delay.

### 001481

#### Assessment of the quality of life 9 months after discharge from ICU in a Third Level Hospital

C. Fuster, C. Murcia, S. Foradada Ubach, C. Lorencio, JM. Sirvent Calvera
Intensive care medicine, Hospital Universitari de Girona Dr Josep Trueta, Girona, Spain
**Correspondence:** C. Fuster
*Intensive Care Medicine Experimental* 2019, **7(Suppl 3):**001481

**INTRODUCTION.** The improvement in the treatment of critical pathologies has increased the survival of patients admitted to intensive care units. Secondarily, some of these patients present psychic and emotional physical alterations encompassed in Post Uci syndrome (PICS)
**OBJECTIVES.** To know the quality of life of patients who survive an ICU admission nine months after discharge through the SF-36 survey to establish the incidence of the post-ICU syndrome in our intensive care unit.Relate these values with the pre-existing clinical situation to obtain risk factors associated with this syndrome.To assess if there are intra-ICU interventions that favour the appearance of the Post-ICU Syndrome.
**METHODS.** Prospective and longitudinal observational study of a cohort of patients during the months of March and April 2018 in a polyvalent ICU.We recorded demographic data (sex, age, weight), comorbidity (Barthel, Charlson), psychopathology (psychiatric history, psychotropic, treatment and toxic habits), as well as the reason for admission to our unit and associated severity (APACHE II and SAPS II).The days of admission to the ICU, death in ICU or hospital, the need for mechanical ventilation and complications (pneumonia associated with mechanical ventilation, cognitive impairment and pain at discharge) were documented.We recorded interventions performed intra-ICU such as treatment with benzodiazepines and its maximum dose, corticosteroids, muscle relaxants or anxiolytics by nasogastric tube.In a second phase, 9 months after discharge, patients who could be contacted by telephone were evaluated,discarding the deceased patients and those residing abroad, and SF-36 survey was conducted for the evaluation of the quality of life (by pone)
**RESULTS.** Out of a total of 50 patients, the survey could be performed on 29, of which 24 answered it. The overall results of the SF-36 survey showed that 95% of our patients obtained values between 56.10 and 75.40, so our patients nine months after discharge had a positive state of health. No intra-ICU intervention was associated with a worse SF-36 score. The only item prior to admission that is related in a statistically significant way to a worse score of SF 36 is previous psychiatric treatment.
**CONCLUSION.** The post-ICU syndrome is a low-frequency entity in our unit.
Although the sample from our study is small, we cannot demonstrate that any intra-ICU factor influences the presence of a post-ICU syndrome. The only factor that is related in a statistically significant way to the presence of the post-ICU syndrome, is previous psychiatric treatment

### 000018

#### Assessment of right ventricular function and acute pulmonary hypertension in patients with pulmonary embolism admitted to the intensive care unit: relationship between radiology, echocardiography and cardiac biomarkers. A retrospective cohort study

D. Pérez-Torres[1], V. Fraile-Gutiérrez[2], E. Prol-Silva[2], JA. De Ayala-Fernández[2], C. Díaz-Rodríguez[2], GJ. Posadas-Pita[2], P. Blanco-Schweizer[2], JJ. Sanz-Hernán[2], PM. Enríquez-Giraudo[2]
[1]Intensive care department, Hospital Universitario Río Hortega, Valladolid, Spain; [2]Department of intensive care medicine, Hospital Universitario Río Hortega, Valladolid, Spain
**Correspondence:** D. Pérez-Torres
*Intensive Care Medicine Experimental* 2019, **7(Suppl 3):**000018

**INTRODUCTION.** Pulmonary embolism (PE) may lead to the release of brain natriuretic peptide (BNP) due to right ventricular (RV) pressure overload, or troponin (Tn) due to transmural RV infarction with patent coronary arteries. Although cardiac biomarkers (CB) have been associated with worse prognosis, correlation with imaging techniques might not be strong.
**OBJECTIVES.** To describe the relationship between angio-CT, transthoracic echocardiography (TTE) and CB in the assessment of RV function and acute pulmonary hypertension (aPH) in patients with PE admitted to the Intensive Care Unit (ICU).
**METHODS.** We conducted a retrospective observational study in the ICU of a single university hospital. All the patients who were admitted to the ICU with PE as a primary diagnosis were included, over an 8-year period. Angio-CT, TTE and admission CB were recorded. Mann-Whitney U test was applied, as CB were not normally distributed.
**RESULTS.** A total of 82 patients were included, 43% male, aged 64 (44-77), APACHE-II 12 (8-17), 42% fibrinolytic therapy, 15% hospital mortality. Angio-CT, TTE and CB were performed in 92%, 84% and 100% of the patients, respectively. Thrombus was located in main pulmonary arteries (74%) or lobar/distal arteries (26%). TTE detected 52% RV systolic dysfunction and 59% tricuspid regurgitation, with an estimated pulmonary artery systolic pressure (PASP) of 53±15 mmHg. 48.4% of patients with thrombus in main or lobar arteries developed aPH, whereas no patient developed aPH with more distal thrombus (OR 14, 95%CI 0.8-257.1, p=0.07). TTE findings and their relationship with CB, expressed as P50 (P25-P75), are summarized in the Tables.
A cut-off point of troponin I ≥1 ng/mL showed 89% specificity, 18% sensitivity (53% NPV and 37% PPV) for the diagnosis of right ventricular disfunction.
**CONCLUSION.** Patients with PE in main or lobar arteries on angio-CT were more likely to develop aPH. In our series, CB did not correlate with RV function or aPH, although patients with highly abnormal levels of troponin were more likely to develop RV dysfunction. Performance of a TTE may be necessary to diagnose RV dysfunction or aPH in the setting of acute PE.

**Table 1 (abstract 000018).** Relationship between RV systolic function assessed by TTE and cardiac biomarkers

| | TAPSE ≥17 mm | TAPSE <17 mm | p |
|---|---|---|---|
| Tn I (ng/mL), n=55 | 0.51 (0.09-0.77) | 0.56 (0.15-1.6) | 0.27 |
| High-sensitivity Tn I (ng/L), n=9 | 12 (5-20) | 86 (20-150) | 0.24 |
| BNP (pg/mL), n=30 | 198 (124-810) | 112 (45-636) | 0.37 |

**Table 2 (abstract 000018).** Relationship between PASP assessed by TTE and cardiac biomarkers

| | PASP <30 mmHg | PASP ≥30 mmHg | p |
|---|---|---|---|
| Tn I (ng/mL), n=55 | 0.15 (0.02-0.3) | 0.16 (0.04-0.71) | 0.42 |
| High-sensitivity Tn I (ng/L), n=9 | 86 (20-150) | 12 (3-20) | 0.14 |
| BNP (pg/mL), n=30 | 150 (70-703) | 320 (90-504) | 0.93 |

## HSRO - Preventing adverse events

### 001490

#### Readmission in Intensive Care - Review to Improve

P. Campos, C. Torrão, D. Mano, R. Antunes, T. Cardoso, I. Aragão
Unidade de cuidados intensivos polivalente, Centro Hospitalar e Universitário do Porto, Porto, Portugal, Portugal
**Correspondence:** P. Campos
*Intensive Care Medicine Experimental* 2019, **7(Suppl 3):**001490

**INTRODUCTION.** The readmission rate in the intensive care unit (ICU) is associated with an increased mortality risk and is identified as a quality indicator. Several predictors of readmission are described in the literature, such as age, gender, severity of disease, type of admission, comorbidities and length of stay in ICU. However, these factors are not modifiable. Therefore, an appropriate analysis of potentially modifiable factors that may influence the readmission of these patients is lacking.
**OBJECTIVES.** This study aims to determine the rate of readmission, analyze the population of patients readmitted, and evaluate the determinants of the readmission rate.
**METHODS.** Retrospective and observational study conducted at Centro Hospitalar e Universitário do Porto, from January, 2016 to December, 2018.
All patients discharged alive from their first ICU admission were included. The exclusion criteria were age bellow 18 years, not at risk of readmission in the same hospital, due to transference to another center and patients with limitation of care after ICU discharge. Readmission to the ICU was defined as any return of a patient to the ICU after ICU discharge during the same hospitalization. A multivariate logistic regression analysis for determinants of readmission at any time during the same hospital episode was performed.
**RESULTS.** A total of 750 patients were included, of which 54 were readmitted during the same hospital episode (7.2%).
When conducting the univariate analysis, the factors independently associated with readmission were admission from emergency service and operating room; admission due to urgent surgery or medical problem without infection; confirmed infection at admission; lower Glasgow Coma Scale (GCS) at admission; acute kidney injury (AKI) during ICU stay; fever, lower GCS and delirium at ICU discharge; and higher Stability and Workload Index for Transfer (SWIFT) Score. In a multivariate model the retained variables were SWIFT score, AKI during ICU stay, lowest GCS at admission, confirmed infection at admission and operating room source of admission. The overall model with readmission at any time during the same hospital episode had a moderate performance with the Area Under an ROC Curve (AUC) of 0.75, 95% CI, 0.686–0.824; $p < 0.001$.
**CONCLUSION.** Several factors can be related to readmission in ICU. In our study, the variables associated with readmission were SWIFT score, AKI during ICU stay, lowest GCS at admission, confirmed infection at admission and operating room source of admission. These variables are patient related and non-modifiable, showing that in our population this indicator is not very useful in quality of care and is not probably potentially modifiable.

**REFERENCE(S)**

1. M.J. Al-Jaghbeer et al.; Incidence and Etiology of Potentially Preventable ICU Readmissions; Critical Care Medicine; 2016 Sep; 44(9):1704–1709.
2. A.L. Woldhek et al.; Readmission of ICU patients: A quality indicator?; J Crit Care. 2017 Apr;38:328-334.

### 001493

#### Intensive Care Unit: Early Readmission

P. TRAVASSOS, MS. Maiko, CL. Agnes, C. Miguel, PM. Maria, CV. Viviane, SOR. Salomón
NEUROCRITICAL CARE UNIT, HOSPITAL BP - A BENEFICÊNCIA PORTUGUESA DE SÃO PAULO, SÃO PAULO, Brazil
**Correspondence:** P. TRAVASSOS
*Intensive Care Medicine Experimental* 2019, **7(Suppl 3):**001493

**INTRODUCTION.** The Intensive Care Unit (ICU) occupies 7% of the hospital beds, representing 20-30% of the hospital cost, equivalent to 62 billion dollars per year in the United States (REF). Studies have demonstrated a relationship between discharge criteria, length of stay and patient outcome (refs), so it is in the interest of physicians and managers to study the topic to avoid excessive patient time in the ICU and the scheduling of expenditures for rehospitalization, both related to increased mortality.

**OBJECTIVES.** To evaluate the early rehospitalization rate and the main associated characteristics in Neurological ICU in the period of 4 years, from 2014 to 2018.

**METHODS.** A review of all early rehospitalizations (<48 hours) was carried out in the period from 2014 to 2018 through a database of the hospital management system and evaluated compliance with the discharge criteria according to the ICU discharge protocol and its correlation with readmission.

**RESULTS.** In the analyzed period, 9,319 patients were admitted to the unit with 25 readmissions (0.32%), 7 in 2014, 7 in 2015, 4 in 2016 and 2 in 2017 and 5 in 2018. Of the 25 patients, only 1 (4%) did not present criteria according to ICU discharge protocol. Patients who were readmitted had increased length of stay when compared to the unit profile (8.4 days x 5.6 days), and there was no correlation with increased mortality in the analyzed sample. Average age: 64.78 years Occupancy Rate: 88.14% SAPS 3: 43.41 SOFA: 2.28. Surgical: 39.57%

**CONCLUSION.** Despite the high complexity, there is a low rate of early rehospitalization in the unit, which evidences a safe process of high and quality care.

### 001501

#### Dark Side of The Moon: Chloride

G. Kir[1], S. Uğur[1], I. Erus[1], OE. Akbulut[2], R. Yılmaz[3], S. Asar[3], Z. Cukurova[3], E. Senturk[1], OG. Hergunsel[3], N. Cakar[1]
[1]Anesthesiology and Intensive Care Unit, Koç University Hospital, Istanbul, Turkey; [2]Anesthesiology and intensive care unit, American Hospital, Istanbul, Turkey; [3]Anesthesiology and intensive care unit, Bakırköy Sadi Konuk Education and Research Hospital, Istanbul, Turkey, Turkey
**Correspondence:** G. Kir
*Intensive Care Medicine Experimental* 2019, **7(Suppl 3):**001501

**INTRODUCTION.** Several studies have evaluated the impact of sodium disturbances on outcomes in critically ill so far but although chloride (Cl−) abnormalities are also very common, the number of studies reporting the relevance of dyschloremia and its association with intensive care (ICU) outcomes, are still limited. We aimed to investigate the prevalence of dyschloremia at the time of ICU admission and its relation with severity of illness and outcome implications of dyschloremia in critical care settings.

**OBJECTIVES.** The primary objective of our study was to evaluate the incidence of dyschloremia in critically ill and its relationship with ICU mortality and length of ICU stay, while the association between dyschloremia and severity of illness and organ failure were the secondary objectives.

**METHODS.** Non-surgical, adult patients who required ICU for more than 48 hours were included in our retrospective study, conducted in three ICUs, between 2015-2018. Demographic data, Acute Physiology and Chronic Health Evaluation (APACHE)II and Sequential Organ Failure Assessment (SOFA) scores, admission Cl − levels and blood gas analyses were abstracted from clinical information systems (MetaVision, iMDsoft and Consult, Orion Health) within first 24 hours of ICU admission. Patients were classified into three groups (Cl*normo*=97-108 mmol/L, Cl*hypo*<97 mmol/L and Cl*hyper*>108 mmol/L) accordingly their worst Cl− values measured within 24 hours. One-way ANOVA, Chi-square, Kruskal Wallis tests were used to analyze data while the association between ICU mortality and chloride levels was examined using multivariate logistic regression. (p<0.005 was considered to be statistically significant)

**RESULTS.** Of 3985 patients, 909(22.8%) were hyperchloremic and 475(11.9%) were hypochloremic. Cl*hyper* had higher ICU mortality (p<0.001; p=0.008, respectively) and longer length of ICU stay (p=0.001; p<0.001, respectively) when compared to Cl*normo* and Cl*hypo*. APACHE II scores were higher in Cl*hypo* when compared to Cl*normo* and Cl*hyper*. (p<0.001; p<0.001, respectively), on the other hand SOFA scores were higher in Cl*hyper* when compared to Cl*normo* and Cl*hypo* (p<0.001; p<0.001, respectively). *(Table 1)* We also evaluated baseline sodium levels in these patients and found an statistically significant association between dysnatremia and higher ICU mortality rates; while length of ICU stay was not associated with sodium disturbances. *(Table 2)* Multivariate analysis showed that dyschloremia and dysnatremia was independently associated with increased ICU mortality and longer length of ICU stay (OR 0.99, 95% CI 0.98-1; p=0.02, OR 1.01, 95% Cl 1-1.02; p=0.03, respectively) as well as APACHE II (OR 0.97, 95% Cl 0.96-0.98; p<0.001) and SOFA scores (OR 0.88, 95% Cl 0.86-0.9; p<0.001).

**CONCLUSION.** Chloride disturbances are associated with severity of illness, organ failure and poor patient outcomes in critically ill. This study suggests that the both sides of the moon is the real picture to be seen by underlining the clinical importance of chloride.

**Table 1 (abstract 001501).** Characteristics and outcomes of dyschloremic patients

| | Hyperchloremia (Cl*hyper*) >108 mmol/L N=909(22.8%) | Normochloremia (Cl*normo*) 97-108 mmol/L N=2601(65.3%) | Hypochloremia (Cl*hypo*) <97 mmol/L N=475(11.9%) | P value (Cl*hyper* vs. Cl*normo*) | P value (Cl*normo* vs. Cl*hypo*) | P value (Cl*hyper* vs. Cl*hypo*) |
|---|---|---|---|---|---|---|
| Age, years | 59.6±21.2 | 64.5±19 | 71.3±16.6 | p<0.001* | p<0.001* | p<0.001* |
| Males (%) | 53.6% | 58.1% | 58.3% | p=0.052 | p=0.901 | p=0.113 |
| APACHE II (mean) | 20.1±7.8 | 20.6±7.9 | 23.4±8.3 | p=0.26 | p<0.001* | p<0.001* |
| SOFA (mean) | 7.7±3.7 | 6.7±3.7 | 6.6±3.4 | p<0.001* | p=0.69 | p<0.001* |
| ICU length of stay, days | 12.6±14.4 | 10.7±14.6 | 9.4±11 | p=0.001* | p=0.17 | p<0.001* |
| ICU mortality, n(%) | 306(33.66) | 707(27.18) | 127(26.74) | p<0.001* | p=0.841 | p=0.008* |

**Table 2 (abstract 001501).** characteristics and outcomes of dysnatremic patients

| | Hypernatremia (Na*hyper*) >145 mmol/L N=454(11.4%) | Normonatremia (Na*normo*) 135-145 mmol/L N=2140(57.5%) | Hyponatremia (Na*hypo*) <135 mmol/L N=1238(31.1%) | P value (Na*hyper* vs. Na*normo*) | P value (Na*normo* vs. Na*hypo*) | P value (Na*hyper* vs. Na*hypo*) |
|---|---|---|---|---|---|---|
| APACHE II (mean) | 23.75±8.52 | 20.05±7.8 | 21.23±7.93 | p<0.001* | p<0.001* | p<0.001* |
| SOFA (mean) | 7.64±3.5 | 6.64±3.68 | 7.24±3.67 | p<0.001* | p=0.107 | P<0.001* |
| ICU length of stay, days | 11.89±15.93 | 10.9±13 | 10.83±15.64 | p=0.363 | p=0.362 | P=0.989 |
| ICU mortality, n(%) | 152(33,50) | 590(25.70) | 398(32.10) | P=0.001* | P=0.604 | P<0.001* |

### 001527

#### Electrical impedance tomography predicts lung strain determined by computed tomography: A translational proof of concept study

R. Cornejo[1], P. Iturrieta[2], T. Milá[3], C. Kajiyama[3], D. Arellano[1], AIJ. Gajardo[1], D. Guiñez[1], L. Lopez[4], R. Brito[1], MA. Cerda[1], M. Lazo[1], S. Gonzalez[1], M. Zavala[1], V. Rojas[1], JN. Medel[1], C. Ramos[4], DE. Hurtado[2], C. Morais[3], A. Bruhn[5], M. Amato,[3]

[1]Hospital clínico, unidad de pacientes críticos, Universidad de Chile, Santiago, Chile; [2]Institute for biological and medical engineering, Pontificia Universidad Catolica, Santiago, Chile; [3]Laboratório de pneumologia lim09, University of Sao Paulo, Sao Paulo, Brazil; [4]Departamento de radiología, hospital clinico universidad de chile, Universidad de Chile, Santiago, Chile; [5]Departamento de medicina intensiva, facultad de medicina, Pontificia Universidad Catolica, Santiago, Chile

**Correspondence:** R. Cornejo

*Intensive Care Medicine Experimental* 2019, **7(Suppl 3):**001527

**INTRODUCTION.** Mechanical ventilation can induce lung injury, phenomenon known as VILI. An important mechanism of the VILI is the excessive global strain. Even more, regional strain is spatially correlated with regional inflammation. Information on regional strain could help to develop protective ventilatory strategies. The standard for this evaluation is computed tomography (CT), which does not allow continuous assessmentand exposes to radiation. Electrical impedance tomography (EIT) is able to monitor changes in regional lung ventilation and it could become a surrogate for CT-measured strain

**OBJECTIVES.** To compare the global and regional strain measured by CT with the change in electrical impedance (ΔZ) adjusted by anthropometric measurements in pigs and ARDS patients

**METHODS.** The study was approved by Ethics Committee. Three pigs, mechanically ventilated using VT of 250 and 500 mL, underwent whole-lung CT at end-inspiration and end-expiration with PEEP 10 and 20 cmH2O, before and after lung injury induction. Eleven ARDS patients also underwent whole-lung CT during at end-expiration and end-inspiration with PEEP 5 and best PEEP according EIT. A biomechanical analysis was employed to construct 3D maps of the volumetric strain. CT and EIT examinations were performed simultaneously. Strain maps were divided into 4 regions of interest "ROI" (Upper and Lower, Right and Left), coinciding spatially with the regions upon which inflation was calculated for EIT. Due to repeated measures in pigs an humans, linear mixed effect models were performed to predict lung strain and aeration measured by CT from ΔZ obtained by EIT, adjusting for known confounders. For all models, individual pigs or humans were considered as random effects, and PEEP as repeated measures. Diagnostic performance of EIT-based predictions of lung strain was evaluated by the Lin's concordance coefficient (LCC) and Bland-Altman Graph

**RESULTS.** In pigs, linear association for global strain was acurate (R = 0.974, p-valor<0,001), and large positive relations were found at different regions of interest. In humans, linear association at global, as well as in many lung ROI were high and positive with R > 0.932. Agreement of lung strain measured by CT and predicted by EIT-based models were excellent in overall terms in humans (LCC >0.95).

**CONCLUSION.** The change in electrical impedance provides a noninvasive assessment of global and regional strain, without radiation at bedside.

1. PROJECT FONDECYT 1161510

### 001530

#### Impact of providing laboratory tests data on clinician ordering behavior in the Intensive Care Unit

MD. Bosque, M. Martínez, S. Barbadillo, J. Lema, R. Tomás, M. Ovejero, A. Olmo

Intensive care medicine department, Hospital Universitario General de Cataluña, Sant Cugat del Vallés, Spain

**Correspondence:** M.D. Bosque

*Intensive Care Medicine Experimental* 2019, **7(Suppl 3):**001530

**INTRODUCTION.** Unnecessary laboratory test ordering in the Intensive Care Unit can be a prominent source of patient discomfort, morbidity, increased workload and wasteful spending.

Providing blood tests order data to physicians has been considered a promising strategy to reduce unnecessary procedures in the intensive care unit but its impact on patterns of care remains unclear.

**OBJECTIVES.** The purpose of this study is to determine whether providing complete and personalized laboratory tests data could change clinicians practice behaviors.

**METHODS.** We conducted a preliminary study in a 16-bed polyvalent intensive care unit from April 2017 to April 2019 with a pre-intervention and post-intervention design.

1. Pre-intervention: during a baseline period (April 2017 through January 2018) a randomized non-consecutive 8 day follow up of the blood tests prescribed was carried out.
2. Intervention (January 2019): the intervention consisted of sending by e-mail to all the Intensive Care physicians relevant information about the prescription of blood tests performed in the last year which included:
   - Number and type of tests ordered by each physician
   - List of laboratory test costs per physician, from highest to lowest.
3. Post-intervention: after the intervention (February through April 2019) a randomized non-consecutive 9 day follow up of the blood tests prescribed was carried out.

Descriptive statistics include frequencies and percentages for categorical variables. Chi- squared test were used to compare variables during pre-intervention and post-intervention periods.

**RESULTS.** Six attending physicians provided input for a total of 229 patient-days. Results are shown in this table:

**CONCLUSION.** There is a global decrease in ordering and prescribing behavior after laboratory information was provided to the Intensive Care physicians.

This intervention may reduce the number of unnecessary blood tests specially in the most critical patients.

**REFERENCE(S)**

1. Mina S. Sedrak, MD et al. Effect of a Price Transparency Intervention in the Electronic Health Record on Clinician Ordering of Inpatient Laboratory Tests. The PRICE Randomized Clinical Trial. JAMA Intern Med. 2017 Jul 1;177(7):939-945
2. Feldman LS, et al. Impact of providing fee data on laboratory test ordering: a controlled clinical trial. JAMA Intern Med. 2013;173(10):903–908.
3. Allan GM, Lexchin J. Physician awareness of diagnostic and nondrug therapeutic costs: a systematic review. Int J Technol Assess Health Care. 2008;24(2):158–165.

**Table 1 (abstract 001530).** See text for description

| | Pre-intervention | Post-intervention | Level of significance (p) |
|---|---|---|---|
| Total number of patients-day | 114 | 117 | |
| Critical patients (%) | 41 (36%) | 35 (30%) | |
| Semi-critical patients (%) | 73 (64%) | 82 (70%) | |
| Total number of unnecessary blood test (%) | 17 (15%) | 10 (8,5%) | P = 0,13 |
| Critical patients (%) | 10 (59%) | 2 (20%) | *P = 0,02** |
| Semi-critical patients (%) | 7 (41%) | 8 (80%) | P = 0,9 |

### 001543

#### Adverse events during intra-hospital transport of critical patients

P. TRAVASSOS, CL. Agnes, MS. Maiko, SF. Luciana, MC. Lígia, MR. Cristiane, RSS. Rosana, CV. Viviane, SOR. Salomón
NEUROCRITICAL CARE UNIT, HOSPITAL BP - A BENEFICÊNCIA PORTUGUESA DE SÃO PAULO, SÃO PAULO, Brazil
**Correspondence:** P. TRAVASSOS
*Intensive Care Medicine Experimental* 2019, **7(Suppl 3):**001543

**INTRODUCTION.** Intra-hospital transport is related to a high incidence of adverse events and negative outcomes. The objective of this work is to describe the incidence of clinical and non-clinical events during in-hospital transport of critical patients and to analyze the associated risk factors

**OBJECTIVES.** To describe the incidence of clinical and non-clinical events during in-hospital transport of critical patients and analyze the associated the risk factors.

**METHODS.** A cohort study with retrospective collection, from October 2016 to October 2017, which analyzed all intrahospital transports for diagnostic and therapeutic purposes in a large hospital, and evaluated the adverse events and related risk factors.

**RESULTS.** : In the period, 1559 intrahospital transports were performed in 1348 patients, with a mean age of 66 ± 17 years, with mean transport time of 43 ± 34 minutes. 19.8% of patients were on vasoactive drugs; 13.7% in sedatives and 10.6% in mechanical ventilation. Clinical events occurred in 117 transports (7.5%) and non-clinical in 125 transports (8.0%). Communication failures were prevalent, however, by applying multivariate analysis, the use of sedatives, noradrenaline, nitroprusside and time of transport were associated with clinical adverse events. Use of dobutamine and transport time have been associated with non-clinical events. At the end of transport, 98.1% of the patients presented clinical conditions unchanged in relation to their baseline state

**CONCLUSION.** Intrahospital transport is related to a high incidence of adverse events, and the time of transport and use of sedatives and vasoactive drugs have been related to these events.

### 001563

#### The current state of critical care ultrasound training in Ukraine

M. Grynovska[1], A. Wong[2]
[1]Anesthesia and intensive care, Ivano-Frankivsk National Medical University and Regional University Teaching Hospital, Ivano-Frankivsk, Ukraine; [2]{street_address}, Guildford, United Kingdom
**Correspondence:** M. Grynovska
*Intensive Care Medicine Experimental* 2019, **7(Suppl 3):**001563

**INTRODUCTION.** Ultrasound (US) is increasingly recognized as an essential tool in the ICU setting. The safety and utility of bedside ultrasonography by an appropriately trained ICU physician has now been well demonstrated.[1] Despite the worldwide routine use of ultrasound in ICUs, a large number of ICU physicians remain unacquainted with US techniques. Following the 2011 international expert statement, the training of every ICU physician should incorporate basic level of critical care ultrasonography (CCUS).[2] We decided to investigate how the evolving CCUS paradigm of ICU physician skills and competencies is applied in our country.

**OBJECTIVES.** To summarize knowledge on the current use of CCUS and attitudes towards its utilization in the ICUs of a university teaching hospital.

**METHODS.** Our study focused on the ICU physicians (n=55) of the university teaching hospital. Data collection was performed with the help of an electronic survey.

**RESULTS.** The response rate of the survey was high (90%). 66% of the respondents were young specialists. The majority (61.7%) had no previous experience in CCUS and only 36.2% were aware of the locally organized training programs. The most useful CCUS modalities reported were pleural/lung US, vascular US for access, FAST, nerve block and vascular US-DVT. All of the respondents recognized the existence of barriers to CCUS training, citing lack of training, curriculum and financial costs as the most significant ones. Although CCUS remains the prerogative of the on-call radiologists, it was not perceived as a training barrier.

**CONCLUSION.** While Ukraine is lacking a formal CCUS training program, there is an unmet demand for it in the ICU. Our survey has demonstrated a prevailing lack of US skills among the young ICU physicians. At the same time there was a general consensus on the list of core US modalities that should be incorporated in such training as well as on the barriers that prevent ICU physicians from mastering them. The young demographics of the study has also highlighted the potential for international training programs and initiatives. Due to the limited number of respondents in our survey, further study across hospitals is required to fill in the knowledge gap on the country-specific state of CCUS training. In future perspective, we hope to impact awareness of the ICU physicians and stimulate the activity of the locally organized training programs. In a broader sense, we expect this study to start a cross-country conversation on the challenges of CCUS standards implementation and the possible ways forward.

**REFERENCE(S)**

1. Beaulieu Y, et al. Chest. 2005 Sep;128 (3):1766-81.
2. International expert statement on training standards for critical care ultrasonography. Intensive Care Medicine 2011; 37: 1077-83.
3. I would like to thank ESICM NEXT Mentoring and my mentor Adrian Wong for supervision and guidance.

### 001569

#### Intensive Care Specific Virtual Reality Exposure Therapy is immersive, feasible and safe to treat psychological post-ICU sequelae; a healthy adult study

JH. Vlake[1], EJ. Wils[1], J. Van Bommel[2], T. Korevaar[3], D. Gommers[2], M. van Genderen[2]
[1]Intensive care, Franciscus Gasthuis, Rotterdam, Netherlands; [2]Intensive care, Erasmus MC, Rotterdam, Netherlands; [3]Internal medicine, environmental epidemiology, Erasmus MC, Rotterdam, Netherlands
**Correspondence:** J.H. Vlake
*Intensive Care Medicine Experimental* 2019, **7(Suppl 3):**001569

**INTRODUCTION.** Virtual Reality Exposure Therapy (VRET) is effective in treating several psychiatric disorders like combat-related posttraumatic stress disorder (PTSD). In recent years, follow-up studies demonstrated that ICU survivors suffer frequently from loss of quality of life due to long term psychiatric morbidity, especially symptoms of PTSD. An effective strategy is currently lacking to prevent and treat psychiatricsequelae in intensive care (ICU) survivors despite it is known that post-ICU patients are motivated to undergo VRET in the aftermath of critical illness (1). To date, the safety and feasibility of ICU specific-VRET is unknown.

**OBJECTIVES.** To evaluate the feasibility, safety, and suitability of a newly developed ICU-specific VRET module compared to 2D exposure in healthy adults.

**METHODS.** Forty-fivehealthy adults were randomly assigned into three groups to receive a newly designed ICU-specific exposure therapy module on different platforms; a 2D flatscreen (n=15; 2D group), VR-goggles (n=15; VR group) and a crossover group that first watched the video on a 2D flatscreen followed by the VR-goggles (n=15; crossover group). Safety was assessed using the cyber sickness questionnaire (SSQ) and by monitoring vital signs (mean arterial pressure, heart rate, respiratory rate and saturation). Feasibility and suitability were assessed through the feeling of presence (immersion) with the Igroup Presence Questionnaire (IPQ).

**RESULTS.** Mean age was 57.7 years (SD 7.1) and 30 out of 45 adults were woman (66.7%). Adults experienced a higher sense of presence using VR (IPQ; VR to 2D group, mean difference [SE], 0.967 [0.345], p=0.01; crossover group, mean difference [SE], 2,787 [0.183], p < 0.001 ) (figure 1).There was no difference in total cyber sickness, nausea or oculomotor score in adults in the crossover group. Comparing the VR group with the 2D group, adults in the VR group showed higher total cyber sickness (mean difference [SE], 2.067 [0.599], p<0.01) and nausea score (mean difference [SE], 0.667 [0.319], p<0.05) (figure 2). All participants scored their cyber sickness as clinically irrelevant and no session was stopped early because of side effects. No differences were found in vital signs during the intervention and between groups.

**CONCLUSION.** We demonstrated that ICU-specific VR exposure is more immersive compared to 2D. This increased feeling of presence (immersion), suggests that VR may be a suitable medium to treat the psychological sequelae of an ICU stay, such as PTSD. Although VR demonstrated to befeasible, safe and well tolerable, one must be aware of the well-known side-effect: cybersickness.

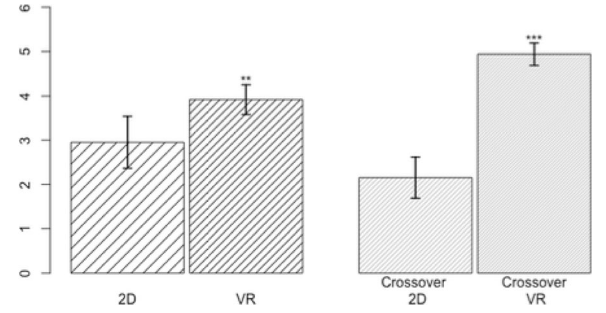

**Fig. 1 (abstract 001569).** Presence

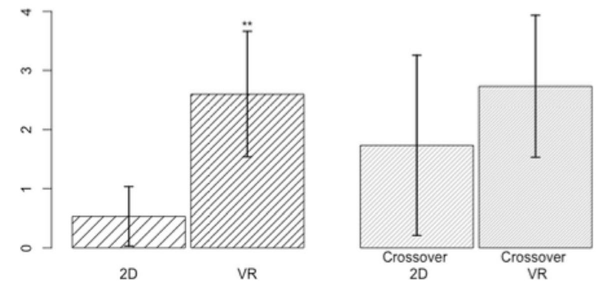

**Fig. 2 (abstract 001569).** Simulator sickness

### 001578

#### Global Challenges in Continuity of Critical Care. A Mixed-Methods Study

A. Casarin[1], E. Ayebale[2], T. Baker[3], J. Mkubwa[4], GS. Shrestha[5], A. Walecka[6], P. Duque[7], N. Kissoon[8], A. Kulkarni[9], A. Kwizera[10], I. Martin-Loeches[11], M. Mer[12], JL. Pinedo[13], L. Sanchez-Hurtado[14], TS. Valley[15], J. Preller[16]
[1]Nihr research design service, University of Hertfordshire, Hatfield, United Kingdom; [2]Department of anaesthesia, Makerere University, Kampala, Uganda; [3]Dept of public health sciences, Karolinska University Hospital, Stockholm, Sweden; [4]Anaesthesia and intensive care department, Princess Marina Hospital, Gaborone, Botswana; [5]Department of anaesthesiology, T. U. Teaching Hospital, Kathmandu, Nepal; [6]Intensive care unit, Royal Free Hospital, London, United Kingdom; [7]Intensive care unit, Gregorio Marañón Hospital, Madrid, Spain; [8]Department of pediatrics and emergency medicine, BC Children's Hospital, Vancouver, Canada; [9]Div of critical care medicine, Tata Memorial Hospital, Mumbai, India; [10]Department of anaesthesia and critical care, Makerere university college of health sciences, Kampala, Uganda; [11]Intensive care unit, St James's University Hospital, Dublin, Ireland; [12]Divisions of critical care and pulmonology, University of the Witwatersrand, Johannesburg, Johannesburg, South Africa; [13]Intensive care unit, Lambayeque Regional Hospital, Chiclayo, Peru; [14]Department critical care medicine, UMAE Specialties Hospital "Antonio Fraga Mouret" National Medical Center, Mexico City, Mexico; [15]Pulmonary and critical care dept, University of Michigan, Ann Arbor, United States of America; [16]John farman intensive care unit, Addenbrookes, Cambridge, UK, United Kingdom
**Correspondence:** A. Casarin
*Intensive Care Medicine Experimental* 2019, **7(Suppl 3):**001578

**INTRODUCTION.** Integrated health services are embedded in a process that provides a continuum of care and have been advocated by the World Health Organisation (WHO) as the vehicle to improve health outcomes worldwide (Regional Office for Europe of the WHO, 2016). The health loss to injuries, conditions and risk factors are quantified using an index called the global burden of disease (GBD). The three leading causes of GBD in the 1990-2016 period have been ischaemic heart disease, cerebrovascular disease, and lower respiratory infections (GBD 2016 DALYs and HALE Collaborators, 2017), all conditions that often need intensive care. The GBD and sequelae in Intensive Care Unit (ICU) survivors depends on the number of critically ill patients, the resources available to treat them and the morbidity protracted after discharge (Stevens et al., 2014). It is hypothesised that low resources limit the possibility of admitting patients to intensive care units and the services offered after ICU discharge.

**OBJECTIVES.**

- To identify how critically ill patients are triaged in Low-Middle-Income Country (LMIC) compared to High-Income Country (HIC) to determine whether triage practice is linked to resource settings;
- To evaluate which post discharge services are available to critically ill patients in LMIC compared to HIC to detect if there is a difference in service provision;

- To explore barriers and facilitators of continuity of critical care and identify potential opportunities for quality improvement initiatives driven by cross-country learning.

**METHODS.** 14 intensive care doctors from Europe, Africa, America and Asia participated in a online survey and five of them in in-depth interviews. The survey included three sections in order to collect data on the services' context, the triage and the post ICU practice. The interviews were conducted to explore barriers and facilitators of ICU triage and post ICU care in different settings. Descriptive statistics and thematic analysis have been used to analyse data. Triangulation has been applied to identify convergence, complementarity or discrepancy between data collected via the two research methods.

**RESULTS.** Nine of the 14 participants who completed the questionnaire described practice in a LMIC, while five respondents provided examples of HIC practice. Globally, there are several models of care: public, governmental funding (6 LMIC and 4 HIC study units); private funding (1 LMIC unit); fee-for-service model where family/relatives contributes (2 LMIC units); access to health insurance (1 HIC unit). According to study results, when family needs to contribute financially there may be no other choice than limit treatment during and after intensive care. Also, lack of standardised end of life procedures in LMIC make triage a challenging process. Post discharge services are limited by resources according to the majority of participants with a tendency to holistic care in the LMIC units. Staffing, bed availability, and infrastructures (equipment, logistics) have been named as potential barriers to triage and discharges from ICU in all resource settings. Outreach services have been advocated for appropriate triage and after discharge monitoring

**CONCLUSION.** Barriers and facilitators of triage are similar between LMIC and HIC study units despite resources available. Capacity of triage and post discharge services are limited in settings where relatives need to contribute to cost of care. Indeed, triage may be easier in the context of low resources because there may be no other choice than refusing admission. Therefore deciding which patient to admit or not should be planned as soon as possible. Outreach and post discharge services could facilitate the prevention of deterioration and therefore help to decrease the global burden of critical illness.

**REFERENCE(S)**

1. Stevens, R., Hart, N. and Herridge, M. (2014) Textbook of Post-ICU Medicine: The Legacy of Critical Care.
2. GBD 2016 DALYs and HALE Collaborators (2017) 'Global, regional, and national disability-adjusted life-years (DALYs) for 333 diseases and injuries and healthy life expectancy (HALE) for 195 countries and territories, 1990-2016: a systematic analysis for the Global Burden of Disease Study 2016', Lancet, 390(10100), pp. 1260-1344.
3. Regional Office for Europe of the World Health Organization (2016) The European Framework for Action on Integrated Health Services Delivery 2016 Available at: http://www.euro.who.int/__data/assets/pdf_file/0020/318323/FAQ-FFA-IHSD.pdf?ua=1.
4. No grant was required for the delivery of this study.

### 001582

#### Experience with Critical Care Admission in a Private ICU, Lagos Nigeria

A. Fadeyi[1], M. Oladimeji[2], G. Asiyanbi[3], S. Olanipekun[1], O. Adekola[1]
[1]Intensive care unit, Onelife Hospital, LAGOS, Nigeria, Federal Republic of; [2]Anesthesia & intensive care unit, Lagos University Teaching Hospital, LAGOS, Nigeria, Federal Republic of; [3]Anaesthesia & intensive care, Lagos University Teaching Hospital, LAGOS, Nigeria, Federal Republic of
**Correspondence:** O. Adekola
*Intensive Care Medicine Experimental* 2019, **7(Suppl 3):**001582

**INTRODUCTION.** The need for critical care service is on the increase; however, ICU services are not readily available and accessible. Lagos, Nigeria with a steaming population of approximately 21 million, who have access to only four government owned ICU centers with 13 beds. This has led to private initiatives in critical care services.

**OBJECTIVES.** We determined the indication and outcome of ICU admission in a private critical care center in Lagos, Nigeria.

**METHODS.** This is a prospective observational study of critically ill patient admitted between January, 2016 and March, 2019. The case note and ICU charts for all patients referred to our center were reviewed.

**RESULTS.** A total of 162 patients were admitted, of whom females were 92 (56.8%), the most common specialist of admission was surgery 50% followed by medicine with 29.2%. The median apache score was 19.8 (25th -75th percentile, 14.5-26.7).

There was delay in referral in 82.2% of patients, with a median period of 34 hours (2-72hours). The most common reason for delay was unavailability of ICU bed space in 63.7% followed by no ICU facility at referral center 26%. The ICU survival at our center is 106 (65.4%).

**CONCLUSION.** There is an urgency need for provision of critical care services to meet the teeming population in Lagos, Nigeria. The most common reason for delay in ICU services is lack of bed space.

**REFERENCE(S)**

1. Anthony A. Iwuafor, Folasade T. Ogunsola, Rita O. Oladele, et al. Incidence, Clinical Outcome and Risk Factors of Intensive Care Unit Infections in the Lagos University Teaching Hospital (LUTH), Lagos, Nigeria. PLoS One 2016; 11(10): e0165242.
2. Adekola O. O, Soriyan OT, Meka I, Akanmu MO, Olanipekun S, Oshodi TAA. The incidence of electrolyte and acid-base abnormalities in critically ill patients using point of care testing (I–Stat portable analyser). Nig Q J Hosp Med. 2012 Apr-Jun; 22(2):103-108.

### 001584

#### Does cognitive, behavioural or mindfulness therapy lead critical care patients to feel more confident in their ability to cope with psychological symptoms?

C. Lambert, J. Preller, Z. Martin
Critical care, Addenbrooke's Hospital, Cambridge, United Kingdom
**Correspondence:** C. Lambert
*Intensive Care Medicine Experimental* 2019, **7(Suppl 3):**001584

**INTRODUCTION.** Critical Care patients often experience pronounced psychological distress during intensive care treatment due to its extremely disempowering nature (e.g. Wade et al., 2013). As such they are highly at risk of developing low self-efficacy regarding influencing negative psychological states. This passive attitude can slow their rate of recovery, and is also strongly predictive of future psychological problems (Wade et al. 2012, Davydow et al. 2009)

**METHODS.** A sample of 64 critical care patients from the Intensive Care and Neurological Intensive Care Units of Addenbrooke's hospital was offered cognitive, behavioural or mindfulness therapeutic input. At the beginning of treatment a focus was agreed with each patient on a main problem area: depression, anxiety or stress (early post-traumatic response, pain response, or agitation). Patients rated their level of confidence that they could improve their psychological symptoms on a 5-point scale at the end of each treatment session. This scale consisted of the options: '1 = low', '2 = quite low', '3 = moderate', '4 = quite high', and '5 = high'. The resulting data was analysed using match-pair T-tests in order to investigate if patients self-rated levels of confidence changed from the beginning to the end of treatment for: (a) all patients in the sample, (b) for patients within each area of psychological distress, and (c) for patients within each modality of treatment (cognitive, behavioural, or mindfulness). Variance amongst means for (b) and (c) was also examined using one-way ANOVA.

**RESULTS.** T-test (a) was significant at $p<0.0001$; the T-tests for (b) and (c) were all significant at $p<0.05$ apart from when applied to those offered behavioural input $p>0.05$; the ANOVA conducted for (b) and (c) were all non-significant apart from when this test was applied to the problem experienced at the end of treatment $p<0.05$.

**CONCLUSION.** The finding that (other than patients offered mainly behavioural treatment) all treatment conditions measured showed a significant increase in self-rated levels of self-efficacy suggests that cognitive and mindfulness techniques are effective for this purpose. These results also indicate that this treatment is very effective in this area for patients overall, and at a lower level of significance, for psychological problems considered individually. However, although the use of behavioural techniques showed a signal for improvement in self-rated patient self-efficacy, this did not reach significance within this cohort, a finding which if substantiated, could indicate that cognitive and mindfulness techniques possess self-efficacy boosting properties for this patient population which behavioural techniques do not stimulate.

The ANOVA results indicate that, both in terms of psychological problem experienced and modality of treatment offered, the patients in this sample do not display statistically significant differences in levels of self-efficacy at the beginning of treatment. This finding maintains to the end of treatment for treatment modality, but does not for problem experienced. This suggests that one or more of the psychological problems responds differently to treatment than the others, which may indicate that patient self-efficacy in this area can be altered more for certain psychological problems than others.

**REFERENCE(S)**

1. Wade, D., Hardy, R., Howell, D., & Mythen, M (2013), Investigating risk factors for psychological morbidity three months after intensive care: a prospective cohort study, Minerva Anestesiol 79(8) 944-63
2. Wade, DM., Howell, DC., Weinman, JA., Hardy, RJ., Mythen, MG., Brew in, CR., Borja-Boluda, S., Matejow sky., CF., & Raine, RA (2012), Investigating risk factors for psychological morbidity three months after intensive care: a prospective cohort study, Critical Care 16(5): R192
3. Davydow , DS., Gifford, JM., Desai, SV., Bienvenu, OJ., & Needham, DM (2009), Depression in general intensive care unit survivors: a systematic review ., Intensive Care Medicine, 35(5) 796 - 809.

### 001589

#### Predictors and consequences of prolonged Intensive Care stay – Real world experience

R. Neto[1], I. Cristina Taveira[2], P. Salvador[3], R. Costa[3], P. Fernandes[1], P. Castelões[1]
[1]Intensive care medicine, Centro Hospitalar de Vila Nova Gaia/Espinho, Vila Nova de Gaia, Portugal; [2]Internal medicine, Unidade Local de Saúde do Litoral Alentejano, Evora, Portugal, Portugal; [3]Internal medicine, Centro Hospitalar de Vila Nova Gaia/Espinho, Vila Nova de Gaia, Portugal
**Correspondence:** R. Neto
*Intensive Care Medicine Experimental* 2019, **7(Suppl 3):**001589

**INTRODUCTION.** Prolonged length of stay (LOS) in the intensive care unit (ICU) is associated with severe morbimortality (1) and high cost of care. Nevertheless, there is no consensus regarding the exact definition of prolonged ICU stay (2), with studies ranging from 3 days to 14 days. There is a lack of evidence regarding even longer periods of stay (30 days), and which factors and critical care outcomes are associated with such duration.

**OBJECTIVES.** 1. Analyse predictors for prolonged ICU stay.

2. Understand the impact of prolonged ICU stay on critical care outcomes.

**METHODS.** Case-control study of the 1509 patients admitted to a polyvalent ICU between 2016-2018. Cases were defined by a LOS in the ICU superior to 30 days.

Forty-two cases were identified and 84 controls were chosen randomly on a 2:1 ratio.

**RESULTS.** During the study period, 2.7% of patients had a prolonged LOS. Median length of stay was 10 days [IQR 7-15] for the control group and 35 [IQR 33-45] for the case group. Average age was 57 years (20-83). There were no statistically significant differences regarding age, gender, time to ICU admission, APACHE II and SAPS II between study groups.

Cases had significantly lower Charlson Comorbidity Index values (p= 0.047), needed more antibiotic regimens (p= 0.047) and longer duration of antibiotic therapy (p<0.01).

They also had significantly longer duration of ventilation (p<0.01). and required more frequently tracheostomy for invasive ventilation weaning (p<0.01; OR 16.0).

Similarly, cases needed more frequently vasopressor support (p=0.07; OR 10.403) and for a longer duration (p<0.01).

The readmission rate was significantly higher in the case group (p=0.01).

Cases also had and days of vasopressor support (p<0.01)

On multivariate analysis, admission because of urgent surgery was a predictor of prolonged ICU stay (p= 0.039; OR 2.47) and an ICU LOS superior to 30 days was a significantly associated with ICU mortality (p=0.045; HR 13.594) in the survival analysis.

Regarding post-discharge outcomes, cases were more prone to be discharged to an institution for continued care and rehabilitation (p= 0.002; OR 4.167). There were no differences in 6-month mortality between study groups (p=0.768). On univariable analysis, the only variable associated with 6-month mortality post-ICU discharge was the need for tracheostomy (p= 0.016; HR 3.871).

**CONCLUSION.** Prolonged ICU stay is associated with a higher rate of tracheostomy and reduced survival in the ICU setting, but similar mortality rate at 6-month post-discharge. Prolonged ICU stay is associated with higher institutionalization rates and, as such, these patients probably require a more intensive rehabilitation care program to ensure adequate quality of life post-ICU stay.

**REFERENCE(S)**

1. Huang YC, Huang SJ, Tsauo JY, Ko WJ. Definition, risk factors and outcome of prolonged surgical intensive care unit stay. Anaesthesia and intensive care. 2010;38(3):500-5.
2. Diab MS, Bilkhu R, Soppa G, Edsell M, Fletcher N, Heiberg J, et al. The influence of prolonged intensive care stay on quality of life, recovery, and clinical outcomes following cardiac surgery: A prospective cohort study. The Journal of thoracic and cardiovascular surgery. 2018;156(5):1906-15.e3.
3. None

### 001620

#### Practice of inhaled bronchodilator therapy during mechanical ventilation in Thailand: a national survey

N. Kongpolprom
Department of internal medicine, Faculty of Medicine, Chulalongkorn University, King Chulalongkorn Memorial Hospital, Bangkok, Thailand
*Intensive Care Medicine Experimental* 2019, **7(Suppl 3):**001620

**INTRODUCTION.** Background: The aerosol therapy during mechanical ventilation is simplified through technological advances however the scientific knowledge about optimal implementation is infrequently applied.

**OBJECTIVES.** To determine the practice and knowledge of inhalation therapy during mechanical ventilation.

**METHODS.** A questionnaire was sent to nurses taking care of mechanically ventilated patients who attended 5 regional respiratory care conferences.

**RESULTS.** Totally, 401 nurses responded to the questionnaire and 84.8% of them were the general nurses working in general medical and surgical wards. Majority of them worked in secondary and tertiary public hospitals with the median experience-year of taking care of ventilated patients of 4[2, 11] and the median numbers of ventilated patients of 15 [4, 30] per month. Of the total responders, 241(60.1%) used metered dose inhaler (MDI) and 295 (73.5%) used nebulizers in mechanical ventilation. Of the 241 nurses who used MDI, only 54% placed the MDIs on the correct position (15 cm. from endotracheal tube). Most of them (93%) did not know how often MDIs needed to be primed. Approximately, 87.5% of them shook MDI before providing to the patients however only 10.6% knew MDI needed to be shaken for 8 actuations. Additionally, 14.5% of them administered MDI at the wrong point of time (at the end of expiration or mid inspiration). Noticeably, 9.2% of them turned off heat humidifier, 28.3% of them did not remove heat moisture exchange and 7.2% of them ignored to check and remove fluid in ventilator circuits before MDI administration. Moreover, 35.4 % did not know an appropriate actuation interval for MDI therapy.

Of the 295 nurses who used jet nebulizers, 70.5% placed the generators on the appropriate position (45 cm. from endotracheal tube), but only 49.5% diluted medication to 4 ml. to maximize aerosol delivery. Additionally, 22.4% of them did not remove heat moisture exchange before nebulization and 24.2% of them used inappropriate flow rates during nebulization.

**CONCLUSION.** The knowledge of inhalation therapy needed to be improved, suggesting the future educational programs focusing on aerosol therapy during mechanical ventilation.

### 001641

#### Point-of-care ultrasound training during anesthesia and critical care residency: preliminary data of a national survey

S. Mongodi[1], F. Bonomi[2], G. Salve[2], A. Orlando[1], S. Pregnolato[2], A. Stella[2], S. Bonaiti[2], A. Colombo[2], E. Santangelo[3], G. Tavazzi[1], L. Vetrugno[4], E. Bignami,[5], R. Vaschetto[3], P. Pelosi[6], F. Mojoli[2]
[1]Department of anesthesia and intensive care, Fondazione IRCCS Policlinico S Matteo, Pavie, Italy; [2]Department of clinical-surgical, diagnostic and pediatric sciences, unit of anaesthesia and inte, The University of Pavia, Pavia, Italy; [3]Department of translational medicine, Università Degli Studi Del Piemonte Orientale, Novara, Italy; [4]Anesthesia and intensive care, Hospital Santa Maria della Misericordia, Udine, Italy; [5]Anesthesia and intensive care, University of Parma, Parma, Italy; [6]Departement of anesthesia and intensive care, University of Genoa, Genova, Italy
**Correspondence:** S. Mongodi,
*Intensive Care Medicine Experimental* 2019, **7(Suppl 3):**001641

**INTRODUCTION.** Point-of-care ultrasound (PoCUS) became increasingly used to assess critically ill patients[1,2]. Appropriate training is required and should integrate anesthesiology and intensive care residency programs. The purpose of this study is to assess the current state of PoCUS training in Anesthesiology and Critical Care residency programs[3].

**OBJECTIVES.** To assess methods, adequacy and limitations of PoCUS teaching during anesthesia and critical care residency in Italy.

**METHODS.** On-line anonymous survey sent to all residents, as approved by the CPAR (Collegio Professori Anestesia Rianimazione), concerning the actual teaching practice for: vascular access (VA), lung ultrasound (LUS), transthoracic echocardiography (TTE); focused-assessment sonography trauma (FAST); transcranial Doppler (TCD); regional anesthesia (RA) and diaphragm ultrasound (DUS).

**RESULTS.** 340 residents from 21/41 universities filled the survey (first year 17.4%, second 20.3%, third 23.8%, fourth 23.5%, fifth 15.0%). Bedside teaching is the most frequent tool for all techniques (Tab.1); frontal lecture is the second one. LUS and DUS teaching more frequently includes research activities. Overall, the most neglected ultrasound technique is FAST. 47.1% of residents never attended an extra-curricular ultrasound course; residents are mentored by attending physicians (73.5%) and/or older residents (46.2%). Acquisition of ultrasound competences is mainly evaluated during bedside activity (64.1%); in only 12.1% a theoretical-practical certification is performed. Ultrasound knowledge is considered extremely important by residents for VA (67.0%), RA (60.9%), FAST (51.8%), TTE (51.2%), LUS (50.3%), DUS (27.9%), TCD (27.6%); the impact of ultrasound technique is mainly perceived to guide procedures (VA and RA) and to improve patient's understanding (LUS, TTE, TCD, DUS, FAST). The training is mainly considered as adequate or more than adequate for VA (62.7%) and RA (42.7%), and nearly sufficient or inadequate for LUS (40.3%), DUS (37.1%) TTE (50.0%), FAST (52.9%) and TCD (44.4%). The main perceived limiting factor is the absence of a standardized didactic process.

**CONCLUSION.** PoCUS teaching is present although not optimal for all the techniques in Italian critical care residency schools. Standardizing resident's ultrasound curriculum is suggested to improve ultrasound teaching.

**REFERENCE(S)**

1. Mojoli F, Am J Respir Crit Care Med 2019; Mar 15;199(6):701-714
2. Zieleskiewicz L, Intensive Care Med 2015 Sep;41(9):1638-47
3. Stolz LA, Acad Emerg Med 2017;24(3):353-361

**Table 1 (abstract 001641).** Teaching instruments adopted for different ultrasound techniques (VA: vascular access; LUS: lung ultrasound; TTE: transthoracic echocardiography; FAST: focused-assessment sonography trauma; TCD: transcranial Doppler; RA: regional anesthesia; DUS: diaphragm ultrasound)

| | Bedside teaching | Online modules | Frontal lectures | Simulation | Research activities | Not faced yet | Nothing |
|---|---|---|---|---|---|---|---|
| VA | **89.9%** | 5.6% | 38.3% | **17.4%** | 7.0% | 3.9% | 3.5% |
| LUS | 77.7% | **10.5%** | **51.9%** | 14.3% | 20.6% | 10.8% | 2.9% |
| TTE | 63.8% | 7.0% | 40.8% | 7.7% | 9.4% | 16.7% | 9.4% |
| FAST | 43.9% | 5.2% | 22.3% | 7.0% | 2.4% | 24.0% | **20.9%** |
| TCD | 46.7% | 6.6% | 29.6% | 3.8% | 7.0% | **26.8%** | 14.3% |
| RA | 78.8% | 6.6% | 49.8% | 13.2% | 7.7% | 10.1% | 4.9% |
| DUS | 53.3% | 7.7% | 35.2% | 10.1% | **21.6%** | 18.5% | 14.6% |

### 001646

#### Immature granulocytes : Early markers for outcome prediction in patients admitted to emergency departement

K. B.ismail, K. Zaouch, Y. Yahia, R. Baccouche, R. Boubaker, H. Maghraoui, K. Mejd
Urgences, Hospital Rabta, Tunis, Tunisia
**Correspondence:** I.K. Ben
*Intensive Care Medicine Experimental* 2019, **7(Suppl 3):**001646

**INTRODUCTION.** The presence of immature granulocytes was originally assigned to the diagnosis of sepsis. Thereafter their rate was correlated with the severity of sepsis. The aim of our study was to assess its prognostic value in patients admitted to emergency department.

**OBJECTIVES.** The aim of our study was to assess its prognostic value in patients admitted to emergency department.

**METHODS.** We conducted a case-control study over a period of two months from march 1st to april 30, 2014. In the group (GI+), we included patients which had immature granulocytes on their blood count. Then, by frequency matching, we included patients of the group (GI-) which had not immature granulocytes. Our primary endpoints were in-hospital mortality and the length of stay in emergency department. Our secondary endpoints were the presence of organ dysfunction and elevation of CRP level over 100 mg/l.

**RESULTS.** We included 122 patients. 61 in the GI+ group and 61 in the GI- group. There were 58 women and 64 men. The sex ratio was 1.1 without difference between the two groups (p=0.46). Sepsis was diagnosed in 84 patients (68.8 %). The two groups had similar past medical history and consultation patterns. Patients in GI+ had Ambulatory Simplified Acute Physiologic Score (ASAPS) significantly higher on admission (5.1 vs 4.1 in GI-, p=0.035) and at least one vital distress at the first examination (p=0.029). Blood glucose level greater than 1.40 g/l was found in 22.1 % of patients. 66.7 % of them belonged to GI+ (p=0.01). In GI+. 45.9% of patients had a creatinine level higher than 17 mg/l (p=0.008, OR =2, [CI=1.17 - 3.41]). Acidemia was found in 90.7 % of patients in GI+ (p=0.008). For an anion gap value greater than 22 mmol/l. the difference was significant in GI+ (p=0.005, OR = 3.1, [CI=1.3 – 6.97]). The average value of ASAT was 50.6 mmol/l in GI+ vs 28.1 mmol/l in GI- (p=0.03). 55.7% of patients in GI+ had a CRP rate greater than 100 mg/l vs 36.1% of patients in GI- (p=0.029). The average length of stay was 78.2 ± 65.7 hours in GI+ vs 54.4 ± 31.9 hours in GI- (p=0.01). Mortality was 16.3% in GI+ vs 8.2% in GI- (p=0.16).

**CONCLUSION.** The presence of immature granulocytes on blood count of patients admitted to emergency department, regardless of diagnosis, is associated with a significantly longer length of stay and a higher number of organ dysfunction. Mortality is twice as high in this group of patients. Immature granulocytes can be a useful prognostic biomarker in emergency department.

### 001656

#### New scoring criteria for ESICM NEXT fellowship evaluation: toward improved equality

M. Greco

*Intensive Care Medicine Experimental* 2019, **7(Suppl 3):**001656

**INTRODUCTION.** ESICM NEXT fellowships are an intense 5 days learning program held at center of excellence. Fellowships are promoted by NEXT, the ESICM Committee representing young members in-training or within 3 years of training, which is responsible for the fellowship application and selection process. As recently published, inequity leads to major loss of potentially important contribution and should be avoided (1). To improve equity in the fellowship application evaluation and to standardize the process, a new scoring system based on 6 criteria was developed through a Delphi-Consensus process (2) on June 2018, and compared with the previous evaluation system.

**OBJECTIVES.** To compare the performance of the new fellowship application scoring system with the previous one.

**METHODS.** Applications of candidates for an ESICM NEXT fellowship were evaluated by two independent raters using the previous scoring criteria, and by two raters per item using new scoring criteria. Data are described as percentage, or median and IQR. Skewness was assessed by skewness and kurtosis test. Interrater agreement was assessed using weighted Cohen's Kappa. Data were specifically assessed in terms of gender and geographical distribution of successful applications. Study obtained IRB approval.

**RESULTS.** A total of 100 applications were included, with 54% male and a median age of 34 (IQR 32-38). Old scoring displayed a right skewed distribution, while the new scoring system was normally distributed (skewness and kurtosis test 0.01 vs 0.6, respectively. Fig. 1). Inter-rater agreement between old and new scoring was 67% (p= 0.001 to refuse agreement by chance), using weighted-Cohen's kappa. New criteria allowed selection of about 30% different applicants compared to old criteria (Fig 2) . Involvement in teaching was the most discriminative variable, followed by quality of published research and quality of recommendation letter. There was no difference in gender (45.6% successful female application over all female application in both scores). There was variation in geographical distribution, with the highest reduction in successful application from Italy and UK.

**CONCLUSION.** The gaussian distribution of new evaluation system, based on more objective criteria selected through a consensus process, reflects a higher discriminative power, compared to the former right skewed distribution. This is mainly related with assessment of personal teaching activity and personal research. The new scoring selected about 30% different candidates compared to the previous system, with no difference in gender selection and no clear pattern in geographical distribution.

##### REFERENCE(S)

1. Weiss B; Task Force and Working Groups for Diversity and Equality of the ESICM. Statement paper on diversity for the European Society of Intensive Care Medicine (ESICM). Intensive Care Med. 2019 Apr 4. [Epub ahead of print] PubMed PMID: 30949715.
2. Jones J, Hunter D. Consensus methods for medical and health services research. BMJ. 1995;311:376–80.
3. Acknowledgment: The authors are grateful to ESICM Diversity Task Force for advice and support

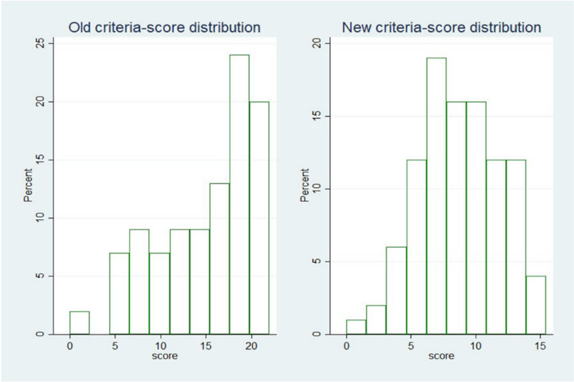

**Fig. 1 (abstract 001656).** See text for description

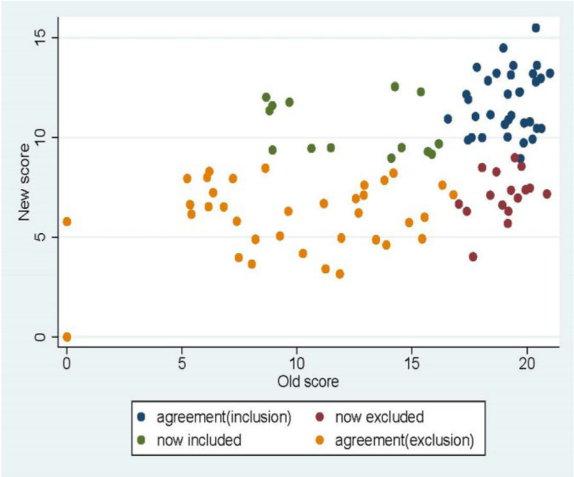

**Fig. 2 (abstract 001656).** See text for description

### 001661

#### National Quality indicator one in Sweden explores resources and compliance to national guidelines 2018

C. Agvald-Öhman[1], L. Engerström[2]

[1]Intensive care, Karolinska University Hospital, Stockholm, Sweden; [2]Department of anasthesia and intensive care, Vrinnevisjukhuset, Gamla Övägen, Norrköping, Sweden, Norrköping, Sweden

**Correspondence:** C. Agvald-Öhman

*Intensive Care Medicine Experimental* 2019, **7(Suppl 3):**001661

**INTRODUCTION.** To measure quality is a delicate task and there are a lot of quality indicators (QI) from different countries. A previous report (1) showed that among 111 QI there was only nine that had a consensual agreement of 90%. The Swedish Intensive Care Registry (SIR) have formed national QI since 2005 and a thorough revision was performed 2014-2016.

**OBJECTIVES.** To describe some data from the new Swedish QI number one, “National guidelines in Intensive Care”.
**METHODS.** The revision of all QI focused on the improving characteristics and (QI 1) is describing nationwide resources and how well the different ICU:s fulfills the goals of the national guidelines in intensive care. Results concerning academic profile, EDIC I+II for the head of the ICU and the level of nurse’s education altogether with number of beds, isolation rooms and staffing ratio was reported 2016 for the first time.
**RESULTS.** The results from 2018 showed and described here, are from all ICU:s that report data to SIR (83/84) in Sweden. In the University ICU:s the head of the ICU had EDIC I+II in 55% and 18/29 (62%) had an academic grade of at least PhD. The guideline stipulates 100%.
In 35 of 83 ICU:s there was a discrepancy between what has been reported by the head of the ICU concerning data delivered to SIR for the eight QI and what was actually found in the database (Table 1).
**CONCLUSION.** To be able to report ICU resources available nationwide including academic profile, education level and how many ICU:s that reach the goals of the guidelines open for public can be a powerful tool for improvement both for the individual ICU and for the intensive care in Sweden.

**REFERENCE(S)**

1. Intensive Care Med. 2012 Apr;38(4):598-605. doi: 10.1007/s00134-011-2462-3. Epub 2012 Jan 26. Prospectively defined indicators to improve the safety and quality of care for critically ill patients: a report from the Task Force on Safety and Quality of the European Society of Intensive Care Medicine (ESICM). Rhodes A1, Moreno RP, Azoulay E, Capuzzo M, Chiche JD, Eddleston J, Endacott R, Ferdinande P, Flaatten H, Guidet B, Kuhlen R, León-Gil C, Martin Delgado MC, Metnitz PG, Soares M, Sprung CL, Timsit JF, Valentin A; Task Force on Safety and Quality of European Society of Intensive Care Medicine (ESICM).
2. The Swedish Intensive Care Registry, SIR
3. None

**Table 1 (abstract 001661).** The discrepancy of reported results according data concerning National Quality Indicators delivered to SIR compared the true result in the database

| ICU Category In % of all ICU:s | Reported result that all data for all QI is delivered to SIR due to head of the ICU | True result found in database |
|---|---|---|
| I 20% | 77% | 47% |
| II 45% | 78% | 35% |
| III 35% | 62% | 17% |

## POIC - Perioperative circulation and biomarkers

### 000627

#### Incidence and clinical implications of late onset hyperlactatemia after cardiac surgery

JA. Sanchez Giralt, B. Muñoz Molina, M. Trigueros Genao, J. Lázaro Gonzalez, F. Suárez Sipmann
ICU, Hospital de La Princesa, Madrid, Spain
**Correspondence:** J.A. Sanchez Giralt

*Intensive Care Medicine Experimental* 2019, **7(Suppl 3):**000627

**INTRODUCTION.** Hyperlactatemia (HL) during cardiac surgery is common, occurring in 10-20% of patients (1). The most studied mechanism is tissue hypoperfusion usually a marker of impaired outcome. There are however, other less well-known causes of perioperatrive HL (2).
Surgical stress response involves metabolic changes aimed at mobilizing different energetic substrates. Lactate is a well-known and important source of energy in different tissues. Most previous studies on HL have focused on the role of intra- or early post-operative hypoperfusion associated HL on morbity and mortality. There are however few studies analyzing the prevalence and clinical significance of delayed HL occurring several hours after surgery in the absence of clinical evidence of shock or tissue hypoperfusion.
**OBJECTIVES.** Analyze the incidence and clinical implications of late onset hyperlactatemia (LOH) after cardiac surgery.
**METHODS.** Observational retrospective study in a tertiary university hospital. We analyzed patients scheduled for cardiac surgery between January 2016 and December 2018. LOH was defined as an increased in lactate ≥3mmol/l after the first 4hours of ICU admission. We excluded patients with intra- or early postoperative HL, those with cardiac arrest, severe arrhythmia or needing reintervention or cardiac assistance in the first 4 hours of ICU admission. We analyzed mortality, prolonged mechanical ventilation, ICU and hospital length of stay (LOS) and occurrence of post-operative adverse events by comparing LOH cases with a matched control population. We also analyzed the presence of hyperglycemia and inflammation as potential mechanisms of LOH.
**RESULTS.** Of the 744 patients analyzed, we detected 55 (7%) cases of LOH that were compared to 52 matched controls. We did not find any differences in mortality (P=0.618 CI95%) and in ICU and hospital LOS (P=0.116 and P=0.063 CI95 respectively). In addition, there were no differences in renal failure, continuous renal replacement, arrhythmias or prolonged ventilation (P=0,77 P=0,16 P=0,14 P=0,44).
We found a significant increase in glucose (p<0,0001) being 52% of those non-diabetics. There was a positive correlation between glucose and lactate levels (r 0.45 Pearson P=0.001 r2 0.21,). Also, we found an increase in leucocytes levels at 4 hours in the LOH group but it was not significative (P=0,29 CI95%).
**CONCLUSION.** LOH is neither associated with a worse prognosis (mortality, ICU and hospital LOS) nor with post-operative adverse events. It seems to represent a secondary postoperative metabolic-inflammatory response that does not require any specific therapeutic intervention other than metabolic control.

**REFERENCE(S)**

1. Lactic Acidosis. N Engl J Med 2014;371:2309-19.
2. Hyperlactatemia and Cardiac Surgery. J Extra Corpor Technol. 2017;49:7–15. The Journal of ExtraCorporeal Technology

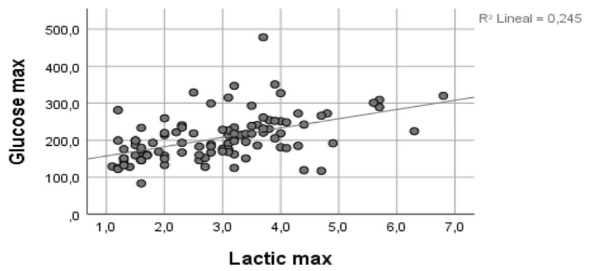

**Fig. 1 (abstract 000627).** See text for description

**Table 3 (abstract 00627).** See text for description

| Variable | Cases | Controls |
|---|---|---|
| Mortality | 5,5% | 1,9% |
| ICU LOS | 4,77d (2,5-6,9) | 2,8d (1,7-3,8) |
| Hospital LOS | 8,8d(7,5-10,2) | 10,7d(8,4-12,9) |
| Glucose | 233mg/dl(216-251) | 179mg/dl(165-192) |
| Leucocytes 4 hours | 14,6mil/mm$^3$(13-15) | 13mil/mm$^3$(12-14) |
| Prolonged MV(>48h) | 7,3% | 11,5% |
| Arrhythmia | 21,8% | 34,6% |
| ARF | 25,5% | 23,1% |
| CRR | 2% | 0% |

### 000654

#### Sublingual microcirculation differences in dialysis patients, kidney transplant recipients and healthy volunteers shown using a new automated analysis software called MicroTools

TYC. Yeh[1], M. Hilty[2], O. Dilken[2], MK. Tsai[3], CM. Liu[1], SH. Chen[1], D. Gommers[2], C. Ince[2]
[1]Department of anesthesiology, National Taiwan University Hospital, Taipei, Taiwan; [2]Department of intensive care, Erasmus MC, Rotterdam, Netherlands; [3]Department of surgery, National Taiwan University Hospital, Taipei, Taiwan
**Correspondence:** T.Y.C. Yeh
*Intensive Care Medicine Experimental* 2019, **7(Suppl 3):**000654

**INTRODUCTION.** Kidneys are important organs in regulation of vascular tone via several circulating systemic mediators Chronic Renal Disease (CRD) has systemic effects, including accelerated atherosclerosis, affecting also the microcirculation (1). We made sublingual IDF imaging video sequences analyzed by new automated analysis software to investigate the microcirculation called MicroTools deveoped by us (2) in healthy volunteers, dialysis patients, and kidney transplant recipients.

**METHODS.** This study was registered at the ClinicalTrials.gov Protocol registration system (ID: NCT02412839 and NCT02940275). A total of 270 patients were recruited in the study with different groups, yielding a total of 1983 videoclips. Analysis of the image sequences was performed by our newly developed automatic software called Microtools. To validate the use of this software on the present data set, two researchers compared image from randomly selected 17 patients independently from each other using the gold standard AVA analysis and showed agreement between their values. Then these results were run through MicroTools for analysis. Bland Altman analysis between AVA data and MicroTools data showed agreement within levels of agreement. After this validation step, 1373 videoclips out of 1983 were excluded due to poor quality based on the existing criteria. The remaining eligible 610 videoclips were run through the MicroTools software providing the following parameters: Total Vessel Density (TVD), Functional Capillary Density (FCD), Capillary Blood Volume (CBV). Quantitative RBC velocity was calculated from Space Time Diagrams of each capillary in a videoclip provided by MicroTools, conform the international consensus.

**RESULTS.** TVD and FCD of Dialysis patients was significantly lower compared to healthy volunteers, and renal transplantation reverted this vascular reaction. Systemic vasoconstriction was shown in reduced capillary blood volume in dialysis patients, which was again improved by renal transplantation. Mean Red Blood Cell Velocity was increased both dialysis and renal transplantation patients, suggesting permanent structural change in extended disease progress.

**CONCLUSION.** CRD affects systemic microcirculation and these effects are relieved to some extent by renal transplantation.

**REFERENCE(S)**

1. Yu-Chang Yeh et al. European Journal of Clinical Investigation 2017; 47(9):630-637
2. Hilty MP et al. Intensive Care Med Exp 2018; 6: 172–173
3. National Taiwan University Hospital grant: NTUH 105-A125

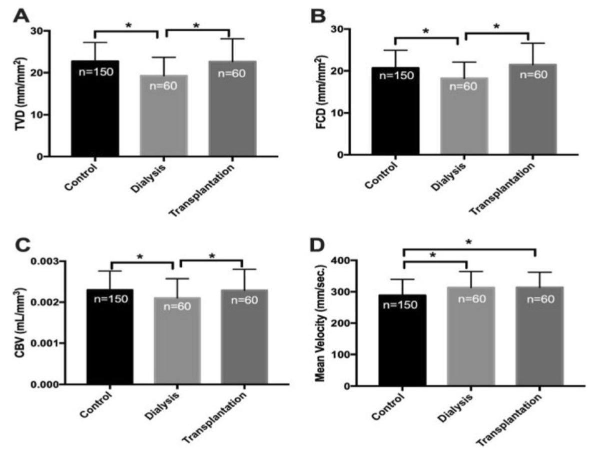

**Fig. 1 (abstract 000654).** See text for description

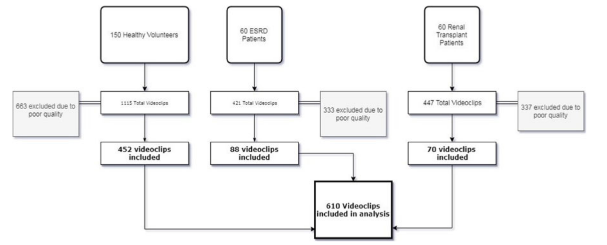

**Fig. 2 (abstract 000654).** See text for description

### 000691

#### Methylene blue in vasoplegic patients after cardiac surgery

E. Bucio[1], EA. LAZCANO DIAZ[2], NA. Chávez Ponce[3], F. Baranda[4]
[1]Terapia intensiva cardiovascular, Instituto Nacional de Cardiología "Ignacio Chávez", México City, Mexico; [2]Cardiovascular intensive therapy, National Institute of Cardiology Ignacio Chavez, Ciudad de México, Mexico; [3]Terapia Intensiva Cardiovascular, Instituto Nacional de Cardiología "Ignacio Chávez", Mexico City, France; [4]Terapia intensiva cardiovascular, Instituto Nacional de Cardiología "Ignacio Chávez", Ciudad de México, Mexico
**Correspondence:** E.A. LAZCANO DIAZ
*Intensive Care Medicine Experimental* 2019, **7(Suppl 3):**000691

**INTRODUCTION.** The vasoplegic syndrome is a condition characterized by low systemic vascular resistance, low blood pressure, as well as normal or elevated cardiac output. It has been found in many contexts, including postoperative state. The pathophysiology coincides with the activation of different intrinsic vasodilation pathways, as well as the poor response to intravenous fluids and vasopressors. [1] The importance of Vasoplegia in Cardiac surgery with extracorporeal circulation lies in its high frequency, its high morbi-mortality, as well as its association with multiple organ failure. [2] Despite being a relatively common syndrome in critical patients, the absence of a definition makes its study, and management more difficult. Historically the cornerstone for the management of vasoplegia has been based on hemodynamic support with vasopressors, giving the best profile to the vasopressin. [3] However, with advances in the study of the pathophysiology, the methylen blue has been proposed as an alternative management. The action of methylene blue is based on the inhibition or limitation of the inflammatory response by the blockade of nitric oxide. There is evidence that suggest that the use of methylene blue as management of vasoplegia after cardiac surgery reduces morbidity and mortality, as well as the time of vasoplegia. [4].

**OBJECTIVES.** To assess the effect of methylene blue in terms of morbidity and mortality in patients who have undergone cardiac surgery complicated with vasoplegic syndrome.

**METHODS.** A retrospective, observational study was conducted. The data of the patients diagnosed with vasoplegic shock , was collected between 2014 and 2018. Those patients were divided in two groups, those who were treated with methylene blue (MB) 2mg/kg, and those who did not received it (they receive the standard treatment ov vasoplegic shock). The criteria used to determine vasoplegic syndrome was hypotension <65mmHg, vascular systemic resistance <1600 dynes, cardiac index >2.2, high doses of vasopressor needed. The exclusión criteria includes patients who did not fulfill the criteria for vasoplegic syndrome, and those with incomplete clinical record. The information was analyzed using STATA 12.1, a p value of <0.05 was used as significant.

**RESULTS.** A total of 50 patients were collected. In the MB group the mean age was 59.6 years SD 14.37, the 59.26% were male, 40.74% had hypertension, 14.81% had diabetes mellitus type II (DM), 22.2% had atrial fibrillation, and 51.85% underwent non elective surgery. In the non MB group the mean age was of 58.4 years SD 13.6, 73.91% were male, 43.48% had hypertension, 34.78% had DM, 4.35% had atrial fibrillation, and 73.91% were of non elective surgery. Non of this data had significant difference. A Pearson correlation did not show any significant difference between the 2 groups regarding mortality, duration of the vasoplegia, neumonia, mediastinitis, reexploration, and lenght of stay.

**CONCLUSION.** Our results suggest that the use of methylene blue as a management of vasoplegic syndrome in post-operative patients of cardiac surgery, did not reduce mortality, duration of vasoplegia, length of stay in intensive care, days of in-hospital stay, or postoperative complications.

**REFERENCE(S)**

1. Kimmoun A, Ducrocq N, Levy B. Mechanisms of vascular hyporesponsiveness in septic shock. Curr Vasc Pharmacol. 2013;11:139–49.
2. Síndrome vasopléjico Janet Silvia Aguirre-Sánchez,1 Eduardo Bucio-Reta,2 Leonel MartínezRamírez,2 Drander M. López-Pineda,2 Luis Efren Santos-Martínez,2 Fernando Florez-Figueroa.
3. Ludhmila Abrahao Hajjar, M.D., Ph.D.; Jean Louis Vincent, M.D., Ph.D.; Filomena Regina Barbosa Gomes Galas, M.D., Ph.D.; Andrew Rhodes, M.D., Ph.D.; Giovanni Landoni, M.D.; et al .Vasopressin versus Norepinephrine in Patients with Vasoplegic Shock after Cardiac Surgery: The VANCS Randomized Controlled Trial.
4. Methylene Blue Reduces Mortality and Morbidity in Vasoplegic Patients After Cardiac Surgery Ricardo L. Levin, MD, Marcela A. Degrange, MD, Gustavo F. Bruno, MD, Carlos D. Del Mazo, MD, Daniel J. Taborda, MD, Jorge J. Griotti, MD, and Fernando J. Boullon, MD Division of Cardiovascular Surgery, Navy Hospital, French Hospital, Swiss-Argentine Clinic, Argentine Institute of Diagnosis and Saint Elizabeth Clinic, Buenos Aires, Argentina.

### 000705

#### Clonidine as a sedative in the intensive care: Optimizing dosing regimens with population pharmacokinetics

M. Cloesmeijer[1], H. van den Oever[2], M. Zeeman[3], A. Kruisdijk-Gerritsen[2], R. Mathôt[1], M. Arbouw[4]
[1]Department of hospital pharmacy-clinical pharmacology, Amsterdam University Medical Centres, Amsterdam, Netherlands; [2]Intensive care unit, Deventer Hospital, Deventer, Netherlands; [3]Department of clinical geriatrics, Deventer Hospital, Deventer, Netherlands; [4]Department of clinical pharmacy, Deventer Hospital, Deventer, Netherlands
**Correspondence:** H. van den Oever
*Intensive Care Medicine Experimental* 2019, **7(Suppl 3):**000705

**INTRODUCTION.** Clonidine is an α2-agonist that is registered for the treatment of hypertension. It is also used as an off-label drug for sedation in the intensive care unit (ICU). However, no standardized dosing regimens are available for this indication [1]. Based on previous studies [2], we defined a target plasma concentration for sedation between 1.5 to 4.0 μg/L.

In order to achieve clonidine levels in the target range, we did a population pharmacokinetic study in ventilated and sedated patients, using various doses of clonidine.

**OBJECTIVES.** To develop a population pharmacokinetic (PK) model to optimize dosing regimens for clonidine as a sedative.

**METHODS.** Mechanically ventilated patients receiving intravenous sedation were included after informed proxy consent. Clonidine was added to standard sedation. Three cohorts of eight patients received continuous infusions of intravenous clonidine in doses of 600, 1200, and 1800 μg/24 h for seven days or less. The second half of each cohort received a loading dose of 300, 600 and 900 μg of clonidine, respectively, infused over 4 h. Biometric and biochemical data were recorded and plasma was sampled at fixed times.

A population nonlinear mixed effects model was constructed using NONMEM 7.3. One, two and three compartment models were tested. The following covariates were tested on the PK parameters: body weight, body surface area, body mass index, age, gender, creatinine clearance, albumin, bilirubin and time after start infusion. After univariate selection, covariates were liberally introduced into the multivariate model using linear and power functions, and then stepwise eliminated using more rigid criteria. The stability of the model was tested by goodness-of-fit plots, bootstrapping and a visual predictive check. After modelling, Monte Carlo simulations were performed to find optimal dosing regimens.

**RESULTS.** We collected 287 plasma samples from 24 patients. Ages ranged from 25 to 83 years (median 67); body weights ranged from 53 to 113 kg (median 84).

A two-compartment model fitted the concentration-time data best. Time had a significant influence on the clearance (CL), which increased with 0.21% per hour. Body weight was a significant covariate on the central volume of distribution (V1), using allometric scaling.

Population PK parameters (relative standard errors) were: CL 17.1 L/h (10%), V1 123 L/70kg (33%), Q (inter-compartmental CL) 84.4 L/h (31%), and V2 (peripheral volume of distribution) 179 L (18%).

Simulations with the final model showed that a continuous infusion of 1200 μg/day resulted in 90% of the population reaching target concentrations during steady state, irrespective of body weight or kidney function.

Without a loading dose, 50% of the population reached the target concentration in 14 hours. After a 450 μg loading dose infused over 4 hours, 50% of patients reached the target concentration within 4 hours. Loading doses below 300 μg had little effect on the time to reach target concentration. After 4 days of continuous infusion, serum half-life was 12 hours.

**CONCLUSION.** The developed population PK model allowed the optimization of dosing regimens for clonidine as a sedative agent in ICU patients.

**REFERENCE(S)**

1. Neth J Crit Care 21, 6-13 (2015)
2. Clin Pharmacol Ther 21, 593-601 (1977)

### 000712

#### Delirium in the intensive care unit: analysis of non-pharmacological measures and risk factors

M. Bersaneti[1], I. Whitaker[2]
[1]Instituto Sírio-Libanês de Ensino e Pesquisa, São Paulo, Brazil; [2]Escola paulista de enfermagem, UNIFESP, São Paulo, Brazil
**Correspondence:** M. Bersaneti
*Intensive Care Medicine Experimental* 2019, **7(Suppl 3):**000712

**INTRODUCTION.** The recommended strategy for the prevention and treatment of delirium are non-pharmacological approaches combined and focused on reducing modifiable risk factors in order to improve and optimize patients' cognition, sleep, mobility, hearing and vision. The use of multiple combined strategies is a plausible recommendation, since the etiology of delirium is multifactorial.

**OBJECTIVES.** To verify the impact of non-pharmacological interventions in the development of delirium in the ICU, according to the hypothesis that natural light, mobilization, presence of family member and absence of physical restriction reduce the risk of delirium.

**METHODS.** Prospective and longitudinal study. Inclusion criteria: patients over 18 years old, hospitalized in the ICU for a period longer than 24 hours. Exclusion criteria: patients with delirium at the time of admission to the ICU, those with a stay in the unit less than 24 hours, transferred to another institution, as well as those that could not be reliably assessed for delirium, such as sustained coma , inability to understand the predominantly spoken language in the ICU, aphasia, or severe auditory or visual disturbances. On a daily basis, the patients were evaluated using CAM-ICU, applied in two moments. To verify the predictive factors of delirium, a logistic regression model was constructed, with odds ratio calculation and respective 95% confidence intervals. We included in the multivariate model all variables that had p <0.10 in the univariate analysis

**RESULTS.** The study sample consisted of 356 patients, 55.9% were males and the median age was 69 years, with a predominantly independent functional capacity (89.0%), admitted to the ICU more frequently from the surgical center (49, 4%) and emergency room (27.8%), with median SAPS3 of 39 points, median SOFA of 2 points and ICC of 2 points. Of the total number of patients, delirium was observed in 64 patients (18.0%) during ICU stay. Natural light, present in 18.8% of the sample, was not associated with the occurrence of delirium (p = 0.297). Physical restraint was observed in 12.4% of the sample. There was an association between physical restraint and occurrence of delirium (p <0.001). After adjustment of covariates (age, gender and mechanical ventilation), the multivariate analysis revealed that age, type of patient, SOFA, family presence (OR=0,15; 95% CI = 0,06-0,41; p<0,001) and mobilization (OR=0,07; 95% CI = 0,03-0,16; p<0,001) were independent variables associated with the development of delirium.

**CONCLUSION.** Mobilization and presence of family members are protective factors for the development of delirium.

### 000725

#### Effect of intravenous morphine bolus on respiratory drive in ICU patients

N. Dey[1], LP. Thomsen[2], S. Larraza[3], M. Nygaard[1], D. Lodahl[4], R. Winding[1], SE. Rees[2], DS. Karbing[2]
[1]Department of anaesthesia and intensive care, Regions Hospital Herning, Herning, Denmark; [2]Respiratory and critical care group (rcare), department of health science and technology, Aalborg University, Aalborg, Denmark; [3]Departamento de ingeniería biomédica, vicerrectoría de ciencias de la salud, Universidad de Monterrey, San Pedro Garza García, Mexico; [4]Department of anaesthesia and intensive care, Aalborg University Hospital, Aalborg, Denmark
**Correspondence:** D.S. Karbing
*Intensive Care Medicine Experimental* 2019, **7(Suppl 3):**000725

**INTRODUCTION.** To reduce patient discomfort and pain in ICU, intravenous opiates, e.g. morphine bolus, are used routinely (1). These analgesics have side effects, one of the most potent is respiratory inhibition (2).

**OBJECTIVES.** To measure acute changes in respiratory drive in response to a morphine bolus.

**METHODS.** Preliminary results for 12 ICU patients on weaning mechanical ventilation modes in prospective observational study. Informed consent and ethical approval were obtained in all cases. Arterial blood gases, ventilation and respiratory drive were measured prior to and 15 minutes following an intravenous 5 mg morphine bolus. Respiratory drive parameters of central chemoreflex threshold in hydrogen ion concentration (TC) and cerebrospinal fluid strong ion difference (SIDcsf) were estimated using a model-based method (3) implemented in a decision support system (Beacon Caresystem, Mermaid Care, Nørresundby, Denmark). This system estimated pulmonary gas exchange parameters (shunt and ventilation/perfusion mismatch) prior to morphine bolus for use in respiratory drive measurement (3). Pre- and post-morphine measurements were compared using paired t-test or Wilcoxon signed rank test, according to normality as assessed by Shapiro-Wilk test.

**RESULTS.** Pre-morphine levels and (average ± SD) or (median (interquartile range)) change from pre- to post-morphine bolus were for SIDcsf 31.9 (0.3 ± 1.0) mmol/L, TC 41.1 (-0.2 ± 2.3) nmol/L, respiratory rate (RR) 27.8 (-3.3 ± 4.8) min-1, alveolar ventilation (VA) 7.6 (-0.4 ± 1.2) L/min, minute ventilation 13.7 (-1.1 ± 2.3) L/min, tidal volume 515 (30 (-14 – 134)) mL, effective respiratory system compliance 39.8 (-0.2 ± 4.5) ml/cm H2O, end-tidal CO2 3.7 (0.1 (-0.1 – 0.4)) %, CO2 production 291 (-8 ± 31) mL/min, O2 consumption 333 (3 ± 41) mL/min, arterial PCO2 5.03 (0.19 ± 0.34) kPa, pH 7.45 (-0.01 ± 0.02) and PO2 12.8 (-0.8 ± 3.0) kPa. Significant RR reduction followed morphine bolus (p = 0.036). Although no systematic changes were observed in other variables (p > 0.05), most patients showed a VA reduction, increasing arterial PCO2 and decreasing pH. Largest changes in VA were associated with opposite changes in TC.

**CONCLUSION.** Predominant acute response to morphine bolus was a reduction in RR. Larger studies are necessary to understand the different observed individual responses.

##### REFERENCE(S)

1. Strøm T et al. A protocol of no sedation for critically ill patients receiving mechanical ventilation: a randomised trial. Lancet. 2010;375(9713):475-80.
2. Macintyre PE et al. Opioids, ventilation and acute pain management. Anaesth. Intensive Care. 2011;39(4):545-58.
3. Larraza S et al. A mathematical model approach quantifying patients' response to changes in mechanical ventilation: Evaluation in pressure support. J Crit Care. 2015;30(5):1008-15.
4. Mermaid Care supplied Beacon Caresystem and related one-use items. SER and DSK are minor shareholders and perform consultancy for Mermaid Care.

### 000743

#### Age and Outcome: ICU Admissions in the Elderly post Emergency Laparotomy

M. De Bono[1], N. Singh[2]
[1]Intensive care medicine, King's College London, London, United Kingdom; [2]Intensive care medicine, King's College Hospital, London, United Kingdom
**Correspondence:** M. De Bono
*Intensive Care Medicine Experimental* 2019, **7(Suppl 3):**000743

**INTRODUCTION.** More elderly patients are receiving emergency treatment and undergoing unplanned surgical procedures with increasing comorbidities and infirmity. Risk stratification tools are utilized to predict outcomes. P-Possum is used by the National Emergency Laparotomy Audit (NELA) database in the UK. However, these measures have been criticized for not adequately quantifying frailty and morbidity burden of an elderly population. It remains unclear if surgical and ICU management are beneficial in consideration of long-term outcomes in this population.

**OBJECTIVES.** This evaluation of service aims to view 6 and 12 month mortality for our elderly patients and assess predictive scores currently used. Secondary objectives include the age stratification of post-operative recovery in terms of length of stay on the ICU, duration of ventilation and necessity for organ support.

**METHODS.** All patients older than 70 admitted to ICU after an emergency laparotomy at a British district general Hospital between August 2015 and November 2017 were included. Data was collected from the NELA Database, ICU charts and electronic patient records. Survivorship was measured at 6 and 12 months. T-Test analysis was performed between surviving and non-surviving groups, then stratified into decades (patients 70, 80, and 90) and ANOVA analysis was performed.

**RESULTS.** 98 patients were identified, of which 18 were excluded due to incomplete documentation. Of the 80 remaining 63 (79%) survived to both 6 and 12 months. The average age of the surviving group was older than the non-surviving group and lacked significance (81.8.4±6.2 yrs vs. 80.5±5.5 yrs; p=0.43). All patients, (n=8) older than 90 were alive at 12 months.

NELA's measure of Operative Severity did not yield a significance in mortality (p=0.16). Other measures employed by NELA were found to be more predictive. Physiology Severity Score (P<0.005) and P-Possum Mortality (18.0±20.6% vs. 34.0±27.4%; p<0.01, 95% CI 3.9 to 28.1%) were good markers of outcome irrespective of age. Length of time mechanically ventilated was associated with poor outcome (1.4±2.3 days vs. 5.1±8.0 days; p<0.01, 95% CI 1.4 to 5.9 days) but not duration of ICU stay (P=0.13). ANOVA stratification between decades saw a reduced length of stay on ICU (F=4.77; P<0.05) and number of days ventilated (F=3.60; P<0.05) as age increased.

**CONCLUSION.** Our results reveal that for patients over the age of 70 admitted to the ICU, age itself is not predictive of outcome. This group demonstrated a 12 month survival of almost 80%. Therefore, in our institution very elderly patients who have been selected for emergency operations can expect to do well after surgery and ICU admission. Future work should be carried out to describe the features of patients declined for surgery.

### 000755

#### Transfusion practice in the non-bleeding critically ill; an international online survey – The TRACE Survey

S. de Bruin[1], T. Scheeren[2], J. Bakker[3], A. Vlaar[1]

[1]Department of intensive care medicine, Academic Medical Centre, Amsterdam, Netherlands; [2]Department of anaesthesiology, University Medical Centre Groningen, Groningen, Netherlands; [3]Department of intensive care medicine, Erasmus University Medical Center, Rotterdam, Netherlands

**Correspondence:** S. de Bruin

*Intensive Care Medicine Experimental* 2019, **7(Suppl 3):**000755

**INTRODUCTION.** The last decade several randomized controlled trials (RCTs) studied different transfusion strategies in critically ill patients. In several studies the safety of restrictive transfusion strategies was proven. Due to the lack of international transfusion guidelines specific for the intensive care unit (ICU), we hypothesized that a large heterogeneity in transfusion practice in this patient population exists.

**OBJECTIVES.** To quantify the variety of current transfusion practices and identify knowledge gaps in critically ill non-bleeding patients.

**METHODS.** An online, anonymous, worldwide survey among physicians working in the intensive care (intensivists and non-intensivists) was performed, in which red blood cell (RBC), platelet and plasma transfusion practices were questioned, including questions regarding transfusion thresholds, transfusion triggers and blood conservations measures. Furthermore, the presence of a hospital or ICU specific transfusion guideline was asked. Only completed surveys were analysed.

**RESULTS.** In total 947 respondents participated of which 725 could be analysed. Hospital transfusion protocol available in their ICU was reported by 53% of the respondents. Only 29% of the respondents used an ICU specific transfusion guideline. The applied haemoglobin (Hb) threshold for the general non-bleeding ICU population was 7 g/dL IQR (7-7 g/dL). For subpopulations including patients on ECMO and patients with traumatic brain injury the highest variance was observed (8 g/dL IQR (7.0-9.0 g/dL)). For patients with acute coronary syndrome (ACS) highest Hb threshold was applied (9.0 g/dL IQR (8-9.7 g/dL)). Thrombocytopenia was treated at a median level of 20x109 cells/L IQR (10-25 x109 cells/L). However, prior invasive procedures significantly higher platelet count thresholds were applied (p< 0.001). In non-bleeding critically ill patients with a vitamin K induced international normalized ratio (INR) >1.5, 43% and 57% of the respondents would consider plasma transfusion without any upcoming procedures or prior a planned invasive procedure respectively. Finally, doctors with base specialty anaesthesiology transfused critically ill patients more liberally compared to internal medicine physicians.

**CONCLUSION.** Current RBC transfusion practice in the general non-bleeding ICU population is restrictive while for different subpopulations higher Hb thresholds are being used. There is a high need for well powered randomised trials investigating the optimal Hb threshold for subpopulations in the critically ill. Furthermore, current practice in plasma and platelet transfusion is liberal and local transfusion guidelines are lacking in the majority of the ICUs.

### 000766

#### A low Fibrinogen plasma level is associated with early postoperative bleeding in Cardiac Surgery: a retrospective monocentric cohort study

E. besnier[1], P. Schmidely[1], L. Todesco[1], Y. Sirejacob[2], C. Thill[2], B. Dureuil[1], V. Compère[3]

[1]Departement of Anesthesia and Critical Care, Hospital Center University De Rouen, Rouen, France; [2]Departement of biostatistics, Hospital Center University De Rouen, Rouen, France; [3]Department of anesthesiology and intensive care, Rouen University Hospital, Rouen, France

**Correspondence:** E. besnier

*Intensive Care Medicine Experimental* 2019, **7(Suppl 3):**000766

**INTRODUCTION.** Cardiac surgery is at high risk of bleeding, inducing additional morbidities and costs. Post-operative fibrinogen plasma levels have been described as associated with enhanced bleeding but very few data are available for pre-operative leves, whereas its correction could may prevent the occurence of bleeding.

**METHODS.** Retrospective cohort study at Rouen University Hospital from January 2016 to July 2018 including patients scheduled for a cardiac surgery with cardiac bypass. EB was defined as a blood loss > 1.5 mL/kg/hour during the first 6 postoperative hours or a need for reoperation [1]. Preoperative, perioperative and postoperative data were collected. Results are presented as medians with first and third quartiles. Comparisons between EB group and no-EB group were realized using a Mann and Whitney test or a $\chi^2$. A multivariable analysis was then realized using a logistic regression model (backward process). P < 0.05 was considered as significant.

**RESULTS.** 1822 patients were included, 107 showed significant bleeding (EB group).

Preoperative data: No difference was observed between no-EB and EB groups concerning age, gender, preoperative cardiac and renal functions, antiplatelet therapies. A significant difference was observed for Body Mass Index (BMI) (27.8 [24.7-31.2] vs 25.3 [22.1-28.4] kg/m$^2$, p<0.0001), past history of cardiac surgery (4.8 vs 13.1%, p=0.01), anti-vitamin K therapy (10.7 vs 18.7%, p=0.02), prothrombin time (PTT) ratio (91 [80-100] vs 85 [70-100]%, p=0.001), platelets count (221 [185-263] vs 200 [160-243] G/L, p=0.001) and fibrinogen plasma level (3.5 [3.1 vs 4.0] vs 3.3 [2.6-4.1], p=0.04).

Peroperative data: excessive duration of cardiac bypass (80 [60-112] vs 118 [79-155] min, p<0.0001] in EB group.

Postoperative data: rised PTT (59 [54-65] vs 55 [48-62]%, p<0.0001), diminushed platelets count (145 [119-177] vs 133 [104-170] G/L, p< 0.0001) and fibrinogen plasma level (2.5 [2.1-3.0] vs 2,3 [1.9-2.7] g/L, p<0.0001) in EB group. EB group presented more frequent complications: higher plasma troponin (969 [610-1835] ng/dL vs 607 [385-1032], p<0.0001) and lactate (2.3 [1.7-3.5] vs 1.7 [1.2-2.4], p<0.0001), kidney failure (KDIGO score ≥ 1 : 17 vs 8%, p=0.003). ICU Length of stay and mortality were higher (5 [3-8] vs 3 [2-5] days, p=0.002 and 5 vs 1%, p=0.02).

Elements independently associated with a lower risk of EB were BMI ≥ 30 kg/m$^2$ (OR 0.46 IC95% [0.45-0.78] p=0.006) and preoperative fibrinogen (OR 0.98 IC95% [0.95-1.00] for each 0.1 g/L, p=0.04). Cardiopulmonary bypass was independently associated with a greater risk of EB (OR 1.11 IC95% [1.07-1.15] for each 10 min, p<0.0001). A preoperative fibrinogen level greater than 3.035 g/L (Se 45%, Sp 71%) appear to be protective factor against EB (Area Under the Curve 0.557 [0.492-0.623]).

**CONCLUSION.** Low levels of fibrinogen before cardiac surgery is associated with an excessive postoperative bleeding. Further randomized study may explore the effect of pre-emptive administration of exogenous fibrinogen to prevent excessive bleeding during cardiac surgery.

### 000779

#### The use of a machine-learning based algorithm to reduce hypotension during surgery: a randomised clinical trial

M. Wijnberge[1], B. Geerts[1], N. Lemmers[1], L. Hol[1], M. Hollmann[1], A. Vlaar[2], D. Veelo[1]
[1]Department of anaesthesia, Academic Medical Centre, Amsterdam, Netherlands; [2]Department of intensive care medicine, Academic Medical Centre, Amsterdam, Netherlands
**Correspondence:** M. Wijnberge
*Intensive Care Medicine Experimental* 2019, **7(Suppl 3):**000779

**INTRODUCTION.** Intraoperative hypotension (IOH) is a frequently occurring event associated with an increased morbidity and mortality.[1,2] Current treatment of IOH is mostly reactive. A machine-learning algorithm was developed to predict IOH minutes before the blood pressure actually decreases. This algorithm, the hypotension prediction index (HPI), was internally and externally validated with good sensitivity and specificity.[3] However, no randomised studies testing the effectivity of HPI to prevent IOH have been performed. We hypothesise, that use of IOH will reduce the time weighted average (TWA) spent in hypotension during surgery.

**OBJECTIVES.** Our primary objective was to determine whether the use of a machine-learning based algorithm can reduce IOH, measured as the TWA during surgery. TWA combines both the time and depth of IOH.[4] Our secondary objectives were to assess whether the use of this algorithm increases the TWA of hypertension and whether it increases the cumulative dose of vasopressors and fluids given during anaesthesia. We also assessed the compliance with the algorithm by the number of protocol violations.

**METHODS.** This was a single center trial in a tertiary academic center consisting of two phases, an observational phase and a randomised clinical trial phase. Adult patients (aged 18 years and older) scheduled to undergo an elective non-cardiac surgical procedure under general anaesthesia and requiring an arterial line were eligible for inclusion. IOH was defined as a MAP < 65 mmHg for more than 1 minute. A HPI alarm was defined as a HPI value above 85%.

**RESULTS.** A total of 100 patients were included between November 2017 and January 2019. The TWA of IOH in the intervention group was 0.13 compared to 0.46 in the control group, p=0.001. Patients suffered from IOH 3.3% versus 10.5 %, p<0.001 of their duration of surgery. The TWA of hypertension was 0.09 versus 0.06, p=0.548. Patients experienced hypertension 1.67% versus 0.98%, p=0.379 of their duration of surgery. Neither the cumulative dose of vasoactive medication given nor fluid balance was significantly different between groups. 80% of all HPI alarms were treated according to protocol.

**CONCLUSION.** This is the first randomised clinical trial employing a machine-learning based tool in the field of anaesthesia. Use of the algorithm resulted in less hypotension without an increase in hypertension or the cumulative of vasoactive medication and fluids given during anaesthesia. Future studies should assess clinical endpoints.

**REFERENCE(S)**

1. Bijker JB, van Klei WA, Kappen TH, van Wolfswinkel L, Moons KG, Kalkman CJ. Incidence of intraoperative hypotension as a function of the chosen definition: literature definitions applied to a retrospective cohort using automated data collection. Anesthesiology. 2007;107(2):213-220.
2. Futier E, Lefrant JY, Guinot PG, et al. Effect of Individualized vs Standard Blood Pressure Management Strategies on Postoperative Organ Dysfunction Among High-Risk Patients Undergoing Major Surgery: A Randomized Clinical Trial. JAMA. 2017;318(14):1346-1357
3. Hatib F, Jian Z, Buddi S, et al. Machine-learning Algorithm to Predict Hypotension Based on High-fidelity Arterial Pressure Waveform Analysis. Anesthesiology. 2018;129(4):663-674.
4. Salmasi V, Maheshwari K, Yang D, et al. Relationship between Intraoperative Hypotension, Defined by Either Reduction from Baseline or Absolute Thresholds, and Acute Kidney and Myocardial Injury after Noncardiac Surgery. Anesthesiology. 2017;126(1):47-65.
5. This work is supported by Edward Lifesciences.

### 000806

#### Cytoreductive Surgery and HIPEC Patients Admitted in UCI

C. Joya Montosa[1], H. Molina Díaz[1], Á. Ortega Guerrero[1], MP. Benítez Moreno[2]
[1]Intensive care unit, Hospital Quirónsalud Málaga, Málaga, Spain; [2]Intensive care unit, Hospital Regional Universitario de Málaga, Málaga, Spain
**Correspondence:** C. Joya Montosa
*Intensive Care Medicine Experimental* 2019, **7(Suppl 3):**000806

**INTRODUCTION. Objetives**: Presenting clinical and epidemiological data of patients with cytoreductive surgery and hyperthermic intraperitoneal chemotherapy who are admitted in ICU.

**METHODS.** Retrospective, descriptive study of patients admitted in ICU from January 2018 to December 2018. Clinical, epidemiological and result-related variables were analyzed. Quantitative variables are expressed as mean and standard deviation, while qualitative variables are expressed as ratios and absolute value.

**RESULTS.** A total number of 14 patients took part in this study; mean age was 57.71± 8.90 years, 78.6% were females. APACHE II at the admission was 17.21 ± 6. SOFA 3 6.85±2.95

78.6% of the patients presented comorbidities; the most frequent was high blood pressure (28.6%), followed by dyslipidemia (21.4%) and smokers (14.3%). 57% had chronic disease: Pulmonary 14.3%(OCPD and asthma), Cardiac 7.1%, and others 42.9%.

Gynecological tumors were the most usual, 57.2% (6 ovarian cancer, 2 uterine cancer); follow by gastrointestinal tumor, 42.8% (3 colon adenoacarcinoma, 3 gastric tumor) Metastasis were presented in 28.6%, been hepatic location the most frequent (75%).

Cytorreduction were successful in all of the patients. Peritonectomy was done in 78.6% of the cases, Omentectomy in 35.7%. 71.4% of the patients need intestinal resection, 21.4% gastrectomy, 14.3% cholecystectomy, and 7.1% appendicectomy. In one case spleen removal was needed, and another patient needed nephrectomy. Oophorectomy was done in 28.6% of the women and same frequency was observed in hysterectomys. Cytoreduction at other levels, such as lymph nodes, pancreas or muscles, was done in 57.1% of the patients. The most used chemotherapic drug was Paclitaxel(50%), next was Cisplatin combined with Doxorrubicin(14.3%). Mitomycin, Fluorouracil and Oxaliplatin were used only once. The treatment was kept and average of 53.57± 12.77 minutes, with a mean temperature of 41.92 ± 1.07 ºC.

At the ICU admission, in the firsts few hour 64.3% of the patients needed *Norepinephrine* and intensive fluid resuscitation. Amoxicilin-Clavulanic was the most frequent antibiotic used as prophylaxis (92.9%). Early complications were found in 50% of the cases, the main one was hemodynamic shock (50%). In 14.3% of the cases ARF was observed, as well as pleural effusion and infections. Two patients had wound dehiscence. And other two needed re-intubation and mechanical ventilation support; CVVHDF was needed in 14.3%.

Length of stay in UCI was 7.78±7.07 days. Three patients were re-entry due to wound dehiscence, two of them with sepsis associated, none of them present renal failure neither respiratory failure; with an average UCI stay of 7.33± 3.51 days. The average hospital stay was 18.88±10.66 days.

**CONCLUSION.** The profile of the patient admitted to the ICU, after Cytoreductive surgery and HIPEC, was a 58 year old female, with comorbidities and gynecological or gastrointestinal cancer; who need Norepinephrine and intensive fluid resuscitation due to initial shock, as well as prophylactic treatment with Amoxicillyn-Calvulanic; with good progress in 24h and without needing to be re-admitted in ICU

### 000812

#### Pro-resolution mediators Resolvin D1 and D2 after left ventricular assist device implantation

E. Hittesdorf[1], R. Whittington[1], K. Maddipati[2], G. Wagener[1]
[1]Anesthesiology, Columbia University, New York, United States of America; [2]Lipidomics core facility, Wayne State University, Detroit, United States of America
**Correspondence:** G. Wagener
*Intensive Care Medicine Experimental* 2019, **7(Suppl 3):**000812

**INTRODUCTION.** Resolution of acute inflammation requires a newly discovered group of lipid Specialized Pro-resolving mediators (SPMs) derived from omega-3 fatty acids to control the inflammatory response and avoid transition to chronic inflammation. Little is known about the kinetics of SPMs in response to an acute inflammatory insult in humans. Few surgeries are associated with more inflammation than left ventricular assist device (LVAD) implantations.
**OBJECTIVES.** The aim of this study was to determine if and when the SPMs Resolvin D1 and D2 are detectable after a major inflammatory insult.
**METHODS.** After IRB approval/consent, the plasma of 10 patients undergoing LVAD implantations was analyzed for resolvin D1 and D2 using liquid chromatography and mass spectrometry (LC-MS) preoperatively and then daily until postoperative day 10. Arterial lactate concentrations were determined as an indicator of inflammation.
**RESULTS.** Ten patients were studied from August to October 2018. Resolvin D1 was 827 +/- 187 pg/mL at baseline, decreased initially to then 327 /- 185 pg/mL and then increased again with a peak on postoperative day 4 of 960 +/- 308 pg/mL. Mean Resolvin D2 levels were 169 +/- 44 pg/mL at baseline and fluctuated around 180 to 340 pg/mL postoperatively with no discernable trend. The lactate concentrations were elevated preoperatively and decreased to normal levels within 4 days.
**CONCLUSION.** We were able to detect SPMs after acute inflammation: Resolvin D1 peaked at day 4 at a time when inflammation decreased as indicated by normalizing lactate concentrations. However, Resolvin D2 remained unchanged throughout the postoperative period.
Patients undergoing LVAD implantation are in end-stage heart failure with other end-organ failures and, therefore, already have high levels of inflammation prior to surgery. Thus, it may be more difficult to identify a pattern of resolution.

**REFERENCE(S)**

1. J Lipid Res. 2017 Dec;58(12):2275-2288.
2. J Clin Invest. 2018 Jul 2;128(7):2657-2669
3. This study was supported by a intramural grant of the Department of Anesthesiology, Columbia University

### 000813

#### Circadian Rhythm Disturbance in Intensive Care and Long-term Cognitive Outcomes

R. Cusack, O. Tujjar
Anaesthesiology and intensive care, Sligo University Hospital, Sligo, Ireland
**Correspondence:** R. Cusack
*Intensive Care Medicine Experimental* 2019, **7(Suppl 3):**000813

**INTRODUCTION.** Patients admitted to the intensive care unit (ICU) are subject to sleep disturbance[Devlin et al, 2018]. Inadequate quality sleep or restlessness at night requiring sedation is a marker of circadian dysfunction in critical illness[Drouot et al, 2008; Pandharipande PP, et al., 2013]. Circadian rhythm disruption is increasingly implicated as a key factor leading to ICU delirium[Madrid-Navarro CJ,et al., 2015]. ICU-related delirium has been associated with ongoing sleep disturbance and cognitive morbidity after critical illness[Orwelius L., 2008].
**OBJECTIVES.** The aim of this study was to evaluate the risk factors associated with long term cognitive dysfunction in patients after ICU admission.
**METHODS.** We conducted a retrospective review of patients admitted to our ICU from January to December 2018. Inclusion criteria were age >18 years, Length of stay>48 hours, mechanical ventilation and use of sedatives. Demographics, comorbidities and known risk factors for ICU-delirium were collected. Patients' overnight state, sleep quality and symptoms of delirium, as documented by nurses each day were also recorded. Patients or Next of Kin were then contacted by telephone for interview, when consent was obtained. Health-related Quality of Life (HRQoL) and Post-traumatic Stress Disorder (PTSD) screening Questionnaires were administered over the phone.
**RESULTS.** Of the 87 patients who met the inclusion criteria, 24 did not survive ICU, 6 were transferred elsewhere and 12 deceased since discharge. Therefore 45 were available for phone interview, with a 62% response rate and one declining consent. Of the 27 patients included, 11 had Mental Component HRQoL scores at 6 months below the population average (42±7 vs 58±3). Patients with lower Mental Component HRQOL scores at 6 months had a higher rate of blood transfusions (6/11 vs 3/16, p=0.056), and circadian rhythm disruption (5/11 vs 14/16, p=0.018) in ICU. They also presented a higher rate of PTSD screen score at 12 months (5 [3,7] vs 1 [0,1.25], p=0.011).
**CONCLUSION.** Long-term cognitive dysfunction is common after ICU admission. These patients had circadian rhythm disturbance in ICU and were transfused more frequently. They were also more prone to develop long-term PTSD. This small retrospective study encourages further investigation of this interesting relationship.

**REFERENCE(S)**

1. Devlin JW, et al. Clinical Practice Guidelines for the Prevention and Management of Pain, Agitation/Sedation, Delirium, Immobility, and Sleep Disruption in Adult Patients in the ICU.Crit Care Med. 2018 Sep;46(9):e825-e873.
2. Drouot X1, et al.Sleep in the intensive care unit. Sleep Med Rev. 2008 Oct;12(5):391-403
3. Madrid-Navarro CJ,et al.Disruption of Circadian Rhythms and Delirium, Sleep Impairment and Sepsis in Critically ill Patients. Potential Therapeutic Implications for Increased Light-Dark Contrast and Melatonin Therapy in an ICU Environment.Curr Pharm Des. 2015;21(24):3453-68.
4. Orwelius L. Prevalence of sleep disturbances and long term reduced health related quality of life after critical care: a prospective multicentre cohort study. Crit Care 2008; 12 (4) R97
5. Pandharipande PP, et al. Long-term cognitive impairment after critical illness. N Engl J Med.2013;369(14):1306-16

### 000835

#### Safety and Tolerability of the Use of Immersive Virtual Reality in Mechanically Ventilated Patients for Neurocognitive Stimulation

J. Quah[1], I. Mardianah[2], T. Poh Choo[2], K. Serena[2], H. Agnes[3], L. Noelle[3], R. Jagadesan[4]
[1]Respiratory and Critical Care Medicine , Changi General Hospital, Singapore, Singapore; [2]Department of nursing, Changi General Hospital, Singapore, Singapore; [3]Department of anaethesiology, Changi General Hospital, Singapore, Singapore; [4]Respiratory and critical care medicine, Changi General Hospital, Singapore, Singapore
**Correspondence:** J. Quah
*Intensive Care Medicine Experimental* 2019, **7(Suppl 3):**000835

**INTRODUCTION.** New or worsening cognitive impairment occurs in up to 58% of survivors of critical illnesses and is long-lasting, with significant disability and socioeconomic cost[1]. There are currently no known interventions that reduce the incidence of cognitive impairment after critical illnesses. Immersive Virtual Reality (IVR) is the use of technology to create a perception of presence in a three-dimensional, computer-generated interactive simulated environment[2]. A prior clinical study has demonstrated potential efficacy in cognitive rehabilitation of severe traumatic brain injury [3,4]. Clinical data on the use of virtual reality in the critical care unit is limited to case report[5], paediatric population[6] or non-immersive virtual reality[7]. It is not known if the use of IVR is safe and tolerable in mechanically ventilated adult patients.

**OBJECTIVES.** The primary aim of this study is to evaluate the safety and tolerability of immersive virtual reality for potential early neurocognitive stimulation in critically-ill, mechanically ventilated patients.The secondary aim of the study is to evaluate the potential of 4-channel electroencephalogram (EEG) headbands in detecting differences in attention during the use of immersive virtual reality.

**METHODS.** Patients aged above 21, admitted to the intensive care unit for acute respiratory failure or septic shock, were evaluated for recruitment from 1st July 2018 to 1st April 2019. Recuited patients were randomised into the control or intervention groups. Patients in the intervention group would have two 15-minute sessions of IVR daily for up to a maximum of 3 days. The IVR videos were specially curated for this study and comprised of a combination of nature scenary coupled with soothing classical music. In both intervention and control groups, a 4-channel EEG headband would be applied over the subject's forehead. Assessment of safety involved monitoring for physiological derangements in heart rate, respiratory rate, pulse oximetry and blood pressure during the IVR session, compared with the control group. Assessment of tolerability involved monitoring for increased agitation.

**RESULTS.** 6 patients were randomised into the intervention group and 5 patients were in the control group. There were no significant differences between both groups in the baseline variables such as age, gender, race, Charlson Co-morbidity Index, APACHE II scores. The patients in the control group required more days of mechanical ventilation compared to the intervention group (5.4 ± 3.6 days vs. 2.8 ± 1.2 days). Use of vasopressors, renal replacement therapy and sedatives were similar in both groups. 2 of 6 patients in the intervention group had delirium during and after the ICU stay compared with 1 of 5 patients in the control group. The intervention group completed an average of 1.3 ± 0.8 IVR sessions compared to the control group, which completed 3.0 ± 1.9 session. In the intervention group, 83.3% (5 of 6) patients did not have significant physiological derangements compared to 100% (5 of 5) patients in the control group. However, 50% (3 of 6) patients in the intervention group demonstrated increased agitation compared to 20% (1 of 5) patients in the control group.

**CONCLUSION.** The use of IVR in mechanically ventilated patients is safe, however, it may not be tolerated in some patients, resulting in increased agitation. It is uncertain if it can provide neurocognitive stimulation, further correlation with EEG data is required.

**REFERENCE(S)**

1. Long-Term Cognitive Impairment after Critical Illness. New England Journal of Medicine. 2014;370(2):184-186. doi:10.1056/nejmc1313886.
2. Pourmand A, Davis S, Lee D, Barber S, Sikka N. Emerging Utility of Virtual Reality as a Multidisciplinary Tool in Clinical Medicine. Games for Health Journal. 2017. doi:10.1089/g4h.2017.0046.
3. Dvorkin AY, Ramaiya M, Larson EB, et al. A "virtually minimal" visuo-haptic training of attention in severe traumatic brain injury. J Neuroeng Rehabil. 2013;10:92
4. Zanier ER, Zoerle T, Di Lernia D, Riva G. Virtual Reality for Traumatic Brain Injury. Front Neurol. 2018;9:345. Published 2018 May 16. doi:10.3389/fneur.2018.00345
5. Blair GJ, Kapil S, Cole SP et al. Virtual reality use in adult ICU to mitigate anxiety for a patient on V-V ECMO. J Clin Anesth 2019; 55:26-27.
6. Badke CM, Essner BS, O'Connell M, et al. An Innovative Virtual Reality Experience in the PICU: A Pilot Study. Pediatr Crit Care Med. 2019 Mar 27. doi: 10.1097/PCC.0000000000001917.
7. Turon M, Fernandez-Gonzalo S, Jodar M, et al. Feasibility and safety of virtual-reality-based early neurocognitive stimulation in critically ill patients. Ann Intensive Care. 2017;7(1):81. doi:10.1186/s13613-017-0303-4
8. Ministry of Health (Singapore) Healthcare Research Scholarship, Master of Clinical Investigation (MCI) 2017

### 000919

#### Intra-abdominal pressure as a decisive variable in the management of critical acute pancreatitis admitted to an Intensive Care Unit (medical) in an eight-year period ( 2011-2018) in a second-level university Hospital

JL. Martinez Melgar[1], E. Moreno Lopez[2], I. Gallego Barbachano[1], E. Sanmartin Mantiñan[1], A. Ortega Montes[1], JI. Cenoz Osinaga[1], JV. Bravo Doviso[1], T. Sanchez De Dios[1], A. Pais Almozara[1], P. Posada Gonzalez[1]

[1]Intensive care unit, Complexo Hospitalario De Pontevedra, Pontevedra, Spain; [2]Anesthesia and perioperative care, Compliexo Hospitalario Universitario de Ferrol, Ferrol, Spain

**Correspondence:** E. Moreno Lopez

*Intensive Care Medicine Experimental* 2019, **7(Suppl 3):**000919

**INTRODUCTION.** Acute pancreatitis (AP) can lead to an increase in the intra-abominal pressure (IAP) that, if it is not recognized, can develop an abdominal compartment sindrome (ACS), causing multiple organ failure and a high mortality rate.

**OBJECTIVES.** To evaluate if intra-abdominal pressure (IAP) can determine the therapeutic attitude and contribute to increase the survival rate in patients diagnosed with critical acute pancreatitis admitted to a medical ICU in a second-level Hospital in an eight-year period (January 2011- December 2018)

**METHODS.** Descriptive and retrospective study of patients with acute pancreatitis admitted to a medical ICU in eight years ( 2011-2018). Acute pancreatitis was classifed accordig to the determinant-based classification of acute pancreatitis severity published in Annals of Surgery$_1$ : mild-moderate (AP-MM), severe (AP-S), critical (AP-C). In critical acute pancreatitis the following parameters were analyzed: gender, age, etiology, APACHE II score (within 24 hours of admission), Balthazar CT severity index, type of early nutrition (within 48 hours), multiple organ failure (MOF): respiratory ( Rp: PaO2/FiO2 ratio<300), cardiovascular ( Cv: inotropic support required), renal ( R: creatinine >2 mg/dL), need for mechanical ventilation ( MV > 5 days), renal support (continuous renal replacement therapy (CRRT)), IAP value$_2$ ( maximum value maintained for at least 12 hours), laparotomy performed ( Lap-P) : if IAP>30 mmHg and MOF (> 1), laparotomy not-perfomed ( Lap-noP): IAP<30 mmHg and MOF ( >1), and mortality rate. Statistical analysis: quantitative variables are expressed as mean and standard deviation (SD) and qualitative variables as percentages (%). The difference between qualitative variables are expressed using chi-square test and quantitative using an ANOVA analysis.

**RESULTS.** 88 patients diagnosed with AP were admitted: type AP-MM 26, type AP-S 30 and type AP-C 32. Of critical AP: 21 male (11 female), age: 59,5±15,3 years old, etiology: gallstone-related 15, alcohol abuse 10, idiopathic 4 and other 3. APACHE II score: 14,7±6,3, Balthazar: 6,1±2,5, nutrition: parenteral 15, enteral 10, mixed 4, none 3. Need for mechanical ventilation: 20 ( 62,5%) and CRRT required: 12 (37,5%). In group Lap-P (20 patients): 8 with Rp+CV, 8 with Cv+R and 4 with Rp+Cv+R. In the Lap-noP group (12 patients): 6 with Rp+Cv, 3 with Cv+R and 3 with Rp+Cv+R. Mortality in Lap group: 6 patients ( 1 Rp+Cv, 3 Cv+ R, 3 Re+Cv+R), and in group Lap-noP: 4 patients ( 1 Rp+Cv, 2 Cv+R, 1 Rp+Cv+R).

**CONCLUSION.** According to our series, in group Lap-P, 80% of the patients with critical AP had failure of at least two organs, whilst the Lap-noP had 75%. In terms of mortality rate, it was 30% in the Lap-group compared with 33,3% in the Lap-noP group

**REFERENCE(S)**

1. Dellinger EP, Forsmark CE, Layer P, Lévy P, Maraví-Poma E, Petrov MS, Shimosegawa T, Siriwardena AK, Uomo G, Whitcomb DC, Windsor JA; Pancreatitis Across Nations Clinical Research and Education Alliance (PANCREA). Determinant-based classification of acute pancreatitis severity: an international multidisciplinary consultation. Ann Surg. 2012 Dec;256(6):875-80
2. Malbrain ML, Cheatham ML, Kirkpatrick A, Sugrue M, Parr M, De Waele J, Balogh Z, Leppäniemi A, Olvera C, Ivatury R, D'Amours S, Wendon J, Hillman K, Johansson K, Kolkman K, Wilmer A. Results from the International Conference of Experts on Intra-abdominal Hypertension and Abdominal Compartment Syndrome. I. Definitions. Intensive Care Med. 2006 Nov;32(11):1722-32

### 000932

#### Delirium In ICU Patients With Malignancy: Patient Characteristics, Resource Utilization and Outcomes

M. Sieber[1], A. Rudiger[2], R. Schüpbach[1], B. Krüger[2], M. Schubert[3], D. Bettex[2]
[1]Insitute of Intensive Care, University Hospital of Zürich, Zürich, Switzerland; [2]Cardio-surgical intensive care unit, institute of anesthesiology, University Hospital of Zürich, Zürich, Switzerland; [3]School of health professions, institute of nursing, Zurich University of Applied Sciences, Winterthur, Switzerland
**Correspondence:** M. Sieber
*Intensive Care Medicine Experimental* 2019, **7(Suppl 3):**000932

**INTRODUCTION.** Delirium in general intensive care unit (ICU) patients has been associated with prolonged ICU and hospital length of stay (LOS), more ventilator days, increased short- and long-term mortality and long-term cognitive impairment [1-3]. However, knowledge on delirium in oncological ICU patients is scarce.
**OBJECTIVES.** To assess the frequency of delirium and its impact on resource utilizations and outcomes in ICU patients with malignancy.
**METHODS.** This retrospective, single-center longitudinal cohort study included all patients with malignancy admitted to ICUs of a University Hospital during one year. Delirium was diagnosed by an Intensive Care Delirium Screening Checklist (ICDSC) score ≥ 4. Group comparisons were made with Fisher's exact and Mann-Whitney U tests. Multivariate analysis was performed with binary logistic regression, Cox regression with hazard ratios < 1 indicating longer LOS, and multiple linear regression. Results are given as number (percentage) and median (interquartile range).
**RESULTS.** Of the 488 ICU patients with malignancy, 176/488 (36%) developed delirium during their ICU stay. Delirium showed high frequencies in patients with hepatic (13/21 [62%]) and lung malignancies (29/65 [45%]) as well as lymphomas (7/15 [47%]). In addition, it was particularly frequent in patients from thoracic (27/47 [57%]) and abdominal surgery (54/136 [40%]) and internal / general medicine (16/29 [55%]). Delirious patients had higher age (66 [55-72] vs 61 [51-69] years, p = 0.001), Charlson Comorbidity Index (4 [2-8] vs. 4 [2-8]), p = 0.034) and SAPS II (41 [27-68] vs 24 [17-32], $p < 0.001$), and more often a sepsis (26/176 [15%] vs 6/312 [1.9%], $p < 0.001$) and a shock (30/176 [6.1%] vs 6/312 [1.9%], $p < 0.001$). Univariate and multivariate analysis show that delirium was independently associated with longer LOS in ICU (HR [95% CI] 0.295 [0.234-0.371], $p < 0.001$) and hospital (HR [95% CI] 0.619 [0.500-0.765], $p < 0.001$), as well as higher ICU nursing workload (B [95% CI] 1.917 [1.665-2.206], $p < 0.001$) and ICU (B [95% CI] 2.077 [1.811-2.382], $p < 0.001$) and total costs per case (B [95% CI] 1.442 [1.301-1.597], $p < 0.001$). However, while being a strong marker of in-hospital mortality in univariate analysis (OR [95% CI] 5.909 [2.872-12.160], $p < 0.001$), delirium did not independently increase in-hospital mortality in multivariate analysis (OR [95% CI] 2.263 [0.925-5.537], $p = 0.074$).
**CONCLUSION.** In ICU patients with malignancy, delirium was a frequent complication independently associated with high resource utilizations, but it was not an independent risk factor of in-hospital mortality.

##### REFERENCE(S)

1 Ely EW, Shintani A, Truman B, Speroff T, Gordon SM, Harrell FE, Inouye SK, Bernard GR, Dittus RS, (2004) Delirium as a predictor of mortality in mechanically ventilated patients in the intensive care unit. JAMA 291: 1753-1762
2. Salluh JI, Wang H, Schneider EB, Nagaraja N, Yenokyan G, Damluji A, Serafim RB, Stevens RD, (2015) Outcome of delirium in critically ill patients: systematic review and meta-analysis. BMJ 350: h2538
3. Pandharipande PP, Girard TD, Jackson JC, Morandi A, Thompson JL, Pun BT, Brummel NE, Hughes CG, Vasilevskis EE, Shintani AK, Moons KG, Geevarghese SK, Canonico A, Hopkins RO, Bernard GR, Dittus RS, Ely EW, Investigators B-IS, (2013) Long-term cognitive impairment after critical illness. N Engl J Med 369: 1306-1316
4. None

### 000939

#### Effect of red blood cell transfusion on endothelial cell activation and coagulation in the critically ill

L. van Manen[1], ME. van Hezel[1], M. Boshuizen[1], M. Straat[1], A. Spoelstra-de Man[2], R. Van Bruggen[3], NP. Juffermans[1]
[1]Department of intensive care, Amsterdam University Medical Centers, Location AMC, Amsterdam, Netherlands; [2]Department of intensive care, Amsterdam University Medical Centers, Location VUmc, Amsterdam, Netherlands; [3]Department of blood cell research, Sanquin Blood Supply, Amsterdam, Netherlands
**Correspondence:** L. van Manen
*Intensive Care Medicine Experimental* 2019, **7(Suppl 3):**000939

**INTRODUCTION.** Red blood cell (RBC) transfusion is a frequently applied intervention at the intensive care unit. However, transfusion is associated with adverse outcome including organ failure and thrombo-embolic events, the mechanism of which is not known, but may be related to the underlying disease severity.
**OBJECTIVES.** The aim of this study was to investigate the effect of RBC transfusion on endothelial cell activation and coagulation markers in critically ill patients.
**METHODS.** In 74 non-bleeding patients admitted to the ICU of a tertiary academic hospital receiving one RBC unit, biomarkers of endothelial cell activation, inflammation and coagulation (von Willebrand Factor (vWF) antigen, soluble ICAM-1, soluble thrombomodulin (sTM), soluble syndecan-1, TNFa, IL-6, d-dimer, fibrinogen, APTT and PT) were measured before transfusion, at one hour after transfusion and 24 hours after transfusion. The impact of disease severity was assessed by comparing septic and non-septic patients and by correlation of biomarkers with organ injury score (SOFA).
**RESULTS.** Included patients were predominantly male (55%) with a median age of 63 years. 55% of the patients was septic according to Sepsis-3 criteria. Levels of vWF antigen, soluble ICAM-1, sTM, fibrinogen and d-dimer were already high at baseline. VWF antigen levels increased significantly 24 hours after RBC transfusion (478% vs 526%, p = 0.02). The other biomarkers did not change. Post transfusion change of the measured biomarkers was not different between septic and non-septic patients and was not correlated with SOFA score.
**CONCLUSION.** RBC transfusion in critically ill patients was associated with an increase in circulating vWF antigen levels, suggesting a further increase in activation status of the endothelium, a finding that was independent of sepsis status or organ injury level.

1. Lisa van Manen was supported by a grant from the Dutch Ministry of Health awarded to Robin van Bruggen

## SIS - Sepsis evaluation and treatment

### 000553

#### The effects of intravenous immunoglobulin in critically ill patients with panperitonitis

H. Shim[1], JY. Jang[1], YU. Choi[1], J. Kim[1], TH. Go[2], KS. Bae[1]
[1]Surgery, Severance Hospital, Yonsei University Wonju Christian Hospital, Weonju, Republic of Korea; [2]Center of biomedical data science, Severance Hospital, Yonsei University Wonju Christian Hospital, Weonju, Republic of Korea
**Correspondence:** H. Shim
*Intensive Care Medicine Experimental* 2019, **7(Suppl 3):**000553

**INTRODUCTION.** In recent, intravenous immunoglobulin(IVIG) has been used as one of the adjunctive therapy for septic patients. Although IVIG is not recommended in the Survival Sepsis guideline, it has still debate to be estimated as an effective treatment for sepsis.
**OBJECTIVES.** The authors tried to evaluate the effect of IVIG in the critically ill patient who got abdominal surgery due to pan-peritonitis.
**METHODS.** It is a retrospective study analyzing 646 patients' electrical medical record from March 2013 to June 2018. The patients who had been diagnosed as secondary pan-peritonitis and managed in the ICU after getting emergency operation were indicated in this study. In this population, the comparison was made between patients who were injected with IVIG and those who did not administer IVIG. As outcome variables, mortality curve, 30-day mortality, hospital mortality and ICU duration were evaluated in each group. Of the 576 patients who were operated for pan-peritonitis during the study period, 169 were analyzed, excluding 307 patients who had left ICU within three days due to minor severity and 100 patients with contraindications. 44 patients were injected with IVIG and 125 patients were treated with conventional management without IVIG. After propensity scoring matching adjusted by APACHE II score and SOFA score, IVIG group (34 patients) and non-IVIG group ( 34 patients) were compared to each other.
**RESULTS.** There was no significant different outcome between the IVIG group and non-IVIG group however the hazard ratio of the non-IVIG group was higher than the IVIG group significantly in mortality and hospital mortality. (Overall mortality, Crude HR 1.68 (0.68~4.12), Adjusted HR 1.75 (0.69~4.44); Hospital mortality, Crude HR 1.83 (0.61~5.47), Adjusted HR 2.12 (0.68~6.67)) In the survival curve, there was a trend to separate completely between the IVIG group and non-IVIG group as the time went on. (Overall mortality, P=0.105; Hospital mortality, p=0.093)
**CONCLUSION.** In septic patients with pan-peritonitis, the use of IVIG after proper surgery and the use of antibiotics could affects the risk of overall mortality and hospital mortality.

**REFERENCE(S)**

1. The Intravenous immunoglobulin Collaborative Study Group. Prophylactic intravenous administration of standard immune globulin as compared with coe-lipopolysacharide globulin in patients at high risk of postsurgical infection. N Engl J med 1992;327:234-40
2. Marissa MA, Mary Ann DL, Leonila FD, et al. Intravenous immunoglobulin for treating sepsis, severe sepsis and septic shock. Cochrane database of systemic reviews 2013, Issue 9, Art. No.:CD001090
3. Turgeon AF, Hutton B, Fergusson DA, et al. Metaanalysis; IV immunoglobulin in critically ill patients with sepsis. Ann intern Med. 2007;146:193-203
4. B Laupland K. Polyclonal intravenous immunoglobulin for the prophylaxis and treatment of infection in critically ill adults. Can J Infect Dis. 2002 Mar;13(2);100-6
5. Taccone FS, Stordeur P, De Backer D, et al. Gamma-globulin levels in patients with community-acquired septic shock. Shock 2009 Oct;32(4):379-85
6. Lyon JM, Davis C, Rieman MT et al. Prophylactic intravenous immune globulin and polymixin B decrease the incidence of septic episodes and hospital length of stay in severely burned children. J Burn Care Res. 2006 Nov-Dec;27(6):813-8.

### 000557

#### Should blood cultures be done for all patients who are admitted to the Intensive Care Unit with septic shock?

L. Denholm[1], S. Drysdale[2], A. Puxty[2]
[1]NHS GGC, Glasgow, United Kingdom; [2]Intensive care unit, NHS GGC, Glasgow, United Kingdom
**Correspondence:** L. Denholm
*Intensive Care Medicine Experimental* 2019, **7(Suppl 3):**000557

**INTRODUCTION.** Blood cultures are part of the septic shock protocol for patients admitted to the Intensive Care Unit (ICU) at Glasgow Royal Infirmary (GRI) Hospital. This aims to identify causative organisms quickly and guide or refine antibiotic therapy. However, patients with sepsis should rarely be admitted to ICU without blood cultures and antibiotic therapy initiated. As per surviving sepsis guidelines, these cultures should have been carried out prior to treatment with antibiotics. Recent antibiotic therapy reduces positive culture rates, therefore it's possible that repeating cultures does not contribute to patient care.
**OBJECTIVES.** To evaluate the clinical value in carrying out blood cultures routinely in all septic shock patients and to determine the frequency at which new causative organisms idetntified.
**METHODS.** This was a retrospective analysis of 241 patients with vasopressin dependent septic shock admitted between Jan 17-Nov 18 to our ICU. Mean age of the cohort was 60.8 yrs (range 19-92) and 56% were male. We recorded blood cultures performed up to 24hrs pre and 24hrs post ICU admission.
**RESULTS.** Of 241 patients, 144 (59.7%) had cultures prior to ICU admission. 45 (31.25%) were positive. Post ICU admission, 115 (47.7%) patients had blood cultures, 13 (11.4%) of these were positive for an organism.
A total of 65 (56.5%) out of 115 patients who were cultured post-ICU admission had not had cultures previously. 8 (6.9%) of these cultures grew micro-organisms. Three (2.6%) patients had cultures which reported no growth prior and became positive following admission cultures. Two (1.7%) patients had blood cultures which were positive before and after admission, however the second set of cultures did not provide any new information only confirming the initial result. 30 (12.45%) of 241 patients had no cultures taken.
**CONCLUSION.** While new information was gained from repeat cultures the yield was low. There remains a significant number of patients who don't have cultures taken prior to ICU admission. Given that septic patients' outcomes are time sensitive and early cultures are more likely to be positive, efforts to improve yield should be concentrated here. This project supports the notion that there is value in post-ICU admission blood cultures, though the number of new positive results is relatively low.

1. https://www.nice.org.uk/guidance/NG51

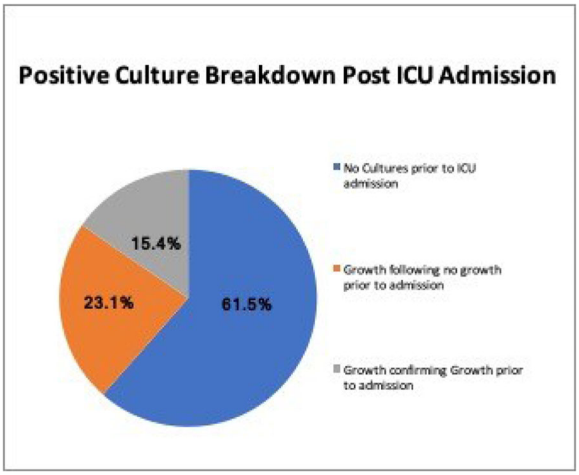

**Fig. 1 (abstract 000557).** See text for description

### 000577

**Evaluation of risk factors for the development of Carbapenem Resistance among Gram-Negative Bacilli**

DN. Mukherjee[1], S. Seal[2]
[1]Clinical microbiology & id, Woodlands, Kolkata, India; [2]Clinical microbiology, Woodlands , Kolkata, India
**Correspondence:** D.N. Mukherjee
*Intensive Care Medicine Experimental* 2019, **7(Suppl 3):**000577

**INTRODUCTION.** Carbapenem resistance among Gram-negative bacilli (CR-GNB) has been increasing over the past several decades, particularly in *Enterobacteriales* species. Infections due to CR-GNR are increasing in frequency and result in high morbidity and mortality. Overuse/misuse of carbapenems as initial antibiotic therapy is a risk factor to develop carbapenem resistance in hospitalized patients.

**OBJECTIVES.** To find out the correlation between early carbapenem exposure and other risk factors in the development of carbapenam resistance amongst Gram negative bacilli.

**METHODS.** We have conducted a retrospective study of all patients with positive cultures from any source over a 2-year period at a tertiary care hospital from 2016 to 2018 to develop a comprehensive model for risk of infection or colonization with CR-GNB, with separate analyses for Ertapenem resistant (ER-GNB) and antipseudomonal carbapenem resistant (ACR-GNB) isolates. Routine susceptibility testing was performed by the either the CLSI reference broth microdilution method (BMD) or using a Vitek 2. All antimicrobial susceptibility data were interpreted using current CLSI breakpoints for carbapenems.

**RESULTS.** A total of 2450 GNB isolates from 1320 patients were analyzed, of which 412 were CR (ER 130 and ACR 282). The majority of ER-GNB were *Klebsiella* species, whereas the most common ES-GNB were *Escherichia coli*; the most common ACR-GNB were *Acenetobacter* species. Risk factors for CR-GNB were, receipt of any carbapenem in the prior 30 days, any surgical intervention in recent past, admission from another health care facility, ventilation at any point before culture during the index hospitalization, any invasive device including foley's catheterization and receipt of any anti-MRSA agent in the prior 30 days.

**CONCLUSION.** Initial choice of antibiotic remains a challenging and high-stakes decision. Early exposure with carbapenem is a risk factor for development of CR-GNB infection in later days of hospitalization. Delayed antimicrobial therapy in carbapenem resistant infections has higher mortality, highlighting the need for rapid identification of patients at high risk for CR-GNB. Rational antibiotic therapy and antimicrobial stewardship is the need of the hour.

**REFERENCE(S)**

1. Papadimitriou-Olivgeris M, Marangos M, Christofidou M, Fligou F, Bartzavali C et al. Risk factors for infection and predictors of mortality among patients with KPC-producing Klebsiella pneumoniae bloodstream infections in the intensive care unit. Scand J Infect Dis 2014;46:642–648.
2. Vincent JL, Rello J, Marshall J, Silva E, Anzueto A et al. International study of the prevalence and outcomes of infection in intensive care units. JAMA 2009;302:2323–2429.
3. N.A

### 000581

**The combination of cold exposure training and a breathing technique attenuates the inflammatory response in humans in vivo**

J. Zwaag, N. Rick, P. Pickkers, M. Kox
Intensive care, Radboud University Medical Center, Nijmegen, Netherlands
**Correspondence:** J. Zwaag
*Intensive Care Medicine Experimental* 2019, **7(Suppl 3):**000581

**INTRODUCTION.** We previously showed that an intervention developed by 'Iceman' Wim Hof enables voluntary activation of the sympathetic nervous system, reflected by profoundly increased plasma adrenaline levels, which subsequently caused profound attenuation of the inflammatory response during experimental human endotoxemia. The intervention consisted of a training program encompassing several elements that may have contributed to the observed effects, namely two different breathing techniques performed during the endotoxemia experiments, and exposure to cold in the days before endotoxemia.

**OBJECTIVES.** For possible future clinical application, we aimed to determine the contribution of the different elements of the training program to the effects observed in our previous study. Furthermore, we assessed if the duration of the training is of relevance, and whether it can be provided by an independent trainer instead of Mr. Hof.

**METHODS.** To determine the optimal breathing technique to induce adrenaline release, 40 healthy male volunteers were randomized to training in both techniques (cycles of hyperventilation followed by either muscle tightening for 10 seconds or breath retention for several minutes) for either 4 days or 1 hour, by either Mr. Hof or an independent trainer. In a subsequent endotoxemia study, 48 healthy male volunteers were randomized to four groups: 4-day cold exposure training (CLD group), training in the optimal breathing technique (BRT group), a combination of both interventions (CBR group), or no intervention (CON group). The CLD and CBR groups were subjected to cold exposure in snow and water of 1 oC. All 48 subjects were subsequently challenged with 2 ng/kg LPS to induce endotoxemia.

**RESULTS.** In the first part of the study, arterial blood saturation levels and pO2 were significantly lower when subjects performed the breathing technique with breath retention (both $p<0.001$), but plasma adrenaline levels increased to a similar extent immediately after initiation of both breathing techniques (0.48 nmol/L [-0.01-1.2] vs. 0.50 nmol/L [0.25-0.91] upon the breath retention and muscle tightening techniques, respectively). No effects of training duration or trainer were observed on adrenaline levels or any other parameters. In the second part of the study, we therefore employed a short training in the breathing technique without breath retention, and all training procedures were provided by an independent trainer. Peak endotoxin-induced flu-like symptom scores were significantly lower in the CLD group (4.1±0.6 vs. 7.9±1.4 in the CON group, p=0.02), and resolved more rapidly. In the CBR group, plasma levels of pro-inflammatory cytokines were attenuated (TNF-α: -33%, p=0.03, IL-6: -58%, p=0.03), whereas concentrations of the anti-inflammatory cytokine IL-10 were increased by 73% (p=0.02) compared with the CON group. In the BRT group, similar, but less pronounced effects on plasma cytokines were observed, whereas no differences in cytokine levels were observed between the CLD and CON groups.

**CONCLUSION.** The combination of cold exposure and the breathing technique without breath retention attenuates the *in vivo* inflammatory response most potently. These results demonstrate that the immunomodulatory effects of the intervention developed by Wim Hof can be reproduced making use of short standardized protocols and an independent trainer.

### 000589

**Site of infection affects the prognosis performance of endothelial biomarkers to predict clinical deterioration of septic patients in Emergency Department**

T. Lafon[1], MA. Cazalis[2], A. Desachy[3], V. Gissot[4], T. Daix[5], F. Verschuren[6], K. Tazarourte[7], PF. Laterre[8], C. Vallejo[9], B. François[5]
[1]Inserm cic 1435 / service d'accueil des urgences, C.H.U de Limoges, Limoges, France; [2]Diagnostic discovery department, bioMérieux - Campus De L'étoile, Marcy-l'Étoile, France; [3]Réanimation polyvalente, C.H.U de Limoges, Limoges, France; [4]Inserm cic 1415, Chru Hôpitaux De Tours, Hospital Bretonneau, Tours, France; [5]Inserm cic 1435 / réanimation polyvalente, C.H.U de Limoges, Limoges, France; [6]Urgences, Cliniques universitaires Saint-Luc UCLouvain, Woluwe-Saint-Lambert, Belgium; [7]Service d'accueil des urgences, Hospital Édouard Herriot, Lyon, France; [8]Intensive care unit, Cliniques Universitaires Saint-Luc, Brussels, Belgium; [9]Inserm cic 1435, C.H.U de Limoges, Limoges, France
**Correspondence:** T. Lafon
*Intensive Care Medicine Experimental* 2019, **7(Suppl 3):**000589

**INTRODUCTION.** Early prognostic assessment of septic patients in the Emergency Department (ED) is crucial. Therefore, identification of patients at high risk of organ failure or shock is key to prevent deterioration and reduce mortality. Currently, no prognostic tool seems efficient to identify these patients. The infection site could represent a major factor of heterogeneity in the prognostic performance of biomarkers in septic patients.

**OBJECTIVES.** To evaluate the prognostic performances of biomarkers to predict the clinical deterioration of patients with sepsis in ED according to their site of infection.

**METHODS.** TRIAGE was an international multi-centre (France and Belgium) prospective observational study (ClinicalTrials.gov: NCT02739152) designed to evaluate a panel of prognostic biomarkers in adult septic patients admitted in ED (SIRS criteria). Blood samples were collected at 0, 6 and 24 hours after ED inclusion. Main outcome was subsequent deterioration (death, ICU admission, 1-point increase of SOFA score) within 72 hours. The diagnosis of sepsis and the evolution criteria were centrally validated by an independent adjudication committee of sepsis experts. The prognostic performances of endothelial biomarkers (sVEGFR2, sUPAR) were assessed according to the site of infection using logistic regression models. AUC were calculated using the DeLong method.

**RESULTS.** Overall 462 patients were analysed, 124 patients were confirmed as deterioration and 338 patients without deterioration. Sites of infection were mainly lungs (29%), urinary tract (27%) and abdomen / pelvis (25%). Patients with pulmonary infection were significantly more severe (qSOFA, and Charlson score) ($p<0.001$) than the other patients. These patients were also the ones who deteriorated the most within 72 hours and had a higher D28 mortality ($p=0.0047$). Expression of biomarkers was significantly associated with the risk of deterioration regardless of the site of infection (Lung: OR = 1.9 [1,24-2.86], Urinary: OR = 2.6 [1.3-5.82], Abdomen-pelvis: OR = 2.4 [1.26-4.97], Others: OR = 1.67 [1.05-2.74], $p<0.05$). Nevertheless, the predictive performance of short-term deterioration by the biomarkers was higher in patients with urinary and abdominopelvic infections (AUC = 0.70, sp=51, NPV=95 and AUC = 0.81, sp=51, NPV=93, respectively) compared to lung infections and other sites of infection (AUC = 0.66, sp=19, NPV=79).

**CONCLUSION.** Although biomarkers were associated with a risk of deterioration of septic patients, the predictive performance of sVEGFR2 and sUPAR was significantly lower in patients with pulmonary infection when compared to those with urinary tract or abdominopelvic infection.

### 000593

#### Prognostic performance comparison between clinicians and endothelial biomarkers to predict the deterioration of septic patients in Emergency Department

T. Lafon[1], MA. Cazalis[2], A. Baisse[3], C. Vallejo[4], K. Tazarourte[5], PF. Laterre[6], V. Gissot[7], B. François[8]

[1]Inserm cic 1435 / service d'accueil des urgences, C.H.U de Limoges, Limoges, France; [2]Diagnostic descoveery department, bioMérieux - Campus De L'étoile, Marcy-l'Étoile, France; [3]Service d'accueil des urgences, C.H.U de Limoges, Limoges, France; [4]Inserm cic 1435, C.H.U de Limoges, Limoges, France; [5]Service d'accueil des urgences, Hospital Édouard Herriot, Lyon, France; [6]Intensive care unit, Cliniques Universitaires Saint-Luc, Brussels, Belgium; [7]Inserm cic 1415, Chru Hôpitaux De Tours, Hospital Bretonneau, Tours, France; [8]Inserm cic 1435 / réanimation polyvalente, C.H.U de Limoges, Limoges, France

**Correspondence:** T. Lafon

*Intensive Care Medicine Experimental* 2019, **7(Suppl 3):**000593

**INTRODUCTION.** Accurate prognostic assessment of septic patients is challenging in the emergency department (ED). Identification of patients at high risk of organ failure or shock could help to prevent deterioration and reduce mortality. Clinician's assessment is based on initial severity, scoring, social context, hospital bed capacity, and on personal experience. The performance of emergency physicians in predicting septic patient's outcome has been scarcely described, and the additional value of a prognostic biomarker has not often been evaluated in the ED.

**OBJECTIVES.** To calculate the prognostic performance of emergency physicians to predict clinical deterioration of septic patients during their initial management in the ED and to evaluate if adding biomarkers information could improve this clinical prediction.

**METHODS.** This is an ancillary study of the TRIAGE study (ClinicalTrials.gov: NCT02739152) designed to evaluate a panel of prognostic endothelial biomarkers (sVEGFR2 and sUPAR) in a cohort of adult septic patients admitted to the ED. The analysis was performed on non-severe patients (SOFA<2) of two teaching hospitals. The risk of clinical deterioration was assessed by an adjudication committee composed of three independent emergency physicians (blinded from deterioration outcome) according to the emergency medical records and the first conventional biological and imaging results. This first judgement allowed to calculate the clinical emergency physician's performance. Then, adjudicators were unblinded from the results of biomarkers (which helped classifying patients into two groups: "high risk" or "low risk") and were asked to keep or revise their first judgement. This second judgement allowed assessing the additional value of biomarkers. Finally, the performance of biomarkers alone was calculated.

**RESULTS.** Analyses were performed on 145 patients (age = 50±20 yr; Charlson score: 1.7 [0-3]; SOFA score: 0.5 [0-1]; lactates: 2.03 [1.17-2.41]; site of infection: pulmonary 12.4%, urinary 32.4%, abdomino-pelvic 34.5% and 30 patients deteriorated (21%). The clinical performance of emergency physicians to predict deterioration was: Sensitivity=80; Specificity=21; Negative Predictive Value=80; Positive Predictive Value=21. Adding the biomarkers improved the clinical prognostic performance of emergency physicians (Sensitivity=90; Specificity=19; Negative Predictive Value =88; Positive Predictive Value =23). Biomarker alone was the best predictor of deterioration (Sensitivity =93; Specificity =50; Negative Predictive Value =97; Positive Predictive Value =33).

**CONCLUSION.** This study confirms that predicting the clinical deterioration of septic patients in the ED remains challenging. Adding prognostic biomarkers (sVEGFR2 and sUPAR) to clinical evaluation could be helpful in early assessing the risk of deterioration of septic patients, and safely ruling out patients after ED admission due to its high negative predictive value.

### 000645

#### Short-term hemodynamic response to calcium supplementation in patients with refractory septic shock

H. Chang[1], TG. Shin[2]

[1]samsung medical, Seoul, Republic of Korea; [2]Emergency medicine, Sungkyunkwan University School of Medicine, Seoul, Republic of Korea

**Correspondence:** H. Chang

*Intensive Care Medicine Experimental* 2019, **7(Suppl 3):**000645

**INTRODUCTION.** Calcium can be considered an adjunctive rescue therapy for refractory septic shock, but research on calcium use is limited. In this study we aimed to investigate short-term hemodynamic response after calcium supplementation in patients with refractory septic shock.

**METHODS.** This was a single-center, retrospective study of patients who presented to the emergency from October 2014 through February 2018. Patients with refractory septic shock requiring norepinephrine-equivalent (NE) dose ≥ 0.5 μg/kg/min and receiving calcium supplementation were included. Patients were categorized into a response group and no-response group based on the changes in NE doses and MAP after calcium administration. A multivariable logistic regression model was used to evaluate the association between hemodynamic response and 28-day survival.

**RESULTS.** A total of 66 patients were included. Among them, the response group included 34 (52%) patients and the no-response group 31 (48%) included 17 patients who deteriorated and 15 patients who had no significant changes in clinical status. The 28-day mortality was 44% in the response group and 63% in the no-response group ($P$ = 0.14). Multivariable analysis revealed that the response group had a significant association with lower 28-day mortality (the adjusted OR, 0.23; 95% CI, 0.06–0.89; $P$ = 0.03).

**CONCLUSION.** Short-term hemodynamic changes after calcium administration were variable in patients with refractory septic shock. Hemodynamic improvement after calcium supplementation was associated with better survival. Further studies are needed to determine when calcium should be considered and how it impacts patient-centered outcomes in refractory septic shock.

### 000664

#### Lower Serum TWEAK Concentration is a Biomarker for Mortality in Community-Acquired Pneumonia

LF. Reyes[1], N. Gonzalez-Juarbe[2], D. Parra-Tanoux[3], M. Sáenz-Valcárcel[3], M. Rodríguez[4], L. Claverias[5], S. Trefler[4], M. Bodí[4], J. Marin-Corral[6], A. García-España[7], A. Rodríguez[4]
[1]Critical care medicine - infectious diseases department, Universidad de la sabana, Bogotá, Colombia; [2]Infectious diseases department, J. Craig Venter Institute, Rockville, United States of America;
[3]Student, Universidad de La Sabana, Bogotá, Colombia; [4]Critical care department, Hospital Universitari de Tarragona Joan XXIII, Tarragona, Spain; [5]Critical care department, Hospital Verge de la Cinta, Tortosa, Spain; [6]Critical care department, IMIM, Barcelona, Spain; [7]Cell biology department, Instituto de Investigación Sanitaria Pere Virgili, Tarragona, Spain
**Correspondence:** L.F. Reyes
*Intensive Care Medicine Experimental* 2019, **7(Suppl 3):**000664

**INTRODUCTION.** Community-acquired pneumonia (CAP) is the primary cause of infectious death in developing countries. During CAP, infected patients initiate immune responses that promote local and systemic inflammation. While several proinflammatory molecules have been described and linked to a greater risk of complications, longer hospital stays and mortality; novel biomarkers are still required for superior identification of disease severity. TNF-like weak inducer of apoptosis (TWEAK) is a member of the TNF-alpha superfamily, involved in immune response, cell growth, angiogenesis, NF-kB activation and apoptosis induction in tumor cells. It is known that serum-TWEAK promotes inflammation in diseases such as multiple sclerosis, atherosclerosis and pancreatitis. Importantly, its role in disease severity of CAP patients is still unknown. Therefore, this study aims to determine whether there is a relationship between serum concentration of TWEAK and prognosis in CAP patients.

**OBJECTIVES.** To determine the relationship of serum concentration of tumor necrosis factor (TNF)-like weak inducer of apoptosis (TWEAK) and mortality in community-acquired pneumonia (CAP) patients.

**METHODS.** This is a multicenter 2-year cohort study in Spain, designed to better understand the role of sTWEAK concentrations in CAP patients. A total of forty-three patients were enrolled in two University hospitals (10 healthy users, 10 uninfected controls and 23 CAP patients). sTWEAK was measured within the first 24 hours of ICU admission. Samples were collected and stored for laboratory analyses. To detect sTWEAK in human samples, we used a commercially available ELISA kit following manufacture's instructions. Demographic patients' characteristics and ICU mortality were prospectively collected. Descriptive statistics and logistical regressions were used to assess the proposed aims.

**RESULTS.** In comparison to healthy volunteers, patients admitted to the hospital (both, infected and non-infected) had lower level of sTWEAK. During hospital admission, 7 (17%) patients died. Patients whom died during ICU stay had significantly lower levels of sTWEAK when comparing with patients whom survived (Median [IQR]; 509.35 [357.49, 953.92] Vs 1103.03 [716.93, 1663.16]; p=0.015). In contrast, patients that developed shock did not have different concentrations of sTWEAK (Median [IQR]; 1008.04 [531.87, 1390.80] Vs 1062.29 [575.24, 1598.83], p=0.84).

**CONCLUSION.** To our knowledge, this is the first study to show a role for sTWEAK in the morbidity and mortality of CAP patients. Here we show that CAP patients with lower systemic sTWEAK had increased mortality rate, suggesting a beneficial role for sTWEAK in the host response against infection. This biomarker may identify patients at risk of developing severe CAP and subsequently died in the ICU. However, further studies are needed to confirm these findings.

**REFERENCE(S)**

1. (Roderburg et al.) Roderburg, C., F. Benz, F. Schüller, I. Pombeiro, H.-J. Hippe, N. Frey, C. Trautwein, T. Luedde, A. Koch, F. Tacke & M. Luedde Serum Levels of TNF Receptor Ligands Are Dysregulated in Sepsis and Predict Mortality in Critically Ill Patients.
2. (Ebrahimi et al. 2018) Ebrahimi, F., S. Giaglis, S. Hahn, C. A. Blum, C. Baumgartner, A. Kutz, S. V. van Breda, B. Mueller, P. Schuetz, M. Christ-Crain & P. Hasler (2018) Markers of neutrophil extracellular traps predict adverse outcome in community-acquired pneumonia: secondary analysis of a randomised controlled trial. Eur Respir J, 51.
3. (Chicheportiche et al. 1997) Chicheportiche, Y., P. R. Bourdon, H. Xu, Y. M. Hsu, H. Scott, C. Hession, I. Garcia & J. L. Browning (1997) TWEAK, a new secreted ligand in the tumor necrosis factor family that weakly induces apoptosis. J Biol Chem, 272, 32401-10.
4. Saitoh, Tatsuya & Nakayama, Masafumi & Nakano, Hiroyasu & Yagita, Hideo & Yamamoto, Naoki & Yamaoka, Shoji. (2003). TWEAK induces NF-kappa B2 p100 processing and long lasting NF-kappa B activation. The Journal of biological chemistry. 278. 36005-12. 10.1074/jbc.M304266200.

### 000703

#### SEPSIS SAVER. Preliminary results of a pilot implementation of a new automatic electronic alert for early detection of sepsis based on Sepsis 3 definition

R. Zaragoza[1], S. Sancho[1], V. Ramirez[1], O. Badallo Arévalo[2], S. Ossa[2], MD. Valle[2], K. Torres[1], S. Ribes[1], I. Sotos[1], M. Borges[3]
[1]Intensive care Unit, Hospital Universitario Doctor Peset, Valencia, Spain; [2]Icu, Hospital Universitario de Burgos, Burgos, Spain; [3]Icu, Hospital Son Llàtzer, Palma, Spain
**Correspondence:** R. Zaragoza
*Intensive Care Medicine Experimental* 2019, **7(Suppl 3):**000703

**INTRODUCTION.** Although the role of automatic alerts for early detection of sepsis remains unclear, it is true that majority of electronic alerts for early detection of sepsis are based on sepsis 2 definitions. No data about alerts based on sepsis 3 definition has been provided.

**OBJECTIVES.** The aims of this study were to describe SEPSIS SAVER, a new electronic alert based on sepsis 3 definition; to compare it with an electronic alert based on sepsis 2 and to know its sensiblity, specificity, negative predictive value (NPV) and positive predictive value (PPV).

**METHODS.** SEPSIS SAVER is a new electronic tool designed by a multidisciplinary group composed by members of three sepsis units of three teaching hospitals based on sepsis 3 definition.

During a month period (March 2018) it has been implemented as a pilot in one of the hospitals and has been prospectively evaluated. This unique center has been working with a previous electronic sepsis alert based in sepsis 2.0 six years ago.

Clinical and microbiological variables were recorded. Any patient admitted at Emergency room was potentially eligible using the two sistems simultaneously. Sensibility, specificity, NPV and PPV were calculated for both alerts using sepsis team criteria as Gold standard.

**RESULTS.** Among 1378 electronic activations, 58 (4,2%) were confirmed as a sepsis severe/ septic shock (sepsis 2) or sepsis (sepsis 3) by the members of sepsis team. It means a rate of 22,8 episodes/ 100.000 inhabitants a month . The most frequent sources of infections were the respiratory focus (39%) following by urinary (26.6%) and abdominal (18.5%). Only 18.9% of patients were admitted to ICU. Global mortality was 18.9%. The majority of episodes were community adquired (85%). Forty episodes were detected by the two alerts. SEPSIS SAVER detected 46 true episodes with 12 false negative results compared with the tool based on sepsis 2 definitions which detected at the same time 52 episodes with 6 false negative results. Sensibility, Specificity, NPV and PPV of SEPSIS SAVER were 79,3%; 50,6%; 3,3% and 99,1% respectively. Sensibility, Specificity, NPV and

PPV of sepsis 2.0 alert were 89.6%; 49.1%; 3.7% and 99.5% respectively

**CONCLUSION.** SEPSIS SAVER showed an adequate capability to detect sepsis as well as did alert based on sepsis 2 definition. Both tools need to be modified in order to improve their specificity with new definitions

### 000706

#### Epidemiology and outcomes of septic shock in Southern Brazil

F. Suparregui Dias, S. Redaelli, P. Gottardo, N. D'arrigo, L. Becker, L. Zerman, F. Canever, V. Daniel
Intensive Care Unit, Nossa Senhora de Pompéia Hospital, Caxias do Sul, Brazil
**Correspondence:** F. Suparregui Dias
*Intensive Care Medicine Experimental* 2019, **7(Suppl 3):**000706

**INTRODUCTION.** Sepsis is a major health issue in Brazil and, is increasing death rate in recent years[1]. A comparison between our country and England showed that crude hospital mortality in sepsis has no difference[2]. The incidence of septic shock (SS) in a French study was 13.7% and the 28 day mortality rate was 42%[3], characterizing a high-risk death population.

**OBJECTIVES.** To identify the epidemiological profile, resource utilization and mortality in SS patients admitted to a single center ICU, in Southern Brazil.

**METHODS.** All patients that presented SS criteria (need of vasopressor therapy despite adequate fluid resuscitation to maintain MAP ≥ 65 mmHg) during ICU stay were enrolled in the study. Data collected prospectively were: age, gender, comorbidities [diabetes mellitus (DM), hypertension, cancer and chronic renal disease (CRD)], site of infection, SAPS 3 and SOFA at admission. Resource utilization was considered mechanical ventilation (MV), invasive hemodynamic monitoring (IHM) and renal replacement therapy (RRT). ICU and hospital mortality were reported. Continuous variables were presented as mean±SD, using Student t test for comparison and categorical variables presented as percent. Mortality prediction was done using the receving characteristic curve (ROC).

**RESULTS.** Between February 2012 and December 2018, 2707 patients were ICU admitted in ICU and 315 (11.6%) presented SS. Mean age was 63,2±15,6 yrs and 56,8% were male. DM, hypertension, cancer and CRD were present in 21.3%, 42.5%, 15.2% and 6.3%, respectively. Average SAPS 3 and SOFA were 66.3±13.7 and 8.4±3.0. The use of MV, IHM and RRT was 93.7%, 7.6% and 15.9%, respectively. Mortality in ICU was 43.5% and in hospital 55.6%. Factors associated with ICU mortality were cancer (OR 1.429; CI 95% 1.081-1.889), CRD (OR 1.546; CI 95% 1.091-2.191), MV (OR 4.576; CI 95% 1.222-17.142) and RRT (OR 1.55; CI 95% 1.192-2.015).

**CONCLUSION.** In Southern Brazil, SS incidence and mortality is similar to the recent published data. The presence of comorbidities and severity of organ dysfunction increase the mortality risk in a similar rate of observational studies. These patients demand an important resource use, mainly MV and RRT.

**REFERENCE(S)**

1. Taniguchi LU, Bierrenbach AL, Toscano CM, Schettino GPP, Azevedo LCP. Sepsis-related death in Brazil: an analysis of the national mortality registry from 2002 to 2010. Crit Care 2014, 18:608.
2. Ranzani OT, Shankar-Hari M, Harrison DA, Rabello LS, Salluh JIF, Rowan KM et al. A comparison of mortality from sepsis in Brazil and England: the impacto f heterogeneity in general and sepsis-specific patient characteristics. Crit Care Med 2019;47:76-84.
3. Quenot JP, Binquet C, Kara F, Martinet O, Ganster F, Navellou JC et al. The epidemiology of septic shock in Fench intensive care untis: the prospective multicenter cohort EPISS study. Crit Care 2013, 17:R65.

### 000710

#### Analysis of the kinetics of Procalcitonin in multidrug-resistant bacteria

I. Huespe[1], J. Sinner[1], E. Prado,[1], I. Staneloni[2], L. Denaday,[3], M. Gimenez[3], E. San Roman[1]
[1]Intensive Care Unit, Hospital Italiano de Buenos Aires, CAPITAL FEDERAL, Argentina; [2]Infectology, Hospital Italiano de Buenos Aires, CAPITAL FEDERAL, Argentina; [3]Central laboratory, Hospital Italiano de Buenos Aires, CAPITAL FEDERAL, Argentina
**Correspondence:** I. Huespe
*Intensive Care Medicine Experimental* 2019, **7(Suppl 3):**000710

**INTRODUCTION.** Emergence of multidrug-resistant bacteria (MDR) are facilitated by the abusive use of antibiotics. Multiple clinical trials have evaluated the usefulness of procalcitonin (PCT) to guide the initiation and completion of antibiotic therapy. However, they included mainly patients with sepsis of the community, with few MDR isolates.

**OBJECTIVES.** Investigate the kinetic of PCT and the time necessary to decrease it in MDR infections.

**METHODS.** Retrospective observational study, PCT curves of all bacterial rescues of ventilation associated pneumonia (VAP) and catheter-related bloodstream infections (CRBSI) were analyzed during the period from November of 2016 to May of 2018 in the intensive care unit of the Hospital Italiano de Buenos Aires, Argentina. Patients with an infectious intercurrence during the antibiotic course were excluded from the study together with those dead before the expected decrease in PCT or with chronic infections.

**RESULTS.** During the period analyzed, 73 bacterial rescues were recovered. 47 patients were excluded from the analysis due to the exclusion criteria. 12 patients with sensitive bacteria (SB) and 14 patients with MDR were selected for the final analysis, without significant differences between population. Patients with CRBSI generated by MDR showed significantly higher maximum values of PCT: MDR mean 39 ug/l (SD+/-30) vs. SB mean 10.7 ug/l (SD+/-11), (p= 0.02). The time elapsed from the detection of the bacteria to the decrease of 80% or values lower than 0.5 ug/l, with effective antibiotic treatment, was in MDR 7.2 (SD+/-2.9) days vs. 5 (SD+/-1.8) days in SB with a significant difference (p=0.03). The PCT decrease was exponential in both groups, with quicker decrease in SB than in MDR (Figure 1).

**CONCLUSION.** The inflammatory response measured by PCT in MDR was greater and longer, even with an effective antibiotic treatment, which could be due to greater virulence. However, the decline occurs before conventional antibiotic schemes are completed. In this sense it is necessary to study the potential application of antibiotic protocols guided by PCT to this population.

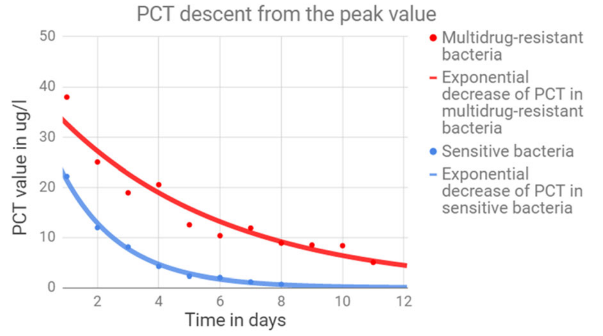

**Fig. 1 (abstract 000710).** See text for description

### 000727

#### Immunomonitoring of monocytic HLA-DR and neutrophilic CD64 expression and Sepsis Index as predictive biomarkers of infection and sepsis

A. Herraiz,[1], B. Quirant[2], E. Lucas[2], EM. Martínez-Cáceres[2], O. Plans[1], E. Argudo[1], V. Philibert[1], F. Arméstar[1]
[1]Intensive care unit, Hospital Germans Trias i Pujol, Badalona, Spain; [2]Immunology, Hospital Germans Trias i Pujol, Badalona, Spain
**Correspondence:** A. Herraiz,
*Intensive Care Medicine Experimental* 2019, **7(Suppl 3):**000727

**INTRODUCTION.** Sepsis is characterized by a simultaneous imbalance of hyperinflammation and immunosuppression. The decreased expression of HLA-DR molecules on circulating monocytes (mHLA-DR) has been associated with anti-inflammatory immune response, referred to as "immunoparalysis" status. On the other hand, in a proinflammatory scenario, neutrophils can increase the expression of CD64 molecules (nCD64), emerging as a potential biomarker in the diagnosis of sepsis. Sepsis Index (SI), which is defined as the ratio between nCD64 and mHLA-DR, is considered a novel parameter in prognostication of sepsis.
**OBJECTIVES.** This study aims to evaluate the behavior of the nCD64, mHLA-DR and SI biomarkers in critically ill patients and their association with the development of infection and sepsis.
**METHODS.** A total of 77 critically ill neurological patients were recruited for two years (2015-2016) from the Intensive Care Unit (ICU) of Germans Trias i Pujol Hospital, Spain. Infection was ruled out in all patients enrolled in the study at the time of admission. Patients under 18 years of age or with an ICU stay of less than 24 hours were excluded of the present study. The nCD64, mHLA-DR and SI biomarkers were analyzed in a standardized flow cytometry protocol at different times after admission. The study was previously approved by the Hospital Ethics Committee.
**RESULTS.** A total of 55 patients developed infection (infected group); the other 22 patients did not develop infection (non-infected group). No significant differences were detected in mortality between groups (p=ns). Nevertheless, SOFA score, days of mechanical ventilation and stay in ICU were higher in infected patients (p<0'05). In the first 24 hours after admission, no differences were observed in the percentage of mHLA-DR and nCD64 between groups. On the third day after admission, however, infected patients showed lower percentage of mHLA-DR, higher expression of nCD64 and an increased Sepsis Index (p<0'05).
**CONCLUSION.** Patients who become infected are in a state of immunosuppression, since they express lower levels of mHLA-DR. These results suggest that nCD64, mHLA-DR and SI could be considered useful biomarkers to evaluate the susceptibility to develop infection in critically ill patients.

##### REFERENCE(S)

1. Pradhan R, Jain P, Paria A, et all. Ratio of neutrophilic CD64 and monocytic HLA-DR: A novel parameter in diagnosis and prognostication of neonatal sepsis. Cytometry B Clin Cytom. 2016 May;90(3):295-302.
2. Tang Z, Qin D, Tao M, et all. Examining the utility of the CD64 index compared with other conventional indices for early diagnosis of neonatal infection. Sci Rep. 2018 Jul 3;8(1):9994.

### 000740

#### Alterations of retinal vessels in patients with sepsis

J. Simkiene[1], Z. Pranskuniene[2], D. Sokas[3], M. Patasius[3], A. Pranskunas[1]
[1]Intensive care medicine, Lithuanian University of Health Sciences, Kaunas, Lithuania; [2]Drug technology and social pharmacy, Lithuanian University of Health Sciences, Kaunas, Lithuania; [3]Biomedical engineering institute, Kaunas University of Technology, Kaunas, Lithuania
**Correspondence:** A. Pranskunas
*Intensive Care Medicine Experimental* 2019, **7(Suppl 3):**000740

**INTRODUCTION.** The retina is a window where the microvascular profile can be imaged directly and non-invasively at the bedside. Sepsis is associated with a decrease in microvascular density and its severity is related to mortality [1]. However, features of retinal vessels calibers and density during sepsis are not fully characterized.
**OBJECTIVES.** To compare the retinal vasculature between septic patients and age matched healthy volunteers.
**METHODS.** Prospective observational study from January 2018 to April 2019 in a third-level ICU. We performed a single fundus imaging using a hand-held digital fundus camera (Aurora, Optomed Oy, Finland) in patients with sepsis or septic shock (n=37) during first 24 hours after ICU admission and compared these data with age-matched healthy controls (n = 20). Image analysis was performed using ARIA software. The average retinal arteriolar and venular caliber were calculated and summarized as the central retinal arteriolar equivalent (CRAE) and central retinal venular equivalent (CRVE). Arteriole–venular ratio (AVR) defined as the ratio of CRAE to CRVE. The density of manually segmentated retinal vessels was calculated using ImageJ software: vascular length density = skeletonized vessel area/ total area * 100%. Data are reported as medians with 25th and 75th percentiles.
**RESULTS.** Out of 37 patients, 35 (95%) were in septic shock, 37 (100%) required mechanical ventilation. Median APACHE II and SOFA scores were 16 (12-20) and 9 (7-11), respectively. Mean arterial pressure (MAP) and cardiac index (CI) were 74 (64-81) mmHg and 2.9 (2.1-4.1) L/min/m2, respectively, and the median norepinephrine (NE) dose was 0.24 (0.10-0.33) mcg/kg/min. Patients with sepsis had a median CRAE of 161.8(146.6-183.2) μm compared with 146.3(141.9-157.7) μm in healthy subjects (p = 0.008). Their median CRVE was 257.6(225.0-286.7) μm compared with 243.7(215.1-272.2)μm in healthy subjects (p = 0.221) and AVR was 0.6(0.6-0.7) compared with 0.6(0.5-0.7) in healthy subjects (p=0.508). We found significant correlation between CRAE and diastolic blood preesure (r=0.35, p=0.047), between arterial tortuosity and systolic blood pressure (r=-0.36, p= 0.042). Patients with sepsis showed a significant decrease in retinal vascular lenght density compared with healthy subjects (p=0.001).
**CONCLUSION.** We found retinal signs of arteriolar vasodilation and decreased retinal vascular density in septic patients.

##### REFERENCE(S)

1. De Backer D et al. Crit Care Med 2013; 41:791–799.
2. This research was funded by a grant (No. S-MIP-17-16) from the Research Council of Lithuania.

### 000741

#### Time evolution of retinal microvascular changes in patients with sepsis

J. Simkiene[1], Z. Pranskuniene[2], J. Trumpaitis[3], M. Patasius[4], A. Pranskunas[1]
[1]Intensive care medicine, Lithuanian University of Health Sciences, Kaunas, Lithuania; [2]Drug technology and social pharmacy, Lithuanian University of Health Sciences, Kaunas, Lithuania; [3]Ophthalmology, Lithuanian University of Health Sciences, Kaunas, Lithuania; [4]Biomedical engineering institute, Kaunas University of Technology, Kaunas, Lithuania
**Correspondence:** A. Pranskunas
*Intensive Care Medicine Experimental* 2019, **7(Suppl 3):**000741

**INTRODUCTION.** The retina is a unique site where the microcirculation can be imaged directly at the bedside and non-invasively, providing an opportunity to study changes in the microvasculature relating to the development of hemodynamic state. Altered microcirculation is cornerstone of sepsis and its severity is related to mortality [1]. However, changes of retinal vessels during course of sepsis and their relationship to hemodynamics and outcomes are not fully characterized.
**OBJECTIVES.** To evaluate changes in retinal microvasculature during 24 hours period of time and compare it with hemodynamic profile and outcomes in patients with sepsis or septic shock.
**METHODS.** Prospective observational study from January 2018 to April 2019 in a third-level ICU. We performed retinal imaging using a hand-held digital fundus camera (Aurora, Optomed Oy, Finland) and systemic hemodynamic measurements at three time points: at baseline (earlier than 24 hours after arrival to ICU), 6 hours and 24 hours later. Retinal image analysis was performed using ARIA software. The average retinal arteriolar and venular caliber were calculated and summarized as the central retinal arteriolar equivalent (CRAE) and central retinal venular equivalent (CRVE). Arteriole–venular ratio (AVR) defined as the ratio of CRAE to CRVE. The density of manually segmentated retinal vessels was calculated using ImageJ software: vascular density = vessel area/total area * 100% and vascular length density = skeletonized vessel area/total area * 100%. Data are reported as medians with 25th and 75th percentiles.

**RESULTS.** Thirty seven patients with median age of 66(54-77) were included. 35 (95%) were in septic shock, 37 (100%) required mechanical ventilation. Median APACHE II and SOFA scores were 16 (12-20) and 9 (7-11), respectively. We compared data between survivors (n= 17) and non-survivors (n=20). We found significant difference in AVR between survivors and non-survivors during first measurement 0.6(0.5-0.6) vs 0.7(0.6-0.8),(p=0.008) as after 6 hours (p=0.041) and 24 hours (p=0.029). We detected significant difference in retinal vessel density (p=0.015) and vessel length density (p=0.05) during second measurement (6 hours after first measurement). We didn't find the difference in mean arterial pressure between survivors and non-survivors during all three measurements.

**CONCLUSION.** We found an association between retinal arteriole-venular ratio, retinal vascular density and mortality in sepsis and septic shock.

**REFERENCE(S)**

1. De Backer D et al. Crit Care Med 2013; 41:791–799.

2. This research was funded by a grant (No. S-MIP-17-16) from the Research Council of Lithuania.

### 000747

#### Evaluation of cardiac function by critical care echocardiography in septic patients

B. Leone, A. Sosa, D. Ivulich, J. Roberti, F. Deketele, A. Matarrese, J. Weith, JJ. Folco, J. Osatnik

Intensive care unit, Hospital Alemán (German Hospital of Buenos Aires), Buenos Aires, Argentina

**Correspondence:** J. Roberti

*Intensive Care Medicine Experimental* 2019, **7(Suppl 3):**000747

**INTRODUCTION.** The cardiovascular system is affected by sepsis and is associated with high mortality in seriously ill patients (1). Myocardial dysfunction occurs in half of septic shock patients despite normal or high cardiac output. Echocardiography and cardiac biomarkers are useful to evaluate cardiac dysfunction (2, 3).

**OBJECTIVES.** To identify if cardiac dysfunctions and cardiac markers are associated with ICU mortality in septic patients.

**METHODS.** This is the first phase of a larger study to evaluate the predictive value of bedside echocardiography at ICU setting. We assessed a cohort of septic and septic shock patients. Systolic and diastolic dysfunction were defined as ejection fraction function: normal >55%, mild deterioration 45-54%, moderate 30-44% and severe <30%. Sepsis and septic shock were defined according to Sepsis 3 criteria (4). SOFA and APACHE-II scores were calculated on the day of admission with the diagnosis of sepsis.

**RESULTS.** 59 patients were included. Mean age was 67.3±16.6 years, 27(45.8%) were women. Median time in ICU was 8 (range, 2-49) days. ICU mortality was 22.0% (n=13). Thirty-three (56%) had septic shock. Thirty-eight (66.7%) patients showed diastolic dysfunction (DD), 13 (22%) showed moderate systolic dysfunction (SD) and 1(1.7%) patient had severe SD. SOFA score at admission and maximum SOFA were significantly higher in non-survivors than in survivors: 8.6±4.0 vs. 5.7± 2.1, P=0.0009, respectively and 12.1±4.6 *vs.* 7.0±2.6, P=0.000, respectively. Apache was 21.3±7.9 vs. 15.9±14.2 in non-survivors vs. survivors, respectively, P<0.05. Fifty-one (86.4%) patients received vasopressors and 40(60.8%) were mechanically ventilated. Of 41 patients who had systolic or diastolic dysfunction, 9(19.5%) patients died. Comparing markers in those patients with any dysfunction vs. patients with no dysfunction, ProBNP was 111.8±223.1 ng/ml *vs.* 188.2±336.4 ng/ml (P>0.05), troponin-T was 91.9±121.5 *vs.* 117.9± 197.4 ng/ml (P>0.05), and procalcitonin was 25.1±41.7 *vs.* 22.6±34.5 ng/ml (P>0.05).

**CONCLUSION.** In our preliminary analysis of a small cohort, dysfunction was not associated with mortality. SOFA score was associated with mortality. Research of a larger sample and robust analysis is necessary to assess the predictive value of echocardiographic tools.

**REFERENCE(S)**

1. Singer M, Deutschman CS, Seymour CW, Shankar-Hari M, Annane D, Bauer M, et al. The Third International Consensus Definitions for Sepsis and Septic Shock (Sepsis-3). JAMA. 2016;315(8):801-10.
2. Celes MR, Prado CM, Rossi MA. Sepsis: going to the heart of the matter. Pathobiology. 2013;80(2):70-86.
3. Sturgess DJ, Marwick TH, Joyce C, Jenkins C, Jones M, Masci P, et al. Prediction of hospital outcome in septic shock: a prospective comparison of tissue Doppler and cardiac biomarkers. Crit Care. 2010;14(2):R44.
4. Christian SA, Schorr C, Ferchau L, Jarbrink ME, Parrillo JE, Gerber DR. Clinical characteristics and outcomes of septic patients with new-onset atrial fibrillation. J Crit Care. 2008;23(4):532-6.

### 000750

#### New Onset Atrial Fibrillation in Septic Patients in an Intensive Care Unit in Argentina

J. Weith[1], D. Ivulich[1], A. Sosa[1], F. Deketele[1], J. Roberti[2], B. Leone[1], JJ. Folco[1], A. Matarrese[1], J. Osatnik[1]

[1]Intensive care unit, Hospital Alemán (German Hospital of Buenos Aires), Buenos Aires, Argentina; [2]Intensive Care Unit, Hospital Alemán (German Hospital of Buenos Aires), Buenos Aires, France

**Correspondence:** J. Roberti

*Intensive Care Medicine Experimental* 2019, **7(Suppl 3):**000750

**INTRODUCTION.** Patients with sepsis have a high risk of developing new onset arrhythmias, such as atrial fibrillation (AF). Previous reports have shown that AF is associated with adverse clinical outcomes in these patients.

**OBJECTIVES.** To assess echocardiographic and clinical variables of patients with sepsis and new onset AF, admitted to a surgical medical intensive care unit.

**METHODS.** We prospectively assessed a cohort of septic and septic shock patients with bedside echocardiography. Atrial depolarization speed was measured in all patients by tissue Doppler echocardiography (TDE). Sepsis and septic shock were defined according to Sepsis 3 criteria. SOFA and APACHE-II scores were calculated on the day of admission with the diagnosis of sepsis.

**RESULTS.** A cohort of 27 patients was evaluated. Five (18.5%) patients presented with AF. SOFA scores at admission were comparable in patients with AF versus patients with no AF (9.2 ± 4.8 vs. 6.5 ± 1.8, p=0.0445). Left atrial area was higher in patients with AF compared to those without AF (21.7 ± 2.0 vs. 18.0 ± 3.5cm2, p=0.029). Left atrial volume was also higher in AF patients (69.4 ± 3.6 vs. 47.8 ± 14.9cm3, p=0.026). Right atrial area was 16.2 ± 1.1cm2in AF patients and 16.1 ± 3.3cm2in patients without AF (p=0.982). Right atrial volume was also similar between both groups (45.3 ± 21.7cm3vs. 38.2 ± 15.5cm3, p=0.44). Atrial depolarization speed was 127 ± 29.1ms in patients with AF and 141.9±37.9ms in patients without AF (p=0.42). Mortality during ICU stay was 2/5 (40%) in patients with AF and 2/22(9.1%) in patients without ICU (p=0.144).

**CONCLUSION.** Atrial fibrillation is a relatively common complication in septic patients. Although septic patients with AF showed unique echocardiographic features, mortality was not significantly higher as compared to thosewithout AF. Larger studies are needed to assess the clinical impact of AF in septic patients.

**REFERENCE(S)**

1. Seymour CW, Liu VX, Iwashyna TJ, Brunkhorst FM, Rea TD, Scherag A, et al. Assessment of Clinical Criteria for Sepsis: For the Third International Consensus Definitions for Sepsis and Septic Shock (Sepsis-3). JAMA. 2016;315(8):762-74.

2. Kanjanahattakij N, Rattanawong P, Krishnamoorthy P, Horn B, Chongsathidkiet P, Garvia V, et al. New-onset atrial fibrillation is associated with increased mortality in critically ill patients: a systematic review and meta-analysis. Acta Cardiol. 2018:1-8.

### 001747

#### Sepsis in cancer patients – a nationwide study on aetiology and outcome

E. Vesteinsdottir[1], GH. Sigurdsson[1], I. Kristinsdottir[2], MI. Sigurdsson[1], A. Blondal[3], S. Karason[1]
[1]Anaesthesia and intensive care, Landspitali- The National University Hospital of Iceland, Reykjavík, Iceland, Iceland; [2]Faculty of medicine, University of Iceland, Reykjavík, Iceland, Iceland; [3]Anaesthesia and intensive care, Akureyri Hospital, Akureyri, Iceland, Iceland
**Correspondence:** E. Vesteinsdottir
*Intensive Care Medicine Experimental* 2019, **7(Suppl 3):**001747

**INTRODUCTION.** Recent advances in oncology have led to better long-term survival and therefore the benefit of admitting patients with advanced cancer to the intensive care unit (ICU) may be increasing. A leading cause of admission of cancer patients to the ICU is sepsis, so it is important to understand its outcomes.

**OBJECTIVES.** To describe the incidence, aetiology and outcomes of cancer patients admitted to ICUs with sepsis.

**METHODS.** All adult admissions to all Icelandic intensive care units (ICU) during six whole calendar years (2006, 2008, 2010, 2012, 2014 and 2016) were screened for severe sepsis or septic shock using the ACCP/SCCM criteria. Demographics and clinical course of sepsis patients with cancer were compared with data from 731 sepsis patients without cancer.

**RESULTS.** During the study period 240 cancer patients were admitted to the ICUs with severe sepsis or septic shock (25% of all sepsis patients). Their mean age was higher (67 vs. 64 years, p = 0.005) and they were more likely to be male (62% vs 55%, p = 0.045) compared with sepsis patients without cancer. Of the patients with cancer, 104 patients had solid tumours, 71 solid tumours with metastases, 34 leukaemia and 31 lymphoma. The most frequent source of infection in patients with solid tumours with or without metastases was abdominal, but the lungs were the most common infection site in patients with lymphoma and leukaemia. Infections were more likely to be hospital-acquired in cancer patients compared with other sepsis patients (51% vs. 18%, p < 0.001) and the incidence of resistant pathogens was higher (11% vs 7%, p = 0.03). Patients with metastases and leukaemia were less likely to receive invasive ventilation (32% and 35% respectively) than other sepsis patients (51%, p < 0.001) and their length of stay in the ICU was shorter (median 2 days vs 4 days, p < 0.001). Treatment limitations were more commonly applied in cancer patients than other sepsis patients (35% vs 20%, p < 0.001). Both ICU (24% vs. 13%, p < 0.001), hospital (45% vs. 25%, p < 0.001), and one-year (66% vs. 32%, p < 0.001) mortality was higher in cancer patients compared with other sepsis patients.

**CONCLUSION.** Cancer is a common co-morbidity in patients admitted to ICUs with sepsis. The length of stay in the ICU for cancer patients is short, limitations of treatment common and both short- and long-term mortality is high. This suggests that a trial of ICU admission is commonly applied with a change towards palliation for patients that do not respond to treatment. The survival rates in this study are similar as in recent studies.

## POIC - Perioperative journey, from delirium to infections

### 001306

#### Differences between local versus manufacturer reference ranges of the platelet function test ROTEM®-platelet (impedance aggregometry assay)

A. Diaz-Martin, D. Cuenca-Apolo, V. Arellano-Orden, M. Casado-Mendez, S. Leal-Noval
I.C.U., H.U. Virgen del Rocio, Seville, Spain
**Correspondence:** A. Diaz-Martin
*Intensive Care Medicine Experimental* 2019, **7(Suppl 3):**001306

**INTRODUCTION.** Platelet functionality test are being ingreasingly used in critically ill patients. However local reference values has been poorly investigated.

**OBJECTIVES.** To establish local reference range in the use of the platelet functionality aggregometry test ROTEM®-platelet, and to compare them with the reference values provided by manufacturer.

**METHODS.** Prospective study (ABCD study, NCT:026552897) funded by Carlos III Institute (PI15/00512). Forty consecutive healthy volunteers selected from the Blood and Tissue Bank of the hospital, after obtaining their informed consent, were included. Subjects with known coagulopathy and/or on antiagregant/anticoagulant therapy were excluded. Blood samples were taken for standard coagulation tests, ROTEM® and platelet function tests (ROTEM®-platelet and PFA®-100 Col-Epi).

The following ROTEM®-platelet tests were performed: ARATEM (activation with arachidonic acid), ADPTEM (activation of the ADP receptor) and TRAPTEM (activation with thrombin activating peptide). The parameters determined in each test were the area under the curve (AUC, ohm*min), maximum amplitude at 6 minutes (A6, ohm) and the maximum slope (MS, ohm/min). The reference ranges were expressed by 2.5-97.5% percentiles, according to the recommendations of the Clinical and Laboratory Standards Institute, NCCLS-Guidelines.

**RESULTS.** Forty subjects [men 19 (47.5%), age 58.5 (56.25-60)] were included. Table depicts the local tests values outside of manufacturer´s range.

**CONCLUSION.** The local values of the impedance aggregometry ROTEM®-platelet differ significantly from the manufacturer's reference range, underlining the need to establish local reference values. This could have important consequences related to platelet transfusion and antiplatelet drug management.

1. This study has been supported by Fondo de Investigacion Sanitaria (FIS), Carlos III Institute (PI15/00512), Ministry of Health, Government of Spain, and European Regional Development Fund (ERDF).

**Table 1 (abstract 001306).** Reference range values of ROTEM®-PLT and PFA®-100

| | ARATEM AUC | ARATEM A6 | ARATEM MS | ADPTEM AUC | ADPTEM A6 | ADPTEM MS | TRAPTEM AUC | TRAPTEM A6 | TRAPTEM MS | PFA®-100 Col-Epi |
|---|---|---|---|---|---|---|---|---|---|---|
| Local reference values | 11-99 | 2-27 | 2-9 | 24-128 | 8-32 | 2-11 | 61-163 | 15-39 | 5-14 | 95-217 |
| Manufacturer´ reference values | 70-153 | 19-41 | 6-13 | 56-139 | 16-38 | 4-11 | 61-156 | 15-36 | 5-14 | 85-165 |
| Number of tests outside below normal range | 24 (60%) | 26 (65%) | 20 (50%) | 13 (32.5%) | 11 (27.5%) | 5 (12.5%) | 0 | 0 | 0 | 0 |
| Number of tests outside above normal range | 0 | 0 | 0 | 0 | 0 | 0 | 1 (2.5%) | 1 (2.5%) | 0 | 2 (5%) |

### 001347

#### Single-dose Ketamine Has Long-lasting Effects on EEG

V. Moll[1], M. Kreuzer[2], P. Garcia[3]
[1]Department of anesthesiology, Emory University Hospital Midtown, Atlanta, United States of America; [2]Department of anesthesiology and critical care, Technische Universität München, München, Germany; [3]Department of anesthesiology, Columbia University, New York, United States of America
**Correspondence:** V. Moll
*Intensive Care Medicine Experimental* 2019, **7(Suppl 3):**001347

**INTRODUCTION.** As a WHO essential medication, ketamine remains clinically significant for sedation, anesthesia, and analgesia. Although ketamine has shown promise in treating psychiatric illness, and for acute treatment of delirium 1–4 its utility for preventing postoperative delirium is controversial. It has been shown in one small RCT in cardiac surgery patients to reduce the incidents of delirium5; a larger RCT in a heterogeneous group of surgical patients failed to reproduce this effect6. Spectral features of the electroencephalogram, EEG have recently been associated with delirium7 yet, the effects of ketamine on these features remain largely unexplored.

**OBJECTIVES.** We present an initial characterization of the neurophysiologic effects of single-dose ketamine in patients undergoing extensive surgery with general anesthesia for treatment of head/neck cancer. This is the initial exploration of preliminary EEG results from a larger study intended to investigate postoperative delirium in these patients.

**METHODS.** We prospectively randomized patients into a placebo, 0.5mg/kg (low) and 1mg/kg (high) ketamine group. Patients received the study drug after induction. 5-channel EEG was recorded using a SEDLine monitor (Masimo, Irvine, CA) from frontal positions at a sample rate of 250 Hz. In order to evaluate the influence of single dose ketamine we only analyzed the EEG from 4h after the start of EEG recording at induction until 15 minutes before the end of the EEG recording at the end of the procedure.

**RESULTS.** Out of 29 enrolled patients, 8 were excluded due to persistent EEG signal distortion. The analysis included 3 groups: placebo-10, low-ketamine (LK)- 6 and high-ketamine (HK) 5 patients. We found a significant effect of the HK on spectral EEG features for up to 4h. Alpha and beta band total power were significantly higher in the HK group compared to the placebo group (alpha KW: p=0.014, Chi-Sq=8.53; beta KW: p=0.031, Chi-Sq=7.01, Fig1A-B) Examination of relative band power (normalized to total power) showed a significant difference for the relative alpha power (KW: p=0.0259; Chi-sq: 7.31), but not for the relative beta-band (KW: p=0.0852; Chi-sq: 4.39) (Fig1C-D). The power spectral density (PSD) and normalized power spectral density (nPSD) plots in Fig2A-B show the difference in the spectral power (PSD, A) and composition (nPSD, B) between the placebo and the HK group. AUC graph presents the AUC (o) and the 95% confidence interval (x). Black dots indicate a significant effect, defined by a 95% Ci not containing 0.5. Grey dots indicate a (non-significant) AUC<0.3, but an AUC <0.3 may indicate a clinically relevant effect8.

**CONCLUSION.** Our preliminary results of a prospective randomized study investigating the effects of ketamine on EEG and delirium reveal that a single dose of subanesthetic ketamine has lasting effects on the EEG (240 min). As enrollment progresses the effects of these spectral features on delirium risk will be determined.

**REFERENCE(S)**

1. Burnett, A. M., Salzman, J. G., Griffith, K. R., Kroeger, B. & Frascone, R. J. The emergency department experience with prehospital ketamine: a case series of 13 patients. Prehosp. Emerg. Care 16, 553–559 (2012).
2. Hopper, A. B. et al. Ketamine use for acute agitation in the emergency department. J. Emerg. Med. 48, 712–719 (2015).
3. Murrough, J. W. et al. Antidepressant efficacy of ketamine in treatment-resistant major depression: a two-site randomized controlled trial. Am. J. Psychiatry 170, 1134–1142 (2013).
4. Berman, R. M. et al. Antidepressant effects of ketamine in depressed patients. Biol. Psychiatry 47, 351–354 (2000).
5. Hudetz, J. A. et al. Ketamine attenuates delirium after cardiac surgery with cardiopulmonary bypass. J. Cardiothorac. Vasc. Anesth. 23, 651–657 (2009).
6. Avidan, M. S. et al. Intraoperative ketamine for prevention of postoperative delirium or pain after major surgery in older adults: an international, multicentre, double-blind, randomised clinical trial. Lancet 390, 267–275 (2017).
7. Hesse, S. et al. Association of electroencephalogram trajectories during emergence from anaesthesia with delirium in the post-anaesthesia care unit: an early sign of postoperative complications. Br. J. Anaesth. (2018).
8. Mandrekar, J. N. Receiver operating characteristic curve in diagnostic test assessment. J. Thorac. Oncol. 5, 1315–1316 (2010).

1. This study is internally supported by the Department of Anesthesiology, Emory University, Atlanta, GA.

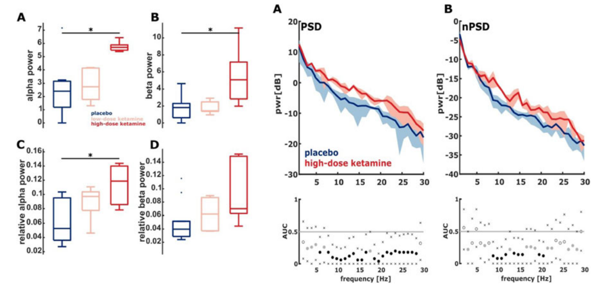

**Fig. 1 (abstract 001347).** See text for description

### 001380

#### Delirium management in a Tunisian Intensive Care unit

S. Kortli[1], I. Ben Saida[2], H. Zorgati[1], N. Fraj[2], N. Kacem[1], W. Zarrougui[2], MA. Boujelbèn[1], M. Boussarsar[2]

[1]Medical intensive care unit, Farhat hached university hospital, Sousse, Tunisia; [2]Medical intensive care unit, farhat hached university hospital, Université de Sousse, Faculté de Médecine de Sousse, LR N° LR12SP09.Heart Failure, Sousse, Tunisia

**Correspondence:**

*Intensive Care Medicine Experimental* 2019, **7(Suppl 3):**001380

**INTRODUCTION.** Delirium is associated with poor outcomes and is an independent predictor of mortality in intensive care patients. Early recognition and management of delirium may improve prognosis of delirious patients.

**OBJECTIVES.** to describe our experience in the management of delirious patients.

**METHODS.** A prospective cohort observational study conducted over nine months duration from October 2017 to June 2018 including all patients admitted in a 9-bed medical ICU for more than 24 hours. Patients who were deaf or unable to speak or understand were excluded. All included patients were screened by psychiatrist for delirium using DSM-V (Diagnostic and Statistical Manual of Mental Disorders, Fifth Edition) criteria.

**RESULTS.** During the study period, 137 patients were included. Patients' characteristics were : median age, 60[49-68] years ; female, 29(27.9%) ; median Charlson comorbidity index, 3[2-5] ; median SAPSII, 27[22-33.5] ; invasive mechanical ventilation (IMV), 57(41.6%), remifentanil use, 67(51.1%) ; midazolam use, 24(17.5%) and vasopressors use, 52(38%).

Using DSM-V criteria, 46(33.6%) had delirium. The most common subtype of delirium seen was hypoactive type, 27(58.6%), followed by hyperactive subtype, 15(32.6%) and few patients had mixed subtype of delirium, 4(8.87%).

Management in all delirious patients included non-pharmacologic procedures when appropriate to decrease or treat the modifiable risk factors : Assess patient for common causes of delirium (hypoxia, hypercapnia, electrolyte imbalance, nicotine withdrawal, medications or infection) ; early ambulation ; encourage family to stay with patient during day as much as possible to improve orientation ; encourage social interaction and family visits during visiting hours and try to maintain sleep-wake cycle by limiting unnecessary awakenings. All these interventions were systematic. When insufficient, pharmacologic and other procedures were performed : antipsychotic medication (haloperidol), 17 (36.9%) ; antidepressants medication, 7(15.22%) ; sedation, 9(18.8%) and physical restraint, 6(15.22%). For 6 patients, neither pharmacologic nor physical restraint were performed.

**CONCLUSION.** The first-line of management of delirium was non-pharmacological. Haloperidol seems to be overused as a result to some physicians'beliefs.

### 001383

#### Efficacy of prothrombin complex concentrate (PCC) for the treatment of bleeding: A systematic review and meta-analysis

D. van den Brink[1], M. Wirtz[2], A. Serpa Neto[3], V. Viersen[4], J. Binnekade[1], NP. Juffermans[1]
[1]Department of intensive care medicine, Amsterdam UMC, locatie AMC, Amsterdam, Netherlands; [2]Deparment of trauma surgery/intensive care medicine, Amsterdam UMC, locatie AMC, Amsterdam, Netherlands; [3]Department of critical care medicine, Hospital Israelita Albert Einstein, São Paulo, Brazil; [4]Department of anesthesiology, Amsterdam UMC, locatie AMC, Amsterdam, Netherlands
**Correspondence:** D. van den Brink
*Intensive Care Medicine Experimental* 2019, **7(Suppl 3):**001383

**INTRODUCTION.** Bleeding often results in coagulopathy through several mechanisms, including consumption of clotting factors. Prothrombin complex concentrate (PCC) contains either three or four of the vitamin K-dependent coagulation factors and is increasingly being used to correct coagulopathy in bleeding patients. However, a summary of the efficacy of PCC to treat bleeding is lacking.

**OBJECTIVES.** The aim of this systematic review and meta-analysis is to evaluate the efficacy of PCC administration in bleeding patients.

**METHODS.** PubMed, EMBASE and CINAHL were searched for studies investigating the efficacy of PCC to treat bleeding in adult patients and providing data on either mortality, red blood cell (RBC) utilization, blood loss or thromboembolic (TE) events. Data were pooled using Mantel-Haenszel random effects meta-analysis or inverse variance random effects meta-analysis.

**RESULTS.** From 3944 identified studies, 14 observational studies were included (with a total of 2187 patients). Of these, 7 studies included patients undergoing cardiac surgery, 4 included trauma patients, 2 included patients undergoing liver surgery and 1 study included bleeding patients due to various causes. In all patient groups taken together, PCC administration was not associated with reduced mortality (odds ratio = 0.95; CI = 0.71 − 1.28; p = 0.75; I2 = 0%). Patients receiving PCC required significantly less RBC units (mean difference = -1.80; CI = -3.22 - -0.38; p = 0.01; I2 = 92%) compared to patients not receiving PCC. Cardiac surgery patients had significantly lower volumes of blood loss (mean difference = -384; CI = -640 - -128, p = 0.003, I2 = 81%) when receiving PCC. In all patient groups together, PCC administration was not associated with TE event rate (odds ratio = 1.13; CI 0.80 -1.60; p = 0.48, I2 = 0%).

**CONCLUSION.** PCC was associated with a reduction in blood loss in cardiac surgery patients and with a decreased RBC transfusion rate across a wide range of bleeding patients, without effecting mortality.

### 001394

#### Use of Blood Products in Severe Trauma Patients

S. Ramiro González[1], R. Prieto Jurado[1], A. López Fernández, A. Iglesias Santiago[1], F. Guerrero López[1], F. Pino Sánchez[1]
[1]Critical care department, Hospital Virgen de las Nieves, Granada, Spain
**Correspondence:** S. Ramiro González
*Intensive Care Medicine Experimental* 2019, **7(Suppl 3):**001394

**INTRODUCTION.** Hemorrhage is the most common cause of shock in trauma patients, and the volume replacement, including the transfusion of blood and blood products, is an important part of treatment to maintain tissue perfusion and oxygenation. We present a descriptive study that analyzes the use of blood products in these patients.

**OBJECTIVES.** To describe the use of blood products in trauma patients as well as their correlation with severity scores, intensive care unit (ICU) stay and mortality and incidence of multiple organ dysfunction syndrome (MODS).

**METHODS.** Data include all trauma patients admitted at intensive care unit from January 2018 to March 2019, who were followed until hospital discharge or death.

Descriptive statistics, quantitative variables were presented as mean values including standard deviation, or median values including interquartile range as was appropriate. Categorical variables were presented as counts and percentages. Bivariate statistics are analyzed with Student's t test for independent variables with normal distribution, U Mann-Whitney if non-normal distribution and Chi-squared test for qualitative variables association.

**RESULTS.** 157 trauma patients were included during the period under review. Within the study population, 11 (7%) and 9 (5.7%) patients were previously under antiaggregant and anticoagulant treatment respectively. 44% presented hemodynamic instability and 21.7% coagulopathy associated with trauma. 4 (2'5%) patients developed massive hemorrhage and 5 (3'2%) required arteriography and endovascular embolization.

48 patients (30.6%) needed transfusion of blood products: 42 patients required red blood cells concentrate transfusion during the first day [3.5 (2, 5.3)], 22 patients were administered fresh frozen plasma [950 (595, 1275)]cc and 23 patients were transfused with platelets [2 (1, 2)] pool. Twenty-five patients received prothrombin complex [1300 (700, 1650)]U, 29 patients received fibrinogen [2 (2, 3)]grams, and tranexamic acid was used in 34 patients.

The mean stay at ICU was 5'04 (2.4, 12.05) days. 13.4% of patients died during ICU stay and 16.2% of patients during hospital stay. 21 patients developed MODS; 20 of them during the first three days (12.7%). In our sample, there is statistically significant association between transfused patients and severity according injury indicators (ISS and NISS) and APACHE II. In addition, the incidence of MODS, mortality in the ICU and hospital mortality were also higher in transfused patients. On the other hand, no statistically significant differences were found in gender, age, previous coagulopathy, incidence of nosocomial infection, ICU stay or hospital stay.

**CONCLUSION.** According to our experience, there is a statistically significant association between the need of transfusions and severity according to the injury indicators; as well as an increase in mortality and the development of MODS in the transfused patients.

### 001427

#### Thyroid function in adults patients undergoing cardiac surgery: a pilot study

D. Berbel-Franco[1], G. Muñoz-Del Rio[1], JC. Lopez-Delgado[1], P. Alia-Ramos[2], A. Padro-Miquel[2], M. Dastis-Arias[2], PA. Revelo-Esquibel[1], RE. Avila-Espinoza[1], P. Berbel-Navarro[3], R. Mañez-Mendiluce[1], M. Potocnik[4]
[1]Intensive care, Hospital Universitari de Bellvitge, Barcelona, Spain;
[2]Laboratory dpt., Hospital Universitari de Bellvitge, Barcelona, Spain;
[3]Medicine school, Universidad Miguel Hernández, Alicante, Spain;
[4]Cardiac surgery dpt., Hospital Universitari de Bellvitge, Barcelona, Spain
**Correspondence:** J.C. Lopez-Delgado
*Intensive Care Medicine Experimental* 2019, **7(Suppl 3):**001427

**INTRODUCTION.** There is an interaction between thyroid hormones, the immune and inflammatory response and illness severity, leading to lower levels in the setting of critical illness. Cardiac surgery represents an important surgical injury associated with non-thyroidal Illness syndrome (NTIS). However, little is know about NTIS in this scenario.

**OBJECTIVES.** Evaluate thyroid function in adult patients undergoing cardiac surgery. We also correlated thyroid function with parameters related with inflammatory response.

**METHODS.** Demographical and surgical characteristics, laboratory results and outcomes were registered. Blood samples were collected before surgery, during first 24h ICU admission,and at day 3 and 5. A serum thyroid profile, including thyroid-stimulating hormone(TSH), free(fT4) and total(tT4) thyroxine, free(fT3) and tota(tT3) triiodothyronine and reverse triiodothyronine (rT3), was analyzed. Drugs potentially having an effect on thyroid function were collected. An statistical analysis was performed to evaluate thyroid function over time.

**RESULTS.** 10 patients aged 66.7±4.5years were included, mean BMI:29.9±5.6Kg·m-2 and APACHE II:15.8±4 were included. 6 patients underwent valve procedures(VP) and 4 coronary artery bypass graft(-CABG), with a mean CBP time 102±23min. 1 patient died during ICU stay and 3 required vasopressor and inotrope support for over 24h. Statistically significant changes (P<0.001) were observed during the first 24h of ICU admission on TSH, fT3, tT3 and rT3 levels. TSH levels dropped in the first 24h, returning to baseline afterwards, whereas fT3 and tT3 levels remained low. tT4 levels were normal, resulting on a tT4/tT3 drop of 39.4±10.0% in the first 24h. On the other hand, rT3 levels increased 46.6±15.5% above baseline levels and remained high during the observation period. No statistical correlations with inflammation or tissue hypoperfusion markers (leukocytes, lymphocytes, lactate and C-reactive protein were observed.

**CONCLUSION.** Our pilot study showed that NTIS may develop soon after cardiac surgery with a lasting effect over T3 levels. The effect of this phenophenon and their influence over outcomes should be evaluated when increasing our sample of patients.

### 001432

#### Postoperative complications in cardiac surgery during ICU stay

E. Rosas Carvajal[1], VA. Hortigüela Martín[1], R. Hernandez Estefanía[2], G. Aldamiz Echevarria Del Castillo[2], MP. Calderón Romero[2], A. Donado Miñambres[2], A. Heredero Jung[2], Á. Vidal González[1], Al. Tejero Redondo[1], D. Robaglia[1], LM. Polanco Mahecha[1], M. Pérez Márquez[3], C. Pérez Calvo[1]
[1]Intensive care unit, Hospital Universitario Fundación Jiménez Díaz, Madrid, Spain; [2]Cardiac surgery, Hospital Universitario Fundación Jiménez Díaz, Madrid, Spain; [3]Intensive care unit, Hospital Universitario Rey Juan Carlos, Madrid, Spain
**Correspondence:** E. Rosas Carvajal
*Intensive Care Medicine Experimental* 2019, **7(Suppl 3):**001432

**INTRODUCTION.** Cardiac surgery is one of the most frequently performed surgical procedures in the world. Regardless of the procedure performed, the final results depend to a great extent on an optimal postoperative care.

**OBJECTIVES.** Describe the immediate postoperative complications in cardiac surgery patients during their ICU stay.

**METHODS.** Prospective and observationaly, all patients undergoing cardiac surgery in the year 2018 in Fundación Jiménez Díaz and Rey Juan Carlos Hospitals in Madrid were recruited.

Demographic variables (age, sex), baseline data (heart rate, EuroSCORE II, NYHA), surgery data (type of intervention, CPB and ischemia times) and immediate postoperative data (heart rithm, time of mechanical ventilation, time of ICU stay and re-intervention) were collected.

**RESULTS.** 293 patients were recruited (61.4% males) with a mean age of 65.6 ± 11 years and EuroSCORE II mean of 5.07 ± 9.39.

The type of intervention was: valve replacement 47.3%, coronary revascularization surgery (CRC) 31.4%, CRC + valvuloplasty 3.4% and other 7.8%. 8.2% of the interventions were emergent or urgent, 26.1% preferred and 65.7% scheduled.

The most frequent complications were cardiac conduction disorders (18.6%): 3° atrio-ventricular block, 2.4% and *de novo* atrial fibrillation 15.5%; with a need for a permanent pacemaker (MP) in 2% of the total. Only 1% (3 patients) presented postoperative acute coronary syndrome.

There was an acute renal failure rate of 8.5% with need for renal replacement therapy of 48% (4.1% of the total) and none required chronic dialysis.

We only found 6.5% of neurological complications: 73.7% diffuse (42.3% encephalopathy, 15.8% seizures, 15.8% delirium) and 26.3% focal. In 10.4% of them there was permanent neurological deficit (0.7% of the total).

6.1% of the total patients required prolonged MV (>96 hours) of which 38.9% ended in tracheostomy (2.4% of the total).

There were infectious complications in 4.1% of the patients (50% pneumonia, 16.7% urinary, 16.7% surgical wound, 8.3% mediastinitis and 8.3% catheter bacteremia).

3.4% of the patients presented pleural effusion requiring drainage, 0.7% pneumothorax, and 0.3% hemopneumothorax.

The average stay in the ICU was 86 hours, the overall mortality was 3.6% and the reoperation rate was 3.4%.

**CONCLUSION.** The results of cardiac surgery postoperatory in our sample are excellent with a mortality rate below what was expected (according to EuroSCORE II). The complications are diverse and expected, but most are resolved in the immediate postoperative period without sequelae.

**REFERENCE(S)**

1. Aneman A, Brechot N, Brodie D, Colreavy F, Fraser J, Gomersall C, McCanny P, Moller-Sorensen PH, Takala J, Valchanov K, Vallely M. Advances in critical care management of patients undergoing cardiac surgery. Intensive Care Medicine. 2018 Jun;44(6):799-810.
2. Nashef SA, Roques F, Sharples LD, Nilsson J, Smith C, Goldstone AR, Lockowandt U. EuroSCORE II. European Jorunal of Cardiothoracic Surgery. 2012 Apr;41(4):734-44
3. The authors declare no conflict of interest.

### 001450

#### Enteral nutrition in ECMO: a pilot study

G. Muñoz-Del Rio[1], E. Casanova-Gongora[2], D. Berbel-Franco[1], PA. Revelo-Esquibel[1], RE. Avila-Espinoza[1], J. Puentes Yáñez[3], F. Esteve-Urbano[1], JC. Lopez-Delgado[1], E. Periche-Piedra[1]
[1]Intensive care, Hospital Universitari de Bellvitge, Barcelona, Spain; [2]Intensive care, Hospital Mutua de Terrassa, Terrassa, Spain; [3]Intensive care, Hospital Universitario de Bellvitge, Barcelona, Spain, Spain
**Correspondence:** J.C. Lopez-Delgado
*Intensive Care Medicine Experimental* 2019, **7(Suppl 3):**001450

**INTRODUCTION.** ECMO post-cardiac surgery is a rescue therapy in the low cardiac output and in the postcardiotomy refractory shock. Nowadays, it exists controversy about what is the best approach for nutritional support in these patients.

**OBJECTIVES.** The objective of our study was to evaluate how we perform enteral nutrition (EN) in this group of patients.

**METHODS.** Prospective observational study from November 2016 to March 2018. Preoperative, intraoperative variables, prognosis scores, postoperative complications and in-hospital and long-term mortality were collected, as well as the NE administered during the first week of admission to the ICU.

**RESULTS.** 16 patients were included with a mean age 61.1±10.3 years; 62.5%(10) were male; BMI:27.6 ± 4.1Kg•m-2; APACHE II: 18±8; SOFA: 9.3±3.4. 56.25%(9) were coronary revascularization and 31.25%(5) were valvular surgeries. Hospital mortality was 37.5% (6) and 1-year mortality was 43% (7). The ECMO was initiated during the first 72h after admission to the ICU post-surgery with a duration of 6±3.2 days. The complications were: bleeding 37.5%(6), re-exploration 31.25%(5) and sepsis 50%(8). None thrombosis was observed. The EN was started in 12 patients, starting early (<24h) in 50%. They received an average of 11.75±5.1 Kcal/Kg/d and 0.62±0.26 g/Kg/d. In 50% of patients EN was interrupted >24h during ICU stay due to complications from their cardiovascular status. In those patients were EN was interrupted a lower caloric (15.48±3.6 vs. 8.02±3.2 Kcal/Kg/d; *P=0.004*) and protein (0.81±0.16 vs. 0.43±0.18 g/Kg/d; *P=0.004*) nutritional intake was shown. Lactate on the 3rd day in patients in whom the EN was interrupted was higher (1.8±0.4 vs. 1.9± 0.9 mmol/L, *P=0.012*).

**CONCLUSION.** Patients with post-cardiotomy ECMO receive inadequate nutritional support in our study. Strategies to improve nutritional support, such as supplementary parenteral nutrition among others, may be evaluated in future studies.

**REFERENCE(S)**

1. Periche-Pedra E, et al. Outcomes of extracorporeal membrane oxygenation in adult patients with hypoxemic respiratory failure refractory to mechanical ventilation. Respir Med Case Rep. 2018;25:220-224.

2. Flordelís Lasierra JL, et al. Early enteral nutrition in patients with hemodynamic failure following cardiac surgery. JPEN J Parenter Enteral Nutr. 2015;39:154-62.

### 001458

#### The influence of nutritional risk on patients who undergo cardiac surgery

G. Muñoz-Del Rio[1], D. Berbel-Franco[1], PA. Revelo-Esquibel[1], RE. Avila-Espinoza[1], JC. Lopez-Delgado[1], F. Esteve-Urbano[1], S. Gonzalez-Del Hoyo[1], N. Latorre-Feliu[1], M. Martinez-Medan[1], C. Sanz-Mellado[1], N. Rodriguez-Perez[2]
[1]Intensive care, Hospital Universitari de Bellvitge, Barcelona, Spain; [2]Statistical dpt., IDIBELL, Barcelona, Spain
**Correspondence:** J.C. Lopez-Delgado
*Intensive Care Medicine Experimental* 2019, **7(Suppl 3):**001458

**INTRODUCTION.** Nutritional status influences outcomes in critically ill and new guidelines do not recommend surgery in patients with malnutrition.

**OBJECTIVES.** To evaluate the influence of nutritional risk on the results and prognosis of patients undergoing cardiac surgery.

**METHODS.** Prospective observational study from 2011 to 2015. Post-operative nutritional risk was evaluated through the Nutrition Risk in the Critically Ill (NUTRIC) Score. Likewise, preoperative, intraoperative variables, prognostic scores, postoperative complications and in-hospital and long-term mortality were collected.

**RESULTS.** 2809 patients were included: mean age of 64.6±11.5 years, 63.9% (n=1796) were men, BMI: 27.9±4.2Kg·m-2, APACHE II:12.9± 5.3.The 51.7% were valvular surgeries, 32.3% were coronary, 6.8% were mixed and 9.2% were another type of cardiac surgery. In-hospital mortality was 5.8% (163). According to the NUTRIC Score, 72.3% (2032) of the patients had a low nutritional risk, while 27.7% (777) had a high nutritional risk. The univariate analysis between subgroups identified greater postoperative complications and mortality in the subgroup with greater nutritional risk. The multivariate analysis showed that a high nutritional risk was associated with an older age (OR: 1.107; 95% CI:1.080-1.134;P <0.001), to a higher creatinine before surgery (OR:1.009; 95% CI:1006-1.013; P <0.001), in the presence of atrial fibrillation pre-surgery (OR: 1.614; 95% CI: 1.085-2.399; P = 0.018) and smoking (OR: 1.777; 95% CI: 1.076-2.934; P=0.025), while higher preoperative cholesterol (OR: 0.827, 95% CI: 0.692- 0.987; P < 0.001) and lymphocytes (OR: 0.694; 95% CI: 0.522- 0.924; P <0.001) were both associated with a lower risk. The same analysis revealed that a higher nutritional risk was associated with a greater need for transfusions (OR: 1.092, 95% CI: 1006-1.185, P = 0.036), a higher incidence of low postoperative cardiac output (OR: 3,351; 95 % CI: 2.030-5.531; P<0.001) as well as a longer time in vasoactive drugs (OR: 1002, 95% CI: 1001-1,004, P = 0.008) and worse renal function (OR: 1006, 95% CI : 1.003-1.010; P = 0.008). Long-term mortality was evaluated in 2715 patients: those with higher nutritional risk had worse survival (87.8% vs.70%; P(LogRank) <0.001) and a higher risk in the Long-term mortality (HR: 4.369; 95% CI: 3.075-6.206; P <0.001).

**CONCLUSION.** A higher nutritional risk evaluated according to the NUTRIC Score was associated with greater postoperative complications after cardiac surgery, as well as a worse long-term survival in our population.

### 001463

#### High risk Pancreaticoduodenectomy patients admitted in Intensive Care for perioperative management. Retrospective cohort study from a high-volume center

R. Pereira[1], IF. Barros[2], TM. Marques[3], I. Marta[4], AT. Ferreira[3], T. Sequeira[1], J. Paulino[5], MF. Pinto[6], N. Germano[1]
[1]UCIP 7, Hospital Curry Cabral, CHULC, Lisboa, Portugal; [2]Cirurgia geral, Hospital Curry Cabral, CHULC, Lisboa, Portugal; [3]Unidade funcional medicina interna 4, Hospital Santa Marta, CHULC, Lisboa, Portugal; [4]Unidade funcional medicina interna 1.4, Hospital São José, CHULC, Lisboa, Portugal; [5]Cirurgia hepato-bilio-pancreática e transplantação, Hospital Curry Cabral, CHULC, Lisboa, Portugal; [6]Microbiologia, Hospital D. Estefânia, CHULC, Lisboa, Portugal
**Correspondence:** R. Pereira
*Intensive Care Medicine Experimental* 2019, **7(Suppl 3):**001463

**INTRODUCTION.** The development of postoperative infectious complications is a major determinant of outcome in pancreaticoduodenectomy (PD) patients.

Patients with preoperative biliary drainage (PBD) are at high risk for positive intraoperative bile cultures, infectious complications and antibiotic resistances.

A short postoperative course of antimicrobial therapy in patients at high risk for biliary contamination has been shown to reduce infectious complications after PD.

PD patients at our institution are admitted in ICU for post-surgical care and monitoring. PBD stents are systematically retrieved during surgery for microbiological culture. Those with PBD are considered high risk patients and undergo a 5-day protocol course of post-surgery empirical antibiotic therapy with piperacillin-tazobactam.

**OBJECTIVES.** To compare clinical and microbiological results between PBD vs non-PBD PD patients and to analyze antibiotic resistance patterns.

**METHODS.** Retrospective cohort study of consecutive PD patients admitted at our ICU for post-operative monitoring and care between January 2015 and April 2018.

Median and interquartile (IQR) values are presented and Mann-Whitney and Chi-square tests were used to compare group results.

**RESULTS.** A total of 313 PD patients were analyzed.

Patient characteristics: median age was 68 years (59-74) and 58,8 % were male. Median Body Mass Index was 24,9 (22,3-27,6), Charlson Comorbidity score was 5 (4-6) and ICU admission-day SOFA was 2 (1-5). Histologic results confirmed malignancy in 81% of patients.

Overall outcomes: median Clavien-Dindo score was 0 (0-2) and 30-day mortality rate was 5,4%.

PBD stent was present in 44,4% (n=139) patients.

When comparing PBD vs. non-PBD groups no differences were found between age (p=0,23), Body Mass Index (p=0,39), Charlson Comorbidiity score (p=0,32), admission-day SOFA score (p=0,20) nor Clavien-Dindo score (p=0,33) or 30-day mortality (p=0,82).

A total of 134 PBD stent cultures revealed 122 microorganisms isolated from 66 (47,4%) patients and 68 (48,9%) had polimicrobian cultures. E. faecalis (22%), E. coli (16%) E. cloacae (13%) and K pneumoniae (12%) were the most frequent bacteria and 7% were ESBL. Antibiotic testing (n=115) reported resistance rates of 21% to piperacillin-tazobactam and 19% to ampicillin plus cefotaxime combination.

**CONCLUSION.** The presence of PBD in PD high risk patients was not associated with worse outcomes.

E. faecalis was the most frequent bacteria isolated from PBD cultures. Overall antibiotic resistance rates from PDB stent cultures were similar between piperacillin-tazobactam and the combination of ampicillin plus cefotaxime.

Ampicillin plus cefotaxime may be an alternative choice for post-operative empirical antibiotherapy in PD high risk patients with PBD.

#### REFERENCE(S)

1. Sourrouille, I. et al. Five days of postoperative antimicrobial therapy decreases infectious complications following pancreaticoduodenectomy in patients at risk for bile contamination. HPB 15, 473–480 (2013).
2. De Pastena, M. et al. Postoperative infections represent a major determinant of outcome after pancreaticoduodenectomy: Results from a high-volume center. Surgery 162, 792–801 (2017).

### 001483

#### Using Near-Infrared Spectroscopy for detection brain and lower limb complication during Venoarterial Extracorporeal Membrane Oxygenation (ECMO)

A. Kogan[1], Y. Kassif[2], E. Ram[2], S. Gezunterman[3], S. Amunz[2], Y. Peled[4], E. Zuroff[2], L. Sternik[2]
[1]Cardiac Surgery ICU, Sheba Medical Center, Sakler Faculty of Medicine, Tel Aviv University, Ramat Gan, Israel; [2]Department of cardiac surgery, Sheba Medical Center, Ramat Gan, Israel; [3]Cardiac surgery icu, Sheba Medical Center, Ramat Gan, Israel; [4]Institute of cardiology, Sheba Medical Center, Ramat Gan, Israel
**Correspondence:** A. Kogan
*Intensive Care Medicine Experimental* 2019, **7(Suppl 3):**001483

**INTRODUCTION.**
**Introduction:** Progress in near-infrared spectroscopy (NIRS) technology seem to have improved its accuracy for continuous, non-invasive monitoring of regional tissue oxygenation and may provide clinicians with an additional information to achieve patient safety.

**OBJECTIVES.** Veno-arterial (VA) extracorporeal membrane oxygenation (ECMO) may result arterial blood mixing and delivery of hypoxic blood to the brain. Additionally, VA-ECMO by femoral artery cannulation may compromise perfusion to the lower limbs. Oxygen saturation measured by near-infrared spectroscopy (NIRS) can be used to monitor brain and lower limb oxygenation in the patients treated by VA-ECMO.

**METHODS.** We retrospectively analyzed prospectively collected data of the patients, who treated by veno-arterial ECMO, using femoral vessels approach, between 01.2015 and 12.2018 in large tertiary center. All patients were continuously monitored using the (INVOS™ 5100C Cerebral/Somatic Oximeter) tissue oximeter. Sensors were placed on the patients' foreheads and on the lower limbs. NIRS tracings were recorded, analyzed, and correlated with clinical events.

**RESULTS.** Ninety-five patients (age 22-78) were monitored with NIRS, and 83 patients (87.4%) has distal lower limb perfusion. Eight patients (8.4%), 2 with distal limb perfusion and 6 without, had unilateral persistent decreasing lower limb oximetry (StO2) below 40% and clinical presentation of lower-limb ischemia: cold limb, mottled skin and pulseless Doppler ultrasound. In 7 patients were performed surgical placement a distal perfusion cannula and in one patients surgical placement and trombectomy from lower limb. Eleven patients (11.6%) had a significant, below 40% and longer than 30 minutes drop in cerebral oximetry (StO2). In 9 patients (9.5%) interventions, which involved increasing pressure, oxygenation, and/or ECMO flow corrected the underlying ischemia. Two patients (2.2%) required further diagnostic intervention and were found to have a cerebrovascular accident (CVA).

**CONCLUSION.** Use of near-infrared spectroscopy (NIRS) in the patients treated by ECMO is important in detecting, prevention and treatment ischemic cerebral and peripheral vascular events.

### 001511

#### Infection in Toxic Epidermal Necrolysis: A 13-year trial from a Burn Unit

JF. Martins[1], I. Coutinho[2], R. Meireles[3], C. Silva[1], E. Sousa[1], L. Cabral[3], P. Martins[1]
[1]Intensive care unit, Centro Hospitalar e Universitário de Coimbra, Coimbra, Portugal; [2]Immunoallergology department, Centro Hospitalar e Universitário de Coimbra, Coimbra, Portugal; [3]Burn unit, Centro Hospitalar e Universitário de Coimbra, Coimbra, Portugal
**Correspondence:** J.F. Martins
*Intensive Care Medicine Experimental* 2019, **7(Suppl 3):**001511

**INTRODUCTION.** Toxic Epidermal Necrolysis (TEN) is a rare drug-related life-threatening acute dermatological condition. The devitalization of epidermis and immunosuppression predispose patients to secondary infection that are the major cause of morbidity and mortality. The mortality rate is higher mainly in those requiring intensive care admission (REF).

**OBJECTIVES.** Characterization of the rates and types of infection-microbials in TEN patients, aged >18 years, admitted to a Burn Unit (BU) of a tertiary hospital, between 2005 and 2017.

**METHODS.** Retrospective, descriptive and inferential study, evaluating: age; gender; onset of symptoms to admission on BU; suspected etiologies of TEN; percentage of epidermal detachment (% of Total Body Surface Area, TBSA); severity of TEN assessed by the SCORTEN score, microbial infections identified by culture; nosocomial pneumonia (NP); Systemic therapy (corticosteroids, Plasmapheresis, intravenous immunoglobulin (IVIG)]; invasive procedures - mechanical ventilation (MV) or hemodialysis (HD) and BU length of stay. The primary outcome was in-hospital mortality.

**RESULTS.** A total of 29 patients were included, 58.6% (n=17) female, with median age of 65.7 years (min: 22.0; max: 91.0). The median SCORTEN score and TBSA were 3.0 and 57.3%, respectively. Alopurinol was the most frequent agent identified (24.1%, n=7) followed by Amoxicilin-Clavulanate, Carbamazepine and Hydantoins with 6.9% (n=2), each one. Etiological agent was not identified in 31.0% of patients. The mucous membranes were involved in 89.7% (n=26) of patients, with the oral mucosa being the most frequent (69.0%, n=20). The incidence of NP was 44.8% (n=13) and the most common agent identified was Methicillin-sensitive *Staphylococcus aureus (MSSA)* with 10.3% (n=3). MSSA was also the most commonly isolated agent in surgical wound infections (17.2%; n=5). Bacteriemia occurred in 58.9% of the patients. 51.7% of patients were under MV (n=15), 65.5% (n=19) of patients were submitted to plasmapheresis, 44.8% (n=13) to IGIV and 10.3% (n=3) to HD. The median BU length of stay was 10.0 days (min: 2, max: 42) and in-hospital mortality was 48.3% (n= 14). There was a statistically significant relationship between age and in-hospital mortality (p=0.02), with a worst prognosis in older patients. The study demonstrated a high variability in the number of infections, and there was no association with regard to mortality (p= 0.275). MV was associated to a higher mortality rate (66.6%, n=10) on this population (p=0.02).

**CONCLUSION.** Compared with other reported studies of secondary infection in TEN patients, our study had a lower rate of bacteremia. This finding suggests a response regarding the infection control measures applied in the study population. Despite that, the numbers of multi resistant microorganisms in this series corroborates the need for an early diagnosis and timely referral of these patients to specialized units.

#### REFERENCE(S)

1. de Prost N, Ingen-Housz-Oro S, Duong Ta, et al. Bacteremia in Stevens-Johnson syndrome and toxic epidermal necroly- sis: epidemiology, risk factors, and predictive value of skin cultures. Medicine (Baltimore) 2010;89:28–36
2. Revuz J, Penso D, Roujeau JC, et al. Toxic epidermal necrolysis. Clinical findings and prognosis factors in 87 pa- tients. Arch Dermatol 1987;123:1160–5

### 001525

#### The use of HPI (Hypotension probability indicator) during major intracranial surgery; preliminary results of a prospective randomized trial

J. Pouska, V. Cerveny, J. Zatloukal, J. Kletecka, J. Beneš
Department of anaesthesiology, resuscitation and intensive care, University Hospital in Pilsen, Plzeň, Czechia, Czech Republic
**Correspondence:** J. Pouska
*Intensive Care Medicine Experimental* 2019, **7(Suppl 3):**001525

**INTRODUCTION.** Based on our current knowledge, intraoperative hypotension is common and may have detrimental effect to patients outcome. HPI (Hypotension probability indicator) is new concept of artificial intelligence application in clinical medicine. It is based on machine learning technique analysis of invasive arterial pressure curve. Development of future hypotensive event (within 5 minutes time) should be predicted with reasonable sensitivity and specificity according to the manufacturer data. To which extent HPI may improve patient care has not been established yet.
**OBJECTIVES.** Major objective of this study was to assess the use of HPI to avoid hypotension in major intracranial surgery.
**METHODS.** Randomized prospective study performed in a University hospital approved by its local ethics committee. Patients undergoing major supratentorial tumour resection with ASA physical status 1-3 were enrolled and randomly (1:1 randomization) assigned to interventional (group A) or control group (group B). Anaesthesia was maintained by propofol and remifentanil. Haemodynamic monitor EV 1000 (Edwards Lifesciences Inc., Irvine, CA, USA) was used for goal directed haemodynamic therapy in both groups with predetermined mean arterial pressure (MAP) target of more than 65mmHg; fluids, vasopressors and inotropes were used based on predefined protocol. In the interventional group, HPI value of more than 85% was considered significant for prediction hypotensive event and used to prompt the appropriate treatment. The HPI value was covered for the treating anaesthesiologist in the control group.
The haemodynamic data were collected in 20-seconds intervals; hypotension and severe hypotension were defined as mean arterial pressure below 65 mmHg and 55mmHg. The cardiac index below 2,0l/min/m2 was deemed as hypoperfusion. Number of events and their duration were used as primary indicators. Because the first 30 minutes after induction are extremely turbulent, a separate maintenance phase of anaesthesia was evaluated too.
**RESULTS.** We enrolled 40 patients in our study, 20 in each group. Analysis of the whole anaesthesia time showed only slight trend towards lower hypotension and severe hypotension related burden without any statistical significance (Table 1). During the maintenance phase the number of patients free of hypotension (10 pts.in Group A vs. 4 pts.in Group B, p=0,047) was significantly lower.
**CONCLUSION.** Based on our data it seems that the inclusion of HPI into a goal directed treatment strategy could lower the incidence of hypotension within maintenance phase of anaesthesia.

**Table 1 (abstract 001525).** See text for description

| | Group A (intervention) | Group B (control) | P value |
|---|---|---|---|
| MAP<65(mmHg) time in (min) | 6,6±8,2 | 9,6±11,6 | 0,34 |
| Number of no MAP<65(mmHg) (pts) | 4 | 2 | 0,38 |
| Hypotensive events (No) | 4,5±1,3 | 5,2±0,9 | 0,69 |
| CI < 2,0 (l/min/m2) time in (min) | 105,3±134,4 | 77,9±82,4 | 0,44 |
| Hypoperfusion events (No) | 19,5±3,9 | 22,0±27,6 | 0,73 |

### 001532

#### What is the perfect place for sevoflurane in our ICUs?

K. Donadello, S. Simari, E. Bonora, M. Citino, G. Cogo, V. Schweiger, E. Polati
Anesthesia and intensive care b unit, University of Verona, AOUI-University Hospital Integrated Trust of Verona, Verona, Italy
**Correspondence:** K. Donadello
*Intensive Care Medicine Experimental* 2019, **7(Suppl 3):**001532

**INTRODUCTION.** Analgesia and sedation are highly discussed components of critically ill patient treatment, aimed at minimizing patient discomfort and modulating the stress response for the least period of time. ICU admission for post-operative patients can be scheduled preoperatively or a consequence of intra-operative complications. Post-operative sedation is maintained until haemodynamic stability, adequate respiratory exchanges and necessary instrumental investigations.
**OBJECTIVES.** The aim of this study was the evaluation of the sedative effect of inhaled sevoflurane and intravenous hypnotic agents (propofol and midazolam) in postoperative ICU sedation after major maxillofacial surgery. We related their use to hemodynamic stability, times of awakening and ICU discharge, onset of adverse events
**METHODS.** Retrospective matched-controlled analysis of prospective, non-randomized data comparing sevoflurane-SEVO group-MIRUS system (courtesy of PALL Medical) and intravenous propofol and midazolam-TIVA group. Inclusion criteria: Postoperative patients of maxillofacial oncologic surgery with vascularized flap, Age> 18 years, Sedation required for> 6 hours. Exclusion criteria: Pregnant Patients, Controindication to the use of halogenates, Neurological and/or psychiatric disorders in chronic pharmacological therapy. Recorded parameters: BIS value, Hemodynamic data, Respiratory parameters, Sedative/analgesic infusions, Inotropic/VP infusion, Hourly administration of fluids, lab and blood gas analysis. We defined: Time of awakening (minutes from the suspension of spontaneous breathing-extubation or breath by tracheostomy), Time of ICU discharge, Onset of adverse events.
**RESULTS.** 40 consecutive patients were evaluated for the study(20 SEVO and 20 TIVA), 4 were excluded from the analysis for postoperative surgical complications(1 group SEVO and 3 group TIVA). The study population was homogeneous and comparable. All patients were in a medium-high age group (67 ± 12 SD and 64 ± 10 SD years) and were in normal weight. Surgery duration was similar (10h 07 '± 1h 17' DS SEVO and 11h 10 '± 2h 20' DS TIVA). No substantial differences in intraoperative management occurred, as all patients were managed according to our Institution procedural protocol. Postoperative management revealed no statistically significant differences for hemodynamic stability, duration of sedation (12.9 ± 1.6 SEVO and 14.4 ± 2.2 TIVA) and administered post-operative fluids. The times of awakening to the suspension of sedation were significantly lower in SEVO patients (27 '± 16' SD) compared to TIVA ones (105 '± 56'DS) with a statistically significant difference (p <0.05). There was no significant difference in the time of ICU discharge and in the total time from the suspension of sedation to discharge. There were no significant differences for complications during sedation and on awakening.
**CONCLUSION.** Sevofluorane can be effective in guaranteeing the sedation depth required for post-operative complex maxillofacial surgery patients, allowing faster recovery and extubation times than intravenous sedation, maintaining adequate hemodynamic stability without relevant adverse events.

### 001541

#### The effect of restricted versus liberal fluid regimen on outcome in living donor related liver transplantantion recipients

HK. Atalan[1], B. Gucyetmez[2], A. Kargi[3], S. Aslan[3], S. Yazar[3], KY. Polat[3]
[1]Anesthesiology department, Memorial Atasehir Hospital, Istanbul, Turkey; [2]Anesthesiology and reanimation, Acıbadem Mehmet Ali Aydınlar University, School of Medicine, Istanbul, Turkey; [3]Organ transplantation department, Memorial Atasehir Hospital, Istanbul, Turkey
**Correspondence:** H.K. Atalan
*Intensive Care Medicine Experimental* 2019, **7(Suppl 3):**001541

**INTRODUCTION.** Recent studies suggests the use of restrictive fluid regimen in major abdominal surgeries to aviod postoperative complications such as acute kidney injury (AKI) or prolonged length of hospital stay (LOS Hospital) (1). Its known that intraoperative fluid management impacts outcome in living donor related liver transplant (LDLT) patients (2).
**OBJECTIVES.** The aim of this study is to evaluate the effects of restrictive versus liberal fluid regimens on outcome in LDLT recipients.
**METHODS.** We retrospectively evaluated the adult patients who underwent LDLT surgery between january 2015 and january 2017. Restricted group has consisted of patients who received <8ml/kg/h fluid, while liberal group has consisted of patients who received ≥8ml/kg/h fluid. Demographic data, surgery time, clamping time, diuresis, noradrenalin requirement, total administered fluid, operation fluid balance, central venous pressure (CVP), blood loss, erithrocyte suspension (ES) requirement, pre and postoperative lactate levels, pre and postoperative serum creatinin levels, delta creatinin, length of intensive care unit stay (LOS ICU), LOS-hospital, renal replacement therapy (RRT) requirement, readmission to ICU, reoperation, ICU and first year mortality rates were recorded.
**RESULTS.** The baseline demographic and clinic characteristics were similar in both groups. The operation fluid balance, postoperative CVP, intraoperative diüresis, intraoperative blood loss, ES requirement, noradrenalin requirement, postoperative serum lactate level and LOS-hospital were signifcantly higher in liberal group (P<0.05 for all). Postoperative 1st day serum creatinin was higher in restricted group (P<0.05), whereas need for RRT, increase in postoperative 7th day serum creatinin levels, ICU and first year mortality rates were similar between two groups.
**CONCLUSION.** Intraoperative restricted fluid regimen seems to decrease the LOS-hospital and ES requirement without negative effect on outcome compared to liberal fluid regimen in LDLT recipients.

**REFERENCE(S)**

1. Nisanevich V, Felsenstein I, Almogy G, et al. Effect of intraoperative fluid management on outcome after abdominal surgery. Anesthesiology 2005;103:25-32
2. Taura P, Martinez-Palli G, Blasi A, Rivas E, Beltran J, Balust J. Intraoperative management of high-risk liver transplant recipients: concerns and challenges. Transplant Proc. 2016; 48:2491-4

### 001546

#### Clinical profile of patients with diffuse alveolar hemorrhage in the course of systemic autoimmune diseases treated in the ICU

K. Polok, J. Górka, J. Fronczek, A. Włudarczyk, W. Szczeklik
Intensive care and perioperative medicine, Jagiellonian University Medical College, Kraków, Poland
**Correspondence:** K. Polok
*Intensive Care Medicine Experimental* 2019, **7(Suppl 3):**001546

**INTRODUCTION.** Patients with systemic autoimmune diseases pose a particular challenge for clinicians involved in critical care due to involvement of multiple organs and severe side effects of immunosuppressive drugs. Diffuse alveolar haemorrhage (DAH) is one of the most serious and life-threatening manifestations, typically present in patients with autoimmune diseases. Available data concerning clinical profile of patients with DAH treated in intensive care units remains scarce.
**OBJECTIVES.** We aimed to describe patients with systemic autoimmune disease who developed DAH and were treated in the intensive care unit in terms of baseline demographic and clinical features as well as mortality.
**METHODS.** In this retrospective cohort study performed in the Intensive Care Unit of University Hospital in Krakow (Poland) we included 21 patients treated for the first time in the ICU due to autoimmune diseases with diagnosis of DAH. For each patient APACHE II, APACHE III, SAPS II and SOFA scores were calculated on the admission. Mortality was assessed during ICU stay and during 5-year follow-up based on telephone calls and available data from national health provider registry.
**RESULTS.** Median age of the patients accounted to 53 (18-78) years. Majority (15/21; 71.4%) of patients were females. The most common diagnoses were granulomatosis with polyangiitis (38.1%), systemic lupus erythematosus (23.8%) and microscopic polyangiitis (14.3%). Most of the patients required mechanical ventilation (85.7%), renal replacement therapy (57.1%) and blood products transfusions (71.4%). Mortality in the ICU was 52.4%, while in both 1-year and 5-year follow-up it accounted to 76.2%.
**CONCLUSION.** Our study shows that among patients with systemic autoimmune diseases, DAH is diagnosed most typically in patients with primary systemic vasculitides such as granulomatosis with polyangiitis and microscopic polyangiitis. These patients often require advanced therapeutic measures and are characterized by high short- and long-term mortality.

### 001555

#### Comparison of preoperative levels of coagulation & fibrinolysis markers between patients undergoing vascular surgeries due to peripheral artery disease and abdominal aortic aneurysm

K. Polok, J. Górka, J. Fronczek,, W. Szczeklik
Intensive care and perioperative medicine, Jagiellonian University Medical College, Kraków, Poland
**Correspondence:** K. Polok
*Intensive Care Medicine Experimental* 2019, **7(Suppl 3):**001555

**INTRODUCTION.** Myocardial injury after noncardiac surgery (MINS) is common and significantly affects patients` outcomes. Its etiology is uncertain, however available studies suggest association between activity of hemostatic and fibrinolytic systems and incidence of MINS.
**OBJECTIVES.** We aimed to compare patients undergoing vascular surgeries due to peripheral artery disease (PAD) and abdominal aortic aneurysm (AAA) in terms of preoperative levels of coagulation and fibrinolysis markers.
**METHODS.** Patients at the age of 45 years or older, who received general or regional anesthesia for vascular surgery and stayed overnight in the hospital. We gathered demographic and clinical data on the patients. Coagulation (fibrinogen, factor VIII [FVIII], von Willebrand Factor:CoR [vWF:CoR], antithrombin III [ATIII]), fibrinolysis (D-dimer, plasmin-antiplasmin complexes [PAP], tissue plasminogen activator [tPA]) and inflammation (soluble CD40 ligand [sCD40L]) markers` levels were measured before surgery.
**RESULTS.** The study group consisted of 131 patients at the mean age of 68.3 years. History of smoking and diabetes were more common in the PAD group. Platelet count (209.5 vs. 250.5, p=0.001) as well as level of fibrinogen (4.1 vs. 5.4, p<0.001), FVIII (141.9 vs. 176.9, p<0.001), vWF:CoR (152.3 vs. 188.9, p=0.009) and sCD40L (7936.6 vs. 9016.0, p=0.005) were higher in the PAD group. The d-dimer level was higher (2590.5 vs. 808.0, p<0.001) in the AAA group. We did not observe statistically significant differences in ATIII, tPA and PAP levels
**CONCLUSION.** Our results suggest more pronounced activation of coagulation in patients undergoing surgeries for PAD compared to those with AAA. Together with relative hypofibrinolysis, it may put this group of patients at higher risk for cardiovascular complications. However, differences in baseline clinical characteristics may partially explain these differences. Further studies, performed on larger study groups, utilizing dynamic assessment of coagulation and fibrinolysis, are warranted.

**REFERENCE(S)**

1. Polok K, Górka J, Fronczek J, et al. Impact of arterial procedures on coagulation and fibrinolysis – a pilot study. Brazilian J Cardiovasc Surg. 2019;In press.

2. Górka J, Polok K, Iwaniec T, et al. Altered preoperative coagulation and fibrinolysis are associated with myocardial injury after non-cardiac surgery. Br J Anaesth. 2017;118(5):713-719. doi:10.1093/bja/aex081
3. This work was supported by a grant from the Polish Ministry of Science and Higher Education, Hoża 20 St., Wspólna 1/3 St., 00-529 Warsaw. Tel. (+48 22) 52 92 718. Grant number NN402083939 and DI2011023141

## INF - Infection prevention and diagnosis: New strategies and ways of improvement

### 001684

#### Effect of antimicrobial stewardship program on carbapenems consumption and Klebsiella resistance in intensive care units of a large rural hospital in Egypt, Menoufia

A. Samir[1], H. Elsawah[1], M. Elrazzaz[1], K. Taema[2], A. Ramadan[1], A. Elnaggar[1]
[1]Clinical pharmacy, Elaraby Hospital, Ashmun, Egypt; [2]Critical care medicine, Cairo University, Faculty Of Medicine, Kasr Al Ainy, Cairo, Egypt
**Correspondence:** H. Elsawah
*Intensive Care Medicine Experimental* 2019, **7(Suppl 3):**001684

**INTRODUCTION.** Because of high rates of prevalence and related mortality, Klebsiella species and their resistance need to be addressed in many rural hospitals of Egypt. Antimicrobial stewardship program (ASP) based on clinical pharmacy services is required to optimize antibiotics use especially broad spectrum ones.
**OBJECTIVES.** to measure the impact of ASP on rational antibiotics use and bacterial resistance
**METHODS.** Carbapenems consumption in intensive care units (ICUs) was recorded by defined daily dose (DDD) per 1000 patients prospectively from April 2017 to January 2019. Klebsiella species isolates and their antimicrobial susceptibility were recorded from October 2017 to November 2018. The ASP started on the first of March 2018 and the effects of the program on the following were measured and compared with the data collected before: (1) carbapenems-resistant Klebsiella prevalence, (2) carbapenems consumption in ICUs, and (3) probability of Klebsiella to be in a lower or a higher category of resistance based on classification of Klebsiella resistance into three categories; carbapenems resistant, carbapenems only sensitive and cephalosporins sensitive . The effect of the program was measured in ICUs of El-Araby hospital.
**RESULTS.** SPSS 25 software was used. Carbapenems consumption after starting the ASP was significantly lower than that consumed before (0.98 vs. 1.51 DDD per 1000 patients respectively, P-value=0.02). The prevalence of carbapenems-resistant Klebsiella was significantly lower than the prevalence before the program (48.6% vs. 85.25% respectively, P-value=.000) with odds ratio (OR) =0.164 (.075-.357). The probability of being in a higher category of Klebsiella resistance was significantly decreased after implementation of the program with OR = 6.3 (2.88 – 13.73). The probability of being in a higher category of Klebsiella resistance was also decreased with the decrease in carbapenems consumption with OR = 3.17 (1.367 – 7.345).
**CONCLUSION.** The antimicrobial stewardship program aiming to reduce unnecessary carbapenems use is extremely important to reduce prevalence of highly resistant strains of Klebsiella in similar hospitals.

##### REFERENCE(S)

1. Microbiology lab. and infection control department at Elaraby hospital
2. ICU department at Elaraby hospital

### 000790

#### Surveillance Results of Central Line Associated Bloodstream Infection Rate in Tertiary Level Intensive Care Unit

H. Basar[1], FS. Erdinc[2], C. Kaymak[1], CA. Hatipoglu[2], M. Kotanoglu[1], GT. Ertem[2], A. Ozcan[1], S. Kinikli[2]
[1]Department of anesthesiology and reanimation, University of Health Sciences, Ankara Health Application and Research Center, Ankara, Turkey; [2]Department of clinical microbiology and infectious diseases, University of Health Sciences, Ankara Health Application and Research Center, Ankara, Turkey
**Correspondence:** C. Kaymak
*Intensive Care Medicine Experimental* 2019, **7(Suppl 3):**000790

**INTRODUCTION.** Central line (central venous catheter) associated bloodstream infections (CLA-BSI) which are one of the healthcare-associated infections pose a great threat to patient safety. Patients followed in intensive care units (ICU) can be colonized with resistant microorganisms during their ICU stay and as a result, they have an increased risk of hospital acquired infections including CLA-BSI. In this reason, ICU needs to be monitored for nosocomial infections.
**OBJECTIVES.** The aim of this study was to evaluate the CLA-BSI rate, invasive device use rate and causative microorganisms isolated in these infections, between 2014 and 2018.
**METHODS.** This study was conducted in an Anesthesiology and Reanimation Intensive Care Unit. Five year's surveillance data was evaluated in this study. CLA-BSI rate per 1000 central venous catheter-days and device utilization ratios are retrospectively evaluated (CLA-BSI rate = CLA-BSI number / central venous catheter-days x 1000). Microbiological culture results of invasive device associated infections were also evaluated. Hospital acquired infection definitions are made according to the US Centers for Disease Control and Prevention (*CDC*) criteria.
**RESULTS.** A total of 2944 patients were enrolled. Central venous catheter usage ratio was 74.8% in the ICU for 28.721 catheter days. The mean rate of central venous catheter associated bloodstream infection was 6.38. Between 2014 and 2018, the number of central venous catheter-associated bloodstream infections was 21, 17, 48, 48, and 57, respectively. When compared with 2014, the CLA-BSI rate was slightly increased in 2018 (6.1 and 7.8 respectively). Candida spp. (25.6%) was the most frequently detected agent between the years of 2014-2016, while it was Klebsiella spp. (21.5%) in 2017-2018.Generally, Klebsiella spp. (20.1%), Candida spp. (19.6%) and Enterococcus spp. (16.6%) were mostly detected in the five-year evaluation.
**CONCLUSION.** Use of central venous catheter, increases the incidence of invasive device associated infection rate in ICU. The high mortality rates in patients with invasive device could be related to high infection rates and inappropriate antibiotic use. Incidence of infections can be reduced by education of health care givers and use of barrier preventive methods.

##### REFERENCE(S)

1. Bouza E, et al. Clin Microbiol Infect. 2004; 10(9):838-42.
2. None

### 000808

#### Peripheral intravenous catheters- The hidden danger of hospital aquired infections?

B. Røtvold[1], LH. Høvik,[2], KH. Gjeilo,[3], JK. Damås[4], E. Solligård,[5], LT. Gustad[5]
[1]Anaestesia, Nord-Trøndelag Hospital Trust, Levanger, Norway; [2]Clinic of anaesthesia and intensive care, St. Olav's University Hospital, Trondheim, Norway; [3]Department of cardiothoracic surgery, department of cardiology and national cometence centre for, St Olav's Hospital HF, Trondheim, Norway; [4]Department of circulation and medical imaging, Norwegian University of Science and Technology, Trondheim, Trondheim, Norway; [5]Department of circulation and medical imaging, Norwegian University of Science and Technology, NTNU, Trondheim, Norway
**Correspondence:** L.T. Gustad
*Intensive Care Medicine Experimental* 2019, **7(Suppl 3):**000808

**INTRODUCTION.** Insertion and management of peripheral intravenous catheters (PIVCs) is a daily routine for nurses. However, PIVCs can pose serious threats to patient safety and can cause catheter associated blood stream infections if not cared for properly (1). Thus, hospitals have guidelines for PIVC care, but do the nurses follow them?
**METHODS.** We aimed to measure if the nurses followed procedure for good PIVC care.
**RESULTS.** At baseline all PIVCs had at least one deviation from procedure, whilst at first follow-up 8 PIVCs an 6 at second follow-up were perfect. At baseline the average PIVC had median 4 deviations from procedure (95%CI 3.7-5.1%) whilst the average PIVC score fell to 2 deviations (95% CI 1.1-2.2) on first and 1 deviation after second measurement after intervention, p<0.001. At second measurement 1 PVK had a score of 7, and this patient and the PVK had just arrived from another hospital.
**CONCLUSION.** Nurses do not automatically follow PIVC guidelines. Improvement processes to improve PIVC care are needed. A 30 minutes training session gave good effect, which points to a high motivation for good PIVC care among the nurses. Since we developed the PIVC quality instrument and only one nurse had the measurement responsibility, the instrument needs testing in different settings, and the interrater reliability needs to be established, as LH Høvik et al recently have done (2).

**REFERENCE(S)**

1.Mermel
LA. Short-term Peripheral Venous Catheter-Related Bloodstream Infections: A Systematic Review. Clin Infect Dis. 2017;65(10):1757-62.
2 Høvik LH, Gjeilo KH, Lydersen S, Rickard CM, Røtvold B; Damås JK, Solligård E, Gustad LT Monitoring quality of care for Peripheral Intravenous Catheters; feasibility and reliability of the Peripheral Intravenous Catheters mini Questionnaire (PIVC-miniQ). Abstract sent to ESICM Lives 2019.

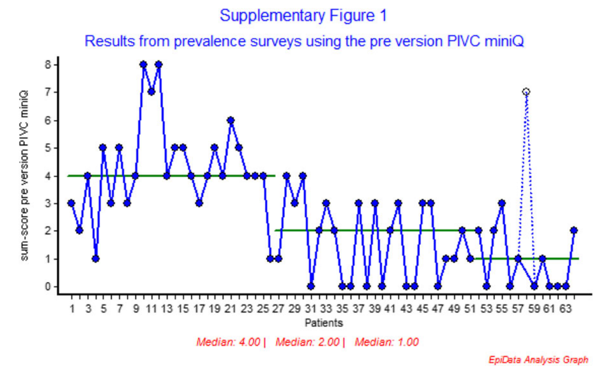

**Fig. 1 (abstract 000808).** Results from prevalence surveys using the pre version PIVC miniQ

### 001492
## Risk Factors and Causative Microorganisms of Cerebrospinal Fluid Drain Infections

E. Drosos[1], G. Bouboulis,[2], M. Nepka,[3], I. Karaminas,[2], P. Giannopoulou,[3], C. Vrettou[2]
[1]Department of neurosurgery, Evaggelismos General Hospital, Athens, Greece; [2]First department of intensive care, Evaggelismos Hospital, University of Athens Medical School, Athens, Greece; [3]Department of microbiology, Evaggelismos Hospital, Athens, Greece
**Correspondence:** C. Vrettou
*Intensive Care Medicine Experimental* 2019, **7(Suppl 3):**001492

**INTRODUCTION.** Risk factors related to CSF infection in patients with CSF drains include drain days, drain insertion conditions, opening and manipulation, diabetes mellitus and CSF leak (1). In most of these studies and with one exception known to us (2) the role of pre-existing extracranial infection has not been addressed.
**OBJECTIVES.** To identify risk factors related to the emergence of cerebrospinal fluid drain (CSFD) infections and to describe the microorganisms cultured in the CSF compared to those cultured in other specimens.
**METHODS.** We designed a retrospective single center cohort study. We reviewed the electronic hospital records of all ICU admissions to identify the eligible neurosurgical cases admitted between January 1st 2011 and December 31st 2018. Eligibility criteria for the study participants were: presence of a CSF drain during ICU stay, time lapsed from drain insertion to ICU admission < 24 hours, no previous diagnosis of CSF or any other infection either hospital or community acquired, and length of ICU stay > 24 hours. Patients' demographic and clinical characteristics were recorded, as was microbiology laboratory data. We used non-parametric statistical methods to identify possible risk factors for CSFD infection.
**RESULTS.** We identified 799 neurosurgical admissions of which 70 fulfilled the inclusion criteria. The figure shows the relative frequencies of different pathogens in the form of comparative pie charts [A: CSF (inner disc) vs blood (annulus); B: CSF vs bronchial; C: CSF vs anywhere else, including bronchial, blood, urine, and wound swab samples]. Acinetobacter baumannii was the most frequent single pathogen isolated in the CSF. The relative frequencies of pathogens in the CSF did not differ significantly from those of pathogens in other specimens ($p = 0.3699$). The presence of previous bacteraemia was the only statistically significant risk factor for CSFD infection in our population OR = 3.274, CI: (1.082-10.71), $p = 0.0274$
**CONCLUSION.** Our data suggests that, in an ICU population, the distribution of pathogens causing CSFD infections is similar to the overall distribution of pathogens cultured in other specimens. The most frequently isolated pathogens were Gram negative Enterobacteriaceae. Among them, the most frequent one was A. Baumannii. In our population, previous bakcteraemia was the only risk factor for CSFD infection. This finding warrants further investigation.

**REFERENCE(S)**

1. Acta Neurochir (Wien) 161 (3): 517-524, 2019.
2. Crit Care Med 43 (8):1630-1637, 2015.
3. There was no grant support for this research.

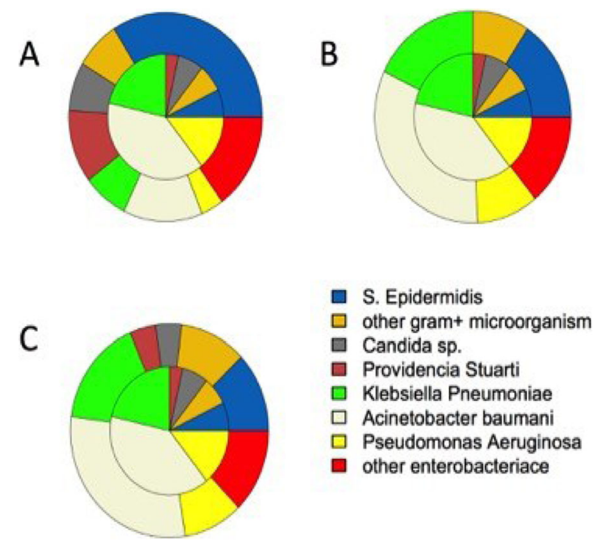

**Fig. 1 (abstract 001492).** See text for description

### 000554

#### Direct immunofluorescence as a diagnostic tool for Pneumocystis jirovecii pneumonia in non-HIV infected Intensive Care Unit Patients

A. Tsimogianni[1], S. Georgiou[1], G. Katsagani[2], F. Kaminari[2], V. Chantziara[3], E. Chinou[4], G. Krimpeni[1]
[1]Intensive Care Unit, Saint Savvas Hospital, Athens, Greece; [2]Intensive care unit, Saint Savvas, Athens, Greece; [3]Intensive care unit, Saiint Savvas Hospital, Athens, Greece; [4]Microbiology dept, Saint Savvas Hospital, Athens, Greece
**Correspondence:** A. Tsimogianni
*Intensive Care Medicine Experimental* 2019, **7(Suppl 3):**000554

**INTRODUCTION.** The incidence of *Pneumocystis* pneumonia (PCP) in non-HIV individuals is increasing. These patients typically present with severe disease and may require admission to the Intensive Care Unit (ICU). Modern diagnostic techniques are not widely available and prompt administration of appropriate treatment is essential.

**OBJECTIVES.** To examine the underlying diseases in non-HIV patients with PCP and the diagnostic yield of direct immunofluorescence (DFA).

**METHODS.** From June 2016 to March 2019 all cases with confirmed PCP pneumonia admitted to ICU of Saint Savvas Hospital were identified. Inclusion criteria were: diffuse pneumonia requiring intubation, absence of HIV antibodies and detection of PCP morphological structures with DFA. Bronchial samples were obtained via aspiration and examined with DFA by the same experienced microbiologist. Patients' demographic data, their underlying diseases-risk factors for PCP, their inflammatory markers as well as co-infection with bacterial strains were recorded. The number of bronchial samples required to make the diagnosis and their short-term prognosis- ICU discharge or death were also recorded. All patients received trimethoprim-sulfamethoxazole (TMP/SMX) and steroids, initiated when PCP pneumonia was suspected.

**RESULTS.** Overall, 9 cases with documented PCP pneumonia were recorded aged from 36 to 72, 3 males. Nobody was receiving TMP/SMX prophylaxis. Due to severe hypoxemia none of the patients underwent bronchoscopy to obtain bronchoalveolar lavage. In 4 patients (44,4 %) the first bronchial sample was diagnostic for PCP, in 3 patients (33.3% ) PCP was detected in the second sample and in 2 cases (22.2%) only the third sample confirmed the diagnosis. The sensitivity of the method on bronchial aspirates was only 44.4%. Seven patients had solid tumors but only one was receiving concurrent chemotherapy. One had multiple myeloma with recent autologous bone marrow transplantation and one had scleroderma on 5 mg prednisolone daily. Five patients had concurrent blood stream or respiratory infection with gram negative bacteria. 7/9 patients survived, and were discharged from ICU (77.7% survival).

**CONCLUSION.** As the accuracy of staining methods is highly dependent on the quality of respiratory specimen, when bronchial aspirates are examined especially in non-HIV-patients who have lower burden of PCP, more than one bronchial samples may be required to increase the diagnostic yield of DFA.

##### REFERENCE(S)

1. Cooley L, Dendle C, Wolf J et al. Consensus guidelines for diagnosis, prophylaxis and management of PCP pneumoniae in patients with haematogical and solid malignancies. Internal Medicine Journal 2014 44:1350-1363.
2. None

### 001548

#### Accelerate Pheno System utility compared to conventional culture-based methods in bloodstream infections

I. Cruz Valero[1], PA. Carranza[1], AME. Poyatos[1], J. Tejero Aranguren[1], MT. Cruces Moreno[1], ME. Yuste Ossorio[1], M. Colmenero Ruíz[1]
[1]Intensive care unit, Hospital Universitario San Cecilio, Granada, Spain
**Correspondence:** I. Cruz Valero
*Intensive Care Medicine Experimental* 2019, **7(Suppl 3):**001548

**INTRODUCTION.** In order to improve the selection of antibiotics, doses and infusion strategies, we wanted to describe the value of the Accelerate PhenoTest kit, approved by the Food and Drug Administration (FDA) as a method that can be useful to speed up microbial identification and to predict antimicrobial susceptibility in patients with bacteraemia compared to the reference standard that continues being the blood culture.

**METHODS.** observational, descriptive study. We included the patients admitted to the Intensive Care Unit and Infectious Diseases Unit, with clinical suspicion of bloodstream infection, from January to June 2018. Antimicrobial susceptibility testing (AST) results were analysed for essential agreement and for categorical agreement. Results based on EUCAST 2016 breakpoints.

**RESULTS.** 31 samples were evaluated, being excluded two of them. About the identification performance, 15 of them were gram- positive bacteria, 13 gram-negative bacteria (achieving a sensitivity and specificity of 100%) and one of the microorganisms was outside the PhenoTest panel, so the kit coverage was finally 96.6%. We also found an essential and categorical agreement of 93.5% and 92.9%, respectively, compared with the routine methods and we found the AST discrepancies in gram-negative bacteria. The potential on-average hours saved to AST was 23,4h compared to current techniques.

**CONCLUSION.** The coverage panel of the Accelerate PhenoTest kit was 96.6%. The essential and categorical agreement were above 90% and the results were obtained with a saved time of 23,4 hours compared to routine methods.

##### REFERENCE(S)

1. Peker, N., Couto, N., Sinha, B., and Rossen, J. W. (2018). Diagnosis of bloodstream infections from positive blood cultures and directly from blood samples: recent developments in molecular approaches. Clin. Microbiol. Infect. 24, 944–955. doi: 10.1016/j.cmi.2018.05.007.
2. Patel JB, Sharp S, Novak-Weekley S (2013) Verification of antimicrobial susceptibility testing methods: a practical approach. Clin Microbiol Newsl 35:103–109
3. Marschal M, Bachmaier J, Autenrieth I, Oberhettinger P, Willmann M, Peter S. 2017. Evaluation of the Accelerate Pheno system for fast identification and antimicrobial susceptibility testing from positive blood culture in Gram-negative bloodstream infection. J Clin Microbiol 55: 2116–2126.

### 000692

#### A Review of Adherence to Endotracheal Cuff Pressure Guidelines

L. Hill
Intensive Care unit, East Surrey Hospital, Redhill, United Kingdom
**Correspondence:** L. Hill
*Intensive Care Medicine Experimental* 2019, **7(Suppl 3):**000692

**INTRODUCTION.** Ventilator associated pneumonia (VAP) is the leading cause of death amongst hospital acquired infections (4,). Guidelines for the Provision of Intensive Care Services (GPICS) (2015) states its prevention as an important and cost-effective healthcare goal. Studies recommend several components should be adhered to help prevent VAP; including maintaining endotracheal tube cuff pressure to reduce micro aspiration.As part of the local Intensive care unit's ventilator care bundle it prompts Nursing staff to check cuff pressure 4 hourly, as per Department of Health (2011). GPICS (2015) recommend cuff pressure is kept above 20 cmH20, however over inflation of the cuff can cause ulceration or fistulation (2).

**OBJECTIVES.** To audit cuff pressure monitoring for endotracheal tubes in patients who are mechanically ventilated.

**METHODS.** A retrospective audit was conducted between the 22/6/18 and 1/8/18. 256 recordings of cuff pressures were analysed in total. 15 Patients were included with a total of 44.5 ventilated days, with stays ranging from 0.5-6 days. Each day was split into 2 x 12-hour shifts. Cuff pressures were recorded into time zones per shift 2-4 hourly, 4-6 hourly, 6-8 hourly, 8-10 hourly, once per shift and never on shift, with a total of 89 recordings taken. Cuff pressure was also recorded and split into groups of <20cmH20, 20-30cmH20 and >30cmH20.
**RESULTS.** 68.5% of the time recommendations were met and cuff pressures checked 2-4 hourly. 31.5% of the time the standards expected were not being met related to timely recordings. 10.11% of the time cuff pressure was never checked on shift. 96.25 % of recorded cuff pressures were between the recommended pressures of 20-30 cmH20. However 3.75% of the time pressure was >30 cmH20 and never recorded <20 cmH20.
**CONCLUSION.** Despite 68.5% of the time standards being met relating to timing of cuff pressure measurement, the aim should be 100% to ensure the best quality care is being provided. Improvements to education of staff surrounding good cuff pressure maintenance and monitoring has now been introduced. Highlighting of the cuff pressure box every four hours has been introduced on the nursing observations charts to act as a visual prompt to staff to check the cuff pressure. These changes are now in place and there is a plan to re-audit in 6 months to see if improvement has been made.

**REFERENCE(S)**

1. Department of Health (2011) High impact intervention care bundle to reduce ventilator-association pneumonia. [Online] Available at: http://webarchive.nationalarchives.gov.uk Accessed: 24.08.2018.
2. Gurman, G., Efrati, S., Deutsch, I. (2012) Endotracheal tube cuff- small important part ofa big issue. Journal of Clinical Monitoring and Computing. 26(1) PP. 53-60.
3. GPICS (2015) Guidelines for provision of intensive care services. [online] Available at: https://www.ficm.ac.uk/sites. Accessed: 24.08.2018.
4. Hellyer, p., Ewan, V., Wilson, P., Simpson, J. (2016) The intensive care society recommended bundle of interventions of prevention of ventilator-associated pneumonia. Journal of Intensive care Society. 17(3) PP. 238-243.
5. Simpson, T., Palazzo,s., Schnapp, L. (2011) Biomarkers for ventilator-associated pneumonia: Review of literature. Heart and Lung: The Journal of Acute and Critical Care. 40(4) PP. 293-298.

#### 000720

#### Rapid Response Teams and hospital/ICU mortality rate in Oncology patients

CM. Park[1], J. Lee[2]
[1]The Catholic University of Korea Seoul St Mary's Hospital, Seoul, Republic of Korea; [2]Division of pulmonary, allergy and critical care, seoul st. mary's hospital, college of medicine, The Catholic University of Korea Songeui Campus, Seoul, Republic of Korea
**Correspondence:** C.M. Park
*Intensive Care Medicine Experimental* 2019, **7(Suppl 3):**000720

**INTRODUCTION.** Although rapid response teams (RRTs) are widely used at many hospitals, their effectiveness in reducing hospital mortality is uncertain. Improving the understanding of RRTs and their utilization in specific patient populations, such as cancer patients, might be helpful to improve the outcome and optimize RRT utilization
**METHODS.** A retrospective cohort study of hospitalized patients requiring RRT activation over 3-year period from January 2016 to December 2018 in a single academic medical center in Seoul, Republic of Korea.
**RESULTS.** Of the 274 patients, 104 were cancer patients. Cancer patients had a significantly higher Charlson Comorbidity Score (5.0 vs 8.0. p < 0.001). Also, cancer patients had significantly higher APACHEII (14.0 vs. 17.5, p < 0.001) and MEWS (6.0 vs. 7.0, p < 0.001). Proportion of patients requiring ICU transfer was significantly higher in cancer patients (52.9% vs. 38.2%, p = 0.001). Cancer patients had a significantly higher in-hospital mortality compared with other patients (44.2% vs. 11.8%, p < 0.001). The survival rate was 75.9% and survival was associated with cancer and Charlson Comorbidity Score. The utilization of RRT resources during the study period was also much higher for cancer patients with 2.34 activations per 1,000 patient discharges compared with 0.78 per 1,000 patient discharges for the other patients.
**CONCLUSION.** Oncology patients requiring rapid response team activation have a significantly higher in-hospital mortality and ICU mortality rate. Presence of cancer was the most important factor related to survival. In addition, cancer patients also utilize RRT resources at a much higher rate.

### SIS - Sepsis pathophysiology and management

#### 001545

#### Serial C-Reactive Protein (CRP) levels do not correlate with antibiotic efficacy in critically ill patients with bacteremia

J. Inglez[1], S. Branco Ribeiro[2], J. Lage[1], J. Monteiro[1], C. Granja[2], A. Binnie,[2]
[1]Department of biomedical sciences and medicine, University of Algarve, Faro, Portugal; [2]Emergency and intensive care medicine department, Algarve University Hospital Centre, Faro, Portugal
**Correspondence:** S. Branco Ribeiro
*Intensive Care Medicine Experimental* 2019, **7(Suppl 3):**001545

**INTRODUCTION.** Antibiotic resistance is an increasing problem in intensive care units and inadequate empiric antibiotic therapy is associated with increased mortality. Biomarkers of treatment efficacy can theoretically be used to determine adequacy of antibiotic therapy prior to culture results being available. The aim of this study was to investigate the relationship between C-Reactive Protein (CRP) levels and (a) appropriateness of antibiotic therapy and (b) the presence of an untreated infectious focus in ICU patients with culture-confirmed bacteremia.
**OBJECTIVES.** To investigate the relationship between CRP levels and (a) appropriateness of antibiotic therapy and (b) the presence of an untreated infectious focus in ICU patients with culture-confirmed bacteremia.
**METHODS.** This retrospective observational study included all critically ill patients with culture-confirmed bacteremia admitted to the intensive care of the Algarve University Hospital Centre in Faro, Portugal from January to December 2018. Patients who did not receive antibiotic therapy were excluded from the study. Appropriateness of empiric antibiotic therapy was determined based on final culture and sensitivity results. Bacteremia episodes were designated as “complicated” or “simple” based on the presence or absence of an untreated or untreatable source (eg intra-abdominal abscess, infected hardware). CRP levels and standard clinical and biological variables were collected for 12 days, starting from the day prior to blood culture draw until 10 days afterwards.
**RESULTS.** A total of 59 culture-confirmed bacteremia episodes were identified. CRP concentrations on the day of positive culture varied widely from 9 mg/L to 480 mg/L. Out of the 59 patients in the cohort, a total of 38 (64.4%) patients received adequate empiric antibiotic therapy (as determined by final culture results) on the day of positive blood culture. CRP levels showed an overall negative trend from Day 0 (day of positive culture) to Day 10 irrespective of the adequacy of empiric antibiotic therapy. The presence of a “complicated” infection also had no significant impact on CRP trend. However, in multivariate regression analysis time to adequate antibiotic therapy was an important determinant of patient mortality, reinforcing the need for early appropriate antibiotics.
**CONCLUSION.** Although some studies have suggested that CRP is a useful marker of antibiotic adequacy, our results do not support this conclusion. Instead, we find that CRP levels show a similar downward trend from Day 0 to Day 10 in bacteremic patients, irrespective of the adequacy of empiric antibiotics. Further studies are needed to determine whether other biomarkers show greater utility than CRP in determining the efficacy of antibiotic therapy in this population.

**REFERENCE(S)**

1. Schmit, X. & Vincent, J. L. The time course of blood C-reactive protein concentrations in relation to the response to initial antimicrobial therapy in patients with sepsis. Infection 36, 213–219 (2008).
2. Póvoa, P., Teixeira-Pinto, A. M. & Carneiro, A. H. C-reactive protein, an early marker of community-acquired sepsis resolution: A multi-center prospective observational study. Crit. Care 15, (2011).
3. Vincent, J. & Beumier, M. Diagnostic and Prognostic Markers in Sepsis The Present : Biomarkers of Sepsis. 11, 265–275 (2014).
4. Garnacho-Montero J, Garcia-Garmendia JL, Barrero-Almodovar A, Jimenez-Jimenez FJ, Perez-Paredes C, Ortiz-Leyba C. Impact of adequate empirical antibiotic therapy on the outcome of patients admitted to the intensive care unit with sepsis. Crit Care Med. 2003 Dec;31(12):2742-51.

### 001551

#### Gastrointestinal motility disorders as a risk factor for sepsis in patients with major trauma

J. Schäper[1], F. Semper[1], M. Quintel[1]
[1]Department of anesthesiology, emergency and intensive care medicine, University Hospital Göttingen - University Medical Center Göttingen, Göttingen, Germany
**Correspondence:** J. Schäper
*Intensive Care Medicine Experimental* 2019, **7(Suppl 3):**001551

**INTRODUCTION.** The gut as a motor of sepsis hypothesis is an accepted hypothesis for the pathogenesis of systemic inflammation in critically ill patients. Impaired gastrointestinal motility may be a surrogate parameter for intestinal failure including gut barrier failure.
**OBJECTIVES.** This retrospective analysis in patients with major trauma tested different clinical variables of gastrointestinal motility as independent risk factors for sepsis and death on ICU.
**METHODS.** Retrospective data analysis of clinical variables from a patient data management system (IntelliSpace Critical Care & Anesthesia, ICCA™). Identification of 424 patients with major trauma (ISS>16) treated on the ICU in the timespan of Jan 2010 - Oct 2014 using the search items polytrauma and PTR. Chronologic analysis of 100 patients with respect to clinical variables of gastrointestinal motility (time until 50% of nutritional needs were achieved, gastric residual volume, time until first defecation) were analysed on days 0, 1, 2, 3, 5, 7, 10, and 14 as well as outcome parameters (sepsis, death on ICU). Statistics: multivariate analysis of prior evaluated variable-candidates for dichotomous outcome measures sepsis and death on ICU. Comparison of variables of patients with gastrointestinal motility disorders with and without sepsis. Group comparison with Mann-Whitney U test. Significance level at p<0.05.
**RESULTS.** Patients with a long duration to achieve 50% of nutritional needs were at a higher risk of developing sepsis (odds ratio 0.94; CI 0.89/0.99; p=0.03). This also applied for severe sepsis and septic shock. As risk factors for death on ICU old age (odds ratio 0.92; CI 0.86/0.98; p=0.01) and presence of brain trauma (odds ratio 38.47; CI 1.73/854; p=0.02) could be determined. Patients with sepsis needed more time until first defecation (173 ± 78 vs. 126 ± 77 h (mean ± SD) p=0.009) and until 50% of nutritional needs were achieved (146 ± 87 vs. 98 ± 51 h (mean ± SD), p=0.012)
**CONCLUSION.** In this ICU-cohort of patients with major trauma variables of gastrointestinal motility served as risk factors for sepsis, severe sepsis and septic shock. Gastrointestinal motility disorders were more pronounced in patients with sepsis compared to patients without sepsis.

### 001564

#### Prediction of sepsis-induced worsening of hemodynamics 6 hours in advance through machine learning

G. Angelotti[1], S. Falini[1], M. Greco[1], P. Morandini[2], A. Chiti[3], M. Cecconi[1], R. Barbieri[2]
[1]Anesthesia and intensive care, Humanitas Research Hospital, Milano, Italy; [2]Electronics, information and bioengineering, Politecnico di Milano, Milano, Italy; [3]Nuclear medicine, Humanitas Research Hospital, Milano, Italy
**Correspondence:** S. Falini
*Intensive Care Medicine Experimental* 2019, **7(Suppl 3):**001564

**INTRODUCTION.** Hemodynamic status of critically ill patients typically declines during sepsis and requires vasopressor support. Machine learning models have been recently implemented for a variety of ICU tasks with considerable success. A model capable of forecasting hemodynamic changes of septic patients could be critical in stratifying clinical intervention. Along this rationale, we hypothesize that the most predictive features would be those related to heart rate and blood pressure measurements, i.e. systolic (SP), diastolic (DP), mean (MP), or pulse pressure (PP).
**OBJECTIVES.** The purpose of the analysis was to create a prediction model for worsening of hemodynamic status in septic patients by using data from the first 6 hours after ICU admission.
**METHODS.** Data was pooled from the Medical Information Mart for Intensive Care III (MIMIC-III)(1) database. Patients in the 18-89 age range fulfilling the international consensus sepsis-3 criteria(2,3) were included. An increment of vasopressor equivalents (VPE) above 0.2 mcg/kg/min over the following 6 hours was chosen as indication of worsening of hemodynamic status. Firstly, a "null" logistic regression model using clinical data relative to the first 6 hours of ICU stay was trained to formulate predictions. Secondly, SP, DP, MP and PP were each integrated in the model and assessed for performance. Finally, a model with all pressure measurements was built and a feature selection process (LASSO) was run to yield only the most indicative. Performance was evaluated using the area under the receiver operator characteristic curve (AUC), recall and F1 score.
**RESULTS.** 5778 ICU stays were evaluated, of which 4370 (76%) do not receive vasopressors in the first 6 hours. The described increment in VPE was observed in 402 (6.9%) patients. Despite thelow prevalence, the models achieved moderate performance. The addition of any pressure measurement only slightly improved the AUC of the null model (0.84 vs 0.82) while it did improve recall, especially when using MP (0.83 vs 0.75). In all models, Glasgow coma scale and peripheral oxygen saturation were the the highest graded features; SP was also particularly highly ranked in its model, and MP and PP in an intermediate fashion. Likewise, the LASSO model selected SP as the most predictive feature.
**CONCLUSION.** Increment of vasopressor dose, as a marker of worsening of hemodynamic status, could be predicted 6 hours in advance using clinical data with moderate accuracy. SP was revealed as the most predictive of all pressure measurements, although it did not markedly improve accuracy of the model itself.

##### REFERENCE(S)

1. Johnson AEW, Aboab J, Raffa JD, Pollard TJ, Deliberato RO, Celi LA, et al. A Comparative Analysis of Sepsis Identification Methods in an Electronic Database. Crit Care Med. 2018 Apr;46(4):494–9.

2. Singer M, Deutschman CS, Seymour CW, Shankar-Hari M, Annane D, Bauer M, et al. The Third International Consensus Definitions for Sepsis and Septic Shock (Sepsis-3). JAMA. 2016 Feb 23;315(8):801–10.
3. Johnson AEW, Pollard TJ, Shen L, Lehman L-WH, Feng M, Ghassemi M, et al. MIMIC-III, a freely accessible critical care database. Sci Data. 2016 May 24;3:160035.

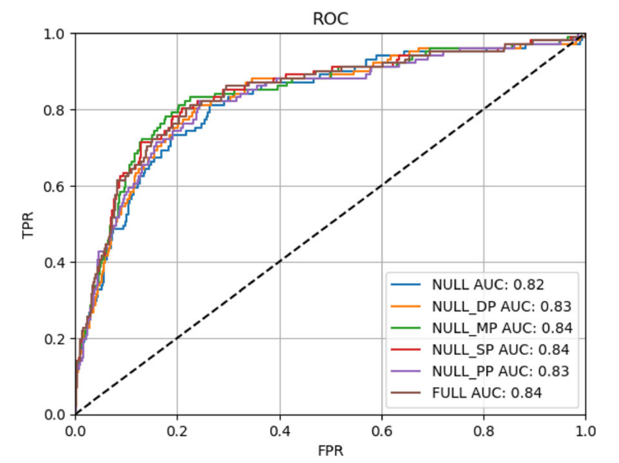

**Fig. 1 (abstract 001564).** See text for description

### 001565

#### New guidelines in sepsis animal modeling: about antimicrobial therapy end points in cecal ligation puncture in rats

P. Vaittinada Ayar[1], H. Jacquier[2], B. Deniau[3], A. Mebazaa[3], A. Blet[3]
[1]Emergency department , Beaujon Hospital, Clichy, France; [2]Laboratory of microbiology, department of infectious agents, Lariboisière Hospital, Paris, France; [3]Anesthesiology and intensive care, Lariboisière hospital, Paris, France
**Correspondence:** P. Vaittinada Ayar
*Intensive Care Medicine Experimental* 2019, **7(Suppl 3):**001565

**INTRODUCTION.** Sepsis is the most preventable cause of death worldwide,whichis estimated between six to nine million deaths every year [1]. Experimental studies are useful for a better understanding of this pathology, but require to follow new guidelines of animal modeling to fit with a clinical relevance.

**OBJECTIVES.** We aimed to explore (A) what is realized in articles published in 2018 using a cecal ligation puncture (CLP) model in rat, (B) if the instructions about antimicrobial therapy fit with microbiology in a peritonitis model based on CLP.

**METHODS.** (A) The review was performed on Pubmed using as keywords "CLP" and "RAT"for the year 2018, and selected English-written papers. We checked if animals received antibiotics and if bacteriological documentation was available before treatment. (B) We explored bacteria involved in the sepsis, using peritoneal fluid and blood cultures, at least 16 hours between CLP and sacrifice.

**RESULTS.** (A) Ninety-five studies were found. Among them 9 were excluded as they had a different meaning for CLP. Only in 11 cases (12%) antibiotics were used and mainly ceftriaxone at the dosage of 30mg/kg. None of the studies identified the pathogen before treatment.(B) In our series of eleven rats, we found mainly *Escherichia coli, Enterococcus faecalis, Lactobacillus murinus* in peritoneal fluid (fig. 1) and *Escherichia coli, Enterobacter cloacae, Enterococcus faecalis* in blood (fig. 2). All the bacteria exhibit a wild type phenotype for antimicrobial agent susceptibility.

**CONCLUSION.** These new recommendations provide a better match between experimental and clinical approaches, and improve translation of pre-clinical findings. Our findings suggest a better adequacy of antimicrobial treatment to pathogens and to the animal. More specific investigations are required to explore the bacterial diversity in peritoneal fluid and blood culture.

**REFERENCE(S)**

1. Fleischmann C et al. Am J Respir Crit Care Med 2016; 193(3): 259-72.
2. none

### 001597

#### Meropenem concentration before and during continuous veno-venous hemodialysis with regional citrate anticoagulation in critically ill patients with sepsis

I. Nowak[1], J. Górka[1], K. Polok[1], J. Fronczek,[1], A. Gielicz,[2], B. Seczyńska[1], M. Czuczwar[3], W. Szczeklik[1]
[1]Intensive care and perioperative medicine, Jagiellonian University Medical College, Kraków, Poland; [2]Department of medicine, Jagiellonian University Medical College, Kraków, Poland; [3]2nd department of anaesthesiology and intensive therapy, Medical University of Lublin, Lublin, Poland
**Correspondence:** J. Górka
*Intensive Care Medicine Experimental* 2019, **7(Suppl 3):**001597

**INTRODUCTION.** Optimal drug dosing is crucial in the therapeutic process. It plays a particularly important role in critically ill patients, especially those with severe sepsis, when proper antibiotics dosing is essential. The influence of renal replacement therapy on meropenem concentration in septic patients is largely unknown.

**OBJECTIVES.** The aim of this study was to determine the effect of continuous veno-venous hemodialysis (CVVHD) with regional citrate anticoagulation on meropenem concentrations in patients with acute kidney injury due to sepsis.

**METHODS.** 15 patients with acute kidney injury in the course of severe sepsis with clinical necessity for CVVHD according to KDIGO guidelines were enrolled in a prospective observational trial. Meropenem was administered by a continuous infusion, 2g every 8 hours. Meropenem concentrations were measured in blood plasma using high performance liquid chromatography coupled with tandem mass spectrometry. Blood samples were obtained prior and then 6 times after introducing CVVHD with regional citrate anticoagulation (RCA-CVVHD).

**RESULTS.** Median APACHE IV score was 118 points (IQR 97-134) and the median SOFA score on the day of admission was 19.5 (18-21). There were no significant differences in plasma meropenem concentrations measured directly prior to RCA-CVVHD and in the first 450 minutes of the procedure. Drug concentration reached its peak 2 hours after start of the infusion and then steadily declined.

**CONCLUSION.** Continuing high dose meropenem (2g every 8 hours) after introducing RCA-CVVHD ensures similar concentrations of meropenem both prior and after introducing RCA-CVVHD.

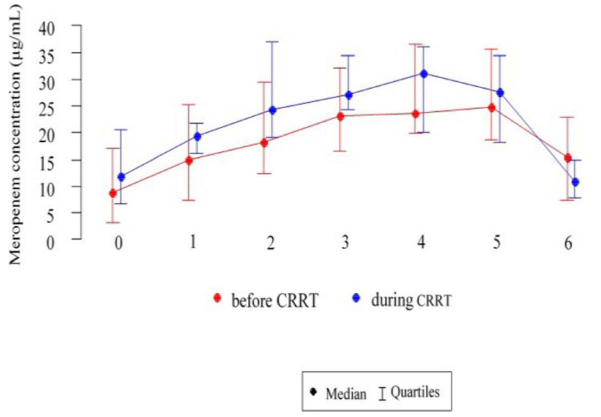

**Fig. 1 (abstract 001597).** See text for description

### 001598

#### Iron metabolism parameters and ICU mortality in septic patients

A. Brandtner[1], C. Pfeifhofer-Obermair[2], M. Nairz[2], GF. Lehner[1], G. Weiss[2], M. Joannidis[1]
[1]Division of intensive care and emergency medicine, department of internal medicine i, Medical University Innsbruck, Innsbruck, Austria; [2]Department of internal medicine ii (infectious diseases, immunology, rheumatology, pneumology), Medical University Innsbruck, Innsbruck, Austria
**Correspondence:** A. Brandtner
*Intensive Care Medicine Experimental* 2019, **7(Suppl 3):**001598

**INTRODUCTION.** The course and outcome of infections is a dynamic interplay between the host and the pathogen. Central to this interplay is the struggle for iron, a micronutrient essential to both the mammalian host and virtually all microbes.(1,2)

**OBJECTIVES.** The aim of this study is to test whether markers of iron metabolism predict patient outcome in a cohort of sepsis patients.

**METHODS.** Patients admitted with sepsis to the ICU were consecutively enrolled from January 2018 to March 2019. Sepsis was defined as fulfilment of Sepsis-3 criteria, patients were enrolled within 24 hours after admission or diagnosis of sepsis respectively. Patients with haemato-oncologic disorders and patients with prior transfusions were excluded from analysis. Serum ferritin, transferrin and iron, as well as interleukin-6, procalcitonin, c-reactive protein and SOFA scores were obtained at the day of study inclusion. Patient outcome was defined as ICU mortality or discharge. Predictor variables were tested for a significant difference between survivors and non-survivors and imputed into a multiple logistic regression model. Predictors were tested for correlation with markers of inflammation. Statistical analyses were performed in R, results are presented as mean ±standard deviation. A p-value of >0.05 was considered as statistically significant.

**RESULTS.** Of 31 included patients, 4 were excluded due to prior transfusion of blood products. Of the remaining 27 patients, four were excluded due to presence of an active haematologic disorder and one due to missing iron parameters. 27% of patients were female, mean age was 65 ±14 years with a mean SOFA score of 12 ±3. 64% of patients survived. Iron levels of survivors vs. non survivors differed significantly (4.87 ±2.47 vs. 7 ±5mmol/L; p = 0.01), as did levels of ferritin (median 700 vs. 302µg/L; p < 0.0001) and transferrin (121.64 ±46.67 vs. 87.75 ±58.86 mg/dL; p < 0.0001) between surviving and deceased patients. Multiple logistic regression analysis of these parameters against ICU mortality did select transferrin as best predictor with an AUC of 0.66. Correlation analysis of transferrin and markers of inflammation did not reveal significant associations.

**CONCLUSION.** Parameters of iron metabolism differ significantly between survivors and non-survivors of sepsis in our cohort. Transferrin levels are inversely correlated with ICU mortality, but no statistical significant correlation could be shown with parameters of inflammation.

##### REFERENCE(S)

1. Lan, P. et al. High Serum Iron level is Associated with Increased Mortality in Patients with Sepsis. Sci. Rep. 8, 11072 (2018).
2. Nairz, M. & Weiss, G. 5. Infections Associated with Iron Administration. in Essential Metals in Medicine: Therapeutic Use and Toxicity of Metal Ions in the Clinic 123–156 (De Gruyter, 2019). doi:10.1515/9783110527872-011

### 001608

#### "Resistencia Zero" as a trend monitor over the last years in accordance with the isolation of multiresistant bacteria in UCI

A. López Fernández[1], A. Ruiz Perea[1], R. Prieto Jurado[2], S. Ramiro González[2], MDM. Jiménez Quintana[1], J. Machado Casas[1]
[1]Medicina intensiva, Hospital Universitario Virgen de las Nieves, Granada, Spain; [2]Intensive care unit, Virgen de las Nieves Hospital, Granada, Spain
**Correspondence:** A. López Fernández
*Intensive Care Medicine Experimental* 2019, **7(Suppl 3):**001608

**INTRODUCTION.** The increase of antibiotic resistance is a serious threat to public health throughout the world that enhances the costs of health care the rate of treatment failure and mortality. The main objective of the "Resistencia Zero" (RZ) program is to reduce by 20% the rate of patients in whom one or more multiresistant bacteria of nosocomial origin are identified during ICU stay

**OBJECTIVES.** To describe, according to the origin, the trend in the pattern of multiresistant germs in a medical ICU based on the results of the RZ.

**METHODS.** Descriptive study of the quantity of multiresistant germs acquired in our ICU with regards to the total of multiresistant germs acquired during ICU stay and out of ICU from 2008 to 2018, in a medical ICU of 26 beds. Data collected from the ENVIN-HELICS study and expressed as a percentage.

**RESULTS.** During the period under review, a total of 81 multiresistant germs were registered. The following results are described: 14 MRSA (8 of them were adquired at ICU); 2 Enterococcus resistant to Vancomycin (1 was adquired at ICU); 13 Pseudomonas multiresistant (7 were adquired at ICU); 19 Acinetobacter resistant to Imipenem (12 were adquired at ICU); 21 Enterobacteria-ESBL (9 were adquired at ICU); 12 BGN producers of Carbapenemase (6 were adquired at ICU).

According to whether the isolation of the germ was prior to admission to the ICU or during ICU stay, we obtain the following annual results.

**CONCLUSION.** We observe a change in the trend of MR isolations during the last decade. At first, most isolates were originated at ICU but during the last years the origin of the majority of isolations is out of ICU. These data are consistent with those of the RZ program nationwide. Antibiotic policies at ICU and the Zero Projects play a key role in the decrease of MR isolates within the ICUs.

**Table 1 (abstract 001608).** See text for description

| Year | Total Multiresistant | Upon Admission to the ICU | Acquired in ICU |
|---|---|---|---|
| 2008 | 6 | 0 (0%) | 6 (100%) |
| 2009 | 4 | 0 (0%) | 4 (100%) |
| 2010 | 2 | 1 (50%) | 1 (50%) |
| 2011 | 15 | 3 (20%) | 12 (80%) |
| 2012 | 5 | 1 (20%) | 4 (80%) |
| 2013 | 5 | 2 (40%) | 3 (60%) |
| 2014 | 8 | 7 (87,5%) | 1 (12,5%) |
| 2015 | 6 | 3 (50%) | 3 (50%) |
| 2016 | 6 | 3 (50%) | 3 (50%) |
| 2017 | 10 | 7 (70%) | 3 (30%) |
| 2018 | 14 | 11 (78,57%) | 3 (21,43%) |

### 001639

#### Preadmission Use of Beta Blockers Is Associated With Improved Mortality in Patients With Sepsis and Septic shock

M. Harazim, M. Nalos, M. Matejovic
1st medical department, University Hospital in Pilsen, Pilsen, Czech Republic
**Correspondence:** M. Harazim
*Intensive Care Medicine Experimental* 2019, **7(Suppl 3):**001639

**INTRODUCTION.** The beta-adrenergic system is a powerful modulator of the immune and cardiovascular response to sepsis and inflammatory cytokine production. Recent data suggest a potential beneficial role of prior β-blockers use in patients with sepsis. The benefit from beta-blockers in sepsis may not be only derived from a class-specific effect. We sought to assess impact of premorbid β-blocker use on mortality within 28 days of ICU admission.

**METHODS.** We conducted a retrospective cohort study of patients with sepsis and septic shock admitted to the medical ICU from January 1, 2014, to December 31, 2018. Chronic beta-blockers use was defined to have a previous prescription for β-blocking agent for 3 or more months prior to ICU admission. Demographics, initial vital signs, the source of infection, 28-day mortality, SOFA and APACHE II were obtained from the electronic database. Adjustment for age, gender, and severity of illness was performed.
**RESULTS.** We identified 784 patients with sepsis and septic shock, 290 (37%) of whom had a previous prescription for β -blockers before hospital admission. Common medications used by patients were metoprolol (54 %) and bisoprolol (22 %). The median age was 67 years (IQR 57- 76), 456 (58%) of patients were males. Patients who were on chronic β-blockers had lower 28-day mortality (20,8%) compared to patients who were not (31,5%), *(p=0.05)*. After adjusting for age, gender, and severity of illness using SOFA and APACHE II scores, the mortality was significantly lower in those on β-blockers *(p<0.05)*. There were no statistically significant association in admission heart rate in both groups.
**CONCLUSION.** Long-term β-blocker therapy decreases 28-day mortality of ICU patients with sepsis and septic shock.

### 001652

#### Usefulness of SEPSIS-3 in diagnosing and predicting mortality of ventilator-associated lower respiratory tract infections

A. Gaudet[1], M. Devos[1], S. Keignart[2], O. Pouly[1], S. Lecailtel[3], S. Nseir[1]
[1]Icu, Chu De Lille, Lille, France; [2]Icu, Ch De Valenciennes, Valenciennes, France; [3]Icu, Boulogne-sur-Mer Hospital Center, Boulogne-sur-Mer, France
**Correspondence:** S. Nseir
*Intensive Care Medicine Experimental* 2019, **7(Suppl 3):**001652

**INTRODUCTION.** SEPSIS-3 was recently reported and validated in patients admitted to the ICU with sepsis. No study to date has evaluated the usefulness of this definition in patients with ICU-acquired infections.
**OBJECTIVES.** The aim of this study is to determine the accuracy of SEPSIS-3 in diagnosing and predicting mortality of ventilator-associated lower respiratory tract infections (VA-LRTI).
**METHODS.** Prospective observational study, conducted in a single mixed ICU during a 2-year period. All consecutive patients with VA-LRTI were included. SEPSIS-3 criteria were considered as present if an increase of total SOFA score ³2, between 48h before and the day of VA-LRTI diagnosis, was observed. VA-LRTI were defined as ventilator-associated pneumonia (VAP), and ventilator-associated tracheobronchitis (VAT). Only first episodes of VAP or VAT with quantitative microbiological confirmation were analyzed. Sensitivity (Se), specificity (Sp), positive predictive value (PPV), and negative predictive value (NPV) for accuracy of SEPSIS-3 in diagnosing or predicting mortality of VA-LRTI were calculated.
**RESULTS.** 206 patients with VA-LRTI (136 VAP, and 70 VAT) were included. SEPSIS-3 criteria were present in 53 (39%), and 6 (8.5%) patients with VAP, and VAT; respectively (p<0.001, OR 6.8 [95%CI 2.8-16.8]). No significant difference was found in ICU-mortality between patients who presented the criteria of SEPSIS-3, and those who did not (24 of 59 (41%) vs 40 of 147 (27%), p=0.059).
**CONCLUSION.** Accuracy of SEPSIS-3 was moderate in diagnosing VAP, and low in predicting mortality among patients with VA-LRTI. The high positive predictive value of SEPSIS-3 in diagnosing VAP might be helpful in differentiating VAP from VAT.

**Table 1 (abstract 001652).** Accuracy of SEPSIS-3 in diagnosing and predicting mortality of VA-LRTI

| | Se | Sp | NPV | PPV |
|---|---|---|---|---|
| VAP diagnosis, % | 39 | 91 | 44 | 89 |
| Mortality of VA-LRTI, % | 38 | 75 | 41 | 73 |

### 001653

#### Impact of oral Midodrine in weaning vasoactive infusions in patients admitted with septic shock

M. Fakher[1], A. Mohamed[2], K. Mashoor[1], A. Alsharif[1]
[1]Critical care, Cairo University, Faculty Of Medicine, Kasr Al Ainy, cairo, Egypt; [2]Critical care, cairo university, Taizz, Yemen
**Correspondence:** M. Fakher
*Intensive Care Medicine Experimental* 2019, **7(Suppl 3):**001653

**INTRODUCTION.** Intravenous vasoactive agents are key component in management of persistant hypotension in septic shock after adequete fluid resusitation ,yet they carry risk of many complications related to the drug complication and acsess etc..(1) The use of oral Midodrine is hypotheised to decresase the duration of vasoactive infusion and help their weaning.
**OBJECTIVES.** Evaluation of the role of midodrine in weaning of vasopressor infusions in patients admitted to the ICU with septic shock and its impact on outcome.
**METHODS.** This was a prospective randomized control study performed in a medical intensive care unit (ICU). All study subjects had a diagnosis of septic shock requiring at least 24 hours of IV vasopressors and demonstrated clinical stability with stable or decreasing doses of IV vasopressors. The two groups compared were those who received IV vasopressors only(30 patients) and those who received IV vasopressors with adjunctive midodrine 10 mg every eight hours(30 patients).Research and Ethics comittee approval was taken prior to performing the study and a written consent from each patient and or his next of kin before participating in the study.
**RESULTS.** There was no difference between both groups regarding demographics ,baseline charechteristics and source of sepsis (age, gender, APACHE 2 score and SOFA score on admission etc..).
Study findings showed that the IV vasopressor duration (hours) and IV vasopressor weaning duration (hours) were lower in the midodrine group when compared to IV vasopressor only group but the difference is statistically insignificant (139.03±46.95 VS 141.90±79.77 and 62.37±14.94 VS 74.20±36.78 p value 0.4.0.11 respectively).
Regarding the effect of midodrine on MAP, we found that despite the decrease of vasopressor dose in the midodrine group, the MAP in this group had significantly improved from 84.50 mmHg at the time of midodrine initiation to 88.03 mmHg after 24hours of midodrine initiation (p=0.04), this was statiscally higher than group A not using Midodrine.
Concerning the effect of midodrine on the outcome of septic patients, our study showed that there was no significant difference between both groups regarding the ICU length of stay and ICU mortality, reinstitution of an IV vasopressor after successful discontinuation and survival rate.
**CONCLUSION.**

- Using midodrine in septic shock patients doesn't reduce duration of IV vasopressor infusion and weaning during the recovery phase of septic shock.
- Using midodrine as an adjunct to vasoactive agents has no impact on overall survival, length of ICU stay and ICU mortality.
- Administration of midodrine improved mean arterial blood pressure MAP.

**REFERENCE(S)**

1. There is no specific research grant received by the authors.
2. (1)6. Shankar-Hari M, Phillips GS, Levy ML, Seymour CW, Liu VX. Developing a New Definition and Assessing New Clinical Criteria for Septic Shock: For the Third International Consensus Definitions for Sepsis and Septic Shock (Sepsis-3). JAMA. 2016; 315(8): 775-87.

### 001670

#### The impact of antibiotics on mitochondrial function in immune cells

M. Khpal, N. Arulkumaran, M. Singer
Bloomsbury institute of intensive care medicine, University College London, London, United Kingdom
**Correspondence:** M. Khpal
*Intensive Care Medicine Experimental* 2019, **7(Suppl 3):**001670

**INTRODUCTION.** Sepsis is associated with both mitochondrial and immune cell dysfunction (1-3). Antibiotics are known to impair mitochondrial function in non-immune cells (4-7). We hypothesise that antibiotics impair immune cell mitochondrial function, contributing to immune cell dysfunction in sepsis.

**OBJECTIVES.** To investigate the impact of antibiotics on mitochondrial function in immune cells *ex vivo*.

**METHODS.** Healthy volunteer immune cells were incubated in Hanks buffered saline solution (37oC, 21% O2, 5% CO2) with one of five antibiotics (amoxicillin, ciprofloxacin, gentamicin, meropenem and teicoplanin) at 5mcg/mL for 5 hours. Cells were subsequently incubated with fluorochromes (Thermo Fisher Scientific, UK) for flow cytometric assessment of mitochondrial membrane potential (Dψm) (using TMRM at 25nM), mitochondrial reactive oxygen species (Mitosox Red at 2mM), and cell death (far red cell viability stain at 1mM) using the LSR Fortessa flow cytometer (BD Biosciences). The two main cell populations, granulocytes and peripheral blood mononuclear cells (PBMCs) were identified by gating on characteristic forward and side scatter profiles. All data were collected from 3–5 individual experiments expressed as mean ± standard error of mean (SEM) compared to untreated cells. Flow cytometric data were analysed using FlowJo version 10.0 (Tree Star Inc, USA). Statistical data analysed using a non-parametric Kruskal Wallis with post-hoc Dunn's test using GraphPad Prism v5 (San Diego, USA).

**RESULTS.** At 5mcg/ml, gentamicin and teicoplanin increased granulocyte mitochondrial ROS production by 22% (p=0.026) and 44% (p=0.012) respectively, compared to untreated cells. At 15mcg/ml, teicoplanin increased granulocyte mitochondrial ROS production by 43% (p=0.041). In PBMC population, ciprofloxacin, gentamicin and meropenem increased mitochondrial ROS production at 5mcg/ml by 13% (p=0.0014), 21% (p=0.0148) and 9% (p=0.0296) respectively. At 15mcg/ml, ciprofloxacin, gentamicin and meropenem further increased mitochondrial ROS production by 75% (p=0.0014), 31% (p=0.026) and 61% (p=0.0369) respectively, compared to untreated cells.Mitochondrial membrane potential (MMP) in granulocytes was not altered when treated with 5mcg/ml antibiotic concentrations (p=0.1585). At 15mcg/ml, teicoplanin induced a reduction in MMP (p=0.006). However, in PBMCs, MMP was unaffected by antibiotics at both 5 and 15mcg/ml (p=0.1722 and p = 0.1436, respectively.Cell viability was unaffected by antibiotic exposure (p=0.98 for granulocytes, p=0.87 for PBMCs compared to untreated cells).

**CONCLUSION.** A number of antibiotics cause direct mitochondrial dysfunction in immune cells *ex-vivo*. This may explain, in part, the immune dysfunction seen in septic patients.

**REFERENCE(S)**

1. Biswas SK, Lopez-Collazo E. Endotoxin tolerance: new mechanisms, molecules and clinical significance. Trends Immunol. 2009;30(10):475-87.
2. Brealey D, Brand M, Hargreaves I, Heales S, Land J, Smolenski R, et al. Association between mitochondrial dysfunction and severity and outcome of septic shock. The Lancet. 2002;360(9328):219-23.
3. Delano MJ, Ward PA. The immune system's role in sepsis progression, resolution, and long-term outcome. Immunol Rev. 2016;274(1):330-53.
4. Jiang S, Li T, Zhou X, Qin W, Wang Z, Liao Y. Antibiotic drug piperacillin induces neuron cell death through mitochondrial dysfunction and oxidative damage. Can J Physiol Pharmacol. 2018;96:562-8.
5. Pious DP, Hawley P. Effect of Antibiotics on Respiration in Human Cells. Pediat Res. 1972;6:687-92.
6. Tune BM, Hsu C. Renal Mitochondrial Toxicity of Beta-Lactam Antibiotics: In Vitro Effects of Cephaloglycin Imipenem. J Am Soc Nephrol. 1990;1:815-21.
7. Kalghatgi S, Spina CS, Costello JC, Liesa M, Morones-Ramirez JR, Slomovic S, et al. Bactericidal antibiotics induce mitochondrial dysfunction and oxidative damage in Mammalian cells. Sci Transl Med. 2013;5(192):192ra85.

8. Nish Arulkumaran- ESICM NEXT start-up grant (2018)

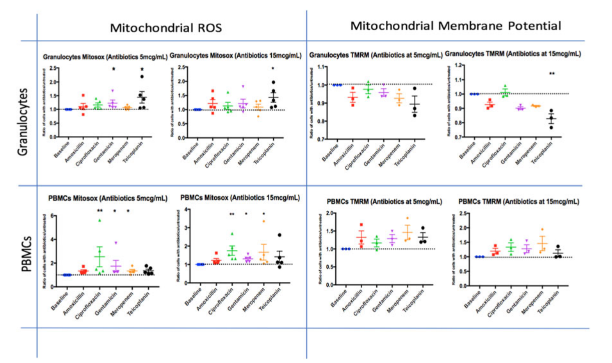

**Fig. 1 (abstract 001670).** See text for description

### 001685

#### Optimal Timing for Vasopressor Initiation in Septic Shock : Revisiting the Surviving Sepsis Campaign guidelines using Machine Learning

A. Waschka[1], M. Komorowski[2], A. Hubbard[3], R. Pirracchio[4]

[1]Department of statistics, UC Berkeley, Berkeley, United States of America; [2]Department of surgery and cancer faculty of medicine, Imperial College London, London, United Kingdom; [3]Biostatistics, UC Berkeley, Berkeley, United States of America; [4]Anesthesia and perioperative care, UCSF, San Francisco, United States of America

**Correspondence:** R. Pirracchio

*Intensive Care Medicine Experimental* 2019, **7(Suppl 3):**001685

**INTRODUCTION.** The Surviving Sepsis Campaign [1] recommends to administer 30 ml/kg of fluid within 3 hours following the onset of hypotension, before introducing vasopressors. However, evidence supporting this recommendation is lacking.

**OBJECTIVES.** Determine i) the optimal timing and ii) the optimal fluid volume before vasopressor introduction in septic shock.

**METHODS.** We used the data from the MIMIC II dataset. (2) To be included, patients had to present an acute hypotensive episode of septic origin and a lactate level > 2 mmol/l. The primary outcome measure was hospital mortality. We used a machine learning approach accounting for time-varying confounders called longitudinal targeted maximum likelihood estimation. (3)

**RESULTS.** 933 patients were included: SOFA 7; arterial lactate 3.3 mmol/L, average hospital mortality rate 11.5%. Hospital mortality was significantly minimized when vasopressors where introduced by hour 3 following the onset of hypotension. When compared to 30 ml/kg, switching to vasopressors after 5 or 10 ml/kg was associated with reduced mortality (Fig. 1).

**CONCLUSION.** In septic shock, administering 5-10 ml / kg within 3 hours after the onset of hypotension appears to maximize the chance of survival.

**REFERENCE(S)**

1. Rhodes A, et al. Surviving Sepsis Campaign: International Guidelines for Management of Sepsis and Septic Shock: 2016. Intensive care Med March 2017, Volume 43, Issue 3, pp 304–377
2. Saeed M, Villarroel M, Reisner AT, et al. Multiparameter Intelligent Monitoring in Intensive Care II: A public-access intensive care unit database*. Crit Care Med. 2011;39(5):952-960. doi:10.1097/CCM.0b013e31820a92c6
3. None
4. Schwab J, Lendle S, Petersen M, van der Laan M (2017). ltmle: Longitudinal Targeted Maximum Likelihood Estimation. R package version 1.0-0,

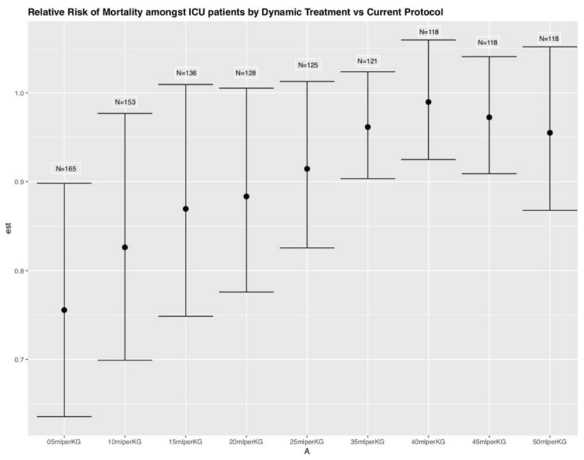

**Fig. 1 (abstract 000685).** Relative risk of mortality amongst ICU patients by Dynamic treatment vs current protocol

### 001692

#### Pentraxin 3: which role in sepsis?

G. Scarpati, I. Russo, E. Della Rocca, L. Ferrara, O. Piazza
Anaesthesia and intensive care, University of Salerno, Baronissi, Italy
**Correspondence:** G. Scarpati
*Intensive Care Medicine Experimental* 2019, **7(Suppl 3):**001692

**INTRODUCTION.** Pentraxin-3 (PTX3) is a molecule of the humoral pattern recognition arm of the innate immune system, as complement components, collectins, and ficolins [1]. PTX-3 has been appreciated for its diagnostic value for sepsis and septic shock.
**OBJECTIVES.** This study evaluated the accuracy of PTX3, Endocan, Nitric Oxide (NO) and Presepsin as biomarkers of severity and prognosis in patients with sepsis/septic shock.
**METHODS.** We enrolled 12 sepsis patients admitted to our ICU. Blood samples for measuring PTX3, NO, Endocan, and Presepsin were taken within 12 hours from diagnosis and repeted for the next three days. A group of patients admitted in ER with hip fracture was used as controls. Mortality at 28-day was considered as outcome.
**RESULTS.**
Serum levels of NO, Endocan, and Presepsin in septic patients were higher than control group, but PTX3, which was higher in the hip fracture group. Endocan and Presepsin day 1 concentrations in non survivors were higher than the concentrations measured in survivors. At day 1, NO and PTX3 concentrations were higher in surviving patients.
**CONCLUSION.** Sepsis patients had higher concentrations of Nitric Oxide, Endocan and Presepsin than those with hip fracture. Beyond its role as the first line of resistance against pathogens, innate immunity is involved in initiating the process of tissue repair. PTX3 appears to be more closely related to endothelial damage, prevalent in patients with femur fractures. Furthermore, some authors suggest a protective role of PTX3 and NO [2].
Models based on the combination of several biomarkers provide a novel and useful strategy to overcome the limited performance of one parameter for a given outcome. Hopefully, a new mortality prediction model could improve the prediction of sepsis related mortality by combining PTX3, NO, endocan and presepsin.

##### REFERENCE(S)

[1] Carrizzo A, Procaccini C, Lenzi P, Fusco C, Villa F, Migliarino S, De Lucia M, Fornai F, Matarese G, Puca AA, Vecchione C. PTX3: an inflammatory protein modulating ultrastructure and bioenergetics of human endothelial cells. Immun Ageing. 2019 Feb 2;16:4
[2] Daigo K, Hamakubo T. Host-protective effect of circulating pentraxin 3 (PTX3) and complex formation with neutrophil extracellular traps. Front Immunol. 2012 Dec 13;3:3

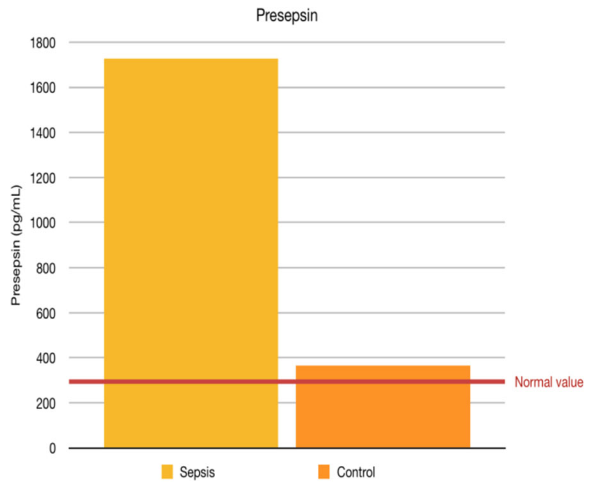

**Fig. 1 (abstract 001692).** See text for description

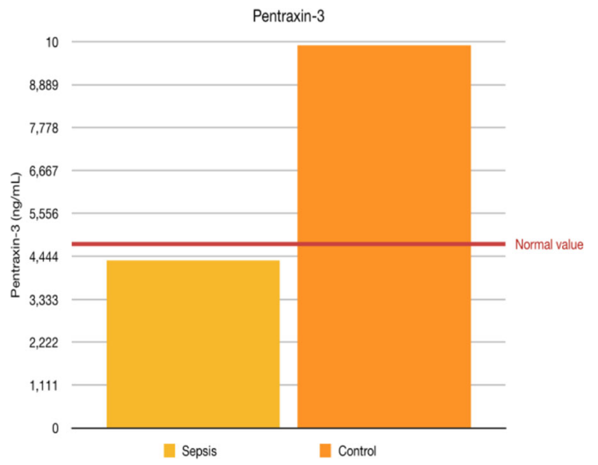

**Fig. 2 (abstract 001692).** See text for description

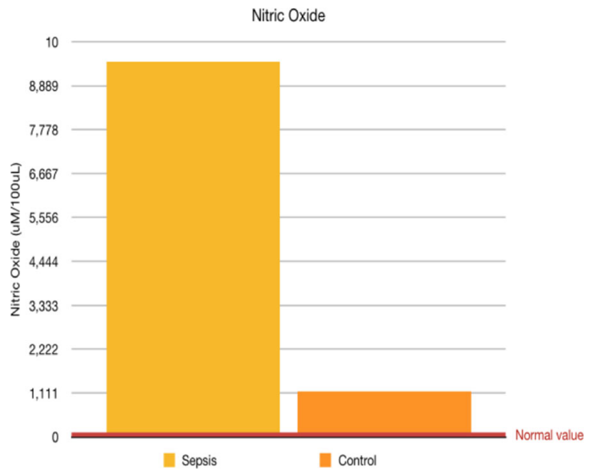

**Fig. 3 (abstract 001692).** See text for description

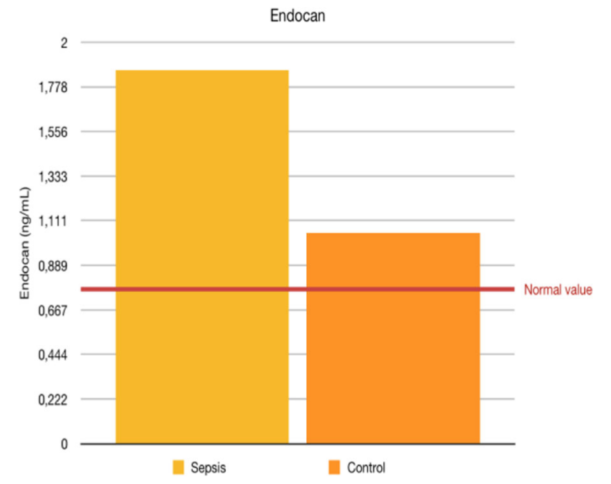

**Fig. 4 (abstract 001692).** See text for description

### 001719

#### IgM-enriched IgG as adjuvant therapy to treat sepsis and septic shock in the critically ill: a proscpective controlled randomised trial

I. Cigada[1], S. Santini[1], A. Corona[1]
[1]Intensive care medicina, ASST Fatebenefratelli Sacco, Milano, Italy
**Correspondence:** A. Corona
*Intensive Care Medicine Experimental* 2019, **7(Suppl 3):**001719

**INTRODUCTION.** Sepsis is responsible of both an immune hyperactivity damage from inflammation and immune suppression and paralysis. A few studies support the role of IgM enriched immunoglobulins G as adjunctive of antimicrobial treatment

**OBJECTIVES.** To assess the efficacy of IgM enriched IgG as adjuvant therapy in treating sepsis in the critically ill.

**METHODS.** A PCRT was set and performed, since 12/2016 to 01/04/2019; patients experiencing a septic shock - admitted with a first 24h SAPS II > 25, associated with a SOFA-score > 4 were randomised in two group: (A) undergoing treatment with IgM-e-IG, given for three days at the total dosage of 500 mg/kg plus conventional antimicrobial therapy; (B) undergoing conventional antimicrobial therapy. The therapy response was based on clinical, microbiological and rheological data.

**RESULTS.** During the study period 23 and 22 patients were respectively recruited in the two groups; no differences were found in median age [61 (55-78) vs. 63 (58-77) p=0.662], ICU length of stay [14 (5-19) vs. 15 (6-18) p=0.462]; duration of mechanical ventilation [7 (5-8) vs. 8 (5-9) p=0.348] and antimicrobial treatment [9 (8-11) vs. 11 (12-14) p=0.552]. PCT, CRP, WBC, Lactates and SOFA score were daily measured and computed the differences between day of stopping and starting IM-enriched IgG (For the patients of group B, such parameters ware considered over the same 72 hrs period. No differences were found in the trend of all parameters but SOFA [3.7 (2-5) vs. 0.8 (0-2) p=0.025], indicating a quicker and more timely recovery from sepis related organ failure in the group A. The VLAD computed on the basis of SMR in the two group showed a higher number of saved lives in the treatment group (4.7 vs. 0.8, p=0.002). No differences in survival was found by Kaplan Meier analysis although at 28 days all patients of the treatment group were still alive.

**CONCLUSION.** IgM enriched immunoglobulins G as adjunctive of antimicrobial treatment may have a role in improving patient outcome, however PRCT should be prosecuted up to proper determined sample size.

##### REFERENCE(S)

1. Cavazzuti I, Serafini G, Busani S, Rinaldi L, Biagioni E, Buoncristiano M, Girardis Early therapy with IgM-enriched polyclonal immunoglobulin patients with septic shock. Intensive Care Med. 2014 Dec;40(12)
2. None

### 001746

#### The Efficacy of Polymyxin-B Hemoperfusion in Critically Ill Patients with Sepsis in Thailand (Thai PMX-registry)

K. Trongtrakul[1], W. Ponsittisak[1], N. Srisawat[2], C. Sittipunt[2], K. Noppakun[3], A. Limsukon[3], K. Chittawatanarat[4], K. Surasit[5], A. Nongnuch[6]
[1]Internal Medicine, Faculty of Medicine Vajira, Bangkok, Thailand; [2]Internal medicine, Faculty of Medicine Chulalongkorn University, Bangkok, Thailand; [3]Internal medicine, Faculty of Medicine Chiang Mai University, Chiang Mai, Mueang Chiang Mai District, Chiang Mai, Thailand, Thailand; [4]Surgery, Faculty of Medicine Chiang Mai University, Chiang Mai, Mueang Chiang Mai District, Chiang Mai, Thailand, Thailand; [5]Internal medicine, Nakornping Hospital, Chiang Mai, Mueang Chiang Mai District, Chiang Mai, Thailand, Thailand; [6]Internal medicine, Faculty of Medicine Ramathibodi Hospital, Mahidol University, Bangkok, Thailand, Thailand
**Correspondence:** K. Trongtrakul
*Intensive Care Medicine Experimental* 2019, **7(Suppl 3):**001746

**INTRODUCTION.** Polymyxin B hemoperfusion (PMX-HP) is one of a new blood purification therapy for sepsis. PMX-HP can remove circulating endotoxin particularly in gram-negative infection which potentiates improving clinical outcomes.

**OBJECTIVES.** To evaluate the efficacy of polymyxin-B hemoperfusion therapy over conventional treatment for sepsis.

**METHODS.** An ambi-directional cohort was conducted. PMX-HP arm was collected prospectively in septic patients during 2017-2018 from 5 ICUs in Thailand. PMX-HP treatment was performed 2 hours for 2 consecutive days. South East Asia-Acute Kidney Injury (SEA-AKI) Study dataset was used as a historical control arm (collected during 2013-2015 from 17 ICU-centers in Thailand, n=5,476). Propensity score match and survival analyses were used to identify 28-day mortality between patient with and without PMXB-HP therapy.

**RESULTS.** A total of 1,358 septic cases were enrolled (PMX-HP, n=30 and SEA-AKI Study, n=1,328). Comparing patients who treated with vs without PMX-HP therapy, 28-day mortality was 14/30 (46.7%) vs 318/1,328 (24.0), respectively, HR = 0.971; 95%CI, 0.567-1.663, p = 0.916. However, there were multiple differences in patient baseline characteristics, including pre-existing diseases (diabetes mellitus, hypertension, and coronary heart disease); severity of illness (APACHE-II and SOFA score); and usage of organ support (ventilatory support, vasopressor, and renal replacement therapy). Although all baseline variables were similar after matching by propensity score, 28-day mortality remained no significant difference ( 11/21 (52.4%) vs 8/21 (38.1%), HR=0.938; 95%CI, 0.377-2.334, p = 0.891).

**CONCLUSION.** In septic patients treated with PMX-HP matched to conventional therapy, no significant differences in 28-day mortality were found.

1. We would like to thank for Thai Society of Critical Care Medicine who initiated to conduct this study and Toray Industries who provided PMX cartridges.

## ARF - Acute respiratory failure 4

### 000899

#### Prone positioning decreases macrophage recruitment in ventral lung regions of animals with experimental acute respiratory distress syndrome

L. Bitker[1], N. Costes[2], D. Le Bars[3], M. Orkisz[4], M. Mezidi[1], JC. Richard[1]
[1]Médecine intensive et réanimation, Hospital La Croix-Rousse - Hcl, Lyon, France; [2]Pet/ct, Cermep - Imagerie du vivant, Bron, France; [3]Radiochemistry, Cermep - Imagerie du vivant, Bron, France; [4]Creatis, CREATIS, Villeurbanne, France
**Correspondence:** L. Bitker
*Intensive Care Medicine Experimental* 2019, **7(Suppl 3):**000899

**INTRODUCTION.** Prone positioning (PP) decreases mortality of moderate to severe acute respiratory distress syndrome (ARDS) [1]. This benefit may be related to the prevention of ventilation-induced lung injury (VILI) by PP. More precisely, it remains unclear whether PP

modulates the deleterious inflammatory response associated with VILI, namely *biotrauma*. [11C]-PK11195 is a positron emission tomography (PET) radiotracer that allows the non-invasive quantification of lung macrophage recruitment in the lungs, in combination with lung computed tomography (CT) evaluation.

**OBJECTIVES.** To evaluate the effects of PP on VILI surrogates and macrophage lung recruitment quantified by [11C]-PK11195 lung uptake in PET/CT, in an experimental model of ARDS.

**METHODS.** Experimental ARDS was performed in 9 pigs (weight, 27 [interquartile range, 25–29] kg) in supine position (SP), by means of tracheal instillation of hydrochloric acid. Animals were under general anesthesia, neuromuscular blockade, and protective ventilation (tidal volume 6 ml/kg, positive end-expiratory pressure 5 cmH2O) [2]. Respiratory mechanics, including esophageal pressures, were recorded. Ventilation settings were not modified thereafter. Immediately after completion of experimental ARDS, animals were randomized to be prone positioned or to remain in SP, during 4 hours. Coupled PET/CT acquisitions were performed immediately before (T1), and after 4 hours of experimental ARDS (T2). Non-invasive measurements in PET/CT were performed on the whole lungs, and by dividing the lungs into 2 regions-of-interest (ROI) defined equally along the ventrodorsal axis. [11C]-PK11195 was quantified using the standardized uptake value (SUV), corrected for tissue density in the corresponding CT ROI. Collapsed lung volume corresponded to the non-aerated volume of gas fraction in a given ROI.

**RESULTS.** PP was performed in 5 animals, and SP in 4. The PaO2/FiO2 ratio was 143 [90–169] at 30 minutes of ARDS onset. Experimental ARDS increased the elastance-derived transpulmonary pressure (ΔPL) between T1 and T2 (8 [7–10] to 15 [13–18] cmH2O, *P*<0.01), and CT-derived lung weight (427 [409–508] to 608 [572–642] g, *P*<0.01). At T2, PP animals had lower ΔPL compared to SP animals (13 [12–14] vs. 20 [17–23] cmH2O, *P*= 0.03). PP decreased collapsed lung volume in the dorsal region at T2, compared to SP (12 [9–22] vs. 262 [204–275] ml, *P*=0.02), by redistributing it to ventral regions (144 [140–169] vs. 13 [9–24] ml, *P*=0.02). At T2, [11C]-PK11195 SUV of the whole lung of PP animals was 1.3 [1.2–1.3], and 1.4 [1.2–1.7] in SP animals (*P*=0.73). However, we observed a lower [11C]-PK11195 SUV at T2 in the ventral region of PP animals, compared to the ventral region of SP animals (1.3 [1.2–1.4] vs. 1.6 [1.4–1.9], *P*=0.03). No difference existed in dorsal regions (1.4 [1.2–1.5] vs. 1.4 [1.2–1.6], *P*=0.55).

**CONCLUSION.** PP improved respiratory mechanics, redistributed collapsed lung volume, and decreased [11C]-PK11195 lung uptake (and presumably macrophage lung recruitment) in the ventral lung regions of animals with experimental ARDS.

**REFERENCE(S)**

1. The Acute Respiratory Distress Syndrome Network, (2000) Ventilation with lower tidal volumes as compared with traditional tidal volumes for acute lung injury and the acute respiratory distress syndrome. N Engl J Med 342: 1301-1308
2. Guerin C, Reignier J, Richard JC, Beuret P, Gacouin A, Boulain T, Mercier E, Badet M, Mercat A, Baudin O, Clavel M, Chatellier D, Jaber S, Rosselli S, Mancebo J, Sirodot M, Hilbert G, Bengler C, Richecoeur J, Gainnier M, Bayle F, Bourdin G, Leray V, Girard R, Baboi L, Ayzac L, Group PS, (2013) Prone positioning in severe acute respiratory distress syndrome. N Engl J Med 368: 2159-2168
3. La Fondation pour l'Avenir

### 000902

#### The impact of non-invasive ventilation (NIV) utilisation in severe community acquired pneumonia: a retrospective observational analysis from a tertiary-centre

S. Yadollahi[1], A. Fahmy[2], J. Finnity[2], W. Pamela[3], A. Dushianthan[3]
[1]University Hospital Southampton NHS Foundation Trust, Southampton, United Kingdom; [2]Emergency department, Derby Royal Hospital, Derby, UK, United Kingdom; [3]General intensive care unit, University Hospital Southampton NHS Foundation Trust, Southampton, United Kingdom
**Correspondence:** S. Yadollahi

*Intensive Care Medicine Experimental* 2019, **7(Suppl 3):**000902

**INTRODUCTION.** Severe community acquired pneumonia (sCAP) frequently requires admission to the intensive care unit (ICU) admission and is associated with significant morbidity and mortality1. The use of non-invasive ventilation (NIV) in this setting is controversial and may be associated with worse outcomes2. Characterisation of parameters predictive of NIV failure may be of value in risk stratification to minimise increased risk of subsequent mechanical ventilation, or death3.

**OBJECTIVES.** Clarify the role and impact of NIV in sCAP cohort and identify factors that might allow earlier detection of NIV failure.

**METHODS.** Retrospective, observational study of consecutive admissions to ICU with sCAP from January 2016 to May 2017. sCAP defined as CAP requiring critical care management. Adequate NIV trial classified as minimum of two hours. NIV cohort was sub-divided into three sub-sets for analysis; NIV as ceiling of care, NIV failure requiring invasive mechanical ventilation (IMV) and NIV success so avoiding intubation. Student t-test utilised in statistical analysis.

**RESULTS.** Total of 201 sCAP patients identified over the study period. NIV was utilised in 121 (60%) cases with immediate IMV required in 54 (27%) cases and no NIV support required in 26 (13%) cases. Successful NIV use was associated with reduced length of ICU (4 days)and hospital (14 days) stay and reduced long-term (6 month) mortality. In the NIV group, partial pressure of oxygen to fraction of inspired oxygen (Pa02/Fi02) ratio improvement in 2 hours of ICU admission was associated with increased hospital and long-term survival. NIV failure cohort had a reduced Pa02/Fi02ratio 2-hour improvement (50% versus 69% in NIV success cohort). Daily sequential organ failure assessment (SOFA) scores over initial 72 hours of admission highlighted daily improvement in the whole cohort. However, worsening SOFA scores at 24-hours were identified in the NIV failure cohort (7.84.4 versus 3.52.0, p-value <0.0001). Average time to intubation in this failed NIV cohort was 25 hours (30 hours).

**CONCLUSION.** Utilisation of NIV in sCAP patients can be successfully employed with good clinical outcomes in selective patients. Failure of NIV is associated with poor short- and long-term outcomes. Worsening Pa02/Fi02ratios within 2 hours of ICU admission and deteriorating SOFA scores are associated with NIV failure. Further large, prospective studies are required to validate clinical indicators of NIV failure.

**REFERENCE(S)**

1. Woodhead M, et al. Community-acquired pneumonia on the intensive care unit: secondary analysis of 17,869 cases in the ICNARC Case Mix Programme Database. Critical Care. 2006; 10(Suppl 2): S1.
2. Murad A, et al. The role of non-invasive positive pressure ventilation in community-acquired pneumonia. Journal of Critical Care. 2015; 30(1): 49-54.
3. Nicolini A, et al. Noninvasive ventilation for acute respiratory failure due to community- acquired pneumonia: A concise review and update. Community Acquir Infect. 2015; 2: 46-50.
4. None.

### 000912

#### Predictive model of mortality in patients with tracheotomy

A. Estrella Alonso[1], N. Arriero Fernández[1], Z. Eguileor Marín[1], MA. Tirado Fernández[1], JE. Romo Gonzales[1], G. Jimenez Puente[1], C. Marian Crespo[1], A. Albaya Moreno[1], JA. Silva Obregón[1], S. Saboya Sanchez[2]
[1]Intensive care, Hospital Universitario de Guadalajara, Calle Donante de Sangre, Guadalajara, Spain, Guadalajara, Spain; [2]Intensive care, Hospital Puerta de Hierro-Majadahonda, Majadahonda, Spain
**Correspondence:** Z. Eguileor Marín

*Intensive Care Medicine Experimental* 2019, **7(Suppl 3):**000912

**INTRODUCTION.** Tracheotomy is a usual technique in patients with invasive mechanical ventilation in intensive care units (ICU)

**OBJECTIVES.** To analyze the variables related to 30-day mortality of patients who underwent tracheotomy during ICU stay, and create a predictor model of mortality.

**METHODS.** Observational and retrospective study conducted between may-2014 and October-2018 that included all patients who underwent tracheotomy in ICU. Exclusion criteria: tracheotomy before the admission and re-admitted patients. There were collected demographic (age, gender) data, clinical (co-morbidity) data and severity scores, related to invasive mechanical ventilation (IMV) and tracheotomy, and to ICU and hospital stay.

Fragility before the hospital admission was evaluated by the Clinical Frailty Scale (CFS) (Fragile CFS ≥ 5). We describe categorical variables as frequency and percentages; and numerical variables as median and interquartile range (IR). We compared percentages using chi-square or Fisher exact test, and continuous variables with t-Student or U Mann-Whitney tests. Multivariate analysis conducted by cox regression model.

**RESULTS.** Of the 2630 patients admitted to ICU, 105 were analyzed. 30-d mortality was 32.4%. There were no significant differences comparing by sex or co-morbidities. Age, severity scores, fragility (CFS) and limitations of life support techniques (LLST) were significantly higher among dead patients. Reintubation, days with IMV, days in spontaneous ventilation (SV), % of time with tracheotomy in SV, ICU and hospital stay were significantly lower among dead patients. Dead patients underwent tracheotomy earlier (table 1). Multivariate analysis showed that age, fragility, day of tracheotomy, disconnection from IMV and % of time with tracheotomy in SV, were independent predictive factors of 30-d mortality (table 2), creating a mortality predictive normogram (Figure 1).

**CONCLUSION.** Age, severity scores and fragility were mortality associated factors in patients with tracheotomy, who also died before. Fragility, day of tracheotomy, disconnection from IMV and % of time without IMV were 30-d mortality independent predictive factors since de ICU admission. Our predictive model could be useful to decide the LLST.

**Table 1 (abstract 000912).** See text for description

| Variables | N= 105 | 30-d mortality (n=34) | 30-d survival (n=71) | OR (IC 95%) | p valour |
|---|---|---|---|---|---|
| *Age (years)* | 69,56 (61,98-77,34) | 68,5 (58,0-74,3) | 74,9 (66,4-79,4) | 1,04 (1,00-1,09) | **0,04** |
| *Sex* | | | | | |
| *Men* | 75 (71,4) | 22 (64,7) | 53 (74,6) | 0,62 (0,26-1,51) | 0,29 |
| *Women* | 30 (28,6) | 12 (35,3) | 18 (25,3) | | |
| *Co-morbidities number* | 3,00 (2,00-4,00) | 3,0 (2,0 – 4,0) | 2,0 (2,0 – 4,0) | 1,11 (0,85-1,45) | 0,44 |
| *COPD* | 18 (17,1) | 6 (17,6) | 12 (16,9) | 1,05 (0,36-3,10) | 0,92 |
| *Severe cardiomiophathy (NYHA III-IV)* | 5 (4,8) | 3 (8,8) | 2 (2,8) | 3,34 (0,53-20,99) | 0,20 |
| *Onco-hematological* | 24 (22,9) | 11 (32,4) | 13 (18,3) | 2,13 (0,84-5,45) | 0,11 |
| *CFS* | 3,00 (3,00-4,00) | 4,0 (3,0 – 5,0) | 3,0 (3,0 – 4,0) | 1,44 (1,02-2,03) | **0,04** |
| *Fragility (CFS ≥ 5)* | | | | | |
| *Fragile* | | 9 (24,5) | 8 (11,3) | 2,83 (0,98-8,17) | 0,05 |
| *No fragile* | | 25 (73,5) | 63 (88,7) | | |
| *APACHE II* | 23,00 (17,00-28,00) | 24,5 (18,5 – 30,5) | 22,00 (17,0 – 26,0) | 1,06 (1,01-1,12) | **0,03** |
| *APACHE II without age* | 19,00 (12,00-23,00) | 19,5 (14,5 – 25,5) | 18,0 (11,0 – 22,0) | 1,05 (0,99-1,11) | 0,07 |
| *Reintubation* | 28 (26,7) | 4 (11,8) | 24 (33,8) | 0,26 (0,08-0,83) | **0,02** |
| *ARDS* | 37 (35,2) | 11 (32,3 %) | 26 (36,6 %) | 0,83 (0,35-1,97) | 0,67 |
| *Days in MV* | 15 (8,62-20,10) | 12,7 (8,1 – 16,5) | 15,8 (9,0 – 24,1) | 0,94 (0,89-0,99) | **0,02** |
| *Day of tracheotomy* | 10,00 (7,00-13,00) | 8,5 (5,0 – 11,1) | 11,0 (8,0 - 14,0) | 0,89 (0,81-0,98) | **0,02** |
| *% t with tracheotomy* | 50,87 (36,11-65,64) | 49,1 (33,9 – 61,9) | 51,0 (38,2 – 66,9) | 0,99 (0,97-1,01) | 0,43 |
| *t. disconnected from IMV (days)* | 4,25 (1,29-7,35) | 0 (0 - 3,6) | 5,2 (2,8 – 8,5) | 0,65 (0,53-0,79) | **<0,01** |
| *% t. in spontaneous ventilation* | 44,20 (9,14-67,92) | 0 (0 – 43,8) | 60,0 (29,0 – 76,1) | 0,96 (0,95-0,98) | **<0,01** |
| *ICU deconulattion* | 43 (41) | 0 | 43 (60,6 %) | | 0,99 |
| *ICU stay (days)* | 21,83 (15,02- 32,37) | 15,0 (10,6 – 19,7) | 25,0 (18,1 – 33,9) | 0,88 (0,83-0,93) | **<0,01** |
| *Hospital stay (days)* | 37,67 (23,52-56,79) | 20,5 (14,0 – 28,7) | 44,2 (36,3 – 76,4) | 0,92 (0,89-0,96) | **<0,01** |
| *LLST* | 35 (33,3) | 22 (64,7) | 12 (16,9 ) | 8,18 (3,24-20,64) | **<0,05** |

Table1. Qualitative variables as n (%); Quantitative variables as: Mean ± SD * or asMedian (IR) *
*Abbreviation: OR, Odds Ratio; CI, Confidence interval; NYHA, New York Heart Association; COPD, chronic obstructive pulmonary disease; APACHE, Acute Physiology and Chronic Health Evaluation; IMV, invasive mechanical ventilation; ICU, Intensive Care Unit; t, Time (days)*

**Table 2 (abstract 000912).** See text for description

| Variables | UNIVARIATE HR (IC 95%) | p valour | MULTIVARIATE HR (IC 95%) | p valour |
|---|---|---|---|---|
| Age | 1,041 (1,004 – 1,079) | 0,028 | 1,036 (1,005 – 1,067) | 0,023 |
| Sex | 0,589 (0,291 – 1,191) | 0,141 | - | - |
| Fragility (CFS ≥ 5) | 2,304 (1,075 – 4,938) | 0,032 | 3,802 (1,585 – 9,122) | 0,003 |
| COPD | 0,949 (0,393 – 2,293) | 0,907 | - | - |
| Cardiomiophaty (NYHA III-IV) | 0,404 (0,123 – 1,323) | 0,134 | - | - |
| Onco/haematological | 0,599 (0,292 -1,229) | 0,162 | - | - |
| Co-morbidity number | 1,066 (0867 – 1,311) | 0,545 | - | - |
| Apache II (Age) | 1,037 (0,994 – 1,082 | 0,096 | - | - |
| Re-intubation | 3,240 (1,141 – 9,201) | 0,027 | - | - |
| % *t* in IMV | 1,056 (1,033 – 1,079) | < 0,001 | 1,033 (1,004 – 1,062) | 0,025 |
| Day of tracheotomy | 0,889 (0,823 – 0,960) | 0,003 | 0,915 (0,849 – 0,986) | 0,020 |
| Disconnection | 14,495 (7,089 – 29,641) | < 0,001 | 0,164 (0,049 – 0,546) | 0,003 |
| ICU decanulattion | 69,370 (4,339 – 1109,40) | 0,003 | - | - |

*Table 2. Predictive variables of mortality. Multivariate analysis by Cox regression.*
*Abbreviation: OR, Odds Ratio; CI, Confidence Interval; NYHA, New York Heart Association; COPD, chronic obstructive pulmonary disease ; APACHE, Acute Physiology and Chronic Health Evaluation; IMV, invasive mechanical ventilation ; ICU, Intensive Care Unit; t, Time (days)*

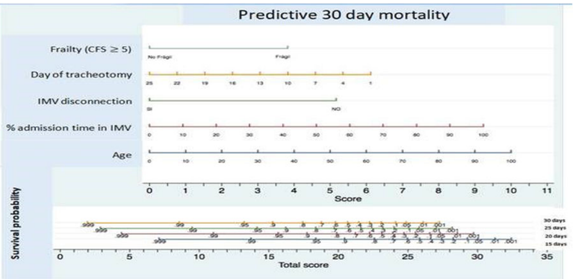

**Fig. 1 (abstract 000912).** See text for description

### 000918

#### Standardizing lung protective mechanical ventilation on ECMO: Automatic, closed loop ventilation in patients with ARDS or cardiogenic shock

U. Wenger[1], MP. Hilty[2], PD. Wendel Garcia[2], H. Nater[2], D. Stark[2], M. Maggiorini[3]
[1]Intensive care unit, University Hospital of Basel, Basel, Switzerland; [2]Institute of intensive care medicine, University Hospital of Zurich, Zürich, Switzerland, Switzerland; [3]Institute of intensive care medicine, University Hospital of Zurich, Zürich, Switzerland
*Intensive Care Medicine Experimental* 2019, **7(Suppl 3):**000918

**INTRODUCTION.** Extracorporeal membrane oxygenation (ECMO) has increasingly found its place in the treatment of severe acute respiratory failure, as well as severe circulatory collapse. Mechanical ventilation and ECMO are both technologies interacting on gas exchange. Nevertheless, besides a consensus paper [1], no evidence-based guidelines regarding protective lung ventilation on ECMO exist to date. Mechanical Ventilation with Intellivent-ASV, an algorithm driven, closed loop system, provides an opportunity to standardize ventilation on ECMO.

**OBJECTIVES.** To propose and validate lung protective ventilation with a closed loop ventilation mode in patients with ECMO due to ARDS or cardiogenic shock.

**METHODS.** We retrospectively analyzed mechanically ventilated patients on ECMO for cardiogenic shock or ARDS admitted to the medical ICU of the University Hospital Zurich from March 2016 on until May 2018. All patients were initially ventilated with the dual positive airway pressure (DuoPAP) mode and were then switched to Intellivent-ASV+®. Every eight hours ECMO settings, ventilation parameters and blood gas analyses were collected. Driving pressure was calculated as the difference between peak pressure and PEEP.

**RESULTS.** 62 patients were included into the study, of which 43 had a cardiogenic shock and 19 had an ARDS, the ICU mortality for both groups was 27.4%, population characteristics were homogeneous. Patients of both groups were on average on ECMO for 11.6 days and ventilated for 9.9 days on Intellivent-ASV+®. After switch to Intellivent ASV, during the first 72 hours, at constant blood flow on ECMO, ASV-Intellivent mode decreased mean FiO2 by 25% while keeping PEEP constant. Regarding ventilation, keeping airflow on ECMO constant over time, Intellivent-ASV+® led to a significant decrease in mean peak (7%) and driving pressure (14%) ($p < 0.0001$), while tidal volumes stayed below 6 in both groups and pCO2 remained constant. On steady state after switch to Intellivent-ASV+® the driving pressure was 13.12±0.67 kPa, despite significant differences in lung compliance 37.28±3.75 ml/mbar (cardiogenic shock) versus 28.46±5.26 ml/mbar (ARDS).

**CONCLUSION.** In this retrospective analysis we, for the first time, show that the automated mechanical ventilation algorithm Intellivent-ASV+® can safely ventilate patients with either ARDS or Cardiogenic Shock on ECMO.

### 000925

#### The use of ROTEM to guide anticoagulation during extracorporeal life support: a prospective observational study

C. Fornasari, M. Giani, V. Russotto, M. Pozzi, P. Mastropasqua, L. Avalli, R. Rona, G. Foti

Department of emergency and intensive care, ASST Monza, University of Milano Bicocca, Monza, Italy

**Correspondence:** C. Fornasari

*Intensive Care Medicine Experimental* 2019, **7(Suppl 3):**000925

**INTRODUCTION.** Optimal anticoagulation monitoring in patients with extracorporeal membrane oxygenation (ECMO) is controversial, but fundamental to avoid haemorrhagic and thromboembolic complications. In addition to conventional coagulation tests (ACT, aPTT), there is growing interest in the use of viscoelastic haemostatic assays (VHA) for management of anticoagulation, in particular of tromboelastography (TEG) [1,2]. However, evidence on the use of rotational tromboelastometry (ROTEM) for this purpose is lacking.

**OBJECTIVES.** The aim of our study is to evaluate ROTEM compared to TEG and conventional coagulation assays to monitor anticoagulation with unfractionated heparin (UFH) during ECMO.

**METHODS.** We conducted a prospective, observational, monocentric study. We included patients admitted to intensive care unit at ASST Monza in ECMO support for either respiratory (Veno-Venous) or cardiac failure (Veno-Arterial), anticoagulated with UFH. Conventional coagulation tests and viscoelastic assays were performed daily, as per clinical practice. We focused our statistical analysis on reaction time parameter (R, min) for Kaolin TEG, and clotting time (CT, sec) INTEM for ROTEM. Test results were correlated with UFH infusion rate (UI/die) and conventional coagulation test results at the time of sampling.

**RESULTS.** We included twelve patients in ECMO support; 41 data points were available for the analysis. CT INTEM parameter showed a moderate correlation with both UFH dosing and standard coagulation tests (see Figure 1), while correlation coefficients for R-TEG K with other variables were poor.

**CONCLUSION.** CT INTEM parameter shows moderate correlation with UFH infusion rate and other coagulation tests. Further studies are needed to identify an appropriate anticoagulation target for ROTEM during ECMO support.

**REFERENCE(S)**

1. Extracorporeal Life Support Organization, "ELSO Anticoagulation Guideline." 2014.
2. Panigada M et Al. Thromboelastography-based anticoagulation management during extracorporeal membrane oxygenation: a safety and feasibility pilot study. Ann Intensive Care. 2018 Jan 16;8(1):7 .

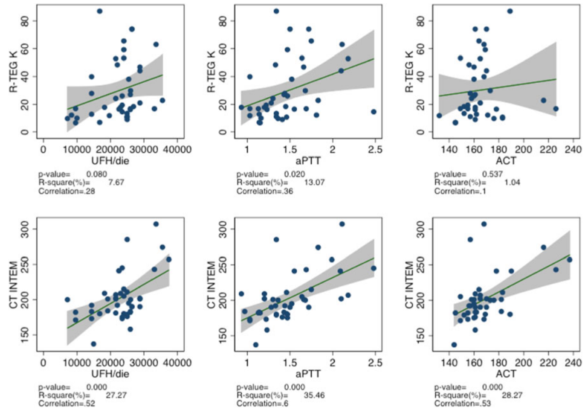

**Fig. 1 (abstract 000925).** Correlation between CT INTEM / R-TEG K and UFH infusion, aPTT and ACT

### 000929

#### Risk factors for adverse events of muscle paralysis in patients under mechanical ventilation

S. Kim, S. Park, S. Kim, B. Shin, JH. Lee, MK. Lee, SH. Kim, WY. Lee, SJ. Yong, SJ. Lee

Internal medicine, Yonsei University Wonju College of Medicine, Wonju, Republic of Korea

**Correspondence:** S.J. Lee

*Intensive Care Medicine Experimental* 2019, **7(Suppl 3):**000929

**INTRODUCTION.** Muscle paralysis during mechanical ventilation (MV) treatment decreases the lung injury through better synchrony and improves survival ultimately. However, some patients have a progress toward development of severe respiratory acidosis and sometimes encounter hemodynamically instability. Contrary to a well-known knowledge of intensive care unit (ICU)-acquired weakness induced by prolonged use of neuromuscular blocking agents (NMBA), respiratory acidosis immediately after infusion of NMBA has not been reported.

**OBJECTIVES.** We investigated to reveal the risk factors for post-NMBA respiratory acidosis in patients under mechanical ventilation who started cisatracurium just before the event.

**METHODS.** Following a retrospective review of the medical records of patients admitted to the ICU of our medical center from January to October 2017, we included adults who received cisatracurium during MV. Data of vital signs, arterial blood gas analysis (ABGA), and parameters of the ventilator before and after NMBA infusion were recruited. Respiratory acidosis developed after cisatracurium infusion was defined discretionally as decreased arterial pH more than 0.2.

**RESULTS.** The mean age of the patients was 64 years and 67% (n = 172) was male. Of total 256 Patients, 24 patients (9.4%) showed a progression of acidosis. Among them, seven patients' blood pressure dropped immediately after NMBA infusion and vasopressor was needed. In univariate analysis, a higher pH, lower arterial partial pressure of carbon dioxide ($PaCO_2$), and higher pulse rate (PR) measured before NMBA infusion were associated with progression of respiratory acidosis. Emphysema was more common in patients with respiratory acidosis. On the other hand, disease severity presented by APACHE II score and pre-NMBA tidal volume based on patients' efforts are not related with a progression of respiratory acidosis. In multiple logistic regression model including variables of age, sex, emphysema, pre-NMBA pH, pre-NMBA $PaCO_2$, and pre-NMBA PR, emphysema (OR, 4.11; 95% CI 1.21–13.96), higher pH (OR, 3.10 per 0.1; 95% CI 1.67–

5.75), lower PaCO2 (OR, 0.90; 95% CI 0.83–0.98) and higher PR (OR, 1.03; 95% CI 1.01–1.05) are associated with progression of respiratory acidosis after NMBA infusion.

**CONCLUSION.** Emphysema, higher pH, lower PaCO2 and higher PR are risk factors for post-NMBA respiratory acidosis. It suggested patients with respiratory failure who showed active cardiopulmonary compensation especially in those with emphysema might have a risk for a progression of respiratory acidosis after muscle paralysis.

### 000931

#### Residual alveolar overdistension and collapse at electrical impedance tomography-guided positive end-expiratory pressure in patients with and without ARDS

T. Becher[1], M. Dargvainis,[1], D. Hassel,[1], N. Weiler,[1], I. Alkatout,[2], H. Ohnesorge,[1], I. Frerichs,[1]

[1]Klinik für anästhesiologie und operative intensivmedizin, Universitätsklinikum Schleswig-Holstein, Campus Kiel, Kiel, Germany; [2]Klinik für gynäkologie und geburtshilfe, Universitätsklinikum Schleswig-Holstein, Campus Kiel, Kiel, Germany

**Correspondence:** T. Becher

*Intensive Care Medicine Experimental* 2019, **7(Suppl 3):**000931

**INTRODUCTION.** Electrical impedance tomography (EIT) can assess alveolar overdistension and collapse during a decremental positive end-expiratory pressure (PEEP) trial in mechanically ventilated patients [1]. Using this approach, it is possible to identify the “best compromise” PEEP at which overdistension and collapse are minimized as shown in patients with acute respiratory distress syndrome (ARDS) [2] and in patients during general surgery [3]. However, even with the “best compromise” PEEP, a variable amount of simultaneous overdistension and collapse remains. We hypothesized that the sum of overdistension and collapse detected by EIT at the “best compromise” PEEP is higher in patients with ARDS than in lung-healthy control patients during general surgery and might be a marker of disease severity.

**OBJECTIVES.** To investigate whether the sum of residual overdistension and collapse at the “best compromise” PEEP is higher in patients with ARDS than in patients without pulmonary pathology.

**METHODS.** We compared EIT data sets of 12 patients with ARDS (5 female, age: 67±11 years, height: 174±9 cm, weight: 79±22 kg, PaO2/FiO2 at baseline: 151±38 mmHg) to 8 patients without pulmonary pathology (all female, age: 38±19 years, height: 169±5 cm, weight: 77±21 kg) undergoing general anesthesia for laparoscopic gynecological surgery. Decremental PEEP trials were carried out in all patients during pressure-controlled ventilation with constant driving pressure. In control patients, one PEEP trial was performed after induction of general anesthesia in the supine position and another one during capnoperitoneum in the Trendelenburg position. All PEEP trials were analyzed as described in [1] and “best compromise” PEEP was defined as the value associated with equally balanced overdistension and alveolar collapse. Residual overdistension and collapse was calculated as the sum of overdistension and collapse at the “best compromise PEEP”. Data sets were compared by one-way analysis of variance with Bonferroni post test. Written informed consent was obtained from control patients and from the legal representatives of ARDS patients. Numerical values are presented as mean±SD.

**RESULTS.** In ARDS patients, we identified 14.5±7.1% of residual overdistension and collapse at the “best compromise” PEEP of 13.3±2.4 mbar. In control patients, the sum of residual overdistension and collapse was 6.3±3.8% at the “best compromise” PEEP of 7.3±2.4 mbar in supine position and 3.4±3.5% at 18.0±2.1 mbar in the Trendelenburg position with capnoperitoneum. The difference between ARDS and lung-healthy patients was statistically significant both for supine (p<0.01) and Trendelenburg positions (p<0.001).

**CONCLUSION.** Our preliminary results imply that the sum of residual alveolar overdistension and collapse at the “best compromise” PEEP level is higher in patients with ARDS than in control patients both during ventilation in the supine and in the Trendelenburg position with capnoperitoneum. This supports the hypothesis that this measure might serve as a marker of disease severity in mechanically ventilated patients.

**REFERENCE(S)**

[1] Costa et al., Intensive Care Med (2009) DOI: 10.1007/s00134-009-1447-y

[2] Heines et al., J Clin Monit Comput (2018) DOI: 10.1007/s10877-018-0164-x

[3] Pereira et al., Anesthesiology (2019) DOI: 10.1097/ALN.0000000000002435

### 000933

#### Development and characterisation of novel animal models of Acute Respiratory Distress Syndrome (ARDS) endotypes

K. Wildi[1], J. Millar[2], N. Bartnikowski[1], S. Pedersen[1], N. Obonyo[1], G. Li Bassi[1], S. Colombo[1], S. Rozencwajg[1], M. Bouquet[1], D. McAuley[2], A. Pesenti[3], J. Suen[1], L. Pugh[4], J. Fraser[1]

[1]CCRG, Clinical Sciences, The Prince Charles Hospital, Chermside, Australia; [2]Institute of experimental medicine, Queen's University Belfast, Belfast, United Kingdom; [3]Policlinico, Policlinico of Milan, Milano, Italy; [4]Medicine, St Andrew's War Memorial Hospital, Spring Hill, Australia

**Correspondence:** K. Wildi

*Intensive Care Medicine Experimental* 2019, **7(Suppl 3):**000933

**INTRODUCTION.** Acute Respiratory Distress Syndrome (ARDS) still remains a serious pulmonary condition with substantial mortality and without specific therapeutic options. Retrospective analysis of randomized controlled trials have identified the presence of two distinct phenotypes, based on clinical and biological variables (1–3), namely a hypo-inflammatory (P1) and a hyper-inflammatory (P2) endotype. The development of clinically relevant models to replicate these endotypes, will likely enable better understanding of these endotypes.

**OBJECTIVES.** We aimed to develop an ovine model of P1 and P2 ARDS endotypes and to characterize clinical and biological features, opening the pathway for further prospective evaluation of endotype-specific treatment options.

**METHODS.** We studied 20 anesthetized sheep on mechanical ventilation up to 6 hours, and randomized into three distinct groups: 1) the oleic acid (OA) only group received an intravenous infusion of OA to achieve a PaO2/FiO2 ratio <100 mmHg (n=8); 2) the OA-IV-LPS group received an OA infusion and intravenous infusion of lipopolysaccharide (n=5) and; 3) the OA-IP-LPS group received an OA infusion and intrapulmonary infusion of LPS (n=7). Pulmonary, hemodynamic and laboratory parameters were assessed hourly. In addition, interleukin (IL) -6, -8 and -10 were quantified in serum and bronchoalveolar lavage (BAL).

**RESULTS.** Severe impairment in PaO2/FiO2 ratio was found in all study groups (Figure 1). We found a) more hemodynamic and pulmonary impairment b) decreased albumin and platelets levels and c) upregulation of inflammatory (IL-6 and IL-8) and anti-inflammatory cytokines (IL-10) in serum and BAL only in OA-IV-LPS in comparison with the other two groups.

Therefore, the OA-IV-LPS model may mimic a pattern closest to the human P2 endotype and the OA only model the P1 endotype.

**CONCLUSION.** We developed innovative models of ARDS that mimic clinical and inflammatory status of human P1-P2 endotypes, while characterized by similar severity of pulmonary dysfunction. These animal models could be applied to explore in detail mechanical and biological differences between ARDS subphenotypes and identify endotype-specific treatment options.

**REFERENCE(S)**

1. Delucchi K, Famous KR, Ware LB, Parsons PE, Taylor Thompson B, Calfee CS. Stability of ARDS subphenotypes over time in two randomised controlled trials. Thorax. 2018;73:439-445.
2. Famous KR, Delucchi K, Ware LB, et al. Acute respiratory distress syndrome subphenotypes respond differently to randomized fluid management strategy. American Journal of Respiratory and Critical Care Medicine. 2017;195(3):331-338.
3. Calfee CS, Delucchi K, Parsons PE, Taylor B, Ware LB, Matthay MA. Latent Class Analysis of ARDS Subphenotypes: Analysis of Data From Two Randomized Controlled Trials. Lancet Respir Med. 2014;2(8):611-620.
4. The Prince Charles Hospital Foundation

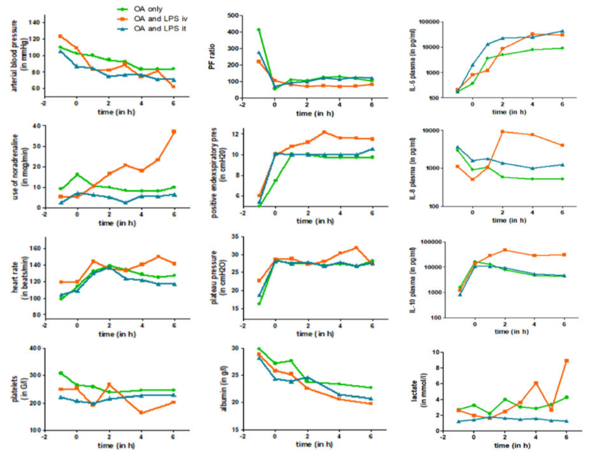

**Fig. 1 (abstract 000933).** See text for description

### 000946

#### Early factors associated with increased risk for intubation in spontaneously breathing patients with sepsis and septic shock

I. Marongiu[1], T. Mauri[1], E. Carlesso[1], A. Rundo[2], A. Luzi[2], F. De Sanctis[2], S. Spadaro[3], A. Bellonzi[3], S. Bertacchini[3], A. Sicignano[1], M. Savioli[1], G. Grasselli[1], E. Spinelli[1], A. Pesenti[1]
[1]Department of anesthesiology, Fondazione IRCCS Ca'Granda Ospedale Maggiore Policlinico, Milano, Italy; [2]Department of anesthesiology, Ospedale di Belcolle, Viterbo, Italy; [3]Department of anesthesiology, Ospedale Sant'Anna, Ferrara, Italy
**Correspondence:** I. Marongiu
*Intensive Care Medicine Experimental* 2019, **7(Suppl 3):**000946

**INTRODUCTION.** Systemic inflammation and metabolic acidosis, which characterize patients with sepsis and septic shock, can increase the spontaneous respiratory drive and effort and the risk of patient self-inflicted lung injury, with potentially detrimental effects on outcome[1]. A retrospective study reported that septic shock patients intubated during their intensive care unit (ICU) stay had fewer 28-day alive without organ support, as compared to patients already intubated upon admission[2].

**OBJECTIVES.** To compare ICU mortality in a large cohort of patients with sepsis/septic shock classified as: already intubated upon admission (early intubation) vs. intubated during ICU stay (late intubation) vs. never intubated (no intubation). To identify early factors associated with risk for late intubation.

**METHODS.** We included 1425 patients with sepsis (824) and septic shock (601), consecutively admitted to three tertiary level general ICUs in Italy between 2014-2018. Patients data and outcomes were prospectively collected in a centralized clinical registry (PROSAFE Giviti electronic case report form). Categorical variables are expressed as counts (%) and compared with Chi-square or Fisher's exact test. Median (IQR) was used for continuous variables and differences in distributions were assessed by ANOVA on ranks with Dunn's post-hoc test.

**RESULTS.** Patients with late intubation were 339 (24%), early intubation 878 (61%), no intubation 208 (15%). Patients in the late intubation group had significantly higher hospital mortality than patients in the other two groups (47% late vs. 31% early vs. 10% no, p<0.0001). Table 1 shows differences between groups at ICU admission: patients with late intubation presented more organ failures, lower GCS, higher SOFA and SAPS II scores in comparison to no intubation

In patients with late intubation, upon admission, we also observed significantly lower oxygenation, more severe acute kidney injury, higher bilirubinemia, lower systolic pressure, higher imbalance of acid-base status vs. no intubation (p<0.01 for all). Interestingly, early clinical and physiologic derangements were similar between late vs. early intubation groups.

**CONCLUSION.** Mortality of ICU patients with sepsis and septic shock is significantly higher when intubation occurs after ICU admission. Comparable baseline severity with patients already intubated at admission might suggest a specific role for injurious spontaneous breathing. Early identification of patients at risk for late intubation might prompt prophylactic interventions.

**REFERENCE(S)**

[1] L. Brochard, A. Slutsky, and A. Pesenti, "Mechanical Ventilation to Minimize Progression of Lung Injury in Acute Respiratory Failure," Am. J. Respir. Crit. Care Med., vol. 195, no. 4, pp. 438–442, Feb. 2017

[2] A. Delbove, C. Darreau, J. F. Hamel, P. Asfar, and N. Lerolle, "Impact of endotracheal intubation on septic shock outcome: A post hoc analysis of the SEPSISPAM trial," J. Crit. Care, vol. 30, no. 6, pp. 1174–1178, Dec. 2015.

**Table 1 (abstract 000946).** See text for description

| | Sepsis/septic shock at the admission | No intubation | Early intubation | Late intubation | P value |
|---|---|---|---|---|---|
| Organ failure at admission, N (%) | | | | | |
| 0 | 53 (3%) | 29 (13%) | 19 (2%) | 5 (1%) | <0.0001 |
| 1 | 235 (16%) | 77 (37%) | 110 (12%) | 48 (14%) | |
| 2 | 314 (22%) | 51 (24%) | 199 (22%) | 64 (18%) | |
| >2 | 823 (57%) | 51 (24%) | 550 (62%) | 222 (65%) | |
| GCS, median [IQR] | 15 [10-15] | 15 [15-15] | 14 [9-15]* | 14 [10-15]* | <0.0001 |
| SAPS II, median [IQR] | 48 [36-63] | 35 [25-47] | 50 [37-65]* | 52,5 [40-67]* | <0.0001 |
| SOFA, median [IQR] | 8 [5-12] | 6 [3-8] | 9 [6-12]* | 10 [6-13]* | <0.0001 |

*p<0,05 vs No intubation group

### 000952

#### Bronchoscopy bronchoalveolar lavage in mechanically ventilated patients in the prone position with Acute Respiratory Distress Syndrome

A. Estella[1], M. Gracia Romero,[1], M. Perez Ruiz[1], L. Fernadez Ruiz[1], B. Diez Del Corral[1], S. Moreno Cano[1], B. Gimenez Beltrán[1], M. Recuerda Nuñez,[2]
[1]University hospital of jerez., Intensive care unit, Jerez, Spain; [2]Medicina intensiva, University Hospital of Puerto Real, Puerto Real, Spain
**Correspondence:** A. Estella
*Intensive Care Medicine Experimental* 2019, **7(Suppl 3):**000952

**INTRODUCTION.** Mechanical ventilation in prone position has demonstrated to decrease mortality in patients with severe Acute Respiratory Distress Syndrome (ARDS). It is scarcely documented in the literature clinical experience about use of fiberoptic bronchoscopy (FOB) in prone position , only 9 patients, (1,2). Bronchoalveolar lavage in these conditions are exceptional.

**OBJECTIVES.** The aim of the present study are: to describe the clinical experience in the realization of FOB in critically ill patients ventilated in prone position and to analyze the complications related to the procedure.

**METHODS.** Retrospective study performed in an medical-surgical ICU. Time of study was five years. Inclusion criteria were patients with ARDS and indication of FOB. Informed consent was requested for the performance of the procedure. The following variables were collected: Demographic data, heart rate and arterial pressure during the FOB, ICU lenght of stay, level of positive end-expiratory pressure (PEEP) and categories of ARDS according Berlin classification criteria, complications and mortality. The statistical analysis was performed using SPSS program, the quantitative variables are shown in mean ± standard deviation.

**RESULTS.**

11 FOB were performed during the time of study. Most of patients (81.8%) were indicated for microbiological study performing bronchoalveolar lavage. In one case it was indicated due to complete atelectasis and another case due to pulmonary hemorrhage. The procedure was performed with control pressure ventilator mode with FiO2 of 100% without modifying the previous PEEP. Table 1 shows clinical characteristics. Mean PaO2/FiO2 was 185,1 ± 52,05, resulting between 200 and 300 in 5 patients, between 100 and 200 in 5 cases and < of 100 in one. In most patients (72.7%) no complications related to FOB were observed. 18.2% developed self-limiting supraventricular tachycardia without hemodynamic impact nor needing to increase vasoactives and we observed in 9% transient hypoxemia that was corrected by removing the FOB.

**CONCLUSION.** In our experience the realization of FOB in patients with ARDS ventilated in prone position is a safe procedure and does not need to decrease or to withdraw the previous PEEP.

**Table 1 (abstract 000952).** Clinical Characteristics

| | |
|---|---|
| Age (years) | 61,5±14,7 |
| APACHE II | 18,9±3,8 |
| Vasoactive drug | 54,5% |
| ICU lenght of stay (days) | 15±8 |
| PaO2/FiO2 | 185,1±52 |
| PEEP | 9.7 ± 2.6 |
| Mortality | 54,5% |

### 000958

#### Decline in skeletal muscle mass during veno-venous extracorporeal membrane oxygenation

S. Patel[1], F. Liew,[1], H. Soliman[1], S. Nawab[2], T. Semple[3], J. Hine[3], S. Desai[3], ZA. Puthucheary[4], B. Patel,[1], S. Singh[1]

[1]Adult intensive care unit, Royal Brompton Hospital, London, United Kingdom; [2]School of medicine, Imperial College London, London, United Kingdom; [3]Department of radiology and imaging, Royal Brompton Hospital, London, United Kingdom; [4]Adult critical care unit, Royal London Hospital, London, United Kingdom

**Correspondence:** S. Patel

*Intensive Care Medicine Experimental* 2019, **7(Suppl 3):**000958

**INTRODUCTION.** Intensive care acquired skeletal muscle atrophy (SkMA) during acute respiratory distress syndrome (ARDS) is associated with long-term functional restriction **(1)**. Veno-venous extracorporeal membrane oxygenation (vvECMO) represents the management of extreme cases of ARDS **(2,3)** but the degree of SkMA and the relationship between lung parenchyma recovery in this cohort has not been described.

**OBJECTIVES.** In patients rapidly deteriorating within 24-hours due to ARDS, we will: (i) describe the degree of SkMA and (ii) evaluate relationships between lung parenchyma change and SkMA.

**METHODS.** Patients with ARDS who presented to hospital, received mechanical ventilation (MV) and vvECMO and underwent thoracic and abdominal computed tomography (CT) imaging within 24-hours were included to ensure an initial measurement of muscle mass as close to pre-morbid baseline . A second CT (at variable intervals) whilst receiving vvECMO was also required. 18 patients met the criteria. Images were assessed for proportions of normal lung, ground-glass opacity (TLGG) and lung consolidation (TLCo) with the sum of the scores giving a total lung parenchyma score (TLS) as previously described **(4)**. Skeletal muscle cross-sectional area (SkMCSA) was calculated at the level of the third lumbar vertebrae **(5,6)** and sarcopenia was determined by the muscle index (=SkMCSA/height2; <55.4cm2/m2 for males, < 38.9cm2/m2 for females). Raters were blinded to all identifiable data and high observer reliability was ensured (intra-class correlation coefficients of >0.9).

**RESULTS.** The interval between images ranged from 3 to 25 days. The mean decline in SkMCSA was 14.7±11.1% (p<0.001) - equating to a mean daily decline of 2.2±2.4%. All who were sarcopenic at admission (9/18, 50%) were male. Males also had a mean decline in MI of 9.2±6.4% (p<0.001). SkMA (10% over 7 days, or 1.4%/day **(7)**) was more common in men (58% vs. 33%) but not statistically significant. SkMA was associated with lower TV (r=-0.711, p=0.007), higher BMI (r=-0.603, p<0.01) and admission lactate (r=0.535, p=0.02). Patients who were sarcopenic at admission had a longer ICU length of stay (LoS) and greater duration of mechanical ventilation and vvECMO (Table 1). The median change per day in TLCo and TLS was -1.65 points/day (-0.43 to -3.30) and -0.75 points/day (-1.33 to -2.40) respectively – favouring resolution. The median change in TLGG was +0.45 points/day (0.00 to 1.73) indicating increased GG. There was no significant association between SkMCSA decline and parenchymal changes per day, nor between the absolute/daily change of TLCO, TLGG and TLS and ventilator/vvECMO parameters or ICU length of stay.

**CONCLUSION.** Underlying sarcopenia was significant in those who required vvECMO for severe ARDS within 24-hours of presentation. This led to significant SkMA, with an average decline of 14% over an interval of 3-25 days. Those with sarcopenia had considerably longer ICU LoS and MV and vvECMO duration. The lung parenchyma score needs further validation over longer time periods to understand its utility and its relationship to muscle atrophy and functional restriction in the post-critical illness period.

**REFERENCE(S)**

1. Herridge, M. S. et al. Functional Disability 5 Years after Acute Respiratory Distress Syndrome. N. Engl. J. Med. 364, 1293–1304 (2011).
2. Combes, A. et al. Extracorporeal Membrane Oxygenation for Severe Acute Respiratory Distress Syndrome. N. Engl. J. Med. 378, 1965–1975 (2018).
3. Peek, G. J. et al. Efficacy and economic assessment of conventional ventilatory support versus extracorporeal membrane oxygenation for severe adult respiratory failure (CESAR): a multicentre randomised controlled trial. Lancet 374, 1351–1363 (2009).
4. Lazoura, O. et al. Acute respiratory distress syndrome related to influenza A H1N1 infection: Correlation of pulmonary computed tomography findings to extracorporeal membrane oxygenation treatment and clinical outcome. J. Crit. Care 27, 602–608 (2012).
5. Gomez-Perez, S. L. et al. Measuring Abdominal Circumference and Skeletal Muscle From a Single Cross-Sectional Computed Tomography Image: A Step-by-Step Guide for Clinicians Using National Institutes of Health ImageJ. JPEN. J. Parenter. Enteral Nutr. 40, 308–18 (2016).
6. Shen, W. et al. Total body skeletal muscle and adipose tissue volumes: estimation from a single abdominal cross-sectional image. J. Appl. Physiol. 97, 2333–2338 (2004).
7. Puthucheary, Z. A. et al. Acute Skeletal Muscle Wasting in Critical Illness. JAMA 310, 1591 (2013).
8. Moisey, L. L. et al. Skeletal muscle predicts ventilator-free days, ICU-free days, and mortality in elderly ICU patients. Crit. Care 17, R206 (2013).

1. None to declare.

**Table 1 (abstract 000958).** See text for description

| Admission detail | | | |
|---|---|---|---|
| Male – no. (%) | 12 (67%) | | |
| Age – years | 44±14 | | |
| BMI – kg/m² | 32.1±10.1 | | |
| Infectious Cause of ARDS – no. (%) | 10 (56%) | | |
| Extra-pulmonary ARDS – no. (%) | 3 (17%) | | |
| Lactate (mmol/L) | 3.27±3.41 | | |
| Muscle index (cm²/m²)** | Male: 52.4±8.4<br>Female: 45.3±8.7 | | |
| **Radiological calculations** | **CT Scan 1** | **CT Scan 2** | **p value** |
| $SkM_{CSA}$ – cm² | 151.0±28.7 | 128.0±25.2 | <0.001 |
| Muscle Index (cm²/m²) | Male: 52.4±8.4<br>Female: 45.3±7.8 | Male: 43.2±7.2<br>Female: 40.8±9.4 | <0.001<br>0.07 |
| Sarcopenia – no. (%) | Male: 9/12 (75%)<br>Female: 0/6 (0%) | Male: 9/12 (75%)<br>Female: 2/6 (33%) | |
| TLCo – points* | 31.0 (19.2-41.9) | 10.7 (6.6-27.9) | <0.01 |
| TLGG – points* | 4.5 (1.4-18.3) | 13.2 (2.5-28.7) | <0.01 |
| TLS – points* | 38.9 (27.3-60.9) | 29.0 (14.6-62.8) | 0.16 |
| **Presence of sarcopenia at admission** | **Yes** | **No** | **p value** |
| Mechanical ventilation days – no.* | 35 (17-44) | 21 (8-26) | 0.07 |
| ICU length of stay (days) – no.* | 35 (17-44) | 19 (9-26) | 0.08 |
| Total ECMO days – no.* | 22 (8-29) | 7 (6-18) | 0.31 |

### 000972

#### A physiological systematic review and meta-analysis on positive end expiratory pressure-induced lung recruitment in patients with acute respiratory distress syndrome

E. Turbil,[1], LM. Galerneau[2], A. Koutsoukou,[3], JJ. Rouby,[4], J. Dellamonica,[5], C. Schwebel[2], N. Terzi,[2], C. Guérin[6]
[1]Anesthesiology and intensive care, Università degli Studi di Sassari, Sassari, Italy; [2]Médecine intensive réanimation, C.H.U de Grenoble, La Tronche, France; [3]Respiratory medicine, National and Kapodistrian University of Athens, Athens, Greece; [4]Surgical icu, University Hospitals Pitié Salpêtrière - Charles Foix, Paris, France; [5]Intensive care unit, CHU de Nice, Nice, France; [6]Service de réanimation médicale, hôpital de la croix rousse, Grande Rue de la Croix Rousse, Lyon, France
**Correspondence:** T. Emanuele
*Intensive Care Medicine Experimental* 2019, **7(Suppl 3):**000972

**INTRODUCTION.** Recruited lung volume (Vrec) elicited by positive end expiratory pressure (PEEP) can be measured by using either CT scan or pressure-volume (P-V) curve method.

**OBJECTIVES.** The purpose of this study was to perform a systematic review and meta-analysis of Vrec measured by the P-V curve in patients suffered from Acute Respiratory Distress Syndrome (ARDS). Primary aim was prevalence of recruiters (R). The secondary aims were to compare physiologic data and ICU outcome in R and non-recruiters (NR).

**METHODS.** We conducted a search on PubMed, including all papers published from inception to 30/11/2018 containing the key words: lung recruitment, alveolar recruitment, volume pressure curve, ARDS, PEEP, humans and adult. Two reviewers independently screened all articles. Articles concerning animals, children, case reports and reviews were excluded. We also excluded studies that didn't measure Vrec by PV curve or in which Vrec was not elicited by PEEP. Disagreements were resolved by discussion. Subsequently, same both reviewers extracted data into a pre-defined case record form. The data of Vrec was obtained directly from the studies or from reported graphs using the GetData Gaph Digitizer software to retrieve values or by contacting directly the authors. Extracted data also included the baseline value of anthropometric variable, ARDS cause, physiologic characteristic of patients, ventilator settings, respiratory mechanics. Patient outcome at ICU discharge was also recorded. We used the threshold of Vrec > 150 ml to define R patients. Data are expressed as mean±SD. The meta-analysis was performed using R (meta package). For the continuous variables we used the mean difference and for the binary variable the relative risk (RR) with their confidence intervals (C.I.) between R and NR. A random effects model was applied for pooling the data.

**RESULTS.** From a total of 650 studies, 26 were potentially eligible. After full-text evaluation, 16 articles were kept for the present study and included 316 patients. Vrec was measured between PEEP 5 and 15 cmH2O in 6 papers, from 0 to 15 in 4, from 0 to 10 in 3 and from other range in 3. Vrec averaged 373±146 ml in R and 81±33 ml in NR (P<0.001). The prevalence of R was 71% (0.57; 0.82). The pooled data analysis showed no significant difference between R and NR groups for baseline anthropometric variables, PEEP, tidal volume, PaO2/FIO2, FIO2, Simplified Acute Physiology score 2, ARDS cause, and for days in ARDS before the start of the investigation. However, we found a significantly higher compliance (mean difference 9.40 (C.I. 2.26; 16.53) ml/cmH2O) and significantly lower plateau pressure (mean difference 2.41 (4.11; 0.72) cmH2O) in R than in NR. Finally, mortality at ICU discharge was similar in R and NR: 40.7 (40.0; 41.0) vs: 47.8% (41.0; 55.0) (P=0.40, Fisher exact test), RR 1.11 (0.85-1.45).

**CONCLUSION.** Most of ARDS patients exhibited lung recruitment after increase in PEEP. However, this recruitement did not correlate with any difference in mortality.

**REFERENCE(S)**

1. University of Sassari
2. University of Lyon

### 000974

#### Feasibility and safety of prolonged continuous monitoring with electrical impedance tomography in neonates and infants with respiratory failure

T. Becher[1], M. Miedema[2], M. Kallio[3], M. Rahtu[3], RW. Van Leuteren[2], T. Papadouri[4], C. Karaoli[4], L. Sophocleus[5], AD. Waldmann[6], C. Strodthoff[1], R. Yerworth[7], A. Dupré[8], S. Nordebo[9], D. Khodadad[9], R. Bayford[10], PC. Rimensberger[8], AH. Van Kaam[2], I. Frerichs[1]
[1]Dept. of anesthesiology and intensive care medicine, University Medical Center Schleswig-Holstein, Campus Kiel, Kiel, Germany; [2]Dept. of neonatology, Amsterdam University Medical Centers, Amsterdam, Netherlands; [3]Dept. of children and adolescents, Oulu University Hospital, Oulu, Finland; [4]Neonatal intensive care unit, Archbishop Makarios III Hospital, Nicosia, Cyprus; [5]Dept. of electrical and computer engineering, University of Cyprus, Nicosia, Cyprus; [6]Eit branch, SenTec AG, Landquart, Switzerland; [7]Dept. of medical physics and biomedical engineering, University College London, London, United Kingdom; [8]Pediatric and neonatal intensive care unit, Children's Hospital, University of Geneva, Geneva, Switzerland; [9]Dept. of physics and electrical engineering, Linnaeus University, Växjö, Sweden; [10]Dept. of natural sciences, Middlesex University, London, United Kingdom
**Correspondence:** T. Becher
*Intensive Care Medicine Experimental* 2019, **7(Suppl 3):**000974

**INTRODUCTION.** Multiple studies have shown the potential of electrical impedance tomography (EIT) as a tool for monitoring of regional lung function in neonates and infants with respiratory failure. In this high-risk population, EIT can detect one-sided intubation [1], pneumothorax [2, 3] and identify recruitable atelectasis and overdistension [4]. As all previous studies have used EIT for analyzing short and specific episodes, the feasibility and safety of long-term monitoring with EIT has not yet been established.

**OBJECTIVES.** To assess the feasibility and safety of continuous EIT monitoring for up to 72 hours in neonates and preterm infants at risk for respiratory failure.

**METHODS.** In the ´**C**ontinuous **R**egional **A**nalysis **D**evice for neonate **L**ungs´ (CRADL) study (clinicaltrials.gov NCT02962505), we determined the feasibility and safety of continuous EIT monitoring for up to 72 hours in critically ill neonates and infants. We included patients needing supplemental oxygen, non-invasive or invasive respiratory support who were treated in neonatal or pediatric intensive care units. After obtaining written informed consent from the parents or legal representatives, a 32-electrode EIT belt was placed around the patient's chest and connected to an EIT image acquisition system (Sentec BB2, Landquart, Switzerland). At the bedside, investigators were blinded to the EIT findings but were prompted by the device to reattach the EIT belt if a loss in electrode contact occurred. The maximum duration of EIT examinations was 72 hours, but examinations could be terminated earlier, e.g. if the patients were discharged or transferred to another unit.

The study was conducted between November 2016 and March 2019 at four European university hospitals. As a primary outcome parameter, we assessed the overall percentage of EIT examination time with at least 26 out of 32 electrodes exhibiting sufficient skin contact impedance, the minimum required for reliable EIT image reconstruction with the Sentec BB2 system. Numerical results are presented as mean±SD unless otherwise specified.

**RESULTS.** 200 patients were included in the study. For our preliminary analysis, we included the first 194 data sets of 117 male and 77 female patients with a gestational age of 31±5 (range: 24-42) weeks and postnatal age of 5±15 (range: 0-122) weeks. The most frequent diagnoses explaining the need for respiratory support included prematurity, respiratory distress syndrome, meconium aspiration and bronchopulmonary dysplasia.
The average duration of EIT measurements was 54±21 hours. The percentage of EIT examination time suitable for analyzing as defined in the primary outcome parameter was 84.7±17.6%. No moderate or severe study-related adverse events were recorded. Minor study-related adverse events included reversible redness of skin or imprint of EIT belt on the patient´s skin.
**CONCLUSION.** In the CRADL observational study, continuous EIT measurement for up to 72 hours was feasible and safe in a mixed population of neonatal and pediatric critically ill patients with respiratory failure.

**REFERENCE(S)**
[1] Steinmann D et al. Acta Anesthesiol Scand 2013; 57: 881-887.
[2] Bhatia R et al. Intensive Care Med 2012; 38:308-315.
[3] Miedema M et al. Neonatology 2011; 99:10-13.
[4] Wolf GK et al. Pediatr Crit Care Med 2012;13:509-515.

1. The study was funded by the European Union's Horizon 2020 Research and Innovation Programme (Project CRADL, grant agreement number 668259)

### 000977

#### Implications and description of steroid use in ARDS patients - a 1 year retrospective study

I. Pinheiro[1], G. Bellingan[2], R. Shulman[3]
[1]Faculty of pharmacy, University of Porto; CMORE, Porto, Portugal; [2]Consultant and medical director, critical care directorate, University College London Hospitals, NHS Foundation Trust, London, United Kingdom; [3]Pharmacy department, UCLH, CMORE, London, United Kingdom
**Correspondence:** I. Pinheiro
*Intensive Care Medicine Experimental* 2019, **7(Suppl 3):**000977

**INTRODUCTION.** ARDS remains a major cause of morbidity and mortality and standard of care involves the treatment of the underlying cause and supportive care [1]. The Phase III clinical study of a new potential treatment - Interferon beta-1a [2] - yielded disappointing mortality outcomes, in part thought to be related to the concomitant corticosteroid use. Steroids are commonly used for septic shock (SS) and for several indications associated with ARDS. They have been used in ARDS to improve oxygenation and airway pressures, however the effectiveness on ARDS mortality is debatable [3].
**OBJECTIVES.** We aimed to identify the current ICU practice around steroid use at UCLH in relation to the severity of ARDS as per the international definition and SS.
**METHODS.** Demographic data, physiologic scores and outcomes, from January 2017 to January 2018 of our 45 bed ICUs, were accessed via the Unit's electronic record system Philips Intellivue. ARDS patients were defined as: mechanical ventilation requirement >48h, ARDS diagnosis stated in medical notes and ARDS severity according to the Berlin definition. SS patients were defined as a noradrenaline requirement.
**RESULTS.** 56 patients were documented as having ARDS, with 44 (79%) receiving steroids (28-day mortality - 64% on steroids and 25% no steroids). 28 (50%) of all patients had concomitant SS diagnosis (Table 1).
Of those without SS, only 3 (16%) received steroids for ARDS and 1 (5%) for PCP; 15 (79%) did not have a clear indication but the majority were haematological malignancy patients (HMP). Of those with SS, 10 (40%) received steroids per SS protocol, 2 (8%) per ARDS protocol, 1 (4%) for PCP and 13 (52%) did not have a clear indication but again were commonly HMP.
**CONCLUSION.** Septic shock-related ARDS patients had a higher overall disease severity, mortality and ventilatory support requirement. 79% of patients with ARDS were treated with steroids, mainly not for ARDS indications and appeared to have a higher mortality rate. This high level of steroid use may have implications for trials in ARDS.

**REFERENCE(S)**
1. Acute Respiratory Distress Syndrome. (2012). JAMA, 307(23). doi:10.1001/jama.2012.5669
2. Bellingan, G. et. al. (2017). Comparison of the efficacy and safety of FP-1201-lyo (intravenously administered recombinant human interferon beta-1a) and placebo in the treatment of patients with moderate or severe acute respiratory distress syndrome: study protocol for a randomized controlled trial. Trials, 18(1).doi:10.1186/s13063-017-2234-7
3. Steinberg KP et al. Efficacy and safety of corticosteroids for persistent acute respiratory distress syndrome. N Engl J Med. 2006;354:1671–1684. doi: 10.1056/NEJMoa051693

1. With the support of the Erasmus+ programme of the European Union.

**Table 1 (abstract 000977).** ARDS severity: in non-SS and SS

| | Non-SS N=28 | SS N=28 |
|---|---|---|
| Mild ARDS (N) | 3 (11%) | 2 (7%) |
| Moderate ARDS (N) | 9 (32%) | 9 (32%) |
| Severe ARDS (N) | 16 (57%) | 17 (61%) |
| ICU Length of stay (Mean ± SD, days) | 20.2 ± 18.9 | 16.4 ± 14.7 |
| 28-day mortality (N) | 10 (36%) | 18 (64%) p = 0.056 |
| 28-day ventilator-free days (VFD) (Mean ± SD) | 4.8 ± 6.4 | 2.2 ± 4.5 |

### 001018

#### Left ventricular diastolic dysfunction during the early phase of ARDS: a pilot study

A. Caccioppola[1], V. Galanti[1], M. Guanziroli[1], V. De Giorgis[1], E. Ferrari[1], P. Formenti[2], S. Coppola[2], L. Massironi[3], D. Chiumello[2]
[1]Anesthesia and intensive care, University of Milan, Milano, Italy; [2]Anesthesia and intensiva care, San Paolo, Milano, Italy; [3]Cardiology, San Paolo, Milano, Italy
**Correspondence:** A. Caccioppola
*Intensive Care Medicine Experimental* 2019, **7(Suppl 3):**001018

**INTRODUCTION.** Left ventricular diastolic dysfunction (LVDD) is quite common in critically ill patients. Echocardiography plays a vital role in identifying diastolic dysfunction at the bedside. It has been demonstrated to have a strong association with weaning failure, abrupt pulmonary edema and sepsis outcome.
**OBJECTIVES.** Aim of this study is to investigate the prevalence of LVDD during the early phase of acute respiratory distress syndrome (ARDS) and its relationship with lung mechanical properties.
**METHODS.** We enrolled mechanically ventilated patients within 48 hours after ARDS diagnosis. Demographic characteristics and partitioned respiratory mechanics variables were recorded; CT scan quantitative analysis was performed at two levels of airways pressure for the evaluation of recruitability; functional residual capacity (FRC) was measured by helium technique. Transthoracic echocardiography, including Pulse wave doppler and Tissue doppler imaging, was performed to measure during early left ventricular diastolic phase the velocity of intracardiac blood flow at the tip of mitral valve (E') and the longitudinal excursion of the septal mitral annulus (e'). LVDD diagnosis was made when septal e' < 8 cm/sec.
**RESULTS.** 10 of 16 patients presented a normal LV function [ND], 6 patients (37.5%) had a LVDD diagnosed [DD], 4 patients had a grade II dysfunction. No significantly differences were reported in terms of demographic, hemodynamics, respiratory variables and CT parameters between groups. DD group presented a significantly higher FRC (756 [450 – 878] mL vs. 403 [116 – 490] mL, P = 0.038) but a higher dead space compared to ND. DD patients demonstrated significantly higher 28 days mortality compared with ND (5/6 = 83% vs. 2/10 = 20%, p = 0.035).

**CONCLUSION.** We did not find any relationship between ARDS severity and the LVDD development. The higher FRC and dead space in DD patients could be due to the presence of emphysema in lung parenchyma. The higher 28-day mortality in DD patients should be further investigated.

**REFERENCE(S)**

1. Sanfilippo F, Corredor C, Fletcher N, Landesberg G, Benedetto U, Foex P, Cecconi M. Diastolic dysfunction and mortality in septic patients: a systematic review and meta-analysis. Intensive Care Med. 2015 Jun;41(6):1004-13
2. Moschietto S, Doyen D, Grech L, Dellamonica J, Hyvernat H, Bernardin G. Transthoracic Echocardiography with Doppler Tissue Imaging predicts weaning failure from mechanical ventilation: evolution of the left ventricle relaxation rate during a spontaneous breathing trial is the key factor in weaning outcome. Crit Care. 2012 May 14;16(3):R81

### 001021

#### Dynamic hyperinflation and intrinsic positive end expiratory pressure in ARDS patients

AC. Lusardi[1], A. Caccioppola[1], E. Ferrari[1], V. Galanti[1], V. De Giorgis[1], S. Froio[2], S. Coppola[2], D. Chiumello[2]
[1]Anesthesia and intensive care, University of Milan, Milano, Italy;
[2]Anesthesia and intensiva care, San Paolo, Milano, Italy
**Correspondence:** A. Caccioppola
*Intensive Care Medicine Experimental* 2019, **7(Suppl 3):**001021

**INTRODUCTION.** In patients with acute respiratory failure, changes affecting respiratory mechanical property (compliance and resistance) and the applied ventilatory setting can generate an incomplete deflation of the respiratory system at end-expiration, promoting a dynamic hyperinflation and intrinsic PEEP (iPEEP).

**OBJECTIVES.** Aim of this study was to evaluate in a large population of ARDS patients mechanically ventilated, the presence and the amount of iPEEP, the possible associated factors (patients characteristics and ventilator setting) and the effect of two levels of external PEEP on iPEEP.

**METHODS.** A secondary analysis of patients previously enrolled in 6 published studies. Patients were divided according to the presence of iPEEP.

The iPEEP was computed as the difference between total PEEP measured at the end of an expiratory pause and external PEEP measured at end-expiration of a regular breath. 5 and 15 cmH2O of PEEP were applied. Lung CT scan was performed at 5 and 45 cmH2O of PEEP.

**RESULTS.** 217 sedated and paralyzed patients were enrolled. iPEEP was detected in 87 patients. Eighty-seven patients (40%) had iPEEP with a median value of 1.1 [1.0-2.3] cmH2O at 5 cmH2O of PEEP.

Patients with intrinsic PEEP had a significantly higher body mass index and arterial carbon dioxide partial pressure.

Both at 5 and 15 cmH2O of PEEP the applied tidal volume was significantly lower (480 [430-540] vs 520 [445-600] mL at 5 cmH2O of PEEP; 480 [430-540] vs 510 [430-590] at 15 cmH2O of PEEP) in patients with intrinsic PEEP while respiratory rate was significantly higher (18 [15-20] vs 15 [13-19] bpm at 5 cmH2O of PEEP; 18 [15-20] vs 15 [13-19] bpm at 15 cmH2O of PEEP).

At both PEEP levels, total airway resistance and compliance of the respiratory system were not different among patients with and without iPEEP.

The total lung gas volume and the lung recruitability were not different among patients with and without iPEEP (961 [701-1535]vs 973 [659-1433] mL; 15 [0-32] % vs 22 [0-36] % respectively).

**CONCLUSION.** In our population iPEEP was detected in 40% of ARDS patients. In sedated and paralyzed ARDS patients the amount of intrinsic PEEP during lung protective ventilation is of negligible entity and does not influence gas exchange and respiratory mechanics properties.

**REFERENCE(S)**

1. Richard JC et al. "Influence of respiratory rate on gas trapping during low volume ventilation of patients with acute lung injury". Intensive Care Med 2002 Aug;28(8):1078-83.
2. D. Junhasavasdikul et al., "Expiratory Flow Limitation During Mechanical Ventilation," Chest, vol. 154, no. 4, pp. 948–962, Oct. 2018.

### 001026

#### Effects of super-nasal high flow (S-NHF) on lung volumes and comfort in critically ill hypoxemic patients: preliminary results

G. Montanari[1], F. Dalla Corte[1], G. Grasselli[2], A. Galazzi[2], F. Migliavacca[2], E. Spinelli[2], T. Mauri[2], A. Pesenti[2]
[1]Terapia intensiva universitaria, University Hospital of Ferrara, Cona, Italy;
[2]Department of anesthesiology, Fondazione IRCCS Ca'Granda Ospedale Maggiore Policlinico, Milano, Italy
**Correspondence:** G. Montanari
*Intensive Care Medicine Experimental* 2019, **7(Suppl 3):**001026

**INTRODUCTION.** Nasal high flow (NHF) in hypoxemic patients is usually delivered at flows of 30-50L/min and recent studies [1] showed that higher flow rates may improve the benefit of NHF. However, only one study in healthy volunteers showed that NHF delivered at flows >60 L/min (super-nasal high flow (S-NHF)) is associated with physiologic advantages [2].

**OBJECTIVES.** Aim of this study was to compare differences in oxygenation, end-expiratory lung volume, minute ventilation and patient's comfort by NHF delivered at three different gas flow rates: 0.5 L/min/Kg PBW, 1 L/min/Kg PBW; and 1.5 L/min/Kg PBW (S-NHF).

**METHODS.** we performed a prospective, interventional, randomized cross-over trial in patients with hypoxemia (PaO2/FiO2≤300 mmHg) admitted to the Intensive Care Unit of Ospedale Maggiore Policlinico in Milan. We delivered stable air/oxygen mix to obtain saturation of 90%-96% during all phases. Flow was set as detailed above for 20 minutes. At the end of each phase, data on lung volumes were recorded by EIT (Dräger Medical GmbH, Lubeck, Germany), ABG was performed and comfort was collected by visual numeric scale (0-10). Statistics were performed by one-way repeated measures ANOVA. Normally distributed variables are represented by means and SD, while median [IQR] have been used for non-normal.

**RESULTS.** We enrolled 7 patients (73±11) years old, 4 females,baseline PaO2/FiO2(166±47). During S-NHF, End Expiratory Lung Impedance (DEELI) significantly increased in comparison to lower gas flow rates (p=0.017), indicating larger PEEP effect, and Global Inhomogeneity (GI) of ventilation distribution was reduced. PaO2/FiO2, respiratory rate and corrected minute ventilation (MVcorr), instead, remained stable. Finally, S-NHF was associated with significantly poorer patient's comfort.

**CONCLUSION.** S-NHF might be associated with partial improvement of the physiologic effects of NHF at the expense of lower patient's comfort.

**REFERENCE(S)**

1. Groves N et al. High flow nasal oxygen generates positive airway pressure in adult volunteers. Aust Crit Care. 2007
2. Parke RL, et al Effect of Very-High-Flow Nasal Therapy on Airway Pressure and End-Expiratory Lung Impedance in Healthy Volunteers. Respir Care. 2015

**Table 1 (abstract 0001026).** Physiological effects of S-NHF

| NHF L/kg PBW | 0.5 | 1 | 1,5 | p-value |
|---|---|---|---|---|
| Set flow, L/min | 32±3 | 63±5 | 95±14 | - |
| PaO2/FiO2 | 184 [104-248] | 179 [140-264] | 181 [142-267] | 0.192 |
| Comfort, 0-10 VNS | 7±0.0 | 7±1 | 5±1.5*$ | **0.018** |
| RR, bpm | 19±7 | 18±5 | 18±5 | 0.280 |
| MVcorr (AU/min) | 41872±22237 | 46136±22586 | 47813±17078 | 0.363 |
| DEELI (AU) | # | 707±763 | 1012±1074* | **0.017** |
| GI, % | 61±14 | 58±13* | 56±13* | **0.002** |

$ p<0.05 vs. HFNC **1 mL/min**by post-hoc Bonferroni test;* p<0.05 vs. HFNC **0,5 mL/min**by post-hoc Bonferroni test; # Reference group

## CD - Haemodynamic monitoring and outcome

### 000567

#### Abnormal lactate after resuscitation from cardiac surgery is associated with impaired microcirculatory convective blood flow and diffusive capacity

J. Greenwood[1], D. Jang[2], J. Gutsche[3], J. Horak[3], M. Acker[4], J. Bakker[5], B. Abella[2]
[1]Departments of emergency medicine, anesthesiology & critical care, Perelman School of Medicine at the University of Pennsylvania, Philadelphia, United States of America; [2]Department of emergency medicine, Perelman School of Medicine at the University of Pennsylvania, Philadelphia, United States of America; [3]Department of anesthesiology & critical care, Perelman School of Medicine at the University of Pennsylvania, Philadelphia, United States of America; [4]Department of surgery, division of cardiovascular surgery, Perelman School of Medicine at the University of Pennsylvania, Philadelphia, United States of America; [5]Department of intensive care medicine, Erasmus University Medical Center, Rotterdam, Netherlands
**Correspondence:** J. Greenwood
*Intensive Care Medicine Experimental* 2019, **7(Suppl 3):**000567

**INTRODUCTION.** Cardiac surgery with cardiopulmonary bypass (CPB) is associated with significant microcirculatory impairment that may impact patient outcomes.1 Post-operative resuscitation routinely targets normalized systemic hemodynamic goals to achieve adequate tissue perfusion. Functional microcirculatory derangements are well described in sepsis, but poorly understood after cardiac surgery. Incident dark field videomicroscopy (IDF) allows for high resolution, real-time functional assessment of human microcirculation.

**OBJECTIVES.** To evaluate changes in microcirculatory function after resuscitation from cardiac surgery compared to baseline measurements when targeting normalization of systemic hemodynamic indices and identify phenotypes of microcirculatory dysfunction when compared with arterial lactate levels.

**METHODS.** These are preliminary results from an ongoing prospective, observational, single center study of elective CABG or valvular surgery patients. Sublingual microcirculation measurements (CytoCam, Braedius Medical BG, the Netherlands) were obtained in the pre-operative ward and in ICU 2-4 hours post-op once hemodynamic indices were normalized. Measurements were required to have a quality score < 10. Microcirculation videos were analyzed manually, measuring criteria based on ESICM 2nd consensus recommendations.3

**RESULTS.** We enrolled 20 subjects, median (IQR) age 63 years (55-71), 85% male, cardiopulmonary bypass time 115 min (82-143). Post-operative macrocirculatory indices were MAP 75 mmHg (70-83), CI 2.3 L/min/m2 (2.0-2.9), CVP 10 (7-12). Post-operative SvO2 was 72% (62-77), and arterial lactate 5.0 mmol/dL (2.1-8.6). Compared to pre-operative measurements, post-operative convective flow was significantly impaired. MFI (AU) 2.9 (±0.1) vs. 2.2 (±0.5), p<0.0001, PPV (%) 93.7 (±3.2) vs. 75.1 (±12.8), p<0.0001, with significant heterogeneity, HI (AU) 0.1 (±0.1) vs. 0.6 (±0.3), p<0.0001. Diffusive capacity was significantly impaired, PVD (mm/mm2) 21.5 (±3.3) vs. 15.8 (±3.9), p< 0.0001, and TVD (mm/mm2) 21.2 (± 4.6) vs. 19.1 (± 3.9) p≤0.05.

**CONCLUSION.** An elevated lactate level after resuscitation from cardiac surgery is associated with a significant reduction in both microcirculatory convective blood flow and diffusive capacity.

**REFERENCE(S)**

1. den Uil C, Lagrand W, Spronk P, et al. Sublingual microcirculation is impaired during cardiopulmonary bypass in cardiac surgery. Crit Care. 2007;11(Suppl 2):P264.
2. Hessler M, Arnemann PH, Zamit F, et al. A new complimentary web-based tool for manual analysis of microcirculation videos: Validation of the Capillary Mapper against the current gold standard AVA 3.2. Microcirculation. 2018;25(8):e12505.
3. Ince C, Boerma EC, Cecconi M, et al. Second consensus on the assessment of sublingual microcirculation in critically ill patients: results from a task force of the European Society of Intensive Care Medicine. Intensive Care Med. 2018;44(3):281-299.
4. Penn Acute Research Collaboration (PARC)
5. European Society of Intensive Care Medicine NEXT Fellowship
6. University of Pennsylvania Abramson Critical Care & Resuscitation Research Fund
7. University of Pennsylvania Center for Resuscitation Science

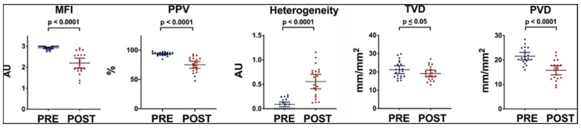

**Fig. 1 (abstract 000567).** Microcirculation measurements preop (blue) and postop (red). Changes in convective blood flow (MFI, PPV, HI) and diffusive capacity (TVD, PVD)

### 000653

#### Hyperlactatemia after cardiac surgery impacts on hospital mortality and prolonged ICU stay in dialysis-dependent patients: a multicenter retrospective study

M. Ezaka[1], A. Tsukamoto[2], K. Matsuo[3], T. Tomioka[2], K. Yamaoka[4], A. Nemoto[4], M. Matsuura[4], N. Kin[1]
[1]Department of anesthesiology, New Tokyo hospital, Matsudo, Japan; [2]Department of anesthesiology, Saitama Red Cross Hospital, Saitama, Japan; [3]Department of intensive care unit, New Tokyo hospital, Matsudo, Japan; [4]Graduate school of public health, Teikyo University, Tokyo, Japan
**Correspondence:** M. Ezaka
*Intensive Care Medicine Experimental* 2019, **7(Suppl 3):**000653

**INTRODUCTION.** Cardiac surgery in dialysis-dependent patients is associated with high mortality and morbidity (1). Lactate is a well-known marker of ischemia and a prognostic indicator, and kidney and liver play the main role in lactate metabolism (2). Although several studies proved the association between hyperlactatemia and prognosis in cardiac surgery (3), the prognostic relevance of lactate levels in dialysis-dependent patients is not well characterized.

**OBJECTIVES.** We aimed to evaluate the prognostic value of hyperlactatemia on the mortality and length of intensive care unit (ICU) stay in dialysis-dependent patients who had undergone cardiac surgery.

**METHODS.** This was a multicenter retrospective study and we included dialysis-dependent patients who underwent cardiac surgery with cardiopulmonary bypass between January 2014 and December 2018 at two mid-size private hospitals. Lactate data were collected at three time-points: at ICU admission (T1); peak level within 24 hours after the operation (T2); and 24 hours after the operation (T3). Lactate clearance was calculated using the following formula: (T2 lactate −T3 lactate)/T2 lactate × 100. We also obtained patient characteristics, hospital mortality, length of ICU stay, and days to return to dry weight after the operation. Prolonged ICU stay was defined as the 70th percentile of subjects. Using ROC curve analysis, we examined which point of lactate predicted hospital mortality and prolonged ICU stay. All statistical analyses were performed using software R (Ver.3.4.2).

**RESULTS.** We enrolled 122 dialysis-dependent patients. The median age was 73 (IQR 69-78) years, and hyperlactatemia was observed in 97 (79.5%) patients. Hospital mortality was 11.5%, median ICU stay was 7 (IQR 5.0-10.8) days, and the median days to return to dry weight were 8 (IQR 5.0-12.3) days. Prolonged ICU stay was defined as ≧10 days by the 70th percentile of ICU stay. On ROC-AUC analysis, T2 lactate showed the strongest association with hospital mortality (AUC 0.848) and the cutoff level was 10.6 mmol/L; T1 lactate showed the strongest association with prolonged ICU stay (AUC 0.778), and the cutoff level was 2.0 mmol/L.

**CONCLUSION.** Peak lactate level within 24 hours after the operation was associated with hospital mortality and lactate at the ICU admission was associated with prolonged ICU stay.

REFERENCE(S)

(1) Minton J et al. Hyperlactatemia and Cardiac Surgery. J Extra Corpor Technol. 2017;49(1):7-15.
(2) Bellomo R. Bench-to-bedside review: lactate and the kidney. Crit Care. 2002;6(4):322-6.
(3) Evans AS et al. Prognostic Value of Hyperlactatemia and Lactate Clearance After Mitral Valve Surgery. J Cardiothorac Vasc Anesth. 2018;32(2):636-643

### 000665

#### Retrospective study of Clinical profile and Outcomes of Arrhythmias in patients admitted to Medical ICU

K. Khatib[1], A. Chavan[1], S. Dixit[2], H. Dongare[3]
[1]Medicine, Smt. Kashibai Navale Medical College, Pune, India; [2]Critical care, MJM Hospital, Pune, India; [3]Anaesthesia, Smt. Kashibai Navale Medical College , Pune, India
**Correspondence:** K. Khatib

*Intensive Care Medicine Experimental* 2019, **7(Suppl 3):**000665

**INTRODUCTION.** Arrhythmias are common clinical events in the intensive care unit (ICU) and its impact on continuum of critical illness is unclear.

**OBJECTIVES.** To study type of arrhythmias in critically ill patients and its association with diagnoses, duration of ICU stay and clinical outcome.

**METHODS.** Retrospective study of 360 patients admitted to medical ICU over 3 months period. Socio-demographic data, associated co-morbidities, arrhythmias identified and interpreted, ICU diagnoses and various clinical outcomes [mortality, ICU-length of stay (ICU-LOS)] were studied.

Inclusion criteria: age > 18yrs

Exclusion criteria: Arrhythmias and other waveforms present during ACLS or CPR.

**RESULTS.** Out of the 360 patients admitted to ICU during study period 50pts (13.88%) had arrhythmias , with 46 pts having 1 event , 2 pts having 2 events and 2 pts having 3 events. Of these 50 pts, 61% patients had supra-ventricular Arrhythmias, 18% patients had ventricular Arrhythmias and 21% patients had premature complexes. The socio-demographic profile and select clinical variables of patients is shown in Table 1. Most Arrhythmias (76% events) occurred in first 3 days of ICU stay, Atrial fibrillation being the most common. Patients with dys-electrolytemia and myocardial infarction upon admission had increased risk of developing Supra-ventricular arrhythmias. Development of Ventricular arrhythmias was not found to be associated with any particular clinical mediator. Patients with Ventricular arrhythmias had increased risk of in-hospital mortality (P<0.05). Patients with Supra-ventricular Arrhythmias were associated with increased duration of ICU stay (5.2days) as compared to patients with premature complexes and ventricular arrhythmias, though not clinically significant (p= 0.06).

**CONCLUSION.** Arrythmias occurring in critically ill patients increase in-hospital mortality and ICU-LOS.

REFERENCE(S)

1. Valderrábano RJ, Blanco A, Santiago-Rodriguez EJ, Miranda C, Rivera-Del Rio Del Rio J, Ruiz J, et al. Risk factors and clinical outcomes of arrhythmias in the medical intensive care unit. J Intensive Care 2016;4:9.
2. Reinelt P, Karth GD, Geppert A, Heinz G. Incidence and type of cardiac arrhythmias in critically ill patients: A singal center experience in medical-cardiological ICU. Intensive Care Med 2001; 27(9):1466-73.
3. Goodman S, Weiss Y, Weissman C.. Update on cardiac arrhythmias in the ICU. Curr Opin Crit Care. 2008(14):549–54.
4. Tarditi DJ, Hollenberg SM. Cardiac arrhythmias in the intensive care unit. Seminars in Respiratory and Critical Care Medicine. 2006;27(3):221–229.
5. NIL

**Table 1 (abstract 000665).** Socio-demographic profile and select clinical variables of patients

| | | ARRYTHMIAS SVA+VA(n=38) | PREMATURE COMPLEX( n=12) | P value |
|---|---|---|---|---|
| AGE(median)IQR | 54(18-81) | 56 (18-79) | 38(28-52) | <0.05 |
| Male % | 34 (68%) | 26 (68.4%) | 8 (66.66%) | 0.12 |
| Pre-morbid | | | | |
| DM | 13( 26%) | 11 (28.9%) | 2 (16.66%) | 0.07 |
| HTN | 12 (25%) | 10 (26.3%) | 2 (16.66%) | 0.06 |
| IHD | 10 (20%) | 10 (26.3%) | 0 | 1.20 |
| CVA | 4 (8%) | 4 (10.52%) | 0 | 1.25 |
| CKD | 9 (18%) | 9(23.68%) | 0 | 1.15 |
| ICU STAY (median) | 3.5 DAYS | 5.2 DAYS | 4.0 DAYS | 0.06 |
| MORTALITY | 12(25%) | 10 (26.3%) | 2 (5.26%) | 0.05 |

### 000666

#### Impact of availability of percutaneous coronary intervention during 24 hours for all STEMI in our center of reference in Huelva province

E. Recart Batrakova, M. Morales, GA. Tristancho, P. Ponce, P. Oliva, F. Rivas, M. Márquez, F. Cabeza, P. Domínguez, P. Ortega, I. Villa, C. Jiménez, MA. Herrero, E. Pino
Critical Care Unit, Hospital Universitario Juan Ramón Jiménez, Huelva, Spain
**Correspondence:** E. Recart Batrakova

*Intensive Care Medicine Experimental* 2019, **7(Suppl 3):**000666

**INTRODUCTION.** Primary coronary intervention (PCI) in STEMI has advantage over fibrinolysis in terms of mortality and complications. Hospitals implement its availability during 24 hours for all patients with STEMI.

**OBJECTIVES.** Evaluate the impact of disponilility of PCI during 24 hours for all STEMI treated in our hospital, Juan Ramón Jiménez Hospital

**METHODS.** Analytical descriptive study of prospective cohort of regional database of Andalusia (ARIAM) of patients with STEMI admitted to Intensive Care Unit between January 2017 and January 2019. From June 2018 the PCI was available 24 hours, being all patients with STEMI referred to our hospital and indicated by the emergency doctor in charge of patient. Prior to this, the indication of PCI was based on classical criteria, indicated by intensivist in case of cardiogenic shock, contraindication or failed of fibrinolysis in of-time work and like fist election in-time work. We used SPSS statistical software with chi-square for frequency analysis and t student for medians. The significant result was defined as p<0,05.

**RESULTS.** There are 206 patients, 82,1% males, median age of 62 years. The most frequent presentation was anterior AMI (43,5%), Killip I (62,8%) with LVEF 46,5%. The 53,9% was hypertensive, 48,5% smokers, 44,2% dyslipidemics, 33,5% diabetics and 19,4% chronic ischemic heart disease. The 44% presented complications, and they are cardiorespiratory arrest (10,6%), cardiogenic shock (9,7%), hypotension (7,7%), complete node-atrial block (4,8%), coronary dissection (3,9%), ventricular tachycardia with pulse (3,9%), stent thrombosis (2,9%) and haemorrhage (0,5%). The 84 patients presented before 24 hours disponilility of PCI and 122 patients after this period. The group of pre-disponilility period, had significantly difference (p<0,05), with predominance of inferior (36,9 vs. 22,1%) and undetermined AMI (3,6 vs 0%) and presented more Killip III (14,3 vs 4,9%).

The other characteristics was similar between the groups. The implementation of PCI 24 hours shown reduction of hypotension (14,3 vs 3,3%, p= 0,003), statistically significant, and, in time from first medical contact to PCI (175 vs 136 minutes, p=0,07) and from first electrocardiogram to PCI (156 vs 118 minutes, p=0,06), in overall mortality (6,0 vs 2,4%, p=0,2) with related to myocardial ischemia (4,8 vs 1,6%, p=0,19) and cardiogenic shock (13,1 vs 7,4%, p=0,17), but non-statistically significant.

**CONCLUSION.** The availability of PCI 24 hours has significantly affected the reduction of several factors that coan influence in unforable evolution of patients with AMI as hypotension or worse killip. Its implementation has improved time from first medical contact to PCI and reduction of global mortality but in a trend non-statistically significant. Its possible needs more following in time of our population to see more changes in outcomes.

**REFERENCE(S)**

1. Huynh T, Perron S, O'Loughlin J, Joseph L, Labrecque M, Tu JV, Théroux P. Comparison of primary percutaneous coronary intervention and fibrinolytic therapy in ST-segment-elevation myocardial infarction: bayesian hierarchical meta-analyses of randomized controlled trials and observational studies. Circulation 2009;119(24):3101
2. Nguyen B, Fennessy M, Leya F, Nowak W, Ryan M, Freeberg S, Gill J, Dieter RS, Steen L, Lewis B, Cichon M, Probst B, Jarotkiewicz M, Wilber D, Lopez JJ. Comparison of primary percutaneous coronary intervention in patients with ST-elevation myocardial infarction during and prior to availability of an in-house STEMI system: early experience and intermediate outcomes of the HARRT program for achieving routine D2B times <60 minutes. Catheter Cardiovasc Interv 2015 Aug;86(2):186-96.
3. Ibanez B, James S, Agewall S, Antunes MJ, Bucciarelli-Ducci C, Bueno H, Caforio ALP, Crea F, Goudevenos JA, Halvorsen S, Hindricks G, Kastrati A, Lenzen MJ, Prescott E, Roffi M, Valgimigli M, Varenhorst C, Vranckx P, Widimský P; ESC Scientific Document Group. 2017 ESC Guidelines for the management of acute myocardial infarction in patients presenting with ST-segment elevation: The Task Force for the management of acute myocardial infarction in patients presenting with ST-segment elevation of the European Society of Cardiology (ESC). Eur Heart J 2018 Jan 7;39(2):119-177

### 000749

#### A comparative study of three therapeutic strategies in patients admitted to the ICU due to symptomatic bradycardia: temporary transvenous pacemaker (TTVP) vs chronotropic drugs (C+) vs observation (OBS)

N. Arriero Fernández[1], JE. Romo Gonzales[1], P. Gallardo Culebradas[1], Z. Eguileor Marín[1], A. Estrella Alonso[1], M. Torralba Gonzalez De Suso[1], MA. Tirado Fernández[1], JA. Silva Obregón[1], S. Arriero Fernández[2], P. Revuelta[1], E. Quiros Oyaguez[1], E. Yañez Parareda[1], A. Albaya Moreno[1], C. Benito Punzel[1], R. Viejo Moreno[1], P. Rojo Villar[1], R. Torres Sánchez Del Arco[1], JM. Román Sánchez[1], MÁ. Navarro Nicuesa[1], C. Marian Crespo[1]

[1]Intensive care, Hospital Universitario de Guadalajara, Calle Donante de Sangre, Guadalajara, Spain, Guadalajara, Spain; [2]Psychiathy, Sanatorio Privado Neuropsiquiatrico Doctor León, Plaza Mariano de Cavia, Madrid, Spain, Madrid, Spain

**Correspondence:** N. Arriero Fernández

*Intensive Care Medicine Experimental* 2019, **7(Suppl 3):**000749

**INTRODUCTION.** Admission for symptomatic bradycardia in the ICU is frequent and the initial therapeutic option, while waiting for permanent pacemaker implantation, depends on the severity of symptoms, the center's infrastructure, and the personnel who perform the care. This study tries to describe the clinical outcome of the three most frequently used therapeutic options before definite pacemaker implantation: temporary transvenous pacemaker (TTVP), chronotropic drugs (C+) and observation (OBS).

**OBJECTIVES.** To analyze the characteristics and clinical outcomes of three initial therapeutic options (TTVP, C+, and OBS) in patients admitted to the ICU due to symptomatic bradycardia.

**METHODS.** A retrospective cohort study of patients admitted to the ICU due to symptomatic bradycardia [symptomatic atrioventricular block (sAVB), sinus node dysfunction (SND), hyperkalemia (HK), drug overdose (OD), etc.] from January 2014 to December 2018.

We collected demographic data: age and sex; and clinical variables: APACHE II, diagnosis at admission, low cardiac output, agitation, time from ICU admission to permanent pacemaker implantation (TPPM), length of stay in ICU (LSICU) and in the hospital (LSH); the last three measured in days.

The demographic variables were compared by χ2 and the quantitative ANOVA or nonparametric test (Kruskal-Wallis).

**RESULTS.** We analyzed 137 patients divided into 3 groups: TTVP: 86, C+: 19, OBS: 32. Of these 54% were men; median age 80.5 (IQR: 72.5-85.5).

The APACHE II median was 14 (IQR: 10-17) with no significant differences (NSD) between the 3 groups (p = 0.200).

The most frequent diagnosis was sAVB (82.5%), with NSD in the type of bradycardia between the 3 groups (p = 0.146).

There where NSD in low cardiac output between the 3 groups (p = 0.535).

Agitation was more frequent in the TTVP (22.1%) and C+ (26,3%) groups compared with OBS (3.1%), although with NSD (p=0,037).

The TPPM (days) was 0.85 (0.61-1.81) in TTVP; 0.88 (0.73-1.02) in C+; and 0.83 (0.6-1.75) in OBS; with NSD between the 3 groups (p = 0.275).

The LSICU (days) was significantly larger in TTVP: 1.65 (1-2.38) in TTVP; 1.1 (1.03-2.17) in C+; 1.06 (0.9-1.35) in OBS (p = 0.028).

The LSH (days) was 4.8 (3.91 - 5.69) in TTVP; 4.78 (1.96-7.6) in C+; 3.83 (3.03-4.63) in OBS; with NSD between the 3 groups (p = 0.334).

Four patients in the OBS group (12.5%) and two patients in the C+ group (10.53%) required an urgent placement of TTVP.

**CONCLUSION.** In our series, there were no significant differences between the three groups in any of the studied variables, except for LSICU days, in which case it was significantly larger in TTVP.

There was a higher incidence of agitation in the TTVP and C + group, although with no significant difference.

**REFERENCE(S)**

1. To my mentor, Javier Romo

### 000753

#### Comparison of noninvasive and invasive arterial blood pressure monitoring

R. Grišiūtė[1], L. Balčiūnas[2]

[1]Vilnius University, Vilnius, Lithuania; [2]Center of anaesthesiology, intensive therapy, and pain management, Vilnius university hospital Santaros klinikos, Vilnius, Lithuania

**Correspondence:** R. Grišiūtė

*Intensive Care Medicine Experimental* 2019, **7(Suppl 3):**000753

**INTRODUCTION.** Peripheral arterial catheters are invasive hemodynamic monitoring devices used in intensive care unit and are considered the gold standard in blood pressure monitoring. Although even if the risk of complication is low, arterial catheters are not harmless and can result in ischemia, infection and thrombosis.

**OBJECTIVES.** We carried out this prospective analysis to evaluate the hypothesis that non-invasive blood pressure (NIBP) monitoring would be as accurate as invasive blood pressure (IABP) monitoring in certain situations. We also evaluated whether the deviation of pressure transducer would affect the results of IABP.

**METHODS.** For the NIBP measurements, oscillometric blood pressure measurements were performed; cuff size was selected on the basis of the patient's limb circumference. For the IABP measurements, a 20-G arterial catheter was present in the radial artery. The transducer system was connected to the arterial catheter. The pressure transducer was placed at the level of the heart and zero-calibrated to atmospheric pressure. For the study it was elevated 10cm upward and downward from zero. The correlations between invasive and noninvasive values were assessed using Pearson correlation coefficient. Agreements between invasive and noninvasive blood pressure methods were assessed using Bland–Altman analysis and t-test. All summarized values are presented as mean ± SE. P-value of less than 0,05 was considered statistically significant.

**RESULTS.** In total, 80 patients were analyzed the study included 3 different groups: control, arrhythmia, vasopressor. The mean age was 59,1±17,0. The mean BMI was 27,4±4,6 kg/m2. The correlation coefficients between invasive and noninvasive devices for systolic, diastolic, and mean arterial pressure were all significantly correlated with P-values of less than 0,0001. The mean biases for IABP and NIBP with brachial cuff SAP were 0,36±11,5 mmHg, DAP –6,25±5,96mmHg, and MAP -5,53±5,5mmHg although with thigh cuff SAP were -6,15±10,2 mmHg, DAP -5,11±6,9mmHg, and MAP -6,4±5,6. The mean biases for mean IABP and NIBP in control group were -5,4±5,48mmHg (brachial), -5,78±5,77mmHg (thigh), in group with arrhythmias -4,75±6,14mmHg; -6,35±5,46mmHg, in group with vasopressor dose 0,1-0,2mcg/kg/min were -5,69±4,4mmHg; -6,3±4,1mmHg, and with dose >0,2mcg/kg/min were -8,14±6,17mmHg; -10,29±6,97mmHg. The correlation coefficients between invasive arterial pressure before and after pressure transducer ascending 10cm and descending 10cm from zero were all significantly correlated with P-values of less than 0,001. The mean biases for calibrated IABP and 10cm higher IABP transducer was 7,85±2,6mmHg and for 10cm lower was -7,59±2,56mmHg.

**CONCLUSION.** NIBP monitoring is not as accurate as IABP monitoring in patients especially with high vasopressor doses. Although NIBP monitoring is more accurate than erroneously calibrated IABP in most cases.

**REFERENCE(S)**

1. Araghi A, Bander JJ, Guzman JA. Arterial blood pressure monitoring in overweight critically ill patients: invasive or noninvasive? Crit Care 2006; 10:R64.
2. Scheer B, Perel A, Pfeiffer UJ. Clinical review: complications and risk factors of peripheral arterial catheters used for haemodynamic monitoring in anaesthesia and intensive care medicine. Crit Care 2002; 6:199–204.
3. Mermel LA. Arterial catheters are not risk-free spigots. Crit Care Med 2008;36:620–622.
4. Lakhal K, Ehrmann S, Martin M, et al. Blood pressure monitoring during arrhythmia: agreement between automated brachial cuff and intra-arterial measurements. Br J Anaesth. 2015;115(4):540-549.
5. Lakhal K, Macq C, Ehrmann S, et al. Noninvasive monitoring of blood pressure in the critically ill: reliability according to the cuff site (arm, thigh, or ankle). Crit Care Med. 2012;40(4):1207-1213.
6. Chatterjee A, DePriest K, Blair R, et al. Results of a survey of blood pressure monitoring by intensivists in critically ill patients: a preliminary study. Crit Care Med. 2010;38(12):2335-2338.

### 000827

#### Differences in new onset and chronic atrial fibrillation in a cohort of critically ill patients

R. Carvalho De Menezes[1], I. Bonifácio Brige Ferreira[2], M. Lisboa Otero[1], G. Andrade Agareno[3], A. Araujo Oliveira[2], L. Pamplona Neto[1], S. Agareno De Souza Filho[1], NM. Filgueiras Filho[3]
[1]Critical care, Hospital da Cidade, Salvador, State of Bahia, Brazil, Brazil; [2]Medicine, Bahia State University, Salvador, Brazil; [3]Medicine, Salvador University, Campus Teacher Barros, Salvador, Brazil
**Correspondence:** N.M. Filgueiras Filho
*Intensive Care Medicine Experimental* 2019, **7(Suppl 3):**000827

**INTRODUCTION.** Atrial fibrillation (AF) is a common arrhythmia in Intensive Care Units (ICUs). It is suggested that different mechanisms related to patients previously diagnosed with AF are related to the development of this pathology during ICU stay. Thus, the present study aims to compare the profile of patients and factors associated with new onset and chronic AF.

**OBJECTIVES.** To assess the incidence and differences of new onset and chronic AF in patients admitted to the general ICU.

**METHODS.** Retrospective cohort of patients with AF, admitted to an adult general ICU between August/2015 and August/2018. Data was prospectively extracted from patient records. The D'agostino test was used to determine the normality of the studied variables and to evaluate the difference between means and risk, the student`s T-test and Chi-square test were respectively used.

**RESULTS.** 2401 patients were admitted, of whom 247 (10.29%) presented AF during their hospitalization. Patients with chronic AF represented 61.13% (n = 151) of our population, with a mean age of 77.59 ± 11.10 and mean SAPS3 score 51.94 ± 9.65. The majority were women [n = 131, (86.75%)], and, of these 26.49% (n=40) presented associated with cardiovascular diseases. New onset AF (NoAF) corresponded to 38.86% (n = 96), with female gender predominating 57.29% (n = 55), mean age = 76.53 ± 15.02, BMI 26.24 ± 6.01, SAPS 3 = 51.08 ± 12.57 and of those 39.58% (n = 38) presenting with cardiovascular diseases. There was identified a higher risk for the development of NoAF in patients with cardiovascular diseases [OR (95% CI) 1.8 (1.053-3.139); p = 0.031]. Mean arterial pressure (MAP) was higher in patients with NoAF (95.0 ± 21.8 vs 103.1 ± 22.6, p = 0.01), as well as C-reactive protein (CRP) = 86, 5 ± 68.1 vs 109.1 ± 72.9; p = 0.037. There were no differences between the groups (chronic / NoAF) regarding outcome variables, such as length of hospital stay, use of vasoactive drugs or mechanical ventilation, changes in sodium, potassium and mortality.

**CONCLUSION.** In this cohort of critically ill patients, the incidence of AF was similar with literature. Demographic characteristics were similar in the new onset and chronic AF groups, but patients with cardiovascular diseases had higher incidence of NoAF. Higher MAP and CRP values was identified in NoAF patients compared with chronic AF patients.

### 000857

#### A National Study of Distress among those with Acute Coronary Syndrome

F. DeKeyser Ganz[1], O. Raanan[2]
[1]School of nursing, Hadassah Hebrew University, Jerusalem, Israel; [2]School of nursing, Sheba Medical Center, Ramat Gan, Israel
**Correspondence:** F. DeKeyser Ganz
*Intensive Care Medicine Experimental* 2019, **7(Suppl 3):**000857

**INTRODUCTION.** Little is known about the level of distress experienced by patients hospitalized with Acute Coronary Syndrome (ACS)

**OBJECTIVES.** To describe levels of physical, psychological and spiritual distress among patients admitted to a hospital with Acute Coronary Syndrome (ACS)

**METHODS.** Every Israeli patient admitted to a hospital over a 2 month period with ACS (n=1958) was included in a bi-annual, national, two month prevalence study, Acute Coronary Syndrome Israel Study (ACSIS). Clinical medical data were collected for the entire sample. A convenience sub-sample of these patients (n=990) completed the Distress Thermometer. Demographic and clinical data were taken from the patient's medical record.

**RESULTS.** The mean overall level of distress was 4.6/10 (Median= 5, *S.D.* = 3.5). The most common source of physical distress was discomfort (n=527, 54.7%), followed by fatigue (n=598, 62%). Most were worried (n=506, 53%) with many reporting feelings of depression (n=139, 15%) or sadness (n=238, 25%). Few reported having spiritual or religious concerns (n=18, 2%).

**CONCLUSION.** Patients admitted to a coronary care unit with ACS experienced moderate levels of distress, from both physical and psychological sources. While previous reports have described distress after discharge, this study provides evidence that such responses are already present in the immediate, acute phase of illness.

**REFERENCE(S)**

1. Acute Coronary Study Israel

### 000883

#### Hydrogen selenide as the 4th gasotransmitter: a metabolic modulator with potential therapeutic utility in acute/critical illness states

K. Samra, M. Singer, A. Dyson
University college london, Bloomsbury Institute of Intensive Care Medicine, London, United Kingdom
**Correspondence:** A. Dyson
*Intensive Care Medicine Experimental* 2019, **7(Suppl 3):**000883

**INTRODUCTION.** The endogenous 'gasotransmitters' – nitric oxide (NO), carbon monoxide (CO) and hydrogen sulphide – are anti-inflammatory, directly inhibit oxidative phosphorylation, and depress metabolic activity. They have potential therapeutic utility in hypoxia, ischaemia/reperfusion and circulatory shock [1]. Selenium is an essential micronutrient and an important antioxidant [2]. Its physiological derivative, hydrogen selenide (comprising gaseous H2Se and the anion, HSe-) was recently proposed as the fourth gasotransmitter [3]. It offers potential as a metabolic modulator *and* antioxidant therapy in critical illness.

**OBJECTIVES.** To characterise the basic salt, sodium hydrogen selenide (NaHSe), and investigate its effects on mitochondrial function.

**METHODS.** Sodium hydrogen selenide (NaHSe) was synthesized as described [4]. H2Se gas liberated by the salt was measured using a commercially available detector. Chemical reduction of oxygen to water was assessed in closed chambers. Effects of NaHSe on oxidative metabolism *ex vivo* was assessed by oxygen consumption (soleus muscle) and cytochrome C oxidase activity (liver). Sodium hydrogen sulfide (NaHS), K-cyanide (KCN) and the reducing agent, sodium dithionite, acted as positive controls. *In vivo* pharmacology of intravenous NaHSe was assessed in anaesthetised, instrumented rats.

**RESULTS.** H2Se gas was detectable from vials containing dissolved NaHSe (Fig 1A). Selenide was a powerful reducing agent (Fig 1B) and dose-dependently decreased muscle oxygen consumption (VO2) (Fig 1C), and inhibited cytochrome C oxidase activity (Fig 1D). Hydrogen selenide was less potent than both sulfide and cyanide (Fig 1C, D), and its activity on mitochondrial function was transient (Fig 1D). *In vivo*, hydrogen selenide exhibited a striking effect on heart rate (Fig 1E).

**CONCLUSION.** Hydrogen selenide caused significant (and, desirably, transient) metabolic effects via inhibition of cytochrome C oxidase (mitochondrial complex IV). In keeping with the known activity of other gaseous mediators (NO, CO, sulfide) as inhibitors of complex IV, hydrogen selenide can indeed be considered the fourth gasotransmitter. The metabolic and antioxidant effects of hydrogen selenide warrant further investigation as a therapeutic agent.

**REFERENCE(S)**

[1] Szabo C. Sci. Transl. Med., 2010; 2:54–59.
[2] Fairweather-Tait SJ et al. Antioxid Redox Signal. 2011; 14:1337–1383.
[3] Hartmann C et al. Front. Physiol. 2017; 8:691.
[4] Klayman DL, Griffin TS. J Am Chem. Soc 1973; 95:197–199.

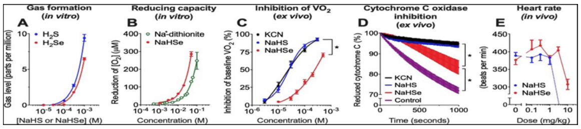

**Fig. 1 (abstract 000883).** Gas formation, redox activity and pharmacology of NaHSe. *p<0.05; overall 2-way RM ANOVA. Data; mean ± SEM, n=3-8/group. Doses >1 mg/kg NaHS causes mortality (blue dotted line in E).

### 000967

#### Ventilator-Free Time During Veno-Arterial Extracorporeal Membrane Oxygenation Is An Independent Predictor Of Hospital Discharge Outcome

A. Prasad[1], K. Singbartl[2]
[1]Anesthesiology, Penn State Health Milton S. Hershey Medical Center, Hershey, United States of America; [2]Critical Care Medicine, Mayo Clinic Hospital, Phoenix, United States of America
**Correspondence:** K. Singbartl

*Intensive Care Medicine Experimental* 2019, **7(Suppl 3):**000967

**INTRODUCTION.** Peripheral veno-arterial extracorporeal membrane oxygenation (pVA-ECMO) has become an accepted treatment option for advanced cardiogenic shock, allowing myocardial recovery as well as limiting or even reversing secondary end-organ damage.[1] Respiratory management of patients on pVA-ECMO-ECMO, in particular extubation and spontaneous breathing without ventilator support, remains controversial.

**OBJECTIVES.** We therefore developed a prediction model to study the effect of spontaneous breathing without ventilator support (ventilator-free time) during pVA-ECMO on hospital discharge outcome.

**METHODS.** After IRB approval, we retrospectively analyzed data from 265 patients undergoing pVA-ECMO for cardiogenic shock with or without concomitant acute respiratory failure. We classified hospital discharge outcome as unfavorable (death/hospice), intermediate (skilled nursing facility/other acute care hospital), and favorable (rehabilitation facility/home). We extracted patient demographics, clinical findings, and laboratory results from the electronic medical records. Statistical analyses included Wilcoxon test, chi-square test, Fisher's exact test as well as machine learning based analytics for model development. P <0.05 was considered statistically significant. Data are given as median (interquartile range).

**RESULTS.** Hospital discharge outcome of all patient was as follows: 41.6% unfavorable, 22.3% intermediate, and 36.2% favorable. Overall hospital length of stay was 21.6 days (12.1-37.9); pVA-ECMO duration was 7.9 days (4.3-16.7). 26.5 % of all patients were ventilator-free during 29.8% (10.9-51.7) of their time on pVA-ECMO. Overall vascular complication rates were low and statistically not significantly different between patients who were extubated and those who were not. After variable selection by means of generalized regression with machine learning validation, we fitted a proportional odds model to predict hospital discharge outcome with the following independent variables: age, race, cardiovascular SOFA scores, respiratory SOFA scores, lactate levels, need for durable mechanical circulatory support after pVA-ECMO, and ventilator-free time (as % of pVA-ECMO duration). In addition to age, need for durable mechanical circulatory support after pVA-ECMO, cardiovascular SOFA score at the end of pVA-ECMO, and respiratory SOFA score at the beginning of pVA-ECMO, ventilator-free time emerged as a significant predictor for hospital discharge outcome [odds ratio 1.18 (95% CI 1.04-1.34) per 10% increase, p=0.011].

**CONCLUSION.** Analysis of our cohort suggests that patients can be safely extubated during pVA-ECMO without any relevant adverse effects. Moreover, our data allows us to hypothesize that ventilator-free time during pVA-ECMO is an independent predictor of hospital discharge outcome.

**REFERENCE(S)**

1. Ghodsizad A, et al. (2014) Curr Opin Cardiol 29:275.

### 000996

#### The impact of exosome from stem cell-derived on pulmonary artery hypertension

MC. Shen[1], SR. Wann[2], YT. Chang[3], CP. Liu[1], PL. Chi[4], WC. Huang[1]
[1]Critical care medicine and cardiovascular center, Kaohsiung Veterans General Hospital, Kaohsiung, Taiwan; [2]Kaohsiung veterans general hospital,pingtung branch,pintung, Kaohsiung Veterans General Hospital,Pingtung Branch,Pintung, Kaohsiung, Gushan District, Kaohsiung City, Taiwan, Taiwan; [3]Department of emergency, Kaohsiung Veterans General Hospital, Kaohsiung, Gushan District, Kaohsiung City, Taiwan, Taiwan; [4]Department of medical education and research, Kaohsiung Veterans General Hospital, Kaohsiung, Taiwan
**Correspondence:** W.C. Huang

*Intensive Care Medicine Experimental* 2019, **7(Suppl 3):**000996

**INTRODUCTION.** In pulmonary arterial hypertension (PAH), abnormal proliferation of vascular smooth muscle cells, inflammation and pulmonary vascular remodeling are prominent features. Severalstudieshave demonstrated that both hypoxia-induced factors-1 (HIF-1) and p21-activated kinase-1 (PAK-1) are involved in the regulation of cell growth, leading to vascular remodeling.Our previous study showed that intraperitoneal administration ofinduced pluripotent stem cells (iPSCs) improves the function of hemodynamics in lung of monorcotaline (MCT) or hypoxia-induced PAH rats. In recent years, therapeutic benefit of iPSCs may be mediated by a paracrine effect of exosomes.However,the underlying mechanisms mediating these protective effects remain obscured.

**OBJECTIVES.** To determine whether iPSC-derived exosomes (iPSC-Exos) treatment can improve chronic hypoxia-induced PAH and vascular remodeling in rats, as well as to identify the mechanisms underlying these effects.

**METHODS.** iPSC-Exos were intraperitoneal injected daily to a chronic hypoxia-induced PAH rat model which under 10% Oxygen concentration for 8 weeks. The protocol of iPSC-Exos were designated as (i) prevention by the provision of iPSC-Exos treatments on same time with under hypoxia condition, or (ii) reversal by administration of iPSC-Exos treatments after hypoxia situation for 4 weeks. The phenomenon of vascular remodelingwere assessed by immunohistochemical and Elastic Van Gieson staining.Hypoxia-induced HIF-1a and PAK1 expression were determined by Western blotting and qPCR.Furthermore, MTT assay and BrdU staining, and TUNEL assay were used to analyze hypoxia-induced pulmonary smooth muscle cells (PASMCs) proliferation and apoptosis, respectively.

**RESULTS.** The *in vivo* study demonstratedthat administration of iPSC-Exos group decreases the hemodynamic values of RVSP and ameliorates the lumen diameter and wall thickening of pulmonary arterioles in hypoxia-induced PAH.Histological examination of lung tissue showed that the levels of HIF-1a and PAK1 was lower in the iPSC-Exos group than the no-treatment group. Administration ofiPSC-Exossignificantly inhibited hypoxia-induced HIF-1a and PAK1 expression inlung tissue lysates revealed by Western blotting and qPCR.Similar to the results observed in vivo,treatment of PASMCs with iPSC-Exossignificantly inhibitedhypoxia-induced HIF-1a and PAK1 protein and mRNA expression.By MTT assay and BrdU staining, the proliferation of PASMCs induced by hypoxia was significantly inhibited by pretreatment with iPSC-Exos. By TUNEL assay, iPSC-Exos promoted PASMCs apoptosis in response to hypoxia stimulation.

**CONCLUSION.** Our findings showed that iPSC-Exos exerts a protective effect on pulmonary vascular remodeling in hypoxia-treated PAH model through down-regulation of HIF-1a and PAK1,leading to ameliorate hemodynamic values of RVSP. In PASMCs, iPSC-Exosinhibits hypoxia-induced HIF-1a and PAK1 signaling pathway and blocks excessive cell proliferation. iPSC-Exos also prevents the resistance of PASMCs to apoptosis. These finding may provideuseful information about iPSC-Exos as a new therapeutic strategy for PAH diseases.

**REFERENCE(S)**

1. Nicolls MR et al. New modelsof pulmonary hypertension based on VEGF receptor blockadeinducedendothelial cell apoptosis. Pulm Circ. 2012;2:434-442.

2. Supported by research grant from Kaohsiung Veterans General Hospital.

### 001035

#### Fluid loading reduces EIT-derived end-expiratory lung impedance in critically ill patients

M. Umbrello[1], AC. Lusardi[1], P. Formenti[1], M. Guanziroli[1], M. Gotti[1], S. Froio[1], S. Coppola[1], D. Chiumello[2]

[1]UO Anestesia e Rianimazione, Ospedale San Paolo - Polo Universitario, ASST Santi Paolo e Carlo, MILANO, Italy; [2]Dipartimento di scienze della salute, Università degli Studi di Milano, Milano, Italy

**Correspondence:** M. Umbrello

*Intensive Care Medicine Experimental* 2019, **7(Suppl 3):**001035

**INTRODUCTION.** Acute circulatory failure is a life-threatening condition. Fluid loading aims to increase cardiac output and restore organ perfusion. Only 50% of patients are fluid responders, while the remainder are at risk of volume overload (peripheral and pulmonary edema).

Electrical impedance tomography (EIT) shows lung impedance as determined by small electrical currents. An increase in intrapulmonary gas volume increases impedance, while an increase in blood or fluid volume, lowers it. In healthy volunteers, saline administration led to a decrease in lung impedance.

**OBJECTIVES.** Aim of the present study was to assess the effect of a fluid challenge on EIT-derived lung impedance in critically ill patients with acute circulatory failure.

**METHODS.** Hemodynamic and respiratory variables, blood samples, cardiac ultrasound and EIT measurements were recorded before a 5 ml/kg fluid challenge, and repeated at the end of fluid infusion and 20 minutes after. As a surrogate for stroke volume, the pulsed-wave Doppler examination of the left-ventricular outflow tract velocity-time integral (VTI) was measured; an increase >15% of VTI identified fluid responders. Factorial analysis of variance for repeated measures was used to compare the values obtained during each study phase.

**RESULTS.** We enrolled 13 patients (8 males, age 70±10 years, BMI 26±4 kg/m2). The average volume of the fluid challenge was 280±90 ml. 5 patients were fluid responders (38.5%).

Systolic blood pressure significantly increased with fluid challenge (105±13 vs. 119±24 vs. 121±26 mmHg, p=0.005), as did the VTI (19.5±4.2 vs. 22.1±5.6 vs. 21.9±5.4 mm, p=0.002). End-expiratory lung impedance significantly decreased after fluid administration: 1700±814 vs. 431±436 vs. 207±202 arbitrary units, p<0.001. The decrease in lung impedance was associated with worsening oxygenation (R=0.560, p=0.047) (Figure 1).

**CONCLUSION.** Electrical impedance tomography, thanks to its high temporal resolution, could be a valuable tool to assess the effect of fluid loading on lung water.

**REFERENCE(S)**

1. Campbell JH, Clinical Science 1994; 87: 97-101

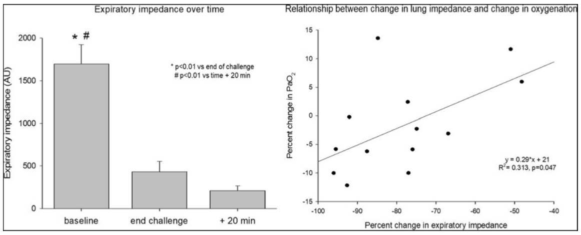

**Fig. 1 (abstract 001035).** See text for description.

### 001043

#### Corrected Carotid Flow Time Assessed by Novice Sonologists Fails to Predict Fluid Responsiveness in Intensive Care Unit Patients

A. Abbasi[1], A. Nader[1], M. Nayeemuddin[1], A. Schick[2], T. Lopardo[3], G. Phillips[4], R. Merchant[5], M. Levy[1], M. Blaivas[6], K. Corl[1]

[1]Division of pulmonary, critical care and sleep medicine, Warren Alpert Medical School at Brown University, Providence, United States of America; [2]Department of emergency medicine, Warren Alpert Medical School of Brown University, Providence, United States of America; [3]Warren alpert medical school, Brown University, Providence, United States of America; [4]Center for biostatistics, department of biomedical informatics, The Ohio State University, Columbus, Ohio, USA, United States of America; [5]Department of emergency medicine, Harvard Medical School, Boston, United States of America; [6]Department of medicine, University of South Carolina School of Medicine, Columbia, United States of America

**Correspondence:** A. Abbasi

*Intensive Care Medicine Experimental* 2019, **7(Suppl 3):**001043

**INTRODUCTION.** Accurately predicting fluid responsiveness in the critically-ill remains a challenge. Point-of-care ultrasound assessment of corrected carotid flow time (cFT) has been proposed as a feasible, non-invasive means of determining fluid responsiveness.1Prior research found a change in cFT of 7ms following a passive leg raise, as measured by expert sonologists, was able to predict fluid responsiveness in patients with undifferentiated shock.2

**OBJECTIVES.** We aimed to prospectively evaluate the ability of point-of-care ultrasound assessment of cFT performed by novice sonologists enrolled in a medical critical care fellowship, to predict fluid responsiveness in critically-ill intensive care unit patients.

**METHODS.** Three novice sonologists performed point-of-care ultrasound assessments of cFT in 115 intensive care unit patients with suspected volume depletion admitted at two urban academic medical centers from November 2016 to July 2018. The novice sonologists received a 6-hour training session and were required to demonstrate proficiency in 10 proctored exams. They performed assessments of cFT in study participants using a Sonosite Edge (Bothell, WA) at baseline and following a 500 mL 0.9% normal saline intravenous bolus. Fluid responsivenesswas defined as a ≥ 10% increase in cardiac index following the fluid bolus, as measured using bioreactance (NICOMTM, Cheetah Medical, Tel Aviv, Israel). The change in cFT was then compared to this fluid responsiveness definition. Novice sonologists were blinded to the NICOM results.

**RESULTS.** Characteristics of the study participants: median age of 53 years old, APACHE II score of 15, BMI of 27 kg/m3, and 57 (50%) were fluid responders. Diagnosis at hospital discharge included; severe sepsis/septic shock (29%), DKA/HHS (31%), GI hemorrhage (12%) and alcohol withdrawal (5%). The optimum change in cFT of 6ms performed poorly at predicting fluid responsiveness: AUC-ROC of 0.53 (95% CI: 0.41-0.63), sensitivity of 55% (95% CI: 43%-67%), specificity of 54% (95% CI: 42%-67%), and positive and negative likelihood ratios 1.21 (95% CI: 1.19-1.22) and 0.82 (95% CI: 0.81-0.84), respectively.

**CONCLUSION.** Our study does not support the use of point-of-care ultrasound assessment of cFT to predict fluid responsiveness in intensive care unit patients by novice sonologists. Further research examining the relationship between point-of-care ultrasound proficiency and cFT is warranted.

**REFERENCE(S)**

1. Marik PE, Levitov A, Young A, Andrews L. The use of bioreactance and carotid Doppler to determine volume responsiveness and blood flow redistribution following passive leg raising in hemodynamically unstable patients. Chest 2013;143:364-70.
2. Barjaktarevic I, Toppen WE, Hu S, et al. Ultrasound Assessment of the Change in Carotid Corrected Flow Time in Fluid Responsiveness in Undifferentiated Shock. Crit Care Med 2018;46:e1040-e6.

### 001048

#### Association between extravascular lung water index and fluid balance in critical care patients

S. Sosa-Santos, LA. Gorordo-Delsol, JC. Gasca-Aldama, AH. Morales-Morales, KJ. Castillo-Medrano, ML. Pacheco-Rivera, D. Sanabria-Cordero, A. Rodríguez-Peredo, JA. Zepeda-Pérez, LE. Gaytán-Medina, I. Maldonado-Beltrán, SE. Zamora Gómez, JA. Castañon-Gonzalez, NI. Medveczky-Ordoñez

Adult intensive care unit, Hospital Juárez de México, Ciudad de México, Mexico

**Correspondence:** L.A. Gorordo-Delsol

*Intensive Care Medicine Experimental* 2019, **7(Suppl 3):**001048

**INTRODUCTION.** In critical care patients the positive fluid balance is associated with highly mortality (HR 1.014 ml/kg)1, therefore the fluid administration, despite being an essential treatment for septic shock patients, must be assessed and individualized. Furthermore in the critical care patients with acute respiratory distress syndrome (ARDS), the extravascular lung water index (EVLWi) has been an independent prognostic factor2. In ARDS patients the fluid administration based in EVLWi meditions is safe and lead to a less fluid balance acummulation, mortality, mechanic ventilation days and UCI stay days3.

**OBJECTIVES.** To determinate the association between fluid balance and ELVWi

**METHODS.** Retrospective, cohort, analytical study establishing the association between daily fluid balance, changes in EVLWi and mortality in ICU at 24, 48 and 72 hours.

**RESULTS.** We collected data from 20 patients with EVLWi and fluid balance daily monitoring for the first 3 days, we established an EVLWi cutoff value of >11 ml/kg, and was associated with a higher mortality; on the first 24 hours with an OR 36 (95% IC 2.7210 a 476.2989, p = 0.0065), at 48 hours OR 20 (95% IC 1.4161-282.4627, p = 0.0266) and at 72 hours OR 19.2857 (95% IC 0.7977-466.2641 p = 0.0686). Fluid balance cutoff value was established at ≥ 3.5 L, without association with mortality OR 0.0877 (95% IC 0.0041-466.2647 p = 0.0686) at day 1, OR 0.4667 95% CI 0.0369 – 5.9028 p=0.5561)at day 2 and OR 0.5000 95% CI 0.0374-6.6838 p= 0.6003) at day 3. The association between fluid balance and EVLWi results in a Pearson's R2= 0.01269.

**CONCLUSION.** EVLWi was associated with an increased mortality, as shown in previous studies3, unlike fluid balance ≥3.5 L, that shows no association with mortality, we believed this results from the management and neutralization of cumulative fluid balance during the UCI stay. We did not find correlation between fluid balance and EVLWi. We believed that lower values of fluid balance and EVLWi maybe correlated with improvement and good response to treatment.

**REFERENCE(S)**

1. Acheampong A, Vincent, JL. A positive fluid balance is an independent prognostic factor in patients with sepsis. Critical care 2015;19(1), 251.
2. Sakka SG, Klein M, Reinhart K, Meier-Hellmann A. Prognostic Value of Extravascular Lung Water in Critically Ill Patients. CHEST. 2002; 122(6), 2080–2086.
3. Cordemans C, De Laet I, Van Regenmortel N, Schoonheydt K, Dits H, Huber W, et al. Fluid management in critically ill patients: the role of extravascular lung water, abdominal hypertension, capillary leak, and fluid balance. Annals of intensive care, 2012;2(Suppl 1):S1.

4. No grant or financing were received

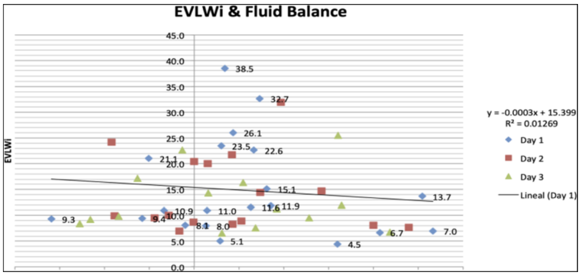

**Fig. 1 (abstract 001048).** See text for description.

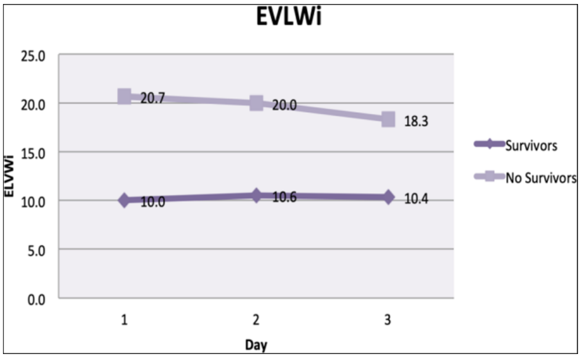

**Fig. 2 (abstract 001048).** See text for description.

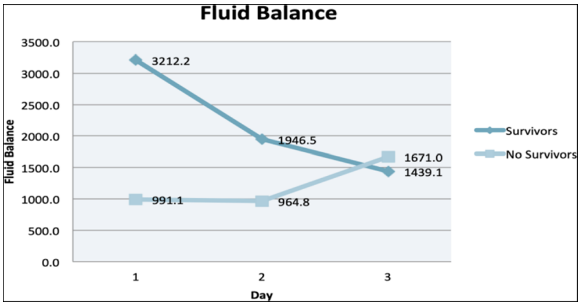

**Fig. 3 (abstract 001048).** See text for description.

### 001063

#### Quality of Life and Survival after Transcatheter Aortic Valve Implantation (Transfemoral TAVI)

CM. Rodríguez Mejías, L. Olivencia Peña, P. Castán Ribas, A. López Fernández, JP. Valencia Quintero, MJ. García Delgado
Medicina Intensiva, Hospital Universitario Virgen de las Nieves, Granada, Spain
**Correspondence:** C.M. Rodríguez Mejías
*Intensive Care Medicine Experimental* 2019, **7(Suppl 3):**001063

**INTRODUCTION.** Aortic stenosis is the most frequent valvular disease in adults, its main etiology being degenerative. The gold standard treatment is the surgical valvular replacement. Although there are non-candidates because of the high surgical risk, using less invasive methods such as transfemoral TAVI.

**OBJECTIVES.** To analyze survival, degree of functionality and quality of life after implantation of transfemoral TAVI.

**METHODS.** Longitudinal, unicentric study, based on the ARIAM prospective registry of cardiac surgery. Including 77 patients consecutively submitted to transfemoral TAVI from January 2015 to December 2016. Follow-up time from 1.5 to 3.5 years, made by telephone. Descriptive analysis of baseline demographic variables: age, sex, comorbidities, surgical risk (EuroSCORE) and NYHA grade. Survival by the Kaplan-Meier method, quality of life with the SF-12 Health Survey questionnaire and post-TAVI functional assessment according to NYHA. We used non-parametric Wilcoxon test for NYHA pre and post, Cox regression for factors associated with survival and multivariate logistic regression model for factors associated with quality of life.

**RESULTS.** The mean age was 78.51 ± 8.70 years, with 53.2% women, NYHA of 2.48 ± 0.66, EuroSCORE of 15.23 ± 9.20 and EuroSCORE 2 of 3.64 ± 2.96. The survival within the first month was 98.70% (95% CI: 96-100%), 90.90% at 6 months (95% CI: 85-96%), 80.52% (95% CI : 72-88%) after one year, 72.0% (95% CI: 63-80%) at two years and 60.30% (95% CI: 50-69%) after three years. Twenty five patients died, being the most frequent cause heart failure for 16% of them and cerebral hemorrhage for 16%. Forty seven patients answered to the quality of life questionnaire. Our SF-12 scores were below those for the Spanish population above ≥75 year. There was a small difference in the physical summary component and a large difference in the mental summary component. Functional capacity results show recovery (NYHA I) in only 17% of patients, with no association in the explored variables associated with survival after TAVI (age, sex and NYHA).

**CONCLUSION.** The implantation of transfemoral TAVI in our center shows very good results in terms of survival in the short and medium terms. Our results for gain in quality of life and functional recovery are comparable to previously reported results.

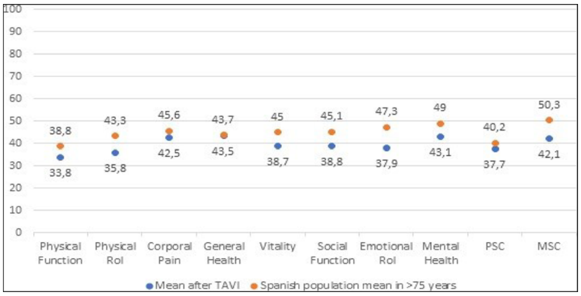

**Fig. 1 (abstract 001063).** See text for description

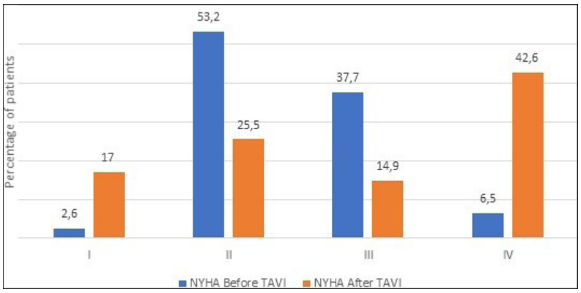

**Fig. 2 (abstract 001063).** See text for description

### 001066

#### The impact of atrial fibrillation on the cardio-circulatory performance in critically ill: An analysis of a prospectively maintained database on transpulmonary thermodilution and pulse contour analysis

W. Huber, F. Weikl, G. Batres-Baires, U. Mayr, A. Herner, S. Schreiber, R. Schmid, T. Lahmer
Medizinische klinik und poliklinik ii, Klinikum rechts der Isar; Technische Universität München, Munich, Germany
**Correspondence:** W. Huber
*Intensive Care Medicine Experimental* 2019, **7(Suppl 3):**001066

**INTRODUCTION.** Atrial fibrillation (AF) is one of the most frequent arrhythmias in the ICU. The prevalence in non-cardiac ICUs is given with up to 10%. While the incidence of new-onset AF and the risk of neurological sequelae is well investigated, little is known about the impact of AF „per se" on the cardio-circulatory performance in critically ill patients.

**METHODS.** Therefore, we analysed a prospectively maintained database on advanced haemodynamic monitoring with transpulmonary thermodilution (TPTD) and pulse contour analysis (PCA) (PiCCO; Pulsion; Germany). Primary endpoint: Comparison of haemodynamic parameters between patients with SR and AF. The final analysis was restricted to patients with jugular/subclavian CVC, since femoral CVC indicator injection might result in overestimation of global end-diastolic volume index GEDVI. Statistics: Wilcoxon-test for unpaired samples. ROC-analysis and multiple regression analysis regarding CI≤2.5L/min/m$^2$. IBM SPSS 25.

**RESULTS.** The database included 19,025 TPTDs in 1,067 patients. AF and sinus rhythm (SR) were documented in 2925 (15.4%) and 15,653 (84%) of the TPTDs, respectively. Among the 11,297 TPTDs in patients with jugular or subclavian CVC, 1,771 (15.7%) TPTDs were performed under AF and 9,306 (82.7%) under SR. 61% of the patients were male, 39% female. The underlying diseases were sepsis (30%), liver cirrhosis (26%), GI-bleeding (7%), ARDS/pneumonia (10%), cardiogenic (7%), pancreatitis (3%), various (17%). Biometrics: Measurements under AF were more frequent among women compared to men (44% vs. 38%; p<0.001). Patients with AF were older (69±9 vs. 61±13 years; p< 0.001), smaller (171±10 vs. 173±8cm; p<0,001) and had higher weight (91±28 vs. 80±25kg; p<0.001) and BMI (33±13 vs. 26±7kg/m$^2$; p<0.001). Patients with AF had a higher heart rate (100±21 vs. 91± 18/min; p<0.001) and CVP (17±6 vs. 15±7 mmHg; p<0.001), but slightly lower MAP (82±14 vs. 83±15mmHg; p=0.039). Global end-diastolic volume index GEDVI was substantially higher in patients with AF (914±210 vs. 816±188mL/m$^2$; p<0.001). As expected, stroke volume variation SVV was markedly higher in patients with AF (21±7 vs. 13±7%; p<0.001). Contractility was moderately impaired under AF: CI (3.3±1.2 vs. 4.4±1.4/min/m$^2$; p<0.001), stroke volume index SVI (35±13 vs. 49±16mL/m$^2$; p<0.001) and dPmax (1392±523 vs. 1446± 596mmHg/s; p=0.007) were lower under AF compared to SR. General cardiac performance as measured by cardiac power index CPI was lower for patients with AF (0.60±0.23 vs. 0.81±0.30W/m$^2$; p=0.331). Extravascular lung water was slightly higher under AF (12±5 vs. 11± 5mL/kg; p<0.001). By contrast, pulmonary vascular permeability index

PVPI (2.1±0.9 vs. 2.1±1.1; p=0.987) was comparable for measurements with AF and SR.
AF (ROC-AUC=0.639; p<0.001), low GEDVI (AUC=0.621; p<0.001) and low heart rate (AUC=0.532; p=0.002), but not CVP (AUC=0.515; p=0.149) predicted a CI≤2.5L/min/m². In multivariate analysis AF, low HR and low GEDVI were independently associated with a CI≤2.5L/min/m².
**CONCLUSION.** AF resulted in a moderate reduction of SVI and CI. Heart rate and GEDVI were slightly elevated. General cardiac performance with AF measured by CPI was reduced by 26%.

#### 001071

#### Association of the contractility index "dPmax" with other haemodynamic parameters and biometric data: Analysis of a prospectively maintained database including 19,025 transpulmonary thermodilution measurements in 1067 patients

W. Huber, S. Schikora, A. Herner, S. Rasch, U. Mayr, S. Schreiber, G. Batres-Baires, R. Schmid, T. Lahmer
Medizinische klinik und poliklinik ii, Klinikum rechts der Isar; Technische Universität München, Munich, Germany
**Correspondence:** W. Huber
*Intensive Care Medicine Experimental* 2019, **7(Suppl 3):**001071

**INTRODUCTION.** dPmax is defined as the maximum increase in arterial pressure per time and can be measured invasively by catheterization or noninvasively by Doppler-technique. Left ventricular and aortic dPmax have been suggested as an index of cardiac performance. However, the usefulness of dPmax is controversely discussed, since it might also depend on preload, afterload, heart rate and myocardial hypertrophy.
Several haemodynamic monitoring devices provide continuous dPmax. Surprisingly, there is a lack of systematic data on the association with other haemodynamic parameters as well as with biometric data. Therefore, we analyzed a large prospectively maintained database on transpulmonary thermodilution and pulse contour analysis (PiCCO; Pulsion; Germany).
**METHODS.** Analysis of a database including 19,025 triplicate measurements in 1067 patients. Statistics: Spearman correlation, Wilcoxon test, multivariate regression, ROC-analysis. IBM SPSS 25.
**RESULTS.** 61% of the patients were male, 39% female. Age 63±13 years, height 172±8cm, weight 80±23kg. The underlying diseases were sepsis (30%), liver cirrhosis (26%), GI-bleeding (7%), ARDS/pneumonia (10%), cardiogenic (7%), pancreatitis (3%), various (17%).
Among biometric parameters, dPmax had a slight, but significant association with low weight (r=-0.099; p<0.001), small height (r=-0-095; p<0.001) and lower age (r=-0.015; p=0.043). Women had significantly higher dPmax (1473±593 vs. 1381±555 mmHg/s; p<0.001). In multivariate analysis ($R^2$=0.019), dPmax was independently associated with low weight (T=-11.3), small height (T=-10.0) and young age (T=-6.1; all p<0.001).
Preload: dPmax correlated with low GEDVI (r=-0.195, p<0.001), low CVP (r=-0.018; p=0.039) and high SVV (r=0.195; p<0.001). In multivariate analysis ($R^2$=0.047), dPmax was independently associated with low GEDVI (T=-2.22) and high SVV (T=6.1; all p<0.001).
With regard to parameters of afterload, dPmax was associated with low SVRI (r=-0.154; p<0.001) but not with MAP (r=0.010; p=0.282).
With regard to contractility, dPmax significantly correlated with CI (r=0.194; p<0.001) and SVI (r=0.082; p<0.001). The strongest univariate association of dPmax was found with pulse pressure PP (r=0.601; p<0.001).
Finally, dPmax was associated with heart rate (r=0.161; p<0.001) and sinus rhythm (vs. atrial fibrillation; r=0.020; p=0.006), but not with extravascular lung water index EVLWI (r=0.001; p=0.891).
In a combined regression analysis ($R^2$=0.673), dPmax was independently associated with pulse pressure (T=38.5), low SVRI (T=-6.1), low preload (GEDVI: T=-5.0); SVV: (T=4.3), high heart rate (T=5.1) and high weight (T=3.1; p=0.002; all other p-values <0.001).
In ROC-analysis, low dPmax (AUC=0.637; p<0.001) provided the largest AUC to predict CI≤2.5L/min*m² compared with low GEDVI (AUC=0.618; p<0.001), low PP (AUC=0.602; p<0.001) and low heart rate (AUC=0.538; p=0.001), whereas CVP was not predictive.
**CONCLUSION.** dPmax is not substantially influenced by biometry, MAP, rhythm and CI. The main determinants are pulse pressure, low peload (GEDVI) and low afterload (SVRI).

### HSRO - What can we do better?

#### 000507

#### Performance of gastric ultrasonography to validate the positioning of gastric tube in intensive care

J. Abily[1], S. Derville[1], C. Lemaître[2], D. Carpentier[3], L. Lagache[3], C. Girault[4], S. Grangé[3], V. Scherrer[1], V. Compère[1], F. Tamion[3], G. Beduneau[4]
[1]Department of Anesthesiology and Intensive Care, Rouen University Hospital, Rouen, France; [2]Department of hepatogastroenterology, Rouen University Hospital, Rouen, France, France; [3]Medical intensive care unit, Rouen University Hospital, Rouen, France; [4]Medical intensive care unit and unirouen ea 3830, Rouen University Hospital and Normandy University, Rouen, France
**Correspondence:** J. Abily
*Intensive Care Medicine Experimental* 2019, **7(Suppl 3):**000507

**INTRODUCTION.** Gastric tubes (GT) are common in intensive care units (ICU) used for enteral feeding, administration of drugs or aspiration of the digestive tract. Current GT have excellent clinical tolerance. Although a malposition may appear as rare, it may have serious consequences that could lead to patient's death. The only method recommended confirming correct positioning of the GT is chest x-ray. However, restrictive strategies for prescribing chest X-rays are being developed to limit their negative effects (irradiation, accidental removal of medical devices, microbial spread, cost). We hypothesized that ultrasonography (US), now widely used in ICU, would allow the evaluation of the correct positioning of the GT.
**OBJECTIVES.** The aim of this study was to evaluate the performance of gastric US for the validation of the good positioning of the GT.
**METHODS.** We carried out a prospective, bicentric study in Medical and Cardiosurgical ICU in Rouen University Hospital. All patients with new placement of GT, except patients in the post-operative period of visceral surgery, were included. For each inclusion we compared the results of a gastric US to the interpretation of a chest x-ray, to validate the good positioning of the GT.
**RESULTS.** Two hundred and nineteen US were performed from July 2016 to August 2018. In 160 cases, US concluded that the gastric tube was correctly positioned. In 59 cases, US did not visualize the tube in gastric area. Among these 59 cases, only 12 cases of malposition were detected by the chest x-ray. The sensitivity and specificity of gastric US were 0.77 [0.71; 0.82] and 1 [0.75; 1]. Positive and negative predictive values were 1 and 0.20, respectively. The median duration of US was 3 minutes [1; 10]. The median time spent before US was performed was 60 minutes [0; 1275]. It was 150 minutes [0; 1900] before the x-ray was performed which is a significant difference (p<0,0001). US was performed 50 minutes [-781; 1630] earlier than chest x-ray after gastric tube insertion.
**CONCLUSION.** Our results suggested a good performance of gastric US to check the positioning of the gastric tube. This result must be interpreted with caution because of a low power of the study. A multicentre study with greater power would be necessary to validate this practice.

**REFERENCE(S)**

1. Chenaitia H, Brun P-M, Querellou E, Leyral J, Bessereau J, Aimé C, et al. Ultrasound to confirm gastric tube placement in prehospital management. Resuscitation. 1 avr 2012;83(4):447-51
2. Timsit JF, Farkas JC, Boyer JM, Martin JB, Misset B, Renaud B, Carlet J. Central vein catheter-related thrombosis in intensive care patients: incidence, risks factors, and relationship with catheter-related sepsis. Chest. 1998; 114:2017–2213
3. Lichtenstein D, Meziere G, Bidermann P, Gepner A. The comet-tail artifact: an ultrasound sign ruling out pneumothorax. Intensive Care Med. 1999; 25:383–388

4. Bankier AA, Wiesmayr MN, Henk C, et al. Radiographic detection of intrabronchial malposition of nasogastric tubes and subsequent complications in intensive care unit patients. Intensive Care Med. 1997;23:406–410
5. Gubler C, Bauerfeind P, Vavricka SR, Mullhaupt B, Fried M, Wildi SM. Bedside sonographic control for positioning enteral feeding tubes: a controlled study in intensive care unit patient. Endoscopy. 2006;38(12):1256–1260

**Table 1 (abstract 000507).** Patients characteristics

| Characteristics | N=219 |
|---|---|
| SAPS II | 46 [10; 102] |
| BMI | 25,4 [11,8; 50,8] |
| Number of chest X-rays only prescribed for checking GT positioning* | 52/102 |
| Complications related to GT positioning | 0 |

SAPS II (Simplified Acute Physiology Score and the BMI (Body Mass Index) were expressed using a median [min-max°. Complications were expressed in absolute values. *N = 102, this data having been collected only during the second course of the study

### 000513

#### Functional trajectory and outcome: the missing link in frailty studies?

J. Gross, D. Chauhan, K. Dombrowsky, J. Borkowski
Intensive care, London North West University Healthcare NHS Trust, London, United Kingdom
**Correspondence:** J. Gross

*Intensive Care Medicine Experimental* 2019, **7(Suppl 3):**000513

**INTRODUCTION.** Frailty characterises increased vulnerability to a dramatic decline in functional status from a relatively minor stressor. There is increasing evidence that frailty is associated with lower survival and poorer non-mortality outcomes following ICU admission(1). However, the majority of published frailty studies in critical care focus on a patient's frailty status at a single point in time - usually just prior to hospital or ICU admission. Trajectory may also play an important role(2).

**OBJECTIVES.** To explore the range of frailty status in patients admitted to ICU

To investigate the association of frailty and worsening trajectory with survival outcome

**METHODS.** Single centre observational study over a 13-month period from 1/10/2017 to 31/10/2018. Worsening functional trajectory was defined by a single Y/N question as to whether there was any functional deterioration in the 1-year preceding ICU admission. Frailty was defined by scores of ≥5 on the Rockwood Clinical Frailty Scale (RCFS).

**RESULTS.** Of 916 patients that were admitted to ICU during the study period, median age was 67 (age range 16-93) with 516 patients ≥65 years of age. Frailty and trajectory data was collected for 634 and 629 patients respectively (349 and 346 in ≥65 years subgroup). From this cohort, prevalence of frailty was 25.9% overall which increased to 34.7% for those ≥65 years. The range of frailty scores is shown (figure).

39.4 % patients (48.8% age ≥65 years) showed a worsening functional trajectory in the 1 year lead up to ICU admission.

There was a significant association between frailty and in-hospital mortality. Chi-squared=18.397, P<0.0001 (Chi-squared=14.284, P= 0.0002 age ≥65 years)

Worsening trajectory was also significantly associated with in-hospital mortality Chi-squared=24.125, P<0.0001 (Chi-squared= 11.726, P=0.0002 age ≥65)

Functional deterioration within last 12 months showed much stronger correlation with mortality outcome (R=-0.93181, AUCROC = 0.613) compared with frailty (R= -0.85117, AUCROC = 0.588)

**CONCLUSION.** This study shows a significant proportion of patients have a functional deterioration in the 1 year preceding ICU admission which is more strongly correlated with mortality outcome compared with frailty status. The concept of functional trajectory warrants further exploration in future studies.

**REFERENCE(S)**

1. Muscedere J et al. The impact of frailty on intensive care unit outcomes: a systematic review and meta-analysis. Intensive Care Med. 2017;43(8):1105-1122. doi:10.1007/s00134-017-4867-0
2. Ferrante LE et al. Functional trajectories among older persons before and after critical illness. JAMA Intern Med. 2015. doi:10.1001/jamainternmed.2014.7889

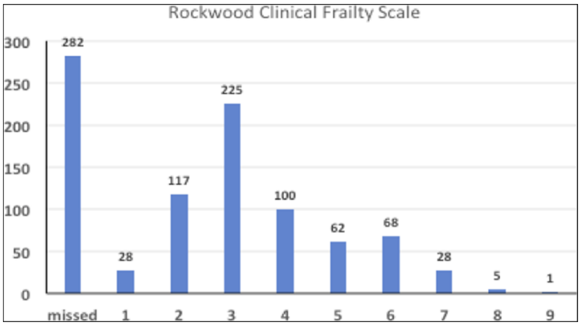

**Fig. 1 (abstract 000513).** See text for description

### 000523

#### Phase Angle and Cumulative Fluid Balance as Prognostic Markers of ICU Mortality

M. Jauniskyte[1], G. Sostakaite[2], A. Klimasauskas[3]
[1]Faculty of Medicine of Vilnius University, Vilnius, Lithuania; [2]Centre of anaesthesiology, intensive therapy and pain management, Vilnius University Hospital Santaros Klinikos, Vilnius, Lithuania; [3]Clinic of anaesthesiology and intensive care, Vilnius University Faculty of Medicine , Vilnius, Lithuania
**Correspondence:** M. Jauniskyte

*Intensive Care Medicine Experimental* 2019, **7(Suppl 3):**000523

**INTRODUCTION.** Low phase angle (PA) values and persistence of a positive fluid balance (FB) are associated with an increased risk of long-term mortality of intensive care unit (ICU) patients.

**OBJECTIVES.** We sought to assess the prognostic value of PA and FB for mortality in critically ill patients.

**METHODS.** Fat-free tissue mass was assessed by measuring the 50-kHz phase angle on the first, fifth and seventh day. The inclusion criteria were: length of ICU stay of ≥7 days; SOFA score of ≥ 3. FB was calculated at 24, 72 hours and at 7 days upon ICU admission. We collected demographic and clinical characteristics of the patients. The APACHE II and SOFA scores were employed to evaluate the severity of illness and the degree of organ dysfunction respectively. We documented the highest vasopressor dose administered within the first 24 and 72 hours of ICU stay. Binary logistic regression analysis was performed to identify independent risk factors associated with ICU mortality.

**RESULTS.** A total of 90 patients admitted to the ICU were included in the present study. 33 patients died during ICU stay. Age 56± 15 y., male 71%, APACHE II 19±7 and SOFA 8±3. There was no statistical difference of gender distribution, APACHE II and SOFA score averages between the outcome groups. Patients in the non-survivor group were significantly older (53,5±14,5 vs. 60±15,1, P=0,045) and had a lower BMI (30,5±6,7; 27,2±4,7 P=0,018) compared to the survivor group. The PA at day 1 and 7 was lower in non-survivors (3,4°± 1,3° vs. 4,0°± 1,2°, P = 0.014 vs. 3,0°± 0,9° vs. 4,1°± 1,4 P= < 0.001). Relative to survivors, non-survivors and patients with lower PA had greater cumulative FB on day 7 - 4,9l±5,5 vs. 0,1l±5,0, (P=< 0.001) and 4,9l±5,4 vs. 0,6l±5,6 (P=0,001) respectively. Mean vasopressor dose during the first 72 hours upon admission was significantly higher in non-survivors (0,17 mcg/kg/min±0,19 vs. 0,08 mcg/kg/min ±0,96, P=0,016). The results of binary logistic regression revealed that greater positive FB during the first week of ICU stay was independently associated with increased mortality (OR 2,2 [1.15- 4,22], P=0,017).

**CONCLUSION.** Fluid accumulation during the first week of ICU stay impacts the PA value and is an independent predictor of mortality. PA at admission is lower in non-survivor. PA is a good prognostic marker of mortality for critically ill patients.

**REFERENCE(S)**

1. Balakumar V, Murugan R, Sileanu FE, Palevsky P, Clermont G, Kellum JA. Both Positive and Negative Fluid Balance May Be Associated with Reduced Long-term Survival in the Critically Ill. Crit Care Med. 2017 m.;45(8):749–57
2. Thibault R, Makhlouf A-M, Mulliez A, Cristina Gonzalez M, Kekstas G, Kozjek NR, et al. Fat-free mass at admission predicts 28-day mortality in intensive care unit patients: the international prospective observational study Phase Angle Project. Intensive Care Med. 2016 m. ;42(9):1445–53

### 000536

#### Review of referrals to Intensive Care Unit (ICU) in a British District General Hospital (DGH)

J. Herzig, E. Landymore, M. Alice, T. Samuels
Critical care, East Surrey Hospital, Redhill, United Kingdom
**Correspondence:** J. Herzig
*Intensive Care Medicine Experimental* 2019, **7(Suppl 3):**000536

**INTRODUCTION.** The NCEPOD and NICE reports state that consultant physicians should be involved in referral of patients to critical care. It is also stated that it is inappropriate for referrals to be made at junior doctor level. Referrals require the doctor to leave ICU in order to assess patients on general wards. Appropriate referrals make the best use of ICU resources and prevent delays in treatment.

**OBJECTIVES.** To review the degree of consultant involvement in the referral and admission process and to ascertain reasons for deeming a referral inappropriate.

**METHODS.** Prospective review of ICU referrals from 01/01/18 to 19/02/18 to a 16 bed adult general ICU. Data was collected by the receiving doctor including; time, demographics, referrer grade, specialist involvement and whether the patient was admitted or not (with reasoning). Data was transcribed for analysis using Microsoft Excel.

**RESULTS.** Data came from 64 referrals over a period of 50 days. The incidence of consultant level referral was 14.1%, with the majority of referrals, 67.1%, originating from non-consultant grades. Allied health professionals and cardiac arrests accounted for 18.8% of referrals. A higher proportion of junior doctor referrals were associated with patients that were not admitted (36.2%) than those that were (24.8%). Referring team consultant awareness occurred in 48.4% of referrals, with an a higher (60.7%) awareness in patients subsequently admitted to ICU than in those then not admitted. Reasons for non-admission to ICU following referral predominantly fell into three categories; no requirement for organ support/invasive monitoring (36.1%), sub-optimisation (27.8%) and inappropriate/irreversible pathology (22.2%). A number of patients were discussed with an ICU doctor but not formally referred for bedside review (8.3%).

**CONCLUSION.** The majority of referrals were from non-consultant grades and in over half the referrals there was no referring team consultant involvement. Incidence of admission to ICU was higher with referring team consultant awareness, suggesting that more appropriate referrals were being generated. Senior involvement, such as that stated in NICE guidance, may result in better optimisation, decision making and escalation planning. Further correlation to mortality data and Apache 2 scores is planned. It is more difficult for escalation decisions to be made out of hours by the critical care team, rather than by the parent team who have been caring for the patient over time. Despite guidance from NCEPOD and NICE there is a continued trend of low referring team consultant level involvement in decision making for patients being referred to ICU. This is potentially due to culture differences in consultant working styles across speciality disciplines. Education, from junior doctor to consultant may be useful in improving senior involvement in decision making for complex and critically unwell patients. Particularly, focussed education at non-consultant level may breed a culture change in the future consultant body.

**REFERENCE(S)**

1. Acutely ill adults in hospital: recognising and responding to deterioration; NICE CG50, July 2007
2. NCEPOD 2005 Report - Chairman Dr Peter Simpson; https://www.ncepod.org.uk/2005report/NCEPOD_Report_2005.pdf

### 000547

#### Duplex Carotid ultrasound assessment of volume status in critical patients : a systematic review

N. parenti[1], ML. Bacchi Reggiani[2], C. Palazzi[3], AM. Pizzini[1], A. Vegetti[3], F. Ferrara[3], F. Pileri[3], E. Romboli[1], F. Agrusta[3], C. Giannoni[1], C. Cavoli[1], S. Zaccaroni[1], C. Cicognani[1], ML. Cipollini[1], L. Baldini[1], A. Pietrangelo[3], M. Silingardi[1]
[1]Internal medicine, Ospedale Maggiore Carlo Alberto Pizzardi, Bologna, Italy; [2]Statistics, Alma Mater Studiorum - Università di Bologna, Bologna, Italy; [3]Internal medicine, University of Modena and Reggio Emilia, Modena, Italy
**Correspondence:** N. parenti
*Intensive Care Medicine Experimental* 2019, **7(Suppl 3):**000547

**INTRODUCTION.** Bedside ultrasonography is a non-invasive, convenient method for volume status evaluation. Recently, Duplex common carotid artery evaluation was reported to predict fluid responsiveness in critical patients. But there is no consensus about optimal index evaluation and very few data on spontaneously breathing patients.

**OBJECTIVES.** To check the validity of Duplex Carotid ultrasonography evaluation in predicting fluid responsiveness and volume status and to assess their correlation with Cardiac Output (CO) in spontaneously breathing patients.

**METHODS.** This review was based on the PRISMA guideline . The systematic search of the literature published from 1941 through 31 December 2018 explored the PubMed, Cochrane Library, Web of Knowledge and Scopus databases. Inclusion criteria were studies who investigated Carotid Ultrasonography correlation with CO, its reliability and accuracy in predicting fluid responsiveness and volume status in adult (>18yrs) spontaneously breathing patients . Two researchers selected studies using inclusion criteria and then assessed their quality using the QUADAS-2 guidelines. The key words for literature search were: fluid responsiveness, spontaneously breathing, ultrasonography, ultrasound echography, volume status, cardiac output.

**RESULTS.** We collected 683 studies: 673 didn't meet inclusion criteria. 10 studies were included in the final analysis. Duplex common carotid artery measures (Corrected Carotid Flow Time, CCFT; Carotid blood flow CBF; variation of carotid artery blood flow peak velocity, Δ V peak) showed an inter-rater agreement range from good to excellent . The CCFT and CBF showed a good correlation with CO but there was a wide range of performance with a range of "r" value from 0,29 to 0,66 . We found only one study which tested the CCFT 's validity in predicting fluid responsiveness: a cut-off value 349 msec has a good accuracy (AUC = 0.84) . Many studies on healthy volunteers showed an increase of CCFT after Passive Leg Raise . The quality of studies were low : many reports had an high risk of Bias in the Reference Standard and index test according to QUADAS-2 guidelines. The main limit of this review is the heterogeneity in design, population and setting of studies included.

**CONCLUSION.** There is only one study investigating duplex carotid ultrasonography's validity in predicting fluid responsiveness in spontaneously breathing critical patients. The quality of the studies included in this systematic review was low. CCFT and CBF seem to correlate with Cardiac Output in healthy volunteers.

### 000552

#### The Sabadell Score as an independent predictor of long term outcomes following intensive care unit admission in a UK teaching hospital

V. Penston[1], B. Morton[1], D. Hungerford[2], P. Mchale[3], G. Dempsey[1]
[1]Critical Care Unit, Aintree University Hospital, Liverpool, United Kingdom; [2]Institute of infection and global health, University of Liverpool, Liverpool, United Kingdom; [3]Department of public health and policy, institute of psychology, health and society, University of Liverpool, Liverpool, United Kingdom
**Correspondence:** V. Penston
*Intensive Care Medicine Experimental* 2019, **7(Suppl 3):**000552

**INTRODUCTION.** The long-term prognosis of post-intensive care unit (ICU) patients is worse than that of the general population. [1] They suffer a significant healthcare burden with many survivors requiring ongoing medical care for over two years. [2] Current scoring systems focus primarily on predicting ICU death and in-hospital deterioration. The Sabadell score has previously been shown to be predictive of survival to hospital discharge. [3,4]

**OBJECTIVES.** To investigate the Sabadell score as a predictor of long-term outcomes following ICU discharge.

**METHODS.** We performed a single centre, observational study at University Hospital Aintree. All patients discharged alive from the ICU between September 2011 and December 2017 were stratified according to the Sabadell score as descrived previously [3,4] and scores recorded along with data including demographics, Acute Physiology and Chronic Health Evaluation II (APACHE II) score and diagnosis. Hospital electronic records were reviewed in July 2018 to determine long-term outcomes.

**RESULTS.** A total of 5953 patients were included in the analysis. Overall in-hospital mortality following ICU discharge was 5.6%. Median survival was 2.2 years in those with a Sabadell score of 2 and 12 days in those with a score of 3. Sabadell score was strongly related to survival (p<0.001) (see figure 1), demonstrating effective discrimination at all time intervals up to five years (area under the receiver operating characteristic curve >0.7). Increasing age and APACHE II score were also associated with mortality (p<0.001).

**CONCLUSION.** The Sabadell score is an effective predictor of long term outcome after ICU discharge with significantly reduced survival in patients classified as Sabadell score of 2 and 3. The score may be used to identify patients more likely to deteriorate following discharge, in order to target early intervention aiming to reduce poor outcomes and, where appropriate, to facilitate timely, informed discussions regarding advance care planning.

**REFERENCE(S)**

1. Gayat E, Cariou A, Deye N et al. Determinants of long-term outcome in ICU survivors: results from the FROG-ICU study. Crit Care 2018;22(1):8
2. Desai S, Law T, Needham D. Long-term complications of critical care. Crit Care Med 2011;39(2)371-379
3. Fernandez R, Baigorri F, Navarro G, Artigas A. A Modified McCabe score for stratification of patients after intensive care unit discharge: the Sabadell score. Crit Care 2006;10(6):R179
4. Fernandez R, Serrano JM, Umaran I et al. Ward Mortality after ICU discharge: a multicentre validation of the Sabadell score. Intensive Care Med 2010;36(7):1196- 1201

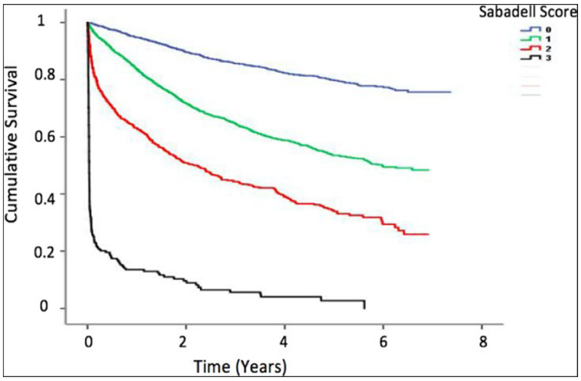

**Fig. 1 (abstract 000552).** See text for description

### 000561

#### Escala de valoración actual del riesgo de desarrollar úlceras por presión en cuidados intensivos – EVARUCI translated to portuguese: concurrent converging validation and correlation with illness severity

F. Wenzel, M. Fernandes Cremasco de Souza, SSV. Zanei, I. Whitaker
Escola paulista de enfermagem, UNIFESP, São Paulo, Brazil
**Correspondence:** M. Fernandes Cremasco de Souza
*Intensive Care Medicine Experimental* 2019, **7(Suppl 3):**000561

**INTRODUCTION.** Pressure injuries (PI) are a global health issue of multifactor and complex causes, being a problem for health services with physical, psychological and economic implications, given that they cause pain and suffering, and are the gateway for infections. The occurrence of PI in hospitalized patients results on an increase in the hospitalization period, being connected to high morbidity and mortality rates and, consequently, increasing direct and indirect spending. In 2001, a group of Spanish specialists proposed the Escala de Valoración Actual del Riesgo de desarrollar Úlceras por Presión en Cuidados Intensivos (EVARUCI) considering the most frequent known risk factors to which critical patients are exposed and the opinion of health professional about which would be the most frequent risk factors in ICU patients. EVARUCI was translated and adapted to Brazilian Portuguese, having satisfactory results concerning the internal coherence and inter-examiner reliability. To continue the validation process of the Portuguese version, this study was performed, aiming to widen the analysis of the scale's performance and to obtain another option for the evaluation of PI risk specifically for critical patients.

**OBJECTIVES.** To analyze the concurrent converging validation of EVARUCI and its correlation with illness severity in ICU patients.

**METHODS.** This methodological study was conducted in three ICU of the University Hospital of the Universidade Federal de São Paulo, Brazil. The inclusion criteria of patients to the sample were age ≥ 18 years old, absence of PI and length of ICU stay > 24 hours. The Braden Scale was used for concurrent converging validation, and Simplified Acute Physiology Score (SAPS 3) for correlation with the illness severity. Both, Braden Scale and SAPS 3 were considered as gold standart and it was applied simultaneously. The analysis was made applying the Spearman's Correlation Test.

**RESULTS.** 324 patients were includes in the sample. 49.4% were female and the average age of patient was 58 years old (18-95; SD 19.2). 53.7% were surgical patients and 85.8% were discharged from ICU. A strong significant correlation was observed between Braden and EVARUCI scores (r = - 0.778 e p<0.001). EVARUCI's values moderately and significantly correlated with those from SAPS 3 score (r = 0.508 e p<0.001).

**CONCLUSION.** EVARUCI scores correlated with Braden Scale scores indicating its possibility of use to evaluate PI risk in critical care patients. The correlation between EVARUCI and SAPS 3 indicated that the greater the illness severity the greater the risk for PU.

**REFERENCE(S)**

1. Cremasco MF, Wenzel F, Zanei SSV, Whitaker IY. Pressure ulcers in the intensive care unit: The relationship between nursing workload, illness severity and pressure ulcer risk. Journal of Clinical Nursing 2012; 22(15-16).
2. Hyun S, Vermillion B, Fall Monica, Li X, Kaewprag P, Moffatt-Bruce S, Lenz ER. Predictive Validity of the Braden Scale for Patients in IntensiveCare Units. Am J Crit Care. 2013 November; 22(6): 514–520.
3. Lospitao-Gómez S, Sebastian-Viana, González-Ruíz JM, Álvarez-Rodriguez J. Validity of the current risk assessment scale for pressure ulcer in intensive care (EVARUCI) and Norton-MI scale in critically ill patients. Applied Nursing Research 2017; 38:76-82.
4. Souza MFC, Zanei SSV, Whitaker IY. Risk of pressure injury in the ICU: transcultural adaptation and reability of EVARUCI. Acta paul. Enferm. 2018;31(2):201-8
5. Pender LR, Frazier SK. The relationship between dermal pressure ulcers, oxygenation and perfusion in mechanically ventilated patients. Intensive Crit Care Nurs. 2005; 21:29-38.
6. Deng X, Yu T, Hu A. Predicting the Risk for Hospital-Acquired Pressure Ulcers in Critical Care Patients. CriticalCareNurse 2017; 37(4).

### 000563

#### Usefulness of the Cuff Leak Test in Patients Undergoing Cervical Spine Surgery

HJ. SUH[1], EY. Kim[1], GJ. Seo[1], SB. Hong[2], Y. Koh[2], CM. Lim[2], JW. Huh[2]
[1]Respiratory care services, Asan Medical Center, Seoul, Republic of Korea; [2]Department of pulmonary and critical care medicine, Asan Medical Center, Seoul, Republic of Korea
**Correspondence:** H.J. SUH

*Intensive Care Medicine Experimental* 2019, **7(Suppl 3):**000563

**INTRODUCTION.** Upper airway oedema following cervical spine surgery can lead to serious complications such as respiratory failure after extubation. The cuff leak test (CLT) has been performed on patients undergoing cervical spine surgery for evaluating the success of extubation because the CLT is used to predict the occurrence of post-extubation laryngeal oedema and stridor. However, the cut-off value of the CLT differs between different studies and there is controversy about its usefulness.

**OBJECTIVES.** This study aimed to evaluate the usefulness of the CLT in patients undergoing cervical spine surgery, as the index of successful extubation.

**METHODS.** We retrospectively reviewed the medical records of patients who underwent a CLT after cervical spine surgery from January 2017 to December 2018. Data from 84 patients were analysed, except for patients in whom the CLT failed or those with incomplete records. The CLT was performed by three respiratory care clinical nurse specialists and values measured using a mechanical ventilator or a wright respirometer. In this study, the cut-off value of CLT for successful extubation was 15% based on the previous studies.

**RESULTS.** All 84 patients had successful extubation. One patient with underlying asthma had a post-extubation stridor and this patient had a cuff leak of 15.7%. Regarding the CLT results, 51 patients (60.7%) had an initial leak of less than 15% and 33 patients (39.3%) had an initial leak of more than 15%. The average leak in the group with initial leak <15% was 6.1% and that in the other group was 30.2%. In the initial leak<15% group, the proportion of women (56.9% vs 27.3%, $p$ = 0.008) and obesity (19.4 ± 19.7% vs 11.0 ± 15.5%, $p$ = 0.042) were higher than those in the initial leak≥15% group. In the initial leak<15% group, the intensive care unit (ICU) length of stay was increased by 1 day (3.3 ± 1.3 days vs 2.3 ± 0.7 days, $p$ < 0.001) and intubation days by 0.9 day (3.2 ± 1.1 days vs 2.3 ± 0.7 days, $p$ < 0.001). There was no significant difference in the use of steroids before and after the CLT and the method of CLT. Thirty-two patients (62.7%) in the initial leak<15% group had CLT results more than 2 times. In this group, the leak just before extubation showed a significant increase compared to the initial leak (5.2 ± 4.7% vs 16.3 ± 11.1%, $p$ < 0.001). Among patients with an initial leak of less than 15%, the intubation days were increased by 1.6 days (3.8 ± 0.8 days vs 2.2 ± 0.8 days, $p$ < 0.001) and ICU length of stay increased by 1.6 days (3.9 ± 1.0 days vs 2.3 ± 1.0 days, $p$ < 0.001) in the group with CLT more than 2 times compared to the group of single CLT.

**CONCLUSION.** Earlier successful extubation was performed when the cut-off value of CLT was more than 15% in patients undergoing cervical spine surgery. However, it is necessary to adjust the cut-off value considering factors such as female sex and obesity.

**REFERENCE(S)**

1. Zhou T, Zhang HP, Chen WW, Xiong ZY, Fan T, Fu JJ, Wang L, Wang G (2011) Cuff-leak test for predicting postextubation airway complications: a systematic review. J Evid Based Med. 4(4):242-254
2. None. No funding to declare.

### 000575

#### The effects of a daily checklist on ICU patients

S. Venturelli, E. Munari, L. Serio, M. Girardis
Intensive care unit, Policlinico of Modena University Hospital of Modena, Modena, Italy
**Correspondence:** S. Venturelli

*Intensive Care Medicine Experimental* 2019, **7(Suppl 3):**000575

**INTRODUCTION.** The World Health Organisation has established that medical errors are the 14thcause of mortality and morbidity worldwide. Due to the specific characteristics of critically ill patients, ICU setting is prone to errors. The use of a checklist has been advocated as a useful tool for reducing the occurrence of errors (1).

**OBJECTIVES.** The objective of our quasi-experimental study is to evaluate whether the use of a daily checklist could improve the clinical outcomes of critically ill patients admitted to ICU with resident program (2,3).

**METHODS.** In 2018 a specific checklist was developed and applied during the morning ICU round to all adult patients admitted to our ICU with an expected length of stay > 24h. To evaluate the effects of check-list use, ICU mortality and length of stay, occurrence of ventilator associated pneumonia (VAP) and catheter-related bloodstream infections (CR-BSI), mechanical ventilation (MV) and central venous catheter (CVC) free days were compared between patients admitted to ICU in 2018 (intervention group) and in the two years before (control group).

**RESULTS.** 264patients in the first nine months of 2018 and 838 in the 2 years before were admitted to ICU and evaluated in the study. No significant differences in age, sex, GCS and SOFA score were observed between the 2 groups. In the intervention group, SAPS II score (44,6 ± DS 19,4) was higher than in the 2 previous years (42,3 ± 19,4). No differences in ICU mortality, ICU length of stay and MV free-days were observed between groups. Differently, incidence of VAP and CR-BSI had a trend in favor of intervention group (1,9% and 1,5% vs 2,7% and 2,3 %) and CVC free-days increased (p<0,05) in 2018 (3 days) compared to other 2 years (2,2 days).

**CONCLUSION.** Our data indicated that the daily use of a check-list in ICU setting with resident program may improve patients' management with reduction of healthcare infections and use of central venous catheter.

**REFERENCE(S)**

1. DuBose JJ, Inaba K, Shiflett A, et al: Measurable outcomes of quality improvement in the trauma intensive care unit: The impact of a daily quality rounding checklist. J Trauma 2008; 64:22–27
2. Resar R, Pronovost P :Using a bundle approach to improve ventilator care processes and reduce ventilator-associated pneumonia. Jt Comm J Qual Patient Saf. 2005 May;31(5):243-8
3. Centofanti JE, Dual E et al: Use of a Daily Goals Checklist for Morning ICU Rounds: A Mixed-Methods Study*. Crit Care Med. 2014 Aug;42(8):1797-803

### 000598

#### Improving Targeted Temperature Management (TTM) in the Neurointensive Care Unit (NICU)

C. Gu[1], YL. Wong,[2], J. Jensen Ng[2], Q. Chen[2], ML. Lim[1]
[1]Nursing service, Tan Tock Seng Hospital, Singapore, Singapore; [2]Anaesthesiology, intensive care and pain medicine, Tan Tock Seng Hospital, Singapore, Singapore
**Correspondence:** C. Gu
*Intensive Care Medicine Experimental* 2019, **7(Suppl 3):**000598

**INTRODUCTION.** Fever is predictive of higher mortality and poorer neurological outcome [1-3] in patients with subarachnoid haemorrhage (SAH), intracerebral haemorrhage (ICH) and traumatic brain injury (TBI). In accordance with expert panel guidelines [4], normothermic TTM should be instituted to improve intracranial pressure control and outcomes.

Retrospective data (Jan-Mar 2018) from our NICU showed that 33% of our target population had persistent fever defined as a core body temperature of ≥38°C for ≥4 hours. Only 48.4% of all febrile patients had hourly temperature monitoring despite an existing protocol.

**OBJECTIVES.** Our project aims to reduce the incidence of persistent fever in patients with SAH, ICH and TBI.

**METHODS.**

A multidisciplinary committee comprising doctors, nurses, pharmacists and support staff was convened.

Macro- and micro-processes of current temperature management were mapped and barriers to existing practices were identified.

An ishikawa diagram was produced using root cause analysis.

Three main causes were identified using Pareto Chart of team votes:

1) Lack of standardised anti-shivering protocol

A new protocol was developed based on existing international guidelines [5].

2) Lack of awareness regarding importance of fever management

We promoted knowledge and skills acquisition through in-service training, roll-call and handovers for nurses. New medical officers were educated during orientation.

3) Lack of effective cooling devices

An auto-feedback temperature regulated machine was put on trial. This device allowed reliable temperature control with minimal increase in staff effort.

**RESULTS.** Since the commencement of an iterative Plan-Do-Study-Act (PDSA) Cycle, there has been increased compliance in hourly temperature monitoring.

After 6 months, the median incidence of persistent fever had reduced from 31.8% to 19%.

TTM can potentially decrease ICU LOS by 3.2 days [6]. Based on a census of 400 patients/year and the 10% reduction we have achieved in patients with persistent fever, the annual cost savings would be $42k-$133k.

**CONCLUSION.** TTM requires education and engagement of a multidisciplinary team. It is a step towards improved patient outcomes and significant cost savings. If further extended to patients with ischemic stroke, we can broaden the spectrum of patients who will benefit from this initiative.

##### REFERENCE(S)

[1] Oliveira-Filho J et al. Fever in SAH: relationship to vasospasm and outcome. Neurology 2001;56:1299–304.
[2] Schwarz S et al. Incidence and prognostic significance of fever following ICH. Neurology 2000;54:354–61.
[3] Bao L et al. Fever burden is an independent predictor for prognosis of TBI. PLoS ONE 2014;9:e90956.
[4] Alain Cariou et al. TTM in the ICU: guidelines from a French expert panel. Ann. Intensive Care 2017;7:70.
[5] Choi HA et al. Prevention of shivering during therapeutic temperature modulation: The Columbia anti-shivering protocol. Neurocrit Care. 2011 Jun;14(3):389-94. doi: 10.1007/s12028-010-9474-7.
[6] Diringer MN et al. Elevated body temperature independently contributes to increased length of stay in neurologic intensive care unit patients. Crit Care Med. 2004 Jul;32(7):1489-95.
[7] This initiative was supported by TTSH hospital quality improvement department. The authors declare no conflicts of interest.

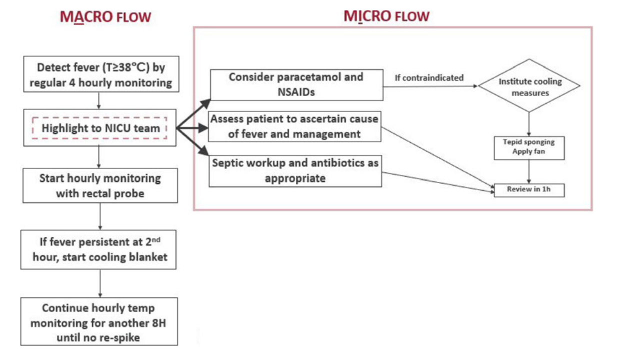

**Fig. 1 (abstract 000598).** See text for description

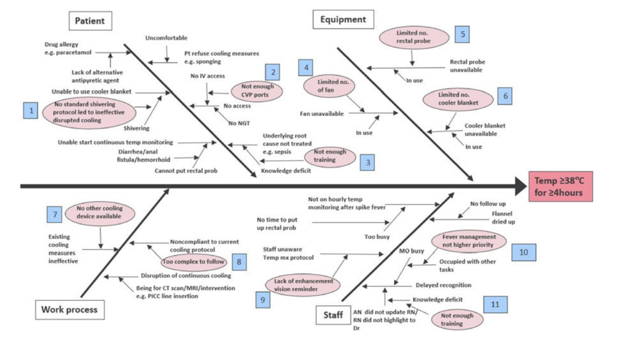

**Fig. 2 (abstract 000598).** See text for description

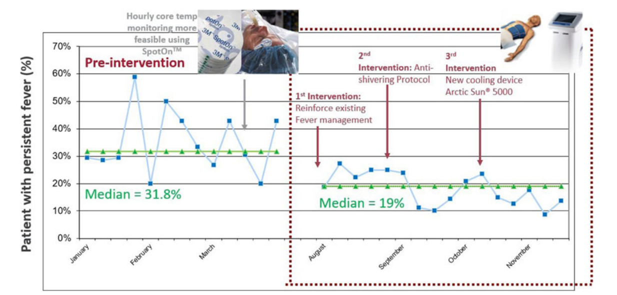

**Fig. 3 (abstract 000598).** See text for description

### 000618

#### Medical criteria for the eligibility of patients to intermediate care units in France: a Delphi national survey

B. Misset[1], R. Boulkedid[2], C. Baillard[3], P. Aegerter[4], B. Guidet[5], C. Alberti[2], M. Beaussier[6]
[1]Department of Intensive Care, CHU de Liège, Avenue de L'Hòpital, Liège, Belgium, Liège, Belgium; [2]Epidemiology, Assistance Publique - Hôpitaux de Paris, Hôpital Robert Debré, Paris, France; [3]Anesthesia and intensive care, Assistance Publique – Hôpitaux de Paris, Hôpital Cochin, Paris, France; [4]Clinical research, Assistance Publique – Hôpitaux de Paris, Hôpital Ambroise Paré, Boulogne-Billancourt, France; [5]Réanimation Médicale, Hôpital Saint-Antoine, Paris, France; [6]Anesthesia, Institut Mutualiste Montsouris, Paris, France
**Correspondence:** B. Misset
*Intensive Care Medicine Experimental* 2019, **7(Suppl 3):**000618

**INTRODUCTION.** In the French healthcare system, intermediate care units (ItCU) were implemented in 2002 under specific legal and financial regulations. Their goal is to admit patients whose acute severity requires continuous surveillance of vital functions but no organ supply. Their use varies among hospitals, depending on the availability of regular ICUs, of emergency department or surgical activities. Some of them may be developed only because their funding is higher than that of regular medical or surgical ward.
**OBJECTIVES.** The aim of this study was to define criteria for intermediate care units, as the first part of a research project designed to establish an admission score to help physicians in the triage process.
**METHODS.** 189 physicians were selected because they had been suggested as experts by French societies of Intensive care, anesthesiology, emergency medicine or surgery, and were working either in public or private hospitals. They were sent a series of 107 criteria, split into 5 categories: chronic disease (n = 29), acute dysfunction (n = 26), etiology of acute disease or specific pathway (n = 35), nursing work-load (n = 11) and hospital organizational aspects (n = 6). Through Delphi analysis, the responders had to assess each item for validity and feasibility with a 0 to 9 Lickert scale. Criteria with a median validity and feasibility over 7 and a concordant response in at least 60% of the responders on the first survey were kept definitively. The other criteria were sent for a second Delphi round. Criteria had to be considered sufficient for ItCU admission either alone or in combination, and had to have the same minimal levels of validity, feasibility and concordance than for the first Delphi round.
**RESULTS.** 81 out of 189 physicians participated in the first round and 62 out of 81 in the second round. They worked mostly in public hospitals (78%) and were aged 54 [47-59] years. 52/107 criteria were kept from the 1st round. Out of the remaining 55 criteria, 11 were kept after the 2nd round and the other ones were definitively discarded. The categories of the kept items after rounds 1 and 2 were: chronic disease (round 1/2 : 1/5), acute dysfunction (26/2), etiology of acute disease (29/2), nursing work-load (3/0) and organizational aspects (3/2).
**CONCLUSION.** In our series or French physicians, criteria for medical eligibility to an ItCU admission are mostly indicators of acute dysfunction and causal acute disease. These criteria will be used in a multicenter prospective study to build and validate a score for admission eligibility.

##### REFERENCE(S)

1. French Ministry of Health

### 000620

#### Outcomes of patients with Learning Disabilities admitted to the Critical Care Unit of a UK District General Hospital: 2012-2017

B. Hopkins[1], M. Spivey[1], R. Mawer[2], W. Woodward[1]
[1]Critical care unit, Royal Cornwall Hospital, Truro, United Kingdom; [2]Department of anaesthetics, Royal Cornwall Hospital, Truro, UK, United Kingdom
**Correspondence:** B. Hopkins
*Intensive Care Medicine Experimental* 2019, **7(Suppl 3):**000620

**INTRODUCTION.** Learning Disability (LD) describes an individual's "reduced intellectual ability and difficulty with everyday activities – which affects someone for their whole life". LD patients have a shorter life expectancy and a higher associated comorbidity e.g. obesity, epilepsy and swallowing problems (1). A UK confidential inquiry in 2013 into premature deaths of LD patients (CIPOLD) identified problems with delayed diagnosis, treatment and advanced health care planning as contributory factors (2). There is a paucity of data examining these healthcare outcomes with relation to Critical Care and so a retrospective study was undertaken to assess this relationship.
**OBJECTIVES.** To identify the number of admissions of patients with LD to Critical Care and conduct an initial review of health outcomes. Primary Outcome Measure: Survival to discharge from Critical Care. Secondary Outcome Measures: Length of Stay, Survival to Hospital discharge.
**METHODS.** A retrospective observational study was performed of all patients with LD admitted to the 15-bed Critical Care Unit, Royal Cornwall Hospital from 01/01/2012 - 31/12/2017. Patients with a registered LD are recorded on the hospital's central database, an automated search identified those admitted to Critical Care in the time period. Patient specific data was captured by a manual review of the electronic patient record (Philiips ICCA).
**RESULTS.**
**CONCLUSION.** Patients with LD represent only 1% of the Critical Care population, yet 1.8% of the general population. Our data suggests LD patients present at a much younger age; 20% of our LD patient cohort were children, compared to only 4.6% of the non-LD patient cohort. Despite comparable illness severity scores, the average length of stay for LD patients was twice that of non-LD patients, reflecting perhaps their complex health and social care needs. Furthermore 25% of LD patients were admitted more than once, compared to just 8% of the non-LD patient group. In our cohort, contrary to what might be expected by existing data regarding outcomes for LD patients, mortality was found to be lower. This might reflect the younger age of the LD cohort, or perhaps lower mortality is explained by higher readmission rates; but this unexpected result demonstrates the importance of further study in this area.
These issues highlight the importance of individualised multidisciplinary care, advanced care planning and treatment escalation discussions, with patient support by specialist LD Liason teams for individuals who may well present on multiple occasions to Critical Care. This study identifies the need for further research in this area, to establish areas for improvement in Critical Care for this vulnerable patient group.

##### REFERENCE(S)

1. MENCAP. 2019. What is a Learning Disability?. [ONLINE] Available at: https://www.mencap.org.uk/learning-disability-explained/what-learning-disability. [Accessed 13 April 2019].
2. Public Health England, (2018). Learning Disabilities: applying All Our Health [ONLINE] Available at: https://www.gov.uk/government/publications/learning-disability-applying-all-our-health/learning-disabilities-applying-all-our-health. [Accessed 28 April 2019]

**Table 1 (abstract 000620).** See text for description

| | LD Patients | Non-LD Patients |
|---|---|---|
| Admissions | 49 | 5273 |
| Number of patients | 33 | 4727 |
| Mean Age (Yrs) | 29.3 | 59.8 |
| Children (%) | 20.4 | 4.6 |
| Male: Female (%) | 57 : 43 | 54 : 46 |
| Unit Mortality (%) | 4 | 12.5 |
| Hospital Mortality (%) | 2 | 5 |
| Mean Length of Stay | 10.3 | 4.4 |
| Mean Admission APACHE II Score | 11.2 | 14.9 |
| Mean Admission ICNARC Score | 18.3 | 16.6 |

### 000623

#### Clinical Frailty Score (CFS): is there a magic number? An observation of mortality in relation to score on the CFS in patients in the Intensive Care Unit (ICU)

T. Sanderson[1], M. Alice[2], S. Ranjan[2], P. Morgan[2], T. Samuels[2]
[1]Intensive Care Unit, East Surrey Hospital, London, United Kingdom; [2]Critical care, East Surrey Hospital, Redhill, United Kingdom
**Correspondence:** T. Sanderson
*Intensive Care Medicine Experimental* 2019, **7(Suppl 3):**000623

**INTRODUCTION.** A growing body of evidence shows that frailty is an independent risk factor for hospital mortality (1, 2). Frailty has also been associated with worse ICU outcomes including increased mortality risk (3, 4). This knowledge base highlights the value of the CFS as a tool included in ICU admission screening. Clinicians have been shown to be accurate assessors of CFS (5), but is there evidence for a "critical value" on the CFS that could increase its utility as part of admission screening by more easily and tangibly predicting increased mortality risk?

**OBJECTIVES.** To review CFS scores in relation to mortality rates to assess evidence for a critical CFS value in predicting mortality, in order to practically aid ICU admission screening.

**METHODS.** Electronic patient records at 1 district general hospital were interrogated to identify all patients admitted to the ICU with a recorded CFS. Data were collected on CFS at ICU admission, ICU outcome ("Improved", "No change", "Worse", "Death") and ultimate hospital outcome ("Lived" or "Died"). Statistical analysis was carried out using R version 3.5.3 (including packages *ggplot2*).

**RESULTS.** A total of 457 patients were identified as having CFS scores on recorded on ICU admission (from 5/10/15-23/1/19), making 3.3% of the 13,882 patients admitted to the ICU during this time period. The Kruskal-Wallis test showed a significant difference in CFS scores between the 4 categories of ICU outcome ($p = 0.02572$). Pairwise comparison using the Wilcoxon rank sum test demonstrated that this significance lay between the "improved" and "died" groups only ($p = 0.019$). Median CFS scores in these groups were 3 (*IQR: 2-4*) and 4 (*IQR: 3-6*) respectively. In ultimate hospital outcome, an unpaired Wilcox test found a significant difference in CFS between the "lived" and "died" groups ($p = <0.001$). Median CFS scores in these two groups were 3 (*IQR: 2-4*) and 4 (IQR: *3-6*) respectively.

**CONCLUSION.** This retrospective, observational study further adds to the evidence base that CFS is associated with ICU and hospital mortality. The small proportion of ICU admissions that recorded CFS demonstrated a current inconsistency in its use during admission screening, possibly uncovering lack of clinicians' confidence in the role of CFS in mortality risk prediction. We found a significant difference in the CFS values between patients who survived ICU or hospital admission and those who did not. These CFS scores may represent critical CFS values at which point ICU or hospital mortality becomes more likely. In a "real-world" setting this could suggest that CFS above a certain level may be a poor prognostic factor of mortality. The findings of this small study may suggest that CFS could be incorporated into well-established prognostic tools, such as the APACHE system. If studied further, the role of the CFS in admission screening could be better defined; allowing clinicians to have another pragmatic, tangible tool to aid them in the real-world when screening patients for mortality risk during ICU admission.

##### REFERENCE(S)

1) Rockwood, K. (2005). A global clinical measure of fitness and frailty in elderly people. Canadian Medical Association Journal, 173(5), pp.489-495.
2) Wallis, S., Wall, J., Biram, R. and Romero-Ortuno, R. (2015). Association of the clinical frailty scale with hospital outcomes. QJM, 108(12), pp.943-949.
3) Muscedere, J., Waters, B., Varambally, A., Bagshaw, S., Boyd, J., Maslove, D., Sibley, S. and Rockwood, K. (2017). The impact of frailty on intensive care unit outcomes: a systematic review and meta-analysis. Intensive Care Medicine, 43(8), pp.1105-1122.
4) Flaatten, H., De Lange, D., Morandi, A., Andersen, F., Artigas, A., Bertolini, G., Boumendil, A., Cecconi, M., Christensen, S., Faraldi, L., Fjølner, J., Jung, C., Marsh, B., Moreno, R., Oeyen, S., Öhman, C., Pinto, B., Soliman, I., Szczeklik, W., Valentin, A., Watson, X., Zaferidis, T. and Guidet, B. (2017). The impact of frailty on ICU and 30-day mortality and the level of care in very elderly patients (≥ 80 years). Intensive Care Medicine, 43(12), pp.1820-1828.
5) Shears, M., Takaoka, A., Rochwerg, B., Bagshaw, S., Johnstone, J., Holding, A., Tharmalingam, S., Millen, T., Clarke, F., Rockwood, K., Li, G., Thabane, L., Muscedere, J., Stelfox, H. and Cook, D. (2018). Assessing frailty in the intensive care unit: A reliability and validity study. Journal of Critical Care, 45, pp.197-203.

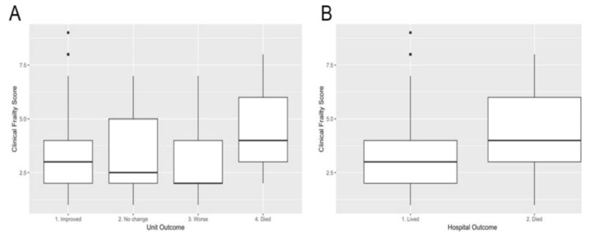

**Fig. 1 (abstract 000623).** See text for description

### 000634

#### Central venous oxygen saturation (ScvO2), lactic acid (LA) and base excess (BE) as determinants of mortality in critically ill patients: A prospective cohort study

M. Torres[1], JC. Torres[1], F. Jaimes[2]
[1]Critical care, SERVIUCIS SA, Medellín, Colombia; [2]Internal medicine, Universidad de Antioquia, Medellin, Colombia
**Correspondence:** F. Jaimes
*Intensive Care Medicine Experimental* 2019, **7(Suppl 3):**000634

**INTRODUCTION.** In critically ill patients, tissue deficit of oxygen can generate hyperlactatemia, acidosis and a dysfunction in venous oxygen saturation, all of them at different body levels. This "dysoxic" disorder is associated with a high mortality rate but a broad assessment of the full state of hypoperfusion, in different categories of critically ill patients, has not been made.

**OBJECTIVES.** To determine the association between several combinations of LA, BE and ScvO2 -at admission to the intensive care unit (ICU) and 12, 24 and 48 hours afterwards- and the probability of hospital death in critical patients admitted to the ICU.

**METHODS.** Prospective cohort study of patients older than 16 years admitted to polyvalent ICUs of two university hospitals in Antioquia, Colombia, between August 2014 and May 2017. Those with cancer, liver failure, expected ICU length-of-stay less than 48 hours or death within 24 hours of admission were excluded. Several logistic regression models were fitted for the analysis, including the main perfusion variables (LA, BE and ScvO2) and their temporal kinetics, as well as acknowledged confounding covariates of clinical importance for the outcome hospital mortality rate.

**RESULTS.** The cohort was composed of 1008 patients, the average age was 55 ± 21 years, 57% (n=575) were men, 85% (n=855) required mechanical ventilation, 56% (n=561) were admitted due to medical problems, 21% (n=211) were post-operative and 23. 4% (n= 236) were trauma patients. At ICU admission, the median SOFA score was 7 (IQR=5;10), the median APACHE-II was 19 (IQR=15;23) and the median LA, BE and ScvO2 were 2,3 (IQR=1,4;4), -5.9 (IQR=-9,6;2,3) and 72,8 (IQR=66,2;78,7), respectively; with an overall hospital mortality of 19.6% (n=198). The multivariate analysis found that, among several potential combinations, only the persistence of abnormal LA and BE at 24 hours after admission to the ICU were associated with a higher mortality (LA > 2 mmol/L, OR = 3,06; 95% CI = 2,05 – 4,61 and BE < -2mm/L, OR 1,56; 95% CI = 1,05-2,32).

**CONCLUSION.** Twenty four hours after admission to the ICU, the persistence of hyperlactatemia and metabolic acidemia (LA> 2 mmol/L and BE < -2mmol/L) indicate the greater risk of mortality in critically ill patients. The abnormality in ScvO2, however, loses clinical importance early in this population.

### 000642

#### Characteristics and Outcomes of Cancer Patients Admitted to the ICU in a Private Hospital in Mexico

RC. Miranda Ackerman[1], IA. Espinoza Mercado[1], JA. Lagunas Fuentes[1], F. Bermúdez Temes[1], BB. Torres Orozco[1], G. Vizcaíno Salazar[1], R. Rouzaud[1], A. González Ojeda[2]
[1]Critical care, Hospital San Javier Guadalajara, Guadalajara, Mexico; [2]General surgery, Centro Médico Puerta de Hierro , Zapopan, Jalisco, Mexico, Mexico
**Correspondence:** R.C. Miranda Ackerman
*Intensive Care Medicine Experimental* 2019, **7(Suppl 3):**000642

**INTRODUCTION.** Mexico is experiencing an epidemiological transition that is adversely impacting and pushing for restructuralization in the healthcare industry. Only a few health institutions in Mexico (public or private) disclose their ICU data. There is a lack of nationwide data to assess the impact of cancer patients admitted to the ICU in our country. Cancer outcomes are important to understand its influence in the health care system. Therefore, it is necessary to standardise statistical cancer-related data across countries, institutions and ICUs.

**OBJECTIVES.** This study aims to scrutinise the outcomes of cancer patients admitted to the ICU in a private hospital in Mexico through statistical analysis.

**METHODS.** Retrospective, observational and descriptive study conducted in the ICU of a private teaching hospital in Guadalajara, Mexico. From January 2017 through December 2018, a total of 201 admissions related to cancer were registered. We enrolled 162 patients, fifteen years of age or older who had cancer and were admitted to the ICU. Patients who were bedridden or receiving palliative care were not excluded.

**RESULTS.** A total of 162 patients were included [56.2% (n=91) were men and 43.8% (n=71) were women], representing 28.2% of the total ICU population. Mean age was 61.27 ± 15.26 years and mean length of stay was 9.6 ± 31.9 days. The most common malignancies were Lung Cancer 18.5% (n=30), Colorectal Cancer 16% (n=26) and Pancreatic Cancer 13.6% (n=22). Most of the patients were admitted for the Oncology service because of complications of its underlying malignant disease 48.1% (n=78). 41.4% (n=67) of the patients were known to have metastases and 59.9% (n=97) underwent surgery. Nearly half of the patients admitted to the ICU presented chronic degenerative conditions 47.5% (n=77) [Hypertension 28.4% (n=46) followed by Type 2 Diabetes Mellitus (T2DM) 19.8% (n=32)]. During their stay in ICU, 14.8% (n=22) required Mechanical Ventilation (MV) and 8.1% (n=12) died ($p< 0.001$). ICU mortality rate was 14.2% (n=23).

**CONCLUSION.** Cancer incidence in ICU is high and it is common that these patients present with comorbidities such as Hypertension and T2DM. In our study mean length of stay was four days less on average compared to data found in prior studies, this could be due to the improvement in cancer management and critical care in general.
Most common primary tumor was lung cancer, followed by colorectal cancer and pancreatic cancer. These same malignancies were more likely to cause readmission to the ICU, this could be due to metastases, however, we did not find any association between metastases, previous ICU admission and mortality.
MV has been associated with higher mortality rates in numerous studies. In our study only a few patients required MV but it was still associated with a higher mortality rate than non-intubated patients.
Further research in specific groups is required to clarify the variables that contribute to the complexity of the associations between cancer and co-morbidities, its complications, and its impact in outcomes.

##### REFERENCE(S)

1. Ríos PR, Rivera AG, Cervantes FS, Martínez PM. Tendencia de la mortalidad por cáncer en México: 1990-2012. Evid Med Invest Salud. 2015; (8)1: 5-15.
2. Romano AM, Gade KE, Nielsen G, Havard R, Harrison JH, Barclay J, et al. Early Palliative Care Reduces End-of-Life Intensive Care Unit (ICU) Use but Not ICU Course in Patients with Advanced Cancer. The Oncologist. 2017 Mar;22(3):318–23
3. Azoulay E, Schellongowski P, Darmon M, Bauer PR, Benoit D, Depuydt P, et al. The Intensive Care Medicine research agenda on critically ill oncology and hematology patients. Intensive Care Medicine. 2017 Sep;43(9):1366–82.
4. Torres VBL, Vassalo J, Silva UVA, Caruso P, Torelly AP, Silva E, et al. Outcomes in Critically Ill Patients with Cancer-Related Complications. Pacheco AG, editor. PLOS ONE. 2016 Oct 20;11(10):e0164537.
5. Fisher R, Dangoisse C, Crichton S, Whiteley C, Camporota L, Beale R, et al. Short-term and medium-term survival of critically ill patients with solid tumours admitted to the intensive care unit: a retrospective analysis. BMJ Open. 2016 Oct;6(10):e011363.
6. Cook D, Rocker G. Dying with Dignity in the Intensive Care Unit. New England Journal of Medicine. 2014 Jun 26;370(26):2506–14.
7. Ríos PR, Rivera AG, Cervantes FS, Martínez PM. Tendencia de la mortalidad por cáncer en México: 1990-2012. :11.
8. Schellongowski P, Sperr WR, Wohlfarth P, Knoebl P, Rabitsch W, Watzke HH, et al. Critically ill patients with cancer: chances and limitations of intensive care medicine—a narrative review. ESMO Open. 2016 Sep;1(5):e000018.
9. Shimabukuro-Vornhagen A, Böll B, Kochanek M, Azoulay É, von Bergwelt-Baildon MS. Critical care of patients with cancer. CA: A Cancer Journal for Clinicians. 2016 Nov 12;66(6):496–517.
10. Aldaco-Sarvide F, Pérez-Pérez P, Cervantes-Sánchez G, Torrecillas-Torres L, Erazo-Valle-Solís AA, Cabrera-Galeana P, et al. Mortalidad por Cáncer en México: actualización 2015. Gaceta Mexicana de Oncología. 2019 Apr 9;17(1):579.
11. The authors are grateful to Dr José Francisco González Sandoval for assisting with the data collection.

### 000644

#### How useful are physiological scoring systems in prolonged critical illness?

J. Gross, M. Dhankhar, K. Dombrowsky, J. Borkowski
Intensive care, London North West University Healthcare NHS Trust, London, United Kingdom
**Correspondence:** J. Gross
*Intensive Care Medicine Experimental* 2019, **7(Suppl 3):**000644

**INTRODUCTION.** Increasing numbers of patients who are admitted to the ICU are older and have co-existing chronic diseases and frailty. Whilst acute illness is strongly associated with mortality risk, there is increased recognition that chronic disease and functional status plays an important role in mortality and long-term ICU outcomes1. This questions the suitability of using physiological scoring systems that largely focus on acute physiology. Recent evidence suggests antecedent factors play a more important role in outcome prediction than acute illness factors for those with prolonged ICU stay2.

**OBJECTIVES.** To compare the performance of APACHE II and SOFA scores in patients with shorter versus more prolonged ICU admission.

**METHODS.** Retrospective analysis of all patients admitted to the ICU at our institution between 1/10/2017 and 30/10/2018. SOFA and APACHE II scores were calculated on admission to the ICU. The discrimination ability of each scoring system for in-hospital mortality (defined by AUROC) was compared in those with short (ICU stay of ≤5 days) versus prolonged (ICU stay of ≥6 days) ICU admission.

**RESULTS.** Over the 13-month period, 916 patients were admitted to the ICU. Median age was 67 years (age range 16-93). From those admitted, APACHE II and SOFA scores were calculated for 601 patients. From this cohort, 307 and 294 patients had short and prolonged ICU stays respectively. For those with short ICU stays, the AUROC for APACHE II and SOFA scores were 0.83 and 0.84 respectively. For those with prolonged ICU stay, the AUROC for APACHE II and SOFA were 0.57 and 0.60 respectively.

**CONCLUSION.** This study shows that the performance of APACHE II and SOFA scores weakens for patients with more prolonged ICU admission. Because APACHE II and SOFA scoring systems are largely based on acute physiology, there may be a greater role for nonphysiological factors (e.g. frailty, chronic disease and functional status) in patients that survive beyond the initial catabolic phase of critical illness. Future studies should explore the relevance of these factors in prolonged critical illness.

#### REFERENCE(S)

1. Krinsley JS et al Pre-admission functional status impacts the performance of the APACHE IV model of mortality prediction in critically ill patients. Crit Care. 2017;21(1):1-9. doi:10.1186/s13054-017-1688-z
2. Bagshaw SM et al. Timing of onset of persistent critical illness: a multicentre retrospective cohort study. Intensive Care Med. 2018;44(12):2134-2144. doi:10.1007/s00134-018-5440-1

### 000646

#### Errors of Omission and Commission in Cardiac Arrest Care in the Intensive Care Setting

HS. Gill[1], S. Srikanth[1], P. Bharati[1], E. Lindgren[2], AD. Steele[2], G. Chakraborti[1], MS. Braga[3]
[1]Critical care medicine, Dartmouth Hitchcock Medical Center, Hanover, United States of America; [2]Critical care medicine, Geisel School of Medicine, Hanover, United States of America; [3]Pediatric critical care medicine, Dartmouth Hitchcock Medical Center, Hanover, United States of America
**Correspondence:** H. Singh Gill
*Intensive Care Medicine Experimental* 2019, **7(Suppl 3):**000646

**INTRODUCTION.** Cardiopulmonary arrest's (CPA's) in the intensive care unit (ICU) are common (1) and guided by advanced cardiovascular life support (ACLS) algorithms. Protocol violations are anecdotally common and their incidence or relation to return of spontaneous circulation (ROSC) in the ICU setting are unknown.

**OBJECTIVES.** To establish the categories and frequency of errors of omission and commission during CPA in the ICU setting and its relationship with CPA outcomes.

**METHODS.** We retrospectively analyzed CPA data entered into the Get with the Guidelines registry from 2015 to 2017. Inclusion criteria: Age above 18, non-traumatic cardiac arrest in the ICU setting. Exclusion criteria: cardiac surgical patients or patients missing data points. Errors of omission were drugs or procedures which were omitted during a CPA but would have been recommended by ACLS algorithms. Errors of commission were drugs or procedures that were committed during a CPA but were not recommended by ACLS algorithms. CPA's with ventricular tachycardia(VT) or ventricular fibrillation(VF) were pooled as were CPA's with asystole and pulseless electrical activity (PEA) for analysis.

**RESULTS.** Of 140 CPA's that met inclusion criteria, 14 were excluded due to missing data fields. Of the final 126 CPA's studied, 103 had asystole/PEA with 65 attaining ROSC and 18 being alive at hospital discharge. Of 23 CPA's with VT/VFib, 20 attained ROSC and 10 were alive at hospital discharge. Mean CPA duration for those achieved ROSC vs those that didn't achieve ROSC were 8.46 minutes and 31.28 minutes respectively.Overall, as errors of omission and commission increased, the percent of patients who attained ROSC decreased. The number of patients with no protocol violations during a CPA was 13.Errors of commission had a statistically significant odds ratio (OR) of 0.52 (95% CI: 0.38 to 0.72) for ROSC during a CPA. Use of sodium bicarbonate (OR-0.22,95% CI: 0.08 to 0.60), calcium chloride (OR-0.25,95% CI: 0.09 to 0.69) and atropine (OR-0.50,95% CI: 0.12 to 2.00) were the most common errors of commission.Errors of omission also had a statistically significant odds ratio (OR) of 2.25 (95% CI: 1.28 to 3.98) for ROSC during a CPA. Errors of omission studied were rate and depth of chest compressions and compression fractions. Mean depth of chest compressions, rate of chest compressions and compression fractions for patients who achieved ROSC vs those that didn't achieve ROSC were 2.52 vs 2.49 inches, 113.55 vs 115.85 per minute and 0.43 vs 0.72 respectively.-Vasopressor infusions initiated prior to but continued during a CPA were also analyzed.This intervention was neither an error of commission or omission but had a statistically insignificant OR of 0.75 (95% CI: 0.52 to 1.10) for attaining ROSC during a given CPA.

**CONCLUSION.** Errors during CPA in the ICU setting were common.Errors of commission,namely sodium bicarbonate and calcium chloride use were statistically significant in decreasing the likelihood of attaining ROSC and likely represent older clinical practices.Errors of omission did not decrease the likelihood of attaining ROSC but effects may be statistically misleading owing to effects of dynamic feedback during CPR.Vasopressor infusions initiated prior to but continued during a CPA decreased the likelihood of ROSC and were almost statistically significant.

#### REFERENCE(S)

1. Ornato JP, Peberdy MA, Reid RD, Feeser VR, Dhindsa HS. Impact of resuscitation system errors on survival from in-hospital cardiac arrest. Resuscitation. 2012; 83(1):63-69.

## TEM - Cardiac arrest and post resuscitation care

### 001283

#### The impact of multidisciplinary targeted temperature management strategies on outcome of patients after cardiac arrest

TH. Hsu[1], YT. Chang[1], KC. Lin[2], CC. Yen[3], WC. Juang[1], YS. Chen[4], WC. Huang[5], CP. Liu[6]
[1]Department of emergency, Kaohsiung Veterans General Hospital, Kaohsiung, Gushan District, Kaohsiung City, Taiwan, Taiwan; [2]Department of critical care medicine and cardiovascular center, Kaohsiung Veterans General Hospital, Kaohsiung, Taiwan; [3]Kaohsiung veterans general hospital, Department of Emergency, Kaohsiung, Gushan District, Kaohsiung City, Taiwan, Taiwan; [4]Department of internal medicine, Kaohsiung Veterans General Hospital, Kaohsiung, Gushan District, Kaohsiung City, Taiwan, Taiwan; [5]Critical care medicine and cardiovascular center, Kaohsiung Veterans General Hospital, Taipei, Taiwan; [6]Critical care medicine and cardiovascular center, Kaohsiung Veterans General Hospital, Kaohsiung, Taiwan
**Correspondence:** W.C. Huang
*Intensive Care Medicine Experimental* 2019, **7(Suppl 3):**001283

**INTRODUCTION.** Cerebral reperfusion injury occurs when cerebral blood flow is restored after cardiac arrest and resuscitation. Increased intracellular levels of glutamate, an excitatory neurotransmitter released from presynaptic terminals, activate ion-channel complexes that cause calcium to shift from the extracellular to the intracellular fluid, leading to the accumulation of oxygen free radicals and the activation of degradative enzymes. In addition, cerebral hemodynamics may remain abnormal after resuscitation from cardiac arrest. Several studies mentioned that induced hypothermia improves outcomes in patients with comatose after resuscitation from out-of-hospital cardiac arrest(OHCA). The aim of this study is to investigate the impact of multidisciplinarytargeted temperature management(TTM) strategies on outcome of patients after cardiac arrest.

**METHODS.** From May 2017 to December 2018, a multidisciplinary CQI team including emergency physician, intensivists, cardiologists, nurses, and quality management team was organized in a tertiarymedical center in Taiwan. The key interventions includedemergent department (ED) activation system, nursing staffs reminding protocol, TTM share decision making protocol, green channel for rapid computerized tomography examination, TTM ED prescription formula, TTM ED quick laboratory and treatment package, comprehensive critical care system and TTM simulation training. The patientswere divided into three groups: pre-interventionalgroup from Jan to Oct 2017, interventional groupOct 2017 to July 2018 and post-interventional group from Aug to December 2018.

**RESULTS.** After implementing multidisciplinary TTM strategies since 2017, the rate of patients with cardiac arrest received TTMincrease from 0% to 33.3% and finally achieved 86.6% ($p<0.001$). The rate of TTM recognition also improved from 50% to 90% in physicians and from 66% to 90% in nursing staffs. Return of spontaneous circulation (ROSC) to targeted temperature time decreased from 540 to 270 minutes.

The survival rate of OHCA with ROSC improved from 22.7% to 25% and the survival rate of OHCA with ROSC received TTM therapy could increase up to 48.4%.

**CONCLUSION.** This study demonstrated multidisciplinary TTM strategies CQI program could improve rate of patients with cardiac arrest received TTM therapy, rate of TTM recognitionin physicians and nursing staffs, ROSC to targeted temperature time, and further survival of cardiac arrest patients.

### 001295

#### Refractory cardiac arrrest treated with extracorporeal cardiopulmonary resuscitation, 2-year follow-up

A. Krüger, P. Ostadal, M. Janotka, J. Naar, D. Vondrakova, P. Neužil
Cardiovascular center, Na Homolce Hospital, Prague, Czech Republic
**Correspondence:** A. Krüger
*Intensive Care Medicine Experimental* 2019, **7(Suppl 3):**001295

**INTRODUCTION.** Extracorporeal cardiopulmonary resuscitation (ECPR) has been introduced as a life-saving procedure in refractory cardiac arrest.

**METHODS.** Eligible patients for this analysis had to undergo ECPR after unsuccessful cardiopulmonary resuscitation with a minimum of three defibrillation attempts. For extracorporeal life support (ECLS) was used Cardiohelp system (Maquet-Cardiopulmonary-AG, Hirrlingen, Germany) or Levitronix CentriMag blood pump (Levitronix LLC, Waltham, MA, USA). LUCAS II (Physio-Control, Lund, Sweden) system was used for chest compressions during cardiac arrest. The relations of blood lactate and pH levels, measured before ECPR insertion and after 24 hours as well as comorbities (diabetes, hypertension, BMI) to the clinical outcomes at 2 years were evaluated.

**RESULTS.** We analyzed data from 49 patients treated with ECPR for refractory cardiac arrest. The mean age of our patients was 59 years. Out-of-hospital cardiac arrest (OHCA) occurred in 25 patients, 24 patients suffered from in-hospital arrest (IHCA). Baseline value of lactate was 11.57 ± 4.22 mmol/l, initial pH 6.95 ± 0.31. In comparison with survivors, patients who died had significantly higher initial lactate levels (12.05 ± 0.81 vs. 8.01 ± 0.77; P< 0.05). Moreover, survivors had significantly lower lactate levels after 24 hours (7.39 vs 2.56) and lower BMI (27.4 vs 31.2; P< 0.05). Diabetes or hypertension in our group have no influence on the mortality. The difference of mortality in the group of OHCA or IHCA was also not significant. 32% patients survived one month with good neurological outcome (CPC 1-2), 30% six months, 23% one year and 21% two years.

**CONCLUSION.** ECPR give the last chance to survive refractory cardiac arrest. The levels of blood lactate are significantly associated with clinical outcomes of ECPR. Obesity was associated with significantly higher mortality in our group.

**REFERENCE(S)**

1. The study was supported by an Institutional grant MH CZ-DRO (Na Homolce Hospital - NNH), IG 150501

### 001317

#### Urinary L-FABP point of care kit is useful for predicting prognosis of refractory ventricular fibrillation patients treated with extracorporeal cardiopulmonary resuscitation

A. Tsuruoka, H. Rinka, T. Miyaichi, H. Arimoto, K. Shigemitsu, T. Yamashita, T. Yoshino, A. Kawamoto, R. Son
Emergency and critical care medical center, Osaka City General Hospital, Osaka, Japan
**Correspondence:** A. Tsuruoka
*Intensive Care Medicine Experimental* 2019, **7(Suppl 3):**001317

**INTRODUCTION.** In recent years, novel AKI biomarkers, such as NGAL, KIM-1, IL-18, L-FABP, IGFBP7, TIMP2 have been discovered and validated to improve early detection for progressive renal failure, need for renal replacement therapy (RRT), or death. Last year, we reported the usefulness of urinary L-FABP for the predictive marker of RRT requirement in critical ill patients and prognosis in patients with septic shock. We are focusing on urinary L-FABP for the diagnostic biomarker of AKI as well as the prognostic marker in critical ill patients.

**OBJECTIVES.** This study was to evaluate whether urinary L-FABP POC kit can predict mortality of refractory ventricular fibrillation (VF) patients treated with extracorporeal cardiopulmonary resuscitation (ECPR).

**METHODS.** This was a single-center retrospective observational study of refractory VF patients treated with ECPR admitted in our intensive care unit (ICU) from December 2017 to January 2019. A total of 16 patients were screened and 14 patients were included, 2 patients died within 24 hours after admission were excluded. We examined the relation of urinary L-FABP POC kit data at ICU admission and 28-day mortality and 90-day mortality.

**RESULTS.** A total of 14 patients were included. Median age was 60 years (IQR 51-67) and all patients were male. Median SOFA score and APACHE2 score were 12 (IQR 10-14) and 35 (IQR 26-38), respectively. Median urinary L-FABP (ng/ml) value was 804.5 (IQR 172.9-3944.3). 12 patients (85.7%) acquired AKI based on KDIGO criteria. All patients had received mechanical ventilation and 6 patients (42.9%) had received RRT. 28-day and 90-day mortality was 42.8% and 64.3%, respectively. The patients were divided into 2 groups according to the results of urinary L-FABP POC kit (group L: negative or slightly positive, group H: strongly positive). The patients who received RRT was 33.3% in group H and 60.0% in group L (p=0.58). There was no significant difference between the groups about initiation of RRT. 28-day mortality was 62.5% in group H and 11.7% in group L. 90-day mortality was 88.9% in group H and 20.0% in group L. Kaplan-Meier curves showed 28-day and 90-day mortality were higher in group H than in group L (p=0.167 and 0.021, respectively).

**CONCLUSION.** Urinary L-FABP POC kit can predict long term prognosis of refractory VF patients treated with ECPR.

**REFERENCE(S)**

1. L-FABP POC kit was provided by CMIC Holdings Co., Ltd.
2. J Korean Med Sci. 2013; 28(1): 100-105.
3. Kidney Int. 2015; 87(3): 640-648.

### 001318

#### Quality of Targeted Temperature Management after out-of-hospital cardiac arrest: an analysis of the TTH48 study

C. De Fazio[1], M. Skrifvars[2], E. Søreide[3], J. Creteur[1], A. Grejs[4], H. Kirkegaard[5], FS. Taccone[1]
[1]Department of intensive care, Erasme Hospital, Université Libre de Bruxelles, Brussels, Belgium; [2]University of helsinki and helsinki university hospital, Perioperative, intensive care and pain medicine, Helsinki, Finland; [3]Critical care and anaesthesiology research group, Stavanger University Hospital, Stavanger, Norway; [4]Department of intensive care, Aarhus University Hospital, Aarhus, Denmark; [5]Research center for emergency medicine, department of emergency medicine and department of clini, Aarhus University Hospital and Aarhus University, Aarhus, Denmark
**Correspondence:** C. De Fazio
*Intensive Care Medicine Experimental* 2019, **7(Suppl 3):**001318

**INTRODUCTION.** Prolonged duration of targeted temperature management (TTM) after out-of-hospital cardiac arrest does not improve neurological outcome.

**OBJECTIVES.** To describe the quality of TTM after out-of-hospital cardiac arrest according to the duration of cooling.

**METHODS.** A retrospective analysis of data from the TTH48 study (NCT01689077), which compared whether TTM at 33°C for 48 hours results in better neurologic outcomes (i.e. good neurological outcome defined by Cerebral Performance Category of 1-2) when compared with standard 24-hour duration. Admission temperature, speed of cooling and rewarming rates were collected in each patient. Precision was assessed by measuring temperature variability (TV), i.e. the standard deviation (SD) of all temperature measurements in the cooling phase. Overcooling and overshoot were defined as one temperature <32°C or >35°C during the cooling phase, respectively. Post-cooling fever was defined as a body temperature exceeding 38.5°C.

**RESULTS.** A total of 352 patients were analyzed in this study; of those, 175 (50%) were treated with TTM for 48 hours. The proportion of patients treated with intravascular catheter was similar between 48-h and 24-h groups (65% vs. 59%; p=0.22). Time from arrest to target (≤34°C) was significantly shorter for patients treated with TTM for 48-h (5.2 [3.9-6.9] vs. 6.0 [4.4-8.4] hours,p= 0.02), with similar body temperature on admission (35.2 [34.3-35.8] vs. 35.0 [34.4-35.7] °C;p=0.44). Temperature variability was similar between TTM for 48-h and 24-h (0.66 [0.46-0.91] vs.0.69 [0.49-0.91]°C; p=0.59), as was rewarming rate (0.34 [0.24-0.46]°C/hour vs. 0.35 [0.22-0.47]°C/hour; p=0.96). The number of patients with overcooling (11% vs. 14%) or overshoot (29%vs. 23%) was also similar between groups. However, the proportion of time outside the target ranges was 13 [5-32]% in the 48-h and 18 [8-37]% in the 24-h groups (p=0.049). The proportion of patients with fever was significantly lower in the 48-h groups than the other (52% vs. 64%; p=0.03).

**CONCLUSION.** TTM for 48 hours was associated with a lower proportion of time with body temperature outside ranges and a lower occurrence of post-cooling fever.

### 001407

#### Hypothermic cardiac arrest patients can tolerate prolonged CPR

K. Maekawa, M. Hayakawa, T. Yoshida, S. Tahara, Y. TAKAHASHI, T. Tsuchida, T. Saito, K. Katabami, T. Wada
Department of emergency medicine, Hokkaido university hospital, Sapporo, Japan
**Correspondence:** K. Maekawa
*Intensive Care Medicine Experimental* 2019, **7(Suppl 3):**001407

**INTRODUCTION.** Resuscitation guidelines suggest that patients with hypothermic cardiac arrest should receive prolonged CPR because reduced core temperature is postulated to result in a neuroprotective effect, in the absence of clinical evidence.

**OBJECTIVES.** To investigate whether patients with hypothermic cardiac arrest can tolerate prolonged CPR and gain a better neurological outcome compared with patients with other etiology of cardiac arrest.

**METHODS.** We performed retrospective analysis using data from Japan's nationwide out-of-hospital cardiac arrest (OHCA) registry, which includes all patients with OHCA transported to the hospital by emergency medical services from January 2013 through December 2016. Patients with CPR duration > 20 min were stratified into hypothermic arrest group and non-hypothermic arrest group, depending on etiology of cardiac arrest. The relationship of CPR duration with neurologically intact survival (defined as cerebral performance category 1 or 2) at 1 month after cardiac arrest was assessed using multivariable logistic regression.

**RESULTS.** Of the eligible 310161 OHCA patients with CPR duration > 20 min, 398 were stratified in hypothermic arrest group and 309763 in non-hypothermic arrest group. Intact survival rate decreased linearly 8.9% per 10 min of CPR duration in non-hypothermic arrest group, but there was no relationship between CPR duration and intact survival in hypothermic arrest group. Compared with non-hypothermic arrest group, hypothermic arrest group had higher adjusted probabilities of intact survival through entire CPR duration; 6.8% vs. 0.84% for 21-30 min of CPR duration, 3.7% vs. 0.75% for 31-40 min, 7.0% vs. 0.7% for 41-50 min, 7.0% vs. 0.67% for 51-60 min and 7.4% vs. 0.68% for > 61 min. Adjusted odds of intact survival for hypothermic arrest were 7.0 (95%CI, 3.7-13.3) for 21-30 min, 7.6 (2.2-25.8) for 31-40 min, 36.9 (12.7-107.4) for 41-50 min and 11.5 (2.2-61.9) for 51-60 min, but no significant odds ratio was found for >61 min.

**CONCLUSION.** Patients with hypothermic cardiac arrest tolerated prolonged CPR. The therapeutic time window of CPR duration might be extended up to 60 min in hypothermic OHCA setting.

### 001413

#### Post-cardiac arrest neuroprognostication: retrospective evaluation of single-centre practice

N. Bolding, E. Svoren-Jabalera, J. Lambert, K. Brown, N. Sudhan
Critical care, Queens Hospital Romford, London, United Kingdom
**Correspondence:** N. Sudhan
*Intensive Care Medicine Experimental* 2019, **7(Suppl 3):**001413

**INTRODUCTION.** The correct identification of patients that are not going to recover consciousness after cardiac arrest is not only clinically challenging but is associated with important ethical and legal implications.

Current guidelines advocate the combination of bedside clinical neurological examination, imaging (CT and MRI) in addition to complex neurophysiological investigations (e.g. SSEPs) and biomarkers (1). These last investigations are not normally available in most centres in the UK. We communicate our appraisal of current practice.

**OBJECTIVES.** To provide further insight into how patients admitted post cardiac arrest are being neuroprognosticated in our general intensive care unit.

**METHODS.** The clinical records of 42 patients admitted to Intensive Care post cardiac arrest over a period of 12 months were revised (1st January 2018–1st January 2019).

**RESULTS.** Out of the 42 patients, thirty-one (74%) were admitted after in hospital cardiac arrest and twenty five (60%) died within first 72 hours. Seventeen (40%) patients underwent post-cardiac arrest prognostication after a period of 72 hours. Sixteen (94%) had a head CT within 24 hours of admission and six (35%) a second CT scan the third day post cardiac arrest. Eight (47%) underwent EEG assessment to neuroprognosticate.

Prognostication was based on bedside clinical examination in all patients (pupillary reactivity and abnormal motor responses). Even though nurse records were complete in all the 17 cases (hourly documentation of pupillary and motor response), we found that the medical clinical notes did not have the same consistency. Furthermore, in the notes of 13 (68%) patients the 24 hours' pupillary reactivity were not stated.

**CONCLUSION.** This assessment of our current practice showed that prognostication of patients post cardiac arrest relies on clinical assessment. In addition to a not protocolised use of auxiliary tests, medical notes documentation need improvement. We should adjust our practice to international recommendations.

##### REFERENCE(S)

(1) European Resuscitation Council and European Society of Intensive Care Medicine. Resuscitation 2014; 85:1779-1789

### 001437

#### Sex differences in prognostic value of neuron-specific enolase in cardiac arrest survivors: results from a prospective registry

D. Vondrakova[1], A. Krüger[1], M. Janotka[1], J. Naar[1], D. Vondrackova[2], P. Neužil[1], P. Ostadal[1]
[1]Cardiovascular center, Na Homolce Hospital, Prague, Czech Republic; [2]Department of neurology, Na Homolce Hospital, Prague, Czech Republic
**Correspondence:** D. Vondrakova
*Intensive Care Medicine Experimental* 2019, **7(Suppl 3):**001437

**INTRODUCTION.** Neuron-specific enolase (NSE) is a well established additional prognostic marker in cardiac arrest survivors. However, the prognostic value of NSE in specific populations remains not fully understood.

**OBJECTIVES.** The aim of our study was to compare the prognostic value of NSE in male and female cardiac arrest survivors at specific time intervals from collapse.

**METHODS.** We analyzed data from a single-center prospective registry of out-of-hospital cardiac arrest survivors admitted between 2010 and 2017 to the tertiary cardiovascular center. All subjects were treated with endovascular hypothermia (33°C for 24 hours). NSE was measured at 1, 2, 3, and 4 days from collapse. Clinical outcomes were assessed according to the Cerebral Performance Category (CPC) at 30 days (good neurologic outcome was defined as CPC 1-2). Prognostic value of NSE for prediction of 30-days outcomes was determined using ROC analysis.

**RESULTS.** A total of 271 individuals were included into the study, 77 female (28%) and 194 male (72%). Female (F) and male (M) population were comparable regarding age, time to return of spontaneous circulation, baseline lactate and pH, and survival with good neurological outcome (57% vs. 61%, P=0.53); however, women had significantly more frequently non-shockable rhythm (43% vs. 19%, P< 0.001). Area under the ROC curve (AUC) at day 1 was 0.78 (P<0.001) in the F group and 0.74 (P<0.001) in the M group. At day 2 the AUC was only 0.85 (P<0.001) in F group and 0.92 (P<0.001) in M group. At day 3 the AUC was only 0.86 (P<0.001) in F group, whereas in M group we observed the highest AUC value (0.94, P<0.001). The highest AUC value in F group was found at day 4 (0.93, P<0.001), corresponding with the AUC of 0.91 (P<0.001) in the M group. NSE values >39.9 mcg/L recorded at any time in the F group were associated with poor prognosis with 100% specificity and 61% sensitivity. On the other hand, the threshold for poor prognosis with 100% specificity in the M group was >57.2 mcg/L (with 52% sensitivity).

**CONCLUSION.** Our results indicate marked sex-differences in the prognostic value of NSE in cardiac arrest survivors. Whereas in women were the prognostic values lower at days 2 and 3 lower in comparison to men and the highest prognostic value was observed 4 days after collapse with lower threshold for poor prognosis, in male was the highest prognostic values of NSE 3 days form collapse with higher threshold for poor prognosis.

**REFERENCE(S)**

1. The study was supported by an Institutional grant MH CZ - DRO (Nemocnice Na Homolce - NNH, 00023884), IG1505

**001496**

**Prognostic value of neuron-specific enolase in cardiac arrest survivors is higher in shockable initial rhythm: results from a prospective registry**

D. Vondrakova[1], A. Krüger[1], M. Janotka[1], J. Naar[1], D. Vondrackova[2], P. Neužil[1], P. Ostadal[1]
[1]Cardiovascular center, Na Homolce Hospital, Prague, Czech Republic; [2]Department of neurology, Na Homolce Hospital, Prague, Czech Republic
**Correspondence:** D. Vondrakova
*Intensive Care Medicine Experimental* 2019, **7(Suppl 3):**001496

**INTRODUCTION.** Neuron-specific enolase (NSE) is a well established additional prognostic marker in cardiac arrest survivors. However, the prognostic value of NSE in specific populations remains not fully understood.

**OBJECTIVES.** The aim of our study was to compare the prognostic value of NSE in groups with shockable and non-shockable initial rhythm at specific time intervals from collapse.

**METHODS.** We analyzed data from a single-center prospective registry of out-of-hospital cardiac arrest survivors admitted between 2010 and 2017 to the tertiary cardiovascular center. All subjects were treated with endovascular hypothermia (33°C for 24 hours). NSE level was measured at 1, 2, 3, and 4 days from collapse. Clinical outcome was assessed according to the Cerebral Performance Category (CPC) at 30 days (good neurologic outcome was defined as CPC 1-2). Prognostic value of NSE for prediction of 30-days outcomes was determined using ROC analysis.

**RESULTS.** A total of 271 individuals were included into the study, 201 patients (74%) had shockable (S) initial rhythm and 70 subjects (26%) had non-shockable (NS) initial rhythm. Patients in the S group were younger (mean age 62 vs. 70 years, P<0.001), more frequently male (78% vs. 53%, P<0.001), had lower baseline lactate (P<0.05), and had better survival with good neurological outcome (67% vs. 41%, P<0.001); time to return of spontaneous circulation was comparable (21 min vs. 18 min, P=0.09). Area under the ROC curve (AUC) at day 1 was 0.70 (P<0.001) in the S group and 0.86 (P<0.001) in the NS group. At day 2 the AUC was 0.92 (P<0.001) in S group and only 0.84 (P<0.001) in NS group. At day 3 the AUC was 0.95 (P<0.001) in S group and only 0.84 (P<0.001) in NS group. At day 4 the AUC was 0.93 (P<0.001) in S group and 0.90 (P<0.001) in NS group. NSE values >50.2 mcg/L recorded at any time in the S group were associated with poor prognosis with 100% specificity and 55% sensitivity. On the other hand, the threshold for poor prognosis with 100% specificity in the NS group was >57.2 mcg/L (with 56% sensitivity).

**CONCLUSION.** Our results indicate that initial rhythm influences the prognostic value of NSE in cardiac arrest survivors. In patients with non-shockable initial rhythm the prognostic values of NSE were generally lower with the highest value 4 days after collapse.

**REFERENCE(S)**

1. The study was supported by an Institutional grant MH CZ - DRO (Nemocnice Na Homolce - NNH, 00023884), IG1505

**001610**

**Surviving a cardiac arrest: What happens after the admission in the Intensive Care Unit?**

R. Menezes Fernandes[1], D. Nuñez[2], N. Barbancho[2], N. Marques[1], C. Dias[3], C. Granja[2]
[1]Cardiology, Centro Hospitalar Universitário do Algarve, Faro, Portugal; [2]Intensive care department, Centro Hospitalar Universitário do Algarve, Faro, Portugal; [3]Medcids – department of community medicine, health information and decision, CINTESIS - Center for Health Technology and Services Research, Porto, Portugal, Portugal
**Correspondence:** R. Menezes Fernandes
*Intensive Care Medicine Experimental* 2019, **7(Suppl 3):**001610

**INTRODUCTION.** Successfully resuscitated patients from a cardiac arrest by out or in-hospital emergency teams are admitted to the Intensive Care Unit (ICU) for post-resuscitation care. However, the prognosis of these patients remains dismal, with only a minority surviving to hospital discharge.

**OBJECTIVES.** To characterize the population admitted to the ICU after successful reanimation from a cardiac arrest, and to analyse the factors associated to the clinical outcomes.

**METHODS.** A retrospective, descriptive and correlational study was performed encompassing patients admitted in the first 24h after a cardiac arrest in an ICU, from 1st January 2014 to 31st December 2018. Through consultation of electronical clinical records, we analysed patient's demographic factors, characteristics of the cardiac arrest, immediate and in-hospital approach, mortality and neurologic (evaluated by Cerebral Performance Category (CPC) Scale) outcomes. Correlation between variables was performed by Chi-square test and Mann-Whitney test, with a significance level of 95%. Independent predictors of clinical outcomes were identified through a binary logistic regression analysis, considering p=0,05. Statistical analysis was conducted using SPSS 23.0.

**RESULTS.** During the defined period, 187 patients were admitted after a cardiac arrest, 65% of whom were male. The median age was 67 years and 39% suffered an out-of-hospital cardiac arrest. 87% had an initial non-shockable rhythm and the most frequent presumed etiology was cardiac (31%). Glasgow coma scale after return of spontaneous circulation was higher in patients that suffered an in-hospital cardiac arrest (p= 0,003) and in patients that survived to discharge (p=0,003). The in-hospital mortality rate was 63%, with most of the deaths associated with initial non-shockable rhythms (93%; p=0,001), higher severity indices (p< 0,001), longer invasive mechanical ventilation (IMV) and vasopressor support (p<0,001). Furthermore, basic life support (BLS) was more frequently initiated in patients who survived to discharge (p=0,027). SOFA score at the admission was statistically significantly higher in males (p=0,007). 30% of the survivors had significant neurologic dysfunction at hospital discharge (CPC 3 and 4). Non-shockable rhythms (p=0,018), non-immediate initiation of BLS (p=0,01), higher SAPS II score (p<0,001) and higher relative duration of vasopressor support (p=0,008) were independent predictors of in-hospital mortality. The absence of post-cardiac arrest shock (p=0,016) and epileptic seizures (p=0,026) were independent predictors of worse neurologic outcome.

**CONCLUSION.** This study confirms the importance of immediate initiation of BLS and, consequently, the need for training out and in-hospital population. Patients with early neurologic dysfunction but no cardiovascular dysfunction may exhibit a worse neurologic outcome.

### 001666

#### Effect of Different Methods of Cooling for Targeted Temperature Management on Outcome after Cardiac Arrest: A Systematic Review and Meta-Analysis

L. Calabro[1], W. Bougouin[2], A. Cariou[3], C. De Fazio[4], M. Skrifvars[5], E. Søreide[6], J. Creteur[7], H. Kirkegaard[8], S. Legriel[9], JB. Lascarrou[10], B. Mégarbane[11], N. Deye[11], FS. Taccone

[1]Intensive care, Hospital Erasme, Bruxelles, Belgium; [2]Intensive care, Université Paris Descartes, Paris, France; [3]Medecine intensive reanimation, Hospital Cochin, Paris, France; [4]Department of intensive care, Erasme Hospital, Université Libre de Bruxelles, Brussels, Belgium; [5]University of helsinki and helsinki university hospital, Perioperative, intensive care and pain medicine, Helsinki, Finland; [6]Critical care and anaesthesiology research group, Stavanger University Hospital, Stavanger, Norway; [7]Soins intensif, ULB Erasme, Anderlecht, Belgium; [8]Research center for emergency medicine, department of emergency medicine and department of clini, Aarhus University Hospital and Aarhus University, Aarhus, Denmark; [9]Medical surgical intensive care, General Hospital Center, Versailles, France; [10]Médecine Intensive Réanimation, Nantes University Hospital Hotel-Dieu, Nantes, France; [11]Medical and toxicology intensive care unit, Hospital Lariboisière, Paris, France

**Correspondence:** L. Calabro

*Intensive Care Medicine Experimental* 2019, **7(Suppl 3):**001666

**INTRODUCTION.** Although targeted temperature management (TTM) is recommended in comatose survivors after cardiac arrest (CA), the optimal method to deliver TTM remains unknown. We performed a meta-analysis to evaluate the effects of different TTM methods on survival and neurological outcome after adult CA.

**METHODS.** We searched on MEDLINE/PubMed database until 22/02/2019 for comparative studies that evaluated at least two different TTM methods in CA patients. Data were extracted independently by two authors. We used QUADAS-2 tool for assessing risk of bias of each study. The Jadad quality scoring system was used to assess the methodological quality of randomized clinical trials (RCT). Primary outcome was the occurrence of unfavorable neurological outcome (UO); secondary outcomes included overall mortality.

**RESULTS.** Our search identified 6886 studies; 22 studies (n=8027 patients) were included in the final analysis. When compared to surface cooling, core methods showed a lower probability of UO (OR 0.85 [95% CIs 0.75-0.96]; p=0.008) but not mortality (OR 0.88 [95% CIs 0.62-1.25]; p=0.21). No significant heterogeneity was observed among studies. However, these effects were observed in the analyses of non RCTs. A significant lower probability of both UO and mortality were observed when invasive were compared to non-invasive TTM methods and when temperature feed-back devices (TFD) were compared to non-TFD methods. These results were significant particularly in non RCTs.

**CONCLUSION.** Although existing literature is mostly based on retrospective or prospective studies, specific TTM methods (i.e. core, invasive and with TFD) were associated with a lower probability of poor neurological outcome when compared to other methods in adult CA survivors (CRD42019111021).

### 000360

#### Hydrocortisone may ameliorate post-arrest myocardial mitochondrial injury: an animal study

SN. Wu, CH. Huang, WJ. Chen, WT. Chang, CK. Wu, MS. Tsai

Department of emergency medicine, National Taiwan University Hospital and College of Medicine, Taipei, Taiwan

**Correspondence:** S.N. WU

*Intensive Care Medicine Experimental* 2019, **7(Suppl 3):**000360

**INTRODUCTION.** Steroid administration during post-arrest period has been reported to improve myocardial dysfunction and hemodynamic following return of spontaneous circulation (ROSC) in cardiac arrest survivors. However the mechanism how post-arrest steroid use ameliorate myocardial injury in cardiac arrest survivors remains largely unknown.

**OBJECTIVES.** To evaluate the effect of post-arrest hydrocortisone administration on myocardial mitochondrial injury and dysfunction in a rat model of ventricular fibrillation (VF) cardiac arrest model.

**METHODS.** VF cardiac arrest was induced and left untreated for 5 minutes in adult male wistar rats. Cardiopulmonary resuscitation and electric shocks were then applied to achieve ROSC. Successfully resuscitated animal were randomized into steroid and non-steroid groups. The steroid group received intravenous hydrocortisone of 8 mg/kg immediately after ROSC and the non-steroid group received saline as placebo. The histology and ultrastructural changes of myocardium, myocardial mitochondrial function, and survival and neurological function at 72nd hour following ROSC were compared between groups.

**RESULTS.** There were 12 animals in each group. Within 4 hours following ROSC, the steroid group had significantly better systolic function (dp/dt) and cardiac output than the non-steroid group. At 4th hour following ROSC, the histology examination and transmission electron microscopy demonstrated less myocardial damage and mitochondrial injury in the steroid group. Besides, steroid prevented the acceleration of Ca2+-induced mitochondrial swelling (mitochondrial permeability transition pore opening) as noted in the non-steroid group. As compared to the non-steroid group, the steroid group also had a significantly higher 72 hour survival rate (91.7% v.s. 50%, $p$ = .013) and higher percentage of good neurological function (75% v.s. 25%, $p$ = .02) at 72nd hour.

**CONCLUSION.** Post-arrest hydrocortisone administration mitigates myocardial damage, ameliorates cardiac mitochondrial injury and improves 72-hour survival and neurological recovery in a rat model of VF cardiac arrest.

### 000384

#### The influences of S-ketamine and alfentanil on lactate, blood glucose, acid-base status and early survival in rats after asphyxia cardiac arrest: pilot study

A. Konkayev[1], N. Akhatov[2], D. Kanzhigalin[3], M. Konkayeva[4]

[1]Anaesthesiology and intensive care, Astana Medical University, Nur-Sultan, Kazakhstan; [2]Anesthesilogy, Astana Medical University, Nur-Sultan, Kazakhstan; [3]Anaesthesiology, Astana Medical University, Nur-Sultan, Kazakhstan; [4]Infectious diseases, Astana Medical University, Beibitshilik Street, Nur-Sultan, Kazakhstan, Nur-Sultan, Kazakhstan

**Correspondence:** A. Konkayev

*Intensive Care Medicine Experimental* 2019, **7(Suppl 3):**000384

**INTRODUCTION.** Acute hypoxia results in uncontrolled release of glutamate and the consequent stimulation of N-methyl-D-aspartate (NMDA) receptors, which affects the homeostasis (1). A potential therapeutic approach to prevent this sequence of events is a blockade of NMDA receptors. Meanwhile, in different models of acute hypoxia, activation of opioid receptors demonstrates significant cardio- and neuroprotective effects with a consequent increase in animal survival (2).

**OBJECTIVES.** Thus, we aimed to test the effects of S-ketamine and alfentanil on lactate, blood glucose, acid-base status and early survival in rats after asphyxia cardiac arrest (ACA).

**METHODS.** After instrumentation under anaesthesia with Isoflurane, Wistar rats (n=26) weighing between 350–400 g were assigned to three groups where: 1. Alfentanil 2.5 mcg/kg iv (n=12) was given 10 min before ACA; 2. S-ketamine 40 mg/kg iv (n=7) was given 10 min before ACA; 3. Control (n=7), the same amount of NaCl 0,9% iv was given 10 min before ACA. The rats were asphyxiated by clamping the endotracheal tube at the end of expiration for 5 min. Resuscitation was initiated by an injection of epinephrine (0.02 mg/kg, iv), followed by manual thoracic compressions (180 compressions/min) and mechanical ventilation (21% O2, 80 breaths/min). Invasive mean arterial pressure (MAP) was recorded at the baseline (BL), injections (Inj), every 1 min during ACA (As) and every 5 min in post-resuscitation (PR) period. Blood gas samples, blood for lactate and glucose were taken at the BL and 20 min at the PR period. Early survival was determined at the 60 min after ACA.

**RESULTS.** Selected groups of animals did not differ on the pre-resuscitation parameters. No differences in blood glucose level and survival between the rats was found at the postresuscitation period. The rats pre-treated by S-ketamine got significantly higher production of lactate (11,4±1,4 mmol/l vs 2,8±1,3 mmol/l, p < 0,002) when compared to the rats treated by alfentanil. Base deficit was significantly lower in group with using of alfentanil (-1.5±1,3 vs -16,5±3,2, p < 0,001) when compared to the rats treated by S-ketamine.

**CONCLUSION.**

Pre-treatment with alfentanil attenuated significantly disturbances in acid-base balance and production of lactate after ACA when compared to the rats pre-treated by S-ketamine.

**REFERENCE(S)**

1. Nolan J.P. et al. Post-cardiac arrest syndrome: epidemiology, pathophysiology, treatment, and prognostication. Resuscitation 2008; 79 (3): 350-379. 2. Bell JD. In Vogue: Ketamine for Neuroprotection in Acute Neurologic Injury. Anesth Analg. 2017 Apr;124(4):1237-1243.

2. The authors are grateful to colleagues V.Kuklin and T.Kondratiev for facilitating this work.

### 000512

#### Post-resuscitation treatment with inhaled argon improves outcome after a prolonged untreated cardiac arrest in a porcine model

F. Fumagalli[1], D. Olivari[1], D. De Giorgio[1], D. Novelli[1], L. Staszewsky[1], D. Zani[2], G. Babini[3], E. Scanziani[4], R. Latini[1], G. Ristagno[3]

[1]Cardiovascular research, Istituto di Ricerche Farmacologiche Mario Negri IRCCS, Milano, Italy; [2]Polo veterinario di lodi, University of Milan, Milano, Italy; [3]Fisiopatologia medico-chirurgica e dei trapianti, University of Milan, Milano, Italy; [4]Medicina veterinaria, University of Milan, Milano, Italy

**Correspondence:** F. Fumagalli

*Intensive Care Medicine Experimental* 2019, **7(Suppl 3):**000512

**INTRODUCTION.** Treatment of post cardiac arrest (CA) syndrome represents a clinical priority. Argon represents an attractive therapeutic option.

**OBJECTIVES.** The purpose of this study was to determine efficacy, safety and the most effective dose of argon in a porcine model of high-severity CA and cardiopulmonary resuscitation (CPR).

**METHODS.** Left anterior descending coronary artery was occluded in 36 pigs, and ventricular fibrillation was induced and left untreated for 12 min. CPR was then performed for 5 min prior to defibrillation. Following successful resuscitation, animals were subjected to 4 hr ventilation with (a) 70% argon - 30% $O_2$ (n=10); (b) 50% argon - 50% $O_2$ (n=10); (c) 70% $N_2$ - 30% $O_2$ (n=10). Hemodynamic parameters were monitored and serial blood samples were obtained for blood gas analysis. Animals were observed up to 96 h for assessment of survival and neurological recovery.

**RESULTS.** Ten animals of 12 were successfully resuscitated in each group. After resuscitation, animals treated with Argon 70% showed higher systolic, mean and diastolic arterial pressure compared to controls and to the Argon 50% treated animals (p<0.05). Ventilation with argon did not have any detrimental effects on respiratory gas exchange during the 4 hr ventilation. There was no statistically significant difference in the number of the resuscitated animals survived up to 96 hrs. However, 6 (60 %) and 8 (80 %) resuscitated animals in the Argon 50% and Argon 70% groups respectively achieved a complete neurological recovery (Overall Performance Categories, OPC=1 or 2), whereas only 3 (30%) animals survived up to 96 hours with a complete neurological recovery in the control group (p<0.0001).

**CONCLUSION.** Argon prevented post CA brain injury in swine model of severe CA, without detrimental effects on hemodynamics and respiratory gas exchanges. Beneficial effects was higher at a concentration of 70% than of 50%.

**REFERENCE(S)**

1. Postresuscitation treatment with argon improves early neurological recovery in a porcine model of cardiac arrest. Ristagno G, et al. Shock. 2014 Jan;41(1):72-8. doi: 10.1097/SHK.0000000000000049

2. This project was supported by the Italian Ministry of Health and Fondazione Sestini.

### 000676

#### Hemoglobin A1c is related to 6-month survival on patients who survived after out-of-hospital cardiac arrest

JH. Lee, SO. Joo

Emergency medicine, Uijeongbu St. Mary's Hospital, The Catholic University, Euijeongbu, Republic of Korea

**Correspondence:** J.H. Lee

*Intensive Care Medicine Experimental* 2019, **7(Suppl 3):**000676

**INTRODUCTION.** Out-of-hospital cardiac arrest(OHCA) is associated with poor survival and can be considered a major global health problem. The early determination of neurologic outcomes is an essential element of risk stratification to identify aggressive treatment of OHCA patients. Hemoglobin A1c(HbA1c) serves as a surrogate marker of glycemic control and is a key risk indicator for diabetes associated microvascular and macrovascular complications and mortality.

**OBJECTIVES.** This study aimed to investigate the association of HbA1c level and out-of-cardiac arrest patients who underwent hypothermic-targeted temperature management.

**METHODS.** This retrospective observational study included adult out-of-hospital cardiac arrest who underwent hypothermic-targeted temperature management from December 2011 to February 2019. Serum HbA1c was measured immediately after return of spontaneous circulation. The primary outcome was 6-month survival.

**RESULTS.** In total, 282 patients were analyzed. Among those, eighty-three patients survived six months. The mean HbA1c in patients surviving six months was 5.67%±SD, which was significantly lower than in patients who did not survive six months (6.21%±SD, P<0.001). The AUC calculated on ROC analysis predicting six month survival was 0.66 (95% confidence interval, 0.60 to 0.71; P<0.001), with a sensitivity of 72% and specificity of 57% at a cut-off of 5.4%. In multivariable logistic regression model, HbA1c level ≥ 5.4% (OR 5.61, 95% CI 2.46-12.81, P<0.01), non-cardiac cause (OR 3.21, 95% CI 1.29-8.01, P=0.01), non-shockable rhythm (OR 2.43, 95% CI 1.02-5.78, P=0.04), non-witness (OR 2.75, 95% CI 1.29-5.88, P=0.01), older age (OR 1.05, 95% CI 1.03-1.08, P<0.01), longer anoxic time (OR 1.08, 95% CI 1.06-1.11, P<0.01) as independent factors of 6-month survivor. The AUC calculated on ROC analysis of multivariable logistic regression model was 0.89 (95% CI 0.85-0.93, P<0.01).

**CONCLUSION.** This study showed that HbA1c of out-of-cardiac arrest(-OHCA) was associated with survival in patients underwent hypothermic-targeted temperature management. But its sensitivity and specificity are unsatisfactory for a single prognostic marker. HbA1c should be considered as an additional modality for prognostification and not as a single reliable variable. Further large multicenter studies are needed to evaluate the utility of HbA1c for early prognostic factor.

**REFERENCE(S)**

[1] Roger VL, Go AS, Lloyd-Jones DM, et al. Heart disease and stroke statistics: 2011 update: a report from the American Heart Association. Circulation 2011;123:e18-209.

[2] Korea Centers for Disease Control and Prevention. EMS-based database for medical record review of out-of-hospital cardiac arrest and mass casualty incident injury. Cheongju: Korea Centers for Disease Control and Prevention; 2011.

[3] Kim SH, Choi SP, Park KN, et al. Association of blood glucose at admission with outcomes in patients treated with therapeutic hypothermia after cardiac arrest. Am J Emerg Med 2014.

[4] Kucharska-Newton AM, Couper DJ, Pankow JS, Prineas RJ, Rea TD, Sotoodehnia N, et al. Diabetes and the risk of sudden cardiac death, the Atherosclerosis Risk in Communities study. Acta Diabetol 2010;47(Suppl. 1):161–8.

[5] none

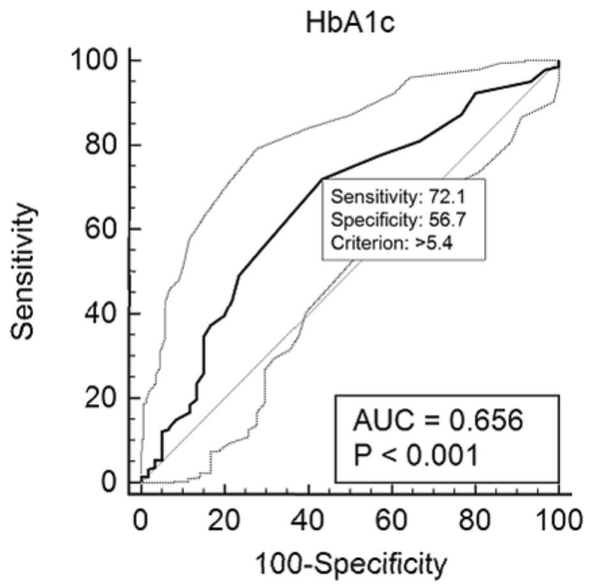

**Fig. 1 (abstract 000676).** See text for description

### 001173

#### Role of venous return in the resolution of obstructive shock

A. Hana[1], P. Werner-Moller[2], J. Takala[1], S. Jacob[1], D. Berger[1]
[1]Department of intensive care medicine, Inselspital, Bern University Hospital, University of Bern, Bern, Switzerland; [2]Department of anaesthesiology and intensive care medicine, Sahlgrenska University Hospital, University of Gothenburg, Gothenburg, Sweden
**Correspondence:** A. Hana
*Intensive Care Medicine Experimental* 2019, **7(Suppl 3):**001173

**INTRODUCTION.** Severe obstructive shock with cardiac arrest leads to a decline in cardiac output of primarily the right heart chamber. We hypothesize, that with return of spontaneous circulation (ROSC) after an obstructive cardiac arrest, the right heart generates cardiac output several heartbeats before the left heart, thereby proving the primary role of venous return for the reconstitution of the circulation.
**OBJECTIVES.** Description of the time course of stroke volume generation of right and left heart after resolution of an obstructive cardiac arrest to clarify the role of venous return in the achievement of ROSC.
**METHODS.** This abstract is based on data from a previous study (1). We induced obstructive circulatory arrest in ten anesthetized pigs (equipped with an ultrasonic flow probe on the pulmonary artery and a pressure catheter in the carotid artery) by balloon occlusion of the right atrium (inflatable high-compliance balloon) over one minute in five experimental conditions (PEEP5, PEEP10, euvolemia, bleeding and hypervolemia). Pulmonary artery pressure and flow tracings confirmed circulatory arrest and ROSC. Single heartbeats were analysed during balloon inflation to create circulatory standstill and then deflation of the balloon after one minute to achieve ROSC. ROSC was defined as the earliest heart beat from which a steady rise in PA flow over the succeeding beats could be observed. After ROSC, PA flow increases by 200%, MAP increases by 10% and arterial stroke volume (calculated from pulse contour) increases by 50% were considered clinically significant. The number of beats until these values were achieved were counted. Comparisons were done with Friedman's ANOVA on ranks and post-hoc Tukey's test and data presented as median (range).
**RESULTS.** We achieved ROSC in 43/50 conditions after 46 (21-84) sec with a flow in the pulmonary artery of 92 (-18-800) ml/min and a mean arterial pressure of 32 (18-51) mmHg. During arrest, MAP increased by 8 (1.9-10) mmHg due to increased sympathetic activity occurring 19-22 sec after arrest (1), while no significant change in PA flow was observed. After ROSC pulmonary flow doubled after 2 (1 to 20) heartbeats, whereas MAP increased 10% only after 7 (1 to 20) beats and arterial stroke volume increased 50% only after 15 (1 to 20) beats, p < 0.001 for all comparisons. Arterial stroke volume and ABP increased by 50% or 10% after 120 (0 to 339) ml or 3.2 (0 to 9) ml/kg and 41 (0 to 333) ml or 1 (0 to 9) ml/kg flow through the pulmonary artery, respectively. Central venous pressure consistently and immediately dropped after ROSC.
**CONCLUSION.** We interpret the time delay between the increase in pulmonary artery flow and arterial pressure and stroke volume as proof of the role of venous return for the recovery from obstructive shock. This is supported by the immediate drop of central venous pressure after ROSC. To what extent these findings may be generalized to non-obstructive forms of cardiac arrest needs further investigation.

##### REFERENCE(S)

1. Berger D, Moller PW, Weber A, Bloch A, Bloechlinger S, Haenggi M, Sondergaard S, Jakob SM, Magder S, and Takala J. Effect of PEEP, blood volume, and inspiratory hold maneuvers on venous return. Am J Physiol Heart Circ Physiol. 2016 Sep 1;311(3):H794-806. doi: 10.1152/ajpheart.00931.2015. Epub 2016 Jul 15.

### 001610

#### CO2 oscillation during Cardiopulmonary Resuscitation: mechanical versus manual chest compression in a porcine model of cardiac arrest

A. Magliocca[1], E. Rezoagli[2], G. Bellani[2], G. Ristagno[3]
[1]Mario Negri Institute for Pharmacological Research, Milan, Italy; [2]School of medicine and surgery, University of Milano-Bicocca, Monza, Italy; [3]Fisiopatologia medico-chirurgica e dei trapianti, University of Milan, Milano, Italy
**Correspondence:** A. Magliocca
*Intensive Care Medicine Experimental* 2019, **7(Suppl 3):**001610

**INTRODUCTION.** Capnographic CO2 oscillations have been described during Cardiopulmonary Resuscitation (CPR).
The Airway Opening Index (AOI) has been recently proposed to quantify the CO2 oscillations at capnography during chest compressions (CCs).(1) It has been proposed that a low oscillating EtCO2 during CCs is a result of airway collapse, which obstructs the expiratory airflow. The aim of this study is to assess the change of AOI over time during 18 minutes of CPR in a porcine model of cardiac arrest (CA). We will assess the relationship between manual and mechanical CCs, the compliance of the respiratory system (Cpl,rs) and the duration of CPR with the AOI.
**METHODS.** Adult male swine were randomized to 18 min of CPR with mechanical (LUCAS® 3.0) or manual CCs after 2 minutes of CA. Mechanical ventilation was provided with tidal volume 500 ml, 10 bpm, FiO2 1.0, ZEEP). Hemodynamic parameters, EKG, SpO2, EtCO2were continuously recorded. Cpl,rs was assessed after return of spontaneous circulation (ROSC). The AOI was computed as ΔCO2/CO2max and the values were averaged on the number of CCs (n=6) during each minute of CPR.
**RESULTS.** The mean AOI was significantly higher in the manual CCs group compared to the mechanical group(58±5% *vs* 24±3%, p< 0.0001 **Fig 1a**). The AOI was higher in the manual CCs group compared to the mechanical group throughout the time of CPR. No decrease of AOI has been identified over time within each group (**Fig 1b**). The mean value of AOI measured at the end of CPR (minute 18) showed a good correlation with Cpl,rs (r=0.749, p=0.0006, Fig **1c**).
**CONCLUSION.** In a randomized porcine model of CA, AOI was significantly higher in the manual compared to the mechanical CCs group during the all 18 minutes of CPR. Cpl,rs was strongly associated with the AOI at the end of CPR.

##### REFERENCE(S)

1. Grieco DL, Brochard L, Drouet A, Telias I, Delisle S, Bronchti G, et al.; CAVIAR Group. Intrathoracic airway closure impacts CO2 signal and delivered ventilation during cardiopulmonary resuscitation. Am J Respir Crit Care Med [online ahead of print] 26 Sep 2018; DOI: 10.1164/rccm.201806-1111OC

#### 001418

**Bosutinib reduces resuscitation volume and pulmonary vascular permeability in a rat polytrauma transfusion model**

D. Kleinveld[1], L. Botros[2], A. Maas[1], J. Aman[2], P. Hordijk[2], M. Hollmann[3], N. Juffermans[1]
[1]Intensive care and laboratory of experimental intensive care and anesthesiology, Amsterdam UMC - Location AMC, Amsterdam, Netherlands; [2]Physiology and pulmonary medicine, Amsterdam UMC - Location VUMC, Amsterdam, Netherlands; [3]Anesthesiology and laboratory of experimental intensive care and anesthesiology, Amsterdam UMC - Location AMC, Amsterdam, Netherlands
**Correspondence:** D. Kleinveld
*Intensive Care Medicine Experimental* 2019, **7(Suppl 3):**001418

**INTRODUCTION.** Traumatic bleeding is characterized by trauma-induced coagulopathy and enhanced endothelial permeability. Currently, transfusion strategies have been adopted using a balanced component strategy to prevent or treat coagulopathy. Interventions aimed at reducing endothelial permeability may further improve outcome. Tyrosine kinase inhibitors such as bosutinib have the potential to reduce vascular permeability by increasing intercellular endothelial adhesion molecules.

**OBJECTIVES.** To examine whether bosutinib in combination with a balanced component strategy reduces endotheliopathy, thereby limiting transfusion requirements and organ failure, in a rat polytrauma transfusion model.

**METHODS.** Blood products were made using syngeneic donor rats according to national blood bank standards. Spraque Dawley rats (n=13 per group) were sedated and mechanically ventilated. Trauma was induced by crush injury to both the small intestine and liver and by a femur fracture. Following trauma, rats were exsanguinated until a MAP of below 40 mmHg, lasting 1 hour. Subsequently, rats were randomized to receive resuscitation with red blood cells, plasma and platelets in a 1:1:1 ratio with either bosutinib or vehicle or only Ringer's Lactate (RL) infusion. Resuscitation was stopped when MAP was 60 mmHg. After six hours, organs were harvested for wet/dry (W/D) ratio. Data are median with interquartile range. A p-value of less than 0.05 was considered to be statistically significant. Bonferroni corrections were applied to correct for multiple testing.

**RESULTS.** The model resulted in shock with increased lactate levels in all animals. Mortality only occurred in RL infused rats (13%). The amount of transfusion or infusion needed to obtain a MAP of 60 mmHg was significantly lower in the bosutinib treated animals (1.0 [1.0 – 1.2] ml) when compared to vehicle (2.0 [2.0 – 2.6] ml) and RL controls (4.0 [4.0 - 5.0] ml, p<0.001). Also, at the end of the experiment, lactate levels were lower in the bosutinib group (2.9 [1.7 – 4.8]) compared to vehicle (6.2 [3.1 – 14.1], p=0.058) and RL controls (11.7 [4.9 – 16.6] mmol/L, p=0.004). Bosutinib resulted in significant lower lung W/D ratios (5.1 [4.6 – 5.2]) when compared to vehicle (5.7 [5.4 – 6.0], p=0.003) and RL controls (5.5 [5.3 – 5.7], p=0.012). Kidney W/D ratios were similar.

**CONCLUSION.** In this model, bosutinib reduces resuscitation volume and results in a better stabilized shock as well as a reduction in pulmonary edema. Whether bosutinib improves outcome should be determined in a trial in patients with traumatic bleeding.

## SIS - Sepsis management and prognostication

#### 000759

**Nationwide Age-specific Infection and Sepsis Mortality in Hospitalized Children in Germany**

C. Fleischmann[1], A. Mikolajetz[2], S. Born[1], D. Schwarzkopf[1], D. Thomas-Rueddel[2], K. Reinhart[1]
[1]Center for sepsis control and care, Jena University Hospital, Jena, Germany; [2]Department for anesthesiology and intensive care medicine, Jena University Hospital, Jena, Germany
**Correspondence:** C. Fleischmann
*Intensive Care Medicine Experimental* 2019, **7(Suppl 3):**000759

**INTRODUCTION.** As most severe complication of infection, sepsis is a major health threat for vulnerable populations such as infants or children with comorbidity. Every year, sepsis affects more than 4 million children worldwide, of which 9-20% die (Fleischmann-Struzek et al. 2018). For Germany, data on nationwide and population-level age-specific pediatric sepsis mortality is scarce.

**OBJECTIVES.** We aimed to assess infection and sepsis mortality in hospital-treated children in different age groups between 2010-2015 based on representative hospital discharge data in Germany.

**METHODS.** Patients <18 years with infection and sepsis were identified in a nation-wide database (DRG statistics) between 2010-2015 based on ICD-10 coded primary and secondary hospital discharge diagnoses. The DRG statistics includes nearly complete inpatient data from German hospital except for military or psychiatric hospitals and contains more than 18 million annual hospitalizations. Sepsis was defined as organ failure due to infection and was identified by ICD-10-codes R65.1 and R57.2. To identify infection-related hospitalizations, ICD-9 infection codes were adapted from Angus et al. and translated into ICD-10 (Angus et al. 2001).

**RESULTS.** Between 2010 and 2015, 2.54 million children with infectious diseases were treated in German hospitals. Overall in-hospital mortality was 0.2%, which corresponds to a mortality rate of 7 deaths per 100 000 children. Highest mortality rates were found in children <1 years (0.5%); in all other age groups, mortality ranged between 0.1-0.2%. Among hospitalized children with infection, 10,975 children had sepsis (0.43%) with an overall mortality of 17.2% (2.4 deaths/100 000 children). Mortality peaked in newborns (18.7%) and was lowest in 2-5year old children (14.5%). Approx. 25% and 8% of all decedents <17 years in Germany received an infection and sepsis code during their terminal hospitalization, respectively.

**CONCLUSION.** Sepsis is an important cause of death in children in Germany. Mortality from pediatric sepsis is higher in Germany than in other high-income countries such as the US with an observed sepsis mortality of 11.8% in children between 2014 and 2016 (Evans et al. 2018). This underlines the need for targeted prevention strategies and quality improvement initiatives in Germany as demanded by the WHO resolution on sepsis (WHA Resolution 2017).

**REFERENCE(S)**

1. http://apps.who.int/gb/ebwha/pdf_files/WHA70/A70_R7-en.pdf
2. Evans et al. JAMA. 2018 Jul 24;320(4):358-367.
3. Angus et al. Crit Care Med. 2001 Jul;29(7):1303-10.
4. Fleischmann-Struzek et al. Lancet Respir Med. 2018 Mar;6(3):223-230.

#### 000765

**Hypertonic Sodium Lactate Improves Sepsis induced alterations of Microcirculation and Cardiac function**

E. besnier[1], D. Coquerel[2], G. Kouadri[3], R. Favory[4], T. Duburcq[4], O. Lesur[2], P. Mulder[5], F. Tamion[6]
[1]Departement of Anesthesia and Critical Care, Hospital Center University De Rouen, Rouen, France; [2]Icu, CRCHUS, Sherbrooke, Canada; [3]Departement of anesthesiology and critical care, Hospital Center University De Rouen, Rouen, France; [4]Intensive care unit, Chu De Lille, Lille, France; [5]Inserm u1096, Université De Rouen, Rue Martainville, Rouen, France, Villeneuve-d'Ascq, France; [6]Medical intensive care unit, Rouen University Hospital, Rouen, France
**Correspondence:** E. besnier
*Intensive Care Medicine Experimental* 2019, **7(Suppl 3):**000765

**INTRODUCTION.** Hypertonic sodium lactate have recently shown interesting effects for fluid management in endotoxemia models1. We therefore evaluated its effects on microcirculation, capillary leakage, cardiac function and different biological parameters in a sepsis rat model of caecal ligation and puncture (CLP).

**METHODS.** 30 rats were randomized in 3 groups (n=10 per group): Sham; CLP-NaCl 0.9%; CLP-Lactate 11.2%. 2.5 mL/kg/h of fluids were intravenous administrated from the CLP procedure and during 18 hours. We then evaluated mesenteric microcirculation (laser speckle imager), cardiac function (echocardiography and pressure-volume (PV) loops) and inflammation (uremia, VEGF-A, IL-1β, IL-10, TNFα).

Capillary leakage was explored using Blue Evans extravasation in the lung and gut on 5 additional rats. Results are expressed as medians with interquartiles and comparisons versus CLP-NaCl were realized using Kruskall-Wallis or ANOVA test.

**RESULTS.** CLP-lactate presented an improved mesenteric microcirculation (735.5 [407.4-878.8] vs 240.6 [209.3-390.8]UI/pixel, p=0.0006) with higher cardiac output (0.34 [0.28-0.43] vs 0.14 [0.10-0.18] mL/min, p<0.0001) and higher left ventricular shortening fraction (55.2 [46.2-73.2] vs 39.1 [32.9-51.8] %, p=0.009). PV loops also showed raised dP/dt max slope (6.3 [3.3-12.1] vs 2.7 [2.0-3.9] mmHg/ms, p=0.04), suggesting an improved inotropism, and a lower Left Ventricular End-Diastolic Pressure-Volume Relation (1.9 [1.1-2.3] vs 3.0 [2.2-3.7] RVU/mmHg, p=0.005), suggesting an improvement in diastolic function independently from preload. Mean arterial pressure between CLP-NaCl and CLP-Lactate was similar at the end of infusion. Evans Blue diffusion was reduced in the gut and the lung for CLP-lactate (37.2 [31.0-43.3] vs. 112.7 [63.3-141.6], p=0.03 and 107.5 [82-174.3] vs. 272.7 [221.8-444.5] ng EB/mg of tissue, p=0.006). Plasma levels of lactate and 3OH-butyrate were higher in CLP-lactate (6.03 [3.08-10.3] vs. 3.19 [2.42-5.11] mmol/L, p=0.04; 400 [174-626] vs. 189 [130-301] μmol/L, p=0.03). Inflammatory response was reduced in CLP-lactate (IL-1β: 172.2 [119.0-446.3] vs. 927.7 [244.8-1470] pg/mL, p=0.004; TNFα: 17.9 [12.5-50.3] vs. 53.9 [30.8-85.6] pg/mL, p=0.005; IL-10: 351.6 [267.0-918.6] vs. 904.5 [723.1-1243] pg/mL) as well as VEGA-A plasma levels (198.2 [185.3-250.0] vs. 260.7 [249.8-268.9] pg/mL, p=0.009).

**CONCLUSION.** Hypertonic lactate fluids protect against cardiac dysfunction, mesenteric microvascular alteration and capillary leakage during sepsis, in association with a significant reduction in inflammatory process and kidney failure.

**REFERENCE(S)**

1. Duburcq T, Favory R, Mathieu D et al. Hypertonic sodium lactate improves fluid balance and hemodynamics in porcine endotoxic shock. Crit Care 2014;18:467.

### 000778

#### Clinical outcomes of dynamic ultrasound-guided versus static central venous pressure-guided fluid resuscitation in patients with sepsis and/or septic shock in Thammasat University Hospital

T. Sricharoenchai[1], P. Saisirivechakun,[2]
[1]Division of pulmonary and critical care medicine, department of medicine, thammasat university, 99/209 Moo 18 Paholyotin Road, Klong Nueng, Klong Luang, Pathum Thani, Thailand; [2]Department of medicine, thammasat university, 99/209 Moo 18 Paholyotin Road, Klong Nueng, Klong Luang, Pathum Thani, Thailand
**Correspondence:** T. Sricharoenchai
*Intensive Care Medicine Experimental* 2019, **7(Suppl 3):**000778

**INTRODUCTION.** Intravenous fluid resuscitation was the mainstay treatment in early goal-directed therapy for sepsis, which measured static central venous pressure (CVP) in first 6 hours (hrs)1. Although several studies demonstrated that dynamic indices were more accurate than static indices for prediction of fluid responsiveness2, no landmark studies compared the efficacy of both types of indices for clinical outcomes.

**OBJECTIVES.** The primary objective is to compare the 30-day all-cause mortality rate of septic patients in whom fluid resuscitation was guided by dynamic inferior vena cava (IVC) diameter with static CVP measurement. The secondary objectives are to compare the proportions of patients achieving ≥1 macrovascular target(s), i.e., mean arterial pressure ≥65 mmHg and urine output ≥0.5 mL/kg/hr; and ≥1 microvascular target(s), i.e., central venous oxygen saturation (ScvO2) ≥70% and lactate clearance ≥10%, which were assessed at 6 hrs after treatment of sepsis, between those using dymamic and static parameters.

**METHODS.** A Single-blinded randomized controlled trial was conducted in Thammasat University Hospital between August 2016 and March 2019. The patients were stratified by APACHE-II (<25 or ≥25) and randomized using block of 2 and 4, to either dynamic IVC diameter measurement by ultrasound (US) or static CVP measurement. We recorded clinical parameters at baseline, during resuscitation and until completion of 6 hrs, including the vital status at 30 days, macrovascular and microvascular targets.

**RESULTS.** A total of 91 patients was enrolled, but one withdrew. Forty-three patients were randomized to dynamic IVC diameter measurement, while 47 patients were randomized to static CVP measurement. The baseline characteristics between two groups were not different. The 30-day mortality rates between IVC-guided group and CVP-guided group were not different (39.5% vs. 44.7%, p=0.622). The proportions of patients who achieved ≥1 macrovascular target(s) at 6 hrs were not different between both groups (65.1% vs. 63.8%, p=0.899). The proportions of patients achieving ≥1 microvascular target(s) at 6 hrs were also not different between both groups (83.7% vs. 66.0%, p=0.054), but the proportion of patients in IVC-guided group achieving ScvO2 ≥70% was higher than that of the other (77.1% vs. 48.6%, p=0.013).

**CONCLUSION.** Dynamic US-guided fluid resuscitation does not affect the mortality of patients with sepsis, however, it may help more patients achieve ScvO2 ≥70%, compared to static CVP-guided resuscitation.

**REFERENCE(S)**

1. Rivers E, Nguyen B, Havstad S, et al. Early goal-directed therapy in the treatment of severe sepsis and septic shock. N Engl J Med 2001;345:1368-77.
2. Rocha LL, Pessoa CM, Corrêa TD, et al. Current concepts on hemodynamic support and therapy in septic shock. Braz J Anesthesiol 2015;65:395-402.

### 000809

#### Prognostic value of cellular markers in sepsis: extracellular DNA traps and platelet count relation

A. Gur'ev[1], D. Mosalskaia[1], A. Lopatin[2], A. Volkov[3]
[1]Scientific-research laboratory, M.F.Vladimirsky Moscow Regional Clinical and Research Institute (MONIKI), Moscow, Russia; [2]Intensive care unit, M.F.Vladimirsky Moscow Regional Clinical and Research Institute (MONIKI), Moscow, Russia; [3]Head, Medtechnopark Ltd., Moscow, Russia
**Correspondence:** A. Gur'ev
*Intensive Care Medicine Experimental* 2019, **7(Suppl 3):**000809

**INTRODUCTION.** Thrombosis in critical states is known to be connected with the formation of Extracellular Traps (ETs), which are produced by blood phagocytes [1]. ETs activate platelets and visa versa: activated platelets prime phagocytes to undergo ETosis.

Development of common methods of ETosis determination in blood is actual. We developed the method for the assessment of phagocyte circulating in blood that are transformed during ETosis, using standardized blood smears [2]. Method implies preparation of smears with monolayer of cells with strictly specified parameters, providing the standard conditions for heterogenic lysis of circulated phagocytes, transformed during ETosis.

**OBJECTIVES.** The aim of the study is to evaluate the connection between ETs level and hemostasis indicators with sepsis outcome.

**METHODS.** ETs levels in standardized blood smears [2], platelets count and antithrombin III (AT III) activity in blood were determined for 73 patients in intensive care unit with verified sepsis in dynamics, using hematological analyzer PENTRA 120 (Horiba Medical, France) and ACL TOP analyzer (Instrumentation Laboratory, USA).

**RESULTS.** Among septic patients survival rate was 60% (29 cases of lethal outcome and 44 cases of recovery). We compared maximal ETs level during all time of observation of each patient; at this moment hemostasis indicators were assessed. In group with lethal outcome ETs level was in average – 24.6% (95% CI: 17.7-31.5%), platelet count – 152×10^9/L (95% CI: 112-192×10^9/L), AT III activity – 67.5% (95% CI: 58-77%). In recovery group ETs level was in average – 9.2% (95% CI: 7.6-10.8%), platelet count – 272×10^9/L (95% CI: 223-321×10^9/L), AT III activity – 77.4% (95% CI: 70-84%). The differences in ETs levels and platelet counts between the groups were statistically significant according to Mann-Whitney U-test (fig. 1). Thrombocytopenia (<120×10^9/L) was observed in 48.3% cases of lethal outcome and 13.6% cases of recovery.

**CONCLUSION.** Maximal level of extracellular phagocyte traps as well as platelet count significantly differ in the groups of recovered and died septic patients, they both are significant mortality predictors, while antithrombin III is not. These preliminary data ensure us in the significance of future studies of the connection between considered cellular markers in dynamics.

**REFERENCE(S)**

1. Boeltz S, Amini P, Anders HJ et al. To NET or not to NET:current opinions and state of the science regarding the formation of neutrophil extracellular traps. Cell Death Differ. 2019 Mar;26(3):395-408.
2. Mosalskaya DV, Gur'ev AS, Lopatin AF. Prognostic value of DNA-traps level in blood smears from patients with sepsis. Intensive Care Medicine Experimental 2018, 6(Suppl 1), p.7.

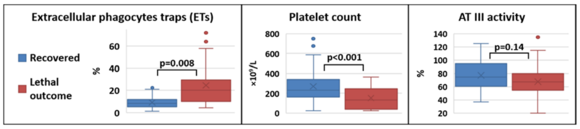

**Fig. 1 (abstract 000809).** The comparison of ETs level, platelets count and AT III activity.

### 000820

#### Presepsina as a biomarker of diagnostic and prognostic of sepsis in intensive care patients

J. Asparch[1], I. Huespe[1], J. Sinner[1], S. Venuti[1], E. Prado[1], S. Terrasa[2], M. Gimenez[3], G. Jimenez[3], E. San Roman[1]

[1]Intensive care unit, Hospital Italiano de Buenos Aires, CAPITAL FEDERAL, Argentina; [2]Internal medicine, Hospital Italiano de Buenos Aires, CAPITAL FEDERAL, Argentina; [3]Central laboratory, Hospital Italiano de Buenos Aires, CAPITAL FEDERAL, Argentina

**Correspondence:** I. Huespe

*Intensive Care Medicine Experimental* 2019, **7(Suppl 3):**000820

**INTRODUCTION.** Sepsis is the primary cause of mortality in intensive care units. It can be treated effectively, but it is essential treated it early. However, differentiate sepsis from non-infectious SIRS at the early stage has become a challenge. In this context, presepsin is a new biomarker with better sensitivity and specificity in the diagnosis of sepsis that other biomarkers.

**OBJECTIVES.** Evaluate the sensibility and specificity of presepsin for the diagnosis of positive blood cultures and the prognostic value in patients with suspected sepsis.

**METHODS.** Prospective study, carry on in the ICU of the Hospital Italiano de Buenos Aires, Argentina, between August 2015 and March 2017. Patients in ICU with more than 48 hours of hospitalization, with new diagnosis of suspected sepsis, have been included. Patients with any antibiotic treatment or SIRS of any cause until three days before the inclusion were excluded and so were burned, immunodepleted, pregnancy and subjects who have a do not resuscitate status. Presepsin were obtained at suspect sepsis, 24 and 72 hours after.

**RESULTS.** 144 patients have been recruited. Mean age was 64 and mean APACHE II 15. The main infectious sources were respiratory (33%) and abdominal (14%). 28 blood cultures (BC) were positive (19%), with 19 rescues of gram-negative bacilli and 9 gram-positive cocci. Baseline presepsin in patients with positive BC was greater (1655 pg/ml, SD+/-1137) than patients with negative BC (mean 1015 pg/ml, SD+/-893), (p=<0.001) (Figure 1). In patients with renal failure (RF), values of presepsin did not have significative differences (positive cultures: mean of 2520 pg/ml, SD+/-3025; negative cultures: mean of 2789 pg/ml, SD+/-2476), (p=0.49). The cut point of 400 pg/ml had the best relationship between sensitivity (96.4%) and specificity (19.8%), with an area under the ROC curve (AUC) of 0.72. In patients with RF, the AUC was 0.59. Global mortality in UCI was 21% (31 patients). Values of basal presepsin, at 24 and 72 hs had a significative correlation with the probability of death in UCI (Figure 2). Progression of presepsin between basal values and 72 hs values did not correlate with the probability of death (in 41% of patients that death in ICU the presepsin values decreased at 72 hs, against 49% in patients that survive).

**CONCLUSION.** Presepsin has good sensitivity for the diagnosis of positive BC in ICU-sepsis, but only in patients without RF. That is a significant problem because it is a common comorbidity in ICU patients. Also, presepsin values had a good correlation with the risk of dying in ICU.

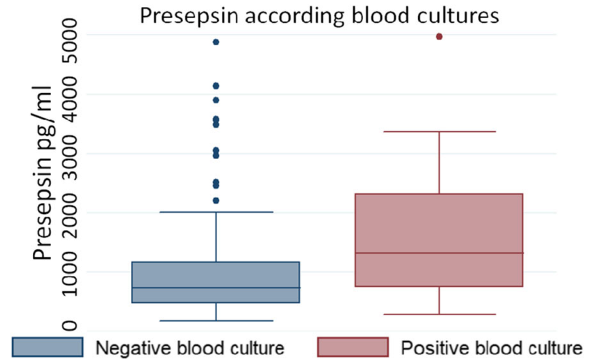

**Fig. 1 (abstract 000820).** See text for description.

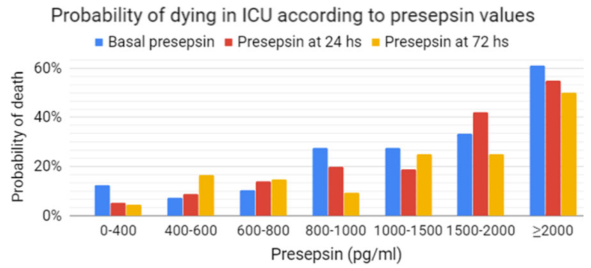

**Fig. 2 (abstract 000820).** See text for description.

### 000841

#### Monitoring tissue antibiotic concentrations with microdialysis: Determination of in-vivo tissue meropenem recovery

K. Tam[1], I. Longo[2], D. Brealey[3], M. O'connell[4], M. Singer[5]

[1]Intensive care medicine, University College London, London, United Kingdom; [2]Anaesthesia and intensive care medicine, Azienda Sanitaria Universitaria Integrata di Trieste, Trieste, Italy; [3]Critical care, UCL Hospitals NHS Foundation Trust, London, United Kingdom; [4]Director, Probe Scientific Ltd, Thurleigh, Bedford, UK, Thurleigh, United Kingdom; [5]Bloomsbury institute of intensive care medicine, University College London, London, United Kingdom

**Correspondence:** K. Tam

*Intensive Care Medicine Experimental* 2019, **7(Suppl 3):**000841

**INTRODUCTION.** Therapeutic drug monitoring of antibiotics is very limited at present and no bedside test is available to offer rapid results. Tissue microdialysis (MD) is a minimally invasive technique that measures only the free (unbound), active fraction of antibiotics in the interstitial fluid of target tissues [1,2]. However, concentration of drug within the microdialysate samples reflects only a fraction of the total unbound concentration in that tissue. This ratio is called relative recover [3]. For accurate determination of true tissue concentration, the analyte concentration measured in microdialysate must be corrected to the relative recovery (%).

**OBJECTIVES.** The aims of this study are to describe in-vivo microdialysis recovery of meropenem in multiple tissues in healthy rats ,and to compare these levels against those found in early and late stages of sepsis.

**METHODS.** Healthy male Wistar rats (300-350g), were allocated into three study groups: sham, 6 hour sepsis and 24 hour sepsis. Sepsis was induced by faecal slurry injected intraperitoneally. MicroEye® probes (Probe Scientific Ltd, Bedford, UK) were implanted into 6 tissues: muscle, subcutaneous, liver, peritoneal cavity, trachea and jugular vein. Probes were perfused with meropenem 10 μg/mL at a rate of 1 μL/min. Microdialysate from each tissue was sampled every 10 minutes for 120 minutes' duration. Meropenem concentration was determined using High Performance Liquid Chromatography (HPLC, Agilent 1260II). MD recovery was calculated as [(Cin-Cout/Cin)*100) [3]. ANOVA with post-hoc Dunn's test were used to seek statistical significance.

**RESULTS.** MD recovery in most tissues in sham rats ranged from 35-41%, except for muscle (23%, p<0.0001) and trachea (27%, p<0.05). At 6 hours' sepsis, recoveries were increased to 35-50% (p<0.05), except endotracheally (9%, p<0.0001). At 24 hours' sepsis, recoveries were significantly lower in all tissues (9-34%, p<0.0001) [Figure 1].

**CONCLUSION.** We conclude that MD drug recovery in tissues are significantly different in health and at different timepoints in sepsis. This highlights the importance of in-vivo determination of drug recovery prior to conducting a MD study, to avoid inaccuracies in true tissue drug concentration measurement.

**REFERENCE(S)**

[1] Shippenberg TS, Thompson AC. Overview of microdialysis. Curr Protoc Neurosci. 2001; Chapter 7: Unit7.1.

[2] Ungerstedt U, Pycock C. Functional correlates of dopamine neurotransmission. Bull. Schweiz. Akad. Med. Wiss. 1974; 30:44–55.

[3] Marchand S, et al. Microdialysis as a way to measure antibiotics concentration in tissues Pharmacol Res 2016; 111:201–7

[4] Collaborate to Innovate/ EU ERDF grant from MedCity London

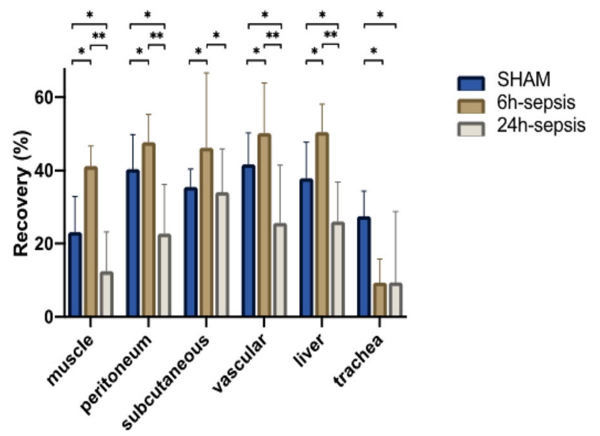

**Fig. 1 (abstract 000841).** See text for description

### 000850

#### The feasibility of end-inspiratory or end-expiratory occlusion to predict fluid responsiveness for elder septic patients

J. Sun, K. Fang, Z. Shi, J. Zhang
Intensive care unit, Hangzhou Red Cross Hospital, Hangzhou, China
**Correspondence:** J. Sun

*Intensive Care Medicine Experimental* 2019, **7(Suppl 3):**000850

**INTRODUCTION.** Predicting fluid responsiveness for elder patients, especially with acute respiratory distress syndrome or arrhythmia or severe cardiac dysfunction, is still a daily challenge in intensive care unit. Based on heart-lung interaction, cardiac output (CO) will be changed derived from occlusion of respiratory.

**OBJECTIVES.** The study was aimed to explore the feasibility of occlusion of end-inspiratory or end-expiratory in prediction of fluid responsiveness for elder septic patients.

**METHODS.** 30 elder septic hypotension patients with mechanical ventilation were enrolled. The ratio of positive fluid responsiveness (PFR) and negative fluid responsiveness (NFR) was 1:1. After analgesia and sedation, all of them were suffered end-inspiratory/end-expiratory occlusion for 15s. Parameters of CO (CI/SVI and LVOT-VTI/Vmax) were dynamic monitored via noninvasive pulse contour and echocardiography. PFR were identified as △CI≥15% after 500ml saline of fluid challenge. T-test or Mann-Whitney test and receiver operating characteristic curve (ROC) were applied for data analysis, and then calculated the diagnostic parameters with regarding to the cutoffs.

**RESULTS.** Occlusion of end-expiratory increased CO significant in PFR patients than negative patients; and vice versa, occlusion of end-inspiratory decreased CO significant in PFR than NFR. ROC for predicting PFR showed that CO parameters had considerable predictive value (C-statistics, 0.849-0.951). In addition, CI/SVI decreased ≥5.52%/6.52 or LVOT-VTI/Vmax decreased ≥7.43%/4.24% derived by occlusion of end-inspiratory and CI/SVI increased ≥4.52%/9.52 or LVOT-VTI/Vmax increased ≥8.59%/3.88% derived by occlusion of end-expiratory had 100% reliability to predict PFR in our elder septic patients.

**CONCLUSION.** Occlusion of end-inspiratory or end-expiratory may be used as a promising method to predict fluid responsiveness for elder septic patients.

**REFERENCE(S)**

1. Jozwiak M, Depret F, Teboul J L, et al. Predicting Fluid Responsiveness in Critically Ill Patients by Using Combined End-Expiratory and End-Inspiratory Occlusions With Echocardiography[J]. Crit Care Med. 2017, 45(11): e1131-e1138.

2. Zhejiang Provincial Health Department foundation

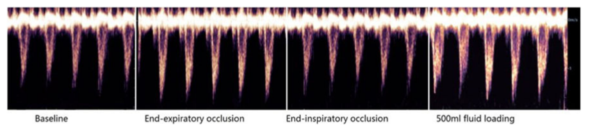

**Fig. 1 (abstract 000850).** Doppler spectrums of left ventricular outflow derived by echocardiography. LVOT-VTI/Vmax was hand-drawn from the spectrum. (Jozwiak M, et al. Crit Care Med. 2017)

### 000858

#### Fluid responsiveness assessment during early septic shock resuscitation. Secondary analysis of ANDROMEDA-SHOCK trial

E. Kattan[1], G. Ferri[2], G. Ospina-Tascon[3], AB. Cavalcanti[4], LP. Damiani[4], E. Estenssoro[5], A. Dubin[6], J. Hurtado[7], G. Friedman[8], R. Castro[1], L. Alegria[1], JL. Teboul[9], M. Cecconi[10], J. Bakker[11], G. Hernandez[1]
[1]Departamento de Medicina Intensiva, Pontificia Universidad Catolica de Chile, Santiago, Chile; [2]Unidad de cuidados intensivos, Hospital Barros Luco Trudeau, Santiago, Chile; [3]Department of intensive care medicine, Fundación Valle del Lili, Universidad ICESI, Cali, Colombia; [4]Hcor research institute-hospital do coração, Faculdade de Medicina da Universidade de São Paulo, São Paulo, Brazil; [5]Unidad de terapia intensiva, Hospital Interzonal de Agudos San Martín de La Plata, La Plata, Argentina; [6]Unidad de terapia intensiva, Sanatorio Otamendi, Buenos Aires, Argentina; [7]Intensive Care Unit, Hospital Español-ASSE, Montevideo, Uruguay; [8]Post-graduation program in pneumological sciences, department of internal medicine,, School of Medicine, Universidade Federal do Rio Grande do Sul, Porto Alegre, Brazil; [9]Service de réanimation médicale, Hôpital de Bicêtre, Université Paris-Sud, Le Kremlin-Bicêtre, France; [10]Humanitas clinical and research center, Department of Biomedical Sciences, Humanitas University, Milan, Italy; [11]{street_address}, Rotterdam, Netherlands
**Correspondence:** E. Kattan

*Intensive Care Medicine Experimental* 2019, **7(Suppl 3):**000858

**INTRODUCTION.** In septic shock patients, fluid therapy has been fostered as one of the pillars of resuscitation. Inadequate fluid resuscitation can lead to tissue hypoperfusion, while indiscriminate fluid administration can induce pulmonary edema, abdominal hypertension, acute kidney injury, among other complications. Fluid responsiveness (FR) tests are dynamic bedside evaluations that allow identifying the position of the patient in the cardiac function curve(1).

Despite that these tests are widely available, extensively described in the literature and almost cost-free, recent surveys have shown that the minority of intensive care practitioners effectively use them to guide fluid therapy(2).

In the recently published ANDROMEDA-SHOCK trial, a comprehensive resuscitation protocol was applied to early septic shock patients with two different perfusion targets (lactate and capillary refill time). The protocol included the evaluation of FR as a mandatory aspect of the hemodynamic profiling of patients(3)

**OBJECTIVES.** To determine the use of FR testing, its' impact on fluid therapy and on perfusion endpoints during initial septic shock resuscitation of patients included in ANDROMEDA-SHOCK trial.

**METHODS.** A secondary analysis of ANDROMEDA-SHOCK study was performed. 424 patients were divided into three groups according to baseline FR status: Responders (FR+), non-responders (FR-) and not analyzable. The first two groups were considered the sample size for this study. Macrohemodynamic, fluid therapy and perfusion variables were compared in both groups during the first 24-h of the study protocol. Non-parametric testing was used.

**RESULTS.** At ICU admission, FR could be determined in 82% of patients, while 25% of patients enrolled were FR– after pre-ICU fluid loading. The most frequent methods used to determine FR were pulse pressure variation (43%), passive leg raising (42%) and inferior vena cava distensibility (10%).

Both groups presented similar baseline demographic, severity and macrohemodinamic characteristics. FR+ had higher CRT at baseline. (Table 1)

FR+ received significantly more fluid bolus than FR– during the first 2 hours (1000[500-1500] vs 0[0-0], p<0.001) and 8 hours (1500 [1000-2500] vs 0[0-500], p<0.001) of protocol. Figure 1 shows the evolution of FR in both groups.

**CONCLUSION.** During initial resuscitation of septic shock patients, withholding fluids in FR– patients doesn't seem to delay hypoperfusion resolution. Evolution of FR is dynamic and should be assessed at every clinical evaluation to avoid unnecessary fluid loading and potential adverse effects.

**REFERENCE(S)**

1. Monnet X, Marik PE, Teboul JL. Prediction of fluid responsiveness : an update. Ann Intensive Care. 2016:1-11
2. Cecconi M, Wilkman E, Rocca G Della, Sander M, Backer D De. Fluid challenges in intensive care : the FENICE study. Intensive Care Med. 2015;41(9):1529-1537.
3. Hernandez G, Ospina-Tascon G, Petri Damiani L, et al. Effect of a Resuscitation Strategy Targeting Peripheral Perfusion Status vs Serum Lactate Levels on 28-Day Mortality Among Patients With Septic Shock. The ANDROMEDA-SHOCK Randomized Clinical Trial. JAMA. 2019;321(17):654-664.

**Table 1 (abstract 000858).** See text for description

| | Fluid Responder | Non Fluid Responder | p |
|---|---|---|---|
| N° | 242 | 106 | |
| Age (years) | 63 [50-74] | 66 [53-75] | 0.56 |
| APACHE score | 23 [18-29] | 21 [15-27] | 0.09 |
| SOFA score | 10 [7-12] | 9 [7-12] | 0.52 |
| MAP (mmHg) | 66 [60-75] | 67 [62-78] | 0.2 |
| CVP (mmHg) | 9 [5-12] | 10 [7-14] | 0.001 |
| Pre-protocol fluids (ml) | 2000 [1194-2643] | 2000 [1200-2500] | 0.9 |
| Norepinephrine dose (mcg/kg/min) | 0.22 [0.1-0.4] | 0.21 [0.12-0.4] | 0.8 |
| Basal Lactate (mmol/L) | 3.8 [2.8-5.5] | 3.6 [2.8-5.5] | 0.4 |
| Basal CRT (s) | 5 [4-6] | 4 [3-6] | 0.002 |

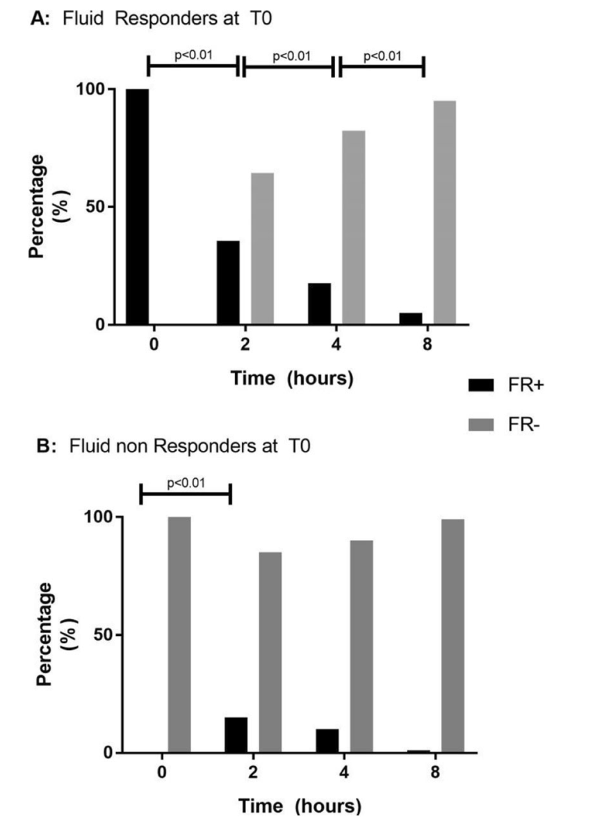

**Fig. 1 (abstract 000858).** See text for description

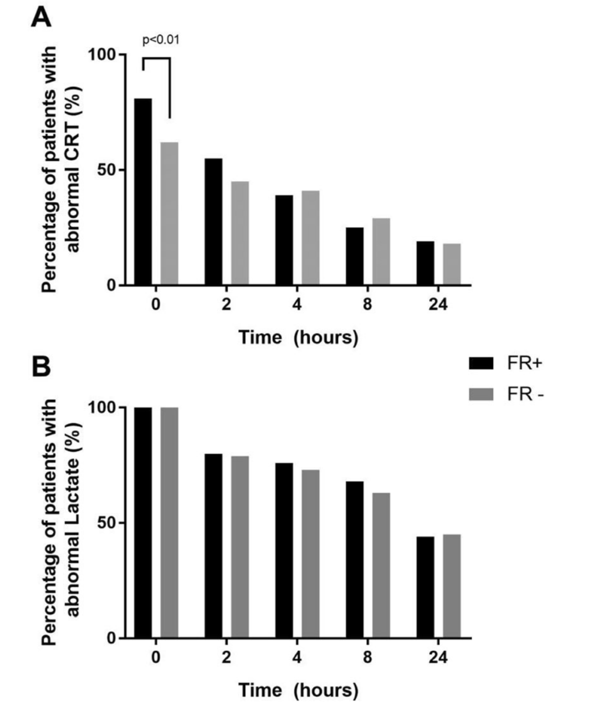

**Fig. 2 (abstract 000858).** See text for description

### 000886

#### Prediction of secondary infections occurrence with biomarkers during sepsis: a non-supervised analysis of the Captain cohort

B. Misset[1], F. Philippart[2], V. Moucadel[3], M. Parlato[4], C. Fitting[4], JP. Bedos[5], JL. Diehl[6], O. Hamzaoui[7], D. Annane[8], D. Journois[6], JM. Cavaillon[4], J. Coste[9], C. Joel[9]

[1]Department of Intensive Care, CHU de Liège, Avenue de L'Hòpital, Liège, Belgium, Liège, Belgium; [2]Intensive care, Groupe hospitalier Paris Saint-Joseph, Paris, France; [3]Medical diagnostics discovery development, bioMérieux, Lyon, France; [4]Cytokines et inflammation, Institut Pasteur, Paris, France; [5]Intensive care, C.H. de Versailles, Le Chesnay, France; [6]Intensive care, Assistance Publique - Hôpitaux de Paris, Hôpital Européen Georges Pompidou, Paris, France; [7]Intensive care, Assistance Publique - Hôpitaux de Paris, Hôpital Antoine Béclère, Clamart, France; [8]Intensive care, Assistance Publique - Hôpitaux de Paris, Hôpital Raymond Poincaré, Garches, France; [9]Biostatistics and clinical epidemiology, Assistance Publique – Hôpitaux de Paris, Hôpital Cochin, Paris, France

**Correspondence:** B. Misset

*Intensive Care Medicine Experimental* 2019, **7(Suppl 3):**000886

**INTRODUCTION.** Sepsis involves inflammatory and immunity mediation. Several mediators, alone or in combination, were proposed to characterize individual response, but none was proven to have good external validity.

**OBJECTIVES.** The aim of this work was to establish whether some combinations allow to predict occurrence of nosocomial infections in patients with sepsis, using the data collected in the Captain multicenter cohort which method and first results were previously published (1).

**METHODS.** The Captain cohort included 279 patients prospectively at the time of sepsis criteria, 188 (67%) of whom having confirmed infection. Community acquired pneumonia was causal in 70% of infections. SAPS2 score = 55 [50-61], age = 65 years [57-78], male sex = 64 %. 203 patients followed for more than 3 days, in whom usual ICU clinical and biological parameters were collected, as well as 29 plasma biomarkers and 10 leucocyte associated RNAs. Patients were classified according to the occurrence of pneumonia and/or bacteremia after day 5. A non-supervised principal component analysis of the maximal values of biomarkers assessed on first 2 days of sepsis, and a Varimax rotation was performed on the selected components using the SAS software.

**RESULTS.** Out of 203 patients, 33 developed pneumonia and/or bacteremia after day 5. Five components explain 57 % of the variance of the biomarkers on the entire cohort. The second component of the Varimax rotation is significantly linked to pneumonia occurrence ($p = 0.02$) or bacteremia ($p = 0.02$). Those biomarkers which are determinants of this component are HLA-DR RNA (correlation coefficient 0.79), CD74 RNA (0.76), CRP (-0.62), CX3CR1 RNA (0.63), CD3D RNA (0.59), Matrix Metallo Proteinase-8 (MMP8) (-0.58), IL-10 RNA (-0.57) and Pancreatic Stone Protein (PSP) (-0.50).

**CONCLUSION.** In our cohort, using a non supervised analysis, we could detect a biomarker association which predicts secondary infection occurrence. Some of these markers are among those which are regularly considered as describers of the peripheral alteration of the immune system observed during sepsis.

##### REFERENCE(S)

1. Parlato et al, Intensive Care Medicine (2018) 44: 1061-70.
2. bioMérieux
3. French Ministry of Health

### 000891

#### Fatty acids modulate cytokine secretion in an in vitro model of sepsis

V. Peters[1], M. Singer[2], T. Roger[3]

[1]University College London, Bloomsbury Institute of Intensive Care Medicine, London, United Kingdom; [2]University College London, Bloomsbury Institute of Intensive Care Medicine, London, United Kingdom; [3]Infectious diseases service, Lausanne University Hospital, Lausanne, Switzerland

**Correspondence:** V. Peters

*Intensive Care Medicine Experimental* 2019, **7(Suppl 3):**000891

**INTRODUCTION.** Sepsis represents life-threatening organ dysfunction caused by a dysregulated host response to infection [1]. Cardiovascular, neural, coagulation, hormonal, bioenergetic, metabolic, inflammatory and immune pathways are variably deranged [2]. Depending on their hydrocarbon chain lengths, degree of unsaturation, number, position and orientation of their double bonds, fatty acids (FAs) have differential effects on immune functions [3].

**OBJECTIVES.** This study aims to determine the immunomodulatory effects of palmitate (long-chain, saturated), alpha-linolenic acid (ALA) (long-chain, unsaturated) and butyrate (short-chain, saturated) in human peripheral blood mononuclear cells (PBMCs) exposed to lipopolysaccharide (LPS) or clinical strains of bacteria.

**METHODS.** PBMCs from 5 healthy donors were cultured in RPMI 1640 GlutaMAX medium containing 10% FCS and 1% pen-strep at 37°C in 5% CO2. PBMCs were incubated overnight with LPS (100 ng/ml), heat-killed *Staphylococcus aureus* or *Escherichia coli* O18 (PBMC:bacterium ratio 5:1) in the presence of palmitate, butyrate or ALA (0.4, 1.8 or 0.3 mM, respectively - concentrations based on physiological and sepsis range ([4,5] (unpublished data)). Supernatants were collected to quantify IL-6, IL-10 and TNF concentrations by ELISA. The MTT assay was used to assess metabolic activity. Non-parametric one-way ANOVA tests with Dunn's correction were used for statistical analyses.

**RESULTS.** PBMCs stimulated with bacteria or bacterial product showed a metabolic activity similar to controls (data not shown) but secreted appreciable levels of cytokines contrary to unstimulated cells. FAs variably affected cytokine secretion [Figure 1]. Palmitate strongly reduced IL-10 secretion, butyrate markedly inhibited all cytokines, whereas ALA increased bacteria-induced IL-6 secretion but inhibited IL-10.

**CONCLUSION.** Palmitate, butyrate and ALA display proinflammatory, immunosuppressive and mixed effects, respectively. The variable immune effects suggest that FA supplementation should be tailored according to the patient's inflammatory phenotype.

##### REFERENCE(S)

1. Singer M et al. JAMA 2016; 315:801-810
2. Tidsw ell R, Singer M. Clin Med 2018; 18.1: 62–68
3. Boisramé-Helms J et al. Prog Lipid Res 2015; 60:1-16
4. Abdelmagid et al. PloS One 2015; 10.2: e0116195
5. Cummings et al. Gut 1987; 28.10: 1221-1227
6. Funded by a Marie-Curie EU Horizon 2020 'European Sepsis Academy' PhD studentship

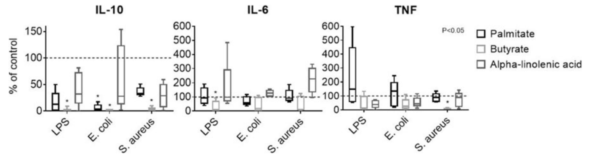

**Fig. 1 (abstract 000891).** Impact of palmitate, butyrate and alpha-linolenic acid on cytokine secretion by human PBMCs exposed to LPS and bacteria

### 000935

#### De-escalation treatment for methicilin-resistant Staphylococcus aureus (MRSA) in culture-negative community or nosocomial extra-ICU infections

ML. Cantón Bulnes, J. Garnacho-Montero

Intensive Care, Hospital Universitario Virgen Macarena, Sevilla, France

**Correspondence:** M.L. Cantón Bulnes

*Intensive Care Medicine Experimental* 2019, **7(Suppl 3):**000935

**INTRODUCTION.** In sepsis and septic shock administration of broad-spectrum antibiotics is the mainstay of therapy. De-escalation (DE) of antimicrobials has been proposed as a safe strategy associated with a lower mortality. This approach most commonly occurs with the positive culture results. However, for many patients with sepsis, cultures remain negative, making de-escalation to a targetable pathogen difficult.

**METHODS.** All patients admitted from January 2012 to December 2018 were included. This is a retrospective analysis of a prospectively collected database (Spanish national registry ENVINHELICS). At ICU admissions, all patients are screened for the nasal MRSA carriage. DE was defined as anti-MRSA agent discontinuation within 4 days of initiation. We analysed variables concerning demographic characteristics, comorbidities, clinical presentation, duration of mechanical ventilation, need of renal replacement therapy, ICU and hospital length of stay and mortality. Acute Physiology and Chronic Health Evaluation II (APACHE II) score and Glasgow Coma Scale (GCS), were used to asses baseline severity of illness.

Results are expressed in mean value and standard deviation (SD) for numerical variables and percentage over total patients for categorical variables. Analysis was performed using chi-square or exact Fisher test for qualitative variables and Student t test or Mann-Whitney U test for quantitative variables when appropriate. P values of less than 0.05 were regarded as statistically significant.

**RESULTS.**

Of 9342 patients analysed, 3043 fulfilled criteria of sepsis: 1527 had positive cultures and 1516 all cultures were negative. Of them, 141 were excluded for being positive for nasal MRSA carriage. Finally, 103 patients were analyzed (59 received empirically vancomycin and 44 linezolid), 36 patients who had anti-MRSA agent de-escalated (vancomycin in 22 and linezolid in 14), and 67 patients in whom de-escalation did not occur. The main infection foci were pneumonia and soft tissue infection in both groups. The results are shown in tables 1 and 2.

**CONCLUSION.** Anti-MRSA agent can be safely accomplished in culture-negative community or nosocomial extra-ICU sepsis and septic shock in non-MRSA nasal carriers.

**REFERENCE(S)**

1. Garnacho-Montero J, Gutiérrez-Pizarraya A, Escoresca-Ortega A et al. De-escalation of empirical therapy is associated with lower mortality in patients with severe sepsis and septic shock. Intensive Care Med. 2014 Jan;40(1):32-40.
2. Kaki R, Elligsen M, Walker S, et al. Impact of antimicrobial stewardship in critical care: a systematic review. J Antimicrob Chemother. 2011 Jun; 66(6):1223–230.
3. Masterton RG. Antibiotic de-escalation. Crit Care Clin. 2011 Jan; 27(1): 149–62.
4. Cowley MC, Ritchie DJ, Hampton N et al. Outcomes Associated With De-escalating Therapy for Methicillin-Resistant Staphylococcus aureus in Culture-Negative Nosocomial Pneumonia. Chest. 2019 Jan;155(1):53-59.

**Table 1 (abstract 000935).** See text for description

| | De-escalation (n=36) | No de-escalation (n= 67) | *P* |
|---|---|---|---|
| Age | 55.92±13.64 (50.29-61.55) | 61.71±12.71(57.89-65.53) | 0.38 |
| APACHE II | 17.78±7.20 (15.34-20.21) | 18.48±7.97 (16.53-20.42) | 0.70 |
| GLASGOW | 12.89±4.58 (11.34-14.44) | 13.54±3.24 (12.75-14.33) | 0.40 |
| Infection origin | | | 0.22 |
| Community | 19 (52.8%) | 27 (40.3%) | 0.22 |
| Extra-ICU | 17 (47.2%) | 40 (59.7%) | 0.22 |
| Inflammatory response | | | **<0.0005** |
| Sepsis | n=1 (2.8%) | n=26 (38.8%) | **<0.0005** |
| Severe sepsis | n=23 (63.9%) | n=30 (44.8%) | 0.06 |
| Septic Shock | n=12 (33.3%) | n=11 (16.4%) | **0.04** |
| Comorbilities | | | |
| DM | n=5 (13.9%) | n=15 (22.4%) | 0.29 |
| COPD | n=5 (13.9%) | n=5 (7.5%) | 0.31 |
| Neoplasia | n=11 (30.6%) | n=15 (22.4%) | 0.36 |
| Neutropenia | n=2 (5.6%) | n=9 (13.4%) | 0.32 |
| Immunocompromised | n=7 (19.4%) | n=9 (13.4%) | 0.42 |
| RCD | n=2 (5.6%) | n=7 (10.4%) | 0.48 |

**Table 2 (abstract 000935).** See text for description

| | De-escalation (n=36) | No de-escalation (n= 67) | *P* |
|---|---|---|---|
| Duration of MV (days) | 12.48±17.13 (5.41-19.55) | 10.73±15.66 (6.03-15.44) | 0.66 |
| ICU-adquired infection | n=6 (16.7%) | n=7 (10.4%) | 0.37 |
| CRRT | n=5 (13.9%) | n=8 (11.9%) | 0.77 |
| ICU stay (days) | 16.33±18.68 (10.01-22.66) | 12.42±9.15 (10.19-14.65) | 0.15 |
| Hospital stay (days) | 37.94±28.77 (28.21-47.68) | 41.70±47.49 (30.12-53.29) | 0.66 |
| ICU mortality | n=4 (11.1%) | n=10 (14.9%) | 0.76 |
| Hospital mortality | n=5 (13.9%) | n=13 (19.4%) | 0.48 |

### 000950

#### Time response of perioperative sepsis markers in a close-meshed investigation in elective cardiac surgical patients

J. Puchinger[1], S. Ryz[1], L. Nixdorf[1], M. Edlinger-Stanger[1], A. Lassnigg[1], M. Hiesmayr[1], A. Spittler[2], MH. Bernardi[1]
[1]Division of cardiac thoracic vascular anaesthesia and intensive care medicine, Medical University of Vienna, Wien, Austria; [2]Core facilities, core facility flow cytometry, Medical University of Vienna, Wien, Austria
**Correspondence:** M.H. Bernardi
*Intensive Care Medicine Experimental* 2019, **7(Suppl 3):**000950

**INTRODUCTION.** An early diagnosis of sepsis can significantly affect outcome.[1] However, up to date there is still a lack of understanding in time response of sepsis markers after cardiac surgery.

**OBJECTIVES.** We investigated in a close-meshed analysis differences in the perioperative time response of Interleukin-6 (IL-6), Procalcitonin (PCT) and C-reactive protein (CRP) during and after elective cardiac surgery in patients developing postoperative sepsis or not.[2]

**METHODS.** A prospective cohort study in 100 patients undergoing elective cardiac surgery with cardiopulmonary bypass (CPB) at the Medical University of Vienna was performed. IL-6 and PCT were measured simultaneously and consecutively at 19 timepoints until the 10th postoperative day (POD). Additionally, CRP was measured from POD 1 to 10. Patients were divided into two groups, Sepsis and no-Sepsis, according to Sepsis-3 guidelines.[3]

**RESULTS.** 96 patients (four patients dropped out for analysis), with a mean age of 67.4±11.2 years were analysed. CABG, valve and combined procedures were performed in 16.7% (N=16), 28.1% (N=27) and 55.2% (N=53), respectively. Sepsis was found in 8.3% of patients (N=8). Mean SOFA score at ICU admission was 8.6±1.8 in septic and 7.7±2.3 in patients without sepsis (*P*=0.1905). Septic patients had a prolonged ICU stay (Sepsis: 8.1±4.2 days vs. no-Sepsis: 2.5±2.1 days; *P*=0.0062). IL-6 increased continuously during the procedure with reaching a plateau 2 to 6 hours after CPB until POD 1 in both, patients with and without postoperative sepsis. Four hours after CPB we found significantly different median IL-6 values between both groups (393.9 [IQR 179.9; 484.0] pg/ml, 164.70 [IQR 108.80; 251.00] pg/ml, respectively; *P* = 0.0374). PCT began to increase four hours post CPB with reaching a peak on POD 1 in both groups (Sepsis: 1.91 [IQR 1.01; 5.00] ng/ml vs. no-Sepsis: 0.37 [IQR 0.21; 0.76] ng/ml; *P*= 0.0066). Patients without Sepsis returned to baseline earlier, compared to those with postoperative Sepsis. We found significantly different median PCT values 6 hours post CPB between both groups (0.57 [IQR 0.2725; 1.16] ng/ml, 0.17 [IQR 0.09; 0.34] ng/ml, respectively, *P=0.01648*). Median CRP levels reached a peak on POD 3 in both groups, whereas the difference did not reach statistical significance (23.57 [IQR 19.48; 27.6] mg/dl, 17.22 [IQR 12.97; 21.87] pg/ml, respectively, *P=0.05525*).

**CONCLUSION.** Earlier increases in IL-6 were found in patients developing later postoperative sepsis, compared with more traditional used inflammation parameters. Nevertheless, rapid changes in IL-6 plasma concentrations make it difficult to detect peak values in contrast to PCT. Therefore, increased IL-6 concentrations may earlier indicate the risk of postoperative sepsis, bu t strong inter-individual differences make it difficult for routine clinical use.

**REFERENCE(S)**

1. Howitt SH et al. Incidence and outcomes of sepsis after cardiac surgery as defined by the Sepsis-3 guidelines. Br. J Anaesth 2018;120:509–16.
2. Rhodes et al. Surviving Sepsis Campaign: International Guidelines for Management of Sepsis and Septic Shock: 2016. Int Care Med 2017;43:304–77.
3. Singer M et al. The Third International Consensus Definitions for Sepsis and Septic Shock (Sepsis-3). JAMA 2016;315:801–10
4. Materials and resources for this study have been partly financially supported by Roche Diagnostics GmbH

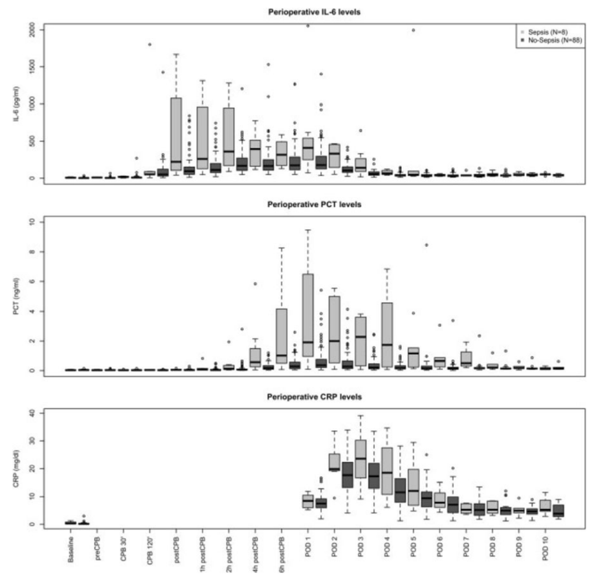

**Fig. 1 (abstract 000950).** See text for description

### 000954

#### Myristic acid and its significance in sepsis diagnosis (pilot study - preliminary results)

R. Zazula[1], M. Prucha[2], F. Pehal[2], K. Dryahina[3], M. Moravec[4], M. Müller[1], T. Nejtek[4]

[1]Department of anesthesiology and intensive care, First Faculty of Medicine, Charles University and Thomayer Hospital, Prague, Czech Republic; [2]Department of clinical biochemistry, hematology and immunology, Na Homolce Hospital, Prague, Czech Republic; [3]Department of chemistry of ions in gaseous phase, J. Heyrovský Institute of Physical Chemistry, Academy of Sciences of the Czech Republic, Prague, Czech Republic; [4]Faculty of military health sciences, University of Defence, Hradec Králové, Czech Republic

**Correspondence:** R. Zazula

*Intensive Care Medicine Experimental* 2019, **7(Suppl 3):**000954

**INTRODUCTION.** Clinical features are still the cornerstone in diagnosis of sepsis, nevertheless, there are various biomarkers which can help physicians to confirm or question sepsis. Study of Kauppi et al. identified myristic acid as a metabolite with highest predictive value in the metabolome of septic patients with bacteremia, with sensitivity 1,00 (95% CI 0,85-1,00) and specificity 0,95 (95% CI 0,74-0,99) [1]. Cambiaghi et al. observed its significant decrease in non-responders to the treatment of septic shock [2].

**OBJECTIVES.** Authors present pilot study of correlation of myristic acid serum levels with diagnosis of sepsis and its comparison with control group.

**METHODS.** The levels of myristic acid, were determined by gas chromatography/mass spectrometry (GC/MS) method in deep-frozen serum samples in the first five days following sepsis diagnosis.

Kruskal-Wallis test was used for evaluation of statistical differences between sepsis group in each time interval and control group. Wilcoxon signed-rank test was used to evaluate the statistical significance of differences in values between time intervals.

**RESULTS.** Values of myristic acid levels are presented as a median and interquartile range - IQR (1st quartile; 3rd quartile).

In healthy subjects ($n = 66$) myristic acid concentration was 18.9 µmol/l (16.2; 22.8), in septic patients ($n = 25$) in time T0 56.4 µmol/l (34.9; 80.9) with decreasing level trend until day 5. Myristic acid levels were in all measurements in septic patients significantly higher than levels in healthy controls ($p < 0.001$). Statistical significance of difference in myristic acid levels in individual measurements in septic group is shown in Figure 1.

**CONCLUSION.** Preliminary results show elevated concentration of myristic acid (vs. control group) at the time of sepsis diagnosis with gradual decrease towards day 5 from sepsis onset.

We conclude that myristic acid may be a promising biomarker in the early identification of septic patients. It is currently being systematically evaluated [3].

**REFERENCE(S)**

1. Kauppi AM, Edin A, Ziegler I, et al (2016) Metabolites in Blood for Prediction of Bacteremic Sepsis in the Emergency Room. PloS One 11:e0147670. https://doi.org/10.1371/journal.pone.0147670
2. Cambiaghi A, Pinto BB, Brunelli L, et al (2017) Characterization of a metabolomic profile associated with responsiveness to therapy in the acute phase of septic shock. Sci Rep 7:9748. https://doi.org/10.1038/s41598-017-09619-x
3. ClinicalTrials.gov Identifier: NCT03314831

4. Supported by MH CZ – DRO (Thomayer Hospital – TN 00064190)

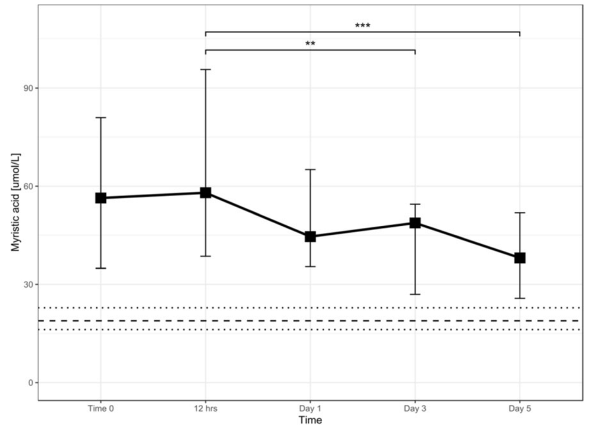

**Fig. 1 (abstract 000954).** See text for description

### 000969

#### Impact of hyperoxemia on mortality during septic shock: retrospective study on the MIMIC-III database

T. Clavier, B. Popoff, B. Dureuil, E. Besnier, B. Veber

Department of Anesthesiology and Critical Care, Rouen University Hospital, Rouen, France

**Correspondence:** T. Clavier

*Intensive Care Medicine Experimental* 2019, **7(Suppl 3):**000969

**INTRODUCTION.** Recent studies have shown excess mortality in intensive care unit (ICU) patients with hyperoxemia, particularly after stroke or cardiac arrest [1]. The effect of hyperoxemia in sepsis is unknown and few studies have been conducted on the subject [2]. The objective of our study was to evaluate the impact of hyperoxemia on mortality during septic shock.

**METHODS.** Design of a retrospective study using the Medical Information Mart for Intensive Care III (MIMIC-III) database provided by the Massachusetts Institute of Technology (MIT) with the approval of the MIT Ethics Committee and the Beth Israel Deaconess Medical Center in Boston. The inclusion criteria were: age ≥ 18 years admitted in polyvalent surgical or medical ICU, SEPSIS 3 septic shock criteria, and ICU stay with invasive ventilation ≥ 24 hours after admission. O2 exposure was defined by the average PaO2 over the first 24 hours of ICU stay with distinction between two groups: normoxemia (PaO2 70-120 mmHg) and hyperoxemia (PaO2 > 120 mmHg). The primary endpoint was ICU mortality. The variables are reported as mean (standard deviation) or median [interquartile range]. Univariate analysis was performed by Student or Wilcoxon tests for continuous variables and Pearson Chi2 tests for categorical variables. A multivariate analysis was performed by logistic regression adjusting for age, gender, and clinical or demographic parameters with p < 0.1 between groups. Gross and adjusted Odds Ratios could thus be calculated. The analyses were carried out bilaterally, taking a significance threshold of p < 0.05.

**RESULTS.** Between 2001 and 2012, 628 patients were included, 225 in the normoxemia group and 403 in the hyperoxemia group. The epidemiological data are presented in Table 1. The mean PaO2 in the normoxemia group was 99.8 ± 12.8 mmHg compared to 166.2 ± 41.4 mmHg in the hyperoxemia group. Hyperoxemia was associated with lower ICU mortality in univariate analysis (34.2% versus 42.7%, OR = 0.69 [0.50-0.98]; p = 0.045) but not in multi-variate analysis (OR = 0.94 [0.65-1.35]; p = 0.72). The parameters associated with mortality in multivariate analysis were initial severity (SAPS II, OR = 1.02 [1.00-1.04]; p = 0.024 and APACHE III, OR = 1.01 [1.00-1.03]; p = 0.022) and duration of norepinephrine treatment (1.00 OR = [1.00 - 1.01]; p = 0.003).

**CONCLUSION.** In this population of patients with septic shock, hyperoxemia was not an independent factor of mortality. These data are in contradiction with those previously published in other ICU patient populations. Thus, a prospective study in septic patients with a direct analysis of the impact of hyperoxemia appears necessary.

**REFERENCE(S)**

1. You J, Fan X, Bi X, et al (2018) Association between arterial hyperoxia and mortality in critically ill patients: A systematic review and meta-analysis. J Crit Care 47:260–268.
2. Demiselle J, Wepler M, Hartmann C, et al (2018) Hyperoxia toxicity in septic shock patients according to the Sepsis-3 criteria: a post hoc analysis of the HYPER2S trial. Ann Intensive Care 8:90.

### 000973

#### Non-carbonic buffer power of whole blood and isolated plasma of septic patients and healthy controls: an in-vitro acid-base study

T. Langer[1], S. Brusatori[2], E. Carlesso[1], A. Mauro[1], P. Brambilla[2], A. Zanella[1], G. Grasselli[1], A. Pesenti[1]
[1]Department of pathophysiology and transplantation, University of Milan, Milano, Italy; [2]Dipartimento di anestesia-rianimazione e emergenza urgenza, Fondazione IRCCS Ca' Granda Ospedale Maggiore Policlinico, Milano, Italy
**Correspondence:** S. Brusatori

*Intensive Care Medicine Experimental* 2019, **7(Suppl 3):**000973

**INTRODUCTION.** Acute alterations of acid-base equilibrium are common in septic patients.[1] Non-carbonic buffers (albumin and phosphates in plasma with the addition of hemoglobin in whole blood) contribute protecting the human body against metabolic acid-base disorders, while they are the only buffer system during respiratory derangements. The ability of these molecules to minimize pH variations due to acute $pCO_2$ changes has been defined as non-carbonic buffer power (β).[2] Septic patients have significantly lower values of hemoglobin and albumin, i.e., the major non-carbonic buffers of whole blood.[3] Moreover, albumin is a macromolecule whose structure might change significantly during pathological conditions such as inflammation.[4] It is currently unknown if and how pathological changes of protein structure affect its buffering function.

**OBJECTIVES.** To compare the β of blood and plasma of septic patients and healthy volunteers and compute the molar β of plasma (βmol PL) in order to evaluate the buffering capacity of albumin.

**METHODS.** Whole blood and isolated plasma collected from septic patients and healthy controls were tonometered at 2, 5, 12 and 20% of $CO_2$ in air. Blood gases, pH, albumin and hemoglobin were measured. For blood and plasma of both groups, variations of [$HCO_3$-] over pH were modeled according to a polynomial multilevel model with random intercept at subject level and random slope at pH level. For each model, a function describing β was obtained as β(pH)=–d[$HCO_3$-]/dpH, and a representative β at pH=7.40 was calculated. The same analysis was performed after normalizing [$HCO_3$-] variations for millimolar albumin concentration (βmmol PL). Patients and controls were compared by t-test or rank-sum test, as appropriate. Data are reported as mean ± SD or as median and interquartile range.

**RESULTS.** Eighteen septic patients (61% with septic shock [1]) and 18 controls were studied. Patients had lower albumin and hemoglobin than controls (3.0 [2.8-3.2] *vs.* 4.7 [4.6-4.9] g/dL, p<.001 and 10.5±0.8 *vs.* 14.3±1.0 g/dL, p<.001, respectively). The β of both blood and plasma of septic patients was significantly lower as compared to healthy volunteers (Fig.1). Furthermore, the βmmol PL of septic patients was also significantly lower (Fig.2).

**CONCLUSION.** Septic patients are less protected against acid-base derangements, as the β of both blood and plasma are significantly reduced. Besides lower buffer concentrations, it is conceivable that the buffering function of albumin is altered during sepsis.

**REFERENCE(S)**

1. Singer M JAMA 315:801-81, 2016
2. Van Slyke DD J Biol Chem 52:525-570, 1922
3. Vincent JL et al. JAMA 288:1499-1507, 2002
4. Roche M et at. FEBS Lett 582:1783-7, 2008

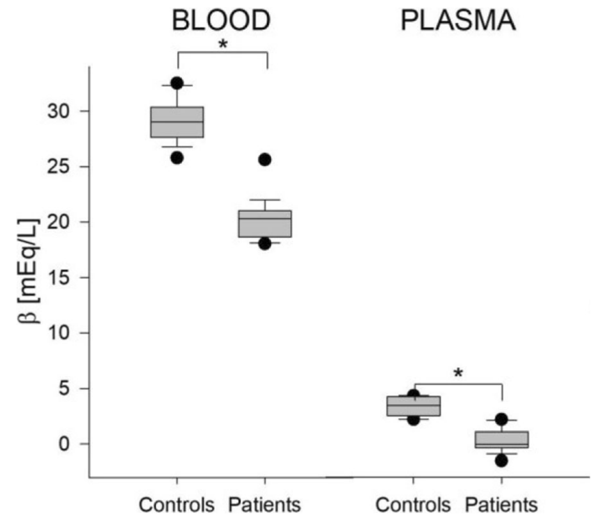

**Fig. 1 (abstract 000973).** See text for description

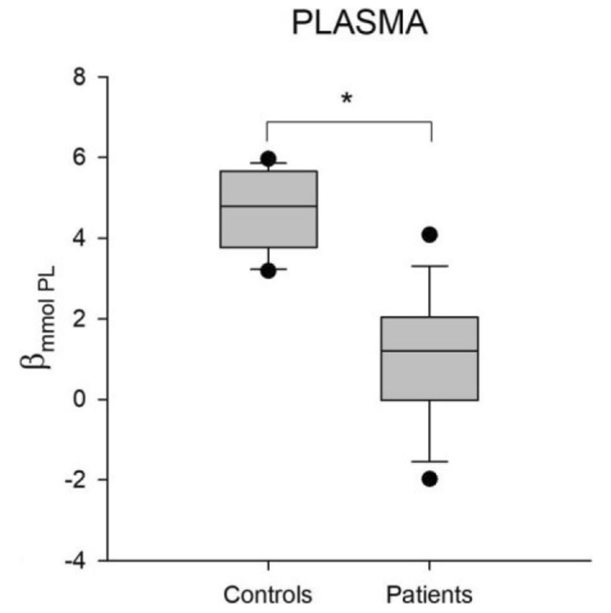

**Fig. 2 (abstract 000973).** See text for description

### 000975

#### Antibiotic Administration in the Emergency Department for Suspected Sepsis: An Evaluation of Unintended Consequences

J. Lee[1], A. Abbasi[1], R. Greene[2], P. Chen[3], C. Mailloux[4], A. Shick[5], N. Hudepohl[5], G. Baird[6], L. Mermel[7], M. Levy[1]
[1]Division of pulmonary, critical care and sleep medicine, Warren Alpert School of Medicine at Brown University, Providence, United States of America; [2]Department of pharmacy, Rhode Island Hospital, Providence, United States of America; [3]Department of respiratory diseases, Shenzhen Third People's Hospital, Shenzhen, China; [4]Lifespan corporate services, Rhode Island Hospital, Providence, United States of America; [5]Emergency department, Rhode Island Hospital, Providence, United States of America; [6]Lifespan biostatistics core at rhode island hospital, Rhode Island Hospital, Providence, United States of America; [7]Deptartment of epidemiology & infection control, Brown University, Providence, United States of America
**Correspondence:** J. Lee
*Intensive Care Medicine Experimental* 2019, **7(Suppl 3):**000975

**INTRODUCTION.** Sepsis is the leading cause of death in U.S. hospitals, and the second most common cause of 30-day hospital-readmission. The "sepsis bundle" was developed by the Surviving Sepsis Campaign with the goal to reduce mortality and morbidity from sepsis. Sepsis bundle compliance is associated with lower risk and reliability-adjusted mortality. A recent update to the Surviving Sepsis Campaign guidelines, including a 'hour-1-bundle' recommends the administration of broad spectrum antibiotics to patients presenting to the emergency department (ED) with possible sepsis. This has raised concerns over possible unintended consequences of inappropriate antibiotic administration in non-septic patients, such as higher rates of *Clostridium difficile* infection (CDI).

**OBJECTIVES.** We aimed to retrospectively assess the incidence of CDI in non-septic patients who received antibiotics in the ED for suspected sepsis. We hypothesized that early antibiotic administration combined with antibiotic stewardship would not be associated with an increased risk of CDI.

**METHODS.** This was a single center, retrospective cohort study. All adult patients admitted from the ED from 2017 to 2018 were included in the analysis. Patients were identified that (i) presented with two or more criteria of the systemic inflammatory response syndrome (SIRS), (ii) received intravenous antibiotics in the ED, (iii) received intravenous antibiotics for less than and more than 48 hours, (iv) discharged from the hospital with and without a diagnosis of sepsis, and (v) diagnosed with CDI within two months of admission.

**RESULTS.** 25,863 non-trauma patients were admitted from the ED during the study period. 7,432 (28.7%) patients received antibiotics in the ED, of which 4,259 (57.3%) patients presented with two or more SIRS criteria and 1372 (32.2%) patients presented with two or more SIRS criteria and were discharged with a diagnosis of sepsis. Of 4,259 patients that presented with two or more SIRS criteria and received antibiotics in the ED, 1,387 (29.2%) patients had antibiotics discontinued within 48 hours. The CDI incidence of non-septic patients administered antibiotics in the ED after presenting with two or more SIRS criteria, and had antibiotics discontinued within 48 hours was 0.67%, compared to 2.29% in those in whom antibiotics were not discontinued ($p$ = 0.0028). In comparison, the CDI incidence of septic patients administered antibiotics in the ED after presenting with two or more SIRS criteria and treated with antibiotics for more than 48 hours was 5.1% ($p$ < 0.001). The CDI incidence in patients not administered any antibiotics throughout their emergency department or inpatient stay was 0.33% ($p$ = 0.1).

**CONCLUSION.** With appropriate antibiotic stewardship, the administration of antibiotics to non-septic patients in the emergency department does not increase the incidence of CDI.

### 000983

#### Metabolism of tryptophan and kynurenine in septic patient: association with the hypotension severity and prognosis

S. Redaelli[1], F. Nespoli[1], F. Fumagalli[2], M. Magnoli[2], L. Ruggeri[2], G. Ristagno[3]
[1]School of medicine and surgery, University of Milano-Bicocca, Monza, Italy; [2]Cardiovascular research, Istituto di Ricerche Farmacologiche Mario Negri IRCCS, Milano, Italy; [3]Fisiopatologia medico-chirurgica e dei trapianti, University of Milan, Milano, Italy
*Intensive Care Medicine Experimental* 2019, **7(Suppl 3):**000983

**INTRODUCTION.** Indoleamine 2,3 dioxygenase 1 (IDO1) metabolizes tryptophan (TRP) through the kynurenine (KYN) pathway (KP) and it is induced when systemic inflammation is present, as in sepsis. The upregulation of the KP has been identified as a hypotension culprit in *in vivo* models of sepsis and septic shock [1] and associated to increased mortality in critically-ill patients [2].

**OBJECTIVES.** We investigated the KP in septic patients to determine if it was involved in sepsis severity. We evaluated the association between IDO1 activity and the severity of sepsis-induced hypotension and we analyzed the association between the plasma concentration of KP metabolites and mortality, both at ICU discharge and at 90 days.

**METHODS.** We performed analysis on a subgroup of patients ($n$=100, 50 survived and 50 deceased at ICU discharge), previously enrolled in the randomized controlled trial Albumin Italian Outcome Sepsis [3]. TRP, KYN, kynurenic acid (KYNA) and 3-hydroxyantranilic acid (3-HAA) concentration were determined through liquid chromatography mass spectrometry on ICU day 1, 2 and 7. We used KYN/TRP ratio as a surrogate of IDO1 activity. We considered the *inotropic score* (*IS*) (dopamine + adrenaline x 100 + noradrenaline x 100 [µg/kg/min]) as the expression of pressors need for keeping a mean arterial pressure above 65 mmHg. We applied univariate and multivariate models adjusted for age, sex, sepsis source, SOFA and SAPS II at ICU admission to determine associations among variables.

**RESULTS.** TRP concentration was halved at ICU admission, while KYN, KYNA, and 3-HAA were increased by 2-10 times, compared to the physiological values. TRP decrease suggested IDO1 activation, as confirmed by the sevenfold increase of the KYN/TRP ratio. IDO1 upregulation and TRP catabolism remained unchanged during all the 7 days, as shown by constantly high KYN, KYNA, 3-HAA and KYN/TRP ratio. *IS* increased by 1.68 points [95%CI 1.18–2.41, $P$=0.006] for each twofold KYN/TRP ratio increment. KYN and its metabolites were increased in the deceased with respect to the survivors, either at ICU discharge or at 90 days ($P$<0.05). The univariate model showed KYN and KYNA associated to ICU discharge mortality ($P$<0.01), whereas KYN, KYNA and 3-HAA associated to 90-day mortality ($P$<0.01). The multivariate model showed KYNA associated to ICU discharge mortality (HR 1.30, [95%CI 1.10–1.54, *P*=0.002] for each twofold concentration increase). Moreover, KYN, KYNA and 3-HAA were independent predictors of 90-day mortality and the hazard ratios were 1.50 [95%CI 1.06–2.13, *P*=0.02], 1.27 [95%CI 1.08–1.49, *P*=0.004] and 1.23 [95%CI 1.04–1.46, *P*=0.02] respectively, for each twofold increase of the metabolites concentration.

**CONCLUSION.** The KP was upregulated in septic patients. IDO1 activation was associated to the severity of sepsis-induced hypotension and KYN, KYNA and 3-HAA high concentrations were independent risk factors of mortality in septic patients.

**REFERENCE(S)**

1. Wang Y, et al: Kynurenine is an endothelium-derived relaxing factor produced during inflammation. Nat Med 2010; 16:279 –285.
2. Huttunen R, et al. High activity of indoleamine 2,3 dioxygenase enzyme predicts disease severity and case fatality in bacteremic patients. Shock. 2010; 33(2):149–54.
3. Caironi P, et al: Albumin replacement in patients with severe sepsis or septic shock. N Eng J Med; 2014 Apr 10; 370(15):1412-21.

## NAHP - Improving outcomes in ICU populations 1

### 000012

#### TLR-2 and TLR-4 predicts early mortality in pediatric patients with spinal muscular atrophy treated with noninvasive positive pressure ventilation

D. Dmytriiev
Grushevskogo 21/15, Vinnytsia, Ukraine

*Intensive Care Medicine Experimental* 2019, **7(Suppl 3):**000012

**INTRODUCTION.** Patients with **spinal** muscular atrophy (SMA) who are unresponsive to appropriate medical treatment, are often treated with Noninvasive PositivePressure Ventilation (NPPV). Clinical predictors of the outcome of this treatment are scarce. Therefore, we evaluated the role of the biomarkers TLR-2 and TLR-4 in predicting 90-day mortality in patients with SMA who receive treatment with NPPV.

**METHODS.** The study population were 17 patients treated with NPPV for SMA. Clinical and background data was registered and blood samples taken for analysis of inflammatory biomarkers. TLR-2 and TLR-4 were selected for analysis, and related to risk of 90-day mortality (primary endpoint) using Cox proportional hazard models adjusted for gender, age and various clinical parameters.

**RESULTS.** Of the 17 patients, there were 2 subgroup in regards to primary diagnosis: **spinal** muscular atrophy TYPE 1 (SMATYPE1, n = 17), **spinal** muscular atrophy TYPE 2 (SMATYPE2, n = 7). There was significant difference in the basic characteristic of the subgroups, but not in the clinical parameters that were used in treatment decisions. 10 patients died within 90 days of admission (58,82%). The Hazard Ratio for 90-days mortality per 1-SD increment of TLR-4 was 4.498 (95% CI 2.34-8.04, p < 0.001). When TLR-4 values were divided into tertiles, the highest tertile had a significant association with 90 days mortality, HR 10.012 (95% CI 1.29-78.17, p for trend 0.022), compared with the lowest tertile. This correlation was maintained when the largest subgroup with SMATYPE1 was analyzed. TLR-2 was correlated in the same way, but when put into the same model as TLR-4, the significance disappeared.

**CONCLUSION.** TLR-4 is a target to explore further as a predictor of 90 days mortality, in patients with SMA treated with NPPV.

### 000041

#### ICU patients' and relatives' experiences of a peer support group

C. Jones[1], R. Endacott[2], P. Gibb[1]
[1]None, ICUsteps Peer Support Charity, London, United Kingdom; [2]School of nursing and midwifery, University of Plymouth, Plymouth, United Kingdom
**Correspondence:** C. Jones

*Intensive Care Medicine Experimental* 2019, **7(Suppl 3):**000041

**INTRODUCTION.** Patients recovering from critical illness involving a stay in an Intensive Care Unit (ICU) and their family and friends may struggle when they go home from hospital. There can be significant physical, psychological and cognitive sequelae for patients (1). A recent study of 4943 ICU survivors found that 46% fulfilled the criteria for anxiety, 40% for depression and 22% for post traumatic stress disorder (PTSD), whilst 18% met the caseness threshold for all three psychological conditions (2). Relatives can also suffer from psychological problems, referred to as post intensive care syndrome (PICS) and post intensive care syndrome – family (PICS-F) (3). ICUsteps started at one peer support group in Milton Keynes, UK, and has grown to 23 support groups around the UK and Ireland. Anecdotally patients and families report finding it helpful to attend a support group meeting but as yet no research has been undertaken examining experiences of attending.

**OBJECTIVES.** The aim of the study was to explore the positive and negative experiences of attending a peer support group meeting.

**METHODS.** A survey was undertaken to explore reasons for attending a support group, benefits and negative aspects of attending. ICUsteps has a system already established for advertising studies for external researchers from across the English speaking world. This involves the study being advertised by email to a list of research volunteers (both ex-patients and family) and, where needed, to the organisers of the 23 ICUsteps support groups. This system was used to advertise this study and questionnaires sent out by email to respondents. The results were analysed using qualitative thematic methods.

**RESULTS.** A total of 40, 37 ex-ICU patients and 3 family members, returned the questionnaire. Thematic analysis resulted in six benefits:- 1. Being able to talk to others who have been through the same experience 2. Normalisation of physical and psychological problems 3. Getting information about ICU and recovery 4. Making new friendships 5. Getting support from others 6. Giving something back for all their care. Over half of the respondents, including the 3 relatives, (n= 26) reported no negative aspects of attending support groups meetings. The remaining 14 patients identified some negative aspects. 1. Feeling overwhelmed revisiting old memories. 2. Feeling left behind or sidelined. 3. Finding the time or place of the meeting difficult. 4. Finding it difficult to see others in the group being upset. 5. Finding it difficult to ensure everyone had time to talk. 6. Feeling really angry. While all the respondents felt they had benefited from attending, 42% reported some negative experiences that would need addressing by group organisers

**CONCLUSION.** Peer support works for some but not all. This is the first research to look at the benefits and negative aspects of attending an peer support group meeting. While some patients, families and friends thrive in a group situation, others may need one to one support and others may find the information available on the internet meets their needs. Although the survival rate from critical illness has significantly improved, there remain unanswered questions about how peer support can best enhance survivorship.

**REFERENCE(S)**

1. Hatch et al. Anxiety, Depression and Post Traumatic Stress Disorder after critical illness: a UK-wide prospective cohort study. Crit Care (2018) 22:310
2. Needham et al. "Improving long-term outcomes after discharge from intensive care unit: report from a stakeholders' conference". Crit Care Med. 2012;40(2):502–509
3. Davidson et al. Family response to critical illness: postintensive care syndrome-family. Crit Care Med. Feb 2012;40(2):618-624

4. Thank you to all the ICUsteps support group attendees who helped with the study

### 000113

#### Outcomes of patients with severe bronchopulmonary dysplasia requiring prolonged mechanical ventilation beyond term corrected gestational age who were transferred to the pediatric intensive care unit

YH. CHOI, DP. June
Department of pediatrics, Seoul National University Hospital, Seoul, Republic of Korea
**Correspondence:** Y.H. CHOI

*Intensive Care Medicine Experimental* 2019, **7(Suppl 3):**000113

**INTRODUCTION.** Some of preterm infants with severe bronchopulmonary dysplasia (sBPD) may require long-term mechanical ventilation beyond term corrected gestation age (CGA), and therefore continue to care after referral to paediatric intensive care unit (PICU). At referral unit, deciding final respiratory support of those patient is the focus of strategy to shift toward less intensive care, rather than routine neonatal care. However, since the number of this patient population is small at any single center, there is very little data regarding the overall outcomes and consideration factors for determining final respiratory support

**OBJECTIVES.** Our aims was to define the character and outcomes of preterm infants with sBPD who was required for referral to PICU due to weaning failure beyond 40 weeks CGA, and to determine the clinical factors associated with decision timing of final respiratory support.

**METHODS.** This study is a retrospective review of preterm infants with sBPD, who was transferred to PICU of a tertiary hospital from nationwide neonatal intensive care unit (NICU) because of weaning failure between January 1, 2014 and September 30, 2018.

**RESULTS.** Of 14 eligible patients, the median gestational age at birth was 26 (25-29) weeks, and birth weight was 875 (642.5-855.0) g. The median (range) CGA at referral was 47 (43-55) weeks and the median length of stay in previous NICU was 154 (10.8-202.3) days after birth. All of patients had endotracheal tube, and 12 patients (85.7 %) required mechanical ventilation at the time of referral. After referral, based on multidisciplinary approach to chronic respiratory failure, the following problems was found that make wean difficult; large airway malacia, 7 (50.0%), peripheral airway obstruction, 6 (42.9 %), significant upper airway obstruction, 3 (21.4 %), and pulmonary arterial hypertension were discovered 8 (57.1 %) respectively. Finally, 8 of 14 patients (57.2%) was successfully extubated without tracheostomy, and remaining 6 patients underwent tracheostomy. Final respiratory support of all patients was determined at median 56 (48-63) weeks CGA. One patient was died after tracheostomy, and the patients requiring tracheostomy had a significantly longer stay in PICU during admission than patients without tracheostomy (187.63 vs. 297.67 days, p=0.033). Multivariable analysis showed that length of stay in previous NICU (p<0.05) and tracheostomy placement (p=0.021) were each associated with the decision time of final respiratory support. Until 56-57 weeks CGA, the 75th percentile of the patient not requiring tracheostomy was liberated from mechanical ventilation. On the other hands, less than 25th percentile of the patient who eventually failed to weaning process underwent tracheostomy until that time (Log rank test p=0.008).

**CONCLUSION.** There was not uncommon to be successful extubation after 40 weeks CGA among preterm infant with BPDs requiring prolonged mechanical ventilation. However, in patients who finally underwent tracheostomy, decision of respiratory support tended to be delayed and the overall outcomes was poor.

### 000121

#### Positive impact od sedation standarization on pediatric intensive care unit patients' outcome

T. Hazwani, Y. Kazzaz, F. Dawoud
Pediatric Intensive Care, King Abdulaziz Medical City, Riyadh, Saudi Arabia
**Correspondence:** T. Hazwani
*Intensive Care Medicine Experimental* 2019, **7(Suppl 3):**000121

**INTRODUCTION.** Optimal sedation for mechanically ventilated pediatric patients is integral to the practice of pediatric critical care, because both under- and oversedation may have negative effects while achieving a good level of sedation and analgesia will reduce anxiety, pain, and agitation, enhance synchronization with mechanical ventilation (1-4). Accidental extubation events were reported many times in our Pediatric Intensive Care Unit (PICU), root cause analysis identified sedation is an issue. There is protocol to evaluate the pain and sedation level in our PICU, but there is a lack of standardized practice of sedation strategy for those patients, which may lead to increase mechanical ventilation days and its associated complications (4-8).

**OBJECTIVES.** Pediatric Intensive Care Unit in King Abdulaziz Medical City, Riyadh, KSA is 25-bed capacity, Sedation and analgesia are necessary components in our patients, especially those requiring mechanical ventilation. The aim of this project was to develop and implement guidelines for sedation management in PICU and evaluate the impact of these as a part of quality and patients' safety program.

**METHODS.** This project used a pre-post design for data collection. All PICU ventilated patients receiving continuous sedation infusion were enrolled over three months for baseline data collection.

Guidelines for sedation and analgesia management for critically ill children including algorithm and assessment tools have been implemented. One month post-implementation data has been collected for three months. In addition to key outcome variables (length of stay, ventilation time, sedation duration, successful Extubation rate, unplanned Extubation rate), process measures data (Sedation boluses use average, Percentage of patients under sedated, Percentage of patients over sedated) were evaluated. A sample of patients post implementation: control phase has been collected and analyzed every two months for one year.

**RESULTS.** 37 patients have been enrolled in the pre-implementation phase, and 36 patients also included in the post-implementation phase, while 19 patients had been included in the control phase. The decrease of sedation boluses requirement average prior to implementation from 7.7 Bolus/patient/day to 5.7 post-implementation, with a remarkable decrease in the percentage of high pain score (above 4) per day from 19.0% to 15.4% (Graph1) approached significance. As well, there was observed a decrease in sedation assessment average by bedside nurses from average 18.9 of pain assessment/patient/day to 14.1 of pain assessment/patient/day (Graph 2). Decrease sedation days is noted in results, in the control phase, but this needs further evaluation (Graph 3).

**CONCLUSION.** The sedation standardization project demonstrated better pain and sedation control by achieving a target comfort level with fewer sedation boluses, which has a positive impact on patient's outcome in PICU. This standardization reflected also on bedside nurses' work as the sedation assessment become less frequent, and more organized overall. The importance of the findings of this project indicates that guidelines can be used to manage the PICU patient's comfort and pain without compromising the quality of care.

**REFERENCE(S)**

1. Vet NJ, Ista E, de Wildt SN, van Dijk M, Tibboel D, de Hoog M. Optimal sedation in pediatric intensive care patients: a systematic review. Intensive Care Med 2013;39:1524-34.
2. Curley MA, Wypij D, Watson RS, et al. Protocolized sedation vs usual care in pediatric patients mechanically ventilated for acute respiratory failure: a randomized clinical trial. JAMA 2015;313:379-89.
3. Deeter KH, King MA, Ridling D, Irby GL, Lynn AM, Zimmerman JJ. Successful implementation of a pediatric sedation protocol for mechanically ventilated patients. Crit Care Med 2011;39:683-8.
4. Playfor S, Jenkins I, Boyles C, et al. Consensus guidelines on sedation and analgesia in critically ill children. Intensive Care Med 2006;32:1125-36.
5. Carnevale FA, Razack S. An item analysis of the COMFORT scale in a pediatric intensive care unit. Pediatr Crit Care Med 2002;3:177-80.
6. Ista E, van Dijk M, Tibboel D, de Hoog M. Assessment of sedation levels in pediatric intensive care patients can be improved by using the COMFORT "behavior" scale. Pediatr Crit Care Med 2005;6:58-63.
7. Johansson M, Kokinsky E. The COMFORT behavioural scale and the modified FLACC scale in paediatric intensive care. Nurs Crit Care 2009;14:122-30.
8. Best KM, Asaro LA, Franck LS, Wypij D et al: Patterns of Sedation Weaning in Critically Ill Children Recovering From Acute Respiratory Failure. Pediatr Crit Care Med 2016, 17(1):19-29.
9. NA

### 000153

#### Prognostic Factors of ICU Mortality in Pediatric Hemato-oncology Patients with Pulmonary Complications

K. Kim, Y. Nali, Y. Jong-Seo
Department of pediatrics, College of Medicine, The Catholic University of Korea, Seoul, Republic of Korea
**Correspondence:** K. Kim
*Intensive Care Medicine Experimental* 2019, **7(Suppl 3):**000153

**INTRODUCTION.** The objective of this study was to determine prognostic factors related with mortality in pediatric hemato-oncology patients admitted for pulmonary complications on the ICU.

**METHODS.** This was a retrospective cohort study of patients below 21 years old with underlying hemato-oncologic diseases admitted for pulmonary complications at the ICU of a tertiary referral hospital in Korea between April 2009 and March 2017. Patients admitted for perioperative management or non-pulmonary complications were excluded. Demographics, laboratory parameters, and clinical parameters such as Glasgow Coma Scale (GCS), the pediatric version of the Sequential Organ Failure Assessment (pSOFA) score and the Pediatric Logistic Organ Dysfunction (PELOD) score, etc., were extensively reviewed.

**RESULTS.** A total of 110 pediatric hemato-oncology patients were admitted at the ICU for pulmonary complications. The median age was 13 (IQR, 8–16) years old, and 62 (56.3%) were boys. The median duration of ICU hospitalization was 8 (IQR, 4.25–16) days, and 45 patients (40.9%) were applied mechanical ventilation. The mortality rate was 59.1% (65/110 patients). Factors with a significant association to increased mortality in a multivariable logistic regression analysis were as follows: low GCS scores, low SpO2/FiO2 ratio, low hematocrit levels, and increasing total bilirubin levels. The pSOFA score and PELOD score assessed on the third day of admission had significant discrimination for in-ICU mortality with an area under the curve of 0.87 (95% CI, 0.80-0.95) and 0.83 (95% CI, 0.74-0.92), respectively.

**CONCLUSION.** The GCS score, SpO2/FiO2 ratio, hematocrit level, and total bilirubin level, pSOFA scores, and PELOD scores are useful factors for the prediction of an increased risk for mortality in pediatric hemato-oncology patients with pulmonary complication.

### 000154

#### Relatives' diaries for critically ill patients prevents distress in relatives. A mixed methods analysis

A. Højager Nielsen[1], S. Angel[2], TB. Hansen[3], I. Egerod[4]
[1]Anesthesiology, Intensive Care, Regionshospitalet Holstebro, Holstebro, Denmark; [2]Department of public health, Aarhus University, Aarhus, Denmark; [3]University clinic of hand, hip and knee surgery, Regionshospitalet Holstebro, Holstebro, Denmark; [4]Department of neuroanaesthesiolgoy, Rigshospitalet, København, Denmark
**Correspondence:** A. Højager Nielsen
*Intensive Care Medicine Experimental* 2019, **7(Suppl 3):**000154

**INTRODUCTION.** Critically ill patients and their closest relatives often suffer from post traumatic stress, anxiety and depression after the patient is discharged from the intensive care unit (1). This may be related to the traumatic situation of being critically ill and fighting for survival. Intensive care unit diaries have been proposed to help relatives and patients to process the experience and find meaning.

**OBJECTIVES.** To achieve a more comprehensive understanding of how relatives' diaries for intensive care unit patients work.

**METHODS.** A convergent mixed methods analysis (2) of data from a randomized controlled trial exploring the effect of an intensive care unit diary on posttraumatic stress symptoms in patients and relatives (3) and two hermeneutic phenomenological studies of patients' and relatives' perceptions and uses of the diary after discharge from the hospital (4,5).

**RESULTS.** The mixed methods analysis expanded the results of the underlying studies by identifying that writing a diary is a preventive measure against posttraumatic stress symptoms for relatives. The process of expressing feelings and thoughts in the critical situation combined with an opening for discussing the difficult time with the patient after discharge and having this experience acknowledged by the patient may have resulted in the lower levels of posttraumatic stress symptoms found in relatives who wrote a diary. For patients, the diary did not prevent posttraumatic stress symptoms but helped the patient interpret fragmented and disturbed memories of the intensive care unit into a coherent story within the framework of the relatives' diary. This strengthened the bonds between patients and relatives. However, some patients do not welcome the diary and want only to look forward.

**CONCLUSION.** Writing a diary supports the relative and patients during recovery and furthermore prevents posttraumatic stress symptoms in relatives. Offering the relative a diary should take relatives preferences into account and guide relatives on how to or whether to share the diary with the patient.

**REFERENCE(S)**

(1) Needham DM et al. Improving long-term outcomes after discharge from intensive care unit: report from a stakeholders' conference. Crit Care Med 2012 Feb;40(2):502-509.
(2) Tashakkori,A., Teddlie,C. editor. SAGE Handbook of Mixed Methods in Social & Behavioral Research. Thousand Oaks: SAGE; 2010.
(3) Nielsen AH et al. The effect of family-authored diaries on posttraumatic stress disorder in intensive care unit patients and their relatives: A randomised controlled trial (DRIP-study). Aust Crit Care 2019 Feb 19.
(4) Nielsen AH et al. Intensive care unit diaries: Developing a shared story strengthens relationships between critically ill patients and their relatives: A hermeneutic-phenomenological study. Int J Nurs Stud 2019 Jan 26;92:90-96.
(5) Nielsen AH et al. Structure and content of diaries written by close relatives for intensive care unit patients: A narrative approach (DRIP study). J Adv Nurs 2019 Jan 21.

The research was funded by Aarhus University, Department of Anaesthesiology Regional Hospital Holstebro and Health Research Fund of Central Denmark Region

### 000207

#### Vitamin A deficiency in critically ill children with sepsis

X. Zhang[1], S. Chen[1], Y. Ji[2], K. Yang[2]
[1]Department of critical care medicine, West China Hospital, Chengdu, China; [2]Department of pediatric surgery, West China Hospital, Chengdu, China
**Correspondence:** X. Zhang
*Intensive Care Medicine Experimental* 2019, **7(Suppl 3):**000207

**INTRODUCTION.** Data that indicate vitamin A status in critically ill children with sepsis are sparse. The association between serum vitamin A levels and the clinical outcomes of sepsis has not been well assessed.

**OBJECTIVES.** The aim of this study was to assess the prevalence of vitamin A deficiency in critically ill children with sepsis and to reveal its association with the clinical outcomes.

**METHODS.** Critically ill children with sepsis admitted to the studied pediatric intensive care unit (PICU) were engaged in this prospective study. Sex- and age-matched approximate-health children form the Department of Pediatric Surgery were enrolled as the control group. Blood samples were collected from all patients in the first 24 hours of admission for measurement of serum vitamin A status. We compared vitamin A status between sepsis group and control group. Then, we compared the clinical characteristics of two the subgroups of septic patients with vitamin A deficiency and those without vitamin A deficiency. Univariate and multivariable methods were used to evaluate the association between vitamin A deficiency and septic shock.

**RESULTS.** One hundred and sixty septic children, and forty-nine approximate-health children were enrolled in this study. Serum VA levels in sepsis group were significantly lower than control group (0.19±0.11 vs 0.34±0.12, p<0.001). Vitamin A deficiency was found in 94 (58.8%) subjects in studied group and 6 (12.2%) subjects in the control group ($P$<0.001). In septic patients, 28-day mortality and hospital mortality in patients with vitamin A deficiency were higher than that in patients without vitamin A deficiency, but the difference was not significant ($P$>0.05). Vitamin A deficiency was associated with septic shock with an unadjusted odds ratio (OR) of 3.297 (95% confidence interval (CI), 1.169 to 9.300; $P$=0.019). In a logistic model, vitamin A deficiency (OR, 4.630; 95% CI, 1.027-20.866; $P$=0.046), procalcitonin (OR, 1.029; 95% CI, 1.009-1.048; $P$=0.003), and the Pediatric Risk of Mortality scores (OR, 1.132; 95% CI, 1.009-1.228; $P$= 0.003) were independently associated with septic shock.

**CONCLUSION.** The prevalence of vitamin A deficiency was high in children with sepsis. Serum vitamin A levels were independently associated with septic shock.

**REFERENCE(S)**

1. This project was supported by grants from the National Natural Science Foundation of China (Grants 81400862 and 81401606).

### 000216

#### Nurses' wellbeing in neonatal and pediatric intensive care units and its relations with perceived nursing sensitive outcomes

A. Bagnasco[1], N. Dasso[1], S. Rossi[2], G. Catania[1], M. Zanini[1], G. Aleo[1], L. Sasso[1]
[1]Department of health science, University of Genoa Department of Health Science, Genova, Italy; [2]University of Genoa Department of Health Science, Genova, Italy
**Correspondence:** S. Rossi
*Intensive Care Medicine Experimental* 2019, **7(Suppl 3):**000216

**INTRODUCTION.** In pediatric health care, moderately high levels of nursing burnout risk were found (Pradas-Hernàndez et al., 2018), with potential negative impacts on professional wellbeing and patient care (Tawfik et al., 2017). The quality of care has been investigated in several studies, analysing nurses' perception about the risk of adverse events, highlighting how this perception matched the actual incidence of the adverse events (Winning et al., 2018).
**OBJECTIVES.** To investigate the relationship between nursing burnout and the perception of the risk of adverse events in Newborn and Pediatric Intensive Care Units (NICUs and PICUs) in Italy.
**METHODS.** Data were extracted from RN4CAST@IT-Ped, a larger cross-sectional observational study involving nurses and pediatric nurses, through convenience sampling of 13 hospitals affiliated to the Italian Pediatric Hospitals Association. Burnout was investigated using the Maslach Burnout Inventory, and the nurses' perception of the risk of adverse events was assessed through questions regarding the frequency of six adverse events. Data were analysed through descriptive analysis and logistic regression with IBM SPSS 22.0 software.
**RESULTS.** The responses of 451 NICU and PICU nurses were analyzed. An increase in 'Emotional Exhaustion' increased nurses' perception of the risk of medication errors ($p$=0.002; OR=1.081) and of the onset of pressure ulcers ($p$=0.004; OR=1.051). An increase in 'Depersonalisation' increased nurses' perception of the risk of pressure ulcers ($p$=0.040; OR=1.079) and pneumonia ($p$=0.018; OR=1.097).
**CONCLUSION.** Develop improvement interventions to increase staff wellbeing and patient safety in the health care system based upon our results, which are consistent with international literature, could improve the overall quality of nursing care in NICU and PICU.

**REFERENCE(S)**

1. Winning AM, Merandi JM, Lewe D, Stepney LMC, Liao NN, Fortney CA, Gerhardt CA. The emotional impact of errors or adverse events on healthcare providers in the NICU: The protective role of coworker support. J Adv Nurs. 2018 Jan;74(1):172-180.
2. Tawfik DS, Sexton JB, Kan P, Sharek PJ, Nisbet CC, Rigdon J, Lee HC, Profit J. Burnout in the neonatal intensive care unit and its relation to healthcare-associated infections. J Perinatol. 2017 Mar;37(3):315-320.
3. Pradas-Hernández L, Ariza T, Gómez-Urquiza JL, Albendín-García L, De la Fuente EI, Cañadas-De la Fuente GA. Prevalence of burnout in paediatric nurses: A systematic review and meta-analysis. PLoS One. 2018 Apr 25;13(4):e0195039.
4. We thank the Italian Association of Pediatric Hospital to funding the study.

### 000217

#### Improving information-giving to critical care patients to guide post discharge rehabilitation: a quality improvement project

A. Fardanesh[1], S. Stavropoulou-Tatla[1], O. Grassby[1], S. Elliott[2]
[1]School of medicine, King's College London, London, United Kingdom; [2]Therapy, Medway NHS Foundation trust, Gillingham, United Kingdom
**Correspondence:** A. Fardanesh
*Intensive Care Medicine Experimental* 2019, **7(Suppl 3):**000217

**INTRODUCTION.** ICU survivors have a 1-year mortality rate of 30% (1), and a reduced quality of life associated with post-ICU syndrome (2); a triad of cognitive decline, physical weakness and psychiatric disorders. Early rehabilitation improves outcomes, leading to greater independence. The NICE CG83 guidelines instruct the provision of rehabilitation information to critical care patients on discharge. Currently, only a third of UK trusts meet these guidelines (3).
**OBJECTIVES.** Within 20 weeks, we aimed to achieve 100% patient and therapist satisfaction with the rehabilitation information given to patients at risk of physical morbidity on discharge from critical care at Medway Maritime hospital.
**METHODS.** Critical care patient and therapist satisfaction was assessed using questionnaires at baseline and after each PDSA cycle. In PDSA1, a generalised rehabilitation information booklet was introduced. In PDSA2, a personalised rehabilitation plan for pre-discharge completion by the therapists was added.
**RESULTS.** A shift was observed in critical care patient satisfaction scores, indicating a significant change in the median from 20% at baseline to 70% after PDSA2. This was also reflected in the therapist satisfaction scores which increased significantly from 60% at baseline to 80% after PDSA2.
**CONCLUSION.** The introduction of a generalised information booklet, supplemented with a personalised recovery plan, is an effective way of increasing critical care patient and therapist satisfaction with post-discharge rehabilitation information provision. This should translate to greater critical care patient engagement with rehabilitation and improved long-term outcomes. To further increase satisfaction, the addition of psychiatric input to the booklet is currently underway.

**REFERENCE(S)**

1. Steenbergen S, Rijkenberg S, Adonis T, Kroeze G, van Stijn I, Endeman H. Long-term treated intensive care patients outcomes: the one-year mortality rate, quality of life, health care use and long-term complications as reported by general practitioners. BMC anesthesiology. 2015 Dec;15(1):142.
2. Rawal G, Yadav S, Kumar R. Post-intensive care syndrome: An overview. Journal of translational internal medicine. 2017 Jun 30;5(2):90-2.
3. Berry A, Cutler LR, Himsworth A. National survey of rehabilitation after critical illness. Journal of the Intensive Care Society. 2013;14(4):334-9.

### 000242

#### The outcomes compared inter-hospital transferred children with non-transferred children: a multicenter retrospective cohort study

Y. Enomoto[1], T. Abe[2], K. Takahiro[3], Y. Inoue[1], T. Nanako[4], Jarpac Study Group[5]
[1]Emergency care and Critical care medicine, University of Tsukuba Tsukuba Campus, Tsukuba, Japan; [2]General medicine, Juntendo University, Bunkyo City, Japan; [3]Pediatrics, University of Tsukuba Hospital, Tsukuba, Japan; [4]Department of health services research , faculty of medicine, University of Tsukuba Tsukuba Campus, Tsukuba, Japan; [5]Department of social medicine, National Center for Child Health and Development Hospital, Setagaya City, Japan
**Correspondence:** Y. Enomoto
*Intensive Care Medicine Experimental* 2019, **7(Suppl 3):**000242

**INTRODUCTION.** Pediatric intensive care units (PICU) has been established in many developed countries in order to provide specific intensive cares for children. However, inter-hospital transport to PICU has been still needed because PICU has been scarce even in developed countries. Deterioration of outcomes associated with inter-hospital transport has been concerned [1].
**OBJECTIVES.** The aim of this study was to evaluate whether inter-hospital transport for severe pediatric patients was associated with in-hospital mortality.
**METHODS.** This study was a multicenter retrospective cohort study. Data were derived from the Japanese Registry of Pediatric Acute Care (JaRPAC) from 2013 to 2017. Inclusion criteria for this study were patients who aged 16 years and under and admitted to the PICU. Patients after elective surgery were excluded. The primary outcome was in-hospital mortality. The secondary outcome was deterioration of pediatric cerebral performance category (PCPC) at PICU discharge compared with at admission. Collected data included institution type of referral hospital, patient's baseline characteristics, supportive therapies, PCPC, in-hospital mortality, and length of PICU stay. We defined the patients admitted PICUs from their hospitals as "In-House" group and transferred from others (referring hospitals) as "Transfer" group. To adjust for inherent characteristics, severity, and diagnosis at admission, we conducted a propensity score matched analysis.

**RESULTS.** A total of 4,011 patients were eligible for our study criteria. Among those, The Transfer group was 1,603 children and the In-House group was 2,408 children. The Transfer group was higher PIM2 score than the In-House group. Transfer group needed more mechanical ventilation, inhaled nitric monoxide, extracorporeal membrane oxygenation, and continuous hemodiafiltration. ICU length of stay was longer in the Transfer group (median) than that in the In-House group (median [IQR]5 [3–9] vs. 3 [2–7], P<0.001). Crude in-hospital mortality was 27/1603 (1.7%) in the Transfer group and 62/2408 (2.6%) in the In-House group (*P*=0.06), In the propensity matched cohort, the Transfer group was not associated with in-hospital mortality compared with the In-House group (12/913 (1.3%) vs 22/913 (2.4%), P=0.08) Moreover, the Transfer group was not associated with deterioration of PCPC compared with the In-House group (84/913 (9.2%) vs 69/913 (7.6%), P=0.21)

**CONCLUSION.** Inter-hospital transport was not associated with unfavorable outcomes among children who admitted to PICU. Transport of severe pediatric patients to the PICU may be feasible.

**REFERENCE(S)**

1. Singh JM, Gunz AC, Dhanani S, et al.: Frequency, Composition, and Predictors of In-Transit Critical Events During Pediatric Critical Care Transport. Pediatr Crit Care Med 2016; 17:984–991
2. none

### 000287

#### Nutritional Status and Organ Dysfunction in Critically Ill Children

P. Hong-Zhu, A. Marroquín, J. Silva-Gburek, Y. Desai, S. Rodriguez, T. Fogarty, L. Shekerdemian, JA. Coss-Bu
Pediatrics, critical care section, Baylor College of Medicine, Texas Children's Hospital, Houston, United States of America
**Correspondence:** J.A. Coss-Bu
*Intensive Care Medicine Experimental* 2019, **7(Suppl 3):**000287

**INTRODUCTION.** The Pediatric Sequential Organ Failure Assessment (pSOFA) scoring system is based on the Multiple Organ Dysfunction Syndrome and it has been shown to be useful in predicting outcome in critically ill children. Also, nutritional status on admission to the pediatric intensive care unit (PICU) has been associated with outcomes in critically ill children.

**OBJECTIVES.** To assess nutritional status and organ dysfunction by pSOFA on children on admission to the PICU and its association with outcomes.

**METHODS.** Retrospective cohort study of children admitted to the PICU at Texas Children's Hospital (01/2016-06/2016) with a length of stay (LOS) > 3 days; nutritional status assessed by weight for length/Body mass index (WFL/BMI: acute malnutrition), z-scores by WHO (0-24 months) and CDC (2-19 yrs), respectively. Malnutrition defined as mild, and moderate-severe if z-scores were < -1,< -2, respectively. Hospital and PICU LOS, risk of mortality (ROM) by the Pediatric Index of Mortality 2 (PIM2), and mortality were obtained. Organ dysfunction was defined as pSOFA score > 5, and were collected on day 1.

**RESULTS.** A total of 358 children (202 males/156 females) were included; with age 2.6 yr. (0.7-10.3; median (25-75th IQR)). PICU LOS, 7.1 (4.7-11.4) days, Hospital LOS, 19 days (11-42); PIM2 ROM (%), 3.3 (1.0-5.0), PSOFA score on day 1 was 5 (4-7), for a 46% prevalence of organ dysfunction. WFL/BMI z-scores of 0.08±1.34 (SD); the prevalence of acute malnutrition was 25.1%. A total of 294 (82%) patients required mechanical ventilation on admission to the PICU and mortality was 7.5%. Patients with organ dysfunction on day 1 vs. pts without organ dysfunction had: PICU and Hospital LOS of 8.1 days (4.9-13.3) vs 6.8 days (4.4-10.3) (p< 0.05) and 21 days (11-47) vs 17 days (11-29) (p=0.1796), respectively; PIM2 ROM (%) of 3.86 (1.37-8.09) vs 2.91 (0.92-4.43) (p<0.0001); and were associated with older age (> 2 years) Odds ratios (OR) (95% CI) 1.56 (1.02-2.38) (p<0.05); need of ventilatory support on admission, 2.80 (1.53-5.10) (p < 0.001) and mortality 2.50 (1.09-5.74) (p < 0.05). Patients with underweight and overweight/obesity vs. normal nutritional status were more likely to have organ dysfunction; (OR) (95% CI); 2.73 (1.37-5.40) and 2.05 (1.29-3.26) (all p<0.005), respectively.

**CONCLUSION.** Organ dysfunction was prevalent in critically ill children and was associated with longer PICU length of stay, higher severity of illness, older age, need of ventilator support and mortality. One out of every 4 children had malnutrition and malnourished children were more likely to have organ dysfunction on admission to PICU.

**REFERENCE(S)**

1: Matics TJ, Sanchez-Pinto LN. Adaptation and Validation of a Pediatric Sequential Organ Failure Assessment Score and Evaluation of the Sepsis-3 Definitions in Critically Ill Children. JAMA Pediatr. 2017 Oct 2;171(10):e172352.
2: Section of Critical Care, Baylor College of Medicine and Texas Children's Hospital.

### 000289

#### Body Mass Index (BMI) and outcomes in critically ill children

A. Marroquín, P. Hong-Zhu, J. Silva-Gburek, Y. Desai, S. Rodriguez, T. Fogarty, L. Shekerdemian, JA. Coss-Bu
Pediatrics, critical care section, Baylor College of Medicine, Texas Children's Hospital, Houston, United States of America
**Correspondence:** J.A. Coss-Bu
*Intensive Care Medicine Experimental* 2019, **7(Suppl 3):**000289

**INTRODUCTION.** The prevalence of overweight and obesity in hospitalized US children has been increasing in the last decade and obesity has been associated with poor outcomes.

**OBJECTIVES.** To assess nutritional status on children on admission to the pediatric intensive care unit (PICU) and its association with outcomes.

**METHODS.** Retrospective cohort study of critically ill children < 5 yr of age admitted to the PICU at Texas Children's Hospital (01/2016-12/2017); nutritional status assessed by weight for length/Body mass index (WFL/BMI: z-scores by WHO. The BMI was categorized as: normal (NOR), underweight (UND), (BMI < 2 SD); overweight (OVE), (BMI > 2 SD), and obese (OBE), (BMI > 3 SD). Hospital and PICU length of stay (LOS), risk of mortality (ROM) by the Pediatric Risk of Mortality (PRISM III), ethnicity, and mortality were obtained.

**RESULTS.** A total of 2101 children (1210 males/891 females) were included; with age 1.17 yr (0.41-2.56; median (25-75th IQR)). PICU LOS: 2.10 (0.99-6.16) days, Hospital LOS: 10 days (4-22); PIM2 ROM (%) 1.14 (0.75-3.86). The prevalence and median age for NOR, UND, OVE and OBE was: 58.6%, 11.1%, 17.4%, and 12.8%, respectively, and 1.1 yr. (0.4-2.5), (median (25-75th IQR)), 0.7 yr. (0.2-2.6), 1.3 yr. (0.7-2.6), and 1.3 yr. (0.5-2.7), respectively (p<0.0001). The Hispanic group vs. the Caucasian and African-American, and Asian groups had the highest prevalence of UND, OVE, and OBE at: 35.9%, 42.9%, and 48%, respectively (p<0.005). The PICU and Hospital LOS for NOR, UND, OVE, and OBE groups were: 2 (1-6), 2.5 (1-7), 2 (1-6), and 2.4 (1-8), respectively; (p<0.05); and 10 (4-22), 11(5-23), 10 (5-20), and 11 (5-28), respectively. Children with UND, OVE, and OBE vs. the normal BMI group were associated with a higher PRISM III ROM (%) and need of ventilator support on admission; Odds ratios (OR) (95% CI) 1.10 (0.79-1.53), (p=0.5750), 1.53 (1.18-1.99) (p<0.005), and 1.64 (1.22-2.19) (p< 0.005), respectively; and 1.24 (0.93-1.67) (p=0.1472), 0.94 (0.74-1.19), and 1.34 (1.01-1.78) (p<0.05), respectively. Hospital Mortality was 4.05% and was not associated with BMI status.

**CONCLUSION.** More than half of the patients had a normal BMI and almost a third were overweight or obese on admission to PICU. Younger children were underweight and older children were overweight or obese. The Hispanic group had a higher prevalence of underweight, overweight and obesity compared to the other ethnic groups.

Overweight and obese children were more likely to have a higher risk of mortality on admission and the obese group was associated with need of ventilator support on admission. Underweight children had a longer PICU LOS. Mortality was not associated with BMI status.

**REFERENCE(S)**

1. World Health Organization. World Health Organization Child Growth Standards. 2006.
2. National Center for Health Statistics (NCHS), Data Brief, No 219, Nov. 2015.
3. Section of Critical Care, Baylor College of Medicine and Texas Children's Hospital.

### 000293

#### Assessment of ICU-acquired weakness and its functional consequences

V. Ferrer Ayats[1], C. Espinal Sacristan[1], N. Ridao Sais[1], A. Betriu Samperi[1], G. Pérez Tejero[1], S. Fernandez-Gonzalo[2], S. Pozo Fernández[2], J. Estela Esteve[1], E. Jové Ponseti[1], M. Santé Roig[1], G. Gomà[2], G. Navarra Ventura[2], M. Rodríguez Alejo[1], J. Mesquida[1]
[1]Àrea de crítics, Parc Taulí Hospital Universitari, Sabadell, Spain; [2]Àrea de crítics, Parc Tauli Hospital Universitari. Institut d'Investigació i Innovació Parc Taulí (I3PT)., Sabadell, Spain
**Correspondence:** V. Ferrer Ayats
*Intensive Care Medicine Experimental* 2019, **7(Suppl 3):**000293

**INTRODUCTION.** ICU-acquired weakness (ICU-AW) is a widespread involvement of diffuse and symmetrical muscular weakness that can develop among ICU patients without any other cause that can justify it. The incidence is different according to some studies, 25-50% in ventilated patients, reaching 50-100% in septic patients, and it is associated with an incomplete and variable functional recovery.

**OBJECTIVES.** 1- To analyze the incidence of ICU-AW in critically ill patients receiving invasive mechanical ventilation (IMV) for at least 24 hours.
2- To analyze the functional consequences of ICU-AW at ICU discharge and one month later.

**METHODS.** Single-center, prospective study (May-December 2017). Inclusion criteria: adult critically ill patients, who required>24h of IMV. Exclusion criteria: previous Barthel score <70, any neurological disease, trauma patients, and prior cognitive disease.
Patients were consecutively recruited at ICU admission. ICU-AW and functional capacity were assessed at ICU discharge, and one month later. Three groups were pre-defined according to the Medical Research Council scale (MRC): MRC> 48, Non-ICU-AW; MRC 36-48, mild-moderate ICU-AW; MRC < 36, severe ICU-AW. Muscle strength was also evaluated with dynamometry (handgrip strength), ambulation ability with the Functional Ambulation Categories (FAC) scale, and functional independence was assessed using the Barthel index for activities of daily living.
A descriptive analysis is presented (median, inter-quartile range and/or percentages). Comparisons among groups were performed using non-parametric tests.

**RESULTS.** We included 52 patients, 46% were women, mean age 69 (51 -74) years old. In 77% of patients, the reason for admission in the ICU were urgent medical conditions. The incidence of ICU-AW was 63.5%, severe in 11.5% of cases. As compared to Non-ICU-AW, patients with ICU-AW showed lower strength in handgrip, lower Barthel Index and lower walking capacity at discharge from ICU, and one month later. These functional alterations were significantly more pronounced in the group of severe ICU-AW. Patients with MRC <36 showed severe functional limitations with values of handgrip 10.5% (9.5-30.6) as compared to healthy population, Barthel Index values of 10 (8.7-17.5), and none of them were able to walk autonomously. At one month of ICU discharge, the Non-ICU-AW group showed normal functional capacity values while severe limitations persisted in the MRC group <36.

**CONCLUSION.** Incidence of ICU-AW is high in patients requiring IMV, and its functional impact is associated to its severity.

### 000328

#### Effects of Patient Monitor Alarm Management Education for Intensive Care Unit Nurses

JS. Hong[1], GM. Seong[2]
[1]Department of nursing, Jeju National University Hospital, Cheju, Republic of Korea; [2]Department of internal medicine, Jeju National University Hospital, Cheju, Republic of Korea
**Correspondence:** G.M. Seong
*Intensive Care Medicine Experimental* 2019, **7(Suppl 3):**000328

**INTRODUCTION.** With the development of medical technology, clinical alarms from diverse medical devices, are becoming a new issue in intensive care units (ICU). An excessive number of alarms will reduce the efficiency of medical staff, cause alarm fatigue and compromise patient safety.
Among the devices of the ICU, the patient monitor is known to generate the most alarms.

**OBJECTIVES.** To examine the effects of patient monitor alarm management education on nurses' performances of alarm management, frequency of false alarms, and alarm fatigue.

**METHODS.** A quasi-experimental study was conducted on 19 nurses in ICU at a single teaching hospital.
A preliminary survey was conducted on the frequency and type of ICU alarm and We analyzed the frequency of false alarms and the effect of alarm fatigue after patient monitor alarm management education.

**RESULTS.** After the intervention, nurse performance of alarm management was increased from 17.95±1.47 to 23.84±5.40 ($t$ = -5.68, $p$ = 0.001), and the frequency of false positive alarms also decreased significantly from 4.42±5.01 to 0.83±1.60 (Z = -4.42, $p$ < 0.001). There was no significant difference in the frequency of valid alarms. (1.15±2.02 vs. 1.06±2.07; Z = -0.16, $p$ = 0.870). The nurse 's alarm fatigue level also decreased significantly after education (25.84±4.59 vs. 21.10±6.09; $t$ = 2.71, $p$ = 0.014)

**CONCLUSION.** The patient monitor alarm management education proved to have beneficial effects on the level of performance of alarm management and alarm fatigue of the nurses. In addition, the frequency of false positive alarms is reduced, but there is no difference in the frequency of false alarms, which is helpful for safe patient monitoring.

### 000333

#### ICU visiting policies: the perspective of the patients and the nurses

A. Glotta[1], C. Duca[1], MP. Pollizzi[2], M. Bianchi[3]
[1]Intensive care unit, Clinica Luganese Moncucco, Lugano, Switzerland; [2]Nurse direction, Clinica Luganese Moncucco, Lugano, Switzerland; [3]Business economics, health and social care, University of Applied Sciences and Arts of Southern Switzerland, Manno, Switzerland
**Correspondence:** A. Glotta
*Intensive Care Medicine Experimental* 2019, **7(Suppl 3):**000333

**INTRODUCTION.** The visits that patients receive promote feelings of support, help and reassurance. On the other hand, there is little literature regarding the wishes of patients on intensive care unit (ICU) visits. In recent years is the trend to liberalize access for visits to patients admitted to the ICU. Experience has demonstrated that neither the patient's clinical situation nor the structural barriers of the ICU impede restriction-free visits, but it is due mainly to cultural factors.

**OBJECTIVES.** Explore the topic of visits during ICU hospitalization by analysing patients' wishes and the nurses' points of view.

**METHODS.** Qualitative research with thematic analysis of the collected data was done in an ICU. The sample involved in the study was 8 patients and 12 nurses. Informed consent was signed by everyone. Data were collected through 8 semi-structured interviews for patients and 2 focus groups (FG) for nurses. The data analysis was performed with the NVivo 10 software.

**RESULTS.** From the analysis of the data have been identified 22 nodes from interviews and 28 nodes from FG. The nodes have been grouped into themes and macro-themes. It emerged:
2 macro-themes from interviews:

Patient experiences related to visits: highlights the presence of strong emotions, often negative, sometimes in conflict with each other.
Patients' thoughtson visits: ideas and advice for nurses who could help the patient in managing visits or making changes to approach.
3 macro-themes from FG:
Patient visits seen by nurses: the behaviours, seen and interpreted by the nurses, that the patients showed with their relatives or staff, the perceptions during the visits, the patients' experience or wishes.
Patients' visit experiences seen by nurses: the team's perspective was privileged then attitudes, behaviours and perceptions regarding patient visits were included.
Needs/suggestions offered by nurses: the improvement ideas emerged from the team compared to those derived from the patients tofully understand the problem.

**CONCLUSION.** The ICU is seen by patients and nurses as a place where the management of emotions and feelings is complicated. The patients want the visits because they help to face emotions and to overcome the physical and psychological insult created in ICU. The visits maintain contact with the world outside.
The nurses emphasize the importance of clear information to regulate the access.
It would be desirable during the admissions in ICU prompted patients their wishes, wherever possible, about the visits and that this cannot be taken for granted and absolutely necessary.
Ideally, every structure, follow a path of awareness of this issue in order to adequately support the wishes of patients without neglecting the suggestions and needs of healthcare staff.

**REFERENCE(S)**

1. Chapman, D. K. (2016). Satisfaction with Elimination of All Visitation Restrictions in a Mixed-Profile Intensive Care Unit. American Journal of Critical Care, 25(1), 46–50.

### 000343

#### Quality improvement projects for time to antibiotics of septic patient's in the emergency department

C. HATOZAKI[1], H. Sakuramoto[2], M. Okamoto[1], H. Nakajima[1], N. Shimojo[3], Y. Inoue[3]
[1]Intensive care unit, University of Tsukuba Hospital, Tsukuba, Japan; [2]Department of adult health nursing, College of Nursing, Ibaraki Christian University, Hitachi, Japan; [3]Department of emergency and critical care medicine, faculty of medicine, University of Tsukuba , tsukuba, Japan
**Correspondence:** C. HATOZAKI
*Intensive Care Medicine Experimental* 2019, **7(Suppl 3):**000343

**INTRODUCTION.** Prevalence of sepsis is high, affecting millions of people around the world each year. Previous studies demonstrate that early antibiotics administration directly reduces mortality. However, because sepsis is sometimes hard to diagnose promptly, the initial treatment is likely to be delayed. For break through the current situation, the qSOFA was induced as a rapid screening tool before the ICU to facilitate the identification of patients with suspected infection in the SSCG 2016.

**OBJECTIVES.** The objective of this study is to evaluate the impact of a nurse-initiated quality improvement (QI) by the primary screening using qSOFA on the sepsis management at the ER and improvement of patient's outcome.

**METHODS.** We conducted a before and after study. Patient older than 20 yrs who were suspected infection in ER were enrolled this study. Phase 1: In the before phase, consecutive eligible patients were enrolled from February 2017 to July 2017. Subsequently, educational intervention for sepsis treatment was provided to the all nurses from August 2017 to October 2017. Phase 2: From November 2017 to April 2018, nurses performed primary screening with qSOFA to make a diagnosis of sepsis for patients who were suspected infection, and the results were reported to doctors and positively proposed for blood cultures obtained and administration of antibiotics. Phase 3: From May 2018 to October 2018, ER physicians were incorporated to this study. Primary outcome was the antibiotic administration rate after visiting the ER, and secondary outcome was the 28th day mortality.

**RESULTS.** A total of 592 patients were included in this study (table1). The antibiotic administration rate after visiting the ER was improved significantly as the phases went on. The time to the initial antibiotic administration after visiting the ER was significant improved as the phases went on (table2). In multivariable logistic regression analysis, intervention for nurses was independently associated with antibiotic administration rate. 28 th day mortality were not significant between each phase, and there was no association between early antibiotic administration and 28 th day mortality.

**CONCLUSION.** A nurse-initiated QI improved the early administration of antibiotic for the patients with suspected sepsis. However, there was no association between early antibiotic administration and mortality.

**REFERENCE(S)**

1. Friedrich, M. E. et al. Time to Treatment and Mortality during Mandated Emergency Care for Sepsis. N Engl J Med 2017, 2235–2244 (2017).
2. Bruce HR, Maiden J, Fedullo PF, Kim SC. Impact of nurse-initiated ED sepsis protocol on compliance with sepsis bundles, time to initial antibiotic administration, and in-hospital mortality. J Emerg Nurs 2015; 41: 130-137
3. none

**Table 1 (abstract 000343).** Baseline characteristics

| | Phase1 n=263 | Phase2 n=182 | Phase3 n=147 | P value |
|---|---|---|---|---|
| Age mean ± SD | 66.2 ± 17.0 | 67.8 ± 16.2 | 67.0 ± 16.2 | 0.621 |
| Male n (%) | 129 (49) | 100 (54.9) | 85 (57.8) | 0.194 |
| Charlson Risk Index median(IQR) | 2 (0-6) | 2 (0-3) | 2 (0-7) | 0.133 |
| Source of infection n (%) | | | | 0.107 |
| Urinary | 37 (14) | 27 (15) | 22 (15) | |
| Respiratory | 71 (27) | 71 (39) | 34 (23) | |
| Gastrointestinal | 25 (10) | 16 (9) | 13 (9) | |
| Abscess | 7 (3) | 3 (2) | 4 (3) | |
| Hepatobiliary | 50 (19) | 24 (13) | 22 (15) | |
| Other | 48 (18) | 32 (18) | 39 (27) | |
| MAP≤65mmHg on arrival n (%) | 23 (9) | 22 (12) | 14 (10) | 0.569 |
| Screening rate of qSOFA (%) | 79.8 | 90.7* | 89.8* | 0.002 |

* $p<0.05$ vs. Phase1

**Table 2 (abstract 000343).** Clinical outcomes

| | Phase1 n=263 | Phase2 n=182 | Phase3 n=147 | P value |
|---|---|---|---|---|
| Antibiotics administered n (%) | | | | |
| Within 1 hour | 12 (4.6) | 14 (7.7) | 23 (15.6)* | 0.000 |
| Within 3 hour | 89 (33.8) | 76 (41.8) | 73 (49.7)* | 0.007 |
| Within 6 hour | 199 (75.7) | 152 (83.5) | 131 (89.1)* | 0.002 |
| Time to initial antibiotic (min) mean±SD | 230±102 | 213±104 | 185±107*† | 0.000 |
| 28-day mortality n (%) | 17 (6.5) | 22 (12.1) | 8 (5.4) | 0.057 |

* $p<0.05$ vs. Phase1
† $p<0.05$ vs. Phase2

### 000396

#### Early Mobilization reality

M. Magret, P. Perelló, S. Manrique, J. Gómez, J. Mariné, D. Moya, I. Reynalds, A. Arasa, MT. Cabas, Z. Ramos, M. Bodí
Intensive care unit, Hospital Universitari de Tarragona Joan XXIII, Tarragona, Spain
**Correspondence:** M. Magret
*Intensive Care Medicine Experimental* 2019, **7(Suppl 3):**000396

**INTRODUCTION.** The implementation of an early mobility (EM) protocol in an ICU is a difficult process that requires change in culture and the overcoming of many barriers. But do we really know what the actual implementation of a protocol is to assess its impact? Clinical information systems (CIS) can help us in this situation to detect the different problems in adherence to a protocol.

**OBJECTIVES.** To evaluate the adherence to the protocol of early mobilization (EM) in an ICU through the Clinical Information System (CIS).
**METHODS.** Descriptive study in a polyvalent ICU of 30 beds during a period of two years (January 2017-December 2018). Patients who met the criteria for inclusion in the EM protocol were included. The analyzed variables: sex, age, APACHE II, reason for admission, length of ICU stay, proportion of EM days, EM start day, Medical Research Council (MRC) and EM level. The reasons why EM was not performed were analyzed. The data was extracted from the CIS by means of extraction, transformation and loading (ETL) processes with the Qlikview® software tool. The mining of the data was done with R.
**RESULTS.** Of the 1961 patients admitted to the ICU in the study period, 819 (41.76%) required mechanical ventilation (MV), of which 510 received MV > 48h and 272 fulfilled the inclusion criteria, representing a total of 3940 stays. The majority were men (72%), with a median age of 63 (53-72) years, a median APACHE II of 24 (18-29), a median SOFA of 7 (5-9) and a median LOS of 10 (6.8-17.3) days. The most frequent reason for admission was respiratory failure 21.7%, followed by monitoring and post-operative monitoring 20.22% and septic shock 19.12%. The average ratio of EM days to total stay was 25%. The causes why it was not carried out were: 47.2% for not meeting the criteria of clinical stability, 38.9% for being weekend, 4.4% for lack of physiotherapist, 3.1% because patient was out of ICU or rejecting physiotherapy and 6.4% the cause is unknown. In 26% of patients with inclusion criteria it was never started; 58% did not meet criteria for clinical stability, 1% for lack of physiotherapist, 28% for being weekend and in 16% the cause is unknown. The median MP start day was 5.8 days and the patients who were not started had an average stay of 6.13 days. We analyzed the number of patients who underwent the MRC and the level of MP they reached, at 96 (35.29%) and 151 (65.51%) patients respectively. Of the 151, the proportion of patients who arrived at each level at ICU discharge were: 11.26% (17) level I, 21.85% (33) level II, 7.95% (12) level III, 20.53% (31) level IV and 38.41% (58) level V.
**CONCLUSION.** The evaluation of the process allowed us to analyze the adherence to the MP protocol observing that its implementation is 74%. The results allow us to identify the improvement actions, adjust the indications and monitor their impact.

**REFERENCE(S)**

1. Bailey P, Thomsen GE, Spuhler VJ et al. Early activity is feasible and safe in respiratory failure patients. Crit Care Med 2007;35:139-45

## POIC - Useful trials in perioperative medicine

### 000043

#### Results of Multicenter Retrospective Cohort Study in Patients with Anti-Neutrophil Cytoplasmic Antibody Associated Vasculitis Requiring Follow-up in Intensive Care Unit

U. Özdemir[1], E. Ortac Ersoy[2], RC. Yüksel[3], E. Kaya[4], G. Aygencel[1], M. Türkoğlu[1], A. Topeli[2], M. Güven[3], M. Sungur[3], N. Defne Altıntaş[4]
[1]Division of critical care medicine, department of internal medicine, Gazi University School of Medicine, Ankara, Turkey; [2]Division of critical care medicine, department of internal medicine, Hacettepe University School of Medicine, Ankara, Turkey; [3]Division of critical care medicine, department of internal medicine, Erciyes University School of Medicine, Kayseri, Turkey; [4]Division of critical care medicine, department of internal medicine, Ankara University School of Medicine, Ankara, Turkey
**Correspondence:** U. Özdemir
*Intensive Care Medicine Experimental* 2019, **7(Suppl 3):**000043

**INTRODUCTION.** Due to the side effects of the given different immuno-suppressive treatments for ANCA-associated vasculitis (AAV) or disease activation, these patients may deteriorate rapidly and intensive care unit (ICU) follow-up can be required (1). There is a need for a scoring system that can predict the ICU prognosis of patients with AAV, but the literature has limited data related with it.
**OBJECTIVES.** For this reason, the prognostic importance of clinical scoring systems was investigated in patients who had AAV activation.
**METHODS.** All adult patients who had to be followed up in ICU due to AAV activation in the last ten years in four reference university hospitals in Turkey were included in this study. Demographic information, immunosuppressive therapies, Birmingham Vasculitis Activity Score (BVAS) at the time of vasculit diagnosis and BVAS, APACHE II, SOFA and SAPS II scores at the ICU admission, treatments and clinical data were recorded in all patients.
**RESULTS.** Total 34 patients from 4 study centers were included. The mean age was 60.5 (42.2-70.2) years, 64.7% of patients were male. The most common causes of ICU admission were infection with 94.1 percent (82.4% pneumonia) and massive hemoptysis (58.8%). Twenty patients (58.8%) died in ICU follow-up. All non-survivors had septic shock. The most commonly identified bacteria related with septic shock were multidrug resistant Acinetobacter baumannii (%35) and Escheria coli (%30). There was a significant difference between survivors and non-survivors according to APACHE II (p=0.003) and SAPS II (p=0.045) scores, serum ALT levels (p=0.012) and platelet counts (p= 0.001). But, there was no significant difference according to BVAS (p= 0.545) and SOFA (p=0.099) scores. APACHE II score was found to be an independent risk factor for ICU mortality in logistic regression analysis (RR=1.239, CI=1.021-1.503, p=0.030). When the ROC curve analysis was performed, APACHE II score greater than 20.5 could predict ICU mortality with 80% specificity and 70% sensitivity in this patient group (AUC=0.8, p=0.004, like hood ratio=2.6).
**CONCLUSION.** APACHE II score can be used as a predictor for ICU mortality in AAV patient group. This result suggests that mortality of patients with AAV is caused by other problems that may occur during follow-up in ICU instead of disease activation.

**REFERENCE(S)**

1. Frausova D, Brejnikova M, Hruskova Z, Rihova Z, Tesar V. Outcome of thirty patients with ANCA-associated renal vasculitis admitted to the intensive care unit. Ren Fail. 2008;30:890-895. doi:10.1080/08860220802353892.
2. No financial support or grant was received for this study.

### 000059

#### The Use of Serum Albumin as a Non Respiratory Predictor of Outcome of Weaning in Critically Ill Patients

MJ. Canillas-Amancio[1], ME. Blanco-Limpin[1], A. Guzman-Banzon[1], E. Aventura[2]
[1]Pulmonary and Critical Care Division, Philippine Heart Center, Quezon City, Philippines; [2]Pulmonary medicine, The Medical City, Pasig, Philippines
**Correspondence:** M.J. Canillas-Amancio
*Intensive Care Medicine Experimental* 2019, **7(Suppl 3):**000059

**INTRODUCTION.** Serum albumin is the most abundant protein and is essential in maintaining the oncotic pressure needed for proper distribution of body fluids between blood vessels and body tissues. Serum albumin concentration is altered in critically ill patients especially with septic shock and after major surgery. Elevated hospital morbidity and mortality has been linked with hypoalbuminemia, which has been recognized to be a dependable prognostic indicator among critically ill patients.
**METHODS.** This was a prospective cross sectional study done at the intensive care unit of the Philippine Heart Center. Patients' ≥19 years old that require the use of invasive mechanical ventilation were included in the study. Demographic profile and clinical characteristics were recorded. Blood extraction for albumin determination was collected 24 hours after attachment to mechanical ventilator and prior to weaning. Patients outcome was categorized into; Group 1-short weaning, patients who had successful weaning within 24 hours; Group 2-difficult weaning, patients who completed weaning more than 24 hours but less than 7 days; Group 3, prolonged weaning, patients who completed weaning from ventilator after 7 days and Group 4, no weaning, patients with no weaning attempt in 4 weeks or separation attempt failed.
**RESULTS.** 120 patients were included in the study who met the selection criteria. Patients with higher albumin level had a shorter duration of weaning (group 1) while a lower albumin concentration upon recruitment was shown in groups 2, 3 and 4 compared to prior to extubation (p-value 0.000, 0.000, and 0.029 respectively). Serum albumin of 27.9 g/L has shown to predict the weaning outcome in mechanically ventilated patients (82.47% sensitivity and 86.96% specificity). Those with lower albumin levels have a higher SOFA score (r= 0.4256; p-value <0.001) thus indicating a higher relative probability of mortality.

**CONCLUSION.** Serum albumin has been recognized as a clinical non-respiratory marker which provides a good predictor of weaning in mechanically ventilated critically ill patients. An admitting albumin level of >27.9 g/L has shown to predict the weaning success among critically ill patients with a sensitivity of 82.47% and specificity of 86.96%. While the association between the decrements in albumin concentration and higher SOFA score predicts a higher relative probability of mortality.

### 000073

#### The usefulness of the E-PRE-DELIRIC model in Japan

M. Kitayama[1], K. Kitaura[2], A. Kudou[2], T. Taguchi[2], T. Taniguchi[1]
[1]Department of anesthesiology and intensive care medicine, Kanazawa University, Kanazawa, Japan; [2]Nursing department, Kanazawa Medical University Hospital, Uchinada, Japan
**Correspondence:** M. Kitayama
*Intensive Care Medicine Experimental* 2019, **7(Suppl 3):**000073

**INTRODUCTION.** Delirium, acute consciousness disturbance with variable course of impaired cognitive function, is common in ICU. It is very important to prevent early onset of delirium. The E-PRE-DELIRIC model has recently received great attention as a scale to predict delirium at the start of ICU stay. The E-PRE-DELIRIC model consists of nine predictors assessed on ICU admission. It is studied to verify its usefulness is various western populations. However, its usefulness has not been established in East Asians.

**OBJECTIVES.** We examined the E-PRE-DELIRIC model in a single facility to clarify usefulness in Japan.

**METHODS.** The present study was designed as a retrospective cohort study in a single center. Enrolled patients were all adult over the age of 18 years. For evaluation of delirium, CAM-ICU was used. Patients who had delirium at ICU entry and who cannot be evaluated as delirium were excluded. The E-PRE-DELIRIC model was calculated and subjected to following analysis. Patients were divided two groups: delirium and non-delirium groups. Primary outcome was to determine the cut off values of the E-PRE-DELIRIC model. Secondary outcomes were to compare each factor of the E-PRE-DELIRIC model between two groups.

**RESULTS.** We enrolled 740 subjects, 137 for delirium and 603 for non-delirium. The incidence of delirium was 18%.

The cut off value of The E-PRE-DELIRIC model score 21 could predict delirium development with 0.871 and 0.606 as specificity and sensitivity, respectively, and AUC was 0.792 (95%CI 0.749 to 0.835). A comparison between the two groups of each factor is shown in the table. Logistic analysis indicated that cut off value was independently associated with 1.10 times ( 95%CI 1.05 to 1.15, p<0.001 )

**CONCLUSION.** The E-PRE-DELIRIC model could predict delirium development in Japanese ICU patients, but its sensitivity remains modest.

**Table 1 (abstract 000073).** See text for description

| | Delirium (N= 137) | Non delirim (N= 603) | p value |
|---|---|---|---|
| Age, median (IQR, range) | 77 (70 – 85) | 70 (63 – 78) | <0.001 |
| History of cognitive impairment (%) | 17 (12) | 12 (1) | <0.001 |
| History of alcohol abuse | 2 (1) | 6(0.9) | 0.64 |
| Admission category (%) | | | |
| Surgery | 28 (20) | 247 (40) | |
| Medical | 108 (78) | 354 (58) | |
| Trauma | 1 (0.7) | 2 (03) | |
| Urgent admission (%) | 120 (87) | 385 (63) | <0.001 |
| MAP at the time of ICU admission | 70 (63 – 78) | 77 (70 – 85) | 0.02 |
| Use of corticosteroids (%) | 9 (6) | 12 (1) | <0.01 |
| Respiratory failure (%) | 38 (27) | 34 (5) | <0.001 |
| BUN at time of ICU admission | 8.9 (6 – 15.3) | 6.7 (5 – 9.9) | <0.001 |
| The E-PRE-DELIRIC model cut off | 23 (16 – 38) | 14 (8 – 19) | <0.001 |

### 000094

#### Presence and importance of the agitation psychomotor on the maxillofacial surgery postoperative

R. Padilla[1], M. Dalorzo[1], F. Moran[1], M. Sanchez Casado[1], R. Martin[2]
[1]Icu, Virgin Health Hospital, Toledo, Spain; [2]Maxillofacial surgery, Virgin Health Hospital, Toledo, Spain
**Correspondence:** R. Padilla
*Intensive Care Medicine Experimental* 2019, **7(Suppl 3):**000094

**INTRODUCTION.** Patients undergoing elective and major maxillofacial surgery are one of the groups of greater risk of psychomotor agitation. It is associated with a greater frequency of complications, longer stay in ICU and hospital stay.

**OBJECTIVES.** Evaluate the presence of the psychomotor agitation in the postoperative of maxillofacial surgery and it repercussion in clinical evolutive variables.

**METHODS.** Retrospective revission in the clinical histories of the patients entered into ICU in the postoperative of mayor maxillofacial surgery in the last 6 years.

It was recorded basal variables, personal history, clinics and evolutive. To determinate the agitation it was used Riker scale and AgitatedBehaviorScales (ABS) scale. We define psychomotor agitation as a ABC value bigger than or equal to 21. The cuantitative data are expressed like medium (interquartile range) and cathegorics like counting (percentage).

**RESULTS.** We got 60 patients, of wich 86,7% were males, with ages of 60,5 (54-65,5) years. 28 of them, (46,7%), presented agitation in ICU. The agitation appeared in the 2nd (1,5-3,5) days of evolution, at the beginning of 1st weaning (1-2 days), and last 3 (2-3) days. They presented a result in Riker's agitation scales 6 (5-7); ABS 42 (25-51). The treatment used were benzodiacepines (96,4%), tiaprizal (82,1%), haloperidol (71,4%), physical restrictions (53,6%), b-blockers (35,7%), other atypical antipsychotics (25%) and dexmedetomidine (21,4%), associating some of them in the most of the cases.

If there are agitation in ICU, is associated with the most presence of agitation at plant (3,6% vs 32%; p=0,006), so that the 88,9% of the patients with agitation at plant, have presented it before in ICU.
**CONCLUSION.** The presence of agitation on maxillofacial surgery postoperative is very frequent, affecting almost half of the patients, and with high intensities, presenting a start pattern from the awakening, and that usually needs a combination of some drugs. That presence predict the repetition of the agitation at plant in the same patient.

**REFERENCE(S)**

1. Fields A, Huang J, Schroeder D, Sprung J, Weingarten T. Agitation in adults in the postanaesthesia care unit after general anaesthesia. Br J Anaesth. 2018;121(5):1052-1058.
2. Chen ZF1, Chen YK, Guo Y, Jiang H. [Effect of dexmedetomidine on emergence agitation after oral and maxillofacial surgery]. Shanghai Kou Qiang Yi Xue. 2013;22(6):698-701.
3. Chen JW, Lv X, Zhang L, Chen ZF. [Effects of remifentanil and dexmedetomidine on recovery profiles after oral and maxillofacial surgery]. Shanghai Kou Qiang Yi Xue. 2016;25(1):101-4.
4. Curiel Balsera E, Prieto Palomino MA, Muñoz Bono J, Arias Verdú MD, Mora Ordóñez J, Quesada García G. [Decision on the time for post-operative extubation of maxillofacial surgery patient in the intensive care unit]. Med Intensiva. 2009 Mar;33(2):63-7.
5. Curiel Balsera E, Macias Guarasa I, Prieto Palomino MA, Muñoz Muñoz JL, Salguero Piedras M, Chaparro Sánchez MJ. Eficacia y seguridad de la analgesia con remifentanilo en el postoperatorio inmediato de cirugía maxillofacial. Med Intensiva. 2012;36(2):154-5.
6. Terada Y, Inoue S, Konda M, Egawa J, Ueda J, Kirita T et al. Effects of deep sedation under mechanical ventilation on cognitive outcome in patients undergoing surgery for oral and maxillofacial cancer and microvascular reconstruction. Med Intensiva. 2019;43(1):3-9.
7. Devlin JW, Skrobik Y, Gélinas C, Needham DM, Slooter AJC, Pandharipande PP et al. Clinical Practice Guidelines for the Prevention and Management of Pain, Agitation/Sedation, Delirium, Immobility, and Sleep Disruption in Adult Patients in the ICU. Crit Care Med. 2018;46(9):e825- e873.
8. Corrigan JD. Development of a scale for assessment of agitation following traumatic brain injury. J Clin Exp Neuropsychol. 1989;11:262-77.
9. Riker RR, Fraser GL, Simmons LE, Wilkins ML. Validating the sedation-agitation scale with the bispectral index and visual analog scale in adult ICU patients after cardiac surgery. Intensive Care med. 2001; 27(4):853-8.
10. Zhu Y, Wang G, Liu S, Zhou S, Lian Y, Zhang C et al. Risk factors for postoperative delirium in patients undergoing major head and neck cancer surgery: a meta-analysis. Jpn J Clin Oncol. 2017;47(6):505-511.

### 000097

#### Preoperative Venoarterial Extracorporeal Membrane Oxygenation (ECMO) improves mortality in advanced structural heart disease complicated by cardiogenic shock

A. Kogan[1], J. Tamer[2], E. Ram[2], S. Amunz[2], Y. Kassif[2], L. Sternik[2]
[1]Cardiac surgery icu, Sheba Medical Center, Sakler Faculty of Medicine, Tel Aviv University, Ramat Gan, Israel; [2]Department of cardiac surgery, Sheba Medical Center, Ramat Gan, Israel
**Correspondence:** A. Kogan
*Intensive Care Medicine Experimental* 2019, **7(Suppl 3):**000097

**INTRODUCTION.** Cardiac surgery for structural heart disease has poor outcomes in the presence of cardiogenic shock and/or multisystem organ failure. The mortality rate remain high, despite progress in pharmaceutical therapy, invasive cardiology, and surgical techniques.
**OBJECTIVES.** We applied venoarterial extracorporeal membrane oxygenation (ECMO) as a bridge to surgery to restore end-organ function and resuscitate patients before high-risk cardiac operation.
**METHODS.** We reviewed all patients with structural heart disease and cardiogenic shock who have been admitted to our institute during one-year period (2018) and have been resuscitated by ECMO preoperatively. Nine patients were included in to present study.
**RESULTS.** Mean age was 52 ± 17 years. Before surgery, patients were placed on ECMO for 3.7 days (average 2-7 days). Comorbidities included acute renal failure (n =7), inotropic support (n = 9), intra-aortic balloon pump (n = 5) and acute liver injury (n=3). With ECMO support, vasopressor requirement decrease, organ perfusion improved with decreased lactate levels from 67 mg/dL mmol/L to 19 mg/dL, (p = 0.01), normalized arterial pH (7.24 vs. 7.38 ($p < 0.04$) and mean arterial pressure (64 mmHg vs. 83 mmHg, $p < 0.01$).Follow, patients were undergoing different surgical procedures: AVR for severe aortic stenosis (n=1), MVR for severe mitral regurgitation (n=4), TVR+PFO closure (n=1) and closure of acute post-infarct ventricular septal defect (n=3). Average length of follow-up is 4 months, with 2 patient deaths at 12 and 7 days and the rest of the patients survived. Complications included acute kidney injury - yet no patients required dialysis at discharge- and one patient was underwent above knee amputation as a result of lower limb ischemia.
**CONCLUSION.** ECMO can be used as a bridge to heart valve or septal defect surgery in severely decompensated patients, suffered from cardiogenic shock. Through recovery of end-organ function, ECMO may allow surgical correction of structural heart disease in patients considered inoperable or convert a salvage situation to an elective operation.

### 000102

#### Central venous-to-arterial carbon dioxide difference and pulse pressure variation as therapeutic targets during elective major gastrointestinal surgeries does impact survival

L. Prado[1], F. Lobo[1], D. Espada[1], BAGS. Salvetti[1], B. Neves[1], N. de Oliveira[2], SM. Lobo[2]
[1]Anesthesiologist, Hospital de Base, São José do Rio Preto, Brazil; [2]Intensivist, Hospital de Base, São José do Rio Preto, Brazil
**Correspondence:** L. Prado
*Intensive Care Medicine Experimental* 2019, **7(Suppl 3):**000102

**INTRODUCTION.** In the next decades an expansion of surgical services to address unmet needs is likely to increase total global deaths, of which 1.9 million would be in low-middle income countries[1]. Current evidence suggests that intraoperative management of major elective gastrointestinal surgery should consider cardiac output (CO) monitoring and goal-directed therapy[2]. However, cost restrains may determine low adherence to these practices in low resource settings. We hypothesized that central veno-arterial difference of CO2 [P(v-a)CO2] could serve as a surrogate for CO monitoring during GDT.
**OBJECTIVES.** We aimed to evaluate the impact of the use of P(v-a)CO2 as a surrogate of cardiac output in an algorithm of GDT on major complications and 90-day mortality.
**METHODS.** The study was a quasi-randomized controlled and explanatory trial performed in a tertiary university hospital in patients undergoing elective major gastrointestinal surgeries. In the interventional group a treatment algorithm aiming to keep mean arterial pressure > 65 mmHg, SpO2 > 94%, CO2 gap lower than 6 mmHg and pulse-pressure variation (PPV) lower than 13% using fluids, dobutamine and noradrenaline was applied in addition to the standard practice. The control group comprised all the consecutive patients that received traditional standard of care from the same team of surgeons and anesthesiologists in the period before the intervention phase.
**RESULTS.** A total of 204 patients were included in this study; 102 in each group. Measurements of P(v-a)CO2 were lower in the GDT group than in Control group at T0 (30-60 min after induction): 5.9 ± 2.1 vs. 6.7 ± 2.7 mmHg, p=0.031; T1 (2 h ± 30min): 6.4 ± 2.6 vs. 7.0 ± 2.3 mmHg, p = 0.033 and T2 (4 h ± 30min):5.9 ± 2.1 vs. 7.1 ± 3.4 mmHg, p = 0.036. Central venous saturation (ScvO2) was significantly higher throughout the intraoperative period in the GDT group, and there was a moderate correlation between P(v-a)CO2 and ScvO2 ($P<0.001$ at all-time points and at ICU admission). The number of patients with major complications was lower in GDT group than in the control group and there was a significant decrease in 90-day mortality in the GDT group (22.5% vs. 9.8%, p=0.014).
**CONCLUSION.** Minimization of P(v-a)CO2 and PPV as therapeutic targets during elective major gastrointestinal surgeries seems to determine better postoperative outcomes. Testing lower cost alternatives during GDT is extremely important for low-resource settings and countries and its value should be evaluated in prospective randomized trials.

**REFERENCE(S)**

1. Nepogodiev D, Martin J, Biccard B, Makupe A. Correspondence: Global burden of postoperative death. The Lancet Vol 393 February 2, 2019
2. Gillies MA, Sander M, Shaw A, Wijeysundera DN, Myburgh J, Aldecoa C, Jammer I,Lobo SM, Pritchard N, Grocott MPW, Schultz MJ, Pearse RM. Current research priorities in perioperative intensive care medicine. Intensive Care Med. 2017;43(9):1173-1186.Remove

### 000104

#### Vasopressor use after surgery. A survey of ESICM and ESA members for the SQUEEZE study

B. Creagh-Brown[1], H. Wunsch[2], L. Forni[3], R. Moonesinghe[4], I. Jammer
[1]Intensive care and perioperative medicine, Royal Surrey County Hospital, Guildford, United Kingdom; [2]Department of anesthesia and the interdepartmental division of critical care, Sunnybrook Health Sciences Centre, Toronto, Canada; [3]Intensive care medicine, Royal Surrey County Hospital, Guildford, United Kingdom; [4]Anaesthetics and perioperative medicine, University College Hospital, London, United Kingdom
**Correspondence:** B. Creagh-Brown
*Intensive Care Medicine Experimental* 2019, **7(Suppl 3):**000104

**INTRODUCTION.** Postoperative hypotension is a common occurrence following major non-cardiac surgery. There is evidence of substantial variation in the management of postoperative hypotension between centres, countries and continents. In preparation for an international prospective observational cohort study concerning the use of postoperative vasopressor infusions ('**Squeeze**'), we surveyed clinicians from perioperative and intensive care medicine regarding use of vasopressors after surgery.
**METHODS.** With the assistance of staff and resources of ESA and ESICM we shared a link to a SurveyMonkey questionnaire of our design. Questions in the survey included location of vasopressor use after surgery, frequency of vasopressor use, and choice of vasopressors after surgery. Ethical approval was not required.
**RESULTS.** Between July 2018 and Feb 2019, we received 2052 complete responses with 97.5% of respondents regularly, or occasionally, taking care of postoperative non-cardiac patients. Respondents were from 102 countries, with the greatest number of replies from Germany, UK, Spain and Italy (each >100 replies). 27.3% worked in hospitals with 250-499 beds, 33.7% with 500-999 beds and 19.8% >1000 beds.
Respondents answered "Non-cardiac surgery patients receive vasopressor infusions after surgery:" 'occasionally' in 58%, and 'frequently' in 22%. Postoperative patients received infusions of vasopressors in a range of differently described environments including PACU, ICU, and HDU; only 7.2% responded that vasopressors could be used on a normal postoperative ward. Respondents answered "Non-cardiac surgery patients receive vasopressor infusions after surgery:" 'occasionally' in 58%, and 'frequently' in 22%.
The most common choice of vasoactive drug used was noradrenaline (used always or frequently by 76%) and phenylephrine (used always or frequently by 18%)"
**CONCLUSION.** Vasopressor infusions are commonly used in patients following surgery. There is variation in choice of drug and location of treatment. Patient-level data are needed to better understand practice patterns and outcomes associated with vasopressor use.
**SQUEEZE WILL LAUNCH EARLY 2020 - PLEASE REGISTER YOUR INTEREST**

**REFERENCE(S)**

1. ESA CTN Award 2018

### 000116

#### Effect of non-sedation on cognitive function in survivors of critical illness

HK. Nedergaard[1], HI. Jensen[1], M. Stylsvig[2], HT. Olsen[3], S. Korkmaz[4], T. Strøm[5], P. Toft[5]
[1]Department of anesthesiology and intensive care, Lillebælt Hospital, Kolding, Kolding, Denmark; [2]Neuropsychology, Neuropsychological Clinic, Odense, Denmark; [3]Department of anesthesiology and intensive care, Odense University Hospital, Svendborg, Svendborg, Denmark; [4]Department of business and economics, University Of Southern Denmark, Odense, Denmark; [5]Department of anesthesiology and intensive care, Odense University Hospital, Odense, Denmark
**Correspondence:** H.K. Nedergaard
*Intensive Care Medicine Experimental* 2019, **7(Suppl 3):**000116

**INTRODUCTION.** Critical illness can cause severe cognitive impairments. Studies frequently find more than half of critical illness survivors to have substantial cognitive impairments, often lasting for years or for a lifetime, significantly affecting quality of life (1). No effective preventive measures have been established. Delirium seem to be the most important risk factor for long-term cognitive impairment. Less sedation is encouraged and might lead to less delirium and less cognitive impairments following critical illness.
**OBJECTIVES.** The objective was to assess the effect of non-sedation versus sedation with a daily wake-up call during mechanical ventilation on cognitive function in adult survivors of critical illness.
**METHODS.** Single center sub-study of the multicenter, randomized NONSEDA trial. Participants were randomized within the first 24 hours from intubation to either non-sedation with sufficient analgesia or to light sedation with a daily wake-up call during mechanical ventilation. Three months after ICU-discharge survivors were tested for cognitive function in-person by a neuropsychologist.
**RESULTS.** A total of 205 critically ill, orally intubated and mechanically ventilated adults were included; 118 patients survived to follow-up and 89 participated (75%). The participating survivors in the two groups did not differ regarding baseline data or premorbid cognitive impairments. Sedated patients had received more sedatives, whereas doses of morphine and antipsychotics were equal. We found more patients with delirium in the sedated group (96% versus 69% of patients, p=0.002) and increased duration of delirium in sedated patients (median 5 days versus 1, p< 0.001). Delirium sub-types were equally distributed between groups, with hypoactive delirium most frequent (61%), followed by mixed delirium (39%). Primary outcome: No significant difference was found in number of patients with mild/moderate cognitive impairments (non-sedated: 6 patients vs sedated: 4) or severe cognitive impairments (non-sedated: 16 patients vs sedated: 17, p=0.71). Two hypothetical worst-case scenarios where all survivors and all included patients, respectively, who had not participated in the follow-up assessment were assumed to have severe cognitive impairments, were analyzed, but still no difference between groups was found (p=0.98 and 0.87, respectively). Secondary outcomes were 1) cognitive test scores, and 2) effect of delirium on cognitive function, where no differences were found between groups in either. In both groups a wide array of cognitive domains was found to be affected, and in both groups visuo-spatial construction was most seriously affected, followed by executive function and then visual learning/memory. The distribution of affected domains did not vary between groups (p=0.64).
**CONCLUSION.** Non-sedation did not affect cognitive function three months after ICU-discharge.

**REFERENCE(S)**

1: Girard et al, Critical illness Brain Injury. Annual Review of Medicine 2016;67:497-513.

### 000117

#### Effect of non-sedation on physical function in survivors of critical illness

HK. Nedergaard[1], Hl. Jensen[1], HT. Olsen[2], T. Strøm[3], JT. Lauridsen[4], G. Sjøgaard[5], P. Toft[3]
[1]Department of anesthesiology and intensive care, Lillebælt Hospital, Kolding, Kolding, Denmark; [2]Department of anesthesiology and intensive care, Odense University Hospital, Svendborg, Svendborg, Denmark; [3]Department of anesthesiology and intensive care, Odense University Hospital, Odense, Denmark; [4]Department of business and economics, University Of Southern Denmark, Odense, Denmark; [5]Department of sport science and clinical biomechanics, University Of Southern Denmark, Odense, Denmark
**Correspondence:** H.K. Nedergaard
*Intensive Care Medicine Experimental* 2019, **7(Suppl 3):**000117

**INTRODUCTION.** Critical illness can cause impaired physical function and lead to dependence of help in everyday activities. In recent years, intensive care unit (ICU) acquired weakness and its possible prevention have received much attention, although the pathophysiology is still not clear. Generally, lower levels of sedation, focus on pain relief, patient comfort and early mobilization (known as the eCASH concept) are encouraged (1).
**OBJECTIVES.** The objective of this study was to assess the effect of non-sedation on physical function.
**METHODS.** This study was a single-center sub-study of the NONSEDA trial, a multicenter randomized trial, assessing harms and benefits of non-sedation versus sedation with a daily wake-up call during mechanical ventilation in critically ill adults. All patients from one NONSEDA trial-site (the ICU in Kolding, Denmark) were included. At extubation, ICU discharge and 3 months after ICU discharge survivors were assessed for physical function.
**RESULTS.** A total of 205 patients were included, 118 survived to follow-up and 116 participated (98%) to the extent they were able. The two groups did not differ significantly regarding baseline data. Sedated patients had received more sedatives, but doses of morphine, corticosteroids and neuromuscular blocking agents were equal, as was mean blood glucose. Primary outcome: Three months after ICU-discharge, health-related quality of life (SF-36, physical component score) did not differ significantly between groups (non-sedated 38.3 vs sedated 36.6, p= 0.3), but a tendency to better function in activities of daily living (Barthel Index) was found in the non-sedated group (19.5 vs 18, p=0.08). Secondary outcomes: Non-sedated patients had a significantly better Barthel Index at ICU-discharge (median 7 vs 4, p=0.01). There were no differences between groups regarding handgrip strength, walking distance, muscle size or biomechanical data on lower extremity function 3 months after ICU discharge. Explorative outcomes: Handgrip strength at extubation and ICU-discharge was significantly better in non-sedated patients.
**CONCLUSION.** Non-sedation did not lead to significantly better quality of life or better function in activities of everyday living 3 months after ICU discharge, however non-sedated patients generally had a better physical function, especially in early phases (extubation and ICU discharge).

##### REFERENCE(S)

1: Vincent JL, Shehabi Y, Walsh TS, et al. Comfort and patient-centred care without excessive sedation: the eCASH concept. Intensive Care Med 2016;42:962–71.

### 000145

#### Intermediate analysis: Dexmedetomidine Versus Standard Clinical Practice During Non Invasive Mechanical Ventilation (DEX-SCP-NIV)

A. Vallejo de la Cueva[1], A. Quintano Rodero[1], I. Perez Francisco[2], D. Iglesias Posadilla[3], JL. Espinosa Berenguel[4], Cl. Bermal Matilla[5], A. Lopez Picado[6], H. Barrasa Gonzalez[1], S. Castaño Avila[1], F. Fonseca San Miguel[1]
[1]Intensive Care unit, University Hospital of Araba, Vitoria-Gasteiz, Spain; [2]Investigation unit. bioaraba, University Hospital of Araba, Vitoria-Gasteiz, Spain; [3]Intensive care unit, University Hospital of Burgos, Burgos, Spain; [4]Intensive care unit, Hospital General Universitario Reina Sofía, Murcia, Spain; [5]Intensive care unit, Miguel Servet University Hospital, Zaragoza, Spain; [6]Fundación de investigación biomédica, Hospital Clinico Universitario San Carlos, Madrid, Spain
**Correspondence:** A. Vallejo de la Cueva
*Intensive Care Medicine Experimental* 2019, **7(Suppl 3):**000145

**INTRODUCTION.** Success of NIV mainly depends on patient's cooperation with the technique. Use of analgesic and sedative drugs during NIV is not a common practice due to the high risk of respiratory depression. Dexmedetomidine (DEX) has a sedative, analgesic and anxiolytic effect and preserves oropharyngeal reflexes. Due to those effects it could became the ideal sedative drug for patients comfort under NIV
**OBJECTIVES.** to compare effectiveness of DEX regarding SCP in ARF under NIV. It will be assess tolerance, patient´s degree of satisfaction, safety profile and acute respiratory failure evolution
**METHODS.** A phase IV multicentre, randomised, open label, parallel-group, controlled clinical trial, from 1/12/16 to 31/10/18. Approved by Ethics Committee. Clinical Trials NCT02958150. **Inclusion Criteria**: >18 years, ARF secondary to Pulmonary Oedema, pneumonia or high risk of post-extubation failure. **Exclusion Criteria**: ARF needing intubation, HR<50, AVB 2º-3º, neurocritical patients. Drug allergy. **Groups**: SCP: no drugs or midazolam (0,03-0,05 mg/kg) and/or propofol (0,25 mg/kg iv), morphine (3-5mg) and/or fentanyl (50-100µg) bolus or remifentanil 6 µg/kg/h. DEX: 0,4-1,4µg/kg/h para RASS(0 -1) ± SCP iv bolus. Categorical variables as percentages % and compared using X2 o Fisher, continuous variables as mean ±SD/median IQR and compared using two samples T-test. P<,05.
**RESULTS. DEX vs SCP**: *N*= 22 vs 27. *Age (years)* 72.7±8.3 vs 69.0±11.5 (p.23). *Female %:* 33.3 vs 37.0 (p=1). *ARF etiology %*: COPD/ Heart failure: 41vs 37; Pneumonia 32 vs 26; Post-extubation/others 27vs 37 (p.76). *Oxygen support pre-NIV Fi02>50* % 52.6 vs 61.9 (p.75). *SOFA score*: 6.48±4,4 vs 6.19±3.5 (p.80). Non-respiratory dysfunctions 85.7 vs 100. Other sedatives/analgesics administered: DEX/SCP None 59.1 vs 57.7 (p.92). Dexmedetomidine dose: 0.87±0.33 µg/kg/h
**NIV Tolerance %:** Nausea 4.8 vs 0 (p.48). Aspiration pneumonia 0 vs 0 (p=1). CAM-ICU+: 14.3 vs 32.0(p.16). Agitation (RASS>+2) 28.5 vs 19.2 (p.45). Interface intolerance 19.0 vs 11.5(p.68). VNS ≥4 14.3 vs 19,2 (p.71). **Adverse effects %:** Respiratory depression 0 vs 0. HR<60 28.5 vs 16.0(p.30). HR<40 solved with reduction of perfusion 9.5 vs 0 (p.20). SBP<80 28.5 vs 16.0(p.30). TAS>160 4.7 vs 4.0 (p=1). FC>140 8.0 vs 0 (p.49) **Patient satisfaction %**: *Rested during ventilation:* agree (A) 87.5 vs 25.0 (p<.00); *Comfortable* (A) 80.0 vs 36.6 (p.02). Unpleasant memories of ventilation (A) 21.4 vs 27.0 (p=1). *Sedated with the same drug?* (A) 85.7 vs 44.4 (p.06). **Dyspnea** (2point decrease)**%:** pre-1hpostNIV: 95.4 vs 77.7 (p.11). pre-6hpostNIV: 95.4 vs 88.8 (p.61). **ARF course:** pC02 pre 50.8±16.2 vs 54.7±12.5 (p.36). 6h post 45 (34.5-49) vs 46 (38-55) (p.31)**. Pa02/Fi02** preNIV144,8 vs 148,6 (p.87). P/F 1hpostNIV 197 (155-235.25) vs 213.5 (147.25-269.75) (p.64). P/F 24h postNIV 226.5 (208.75-304) vs 187 (147-257) (p.01). **Hours of NIV**: 15 (9.5-25.5) vs 18 (8.1 -39) (p.44). **ICU stay** (d): 5 (3-9) vs 8 (3-15) (p.25). **Endotracheal intubation** %: 23,8 vs 20.0 (p.75)**. ICU mortality** % 15.7 vs 19.0 (p=1)
**CONCLUSION.** DEX vs SCP provided higher degree of satisfaction and better oxygenation to patients under NIV with ARF without improving side effects and percentage of intubation or ICU mortality. Use of DEX vs SCP could reduce ICU stay and NIV duration

##### REFERENCE(S)

1. Devlin JW, Al-Qadheeb NS, Chi A, et al. Efficacy and safety of early dexmedetomidine during noninvasive ventilation for patients with acute respiratory failure: a randomized, double-blind, placebo-controlled pilot study. Chest. 2014;145:1204-12.
2. Takasaki Y, Kido T, Semba K. Dexmedetomidine facilitates induction of noninvasive positive pressure ventilation for acute respiratory failure in patients with severe asthma. J Anesth. 2009;23:147-50.
3. Orion Pharma for suppling us Dexdor.

### 000162

#### Differences in 90-day mortality of delirium subtypes in the intensive care unit: a retrospective cohort study

P. Rood, F. Van De Schoor, K. Van Tertholen, P. Pickkers, M. van den Boogaard
Intensive care, Radboud University Medical Center, Nijmegen, Netherlands
**Correspondence:** P. Rood
*Intensive Care Medicine Experimental* 2019, **7(Suppl 3):**000162

**INTRODUCTION.** Many intensive care unit (ICU) patients suffer from delirium which is associated with deleterious short-term and long-term effects, including mortality.

**OBJECTIVES.** We determined the association between delirium and the different delirium subtypes and 90-day mortality.

**METHODS.** Retrospective cohort study of ICU patients admitted between 2015-2017. Delirium, including its subtypes (i.e. hyperactive, hypoactive, mixed and rapidly reversible delirium), was determined using the confusion assessment method-ICU (CAM-ICU) and Richmond agitation sedation scale (RASS-)scores. Exclusion criteria were insufficient assessments and persistent coma. Cox-regression analysis was used to determine associations of delirium subtypes with 90-day mortality, including relevant covariates (i.e. APACHE-IV, length of ICU stay and mechanical ventilation).

**RESULTS.** 7,362 ICU patients were eligible of whom 6,323 (85.9%) were included. A total of 1,600 (25%) patients were prevalent ICU delirium cases of which the mixed subtype occurred the most frequent (36%). The crude hazard ratio (HR) for overall prevalent delirium with 90-day mortality was 2.84 (95%CI:2.32-3.49), and the adjusted HR 1.29 (95%CI:1.01-1.65). The adjusted HR for 90-day mortality was 1.57 (95%CI:1.51-2.14) for the mixed subtype, 1.40 (95%CI:0.71-2.73) for hyperactive 1.31 (95%CI:0.93-1.84) for hypoactive and 0.95 (95%CI:0.64-1.42) for the rapidly reversible delirium subtype.

**CONCLUSION.** After adjusting for covariates, including the competing risk factor length of ICU stay, only the mixed delirium subtype was significantly associated with 90-day mortality.

### 000163

#### Prophylactic haloperidol effects on long-term Quality of Life in critically ill patients at high risk for delirium: results of the REDUCE study

P. Rood[1], M. Zegers[1], A. Slooter[2], H. Van Der Hoeven[1], P. Pickkers[1], M. van den Boogaard[1]
[1]Intensive care, Radboud University Medical Center, Nijmegen, Netherlands; [2]Intensive care, University Medical Center Utrecht, Utrecht, Netherlands
**Correspondence:** P. Rood
*Intensive Care Medicine Experimental* 2019, **7(Suppl 3):**000163

**INTRODUCTION.** Delirium is a frequently occurring syndrome which burdens Intensive Care Unit (ICU) patients. The effect of prophylactic haloperidol use on long-term quality of life is unknown.

**OBJECTIVES.** To evaluate the effects of prophylactic haloperidol use on long-term quality of life in critically ill patients at high risk for delirium, and to explore which factors are associated with change in quality of life.

**METHODS.** A pre-planned secondary analysis of long-term outcomes of the REDUCE study was conducted. In this multicenter randomized clinical trial, non-delirious ICU patients were assigned to the prophylactic haloperidol or placebo group. Long-term outcomes were assessed using the Short Form-12 questionnaire at ICU admission (baseline), and after 1 and 6 months. Quality of life was summarized in the physical component summary (PCS) score and mental component summary (MCS) score. Differences between the haloperidol and placebo group, and factors associated with changes in quality of life were analyzed.

**RESULTS.** Of 1789 study patients, 1245 ICU patients were approached of which 887 (71.2%) responded. Long-term quality of life did not differ between the haloperidol and placebo group mean (±SD) PCS score 39.3±11.0 and 38.9±10.6, respectively; P=0.35, and mean MCS score 50.3±10.1 and 51.1±10.0, respectively; P=0.68). Factors associated with physical decline after 6 months were age (odds ratio [OR] 1.02; 95%CI:1.01-1.04), medical admission (OR 2.08; 95%CI:1.39-3.10), trauma admission (OR 5.34; 95%CI:1.65-17.27), baseline PCS score (OR: 1.06; 95%CI:1.04-1.08) and number of sedation-induced-coma days (OR 1.15; 95%CI:1.05-1.25). Factors associated with mental decline after 6 months were age (OR 1.03; 95%CI:1.01-1.04), medical admission (OR 2.09; 95%CI:1.43-3.03), baseline MCS score (OR 1.03; 95%CI:1.01-1.05) and the number of sedation-induced-coma days (OR 1.09; 95%CI:1.01-1.17).

**CONCLUSION.** Prophylactic haloperidol use does not affect long-term quality of life in critically ill patients at high-risk for delirium. Several factors, including the modifiable factor number of sedation-induced-coma days, are associated with decline in long-term outcomes.

**REFERENCE(S)**

1. Dutch government organization ZonMw

### 000179

#### Increased ventilatory drive during exercise may predict prolonged air leak after pulmonary lobectomy

K. Brat[1], M. Chobola[2], P. Homolka[3], M. Heroutova[1], M. Benej[4], L. Mitas[5], T. Horvath[5], I. Cundrle[2]
[1]Department of respiratory diseases, University Hospital Brno, Brno-Bohunice, Czech Republic; [2]Department of anesthesiology and intensive care, St. Anne's University Hospital Brno, Brno, Czech Republic; [3]Department of sports medicine and rehabilitation, St. Anne's University Hospital Brno, Brno, Czech Republic; [4]First department of surgery, St. Anne's University Hospital Brno, Brno, Czech Republic; [5]Department of surgery, University Hospital Brno, Brno-Bohunice, Czech Republic
**Correspondence:** I. Cundrle
*Intensive Care Medicine Experimental* 2019, **7(Suppl 3):**000179

**INTRODUCTION.** Pleural adhesions, upper lobe resections and low forced expiratory volume in one second (FEV1) were proposed as predictors of prolonged air leak after lung resection surgery. Increased ventilatory drive may be associated with dynamic hyperinflation and may therefore also contribute to the development of prolonged air leak after lung resection surgery.

**OBJECTIVES.** We hypothesized increased ventilatory drive, characterized by the slope of minute ventilation to carbon dioxide output (VE/VCO2), predicts prolonged air leak after pulmonary lobectomy. Accordingly, aim of this study was to compare ventilatory and gas exchange parameters during exercise in patients with and without air leak after pulmonary lobectomy.

**METHODS.** Consecutive lung resection (lobectomy) candidates were recruited in this prospective bicenter study. All of the recruited patients underwent pulmonary function tests and cardiopulmonary exercise testing prior surgery. Prolonged air leak was defined as presence of air leak from the chest tube on the 5th postoperative day. Student t-test, Mann-Whitney U test and two-tailed Fisher exact test were used for comparison. Stepwise logistic regression analysis was performed for evaluation of parameters association with postoperative prolonged air leak. Data are summarized as mean ± SD; $p < 0.05$ was considered as significant.

**RESULTS.** A total of 96 patients were included. Prolonged air leak was observed in 28 (29%) of patients. Between groups, there was no significant difference in age, sex, ASA class, type of surgery (thoracotomy/video-assisted thoracoscopic surgery) and side of surgery (right/left lung; upper/lower lobes). Patients with prolonged air leak had significantly longer hospital (14 ± 7 vs. 9 ± 6 days; p<0.01) and intensive care unit length of stay (7 ± 5 vs. 5 ± 4 days; p<0.01), lower FEV1 (82 ± 21 vs. 91 ± 18 %; p=0.03), longer duration of surgery (210 ± 67 vs. 187 ± 72 min; p=0.04), more frequent pleural adhesions (50% vs. 21%; p=0.01) and higher VE/VCO2 slope (35 ± 7 vs. 30 ± 5; p<0.01). Stepwise logistic regression showed only presence of pleural adhesions (OR= 3.9; 95% CI 1.4-10.9; p=0.01) and VE/VCO2 (OR 1.1; 95% CI 1.0-1.2; p<0.01) to be independently associated with the prolonged air leak. VE/VCO2 values above 45 were highly specific for the prediction of prolonged air leak (specificity 96%, sensitivity 18%).

**CONCLUSION.** Increased ventilatory drive during exercise (VE/VCO2 slope) may predict prolonged air leak after pulmonary lobectomy.

### 000183

#### Prognostic value of procalcitonin after gynecological surgery: a preliminary retrospective cohort study

Y. Fujita, M. Okumura, A. Hashimoto, Y. Sato, Y. Fujiwara
Anesthesiology and intensive care medicine, Aichi Medical University Hospital, Nagakute, Japan
**Correspondence:** Y. Fujita
*Intensive Care Medicine Experimental* 2019, **7(Suppl 3):**000183

**INTRODUCTION.** Procalcitonin (PCT) is a well-known prognostic marker after elective cardiac surgery. Klingele et al. (1) demonstrated that measurement of PCT seems to be useful for identification of patients at risk of delayed complications despite an initially uneventful postoperative course. However, the prognostic value of PCT after non-cardiac surgery remains unknown.

**OBJECTIVES.** We chose gynecological surgery as a non-cardiac surgery with which to evaluate whether PCT is useful for predicting a risk of delayed complications.

**METHODS.** In total, 1,015 patients who underwent gynecologic surgery in our institution were retrospectively evaluated. The serum PCT concentration was measured the morning after surgery. All patients were screened for the occurrence of delayed complications. Delayed complications were defined as in-hospital death, intensive care unit readmission, or a prolonged hospital stay (>10 days). Odds ratios with 95% confidence intervals were calculated by logistic regression analysis and adjusted confounders. The confounders used to calculate the odds ratios were age, sex, diabetes mellitus, hypertension, past stroke, coronary artery disease, hepatic disease, renal disease, past cancer, active cancer, chronic obstructive disease, allergy, estimated glomerular filtration rate, American Society of Anesthesiologists physical status, and emergency operation.

**RESULTS.** Among 1,015 patients, 101 developed delayed complications. The PCT level was significantly higher in these 101 patients (1.01ng/ml) than in the remaining 914 patients (1.01 vs. 0.31 ng/ml, respectively; $p = 0.0089$). We divided the patients into two groups using the normal cutoff level for PCT (0.5 ng/ml). Patients with a PCT level of >0.5 mg/ml on the first postoperative day had a highly increased risk of delayed complications (adjusted odds ratio, 5.73; 95% confidence interval, 3.57–9.20; $p < 0.0001$).

**CONCLUSION.** In our preliminary study, a single measurement of PCT seems to be a useful marker to identify patients at risk of delayed complications after gynecological surgery.

##### REFERENCE(S)

1. Klingele M, Bomberg H, Schuster S. et al. Prognositic value of procalcitonin in patients after elective cardiac surgery: a prospective cohort study. Ann. Intensive Care (2016) 6:116

### 000185

#### Paralyzed with fear: 2019 perspective

D. Epstein[1], E. Marcusohn[1], Y. Steinfeld[2], H. Ammouri[3], A. Miller[3]
[1]Department of internal medicine "b", Rambam Healthcare Campus, Haifa, Israel; [2]Orthopedic surgery division, Rambam Healthcare Campus, Haifa, Israel; [3]Medical intensive care unit, Rambam Healthcare Campus, Haifa, Israel
**Correspondence:** D. Epstein
*Intensive Care Medicine Experimental* 2019, **7(Suppl 3):**000185

**INTRODUCTION.** Pain management and sedation are important aspects in the treatment of hospitalized patients, especially those mechanically ventilated. In many hospitals, such patients are treated not only in intensive care units, but also in other wards. In the nineteen eighties, numerous studies demonstrated a wide array of misconceptions and inadequate knowledge related to commonly used sedative, analgesics and muscle relaxants which may prevent appropriate treatment. Since these publications, multiple studies have shown that appropriate sedation and analgesia are associated with improved clinical outcomes, educational programs were developed and guidelines published. Whether the personnel's knowledge kept up with these changes is unknown.

**OBJECTIVES.** To determine the current rate of misconceptions and knowledge gaps regarding commonly used sedative, analgesic and neuromuscular drugs.

**METHODS.** We conducted a prospective, observational, cross-sectional e-questionnaire survey among physicians and nurses who routinely treat mechanically ventilated patients. The study was performed in Rambam Health Care Campus ,Haifa, Israel, a 1000-bed academic hospital, serving a population of over two million residents.

**RESULTS.** 355 questionnaires were returned. The response rate was 42.49% (181/ 426) for physicians and 30.05% (174/ 579) for nurses. Only 82.54% knew that midazolam has no analgesic effect. Only 71-72% were familiar with the sedative effect of opiates. 27% believed that propofol has analgesic properties. 30.52% thought that rocuronium has a sedative effect and 10.17% believed that it has analgesic proporties. As much as 40% of emergency room personnel and 17% of ICU staff believed that muscle relaxants have a sedative effect.

**CONCLUSION.** Our findings demonstrate that although a lot has been done during the last decades in order to improve the treatment of critically ill patients, the rate of misconceptions regarding pharmacological characteristics of commonly used drugs is unacceptably high. These basic misconceptions are in contrary with the guidelines recommending an analgesia- first sedation strategy and may lead medical personnel to provide excessively deep sedation and lack of analgesia on one hand, and to treat patients receiving opiates with unnecessary midazolam or propofol, on the other hand. Using muscle relaxants without concomitant sedatives or analgesics may cause a situation in which patients are aware of their surroundings and feel pain without having the ability to express it. This terrifying scenario is one of the biggest fears people have when they are about to undergo a medical procedure.

Our findings call for immediate action to improve patients' care in our hospital and other institutes. It seems crucial to promote new educational programs, protocols, and guidelines in ICU and other wards. More rigid "safeguards" such as daily clinical pharmacist review of sedatives, analgesics and paralytics administered as well as incorporation of automated clinical decision support tools into an electronic health record system of mechanically ventilated patients should be considered.

##### REFERENCE(S)

1. Shehabi Y, Bellomo R, Mehta S, Riker R, Takala J. Intensive care sedation: the past, present and the future. Crit Care. 2013 Jun 13;17(3):322.
2. Loper KA, Butler S, Nessly M, Wild L. Paralyzed with pain: the need for education. Pain. 1989;37(3):315–6.
3. Paralysed with fear. Lancet (London, England). 1981 Feb 21;1(8217):427.
4. Miller-Jones CMH, Williams JH. Sedation for ventilation. Anaesthesia. 1980 Nov 1;35(11):1104–7.

5. Patel SB, Kress JP. Sedation and analgesia in the mechanically ventilated patient. Vol. 185, American Journal of Respiratory and Critical Care Medicine. 2012. p. 486–97.
6. Devlin JW, Skrobik Y, Gélinas C, Needham DM, Slooter AJC, Pandharipande PP, et al. Clinical Practice Guidelines for the Prevention and Management of Pain, Agitation/Sedation, Delirium, Immobility, and Sleep Disruption in Adult Patients in the ICU. Crit Care Med. 2018 Sep;46(9):e825–73.
7. Sigakis MJG, Bittner EA. Ten myths and misconceptions regarding pain management in the ICU. Crit Care Med. 2015;43(11):2468–78.
8. Hersch M, Sonnenblick M, Karlic A, Einav S, Sprung CL, Izbicki G. Mechanical ventilation of patients hospitalized in medical wards vs the intensive care unit—an observational, comparative study. J Crit Care. 2007 Mar;22(1):13–7.
9. Mehta AB, Syeda SN, Wiener RS, Walkey AJ. Epidemiological trends in invasive mechanical ventilation in the United States: A population-based study. J Crit Care. 2015 Dec;30(6):1217–21.
10. This research did not receive any specific grant from funding agencies in the public, commercial, or not-for-profit sectors.

### 000194

#### Intraoperative fluid management in patients undergoing renal transplant surgery.Comparison of pulse pressure variation with central venous pressure

A. Hazarika[1], G. Kannan[1], K. Kajal[1], I. Sen[1], S. Sethi[1], S. Singh[2]
[1]Anaesthesia and intensive care, Post Graduate Institute of Medical Education & Research, Chandigarh, Chandigarh, India; [2]Renal transplant surgery, Post Graduate Institute of Medical Education & Research, Chandigarh, Chandigarh, India
**Correspondence:** A. Hazarika
*Intensive Care Medicine Experimental* 2019, **7(Suppl 3):**000194

**INTRODUCTION.** Renal transplant (RT) is considered the treatment of choice in End-Stage Renal disease(ESRD).Besides graft characteristics, an important factor in determining immediate graft function is the intravascular volume maintained during surgery.(1) Infusion of fluid targeting Central venous pressure(CVP) values are routinely used. The controversies of CVP to accurately predict fluid status initiated the use of dynamic parameters of fluid responsiveness like Pulse Pressure Variation (PPV).Our study was to determine the utility of PPV to guide fluid therapy during RT.

**OBJECTIVES. Primary aim-**To determine the ability of PPV guided fluid therapy as an alternative to CVP in RT surgeries by comparing the total volume of fluid administered during surgery.

**Secondary aim-**To compare the adequacy of tissue perfusion by measuring blood lactate levels and to find out the volume of fluid administered in two groups in the post-operative period.

**METHODS.** Consenting ESRD patients aged 18-60 years scheduled for RT receiving kidney either living donors or from cadavers were randomly allocated in one of the two groups -Group C (CVP assisted fluid therapy) and Group P (fluid therapy by PPV).General anesthesia was administered.Fluid resuscitation in group P was to maintain a PPV of <6% at the time of declamping and in group C, fluid infusion was done to target CVP of 10-12 mm of Hg at the same time point. Post operatively signs of fluid overload such as conjunctival edema and fluid requirement for the first 24 and 48 hours were noted. Urine output,urea,and serum creatinine values in the first 24 and 48 hours were also obtained. A sample size of 70 (35 patients in each group) was calculated assuming a 30% effect size and 80% power at 5% level of significance for the mean outcome of the volume of crystalloids administered.

**RESULTS.** Out of 77 randomised patients,70 completed the study-Group P (*n*-35) and Group C (*n*-35) (Figure1).The total volume of crystalloids infused intra-operatively was 1345.71±337.24 (mean±SD) ml in Group P which was 29% lesser than 1901.4 ± 379.34 (mean±SD) ml in Group C ($p = 0.000$). Intra operative lactate levels and postoperative fluid, urine output, serum urea, and creatinine levels were comparable (Figure2). Conjunctival edema was significantly higher in group C.

**CONCLUSION.** Administering fluid guided by PPV decreases the intraoperative fluid requirement by 29 % when compared to CVP guided therapy with no adverse effect on immediate graft function. This modality of fluid therapy can be a judicious alternative in RT surgery.

**REFERENCE(S)**

1. PGIMER, CHANDIGARH
2. Othman MM, Ismael AZ, Hammouda GE. The impact of timing of maximal crystalloid hydration on early graft function during kidney transplantation..Anesthesia and analgesia. 2010;110(5):1440-6.

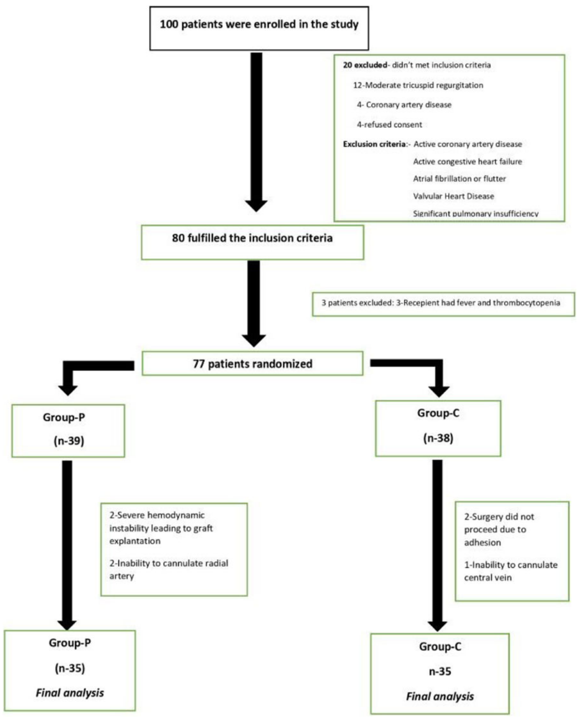

**Fig. 1 (abstract 000194).** CONSORT flow diagram of the randomised trial of two group

| Variables | Group P (Mean±S.D.) | Group C (Mean±S.D.) | *p*-value |
|---|---|---|---|
| **Values** | | | |
| Baseline | 5.14±2.18% | 9.65±2.58 mmHg | |
| After declamping | 4.37±2.32% | 11.91±2.02 mmHg | |
| **Intra-op Fluid Balance** | | | |
| Total Fluid (ml) | 1345.71±337.24 | 1901.4±379.33 | 0.00 |
| Total Blood Loss (ml) | 322.86±117.63 | 309.14±115.46 | 0.58 |
| **Blood lactate levels** (mmol $L^{-1}$) | | | |
| After de-clamping | 1.39±0.62 | 1.38±0.59 | 0.95 |
| **After de-clamping** | | | |
| Mean arterial pressure(**MAP**) Hg | 93.28±12.41 | 92.51±13.19 | 0.92 |
| **Arterial Blood values** | | | |
| pH | 7.348±.060 | 7.332±.048 | 0.21 |
| $HCO_3$ (mmol $L^{-1}$) | 20.18±2.27 | 20.22±2.50 | 0.94 |
| Base deficit | -4.99±2.95 | -5.12±2.28 | 0.85 |
| **Post operative fluid requirement (ml)** | | | |
| POD – 1 | 12383.86± 4124.28 | 11539.43± 3604.29 | 0.37 |
| POD – 2 | 6911.57± 2309.94 | 6993.14± 2434.68 | 0.88 |
| **Urine output (ml)** | | | |
| POD --1 | 11414.29± 4546.48 | 11122.14± 4398.61 | 0.79 |
| POD-- 2 | 6891.43± 2237.94 | 6579.71± 2070.44 | 0.55 |
| **Urea (mg $dl^{-1}$)** | | | |
| POD—1 | 68.97±22.38 | 74.77±24.71 | 0.30 |
| POD—2 | 50.69±24.13 | 52.86±20.24 | 0.68 |
| **Creatinine (mg $dl^{-1}$)** | | | |
| POD—1 | 4.89±1.55 | 5.42±1.43 | 0.13 |
| POD—2 | 2.99±1.29 | 3.45±1.37 | 0.15 |

POD- post operative day; $p<0.05$ is significant

**Fig. 2 (abstract 000194).** Showing the variables observed between the groups perioperatively

### 000167

#### Incidence of ICU and hospital discharge with newly administered antipsychotics in the ICU

J. Lambert[1], J. Fierens[1], P. Depuydt[2], W. Vandenberghe[2], K. Colpaert[1]
[1]Faculty of medicine and health sciences, Ghent University, Ghent, Belgium; [2]Intensive care, Ghent University Hospital, Gent, Belgium
**Correspondence:** K. Colpaert
*Intensive Care Medicine Experimental* 2019, **7(Suppl 3):**000167

**INTRODUCTION.** Delirium is a frequent problem is the intensive care unit (ICU) and is associated with adverse outcomes, such as longer hospital length of stay, cognitive impairment and potentially higher mortality. Haloperidol and atypical antipsychotics are often used to treat delirium in the ICU. Antipsychotics can lead to severe adverse effects, such as arrhythmia, pneumonia, orthostatic hypotension and falling. When antipsychotics are administered for delirium in the ICU, patients are at risk of having antipsychotics continued at ICU and hospital discharge, even though they are no longer indicated. No European data on hospital discharge of patients with newly administered antipsychotics is available.

**OBJECTIVES.** This study aims to determine the incidence of ICU and hospital discharge with newly administered antipsychotics for ICU delirium, to identify risk factors for discharge with antipsychotics and to evaluate follow-up of patients with antipsychotics upon ICU and hospital discharge.

**METHODS.** This retrospective observational study was performed in a tertiary care center. Patients older than 18 years who received antipsychotics for delirium in the ICU during 2016 were included. Preliminary risk factors were determined by performing chi-square tests for categorical variables and univariate binary logistic regression tests for continuous variables. Preliminary risk factors were included in a multivariate binary logistic regression model to identify independent risk factors. Results are expressed as odds ratios with 95% confidence intervals.

**RESULTS.** A total of 196 patients met the inclusion criteria, of which 104 (53.1%) were discharged from the ICU with continued antipsychotics, and 41 (20.9%) patients were discharged from the hospital with continued antipsychotics. At ICU discharge, continuation of antipsychotics was independently associated with a lower Charlson comorbidity index (0.84 [0.73 – 0.97]), treatment with quetiapine (7.14 [3.06 – 16.67]), less days between antipsychotic treatment initiation and ICU discharge (0.95 [0.91 – 0.99]), and a higher ICDSC score at ICU discharge (1.21 [1.04 – 1.39]). At hospital discharge, admission to the medical ICU (2.97 [1.37 -6.41]) and treatment with quetiapine (5.81 [1.63 – 20.83]) were independently associated with continuation of antipsychotics. Sixteen (39.0%) patients who were discharged from the hospital with continued antipsychotics had no mentioning of these antipsychotics in their hospital discharge letter. All of these patients were discharged from a surgical ward.

**CONCLUSION.** One out of five patients were discharged from the hospital with continued antipsychotics. This result is comparable to previous, albeit all US, studies. There is a lack of systematic follow-up of antipsychotics. Hospital policies should implement systematic strategies for better follow-up of antipsychotics at transitions of care.

## NIC - Neurocritical care 2

### 000065

#### Cooling methods in exertional heatstroke; combined methods with an extra-cooling device or conventional approaches

Y. Iwasaki, K. Noguchi, K. Kasai, K. Hirukawa, M. Kawakami
Emergency and Critical Care Center, Ome Municipal General Hospital, Tokyo, Japan
**Correspondence:** Y. Iwasaki
*Intensive Care Medicine Experimental* 2019, **7(Suppl 3):**000065

**INTRODUCTION.** Heatstroke is a life-threatening illness which occurs in hot environment, especially in the summertime. The summer of 2018 was remarkable, because emergency transport to medical facilities of heat-related illness casualties hit the highest record in history at Tokyo, Japan.

Rapid and effective cooling is essential for victims of heatstroke. However, there is little clinical evidence about treatment with new cooling devices.

We compared combined methods with an extra-cooling device (Arctic Sun 5000®, Medivance, USA) to conventional approaches (e.g. lukewarm water spray, electric fan and cooling fluid infusion) for treatment with heatstroke.

**METHODS.** Patients (aged >16 years) with heat-related illness transported to our emergency department (ED) via emergency medical service (EMS) from June to September of 2018 were included in this study.

We used the Japanese Association for Acute Medicine (JAAM) criteria for definition and classification (grade I to III) of heat-related illness. Treatment and clinical outcome of exertional heatstroke cases were examined. Data was derived from prehospital records and electronic health record (EHR).

**RESULTS.** The total number of emergency transport was 1764. Fifty-one (2.9%) patients presented to ED via EMS with symptoms of heat-related illness.

With the JAAM criteria, the number of grade III (severe) heat-related illness that corresponds to heatstroke was 9 (18%). Among them, 4 (7.8%) cases were diagnosed with exertional heatstroke.

With severe exertional heatstroke, three (5.9%) presented with hyperthermia (deep body temperature over 40 C). One was treated by combined methods with Arctic Sun 5000®, and the other two were treated by conventional approaches. All of three case were treated under mechanical ventilation and use of sedatives and muscle relaxants at intensive care unit (ICU).
The time required for reaching to target body temperature was shorter with combined methods than with conventional approaches. Also, no rebound of body temperature was observed with combined methods with Arctic Sun 5000®.
Arctic Sun 5000® was considered minimally invasive and easy to introduce at ED. Overall clinical courses were fine without neurological dysfunction.
**CONCLUSION.** Treatment with extra-cooling devices combined with conventional approaches may be beneficial to patients with exertional heatstroke. Further investigations including large clinical trials are required.

**REFERENCE(S)**

1. Gaudio FG, Grissom CK. Cooling Methods in Heat Stroke. J Emerg Med. 2016 Apr;50(4):607-16. doi: 10.1016/j.jemermed.2015.09.014. Review.
2. Hifumi T, et al. Heat stroke. J Intensive Care. 2018 May 22;6:30. doi: 10.1186/s40560-018-0298-4. Review.

### 000178

#### International prospective observational StudY on iNtrAcranial PreSsurE in intensive care (ICU): The SYNAPSE-ICU Study. Preliminary data

C. Bonetti[1], F. Elli[1], C. Robba[2], A. Vargiolu[3], C. Iaquaniello[1], G. Citerio[1]
[1]School of medicine and surgery, University of Milan-Bicocca, Milan, Italy; [2]Department of anesthesia and intensive care, San Martino Policlinico Hospital, IRRCS for Oncology, Genova, Italy; [3]Neurointensive care, department of emergency and intensive care, Ospedale San Gerardo di Monza, Monza, Italy
**Correspondence:** C. Bonetti
*Intensive Care Medicine Experimental* 2019, **7(Suppl 3):**000178

**INTRODUCTION.** Increased intracranial pressure (ICP) is one of the major clinical complications of acute brain injuries (ABIs) and correlates with poor outcome. ICP monitoring (ICPm) is the most common neuromonitoring modality used in intensive care units (ICUs). The indications for ICPm are mostly based on traumatic brain injury (TBI), whereas uncertainties remain for ICPm in non-TBI (acute subarachnoid haemorrhage (SAH) and intracerebral haemorrhage (ICH)). Moreover, practice about indications and use of ICPm in patients with ABIs is highly variable in high-income countries (HICs), while data on ICPm in low- (LICs) and middle-income countries (MICs) is scarce or inconsistent.
**OBJECTIVES.** SYNAPSE-ICU is an international, prospective, observational, cohort study (NCT03257904) designed to describe the current practice of ICPm using a worldwide sample. Aim is to quantify practice variations in ICPm and management in HICs, MICs and LICs, and to provide a correlation between ICPm and neurological clinical outcome (GOSE: Glasgow Outcome Scale Extended).
**METHODS.** From March 2018 to April 2019, all patients fulfilling the following inclusion criteria were recruited: age >18 years; ABI due to primary haemorrhagic stroke or TBI; Glasgow Coma Score (GCS) with Motor score (M) ≤ 5 at ICU admission or within the first 48 hours. Data related to clinical examination and to ICP interventions was recorded at ICU admission, at day 1, 3 and 7. GOSE was collected at discharge from ICU, from hospital and at 6-month follow-up.
**RESULTS.** To date, 41 countries around the world enrolled 2302 patients in 143 active sites (95 in Europe, 28 in America, 15 in Asia, 3 in Australia and 2 in Africa).
We described the main characteristics of the first 1000 recruited patients (68.0 % males, 32.0 % females, mean age 55.0 years ± 19.2) enrolled in HICs (87.5%), in upper-MICs (U-MICs) (6.7%) and in lower-MICs (L-MICs) (5.8%). Primary diagnosis was TBI in 56.0% of patients and non-TBI in 44.0% of them (19.1% SAH and 24.9% ICH). In the first week of ICU stay, ICP was measured in 585 patients (90.8% in HICs, 5.1% in U-MICs, 4.1% in L-MICs), whereas ICPm was never applied in 415 patients. ICPm patients had a 6-months lower mortality compared to no ICPm ones (40.0% vs 56.1%).
**CONCLUSION.** The high number of enrolled patients and the distribution of active ICUs will represent the worldwide variability in ICPm practice variations. Further analysis will be presented at the end of the study period.

**REFERENCE(S)**

1. The study received an award from the ESICM, and it is inserted in the ESICM research portfolio.

### 000188

#### Ventilatory Strategies in Patients with Severe Traumatic Brain Injury - Survey (VENTILO)

E. Picetti[1], FS. Taccone[2], G. Citerio[3], P. Pelosi[4], M. Oddo[5], C. Robba[6]
[1]Department of anesthesia and intensive care, Parma University Hospital, Parma, Italy; [2]Department of intensive care, Erasme Hospital, Brussels, Belgium; [3]Neurointensive care unit, Ospedale San Gerardo di Monza, Monza, Italy; [4]Departement of anesthesia and intensive care, University of Genoa, Genoa, Italy; [5]Department of intensive care medicine, CHUV-Lausanne University Hospital, Lausanne, Switzerland; [6]Department of anesthesia and intensive care, University of Genoa, Genoa, Italy
**Correspondence:** E. Picetti
*Intensive Care Medicine Experimental* 2019, **7(Suppl 3):**000188

**INTRODUCTION.** Severe traumatic brain injury (TBI) patients may develop acute respiratory failure or acute respiratory distress syndrome (ARDS) (1-2). Optimal ventilatory strategies in this setting are not well established.
**OBJECTIVES.** To survey practices in the respiratory management of adult TBI patients with and without ARDS.
**METHODS.** An electronic questionnaire, including 40 items and 3 different clinical scenarios (PaO2/FiO2: >300, 150-300, <150), was available on the European Society of Intensive Care Medicine (ESICM) website between November 2018 and March 2019. The survey was endorsed and promoted by ESICM.
**RESULTS.** Respondents (RSP) were 687 [472 (69%) from Europe]; mainly intensivists [328 (48%)] and anesthesiologists [206 (30%)]. A standard protocol for mechanical ventilation in TBI patients was utilized by 277 (40%) RSP and a specific weaning protocol by 198 (30%). The most frequently reported ventilator settings and respiratory targets according to acute lung injury severity are summarized in Table 1. The most frequent rescue strategies utilized in case of refractory hypoxemia despite conventional ventilator settings are represented by neuromuscular blocking agents [406 (88%)], recruitment maneuvers [319 (69%)] and prone position [292 (63%)].
**CONCLUSION.** Different practices on respiratory management of adult TBI patients with and without ARDS are identified in this survey. These findings may be helpful to define future investigations in this topic.

**REFERENCE(S)**

1) Asehnoune K et Al. Respiratory management in patients with severe brain injury. Crit Care 2018; 22: 76. 2) Frisvold SK et Al. What respiratory targets should be recommended in patients with brain injury and respiratory failure? Intensive Care Med. 2019 Feb 18.
2) The Survey was endorsed and promoted by ESICM.

**Table 1 (abstract 000188).** Ventilator settings and respiratory targets in severe TBI patients according to acute lung injury severity

| PaO2/ FiO2 | TV (ml/kg PBW) | highest PEEP no IH (cmH2O) | highest PEEP IH (cmH2O) | PaCO2 no IH (mmHg) | PaCO2 IH (mmHg) | PaO2 (mmHg) | SpO2 (%) |
|---|---|---|---|---|---|---|---|
| 1) > 300 | 6-8 [433 (72%)] | 15 [182 (72%)] | 5 [163 (27%)] | 36-40 [308 (51%)] | 36-40 [260 (43%)] | 81-100 [345 (57%)] | > 95 [311 (52%)] |
| 2) 150-300 | 6-8 [331 (62%)] | 15 [218 (41%)] | 10 [171 (32%)] | 36-40 [250 (47%)] | 36-40 [262 (49%)] | 81-100 [283 (53%)] | 92-94 [258 (48%)] |
| 3) < 150 | 4-6 [252 (53%)] | 15 [239 (50%)] | 10 [158 (33%)] | 36-40 [175 (37%)] | 36-40 [224 (47%)] | 81-100 [218 (45%)] | 92-94 [227 (47%)] |

*Abbreviations*: *TV* tidal volume, *PBW* predicted body weight, *PEEP* positive end-expiratory pressure, *IH* intracranial hypertension, *PaCO2* partial pressure of arterial carbon dioxide, *SpO2* arterial blood oxygen saturation

### 000234

#### Platelet distribution width as a predictor for in-hospital mortality in intracranial hemorrhage

AY. Wang[1], CK. Chang[2]
[1]Department of Critical Care Medicine, Taipei Medical University Hospital, Taipei City, Taiwan; [2]Department of neurosurgery, Shuangho Hospital, New Taipei City, Taiwan
**Correspondence:** A.Y. Wang
*Intensive Care Medicine Experimental* 2019, **7(Suppl 3):**000234

**INTRODUCTION.** Platelet distribution width (PDW) reflects the variation and heterogeneity of platelet size, and elevation of PDW indicators of platelet activation. Elevated PDW was associated with poor prognosis in thromboembolic diseases. However, little is known about the association between platelet activation and clinical outcome of intracranial hemorrhage.

**OBJECTIVES.** To study the role of PDW as a prognostic marker of acute intracranial hemorrhage.

**METHODS.** This was a retrospective cohort study conducted in emergency department intensive care units between April 2015 and December 2016. Patients admitted with diagnosis of acute traumatic and non-traumatic intracranial hemorrhage were included. The demographic data, hemorrhage type, platelet count, mean platelet volume (MPV), PDW were compared between survivors and nonsurvivors.

**RESULTS.** A total of 120 patients with acute intracranial hemorrhage were included, 65% (78/120) were traumatic cause. In our cohort, overall mortality was 15.4% (16/104). There was no difference in age, gender, antiplatelet, anticoagulant use, platelet count and MPV level between survivors and non-survivors. In multivariate logistic regression model, PDW was an independent predictor for in-hospital mortality (Odds ratio = 6.07; 95% confidence interval (CI.)=[1.17-31.41], p = 0.031) after adjusting APACHE II score, GCS, mechanism and hemorrhage type. The receiver-operating characteristic area under the curve (AUC) of PDW was 0.71 (95% CI=[0.55-0.87], p=0.006). The PDW level is lowest in aneurysmal SAH group and highest in spontaneous ICH group.

**CONCLUSION.** PDW is simple, inexpensive and available in routine blood sampling. Our study revealed the potential value of PDW as a predictor for in-hospital mortality in intracranial hemorrhage.

### 000240

#### Incidence and characteristics of Takotsubo cardiomyopathy in subarachnoid haemorrhage: a prospective study

D. Szántó[1], J. Gál[1], L. Fülöp[2], A. Szegedi[2], B. Fülesdi[1], C. Molnár[1]
[1]Department of anesthesiology and intensive care, University of Debrecen, Debrecen, Hungary; [2]Department of cardiology and cardiac surgery, University of Debrecen, Debrecen, Hungary
**Correspondence:** D. Szántó
*Intensive Care Medicine Experimental* 2019, **7(Suppl 3):**000240

**INTRODUCTION.** Takotsubo cardiomyopathy (TTC) is an acute, usually reversible heart failure syndrome, precipitated by emotional or physical stressors. The initial presentation of TTC has similar features to acute coronary syndrome, however, coronary angiography usually shows an absence of significant coronary artery disease. TTC has been previously described as a notable complication of SAH because of its impact on cerebral blood flow.

**OBJECTIVES.** The aim of our prospective observational study was to investigate the incidence, predisposing factors and cardiac biomarkers of TTC associated with SAH. Our secondary goal was to evaluate its effect on outcome.

**METHODS.** This study was conducted in our neurosugical intensive care unit between March 2015 and June 2018 (Clinical trials reg. Nr: NCT02659878). We enrolled non-traumatic SAH patients without a history of cardiac disease, who were admitted within 48 hours from symptom onset. On admission we noted the severity of the haemorrhage (modified Fisher score) and neurological state (Hunt-Hess and WFNS scores) and transthoracic echocardiogram was performed. Patients with wall motion abnormality (WMA) were diagnosed as TTC, TTC patients with ejection fraction lower than 40% were classified as severe TTC (sTTC), patients without WMA served as control group (CG). Cardiac necroenzyme detection and transcranial colour duplex was performed on a daily basis. Each patient went through follow-up echocardiograms. We evaluated Glasgow Outcome Scale (GOS) and Barthel Scale (BS) 30 days and 6 months after the onset of SAH.

**RESULTS.** During the study period 136 patients fulfilled inclusion criteria. Incidence of TTC was 28,7% (n=39), sTTC was found in 8,1% (n=11) of the cases. TTC was more common among females than males (female/male: CG 50/47 vs. TTC 30/9, p=0,007). Higher modified Fisher score was more frequent in the TTC group (modified Fisher>2: TTC 32/39 vs. CG 54/97; p=0,004). Higher Hunt-Hess (H-H>3: sTTC 8/11 vs. CG 29/97; p=0,012) and WFNS-score (WFNS>3: sTTC 9/11 vs. CG 29/97; p=0,002) was characteristic for sTTC. Serious vasospasm had higher incidence in the sTTC group (sTTC 3/11 vs. CG 5/97; p=0,04). We found significantly elevated cTnT and NT-proBNP levels in TTC patients. On 1 and 6 month follow-up sTTC was related to increased mortality (sTTC vs. CG: 7/11 vs. 15/95, p<0,001; 8/11 vs. 24/93, p=0,004) and lower GOS score (GOS<4: sTTC vs. CG 11/11 vs 44/95, p=0,002; 9/11 vs. 32/93, p=0,007). At 30th day lower quality of life was observed in the sTTC group (BS<50: sTTC 4/4 vs. CG 23/79, p=0,016).

**CONCLUSION.** TTC is a common cardiac complication of SAH, especially in serious SAH with severe neurological symptoms. cTnT and NT-proBNP properly signs the presence of TTC. The harmful effect of TTC on cerebral circulation may contribute to increased mortality and disability.

##### REFERENCE(S)

1. Hungarian Brain Research Program [grant number: 2017-1.2.1-NKP-2017-00002]

### 000252

#### Epidemiology, Treatment and Outcome of Subarachnoidal Haemorrhage: a retrospective cohort study

H. Vanoverschelde[1], L. Capiau[1], A. Decruyenaere[2], J. Decruyenaere[3], W. Pype[1], K. Colpaert[3]
[1]Faculty of medicine and health sciences, Ghent University, Ghent, Belgium; [2]Internal medicine, Ghent University Hospital, Ghent, Belgium; [3]Intensive care, Ghent University Hospital, Ghent, Belgium
**Correspondence:** K. Colpaert
*Intensive Care Medicine Experimental* 2019, **7(Suppl 3):**000252

**INTRODUCTION.** Spontaneous subarachnoid haemorrhage (SAH) is frequently a devastating type of acute cerebral bleeding which, despite continuous improvements in treatment and care, often remains a disease with high mortality and poor functional outcome.

**OBJECTIVES.** To understand and map all contributing factors regarding epidemiology, treatment with outcome in patients with both aneurysmal SAH (aSAH) and non-aneurysmal SAH (non-aSAH).

**METHODS.** This retrospective cohort study was carried out in a tertiary university hospital. Data regarding demographics, Intensive Care Unit (ICU) stay and functional outcome were collected for all patients during a period of eleven years (2007-2017). Univariate logistic regression was carried out in order to assess the influence of each variable on the occurrence of vasospasm/delayed cerebral ischaemia, the ICU and hospital survival. Univariate multinominal logistic regression was used for the analysis on the modified Rankin Scale (mRS), a functional outcome scale taken five to eight months after SAH.

**RESULTS.** A total of 498 SAH patients were identified, of which 421 were diagnosed with aSAH. Female gender, being an active smoking, problematic alcohol use and hypertension are all more prevalent in aSAH patients than in non-aSAH patients. 65.1% of aSAH and 23% of non-aSAH patients had polyuria, which was significantly associated with vasospasm. 24.5% of aSAH and 5.3% of non-aSAH patients experienced vasospasm, which was significantly related to poorer outcome. Most aSAH patients (88.8%) were treated with coiling, versus only 5.5% who were clipped. Epileptic insults occurred in 12.3% of aSAH opposed to 2.3% in non-aSAH patients. Infections occurred in 50.1% of aSAH, and 20% of non-aSAH patients. Location of the aneurysm at the posterior communicating artery, and hyponatraemia (Na<135 mmol/L) during ICU stay were associated with a better ICU and hospital outcome. Hypernatraemia (Na>150mmol/L) and increased lactate levels (at admission, at 48 hours) were associated with poor outcome. The hospital mortality of 133 patients (26.7%) presenting with poor grade SAH (Hunt & Hess scale 4-5) was 38.3%. Overall mortality was 15.5%. Hospital survival decreased substantially in patients over 80 years. Most aSAH patients (68.3%) had a good functional outcome (mRS 0-2).

**CONCLUSION.** Patients with SAH still have a substantial mortality and often a poor functional outcome. aSAH and non-aSAH patients demonstrated distinct characteristics in epidemiology and outcome. Analysis identified multiple variables which were associated with vasospasm, ICU and hospital survival and functional outcome.

### 000323

#### Serum lactate level upon admission to the neuro-intensive care unit and 90-day mortality: A retrospective association study

C. Hyunhee[1], TK. Oh[2], S. In-Ae[1], I. Chami[3]
[1]Anesthesiology and pain medicine, Seoul National University Bundang Hospital, Seongnam, Republic of Korea; [2]Anesthesiology, Seoul National University Bundang Hospital, Seongnam, Republic of Korea; [3]Surgery, Seoul National University Bundang Hospital, Seongnam, Republic of Korea
**Correspondence:** T.K. Oh
*Intensive Care Medicine Experimental* 2019, **7(Suppl 3):**000323

**INTRODUCTION.** The serum lactate level is a useful predictor of mortality in critically ill patients. However, little is known about the association between the serum lactate level and mortality in patients admitted to neuro-intensive care units (NCUs).

**OBJECTIVES.** The present study aimed to investigate the association between the initial lactate level and 90-day mortality in NCU patients.

**METHODS.** This retrospective observational study was conducted by reviewing the medical records of adult (age ≥18 years) patients admitted to the NCU at a single tertiary care academic hospital during 2013–2017. The initial lactate level (mmol L-1) was defined as the serum lactate level measured within 6 hours following NCU admission.

**RESULTS.** The final analysis included 2,737 patients, of whom 280 (10.2%) died within 90 days of NCU admission. In a receiver operating characteristic (ROC) analysis, the estimated area under the curve (AUC) for the initial lactate level in predicting overall 90-day mortality was 0.55 [95% confidence interval (CI): 0.52–0.59]. The corresponding values for neurologic and non-neurologic disease-related 90-day mortality were 0.76 (95% CI: 0.71–0.82) and 0.49 (95% CI: 0.45–0.53), respectively. In a multivariable Cox regression analysis, a 1-mmol L-1 increase in the initial lactate level was associated with 1.17- and 1.22-fold increases in overall and neurologic disease-related 90-day mortality, respectively, but not with non-neurological disease-related 90-day mortality (*P*=0.422).

**CONCLUSION.** Elevated lactate levels were related with an increase in overall 90-day mortality among NCU patients. This association was specifically attributed to neurologic disease-related 90-day mortality.

### 000329

#### Optic nerve sheath diameter differences between sex and age in healthy volunteers and traumatic brain injured patients

K. Chandrapatham[1], D. Cardim[2], F. Corradi[3], J. Donnelly[4], DN. Anna[5], A. Bertuccio[6], P. Pelosi[1], P. Hutchinson[7], M. Czosnyka[8], C. Robba[5]
[1]Department of surgical sciences and integrated diagnostics, University of Genoa, Genova, Italy; [2]Department of anesthesiology, pharmacology and therapeutics, vancouver general hospital, University of British Columbia, Vancouver, Canada; [3]Department of surgical, medical and molecular pathology and critical care medicine, University of Pisa, Pisa, Italy; [4]Department of anesthesiology, University of Auckland, Auckland, New Zealand; [5]Department of anesthesia and intensive care, IRCCS AOU San Martino, Genova, Italy; [6]Department of neurosurgery, San Cesare, Arrigo, Antonio, Biagio Hospital, Alessandria, Italy; [7]Department of neurosurgery, University of Cambridge, Cambridge, United Kingdom; [8]Brain physics laboratory, division of neurosurgery, department of clinical neurosciences, University of Cambridge, Cambridge, United Kingdom
**Correspondence:** K. Chandrapatham
*Intensive Care Medicine Experimental* 2019, **7(Suppl 3):**000329

**INTRODUCTION.** The optic nerve sheath diameter (ONSD) is considered an indirect marker for intracranial pressure (ICP). Many studies investigated the normal range of ONSD, the cut-off for increased ICP and what can affect these values including age and sex. However, questions remain about the differences of ONSD versus age and sex, among healthy and brain injured population.

**OBJECTIVES.** The aim of this study is to investigate the values of ultrasonographic ONSD in healthy volunteers of different sex and age and to assess whether there are differences in sex and age in a cohort of brain injured patients.

**METHODS.** We prospectively recruited 122 healthy volunteers undergoing pre-assessment evaluation and compared age/sex dependence of ONSD to 95 patients aged >18 years old with severe traumatic brain injury (TBI) requiring intubation and invasive ICP monitoring. The two groups were stratified for sex and age. Age was divided into 3 subgroups (18-44 years; 45-64 years; >65 years).

**RESULTS.** In healthy volunteers, ONSD was significantly different between males and females (mean ONSD 4.26 vs 4.00 mm, [p = 0.01]). ONSD showed a statistically significant correlation with age (R=0.50, p <0.0001). In TBI patients, no differences in ONSD were found for sex and the correlation between age and ONSD was non-significant (R=0.13 p = 0.20).

**CONCLUSION.** ONSD correlates with age and is significantly different between males and females in healthy volunteers. However, these findings do not persist in TBI patients.

**REFERENCE(S)**

1. Robba C. et al. Optic nerve sheath diameter measured sonographically as non-invasive estimator of intracranial pressure: a systematic review and meta-analysis. Intensive Care Med. 2018 Aug 17
2. Nil

### 000359

#### Diagnostics delirium in patients with stroke with speech disorders

A. Gritsan[1], N. Dovbish[1], D. Kurnosov[1], A. Danilovich[2], O. Chernetsky[2]
[1]Anaesthesiology and intensive care, Krasnoyarsk State Medical University, Krasnoyarsk Regional Clinical Hospital, Krasnoyarsk, Russia; [2]Anaesthesiology and intensive care, Krasnoyarsk Regional Clinical Hospital, Krasnoyarsk, Russia
**Correspondence:** A. Gritsan
*Intensive Care Medicine Experimental* 2019, **7(Suppl 3):**000359

**INTRODUCTION.** The incidence of delirium in the ICU ranges from 45% to 87%. Several scales are used to assess the presence of delirium among which two are most widely used – Confusion Assessment Method-ICU (CAM-ICU) and Intensive Care Delirium Screening Checklist (ICDSC).

**OBJECTIVES.** To assess the frequency of manifestation and severity of agitation/sedation in patients with strokes with speech and without speech disorders; estimate the frequency of occurrence of delirium and its types.

**METHODS.** We conducted an observational study of 80 patients admitted to the stroke unit. In the first 24-48 hours from the moment of arrival, the presence of delirium was evaluated on the ICDSC scale. The degree of depression of consciousness was assessed according to the CGS and FOUR scales. The degree of agitation/sedation was assessed according to the RASS scale. The patients were divided into 2 groups - the 1st group (34 cases) of patients who had speech disorders in the form of total aphasia or motor/sensory aphasia and the 2nd group - patients without speech disorders (46 cases).

**RESULTS.** The degree of depression of consciousness from CGS and FOUR, the degree of sedation / arousal on the RASS scale are presented in Table.

In 1 group agitation (RASS +1 and more) was observed in 20 (59%) patients, in 2 group agitation was observed in 13 (28%) patients. We noted a statistically significant (φ = 2.768, p≤0.01) increasing in the frequency of occurrence of agitation in 1group assessed by the RASS scale and a statistically significant decreasing in the level of consciousness which was evaluated by the CGS scale. A more pronounced degree of depression of consciousness in patients when evaluated on the CGS scale compared to assessing the level of consciousness in them on the FOUR scale can be explained by the presence of verbal contact in the CGS scale, which is absent in the FOUR scale. In 2 group, where it was possible to assess the presence of delirium on the ICDSC scale, subsyndromal delirium was detected in 18 (40%) patients, delirium was diagnosed in 13 (29%) patients. At the same time, hyperactive delirium was diagnosed in 6 (14%) patients, in 7 (15%) - mixed delirium. Hypoactive delirium in patients was not observed by us.

**CONCLUSION.** The presence of speech disorders is the significant factor complicating the assessment of presence of delirium in patients with stroke. A statistically significant increase in the level of agitation in the assessment on the RASS scale in patients with speech disorders can be regarded as manifestations of delirium in patients of this group based on the frequency of delirium in patients of 2 group with agitation.

**Table 1 (abstract 000359).** Level of consciousness, the presence of sedation/agitation, Me [25; 75]

| | GCS | FOUR | RASS |
|---|---|---|---|
| 1 group | 14 [11;15] | 16 [12;16] | 1 [-1;2] |
| 2 group | 15 [14;15] | 16 [14;16] | 0 [-2;0] |
| p | ≤ 0.05 | ≥0.05 | ≤0.05 |

### 000368

#### Evolution according to age of acute ischemic stroke patients treated with thrombectomy

S. Alcántara Carmona[1], L. Esteban García[2], N. Martinez Sanz[1], M. Pérez Redondo[1], B. Balandín Moreno[1], M. Valdivia De La Fuente[1], I. Fernández Simón[1], ML. Pérez Pérez[1]

[1]Intensive care, Hospital Puerta de Hierro-Majadahonda, Majadahonda, Spain; [2]Radiology, Hospital Puerta de Hierro-Majadahonda, Majadahonda, Spain

*Intensive Care Medicine Experimental* 2019, **7(Suppl 3):**000368

**INTRODUCTION.** Age is not an exclusion criterion for thrombectomy (TRB) in acute ischemic stroke (AIS).

**OBJECTIVES.** To compare six-month mortalities and neurological statuses of AIS patients of different ages treated with TRB.

**METHODS.** Retrospective study (October 2013–July 2017) including AIS patients treated with TRB admitted to our ICU. Patients were divided in three groups based on age: group A (<65 years); group B (65-79 years) and group C (≥ 80 years). Complete reperfusion was defined by a IIb/III punctuation in the modified treatment in cerebral infarction score (mTICI). A difference ≥ 4 points between initial and before discharge National Institutes of Health Stroke Scale (NIHSS) scores was considered as favorable in-hospital neurological evolution. A modified Rankin Scale (mRS) score between 0–2 at six months was acknowledged as good neurological status. Univariate analysis: X2 or Fishers test (categorical) and Kruskall-Wallis (numerical). Multivariate analysis: logistic regression.

**RESULTS.** One hundred and sixty-five patients were included: 64 in group A; 66 in B and 35 in C. We found no differences in the prevalence of previous ischemic/hemorrhagic stroke, initial NIHSS, basilar artery involvement or initial CT findings evaluated by the Alberta Stroke Program Early CT Score (ASPECTS). Patients ≥ 65 years had more comorbidities [A: 2 (1-3) vs. B and C: 2 (2-3); p < 0.05) and higher APACHE II scores [A: 9 (7-12) vs. B and C: 13 (11-17); p < 0.001].

Regarding treatment characteristics and patient's evolution, the main reason for TRB across groups was thrombolysis failure and stent retriever was the most used technique. The interval symptom onset to reperfusion (min) was similar between groups [A: 285 (241 – 382) vs. B: 305 (252 – 355) vs. C: 313 (270 – 360); NS] as was the rate of complete reperfusion [A: 43 (78.2%) vs. B: 53 (82.2%) vs. C: 21 (63.6%)]. Even though no statistical differences were found, patients in group C had a longer AIS duration and a lower reperfusion rate. TRB was not feasible due to anatomical difficulties in seven patients (20%) in group C as compared to 1 (1.6%) and 2 (3%) patients in groups A and B. Forty-four patients (75.9%) in group A, 39 (72.2%) in B and 18 (64.3%) in C achieved a favorable neurological in-hospital evolution (NIHSS ≥ 4; NS).

When looking at 6-month mortality, after adjusting by confounding factors (number of comorbidities, APACHE II, initial NIHSS, basilar artery involvement, initial ASPECTS, interval symptom onset to reperfusion, TRB complications, hemorrhagic transformation, use of vasoactive drugs and mTICI < IIb), only patients below 65 years had a higher probability of survival [OR 0.04 (IC95 0.00 – 0.77); p = 0.03]. No differences were found when studying mRS (0-2) at six-months [group A: OR 0.24 (IC95 0.04 – 1.32); p = 0.10 and group B: OR 0.57 (IC95 0.11 – 2.80); p = 0.49. Reference category: Group C].

**CONCLUSION.** In our series, patients below 65 years of age with an AIS treated with TRB had better chances of six-month survival when compared to elder patients, but no differences in neurological outcomes at six-months were demonstrated. We believe age should be taken into account when TRB is being considered as part of the treatment for AIS.

**REFERENCE(S)**

1. Powers WJ et al. 2018 Guidelines for the Early Management of Patients With Acute Ischemic Stroke: A Guideline for Healthcare Professionals From the American Heart Association/American Stroke Association. Stroke. 2018 Mar;49(3):e46-e110.

### 000377

#### Gastrointestinal dysmotility and nutritional impairment in patients with Intensive Care Unit Acquired Weakness

F. Klawitter[1], R. Patejdl[2], DA. Reuter[1], J. Ehler[1]

[1]Department of anaesthesia and intensive care medicine, University Medical Center Rostock, Rostock, Germany; [2]Institute of physiology, University Medical Center Rostock, Rostock, Germany

**Correspondence:** F. Klawitter

*Intensive Care Medicine Experimental* 2019, **7(Suppl 3):**000377

**INTRODUCTION.** Intensive Care Unit Acquired Weakness (ICUAW) is a common complication affecting peripheral nerves and muscles resulting in severe immobility and prolonged intensive care treatment with increased mortality [1]. Limited data are available for a possible impairment of the enteric nervous system in this generalized neuromuscular dysfunction. Furthermore, the relevance of ICUAW for gastrointestinal dysmotility and impaired enteral nutrition was not studied before.

**OBJECTIVES.** We investigated gastrointestinal function and nutritional status in patients with and without ICUAW.

**METHODS.** In this retrospective analysis, we included critically ill patients (main inclusion criterion: SOFA-Score ≥ 8 for three consecutive days after intensive care unit (ICU) admission; main exclusion criteria: pre-existing neuromuscular disease or gastrointestinal dysfunction, present abdominal surgery) on two perioperative ICUs. The Medical Research Council-Sum Score (MRC-ss) was used to diagnose ICUAW (MRC-ss < 48 points) [2]. Within a 14-day observational period the following parameters for gastrointestinal function and nutritional status were collected: gastric residual volume (GRV), administered doses of laxatives, defecation frequency and days with enteral tube feeding.

**RESULTS.** Thirty patients were enrolled for an analysis. ICUAW was diagnosed in 14 patients (mean age 68.17 ± 11.3 years; APACHE-II 26.1 ± 3.5), 16 patients were ICUAW negative (mean age 59.75 ± 15.5 years, p=0.19; APACHE-II 22.9 ± 6.2, p=0.09). Patients with ICUAW were significantly longer sedated and mechanically ventilated. The mean cumulative GRV was higher in patients with ICUAW (1797.9 ± 1896.9 ml/patient vs. 835.9 ± 1101.2 ml/patient, p=0.058). Patients with ICUAW needed higher cumulative doses of laxatives (lactulose: 103.4 ± 41.3 g vs. 69.2 ± 57.2 g, p=0.07; sodium picosulfate: 60 ± 16.4 mg vs. 34.7 ± 28.3 mg, p=0.013; suppositories: 29 vs. 9, p=0.009) and the cumulative time of administration was longer (lactulose: 123 vs. 91 days, p<0.001; sodium picosulfate: 112 vs. 75 days, p<0.001). Patients with ICUAW were more frequently depended on enteral tube feeding after extubation (cumulative: 79/103 days [76.7%] vs. 42/172 days [24.4%], p<0.001) and after 14 days (11/14 [78.6%] ICUAW positive patients vs. 3/16 [18.8%] ICUAW negative patients, p<0.01). The cumulative defecation frequency was similar between both groups (87/195 days [44.6%] vs. 86/222 days [38.7%], p=0.26).

**CONCLUSION.** Gastrointestinal dysmotility was more frequent in critically ill patients with ICUAW and might result from an impairment of the enteric nervous system. Prolonged dependency of enteral tube feeding might point to a higher risk of nutritional impairment in patients with ICUAW, which should be further investigated.

**REFERENCE(S)**

[1] Greet Hermans and Greet Van den Berghe 2015. Clinical review: intensive care unit acquired weakness.Crit Care. 19(1): 274.
[2] Stevens et al 2009. A framewok for diagnosing and classifying intensive care unit-acquired weakness. Crit Care Med. 37 (10 Suppl):S299-308.

None.

### 000378

#### Effect of sedative drugs on outcome in traumatic brain injury patients with intracranial pressure monitoring: a CENTER-TBI study

M. Carbonara[1], B. Gravesteijn[2], T. Zoerle[1], F. Ortolano[1], T. Birg[1], G. Citerio[3], R. Helbok[4], A. Chieregato[5], N. Stocchetti[1], HF. Lingsma[2]
[1]Neurointensive care unit, Fondazione IRCCS Ca'Granda Ospedale Maggiore Policlinico, Milano, Italy; [2]Center for medical decision sciences, department of public health, Erasmus University Medical Center, Rotterdam, Netherlands; [3]School of medicine and surgery, University of Milano-Bicocca, Monza, Italy; [4]Department of neurology, University of Innsbruck, Innsbruck, Austria; [5]Neurointensive care, ASST Great Metropolitan Niguarda, Milano, Italy
**Correspondence:** M. Carbonara
*Intensive Care Medicine Experimental* 2019, **7(Suppl 3):**000378

**INTRODUCTION.** Sedation is a first-line therapy used in intensive care unit (ICU) to control raised intracranial pressure in severe traumatic brain injury (TBI) patients. However, it remains unclear what the effect of different sedative drugs is on outcome. This analysis of the CENTER-TBI study uses comparative effectiveness research to fill this knowledge gap.

**METHODS.** The CENTER-TBI study (clinicaltrials.gov registration NCT02210221) is a prospective observational longitudinal cohort study including patients with TBI from 65 centers across Europe. Data were extracted from the CENTER-TBI database v1.1 with Neurobot v2.6. We included all patient who were admitted to the ICU with ICP monitoring and who had more than one day of mechanical ventilation. For every patient, the primary sedative (the sedative that the patient received most of the days) was selected. We focused on midazolam and propofol, since these were the most frequently used.

**RESULTS.** 4509 patients were included in the CENTER-TBI study: we selected 611 patients who received propofol, and 451 patients who received midazolam. There was a large variation between centers in choice of sedative drugs and length of sedation. The length of ICU stay was 0.84 time shorter (95% CI: 0.81 - 0.87) before adjustment, and 0.83 (95% CI: 0.80 - 0.86) after adjustment for major confounders compared to midazolam. Total adjusted hospital length of stay was similarly 0.97 time shorter (95% CI: 0.95 - 1.00) for propofol versus midazolam patients. The OR of propofol for a better functional outcome was 1.10 (95% CI: 0.83 - 1.45) before adjustment, and 0.93 (95% CI: 0.69 - 1.26) after adjustment.

**CONCLUSION.** There is large variation in length of sedation and choice of sedative drugs among European neurotrauma centers. Propofol is associated with a shorter ICU and hospital length of stay but similar functional outcome as midazolam.

**REFERENCE(S)**

1. CENTER-TBI (clinicaltrials.gov NCT02210221) was supported by the European Union 7th Framework program (EC grant 602150)

### 000408

#### Measurement of the diameter of the optic nerve sheath by Axial Tomography an Ocular Ultrasound: Non-Invasive evaluation of intracranial pressure

MJ. Dominguez Rivas[1], J. Navarro[2], ML. Carmona[1], S. Fernández[1], A. Casas[1], I. Valiente Aleman[1]
[1]Medicina intensiva, University Hospital of Puerto Real, Puerto Real, Spain; [2]Radiology, University Hospital of Puerto Real, Puerto Real, Spain
**Correspondence:** M.J. Dominguez Rivas
*Intensive Care Medicine Experimental* 2019, **7(Suppl 3):**000408

**INTRODUCTION.** Measurement of optic nerve sheath (ONSD) using ocular sonography is an accurate, non-invasive technique for the detection of intracranial hypertension (> 20 mmHg) in a heterogeneous group of patients with acute brain injury when performed by an experienced operator. ONSD ≥0.48 cm has the greatest accuracy. In the same way the Axial Tomography proves to be a good technique for demostrate intracranial hypertension with the increase ONSD. At present, these techniques are increasingly used in neurocritical patients.

**OBJECTIVES.** To determine the values of the diameter of the optic nerve sheath (ONSD) by computerized tomography (CT) and ocular ultrasound (OU), in neurocritical patients admitted to a polyvalent ICU without neurosurgery. ONSD values of intracranial hypertension are considered by CT> 5mm and by OU> 4.8mm (measured both at 3mm from the posterior wall of the eyeball).

**METHODS.** Cross-sectional prospective study from January 2017 to December 2018, in patients admitted consecutively with neurological pathologies, performing measurements of the ONSD by CT (SIEMENS, WW 2; WL 98; thickness of the cut 2mm) to 3mm of the posterior wall of the eyeball, and measurements of ONSD by OU (ESAOTE ultrasound, linear probe 7.5MHz) to 3mm of the posterior wall of the eyeball. Epidemiological variables are studied, and ONSD (bilateral) measurements are compared by both CT and OU. Statistical study with IBM SPSS statistics 20.0, determination of the T-Student for independent samples and Pearson correlation.

**RESULTS.** A total of 70 patients with a mean age of 65.23 years (35-86), males 45 (64.28%) were collected. Causes of admission: hemorrhagic stroke-17 (24,28%) Ischemic stroke-22 (31,42%) postanoxic encephalopathy-9 (12,86%) Meningitis-12 (17,14%) and Others-10 (14,28%). Mean initial GCS: 6,66 (3-13). They required mechanical ventilation: 44 (62,85%), APACHE II medium: 19,85 ± 7,14 (8-35). Deaths: 25 (35,71%).

**CONCLUSION.**

- In our patients there are no statistically significant differences when measuring ONSD by CT and by OU.
- There is a very good correlation in ONSD measurements by CT and OU (the correlation is significant at the bilateral 0.01 level) and they are good methods, to evaluate intracranial hypertension non-invasively.

**REFERENCE(S)**

1. Ueda T., Ishida E., Kojima Y., et al. Sonographic optic nerve sheath diameter: a simple and rapid tool to assess the neurologic prognosis after cardiac arrest. J Neuroimaging (2105) 25; 927-930.
2. Venkatakrishma R., Vanaman M., James Fletcher J., Lee Jacob T. Optic nerve ultrasound for the detection of raised intracranial pressure. Neurocrit Care (2011)15; 506-515.
3. Wang L., Chen L., Chen Y., et al. Ultrasonography assessments of optic nerve sheath diameter as a noninvasive and dynamic method of detecting changes in intracranial pressure. Jama Ophthalmology (2018)

**Table 1 (abstract 000408).** See text for description

| DATABASE | RIGHT EYE | | | LEFT EYE | | |
|---|---|---|---|---|---|---|
| | Mean ± SD | Min | Max | Mean ± SD | Min | Max |
| ONSD- CT (3mm) | 5,47 ±1,04 | 2,96 | 8,6 | 5,51 ±1,14 | 3,6 | 8,5 |
| ONSD- OU (3 mm) | 5,35 ±0,66 | 3,78 | 7,1 | 5,39±0,70 | 3,8 | 7,1 |

### 000419

#### Level of Low Blood Pressure Associated with Poor Functional Outcomes after Subarachnoid Hemorrhage

M. Hravnak[1], E. Crago[1], Y. Chang[2], T. Lagatutta[1], AM. Fisher[1], K. Yousef[3], M. Pinsky, RM. Friedlander[2]
[1]School of nursing, University of Pittsburgh, Pittsburgh, United States of America; [2]School of medicine, University of Pittsburgh, Pittsburgh, United States of America; [3]School of nursing, University of Pittsburgh, Pittsburgh PA, United States of America
**Correspondence:** M. Hravnak
*Intensive Care Medicine Experimental* 2019, **7(Suppl 3):**000419

**INTRODUCTION.** Patients with aneurysmal subarachnoid hemorrhage (aSAH) frequently display elevated cardiac troponin-I (cTnI), often with cardiac transient regional wall motion abnormalities, both of which often resolve in the days after bleed. However, the peak level of cTnI associated with poor functional outcomes is not explicated.
**OBJECTIVES.** To evaluate the cut-point value of peak cTnI associated with poor functional outcomes as measured by the modified Rankin Score (mRS) at hospital discharge, and at 3 and 12 months after aSAH.
**METHODS.** We recruited 175 aSAH patients (inclusion: ages 21-75 years, Fisher grade >1; exclusions: chronic neurological deficit, history of myocardial infarction). Serum cTnI was measured daily, and the patients peak value from days 1-3 used in the analysis. mRS was obtained by trained study staff at hospital discharge by chart review, and at 3 and 12 months by interview, and dichotomized as good (scores 0-3) and poor (scores 4-6). Descriptive statistics were obtained, and the cTnI cut-point value associated with poor outcomes at the 3 time points estimated using Receiver Operating Characteristic Curve where the optimal cut-point maximized the sum of sensitivity and specificity for all possible values of the cut point.
**RESULTS.** The sample of 175 patients was primarily female (72%), with a mean age of 53 years (SD 11), and Hunt and Hess grade mean 2.9 (SD 0.7). As shown in Table 1, mean values of cTnI for patients with poor outcome were generally greater that 1.0 ng/ml (poor outcome pateints cTnI means of 1.78, 1.19 and 1.27ng/ml at discharge, 3 and 12 months respectively). Standard deviations were large, while median values for cTnI for patients with poor outcome were 0, 0.17 and 0.31 at discharge, 3 and 12 months. Cuttpoint values of cTnI associated with poor outcome were quite low, with poor to moderate sensitivity but good specificity (discharge cTnI cutpoint 0.33ng/ml, sensitivity 0.343, specificity 0.922; 3 month cTnI threshhold 0.28ng/ml, sensitivity 0.455, specificity 0.810; 12 month cTnI threshhold 0.28ng/ml, sensitivity 0.523, specificity 0.802). Of note, sensitivity of cTnI threshhold values associated with poor outcome improved slightly as time from injury lengthened.
**CONCLUSION.** All patients with a cTnI above 0.28ng/ml during days 1-3 after aSAH have a greater propensity towards poorer outcomes, but sensitivity for prognostic purposes is low.

**REFERENCE(S)**

1. NIH R01NR014221

**Table 1 (abstract 000419).** Mean peak cTnI values associated with good and poor functional ability as measured by mRS after aSAH

| Day 1-3 Peak Troponin by mRS *(poor vs. good)* | | | | | |
|---|---|---|---|---|---|
| | | Day 1-3 peak troponin | | | |
| | | *n* | *mean* | *SD* | *median* |
| mRS at discharge | *Good < 4* | 77 | 0.27 | 0.92 | 0 |
| | *Poor >= 4* | 99 | 1.78 | 5.29 | 0 |
| mRS at 3 months | *Good < 4* | 137 | 0.90 | 3.63 | 0 |
| | *Poor >= 4* | 44 | 1.19 | 2.24 | 0.17 |
| mRS at 12 months | *Good < 4* | 131 | 0.88 | 2.87 | 0 |
| | *Poor >= 4* | 44 | 1.27 | 2.23 | 0.31 |

### 000465

#### Anticoagulant and antiplatelet use in patients admitted to ICU with haemorrhagic stroke

B. Narayan[1], A. Goodliff[2], T. Holzmann[1]
[1]Critical care, Salford Royal NHS Foundation Trust, Manchester, United Kingdom; [2]Medicine, Salford Royal NHS Foundation Trust, Manchester, United Kingdom
**Correspondence:** B. Narayan
*Intensive Care Medicine Experimental* 2019, **7(Suppl 3):**000465

**INTRODUCTION.** Anticoagulation and antiplatelet medications are beneficial in the treatment/prevention of a range of conditions. An estimated 1-2% of the UK population is therapeutically anticoagulated. Recent evidence has demonstrated superiority of direct oral anticoagulant drugs (DOACs) over warfarin and there has been a sharp increase in the use of these medications. This is not without risk and European guidelines recommend a bleeding risk assessment (e.g. HAS-BLED score). It is estimated that DOACs will account for 5% of the NHS drug budget in 2020. This means that it is highly relevant to evaluate real-world data focussing on adverse events associated with these drugs, particularly intracranial haemorrhage. There have also been recent updates to evidence and guidance regarding aspirin, making it relevant to compare events in patients taking antiplatelet drugs.
**OBJECTIVES.** To assess prior anticoagulant or antiplatelet medication use in patients admitted with haemorrhagic stroke in a neurosciences critical care unit.
**METHODS.** Retrospective search of the local critical care admissions database covering the period April 2017 to March 2019. Patients admitted with diagnosis codes “Intracerebral haemorrhage" ("supratentorial" or "posterior fossa") were included. Subarachnoid haemorrhage and traumatic injury were excluded. Of the 178 records, 69 were excluded for secondary causes of bleeding or duplicate entries, resulting in 109 patients included in the analysis.
**RESULTS.** 26/109 (24%) of identified patients were on anticoagulant or antiplatelet therapy prior to admission. Of these, 14 (13%) were anticoagulated (7 DOAC, 6 warfarin, 1 heparin) and 14 (13%) were on an antiplatelet drug (11 aspirin [1 also on ticagrelor], 3 clopidogrel). 2 patients were on combination anticoagulation/antiplatelet therapy.

Indications for anticoagulation included atrial fibrillation (AF), venous thromboembolism (VTE) and metallic heart valve. Indications for antiplatelet therapy included coronary artery disease, stroke or transient ischaemic attack (TIA). 2 patients on aspirin had no clear indication to be on antiplatelet therapy. Mortality was 46% and not significantly different between patients on anticoagulant/antiplatelet therapy and those who were not (p=0.38), or between patients on aspirin versus DOAC (p=1.0).

**CONCLUSION.** A quarter of patients admitted to our ICU with haemorrhagic stroke were taking anticoagulant or antiplatelet medications. Mortality was high and not significantly different between patient groups. Aspirin use was more frequent than DOACs. This is perhaps surprising given that DOACs confer a higher intracranial bleeding risk, and recent trends of increasing DOAC use. One explanation is that patients on aspirin also have comorbidities that put them at particularly high risk of haemorrhagic stroke, and the likelihood that aspirin is still more frequently prescribed (for a variety of indications) than DOACs. Two patients on aspirin had no clear reason to be taking it. This highlights the importance of balancing risks and benefits, particularly with aspirin, which has been shown not to be of benefit in the primary prevention of cardiovascular disease, or for stroke prevention in AF. As use of DOACs increases, and specific reversal agents become more readily available, it will be interesting to observe for potential changes in rates and patterns of haemorrhagic stroke.

**REFERENCE(S)**

1. National Institute of Clinical Excellence: Support for commissioners using the quality standard for venous thromboembolism in adults: diagnosis and management March 2013 (updated: April 2016)
2. J Campbell Cowan, Jianhua Wu, Marlous Hall, Andi Orlowski, Robert M West, Chris P Gale, A 10 year study of hospitalized atrial fibrillation-related stroke in England and its association with uptake of oral anticoagulation, European Heart Journal, Volume 39, Issue 32, 21 August 2018, Pages 2975–2983, https://doi.org/10.1093/eurheartj/ehy411
3. Burn J, Pirmohamed M Direct oral anticoagulants versus warfarin: is new always better than the old? Open Heart 2018;5:e000712. doi: 10.1136/openhrt-2017-000712
4. Huang W, Singer DE, Wu Y, et al. Association of Intracranial Hemorrhage Risk With Non–Vitamin K Antagonist Oral Anticoagulant Use vs Aspirin Use: A Systematic Review and Meta-analysis. JAMA Neurol. 2018;75(12):1511–1518. doi:10.1001/jamaneurol.2018.2215
5. Kirchhof P, Benussi S, Kotecha D, et al ESC Scientific Document Group, 2016 ESC Guidelines for the management of atrial fibrillation developed in collaboration with EACTS, European Heart Journal, Volume 37, Issue 38, 7 October 2016, Pages 2893–2962, https://doi.org/10.1093/eurheartj/ehw210

### 000538

#### Arterial and venous cerebrovascular flow velocities differences between sex and age in healthy volunteers and traumatic brain injured patients: a prospective observational study

K. Chandrapatham[1], D. Cardim[2], F. Corradi[3], M. Sekhon[4], D. Griesdale[2], P. Hutchinson[5], M. Czosnyka[6], C. Robba[7]

[1]Department of surgical sciences and integrated diagnostics, University of Genoa, Genova, Italy; [2]Department of anesthesiology, pharmacology and therapeutics, vancouver general hospital, University of British Columbia, Vancouver, Canada; [3]Department of surgical, medical and molecular pathology and critical care medicine, University of Pisa, Pisa, Italy; [4]Division of critical care medicine, department of medicine, vancouver general hospital, University of British Columbia, Vancouver, Canada; [5]Department of neurosurgery, University of Cambridge, Cambridge, United Kingdom; [6]Department of clinical neurosciences, brain physics laboratory, division of neurosurgery, University of Cambridge, Cambridge, United Kingdom; [7]Department of anesthesia and intensive care, IRCCS AOU San Martino, Genova, Italy

**Correspondence:** K. Chandrapatham

*Intensive Care Medicine Experimental* 2019, **7(Suppl 3):**000538

**INTRODUCTION.** Transcranial Doppler (TCD) offers a view of brain hemodynamics through the measurements of Flow Velocities (FV) on cerebral arteries and vein system and the assessment of advanced parameters such as autoregulation, compliance and intracranial pressure. These parameters may vary with age and sex. Nowadays few studies describe normal ranges of flow velocities between different gender and ages especially involving the poorly explored venous compartment and the coupling between venous and arterial compartment.

**OBJECTIVES.** The aim of this study is to determine the normal range and coupling of flow velocities in the Straight Sinus and in the Middle Cerebral Artery according to sex and age.

**METHODS.** We prospectively recruited 122 healthy volunteers undergoing pre-assessment evaluation and 95 patients aged >18 years old with severe traumatic brain injury (TBI) requiring intubation and invasive ICP monitoring. The two groups were stratified for sex and age. Age was divided into 3 subgroups (18-44 years; 45-64 years; >65 years). Coupling between arterial and venous flow was assessed within different age groups and sex and between the two cohorts.

**RESULTS.** We found a significant correlation between arterial and venous systolic flows (FVs vs FVVs) in the whole population (R= 0.3953745; p-value < 0.0001) as well as in the females and males subgroups, suggesting the presence of physiologic coupling between arterial and venous flow in healthy volunteers. This correlation was significant even considering the effect of age. In TBI patients, no correlation between FVs and FVVs was found.

**CONCLUSION.** Arterial-venous coupling can be studied using TCD. This coupling is lost in TBI patients, maybe for the compression of the sinuses in high ICP and decoupling of flow from metabolism in the injured brain. Venous TCD could open new scenarios in the comprehension of brain physiopatology.

**REFERENCE(S)**

1. Schoser BGH et al. The impact of raised intracranial pressure on cerebral venous hemodynamics: a prospective venous transcranial Doppler ultrasonography study. J Neurosurg 1999
2. Nil

## CD / ETH / POIC - Perioperative haemodynamic and ethical dilemmas

### 000218

#### Safety and efficacy evaluation of the pharmacoinvasive therapy protocol in Campo de Gibraltar. ARIAM ANDALUCIA

I. Diaz-Torres[1], M. Recuerda Nuñez[1], S. Fernandez Coello[1], MJ. Dominguez Rivas[1], D. Fortet Cortes[1], R. Jimenez Gomez[1], A. Fregosi[2], X. Romani[3], JP. Benalcazar Arias[1], JC. Rodriguez Yañez[1], I. Valiente Aleman[1]

[1]Medicina intensiva, University Hospital of Puerto Real, Puerto Real, Spain; [2]Medicina intensiva, Hospital Universitario Punta de Europa, Algeciras, Spain; [3]Medicina intensiva, Hospital Comarcal La Línea de la Concepción, La Línea de la Concepción, Spain

**Correspondence:** J.C. Rodriguez Yañez

*Intensive Care Medicine Experimental* 2019, **7(Suppl 3):**000218

**INTRODUCTION.** Primary percutaneous coronary intervention (pPCI) is the ideal method of reperfusion for acute STEMI. Although, in some areas it is very difficult to implement pPCI between two hours, and it is well-known that 'Time is myocardium'.

**OBJECTIVES.** To analyze the safety and efficacy of the pharmacoinvasive therapy protocol in a setting without primary percutaneous coronary intervention (PCI), Campo de Gibraltar (CG) area.

**METHODS.** Multicentre observational prospective study of consecutive patients with STEMI treated with fibrinolysis in the CG area and transferred to our intensive care unit for coronarography and PCI between January 1, 2012 and March 31, 2018, included in the ARIAM ANDALUCIA registry. We considered as effective fibrinolysis ( EffFx) those with post-treatment TIMI 3 flow grade and early fibrinolysis (EarlyFx)those given in the first 3 hours from symptom onset.

Haemorrhagic complications were evaluated according to the Bleeding Academic Research Consortium classification.
**RESULTS.** 437 patients were included, mean age (sd) 59.79 (11.89), 242 were inferior STEMI and 194 anterior STEMI. Culprit arthery : none 44, RCA 192, DCA 163 and CXA 38.Distribution (n) of Killip grades I, II, III and IV were 383, 10, 13 and 31 respectively. Mean GRACE (sd) was 149.59(33.49)
80.3% of fibrinolysis treatments were given early and 79.4% in hospital, with a median symptoms-to-needle time of 95 minutes. TIMI 3 was achieved in 79.17% of the cases. 264 coronarographies were performed in the first 24 hours (76 rescue-PCI) and 188 in 48-72 horus. PCI were effective in 382 (87.8%) cases. In 48 (11 %) was not performed because of MINOCA, and 0.9% failed. . EffFx was associated with EarlyFx (p<0.001). Complication rates were 1.83% PCI-related and 2.28% haemorrhagic.
Procedural mortality was 2.3%, and mortality at 30 days was 3.1%.
**CONCLUSION.** The efficacy of both prehospital and in-hospital fibrinolysis in CG health services was related to its early administration. The Hemodynamics unit in the Puerto Real University Hospital ensured the success of the procedure. Pharmocoinvasive therapy with fibrinolysis and PCI has demonstrated to be a safe and effective alternative to primary PCI for CG patients unable to achieve a symptoms-to-ballon time lesser than 180 minutes.

**REFERENCE(S)**
1. Arbel Y ,Ko T , Yan A , Cantor W, Bagai A and TRANSFER-AMI Trial Investigators. Long-Term Follow-up of the Trial of Routine Angioplasty and Stenting After Fibrinolysis to Enhance Reperfusion in Acute Myocardial Infarction(TRANSFER-AMI). Can J Cardiology 2018; 34;736-743.
2. Armstrong PW, Gershlick A, Goldstein p, et al. for the STREAM Investigative Team. Fibrinolysis or primary PCI in ST-segment elevation myocardial infarction. N Engl J Med 2013;368:1379-1387.
3. To memory of Javier Lozano Cid MD who performed numerous coronary interventions with excellence.

### 001398

#### Speckle tracking-derived indices of left ventricular systolic function depend on cardiac preload only in case of preload responsiveness

S. Carelli[1], R. Shi[1], JL. Teboul[1], D. Chemla[2], N. De Vita[1], F. Gavelli[1], A. Pavot[1], W. Mongkolpun[1], C. Richard[1], X. Monnet[1]
[1]Service de médecine intensive-réanimation, Hôpital de Bicêtre, Hôpitaux universitaires Paris-Sud, AP-HP, Le Kremlin-Bicêtre, France; [2]Service de physiologie, Hôpital de Bicêtre, Hôpitaux universitaires Paris-Sud, AP-HP, Le Kremlin-Bicêtre, France
**Correspondence:** S. Carelli
*Intensive Care Medicine Experimental* 2019, **7(Suppl 3):**001398

**INTRODUCTION.** Left ventricle (LV) global longitudinal peak systolic strain (GLS) and peak systolic strain rate (SSR) are validated speckle tracking echocardiography indices of systolic function. They have been shown to be affected by loading conditions in cardiac and critically ill patients. Nevertheless, we made the hypothesis that patients with and without preload responsiveness behave differently due to the effects of the Starling law. In patients with preload responsiveness, indices of systolic function should increase because the stretching of the LV fibers with fluid loading enhances their shortening. This should not occur in preload unresponsive patients.
**METHODS.** We included septic shock patients undergoing a passive leg raising (PLR) test for clinical purposes. GLS and SSR were assessed before and during the PLR test and compared. The LV GLS and SSR were calculated as the average of the value obtained in the 4-chamber view and the value obtained in the 2-chamber and/or the 3-chamber view. Preload responsiveness was defined by a PLR-induced increase in cardiac output ≥10%.
**RESULTS.** We performed 24 measurements in 15 patients (SAPSII at admission = 46±13, SOFA at inclusion = 10±3, pneumonia in 67% of cases). PLR revealed 8 preload responder cases (R) and 16 preload non-responder cases (NR). In both groups, LV end-diastolic volume increased significantly during PLR (from 67 [57;85] to 75 [67;92] mL in R and from 62 [56;87] to 73 [67;92] mL in NR). During PLR, cardiac output increased by 21±9% in R and by 5±3% in NR. In R, GLS improved from -12.1 [-11.3;-16.4] to -13.7 [-12.1;-18.9]% (p=0.02). Similarly, SSR rose from -1.1 [-0.9;-1.2] to -1.2 [-1.1;-1.3] 1/s (p=0.03). In NR, GLS and SSR did not change significantly (p=0.30 and p=0.50, respectively).
**CONCLUSION.** The preliminary data of this ongoing study show that GLS and SSR depend on LV preload only in R. This suggests that they increase as a result of the Starling law.

### 000039

#### A survey on the perspective of end-of-life care in critically ill patients by Anaesthesiology senior residents

YL. Lee[1], SY. Ng[2]
[1]Outram Road, Singapore General Hospital, Singapore, Singapore, Singapore; [2]Surgical intensive care, Outram Road, Singapore General Hospital, Singapore, Singapore, Singapore
**Correspondence:** Y.L. Lee
*Intensive Care Medicine Experimental* 2019, **7(Suppl 3):**000039

**INTRODUCTION.** Poor quality end-of-life(EOL) care is associated with psychological distress among dying patients and their families,and this has been contributed by the inadequacies in physician education and training.We therefore attempted to determine the competency levels of Anaesthesiology trainees,who manage surgical intensive care patients.
**METHODS.** A self-rated questionaire modified from Weissman[1] was used to evaluate the competency of physicians in managing 1)patient-family interactions,2)EOL patient care issues and 3)terminal discharge procedures was administered to all Anaesthesiology senior residents.
**RESULTS.** 34/51(67%) trainees responded to the survey with their baseline demographics in table 1.The comfort level of trainees on patient-family and physician-physican interactions and their proficiencies in EOL care issues are found in Figures 1 and 2.Trainees were generally comfortable discussing all aspects of treatment withdrawal with family over areas ranging from ventilation,transfusions,-feeding,RRT to vasopressor.
In decisions made by the medical team for a terminally-ill DNR(do-not-resuscitate) patient with no decision-making capacity,the trainees felt that the withdrawal of feeding/hydration was associated with violation of ethical and/or personal beliefs.In contrast,trainees had less issues when these decisions were made by the surrogate decision maker.
**CONCLUSION.** While our trainees are comfortable in basic EOL care,-most discomfort arises from conflicts management with other team members,families or personal values and beliefs.This could be as our current training system focuses largely on active management but does not prepare the trainee on how to transition to palliative care.This survey helps to identify the gaps in our training system and can help us to improve it.

### 001276

#### The use of Vasopressors and Opioids in patients admitted to Intensive Care post-op Elective Gynaecological surgery

W. Saghir, M. White, R. Lewis
Anaesthetics & intensive care, Ipswich Hospital NHS Trust, Ipswich, United Kingdom
**Correspondence:** W. Saghir
*Intensive Care Medicine Experimental* 2019, **7(Suppl 3):**001276

**INTRODUCTION.** Opioid analgesics are commonly added to intrathecal bupivacaine to improve patient comfort during and after elective gynaecological procedures. The National Institute for Clinical Excellence (NICE) recommends diamorphine 0.3–0.4 mg for analgesia after elective Caesarean section in preference to morphine. Risk factors for development of respiratory depression include long-acting sedatives, known respiratory disease, increasing age and positivepressure ventilation. In addition to this the incidence of nausea, vomiting and pruritus is proportional to the dose of diamorphine used.

**OBJECTIVES.** We sought to discover if the quantity of opioid added to the spinal for elective gynaecological surgery influenced post-op respiratory function and requirement for vasopressors on intensive care.

**METHODS.** A retrospective cohort approach was used. The anaesthetic documentation of all patients admitted to intensive care post op gynaecological surgery over six and a half months was examined. Eighteen patients met the inclusion criteria. The age ranged from 43 – 91 years old. The average age was 71 years.

**RESULTS.** There were 94% planned admissions and 6% were unplanned. Sixty-seven percent of patients had had a Total Abdominal Hysterectomy + Bilateral Salpingo-oophorectomy. Seventeen percent of surgeries were complex involving the input of other specialties. ASA ranged from 2-4. Sixty-one percent of patients had both a general anaesthetic (GA) and spinal. Seventeen percent had GA only whereas 11% had GA and epidural. Of the anaesthetic charts that were available, Diamorphine was used in the spinals of 69% of patients. Doses ranged from 100 mcg – 1mg with an average dose of 780mcg. In 44% of patients, an additional opioid was used intraoperatively.

The CO2 on admission to intensive care was raised in 39% of patients. Metaraminol was required in 22% of patients. One patient required CPR immediately post-op. Average length of stay was 1 day.

**CONCLUSION.** There was a positive correlation between the dose of intrathecal diamorphine used and post-op CO2 on admission to intensive care. There was no correlation between intrathecal diamorphine dose and need for vasopressors on admission.

**REFERENCE(S)**

1. Wilkinson, D., Curtis, M., Natarajan, M. and Watters, M., 2005. Safety of high dose intrathecal diamorphine in non-obstetric patients: A-738. European Journal of Anaesthesiology (EJA), 22, pp.190-191.
2. Saravanan, S., Robinson, A.P.C., Dar, A.Q., Columb, M.O. and Lyons, G.R., 2003. Minimum dose of intrathecal diamorphine required to prevent intraoperative supplementation of spinal anaesthesia for Caesarean section. British journal of anaesthesia, 91(3), pp.368-372.
3. No grant was used for this project

### 001459

#### Preoperative iron deficiency and transfusions in cardiac surgery patients

M. Ocon Lopez[1], R. Amezaga Menendez[1], J. Colomina Climent[1], MA. Rodriguez Yago[1], C. Gomez Cobo[2], L. Vidal Bonet[3], M. Riera Sagrera[1]
[1]Intensive care medicine department, Hospital Universitari Son Espases, Palma de Mallorca, Spain, Spain; [2]Clinical analysis laboratory, Hospital Universitari Son Espases, Palma de Mallorca, Spain, Spain; [3]Cardiac surgery department, Hospital Universitari Son Espases, Palma de Mallorca, Spain, Spain
**Correspondence:** J. Colomina Climent
*Intensive Care Medicine Experimental* 2019, **7(Suppl 3):**001459

**INTRODUCTION.** Anemia is often found among patients undergoing cardiac surgery and it is associated with an increased risk of perioperative complications [1]. The most frequent cause of anemia is iron deficiency (ID), and it may have a role in transfusions requirements and clinical outcomes in these patients [2].

**OBJECTIVES.** To analyze the prevalence of preoperative iron deficiency in cardiac surgery patients, its association with preoperative anemia and packed red blood cells transfusions.

**METHODS.** Retrospective, observational, cohort study in which all adult patients undergoing elective or urgent cardiac surgery at Son Espases University Hospital were included from April to November 2018.

Anemia was defined as a hemoglobin (Hb) <12/13 g/dL (women/men) and iron deficiency as ferritin <30 μg/L or ferritin <100 μg/L + Transferrin saturation index (TSI) <20%.

Preoperative clinical variables, transfusions performed, hospital length of stay and global mortality were analyzed. Patients without preoperative iron profile study were excluded.

Quantitative variables are expressed as mean ± standard deviation (SD) and the Student's t-test was used to compare them. Those that do not follow a normal distribution, are expressed as median and interquartile range (IQR) and were compared using the Mann-Whitney U test. The qualitative variables were expressed as absolute value and percentage (%) and analyzed with Fisher's exact test. To asses the transfusion requirements, patients were stratified according to the presence or not of preoperative anemia.

**RESULTS.** From the initial sample of 231 patients, 48 were excluded. The most relevant baseline characteristics and clinical results are represented in **Table 1**. ID was present in 70 patients (30.3%), associated with anemia in 32 of them (45.7%) and without it, in 38 cases (54.35%). ID was more common in women and was associated with lower presurgical hemoglobin levels (p = 0.002). Preoperative ID anemia diagnosis was stablished only in 22 (9.5%) patients and 19 (8.2%) of them were treated previously with iron supplements. Patients with ID, stratified by preoperative anemia, received more packed red blood cells transfusions (p = 0.000) (**Figure 1**).

**CONCLUSION.** Preoperative ID is common in cardiac surgery patients, even in those without preoperative anemia. ID is associated with a greater amount of transfusions. Treating the ID could be a strategy to reduce preoperative anemia and the need of transfusions in this population.

**REFERENCE(S)**

1. Kulier A, Levin J, Moser R, Rumpold-Seitlinger G, Tudor I, et al. Impact of preoperative anemia on outcome in patients undergoing coronary artery bypass graft surgery. Circulation 2007; 31; 116(5):471-9.
2. Abraham J, Sinha R, Robinson K, et al. Aetiology of Preoperative Anaemia in Patients Undergoing Elective Cardiac Surgery-the Challenge of Pillar One of Patient Blood Management. Anaesth Intensive Care 2017; 45(1):46-51.

**Table 1 (abstract 001459).** Baseline characteristics and clinical results

| | No iron deficiency | Iron deficiency | p |
|---|---|---|---|
| **Age (years)** | 68,14±11,42 | 69,44±10,84 | 0,42 |
| **Gender** | | | |
| **Women, n (%)** | 50(31,1%) | 29(41,4%) | 0,08 |
| **NYHA** | | | |
| **I** | 44(27,3%) | 11(15,7%) | |
| **II** | 73(45,3%) | 31(44,3%) | |
| **III** | 43(26,7%) | 26(37,1%) | |
| **IV** | 1(0,6%) | 2(2,9%) | 0,10 |
| **EuroSCORE II** | 2,05(1,13-4,68) | 2,14(1,01-3,73) | NS |
| **Surgical priority** | | | |
| **Urgent** | 50(31,1%) | 14(20,0%) | 0,11 |
| **Surgery type** | | | |
| **Isolated coronary bypass** | 51(31,7%) | 23(32,9%) | |
| **Valvular** | 63(39,1%) | 24(34,3%) | |
| **Coronary + valvular** | 32(19,9%) | 17(24,3%) | |
| **Aorta** | 12(7,4%) | 3(5,3%) | |
| **Other** | 3(1,9%) | 3(4,3%) | 0,75 |
| **Creatinine clearance** | | | |
| **< 60 ml/min** | 29(18,0%) | 15(21,4%) | 0,59 |
| **Postoperative Creatinine (mg/dL)** | 1,18±1,22 | 1,07±0,39 | 0,45 |
| **Preoperative Hemoglobin (g/dL)** | 13,43±1,77 | 12,64±1,63 | **0,002** |
| **Length of ICU stay (days)** | 2(2-3) | 2(2-3) | NS |
| **Length of hospital stay (days)** | 7(6-10) | 7(6-10) | NS |
| **Packed Red blood cells transfusions, n (%)** | 96(59,6%) | 46(65,7%) | 0,46 |
| **Perioperative bleeding, n (%)** | 9(5,6%) | 4(5,7%) | 1,0 |
| **Global mortality, n (%)** | 2(1,2%) | 2(2,9%) | 0,59 |

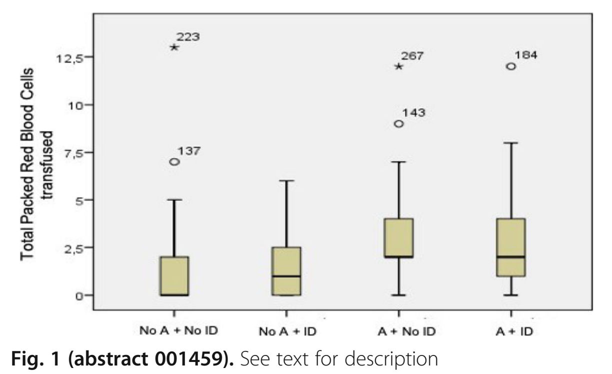

**Fig. 1 (abstract 001459).** See text for description

### 001588

#### Usage of psychotropic drug therapy in a polyvalent ICU: a retrospective study

L. Lopez, D. Díaz Diaz, M. Villanova, A. Martinez De La Gandara, T. Fariña, E. Palencia
Intensive care unit, Hospital Universitario Infanta Leonor, Madrid, Spain
**Correspondence:** L. Lopez
*Intensive Care Medicine Experimental* 2019, **7(Suppl 3):**001588

**INTRODUCTION.** Acute brain dysfunction is very common in critical care patients, and may manifest as delirium, agitation, anxiety or coma. Other psychological conditions can develop in ICU patients: generalized anxiety, sleep disorders, alcohol/drug withdrawal syndromes, depressant symptoms, or previous psychiatric conditions among others. Because of it, the use of psychotropic drugs (PD) is common in ICU setting.
**OBJECTIVES.** To identify the different PDs used in a community hospital ICU. We analyze the use of different antipsychotics, BZDs, antidepressants and also alpha2 agonists. We did not include other sedative drugs.
**METHODS.** Retrospective study in a cohort of patients consecutively admitted to the ICU for more than 24 hours during 2018. We collected demographic data, reason for ICU admission, ICU LOS, CAM-ICU, mechanical ventilation (MV), use of the different PDs, duration of treatment (DOT) and the main indication.
**RESULTS.** Total of 325 patients (pts) were admitted in our ICU during 2018. PDs were used in 179 (55%). Average age was 65.3 years (SD 14.1), 53.6% were males. Mean (SD) ICU LOS 9.6 days (13.8). 80 patients (44.7%) had MV. CAM-ICU + in 43 patients (24%). Main results:

1. **Dexmedetomidine**: 23 pts (12,8%), alone or with more PD in 16 pts (72,8%). Mean (SD) DOT 3.7 days (3.3). CAM-ICU+ in 13 (59%). Mean (SD) dose: 0.40 (0.16) to 1.01 ( 0.42) mcg/kg/h. Indication: sedation (86.4%)
2. **Clonidine**: 11 pts (6.1%). Mean (SD) DOT 8.2 days (7.8). CAM-ICU+ in 7 (63%). Indication: withdrawal (36.4%), agitation/delirium (54.6%)
3. **Haloperidol:** 53 pts (29.6%). Mean (SD) daily dose. 5.1 (2.1) to 7.9 (4.4) mg
   Mean (SD) DOT 2.6 (2.4) days. CAM-ICU + in 34 (79.1%). Indication: agitation (82.7%)
4. **Quetiapine:** 59 pts (33%). Mean (SD) daily dose: 35.5 (35) to 83 (98) mg
   Mean (SD) DOT 6.9 days (8.6). CAM-ICU + in 37 pts (62.7%). Indication: Delirium (93%)
5. **Other antipsychotics:** 27 pts (15%). Tiapride 18 pts (10%), Olanzapine 5 pts (3%), Others 4 pts (2.5%). Indication: withdrawal 15 (58%), agitation/delirium 3 (8%). Mean (SD) DOT 2.7 (1.3)
6. **Benzodiazepines:** 116 pts (64.8%). Lorazepam 64 (36%), Clorazepate 21 (12%), Bromazepam 16 (9%), Diazepam 6 (3.5%), others 9 (4.5%). CAM ICU + in 43 pts (51.2%). Mean (SD) DOT 2 (1.8) days. Indication: hypnotic 65 (56%), anxiety 28 (24%), withdrawal 8 (7%), others 15 (13%)
7. **Antidepressants:** 35 pts (19.6%): Mirtazapine 17 (9.5%), Citalopram 5 (3%), Sertraline 5 (3%), others 8 (4.5%). Mean (SD) DOT 7.4 days (0.7). Indication: Usual medication 20 (57%), depression 9 (26%), hypnotic 6 (17%)

**CONCLUSION.** In our ICU DEX is used mainly for transition sedation. Delirium was usually treated with quetiapine and agitation with haloperidol. BZPs and antidepressants were also widely used, confirming the existence of a gap between Guidelines and the routine ICU clinical practice. Further investigations are required for the use of psychotropic drugs in the ICU.

### 001737

#### Study on prevalence and management of pain in an intensive care unit

C. Mata Martínez, A. Garrido, J. Cedeño Mora, S. Casanova Prieto, B. Moreno Rivero, E. Bermejo López, C. Herrera Alonso, C. Diez Saenz, C. Sotillo
Intensive Care Medicine, Gregorio Marañón Hospital, Madrid, Spain
**Correspondence:** C. Mata Martínez
*Intensive Care Medicine Experimental* 2019, **7(Suppl 3):**001737

**INTRODUCTION.** Management of pain in critical patients is one of the most important part of the treatment and it is necessary to improve it in order to get better prognosis.
**OBJECTIVES.** To describe the prevalence of pain, its quantification and the analgesic regimens in patients admitted to an Intensive Care Unit (ICU)
**METHODS.** Weekly registration of patients to an ICU over a month, type of admission, analgesic regimen and assessment of acute pain episodes through Visual Analog Scale (VAS), Behaviour Indicators of Pain, and Richmond Agitation Sedation Scale (RASS) as apropiate, as well as pharmacological and non-pharmacological interventions.
**RESULTS.** In a total of 92 patients, of which 62% of them were males, with an average age of 61.9 +-15.1 years, main causes of admission were: respiratory failure (50%), neurological disabilities (35.6%) and shock (10.9%). Attending to severity, the average APACHE II was 16.13+-6.2 (2.31) and SOFA 5.6 +- 3.3. 61% of the patients were awaken and able to self-report pain and 39% of them were under sedation (25% had light sedation (RASS-2); 16,7% moderate (RASS-3); 25% deep (RASS-4) and 33% very deep (33%)). 70% of patients required Mechanical Ventilation (MV) (Invasive MV 78.5% and Non Invasive MV 21.5%) and 26.1% were tracheostomized. Attending to analgesic regimen: 98.9% had a established treatment: 53.3% of them had a fixed regimen plus adjuvant therapy during episodes of acute pain; 28.3% fixed regimen without adjuvant therapy; 17.4% were only prescribed in case of pain and 1.1% had no analgesic regimen. Acetaminophen was the most frequently prescribed analgesic (91.3%), followed by opiods (61%): (fentanyl 48.2%, morphine 42.9% and remifentanill 7.1%), metamizole (54.3%) and NSAIDs (3,3%).
Attending to pain: All of patients had at least one measurement of pain daily. There was also a explicit reference of that in 68% of nursery comments and in 76,1% of medical comments. 41% of patients did not show pain, 41,3% of them showed mild pain, and 19,6% registered relevant pain, being intense in 6% of them. 95% of these episodes were solved by adjuvant medication and 5% of them by other non pharmacological interventions. Regarding non pharmacological interventions: calm environment was detected in 85% of the registrations, adequate light in 96,7%, proper sound in 74% and pleasant termperature in 85%. Every patient had postural changes during the day (81,5% of them had more than five a day). Only 20,5% of patients were under physical therapy. Pain episodes were more frequent in patients with respiratory failure (30%) or shock (21%), specially in those without MV. Existence of tracheostomy did not imply more pain. Awaken and conscious patiens had higher risk of suffering pain episodes (RR 4). Physical therapy was related to more pain episodes, but it was not statistically significant. Environmental factors are not related to more pain episodes. Attending analgesic regimen, the one prescribed just in case of pain was related to more pain episodes (RR 2,4). Patients with higher risk of episodes of moderate or severe pain includes: those conscious and able to self-report pain, those with analgesic regimen conditioned to pain, those with more than 2 analgesic, those without MV and those not mobilized. This explanatory model shows a high discriminative power (AuROC 0.91, CI 0.84-0.97).
**CONCLUSION.** Pain monitoring and analgesic regimens are adequate, as well as mobilization of patients, with fewer episodes of severe pain. Physical therapy is not sufficient. Environmental factors are not related to severe pain. Patients with higher risk of pain are those conscious and collaborative, with regimens conditioned to pain, without MV and not mobilized.

**REFERENCE(S)**

1. E. Celis-Rodríguez, C. Birchenall, M.A. de la Cal, G. Castorena Arellano, A. Hernández, D. Ceraso, et al. Guía de práctica clínica basada en la evidencia para el manejo de la sedoanalgesia en el paciente adulto críticamente enfermo. Med Intensiva, 37 (2013), pp. 519-574
2. J. Barr, G.L. Fraser, K. Puntillo, E.W. Ely, C. Gélinas, J.F. Dasta, et al. Clinical practice guidelines for the management of pain, agitation, and delirium in adult patients in the intensive care unit. Crit Care Med, 41 (2013), pp. 263-306

## Added information through meta-analyses

### 000776

#### Erythropoiesis stimulating agents as replacement therapy for blood transfusions in critically ill patients with anemia, a systematic review with meta-analysis

M. Wijnberge[1], S. Rellum[2], S. de Bruin[1], M. Cecconi[3], S. Oczkowski[4], A. Vlaar[1]
[1]Department of intensive care medicine, Academic Medical Centre, Amsterdam, Netherlands; [2]Department of anesthesiology, Academic Medical Centre, Amsterdam, Netherlands; [3]Department of intensive care medicine, Humanitas Medical Care, Milano, Italy; [4]Department of medicine and department of health research methods, evidence and impact, McMaster University, Hamilton, Canada
**Correspondence:** M. Wijnberge

*Intensive Care Medicine Experimental* 2019, **7(Suppl 3):**000776

**INTRODUCTION.** Anemia is common in critically ill patients admitted to the intensive care unit (ICU). [1,2] The etiology is usually multifactorial, including a shortened red blood cell lifespan and impaired erythropoiesis. [3-5] Currently the most common therapy of anemia in ICU patients is red blood cell (RBC) transfusion which is known to be associated with adverse events. [6,7] Erythropoietin stimulating agents (ESA) could potentially reduce the need for RBC transfusion.

**OBJECTIVES.** The aim of this systematic review with meta-analysis is to summarize current evidence on the use of ESA in critically ill patients with anemia. Our primary outcomes are difference in RBC transfusion frequency and change in hemoglobin (Hb) concentration. Our secondary outcomes include adverse events, and mortality.

**METHODS.** A comprehensive literature search was performed in EMBASE, Cochrane and PubMed databases up to September 2018. We restricted inclusion to original studies with adult patients (>18 years of age) in which ESA were given as treatment for anemia of the critically ill. Case reports, case series, conference abstracts and literature studies were excluded. Articles were independently reviewed by two authors for inclusion and data extraction. A third author performed quality assessment for each outcome using the GRADE approach. [8] A random-effects model was used for the meta-analysis.

**RESULTS.** A total of 842 articles were identified of which 18 articles met the inclusion criteria for the qualitative synthesis. Eight of these studies were randomized clinical trials (RCT), with a total of 3387 patients. These studies were included in the meta-analysis. Comparing ESA versus control group, there was a small reduction in the proportion of patients who received one or more RBC transfusions (RR 0.88; CI 0.78-1.00, moderate certainty). The change in Hb concentration was trivial (mean difference -0.31 g/dL; CI -0.51 to - 0.05, high certainty). The number of serious adverse events (RR 1.02; 0.90-1.15, low certainty) and the overall short-term mortality were similar (RR 0.80; CI 0.61-1.05, low certainty). Subgroup analysis for trauma patients showed lower mortality rates in the ESA group (RR 0.49; CI 0.32-0.75, moderate certainty). If real, this advantage is not due to an increase in Hb since there is no reduction in RBC transfusions in the trauma population. The price of recombinant erythropoietin is around $0.0119 per unit. With an average of 40,000 units per dose, the price of one single dose is circa $450. [9]

**CONCLUSION.** Use of ESA resulted in a small reduction in proportion of patients transfused and a trivial increase in hemoglobin concentration. It is debatable whether these changes are clinically relevant. There was no overall difference in adverse events or mortality. Based on this meta-analysis and taking into account the cost of ESA there is insufficient evidence to recommend use of ESA as substitute for RBC transfusion in current standard treatment of anemia of the critically ill.

**REFERENCE(S)**

1. Corwin HL, Gettinger A, Pearl RG, et al. The CRIT Study: Anemia and blood transfusion in the critically ill–current clinical practice in the United States. Crit Care Med. 2004;32(1):39-52.
2. Retter A, Wyncoll D, Pearse R, et al. Guidelines on the management of anaemia and red cell transfusion in adult critically ill patients. Br J Haematol. 2013;160(4):445-464.
3. Rogiers P, Zhang H, Leeman M, et al. Erythropoietin response is blunted in critically ill patients. Intensive Care Med. 1997;23(2):159-162.
4. Walsh TS, Saleh EE. Anaemia during critical illness. Br J Anaesth. 2006;97(3):278-291.
5. Hayden SJ, Albert TJ, Watkins TR, Swenson ER. Anemia in critical illness: insights into etiology, consequences, and management. Am J Respir Crit Care Med. 2012;185(10):1049-1057.
6. Salpeter SR, Buckley JS, Chatterjee S. Impact of more restrictive blood transfusion strategies on clinical outcomes: a meta-analysis and systematic review. Am J Med. 2014;127(2):124-131 e123.
7. Vincent JL, Jaschinski U, Wittebole X, et al. Worldwide audit of blood transfusion practice in critically ill patients. Crit Care. 2018;22(1):102.
8. Guyatt GH, Oxman AD, Vist GE, et al. GRADE: an emerging consensus on rating quality of evidence and strength of recommendations. BMJ. 2008;336(7650):924-926.
9. Christmas, A.B., et al., Removal of erythropoietin from anaemia trauma practice guideline does not increase red blood cell transfusions and decreases hospital utilization costs. Injury, 2009. 40(12): p. 1330-5.

10. No funding was received

### 000787

#### Extravascular lung water levels and kinetics are associated with mortality – A systematic review and meta-analysis

F. Gavelli[1], P. Mercado[1], JL. Teboul[1], V. Girotto[1], M. Jozwiak[1], MS. Chew[2], W. Huber[3], MY. Kirov[4], VV. Kuzkov[4], MLNG. Malbrain[5], J. Mallat[6], SG. Sakka[7], R. Shi[1], T. Tagami[8], C. Richard[1], X. Monnet[1]
[1]Service de médecine intensive - réanimation, Hôpital de Bicêtre, Université Paris-Sud, Le Kremlin-Bicêtre, France; [2]Department of anaesthesia and intensive care, medical and health sciences, Linköping University Hospital, Linköping, Sweden; [3]Ii medizinische klinik und poliklinik, Klinikum rechts der Isar der Technischen Universität München, München, Germany; [4]Department of anesthesiology and intensive care medicine, Northern State Medical University, Arkhangelsk, Russia; [5]Intensive care unit, University Hospital Brussels (UZB), Jette, Belgium; [6]Department of anesthesiology and critical care medicine, Schaffner Hospital, Lens, France; [7]Department of anesthesiology and operative intensive care medicine, Cologne Merheim Medical Center, University of Witten/Herdecke, Cologne, Germany; [8]Department of emergency and critical care medicine, Nippon Medical School Musashikosugi Hospital, Kanagawa, Japan

*Intensive Care Medicine Experimental* 2019, **7(Suppl 3):**000787

Correspondence: F. Gavelli
*Intensive Care Medicine Experimental* 2019, **7(Suppl 3):**000787

**INTRODUCTION.** The role of extravascular lung water (EVLW) measured by transpulmonary thermodilution (TPTD) in the management of critically ill patients remains a matter of debate. We performed a systematic review and meta-analysis of studies assessing the effects of elevated TPTD-estimated EVLW on mortality in critically ill patients with/without septic shock with/without acute respiratory distress syndrome (ARDS).

**METHODS.** MEDLINE, EMBASE and Cochrane Database were screened for original articles. Random effects meta-analyses estimated the pooled relative risk of death associated with elevated EVLW, and the pooled area under the receiver operating characteristics curve (AUROC) of elevated EVLW as a predictor of mortality. Missing data were provided by the authors of the original studies.

**RESULTS.** Fifteen studies (1086 patients) were included. Elevated EVLW levels were associated with a pooled relative risk for mortality of 3.33 [2.21-5.03]. The pooled AUROC, estimated from 13 studies, was 0.84±0.04. The pooled sensitivity and specificity were 70[65-74]% and 69[65-73]%, respectively. Both the baseline and the maximal EVLW values were significantly different among survivors and non-survivors, as well as EVLW variation over time. Subgroup analyses comparing indexation of EVLW to actual vs. predicted body weight and specific populations of ARDS patients vs. other ones were consistent with primary analysis. According to the multivariable analysis performed in seven studies, the odds ratio of elevated EVLW for mortality ranged from 1.01 to 6.21.

**CONCLUSION.** The level of EVLW measured by TPTD and its changes over time are associated with mortality in critically ill patients, which may emphasize its clinical value.

**REFERENCE(S)**

1. Jozwiak M, Teboul J-L, Monnet X (2015) Extravascular lung water in critical care: recent advances and clinical applications. Ann Intensive Care 5:38.
2. Monnet X, Teboul J-L (2017) Transpulmonary thermodilution: advantages and limits. Crit Care 21:147.

### 000685

#### Effects of different early mobilization initiative time on the ICU mechanical ventilated patients :a network meta-analysis

N. Ding[1], Z. Zhigang[2], Z. Caiyun[2], Y. Li[3], W. Yuchen[2], J. Biantong[4], J. Lingjie[5]
[1]Lanzhou university, NO, Lanzhou, China; [2]Department of icu, The First Hospital of Lanzhou University, Lanzhou, China; [3]Lanzhou university, Lanzhou university, Lanzhou, China; [4]School of nursing, lanzhou university, Lanzhou University, Lanzhou, China; [5]The first hospital of lanzhou university, Lanzhou University, Lanzhou, China
**Correspondence:** N. Ding
*Intensive Care Medicine Experimental* 2019, **7(Suppl 3):**000685

**INTRODUCTION.** Early mobility can improve patients' ability of activity and muscle strength, reduce duration of mechanical ventilation and improve patients' life quality after discharge,and the adverse events of early mobility were less than 1%.there is no uniform definition of early mobilization initiative time up to now which ranging from duration of mechanical ventilation less than 24 hours to more than one week of ICU admitted which hinder the widespread implementation of early mobilization in clinic.Therefore,Determine a initiative time to start early mobility is very important

**OBJECTIVES.** To evaluate the effects of different early mobilization initiative time on the intensive care unit(ICU) mechanical ventilated patients

**METHODS.** Chinese Biomedical Literature Database(CBM),Chinese Knowledge Infrastructure(CNKI),Wanfang Data,PubMed,Cochrane library,Web of Science,EMbase and other sources were carried out up to October,2018 about Randomized control trials(RCTs) examining the effects of early mobilization on ICU mechanical ventilated patients.Two investigators independently screened literature,extracted data and evaluated the risk of bias.Outcomes were incidence of ICU acquired weakness(ICU-AW),duration of mechanical ventilation and ICU length of stay.Network commands of mvmeta package in Stata 13.0 were used to perform statistical analyses

**RESULTS.** A total of fifteen RCTs involving 1,726 patients and eight kinds of initiative time to start early mobilization.The network meta-analysis showed that,on the incidence of ICU-AW,there were significant difference between early mobilization began on duration of mechanical ventilation≤24h,48h<duration of mechanical ventilation≤72h,72h<duration of mechanical ventilation≤96h compared with usual care,and no difference between other comparisons;The ranking results were,72h< duration of mechanical ventilation≤96h,duration of mechanical ventilation≤24h,48h<duration of mechanical ventilation≤72h,24h<duration of mechanical ventilation≤48h,usual care.On mechanical ventilation time,duration of mechanical ventilation≤24h,24h<duration of mechanical ventilation≤48h,48h<duration of mechanical ventilation≤72h,duration of mechanical ventilation>96h were all superior to ICU admitted>5 days and usual care,and no difference between other comparisons;The ranking results were,48h<duration of mechanical ventilation≤72h,duration of mechanical ventilation≤24h,24h<duration of mechanical ventilation≤48h,ICU admitted>7 days,duration of mechanical ventilation>96h,usual care,ICU admitted>5 days.And there were no difference between the 8 kinds of initiative time on decreasing ICU length of stay.

**CONCLUSION.** Based on the Network meta-analysis and ranking results,early mobilization start on duration of mechanical ventilation< 24h and 48h<duration of mechanical ventilation≤72h were superior to other time on improving the effects of ICU mechanical ventilated patients.

### 000784

#### High Flow Nasal Cannula use compared to conventional oxygen therapy in the peri-intubation period: A systematic review and meta-analysis

D. Chaudhuri[1], D. Granton[2], D. Wang[3], S. Maggiore[4], S. Einav[5], L. Brochard[6], Y. Helviz[5], T. Mauri[7], J. Mancebo Cortes[8], JP. Frat[9], S. Jog[10], G. Hernandez[11], C. Hodgson[12], S. Jaber[13], KEA. Burns[14], B. Rochwerg[1]
[1]Critical care, McMaster University, Hamilton, Canada; [2]Medicine, McMaster University, Hamilton, Canada; [3]Medicine, Western University, London, Canada; [4]Anesthesiology, D'Annunzio University of Chieti–Pescara, Chieti, Italy; [5]General intensive care unit, Shaare Zedek Medical Center, Jerusalem, Israel; [6]Interdepartmental division of critical care- university of toronto, Hospital St. Michael and Keenan research center, Toronto, Canada; [7]Department of anesthesiology, Fondazione IRCCS Ca'Granda Ospedale Maggiore Policlinico - Servizio Beni Culturali, Milano, Italy; [8]Servei de medicina intensiva, Hospital Universitari Sant Pau, Barcelona, Spain; [9]Medecine intensive reanimation, Poitiers University Hospital, Poitiers, France; [10]Adult intensive care, Pune University, Pune, India; [11]Critical care department, Virgen de la Salud University Hospital, Toledo, Spain; [12]Department of epidemiology and preventive medicine, Monash University, Melbourne, Australia; [13]DAR B, Hôpital Saint Eloi, Montpellier, France; [14]Critical care, St. Michael's Hospital, Toronto, Canada
**Correspondence:** D. Chaudhuri
*Intensive Care Medicine Experimental* 2019, **7(Suppl 3):**000784

**INTRODUCTION.** High flow nasal cannula (HFNC) may be beneficial to patients in the pre-oxygenation and peri-intubation stages of orotracheal intubation by preventing hypoxemia. Despite a number of small randomized controlled trials (RCTs) examining this question, a comprehensive synthesis of the available data has not been conducted previously. Our objective was to conduct a systematic review and meta-analysis examining the role of HFNC in the peri-intubation period

**METHODS.** We performed a comprehensive search of relevant databases (MEDLINE, EMBASE and Web of Science) screening for randomized control trials that compared HFNC oxygen to any other non-invasive modality of oxygen delivery in the peri-intubation time period. Our primary outcome was severe desaturation (defined as SpO2 reading less than 80% during intubation procedure). Other outcomes included short-term mortality, peri-intubation complications, apneic time, and PaO2 before and after intubation. We performed meta-analyses using a random effects model. *A priori* we assessed for predefined subgroup effects based on patient population, risk of bias, and the comparator used. We assessed risk of bias (ROB) of individual RCTs using a modified Cochrane risk of bias tool and assessed overall certainty of pooled estimates using the Grading of Recommendations, Assessment, Development and Evaluation (GRADE) framework.

**RESULTS.** We identified 9 RCTs with a total of 917 patients that met eligibility criteria; 4 RCTs examined HFNC in pre-operative patients and 5 RCTs studied HFNC in acutely hypoxemic critically ill patients.

Two studies compared HFNC to non-invasive ventilation (NIV) while the remaining trials compared HFNC to facemask with bag mask ventilation. HFNC showed no effect on the incidence of peri-intubation hypoxemia (relative risk [RR] 0.93, 95% confidence interval [CI] 0.59 to 1.44, 0.9% absolute risk reduction, 95% CI 5.5% reduction to 5.9% increase; moderate certainty) when compared to NIV or conventional oxygen therapy and no effect on 28-day mortality (RR 0.91, 95% CI 0.72 to 1.12, 3.0% absolute risk reduction, 95% CI 9.3% reduction to 3.9% increase, moderate certainty). HFNC also showed no effect on serious peri-intubation complications (RR 0.87, 95% CI 0.70 to 1.08, 1.4% absolute risk reduction, 95% CI 5.0% reduction to 1.0% increase; low certainty,?) and ICU length of stay (mean difference [MD] 1.15 days less, 95% CI 2.45 days less to 0.16 days more; moderate certainty). Finally, there was no effect of HFNC on PaO2 measured after induction and pre-oxygenation (MD 2.7 mm Hg higher, 95% CI 4.1 mm Hg lower to 9.6 mm Hg higher, moderate certainty), or measured after intubation (MD 30.0 mm Hg higher, 95% CI 8.5 mm Hg lower to 68.6 mm Hg higher, moderate certainty) or in overall apneic time (MD 12.3 seconds higher with HFNC, 95% CI 11.9 seconds lower to 36.6 seconds higher, low certainty) as compared to conventional oxygen therapy or NIV. There were no credible subgroup effects.

**CONCLUSION.** We did not find evidence of an effect of HFNC on the incidence of hypoxemia, complications, oxygenation, ICU LOS, or overall survival when used in the peri-intubation period.

## CD - Haemodynamics

### 000658

#### Comparison between femoral vein diameter and inferior vena cava diameter by ultrasound in estimation of central venous pressure in mechanically ventilated patients

D. zidan[1], A. Baess[2]
[1]University Hospital, Alexandria, Egypt; [2]Chest disease, University Hospital, Alexandria, Egypt
**Correspondence:** D. zidan
*Intensive Care Medicine Experimental* 2019, **7(Suppl 3):**000658

**INTRODUCTION.** Bedside ultrasonography is used as a noninvasive method for hemodynamic monitoring, Evaluation of inferior vena cava diameter by sonography provide an alternative tool for evaluation of intravascular volume. Femoral vein is a superficial compliant vessel, images can be easily obtained.

**METHODS.** We enrolled 100 adult patients in this study. During measurement, patient was in supine position. The CVP was uniformly measured at end of expiration and the pressure transducer having been zeroed at the level of mid axillary line. IVC image was obtained using General Electric ultrasound machine and 3.5 MHz convex probe. The transducer is placed in a vertical plane in the subxiphoid view. The intrahepatic portion of the IVC was visualized as it entered the right atrium. Approximately 3–4 cm from the junction of the IVC and right atrium the IVC diameter was obtained. Using M mode, the maximum and minimum diameter during inspiration and expiration diameter were recorded respectively In order to get the FVD, we scanned the femoral triangle starting at the inguinal crease using a linear array transducer (5-10 MHZ). FVD was measured just caudal to the sapheno-femoral junction.

**RESULTS.** , mean age was 56 y, mean CVP was 6 cm H2O, mean IVC diameter during inspiration was 15 cm, while during expiration was 13.6 cm and the of femoral vein diameter was 8.2 cm. There were significant correlations between CVP , IVC diameter ( Insp, Exp)and FVD. CVP correlation with FVD was 0.59, IVC diameter during inspiration with FVD was 0.41 and IVC diameter during expiration was 0.42

**CONCLUSION.** FVD can be used as a noninvasive alternative method to assess intravascular volume status.

##### REFERENCE(S)

1- Boldt J, Lenz M, Kumle B. Volume replacement strategies on ICUs: results from a postal survey. Intensive Care Med 1998;24:147-151.
2- Citilcioglu S, Sebe A, Oguzhan A, Icme F, Avci A, Gulen A. The relationship between inferior vena cava diameter measured by bedside ultrasonography and central venous pressure value. Pak J Med Sci 2014, 30: 310-16.
3- Cho R, Williams D, Leatherman J. Measurement of Femoral Vein Diameter by Ultrasound to Estimate Central Venous Pressure. Annals ATS 2015,10 : 1-15.
4- Citilcioglu S, Sebe A, Ay M, Icme F, Avci A , Gulen M, etal. The relationship between inferior vena cava diameter measured by bedside ultrasonography and central venous pressure value. Pak J Med Sci 2014, 30 : 2.
5- Malik A , Akhtar A, Saadat S , Mansoor S. Predicting central venous pressure by measuring femoral venous diameter using ultrasonography. Cureus 2016; 8(11): e893

### 000917

#### Outcomes and complications of postoperative right ventricular failure after heart transplantation with ventricular assist device support

S. Escalona-Rodriguez[1], N. Palomo-López[1], A. Escoresca-Ortega[1], A. Adsuar-Gomez[2], M. Porras-Lopez[1], Y. Corcia-Palomo[1], R. Martin-Bermudez[1], L. Martin-Villen[1]
[1]Critical care unit, Virgen del Rocio University Hospital, Seville, Spain; [2]Cardiac surgery, Virgen del Rocio University Hospital, Seville, Spain
**Correspondence:** S. Escalona-Rodriguez
*Intensive Care Medicine Experimental* 2019, **7(Suppl 3):**000917

**INTRODUCTION.** Right ventricular (RV) failure is one of the most serious complications after heart transplantation (HTx). It is an independent risk factor of mortality. Ventricular Assist Devices (VAD) must be considered if conservative medical management fails.

**OBJECTIVES.** Describe our series of patients with RV failure after HTx that required VAD implantation.

**METHODS.** We performed a retrospective study from January 2011 to March 2019 in a third-level ICU with heart transplantation program. All patients that required VAD after RV failure followed by HTx were included. We examined demographics, previously hospitalized in ICU, preoperative VAD implantation, postoperative complications, and survival rate. Absolute number and frequency described qualitative variables and median and interquartile range (IR) described quantitative variables.

**RESULTS.** We collected a total of 10 patients. Demographic data is shown in Table 1. Causes of heart failure were dilated cardiomyopathy (4 cases), congenital cardiomyopathy (2 cases), ischemic cardiomyopathy (2 cases), hypertrophic (1 case) and restrictive cardiomyopathy (1 case). Six patients were transferred from the theatre, 2 from the ward, 1 from the emergency room and 1 from another centre. Five patients were hospitalized in ICU while waiting for transplantation, of whom 4 needed a pre-transplantation VAD: CentriMag Right VAD in 2 patients, central ECMO in 1 patient and Peripheral ECMO in 1 patient. The causes of hospitalization in ICU previous to HTx were acute decompensated heart failure in 2 cases, ischemic cardiogenic shock in 1 case and cardiac electric storm in 1 case. The most frequent VADs implanted after HTx were peripheral ECMO in 6 patients, followed by CentriMag Right Ventricular Assist Device and central ECMO in 2 patients each. VAD was placed as a bridge to recovery in all cases. The most frequent postoperative complications were acute kidney injury with renal replacement therapy (7 cases), critical illness polyneuropathy (6 cases) and stroke (2 cases). The overall mortality was 40%.

**CONCLUSION.** Admission in ICU before heart transplantation and VAD implantation is common in our series, being acute decompensated heart failure the most habitual cause of hospitalized, and CentriMag Right VAD the most frequent device placed. The most frequent VAD placed after RV failure followed by HTx was peripheral ECMO. More data is needed to gain a deeper understanding of these results.

##### REFERENCE(S)

1. Severe right ventricular dysfunction is an independent predictor of pre- and post-transplant mortality among candidates for heart transplantation. Ravis E et al. Arch Cardiovasc Dis. 2017 Mar;110(3):139-148.

2. Mechanical support for isolated right ventricular failure in patients after cardiotomy. Moazami N, Pasque MK, Moon MR, Herren RL, Bailey MS, Lawton JS, Damiano RJ Jr. J Heart Lung Transplant. 2004 Dec;23(12):1371-5.
3. Right ventricular failure following heart transplantation – recovery after extended mechanical support. Yerebakan C, Buz S, Huebler M, Weng Y, Lehmkuhl H, Hetzer R. J Card Surg. 2008 Sep-Oct;23(5):578-80
4. 10 tips for intensive care management of transplanted heart patients. H Moller-Sorensen, H M.Norum, S.E, Ricksten. Intensive Care Med (2019) 45:374-376.

**Table 1 (abstract 000917).** See text for description

| | |
|---|---|
| Male - n (%) | 8 (80%) |
| Age - median (IR) | 52 (31.75-57) |
| Smoking habits - n (%) | 5 (50%) |
| Alcoholism - n (%) | 4 (40%) |
| Hypertension - n (%) | 4 (40%) |
| Diabetes mellitus - n (%) | 3 (30%) |
| Chronic kidney disease - n (%) | None |
| Cirrhosis - n (%) | None |
| LVEF - median (IR) | 31.7 (24.5-36.25) |

### 001305

#### Heart transplant in Congenital heart diseases...a challenge!

N. Palomo-López[1], S. Escalona-Rodríguez[2], A. Adsuar-Gómez[3], A. Herruzo-Avilés[2], R. Hinojosa-Pérez[2], A. Escoresca-Ortega[2], M. Porras-López[2], Y. Corcia-Palomo[2], L. Martin-Villén[2]
[1]Critical care unit, Virgen del Rocío University Hospital, Sevilla, Spain; [2]Critical care, Virgen del Rocio Hospital, Sevilla, Spain; [3]Cardiac surgery, Virgen del Rocio Hospital, Sevilla, Spain
**Correspondence:** N. Palomo-López
*Intensive Care Medicine Experimental* 2019, **7(Suppl 3):**001305

**INTRODUCTION.** Heart transplant in Congenital Heart diseases is challenging, but it is the last option when the heart failure is end.stage.

**OBJECTIVES.** To describe Heart transplant in congenital Heart Disease patients in a third Level Hospital

**METHODS.** Retrospective observational study from 1997 to 2019 in a third-level cardiovascular ICU. We included all patients with Congenital Heart diseases who had a heart transplant. We included demographic data, cardiac catheterization data [pulmonary artery median pressure (PAPm), wedge pressure (PWP), pulmonary vascular resistance (PVR)]. Heart failure stage according to NYHA and FEVI, renal failure and liver failure before the transplantation. Complications during their stay in ITU after transplant were collected: any infection, renal failure develop, ECMO support needed, and global survive. Absolute number and frequency described qualitative variables and median and interquartile range (IR) described quantitative variables

**RESULTS.** We collected 14 patients, median age was 25,5 (20,7-32,25), 57,1% were males. 100% had a NYHA ³3 before transplant, Median FEVI was 37 (22,5-55). Cardiac catheterization values were: median PAPm was 19 (12-22,5); median PWP was 16 (13,5-19,5), median RVP 1,83 (1-4). During their stay in ITU 54,5%(6) presented an infection being respiratory tract infection the most frequent (50%). The 21,4 %(3) needed RRT and 36,4% (4) presented liver failure. 28,6 %(4) develop graft failure, which needs ECMO support, with a median of 6 days (1-17,5) and the mortality in that Group was 50%. Our global survival was 78,6 %(11)

**CONCLUSION.** The most frequent complication was the infection Mortality in those who develop graft failure and need ECMO support is high.
Heart transplant in this population is a valid option with good global results.

### 001322

#### Veno-arterial extracorporeal life support (ECLS) as rescue therapy in a mixed medical and surgical cardiac ICU

M. Stefan[1], M. Luchian[1], I. Marinica[1], A. Paunescu[1], L. Valeanu[2], VA. Iliescu[3], S. Bubenek[2], D. Filipescu[1]
[1]Department of anesthesia and intensive care 2, Institute for Cardiovascular Diseases C.C. Iliescu, Bucureşti, Romania; [2]Department of anesthesia and intensive care 1, Institute for Cardiovascular Diseases C.C. Iliescu, Bucureşti, Romania; [3]Department of cardiac surgery 2, Institute for Cardiovascular Diseases C.C. Iliescu, Bucureşti, Romania
**Correspondence:** M. Stefan
*Intensive Care Medicine Experimental* 2019, **7(Suppl 3):**001322

**INTRODUCTION.** Mortality in cardiogenic shock remains high, despite advances in treatment, including mechanical circulatory support, using ECMO or other alternative systems (1).

**METHODS.** We performed a retrospective study, analysing the activity in cardio-pulmonary support via Extracorporeal Membrane Oxygenation (ECMO), from November 2015 to October 2018, in a mixed medical and surgical cardiac ICU. Data were collected from electronic and hard-copy archives.

**RESULTS.** Twelve patients were treated with ECMO (CardioHelp System, Maquet). All patients had a veno-arterial (VA) ECMO implanted, using a percutaneous, femoro-femoral technique, with a posterior tibial reperfusion cannula, for limb ischemia prevention. Left ventricular (LV) unloading was attempted using an intra-aortic balloon pump (IABP) on the contra-lateral leg. Implantation was done using either ultrasonographic or fluoroscopic guidance.
Refractory cardiogenic shock was the indication for ECLS in all cases: seven patients with post-cardiotomy shock, two patients with fulminant acute myocarditis, and three with ST-elevation myocardial infarction (STEMI), of which one had a mechanical complication – septal ventricular rupture.
Cardiac dysfunction was severe in all cases, either due to LV dysfunction (ejection fraction <25%), RV dysfunction or both, after attempting pharmacologic optimization and, in most cases, IABP. Mean serum lactate at the moment of implantation was 9,4 mmol/L (SD= 4,73).
Monitoring included lactate dynamics, continuous SvO2, daily echocardiography, either transthoracic or transoesophageal, near-infrared spectroscopy and a protocol of classic coagulation tests and viscoelastic point-of-care tests. Adequate multi-organ support was obtained in 11/12 patients.
ECMO support was maintained for a median of 12 days, with a minimum of 6 days and a maximum of 30 days. Seven out of twelve patients were successfully weaned off ECMO (bridge to recovery), another was bridged to long-term mechanical assistance via a left ventricular assist device (LVAD), using an echocardiography guided weaning protocol, and four patients died while on ECMO.
Five patients survived to discharge, three following cardiac surgery and two after recovering from myocarditis.
Complications related to cannulation or limb ischemia did not occur in any of the patients. One patient suffered a severe septic complication - acute gangrenous cholecystitis and underwent abdominal surgery while on ECMO. The same patient required oxygenator replacement due to fibrin deposition.

**CONCLUSION.** ECLS is a technique for multi-organ support in shock in the most severe of cases. In a case series of twelve patients with cardiogenic shock due to STEMI, myocarditis or after cardiac surgery treated with ECMO as a rescue therapy we report a 67% weaning success and 42% survival to discharge Careful monitoring and protocolized weaning can lead to positive outcomes in shock, despite severe illness (mean lactate 9,4 mmol/L).

**REFERENCE(S)**

1. Hajjar LA, Teboul JI. Mechanical Circulatory Support Devices for Cardiogenic Shock: State of the Art. Critical Care. 2019;23:76.

### 000278

#### Percutaneous coronary intervention vs. fibrinolysis in acute coronary syndrome

I. Fernández, A. Alvarez, R. Torcuato, M. Salgado, P. Cobo, A. Ubeda
Intensive care unit, Hospital Point Europe, Algeciras, Spain
**Correspondence:** A. Ubeda
*Intensive Care Medicine Experimental* 2019, **7(Suppl 3):**000278

**INTRODUCTION.** Acute coronary syndrome (ACS) frequently requires Intensive Care Unit (ICU) admission. Percutaneous coronary intervention (PCI) has relegated systemic fibrinolysis as a second line intervention. Delay in time to start the treatment may lead to cardiac complications.

**OBJECTIVES.** To analyze the differences observed in patients with ACS with ST-segment elevation (STEMI), depending on reperfusion treatment used patients in the ICU of Hospital Punta de Europa using ARIAM database.

**METHODS.** Retrospective descriptive analysis on a prospective cohort, performed in a 12-bed ICU for 6 years (2013-2018). Two groups were compared: fibrinolysis vs. PCI. Demographic variables, comorbidities, risk factors, initial Killip, extension, TIMI, GRACE, CRUSADE have been registered. Statistical analysis: categorical (frequencies and percentages) and numerical variables (mean and standard deviation). Comparisons: test of X2 (percentages), Student's t (means). Statistical significance with p <0.05.

**RESULTS.** 293 patients diagnosed with STEMI were included. Those patients were compared according to the reperfusion strategy used: fibrinolysis n = 247 (84.3%) vs. PCI n = 46 (15.6%). There were no differences in terms of age, sex, cardiovascular risk factors or comorbidities. Creatinine (1.04 [± 0.27] vs. 1.21 [± 0.56], p <.001), TIMI (3.06 [± 2.45] vs. 3.88 [± 3.22], p=.024), CRUSADE (27.69 [± 14.42] vs 32.62 [± 20.03], p=.036). Use of clopidogrel during ICU stay: 42 (17%) vs. 1 (2.2%) p=.009. Infectious complications 2 (0.8%) vs. 3 (6.5%) p=.006. Transthoracic echocardiography 56 (23%) vs. 2 (4.3%) p=.004, defibrillation 8 (3.3%) vs 5 (10.9%), p=.022, invasive mechanical ventilation (IMV) 6 (2.5%) vs. 4 (8.7%) p=.033. Destination after ICU discharge (p <.001): hospital ward 217 (87.9%) vs. 31 (67.4%), another hospital 17 (6.9%) vs. 11 (23.9%), death 12 (4.9%) vs. 3 (6.5%). Initial TIMI: 0 66 (24.1%) vs. 19 (9.5%), I 98 (35.8%) vs. 80 (40.2%), II 70 (25.5%) vs 65 (32.7%), III 40 (14.6%) vs. 35 (17.6%), p=.001. ICU mortality: 4.9% vs 6.5%, p=.713. First contact-ICU income (minutes) 91.1 ± 150.4 vs. 172.2 ± 116.4, p=.005. ICU length of stay (LOS) (days): 3.4 ± 3.5 vs. 2.1 ± 3.2, p=.008.

**CONCLUSION.** No significant differences were found in terms of risk factors, comorbidities, non-infectious complications and mortality. Patients treated with primary PCI experienced significantly shorter ICU LOS.

### 000279

#### Heat generated by a continuous cardiac output pulmonary artery catheter: a possible cause of thrombocytopenia in ICU patients?

N. Joxhorst[1], C. Barends[1], M. Nijsten[2]
[1]Department of anaesthesiology, University Medical Center Groningen, Groningen, Netherlands; [2]Department of critical care, University Medical Center Groningen, Groningen, Netherlands
**Correspondence:** N. Joxhorst
*Intensive Care Medicine Experimental* 2019, **7(Suppl 3):**000279

**INTRODUCTION.** We have previously observed a potential association between thrombocytopenia and the presence of a continuous cardiac output pulmonary artery catheter (CCO-PAC). Once the CCO-PAC was removed, platelet counts increased [1]. A potential explanation is the extraordinary sensitivity of platelets to intermittent heat pulses produced by the thermofilament of the CCO-PAC. It is known that platelets are more easily damaged by temperatures >42◦C than erythrocytes or leukocytes [2].

**OBJECTIVES.** The main objective of our initial experiment was to measure the temperatures produced by the thermofilament of the CCO-PAC under simulated blood flow.

**METHODS.** We constructed a circular tubing system, with a mechanical one-way valve, which was able to replicate pulsatile flow of water at 37◦C, in which the CCO-PAC was centrally positioned, along with a thermistor with a high sensitivity and a high time resolution. A compatible monitor (Edwards Lifesciences) was connected to the CCO-PAC and to an ECG-simulator. The pulsatile blood flow and ECG-simulator generated frequencies were both set at 60 /min. Once the CCO-PAC system generated regular heat pulses compatible with actual continuous cardiac output measurements, we started recording the temperature generated by the thermofilament. Under this simulated blood flow, temperature was measured both internally in the distal lumen halfway the thermofilament and external to the CCO-PAC directly in contact with the thermofilament.

**RESULTS.** It took the monitor about 10 minutes to calibrate and measure a cardiac output. During stable CO measurements we observed a constant repetitive pattern in de temperatures generated by the catheter. At a 'blood' temperature of 37◦C we measured rapidly varying temperatures inside and directly near the thermofilament. Such pulses were generated approximately 4 times /min. Once a pulse terminated, the temperature rapidly dropped to the temperature of the surrounding fluid. The surface temperature was 8◦C above the 'blood' temperature for 35% of the cycle, equivalent to 45◦C at a blood temperature of 37◦C.

**CONCLUSION.** Under simulated conditions catheter temperatures >42◦C were observed for a considerable fraction of the CCO-PAC heat-pulse cycle. We believe the sustained emission of thermal pulses may be a key contributor to thrombocytopenia in patients with this catheter in situ. Further studies could confirm and further analyze this phenomenon.

##### REFERENCE(S)

[1] Zeedijk EJ, Ismael F, CR Barends, Nijsten MW. Temporal and quantitative impact of removal of a pulmonary artery catheter on platelet counts. Abstract ESICM Barcelona, 2010.
[2] White JG. Effects of heat on platelet structure and function. Blood 1968;32:324-335.
[3] No funding from external parties was used for our experiments.

### 000612

#### Diagnosis of Acute Myocardial Infarction after Coronary Artery Bypass Graft (CABG) Surgery: A Systematic Review

A. Shoala
Cardiac icu, national heart institute, cairo, Egypt
*Intensive Care Medicine Experimental* 2019, **7(Suppl 3):**000612

**INTRODUCTION.** Myocardial infarction after coronary artery bypass grafting is a serious complication and one of the most common causes of perioperative morbidity and mortality. Multiple mechanisms have been proposed to explain myocardial injury after CABG. Diagnosis will be established according to creatine kinase (CK) values more than five times the 99th percentile of the normal reference range during the first 72 hours following CABG, (or Troponin or CKMB more than ten time increase) when associated with the appearance of new pathological Q-waves or new left bundle-branch block (LBBB), or angiographically documented new graft or native coronary artery occlusion, or imaging evidence of new loss of viable myocardium, should be considered as diagnostic of a CABG related MI.

**OBJECTIVES.** : to identify the methods of diagnosis of post coronary artery bypass graft (CABG) acute myocardial infarction.

**METHODS. Data sources:** MEDLLINE (PubMed), EMBASE, Google Scholar and the Cochrane Library and all materials available in the internet till 2017.

**Study selection:** this search presented 23 eligible studies which studied the diagnostic methods for acute myocardial infarction after coronary artery bypass graft (CABG) surgery.

**Data extraction:** if the studies did not fulfill the inclusion criteria, they were excluded. The methodological quality of included studies was assessed using an adjusted QUADAS-tool.

**Data synthesis:** comparisons was made by structured review with the results tabulated.

**RESULTS.** Troponin I and T can both be used to indicate myocardial damage, with the level correlating well with the level of injury. However until issues such as a 'gold standard' for peri-operative MI are addressed, one single cut-off point cannot be recommended for either test.

**CONCLUSION.** Troponin I and T can both be used to indicate myocardial damage, with the level correlating well with the level of injury. However until issues such as a 'gold standard' for peri-operative MI are addressed, one single cut-off point cannot be recommended for either test.

**REFERENCE(S)**

1. Danese E and Montagnana M. An historical approach to the diagnostic biomarkers of acute coronary syndrome. Ann Transl Med 2016; 4(10):194. Dianne VB, Bas van Zaane, Marc PB, et al. Implementation of the Third Universal Definition of Myocardial Infarction after Coronary Artery Bypass Grafting: ASurvey Study in Western Europe. J Am Heart Assoc. 2015; 114: 1100-1161. Pretto P, Gerez FM, Andressa B, et al. Perioperative myocardial infarction in patients undergoing myocardial revascularization. Rev Bras Circ Cardiovasc. 2015; 30: 1678 -1686.
2. i would like to express my deepest appreciation to all those who provided me the possibility to complete this results A special gratitude I give to prf dr sameh mishel prof of anathesia and intensive care at ain shams university

#### 000630

#### Haemodynamic assessment by echocardiography and cardiac bioimpedance: a pilot study

V. Losada Martínez[1], G. Ferrigno Bonilla[1], A. Tejero Pedregosa[1], A. González Salamanca[1], D. Monge Donaire[1], N. Rodrigo Castroviejo[1], R. Beltrán Bernáldez[1], S. Cortés Díaz[1], C. Tarancón Maján[1], A. Marcos Gutiérrez[1], C. Ochoa Sangrador[2]

[1]Intensive care, Hospital Virgen De La Concha, Zamora, Spain; [2]Research support unit, Hospital Virgen De La Concha, Zamora, Spain

**Correspondence:** A. Tejero Pedregosa

*Intensive Care Medicine Experimental* 2019, **7(Suppl 3):**000630

**INTRODUCTION.** The knowledge and monitoring of cardiac output give us fundamental information in critically ill patients. Fick's method, previously used to calculate it, has been replaced in clinical practice by minimally (transpulmonary thermodilution) or non-invasive techniques (echocardiography, bioimpedance) obtaining cardiac output measurements in a patient-safe, fast and reliable way (1,2).

**OBJECTIVES.** To compare the estimation of stroke volume and cardiac output by non-invasive methods in critical patients. To determine if there is a correlation between indicators of cardiac preload and systolic function between both methods.

**METHODS.** Prospective observational study. An echocardiographic study and bioimpedance monitoring were performed in adult patients admitted to ICU. Stroke volume (SV) and cardiac output (CO) values were collected; systolic function parameters: aortic acceleration index (AI); heather index (HI); aortic velocity index (VI); preload parameters: end-diastolic volume (EDV); inferior cava vein diameter (ICV); thoracic fluid content (TFC) were also retrieved. Correlations between parameters were scanned.

**RESULTS.** 7 adult males were studied, 57,1% receiving invasive mechanical ventilation and 28,6% vasoactive support. The demographic characteristics and the hemodynamic study are shown in the table.

000840

No correlation was observed between values of stroke volume and cardiac output nor between parameters of contractility and cardiac preload. Determination of stroke volume and cardiac output by bioimpedance tends to overestimate values respect to ultrasonography: +9,7 (-2,5 a 21) ml (p= 0,1); +0,6 (-0,6 a 1,3) l/min (p=0,07).

**CONCLUSION.** In our sample, there is no correlation in serial haemodynamic assessment by echocardiography and bioimpedance. Cardiac bioimpedance may overestimate stroke volume and cardiac output relative to echocardiography.

**REFERENCE(S)**

1. Estimating cardiac output. Utility in the clinical practice. Available invasive and non-invasive monitoring. García X, Mateu L, Maynar J, Mercadal J, Ochagavía A, Ferrandiz A. X.García, L.Mateu, J.Maynar, J.Mercadal, A.Ochagavia, A.Ferrandiz. Med Intensiva. 2011 Dec;35(9):552-61.
2. Cardiac output monitoring: a contemporary assessment and review. Crit Care Med. 2015 Jan;43(1):177-85.

**Table 1 (abstract 000630).** See text for description

| Age | APACHE II | SV echo | SV BP | CO echo | CO BP | EDV | IVC | TFC | LVEF | AI | HI | VI |
|---|---|---|---|---|---|---|---|---|---|---|---|---|
| 58,6 (40,1-77) | 17(7,7-26,3) | 53,1(41,4-64,9) ml | 62,9(54,3-71,4) ml | 3,4(2,6-4,2)l/min | 4(3,3-4,7) l/min | 104,3(75,9-132,6) ml | 2(1,7-2,3)cm | 42,8(32-52,6)l/kOhm | 52,3(43,5-61)% | 64,6(47,1-80,1)l/100/s2 | 5,2(4,1-6,4)Ohm/s2 | 26,6(22,8-30,3)1/1000/s |

## AKI - Critical care nephrology 2

#### 000795

#### Age does not independently predict one-year survival in patients undergoing renal replacement therapy: a dual-centre observational study

D. Roberts, L. Hodgson

Intensive care, Worthing Hospital, Worthing, United Kingdom

**Correspondence:** D. Roberts

*Intensive Care Medicine Experimental* 2019, **7(Suppl 3):**000795

**INTRODUCTION.** In the UK and other Western countries, the proportion of elderly patients admitted to intensive care units (ICUs) is rising (1-3). The challenge of determining which treatments will be beneficial to this older cohort is significant(4) and one specific decision, undertaking renal replacement therapy (RRT) holds a delicate balance of risks and benefits. The South Coast of England where this study was performed has a population significantly older than the UK average with 14% over 70 years of age(2), therefore well placed to examine this issue.

**OBJECTIVES.** To understand contemporaneous, longer-term outcomes for older people undergoing acute renal replacement therapy (RRT) in the intensive care unit (ICU) setting.

**METHODS.** A retrospective observational cohort analysis of data collected (2013-2018) on adult patients commenced on RRT in two non-specialist UK ICUs (consisting of a 12-bed and a 10-bed unit) using data collected as part of routine care. Baseline characteristics and outcomes between patients younger or older than 75 were compared.

**RESULTS.** After excluding cases previously on long-term dialysis (n=65) the cohort included n=698 admissions with a median age 69 (IQR 57-77), 33% (n=231) were ≥75 years old and 17% (n=121) were ≥80. Mechanical ventilation was required in 62% and cardiovascular support in 56%. Median ICNARC score was 28 (23-35) and APACHE II score 23 (18-28).

Between those aged <75 and ≥75 there was no significant mortality difference in ICU (42.4% vs 46.3%, OR 1.17 (95% CI 0.85-1.61), P=0.332), in-hospital (47.8% vs 55.0% OR 1.34 (0.97-1.83), P=0.077) and at one year (58.9% vs 63.3% OR 1.21 (0.85-1.71), p=0.329), respectively. In logistic regression age was not an independent predictor of 1-year mortality whilst lactate, and requirement for cardiovascular support were.

**CONCLUSION.** 1-year mortality in patients who have required RRT is substantial, however age alone did not predict survivors.

**REFERENCE(S)**

1. Bagshaw SM, Webb SAR, Delaney A, George C, Pilcher D, Hart GK, et al. Very old patients admitted to intensive care in Australia and New Zealand: a multi-centre cohort analysis. Critical care (London, England). 2009;13(2):R45-R.
2. Ihra GC, Lehberger J, Hochrieser H, Bauer P, Schmutz R, Metnitz B, et al. Development of demographics and outcome of very old critically ill patients admitted to intensive care units. Intensive Care Med. 2012;38(4):620-6.
3.Docherty
AB, Anderson NH, Walsh TS, Lone NI. Equity of Access to Critical Care Among Elderly Patients in Scotland: A National Cohort Study. Crit Care Med. 2016;44(1):3-13.
4.Rodríguez-Molinero
A, López-Diéguez M, Tabuenca AI, Juan J, Banegas JR. Physicians' impression on the elders' functionality influences decision making for emergency care. The American journal of emergency medicine. 2010;28(7):757-65.

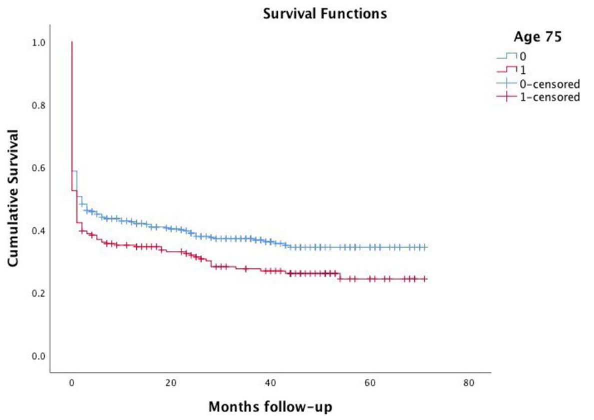

**Fig. 1 (abstract 000795).** Kaplan-Meier survival Blue line <75 years age Red ≥75 Log-rank P=0.023

### 000800

#### Proposal for a modified prognostic nutritional index of acute kidney injury within 1-week in patients underwent liver transplantation

HW. Chung[1], SH. Hong[2], MS. Chae[2], CS. Park[2], JH. Choi[2], HS. Chung[1]
[1]Department of anesthesiology and pain medicine, EunPyeong St.Mary's Hospital, College of Medicine, The Catholic University of Korea, Seoul, Republic of Korea; [2]Department of anesthesiology and pain medicine, Seoul St. Mary's Hospital, College of Medicine, The Catholic University of Korea, Seoul, Republic of Korea
**Correspondence:** H.W. Chung
*Intensive Care Medicine Experimental* 2019, **7(Suppl 3):**000800

**INTRODUCTION.** Acute kidney injury (AKI) is one of the common complications after liver transplantation (LT) which associated with increased mortality, long-term risk of chronic kidney diseases, and prolongated length of hospital stay. Nutritional status, especially prognostic nutritional index (PNI), has been used as a predictive value for postoperative complications. However, there is a lack of prognostic value applied for PNI to the prognosis of LT. We suggested a new predictive model modified by PNI and investigated which model has the highest predictive accuracy for AKI in patients who underwent LT

**METHODS.** Data from a total of 423 patients underwent LT were collected retrospectively. Based on Acute Kidney Injury Network (AKIN) criteria, the patients in the AKI group are confirmed to AKI within 1-week after LT. PNI was calculated as 10 X serum albumin (g/dL) + 0.005 X total lymphocyte count (per mm3). After the univariate analysis, multivariate statistical adjustment for post-operative variables of AKI was performed. As a new predictive model was developed. It was called the modified PNI model (mPNI). The mPNI was calculated as 6.9 X serum bilirubin (mg/dL) + 134 X INR – 29 X PNI + 250. The individual diagnostic accuracy in AKI within 1-week after LT was evaluated using the area under receiver operating characteristic curve (AUC).

**RESULTS.** 54 patients (12.7 %) were diagnosed AKI within 1-week after LT. The mPNI has the highest predictive accuracy (AUC = 0.810, Table 1.). The MELD score and PNI were 0.793 and 0.712, respectively. The INR and serum bilirubin were 0.705 and 0.637, respectively. The mPNI was the highest predictive value for development of AKI within 1-week after LT.

**CONCLUSION.** The mPNI is the highest prognostic model for AKI within 1-week in patients who underwent LT (AUC = 0.810).

**REFERENCE(S)**

1. The predictive value of the prognostic nutritional index for postoperative acute kidney injury in patients undergoing on-pump coronary bypass surgery
2. Acute kidney injury after liver transplantation: Recent insights and future perspectives

**Table 1 (abstract 000800).** Receiver operating characteristic curve

| | Area | 95%CI | Palue |
|---|---|---|---|
| mPNI | 0.810 | 0.740-0.879 | <0.001 |
| PNI | 0.712 | 0.625-0.799 | 0.044 |
| MELD | 0.793 | 0.722-0.865 | <0.001 |
| INR | 0.705 | 0.630-0.781 | <0.001 |
| Serum Bilirubin | 0.637 | 0.560-0.714 | 0.001 |

### 000953

#### The association between Acute Kidney Injury (AKI) and Intensive Care Unit (ICU) acquired hypernatremia

M. te Pas[1], E. Mestrom[1], J. Van Der Stam[2], V. Scharnhorst[2], A. Bindels[1]
[1]Intensive care, Catharina Ziekenhuis, Eindhoven, Netherlands; [2]Clinical laboratory, Catharina Ziekenhuis, Eindhoven, Netherlands
**Correspondence:** M. te Pas
*Intensive Care Medicine Experimental* 2019, **7(Suppl 3):**000953

**INTRODUCTION.** Intensive care unit (ICU) acquired hypernatremia is a highly prevalent condition in critically ill patients and is associated with increased mortality and length of stay. (1) The kidneys play an important role in the volume and osmolality regulation, and are therefore a possible key player in sodium derangements. Approximately 30% of ICU admissions is complicated by acute kidney injury (AKI). (2) Therefore, this study focused on AKI in the development of hypernatremia.

**OBJECTIVES.** This study aimed to provide insight in the effect of AKI on the development of ICU acquired hypernatremia.

**METHODS.** A retrospective comparative analysis was performed using prospectively collected data of ICU patients in a tertiary referral hospital. All adult patients requiring ICU admission more than 48hours were identified and included between April and December 2018. Urine samples were collected and analyzed for electrolytes and osmolality. Additional serum osmolality analyses were performed. Further data collection consisted of detailed fluid balances and medication use.

**RESULTS.** Of the 199 included patients, 85 (43%) developed hypernatremia. According to the RIFLE criteria, 1 (1%) patient was at **R**isk for acute kidney injury, 21 (25%) patients developed kidney **I**njury and 20 (24%) patients had acute kidney **F**ailure in the hypernatremia group. The proportion of patients with AKI was not significantly different between the two groups. However, when comparing the median maximum level of serum creatinine (128,0 µmol/L versus 92,6 µmol) and serum urea (14,0 mmol/L versus 9,7 mmol/L) between hypernatremic patients and normonatremic patients during ICU stay, a significant difference was observed (P<0,001 and P<0,001). The total median salt intake was 26 gram in the normonatremia group and 40 gram in the hypernatremia group (P=0,004). A significant difference was found in median serum osmolality levels between the two groups resulting in 298 mOsm/kg in the normonatremia group and 319 mOsml/kg in the hypernatremia group (P<0,001). The urine-osmolality of the normonatremia group was 695 mmol compared to 612 mmol in the hypernatremia group.

**CONCLUSION.** Patients with hypernatremia did not develop AKI more frequently than patients without hypernatremia. However, the higher levels of serum creatinine and serum urea in the hypernatremia group suggest impaired renal function. In addition, decreased urine osmolality was found in the hypernatremia group despite an (iatrogenic) increased sodium intake. Therefore, a role for the kidneys in the development of hypernatremia is not to be ruled out yet.

**REFERENCE(S)**

1. Waite MD, Fuhrman SA, Badawi O, Zuckerman IH, Franey CS. Intensive care unit-acquired hypernatremia is an independent predictor of increased mortality and length of stay. J Crit Care. 2013;28(4):405-12.
2. Kes P, Basic Jukic N. Acute kidney injury in the intensive care unit. Bosn J Basic Med Sci. 2010;10 Suppl 1:S8-12
3. No grant acknowledgements to declare.

### 000963

#### An equation to estimate glomerular filtration rate in critically ill patients using the novel biomarker proenkephalin

R. Beunders[1], R. van Groenendael[2], P. Vart[3], S. Joachim[4], P. Pickkers[1]
[1]Intensive care, Radboud University Medical Center, Nijmegen, Netherlands; [2]Anesthesiology, pain and palliative medicine, Radboud University Medical Center, Nijmegen, Netherlands; [3]Department for health evidence, Radboud University Medical Center, Nijmegen, Netherlands; [4]Sphingotec gmbh, Sphingotec GmbH, Hennigsdorf, Germany
**Correspondence:** R. Beunders
*Intensive Care Medicine Experimental* 2019, **7(Suppl 3):**000963

**INTRODUCTION.** Deterioration of kidney function in critically ill patients is independently associated with impaired outcome. Diagnosis of acute kidney injury (AKI) is currently hampered by the lack of accurate markers for timely assessment of kidney function. Conventional creatinine-based methods to estimate glomerular filtration rate (GFR) are notoriously insensitive, late, and inaccurate. The gold standard methods to measure GFR using exogenously administered substances, such as inulin or iohexol, are not used in clinical practice because they are labour-intensive and expensive. A promising novel candidate to assess kidney function is plasma proenkephalin (PENK), a stable byproduct of the endogenous opioid enkephalin.

**OBJECTIVES.** 1) To determine to what extent PENK reflects the GFR in a cohort of critically ill post cardiac surgery patients that are prone to develop AKI. 2) To develop an equation to estimate the GFR using PENK plasma concentration.

**METHODS.** We prospectively included 180 patients undergoing cardiac surgery. Post surgery, patients received an intravenous bolus of iohexol, and plasma iohexol concentrations were determined in four samples obtained during the subsequent hours. The true GFR was determined using the slope-intercept method and the Brøchner-Mortensen correction [1]. Plasma PENK concentrations were determined using the Sphingotec immunoassay [2]. Using linear regression model, three equations were developed in a block randomized training sample of the cardiac surgery cohort. 1) PENK only; 2) age, gender and PENK; 3) age, gender, PENK and creatinine. Bias, precision and accuracy were assessed and results were compared with the currently used Modification of Diet in Renal Disease (MDRD) equation.

**RESULTS.** Median of the true GFR was 81 [IQR: 69-90] mL/min/1.73m². Results of the equations are listed in Table 1. The mean bias of the MDRD was significantly different compared to the true GFR (p<0.0001), while the mean bias of the equation of PENK was not (p=0.47). The equation using only PENK was significantly more accurate compared to the MDRD (p=0.02), when the covariates age, gender and creatinine were added, accuracy improved (p=0.0003).

**CONCLUSION.** Our study underscores the inaccuracy of conventional creatinine-based methods to estimate GFR in critically ill patients, causing misclassification of kidney function. Furthermore, we demonstrate that a single plasma PENK determination correlates with the true GFR in cardiac surgery patients and the PENK equation to estimate GFR performs better compared to the MDRD. Therefore, PENK has potential as an accurate and feasible marker to assess kidney function in critically ill patients. External validation in additional critically ill patient cohorts is warranted.

**REFERENCE(S)**

1. Donato, L.J., et al., Analytical performance of an immunoassay to measure proenkephalin. Clin Biochem, 2018. 58: p. 72-77.
2. Brochner-Mortensen, J., A simple method for the determination of glomerular filtration rate. Scand J Clin Lab Invest, 1972. 30(3): p. 271-4.

**Table 1 (abstract 000963).** Equations compared to the *true GFR* calculated using plasma clearance of iohexol

| Equation | *R2* | Mean bias | SD | Accuracy |
|---|---|---|---|---|
| Modification of Diet Renal Disease | 0.57 | -13.1 | 17.8 | 83% |
| PENK | 0.63 | -0.9 | 16.7 | 91% |
| PENK, age, gender | 0.69 | -2.5 | 15.6 | 93% |
| PENK, age, gender, creatinine | 0.77 | -3.9 | 13.6 | 95% |

*R2* R-squared; *Mean bias* and *SD* in mL/min/1.73m2, *SD* standard deviation

### 000984

#### Striking persistency of kidney hemodynamic dysfunction in sepsis survivals

AMA. Liberatore[1], YR. Kang[2], MN. Nakamae[3], RB. Souza[4], SA. De Moura[5], IHJ. Koh[6]
[1]Cirurgia, Universidade Federal de São Puo, Sao Paulo, Brazil; [2]Cirurgia, Unversidade Federal de São Paulo, São Paulo, Brazil; [3]Cirugia, Universidade Federal de São Paulo, São Paulo, Brazil; [4]Genetics and evolutionary biology, University of São Paulo, São Paulo, Brazil; [5]Cirurgia, Universidade Federal de São Paulo, São Paulo, Brazil; [6]Surgery, Universidade Federal de São Puo, Sao Paulo, Brazil
**Correspondence:** I.H.J. Koh
*Intensive Care Medicine Experimental* 2019, **7(Suppl 3):**000984

**INTRODUCTION.** AKI associated with severe sepsis has a significant increase in mortality [1] and it is not known whether there is continuity of renal injury in the post sepsis recovery period. The fact is that survivors have 2.5 times more likely to be readmitted to the hospital for AKI within 90 days than patients without sepsis [2].

**OBJECTIVES.** Evaluate the kinetics of renal hemodynamics alterations in animals surviving from severe sepsis.

**METHODS.** Following DL50 sepsis model induction (2mL *E.coli* 108 CFU/mL, iv.) in adult Wistar female rats, the surviving animals were monitored to macro (MAP, HR and abdominal aorta blood flow by Transonic, TS420 transit-time flowmeter), regional (renal artery and vein by Transonic, TS420 transit-time flowmeter), and micro hemodynamics (kidney cortical microcirculation by SDF videomicroscopy). Animals were monitored at 6 hours, 1month, and 3 month post-sepsis periods. Naïve (N) animals were used as control. (n=4-5/period). All rats were euthanized at the end of experiments.

**RESULTS.** There was a stability of MAP at normal levels in post-sepsis periods. Only in the hypodynamic phase of acute sepsis (S-6h) did a significant reduction occur. Variations of HR (250-400 bpm) were dependent on aortic flow, being higher when the flow drop was observed in the S-6h, denoting to be a parameter more related to hemodynamic adjustment and unrelated to the post- sepsis clinical condition. Moreover, the significantly diminished renal vessels blood flow (fig.1) suggests the kidney hypoperfusion state with microcirculatory dysfunction showed by SDF (fig. 2).

**CONCLUSION.** In conclusion, renal damage in severe sepsis seems to improve partially in the post-sepsis period, but the persistence of the hypoperfusion may predispose to organ dysfunction in severe sepsis survivors.

**REFERENCE(S)**

1. Lameire NH, Bagga A, Cruz D et al. Acute kidney injury: an increasing global concern Lancet 2013, 13: 170-179
2. Prescott HC, Langa KM, Iwashyna TJ. Readmission diagnoses after hospitalization for severe sepsis and other acute medical conditions. JAMA 2015;313:1055-7
3. Grant 2017/21052-0, São Paulo Research Foundation (FAPESP).

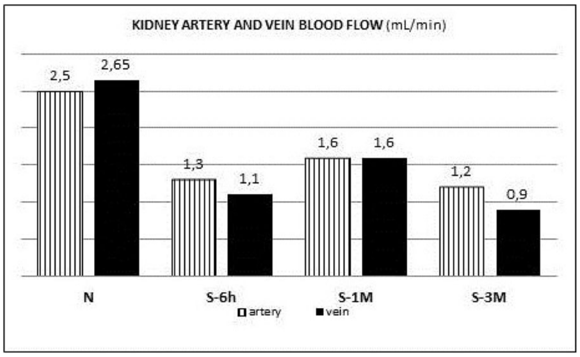

**Fig. 1 (abstract 000984).** Renal artery and vein blood flow following sepsis induction. S=sepsis, h=hours, M=month, N=name

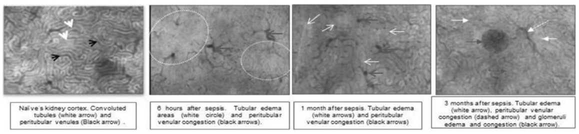

**Fig. 2 (abstract 000984).** SDF images of the kidney cortex following the sepsis showing microcirculatory injuries up 3 months post-sepssis.

### 001096

#### Cardiac and renal effects of remote ischaemic preconditioning on diabetic patients and the relationships with antioxidant and inflammatory markers

M. Galindo Martínez[1], MD. Rodríguez Mulero[1], L. Martínez Gascón[2], R. Jiménez Sánchez[3], L. Tárraga García[3], S. Sánchez Argente Del Castillo[3], J. Batllés Muñoz De Escalona[3], JF. Murcia Payá[3], MD. Albaladejo Otón[2], JM. Allegue Gallego[3], MC. Ortiz Ruiz[4], F. Rodríguez Mulero[4]

[1]Intensive care. instituto murciano investigación biosanitaria, Hospital General Universitario Santa Lucía, Cartagena, Spain; [2]Análisis clínicos, Hospital General Universitario Santa Lucía, Cartagena, Spain; [3]Intensive care, Hospital General Universitario Santa Lucía, Cartagena, Spain; [4]Dpto fisiología. instituto murciano investigación biosanitaria, University of Murcia, Murcia, Spain

**Correspondence:** M. Galindo Martínez

*Intensive Care Medicine Experimental* 2019, **7(Suppl 3):**001096

**INTRODUCTION.** Remote ischemic preconditioning (RIPC) has demonstrated beneficial effects on acute kidney injury (AKI) and/or cardiac function after coronary angiography (CA). These protective effects have been partly explained by enhanced of the antioxidant or anti-inflammatory mechanisms. However, the effects by RIPC might be attenuated in diabetic patients, a population with enhanced of pro-inflammatory and pro-oxidant pathways.

**OBJECTIVES.** To evaluate the effects of RIPC on the myocardial and renal function, as measured by plasma Troponin I, N-terminal prohormone of brain natriuretic peptide (NT-proBNP), creatinine (Cre) and cystatin C (Cysc C) levels (an early marker of glomerular function), as well as plasma levels of the antioxidant enzyme Heme oxygenase-1 (HO-1), the acute phase reactants increasing during inflammation (ferritin) and protein C reactive (PCR), and the ratio of neutrofile/linfocites (NLR).

**METHODS.** A prospective, randomized, single-center trial was conducted at the St. Lucía University Hospital (Cartagena, Spain). Diabetic patients admitted to medical intensive care unit, with an acute coronary syndrome requiring standard CA, were enrolled and randomly assigned to an RIPC group (n = 36) or a control group without RIPC (n = 35). In the RIPC group, four cycles of 5-min of ischemia and 5-min of reperfusion were applied to the right upper limb by a cuff inflated to 50 mmHg over the systolic blood pressure. We recorded demographical, clinical and analytical data before and after 24, 48 and 72 hours of CA procedure. We analyzed qualitative variables with percentages by categories, and square Chi; quantitative variable with median ± standard deviation and T student; or median, interquartile rank and U- Mann Whitney, depending on statistical distribution

**RESULTS.** Patients receiving RIPC showed lower levels of NT-proBNP (pg/ml) at 72 hours (4010±6600) compared to those in the control group (6373±9600), p<0.05, without any differences in the troponin I peak. Regarding to the renal function, the increases in Cre (mg/dl) and Cysc C (mg/L) before and after 72 hours of CA were similar between groups (Cre RIPC; 1.05±0.4 vs 1.37±1 and control; 1.10±0.5 vs 1.17±0.6 and Cysc C RIPC; 1.16±0.5 vs 1.35±0.8 and control; 1.28±0.6 vs 1.38±0.6). Importantly, changes before and after 48 hours in ferritin (ng/ml) (RIPC; 175±176 vs 218±161 and control; 115±98 vs 145± 92); HO-1 (ng/mL) (RIPC; 0.72±1.5 vs 1.36±1.5 and control; 0.77±1.5 vs 1.56±2.2); PCR (mg/dL) (RIPC; 4.25±4.3 vs 5.27±4.2 and control; 4.90±6.1 vs 3.96±3.7); and NLR (RIPC; 0.70±0.1 vs 0.70±0.1, control; 0.72±0.1 vs 0.70±0.1); were similar in both groups.

**CONCLUSION.** The RIPC maneuver before CA in diabetic patients demonstrated favorable effects on cardiac function without improving the renal parameters. These effects could not be attributed to increase in the antioxidant or inflammatory mechanisms analyzed. Alternative pathways underlying these effects merit further investigation.

### 001129

#### A novel technique to increase extracorporeal citrate clearance to achieve regional anticoagulation of high blood flows

A. Zanella[1], L. Vivona[1], A. Galli[1], M. Battistin[2], O. Biancolilli[2], D. Dondossola[3], S. Colombo[1], C. Anzanello[1], I. Protti[1], M. Busana[1], A. Pesenti[1]

[1]Department of pathophysiology and transplantation, University of Milan, Milano, Italy; [2]Department of anesthesiology, Fondazione IRCCS Ca'Granda Ospedale Maggiore Policlinico, Milano, Italy; [3]Liver transplant and general surgery unit, Fondazione IRCCS Ca'Granda Ospedale Maggiore Policlinico, Milano, Italy

**Correspondence:** L. Vivona

*Intensive Care Medicine Experimental* 2019, **7(Suppl 3):**001129

**INTRODUCTION.** Regional anticoagulation allows to perform extracorporeal treatments avoiding the complications of systemic anticoagulation. Citrate-based regional anticoagulation is the most widespread technique but, due to limited citrate clearance, may be applied only to low extracorporeal blood flows (i.e. < 200 ml/min). We developed a novel technique to highly increase extracorporeal citrate clearance, thus achieving regional anticoagulation of higher blood flows.

**METHODS.** Five healthy swine (42±3 Kg) were sedated, mechanically ventilated and connected to a prototype extracorporeal circuit for continuous renal replacement therapy featuring a citrate-removal stage based on absorbent materials and replacement fluids. Blood flow was 500 ml/min. Sodium citrate was continuously infused at the circuit inlet (5 mmol/L). Heparin was continuously infused. Citrate concentration and Heparinase Kaolin thromboelastography (HK-TEG) were measured on arterial blood, extracorporeal blood downstream the citrate infusion port and downstream the citrate-removal stage.

Samples were collected at baseline, 2, 8, 15, 30, 45, 60, 90, and 120 minutes for citrate and every 30 min for HK-TEG. Calcium chloride was infused to maintain systemic ionized calcium within physiological range. The experiment lasted 2 hours.

**RESULTS.** Citrate concentrations in blood samples were stable within the first 30 minutes than started to slowly increase (Figure-left). The efficacy of the citrate-removal stage in removing citrate was 88% at 30 minutes and decreased down to 48% at the end of the study (Figure-right) due to loss of efficiency of the absorbent materials. During the whole experiment, HK-TEG in artery showed normal coagulation: Reaction time (R) was 8.6 ±1.3 minutes, with Maximum Amplitude (MA) of 72.2±6.2 mm. In the extracorporeal circuit, HK-TEG showed no sign of clot formation R>60 min, MA=0 mm.

**CONCLUSION.** The tested prototype effectively removed the infused citrate required to anticoagulate 500 ml/min of extracorporeal blood flow.

**REFERENCE(S)**

1. This study was funded by the Italian Ministry of Health: Project Code: GR-2013-02356711; Project Title: Optimization of extracorporeal carbon dioxide removal through blood acidification: development of new technologies; Project Type: Young Researcher.

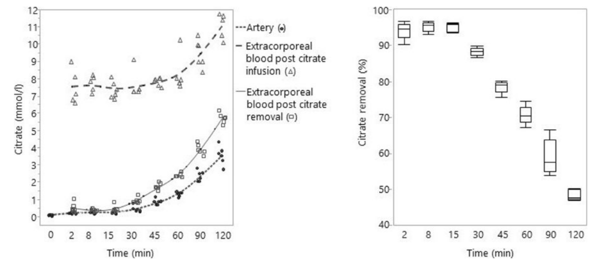

**Fig. 1 (abstract 001129).** See text for description

### 001138

#### Use of cardiac biomarkers as predictors of acute renal injury in cardiac surgery: Prospective Cohort Study

P. Palacios Moguel, OE. Palacios Calderon, A. Aisa Alvarez
Intensive care unit, The American British Cowdray Medical Center, Mexico City, Mexico
**Correspondence:** O.E. Palacios Calderon
*Intensive Care Medicine Experimental* 2019, **7(Suppl 3):**001138

**INTRODUCTION.** Acute kidney injury is common after cardiac surgery and is associated with postoperative mortality. Cardiac biomarkers are more accessible and can help to predict this complication.

**OBJECTIVES.** To investigate relationship between cardiac biomarkers and postoperatively acute kidney injury (AKI) in adults undergoing cardiac surgery1.

**METHODS.** Prospective cohort study in post-operated cardiac surgery adults. We measured the following cardiac biomarkers in the first 6 hours of the postoperative period: B-type natriuretic peptide (BNP), ultrasensitive troponin I (usTI), creatine phosphokinase (CPK), creatine phosphokinase MB (CPK-MB) and myoglobin.

**RESULTS.** 63 patients evaluated, 31 (49.2%) had AKI. Patients with AKI had significantly higher levels of BNP, usTI, CPK-MB, and myoglobin. Myoglobin had a good area under the curve (AUC 0.81, 95% CI 0.66-0.95), followed by usTI (AUC 0.71, 95% CI 0.58-0.92) and BNP (AUC 0.71 IC 95% 0.53-0.88) to detect AKI. Myoglobin levels ≥731 ng/mL had a positive likelihood ratio (7.73, 95% CI 1.04-57.24). the time of extracorporeal circulation (Adjusted Odds-ratio 6.11, 95% CI 1.55 to 9.55) and myoglobin elevation (Adjusted Odds-ratio 1.00, 95% CI 1.00 to 1.01) were independent predictors of AKI.

**CONCLUSION.** The postoperatory levels of cardiac biomarkers are significantly higher in patients with AKI. Extracorporeal circulation (CCE) time and myoglobin elevation were shown to be predictors of AKI. These biomarkers may be suitable for identifying patients at high risk for AKI after cardiac surgery.

**REFERENCE(S)**

1. Emilie P. Belley-Cote, MD, Chirag R. Parikh, MD, PhD, Colleen R. Shortt, et al. Association of cardiac biomarkers with acute kidney injury after cardiac surgery: A multicenter cohort study, The Journal of Thoracic and Cardiovascular Surgery c Volume 152, Number 1. 2016.

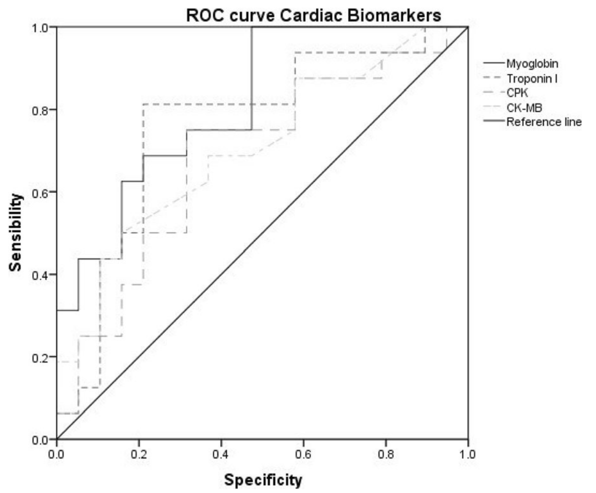

**Fig. 1 (abstract 001138).** See text for description

### 001145

#### Carbon dioxide production derived resting energy expenditure in pediatric continuous renal replacement therapy (CRRT)

M. Vega[1], T. Fogarty[2], P. Srivaths[1], T. Mottes[1], J. Angelo[1], JA. Coss-Bu[2], A. Akcan Arikan[3]
[1]Pediatric nephrology, Baylor College of Medicine, Houston, United States of America; [2]Pediatrics, critical care section, Baylor College of Medicine, Texas Children's Hospital, Houston, United States of America; [3]Pediatrics, critical care and nephrology section, Baylor College of Medicine, Texas Children's Hospital, Houston, United States of America
**Correspondence:** A. Akcan Arikan
*Intensive Care Medicine Experimental* 2019, **7(Suppl 3):**001145

**INTRODUCTION.** Indirect calorimetry (IC) is the gold standard for estimating energy needs in critically ill children. There is no reported IC data in children receiving CRRT. The modified-Weir equation using real-time carbon dioxide production (VCO2 REE) collected via standard clinical ventilators might more accurately define energy needs without the expense of IC or the inaccuracy of predictive equations.

**OBJECTIVES.** This pilot feasibility study investigates whether VCO2 REE could be used in pediatric CRRT patients.

**METHODS.** We investigated if a detectable change in VCO2 occurs after CRRT circuit start and compared daily VCO2 REE to calculated REE. Patients were classified as hypo or hyper metabolic based on the VCO2 REE/calculated REE ratio (<0.9, >1.1, respectively). Continuous VCO2 data were obtained from the Sickbay database (Medical Informatics Corp, Houston TX). Data were sampled prior to initiation of CRRT (baseline) and then every 24 hours for a total of five days or until ventilator discontinuation.

**RESULTS.** There was no difference in VCO2 between baseline and first hour of CRRT. Median VCO2 REE was 903 cal/d (IQR 554-1600) and calculated REE was 874 cal/d (IQR 606-1427) (n=9). There was a moderate correlation between baseline VCO2 REE and calculated REE (r=0.75, p=0.02) but this relationship was lost after day 2 of CRRT. Patients displayed dynamic characteristics throughout the study period transitioning between hypo and hypermetabolic. Hypometabolic patients had lower VCO2 REE (300 cal/d, 95% CI 125-475) and hypermetabolic patients higher (619 cal/d, 95% CI 440-799) than normometabolic patients. On any given day 1/3 of patients were hypermetabolic but individual patients varied daily. Longitudinal data were analyzed using mixed-effects multilevel modeling. Controlling for metabolic state (hypo or hyper) and baseline values, VCO2 REE increased daily by 44.7 cal (95%CI 20.2-69.1). Calculated REE overestimated VCO2 REE at baseline, and underestimated it after CRRT start (VCO2 REE baseline 594 cal/d (IQR 523-1299), day 5 927 cal/d (IQR 554-1251)). There was an interaction between CRRT day and metabolic state. Approximately 50% of the variation in VCO2 REE across the study population was due to patient level differences.

**CONCLUSION.** Pediatric CRRT patients have dynamic metabolic needs that are poorly approximated with predictive formulas. If validated in larger studies, VCO2 derived REE could bridge an important lacuna by addressing dynamic energy demands of pediatric CRRT patients, therefore, allowing for individualized nutrition prescriptions.

### 001209

#### Comparison of HES and PEGylated-carboxyhemoglobin on renal oxygenation and function in IR-induced AKI

B. Ergin[1], P. Guerci[2], C. Ince[3]
[1]Deaprtment of intensive care, Erasmus Medical Center, Amsterdam, Netherlands; [2]Département d'anesthésie-réanimation, institut lorrain du cœur et des vaisseaux, CHRU Nancy, Nancy, France; [3]Department of intensive care, Erasmus University Medical Center, Rotterdam, Netherlands
**Correspondence:** B. Ergin
*Intensive Care Medicine Experimental* 2019, **7(Suppl 3):**001209

**INTRODUCTION.** Ischemia/reperfusion (I/R) is one of the main causes of acute kidney injury (AKI), which is characterized by sterile inflammation and oxidative stress leading to perturbation of the microcirculation and tissue oxygenation.

**OBJECTIVES.** The primary outcome of this study was to improve microcirculatory perfusion and oxygenation by use of a hemoglobin-based oxygen carrier (PEG-COHb) or a colloid (HES-RA) to provide an improved organ perfusion, oxygenation and function in I/R induced renal microcirculatory injury in the rat.

**METHODS.** 36 fully instrumented, mechanically ventilated and anesthetized male *Wistar albino*rats were split into 4 groups consisting of control (C), I/R (45min. aortic occlusion and 1 hour reperfusion without resuscitation), I/R group resuscitated with either PEG-COHb or HES-RA at a dose of 5 ml.kg-1h-1. Systemic and renal hemodynamic, renal oxygenation, blood gas, electrolytes and blood chemistry were measured.

**RESULTS.** All of the fluids used in this study improved the MAP similarly compared to the C and I/R groups (p<0.05, respectively). CVP level was significantly increased by the PEG-COHb and in the IR group (p<0.05 vs. C) (Fig 1). Plasma lactate level was normalized only by HES-RA resuscitation (p<0.05 vs. C and I/R). RBF, cortical mPO2, Clcrea and urine output was depleted after the IR insult but was improved by PEG-COHb and HES resuscitation at T3 (p<0.05 vs. IR). DO2ren and VO2ren were increased by PEG-COHb and HES at T3 (p< 0.05 vs. IR) (Fig 2). PEG-COHband HES-RA provided efficient buffer capacity to maintain the plasma HCO3- and anion gap to correct the pH (p<0.05 vs. C and IR). Additionally, plasma proADMA, inflammation markers and tissue damage score were also comparable in between the PEG-COHb and HES-RA groups (p<0.05 vs. I/R).

**CONCLUSION.** To conclude, PEG-COHb and HES-RA showed similar beneficial effects on the systemic hemodynamic, renal blood flow, renal oxygenation and oxygen delivery resulting in better renal function.

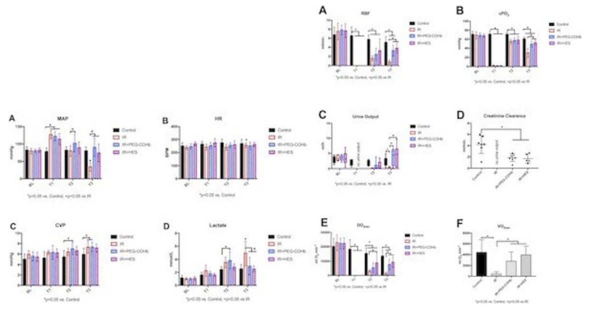

**Fig. 1 (abstract 001209).** See text for description

### 001212

#### The 6-h creatinine clearance in the ICU contributes to proper antibiotic dosage

E. Nanou[1], E. Tsigou[1], E. Boutzouka[1], T. Katsoulas[2], V. Psallida[1], V. Zidianakis[1], G. Fildissis[1]
[1]Icu, Agioi Anargiroi Hospital, Athens, Greece; [2]Nursing department, National & Kapodistrian University of Athens, Athens, Greece
**Correspondence:** E. Tsigou
*Intensive Care Medicine Experimental* 2019, **7(Suppl 3):**001212

**INTRODUCTION.** Seriously ill pts receive a great spectrum of antibiotics, whose dose is usually determined by estimated creatinine clearance (CrCl). Given the bad performance of the various equations for GFR estimation, we decided to evaluate calculated CrCl as a means to more objectively adjust antibiotics in ICU pts.

**METHODS.** We retrospectively compared 6-hour creatinine clearance (CrCl-6h), with the Cockroft–Gault equation (Ck-G) concurrently estimated, and recorded clinical characteristics and the changes in antibiotic dosage that were attributed to abnormal values of the parameters. CrCl was calculated as [Cr urine (mg/dl)× urine volume (ml)× / Crs (mg/dl)× minutes] and the Ck-G was calculated as [(140-age)× (weight)×(0.85 females) / 72× Crs].

Results are shown as mean±SD or as median and interquartile ratio. Comparisons between groups were made by Kruskal-Wallis test, applying SPSS 21.

**RESULTS.** In 47 randomly selected pts (age 71.57±12.44, male 22, APACHE II score 23±8.71, SOFA score 9.02±3.08, length of stay 19.89±15.03 days, mortality 40.4%) 89 recordings were evaluated. The Ck-G was 50.49 (47.36) ml/min and the CrCl-6h was 34.4 (37.37) ml/min. The therapeutic amendment according to calculated and estimated CrCl is shown in table 1.

**CONCLUSION.** The use of calculated 6-h led to antibiotics' dose amendment in almost half of cases, that in the majority had to do with dose reduction. Six-hour CrCl proved more reliable of the estimated CrCl, is easily measured regularly and can be used in order to avoid nephrotoxic doses of antibiotics or undertreatment of infections.

**Table 1 (abstract 001212).** See text for description

| | No modification N=50 (56.2%) | Dose reduction N=27 (30.3%) | Dose increase N=12 (13.5%) | p |
|---|---|---|---|---|
| CrCl-6h (ml/min) | 37.85 (52.13) | 27.93 (34.05) | 35.16 (29.13) | 0.03 |
| Ck-G (ml/min) | 50.18 (43.96) | 56.73 (42.26) | 31.52 (34.46) | 0.187 |
| Difference [(Ck-G) – (CrCl-6h)] | 5.68 (24.05) | 19.03 (26.55) | -9.33 (28.37) | < 0.001 |

### 001223

#### The Association between Mean Perfusion Pressure and Creatinine Serum In Intensive Care Unit

B. Lubis[1], P. Amelia[2], AH. Nasution[1], T. Hamdi[1]
[1]Anasthesiology department, Faculty of Medicine, Universitas Sumatera Utara, Medan, Indonesia; [2]Child health department, Faculty of Medicine, Universitas Sumatera Utara, Medan, Indonesia
**Correspondence:** B. Lubis
*Intensive Care Medicine Experimental* 2019, **7(Suppl 3):**001223

**INTRODUCTION.** Mean perfusion pressure (MPP) is the difference between mean arterial pressure (MAP) and central venous pressure (CVP). The difference in both pressures is also related to the patient mortality rate in the ICU. Patients with low MPP were also found to have high rates of renal failure. Patients with renal failure in the ICU have a high mortality rate as well. We expacted that by maintaining a normal MPP value, incidence of acute kidney injury decrease and the mortality rate will decrease. We hoped that calculating MPP can be an effective way to manage ICU patients and early detection for acute kidney injury (AKI). By measuring blood pressure and CVP we can easier to detect patient with acute kidney injury.

**OBJECTIVES.** The main purpose of this study is to assess for an association between Mean Perfusion Pressure, creatinine serum, and cumulative fluid balance in Intensive Care Unit (ICU).

**METHODS.** A cohort study was conducted from January to December 2017 in ICU Adam Malik Hospital Medan. Patients with age over 18 years admitted to our ICU were included. The demographic data, MPP, creatinine serum, and cumulative fluid balance were compared.

**RESULTS.** During the study period, 74 patients were admitted. 58.1% were male with mean age 45.5 ± 17.3 years old. There was association between Mean Perfusion Pressure with increasing creatinine serum in ICU (p=0.012). But there was no association between Mean Perfusion Pressure with cumulative fluid balance in ICU (p=0.203).

**CONCLUSION.** There was a correlation between Mean Perfusion Pressure and increasing of creatinine serum. Mean Perfusion Pressure can be a simple sign for acute kidney injury (AKI). Further study with a bigger sample is needed to explore the accuracy of MPP in ICU.

**REFERENCE(S)**

1. Ostermann M, Hall A, Crichton S. Low mean perfusion pressure is a risk factor for progression of acute kidney injury in critically ill patients – A retrospective analysis. BMC Nephrology. 2017; 18:151.
2. Patel R, Solanski A, Patel H, Pandya H, Patel J, Patel S. Does mean perfusion pressure during cardiopulmonary bypass affect renal function? J of Evidence Based Med & Hlthcare. 2015; 2(41): 6946-53.
3. Panwar R, Lanyon N, Davies AR, Bailey M, Pilcher D, Bellomo R. Mean perfusion pressure deficit during the initial management of shock—an observational cohort study. J Crit Care. 2013.
4. Amelia P, Lubis M, Mutiara E, Trisnawati Y. Acute kidney injury and mortality in critically ill children. Paediatr Indones. 2014;54:251-5.
5. Damman K, Deursen VM, Navis G, Voors AA, Veldhuisen DJ, Hillege HL. Increased central venous pressure is associated with impaired renal function and mortality in a broad spectrum of patients with cardiovascular disease. Journal of the American College of Cardiology. 2009; 53 (7): 582-8.
6. Boyd JH, Forbes J, Nakada T, Walley KR, Russell JA. Fluid resuscitation in septic shock: A positive fluid balance and elevated central venous pressure are associated with increased mortality. Critical Care Medicine. 2011; 39 (2):1-7.
7. Long Y, Su L, Zhang Q, Zhou X, Wang H, Cui N, et all. Elevated mean airway pressure and central venous pressure in the first day of mechanical ventilation indicated poor outcome. Critical Care Medicine. 2017; 1-8.
8. This research was supported by TALENTA 2019 from Universitas Sumatera Utara.

### 001265

#### Cardiorenal syndrome type 1 needing renal replacement therapy: a retrospective cohort study

TM. Huang[1], V. Wu[1], TS. Chu[2]
[1]Attending Physician, National Taiwan University Hospital and College of Medicine, Taipei City, Taiwan; [2]Professor, National Taiwan University Hospital and College of Medicine, Taipei City, Taiwan
**Correspondence:** T.M. Huang
*Intensive Care Medicine Experimental* 2019, **7(Suppl 3):**001265

**INTRODUCTION.** Cardiorenal syndrome type 1 (CRS-1) refers to syndromes denoting acute renal dysfunction caused by acute cardiac dysfunction or related to cardiac procedures. Patients with CRS-1 who need renal replacement therapy may suffer from a variety risk for adverse outcomes, including mortality and increased length of stays. It is unknown the impacts of the etiologies of CRS-1 on patients' outcomes.

**METHODS.** This was a nationwide sampling retrospective cohort study based on a clinical trial consortium in Taiwan. Up to January of 2016, 30 hospitals have joined this consortium. We enrolled all patients with acute dialysis in these hospitals and followed them up to 90 days after dialysis. The primary outcome was 90 days' all cause mortality and the secondary outcome was free from dialysis and length of stay.

**RESULTS.** A total of 1075 patients were in the final analysis. The mortality rates of patients with CRS-1 were listed in Table 1. Patients who were dialyzed for reasons other than CRS-1 had a mortality rate of 59.69%. After adjusting co-morbidities and acute physiology parameters, patients with CRS-1 after cardiogenic shock have a hazard ratio (HR) of 1.76 (1.06-2.93) to die within 90 days than patients without CRS-1; patients with acute decompensated heart failure (ADHF) had a more favorable 90 days' survival than non-CRS-1 patients, with an HR of 0.66 (0.46-0.96). Patients with cardiogenic shock were more likely to be dialysis dependence, with an HR of 1.78 (1.07-2.97). Patients with CRS-1 after cardiac surgery or ADHF were more likely to be off dialysis, [HR=0.53 (0.30-0.94) and 0.65 (0.45-0.94)]

**CONCLUSION.** Among patients with CRS-1 needing dialysis. The outcomes differed in terms of etiologies of cardiorenal syndrome. The discussion of CRS-1 outcomes should include the etiologies.

**Table 1 (abstract 001265).** 90 days' Outcomes of CRS-1 needing dialysis

| | 90 days' Mortality | 90 days' Free From RRT | Length of Stay (ICU) | Length of Stay (Hospital) |
|---|---|---|---|---|
| Cardiorenal Syndrome type 1 | 158 (40.5%) | 85 (29.11%) | | |
| Dissected aorta | 7 (53.85%) | 4 (30.77%) | 19.69 | 31.00 |
| Cardiac surgery | 12 (38.71%) | 14 (45.16%) | 25.87 | 33.03 |
| Cardiogenic shock | 16 (72.73%) | 4 (18.18%) | 19.55 | 16.36 |
| Cardiac arrest | 52 (73.23%) | 14 (19.72%) | 16.01 | 20.38 |
| Bradycardia | 3 (37.5%) | 8 (62.5%) | 3.88 | 14.25 |
| Acute decompensated heart failure | 32 (41.56%) | 28 (36.36%) | 14.49 | 24.09 |
| Acute coronary syndrome (ACS) | 36 (52.17%) | 16 (23.19%) | 12.09 | 16.71 |
| Non-Cardiorenal Syndrome type 1 | 468 (59.69%) | 214 (27.30%) | 19.7 | 26.40 |

### 001268

#### Early diagnosis of Acute Kidney Injury in patients with sepsis in the ED

D. Paz
Intensive Care Unit, Angeles Lomas Hospital, Mexico City, CDMX, Mexico, Mexico
*Intensive Care Medicine Experimental* 2019, **7(Suppl 3):**001268

**INTRODUCTION.** The incidence of sepsis and acute kidney injury in critical patients is gradually increasing, both indicate a poor prognosis with higher morbidity and mortality. Failure on acute diagnosis may delay the onset of an adequate therapy which may cause an unfavorable outcome, such as the need for renal replacement therapy, generating high costs to the health system. The validation of new methods that identify the presence of acute kidney injury in its early stages is needed.

**OBJECTIVES.** To demonstrate which is the earliest marker for the diagnosis of acute kidney injury in patients with sepsis, including creatinine, cystatin C, urinary sediment and N-GAL.

**METHODS.** A prospective, longitudinal and descriptive study is presented, with data obtained from patients over 18 years of age, with sepsis, who attended to the American British Cowdray Medical Center Emergency Department, between July 2017 and July 2018.

**RESULTS.**

Cylinders in urine and cystatin C had the same capacity to detect cases of acute kidney injury on admission (sensitivity 71.4% and specificity 77.8%).

N-GAL showed a high sensitivity (greater than 85%) and a specificity of 100% at hospital admission and also maintained a high sensitivity of 87.5%, although its specificity was reduced due to transitions between patients from sick to healthy.

**CONCLUSION.** In this study we found that N-GAL is the most reliable biomarker for the diagnosis and prognosis of acute kidney injury compared to creatinine, cystatin C and urinary sediment. Its urinary levels increased much earlier. Without being modified by the patient's previous condition.

**REFERENCE(S)**

1. I thank Alfredo Sierra MD, Jean Paul Vazquez MD and Erick Vidal MD for their support. Thanks for letting me be part of your ICU group at Angeles Lomas Hospital.
2. Dai X, Zeng Z, Fu C, Zhang SA, Cai Y, Chen Z. Diagnostic value of neutrophil gelatinase-associated lipocalin, cystatin C, and soluble triggering receptor expressed on myeloid cells-1 in critically ill patients with sepsis-associated acute kidney injury. Critical Care. 2015;19(1).
3. Medi| B, Rovcanin B, Vujovic KS, Obradovic D, Duric D, Prostran M. Evaluation of Novel Biomarkers of Acute Kidney Injury: The Possibilities and Limitations. Current Medicinal Chemistry. 2016;23(19):1981–97.
4. Schrezenmeier E, Barasch J, Budde K, Westhoff T. Biomarkers in acute kidney injury, pathophysiological basis and clinical performance. Acta Physiol. 2017Mar;213(3):554–72.
5. Beker M, Corleto M, et.al. Novel acute kidney injury biomarkers: their characteristics, utility and concerns. International Urology and Nephrology https://doi.org/10.1007/s11255-017-1781-x
6. Andreucci M, Faga T, Pisani A, Perticone M. The ischemic/nephrotoxic acute kidney injury and the use of renal biomarkers in clinical practice. European Journal of Internal Medicine. 2017;39:1–8.
7. Somma D, Magrini L. Additive value of blood neutrophil gelatinase-associated lipocalin to clinical judgement in acute kidney injury diagnosis and mortality prediction in patients hospitalized from the emergency department. Critical Care Medicine. 2013;17(1):186–91.
8. Kustan P, Horvath-Szalai Z, Muhl D. Nonconventional markers of sepsis. EJIFCC. 2017May1;2:122–33.
9. Deutschman CS, Singer M. Definitions for Sepsis and Septic Shock—Reply. Jama. 2016;316(4):801–10.
10. Levy MM, Evans LE, Rhodes A. The Surviving Sepsis Campaign Bundle. Critical Care Medicine. 2018;46(6):997–1000.
11. Kashani K, Cheungpasitporn W, Ronco C. Biomarkers of acute kidney injury: the pathway from discovery to clinical adoption. Clinical Chemistry and Laboratory Medicine (CCLM). 2017;55(8):1074–89.

**Table 1 (abstract 001268).** Baseline sociodemographic characteristics of the study patients

| Caracteristic | Total (n=23) | AKI present(n=14) | AKI absent(n=9) | p Value |
|---|---|---|---|---|
| Serum Creatinine(mg/dL) | 2.3±3.4 | 3.2±4.1 | 0.8±0.2 | <0.001 |
| Median (IQR) | 1.5 (0.8,2.1) | 1.75 (1.6,2.4) | 0.8 (0.6,0.9) | |
| N-GAL (ng/dL) | 1003.9±1738.5 | 1629.3±2006.8 | 31.3±16.9 | <0.01 |
| Median (IQR) | 125 (25,1485) | 801.5 (159.3,3103.0) | 25 (23,41) | |
| Cistatina C (mg/L) | 2.4±1.5 | 2.7±1.5 | 1.9±1.6 | 0.051 |
| Median (IQR) | 2.1 (1.2,3.1) | 2.5 (1.8,3.1) | 1.2 (0.9,1.7) | |
| Cilinders in urine | | | | 0.029 |
| Absent | 11 (47.8) | 4 (28.6) | 7 (77.8) | |
| Present | 12 (52.2) | 10 (71.4) | 2 (22.2) | |

*AKI* Accute Kidney Injury, *IQR* Interquartile range, *SD* Standard Deviation
Categorical variables are presented in frequency and percentages; Continuous variables are presented as mean ± SD. P value for the comparison between LRA present vs absent

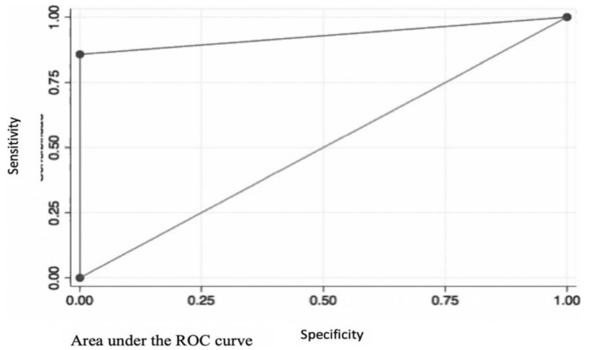

**Fig. 1 (abstract 001268).** Sensitivity and specificity. Area under the ROC curve for serum N-GAL in relation to the baseline diagnosis of acute renal injury

### 001299

#### The use of bioelectrical impedance vector analysis (BIVA) guide for fluid elimination in critically ill patients undergoing renal replacement therapy

P. Nuchpramool, R. Ratanarat, V. Viarasilpa
Dept of medicine, siriraj hospital, mahidol university, Division of Critical Care, Bangkok, Thailand
**Correspondence:** R. Ratanarat
*Intensive Care Medicine Experimental* 2019, **7(Suppl 3):**001299

**INTRODUCTION.** Fluid restriction and elimination during stabilization and de-escalation phases of fluid therapy are the keys to reduce mortality. Using bioelectrical impedance vector analysis (BIVA), the body-composition measuring equipment for excessive body fluid evaluation guiding the negative fluid balance, may reduce mortality in critically ills undergoing renal replacement therapy.

**OBJECTIVES.** To compare 28-day mortality between using BIVA-guided management and using standard clinical management. The secondary outcomes were ventilator-free days, ICU and hospital mortality, ICU and hospital length of stay, ventilator-associated pneumonia occurrence, incidence of hemodynamic instability during RRT (HIRRT) and renal recovery.
**METHODS.** In this randomized control trial, the medical intensive care unit (MICU) patients who had stable hemodynamics and required renal replacement therapy for fluid removal, were assigned to either the BIVA and the control groups. In both groups, the rate of fluid removal and vasopressor requirement were adjusted according to hemodynamics and clinical needs. Physician's fluid management decision depended on only standard clinical data for the control group, and on additional BIVA-derived values for the BIVA group in order to achieve %BIVA hydration of 72.4-74.3% and phase-angle of more than 5.5, respectively.
**RESULTS.** Of 36 enrolled patients, 17 patients (47.2%) were assigned to the BIVA group. Most of the reasons for MICU admission (77.8%) were sepsis/septic shock. Baseline characteristics of the patients in the two groups were similar. Mean APACHE II score at ICU admission in both groups were not different (30.5±12.5 in BIVA,29.8±8.2 in control, $p$ = 0.85). Before enrollment, both groups were comparable in percentage of fluid accumulation (%FA) [11.3% (6.3,21.5) in BIVA group, 10.7% (5.7,19.2) in control group, $p$= 0.66)]. Use of add-on BIVA to standard treatment did not provide beneficial effect in rate of fluid removal indicating by body weight and %FA. The mortality from any cause by day 28 was 47% (8/17) in BIVA and 52.6% (10/19) in control group ($p$ = 0.78). There was no statistically significant difference between the two groups in ICU and hospital mortality, ICU and hospital length of stay, and ventilator free day. For safety and complication, HIRRT and vasopressor requirements were comparable.
**CONCLUSION.** Using add-on BIVA guide fluid removal to standard therapy in critically-ill patient who required renal replacement therapy does not associate with 28-day mortality. Further studies are required due to small sample size in this study.

### 001345

#### Renal Biomarkers can predict Progression and maladaptive repair after AKI in Critically Ill Cirrhotics

R. Maiwall, P. Jain, S. Sarin
Hepatology, Institute of Liver and Biliary Sciences, New Delhi, India
**Correspondence:** R. Maiwall
*Intensive Care Medicine Experimental* 2019, **7(Suppl 3):**001345

**INTRODUCTION.** Bacterial infections and systemic inflammation(SIRS) are the major precipitants of organ failure including acute kidney injury (AKI) in critically ill(CIC) and predispose to increased progression to Chronic Kidney Disease(CKD). Timely identification of AKI is therefore an unmet need. Biomarkers can help in differential diagnosis and prediction of the trajectory of AKI course. We evaluated Cystatin C(CysC) and urinary neutrophil-gelatinase-associated lipocalin (NGAL) as biomarkers of glomerular and tubular injury respectively in predicting a progressive AKI and maladaptive repair in CIC.
**METHODS.** CIC admitted to the Liver ICU were prospectively followed up. Baseline serum creatinine, history of prior AKI (past 3 months) and precipitant for AKI was recorded at admission. Progressive AKI was defined at day 7 as an increase of serum creatinine by 0.3 mg/dl from baseline or percentage increase of more than 50% or an increase in AKI stage or requirement of dialysis.
**RESULTS.** A total of 519 CIC, mean age 48.4±11.4 years, 86% males were followed for 26(8-97) days. The median serum NGAL and CysC was 759(223-1230) ng/ml and 2.1(1.5-2.7)mg/l respectively. SIRS was seen in 84%, sepsis in 58%, prior AKI in 18%. AKI at admission was present in 60% (Stage 1:2:3 43%vs 14%vs 43%) of which 48% recovered and 52% had progressive AKI. Sepsis was the commonest (46%) cause of AKI. At day 7, 317(61%) patients had progressive AKI of which 51% had new onset AKI. Of these, 56% had HRS, 10% prerenal AKI and remaining had acute tubular necrosis (ATN). The levels of NGAL and CysC were higher in patients in sepsis vs non-sepsis AKI (< 0.001), correlated with the severity of AKI stage (p<0.001), response to terlipressin (p<0.001), and recovery of AKI (p<0.001) but could not differentiate HRS from ATN. On multivariate analysis (OR,95% CI), u-NGAL (2.53,1.6-4), Cyst C (1.9, 1.1-3.4) and prior AKI ( 3.15,1.36-7.3) significantly predicted progressive AKI which in-turn was associated with higher mortality (HR 2.32,95%CI 1.8-3.1). Further, of all patients with progressive AKI, 144(45%) had transition to chronic kidney disease which signifies maladaptive repair. The levels of NGAL(OR 1.9,1.3-2.9) and Cyst C(OR 1.9,1.3-2.5) could also predict the development of end stage renal disease (ESRD) in CIC.
**CONCLUSION.** Almost two-thirds of CIC have AKI at presentation, which in majority is a result of sepsis, is progressive and is associated with worse outcome. u-NGAL and CysC can accurately predict progression of AKI and maladaptive repair with development of ESRD, and can help in stratifying patients for early therapeutic intervention. Prior AKI itself predisposes to AKI progression in CIC and should be carefully assessed in readmissions.

### 001469

#### SARF-AKI: Severe acute respiratory failure related acute kidney injury, a large retrospective analysis

L. Teresa, J. Wilson, S. Matharu, S. Ledot, J. Doyle
Adult intensive care unit, Royal Brompton, London, United Kingdom
**Correspondence:** J. Doyle
*Intensive Care Medicine Experimental* 2019, **7(Suppl 3):**001469

**INTRODUCTION.** Veno-venous extracorporeal membrane oxygenation (VV-ECMO) in adults is an established therapy used to treat severe acute respiratory failure (SARF). Acute Kidney injury (AKI) is a major predictor of morbidity & mortality in critical illness, indeed a single episode of AKI confers an increased long-term mortality. Recently the characteristics of AKI induced by differing conditions has been better elucidated i.e. sepsis associated-AKI. Studies suggest renal replacement therapy (RRT) is required in 60% of patients on ECMO (1). However, to date there is a paucity of evidence in this area. Some data suggests the use of RRT in ECMO patients is not associated with an increased mortality (2). Whilst other data shows higher mortality & prolonged mechanical ventilation (3). There is no doubt AKI influences ECMO patient outcomes, we hope to add significantly to this emerging body of evidence.
**METHODS.** Retrospective analysis of consecutive patients on VV-ECMO for SARF over a 9 year period 01/2010-02/2019. Factors contributing to AKI, the need for RRT & duration of ECMO were recorded. Outcomes included; AKI stage by both creatinine and urine output criteria (as defined by KDIGO), need for RRT during the critical illness, persistent AKI on discharge and 28, 90 day & hospital mortality. These outcomes were further stratified by age group.
**RESULTS.** Data was obtained from 384 patients out of a total of 447 consecutive SARF patients from 01/2010 – 02/2019. Demographics as per (table 1). **Incidence of AKI**: As calculated by creatinine criteria; AKI1 32.8%, AKI2 9.6%, AKI3 52.3%. We also report the incidence of AKI as calculated by urine output criteria; AKI1 41.4%, AKI2 3.6%, AKI3 52.3%. **Need for RRT**: 50% of patients required RRT, with higher rates in increasing age groups (40.3% in <30s, 46% in 30-50 years and 58.6% in >50s). **Persistent AKI**: Overall, of the patients who received RRT and survived, 9.3% of them had persistent versus 0% for AKI 1/2. Hospital mortality: 24.4% for the 384 patients. **Mortality:** in patients who needed RRT was 61/192 (31.8%) compared with 15/66 (22.7%) in those with AKI 1,2&3 (without RRT) and 17/126 (13.5%) in those with no AKI. Mortality rates for ECMO patients with RRT compared to those without RRT are significant (p0.05). Mortality is further classified by age (table 2).
**CONCLUSION.** We report on one of the largest European data sets to date for VV-ECMO patients and for the first time on AKI as defined by KDIGO urine output criteria. The incidence of SARF-AKI for patients requiring ECMO is expectedly high. However reassuringly there is a relatively low occurrence of persistent AKI amongst ECMO survivors suggesting capacity for organ recovery. Additionally, the risk of mortality for those with AKI stage 1/2 is not different to those without AKI. RRT requirement confers a significantly increased mortality risk, this is compounded in younger age groups which is of concern given this patient population.

**REFERENCE(S)**

1. Chen et al, 2018. Long-term outcomes after extracorporeal membrane oxygenation in patients with dialysis-requiring acute kidney injury: A cohort study. PLoS ONE 14(3): e0212352. Chen S-W, Lu Y-A, Lee C-C, Chou A-H, Wu VC-C, Chang S-W, et al. (2019) First Published: March 13, 2019 https://doi.org/10.1371/journal.pone.0212352
2. Antonucci et al, 2016 The Impact of Renal Failure and Renal Replacement Therapy on Outcome During Extracorporeal Membrane Oxygenation Therapy. Artificial Organs; Volume40, Issue8. August 2016 Elio Antonucci, Irene Lamanna, David Fagnoul, Jean-Louis Vincent, Daniel De Backer, Fabio Silvio Taccone First published: 02 May 2016 https://doi.org/10.1111/aor.12695
3. Delmas et al, 2018 3-Month prognostic impact of severe acute renal failure under veno-venous ECMO support: Importance of time of onset. Journal of Critical Care 44 (2018) 63-71. https://doi.org/10.1016/j.jcr.2017.10.022
4. Nil funding received

**Table 1 (abstract 001469).** Demographics for total 384 Patients.

| | | Male | Female | Total | | | Male | Female | Total |
|---|---|---|---|---|---|---|---|---|---|
| Sex | | 220 | 164 | 384 | CKD Stages On admission | G1 | 61 | 62 | 123 |
| | | | | | | G2 | 42 | 21 | 63 |
| Age Group | <30 | 34 | 38 | 72 | | G3a | 21 | 20 | 41 |
| | 30-49 | 89 | 66 | 155 | | G3b | 31 | 28 | 59 |
| | >50 | 97 | 60 | 157 | | G4 | 55 | 27 | 82 |
| | | | | | | G5 | 10 | 6 | 16 |

**Table 2 (abstract 001469).** % Death per Age group and AKI Group.

| | <30 | 30-49 | >50 |
|---|---|---|---|
| No AKI | 2/34 (5.8%) | 6/55 (10.9%) | 9/37 (24.3% |
| AKI 1-3 | 0/9 (0%) | 9/29 (31%) | 6/28 (21.4%) |
| AKI with RRT | 29 (31%) | 10/71 (26.8%) | 33/93 (35.8%) |

*CI* confidence interval, *C/T* ceftolozane/tazobactam, *ITT* intent-to-treat, *MEM* meropenem, *SOFA* sequential organ failure assessment, *TOC* test-of cure
[a] Positive differences are in favor of C/T, negative differences are in favor of MEM. *b* Unstratified Newcombe CIs

## ARF - Acute respiratory failure 5

### 000193

#### Does Extracorporeal Membrane Oxygenation improve prognosis of Severe Acute Respiratory Distress Syndrome: A Systematic Review and Meta-analysis

D. xu
Department of critical care medicine, Zhongnan Hospital of Wuhan University, Wuhan, China
*Intensive Care Medicine Experimental* 2019, **7(Suppl 3):**000193

**INTRODUCTION.** The effect of extracorporeal membrane oxygenation (ECMO) on mortality in patients with severe acute respiratory distress syndrome(ARDS) remains controversial. To evaluate the use of ECMO for ARDS on prognosis in critically ill patients, we systematically reviewed the literature.

**OBJECTIVES.** Due to the heterogeneity of the study population and ECMO treatment, the existing clinical results of current studies on the prognosis of ARDS patients are inconsistent. Therefore, we use the quantitative evaluation method of meta-analysis to increase the total sample size and improve statistical efficiency.

**METHODS.** Databases from Pubmed, MEDLINE, EMBASE, and Cochrane were queried for studies on ECMO in patients with ARDS from 1948 through to November 2018. Databases from Pubmed, MEDLINE, EMBASE, and Cochrane were queried for studies on ECMO in patients with ARDS from 1948 through to November 2018.

**RESULTS.** Ten studies (three RCTs and seven observational studies) met our inclusion criteria in this meta-analysis, 1562 patients were included. Pooled analysis suggested that ECMO does not increase mortality in patients with ARDS in both the RCTs, (RR=0.83; 95%CI, [0.66-1.03]; P=0.32; $I^2$=12%) and the observational studies (RR=1.21 95%CI, [0.81-1.80]; P<0.01; $I^2$= 79%). These findings were also consistent across all subgroup analyses from observational studies. Four studies included data for the incidence of neurologic complications in the ECMO group, in which intracranial hemorrhage is the most frequent neurologic complication.

**CONCLUSION.** Our meta-analysis indicates that no statistically significantly higher mortality was associated with the application of ECMO in patients with ARDS. However, this finding largely relies on data from observational studies and is potentially subject to selection bias; hence, high-quality and adequately powered RCTs are warranted. Moreover, venovenous ECMO was also associated with a moderate risk of neurologic complications, and intracranial hemorrhage is rather common among patients receiving ECMO.

### 000211

#### Impact of mechanical power on mortality in patients with invasive mechanical ventilation

L. Delgado Baldazo, ACO. Antonio, HLA. Sánchez, TS. Otoniel, EC. Alejandro, SR. Esteban
Intensive care unit, Centro Medico Nacional La Raza, Atzcpotzalco, Mexico
**Correspondence:** L. Delgado Baldazo
*Intensive Care Medicine Experimental* 2019, **7(Suppl 3):**000211

**INTRODUCTION.** Protective mechanical ventilation (low tidal volume, plateau pressure <30 cmH2O, and high positive end expiratory pressure) has been proposed as a strategy to reduce the risk of ventilator induced lung injury (VILI). Mechanical power (MP) is a relatively new concept that takes into account variables that can cause VILI and that are not included in the protective strategy. Elevated MP could have a deleterious impact on the outcome of patients, so it should be monitored.

**OBJECTIVES.** To determine if elevated MP increases mortality in patients with invasive mechanical ventilation (MV).

**METHODS.** A prospective study was conducted in patients with MV for >24 continuous hours during their stay in the Intensive Care Unit (ICU). The MP was measured four times a day for the duration of the MV and the mean MP was calculated. Elevated MP was defined as a mean value >12 J/min. Mortality in the ICU was compared between patients with normal and elevated MP. The discriminative capacity for death of the mean MP was measured by analysis of the ROC curve. We identified independent risk factors for death through bi and multivariate logistic regression analysis. A p value <0.05 was considered statistically significant.

**RESULTS.** Eighty-seven patients were analyzed, 51.7% were males, mean age of 54.4 years. Twenty-nine (33.3%) patients had elevated MP. Mortality was significantly higher in patients with elevated MP (44.8%) compared to those with normal MP (8.6%), with p = 0.001. The area under the ROC curve of the MP for mortality was 0.799 (95%CI 0.674 - 0.923, p = 0.001). In the multivariate analysis, elevated MP showed RR of 1.460 (95%CI 1.091 - 1.954, p = 0.011).

**CONCLUSION.** The frequency of elevated MP is high. Mortality of patients with elevated MP is higher than that of those with normal MP. MP has adequate discriminative capacity to predict mortality and is an independent risk factor for death in patients with MV.

**REFERENCE(S)**

1. Pierson DJ. Patient-ventilator interaction. Respir Care. 2011 Feb;56(2):214-28.

2. Gramlich TA. Establishing the Need for Mechanical Ventilation. In: Cairo JM. Pilbeam's mechanical ventilation: Physiological and clinical applications. 5th ed. St. Louis, MO: Elsevier; 2012. pp. 48-62.
3. Schneider J, Sweberg T. Acute respiratory failure. Crit Care Clin. 2013 Apr;29(2):167-83.
4. Nieman GF, Satalin J, Andrews P, Aiash H, Habashi NM, Gatto LA. Personalizing mechanical ventilation according to physiologic parameters to stabilize alveoli and minimize ventilator induced lung injury (VILI). Intensive Care Med Exp. 2017 Dec; 5:8.
5. Rahaman U. Mathematics of ventilator-induced lung injury. Indian J Crit Care Med. 2017 Aug;21(8):521-24. doi: 10.4103/ijccm.IJCCM_411_16.
6. Tonetti T, Cressoni M, Collino F, Maiolo G, Rapetti F, Quintel M. Volutrauma, Atelectrauma, and Mechanical Power. Crit Care Med. 2017 Mar;45(3):e327-e328.
7. Cressoni M, Cadringher P, Chiurazzi C, Amini M, Gallazzi E, Marino A, et al. Lung inhomogeneity in patients with acute respiratory distress syndrome. Am J Respir Crit Care Med. 2014 Jan;189(2):149-58. doi: 10.1164/rccm.201308-1567OC
8. Marini JJ. Dissipation of energy during the respiratory cycle: conditional importance of ergotrauma to structural lung damage. Curr Opin Crit Care. 2018 Feb;24(1):16-22. doi: 10.1097/MCC.0000000000000470.

### 000214

#### Effects of levosimendan on diaphragm contractile efficiency in patients weaning from mechanical ventilation

L. Roesthuis[1], H. Van Der Hoeven[1], C. Sinderby[2], C. Ottenheijm[3], L. Brochard[4], J. Doorduin[5], L. Heunks[6]

[1]Department of intensive care medicine, Radboud University Medical Center, Nijmegen, Netherlands; [2]Keenan research centre for biomedical science - ibest - medicine, St. Michael's Hospital Li Ka Shing Knowledge Institute-Ryerson University-University of Toronto, Toronto, Canada; [3]Department of physiology - department of cellular and molecular medicine, Amsterdam UMC, locatie VUmc - University of Arizona, Amsterdam, Netherlands; [4]Interdepartmental division of critical care- university of toronto, Hospital St. Michael and Keenan research center, Toronto, Canada; [5]Donders institute for brain, cognition and behaviour, department of neurology, Radboud University Medical Center, Nijmegen, Netherlands; [6]Department of intensive care medicine, Amsterdam UMC, locatie VUmc, Amsterdam, Netherlands

**Correspondence:** L. Roesthuis

*Intensive Care Medicine Experimental* 2019, **7(Suppl 3):**000214

**INTRODUCTION.** Respiratory muscle weakness frequently develops in ventilated critically ill patients and is associated with adverse outcome, including difficult weaning from mechanical ventilation. Today, no drug is approved to improve respiratory muscle function in these patients. Previously, we have shown that the calcium sensitizer levosimendan improves calcium sensitivity of human diaphragm muscle fibers (from healthy and patients with chronic obstructive pulmonary disease) *in vitro* [1] and contractile efficiency of the diaphragm in healthy subjects *in vivo* [2].

**OBJECTIVES.** To investigate the effects of levosimendan on diaphragm contractile efficiency in mechanically ventilated patients.

**METHODS.** In a double-blind randomized placebo-controlled trial mechanically ventilated patients performed two continuous positive airway pressure (CPAP) trials for 30-minutes each with 5 hour interval. After the first CPAP trial, study medication (either levosimendan 0.2 µg/kg/min continuous infusion or placebo (equal volume)) was administered. During the CPAP trials electrical activity of the diaphragm (EAdi), transdiaphragmatic pressure (Pdi) and tidal volume (TV) were continuously measured. Neuromechanical efficiency (primary outcome parameter; computed as ΔPdi/ΔEAdi) and neuroventilatory efficiency (computed as TV/ΔEAdi) were calculated.

**RESULTS.** Thirty-nine patients were included in the study. Neuromechanical efficiency was not different during the CPAP trial after levosimendan administration compared to the CPAP trial before study medication (Figure 1). ΔEAdi and tidal volume were higher only after levosimendan administration, therefore resulting in no difference in neuroventilatory efficiency between the CPAP trial before and after study medication (Figure 1). ΔPdi was higher in both groups in the CPAP trial after study medication compared to the CPAP trial before study medication

**CONCLUSION.** Levosimendan does not improve diaphragm contractile efficiency in mechanically ventilated patients. The effects of levosimendan on weaning outcome needs to be established.

**REFERENCE(S)**

1. van Hees, H.W., P.N. Dekhuijzen, and L.M. Heunks, Levosimendan enhances force generation of diaphragm muscle from patients with chronic obstructive pulmonary disease. Am J Respir Crit Care Med, 2009. 179(1): p. 41-7.
2. Doorduin J, Sinderby CA, Beck J, Stegeman DF, van Hees HW, van der Hoeven JG, Heunks LM. The calcium sensitizer levosimendan improves human diaphragm function. American journal of respiratory and critical care medicine 2012; 185: 90-95.

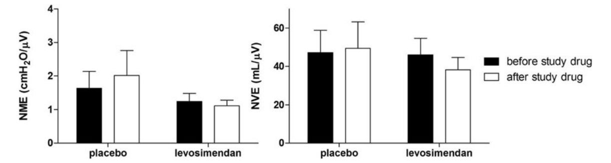

**Fig. 1 (abstract 000214).** See text for description

### 000221

#### The effect of high flow nasal oxygen in hematological malignancy patients with acute hypoxemic respiratory insufficiency: Prospective, single center, randomized controlled trial

N. Mendil[1], S. Temel[1], RC. Yüksel[1], K. Gundogan[1], B. Eser[2], L. Kaynar[2], M. Sungur[1]

[1]Division of critical care medicine, department of internal medicine, Erciyes University School of Medicine, Kayseri, Turkey; [2]Division of hematology, department of internal medicine, Erciyes University, Kayseri, Turkey

**Correspondence:** M. Sungur

*Intensive Care Medicine Experimental* 2019, **7(Suppl 3):**000221

**INTRODUCTION.** Acute respiratory failure is the leading cause for intensive care unit admission for hematological patients. High flow nasal canula devices (HFNC) can deliver oxygen up to 60 L/min with active heated humidification. HFNC was proved to be beneficial in various forms of acute respiratory failure patients.

**OBJECTIVES.** We hypothesized that HFNC is beneficial for mild respiratory insufficiency in hematological malignancy patients.

**METHODS.** The primary goal of the study to show if HFNC can decrease need for invasive and non-invasive ventilation as well as comfort VAS and dyspnea VAS. Eligible patients were hematological malignancy at our hematogy ward regardless of time from diagnosis and bone marrow transplant patients with signs of respiratory distress or labored breathing and SaO2 less than 92% for at least one hour on room air or PaO2/FiO2 less than 300 mmHg. All randomized patients were included into the final analysis. The patients were randomized to either standard medical treatment or HFNC treatment groups

**RESULTS.** Total of one hundred, 34 female and 66 male patients were included into the study. Mean age was 56±15. Mean APACHE II score was 17±5. The most common reason for respiratory insufficiency was pneumonia with 74 patients. HFNC group was consisted of 51 patients and standard treatment group 49 patients. Endotracheal intubation was required in 16 (33.3%) patients in standard group and 24 (48.0%) patients in HFNC group (p=0.15). A total of 20 (40.8%) patients in standard group and 21 (41.2%) patients in HFNC group received non-invasive mechanical ventilation (p=0.97). Median VAS comfort score, VAS dyspnea score and VAS thirsty score at 2nd and 24th hours were not different between groups. Baseline median P/F ratio was 276 (min-max: 190 – 295) in standard group and 257 (min-max: 209 – 295) in HFNC group (p=0.07). P/F ratios were similar between the groups throughout the study period. Median hospital length of stay was 36 (min-max: 3 – 130) days in standard group and 28 (min-max: 3 – 126) days in HFNC group (p=0.54). Twenty-eight day mortality rate was 18 (36.7%) in standard group and 23 (45.0%) in HFNC group (p=0.42).
**CONCLUSION.** HFNC is not superior to standard medical treatment in respiratory insufficient of hematological malignancy patients.

### 000243

#### Seven years after "The Berlin Definition of ARDS", what is our experience and what has it left to us?

A. Agrifoglio, L. Cachafeiro, E. Herrero, M. Hernández, M. Sánchez, JM. Añón, A. García De Lorenzo
Department of Intensive Care Medicine, Hospital La Paz-madrid, Madrid, Spain
**Correspondence:** A. Agrifoglio
*Intensive Care Medicine Experimental* 2019, **7(Suppl 3):**000243

**INTRODUCTION.** Acute respiratory distress syndrome (ARDS) is a life-threatening condition of critically ill patients, characterized by poor oxygenation and associated with capillary endothelial injury and diffuse alveolar damage. The proposed **"Berlin Definition of ARDS"**, published in 2012, predicted mortality ever-so-slightly better than the existing ARDS criteria (created at the 1994 by American-European Consensus Conference/AECC).
**OBJECTIVES.** The aim of this study was to find a new approach from the impact of ARDS using the Berlin Definition and to analyze APACHE II score, days of mechanical ventilation (MV), duration of ARDS and mortality in critical ill patients in an ICU of a University Hospital.
**METHODS.** We collected prospectively from January 2015 to December 2018 and regarded all patients undergoing MV for 48 hours or more and who met the Berlin criteria of ARDS. All patients were ventilated according to the *ARDS.Net protocol* and we focused the analysis on the **moderate** and **severe** ARDS, excluding the mild patients (corresponding to the old AECC definition of acute lung injury - ALI) with PaO2/FIO2 ≤ 300 mmHg. We also conducted a subgroup analysis between *survivors* and *non-survivors patients*.
**RESULTS.** During this period 2219 patients were admitted in the ICU, 757 were under MV for at least 48 hours and ***117 had ARDS criteria.*** Patients with **moderate ARDS** 60%: 55% male, median age 61 years, APACHE II mean 21.79 (95% CI=20.21-23.27), mean duration MV 21.84 days (95% CI=18.18-27.60), ARDS duration 10.87 days (95% CI= 7.83-13.90), average stay 26.88 days, mortality 51.4%. Patients with **severe ARDS** 40%: 66% male, median age 49 years, APACHE II mean score 23.79 (95% CI=20.62-25.75), mean duration MV 17.25 days (95% CI=13.01-24.03), ARDS duration 13.33 days (95% CI=8.72-15.58), average stay 21.65 days, mortality 48.8% with no statistically significant difference with moderate ARDS (*p*=0.72). Leading cause of death was refractory multiorgan failure (60%).
**CONCLUSION.** Using the Berlin criteria of our ARDS patients we found no statistically significant differences in relation to the variables of interest to analyze in the two groups. In the analysis by subgroups, patients with severe ARDS who survive at the ICU admission have a longer duration of ARDS, which means more days on MV and therefore increased ICU stay.

##### REFERENCE(S)

1. Ranieri VM, Rubenfeld GD, Thompson BT, et al. Acute Respiratory Distress Syndrome: The Berlin Definition. JAMA;307(23):2526-33.

**Table 2 (abstract 000243).** Subgroup analysis: Severe ARDS patients

| VARIABLE | SURVIVORS | NON-SURVIVORS | *p* value |
|---|---|---|---|
| APACHE II | 20.08 ± 8.88 | 25.33 ± 5.33 | 0.671 |
| MV (days) | 26.95 ± 18.01 | 11.22 ± 11.31 | **0.001** |
| ARDS (days) | 16.72 ± 10.11 | 8.22 ± 8.91 | **0.031** |
| ICU stay (days) | 33.12 ± 19.87 | 11.88 ± 9.61 | **0.001** |

**Table 1 (abstract 000243).** Subgroup analysis: Moderate ARDS patients

| VARIABLE | SURVIVORS | NON-SURVIVORS | *p* value |
|---|---|---|---|
| APACHE II | 21.08 ± 6.96 | 25.83 ± 6.11 | 0.006 |
| MV (days) | 26.53 ± 29.88 | 21.22 ± 14.33 | 0.577 |
| ARDS (days) | 12.09 ± 7.09 | 15.22 ± 11.88 | 0.108 |
| ICU stay (days) | 31.12 ± 29.87 | 25.88 ± 17.61 | 0.124 |

### 000282

#### Bench assessment of endotracheal tube clamping to avoid leak at the time of disconnection for changing an intensive care unit ventilator

E. Turbil[1], LM. Galerneau[2], B. Louis[3], C. Schwebel[2], C. Guérin[4], N. Terzi[2]
[1]Anesthesiology and intensive care, Università degli Studi di Sassari, Sassari, Italy; [2]Médecine intensive réanimation, C.H.U de Grenoble, La Tronche, France; [3]Inserm 955, IMRB, Creteil, France; [4]Service de réanimation médicale, hôpital de la croix rousse, Grande Rue de la Croix Rousse, Lyon, France
**Correspondence:** T. Emanuele
*Intensive Care Medicine Experimental* 2019, **7(Suppl 3):**000282

**INTRODUCTION.** Moving from ICU ventilator to transportation ventilator is commonly performed by clamping endotracheal tube (ETT) to prevent lung derecruitment. Whether or not this clamping is effective in preventing derecruitment has not previously been investigated.
**OBJECTIVES.** The primary objective of this bench study is to evaluate if this procedure is effective to prevent air leakage. A second objective is to evaluate the role of different clamps and ETT .
**METHODS.** Elysee 350 ventilator was connected via ETT cuffed inflated to a lung model set in passive condition with compliance 40 ml/cmH2O and resistance 10 cmH2O/L/s. Ventilator was set in volume-controlled mode, tidal volume 400 ml, respiratory rate 21 breaths/min and positive end expiratory pressure 15 cmH2O. Airway pressure (Paw) and flow were measured between ETT distal end and lung model and sent to Biopac 150 datalogger. Three kinds of ETT (nasal, oral, reinforced) of same 8mm internal diameter and three kinds of clamps (plastic, metallic, ECMO) were tested. A single tube was used once for each clamp. Absence of leak in the whole setup was checked. Then after 4 breaths, an end-expiratory occlusion was done during which ETT was clamped. Then, the ventilator was disconnected from the ETT whilst Paw and flow were continuously recorded for 30 sec. This procedure was repeated three times for every combination of ETT and clamp. Leak after disconnection was defined as flow in the expiratory direction and Paw drop. Primary endpoint was Paw decay during 5 sec after disconnection and secondary endpoint volume lost over this period. Data are shown as median (1st-

3rd quartiles) and analyzed by ANOVA and pairwise comparisons with Holm procedure.

**RESULTS.** Every ETT-clamp combination was leaky. The magnitude of leak is shown in table 1. There was a significant effect of both ETT and clamp and a significant interaction between them. ECMO clamp performed better than the two others for the nasal ETT only. For the two other ETT there were no statistical difference between ECMO and metallic clamp. Plastic clamp had the worst performance in every case.

**CONCLUSION.** Any clamping ETT procedure was leaky. ECMO clamp performed the best.

**REFERENCE(S)**

1. University of Lyon
2. University of Sassari

**Table 1 (abstract 000282).** Decay in Paw and volume lost 5 seconds after disconnection of ETT once clamped across ETT and clamps

| ETT* ** | Clamp* ** | Paw decay (cmH2O/s) | Volume lost (L) |
|---|---|---|---|
| Nasal | ECMO | 0.00 (-0.06;0.04) | -0.027(-0.028; -0.026) |
| | metallic | -3.35 (-3.43;-3.16) † | -0.13 (-0.15; -0.12) † |
| | plastic | -14.62(-14.66;-14.40)†$ | -0.65(-0.65; -0.64)†$ |
| Normal | ECMO | 0.00 (-0.73;0.08) | -0.030 (-0.056; 0.03) |
| | metallic | -3.105 (-3.33;-2.23) | -0.13 (-0.13;-0.096) |
| | plastic | -15.525(-17.77;-14.99)†$ | -0.72 (-0.79;-0.68) †$ |
| Reinforced | ECMO | -0.37 (-0.39;-0.37) | -0.042(-0.042;-0.040) |
| | metallic | -1.39 (-1.63;-1.06) | 0.070 (-0.077;-0.060) |
| | plastic | -17.49(-17.99;-17.078)†$ | -0.84 (-0.85;-0.79) †$ |

*P<0.001 ** P<0.001 for interaction between ETT and clamp. † P<0.001 vs. ECMO $P<0.001 vs.metallic

### 000295

#### Changes in tracheal intubation practice and complications over a ten-year period in an Intensive Care Unit in Scotland

AA. Ahmad, S. Elawad, Y. Neil, R. David

Intensive care, Royal Infirmary Edinburgh, Edinburgh, United Kingdom

**Correspondence:** S. Elawad

*Intensive Care Medicine Experimental* 2019, **7(Suppl 3):**000295

**INTRODUCTION.** A nationwide study performed in Scotland in 2009 reported the practice and complications of tracheal intubation in Intensive Care Units (ICU)1. Since then new guidelines 2,3 and trials 4 have been published. Anecdotally we have noticed changes in practice in our unit related to drug choice and use of videolaryngoscopy. To determine the extent of change we performed a pilot study in the Edinburgh Royal Infirmary, Scotland (RIE).

**OBJECTIVES.** To determine how intubation practice has changed in our ICU since 2009 and whether such change might warrant repeating a nationwide study.

**METHODS.** We conducted a prospective, observational study in critically ill adult patients requiring tracheal intubation over a three month period in 2018. We used an anonymised proforma to collect data including; timing and urgency of intubation, drugs used, number of attempts and complications. We used RIE specific data from the 2009 study for comparison and the Chi-square test for statistical analysis.

**RESULTS.** Data from 45 intubations were analysed. Forty-three intubations (95%) were performed by doctors with more than 24 months anaesthetic experience. Intubation was successful at the first attempt in 42 (89%). There was a significant change in drugs used for induction (p<0.001) and paralysis (p=0.03); in 2009 propofol (61%) and suxamethonium (58%) were commonest, and in 2018 ketamine (36%) and rocuronium (91%) were commonest [Figure1]. Use of videolaryngoscopy increased significantly from 0.7% in 2009 (nationwide data) to 27% in 2018 [Figure2, p<0.0001]. Severe hypoxaemia (SpO2<80%) occurred in significantly fewer patients in 2018 (9.5% v 22%, p=0.03), and hypotension appeared to occur less frequently but this was not statistically significant (16% v 21%, p=0.54) [Figure3].

**CONCLUSION.** Drugs used for intubation and laryngoscopy technique have changed, and intubation-related morbidity has decreased significantly in our ICU in the past ten years. Our pilot study suggests that a nationwide study should be repeated.

**REFERENCE(S)**

1. Casey JD, Janz DR, Russell DW, et al. Bag-mask ventilation during tracheal intubation of critically ill adults. N Engl J Med 2019; 380:811-821
2. Frerk C, Mitchell VS, McNarry AF, et al. Difficult Airway Society 2015 guidelines for management of unanticipated difficult intubation in adults. Br J Anaesth 2015;115:827e48
3. Higgs A, McGrath BA, Goddard C, et al. Guidelines for the management of tracheal intubation in critically ill adults. Br J Anaesth 2018;120:323-352
4. Simpson GD, Ross MJ, McKeown DW, Ray DC. Tracheal intubation in the critically ill: a multi-centre national study of practice and complications. Br J Anaesth 2012;108:792–799
5. No grants have been awarded for this study

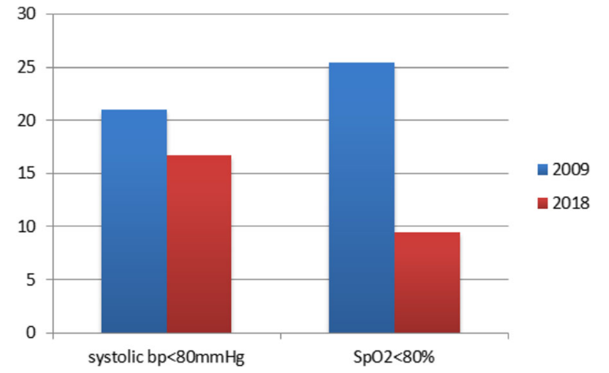

**Fig. 1 (abstract 000295).** See text for description

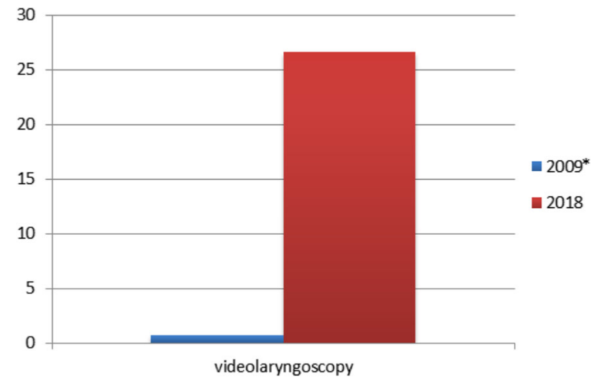

**Fig. 2 (abstract 000295).** See text for description. *nationwide 2009 data

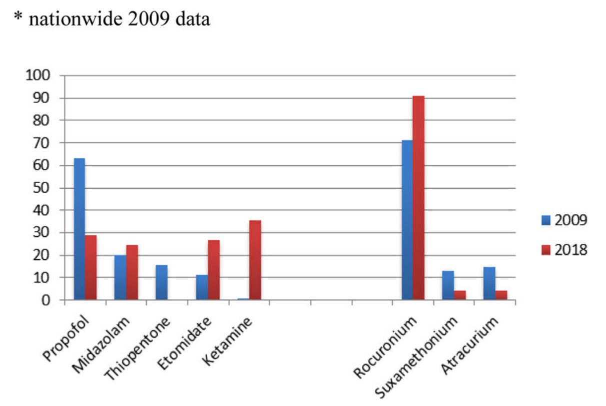

**Fig. 3 (abstract 000295).** See text for description

### 000296

#### Improving non-invasive ventilation practice in St John's Hospital

S. Elawad[1], O. Fiona[2]
[1]Intensive care, Royal Infirmary Edinburgh, Edinburgh, United Kingdom; [2]Respiratory medicine, St John's Hospital at Howden, Howden, United Kingdom
**Correspondence:** S. Elawad
*Intensive Care Medicine Experimental* 2019, **7(Suppl 3):**000296

**INTRODUCTION.** Acute exacerbation of chronic obstructive pulmonary disease (COPD) is one of the most common reasons for hospital admissions in the UK. Non-invasive ventilation (NIV) has revolutionised the management of acute hypercapnic respiratory failure and has become one of the major advances in medicine for the last decades given that it is used correctly with effective settings. Recently released BTS and NCEPOD guidelines showed the delivery of NIV in the acute setting is frequently sub-optimal [1,2]. This was also the case in St John's Hospital.

In St John's Hospital NIV is managed by medical staff in high dependency area which is part of the medical admission unit. Our aim was to audit the current practice in comparison with the British Thoracic Society (BTS) quality standards [2] focusing on and aiming to improve ventilator's settings. Also, we aimed to formalise a hospital protocol for NIV. We have also looked at our escalation plan in treatment failure.

**METHODS.** For each round, non-identifiable patient data was collected prospectively aiming for at least 20 patients. We obtained verbal consent to look in to patient's notes. We used quality improvement methodology and created an audit tool using the BTS template example. The first round was carried out between November 2017 and February 2018 where we collected the data for 20 patients and analysed the results. We presented the findings in our local hospital medical teaching day and introduced the hospital protocol. We collected the data in the second round between February and July 2018 for 18 patients and analysed the results.

**RESULTS.** The total number of patient's data collected in both rounds 38. In both rounds patient mix was similar. We have been consistently managing to obtain CXRs and initial blood gases before starting non-invasive ventilation 38 (100%). In the first round 13 (65%) reached recommended setting of IPAP 16-22 compared to 14 (78%) in the second round. On the other hand escalation decisions were better in the first rounds were 18 (90%) of patients had a resuscitation decisions made and 14 (80%) had clear escalation plans before starting NIV compared to 15 (83%) in the second rounds.

**CONCLUSION.** Having a protocol to guide the use and modification of NIV settings along with ensuring the medical and nursing staffs' familiarity with the protocol helps in providing effective care. There is still scope for improvement in escalation decisions. Also, one of the issues we encountered was effective documentation which would be a further project to undertake.

**REFERENCE(S)**

1. BTS/ICS Guidelines for the Ventilatory Management of Acute Hypercapnic Respiratory Failure in Adults April 2016.
2. NCEPOD Report: Inspiring Change - A review of the quality of care provided to patients receiving acute non-invasive ventilation July 2017
3. British Thoracic Society Quality Standards for acute non-invasive ventilation in adults Feb 2018.
4. No grants have been awarded for this study

### 000310

#### Bronchoscopy in critical care: experience in 443 procedures in Hospital Italiano de Buenos Aires

I. Carboni Bisso, C. Videla, S. Di Stefano, M. Las Heras, JM. Dianti, E. San Román
Unidad de terapia intensiva adultos, Hospital Italiano de Buenos Aires, Ciudad Autónoma de Buenos Aires, Argentina
**Correspondence:** I. Carboni Bisso
*Intensive Care Medicine Experimental* 2019, **7(Suppl 3):**000310

**INTRODUCTION.** Bronchoscopy is frequently performed in intensive care units (ICU) for therapeutic and diagnosis purposes. The technique is relatively easy to perform bedside, poses few complications and avoids potentially dangerous patient transfer outside the ICU. As a result, bronchoscopy is presently considered to be an essential tool in critical care.

**OBJECTIVES.** Assessment of main indications, clinical outcomes and complications associated with bronchoscopy in critically ill patients.

**METHODS.** A retrospective, single-center observational study over a period of 2 years (2017- 2019) was carried out in 215 patients undergoing bronchoscopy during their stay in the ICU.

**RESULTS.** A total of 443 bronchoscopies were carried out. Most of the procedures (86%) were performed in mechanically ventilated patients. The average patient age was 52.1 years. The most frequent indication was flexible bronchoscopy (FB) to perform the diagnosis confirmation of initially suspected pneumonia (38.8% [172]), with positive bronchoalveolar lavage (BAL) findings in 25.3%, followed by endoscopic control of percutaneous tracheostomy confection (31.6% [140]), direct evaluation of the airway (17.6% [78]) and bronchial toilette (9.0% [40]). Other indications, such as rigid bronchoscopy in a patients with acute tumor obstruction of the bronchial tree (1.6% [7]) and pulmonary cryobiopsy (1.4% [6]) were performed. Main complication associated with the procedure was bleeding (3.6% [16]). In most of the cases it was considered mild bleeding (2.0% [9]) and did not require any particular treatment for their control. Minor complications were transient hypoxemia (0.7% [3]) and hypotension (0.5% [2]). No serious events such as pneumothorax, arrhythmias or hemodynamic collapse were recorded.

**CONCLUSION.** Microbiological diagnosis of pneumonia with BAL and endoscopic control of percutaneous tracheostomy are the most frequent indications in critically ill patients. FB performed by an intensivist in ICU is a safe procedure, with low incidence of complications.

**REFERENCE(S)**

1. Ranzani OT, Senussi T, Idone F, et al. Invasive and non-invasive diagnostic approaches for microbiological diagnosis of hospital-acquired pneumonia. Critical Care. 2019;23(1). doi:10.1186/s13054-019-2348-2

2. Mehta Y, Mehta C. Percutaneous tracheostomy. Annals of Cardiac Anaesthesia. 2017;20(5):19. doi:10.4103/0971-9784.197793
3. Dellinger RP, Bandi V. Fiberoptic Bronchoscopy in The Intensive Care Unit. Critical Care Clinics. 1992;8(4):755-772. doi:10.1016/s0749-0704(18)30224-0
4. We would like to thank Fundación de Cuidados Críticos del Hospital Italiano de Buenos Aires (FCC-HIBA), Victoria Ruiz, Ignacio Fernández Ceballos, Ana Montserrat Rivera, Manuel Tisminetzky and Servicio de Neumonología del Hospital Italiano for their invaluable collaboration in this work.

### 000311

#### Supportive interventions to improve PaO2/FiO2 ratio in neurologically dead lung donors

S. Demers-Marcil[1], AJ. Frenette[2], M. Albert[2], P. Bellemare[2], F. Bernard[2], YA. Cavayas[2], AM. Lagacé[3], K. Serri[2], V. Williams[2], D. Williamson[2], P. Marsolais[2], E. Charbonney[2]
[1]Pneumology, Université de Montréal, Montreal, Canada; [2]Critical care, Hôpital du Sacré-Coeur de Montreal, Montréal, Canada; [3]Nursing management, Hôpital du Sacré-Coeur De Montréad, Montreal, Canada
**Correspondence:** S. Demers-Marcil
*Intensive Care Medicine Experimental* 2019, **7(Suppl 3):**000311

**INTRODUCTION.** Pulmonary transplantation is life changing for patients with incurable lung disease. In potential donors, several supportive interventions are performed, in order to improve the yield of lungs available for transplantation. One purpose of these interventions is to reverse pulmonary atelectasis associated with the brain death process and to improve oxygenation; PaO2/FiO2 (P/F) ratio is a key parameter in the acceptability of donor's lungs. Protective ventilation and recruitment manoeuvres are two interventions that have already shown some benefit (1,2).
**OBJECTIVES.** Our objective was to describe the ventilatory and recruitment interventions performed in potential lung donors in our centre and their association with P/F ratio improvement.
**METHODS.** We performed a retrospective study between June 2013 and May 2018 at the Sacré-Cœur Hospital organ procurement centre. For this first analysis, we included all neurologically dead donors who successfully donated their lungs. We used data from our prospective database and completed data collection retrospectively from medical records. We recorded data on interventions potentially influencing P/F ratio: duration of donor support, initial tidal volume, positive end-expiratory pressure (PEEP) applied, recruitment manoeuvres, bronchoscopies, intrapulmonary percussive ventilation, and the use of diuretics. We calculated the P/F ratio (at 100% FiO2) and static lung compliance changes for each patient, between the neurological death (ND) and the last value before lung harvesting.
**RESULTS.** Of 268 potential donors, 222 were declared neurologically dead and 184 had organs harvested. We included 89 patients in whom lungs were retrieved for transplantation. Mean age was 46±18 years and 37% were women. Mean duration of support was 33±20h and half of them received at least one dose of diuretic. Initial protective tidal volume (≤8mL/kg predicted body weight) was applied to 42% of patients and at least one recruitment manoeuvre (median 2 [0 -10]) was performed in 69% of them. The median PEEP was 8 (range 5-12). Median number of bronchoscopies post ND was 1 (range 1-8) and 50 patients (56%) received intrapulmonary percussive ventilation. Between the ND and harvesting, the median change in compliance was 4 mL/cmH2O (range -25 to 48) and the median change in P/F ratio was 93 (range -176 to 277). Exposure to percussive ventilation, adjusted for age, PEEP, protective ventilation and the number of recruitments, was the only intervention independently associated with P/F ratio improvement (OR: 4.5, 95% CI 1.7-11.6).
**CONCLUSION.** In our institution, less than half of lung donors were ventilated with protective tidal volume. Percussive ventilation was associated with significant improvement in oxygenation. Further prospective studies are needed to validate these findings.

**REFERENCE(S)**
1. Miñambres E. J Heart Lung Transplant. 2014 Feb;33(2):178-84
2. Mascia L. JAMA. 2010 Dec 15;304(23):2620-7.

### 000312

#### Respiratory mechanics under paralysis and spontaneous breathing in mechanically ventilated ARDS/acute hypoxemic respiratory failure patients

WC. Lin, CC. Hsieh, JC. Lee, CW. Chen
Division of critical care medicine, department of internal medicine, National Cheng Kung University Hospital, Tainan, Taiwan
**Correspondence:** C.W. Chen
*Intensive Care Medicine Experimental* 2019, **7(Suppl 3):**000312

**INTRODUCTION.** Accurate measurement of respiratory mechanics is important because end-inspiratory plateau pressure (Ppla), positive end-expiratory pressure (PEEP) and tidal volume (Vt) remained the core parameters to be monitored in mechanically ventilated ARDS patients. It is easy to have reliable measurement of respiratory mechanics when patients are paralyzed but may be problemic when respiratory efforts are present. A clean Ppla was proposed to be trustable. This proposal was not validated in mechanically ventilated ARDS or acute hypoxemic respiratory failure patients under pressure control ventilation (PCV).
**OBJECTIVES.** A crossover study for comparison of respiratory mechanics measured during paralysis and spontaneous breathing in mechanically ventilated patients under PCV
**METHODS.** Twenty-four patients with ARDS or acute hypoxic respiratory failure were included in this study over a two-year period. Esophgeal pressure monitoring was placed in all cases. Tidal volume (Vt), total PEEP (PEEPt), airway driving pressure (ΔP), transpulmonary driving pressure (ΔPL), respiratory system compliance (Crs), lung compliance (CL), chest wall compliance (Ccw) were calculated with performance of end-inspiratory occlusion. A clean Ppla was defined as no steadily increasing or decreasing Ppla and Ppla with periodic oscillations less than 2–3 cmH2O. Data are presented as mean ± SD. Mann Whitney test and Spearman's rank correlation test were used for statistical analysis.
**RESULTS.** Fourteen patients have reliably measured respiratory mechanics during both paralysis and spontaneous breathing under PCV. Respiratory mechanics measured under paralysis and spontaneous breathing were: Vt: 468 ± 77 vs. 519 ± 90 ml, p = 0.11; PEEPt: 11.4 ± 3.4 vs. 10.2 ± 2.2 cmH2O, p = 0.42; ΔP : 12.4 ± 2.2 vs. 13.0 ± 2.0 cmH2O, p = 0.60; ΔPL: 8.5 ± 2.1 vs. 9.6 ± 2.7 cmH2O, p = 0.28; Crs: 38.9 ± 8.5 vs. 41.1 ± 9.6 ml/cmH2O, p = 0.70; CL: 58.8 ± 19.2 vs. 58.8 ± 19.9 ml/cmH2O, p = 0.87; Ccw: 130.3 ± 40.2 vs 213.1 ± 179.0 ml/cmH2O, p = 0.12. Spearman's rank correlation test revealed high correlation in measured Crs (r = 0.85, p < 0.0001) and CL (r = 0.94, p < 0.0001) between paralysis and spontaneous breathing in individual patient, but poor correlation for Ccw (r = 0.433, p = 0.12).
**CONCLUSION.** Our findings suggest a clean Ppla during PCV is reliable for measurement of Crs and CL, but not for Ccw in spontaneously breathing patients.

**REFERENCE(S)**
1. Vaporidi K, Prinianakis G, Georgopoulos D and Guérin C (2019) Assessment of respiratory mechanics during pressure support ventilation? Caution required. Intensive Care Med (2019) 45:299–300.
2. Bellani G, Graselli G, Teggia-Droghi M, Mauri T, Coppadoro A, Brochard L and Pesenti A (2016) Do spontaneous and mechanical breathing have similar effects on average transpulmonary and alveolar pressure? A clinical crossover study. Critical Care (2016) 20:142
3. These cases are enrolled in the BEARDS project

### 000314

#### Patient-ventilator asynchrony in brain-injured patients: a prospective observational study

L. X-Y, H. X, JX. Zhou
Critical care medicine, Capital Medical University, Beijing, China
**Correspondence:** J.X. Zhou
*Intensive Care Medicine Experimental* 2019, **7(Suppl 3):**000314

**INTRODUCTION.** It has been reported that patient-ventilator asynchrony is associated with adverse clinical outcomes [1]. However, asynchrony is poorly defined in brain-injured patients. This study aimed to investigate the incidence and risk factors of asynchrony in this population.

**METHODS.** In this prospective observational study (NCT03212482), adult patients with brain injury and mechanical ventilation were included. Exclusion criteria were age < 18 yrs, without spontaneous breathing, and presenting with agitation. After enrollment, esophageal pressure (Pes) monitoring was established using standard method [2]. A dedicated acquisition system was used to obtain data of flow, airway pressure and Pes, which were collected for 15 min at 6-h intervals for 72 h. Four types of asynchrony were confirmed by Pes tracing according to previous reported definitions, which included ineffective-trigger, double-trigger, premature-cycle and delayed-cycle [1]. The asynchrony index was calculated as previous report [3]. Fifteen-min recording datasets with asynchrony index ≥ 10% were defined as asynchronous datasets, whereas patients with asynchrony index ≥ 10% for the sum of all datasets were defined as asynchronous subjects. Demographics data and type of injury were documented. Prior to each recording period, data of ventilator settings, blood gas, level of consciousness and the use of sedation and paralysis were collected. Clinical outcomes were followed up to hospital discharge or death. Comparisons between the asynchronous and non-asynchronous datasets and subjects were performed using t test or Mann-Whitney U test, as appropriate.

**RESULTS.** We enrolled 30 patients with mean age of 53 ± 17 yrs and 60% male. A total of 311 fifteen-min datasets were collected yielding 87,260 breaths, in which 11,601 (13.3%) asynchronous breaths were identified. The most common type of asynchrony was ineffective-trigger (61.8% of total asynchronous breaths), followed by delayed-cycle (21.9%), premature-cycle (12.8%) and double-trigger (3.5%). There were 91 (29.3%) datasets with asynchrony index ≥ 10%, and 11 (36.7%) patients had total asynchrony index ≥ 10%. A more severe consciousness impairment, more frequent use of synchronized intermittent mandatory ventilation, higher tidal volume and respiratory rate, and more frequent use of sedation and paralysis were found in the asynchronous datasets (Table 1). More patients with traumatic brain injury was found in the asynchronous subjects (Table 2). Adverse clinical outcomes were found in asynchronous subjects (Table 2).

**CONCLUSION.** Patient-ventilator asynchrony was prevalent among brain-injured patients. Level of consciousness, and the ventilator mode and settings, as well as injury type were associated with asynchrony. Clinical outcomes were worsen in asynchronous patients.

##### REFERENCE(S)

1. Pham T, Telias I, Piraino T, et al. Asynchrony consequences and management. Crit Care Clin 2018; 34: 325-41.
2. Sun XM, Chen GQ, Huang HW, et al. Use of esophageal balloon pressure-volume curve analysis to determine esophageal wall elastance and calibrate raw esophageal pressure: a bench experiment and clinical study. BMC Anesthesiology 2018; 18: 21.
3. Thille AW, Rodriguez P, Cabello B, et al. Patient-ventilator asynchrony during assisted mechanical ventilation. Intensive Care Med 2006; 32: 1515-1522.
4. The work is supported by a grant from the Beijing Municipal Science and Technology Commission (No. Z161100000116081).

**Table 1 (abstract 000314).** Variables in asynchronous and non-asynchronous datasets

| Variables | Asynchronous datasets (n=91) | Non-asynchronous datasets (n=220) | p |
|---|---|---|---|
| GCS≤8, n (%) | 41 (45) | 90 (41) | 0.000 |
| SAS | 3 (3, 3) | 3 (3, 3) | 0.793 |
| MV modes with SIMV, n (%) | 29 (32) | 33 (15) | 0.024 |
| Ventilator settings and monitoring | | | |
| Tidal volume, ml | 510 (443, 600) | 470 (418, 560) | 0.033 |
| Respiratory rate, breaths/min | 16 (15, 19) | 15 (12, 17) | 0.000 |
| PEEP, $cmH_2O$ | 5 (5, 6) | 5 (5, 7) | 0.446 |
| Arterial blood gas | | | |
| pH | 7.47 (7.44, 7.50) | 7.45 (7.38, 7.51) | 0.181 |
| $PaO_2$, mmHg | 116 (87, 146) | 123 (93, 179) | 0.192 |
| $PaCO_2$, mmHg | 35 (33, 38) | 36 (30, 40) | 0.937 |
| Sedation and paralysis, n (%) | 31 (14) | 30 (33) | 0.000 |

Continuous data are expressed as median (interquartile range).
GCS=Glasgow Coma Scale; SAS=Sedation Agitation Scale; MV=mechanical ventilation;
SIMV=synchronized intermittent mandatory ventilation; PEEP=positive end-expiratory pressure;
$PaO_2$=partial pressure of oxygen in arterial blood; $PaCO_2$= partial pressure of carbon dioxide in arterial blood

**Table 2 (abstract 000314).** Variables in asynchronous and non-asynchronous subjects

| Variables | Asynchronous subjects (n=11) | Non-asynchronous subjects (n=19) | p |
|---|---|---|---|
| Age, yrs | 63±21 | 52±18 | 0.112 |
| Male, % | 7 (63.6) | 11 (57.9) | 0.534 |
| MV modes with SIMV, n (%) | 29 (32) | 33 (15) | 0.024 |
| GCS at study entry | 510 (443, 600) | 470 (418, 560) | 0.033 |
| Type of injury, n (%) | | | 0.014 |
| TBI | 2 (18.2) | 2 (10.5) | |
| Stroke | 3 (27.3) | 5 (26.3) | |
| After elective craniotomy | 5 (45.5) | 11 (57.9) | |
| Other | 1 (9.1) | 1 (5.3) | |
| MV duration, days | 6 (4, 7) | 5 (4, 6) | 0.042 |
| ICU LOS, days | 8 (7, 10) | 8 (6, 9) | 0.033 |
| ICU mortality, n (%) | 2 (18.2) | 0 (0) | 0.002 |
| Hospital mortality, n (%) | 3 (27.3) | 2 (10.5) | 0.002 |

Continuous data are expressed as mean±SD or median (interquartile range).
GCS=Glasgow Coma Scale; TBI=traumatic brain injury; MV=mechanical ventilation; ICU=intensive care unit; LOS=length of stay

### 000330

#### High-flow oxygen through Nasal Cannula versus Continuous Positive Airway Pressure in moderate Acute Respiratory Distress Syndrome due to pneumonia

M. Giovini, M. Barbera, E. Antonucci
Intermediate care unit, Ospedale "Guglielmo da Saliceto", Piacenza, Italy
**Correspondence:** E. Antonucci
*Intensive Care Medicine Experimental* 2019, **7(Suppl 3):**000330

**INTRODUCTION.** Pneumonia can progress in acute respiratory distress syndrome (ARDS) that usually shows poor outcomes in critically ill patients. If on one hand the management of severe ARDS is extensively debated in the literature, on the other hand the treatment of moderate ARDS is not well standardized. In this perspective, oxygenation modalities (OM) such as continuous Positive Airway Pressure (C-PAP) and High Flow Oxygenation through Nasal Cannula (HFO) could be useful in adult patients with moderate ARDS due to pneumonia, but no study has ever compared helmet c-PAP and HFO in this field.

**METHODS.** We performed a retrospective analysis, reviewing all patients with moderate ARDS due to pneumonia and admitted to our Intermediate Care Unit (IMCU) from December 2016 to December 2018. The inclusion criteria were age ≥ 18 years old; moderate ARDS classified by Berlin definition; oxygenation with HFO or helmet C-PAP (stand-alone system) for 2 consecutive days at least. Exclusion criteria were history of chronic obstructive pulmonary disorder (COPD) or other chronic respiratory disorders; lung cancer; acute cardiogenic edema; severe neutropenia; circulatory failure. We analyzed two groups (C-PAP and HFO) matched for age, SAPS2 score, SOFA score and PaO2/FiO2 ratio before OM. We also collected demographic characteristics, settings of OM and we investigated rates of ETI, IMCU mortality and mortality at 90 days.

**RESULTS.** We reviewed 54 patients and 20/54 respected the inclusion criteria (10 in HFO group; 10 in CPAP group). The two groups were well matched (PaO2/FiO2 ratio before OM: 108 ± 39 (C-PAP), 119 ± 35 (HFO) p= 0.5; SAPS 2 score: 38 ± 14 (C-PAP), 30 ± 11 (HFO) p= 0.2; SOFA score: 5 ± 2 (C-PAP), 4 ± 2 (HFO) p= 0.4; age: 67 ± 14 (C-PAP), 60 ± 19 (HFO) p= 0.4). We found no significant difference in ETI rate [HFO 3/10 (30%), C-PAP 3/10 (30%)], IMCU mortality [HFO 1/10, C-PAP 2/10, OR= 2.25, 95% CI: 0.17-29.77, p= 0.5] and mortality at 90 days [HFO 2/10, C-PAP 3/10, OR = 1.7, 95% CI: 0.22-13.41, p= 0.6] (see Figure). Interestingly, C-PAP induced a higher increase of PaO2/FiO2 ratio than HFO (although not statistically significant, p=0.1) without effects on ETI and mortality rates.

**CONCLUSION.** In adult patients with moderate ARDS due to pneumonia, treatment with helmet C-PAP or HFO did not result in significantly different ETI and mortality rates.

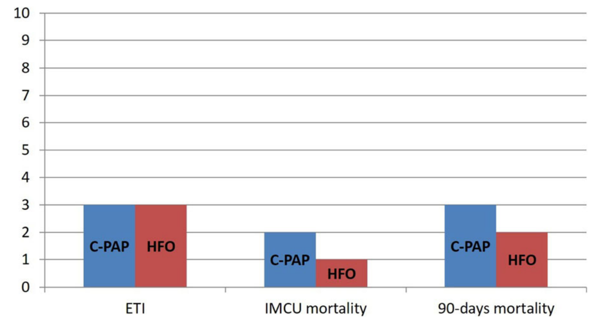

**Fig. 1 (abstract 000330).** See text for description

### 000339

#### Impact of prone positioning on ventilation homogeneity evaluated by Electrical impedance tomography in ARDS patients

A. Shono[1], T. Miho[2], S. Yuri[2], T. Kotani[2]
[1]Anesthesiology, Shimane University Hospital, Izumo, Japan; [2]Intensive care medicine, Showa University Hospital, Tokyo, Japan
**Correspondence:** A. Shono
*Intensive Care Medicine Experimental* 2019, **7(Suppl 3):**000339

**INTRODUCTION.** Prone positioning more than 10 hours improves oxygenation and mortality in patients with moderate to severe acute respiratory distress syndrome (ARDS). Homogenization of lung ventilation in prone positioning establishing more uniform distribution of tidal breath is the possible mechanism contributing to better outcome by alveolar recruitment and prevention of ventilator-induced lung injury. However, the time course of homogenization of ventilation has not been investigated. Electrical impedance tomography (EIT), which is the real-time monitoring tool of ventilation, has been demonstrated to be used for the evaluation of ventilation homogeneity in different body positions.

**OBJECTIVES.** The objective of this study was to examine the impact of prone positioning on ventilation homogeneity evaluated by EIT in ARDS patients.

**METHODS.** In this retrospective study, we performed off-line analysis of EIT data previously recorded before (in supine position, SP) and after taking prone position (PP) in clinical settings. In each PP session, ventilation homogeneity was evaluated using EIT measures; the proportion of ventilation distribution (ventral and dorsal lung parts, expressed in percentage to the tidal volume=100%), spatial dispersion and temporal delay in regional distribution of tidal breath (global inhomogeneity index: GI index, regional ventilation delay: RVD). These EIT measures and oxygenation were compared to the data measured in SP immediately before PP. Off-line analysis was performed by a blinded researcher. For the comparison between before and after PP, paired t-test or Wilcoxon rank sum test were used for statistical analysis as appropriate.

**RESULTS.** The EIT data of seven PP sessions in five patients (three primary and two secondary ARDS, mean age 66) were analyzed. Mean time to EIT measurement from the initiation of PP was 7 ± 2 hours (mean ± SD). The ventilator mode and/or pressures were changed in three PP sessions. Ventilator mode was changed from assist control to airway pressure release ventilation in one session and inspiratory pressures in assist control were increased in two sessions after taking PP. The mean proportion of ventilation in dorsal lung region increased from 44 ± 9% to 56 ± 9% (P=0.010) in PP, showing a beneficial effect of lung recruitment in dorsal part in 6 PP sessions. GI index and RVD significantly reduced after taking PP, indicating more homogeneous ventilation was achieved compared to SP. The oxygenation improved in 6 PP sessions. Significant increase in mean PaO2/FIO2 was demonstrated after taking PP (SP;130 ± 65 mmHg vs PP; 202 ± 98 mmHg, P=0.020). All patients were successfully weaned from the ventilator. Four patients have survived and discharge from the hospital.

**CONCLUSION.** In our cohort relatively short period application of prone positioning provided more homogeneous ventilation compared to supine position, which might result in better oxygenation.

##### REFERENCE(S)

1. Guérin C, Reignier J, Richard J-C, Beuret P, Gacouin A, Boulain T, Mercier E, Badet M, Mercat A, Baudin O, et al.; PROSEVA Study Group. Prone positioning in severe acute respiratory distress syndrome. N Engl J Med 2013;368(23):2159–2168.
2. Luciano Gattinoni, Paolo Taccone, Eleonora Carlesso, and John J. Marini. Prone Position in Acute Respiratory Distress Syndrome Rationale, Indications, and Limits. Am J Respir Crit Care Med 2013;188(11):1286–1293

### 000351

#### Lung ultrasound score to predict the failure of non-invasive respiratory support in hypoxemic patients

S. Mongodi[1], S. Pregnolato[2], G. Salve[2], A. Orlando[1], E. Santangelo[3], S. Bonaiti[2], A. Stella[2], A. Colombo[2], O. Dransart-Rayé[4], GA. Iotti[1], R. Vaschetto[3], B. Bouhemad[4], F. Mojoli[2]
[1]Department of anesthesia and intensive care, Fondazione IRCCS Policlinico S Matteo, Pavie, Italy; [2]Department of clinical-surgical, diagnostic and pediatric sciences, unit of anaesthesia and inte, The University of Pavia, Pavia, Italy; [3]Department of translational medicine, Università Degli Studi Del Piemonte Orientale, Novara, Italy; [4]Department of anaesthesiology and intensive care, Chu Dijon, Dijon, France
**Correspondence:** S. Mongodi
*Intensive Care Medicine Experimental* 2019, **7(Suppl 3):**000351

**INTRODUCTION.** Lung ultrasound (LUS) score allows reliable quantification of loss of aeration[1]; it has already been applied to PEEP-induced recruitment, ARDS lung healing, weaning from mechanical ventilation and VAP recovery[2].

**OBJECTIVES.** To determine if LUS score early identifies responders to non-invasive respiratory support.

**METHODS.** Prospective observational multicenter international study (Pavia, Novara, Dijon). LUS score (from 0-normal aeration to 3-complete loss of aeration computed in 6 standard regions per hemithorax–2 anterior, 2 lateral, 2 posterior) was measured before and after 2 hours of non-invasive support (high-flow nasal cannula-HFNC, continuous positive airways pressure-CPAP, non-invasive ventilation-NIV) in hypoxemic patients (PaO2/FiO2<300). Non-responders required intubation within 48 hours.

**RESULTS.** 31 non-invasive supports in 29 patients (males 13, age 71.5± 13.4 yo, BMI 27.1±4.5 kg/m2, SAPS2 35.0 [29.0-47.0]), admitted to ICU from ED (15), OR (9) and medical ward (5) for acute respiratory failure (15), major surgery (9), neurological diseases (3), HELLP syndrome (1), cardiac arrest (1). They required non-invasive respiratory support for weaning failure (7), pneumonia (7), ARDS (6), derecruitment (6), pulmonary edema (5). The respiratory support was helmet CPAP (26), HFNC (3), helmet (1) and mask (1) NIV; 7 failed (22.6%).

Failing and non-failing patients didn't significantly differ before non-invasive respiratory support in term of PaO2/FiO2, respiratory rate, pH and PaCO2; LUS score similarly was not significantly different (Tab.1). After 2 hours of support, failing patients were significantly more hypoxemic and hypocapnic, despite pH and respiratory rate were not significantly different from non-failing ones. They also had significantly higher LUS score. A LUS score ≥17 predicted the failure of non-invasive respiratory support with 100% sensitivity and 83.3% specificity, while a global LUS score ≤12 predicted the success of non-invasive respiratory support with 100% sensitivity and 85.7% specificity.

**CONCLUSION.** LUS score early identifies responders to non-invasive respiratory support.

**REFERENCE(S)**

1. Chiumello D. et al. Crit Care Med 2018: 46(11):1761-1768
2. Mojoli F. et al, Am J Resp Crit Care Med 2019:15;199(6):701-714

**Table 1 (abstract 000351).** Failing and non failing patients features before (T0) and after 2 hours (T1) of non-invasive respiratory support

| | Non failing (24) | Failing (7) | p value |
|---|---|---|---|
| T0: PaO2 / FiO2 | 140.7±62.0 | 139.4±56.3 | 0.959 |
| T0: PaCO2 | 43.5±13.3 | 33.0±7.3 | 0.057 |
| T0: pH | 7.4±0.1 | 7.4±0.0 | 0.517 |
| T0: Respiratory rate | 24.5±7.2 | 26.3±5.7 | 0.560 |
| T0: Lung ultrasound score | 15.3±5.2 | 17.4±6.1 | 0.373 |
| T1: PaO2 / FiO2 | 192.2±55.4 | 134.0±48.7 | **0.0181** |
| T1: PaCO2 | 41.9±10.1 | 32.8±6.0 | **0.0308** |
| T1: pH | 7.4±0.1 | 7.4±0.1 | 0.0880 |
| T1: Respiratory rate | 21.5±6.2 | 26.9±5.7 | 0.0513 |
| T1: Lung ultrasound score | 11.7±5.3 | 17.3±5.7 | **0.021** |

### 000357

#### Use of high flow oxygen therapy in acute respiratory failure with moderate hypercapnia

V. Losada Martínez[1], G. Ferrigno Bonilla[1], A. Tejero Pedregosa[1], D. Monge Donaire[1], N. Rodrigo Castroviejo[1], R. Beltrán Bernáldez[1], A. González Salamanca[1], S. Cortés Díaz[1], C. Tarancón Maján[1], A. Marcos Gutiérrez[1], C. Ochoa Sangrador[2]

[1]Intensive care, Hospital Virgen De La Concha, Zamora, Spain; [2]Research support unit, Hospital Virgen De La Concha, Zamora, Spain

**Correspondence:** A. Tejero Pedregosa

*Intensive Care Medicine Experimental* 2019, **7(Suppl 3):**000357

**INTRODUCTION.** In the last decade, high-flow nasal oxygen therapy (HFNO) has appeared as an intermediate alternative ventilatory support between conventional oxygen and mechanical ventilation. Its efficacy has been demonstrated in patients with respiratory failure due to different etiologies (1-2). Patients treated with HFNO rapidly showed a reduction in respiratory rate and work of breathing, associated with an improvement in functional residual capacity and gas exchange.

**OBJECTIVES.** To analyze the outcome of clinical and gasometric parameters during the first 24 hours of high-flow nasal oxygen therapy administration in patients with acute respiratory insufficiency with and without associated hypercapnia. Identify bad outcome predictors such as: need for mechanical ventilation and mortality.

**METHODS.** Prospective observational study. Adult patients admitted in the ICU to whom high-flow oxygen therapy is administered at the decision of their responsible physician. Demographic data, severity scales, treatment parameters, physiological variables and gasometry were collected.

Gasometric monitoring is performed at 1, 3, 12 and 24 hours after the start of therapy.

**RESULTS.** 45 patients were recruited. Mostly male (73%), the average age was 65.7 (61.7-69.7) years. APACHE II 20.5 (17.7-23.3). Among the causes of acute respiratory failure were found: heart failure (31.1%), pneumonia (22.2%), exacerbated COPD (13.3%), post extubation respiratory failure (11.1%). The therapy was initiated with FiO2 77.8% (70.6-68.5) and flow of 52.6 l/min (51-54.2). 12 (26.7%) patients required mechanical ventilation and mortality in the ICU was 18%.

A statistically significant reduction in respiratory rate was observed: -6 (-7 to -9.5) rpm and cardiac frequency: -13.6 (-7.2 to -20) bpm. An increase in SpO2 was observed: 4.4 (1.7-7)%. No significant changes were observed in pCO2. Similar changes were confirmed in the subgroup of patients with initial pCO2> 45 mmHg. The cardiac frequency was associated with mortality: area under the ROC curve: 0.72 (0.55-0.89), with an HR of 99 bpm being the cut-off point that best discriminated between patients who died and survived.

**CONCLUSION.** High-flow oxygen therapy improved physiological parameters of acute respiratory failure in our sample. Despite no improvement on pCO2, its use seems safe in patients with moderate hypercapnia. Persistent tachycardia could be a predictor of therapeutic failure.

**REFERENCE(S)**

1. High-flow oxygen through nasal cannula in acute hypoxemic respiratory failure. Frat JP, Thille AW, Mercat A, Girault C, Ragot S, Perbet S, Prat G, Boulain T, Morawiec E, Cottereau A, Devaquet J, Nseir S, Razazi K, Mira JP, Argaud L, Chakarian JC, Ricard JD, Wittebole X, Chevalier S, Herbland A, Fartoukh M, Constantin JM, Tonnelier JM, Pierrot M, Mathonnet A, Béduneau G, Delétage-Métreau C, Richard JC, Brochard L, Robert R; FLORALI Study Group; REVA Network. N Engl J Med. 2015 Jun 4;372(23):2185-96.

2. Effect of Postextubation High-Flow Nasal Cannula vs Noninvasive Ventilation on Reintubation and Postextubation Respiratory Failure in High-Risk Patients: A Randomized Clinical Trial. Hernández G, Vaquero C, Colinas L, Cuena R, González P, Canabal A, Sanchez S, Rodriguez ML, Villasclaras A, Fernández R. JAMA. 2016 Oct 18;316(15):1565-1574.

### 000364

#### Efficacy and safety of high-flow nasal oxygen cannula in patients with acute respiratory failure at general internal medicine wards: A prospective cohort study

N. Rittayamai, P. Chuariyakul, N. Promlee, P. Chailard, N. Chierakul
Department of medicine, Faculty of Medicine Siriraj Hospital, Mahidol University, Bangkok, Thailand
**Correspondence:** N. Rittayamai
*Intensive Care Medicine Experimental* 2019, **7(Suppl 3):**000364

**INTRODUCTION.** Oxygen therapy is a mainstay treatment in patients with hypoxemia or after extubation for maintaining adequate oxygenation and reducing work of breathing. Conventional low flow oxygen therapy via simple nasal cannula and oxygen rebreathing mask with bag is commonly used. However, these devices can only provide a maximum flow rate of 15 LPM which may be inadequate for patients with acute respiratory failure. High-flow nasal cannula (HFNC) is a high flow oxygen delivery system that can deliver up to 60 LPM of air-oxygen mixture gas via a wide-bore nasal cannula. It can also adjust oxygen concentration from 21-100% and provide heated and humidified gas. HFNC has been demonstrated several benefits in patients who were admitted in Intensive Care Unit (ICU) with acute hypoxemic respiratory failure and after extubation. Using HFNC outside ICU without close monitoring is growing especially in general ward and emergency department; however, the evidence in terms of efficacy and safety of HFNC in such places is scant.

**OBJECTIVES.** To evaluate efficacy and safety of HFNC in general internal medicine wards and factor associated with failure of HFNC.

**METHODS.** We performed a prospective cohort study during October 2017 to April 2018 in general internal medicine wards at Siriraj Hospital, Bangkok, Thailand. Adult patients who were admitted in general internal medicine wards and received HFNC (Airvo-2TM, Fisher & Paykel Healthcare, Auckland, New Zealand) were enrolled. All included patients were continuously monitored by multidisciplinary care team who were trained and familiar with HFNC device. The primary outcome was the rate of HFNC failure (defined by intubation or receiving non-invasive ventilation). The secondary outcomes included in-hospital mortality, 28-day mortality and factors associated with failure of HFNC.

**RESULTS.** A total of 88 patients were enrolled and 17 of them were patients with do-not-resuscitate (DNR) order. Mean age was 71±15 years old, APACHE II score was 18±6 and SOFA score was 6±3. Lower respiratory tract infection was the leading cause of hospital admission. The main indications of HFNC were acute hypoxemic respiratory failure (63.6%) and prophylactic after extubation (31.8%). Mean duration of HFNC use was 76.6±62.8 hours. Rate of HFNC failure was 39.8% and in-hospital and 28-day mortality were 30.7% and 36.4%, respectively. We performed subgroup analysis by excluding patients with DNR status and demonstrated that the rate of HFNC failure was 25.4% and in-hospital and 28-day mortality were 14.0% and 21.1%, respectively. The main reasons for failure of HFNC were hypoxemia and increased work of breathing. Factors associated with failure of HFNC were presence of comorbidity and respiratory rate on the first day of HFNC use ($P < 0.05$). Overall adverse event of HFNC was 6.8% and all of them were minor adverse event.

**CONCLUSION.** HFCN is safe and effective to use in general internal medicine wards. Presence of comorbidity and higher respiratory rate on the first day are the risk factors of HFNC failure. Appropriate patient selection and monitoring are the key for the success.

**REFERENCE(S)**

1. The authors gratefully acknowledge Ms.Khemajira Karaketklang for the assistance with data analysis and the staffs of Division of Respiratory Diseases and Tuberculosis, Department of Medicine, Faculty of Medicine Siriraj Hospital, Mahidol University for their support and collaboration.
2. Hernandez G, Vaquero C, Colinas L, Cuena R, Gonzalez P, Canabal A, et al. Effect of postextubation high-flow nasal cannula vs noninvasive ventilation on reintubation and postextubation respiratory failure in high-risk patients: A Randomized Clinical Trial. JAMA. 2016;316(15):1565-74.
3. Hernandez G, Vaquero C, Gonzalez P, Subira C, Frutos-Vivar F, Rialp G, et al. Effect of postextubation high-flow nasal cannula vs conventional oxygen therapy on reintubation in low-risk patients: A Randomized Clinical Trial. JAMA. 2016;315(13):1354-61.
4. O'Driscoll BR, Howard LS, Davison AG. BTS guideline for emergency oxygen use in adult patients. Thorax. 2008;63 Suppl 6:vi1-68.
5. Rittayamai N, Tscheikuna J, Praphruetkit N, Kijpinyochai S. Use of high-flow nasal cannula for acute dyspnea and hypoxemia in the Emergency Department. Respiratory care. 2015;60(10):1377-82.
6. Frat JP, Thille AW, Mercat A, Girault C, Ragot S, Perbet S, et al. High-flow oxygen through nasal cannula in acute hypoxemic respiratory failure. The New England journal of medicine. 2015;372(23):2185-96.
7. Papazian L, Corley A, Hess D, Fraser JF, Frat JP, Guitton C, et al. Use of high-flow nasal cannula oxygenation in ICU adults: a narrative review. Intensive care medicine. 2016;42(9):1336-49.

## ARF - Acute respiratory failure 6

### 001436

#### A new mask design with different ports for inflow and outflow gases reduces CO2 rebreathing in healthy volunteers during Non Invasive Ventilation

D. Signori[1], A. Grassi[1], F. Rossi[1], V. Meroni[1], L. Bosa[1], C. Crivellari[1], G. Foti, G. Bellani[2]
[1]School of medicine and surgery, University of Milano-Bicocca, Monza, Italy; [2]Department of emergency and intensive care, school of medicine and surgery, University of Milano-Bicocca, Monza, Italy
**Correspondence:** A. Grassi
*Intensive Care Medicine Experimental* 2019, **7(Suppl 3):**001436

**INTRODUCTION.** $CO_2$ rebreathing is known to be a cause of Non Invasive Ventilation (NIV) failure [1]. Recently we showed in a bench study that a new mask design (DiMax Zero, Dimar srl, Medolla, Italy) with different ports for inflow and outflow gases significantly reduced $CO_2$ rebreathing in different ventilation settings [2].

**OBJECTIVES.** To test wheter a new mask design reduces $CO_2$ rebreathing in healthy volunteers during NIV.

**METHODS.** Young adults with no pulmonary or cardiac comorbidities were eligible. Volunteers underwent NIV through two different masks: a traditional one with a single port for inflow and outflow gases (DiMax) and the new mask with separated ports (Dimax Zero). Six ventilation settings were tested: cPAP (PEEP 5 cmH2O) and Pressure Support Ventilation (PSV, PS Level 5 cmH2O) performed with mechanical ventilator and two different flow-by (8 and 20 l/min); free flow cPAP (PEEP 5 cmH2O) with two different gas flows (60 and 90 l/min). A nasal cannula was inserted in one nostril of the volunteer and connected to a gas analyzer (ADInstruments) to record $CO_2$ concentration along the respiratory cycle (Lab Chart, ADInstruments). An EIT belt (Pulmovista, Draeger) was used to record the change in impedance during tidal breathing, to estimate tidal volume (Vt). Respiratory rate (RR) was also calculated. $CO_2$ during inspiration, RR and and Vt were compared between the two masks in the same ventilation setting and within the same mask in different ventilation settings. ANOVA for repeated measured + post tests was used for statistical analysis. A $p<0,05$ was considered significant.

**RESULTS.** Seven healthy adults were enrolled in the study and gave their informed consent. $CO_2$ during inspiration was significantly lower with DiMax Zero than with DiMax mask (Fig. 1, $p<0,001$ for DiMax Zero vs DiMax in all settings).

The maximum reduction was observed in the free-flow cPAP settings. The different ventilation settings had no influence on inspired CO2 with both masks. The difference in inspired CO2 was associated with a slight, non significant reduction in Vt during cPAP settings with DiMax Zero mask, while there were no differences in Vt during PSV settings nor in respiratory rate in any setting between the two masks (Fig. 2).

**CONCLUSION.** The new mask design with different ports for inhaled and exhaled gases was able to significantly reduce the amount of CO2 rebreathing in all the ventilation settings tested. The sample size has to be increased to highlight if this translate into a reduction in minute ventilation.

**REFERENCE(S)**

1. Signori D et al. Effect of Face Mask Design and Flow-by on Rebreathing During Noninvasive Ventilation. Respir Care. 2019; Epub ahead of print
2. Schettino GPP et al. Position of exhalation port and mask design affect CO2 rebreathing during noninvasive positive pressure ventilation. Crit Care Med 2003; 31(8):2178-2182

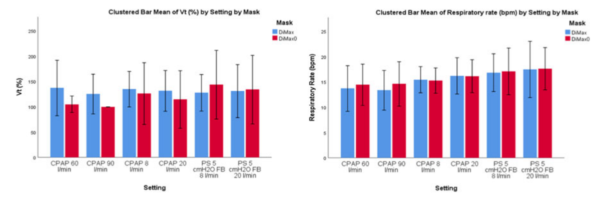

**Fig. 2 (abstract 001436).** See text for description

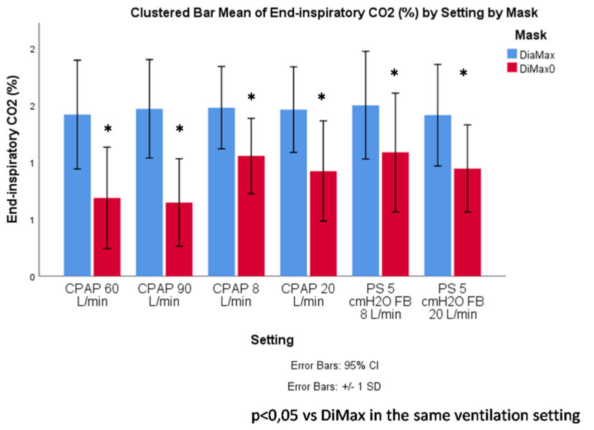

**Fig. 1 (abstract 001436).** See text for description

### 001439

#### Early versus late tracheostomy in neurocritical care patients: Which policy is more advantageous?

M. Sileli, C. Iasonidou, A. Kosmas, E. Lazoudi, E. Siomos, E. Setsidou, N. Kapravelos

B icu, General Hospital “G. Papanikolaou”, Thessaloniki, Greece

**Correspondence:** M. Sileli

*Intensive Care Medicine Experimental* 2019, **7(Suppl 3):**001439

**INTRODUCTION.** No consensus exists regarding optimal timing for performing tracheostomy in mechanically ventilated neurocritical care patients.

**OBJECTIVES.** To evaluate the correlation between timing of tracheostomy and ICU mortality. Secondary end points were tracheostomy effects on: duration of sedation, duration of mechanical ventilation (MV), ventilator-associated pneumonia (VAP) incidence prior and post-tracheostomy and length of ICU stay (LOS) as well.

**METHODS.** Single-center study on adult neurocritical care patients (TBI, aSAH, ICH) admitted to our ICU from January to December 2018. Percutaneous elective tracheostomy was performed in all expected to need >14 days MV due to current neurological status. We assigned patients into two groups: early tracheostomy (ET ≤8 days after endotracheal intubation) and late tracheostomy (LT >8 days). Exclusion criteria were: poor prognosis, use of barbiturates, extubation failure and readmissions. Demographics, diagnosis at admission, severity of illness estimated using the APACHE II and GCS, Charlson comorbidity index (CCI) score, duration of sedation and MV, incidence of VAP, LOS and in ICU mortality were recorded. Student t-test or Mann Whitney U test were used for quantitative data and the Pearson chi-squared test or Fisher's exact test for qualitative data as necessary. The level of significance was set at $P<0.05$.

**RESULTS.** 41 patients were recorded but finally 38 (31.6%TBI, 26.3% aSAH, 42.1% ICH) were included (missing data). Each group consisted from 19 patients. The median time between ICU admission and ET or LT was 6(2-8) and 11(9-16) days respectively. Baseline characteristics (sex, age, GCS, APACHE II) were similar in both ET and LT group ($p>0.05$) except from CCI score (2.37±1.42 vs1.47±1.31; p =0.043) and ICU admission diagnosis (TBI:3vs9, aSAH:1vs9, ICH:15vs1 patients). Total MV and sedation duration were significantly longer in the LT-group (9vs16.1d; p<0.001 and 5.74vs10.42d; p=0.005 respectively) but post-tracheostomy there were no differences neither in sedation (p=0.682) nor in MV duration (p=0.914). Patients with LT had a higher incidence of VAP before tracheostomy (p=0.034) but no significant difference was found in post-tracheostomy MV period (p=0.547). All survivors were completely weaned from MV but none was decannulated at ICU discharge. The ICU LOS and mortality did not differ between the compared groups (p=0.075 and p=0.29 respectively).

**CONCLUSION.** ET reduced sedation requirements, time to wean from MV and VAP incidence in our cohort. No mortality or ICU LOS benefit was found between the compared groups. Our findings are in agreement with previous studies. It seems that tracheostomy policy in neurocritical care patients still remains a challenge and needs to be personalized.

**REFERENCE(S)**

1. Young D, Harrison DA, Cuthbertson BH, Rowan K. Effect of early vs late tracheostomy placement on survival in patients receiving mechanical ventilation: the TracMan randomized trial. JAMA 2013;309:2121-9
2. none

### 001446

#### Effects of spontaneous breathing efforts in regional ventilation distribution and inflammation in acute hypoxemic respiratory failure experimental setting

MC. Bachmann[1], C. Santis[2], V. Oviedo[1], D. Soto[1], P. Cruces[3], F. Diaz[4], M. Goich[5], J. Fuenzalida[5], E. Kattan[6], S. Merino[6], R. Basoalto[7], S. Dubo[8], Y. Jalil[1], ED. Valenzuela Espinoza[1], G. Bugedo[1], A. Bruhn[1], J. Retamal[1]

[1]Departamento de medicina intensiva, Pontificia Universidad Católica de Chile, Santiago, Chile; [2]Hospital barros luco trudeau, Unidad de Cuidados Intensivos, Santiago, Chile; [3]Unidad de pacientes críticos, Hospital El Carmen de Maipú, Santiago, Chile; [4]Unidad de cuidados intensivos pediátricos, Clinica Alemana, Vitacura, Chile; [5]Hospital clínico veterinario, UNAB, Santiago, Chile; [6]Departamento de Medicina Intensiva, Pontificia Universidad Catolica de Chile, Santiago, France; [7]Unidad de cuidados intensivos, Hospital Clínico Universidad Católica, Santiago, Chile; [8]Departamento de kinesiología, Universidad de Concepción, Concepción, Chile, Concepción, Chile

**Correspondence:** M.C. Bachmann

*Intensive Care Medicine Experimental* 2019, **7(Suppl 3):**001446

**INTRODUCTION.** Spontaneous breathing (SB) efforts in acute respiratory distress syndrome patients on mechanical ventilation, has been associated with strong diaphragmatic activity and the generation of excessive strain and high regional pleural pressures in the dependent lung regions [1]. However, these mechanisms may will be present before intubation [2]. We hypothesize that spontaneous breathing efforts during early stages of lung injury may promote high regional strain and further regional lung inflammation and that controlled mechanical ventilation is able to avoid and or reverse and control this inflammation.

**OBJECTIVES.** To determine the effects of spontaneous breathing efforts, during the early stage of lung injury, on regional ventilation distribution and regional inflammation.

**METHODS.** Six pigs (30±5kg) were anesthetized and mechanically ventilated (PEEP 5 cmH2O, VT 8 ml/kg). Lung injury was induced by 2 lavages with 30 ml/kg of warm saline. After the injury, pigs were ventilated 4 hours (T4) in pressure support ventilation (PSV for Vt of 6-8 ml/kg, PEEP between 0 and 5, and FIO2 of 1), and then switched to volume controlled ventilation (VCV) for 4 more hours (T8) using VT of 6 ml/kg, RR of 30 bpm, PEEP of 5 cmH2O, I:E ratio 1:2 and FIO2 of 1. Respiratory and hemodynamic data, as well as regional lung impedance, assessed by EIT (Pulmovista 500, Dräger), were collected at baseline, T4 and T8. BAL were performed at the anterior and posterior lung at T4 and T8 for analysis of inflammatory parameters (IL-8 and TNF-a)

**RESULTS.** VT increased from 8 ml/kg at baseline to 8.2 (7.1-8.8) (p= 0.03) at PSV. During T8, VT decreased to 6 ml/kg (5.4-6.7) (p= 0.001). Mean airway prressure decreases from 9 (6.7-11) to 2 (3.8-1.8) cmH2O (p= 0.001) but when switched to VCV it increased to 11 (8.8-12.8) (p=0.003). EIT analysis showed an increase in global inhomogeneity index (GI) from 1.03 (0.99-1.04) to 1.09 (1.06-1.38)(p=0.04) (Fig. 1A). In addition, when we compared T4 vs T8, tidal variation of impedance (TVI) decreased from 2227 (1520-2933) to 1137 (876-1768) (p=0.03) (Fig. 1B) and impedance ratio (IR) showed an increased from 0.75 (0.3-1.2) to 1.3 (0.5-2.0) (p= 0.03) (Fig. 1C). Regional IL-8 quantification showed greater inflammation in the dependent lung regions during PSV, situation that was reversed with VCV (3670 ± 2310 vs 1603 ± 2311 pg/uL, p= 0.02) (Fig. 2).

**CONCLUSION.** SB efforts during MV were associated with higher VT than controlled ventilation and lung aeration was mainly distributed in the dependent lung regions and these regions presented higher concentration of inflammatory markers, situation that was reversed during CMV.

**REFERENCE(S)**

1. Mascheroni D, Kolobow T, Fumagalli R, Moretti MP, Chen V, Buckhold D. Acute respiratory failure following pharmacologically induced hyperventilation: an experimental animal study. Intensive Care Med. 1988;15:8-14.
2. Yoshida T, Roldan R, Beraldo MA, Torsani V, Gomes S, De Santis RR, et al. Spontaneous Effort During Mechanical Ventilation. Crit. Care Med. 2016;44:e678-88

3. This study was supported by a grant of Fondo Nacional de Desarrollo Científico y Tecnológico (FONDECYT) 1171810.

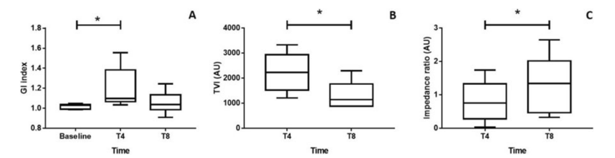

**Fig. 1 (abstract 001446).** See text for description

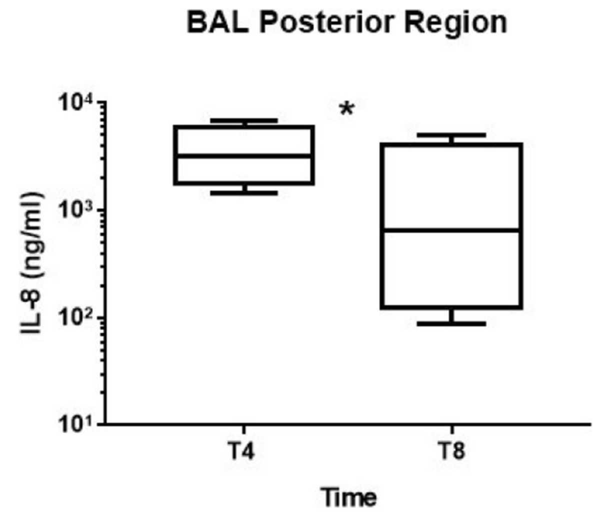

**Fig. 2 (abstract 001446).** See text for description

### 001479

#### Invasive vs. noninvasive ventilation as the initial ventilatory strategy in very elderly patients admitted to intensive care due to community-acquired pneumonia: a multicenter retrospective cohort study

B. Besen[1], O. Ranzani[2], M. Park[1]

[1]Medical intensive care unit, Hospital das Clínicas HCFMUSP - University of Sao Paulo Medical School, Sao Paulo, Brazil; [2]Pulmonary division, Heart Institute (InCor) - Hospital das Clínicas HCFMUSP - University of Sao Paulo Medical School, Sao Paulo, Brazil

**Correspondence:** B. Besen

*Intensive Care Medicine Experimental* 2019, **7(Suppl 3):**001479

**INTRODUCTION.** The very elderly patients (i.e., those aged ≥ 80 years-old) represent an increasing subgroup of patients admitted to intensive care units (ICUs). Community-acquired pneumonia (CAP) is a common reason for ICU admission and how to best provide initial ventilatory support in these patients is unknown.

**OBJECTIVES.** To evaluate if the initial ventilatory strategy is associated with hospital mortality in very elderly patients admitted to the ICU.

**METHODS.** We did a retrospective multicenter cohort study of very elderly patients admitted to the ICU with a diagnosis of CAP from 2009 through 2012. Data was retrieved from the ICU quality database (Epimed Monitor) and checked for consistency. We used logistic regression to evaluate the primary outcome (hospital mortality), after adjusting sequentially for potential confounders chosen from a causal directed acyclic graph. We imputed missing data with multiple imputation by chained equations using the predictive mean matching algorithm. We evaluated ad-hoc defined subgroup effects with interaction terms in the logistic model. Sensitivity analyses were done to assess model consistency regarding the primary outcome.

**RESULTS.** 6,318 very elderly patients were admitted to the 11 ICUs during the study period. 1,713 admissions were due to respiratory causes, of which 678 patients were admitted for CAP. Mean age was 86.3 years (4.7); 288/678 (42.5%) were male. Mean

SAPS 3 score was 63.5 (12.8), with a median SOFA of 4 [2; 7] and a median Charlson comordity score of 1 [1; 3]. 191/678 (28.2%) patients were previouslly bedridden and 253/678 (37.3%) needed some assistance in activities of daily living. 309/678 (45.6%) did not use any MV strategy (no MV group); 232/678 (34.2%) underwent NIV first; 137/678 (20.2%) underwent IMV first. IMV patients were sicker at ICU admission when compared to both NIV and no MV groups (SAPS 3: 76.5 vs. 62.6 vs. 58.4, p-value < 0.001), with no statistically significant differences in the antecedent characteristics (burden of comorbidities and performance status). Hospital mortality was 90/309 (29 %) for no MV; 114/232 (49 %) for NIV; 90/137 (66 %) for IMV. For the comparison IMV vs. NIV, crude OR (95% CI, p-value) was 1.98 (1.28 – 3.07, p = 0.002). After adjusting for age, sex and antecedent characteristics, the aOR was 2.17 (1.38 – 3.42, p = 0.001). After including non-respiratory SOFA in the model, aOR was 1.23 (0.74 – 2.03, p = 0.419). After including respiratory variables (Pao2/Fio2; pH; Pco2) in the model, the aOR was 1.1 (0.65 – 1.85, p = 0.720). Interaction terms for the following characteristics showed no evidence of effect modification: heart failure, chronic obstructive pulmonary disease, bedridden status, non-respiratory SOFA ≥ 4, acute respiratory acidosis and Pao2/Fio2 ≤ 150. Complete case analysis (sensitivy analysis) yielded the same results.

**CONCLUSION.** The initial ventilatory strategy was not associated with hospital mortality in very elderly patients admitted to the ICU due to CAP, regardless of antecedent characteristics. Acuity variables were responsible for the major part of positive confounding for mortality in this population. This suggests a less invasive approach may be sufficient for most patients to provide the benefit of the doubt during an ICU trial for very elderly patients.

### 001504

#### Diaphragm electromyography to estimate transdiaphragmatic pressure

H. de Vries[1], B. Van Der Werff[1], A. Jonkman[1], D. Jansen[2], EC. de Boer[1], A. Spoelstra-de Man[1], L. Heunks[1]

[1]Intensive care, Amsterdam University Medical Center, location VUmc, Amsterdam, Netherlands; [2]Intensive care, Radboud UMC, Nijmegen, Netherlands

**Correspondence:** H. de Vries

*Intensive Care Medicine Experimental* 2019, **7(Suppl 3):**001504

**INTRODUCTION.** The electrical activity of the diaphragm (EAdi) might be a useful parameter to quantify breathing effort in ventilated patients because it is continuously available after placing a dedicated nasogastric catheter. EAdi demonstrated a tight correlation with inspiratory muscle pressure (Pmus) in an earlier study(1). The slope of the relationship between EAdi and Pmus could be predicted by dividing the peak of EAdi by the drop in airway pressure (Paw) during an expiratory hold. However, a narrow range of respiratory muscle effort was evaluated in that study, and the gold-standard parameter for diaphragm effort (the transdiaphragmatic pressure, Pdi) was not obtained.

**OBJECTIVES.** To study the correlation between EAdi and Pdi in the full range of breathing effort in healthy subjects. Second, to assess whether the slope of the correlation between EAdi and Pdi could be predicted by calculating the Paw/EAdi-ratio within each subject.

**METHODS.** Paw, Pdi and EAdi were recorded continuously in 23 healthy subjects. Maximal Pdi against an occluded airway was measured, and subsequently different levels of inspiratory threshold loading were applied ranging from 10% to 80% of maximal Pdi.

**RESULTS.** All subjects successfully completed the study protocol. Four subjects were excluded from analysis for technical reasons. In the remaining 19 subjects, a total of 2327 breaths were analyzed. There was a significant, but poor linear correlation between peak EAdi and peak Pdi when pooling data from all subjects ($R2=0.14$, $p<0.0001$). Correlations between EAdi and Pdi within subjects were much stronger, especially when diaphragm effort above 60% of maximum was excluded ($R2=0.80\pm 0.12$, $p<0.001$). See figure 1 for a representative example. However, the ratio of Paw/EAdi was not significantly correlated to the slope of EAdi and Pdi within each subject ($p = 0.51$).

**CONCLUSION.** A strong linear correlation exists between peak EAdi and Pdi within healthy subjects, up to 60% of maximal effort. The relationship was poor at effort above 60% of maximum, possibly because of accessory muscle recruitment. The ratio of Paw/EAdi did not relate to the slope of Pdi/EAdi within each subject. Further analysis is required to determine whether a more complex model based on EAdi and Paw can reliably estimate transdiaphragmatic pressure.

**REFERENCE(S)**

1. Bellani et al., Estimation of patient's inspiratory effort from the electrical activity of the diaphragm, CCM 2013
2. HdV is supported by a research grant of Amsterdam Cardiovascular Sciences

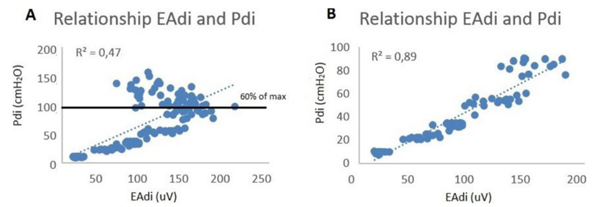

**Fig. 1 (abstract 001504).** Relationship between EAdi and Pdi within one subject. In panel B, breaths above 60% of maximum have been excluded

### 001516

#### Transient receptor potential cation channel vanilloid (TRPV) 1 in ventilator-induced lung injury

L. Michalick[1], W. Liedtke[2], WM. Kuebler[3]

[1]Institute of physiology, Charité - Universitätsmedizin Berlin, Berlin, Germany; [2]Departments of medicine, neurology and neurobiology, Duke University, Durham, United States of America; [3]Institute of physiology, Charité – Universitätsmedizin Berlin, Berlin, Germany

**Correspondence:** L. Michalick

*Intensive Care Medicine Experimental* 2019, **7(Suppl 3):**001516

**INTRODUCTION.** Mechanical ventilation is a life-saving intervention in critically ill patients, but may at the same time cause lung injury characterized by hyperinflammation, endothelial barrier failure, and edema formation. Yet, the exact mechanotransduction mechanisms underlying the effects of mechanical ventilation on the microvascular barrier are still unclear. In previous work, we had identified a critical role of the endothelial $Ca2+$ channel TRPV4 in endothelial barrier failure subsequent to mechanical overventilation. Here, we assessed the role of TRPV1 and its potential association to TRPV4-induced $Ca2+$ signaling *in vivo*, *ex vivo* and *in vitro*.

**METHODS.** Wild type, TRPV4- or TRPV1-deficient mice were ventilated for 2 h with low (7 mL/kg) and high (20 mL/kg) tidal volumes (LVT/HVT) *in vivo*. Endothelial [Ca2+]i in isolated-perfused lungs, inflated with 5 or 15 cmH2O, and in human pulmonary microvascular endothelial cells (HPMVECs) was quantified by real-time imaging with or without inhibition of TRPV1 and TRPV4, or after activation of TRPV1 and/or TRPV4, respectively.

**RESULTS.** TRPV1 or TRPV4 deficiency or inhibition by either SB366791 or HC-067047, respectively, attenuated VILI, and reduced the endothelial [Ca2+]i response to high pressure inflation *ex vivo*, except SB366791, which only diminished the sustained [Ca2+]i, but not the initial Ca2+ influx. *In vitro,* [Ca2+]i measurements in HPMVECs showed a characteristic increase in endothelial [Ca2+]i response to TRPV4 agonism, in which the sustained phase could be blocked by TRPV1 inhibition. TRPV1 agonism alone induced no [Ca2+]i increase; yet, TRPV1 and TRPV4 agonism in combination amplified the TRPV4-induced [Ca2+]i response.

**CONCLUSION.** Here, we demonstrate a critical role for TRPV1 in amplification and sustenance of TRPV4-mediated [Ca2+]i influx. We conclude that both TRPV channels act conjointly to regulate vascular barrier integrity, with TRPV4 causing endothelial leak by triggering an initial [Ca2+]i increase and TRPV1 facilitating the sustenance of the [Ca2+]i response which ultimately results in gap formation and barrier failure. The novel TRPV1/4 signaling axis may present a putative pharmacological target for the prevention or treatment of ventilator-associated lung disease.

### 001526

#### Passive Orthostatism as an Additional Resource for Severe ARDS Treatment

P. TRAVASSOS, DST. ÉRiton, MS. Maiko, CL. Agnes, MC. Lígia, CV. Viviane, SOR. Salomón
NEUROCRITICAL CARE UNIT, HOSPITAL BP - A BENEFICÊNCIA PORTUGUESA DE SÃO PAULO, SÃO PAULO, Brazil
**Correspondence:** P. TRAVASSOS
*Intensive Care Medicine Experimental* 2019, **7(Suppl 3):**001526

**INTRODUCTION.** Acute respiratory distress syndrome (ARDS) is a form of non-cardiogenic pulmonary edema due to alveolar injury secondary to an inflammatory process, which may be both pulmonary and systemic in origin. This syndrome presents as acute hypoxemia with bilateral pulmonary infiltrate, which can not be correlated exclusively with cardiac dysfunction. In this study, we evaluated the use of Passive Ortostatism in ARDS.

**OBJECTIVES.** To analyze the use of the orthostatic board as an auxiliary device for the treatment of severe ARDS by assessing its risks and benefits.

**METHODS.** We selected 91 patients, 43 females and 48 males, hospitalized in a Neurological ICU, between June 2014 and July 2018, in a physiotherapeutic follow-up with diagnosis of severe ARDS. The patients were submitted to orthotics assisted for 40 to 60 minutes and monitored HR, PAM, FR, SatO2 at 30 ° and 60 ° of inclination and the PaO2 / FiO2 ratio after the procedure. The mean number of sessions per patient was 6.6. All patients were undergoing anticoagulation in RASS -5, in the treatment of the cause of ARDS. The mean time of mechanical ventilation was 8.5 days.

**RESULTS.** Among the patients selected, 36.3% presented tachycardia above 115 bpm, requiring intervention in 12.1% and interruption of the procedure in 6.6%. PAM arterial hypotension <65 mmHg was observed in 34.1%, requiring intervention (increase of vasopressor dose and / or change of plank angulation) in 22% and interruption of the procedure in 14.3%. Hypoxemia SatO2 <92% was observed in 8.8%, without interruption, but an improvement in PaO2 / FiO2 was observed in only 95.6% of the patients

**CONCLUSION.** Assisted orthostatism as an auxiliary device for the treatment of severe ARDS was shown to be an alternative, with improvement of PaO2 / FiO2 in 95.6% of the patients, safe and without significant hemodynamic repercussions that could lead to interruption of the procedure

### 001540

#### Feasibility and outcomes of Non-invasive ventilation in a Pediatric Intensive Care Unit in a resource limited setting

N. Ravikumar, R. Aramanadka, TK. Kavitha, M. Jayashree, A. Bansal, AK. Baranwal, N. Karthi[1], SK. Angurana
Pediatric Intensive Care Unit, Post Graduate Institute of Medical Education & Research, Chandigarh, Chandigarh, India
**Correspondence:** N. Ravikumar
*Intensive Care Medicine Experimental* 2019, **7(Suppl 3):**001540

**INTRODUCTION.** Non-invasive ventilation (NIV) is being increasingly used in adults and children both in acute intensive care as well as for chronic home ventilation (1). NIV delivers positive pressure through an interface without endotracheal or tracheostomy tube. The success of NIV in Pediatric ICU (PICU) depends on patient selection, type of interface and close monitoring (2,3). Advantages of NIV are reduced Healthcare associated infection (HCAI), need for sedation and duration of ventilation and PICU stay. In children, acceptability of NIV remains poor due to claustrophobic nature of face masks, high pressure delivered through the interface and gastric distension. Introduction of patient friendly nasal masks, has improved the success of NIV and increased its use in PICU. In settings with limited availability of ventilators, BIPAP machines can serve as a good alternative to deliver NIV.

**OBJECTIVES.** To study the feasibility and outcomes of children requiring non-invasive ventilation

**METHODS.** Retrospective chart review from January 2018 to March 2019 (15 months) of children aged 1 month to 12 years requiring NIV and admitted to PICU of a tertiary care referral hospital. Children on indigenous CPAP and HFNC were not included. Records of children receiving NIV were analysed from patient electronic database for demographic data, primary diagnosis, indication for NIV, type of interface and ventilator used, mode, pressure settings and blood gases prior to initiation of NIV, reasons for NIV failure, sedation use, complications and final outcome (survival or death) on a pre designed proforma. The duration of NIV, need for intubation and length of PICU stay were also analysed.

**RESULTS.** During the study period, 1081 children were admitted to PICU, of which 530 (49%) and 32 (2.9%) required invasive and non-invasive ventilation respectively. Fourteen of the 32 who required NIV, was for post extubation. The median (IQR) age of the study group was 72 (36,108) months. Nasal interface was used in 12 and oronasal face mask in 20; 17 were ventilated using conventional ventilator and 15 through BIPAP machine. Pneumonia with ARDS (n=19) was the most common diagnosis followed by immunocompromised state (n= 6) and tropical fevers with capillary leak (n=5). The commonest indication for NIV was respiratory failure (n=26; 81% ), followed by neuromuscular weakness (n=4;12.5% ) and cardiac failure (n=2; 6.25% ). The mean ± SD PIP/IPAP (Max) was 14.5 ±2.9 and the mean ± SD PEEP/EPAP (Max) was 6.4 ±1.4 cm H2O. The median (IQR) duration of NIV and PICU stay were 3 (2,4) and 7.5 (5,23) days respectively. The failure rate was 11/32 (34%); 6 failed in nasal mask and 5 in oronasal mask. Four (12.5%) children died. Overall survival rate was 87.5%; among survivors, 2 needed long term NIV for neuromuscular weakness and compressive lesion in thorax, 1 child underwent tracheostomy for upper airway obstruction, and 1 child was referred for cardiac transplant. Compared to year 2018, the use of NIV has tremendously increased in 2019 (17 in 12 months;1.8% vs 15 in 3 months; 8.7%) probably due to better awareness and availability of age-appropriate interface.

**CONCLUSION.** NIV is a good modality of ventilation in respiratory failure, cardiac failure and in post-extubation patients.

**REFERENCE(S)**

1. Bonora JP, Frydman J, Retta A, and Canepari A. Post-extubation non-invasive ventilation in the pediatric intensive care unit: a multicenter study. Arch Argent Pediatr. 2018;116(5):333-339.
2. Alith, M.B., Fernandes, L.A., Borges, R.C. et al. Predictive factors of noninvasive ventilation failure in a pediatric ICU. Crit Care. 2015; 19 (2): P65.
3. GBS McNeill, AJ Glossop, Clinical applications of non-invasive ventilation in critical care, Continuing Education in Anaesthesia Critical Care & Pain, Volume 12, Issue 1, February 2012, Pages 33–37

### 001553

#### TiVel. A dual-centre feasibility study for setting tidal volume according to end expiratory lung volume: preliminary results

D. Manzolini[1], A. Grassi[1], C. Giovannoni[1], V. Meroni[1], M. Jankovsky[2], K. Szuldrzynski[3], G. Foti, G. Bellani[4]

[1]School of medicine and surgery, University of Milano-Bicocca, Monza, Italy; [2]Intensive care unit, the centre for extracorporeal therapies, University Hospital, Krakow, Poland; [3]Intensive care unit, the centre for extracorporeal therapies, university hospital, Department of interdisciplinary intensive care jagiellonian university medical college, Krakow, Poland; [4]Department of emergency and intensive care, ASST Monza, University of Milano Bicocca, Monza, Italy

**Correspondence:** A. Grassi

*Intensive Care Medicine Experimental* 2019, **7(Suppl 3):**001553

**INTRODUCTION.** Protective ventilation with low Tidal Volume (TV) improves outcome of acute respiratory distress syndrome (ARDS) patients [1]. Usually TV is set on ideal body weight (IBW) which reflects the size of healthy lung. However, in ARDS the amount of functional parenchyma, the “baby lung”, is reduced proportionally to the severity of disease [2]. In these patients the strain applied to the lung can be determined as TV/EELV (End Expiratory Lung Volume) and an increase in strain is associated with a proinflammatory response [3]. Measuring EELV may allow to set protective TV in order to maintain strain below a safety threshold [4]

**OBJECTIVES.** To verify the clinical feasibility of setting TV=0,25*EELV in ARDS patients.

**METHODS.** ARDS patients defined according to Berlin criteria, intubated and mechanically ventilated for less than 96 hours were enrolled. Exclusion criteria were conditions precluding measurement of EELV (i.e. air leaks, extracorporeal membrane oxygenation), haemodynamic instability, history of COPD, emphysema or pulmonary fibrosis. EELV was measured with the “oxygen wash-in wash-out” technique using GE Carescape R860 ventilator. Target TV (TTV) was set as 0.25*(measured EELV–PEEP related strain), up to 8 ml/kg Ideal Body Weight and respiratory rate (RR) was adjusted to keep baseline pCO2 ±5 mmHg. TTV was maintained for 24 hours unless one of the following occurred: need to increase RR >35 bpm, desaturation requiring a FiO2 change>0,20, development of a plateau pressure >30 cmH2O, need of neuromuscular blocking agents if not already administered. One-way ANOVA for repeated measures and Pearson’s linear correlation where used for statistical analysis.

**RESULTS.** 11 mild to moderate ARDS patients were enrolled (mean P/F ratio was 206±48mmHg). TTV was maintained for 24h in 7 out of 11 patients. 3 patients dropped out because of the shift to assisted ventilation and did not maintain the TTV; one dropped out because of calculated TV >8ml/kg. The mean TTV was 336±94 ml, comparable to clinical baseline TV 371±68 ml (Fig.1 Panel A). Mean Driving Pressure (DP) slightly decreased from 9±1,6 to 8,2±0,8 cmH2O (Fig. 1 Panel B). RR and pH were maintained within the safe limits (Fig. 1 Panel C).

Subsequent analysis revealed a strict correlation between lung strain and DP (Fig. 2, R=0,96).

**CONCLUSION.** These preliminary results show the feasibility of setting the TV as 0.25*(EELV-PEEP related strain) in the majority of patients enrolled. Moreover, DP is a good indicator of strain in ARDS patients.

**REFERENCE(S)**

1. ARDS Network. Ventilation with lower tidal volumes as compared with traditional tidal volumes for acute lung injury and the acute respiratory distress syndrome. N Engl J Med. 2000 May 4;342(18):1301-8
2. Gattinoni L, Pesenti A. The concept of "baby lung". Intensive Care Med. 2005 Jun;31(6):776-84
3. Bellani G et al. Lung regional metabolic activity and gas volume changes induced by tidal ventilation in patients with acute lung injury. Am J Respir Crit Care Med. 2011 May 1;183(9):1193-9.
4. González-López A et al. Lung strain and biological response in mechanically ventilated patients. Intensive Care Med. 2012 Feb;38(2):240-7

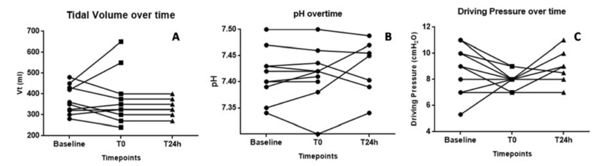

**Fig. 1 (abstract 001553).** See text for description

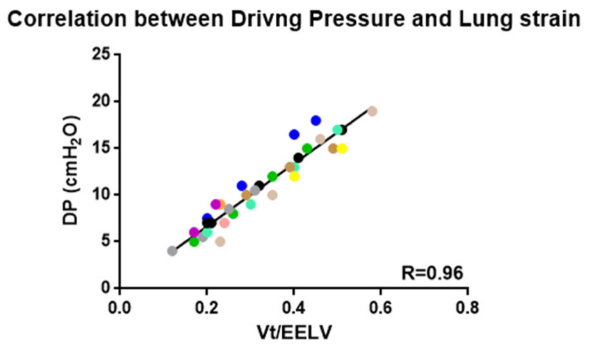

**Fig. 2 (abstract 001553).** See text for description

### 001554

#### Metabolic measures and esophageal measured tension time index in patients successfully passing a SBT

MK. Poulsen[1], S. Spadaro[2], F. Dalla Corte[2], V. Cricca[2], SE. Rees[1], DS. Karbing[1], CA. Volta[2]

[1]Department of health science and technology, respiratory and critical care group (rcare), Aalborg University, Aalborg, Denmark; [2]Intensive care unit, morphology surgery and experimental medicine, University of Ferrara, Ferrara, Italy

**Correspondence:** M.K. Poulsen

*Intensive Care Medicine Experimental* 2019, **7(Suppl 3):**001554

**INTRODUCTION.** Extubation readiness is normally assessed by spontaneous breathing trials (SBT) of 30-120 min (1). Tension time index (TTI) calculated from esophageal pressure (Pes) measurement is the reference technique for assessing breathing effort of a SBT, with a threshold of >0.15-0.18 following a SBT previously shown to accurately describe SBT response (2,3). However, limited data exist to explain why some patients pass a SBT despite TTI>0.15-0.18 (3).

**OBJECTIVES.** To investigate if metabolic measurement may explain SBT success when TTI is elevated.

**METHODS.** After ethical approval and informed consent, four patients (3f/1m, 65±12yrs, 85±7kg), have so far been enrolled. All were difficult to wean patients according to WIND classification (4), on pressure support (PS) ventilation, and eligible for SBT (1). Inspired oxygen fraction was set to 0.4 and a maximal inspiratory pressure effort was performed by 20s inspiratory occlusion at baseline (5). A SBT was then performed using PS=0 cmH2O, and PEEP=5 cmH2O. The SBT was ended after 120 min. Pes, VO2 and VCO2 were measured continuously. SBT outcome was decided according to guidelines (1). Average VO2 and VCO2 were calculated across six 1 min intervals throughout the SBT duration.

**RESULTS.** Throughout the SBT, TTI was close to 0.18 in two cases (high TTI) and ≤0.1 in the remaining two cases (low TTI) (fig 1). Mean VO2 and VCO2 were 295 and 359 mlO2/min and 225 and 290 mlCO2/min in high TTI patients, respectively (fig 1). Mean VO2 and VCO2 were 241 and 206 mlO2/min and 210 and 169 mlCO2/min in low TTI patients, respectively (fig 1). All patients were successfully extubated within 24 hours of the SBT. Figure 1 show mean TTI and VO2 for the four patients at six different time points during a SBT.

**CONCLUSION.** A higher metabolic response was observed in high TTI patients which likely reflect the greater breathing effort. Regardless of the underlying physiological mechanism, these preliminary findings indicate that SBT VO2 may follow TTI, and that there may be different metabolic responses between patients with high vs low TTI during successful SBTs. Further investigation is required to explore the patient characterization potential of VO2.

**REFERENCE(S)**

1) Boles J-M et al. Eur Respir J 2007;29(5):1033-56
2) Vassilakopoulos T et al. Am J Respir Crit Care Med 1998;158:378-385
3) Laghi F et al. Am J Respir Crit Care Med 2003;167:120-127
4) Béduneau G et al. Am J Respir Crit Care Med 2017;195(6):772-83
5) Spadaro S et al. Minerva Anestesiol 2015;81:33-38

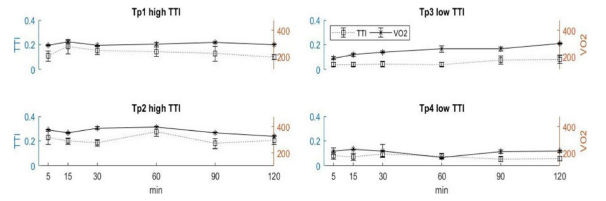

**Fig. 1 (abstract 001554).** See text for description

### 001561

#### Correlation between ultrasound diaphragm thickness and histologic diaphragm cross-sectional area in mechanically ventilated patients

I. Dot Jordana[1], J. Marin-Corral[1], C. Vilà-Vilardell[1], A. Castellví-Font[1], M. Boguñà-Planas[2], J. Boncompte-Torres[3], A. Zapatero-Ferrandiz[2], P. Pérez-Terán[2], JR. Masclans-Enviz[2]

[1]Intensive Care Medicine, Hospital del Mar de Barcelona, GREPAC (Grup d'Investigació en Patologia Crítica) - IMIM, Barcelona, Spain; [2]Universitat de barcelona, GREPAC (Grup d'Investigació en Patologia Crítica) - IMIM, Barcelona, Spain; [3]Barcelona, Universitat Pompeu Fabra, Barcelona, Spain

**Correspondence:** I. Dot Jordana

*Intensive Care Medicine Experimental* 2019, **7(Suppl 3):**001561

**INTRODUCTION.** Ventilator-induced diaphragm dysfunction (VIDD) is highly prevalent in patients under mechanical ventilation (MV). The study of this pathology is difficult because histological samples are difficult to obtain in critically ill patients. Recently, ultrasound measurements have been described as a good method to monitor diaphragm structure but there are no previous studies that correlates histological findings with ultrasound measurements.

**OBJECTIVES.** To correlate the cross-sectional area (CSA) of diaphragmatic muscle fibers of diaphragm muscle biopsy, with the ultrasound measurement of diaphragm thickness (*Tdi*) in mechanically ventilated donor patients.

**METHODS.** We prospectively enrolled 13 adult patients requiring mechanical ventilation (organ donor) who were admitted to the intensive care unit during 2017. Demographic data, clinical data, comorbidities, and severity scores were collected. The diaphragm thickness (*Tdi*) was determined by bedside ultrasonography previous to organ donation, as described previously in literature1. Three measurements were taken by 2 observers and average was performed. Diaphragm(DF) biopsies from DF anterior lateral costal area were obtained from ICU donor patients. Tissue was preserved immediately in alcohol-formol bath to be thereafter embedded in paraffin. Immunohistochemical and morphometric analyses were done: CSA, and proportions of type I(MHCI) and type II(MHCII) diaphragm fibers were assessed. Statistical analysis was performed using Pearson correlation coefficient, significance was set to p<0.05. CEIC PSMAR 2017/7183/I.

**RESULTS.** Muscle and ultrasound samples were obtained from 13 patients. Man were predominant (69,2%), with a mean age of 58(14) years and APACHEII score of 27(5). ICU median stay was 6(3-11) days with a median mechanical ventilation days of 6(3-14). Immunohistochemical and morphometric analyses showed a similar proportion of MHCI (50,5%) and MHCII (49,4%) diaphragm fibers with a CSA of 1480 (660) µm2 and 1722 (1031) µm2 respectively. Mean ultrasound measurement of *Tdi* was 1.68 (0.33)cm, and had a positive and significant correlation with MHCI and MHCII and Cross-sectional area (MHCI: r2 0.765 / p= 0.004; MHCII: r2 0.854 / p=0.000).

**CONCLUSION.** The ultrasound measurement of diaphragm thickness (Tdi) in mechanically ventilated patients has a good correlation with the cross-sectional area (CSA) of diaphragm fiber I and II (MHCI and MHCII).

**REFERENCE(S)**

1. Matamis, D., Soilemezi, E., Tsagourias, M. et al. Sonographic evaluation of the diaphragm in critically ill patients. Technique and clinical applications. Intensive Care Med (2013) 39: 801

### 001573

#### One-year single-centre experience of emergency department endotracheal intubations: a retrospective observational study

F. Fadhlillah[1], S. Bury[2], E. Grocholski[3], M. Dean[4], A. Refson[5]

[1]Emergency and intensive care medicine specialty registrar, London North West University HealthCare NHS Trust, London, United Kingdom; [2]Acute care common stem anaesthetics trainee, London North West University HealthCare NHS Trust, London, United Kingdom; [3]Acute care common stem emergency medicine trainee, London North West University HealthCare NHS Trust, London, United Kingdom; [4]Intensive care consultant, London North West University HealthCare NHS Trust, London, United Kingdom; [5]Emergency medicine consultant, London North West University HealthCare NHS Trust, London, United Kingdom

**Correspondence:** S. Bury

*Intensive Care Medicine Experimental* 2019, **7(Suppl 3):**001573

**INTRODUCTION.** Endotracheal intubation in the critically unwell patient is a life saving procedure, which is frequently performed in the Emergency Department (1). A high level of skill and training is needed to facilitate a controlled, safe environment in which this can be performed. The 4th National Audit Project (NAP4) of the Royal College of Anaesthetists (RCoA) and Difficult Airway Society (DAS) was designed, in part, to look at airway management in the Emergency Department and highlight any deficiencies that could lead to serious harm (2). In direct response to NAP4, the published 2018 guideline on the management of tracheal intubation in critically ill adults recommends the use of a checklist to facilitate intubations (3). Checklists have been shown to significantly reduce mortality globally in surgery and have been adopted in high-pressure environments including in pre-hospital medicine (4,5). This study describes the current practice of endotracheal intubation in a single centre emergency department in a district general hospital in Greater London.

**METHODS.** The study was a retrospective observational study. Using the emergency department's electronic system a search was carried out over a one-year period. Cases were identified if they had been coded as: transferred to another hospital; died in the emergency department; referred to Intensive Care Unit (ICU); admitted to ICU. A total of 1553 notes were reviewed and 94 intubations were identified. Factors studied included: age and sex; indication for intubation; checklist used; if no checklist, were any pre-defined safety measures documented; drugs used; arrival time and complications. No ethical approval was sought, as per institutional guidelines.

**RESULTS.** 94 intubations were identified in the one-year time period, averaging 1.8 intubations in the department per week. The most common indication for intubation was for airway protection (n=35) and 42% of the cases were due to cardiac/respiratory arrest. Only 16% of cases showed evidence of use of a checklist; in the remaining 79 patients, no patients had all the pre-defined safety measures documented. The most commonly used neuromuscular relaxant was found to be rocuronium in 45% of cases. The mean response rate time of a clinician was 10 minutes 16 seconds. There was no significant difference between the response rate times of Emergency Doctors and external physicians (p=0.0477). All intubations were successful, however, 8 complications were reported of which 100% of these were in patients without a checklist.

**CONCLUSION.** This study provides an overview of the intubation practices in a single-centre Emergency Department after the NAP4 recommendations. It has identified poor compliance (16%) to the use of a checklist in endotracheal intubations, despite current guidelines(2). Whilst checklists have been shown to cognitively off load the medical practitioner, there may be many barriers limiting its implementation(6). It adds to the growing call for better provision of care to patients with a deteriorating airway and for the continued auditing of practice. In response to these findings, our institution has introduced a structured teaching programme for staff and will aim to re-study and audit after one year.

**REFERENCE(S)**

1. Butler JM, Clancy M, Robinson N, Driscoll P. An observational survey of emergency department rapid sequence intubation. Emerg Med J. 2001 Sep;18(5):343-348
2. Cook TM, Woodall N, Frerk C; Fourth National Audit Project. Major complications of airway management in the UK: results of the Fourth National Audit Project of the Royal College of Anaesthetists and the Difficult Airway Society. Part 1: anaesthesia. Br J Anaesth. 2011 May;106(5):617-631.
3. Higgs A, McGrath BA, Goddard C, Rangasami J, Suntharalingam G, Gale R, et al. Guidelines for the management of tracheal intubation in critically ill adults. Br J Anaesth. 2018 Feb;120(2):323-352.
4. Haynes AB, Weiser TG, Berry WR, et al. A surgical safety checklist to reduce morbidity and mortality in a global population. N Engl J Med 2009;360:491–9.
5. Burgess MR, Perkins Z A standardised approach to pre-hospital RSI in the UK; utility, governance and content of current pre-induction checklists. From: London Trauma Conference 2014 London, UK. 9-12 December 2014
6. Galloway R, Swann F Introducing checklist for sedation and intubation in the emergency department Emerg. Med. J 2013; 30 : 871

### 001579

#### Patterns of care in patients with community acquired pneumonia and moderate to severe acute respiratory distress syndrome

JA. Escalona Solari[1], E. Henriquez[1], N. Pavez[2], L. Alegria[2], V. Oviedo[2], A. Bruhn[2], J. Retamal[2], G. Bugedo[2]
[1]Departamento de medicina intensiva, Pontificia Universidad Catolica de Chile, Santiago, Chile; [2]Departamento de medicina intensiva, Pontificia Universidad Católica de Chile, Santiago, Chile
**Correspondence:** J.A. Escalona Solari
*Intensive Care Medicine Experimental* 2019, **7(Suppl 3):**001579

**INTRODUCTION.** Severe community pneumonia is the most frequent cause of respiratory failure and acute respiratory distress syndrome (ARDS). The mortality reported in severe forms reaches 40%. Since 2013, a mechanical ventilation protocol (MV) based on the limitation of tidal volume (VT), recruitment maneuvers and high levels of PEEP, the sequential application of neuromuscular blockade (NMB) and prone positioning, and fluid restriction have been applied in our hospital since 2013, leaving extracorporeal oxygenation (ECMO) as a rescue therapy for refractory hypoxemia.

**OBJECTIVES.** To present the results of the application of a protocol of respiratory failure in the management of patients with moderate and severe ARDS.

**METHODS.** Observational clinical study. We analyzed retrospectively from 2013 to 2015, and prospectively since 2016 to all patients admitted with moderate to severe ARDS due to community-acquired pneumonia. Demographic data, infectious agents, PaO2:FiO2 ratio (PaO2/FiO2) on admission and worst values during MV, ventilatory mechanics, treatments received (prone, NMB and/or ECMO). Main outcomes were hospital stay and hospital mortality. Data were analyzed by descriptive statistics, distribution was evaluated according to the Shapiro-Wilk test, and were expressed according to mean ± SD and/or median and p25-75, as appropriate.

**RESULTS.** 59 patients were included (age 49.5 years, 36 women (61%), PaO2/FiO2 at 24 hours was 162 [125 – 197] and worst PaO2/FiO2 96.4 [66.7 – 115.2). Influenza virus was identified In 49% of cases as etiology. Mean VT and PEEP during the first day were 331 [294 – 389] ml (5.7 [5.1 – 6.7] ml/kg IBW), and 13 [12-16] cmH2O, respectively. Forty five (76%) patients received NMB for 4 (1-7) days, 36 (61%) were in prone position for 3 (1-6) days, and 8 (13.5%) received ECMO therapy. From 18 patients transferred from other centers for ECMO, only 4 (22%) were connected to ECMO. The median time in MV and hospital were 15 [9 - 25] and 26 [19 - 38] days, respectively. Five (8.5%) patients died.

**CONCLUSION.** The implementation of a protective ventilation program allows achieving good results, using widely available and low cost therapies. Using prone positioning and NMB allow to rescue a high proportion of patients from ECMO, highlighting the importance of these cost effective therapies.

### 001593

#### High-flow nasal cannula oxygen therapy in adult patients with acute respiratory failure: our initial experience

D. Adrião, R. Neto, AL. Rios, M. Basto, P. Castelões
Department of Intensive Care, Centro Hospitalar Vila Nova de Gaia/Espinho, Vila Nova de Gaia, Portugal
*Intensive Care Medicine Experimental* 2019, **7(Suppl 3):**001593

**INTRODUCTION.** Acute respiratory failure (ARF) is one of the leading causes for ICU admission [1]. Recently, high-flow nasal cannula (HFNC) oxygen therapy has seen increasing use in patients with ARF. The physiological benefits of HFNC include higher and more stable $FiO_2$ values, delivery of heated and humidified gas and reduction of the anatomical dead space and work of breathing. HFNC also generates low levels of positive pressure in the upper airways [1, 2].

**OBJECTIVES.** Describe our ICU initial experience with HFNC oxygen therapy in non-intubated patients with acute respiratory failure and evaluate improvement of oxygenation, the need for intubation and mortality.

**METHODS.** We performed an observational retrospective study involving non-intubated patients with acute respiratory failure treated with HFNC admitted to a polyvalent intensive care unit between January 2017 and March 2019. The HFNC used consisted of an air-oxygen blender with adjustable $FiO_2$ (0.21 to 1.0), delivering a modifiable gas flow up to 60 L/minute (Optiflow; Fisher & Paykel Healthcare, Auckland, New Zealand).

**RESULTS.** Twenty-four patients were included, 19 men (79%), mean age 61 ± 11 years. Mean APACHE II score and SAPS II were 19 ± 8 and 36 ± 11, respectively. The main cause of acute respiratory failure was hospital-acquired pneumonia (n = 8), followed by community-acquired pneumonia (n = 5), extrapulmonary sepsis (n = 3), pneumonia related to imunosupression (n = 2) and aspiration (n = 1).

The mean duration of HFNC oxygen therapy was 56 ± 46 hours and 3 patients received alternating periods with noninvasive ventilation. After 1 hour of therapy, $PaO_2$ increased from 59 ± 9.1 mmHg to 88 ± 33.7 mmHg ($p$ = 0.005) and to 78 ± 33.7 mmHg ($p$ = 0.018) after 6-12h. $PaO_2/FiO_2$ ratio increased from 107 ± 52.4 to 129 ± 76.3 after 1 hour ($p$ = 0.159).

Despite de use of HFNC, in 13 (54%) patients intubation was unavoidable. In this group the mean duration of HFNC was 28 ± 13 hours.

Mean ICU length of stay was 8 ± 4 days and was higher for patients who required intubation (14 ± 8 days). Eight patients died in the ICU, in 7 of which was decided not to reanimate or intubate due to extensive comorbidity. The overall mortality at 90 days was 38% (n = 9). No statistical significance was found between oxygen saturation, $PaO_2$, $PaCO_2$, $PaO_2/FiO_2$ ratio or pH prior to HFNC therapy and mortality.

**CONCLUSION.** Few studies have examined large cohort of patients with *de novo* acute respiratory failure and the results suggesting a reduction in the intubation rate with HFNC remain controversial [3].

Although hypoxemia was improved in the first hours, a high proportion of patients still required intubation. Further studies are needed to clarify the clinical circumstances in which HFNC should be used.

**REFERENCE(S)**

1. Papazian L, et al. Use of high-flow nasal cannula oxygenation in ICU adults: a narrative review. Intensive Care Med. 2016; 42(9):1336-49.
2. Frat JP, et al. High-flow oxygen through nasal cannula in acute hypoxemic respiratory failure. N Engl J Med. 2015; 4;372(23):2185-96.
3. Helviz Y, Einav S. A Systematic Review of the High-flow Nasal Cannula for Adult Patients. Crit Care. 2018; 20;22(1):71.

### 001595

#### Impact of fluid administration in an experimental model of VILI

F. Vassalli[1], M. Bonifazi[2], F. Romitti[2], M. Busana[2], I. Pasticci[2], L. Giosa[2], MM. Macrì[2], M. Quintel[2], L. Gattinoni[2]

[1]Göttingen university, Göttingen, Germany; [2]Department of anesthesiology, emergency and intensive care medicine, University Hospital Göttingen - University Medical Center Göttingen, Göttingen, Germany

**Correspondence:** F. Vassalli

*Intensive Care Medicine Experimental* 2019, **7(Suppl 3):**001595

**INTRODUCTION.** Non-cardiogenic pulmonary edema and increased lung weight are hallmarks of ARDS, as a consequence lung weight and wet-to-dry ratio are often used as markers of lung damage in animal experimental models of ARDS. However, fluid management may have a profound impact on these indicators of lung damage.

**OBJECTIVES.** To describe the impact of different fluid administration strategies on lung weight and wet-to-dry ratio in two series of experiments on ventilator-induced lung injury in the framework of mechanical power

**METHODS.** In the first series of experiments, 36 healthy piglets (23.3 ± 2.3 kg) were mechanically ventilated for 52 hours with a mechanical power of 26.6 ± 12.1 J/min. In the second series, 42 healthy piglets (24.2 ± 2 kg) were mechanically ventilated for 48 hours with a mechanical power of 21.8 ± 8.3 J/min. Due to institutional policy, the sedation with propofol was increased from 6-9 mg/kg/h in the first series to 10-12 mg/Kg/h in the second series, while sufentanil (from 2-3 μg /kg/ h to 1.5-2.5 μg /kg/ h) and midazolam (from 1.5-2.5 mg/kg/h to 0.8-1.5 mg/kg/h) were reduced. Accordingly, we modified the fluid management strategy in order to attain a mean arterial pressure of 60 mmHg: the first animals received 1-2 ml/kg/h of maintenance Stereofundin 1:1 and 150 ± 232 ml/h of Gelafundin 4%, the second group received a maintenance of 3-4 ml/kg/h and 647 ± 411 ml/h of colloids. Norepinephrine was started in case of persistent hypotension, epinephrine could also be added, but only in the second group.

**RESULTS.** The total amount of administered fluids was 4119 ± 910 ml in the first series and 9976 ± 2376 ml in the second series (see figure 1), at the end of the experiment the pigs gained 2.4 ±1.6 kg and 5.7 ±2.7 kg respectively (p < 0.001 for both). There was no difference in norepinephrine (0.13 ± 0.21 vs 0.14 ± 0.21 mcg/kg/min), while epineprhine was 0.03 ± 0.07 mcg/kg/min in the second group. Lung weight and wet-to-dry ratio were higher in the second group (616.6 ±142 and 6.95± 1.1) when compared to the first group (371.2 ±105 and 6.26 ±0.6) (p < 0.001 for both). See figure 2. Mechanical power, instead, was higher in the first group (p = 0.049).

**CONCLUSION.** Propofol increase, despite decrease in other sedative and analgesic agents, lead to increased need of fluids to maintain hemodynamic stability in this animal experimental model. Lung weight and wet-to-dry ratio were increased in the group with liberal fluid strategy, despite a lower mechanical power. The assessment of lung damage via surrogates of lung edema should take into account the amount of administered fluids. Sedation policies should be standardized to allow a better comparison among different experiments.

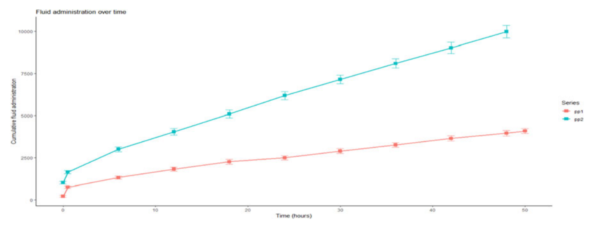

**Fig. 1 (abstract 001595).** See text for description

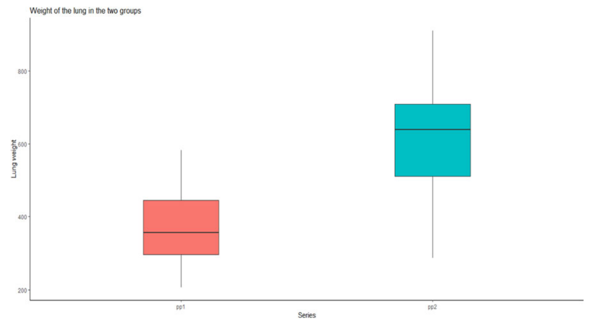

**Fig. 2 (abstract 001595).** See text for description

### 001605

#### Role of hypercapnia in LPS injured human primary alveolar cells

J. Bringue Roque[1], M. Camprubí-Rimblas[2], L. Morales-Quinteros[3], A. Artigas[4]

[1]Institut d'investigació i innovació parc tauli (i3pt), Universitat Autònoma de Barcelona, Barcelona, Spain; [2]Institut d'investigació i innovació parc tauli (i3pt), Universitat Autònoma de Barcelona, Cerdanyola del Vallès, Spain; [3]Intensive care, Hospital Universitari Sagrat Cor - Grup Quirónsalut, Barcelona, Spain; [4]Critical care center, Universitat Autònoma de Barcelona - UAB, Sabadell, Spain

**Correspondence:** J. Bringue Roque

*Intensive Care Medicine Experimental* 2019, **7(Suppl 3):**001605

**INTRODUCTION.** In patients with acute respiratory distress syndrome (ARDS) lung-protective ventilationstrategies using low tidal volume and low airway pressure ameliorated outcome although they lead tohypercapnia. Controversial effects of hypercapnia in ARDS patients are nowadays being discussed

**OBJECTIVES.** Evaluate the effect of hypercapnia on inflammatory response, mediators of recruitment and apoptosis in human primary alveolar cells (hATII) injured with lipopolysaccharide (LPS)

**METHODS.** hATII from pulmonary biopsies of patients that underwent lobectomy were isolated and incubated at 37ºC with 5% CO2 (Normocapnia) or 15% CO2 (Hypercapnia; pCO2=80 mmHg) with or without 15mM THAM buffer to maintain pH at 7.4. All groups were injured with 100 ng/ml LPS. Pro-inflammatory molecules (IL-1β, iNOS), mediators of cellular recruitment (IL-8, CCL-2) and apoptosis (caspase-3) were analyzed

**RESULTS.** In hATII injured with LPS the expression of mediators of neutrophils (IL-8) and monocytes (CCL-2) recruitment was decreased when cells were exposed to hypercapnia. No effects of hypercapnia were found when analyzing pro-inflammatory markers and apoptosis

**CONCLUSION.** Hypercapnic conditions in hATII cells injured with LPS reduced the expression of mediators of recruitment of pro-inflammatory cells but did not affect pro-inflammatory molecules nor apoptosis. There is a need to better understand the biological and physiological effects of hypercapnia

### 001606

#### A dual-centre cohort study of patients with interstitial lung disease admitted to intensive care

M. Bridgett[1], D. Jennings[2], H. Roth[3], L. Hodgson[4]
[1]Respiratory, Worthing, Worthing, United Kingdom; [2]Respiratory, St Richard, Chichester, United Kingdom; [3]Respiratory, Worthing Hospital, Worthing, United Kingdom; [4]Intensive care, Worthing Hospital, Worthing, United Kingdom
**Correspondence:** L. Hodgson
*Intensive Care Medicine Experimental* 2019, **7(Suppl 3):**001606

**INTRODUCTION.** A recent systematic review of interstitial lung disease (ILD) cases admitted to ICU found high short-term mortality, though few studies reported longer-term outcomes. We examined short- and long-term mortality in an ICU population of patients with ILD.

**METHODS.** To examine associations of short and longer term mortality we conducted a retrospective cohort study at two non-specialist UK ICUs (consisting of a 12-bed and a 10-bed unit) of consecutive adults admitted for respiratory failure with ILD (April 2008- January 2019).

**RESULTS.** 84 patients were included with median age 72 (IQR 62-78), 57% male and unit stay was 5 (2-9) days. Mechanical ventilation (MV) was performed in 43%. Median ICNARC score was 18 (14-27) and APACHE II 18 (14-23). Amongst subtypes idiopathic pulmonary fibrosis (IPF) and connective-tissue disease associated ILD (CTD-ILD) were most common (23% and 18% respectively). ICU mortality was 60% and one-year mortality 70%. MV was associated with a significant increase in ICU mortality (86.1% vs 39.6%, odds ratio (OR) 9.5 (3.1-28.6), P <0.001). Of the 4/36 MV patients (11%) alive at 1-year, 2 were classed IPF, 1 NSIP and 1 drug-induced. Of the patients who did not require MV 44% remained alive at 1-year follow-up. On Kaplan-Meier survival analysis MV was associated with a shorter duration of survival (see figure). There was no significant difference in survival between ILD subtypes.

**CONCLUSION.** In this dual-centre ICU cohort ILD was associated with significant short and longer-term mortality as previously described, however a small number of patients who were ventilated remained alive on longer-term follow-up.

**REFERENCE(S)**

1. Huapaya, JA et al. Risk factors for mortality and mortality rates in interstitial lung disease patients in the intensive care unit. European Respiratory Review. 2018 Nov 21;27(150). pii: 180061. doi: 10.1183/16000617.0061-2018.

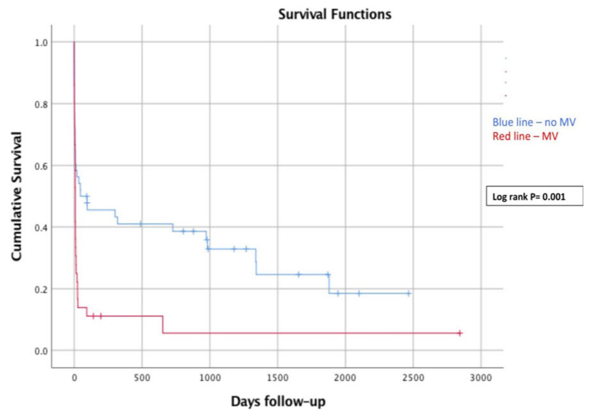

**Fig. 1 (abstract 001606).** See text for description

## INF - New insights in specific infectious situations

### 000150

#### The prognostic role of infectious complications after Subarachnoid Hemorrhage

E. Bogossian[1], J. Creteur[1], JL. Vincent[2], FS. Taccone[1], L. Attanasio[1]
[1]Soins intensif, ULB Erasme, Anderlecht, Belgium; [2]Soins intensif, ULB Erasme, Brussels, Belgium
**Correspondence:** E. Bogossian
*Intensive Care Medicine Experimental* 2019, **7(Suppl 3):**000150

**INTRODUCTION.** Subarachnoid hemorrhage (SAH) is associated with high morbidity[1,2].Among all complications, infections, in particular if hospital-acquired, could represent an important cause of death in SAH patients[3]. However, few data on this issue are available.

**OBJECTIVES.** The aim of this study was to describe infectious complications in SAH patients and to evaluate their impact on outcome.

**METHODS.** Single-center cohort study including all SAH patients admitted from January 2011 to December 2016, who stayed in the ICU for at least 24 hours. Infection diagnosis was retrieved from medical files; CNS infections were not included. Multivariable logistic regression analysis was performed to identify risk factors for development of infection, for ICU mortality and unfavorable neurological outcome (UO) at 3 months, defined as a Glasgow Outcome Scale of 3-5.

**RESULTS.** Of the 250 SAH patients without vetriculitis treated, 70 (28%) developed at least one infection; the most frequent site of infection was respiratory (57.1%), primary blood stream (16%) and urinary tract infections (15.7%). Twenty nine patients (41.4%) had at least on episode of septic shock. ICU mortality rate was similar between infected and non-infected patients (35.7% vs. 27.2%, p=0.42 but infected patients had a higher UO rate were – 60.0% vs. 33.3%, p= 0,001. ICU length of stay [OR 1.14 (CI 95% 1.07-6.58)], WFNS score of 4-5 [OR 5.34 (CI 95% 1.40-20.33)], delayed cerebral ischemia [OR 3.75 (CI 95% 1.47-9.60)], seizures [OR 2.78 (CI 95% 1.17-6.58)] and fever [OR 8.69 (CI 95% 3.45-21.86)] were independently associated with the occurrence of infections. Infection was independently associated with ICU mortality (OR 3.93 CI 95% 1.31-11.80). The development of sepsis [OR 25.22 (CI 95% 1.75-363.19)] was independently associated with UO.

**CONCLUSION.** Infection is a common complication in SAH patients. Sepsis is independently associated with poor long-term neurological outcome.

**REFERENCE(S)**

1. Dettenkofer, M., et al., Surveillance of nosocomial infections in a neurology intensive care unit. J Neurol, 2001. 248(11): p. 959-64.
2. Connolly, E.S., Jr., et al., Guidelines for the management of aneurysmal subarachnoid hemorrhage: a guideline for healthcare professionals from the American Heart Association/american Stroke Association. Stroke, 2012. 43(6): p. 1711-37.
3. Mozaffarian, D., et al., Heart disease and stroke statistics–2015 update: a report from the American Heart Association. Circulation, 2015. 131(4): p. e29-322. 2

### 000171

#### Preliminary prospective study of veno-arterial extracorporeal membrane oxygenation device's bacterial colonization

S. Pons[1], C. Rodriguez[2], D. Lobo[1], M. Martin[1], G. Gricourt[2], F. Cook[1], R. Mounier[1]
[1]Polyvalent surgical icu, Hôpital Henri-Mondor Ap-Hp, Créteil, France; [2]Virology, Hôpital Henri-Mondor Ap-Hp, Créteil, France
**Correspondence:** S. Pons
*Intensive Care Medicine Experimental* 2019, **7(Suppl 3):**000171

**INTRODUCTION.** Veno-arterial extracorporeal membrane oxygenation (VA ECMO) is a medical device complicated with high rates of mortality and morbidity, especially infectious complications occurring in 25% of patients (1-3).

**OBJECTIVES.** To study the bacterial colonization and biofilm present on VA ECMO's cannulae and oxygenator membranes and to determine the potential use of advanced techniques in daily clinical practice.

**METHODS.** A preliminary monocentric prospective physiological study was performed at Henri Mondor Hospital, France. Samples of both arterial and venous cannulae and oxygenator membranes were aseptically collected from patients under VA ECMO support, at the time of ECMO's weaning or immediately after patient's death. Each sample was analyzed by three different methods in order to detect bacterial colonization: standard culture, scanning electron microscopy (SEM) and 16S rRNA gene-based metagenomic analysis (next generation sequencing).

**RESULTS.** Overall, ten patients were included in the study. The median time of ECMO support was 10 [7-20] days. Seven (70%) patients developed an infection during VA ECMO support, including two (20%) patients with a bloodstream infection. In culture, three on nine (33%) arterial cannulae and 3/9 (33%) venous cannulae were positive. In SEM, all the oxygenator membranes had bacterial biofilm on their surface while being all negative in culture. Seven on nine (78%) arterial cannulae and 7/9 (78%) venous cannulae had also bacterial biofilm on their surfaces in SEM. In metagenomic analysis, 7/9 (78%) arterial cannulae, 4/9 (44%) venous cannulae and 8/9 (89%) membranes were positive. However, four (44%) negative venous cannulae in metagenomics analysis were considered positive in SEM. Bacterial species richness was significantly different between oxygenator membranes and arterial/venous cannulae ($p<0,01$). Indeed, Faith's phylogenetic diversity was 71 [58-78] on membranes; 112 [100-117] on arterial cannulae ($p<0,01$) and 98 [80-118] on venous cannulae ($p=0,01$). Antibiotics therapy during ECMO support did not modify Faith's phylogenetic diversity (81 [39-150] without antibiotics and 95 [77-108] with antibiotics; $p=0,79$). There was no significant difference of bacterial abundance between the different samples' types (membranes 0,63 [0,62-0,71]; arterial cannulae 0,71 [0,67-0,72]; venous cannulae 0,68 [0,62-0,70]; $p=0,53$). Bacterial colonization was mostly found on oxygenator membranes followed by arterial cannulae and then venous cannulae. SEM was the most powerful tool to detect bacterial colonization. ECMO colonization was systematically detected in patients with bloodstream infection but also in five (50%) patients without infection.

**CONCLUSION.** Bacterial colonization's detection by standard culture is often negative but SEM and 16S rRNA gene-based metagenomic analysis improve biofilm detection on ECMO's cannulae and oxygenator membranes, while not being used in this indication in daily clinical practice. More patients would be necessary to better define the bacterial colonization's role in the occurrence of infectious complications and to precisely describe the microorganisms responsible for colonization.

**REFERENCE(S)**

1. Combes A, Leprince P, Luyt C-E, Bonnet N, Trouillet J-L, Léger P, et al. Outcomes and longterm quality-of-life of patients supported by extracorporeal membrane oxygenation for refractory cardiogenic shock. Crit Care Med 2008. 36(5):1404–11.
2. Bizzarro MJ, Conrad SA, Kaufman DA, Rycus P, Extracorporeal Life Support Organization Task Force on Infections, Extracorporeal Membrane Oxygenation. Infections acquired during extracorporeal membrane oxygenation in neonates, children, and adults. Pediatr Crit Care Med J 2011. 12(3):277–81.
3. Schmidt M, Brechot N, Hariri S, Guiguet M, Luyt CE, Makri R, et al. Nosocomial Infections in Adult Cardiogenic Shock Patients Supported by Venoarterial Extracorporeal Membrane Oxygenation. Clin Infect Dis 2012. 55(12):1633–41.

### 001476

#### Evolution of HIV patients in Spanish ICUs in the last 30 years

VA. Hortigüela Martín, E. Rosas Carvajal, D. Robaglia, LM. Polanco Mahecha, JJ. Paez Vargas, AM. Ioan, N. Arias Martínez, Á. Vidal González, AI. Tejero Redondo, C. Pérez Calvo
Intensive care unit, Hospital Universitario Fundación Jiménez Díaz, Madrid, Spain
**Correspondence:** E. Rosas Carvajal
*Intensive Care Medicine Experimental* 2019, **7(Suppl 3):**001476

**INTRODUCTION.** HIV is a pathology that has evolve much in the past decades with the introduction of new antiretroviral treatments.

**OBJECTIVES.** Compare today's Spanish HIV patients ICU admissions with the 90's ones.

**METHODS.** Observational and retrospective study where we included all HIV patients admitted in the ICU of Jiménez Díaz Fundation from Madrid (Spain) between 2013 and 2018 [actual sample (AS)].

We collected demographics parameters (age, sex), ICU cause of admission, time of diagnosis of HIV (before/during ICU stay), severity scores (APACHE II), lenght of ICU stay and ICU mortality.

Then we compared this information with the existing bibliography, specifically with the data published by Choperena et cols (1) from the Arantzazu Hospital in San Sebastián (Spain) between 1985 and 1997 [reference sample (RS)].

**RESULTS.** We collected 122 HIV patients (2% of the total admitted on ICU). The demographic characterictics are compared on the table bellow. Sexual was the most frequent mechanism of infection. 12 patients were admitted on AIDS state.

The most common causes of admission in the AS were: infections (31.2%), intoxications (27.9%) and scheduled surgery (17.1%), mostly cardiac surgery in 7.3%. However, in the RS, the vast majority of the patients were admitted for an infection.

The HIV primodiagnosis was performed in the ICU in 11.5% of the cases on the AS and the majority of this group was admitted for pneumonia due to penumocystis jirovecii (71.4% with $p=0.013$) with a longer ICU stay (5,1 vs 14,3 days, $p=0.002$) but without more mortality associated. On the contrary, in the RS the HIV diagnosis was made during ICU stay in 20% of the patients. There has been a remarkable reduction in ICU stay and mortality between the AS and the RS.

**CONCLUSION.** HIV is common desease in today's Spanish ICUs patients and is associated to infections and intoxications in a remarkable way. We have found that nowadays' HIV patients are admitted in ICU for causes independent from their basal disease, with a similar prognostic to the general population, except in patients with a poor control of HIV.

**REFERENCE(S)**

1. G. Choperena. One-year survival of HIV-positive patients admitted to the intensive care unit before the era of combined antiretroviral therapy (Pre-HAART). Med Intensiva, 2001 January; 25(7): 263-268.
2. The authors declare no conflict of interest.
3. Vidal-Cortés P, Álvarez-Rocha LA, Fernández-Ugidos P, Pérez-Veloso MA, Suárez-Paul IM, Virgós-Pedreira A, Pértega-Díaz S, Castro-Iglesias ÁC. Epidemiology and outcome of HIV-infected patients admitted to the ICU in the current highly active antiretroviral therapy era. Medicina Intensiva. 2019 Apr 7.

**Table 1 (abstract 001476).** See text for description

| | 2013-2018 (5 years) *Actual sample* | 1985-1997 (12 years) *Reference sample* |
|---|---|---|
| N | 122 | 102 |
| Males | 88.5% | 70% |
| Average age | 46.8 ± 11.6 | 32 |
| AIDS state | 9.8% | 65% |
| APACHE II | 16.1 ± 8.4 | 21.27 ± 7.18 |
| Infection as cause of admission on ICU | 31.2% | 60% |
| Primodiagnosis on ICU | 11.5% | 20% |
| Average stay on ICU | 6.1 ± 8.9 days | 8.9 ± 9.1days |
| Mortality on ICU | 16.4% | 48% |

### 001486

#### 2019 Influenza outbreak: overview of patients admitted to our hospital and ICU

J. Tejero Aranguren[1], MT. Cruces Moreno[1], O. Moreno Romero[2], I. Cruz Valero[1], A. Carranza Pinel[3], ME. Yuste Ossorio[1], M. Colmenero Ruíz[1]
[1]Intensive care unit, Hospital Universitario San Cecilio, Granada, Spain; [2]Intensive care unit, Regional Hospital Santa Ana de Motril, Motril, Spain; [3]Intensive care unit, Hospital Universitario Clínico., Granada, Spain
**Correspondence:** J. Tejero Aranguren
*Intensive Care Medicine Experimental* 2019, **7(Suppl 3):**001486

**INTRODUCTION.** Seasonal influenza causes high morbidity worldwide and high mortality in develope countries. While most influenza patients have a self-limited respiratory illness, 5-10% of hospitalized patients develop severe disease requiring Intensive Care Unit (ICU) admission (1,2).

**OBJECTIVES.** Describe the hospitalization rate and ICU admission rate, of patients who came to the emergency room and were diagnosed with influenza A, during Influenza Epidemic 2019. Describe the clinical characteristics of both groups.

**METHODS.** Descriptive analysis of patients admitted for Influenza A in two hospitals of Granada, Spain, during the Influenza Epidemic in 2019. The data have been obtained from the microbiology registry as well as from ENVIN-HELICS registry of Spanish Society of Intensive Care. We evaluated the clinical records of all the patients with positive rapid diagnostic testing of influenza A in this period, who were hospitalized. We recorded the epidemiological characteristics, comorbidities and mortality among patients admitted to the ICU and those admitted to other wards.

**RESULTS.** Rapid diagnostic testing on Influenza was performed in 1550 patients, 204 patients were positive for Influenza A. Finally, 111 patients required hospital admission, 51.8% were women (2.7 pregnant women) and 48.2% men with an average age of 70.24 years (27-99). In 42.7% of the cases, the patients were smokers or ex-smokers and 12.7% were obese. 69.1% of the patients suffered multiple pathologies; 10.9% had no personal medical history of interest. 24.5% of cases had oncohematologic disease, 29.6% had corticoid therapy and 18.5% chemotherapy. In 70% of cases the clinic debut was fever and in 68.2% they also associated dysnea. 27.3% associated bacterial infection. 43.3% of the cultures insolated Streptococcus pneumoniae followed by Staphylococcus (10%) and Pseudomonas (10%). 64 patients received antibiotic treatment associated with olsentamivir, with the most commonly used combinations being ceftriaxone + azithromycin (28.12%), levofloxacin (28.12%) and ceftriaxone + levofloxacin (17.18%).

Twenty one patients required admission to Intensive Care Unit: In this group 47.6% requiered High Flow Nasal Canula or noninvasive ventilation and 43.3% required mecanic ventilation of which 90% needed muscle relaxation and 50% tracheotomy. The mean SOFA upon admission was 4.67 (1-14) and APACHE 15.86 (5-25). Of the patients admitted to ICU, 38.1% had mild Acute Respiratory Distress Syndrome (ARDS) , 33.3% had moderate ARDS and 23.8% had severe ARDS. 19 patients (80%) were suffered an infection associated with mechanic ventilation: 50% tracheobronchitis and 30% pneumonia. Two patients died during their stay in ICU.

**CONCLUSION.** Half of the patients who came to the emergency department for Influenza A needed hospital admission, 18.9% required admission to ICU. The rest of clinical and therapeutic characteristics are similar to those of previous years.

**REFERENCE(S)**

1. Arcos González P, Pérez García S, Castro Delgado R. Potential role of Emergency Medical System call centres in epidemiological surveillance of seasonal influenza. Cent Eur J Public Health. 2019 Mar;27(1):64-67
2. Beumer MC, Koch RM, van Beuningen D, OudeLashof AM, van de Veerdonk FL, Kolwijck E, van der Hoeven JG, Bergmans DC, Hoedemaekers CWE. Influenza virus and factors that are associated with ICU admission, pulmonary co-infections and ICU mortality. J Crit Care. 2019 Apr;50:59-65
3. The authors declare that they have not received any funding.

### 001502

#### Invasive aspergillosis in non-neutropenic critically ill patients: clinical presentation, underlying conditions and outcome

M. Miranda, A. Catarino, V. Ferreira, S. Teixeira, A. Marques, J. Janeiro, P. Martins
Intensive Care Unit, Centro Hospitalar e Universitário de Coimbra, Coimbra, Portugal
**Correspondence:** M. Miranda
*Intensive Care Medicine Experimental* 2019, **7(Suppl 3):**001502

**INTRODUCTION.** Invasive aspergillosis (IA) is a fungal infection that mainly affects immunocompromised patients. However, non-neutropenic patients in the Intensive Care Unit (ICU) population have shown an increasing risk profile for aspergillosis mainly due to underlying comorbidities such as chronic obstructive pulmonary disease (COPD), malignancy and prolonged corticosteroid use. In the ICU setting, IA remains difficult to diagnose and problematic to treat.

**OBJECTIVES.** The aim of this study was to determine the incidence, risk factors and outcome of patients with *Aspergillus* colonization or IA in our ICU.

**METHODS.** A 10-year retrospective study including all patients with a positive *Aspergillus* culture during ICU stay was performed. Cases were classified as *Aspergillus* colonization, putative IA and proven IA according to validated criteria. Clinical records were reviewed in order to obtain demographic, microbiological and diagnostic data as well as underlying conditions of these patients.

**RESULTS.** A total of 87 patients were included, 51 (58.6%) of whom were colonized, 34 (39.0%) had putative IA and 2 (2.3%) had proven IA. The lung was the most frequent site of infection (98.8%) and *Aspergillus fumigatus* the most commonly isolated species (55.2%). COPD and liver disease were the most frequent comorbidities, equally seen in both groups. Patients with IA had higher incidences of malignancy (both solid tumors and haematological malignancy) and history of glucocorticoid treatment.
Compared with other patients, they were more frequently diagnosed with sepsis and acute respiratory distress syndrome (ARDS) on ICU admission and more frequently received renal replacement therapy (RRT) during the ICU stay.
IA patients had increased length of stay (25.2 versus 16.1 days, p< 0.05) and higher ICU mortality (31.3% among colonized patients and 44.4% in those with IA, p <0.05) but there was no difference concerning in-hospital mortality (47.1% and 50%, respectively).
**CONCLUSION.** IA among critically ill patients is associated with high mortality. Patients diagnosed with proven or putative IA had greater severity of illness and more frequently needed RRT than those with *Aspergillus spp* colonization.

**REFERENCE(S)**

1. Cuenca-Estrella et al, Defining standards of CARE for invasive fungal diseases in the ICU, J Antimicrob Chemother 2019; 74 Suppl 2: ii9–ii15
2. Taccone et al., Epidemiology of invasive aspergillosis in critically ill patients: clinical presentation, underlying conditions, and outcomes, Critical Care (2015) 19:7
3. None

### 001609

#### Clinical differences of severe influenza infection among healthcare workers compared to general population

S. Barbadillo[1], F. González de Molina[2], F. Alvarez-Lerma[3], S. González Marcos[4], ML. Martínez[1], C. Hermosa[5], JC. Pozo Laderas[6], J. Bonastre[7], B. Sánchez González[2], J. Trenado Alvarez[2], MD. Bosque[1]
[1]Intensive care medicine department, Hospital Universitario General de Cataluña, Sant Cugat del Vallés, Spain; [2]Intensive care medicine, Mútua Terrassa University Hospital, Terrassa, Spain; [3]Intensive care unit, Hospital del Mar, Barcelona, Spain, Spain; [4]Workplace health and safety department, Mútua Terrassa University Hospital, Terrassa, Spain; [5]Intensive care medicine, Hospital Universitario del Henares, Coslada, Spain, Spain; [6]Intensive care medicine, Hospital Universitario Reina Sofía, Córdoba, Spain, Spain; [7]Intensive care medicine, Hospital Universitario y Politécnico La Fe, Valencia, Spain, Spain
**Correspondence:** F. González de Molina
*Intensive Care Medicine Experimental* 2019, **7(Suppl 3):**001609

**INTRODUCTION.** Influenza infection causes severe morbidity and mortality around the world. There are limited data about severe influenza infection among healthcare workers compared to the general population.
**OBJECTIVES.** Our aim was to determine clinical differences between health-care workers and general population with severe influenza infection admitted to the intensive care units.
**METHODS.** Prospective, observational, multicenter study conducted in 148 Spanish ICUs from 2009 to 2017. Healthcare workers with severe influenza infection were compared with the general population. All serotypes were confirmed using RT-PCR at ICU admission. Patients´ demographic, clinical, radiologic features, laboratory values, ICU and hospital length of stay (LOS) and outcomes were recorded. Influenza vaccination status is reported. Discrete variables are expressed as counts (percentage) and continuous variables as medians with 25th to 75th interquartile range (IQR). Differences between groups were assessed using the x2 test and the Fisher exact test for categoric variables and Mann-Whitney U test for continuous variables.
**RESULTS.** Of 4175 patients with confirmed influenza infection at ICU admission, 2915 of working-age adults were selected. A total of 47 healthcare workers were compared to 2864 non-healthcare professionals. There was a less proportion of male among healthcare workers (58.1% vs 36.2%, P =0.003), and less overall comorbidities (69,0% vs 53.2%, P =0.020). Healthcare workers had a higher history of asthma (21.3% vs 9.8, P=0.009) and less COPD (6.4% vs 14.4%, P=0.048), and heart failure (0% vs 7.7%, P= 0.047). There are no statistical differences in other variables related to clinical influenza infection and outcome between groups. There are no differences in MODS, mechanical ventilation, RRT, cobacterial infection or hospital-acquired pneumonia, influenza serotype (A/H1N1pdm8, 78.2% vs 76.6%, P=0.758) or proportion of vaccinated patients (4.3% vs 5.9%, P=0.917). Vaccinated rate among healthcare workers reported by Spanish Ministry of Health and Social Policy is 30%. No difference in ICU LOS, hospital LOS or mortality was observed.
**CONCLUSION.** The clinical characteristics, evolution and outcome of healthcare workers with severe influenza admitted to ICU is similar to the general working population.

**REFERENCE(S)**

1. https://www.mscbs.gob.es/profesionales/saludPublica/prevPromocion/vacunaciones/Vacunacion_Gripe.htm
2. Grupo Español de Trabajo de Gripe Grave A (SEMICYUC)

### 000454

#### Characteristics and outcomes of Stenotrophomonas maltophilia hospital-acquired pneumonia in Intensive Care Unit: a multicenter retrospective study

P. Guerci[1], H. Bellut[2], M. Mokhtari[1], J. Gaudefroy[3], N. Mongardon[4], C. Charpentier[5], G. Louis[6], P. Tashk[7], C. Dubost[8], E. Novy[1], S. Ledochowski[9], A. Kimmoun[10], T. Godet[11], J. Pottecher[3], JM. Lalot[12], D. Hajage[13], A. Bouglé[2]
[1]Département d'anesthésie-réanimation, institut lorrain du cœur et des vaisseaux, CHRU Nancy, Nancy, France; [2]Département d'anesthésie-réanimation, Groupe Hospitalier Pitié-Salpêtrière, Assistance Publique - Hôpitaux de Paris, Paris, France; [3]Service d'anesthésie-réanimation chirurgicale, Hospital Hautepierre Hospitals Academics De Strasbourg, Strasbourg, France; [4]Service d'anesthésie-réanimation, Hôpital Henri Mondor, Assistance Publique - Hôpitaux de Paris, Créteil, France; [5]Réanimation chirurgicale polyvalente, hôpital central, CHRU Nancy, Nancy, France; [6]Réanimation polyvalente, Hôpital de Mercy, CHR Metz Thionville, Metz, France; [7]Département d'anesthésie-réanimation, Hôpital Bichat, Assistance Publique - Hôpitaux de Paris, Paris, France; [8]Réanimation polyvalente, Hôpital d'Instruction des Armées Bégin, Saint-Mandé, France; [9]Service de réanimation polyvalente, Groupement Hospitalier Nord Dauphiné- Centre Hospitalier Pierre Oudot, Bourgoin-Jallieu, France; [10]Réanimation médicale, institut lorrain du cœur et des vaisseaux, CHRU Nancy, Nancy, France; [11]Réanimation adultes et soins continus, pôle de médecine péri-opératoire, Hôpital Estaing, CHU Clermont-Ferrand, Clermont-Ferrand, France; [12]Service d'anesthésie-réanimation, réanimation polyvalente, Centre Hospitalier Emile Durkheim, Épinal, France; [13]Department of statistics, University Hospitals Pitié Salpêtrière - Charles Foix, Paris, France
**Correspondence:** A. Bouglé
*Intensive Care Medicine Experimental* 2019, **7(Suppl 3):**000454

**INTRODUCTION.** There is little descriptive data on Stenotrophomonas maltophilia hospital-acquired pneumonia in critically ill patients. However, this non-fermenting Gram negative bacteria is one of the tenth most isolated bacteria in intensive care units. Due to its unique resistance profile to antimicrobial agents, the optimal modalities of antimicrobial therapy remain to be determined.
**OBJECTIVES.** Our objective was to describe the epidemiology and prognostic factors associated with Stenotrophomonas maltophilia pneumonia, focusing on antimicrobial therapy.
**METHODS.** This nationwide retrospective study included all patients admitted to 25 French intensive care units between 2012 and 2017 with hospital-acquired Stenotrophomonas maltophilia pneumonia. The primary endpoint was time to in-hospital death. The secondary endpoints included microbiologic effectiveness and antimicrobial therapeutic modalities such as, delay to appropriate antimicrobial treatment, mono versus combination therapy and duration of antimicrobial therapy.

**RESULTS.** Of the 282 patients included, 84% were intubated at Stenotrophomonas maltophilia pneumonia diagnosis for a median duration of 11 [5-18] days. The median Simplified Acute Physiology Score II was 47 [36–63] and the in-hospital mortality was 49.7%. Underlying chronic pulmonary comorbidities were present in 14.1% of cases. Empirical antimicrobial therapy was microbiologically defined as effective on Stenotrophomonas maltophilia according to susceptibility patterns in only 30% of cases. Delay to appropriate antimicrobial treatment had, however, no significant impact on the primary endpoint. Survival analysis did not show any benefit from combination antimicrobial therapy (HR=1.27, 95%CI [0.88; 1.83]; p=0.20) or prolonged antimicrobial therapy for more than 7 days (HR=1.06, 95%CI [0.6; 1.86]; p=0.84).

**CONCLUSION.** Stenotrophomonas maltophilia hospital-acquired pneumonia occurred in severe, long-stay intensive care patients who mainly required prolonged invasive ventilation. Empirical antimicrobial therapy was barely effective while antimicrobial treatment modalities may have no significant impact on hospital survival.

**REFERENCE(S)**

1. The authors would like to thank the AZUREA network for its support.

### 000514

#### Incidence and etiology of ventilator associated tracheobronchitis: a prospective study in intensive care units of two tertiary care hospitals

DN. Mukherjee[1], S. Seal[2], H. Dasgupta[3]
[1]Clinical microbiology & id, Woodlands, Kolkata, India; [2]Clinical microbiology, Woodlands , Kolkata, India; [3]Pulmonology, Woodlands , Kolkata, India
**Correspondence:** D.N. Mukherjee
*Intensive Care Medicine Experimental* 2019, **7(Suppl 3):**000514

**INTRODUCTION.** Ventilator-associated tracheobronchitis (VAT) is an infective complication of mechanical ventilation. In the Intensive Care Units (ICUs), VAT is a relatively common problem but in comparison to ventilator-associated pneumonia (VAP), much less data are available on VAT and its management. The aim of this study was to determine the frequency, etiology, microbiological sensitivity and outcomes of nosocomial tracheobronchitis (NTB) in mechanically ventilated patients admitted in intensive care unit.

**OBJECTIVES.** To find out the incidence of ventilator associated tracheobronchitis in ventilated patients.

To map the microbiological profile of ventilator associated tracheobronchitis in the region

**METHODS.** A prospective observational study was conducted in two medical Intensive Care Unit (ICU), in Kolkata, India from March 2016 to April 2017. All the patients on mechanical ventilator for more than 48 hours in the ICU were evaluated according to the criteria for the diagnosis of nosocomial tracheobronchitis (NTB). Out-comes of the patients were measured in terms of development of nosocomial pneumonia after NTB, length of ICU stay, duration of mechanical ventilation and mortality in the ICU.

**RESULTS.** Four hundred and sixteen patients were evaluated for this study. Nosocomial tracheobronchitis was diagnosed in 48 patients. The frequency of NTB was 11.5%. Nine types of micro-organisms were identified, 42% cases were poly-microbial, while in the remaining 58% cases single organism was isolated. The most common organism was Klebsiella spp (56%), followed by Acenetobacter spp (20%) and Pseudomonas aeruginosa (8%). The majority patients who developed VAT had underlying co-morbidities. The mean time to develop VAT from the time of mechanical ventilation was 6.8 days and from time of ICU admission was 9 days, respectively. More than ninety percent of isolated GNB were multidrug resistant.

**CONCLUSION.** VAT is a common healthcare-associated infection caused mostly by MDR Gram-negative bacteria that significantly affects the development of pneumonia and length of ICU stay for the patients. Monitoring and active surveillance are required to detect VAT at the earliest to initiate appropriate isolation measures and therapy for better outcome.

**REFERENCE(S)**

1. Craven DE, Hjalmarson KI. Ventilator-associated tracheobronchitis and pneumonia: Thinking outside the box. Clin Infect Dis 2010;51 (Suppl 1):S59-66.
2. Dallas J, Skrupky L, Abebe N, Boyle WA 3rd, Kollef MH. Ventilator-associated tracheobronchitis in a mixed surgical and medical ICU population. Chest 2011;139:513-8.
3. N.A

### 000724

#### Incidence, characteristics and outcomes of adult patients admitted to intensive care units in Australia and New Zealand for a skin or soft tissue infection between 2006-2017

M. Bekker[1], S. Rai[2], S. Arbous[3], E. Georgousopoulou[4], D. Pilcher[5], FV. Haren[4]
[1]University, Leiden University Medical Center (LUMC), Leiden, Netherlands; [2]Intensive care unit, Canberra Hospital, Garran, Australia; [3]Intensive care, Leiden University Medical Center (LUMC), Leiden, Netherlands; [4]Medical school, The Australian National University, Canberra, Australia; [5]Australian and new zealand intensive care society, Centre for Outcome and Resource Evaluation, Melbourne, Australia
**Correspondence:** M. Bekker
*Intensive Care Medicine Experimental* 2019, **7(Suppl 3):**000724

**INTRODUCTION.** Skin and soft tissue infections (SSTIs) are usually mild infections, but when severe, they can lead to intensive care unit (ICU) admission. There is limited published data on the epidemiology of SSTIs necessitating ICU admission.

**OBJECTIVES.** To describe the incidence, characteristics and outcomes of critically ill adult patients admitted to the ICU for a SSTI, as well as independent predictors of outcomes.

**METHODS.** A registry-based retrospective cohort study using data submitted to the Australian and New Zealand Intensive Care Society Adult Patient Database for admissions between 2006 and 2017. Inclusion criteria: primary diagnosis of SSTI and age ≥16 years. Exclusion criteria: ICU readmissions (during the same hospital admission) and transfers from ICUs from other hospitals. Primary outcome was in-hospital mortality, secondary outcomes were ICU mortality and ICU and hospital length of stay (LOS). Furthermore, independent predictors of these outcomes were determined.

**RESULTS.** Of 1,470,197 first admissions at ICU between 2006 and 2017, 10,962 patients (0.7%) were admitted because of SSTI. Of these, 5,706 (52.1%) were medical and 5,256 (47.9%) surgical patients. Of all medical patients, 3,682 (64.5%) had sepsis. Comorbidities were present in 25.2% of the study sample. In-hospital mortality was 9.1% (n=991), which accounted for a 0.07% SSTI in-hospital mortality rate for all first admissions at ICU between 2006-2017. Incidence of ICU admissions increased between 2006-2017 from 0.4% to 0.9%, but in-hospital mortality decreased from 16.1% to 6.8%. Median ICU LOS was 2.1 days (IQR: 3.4), median hospital LOS was 12.1 days (IQR: 20.6). ICU LOS remained stable between 2006-2017 (2.0 to 2.1 days), whereas hospital LOS decreased from 15.7 to 11.2 days. Predicting factors for in-hospital mortality were: ANZROD [1] score (OR: 1.07; CI (1.05, 1.09); $p<0.001$), any comorbidity except diabetes (OR: 2.00; CI

(1.05, 3.79); p=0.035) and admission through an emergency response call (OR: 2.07; CI (1.03, 4.16); p=0.041).

**CONCLUSION.** SSTIs are uncommon as primary ICU admission diagnosis. While the incidence of ICU admissions for SSTI has increased, mortality and hospital LOS have decreased over the last decade.

**REFERENCE(S)**

1. Paul E, Bailey M, Pilcher D: Risk prediction of hospital mortality for adult patients admitted to Australian and New Zealand intensive care units: development and validation of the Australian and New Zealand Risk of Death model. Journal of critical care 2013, 28(6):935-941.
2. The authors received no financial support for the research, authorship, and publication of this article.

### 000861

#### Pneumocystis pneumonia critical care management and predictors of mortality

J. Serodio[1], L. Melo[1], A. Graça[2], P. Fortuna[2], AV. Santos[2], L. Bento[2]
[1]Department of internal medicine iv, Hospital Prof. Dr. Fernando Fonseca, Amadora, Portugal; [2]Unidade de urgência médica, Hospital S. José, Centro Hospitalar Universitário Lisboa Central, Lisboa, Portugal
**Correspondence:** J. Serodio
*Intensive Care Medicine Experimental* 2019, **7(Suppl 3):**000861

**INTRODUCTION.** Pneumocystis pneumonia (PCP) often presents with severe respiratory failure leading to intensive care admission and organ support. Patterns of disease have been changing as PCP is increasingly recognized among non-HIV Immunosuppressed patients. Little is known about critical care management of these patients.

**OBJECTIVES.** Our aim was to evaluate intensive care management of severe PCP and to identify clinical predictors of mortality.

**METHODS.** A retrospective cohort study was performed that included all patients with severe PCP from 2005 to 2018 (14 years) admitted to an intensive care unit of an university hospital. Patients were characterized according to immunosuppression status into HIV-positive and HIV-negative. Clinical variables at admission and during intensive care unit stay were assessed to identify clinical predictors of in-hospital mortality.

**RESULTS.** Forty-three patients with PCP were included with median age 53 (41-65) years and male sex predominance (71%). HIV-negative patients were responsible for 21 (49%) cases of PCP. Between 2005-2010 HIV was responsible for all cases of PCP whereas HIV-negative represented 64% of PCP from 2011-2018 (p=0,001). Most HIV-negative patients had recognizable causes of immunosuppression: 15 patients with hematologic cancer, 3 with solid organ cancer and 3 with autoimmune disease. HIV-negative patients were significantly older (63 [56-74] vs. 43 [33-53] years, p=0,0001), presented shorter duration of symptoms (3 [2-6] vs. 15 [7-31], p=0,0002), had higher APACHEII (24 [23-30] vs. 20 [15-24], p=0,0143) and increased in-hospital mortality (61% vs. 36%, p=0,094), but equal pO2/FiO2 on admission (113 [84-133] vs. 101 [71-151] mmHg, p=0,794) and lactate (1,9 [1,3-2,6] vs. 1,6 [1,2-2,1] mmol/L, p=0,243). Non-invasive ventilation (NIV) or high-flow nasal oxygen (HFNO) were attempted on 11 (26%) patients. Failure of NIV or HFNO was high in both groups (HIV-negative 57% vs HIV-positive 75%, p=0,473). The rate of mechanical ventilation was equal in both groups (81% vs 82%). Failure of NIV or HFNO was not significantly associated with mortality.

On univariate analysis, predictors of in-hospital mortality were need for mechanical ventilation (OR 25,2 [95% CI 1,35 – 471,5], p= 0,031), renal replacement therapy (OR 6,96 [95% CI: 1,57-30,86], p=0,011) and age (OR 1,07 [95% CI 1,02-1,12], p=0,006).

**CONCLUSION.** Severe PCP predominantly affects HIV-negative immunosuppressed patients during last years. HIV-negative patients are older, have more abrupt disease onset and seem to have increased mortality. Increased mortality might be associated with increasing age, comorbidities and need for organ support. Need for mechanical ventilation and renal replacement therapy were the stronger predictors of mortality. Use of NIV or HFNO was associated with a high percentage of progression to mechanical ventilation but without increased mortality keeping the non invasive strategy as an option in this patients.

**REFERENCE(S)**

1. Monnet X, Vidal-Patiot E, Osman D, et al. Critical care management and outcome of severe Pneumocystis pneumonia in patients with and without HIV infection. Critical Care 2008; 12:R28.
2. Akgün KM, Pisani M, Crothers K. The Changing Epidemiology of HIV-Infected Patients in the Intensive Care Unit. J Intensive Care Med 2011 26: 151.
3. Roux A, Canet E, Valade S, et al. Pneumocystis jirovecii Pneumonia in Patients with or without AIDS, France. Emerg Infect Dis. 2014; 20:1490-1497.

### 000914

#### Extracorporeal membrane oxygenation support to handle patients with Hantavirus Cardiopulmonary syndrome: a 3 – Years experience on a public ICU of Chile

P. Fernandez[1], N. Pavez[1], J. Yañez[1], P. Sanchez[1], F. Fasce[1], H. Juan[2], D. Ponce[1], B. Nahuelpan[1], J. Lastra[1], R. Colima[1], M. Hernandez[1], C. Stehr[1], L. Ferreira[2]
[1]Departamento de medicina interna, Universidad de Concepcion, Concepcion, Chile; [2]Hospital guillermo grant benavente, Unidad de Cuidados Intensivos, Concepcion, Chile
**Correspondence:** N. Pavez
*Intensive Care Medicine Experimental* 2019, **7(Suppl 3):**000914

**INTRODUCTION.** Hantaviral infection is a severe disease caused primarily by inhalation of contaminated particles of infected rodents. Most deaths are caused by a combination of progressive respiratory and cardiac failure, which is called the cardiopulmonary syndrome. In 2016, our institution introduced the use of extracorporeal membrane oxygenation (ECMO) as rescue therapy in patients who developed cardiopulmonary syndrome and refractory shock or severe hypoxemia.

**OBJECTIVES.** The purpose of this abstract is to describe patients, complications and evaluate the outcomes of this therapy.

**METHODS.** Retrospective data from ECMO supported patients was analyzed. Continuous variables were expressed as mean ± standard deviation, and categorical variables were expressed as the absolute number and proportions (%). Comparisons between survivors and non-survivors were made with the Mann-Whitney U test. A descriptive analysis was performed.

**RESULTS.** A total of 19 patients were described. Table 1 resumes demographic characteristics and severity scores at admission, remarking SOFA 11±2,2 and APACHE II of 18,4±7. Before connection, cardiac index was 1,95±0,3, PaO2/FiO2 111±37 and lactate 3,45±2,1. Table 2 highlights other pre ECMO status. SOFA score was significantly different between survivors and non-survivors (figure 1). The most common complication was acute kidney failure. A tendency to statistical difference was seen on age, APACHE II and initial PaO2/FiO2. Cardiac output, time of ECMO and distance to ECMO center did not show any clinical difference. In-hospital survival was 68,4%.

**CONCLUSION.** We described the results of a new developed ECMO program focusing on the outcomes of Hantavirus patients, showing an important survival in patients whose mortality remained extremely high before support therapy.

**REFERENCE(S)**

1. Wernly J, Dietl C, et al. Extracorporeal membrane oxygenation support improves survival of patients with Hantavirus cardiopulmonary syndrome refractory to medical treatment. Eur J Cardiothorc Surg (2011) 40(6):1334 – 40.
2. none.

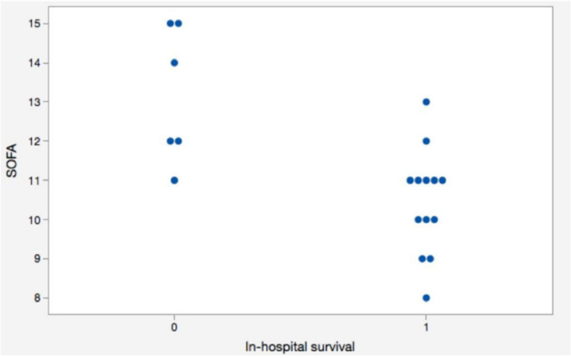

**Fig. 1 (abstract 000914).** See text for description

**Table 1 (abstract 000914).** Demographic characteristics and baseline severity scores

| | |
|---|---|
| **Patients** (n) | 19 |
| **Sex** (w) | 8 |
| **Age** | 41,5±16 |
| **SOFA** | 11±2,2 |
| **APACHE II** | 18,4±7 |
| **IMC (kg/m2)** | 26±3,6 |
| **Distance (km)** | 160 [98 - 542] |

**Table 2 (abstract 000914).** Baseline characteristic before ECMO

| | |
|---|---|
| **Lactate** (mmol/L) | 3,1±2,4 |
| **CI (L/min/m2)** | 1,95±0,3 |
| **PaO2/FiO2** | 111±37 |
| **Murray score** | 3,34±0,36 |
| **HTC %** | 43,7±8,6 |
| **PLT** | 50600±25000 |
| **VM before ECMO (h)** | 12 [8 - 36] |
| **ECMO (h)** | 97 [76 - 145] |

**Table 3 (abstract 000914).** Complications during ECMO therapy

| **Complications** | |
|---|---|
| **AKI** | 16/19 |
| **RRT** | 9/19 |
| **Hemorrhage** | 6/19 |
| **Severe hemorrhage** | 2/6 |
| **Ischemia** | 3/19 |
| **VAP** | 3/19 |

**Table 4 (abstract 000914).** Outcomes.

| | |
|---|---|
| ECMO time (h) | 97 [76 - 145] |
| Tracheostomy | 5 / 19 |
| VM (d) | 9 [7 - 20] |
| ICU (d) | 13 [8 - 24] |
| ICU survival | 68,40 % |
| Hosp survival | 68,40 % |

### 001132

#### Impact of chronic obstructive pulmonary disease on incidence, characteristics and outcome of ventilator-associated lower respiratory tract infections

M. Houard[1], P. Boddaert[1], S. Nseir[1], I. Martin-Loeches[2], P. Povoa, A. Rodriguez[3], J. Salluh[4], A. Rouzé[1]
[1]Icu, Chu De Lille, Lille, France; [2]School of medicine, Trinity College Dublin, Dublin, Ireland; [3]Uci, Hospital Universitari de Tarragona Joan XXIII, Tarragona, Spain; [4]Institute for research and education, D'Or, Rio de Janeiro, Brazil
**Correspondence:** S. Nseir
*Intensive Care Medicine Experimental* 2019, **7(Suppl 3):**001132

**INTRODUCTION.** Previous studies reported conflicting results regarding the impact of COPD on ventilator-associated pneumonia (VAP) incidence.
**OBJECTIVES.** To determine the impact of COPD on incidence, characteristics and outcomes of ventilator-associated lower respiratory tract infections (VA-LRTI).
**METHODS.** Retrospective analysis of prospective observational multinational TAVeM database (1). From September 2013 to July 2014, data were obtained from 2960 consecutive patients older than 18 years who received invasive mechanical ventilation (MV) for ≥48 hours. COPD patients (n=494) were compared to non COPD patients (n=2466). The diagnosis of ventilator-associated tracheobronchitis (VAT) and VAP was based on clinical, microbiological and radiological criteria.
**RESULTS.** VAP and VAT incidences were not significantly different between COPD and non COPD patients (12% *versus*13%, *p*=0.931, and 13% *versus*10%, *p*=0.093, respectively). Among patients with VA-LRTI, *Escherichia coli*and *Stenotrophomonas maltophilia*were significantly more frequent in COPD patients as compared with non COPD patients. The rate of multidrug-resistant bacteria was similar between the two groups. Appropriate antibiotic treatment was not significantly associated with progression from VAT to VAP among COPD patients who developed VAT, unlike non COPD patients.MV duration, ICU and hospital length of stay were significantly longer in patients with VAT or VAP as compared to patients without VA-LRTI, in both COPD and non COPD patients. Among patients who developed VAT, COPD patients exhibited significantly longer MV duration and ICU length of stay, as compared to non COPD patients (17 [9-30]) *versus*13 days [8-21], *p*= 0.042 and 24 [17-39] *versus*20 days [14-31], *p*=0.042, respectively). No significant difference regarding ICU mortality was found between COPD and non COPD groups in patients with VAT, VAP or without VA-LRTI.
**CONCLUSION.** Incidence of VA-LRTI was similar in both COPD and non COPD population, but VAT was associated with significantly longer duration of MV and ICU length of stay among COPD patients as compared to non COPD patients. Appropriate antibiotic treatment did not reduce the progression from VAT to VAP among COPD patients.

**REFERENCE(S)**

1. Martin-Loeches I, et al. Incidence and prognosis of ventilator-associated tracheobronchitis (TAVeM): a multicentre, prospective, observational study. Lancet Respir Med. 2015;3:859-68.

### 001273

#### Incidence of Healthcare associated infections in the ICU: type, pathogens and mortality

KJ. Castillo-Medrano[1], LA. Gorordo-Delsol[1], O. Sosa-Hernández[2], JA. Castañon-Gonzalez[1], SE. Zamora Gómez[1], MA. Espino-áNgeles[1], ML. Pacheco-Rivera[1], S. Sosa-Santos[1], NI. Medveczky-Ordoñez[1], JC. Gasca-Aldama[1], AH. Morales-Morales[1], A. Rodríguez-Peredo[1], JA. Zepeda-Pérez[1], D. Sanabria-Cordero[1], I. Maldonado-Beltrán[1], LE. Gaytán-Medina[1]
[1]Adult intensive care unit, Hospital Juárez de México, Ciudad de México, Mexico; [2]Hospital epidemiological surveillance unit, Hospital Juárez de México, Mexico City, Mexico
**Correspondence:** L.A. Gorordo-Delsol
*Intensive Care Medicine Experimental* 2019, **7(Suppl 3):**001273

**INTRODUCTION.** A quarter of HAI's will be produced in the ICU, are an important cause of complications, prolonged ICU stay, increased costs and mortality (1-2)
**OBJECTIVES.** To describe the incidence and characteristics of infections associated with health care in the ICU.
**METHODS.** A retrospective, observational study of all patients admitted to the ICU of the Hospital Juárez de México between January 2013 and December 2018, who had a diagnosis of HAI's acquired in the ICU, was registered type of infection, pathogen and outcome.
**RESULTS.** We analyzed 1727 patients admitted to the ICU, of whom 479 met criteria for HAI's and 316 had at least one positive microbiological isolation. The incidence of infections associated with health care, acquired in the ICU was 44.03, 41.38, 31.50, 21.77, 20.81, 15.01% during 2013, 2014, 2015, 2016, 2017 and 2018 respectively, observing an increase in the number of patients attended in ICU with a constant and significant reduction of HAI's in ICU; the average time between admission to the ICU and the presentation of HAI's was 12 days (IQR 8-20 days). The ventilator associates pmneumonia had incidence of 60.12%, other pneumonias 9.39% and non cathether related bacteriemia 8.97%. It was found that A. baumannii (n = 128 cases) as a risk factor for mortality in ICU with OR = 1.0215 (95% CI 0.5455 to 1.9127, p = 0.9470) and of Pseudomonas sp. There were 91 cases OR 1.1250 (95% CI 0.5639 to 2.2445, p = 0.7382) in comparison to other pathogens.
**CONCLUSION.** VAPs are the most common cause of HAIs in ICU, pathogens such as A. baumannii and Pseudomonas are common in our environment, however, they do not seem to increase mortality in relation to other pathogens (1-3).

**REFERENCE(S)**

1. IDSA. Management of Adults With Hospital-acquired and Ventilator-associated Pneumonia: 2016 Clinical Practice Guidelines by the Infectious Diseases Society of America and the American Thoracic Society. Clin Infect Dis 2016;63(5):e61–e111
2. Salgado, MR. Frequency of healthcare associated infections in the main healthcare systems of Mexico. National Commission of Medical Arbitration. Mexico: CONAMED BULLETIN - PAHO 2018;3(17):1
3. No financial support or grants were received for this manuscript

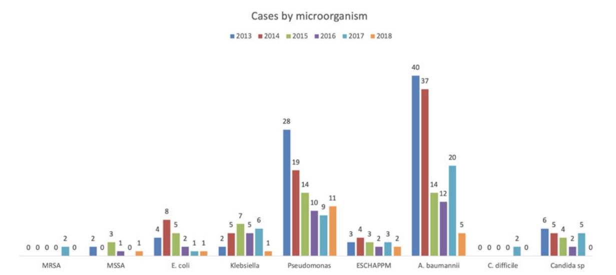

**Fig. 1 (abstract 001273).** See text for description

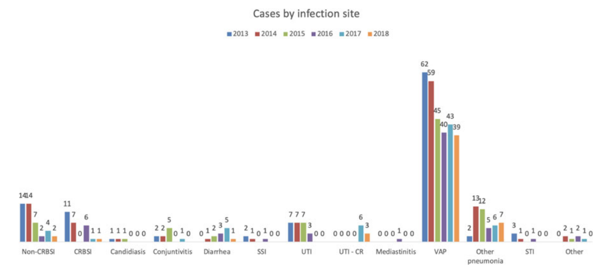

**Fig. 2 (abstract 001273).** See text for description

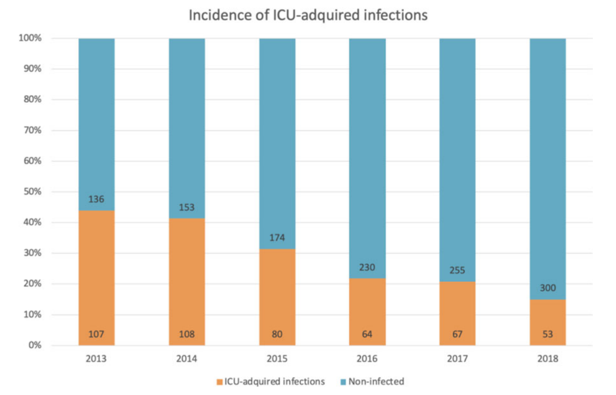

**Fig. 3 (abstract 001273).** See text for description

### 001286

#### Clostridioides difficile infections (CDI) in the ICU: timing, severity, treatment and outcomes

J. Dionne[1], J. Johnstone[2], EH. Duan[1], L. Saunders[3], D. Heels-Ansdell[3], W. Alhazzani[1], D. Cook[1]
[1]Department of medicine/department of health research methods, evidence and impact, McMaster University, Hamilton, Canada; [2]Public health, Public Health Ontario, Toronto, Canada; [3]Department of health research methods, evidence and impact, McMaster University, Hamilton, Canada
**Correspondence:** J. Dionne
*Intensive Care Medicine Experimental* 2019, **7(Suppl 3):**001286

**INTRODUCTION.** The timing, severity and treatment of healthcare-associated CDI are not well described in critically ill patients.

**OBJECTIVES.** The objectives of this nested cohort study are: 1) the incidence of CDI and timing of CDI (pre-ICU, in ICU, post-ICU); 2) severity of CDI based on the Infectious Diseases Society of American/ Society for Healthcare Epidemiology of American (IDSA/SHEA), American College of Gastroenterology (ACG) and European Society of Clinical Microbiology and Infectious Diseases (ESCMID) scores; 3) complications and mortality; 4) treatment for CDI in the ICU.

**METHODS.** This multicenter study was nested within a clinical trial testing *Lactobacillus rhamnosus* GG (Culturelle, Locin Industries Ltd) versus placebo to reduce ventilator associated pneumonia and other ICU-acquired infections. Research Coordinators completed a case report form for any patient who was suspected to have CDI. All CDI parameters were adjudicated in duplicate with respect to timing of the infection, severity, treatment, and complications; any adjudicator disagreement was resolved by a third adjudicator.

**RESULTS.** 64 of 1,766 patients (3.6%) developed CDI; 7 of whom (0.4%) had CDI at ICU admission (pre-ICU), while 41/1766 (2.3%) developed CDI in the ICU (ICU-acquired), and 16/1766 (0.9%) developed CDI after post-ICU discharge (post-ICU). The incidence rate of ICU-acquired CDI was 1.4/1000 patient-days. CDI severity in the ICU was mostly mild/moderate according to these criteria: 45/64 (70.3%) by IDSA/SHEA; 49/64 (76.6%) by ACG; 53/64 (82.8%) by ESCMID. Fewer patients developed severe CDI (12/64, 18.8% IDSA/SHEA; 6/64, 9.4% ACG; 11/64,17.2% ESCMID) and severe, complicated CDI was rare (7/ 64, 10.9% IDSA/SHEA; 9/64, 14.1% ACG). Complications attributed to CDI were frequent: 17/64 (26.6%) patients had septic shock, 12/64 (18.8%) developed new end-organ failure and 1 (1.6%) had toxic megacolon necessitating colectomy. ICU and hospital mortality was 338/1766 (22.0%) and 501/1765 (28.4%), respectively. ICU and hospital mortality based on IDSA/SHEA severity was: mild/moderate: 7/ 45 (15.6%), 14/45 (31.1); severe: 4/12 (33.3%), 4/12 (33.3%); severe and complicated: 3/7 (42.9%) and 4/7 (57.1%), respectively. Most patients with ICU-acquired CDI in our cohort received metronidazole [total 45/64 (70.3%)] either orally [42/64 (65.6%)] or intravenously [21/64 (32.8%)], and 40/64 (62.5%) received vancomycin orally [39/64 (60.9%)] or rectally [2/64 (3.1%)]. Fidaxomicin wasn't prescribed, but 3/64 (4.7%) patients underwent fecal transplantation.

**CONCLUSION.** CDI was more likely to be acquired in the ICU than to be pre-existing or develop following recovery from critical illness. Most cases were mild-moderate in severity; treatment primarily involved metronidazole, consistent with prior CDI guidelines for first episodes of non-severe CDI. CDI remains a concerning clinical problem; further research should focus on optimal risk stratification and treatment.

**REFERENCE(S)**

1. JC Dionne holds a Physicians Services Incorporated (PSI) Research Trainee Award and a Canadian Association of Gastroenterology PhD Studentship Award. D Cook holds a Chair of the Canadian Institutes of Health Research. The study also received funding from Canadian Institutes of Health Research, PSI, and the Canadian Frailty Network.

### 001003

#### Effectiveness of a hand hygiene promotion program in a regional public hospital in rural Cambodia

YT. Lin[1], YH. Chiou[1], WS. Liu[2], CH. Li[3], YP. Su[2], MF. Cheng[4], WH. Lu[1], SC. Fu[2], HT. Chen[2], HL. Chen[2], YH. Huang[2], IC. Tsai[2], FC. Chen[5], HJ. Lu[5], WC. Juang[6], HY. Lin[6], FY. Shen[7], CP. Liu[8], WC. Huang[9]
[1]Department of pediatrics, Kaohsiung Veterans General Hospital, Kaohsiung, Taiwan; [2]Department of nursing, Kaohsiung Veterans General Hospital, Kaohsiung, Taiwan; [3]Nursing school, Fooyin University, Kaohsiung, Taiwan; [4]Kaohsiung veterans general hospital, Department of Pediatrics, Kaohsiung, Taiwan; [5]Department of infection control, Kaohsiung Veterans General Hospital, Kaohsiung, Taiwan; [6]Department of quality management center, Kaohsiung Veterans General Hospital, Kaohsiung, Taiwan; [7]Department of medical affair administration, Kaohsiung Veterans General Hospital, Kaohsiung, Taiwan; [8]Critical care medicine and cardiovascular center, Kaohsiung Veterans General Hospital, Kaohsiung, Taiwan; [9]Critical care medicine and cardiovascular center, Kaohsiung Veterans General Hospital, Taipei, Taiwan
**Correspondence:** W.C. Huang
*Intensive Care Medicine Experimental* 2019, **7(Suppl 3):**001003

**INTRODUCTION.** Hand hygiene is the most important infection control intervention that has proved to decrease the risk of hospital-acquired infections in intensive care units or hospitals. However, proper hand hygiene implementation in healthcare institutions still faces various challenges in many developing countries due to limited resources and non-availability of hand hygiene infrastructure.

**OBJECTIVES.** We aimed to assess the effectiveness of environmental and behavioral changes before and after the implementation of a hand hygiene promotion program in a rural public hospital in Cambodia.

**METHODS.** This project was operated the cooperation by Kaohsiung Veterans General Hospital (KVGH), Taiwan, and carried out in a regional public hospital, namely, Bati Referral Hospital (BRH), in Bati District, Takeo Province, Cambodia during May 2017 to December 2018. A baseline survey was conducted before the implementation andcontinuous quality improvement program were used to analyze and solve the problems.

**RESULTS.** A total of 42 health care workers participated in the project, among these ten were chosen as auditors. The rate of technical practice of hand hygiene was 0% at baseline and rose to 95.6% (p< 0.001) in the follow up assessment. The rate of auditors was from 0% to 100%. Compliance rates of moment one (before touching patients) and moment four (after touching patient) of hand hygiene improved from 0% to 100%. The prevalence ratesof diarrhea and pneumonia decreased from 4.15% to 3.78% and 4.8% to 4.4%, respectively. The length of admission decreased from 3 to 2.7 days.

**CONCLUSION.** This program showed continuous quality improvement program could establish a pilot hand hygiene promotion program and system in a rural hospital in where hand hygiene program was never implemented in a developing country. Availability of hand hygiene equipment, periodical training and evaluation, and managerial empowerment are the key factors to ensure long-term sustainability. In addition, commitment and support by government and hospital authorities arealso crucial for successfulimplementation.

**REFERENCE(S)**

1. Phan HT, Tran HTT, Tran HTM, Dinh APP, Ngo HT, Theorell-Haglow J, Gordon CJ. An educational intervention to improve hand hygiene compliance in Vietnam. BMC Infect Dis. 2018 Mar 7;18(1):116
2. Pfäfflin F, Schmidt N, Tufa TB, Feldt T. Implementation of the WHO multimodal Hand Hygiene Improvement Strategy in a University Hospital in Central Ethiopia. Antimicrob Resist Infect Control. 2015;4(Suppl 1):153.
3. Wilkinson MAC, Kiernan MA, Wilson JA, Loveday HP, Bradley CR. Assessment of the efficacy of a patient hand wipe: development of a test method. J Hosp Infect. 2018 Apr;98(4):339-344. https://doi: 10.1016/j.jhin.2017.08.013
4. Hoang D, Khawar N, George M, Gad A, Sy F, Narula P. Video didactic at the point of care impacts hand hygiene compliance in the neonatal intensive care unit (NICU). J Healthc Risk Manag. 2018 Apr;37(4):9-15. https://doi: 10.1002/jhrm.21314
5. Martischang R, Pires D, Masson-Roy S, Saito H, Pittet D. Promoting and sustaining a historical and global effort to prevent sepsis: the 2018 World Health Organization SAVE LIVES: Clean Your Hands campaign. Crit Care. 2018 Apr 13;22(1):92. https://doi: 10.1186/s13054-018-2011-3
6. Tartari E, Abbas M, Pires D, de Kraker MEA, Pittet D. World Health OrganizationSAVE LIVES: Clean Your Hands global campaign-'Fight antibiotic resistance-it's in your hands'. Clin Microbiol Infect. 2017 Sep;23(9):596-598. https://doi: 10.1016/j.cmi.2017.04.021

### 000337

#### Prognostic factors in critically ill oncological patients admitted in a tertiary ICU: The Vall d'Hebron Intensive Care Department/Vall d'Hebron Institute of Oncology cohort

I. Romera[1], J. Assaf[2], A. Pacheco[1], N. Saoudi[2], B. Encina[1], D. García[2], C. Díaz[1], A. García[1], E. Elez[2], R. Ferrer Roca[1]
[1]Intensive care department, Vall d'Hebron University Hospital. SODIR. VHIR, Barcelona, Spain; [2]Oncology department, Vall d'Hebron University Hospital. VHIO, Barcelona, Spain
**Correspondence:** C. Díaz
*Intensive Care Medicine Experimental* 2019, **7(Suppl 3):**000337

**INTRODUCTION.** The number of patients with cancer who receive intensive care treatment is growing. However, the prognosis of these patients remains poor with a mortality after ICU admission of around 50%. So, efforts are needed in order to improve their outcome.

**OBJECTIVES.** Our aim was to describe the cohort of patients with solid organ cancer admitted to a tertiary hospital ICU and analyze factors that are likely to affect their survival.

**METHODS.** Retrospective study, including adult (+18 years) patients with solid organ cancer who required admission to ICU between 2010 and 2016. X-Square, Fisher´s test, T test, U Mann-Whitney and logistic regression were employed as required. Quantitative variables are reported as median (IQR) and categorical as frequency (%).

**RESULTS.** One hundred and fifty patients were included, 77 (51%) female, with a median age of 62 (52-70) years. Lung cancer was the most frequent one, being present in 34 (25%), followed by colon 20 (13%), breast 13 (9%) and ovary 13; (9%). Metastatic disease affected to 84 (58%). The functional status was ECOG 0 in 12 (8%), ECOG 1 in 74 (51%), ECOG 2 in 56 (39%) and ECOG 3 in 3 (2%). The main indications for ICU admission were respiratory failure in 55 (37%), shock in 31 (21%), post urgent surgery in 29 (20%) and central nervous system involvement in 10 (7%). SOFA score at admission was 6 (4-9) Neutropenia was present in 19 (13%). Ninety-two (62%) required vasoactive drugs (VAD), 55 (37%) high flow nasal cannula (HFNC), 66 (44%) mechanical ventilation (MV), and 13 (9%) renal replacement therapy (RRT). ICU mortality affected to 38 (26%) and hospital mortality to 66 (44%). We identified the following factors related to hospital mortality in the univariate analysis: SOFA score on admission (OR 1.1, IC95% 1-1.3, p<0.004), no improvement in SOFA score at day 5 (OR 6.8, IC95% 2.1-22.3, p<0.002), need of MV (OR=5.5, IC95%:2.7-11.2, p< 0.0001), VAD (OR=2.2, IC95%:1.1-4.3, p=0.03). We did not find any differences in mortality according to the need of RRT, ECOG status, the presence of metastatic disease or neutropenia. In the multivariate analysis, MV (OR=6.8, IC95%:1.4-32.2, p=0.016) and no improvement in SOFA score at day five (OR 6.7 IC95%:1.7-27: p=0.007) maintained statistical significance.

**CONCLUSION.** Despite improvement in cancer therapies and critical care management, mortality is still high in oncologic patients admitted to ICU. Organ failure and need of mechanical ventilation is related to mortality, so these patients could benefit of an earlier admission. Patients with no improvement in organ dysfunction at day 5 have a greater mortality, so this information could be useful in the decision process during an ICU trial.

### 001557

#### Characteristics of ICU admissions of adult patients with B-cell malignancies treated with ARI-0001 cells (an academic CART-19)

P. Castro Rebollo[1], V. Ortiz-Maldonado[2], S. Fernandez[1], A. Téllez[1], F. Seguí[1], M. Caballero-Baños[3], M. Castella[4], T. Baumann[2], J. Cid[5], M. Lozano[5], I. Jordan[6], J. Esteve[2], E. Trias[7], J. Yagüe[4], M. Rovira[2], JM. Nicolás[1], Á. Urbano-Ispizua[2], M. Juan[3], S. Rives[8], J. Delgado[2]
[1]Medical Intensive Care Unit, Hospital Clínic of Barcelona, Barcelona, Spain; [2]Hematology department, Hospital Clínic of Barcelona, Barcelona, Spain; [3]Immunology platform clínic-sant joan de déu, Hospital Clínic of Barcelona, Barcelona, Spain; [4]Immunology department, Hospital Clínic of Barcelona, Barcelona, Spain; [5]Apheresis unit, Hospital Clínic of Barcelona, Barcelona, Spain; [6]Intensive care unit, Hospital Sant Joan de Déu, Barcelona, Spain; [7]Banc de sang i teixits, Hospital Clínic of Barcelona, Barcelona, Spain; [8]Hematology department, Hospital Sant Joan de Déu, Barcelona, Spain
**Correspondence:** P. Castro Rebollo
*Intensive Care Medicine Experimental* 2019, **7(Suppl 3):**001557

**INTRODUCTION.** Redirecting T-cell cytotoxic specificity through chimeric antigen receptor gene transfer (CAR-T cells) has become a major breakthrough in cancer immunotherapy, especially against relapsing/refractory CD19 hematologic neoplasms as acute lymphoid leukemia (ALL) or non-Hodgkin lymphoma (NHL). Significant toxicity is associated with this therapy, requiring sometimes ICU admission. The first academic pilot clinical trial (clinicaltrials.gov NCT03144583) using our fully academic (A3B1:CD8:4-1BB:CD3Z) CART-19 (ARI-0001 cells) was approved by the Spanish Agency of Medicines on May/2017

**OBJECTIVES.** To report the rate and characteristics of ICU admissions of patients treated with ARI-0001 cells

**METHODS.** Patients with NHL and ALL who had failed standard available therapy have been included in the trial. The primary objective of the study was safety, and secondary objectives were response rate and its duration. After conditioning with fludarabine (90 mg/m2) and cyclophosphamide (900 mg/m2), we infused 0.4-5 x106ARI-0001 cells /kg, either as a single infusion or in 2-3 fractions. Data are expressed in median (range) and n (percentage)

**RESULTS.** As of April/2018, 25 adult patients have been infused (3 of them twice and 1 three times), 14 as a single infusion and 11 as fractioned aliquots. Seven patients (28%) have been admitted to the ICU (1 patient was admitted twice). The median age was 27 years (19-54), 4/7 were men. Four had refractory ALL and three NHL, with a median of 4 previous lines of treatment (4-6). Five of them also had been treated with hematopoietic stem cell transplant (3 allogeneic). All 7 needed bridging chemotherapy between apheresis and CART-19 infusion. Five patients received the treatment as a single infusion and two as fractioned aliquots (one of them only received 40% of the planned dose). Reasons for ICU admission were cytokine releasing syndrome (CRS) (4 patients), neurotoxicity (2 patients), infection (2 patients) and non-CRS related hypotension (1 patient). One patient was admitted previously to infusion because of respiratory failure due to illness progression. Median APACHE II and SOFA were 15 (13-22) and 4 (2-8), respectively. Organ support was needed in 4/7 patients (vasopressors 4/7, mechanical ventilation 3/7, renal support therapy 3/7). Regarding CRS, severity was measured by Lee scale. One patient died (grade 5) and 2 and 1 patient had grade 3 and 2, respectively, all of them starting the day of infusion. Tocilizumab was administered in these 4 cases. Regarding neurotoxicity, the maximum grade was 3 (1 patient) and 1 (1 patient). Three patients died in the ICU, due to refractory CRS (1 patient), CRS + *Clostridium difficile* infection (1 patient) and disease progression (1 patient). The median length of stay in ICU was 4 days (2-34)

**CONCLUSION.** Rate of ICU admission in patients treated with ARI-0001 cells was 28%, mainly due to CRS. Fractioned infusion dose seemed associated with a lower ICU admission rate

**REFERENCE(S)**

1. Azoulay, E., Shimabukuro-Vornhagen, A., Darmon, M., & Bergwelt-Baildon, von, M. (2019). Critical Care Management of Chimeric Antigen Receptor-T Cells-Related Toxicity: Be Aware and Prepared. American Journal of Respiratory and Critical Care Medicine, rccm.201810–1945ED–11
2. Lee, D. W., Santomasso, B. D., Locke, F. L., Ghobadi, A., Turtle, C. J., Brudno, J. N., et al. (2019). ASTCT Consensus Grading for Cytokine Release Syndrome and Neurologic Toxicity Associated with Immune Effector Cells. Biology of Blood and Marrow Transplantation, 25(4), 625–638
3. Neelapu, S. S., Tummala, S., Kebriaei, P., Wierda, W., Gutierrez, C., Locke, F. L., et al. (2017). Chimeric antigen receptor T-cell therapy — assessment and management of toxicities. Nature Reviews Clinical Oncology, 15, 47–62
4. Gutierrez, C., McEvoy, C., Mead, E., Stephens, R. S., Munshi, L., Detsky, M. E., et al. (2018). Management of the Critically Ill Adult Chimeric Antigen Receptor-T Cell Therapy Patient. Critical Care Medicine, 46(9), 1402–1410
5. Generalitat de Catalunya
6. FEHH
7. CatSalut
8. ISCIII
9. Fundació Gloria Soler
10. Proyecto ARI
11. Lee DW, Gardner R, Porter DL, Louis CU, Ahmed N, Jensen M, et al. (2014). Current concepts in the diagnosis and management of cytokine release syndrome. Blood, 124(2):188-95

## CD - Cardiovascular assessment and support strategies

### 001078

#### Does an incremental titration of norepinephrine (NE) impair stroke volume in fluid-unresponsive septic shock patients?

ED. Valenzuela Espinoza[1], D. Carpio[1], R. Fernández[2], L. Gabrielli[2], A. Bruhn[1], G. Hernández[1], J. Bakker[3]

[1]Departamento de medicina intensiva, Pontificia Universidad Católica de Chile, Santiago, Chile; [2]Departamento de cardiología, Pontificia Universidad Católica de Chile, Santiago, Chile; [3]Department of intensive care medicine, Erasmus University Medical Center, Rotterdam, Netherlands

**Correspondence:** E.D. Valenzuela Espinoza

*Intensive Care Medicine Experimental* 2019, **7(Suppl 3):**001078

**INTRODUCTION.** Current guidelines recommend to increase mean arterial pressure (MAP) in previously hypertensive septic shock patients to higher levels (80-85 mmHg) when tissue hypoperfusion has not been corrected with the initial fluid resuscitation and restoration of MAP to 65 mmHg. The impact of incremental NE titration on cardiac function depends on the balance between the effects on preload, contractibility and afterload. As NE may both increase venous return and afterload, a particular concern is a potential decrease in stroke volume (SV) in fluid-unresponsive patients.

**OBJECTIVES.** To determine the impact of an increase in MAP from 65 to 85 mmHg by incremental NE titration on SV and several parameters of cardiac performance in previously hypertensive fluid-unresponsive patients.

**METHODS.** We evaluated patients in whom the attending doctor decided to increase MAP from 65 to 85 mmHg because of persistent hypoperfusion. A comprehensive echocardiographic assessment was performed at baseline (B1), after reaching the MAP target (MAP 85), and after return to baseline MAP (B2). A SV responder was defined as patients that increased SV by >10% at MAP 85. Mean Systemic Filling Pressure (Pmsf) and pressure for venous return (Pvr) were calculated. Preload, contractility and afterload variables were assessed. Statistical analysis was performed with Wilcoxon signed-rank test, $P < 0.05$ was considered as significant.

**RESULTS.** Seven mechanically ventilated patients (age 71± 8 yr, APACHE II 22±7, SOFA on admission 10 ± 2) were included in this preliminary report. Hemodynamic variables are shown in table 1. No patient decreased SV after reaching MAP 85. Three patients were SV-responders this was associated with an increase in Pmsf and Pvr accompanied by a direct increase in left ventricular contractility as assessed by ventricular elastance.

**CONCLUSION.** In previously hypertensive and currently fluid unresponsive septic shock patients in whom MAP was increased from 65 to 85mmHg stroke volume did not decrease despite the increased afterload due to an increase in left ventricular contractility.

**REFERENCE(S)**

1. Chen CH, Fetics B, Nevo E, Rochitte CE, Chiou KR, Ding PA, et al. Noninvasive single-beat determination of left ventricular end-systolic elastance in humans. J Am Coll Cardiol. 2001;38(7):2028-34.
2. Parkin WG, Leaning MS. Therapeutic control of the circulation. J Clin Monit Comput. 2008;22(6):391-400.

3. Maas JJ, Pinsky MR, de Wilde RB, de Jonge E, Jansen JR. Cardiac output response to norepinephrine in postoperative cardiac surgery patients: interpretation with venous return and cardiac function curves. Crit Care Med. 2013;41(1):143-50.

**Table 1 (abstract 001078).** Hemodynamics variables

| | Baseline 1 | MAP 85 | Baseline 2 |
|---|---|---|---|
| MAP, mmHg | 66 [63,68] | 84[83,87]* | 66[63,67]† |
| NE, mcg/kg/min | 0.17[0.10,0.37] | 0.45[0.14,0.61]* | 0.32[0.08,0.42]† |
| HR, beats/min | 96[78,105] | 95[82,101] | 97[79,113] |
| CVP, mmHg | 8[6,10] | 9[6,11] | 6[5,9] † |
| Pmsf, mmHg | 16.8[14.0,18.6] | 18.7[17.2,19.3]* | 15.7[14.9,18.2] † |
| Pvr, mmHg | 7.9[6.7,10.6] | 8.7[7.7,12.3]* | 8.5[7.2,11.5]* |
| CO, $L \cdot min^{-1}$ | 5.0[3.6,6.9] | 4.8[4.0,7.6] | 5.2[4.1,7.7] |
| RVR, $mmHg \cdot min \cdot mL^{-1}$ | 1.62[1.45,2.01] | 1.8[1.48,2.01]* | 1.62[1.39,1.84] † |
| SVR, $mmHg \cdot min \cdot mL^{-1}$ | 864[665,1478] | 1270[745,1555]* | 896[563,1227] † |
| Aortic Elastance | 2.47[2.06,2.56] | 3.02[2.60,3.05]* | 2.13[2.03,2.46]† |
| Echocardiography assessment of contractility | | | |
| LV EF, % | 66[44,69] | 66[48,68] | 63[44,67] |
| LV EndSyst-Elastance | 1.86[1.55,2.27] | 3.03[1.84,3.12]* | 1.81[1.22,2.05] † |

Data are shown as median [IQR 25,75]. Legend: MAP, mean arterial pressure; NE, Norepinephrine; HR, heart rate; CVP, central venous pressure; Pmsf, mean systemic filling pressure; Pvr, driving pressure for venous return; CO, cardiac output; RVR, resistance to venous return; SVR, systemic vascular resistance; EF, ejection fraction; LV EndSyst, left ventricle end systolic. * p<0.05 vs. Baseline1, † p<0.05 vs. MAP 85.

### 001108

#### Skin blood flow and thenar tissue oxygenation are not correlated to hemodynamic parameters in patients with circulatory shock

P. Bakos, W. Mongkolpun, E. Cavalcante dos Santos, FS. Taccone, J. Creteur
Department of intensive care, Erasme Hospital, Brussels, Belgium
**Correspondence:** P. Bakos
*Intensive Care Medicine Experimental* 2019, **7(Suppl 3):**001108

**INTRODUCTION.** Despite hemodynamic stabilization after resuscitation, tissue hypoperfusion may persist in circulatory shock (CS) patients. Volume expansion (VE) and increasing mean arterial pressure (MAP) are two of the strategies to correct tissue hypoperfusion.

**OBJECTIVES.** We hypothesized that a VE or a change in MAP by norepinephrine (NE) titration would affect skin blood flow (SBF) and thenar muscle oxygenation (StO2).

**METHODS.** Hemodynamically stabilized patients with CS undergoing a VE or an adjustment of MAP were included. CS was defined as the presence of NE to maintain MAP $\geq$ 65 mmHg with $\geq$ 1 signs of tissue hypoperfusion (oliguria, alteration of consciousness, mottled skin) or lactate $\geq$ 2 mmol/L. VE (crystalloid solution administration at 500 ml/30 minutes infusion rate) and the adjustment of MAP (ΔNE) (an increase or decrease in NE infusion rate $\geq$ 20%) were decided by the attending physician.

SBF was measured (Periflux 5000, Perimed; index finger, Perfusion unit: PU) before (T0) and after (T1) VE or ΔNE. Thenar StO2 was measured at T0 and T1 by near-infrared spectroscopy (NIRS; InSpectra TM Model 325). A vaso-occlusive test (VOT) of 3 minutes was also performed at T0 and T1 to calculate the StO2 ascending slope (AscStO2) (%/min) following the end of the VOT. Hemodynamic variables and lactate levels were obtained at the same times as the measurements. The changes in SBF (ΔSBF) and AscStO2 were calculated as relative percent changes. Data are reported as medians with 25th and 75th percentiles.

**RESULTS.** 19 patients with CS (septic (8), cardiogenic (10), hypovolemic (1)) were studied. APACHE score at admission was 19 (17-28), SOFA score at inclusion time was 11 (8-12). At baseline there were no differences in MAP (p=0.6), cardiac index (CI) (p=0.7), lactate levels (p=0.7), StO2 (p=0.6) and AscStO2 (p=0.3) between VE patients (n = 9) and ΔNE patients (n = 10). SBF was lower in VE patients than in ΔNE patients (20 (12-121) vs 102 (64-201) p=0.04, respectively).

Hemodynamic effects of VE and ΔNE are reported in the Table. SBF and StO2 did not change, AscStO2 increased in VE patients but not in ΔNE patients (Table). Changes in CI were not correlated to ΔSBF (r=0.02; p=0.4) or ΔAscSto2 (r=0.29; p=0.5). Also, changes in MAP were not correlated to ΔSBF (r=0.05; p=0.8) or ΔAscSto2 (r=-0.04; p=0.8).

**CONCLUSION.** Changes in hemodynamic parameters during a VE or a change in MAP target are not correlated to changes in skin blood flow or thenar muscle tissue oxygenation.

**Table 1 (abstract 001108).** See text for description

| | Total (N=19) | | | VE (N=9) | | | ΔNE (N=10) | | |
|---|---|---|---|---|---|---|---|---|---|
| | Before | After | p | Before | After | p | Lower NE infusion rate | Higher NE infusion rate | p |
| MAP (mmHg) | 69 (65-73) | 78 (73-86) | <0.01 | 71 (66-81) | 75 (73-86) | 0.2 | 69 (65-70) | 80 (75-81) | <0.01 |
| CI (L/min/m2) (N=12) | 2.6 (2.1-2.9) | 2.7 (2.3-3.0) | 0.1 | 2.6 (2.1-2.9) | 2.7 (2.2-3.0) | 0.1 | 2.6 (2.2-4.0) | 2.8 (2.4-4.0) | 0.2 |
| SBF (PU) | 104 (19-164) | 71 (23-149) | 0.4 | 20 (12-121) | 30 (15-87) | 0.8 | 130 (82-194) | 96 (64-203) | 0.4 |
| StO2 | 74 (70-80) | 73 (69-80) | 0.7 | 78 (66-83) | 75 (73-83) | 0.2 | 74 (70-79) | 73 (66-80) | 0.7 |
| AscStO2 (%/min) | 87 (67-170) | 104 (85-154) | 0.6 | 59 (56-99) | 95 (85-121) | 0.04 | 103 (80-170) | 106 (86-154) | 0.4 |

### 001115

#### A retrospective audit of the de-prescribing of anti-arrhythmic and anti-coagulant medications in patients with new-onset atrial fibrillation (AF) upon discharge from Critical Care

B. Salmon[1], I. Welters[2], B. Johnston[2]
[1]Liverpool medical school, University of Liverpool, Liverpool, United Kingdom; [2]Critical care, Royal Liverpool and Broadgreen University Hospitals NHS Trust, Liverpool, United Kingdom
**Correspondence:** B. Salmon
*Intensive Care Medicine Experimental* 2019, **7(Suppl 3):**001115

**INTRODUCTION.** AF is the most common arrhythmia seen in Critical Care and is associated with poorer long-term outcomes and an increased incidence of permanent AF. The follow-up of patients with new-onset AF (NOAF) in Critical Care may help to determine the long-term risk of recurrence of AF.

Amiodarone and beta-blockers are commonly used to treat AF in critically ill patients. Long-term use of these medications has been associated with adverse effects. Anticoagulant therapy also has well established bleeding risks. The long-term use of these medications is currently not recommended for patients who solely develop episodes of AF as part of their critical illness.

**OBJECTIVES.** The primary outcome measure was the number of patients with NOAF that were started on an anti-arrhythmic and/or an anti-coagulant and discharged home on these medications.

Secondary outcome measures included the number of patients that were reviewed by cardiology as an inpatient, the number that were still in AF at discharge and the number of patients with follow-up arranged upon their discharge from hospital.

**METHODS.** 95 critically ill patients were found to have AF in 2017. 26 patients who were treated for NOAF in critical care and survived their admission were included in this analysis.

Electronic clinical notes, referrals and discharge summaries were reviewed to identify pre-hospital and discharge medications, AF status and follow-up arrangements at discharge.

**RESULTS.** No patients were discharged home on amiodarone. However, 12 patients were discharged home on bisoprolol and only 6 of these patients had follow-up arranged for AF. None of these patients were taking bisoprolol before admission to hospital. Similarly, one patient was discharged home on digoxin without follow-up.

One patient was anti-coagulated for AF and was discharged home on a Direct-Acting Oral anticoagulant (DOAC) with follow-up arranged.

8 of the 26 patients were reviewed by cardiology as inpatients, and only 7 patients were discharged from hospital with follow-up for their AF. Of the 6 patients still in AF at discharge, 4 had planned follow-up.

**CONCLUSION.** In contrast to previous reports, no patients were discharged home on amiodarone, However, a significant number of patients with NOAF in critical care were discharged on bisoprolol. Follow-up for AF was arranged only for two third of patients who developed NOAF during critical illness.

**REFERENCE(S)**

1. Leelathanalerk A, Dongtai W, Huckleberry Y, Kopp B, Bloom J, Alpert J. Evaluation of Deprescribing Amiodarone After New-Onset Atrial Fibrillation in Critical Illness. Am J Med 2017;130(7):864-6.
2. Gialdini G, Nearing K, Bhave PD, Bonuccelli U, Iadecola C, Healey JS, et al. Perioperative atrial fibrillation and the long-term risk of ischemic stroke. Jama 2014;312(6):616-22.
3. None to declare.

### 001116

#### Day case permanent pacing

J. Llabata, J. Ruiz Izquierdo, C. Palencia, ML. Urendes, R. Algarte, B. Sánchez González, J. Trenado Alvarez, F. Jara
Intensive care medicine, Hospital Universitari Mútua Terrassa, Terrassa, Spain
**Correspondence:** B. Sánchez
*Intensive Care Medicine Experimental* 2019, **7(Suppl 3):**001116

**INTRODUCTION.** Implantation of a permanent cardiac pacemaker is a surgical procedure associated with minimal patient discomfort and low percentage of complications in expert centers.

**OBJECTIVES.** To analyze the safety of implantation of a cardiac pacemaker as a day case procedure.

**METHODS.** Retrospective study in a University Hospital from February 2017 to February 2019.Inclusion criteria:over 18 years old, requiring elective pacemaker and no pacemaker dependent. Patients who did not accept the program were hospitalized in the usual way, as well as those who presented some social exclusion criteria.The Pacemaker Unit, dependent from the Critical Care Department and integrated by medical specialists in intensive medicine accredited for the implantation of pacemakers, established a program for the implantation of definitive pacemakers without hospital admission. After the implant, the patients remain under observation for 8 hours. Prior to discharge, chest radiography and pacemaker monitoring was performed. At 24 hours a patient evaluation was carried out by telephone control, at 48 hours by means of an ambulatory face-to-face visit, which repeats after 2-3 months and continuing every 6-9 months.

**RESULTS.** 39 patients included, all of them could be discharged following the protocol. Average age 77 (48-91) years old, 69% male. Pacing Systems: DDDR 79%-VVIR 21%. Indications: Third-degree AV block 26%, Mobitz II 21%, sick sinus syndrome 20%, Atrial fibrillation Blocked 15%. Symptom: syncope 46%, bradycardia 26%, dizziness 18% and heart failure 10%.Etiology was unknown in 95% of cases. 36% of patients were under treatment with oral anticoagulation.The most frequent placement of right ventricular electrode was the septum in a 92%. Any patient presented complications during the procedure or at discharge (table 1); only one complication at 48 hours due to hematoma in the area of the pacemaker, related to oral anticoagulation and ambulatory solved.The implant parameters were the most adequates in all cases (table 1), remaining stable in the subsequent controls. There was only one case with high capture threshold in the atrial lead but remained stable at follow-up.

**CONCLUSION.** Ambulatory implantation of pacemakers has proven to be a safe treatment in our experience.

**Table 1 (abstract 001116).** See text for description

| | Procedure | 48 h control | 2-3 months control | Following controls |
|---|---|---|---|---|
| Complications | 0 | 1 | 0 | 0 |
| Unexpected consultations | 0 | 0 | 0 | 0 |
| Hospital admission related to the pacemaker | 0 | 0 | 0 | 0 |
| Pacemaker normal operation | 100% | 100% | 100% | 100% |
| Ventricular Normal capture threshold | 100% | 100% | 100% | 100% |
| Atrial Normal capture threshold | 95% | 95% | 95% | 95% |
| Normal Ventricular impedance | 100% | 100% | 100% | 100% |
| Normal Atrial impedance | 100% | 100% | 100% | 100% |

### 001135

#### Right ventricular, renal function, and fluids balance analysis after resuscitation in patients on mechanical ventilation

S. Gallego Zarzosa[1], M. López De Olivencia[2], R. De Pablo[2], D. Cabestrero[2], J. Higuera[1]
[1]intensive medicine, Hospital Ramón YCajal, Madrid, Spain; [2]Intensive care medicine, Hospital Ramón YCajal, Madrid, Spain
**Correspondence:** S. Gallego Zarzosa
*Intensive Care Medicine Experimental* 2019, **7(Suppl 3):**001135

**INTRODUCTION.** Hemodynamic optimization is a main goal in the management of critically ill patients. Right ventricular function, renal failure and fluid balance are part of this process. Our goal is to analysis those, after the initial resuscitation and the effects of mechanical ventilation over them.

**METHODS.** A prospective, observational study was performed with all the patients admitted to a Medical Service of Intensive Care of a Tertiary, University Hospital. All patients analyzed required mechanical ventilation as a support for their underling pathology. All consecutive patients admitted to the unit under mechanical ventilation were collected, in the absence of shock, with the expectation of remaining under mechanical ventilation for at least 24 more hours after data collection.

The incidence of right ventricular failure according to the defined parameters, renal failure, and fluid balance were described. We had described its association with mechanical ventilation and mortality.

**RESULTS.** A total of 30 patients were selected. Right ventricular failure was observed in 16.6% of patients (5/30). There was no statistically significant association with the need of tracheotomy, renal failure or mortality. It was not associated with longer average stay, days of mechanical ventilation or higher severity scores.

40% of the patients presented acute renal failure. Renal failure was not associated statistically significant with the need of tracheotomy or failure in scheduled extubation, however, it was associated with higher mortality. Patients requiring mechanical ventilation with normal creatinine values after initial resuscitation, had a mortality rate of 28.5% in comparison to patients who had altered values in which the mortality rate was 77.7%. ($p < 0.018$).

Regarding the post-resuscitation net fluid balance, no statistically significant differences were found in the comparison of means between survivors and non survivors, with renal failure or right ventricular failure. No even was it associated with higher mortality.

**CONCLUSION.** Right ventricular failure, despite not being associated with mortality, days of mechanical ventilation or failure in extubation in a statistically significant way, presents an incidence of 16.6% in patients connected to mechanical ventilation admitted to a polyvalent icu.

Acute renal failure is associated in a statistically significant way with mortality in our sample. We had not found association between fluid overload and renal failure in our sample.

### 001157

#### Arterial pressure waveform distortion: a new dynamic electronic filter for automatic correction of underdamping/resonance artifact. A prospective study

L. Foti[1], G. Villa[2], Z. Ricci[3], S. Romagnoli[2]
[1]School of anesthesia and critical care, university of florence, Careggi, Florence, Italy; [2]Health sciences department, section of anesthesiology, intensive care and pain medicine, Careggi, Florence, Italy; [3]Cardiology and cardiac surgery, pediatric cardiac intensive care unit, Bambino Gesù Children's Hospital, Rome, Italy
**Correspondence:** L. Foti
*Intensive Care Medicine Experimental* 2019, **7(Suppl 3):**001157

**INTRODUCTION.** Invasive blood pressure (IBP) is the gold standard for arterial pressure (AP) monitoring in critically ill patients. Nevertheless, IBP may be affected by underdamping/resonance artifacts, that eventually lead to AP overestimation. In these cases, when a pulse contour method (PCM) is applied, wrong hemodynamic data are also delivered by the monitor. In order to overtake this issue, MostCareUp (a PCM powered by Pressure Recording Analytical Method, PRAM), has been implemented with a dynamic electronic filter (EFMC) that automatically corrects the resonant AP waveform with two modalities: standard and advanced (i.e., higher-intensity).

**OBJECTIVES.** In order to test the EFMC, the electronically corrected AP, the derived cardiac output (CO) and the maximal slope of the systolic upstroke (dP/dtMAX) were compared with respective raw data corrected with the Accudynamic, an adjustable damping device specifically manufactured for normalizing the pressure waveform (in our study: the reference method).

**METHODS.** Systolic AP, CO and dP/dtMAX, were recorded with EFMC disabled (A), standard EFMC (B), advanced EFMC (C), Accudynamic with EFMC disabled (D).

**RESULTS.** Seventy patients with resonant IBP were enrolled (Table 1). According to the Bland-Altman analysis, systolic AP biases (LoAs) (mmHg) were 17.21 (-0.43; 34.87); 13.27 (-2.86; 29.4); 6.68 (-5.18;18.55) in D-A, D-B and D-C conditions respectively. CO biases (LoAs) (L/min) were 4.1 (-1;9.25); 1.19 (-1.1;3.5); 0.3 (-1.1;1.64) in in D-A, D-B and D-C conditions respectively. DP/dtMAX biases (LoAs) (mmHg/ms) were 0.88 (0.09;1.68); 0.28 (-0.04;0.6); -0.04 (-0.4;0.33) in D-A, D-B and D-C conditions respectively.

**CONCLUSION.** The EFMC, especially in advanced modality, efficiently corrects resonant waveforms allowing a correct measurement of AP values and contributing to improve the estimation of exact hemodynamic data.

**REFERENCE(S)**

1. Romagnoli, S., et al., Accuracy of invasive arterial pressure monitoring in cardiovascular patients: an observational study. Crit Care, 2014. 18(6): p. 644.
2. Romagnoli, S. and G. Zagli, Blood pressure monitoring in the ICU. Vol. Oxford textbook of Critical Care. 2015.
3. Romagnoli, S., et al., Dynamic response of liquid-filled catheter systems for measurement of blood pressure: precision of measurements and reliability of the Pressure Recording Analytical Method with different disposable systems. Crit Care, 2011. 26(4): p. 415-22.

**Table 1 (abstract 001157).** See text for description

| | Accudynamic (D) | $EF_{MC}$ disabled (A) | Standard $EF_{MC}$ (B) | Advanced $EF_{MC}$ (C) |
|---|---|---|---|---|
| **Systolic AP (mmHg)** | 137.8 (21.44) 96-202 | 155 (25) 103-214 | 151.07 (23.5) 104-206 | 144.48 (23.08) 97-199 |
| Bias (LoAs) *vs Accudynamic* | | 17.21 (-0.43; 34.87) | 13.27 (-2.86; 29.4) | 6.68 (-5.18-18.55) |
| **CO (L/min)** | 4.54 (0.76) 2.8-6.4 | 8.64 (2.52) 3.7-16.1 | 5.73 (1.19) 3.5-8.5 | 4.81 (0.91) 2.7-6.9 |
| Bias (LoAs) *vs Accudynamic* | | 4.1 (-1;9.25) | 1.19 (-1.1;3.5) | 0.27 (-1.1;1.64) |
| **$dP/dt_{MAX}$ (mmHg/msec)** | 1.08 (0.25) 0.55-1.73 | 1.96 (0.55) 0.97-3.2 | 1.36 (0.27) 0.89-2.08 | 1.04 (0.25) 0.6-1.55 |
| Bias (LoAs) *vs Accudynamic* | | 0.88 (0.09;1.68) | 0.28 (-0.04;0.6) | -0.04 (-0.4;0.33) |

### 001160

#### Intravenous administration of magnesium sulfate may increase successful electrical cardioversion in patients with new onset atrial fibrillation in the intensive care unit

T. Yoshida[1], K. Maekawa[2], K. Moriki[1], S. Tahara[1], M. Takahashi[1], Y. TAKAHASHI[1], T. Tsuchida[3], Y. Sadamoto[1], Y. Homma[1], A. Mizugaki[1], M. Hayamizu[1], T. Oyasu[1], T. Saito[1], K. Katabami[1], T. Wada[3], T. Yoshida[4], S. Uchino[5], M. Hayakawa[1]

[1]Department of emergency medicine, Hokkaido University Hospital, Sapporo, Japan; [2]Kita14joh nishi5 choume ,kita-ku , sapporo city , hokkaido, Department of emergency medicine , Hokkaido university hospital, Sapporo, Japan; [3]Department of anesthesiology and critical care medicine, Hokkaido University Graduate School of Medicine, Sapporo, Hokkaido, Japan; [4]Intensive care unit, department of anesthesiology, Jikei University School of Medicine, Minato City, Japan; [5]Icu, Jikei University School of Medicine, Tokyo, Japan

**Correspondence:** T. Yoshida

*Intensive Care Medicine Experimental* 2019, **7(Suppl 3):**001160

**INTRODUCTION.** Although the potential ability of magnesium administration to prevent and treat arrhythmias has been recognized in clinical setting, limited evidence exists. In this study, we examined whether intravenous administration of magnesium sulfate increases successful electrical cardioversion for new-onset atrial fibrillation (AF) in critically ill patients.

**METHODS.** Atrial Fibrillation Treatment Evaluation Registry in the ICU (AFTER-ICU) was a multicenter, prospective cohort study of adult patient who developed new-onset AF after ICU admission, conducted from April 1, 2017 to March 31, 2018 in 31 Japanese ICUs. In this prospectively planned sub-analysis of the AFTER-ICU study, we assessed the value of magnesium sulfate in facilitating electrical cardioversion. All delivered electrical cardioversions for AF were assigned to the magnesium group (administered within 12 hours before electrical cardioversion) or the control group. The primary outcome was successful cardioversion defined as conversion to sinus rhythm after 30 seconds of electrical cardioversion.

**RESULTS.** Of 158 electrical cardioversions in 52 patients, 19 were assigned to the magnesium group and 139 to the control group. Compared with the control group, the magnesium group was younger (66yr vs. 70yr) and had a lower proportion of male (10.5% vs. 23.7%), but severity of critical illness was similar (APACHE II score, 29 vs. 28). Successful electrical cardioversion was more frequently observed in the magnesium group (42.1% vs. 27.3%), and there was non-significant association between magnesium pre-administration and successful electrical cardioversion (adjusted odds ratio 2.0, 95%CI 0.7-5.5).

**CONCLUSION.** Intravenous administration of magnesium sulfate may increase successful electrical cardioversion in patients with new-onset AF. This study was not powered to detect any associations due to the small sample size and future research is warranted.

### 001170

#### Effectiveness of catheter-directed interventions as an alternative to thrombolysis or anticoagulation in high-risk pulmonary embolism

F. Einarsson[1], J. Oras[2], C. Sandström[3], K. Svennerholm[2], C. Rylander[2]

[1]Medical student, Sahlgrenska University Hospital, Gothenburg, Sweden; [2]Anesthesiology and intensive care medicine, Sahlgrenska University Hospital, Gothenburg, Sweden; [3]Department of radiology, Sahlgrenska University Hospital, Gothenburg, Sweden

**Correspondence:** J. Oras

*Intensive Care Medicine Experimental* 2019, **7(Suppl 3):**001170

**INTRODUCTION.** According to European and American guidelines, intravenous (iv) thrombolysis is the primary treatment for high-risk pulmonary embolism (PE) signified by hemodynamic instability. In recent years, catheter-directed interventions (CDI) has emerged as an alternative in patients with contraindications to thrombolysis. However, there are numerous variants of CDI techniques and their effectiveness is not sufficiently studied.

**OBJECTIVES.** To evaluate the outcome and effectiveness of CDI, measured in terms of survival and reduction of right ventricle distension, as treatment for massive PE compared to iv thrombolysis or anticoagulation only.

**METHODS.** This is a retrospective study from a tertiary hospital where CDI is used for patients with high-risk PE fulfilling all three criteria: (1) distention of the right ventricle (RV); (2) systolic blood pressure <90mmHg or syncope at any time from onset of symptoms; (3) contraindication(s) to or insufficient effect from iv thrombolysis. We included patients from July 2013, when CDI with the Angiojet system as the main technique, was introduced, to December 2018. The control cohort were PE patients from 2006 to 2013, before CDI was introduced, who fulfilled the CDI criteria above but received either heparin only or iv thrombolysis, administered despite contraindications on vital indication.

**RESULTS.** Ninety-day survival was 60% in the CDI (n=25) and 61% (p=0.951) in the control group (n=23). The PESI score did not differ between the groups; CDI 183 ± 46 vs control 168 ± 35 (p=0.211). Median pre-treatment RV/LV ratio was 2.2 [IQR 1.6 – 2.7] in the CDI group which was higher than 1.3 [IQR 1.1 – 1.6] which was found in the control group (p<0.001). When adjusting for PESI-scores and RV/LV ratio, there was no difference between the groups as to survival at 90

days (p=0.529). There were neither any differences in long-term survival between the groups. (p=0.975, Figure). In a linear mixed model, with adjustment for timing of pre- and post-treatment imaging, the decrease of RV/LV ratio was 0.4 units higher per 24 hours in the CDI group (p=0.007).

**CONCLUSION.**

In this retrospective observational study of patients with high-risk PE and contraindications to thrombolysis, treatment by CDI was as efficient as treatment by iv thrombolysis or anticoagulation with regards to survival. However, CDI resulted in faster resolution of RV dilatation. The latter is to be interpreted with caution since the patients in the CDI group had higher pre-treatment RV/LV ratios.

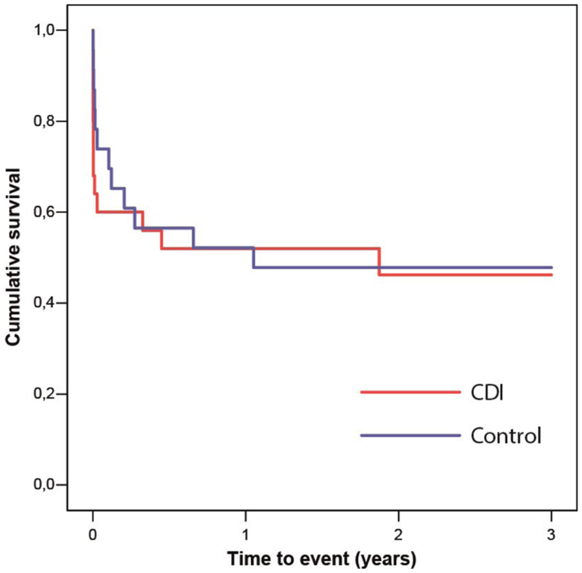

**Fig. 1 (abstract 001170).** See text for description

### 001198

#### The Clinical Characteristics of Stress-induced Cardiomyopathy in Critically ill Patients

S. Park[1], J. Jeong[2], D. Lee[2], H. Lee[1]
[1]Department of internal medicine, Chonbuk National University Hospital, Jeonju, Republic of Korea; [2]Department of nursing, Chonbuk National University Hospital, Jeonju, Republic of Korea
**Correspondence:** S. Park
*Intensive Care Medicine Experimental* 2019, **7(Suppl 3):**001198

**INTRODUCTION.** Stress or Takotsubo cardiomyopathy(SCMP) is a syndrome characterized by transient regional left ventricular dysfunction in the absence of significant coronary artery disease. The clinical presentation of SCMP is similar to that of an acute coronary syndrome. Wall motion abnormalities in patients with SCMP are typically detected by echocardiography.

However, diagnosis of SCMP can be challenging in patients who are being treated for other diseases in the intensive care unit. And the clinical characteristics and risk factors of SCMP among patients in intensive care unit with a non-cardiac diagnosis and no prior history of cardiac disease is not known.

**OBJECTIVES.** The aim of study was to investigate the clinical characteristics of stress-induced cardiomyopathy in critically ill patients.

**METHODS.** We retrospectively assessed the medical records of patients who performed echocardiography (ECG) in the Medical intensive care unit at tertiary university hospital, from January 2015 to December 2018.

Diagnosis of SCMP was based on the following echocardiographic finding: reversible akinesia or dyskinesia of the apical and mid portions of the left ventricle beyond a single major coronary artery vascular distribution.

**RESULTS.** A total 62 patients were performed formal echocardiography. Of the 62 cases, 11 patients were SCMP (17.7%), and 38 patients had diagnosed a sepsis or septic shock.

There was no significant difference between the SCMP positive group and the negative group in baseline characteristics such as age, sex and other clinical features. T-wave inversion on ECG at admission day (8/11 vs. 17/51, p=0.038) was statistically different between two groups (SCMP + vs SCMP -, respectively).

And in subjects who diagnosed sepsis or septic shock (n = 38), initial T-wave inversion on ECG (7/8 vs. 13/30, p=0.045), abnormal troponin level at admission day (7/8 vs. 13/30, p=0.027), and elevated lactate even 48 hours later (3/118 vs. 3/30, p=0.026) were related with the incidence of SCMP.

**CONCLUSION.** In critically ill patients, T-wave inversion on ECG may be a positive SCMP suggesting clue. And also, abnormal troponin level and elevated lactate at 48 hours later could be a good clinical features for suggesting SCMP in sepsis or septic shock patients.

### 001214

#### End-expiratory occlusion test for predicting fluid responsiveness in mechanically ventilated patients: a systematic review and meta-analysis

S. Xiang[1], W. Jianfeng[1], S. Xiaodong[2], C. Minying[1], G. Xiangdong[1]
[1]Critical care medicine, The First Affiliated Hospital, Sun Yat-sen University, Guangzhou, Guangdong, China, China; [2]Zhongshan school of medicine, Sun Yat-sen University,Guangzhou, China, Guangzhou, Guangdong, China, China
**Correspondence:** X. Si
*Intensive Care Medicine Experimental* 2019, **7(Suppl 3):**001214

**INTRODUCTION.** Hypovolemia is a common clinical condition in intensive care units (ICUs) and fluid resuscitation is considered the first line of therapy. Multiple studies have found that only 50% of hemodynamically unstable patients in the ICU respond to volume expansion(VE). It is therefore essential to have reliable tools to predict the efficacy of VE and ultimately distinguish patients who may benefit from VE from those who are unlikely to respond.Over the last decade, new approaches have been developed. Among the tests and indices that have been developed to predict fluid responsiveness, the end-expiratory occlusion(EEO) test has gained considerable attention, especially in mechanically ventilated patients.Following the first study demonstrating the accuracy of the EEO test by Monnet et al, conflicting results concerning the ability of EEO test have been published.

**OBJECTIVES.** We performed a systematic review and meta-analysis of studies investigating the EEO-induced changes in cardiac output (CO) and in arterial pressure as predictors of fluid responsiveness in adults receiving mechanical ventilation.

**METHODS.** MEDLINE, EMBASE, Cochrane Database and Chinese database(China National Knowledge Internet, Wanfang Database) were screened for relevant original and review articles.The meta-analysis determined the pooled area under the ROC curve, the sensitivity, specificity and threshold for the EEO test when assessed with CO and arterial pressure.

**RESULTS.** Thirteen studies enrolling 479 adult patients and 523 volume expansion were included. CO was measured by transpulmonary thermodilution in ten studies, echocardiography in one study, oesophageal Doppler in one study, and calibrated pulse contour analysis in one study. For the EEO-induced changes in CO, the pooled sensitivity was 0.92 (95% CI, 0.85–0.97) and the pooled specificity was 0.90 (95% CI, 0.84–0.994). The pooled AUROC was 0.96 (95% CI, 0.94–0.97). The best threshold was a EEO-induced increase in CO ≥5± 2%. For the EEO-induced changes in arterial pressure (4 studies, 149 volume expansion), the pooled sensitivity was 0.88 (95% CI, 0.63–0.97), the pooled specificity was 0.77 (95% CI, 0.58–0.89) and the pooled AUROC was 0.87 (95% CI, 0.84–0.90). Significant heterogeneity existed among the studies, and meta regression indicated that the patients position for EEO was the major source of heterogeneity. When excluding the only one study in which EEO was performed during the prone position, the pooled sensitivity for the EEO-induced changes in CO (or surrogate) was 0.92 (0.88–0.95) and the pooled specificity was 0.89 (0.83–0.93) in semirecumbent or supine patients.

**CONCLUSION.** EEO-induced changes in CO very reliably predict the response of CO to volume expansion in adults with acute circulatory failure during semirecumbent position or supine position but not during prone position. When EEO effects are assessed by changes in arterial pressure, the sensitivity of the EEO test remains acceptable but its specificity is poor.

**REFERENCE(S)**

1. Monnet X, Osman D, Ridel C, Lamia B, Richard C, Teboul JL(2009).Predicting volume responsiveness by using the end-expiratory occlusion in mechanically ventilated intensive care unit patients.Crit Care Med, Mar;37(3):951-6.
2. Monnet X, Bleibtreu A, Ferré A, Dres M, Gharbi R, Richard C, Teboul JL(2012).Passive leg-raising and end-expiratory occlusion tests perform better than pulse pressure variation in patients with low respiratory system compliance.Crit Care Med, Jan;40(1):152-7.
3. Monnet X, Dres M, Ferré A, Le Teuff G, Jozwiak M, Bleibtreu A, Le Deley MC, Chemla D, Richard C, Teboul JL(2012).Prediction of fluid responsiveness by a continuous non-invasive assessment of arterial pressure in critically ill patients: comparison with four other dynamic indices.Br J Anaesth, Sep;109(3):330-8.
4. Silva S, Jozwiak M, Teboul JL, Persichini R, Richard C, Monnet X(2013).End-expiratory occlusion test predicts preload responsiveness independently of positive end-expiratory pressure during acute respiratory distress syndrome.Crit Care Med, Jul;41(7):1692-701.
5. Guinot PG, Godart J, de Broca B, Bernard E, Lorne E, Dupont H(2014).End-expiratory occlusion manoeuvre does not accurately predict fluid responsiveness in the operating theatre.Br J Anaesth, Jun;112(6):1050-4.
6. Fengming liang, Jun jin, Jie Yan, Liang Dong.Predicting volume responsiveness by using the end -expiratory occlusion in mechanically ventilated patients with septic shock(2015).Chin J Crit Care Med, June, Vol.35, No.6(486-494)
7. Kaizhong Liu, Yongqing Cao, Jue Shen,Jieming Liu(2015).Predictive value of end-expiratory occlusion and pulse contour-derived cardiac index on fluid responsiveness in shock patients with mechanical ventilation. Chin J Crit Care Med(ElectronicEdition),June,Vol.8 No.3 (159-164)
8. Biais M, Larghi M, Henriot J, de Courson H, Sesay M, Nouette-Gaulain K(2017).End-Expiratory Occlusion Test Predicts Fluid Responsiveness in Patients With Protective Ventilation in the Operating Room.Anesth Analg, Dec;125(6):1889-1895.
9. Jozwiak M, Depret F, Teboul JL, Alphonsine JE, Lai C, Richard C, Monnet X(2017). Predicting Fluid Responsiveness in Critically Ill Patients by Using Combined End-Expiratory and End-Inspiratory Occlusions With Echocardiography. Crit Care Med, Nov;45(11):e1131-e1138.
10. Myatra SN, Prabu NR, Divatia JV, Monnet X, Kulkarni AP, Teboul JL(2017).The Changes in Pulse Pressure Variation or Stroke Volume Variation After a "Tidal Volume Challenge" Reliably Predict Fluid Responsiveness During Low Tidal Volume Ventilation.Crit Care Med, Mar;45(3):415-421.
11. Yonis H, Bitker L, Aublanc M, Perinel Ragey S, Riad Z, Lissonde F, Louf-Durier A, Debord S, Gobert F, Tapponnier R, Guérin C, Richard JC(2017).Change in cardiac output during Trendelenburg maneuver is a reliable predictor of fluid responsiveness in patients with acute respiratory distress syndrome in the prone position under protective ventilation.Crit Care, Dec 5;21(1):295.
12. Georges D, de Courson H, Lanchon R, Sesay M, Nouette-Gaulain K, Biais M(2018).End-expiratory occlusion maneuver to predict fluid responsiveness in the intensive care unit: an echocardiographic study.Crit Care, Feb 8;22(1):32.
13. Dépret F, Jozwiak M, Teboul JL, Alphonsine JE, Richard C, Monnet X(2019).Esophageal Doppler Can Predict Fluid Responsiveness Through End-Expiratory and End-Inspiratory Occlusion Tests.Crit Care Med, Feb;47(2):e96-e102.
14. This work was supported by the grants from the Wu Jieping Medical Foundation (320.6750.18037, 320.6750.18068) and Guangdong Science and Technology Planning Project (2017B 020247002)

### 001218

#### Context-sensitive indexation of pulse pressure variation CONSORTION I: Analysis of a prospectively maintained database

W. Huber, C. Schwamberger, A. Herner, U. Mayr, G. Batres-Baires, S. Rasch, S. Schreiber, S. Schikora, R. Schmid, T. Lahmer
Medizinische klinik und poliklinik ii, Klinikum rechts der Isar; Technische Universität München, Munich, Germany
**Correspondence:** W. Huber
*Intensive Care Medicine Experimental* 2019, **7(Suppl 3):**001218

**INTRODUCTION.** Background: Pulse pressure variation (PPV) is a dimension-less parameter calculated from the changes in the arterial pressure curve induced by controlled mechanical ventilation CMV. PPV is a predictor of fluid responsiveness FR. Due to a potential impact of arrhythmia and spontaneous breathing on PPV, its application usually is restricted to patients with both sinus rhythm (SR) and CMV. Several other confounders and "contexts" are considered to impede the use of PPV. However, there is a lack of systematic studies investigating the exact impact of potential cofounders on PPV.

**OBJECTIVES.** We hypothesized that PPV might be adjusted to some of these confounders ("context-sensitive indexation"). Therefore, we analyzed a large prospectively maintained database on haemodynamic monitoring (PiCCO; Pulsion; Germany). The CONSORTION I study investigated the general impact of biometrics, other haemodynamic data and contexts (e.g. type of ventilation, heart rhythm) on PPV in all measurements. The CONSORTION II study investigated the impact of ventilator setting and potential confounders on PPV in the subgroup of measurements with both SR and CMV.

**METHODS.** Analysis of a database including 9001 haemodynamic measurements in 513 critically ill patients. Spearman correlation. Multiple regression analysis. IBM SPSS 25.

**RESULTS.** Biometrics: In the totality of measurements, PPV was slightly associated with age (r=0.162; p<0.001), weight (r=0.119; p<0.001) and height (r=0.068; p<0.001). Men had slightly higher PPV compared to women (11.9±7.7 vs. 10.2±6.6; p<0.001). In multivariate analysis ($R^2$=0.037) regarding biometric data, all biometrics were independently associated with PPV (T-values of 10.3, 8.8, 8.8 and -2.4 for age, weight, male gender and height). Among the „contexts", there was strong impact of the heart rhythm on PPV: Patients with atrial fibrillation AF had a markedly higher PPV compared to patients with sinus rhythm SR (19.9±7.1 vs. 10.2±6.6%; p<0.001). Patients with mechanical ventilation MV (irrespective of assisted or controlled modality) had a lower PPV compared to spontaneously breathing patients (10.5±7.4 vs. 12.4±6.9%; p<0.001). Patients with controlled MV (CMV) hat a lower PPV compared to patients with assisted MV (AMV; 8.9±7.5 vs. 11.3±7.2%; p<0.001).

Among haemodynamic contexts, PPV was most strongly associated with high heart rate (HR; r=0.253; p<0.001), low stroke volume (SV; r=-0.289; p<0.001) and low cardiac output (CO; r=-0.155; p<0.001). There were poor associations with dPmax (r=-0.074; p<0.001), global end-diastolic volume (GEDV; r=0.070; p<0.001) and low CVP (r=-0.027; p=0.023).

In multivariate analysis ($R^2$=0.333), PPV was strongly associated with atrial fibrillation (T=19.0), heart rate (T=11.9), low SV (T=-13.1), spontaneous vs. assisted vs. controlled ventilation (T=10.4) and weight (T=9.2). Furthermore, PPV was associated to low dPmax (T=-6.5), female gender (T=4.2) and age (T=2.9; all p<0.001 except age (p=0.004)).

**CONCLUSION.** Among potential confounders, PPV is most strongly associated to atrial fibrillation, while the impact of assisted or even spontaneous ventilation seems to be lower. A strong association of PPV with low SV and high HR might be in line with the role of PPV as marker of fluid de-pletion. Independent association to age, sex and weight carries the potential for indexation.

### 001222

#### Context-sensitive indexation of pulse pressure variation in patients with sinus rhythm and controlled mechanical ventilation: The CONSORTION II-study (analysis of a prospectively maintained database)

W. Huber, C. Schwamberger, S. Rasch, A. Herner, U. Mayr, G. Batres-Baires, S. Schikora, S. Schreiber, R. Schmid, T. Lahmer
Medizinische klinik und poliklinik ii, Klinikum rechts der Isar; Technische Universität München, Munich, Germany
**Correspondence:** W. Huber
*Intensive Care Medicine Experimental* 2019, **7(Suppl 3):**001222

**INTRODUCTION.** Pulse pressure variation (PPV) is a dimension-less parameter calculated from the changes in the arterial pressure curve induced by controlled mechanical ventilation CMV. PPV is a predictor of fluid responsiveness FR. Due to a potential impact of arrhythmia and spontaneous breathing on PPV, its application usually is restricted to patients with both sinus rhythm (SR) and CMV. Several other confounders and "contexts" are considered to impede the use of PPV. However, there is a lack of systematic studies investigating the exact impact of potential cofounders on PPV.

**OBJECTIVES.** We hypothesized that PPV might be adjusted to some of these confounders ("context-sensitive indexation"). Therefore, we analyzed a large prospectively maintained database on haemodynamic monitoring with the PiCCO-device (Pulsion; Germany). While the CONSORTION I study (Abstract 1218) investigated the general impact of biometrics, other haemodynamic data and contexts (e.g. type of ventilation, heart rhythm) on PPV in **all** measurements, CONSORTION II analyzed the impact of ventilator setting and potential confounders on PPV in the target subgroup of measurements with both SR and CMV.

**METHODS.** Subgroup-analysis (measurements with both SR and CMV (15.5% of all PPV-measurements)) of a database including 9001 haemodynamic measurements in 513 critically ill patients. Spearman correlation. Multiple regression analysis. IBM SPSS 25.

**RESULTS.** Among ventilator settings, PPV was univariately associated with low PEEP (r=-0.198; p=0.006), low mean respiratory pressure P_mean (r=-0.191; p=0.008), high Delta_P (driving pressure; r=0.180; p=0.013), high respiratory rate RR (r=0.191; p=0.008), but neither with tidal volume (TV; r=-0.002; p=0.944) nor with TV/predicted bodyweight (r=0.012; p=0.689). In multivariate analysis restricted to ventilator parameters ($R^2$=0.058), PPV was independently associated to high Delta_P (T=2.2; p=0.028) and low P_mean (T=-2.6; p=0.011), whereas TV/predicted bodyweight slightly failed significance (p=0.054).

In univariate analysis regarding biometric and haemodynamic data, PPV was most strongly associated to low stroke volume (SV; r=-0.427; p<0.001) and heart rate (HR; r=0.355; p<0.001). Furthermore, PPV was weakly correlated to low global end-diastolic volume (GEDV; r=-0.153; p<0.001) and low CVP (r=-0.101; p<0.001).

In a final multivariate analysis ($R^2$=0.363) including age, gender, weight, height, HR, SV, P_peak, P_mean, PEEP, Delta_P, RR, TV/predicted bodyweight, SV and HR, PPV was independently associated only with low P_mean (T=-3.2; p=0.001), heart rate (T=5.2; p<0.001), low SV (T=-3.9; p<0.001), young age (T=-2.9; p=0.004) and high weight (T=2.1; p=0.038), but not with any other ventilatory parameter including Delta_P and TV/predicted bodyweight.

**CONCLUSION.** 1.) Among ventilatory parameters, pressures were more strongly associated with PPV than TV/predicted bodyweight. 2.) PPV increased with higher Delta_P and lower P_mean. 3.) Haemodynamic parameters like high heart rate and low stroke volume as well as biometric data such as age and weight have substantial impact on PPV. Therefore, adjustment to these contexts might improve the use of PPV.

### 001224

#### 24h-treatment for high-volume ultrafiltration and/or CO2-elimination with the ADVOS/HepaWash-device: Impact on haemodynamics in ICU-patients with transpulmonary thermodilution (TPTD) and pulse contour analysis (PCA)-monitoring: The HAEMADVOS-IV-study

W. Huber[1], D. Busch[2], U. Mayr[2], A. Herner[2], G. Batres-Baires[2], S. Rasch[2], S. Schreiber[2], R. Schmid[2], T. Lahmer[2]

[1]Ii medizinische klinik und poliklinik, Rechts der Isar Hospital, München, Germany; [2]Medizinische klinik und poliklinik ii, Klinikum rechts der Isar; Technische Universität München, Munich, Germany

**Correspondence:** W. Huber

*Intensive Care Medicine Experimental* 2019, **7(Suppl 3):**001224

**INTRODUCTION.** Since multi-organ failure MOF is frequent in critically ill patients, apparative multi-organ support is intriguing. The ADVOS-device (ADVanced Organ Support) has been developed for liver support, renal replacement, CO2-elimination and modulation of the acid-base balance. To provide high efficiency, the device is equipped with two dialyzers for blood purification and ultrafiltration as well as with two dialyzers for regeneration of the albumin-containing dialysate. The standard treatment period for liver support is 8 hours. In case of treatment for CO2-elimination and/or large volume ultrafiltration, patients are treated (up to) 24h per session. Previous studies (see ESICM 2018) reported on haemodynamic effects of connection and disconnection as well as ultrafiltration (ISICEM 2019) during 8h-treatment sessions.

**OBJECTIVES.** This study reports on haemodynamic effects of 24h-treatment periods primarily aimed at CO2-elimination and/or high volume ultrafiltration.

**METHODS.** Transpulmonary thermodilution with the PiCCO-device (Pulsion; Germany) was performed immediately before and after connection (T1, T2) with the ADVOS-device and disconnection (T3; T4). The tubing was prefilled with 0.9% saline ("acute connection"). Connection, ADVOS-treatment and disconnection were performed with a blood flow of 100mL/min. Concentrate flow was 160mL/min. The vasopressor dosages were kept stable.

**RESULTS.** We analyzed 20 treatment sessions with a treatment-goal of 24h in 4 patients. Patients (2m; 2f), 49±14 years, APACHE-II-score 24±4, aetiology: 1 alcoholic cirrhosis, 1 non-alcoholic steato-hepatitis, 2 MOF; mechanical ventilation 20/20 (100%) treatments. Mean treatment time was 21±3h. Five of the sessions had to be terminated early (after 12h-19h) due to clotting or organizational reasons (scheduled diagnostic or therapeutic procedures outside the ICU). Net ultrafiltration was not significantly different from the pre-set ultrafiltration goal (3325±2444 vs. 3818±2508mL; p=0.182). 12 sessions were performed for high-volume ultrafiltration ≥4000mL. Also for these sessions the net ultrafiltration was not different from the pre-defined ultrafiltration goal (4621±1550 vs. 5333±778mL; p=0.139), although three of the treatments were stopped early after 15-18h.

Over up to 24h, ADVOS-therapy did not result in significant changes in heart rate (94±16 vs. 94±13/min; p=0.766), MAP (82±11 vs. 76±13mmHg; p=0. ), CVP 29±9 vs. 27±9mmHg; p=0.482), SVRI 1016±297 vs. 1017±368 dyne/cm2/m4; p=0.123), dPmax (1798±727 vs. 1669±557mmHg/s; p=0.374), global end-diastolic volume index GEDVI 760±128 vs. 736±92mL/m$^2$; p=0.515), stroke volume variation SVV (8±3 vs. 9±6%; p=0.541), CI (4.2±0.6 vs. 4.1±0.4L/min/m$^2$; p=0.515), global ejection fraction GEF (29±3 vs. 29±4%; p=0.673), pulmonary vascular permeability index PVPI (2.3±0.6 vs. 2.0±0.6; p=0.068) and cardiac power index CPI (0.73±017 vs. 0.70±0.16W/m$^2$; p=0.493) after disconnection (T4) vs. before connection (T1). Only extravascular lung water index EVLWI slightly increased (13.7±4.8 vs. 12.1±4.9mL/kg; p=0.041). The noradrenaline dosage was comparable after (T4) vs. before connection (T1; 606±357 vs. 600±278µg; p=1.000).

**CONCLUSION.** 24h-treatment with high-volume ultrafiltration and/or CO2-elimination with the ADVOS-device were haemodynamically well tolerated.

### 001229

#### Comparison of Laser Doppler Imaging With Laser Speckle Contrast Imaging For Assessing Microvascular Function in Healthy Volunteers

G. Guven[1], A. Dijkstra[2], Y. Ince[3], J. Montomoli[3], N. Trommel[4], MV. Baar[4], C. Van Der Vlies[4], C. Ince[3]

[1]Intensive care unit, Erasmus Medical Center, Rotterdam, Netherlands; [2]Intensive care unit, Maasstad Hospital, Rotterdam, Netherlands; [3]Translational physiology, Academic Medical Centre, Amsterdam, Netherlands; [4]Burn unit, Maasstad Hospital, Rotterdam, Netherlands

**Correspondence:** G. Guven

*Intensive Care Medicine Experimental* 2019, **7(Suppl 3):**001229

**INTRODUCTION.** Laser Speckle Contrast Imaging (LSCI) and Laser Doppler Imaging (LDI) are non-contact real-time monitoring techniques used to assess the microvascular blood flow of tissue surfaces [1]. These two techniques measure the microcirculation of different skin depth. The LSCI is used to evaluate the skin at a depth of 300 µm whereas LDI can evaluate the depth at 1-1.5 mm [2]. Measurement of different skin depths becomes a critical step in the management of some clinical states as occurs in burn-injured patients. Currently, LDI is the most widely used and validated noninvasive technique for the assessment of burn wounds [3]. However, LDI device is rather costly, cumbersome and has a poor spatial resolution. LSCI measures perfusion in a similar way, but it provides high-quality images with a much higher spatial resolution. Moreover, the use of LSCI is much easier than using LDI [4].

**OBJECTIVES.** In this study, we sought to test the linearity of the LSCI device and LDI device, and assess if these two techniques can be used interchangeably.

**METHODS.** Fifteen healthy, non-smoker volunteers participated in the study. Iontophoresis technique with sodium nitroprusside was performed as a provocation test on the skin of the forearm. All volunteers were measured using LSCI (Perimed AB, Jarfalla, Sweden) and LDI (Moor Instruments, Devon, UK) devices at baseline and then consecutively after initiating the iontophoresis test by. Microcirculatory blood flow was analyzed by the software PIMsoft 1.5 (Perimed AB, Järfälla, Sweden) and MoorLDI2-BI Software Version 4.0. The units of each technique were reported as arbitrary units. Spearman correlation and linear regression were used to compare the values.

**RESULTS.** The baseline microcirculatory blood flow was 61 AU (IQR, 51-70 AU) and 34 AU (IQR, 28-37 AU) using LDI and LSCI devices, respectively. Coefficient of variance (CV) was similar within two devices (CVLDI:19% and CVLSCI: 19.8%). An excellent correlation was detected between the two LSCI devices (R2:0.88, p<0.001) (Figure-1).

**CONCLUSION.** Skin blood flux measured with Perimed LSCI and Moor LDI are highly correlated with each other. The higher resolution and considerably faster scan time of LSCI technique may provide an advantage if used instead of the LDI technique.

**REFERENCE(S)**

1. Tew, G.A., et al., Comparison of laser speckle contrast imaging with laser Doppler for assessing microvascular function. Microvasc Res, 2011. 82(3): p. 326-32.
2. O'Doherty, J., et al., Comparison of instruments for investigation of microcirculatory blood flow and red blood cell concentration. J Biomed Opt, 2009. 14(3): p. 034025.
3. Devgan, L., et al., Modalities for the assessment of burn wound depth. J Burns Wounds, 2006. 5: p. e2.
4. Roustit, M., et al., Excellent reproducibility of laser speckle contrast imaging to assess skin microvascular reactivity. Microvasc Res, 2010. 80(3): p. 505-11.

### 001252

#### Echocardiographic predictors of VA-ECMO weaning in patients with cardiogenic shock

CNJ. Colombo[1], V. Dammassa[1], M. Pozzi[1], M. Belliato[1], G. Via[2], GA. Iotti[3], F. Mojoli[3], G. Tavazzi[3]
[1]Intensive care department, Fondazione Policlinico San Matteo IRCCS, Pavia, Italy; [2]Cardiac anesthesia and intensive care, Cardiocentro Ticino, Lugano, Switzerland; [3]Department of clinical, surgical, diagnostic and pediatric sciences, University of Pavia, Pavia, Italy
**Correspondence:** C.N.J. Colombo
*Intensive Care Medicine Experimental* 2019, **7(Suppl 3):**001252

**INTRODUCTION.** Veno-arterial extracorporeal membrane oxygenation (VA-ECMO) is an advanced treatment for refractory cardiogenic shock (CS) and cardiac arrest (CA). VA-ECMO is used as bridge to recovery, cardiac transplantation or ventricular assist device (VAD). Successful weaning from VA-ECMO is defined as device removal without further requirement for re-cannulation over the following 30 days. There are few data available regarding timing and reliable parameters for the VA-ECMO weaning. Total isovolumic time (t-IVT) is a Pulse Wave Doppler established echocardiographic parameter of systo-diastolic interaction and ventricular efficacy (1). Along with MAPSE (mitral annular systolic plane excursion), it showed to be the most sensitive echocardiographic marker of haemodynamic profile in patients with cardiogenic shock.

**OBJECTIVES.** To evaluate which echocardiographic parameters are able to predict successful VA-ECMO weaning.

**METHODS.** Single-center retrospective observational study of patients with refractory CS and CA who underwent VA-ECMO insertion from January 2013 to December 2017. Demographic, clinical, echocardiographic and outcome data were collected. Primary endpoint was to test the predictive power of echocardiographic indices for VA-ECMO weaning. We measured left ventricular (LV) t-IVT, MAPSE (calculated as mean of septal, lateral, anterior and inferior values), ejection fraction (EF), Echo-derived stroke volume (SV) and cardiac output (CO). Data analysed: time of cannulation (t0), during the first weaning trial after 48 hours (t1) and, in those who survived, at last trial before decannulation (t2).

**RESULTS.** Preliminary results of 46 patients (76% male; 52±12.5 y.o.) underwent VA-ECMO cannulation. 21 (44.6%) patients died within 24 hours; 19 of them had CA. 25 patients undertook weaning trial: 18 were weaned (72%; 39% overall population) and 14 (56%; 30% overall population) were discharged alive from ICU. At time of cannulation, patients successfully weaned form VA-ECMO had shorter t-IVT (23.12 vs 33.6 sec/min, p<0.001) and greater MAPSE (5.82 vs 4.83 mm, p<0.05), SV (15.11 vs 10.42 mL, p<0.05), CO (1.36 vs 0.91 L/min, p<0.05) and EF (11.4 vs 9.04, p<0.05); heart rate between two groups was not significantly different (84.7 vs 95.4, p 0.24). In weaned patients, the variations from t0 to t2 of all these parameters (left ventricular t-IVT, MAPSE, SV, CO and EF) were significative, as shown in table 1.

**CONCLUSION.** The mortality of patients undertaking VA-ECMO remains exceptionally high, especially in patients with refractory cardiac arrest. Amongst all the echocardiographic parameters tested, t-IVT at the baseline was the strongest predictor of VA-ECMO weaning.

**REFERENCE(S)**

1 Duncan AM, Francis DP, Henein MY, Gibson DG. Limitation of cardiac output by total isovolumic time during pharmacologic stress in patients with dilated cardiomyopathy: activation-mediated effects of left bundle branch block and coronary artery disease. J Am Coll Cardiol 2003;41(1):121-8.

**Table 1 (abstract 001252).** *p<0.05, **p<0.001

| | t0 | t1 | t2 |
|---|---|---|---|
| t-IVT (sec/min) | 23.12±0.42 | 19.85±0.41** | 15.87±0.28** |
| MAPSE (mm) | 5.52±0.11 | 6.85±0.13** | 9.18±0.16** |
| CO (L/min) | 1.34±0.11 | 1.74±0.09* | 3.41±0.19** |
| EF (%) | 12.47±0.60 | 14.65±0.76* | 19.82±0.64** |

### 001257

#### The volume of the aorta: a neglected confounding variable in transpulmonary thermodilution

A. Akohov[1], C. Barner[1], S. Grimmer[1], R. Francis[1], S. Wolf[2]
[1]Anesthesiology, Charite University Medicine, Berlin, Germany;
[2]Neurosurgery, Charite University Medicine, Berlin, Germany
**Correspondence:** S. Wolf
*Intensive Care Medicine Experimental* 2019, **7(Suppl 3):**001257

**INTRODUCTION.** Global end-diastolic volume (GEDV) derived by transpulmonary thermodilution is regarded as indicator of cardiac preload. The thermistor catheter required for measurements is usually placed in a femoral artery. A bolus of cold saline injected in a central vein travels through the right heart, arterial and venous vessels of the lung, the left heart, and finally the aorta until detection. Surprisingly, the latter is not considered in the theoretical framework of transpulmonary thermodilution (1).

**OBJECTIVES.** to investigate the impact of the aortic volume on measurements of GEDV.

**METHODS.** We screened our database of patients with transpulmonary thermodilution monitoring for subjects having additionally received a contrast-enhanced thoraco-abdominal computed tomography (CT) scan. From the CT scan we calculated the volume of the aorta, using the aortic valve and the tip of the thermodilution catheter as longitudinal boundaries. The aortic volume was then compared to the GEDV measurement closest in temporal proximity.

**RESULTS.** 88 patients with 103 CT scans were identified. Median aortic volume was 193.78 ml (IQR: 147.15-248.92 ml). Median GEDV was 1305.67 ml (IQR: 1104-1568.77 ml). Average time difference between CT and GEDV measurement was 2 hours (IQR: 1-5 hours). GEDV increased linearly by 2.9 ml (95% CI: 1.8 to 4 ml, p < 0.001) per ml of aortic volume. The variance of thermodilution-derived GEDV explained by aortic volume was 59.3 % (p < 0.001), without significant further relationships to sex, age, body height or weight.

**CONCLUSION.** We provide evidence that the aortic volume is the main source of variance of GEDV in patients measured by single-indicator transpulmonary thermodilution, thus representing a so far unrecognized confounder. Our findings challenge the view of GEDV as preload indicator. In consequence, guiding clinical volume therapy by reference ranges of raw or indexed GEDV may be misleading.

**REFERENCE(S)**

1. (1) Brown LM, Am J Physiol Lung Cell Mol Physiol 297:547-558, 2009
2. none

### 001294

#### Changes in mean systemic filling pressure during a passive leg raising test

JC. Gasca-Aldama, AH. Morales-Morales, SE. Zamora Gómez, LA. Gorordo-Delsol, S. Sosa-Santos, NI. Medveczky-Ordoñez, ML. Pacheco-Rivera, KJ. Castillo-Medrano, I. Maldonado-Beltrán, D. Sanabria-Cordero, A. Rodríguez-Peredo, JA. Zepeda-Pérez, LE. Gaytán-Medina, GD. Hernández-López, JA. Castañon-Gonzalez
Adult intensive care unit, Hospital Juárez de México, Ciudad de México, Mexico
**Correspondence:** S.E. Zamora Gómez
*Intensive Care Medicine Experimental* 2019, **7(Suppl 3):**001294

**INTRODUCTION.** Mean systemic filling pressure (Pmsf) correlates with intravascular volume. Bedside estimation of Pmsf has been described in critically ill patients by three methods(1). Pmsf along with other haemodynamic variables can provide valuable information to correctly understand the cardiovascular status of critically ill patients, and their performance before and after passive leg raising (2), can facilitate the understanding of venus return (3).

**OBJECTIVES.** To determine the grade of correlation bewteen Pmsf and other haemodinamic variables during passive leg raising test (PLRt).

**METHODS.** observational, retrospective, cross-sectional and descriptive study in patients with continuous hemodynamic monitoring during a shock state. Pmsf analogue (4)value before and after a Passive Leg Raising test was analyzed.

**RESULTS.** 62 simultaneous measurements of CO, CVP and MAP were performed, Pmsf was calculated before and after a PLRt. Correlation between Pmsf and CO was Pearson's R = 0.6064 (p≤0.00001) that indicate a moderate to strong relationship between both variables, that support that increases in ΔPmsf will produce increases in ΔCO during PLR (and viceversa). However, Pearson's R = 0.2486 (p= 0.0513), that indicates a poor relationship between both ΔPsma and final CO (after PLRt). Correlation between CVP and CO was Pearson's R = 0.34562 with p = 0.005831, although technically a positive correlation, the relationship between ΔCVP and ΔCO is pretty low.

**CONCLUSION.** We found a significative positive correlation between ΔPmsf and ΔCO, but there was not significative correlation between ΔPVC and ΔCO. ΔPmsf can be useful as a volumen response indicator in selected patients.

**REFERENCE(S)**

1. K. Cooke, R. Sharvill, S. Sondergaard, A. Aneman. Volume responsiveness assessed by passive leg raising and a fluid challenge: a critical review focused on mean systemic filling pressure. Anaesthesia 2017.
2. Wijnberge et al. Estimating mean circulatory filling pressure in clinical practice: a systematic review comparing three bedside methods in the critically ill. Ann. Intensive Care (2018) 8:73.
3. Repess_e X, Charron C, Fink J, et al. Value and determinants of the mean systemic filling pressure in critically ill patients. American Journal of Physiology Heart and Circulatory Physiology 2015; 309: H1003–7.
4. Cecconi M, Aya HD, Geisen M, et al. Changes in the mean systemic filling pressure during a fluid challenge in postsurgical intensive care patients. Intensive Care Medicine 2013; 39: 1299–305.
5. No financial support or grants were received for this manuscript

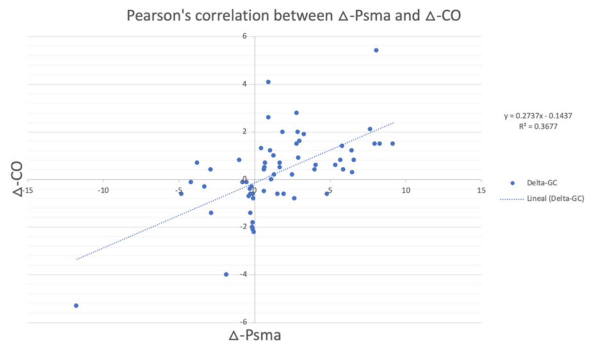

**Fig. 1 (abstract 001294).** See text for description

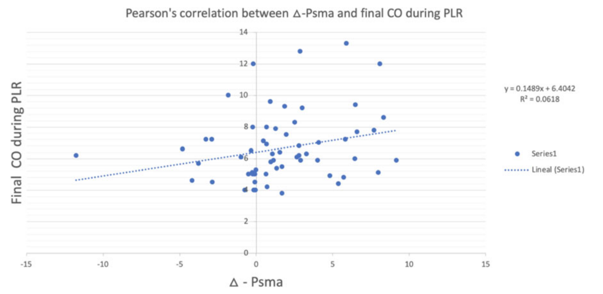

**Fig. 2 (abstract 001294).** See text for description

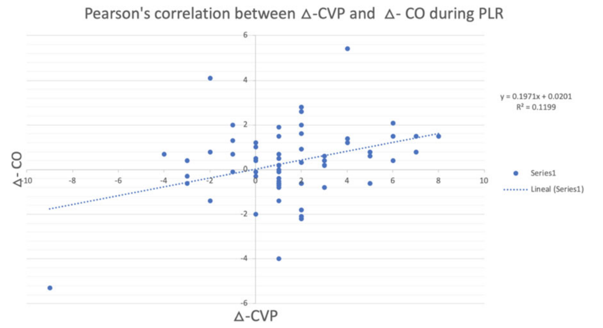

**Fig. 3 (abstract 001294).** See text for description

## HSRO - Goals that improves outcome

### 000662

#### Impact of social deprivation on outcome following admission to the intensive care unit with a cardiac arrest: A retrospective study

K. MOHEE[1], H. Haboubi[2], M. Protty[3], T. Whiffen[4], S. Pillai[1]
[1]Ed major critical care unit, Morriston Hospital, Morriston, United Kingdom; [2]Medical school, Swansea University, Swansea, United Kingdom; [3]Systems immunity university research institute, Cardiff University, Cardiff, United Kingdom; [4]Administrative data research unit, Welsh Government, Cardiff, United Kingdom
**Correspondence:** K. MOHEE
*Intensive Care Medicine Experimental* 2019, **7(Suppl 3):**000662

**INTRODUCTION.** Cardiac arrest (CA) is a leading cause of mortality annually in the USA and Europe. The relationship between socioeconomic status and various components of health is well established.
**OBJECTIVES.** The aim of this study was to investigate the relationship between socioeconomic factors and outcomes following admission to intensive care with cardiac arrest, both in hospital cardiac arrest (IHCA) and out of hospital cardiac arrest (OHCA) using deprivation measures calculated on the basis of domicile postcodes.
**METHODS.** All cardiac arrest cases admitted to our regional intensive care unit in South West Wales, UK between January 2007 and June 2018 were included in this analysis. Data included age, gender, domiciliary postcode, comorbidities, length of stay, follow-up time and survival status. The Welsh Index of Multiple Deprivation, 2014 (WIMD) was used to stratify cases by level of social deprivation according to domiciliary postcodes. Outcomes were investigated using multivariate regression modelling.
**RESULTS.** Of the 1034 (532OHCA and 502 IHCA) patients included in the study, 679 patients were deemed 'less deprived' while 355 patients 'more deprived' according to established definitions. Compared to their 'more deprived' counterparts, patients who were 'less deprived' were older on admission, 66.2 vs 62.1 (p<0.001) and predominantly male (473 vs 205, p<0.001). There were no differences at 7 and 30 days in terms of mortality, p=0.62 and p=0.26 respectively.
In the OHCA cohort, being 'more deprived' showed strong and independent correlations with mortality within the first year after admission to ICU [All: OR 1.71 (95% CI 1.14-2.61), IHCA: OR 1.81 (95% CI: 1.19 – 2.76), OHCA: OR 3.26 (95%CI: 2.12-5.07)]. Conversely, in the IHCA cohort social deprivation was not a risk factor one-year post admission to ICU.
**CONCLUSION.** This is the first study in which social deprivation has been investigated as a risk factor for death following admission to ICU with cardiac arrest in South West of Wales. Residing in a 'more deprived' area in South West Wales following admission an OHCA is associated with an increased risk of death compared to IHCA following one year after admission to ICU.

### 000672

#### Economic burden of cardiac arrest patients admitted in intensive care- A single centre study

K. MOHEE[1], H. Haboubi[2], S. Pillai[1]
[1]Ed major critical care unit, Morriston Hospital, Morriston, United Kingdom; [2]Medical school, Swansea University, Swansea, United Kingdom
**Correspondence:** K. MOHEE
*Intensive Care Medicine Experimental* 2019, **7(Suppl 3):**000672

**INTRODUCTION.** There is a dearth of literature reporting cost analysis of patients admitted to intensive care unit (ICU) post out of hospital cardiac arrest especially within the UK. This is essential for assessment of cost-effectiveness of interventions necessary to allow just allocation of resources within the National Health Service.
**OBJECTIVES.** We aimed at performing a cost analysis of ICU stay as well as hospital stay in both in hospital (IHCA) and out of hospital (OOHCA) cardiac arrest patients admitted to ICU.
**METHODS.** This is a single centre retrospective observational review of Wardwatcher database. All post cardiac arrest patients admitted to a tertiary referral ICU from January 2007 until December 2017 is included. The cardiac arrest patients were grouped as out of hospital cardiac arrest (OHCA) and in hospital cardiac arrest (IHCA).
**RESULTS.** Of 965 consecutive patients admitted 475 were in OHCA group and 490 in IHCA group. The Trust finance department supplied ICU and hospital costs. Estimated cost of an ICU bed was £1159.00 and estimated cost of a ward bed was £327.00 according to the NHS reference costs data 2017-18. Of those patients with return of spontaneous circulation (ROSC) admitted to ICU, survival to hospital discharge in OHCA was 37% while IHCA was 35% survivors at hospital discharge. Mean length of days on mechanical ventilation was higher in the IHCA group compared to the OHCA (5.3 vs 5.1 days, p=0.006). Similarly mean length of ICU stay was longer in the OHCA as compared to IHCA (6.8 vs 8.4 days, p= 0.004) as well as mean length of total stay in hospital was longer in the OHCA as compared to IHCA (16.7 vs 15.0 days, p= 0.03). Total in-hospital costs was significantly higher in OHCA compared to IHCA (£12,796 vs £10,825, p=0.02).
**CONCLUSION.** The costs of in-hospital patient care and mean total length of stay in hospital for ICU admissions following ROSC after OHCA are significantly higher than those of IHCA. This might be due to the fact that OHCA patients have a longer stay in ICU compared to IHCA.

### 000673

#### Is there a "weekend effect" on outcome of patients admitted with cardiac arrest to intensive care?

K. MOHEE[1], H. Haboubi[2], M. Protty[3], S. Pillai[1]
[1]Ed major critical care unit, Morriston Hospital, Morriston, United Kingdom; [2]Medical school, Swansea University, Swansea, United Kingdom; [3]Systems immunity university research institute, Cardiff University, Cardiff, United Kingdom
**Correspondence:** K. MOHEE
*Intensive Care Medicine Experimental* 2019, **7(Suppl 3):**000673

**INTRODUCTION.** It was demonstrated by several studies that patients admitted to hospitals on weekends have a higher mortality rate. The reasons might be multifactorial and may be limited to certain diagnostic groups and admission subtypes.
**OBJECTIVES.** We aimed at investigating whether the outcomes of cardiac arrest patients, both in-hospital (IHCA) and out-of-hospital (OHCA) differed between weekday and weekend admissions at our intensive care unit (ICU).
**METHODS.** This is a single centre retrospective observational study. The database WardWatcher is widely used in United Kingdom (UK) ICU's and the data was collected from this. Cardiac arrest patients who were admitted between January 2007 to July 2018 to a tertiary ICU were included. Death in ICU and death in hospital were used as outcome measure. Logistic regression was used to adjust for potential confounding factors.
**RESULTS.** Of the 1034 patients admitted with cardiac arrest, 629 patients were admitted on a weekday while 405 patients on a weekend. Patients admitted on weekday were older, 65.6 vs 63.5 years old, (P<0.0001), had worse creatinine function, 172.7 vs 167.2 (P< 0.0001). No significant differences in outcome were found between weekday and weekend admissions in rates of death in ITU and death in hospital (P= 0.89 and P = 0.61, respectively) even after adjusting for possible confounding factors.
**CONCLUSION.** The rate of survival to hospital discharge was similar for patients admitted with cardiac arrest on a weekday or weekend even after adjusting for many potentially confounding factors. This study demonstrates that increased mortality rates during weekend admission is limited to certain diagnostic groups and admission subtypes.

### 000682

#### Association between medical aid status and pattern of use and outcomes in ICU: The Korean ICU National Data (KIND) Study

Y. lee[1], K. Danbee[2], G. Eliseo[2], C. Juhee[2], J. Kyeongman[3], RC. Chi[1], HY. Jeong[1], HC. Yang[1], C. Joongbum[1], P. Chi-Min[1], YS. Gee[1]
[1]Department of critical care medicine, Samsung Medical Center, Seoul, Republic of Korea; [2]Center for clinical epidemiology, Samsung Medical Center, Seoul, Republic of Korea; [3]Division of pulmonology and critical care medicine, department of medicine, Samsung Seoul Hospital Samsung Medical Center, Seoul, Republic of Korea
**Correspondence:** Y. lee
*Intensive Care Medicine Experimental* 2019, **7(Suppl 3):**000682

**INTRODUCTION.** Relationship between poverty and worse health outcomes is well established. However, studies examined the association between poverty and mortality among critically ill patients and results were contradictory. Yet, previous studies were conducted with sample size of few selected hospitals. In this study, we evaluated the association between poverty and outcomes including in-hospital mortality, re-admission of intensive care unit (ICU) and emergency room (ER) visit after discharge using national health insurance data.

**METHODS.** This is a retrospective cohort study using the national database from Health Insurance Review and Assessment (HIRA). We identified 1,657,218 patients who were 18 years of age or older, who had a first ICU admission between January 1, 2008 and May 31, 2015 and who was not being poor poverty status during inpatient. Poverty was defined when patients were Medical Aid beneficiaries. We used logistic regression and cox regression to evaluate risk of in-hospital death, re-admission, and ER visit among people with poverty compared to people without poverty.

**RESULTS.** Of total, 11.0% (n=182,091 patients) were patients with poverty. People with poverty were more likely to be female (50.7% vs. 42.2%), had more co-morbidities, and were more likely to be admitted to lower quality hospitals compared to patients without poverty. In -hospital mortality rate among patients with poverty was higher (16.5 %) than patients without poverty (12.2 %). The adjusted OR for in-hospital mortality associated with poverty was 1.20 (95% CI 1.18-1.22) and it remained significant even after matching hospital (OR 1.10; 95% CI 1.09-1.12). Among patients who were discharged without death (n= 1,411,008), patients with poverty were more likely to have re-admissions to ICU (fully-adjusted HR 1.26; 95% CI 1.23, 1.29) and ER visits (fully-adjusted HR 1.28; 95% CI 1.25, 1.32) within a year after discharge compared to patients without poverty.

**CONCLUSION.** Poverty was associated with an increased risk of in-hospital mortality, re-admission to ICU, and ER visits after discharge from ICU. The association was consistent even after controlling quality of hospital. It would be necessary to find out factors associated with worse outcomes among patients with poverty at ICU and ways to intervene them.

### 000688

#### Acuity of Patient Condition Has No Affect on Critically Ill or Injured Adult Patient and Surrogate Decision Maker Opinions of Research

J. Raddatz[1], LA. Sealey[2], KW. Cunningham[3], NR. Shah[3], A. Zykova[3], MR. Nahouraii[3], CA. Becker[4], SL. Davis[4], SL. Evans[3], T. Huynh[3]

[1]Trauma and acute care surgery, F H Sammy Ross Jr Center, Charlotte, United States of America; [2]Surgery, F H Sammy Ross Jr Center, Charlotte, United States of America; [3]Department of surgery, F H Sammy Ross Jr Center, Charlotte, United States of America; [4]Office of clinical and translational research, Atrium Health, Charlotte, United States of America

**Correspondence:** J. Raddatz

*Intensive Care Medicine Experimental* 2019, **7(Suppl 3):**000688

**INTRODUCTION.** Research in the critically ill and injured population often requires time-sensitive enrollment when patients and their families are focused on making important clinical care decisions. Patients with chronic medical conditions may have the opportunity to contemplate clinical choices, and as such more readily consider participation in research trials.

**OBJECTIVES.** To examine if the acuity of illness correlates with patient's or surrogate decision-maker's opinions informing research participation.

**METHODS.** Study participants were identified by admission >24 hours to an intensive care unit (ICU) at an urban tertiary hospital. Decision makers were approached to complete an anonymous electronic survey between 3-7 days following ICU admission. Only the person providing informed consent at the time of survey was asked to participate. Surveys consisted of closed-ended questions and took 5-15 minutes to complete. A full waiver of consent was granted by the IRB. Standard statistical techniques were used for analysis.

**RESULTS.** There were a total of 674 subjects, of which 335 were critically ill or injured, and 339 were surrogate decision makers. Subjects completed surveys from April 2018 to January 2019. Participants were stratified as being admitted to the ICU for either an acute (372) or chronic (302) condition. There was no difference in mean age (56.8, 59.3), gender (60.8% and 60.6% male), or race of patients among the groups. Acute (68.7%) and chronic (71.9%) subjects were equally as open to consider participating in a research study. Acute (43.7%) and chronic (41.7%) subjects also had similar, but reduced interest (p<0.001) in co-enrolling in multiple research studies. Furthermore, both groups were equally more interested (p<0.001) in participating in a research study if it may potentially benefit the patient directly (79.3%, 78.8%) vs. benefiting others (57.4%, 56.6%).

**CONCLUSION.** Acuity of illness does not affect decision makers' opinions of research. Critically ill and injured patients and their surrogate decision makers are collectively more open to considering one research study vs. multiple research studies, suggesting that investigators should prioritize research in a manner that reduces participant burden. Further, such prioritization may include potential direct benefit to study participants. Researchers should not approach patients with acute vs. chronic conditions differently regarding research participation.

### 000694

#### Daily Ultrasound-screening for Central Venous Catheter-related Thrombosis (DUCT) in the Critically Ill-patients: a Multicenter Prospective Study

W. Chunshuang, L. Shaoyun, Z. Mao

Department of emergency medicine, 2nd Affiliated Hospital, Zhejiang University School of Medicine, Hangzhou, China

**Correspondence:** Z. Mao

*Intensive Care Medicine Experimental* 2019, **7(Suppl 3):**000694

**INTRODUCTION.** Central venous catheters (CVCs) are frequently used in intensive care units (ICUs) for variety of indications. CVC-related thrombosis (CRT) is a potentially serious complication and may contribute to some adverse effects, including pulmonary embolism, CVC dysfunction, infection or loss of central venous access [1]. Preliminary observation studies showed that CRT was mostly asymptomatic, and the incidence was not low. However, there is a lack of medical literature about the incidence and characteristics of CRT in ICU [2,3].

**OBJECTIVES.** This is a multi-center, large-sample prospective study to identify the incidence, occurrence time, risk factors, treatment of CRT and outcome in ICU patients, so as to provide a basis for further optimization of CRT screening protocol and prevention.

**METHODS.** We prospectively included patients with CVCs and collected patient characteristics in 28 Chinese ICUs during 9-month study period. Compression test and duplex Doppler ultrasound screening of catheter-veins were systematically performed prior to insertion, 12h after insertion, and then, daily until removal of CVC or patient discharge. In addition, CVC characteristics, catheterization, CRT characteristics, treatment and complications of CRT were also daily recorded.

**RESULTS.** There were 1479 CVCs placed during the study period in 1357 subjects. Of these, 740 (50.03%) were inserted into the internal jugular veins, 413 (27.92%) in the subclavian veins, 297 (20.08%) in femoral vein, and 29 (1.97%) in axillary vein. The incidence of CVC-related thrombosis was 15.28% (n=226). Univariate and multivariate analysis demonstrated that CRT is associated with the major diagnosis, ICU length of stay, place of catheterization, length of catheterization, and the catheterization location ($p \leq 0.001$). Notably, thrombosis can occur at any time from the first day after insertion to 3 days after removal, with a median of 4 days (IQR, 2-7). 88.94% of thrombosis occurred within a week after insertion. 77.43% of CVCs with CRT injected blood or blood products. In addition, the treatments of CRT were uniformity. Of these, 21.90% of patients with CRT are treated with anticoagulation, but the type, dose and duration of drugs were highly variable. 19.91% of CVCs with CRT were removed immediately, and the rest were no interventions for the thrombosis (Table 1).

**CONCLUSION.** Central venous catheters-related thrombosis is a relatively common complication in ICU. It is most associated with the major diagnosis, ICU length of stay, place of catheterization, length of catheterization, and the catheterization location. It may occur at any time after catheterization. Thus, a protocol of routine ultrasound screening for CRT after CVC needs to be developed.

### REFERENCE(S)

[1] Parienti JJ, Mongardon N, Megarbane B, et al. Intravascular Complications of Central Venous Catheterization by Insertion Site[J]. N Engl J Med, 2015, 373(13): 1220-1229.

[2] Joynt GM, Kew J, Gomersall CD, et al. Deep venous thrombosis caused by femoral venous catheters in critically ill adult patients[J]. Chest, 2000, 117(1): 178-183.

[3] Faustino EV, Spinella PC, Li S, et al. Incidence and acute complications of asymptomatic central venous catheter-related deep venous thrombosis in critically ill children[J]. J Pediatr, 2013, 162(2): 387-391.

[4] Welfare scientific research project of Zhejiang Province (NO. LGF18H150003)

[5] Key joint research project of Chinese Ministry of Health & Zhejiang Province (NO. 2018271879)

[6] 2015 Welfare Scientific Research Project from the Chinese Ministry of Health (2015SQ00050)

**Table 1 (abstract 000694).** Descriptive of Patents with and without CRT

| | Mean±SD or median (IQR) or count (percent) | | | Bivariable logistic regression | |
|---|---|---|---|---|---|
| | Without CRT (n=1253) | CRT (n=226) | *P*-value | Odds Ratio (95% CI) | *P*-value |
| Patient characteristics | | | | | |
| Demographics | | | | | |
| Age | 60.42±17.98 | 62.8±16.38 | 0.063 | | |
| Sex, male | 826 (65.92%) | 147 (65.04%) | 0.798 | | |
| BMI | 22.61±3.12 | 22.72±3.37 | 0.636 | | |
| APPACHE II | 18 (11, 24) | 17 (12, 21) | 0.190 | | |
| Medical history | | | 0.104 | | |
| Chronic heart failure | 95 (7.58%) | 18 (7.96%) | | | |
| Chronic renal failure | 79 (6.30%) | 12 (5.31%) | | | |
| Chronic obstructive pulmonary disease | 119 (9.50%) | 37 (16.37%) | | | |
| Cerebral infarction | 65 (5.19%) | 16 (7.08%) | | | |
| Diabetes Mellitus | 143 (11.41%) | 22 (9.73%) | | | |
| Active cancer | 55 (4.39%) | 9 (3.98%) | | | |
| Venous thrombosis | 6 (0.48%) | 4 (1.77%) | | | |
| Atrial fibrillation | 56 (4.47%) | 12 (5.31%) | | | |
| Major diagnosis | | | <0.001* | | |
| Respiratory failure | 134 (10.69%) | 45 (19.91%) | | Ref | Ref |
| Intoxication | 55 (4.39%) | 6 (2.65%) | | 0.415 (0.145, 1.187) | 0.101 |
| Multiple organ dysfunction syndrome | 84 (6.70%) | 3 (1.33%) | | 0.146 (0.043, 0.499) | 0.002* |
| Multiple trauma | 382 (30.49%) | 68 (30.09%) | | 0.544 (0.330, 0.895) | 0.016* |
| Serious infection | 136 (10.85%) | 32 (14.16%) | | 0.792 (0.450, 1.393) | 0.441 |
| Cardiovascular or cerebrovascular diseases | 206 (16.44%) | 39 (17.26%) | | 0.638 (0.373, 1.091) | 0.110 |
| cardiopulmonary resuscitation | 74 (5.91%) | 15 (6.64%) | | 0.723 (0.355, 1.473) | 0.422 |
| other | 182 (14.53%) | 18 (7.96%) | | 0.340 (0.178, 0.650) | 0.002* |
| Intensive care unit length of stay (days) | 7 (3, 12) | 10 (6, 17) | <0.001* | 1.036 (1.022, 1.050) | <0.001* |
| CVC characteristics | | | | | |
| Number of lumens | | | 0.078 | | |
| 1 | 175 (13.97%) | 31 (13.72%) | | | |
| 2 | 811 (64.72%) | 152 (67.26%) | | | |
| 3 | 163 (13.01%) | 35 (15.49%) | | | |
| other | 104 (8.30%) | 8 (3.54%) | | | |
| Place of Catheterization | | | 0.001* | | |
| Other hospital | 68 (5.43%) | 28 (12.39%) | | Ref | Ref |
| Emergency room | 111 (8.86%) | 21 (9.29%) | | 0.616 (0.304, 1.249) | 0.179 |
| During operation | 332 (26.50%) | 56 (24.78%) | | 0.328 (0.183, 0589) | <0.001* |
| Intensive care unit | 742 (59.22%) | 121 (53.54%) | | 0.387 (0.224, 0.667) | 0.001* |
| Side | | | 0.118 | | |
| Left | 94 (7.50%) | 26 (11.50%) | | | |
| Right | 1159 (92.50%) | 200 (88.50%) | | | |
| Catheterization location | | | <0.001* | | |
| Internal jugular | 577 (46.05%) | 163 (72.12%) | | Ref | Ref |
| Subclavian | 381 (30.42%) | 32 (14.16%) | | 0.246 (0.159, 0.382) | <0.001* |
| Femoral | 274 (21.87%) | 23 (10.18%) | | 0.580 (0.317, 1.061) | 0.077 |
| Axillary vein | 21 (1.68%) | 8 (3.54%) | | 1.669 (0.681, 4.087) | 0.262 |
| Length of catheter (cm) | 14.71±2.71 | 13.76±1.98 | <0.001* | 0.834 (0.758, 0.918) | <0.001* |
| Duration in days | 6 (4, 10) | 7 (5, 10) | 0.025* | | |
| Catheterization in ICU | | | | | |
| Catheterized by | | | 0.602 | | |
| Attending | 380 (52.20%) | 56 (47.46%) | | | |
| Resident | 288 (39.56%) | 54 (45.76%) | | | |
| Other doctor | 60 (8.24%) | 8 (6.78%) | | | |
| Ultrasonic localization | 420 (58.01%) | 69 (58.47%) | 0.995 | | |
| Ultrasonic guidance | 216 (29.88%) | 36 (30.51%) | 0.980 | | |
| Puncture times | | | 0.010* | | |
| 1 | 476 (66.02%) | 60 (50.85%) | | | |
| 2 | 166 (23.02%) | 39 (33.05%) | | | |
| 3 | 63 (8.74%) | 17 (14.41%) | | | |
| >3 | 16 (2.22%) | 2 (1.69%) | | | |
| Venous penetration | 50 (6.92%) | 9 (7.63%) | 0.960 | | |
| Arterial puncture | 47 (6.50%) | 3 (2.54%) | 0.241 | | |
| Days from CVC placement to CRT diagnosis | — | 4 (2, 7) | | | |

SD, standard deviation; IQR, interquartile range; CI, confidence interval; BMI, body mass index; APACHE, acute physiology and chronic health evaluation; CVC, central venous catheter; ICU, intensive care unit; CRT, catheter-related thrombosis; Ref, reference ; * Denotes statistically significant association(s).

### 000700

### Risk Factors for Failing Bridge Therapy with High Flow Nasal Cannula During Weaning of Invasive Mechanical Ventilation

K. Carvajal-Canizales[1], S. Cardona-Marín[1], LP. Sua[2], S. Garay[2], V. Ortiz-Perez[3], I. Chezzi-Guerra[3], P. Marcela[1], R. Buitrago[1], LF. Reyes[4]

[1]Critical care medicine department, Universidad de La Sabana, Bogotá, Colombia; [2]Critical care medicine department, Fundación clínica shaio, Bogotá, Colombia; [3]Student, Universidad de La Sabana, Bogotá, Colombia; [4]Critical care medicine - infectious diseases department, Universidad de la sabana, Bogotá, Colombia

**Correspondence:** L.F. Reyes

*Intensive Care Medicine Experimental* 2019, **7(Suppl 3):**000700

**INTRODUCTION.** Up to 2/3 of patients admitted to the intensive care unit (ICU) will develop acute respiratory failure (ARF) during their hospitalization, and thus, will require ventilatory support. Invasive mechanical ventilation (IMV) is the most frequently used ventilatory support strategy in the ICU. Once patients achieve clinical stability, clinicians face an important challenge when attempting ventilatory weaning; because around 13% of patients will need re-intubation, which increases morbidity and mortality. High Flow Nasal Cannula (HFNC) is a recently introduced ventilatory strategy used in patients with hypoxemic ventilatory failure. HFNC has been successfully used as initial ventilatory support and for weaning in several studies. Whereas bridge therapy with HFNC is a proved strategy, many patients will still require re-intubation within the first 48 hours of extubation; and risk factors for HFNC failure are controversial. Therefore, the goal of this study is to identify risk factors that could predict extubation failure in spite of using HFNC.

**OBJECTIVES.** To determine the risk factors associated with High Flow Nasal Cannula (HFNC) treatment failure during ventilatory weaning.

**METHODS.** This is a 2-year retrospective observational cohort study conducted in a hospital in Bogota, Colombia; between January 2016 and June 2018. All adult patients receiving IMV for more than 12 hours, who were posteriorly extubated and treated with HFNC as bridge therapy were included. HFNC failure was defined as the need for non-invasive mechanical ventilation (NIMV) or re-intubation within 48 hours after extubation. Difficult weaning group was defined as patients who had fail the extubation process or did not pass weaning tests. Descriptive statistics, parametric, non-parametric and logistic regressions were used to determine risk factors for HFNC failure. All statistical analyzes were performed using IBM SPSS statistics, version 22.0 Armonk, NY: IBM Crop.

**RESULTS.** A total of 109 patients were included in the study. 23 (21.1%) patients developed HFNC failure after weaning of IMV. As expected, patients who require vasopressors during ICU admission (52.2% Vs 10.5%, p=<0.01) and inotropes (34.8% Vs 10.5%, p=0.01) were more likely to present HFNC failure. Patients who fail HFNC were most likely to be in the difficult weaning group (60.9% Vs 30.2%, p=<0.01) and had higher respiratory rate before extubation (Median, Interquartile Range; 22 breaths per minute (BPM) [IQR 18-25] Vs 18 BPM [IQR 16-21], p=0.04). No differences were found among groups regarding gasometrical variables prior extubation, days of mechanical ventilation, ICU length of stay nor ICU mortality.

**CONCLUSION.** Bridge therapy with HFNC is a safe method for ventilatory weaning. We have identified 2 novel risk factors (weaning type and higher respiratory rate) that were independently associated with HFNC failure. Further studies are needed to confirm these findings.

### REFERENCE(S)

1. Duan J, Han X, Bai L, et al: Assessment of heart rate, acidosis, consciousness, oxygenation, and respiratory rate to predict noninvasive ventilation failure in hypoxemic patients. Intensive Care Med 2017; 43:192–199.
2. Thille AW, Boissier F, Ghezala HB, Razazi K, Mekontso Dessap A, Brunson C. Risk factors for and prediction by caregivers of extubation failure in ICU patients: a prospective study. Crit Care Med. 2015;43:613–20.
3. Timothy Miu, Aaron M Joffe DO, N David Yanez, Nita Khandelwal, Armagan HC Dagal, Steven Deem, and Miriam M Treggiari. Predictors of Reintubation in Critically Ill Patients. Respir Care 2014;59(2):178 –185.
4. Coplin WM, Pierson DJ, Cooley KD, et al. Implications of extubation delay in brain-injured patients meeting standard weaning criteria. Am J Respir Crit Care Med 2000; 161: 1530–1536
5. Chastre J, Fagon JY. State of the art: ventilator-associated pneumonia. Am J Respir Crit Care Med 2002; 165:867–903
6. Cook DJ, Walter SD, Cook RJ, et al. Incidence of and risk factors for ventilator-associated pneumonia in critically ill patients. Ann Intern Med 1998; 129:433–440
7. Manthous CA. The anarchy of weaning techniques. Chest 2002; 121:1738–1740
8. Epstein SK. Decision to extubate. Intensive Care Med 2002; 28:535–546
9. MacIntyre NR, Cook DJ, Ely EW, et al. Evidence-based guidelines for weaning and discontinuing ventilatory support. Chest 2001; 120:375S–395S
10. Epstein SK, Ciubotaru RL, Wong JB. Effect of failed extubation on the outcome of mechanical ventilation. Chest 1997; 112:186–192

### 000709

#### Bariatric surgery and utilisation of critical care resources: a 12 year experience

J. Green[1], N. Daines[1], K. Fraser[1], C. Pring[2], M. Margarson[1]
[1]Department of Anaesthetsia and Critical Care, St Richard's Hospital, Chichester, United Kingdom; [2]Department of surgery, St Richard's Hospital, Chichester, United Kingdom
**Correspondence:** J. Green
*Intensive Care Medicine Experimental* 2019, **7(Suppl 3):**000709

**INTRODUCTION.** Morbidly obese patients attending for bariatric surgery are at increased risk of complications in the peri-operative period due their associated medical co-morbidities (1) (2). Our NHS hospital has performed over 4,000 bariatric surgical procedures since the service started 12 years ago and we sought to analyse the impact that this has had on critical care.

**OBJECTIVES.** - To identify what proportion of all bariatric cases were admitted to the intensive care unit, along with details surrounding the support required and overall outcome.

- To compare these details to the total ICU workload and thereby demonstrate the overall impact on the service.

**METHODS.** We have maintained a single site bariatric anaesthesia airway database since 2006, which was checked for completeness by cross-referencing against Hospital Episode Statistics surgical codes. This was then matched against an ICU dataset of all admissions under the names of the four surgeons who admitted all bariatric surgery performed between 2007 and 2018. Cases admitted following primary bariatric surgery, or procedures related to any previous bariatric surgery, were included. Cases were individually reviewed to verify that they were genuinely associated with the inclusion criteria. Key parameters of interest including type of admission, system and level of organ support, duration of that support and the final outcome were obtained.

**RESULTS.** Over a 12 year period, out of a total of 4405 bariatric procedures, a cohort of 193 (4.4%) patients required admission. These cases utilised 533 bed days which accounted for 1.51% of all ICU bed days in the same period. 36 (19%) of this cohort received level 3, i.e. advanced ICU support, which utilised 0.78% of all ICU level 3 bed days in that period. 81% were level 2 only (HDU) and utilised 2.44% of all level 2 bed days. 53% were planned admissions, 32% emergency surgery and 15% were admitted from wards. APACHE II predicted 30 day mortality was 10.2 deaths; the actual was 6, giving a standardised mortality ratio of 0.59. In 2011 a new model of multi-disciplinary pre-assessment was introduced where all patients were screened by a team involving anaesthetic input before surgery was offered. Since that change, our rate of admission of level 3 patients has seen a six-fold reduction.

**CONCLUSION.** A mature Bariatric service is responsible for a small proportion of the overall workload in our intensive care unit, between 0.5 and 1% annually. Patients who are admitted tend to be older, predominantly male and lengths of stay are usually very short. Critical care units in a centre that is to introduce such a service may find an initial higher workload, but with agreed and sensible pre-assessment pathways the number of patients requiring advanced ICU support will be very low.

##### REFERENCE(S)

1. Cendán, J. C., Abu-aouf, D., Gabrielli, A., Caruso, L. J., Rout, W. R., Hocking, M. P., & Layon, A. J. (2005). Utilization of intensive care resources in bariatric surgery. Obesity Surgery, 15(9), 1247–1251.
2. Morgan, D. J. R., Ho, K. M., Armstrong, J., & Baker, S. (2015). Incidence and risk factors for intensive care unit admission after bariatric surgery: a multicentre population-based cohort study. British Journal of Anaesthesia, 115(6), 873–882.

### 000711

#### Determining the Prevalence and Impact of ICU Goals of Care Decisions That Reflect Discordance Between Surrogate Choice and Patient Preferences: Report from a Multi-Center RCT

M. Olsen[1], B. Wendtlandt[2], T. Hough[3], D. White[4], D. Jones[5], J. Kahn[6], C. Lewis[7], L. Hanson[2], C. Cox[8]
[1]Biostatistics, Duke University, Durham, United States of America; [2]Medicine, UNC Chapel HIll, Chapel Hill, United States of America; [3]Medicine, University of Washington, Seattle, United States of America; [4]Critical care, University of Pitt, Pittsburgh, United States of America; [5]Medicine, Duke University, Durham, United States of America; [6]Critical care, University of Pittsburgh, Pittsburgh, United States of America; [7]Medicine, University of Colorado, Denver, CO, United States of America; [8]Critical Care Medicine, Duke University, Durham, NC, USA, Chapel Hill, United States of America
**Correspondence:** C. Cox
*Intensive Care Medicine Experimental* 2019, **7(Suppl 3):**000711

**INTRODUCTION.** Little is known about the prevalence of critically ill patient-surrogate discordance for treatment goals in the moment, or about the clinical implications of this discordance. In a recent multicenter RCT testing a web-based personalized decision aid (DA) for surrogates of prolonged mechanical ventilation patients, the DA displayed the goal of treatment most concordant with patient values based on surrogates' reports of patient preferences.1 The unique design of the DA captured intervention surrogates' adjustment of the calculated goal of treatment option on their digital device at the moment of decision making.

**OBJECTIVES.** To identify characteristics of surrogates who chose discordant treatment goals and to determine the impact on patient outcomes of surrogate decisions that appeared discordant from patient preferences.

**METHODS.** 276 participants including all 138 primary decision makers and 138 patients ventilated for ≥10 days from the RCT's decision aid intervention group. We used chi square and t-tests to compare baseline and 1-week surrogate (Hospital Anxiety and Depression Scale, PTSS, clinician-surrogate prognostic concordance score) as well as patient outcomes (days of ventilation, ICU care, hospitalization; 6-month mortality) by decisional groups (surrogate-patient decisional discordance vs. surrogate-patient decisional concordance).

**RESULTS.** Of 129 (93%) surrogates witht complete data, 56 (43%) chose a goal of care that was discordant overall with patient preferences. 20 (11%) were markedly different than patient preferences (i.e., change among the three main choices [comfort, aim for survival but avoid prolonged care, aggressive care). 55 (98%) discordant decisions were related to a desire for more aggressive care than patients preferred.

We found no statistically significant associations between decisional discordance and either surrogate characteristics (age, race, gender, education, medical comprehension, employment, relation to patient, religion, distress level, amount of decisional conflict) or patient (demographics, APACHE II, diagnosis, comorbidities, medical vs. surgical ICU) factors ($p$ >0.05).

Compared to concordant dyads, discordance was not associated with differences at 1 week in change in either prognostic discordance with physicians or symptoms of distress (depression / anxiety / PTSD symptoms; all $p$ >0.05). Compared to concordant patients, discordant patients had numerically greater mean days of ventilation (18.0 [31.6] vs. 13.9 [15.5]), ICU care (23.2 [33.2] vs. 18.7 [16.3]), and hospitalization (45.6 [36.4] vs. 41.3 [27.8])—though identical 6-month mortality (39.3% vs. 37.0%); all $p$ <0.05.

**CONCLUSION.** Surrogates commonly make goals of care decisions that are discordant with patient preferences they themselves report, yet we identified no characteristics that separated them from concordant surrogates. Patients linked to discordant surrogates had longer use of life support and greater resource utilization, though no improvement in mortality compared to concordant patients. These exploratory findings suggest that it may be particularly important to focus future palliative care and decision support interventions on surrogate decision makers who choose goals of care that are not aligned with patient preferences.

**REFERENCE(S)**

1. Cox CE, White DB, Hough CL, Jones DM, Kahn JM, Olsen MK, Lewis CL, Hanson LC, Carson SS. Effects of a Personalized Web-Based Decision Aid for Surrogate Decision Makers of Patients With Prolonged Mechanical Ventilation: A Randomized Clinical Trial. Annals of Internal Medicine. Jan 29, 2019. doi: 10.7326/M18-2335. [epub ahead of print]
2. National Institutes of Health grant R01 HL109823

### 000718

#### Use of landmark method versus ultrasound for central venous catheterization: A questionnaire based survey

K. Khatib[1], S. Dixit[2]
[1]Medicine, Smt. Kashibai Navale Medical College, Pune, India; [2]Critical care, MJM Hospital, Pune, India
**Correspondence:** K. Khatib
*Intensive Care Medicine Experimental* 2019, **7(Suppl 3):**000718

**INTRODUCTION.** Though ultrasonography (USG) guided central venous catheter (CVC) insertion has been advocated by critical care societies and hospitals, it has still not become the norm in 'real life' scenario in the ICUs. Lack of training in use, ability to handle, and availability of USG machines have been demonstrated to be the reasons for the same.
**OBJECTIVES.** We conducted a survey to assess the use of USG during CVC insertion.
**METHODS.** A survey comprising 10 questions was designed and mailed electronically to 386 physicians working in various ICU in our district. The questionnaire comprised queries regarding aspects of USG training, use and opinion of USG during/after CVC insertion and frequency of use, need for further training, etc.
**RESULTS.** Of the 190 questionnaires returned, 60% were consultant physicians, while 40% were residents. 28% were working in rural locations. About 56% of doctors reported training in the use of USG, while 46% used it routinely for CVC insertion (always 30%, almost always 66%).
The main reasons for non-use of USG during CVC insertion were i) lack of adequate training (50%), ii) no need of USG guidance during CVC insertion (54%), iii)landmark technique equally good for CVC insertion (61%),iv) other nonspecified reason (8%).
All respondents thought that landmark technique should be taught to trainee doctors.
Almost all resident doctors (96%) as compared to seniors declared that they use USG (always, almost always) for CVC insertion.
**CONCLUSION.** Despite various guidelines and studies demonstrating superiority of USG guidance during CVC insertion, its use is still not widespread and enough. Training and use of USG needs major improvement amongst the ICU doctors in our district.

**REFERENCE(S)**

1. Maizel J, Bastide MA, Richecoeur J, Frenoy E, Lemaire C, Sauneuf B, Dupont H, Tamion F, Nseir S, Du Cheyron D. Practice of ultrasound-guided central venous catheter technique by the French intensivists: a survey from the BoReal study group. Ann Intensive Care. 2016;6:76.
2. Soni NJ, Reyes LF, Keyt H, Arango A, Gelfond JA, Peters JI, Levine SM, Adams SG, Restrepo MI. Use of ultrasound guidance for central venous catheterization: a national survey of intensivists and hospitalists. J Crit Care. 2016;36:277–83.
3. Buchanan MS, Backlund B, Liao MM, Sun J, Cydulka RK, Smith-Coggins R, Kendall J. Use of ultrasound guidance for central venous catheter placement: survey from the American Board of Emergency Medicine Longitudinal Study of Emergency Physicians. Acad Emerg Med. 2014;21:416–21.
4. McGrattan T, Duffty J, Green JS, O'Donnell N. A survey of the use of ultrasound guidance in internal jugular venous cannulation. Anaesthesia. 2008;63:1222–5.
5. Saugel B, Scheeren TWL, Teboul JL. Ultrasound-guided central venous catheter placement: a structured review and recommendations for clinical practice. Crit Care. 2017;21(1):225.
6. NIL

### 000721

#### Retrospective study of Outcomes in Prolonged stay critically ill patients in an Indian ICU

K. Khatib[1], S. Dixit[2], A. Chavan[1]
[1]Medicine, Smt. Kashibai Navale Medical College, Pune, India; [2]Critical care, Sanjeevan Hospital, Karve Road, Pune, Pune, India
**Correspondence:** K. Khatib
*Intensive Care Medicine Experimental* 2019, **7(Suppl 3):**000721

**INTRODUCTION.** Many patients admitted to ICU require a prolonged stay in an intensive care unit (ICU) as they recover from their critical illness. These patients have been reported to have increased mortality and morbidity.
**OBJECTIVES.** To study the incidence, characteristics and outcomes of prolonged stay critically ill patients admitted to our ICU.
**METHODS.** Retrospective analysis of patients admitted to our adult ICU (Total- 40 bedded; 20 bed medical plus cardiac ICU, 20 bed surgical ICU) during last 2 years. All patients with ICU stay > 21 days (due to mechanical ventilation and/or for hemodynamic monitoring) were included in the study. Prolonged stay critically ill patients were classified according to their location in ICU and reason for ICU admission, as medical or surgical prolonged stay critically ill patients. The main endpoints of the study were ICU and hospital length of stay (LOS), and mortality.
**RESULTS.** Of the total 1097 patients admitted to the ICU during the period of study, 67 patients were included in the study, for an incidence of 6.1% (Medical patients- 4.6%; Surgical patients- 7.9%). The most common diseases at ICU admission were stroke and neuromuscular disease in medical patients and intestinal diseases (obstruction or perforation) in surgical patients. Mean APACHE II score on admission in the medical and surgical patients were 17 (IQR 11-25) and 19 (IQR 14-28) (p=0.88). There was no significant difference in age, gender, tracheostomy, use of mechanical ventilation and vasopressors between the two groups of patients (p> 0.05 for all). Surgical prolonged stay critically ill patients had more episodes of hospital acquired infections as compared to medical prolonged stay critically ill patients (p<0.05).
Mean LOS in ICU for medical and surgical prolonged stay critically ill patients were 26.6 and 33.6 days, respectively (p< 0.001). Mean LOS in hospital for medical and surgical prolonged stay critically ill patients was 28.5 and 39.2 days, respectively (p< 0.001). The mortality for medical and surgical prolonged stay critically ill patients was 50% and 44%, respectively (p=0.08).
**CONCLUSION.** A small component of critically ill patients admitted to ICU will turn into prolonged stay critically ill patients, requiring ICU care for a longer period of time. Prolonged stay critically ill patients due to surgical indications for admission to ICU have more hospital acquired infections, longer ICU and hospital LOS but no significant increase in mortality as compared to prolonged stay critically ill patients due to medical indications for admission to ICU. More and larger studies are required to further analyse prolonged stay critically ill patients.

**REFERENCE(S)**

1. Arabi Y, Venkatesh S, Haddad S, Al Shimemeri A, Al Malik S: A prospective study of prolonged stay in the intensive care unit: predictors and impact on resource utilization. Int J Qual Health Care 2002, 14: 403-410.
2. Williams TA, Ho KM, Dobb GJ, et al. Effect of length of stay in intensive care unit on hospital and long-term mortality of critically ill adult patients. Br J Anaesth. 2010;104(4):459–64.
3. NIL

### 000723

#### Feasibility and safety of bedside watershed-detection by contrast-enhanced ultrasound in patients receiving veno-arterial extracorporeal membrane oxygenation

N. Buchtele[1], M. Schwameis[2], C. Schörgenhofer[1], P. Schellongowski[3], C. Weiser[2], A. Spiel[2], T. Staudinger[3]
[1]Department of clinical pharmacology, Medical University of Vienna, Vienna, Austria; [2]Department of emergency medicine, Medical University of Vienna, Vienna, Austria; [3]Department of medicine i, Medical University of Vienna, Vienna, Austria
**Correspondence:** N. Buchtele
*Intensive Care Medicine Experimental* 2019, **7(Suppl 3):**000723

**INTRODUCTION.** In veno-arterial extracorporeal membrane oxygenation (VA ECMO) with femoro-femoral cannulation the watershed is the location where retrograde ECMO-perfusion and antegrade cardiac perfusion encounter (figure 1). To date, angiography or contrast enhanced computed tomography is the only available method to determine the watershed location, which has only been described in several case reports (1-3).

**OBJECTIVES.** We hypothesized that contrast-enhanced ultrasound is a suitable method to detect the watershed at the bedside and examined its feasibility and safety.

**METHODS.** This prospective study included consecutive adult patients undergoing emergency femoro-femoral VA ECMO cannulation. The primary outcome measure was the feasibility of contrast-enhanced ultrasound for bedside detection of the watershed. The Secondary outcome was the safety of the procedure.

1ml of contrast media (Sonovue) was administered via the venous drainage ECMO cannula while transabdominal and transesophageal ultrasound was performed to concurrently display the abdominal and the thoracic aorta. Application of contrast media was repeated until the watershed was detected or the maximum volume of 4ml was reached.

Safety values obtained included hemodynamic values (mean arterial pressure, mmHg; heart rate, bpm; SpO2, %; paO2, mmHg; left and right if applicable; paCO2, mmHg), respirator settings (FiO2, %; peak pressure, mbar; tidal volume, ml), neurologic assessment (examination of pupils and computed tomography imaging of the brain if appropriate) and ECMO settings (FiO2, %; sweep gas flow, l/min; pump speed, rpm; blood flow, l/min). Patients were assessed for safety parameters as well as ventilatory demand, hemodynamic instability, arrhythmias, anaphylactic reactions or death at baseline, 5min, 15min, 2h and 6h after contrast media administration. Patients were followed up until hospital discharge or death.

**RESULTS.** Between October 2018 and February 2019 10 patients (90% male, mean age 52 years) were enrolled at three intensive care units at the Medical University of Vienna. The watershed was detected in the abdominal aorta in five patients (figure 2). In the remaining patients continuous blood flow was visible in all examined areas, suggesting the watershed located at the level of the aortic arch. No adverse events in close temporal relationship to contrast media administration were observed.

**CONCLUSION.** Contrast-enhanced ultrasound appears to be a technically feasible and safe method to determine the location of the watershed at the bedside in adult patients on VA ECMO with femoro-femoral cannulation.

**REFERENCE(S)**

1. Cove ME. Disrupting differential hypoxia in peripheral veno-arterial extracorporeal membrane oxygenation. Crit Care. 2015;19:280.
2. Hoeper MM, Tudorache I, Kuhn C, Marsch G, Hartung D, Wiesner O, et al. Extracorporeal membrane oxygenation watershed. Circulation. 2014;130(10):864-5.
3. Napp LC, Brehm M, Kuhn C, Schafer A, Bauersachs J. Heart against veno-arterial ECMO: Competition visualized. Int J Cardiol. 2015;187:164-5.
4. This study was kindly supported by the Austrian Society for Internal Medicine and General Intensive Care and Emergency Medicine (ÖGIAIN)

### 000737

#### Nursing Staff Experience of a Critical Care Rotation Programme

K. Russell
Critical Care, Charing Cross Hospital, London, United Kingdom
**Correspondence:** K. Russell
*Intensive Care Medicine Experimental* 2019, **7(Suppl 3):**000737

**INTRODUCTION.** Within healthcare many multidisciplinary teams rotate to gain wider clinical experience, develop skills and promote professional development, however within nursing in the UK rotation programmes are not as widely established. The Department of Health (2001) advocated for the introduction of rotation programmes within Critical Care, as an initiative to increase recruitment and retention. Prior studies looking into rotation programmes found that staff expressed development of their clinical skills and knowledge (Chen *et al.,* 2015; Lawton, 2006), which in turn lead to career development opportunities (Järvi and Uusitalo, 2004; Fujino and Nojima, 2005; Richardson *et al.,* 2003). However, it was also recognised that individuals can find rotating daunting when required to work in an environment or speciality in which they are unfamiliar (Richardson *et al.,* 2003). The purpose of this study was to explore nurses experience of the Critical Care Rotation Programme based in a London Trust.

**OBJECTIVES.** To explore nurses experience of the Critical Care Rotation Programme, specifically looking at pre-rotation preparedness, the rotation experience and post-rotation reflection.

**METHODS.** A phenomenological design was used to explore attitudes, experiences and opinions (Parahoo, 2006). An evaluative study was developed in the form a 'service evaluation', whereby ethical approval was sort from the Trust and the researchers University of study. A mixed-methods design was adopted to explore both qualitative and quantitative data; comprising of an electronic questionnaire and semi-structured interviews. Forty-four individuals completed the Critical Care Rotation Programme within 2018 and were invited to participate; 21 (47.7%) electronic questionnaire responses were received. A further 10 participants were interviewed to gain further narrative of their experiences. The data was evaluated using thematical analysis (Braun and Clarke, 2006).

**RESULTS.** The recognised benefits of the rotation programme included improving clinical knowledge and specialist skills, enhancing interdepartmental teamworking and communication, and the development of global awareness and leadership skills. Day to day support from staff and mentors was highly valued and attributed to an overall better experience. However, apprehensions prior to the rotation where common; which included fears surrounding working in a new environment and team. Disadvantages of the programme largely focused on logistical issues such as issues with security access to the rotation site and an inadequate induction period. Operational issues were reported including patient allocation and limited access to rotation mentors. However, overall all of the participants valued the experience.

**CONCLUSION.** The evidence from this study suggests that the Critical Care Rotation Programme is a valuable strategy for promoting learning, enhancing interdepartmental relationships which promote cross-site working, and encouraging professional development and career progression. In order to improve the programme for 2019; a new mentoring strategy has been introduced to provide improved support. There is limited prior research in this area, and due to the small sample included in this study, more research would need to be conducted in the field before conclusions can be drawn to influence general practice.

**REFERENCE(S)**

1. Braun, V. and Clarke, V. (2006) 'Using thematic analysis in psychology', Qualitative Research in Psychology, 3(2), pp. 77-101.
2. Chen, S., Wu, W., Chang, C. and Lin, C. (2015) 'Job rotation and internal marketing for increased job satisfaction and organisation commitment in hospital nursing staff', Journal or Nursing Management, 28, pp. 297-306.
3. Department of Health (2001) 'The Nursing Contribution to the Provision of Comprehensive Critical Care for Adults'. Available at: http://anaesthesiaconference.kiev.ua/downloads/nursing%20contribution%20to%20comprehensive%20critical%20care_2005.pdf (Accessed 10th October 2018).
4. Fujino, M. and Nojima, Y. (2005) 'Effects of ward rotation on subsequent transition process of Japanese clinical nurses', Nursing and Health Sciences, 7, pp. 37-44.
5. Järvi, M. and Uusitalo, T. (2004) 'Job rotation in nursing: a study of job rotation among nursing personnel from the literature and via a questionnaire', Journal of Nursing Management, 12, pp. 337-47.
6. Lawton, S., Gent, P., Pearson, E., Barclay, H. and Reid, C. (2006) 'Crossing boundaries: a staff nurse exchange initiative', Nursing Management, 13(7), pp. 28-31.
7. Parahoo, K. (2006) Nursing Research: Principles, Process and Issues. 2nd edition. New York: Palgrave Macmillan.
8. Richardson, A., Douglas, M., Shuttler, R. and Hagland, M. R. (2003) 'Critical care staff rotation: outcome of a survey and pilot study', Nursing in Critical Care, 8(2), pp. 84-89.

### 000751

#### Results of the application of a clinical alert system for the early detection of in-hospital critical patients

D. Díaz Díaz, G. Gonzalo Somoza, M. Villanova, A. Martinez De La Gandara, L. Lopez, G. Andrade, B. Bueno Garcia, R. Garcia Gigorro, T. Fariña, E. Palencia
Intensive care units, Hospital Universitario Infanta Leonor, Madrid, Spain
**Correspondence:** D. Díaz Díaz

*Intensive Care Medicine Experimental* 2019, **7(Suppl 3):**000751

**INTRODUCTION.** Early identification of hospitalized patients at risk to develop clinical worsening would reduce the number of preventable deaths and admission at the Intensive Care Unit (ICU).

**OBJECTIVES.** To implement, in clinical practice, an early alert system based on clinical and laboratory findings aimed at establishing an effective communication between ICU physicians and the wards physicians and to evaluate its usefulness to detect and to prevent the avoidable clinical deterioration in hospitalized patients.

**METHODS.** Retrospective analysis of the electronic medical charts of patients older than 18 years hospitalized in any medical or surgery ward during the study period (January-December 2018). We collected, within the 6 hours prior to the consultation of the system, the following variables: age, sex, patient location, heart rate (HR) (<40 lpm; >130 lpm), respiratory rate (RR) (< 10 breaths/min;> 30 breaths/min.), systolic blood pressure (SBP) < 85 mmHg, partial oxygen saturation (SaO2)< 85%, pH < 7,30, PaCO2 > 65 mmHg and Lactate > 4 mmol/L. Patient´s charts showing alteration of one or more variables prompt a full evaluation of the clinical chart by the ICU physician and an on-site intervention with treatment reorientation or intensification or immediate transfer to ICU or participation in the decision to life support therapy limitation (LSTL).

**RESULTS.** 1189 records were evaluated. The mean age of patients was 71 years (SD 2.4), 52.34% were males. The hospital wards where more alerts were detected were the emergency room (n=462; 38%), internal medicine ward (n=396; 32.5%), oncology ward (n=68;5.6%), pneumology ward (n=50;4.1%), surgery (n=34;2.9%), digestive ward (n=35;2.6%), traumatology (n=31; 2.5%) and gynaecology-obstetrics (n=26;2.1%). A single altered variable was detected in 94.2% of the alerts, being the most frequent the SBP< 85mmHg (26.1%),SaO2 < 85%(23%), lactate > 4mmol/L (13%), HR> 130 lpm (12.5%), pCO2 > 65 mmHg (11.4%), RR > 30 breaths/min. (7.6%) and pH < 7.30 (5.12%). Up to 71 patients (5.8%) showed more than two altered variables being the most frequent combination pCO2/Ph (n=31). In-situ evaluation and treatment were done in 167 cases (13.74%), and only 15 patients were considered candidates to immediate admission at ICU (1.25%), being alterations in SBP and in SaO2 the main causes for ICU admission. In the remainder patients an intensification of treatment consisting in the start or adjustment of non-invasive positive pressure ventilation and initiation or intensification of fluids therapy was recommended (67.5%). In up to 5.45% of the patients the consensus decision was to not transfer to the ICU, in up to 12.75% of the patients the patient´s physician had already decided to LSTL.

**CONCLUSION.** This alert system allowed the early detection of patients at risk of clinical deterioration. Despite it is a consuming-time task for ICU physicians, it allows to participate, together with the patient´s physician, in the optimization of the treatment as well as in the decisions of LSTL ,and reduce the number of admissions at ICU.

### 000767

#### The Respiratory Rate–Oxygenation (ROX) index Predict Failure of Postextubation High-Flow Nasal Cannula (HFNC) Therapy in ICU Patients

LF. Reyes[1], AJ. Arango[2], D. Barros-Toro[2], S. Cardona-Marín[3], K. Carvajal-Canizales[3], P. Marcela[3], R. Buitrago[3], A. Rodríguez[4]
[1]Critical care medicine - infectious diseases department, Universidad de la sabana, Bogotá, Colombia; [2]Student, Universidad de La Sabana, Bogotá, Colombia; [3]Critical care medicine department, Universidad de La Sabana, Bogotá, Colombia; [4]Critical care department, Hospital Universitari de Tarragona Joan XXIII, Tarragona, Spain
**Correspondence:** L.F. Reyes

*Intensive Care Medicine Experimental* 2019, **7(Suppl 3):**000767

**INTRODUCTION.** Up to 60% of the patients hospitalized in the ICU require ventilatory support, being invasive mechanical ventilation (IMV) the most frequently used therapy. Patients treated with IMV are at risk of developing adverse events such as barotrauma, infections, among others. Re-intubation is needed in 13% of cases, with associated increased morbidity and mortality. Several scores and strategies have been developed to prevent reintubation. However, their efficacy and clinical utility is still being debated. HFNC is a relatively new ventilatory support therapy that allows administration of high-flow (up to 60Lts/min) humidified gas through a nasal cannula with 100% O2. This technology has shown a lower risk of complications (sedation is not required) and provides comfort to patient. HFNC has been successfully used for hypoxemic respiratory failure and during weaning of mechanical ventilation (bridge therapy). Nonetheless, there are some patients that fail HFNC therapy and require re-intubation, but these patients have not been well characterized. The respiratory rate–oxygenation (ROX) index, defined as the ratio of SpO2/FiO2 to respiratory rate, was recently validated to identify patients at risk of HFNC failure in pneumonia patients. Therefore, we hypothesize that ROX Index could accurately predict extubation failure in patients treated with postextubation HFNC.

**OBJECTIVES.** To investigate the accuracy of ROX Index to identify patients at risk of failing postextubation High-Flow Nasal Cannula (HFNC) therapy in intensive care unit (ICU).

**METHODS.** This is a 2-year retrospective observational study conducted in a reference hospital in Bogotá, Colombia. All patients in whom postextubation HFNC therapy was used as bridge to extubation were included in the study. The ROX index was calculated prior to HFNC treatment to assess the risk of postextubation HFNC failure. Secondary, we determine whether ROX index could predict ICU mortality. The cohort was stratified among patients who developed HFNC failure to compare patients characteristics and statistical analyses.

**RESULTS.** A total of 162 mechanical ventilated patients were included in the study, 38 patients developed HFNC failure and 28

patients died during ICU stay. After adjusting for comorbid conditions and diseases severity, ROX index was statistically lower in patients whom fail HFNC therapy (median [IQR], 10.00 [6.78] Vs 12.65 [5.47], p=0.006). However, ROX was not different in patients whom died during ICU stay (11.59 [8.70] Vs 12.37 [5.23], p=0.3).

**CONCLUSION.** The ROX index is an easy to calculate score that could identify patients at higher risk of HFNC failure during postextubation treatment. A prospective study is needed to confirm the utility of this index in ICU patients treated with HFNC as bridge therapy.

**REFERENCE(S)**

1. Masclans JR, Pérez-Terán P, Roca O. The role of high flow oxygen therapy in acute respiratory failure. Med Intensiva 2015;39(8):505-515.
2. Thille AW, Boissier F, Ben Ghezala H, Razazi K, Mekontso-Dessap A, Brun-Buisson C. Risk factors for and prediction by caregivers of extubation failure in ICU patients: a prospective study. Crit Care Med 2015;43(3):613-620.
3. Roca O, Messika J, Caralt B, García-de-Acilu M, Sztrymf B, Ricard JD, et al. Predicting success of high-flow nasal cannula in pneumonia patients with hypoxemic respiratory failure: The utility of the ROX index. J Crit Care 2016;35:200-205.
4. Thille AW, Harrois A, Schortgen F, Brun-Buisson C, Brochard L. Outcomes of extubation failure in medical intensive care unit patients. Crit Care Med 2011;39(12):2612-2618.
5. Roca O, Caralt B, Messika J, Samper M, Sztrymf B, Hernández G, et al. An Index Combining Respiratory Rate and Oxygenation to Predict Outcome of Nasal High Flow Therapy. Am J Respir Crit Care Med 2018.
6. Lodeserto FJ, Lettich TM, Rezaie SR. High-flow Nasal Cannula: Mechanisms of Action and Adult and Pediatric Indications. Cureus 2018;10(11):e3639.
7. Hernández G, Vaquero C, Colinas L, Cuena R, González P, Canabal A, et al. Effect of Postextubation High-Flow Nasal Cannula vs Noninvasive Ventilation on Reintubation and Postextubation Respiratory Failure in High-Risk Patients: A Randomized Clinical Trial. JAMA 2016;316(15):1565-1574.
8. Huang HW, Sun XM, Shi ZH, Chen GQ, Chen L, Friedrich JO, et al. Effect of High-Flow Nasal Cannula Oxygen Therapy Versus Conventional Oxygen Therapy and Noninvasive Ventilation on Reintubation Rate in Adult Patients After Extubation: A Systematic Review and Meta-Analysis of Randomized Controlled Trials. J Intensive Care Med 2018;33(11):609-623.
9. Ni YN, Luo J, Yu H, Liu D, Liang BM, Yao R, et al. Can high-flow nasal cannula reduce the rate of reintubation in adult patients after extubation? A meta-analysis. BMC Pulm Med 2017;17(1):142.

#### 000152

#### Implementation and evaluation of a training curriculum in intensive care

M. Spacek[1], C. Prautzsch[1], J. Mehrholz[2]
[1]Intensive care unit, KLINIK BAVARIA Kreischa, Kreischa, Germany; [2]Scientific institut, KLINIK BAVARIA Kreischa, Kreischa, Germany
**Correspondence:** M. Spacek
*Intensive Care Medicine Experimental* 2019, **7(Suppl 3):**000152

**INTRODUCTION.** At many intensive care units there is need in training for decision-making for medical staff especially for the novice physicians.

**OBJECTIVES.** The aim of this study was therefore to develop and implement a training curriculum for novice physicians at intensive care. The training curriculum, which incorporates different medical specialities, aims to increase the physician and patient safety and reliability in decision-making.

**METHODS.** The present study was carried out in one of the largest German interdisciplinary intensive care and weaning centres, the Klinik Bavaria Kreischa in Saxony.

In the first step a survey was send out to all physician in intensive care to analyse job satisfaction and clinical inconveniencies with medical training.

In the second step a first concept of the training curriculum was developed and then implemented clinically for 6 months, according to principles of the PDCA-cycle (plan, do, check, act). We developed a three-level training curriculum (level A-C). Where the level A is aimed on beginners with a focus on the basics of work in intensive care and aims to achieve the knowledge level for the on-call duty. The level B is aimed on the advanced staff and wants to gain deeper specialist knowledge. The level C is specialised to expand the expertise with the goal of leading the emergency team. Each level includes a theoretical part, practical part and hands-on with medical equipment.

More than 15 months after the pilot-implementation, a follow-up survey was send out. It focused on overall job satisfaction, training in intensive care and preparation and safety and reliability in decision-making.

**RESULTS.** In the first round 27 physicians were surveyed with a response rate of 86%. Our first survey showed a large range of satisfaction with the training at intensive care. We concluded that there was a need in basic knowledge and technical skills for beginners (level A) and for advanced staff (level B and C) in emergency management.

In December 2018, we surveyed 20 physicians with a response rate of 83%. Although we found only a slightly higher general satisfaction (from 7.03 to 7.56 points out of 10), we showed a statistically significant improvement in the satisfaction of training at intensive care (from 6.62 to 7.19, p <0.001), better preparation for on-call duty (from 5.38 to 7.25, p <0.0031) and a small improvement in reliability and safety during on-call duty (from 6.76 to 7.00).

**CONCLUSION.** We conclude that our specific training curriculum is very useful for a standardized and routinely training of physicians at intensive care.

**REFERENCE(S)**

1. There was no funding for this research

### NIC - Acute brain injury

#### 000545

#### Agreement analysis of transcutaneal CO2 partial pressure in neurocritical patients with mechanical ventilation

A. Fuentes Calatayud[1], FA. López[1], LF. Guerrero[2]
[1]Intensive care, University Hospital Complex of Granada, Granada, Spain; [2]Neurocritical and trauma intensive care unit, University Hospital Complex of Granada, Granada, Spain
**Correspondence:** A. Fuentes Calatayud
*Intensive Care Medicine Experimental* 2019, **7(Suppl 3):**000545

**INTRODUCTION.** In neurocritical ill patients, a narrow control of the carbonic anhydride partial pressure (pCO2) must be done as a first level step to control intracraneal pressure elevation. A continuous monitoring of pCO2 would help to keep the values in optimal ranges and would reduce the necessity of arterial gasometries.

**OBJECTIVES.** To analyze the agreement in the values of CO2 partial pressure measured by a transcutaneal monitor (PtcCO2) with the values measured in arterial gasometries samples in patients with mechanical ventilation joined in a neurocritical ICU.

**METHODS.** We made an observational study in which were included 6 patients with invasive mechanical ventilation joined in our ICU. We measured PtcCO2 by SenTec® monitor and simutaneous arterial gasometry in several moments of their evolution. The continuos variables are described with averages and typical deviation. We calculated the Pearson correlation coefficient (r) and the coefficient of intraclass correlation (ICC) with a confidence interval (CI) of 95% between PaCO2 and PtcCO2 as agreement measure with Landis and Koch interpretation. We considered statistically significant $p < 0.05$. Bland and Altman analysis was applied for the dispersion data.

**RESULTS.** 36 samples from the 6 patients were included. The values obtained of PaCO2 were 42.05 ± 3.34 mm Hg and of PtcCO2 were 42.56 ± 3.68 mmHg. Due to correlation and concordance, the results were for PaCO2 and PtcCO2, r = 0,843 (p< 0.001) and ICC = 0,91 (CI 95% 0.83 , 0.96). In 95% of the cases the discrepancies are included between -3.48 y 4.48 mmHg. Bland and Altman analysis did not show significant dispersion data. It was showed that SenTec® monitor overvalued the values of PaCO2 in 0.5 mmHg.

**CONCLUSION.** The agreement between the measures of CO2 is almost perfect. The unconformities of the monitor in the measure of PtcCO2 compared with PaCO2 in arterial gasometry do not have clinical signification. The values obtained with PtcCO2 monitor seems reliables.

REFERENCE(S)

1. No grants received from any source. We thank SenTec® for lenting us the PtcCO2 monitor for make the study.

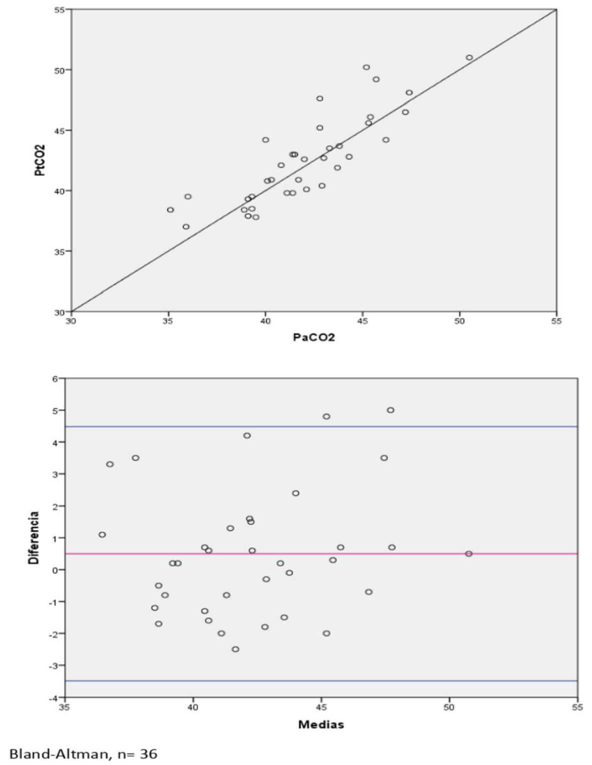

**Fig. 1 (abstract 000545).** See text for description

### 000568

#### Patient State Index, Suppression Rate and association with survival in ICU

E. Monares Zepeda[1], EA. Ojeda Izquierdo[2], MCA. Galindo[3], LM. Cruz Gomez[2], JO. Guamán Crespo[2], R. Lozano Zúñiga[2], A. Garza de la Maza[2]
[1]Head of the department of critical care medicine, Hospital San Angel Inn Universidad, Mexico City, Mexico; [2]Department of critical care medicine, Hospital San Angel Inn Universidad, Mexico City, Mexico; [3]Head of the clinical nutrition department, Hospital San Angel Inn Universidad, Mexico City, Mexico
**Correspondence:** E.A. Ojeda Izquierdo
*Intensive Care Medicine Experimental* 2019, **7(Suppl 3):**000568

**INTRODUCTION.** The Patient´s State Index (PSI) is a useful form of continuous neuromonitoring in the Intensive Care Unit (ICU). Suppression rate (SR) is a measurement of the suppression of brain electrical activity. Due to its good correlation with the neurological status of patients in the ICU, we believe they are associated with mortality and neurological damage in critically ill patients.

**OBJECTIVES.** Establish if there is a relationship between the Patient´s State Index (PSI) and Suppression rate (SR)values with mortality in intensive care patients in a given period.

**METHODS.** Retrospective study. From January 2016 to November 2017 all patients admitted to intensive therapy under mechanical ventilation were included, continuous qualitative electroencephalography was performed, PSI values and SR were recorded, along with epidemiological variables and SOFA score upon admission. The sample was divided into survivors and non-survivors. Primary objective: association of the PSI and SR with mortality at 28 days

**RESULTS.** A total of 46 patients were included, 74% of survivors. The diagnosis of septic shock was the most observed. There were no differences in the time of need for mechanical ventilation or in the use of analgesics or sedatives between the groups. The ROC curve in relation to 28-day mortality shows an AUC for PSI of 0.813 (95% CI: 0.650-0.975, p = 0.001), and for SR of 0.857 (95% CI: 0.734-0.980, p=0.001) for detection of survival. The cut point of PSI of 30.5 has a sensitivity of 88.2% and specificity of 83.3%, and the cut point of SR of 2 has 92.3% and 67% of sensitivity and specificity respectively.

**CONCLUSION.** Under a sedation protocol necessary to achieve minimum RASS 0 to -1, PSI values lower than 30 and SR greater than 2 are auxiliary in the analysis of the mortality risk of the patient in critical condition.

### 000585

#### Incidence, risk factors, and effects on outcome of ventilator associated pneumonia in patients with traumatic brain injury: data from the CENTER TBI study

F. Cipulli[1], C. Robba[2], E. Banzato[1], F. Fossi[1], C. Iaquaniello[1], F. Graziano[1], E. Wiegers[3], A. Vargiolu[4], P. Rebora[1], G. Citerio[1]
[1]School of medicine and surgery, University of Milano-Bicocca, Milano, Italy; [2]Department of anaesthesia and intensive care, IRCCS AOU San Martino, Genova, Italy; [3]Department of public health, Erasmus University Medical Center, Rotterdam, Netherlands; [4]Neurointensive care, department of emergency and intensive care, Ospedale San Gerardo di Monza, Monza, Italy
**Correspondence:** C. Iaquaniello
*Intensive Care Medicine Experimental* 2019, **7(Suppl 3):**000585

**INTRODUCTION.** Traumatic brain injured (TBI) patients are at high risk for respiratory complications and in particular of ventilator-associated pneumonia (VAP). Aim of this study is to evaluate the occurrence of VAP after TBI and its effect on patients' outcome as well as hospital and intensive care unit (ICU) stay.

**METHODS.** The CENTER-TBI study (clinicaltrials.gov registration NCT02210221) is a prospective observational longitudinal cohort study including patients with TBI from 65 centers across Europe. Data were extracted from the CENTER-TBI database v1.1 with Neurobot v2.6. We focused on patients with an ICU length of stay >72 hours who were intubated (or mechanically ventilated) for at least 48 hours.

**RESULTS.** 4509 patients were included in the CENTER-TBI study, 2138 were admitted to the ICU and we focused on 917 patients fulfilling the inclusion criteria. Of these, 188 (20.5%) developed a VAP at a median time of 4.5 days (IQR: 3-7 days) from intubation.

Patients who developed VAP were younger (median age 39.5 vs 51 y/o, p<0.001), with a higher incidence of alcohol abuse (35% vs 28%, p=0.098), thoracic trauma (53% vs. 43.5% p=0.021), and more frequent neuroworsening episodes during ICU stay (44% vs 35%, p=0.039). VAP cohort had a longer duration of mechanical ventilation (median 15 vs 9 days, p<0.001) and ICU stay (median 20 vs 13 days, p<0.001) and a higher incidence of tracheostomies (55 vs. 34%). VAP cohort had lower ICU mortality (7.4% vs 16% p=0.004) as well as a lower six months mortality (16.4% vs 26%, p=0.012). Neurological outcome was similar in the two groups (GOSE ≤ 4 in 51.5% vs 58%, p= 0.154).

**CONCLUSION.** VAP occurs very often in intubated patients after TBI and has an important effect on ICU stay. However, the development of VAP does not seem to have a detrimental effect on mortality and neurological outcome.

REFERENCE(S)

### 000594

#### Prevalence and timing of tracheostomy in traumatic brain injured patients: a secondary analysis from the CENTER-TBI study

F. Cipulli[1], C. Robba[2], F. Graziano[1], F. Fossi[3], C. Iaquaniello[1], E. Banzato[1], S. Galimberti[1], E. Wiegers[4], N. Stocchetti[5], A. Vargiolu[6], G. Citerio[1]
[1]School of medicine and surgery, University of Milano-Bicocca, Milano, Italy; [2]Department of anaesthesia and intensive care, IRCCS AOU San Martino, Genova, Italy; [3]Department of, University of Milano-Bicocca, Milano, Italy; [4]Dept. of public health, Erasmus University Medical Center, Rotterdam, Netherlands; [5]Neurointensive care unit, Fondazione IRCCS Ca'Granda Ospedale Maggiore Policlinico, Milano, Italy; [6]Neurointensive care, department of emergency and intensive care, Ospedale San Gerardo di Monza, Monza, Italy
**Correspondence:** F. Fossi
*Intensive Care Medicine Experimental* 2019, **7(Suppl 3):**000594

**INTRODUCTION.** In traumatic brain injured (TBI), tracheostomy can facilitate the weaning process and can potentially result in fewer days in the intensive care unit (ICU). However, the optimal timing for tracheostomy placement remains uncertain. The aims of our study are to describe the prevalence and timing for tracheostomy in TBI patients and its effects on patients' outcome.

**METHODS.** The CENTER-TBI study (clinicaltrials.gov registration NCT02210221) is a prospective observational longitudinal cohort study including patients with TBI from 65 centers across Europe. Data were extracted from the CENTER-TBI database v1.1 with Neurobot v2.6. We focused on patients with an ICU stay >72h. Tracheostomy was defined as early (≤7 days from admission) or late (>7).

**RESULTS.** 4509 patients were included in the CENTER-TBI study, 2138 were admitted to the ICU and we focused on 1361 patients fulfilling the inclusion criteria. Of these, 436 (32%) had a tracheostomy after a median of 12 days (IQR=6-20 days). Patients who underwent tracheostomy had a more severe TBI (71.5% vs 52.7%, p<0.001) and more frequent episodes of prehospital hypoxia (19.6 vs 13%, p=0.003) and hypotension (21 vs 12.2%, p<0.001), as well as a higher number of extracranial injuries, in particular thoracic trauma (47.7% vs 36.6%, p<0.001).

The decision for late (58%) or early (42%) tracheostomy was strongly influenced by country and treating center (p<0.001). Respiratory failure (52 vs 41%, p=0.029) and ventilator associated pneumonia (40 vs 26.5%, p=0.006) were more common in patients who underwent late tracheostomy. Tracheostomy was associated with longer ICU length of stay (median 24 vs 15 days, p<0.001) and duration of mechanical ventilation (median 19 vs 12 days, p<0.001). Effect on ICU mortality (4.3 vs 7.1%, p=0.302) or 6 months neurological outcome (GOSE ≤ 4: 64.2 vs 68.4%, p=0.44) was not significant.

**CONCLUSION.** Tracheostomy is commonly performed after TBI. Tracheostomy policies are center dependent and are more frequent in more severe TBI and in patients with respiratory complications. The timing for tracheostomy seems to have effect on ICU length of stay and duration of mechanical ventilation, but no effect on patients' outcome.

### 000622

#### Continuous EEG in critical patients with acute cerebral damage. Markers to guide monitoring

M. Sánchez Galindo[1], L. López Viñas[2], A. Canabal Berlanga[1], LC. Vega Zelaya[2], J. Pastor Gómez[2]
[1]ITU, Hospital de La Princesa, Madrid, Spain; [2]Neurophisiology, Hospital de La Princesa, Madrid, Spain
**Correspondence:** M. Sánchez Galindo
*Intensive Care Medicine Experimental* 2019, **7(Suppl 3):**000622

**INTRODUCTION.** Seizures are common entities in patients with acute brain damage which cast their prognosis. **Continuous EEG** has become a tool of increasing use for its detection but it implies an infrastructure and costs that make necessary more data that allow us to identify high risk patient who benefit most from this monitoring, making its use profitable.

**OBJECTIVES.** To study the **prevalence** of seizures as well as the **morbidity** and **mortality** associated with them in patients with acute brain damage. To study the relationship between the presence of seizures and different clinical and electroencephalographic variables in order to find **predictive factors** that guide us in our clinical practice.

**METHODS.** It is a **cross-sectional study** in 74 patients **(n = 74)** admitted to ICU with diagnosis of acute brain damage and subjected to study by continuous EEG in the period between **2015-2018.** It has been carried out a descriptive analysis of **prevalence,** main characteristics and **morbimortality** of seizures. Likewise, it has been measured the **association** of seizures with different **clinical** (sex, age, etiology, history of epilepsy, presence of clinical crises, anti-epileptic prophylaxis) and **electroencephalographic variables**(presence of periodic discharges, irritating activity and rhythmic patterns).For this purpose,-descriptive statisticians,Chi-square Test,exact Fisher Test and correlation of Spearman and Pearson have been used, with a statistical significance level p > 0.05.

**RESULTS. Seizures** were observed in **59%** of the analyzed patients, 77% of whom presented as an epileptic status. The average **time to the onset** of seizures is **17.9 hours** and the average **time of crisis control** is **51.8** hours. An average of **two drugs** per patient was necessary for the control of seizures, being the **most frequently used** the **Levetiracetam** (73%). Patients who present seizures had a **worse functional prognosis** at the time of hospital discharge, with a score between 4 and 6 in the Rankin scale in 80% of the cases and a significantly **higher** both **intrahospital** (48% vs 17%) **and 6-month mortality** (50% vs 20%) (p < 0.05, OR = 4 (1.4-11.7)). With regard to the presence of possible **predictor factors** we have found that seizures **prevalence** is **greater** in patients with **higher age** (Average age: 62 years vs. 53 years) or with a history of **clinical seizures** (84% vs 47%)(p = 0,002 OR 5.9) and **lower** in those who have received **anti-epileptic prophylaxis** (43% vs 70%) (P = 0.02 OR 0.32). It is worth highlighting the increased risk of seizures in patients with **periodic discharges**, considering it as the **main electroencephalographic marker**. The presence of seizures is greater when the discharges are widespread (GPLDs) and it increases proportionally to the frequency with which these periodic discharges appear.

**CONCLUSION.** Epileptic seizures are a common entity in patients with acute brain damage associated with an important morbimortality so that it is important their early detection and treatment. Given the average time of onset of seizures, we think that continuous EEG is a very useful tool because of its greater sensitivity but its high costs make us neccessary to guide by markers that allow to select high-risk patients. In this sense, the upper ages, the presence of clinical seizures, the absence of prophylaxis or the appearance of electroencephalographic patterns such as periodic discharges seem to have an important predictive value.

##### REFERENCE(S)

1. Han-Tao Li a, Tony Wua, Wey-Ran Linb, Wei-En Johnny Tsenga, Chun-Wei Changa, Mei-Yun Chenga, Hsiang-Yao Hsieha, Hsing-I Chianga, Chih-Hong Leea, Bao-Luen Changa, Siew-Na Lima. Clinical correlation and prognostic implication of periodic EEG patterns: A cohort study,Epilepsy Research 131 (2017) 44–50
2. Alvarez V., Rodriguez Ruiz A.A., LaRoche S., Hirsch L.J., Parres C., Voinescu P.E., Fernandez A., Petroff O.A., Rampal N., Haider H.A., Lee J.W.The use and yield of continuous EEG in critically ill patients: A comparative study of three centers, Clinical Neurophysiology 128 (2017) 570–578

3. Struck AF; Osman G; Rampal N; Biswal S; Legros B; Hirsch LJ; Westover MB; Gaspard N. Time-dependent risk of seizures in critically ill patients on continuous electroencephalogram. Ann Neurol. 2017; 82(2):177-185
4. Hilkman D, van Mook W, van Kranen-Mastenbroek V.Continuous electroencephalographic-monitoring in the ICU: an overview of current strengths and future challenges, Current Opinion in Anaesthesiology 2017, 30 (2): 192-199
5. Lin L, Drislane FW. Lateralized Periodic Discharges: A Literature Review, J Clin Neurophysiol 2018;35: 189–19
6. Newey CR, Kinzy TG, Punia V, Hantus S. Continuous Electroencephalography in the Critically Ill: Clinical and Continuous Electroencephalography Markers for Targeted Monitoring, J Clin Neurophysiol 2018;0: 1–7

### 000697

#### Epileptic seizures as a complication in cerebral hemorrhage surgically treated in ICU patients

MM. Gordillo-Resina[1], MC. Molina De la torre[1], E. Aguilar-Alonso[2], D. Arias-Verdú[3], E. Castillo-Lorente[4], JM. Mora Ordóñez[3], R. Rivera Fernandez[1], MA. Arraez-Sanchez[3]
[1]Intensive care, Hospital of Jaen, Jaén, Spain; [2]Hospital Cabra, Cabra, Spain; [3]Intensive care, Hospital Carlos Haya, Málaga, Spain; [4]Intensive care, Hospital Universitario Virgen de las Nieves , Granada, Spain
**Correspondence:** E. Aguilar-Alonso
*Intensive Care Medicine Experimental* 2019, **7(Suppl 3):**000697

**INTRODUCTION.** To evaluate the presence of epileptic seizures and surgical treatment in patients with supratentorial spontaneous non-traumatic intracerebral hemorrhage (ICH) and intraventricular hemorrhage (IVH).

**METHODS.** During the years 2009 to 2012, ICU admitted patients from three Spanish hospitals with Neurosurgery Services with the diagnosis of supratentorial ICH and IVH were studied. The effect of the surgical intervention over these patients was studied. Using a propensity index, 26 patients who underwent surgery were paired with the same number of non-operated patients.

**RESULTS.** 163 patients with supratentorial and intraventricular ICH were admitted. These patients presented in the admission a Glasgow 8 + 4, APACHE-II 21.42 + 7.55 and an ICH score of 2.75 + 0.9 with a predicted mortality (30 days) of 65% and with a mortality of 59.5%. At admission, 13.5% had bilateral arreactive mydriasis. 23.3% (n=38) of patients underwent surgery. Mortality of those who underwent surgery was 34.2% and of the non-operated patients it was 67.2% (p <0.001). In the multivariate analysis, OR for surgical intervention was 0.14 (0.05-0.41).Subsequently, 26 surgically operated patients were matched with further 26 non-surgically treated patients, based on a propensity index.This index was calculated based on: age, presence of pupillary anomalies, size and location of the hematoma and Glasgow on admission. The surgical and non-surgical patients presented similar characteristics (age, Glasgow, ICH score, APACHE II, volume and location of the hematoma) and none presented bilateral mydriasis at admission.The hospital mortality of the 26 operated was 30.8% and of the 26 not operated was 65.4%, (p = 0.001) and OR 0.23; CI: 95%: 0.07-0.75.Afterwards, these 52 paired by propensity index patients were tracked for 6-9 years and mortality was 57.7% in those operated, and 73.1% in those not operated on.Of the 26 patients non-surgically treated, 6 were alive and none had epileptic seizures, and of the 26 who were surgically treated 11 were alive and had epilepsy 6 of them (54.5%), (p = 0.017).

**CONCLUSION.** Patients admitted to the ICU for non-traumatic cerebral hemorrhage open to ventricle who undergo surgery have lower mortality than those who have not undergone surgery, but have epileptic disorders with a frequency much higher than those who have not undergone surgery

### 000756

#### Accuracy of Brain Multimodal Monitoring to Detect Cerebral Hypoperfusion After Traumatic Brain Injury

T. Gargadennec[1], G. Ferraro[2], S. Schuind[3], N. Sadeghi[4], M. Van Wettere[4], J. Creteur[2], FS. Taccone[2]
[1]Département d'anesthésie réanimation, University Hospital of Brest, Brest, France; [2]Soins intensifs, ULB Erasme, Anderlecht, Belgium; [3]Service de neurochirurgie, ULB Erasme, Anderlecht, Belgium; [4]Radiologie, ULB Erasme, Anderlecht, Belgium
**Correspondence:** T. Gargadennec
*Intensive Care Medicine Experimental* 2019, **7(Suppl 3):**000756

**INTRODUCTION.** Brain multimodal monitoring including intracranial pressure (ICP) and brain tissue oxygen pressure ($PbtO_2$) is more accurate than ICP alone in detecting cerebral hypoperfusion after traumatic brain injury (TBI). No data are available for the role of $PaO_2$ changes on $PbtO_2$ in this setting.

**OBJECTIVES.** To examine the accuracy of ICP, brain tissue $PO_2$ and the oxygen ratio (OxR) in detecting cerebral hypoperfusion in patients with acute brain injury.

**METHODS.** Single-center study including patients with TBI, subarachnoid hemorrhage (SAH) and intracranial hemorrhage (ICH) undergoing cerebral blood flow (CBF) measurements using perfusion CT scan (CTP) concomitantly to ICP and $PbtO_2$ monitoring. Before CTP, $FiO_2$ was increased directly from baseline to 1.0 for a period of 20 minutes under stable conditions to test the $PbtO_2$ catheter as standard of care. The difference between $PbtO_2$ and $PaO_2$ at the end of test vs baseline ($\Delta PbtO_2$ and $\Delta PaO_2$, respectively) were collected and the OxR calculated as $\Delta PbtO_2/\Delta PaO_2$. Regional CBF (rCBF) was measured using CTP in the tissue area around intracranial monitoring. The accuracy of different monitoring tools to predict cerebral hypoperfusion (i.e. CBF < 35 mL/100 g.min) was assessed using area under the receiver-operating characteristic curves (AUC).

**RESULTS.** 51 CTP were performed in 33 patients (age 52 [41-65] years – TBI, n=12; SAH, n=19; ICH, n=2). Median ICP, $PbtO_2$, OxR and rCBF were 13 [9–18] mmHg, 21 [19-23] mmHg, 0.25 [0.16–0.30] and 30.0 [15.8–37.4] mL/100g.min. 38 (69%) patients had cerebral hypoperfusion; rCBF correlated with ICP ($r^2$=0.12; p=0.01), $PbtO_2$ ($r^2$=0.22; p<0.001) and OxR ($r^2$=0.36; p<0.001). Compared with ICP alone (AUC = 0.73 [95% CI, 0.59–0.86]), monitoring ICP + $PbO_2$ (AUC=0.81 [0.69–0.92]) or ICP + $PbtO_2$ + OxR (AUC=0.84 [0.72–0.95]) were more accurate in predicting cerebral hypoperfusion.

**CONCLUSION.** The combination of ICP and $PbtO_2$ monitoring provides a better detection of cerebral hypoperfusion than ICP alone in patients with acute brain injury.

### 000774

#### Effects of Age on Mortality in patients with Non-Traumatic Subarachnoid Haemorrhage on a Neurocritical Care Unit

J. Sokhi, V. Louma, G. Bird, S. Bapat
The national hospital for neurology and neurosurgery, University College London Hospital, London, United Kingdom
**Correspondence:** J. Sokhi
*Intensive Care Medicine Experimental* 2019, **7(Suppl 3):**000774

**INTRODUCTION.** Mortality rates from non-traumatic subarachnoid haemorrhage (ntSAH) in the UK are declining (9/100,000)(1). How this trend correlates with age is unclear. This observational study aimed to quantify, neurocritical care unit (NCCU) mortality, complications and functional outcome by age groups.

**METHODS.** A retrospective, electronic case note review of all patients admitted with ntSAH to NCCU between 2014-17. 241 patients were analysed. Functional outcome was defined as good, modified Rankin Scale (mRS 0/1/2/3) and poor (mRS 4/5/6) at 3 months. The presenting grade of ntSAH was defined by the World Federation of Neurological Surgeons scale (WFNS), good was WFNS 1/2/3 and poor

WFNS 4/5. On NCCU, the rates of acute kidney injury (AKI - KDIGO criteria), ventilation, infection and vasospasm were analysed for each age group. Data was analysed using GraphPad Prism by the $\chi 2$ test and length of stay (LOS) by the mean average.

**RESULTS.**

Overall NCCU mortality was 18.3% in keeping with other NCCUs (2). NCCU mortality across each age group was similar, except for the observed increase in the 80-89 year olds (p= 0.74). Hospital mortality at 6 months, increased with age but was not statistically significant (p= 0.14). The mean LOS in NCCU was 12 days, with no significant variation with age. The mean LOS in hospital was 34 days. There was a non-significant (p=0.12), increasing trend towards poor outcomes with advancing age.

**CONCLUSION.** Elderly patients' required more frequent mechanical ventilation (p=0.03) and had an increased risk of AKI (p=0.05). This is possibly due to reduced physiological reserve and a higher grade of ntSAH, WFNS 4/5 (p=0.03). There was no difference in the incidence of infection (p=0.17) and vasospasm (p=0.11) across age groups.

Surprisingly, advancing age showed no significant correlation with increased mortality or NCCU LOS, although the numbers in the elderly groups were smaller. Possible explanations include; centralisation of neurosurgical services with faster diagnostics, less invasive interventions, specialised multidisciplinary critical care teams and holistic critical care bundles (e.g. for ventilated patients and management of AKI). However, there was a trend towards poorer functional outcomes and increased hospital LOS with increasing age.

**REFERENCE(S)**

1. Mukhatar TK et al: The falling rates of hospital admission, case facility, and population-based mortality for subarachnoid haemorrhage in England, 1999-2010: J Neurosurg 2016:125:698-704
2. Lantigua et al: Subarachnoid Haemorrhage: who dies and why? Critical Care 2015:19:309
3. Nil

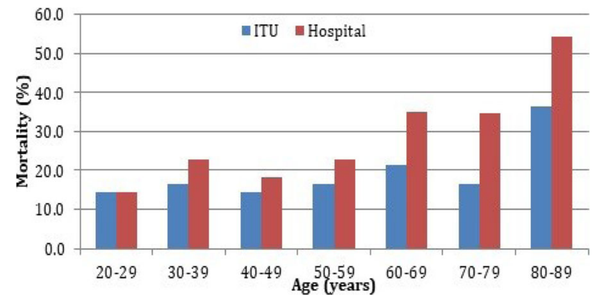

**Fig. 1 (abstract 000774).** See text for description

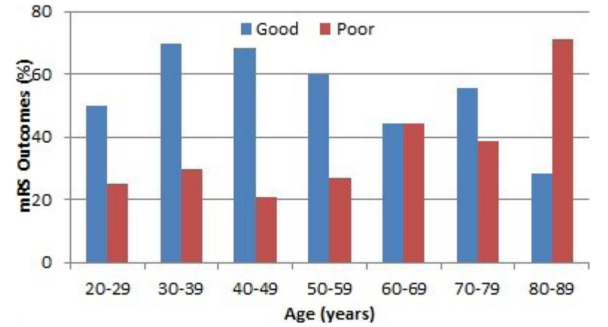

**Fig. 1 (abstract 000774).** Mortality vs age group

### 000798

#### Sedation policy in traumatic brain injury patients without intracranial pressure monitoring: a CENTER-TBI study

B. Gravesteijn[1], M. Carbonara[2], T. Zoerle[2], F. Ortolano[2], T. Birg[2], G. Citerio[3], R. Helbok[4], A. Chieregato[5], HF. Lingsma[1], N. Stocchetti[2]
[1]Center for medical decision sciences, department of public health, Erasmus University Medical Center, Rotterdam, Netherlands; [2]Neurointensive care unit, Fondazione IRCCS Ca'Granda Ospedale Maggiore Policlinico, Milano, Italy; [3]School of medicine and surgery, University of Milano-Bicocca, Monza, Italy; [4]Department of neurology, University of Innsbruck, Innsbruck, Austria; [5]Neurointensive care, ASST Great Metropolitan Niguarda, Milano, Italy
**Correspondence:** M. Carbonara

*Intensive Care Medicine Experimental* 2019, **7(Suppl 3):**000798

**INTRODUCTION.** Sedation is used in traumatic brain injury (TBI) patients admitted to intensive care unit (ICU) without intracranial pressure (ICP) monitoring to allow invasive treatments as mechanical ventilation and improve comfort. However, it remains unclear what the effect of prolonged sedation is and whether different sedative drugs have different effect on outcome in this population. This analysis of the CENTER-TBI study uses comparative effectiveness research to fill this knowledge gap.

**METHODS.** The CENTER-TBI study (clinicaltrials.gov registration NCT02210221) is a prospective observational longitudinal cohort study including patients with TBI from 65 centers across Europe. Data were extracted from the CENTER-TBI database v1.1 with Neurobot v2.6. We included all patient who were admitted to the ICU, without ICP monitoring and who had more than one day of mechanical ventilation. For every patient, the primary sedative (the sedative that the patient received most of the days) was selected. We focused on midazolam and propofol, since these were the most frequently used.

**RESULTS.** 4509 patients were included in the CENTER-TBI study: we selected 265 patients who received propofol and 101 patients who received midazolam as primary sedative. There was a large variation between centers in the cumulative days of sedation. Propofol increased the number of sedation days 4.00 times (95% CI: 1.62 - 9.89) when adjusted for patient characteristics. Nevertheless, unadjusted total ICU stay was 0.83 (95% CI: 0.77 - 0.90) times shorter when propofol was used, and the adjusted was 0.79 (95% CI: 0.73 - 0.86) times shorter. The unadjusted OR for a better functional outcome was 1.29 (95% CI: 0.80 - 2.09), and the adjusted was 1.15 (95% CI: 0.65 - 2.04).

**CONCLUSION.** There is large variation in the number of days during which TBI patients are sedated. The number of sedation days is often larger when propofol is primarily used. However, propofol is associated with a shorted length of ICU stay. This study did not find evidence that this improves outcome.

**REFERENCE(S)**

1. CENTER-TBI (clinicaltrials.gov NCT02210221) was supported by the European Union 7th Framework program (EC grant 602150)

### 000802

#### Incidence of early hyperemia post primary craniectomy in severe traumatic brain injury measured by Doppler

B. Romima, M. Victor, C. Nestor, Z. Carlos, MF. Maria, R. Avila
Critical care, Provincial Hospital Dr. José María Cullen, Santa Fe, Argentina
**Correspondence:** R. Avila

*Intensive Care Medicine Experimental* 2019, **7(Suppl 3):**000802

**INTRODUCTION.** The different hemodynamic phases in cerebral blood flow (CBF) after traumatic brain injury (TBI) have previously been characterized. Moreover, a hyperemic pattern by Doppler is associated with worse clinical outcomes. Some patients with severe TBI require a craniectomy as part of their treatment. It is not well understood how a craniectomy can affect CBF in this population.

**OBJECTIVES.** We sought to determine the cerebral blood flow velocities with transcranial Doppler during the first 24 hours in patients with severe TBI looking at how craniectomy affects these parameters.

**METHODS.** We retrospectively analyzed our database for transcranial Doppler procedures (TCD) performed during the first 24 hours of admission on patients with a diagnosis of severe TBI from July 2018 to March 2019. All studies measured middle cerebral artery (MCA) median blood flow velocity (MBFV) and accordingly, the results were classified in three patterns: normal, hyperemia and oligohemia. We compared the incidence of early hyperemia among patients that received primary craniectomy plus medical treatment or medical treatment alone.

**RESULTS.** Thirty-one patients were identified, of which 83.8% were male with a mean age 29 ± 15 years old and a median Marshall score of 2. The mean MBFV was 55 ± 17.7 cm/sec, ( 57.4 ±19.6 cm/sec in the left MCA and 52.7 ± 22.6 cm/sec in the right (p=0.2599) ). Eleven patients (35.5%) underwent primary craniectomy. There was no difference in MBFV between patients who underwent craniectomy and those who did not (52.6 vs 56.4 cm/sec, p=0.5572). There was no difference in the incidence of early hyperemia in patient that required primary craniectomy compared to those who did not (p= 0.999)

**CONCLUSION.** In patients admitted with severe TBI to whom the craniectomy was performed, we found no difference in the presence of early hyperemia as well as other parameters measured by TCD.

**REFERENCE(S)**

1. Bor-Seng-Shu E, Hirsch R, Teixeira MJ, De Andrade AF, Marino R Jr. (2006). Cerebral hemodynamic changes gauged by transcranial Doppler ultrasonography in patients with posttraumatic brain swelling treated by surgical decompression. Journal of Neurosurgery JNS, 104(1), 93-100.
2. Ojha B, Jha D, Kale S, Mehta V. Trans-cranial Doppler in severe head injury: Evaluation of pattern of changes in cerebral blood flow velocity and its impact on outcome. Surg Neurol. 2005 Aug;64(2):174-9

### 000822

#### Continuous processed EEG monitoring for sedation optimization in ICU

M. Marchesi, F. Terranova, S. Sala, M. Brognoli, GP. Nocivelli, R. Bertuetti, E. De Peri, F. Rasulo

Neuro critical care, Spedali Civili University affiliated Hospital of Brescia, Brescia, Italy

**Correspondence:** M. Marchesi

*Intensive Care Medicine Experimental* 2019, **7(Suppl 3):**000822

**INTRODUCTION.** Sedation is a significant part of medical treatment in ICU patients. Patients exposed to a too light sedation can develop agitation, pain, fear, adverse events as devices removal and respiratory distress, which can lead to post-traumatic stress disorder.A too deep sedation is associated with a longer time of mechanical ventilation and therefore of extubation, lung injury, infections, neuromuscular disease and delirium. A longer duration of ICU hospitalization and an increase of morbility incidence and mortality are also demonstrated. Many patients spend a considerable amount of time in a non-optimal sedation level. A continuous monitoring system of the sedation level is therefore necessary to improve clinical evaluation. Different pEEG analyses have been studied to monitoring sedation depth, Next Generation SedLine (Masimo) has shown a strong correlation with other monitoring systems, resulting more sensitive and less susceptible to interferences.

**OBJECTIVES.** The goal of our study was to evaluate the incidence of non-optimal sedation (under and over sedation) comparing the parameters expressed from NGSedLine with clinical evaluations.

**METHODS.** We have studied a cohort of patients admitted to the ICU of Spedali Civili of Brescia University Hospital requiring continuous sedation for more than 12 hours. In addition to standard monitoring, the patients have been studied using Next Generation Sedline (Masimo), which provides a continuous 4 channel pEEG monitoring. Derived parameters were: PSI (Patient State Index), SEFL, SEFR, EMG, ARTF, DSA and Suppression Ratio (SR). Sedation depth was evaluated through Richmond-Agitation-Sedation Scale (RASS). Non-optimal sedation has been evaluated by confronting RASS and PSI values.

**RESULTS.** Clinical data of our patients are shown in Table 1, while a summary of collected data is shown in Table 2.Average artefacts percentage was less than 10% in 32 of 34 patients. Accuracy is also confirmed by the analysis of EMG data: 2 patients of 34 spent more than half of the time of sedation with an EMG value of 30 or less.Collected data showed high incidence of oversedation: SR analysis showed that all patients but one had at least one episode of burst suppression and that 20 of our 34 patients spent more than 10% of total time with a high level of SR and 16 of our patients spent more than half the time with a level of PSI lower than 30, confirming an important oversedation. Also SEFL and SEFR were lower than the expected: graphic 2 shows that the average values were both lower than 10 in 18 of 34 patients.

**CONCLUSION.** Non-optimal sedation is an unsolved problem in ICU, affecting many patients, with a major incidence of over-sedation compared to under-sedation. Association of EEG-based monitoring systems to clinical evaluations can optimize sedation in critically-ill patients.

**REFERENCE(S)**

1. Drover D, Ortega HR. Patient state index. Best Pract Res Clin Anaesthesiol. 2006 Mar;20(1):121-8. PubMed PMID: 16634419
2. Soehle M, Kuech M, Grube M, Wirz S, Kreuer S, et al. Patient state index vs bispectral index as measures of the electroencephalographic effects of propofol. Br J Anaesth. 2010 Aug;105(2):172-8. PubMed PMID: 20587537
3. Adesanya AO, Rosero E, Wyrick C, Wall MH, Joshi GP. Assessing the predictive value of the bispectral index vs patient state index on clinical assessment of sedation in postoperative cardiac surgery patients. J Crit Care. 2009 Sep;24(3):322-8. PubMed PMID: 19540087
4. Payen JF, Chanques G, Mantz J, Hercule C, Auriant I, et al. Current practices in sedation and analgesia for mechanically ventilated critically ill patients: a prospective multicenter patient-based study. Anesthesiology. 2007 Apr;106(4):687-95; quiz 891-2. PubMed PMID: 17413906
5. Martin J, Franck M, Fischer M, Spies C. Sedation and analgesia in German intensive care units: how is it done in reality? Results of a patient-based survey of analgesia and sedation. Intensive Care Med. 2006 Aug;32(8):1137-42. PubMed PMID: 16741692
6. Kress JP, Pohlman AS, O'Connor MF, Hall JB. Daily interruption of sedative infusions in critically ill patients undergoing mechanical ventilation. N Engl J Med. 2000 May 18;342(20):1471-7. PubMed PMID: 10816184
7. Treggiari MM, Romand JA, Yanez ND, Deem SA, Goldberg J, et al. Randomized trial of light versus deep sedation on mental health after critical illness. Crit Care Med. 2009 Sep;37(9):2527-34. PubMed PMID: 19602975
8. Girard TD, Kress JP, Fuchs BD, Thomason JW, Schweickert WD, et al. Efficacy and safety of a paired sedation and ventilator weaning protocol for mechanically ventilated patients in intensive care (Awakening and Breathing Controlled trial): a randomised controlled trial. Lancet. 2008 Jan 12;371(9607):126-34. PubMed PMID: 18191684
9. Jackson DL, Proudfoot CW, Cann KF, Walsh TS. The incidence of sub-optimal sedation in the ICU: a systematic review. Crit Care. 2009;13(6):R204. PubMed PMID: 20015357; PubMed Central PMCID: PMC2811948

**Table 1 (abstract 000822).** A summary of the characteristics of studied patients

| | REASON FOR HOSPITALIZATION | AGE | SEX | | REASON FOR HOSPITALIZATION | AGE | SEX |
|---|---|---|---|---|---|---|---|
| **Patient 1** | Respiratory failure | 68 | M | **Patient 18** | Respiratory failure | 75 | F |
| **Patient 2** | Respiratory failure, hypercapnic acidosis | 69 | M | **Patient 19** | Post-cholecystectomy | 58 | F |
| **Patient 3** | Laryngeal-tracheal trauma | 19 | M | **Patient 20** | Respiratory failure | 56 | M |
| **Patient 4** | Laryngeal-tracheal trauma | 19 | M | **Patient 21** | Respiratory failure, subarachnoid hemorrhage, intraparenchimal hemorrhage | 55 | M |
| **Patient 5** | Precipitation polytrauma | 64 | M | **Patient 22** | Subarachnoid hemorrhage | 63 | M |
| **Patient 6** | Street polytrauma | 39 | M | **Patient 23** | Respiratory failure | 74 | M |
| **Patient 7** | Spontaneous subarachnoid hemorrhage, intraparenchimal bleeding | 49 | M | **Patient 24** | trauma da arma da fuoco | 44 | M |
| **Patient 8** | crush polytrauma | 46 | M | **Patient 25** | Subarachnoid hemorrhage | 34 | F |
| **Patient 9** | Post-traumatic intracranial bleeding | 60 | M | **Patient 26** | Respiratory failure | 79 | M |
| **Patient 10** | Post-broken AAA substitution, candida sepsis | 86 | F | **Patient 27** | Respiratory failure | 81 | M |
| **Patient 11** | Respiratory failure in obese patient | 58 | M | **Patient 28** | Post-operative monitoring | 76 | M |
| **Patient 12** | Upper digestive tract bleeding, metabolic unbalance and hepatic failure | 44 | M | **Patient 29** | Septic shock | 79 | M |
| **Patient 13** | Precipitation polytrauma | 41 | M | **Patient 30** | Respiratory failure | 81 | M |
| **Patient 14** | Right MCA thrombosis | 50 | M | **Patient 31** | Post-operative monitoring | 76 | M |
| **Patient 15** | subarachnoid hemorrhage caused by MCA aneurysm rupture | 48 | F | **Patient 32** | Status epilepticus | 46 | F |
| **Patient 16** | Respiratory failure | 64 | M | **Patient 33** | Respiratory failure | 79 | F |
| **Patient 17** | Polytrauma | 42 | M | **Patient 34** | Post-operative monitoring | 55 | M |

**Table 1 (abstract 000822).** A recap of the data collected by the SedLine monitoring system

| | Average | | | | SR-Time | PSI-Time | | | EMG-Time | | |
|---|---|---|---|---|---|---|---|---|---|---|---|
| | PSI | ARTFs | SEFL | SEFR | Value > 2 | 100-60 | 60-30 | 30-0 | 100-70 | 70-40 | 40-0 |
| Patient 1 | 45 | 1 | 13.1 | 13.9 | 15,16 | 21,79% | 45,96% | 32,26% | 2,32% | 8,49% | 89,19% |
| Patient 2 | 60 | 1 | 8.8 | 8.5 | 7,65 | 47,76% | 46,29% | 5,94% | 7,23% | 9,58% | 83,19% |
| Patient 3 | 71 | 1 | 16.2 | 15.9 | 6,4 | 90,38% | 9,60% | 0,00% | 6,20% | 15,11% | 78,69% |
| Patient 4 | 58 | 0 | 17.7 | 18.2 | 13,11 | 67,21% | 19,99% | 12,78% | 0,29% | 2,17% | 97,54% |
| Patient 5 | 83 | 6 | 18.6 | 5.5 | 0 | 95,23% | 1,76% | 3,10% | 37,05% | 39,01% | 23,94% |
| Patient 6 | 33 | 0 | 3.2 | 3.4 | 27,26 | 18,58% | 6,12% | 75,33% | 10,62% | 6,54% | 82,84% |
| Patient 7 | 34 | 0 | 13.2 | 5.8 | 0,3 | 14,45% | 19,67% | 65,92% | 1,82% | 4,07% | 94,11% |
| Patient 8 | 72 | 0 | 15.3 | 12.3 | 1,58 | 85,98% | 12,70% | 1,32% | 0,19% | 1,90% | 97,91% |
| Patient 9 | 43 | 0 | 3.0 | 2.8 | 5,75 | 33,93% | 6,99% | 59,10% | 0,42% | 11,07% | 88,51% |
| Patient 10 | 62 | 14 | 10.0 | 8.9 | 17,91 | 62,24% | 1,56% | 36,16% | 25,47% | 34,27% | 40,26% |
| Patient 11 | 32 | 0 | 6.3 | 6.8 | 19,3 | 8,34% | 30,61% | 61,09% | 0,03% | 0,06% | 99,91% |
| Patient 12 | 68 | 0 | 12.1 | 14.6 | 21,08 | 70,24% | 8,27% | 21,46% | 32,78% | 18,83% | 48,39% |
| Patient 13 | 36 | 0 | 6.6 | 7.1 | 0,21 | 15,50% | 33,04% | 51,39% | 2,15% | 13,78% | 84,07% |
| Patient 14 | 62 | 0 | 16.2 | 8.0 | 9,4 | 59,79% | 20,41% | 19,85% | 7,26% | 23,53% | 69,21% |
| Patient 15 | 24 | 0 | 5.2 | 3.2 | 81,93 | 2,97% | 2,14% | 94,88% | 1,02% | 1,84% | 97,14% |
| Patient 16 | 35 | 1 | 5.1 | 5.2 | 48,02 | 22,04% | 8,82% | 69,13% | 2,60% | 16,04% | 81,36% |
| Patient 17 | 69 | 1 | 9.8 | 6.8 | 18,42 | 83,79% | 7,69% | 8,51% | 0,02% | 2,20% | 97,78% |
| Patient 18 | 35 | 1 | 5.9 | 6.0 | 13,6 | 15,30% | 9,15% | 75,55% | 2,95% | 18,88% | 78,17% |
| Patient 19 | 75 | 7 | 14.9 | 13.8 | 8,48 | 84,12% | 14,32% | 1,56% | 22,03% | 15,14% | 62,83% |
| Patient 20 | 30 | 1 | 8.8 | 7.3 | 27,21 | 7,51% | 20,02% | 72,47% | 0,45% | 0,87% | 98,68% |
| Patient 21 | 66 | 0 | 11.2 | 6.8 | 32,08 | 73,81% | 6,81% | 19,39% | 2,31% | 14,90% | 82,79% |
| Patient 22 | 40 | 1 | 10.6 | 9.9 | 10,47 | 10,38% | 65,34% | 24,28% | 0,17% | 2,00% | 97,83% |
| Patient 23 | 22 | 0 | 3.7 | 3.9 | 12,25 | 1,53% | 6,20% | 92,27% | 0,02% | 0,05% | 99,93% |
| Patient 24 | 34 | 2 | 3.6 | 5.0 | 25,08 | 17,87% | 12,22% | 69,94% | 1,28% | 4,12% | 94,60% |
| Patient 25 | 83 | 4 | 19.8 | 22.6 | 3,62 | 94,13% | 0,67% | 5,19% | 83,52% | 6,85% | 9,63% |
| Patient 26 | 44 | 2 | 8.4 | 8.2 | 14,14 | 33,18% | 2,93% | 63,93% | 16,10% | 13,00% | 70,90% |
| Patient 27 | 24 | 0 | 9.7 | 0.5 | 73,74 | 22,25% | 11,38% | 87,13% | 0,06% | 0,76% | 99,18% |
| Patient 28 | 24 | 1 | 5.4 | 5.2 | 4,11 | 2,41% | 2,78% | 94,79% | 0,00% | 0,06% | 99,94% |
| Patient 29 | 83 | 1 | 4.4 | 5.4 | 8,69 | 94,45% | 1,62% | 3,92% | 56,38% | 25,13% | 18,49% |
| Patient 30 | 41 | 3 | 6.0 | 5.6 | 43,34 | 30,45% | 8,88% | 60,61% | 6,53% | 9,44% | 84,03% |
| Patient 31 | 51 | 0 | 11,7 | 16 | 10,63 | 46,27% | 4,07% | 49,61% | 24,99% | 24,83% | 50,18% |
| Patient 32 | 67 | 0 | 16.6 | 17.6 | 7,87 | 68,77% | 27,22% | 4,04% | 11,17% | 9,91% | 78,92% |
| Patient 33 | 43 | 25 | 11 | 9,1 | 10,7 | 32,59% | 4,94% | 62,48% | 43,67% | 3,58% | 52,76% |
| Patient 34 | 49 | 3 | 16,3 | 17,2 | 4,55 | 11,96% | 95,60% | 1,40% | 2,95% | 4,42% | 92,63% |

### 000832

#### Prognostic value of endothelial progenitor cells after aneurysmal subarachnoid hemorrhage (EVAPROPEC study)

L. Carteron, I. Fouet, E. Samain, S. Pili-Floury, G. Besch
Anesthesiology and intensive care medicine, CHRU de Besançon, Besançon, France
**Correspondence:** L. Carteron
*Intensive Care Medicine Experimental* 2019, **7(Suppl 3):**000832

**INTRODUCTION.** Aneurysmal subarachnoid hemorrhage (aSAH) is a devastating condition with high morbidity and mortality. Physiopathological mechanisms leading to long term outcome are still partially unexplained. Previous studies showed an association between plasma levels of Endothelial Progenitor Cells (EPC) and outcome after acute brain injury (stroke, traumatic brain injury)[1]. The link between EPC and aSAH, complicated or not of cerebral vasospasm (CVS), has not been studied yet. We previouly showed a different EPC kinetic after serious or non serious aSAH[2], and in patients with or without CVS[3].

**OBJECTIVES.** To compare plasma level of EPC 3 days after aSAH according to 1-year outcome.

**METHODS.** Prospective monocentric EVAPROPEC cohort (NCT01773200) including every adult patient admitted for aSAH, after obtaining patient's or next-of-kin's consent, or after emergency inclusion as allowed by local ethic committee. Plasma levels of CD34+/CD133+ and CD34+/CD133+/VEGFR2+ EPC (expressed as stained cells per 100 000 mononuclear cells) were measured by flow cytometry after isolation of mononuclear cells by a Ficoll gradient and surface staining on blood samples collected at admission (D0), and D3, D6, D10, D14, D21. 1-year neurologic outcome was evaluated by Glasgow Outcome Scale (GOS) assessed by a standardized questionnaire. EPC levels at D3 (principal judgement criterion), the maximal variation of EPC level between D0 and D21, and the proportion of patients with a significant mobilization of EPC (defined as an EPC level increase > 100% compared to D0 level) (secondary judgement criteria), were compared between a "bad outcome" (GOS 1-3) and a "good outcome" (GOS 4-5) group using a Mann-Whitney test (significance was defined by a p value < 0.05). Results are expressed as median [25th-75th percentiles] or n(%).

**RESULTS.** 90 patients: 34 with a "bad outcome" and 46 with a "good outcome" (10 lost of view). Demographical data are presented in table. D3 plasmatic level of CD34+/CD133+ EPC (46 [26-121] vs. 89 [42-143], p=0.22) or CD34+/CD133+/VEGFR2+ EPC (8 [3-31] vs. 18 [1-46], p=0.47] (figure), maximal variation of CD34+/CD133+ EPC (9 [0-48] vs. 30 [0-103], p=0.48) or CD34+/CD133+/VEGFR2+ EPC (4 [0-73] vs. 4 [0-47], p=0.89), the proportion of patients with a significant mobilization of CD34+/CD133+ EPC (29% vs. 29%, p=1) or CD34+/CD133+/VEGFR2+ EPC (38% vs. 35%, p=1) were not significantly different between both groups.

**CONCLUSION.** Endothelial progenitor cells level and kinetic do not seem associated with 1-year outcome after aneurysmal subarachnoid hemorrhage.

**REFERENCE(S)**

[1] Brain Injury 2012; 26; 291-297
[2] SFAR 2014 Abstract R042
[3] SFAR 2016 Abstract MED-1317
[4] The authors thank Barbara Dehecq for technical support, and Gaëlle Amiotte & Lucie Vettoretti for unvaluable assistance.

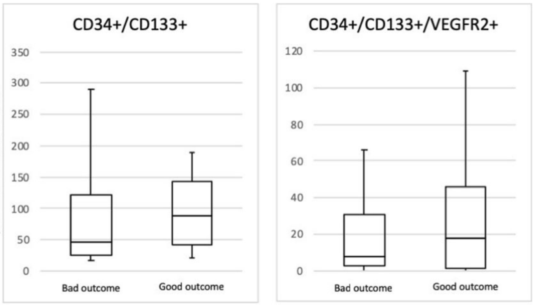

| | Bad outcome | Good outcome | p |
|---|---|---|---|
| Age, years | 62 [54-70] | 52 [45-60] | 0.0001 |
| WFNS | 4 [4-5] | 1 [1-3] | <.0001 |
| Fisher | 4 [4-4] | 3 [2-4] | <.0001 |
| Cerebral vasospasm | 14 (19%) | 7 (10%) | 0.01 |
| ICU length of stay, d | 14 [6-24] | 6 [3-12] | 0.01 |
| Length of stay | 29 [14-64] | 18 [12-28] | 0.08 |

**Fig. 2 (abstract 000832).** mRS outcome vs age group

### 000853

#### Early endovascular treatment of acute ischemic stroke. Our experience after the publication of new guidelines

AM. Domínguez-Berrot[1], M. González-Vaquero[1], J. Valdivia-Ruiz[1], ME. Vallejo-Pascual[2], AM. Fernández-Martínez[3]
[1]Intensive care unit, COMPLEJO ASISTENCIAL UNIVERSITARIO DE LEÓN, León, Spain; [2]Economía y estadística, University of León, León, Spain; [3]Neuro/radiología intervencionista, COMPLEJO ASISTENCIAL UNIVERSITARIO DE LEÓN, León, Spain
**Correspondence:** A.M. Domínguez-Berrot
*Intensive Care Medicine Experimental* 2019, **7(Suppl 3):**000853

**INTRODUCTION.** Early endovascular treatment of patients with acute ischemic stroke has changed according to the new recommendations published in 2018, mainly focused on an earlier approach over a longer period for patients with or without thrombolytic therapy

**OBJECTIVES.** Primary outcome: to compare the outcomes at discharge and at 90 days of patients admitted to our ICU over two periods of time (2018 and pre-2018). Secondary outcomes: 1. To discover whether global time (minutes from the beginning of the symptoms until the end of the procedure) is shorter. 2. To analyse whether any differences exist in the use of vasoactive drugs (antihypertensive therapy / amines).

**METHODS.** Retrospective study. Review of medical records. Patients with AIS admitted to our ICU for endovascular treatment are divided into 2 groups: G1 (year 2018); G2 (years 2015-2017). The recorded variables include (among others): demographic data, cardiovascular risk factors, previous treatment with antiplatelet agents/oral anticoagulants, outcome at discharge and after 3 months using modified Rankin Scale (mRS-d, mRS-3).

Mann-Whitney *U*-test (for independent samples) and Wilcoxon test (for related samples) were used to compare medians. The Student test was used to compare differences in time between the two groups. Pearson's chi-square test and Fisher's exact test were used to analyse differences in vasoactive drug use.

**RESULTS.** A total of 103 patients were included: 63 in G1 and 40 in G2. The demographic data, risk factors and NIHSS on admission did not differ, despite the difference in sample sizes.

Primary outcome: there were no statistically significant differences in mRS-d between G1 and G2, and 50% of patients have mRS-3 $\leq$ 2. Nonetheless, though the difference is not statistically significant, outcome at discharge is better in G1, given that 25% of the patients have mRS-d $\leq$1.

Outcome after three months was compared with that at discharge in each group separately (excluding patients with mRS-d=6, as improving is impossible for them). We found a statistically significant improvement in both groups, which was higher in G1. 50% of patients in G1 have mRS-3 $\leq$ 1, while 50% of those in G2 achieve mRS-3 $\leq$ 2. Furthermore, 25% of patients in G1 have mRS-3 = 0, and this is not achieved in G2.

Secondary outcomes: 1. Time is shorter, but the difference is not statistically significant. There may be external factors which bias this parameter. 2. We have used fewer vasoactive drugs in G1 than in G2, and the difference is statistically significant.

**CONCLUSION.** CONCLUSIONS

1.- The introduction of new recommendations for AIS endovascular treatment has improved the outcome of our patients. 2.- Thanks to early endovascular therapy, as a first line treatment for AIS, 25% of patients have no neurological sequelae. 3.- Although the time period is shorter, we have not found any statistically significant differences, probably due to several external factors. 4.-The use of vasoactive drugs has decreased upon the introduction of the new guidelines

**REFERENCE(S)**

1. 2018 guidelines for the early management of patients with acute ischemic stroke. Powers et al. Stroke 2018:49
2. No grants have been received

### 000862

#### Hyperoxia in Traumatic Brain Injury. Data from Center-TBI

A. Mazeraud[1], C. Robba[2], C. Iaquaniello[3], E. Banzato[3], E. Wiegers[4], A. Vargiolu[5], G. Citerio[6]

[1]Neurointensive Care, CHSA, Paris, France; [2]Department of anaesthesia and intensive care, IRCCS AOU San Martino, Genova, Italy; [3]School of medicine and surgery, University of Milano-Bicocca, Milano, Italy; [4]Dept. of public health, Erasmus University Medical Center, Rotterdam, Netherlands; [5]Neurointensive care, department of emergency and intensive care, Ospedale San Gerardo di Monza, Monza, Italy; [6]School of medicine and surgery, University of Milano-Bicocca, Monza, Italy

**Correspondence:** A. Mazeraud

*Intensive Care Medicine Experimental* 2019, **7(Suppl 3):**000862

**INTRODUCTION.** Controversial evidences exist concerning the effect of hyperoxia on outcome after traumatic brain injury in ICU.

**OBJECTIVES.** We aimed at evaluating the effect of transient and prolonged hyperoxia on outcome.

**METHODS.** The CENTER-TBI study is a prospective observational longitudinal cohort study including patients with TBI from centers across Europe. Data were extracted from the CENTER-TBI database v1.0 with Neurobot v2.6. We focused on patients receiving mechanical ventilation in ICU > 24 hours. We analyzed previously published predictors of outcome: age, preinjury state, ISS score, Glasgow motor score, pupils' examination, imaging results and secondary insults such as hypoxia, hypotension, unwanted hypocapnia, seizures, highest blood glucose level (BGL) at day 1, lowest hemoglobin level at day 1, raised ICP episode and length of stay.

Transient hyperoxia was defined as a single blood gas analysis (BGA) PaO2 >100 (THMild) mmHg and >300 mmHg (THsevere) and prolonged hyperoxia as the daily lowest BGA PaO2 >100 mmHg (PHmild) and 300 mmHg (PHsevere). The main outcome was a negative outcome at 6 months (Glasgow outcome scale extended, GOSe $\leq$4). Chi square tests and fisher tests were used to compare variables between good and bad outcome groups.

**RESULTS.** 4509 patients were included in the CENTER-TBI study, 2138 were admitted in the ICU and we focused on the 1099 receiving MV for more than 24 hours. 771/1099 (70.2%) patients were exposed at least once to PHmild whereas 2487 episodes of PHmild occurred during the entire stay, 908 (36.5%) of which during the first 48 hours. Only 26 episodes of PHsevere occurred in 23/1099 (2.1%) patients, 18 of which in the first 48 hours. 112/214 patients that died before day 4 had an episode of PHmild *vs.* 289/885 (p<.0001).

1061/1099 patients were exposed to THMild whereas 6373 episodes of THMild occurred, 1808 (26.3%) of which during the first 48 hours. 213/1099 patients presented 253 episodes of THsevere, 168(66.8%) of which in the first 48 hours. 200/214(93.4) patients that died before day 4 had an episode of THMild vs. 847/884(95.8) and 40/214 vs 136/884 (p=.280) for THsevere.

THMild or THsevere were not associated with GOSE in univariate analysis (p=0.259, p=.566) nor PHmild or PHsevere (p=.258, p=.056)

**CONCLUSION.** Hyperoxia is frequent in ICU after TBI but it is not associated with worse outcome.

**REFERENCE(S)**

### 000870

#### Association of Hospital-level Intracranial Pressure Monitoring Utilization for Severe Traumatic Brain Injury with Clinical Outcome: a post hoc analysis of a multicenter, prospective registry

T. Okazaki[1], K. Kenya[2], K. Yasuhiro[2]

[1]Emergency Medical Center, Kagawa University Hospital, Miki, Japan; [2]Emergency medical center, Kagawa University Hospital, Miki, Japan

**Correspondence:** T. Okazaki

*Intensive Care Medicine Experimental* 2019, **7(Suppl 3):**000870

**INTRODUCTION.** Severe traumatic brain injury management guidelines recommend intracranial pressure monitoring. However, the use of hospital-level intracranial pressure monitoring varies greatly, and the association between hospital-level intracranial pressure monitoring utilization and clinical outcomes remains unknown.

**OBJECTIVES.** To examine whether severe traumatic brain injury patients treated at hospitals with higher intracranial pressure monitoring utilization rates have better functional outcomes, based on data from the Japan Neurotrauma Data Bank Project 2015.

**METHODS.** In this post hoc analysis of a nationwide prospective registry of patients admitted between April 2015 and March 2017, the primary exposure was hospital-level intracranial pressure monitoring utilization. Patients treated at hospitals where intracranial pressure monitoring was used for monitoring >80% of severe traumatic brain injury patients were assigned to a “high” group and all other patients to a “low” group. The secondary exposure was patient-level intracranial pressure monitoring utilization (intracranial pressure monitoring group vs non-intracranial pressure monitoring group). The primary endpoint was a 6-month favorable functional outcome, defined as a Glasgow Outcome Scale score of 4 or 5.

**RESULTS.** Intracranial pressure monitoring utilization at each hospital ranged from 0%to 100%. Overall, 459 patients from 25 hospitals were included, with 59 (12.9%) of them assigned to the high group. Multiple logistic regression model showed that patients in the high group had significantly better functional outcome than those in the low group (adjusted odds ratio 2.77; 95% confidence interval, 1.30–5.92; p = 0.009). However, patients in the intracranial pressure monitoring group at the patient-level analysis did not exhibit any clear association with a specific functional outcome.

**CONCLUSION.** Treatment at hospitals with higher intracranial pressure monitoring utilization for severe traumatic brain injury can be associated with better functional outcomes.

**REFERENCE(S)**

1. Suehiro E, Fujiyama Y, Koizumi H, et al. Directions for use of intracranial pressure monitoring in treatment of severe traumatic brain injury using data from the Japan Neurotrauma Data Bank. J Neurotrauma. 2017;34:2230–2234.
2. Dawes AJ, Sacks GD, Cryer HG, et al. Compliance with evidence-based guidelines and interhospital variation in mortality for patients with severe traumatic brain injury. JAMA Surg. 2015;150:965–972.
3. Alali AS, Fowler RA, Mainprize TG, et al. Intracranial pressure monitoring in severe traumatic brain injury: results from the American College of Surgeons Trauma Quality Improvement Program. J Neurotrauma. 2013;30:1737–1746
4. Carney N, Totten AM, O'Reilly C, et al. Guidelines for the management of severe traumatic brain injury. Neurosurgery. 2017;80:6–15.
5. The Japan Neurotrauma Data Bank Project 2015 was supported by the Japan Society of Neurotraumatology. The authors received no financial support for the current post hoc analysis.

### 000879

#### Is the depth of anemia during the first 8 days of an ICU stay associated with the functional outcome of severe brain injured patients? a prospective cohort study

M. Leger[1], A. Roquilly[2], T. Gaillard[1], R. Cinotti[2], K. Asehnoune[2], S. Lasocki[3]
[1]Departement anesthesie réanimation, centre hospitalier universitaire d'Angers, Angers, France; [2]Anesthesie reanimation, Nantes University Hospital Hotel-Dieu, Nantes, France; [3]Anesthésie-réanimation, C.H.U. d'Angers, Angers, France
**Correspondence:** S. Lasocki
*Intensive Care Medicine Experimental* 2019, **7(Suppl 3):**000879

**INTRODUCTION.** Anemia is considered as one of the factor that could aggravate brain injuries after traumatic or non-traumatic lesions. However, the “safe” hemoglobin threshold is not clearly defined in this particular population [1].

**OBJECTIVES.** The objective of this study is to evaluate the association between the intensity of anemia (defined as the proportion of time spent under different hemoglobin (Hb) thresholds) and functional outcome at 3 months in brain damaged patients.

**METHODS.** Prospective observational study based on data from the ATLANREA cohort (traumatic or non-traumatic brain-injured patients hospitalised in intensive care), restricted to the centers of Nantes and Angers, between 2013 and 2018. From the Hb dosages of the first 8 days, an area index has been calculated by dividing the area representing the time spent with Hb values below a threshold by the total monitored time. The area below the various thresholds (10 g/dl, 9 g/dl, 8 g/dl and 7 g/dl) has been calculated using the trapezoid method (figure). Functional status at 3 months has been determined by the dichotomized GOS score (unfavourable prognosis if death, vegetative state or severe deficiency). We evaluated the judgment criterion through multivariate logistic regression by adjusting on the variables significantly associated in univariate with functional outcome (centre, age, sex, traumatic entity or not, number of pack blood cells unit transfused, initial GCS, mydriasis on admission, smoking status and SOFA score, Hb, urea, fibrinogen on admission). The statistical analyses were performed with software R (version 3.4.0).

**RESULTS.** Among the 585 patients studied (mean age: 49±21 years, women: 177 (30%), SOFA: 8±3; initial Hb level: 11±3 g/dl; initial GCS: 7±3), 394 (67%) had a traumatic brain injury. We recovered 4258 Hb dosages during the first 8 days (mean 0.90 ± 0.48/patient/day). During this period, 340 patients (58%) had at least one Hb value < 10 g/dl, 252 (43%) < 9 g/dl, 153 (26%) < 8 g/dl and 65 (11%) < 7 g/dl. 222 patients (38%) had an unfavourable outcome at 3 months, with a significant association for the four different hemoglobin thresholds (p-value < 0.01 for each). After adjusting for confounding factors, Hb area indexes are not associated with functional outcome at 3 months, regardless of the specified threshold (p-value: 0.746 for 10g/dl; 0.819 for 9g/dl ; 0.435 for 8g/dl ; 0.128 for 7g/dl).

**CONCLUSION.** There is no apparent association between the intensity of anemia during the first 8 days of resuscitation and functional development at 3 months in patients who have undergone a brain injury. A lack of power may explain the absence of any negative impact below the 7g/dl threshold.

**REFERENCE(S)**

[1] Mueller et al. JAMA. 2019

### 000888

#### Interaction effects of arterial oxygen and circulatory shock in neurologically ill mechanically ventilated patients

J. Humaloja[1], E. Litonius[2], R. Rahul[3], E. Wilkman[2], PT. Pekkarinen[2], S. Bendel[4], M. Reinikainen[4], MB. Skrifvars[1]
[1]Department of emergency care and services, University of Helsinki and Helsinki University Hospital, Helsinki, Finland; [2]Division of intensive care medicine, department of anaesthesiology, intensive care and pain medi, Helsinki University Hospital, Helsinki, Finland; [3]Department of neurosurgery, Helsinki University Hospital, Helsinki, Finland; [4]Department of anaesthesiology and intensive care, Kuopio University Hospital , Kuopio, Finland
**Correspondence:** J. Humaloja
*Intensive Care Medicine Experimental* 2019, **7(Suppl 3):**000888

**INTRODUCTION.** Circulatory shock results in impaired tissue oxygenation and brain ischemia.[1] Whether this can be alleviated with higher blood oxygen content is unknown. [2]

**OBJECTIVES.** We studied the interaction between the severity of circulatory shock and blood oxygen content in patients treated in the intensive care unit (ICU) with neurologic injury.

**METHODS.** The Finnish Intensive Care Consortium database was screened for mechanically ventilated neurologically ill adult patients treated in tertiary hospitals ICUs in years 2003–2013. Admission diagnoses included traumatic brain injury (TBI), cardiac arrest (CA), subarachnoid haemorrhage (SAH), intracerebral haemorrhage (ICH) and stroke. The primary endpoint was neurological outcome one year after intensive care admission. Independence in self-care was defined as favourable outcome and disability or death as unfavourable outcome. Patients were defined as disabled if they were granted a permanent disability allowance by the public social insurance institution at the time of data collection (September 2016). Patients already disabled before ICU admission were excluded from the study. The primary exposure of interest was arterial PaO2 recorded in connection with the lowest measured PaO2/FiO2 ratio during the first 24 hours of ICU admission. A compromised haemodynamic state was determined by a cardiovascular SOFA subscore of 4 during the first 24 h of ICU admission. The association between outcome and predictors were analysed by multiple logistic regression. The association of oxygen exposure and haemodynamic state with outcome was inspected with interaction term PaO2*SOFA subscore of 4 as a confounding factor. Additionally, age, gender and modified APACHE II score (excluding points for oxygenation) and type of admission were included in the model.

**RESULTS.** The study population included a total of 10,143 cases of whom 2,788 (28%) were treated for TBI, 3,390 (33%) for CA, and 3965 (39%) for stroke (SAH 1684 [17%], ICH 1587 [15%] and, ischemic 694 [7%]). Of all cases, 4,056 (40%) survived as independent. Poor haemodynamic state (SOFA cardiovascular subscore of 4) was not an independent predictor of favourable neurological outcome (odds ratio [OR] 1.00: 95% confidence interval [CI] 0.75–1.32). We found no association between PaO2 level and favourable neurological outcome, (OR 1.01: 95% CI 0.99–1.02) and the interaction PaO2*SOFA subscore 4 remained insignificant.

**CONCLUSION.** In mechanically ventilated patients treated after TBI, CA or stroke (SAH, ICH or ischemic) in the ICU there was no association between arterial oxygen level and circulatory shock with long-term neurological outcome.

**REFERENCE(S)**

[1] Lefer AM, Lefer DJ. Pharmacology of the Endothelium in Ischemia-Reperfusion and Circulatory Shock. Annu Rev Pharmacol Toxicol 1993;33:71–90. doi:10.1146/annurev.pa.33.040193.000443.

[2] Calzia E, Asfar P, Hauser B, Matejovic M, Ballestra C, Radermacher P, et al. Hyperoxia may be beneficial. Crit Care Med 2010;38:S559–68. doi:10.1097/CCM.0b013e3181f1fe70.

## SIS - Sepsis and antibiotic management

### 000997

#### Interleukin-36β activates autophagy of CD4+CD25+ regulatory T cell and inhibits its immunosuppressive activity in sepsis

Y. Ge[1], M. Huang[1], N. Dong[2], YM. Yao[2]

[1]Department of general intensive care unit, The Second Affiliated Hospital of Zhejiang University School of Medicine, Hangzhou, China; [2]Trauma research center, Fourth Medical Center of the Chinese PLA General Hospital, Beijing, China

**Correspondence:** Y. Ge

*Intensive Care Medicine Experimental* 2019, **7(Suppl 3):**000997

**INTRODUCTION.** Sepsis involves a dysregulated host response to infection, leading to a high mortality rate. As part of this dysregulation, CD4+CD25+ regulatory T cells (Tregs)play an essential role in sepsis-induced immunosuppression. Here, we studied the effects of interleukin (IL)-36 cytokines, which are newly described members of the IL-1 cytokine family, on CD4+CD25+ Tregs and their underlying mechanism in sepsis.

**METHODS.** Our study was designed to investigate the impacts of IL-36 cytokines on murine CD4+CD25+ Tregs in presence of lipopolysaccharide (LPS) and in a mouse model of sepsis induced by caecal ligation and puncture (CLP). IL-36-activated autophagy was evaluated by autophagy markers (LC3-II, Beclin1, p62), autophagosome formation and autophagic flux.

**RESULTS.** We provide evidence that IL-36α, IL-36β, and IL-36γ are expressed in murine CD4+CD25+ Tregs. Stimulation of CD4+CD25+ Tregs with LPS markedly upregulated the expression of these cytokines, particularly IL-36β. IL-36β strongly suppressed CD4+CD25+Tregs under LPS stimulation and in septic mice challenged with CLP, resulting in the amplification of Th1 responses and the proliferation of effector T cells. Mechanistic studies revealed that IL-36β triggered autophagy of CD4+CD25+ Tregs. These effects were significantly attenuated in the presence of the autophagy inhibitor 3-methyladenine (3-MA) or Beclin1 knockdown. Additionally, early IL-36βadministration reduced the mortality rate of CLP mice. Depletion of CD4+CD25+Tregs before the onset of sepsis obviously abrogated IL-36β-mediated protection against sepsis.

**CONCLUSION.** These findings suggest that IL-36β diminishes the immunosuppressive activity of CD4+CD25+ Tregs by activating the autophagic process, thereby contributing to improvement of the host immune response and prognosis in sepsis.

**REFERENCE(S)**

1. Shankar-Hari M, Phillips GS, Levy ML, Seymour CW, Liu VX, Deutschman CS, Angus DC, Rubenfeld GD, Singer M, Sepsis Definitions Task Force: Developing a new definition and assessing new clinical criteria for septic shock: for the third international consensus definitions for sepsis and septic shock (sepsis-3). JAMA 2016; 315: 775-787.
2. Angus DC, Van der Poll T: Severe sepsis and septic shock. N Engl J Med 2013; 369: 840-851.
3. Hotchkiss RS, Moldawer LL, Opal SM, Reinhart K, Turnbull IR, Vincent JL: Sepsis and septic shock. Nat Rev Dis Primers 2016; 2: 16045.
4. Savelkoel J, Claushuis TAM, van Engelen TSR, Scheres LJJ, Wiersinga WJ: Global impact of world sepsis day on digital awareness of sepsis: an evaluation using Google Trends. Crit Care 2018; 22: 61.
5. Hotchkiss RS, Monneret G, Payen D: Sepsis-induced immunosuppression: from cellular dysfunctions to immunotherapy. Nat Rev Immunol 2013; 13: 862-874.
6. Delano MJ, Ward PA: Sepsis-induced immune dysfunction: can immune therapies reduce mortality? J Clin Invest 2016; 126: 23-31.
7. Fontenot, JD, Gavin, MA, Rudensky, AY: Foxp3 programs the development and function of CD4+CD25+ regulatory T cells. Nat Immunol 2003; 4: 330-336.
8. McHugh RS, Whitters MJ, Piccirillo CA, Young DA, Shevach EM, Collins M, Byrne MC: CD4+CD25+ immunoregulatory T cells: gene expression analysis reveals a functional role for the glucocorticoid-induced TNF receptor. Immunity 2002; 16: 311-323.
9. Takahashi T, Tagami T, Yamazaki S, Uede T, Shimizu J, Sakaguchi N, Mak TW, Sakaguchi S: Immunologic self-tolerance maintained by CD25+CD4+ regulatory T cells constitutively expressing cytotoxic T lymphocyte-associated antigen 4. J Exp Med 2000; 192: 303-310.
10. Caramalho I, Lopes-Carvalho T, Ostler D, Zelenay S, Haury M, Demengeot J: Regulatory T cells selectively express toll-like receptors and are activated by lipopolysaccharide. J Exp Med 2003; 197: 403-411.
11. Chen ML, Pittet MJ, Gorelik L, Flavell RA, Weissleder R, von Boehmer H, Khazaie K: Regulatory T cells suppress tumor-specific CD8 T cell cytotoxicity through TGF-beta signals in vivo. Proc Natl Acad Sci U S A 2005; 102: 419-424.
12. Zhou F, Zhang GX, Rostami A: LPS-treated bone marrow-derived dendritic cells induce immune tolerance through modulating differentiation of CD4+ regulatory T cell subpopulations mediated by 3G11 and CD127. Immunol Res 2017; 65: 630–638.
13. Okeke EB, Okwor , Mou Z, Jia P, Uzonna JE: CD4+CD25+ regulatory T cells attenuate lipopolysaccharide-induced systemic inflammatory responses and promotes survival in murine Escherichia coli infection. Shock 2013; 40: 65-73.
14. Rubtsov YP, Rasmussen JP, Chi EY, Fontenot J, Castelli L, Ye X, Treuting P, Siewe L, Roers A, Henderson WR Jr, Muller W, Rudensky AY: Regulatory T cell-derived interleukin-10 limits inflammation at environmental interfaces. Immunity 2008; 28: 546-558.
15. Collison LW, Workman CJ, Kuo TT, Boyd K, Wang Y, Vignali KM, Cross R, Sehy D, Blumberg RS, Vignali DA: The inhibitory cytokine IL-35 contributes to regulatory T-cell function. Nature 2007; 450: 566-569.
16. Thornton AM, Shevach EM: CD4+CD25+ immunoregulatory T cells suppress polyclonal T cell activation in vitro by inhibiting interleukin 2 production. J Exp Med 1998; 188: 287-296.
17. Pandiyan P, Zheng L, Ishihara S, Reed J, Lenardo MJ: CD4+CD25+Foxp3+ regulatory T cells induce cytokine deprivation-mediated apoptosis of effector CD4+ T cells. Nat Immunol 2007; 8: 1353-1362.
18. Turnquist HR, Zhao Z, Rosborough BR, Liu Q, Castellaneta A, Isse K, Wang Z, Lang M, Stolz DB, Zheng XX, Demetris AJ, Liew FY, Wood KJ, Thomson AW: IL-33 expands suppressive CD11b+ Gr-1(int) and regulatory T cells, including ST2L+ Foxp3+ cells, and mediates regulatory T cell-dependent promotion of cardiac allograft survival. J Immunol 2011; 187: 4598-4610.
19. Ryba-Stanisławowska M, Buksa L, Brandt A, Juhas U, Mysliwiec M: IL-33 improves the suppressive potential of regulatory T cells in patients with type 1 diabetes. Diabetes Res Clin Pract 2017; 128: 67-73.
20. Tripodi D, Conti F, Rosati M, Maccauro G, Saggini A, Cianchetti E et al: IL-36 a new member of the IL-1 family cytokines. J Biol Regul Homeost Agents 2012; 26: 7-14.
21. Towne JE, Renshaw BR, Douangpanya J, Lipsky BP, Shen M, Gabel CA et al: Interleukin-36 (IL-36) ligands require processing for full agonist (IL-36α, IL-36β, and IL-36γ) or antagonist (IL-36Ra) activity. J Biol Chem 2011; 286: 42594-42602.
22. Carrier Y, Ma HL, Ramon HE, Napierata L, Small C, OToole M et al: Inter-regulation of Th17 cytokines and the IL-36 cytokines in vitro and in vivo: implications in psoriasis pathogenesis. J Invest Dermatol 2011; 131: 2428-2437.
23. Gresnigt MS, van de Veerdonk FL: Biology of IL-36 cytokines and their role in disease. Semin Immunol 2013; 25: 458-465.
24. Vigne S, Palmer G, Martin P, Lamacchia C, Strebel D, Rodriguez E et al: IL-36 signaling amplifies Th1 responses by enhancing proliferation and Th1 polarization of naive CD4+ T cells. Blood 2012; 120: 3478-3487.
25. Foster AM, Baliwag J, Chen CS, Guzman AM, Stoll SW, Gudjonsson JE et al: IL-36 promotes myeloid cell infiltration, activation, and inflammatory activity in skin. J Immunol 2014; 192: 6053-6061.
26. Lamacchia C, Palmer G, Rodriguez E, Martin P, Vigne S, Seemayer CA et al: The severity of experimental arthritis is independent of IL-36 receptor signaling. Arthritis Res Ther 2013; 15: R38.
27. Dietrich D, Gabay C: Inflammation: IL-36 has proinflammatory effects in skin but not in joints. Nat Rev Rheumatol 2014; 10: 639-640.
28. Tao X, Song Z, Wang C, Luo H, Luo Q, Lin X et al: Interleukin 36α attenuates sepsis by enhancing antibacterial functions of macrophages. J Infect Dis 2017; 215: 321-332.
29. Jia W, He YW: Temporal regulation of intracellular organelle homeostasis in T lymphocytes by autophagy. J Immunol 2011; 186: 5313–5322.
30. Lu Y, Gao J, Zhang S, Gu J, Lu H, Xia Y et al: miR-142-3p regulates autophagy by targeting ATG16L1 in thymic-derived regulatory T cell (tTreg). Cell Death Dis 2018; 9: 290.
31. Alissafi T, Banos A, Boon L, Sparwasser T, Ghigo A, Wing K et al: Tregs restrain dendritic cell autophagy to ameliorate autoimmunity. J Clin Invest 2017; 127: 2789-2804.
32. Kabat AM, Harrison OJ, Riffelmacher T, Moghaddam AE, Pearson CF, Laing A et al: The autophagy gene Atg16l1 differentially regulates Treg and TH2 cells to control intestinal inflammation. Elife 2016; 5: e12444.
33. Ge Y, Huang M, Yao YM: Autophagy and proinflammatory cytokines: interactions and clinical implications. Cytokine Growth F R 2018; 43: 38-46.
34. Cohen J, Vincent JL, Adhikari NK, Machado FR, Angus DC, Calandra T et al: Sepsis: a roadmap for future research. Lancet Infect Dis 2015; 15: 581-614.
35. Venet F, Chung CS, Monneret G, Huang X, Horner B, Garber M et al: Regulatory T cell populations in sepsis and trauma. J Leukoc Biol 2008; 83: 523-535.
36. Jensen IJ, Sjaastad FV, Griffith TS, Badovinac VP: Sepsis-induced T cell immunoparalysis: the ins and outs of impaired T cell immunity. J Immunol 2018; 200: 1543-1553.
37. Lewis AJ, Billiar TR, Rosengart MR: Biology and metabolism of sepsis: innate immunity, bioenergetics, and autophagy. Surg Infect 2016; 17: 286–293.
38. Zhang L, Ai YH, Tsung A: Clinical application: restoration of immune homeostasis by autophagy as a potential therapeutic target in sepsis (Review). Exp Ther Med 2016; 12: 1159-1167.
39. Henry CM, Sullivan GP, Clancy DM, Afonina IS, Kulms D, Martin SJ: Neutrophil-derived proteases escalate inflammation through activation of IL-36 family cytokines. Cell Rep 2016; 14: 708-722.
40. Macleod T, Doble R, McGonagle D, Wasson CW, Alase A, Stacey M et al: (2016). Neutrophil elastase-mediated proteolysis activates the anti-inflammatory cytokine IL-36 receptor antagonist. Sci Rep 2016; 6: 24880.
41. Medina-Contreras O, Harusato A, Nishio H, Flannigan KL, Ngo V, Leoni G et al: Cutting edge: IL-36 receptor promotes resolution of intestinal damage. J Immunol 2016; 196: 34-38.
42. He Q, Chen HX, Li W, Wu Y, Chen SJ, Yue Q et al: IL-36 cytokine expression and its relationship with p38 MAPK and NF-κB pathways in psoriasis vulgaris skin lesions. J Huazhong Univ Sci Technolog Med Sci 2013; 33: 594-599.
43. Penha R, Higgins J, Mutamba S, Barrow P, Machida Y, Foster N: IL-36 receptor is expressed by human blood and intestinal T lymphocytes and is dose-dependently activated via IL-36β and induces CD4+ lymphocyte proliferation. Cytokine 2016; 85: 18-25.
44. Ahsan F, Maertzdorf J, Guhlich-Bornhof U, Kaufmann SHE, Moura-Alves P: IL-36/LXR axis modulates cholesterol metabolism and immune defense to mycobacterium tuberculosis. Sci Rep 2018; 8: 1520.
45. Zhang J, Yin Y, Lin X, Yan X, Xia Y, Zhang L et al: IL-36 induces cytokine IL-6 and chemokine CXCL8 expression in human lung tissue cells: implications for pulmonary inflammatory responses. Cytokine 2017; 99: 114-123.
46. Sridhar S, Botbol Y, Macian F, Cuervo AM: Autophagy and disease: always two sides to a problem. J pathol 2012; 226: 255-273.
47. Choi AM, Ryter SW, Levine B: Autophagy in human health and disease. N Engl J Med 2013; 368: 651–662.
48. Chen YQ, Klionsky DJ: The regulation of autophagy–unanswered questions. J Cell Sci 2011; 124: 161-170.
49. Watanabe E, Muenzer JT, Hawkins WG, Davis CG, Dixon DJ, McDunn JE et al: Sepsis induces extensive autophagic vacuolization in hepatocytes: a clinical and laboratory-based study. Lab Invest 2009; 9: 549-561.
50. Ho J, Yu J, Wong SH, Zhang L, Liu X, Wong WT et al: Autophagy in sepsis: degradation into exhaustion? Autophagy 2016; 12: 1073-1082.
51. Patil NK, Bohannon JK, Sherwood ER: Immunotherapy: a promising approach to reverse sepsis-induced immunosuppression. Pharmacol Res 2016; 111: 688-702.
52. Yadav H, Cartin-Ceba R: Balance between hyperinflammation and immunosuppression in sepsis. Semin Respir Crit Care Med 2016; 37: 42.
53. Fattahi F, Ward PA: Understanding immunosuppression after sepsis. Immunity 2017; 47: 3.
54. Venet F, Rimmelé T, Monneret G: Management of sepsis-induced immunosuppression. Crit Care Clin 2018; 34: 97.
55. Ohsumi Y: Historical landmarks of autophagy research. Cell Res 2014; 24: 9-23.
56. This work was supported by grants from the National Natural Science Foundation (81873946, 81730057) and the National Key Research and Development Program of China (2017YFC1103302).

### 000998

**Interleukin-38 protects against sepsis by augmenting immunosuppressive activity of CD4+CD25+ regulatory T cells**

Y. Ge[1], M. Huang[1], N. Dong[2], YM. Yao[2]
[1]Department of general intensive care unit, The Second Affiliated Hospital of Zhejiang University School of Medicine, Hangzhou, China; [2]Trauma research center, Fourth Medical Center of the Chinese PLA General Hospital, Beijing, China
**Correspondence:** Y. Ge
*Intensive Care Medicine Experimental* 2019, **7(Suppl 3):**000998

**INTRODUCTION.** Naturally occurring CD4+CD25+regulatory T cells (Tregs)are required to limit immune-induced pathology and to maintain homeostasis during the early phase of sepsis. This study aimed to investigate the role of interleukin (IL)-38, a newly described member of the IL-1 cytokine family, in mediated immune response of CD4+CD25+Tregs in sepsis.

**METHODS.** Primary CD4+CD25+Tregs isolated from BALB/c mice were incubated with lipopolysaccharide (LPS), recombinant murine IL-38 (rmIL-38), and anti-IL-38 antibody. A mouse septic model induced by cecal ligation and puncture (CLP) was treated with rmIL-38

or anti-IL-38 antibody. The effects of IL-38 on activity of CD4+CD25+Tregs and survival rate of mice were assessed.

**RESULTS.** Expressions of IL-38 and its receptor were detected in murine CD4+CD25+Tregs. Stimulation of CD4+CD25+Tregs with LPS markedly up-regulated the expression of IL-38. Treatment with rmIL-38 dramatically enhanced the immunosuppressive activity of CD4+CD25+Tregs after LPS stimulation as well as in septic mice induced by CLP, resulting in amplification of helper T cell (Th) 2 response and reduction in the proliferation of effector T cells. These effects were robustly abrogated when anti-IL-38 antibody was administered.Administrationof rmIL-38 improved the survival rate of CLP mice. In addition, CD4+CD25+Tregs depletion before the onset of sepsis obviously abolished IL-38-mediated protective response.

**CONCLUSION.** IL-38 enhances the immunosuppressive activity of CD4+CD25+Tregs, which might contribute to the improvement of host immune function and prognosis in the setting of sepsis.

**REFERENCE(S)**

1. Spence A, Klementowicz JE, Bluestone, JA, Tang Q. Targeting Treg signaling for the treatment of autoimmune diseases. Curr Opin Immunol 2015; 37: 11-20.
2. Schreiber TH, Wolf D, Tsai MS, Chirinos J, Deyev VV, Gonzalez L, Malek TR, Levy RB, Podack ER. Therapeutic Treg expansion in mice by TNFRSF25 prevents allergic lung inflammation. J Clin Invest 2010; 120: 3629-3640.
3. Hanidziar D, Koulmanda M. Inflammation and the balance of Treg and Th17 cells in transplant rejection and tolerance. Curr Opin Organ Transplant 2010; 15: 411-415.
4. Cohen JL, Trenado A, Vasey D, Klatzmann D, Salomon BL. CD4+CD25+ immunoregulatory T cells: new therapeutics for graft-versus-host disease. J Exp Med 2002; 196: 401-406.
5. Fontenot JD, Gavin MA, Rudensky AY. Foxp3 programs the development and function of CD4+CD25+ regulatory T cells. Nat Immunol 2003; 4: 330-336.
6. McHugh RS, Whitters MJ, Piccirillo CA, Young DA, Shevach EM, Collins M, Byrne MC. CD4+CD25+ immunoregulatory T cells: gene expression analysis reveals a functional role for the glucocorticoid-induced TNF receptor. Immunity 2002; 16: 311-323.
7. Takahashi T, Tagami T, Yamazaki S, Uede T, Shimizu J, Sakaguchi N, Mak TW, Sakaguchi S. Immunologic self-tolerance maintained by CD25+CD4+ regulatory T cells constitutively expressing cytotoxic T lymphocyte-associated antigen 4. J Exp Med 2000; 192: 303-310.
8. Zhou F, Zhang GX, Rostami A. LPS-treated bone marrow-derived dendritic cells induce immune tolerance through modulating differentiation of CD4+ regulatory T cell subpopulations mediated by 3G11 and CD127. Immunol Res 2017; 65: 630-638.
9. Chen ML, Pittet MJ, Gorelik L, Flavell RA, Weissleder R, von Boehmer H, Khazaie K. Regulatory T cells suppress tumor-specific CD8 T cell cytotoxicity through TGF-beta signals in vivo. Proc Natl Acad Sci USA 2005; 102: 419-424.
10. Rubtsov YP, Rasmussen JP, Chi EY, Fontenot J, Castelli L, Ye X, Treuting P, Siewe L, Roers A, Henderson WR Jr, Muller W, Rudensky AY. Regulatory T cell-derived interleukin-10 limits inflammation at environmental interfaces. Immunity 2008; 28: 546-558.
11. Collison LW, Workman CJ, Kuo TT, Boyd K, Wang Y, Vignali KM, Cross R, Sehy D, Blumberg RS, Vignali DA. The inhibitory cytokine IL-35 contributes to regulatory T-cell function. Nature 2007; 450: 566-569.
12. Guillonneau C, Bézie S, Anegon I. Immunoregulatory properties of the cytokine IL-34. Cell Mol Life Sci 2017; 74: 2569-2586.
13. Thornton AM, Shevach EM. CD4+CD25+ immunoregulatory T cells suppress polyclonal T cell activation in vitro by inhibiting interleukin 2 production. J Exp Med 1998; 188: 287-296.
14. Pandiyan P, Zheng L, Ishihara S, Reed J, Lenardo MJ. CD4+CD25+Foxp3+ regulatory T cells induce cytokine deprivation-mediated apoptosis of effector CD4+ T cells. Nat Immunol 2007; 8: 1353-1362.
15. Okeke EB, Okwor I, Mou Z, Jia P, Uzonna JE. CD4+CD25+ regulatory T cells attenuate lipopolysaccharide-induced systemic inflammatory responses and promotes survival in murine Escherichia coli infection. Shock 2013; 40: 65-73.
16. Caramalho I, Lopes-Carvalho T, Ostler D, Zelenay S, Haury M, Demengeot J. Regulatory T cells selectively express Toll-like receptors and are activated by lipopolysaccharide. J Exp Med 2003; 197: 403-411.
17. Prescott HC, Angus DC. Enhancing recovery from sepsis: a review. JAMA 2018; 319: 62-75.
18. Cecconi M, Evans L, Levy M, Rhodes A. Sepsis and septic shock. Lancet 2018; 392: 75-87.
19. Cabrita J, Pinheiro I, MenezesFalcão L. Rethinking the concept of sepsis and septic shock. Eur J Intern Med 2018; 54:1-5.
20. Savelkoel J, Claushuis TAM, van Engelen TSR, Scheres LJJ, Wiersinga WJ. Global impact of world sepsis day on digital awareness of sepsis: an evaluation using Google Trends. Crit Care 2018; 22: 61.
21. Hotchkiss RS, Monneret G, Payen D. Sepsis-induced immunosuppression: from cellular dysfunctions to immunotherapy. Nat Rev Immunol 2013; 13: 862-874.
22. Delano MJ, Ward PA. Sepsis-induced immune dysfunction: can immune therapies reduce mortality? J Clin Invest 2016; 126: 23-31.
23. Cohen J, Vincent JL, Adhikari NK, Machado FR, Angus DC, Calandra T, Jaton K, Giulieri S, Delaloye J, Opal S, Tracey K, van der Poll T, Pelfrene E. Sepsis: a roadmap for future research. Lancet Infect Dis 2015; 15: 581-614.
24. Jensen IJ, Sjaastad FV, Griffith TS, Badovinac VP. Sepsis-induced T cell immunoparalysis: the ins and outs of impaired T cell immunity. J Immunol 2018; 200: 1543-1553.
25. Venet F, Chung CS, Monneret G, Huang X, Horner B, Garber M, Ayala A. Regulatory T cell populations in sepsis and trauma. J Leukoc Biol 2008; 83: 523-535.
26. Patil NK, Bohannon JK, Sherwood ER. Immunotherapy: a promising approach to reverse sepsis-induced immunosuppression. Pharmacol Res 2016; 111: 688-702.
27. Yadav H, Cartin-Ceba R. Balance between hyperinflammation and immunosuppression in sepsis. Semin Resp Crit Care 2016; 37: 42-50.
28. Fattahi F, Ward PA. Understanding immunosuppression after sepsis. Immunity 2017; 47: 3-5.
29. Venet F, Rimmelé T, Monneret G. Management of sepsis-induced immunosuppression. Crit Care Clin 2018; 34: 97-106.
30. Ge Y, Huang M, Yao YM. Recent advances in the biology of IL-1 family cytokines and their potential roles in development of sepsis. Cytokine Growth F R, 2019, 45: 24-34.
31. van de Veerdonk FL, de Graaf DM, Joosten LA, Dinarello CA. Biology of IL-38 and its role in disease. Immunol Rev 2018; 281: 191-196.
32. Garraud T, Harel M, Boutet MA, Le Goff B, Blanchard F. The enigmatic role of IL-38 in inflammatory diseases. Cytokine Growth F R 2018; 39: 26-35.
33. van de Veerdonk FL, Stoeckman AK, Wu G, Boeckermann AN, Azam T, Netea MG, Joosten LA, van der Meer JW, Hao R, Kalabokis V, Dinarello CA. IL-38 binds to the IL-36 receptor and has biological effects on immune cells similar to IL-36 receptor antagonist. Proc Natl Acad Sci USA 2012; 109: 3001-3005.
34. Rudloff I, Godsell J, Nold-Petry CA, Harris J, Hoi A, Morand EF, Nold MF. Brief report interleukin-38 exerts ant-inflammatory functions and is associated with disease activity in systemic lupus erythematosus. Arthritis Rheumatol 2015; 67: 3219-3225.
35. Mora J, Schlemmer A, Wittig I, Richter F, Putyrski M, Frank AC, Han Y, Jung M, Ernst A, Weigert A, Brüne B. Interleukin-38 is released from apoptotic cells to limit inflammatory macrophage responses, J Mol Cell Biol 2016; 8: 426-438.
36. Boutet MA, Najm A, Bart G, Brion R, Touchais S, Trichet V, Layrolle P, Gabay C, Palmer G, Blanchard F, Le Goff B. IL-38 overexpression induces anti-inflammatory effects in mice arthritis models and in human macrophages in vitro. Ann Rheum Dis 2017; 76: 1304-1312.
37. Yuan X, Li Y, Pan X, Peng X, Song G, Jiang W, Gao Q, Li M. IL-38 alleviates concanavalin A-induced liver injury in mice. Int Immunopharmacol 2016; 40: 452-457.
38. Chu M, Chu IM, Yung EC, Lam CW, Leung TF, Wong GW, Wong CK. Aberrant expression of novel cytokine IL-38 and regulatory T lymphocytes in childhood asthma. Molecules 2016; 21: 933-947.

39. Chu M, Tam LS, Zhu J, Jiao D, Liu H, Cai Z, Dong J, Kai Lam CW, Wong CK. In vivo anti-inflammatory activities of novel cytokine IL-38 in Murphy Roths Large (MRL)/lpr mice. Immunobiology 2017; 222: 483-493.
40. Yuan XL, Li Y, Pan XH, Zhou M, Gao QY, Li MC. Production of recombinant human interleukin-38 and its inhibitory effect on the expression of proinflammatory cytokines in THP-1 cells. Mol Biol (Mosk) 2016; 50: 466-473.
41. Jiang K, Guo Y, Xiao L, Pan W, Ma J. Dendritic cells should not be overlooked when studying the effect of IL-38 administration in arthritis. Ann Rheum Dis 2018; 77: e12.
42. Takenaka SI, Kaieda S, Kawayama T, Matsuoka M, Kaku Y, Kinoshita T, Sakazaki Y, Okamoto M, Tominaga M, Kanesaki K, Chiba A, Miyake S, Ida H, Hoshino T. IL-38: a new factor in rheumatoid arthritis. Biochem Biophys Rep 2015; 4: 386-391.
43. Xu F, Lin S, Yan X, Wang C, Tu H, Yin Y, Cao J. Interleukin 38 protects against lethal sepsis. J Infect Dis 2018; 218: 1175-1184.
44. This work was supported by grants from the National Natural Science Foundation of China (81873946, 81730057, 81842025), the Key Project of Military Medical Innovation Program of Chinese PLA (No. 18CXZ026), and the National Key Research and Development Program of China (2017YFC1103302).

### 000999

#### Rapid Response System Improves Sepsis Bundle Compliances & Survival in Patients with Septic Shock for 10 Years

S. Choi[1], J. Son[1], J. Lee[1], Y. Shin[1], J. Lee[1], Y. Jung[1], E. Choi[1], DH. Kim[1], UJ. Go[1], HY. Oh[1], JW. Huh[2], SB. Hong[2]
[1]Medical alert team, Asan Medical Center, Seoul, Republic of Korea; [2]Department of pulmonary and critical care medicine, Asan Medical Center, Seoul, Republic of Korea
**Correspondence:** S. Choi
*Intensive Care Medicine Experimental* 2019, **7(Suppl 3):**000999

**INTRODUCTION.** Sepsis should be treated as medical emergencies and early management for sepsis is reported to be associated with improving patient outcomes. Patients on the ward can develop to septic shock at any time during their hospitalization. Therefore, it is very important to identify septic patient on the ward and to perform optimal treatment quickly. Rapid response systems (RRS) are providing optimal care for sepsis on the general ward. Although studies are often performed for early detection of sepsis on ward, there is a little published data on management factors of sepsis on ward.
**OBJECTIVES.** To study between the outcomes and bundle compliance of patient with septic shock managed by rapid response system for 10 years
**METHODS.** Retrospective cohort study of 935 patients with septic shock who were activated by RRS at an academic, tertiary care hospital in Korea from 2008 to 2017
**RESULTS.** We provided sepsis management by RRS during study period. Of the 935 enrolled patients, the compliances of each sepsis bundle were high (87.8%-99.5%) but the overall success rate of bundle was shown low (73.4%). Compliance rate for achieving overall sepsis bundle was increased and the 28 day mortality was continuously decreased for 10 years in figure 1. Of the 935 enrolled patients, 686 (73%) completed all sepsis bundle and 248(27%) were incomplete. We analyzed the two groups according to whether they completed sepsis bundle overall or not. Baseline characteristics were no difference between two groups. Bundle complete group showed lower 28-day mortality than incomplete bundle group (43.5% vs. 52.6%; *p*=0.001) and in the multivariate logistic regression model, 28 day mortality was significantly related to complete of all bundle (adjusted odds ratio, 0.50; 95% CI, 0.32-0.78; *p*=0.003).
**CONCLUSION.** Rapid response system provides improving sepsis bundle compliances & survival in patients with septic shock on the general ward.

**REFERENCE(S)**

1. This research was supported by a grant of the Korea Health Technology R&D Project through the Korea Health Industry Development Institute (KHIDI), funded by the Ministry of Health & Welfare, Republic of Korea (HI18C0599).
2. Levy, M. M., Rhodes, A., Phillips, G. S., Townsend, S. R., Schorr, C. A., Beale, R., ... & Dellinger, R. P. (2014). Surviving Sepsis Campaign: association between performance metrics and outcomes in a 7.5-year study. Intensive care medicine, 40(11), 1623-1633.
3. Scheer, C. S., Fuchs, C., Kuhn, S. O., Vollmer, M., Rehberg, S., Friesecke, S., ... & Hahnenkamp, K. (2017). Quality improvement initiative for severe sepsis and septic shock reduces 90-day mortality: a 7.5-year observational study. Critical care medicine, 45(2), 241-252.

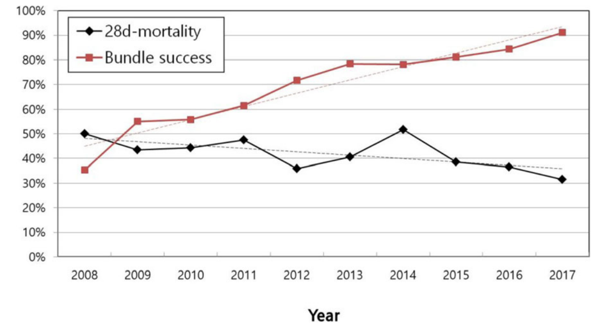

**Fig. 1 (abstract 000999).** See text for description

### 001001

#### Benefit of Meropenem Therapeutic Drug Monitoring in Critically Ill Patients with Sepsis

B. Meyer[1], U. Tröger[2], S. Lohmeier[1], S. Bode-Böger[2], M. Hansen[1], A. Schmeißer[1], R. Braun-Dullaeus[1], I. Tanev[1]
[1]Department of cardiology, University Hospital Magdeburg, Magdeburg, Germany; [2]Institute of clinical pharmacology, University Hospital Magdeburg, Magdeburg, Germany
**Correspondence:** B. Meyer
*Intensive Care Medicine Experimental* 2019, **7(Suppl 3):**001001

**INTRODUCTION.** Meropenem is a cornerstone in the antibiotic treatment of critically ill patients with sepsis. However, complex pathophysiologic mechanism in sepsis lead to a wide variability of plasma levels of any given drug. Subtherapeutic dosing can lead to treatment failure and antimicrobial resistance while overdosing is associated with toxic side effects. Individualized treatment guided by therapeutic drug monitoring (TDM) might be an effective means to overcome this dilemma.
**OBJECTIVES.** The primary aim of this study was to assess the effect of therapeutic drug monitoring on the use of meropenem in critically ill patients. The secondary aim was to assess the impact of meropenem TDM on Intensive Care Unit (ICU) length of stay and survival.
**METHODS.** A total of 247 septic patients treated with meropenem for at least 5 days were included. Baseline characteristics, total doses of meropenem and duration of meropenem treatment were recorded. TDM was done by an attending clinical pharmacologist. Plasma levels of meropenem were measured by use of HPLC. Dosing recommendations were based on microbiologic data were available. In all patients ICU length of stay and ICU mortality were recorded. Statistical analyses were performed by SPSS.
**RESULTS.** A total of 101 patients received standard doses of meropenem, 146 patients had an individualized treatment by means of TDM. In patients with TDM, meropenem doses were higher (2731.2 ± 1092.1 mg vs 2327.1 ± 652.7 mg, p < 0.001) and treatment duration was longer (14.0 ± 9.7 days vs. 10.5 ± 4.7 days, p < 0.001) than in patients with standard therapy, respectively. An average of 3.2 meropenem plasma level measurements was done in the TDM group. Dose modifications were 1.6 in the TDM group vs. 0.8 in the standard group (p<0.001). ICU length of stay was similar between the two groups (14.7 ± 10.7 vs. 14.6 ± 9.8 days for TDM vs. standard group, respectively). However, ICU mortality was lower in the TDM group compared to the standard group (20 patients (14%) vs. 24 patients (24%), p=0.042).

**CONCLUSION.** Bearing in mind the high variability of meropenem plasma levels in critically ill patients and patients with sepsis, risk of underdosing is evident. The same is true for overdosing and unnecessary antibiotic therapy. TDM is a potent option to maximize treatment effects and to avoid resistance and toxic side effects.

In our cohort of patients with sepsis, TDM led to higher meropenem doses and longer treatment duration. Importantly, in an exploratory analysis, a lower mortality was seen in our cohort of critically ill patients within the TDM treated group when compared to a standard non-TDM group.

### 001002

#### Acute lung injury could be alleviated by conditional medium from induced pluripotent stem cellsin sepsis rodent model

YT. Chang[1], PL. Chi[2], WC. Huang[3], SR. Wann[4], MC. Shen[5], CP. Liu[5]
[1]Department of emergency, Kaohsiung Veterans General Hospital, Kaohsiung, Gushan District, Kaohsiung City, Taiwan, Taiwan; [2]Department of medical education and research, Kaohsiung Veterans General Hospital, Kaohsiung, Taiwan; [3]Critical care medicine and cardiovascular center, Kaohsiung Veterans General Hospital, Taipei, Taiwan; [4]Kaohsiung veterans general hospital,pingtung branch,pintung, Kaohsiung Veterans General Hospital,Pingtung Branch,Pintung, Kaohsiung, Gushan District, Kaohsiung City, Taiwan, Taiwan; [5]Critical care medicine and cardiovascular center, Kaohsiung Veterans General Hospital, Kaohsiung, Taiwan
**Correspondence:** W.C. Huang
*Intensive Care Medicine Experimental* 2019, **7(Suppl 3):**001002

**INTRODUCTION.** It is a challenging issue to reduce mortality of sepsisin intensive care units. After decades of research, and numerous preclinical and clinical trials, sepsis remains without a specific and effective pharmacotherapy.In the last few years, cell therapies gained potential as a therapeutic treatment for various diseases. However, there are still many problems that limit the therapeutic use of stem cells, including immunological rejection phenomena and unwanted cells differentiation.In recent years, researchers have found that therapeutic activity of *induced pluripotent stem cells* may be mediated by a paracrine effect.

**OBJECTIVES.** In this study, we investigated the potential protective effects of iPSCs-conditioned media (iPSCs-CM) on E.coli-induced lung injury in rats and LPS/IFN-g-induced inflammatory responses in murine macrophage RAW 264.7 cells, and identified the mechanisms underlying these effects.

**METHODS.** Rats underwent intraperitoneal inoculation with 109 CFU of Escherichia coli isolated from patient. Animals were randomized to receive (i)co-administration of conditioned medium from iPSCsin combination withceftriaxone (ii) IV administration of conditioned medium from iPSCs following 4 hr of infectionin combination with ceftriaxone; (iii) IV administration of ceftriaxone (30mg/kg)following 8 hr of infection; and (iv) IV instillation of PBS vehicle. Raw 264.7 cells were pretreated with iPSCs-CM for 2 hr prior to LPS/INF-g stimulation or were treated with iPSCs-CM after 1 hr of LPS/TNF-g treatment.Lethality; lung injury scores, including protein leakage and inflammatory cell infiltration; cell count in bronchoalveolar lavage (BAL) fluid;and chemokines and cytokines concentrations in serum were determined to evaluate the effects of iPSCs-conditioned media on sepsis-induced lung injury.

**RESULTS.** Administration of iPSCs-CM in combination with ceftriaxone (iPSCs-CM/ceftriaxone) has a higher survival rate than ceftriaxone treatment alone.iPSCs-CM/ceftriaxone treatment also significantly decreased the cell counts in BAL fluid in comparison to ceftriaxone treatment alone. Histological examination of lung tissue showed that the iPSCs-CM/ceftriaxone group was less than the ceftriaxone group in macrophage infiltration and MMP-9 expression.Serum levels of CXCL2, IL-1b, and IL-6 also showed decreased in E.coli-induced lung injury rat model, as well as in cells treated with LPS/INF-g.Further, expression of NLRP3, ASC, HMGB1, IL1b, IL-6, and CXCL2 mRNA was significantly decreased in iPSCs-CM/ceftriaxone group compared with no treatment. Similar effects of iPSCs-CM on LPS/INF-g-stimulated raw 264.7 cells.

**CONCLUSION.** Our results revealed that treatment of iPSCs-CM ameliorates lung inflammatory responses through inhibiting NLRP3 inflammasome activation, which attenuates lung injury and improves the survival rate.These data suggested that iPSCs-CM may be important in sepsis and shed light on therapeutic strategies.

**REFERENCE(S)**

1. Ratajczak MZ et al. (2012). Pivotal role of paracrine effects in stem cell therapies in regenerative medicine: can we translate stem cell-secreted paracrine factors and microvesicles into better therapeutic strategies? Leukemia26, 1166-1173.
2. Supported by research grant from Kaohsiung Veterans General Hospital.

### 001004

#### High variability of voriconazole plasma levels in critically ill patients with sepsis – benefit of therapeutic drug monitoring

B. Meyer[1], U. Tröger[2], J. Tilsen[1], S. Bode-Böger[2], M. Hansen[1], A. Schmeißer[1], R. Braun-Dullaeus[1], I. Tanev[1]
[1]Department of cardiology, University Hospital Magdeburg, Magdeburg, Germany; [2]Institute of clinical pharmacology, University Hospital Magdeburg, Magdeburg, Germany
**Correspondence:** B. Meyer
*Intensive Care Medicine Experimental* 2019, **7(Suppl 3):**001004

**INTRODUCTION.** Voriconazole is an azole antifungal agent frequently used in the treatment of severely ill patients with suspected or proven fungal infection. While voriconazole has excellent in vitro susceptibility testing, it´s clinical use is limited by high interindividual variability, particularly in critically ill and septic patients.

**OBJECTIVES.** The primary aim of the study was to assess voriconazole plasma levels in critically ill patients with sepsis. The secondary aim was to assess whether therapeutic drug monitoring leads to dose modifications in these patients.

**METHODS.** A total of 92 patients with sepsis and voriconazole treatment were included. All patients received an initial dose of 400 mg voriconazole twice daily. Therapeutic drug monitoring was performed and voriconazole plasma levels were assessed at least once in all patients. Additional determination of plasma levels were ordered if deemed clinically necessary by the treating physicians. Measured patients´ voriconazole plasma levels were classified as within target range (0.5 to 5.0 mg/l depending on the underlying disease), below target range (< 0.5 to 1.0 mg/l) and above target range (>2.5 to 5.0 mg/l).

**RESULTS.** A total of 45 patients (49%) had voriconzole plasma levels within the predefined therapeutic range. Seven patients (8%) had voriconzole plasma levels below target range, 40 patients (43%) had voriconazole plasma levels above the therapeutic range. Thus dose modifications became necessary in more than half of the patients treated with voriconazole as a result of therapeutic drug monitoring.

**CONCLUSION.** Our results show high variability of voriconazole plasma levels in critically ill patients with suspected or proven fungal infection. Standard dosing of voriconazole led to plasma levels within the predefined therapeutic range in only less than half of the patients. Therapeutic drug monitoring is therefore an appropriate tool to avoid treatment failure by underdosing and at the same time toxic overdosing in patients with sepsis.

### 001012

#### Hyperlactatemia in infected patients at admission in intensive care units

C. Mendes Silva[1], JP. Baptista[2], P. Mergulhão[3], F. Froes[4], J. Gonçalves-Pereira[5], JM. Pereira[3], C. Dias[6], JA. Paiva[3]
[1]Intensive Care Medicine Service, Hospital and Universitary Centre of Coimbra, Coimbra, France; [2]Intensive care medicine service, Hospitalar and Universitary Center of Coimbra, Coimbra, Portugal, Portugal; [3]Emergency and intensive care department, Centro Hospitalar de São João, Porto, Portugal; [4]Intensive care unit, Hospital Pulido Valente, Centro Hospitalar Lisboa Norte, Lisboa, Portugal; [5]Intensive care unit, Hospital de Vila Franca de Xira, Vila Franca de Xira, Portugal; [6]Department of community medical information and health decision sciences, Faculty of Medicine, University of Porto, Porto, Portugal
**Correspondence:** C. Mendes Silva
*Intensive Care Medicine Experimental* 2019, **7(Suppl 3):**001012

**INTRODUCTION.** Serum lactate has been incorporated in the new definition of septic shock as a way to identify hypotensive patients at higher risk of death (1). Hyperlactatemia at the time of admission has been shown to be a good prognostic marker (2). The usual cut-off value for hyperlactatemia is 2 mmol/L. Haas et al. (3) found a mortality approaching 80% for patients with lactate levels >10 mmol/L.
**OBJECTIVES.** To evaluate the influence of patient and clinical characteristics on the severity of hyperlactatemia in infected patients at ICU admission and define differences in outcomes.
**METHODS.** Post-hoc analysis on hyperlactatemia in the INFAUCI study, a prospective, observational, cohort, multicenter study, conducted in 14 Portuguese Intensive Care Units (ICU) with data collected between 1 May 2009 and 31 December 2010. Infected patients at admission were divided in three groups, accordingly to the severity of hyperlactatemia at admission, mild (2-3.9 mmol/L), moderate (4.0-9-9 mmol/L) and severe (>10 mmol/L).
**RESULTS.** Of the 3766 patients admitted to the study, 1652 (43.9%) were deemed to be infected on admission. Lactate was measured in 1640 patients, with 934 (57%) having hyperlactatemia, distributed as mild, moderate and severe in 57.0%, 34.4% and 8.7% of the cases. The degree of hyperlactatemia increased significantly with SAPS II, APACHE II score, Charlson Score, chronic liver disease as a comorbidity, presence of septic shock and secondary bacteremia. On the other hand, the degree of hyperlactatemia was significantly inversely associated with chronic respiratory disease as a comorbidity and appropriate antibiotic selection. ICU and hospital length of stay decreased with increased hyperlactatemia due to higher and earlier mortality. ICU mortality rate was 26%, 47% and 68% for mild, moderate and severe hyperlactatemia, which increased to 36%, 55% and 79% for in-hospital mortality, respectively. ICU and in-hospital mortality adjusted for age, SAPS II, APACHE II and Charlson score was correlated with moderate and severe hyperlactatemia but not with mild hyperlactatemia with an OR 2.0 [95% CI 1.4-2.8; p<0.001] and 5.4 [95% CI 2.9-9.9; p<0.001] for ICU mortality and 1.8 [95% CI 1.3-2.5; p<0.001] and 3.8 [95% CI 2.0-7-2; p<0.001] in moderate and severe hyperlactatemia, respectively.
**CONCLUSION.** Hyperlactatemia is a strong predictor of mortality in ICU infected patients. Lactate level correlates independently with ICU and in-hospital mortality for moderate and severe degrees of hyperlactatemia.

**REFERENCE(S)**

(1) Singer M et al. The Third International Consensus Definitions for Sepsis and Septic Shock (Sepsis-3). JAMA. 2016;315(8):801-10;
(2) Nichol AD et al. Relative hyperlactatemia and hospital mortality in critically ill patients: a retrospective multi-centre study. Crit Care. 2010;14(1):R25.
(3) Haas SA et al. Severe hyperlactatemia, lactate clearance and mortality in unselected critically ill patients. Intensive Care Med. 2016;42(2):202-10

### 001020

#### Using longitudinal wall fractional shortening to predict strain in patients with sepsis in the intensive care unit

P. Blixt[1], R. ÅHman[2], AM. ÅStröm[3], J. Engvall[3], M. Chew[2]
[1]Linköping University, Linköping, Sweden; [2]Anopiva, Linköping University, Linköping, Sweden; [3]Klinisk fysiologi, Linköping University, Linköping, Sweden
**Correspondence:** P. Blixt
*Intensive Care Medicine Experimental* 2019, **7(Suppl 3):**001020

**INTRODUCTION.** Left ventricular longitudinal strain (LVLS) may be a better method for detecting systolic dysfunction in critically ill patients. However, LVLS is limited by poor feasibility, dependency on high image quality and availability of analysis in scanners. Recently Huang et al. (1) described a method for estimating LVLS using M-mode derived longitudinal wall fractional shortening (LWFS), where MAPSE is normalized to the ventricle length. LWFS is less dependent on image quality, is more feasible in critically ill patients and may be done at bedside. However, this method requires validation, especially within the setting of septic shock.
**OBJECTIVES.** The aim of this study is to 1) test if LVLS measured using speckle tracking (LVLSspeckle) is predicted by LWFS and MAPSE and to assess 2) agreement between LVLSspeckle & LWFS and 3) agreement between LVLS measurements as predicted by Huang's equation (LVLSHuang) & our equation (LVLSpredicted).
**METHODS.** We retrieved 93 TTE studies from 2 echocardiographic data repositories of patients admitted to ICU with septic shock. LVLS was measured offline (Tomtec Image-Arena Ver. 4.6.4.44) using speckle tracking from the A4C view. MAPSE was measured using the same software and the ventricle length was measured from DICOM-images, to generate LWFS.
**RESULTS.** The correlations between LVLSspeckle and LWFS, LVLSspeckle and MAPSE, and LWFS and MAPSE were good with r-values of 0.77, 0.68 and 0.94, see table 1.
The prediction equation demonstrated a slope close to 1 and an offset of +3.7%, very similar to those reported by Huang et al (1). The bias ±2 SD for LVLSspeckle vs. LVLSpredicted was 0 ±6.0% (see figure 1), for LVLSspeckle vs. LVLSHuang it was 3.2 ±6.2%.
**CONCLUSION.** LWFS is strongly correlated with LVLSspeckle measurements. The utility of LWFS for estimating LVLSspeckle is supported by its excellent accuracy and precision. This strong relationship persisted even when applying the previous equation described by Huang et al. (1) speaking for the validity of the method. Our study supports the use of the simpler LWFS method for the bedside estimation of LVLS in patients with septic shock.

**REFERENCE(S)**

1. Huang SJ, Ting I, Huang AM, Slama M, McLean AS. Longitudinal wall fractional shortening: an M-mode index based on mitral annular plane systolic excursion (MAPSE) that correlates and predicts left ventricular longitudinal strain (LVLS) in intensive care patients. Critical Care 2017;21:292. Doi: 10.1186/s13054-017-1876-x
2. Region Ostergotland County Council

**Table 1 (abstract 001020).** Correlations between LVLSspeckle, MAPSE and LWFS

| | LVLSspeckle | MAPSE | LWFS |
|---|---|---|---|
| LVLSspeckle | 1 | 0.68 | 0.77 |
| MAPSE | 0.68 | 1 | 0.94 |
| LWFS | 0.77 | 0.94 | 1 |

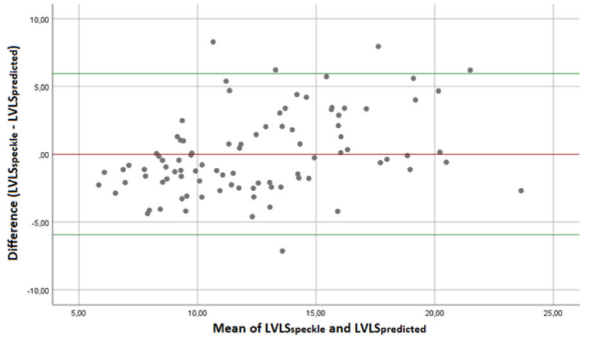

**Fig. 1 (abstract 001020).** Bland and Altman plot.

### 001032

#### Epidemiology of carbapenem-use in initial antibiotic therapy for sepsis in Japanese ICUs: a secondary descriptive study of the FORECAST study

E. Kobayashi[1], A. Shiraishi[2], T. Karumai[1], Y. Hayashi[1], T. Abe, H. Ogura[3], S. Kushimoto[4], S. Gando[5], Y. Otomo[6]
[1]Department of intensive care medicine, Kameda Medical Center, Kamogawa, Japan; [2]Emergency and trauma center, Kameda Medical Center, Kamogawa, Japan; [3]Department of traumatology and acute critical medicine, Osaka University Graduate School of Medicine, Osaka, Japan; [4]Division of emergency and critical care medicine, Tohoku University Graduate School of Medicine, Sendai, Japan; [5]Division of acute and critical care medicine, Hokkaido University Graduate School of Medicine, Sapporo, Japan; [6]Trauma and acute critical care center, medical hospital, Tokyo Medical and Dental University, Tokyo, Japan
**Correspondence:** E. Kobayashi
*Intensive Care Medicine Experimental* 2019, **7(Suppl 3):**001032

**INTRODUCTION.** The carbapenem class of antibiotics are considered as the last resort. However, there is an increasing chance of clinical isolation of carbapenem-resistant gram-negative bacilli globally1). Although administration of broad-spectrum antibiotics, such as carbapenem, within one hour is encouraged in the treatment of sepsis, a carbapenem-sparing strategy to combat antimicrobial resistance is important2).Currently, the epidemiology of carbapenem-use in the initial antibiotic therapy for sepsis in Japanese intensive care units (ICUs) is unknown.

**OBJECTIVES.** To describe the epidemiology of carbapenem-use in initial antibiotic therapy for sepsis in Japanese ICUs.

**METHODS.** Design: Secondary descriptive study of the FORECAST study, which was a multi-center, prospective, cohort study conducted at 59 ICUs in Japan from January 2016 to March 20173).Participants: Patients ≥16 years of age with severe sepsis based on the sepsis-2 criteria were included. Patients whose antibiotic-use data was missing were excluded.Exposure: Patients were classified into two groups based on the presence or absence of carbapenem-use in initial antibiotic therapy.Variables: Patient background and prognostic data were collected for each group. The rate of use of carbapenem in ICUs, which enrolled ≥4 cases, were calculated. The log-Poisson regression mixed-effect model was used to assess the association between initial use of carbapenem and hospital mortality after adjustment for fixed effects of age, baseline SOFA score, random effects of facility, and site of infection.

**RESULTS.** Among the 1184 patients, information of antibiotic-use was available for 1140 patients. The number of patients who were treated with carbapenem was 627 (55.0%). Patients' characteristics and prognosis with or without carbapenem are shown in Table1. Among the 59 ICUs that participated in the study, 48 ICUs enrolled ≥4 cases. In these 48 ICUs, the median carbapenem-use rate was 43.6% [minimum: 20.0, maximum: 100.0, interquartile range: 43.6-67.2] (Fig.1). In the unadjusted and adjusted models with the log-Poisson regression analysis, there was no significant association between carbapenem-use and hospital mortality (unadjusted risk ratio [RR]: 1.25, 95% confidence interval (CI)[0.98, 1.61], p=0.078 and adjusted RR: 1.02, 95% CI [0.77, 1.35], p=0.888).

**CONCLUSION.** Approximately half of the patients with sepsis received a carbapenem-class antibiotic in the initial antibiotic therapy in Japanese ICUs. The rate of carbapenem-use for sepsis varied among ICUs. Carbapenem-use was not associated with hospital mortality.

**REFERENCE(S)**

1)Wilson
APR. J Antimicrob Chemother. 2017;72:2410-7
2)Levy
MM, et al. Intensive Care Med. 2018;44:925-8.
3)Abe
T, et al. Crit Care. 2018;22:322
4)This
study was not supported by any grant.

**Table 1 (abstract 001032).** Patient's characteristics with or without carbapenem (n-1140)

| | with carbapenem n=627 (55.0%) | without carbapenem n=513 (45.0%) |
|---|---|---|
| Age, year old | 72 [64, 81] | 73 [63, 81] |
| Male gender | 380 (60.6%) | 316 (61.6%) |
| BMI | 20 [19.2, 24.4] | 22 [18.8, 25.1] |
| Infection site | | |
| Abdominal | 182 (29.0%) | 115 (22.4%) |
| Respiratory | 159 (25.4%) | 194 (37.8%) |
| Urinary tract | 127 (20.3%) | 86 (16.8%) |
| Skin soft tissue | 75 (12.0%) | 37 (7.2%) |
| Intraveous catheter | 11 (1.8%) | 11 (2.1%) |
| Central nervous system | 10 (1.6%) | 12 (2.3%) |
| Osteoarticular | 8 (1.3%) | 13 (0.6%) |
| Endocardium | 8 (1.3%) | 8 (1.6%) |
| Implant device | 3 (0.5%) | 5 (1.0%) |
| Wound | 4 (0.6%) | 8 (1.6%) |
| Other | 40 (6.4%) | 24 (4.7%) |
| Charlson comorbidity index | 1 [0, 2] | 1 [0, 2] |
| SOFA score | 9 [7, 12] | 8 [5, 11] |
| APACHEII score | 23 [17, 30] | 22 [16, 29] |
| Hospital mortality | 158 (25.2%) | 101 (19.7%) |

Categorical and continuous variables are displayed as count with percentage and median with 25th-75th percentile, respectively.
BMI body mass index
SOFA sequential organ failure assessment score
APACHEII acute physiology and chronic health evaluation scoring system II

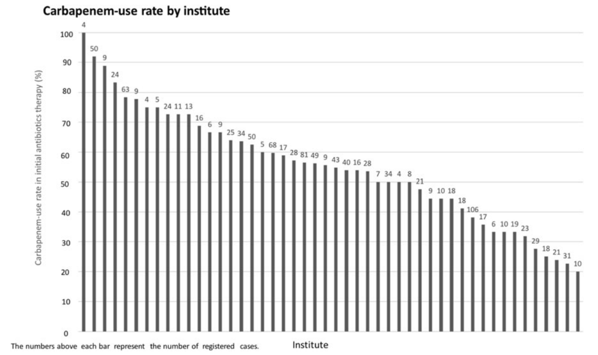

**Fig. 1 (abstract 001032).** Carbapenem-use rate by institute

### 001045

#### The prognostic role of hyponatremia and hypernatremia in sepsis and respiratory tract infection-related sepsis (RTI-r): a sub-group analysis of the Need-Speed trial

F. Gavelli, M. Baldrighi, F. Patrucco, GC. Avanzi, LM. Castello
Dipartimento di medicina traslazionale, Università degli Studi del Piemonte Orientale 'Amedeo Avogadro', Novara, Italy
**Correspondence:** F. Gavelli
*Intensive Care Medicine Experimental* 2019, **7(Suppl 3):**001045

**INTRODUCTION.** Early risk stratification of septic patients presenting to the emergency department (ED) is challenging. Many biomarkers have been studied to help the emergency physician to accomplish this goal, with encouraging results. However, some biomarkers are not readily available in the ED setting. On the other hand, biochemical analyses are routinely performed, giving useful information. The aim of the study was to evaluate the prognostic value of plasma sodium concentration (pNa+) derangements at the ED presentation in a population of septic patients.

**METHODS.** This study is a subgroup analysis of the Need-Speed database, a multicenter observational study that included consecutive patients with suspicious of sepsis admitted to the ED of five Italian hospitals between March 2013 and March 2015 and in which several biomarkers were evaluated. The aim of this subgroup analysis was to evaluate the prognostic role of pNa+ derangements in patients with sepsis and particularly in those in which sepsis was related to respiratory tract infection (RTI-r sepsis). Mortality was evaluated at 7 and 28 days according to the presence of pNa+ derangements (hyponatremia and hypernatremia) and subsequently according to the severity of hyponatremia. Then, the same analysis was performed only in patients with RTI-r sepsis.

**RESULTS.** Among the 1132 patients of Need-Speed, 879 septic patients were included in this analysis. A pNa+ derangement was present in 48.7% of patients (40.3% hyponatremia, 5.7% hypernatremia). Mortality was significantly higher at 7 days in dysnatremics compared to eunatremics (11.9% vs 7.5% p=0.038) but not at 30 days (p=0.082). When patients were analyzed in terms of typology of pNa+ derangements, only hypernatremia resulted significantly associated to mortality, at both end-points (p<0.001). The Cox regression hazard model showed that both hypernatremia and hyponatremia were independent predictors of 7-day mortality (HR 3.51[1.67-7.38] and 1.80[1.02-3.18], respectively) but only hypernatremia remained significant at 30 days (HR 1.94[1.07-3.53]). Survival analysis of non-hypernatremics showed that moderate-to-severe hyponatremia independently predicted mortality both at 7 and 30 days (HR 5.92[2.83-12.39] and 1.84[1.03-3.28], respectively). In patients with RTI-r sepsis (n=549) similar results were observed as for the overall population.

**CONCLUSION.** Both hypernatremia and hyponatremia at ED admission are early predictors of mortality in septic patients. Thus, detection of pNa+ derangements should be part of the initial management and risk stratification of such patients in the ED.

**REFERENCE(S)**

1. Mearelli F, Fiotti N, Giansante C, Casarsa C, Orso D, De Helmersen M, Altamura N, Ruscio M, Castello LM, Colonetti E et al: Derivation and Validation of a Biomarker-Based Clinical Algorithm to Rule Out Sepsis From Noninfectious Systemic Inflammatory Response Syndrome at Emergency Department Admission: A Multicenter Prospective Study. Crit Care Med 2018

### 001052

#### Factors associated with septic shock in patients with nosocomial infection, in an ICU after 7 years of Selective Digestive Decontamination

C. Sánchez Ramírez[1], S. Hípola Escalada[1], M. Cabrera Santana[1], RE. Morales Sirgado[1], MA. Hernández Viera[1], L. Caipe Balcázar[1], CF. Lübbe Vázquez[1], P. Saavedra-Santana[2], S. Ruiz-Santana[1]
[1]Intensive care medicine, University Hospital of Gran Canaria Dr. Negrin, Las Palmas de Gran Canaria, Spain; [2]Mathematics and informatics deparment, University of Las Palmas: , Las Palmas de Gran Canaria, Spain
**Correspondence:** C. Sánchez Ramírez
*Intensive Care Medicine Experimental* 2019, **7(Suppl 3):**001052

**INTRODUCTION.**
Sepsis and septic shock (SS) have been shown to be associated with substantial mortality and can lead to the consumption of a significant amount of health care resources. Recognition of SS risk factors and early intervention of proper broad-spectrum antimicrobial administration may significantly improve the outcome. Selective Digestive Decontamination (SDD) has been associated with reduced ICU mortality and acquired infection rates and its use could also modify SS risk factors.

**OBJECTIVES.** To analyze SS risk factors in patients with nosocomial infection (NI) in an ICU after seven years of SDD.

**METHODS.** Prospective study. Patients with NI were included from October 1, 2010 to September 30, 2018 in a polyvalent ICU of 30 beds. SDD was applied for 7 years, from October 1, 2011 to September 30, 2018. Patients requiring mechanical ventilation for more than 48 hours , an enteral solution and a paste with colistin, tobramycin and nystatin every 8 hours until discharge were applied. Also intravenous cefotaxime were administered during the first 4 days. Rectal and pharyngeal exudates were collected on admission and weekly. The categorical variables were summarized in frequencies and percentages and the numerical variables in means and standard deviations or in medians and interquartile ranges. The percentages were compared with the X2 test or the Fisher exact test, the means with the t-test and the medians with the Wilcoxon test for independent data. A multidimensional logistic analysis was carried out. It was considered significant if p≤.05.

**RESULTS.** Of 7240 patients admitted, 286 (56,7%) out of 504 patients with NIs developed septic shock (SS). In an univariate analysis had statistically significant SS, *Acinetobacter baumannii* NIs (p: 0.009) and multiresistant (MR) *Pseudomonas* NIs (p< 0.001). ICU stay (p: 0.003) and mortality (p< 0.001) were significantly higher in patients who developed SS (Table 1). Independent factors associated with SS were: urgent surgery, renal replacement therapy, nosocomial pneumonia, and secondary bacteremia (Table 2).

**CONCLUSION.**
In an ICU with SDD, factors that were independently associated with SS were: urgent surgery, renal replacement therapy, nosocomial pneumonia, and secondary bacteremia. MR *Pseudomonas* and *Acinetobacter baumannii* NIs were also associated with higher development of SS.

**Table 1 (abstract 001052).** Characteristics of the patients according to presence / absence of septic shock

| | Septic Shock | | |
|---|---|---|---|
| | No N = 318 | Yes N = 286 | P |
| *Age, years* | 61.8 ± 14.6 | 60.5 ± 15.7 | 0.289 |
| *APACHE II score* | 21.6 ± 7.8 | 22.5 ± 7.2 | 0.158 |
| *Sex male* | 186 (58.7) | 210 (73.4) | < 0.001 |
| *SDD* | 259 (81.5) | 235 (82.2) | 0.819 |
| *Trauma patient* | 35 (11.0) | 38 (13.3) | 0.399 |
| *Coronary artery disease patients* | 78 (24.5) | 53 (18.5) | 0.084 |
| *Urgent surgery* | 61 (19.2) | 94 (32.9) | < 0.001 |
| *Immunosuppression* | 31 (9.8) | 29 (10.1) | 0.872 |
| *Neutropenia* | 12 (3.8) | 12 (4.2) | 0.791 |
| *Immunodeppression* | 1 (0.3) | 4 (1.4) | 0.195 |
| *Parenteral nutrition* | 77 (24.2) | 90 (31.5) | 0.047 |
| *Venticular device catheter* | 27 (8.5) | 27 (9.4) | 0.683 |
| *RRT* | 92 (28.9) | 134 (46.9) | < 0.001 |
| *Malnutrition* | 31 (9.8) | 30 (10.5) | 0.684 |
| *Diabetes mellitus* | 93 (29.2) | 96 (33.6) | 0.253 |
| *COPD* | 37 (11.6) | 54 (18.9) | 0.013 |
| *Renal failure* | 64 (20.1) | 77 (26.9) | 0.049 |
| *Cirrhosis* | 15 (4.7) | 18 (6.3) | 0.373 |
| *Neoplasm* | 30 (9.4) | 32 (11.2) | 0.478 |
| *Nosocomial pneumonia* | 86 (27.0) | 141 (49.3) | < 0.001 |
| *Catheter related bacteremia* | 121 (38.0) | 95 (33.2) | 0.216 |
| *Secondary bacteremia* | 68 (21.4) | 90 (31.5) | 0.005 |
| *Urine infection* | 99 (31.1) | 61 (21.3) | 0.006 |
| *ATB 48 h prior admission* | 87 (27.4) | 76 (26.6) | 0.317 |
| *Mortality* | 84 (26.4) | 143 (50.0) | < 0.001 |
| *Acinetobacter baumannii* | 4 (1.3) | 14 (4.9) | 0.009 |
| *MRSA* | 7 (2.2) | 5 (1.8) | 0.69 |
| *ESBL* | 67 (21.1) | 75 (26.2) | 0.136 |
| *MR Pseudomonas* | 12 (3.8) | 35 (12.2) | < 0.001 |
| *MR-GNB* | 15 (4.7) | 18 (6.3) | 0.395 |
| **Admission** | | | 0.073 |
| Medical | 223 (70.1) | 210 (73.7) | |
| Scheduled surgery | 52 (16.4) | 29 (10.2) | |
| Urgent surgery | 43 (13.5) | 46 (16.1) | |
| *ICU days* | 29.0 (15.0 - 45.8) | 35.0 (22.0 - 52.0) | 0.003 |

Data are means SD and frequencies (%)..SDD: selective digestive decontamination; COPD; Crhonic obstructive pulmonary desease; ATB:: antibiotic: RRT: Renal replacement therapy; COPD: chronic obstructive pulmonary disease; MRSA: methicillin resistant Sthaphyloccocus aureus; ESBL: extended spectrum betalactamase; MR: multiresistant; GNB: gram negative bacteria

**Table 2 (abstract 001052).** Multivariate logistic analysis of Septic Shock

| | P | OR (95% CI) |
|---|---|---|
| *Urgent surgery* | < .001 | 2.037 (1.376 ; 3.017) |
| *Renal replacement therapy* | < .001 | 2.006 (1.409 ; 2.858) |
| *Nosocomial pneumonia* | < .001 | 3.489 (2.401 ; 5.070) |
| *Secondary bacteremia* | < .001 | 2.279 (1.520 ; 3.415) |

### 001068

#### Antibiotic de-escalation in a mixed ICU with Selective Digestive Decontamination: relation to ICU mortality

C. Sánchez Ramírez[1], S. Hípola Escalada[1], MA. Hernández Viera[1], M. Cabrera Santana[1], L. Caipe Balcázar[1], RE. Morales Sirgado[1], CF. Lübbe Vázquez[1], F. Artiles Campelo[2], P. Saavedra-Santana[3], S. Ruiz-Santana[1]
[1]Intensive care medicine, University Hospital of Gran Canaria Dr. Negrin, Las Palmas de Gran Canaria, Spain; [2]Microbiology department, University Hospital of Gran Canaria Dr. Negrin, Las Palmas de Gran Canaria, Spain; [3]Mathematics and informatics deparment, University of Las Palmas, Las Palmas de Gran Canaria, Spain
**Correspondence:** C. Sánchez Ramírez
*Intensive Care Medicine Experimental* 2019, **7(Suppl 3):**001068

**INTRODUCTION.** De-escalation (DE) is a strategy to replace empirical broad-spectrum antimicrobial treatment by using a narrower antimicrobial therapy. Some studies has no impact on mortality in patients with sepsis and/or septic shock,.We assess the appropriate use of antibiotics and their DE along 6 years, in an ICU with Selective Digestive Decontamination (SDD).

**METHODS.** In a 30 bed mixed ICU from October 1, 2011 to September 30, 2017 nosocomial infections were prospectively collected. Etiology, inflammatory response to infection, antibiotic treatment (ATB T) and treatment modifications according to culture results, were analyzed. SDD was applied to patients requiring endotracheal intubation over 48 hours.We performed a univariate and a logistic multidimensional DE analysis. P was significant if was ≤ .05.

**RESULTS.** DE was done in 113 out of 338 patients. Demogaphic data is shown in Table 1. Mortality was lower in patients receiving DE ATB (24.8% vs. 46.1%, p< 0.001). In the multivariate study
parenteral nutrition (PN), catheter-related bacteremia (CRB), pneumonia (NN), ICU mortality and septic shock were statistically significant (Table 2).The ATB T was inadequate in 95 infections (16,1%).Targeted therapy was performed in 254 infections (43,1%). Also 87 patients with septic shock had DE.The number of antibiotics used was 1025 and in 158 ATB DE were performed. Fifty-three patients with CRB (46.9%) and 50 with NN (44.2%) had DE. The ATB T was inadequate in 95 infections (16,1%).Targeted therapy was performed in 254 infections (43,1%). Also 87 patients with septic shock had DE. The number of antibiotics used was 1025 and in 158 ATB DE were performed. The most DE ATB were levofloxacin (22,.37%), meropenem (18.3%) and linezolid (15.38%).

**CONCLUSION.** ICU patients who received, compared to those that did not received DE, had a significantly lower mortality. Factors independently associated to DE were in addition to ICU mortality, PN, septic shock, CRB and NN.Inadequate ATB T was applied to 16,1% of NI. Finally, the most commonly DE ATB were levofloxacin.

**Table 1 (abstract 001068).** Characteristics of the patients according De-escalation

| | Total N = 451 | De-escalation No N = 338 | De-escalation Yes N = 113 | P |
|---|---|---|---|---|
| ***Age***, years | 61.1 ± 15.0 | 61.7 ± 14.7 | 59.3 ± 15.9 | 0.135 |
| ***Sex male*** | 299 (66.3) | 222 (65.7) | 77 (68.1) | 0.632 |
| ***Apache-II*** | 22.3 ± 7.6 | 22.7 ± 7.6 | 21.2 ± 7.2 | 0.079 |
| ***Admission*** | | | | 0.184 |
| Medical | 326 (72.4) | 246 (73.0) | 80 (70.8) | |
| Scheduled surgery | 65 (14.4) | 52 (15.4) | 13 (11.5) | |
| Urgent surgery | 59 (13.1) | 39 (11.6) | 20 (17.7) | |
| ***Inflammatory response*** | | | | 0.044 |
| Non Sepsis | 27 (6.0) | 25 (7.4) | 2 (1.8) | |
| Sepsis | 156 (34.6) | 123 (36.4) | 33 (29.2) | |
| Septic shock | 268 (59.4) | 190 (56,2) | 78 (69.0) | |
| ***Trauma patients*** | 51 (11.3) | 33 (9.8) | 18 (15.9) | 0.075 |
| ***Catheter related bacteremia*** | 176 (39.0) | 123 (36.4) | 53 (46.9) | 0.047 |
| ***Secondary bacteremia*** | 108 (23.9) | 89 (26.3) | 19 (16.8) | 0.040 |
| ***Nosocomial pneumonia*** | 159 (35.2) | 109 (32.2) | 50 (44.2) | 0.009 |
| ***Diabetes mellitus*** | 145 (32.1) | 109 (32.2) | 36 (31.9) | 0.939 |
| ***Cirrhosis*** | 25 (5.5) | 23 (6.8) | 2 (1.8) | 0.043 |
| ***COPD*** | 72 (16.0) | 56 (16.6) | 16 (14.2) | 0.545 |
| ***Urinary infection*** | 119 (26.4) | 99 (29.3) | 20 (17.7) | 0.016 |
| ***Chronic renal failure*** | 90 (20.0) | 70 (20.7) | 20 (17.7) | 0.488 |
| ***Neoplasms*** | 47 (10.4) | 35 (10.4) | 12 (10.6) | 0.937 |
| ***Coronary artery disease patients*** | 100 (22.2) | 75 (22.2) | 25 (22.1) | 0.988 |
| ***Renal replacement therapy*** | 275 (61.0) | 201 (59.5) | 74 (65.5) | 0.256 |
| ***Parenteral nutrition*** | 161 (35.7) | 139 (41.1) | 22 (19.5) | < .001 |
| ***Immunodeppression*** | 2 (0.4) | 2 (0.6) | 0 (0.0) | 1 |
| ***Neutropenic patients*** | 15 (3.3) | 12 (3.5) | 3 (2.6) | 0.771 |
| ***Malnutrition*** | 39 (8.7) | 31 (9.2) | 8 (7.1) | 0.493 |
| ***Death*** | 180 (40.6) | 152 (46.1) | 28 (24.8) | < .001 |
| ***ICU days*** | 31 (19 - 49) | 31 (19 - 50) | 34 (20 - 47) | 0.781 |

Data are means ± SD, frequencies (%) and medians (IQR). COPD; Chronic obstructive pulmonary disease;

**Table 2 (abstract 001068).** Multivariante logistic regression for the De-escaltion

| | P | OR (95% CI) |
|---|---|---|
| *Catheter related bacteremia* | 0.003 | 2.150 (1.288 ; 3.587) |
| *Nosocomial pneumonia* | 0.006 | 2.136 (1.245 ; 3.666) |
| *Parenteral nutrition* | 0.003 | 0.434 (0.251 ; 0.750) |
| *Death* | < 0.001 | 0.345 (0.202 ; 0.589) |
| *Septic shock* | 0.013 | 1.920 (1.151 ; 3.205) |

### 001118

#### Mitochondrial derangement contributes to abnormal platelet function in septic patients

E. Greco[1], O. Bosco[2], B. Vizio[2], G. Montrucchio[2], E. Lupia[1]
[1]Emergency medicine department, University of Turin, Torino, Italy; [2]Department of medical science, University of Turin, Torino, Italy
**Correspondence:** E. Greco
*Intensive Care Medicine Experimental* 2019, **7(Suppl 3):**001118

**INTRODUCTION.** Abnormal platelet count and function are markers of poor prognosis in sepsis and may be involved in organ failure. However, underlying mechanisms have not been fully explained. Mitochondrial dysfunction has been widely recognized both in septic patients and laboratory models and has been shown in different organs and blood cells.

**OBJECTIVES.** To evaluate the role of septic plasma in modulating human platelet mitochondrial function in vitro, independently from hemodynamic alterations.

**METHODS.** Platelets were collected from healthy donors. Isolated mitochondria were obtained from platelet-rich plasma (PRP) and protein concentration was measured. 30-mcg samples were incubated with plasma collected from septic patients or healthy donors for 1 minutes at 37°C (N=5 or 6). Afterwards Complex I (CI) and citrate synthase (CS) activity were measured with Sinergy HT microplate reader (Bio-Tek Instruments, Winooski, VT) in duplicate. CI was calculated according to Spinazzi et al. protocol [1] and normalised to CS activity. We also analysed platelet aggregation as a marker of platelet function, with light transmission aggregometry (LTA) after incubation with either septic or control plasma.

**RESULTS.** Complex I activity was significantly reduced after incubation with septic plasma (Figure 1). As previously shown in burned patients [2], we confirmed the priming effect of septic plasma on different agonist involved in platelet aggregation in vitro. Inverse correlation between mitochondrial CI activity and in vitro aggregation was statistically significant (Figure 2).

**CONCLUSION.** Circulating factors in septic plasma affect platelet mitochondrial function. Moreover, mitochondrial derangement correlates with increased aggregation. Microthrombi formation has been suggested as a possible mechanism of organ failure. Protecting against humoral factor could improve haematologic and possibly multi-organ failure in sepsis.

**REFERENCE(S)**

1. Spinazzi M, Casarin A, Pertegato V, Salviati L, Angelini C. Assessment of mitochondrial respiratory chain enzymatic activities on tissues and cultured cells. Nat Protoc. 2012 May 31;7(6):1235-46. doi: 10.1038/nprot.2012.058
2. Lupia, E.; Bosco, O.; Mariano, F.; Dondi, A.E.; Goffi, A.; Spatola, T.; Cuccurullo, A.; Tizzani, P.; Brondino, G.; Stella, M.; et al. Elevated thrombopoietin in plasma of burned patients without and with sepsis enhances platelet activation. J. Thromb. Haemost. 2009, 7, 1000–1008

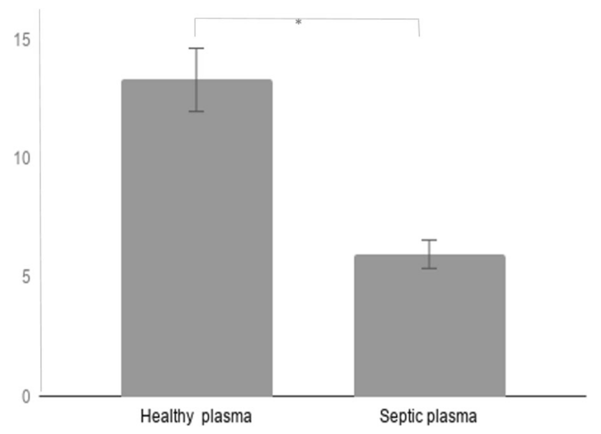

**Fig. 1 (abstract 001118).** See text for description

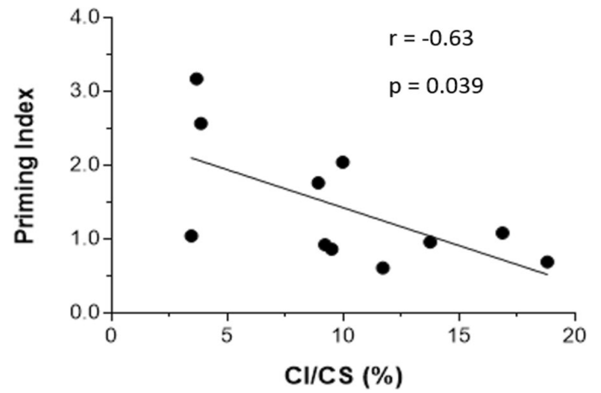

**Fig. 1 (abstract 001118).** See text for description

### 001128

#### "Right ventricular dysfunction in sepsis cardiomyopathy. An unrecognized problem."

I. Laniado[1], BK. Hanumanthu[2], P. Gulani[3]
[1]Internal Medicine, NYC Health + Hospitals/Jacobi, New York, United States of America; [2]Internal medicine, NYC Health + Hospitals/Jacobi, New York, NY, USA, United States of America; [3]Internal medicine, critical care, NYC Health + Hospitals/Jacobi, New York, United States of America
**Correspondence:** I. Laniado
*Intensive Care Medicine Experimental* 2019, **7(Suppl 3):**001128

**INTRODUCTION.** Sepsis induced cardiomyopathy (SICM) is defined as a new onset myocardial dysfunction during sepsis that is reversible within days to weeks, this condition was first described in 1984 by Parker, et al. (1, 2). While the pathophysiology leading to SICM is now known to involve a complex interplay of cytokines, endotoxin, nitric oxide and autonomic dysfunction, it remains a poorly understood entity (3,4). In addition, development of SICM is also known to be a risk factor for increased mortality, yet specific management is not clear (5, 6). SICM does not include right ventricular dysfunction (RVD) in its definition and the presence of RV failure has unclear effects on patient outcomes. We aim to clarify the prevalence of RV dysfunction in SICM.

**METHODS.** A retrospective chart review study was designed to review patients admitted to the medical intensive care unit (ICU) with a diagnosis of sepsis from January 2016 to December 2017. Patients were included if an echocardiogram showing an ejection fraction (EF) ≤ 50% within 72 hours of diagnosis of sepsis was available. A comparison echocardiogram, before or after the sepsis episode, was used to characterize patients into SICM from those with preexisting reduced EF. Patients with acute coronary syndrome or significant valvular dysfunction were excluded. Descriptive statistics and multivariate logistic regression was used to analyze the data

**RESULTS.** We analyzed 580 patient charts, of which 38 met our inclusion criteria, with SICM being diagnosed in 19 patients. Patients with SICM had a mean age of 64 (SD 17.5) and were predominantly male (63%). The prevalence of RVD was 73.6%. Troponin leak was higher in patients with SICM and RVD 1.103 (0.143 – 1.131) vs 0.017 (0.006-0.024), p=0.006. On average, over a day had elapsed from admission to TTE. However, the presence of RVD in patients with SICM did not have a significant effect on mortality.

**CONCLUSION.** Most patients with SICM have biventricular failure upon diagnosis, despite the high prevalence of RVD in our cohort we suspect that this is still underestimating the actual cases of biventricular failure due to delays in obtaining initial TTE. While the presence of RVD did not impact our patient mortality, this might be a result of our limited sample size and its presence might have important implications in management. Its inclusion into diagnostic definitions might increase its recognition and the overall diagnoses made, approaching the actual prevalence of SICM.

**REFERENCE(S)**

1. Jeong, H., Lee, T., Bang, C., Kim, J., & Hong, S. (2018). Risk factors and outcomes of sepsis-induced myocardial dysfunction and stress-induced cardiomyopathy in sepsis or septic shock. Medicine, 97(13), e0263. doi: 10.1097/md.0000000000010263
2. Parker, M. (1984). Profound but Reversible Myocardial Depression in Patients with Septic Shock. Annals Of Internal Medicine, 100(4), 483. doi: 10.7326/0003-4819-100-4-483
3. Sato, R., & Nasu, M. (2015). A review of sepsis-induced cardiomyopathy. Journal Of Intensive Care, 3(1). doi: 10.1186/s40560-015-0112-5
4. de Montmollin, E., Aboab, J., Mansart, A., & Annane, D. (2009). Bench-to-bedside review: β-Adrenergic modulation in sepsis. Critical Care, 13(5), 230. doi: 10.1186/cc8026
5. J. Romero-Bermejo, F., Ruiz-Bailen, M., Gil-Cebrian, J., & J. Huertos-Ranchal, M. (2011). Sepsis-induced Cardiomyopathy. Current Cardiology Reviews, 7(3), 163-183. doi: 10.2174/157340311798220494
6. Hanumanthu BJ, Rosenzvit C, Medhane F, et al. (2018). Differential implications of developing new onset heart failure in contrast to pre-existing heart failure on sepsis related mortality. Circulation, 138:A15570

### 001142

### Detection of sepsis and sepsis shock in hospitalized adult patients using Artificial Intelligence (AI) and Machine Learning (ML) techniques

M. Borges[1], A. Socias[1], A. Castillo[1], M. Aranda[1], C. Pruenza[2], V. Estrada[3], J. Mena[1], J. Diaz[2]
[1]Multidisciplinary sepsis unit, ICU. Hospital Son Llatzer, Palma, Spain; [2]Universidad autonoma madrid, Knowledge Engineering Institute - IIC, Madrid, Spain; [3]Hospital son llatzer, Serviico de Informática, Palma de Mallorca, Spain
**Correspondence:** M. Borges,
*Intensive Care Medicine Experimental* 2019, **7(Suppl 3):**001142

**INTRODUCTION.** Recent studies have shown that the use of AI and ML techniques have improved the detection of sepsis (SE) and septic shock (SS) compared traditional models.

**OBJECTIVES.** Development of predictive models for the detection of sepsis using AI-ML techniques.

**METHODS.** We used AI-ML techniques from historical data from the Electronic Health Record (EHR) in a University Hospital with 450 beds. Retrospective asessed, but the validation of the cases with the diagnosis of SE/SS was made prospectively by the clinical experts of the Multidisciplinary Sepsis Unit within the Code Sepsis Program. We used a SEPSIS.2 definition. The predictive variables that make up the models come from different sources of structured data (clinical, analytical, pharmacological data), as well as from non-structured text from the Triage and Emergency Department (ED) reports. The Mann-Whitney-Wilcoxon test was used to identify statistically significant clinical and analytical variables, with a significance level of 0.01. And to obtain relevant unstructured data Natural Language Processing techniques. The total sample was divided into 2 groups: the 5/7 proportion of the total of randomly selected records constituted the training set and the rest of the records (2/7) formed the test set.

**RESULTS.** From JAN-2014 to OCT-2018, 815,170 records of the EHR have been analyzed. We included 218,562 adult patients from all areas of Hospital (78% from ED) divided into 2 groups: 1) 9301 (4.6%) had SE/SS; and 2) 209,261 (95.4%) who did NOT have sepsis. A total of 3,927 variables have been extracted from the different data sources. By relevance 244 (6.2%) variables associated with SE/SS have been selected. We have analyzed 5 predictive models: Sepsis.2, SEPSIS.2 plus qSOFA, general ML (IIC), ML + Sepsis.2 (BISEPRO), ML + Sepsis.2 + qSOFA (COMBINED), Table I and Figure 1. BISEPRO was the best approximation of the resulting prediction *t*hat offers an AUC-ROC of 0.95 (95% CI 0,94-0,96).

**CONCLUSION.** The use of predictive models based on AI-ML and has provided more dynamic and precision models for the detection of patients with SE/SS.

**REFERENCE(S)**

1. Co-funded by MSD and IDISBA

**Table 1 (abstract 001142).** See text for description

| | SEPSIS.2 | SEPSIS.2+qSOFA | IIC | COMBINED | BISEPRO |
|---|---|---|---|---|---|
| AUC-ROC | 0,89 | 0,88 | 0,94 | 0,94 | 0,95 |
| Sensitivity | 0,97 | 0,98 | 0,93 | 0,93 | 0,94 |
| Specificity | 0,69 | 0,62 | 0,83 | 0,83 | 0,83Figure 1: |

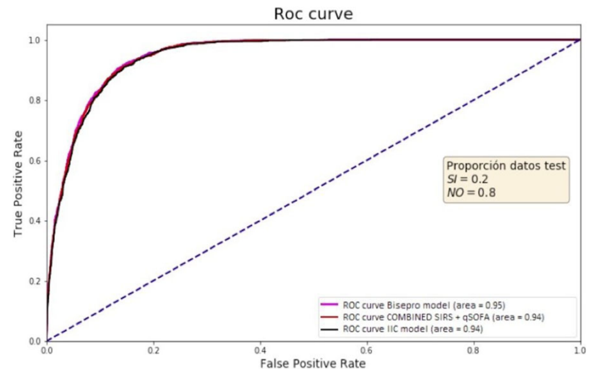

**Fig. 1 (abstract 001142).** See text for description

### 001154

### Prognostic implication and adverse events associated with very high-dose norepinephrine in septic shock patients

C. Barreiros, M. Abecasis, J. Martins, S. Fernandes
Serviço Medicina intensiva, Hospital de Santa Maria, Lisboa, Portugal
**Correspondence:** C. Barreiros
*Intensive Care Medicine Experimental* 2019, **7(Suppl 3):**001154

**INTRODUCTION.** Management of severe septic shock is controversial, particularly regarding the use of corticosteroids, thiamine, high-dose norepinephrine (NE) or combination with other vasopressors. Regarding the administration of NE, there is no clear recommendation for maximum dosage, although certain authors refer that doses above 2 μg/kg/min might be ineffective and associated with adverse drug events.

**OBJECTIVES.** To study the median dose of NE perfusion in patients diagnosed with septic shock, to evaluate the need of very high dose of NE (here defined as dose > 1 μg/kg/min) and assess it in terms of duration, adverse reactions, clinical complications and mortality.

**METHODS.** 31-bed level II-III Intensive Care Unit, tertiary university-affiliated urban hospital. Retrospective analysis and protocol-driven data collection from all patients admitted with a diagnosis of septic shock from 16.09.2018 to 24.01.2019: SAPS II and SOFA scores, hemodynamic variables, adequacy of antimicrobial therapy, associated organ failure (respiratory and need for mechanical ventilation, kidney failure and need for renal replacement therapy), use of adjunctive therapies (hydrocortisone, thiamine), use of albumin and other vasopressors (epinephrine, terlipressin) or inotropes (dobutamine), lactate clearance, fluid balance and ischemic complications. Mortality was the primary clinical outcome. We performed a descriptive statistical analysis using non-parametric tests, as well as regression analysis as appropriate. Significance was defined as a p value < 0.05.

**RESULTS.** In a preliminary analysis we included 62 patients. Median age was 68 years (IQ range: 58-77), median SAPS II was 65 (45-81). Overall mortality was 49.2%. Median maximum dose of NE was 1.4 μg/kg/min (IQ range: 0.7-2.7), administered for a median time of 5 hours (IQ range: 2-7). In the majority of patients, maximum dose of NE was administered during the first day of disease, and titration above 0.5 μg/kg/min was used in 80% of patients. Patients who needed higher doses in order to achieve target perfusion pressure tended to be more severe (Coef. 0.04; *p< 0.001*) and younger (-0.03; *p=0.02*). Subgroup analysis revealed a mortality of 64% in patients treated with NE dose higher than 1.0 μg/kg/min and a mortality of 75% in patients treated with a NE dose higher than 2.0 μg/kg/min. We did not identify higher incidence of splanchnic (OR 1.1; p=0.947) or digital ischemia (OR 1.3, p=0.47) from high dose of NE.

**CONCLUSION.** Septic shock remains a condition with ominous prognosis. Although doses higher than 2.0 μg/kg/min mark a very high risk of death, we still believe that doses in this range could be used for a short period of time, during the first day of disease and with young patients, in order to revert refractory septic shock.

**REFERENCE(S)**

1. Auchet et al. Annals Intensive Care (2017) 7: 43.Outcome of patients with septic shock and high-dose vasopressor therapy

#### 001156

#### Impact of a rapid microbiological diagnostic system on the clinical management of septic patients

P. Carfagna[1], P. Placanica[2], M. D'ambrosio[3], S. Lauri[2], M. Gaudio[2]
[1]Nosocomial infection control unit, A.O. San Giovanni Addolorata, Rome, Metropolitan City of Rome, Italy, Italy; [2]Clinical pathology, A.O. San Giovanni Addolorata, Rome, Metropolitan City of Rome, Italy, Italy; [3]Intensive care unit, A.O. San Giovanni Addolorata, Rome, Metropolitan City of Rome, Italy, Italy
**Correspondence:** P. Carfagna
*Intensive Care Medicine Experimental* 2019, **7(Suppl 3):**001156

**INTRODUCTION.** Prompt diagnosis and initiation of appropriate antimicrobial therapy can improve morbidity and mortality in patients affected by sepsis or septic shock. Rapid diagnostic tests that can accurately identify the pathogen causing an infection and the effective antimicrobials would increase the likelihood that patients are treated appropriately. In addition, rapid diagnostic tests can also be used to help clinicians discontinue or de-escalate antimicrobial therapy in favor of narrower-spectrum options. The Accelerate Pheno system is a new diagnostic device that can provide rapid bacterial identification and antimicrobial susceptibility test results directly from a positive blood culture.

**OBJECTIVES.** To evaluate the role of Accelerate Pheno system in the management of septic patients in a hospital with an antimicrobial stewardship program.

**METHODS.** At San Giovanni Hospital in Rome, positive blood cultures from septic patients were loaded onto the Accelerate Pheno system. The results were immediately communicated to the infectivologist for the consequent therapeutic choices. Results obtained from the Accelerate Pheno system were also compared to those of the usual standard of care.

**RESULTS.** Twenty positive blood cultures taken from septic patients were analyzed with Accelerate Pheno™ system from June '18 to March '19. Results were obtained from the microbiology laboratory after an average of 1.1 days versus an average of 2.95 days using conventional methods. The isolated organisms were: 6 S. aureus (3 MRSA), 6 Enterococcus spp, 5 E.coli (2 ESBL +), 2 K. pneumoniae KPC, 1 coagulase negative Staphylococcus spp. No identification errors or discrepancies in antimicrobial susceptibility test report were observed when compared to conventional methods. The infectivologist was able to establish a targeted therapy in 65% of cases, de-escalate the antibiotic therapy in 25%, and confirm empirical therapy in 10% of cases.

**CONCLUSION.** In septic patients, the Accelerate Pheno™ system seems to represent an effective method to obtain not only a rapid and accurate microbiological diagnosis, but also to optimize antimicrobial therapy within the context of antimicrobial stewardship principles.

**REFERENCE(S)**

1. Charnot-Katsikas A, et al. Use of the Accelerate Pheno System for Identification and Antimicrobial Susceptibility Testing of Pathogens in Positive Blood Cultures and Impact on Time to Results and Workflow. J Clin Microbiol. 2017 Dec 26; 56(1).
2. Lutgring JD, et al. Evaluation of the Accelerate Pheno System: Results from Two Academic Medical Centers. J Clin Microbiol. 2018 Mar 26; 56(4).
3. Singer M, et al. The Third International Consensus Definitions for Sepsis and Septic Shock (Sepsis3). JAMA. 2016; 315(8): 801-10.
4. Study conducted with data from clinical activity.

### HSRO - Modifying the risks of the ICU

#### 001308

#### Lung ultrasound seems to allow a better diagnosis of severe community and nosocomial pneumonia in critical ill paediatric patients. Preliminar data from a randomized clinical trial

S. Bobillo[1], M. Balaguer[1], J. Rodríguez-Fanjul[2], C. Guitart[1], M. Girona[1], A. Solé[1], E. Inarejos[3], FJ. Cambra[1], I. Jordan[4]
[1]Picu, H Sant Joan de Déu, Barcelona, Spain; [2]Picu, H Joan XXIII Tarragona, Tarragona, Spain; [3]Radiology departament, H Sant Joan de Déu, Barcelona, Spain; [4]PICU, H Sant Joan de Déu Barcelona, Barcelona, France
**Correspondence:** I. Jordan
*Intensive Care Medicine Experimental* 2019, **7(Suppl 3):**001308

**INTRODUCTION.** Respiratory infection is a major cause of admission at Pediatric Intensive Care Unit (30-70% of admissions depending on the season). Differential diagnosis between viral and bacterial pneumonia still is a problem in pediatric population: clinical data is unspecific and Torathic X-Ray (TXR) has its limitations. Lung ultrasound (LUS) would have a better sensibility and specificity for pneumonia diagnosis, as it has been demonstrated in adult patients.

**OBJECTIVES.** To compare the usefulness of LUS (sensibility and specificity) with respect to TXR, for the diagnosis of severe community and nosocomial pneumonia. To analyze if LUS implementation could decrease the TXR indication.

**METHODS.** Prospective, randomized, blinded and interventional clinical trial, developed between June 2017-march 2019. Inclusion criteria: patients (7 days-18 years) with suspected community (CP) or nosocomial pneumonia (NP) admitted at PICU. Exclusion criteria: previous severe respiratory disease (bronchopulmonary dysplasia, cystic fibrosis…), immunodeficiency, non-acceptance of informed consent. Pneumonia diagnosis was based on clinical signs, radiological findings (group 1-experimental: LUS initial diagnosis; group 2-control: TXR initial diagnosis) and analytical data. Images were assessed by both, the paediatric investigator who was blind to the LUS or RXT, and the radiologist, blinded to clinical data. Statistics: spps 22.0. Trial design was accepted by the Institutional Research Board and the Ethical Committee.

**RESULTS.** There were recruited 112 cases; males 64 (57.1%), mean age: 21.14 months. Patients randomization: group1-LUS= 59 (52.7%), and group 2-TXR= 53 (47.3%).

Final diagnosis in CP suspicious (n=78) was: bacterial pneumonia in 28 patients, viral pneumonia in 22, and no pneumonia in 20. The sensitivity and specificity of LUS for CP was 98.7% and 87.2%, and 90.2% and 73.3% for TXR. LUS allowed an early diagnosis than the

TXR in 28 patients. The number of TXR in group 1-LUS resulted in 1.8/patient, with respect 2.5 in group 2-TXR, p= 0.075.

NP was confirmed in 21 of the 34 with initial suspicious. Sensitivity and specificity were also higher for group1-LUS than for TXR (96.7% and 85% vs 89.2 and 74%); with an early diagnosis for LUS in 18 patients; and lower rate of TXR in group1-LUS (2.1 vs 2.7, p= 0.084).

**CONCLUSION.** LUS showed better sensitivity and specificity for the diagnosis of CP and NP than TXR in this preliminary analysis. LUS seemed to allow an early diagnosis of pneumonia in some cases. LUS could lead to a lower irradiation of paediatric patients.

### 001331

#### Ventilator Induced Diaphragmatic Dysfunction in ICU: incidence, impact on morbidity, mortality and weaning outcome

D. Panda, B. Manmohan, K. Manoj, KS. Tapas
Critical care medicine, Medanta hospital, Ranchi, India
**Correspondence:** D. Panda
*Intensive Care Medicine Experimental* 2019, **7(Suppl 3):**001331

**INTRODUCTION.** Diaphragmatic dysfunction (DD) has been reported to occur in 40- 60% of patients in ICU.1 This impairs the ability of respiratory pump to compensate the increased demand during acute illness resulting in increased the morbidity and mortality. Early recognition may help to optimize treatment strategy and assist in prognostication.2 Optimal timing of weaning is crucial. Clinical predictions are often incorrect. Ultrasound has emerged as an inexpensive, easily available and reproducible, bed-side tool for assessment of DD.1

**OBJECTIVES.** To assess incidence of DD till first spontaneous breathing trial (SBT), to identify the risk factors and to evaluate its impact on weaning outcome, mechanical ventilation (MV) days, ICU length of stay (LOS) and 28 day mortality.

**METHODS.** A prospective observational, study in an 18 bed multi disciplinary ICU of a tertiary care, teaching hospital between November 2018 to March 2019. All consecutive adult patients requiring MV during the period were included in the study. These patients were screened for DD on the day of intubation and then on every 3rd day till first SBT. They were followed up till 28 days or death. Patients with neuromuscular disorder, thoracic or abdominal surgery and patients extubated or died before 48 hr of MV were excluded. Data on patient demographics, ICU LOS, days on MV, 28 day mortality and result of first SBT were collected. Risk factors analyzed were sepsis on admission, duration of assist control ventilation, sedation and paralytic infusion duration, hyperglycemia requiring insulin infusion, steroid use and use of polymyxins.2,3 DD was assessed by ultrasound measurement of diaphragmatic thickness index (DTI) by a linear high frequency ultrasound probe, placed between 8-9th intercostal space near the zone of apposition of diaphragm.1 All patients were cared following standard ICU protocols and guidelines.

**RESULTS.** 233 patients were screened, 47 were excluded either due to extubation or death before 48 hours. 23 patients left in between the study period. The incidence of DD was 47.8% (n=78/163) using the criteria of DTI < 30%. Out of various risk factors analyzed, increased risk of DD was observed with sepsis on admission (Odds ratio 6) and use of paralytic infusion (odds ratio 16). Patients with DD had higher failure rate on their first SBT (79.4% vs. 34.1%) with relative risk of 2.32. Patients who had DD had higher ICU LOS (9.9+4.3 vs. 7.71+3.8 p=0.04). There was no significant difference in MV days in both the groups. Patients with DD had higher 28 days mortality (26 vs.16 p=0.034)

**CONCLUSION.** The incidence of DD was 47.8% till first SBT**.** Sepsis and use of paralytic infusion were found to be the significant risk factors. Patients with DD had a significantly higher failure rate on first SBT, had a significantly higher 28 days mortality and ICU LOS with no difference in MV days.

**REFERENCE(S)**

1. Gerald S. CHEST 2018; 153 (4): 1040–1051
2. Demoule A, Am J Respir Crit Care Med. 2013; 188 (2): 213-219
3. Martin Dres, Intensive Care Med (2017) 43:1441–1452,

### 001333

#### Mortality from acute pancreatitis may double when waiting for ICU admission

R. Fernandes[1], P. Campelo[2], Al. Rodrigues[1], D. Nuñez[3], C. Dias[4], C. Granja[3]
[1]Internal medicine, Algarve University Hospital - Faro, Faro, Portugal; [2]Gastroenterology, Algarve University Hospital - Faro, Faro, Portugal; [3]Intensive care department, Algarve University Hospital - Faro, Faro, Portugal; [4]Medcids - department of community medicine, health information and decision, Faculty of Medicine of the University of Porto, Porto, Portugal, Portugal
**Correspondence:** R. Fernandes
*Intensive Care Medicine Experimental* 2019, **7(Suppl 3):**001333

**INTRODUCTION.** Acute pancreatitis (AP) is an inflammatory condition of the pancreas with a broad clinical spectrum that can cause local injury, systemic inflammatory response syndrome and organ failure (1). The more severe cases often require management in an intensive care unit and are associated with significant morbidity and mortality (2). The objective of the present study was to analyse the outcomes of patients admitted with AP in the last 5 years to the intermediate and intensive care units (IntCU/ICU) of a single academic medical center.

**METHODS.** A retrospective review of the patients admitted from January 2014 to December 2018 was undertaken. A review of the medical records for each patient was performed.

The outcomes evaluated were mortality, the presence of organ dysfunction and the need for antibiotics.

The variables collected were provenience, time of stay in the emergency department (ED) before admission and severity scores [Ranson, Bedside Index of Severity in AP (BISAP), Acute Physiology, Age Chronic Health Evaluation II (APACHE II), Sequential Organ Failure Assessment (SOFA), CT Severity Index (CTSI) score].

The collected data was analysed using SPSS.

**RESULTS.** Ninety-four patients (male 61, mean ± SD age = 64.6 ± 18years) with AP were admitted to the IntCU/ICU in the 5-year period. The global mortality rate was 22,3%.74.5% of patients came from the ED, 25,5% came from the wards. Patients that came from the wards had a higher mortality rate when compared to those who came from de ED [39,1% vs 18,6% (*p*=0.044)]. The median time in the emergency room prior to admission was 13 hours (IQR 15). We found no statistical significant difference in mortality when evaluating time of stay in the ED (*p*=0.530).

Severity scores had a statistically significant correlation with mortality, with the exception of CTSI [APACHE II ≥ 8 (29,4% vs 3,8%; *p*= 0.008); SOFA ≥ 8 (60% vs 12,2%; *p*<0.001); Ranson ≥ 5 (36,0% vs 11,1%; *p*=0.012); BISAP ≥ 3 (35,9% vs 12,7%; *p*=0.008); CTSI ≥ 4 (33,3% vs 17,9%; *p*=0.129)] and a correlation with at least one organ dysfunction [APACHE II ≥ 8 (*p*<0.001); SOFA ≥ 8 (*p*<0.001); Ranson ≥ 5 (*p*<0.001); BISAP ≥ 3 (*p*<0.001); CTSI ≥ 4 (*p*=0.019)].

After excluding patients that needed antibiotics for other reasons, there was no statistical significant diference in the need for antibiotics for AP complications when analysing CTSI score severity [CTSI ≥ 4 (*p*=0.078)].

**CONCLUSION.** Severity scores were statistically significantly associated with mortality and organ dysfunction. We found no statistical significant difference in mortality when evaluating time of stay in the ED and the need for antibiotics for AP complications when correlated to CTSI score severity. Mortality was twice higher when patients came from wards when compared to those who came from ED – this should draw our attention to this group of patients who may need earlier admission and intervention.

**REFERENCE(S)**

1. Crockett SD, et al. American Gastroenterological Association Institute Guideline on Initial Management of Acute Pancreatitis. Gastroenterology 2018;154:1096–1101. https://doi.org/10.1053/j.gastro.2018.01.032
2. van Dijk SM, et al. Acute pancreatitis: recent advances through randomised trials. Gut 2017 Nov;66(11):2024-2032. doi: 10.1136/gutjnl-2016-313595.

### 001343

#### Health-related quality of life and 6-min walk distance in critically ill survivors of acute exacerbation of COPD

W. Zarrougui[1], A. Khedher[2], H. Zorgati[2], K. Meddeb[1], I. Elmeknassi[2], S. Kortli[2], A. Azouzi[2], I. Ben Saida[1], M. Boussarsar[1]
[1]Medical intensive care unit, farhat hached hospital, Université de Sousse, Faculté de Médecine de Sousse, LR N° LR12SP09.Heart Failure, Sousse, Tunisia; [2]Medical intensive care unit, Farhat hached university hospital, Sousse, Tunisia
**Correspondence:** W. Zarrougui
*Intensive Care Medicine Experimental* 2019, **7(Suppl 3):**001343

**INTRODUCTION.** The 6-min walk test (6MWT) is a validated measure of physical function since the walked distance can reflect the capacity to undertake daily activities after ICU stay for acute exacerbation of COPD (AECOPD). Determination of association between functional variables and health-related quality of life (HRQL) may facilitate interpretation of disease progression and impacts of therapy.
**OBJECTIVES.** To evaluate the association between the health-related quality of life after ICU discharge and the 6MWT in AECOPD ICU admitted patients.
**METHODS.** A prospective observational cohort study was performed in a Tunisian 9-beds tertiary medical ICU between February 2017 and January 2018, including all consecutive survivors of AECOPD. The 6MWT is performed the day of ICU discharge according to the international guidelines. Web-based version of the St George's Respiratory Questionnaire score (SGRQ) was used to measure HRQL. The SGRQ variables (activity, symptoms, impacts) were collected within three months of ICU discharge via phone calls. Data collected : clinical features at admission, severity of illness and ICU course. Walked and predicted distances were calculated. Spearman rank correlation was used to measure the degree of association between mean walked/predicted ratio and the SGRQ score.
**RESULTS.** Among 90 patients admitted for AECOPD during the study period, 62(69%) were included. General characteristics were : age, 66.4±9.5 years; sex ratio, 4,5/1; Charlson index,3.9±1.8; COPD GOLD D, 55(88%); SAPSII score, 29.8±9.9; initial invasive mechanical ventilation, 17 (27.4%); mean length of stay, 13.64±10.09. Mean walked distance, 223±96m; mean ratio walked/predicted distance, 0.41±0.17. Three months after discharge, 58(93%)patients could be interviewed via phone. SGRQ estimation was as follow: median symptoms score, 59.3[43.6-75]; median activity score, 69[53.7-84.4]; median impacts score, 49[23.8-74.2] and median total score, 56.8[63.6-77]. Compared to population norms (Symptoms, 16.1; activity, 16.3; impacts, 8.1 and total score, 12.2). 6MWD was significantly correlated with all components of the SGRQ : symptoms, -0.43(-0.64 to -0.27), p= 0.001; activity, -0.53(-0.62 to -0.19), p<0.0001; impacts, -0.46(-0.69 to-0.32), p< 0.0001 and total -0.49 (-0.62 to -0.24), p< 0.0001.
**CONCLUSION.** 6-min walk distance at ICU discharge was associated with HRQL among survivors of AECOPD patients admitted in a Tunisian medical ICU.

### 001355

#### Lowest admission pH and its association with Mortality in all-cause emergency and Diabetic ketoacidosis patients in a district general Critical Care Unit

D. Wright[1], J. Morris[2], T. Samuels[1], P. Morgan[1], M. Alice[3]
[1]Intensive care, East Surrey Hospital, Redhill, United Kingdom; [2]Intensive care, Croydon University Hospital, Croydon, United Kingdom; [3]Icu, East Surrey Hospital, Redhill, United Kingdom
**Correspondence:** D. Wright
*Intensive Care Medicine Experimental* 2019, **7(Suppl 3):**001355

**INTRODUCTION.** Decreasing pH and advancing age are associated with increased mortality in critically ill patients. Acidosis has various etiologies and is common amongst critically ill patients. It is associated with organ dysfunction and is therefore associated with worse outcomes. Diabetic ketoacidosis (DKA) is a common complication of diabetes mellitus which infrequently requires emergency admission to the critical care unit. The outcome in these patients are better as they are young patients with a greater physiological reserve.
**OBJECTIVES.** The primary aim of the study was to look at mortality related to the lowest initial pH amongst all-cause emergency admissions and DKA emergency admissions to the critical care unit. Secondary outcomes looked at the age-related mortality amongst the higher risk group, that is, pH ≤7.23.
**METHODS.** A retrospective analysis of all-cause emergency admissions to a 16-bed mixed critical care unit between 2013 and 2019 was done using our electronic patient database. Patients aged ≥18 years with an admission pH of ≥6.8 were included in the analysis. A subset of the emergency admissions admitted with DKA were analyzed separately. Wilcoxon rank sum test was used to test for significance using R version 3.5.3 (R Foundation).
**RESULTS.** There was a total of 5,416 all-cause emergency admissions, 2966 (54.8%) of which were male and 1630 (30.1%) were non-survivors. Of the non-survivors, the mean lowest pH was 7.22 SD 0.16 vs 7.32 SD 0.11 in the survivor group (p=<0.0001). Non-survivors were older (mean age 69.8 vs 61.9 years p=<0.0001) than those in the surviving group.
Of those emergency admissions, 99 (1.8%) were admitted with DKA, 10(10.1%) of which were non-survivors. There was no significant difference in pH between the survivors and non-survivors (mean pH 7.15 vs 7.15 p=0.8724). The non-survivors were older than the survivors (63.8 vs 46.4 years p=0.0016).
**CONCLUSION.** PH and age are inversely proportional to mortality amongst all-cause emergency admissions to the critical care unit. In the DKA patients, however, there was no difference in the pH related mortality. This subset was limited by the small sample size and also the group sizes were unequal which may have led to a type II error. In both groups, the mortality was noted to be higher amongst the older patients. DKA typically presents in young patients with good physiological reserve and as such, this raises the question of whether Hyperosmolar Hyperglycaemic States were misdiagnosed as DKA.

**REFERENCE(S)**

1) Samanta S, Singh RK, Baronia AK, et al. Early pH Change Predicts Intensive Care Unit Mortality. Indian J Crit Care Med. 2018.
2) Freire AX1, Umpierrez GE, Afessa B, et al. Predictors of intensive care unit and hospital length of stay in diabetic ketoacidosis. J Crit care. 2002.

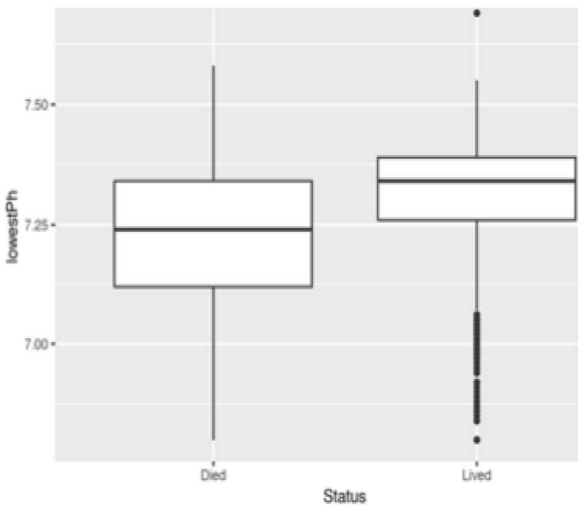

**Fig. 1 (abstract 001355).** Box Plot of lowest pH amongst Emergency Survivors and Non-Survivors

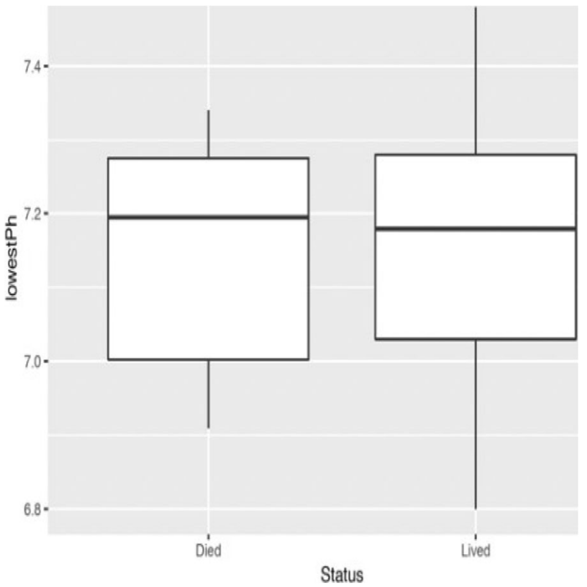

**Fig. 2 (abstract 001355).** Box Plot of lowest pH amongst Emergency DKA survivors and Non-Survivors

### 001356

#### Impact of Ramadan on ICU admission patterns and outcomes

I. Ben Saida[1], H. Kallel[2], S. Chaouch[2], R. Toumi[1], W. Zarrougui[1], M. Boussarsar[1]
[1]Medical intensive care unit, farhat hached university hospital, Université de Sousse, Faculté de Médecine de Sousse, LR N° LR12SP09.Heart Failure, Sousse, Tunisia; [2]Medical intensive care unit, Farhat hached university hospital, Sousse, Tunisia
*Intensive Care Medicine Experimental* 2019, **7(Suppl 3):**001356

**INTRODUCTION.** Fasting Ramadan is a religious obligation for muslims and one of the five pillars of Islam. Even though those unable to fast are exempt, many individuals refuse and chose to fast. This may lead to life threatening conditions and an increase in ICU demand.

**OBJECTIVES.** To investigate the impact of lifestyle changes during Ramadan on ICU admission patterns and outcomes.

**METHODS.** It was a retrospective cohort study carried out in a 9-bed medical ICU of Farhat Hached teaching hospital. Patients who were admitted to the ICU during the month of Ramadan, the preceding month (Chaaban), and the following month (Shawal) over a period of 10 years (2018–2009) were included. Underlying condition, severity of illness, diagnosis at admission, management and outcomes were recorded. Patients were divided into three groups according to the time of presentation in relation to the month of Ramadan. Demographic, clinical features and outcomes were compared between groups.

**RESULTS.** During the review period, 748 patients were included (G1= 257 ; G2=230 and G3=261). Patients' characteristics were : median age 57[35-71]years ; male, 438(58.6 %) ; median SAPSII, 30[20-42] ; median Charlson index, 3[0-5]. The main reason for admission were respiratory disorders, 408(54.4 %).

Patients with chronic kidney disease (CKD) as underling condition and hypovolemic Shock as clinical presentation were more frequently admitted during Ramadan and/or Shawal vs Chaaban respectively (8(3.5%), 19(7.3%), 6(2.3%) ; p=0.016) and (14(6.1%), 13(5%), 4(1.6%) ; p=0.031). Furthermore, patients during Ramadan and/or Shawal were more likely to have inverted urinary sodium to potassium ratio vs Chaaban respectively (94(48.7%), 77(36.8%), 58(28.3%) ; p=0.000). There was no significant difference in length of stay nor in mortality.

**CONCLUSION.** While there were no differences in any studied outcomes in patients admitted to ICU before, during Ramadan or after, there was a significant increase in patients presenting with history of CKD, hypovolemic shock and inverted urinary sodium to potassium ratio.

### 001372

#### Swedish intensive care. Low number of beds, short length of stay. A description of resources and outcomes

L. Engerström[1], C. Agvald-Öhman[2]
[1]Department of anasthesia and intensive care, Vrinnevisjukhuset, Gamla Övägen, Norrköping, Sweden, Norrköping, Sweden; [2]Department of anaesthesia and intensive care, Karolinska University Hospital Huddinge, Stockholm, Sweden
**Correspondence:** L. Engerström
*Intensive Care Medicine Experimental* 2019, **7(Suppl 3):**001372

**INTRODUCTION.** The patients cared for in intensive care and the available resources differs between regions. Swedish intensive care is like in the other Scandinavian countries characterized by short length of stay and the number of intensive care beds is low.

**OBJECTIVES.** The objectives of the study is to describe the characteristics of Swedish intensive care patient characteristics and resources and describe the outcome measured as raw and risk-adjusted mortality.

**METHODS.** Data publicly available from the Swedish intensive care registry (SIR, www.icuregswe.org) for 2018 is presented. SIR covered 81/84 (96 %) of the Swedish general-, pediatric-, burns-, neuro- and cardiothoracic intensive care units, 100 % of the general intensive care units. Mortality data is imported to SIR from the Swedish National Population Register.

**RESULTS.** There were 527 ICU-beds in Sweden in 2018 with enough staff to be used for intensive care. That is 5.2 intensive care beds per 100 000 inhabitants. There were in mean 0.7 nurses and 0.7 assistant nurses per bed. The mean SAPS3 score was 56.2 while the intensive care mortality was 8.9 % and the 30-day mortality 19.2 %. The 30-day mortality rate has previously been shown to be higher than the in-hospital mortality rate. The standardized mortality rate (SMR) using SAPS3 general formulae and 30-day mortality as outcome was 0.63. A Swedish recalibration of the SAPS3 is therefore used for comparisons in Sweden.

**CONCLUSION.** The Swedish intensive care is characterized by low number of beds and high SAPS3 scores, but neither raw- or risk-adjusted mortality rates are high.

**REFERENCE(S)**

1. Strand K, Walther SM, Reinikainen M, et al.: Variations in the length of stay of intensive care unit nonsurvivors in three Scandinavian countries. Crit Care 2010; 14:R175
2. Rydenfelt K, Engerström L, Walther S, et al.: In-hospital vs. 30-day mortality in the critically ill - a 2-year Swedish intensive care cohort analysis. Acta Anaesthesiol Scand 2015; 59:846–858
3. Engerström L, Kramer AA, Nolin T, et al.: Comparing Time-Fixed Mortality Prediction Models and Their Effect on ICU Performance Metrics Using the Simplified Acute Physiology Score 3. Crit Care Med 2016; 44:e1038–e1044
4. Engerström L, Nolin T, Mårdh C, et al.: Impact of Missing Physiologic Data on Performance of the Simplified Acute Physiology Score 3 Risk-Prediction Model. Crit Care Med 2017

### 001384

#### Risk factors of mortality in patients admitted in a medical Intensive Care Unit

N. Fraj[1], O. Ezzi[2], MA. Boujelbèn[3], N. Bouafia[2], K. Meddeb[1], A. Ammar[2], W. Zarrougui[1], A. Ben Cheikh[2], H. Ghali[2], N. Mansour[2], M. Boussarsar[1]
[1]Medical intensive care unit farhat hached university hospital, Université de Sousse, Faculté de Médecine de Sousse, LR N° LR12SP09.Heart Failure, Sousse, Tunisia; [2]Hospital hygiene unit, Farhat Hached University Hospital, Sousse, Tunisia; [3]Medical intensive care unit, Farhat Hached University Hospital, Sousse, Tunisia
**Correspondence:** K. Meddeb
*Intensive Care Medicine Experimental* 2019, **7(Suppl 3):**001384

**INTRODUCTION.** Patients admitted to intensive care unit (ICU) often have at least one or more organ failures that require special care with high mortality risk.
**OBJECTIVES.** The aim of the present study was to determine ICU mortality rate and associated risk factors in a Tunisian medical ICU.
**METHODS.** A prospective observational cohort study over an eighteen-month period from September 15th, 2015 to March 15th, 2017 in a medical intensive care unit of FarhatHachedUniversity Hospital (Sousse-Tunisia). Were included all patients hospitalized for more than 48 hours. Data collected were patients' demographics, clinical characteristics, therapeutics and outcomes. Multivariate analyses were performed to identify risk factors associated with ICU mortality.
**RESULTS.** 258 were included, they were 60.1±17.7yrs mean aged, predominantly male, 161(62.4%); with at least two comorbidities, 145(56.2%); immunocompromised, 26(10.1%); Charlson Score Index>3, 45(17.4%). SAPSII>30 on admission, 119(46.1%); 186(72.1%) needed mechanical ventilation. Median[IQR] mechanical ventilation duration, 7[4-12.5]days with median[IQR] length of stay, 8[4-17]days. Mortality rate was 37.2%. In multivariate analysis, risk factors associated to ICU mortality were the occurrence of healthcare-associated infections (RR,7.1,95%CI,[2.6-19.6]; p<0.0001), and non-invasive ventilation failure (RR,13.0, 95%CI,[4.5-38.0]; p<0.0001).
**CONCLUSION.** The present study conducted in a medical ICU identified healthcare-associated infections and non-invasive ventilation failure as independent risk factors associated with ICU mortality.

### 001386

#### Predictors of 6-month mortality in elderly ICU survivors

H. Zorgati[1], I. Ben Saida[2], N. Fraj[2], MA. Boujelbèn[1], W. Zarrougui[2], K. Meddeb[2], M. Boussarsar[2]
[1]Medical intensive care unit, Farhat hached university hospital, Sousse, Tunisia; [2]Medical intensive care unit, farhat hached university hospital, Université de Sousse, Faculté de Médecine de Sousse, LR N° LR12SP09.Heart Failure, Sousse, Tunisia
*Intensive Care Medicine Experimental* 2019, **7(Suppl 3):**001386

**INTRODUCTION.** Worldwide there is an increase in the life expectancy and thus an increasing of ICU demand for elderly patients. However, little is known about their long-term outcomes after ICU discharge.
**OBJECTIVES.** To evaluate outcomes and risk factors associated with 6-month mortality after ICU discharge in elderly patients.
**METHODS.** It is a mixed method study conducted in a 9-bed medical ICU from January 2016 to January 2017 including all old ICU survivors (≥65years). Information regarding demographic and clinical characteristics were obtained from medical records. At 6 months post-ICU discharge, follow-up was performed by phone-calling patients or their families. Univariate and multivariate analyses were carried out to identify predictors of 6-month mortality after ICU discharge.
**RESULTS.** Among 178 old patients admitted during the study period, 94 patients were discharged alive. Baseline characteristics of elderly ICU survivors were: median age, 74[69-77]years ; median Charlson score, 4[4-5] ; Knauss C or D, 71(75,5%) ; Mac Cabe≥2, 42(44,7%) ; median SAPSII, 32[27-40] ; invasive mechanical ventilation (IMV) on admission 27(28,7%) and vasopressors use, 65(36,5%). The median length of stay was 8[5-15,5]days. Among the elderly ICU survivors, 27 could not be contacted by phone and 21 died within the 6-month period after discharge. Figure 1 displays the Kaplan-Meier survival curve in elderly ICU survivors at 6 months. Univariate analysis identified factors associated to 6-month mortality after ICU discharge respectively: Knauss C or D (95,2% vs 67.4%, p=0.013) ; history of arrhythmia (38,1% vs 10,9%, p=0,023) and prolonged ICU stay(≥15 days) (42.9% vs 17.4%, p=0.026). Multivariate regression model identified the following factors as independently associated to 6-month mortality: Knauss C or D (OR, 9.01 ; 95%CI, [1.03-78.53] ; p=0.026), history of arrhythmia (OR, 6.12 ; 95%CI, [1.42-26.42] ; p=0.015) and prolonged ICU stay (OR, 4.3 ; 95% CI, [1.15-16.21] ; p=0.03).
**CONCLUSION.** Knauss C or D, history of arrhythmia and prolonged ICU stay were identified as risk factors associated with 6-month mortality of elderly ICU survivors.

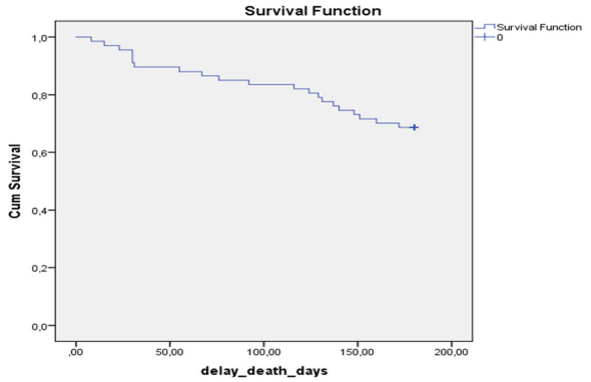

**Fig. 2 (abstract 001386).** kaplan Meier survival in elderly ICU survivors

### 001395

#### Analysis of the intensive care unit bed occupancy and its relationship with the length of stay of admitted patients

L. Esparza[1], D. García De Vicuña[2], F. Mallor[2], C. Azcárate[2], J. Barado[1], A. Orera[1]
[1]Critical care department, Hospital Complex of Navarre, Pamplona, Spain; [2]Department of statistics, computer science and mathematics, UPNA, Pamplona, Spain
**Correspondence:** L. Esparza
*Intensive Care Medicine Experimental* 2019, **7(Suppl 3):**001395

**INTRODUCTION.** Several studies show that in the event of bed shortage, admissions and discharges are triaged, and shortens length of stay (1-5).

**OBJECTIVES.** Relational analysis of the bed occupancy level in the ICU with respect to admissions and discharges and their possible effect on the length of stay of patients.

**METHODS.** An observational, prospective study was performed on patients admitted from October 1, 2015 to April 30, 2018 in the medical-surgical ICU of the Hospital Complex of Navarra, with 24 beds. Variables such as age, APACHE II on admission and type of admission (elective surgical patient, urgent surgical patient, emergency medical patient, polytrauma, hospitalized in medical service, hospitalized in surgical service and others) are also included. Markov chains transition matrices are used to analyze the probabilities of changing the bed occupancy, globally and according to an occupation level. On the one hand, the percentage of each change in the occupation level is calculated with respect to the possible total changes that take place, that is, considering 17 beds occupied, moving to 19 beds in the following day represents 0,74% of the total of all possible changes for all beds and occupations. And on the other hand, the percentage of each change considering only the movements that occur in a given occupation level, of all changes that occur with 17 occupied beds, moving to 19 beds in the next day has a probability of 11,3%. Multivariate analysis of the relationship between the length of the stay and bed occupation at the time of patients' discharge was carried out, adjusting for the rest of variables studied. Statistical significance p < 0,05.

**RESULTS.** We analyzed 2217 patients. Average age of 62,9 years (SD 14,8), APACHE II of 14,2 points (SD 8,1), length of stay of 7,7 days (SD 12,7, median 3,0) and occupation at the time of discharge of 19,4 beds (CI 19,3-19,6, median 20), with 17,2% of > 22 beds occupied and 8,2% of> 23 beds. In the analysis of the probabilities of transitions in bed occupancy, it is observed that, given an initial occupation, it is most likely to stay the next day at that same level. In addition, given an occupation level, there is more probability of admitting a new patient than of discharging up to 21 beds, where the trend is reversed being statistically significant. The multivariate analysis in terms of length of stay was significant only for occupations > 23 beds (-2,4 days, CI -4,5- -0,2). APACHE II (0,2 days, CI 0,1-0,3), urgent surgical patient (3,2 days, CI 1,1-5,3), polytrauma (6,4 days, CI 3,9-9,0) and the admitted medical patient (3,6 days, CI 1,5-5,8) were also significant.

**CONCLUSION.** Above 85% occupancy, bed management is observed, which involves triage of admissions and discharges. The influence of the occupation level on the patient's length of stay is only significant at maximum occupation.

**REFERENCE(S)**

1. Singer DE, Carr PL, Mulley AG, et al: Rationing intensive care–physician responses to a resource shortage. N Engl J Med 1983; 309:1155–1160.
2. Walther SM, Jonasson U: A prospective cohort study of 6-month mortality in a community hospital experiencing a gradual reduction in critical care services. Intensive Care Med 2001; 27:700–705.
3. Sinuff T, Kahnamoui K, Cook DJ, et al: Rationing critical care beds: A systematic review. Crit Care Med 2004; 32:1588–1597.
4. Simchen E, Sprung CL, Galai N, et al: Survival of critically ill patients hospitalized in and out of intensive care units under paucity of intensive care unit beds. Crit Care Med 2004; 32:1654–1661.
5. Santana Cabrera L, Sánchez-Palacios M, Rodríguez González F, et al: [Physicians' attitudes and perceptions regarding the critical care and critical care specialty]. Med Intensiva 2008; 32:319–328.
6. This work is integrated into the development of the research carried out by the DECYL research group within the framework of the project MTM 2016-77015-R.

**Table 2 (abstract 001395).** Transition matrix of the percentages of changes of occupation with respect to the changes that occur.

| BEDS | 10 | 11 | 12 | 13 | 14 | 15 | 16 | 17 | 18 | 19 | 20 | 21 | 22 | 23 | 24 | 25 | 26 |
|---|---|---|---|---|---|---|---|---|---|---|---|---|---|---|---|---|---|
| 10 | 0,00% | 0,00% | 0,00% | 0,00% | 0,00% | 0,00% | 0,00% | 0,00% | 0,00% | 0,00% | 0,00% | 0,00% | 0,00% | 0,00% | 0,00% | 0,00% | 0,00% |
| 11 | 0,00% | 0,21% | 0,00% | 0,00% | 0,00% | 0,11% | 0,00% | 0,00% | 0,00% | 0,00% | 0,00% | 0,00% | 0,00% | 0,00% | 0,00% | 0,00% | 0,00% |
| 12 | 0,00% | 0,00% | 0,74% | 0,11% | 0,11% | 0,00% | 0,00% | 0,00% | 0,00% | 0,00% | 0,00% | 0,00% | 0,00% | 0,00% | 0,00% | 0,00% | 0,00% |
| 13 | 0,00% | 0,11% | 0,00% | 2,02% | 0,21% | 0,11% | 0,11% | 0,00% | 0,00% | 0,00% | 0,00% | 0,00% | 0,00% | 0,00% | 0,00% | 0,00% | 0,00% |
| 14 | 0,00% | 0,00% | 0,00% | 0,11% | 1,91% | 0,32% | 0,21% | 0,21% | 0,00% | 0,11% | 0,00% | 0,00% | 0,00% | 0,00% | 0,00% | 0,00% | 0,00% |
| 15 | 0,00% | 0,00% | 0,11% | 0,00% | 0,21% | 3,50% | 0,32% | 0,11% | 0,21% | 0,00% | 0,00% | 0,00% | 0,00% | 0,00% | 0,00% | 0,00% | 0,00% |
| 16 | 0,00% | 0,00% | 0,00% | 0,32% | 0,32% | 0,21% | 5,41% | 0,42% | 0,21% | 0,42% | 0,00% | 0,00% | 0,00% | 0,00% | 0,00% | 0,00% | 0,00% |
| 17 | 0,00% | 0,00% | 0,11% | 0,00% | 0,00% | 0,21% | 0,21% | 4,78% | 0,42% | 0,74% | 0,11% | 0,00% | 0,00% | 0,00% | 0,00% | 0,00% | 0,00% |
| 18 | 0,00% | 0,00% | 0,00% | 0,00% | 0,00% | 0,00% | 0,32% | 0,42% | 7,32% | 1,17% | 0,42% | 0,21% | 0,00% | 0,00% | 0,00% | 0,00% | 0,00% |
| 19 | 0,00% | 0,00% | 0,00% | 0,00% | 0,11% | 0,00% | 0,32% | 0,64% | 0,96% | 11,46% | 1,49% | 0,21% | 0,32% | 0,11% | 0,00% | 0,00% | 0,00% |
| 20 | 0,00% | 0,00% | 0,00% | 0,00% | 0,00% | 0,00% | 0,32% | 0,11% | 0,32% | 0,64% | 10,51% | 1,06% | 0,53% | 0,42% | 0,11% | 0,00% | 0,00% |
| 21 | 0,00% | 0,00% | 0,00% | 0,00% | 0,00% | 0,00% | 0,11% | 0,00% | 0,21% | 0,64% | 0,42% | 8,39% | 0,96% | 0,11% | 0,11% | 0,00% | 0,00% |
| 22 | 0,00% | 0,00% | 0,00% | 0,00% | 0,00% | 0,00% | 0,00% | 0,00% | 0,21% | 0,21% | 0,64% | 0,74% | 7,32% | 0,64% | 0,00% | 0,11% | 0,00% |
| 23 | 0,00% | 0,00% | 0,00% | 0,00% | 0,00% | 0,00% | 0,00% | 0,00% | 0,00% | 0,21% | 0,21% | 0,21% | 0,32% | 6,26% | 0,85% | 0,11% | 0,11% |
| 24 | 0,00% | 0,00% | 0,00% | 0,00% | 0,00% | 0,00% | 0,00% | 0,00% | 0,00% | 0,00% | 0,21% | 0,00% | 0,32% | 0,32% | 2,76% | 0,11% | 0,11% |
| 25 | 0,00% | 0,00% | 0,00% | 0,00% | 0,00% | 0,00% | 0,00% | 0,00% | 0,00% | 0,00% | 0,00% | 0,00% | 0,11% | 0,32% | 0,00% | 1,49% | 0,00% |
| 26 | 0,00% | 0,00% | 0,00% | 0,00% | 0,00% | 0,00% | 0,00% | 0,00% | 0,00% | 0,00% | 0,00% | 0,00% | 0,00% | 0,11% | 0,00% | 0,11% | 0,42% |

**Table 2 (abstract 001395).** Transition matrix of the percentages of change of occupation with respect to a given accupation level.

| BEDS | 10 | 11 | 12 | 13 | 14 | 15 | 16 | 17 | 18 | 19 | 20 | 21 | 22 | 23 | 24 | 25 | 26 |
|---|---|---|---|---|---|---|---|---|---|---|---|---|---|---|---|---|---|
| 10 | 0,00% | 0,00% | 0,00% | 0,00% | 0,00% | 0,00% | 0,00% | 0,00% | 0,00% | 0,00% | 0,00% | 0,00% | 0,00% | 0,00% | 0,00% | 0,00% | 0,00% |
| 11 | 0,00% | 66,67% | 0,00% | 0,00% | 0,00% | 33,33% | 0,00% | 0,00% | 0,00% | 0,00% | 0,00% | 0,00% | 0,00% | 0,00% | 0,00% | 0,00% | 0,00% |
| 12 | 0,00% | 0,00% | 77,78% | 11,11% | 11,11% | 0,00% | 0,00% | 0,00% | 0,00% | 0,00% | 0,00% | 0,00% | 0,00% | 0,00% | 0,00% | 0,00% | 0,00% |
| 13 | 0,00% | 4,17% | 0,00% | 79,17% | 8,33% | 4,17% | 4,17% | 0,00% | 0,00% | 0,00% | 0,00% | 0,00% | 0,00% | 0,00% | 0,00% | 0,00% | 0,00% |
| 14 | 0,00% | 0,00% | 0,00% | 3,70% | 66,67% | 11,11% | 7,41% | 7,41% | 0,00% | 3,70% | 0,00% | 0,00% | 0,00% | 0,00% | 0,00% | 0,00% | 0,00% |
| 15 | 0,00% | 0,00% | 2,38% | 0,00% | 4,76% | 78,57% | 7,14% | 2,38% | 4,76% | 0,00% | 0,00% | 0,00% | 0,00% | 0,00% | 0,00% | 0,00% | 0,00% |
| 16 | 0,00% | 0,00% | 0,00% | 4,35% | 4,35% | 2,90% | 73,91% | 5,80% | 2,90% | 5,80% | 0,00% | 0,00% | 0,00% | 0,00% | 0,00% | 0,00% | 0,00% |
| 17 | 0,00% | 0,00% | 1,61% | 0,00% | 0,00% | 3,23% | 3,23% | 72,58% | 6,45% | 11,29% | 1,61% | 0,00% | 0,00% | 0,00% | 0,00% | 0,00% | 0,00% |
| 18 | 0,00% | 0,00% | 0,00% | 0,00% | 0,00% | 0,00% | 3,23% | 4,30% | 74,19% | 11,83% | 4,30% | 2,15% | 0,00% | 0,00% | 0,00% | 0,00% | 0,00% |
| 19 | 0,00% | 0,00% | 0,00% | 0,00% | 0,68% | 0,00% | 2,04% | 4,08% | 6,12% | 73,47% | 9,52% | 1,36% | 2,04% | 0,68% | 0,00% | 0,00% | 0,00% |
| 20 | 0,00% | 0,00% | 0,00% | 0,00% | 0,00% | 0,00% | 2,27% | 0,76% | 2,27% | 4,55% | 75,00% | 7,58% | 3,79% | 3,03% | 0,76% | 0,00% | 0,00% |
| 21 | 0,00% | 0,00% | 0,00% | 0,00% | 0,00% | 0,00% | 0,97% | 0,00% | 1,94% | 5,83% | 3,88% | 76,70% | 8,74% | 0,97% | 0,97% | 0,00% | 0,00% |
| 22 | 0,00% | 0,00% | 0,00% | 0,00% | 0,00% | 0,00% | 0,00% | 0,00% | 2,15% | 2,15% | 6,45% | 7,53% | 74,19% | 6,45% | 0,00% | 1,08% | 0,00% |
| 23 | 0,00% | 0,00% | 0,00% | 0,00% | 0,00% | 0,00% | 0,00% | 0,00% | 0,00% | 2,56% | 2,56% | 2,56% | 3,85% | 75,64% | 10,26% | 1,28% | 1,28% |
| 24 | 0,00% | 0,00% | 0,00% | 0,00% | 0,00% | 0,00% | 0,00% | 0,00% | 0,00% | 0,00% | 5,56% | 0,00% | 8,33% | 8,33% | 72,22% | 2,78% | 2,78% |
| 25 | 0,00% | 0,00% | 0,00% | 0,00% | 0,00% | 0,00% | 0,00% | 0,00% | 0,00% | 0,00% | 0,00% | 0,00% | 5,56% | 16,67% | 0,00% | 77,78% | 0,00% |
| 26 | 0,00% | 0,00% | 0,00% | 0,00% | 0,00% | 0,00% | 0,00% | 0,00% | 0,00% | 0,00% | 0,00% | 0,00% | 0,00% | 16,67% | 0,00% | 16,67% | 66,67% |

### 001399

#### Are we documenting enough? Improving documentation for 'out of theatre' emergency endotracheal intubation in large UK District General Hospital

S. Islam, M. Singh, Y. Li

Anaesthetics, Royal Wolverhampton NHS Trust, Wolverhampton, UK, United Kingdom

**Correspondence:** S. Islam

*Intensive Care Medicine Experimental* 2019, **7(Suppl 3):**001399

**INTRODUCTION.** Endotracheal intubation for airway control is performed in emergency situations and should be documented post procedure. The United Kingdom Intensive Care Society (UKICS) recommends using an invasive procedure checklist and post intubation document. Within Royal Wolverhampton NHS Trust exists an emergency intubation checklist, however documentation of events is variable between clinicians. We have created a standardised method of documentation using a 'blue sticker' which follows a similar structure to the UKICS post intubation document. It provides a clear, concise and cost effective method of recording important information regarding intubation which can be easily identified in the case notes with a lower risk of misplacement.

**OBJECTIVES.** Improve use of the emergency intubation checklist and documentation of rapid sequence inductions in a clear and concise manner.

**METHODS.** Retrospective review of 28 case files for emergency intubations from May to July 2018. We assessed nine important categories; Rapid sequence induction (RSI), use of intubation checklist, laryngoscopy grade, endotracheal tube (ETT) parameters (size, length and confirmation), and administered drugs (name, dose and additional). The data was input into a spreadsheet and analysed, producing graphs for interpretation. The project was re-evaluated in February 2019 to measure improvements following the introduction of the sticker. 22 case files were reviewed and anaylsed using the same methodology.

**RESULTS.** Comparisons between the two data sets showed similar indications for intubation. The leading cause was following cardiac arrest (32%) in both sets of data. Documentation of the subcategories all improved individually, with the largest improvements in additional drugs used (41%), drug dose (34%), and tube confirmation (33%). The sticker was used in 23% of the case note after the first round of implementation. The use of the checklist had also improved by 24%. Prior to introduction of the sticker, 11% had no documentation of events, and 0% covered all 9 categories. After implementation, 23% had all 9 categories documented. The lowest number of categories documented was 2 (5%).There was also an improvement in the mean number of categories from 4.35 to 6. The mode and median improved from 5 and 6, to 6, and 5 to 6 respectively.

**CONCLUSION.** Before the project, documentation varied between clinicians, but was poor overall. After introduction of the 'blue sticker', there has been a great improvement. When the sticker is used documentation has been clear and easily identified in the case notes containing all essential information. Improvement in training clinicians to use the resources available will improve this further.

### 001400

#### Incidence and risk factors of healthcare associated infections (HAIs) in a medical ICU

H. Ghali[1], N. Fraj[2], N. Bouafia[1], MA. Boujelbèn[3], O. Ezzi[1], K. Meddeb[2], A. Ammar[1], W. Zarrougui[2], A. Ben Cheikh[1], N. Mansour[1], M. Boussarsar[2]
[1]Hospital hygiene unit, Farhat Hached University Hospital, Sousse, Tunisia; [2]Medical intensive care unit farhat hached university hospital, Université de Sousse, Faculté de Médecine de Sousse, LR N° LR12SP09.Heart Failure, Sousse, Tunisia; [3]Medical intensive care unit, Farhat hached university hospital, Sousse, Tunisia
**Correspondence:** K. Meddeb
*Intensive Care Medicine Experimental* 2019, **7(Suppl 3):**001400

**INTRODUCTION.** Health care associated infections in the intensive care setting (ICU-HAIs) presenta worldwide medical challenge, increasing the need to implement national and international surveillance strategies.

**OBJECTIVES.** The objective of the present study was to determine incidence and risk factors of ICU-HAIs in a Tunisian medical ICU.

**METHODS.** A prospective observational cohort study, going from September 15th, 2015 to March 15th, 2017, in a medical intensive care unit of Farhat Hached UniversityHospital (Sousse-Tunisia). Data collected were patients' demographics, clinical characteristics, therapeutics, HAIs incidence rate. HAI definitions and incidence rates were carried out using the Centers for Disease Control and Prevention National Health-Care Safety Network (CDC/NHSN) recommendations. Univariate and multivariate statistical analyses were carried out to determine ICU-HAIs risk factors.

**RESULTS.** During the study period, 258 patients were included. They were 60.1±17.7yrs mean aged, predominantly male, 161(62.4%); with at least two comorbidities, 145(56.2%); immunocompromised, 26(10.1%); Charlson Score Index >3, 45(17.4%). Antibiotherapy prior to admission, 154(59.7%). SAPSII>30 on admission, 119(46.1%); 186(72.1%) needed mechanical ventilation. Median[IQR]ventilation duration, 7[4-12.5]days; median[IQR] length of stay, 8[4-17]days equating to 3.357 patient-days. Eighty four patients (32.6%) developed 112 HAIs, 33.4 infections/1000days of hospitalization. Ventilator Associated Pneumonia, 24.3VAP/1000ventilator-days; central-venous-catheter-associated infection, 27.5CVC-AI/1000CVC-days. Risk factors associated with acquiring ICU-HAI were length of stay≥7days (RR 9.42, 95%CI[2.33-38.08]; p=0.002), prolonged duration of sedation (RR,1.15, 95%CI,[1.02-1.31]; p=0.021), and non-invasive ventilation failure (RR,2.70,95%CI,[1.009-7.27]; p=0.048).

**CONCLUSION.** The present study showed higher ventilator associated pneumonia and central-venous-catheter-associated infection rates compared to International Nosocomial Infection Control Consortium report for 2010-2015. Length of stay>7days, prolonged sedation and non invasive ventilation failure were independent risk factors associated with acquiring ICU-HAIs.

### 001404

#### Mortality among patients with Health-care associated infections in a Medical Intensive Care Unit

N. Fraj[1], A. Ammar[2], MA. Boujelbèn[3], N. Bouafia[2], K. Meddeb[1], O. Ezzi[2], W. Zarrougui[1], A. Ben Cheikh[2], H. Ghali[2], N. Mansour[2], M. Boussarsar[1]
[1]Medical intensive care unit farhat hached university hospital, Université de Sousse, Faculté de Médecine de Sousse, LR N° LR12SP09.Heart Failure, Sousse, Tunisia; [2]Hospital hygiene unit, Farhat Hached University Hospital, Sousse, Tunisia; [3]Medical intensive care unit, Farhat hached university hospital, Sousse, Tunisia
**Correspondence:** K. Meddeb
*Intensive Care Medicine Experimental* 2019, **7(Suppl 3):**001404

**INTRODUCTION.** Healthcare-associated infections (HAI) are a major cause of morbidity and mortality in patients admitted in intensive care.

**OBJECTIVES.** The aim of the present study was to determine mortality rate and associated risk factors in patients with HAIs in the intensive care unit (ICU).

**METHODS.** A prospective cohort study was conducted in a medical intensive care unit (ICU) at Farhat Hached University Hospital (Sousse-Tunisia), from September 15th, 2015 to March 15th, 2017. Were included all patients hospitalized for more than 48 hours. Surveillance was carried out till discharge or death. Data collected were patients' demographics, clinical characteristics, therapeutics. HAI definitions and incidence rates were carried out using the Centers for Disease Control and Prevention National Health-care Safety Network (CDC/NHSN) recommendations. Logistic regression was performed to identify independent risk factors of mortality in patients with HAIs.

**RESULTS.** 258 patients were included over the study period. They were 60.1±17.7yrs mean aged, predominantly male, 161(62.4%); with at least two comorbidities, 145(56.2%); immunocompromised, 26(10.1%); Charlson Score Index >3, 45(17.4%). Antibiotherapy prior to admission, 154(59.7%).SAPSII>30 on admission, 119(46.1%); 186(72.1%) needed mechanical ventilation. Median ventilation duration, 7[4-12.5] days with median length of stay,8[4 –17] days equating to3.357 patient-days. Eighty four patients (32.6%) developed 112 HAIs, 33.4 infections/1000 days of hospitalization. Ventilator Associated Pneumonia, 24.3 VAP/1000ventilator-days; central-venous-catheter-associated infection, 27.5 CVC-AI/1000CVC-days. Mortality rate in HAIs patients was at 63.1%. Ventilator associated pneumonia was identified by multivariate analysis as anindependent risk factor for HAI-associated mortality (RR, 17.1, 95%CI, [5.1-57.2]; p<0.001).

**CONCLUSION.** The present study showed Ventilator associated Pneumonia to be associated with HAIs mortality the in intensive care.

### 001411

#### Trends in the use of intensive care by very elderly patients in a Tunisian hospital

H. Zorgati[1], I. Ben Saida[2], D. Ben Braiek[1], S. Kortli[1], N. Fraj[2], W. Zarrougui[2], MA. Boujelbèn[1], M. Boussarsar[2]
[1]Medical intensive care unit, Farhat hached university hospital, Sousse, Tunisia; [2]Medical intensive care unit, farhat hached university hospital, Université de Sousse, Faculté de Médecine de Sousse, LR N° LR12SP09.Heart Failure, Sousse, Tunisia
*Intensive Care Medicine Experimental* 2019, **7(Suppl 3):**001411

**INTRODUCTION.** Due to advancements in medical technology and management of illnesses in the past decade, an increasing proportion of critically ill patients are elderly. Given the limited healthcare resources available, admissions to intensive care in very elderly patients are often restricted. The benefits of an ICU hospitalization for those patients remain unclear.

**OBJECTIVES.** To describe trends of mortality of very old patients admitted in ICU during the last 9 years.

**METHODS.** A retrospective observational study was performed in a medical ICU at a tertiary hospital of Farhat Hached from January

2009 to December 2017 and included all patients aged 80 years or more. Comorbid illness, severity at admission, diagnosis, length of stay and outcomes were recorded from medical patients 'charts.

**RESULTS.** During the study period 2352 patients were admitted to ICU. 232(9, 98%) were older than 80 years. Patients demographic and clinical characteristic's were : median age, 83[81-85] ; male, 143(61.6%) ; median SAPS II, 40[33-51] ; median Charlson comorbidity score 5[2-6] ; Knauss C or D, 146(62,9%) ; MacCabe≥2, 193(83,2%) ; invasive mechanical ventilation (IMV), 168(72,4%) ; median duration of IMV, 3[2-8]days ; vasopressors use 154(66,4%) ; median length of ICU stay was 6[3-10]days. The mean reason for admission was respiratory disorder for 139(59, 9%). Overall mortality rate was 59.9%. Figure1 shows trends in mortality rates of ICU admissions and of very elderly ICU patients between 2009 and 2017.

**CONCLUSION.** The use of ICU by very elderly patients was similar throughout the 9-year study period. In-hospital mortality was consistently high in those patients.

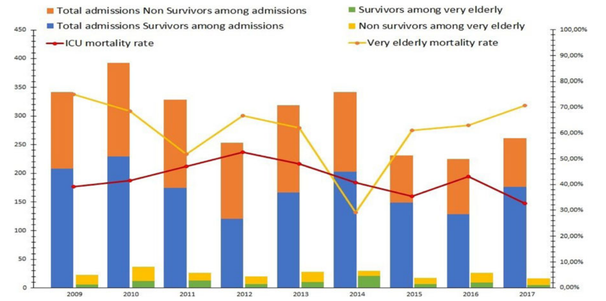

**Fig. 1 (abstract 001411).** Mortality rates over years of ICU admission and very elderly ICU patients

### 001419

#### Community Hospital Contributions to a Multicenter Randomized Trial

J. Tsang[1], A. Binnie[2], EH. Duan[3], P. Archambault[4], N. Zytaruk[3], M. Camargo[1], W. Dechert[3], B. Reeve[3], R. Cirone[3], S. Trop[3], P. Lysecki[3], T. Campbell[3], K. Kavikondala[3], Z. Panchbhaya[3], P. Hosek[3], R. Jesso[3], E. Mcmillan[3], E. Durquet-Deblois[4], J. Johnstone[5], D. Cook[3]
[1]Critical Care, Niagara Health, St. Catharines, Canada; [2]Critical care, William Osler Health System, Toronto, Canada; [3]Department of medicine/department of health research methods, evidence and impact, McMaster University, Hamilton, Canada; [4]Critical care, L'Hotel-Dieu de Levis, Levis, Canada; [5]Public health, Public Health Ontario, Toronto, Canada
**Correspondence:** J. Tsang
*Intensive Care Medicine Experimental* 2019, **7(Suppl 3):**001419

**INTRODUCTION.** Traditionally, health research has been conducted in "academic" or university-affiliated hospitals, whereas most patients receive care in "community" hospitals. However, the line between "academic" and "community" hospitals is blurring in terms of research involvement, as community hospitals increasingly prioritize research engagement in addition to clinical excellence. In this study nested within an international probiotics trial (PROSPECT: Probiotics: Prevention of Severe Pneumonia and Endotracheal Colonization Trial), we describe the contribution of Canadian community hospitals.

**METHODS.** In PROSPECT, we randomized critically ill patients with expected mechanical ventilation of ≥72 hours to receive either 1x10e10 colony forming units (CFU) of *L. rhamnosus* GG or an identical placebo, twice daily, to evaluate the effect of probiotics on infectious outcomes, diarrhea and other clinical endpoints. Trial participation was open to all interested centers. In this study, we compared trial metrics, patient demographics and outcomes between academic and community hospitals using t-tests and Chi-square, based on the first 2/3 of enrolled patients in PROSPECT.

**Trial Registration**: www.clinicaltrials.gov : NCT02462590

**RESULTS.** Of the 44 participating centers, 41 (93.2%) were Canadian and 7 (15.9%) were community ICUs. All academic hospitals were located in urban centres, whereas amongst the 7 community hospitals, only 1 was located in an urban center, 2 were in suburban areas, and the remaining 4 were located in smaller communities near urban centers. Of the 1766 trial patients that were analyzed, 189 (10.7%) were enrolled in community ICUs. Regarding trial metrics, patients in community ICUs were less likely than in academic ICUs to be coenrolled in other randomized trials or observational studies (9.0% vs 23.0%, p<0.001) whereas protocol adherence defined as patients receiving at least one dose of study product were similar between community and academic ICUs (88.1% vs 90.9%). Several patient characteristics were similar between community and academic ICUs, including proportion of female participants (34.9% vs 41.0%, p= 0.117) and illness severity (APACHE II score 21.1 vs 22.2, p=0.075). However, some patient characteristics were different between community and academic ICUs, including mean age (62.6 vs 59.9 years, p=0.036), and medical admission status (93.7% vs 76.5%, p<0.001). Regarding patient outcomes, ICU and hospital mortality rates were higher in community ICUs than in academic ICUs (29.1% vs 21.2%, p= 0.015) and (37.2% vs 27.3%, p= 0.006), respectively, whereas the proportions of patients with *C. difficile* colitis during ICU stay and post ICU stay were similar between community and academic ICUs (1.1% vs 1.8%, p=0.764) and (1.1% vs 0.9%, p = 0.686), respectively.

**CONCLUSION.** Community hospital engagement in ICU research can enrich study populations with more geographically diverse participants, thereby improving the generalizability of results, while simultaneously hastening trial completion.

**REFERENCE(S)**

1. Canadian Institutes of Health Research

### 001425

#### Effect of a multiprofessional structured clinical debriefing tool (TALK®) after non-planned learning events in the safety culture among Intensive Care Unit professionals

P. Castro Rebollo[1], E. León-Castelao[2], C. Diaz-Navarro[3], I. Enjo-Pérez[4], S. Fernandez[1], I. Carmona[1], J. Pérez-Dueñas[1], M. Sanz-Moncusí[5], JR. Alonso[6], R. Ponce-Muñoz[4], M. Olivares-Rojas[4], M. García-Font[4], JM. Nicolás[1]
[1]Medical Intensive Care Unit, Hospital Clínic of Barcelona, Barcelona, Spain; [2]Investigación, desarrollo, innovación educativa. programa integral enfermo crítico y emergencias, School of Medicine and Health Sciences, University of Barcelona, Barcelona, Spain; [3]Anaesthesology department, University Hospital of Wales, Cardiff, United Kingdom; [4]Programa integral enfermo crítico y emergencias, School of Medicine and Health Sciences, University of Barcelona, Barcelona, Spain; [5]Liver intensive care unit, Hospital Clínic of Barcelona, Barcelona, Spain; [6]Emergency department, Hospital Clínic of Barcelona, Barcelona, Spain
**Correspondence:** P. Castro Rebollo
*Intensive Care Medicine Experimental* 2019, **7(Suppl 3):**001425

**INTRODUCTION.** Increasing complexity in the ICU environment represents a safety challenge. Medical errors are frequent, and their consequences can be fatal. One of the main sources of error is interprofessional communication within ICU team. Clinical debriefing, a process of formal reflection about our clinical practice, can improve processes, professional performance and safety culture. However, it is difficult to implement due to lack of knowledge and tools. TALK ® is a multiprofessional structured clinical debriefing tool designed to guide reflection in clinical teams to improve safety.

**OBJECTIVES.** To implement TALK® in the ICU setting and analyze its effect in culture safety.

**METHODS.** We designed a prospective and longitudinal study. TALK® tool was implemented in 2 different ICU in the same University Hospital. The implementation comprised 1h training sessions, focused discussions and distribution of materials (flashcards, posters).

Debriefing was considered a non-mandatory multidisciplinary phenomenon. Debriefing events were systematically registered following an interview with the team at the end of every shift. We also explored leadership and participation in debriefing episodes, improvement decisions agreed upon during debriefing and barriers encountered. Data were collected in 15-day periods for baseline, immediate (q week) and 3 months follow-up. Additional follow-up measurements are planned at 6, 12 and 24 months.

**RESULTS.** Data on 119 baseline and 114 immediate follow-up shifts have been collected. There have been 36 debriefings in 30 shifts among the two periods. Debriefing were performed in 13,5% of the shifts during pre-implementation period(n=16/119), and in 12,3% of the shifts in the post-implementation period (n=14/114) (not significant difference). However, consideration to do debriefing increased from 36,8% to 94% between the pre- and post-implementation period. Not having any issues to discuss was the main reason not to do debriefing in both periods. Debriefing was led mainly by nurses (72,2%), followed by physicians (13,8%), residents 8% and nurse assistants (5%). Three months follow up data results will also be available for presentation at the conference.

**CONCLUSION.** Implementation of TALK® tool was feasible and it was associated to an increase in the consideration to do debriefing during shifts, but not to an increase in performing debriefing in the immediate post-implementation period.

**REFERENCE(S)**

1. Donchin Y, Seagull FJ. The hostile environment of the intensive care unit. Curr Opin Crit Care. 2002;8:316-320
2. St.Pierre M, Hofinger G, Simon R, Buerschaper C. Crisis Management in Acute Care Settings. Berlin, Heidelberg: Springer Berlin Heidelberg; 2011.
3. Rothschild JM, Landrigan CP, Cronin JW, et al. The Critical Care Safety Study: The incidence and nature of adverse events and serious medical errors in intensive care. Crit Care Med. 2005;33:1694-1700
4. Donchin Y, Gopher D, Olin M, et al. A look into the nature and causes of human errors in the intensive care unit. Crit Care Med. 1995;23:294-300
5. Couper K, Salman B, Soar J, Finn J, Perkins GD. Debriefing to improve outcomes from critical illness: A systematic review and meta-analysis. Intensive Care Med. 2013;39:1513-1523
6. Tannenbaum SI, Cerasoli CP. Do team and individual debriefs enhance performance? A meta-analysis. Hum Factors. 2013;55:231-245
7. Diaz-Navarro C, Hadfield A, Pierce S, Leon-Castelao E. Talk Debriefing. http://www.talkdebrief.org
8. H2020-MSCA-RISE: "734753 TALK supporting implementation of clinical debriefing". 2017-2020. Financing entity: European Commission. PI: Cristina Diaz-Navarro.

#### 001297

**Phase angle: a new predictor of mortality in critically ill patients measured by a noninvasive tool, The bioimpedance vector analysis (BIVA)**

V. Viarasilpa, R. Ratanarat, T. Viarasilpa, Y. Apichatbutr, P. Nuchpramool
Dept of medicine, siriraj hospital, mahidol university, Division of Critical Care, Bangkok, Thailand
**Correspondence:** R. Ratanarat

*Intensive Care Medicine Experimental* 2019, **7(Suppl 3):**001297

**INTRODUCTION.** Bioimpedance vector analysis (BIVA) is a noninvasive, and rapid screening tool for estimation of fluid status in different populations, but there are scant data in critically ill patients.

**OBJECTIVES.** To evaluate the relationship of fluid balance assessed by percentage of fluid accumulation (%FA), a conventional tool, and by BIVA-derived variables, and to determine which parameter derived by BIVA is the best for predicting 28-day mortality.

**METHODS.** We conducted a prospective, clinician-blinded, observational study in patients admitted to medical ICUs with an expected length of ICU stay of at least 72 hours. We performed BIVA measurements and cumulative fluid balance calculation and recorded physiological variables on day 0, 3, and 7 after ICU admission. BIVA results included percentage of fat free mass hydration (%FFMH), and phase angle (PhA). The correlation between BIVA-derived values, %FA, and 28-day mortality were analyzed. The parameters that associated with 28-day mortality were identified, and the significant parameters then were analyzed using the area under the ROC (aROC) curve for searching the cutoff value to predict the mortality.

**RESULTS.** Sixty-nine patients were enrolled, with a mean age of 61 ± 21 year, and a mean Acute Physiology and Chronic Health Evaluation II (APACHE II) score of 23 ± 8. The median %FA were 7.04%, 9.9%, and 11.48% on day 0, 3, and 7 after ICU admission. Based on BIVA-derived values, the median %FFMH were 79.8 %, 79.6%, and 79.2%, and the median PhA were 4.9, 4.9, and 5.1, on day 0, 3, and 7 after ICU admission, respectively. According to 28-d mortality, no differences of %FA and BIVA-derived variables, which measured on D0, and D3 after admission between non-survivors and survivors. The median %FA on D7 was higher in non-survivors than in survivor [14.8% (10.9%, 24.1%) vs 6.9% (1.1%, 15.2%), p = 0.003]. The median PhA on D7 of non-survivors was 3.6 (3.2, 4.1), while that of survivors was 5.5 (4.6, 6.4) (p < 0.001). Whereas, the median %FFMH on D7 of non-survivors was not statistically different with that of survivors [79.2% (75.9%, 85%) vs 78.5% (74.4%, 82.3%), p = 0.4]. Phase angle of less than 4.5 on the 7th day after admission could predict 28-d mortality with the OR of 2.1, (95% CI 1.2–3.4, P < 0.001).

**CONCLUSION.** BIVA-derived variables, percentage of fat free mass hydration (%FFMH) and phase angle (PhA) had good correlation with percentage of fluid accumulation (%FA) on day 3 and day 7. Only PhA on the seventh day after admission were associated with the 28-day mortality. PhA of less than 4.5 might be used as cut-off value for guidance of fluid management in critically ill patients.

### SIS - Optimal antibiotic use in sepsis

#### 000363

**An international survey on aminoglycoside practices in critically ill patients: The AMINO III study**

C. Roger[1], B. Louart[1], L. Elotmani[1], G. Barton[2], L. Escobar[3], D. Koulenti[4], J. Lipman[5], M. Leone[6], L. Muller[1], C. Boutin[1], J. Amour[7], I. Banakh[8], J. Cousson[9], J. Bourenne[10], JM. Constantin[11], J. Albanese[12], J. Roberts[13], JY. Lefrant[1]
[1]Department of intensive care medicine, Nimes University Hospital, Nîmes, France; [2]Department of pharmacy, St Helens and Knowsley Hospitals NHS Trust, Liverpool, United Kingdom; [3]Faculty of medicine, Universidad de Chile, Santiago, Chile; [4]The university of queensland centre for clinical research, The University of Queensland, Brisbane, Australia; [5]Department of intensive care medicine, the university of queensland centre for clinical research, The University of Queensland, Brisbane, Australia; [6]Department of anesthesiology and intensive care medicine, University Hospital of Marseille, Marseille, France; [7]Department of intensive care medicine, Pitié-Salpêtrière Hospital, Paris, France; [8]Department of pharmacy, Frankston Hospital, Frankston, Australia; [9]Department of anesthesiology and intensive care medicine, University Hospital of Reims, Reims, France; [10]Department of emergency and intensive care medicine, University Hospital of Marseille, Hôpital de la Timone, Marseille, France; [11]Department of anesthesiology and intensive care medicine, University Hospital of Clermont-Ferrand, Clermont-Ferrand, France; [12]Department of anesthesiology and intensive care medicine, University Hospital of Marseille, Hôpital de la Conception, Marseille, France; [13]The university of queensland centre for clinical research,department of intensive care medicine, The University of Queensland, Brisbane, Australia
**Correspondence:** C. Roger

*Intensive Care Medicine Experimental* 2019, **7(Suppl 3):**000363

**INTRODUCTION.** The optimal dosing strategy of aminoglycosides (AG) remains debated in intensive care units (ICUs).

**OBJECTIVES.** We investigated the international practices of AG regarding dosing and therapeutic drug monitoring in ICU patients.

**METHODS.** A prospective, multicentre, observational, cohort study was conducted in 59 intensive care units (ICUs) across 5 countries. All patients admitted to the ICU and receiving AG therapy for septic shock were included in the present study.

**RESULTS.** We enrolled 931 patients (mean ± standard deviation (SD), age 63 ± 15 years, female 364 (39%), median SAPS II 51

((interquartile (IQR):38-65)) receiving AG as part of empirical (761, 84%) or directed (147, 16%) antimicrobial therapy for sepsis or septic shock. The AG used was amikacin in 625 (67%), gentamicin in 312 (34%) and tobramycin in 15 (2%) patients. Aminoglycosides were mainly used for pulmonary (405, 44%), abdominal (233 (25%), urinary (126 (14%) infections and bacteremia (158, 17.0%). Aminoglycoside resistance was reported in 58 (18%) of documented infections. The median duration of therapy was 2 ((IQR 1-3) days, the median number of AG doses was 2 ((IQR 1-2), the mean dose was 25 ± 6, 6 ± 2, 6 ± 1.6 mg/kg of total body weight for amikacin, gentamicin and tobramycin respectively, and the median dosing interval was 26 (IQR 23.5- 43.5) h. Proportion of changes in median serum creatinine levels during AG therapy were -7% (IQR-24%; 12%). Therapeutic drug monitoring (TDM) of peak and trough concentrations was performed in 445 (48%) and 501 (57%) patients after the first dose. After the first AG dose, only 250 (56%) patients achieved the PK/PD target of Cmax/MIC >8 and 336 (67%) had concentrations above Cmin recommended thresholds. The ICU mortality rate was 27.3% with multivariable analysis showing AG resistant isolate (OR 8.31, 95% CI: 1.31-78.30, p=0.0355), SOFA score (OR 1.54, 95% CI: 1.24-2.03, p=0.0004) and directed antimicrobial therapy (OR 0.08, 95% CI: 0.01-0.70, p= 0.0254) as independent predictors of ICU mortality. We could show no correlation between AG dosing or PK/PD target attainment and clinical outcomes (Table 1).

**CONCLUSION.** Short courses of high AG doses are mainly used in ICU patients with septic shock although wide variability in AG practices is reported. Efforts to optimize the first AG dose and to perform TDM are still needed.

**REFERENCE(S)**

1. We acknowledge endorsement of the study by the ESCMID PK/PD of Anti-Infectives Study Group and the Antimicrobial Use Working group of ESICM.

**Table 1 (abstract 000363).** See text for description

| Variables | OR (95%CI) | p-value |
|---|---|---|
| Age | 1.03 (0.99;1.09) | 0.1923 |
| Sex | 1.31 (0.32;5.91) | 0.7115 |
| BMI | 1.04 (0.92;1.19) | 0.5703 |
| SOFA score | 1.54 (1.24;2.03) | **0.0004** |
| AG resistant isolate | 8.31 (1.31;78.30) | **0.0355** |
| Directed antimicrobial therapy | 0.08 (0.01;0.70) | **0.0254** |
| AG dose in mg/kg | 0.96 (0.89;1.04) | 0.3189 |
| Targeted AG Cmax | 1.18 (0.76;91.92) | 0.1169 |

### 000840

#### Can microdialysis be used as a real-time monitoring tool for different meropenem dosing regimens?

K. Tam[1], I. Longo[2], D. Brealey[3], M. O'connell[4], M. Singer[5]
[1]Intensive care medicine, University College London, London, United Kingdom; [2]Anaesthesia and intensive care medicine, Azienda Sanitaria Universitaria Integrata di Trieste, Trieste, Italy; [3]Critical care, UCL Hospitals NHS Foundation Trust , London, United Kingdom; [4]Director, Probe Scientific Ltd, Thurleigh, United Kingdom; [5]Bloomsbury institute of intensive care medicine, University College London, London, United Kingdom
**Correspondence:** K. Tam
*Intensive Care Medicine Experimental* 2019, **7(Suppl 3):**000840

**INTRODUCTION.** Antibiotic dosing in the critically ill patient is predominantly "one-size-fits-all" despite often grossly deranged pharmacokinetics and pharmacodynamics. Other than intermittent monitoring of aminoglycosides and glycopeptides (mainly for toxicity), there is miminal routine therapeutic drug monitoring (TDM), and none is offered in real time. This leads to dual issues of both under-treatment - post-mortem studies reveal persistent septic foci in >70% cases (1) – or toxicity (2,3). Since only the free (unbound) fraction of an antibiotic is active against bacteria in infected tissue, a tissue-based technique such as microdialysis (MD) may provide real-time monitoring of tissue concentrations (4).

**OBJECTIVES.** This study aims to determine the feasibility of *in vivo*microdialysis, monitoring real-time changes of different dosing regimens of meropenem at six different sites simultaneously, with comparison against direct blood level measurement.

**METHODS.** Healthy male Wistar rats (300-350g) underwent general anaesthesia and surgery for vascular access and MicroEye®(Probe Scientific, Bedford, UK) insertion into 6 sites: muscle, subcutaneous, liver, peritoneal cavity, trachea and jugular vein. The microdialysate flow rate was set at 1 μL/min. Six rats received a meropenem bolus of 30 mg/kg i.v., administered over 30 min; another 6 rats received a continuous meropenem infusion of 2.5 mg/kg/h.Tissue microdialysates and blood were sampled at 10 and 15 minute intervals, respectively for 90 minutes' duration. Meropenem concentrations in all samples were measured using High Performance Liquid Chromatography (HPLC, Agilent 1260II) ANOVA with post-hoc Dunn's tests were used to seek statistical significance.

**RESULTS.** The meropenem bolus generated a similar pattern of microdialysate drug concentration changes over time in all tissues, with no statistically significant difference in area under the curve (AUC). Peak concentrations (Cmax 5.43-15.72 μg/mL were reached in all tissues by 50-60 min (this includes a 20 minute lag-time for dead-space tubing) followed by a gradual decrease to zero at 100 min [Figure 1]. With continuous infusion, steady state was reached in all tissues by 50-60 min (0.33-1.16μg/mL), with liver AUC being significantly lower than venous (22.54 vs 69.22 μg/mL; p=0.008) [Figure 2].

**CONCLUSION.** In conclusion, MicroEye® shows promise as a novel point-of-care tool for monitoring tissue antibiotic concentrations.

**REFERENCE(S)**

[1] Torgersen C, Moser P, Luckner G, Mayr V, Jochberger S, Hasibeder WR & Dünser MW. (2009). Macroscopic postmortem findings in 235 surgical intensive care patients with sepsis.Anaesth Analg. 108(6):1841-7
[2] Roberts, JA. et al. (2011). Antibiotic dosing in the 'at risk' critically ill patient: Linking pathophysiology with pharmacokinetics/ pharmacodynamics in sepsis and trauma patients. Anaesthesiol. 11:3
[3] Roberts, JA. et al. (2014). Variability in protein binding of teicoplanin and achievement of therapeutic drug monitoring targets in critically ill patients: Lessons from the DALI StudyInt J Antimicrob Agents. 43:423-430
[4] Shippenberg TS, Thompson AC. Overview of microdialysis. Curr Protoc Neurosci. 2001; Chapter 7:Unit7.1
[5] Collaborate to Innovate/ EU ERDF grant from MedCity London.

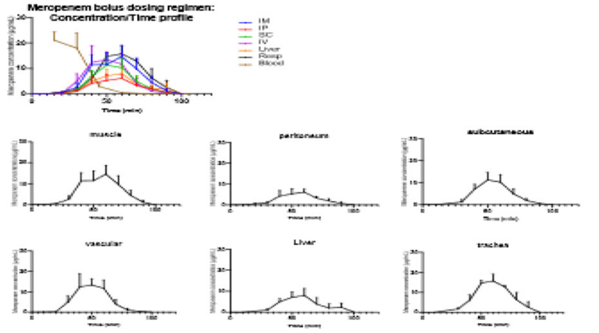

**Fig. 1 (abstract 000840).** Meropenem bolus dosing regimen: concentration/time profile

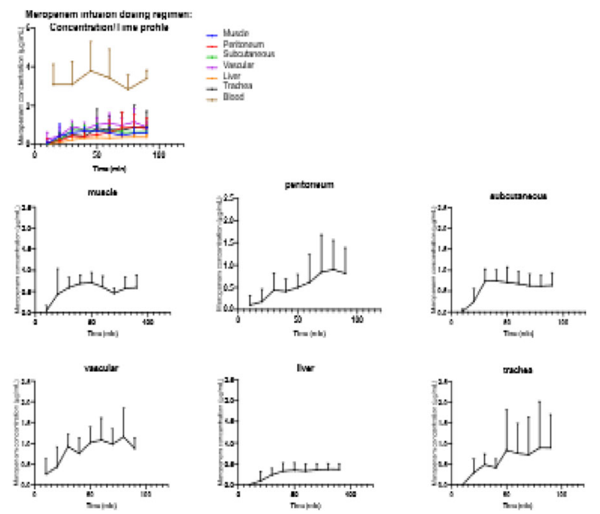

**Fig. 1 (abstract 000840).** Meropenem continuous infusion dosing regimen: concentration/time profile

### 000459

#### A distinctive and differential hemodynamic signature of Apelin-13 and Elabela in healthy sheep and ovine fecal peritonitis

D. Coquerel[1], M. Sage[2], F. Chagnon[1], E. Delile[1], C. Nadeau[2], N. Samson[2], E. Fortin-Pellerin[2], JP. Praud[2], O. Lesur[1]
[1]Icu, CRCHUS, Sherbrooke, Canada; [2]Pediatrie & pharmacologie/ physiologie, CRCHUS, Sherbrooke, Canada
**Correspondence:** O. Lesur
*Intensive Care Medicine Experimental* 2019, **7(Suppl 3):**000459

**INTRODUCTION.** From humans to rodents, cardiac contractility, vascular tones, fluid homeostasis and kidney function are all physiological hallmarks impacted by the engagement of the Apelin-receptor (APJ). Both Apelin-13 (APLN-13) and Elabela (ELA) endogenous APJ agonists exhibit bioactive properties and positive effects to support myocardial function both in endotoxemia and peritonitis models of sepsis in rats [1,2]. Because the apelinergic system stimulation has gained credibility as a potential alternative therapeutic pathway a first pilot study was undergone to evaluate the hemodynamic effects of APLN-13 or ELA perfusion in a clinically relevant large animal model of sepsis.

**OBJECTIVES.** Assess the acute hemodynamic impacts of APLN-13 or ELA infusions in healthy or septic sheep.

**METHODS.** Polymicrobial sepsis was induced by autologous feces injection into the abdominal cavity. Hemodynamics monitoring was assessed by arterial thermodilution (right: Swan-Ganz, left: PiCCO) and Left Ventricle (LV) catheterization to record LV End-Systolic Pressure and dP/dtmax. When shock criteria were reached (decrease of 40% of the baseline (bsl) Mean Arterial Pressure (MAP) and Cardiac Output (CO), SCVO2 <70%, lactatemia> 2 mmol/L) sheep received a Lactate Ringer bolus (30ml/kg in 1hr) before random assignment to a continuous infusion of 5 incremental doses of APLN-13 or ELA perfused in 20 min (from 0.007 to 3.6 μM/kg/hr) or equivalent volume of Normal Saline (NS).

**RESULTS.** APJ expression (qPCR) was confirmed in ovine heart & kidney. APLN-13 infusion was inotropic compared to NS, while ELA decreased cardiac function in healthy sheep (% from bsl at 1.8μM/kg/hr; CO, NS: 6±4%, APLN-13: 26±9% p<0.05 vs NS, ELA: -20±1% p< 0.05 vs NS; dP/dtmax, NS: 7±8%, APLN-13: 52±15% p<0.05 vs NS, ELA: -34±2% p<0.05 vs NS).

Sustained Gram negative bacteremia was documented and shock criteria were met 240±55min after feces injection, along with acute kidney dysfunction (urine output: bsl 108±18 ml/hr vs shock 9±3 ml/hr, p<0.05; creatinine, bsl: 2.7±0.1 mmol/L vs shock: 3.6±0.3 mmol/L, p< 0.05). Without significant impact on MAP, APLN-13 was the only one inotrope in septic sheep (% to shock time-point at 0.7μM/kg/hr; CO, NS: 11±3%, APLN-13: 48±13% p<0.05 vs NS, ELA: -25±11%, ; dP/dtmax, NS: 5±15%, APLN-13: 78±12% p<0.05, ELA: -2±9 %).

**CONCLUSION.** A differential hemodynamic signature of APLN-13 and ELA was observed in healthy and septic sheep opening new physiological questions on the apelinergic system regulatory role in the cardiovascular function. The APLN-13/APJ axis is emerging as a new candidate pathway to manage hemodynamics in septic shock and could be an alternative to catecholamines.

##### REFERENCE(S)

1. PMIDs 1. 28777197, 2. 29347994
2. CRCHUS projet structurant 2018-19, CIHR (# 376770-201610; 398298-201710; 399567-201803).

### 001389

#### Cardiac fibroblastic LRP1-dependent endocytosis of liver angiotensinogen promotes septic cardiac dysfunction via NLRP3 inflammasome pathway

J. Rong[1], Z. Zhang[2], Y. Xu[1], J. Wang[1]
[1]Department of cardiology, cardiovascular key laboratory of zhejiang province, Second Affiliated Hospital, Zhejiang University School of Medicine, Hangzhou, China; [2]Department of intensive care unit, Second Affiliated Hospital, Zhejiang University School of Medicine, Hangzhou, China
**Correspondence:** Y. Xu
*Intensive Care Medicine Experimental* 2019, **7(Suppl 3):**001389

**INTRODUCTION.** Sepsis combined with cardiac dysfunction leads to a significant increase in mortality. The renin-angiotensin system (RAS system) plays an important role in the development of sepsis induced myocardial dysfunction (SIMD), while angiotensinogen (AGT) is the only known substrate of the RAS system, and its effect on SIMD has not been illuminated.

**OBJECTIVES.** To investigate whether inhibition of AGT ameliorates SIMD and explore its potential mechanism.

**METHODS.** Male C57BL/6 mice were intraperitoneally injected by either lipopolysaccharide (LPS) or PBS, and then taken down after 6 hours. Cardiac function was evaluated by echocardiography and the survival rate was monitored every hour for a 72-hour period. Plasma AGT level was measured by ELISA while hepatic and cardiac AGT expression were determined by real-time quantitative PCR (RT-qPCR) and western blot. Expression of proinflammatory cytokine was assessed by RT-qPCR. Combination of AGT and its receptor, low density lipoprotein receptor related protein1 (LRP1), was confirmed by surface plasmon resonance (SPR).

**RESULTS.** LPS injection significantly activated AGT expression both in liver and heart, and also elevated plasma AGT level after 6 hours. We further generated hepatocyte-specific AGT-deficient (hepAGT-/-) mice and cardiomyocyte-specific AGT-deficient (carAGT-/-) mice. Interestingly, we found that hepatic AGT knock-out led to a distinct reduction of AGT expression in liver but in heart it showed a remarkably decreased AGT protein level with a compensatory augment of mRNA level. However in carAGT-/- mice, cardiac AGT expression was restrained in mRNA level but did not change in protein level, while hepatic AGT remained unchanged compared with their wild-type counterparts. These facts implied that cardiac AGT was mainly derived from circulation rather than local synthesis. We then demonstrated that hepatic conditional AGT deletion in mice triggered resistance to septic cardiac dysfunction and lethality accompanied with less cardiac proinflammatory cytokine production, like tumor necrosis factor alpha (TNF-α), Interleukin 1 beta (IL-1β), and Interleukin 6 (IL-6). Contrarily, cardiac conditional AGT deletion in mice had a similar phenotype with wild-types. Furthermore, we identified liver derived AGT entered cardiac fibroblast via LRP1 mediated endocytosis, which in turn activated NLRP3 inflammasome and improved TNF-α, IL-1β as well as IL-6 production.

**CONCLUSION.** Hepatocyte-specific deficiency of AGT ameliorates SIMD via preventing cardiac fibroblastic LRP1-dependent endocytosis and then decreasing NLRP3 inflammasome assembly, which alleviates proinflammatory factors releasing. These findings provide potential therapeutic targets in liver to treat SIMD.

**REFERENCE(S)**

1. Merx MW, Weber C. Sepsis and the heart. Circulation. 2007;116(7):793-802.
2. Salgado DR, Rocco JR, Silva E, Vincent JL. Modulation of the renin-angiotensin-aldosterone system in sepsis: a new therapeutic approach? Expert Opin Ther Targets. 2010;14(1):11-20.
3. Lu H, Cassis LA, Kooi CW, Daugherty A. Structure and functions of angiotensinogen. Hypertens Res. 2016;39(7):492-500.
4. This study was supported by national natural science foundation of China (No.81471889 and No. 81772110 to ZZC, No. 81500176 to XYC).

### 001019

#### Does sepsis affect tissue concentrations of antibiotic? A pre-clinical study with in-vivo monitoring of meropenem using microdialysis

K. Tam[1], I. Longo[2], D. Brealey[3], M. O'connell[4], M. Singer[5]

[1]Intensive care medicine, University College London, London, United Kingdom; [2]Anaesthesia and intensive care medicine, Azienda Sanitaria Universitaria Integrata di Trieste, Trieste, Italy; [3]Critical care, UCL Hospitals NHS Foundation Trust , London, United Kingdom; [4]Director, Probe Scientific Ltd, Thurleigh, United Kingdom; [5]Bloomsbury institute of intensive care medicine, University College London, London, United Kingdom

**Correspondence:** K. Tam

*Intensive Care Medicine Experimental* 2019, **7(Suppl 3):**001019

**INTRODUCTION.** Antibiotic handling is significantly altered in sepsis and other critical illnesses due to changes in metabolism and excretion, protein binding and volumes of distribution [1,2]. This handling will vary markedly between septic patients yet antibiotics are generally given as a 'one-size-fits-all' dosing regimen with fixed adjustments only made for severe renal dysfunction. Monitoring of tissue antibiotic concentrations should enable better titration of dose to avoid under- or over-treatment [3].

**OBJECTIVES.** To compare tissue and blood concentrations of meropenem in sham rats and during the early stage of sepsis (faecal peritonitis) using a type of microdialysis probe, MicroEye®,and compare these levels with that measured in blood and healthy rats.

**METHODS.** Male Wistar rats (300-350g) underwent brief general anaesthesia to enable instrumentation for vascular access. They were randomised to a sham control group or a septic group that received an intraperitoneal injection of faecal slurry to induce peritonitis. Fluid resuscitation was commenced from 2 hours. At 6 hours, the animals were re-anaesthetised and MicroEye® (Probe Scientific Ltd, Bedford, UK) microdialysis catheters were inserted into muscle, subcutaneous tissue, liver, peritoneal cavity, trachea and jugular vein. Meropenem 30mg/kg was administered over 30 min with tissue and blood microdialysates sampled respectively at 10 and 15 min intervals for a total of 90 min. Meropenem concentrations were measured using High Performance Liquid Chromatography (HPLC, Agilent 1260II). Blood samples were also taken for a direct measurement of meropenem levels. ANOVA with post-hoc Dunn's tests were used to seek statistical significance.

**RESULTS.** Following a single bolus of meropenem, there was a similar pattern of microdialysate drug concentration changes over time in all tissues in both sham and septic groups [Figures 1,2]. Peak concentrations were reached by 50-60 minutes in both study groups (this includes a 20 minute lag-time for deadspace tubing. There was no statistically significant difference in the area under the curves (AUC) for meropenem concentration in most tissues, except for trachea ($p$<0.05) and peritoneum (p=0.11) that were higher in the septic group [Figure 3].

**CONCLUSION.** In early sepsis, tissue concentrations were generally similar to sham rats, except within the trachea. Further work is needed to increase sample size and to perform repeat studies at later timepoints.

**REFERENCE(S)**

[1] De Paepe P et al. Pharmacokinetic and pharmacodynamic considerations when treating patients with sepsis and septic shock. Clin Pharmacokinet. 2002; 41:1135-1151

[2] Varghese, JM et al. Antimicrobial pharmacokinetic and pharmacodynamic issues in the critically ill with severe sepsis and septic shock. Crit Care Clin. 2011; 27:19-34

[3] Roberts JA. et al. Therapeutic drug monitoring of antimicrobials. Br J Clin Pharmacol. 2012; 73:27-36

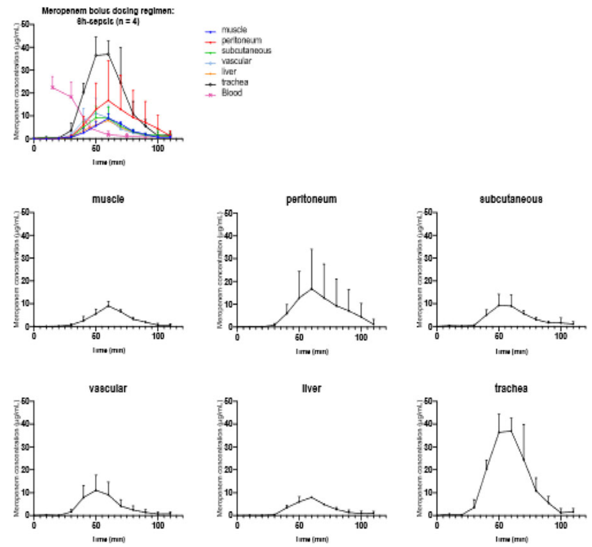

**Fig. 1 (abstract 001019).** Tissue concentration/time profiles following a meropenem bolus in septic rats at 6h

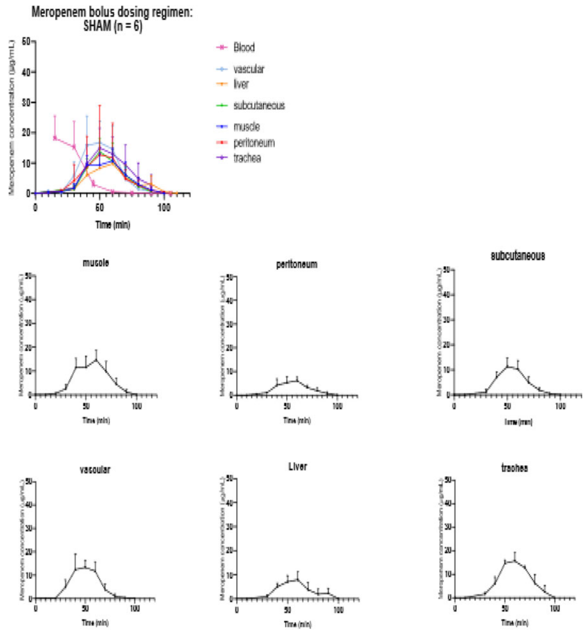

**Fig. 2 (abstract 001019).** Tissue concentration/time profiles following a meropenem bolus in sham rats at 6h

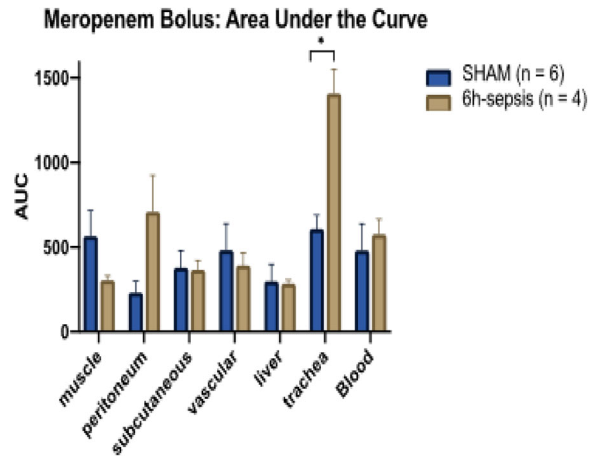

**Fig. 3 (abstract 001019).** Comparison of tissue concentration AUC between 6h-septic and sham-operated rats

### ARF - Of muscles, power, imaging and gas exchange

#### 000732

#### Electrical Impedance Tomography (EIT) reveals occult Pendelluft in difficult to wean patients

A. Coppadoro[1], A. Grassi[2], V. Ormas[2], C. Giovannoni[2], V. Meroni[2], D. Manzolini[2], D. Celsi[2], N. Eronia[1], G. Foti[2], G. Bellani[2]
[1]Anesthesia and Intensive care, Ospedale San Gerardo di Monza, Monza, Italy; [2]School of medicine and surgery, University of Milano-Bicocca, Monza, Italy
**Correspondence:** A. Coppadoro
*Intensive Care Medicine Experimental* 2019, **7(Suppl 3):**000732

**INTRODUCTION.** Pendelluft, defined as movement of gas within the lung from regions characterized by a short time-constant to regions with long time-constant, has been described in animal models of assisted mechanical ventilation and associated with lung tissue overstretching [1]. Due to the different contents of $O_2$ and $CO_2$ as compared to fresh gas, pendelluft might also reduce ventilation efficiency. We hypothesized that during weaning, increased patient's inspiratory effort due to withdrawal of ventilator support might trigger pendelluft phenomenon, exacerbating patient's fatigue.

**METHODS.** We enrolled 18 difficult weaning patients undergoing pressure support ventilation (PSV). Electrical impedance tomography (EIT) signal was recorded while PSV was progressively reduced from clinical level (baseline) to 2 $cmH_2O$ (or the lowest tolerable level); four ventral-to-dorsal lung regions of interest were identified for pendelluft measurement. A regional gas movement (>5ml) occurring in a direction opposite to the global EIT signal was considered diagnostic for pendelluft.

**RESULTS.** Pendelluft was detected by EIT in 8/18 patients; in those patients PSV reduction increased pendelluft (p=0.036), in the others it did not (see Figure). In pendelluft patients, PSV reduction was associated with decreased tidal volumes (from 415 [377-502] to 355 [272-402] mL, p=0.012) and increased respiratory rates (from 23 [15-27] to 29 [20-36] breaths/min, p=0.017), while minute ventilation remained stable (from 9 [6.4-11.3] to 9 [7-11.9] L/min, p=NS). In the no-pendelluft group, the change in tidal volumes was not significant, as were the change in respiratory rate and minute ventilation (all p= NS). $EtCO_2$ increased in the pendelluft group (from 38 [32-43] to 40 [33-49] mmHg, p=0.027), but not in the no-pendelluft group.

**CONCLUSION.** Occult pendelluft can be measured by EIT, and is frequently present in difficult to wean patients. When present, pendelluft increases with the reduction of ventilator support and is associated with a significant change of the ventilatory pattern. Pendelluft-associated ventilatory inefficiency might play a role in weaning failure.

**REFERENCE(S)**

1] Spontaneous effort causes occult pendelluft during mechanical ventilation, Yoshida T et al., Am J Respir Crit Care Med. 2013 Dec 15;188(12):1420-7. doi: 10.1164/rccm.201303-0539OC.
2] UNIMIB department funds

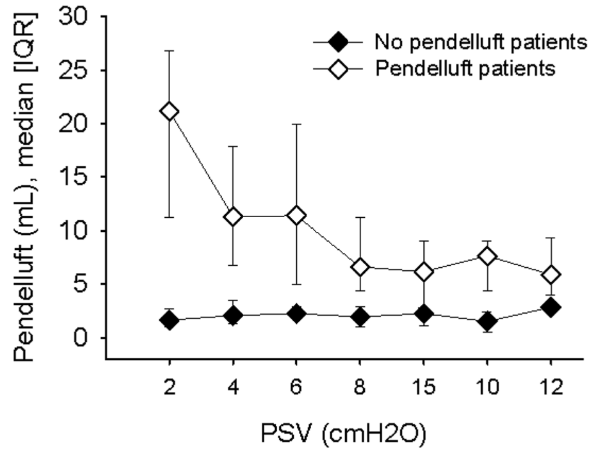

**Fig. 3 (abstract 000732).** See text for description

#### 000965

#### Ultra-protective ventilation without extracorporeal circulation in moderately severe and severe ARDS patients (for the REVA research network)

JC. Richard[1], S. Marque[2], A. Gros[3], M. Muller[4], G. Prat[5], G. Beduneau[6], JP. Quenot[7], J. Dellamonica[8], R. Tapponnier[9], E. Soum[10], L. Bitker[1], J. Richecoeur[11]
[1]Médecine intensive et réanimation, Hospital La Croix-Rousse - Hcl, Lyon, France; [2]Intensive care unit, Centre Hospitalier Sud Francilien, Corbeil-Essonnes, France; [3]Intensive care unit, C.H. de Versailles, Le Chesnay, France; [4]Intensive care unit, C.H. Annecy Genevois, Metz-Tessy, France; [5]Intensive care unit, CHU de Brest, Brest, France; [6]Medical intensive care unit, CHU de Rouen, Rouen, France; [7]Medical intensive care unit, Chu Dijon, Dijon, France; [8]Intensive care unit, CHU de Nice, Nice, France; [9]Intensive care unit, Centre Hospitalier Lyon Sud - HCL, Lyon, France; [10]Medical intensive care unit, CHU de Clermont-Ferrand, Clermont-Ferrand, France; [11]Intensive care unit, Centre hospitalier De Beauvais, Beauvais, France
**Correspondence:** J.C. Richard
*Intensive Care Medicine Experimental* 2019, **7(Suppl 3):**000965

**INTRODUCTION.** Ultra-protective ventilation with tidal volume (VT) reduction below 6 ml/kg predicted body weight (PBW) in severe ARDS may reduce alveolar strain, driving pressure and hence ventilator-induced lung injury, with main drawback of worsening respiratory acidosis. We hypothesized that VT could be reduced down to 4 ml/kg PBW, with clinically significant decrease in driving pressure, without the need for extracorporeal CO2 removal, while maintaining pH in the range targeted in recent randomized controlled trials on ARDS.

**METHODS.** We conducted a non-experimental before-and-after multicenter study on 35 ARDS patients with PaO2/FiO2 ≤ 150 mm Hg, within 24 hours of ARDS diagnosis. After inclusion (H0), VT was reduced to 4 ml/kg PBW and further adjusted to maintain pH ≥ 7.20, respiratory rate was increased up to 40/min and PEEP was set using the PEEP-FiO2 table of the high PEEP arm of the ALVEOLI trial. This strategy was applied until positivity of a PEEP weaning trial. The primary judgment criterion was the driving pressure on day 2 of the study, as compared to study inclusion. Data are presented as median [1st quartile-3rd quartile].

**RESULTS.** One patient's next of kin withdrew consent leaving 34 analyzable patients. Patients' age was 67 [53-73] year, SAPS II amounted to 50 [35-58], 29 patients (85%) had pneumonia as ARDS risk factor, SOFA at inclusion was 13 [11-14], and PaO2/FiO2 at inclusion was 101 [78-132] mm Hg under a PEEP of 10 [8-12] cmH2O. From inclusion to day 2, driving pressure decreased significantly from 12 [9-15] to 8 [6-11] cmH2O, while VT decreased from 6.0 [5.9-6.1] to 4.1 [4.0-4.7] ml/kg PBW. On day 2 of the study, VT was below 4.2 ml/kg in 22 patients (65% [IC95% 48%-79%]), and below 5.25 ml.kg-1 in 30 patients (88 % CI95% [73%-95%]). Time with VT below 4.2 ml/kg and 5.25 ml/kg averaged 2 [0.5-2.0] days and 2 [1-4] days, respectively. Respiratory rate increased significantly from 28 [23-30] /min to 40 [35-40]/min and 37 [30-40]/min on day 2 and 3 of the study, respectively. PEEP was significantly increased from hour 4 to day 3 after inclusion as compared to baseline, while intrinsic PEEP was not significantly modified during the first four days of the study. PaO2/FiO2 and PaCO2 increased significantly from baseline values as early as hour 4 up to day 3 after inclusion, while pH was significantly lower than baseline only at hour 4 and hour 10. Sedation drugs were not significantly modified.

Regarding safety, 2 patients (6%) presented with acute cor pulmonale after inclusion. Right ventricle/left ventricle ratio increased non-significantly from 0.50 [0.50-0.72] at H0 to 0.66 [0.60-0.76] on day 2. Eleven patients (32%) presented with severe mixed acidosis with pH < 7.15. Multivariate analysis identified renal SOFA at inclusion (OR: 1.91 [CI95% : 1.08-3.71] per 1 unit increase) and pH at inclusion (OR: 0.91 [CI95% : 0.80-0.99] per 0.01 unit increase) as variables independently associated with occurrence of any episode of severe mixed acidosis during the study. Fourteen patients (41%) died before day 90 with median delay between inclusion and death amounting to 16 [9-22] days.

**CONCLUSION.** Ultraprotective ventilation may be applied in approximately 2/3 of moderately severe to severe ARDS patients, with a 4 cmH2O median reduction of driving pressure, at the price of severe mixed acidosis in approximately 1/3 of the patients.

**REFERENCE(S)**

1. The study was endorsed by the REVA research network

### 001030

#### Dysfunction of cerebral autoregulation in adult patients treated with veno-venous Extracorporeal Membrane Oxygenation (v-v ECMO) for severe Acute Respiratory Distress Syndrome (ARDS). An observational prospective study

AT. Mazzeo[1], G. Catozzi[1], S. Caccia[1], I. Battaglini[1], V. Fanelli[1], L. Mascia[2]
[1]Department of surgical sciences, anaesthesia and intensive care, University of Turin, Turin, Italy; [2]Department of medico-surgical sciences and biotechnologies, Sapienza University of Rome, Rome, Italy
**Correspondence:** A.T. Mazzeo
*Intensive Care Medicine Experimental* 2019, **7(Suppl 3):**001030

**INTRODUCTION.** Patients with severe ARDS are likely to develop cerebral hemodynamic impairment due to respiratory failure leading to hypoxemia and hypercapnia. ECMO therapy, by restoring normal level of PaO2 and PaCO2, is a life-saving treatment when hypoxemia is refractory to conventional treatment. However, ECMO support itself may cause neurological complications, affecting both long- and short-term outcome.

**OBJECTIVES.** Aim of the study was to evaluate cerebral autoregulation (CA) impairment in ARDS patients undergoing v-v ECMO and to assess whether normalization of arterial blood gases induced by the treatment can restore this dysfunction.

**METHODS.** Adult patients with severe ARDS requiring v-v ECMO were consecutively enrolled in our university hospital. Prior neurological disease and any condition hampering cerebral blood flow measurement were excluded from study. CA was assessed using Mxa, a Transcranial Doppler-derived index of dynamic CA, expressing the correlation between mean flow velocity in middle cerebral artery and mean arterial pressure; a Mxa values >0.20 shows impaired CA. Transcranial Doppler was performed before ECMO institution (PRE), at day 0, 1, 2, 4 and 7 of ECMO therapy and after ECMO removal (POST). Hemodynamic and respiratory parameters were recorded concurrently. Data are presented as median (IQR).

**RESULTS.** We enrolled 31 patients: age 48 (41-57) yrs; male 65%; pneumonia was the cause of ARDS in 94% of patients; ICU length of stay 27 (16-38) days; v-v ECMO duration 13 (8-17) days; APACHE II 26 (23-34); SAPS II 66 (60-72); 28-days mortality 35%. During ICU stay, clinical neurologic complications confirmed at neuroimaging were found in 5 (16%) patients, 3 ischemic and 2 hemorrhagic. Mxa was 0.25 (-0.12-0.50) PRE ECMO, 0.13 (0.04-0.20), -0.05 (-0.08-0.09), -0.03 (-0.19-0.05), 0.04 (-0.04-0.21) and -0.01 (-0.05-0.08) at day 0, 1, 2, 4 and 7 of ECMO therapy, respectively, and 0.04 (0.00-0.18) POST-ECMO, showing a trend toward restoration of CA from PRE-ECMO to day 7 (p=0.063). Furthermore, restored CA was maintained also after ECMO removal. Concomitant values of PaCO2 in mmHg were 54 (44-79) PRE ECMO, 47 (35-54), 46 (42-56), 44 (39-52), 44 (41-52), 44 (39-52) at day 0, 1, 2, 4 and 7 of ECMO therapy, respectively, and 44 (41-49) POST-ECMO; concomitant values of pH were 7.33 (7.16-7.46) PRE-ECMO, 7.42 (7.34-7.46), 7.42 (7.35-7.46), 7.43 (7.39-7.48), 7.42 (7.37-7.47), 7.40 (7.36-7.46) at day 0, 1, 2, 4 and 7 of ECMO therapy, respectively, and 7.43 (7.33-7.46) POST-ECMO. Significant correlations were found between Mxa and values of PaCO2 (p=0.002), pH (p< 0.001) and hemoglobin (p=0.001). A pH value of 7.35 could discriminate between impaired and preserved CA with a sensitivity of 0.88 and a specificity of 0.59.

**CONCLUSION.** In ARDS patients with refractory hypoxia, v-v ECMO therapy through normalization of arterial blood gas levels, may restore CA. Mxa values during ECMO support are significantly correlated with PaCO2, pH and hemoglobin.

### 001233

#### Timing of diaphragmatic and intercostal neural activation during weaning from mechanical ventilation

R. Di Mussi[1], DC. Francesca[1], R. Iannuzziello[1], S. Spadaro[2], G. Frasso[1], F. Murgolo[1], T. Stripoli[1], S. Grasso[1]
[1]University of bari "aldo moro", Department of emergencies and organ transplant, Bari, Italy; [2]Department of morphology, surgery and experimental medicine, University of Ferrara, Ferrara, Italy
**Correspondence:** R. Di Mussi
*Intensive Care Medicine Experimental* 2019, **7(Suppl 3):**001233

**INTRODUCTION.** Surface electromyography allows to detect non-invasively and at the bedside the inspiratory activation of diaphragmatic and intercostal muscles.Our hypothesis was that the timing of neural activation of these muscles during a spontaneous breathing trial could be different in patients successfully extubated and in patients failing weaning.

**OBJECTIVES.** To evaluate the timing of diaphragmatic and intercostal neural activation during weaning from mechanical ventilation in critically ill patients.

**METHODS.** In 24 patients, who underwent at least 72 hours of mechanical ventilation, surface electromyography, s-EMG (Dräger, Lubeck, Germany), was applied for 5-8 minutes during a spontaneous breathing trial (SBT).The delta time between the beginning of diaphragmatic neural activation and the peak of intercostal muscles neural activation was calculated on a breath by breath basis (fig.1). Further, for each patient, we recorded the percentage of breaths in which the neural activation of the intercostal muscles occurred after the peak of the diaphragmatic neural activation (i.e. in the diaphragmatic expiratory phase).

**RESULTS.** Fourteen patients were successfully extubated whereas 10 patients were reintubated within 48 hours.

In failed vs successful weaning the delta time between the peak of diaphragmatic and the intercostal muscles neural activation was respectively 0,63 ± 0,20 and 1 ± 0,39 sec (p < 0.05). (FIG. 2)
The % of intercostal muscles activation occurring in the diaphragmatic expiratory phase was respectively 63,8% ± 21,34% vs 14,26%± 8,22% (p< 0.05) in failed vs successful weaning. (FIG. 2)
**CONCLUSION.** In the present study the delta time between the diaphragm and the intercostal muscles activation and the neural activation of the intercostal muscles during the diaphragmatic expiratory phase were higher in patients failing weaning than in patients successfully weaned from mechanical ventilation.
We speculate that s-EMG may be a promising tool to predict weaning failure in a non-invasive way.

**REFERENCE(S)**

1. Doorduin J1, Roesthuis LH, Jansen D, van der Hoeven JG, van Hees HWH, Heunks LMA., Respiratory Muscle Effort during Expiration in Successful and Failed Weaning from Mechanical Ventilation. Anesthesiology. 2018 Sep;129(3):490-501.

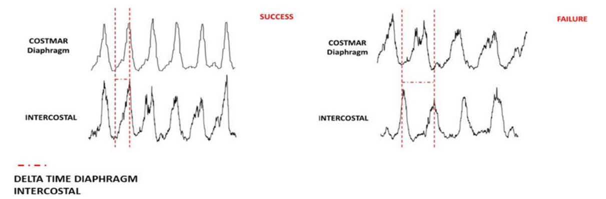

**Fig. 1 (abstract 001233).** See text for description

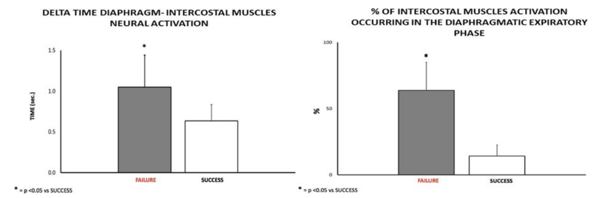

**Fig. 2 (abstract 001233).** See text for description

### 001529

#### Does mechanical ventilation power have an effect on prognosis?

E. Senturk[1], Y. Inel[2], Z. Cukurova[3], S. Ugur[1], S. Asar[3], R. Yılmaz[3], A. Esin[1], GO. Hergünsel[3], S. Erus[4], N. Cakar[1]
[1]Anesthesiology and reanimation, Koc University, Istanbul, Turkey; [2]Anesthesiology and reanimation, American Hospital , Istanbul, Turkey; [3]Anesthesiology and reanimation, Sadi Konuk Research Hospital, Istanbul, Turkey; [4]Thorasic surgery, Koc University , Istanbul, Turkey
**Correspondence:** E. Senturk
*Intensive Care Medicine Experimental* 2019, **7(Suppl 3):**001529

**INTRODUCTION.** Recently, the role of ventilation-associated mechanical ventilation power (MVP) has been emphasized in lung injury. It is argued that MVP correlates with ventilation-induced lung injury (VILI) and prognosis. However, the relationship between MVP and prognosis needs to be further investigated. Electronic recording systems (ERS) can easily calculate mechanical ventilation power by using mechanical ventilator parameters.
**OBJECTIVES.** The aim of this retrospective study was to evaluate the relationship of the first 24 hours MVP with the survival and length of ICU stay.
**METHODS.** ERS data were retrospectively reviewed for patients receiving invasive mechanical ventilation for ≥ 24 hours and ICU stay ≥48 hours between 2015-2018. Patients ≥ 18 years were included. During the first 24 hours, MVP was calculated every 30 minutes and averaged.Formula: MVP(J/minutes)= 0.098 x tidal volume x respiratory rate x ( Ppeak – ½ ΔP)ΔP is driving pressure. Based on a previous study, the patients were classified into two groups (Group 1: MP < 17 J/min, Group 2: MP ≥17 J/min) (ref 1).
Oxygenation, gas exchange values, and APACHE II and SOFA scores in the first 24 hours and survival were recorded. The primary outcome was ICU mortality.
**RESULTS.** A total of 1219 patients were included in the study. A significantly higher mortality rate was detected in Group 2 (p<0.001 Table 1). Demographic data and relation of MVP and driving pressure with mortality are shown in Table 2 and 3.
**CONCLUSION.** Our results suggest that higher baseline MVP values are related with poorer outcome.

**REFERENCE(S)**

1. Ary Serpa Neto et.al. Intensive Care Med (2018) 44:1914–1922 . Mechanical power of ventilation is associated with mortality in critically ill patients: an analysis of patients in two observational cohorts

**Table 1 (abstract 001529).** Driving pressure and Mortality

| | Driving pressure<15 | Driving pressure ≥ 15 | p |
|---|---|---|---|
| Mortality | 60.6 | 64.3 | 0.001 |

**Table 2 (abstract 001529).** Mortality and Power

| | Power <17 | Power ≥ 17 | p |
|---|---|---|---|
| Mortality | 44.9 | 63.5 | 0.01 |

**Table 3 (abstract 001529).** Demographic

| | Power<17 mean | SD | Power ≥ 17 mean | SD | p |
|---|---|---|---|---|---|
| Apache II | 20.8 | 8.9 | 23 | 8.1 | 0.6 |
| SOFA | 8.1 | 3.9 | 8.6 | 3.9 | 0.8 |
| ICU stay hours | 410.6 | 465 | 442 | 444 | 0.5 |
| Gender (Male %) | 59 | | 63 | | 0.3 |
| Predicted body weight | 63 | 9.5 | 64 | 8.9 | 0.07 |

## AKI - Epidemiology and early diagnosis

### 000288

#### PenKid at admission is predictive of outcome in severely ill burn patients: A report from the PRONOBurn cohort

D. François[1], H. Alexa[2], S. Joachim[3], H. Oliver[3], A. Juliette[1], P. Adrien[1], A. Mebazaa[1], L. Matthieu[1]
[1]Department of anaesthesiology, surgical intensive care and burn unit, Saint-Louis Hospital, Paris, France; [2]Department of anesthesia, surgical intensive care, University Hospital of Basel, Basel, Switzerland; [3]Sphingotec gmbh, Sphingotec GmbH, Hennigsdorf, Germany
**Correspondence:** D. François
*Intensive Care Medicine Experimental* 2019, **7(Suppl 3):**000288

**INTRODUCTION.** Proenkephalin A 119-159 (penKid) is a filtration marker and has been proposed as a sensitive biomarker of glomerular function. In this ancillary study of the PRONOburn cohort we evaluated the association of admission plasma penKid with kidney events in severely ill burn patients.
**METHODS.** PRONOburn is a French prospective, observational study designed to investigate factors associated with outcome of burn patients. All consecutive severely burn patients (inclusion criteria: age>18 years, total body surface area burn >15% and non-opposition to participate) admitted to 2 participating burn intensive care units (ICU) in France were included. The primary endpoint of this ancillary study was to evaluate the predictive value of penKid for 90 day mortality. Secondary endpoints were to evaluate the predictive value of penKid for in-ICU AKI, as well as for a combined endpoint (90 mortality and/or in-ICU AKI). AKI was defined using the KDIGO definition. Subclinical AKI was defined as patient without AKI in the first seven days but a penKid concentration>80pmol/L on admission.

**RESULTS.** From April 2014 to April 2016, 109 consecutive patients were enrolled. Mean age was 48 (IQ 33-64) years, mean Sequential related organ failure assessment was 4 (IQ1-4), 67% (N=73) were male. Total burn surface area was 35 (IQR 25-53). Overall 90 day mortality was 27.5 % (N=30), 35.7% (N=39) had AKI during ICU stay and 44% (N=48) had the combined endpoint. Penkid was significantly higher in non-survivors compare to survivors (113.2pmol/L [IQR 57.5-182.1] vs 54.8pmol/L [IQR 36.0-76.7]; p<0.0001) and in patient with in-ICU AKI compare to patient without (91.4pmol/L [IQR 56.5-153.4] vs 53.1pmol/L [IQR 34.4-67.9]; p<0.0001). Penkid provided added value on top of creatinine and SOFA score to predict 90 day mortality (combined c index=0.766 vs. 0.716; p=0.004 and 0.812 vs. 0.762; p<0.001, respectively). PenKid c index to predict in-ICU AKI was 0.755. Penkid added value on top of creatinine and SOFA score to predict the combined endpoint (c index=0.839 vs. 0.799; p=0.006 and 0.878 vs. 0.806; p<0.001, respectively). Day 3 penKid measurements added value on top of admission measurement to detect both mortality and the combined end point. Finally, there was a progressive increase risk of mortality from patients without AKI, sub-AKI and AKI (Survival rate 91% [95% CI 83.8-98.9], 66.7% [95% CI 48.1-92.4] and 40.4% [95% CI 26.1-62.4]; respectively, p<0.0001).

**CONCLUSION.** Admission penKid concentration was associated with risk of mortality in burn patients. It added value on top of creatinine and SOFA to predict 90 day mortality. Sub-AKI detected with penKid was associated with risk of death.

**REFERENCE(S)**

1. We thank the Clinical Research Assistants and Elisabeth Cerrato from the joint research unit HCL/bioMérieux for technical assistance with the samples.
2. This study was supported by a grant from the "Association des Gueules cassées" to Matthieu Legrand. Measurement of penKid was funded by sphingotec.

### 000291

#### Sub-clinical acute kidney injury detection using penKid®: A report from the FROG-ICU and AdrenOSS-1 cohorts

H. Alexa[1], D. François[2], H. Oliver[3], S. Joachim[3], L. Pierre-François[4], G. Etienne[5], A. Mebazaa[2], L. Matthieu[2]
[1]Department of anesthesia, surgical intensive care, University Hospital of Basel, Basel, Switzerland; [2]Department of anaesthesiology, surgical intensive care and burn unit, Saint-Louis Hospital, Paris, France; [3]Sphingotec gmbh, Sphingotec GmbH, Hennigsdorf, Germany; [4]Department of critical care medicine, Cliniques Universitaires Saint-Luc, Woluwe-Saint-Lambert, Belgium; [5]Department of anaesthesiology, surgical intensive care and burn unit, Hospital Lariboisière, Paris, France
**Correspondence:** D. François
*Intensive Care Medicine Experimental* 2019, **7(Suppl 3):**000291

**INTRODUCTION.** Plasma proenkephalin A 119-159 (penKid) is a filtration marker and has been proposed as a sensitive biomarker of glomerular function. As such it was found predictive for septic kidney events including acute kidney injury (AKI) in sepsis. In this ancillary study of the FROG-ICU and AdrenOSS-1 cohorts we evaluated the association between subclinical acute kidney injury (sub-AKI; defined by supra-normal penKid) and outcome.

**METHODS.** FROG-ICU was a European prospective, observational study of 2087 critically ill patients designed to investigate long-term mortality in patients with invasive mechanical ventilation and/or vasopressors or inotropes for more than 24 hours. AdrenOSS-1 was a European prospective, observational study with 583 sepsis and septic shock patients. sub-AKI was defined by an admission penKid concentration above the normal range (i.e. > 80 pmol/L) in patients without acute kidney injury (AKI). AKI was defined by the KDIGO (Kidney Disease: Improving Global Outcomes) criteria.

**RESULTS.** 2015 from the FROG cohort and all 583 patients from the AdrenOSS cohort were eligible for data analysis. 28-day mortality was 22% in both cohorts. 8.6% and 9.4% of patients from the FROG and AdrenOSS cohorts had sub-AKI (11.5% and 17.5% of non-AKI; respectively). Median SOFA score was similar in non-AKI and sub-AKI patients in the FROG cohort, and slightly higher for sub-AKI in AdrenOSS-1 (6 vs 5 points, p=0.011). Compared to non-AKI patients, patients with sub-AKI were more likely to receive hemodynamic support (p<0.001). We found a significant stepwise increase of 28-day mortality risk from non-AKI (9.1%) to sub-AKI (20.5%) to AKI patients (34.9%) in the FROG cohort, and the AdrenOSS cohort (8.7%, 21.1% and 28.7%, respectively). In all patients, admission penKid concentration was associated with 28-day mortality: c index = 0.664 (p<0.001) for penKid and 0.618 (p<0.001) for creatinine in the FROG cohort and 0.661 (p<0.001) for penKid and 0.589 (p=0.006) for creatinine in the AdrenOSS cohort.

**CONCLUSION.** Sub-AKI detected using admission penKid was associated with high cardiovascular failure and short-term mortality in critically ill patients. PenKid better predicted 28-day mortality risk than serum creatinine.

### 000960

#### Proenkephalin, a novel biomarker for kidney function, is earlier in detecting acute kidney injury compared to creatinine

R. Beunders[1], M. Meekes[1], S. Joachim[2], P. Pickkers[1]
[1]Intensive care department, Radboud University Medical Center, Nijmegen, Netherlands; [2]Sphingotec gmbh, sphingotec GmbH, Hennigsdorf, Germany, Hennigsdorf, Germany
**Correspondence:** R. Beunders
*Intensive Care Medicine Experimental* 2019, **7(Suppl 3):**000960

**INTRODUCTION.** Acute kidney injury (AKI) is independently associated with prolonged hospital stay and impaired outcome. Creatinine is most used to assess kidney function. However, following a decrease in glomerular filtration rate, plasma creatinine concentration increase is insensitive and late. This hampers the diagnosis of AKI and research related to therapies. The novel plasma biomarker proenkephalin (PENK), a stable byproduct of the endogenous opioid enkephalin, is currently studied for its reflection of kidney function and thereby detection of AKI. Kinetics of PENK during a period of AKI compared to creatinine are unknown.

**OBJECTIVES.** To determine whether or not the plasma biomarker PENK is able to diagnose AKI earlier than plasma creatinine.

**METHODS.** In this prospective study, all patients admitted to our Intensive Care Unit (ICU) with an ICU duration >1 day and not already fulfilling AKI criteria on admission were included. Plasma creatinine concentrations were determined using daily routine sampling. Residual material was collected and frozen to determine PENK retrospectively using Sphingotec immunoassay [1]. AKI was diagnosed using the KDIGO criteria [2] with the most sensitive (AKI-stage 1) measure of a creatinine concentration rise of 0.3 mg/dL (26.5 μmol/L) within 2 days. One sample t-test was used to assess the difference in time of the increase before and decrease after the occurrence of AKI. Receiver operating characteristics (ROC) curves were created to assess the diagnostic performance of PENK.

**RESULTS.** We included 189 critically ill patients on the ICU, median [IQR] age was 65 [55-72] years, 119 patients (63%) were male. 56 patients (24%) developed AKI during ICU admission. PENK concentrations increased (mean±SEM) 0.4±0.08 day earlier than creatinine ($p<$ 0.0001), figure 1-A. When creatinine decreased, PENK concentrations also decreased 0.7±0.22 day earlier. The area under the ROC curve of the PENK concentration the day before the diagnosis of AKI was 0.82 (95%CI 0.75-0.89), with an optimal cut-off value of 66 pmol/L resulting in sensitivity and specificity of 83% and 75%, respectively, see figure 1-B. The positive and negative likelihood ratio was 3.3 and 0.30, respectively.

**CONCLUSION.** PENK is freely filtrated through the glomerulus without active secretion or resorption and may therefore be capable to detect AKI more swiftly compared to plasma creatinine. Furthermore, PENK plasma concentrations the day before diagnosis of AKI as a diagnostic test is classified as good in an unselected population of critically ill patients.

**REFERENCE(S)**

1. (KDIGO), K.D.I.G.O., Kidney disease: Improving global outcomes (KDIGO) acute kidney injury work group. KDIGO clinical practice guideline for acute kidney injury. Kidney International Supplements, 2012. 2(1): p. 1-138.
2. Donato, L.J., et al., Analytical performance of an immunoassay to measure proenkephalin. Clin Biochem, 2018. 58: p. 72-77.

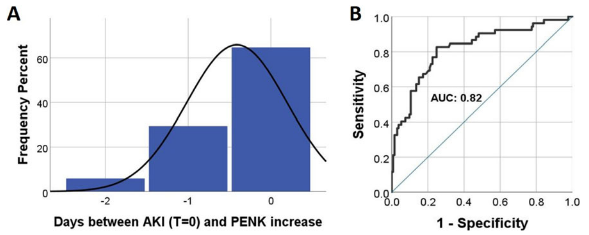

**Fig. 1 (abstract 000960).** See text for description

### 001186

### Signs of venous congestion and Acute Kidney Injury in critically ill patients: a SICS-II substudy

JP. De Bruin[1], R. Wiersema[1], T. Kaufmann[2], RJ. Eck[3], J. Koeze[1], F. Keus[1], ICC. Van Der Horst[1], Sics Study Group[1]

[1]Critical care, University Medical Center Groningen, Groningen, Netherlands; [2]Anaesthesiology, University Medical Center Groningen, Groningen, Netherlands; [3]Internal medicine, University Medical Center Groningen, Groningen, Netherlands

**Correspondence:** R. Wiersema

*Intensive Care Medicine Experimental* 2019, **7(Suppl 3):**001186

**INTRODUCTION.** Venous congestion is considered to play a role in the development of Acute Kidney Injury (AKI). Currently no strict definition for venous congestion exists and all proxies have limitations.

**OBJECTIVES.** To assess whether signs of venous congestion, defined as interstitial pulmonary syndrome and as a non-collapsing inferior vena cava (IVC), both observed by bedside ultrasonography, were associated with the development of AKI in a population of critically ill patients in the Intensive Care Unit (ICU).

**METHODS.** In this sub-study of the Simple Intensive Care Studies-II (NCT03577405)(1), bedside ultrasonography was performed by trained researchers within 24 hours of ICU admission. The number of B-lines (0-5) was assessed according the Bedside Lung Ultrasound in Emergency protocol(2). Pulmonary interstitial syndrome was defined as three or more B-lines in two or more bilateral fields. IVC collapsibility was assessed using the IVC-Collapsibility Index and a value of less than 20% as cut off score for IVC-congestion (accounting for mechanical ventilation where appropriate)(3). Congestion was assessed as a categorical variable (0: no signs of congestion, 1: one of two signs of congestion, and 2: both signs of congestion) and AKI as a binary variable following KDIGO criteria. Via multivariate logistic regression analysis, corrected for age, we determined an association between signs of venous congestion and AKI.

**RESULTS.** From May 15th 2018 to April 8th 2019 a total of 426 patients were included. We excluded 152 patients (36%) with pre-existing renal failure or lack of ultrasonography images. Of the remaining 274 patients, 128 patients (46%) developed AKI within 72 hours of admission. In total 97 patients (35%) had no collapsing IVC, 31 patients (11%) had pulmonary interstitial syndrome and 11 patients (3%) had both (figure 1). Congestion was associated with AKI in univariate analysis, and this association remained after correction for age (OR 1.54, 95%CI 1.01-2.38, p=0.045).

**CONCLUSION.** Interstitial syndrome and/or a non collapsing IVC as signs of venous congestion were associated with AKI in a finding cohort. Bedside ultrasonography measurements of venous congestion early during admission could predict the development of AKI in critically ill patients. Further studies have to elucidate the validity and efficacy of these findings.

**REFERENCE(S)**

1. Wiersema, R. et al. Observational Study Protocol for Repeated Clinical Examination and Critical Care Ultrasonography Within the Simple Intensive Care Studies. J. Vis. Exp. 1–14 (2019). doi:10.3791/58802
2. Lichtenstein, D. A. BLUE-Protocol and FALLS-Protocol Two Applications of Lung Ultrasound in the Critically Ill. Chest 147, 1659–1670 (2015).
3. Stawicki, S. P. et al. Intensivist Use of Hand-Carried Ultrasonography to Measure IVC Collapsibility in Estimating Intravascular Volume Status: Correlations with CVP. J. Am. Coll. Surg. 209, 55–61 (2009
4. Inclusion is ongoing and if accepted for presentation at the ESICM updated results will be presented.

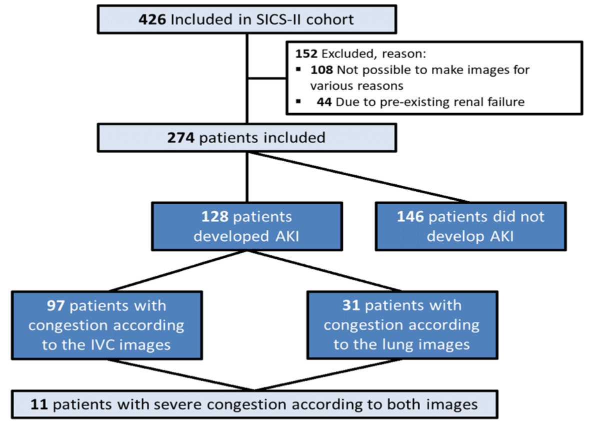

**Fig. 1 (abstract 001186).** See text for description

### 001705

### Temporal change in characteristics and outcomes of acute kidney injury on renal replacement therapy in intensive care units: Analysis of a nationwide administrative database in Japan, 2007–2016

Y. Miyamoto[1], K. Doi[2], M. Iwagami[3], H. Yasunaga[4]

[1]Nephrology, The University of Tokyo, Bunkyo City, Japan; [2]Cc, The University of Tokyo, Tokyo, Japan; [3]Health services research, University of Tsukuba Tsukuba Campus, Tsukuba, Japan; [4]Clinical epidemiology and health economics, The University of Tokyo, Bunkyo City, Japan

**Correspondence:** K. Doi

*Intensive Care Medicine Experimental* 2019, **7(Suppl 3):**001705

**INTRODUCTION.** Acute kidney injury (AKI) is common in critically ill patients and is strongly associated with high mortality. Renal replacement therapy (RRT) is an important supportive therapy for patients with AKI. In particular, continuous renal replacement therapy (CRRT) is used for hemodynamically unstable critically ill patients with loss of kidney function. Previous studies have shown that the overall mortality of patients with AKI requiring CRRT is high—over

50%—indicating that further improvement is necessary for this unacceptable poor outcome.
There have been numerous reports on a recent decrease in mortality among critically ill patients. It is of interest whether the characteristics and outcomes of patients with AKI-RRT have changed over time.

**OBJECTIVES.** To examine recent trends in patient characteristics and mortality in patients with AKI receiving RRT, including CRRT and intermittent RRT (IRRT), in intensive care units (ICUs)

**METHODS.** From the Diagnosis Procedure Combination database in Japan during 6 months (July–December) from 2007 to 2016, patients with AKI who received RRT in ICUs were identified and examined for trends in patient characteristics and overall mortality by RRT modality and main diagnosis category subgroup. Logistic regression analysis was performed to adjust for patient characteristics.

**RESULTS.** Overall, 51,758 patients starting RRT were identified (3.9% of all patients admitted to ICUs; CRRT: 39,471 (76.3%) patients; IRRT: 12,287 (23.7%) patients) in 287 hospitals. The crude in-hospital mortality declined from 44.9% to 36.1% (P for trend <0.001). Compared with 2007, the adjusted odds ratio (aOR) for in-hospital mortality was 0.66 [95% confidence interval (CI): 0.60–0.72] in 2016, and the decreasing trend was observed in both patients starting CRRT (aOR 0.68, 95% CI 0.61–0.75) and IRRT (aOR 0.58, 95% CI 0.45–0.74) and in all subgroups: sepsis aOR, 0.68 (95% CI 0.57–0.81); cardiovascular surgery, 0.58 (0.45–0.76); coronary artery disease, 0.85 (0.60–1.20); noncoronary heart disease, 0.78 (0.65–0.94); central nervous system disorders, 0.42 (0.28–0.62); trauma, 0.39 (0.22–0.72); and others, 0.64 (0.50–0.83).

**CONCLUSION.** This nationwide study confirmed a consistent decline in mortality among patients with AKI on RRT in ICUs, although several patient characteristics were changing over time.

**REFERENCE(S)**

1. This work was supported by grants for Research on Policy Planning and Evaluation from the Ministry of Health, Labour and Welfare, Japan (H29-Policy-Designated-009 and H29-ICT-General-004); the Ministry of Education, Culture, Sports, Science and Technology, Japan (17H04141); and the Japan Agency for Medical Research and Development (AMED).

## DS - From bytes to bedside: Improving intensive care with Data

### 001005

#### Predicting unplanned admissions to intensive care: development and validation of a machine learning model

OC. Redfern[1], MA. Pimentel[2], D. Prytherch[3], P. Meredith[4], DA. Clifton[2], L. Tarassenko[2], PJ. Watkinson[1]
[1]Critical care research group, University of Oxford, Oxford, United Kingdom; [2]Institute of biomedical engineering, department of engineering science, University of Oxford, Oxford, United Kingdom; [3]Centre for healthcare modelling and informatics, University of Portsmouth, Portsmouth, United Kingdom; [4]Research and innovation department, Portsmouth Hospitals NHS Trust, Portsmouth, United Kingdom
**Correspondence:** O.C. Redfern
*Intensive Care Medicine Experimental* 2019, **7(Suppl 3):**001005

**INTRODUCTION.** Unplanned admissions to intensive care units (ICU) are associated with high mortality rates [1]. In the UK, the Royal College of Physicians recommend that patients on general wards with a National Early Warning Score (NEWS) of ≥7 should be reviewed by the critical care team [2]. However, the NEWS only considers vital sign measurements. We recently showed that a simple risk score (LDTEWS:NEWS), which combines NEWS with routine laboratory results, was better able to identify patients who may require critical care [3]. Recently, machine learning algorithms have shown potential to outperform standard statistical approaches when predicting patient outcomes using data from electronic health records [4].

**OBJECTIVES.** To develop and externally validate a risk score to predict unplanned admissions to intensive care using machine learning.

**METHODS.** This was a retrospective cohort study of emergency medical admissions to two large acute hospitals groups (Portsmouth Hospitals, PH and Oxford University Hospitals, OUH). We excluded admissions where the patient was: discharged alive on the day of admission or had no vital signs observations in the 24 hours prior to the initial ICU admission or hospital discharge. Seven vital signs, seven common laboratory tests and the patient's gender were extracted from hospital records as described previously [3]. Using these data, a novel risk score (XgICU) was developed from five years' of admissions to one hospital (PH) using gradient-boosted decision trees (xgboost [5]). Optimal hyper-parameters of xgboost were identified through a random search with five-fold cross-validation on the development cohort. XgICU was externally validated on one year of admissions from OUH. The discrimination of XgICU, NEWS and LDTEWS:NEWS for observations within 24 hours (median 9 ; interquartile range 4-6) of unplanned ICU admission was assessed using the c-statistic.

**RESULTS.** The external validation cohort (OUH) comprised 16,309 admissions (median age = 73 years), of which 159 (1.0%) patients had an unplanned admission to the ICU. The novel risk score (XgICU) showed substantially better discrimination (0.920, 95% confidence interval 0.913–0.927) than both LDTEWS:NEWS (0.894, 0.885–0.904) and NEWS (0.876, 0.865–0.887). At the recommended escalation threshold of ≥7, NEWS identified 53% of observations in the 24-hour window preceding an unplanned ICU admission to be above the threshold. At an equivalent false positive rate (5%), LDTEWS:NEWS was marginally more sensitive (55%), whilst the sensitivity of XgICU was 64%.

**CONCLUSION.** Using the same vital sign and laboratory data as LDTEWS:NEWS, gradient-boosted decision trees were substantially better at identifying observations above the escalation threshold in the 24 hours prior to unplanned transfers to the ICU. If carefully applied, machine learning algorithms could provide powerful tools to aid clinical decision making for critically ill patients.

**REFERENCE(S)**

1. Arulkumaran N, Harrison DA, Brett SJ. Association between day and time of admission to critical care and acute hospital outcome for unplanned admissions to adult general critical care units: cohort study exploring the 'weekend effect'. Br J Anaesth 2017;118:112–22.
2. Royal College of Physicians (London). National Early Warning Score (NEWS) 2: Standardising the assessment of acute-illness severity in the NHS. 2017.
3. Redfern O, Pimentel MAF, Prytherch D, et al. Predicting in-hospital mortality and unanticipated admissions to the intensive care unit using routinely collected blood tests and vital signs: development and validation of a multivariable model. Resuscitation Published Online First: September 2018.
4. Churpek MM, Yuen TC, Winslow C, et al. Multicenter Comparison of Machine Learning Methods and Conventional Regression for Predicting Clinical Deterioration on the Wards. Crit Care Med 2016;44:368–74.
5. Chen T, He T, Benesty M, et al. xgboost: Extreme Gradient Boosting. 2019.
6. This publication presents independent research commissioned by the Health Innovation Challenge Fund (HICF-R9-524; WT-103703/Z/14/Z), a parallel funding partnership between the Department of Health and Wellcome Trust. The views expressed here are those of the authors and not necessarily those of the Department of Health or Wellcome Trust. MAFP is funded by a Drayson Research Fellowship. PJW is supported by the National Institute for Health Research (NIHR), Biomedical Research Centre, Oxford.

### 001163

#### Gender Disparity in Critical Care Publications: A novel female first author index

S. Chary[1], K. Amrein[2], S. Mehta[3], K. Christopher[1]
[1]Division of renal medicine, Brigham and Women"s Hospital, Boston, United States of America; [2]Division of endocrinology and diabetology, Medical University of Graz, Graz, Austria; [3]Department of medicine, University of Toronto, Toronto, ON, Canada
**Correspondence:** K. Christopher
*Intensive Care Medicine Experimental* 2019, **7(Suppl 3):**001163

**INTRODUCTION.** Bibliometric analyses in science show gender disparity exits in publications and citations which are crucial for academic and professional advancement.

**OBJECTIVES.** To summarize the state of gender equality in original critical care research.

**METHODS.** We conducted a bibliometric analysis of original research publications in critical care medicine from 2008-2018 in 11 journals: NEJM, JAMA, Lancet, BMJ, ICM, Critical Care, CCM, CHEST, AJRCCM, Annals of Intensive Care, and Journal of Critical Care. We included only clinical trials, observational studies, systematic reviews, meta-analyses and experimental studies. We utilized PubReMiner to produce full names and country from PubMed. Gender for the first and last authors was assigned manually and by Gender API a web-based gender determination platform. Publication citations were determined via the Web of Science. We defined the novel Female First Author index (FFA index) for an individual last author. FFA Index = (#Female first authors in publications by an individual last author/ #Total publications by the individual last author).

**RESULTS.** 7370 manuscripts that met criteria had first and last author gender determined: 2237 manuscripts with female first authors were identified (30.4%), 1140 manuscripts had female last authors (15.5%). There were 5153 different individual first authors with 34.1% individual female first authors. 19.6% of the 3890 individual last authors were female. The mean impact factor for manuscripts with female first authors was 5.98 and male first authors was 6.27 (ANOVA p= 0.055). This relationship did not change with restriction to higher impact factors (ie >5, 10, or 20). Average citations per year for female first authors was lower compared to those for male first authors at 3.79 vs. 4.63 respectively, (ANOVA p< 0.001). Female last authorship was associated with increased odds of female first authorship. After adjustment for impact factor, journal and year, female last authorship was associated with a nearly 2-fold increase in female first authorship [OR=1.87 (95%CI 1.64, 2.13); P<0.001]. Geographically, only Finland, the Netherlands and Greece approach gender parity in first authorship. In our data, the proportion of female first authorship has not increased over the last 10 years. The mean Female First Author index (FFA index) for all 3890 individual last authors was 30.5%. In male last authors (N=3125), the mean (SD) FFA index was 27.6 (41.2)%. In female last authors (N=763), the mean (SD) FFA index was 42.5 (47.6)%. The gender differences in FFA index were significant ($\chi 2$ = 46.6; P < 0.001). For male last authors with 5 or more senior author manuscripts (N=251), the mean (SD) and median FFA index were 28.7% (26.8%) and 20.0%. For female last authors with 5 or more senior author manuscripts (N=20), the mean (SD) and median FFA index were 47.5% (23.9%) and 46.4%. The gender differences in FFA index in those with 5 or more senior author manuscripts were significant ($\chi 2$ = 12.9; P < 0.001).

**CONCLUSION.** Female representation at prominent authorship positions in critical care publications is still far from achieving gender parity. By creating an authorship index score, we have now established a frame of reference for the advancement of female first authorship. Bridging this authorship gap can advance women in academics towards true gender equality.

**REFERENCE(S)**

1. NIH R01GM115774

### 001277

#### Time-Sensitive Deep Learning for ICU Outcome Prediction Without Variable Selection or Cleaning

J. Deasy[1], P. Liò[1], A. Ercole[2]
[1]Department of computer science, University of Cambridge, Cambridge, United Kingdom; [2]Department of medicine, University of Cambridge, Cambridge, United Kingdom
**Correspondence:** J. Deasy

*Intensive Care Medicine Experimental* 2019, **7(Suppl 3):**001277

**INTRODUCTION.** Accurate prognostication is important for shared decision making as well as for benchmarking. Deep Learning (DL) with many predictors can outperform traditional models (e.g. APACHE II). ICU data is a particular challenge as it consists of multiple investigations, physiological measurements and treatments at different sampling times, any of which may also be subject to error. Furthermore, as ICU admissions are dynamic with prognostic accuracy which changes over time, and current techniques cannot make predictions at arbitrary times, a temporally-sensitive model would help guide optimal decision-making for trials of intensive care.

**OBJECTIVES.** We present a novel DL survival algorithm using the entire multivariable patient time-series, regardless of variable type or frequency and without the need for variable selection or cleaning. Furthermore, we seek to be able to track predicted survival probability over time for optimal prediction.

**METHODS.** Using data from the MIMIC-III dataset, we represent all 57,823 possible event types from the first 48 hours of each patient's ICU stay using a flexible latent space embedding previously described. In this way, an adaptable recurrent DL model with 1,998,828 parameters is also able to learn erroneous data values to be discarded. By collapsing the time-series to arbitrary resolution, we train the model to predict mortality after each hour of the patient's stay.

**RESULTS.** A total of 18,067 patients with 21,110 ICU stays and 208,572,237 events were used for training. The median [IQR] patient age was 65.8 [52.8; 77.8] and there were 2,764 (13.1%) in-hospital deaths. Hourly predictions for patient survival to hospital discharge were made for the first 48 hours of their stay in the ICU. On a 10-fold cross-validation, our model obtains a mean AUROC of 0.90 (95% CI 0.88-0.92) for prediction at 48 hours, exceeding several recent deep learning models. Furthermore, the model is able to track predictions over time and showed that prediction strength improves throughout the first 48 hours after admission - rapidly for the first 12 (to AUROC>0.8) before levelling out at 48 hours.

**CONCLUSION.** Our model is time-sensitive and able to achieve state of the art survival predictions while overcoming the need for data selection or pre-processing. This is a significant advantage when considering the high data density of the ICU. Predictive performance increases over the first 48 hours of the ICU stay, providing a rationale for time-limited trials in the ICU and suggesting that the timing of decision making can be optimised/individualised.

**REFERENCE(S)**

1. A Rajkomar et al. "Scalable and accurate deep learning with electronic health records". In: NPJ Digital Medicine 1.1 (2018), p. 18.
2. This work was supported by the UK Medical Research Council
3. Zhengping Che et al. "Recurrent neural networks for multivariate time series with missing values". In: Scientific reports 8.1 (2018), p. 6085.
4. C. Meiring et al. "Optimal intensive care outcome prediction over time using machine learning". In: PLoS ONE13.11 (2018), e0206862.
5. Hrayr Harutyunyan et al. "Multitask learning and benchmarking with clinical time series data". In: arXiv preprint arXiv: 1703.07771 (2017).
6. Alistair EW Johnson et al. "MIMIC-III, a freely accessible critical care database". In: Scientific data 3 (2016), p. 160035.

### 001375

#### Richt Dose, Right Now: Machine Learning to Predict Creatinine Values in Intensive Care Patients

L. Fleuren[1], A. Hoeijmakers[2], M. Fornasa[2], L. Roggeveen[1], T. Guo[1], PJ. Thoral[1], E. Swart[3], A. Girbes[1], M. Hoogendoorn[4], PWG. Elbers[1]
[1]Intensive care adults, Amsterdam UMC, locatie VUmc, Amsterdam, Netherlands; [2]Intensive care, Pacmed, Amsterdam, Netherlands; [3]Clinical pharmacology, Amsterdam UMC, locatie VUmc, Amsterdam, Netherlands; [4]Computer science, VU, Amsterdam, Netherlands, Netherlands
**Correspondence:** L. Fleuren

*Intensive Care Medicine Experimental* 2019, **7(Suppl 3):**001375

**INTRODUCTION.** Acute kidney injury (AKI) is a frequently observed complication encountered in the intensive care and greatly affects the elimination of commonly prescribed drugs such as antibiotics and anti-convulsants. As defining AKI in clinical practice is challenging, pharmacokinetic models often rely on creatinine values as their input. We hypothesize that predicting creatinine values ahead of time would improve these models and optimize dosing strategies in critically ill patients. Additionally, we foresee that clinicians could use these predictions to guide management. Machine learning provides a novel approach to analyze large quantities of data and improve predictive accuracy. To this point, no machine learning models have focused on the prediction of creatinine values.

**OBJECTIVES.** We set out to predict creatinine as a continuous variable ahead of time to improve pharmacokinetic modeling and guide clinical therapy.

**METHODS.** A retrospective cohort study of 26.251 critically ill adult patients admitted between 2004 and 2017 was conducted in the Amsterdam UMC, The Netherlands. Patients with a stay shorter than 48 hours and medium care patients were excluded from the analyses. A total of 45 features were collected and included patient demographics, device data, laboratory values, and clinical observations. Non-sequential methods (Lasso, Random Forest) and sequential methods (LSTM) were tested. The granularity of data was one hour, more frequent data was condensed by calculating the mean per hour, missing data was imputed using several imputation approaches for the deep learning models (LSTM-naïve, LSTM-Mask, LSTM-Naïve-MFeat, LSTM-Mask-MFeat). Using a train-validation-test split (60-20-20%), a continuous value of creatinine was predicted ahead of time for a 24-hour interval, using 24 hour of previous data as input into the model. The last known creatinine value served as a baseline.

**RESULTS.** A total of 8330 patients fulfilled the inclusion and exclusion criteria (median age 65.9 [IQR 54.1-74.8], 36% female). Of these, 5482 patients developed AKI during their ICU stay, with median creatinine values ranging from 92.0 to 242.0 for non-AKI and KDIGO AKI stage 3 patients respectively. 51.2% of the stage 3 patients received renal replacement therapy. Last creatinine as a baseline resulted in an R2 of 0.80 (SE±0.002). Random forest performed best (R2 0.86, SE±0.001), followed by the LSTM-Mask (R2 0.84, SE±0.002). In the random forest model, last creatinine and creatinine trends, renal replacement therapy, and pH came out as most important predictors.

**CONCLUSION.** In this study we showed machine learning models improve the prediction of continuous creatinine values. Further enhancement of the models is likely to improve performance. Predictions for different time intervals would make the models more suitable for integration in the daily workflow.

### 001637

#### Network topology: a novel method for understanding hospital strain and the role of the ICU

K. Kohler, A. Ercole
University division of anaesthesia, University of Cambridge, Cambridge, United Kingdom
**Correspondence:** K. Kohler
*Intensive Care Medicine Experimental* 2019, **7(Suppl 3):**001637

**INTRODUCTION.** Hospitals can be described as complex adaptive systems, where discrete interventions are often unable to remedy problems. Network science methods provide tools to analyse the architecture of such complex systems. We apply these tools to emergency surgical admissions to give a better understanding of the structure behaviour of our service and will allow us to develop predictive methods for service needs, eg ICU bed capacity.

**METHODS.** We selected all unplanned surgical admissions over a two and a half year period and used the electronic care records to extract the locations of the patients during their admission. From this we constructed a network representation of the care system and added a measure of daily emergency department load to investigate the influence of hospital strain on the system. Here we focused on patients who had a stay in ICU or HDU areas.

**RESULTS.** The graph shows the network representation of the emergency surgical ICU patients with the nodes representing a ward or area. The edges connecting them represent the transfers of patients between the two wards (width proportional to the frequency of transfers). The nodes are coloured by their 'betweenness centrality', a measure of the importance of a node to the function of the system. In this graph ICU is densely connected to most areas of the hospital and is therefore highly sensitive to disruptions or problems elsewhere.

We constructed networks for the busiest and calmest days in the emergency department. The analysis shows that flow through the system is reduced in busy times (lower flow hierarchy) and patients are sent to a larger range of areas from ED (higher ED degrees). Additionally, we have a higher percentage of transfers between different ICU areas during busier times, possibly reflecting increased non-medical transfers.

**CONCLUSION.** The network representation yields the expected results of extensive interconnectedness and the effects of system strain on flow of patients. The increased load in ED may be due to a bottleneck into the hospital or due to higher demand. The next steps will be further analysis of the influence of strain, patient subgroup analysis and the development of predictive tools.

**REFERENCE(S)**

1. Katharina Kohler is funded by a NIHR ACL fellowship
2. Opsahl T, Agneessens F, Skvoretz J. Node centrality in weighted networks: Generalising degree and shortest path. Social Networks 2010. 32, p245-251
3. Braithwaite J. Changing how we think about healthcare improvement, BMJ 2018; 361:k201
4. Strogatz SH. Exploring complex networks. Nature 2001. 410, p268-276

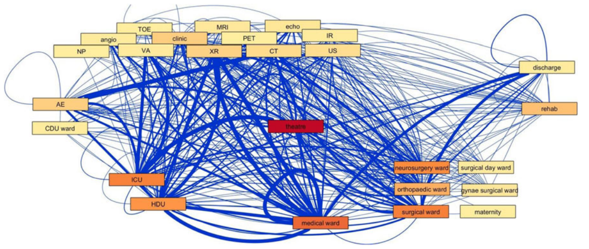

**Fig. 1 (abstract 001637).** See text for description

**Table 1 (abstract 001637).** See text for description

| | ED degrees | Flow Hierarchy | Transfers between ICUs |
|---|---|---|---|
| "Calm" ED | 21 (6 in, 15 out) | 0.049 | 0.089% of all transfers |
| "Busy" ED | 29 (9 in 20 out) | 0.039 | 0.129% of all transfers |

## ETH - End of life care

### 000071

#### Poor agreement between critically ill patients and surrogates opinion on enrollment in clinical research

FJA. Pfeilsticker[1], NS. Campos[1], FG. Aguiar[1], CAS. Siqueri[1], ML. Romagnoli[1], RCDF. Chaves[1], CS. Guimaraes[2], AJ. Pereira[1], RL. Cordioli[1], MSC. Assuncao[1], TD. Corrêa[1]
[1]Intensive care unit, Hospital Israelita Albert Einstein, São Paulo, Brazil; [2]Intensive care unit, Hospital Municipal Vila Santa Catarina, São Paulo, Brazil
**Correspondence:** F. Pfeilsticker
*Intensive Care Medicine Experimental* 2019, **7(Suppl 3):**000071

**INTRODUCTION.** In most emergency situations or severe illness, critical care patients are unable to give consent for enrollment in a clinical trial. In such circumstances, the decision of whether or not to participate in a clinical trial is made by a legal representative (1).

**OBJECTIVES.** To address the willingness of patients admitted to the intensive care unit (ICU) to be enrolled in a scientific study as a volunteer and to assess the agreement between ICU patient's and their legal representative's opinion concerning enrollment in a scientific study.
**METHODS.** This survey was conducted in two hospitals (one private and one public) in São Paulo, Brazil. Adult patients (≥18 years) with preserved cognitive function accompanied by a surrogate admitted to the ICU were eligible for this study. A survey containing 28 questions for patients and 8 questions for surrogates was conducted by the investigator preferably within the first 48 hours of ICU admission. The survey of patients comprised three sections: demographic characteristics, opinion about participation in clinical research andknowledge of the importance of research. The survey of legal representatives contained two sections: demographic characteristics and assessment of the legal representatives' opinion on authorizing patients' to be enrolled in research.
**RESULTS.** Between January 2017 and May 2018, 208 pairs of ICU patients and their respective legal representatives answered the survey. The median (IQR) age of patients was 60 (43-75) years, 49% were female and 56.7% had a higher educational level. The median (IQR) age oflegal representatives was 49 (37-59) years, 66% were female and 68.8% were next of kin (spouse/son/daughter). Of the 208 ICU patients who answered the survey, 73.6% (153/208) would like to be enrolled in study as a volunteer. Of those patients, 65.1% (97/149) would continue participating in a research even if their legal representative did not support their enrollment. The agreement between patients' and surrogates' opinion concerning participation was poor [Kappa=0.11 (IC95% -0.02 to 0.25)]. If a deferred consent were obtained, 69.1% (103/149) of patients would continue participating in the study until its conclusion, and 23.5% (35/149) would allow researchers to use only the already collected data, but would withdraw the study in this occasion.
**CONCLUSION.** The surrogate's opinion reflects poorly on critically ill patient's opinion concerning enrollment in clinical research. To boost patient's recruitment in clinical trials, efforts to improve patients and relative's knowledge of the importance and value of medical research need be made.

**REFERENCE(S)**

1. Jansen T C et al. Deferred consent in emergency intensive care research: what if the patient dies early? Use the data or not? Intensive Care Med (2007) 33:894-900.

### 000324

#### Changes in the use of DNR orders in European Intensive Care Units over 16 years: the Ethicus-1 and Ethicus-2 studies

P. Maia[1], A. Avidan[2], M. Weiss[3], JC. Schefold[4], A. Estella[5], MG. Bocci[6], L. Bento[7], S. Mullick[8], C. Sprung[2]
[1]Intensive care medicine, Hospital Santo António, Porto, Portugal; [2]Anesthesiology and critical care, Hadassah Medical Center, Hebrew University of Jerusalem,Faculty of Medicine, Jerusalem, Israel; [3]Anesthesiology, University Hospital Ulm, Ulm, Germany; [4]Department of intensive care, Inselspital, Bern University Hospital and University of Bern, Bern, Switzerland; [5]University hospital of jerez., Intensive care unit, Jerez, Spain; [6]Department of anesthesiology and intensive care medicine, Fondazione Policlinico Universitario Agostino Gemelli – IRCCS –, Roma, Italy; [7]Unidade de urgência médica, Hospital S. José, Centro Hospitalar Universitário Lisboa Central, Lisboa, Portugal; [8]Department of critical care medicine, Tata Medical Center, New Town, Kolkata, India
**Correspondence:** P. Maia
*Intensive Care Medicine Experimental* 2019, **7(Suppl 3):**000324

**INTRODUCTION.** *Do-not-resuscitate* (DNR) orders apply to the decision to withhold *cardiopulmonary resuscitation* (CPR) and may already occur on ICU admission or when the patient´s condition worsens, alone or with other *limitation of life-sustaining therapy* (LT).
**OBJECTIVES.** To investigate the change in use of DNR orders in European ICUs over 16 years
**METHODS.** All centres that participated in the Ethicus-1 study were invited to participate in a follow-up study (Ethicus-2). Consecutively admitted patients who died or had LT during a 6 month period from September 1, 2015 to September 30, 2016 were prospectively studied. End-of-life Practices (EOLP) and region definitions from the Ethicus-1 study were used and comparisons made [1].
**RESULTS.** Twenty-two ICUs participated in both studies, enrolling patients (Ethicus-2; Ethicus-1): total (1785;2807); Northern Europe (NE) (424; 587); Central Europe (CE) (893; 906); and in Southern Europe (SE) (468; 1314). The use (% Ethicus-2; % Ethicus-1) of DNR orders for the different regions was: NE (93; 80), CE (83; 72), SE (78; 55). When a decision to either WH CPR or WD CPR was made, a DNR order was written in (% Ethicus-2; % Ethicus-1): NE (84; 82), CE (93; 84), SE (37; 32).
**CONCLUSION.** The use and the documentation of DNR orders increased in all European regions ICUs that participated in ETHICUS-1 after 16 years. WH CPR were the first and the most common LT. Written DNR orders and documentation of decisions to WH CPR in the medical record are still not frequent in SE.

**REFERENCE(S)**

1. Sprung CL et al. JAMA 2003; 290:790-797

### 000474

#### Family Crisis and Coping Strategies of Intensive Care Unit Patient's Relatives

M. Gouva[1], Z. Konstanti[1], A. Papathanasiou[2], V. Salma[2], V. Koulouras[2], G. Papathanakos[2]
[1]Department of nursing, research laboratory psychology of patients families and health profession, University of Ioannina, Ioannina, Greece; [2]Intensive care unit, University Hospital of Ioannina, Ioannina, Greece
**Correspondence:** G. Papathanakos
*Intensive Care Medicine Experimental* 2019, **7(Suppl 3):**000474

**INTRODUCTION.** A patient's admission in the Intensive Care Unit (ICU) can lead to intense stress symptoms in the patient's family, resulting in anxiety, depression and emotional distress.
**OBJECTIVES.** To investigate on the coping mechanisms of the ICU patient's family, depending on socio-demographic characteristics and the relative-patient closeness.
**METHODS.** Family members (first degree relatives, close relatives and intimate friends) of patients hospitalized in a University ICU constituted our research material. A questionnaire for sociodemographic data and the Family Crisis Oriented Personal Evaluation Scales (F-COPES) were completed by family members after their beloved one's ICU admission. F-COPES consists of 5 subscales: 'Acquiring social support', 'reframing', 'seeking spiritual support', 'mobilizing family to acquire and seek help' and 'passive appraisal'.
**RESULTS.** Two hundred and twenty-three family members, 81 (36.3%) men and 142(63.7%) women, mean-aged 41.4±11.9 years, corresponded to 147 ICU patients were studied. Family members were children (40.8%), siblings (19.3%), spouses (16.1%), parents (4.9%), other family members (17%) and friends (1.8%). Regarding the strategies used to deal with family crisis, it became evident that the quest for social help (31.27±4.72), the mobilization of the family to get and receive help (14.26±3.17) and the passive attitude (14.04±2.81) were those most commonly used. Gender differences were observed almost in every F-COPES subscale; women achieved higher scores and differences were more prominent in social support subscale (p=.001). First degree relatives exhibited lower scores in 'spiritual support' and 'accept help' subscales (p=.021 and p=.014 respectively). Offspring exhibited lower scores in 'spiritual support' subscale compared with siblings (p=.015) and friends (p=.006) and lower scores in 'accept help' subscale compared with 'friends' (p=.008). Elementary school graduates exhibited higher scores in 'reframing' subscale compared with university and postgraduate students (p=.005 and p=.021 respectively).
**CONCLUSION.** Male spouse and offspring are most disadvantaged among ICU patients' family members in terms of effective coping strategies, while 'reframing' is used less frequently among family members with higher level education.

### REFERENCE(S)

1. McCubbin H LA, Olson D (1987). F-COPES Family Crisis Oriented Personal Scales. In: A. MHT (ed.) Family assessment inventories for research and practice. Madison: WI: University of Wisconsin.
2. Gouva M, Dragioti E, Konstanti Z, Kotrotsiou E & Koulouras V. Translation and Validation of a Greek version of the Family Crisis Oriented Personal Evaluation Scales (F-COPES). Interscientific Health Care. 2016;8:64-72.
3. Azoulay E, Chaize M, Kentish-Barnes N. Involvement of ICU families in decisions: fine-tuning the partnership. Ann Intensive Care. 2014;30:4:37.
4. Acaroglu R, Kaya H, Sendir M, Tosun K, Turan Y. Levels of anxiety and ways of coping of family members of patients hospitalized in the Neurosurgery Intensive Care Unit. Neurosciences (Riyadh). 2008;13(1):41-5.
5. None

### 000791

#### Communication and decision processes in End of Life Decision in German Intensive Care Units – Results from the ETHICUS-2 Study

C. Denke[1], U. Jaschinski[2], R. Riessen[3], S. Bercker[4], C. Spies[1], M. Ragaller[5], M. Weiss[6], K. Dey[7], A. Michalsen[8], J. Briegel[9], A. Pohrt[10], CL. Sprung[11], A. Avidan[12], C. Hartog[1]

[1]Klinik für anästhesie m.s. operative intensivmedizin, Charité – Universitätsmedizin Berlin, Berlin, Germany; [2]Klinik für anästhesiologie und operative intensivmedizin, Universitätsklinik Augsburg, Augsburg, Germany; [3]Medizinische klinik, universitätsklinik Tübingen, Tübingen, Germany; [4]Klinik und poliklinik für anästhesiologie und intensivtherapie, Universitätsklinik Leipzig, Leipzig, Germany; [5]Klinik und poliklinik für anästhesiologie und intensivtherapie, Universitätsklinikum Carl Gustav Carus Dresden, Dresden, Germany; [6]Klinik für anästhesiologie, Universitätsklinikum Ulm, Ulm, Germany; [7]Anästhesiologie, intensivmedizin, schmerztherapie und notfallmedizin, Bundeswehrkrankenhaus Berlin, Berlin, Germany; [8]Klinik für anästhesiologie, intensivmedizin, notfallmedizin und schmerztherapie, Medizincampus Bodensee - Klinik Tettnang, Tettnang, Germany; [9]Klinik für anästhesiologie, Klinikum der Universität, LMU München, München, Germany; [10]Institut für biometrie und klinische epidemiologie, Charité – Universitätsmedizin Berlin, Berlin, Germany; [11]Department of anesthesiology, critical care and pain medicine, hadassah medical center, Hebrew University of Jerusalem, Faculty of Medicine, Jerusalem, Israel; [12]Hebrew university of jerusalem, faculty of medicine, Department of anesthesiology, critical care and pain medicine, hadassah medical center, Jerusalem, Israel

**Correspondence:** C. Hartog

*Intensive Care Medicine Experimental* 2019, **7(Suppl 3):**000791

**INTRODUCTION.** Communication strategies have an important impact on end-of-life decision (EOLD) processes in intensive care units (ICUs) (1), but current communication and decision-making practices in German ICUs are unknown.

**OBJECTIVES.** This study presents data from the subset of German ICUs which participated in the worldwide Ethicus-2 study on end-of-life care practices in ICUs.

**METHODS.** Observational study of consecutive ICU patients who died or had end-of-life decisions (EOLD) to limit therapies during a 6-months period.

**RESULTS.** A total of 1092 patients were included, 13% of patients were mentally competent at the time of EOLD. Advance directives were present in 270 (27%) patients. Discussion of EOLD occurred with 92% of mentally competent patients and shared decision making (SDM) was reported in 90%.

In general, physicians asked for or received information about patient wishes in 816 (78%) cases, most commonly from the family (94%) and sometimes from the patient (18%). However, the question whether patient wishes were followed if they were known was answered positively in 98% of 641 patients, constituting only 59% of the total. Patients with EOLD more often had advance directives (29% vs. 11%, Chi2=12.69, p= 0.00037) and information about patient treatment desires were more frequent (83% vs. 34%), $Chi^2$=118.73, p≤0.0001) than in patients without EOLD.

The question whether physicians discussed EOLD with the family was not answered in 11% of patients. For the remaining patients, physicians reported using SDM in 76%; the family was told the decision in 19% and the family was asked to decide in 5% of patients. Physicians' reasons for not discussing EOLD with the family included unresponsiveness to therapy (38%) or unavailability of family (19%). Agreement with the final EOLD occurred in 99% between physicians and nurses in 98% of cases between health care providers and family.

**CONCLUSION.** In the majority of patients, physicians seek knowledge about patients' treatment wishes, mostly from the family or the patients themselves if those are mentally competent. Advance directives are legally binding in Germany, therefore the fact that every fourth patient with EOL care has an advance directive is noteworthy. Our data suggest that when advance directives are present, they are considered in the process of EOL decision-making. When patient wishes are known, they are usually followed. On the other hand, our data suggest that patient wishes remain elusive in about half of the included ICU patients. Physicians commonly discuss EOL decisions with the family. The most frequent communication model is shared decision-making with the family.

### REFERENCE(S)

1. Cohen S et al. Communication of end-of-life decisions in European intensive care units. Intensive Care Med. 2005 Sep;31(9):1215-21.

### 000815

#### Changes in communication of end-of-life decisions in European intensive care units over 16 years: The Ethicus-2 study

C. Hartog[1], P. Maia[2], C. Danbury[3], S. Mullick[4], CL. Sprung[5], A. Avidan[6]

[1]Anesthesiology and operative intensive care, Charité – Universitätsmedizin Berlin, Berlin, Germany; [2]INTENSIVE CARE MEDICINE, Centro Hospitalar do Porto, Porto, Portugal; [3]Intensive care, Royal Berkshire Hospital, Reading, United Kingdom; [4]Department of critical care medicine, Tata Medical Center, New Town, Kolkata, India; [5]Department of anesthesiology, critical care and pain medicine, hadassah medical center, Hebrew University of Jerusalem, Faculty of Medicine, Jerusalem, Israel; [6]Hebrew university of jerusalem, faculty of medicine, Department of anesthesiology, critical care and pain medicine, hadassah medical center, Jerusalem, Israel

**Correspondence:** C. Hartog

*Intensive Care Medicine Experimental* 2019, **7(Suppl 3):**000815

**INTRODUCTION.** Communication about end-of-life decisions (EOLD) may have changed over time.

**OBJECTIVES.** This study evaluated differences in EOLD in European intensive care units (ICUs) 16 years after the Ethicus-1 study (1,2)

**METHODS.** Consecutively admitted ICU patients who died or had life-sustaining treatment limitations during a self-selected 6-month period from September 1, 2015 to September 30, 2016 were prospectively studied. Previous EOLD definitions from the Ethicus-1 study were used (1) and comparisons made between studies.

**RESULTS.** Twenty-two ICUs participated in both studies. with 1785 patients in Ethicus-2 (424 in North Europe (NE), 893 in Central Europe (CE), and 468 in South Europe (SE) versus 2806 patients in Ethicus-1 (587 in NE, 906 in CE and 1,314 in SE). Comparing Ethicus-1 with Ethicus-2, more patients had capacity to make decisions in Ethicus-2 (4% vs. 16%) and more physicians asked for or received information about patient's treatment desires (20% vs. 47%). This information was elicited more often from patients (11% vs 14%) and less often from the family (93% vs 40%). More physicians had information about patient wishes in Northern and Central Europe (54% and 53,) than in Southern Europe (28%). The most frequent reasons for not discussing EOLD with family were patient's unresponsiveness to therapy (39% vs 17%) or unavailability of family (36% vs. 9%).

**CONCLUSION.** Today, ICU physicians in Europe seem to be better informed about patients' wishes and patients appear to engage more with ICU physicians than 16 years ago. Still, the North-South divide persists. In Northern and Central Europe, every second ICU physician who was responsible for EOLD had asked for or had received information about patients' wishes in contrast to less than a third of ICU physicians in Southern Europe. Cultural differences like patriarchalism or religiosity may play a role.

**REFERENCE(S)**

(1) Sprung CL et al. End-of-life practices in European intensive care units: the Ethicus Study. JAMA. 2003;290:790-7 (2) Cohen, S., et al. Communication of end-of-life decisions in European intensive care units. Intensive Care Med 2005 31: 1215-1221.

## CD - Functional haemodynamic assessment

### 000040

#### Autonomic responses to passive leg raising in healthy volunteers

S. Sondergaard
Regional Hospital Silkeborg, Silkeborg, Denmark
**Correspondence:** S. Sondergaard
*Intensive Care Medicine Experimental* 2019, **7(Suppl 3):**000040

**INTRODUCTION.** Passive leg raising (PLR) has been promoted as an confirmation of volume responsiveness perioperatively and in patients in the intensive care when ΔCO or ΔSV >10-15%. A guideline has been published (1). Still, much needs to be defined: patient characteristics, feasibility of assessment in ICU populations, impact of concurrent morbidity and medication, the role of the homeostatic mechanisms of the body and the physiologic frame necessary to understand and interpret the PLR.

**OBJECTIVES.** The aim was to describe the changes in spectral energy of the sympathetic (SNS) and parasympathetic (PNS) divisions of the autonomic nervous system in healthy subjects performing a passive leg raising maneuvre.

**METHODS.** Eleven healthy subjects passed through 5-10 minute phases of semi-recumbent, passive leg raising and back to semi-recumbent positions while ECG was recorded at 1000 Hz and haemodynamic variables (HR, MAP, SV and CO) were collected by the Finometer. ECGs were characterized in their spectral aspects by Kubios Pro. PNS, SNS indices, spectral (LF, HF, LF/HF) and Finometer variables were analysed with respect to relative and absolute changes through transitions from initial to leg-raised positions and back to initial position, following the suggestions of Monnet & Teboul (1).

**RESULTS.** All subjects increased SV>10% (but not CO) and were by definition volume responsive. Some increased HR and MAP, some decreased. Over all, COs were unchanged by topographical change. In semirecumbent positions (relative hypovolemia) SNS index increased. In PLR (relative hypervolemia) PNS index increased.

**CONCLUSION.** In supposedly normovolemic subjects the gravitational dislocation of central blood volume elicited a response from the divisions of ANS to maintain homeostasis. From a histocentric point, incorporating the Guyton venous return (2) and the Krogh two-compartment venous compartment (3) models, their organisms had no need of increased SV or CO. Homeostatic reflexes worked to adapt the ratio of stressed/unstressed volumes by mobilising from (↗SNS/↘PNS in semirecumbent) or demobilising (↘SNS/↗PNS in PLR) to the splanchnic circulation. This interpretation warrants closer scrutiny of the autonomic nervous system in the context of PLR in the intensive care or perioperative setting.

**REFERENCE(S)**

1. Monnet X, Teboul JL, (2015) Passive leg raising: five rules, not a drop of fluid! Critical care 19:18
2. Krogh A, (1912) The Regulation of the Supply of Blood to the Right Heart. Skandinavisches Archiv Für Physiologie 27: 227-248
3. Guyton AC, (1955) Determination of cardiac output by equating venous return curves with cardiac response curves. Physiological reviews 35: 123-129

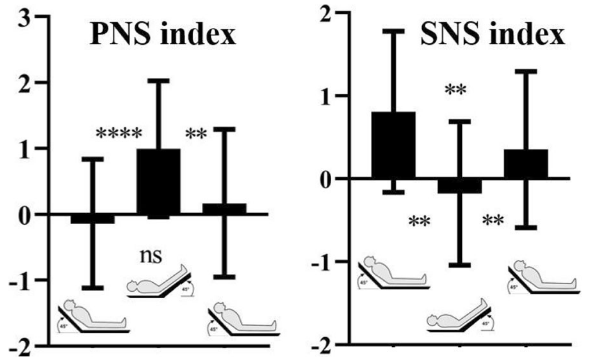

**Fig. 1 (abstract 000040).** See text for description

### 000139

#### Beyond passive leg raising: functional hemodynamic assessment in critically ill and surgical patients. A systematic review and metanalysis

A. Messina[1], A. Dell'anna[2], M. Baggiani[3], F. Torrini[2], G. Maresca[2], V. Bennett[4], L. Saderi[5], G. Sotgiu[5], M. Antonelli[2], M. Cecconi[1]
[1]Anesthesia and intensive care, Humanitas Research Hospital, Rozzano, Italy; [2]Anesthesia and intensive care, Agostino Gemelli University Polyclinic, Roma, Italy; [3]Anesthesia and intensive care, Azienda Ospedaliero Universitaria Maggiore della Carità di Novara, Novara, Italy; [4]Anesthesia and intensive care, St George's Hospital Atkinson Morley Wing, London, United Kingdom; [5]Anesthesia and intensive care, University of Sassari, Sassari, Italy
**Correspondence:** A. Messina
*Intensive Care Medicine Experimental* 2019, **7(Suppl 3):**000139

**INTRODUCTION.** Tailored fluid therapy has received increasing attention in the management of patients with acute circulatory failure in both the intensive care unit (ICU) and operating room (OR). The only physiological reason to give a fluid challenge (FC) to a patient with acute circulatory failure is to increase the stroke volume (SV) ultimately leading to an increase in oxygen transport. However, this is only achieved in approximately 50% of ICU and OR patients. The values of the ventilator-induced dynamic changes in pulse pressure and stroke volume [pulse pressure variation (PPV) and stroke volume variation (SVV), respectively] are often unreliable in a significant number of ICU and OR patients. To overcome these limitations, bedside functional hemodynamic assessment has gained in popularity. The functional hemodynamic test (FHT) called passive leg raise (PLR), has been successfully used since 2009 to assess fluid responsiveness in ICU patients. Since the reliability and the limits of PPV, SVV and PLR in predicting fluid responsiveness has been already extensively investigated in different clinical settings, we conducted a systematic review of the literature and performed a metanalysis aimed at assessing the overall quality, external validation, consistency and risk of bias of the other FHTs available in both the ICU and OR.

**METHODS.** We considered a FHT as a clinical maneuver affecting heart-lung interactions and causing a different hemodynamic response in fluid responders and non-responders. MEDLINE, EMBASE and Cochrane Databases were screened for relevant articles using a FHT, with the exception of the PLR. The QUADAS-2 scale was used to assess the risk of bias of the included studies. In-between study heterogeneity was assessed through the I2 indicator. Bias assessment graphs were plotted and Egger's regression analysis was used to evaluate the publication bias. The metanalysis determined the pooled area under the receiving operating characteristic (ROC) curve, the sensitivity, specificity and threshold for two FHTs: the EEOT (end-expiratory occlusion test) and the mini-fluid challenge (FC).

**RESULTS.** The electronic search identified 7,674 titles. After text selection, 21 studies met the inclusion criteria, 7 performed in the OR and 14 in the ICU between 2005 and 2018. The search included 805 patients and 870 FCs with a median (IQR) of 39 (25-50) patients and

36 (29 - 50) FCs per study. The median fluid responsiveness was 54% (45-59). The pooled area under the ROC curve for the end-expiratory occlusion test (EEOT) was 0.96 (95% CI 0.92-1.00). The pooled sensitivity and specificity was 0.86 (95%CI 0.74-0.94) and 0.91 (95%CI 0.85-0.95) respectively, with a best threshold of 5% (4.0–8.0). The pooled area under the ROC curve for the mini-FC was 0.91 (95% CI 0.85-0.97). The pooled sensitivity and specificity were 0.82 (95%CI 0.76-0.88) and 0.83 (95%CI 0.77-0.89), respectively, with a best threshold of 5.4% (3.0–7.0).

**CONCLUSION.** The EEOT and the mini-FC reliably predict fluid responsiveness in the ICU and OR. Other FHTs have been tested insofar in heterogeneous clinical settings, and, despite promising results, warrant further investigations.

**REFERENCE(S)**

1. None
2. Monnet X, Marik P, Teboul JL: Passive leg raising for predicting fluid responsiveness: A systematic review and meta-analysis. Intensive Care Med 2016;42:1935-1947.
3. Lobo SM, de Oliveira NE: Clinical review: What are the best hemodynamic targets for noncardiac surgical patients? Critical care 2013;17:210.
4. Pinsky MR, Payen D: Functional hemodynamic monitoring. Crit Care 2005;9:566-572.

### 000603

#### A systematic review of practice patterns for treatment of atrial fibrillation in critically ill patients

L. O'Bryan, OC. Redfern, J. Bedford, P. Watkinson
Critical care research group, University of Oxford, Oxford, United Kingdom
**Correspondence:** L. O'Bryan
*Intensive Care Medicine Experimental* 2019, **7(Suppl 3):**000603

**INTRODUCTION.** New-onset atrial fibrillation (NOAF) occurs commonly in critical care and can lead to longer hospital stay, an increased risk of thromboembolic stroke and higher in-hospital mortality.(1) Treatment guidelines for atrial fibrillation (AF) in the general population are less safe and effective when applied to the critically ill patient.(2,3) At present, there are no consensus recommendations for the management of NOAF which are specific to critically ill patients.(4) Previous systematic reviews have identified a paucity of high-quality evidence to inform clinical practice and published studies suggest that current approaches to treatment vary considerably.(1,4)

**OBJECTIVES.** To systematically review published studies that report current practices for management of NOAF in critically ill patients

**METHODS.** We searched EMBASE, MEDLINE and Web of Knowledge for observational studies reporting data related to treatments used for NOAF in critically ill adult patients. The outcome of interest was the treatment(s) given to patients with NOAF. Treatments could be drugs to control cardiac rate or rhythm (including magnesium), DC cardioversion or anticoagulants. Data relating to study design, setting, population, interventions, outcomes and risk of bias were extracted. Risk of bias assessment was performed using an adapted Newcastle-Ottawa Scale.

**RESULTS.** We initially obtained 1,406 search results, of which we included 9 studies with a combined total of 8,689 patients. Of drugs used for rate/rhythm control, amiodarone use was reported most often (in 8 studies) followed by beta-blockers (7 studies), calcium channel blockers (5 studies), digoxin (5 studies) and magnesium (4 studies). Rate of DC cardioversion use was reported in 8 studies. Anticoagulant use was reported in 4 studies. As a proportion of all patients across studies, amiodarone was used in 48.9%, beta-blockers in 36.1%, magnesium in 29.8%, calcium channel blockers in 18.8%, digoxin in 17.8% and DC cardioversion in 16.2%. Anticoagulants were used in an average of 44.0% of patients. When comparing practices between countries, studies from Europe demonstrated higher rates of amiodarone use when compared to studies from the USA. The mean proportion of patients treated with amiodarone in European studies was 69.3%, compared with 47.7% in American studies. In contrast, American studies reported use of either a calcium channel blocker or beta-blocker in 49.7% of patients, compared with 24.2% in European studies.

**CONCLUSION.** There is a clear difference in practice patterns between the USA and Europe in antiarrhythmic use for NOAF management. Even within countries it appears that practices vary significantly. Furthermore the use of anticoagulation varies considerably. Future research should focus on comparing the efficacy of the most commonly used anti-arrhythmic agents including amiodarone, beta-blockers, calcium channel blockers and magnesium. Large cohort studies should test the rates of ischaemic stroke and bleeding associated with anticoagulation in a generally critically unwell population with NOAF.

**REFERENCE(S)**

1. Yoshida T, Fujii T, Uchino S, Takinami M. Epidemiology, prevention, and treatment of new-onset atrial fibrillation in critically ill: A systematic review. J Intensive Care. England; 2015;3(1):19.
2. Walkey AJ, Evans SR, Winter MR, Benjamin EJ. Practice patterns and outcomes of treatments for atrial fibrillation during sepsis a propensity-matched cohort study. Chest. United States; 2016;149(1):74–83.
3. Walkey AJ, Quinn EK, Winter MR, McManus DD, Benjamin EJ. Practice patterns and outcomes associated with use of anticoagulation among patients with atrial fibrillation during sepsis. JAMA Cardiol. 2016;1(6):682–90.
4. Kanji S, Stewart R, Fergusson DA, McIntyre L, Turgeon AF, Hébert PC. Treatment of new-onset atrial fibrillation in noncardiac intensive care unit patients: A systematic review of randomized controlled trials. Crit Care Med. 2008;36(5):1620–4.
5. Peter Watkinson is supported by the NIHR Biomedical Research Centre, Oxford
6. Funding for this project was obtained through the University of Melbourne Global Scholars Award for high achieving students to undertake overseas research.

### 000729

#### Higher skin blood flow is associated with better hemodynamic tolerance to fluid removal in patients with acute respiratory distress syndrome

W. Mongkolpun[1], B. Péter[1], JL. Vincent[2], J. Creteur[1]
[1]Department of intensive care, ULB Erasme, Anderlecht, Belgium; [2]Department of intensive care, ULB Erasme, Brussels, Belgium
**Correspondence:** W. Mongkolpun
*Intensive Care Medicine Experimental* 2019, **7(Suppl 3):**000729

**INTRODUCTION.** A negative fluid balance (NFB) can improve oxygenation in patients with acute respiratory distress syndrome (ARDS) but an excessive NFB can result in hemodynamic instability and tissue hypoperfusion. Skin blood flow (SBF) measurements can provide an indication of tissue perfusion.

**OBJECTIVES.** We hypothesized that SBF measurements could predict hemodynamic tolerance (HT) during induction of a NFB in patients with ARDS.

**METHODS.** We prospectively included hemodynamically stable (mean arterial pressure (MAP) $\geq$ 65 mmHg with no increase in norepinephrine (NE) dose in the last 6 hours) patients with ARDS (Berlin criteria) in whom a strategy of fluid removal was applied to reach a NFB of more than 1 L in 24 hours. SBF was measured (Periflux 5000, Perimed; index finger; Perfusion unit: PU) for 3 min at basal temperature before the NFB strategy (T0) and 24 hours later (T24). Hemodynamic variables, the PaO2/FiO2 ratio, central venous oxygen saturation (ScvO2) and blood lactate concentrations were also recorded at H0 and H24. HT after NFB (H24) was defined as MAP $\geq$ 65 mmHg with no increase in NE dose. In patients with a NFB $\geq$ 1 L at H24, a receiver operating characteristic (ROC) curve was constructed to assess the best predictive values for HT. Data are reported as medians with 25th and 75th percentiles.

**RESULTS.** We studied 35 mechanically ventilated patients with ARDS (pneumonia (29), abdominal infection (5), pancreatitis (1)). Clinical characteristics are shown in Table1; 11/35 patients received continuous hemofiltration. At T0, there were no differences in SOFA score, hemodynamic variables, NE dose, PaO2/FiO2, ScvO2, time from diagnosis or furosemide dose between patients with and without HT (Table 1). At T24, the degree of NFB did not differ significantly between the group with and without HT (1.7 (1.2-2.0) vs 1.3 (1.1-1.9) L,p=0.1). Patients who tolerated a NFB had lower lactate levels (1.1 (0.7-1.2) vs 1.4 (1.0-1.7) mmol/L, p=0.03) and higher SBF (151 (81-194) vs 70 (45-135) PU, p=0.01) than those who did not. MAP, cardiac index (CI) and ScvO2 were similar in patients with and without HT. SBF decreased in patients with and without HT (p<0.01) but was higher at H24 in patients who tolerated the NFB than in those who did not (100 (35-173) vs 35 (22-78) PU, p=0.01). The area under the ROC curve to predict HT after a NFB was higher for baseline SBF (0.92[0.83-0.99]) than for lactate level (0.72 [0.58-0.87]) and ScvO2 (0.69 [0.53-0.85]) (both p=0.03), with a cut-off ≥ 125 PU (sensitivity 90, specificity 93%).

**CONCLUSION.** Baseline SBF can predict good hemodynamic tolerance to a NFB in patients with ARDS.

**Table 1 (abstract 000729).** See text for description

| | All patients (N=35) | NFB with HT (N=18) | NFB without HT (N=17) | p |
|---|---|---|---|---|
| SOFA at T0 | 10 (7-11) | 9 (6-11) | 11 (8-12) | 0.2 |
| MAP at T0 (mmHg) | 71 (69-72) | 72 (71-73) | 69 (68-72) | 0.5 |
| Heart rate at T0 (bpm) | 100 (86-114) | 98 (85-108) | 94 (66-116) | 0.2 |
| Cardiac index at T0 (L/min/m²) (N=27) | 2.7 (2.1-3.0) | 2.7 (2.4-3.1) | 2.5 (2.0-3.0) | 0.7 |
| Norepinephrine dose at T0 (mcg/kg/min) (N=27) | 0.2 (0.03-0.3) | 0.2 (0.04-0.30) | 0.1 (0.03-0.3) | 0.5 |
| PaO2/FiO2 at T0 | 134 (86-193) | 136 (115-200) | 143 (87-198) | 0.4 |
| Time from diagnosis to initial NFB (hours) | 74 (49-96) | 72 (48-96) | 74 (64-94) | 0.6 |
| Furosemide dose during NFB (mg/24 hours) | 80 (70-120) | 80 (80-120) | 80 (60-120) | 0.9 |
| Negative fluid balance at T24 (L) | 1.3 (1.1-2.0) | 1.7 (1.2-2.0) | 1.3 (1.1-1.9) | 0.1 |

#### 001254

#### Monitoring resuscitation in severe sepsis and septic shock patients. Results of the MORESS trial

G. Gruartmoner, J. Mesquida, C. Espinal Sacristan, G. Gomà, X. García, A. Ochagavia, A. Artigas, F. Baigorri

Àrea de crítics, Parc Tauli Hospital Universitari. Institut d'Investigació i Innovació Parc Taulí (I3PT)., Sabadell, Spain

**Correspondence:** G. Gruartmoner

*Intensive Care Medicine Experimental* 2019, **7(Suppl 3):**001254

**INTRODUCTION.** During the resuscitation process of septic shock, use of dynamic parameters over static parameters to assess fluid responsiveness has been proposed. Surprisingly, scarce evidence is available showing a beneficial clinical outcome effect of dynamic parameters over static parameters to guide fluid resuscitation.

**OBJECTIVES.** To evaluate the impact on mortality of a fluid resuscitation protocol based on the use of dynamic parameters compared to static parameters to assess fluid responsiveness in the resuscitation process of severe sepsis and septic shock patients.

**METHODS.** Prospective, randomized, multicenter study. Adult patients with severe sepsis or septic shock, with evidence of tissue hypoperfusion, and under mechanical ventilation. At ICU admission, patients were randomized to one of the two fluid resuscitation protocols: *Control Group*, where fluid resuscitation was guided by Central Venous Pressure (CVP), according to the recommendations of *Surviving Sepsis Campaign 2012*; and *Intervention Group*, where fluid resuscitation was guided by Pulse Pressure Variation (PPV), Systolic Volume Variation (SVV), or Passive Leg Raising (PLR) maneuver. The fluid resuscitation protocol was followed during the first 72h of ICU stay. Main study outcome was 28-day mortality. Baseline patient characteristics, hemodynamic data, metabolic data, fluid balance, organ failure evolution, and ICU/hospital stay were also recorded.

**RESULTS.** The study was interrupted after two years because of low patient inclusion rate. A total of 60 patients were included in 12 centers. 66.6% of the patients were randomized to control group (n=40), and 33.3% to intervention group (n=20). There were no differences in baseline patients characteristics between the two groups. Median age was 62 ± 15 years, 65% male, 82% in septic shock, with initial SOFA score 12 ± 4 points. Lactate at inclusion was 4.7 ± 3.6 mmol/L. Global mortality was 30%, 47% in the control group and 21% in the intervention group ($p$ = 0.1). There were no differences in lactate evolution at 6 and 24 hours of admission. Fluid balance during the study period (72h) was significantly increased in the control group (5812 ± 4818 mL vs 3035 ± 3031 mL, $p$ 0.02).

**CONCLUSION.** A fluid resuscitation protocol based on dynamic parameters to assess fluid responsiveness is associated to a decreased fluid balance in the first 72h of the resuscitation process of septic shock. The inclusion of these dynamic variables may help to limit excessive fluid administration, potentially improving the outcome in this population of patients.

### HSRO - Predicting outcome, what are we missing?

#### 000349

#### Factors associated with changes in frailty one year after ICU admission; a prospective cohort study

W. Geense[1], M. Zegers[1], H. Vermeulen[2], H. Van Der Hoeven[1], M. Van Den Boogaard[1]

[1]Department of intensive care medicine, Radboud University Medical Center, Nijmegen, Netherlands; [2]Department of iq healthcare, Radboud University Medical Center, Nijmegen, Netherlands

**Correspondence:** W. Geense

*Intensive Care Medicine Experimental* 2019, **7(Suppl 3):**000349

**INTRODUCTION.** Frailty significantly impacts ICU patient's outcomes, recovery and healthcare utilization. However, knowledge about changes in frailty after ICU admission and associated factors is limited.

**OBJECTIVES.** To assess frailty before ICU admission and determine differences between frail and non-frail patients; to study changes in frailty in the year after ICU admission and its associated variables.

**METHODS.** Adult patients admitted for ≥12 hours to a university hospital ICU between July 2016 and December 2017 were included (MONITOR-IC study, clinicaltrials.gov NCT03246334). Moribund patients were excluded. Frailty was assessed using the Clinical Frailty Scale (CFS) (range 1 'Very fit' to 9 'Terminally ill') before ICU admission (T0), at hospital discharge (T1), and three (T2) and 12 months (T3) after ICU admission. Baseline differences between 'non frail' (scale 1-4) and 'frail' patients (scale 5-9) were compared. We calculated CFS mean differences between time points. We used multiple linear regression to determine factors associated with frailty change scores over one year (T0-T3).

**RESULTS.** A total of 1300 patients was included: mean age 61 (±14.9 years), 65% male and 66% planned ICU admissions. Median baseline CFS score was 3 [IQR:2-4]. Frail patients (12%) were significantly more often female, lower educated, divorced or widowed, living in a healthcare facility, had more often a chronic condition and an unplanned ICU admission.

At T1 the number of frail patients increased from 12 to 43%. At T2 and T3 this decreased to 15% and 9%. After one year, 30% had a lower CFS score compared to T0 (less frail), 31% a higher score (more frail) and in 39% the score did not change. Factors significantly associated with lower scores after 12 months, were planned admission (B=-0.306), mechanical ventilation (B=-0.320) and higher baseline CFS score (B=-0.711). Factors significantly associated with higher scores were age (B=0.013), living in a healthcare facility before admission (B=1.199), hospital LOS (B=0.023), and being discharged to a nursing home (B=0.861). APACHE IV score, chronic diagnosis, admission type and ICU LOS were not associated with changes in frailty.

**CONCLUSION.** Frailty levels changed in the first year after ICU admission. After one year, one third of the patients were more frail and one third less frail. Several factors, including age, living in healthcare facilities, hospital LOS and discharged to a nursing home were associated with being more frail after one year.

### 001013

#### Severity scoring systems as predictors of acute inpatient mortality; a multicentre comparison of NEWS, SOFA and ICNARC

A. Hughes[1], E. Palmer[2], S. Harris[1]
[1]Critical care department, University College Hospital, Euston Road, London, UK, London, United Kingdom; [2]{street_address}, London, United Kingdom
**Correspondence:** A. Hughes
*Intensive Care Medicine Experimental* 2019, **7(Suppl 3):**001013

**INTRODUCTION.** Early identification of the deteriorating patient is a priority for healthcare professionals. Previous studies have examined the prognostic accuracy of early warning and critical care scoring systems, however comparative data is rare(1,2).

**OBJECTIVES.** Compare the prognostic accuracy of the Intensive Care National Audit & Research Centre (ICNARC) physiology score, the NHS National Early Warning Score (NEWS) and the Sequential Organ Failure Assessment (SOFA) score for predicting acute (7-day) mortality in the deteriorating ward patient.

**METHODS.** Data for this abstract were taken from the (SPOT)light study; a multicentre prospective observational cohort study of the deteriorating ward patient referred for assessment by critical care. From the physiology measurements at ward assessment, the ICNARC physiology, NEWS and SOFA scores were calculated with missing values given zero weights as previously recommended(3–5). Survival status was obtained from the NHS Information Service with acute mortality defined as death within 7 days of the first ward assessment by critical care. Statistical analysis was undertaken using R. Unadjusted logistic regression was used to test for the association between each score as a continuous variable and acute mortality, with odds ratios calculated. Performance of scoring systems was assessed by discrimination using receiver operating characteristic (ROC) curve analysis. ROC curves were plotted and the area under the curve (AUC) calculated using the PRROC package(6).

**RESULTS.** 48 hospitals reported 15,158 visits for ward assessment over 369 study months (mean age 67 years, 51.9% male). 2708 (18%) patients died during the 7-days following ward assessment, 1539 (57%) of these deaths occurred within the first 48 hours. There was a clear correlation between physiological severity and acute mortality using either ward based (NEWS) or critical care scoring systems (SOFA, ICNARC). For predicting acute mortality, the AUC-ROC values (95% CI) were similar between the scoring systems with NEWS 0.67 (0.66-0.68), SOFA 0.65 (0.64-0.67) and ICNARC 0.68 (0.67-0.69).

**CONCLUSION.** The results demonstrate that NEWS, ICNARC and SOFA have equivalent accuracy for predicting acute mortality in the deteriorating ward patient. This result is of important practical value as using simpler scores such as NEWS or SOFA could facilitate greater efficiency when assessing patients in the ward setting.

**REFERENCE(S)**

1. The ability of the National Early Warning Score (NEWS) to discriminate patients at risk of early cardiac arrest, unanticipated intensive care unit admission, and death- ClinicalKey [Internet]. [cited 2019 Apr 13]. Available from: https://www-clinicalkey-com.rsm.idm.oclc.org/#!/content/journal/1-s2.0-S0300957213000026
2. Goulden R, Hoyle M-C, Monis J, Railton D, Riley V, Martin P, et al. qSOFA, SIRS and NEWS for predicting inhospital mortality and ICU admission in emergency admissions treated as sepsis. Emerg Med J EMJ. 2018 Jun;35(6):345–9.
3. Harrison DA, Parry GJ, Carpenter JR, Short A, Rowan K. A new risk prediction model for critical care: the Intensive Care National Audit & Research Centre (ICNARC) model. Crit Care Med. 2007 Apr;35(4):1091–8.
4. National Early Warning Score (NEWS): Standardising the assessment of acute-illness severity in the NHS. 2012 p. 1–30.
5. Vincent JL, Moreno R, Takala J, Willatts S, De Mendonça A, Bruining H, et al. The SOFA (Sepsis-related Organ Failure Assessment) score to describe organ dysfunction/failure. On behalf of the Working Group on Sepsis-Related Problems of the European Society of Intensive Care Medicine. Intensive Care Med. 1996 Jul;22(7):707–10.
6. Grau J, Grosse I, Keilwagen J. PRROC: computing and visualizing precision-recall and receiver operating characteristic curves in R. Bioinformatics. 2015 Aug 1;31(15):2595–7.

### 001660

#### Static Sepsis Mortality 2000-2018 in the State of Victoria, Australia

G. Duke[1], J. Moran[2], J. Santamaria[3], D. Pilcher[4]
[1]Eastern Health Intensive Care Service, Eastern Health, Box Hill, Australia; [2]Intensive care, The Queen Elizabeth Hospital, Woodville South, Australia; [3]Intensive care, St. Vincent's Hospital Melbourne, Fitzroy, Australia; [4]Intensive care department, The Alfred, Melbourne, Australia
**Correspondence:** G. Duke
*Intensive Care Medicine Experimental* 2019, **7(Suppl 3):**001660

**INTRODUCTION.** Improved survival following sepsis has been widely reported and asserted, even for those with severe sepsis treated in an intensive care unit (ICU). These favourable mortality trends are often interpreted as the success of healthcare investment and sepsis research. Reliable interpretation of long-term trends requires an appropriate metric with comprehensive data to prevent misinterpretation.

**OBJECTIVES.** To review temporal trends in sepsis-related incidence and mortality based on a retrospective analysis of a eighteen-year jurisdictional dataset.

**METHODS.** Sepsis-related hospital admissions were electronically identified using international consensus criteria mapped to ICD-10AM codes. Risk-adjustment was derived from available demographic and diagnostic codes. Mixed-effects regression models were employed to identify significant temporal trends.

**RESULTS.** Over eighteen years the population in Victoria (6.2 million) grew by 34.5%. 447,417 sepsis-related records were identified including 80,455 (18.0%) treated in ICU. 44,561 (10.0%) in-hospital deaths were reported. Annual population-derived mortality rate remained unchanged for both total (60.0±3.4 per 10^5, p=0.72) and ICU (19.8±1.6 per 10^5; p=0.73) cohorts. Hospital mortality rates declined significantly (p<0.001) but were static when adjusted for population size (p=0.074) and total sepsis burden (p= 0.14). Total population (VIF=37.6), adult population (VIF=51.0), and hospitla sepsis burden (VIF=24.2) were all collinear with 'year of separation'.

**CONCLUSION.** Sepsis-related incidence is rising but sepsis-related mortality has not declined over the past eighteen years in the State of Victoria. Reports of apparent decline in sepsis mortality rates may be due to rising admissions and incomplete risk-adjustment.

**REFERENCE(S)**

1. Iwashyna TJ, Angus DC. Declining case fatality rates for severe sepsis: good data bring good news with ambiguous implications. JAMA, 2014;311(13):1295–1297.
2. We thank the Victorian Department of Health and Human Services, and clinicians, data collectors, and health information managers for their assistance in collecting and accessing these data.

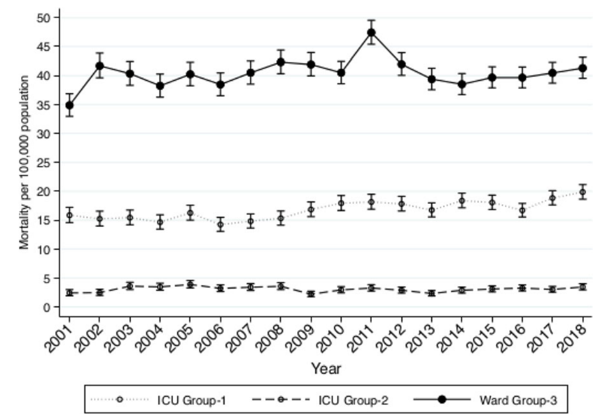

**Fig. 1 (abstract 001660).** See text for description

### 001690

#### Understanding deterioration in hospitalised patients: Application of a failure to rescue score

S. Hadfield[1], R. Loveridge[2], E. Helme[3], D. Hadfield[4], E. Madine[4], V. Metaxa[5], J. Wendon[4], M. Mcphail[4]
[1]Kings College Hospital NHS Foundation Trust, London, United Kingdom; [2]Critical care, Kings College Hospital NHS Foundation Trust, London, United Kingdom; [3]Anaesthetics, Royal Surrey County Hospital, Guildford, United Kingdom; [4]Critical care, King's College Hospital, London, United Kingdom; [5]Intensive care, King's College Hospital, London, United Kingdom
**Correspondence:** S. Hadfield
*Intensive Care Medicine Experimental* 2019, **7(Suppl 3):**001690

**INTRODUCTION.** Mismanagement of deterioration is the most common area of systemic failure in avoidable patient death across the NHS.[1] To address this issue, national guidelines recommend routine analysis of patient safety data to identify areas of service failure and to highlight opportunities for corrective action.[2] We previously described a novel 'failure to rescue' (FR) scoring system that can be applied to unplanned ICU admissions (UPAs) from ward areas, to identify any aspects of care that may have contributed to patient deterioration.[3]
Using data collected over a 4-year period, here we validate this score and explore the association between the FR score, illness severity and patient outcome.
**METHODS.** All adult UPAs in a single university foundation trust between January 2014 and December 2018 were reviewed. As previously described, the FR score consists of 18 questions that objectively appraise four categories of care failure in the pre-ICU admission period.

1. Failure to record: Were frequency of vital signs increased? Were vital signs complete?
2. Failure to recognise: Was deterioration recognised?
3. Failure to escalate: Was the patient escalated, was there evidence of an escalation block?
4. Failure to manage: Where appropriate diagnostic tests performed? Was there timely review?

Scores range 0 to 18.5; higher scores indicate greater failures in care. Additional data collected included: patient demographics, APACHE II score, ICU admission, ICU and hospital mortality.
**RESULTS.** 3112 patients were included; median age 63(IQR 51-73), 59% male, median admission APACHE II scores 19(14-23) with a 66% survival rate to hospital discharge. Median total UPA score was 2(1-4) which was higher in patients who did not survive (p<0.001). Patients who suffered cardiac arrest prior to ICU admission also had higher UPA scores (p<0.001). The majority of patients scored between 0-3; and the most commonly occurring category of care failure was Failure to manage in 20% of cases.
In a multivariate logistic regression model controlling for age, sex and APACHE II score total UPA score retained significance for prediction of hospital mortality (OR 1.040, 95% CI 1.007-1.040, p=0.017, overall p value <0.001). The area under the receiver operating curve for this model was 0.735 (0.718-0.753), p<0.001.
**CONCLUSION.** In this large observational cohort, we have demonstrated the ability of the FR scoring system to identify categories of care failure. We have demonstrated an association between higher scores (greater failures in care) and worse patient outcomes. This system provides critical information about patient deterioration, allowing focused quality improvement projects that may be tracked via FR scores over time.

**REFERENCE(S)**

1. Helme E, Brodrick R, Loveridge R. Tracking failure to rescue in the future hospital, Clinical Medicine 2015; 15 (s4).
2. NHS National Patient Safety Agency. Recognising and responding appropriately to early signs of deterioration in hospitalised patients. London: National Patient Safety Agency; 2007.
3. Donaldson LJ, Panesar SS, Darzi A. Patient-safety-related hospital deaths in England: thematic analysis of incidents reported to a national database, 2010-2012. PLoS medicine 2014; 11: e1001667.

### 001259

#### Two different ultrasound guided techniques for peripheral venous catheter placement – randomized study

R. Skulec[1], P. Vojtisek[1], J. Callerova[2], V. Cerny[1]
[1]Department of Anesthesiology, Perioperative Medicine and Intensive Care, Masaryk Hospital Usti nad Labem, Usti nad Labem, Czech Republic; [2]Pribram, Emergency Medical Service of the Central Bohemian Region, Kladno, Czech Republic
**Correspondence:** R. Skulec
*Intensive Care Medicine Experimental* 2019, **7(Suppl 3):**001259

**INTRODUCTION.** Ultrasound guidance of peripheral venous catheter (PVC) insertion may increase the success rate of the procedure.
**OBJECTIVES.** We performed randomized pre-hospital clinical study to compare two different techniques of ultrasound guided PVC insertion and conventional cannulation technique regarding the success of the first attempt of PVC insertion, the overall success, the number of attempts required for successful PVC placement, the time required to introduce PVC and the incidence of prehospital complications.
**METHODS.** Pre-hospital prospective controlled randomized clinical trial allocated patients treated by emergency medical service to either undergo PVC insertion fully controlled by ultrasound (ultrasound guidance of the tip of the PVC until it penetrates the lumen; Group A); or to undergo PVC insertion partially controlled by ultrasound (target vein identification only; Group B); or to receive PVC by conventional approach without any ultrasound guidance (Group C). The study outcomes were monitored until takeover of the patient to the hospital.
**RESULTS.** A total of 300 adult patients were enrolled. The success of the first attempt (group A: 88%, group B: 94%, group C: 76%, p< 0.001) and overall success rate (A: 99%, B: 99%, C: 90%, p<0.001) were significantly higher in the groups A and B than in the group C without significant differences between the groups A and B. The number of attempts was significantly lower (A: 1.18±0.54, B: 1.05±0.22, C: 1.22±0.57, p<0.001) and the time required for the procedure shorter (A: 75.3±60.6, B: 43.5±26.0, C: 82.3±100.9 s, p<0.001) in the group B than in the groups A and C.
**CONCLUSION.** Pre-hospital ultrasound guidance of PVC placement was associated with higher success rate than conventional method, irrespective of the technique of ultrasound guidance. However, PVC insertion partially controlled by ultrasound was superior to full ultrasound guidance in terms of the time and number of cannulation attempts required.

## POIC - Improving perioperative care

### 000433

#### A comparison of balanced salt solution vs. Normal saline in major abdominal surgery: a quantitative acid base study

K. Kajal[1], S. Joshiya[1], A. Jayant[2], T. Yadav[1], N. Sahni[1]
[1]Anaesthesia and intensive care, Post Graduate Institute of Medical Education & Research, Chandigarh, Chandigarh, India; [2]Anaesthesia and intensive care, Department of Cardiac Anaesthesia, Amrita Institute of Medical Sciences, Kochi, India, jayant.aveek@gmail.com, India
**Correspondence:** K. Kajal
*Intensive Care Medicine Experimental* 2019, **7(Suppl 3):**000433

**INTRODUCTION.** Fluid therapy is a cornerstone of modern medical practice, particularly in acute settings such as intra-operative or intensive care. The acidosis attributed to Normal Saline has a clear dose-dependent effect with larger volumes associated with this phenomenon. However, the exact quantitative impact of this solution when used in a restrictive paradigm is not adequately assessed in the current literature. Thus, this study attempted to assess the effects of vs. Normal Saline when used in restrictive quantities as determined by a predefined the hemodynamic algorithm.

**OBJECTIVES.** To determine the equivalence or otherwise of Normal saline in comparison to balanced salt solution for intra- operative fluid replacement in elective, adult open abdominal surgery on serum chloride level using a restrictive fluid therapy algorithm.

**METHODS.** 120 adult patients were randomized to receive either Normal saline or Plasmalyte as the predominant fluid during surgery using a computer-generated the random number. Patients received a bolus of 150ml of study fluid at induction and 2ml/kg/hr + urine output+ blood loss every hour. Systolic Pressure variation and Pulse Pressure variation was assessed every time the systolic blood pressure < 90 mm Hg or < 20% from baseline whichever was higher using the predefined algorithm. Arterial blood gas samples were repeated every two hours until the end of surgery. Additional samples were also obtained pre-induction and 24 hours after the end of surgery for the biochemistry variables. Previous studies had reported a difference of approximately 6 mEq L-1 in the chloride levels when saline was used in comparison to Plasmalyte at the volumes infused in the study. Assuming an α of 0.05 and a power of 80%, 120 patients were required to demonstrate that the limits of a two-sided 95% confidence interval would exclude a difference in the Chloride concentration of <3 mEq L-1 between the two groups at the conclusion of surgery.

**RESULTS.** 120 patients were enrolled in the study. Fig 1is the CONSORT diagram.

The chloride anion level decreased by a mean of 0.54 ± 3.29 in the Plasmalyte group and increased by mean of -0.43 ± 4.90 in the normal saline group, the difference between the two insignificant (p value=0.22). Subgroup analysis of 50 patients with duration of surgery exceeding 4 hours demonstrated chloride increase until 2 hours in both groups and thereafter a drop was noted by the 4th hour. (FIGURE 2)

Higher total urine output was recorded in Plasmalyte group (p= 0.006, significant) with a mean difference of 69.368 ml, 95% CI (19.0 to 119.7). (FIG 3)

**CONCLUSION.** CONCLUSIONS: Restrictive fluid regimen shows equivalence in the choice of crystalloid fluid (Plasmalyte versus Normal saline) used for intraoperative fluid therapy.

**REFERENCE(S)**
1. PGIMER CHANDIGARH

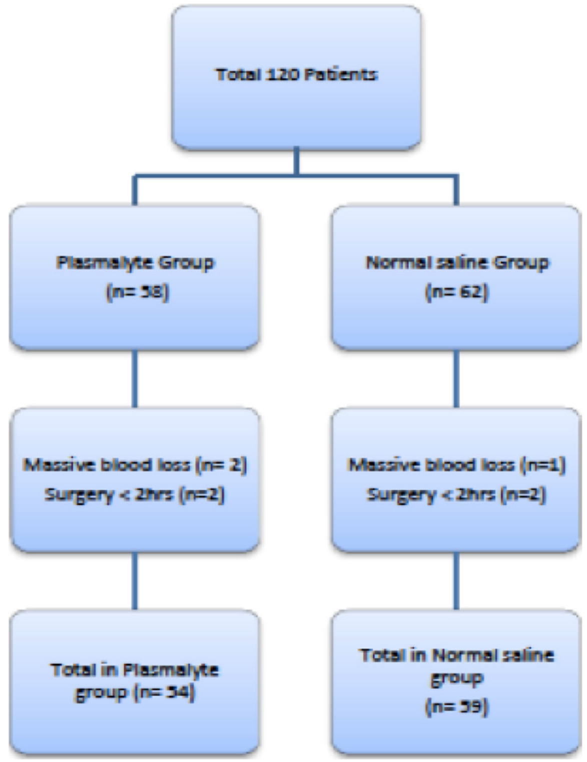

**Fig. 1 (abstract 000433).** Consort diagram showing enrolment of patients in the groups (n=number of patients)

| Duration | Plasmalyte group (n=28) | | | Normal saline group (n=22) | | |
|---|---|---|---|---|---|---|
| | Mean difference during the time interval | p-value | 95% CI | Mean difference during the time interval | p-value | 95% CI |
| 0 – 2 hrs | -1.86 | <0.05 | -3.5 to -0.2 | -0.89 | 0.45 | -2.4 to 0.7 |
| 2 – 4 hrs | 0.32 | 1.0 | -1.1 to 1.7 | 0.15 | 1.0 | -1.3 to 1.6 |
| 0 – 4 hrs | -1.54 | 0.11 | -3.3 to 0.3 | -0.74 | 0.62 | -2.2 to 0.7 |

**Fig. 2 (abstract 000433).** See text for description

### 000704

#### Does MR-proADM predict infectious complications in patients undergoing major abdominal surgery?

W. Gääw Rolander, HA. Andersson, H. Hansson, H. Didriksson, L. Sundin, R. Nordström, MS. Chew
Department of anaesthesia and intensive care, medical and health sciences, Linköping University Hospital, Linköping, Sweden
**Correspondence:** W. Gääw Rolander
*Intensive Care Medicine Experimental* 2019, **7(Suppl 3):**000704

**INTRODUCTION.** Postoperative infections are a major challenge for perioperative medicine. It is a major contributor to morbidity and mortality worldwide. Sensitivity and specificity of traditional biomarkers for predicting and diagnosing infection are often reduced in this setting. Lack of precision combined with the importance of early diagnosis and treatment emphasize the need for more sensitive markers of infection. Adrenomedullin (analyzed indirectly with mid-regional proadrenomedullin, MR-proADM) is a novel peptide that has predominantly vasoactive and immunological properties and seems to be an important prognostic marker for sepsis. However, whether one could use MR-proADM for the prediction and early diagnosis of postoperative infections remains unclear.

**OBJECTIVES.** To investigate the association between elevated levels of MR-proADM perioperatively and infectious complications in the 30 days following surgery.

**METHODS.** This was a preplanned substudy of the Myocardial Injury in Noncardiac Surgery in Sweden (NCT03436238). We enrolled 504 patients undergoing major elective abdominal surgery in a multicenter prospective cohort study. MR-proADM levels were measured preoperatively, immediately postoperatively, and on days 1, 2 and 3 after surgery. Postoperative infections were registered at 30 days after surgery by a blinded assessor. The relationship between MR-proADM levels and the presence of infection was investigated. ROC-curves were constructed in order to obtain a cutoff value maximized for sensitivity.

**RESULTS.** MR-proADM was significantly higher at all postoperative data points across all patients compared to preoperatively. There were significant differences in MR-proADM levels between patients who developed an infectious complication and those who did not. This difference were noted immediately after surgery and at days 2 and 3. A cutoff was obtained from ROC-analysis which revealed that 1.043 nmol/L could significantly discriminate blood infection from no infection groups with a sensitivity of 89 % and specificity of 41 %.

**CONCLUSION.** MR-proADM levels immediately after surgery, at day 2 and day 3 predict infectious complications in the 30 day postoperative time period.

**REFERENCE(S)**

1. Andaluz-Ojeda D, Nguyen HB, Meunier-Beillard N, Cicuendez R, Quenot JP, Calvo D, et al. Superior accuracy of mid-regional proadrenomedullin for mortality prediction in sepsis with varying levels of illness severity. Ann Intensive Care. 2017;7(1):15.
2. Elke G, Bloos F, Wilson DC, Brunkhorst FM, Briegel J, Reinhart K, et al. The use of mid-regional proadrenomedullin to identify disease severity and treatment response to sepsis - a secondary analysis of a large randomised controlled trial. Critical Care. 2018;22(1):79.
3. Kitamura K, Kangawa K, Kawamoto M, Ichiki Y, Nakamura S, Matsuo H, et al. Adrenomedullin: a novel hypotensive peptide isolated from human pheochromocytoma. Biochem Biophys Res Commun. 1993;192(2):553-60.
4. Hirata Y, Mitaka C, Sato K, Nagura T, Tsunoda Y, Amaha K, et al. Increased circulating adrenomedullin, a novel vasodilatory peptide, in sepsis. J Clin Endocrinol Metab. 1996;81(4):1449-53.
5. Temmesfeld-Wollbruck B, Hocke AC, Suttorp N, Hippenstiel S. Adrenomedullin and endothelial barrier function. Thromb Haemost. 2007;98(5):944-51.
6. Andaluz-Ojeda D, Cicuendez R, Calvo D, Largo E, Nogales L, Munoz MF, et al. Sustained value of proadrenomedullin as mortality predictor in severe sepsis. J Infect. 2015;71(1):136-9.
7. NHS. Surgical site infection. Clinical guideline 742008.
8. Perencevich EN, Sands KE, Cosgrove SE, Guadagnoli E, Meara E, Platt R. Health and economic impact of surgical site infections diagnosed after hospital discharge. Emerg Infect Dis. 2003;9(2):196-203.
9. Swedish Research Council
10. Medical Research Council of Southeast Sweden
11. Region Östergotland County Council

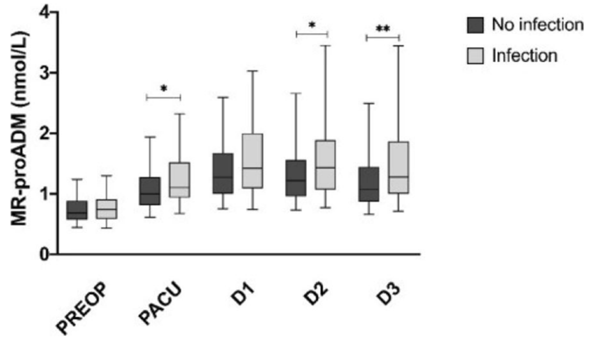

**Fig. 1 (abstract 000704).** See text for description

### 001264

#### Preoperative natriuretic peptides levels and incidence of perioperative atrial fibrillation after non-cardiac surgery: A prospective cohort study

W. Szczeklik[1], J. Fronczek[1], K. Polok[1], J. Górka[1], D. Conen[2], Y. Lemanach[2], F. Mcalister[3], B. Biccard[4], S. Srinathan[5], P. Alonso-Coello[6], D. Heels-Ansdell[7], E. Duceppe[2], P. Devereaux[2]

[1]Intensive care and perioperative medicine, Jagiellonian University Medical College, Kraków, Poland; [2]Population health research institute, McMaster University and Hamilton Health Sciences, Hamilton, Canada; [3]Division of general internal medicine and heart function clinic, University of Alberta, Edmonton, Canada; [4]Department of anaesthesia and perioperative medicine, University of Cape Town and New Groote Schuur Hospital, Cape Town, South Africa; [5]Department of surgery, University of Manitoba, Winnipeg, Canada; [6]Centro cochrane iberoamericano, Instituto de Investigación Biomédica (CIBERESP-IIB Sant Pau), Barcelona, Spain; [7]Department of clinical epidemiology & biostatistics, McMaster University and Hamilton Health Sciences, Hamilton, Canada

**Correspondence:** W. Szczeklik

*Intensive Care Medicine Experimental* 2019, **7(Suppl 3):**001264

**INTRODUCTION.** Perioperative atrial fibrillation (POAF) is associated with significant short and long-term complications.

**OBJECTIVES.** To establish the incidenceof POAF after noncardiac surgery and investigate the association between the preoperative NT-pro-BNP level and POAF.

**METHODS.** The Vascular events In non-cardiac Surgery patIents cOhort evaluatioN (VISION; NCT00512109) Study was an international prospective cohort study of a representative sample of adults ≥45 years of age who underwent non-cardiac surgery. One of the predefinedobjectives was to determine the 30-day incidenceof clinically important POAF (defined as POAFresulting in angina, congestive heart failure, symptomatic hypotension, or requiring treatment). We estimated the relationship between preoperative NT-pro-BNP levels and clinically significantPOAF using multivariable logistic regression models with restricted cubic splines.

**RESULTS.** In 37,664 patients with no prior history of AF, the incidence of POAF was 1.0% (95% CI, 0.9-1.1; 369 events). It was 3.2% (95% CI, 2.3-4.4) in patients undergoing major thoracic surgery, 1.3% (95% CI, 1.2-1.5) in major nonthoracic surgery, and 0.2% (95% CI, 0.1-0.3) in low-risk surgery. NT-pro-BNP measurement was obtained in 9789 individuals before surgery. An increase in the preoperative NT-pro-BNP level from 39 ng/ml (first quartile) to 228 ng/ml (third quartile) was associated with an unadjusted OR for developing POAF of 3.92 (95% CI, 2.32-6.65), and anadjusted OR of 2.55 (95% CI, 1.49-4.36).

**CONCLUSION.** Clinically important POAF was observed in every one hundredth patient having noncardiac surgery. Preoperative NT-pro-BNP levels were strongly associated with an increased risk of POAF.

### 001686

#### Does warmed intravenous fluid reduces shivering after general anesthesia in elderly?

V. GHERGHINA, I. Cindea, A. Balcan
Facultatea de medicina, Universitatea „Ovidius" din Constanța, Constanța, Romania
**Correspondence:** V. GHERGHINA
*Intensive Care Medicine Experimental* 2019, **7(Suppl 3):**001686

**INTRODUCTION.** Shivering is an unpleasant condition which usually occur as a complication after both general and regional anaesthesia in patient underwent either elective or emergency surgery.

The elderly constitute an important risk group due to the physiological alterations that occur in the aging process and contribute to a diminished thermoregulation capacity.

**OBJECTIVES.** Present prospective, randomized, and controlled study was performed to determine the benefits of warmed liquids infusion compared with infusion of liquids kept at room temperature in maintaining normal core temperature and prevent shivering after general anesthesia in elderly.

**METHODS.** After obtaining institutional approval and written informed consent from all patients, 62 patients with American Society of Anesthesiologists physical status of I-III, aged 65 years and above, who were scheduled for elective surgery under general anesthesia, were included in this prospective, randomized study. Exclusion criteria included history of head injury, thyroid disease, severe cardiovascular and respiratory disease, a core temperature of ≥ 37.5°C.

Our patients were randomly allocated to receive crystalloid at room temperature (31 patients, Group A, without infusion warmer) or warmed at 37°C (31 patients, Group B, with infusion warmer) .

Shivering was graded using the Bedside Shivering Assessment Scale.

The occurrence of shivering during surgery and in the recovery room was recorded.

The data obtained were analyzed with SPSS software with a significance level of p.

**RESULTS.** At the end of surgery, core temperature was 36.8 +/- 0.1 degrees C in the group receiving warmed fluids and 35.7 +/- 0.3 degrees C in the control group (P < 0.05).

During recovery, four patient shivered in the group receiving warmed fluids and seventeen in the control group (P < 0.05).

The severity of post-anesthesia shivering was also significantly less in the infusion warmer group (P < 0.05).

**CONCLUSION.** The use of infusion warmer may help in reducing the incidence of hypothermia and shivering after general anesthesia in elderly patients.

Prevention of hypothermia and shivering should be done with multimodal approach, either by pharmacological or non-pharmacological strategies.

Warm IV saline solution given before, during, and after surgery, is the cheapest and easiest way to maintain core temperature, preventing hypothermia and shivering.

### 001743

#### Continuous intravenous infusion of enoxaparin thromboprophylaxis leads to greater prothrombin inhibition compared to standard subcutaneous dosing

A. Vahtera[1], T. Szanto[2], V. Pettilä[3], R. Lassila[4], H. Huhtala[5], A. Kuitunen[1]
[1]Intensive care unit, Tampere University Hospital, Tampere, Finland; [2]Department of clinical chemistry, Helsinki University Hospital, Helsinki, Finland; [3]Perioperative, intensive care and pain medicine, University of Helsinki and Helsinki University Hospital, Helsinki, Finland; [4]Department of hematology and comprehensive cancer center and research program of oncology, Helsinki University Hospital, Helsinki, Finland; [5]Faculty of social sciences, Tampere University, Tampere, Finland
**Correspondence:** A. Vahtera
*Intensive Care Medicine Experimental* 2019, **7(Suppl 3):**001743

**INTRODUCTION.** A standard subcutaneous low-molecular weight heparin (LMWH) thromboprophylaxis yields subtherapeutic level of plasma anti-FXa activity (anti-FXa) in intensive care unit (ICU) patients. Moreover, the ability of the anti-FXa to predict clinical adverse events is limited.

**OBJECTIVES.** To assess coagulation status in mixed ICU patients randomized to receive enoxaparin thromboprophylaxis either as a standard subcutaneous bolus (SCB) or continuous intravenous infusion (CII) for three consecutive days.

**METHODS.** A randomized, single-blind study at two tertiary university hospitals was conducted. Thirty-eight mixed ICU patients were studied by conventional coagulation parameters. Additionally, 18 patients were analyzed for thrombin generation by calibrated automated thrombogram (TG-CAT). Blood samples were taken prior the study start (i.e. baseline), at 51 (i.e. the final peak level of the SCB group) and at 72 (i.e. the final through level of the SCB group) hours from the study start.

**RESULTS.** At baseline, no difference in coagulation status was observed. The inhibition of prothrombin fragments F1+2 (F1+2) was significantly superior at 51 and 72 hours in the CII group compared to the SCB group. Correspondingly, a significant increase in antithrombin (AT 3) level was observed within the CII group whereas the level in the SCB group remained unchanged (Table 1). At 51 hours lag time (7.5 vs. 4.3 min, p <0.05) and time to peak (14.3 vs. 7.7 min, p <0.05) of the TG-CAT analysis were significantly prolonged in the SCB group. At 72 hours peak thrombin was significantly lower in the CII group vs. the SCB group: 364 nM vs. 271 nM, p <.05. The final through level of plasma anti-FXa was higher in the CII group: 0.05 IU/ml vs. 0.12 IU/ml, p=0.067.

**CONCLUSION.** Continuous intravenous infusion of enoxaparin thromboprophylaxis resulted in more prominent inhibition of prothrombin fragments and better preservation of the natural anticoagulant antithrombin level in comparison to standard subcutaneous care.

**REFERENCE(S)**

1. Dörffler-Melly, J, de Jonge, E, Pont, AC et al.: Bioavailability of subcutaneous low-molecular-weight heparin to patients on vasopressors. Lancet 2002; 359:849-850
2. Vahtera A, Vaara S, Pettilä V, Kuitunen A. Plasma anti-FXa level as a surrogate marker of the adequacy of thromboprophylaxis in critically ill patients: A systematic review. Thromb Res. 2016 Mar; 139:10-6

**Table 1 (abstract 001743).** Comparison of Coagulation Parameters within and between Study Groups. Data are presented as median (interquartile range)

| Parameter | Study group | 0 h | | 51 h | | 72 h | |
|---|---|---|---|---|---|---|---|
| AT3, % | SCB | 70 | (63–81) | 70 | (54–82) | 69 | (54–110) |
| | CII | 65 | (52–89) | 79 | (59–94) ✦ | 83 | (64–96) ✦ |
| D-dimer, mg/l | SCB | 5.4 | (1.7–7.0) | 5.5 | (2.5–9.1) | 3.1 | (2.7–7.3) |
| | CII | 3.1 | (1.7–6.1) | 5.2 | (1.8–8.5) | 6.1 | (2.2–7.1) |
| $F_{1+2}$, nM | SCB | 413 | (300–831) | 634 | (501–846) * | 657 | (482–880) * |
| | CII | 438 | (197–731) | 372 | (237–627) * | 374 | (287–531) * |
| anti-FXa, IU/ml | SCB | 0.05 | (0–0.05) | 0.21 | (0.09–0.25) *✦ | 0.05 | (0.01–0.05) |
| | CII | 0.05 | (0–0.05) | 0.10 | (0.05–0.13) *✦ | 0.12 | (0.05–0.17) ✦ |

Abbreviations AT3: Antithrombin 3, F1+2: prothrombin fragment 1+2, SCB: subcutaneous bolus, CII: continuous intravenous infusion

*$P$ <0.05 between groups (SCB vs. CII)

✦ P <0.05 within groups (baseline vs. 51 h or baseline vs. 72 h from the study start, respectively)

## NAHP - Post ICU care: Impact & consequences

### 000057

#### Dealing with the aftermath of critical illness – the ENSURE (ENabling and SUpporting REcovery) intensive care follow up clinic

A. Lockwood, T. Jackson, R. Appelboam
Intensive care, Royal Devon and Exeter Hospital (Wonford), Exeter, United Kingdom
**Correspondence:** R. Appelboam
*Intensive Care Medicine Experimental* 2019, **7(Suppl 3):**000057

**INTRODUCTION.** Patients who survive intensive care are left with long term psychological, physical and cognitive impairments which may persist for up to 15 years following discharge. Collectively, these impairments are known as post intensive care syndrome (PICS), which affects up to 70% of survivors (1). These impairments are not limited to patients alone, with carers also carrying a high burden of psychological and social comorbidity (2).

**OBJECTIVES.** To assess the effectiveness of a 5 week multidisciplinary programme (clinic) for intensive care survivors and their carers on optimising physical and psychological recovery.

**METHODS.** Survivors of intensive care were invited to clinic with their carers, within 3 months of hospital discharge. Patients could also self-refer, or be referred by their family doctor.

On one morning a week, for 5 weeks, a selection of professionals from the multidisciplinary team (Consultant, Physiotherapist, Nurse, Psychologist, Dietician, Occupational Therapist, Pharmacist) attended and delivered a combination of group sessions and 1:1 sessions.

Using hospital data and self-administered questionnaires, information on patient characteristics, health status, and caregiver strain were collected. Patient outcome data were collected using the EQ-5D Health Questionnaire and self-efficacy scores at week 1, week 5, 3 months and 12 months. Carer outcomes were collected using a carer strain index. Professional interventions were recorded, and qualitative outcomes collected using standardised feedback forms.

**RESULTS.** A total of 28 patients have attended in 4 cohorts so far, with 22 family members. 25% self referred or were referred by their doctor. All patients completed their programme. 5 patients (18%) were trauma patients, 13 (46%) were medical, and 10 (36%) were surgical.

**CONCLUSION.** Patients and carers presented to our clinic with evidence of altered health status and carer strain. This multidisciplinary follow up clinic is an effective intervention in providing support and therapy for patients and their carers after intensive care, and results in improvements in self-efficacy and health status. The clinic is received positively by all who attend.

**REFERENCE(S)**

1 Desai SV et al. Crit Care Med. 2011; 39(2):371
2 Cameron JI et al. NEJM. 2016; 374:1831

**Table 1 (abstract 000057).** Patient Characteristics

| | |
|---|---|
| Median age – years (range) | 58.5 (18-83) |
| Number of patients sedated and ventilated (%) | 26 (94) |
| Median ICU length of stay - days (range) | 12.5 (2-54) |
| APACHE Score- mean ±SD | 34.29 (±21.7) |

**Table 2 (abstract 000057).** Patient outcomes

| | Week 1 | Week 5 | 3 months |
|---|---|---|---|
| EQ5D visual analogue score - mean ±SD | 62.0 ±20.8 | 72.9±19.9 | 81.67±12.9 |
| Self efficacy score - mean ± SD | 27.7±7.5 | 29.8±7.3 | 33.5±5.3 |

**Table 3 (abstract 000057).** Other outcomes

| | |
|---|---|
| Carer Strain Index – mean ± SD | 0.44 ± 0.3 |
| Pharmacy interventions - number (%) | 7 (25) |
| Total number of specialist referrals (%) | 21 (75) |
| Median satisfaction scores out of 6 (range) | 6 (5-6) |

### 000215

#### How does healthcare quality influence care left undone in neonatal and paediatric intensive care units?

A. Bagnasco[1], S. Rossi[2], N. Dasso[1], G. Catania[1], M. Zanini[1], G. Aleo[1], L. Sasso[1]
[1]Department of health science, University of Genoa Department of Health Science, Genova, Italy; [2]University of Genoa Department of Health Science, Genova, Italy
**Correspondence:** S. Rossi

*Intensive Care Medicine Experimental* 2019, **7(Suppl 3):**000215

**INTRODUCTION.** Providing high-quality care is a priority worldwide (WHO, 2018). One way of evaluating quality is the investigation of nursing care models. A topic under investigation in the last decade, due to its impact on the quality of health care, is the care left undone phenomenon (Ball et al., 2014). Understanding how healthcare quality is related to care left undone could contribute to quality improvement (Recio-Saucedo et al., 2018).

**OBJECTIVES.** Investigate which nursing staff and work environment variables could influence the prevalence of care left undone in Neonatal and Paediatric Intensive Care Units (NICUs and PICUs).

**METHODS.** We extracted data from the RN4CAST@IT-Ped project, a larger cross-sectional, observational, multicentre Italian study. The project involved 13 Italian hospitals, associated to Italian Association of Paediatric Hospitals, and the NICU and PICU nurses and paediatric nurses was enrolled using convenience sampling. We asked which activities, from a list of 13, were necessary but left undone because of lack of time during the last shift worked. Data were analysed through descriptive and inferential statistics methods (logistic regression), with IBM SPSS 22.0 software.

**RESULTS.** We analyzed the answers of 451 nurses. Poor health care, depersonalization and emotional exhaustion, and intention to leave the job are some variables that could influence the omission of some nursing activities, like surveillance, pain management, information and education, and mobilization (OR min=OR 1,024, $p$=0,026; OR max=OR 2,469, $p$=0,001). A good work environment is a protective factor related to the omission of nursing activities, like the development of nursing care plans (OR=0,512, $p$=0,001).

**CONCLUSION.** Our results are consistent with international literature showing that nurses miss some activities, especially in the presence of some environmental and personal conditions. Understanding the relations that underlay the nursing care left undone could be a starting point to develop improvement measures to both better patient-centred care and quality of health care.

**REFERENCE(S)**

1. World Health Organization. Improving the quality of paediatric care: an operational guide for facility-based audit and review of paediatric mortality. Geneva: World Health Organization; 2018. Licence: CC BY-NC-SA 3.0 IGO.2.
2. Ball JE, Murrels T, Rafferty AM, et al. 'Care left undone' during nursing shifts: associations with workload and perceived quality of care. BMJ Qual Saf.2014;23(2):116-25. doi: 10.1136/bmjqs-2012-001767
3. Recio-Saucedo A, Dall'Ora C, Maruotti A, et al. What impact does nursing care left undone have on patient outcomes? Review of the literature. J Clin Nurs. 2018;27(11-12):2248-2259. doi: 10.1111/jocn.14058

4. We thank the Italian Association of Pediatric Hospital to funding the study.

### 000500

#### Effect of nurse led follow-up consultations to improve sence of coherence in patients discharged after intensive care treatment

Å. Valsø[1], T. Rustøen[1], K. Sunde[2], Ø. Ekeberg[3], M. Cvancarova Småstuen[1], L. Skogstad[4], I. Sechou-Bredal[5], H. Myhren[6], K. Tøien[1]
[1]Department of research and development, division of emergencies and critical care, Oslo University Hospital, Oslo, Norway; [2]Department of anesthesiology, division of emergencies and critical care, Oslo University Hospital, Oslo, Norway; [3]Division of mental health and addiction, Oslo University Hospital, Oslo, Norway; [4]Department of nursing and health promotion, prehospital trauma care – bachelor paramedics, oslom, Oslo Metropolitan University of Oslo, Norway, Oslo, Norway; [5]Unit for breast- and endocrine surgery, Oslo University Hospital, Oslo, Norway; [6]Department of acute medicine, division of medicine, Oslo University Hospital, Oslo, Norway
**Correspondence:** Å. Valsø

*Intensive Care Medicine Experimental* 2019, **7(Suppl 3):**000500

**INTRODUCTION.** Sense of coherence (SOC) relates to the ability to endure stressful life events, and is divided into comprehensibility (SOC C), manageability (SOC MA) and meaningfulness (SOC ME). Low SOC reflects low coping ability and is strongly associated with post-traumatic stress symptoms (PTSS). It is not known whether nurse led follow-up consultations can improve SOC in discharged intensive care unit (ICU) patients with moderate to high PTSS.

**OBJECTIVES.** An a priori defined post hoc analysis of a randomized controlled trial (RCT) on nurse led follow-up consultations in discharged ICU patients with moderate to high levels of PTSS was performed, where both the effect on total SOC (as a composite endpoint), and on each of the three SOC dimensions (SOC C, SOC MA and SOC ME) was evaluated.
**METHODS.** ICU patients ≥18 years admitted to one of five ICUs at Oslo University Hospital (OUH) for ≥24 hours were included. Patients were screened on PTSS (PTSS-10) immediately after ICU discharge, and those with PTSS-10 score ≥25 (clinically relevant symptoms) were randomized to an intervention (IG) or control (CG) group. IG patients received up to three individually adjusted nurse led follow-up consultations immediately after ICU discharge, and one and two months later. Consultations were based on trauma focused cognitive behavioural therapy combined with narrative method to process traumatic memories and achieve coherence of what happened during the ICU stay. The SOC Scale with 13 items on a 1-7 scale measured the total score (range 13-91), but also the score for each of the three dimensions SOC C, SOC MA and SOC ME. This was done immediately after ICU discharge (baseline), and after 3, 6 and 12 months. Possible differences between IG and CG were analysed using linear mixed models for repeated measures.
**RESULTS.** In total, 523 patients were screened and 224 (43%) had PTSS-10 score ≥25 (53% male, median age 52 years) and were randomized into IG (n=111) and CG (n=112). No statistically significant differences were found between IG and CG in total SOC (p= 0.47), SOC C (p=0.58), SOC MA (p=0.17) or SOC ME (p=0.78) over time from baseline to 12 months. During the same time period total SOC increased in both groups, with a non-significant trend towards a different time trajectory in IG and CG (Table).
**CONCLUSION.** Nurse led follow-up consultations in discharged ICU patients with moderate to high risk of PTSS, did not reveal any significant effect on the composite endpoint SOC or on any of the three SOC dimensions.

**REFERENCE(S)**

1. The study received grants from OUH and The Norwegian Nurses Organisation

**Table 1 (abstract 000500).** Estimated means dependent variable: Total Sense of coherence (SOC)

| Time | Intervention Group (IG) | | Control Group (CG) | |
|---|---|---|---|---|
| | Mean | 95% CI | Mean | 95% CI |
| 1 (baseline) | 60.6 | 57.9, 63.2 | 62.3 | 59.6, 65.0 |
| 2 (3 months) | 62.9 | 59.8, 65.9 | 61.4 | 58.3, 64.5 |
| 3 (6 months) | 62.2 | 58.8, 65.7 | 64.7 | 61.2, 68.2 |
| 4 (12 months) | 62.2 | 59.1, 65.3 | 64.9 | 61.7, 68.2 |

### 000586

#### Pain occurrence and associated factors after discharge from the intensive care unit to the hospital ward

K. Tøien[1], Å. Valsø[1], I. Schou-Bredal[2], Ø. Ekeberg[3], L. Skogstad[4], K. Sunde[5], T. Rustøen[1]
[1]Department of research and development, division of emergencies and critical care, Oslo University Hospital, Oslo, Norway; [2]Department of canser, Oslo University Hospital, Oslo, Norway; [3]Division of mental health and addiction, Oslo University Hospital, Oslo, Norway; [4]Department of nursing and health promotion, prehospital trauma care, Oslo Metropolitan University, Oslo, Norway; [5]Department of anesthesiology, division of emergencies and critical care, Oslo University Hospital, Oslo, Norway
**Correspondence:** K. Tøien
*Intensive Care Medicine Experimental* 2019, **7(Suppl 3):**000586

**INTRODUCTION.** Pain has been shown to be a serious and challenging problem for intensive care unit (ICU) patients. Research is, however, scarce on pain related issues in ICU patients immediately after ICU discharge to the ward, with a significant lower nurse – patient ratio compared to the ICU.
**OBJECTIVES.** The aim of this study was to investigate pain intensity and pain interference in ICU patients immediately after ICU discharge, and the association between pain and sense of coherence (SOC, reflecting coping), post-traumatic stress symptoms (PTSS), memories from the ICU stay and demographic and clinical variables. SOC, PTSS, and memories from the ICU stay were hypothesized to be associated with pain after ICU discharge.
**METHODS.** In this a priori defined sub-study of a randomized controlled trial on PTSS in ICU patients, 523 consenting patients aged ≥ 18 years admitted for ≥ 24 hours in one of five ICUs at Oslo University Hospital filled out Brief Pain Inventory (BPI) immediately after ICU discharge. BPI is measuring pain intensity using a numeric rating scale (NRS) from 0 (no pain) to 10 (pain as bad as you can imagine), pain interference with daily life (NRS 0-10) and pain location using a body map. The patients also answered questionnaires measuring PTSS (PTSS-10-I), SOC (SOC-13) and memories from the ICU stay (ICU memory tool). Data analyses were performed using descriptive statistics and multiple linear regression analysis.
**RESULTS.** Among the 523 included patients, 469 (90%) experienced pain, and 280 (54%) reported moderate to severe (NRS 4-10) worst pain intensity. Mean pain intensity and mean pain interference were 5.9 (SD 2.7) and 4.5 (SD 2.7), respectively. The majority of patients reported pain in the abdomen (43%), lower back (28%), and shoulder/forearm (22%). Variables significantly associated with worst pain intensity were; shorter ICU stay (beta -0.06, 95% CI [-0.09, 0.03]), low SOC (beta -0.03, 95% CI [-0.05, -0.00]), scary ICU memories (beta 0.44, CI [-0.25, 0.66]) and having undergone surgery (beta 1.15, 95% CI [0.17, 2.14]).Variables significantly associated with higher pain interference were scary ICU memories (beta 0.17, 95% CI [-0.02, 0.36), higher PTSS (beta 0.09, 95% CI [0.07, 0.11), prior anxiolytic use before hospital admittance (beta 0.53, 95% CI [0.01, 1.05]), trauma admission (beta 1.24, 95% CI [0.55, 1.92]) and regional analgesic use in the ICU (beta 0.53, 95% CI [ 0.06, 1.00]).
**CONCLUSION.** In total, 90% of the patients reported pain immediately after ICU discharge, and more than half of the patients reported moderate to severe pain. In addition, the association between low SOC and pain intensity highlights the importance of coping in patients after an ICU stay. The association between pain interference and prior anxiolytic use, scary memories and PTSS brings attention to the significance of psychological issues when relieving pain in ICU patients after ICU discharge.

**REFERENCE(S)**

1. The study received grants from Oslo University Hospital and The Norwegian Nurses Association

### 000892

#### Physical and Psychological Outcomes of patients discharged from a rehab active Critical Care Unit in the United Kingdom

F. Howroyd, J. Oldhams, A. Waqaar, D. McWilliams
Critical care rehab team, Queen Elizabeth Hospital Birmingham, Birmingham, United Kingdom
**Correspondence:** D. McWilliams
*Intensive Care Medicine Experimental* 2019, **7(Suppl 3):**000892

**INTRODUCTION.** Survivors of critical illness often experience ongoing physical, psychological and cognitive morbidity, collectively known as 'post intensive care syndrome' [1]. These effects can last for months to years after hospital discharge [2]. A recent trial of early and structured rehabilitation within our Intensive Care Unit (ICU) demonstrated a greater intensity of physical rehabilitation than that provided as standard care in previous trials [3]. Currently the psychological status of patients leaving our 'rehab active' ICU is unknown.
**OBJECTIVES.** 1. To identify levels of anxiety, depression, psychological distress and mobility reported at ICU discharge.
2. To explore the impact of mobility levels upon psychological outcomes.

**METHODS.** All patients under the care of a specialist critical care rehabilitation team and surviving to ICU discharge between December 2018 and February 2019 were included in the analysis. Patients were asked to complete the Hospital Anxiety and Depression Scale (HADS) and the Intensive Care Psychological Assessment Tool (IPAT) questionnaires within 48 hours of transfer from ICU to the ward. Manchester Mobility Scores (MMS) were also recorded by the patients' physiotherapist.

**RESULTS.** A total of 33 patients were included in the analysis, with 73% (n = 24) able to mobilise at the point of ICU discharge (defined as an MMS score of 6 or above). 42% (n = 14) of patients demonstrated symptoms of anxiety (HADS score > 7), 58% (n = 19) showed symptoms of depression (HADS score > 7) and 70% (n = 23) showed risk of psychological distress (IPAT score of 7 or above).

A sub analysis of outcomes (see table 1) demonstrated patients with MMS of ≤ 5 were more likely to be anxious (78% vs 29%, p<0.05) and had a longer ward length of stay (38.6 vs 17.5 days, p<0.05). No significant difference was seen for depression or IPAT.

**CONCLUSION.** A high prevalence of psychological morbidity was observed amongst ICU survivors on discharge to the ward. Increased mobility levels at the point of ICU discharge were associated with a reduction in the incidence of anxiety and shorter ward length of stay. However, symptoms of psychological distress were high within both groups. Our results identify the need for more structured follow up and psychological support of intensive care survivors on discharge to the ward.

**REFERENCE(S)**

1. Dinglas, V. D.. Faraone, L. N., Needham, D. M. (2018) Understanding patient-important outcomes after critical illness a synthesis of recent qualitative, empirical, and consensus-related studies, Current Opinion in Critical Care, 24 (5), pp. 401 – 409
2. Herridge MS, Tansey CM, Matte A et al. (2011) Functional disability 5 years after acute respiratory distress syndrome. N Eng J Med. 364(14):1293–304.
3. McWilliams, D., Jones, C., Atkins, et al. (2018) Earlier and enhanced rehabilitation of mechanically ventilated patients in critical care: A feasibility randomised controlled trial. Journal of Critical Care. Apr (44). pp. 407-412
4. n/a

**Table 1 (abstract 000892).** Outcomes on ICU discharge with mobility as an independent variable

| | MMS ≦ 5 | MMS ≧ 6 | p |
|---|---|---|---|
| n | 9 | 24 | - |
| Mean ICU length of stay (days) | 21.8 | 22.1 | 0.839 |
| Mean ward length of stay (days) | 38.6 | 17.5 | <0.05 |
| Anxiety (HADS > 7) | 78%(n=7) | 29%(n=7) | <0.05 |
| Depression (HADS > 7) | 78%(n=7) | 50%(n=12) | 0.118 |
| IPAT score ≧ **7** | 89%(n=8) | 63%(n=15) | 0.127 |

## HSRO - Important outcomes for patients, family and doctors

### 000865

#### Differences in satisfaction among patients and relatives in the Intensive Care Unit

P. Vega Ocaña[1], L. González Bautista[1], JD. Martin Santana[2], C. García Del Rosario[3], JL. Santana Cabrera[1]

[1]Intensive care unit, Hospital Universitario Insular de Gran Canaria, Las Palmas de Gran Canaria, Spain; [2]Economics, Universidad de las Palmas de Gran Canaria, Las Palmas de Gran Canaria, Spain; [3]Quality department, Hospital Universitario Insular de Gran Canaria, Las Palmas de Gran Canaria, Spain

**Correspondence:** P. Vega Ocaña

*Intensive Care Medicine Experimental* 2019, **7(Suppl 3):**000865

**INTRODUCTION.** In the past, quality and improvement in healthcare have focused on what proffesionals think should be valued and have been less interested in what serve userd felt was important. In the Intensive Care Unit, the patient's perspective, as well as their relatives', is central to quality improvement.

**OBJECTIVES.** To evaluate patients and relatives' satisfaction in an Intensive Care Unit (ICU).

**METHODS.** Information was collected from June to September 2018 through a survey adapted to patients and family members. The questionnaire included a 7-score Likert scale conformed by different items aimed at assessing the quality of the service provided to patients in the ICU.

Based on the work of Mora Lourido (2015), we took the dimensions created to measure the technical and structural human quality in the ICU, and we conducted an analysis of means using the student's T, to check the differences in patients and relatives' satisfaction.

**RESULTS.** The measurement scale was consisted of 7 dimensions, whose average values are shown in the attached table.

**CONCLUSION.** There are statistically significant differences in patients and relatives' satisfaction in all dimensions except those related to satisfaction with caretakers. In general, patients are more satisfied tan realtives with the ICU, with the exception of food, which is worse valued by patients. The quality of the ICU has received the highest score by both groups, being the dimension that has more weight in the satisfaction of patients and relatives. On the contrary, satisfaction with food is the item that has received the worst assessment.

**REFERENCE(S)**

1. Mora Lourido CJ. Análisis del impacto de la dirección de recursos humanos orientada a la gestión de la calidad y la excelencia organizativa: una aplicación en los servicios de Medicina Intensiva del Complejo Hospitalario Universitario Insular-Materno Infantil. Tesis doctoral Universidad de Las Palmas de Gran Canaria (2015).

**Table 1 (abstract 000865).** Differences in pacients and relatives' satisfaction in the ICU

| Dimensions | Mean (T.D.) | | *t* (*p*) |
|---|---|---|---|
| | Pacients | Relatives | |
| Global satisfaction | 6.95 (0.21) | 6.59 (0.69) | 5.252 (0.000) |
| Satisfaction with quality of ICU | 6.95 (0.21) | 6.78 (0.38) | 4.103 (0.000) |
| Satisfaction with healthcare personnel | 6.97 (0.16) | 6.89 (0.31) | 2.201 (0.029) |
| Satisfaction with watchmen | 6.95 (0.25) | 6.88 (0.35) | 1.718 (0.087) |
| Satisfacción con instalaciones y equipamiento | 6.93 (0.30) | 6.58 (0.73) | 4.631 (0.000) |
| Satisfaction with food | 5.47 (1.03) | 6.63 (0.66) | -10.045 (0.000) |
| Satisfaction with cleaning | 6.95 (0.25) | 6.85 (0.44) | 2.145 (0.033) |

### 000920

#### Can the traditional morbidity and mortality (M&M) process be used to enhance staff wellbeing on the intensive care unit (ICU)?

A. Cavalier[1]; J. Macallan[1]; M. Alice[1]; T. Samuels[1]; F. Lamb[1]

[1]Critical care, East Surrey Hospital, Redhill, United Kingdom

**Correspondence:** J. Macallan

*Intensive Care Medicine Experimental* 2019, **7(Suppl 3):**000920

**INTRODUCTION.** Intensive care staff wellbeing is important and often overlooked. Emotional distress may result from involvement in traumatic events and witnessing death on the ICU. ICU staff should be offered opportunities to discuss these sensitive and emotional issues. The current morbidity and mortality (M&M) presentation model used in our ICU is only attended by ICU doctors. It is focused on educational and clinical aspects of patient deaths. The national General

Provision of Intensive Care Services guidelines version 2 (GPICSv2) 2018 now includes guidance for staff support (1).

**OBJECTIVES.** To assess whether the current M&M model allows staff wellbeing and emotional welfare to be explored and if this is required on a regular basis.

**METHODS.** ICU doctors of all grades were surveyed; on their attendance at M&M presentations; whether staff wellbeing was discussed; whether they would like this to be reviewed regularly; and whether they felt it would be beneficial for other ICU staff (nurses and therapists) to attend and contribute.

**RESULTS.** Sixteen doctors were surveyed (5 consultants, 6 middle grades and 5 SHOs). 14 of those had attended an ICU M&M presentation in the last 12 months. Of those that had attended, 5 out of 14 felt that the current M&M model allowed staff wellbeing and emotional issues to be discussed. 13 (81%) doctors surveyed would like to have a regular opportunity to discuss such issues. 15 (94%) doctors surveyed felt it would be beneficial for nurses and other MDT members to attend and contribute at the M&M presentation.

**CONCLUSION.** Staff wellbeing impacts upon quality of care (2). Doctors working on our ICU feel that it would be beneficial to incorporate a debriefing section at M&M meetings to discuss emotional welfare and staff wellbeing. Introducing this discussion and inviting all members of the multi-disciplinary team (MDT) may reduce the impact of the 'Second Victim Phenomenon' (3) caused by distressing events. M&M meetings could be improved to provide this opportunity by incorporating regular discussion about emotional distress caused by cases. Although it has been recommended in the consultation document of GPICSv2 (1), we cannot find any large studies in the literature to assess the impact of this. To enable safe facilitation of debrief sessions it is likely to be beneficial for staff members to undertake formal training. Further work is needed to assess the benefit of inviting members of the MDT to M&M. We plan to expand this survey out to all MDT members on our own ICU as well as conducting a larger survey across other intensive care units to assess this. We propose a change in the presentation format to include discussion on staff wellbeing and invite all MDT members to participate in discussion to provide a supportive environment.

**REFERENCE(S)**

1. Intensive Care Society. 2018. Guidelines for the provision of intensive care services Version 2 (draft) [Online]. [Accessed 8 April 2019]. Available from: http://www.ics.ac.uk/ICS/guidelines-and-standards.aspx
2. Shanafelt TD, et al. 2010. Burnout and medical errors among American surgeons. Annals of Surgery. 251(6):995-1000.
3. K Stewart et al. 2015. Supporting "second victims" is a system-wide responsibility. BMJ. 350: h2341.

### 000922

#### Association of airway pressures´ trends with mortality

N. Fraj[1], MA. Boujelbèn[2], W. Zarrougui[1], K. Meddeb[1], D. Ben Braiek[2], H. Zorgati[2], A. Azouzi[2], I. Ben Saida[1], M. Boussarsar[1]

[1]Medical intensive care unit, farhat hached university hospital, Université de Sousse, Faculté de Médecine de Sousse, LR N° LR12SP09.Heart Failure, Sousse, Tunisia; [2]Medical intensive care unit, Farhat hached university hospital, Sousse, Tunisia

**Correspondence:** W. Zarrougui

*Intensive Care Medicine Experimental* 2019, **7(Suppl 3):**000922

**INTRODUCTION.** The evolvement of airway pressures within the MV may reflect the modification of the viscoelastic properties of the respiratory system and hence predict poor outcome.

**OBJECTIVES.** To identify a relationship between airway pressures and mortality in MV patients.

**METHODS.** A retrospective charts' review of MV patients admitted to a medical ICU of Farhat Hached hospital from November 2015 to February 2018. Were collected patients' characteristics at admission and respective airway pressures (Peak, plateau, driving and intrinsic PEEP) at admission and at day 4. Trends of airway pressures (Paw) (Paw at day 4 - Paw at admission). High pressure ratio (HPR) is defined as the number of days spent with high pressures : Peak≥40 and/or plateau ≥30; and/or driving pressure ≥15 and/or; intrinsic PEEP ≥6; divided bylength of stay) and outcomes were recorded from chosen eligible charts. Univariate and multivariate regression analyses were performed to identify factors independently associated with mortality. ROC curves were used to check for the discriminative properties of the significant factors.

**RESULTS.** 304 mechanically ventilated patients were collected within the study period. 199(65%) were non-COPD patients. They were 50± 18 years aged; ARDS, 17(8.5%); pneumonia, 14(7%); Pulmonary edema, 10(5%); SAPS II, 35.1±15.7; pH, 7.31±0.14; PCO2, 42.4±19.9 mmHg; PaO2/FiO2, 210.6±109.2 mmHg; MV duration, 8.9±9.4 days; tracheostomy, 18(9%); length of stay, 10±10 days. Mortality, 114(57%). Median [IOR] trends of Paw : delta Peak, delta plateau, delta driving, delta intrinsic PEEP were respectively; 0[-5,5], 1[-2,3], 0[-2,3], 0[-2,0]. Median HPR, 0.15[0,1]. Univariate analysis and multivariate logistic regression analysis showed an association between Paw trends and mortality : 1) For non-COPD patients; delta Peak, (OR, 1.10; 95%CI, [1.03-1.19]; p=0.007), HPR, (OR, 38.3; 95%CI, [6.4-228.3]; p=0.000), 2) For COPD patients; delta plateau, (OR, 1.11; 95%CI, [1.01-1.22]; p=0.03), HPR (OR, 14.3; 95%CI, [3.6-56.6]; p=0.000). For non-COPD patients, respective ROC AUCs for delta Peak and HPR (0.73, 0.69) confirmed interesting discriminative properties to predict mortality. For COPD patients, respective ROC AUCs for delta plateau and HPR (0.70, 0.75) confirmed interesting discriminative properties to predict mortality.

**CONCLUSION.** The present study demonstrated that airway pressures' trends in MV patients could predict mortality.

### 000944

#### An airway pressures score for the prediction of poor outcomes in mechanically ventilated patients

MA. Boujelbèn[1], N. Fraj[2], W. Zarrougui[2], E. Ennouri[1], D. Ben Braiek[1], H. Zorgati[1], A. Azouzi[1], I. Ben Saida[2], K. Meddeb[2], M. Boussarsar[2]

[1]Medical intensive care unit, Farhat hached university hospital, Sousse, Tunisia; [2]Medical intensive care unit, farhat hached university hospital, Université de Sousse, Faculté de Médecine de Sousse, LR N° LR12SP09.Heart Failure, Sousse, Tunisia

**Correspondence:** W. Zarrougui

*Intensive Care Medicine Experimental* 2019, **7(Suppl 3):**000944

**INTRODUCTION.** Poor outcomes in mechanically ventilated (MV) patients may be related to strenuous viscoelastic properties of the lungs and difficult respiratory mechanics which could be illustrated by elevated airway pressures (Paw).

**OBJECTIVES.** To identify the properties of a validated airway pressures score for the prediction of poor outcomes in mechanically ventilated patient.

**METHODS.** A retrospective chart reviews (from November 2015 to February 2018) of MV patients was conducted in the MICU of Farhat Hached teaching of Sousse, Tunisia. A Mortality prediction score (Paw-MPS) was developed and validated around three items (plateau at day 4 ≥18cmH2O, + 1 point; delta peak ≥2, + 1 point; HPR ≥ 0.34, + 2 points) where delta peak=trends of Ppeak (Ppeak at day 4 of ICU stay - Ppeak upon admission and HPR=number of days spent with high pressures : Peak ≥40 and/or plateau ≥30; and/or driving pressure ≥14; and/or auto-PEEP ≥6cmH2O; divided by length of stay). Poor outcomes were defined as : a ventilator-free days at day 28 (VFDs) = 0 and a composite outcome : death or length of stay ≥ 14 days. ROC curves were used to assess discriminativ e properties of Paw-MPS to predict outcomes.

**RESULTS.** A total of 304 MV patients were included. Their main characteristics were : mean age, 56±18years; male sex ratio, 64.8%(n= 197); mean SAPS II, 34.9±14.3; pH, 7,3±0,1; pCO2, 50±23mmHg; P/F ratio, 204±101 mmHg; AE/COPD, 105(34.5%); ARDS, 25(8.2%); restrictive lung disease, 20(6.6%); pneumonia, 14(4.6%); pulmonary edema, 11(3.6%); median mechanical ventilation duration, 3[6-14] days; tracheostomy, 44(14.5%); median length of stay, 13[6-21] days; median VFDs, 0[0-2] days; mortality, 173(56.9%). Mean plateau at day 4, 20.9±6.6 cmH2O; median delta peak, 0[-5;5] cmH2O; HPR, 0.15[0-0.6]; mean Paw-MPS, 2.2±1.2. For the prediction of composite outcome, the Paw-MPS demonstrated respectively for the whole population, COPD patients and non-COPD patients the following ROC-AUCs : (AUC=0.75; 95%CI, [0.685- 0.809]; p≤10 ); (AUC=0.7; 95%CI, [0.620-0.859]; p=0.002) and (AUC=0.74; 95%CI, [0.666- 0.819]; p≤10 ). For the prediction of zero VFDs, the Paw-MPS demonstrated interesting ROC-AUC only for COPD patients : (AUC=0.71; 95%CI, [0.602- 0.816]; p≤10 ).

**CONCLUSION.** This simple prognostic score based on airway pressures seems to demonstrate interesting discriminative properties to predict poor outcomes of patients requiring invasive mechanical ventilation.

### 001117

#### Predicting hospital mortality in very elderly patients (VEP):preliminary analysis

E. Bertellini[1], G. Melegari[1], A. Marudi[1], M. Pavesi[1], F. Gazzotti[1], A. Barbieri[2]
[1]Anestesia e rianimazione, Ospedale Civile di Baggiovara, Baggiovara, Italy; [2]School of anaesthesia and intensive care, University of Modena and Reggio Emilia, Reggio Emilia, Italy
**Correspondence:** G. Melegari
*Intensive Care Medicine Experimental* 2019, **7(Suppl 3):**001117

**INTRODUCTION.** Nowadays the rate of patients with age ≥ 80 years old (VEP) admitted in intensive care unit (ICU) is increasing. Prognostic scores are necessary to determine the best therapeutic strategy, and when to withdrawal therapies. Recently, it was suggested to use Frailty index in elderly to better predict hospital mortality [1].

**OBJECTIVES.** It was investigated the predicting hospital mortality comparing ROC curve of SAPSII, first SOFA and Frailty index in VEP admitted in ICU.

**METHODS.** There were collected data from all unselected VEP patients for 3 consecutive months in ICU for different reasons: scheduled surgery, sepsis, cardiac arrest, haemorrhage or neurological coma. There were recorded all general data of patients and also measured SAPS II, first SOFA, Frailty index and hospital mortality (28 days mortality). Ethical Committee Modena procedure 184/16.

**RESULTS.** During the study period were admitted 34 patients with mean age of 84.06±0.52, 54.29% medical admissions, 31.43% unscheduled surgery, and 14.29% scheduled surgery. The hospital mortality was the 57.58%, the mean value for the length of stay in ICU was 12.47 days (CI 7.78-17.16). The mean value of SAPSII was 50.18± 4.07, first SOFA 7.0±0.71, Frailty Index 3.87±0.24. SAPSII showed an AUC curve of 0.96 (CI 0.88-0.99), first SOFA AUC 0.90 (CI 0.79-0.99), Frailty index 0.58 (CI 0.39-0.78). SAPSII and first SOFA did not show any difference, but both predicted better than Frailty index with p<0.001.

**CONCLUSION.** Frailty index can assess the resilience of elderly patients, but it does not predict hospital mortality as SAPSII or first SOFA because they measure the impact of multiple organ failure at ICU admission. The sample size of the study is limited, anyway SAPSII and first SOFA show a good predicting performance in VEP with ROC curve ≥0.9. Frailty index does not improve SOFA score predicting performance even if combined with first SOFA [2]. Physicians need easy and powerful prognostic scores, in ICU settings: SAPS and first SOFA represent an useful tools also in elderly.

**REFERENCE(S)**

[1] Flaatten H et al.Intensive Care Med 2017;43:1820-8
[2] Langlias E et al. J Crit Care 2018;46:67-72

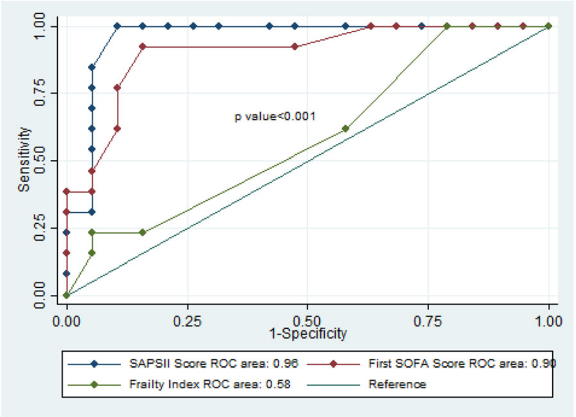

**Fig. 3 (abstract 001117).** ROC curve of SAPSII, first SOFA and Frailty Index

### 001231

#### Reducing mortality of Severe Sepsis and Septic Shock: Results of a 12 years teaching of protocolized management a a University Hospital of a developing country

A. Gajardo, D. Pérez, A. Hernández, F. Cayupi, N. Medel, C. Luengo
Critical care unit, department of medicine, University of Chile Clinical Hospital, Santiago, Chile
**Correspondence:** C. Luengo
*Intensive Care Medicine Experimental* 2019, **7(Suppl 3):**001231

**INTRODUCTION.** Sepsis and septic shock are worldwide health emergencies. Implementation of early, adequate and effective diagnostic and therapeutic measures within a protocolized strategy could ameliorate patients outcomes. We present the experience of a university hospital where a physiological, evidence-based protocolized sepsis management has been progressively implemented and taught to post-graduate physicians and residents over the last 12 years. Constant post-graduate training and a local well spread protocol could create in the institution the ability to diagnose and start resuscitation at the emergency room, operating rooms and wards, before patients arrive at the ICU, improving their outcomes.

**OBJECTIVES.** To compare the mortality of septic patients before and after a teaching program, including a physiological, evidence-based protocol for sepsis management was installed at a university hospital in Chile.

**METHODS.** Retrospective cohort study based on patients medical records. We included patients with severe sepsis or septic shock admitted to ICU in 2 time periods: 2005-2006 (pre-intervention, n= 109) and 2017-2018 (post-intervention, n= 91). We characterized septic patients and assessed outcomes in both periods. To evaluate the interventions' effect on mortality, we used logistic regression models adjusting by sex, age, organic dysfunctions, and lactate levels.

**RESULTS.** Patients in both periods were similar in terms of: age (61.3 ± 17.1 versus (*vs*) 61.4 ± 16.4 years old (yo), p-value= 0.95), sex (45% *vs* 57% men, p-value= 0.09), and number of dysfunctions (3 [IQR 2-3] *vs* 3 [IQR 2-4], p-value= 0.07). The percentage of septic patients admitted directly from the emergency room increased significantly in the post-intervention period compared to the pre-intervention period (41% *vs* 16%, p-value<0.001), and admissions from intermediate care units decreased significantly (67% *vs* 40%, p-value< 0.01). Mortality was higher in the pre-intervention than in the post-intervention period (42.2 *vs* 23.1%, p-value= 0.005). The effect of the intervention was independent of sex, age, organic dysfunctions and lactate levels (OR= 0.36, p-value= 0.007).

**CONCLUSION.** A teaching strategy and implementation of a physiological and evidence-based protocol for sepsis management reduced the mortality of septic patients in our hospital.

**REFERENCE(S)**

1. Romero C, Luengo C, Regueira T, et al. Recomendaciones SOCHIMI para el Manejo Inicial de la Sepsis.Revista Chilena de Medicina Intensiva 2017; Vol 32(2)
2. Gálvez R, Luengo C, Cornejo R, et al. Higher than recommended amikacin loading doses achieve pharmacokinetic targets without associated toxicity. Int J Antimicrob Agents. 2011 Aug;38(2):146-51
3. Hernandez G, Bruhn A, Luengo C, et al. Effects of dobutamine on systemic, regional and microcirculatory perfusion parameters in septic shock: a randomized, placebo-controlled, double-blind, crossover study. Intensive Care Med. 2013 Aug;39(8):1435-43
4. Hernandez G, Luengo C, Bruhn A, et al. When to stop septic shock resuscitation: clues from a dynamic perfusion monitoring. Ann Intensive Care. 2014 Oct 11;4:30
5. Levy MM, Fink MP, Marshall JC, et al. 2001 SCCM/ESICM/ACCP/ ATS/SIS International Sepsis Definitions Conference. Crit Care Med 2003; 31: 1250-56.
6. Rhodes A, Evans LE, Alhazzani W, et al. Surviving Sepsis Campaign: International Guidelines for Management of Sepsis and Septic Shock: 2016. Intensive Care Med 2017; 43: 304-77

### 001393

#### Complications and outcome of patients with severe influenza infection admitted to intensive care unit

O. Kampouropoulou, A. Sakagianni, K. Mandis, S. Michelidou, K. Valakis
Intensive care unit, SISMANOGLIO GENERAL HOSPITAL, ATHENS, Greece
**Correspondence:** A. Sakagianni
*Intensive Care Medicine Experimental* 2019, **7(Suppl 3):**001393

**INTRODUCTION.** Seasonal influenza epidemic constitutes a global health problem with serious complications leading to high morbidity and mortality, particularly among elderly patients and patients with chronic underlying conditions According to WHO, the most effective way to prevent the disease is annual vaccination.

**OBJECTIVES.** To evaluate the complications and outcome of patients with confirmed influenza infection requiring admission to the intensive care unit during 2018-2019 flu season in a tertiary hospital in Greece. We also investigated factors associated with poor prognosis.

**METHODS.** This is a retrospective observational study that included all patients admitted to the ICU of a tertiary hospital for complicated influenza infection, between October 1st 2018 and April 10th 2019. Demographic variables, clinical features, comorbidities, complications and ICU outcome were reviewed.

**RESULTS.** A total of 15 patients were included with a median age of 68 (range 49-87) years. Nine of them (60%) were male. Eleven patients (73%) were infected with influenza A and four patients (27%) with influenza B. 46,6% of the patients (n=7) were vaccinated against influenza. Median length of ICU stay was 17 days (range 4-37) and the ICU mortality rate was 33% (n=5). The median APACHE II score on admission was 25 (range 21-38). The most common underlying diseases of the patients were chronic obstructive pulmonary disease (53%), diabetes mellitus (47%) and coronary artery disease (20%). 60% were smokers and 33% were immunosuppressed (Rheumatoid Arthritis, Chronic Lymphocytic Leukemia, Multiple Sclerosis, Sarcoidosis). The majority of patients presented bacterial superinfections- 40% (n=6) ventilator associated tracheobronchitis or pneumonia and 13% (n=2) secondary bacteremia. More frequent complications were acute kidney injury (AKI) in 8 patients (53%) requiring CRRT in one third of the cases, acute heart failure (n=5, 33%), and rhabdomyolysis (n=5, 33%). Less frequent complications were myopathy/neuropathy (20%), ARDS (13%), encephalitis (7%), pulmonary embolism/DVT (7%), and invasive aspergillosis (7%). Compared with survivors, non-survivors were older (median age 71 vs 63,5), had longer ICU stay (median 26 days vs 13,5d), higher APACHE II score (median 30 vs 26), suffered more infectious complications (80% vs 30%), more cardiac complications (80% vs 20%), and presented AKI in 100% of the cases (vs 30% of survivors). Among survivors 40% (n=4) were vaccinated, and 60% (n=3) among non-survivors.

**CONCLUSION.** Findings from our single center study suggest that patients requiring intensive care management during the recent influenza outbreak, in contrast with previous years, suffered less ARDS and more extra-pulmonary complications. Although our study population is limited, and we cannot extrapolate conclusions, it seems that vaccination did not protect against multiple organ failure and fatal outcomes.

**REFERENCE(S)**

1. None
2. Severe Influenza; Greece 2016–2017: Vaccine Failures in Type A Influenza and Risk Factors for Poor Outcome Theodore Lytras, MD, MPH Agoritsa Baka, MD Anastasia Andreopoulou, MSc Elisavet Mouratidou, RN, MSc Ourania Kalkouni, MD Theano Georgakopoulou, MD, MPH, PhD Sotirios Tsiodras, MD, MSc, PhD Open Forum Infectious Diseases, Volume 4, Issue suppl_1, Fall 2017, Page S583,
3. Blank PR, van Essen GA, Ortiz de Lejarazu R, et al. Impact of European vaccination policies on seasonal influenza vaccination coverage rates: An update seven years later. Hum Vaccin Immunother. 2018;14(11):2706–2714. doi:10.1080/21645515.2018.1489948

### 001536

#### Cognitive and functional status assessment in neurocritical patients accompanied by occupational therapy

P. TRAVASSOS, CL. Agnes, MS. Maiko, A. Rafaella, RL. Camila, AG. Bruna, AG. Bruna, MC. Lígia, CV. Viviane, SOR. Salomón
NEUROCRITICAL CARE UNIT, HOSPITAL BP - A BENEFICÊNCIA PORTUGUESA DE SÃO PAULO, SÃO PAULO, Brazil
**Correspondence:** P. TRAVASSOS
*Intensive Care Medicine Experimental* 2019, **7(Suppl 3):**001536

**INTRODUCTION.** The survivors of diseases, with the presence of impairment of cognitive, psychological and functional, but the reversible potential rooms 5 years after intensive therapy (ICU) therapy has not yet been Characterized and understood. (1) Cognitive dysfunction in this population is characterized by new deficits (exacerbation of preexisting mild deficits) in global cognition, memory, attention / concentration, and executive functions.

**OBJECTIVES.** To assess cognitive and functional status in patients followed by occupational therapy in neurocritical patients.

**METHODS.** A retrospective analysis of the patients hospitalized in a neurological intensive care unit, followed by occupational therapy from March to June 2018, was carried out. The cognitive status was assessed through CAM-ICU (Confusion Assessment Method) and MEEM (Mini Mental State Examination), and functional status by means of the muscular strength degree MRC (Medical Research Council) and MIF (Functional Independence Measure), applied on admission and discharge from the ICU.

**RESULTS.** During the period, 50 patients were followed up, of which 32 were female, with a mean age of 68.4 years and an ICU stay of 5.05 days. The activation of the occupational therapy team occurred in the first 2 days of hospitalization in 80% of the cases. Among the patients evaluated, there was an improvement in cognitive ability in 94.1% of ICU discharge, in relation to admission. The motor capacity showed improvement of 80.4% during the evaluation period.

**CONCLUSION.** Early occupational therapy may favor occupational performance and promote quality of life, exploring the interests, needs, and functional and cognitive abilities of individuals, minimizing the impact of long-term stay in the hospital environment.

## TEM - New clinical aspects in cardiac arrest management

### 000976

#### A prospective assessment of the demographics and neuroprognostication practices of a high volume out-of-hospital cardiac arrest centre

M. Naughton, T. Bagnall, M. Ahmed
Cardiothoracic Intensive Care, St George's Hospital Atkinson Morley Wing, London, United Kingdom
**Correspondence:** M. Naughton
*Intensive Care Medicine Experimental* 2019, **7(Suppl 3):**000976

**INTRODUCTION.** Out-of-hospital cardiac arrest (OHCA) remains a significant cause of mortality[1]. In response to the wide-ranging specialist input required to optimise the care of these patients dedicated centres have been set up with evidence they may improve survival[2]. However, survival rates remain low with considerable ongoing morbidity with respect to neurological recovery.

**OBJECTIVES.** 1. To examine the demographics of arrests presenting to a 'Heart Attack Centre'.

2. To corroborate work investigating prognostic factors for survival to hospital discharge.

3. To examine our neuroprognostication practices.

**METHODS.** All patients following OHCA between October 2018 and March 2018 were included as part of an ongoing prospective registry. Patients were excluded if arrest was secondary to trauma or was iatrogenic. Patient and arrest demographics were collected alongside clinical and investigation findings including age, downtime, initial rhythm, pupil reactivity, corneal reflex, M score and CT and EEG reports.

**RESULTS.** 39 patients were identified. Two patients were excluded as they had traumatic or iatrogenic arrests. 18 of the 37 remaining patients survived to hospital discharge. The table and graphs below demonstrate increased survival to hospital discharge with shockable rhythms and shorter downtimes.

CT heads were performed 27 of 37 cases, with EEGs performed in only 5 of 37 cases. The right sided graph in the figure demonstrates the limited role for CT in prognosis. Furthermore, clinical examination findings which are crucial for neuroprognostication including M score and pupilliary response were only documented 39%-77% of occasions.

**CONCLUSION.** Our findings corroborate previous work that shockable rhythms and short downtimes are good prognostic factors. The higher than previously reported survival rate may lend further support to the role of specialist centres. Despite this, a combination of inadequate documentation and a reliance on limited array of tools for neuroprognostication such CT head alone clearly does not provide reliable accuracy for neuroprognostication. We are therefore implementing a new standardised approach, which incorporates a wider use of multi-modal tools such as quantitative pupillometry and investigations such as neuron specific enolase.

**REFERENCE(S)**
1. Berdowski J, Berg RA, Tijssen JGP, Koster RW. Global incidences of out-of-hospital cardiac arrest and survival rates: systematic review of 67 prospective studies. Resuscitation 2010; 81: 1479–87
2. Apps A, Malhotra A, Mason M, Lane R. Regional systems of care after out-of-hospital cardiac arrest in the UK: premier league care saves lives. J R Soc Med. 2012;105(9):362–364
3. None

| Initial Rhythm | Survived to Hospital Discharge | Did not Survive to Hospital Discharge | Percent Survived to Hospital Discharge |
|---|---|---|---|
| VF/VT | 16 | 8 | 66.67 |
| PEA/Asystole | 2 | 11 | 15.38 |

**Fig. 1 (abstract 000976).** A table showing the number of patients of survived to hospital discharge for shockable and non-shockable rhythms

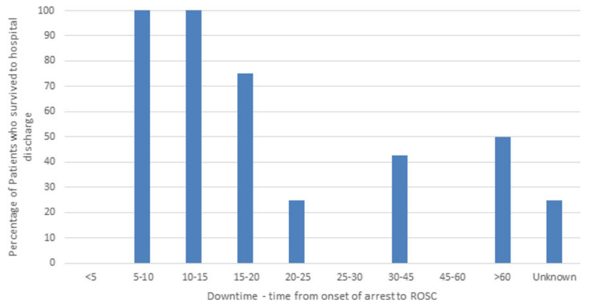

**Fig. 2 (abstract 000976).** The percentage of patients who survived to hospital discharge for a range of downtimes.

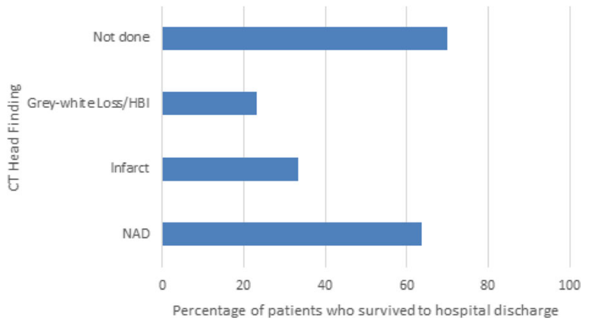

**Fig. 3 (abstract 000976).** The percentage of patients surviving to hospital discharge per CT head report findings

### 001451

#### Post resuscitation predictors of outcome in patients admitted in ICU for Out of Hospital Cardiac Arrest

A. Bagliani[1], G. Tavazzi[2], A. Orlando[3], G. Maggio[3], M. Belliato[3], F. Mojoli[2], G. Iotti[2]
[1]Anestesia e rianimazione scuola di specializzazione, Irccs Policlinico San Matteo, Pavia, Italy; [2]Department of clinical-surgical, diagnostic and pediatric sciences, unit of anaesthesia and inte, The University of Pavia, Pavia, Italy; [3]Department of anesthesia and intensive care, Fondazione IRCCS Policlinico S Matteo, Pavie, Italy
**Correspondence:** A. Bagliani
*Intensive Care Medicine Experimental* 2019, **7(Suppl 3):**001451

**INTRODUCTION.** Cardiac arrest mortality varies from less than 7% to up to 26% according to a number of variables, including time from event to resuscitation and underline cause (1). Post cardiac arrest syndrome treatment plays a pivotal role in the patient's outcome.
**OBJECTIVES.** To evaluate the importance of clinical and biochemical data at the time of admission in ICU and their variations over time in patients admitted for out of hospital cardiac arrest (OHCA).
**METHODS.** Single centre prospective observational study of patients admitted for OHCA. Enrollment period was from January 2018 to January 2019. Primary endpoint was to assess the organ dysfunction in patient with OHCA considering serum lactate (SL), troponin I and neuron-specific enolase (NSE) analysed at the admission in our intensive care unit (t0), after 24 h (t1), 48 h (t2) and 72 h (t3). T test statistic and ANOVA for repeated measurement were performed.
**RESULTS.** Preliminary results of 33 patients were enrolled, the average age was 62 y.o. (± 19.3), male patients where 25 (75.75%). The presentation rhythm was ventricular fibrillation in 19 patients (57.5%), pulseless electrical activity in 7 (21.2%) and asystolia in 4 (12.1%), in 3 patients (9.1%) the initial rhythm was unknown.
Ischemic underlying cause was suspected in 22 patients (66.6%) who underwent coronary angiography. A culprit lesion was found and treated in 13 patients (39,4%)(11 mono and 2 multivessel disease). The putative other causes of CA were: primary ventricular tachyarrhythmias (24.2%), hypoxia (18.2%), chronic heart failure decompensation (6.06 %), intoxication and haemorrhagic shock (12.1%). Six patients have been supported with Veno-Arterial ECMO of whom two were successfully weaned.
15 patients died in the ICU, with a mortality rate of 45.45% among all patients admitted for OHCA. 9 patients died within the first 72 hours for the severity of the anoxic brain damage.
Patients survived had lower SL values at t0 (3.88 ± 1.8 mmol/L) that significantly decreased over time (p 0.002), compared to non-survivor (p 0.65) who had higher values (8.85 ± 5.39 mmol/L) without significant variations (p>0.05). NSE values between survivor (26.9 ± 8.7mcg/L) and non-survivor (128.3 ± 57.7 mcg/L) varies significantly at t0 (p0.001). NSE increased significantly in non-survivors over time (p 0.01) whereas it did not change in those who survived (p 0.5). TNI values and mean temperature obtained during the first 24 hours mild hypothermia (35.2 ° ± 0.4 vs 35° ± 0.41) did not vary significantly amongst groups (p < 0.05).
**CONCLUSION.** Mortality rate in patients experiencing OHCA remains high. SL and NSE values at the admission and SL variation over the observation time showed the best discriminator power between survivor and non-survivor compared with the other studied variables.

**REFERENCE(S)**

1. Survival in Out-of-Hospital Cardiac Arrest After Standard Cardiopulmonary Resuscitation or Chest Compressions Only Before Arrival of Emergency Medical Services. Riva G, Ringh M, Jonsson M et al. Circulation. 2019;139:00–00.

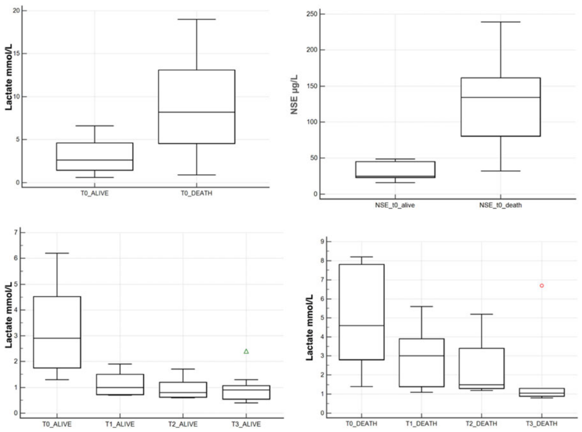

**Fig. 1 (abstract 001451).** See text for description

### 001531

#### Medical Emergency Team activity in intra-hospital resuscitation

D. Rosinha[1], R. Regufe[2], G. Possolo[2], I. Gonçalves[2], R. Ribeiro[2]
[1]ICU, Hospital São Bernardo, Centro Hospitalar de Setúbal, Setúbal, Portugal; [2]Intensive Care, Hospital São Bernardo - Centro Hospitalar Setúbal, Setúbal, Portugal
**Correspondence:** D. Rosinha
*Intensive Care Medicine Experimental* 2019, **7(Suppl 3):**001531

**INTRODUCTION.** Medical Emergency Teams (MET) are constituted by healthcare providers, trained in Advanced Cardiovascular Life Support (ACLS) and Resuscitation. In our institution, these teams are composed by one ICU nurse and a physician with experience in intensive care and/or medical emergency.
The activation criteria includes cardiac arrest or patients deteriorating condition, to any person within the hospital area, either inpatients or others (visitors, staff.). These teams were implemented to respond to critical situations avoiding cardiorespiratory arrest (CRA) or to improve outcomes after CRA.
**OBJECTIVES.** We present a retrospective analysis conducted at Hospital São Bernardo – Centro Hospitalar de Setúbal, Portugal, of the MET's intervention in ACLS from 2013 to 2018. Immediate mortality and intra-hospital mortality post-ACLS were evaluated.
The aim of this work was to compare our results with actual best practice and with our previous analysis to identify our pitffals in ording to improve.
**METHODS.** Data was obtained from the ICU department database, created in 2011 with the purpose of periodic audits. All activations due to or that evolved to cardiac arrest during the team approach, from January 2013 to December 2018, were included.
**RESULTS.** During the study period, there were a total of 262 cardiac arrests, 11,7% of all activations, with an average of 43,7 cases per year.
Time response was around 2 minutes and 36 seconds. The majority of activations were to Level 0 and level 2 wards, summing up to 85,5% of all cases. The most frequent first documented cardiac arrest rhythm was asystole in 53%, followed by pulseless electric activity (PEA) in 31% and pulseless ventricular tachycardia or fibrillation in 16%. An average of 7 cycles of ACLS were performed with a success rate over 40% (between 31 and 50%, through the years). Survival to hospital discharge of all CRA was 16,5% with a survival rate after return of spontaneous circulation (ROSC) of 37,4%
**CONCLUSION.** Our results are in accordance to the best practice in rate of discharge post CRA (between 16-18%) (1,2). Nonetheless the incidence of asystole is still higher than expected (30-40%), which may reflect delayed recognition (3). Despite this, our study, revealed a tendency of improvement along the years.

**REFERENCE(S)**

(1) Long-term survival following in-hospital cardiac arrest: A matched cohort study, ERC, February 2016 vol. 99, Pages 72–78
(2) Strategies for Improving Survival After In-Hospital Cardiac Arrest in the United States: 2013 Consensus Recommendations, Circulation.2013;127:1538-1563
(3) Rhythms and outcomes of adult in-hospital cardiac arrest Crit Care Med. 2010 Jan;38(1):101-8

### 001603

#### Mortality in vaECMO treated Patients Did Not Change Over Time

C. Dietrich[1], U. Jaschinski[2]
[1]Anesthesiology and intensive care medicine, University Hospital, Augbsurg, Germany; [2]Anesthesiology and intensive care, University Hospital Augsburg, Augsburg, Germany, Germany
**Correspondence:** U. Jaschinski
*Intensive Care Medicine Experimental* 2019, **7(Suppl 3):**001603

**INTRODUCTION.** Extracorporal life Support (ECLS) is a rescue strategy with increasing popularity and experienced a sharp increase in performance over the years. Nevertheless, survival in this clinical scenario is still pretty low.

**OBJECTIVES.** In a retsrospective analysis we tried to evaluate the population who might benefit most from ECLS. We speculate that ECLS in CPR may be able to increase hospital survial.

**METHODS.** Retrospective analysis of an institutional data set from 2006-2016

**RESULTS.** 207 patients in cardiogenic shock were scheduled for venous-arterial-ECMO as a ECLS system. Mean age was 63,6 years, 13,3 SD. Overall mortality was 67,6% (140/207). 94 received ECLS during reanimation. Mortality in this group was 69,1%. 113 patients in severe cariogenic shock were provided with an ECLS system and in this group 33,6% survived.

**CONCLUSION.** Mortality in patients with ECLS is still high and in a recently published systemic review 1-year survival was 36,7% (1). These results are in line with our data. Amazing is the fact that survival after cardio-pulmonary resuscitation (CPR) was similar as in non-CPR. In this regard ECLS seems to be beneficial since hospital mortality after in hospital CPR has been reported as high as 85% (2). Mortality after ECLS treatment is still high and the subgroup of patients which will benefit from this invasive procedure mostly are patients during CPR. However, these results have to be confirmed by examining a greater cohort.

**REFERENCE(S)**

1. Ormseth CH et al. (2019) Resuscitation 139:9-16
2. Wilson-Smith et al. (2019) Ann Cardiothorac Surg 8(1):1-8

### 001693

#### A case of intracoronary and endovenous levosimendan as a rescue therapy for refractory cardiac arrest

B. Ferro[1], R. Tofani[1], L. Gargani[2], G. Borelli[3], P. Roncucci[1]
[1]Anestesia e rianimazione, Spedali Riuniti Livorno ATNO ESTAR, Livorno, Italy; [2]Cardiology, CNR PISA, Pisa, Italy; [3]Cardiology, Spedali RIuniti Livorno ATNO ESTAR, Livorno, Italy
**Correspondence:** B. Ferro
*Intensive Care Medicine Experimental* 2019, **7(Suppl 3):**001693

**INTRODUCTION.** Reducing time of cardiac arrest is the goal to obtain better prognosis in every setting from the street to the cardiac catheterization laboratory.

Epinephrine is the standard medication for acls but its effect on both recovery of spontaneous circulation and amelioration of neurological prognosis remains controversial (1). As a consequence there's need to develop pharmacological strategies to reduce time to ROSC and to cerebral reperfusion especially when ECLS is not available.

**METHODS.** We report a case of a 60 years old male patient with refractory cardiac arrest secondary to myocardial infarction unresponsive to common therapies in whom intracoronary injection followed by endovenous infusion of calcium sensitizer levosimendan was able to gain and mantain ROSC

**RESULTS.** The patient was admitted to cardiac catheterization laboratory with diagnosis of cardiogenic shock secondary to acute anterior myocardial infarction. Patient was intubated, an aortic counterpulsation balloon was placed and PTCA of IVA was performed. During procedure numerous episodes of shockable rhythms (ventricular fibrillation and ventricular tachycardia) were treated following ACLS guidelines. After coronary reperfusion an episode of ventricular tachycardia followed by pulseless electric activity of more ten minutes was unresponsive to therapy. A bolus of 10 mcg/kg of levosimendan was injected in the left coronary artery followed by a continuous endovenous infusion (0,2 mcg/kg/min). After 30 seconds ROSC was obtained,, foretold by increasing etCO2 from 10 to 25 mmhg during chest compressions (figure) . Hemodynamic was mantained with low dosage of norepinephrine and continuous infusion of levosimendan. In intensive care unit first Bispectral index was 38. Patients was treated with targeted temperature management and then gradually weaned from vasoactives, aortic counterpulsation and ventilation, recovering good neurological condition and discharged with cpc score of 2.

**CONCLUSION.** To our knowledge, this is the first report of successful reversal of refractory cardiac arrest with intracoronary followed endovenous infusion of levosimendan. Only preclinical studies have analyzed the potentiality of levosimendan in cardiac arrest. Nan et al showed that a combination of levosimendan and epinephrine was superior to epinephrine alone to reverse bupivacaine-induced cardiac arrest in a rat model. (2)

There is evidence that intracoronary injection of levosimendan in cardiac surgery guarantees optimal drug spread, increasing contractility, global cardiac function, coronary perfusion pressure and reducing arrhytmic ischemic events (3). In this case intracoronary administration was possible because arrhytmic events followed coronary reperfusion during catheterization procedure. Intracoronary administration of drug is important to hypothesize the direct effect of levosimendan on arrhytmic unresponsive events and ROSC.

**REFERENCE(S)**

1) Perkins GD, Ji C, Deakin CD, Quinn T, Nolan JP, Scomparin C, Regan S, Long J, Slowther A, Pocock H, Black JJM, Moore F, Fothergill RT, Rees N, O'Shea L, Docherty M, Gunson I, Han K, Charlton K, Finn J, Petrou S, Stallard N, Gates S, Lall R; PARAMEDIC2 Collaborators A Randomized Trial of Epinephrine in Out-of-Hospital Cardiac Arrest. N Engl J Med. 2018 Aug 23;379(8):711-721
2) Nan F, Cai X, Ye Y, Xu X, Li Z, Li M, Chen L. Levosimendan combined with epinephrine improves rescue outcomes in a rat model of lipid-based resuscitation from bupivacaine-induced cardiac arrest. BMC Anesthesiol. 2017 Sep 15;17(1):128
3)Jamali
IN1, Kersten JR, Pagel PS, Hettrick DA, Warltier DC.
Intracoronary levosimendan enhances contractile function of stunned myocardium. Anesth Analg. 1997 Jul;85(1):23-9.

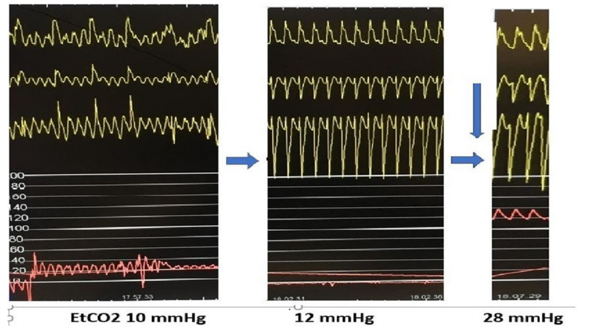

**Fig. 1 (abstract 001693).** See text for description

## INF - Opportunistic infections and onco-haematological patients: Specificities and approaches

### 001341

#### Central venous access in lymphoma patients (pts) with superior vena cava syndrome (SVCS)

M. Spirin[1], G. Galstyan[2], M. Drokov[3]
[1]National Research Center for Hematology, Moscow, 125167, Russian Federation;, Moscow, Russia; [2]Icu, National Research Center for hematology, Moscow, Russia; [3]Hematology, National Research Center for Hematology, Moscow, 125167, Russian Federation;, Moscow, Russia
**Correspondence:** M. Spirin
*Intensive Care Medicine Experimental* 2019, **7(Suppl 3):**001341

**INTRODUCTION. Introduction.** SVCS results from compression of the superior vena cava. Lymphoma - one of the most frequent causes of the malignant SVCS.

**The aim** of the study was to identify the incidence of SVCS in lymphoma pts and features of the central venous access in lymphoma pts with SVCS.

**METHODS. Materials and methods**. 686 lymphoma pts (311 male, 375 female) were included in the prospective study. The pts suffered from Hodgkin's lymphoma (HL) (69 pts) and non-Hodgkin's lymphoma (NHL) (617 pts). In all pts SVCS was diagnosed clinically and was confirmed by CT scan or MRI. In pts with confirmed SVCS the central venous access was provided by non-tunneled central venous catheters (non-tCVCs) (Certofix DUO 7Fr, BBraun) and totally implantable central venous port system (PowerPort, Bard Access System). The indwelling time, the number of the replaced CVCs and the incidence of complications were analyzed. Statistic: SPSS ver.23 (IBM, Chicago, Il., USA). Pearson's chi-squared test and Fisher's exact test were used.

**RESULTS.** . SVCS was revealed in 70 (10.2%) of 686 lymphoma pts (9 pts with HL and 61 pts with NHL). Among lymphoma pts the frequency of SVCS was highest in pts with primary mediastinal large B-cell lymphoma (PMBCL). SVCS was revealed in 49 (51.6%) of 95 PMBCL pts. In SVCS pts venous access was provided by non-tCVC inserted in femoral vein (50 pts) and TIVAPS (20 pts) implanted into v. cava inferior through the femoral vein. TIVAPS reservoirs were implanted on the thigh. During lymphoma treatment most pts with non-tCVCs needed repeated catheterizations: median 3 (1-14) CVCs. Among pts with TIVAPS only in 3 pts TIVAPS needed repeated catheterizations; their TIVAPS were removed due to complications. The median indwelling times were 6 (1-129) catheter days for CVCs and 164 (1-280) catheter days for TIVAPS (p<0.05). Incidences of CVC related bloodstream infections (CRBSI) were 2.1/1000 catheter days for CVCs and 0.1/1000 catheter days for TIVAPS. Incidence of CVC associated thrombosis (CAT) were 4.1/1000 catheter days for CVCs and 0.5/1000 catheter days for TIVAPS. One patient had infection of the subcutaneous pocket (0.3/1000 catheter days). The reasons for non-tCVCs removal were the treatment completion (82.8%) and complications: CRBSI (7.1%), CAT (8.5%), malfunction (1.6%). The reasons for the TIVAPS removal were the treatment completion (85%), CRBSI (5%), infection of the subcutaneous pocket (5%) and CAT (5%).

**CONCLUSION.** SVCS was revealed in 10.2% lymphoma pts. Half of the PMBCL pts had SVCS. In PMBCL pts central venous access CT should be perform after assessment of vena cava superior patency by CT or MRI. In SVCS lymphoma pts implantation of TIVAPS into v. cava inferior is access of choice.

***Key words:****lymphoma, superior vena cava syndrome, central venous catheter, vena cava inferior, totally implantable central venous port system.*

### 001470

#### Mortality analysis of patients with cancer immunotherapy in two critical care units

LE. Varela Sanchez[1], RA. Noguez[2], C. Rubio Madrigal[1], J. Franco Granillo[1], G. Camarena Alejo[1], J. Aguirre Sánchez[1], CR. Gerson[2], AR. Contreras Contreras[1]
[1]Critical care, ABC Medical Center, Mexico City, Mexico; [2]Oncology, ABC Medical Center, Mexico City, Mexico
**Correspondence:** L.E. Varela Sanchez
*Intensive Care Medicine Experimental* 2019, **7(Suppl 3):**001470

**INTRODUCTION.** Cancer immunotherapy is a modality of treatment that can favor survival; some oncological patients undergoing immunotherapy require critical care during the period of their disease. Immunotherapy itself and cancer can carry complications that need different organic supports that some oncologic patients in advanced stages are not typically considered for. Due to multiple factors that can affect the mortality of these patients, we made an analysis of mortality and its associations with organic support in patients that received immunotherapy before or during their stay in the intensive care unit (ICU).

**OBJECTIVES.** Describe the causes of admission to the ICU, causes of mortality, associated factors and the impact of different organic supports on mortality.

**METHODS.** Analysis of a historic cohort of patients with cancer diagnosis in III and IV clinical stages undergoing immunotherapy previous or during their stay in two critical care units.

**RESULTS.** 32 patients with a total of 38 admissions to the ICU, female 28.1%, male 71.9%, average age: 63.2%, pembrolizumab 50%, nivolumab 34.4%, ipilimumab 15.6%. 32% patients needed vasopressors, 9.3% inotropes, 25% renal replacement therapy, 56.2% noninvasive ventilation and 43.7% invasive ventilation. Main cause of admission was initiation of ventilatory support (39.6%); only 21.1% of all admissions were associated with a progression of the oncological disease.

All around mortality was 34.3%, only 15% of the 32 patients were considered to have side effects of immunotherapy. Mortality was mainly associated to the need of ventilatory support yet it was not meaningful in our analysis (p=0.06), the use of renal replacement therapy, vasopressors, inotropes was not directly associated with a higher incidence of mortality. The modality of immunotherapy was not itself associated with more complications or a higher incidence of mortality.

**CONCLUSION.** Patients under cancer immunotherapy are a new group of patients in the intensive care unit. We presented a historic cohort in terminal stage that needed critical care and analyzed mortality and possible associated factors. None of the modalities of organic support and types of immunotherapy were significant factors for mortality.

Although our cohort is small and our study is retrospective, our results suggests that these patients can undergo with critical care without conferring a major mortality factor. More studies are needed to determine if critical care alters the quality of life on these patients.

**REFERENCE(S)**

1. Haanen, J., Carbonnel, F., Robert, C., Kerr, K., Peters, S., Larkin, J. and Jordan, K. (2017). Management of toxicities from immunotherapy: ESMO Clinical Practice Guidelines for diagnosis, treatment and follow-up. Annals of Oncology, 28(suppl_4), pp.iv119-iv142.
2. Carmona-Bayonas, A., Gordo, F., Beato, C., Castaño Pérez, J., Jiménez-Fonseca, P., Virizuela Echaburu, J. and Garnacho-Montero, J. (2018). Intensive care in cancer patients in the age of immunotherapy and molecular therapies: Commitment of the SEOM-SEMICYUC. Medicina Intensiva, 42(6), pp.363-369.
3. Barth, C., Soares, M., Toffart, A., Timsit, J., Burghi, G., Irrazabal, C., Pattison, N., Tobar, E., Almeida, B., Silva, U., Azevedo, L., Rabbat, A., Lamer, C., Parrot, A., Souza-Dantas, V., Wallet, F., Blot, F., Bourdin, G., Piras, C., Delemazure, J., Durand, M., Salluh, J., Azoulay, E. and Lemiale, V. (2018). Characteristics and outcome of patients with newly diagnosed advanced or metastatic lung cancer admitted to intensive care units (ICUs). Annals of Intensive Care, 8(1).
4. Barth, C., Soares, M., Toffart, A., Timsit, J., Burghi, G., Irrazabal, C., Pattison, N., Tobar, E., Almeida, B., Silva, U., Azevedo, L., Rabbat, A., Lamer, C., Parrot, A., Souza-Dantas, V., Wallet, F., Blot, F., Bourdin, G., Piras, C., Delemazure, J., Durand, M., Salluh, J., Azoulay, E. and Lemiale, V. (2018). Characteristics and outcome of patients with newly diagnosed advanced or metastatic lung cancer admitted to intensive care units (ICUs). Annals of Intensive Care, 8(1).

### 001629

#### Cytokine release syndrome and other complications of cancer immunotherapy: a new reason for ICU admission

A. Garcia Roche, C. Díaz, I. Romera, A. Pacheco, R. Ferrer Roca
Intensive care department, Vall d'Hebron University Hospital, Barcelona, Spain
**Correspondence:** A. Garcia Roche
*Intensive Care Medicine Experimental* 2019, **7(Suppl 3):**001629

**INTRODUCTION.** Cancer immunotherapy has emerged as a promising treatment for many tumors. The expanding application of this new treatments to cancer will have profound consequences for the use of intensive care.

**OBJECTIVES.** Our aim is to describe the treatment and clinical evolution of oncohematological patients that required ICU admission after cancer immunotherapy.

**METHODS.** Retrospective study, including adult (+18 years) patients with cancer either hematologic or solid organ cancer, who received immunotherapy and required admission to the ICU between 2017 and 2018. Qualitative variables are expressed as N(%).

**RESULTS.** Ten patients were admitted in this two years period. Four (40%) were men and the median age was 64 years. Seven (70%) had solid tumors and 3 (30%) had hematological malignancies. All of them were at an advanced stage of their cancer disease, and had received at least 3 lines of treatment previously.

The main indication for ICU admission was severe cytokine release syndrome (CRS) in 5 cases (50%): all of them were grade 3, and one with severe neurologic impairment (Glasgow Coma Score 10/15). Regarding the trigger of CRS, in two cases were hematologic patients after CAR-T cell therapy and the other three cases were after bispecific antibodies therapy (BiTe). All patients receive anti-IL6 treatment with Tocilizumab, and two receive corticosteroids also. The CRS was controlled in all five patients and all were discharge from ICU and hospital.

Four (40%) patients were admitted because immune-related events after checkpoint inhibitors: 1 myasthenia-like syndrome, 1 myocarditis, 1 severe pneumonitis and 1 hepatitis. Two required invasive mechanical ventilation support, one high nasal flow oxygen and the other one non-invasive mechanical ventilation. The median SOFA score at admission was 7 points. Management included corticosteroids in all of them, combined with other immunosuppressive therapies (tacrolimus, mycophenolate mofetil) in two of them, combined symptomatic and support treatment. The outcome was poor, three can be discharge from the ICU but only one survive and was discharge from the hospital.

The last patient was a preventive ICU admission to receive Tumor-Infiltrating lymphocytes (TIL) and IL-2 therapy. The patient present capillary leak syndrome with impairment of renal function, pulmonary and neurologic dysfunction (nightmares, hallucinations, somnolence...). The impairments resolved without specific treatment and can complete the treatment and discharge from ICU and hospital.

**CONCLUSION.** Adverse events associated to cancer immunotherapy treatments can be severe and require ICU admission. The treatment of these complications is mainly supportive but in some cases specific treatment is needed. A multidisciplinary approach with early ICU admission is recommended.

### 000717

#### Candidemia in non-neutropenic crtically ill patients

A. Catarino[1], M. Miranda[1], D. Sousa[2], JF. Martins[1], A. Marques[1], P. Martins[1]
[1]Serviço Medicina Intensiva, CHUC- Centro Hospitalar e Universitário de Coimbra, Coimbra, Portugal; [2]Serviço medicina interna, CHUC- Centro Hospitalar e Universitário de Coimbra, Coimbra, Portugal
**Correspondence:** A. Catarino
*Intensive Care Medicine Experimental* 2019, **7(Suppl 3):**000717

**INTRODUCTION.** Candidemia has increased in the last decades in patients admitted to intensive care units (ICU). Candida bloodstream infection is life-threatening, recognition and treatment is frequently delayed, with clinical deterioration and death often preceding the detection of Candida in blood cultures.

Thus, it is crucial to maintain a high level of suspicion. Is important to know the epidemiology of candida infection, risk factors and tools that allow the selection of patients benefiting from empirical / preemptive therapy.

**OBJECTIVES.** Establish the incidence of candidemia; the prevalence and sensitivity pattern of different species; Identify possible risk factors; Evaluate the predictive value of tools such as candida score.

**METHODS.** Retrospective evaluation of the candidemia cases from January 2011 to December 2018, through the consultation of clinical files from the patients hospitalized in our ICU with candida in blood cultures.

Patients with ICU length of stay <24h and haematological malignancies patients were excluded.

Evaluation of risk factors. Candida score calculation. Characterization of the isolated candida species, sensitivity pattern, antifungals used, duration of therapy and outcome.

**RESULTS.** We enrolled 55 non-neutropenic patients with candidemia (54,5% males, average age 63,4±17 years, SAPS II 52,2±16,5). *Candida albicans* was the most prevalent species - 36 cases (65,5%) - followed by C. *parapsilosis* in 11 patients (20%). All *Candida albicans* isolates were susceptible to azoles. Fluconazole resistance rate among the non-albicans candida spp. was 7,3%. An echinocandin was used as empirical antifungal therapy in 61,8% of the cases and fluconazole in 30,9%. ICU mortality was 38,2% and cumulative mortality 30 days after ICU discharge was 47,3%. Candida score was 3,61±1,0. There was no difference in ICU mortality between patients with Candida score >3 or < 3 (p= 0,46). Despite the slight trend to higher mortality rate in patients with C. non-albicans vs albicans, we found no statistical relevance (p 0,09).

**CONCLUSION.** Candidemia in our ICU was mainly caused by *albican* species. Although echinocandins were the antifungal of choice, resistance rate to azoles was low. Our patients had a high candida score and a high overall mortality rate.

##### REFERENCE(S)

1. Piarroux R, Grenouillet F, Balvay P, et al: Assessment of preemptive treatment to prevent severe candidiasis in critically ill surgical patients. Crit Care Med 2004; 32: 2443–2449
2. Blumberg HM, Jarvis WR, Soucie JM, et al: Risk factors for candidal bloodstream infections in surgical intensive care unit patients: The NEMIS prospective multicenter study. Clin Infect Dis 2001; 33:177–186 25.
3. León C1, Ruiz-Santana S, Saavedra P, Almirante B, Nolla-Salas J, Alvarez-Lerma F, Garnacho-Montero J, León MA; EPCAN Study Group. A bedside scoring system ("Candida score") for early antifungal treatment in non-neutropenic critically ill patients with Candida colonization. Crit Care Med 2006; 34:730 –737

### 000678

#### Clinical analysis of critically ill patients with influenza and cryptococcal infections

J. Huang[1], H. Li[1], C. Lan[2], S. Zou[3], L. Chen[1], H. Weng[1]
[1]Respiratory and critical care medicine, Fuzhou Pulmonary Hospital of Fujian, Educational Hospital of Fujian Medical University, Fuzhou, China; [2]Radiology, Fuzhou Pulmonary Hospital of Fujian, Educational Hospital of Fujian Medical University, Fuzhou, China; [3]Clinical laboratory, Fuzhou Pulmonary Hospital of Fujian, Educational Hospital of Fujian Medical University, Fuzhou, China
**Correspondence:** J. Huang
*Intensive Care Medicine Experimental* 2019, **7(Suppl 3):**000678

**INTRODUCTION.** Infections due to *Cryptococcus* species occur globally and can affect both immunocompromised (IC) and non-IC hosts. To date, many cases of cryptococcal infection have been reported, but concomitant influenza and cryptococcal infections are rare.

**OBJECTIVES.** To improve diagnosis and management of critically ill patients with influenza and cryptococcal infections.

**METHODS.** An unusual case of an avian influenza A (H7N9) infection with systemic super-infection with *Cryptococcus neoformans* presenting as ventilator-associated pneumonia (VAP) and bloodstream infection in a previously immunocompetent man during hospitalization was reported and the literatures were reviewed.

**RESULTS.** A review of the medical literature revealed only 3 reported cases regarding concomitant hospital-acquired cryptococcal infection and severe influenza. Adding our case to the review, a total of four cases are summarized and analyzed. All cases occurred in patients with severe influenza virus infection (2 cases with H1N1 and 2 cases with H7N9) from 7 to 25 days after admission. During the four patients' courses, there were 3 cases with cryptococcal pneumonia, 2 cases with cryptococcal fungemia, 1 case with cryptococcal pleurisy and 2 cases with cryptococcal meningitis, and 3 of them presented with disseminated cryptococcal infection. In the 3 cases with cryptococcal pneumonia, 1 case was diagnosed as hospital-acquired pneumonia (HAP), and 2 cases were VAP according to the diagnostic criteria of HAP and VAP. After the anti-fungal treatment, only one case with H1N1 and cryptococcal infection survived, the other 3 cases including our patient all died.
**CONCLUSION.** Cryptococcal infection can occur in patients with severe influenza during hospitalization with a more severe condition, and the clinician should be aware of this infection.

**REFERENCE(S)**

[1] Pappas PG. Cryptococcal infections in non-HIV-infected patients. Trans Am Clin Climatol Assoc. 2013; 124: 61-79.
[2] Wang BQ, Zhang HZ, Fan BJ, et al. Meta-Analysis of Clinical Manifestations of Pulmonary Cryptococcosis in China Mainland. Chinese Journal of Clinical Medicine. 2013;20: 351-4.
[3] Kohno S, Kakeya H, Izumikawa K, et al. Clinical features of pulmonary cryptococcosis in non-HIV patients in Japan. J Infect Chemother. 2015;21:23-30.
[4] Lan CQ, Weng H, Li HY, et al. Retrospective analysis of 117 cases of pulmonary cryptococcosis. Chin J Tuberc Respir Dis. 2016 ;39:862-5.
[5] Lomes NR, Melhem MS, Szeszs MW, Martins Mdos A, Buccheri R. Cryptococcosis in non-HIV/non-transplant patients: A Brazilian case series. Med Mycol. 2016;54:669-76.
[6] Chen XH, Wang Q, Ni FY, et al. A case of severe avian influenza A (H7N9) complicated by pulmonary cryptococcal disease. Chin J Tuberc Respir Dis. 2016;39:558-61.
[7] Hosseinnezhad A, Rapose A. Cryptococcccal meningoencephalitis after H1N1 influenza. BMJ Case Rep. 2012;2012.
[8] Gupta A, Capoor MR, Gupta S, et al. Concomitant Infections of Influenza A H1N1 and Disseminated Cryptococcosis in an HIV Seropositive Patient. J Lab Physicians. 2015;7:134-6.
[9] Chinese medical association severe medicine branch. Guidelines for the diagnosis, prevention and treatment of ventilator-associated pneumonia (2013). Chin J Intern Med. 2013;52:524-43.
[10] Infectious group of Chinese medical association respiratory branch. Guidelines for the diagnosis and treatment of Chinese adults with hospital-acquired and ventilator-associated pneumonia (2018). Chin J Tuberc Respir Dis. 2018;41:255-80.
[11] The work was sponsored by the fund of the Key Clinical Specialty Discipline Construction Program of Fujian, P.R.C. (Minwei medical administration letter [2018] 145) and the Clinical Medicine Center Construction Program of Fuzhou, Fujian, P.R.C. (2018080305).

### 001114

#### Citomegalovirus viremia in immunocompetent patients admitted to a tertiary Intensive Care Unit

A. Pacheco[1], J. Esperalba[2], C. Díaz[1], G. Codina[2], I. Romera[1], A. García[1], R. Ferrer Roca[1]
[1]Intensive care department, Vall d'Hebron University Hospital. SODIR. VHIR, Barcelona, Spain; [2]Microbiology department, Vall d'Hebron University Hospital, Barcelona, Spain
**Correspondence:** C. Díaz
*Intensive Care Medicine Experimental* 2019, **7(Suppl 3):**001114

**INTRODUCTION.** Immunocompetent critically ill patients may be at risk for Citomegalovirus (CMV) reactivation as a result of immunosuppression associated with critical illness. Some studies have related this reactivation with poor outcomes.
**OBJECTIVES.** To describe the clinical profile of immunocompetent patients admitted to a tertiary ICU and tested for detection of CMV viremia, and address the prognostic implications of CMV viremia.
**METHODS.** Retrospective study, including adult (+18 years) immunocompetent patients who were tested for the presence of CMV viremia between 2014 and 2017. The CMV viral load was determined using the real time polymerase chain reaction RealStar® CMV PCR Kit 1.2. (Altona®). X-Square, Fisher´s test, T test, U Mann-Whitney and logistic regression were employed as required. Quantitative variables are reported as median (IQR) and categorical as frequency (%).
**RESULTS.** Eighty-one immunocompetent patients were tested for the presence of CMV viremia, with an age of 62 (43-68) years. Sixty (74%) were men. Sixteen (22%) had cirrhosis, 15 (21%) COPD, 6 (8%) chronic renal failure and 4 (5%) chronic heart failure. Thirty-five (43%) had septic shock and 22 (31%) were surgical patients. Twenty-eight (39%) were treated with high flow nasal cannula (HFNC), 64 (88%) required mechanical ventilation (MV), 52 (72%) vasoactive drugs (VAD) and 22 (31%) renal replacement therapy (RRT). Forty-nine (68%) were discharged from the ICU and 43 (60%) were discharged home. Nineteen (26%) were positive for CMV viremia and 13 (16%) were treated with ganciclovir. Hemoglobin levels, leukocyte count, platelet count, lymphocyte count, aspartate aminotransferase and alanine aminotransferase levels were similar in both groups (Table 1). Patients on VAD (OR: 8.7, IC95%: 1.1-71.1, p=0.043) or RRT (OR: 3.6, IC95%: 1.1-71.1, p=0.043) had a higher risk for CMV viremia, while use of MV (OR: 1.1, IC95%: 0.2-5.9, p=0.925) or HFNC (OR: 0.5, IC95%: 0.2-1.6, p=0.246) was not related. CMV viremia was not associated to ICU (OR: 1.4, IC95%: 0.45-5, p=0.594) or hospital mortality (OR: 1.4, IC95%: 0.5-5, p=0.464), length of mechanical ventilation (OR: 0.9 IC95%: 1-1, p=0.942), length of ICU (OR: 1.0, IC95%: 1-1, p=0.875) or hospital admission (OR: 1, IC95%: 1-1, p=0.837).
**CONCLUSION.** Patients without classical factors for immunosuppresion can suffer CMV viremia, with those on VAD or RRT suffering a higher risk. Despite the well known laboratory abnormalities related to CMV infection, these abnormalities have a great prevalence in ICU patients, so are not useful for suspecting CMV infection. In our cohort, CMV viremia was not related to mortality, length of mechanical ventilation or length of admission.

**Table 1 (abstract 001114).** See text for description

| CMV viremia | Hemoglobin (g/dL) | Platelets (x10E)/L) | Leucocyte (x10E9/L) | Lymphocites (x10E9/L) | AST (UI/L) | ALT (UI/L) |
|---|---|---|---|---|---|---|
| Positive | 8.7 | 131 | 12530 | 1200 | 48 | 37 |
| Negative | 8.6 | 105 | 9620 | 800 | 46 | 29 |
| p | 1 | 0.593 | 1 | 0.134 | 1 | 0.285 |

### 001207

#### Fungal superinfection complicating influenza

R. Knafelj[1], N. Erzen[1], L. Demsar[2], G. Vojka[1], B. Jernej[1], N. Marko[1]
[1]Micu, University Medical Center, Ljubljana, Zaloška cesta, Ljubljana, Slovenia, Ljubljana, Slovenia; [2]Micu, Medicinska fakulteta, Univerza v Ljubljani, Korytkova ulica, Ljubljana, Slovenia, Ljubljana, Slovenia
**Correspondence:** R. Knafelj
*Intensive Care Medicine Experimental* 2019, **7(Suppl 3):**001207

**INTRODUCTION.** Invasive pulmonary aspergilosis (IPA) is usually seen in immunocompromised patients, however over the past years numbers patients with flu and IPA as superinfection is increasing, while data on candida superinfection is scarce. While IPA independently increases mortality in patients with flu effect of candida superinfection on mortality remains unclear.
**OBJECTIVES.** We measured IPA and candida (C.) respiratory tract superinfection incidence in 2018/19 flu season in patients with influenza treated in ICU. Factors and patients characteristics that are linked to fungal superinfection were determined.
**METHODS.** Charts from consecutive patients diagnosed with flu hospitalized in tertiary medical ICU in academic hospital were analyzed. Flu was confirmed using molecular diagnostic test (PCR). Fungal superinfection was confirmed by immunoassays (galactomannan assay for IPA and beta-D-glucan for Candida), and by fungal growth on media plates from speciments. In 3 patients Aspergilus (A) growth was confirmed during bronshocsopy (**Figure 1**).
**RESULTS.** During the 2018/19 influenza season, 40 patients with flu were admitted (23 male, 17 female). In 11 fungal superinfection was confirmed (A. fumigatus in 3, C. albicans in 8 pts). In one patient Aspergilus and Mucor coinfection was demonstrated and in 3 patients more than 1 Candida species was isolated from various sites. None of microorganisms demonstrated significant in vitro resistance. 3 patients with IPA (100%) and 3 (38%) with Candida superinfection died. Selected data are presented in Figure 2. (**Figure 2**).
**CONCLUSION.** Fungal superfection was demonstrated in 27% of our patient with A. fumigatus and C. albicans being predominant species.

Candida and Aspergilus superfection is associated with increased mortality in influenza patients. Metlprednisolone use is associated with increased fungal infection rate. Inhalation corticosteroids use was not associated with increased fungal superinfection. Further investigation is needed for better understanding fungal superinfection in influenza patients.

### REFERENCE(S)

1. European Centre for Disease Prevention and Control. Influenza-associated invasive pulmonary aspergillosis, Europe – 30 November 2018. ECDC: Stockholm; 2018.
2. none

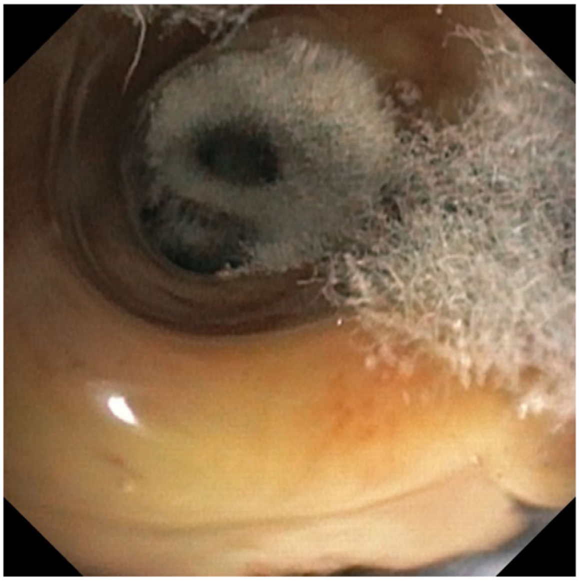

**Fig. 1 (abstract 001207).** See text for description

| | No coinfection | Fungal coinfection | p |
|---|---|---|---|
| patients (N,%) | 29 (73%) | 11 (27%) | / |
| male (N,%) | 16 (55%) | 8 (72%) | 0.31 |
| age | 66±12 | 59±14 | 0.19 |
| APACHE II | 14±5 | 22±5 | 0.02 |
| SOFA | 6±2 | 10±3 | 0.01 |
| inhalation corticosteroids (N,%) | 4 (14%) | 1 (9%) | 0.12 |
| metilprednisolone (N,%) | 7 (24%) | 7 (64%) | 0.02 |
| ECMO (N,%) | 1 (3%) | 6 (54%) | <0.05 |
| survived (N,%) | 24 (82%) | 5 (45%) | 0.02 |
| Died with IPA (N,%) | / | 3 (100%) | / |
| Died with Candida (N,%) | / | 3 (38%) | / |

**Fig. 2 (abstract 001207).** See text for description

### 000683

### Clinical analysis of patients with severe pulmonary tuberculosis presenting as acute respiratory failure and diffuse lung disease

J. Huang[1], H. Li[1], L. Chen[1], C. Lan[2], Q. Lin[3], H. Weng[1]
[1]Respiratory and critical care medicine, Fuzhou Pulmonary Hospital of Fujian, Educational Hospital of Fujian Medical University, Fuzhou, China; [2]Radiology, Fuzhou Pulmonary Hospital of Fujian, Educational Hospital of Fujian Medical University, Fuzhou, China; [3]Pathology, Fuzhou Pulmonary Hospital of Fujian, Educational Hospital of Fujian Medical University, Fuzhou, China
**Correspondence:** J. Huang

*Intensive Care Medicine Experimental* 2019, **7(Suppl 3):**000683

**INTRODUCTION.** Generally, clinical manifestations of pulmonary tuberculosis (PTB) are mild and some were asymptomatic. However, a series of cases of severe PTB characterized by acute respiratory failure and diffuse lung disease have been found in recent years in our hospital. Notably, such patients tend to have a short course of disease and a rapid disease progression with respiratory distress and severe hypoxia, which are similar to severe pneumonia and interstitial pneumonia and it is prone to misdiagnosis.

**OBJECTIVES.** To investigate the clinical features of diffuse type of severe PTB and improve diagnosis and management of the disease.

**METHODS.** A total of 17 cases of severe PTB, which were characterized by acute respiratory failure and diffuse lung disease and confirmed by etiological and (or) pathological examinations , were studied.

**RESULTS.** 11 of 17 cases (64.7%) were males and the patients were aged from 20 to 70 years with a median age of 36 years. Patients with and without immunocompromised underlying disease were 9 cases (52.9%) and 8 cases (47.1%), respectively. The most common symptoms were hyperpyrexia, shortness of breath, cough and sputum coughing. TypeIrespiratory failure with a median oxygenation index of 138mmHg occurred in all the cases. The chest CT manifestations were characterized by diffuse small nodular or miliary nodules, ground-glass opacities (GGOs), consolidations and fibrotic reticular opacities. Among the 17 patients with PTB, the test of the lower respiratory secretions (sputum, alveolar lavage or bronchoscopic brushing smear) showed as followed: 8 cases (47.1%) were positive for acid fast bacillus (AFB) - smear, 11 cases (64.7%) were positive for mycobacterium tuberculosis (MTB) by bacterium culture, 14 cases (82.3%) were positive for MTB DNA by polymerase chain reaction (PCR). Additionally, the strains identification of MTB and the detection of rifampin (RFP) and isoniazid (INH) resistance in MTB were tested by gene chip technology in 6 cases, of which 5 cases (83.3%) were positive and confirmed as MTB complex without RFP and INH resistance. The transbronchial lung biopsy was performed in 8 cases and bone marrow biopsy in 2 cases and all the pathological examination revealed tuberculosis. There were 9 cases (52.9%) with coexisting extrapulmonary tuberculosis and 11 cases (64.7%) with one or more complications including secondary pulmonary infection (7 cases), acute respiratory distress syndrome (6 cases), acute left heart dysfunction (4 cases), hemophagocytic syndrome (3 cases), pneumothorax (3 cases), septic shock (2 cases), etc. 16 of 17 cases (94.1%) were cured with antituberculosis drugs and other therapy except one death after abandoning treatment.

**CONCLUSION.** Diffuse PTB was characterized by acute respiratory failure and could lead to various complications. The chest imaging showed bilateral diffuse infiltration distributing and the disease was easy to be misdiagnosed as interstitial pneumonia and severe pneumonia. In combination with bacteriology, pathology, PCR, gene chip technology and tracheoscopy can significantly improve the diagnosis ability of severe tuberculosis. Majority of patients can be obtained satisfactory results after timely diagnosis and early antituberculous treatment.

### REFERENCE(S)

[1] Huang JB, Weng H, Li HY,et al. A case of severe pulmonary tuberculosis presenting as acute respiratory failure and interstitial pneumoni. Chin J Antituberc 2012; 34: 684-6.
[2] Tuberculosis chapter of Chinese Medical Association. Guidelines for the diagnosis and treatment of tuberculosis. Chin J Tuberc Respir Dis 2001;24:70-4.

[3] Shu XY, Tang L, Sun B. Clinical features of pulmonary tuberculosis patients manifesting respiratory failure in respiratory intensive gave unit. Journal of China-Japan Friendship Hospital 2014; 28(3): 139-42.
[4] Shi JH, Feng RE, Tian XL, et al. Clinical and radiologlcal features of pulmonary tuberculosis manifested as interstitial lung diseases. Chin J Tuberc Respir Dis 2009;32:893-6.
[5] Wu JL, Shen J, Xu K,et al. The value of CT appeanrances of interstitial changes in secondary pulmonary tuberculosis. Chin J Antituberc 2012; 34: 207-11.
[6] Lu XW, Wu JL, Miao YW, et al. High resolution computed tomographic findings of interstitial lesions of the lung in patients with active pulmonary tuberculosis. Journal of Clinical Radiology 2008; 27: 605-8.
[7] The work was sponsored by the fund of the Key Clinical Specialty Discipline Construction Program of Fujian, P.R.C. (Minwei medical administration letter [2018] 145) and the Clinical Medicine Center Construction Program of Fuzhou, Fujian, P.R.C. (2018080305).

## POIC - Pain, blood and brain in the perioperative period

### 000213

#### Clinical characteristics of leptospirosis in an intensive care unit: A 10-year retrospective review at a university hospital in southern Thailand

A. Ajjimarungsri[1], R. Bhurayanontachai[2]
[1]Department of Internal Medicine, Faculty of Medicine, Prince of Songkla University, Hat Yai, Thailand; [2]Division of critical care medicine, department of internal medicine, Faculty of Medicine, Prince of Songkla University, Kho Hong, Thailand
**Correspondence:** A. Ajjimarungsri
*Intensive Care Medicine Experimental* 2019, **7(Suppl 3):**000213

**INTRODUCTION.** Leptospirosis is a common zoonotic infection in Thailand which widely presents as a mild to severe disease. The clinical characteristics of leptospirosis in our hospital were reviewed and the predictive factors of ICU admission were determined.

**METHODS.** A retrospective review of the medical charts was conducted in patients diagnosed as leptospirosis and hospitalized in our university hospital from 2007 to 2017. Patients with a positive test for serum immunofluorescence assay for leptospirosis were defined as definite cases and patients who had a Thai LeptoScore1 ≥4 were defined as possible cases. Patients were divided into ICU admission and non-ICU admission groups. All demographic data, clinical characteristics, and laboratory results were compared between the groups. Logistic regression was introduced to determine the predictive factors of ICU admission.

**RESULTS.** Sixty-eight patients were included. Forty-six (67.6%) patients were definite cases. Thirty-two cases (47.06%) were admitted to ICU and of these 20 (62.5%) had sepsis and septic shock. ICU patients had a shorter onset of symptoms (4.09 ± 1.67 vs. 6.44 ± 4.74 days, p = 0.008), higher Thai LeptoScore (7.64 ± 2.70 vs. 5.65 ± 2.37, p = 0.002), lower hematocrit (32.69 ± 7.50 vs. 36.59 ± 7.90 %, p = 0.04) and platelet count (88.69 ± 92.19 vs. 173.26 ± 150.13 x103/mm3, p = 0.01) and higher serum creatinine (4.24 ± 2.94 vs. 2.80 ± 2.09 mg/dL, p = 0.02) and total bilirubin levels (6.20 ± 6.33 vs. 2.98 ± 3.60 mg%, p = 0.01). ICU patients also had diffuse infiltration on chest x-rays (65.62% vs. 25%, p < 0.001), higher mechanical ventilation requirement (84.38% vs. 5.0%, p < 0.001), higher hemodialysis requirement (37.5% vs. 0%, p < 0.001), and higher inotropic support (90.63% vs. 11.1%, p < 0.001). The predictive factors for ICU admission were Thai LeptoScore (OR 1.36; 95% CI 1.10, 1.70, p < 0.001), requirement for mechanical ventilation (OR 63.24; 95% CI 5.79, 691.39, p < 0.001) and inotropes (OR 53.47; 95% CI 5.45, 524.47, p = 0.006). A Thai LeptoScore ≥7.5 could predict ICU admission (AUROC 0.72; 95% CI 0.59, 0.84, p < 0.001).

**CONCLUSION.** Predictive factors of ICU admission in leptospirosis were a high Thai LeptoScore and the need for mechanical ventilation and inotropes. A Thai LeptoScore ≥7.5 can predict ICU admission in patients with leptospirosis.

**REFERENCE(S)**

1. Sukmark T, Lumlertgul N, Peerapornratana S, Khositrangsikun K, Tungsanga K, Sitprija V, et al. Thai-Lepto-on-admission probability (THAI-LEPTO) score as an early tool for initial diagnosis of leptospirosis: Result from Thai-Lepto AKI study group. PLoS Negl Trop Dis. 2018;12(3):e0006319.

### 000241

#### Enteral free water treatment for ICU acquired hypernatremia – a retrospective study

DV. E.a.j., VDV. P.h.j., L. T.
Intensive care, OLVG location East, Amsterdam, Netherlands
**Correspondence:** E. de vos
*Intensive Care Medicine Experimental* 2019, **7(Suppl 3):**000241

**INTRODUCTION.** ICU acquired hypernatremia (IAH) occurs frequently and is associated with increased morbidity and mortality (1). However the treatment of IAH remains controversial (2) and the relationship between sodium and body fluid volume appears to be complex (3,4).

**OBJECTIVES.** To determine the effect of enteral free water treatment on plasma sodium level in patients with IAH.

**METHODS.** A retrospective single centre study from 2008-2019 was conducted in adult patients with IAH treated with enteral free water. Patients with renal replacement therapy, diabetic ketoacidosis or hyperosmolar hyperglycemic state were excluded. Primary outcome was the change in plasma sodium (in mmol/L) after 5 days treatment (delta sodium). Responders were defined as a decrease in sodium level of ≥5 mmol/l. The volume of enteral free water and total sodium administration were also recorded. Descriptive statistics, Fischer exact and Mann-Whitney U test were used.

**RESULTS.** 409 consecutive patients (Table 1) were included. The median volume of enteral water was 4183 ml (IQR 3296 - 5310) after 5 days and mean sodium decrease was 1.4 mmol (SD 5.0). (Fig. 1). There was no significant correlation between the volume of enteral water and delta sodium r2=0.01 (Fig. 2). Responders (97) vs non-responders (312) did not differ significantly with the administration of furosemide or hydrochlorothiazide concerning sodium change. Responders had a significantly lower sodium intake compared to non-responders (842 mmol vs 894 mmol, p= 0.039).

**CONCLUSION.** The volume of enteral water was unrelated with sodium change over 5 days. The lower sodium intake in responders suggests that restricting salt intake is more effective then enteral free water treatment but further study is needed.

REFERENCE(S)
1. Waite MD, et al. J Crit Care. 2013;28:405-12
2. Hessels L, et al. Ann Intensive Care. 2018;8:97
3. Titze J. Curr Opin Nephrol Hypertens. 2015;24:14-20
4. Quinn JW, et al. SAGE Open Med. 2018 Mar 21;6:2050312118762043
5. None

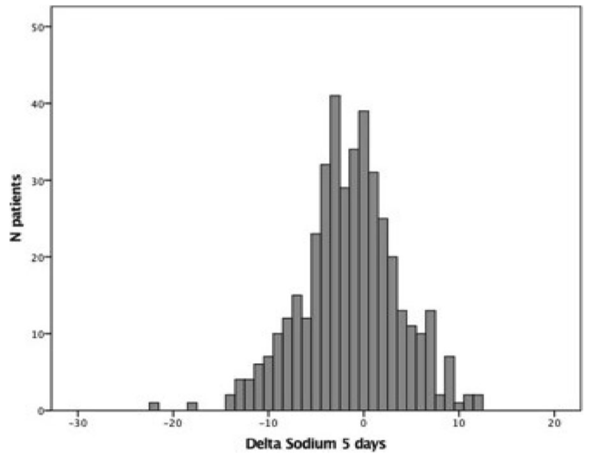

**Fig. 1 (abstract 000241).** See text for description

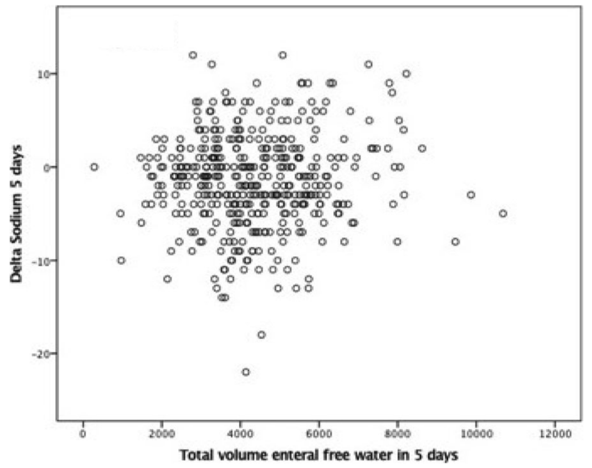

**Fig. 1 (abstract 000241).** See text for description

**Table 1 (abstract 000241).** Patient characteristics (median and IQR)

| | |
|---|---|
| Age (years) | 68 (61-76) |
| Male (N) | 281 (64%) |
| SOFA score | 6 (4-8) |
| APACHE II score | 22 (18-27) |

### 000276

#### Early fibrinogen concentrates administration improve the outcome of the severe trauma patients: A propensity score matched analysis

Y. Itagaki[1], M. Hayakawa[2], K. Maekawa[2], Y. Honnma[2], A. Mizugaki[2], T. Yoshida[2], T. Wada[2], K. Katabami[2], T. Saito[2]
[1]kita11 joh nishi 13 choume chu-o-ku , Sapporo city ,Hokkaido ,Japan, Emergency and critical care center , Sapporo City General Hospital, Sapporo, Japan; [2]Kita14joh nishi5 choume ,kita-ku , sapporo city , hokkaido, Department of emergency medicine , Hokkaido university hospital, Sapporo, Japan
**Correspondence:** Y. Itagaki
*Intensive Care Medicine Experimental* 2019, **7(Suppl 3):**000276

**INTRODUCTION.** Fibrinogen plays an important role in hemostasis in the early phase of trauma. Numerous studies have shown that low fibrinogen levels were associated with hemostatic impairment, massive bleeding, and poor outcomes in severe trauma patients. Because fibrinogen levels deteriorate before other routine coagulation parameters in the early phase of severe trauma, aggressive supplementation of fibrinogen may improve hemostatic function.

**METHODS.** We conducted asingle-center retrospective cohort study with propensity score-matched analysis.Patients with severe trauma (injury severity score ≥ 16) who were admitted to the emergency department (ED) of Hokkaido University Hospital from January 2010 to July 2018 were eligible for inclusion in this study. Exclusion criteria were as follows: age < 18 years, cardiac arrest before arrival to the ED, cervical spinal cord injury not caused by a high-energy accident, and severe burn injury. The patients were divided into the FC and control groups. The FC group included trauma patients who received FC within 1 hour of arrival to the ED. The control group included patients who did not receive FC within 1 hour of arrival to the ED. Trauma and injury severity score, heart rate at admission to the ED, and age were included in the propensity score model. We compared the two groups using propensity score-matching to reduce selection bias and to balance characteristics and clinical variables that could potentially affect outcomes. The primary outcome was in-hospital survival rate.

**RESULTS.** The c-statistic of the propensity score model was 0.734. The Hosmer-Lemeshow chi-squared value was 7.036 (degrees of freedom=8), with a non-significant p value of 0.533, indicating the goodness of model fit. Propensity score-matching created 31 1:1-matched pairs. The characteristics of the two matched groups were appropriately balanced. In-hospital survival rate was higher in the FC group than in the control group (p=0.013 by log-rank test). The amounts of red blood cell and fresh frozen plasmaadministered within 24 hours after admission to the ED in the FC group were significantly higher than those in the control group.

**CONCLUSION.** This propensity score-matched analysis indicates that early administration of FCs improves in-hospital survival rates in severe trauma patients.

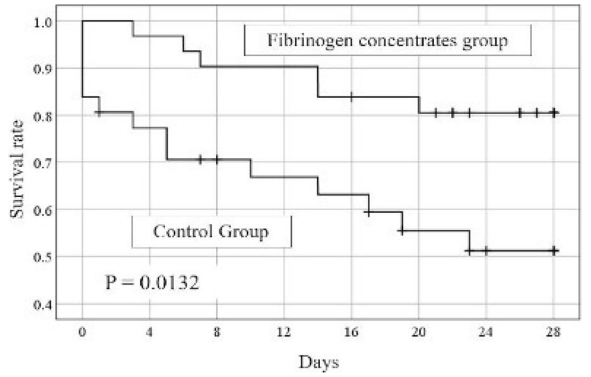

**Fig. 1 (abstract 000276).** See text for description

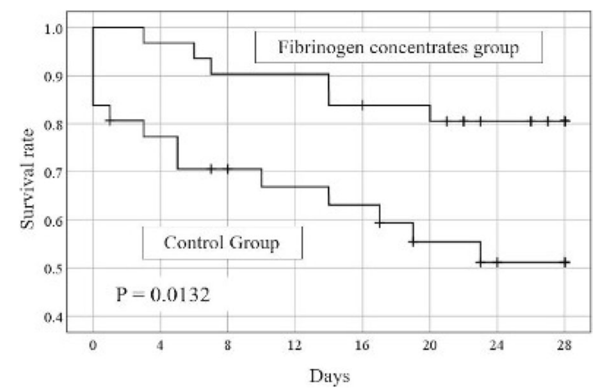

**Fig. 2 (abstract 000276).** See text for description

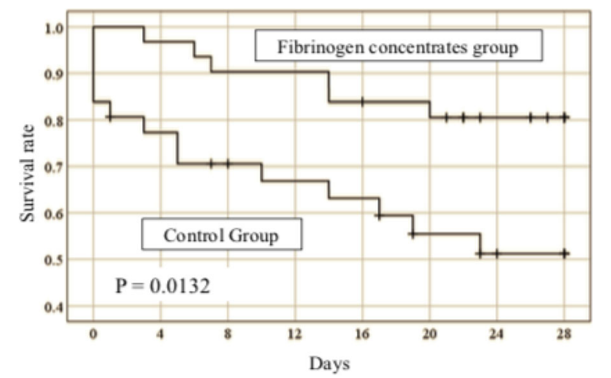

**Fig. 3 (abstract 000241).** See text for description

### 000308

#### Effects of magnesium administration on the occurrence of atrial fibrillation after cardiac surgery: a before-and-after study

E. Osawa[1], S. Cutuli[2], L. Cioccari[3], L. Bitker[4], L. Peck[1], H. Young[1], F. Yanase[1], L. Hessels[1], G. Matalanis[5], G. Eastwood[1], R. Bellomo[1]
[1]Intensive care unit, Austin Hospital, Heidelberg, Australia; [2]Department of anesthesiology and intensive care, Università Cattolica del Sacro Cuore, Facoltà di Medicina e Chirurgia, Roma, Italy; [3]Department of intensive care medicine, University of Bern, Bern, Switzerland; [4]Intensive care unit, Université de Lyon, Lyon, France; [5]Department of cardiac surgery, Austin Hospital, Heidelberg, Australia
**Correspondence:** E. Osawa
*Intensive Care Medicine Experimental* 2019, **7(Suppl 3):**000308

**INTRODUCTION.** Intensive care unit (ICU) magnesium administration for the prevention of atrial fibrillation (AF) after cardiac surgery remains controversial. Previously we demonstrated that a strategy of bolus magnesium delivery followed by a continuous infusion resulted in a more sustained and stable serum magnesium level in comparison to a single bolus. However, the effectiveness of such approach has not been demonstrated in clinical studies.

**OBJECTIVES.** We aimed to evaluate the effectiveness of a post-operative 'bolus + continuous magnesium' strategy on the occurrence of AF (primary outcome) and other relevant clinical outcomes (secondary outcomes).

**METHODS.** We performed a before-and-after study in adult cardiac surgery patients admitted to the ICU of a tertiary metropolitan hospital. We enrolled patients with normal renal function and a serum magnesium level lower than 1.5 mmol/L on ICU arrival. Intervention group patients received a bolus of 10 mmol of magnesium-sulphate over 1 hour followed by a continuous infusion of 1.5 to 3 mmol/h until ICU discharge, aiming at a serum magnesium level of 1.5 to 2 mmol/L. Propensity score matching was performed to select control group patients.

**RESULTS.** We enrolled 99 intervention group patients and 99 control group patients. Both groups had similar baseline characteristics, except for a lower cardiac index at ICU admission in the intervention group. By 28-days after ICU admission, the occurrence of AF was reached by 25 patients (25.3%) in the intervention group and by 40 patients (40.4%) in the control group (OR 0.49 [95% CI: 0.27 to 0.92), P=0.023). The median ICU length of stay was longer in the intervention group than in the before-group (44.8 h [IQR: 22.2 to 68.2] vs. 24.9 h [IQR: 22.0 to 43.7), P=0.0007). There were no significant differences between the two groups regarding other secondary outcomes. A multivariable Cox regression model for 'time to AF' showed that the intervention was associated with reduced incidence of AF (HR 0.45 [95% CI: 0.25 to 0.77], P=0.004), and that the variables age, duration of bypass and requirement of vasopressors > 6 h were associated with increased risk of AF.

**CONCLUSION.** An ICU strategy of bolus followed by continuous magnesium administration after cardiac surgery was associated with a reduction in the occurrence of AF.

##### REFERENCE(S)

1. Osawa EA, Biesenbach P, Cutuli SL, Eastwood GM, Mårtensson J, Matalanis G, Fairley J, Bellomo R. Magnesium sulfate therapy after cardiac surgery: a before-and-after study comparing strategies involving bolus and continuous infusion. Crit Care Resusc. 2018 Sep;20(3):209-216.
2. A financial support for this study was received from Anaesthesia and Intensive Care Trust Fund, Austin Hospital.

### 000340

#### Correlation between Patient State Index and Richmond Agitation-Sedation Scale in sedated Intensive Care Unit patients: A Single-Center, Single-Blind, Prospective, Observational Study

M. Idei, Y. Seino, N. Sato, T. Yoshida, Y. Saishu, K. Fukui, K. Ota, J. Ishikawa, D. Kamei, M. Nakagawa, T. Nomura
Department of intensive care unit, Tokyo Women's Medical University Hospital, Tokyo, Japan
**Correspondence:** M. Idei
*Intensive Care Medicine Experimental* 2019, **7(Suppl 3):**000340

**INTRODUCTION.** The Richmond Agitation-Sedation Scale (RASS) is widely used for assessing sedation level of the ventilated patients in intensive care unit (ICU), even though it has clinical limitations such as subjective and also intermittent measurements. The Patient State Index (PSI) has been a newly introduced processed EEG parameter and enables an objective and continuous monitoring of sedation levels by analyzing electroencephalograms. However, few studies have examined the correlation between PSI and RASS and the usefulness of PSI in assessing sedation levels in ICU patients is unknown.

**OBJECTIVES.** To analyze the correlation between PSI and RASS in critically ill ICU patients and the usefulness of PSI in assessing the sedation level.

**METHODS.** In the medical and surgical ICU at Tokyo Women's Medical University Hospital (Tokyo, Japan), PSI values continuously monitored with SedLine (Masimo, Irvine, CA, USA) and RASS were recorded every 2 hours for 24 hours in adult critically ill patients who required mechanical ventilation for 12 hours or more. Patients with a history of neurosurgery, cerebral infarction, or cerebral hemorrhage were excluded. Propofol, dexmedetomidine, midazolam, and fentanyl were used as sedatives and analgesics. The doses of sedatives and analgesics were adjusted to achieve the target RASS score set for each patient, and the bedside doctors and nurses were blinded to the PSI values. The correlation between PSI and RASS was analyzed by Spearman's rank correlation coefficient. P-value <0.05 was considered significant.

**RESULTS.** In total, 382 PSI and RASS sets were recorded from 50 patients. The patient's age was 65.7 ± 10.3 years (mean ± standard deviation [SD]); the Acute Physiology and Chronic Health Evaluation (APACHE) II score was 15.3 ± 6.0 (mean ± SD); and 92% patients were postoperative. The Spearman's rank correlation coefficient between PSI and RASS was 0.783, and a positive correlation was observed (p < 0.001). Moreover, a RASS score of -3 or less (-3, -4, or -5) was defined as deep sedation and of -2 or more as light sedation. The PSI cutoff value for deep sedation was 53 (Area Under the Curve (AUC): 0.917, sensitivity: 0.824, specificity: 0.901).

**CONCLUSION.** In our prospective, single-blinded observational study, PSI showed a positive correlation with RASS, which is the gold standard for assessing sedation levels, and PSI was highly sensitive and specific in detecting deep sedation. PSI could be useful in assessing sedation levels in critically ill ICU patients.

**REFERENCE(S)**

1. Devlin JW, Skrobik Y, Gélinas C, et. al; Clinical Practice Guidelines for the Prevention and Management of Pain, Agitation/Sedation, Delirium, Immobility, and Sleep Disruption in Adult Patients in the ICU. Crit Care Med. 2018 Sep;46(9): e825-e873.
2. Schneider G, Heglmeier S, Schneider J, et al; Patient State Index (PSI) measures depth of sedation in intensive care patients. Intensive Care Med. 2004 Feb;30(2):213-216.

### 000365

#### Outcomes and complications following cytoreductive surgery and hypertermic intraperitoneal chemotherapy: three year experience

S. Di Stefano[1], C. Videla[1], I. Carboni Bisso[1], M. Las Heras[1], JM. Dianti[1], B. Viaña[2], E. San Román[1]
[1]Unidad de terapia intensiva adultos, Hospital Italiano de Buenos Aires, Ciudad Autónoma de Buenos Aires, Argentina; [2]Servicio de cirugia general, Hospital Italiano de Buenos Aires, Buenos Aires, Argentina
**Correspondence:** S. Di Stefano
*Intensive Care Medicine Experimental* 2019, **7(Suppl 3):**000365

**INTRODUCTION.** Cytoreductive surgery (CRS) with Hyperthermic Intraperitoneal Chemotherapy (HIPEC) is a viable treatment option for subjects with locally advanced abdominal cancer but has high risk complications that make postoperative management challenging for intensivists.

**OBJECTIVES.** To report our experience in the immediate postoperative care of subjects who underwent CRS-HIPEC. Subjects were managed following a protocol which included goal directed fluid therapy and minimally invasive hemodynamic monitoring.

**METHODS.** Retrospective, single-center observational study of patients who underwent CRS-HIPEC over a three year period (2015-2018).

**RESULTS.** A total of 55 patients were included. Mean (SD) age was 52.7 (±12.7) years and 69.1% were women. Mean (SD) Charlson comorbidity index and APACHE II score were 7.4 (±1.2) and 13 (±5.91) respectively. The most common indication was colon cancer (41,8%), followed by appendiceal mucinous neoplasm (25,4%) and ovarian cancer (20%). Mean (SD) PCI was 12.72 (±7.37). Complete cytoreduction (CC-0/1) was achieved in all patients. Mitomycin C was used as the main chemotherapy drug (49,09%) in digestive cancer, followed by oxaliplatin (25,4%) and cisplatin (18,1%) in gynecological cancer. The most frequent complications were anemia (60%) and thrombocytopenia (40%). 34.5% subjects presented acute kidney injury, but only 7 patients needed renal replacement therapy. Other severe complications included ARDS (3.6%) , peritonitis and intraabdominal bleeding (12.7%), subarachnoid hemorrhage (1.8%[n=1]) and pulmonary thromboembolism(1.8%). Mechanical ventilation for more than 24 hours was required by 29% of the subjects and only 3 were tracheostomized. The median (IQR) LOS in the intensive care unit was 6 (5-9) days and the hospital stay of 13(9-22) days. Global ICU mortality was 5.45% [n=3].

**CONCLUSION.** Nevertheless the high rate of complications after CRS-HIPEC surgery, global ICU mortality was low and complications with protocolized fluid management were similar to other series published.

**REFERENCE(S)**

1. Colantonio L. et al. ¨A Randomized Trial of Goal Directed vs Standard Fluid Therapy in Cytoreductive Surgery with Hyperthermic Intraperitoneal Chemotherapy" J Gastrointest Surg. 2015,19:722–729.
2. Padmakumar A. "Intensive Care Management of Patient After Cytoreductive Surgery and HIPEC - A Concise Review." Indian Journal of Surgical Oncology. 2016. 7(2): 244–48
3. Fundación de Cuidados Críticos del Hospital Italiano de Buenos Aires (FCC-HIBA)

### 000375

#### Sodium valproate versus continuous infusion of haloperidol in management of agitated critically ill patients

R. khalil, H. Khalid, A. Kamel, O. Mohamed, S. Mohamed
Critical care, Cairo University, Faculty Of Medicine, Kasr Al Ainy, Cairo, Egypt
**Correspondence:** R. khalil
*Intensive Care Medicine Experimental* 2019, **7(Suppl 3):**000375

**INTRODUCTION.** Agitation occurs in up to 70% of critically ill patients and is a significant source of distress for patients, families, and providers (Fraser GL, et al.2000). Sedatives are administered to 50% of intensive care unit (ICU) patients to alleviate agitation (1). Choice of sedative is complex and largely driven by patient context. No sedative has consistently been shown to be superior to the rest, and alternative agents are greatly needed (2).Most ICU patients, especially those requiring mechanical ventilation, are treated with opioids, propofol, and/or benzodiazepines (1&3). Use of these agents is limited by adverse effects (eg, hemodynamic derangement for safe administration (4). New therapies for treating agitation are rarely introduced into practice, with dexmedetomidine being the most recent in 1999. Consequently, providers have increasingly repurposed older pharmacologic agents as ICU sedatives (eg, clonidine, Phenobarbital, and valproate (depakene) (5 & 6).

Recently, valproate has been administered to critically ill patients to treat agitation and delirium, but there are few published reports to support this practice (7-9). Valproate is an emerging treatment for ICU agitation because it allows patients to interact with their caregivers; can be administered outside of the ICU; has both an intravenous (IV) and enteral formulation; has a low drug acquisition cost; and has not been associated with respiratory depression, hemodynamic derangements, or delirium. In this study we describe the use of depakene & haloperidol for agitation in critically ill patients and examine their safety.

**METHODS.** prospective study on 100 critically ill patients with agitation in kasralainy hospital over period from may 2016 to June 2017.patients were divided in to two groups, each group included 50 patients, 1st group patients received depakene orally and 2nd group patients received haloperidol by i.v infusion for 72h.Richmound agitation sedation score and doses of additional sedative drugs were noted and calculated daily in the first three days.

**RESULTS.** our study showed that valproate was equal in efficacy in controlling agitation; decreasing the RAAS significantly after 48h from initiation (2.52±0.61 Vs 0.28±0.54 with p<0.001) for depakene and (2.6±0.67 Vs 0.34±0.48 with p<0.001) for haloperidol. There was also decrease in the doses of additional sedative drugs used to control agitation (midazolam & propofol) after 48h from drug initiation.-both drugs therapy was associated with decrease in heart rate (89±20 vs. 86.6±13.6 with p=0.002 for valproate and 99.8±23.3 vs. 91±16.7 with p<0.001 for haloperidol) .theyhad no effect on blood pressure. Haloperidol therapy was associated with significant QTc prolongation.

**CONCLUSION.** valproate was equal in efficacy as haloperidol infusion in controlling agitation in ICU and decreasing the doses of additional sedative drugs used after 48h from initiation.

**REFERENCE(S)**

1. Wunsch H, Kahn JM, Kramer AA, Rubenfeld GD. Use of intravenous infusion sedation among mechanically ventilated patients in the United States. Crit Care Med 2009; 37(12):3031–9.
2. Roberts DJ, Haroon B, Hall RI. Sedation for critically ill or injured adults in the intensive care unit: a shifting paradigm. Drugs 2012; 72(14):1881–916.
3. Weinert CR, Calvin AD. Epidemiology of sedation and sedation adequacy for mechanically ventilated patients in a medical and surgical intensive care unit. Crit Care Med 2007; 35(2):393–401.
4. Riker RR, Fraser GL. Adverse events associated with sedatives, analgesics, and other drugs that provide patient comfort in the intensive care unit. Pharmacotherapy 2005; 25(5 Pt 2):8S–18S.
5. Gagnon DJ, Riker RR, Glisic EK, Kelner A, Perrey HM, Fraser GL. Transition from dexmedetomidine to enteral clonidine for ICU sedation: an observational pilot study. Pharmacotherapy 2015; 35(3):251–9.
6. Fraser GL, Riker RR. Phenobarbital provides effective sedation for a select cohort of adult ICU patients intolerant of standard treatment: a brief report. Hosp Pharm 2006; 41:17–23.
7. Bourgeois JA, Koike AK, Simmons JE, Telles S, Eggleston C. Adjunctive valproic acid for delirium and/or agitation on a consultation-liaison service: a report of six cases. J Neuropsychiatry Clin Neurosci 2005; 17(2):232–8.
8. Sher Y, Miller AC, Lolak S, Ament A, Maldonado JR. Adjunctive valproic acid in management-refractory hyperactive delirium: a case series and rationale. J Neuropsychiatry Clin Neurosci 2015; 27(4):365–70.
9. Fitz K, Harding A. Safety and efficacy of valproic acid for treatment of delirium in critically ill patients [abstract]. Crit Care Med 2011; 39(12 Suppl.):239.
10. My dear professors, my family

### 000380

#### Could factor XIII deficiency increase intracerebral hemorrhage?

E. Val-Jordán[1], A. Nebra-Puertas[2], J. Casado-Pellejero[3], N. Fernández-Monsteirín[4], C. Revilla-López[5], G. Jiménez-Jiménez[1], N. Ramón-Coll[1], J. Codina Calero[1], J. Rubio-Ruiz[1], J. Caballero-López[1]

[1]Intensive care department, University Hospital Arnau de Vilanova, Lleida, Spain; [2]Intensive care department, Hospital Universitario Miguel Servet, Zaragoza, Spain; [3]Neurosurgery department, Hospital Universitario Miguel Servet, Zaragoza, Spain; [4]Coagulation department, Hospital Universitario Miguel Servet, Zaragoza, Spain; [5]Statistic department, Hospital Universitario Miguel Servet, Zaragoza, Spain

**Correspondence:** E. Val-Jordán

*Intensive Care Medicine Experimental* 2019, **7(Suppl 3):**000380

**INTRODUCTION.** Intracerebral hemorrhage (ICH), sometimes unexplained, is one of the most feared complication after brain tumor surgery. Blood clot stabilization after surgery depends on factor XIII levels. Acquired or congenital factor XIII deficiency could be associated with hemorrhage after neurosurgery.

**OBJECTIVES.** To analyse the association between perioperative levels of factor XIII and ICH after neurosurgery.

**METHODS.** A prospective, longitudinal, 18-month study was conducted at a single third-level hospital in Spain. The study included all consecutive adults operated on brain tumor during 2013-2014 and postoperatory stay in intensive care unit. Informed consent was required. To evaluate factor XIII three blood samples were taken (A-presurgical or baseline, B-postsurgical and C-24 hours after surgery). Normal range considered was 70-140%. ICH was defined as bleeding that generates radiological signs of intracranial hypertension either by volume or by mass effect on the routine CT scan 24 hours after surgery. Mann-Whitney U Test and T-Test were used to inferential analysis. Ethics Committee approval was obtained.

**RESULTS.** The study included 109 patients. ICH was confirmed in 39 of them (35,78%). The average of factor XIII in sample A was 77,52% in patients without ICH vs 71,2% in patients with ICH, in sample B 70,14% vs 51,57% and in sample C 69,68% vs 52,14%, respectively. Inferential analysis determined significant association between factor XIII-B and C deficiency with ICH (both p0,000) and absence of association in patients without ICH.Their variation, compared to the baseline sample, was also significative (factor XIII A-B p0,000 and factor XIII A-C p0,004).

**CONCLUSION.** Factor XIII deficiency after brain tumor surgery increased ICH.

Further studies are necessary to demonstrate this association, in which case deficiency treatment could become a therapeutic target

**REFERENCE(S)**

1. Odame JE, Chan AK, Wu JK, Breakey VR. Factor XIII deficiency management: a review of the literature.Blood Coagul Fibrinolysis.2014;25(3):199-205
2. Naderi M, Zarei T, Haghpanah S, Eshghi P, Miri-Moghaddam E, Karimi M. Intracranial hemorrhage pattern in the patients with factor XIII deficiency. Ann Hematol. 2014;93(4):693-7
3. Gerlach R, Raabe A, Scharrer I, Meixensberger J, Seifert V. Post-operative hematoma aftersurgery for intracranial meningiomas: causes, avoidable risk factors and clinical outcome.Neurol Res. 2004;26(1):61-6
4. Gerlach R, Raabe A, Zimmermann M, Siegemund A, Seifert V.Factor XIII deficiency and postoperative hemorrhage after neurosurgical procedures.Surg Neurol.2000;54(3):260-4
5. Intensive Care Department, Neurosurgery and Hematology Department at Miguel Servet University Hospital, Intensive Care Department at Arnau de Vilanova University Hospital :

### 000392

#### Circadian melatonin rhythm in critically ill patients randomized to sedation or non-sedation: A NONSEDA substudy

J. Oxlund[1], T. Strøm[2], T. Knudsen[3], P. Toft[2]

[1]Sydvestjysk Sygehus, Esbjerg, Denmark; [2]Department of anesthesiology and intensive care, Odense University Hospital, Odense, Denmark; [3]Medicine, Sydvestjysk Sygehus, Esbjerg, Denmark

**Correspondence:** J. Oxlund

*Intensive Care Medicine Experimental* 2019, **7(Suppl 3):**000392

**INTRODUCTION.** Abolished circadian rhythm is associated with reduced cognitive function, delirium, and in turn increased mortality (1, 3, 6). Sleep disorders and poor sleep quality are frequent complications in the critically ill patient, especially when mechanically ventilated (4,5). The causes are multifactorial, but research suggests that changes in the biological 24-hour clock located in the nuclus suprachiasmaticus of the hypothalamus affect the circadian rhythm (2). Melatonin is secreted from this site and plays a physiological role in the regulation of sleep and daytime rhythm (7). No previous studies have investigated the melatonin level on sedated vs. non-sedated patients. This study investigates circadian melatonin rhythm on the largest population of critically ill patients.

**OBJECTIVES.** To evaluate the melatonin level in critically ill patients included in the NONSEDA trial and to investigate the correlation with delirium.

**METHODS.** This study was conducted as a sub study to the NONSEDA study where critically ill patients were randomized either to non-sedation or sedation with a daily wake-up trail. S-Melatonin was analyzed 3 times a day (14:00, 22:00 and 03:00 o'clock) for 4 days, starting on day 2 after intubation. Delirium was evaluated by CAM-ICU.

**RESULTS.** Seventy-nine of 100 patients included in the NONSEDA study at the Hospital of Southwest Jutland Esbjerg were included.

**CONCLUSION.** Circadian melatonin rhythm in patients allocated to non-sedation was closer to normal after 3 days of intensive care compared to sedated patients. During the study period the risk of delirium was significantly higher in the sedated group (OR 0,2). No correlation between abolished circadian melatonin rhythm and development of delirium was established in this study.

**REFERENCE(S)**

1. Delirium and Circadian Rhythm of Melatonin During Weaning From Mechanical Ventilation: An Ancillary Study of a Weaning Trial. Dessap AM1, Roche-Campo F2, Launay JM3, Charles-Nelson A4, Katsahian S4, Brun-Buisson C5, Brochard L6. Chest: 2015 Nov;148(5):1231-1241. doi: 10.1378/chest.15-0525.
2. Abolished circadian rhythm of melatonin secretion in sedated and artificially ventilated intensive care patients. Ollufson K1, Alling C, Lundberg D, Malmros C. Acta Anaesthesiol Scand. 2004 Jul;48(6):679-84
3. Delirium is associated with increased mortality in the geriatric population. Tosun Tasar P1, Sahın S2, Akcam NO3, Dınckal C4, Ulusoy MG5, Sarıkaya OF4, Duman S4, Akcıcek F2, Noyan A3. Int J Psychiatry Clin Pract. 2018 Sep;22(3):200-205. doi: 10.1080/13651501.2017.1406955. Epub 2017 Nov 27.
4. Bench-to-bedside review: delirium in ICU patients - importance of sleep deprivation. Weinhouse GL1, Schwab RJ, Watson PL, Patil N, Vaccaro B, Pandharipande P, Ely EW. Crit Care. 2009;13(6):234. doi: 10.1186/cc8131. Epub 2009 Dec 7.
5. Circadian disruption of ICU patients: A review of pathways, expression, and interventions. Korompeli A1, Muurlink O2, Kavrochorianou N3, Katsoulas T1, Fildissis G1, Baltopoulos G1. J Crit Care. 2017 Apr;38:269-277. doi: 10.1016/j.jcrc.2016.12.006. Epub 2016 Dec 13.
6. Delirium as a predictor of mortality in mechanically ventilated patients in the intensive care unit. Ely EW1, Shintani A, Truman B, Speroff T, Gordon SM, Harrell FE Jr, Inouye SK, Bernard GR, Dittus RS. JAMA. 2004 Apr 14;291(14):1753-62.
7. Melatonin in Critical Care. Marra A1, McGrane TJ2, Henson CP2, Pandharipande PP3. Crit Care Clin. 2019 Apr;35(2):329-340. doi: 10.1016/j.ccc.2018.11.008. Epub 2019 Jan 30.

### 000394

#### Preoperative nutritional assessment, sarcopenia and the prevalence of refeeding syndrome in patients undergoing pancreatic oncologic surgery

YL. Nguyen[1], M. Rajaona[1], A. Azri[1], A. Dohan[2], S. Gaujoux[3], C. Baillard[1]
[1]Anesthesiology and critical care department, Hospital Cochin, Paris, France; [2]Radiology department, Hospital Cochin, Paris, France; [3]Department of hepato-pancreato-biliary and endocrine surgery, Hospital Cochin, Paris, France
**Correspondence:** Y.L. Nguyen
*Intensive Care Medicine Experimental* 2019, **7(Suppl 3):**000394

**INTRODUCTION.** According to the latest recommendations of ESPEN, one of the following criteria define surgical patients at severe nutritional risk: a weight loss>10-15% within 6 months, BMI<18.5Kg/m2, subjective global assessment Grade C or NRS>5, preoperative serum albumin<30g/L(1). Sarcopenia measurement is now recommended in the nutritional assessment of oncological patients (2). In the setting of pancreatic cancer and surgical resection, patients are at risk to develop endocrine and exocrine pancreatic insufficiency, leading to malnutrition and a higher risk of developing a refeeding syndrome after starting nutritional support. To our knowledge, the prevalence of sarcopenia and refeeding syndrome in this population remains unknown.

**OBJECTIVES.** To describe the perioperative nutritional assessment, the prevalence of sarcopenia and of refeeding syndrome in subjects undergoing pancreatic oncologic surgery.

**METHODS.** A single-center observational study was conducted between July 2018 and March 2019. This study was endorsed by SFAR's CERAR. All subjects undergoing pancreatic oncologic surgery were included. Demographic, clinical, biological and CT data were collected on medical records. Total skeletal muscle surface area was evaluated on a single image at the third lumbar vertebra, using dedicated software, by a single researcher blinded to clinical data. Sarcopenia was defined by a lumber skeletal muscle index (total skeletal muscle surface normalized for stature) of less than 52.4 cm2/m2in men and 38.9 cm2/m2in women. The refeeding syndrome was defined by a hypophosphatemia £0.7 mmol/L occurring between J1 and J7 postoperatively. Data were presented median (IQR).

**RESULTS.** Thirty-two patients were included in our study. The sex ratio was 1/1, aged 67 yo (58; 74), a body mass index of 27 Kg/m2(22; 31), an albumin serum 34g / L (28; 39) and a skeletal muscle index of 48 cm2/m2(39; 54). 15% (n = 5) had preoperative chemotherapy, 3% (n = 1) had radiotherapy, 28% (n = 9) had diabetes, and 13% (n = 4) chronic renal failure. We did not find the global assessment grade or NRS. According to the ESPEN malnutrition assessment criteria, the rates of malnourished patients were 30% (n = 6) for weight loss, 3% (n = 1) for BMI and 33% (n = 1). 7) for serum albumin. According to the evaluation of muscle mass, 47% (n = 15) were sarcopenic. Nearly one third of the patients (n = 9) had preoperative nutritional supplementation. The prevalence of post-operative refeeding syndrome was 59% (n = 19).

**CONCLUSION.** The use of a single clinical or biological criterion to define malnutrition underestimates the prevalence of sarcopenia and the risk of post-operative refeeding syndrome in patients with pancreatic oncologic surgery. The calculation of the skeletal muscle index may help clinicians to optimize perioperative nutritional support management. Refeeding syndrome is common and affects more than one in two patients.

**REFERENCE(S)**

1. Weimann A et al. Clin Nutr Edinb Scotl. 2017
2. Fearon K et al. Lancet Oncol 2011

### 000395

#### Assement of analgesic potency and feasability of serratus plane block in chest trauma patients

P. Brunet, K. Cogne, J. Pottecher, P. Diemunsch
Anesthésie - Réanimation chirurgicale, Hospital Hautepierre Hospitals Academics De Strasbourg, Strasbourg, France
**Correspondence:** P. Brunet
*Intensive Care Medicine Experimental* 2019, **7(Suppl 3):**000395

**INTRODUCTION.** Locoregional analgesia is one of the cornerstones of chest trauma management (epidural and paravertebral block)1. The serratus plane block is a regional analgesia method targeting the thoracic wall2. Several case reports described sustained pain relief in chest trauma patients. In a series of consecutive trauma patients presenting with rib fractures, admitted in an intensive care unit, we studied the effectiveness of the serratus plane block on analgesia, its feasibility and the incidence of complications.

**METHODS.** After the approval from the local ethics committee and informed consent we retrospectively studied the medical records of all consecutive patients presenting with a chest trauma and who benefitted from a serratus plane block according to the local practice between January 2018 and February 2019. The serratus plane block was performed in the supine or lateral position under ultrasound guidance. First, the local anesthetic (Ropivacaïne 0.2%) was instilled in the deep serratus plane followed by the insertion of a perineural catheter. Another instillation was performed in the superficial serratus plane. An elastomeric pump was then connected to the perineural catheter for continous infusion (Ropivacaïne 0.2% 10 mL/h).

**RESULTS.** The records of 13 patients were retrospectively analysed. The median age was 57 years [44;71], all patients were male. The trauma was the result of a car accident for 7 patients and of a fall for 5 patients. The rib fractures were bilateral for 7 patients, 9 patients presented a flail chest, 9 a pneumothorax, 5 an hemothorax. A chest tube was in place for 7 patients. 4 patients benefited from thoracic surgery. All a patients presented with an associated trauma, 8 orthopedic trauma, 4 neurosurgical trauma and 4 abdominal trauma. 9 patients required a surgical procedure. 10 patients received systemic analgesic alone before the serratus plane block, while 3 were under sedation. The values of Visual Analog Scale and morphine consumption are presented in Figure 1.No complication was observed.

**CONCLUSION.** Our results suggest that the serratus plane block is an effective locoregional analgesia method for pain management in chest trauma patients. A randomized clinical trial comparing the serratus plane block with the standard locoregional analgesia methods is needed to define the place of this new analgesic technique in the management of chest trauma patients.

**REFERENCE(S)**

1. Société française d'anesthésie et de réanimation, d'urgence S française de médecine. Traumatisme thoracique : prise en charge des 48 premières heures. Anesthésie & Réanimation. 2015;1(3):272-287. doi:10.1016/j.anrea.2015.01.003
2. Blanco R, Parras T, McDonnell JG, Prats-Galino A. Serratus plane block: a novel ultrasound-guided thoracic wall nerve block. Anaesthesia. 2013;68(11):1107-1113. doi:10.1111/anae.12344
3. Durant E, Dixon B, Luftig J, Mantuani D, Herring A. Ultrasound-guided serratus plane block for ED rib fracture pain control. The American Journal of Emergency Medicine. 2017;35(1):197.e3-197.e6. doi:10.1016/j.ajem.2016.07.021

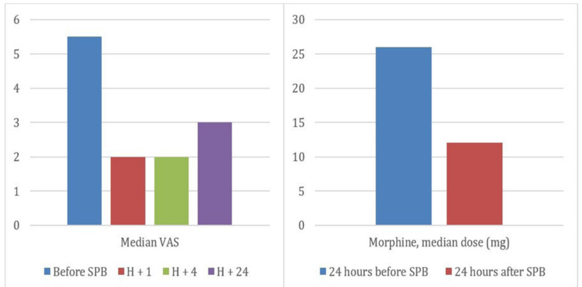

**Fig. 1 (abstract 000395).** See text for description

### 000434

#### Type of volatile anesthetics in the long time general anesthesia for elderly patients does not affect incident postoperative delirium

K. NAKANO, T. Taniguchi
Department of anesthesiology and intensive care medicine, Kanazawa University, Kanazawa, Japan
**Correspondence:** K. NAKANO
*Intensive Care Medicine Experimental* 2019, **7(Suppl 3):**000434

**INTRODUCTION.** Postoperative delirium (PD) deteriorates morbidity, ICU length of stay, prolongation of existing hospitalization, and healthcare costs. Moreover, PD causes delayed functional recovery, cognitive dysfunction, and higher mortality. Several reports showed differences in general anesthesia methods affects incident PD. However, it is unknown whether type of volatile anesthetics affects incident PD.
**OBJECTIVES.** We evaluated retrospectively type of volatile anesthetics in long time general anesthesia for elderly patients affects incident PD.
**METHODS.** This study was a retrospective, single-center, observational study, which was approved by the ethics board of our hospital. 170 patients (110 male, 60 female, average age: 70 years old) aged at least 60, underwent surgery more than 8 hours by the general anesthesia method using volatile anesthetics, and admitted to the ICU at our hospital from April 2017 to September 2018. We excluded patients who underwent cardiac surgery or received total intravenous anesthesia (TIVA). After admission to the ICU, Intensive Care Delirium Screening Checklist (ICDSC) was measured to diagnose PD. Delirium defined four or more points. The subjects were divided into Sevoflurane group (S group) and Desflurane group (D group), and compared. The primary outcome was the difference in the incidence of PD between the two groups. The secondary outcomes were to identify explanatory factors, such as patient factors, surgical factors, and anesthetic factors that influence the onset of PD. The statistical method used the chi-square test or Fisher's exact test for comparison of incident PD between two groups. After univariate analysis, a multivariate analysis was applied to test for independent risk factors for PD. When*P* value was <0.05, the test was considered statistically significant.
**RESULTS.** Data of 94 patients in S, 76 in D groups were analyzed. There were no significant differences in the patients' background between the two groups. Delirium developed in 16 patients. The incidence rates of PD in S and D group were 6.4% and 13.2%, respectively, and there was no significant difference between the two groups (*P*=0.186). With regard to PD onset, a causal relationship was recognized between age (*P*<0.01) and amount of bleeding (*P*= 0.0389).
**CONCLUSION.** In long time general anesthesia for the elderly, the type of volatile anesthetics is not significantly different in the incident PD. Further study is required to confirm the risk of PD with more number of patients and multiple center trial.

**Table 1 (abstract 000434).** See text for description

| Factor | Odds | 95% CI | *P* value |
|---|---|---|---|
| age | 1.35 | 1.15–1.59 | 0.00029 |
| amount of bleeding | 0.998 | 0.996–1.000 | 0.0389 |
| antipsychotics | 4.67 | 0.723–30.20 | 0.106 |
| fentanyl | 1.06 | 0.907–1.24 | 0.460 |
| operative duration | 1.01 | 0.999–1.010 | 0.0911 |
| Respiratory Failure | 0.545 | 0.12–2.47 | 0.431 |
| sex | 1.63 | 0.410–6.46 | 0.489 |
| type of volatile anesthetics | 2.90 | 0.817–10.30 | 0.0996 |

### 000457

#### Goal-directed therapy reduces hemodynamic changes during elective laparoscopic colorectal surgery

V. Panafidina, I. Shlyk
Anesthesiology and intensive care, Pavlov First State Medical University of St. Petersburg, Sankt-Peterburg, Russia
**Correspondence:** V. Panafidina
*Intensive Care Medicine Experimental* 2019, **7(Suppl 3):**000457

**INTRODUCTION.** Goal-directed therapy is considered as an important option in perioperative care. However, most of the studies analyze the postoperative outcomes, but intraoperative hemodynamic changes seem to be a significant factor in development of hypoperfusion during operation. Moreover, laparoscopy effects on hemodynamic parameters are well known and its influence on cardiovascular physiology can be challenging.
**OBJECTIVES.** The aim of this study is to compare two different approaches to hemodynamic and fluid management during elective laparoscopic colorectal surgery.
**METHODS.** 102 patients with curable colorectal cancer were divided in 2 groups: observational (group №1, n = 46) and goal-directed therapy group (group №2, n = 56). All patients have a non-invasive monitoring of hemodynamic (EsCCO, Nihon Kohden, Japan). Management of hemodynamic and fluids in the group №1 were performed according to opinion and agreement of 2 experienced anesthesiologists. Hemodynamic optimization in the group №2 included some steps: first is Trendelenburg maneuver after induction and bolus of 500 ml of balanced crystalloid if stroke volume increased more than 15%, then assessment during pneumoperitoneum and infusion of small (3ml/kg) volume if intraabdominal perfusion pressure (IPP) less than 65 mm Hg, and, if patient is a non-responder, infusion of norepinephrine. Total infusion volume, mean norepinephrine dose, duration of vasopressors, lactate, BE, number of episodes of hypotension (MAP and/or IPP ≤ 65 mm Hg or decrease of SBP more than 20% from baseline), changes in HR more than 30% from baseline were measured. Data was analyzed using IBM SPSS Statistics 20.0. Continuous data variables were compared using *Mann-Whitney* U tests, results are reported as median (Q1; Q3).

**RESULTS.** Groups were similar in terms of demographic characteristics. Fluid volume were significantly less in the group №2: 2590 (2250; 2900) ml vs 3000 (2500; 3312) ml, *Mann-Whitney* U tests, p = 0.005. Norepinephrine time and dosage were similar between two groups. Lactate at the end of operation were lower in the group №2: 1,6 (1,2; 2,1) mmol/l vs 1,05 (0,7; 1,47) mmol/l in the group №1 and 2, respectively, *Mann-Whitney* U tests, p = 0.002. Hypotension were more often in the group №1: 2 (1; 5) episodes vs 1 (0; 1) in the group №2, *Mann-Whitney* U tests, p < 0.0001. Drop of SBP from baseline were significantly higher in the group №1: 35,4% from baseline (13%; 38%) and 21,8% (16,7%; 27,4%) in the group №2, *Mann-Whitney* U tests, p = 0.006.
**CONCLUSION.** Goal-directed hemodynamic and fluid therapy may be beneficial in terms of hemodynamic stability during elective laparoscopic operations in patients with colorectal malignances.

### 000517

#### An Audit Evaluating Current Monitoring Practices of Low Molecular Weight Heparins and Unfractionated Heparin within the Critical Care Setting

A. Main-lan[1], I. Welters[1], J. Hughes[2], P. Brown[3]
[1]Critical care, Royal Liverpool and Broadgreen University Hospitals NHS Trust, Liverpool, United Kingdom; [2]Pharmacy, Royal Liverpool and Broadgreen University Hospitals NHS Trust, Liverpool, United Kingdom; [3]Icnarc, Royal Liverpool and Broadgreen University Hospitals NHS Trust, Liverpool, United Kingdom
**Correspondence:** A. Main-lan
*Intensive Care Medicine Experimental* 2019, **7(Suppl 3):**000517

**INTRODUCTION.** Unfractionated Heparin (UFH) and Low Molecule Weight Heparins (LMWH) are commonly used within critical care to provide therapeutic doses of anticoagulation. Limited evidence is available whether target concentrations are being met in critically ill patients, who are at particular risk of over- or underdosing due to reduction in bioavailability due to vasopressors, lack of weight-adjusted dosing and renal impairment. The Royal Liverpool University Hospital's current protocol demands monitoring of activated partial thromboplastin time (APTT) ratios after 6-8 hours for UFH infusions, and anti-factor Xa (anti-Xa) levels 4 hours post dose for subcutaneous LMWH when prescribed for patients with renal impairment or pulmonary embolism (PE). This audit evaluates whether target concentrations of therapeutic LMWH and UFH were met in patients admitted to the Intensive Care Unit (ICU) and how monitoring was performed in critically ill patients compared to guideline recommendations.
**OBJECTIVES.** Standards set included 100% post-dose monitoring at or within time-limits for all patients - receiving UFH; with renal impairment; with PE. 100% reaching satisfactory anticoagulation - within 24 hours for an UFH infusion; by the end of ICU admission or last dose of UFH/LMWH.
**METHODS.** Data was collected retrospectively over a two-month period. Patients who received therapeutic doses of UFH and LMWH were included. Data collected included anonymised patient demographics, renal function, drug used, indications and monitoring.
**RESULTS.** 144 patients were screened, and 30 patients receiving therapeutic anticoagulation were included in the audit. 86.7% of patients exceeded their ideal body weight. 16.7% had anti-Xa levels or APTT ratios measured within trust-formulary limits. 50% reached therapeutic levels of anticoagulation. 44.4% of patients with PE did not have anti-Xa levels/APTT ratios measured at all. 22.2% reached therapeutic levels of anticoagulation for PE. 77.8% of UFH patients reached therapeutic levels of anticoagulation within 24 hours. None of the patients receiving once daily (OD) LMWH had Anti-Xa levels checked 4 hours post first dose. 14.3% of OD and 12.5% of patients receiving twice daily LMWH reached therapeutic levels of anticoagulation during their admission. 64.3% of patients with acute kidney injury, 20% with chronic kidney disease and 33.3% with both achieved satisfactory levels of anticoagulation.
**CONCLUSION.** Due to inconsistent monitoring, the level of anticoagulation remained uncertain in patients with renal impairment. Patient safety could have been compromised from over- or underdosing. Only 22.2% of PE patients reached therapeutic levels which could exacerbate symptoms. We conclude that tighter adherence of the recommended frequency and timing of monitoring is required to avoid over- or underdosing in critically ill patients.

**REFERENCE(S)**

1. Parker RI. Low-Molecular-Weight Heparin and Anti-Xa Targets in Critically Ill Children: Are We on Target With Our Target?*. Pediatr Crit Care Med 2014;15(7):679-681. Doi:10.1097/PCC.0000000000000185
2. Robinson S, Zincuk A, Strøm T, Larsen TB, et al. Enoxaparin, effective dosage for intensive care patients: double-blinded, randomised clinical trial. Crit Care 2010;14(2):R41. Doi:10.1186/cc8924
3. Dörffler-Melly J, de Jonge E, de Pont A-C, Meijers J, et al. Bioavailability of subcutaneous low-molecular-weight heparin to patients on vasopressors. Lancet 2002;359(9309):849-850. Doi:10.1016/S0140-6736(02)07920-5

### 000518

#### Incidence of Delirium amongst Neurosurgical and Non-Neurosurgical Patients in a Tertiary Trauma Centre

W. Flesher, A. Akbar, S. Kulkarni
Critical care unit, Royal Preston Hospital, Fulwood, United Kingdom
**Correspondence:** W. Flesher
*Intensive Care Medicine Experimental* 2019, **7(Suppl 3):**000518

**INTRODUCTION.** Delirium is a major cause of morbidity within the ICU environment1 and is associated with prolonged stay, longer time on mechanical ventilation2 and all-cause mortality3. In Royal Preston Hospital (RPH), a tertiary centre for neurosurgery in the Northwest of England, it has been anecdotally identified that neurosurgical patients represent a degree of delirium-related morbidity on the ICU disproportionate to the size of the group.
**OBJECTIVES.**

1. To quantify the incidence of delirium in the neurosurgical and non-neurosurgical ICU populations and
2. To audit compliance with existing Trust pathway for management of delirium.

**METHODS.** This was a retrospective audit of all admissions to ICU in RPH between 01/09/2018 and 30/11/2018. Patient notes were analysed to determine the primary reason for admission (whether neurosurgical or not), the number of days spent delirious based off the CAM-ICU assessment, the Richmond Agitation and Sedation Score (RASS), the type of delirium (hypoactive, hyperactive or mixed) and the treatment given (pharmacological or non-pharmacological).
**RESULTS.** 397 patients were included, comprising 346 non-neurosurgical and 51 neurosurgical patients. 100 (28.9%) non-neurosurgical patients and 29 (57%) neurosurgical patients had at least 1 delirious day on the ICU.
Neurosurgical patients represented 37.2% of all delirium days despite their small group size.
Further results of audit will be presented in the poster.
**CONCLUSION.** Neurosurgical patients have a disproportionately higher rate of delirium in this centre. We should consider neurosurgical patients to be an at-risk group for developing acute delirium and have a documented pharmacological and non-pharmacological plan as an anticipatory measure.
Our current statistics use delirium days as an outcome. This is a flawed statistic which is heavily skewed by neurosurgical long-stay patients. A different reporting measure is therefore required for ongoing audit purposes.

**REFERENCE(S)**

1. Ortega DG, Papathanassoglou E, Norris CM. The lived experience of delirium in intensive care unit patients: A meta-ethnography. Australian Critical Care. 2019 Mar 11. Robinson BR, Mueller EW, Henson K, Branson RD, Barsoum S, Tsuei BJ. An analgesia–delirium–sedation protocol for critically ill trauma patients reduces ventilator days and hospital length of stay. Journal of Trauma and Acute Care Surgery. 2008 Sep 1;65(3):517-26.

Chaiwat O, Chanidnuan M, Pancharoen W, Vijitmala K, Danpornprasert P, Toadithep P, Thanakiattiwibun C. Postoperative delirium in critically ill surgical patients: incidence, risk factors, and predictive scores. BMC anesthesiology. 2019 Dec;19(1):39.

#### 000602

#### The use of neuromuscular blocking agent infusion and titration of sedation in a teaching hospital critical care unit

L. Buswell, J. Hanison
Critical care, Manchester Royal Infirmary, Manchester, United Kingdom
**Correspondence:** L. Buswell
*Intensive Care Medicine Experimental* 2019, **7(Suppl 3):**000602

**INTRODUCTION.** Neuromuscular blocking agent (NMBA) infusions in critical care are frequently used in acute respiratory distress syndrome (ARDS) where they provide a mortality benefit in early severe ARDS (1). Administration of such infusions requires a careful approach; monitoring paralysis with peripheral nerve stimulators and a train of four twitches (TOF); and monitoring depth of anaesthesia using clinical judgement and monitors such as Bispectral Index Score (BIS) (2).

**OBJECTIVES.** Firstly, to study rate of use of TOF and BIS monitoring amongst patients receiving NMBA infusion. Secondly, to look at titration of sedation after commencing NMBA infusion and physiological signs that could suggest awareness under anaesthesia.

**METHODS.** A retrospective review was performed of all episodes of cisatracurium infusion in Manchester Royal Infirmary general adult intensive care during a 23 month period. Data was gathered on use of BIS and TOF, looking at titration of sedation and change in physiological parameters after commencing NMBA infusion.

**RESULTS.** 104 patients received NMBA infusion, 18 more than once, with 130 episodes of NMBA infusion included for analysis. BIS monitoring was used in 95% of infusions but TOF monitoring in only 10%. Sedation was reduced after commencing NMBA infusion in 62% of episodes. A trend was seen towards increased sympathetic stimulation in the sedation reduction group. Comparing the group with reduced sedation to the group without reduced sedation, an increase of ≥20% in heart rate was seen in 44% Vs 18% respectively.

Pupillary dilatation was seen in 40% Vs 22% respectively. Similar effects of lesser magnitude were seen with reduction in noradrenaline dose and increase in systolic blood pressure.

**CONCLUSION.** BIS monitoring was used frequently in the unit but use of TOF monitoring was suboptimal; this is of some concern given potential for variations in dose response amongst a heterogeneous critical care population. Sedation was reduced after commencing NMBA infusion in the majority of patients. Although these parameters are non-specific in isolation for predicting awareness, there was a trend towards increased sympathetic stimulation in patients in the sedation reduction group. Given uncertainty about BIS monitoring as a sole indicator of depth of anaesthesia amongst patients undergoing NMBA infusion (3), this raises the question of whether patients are being exposed to the risks of accidental awareness under anaesthesia. A protocol is suggested to combine the use of clinical assessment, BIS and TOF monitoring to safely administer cisatracurium infusions (Fig 1).

**REFERENCE(S)**

1) FICM/ICS guideline development committee. Guidelines on the Management of Acute Respiratory Distress Syndrome 2018.
2) Checketts MR et al. Recommendations for standards of monitoring during anaesthesia and recovery 2015 : AAGBI. Anaesthesia 2015;71:85–93.
3) Murray MJ et al. Clinical Practice Guidelines for Sustained Neuromuscular Blockade in the Adult Critically Ill Patient. Critical Care Medicine 2016;44:2079–103
4) None

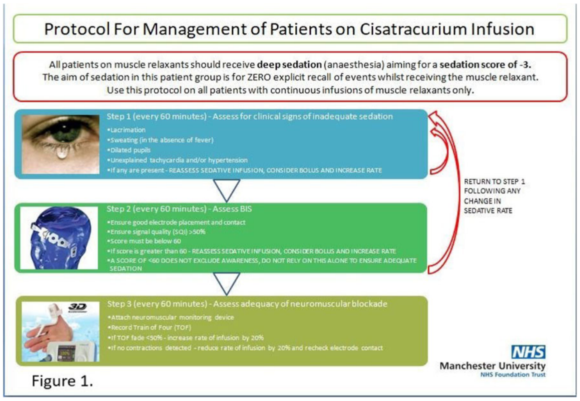

Figure 1.

**Fig. 1 (abstract 000602).** See text for description

## ARF - Acute respiratory failure 7

#### 000621

#### A review of low tidal volume ventilation and 28-day mortality in a UK Intensive Care Unit (ICU) in all mechanically ventilated patients

N. Clayden, A. Rochester, M. Alice, T. Samuels
Intensive care unit, East Surrey Hospital, Redhill, United Kingdom
**Correspondence:** N. Clayden
*Intensive Care Medicine Experimental* 2019, **7(Suppl 3):**000621

**INTRODUCTION.** Low tidal volume (LTV) ventilation in mechanically ventilated patients has been associated with decreased mortality and a decreased number of days of ventilator use in patients with acute respiratory distress syndrome (ARDS).[1, 2] The recommendation is an initial tidal volume of 6ml per kilogram of ideal body weight (IBW).

**OBJECTIVES.** To review the use of LTV ventilation in all mechanically ventilated patients and determine whether there is an association with maintenance of LTV ventilation in the initial 24 hours of ventilation and patient mortality.

**METHODS.** Data were collected retrospectively from all patients who were mechanically ventilated on the intensive care unit at a UK district general hospital over a period of one month (January 2019). Data were collected at regular intervals over the first 24 hours of ventilation. All ventilated patients were included in this study regardless of whether they had a diagnosis of ARDS or not. Patients with inadequate data or being ventilated with airway pressure release ventilation mode (APRV) were excluded. Outcome was measured as 28 day mortality. Statistical analysis was carried out using R version 3.5.3 (R Foundation).

**RESULTS.** Of the 32 patients included in this study 22 patients survived and 10 were deceased at 28 days. The mean age of patients who survived was 60.8 years, compared to the mean age of those who died which was 68.5 years. We found the mean and median ideal body weight (IBW) adjusted tidal volumes were 8.1 and 8.4 ml/kg respectively. The graph illustrates that there is no significant difference between TV (ml/kg) against 28 day mortality ($p = 1.0$ ). It highlights the similar medians between each group and a larger interquartile range in the group of patients that survived.

**CONCLUSION.** In conclusion, in this cohort of patients, during the first 24 hours of admission, we are ventilating slightly beyond the range recommended by ARDSNet of 6-8ml/kg tidal volume. However, we found no association between mortality and tidal volume. Limitations of this analysis include a small sample size. In addition, we have not assessed other aspects of lung protective ventilation, such as driving pressures and plateau pressures, which other studies have found to have an impact on mortality.[3] The original ARDSNet study compared LTV ventilation (6ml/kg of IBW) vs high tidal volume ventilation (12ml/kg of IBW).[1] The PREVENT trial compared LTV and intermediate tidal volume (10ml/kg) in patients without ARDS, finding no difference in the number of ventilator free days.[4] Further studies may be useful to assess the optimal tidal volume in different cohorts of patients, i.e. ARDS and non-ARDS.

**REFERENCE(S)**

1. The Acute Respiratory Distress Syndrome Network. Ventilation with lower tidal volumes as compared with traditional tidal volumes for acute lung injury and the acute respiratory distress syndrome. N Engl J Med 2000;342:1301–1308.
2. Putensen C, et al. Meta-analysis: ventilation strategies and outcomes of the acute respiratory distress syndrome and acute lung injury. Ann Intern Med 2009;151:566-76.
3. Amato MBP et al. Driving Pressure and Survival in the Acute Respiratory Distress Syndrome. N Engl J Med 2015; 372:747-755.
4. Simonis FD, et al. Effect of a Low vs Intermediate Tidal Volume Strategy on Ventilator-Free Days in Intensive Care Unit Patients Without ARDS-JAMA. 2018;320(18):1872-1880.

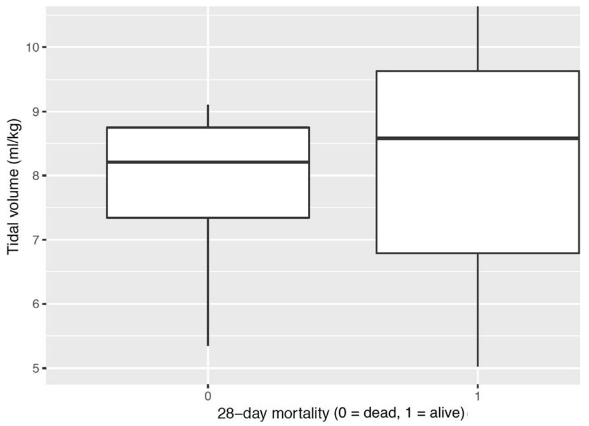

**Fig. 1 (abstract 000621).** See text for description

### 000635

#### Automatic Detection of Reverse Triggering: validation of the a dedicated software

T. Pham[1], J. Montanya[2], T. Piraino[3], R. Magrans Nicieza[2], IG. Telias[1], FL. Damiani[1], R. Coudroy[1], M. Madorno[4], R. Mellado Artigas[1], M. Dres[1], L. Chen[1], JJ. Gu[1], M. Rauseo[1], L. Blanch[2], L. Brochard[1]
[1]Keenan research centre, St Michael's hospital, Toronto, Canada; [2]Institut d'investigació i innovació parc taulí i3pt, Parc Taulí Hospital Universitari, Sabadell, Spain; [3]Department of respiratory therapy, St. Michael's Hospital, Toronto, Canada; [4]Bioengineering, Buenos Aires Institute of Technology, Buenos Aires, Argentina
**Correspondence:** T. Pham
*Intensive Care Medicine Experimental* 2019, **7(Suppl 3):**000635

**INTRODUCTION.** Reverse triggering (RT) is a diaphragmatic contraction triggered by an insufflation initiated by the ventilator. This patient-ventilator dyssynchrony is potentially harmful through increased tidal volume, breath stacking or eccentric diaphragm contraction. The diagnosis at bedside is challenging for the clinician and automatic monitoring seems required.

**OBJECTIVES.** We aimed at validating the accuracy of an automatic detection based on the analysis of ventilator waveforms, (pressure and flow) against a visual assessment using flow, Paw and esophageal pressure signals (Peso).

**METHODS.** An algorithm was created to detect RT based only on flow and airway pressure (Paw) signals. This algorithm has been developed in collaboration between data scientists of Better Care® and researchers of Dr. Brochard's laboratory in Toronto and Parc Tauli University Hospital, using recordings from the BEARDS study having esophageal pressure (NCT03447288).

Two researchers visually reviewed 16 recordings from 16 different patients with ARDS included in an observational physiologic study (BEARDS). Reverse triggering was defined by visual assessment as a patient effort starting less than 1.5 sec after a machine triggered breath and generating a Peso deflection ≥ 2cmH2O. This threshold was chosen has having a potential clinical relevance. The automated algorithm was based on flow and Paw and detected events in the same tracings without using esophageal pressure: we compared automatic detection to visual assessment (gold standard). Sensitivity, specificities, predictive values, accuracy were calculated.

**RESULTS.** We included recordings from 16 patients (mean±SD age of 61±12, SOFA 10±3, 63% male, 81% with pneumonia). At time of recording, they had been intubated for a median [IQR] of 4 [1-7] days and 93% were receiving sedation or analgesia (Midazolam: 56%; Propofol: 19%; opioid: 94%). Their total duration of mechanical ventilation was 9 [7-14] days and 63% were discharged alive from the ICU.

Sixteen recordings were assessed: under pressure control ventilation (n=6), volume control ventilation (n=6) and pressure support ventilation (n=4); 3735 breaths were evaluated of which 752 breaths (20.1%) were considered as RT by visual assessment. The automatic detection had a global accuracy of 95%: sensitivity of 85%, specificity of 97%, positive predictive value of 90% and a negative predictive value of 96%. Individual patients' tracing assessment showed an accuracy of the automatic detection ranging from 77% to 100%.

**CONCLUSION.** An automatic detection algorithm can reliably detect breaths with reverse triggering and a potential clinical relevance using only ventilator waveforms. This is an essential first step to describe and understand the clinical impact of this frequent patient-ventilator dyssynchrony.

**REFERENCE(S)**

1. European Society of Critical Care Medicine (ESICM) Young investigator award 2016
2. Society of Critical Care Medicine (SCCM) - Weil Research Grant 2017
3. Project RTC-2017-6193-1 from the Ministry of Science, Innovation and Universities (Spain)
4. Blanch L, Sales B, Montanya J, et al. Validation of the Better Care system to detect ineffective efforts during expiration in mechanically ventilated patients: a pilot study. Intensive Care Med 2012;38:772–80
5. Akoumianaki E, Lyazidi A, Rey N, et al. Mechanical ventilation-induced reversetriggered breaths: a frequently unrecognized form of neuromechanical coupling. Chest 2013;143:927–38

### 000641

#### A preclinical comparative study of aerosol deposition cartography in the respiratory tract under mechanical ventilation: impact of humidification and nebulizer position in the ventilator circuit

Y. Montigaud[1], A. Louf-Durier[2], Q. Georges[2], L. Leclerc[1], A. Clotagatide[3], N. Prevot[3], J. Pourchez[1], S. Perinel Ragey[2]
[1]Cis, Ecole des Mines de Saint-Étienne, Saint-Étienne, France; [2]Réanimation g, University Hospital of Saint-Etienne, Saint-Priest-en-Jarez, France; [3]Nuclear medicine, University Hospital of Saint-Etienne, Saint-Priest-en-Jarez, France
**Correspondence:** S. Perinel Ragey
*Intensive Care Medicine Experimental* 2019, **7(Suppl 3):**000641

**INTRODUCTION.** Nebulization is widely used in intensive care unit (ICU), by up to 99% of intensivists in an epidemiological study (1). However, even if vibrating mesh nebulizers are actually developed, efficiency of such administration route remain relatively poor, around 20% of nebulized dose under mechanical ventilation (MV) being deposited in the respiratory tract (2). Moreover, aerosol deposition is impacted by numerous factors such as gas humidification, position of the nebulizer in the circuit etc. Consequently, a preclinical *ex vivo* respiratory model was developed to study regional aerosol deposition under MV.

**OBJECTIVES.** To study the impact of humidification and vibrating mesh nebulizer position in the circuit in order to assess and then optimize lung deposition under MV.

**METHODS.** A preclinical *ex vivo* respiratory model was used, under controlled volumetric ventilation. Aerosols were performed with a vibrating mesh nebulizer (Aerogen Solo, Aerogen Ltd, Galway Ireland) with or without humidification. Experiments were performed at least in triplicate for each experimental condition. Without humidification, the nebulizer was placed just after the inspiratory valve. When heated humidifier was used, the nebulizer was placed either previous it or between endotracheal tube (ETT) and the Y piece adapter or 15 cm before this latter one. A quantitative 99mTc-DTPA scintigraphic study was then performed to determine nebulized fraction and deposited fractions on each part of the system and the circuit.

**RESULTS.** Efficiency of the nebulization was achieved with a mean nebulized dose higher than 95% of nominal dose. The respiratory tract deposited fraction of the nebulized dose varied considerably: 18±4% without humidification, and from 25±3% to 57±8% depending of the nebulizer position on the humidified ventilator circuit (*i.e.* 25±3% prior the humidifier, 43±11% between ETT and Y piece adapter and 57±8% upstream the Y piece adapter). These results are strongly correlated to change of aerosol droplet sizes for the different experimental conditions studied. Moreover, the aerosol distribution varied equally in the repartition between tracheal and pulmonary deposition but was not significantly different for the ETT deposition.

**CONCLUSION.** Nowadays, mesh nebulizers allow an increase in nebulized dose under MV, potentially leading to significant improvements of aerosol therapies. Consequently, determining factors as humidification and nebulizer position could have a higher impact on the delivered dose to the respiratory tract. This study showed very important differences in the dose delivered to the respiratory tract with potential significant impacts for patients in ICU.

**REFERENCE(S)**

(1) Ehrmann et al. Intensive care medicine 2010
(2) Dugernier et al Critical care 2015
(3) Ari et al. Respiratory care 2010
(4) Aerogen Ltd graciously provided the nebulizers but was not implicated in the study.

### 000659

#### The impact of introduction of a prone positioning protocol in patients with acute respiratory distress syndrome (ARDS) over an 8 month period in one adult district general hospital (DGH) intensive care unit (ICU)

J. Burns[1], T. Samuels[1], S. Ranjan[1], R. Phelan[1], T. Sanderson[2], M. Alice[3]
[1]Critical care, East Surrey Hospital, Redhill , United Kingdom; [2]Intensive Care Unit, East Surrey Hospital, London, United Kingdom; [3]Intensive care unit, East Surrey Hospital Emergency Room, Redhill, United Kingdom
**Correspondence:** J. Burns
*Intensive Care Medicine Experimental* 2019, **7(Suppl 3):**000659

**INTRODUCTION.** The use of prone positioning for patients with moderate to severe ARDS has been shown to improve oxygenation and survival (1). Despite evidence demonstrating benefit, many patients with ARDS do not undergo prone positioning - one large study found prone position was used in only 16.4% of eligible patients (2). Prohibitive reasons are likely to be multiple and include human factors and lack of a protocolised approach.

**OBJECTIVES.** To review the effect of introducing protocolised practise in prone positioning over an 8 month period in one DGH ICU.

**METHODS.** Following the results of a previous study (3) at the same unit, a protocol was introduced to guide timing and procedure for prone positioning. During the 8 months following the introduction of the protocol, data were collected regarding aetiology of ARDS, severity at initiation of prone position (determined by P/F ratio), arterial blood gas (ABG) results, outcome (Hospital Discharge/ITU Discharge/Death), demographics and adherence to protocol.

**RESULTS.** During the study, 11 patients underwent prone positioning (1.4 episodes per month). In the previous 2 year study (3), there were 14 patients (0.6 episodes per month). 2 patients from this study underwent a second episode of prone position soon after being returned to supine - i.e 13 episodes in total. 11 out of 13 cases (84.6%) were classified as severe ARDS with a P/F ratio of <13.3 kPa. The remaining 2 (15.4%) were moderate ARDS (13.3kPa-26.6kPa). The mean age was 55.3 (SD 10.4 years), 8 male and 3 female. The mean duration of prone position was 15.3 hours (SD 4.1 hours); the protocol targeted 16 consecutive hours of proning. Prone position was implemented as per protocol in 92.3% of cases and intra-proning checks (head turn etc.) were correct for 84.6% of proning episodes. Improvement in oxygenation after 6 hours of prone position was observed in 92.3% of cases. In terms of outcome, 7 (63.6%) patients survived to hospital discharge, 3 (27.3%) survived to ITU discharge and 1 (9.1%) died. In-hospital mortality was 36.6%, which compares with 50% observed in the previous study (3).

**CONCLUSION.** Introduction of a protocol has increased awareness and utilisation of prone positioning in our unit. Protocolisation has also yielded high rates of correct pre-prone and intra-proning checks, meaning there is standardisation of the way prone position is used within the unit. The improvements observed in oxygenation in this patient cohort echo what is also seen in the wider literature (1). Although a reduction in mortality was observed after the introduction of a protocol, given the size of this study this would not be statistically significant. Guidance in the protocol on when to initiate prone positioning may be useful, as the majority of patients (84.6%) underwent prone positioning when ARDS was severe and may have benefitted from earlier proning. A future study involving a larger cohort aiming to prone at an earlier, less severe stage would be useful and may demonstrate improvement in patient outcome.

**REFERENCE(S)**

1. Gattinoni L, Carlesso E, Taccone P, Polli F, Guerin C, Mancebo J. Prone positioning improves survival in severe ARDS: a pathophysiologic review and individual patient meta-analysis. Minerva Anestesiologica. 2010, 76(6): 448-454.
2. Guerin C, Beuret P, Constantin JM, Bellani G, Garcia-Olivares P, Roca O, Meertens JH, Maia PA, Becher T, Peterson J, Larsson A. A prospective international observational prevalence study on prone positioning of ARDS patients: the APRONET (ARDS Prone Position Network) study. Intensive care medicine. 2018, 44(1): 22-37.
3. Nwamarah AF, Lakhani RD, Myers AE, Ranjan S, Samuels TL. A retrospective observational study of prone positioning practices in the management of acute respiratory distress syndrome (ARDS) over a 2 year period in a single adult district general hospital (DGH) intensive care unit (ICU). Intensive Care Medicine Experimental. 2018, 6(2): 0433.

### 000661

#### Effect of Early versus Late Tracheostomy Post-Orthotropic Heart Transplant in Patients Requiring Prolonged Mechanical Ventilation

J. Dutton, M. Morosin, R. Fernandez Garda, M. Zaleska, A. Hurtado-Doce
Department of anaesthesia and intensive care, The Royal Brompton & Harefield N H S Trust, London, United Kingdom
**Correspondence:** J. Dutton
*Intensive Care Medicine Experimental* 2019, **7(Suppl 3):**000661

**INTRODUCTION.** Tracheostomy (TT) is performed in ITU patients requiring prolonged ventilation as a means to facilitate weaning. However, the optimal timing of TT has been debated. The TracMan RCT 2013 failed to demonstrate beneficial effects of early TT on 30-day mortality or ITU length of stay. Orthotopic heart transplant (OHT) patients are often complex and require prolonged mechanical ventilation, yet there is limited data available for this cohort. We wanted to see if there was a benefit to early TT in heart transplant patients who were likely to require prolonged mechanical ventilation.

**METHODS.** We retrospectively reviewed 153 consecutive patients who underwent OHT in our centre between 2013 and 2018. 47 patients required TT. We compared the baseline factors and outcomes of those who underwent early-TT(<10days) versus those who underwent late TT (>10days).

**RESULTS.** Over one third of OHT patients required TT (TT group; n= 47; 31.5%) during their ITU stay. Risk factors for tracheostomy were previous stroke/TIA (OR 3.52;95% 1,41-8.79), left ventricle assist device support prior to OHT (OR2.14; 95% 1.06-4.34; p=0.034), cardiopulmonary bypass time (OR 1.1;95%; 1-1.01; p=0.022). Other baseline factors were normal.

20/47(42.6%) patients underwent early TT, whilst 27/47(57.4%) patients underwent late TT. Mean time from ITU arrival to TT was 6.5± 2.4 days in the early group versus 15.3±6.3 days in the late group(p< 0.001). There were no life-threatening complications related to procedure. Early-TT was more common in female patients with 90% of early-TT being done in female patients(p=0.006), but there were no other differences in baseline factors. Patients who developed primary graft failure were significantly more likely to have a TT, 14.7% versus 51.1%(p<0.001), and it was more likely to be performed late p<0.001. However, total duration of mechanical ventilation (25.4±38.4 vs 35.4± 24.7days; p=0.284), and ITU length of stay was also comparable (early-TT 27.5±27 days vs late-TT 38.1±19.5 days; p=0.122). All-cause early mortality within 30-days of OHT was comparable between early-TT 15% vs Late-TT 7.4% (p=0.438). Although there was a trend to increased 1-year survival in the early-TT group versus the late-TT group, this wasn't significant (25% vs 48.2%; p=0.104).

**CONCLUSION.** The timing of TT in critically unwell patients has been debated, and previous trials have failed to show major benefits to favour early TT. In comparing early-TT versus late-TT, we have found that female patients are more likely to have early-TT, whilst developing PGF would appear to be a factor favouring a decision for late-TT. Despite these differences, there was no difference in over duration of mechanical ventilation, or short-term survival outcomes. Though early-TT appears to show a trend towards improved 1-year survival, the sample size may have been too small to demonstrate significance.

**REFERENCE(S)**

1. Young D, Harrison DA, Cuthbertson BH, Rowan K, TracMan Collaborators. Effect of early vs late tracheostomy placement on survival in patients receiving mechanical ventilation: the TracMan randomized trial. JAMA. 2013 May 22;309(20):2121–9.

### 000726

#### Improved gas exchange and lung aeration in flow-controlled ventilation (FCV) compared to pressure-controlled ventilation (PCV) – A prospective, randomized porcine study

P. Spraider[1], J. Abram[1], G. Putzer[1], B. Glodny[2], J. Martini[1]
[1]Department of anaesthesia and general intensive care medicine, Medizinische Universität Innsbruck, Innsbruck, Austria; [2]Department of radiology, Medizinische Universität Innsbruck, Innsbruck, Austria
**Correspondence:** P. Spraider
*Intensive Care Medicine Experimental* 2019, **7(Suppl 3):**000726

**INTRODUCTION.** Ventilator-induced lung injury significantly increases patient morbidity and mortality. To reduce the underlying pathophysiological mechanisms different ventilation strategies are applied. Flow-controlled ventilation (FCV) is a new ventilation mode to establish a continuous, stable flow throughout the ventilation cycle resulting in a slow and steady change of intrapulmonary pressure during in- and expiration, to minimize applied and also dissipated energy (both related to lung injury) and to individualize ventilation based on measurements of dynamic lung mechanics [1].

**OBJECTIVES.** Aim of this porcine study was to investigate FCV compared to best "standard of care" pressure-controlled ventilation (PCV) with corresponding lung-protective ventilator settings.

**METHODS.** Animals were randomly assigned to FCV or PCV. FCV (with Evone®, Ventinova, Eindhoven, The Netherlands) was established at compliance-guided pressure settings (PEEP and peak), fixed FiO2 of 0.3 and flow adjusted to maintain normocapnia (paCO2 = 35-45 mmHg) at an I:E ratio of 1:1. PCV ventilation (with Evita XL®, Dräger, Lübeck, Germany) was performed with at least a PEEP of 5 mbar adjusted to transesophageal pressure, peak pressure set to achieve a tidal volume of 6 - 8 ml/kg, fixed FiO2 of 0.3 and respiratory rate adjusted to maintain normocapnia at an I:E ratio of 1:1.5. After ventilation for 10 hours a CT-scan was carried out in order to visualize and quantify lung tissue aeration by Hounsfield unit analysis.

**RESULTS.** 12 pigs were enrolled in the study (FCV and PCV n=6). Baseline characteristics were comparable in both groups. Animals in the FCV group showed a significant reduction of respiratory minute volume needed to maintain normocapnia (6.0±0.5 vs. 12.7±0.8 l/min,p<0.0001; fig. 1) thereby achieving significantly better oxygenation (paO2 = 120.0±6.1 vs. 96.6±7.4 mmHg, p=0.0097; fig. 2) compared to the PCV group. CT scans of FCV animals obtained at the end of the experiment showed a significantly higher portion of normally ventilated lung tissue (73.7±3.9 vs. 68.1±5.1 %, p=0.0068) compared to PCV animals.

**CONCLUSION.** In this porcine study FCV significantly improved gas exchange and maintained better aeration of lung tissue.

**REFERENCE(S)**

[1] Barnes T, van Asseldonk D, Enk D: Med Hypotheses 2018;121/Dec.:167-76

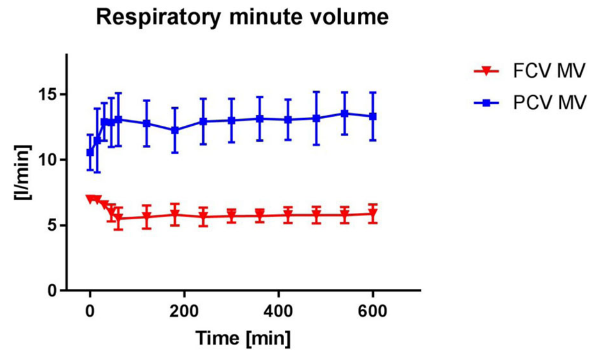

**Fig. 1 (abstract 000726).** See text for description

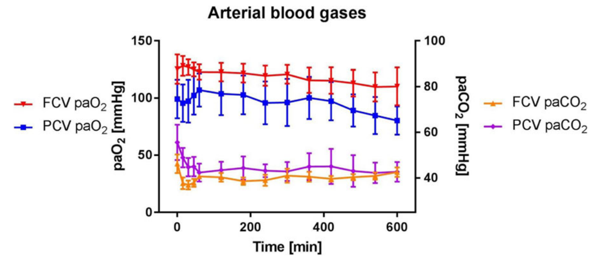

**Fig. 2 (abstract 000726).** See text for description

### 000730

#### Indices of diaphragm ultrasound during voluntary and involuntary cough and extubation outcome: A final report

T. Santanda[1], N. Yasuhiro[1], N. Tadanori[1], F. Yoshihisa[1], T. Shizuka[1], K. Jun[2], S. Shinjiro[3], F. Shigeki[4]
[1]Emergency and critical care medicine, Tokyo Bay Urayasu Ichikawa Medical Center, Urayasu, Japan; [2]Intensive care medicine, Nerima Hikarigaoka Hospital, Nerima City, Japan; [3]Department of anesthesiology, The Jikei University Hospital, Minato City, Japan; [4]Emergency and critical care medicine, St. Marianna University School of Medicine, Kawasaki, Japan
**Correspondence:** T. Santanda
*Intensive Care Medicine Experimental* 2019, **7(Suppl 3):**000730

**INTRODUCTION.** Assessing the ability to clear secretions and protect airway with effective cough is an important part of pre-extubation evaluation in patients who have passed spontaneous breathing trial (SBT). We hypothesized that passive cephalic excursion of diaphragm, peak velocity of the diaphragm, or both during cough expiration measured by ultrasound might predict extubation outcome.
**OBJECTIVES.** The aims of this study are 1) to identify associations of the ultrasonographic indices with simultaneously measured cough peak expiratory flow (CPEF) and 2) to investigate the predictive values of the ultrasonographic indices for extubation outcome in patients who have passed SBT.
**METHODS.** A total of 323 mechanically ventilated patients who passed SBT were enrolled in the study. Diaphragm excursion and max velocity were measured during voluntary and involuntary cough by ultrasound in the supine position. CPEF was also measured using the ventilator flow waveforms simultaneously with the ultrasonographic measurement.
**RESULTS.** Diaphragmatic excursion during voluntary cough has a significant correlation with CPEF (r=0.491, p<0.001). The areas under the curves (AUCs) of diaphragmatic excursion and CPEF during voluntary cough for extubation failure are 0.753 (95% CI 0.623 - 0.894, cutoff value 1.6 cm) and 0.715 (95% CI 0.54 - 0.89, cutoff value 50 L/min) respectively. There was no significant difference in predictive accuracy between diaphragmatic excursion and CPEF (p=0.61). Diaphragmatic excursion during involuntary cough or peak velocity of the diaphragm during voluntary or involuntary cough did not predict extubation outcome.
**CONCLUSION.** Passive cephalic excursion of the diaphragm during voluntary cough measured by ultrasound is as accurate a predictor of extubation outcome as CPEF in patients who have passed SBT.

### 000752

#### Measurement of blood recirculation and adjustment of V-V ECMO blood flow – preliminary data of a prospective clinical trial (BRAVVE)

M. Ruß, L. Seesko[1], T. Busch[2], V. Skrypnikov[1], W. Boemke[1], E. Swenson[3], S. Weber-Carstens[1], R. Francis[1], P. Pickerodt[1]
[1]Department of anesthesiology and intensive care medicine, Charité - Universitätsmedizin Berlin, Berlin, Germany; [2]Department of anesthesiology and intensive care medicine, University of Leipzig Medical Faculty, Leipzig, Germany; [3]Division of pulmonary critical care and sleep medicine, University of Washington, Seattle, United States of America
**Correspondence:** M. Ruß
*Intensive Care Medicine Experimental* 2019, **7(Suppl 3):**000752

**INTRODUCTION.** Veno-venous extracorporeal membrane oxygenation (V-V ECMO) can support arterial oxygenation in case of refractory hypoxemia. With total ECMO blood flow (QB) being delivered through the ECMO return cannula, the fraction of QB recirculating into the drainage cannula constitutes the recirculation fraction (R*f*), which does not contribute to systemic oxygenation and has to be substracted from QB to determine the effective extracorporeal flow (QEFF; QEFF = QB – R*f* x *QB*) [1]. Routine calculation of R*f* is cumbersome and requires mixed venous blood samples. A new ultrasound technique supports bedside measurements of Rf without the need for a pulmonary artery catheter [2].
**OBJECTIVES.** We investigated whether the bedside quantification of R*f* enables the optimization of ECMO blood flow (QB). Theoretically, QB can be reduced by R*f* without compromising systemic oxygenation. Reduction of QB can minimize adverse effects related to very negative inlet pressures like hemolysis or vessel damage.
**METHODS.** In a prospective clinical trial (ClinicalTrials.gov Identifier: NCT03200314), we measured R*f* in patients on V-V ECMO suffering acute respiratory distress syndrome (ARDS) using a saline ultrasound dilution technique. In the case that R*f* was greater than 10%, QB was reduced by the volume flow of R*f*. Following a safety protocol, reductions of QB were stopped when pulse oximetry saturation (SpO2) fell below 90%. Likewise, QB was increased when SpO2 fell below 88%.
**RESULTS.** Eighteen patients were examined (52 ± 16 years old, 2 ± 1 days on ECMO; mean ± SD). Mean QB was 4 ± 0.7 l/min with a R*f* of 15 ± 9 % (0.7 ± 0.48 L/min). R*f* exceeded 10% of QB in 11 patients (21 ± 7%, 0,9 ± 0.46 L/min). QB was reduced in 7 of these 11 patients by 0.6 ± 0.17 L/min (to 3.3 ± 0.77 L/min; p < 0,05) resulting in a new R*f* of 10 ± 5 %. QB reduction resulted in a decrease in arterial oxygenation (before reduction: SpO2 98 ± 2 %, arterial partial pressure of oxygen, PaO2 101 ± 24 mmHg; post reduction: SpO2 96 ± 3 %, PaO2 85 ± 23 mmHg; p < 0.05). Despite a R*f* > 10%, QB was not reduced in 3 patients with SpO2 < 90% and increased in one patient with SpO2 < 88%.
**CONCLUSION.** Ultrasound-based estimation of blood recirculation in a V-V ECMO setting provides an objective measure supporting the reduction of recirculation to optimize ECMO blood flow within safe limits of arterial oxygenation.

##### REFERENCE(S)

1. Abrams D, et al. Recirculation in venovenous extracorporeal membrane oxygenation. ASAIO J. 2015 Mar-Apr;61(2):115-21
2. Darling EM, et al. Use of dilutional ultrasound monitoring to detect changes in recirculation during venovenous extracorporeal membrane oxygenation in swine. ASAIO J. 2006 Sep-Oct;52(5):522-524
3. This study was supported by a grant from the Deutsche Forschungsgemeinschaft to P. Pickerodt and W. Boemke (Pi795/2-2).

### 000782

#### Monitoring respiratory drive during mechanical ventilation from tracheal pressure

JA. Benitez Lozano[1], P. Carmona Sánchez[2], M. Delgado Amaya[1], JM. Serrano Simón[2]
[1]Intensive care medicine, Hospital Regional Universitario de Málaga, Málaga, Spain; [2]Intensive care medicine, Reina Sofia University Hospital, Córdoba, Spain
**Correspondence:** P. Carmona Sánchez
*Intensive Care Medicine Experimental* 2019, **7(Suppl 3):**000782

**INTRODUCTION.** Lung and diaphragmatic injury can be related to an unsuitable respiratory drive (1).
It's usually handled by the airway occlusion pressure or by the esophageal pressure (2).
**OBJECTIVES.** To assess the usefulness of tracheal pressure (Ptrach) to monitoring the respiratory drive.
**METHODS.** We studied 18 patients under pressure support ventilation with different levels of assistance and a maximum sensitivity of the inspiratory trigger. Esophageal (Pes), airways pressure (Paw), Ptrach and flow were registered at 560 Hz. A total of 151 cycles were analyzed. Ptrach was measured using a specific dedicated Intratracheal catheter. P100 was measured in Paw between 0 and 300 milliseconds (ms) after occlusion, selecting the segment with greater slope, at 100 ms. The cycles prior to occlusion were analyzed to measure the Ptrach 100, from nadir to backwards selecting the greater slope segment.
The results are expressed as mean±SD, median (IRQ), or percentage. The comparisons by t-student. Bland-Altman and linear regression analyses were performed.
**RESULTS.**
**CONCLUSION.** Tracheal pressure may be useful for continuous monitoring of respiratory drive. The good fit agrees as the respiratory drive increases.

##### REFERENCE(S)

1, Brochard L. Slutsky A. Pesenti A. Mechanical ventilation to minimize progression of lung injury in acute respiratory failure. Am J Respir Crit Care Med 2017;195:438-442.

2, Whitelaw WA, Drenne JP, Milic-Emili J. Occlusion pressure as a measure of respiratory center output in conscious man. Respiratory Physiology 1975; 23, 181-199.

**Table 1 (abstract 000782).** Agreement of the measurements of P100 between both methods

| P100 ms, cmH2O | | Means (SD) | P | Means Difference (SD | Limits agreement, CI 95% | R2 |
|---|---|---|---|---|---|---|
| All Data | Tracheal Pressure<br>Occlusion Pressure | 2.74 (1.89)<br>2.89 (1.88) | 0.486 | -0.15 (0.41) | -0.98 to 0.67 | 0.95 |
| Relate to > median airway occlusion pressure (1.716 cmH2O) | Tracheal Pressure<br>Occlusion Pressure | 4.50 (1.17)<br>4.58 (1.32) | 0.695 | -0.08 (0.58) | -1.23 to 1.07 | 0.81 |
| Relate to airway occlusion pressure < median (1.716 cmH2O) | Tracheal Pressure<br>Occlusion Pressure | 1.08 (0.25)<br>1.28 (0.24) | < 0.001* | -0.195 (0.20) | -0.6 to 0.21 | 0.44 |

### 000789

#### "Alveolar Tidal Flooding" – a new concept addressing edema liquid shear stress in ventilator-induced lung injury

J. Matuszak, A. Tabuchi, J. Grune, WM. Kuebler
Institute of physiology, Charité – Universitätsmedizin Berlin, Berlin, Germany
**Correspondence:** J. Matuszak
*Intensive Care Medicine Experimental* 2019, **7(Suppl 3):**000789

**INTRODUCTION.** While mechanical ventilation is a mainstay of therapy for the acute respiratory distress syndrome (ARDS), it can simultaneously trigger adverse complications, most prominently ventilator-induced lung injury (VILI). This fatal association necessitates the need for optimized ventilation strategies, and for a better understanding of alveolar dynamics and the mechanisms underlying alveolar epithelial injury in the mechanically ventilated, injured and edematous lung.
**OBJECTIVES.** In previous studies, we observed a variety of abnormal dynamics (alveolar dyskinesias) including pendelluft, alveolar stunning and inversed ventilation, but never anatomical collapse and reopening of alveoli [1, 2]. Therefore, we propose that during ventilation of injured lungs, edema fluid will – according to LaPlace´s law - cyclically shift in and out of the alveolus as a function of ventilation pressure. We suspect that the cyclic flooding of alveoli with edema liquid, termed by us "alveolar tidal flooding" (ATF), rather than anatomical opening-and-collapse of alveoli contributes critically to the development of alveolar damage during VILI by exerting excessive shear stresses upon the alveolar epithelium.
**METHODS.** We tested this concept by direct visualization of alveolar dynamics with lung intravital microscopy (IVM) in a murine model of alveolar edema [3]. Onset, extent, and time course of ATF in injured lungs as well as the cyclic fluid shifts in and out of the alveolar space will be analyzed under a range of ventilation settings with variation of IPP, PEEP, and driving pressure (ΔP). Further the cellular damage exerted by ATF will be assessed with characteristic markers.
**RESULTS.** ATF was detectable in fluid-filled, yet not in control lungs. ATF frequency increased as a function of inspiratory plateau pressure (IPP) at PEEP = 0 cmH20, and decreased in turn with higher PEEP. We compared the effects of three ventilation settings with different IPP and PEEP values (IPP /PEEP: 18/1; 24/7; 30/13 cm H2O) but same driving pressure in a mouse model of edema. In line with the driving pressure hypothesis, no significant differences in wet-to-dry weight ratio, oxygen saturation, serum pH and lung compliance were detected between groups. Further studies comparing different driving pressure, as well as flooded and unflooded lungs are presently ongoing.
**CONCLUSION.** We think understanding of this physiological principle will have direct impact on clinical care in terms of optimized ventilation strategies to prevent ATF.

**REFERENCE(S)**
1. Mertens et al.,Crit Care Med, 2009.
2. Tabuchi et al., Am J Respir Crit Care Med, 2016.
3. Tabuchi et al., J Appl Physiol (1985), 2008.

### 000805

#### Necroptosis activation distinguishes hypo- and hyper-inflammatory ARDS endotypes

R. Handslip[1], S. Mumby[2], C. Calfee[3], K. Delucchi[4], C. O'kane[5], D. Mcauley[5], M. Takata[6], B. Patel[7]
[1]Anaesthetics, pain medicine & intensive care, surgery and cancer, Imperial College London, London, United Kingdom; [2]Airways disease section, national heart and lung institute, Imperial College London, London, United Kingdom; [3]Cardiovascular research institute, University of California, San Francisco, United States of America; [4]Department of psychiatry, University of California, San Francisco, United States of America; [5]Centre for infection and immunity, Queen's University Belfast, Belfast, United Kingdom; [6]Anaesthetics, pain medicine and intensive care, surgery and cancer, Imperial College London, London, United Kingdom; [7]Department of anaesthesia and intensive care, Royal Brompton and Harefield NHS Foundation Trust, London, United Kingdom
**Correspondence:** R. Handslip
*Intensive Care Medicine Experimental* 2019, **7(Suppl 3):**000805

**INTRODUCTION.** Two distinct hypo- and hyper-inflammatory sub-phenotypes have been identified in patients with ARDS. The hyper-inflammatory sub-phenotype is associated with fewer non-pulmonary organ failure free days and increased mortality 1. Necroptosis is a form of programmed cell necrosis mediated through activation of receptor interacting serine/threonine kinase-3 (RIPK3) signalling. Furthermore, necroptosis is associated with greater degree of inflammation through extensive cell lysis and release of damage associated molecular patterns, leading to more severe systemic inflammation.
**OBJECTIVES.** To establish if plasma markers of necrosis and necroptosis are different between the sub-phenotypes in ARDS in the placebo arm of the HARP-2 cohort 2. HARP-2 was a randomised, controlled clinical trial evaluating simvastatin in patients with ARDS.
**METHODS.** We measured RIPK3 (necroptosis marker) and Cytokeratin-18 (CCK18, epithelial necrosis marker) by Sandwich ELISA (Cusabio & Cytokeratin-18, M65 PEVIVA respectively) in blinded samples from 30 patients (N=15 per endotype as defined at ARDS diagnosis) from the HARP-2 clinical trial over a 14-day time course.
**RESULTS.** Levels of RIPK3 and CCK18 were significantly higher in the plasma of the hyper-inflammatory ARDS endotype as compared with the hypo-inflammatory endotype. This elevation was maintained across 14 days.
**CONCLUSION.** We found that markers of necroptosis and epithelial necrosis were raised in patients with ARDS with a hyper-inflammatory endotype. These data implicate necroptosis in the development of the hyper-inflammatory endotype of ARDS. This pathobiological mechanism may provide an opportunity for pharmacological blockade and improved outcomes in this hyper-inflammatory cohort.

**REFERENCE(S)**
1. Calfee CS, Delucchi KL, Sinha P, et al. Lancet Respir Med. 2018 Sep;6(9):691–8. 2. McAuley DF, Laffey JG, O'Kane CM, et al. N Engl J Med. 2014 Oct 30;371(18):1695–703.
2. McAuley DF, Laffey JG, O'Kane CM, et al. N Engl J Med. 2014 Oct 30;371(18):1695–703
3. ESICM Basic Research Award to Dr Brijesh Patel

**Table 1 (abstract 000805).** Data shown as median with interquartile range for epithelial necrosis and necroptosis markers. Comparisons were made between hyper-inflammatory vs hypo-inflammatory groups using Mann Whitney U. N = 15 per group. (**p<0.001 * p<0.02)

| Epithelial Necrosis Cytokeratin-18 (Picograms) | | | Necroptosis RIPK3 (Picograms) | | |
|---|---|---|---|---|---|
| | Hyper-inflammatory | Hypo-inflammatory | | Hyper-inflammatory | Hypo-inflammatory |
| D0 | 1344<br>1496 | 392.1**<br>546.8 | D0 | 8301<br>13791 | 5910<br>9677 |
| D3 | 1471<br>936 | 485.8 **<br>1179.8 | D3 | 9978<br>7596 | 5979**<br>4617 |
| D7 | 1776<br>1772 | 470**<br>1074 | D7 | 17674<br>1244 | 6062**<br>5625 |
| D14 | 1210<br>957.6 | 378.8 *<br>909.6 | D14 | 20361<br>14105 | 7316**<br>5888 |

### 000837

#### Safety of Laryngeal mask airway versus endotracheal tube for bronchoscope guided percutaneous dilatational tracheostomy in critically ill adult patients

K. Tsikritsaki, G. Koukoulitsios, E. Koutrouba, D. Belesiotis, S. Bakouli, M. Anifanti, D. Toumpanakis, I. Tsoni, EM. Papadimitriou, C. Mandila, N. Panagiotopoulou, L. Avramopoulou, A. Dafni, I. Poularas, V. Koutsoukou, V. Romanou, S. Antonopoulou, M. Gianoulopoulou, K. Tsironas, A. Kalogeromitros
Icu, G.Gennimatas General Hospital, Athens, Greece
**Correspondence:** K. Tsikritsaki
*Intensive Care Medicine Experimental* 2019, **7(Suppl 3):**000837

**INTRODUCTION.** Long-term mechanical ventilation is the most common situation where tracheostomy is indicated for patients in Intensive Care Units (ICU). Percuteneous dilatational tracheotomy (PDT) was first described in 1985 and now is a well-established procedure that can be performed at the bedside by an intensivist with less surgical equipment required.

**OBJECTIVES.** The use of a laryngeal mask airway was compared with the existing endotracheal tube in terms of the safety, effectiveness and duration of the procedure. Our aim was to compare the safety and effectiveness of bronchoscope guided percutaneous tracheostomy performance via endotracheal tube (ETT) vesrus Laryngeal Mask Airway (LMA) in critically ill adults patients

**METHODS.** 287 patients 18-83 yrs of age, 132 females and 155 males with body mass index 31±6 kg/m² underwent PDT with bronchoscopy assistance due to prolonged endotracheal intubation between December 2009 and March 2019. The procedures of percuteneous dilatation tracheotomy with guide wire dilator forceps (GWDF) were done bedside with bronchoscopic guidance under general anaesthesia in the (ICU). Operative and post operative complications were observed. In 57 patients (23 females and 29 males) the ETT tube was changed to LMA 20 min before the procedure. The rest of the patients underwent PDT via the pre-existing ETT. Bronchoscope was used in both groups during procedure.

**RESULTS.** Overall complication rate was low and occurred in 56 patients, there was no procedure-related mortality. 18 accidental extubations were noted in PDT via ETT group. Subcutaneous emphysema without pneumothorax occurred in twelve patients ( 2 in LMA group and 10 in ETT group), nine patients had transient hypotension related to sedation (1 in LMA group and 8 in ETT group) and seventeen patients had peristomal oozing (3 in LMA group and 14 in ETT group). The mean time for procedure completion was 15 minutes for the EET group and 12 minutes for the LMA group, and no patient required conversion to surgical tracheotomy

**CONCLUSION.** Use of LMA seems to shorten the duration of the procedure with better visibility field through bronchoscope and this shortens the period during which the airway is insecure. On the basis of the limited number of patients investigated it is not possible to draw any conclusions as to which procedure is superior in terms of the likelihood of occurrence of mortality and adverse events

### 000842

#### CORRELATION OF DIAPHRAGMATIC DISPLACEMENT WITH Pdi AND Pes VALUES IN PATIENTS DURING A T-PIECE WEANING TRIAL

D. Smyrniotis, A. Kefalidou, E. Soilemezi, E. Koco, M. Tsagourias, D. Matamis
Icu, Papageorgiou General Hospital, Thessaloniki, Greece
**Correspondence:** E. Soilemezi
*Intensive Care Medicine Experimental* 2019, **7(Suppl 3):**000842

**INTRODUCTION.** Measurement of esophageal pressure (Pes) allows quantification of respiratory muscle activity, and transdiaphragmatic pressure (Pdi) represents a specific measure of diaphragm contractility. However, acquisition and interpretation of the relevant waveforms remains a complex procedure, limiting wide implementation of the technique in everyday ICU clinical practice. Ultrasonography, on the other hand, is an increasingly popular tool in the diaphragmatic assessment of ICU patients. However, no association has so far been described between Pes or Pdi and sonographically acquired indexes of diaphragmatic function, such as diaphragmatic displacement (DD).

**OBJECTIVES.** The purpose of this study was to investigate a possible correlation between Pes and Pdi values with diaphragmatic displacement, in patients during a T-piece weaning trial.

**METHODS.** In fifteen patients undergoing a T-piece weaning trial, we recorded diaphragmatic displacement along with simultaneous Pdi and/or Pes recording. When assessing displacement along with Pdi recording, we studied two consecutive stages: i) breathing normally, and ii) breathing normally with performance of sniff-like maneuvers. When assessing displacement along with Pes recording, we again studied two different breathing conditions: i) breathing normally, and ii) breathing with resistances of 40cmH2O/L.

**RESULTS.** A total of 1360 breaths were studied at all different breathing conditions (approximately 350 breaths/study condition). Statistical significant correlations were observed in all stages. Pearson correlation coefficient for the Pdi – DD relationship was r=0.57 (p< 0.001) and r=0.67 (p<0.001) for normal breathing and breathing with sniff-like maneuvers respectively. Pearson correlation coefficient for the Pes – DD relationship was r=0.65 (p<0.001) and r=0.8 (p<0.001) for normal breathing and breathing with resistances respectively.

**CONCLUSION.** Our results suggest a statistically significant correlation between the amplitude of diaphragmatic motion and the derived changes in esophageal and transdiaphragmatic pressures. More research is needed to confirm the potential of this non-invasive index of diaphragmatic function to provide information once considered strictly provided by esophageal pressure catheters.

### 000856

#### Estimation of alveolar pressure from continuous monitoring of tracheal pressure during pressure support ventilation

P. Carmona Sánchez[1], JA. Benitez Lozano[2], M. Delgado Amaya[2], JM. Serrano Simón[1]
[1]Intensive care medicine, Reina Sofia University Hospital, Córdoba, Spain; [2]Intensive care medicine, Hospital Regional Universitario de Málaga, Málaga, Spain
**Correspondence:** P. Carmona Sánchez
*Intensive Care Medicine Experimental* 2019, **7(Suppl 3):**000856

**INTRODUCTION.** The evaluation of muscular effort, measured by PTP and Wob, during mechanical ventilation, requires the use of esophageal pressure, scarcely used in clinical practice. We evaluated a new method based on the tracheal pressure, easier to use and, more convenient than esophageal.

**OBJECTIVES.** Assess the usefulness of tracheal pressure (Ptrach) to monitoring Wob and PTP.

We are designed a method for getting muscular inspiratory effort from tracheal pressure, based on respiratory mechanic related to resistances of the endotracheal tube (Rett) and Elastance of the respiratory system (Ers) obtained from the time constant (τ). A new signal is generated as Alveolar pressure (P Alv), by subtraction from the tracheal pressure the product of the instantaneous volume and Ers.

**METHODS.** We have studied 12 patients mechanically ventilated during pressure support ventilation (PSV) at three different levels of assistance. We recorded the signals of Flow (V'), Airway pressure (P Aw), Esophageal pressure (P Eso), Tracheal pressure (P Trach, sampling at 561 Htz. A total of 310 cycles were selected. Pressure drop across the ETT (DPETT) was obtained by subtraction of P trach to Paw. The ratio DPETT change to the corresponding change in flow was calculated to obtain the ETT resistance (Rett) using linear regression. Time constant (τ) was determined by exponential regression from exhaled volume. Ers was calculated as the ratio of the Rett to τ. The work of breathing (WOB, J/L) and effort as product pressure-time product (PTP, cmH2O*s/min) were calculated, according to the P Eso and P Alv. The results are expressed as mean±SD, the comparisons by t-student; the relationship and agreement by linear regression and Bland-Altman analysis.

**RESULTS.**

**CONCLUSION.** This method using tracheal pressure is a good approach to continuous monitoring muscular effort.

**Table 1 (abstract 000856).** Agreement of the measurements between both methods.

| *All Data* | Means (SD) | Means Difference | Limits agreement, CI 95% | Regression equation | R |
|---|---|---|---|---|---|
| WOB_Peso vs WOB_P Alv *(J/L)* | 0.26 (0.12) 0.20 (0.09) | -0.05 (0.07) | -0.19 to 0.08 | WOB P Alv = 0.0455 + (0.613 * WOB Pes+) | 0.83 |
| PTP Peso vs PTP P Alv *(cmH2O*s/min)* | 92.72 (34.56) 72.59 (27.76) | -20.12 (23.21) | -66.53 to 26.29 | Ptp/min Av = 17.236 + (0.597 * Ptp/min Esof) | 0.74 |
| Delta Peso vs Delta P Alv *(cmH2O)* | 7.07 (2.85) 5.61 (2.09) | -1.47 (2.39) | -6.25 to 3.32 | D PAlv = 2,655 + (0,417 * D Pes) | 0.57 |

### 000867

#### Distribution of Lung Involvement Correlated with Response of Prone Positioning and Mortality in Acute Respiratory Distress Syndrome

C. You-Yi, R. Sheng-Yuan, JY. Chien
Internal medicine, National Taiwan University Hospital, Taipei, Taiwan
**Correspondence:** J.Y. Chien
*Intensive Care Medicine Experimental* 2019, **7(Suppl 3):**000867

**INTRODUCTION.** Prone position reduces mortality among patients with moderate to severe acute respiratory distress syndrome. We aimed to evaluate whether the radiographic extent in high resolution computed tomographic (CT) has predictive value for prone positioning response and its role in outcomes prediction.

**METHODS.** This study was conducted in a medical center from October 2014 to June 2016. Patients with moderate to severe acute respiratory distress syndrome underwent prone-position were included. Each lung was divided into three representative levels (apex, hilum and base lung) and each levels was divided into three sections (sternal, central and vertebral part). Each area was rated on a six-point scale of 0–5 for the extent of consolidation or ground glass opacity (GGO). Prone-responders are patients whose PaO2/FiO2 ratio (PF ratio) increases by at least 20% or by ≧ 20mmHg.

**RESULTS.** Among 52 patients, 34 (65.4%) patients was prone responders and 18 (34.6%) patients were non-responsive. The consolidation difference between dorsal and ventral section at basal lung level was higher in prone responder than non-responder (5.3±2.6 vs 2.0±2.5, p<0.001). If the dorsal section had ≧ 4 points of consolidation score than ventral section at basal lung, the sensitivity/specificity/positive-predictive-value/positive-predictive-value of prone response prediction were 73.5%/77.8%/86.2%/60.9%, respectively. Neither prone oxygenation response nor dorsal-ventral consolidation difference at base lung was independently associated with 60-day mortality, but a CT GGO score more than 14 was an independent factor associated with 60-day mortality (Odds ratio =4.46, p=0.030).

**CONCLUSION.** The results indicated that high vertical consolidation difference (≧ 4 points) at base lung level was a helpful predictor in predicting prone response. High extent of GGO change (≧ 14 points) significantly correlated to 60-day mortality in patients with moderate to severe acute respiratory distress syndrome.

### 000889

#### Circulating neutrophil and endothelial-derived microvesicles differentiate ARDS subphenotypes

N. Tirlapur[1], K. O'dea[1], R. Handslip[1], C. Calfee[2], K. Delucchi[3], C. O'kane[4], D. Mcauley[4], B. Patel[5], M. Wilson[1], M. Takata[1]
[1]Anaesthetics, pain medicine and intensive care, Imperial College London, Chelsea & Westminster Campus, London, United Kingdom; [2]Cardiovascular research institute, University of California, San Francisco, San Francisco, United States of America; [3]Department of psychiatry, University of California, San Francisco, San Francisco, United States of America; [4]Centre for infection and immunity, Queen's University Belfast, Belfast, United Kingdom; [5]Anaesthesia and intensive care, Royal Brompton & Harefield NHS Foundation Trust, London, United Kingdom
**Correspondence:** N. Tirlapur
*Intensive Care Medicine Experimental* 2019, **7(Suppl 3):**000889

**INTRODUCTION.** ARDS has recently been proposed to consist of 'hypo-' and 'hyper-inflammatory' subphenotypes. Patients with ARDS with a hyper-inflammatory phenotype have a higher mortality.

Microvesicles (MVs) are plasma membrane-derived extracellular particles of 0.1-1μm size, produced in response to cell activation, and retaining the antigenic characteristics of their parent cells. Circulating MVs in ARDS represent the status of intravascular cell activation, either within the systemic (e.g. from underlying sepsis, trauma or burns) or pulmonary (e.g. from ventilator-induced lung injury) circulation. We hypothesised that ARDS subphenotypes may be differentiated by these circulating MVs.

**OBJECTIVES.** To test our hypothesis, we analysed the profiles of MVs in platelet-poor plasma samples from patients with ARDS from the HARP-2 clinical trial**(1)**, according to their allocation to a hypo- or hyper-inflammatory subphenotype**(2)**.

**METHODS.** Plasma samples from 30 ARDS patients (15 hypo-, 15 hyper-inflammatory as defined at ARDS diagnosis) were analysed at baseline (<48h ARDS onset) and day 7. Samples were analysed in a blinded manner by flow cytometry for endothelial-(CD146+), neutrophil-(CD66b+), monocyte-(CD14+), and total leukocyte-derived (CD45+) MV counts.

**RESULTS.** Endothelial- and neutrophil-derived MVs were significantly increased at baseline in the hyper-inflammatory group. MV counts decreased in both MV subpopulations at day 7 (Table 1).

**CONCLUSION.** These results demonstrate that a significant early release of endothelial- and neutrophil-derived MVs into the circulation occurs with 'hyper-inflammatory' but not 'hypo-inflammatory' ARDS, suggesting that intravascular inflammation, either within the systemic or pulmonary circulation, plays a crucial role in defining ARDS subphenotypes and early ARDS pathophysiology. Further research into the cargoes and biological effects of these MV subpopulations, and the contribution of ventilator-induced pulmonary microvascular inflammation to MV production in ARDS, is warranted.

**REFERENCE(S)**

1. McAuley DF, Laffey JG, O'Kane CM, et al. N Engl J Med. 2014 Oct 30;371(18):1695-703.
2. Calfee CS, Delucchi KL, Sinha P, et al. Lancet Respir Med. 2018 Sep;6(9):691-8.
3. Medical Research Council Clinical Research Training Fellowship

**Table 1 (abstract 000889).** Mean±SD.*****p<0.05,******p<0.01 vs hypo-inflammatory; **$**p<0.05,**$$**p<0.01 vs corresponding baseline by t-test or Mann-Whitney U test. N=15/group

| | BASELINE | | DAY 7 | |
|---|---|---|---|---|
| | Hypo-inflammatory | Hyper-inflammatory | Hypo-inflammatory | Hyper-inflammatory |
| Endothelial-derived MVs/µl | 48.2 ± 38.9 | 92.6 ± 50.7***** | 1.6 ± 6.1**$$** | 4.5 ± 9.7**$$** |
| Neutrophil-derived MVs/µl | 59.5 ± 59.6 | 115.2 ± 49.4****** | 4.0 ± 8.4**$$** | 22.8 ± 30.0**$$** |
| Monocyte-derived MVs/µl | 5.6 ± 7.6 | 12.6 ± 14.5 | 3.7 ± 6.2 | 7.3 ± 8.2 |
| Leukocyte-derived MVs/µl | 210.9 ± 107.5 | 204.8 ± 129.1 | 111.9 ± 84.9**$$** | 109.9 ± 78.2**$** |

### 000893

#### Response in urine output to a small dose of furosemide predicts tolerance to negative fluid balance in acute respiratory failure

J. Kataoka[1], Y. Norisue[1], R. Uchimido[2], S. Fujitani[3]
[1]Intensive care medicine, Tokyo Bay Urayasu Ichikawa Medical Center, Urayasu, Japan; [2]Department of emergency medicine, Beth Israel Deaconess Medical Center, Boston, United States of America; [3]Department of emergency and critical care medicine, St. Marianna University School of Medicine, Kawasaki, Japan
**Correspondence:** J. Kataoka
*Intensive Care Medicine Experimental* 2019, **7(Suppl 3):**000893

**INTRODUCTION.** In hemodynamically stable patients with acute respiratory distress syndrome (ARDS), a conservative fluid management strategy has been shown to improve lung function and shorten the duration of mechanical ventilation (1). However premature initiation of diuresis can increase non-pulmonary organ failure such as acute kidney injury. Previous study demonstrated high E/E' and weight gain predict poor tolerance of negative fluid balance (NFB) defined as development of hypotension or AKI, or need for fluid expansion (2), but no study has investigated whether urine output in response to a small dose of diuresis predicts the poor tolerance of NFB. We hypothesized that urine output after the administration of small dose of diuresis are predictive to the poor tolerance of NFB in patients with acute respiratory failure.
**OBJECTIVES.** We aimed to investigate whether the amount of urine output after a small dose of furosemide administration predicts the poor tolerance of NFB in acute respiratory failure patients.
**METHODS.** This study was a prospective observational study. All mechanically ventilated and hemodynamically stable patients with evaluations of hypervolemia for whom diuresis were planned were included. A small amount of furosemide was administered (10mg for eGFR>50, 20mg for eGFR 30-50), and the urine output was examined for 4 hours, which is a part of the traditional practice in our hospital to predict tolerability to NFB although no study has validated the practice. Before administrating the furosemide, we performed transthoracic echography to investigate several parameters, such as E/E', inferior vena cava diameters. After 4 hours of the meseaurement, the patients were diuresed to achieve an NFB of more than 1000ml in 24 hours with additional doses of furosemide as needed. The primary outcome of this study was the tolerance to NFB. Tolerance to NFB was defined as absence of hypotension, acute kidney injury, or need for fluid expansion.
**RESULTS.** A total of 60 mechanically ventilated patients were included in the intensive care unit at Tokyo Bay Urayasu/Ichikawa medical center. 48 patients (80%) had a tolerance of NFB and 12 patients (20%) did not. The urine output following the furosemide administration in patients who did not have tolerance of NFB showed significantly lower urine output than that of patients with tolerance to NFB in each of the first 4 hours (p<0.01). The area under the receiver operator (AUC) characteristic curves for the urine output in the first 4 hours following FST to predict tolerance to NFB was 0.826. The cutoff point of 500ml has the sensitivity of 0.85, and the specificity of 0.75. The AUC of the urine output was higher than those of E/E' (AUC=0.626) and an amount of positive fluid balance (AUC=0.633).
**CONCLUSION.** The urine output in response to a small dose of furosemide could predict poor tolerance of NFB in patients with acute repiratory failure. Further studies to validate this finding are needed.

**REFERENCE(S)**
(1) Wiedemann HP, et al. Comparison of two fluid-management strategies in acute lung injury. N Engl J Med. 2006 Jun 15;354(24):2564-75.
(2) Allyn J, et al. Echocardiography to predict tolerance to negative fluid balance in acute respiratory distress syndrome/acute lung injury. J Crit Care. 2013 Dec;28(6):1006-10.
(3) None

## TEM - Outcome in clinical and experimental cardiac arrest studies

### 000021

#### The Maximum Diameter of the Left Ventricle May Not Be the Optimum Target for Chet Compression during Cardiopulmonary Resuscitation: A Preliminary Observational Study to Report Clinical Outcomes Challenging the Traditional Assumption

SB. Chon[1], GY. Park[1], WS. Oh[2], S. Kim[1]
[1]Department of emergency medicine, CHA Bundang Medical Center, Seongnam, Republic of Korea; [2]Department of internal medicine, Kangwon National University School of Medicine, Chuncheon, Republic of Korea
**Correspondence:** S.B. Chon
*Intensive Care Medicine Experimental* 2019, **7(Suppl 3):**000021

**INTRODUCTION.** Researchers have assumed that compressing the point (P_max.LV), beneath which the left ventricle (LV) diameter is maximum, would improve cardiopulmonary resuscitation (CPR) outcomes. Defining the mid-sternum, the currently recommended chest compression, as the reference (x=0), the lateral deviation (x_max.LV) of personalized P_max.LV has become estimable using posteroanterior chest radiography (chest_PA) (Fig 1).[Ref. 1]
**OBJECTIVES.** We aimed to investigate whether out-of-hospital cardiac arrest (OHCA) victims, whose x_max.LV was closer to the mid-sternum (where x=0) and thus had their P_max.LV compressed closer during CPR, showed better chances of return-of-spontaneous-circulation and survival-to-discharge (Fig 2).
**METHODS.** A retrospective, cross-sectional study was performed on adult OHCA victims with available chest_PA checked before the cardiac arrest. For each clinical outcome, multivariable logistic regression was performed grouping x_max.LV into tertiles and adjusting the variables selected among the core elements of the Utstein template showing possible differences ($p$>0.10) in univariate analysis.
**RESULTS.** Among 309 cases (age: 65.6±15.8 y, female 106 (34.3%)), 141 (45.6%) achieved ROSC and 48 (15.5%) survived to discharge. Compared with the third tertile of x_max.LV (58~87 mm), its first (-15~47 mm) and second (47~58 mm) tertiles whose P_max.LV were closer to the mid-sternum, were negatively associated with return-of-spontaneous-circulation (OR: 0.461 (0.250, 0.849), $p$=0.013; and OR: 0.429 (0.236, 0.782), $p$=0.006, respectively) and survival-to-discharge (OR: 0.272 (0.086, 0.861), $p$=0.027; and OR: 0.131 (0.035, 0.492), $p$= 0.003, respectively).
**CONCLUSION.** OHCA victims with P_max.LV located closer to the mid-sternum showed worse chances of return-of-spontaneous-circulation and survival-to-discharge. This challenges the traditional assumption identifying P_max.LV as the optimum compression point.

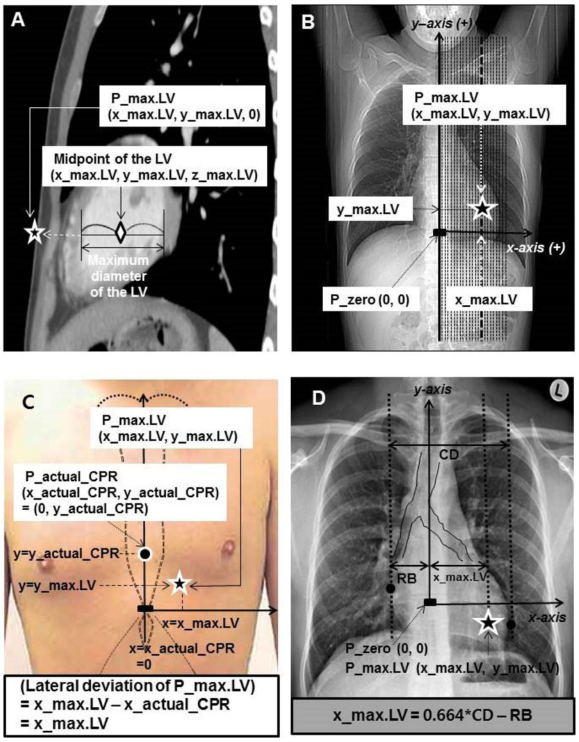

**Fig. 1 (abstract 000021).** See text for description

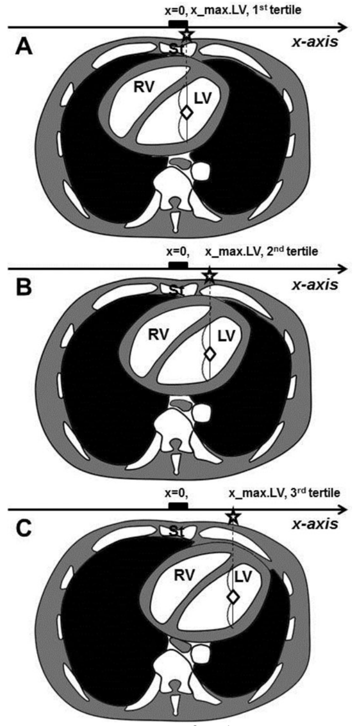

**Fig. 1 (abstract 000021).** See text for description

### 000028

### Can New Wearable Technology significantly increase the Efficacy Cardiopulmonary Resuscitation? A controlled, randomized trial testing

A. Saporito[1], M. Musiari[1], L. Anselmi[1], S. Ceruti[2], X. Capdevila[3], T. Cassina[4]
[1]Anesthesia, EOC Ente Ospedaliero Cantonale, Bellinzona, Switzerland, Bellinzona, Switzerland; [2]Soins Intensifs, Hôpitaux Universitaires de Genève (HUG), Genève, Switzerland;
[3]Anesthesia, Université de Montpellier, Boulevard Henri IV, Montpellier, France, Montpellier, France; [4]Anesthesia, Fondazione Cardiocentro Ticino, Via Tesserete, Lugano, Switzerland, Lugano, Switzerland
**Correspondence:** S. Ceruti
*Intensive Care Medicine Experimental* 2019, **7(Suppl 3):**000028

**INTRODUCTION.** Cardiovascular accidents are the leading cause of death. A cardiopulmonary resuscitation (CPR) of quality has well shown that can reduce the mortality; despite this, survival rate has not changed significantly during last years.

**OBJECTIVES.** Aim of this study is to test a new wearable glove to provide lay people with instructions during out-of-hospital CPR.

**METHODS.** After ethical committee approval, we performed a blinded, controlled trial on an electronic mannequin AmbuMan (Ballerup, Denmark) to test the performance of adult volunteers, non-healthcare professionals performing a simulated CPR both, without and with glove, following the glove instructions. The group without glove, also called "no-glove" is intended as control group. Each compression performed on theelectronic mannequin AmbuMan was recorded by a connected laptop computer, drawing a depth frequency curve over the time. Primary outcome was to compare the accuracy of the two simulated CPR sessions in terms of depth and frequency of chest compression performed by the same lay volunteers. Secondary outcome was to compare the decay of performance and percentage of time in which the candidate performed accurate CPR. Finally, the participants were asked if the glove was useful for CPR maneuvers. The difference between the two groups in regard to change in chest compression depth over time due to fatigue, defined as decay were also analyzed.

**RESULTS.** 571 chest compressions were included: 293 in control group, 278 in glove group (Table 1). Mean depth of compression in the control group was 55.17 mm versus 52.11 mm in the glove-group (p=0.000016). Compressions with an appropriate depth were not statistically different (81.9% vs 73.6%, p=0.017). Mean frequency of compressions in the group with glove was 117.67 rpm vs 103.02 rpm in the control group (p<0.00001). The percentage of compression cycles with an appropriate rate (>100 rpm) was 92.4% in the group with the glove versus 71% in the control group, with an observed difference of 21.4% between the two groups, which was statistically significant (p<0.0001, CI=95%). A mean reduction over time of compressions depth of 5.3 mm (SD 10.28) was observed in the control group versus a mean reduction of 0.83 mm in the group wearing the glove (SD 9.91), but this mean difference in the decay of compressions delivery was not statistically significant (f-ratio=5.680, SS=579.61, df=1, MS=579.61, p=0.018).

**CONCLUSION.** The use of the glove was effective in reducing by more than 20% the inappropriateness of the frequency of chest compressions during CPR. The visual and acoustic feedbacks provided by the device were useful in dictating the correct rhythm for non-healthcare professionals, translating in a significantly more accurate CPR.

**REFERENCE(S)**

1. Krikscionaitiene A., Stasaitis K., Dambrauskiene M., Dambrauskas Z., Vaitkaitiene E., Dobozinskas, P. et al. Can lightweight rescuers adequately perform CPR according to 2010 resuscitation guideline requirements?. Emerg Med J. 2013;30:159
2. Blewer A.L., Buckler D.G., Li J., Leary M., Becker L.B., Shea J.A. et al. Impact of the 2010 resuscitation guidelines training on layperson chest compressions. World J Emerg Med. 2015;6:270
3. Travers A.H., Perkins G.D., Berg R.A., Castren M., Considine J., Escalante R. et al. Part 3: adult basic life support and automated external defibrillation. 2015 international consensus on cardiopulmonary resuscitation and emergency cardiovascular care science with treatment recommendations 2015;132:S51
4. Vadeboncoeur T., Stolz U., Panchal A., Silver A., Venuti M., Tobin J. et al, Chest compression depth and survival in out-of-hospital cardiac arrest. Resuscitation. 2014;85:182
5. Meaney P.A., Bobrow B.J., Mancini M.E., Christenson J., de Caen A.R., Bhanji F. et al. Cardiopulmonary resuscitation quality: [corrected] improving cardiac resuscitation outcomes both inside and outside the hospital: a consensus statement from the American Heart Association. Circulation. 2013;128:417

### 000035

#### Right ventricular dysfunction after out of hospital cardiac arrest

SN. Voorrips, JC. Winkelhorst, IT. Bootsma, F. De Lange, EC. Boerma
Department of intensive care, Medical Centre Leeuwarden, Leeuwarden, Netherlands
**Correspondence:** S.N. Voorrips
*Intensive Care Medicine Experimental* 2019, **7(Suppl 3):**000035

**INTRODUCTION.** In addition to poor neurological outcome, circulatory failure has been described as an important determinant of prognosis after out of hospital cardiac arrest (OHCA). The role of left ventricle dysfunction after cardiac arrest has been established in multiple studies [1]. However, data on right ventricle (RV) dysfunction in this setting remains scarce.

**OBJECTIVES.** To investigate a potential association between RV failure and mortality after OHCA.

**METHODS.** A retrospective single-centred study was conducted. All patients ≥18 years, admitted to the ICU with return of spontaneous circulation (ROSC) after OHCA between 2011 and 2018 were included. By protocol, all OHCA patients were equipped with a pulmonary artery catheter that enables near-continuous assessment of RV ejection fraction (RVEF). (Vigilance® CCO/SvO2/CEDV Monitor, Baxter Healthcare Corporation, Irvine, CA). Resuscitation characteristics, hemodynamic variables and 1-year all-cause mortality were recorded. Primary endpoint of the study was 1-year all-cause mortality in different RVEF-subsets (group A: <20%, group B: 20-30%, group C: >30%). RVEF was calculated as an average over the first 24h of ICU admission. Data are presented as median [IQR].

**RESULTS.** N=238, divided over group A: 47 (20%), group B: 123 (52%), group C: 68 (29%); 77% male, median age 66 [55-73]. Patients with the lowest RVEF had a significantly longer time to ROSC and the incidence of a non-shockable rhythm and adrenaline administration was significantly higher. However, presumed aetiology and the percentage of patients with an intervention of the right coronary artery were not different across groups. 1-year all-cause mortality was significantly different across groups (group A: 64%, group B: 43%, group C: 32%; p<0.01). In a Kaplan-Meier survival analysis there was a significant separation between groups (X2=18.90; p=<0.001; fig.). However, in multivariate analysis, with statistical correction for potential baseline confounders, this association between RVEF (as a continuous variable) and 1-year all-cause mortality was not independent (OR 0.99, CI 0.93-1.06, p=0.81).

**CONCLUSION.** The incidence of patients with an RVEF of <20% after OHCA was 20%. 1-year all-cause mortality was significantly higher in patients with the lowest RVEF in the first 24h after OHCA. However, after correction for potential baseline confounders this association did not remain statistically significant.

##### REFERENCE(S)

1. Tang, W. A. N. C. H. U. N., et al. "Progressive myocardial dysfunction after cardiac resuscitation." Critical care medicine 21.7 (1993): 1046-1050.
2. None applicable

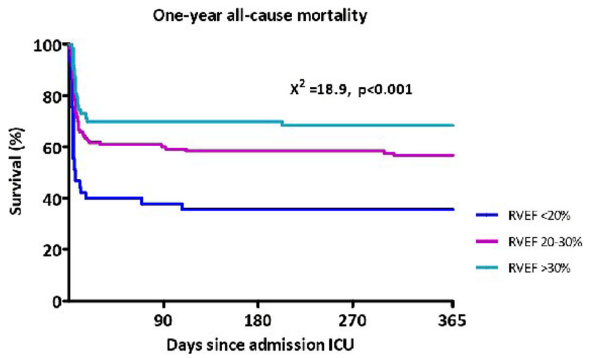

**Fig. 2 (abstract 000035).** See text for description

### 000082

#### Recovery from acute kidney injury as a potent predictor of survival discharge after out-of-hospital cardiac arrest

JH. Oh[1], YS. Park[2], YH. Choi[3], IS. Cho[4], KC. Cha[5], JS. You[2]
[1]Chung-Ang University College of Medicine, Seoul, Republic of Korea; [2]Emergency medicine, Yonsei University College of Medicine, Seoul, Republic of Korea; [3]Emergency medicine, Ewha Womans University Medical Center and Ewha Womans University Mokdong Hospital, Seoul, Republic of Korea; [4]Emergency medicine, Hanil General Hospital, Seoul, Republic of Korea; [5]Emergency medicine, Yonsei University Wonju College of Medicine, Wonju-si, Republic of Korea
**Correspondence:** J.H. Oh
*Intensive Care Medicine Experimental* 2019, **7(Suppl 3):**000082

**INTRODUCTION.** Development of acute kidney injury (AKI) after out-of-hospital cardiac arrest (OHCA) is a well-known predictor for mortality and poor neurological outcomes in both adult and paediatric populations [1,2]. However, the natural course of AKI in terms of its recovery rate after OHCA is uncertain. If we know the natural course of AKI after OHCA, it will be helpful in making appropriate clinical decisions such as determining the initiation time for renal replacement therapy and making a prognosis in OHCA patients with AKI development. We hypothesized that recovery from AKI may affect the survival discharge and neurological status at discharge in OHCA patients treated with targeted temperature management (TTM).

**OBJECTIVES.** This study investigated the clinical course of AKI after OHCA and determined whether recovery from AKI impacted the outcome of OHCA.

**METHODS.** This retrospective multicentre cohort study included adult OHCA patients treated with TTM between January 2016 and December 2017. AKI was diagnosed using the Kidney Disease: Improving Global Outcomes criteria [3]. The primary outcome was the recovery rate after AKI and its association with survival discharge.

**RESULTS.** A total of 3,697 OHCA patients from 6 hospitals were screened and 275 were finally included. AKI developed in 175/275 (64%) patients and 69/175 (39%) patients recovered from AKI. In most cases, AKI developed within 3 days of return of spontaneous circulation [155/175 (89%), median time to AKI development: 1 (1–2) day] and patients recovered within 7 days of return of spontaneous circulation [59/69 (86%), median time to AKI recovery: 3 (2–7) day]. The median duration of AKI was 4 (2–7) days. Duration of AKI was significantly longer in the AKI non-recovery group than in the AKI recovery group [5 (2–9) vs. 1 (1–5) days, $P < 0.001$]. Most patients were diagnosed with AKI Stage 1 initially [120/175 (69%)]. However, the number of Stage 3 AKI patients increased from 30/175 (17%) to 77/175 (44%) after the initial diagnosis of AKI. The rate of survival discharge was significantly higher in the AKI recovery group than in the AKI non-recovery group [45/69 (65%) vs. 17/106 (16%), $P < 0.001$]. Recovery from AKI was a potent predictor of survival discharge in multivariate analysis (adjusted odds ratio, 12.622; 95% confidence interval: 3.390–46.993; $P < 0.001$).

**CONCLUSION.** The recovery rate from AKI after OHCA was 39% and recovery from AKI was a potent predictor of survival discharge in adult OHCA patients treated with TTM.

**REFERENCE(S)**

[1] Sandroni C, Dell'anna AM, Tujjar O, Geri G, Cariou A, Taccone FS. Acute kidney injury after cardiac arrest: a systematic review and meta-analysis of clinical studies. Minerva Anestesiol. 2016;82:989-99.
[2] Cornell TT, Selewski DT, Alten JA, Askenazi D, Fitzgerald JC, Topjian A, et al. Acute kidney injury after out of hospital pediatric cardiac arrest. Resuscitation. 2018;131:63-8.
[3] Section 2: AKI Definition. Kidney Int Suppl (2011). 2012;2:19-36.

[4] This work was supported by the National Research Foundation of Korea (NRF) grant funded by the Korean government (MSIT) (grant no. NRF-2018R1C1B5082969).

### 000088

#### An exploratory, observational study regarding extracorporeal cardiopulmonary resuscitation for non-shockable out-of-hospital cardiac arrest developed after emergency medical service arrival (SOS-KANTO 2012 study report)

T. Yoshida, S. Fujitani, M. Yoshida, K. Tsutsumi, Y. Masui, Y. Taira
Department of emergency and critical care medicine, St. Marianna University School of Medicine, Kawasaki, Japan
**Correspondence:** T. Yoshida

*Intensive Care Medicine Experimental* 2019, **7(Suppl 3):**000088

**INTRODUCTION.** The prognosis of non-shockable out-of-hospital cardiac arrest (OHCA) patients is poorer than that of shockable OHCA patients[1]. The advantage of extracorporeal cardiopulmonary resuscitation (E-CPR) for OHCA has been suggested[2], but as most studies involved shockable OHCA patients with a relatively favorable outcome[3], the usefulness of E-CPR in patients with non-shockable OHCA remains to be clarified.

**OBJECTIVES.** The purpose of this study was to examine the application of percutaneous cardiopulmonary support (PCPS: V-A ECMO) as E-CPR and its outcome-improving effects in patients with non-shockable OHCA developed after EMS arrival.

**METHODS.** Of 16,452 patients accumulated in the SOS-KANTO 2012 study (OHCA patients admitted to 67 emergency hospitals in Kanto region (central Japan) from January 2012 until March 2013 were registered), the subjects (n=531) were selected. We retrospectively analyzed the background and clinical outcomes by dividing the subjects into two groups: the PCPS group and the non-PCPS group.

**RESULTS.** Thirty-eight patients (7.2%) was undertaken PCPS and not for 493 patients (92.8%). On univariate analysis of background factors, the age was significantly younger (p<0.001), ADL before the onset were significantly more favorable (p=0.002), the body weight was significantly heavier (p=0.02), and the interval from onset until hospital arrival was significantly shorter in the PCPS group than in the non-PCPS group (p=0.001). The incidences of ACS, non-ACS cardiac disease, and pulmonary embolism were significantly higher in the PCPS group than in the non-PCPS group (p<0.001, p=0.004, P<0.001). The 3-month survival/favorable cerebral function outcome rates were significantly higher in the PCPS group than in the non-PCPS group (p=0.003, p=0.001). On multivariate analysis corrected for background factors, the execution of PCPS and interval from onset until hospital arrival were independent prognostic factors for favorable cerebral function outcome at 3 months (OR 7.62, 95%CI 1.641 - 35.38 / OR 1.08, 95%CI 1.004 - 1.194).

**CONCLUSION.** In this study, the outcomes at 3 months were significantly more favorable in the PCPS group than in the non-PCPS group, although there were differences in the background factors. Even in patients with non-shockable OHCA, E-CPR may improve the outcome if patients are carefully selected.

**REFERENCE(S)**

[1] Zipes DPL, et al. Braunwald's Heart Disease. 11 ed. 2019. p. 807-47.
[2] Maekawa K, et al. Crit Care Med. 2013;41:1186-96.
[3] Sakamoto T, et al. Resuscitation. 2014;85:762-8.

[4] Grant acknowledgment - None.

### 000119

#### Prognostic value of base excess at admission after out-of-hospital cardiac arrest

J. Grand[1], C. Hassager[2], H. Friberg[3], T. Cronberg[4], N. Nielsen[5], J. Düring[3], J. Kjaergaard[2]
[1]Blegdamsvej 9b, Copenhagen, Denmark; [2]Department of cardiology, Rigshospitalet, København, Denmark; [3]Department of clinical sciences, intensive and perioperative care, Skåne University Hospital, SUS, Malmö, Malmö, Sweden; [4]Department of clinical sciences, neurology, Lund University, Lund, Sweden; [5]Department of clinical sciences, department of anesthesiology and intensive care, Helsingborg Hospital, Helsingborg, Sweden
**Correspondence:** J. Grand

*Intensive Care Medicine Experimental* 2019, **7(Suppl 3):**000119

**INTRODUCTION.** In patients resuscitated from out-of-hospital cardiac arrest (OHCA), severe metabolic acidosis occurs due to whole-body ischemia and anaerobic metabolism. Lactate level at hospital admission is often used as a marker og ischemia and prognosis. However, hyperlactatemia does not alone explain the metabolic acidosis after OHCA, since other metabolic products is formed during ischemia. Base excess, which is an easily obtainable measure of the total metabolic acid-base status, could potentially have additional prognostic information compared with lactate.

**OBJECTIVES.** We aimed to investigate and compare the prognostic value of base excess and lactate at hospital-admission and during intensive care after OHCA.

**METHODS.** We included 939 consecutive, comatose OHCA-patients in the targeted temperature management (TTM)-trial, which randomly allocated patients to TTM at 33°C and 36°C for 24 hours. Base excess and lactate were prospectively measured at hospital admission and at several time points during TTM and mortality was reported in all patients after 180 days. Receiver operation characteristic (ROC) curves for admission base excess and admission lactate were computed in respect to 180-day mortality with calculation of optimal cut-off values to predict ICU mortality. Repeated measurements models were used to assess differences in base excess during TTM between survivors and non-survivors.

**RESULTS.** We excluded 5 (1%) patients with no base excess data which left 934 patients (99%) eligible for analysis. The admission-base excess measurement was missing in 62 (6%) patients. Patients were 64 (±12) years old, 81% were men and 89% had a witnessed OHCA. At hospital admission, mean base excess was -9.09 (±6.15) mmol/L and median lactate was 6.0 (3.0-9.5) mmol/L. Comparing survivors and non-survivors, admission-base excess was less negative in survivors (-7.63±5.59 versus -10.57±6.53, p=0.01). Furthermore, during TTM, not including the admission values, base excess remained significantly less negative in survivors (β = 1.31 [95% confidence limit, 1.85 – 0.78 mmol/L]; pgroup <0.0001). Pairwise comparison of ROC-curves showed no difference in predictive value between base excess (area under the (AU) ROC = 0.66 (95%-CI 0.62-0.69)) and lactate (AUROC = 0.64 (95%-CI 0.61-0.68)) (p = 0.88). The optimal base excess cut-off value to predict mortality was <-9.35 mmol/l (sensitivity 61%, specificity 69%, Youdens Index: 0.28). For lactate the optimal cut-off value to predict mortality was >5.65 mmol/L (sensitivity 63%, specificity 59%, Youdens Index: 0.21). In a multivariate Cox regression model adjusted for confounders including admission-lactate, admission-base excess <-9.35 mmol/L was independently associated with mortality (hazard ratio: 1.83 [95%-CI 0.44-2.32], p<0.0001).

**CONCLUSION.** Admission base excess below a cut off -9.35 mmol/l was independently associated with mortality. Lactate and base excess had identical predictive value and can be used interchangeably in the patient-assessment at hospital-admission after OHCA.

**REFERENCE(S)**

1. The research fund Gangstedfonden and the Research fund of Rigshospitalet has supported this study with unrestricted salary in Dr. Grand's PhD project. Dr. Kjaergaard was supported by unrestricted grants from the Novo Nordisk Foundation: NNF17OC0028706. The TTM-trial was supported by independent research grant from TrygFonden (Denmark) (grant no: 7-12-0454).

### 000223

#### Detecting the appropriate candidates for early coronary angiography and targeted temperature management following out-of-hospital cardiac arrest by sCAHP score

CH. Huang[1], W. Chih-Hung[2], T. Min-Shan[1], W. Shih-Ni[1], W. Chien-Kai[1], WJ. Chen[1]

[1]Emergency medicine, National Taiwan University Hospital, Taipei, Taiwan; [2]Department of emergency medicine, National Taiwan University Hospital and College of Medicine, Taipei, Taiwan

**Correspondence:** C.H. Huang

*Intensive Care Medicine Experimental* 2019, **7(Suppl 3):**000223

**INTRODUCTION.** Choosing the appropriate candidate who can get benefit from early coronary angiography(CAG) and targeted temperature management(TTM) is still challenging. Simplified CAHP (sCAHP) score is a validated tool to predict neurological outcomes following out-of-hospital cardiac arrest (OHCA).

**OBJECTIVES.** To evaluate whether the effect of CAG and TTM is modulated by the sCAHP score in OHCA patients.

**METHODS.** Consecutive OHCA patients were evaluated from 2011 to 2017 in a tertiary medical center. The optimal cutoff value of sCHAP score for neurological prognosis was estimated by generalized additive model (GAMs).

**RESULTS.** There were 412 patients included in the study and 94 patients (22.8%) had favorable neurological outcome with cerebral performance category (CPC) 1-2. The optimal cutoff value of sCAHP for differentiating high risk from low risk was 185 by GAM plot. There was interaction between the sCAHP score and TTM in regression analysis for predicting the favorable neurological outcome. TTM was associated with favorable neurological outcome only in the patients with sCAHP score < 185 (odds ratio (OR) 2.13, 95% confidence interval (CI) 1.10–4.13, *p*=0.02). Improvement of survival outcome was associated with TTM independent of sCAHP scores (OR 2.66, 95% CI 1.54–4.59, *p*<0.001). Early CAG was associated with favorable neurological (OR 4.43, 95% (CI) 2.28–8.60, p<0.001) and survival outcome (OR 3.47, 95% CI 1.93–6.25, p<0.001) irrespective of low or high sCAHP scores.

**CONCLUSION.** Early CAG should be considered for all OHCA patients, irrespective of the immediately predicted neuroprognosis after ROSC. The appropriate selection of candidate for TTM needs further studies, especially for those with worse predicted immediate neuroprognosis.

**REFERENCE(S)**

1. Resuscitation. 2019 Apr;137:133-139.
2. JACC Cardiovasc Interv, 2018. 11(3): p. 249-256
3. NTUH 108-S4237, NTUH 105-FTN12

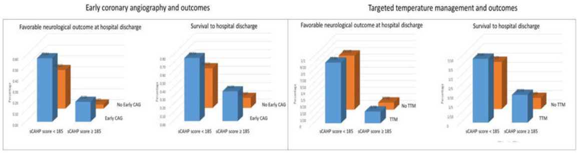

**Fig. 1 (abstract 000223).** See text for description

### 000227

#### Manual Chest compression quality during intra-hospital transportation is not inferior to Corpuls-CPR

G. Jansen[1], K. Kipker[1], E. Latka[2], R. Borgstedt[1], S. Rehberg[1]

[1]Anaesthesiology, Prostestant Hospital of the Bethel Foundation, Bielefeld, Germany; [2]Fachbereich rettungswesen, Studieninstitut für kommunale Verwaltung Westfalen - Lippe, Bielefeld, Germany

**Correspondence:** G. Jansen

*Intensive Care Medicine Experimental* 2019, **7(Suppl 3):**000227

**INTRODUCTION.** High-quality chest compressions (CC) with minimized interruptions are one of the most essential prerequisites for an optimal outcome of resuscitation. Therapy of reversible causes of cardiac arrest, such as coronary angiography, often requires intrahospital transport (IHT) during ongoing resuscitation. There is concern for a relevant decline of compression quality while moving patients. Therefore, automatic CC devices have been developed by the industry. As guidelines only recommend these devices under certain circumstances, such as transport during resuscitation, manual CC remains the gold standard [1].

**OBJECTIVES.** The present study investigated manual CC quality during IHT in two different provider positions in comparison with the automatic CC device Corpuls-CPR.

**METHODS.** 20 paramedics were enrolled into a manikin study (Laerdal Resusci Anne QCPR) with four groups: a reference group (RG) with the provider kneeling beside the manikin on the floor and 2 groups performing CC during a simulated IHT: kneeling in bed beside the patient (group 1, figure 1) or squatting above the patient in bed (group 2,figure 2). Each participant completed each scenario. In comparison, 20 simulated IHT were performed using the Corpuls-CPR (GS Elektromedizinische Geräte G. Stemple GmbH) (group 3, figure 3). Indicators of CC quality (pressure point and depth, compression frequency, complete relief and sufficient pressure depth) were measured as defined in the European Resuscitation Council Guidelines 2015 [1].

**RESULTS.** The RG was able to perform high quality CC. Compared to the RG, there were no statistical differences in CC quality in groups 1-3. Notably, CC quality was similar in the manual groups 1 and 2 as compared to Corpuls-CPR in group 3. Detailed results are shown in table 1.

**CONCLUSION.** Carrying out guideline-compliant CC [1] during IHT is feasible with multiple provider positions. A manual CC in this context is not inferior to a mechanical CC with the Corpuls-CPR. These results suggest that even in the absence of automatic devices high quality CC are possible during intra-hospital transportation.

**REFERENCE(S)**

[1] Perkins GD et al. Resuscitation 95:81-99

[2] We thank Daniel Marx for his wonderful graphics.

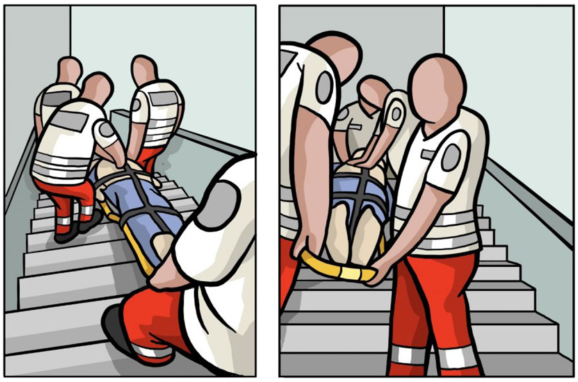

**Fig. 1 (abstract 000227).** See text for description

**Table 1 (abstract 000227).** See text for description

| Group | Reference Group | 1 | 2 | 3 |
|---|---|---|---|---|
| Correct pressure point (%) | 99 ± 0.6 | 85 ± 29 | 86 ± 32 | 100 ± 0 |
| Compression depth (mm) | 57 ± 11 | 54 ± 6 | 56 ± 5 | 53 ± 0 |
| Frequency of compressions (/min) | 114 ± 7 | 116 ± 9 | 116 ± 9 | 109 ± 0 |
| Compressions with complete relief (%) | 86 ± 23 | 74 ± 31 | 70 ± 38 | 100 ± 0 |

### 000231

#### Characterization of Peri-operative Paediatric Cardiac Arrest - A 10-year Review of 22,650 Anaesthesiologic Procedures in a German Tertiary Hospital

G. Jansen[1], E. Lang[1], J. Popp[1], B. Schmidt[1], M. Barthel[2], S. Rehberg[1]
[1]Anaesthesiology, Prostestant Hospital of the Bethel Foundation, Bielefeld, Germany; [2]Paediatric surgery, Prostestant Hospital of the Bethel Foundation, Bielefeld, Germany
**Correspondence:** G. Jansen
*Intensive Care Medicine Experimental* 2019, **7(Suppl 3):**000231

**INTRODUCTION.** Cardiac arrest (CA) in the peri-operative care of paediatric patients is a dreaded event [1,2]. The incidence ranges between 3-21 per 10,000 paediatric anaesthetic procedures, depending on the investigated patient population. [1,2]

**OBJECTIVES.** The aim of our study was to characterize the causes of perioperative paediatric CA at a German tertiary care hospital between 2008-2018.

**METHODS.** The anaesthetic database was screened for perioperative paediatric CA during all anaesthesiologic procedures in patients <16 years of age. We defined the peri-operative period from anaesthesia induction until the end of care of the team of anaesthesists care. CA was defined as the necessity of performing chest compressions. In each case the cause of CA was documented and it was assigned to one of the 3 peri-operative phases: induction of anesthesia, intraoperatively and postoperatively until the end of anaesthesiologists´ care.

**RESULTS.** During the 10 year observation period,18 perioperative paediatric CA were observed in a total of 22650 general anaesthesia procedures according to an incidence of 7,9 ± 4,2 per 10,000 paediatric anaesthetics (CI 95 4,2-11,6). In 17 cases a surgical procedure was performed, while in one case a bronchoscopy was the reason for anaesthesia. Five CA occurred during induction of anaesthesia, eleven intra-operatively and two in the post-operative phase. The causes of CA are displayed in Table 1. Two children died intraoperatively as a result of liver haemorrhage and one child at the post-anaesthesia care unit as a result of malignant hyperthermia. After 30 days seven children with perioperative CA had died during the hospital stay as a result of their underlying condition, nine of the resuscitated children survived.

**CONCLUSION.** Paediatric peri-operative CA is a rare event. During anaesthesia induction, CA mainly occurs in association with a difficult airway. Intraoperatively, acute shock events (haemorrhage, sepsis) and complications with the endotracheal tube are the most common causes of paediatric perioperative CA.

##### REFERENCE(S)

[1] Habre W. et al. Incidence of severe critical events in paediatric anaesthesia (APRICOT): a prospective multicentre observational study in 261 hospitals in Europe. Lancet Respir Med 2017; 5: 412-25
[2] Davis PJ. Et al. Anesthesia-Related Cardiac Arrest in Children with Heart Disease: Data from the Pediatric Perioperative Cardiac Arrest (POCA) Registry Anesthesia & Analgesia: May 2010 - Volume 110 - Issue 5 - p 1376–1382 doi: 10.1213/ANE.0b013e3181c9f927

**Table 1 (abstract 000231).** Characteristics of perioperative paediatric cardiac arrests (n=16)

| Time of occurence | n | Cause of cardiac arrest | n |
|---|---|---|---|
| Induction | 5 | Difficult airway during induction | 4 |
| | | Septic shock | 1 |
| Intraoperative | 9 | Acute haemorrhage | 5 |
| | | Obstructed endotracheal tube | 2 |
| | | Septic shock | 1 |
| | | Dislocated endotracheal tube | 1 |
| | | Tension pneumothorax | 1 |
| | | Vagal | 1 |
| Postoperative | 2 | Malignant Hyperthermia | 1 |
| | | Hypoxemic attack in pneumothorax and Fallot's pentalogy | 1 |

### 000264

#### Multi-vessel versus culprit-only revascularization in cardiac arrest survivors with multi-vessel coronary artery disease: A multi-center cohort study

CK. Wu[1], CW. Sung[2], CH. Huang[1], WJ. Chen[1], SN. Wu[1], WT. Chang[1], T. Ming-Sham[1]
[1]Department of emergency medicine, National Taiwan University Hospital and College of Medicine, Taipei, Taiwan; [2]Department of emergency medicine, National Taiwan University Hospital Hsin-Chu Branch, Hsinchu, Taiwan
**Correspondence:** C.K. Wu
*Intensive Care Medicine Experimental* 2019, **7(Suppl 3):**000264

**INTRODUCTION.** Conflicting results existed as to the recommendation of complete revascularization for multi-vessel coronary artery disease (CAD) in patients with ST-elevation myocardial infarction (STEMI). In survivors successfully resuscitated from cardiogenic arrest with multi-vessel CAD, whether multi-vessel revascularization or culprit-only revascularization benefit outcomes more remains undetermined and worth further investigation.

**OBJECTIVES.** To evaluate whether multi-vessel revascularization following return of spontaneous circulation (ROSC) is associated less in-hospital mortality and poor neurological recovery than culprit-only revascularization.

**METHODS.** Two hundred and seventy-three non-traumatic, adult (ages ≥ 18) cardiac arrest patients with sustained ROSC and underwent emergent coronary angiography (CAG) within 24 hours following cardiac arrest were retrospectively recruited from three hospitals from 2012 to 2017. Patients with definite coronary artery stenosis (< 70%) (n=72) or 1-vessel stenosis (n=74) or failed percutaneous coronary intervention (PCI) (n=37) were excluded. Multi-vessel PCI was defined as revascularization of more than one major vessel during the index CAG, and culprit-only PCI as revascularization of the infarct-related artery only. In-hospital mortality and neurological function at hospital discharge were compared between groups.

**RESULTS.** A total of 90 patients were enrolled in the final analysis and classified into multi-vessel (n=45) and culprit-only (n=45) groups. No significant differences of baseline characteristics, CPR events, and post-arrest care was noted between these two groups. Twenty-five patients (55.6%) in the culprit-only groups failed to survive to discharge while 17 patients (37.8%) in the multi-vessel group did (adjusted HR = 0.54, 95% CI = 0.28 – 0.98, $p$ = 0.04). There were 27 patients (60.0%) in the culprit-only groups discharged with poor neurological recovery (cerebral performance category: 3-5) while 25 patients (55.6%) in the multi-vessel group (adjusted OR = 0.89, 95% CI = 0.29 – 1.98, $p$ = 0.78). Consistent decrease of in-hospital mortality in patients receiving multi-vessel revascularization was noted with CPR duration ≥ 10 mins.

**CONCLUSION.** In cardiac arrest survivors with multi-vessel CAD, multi-vessel revascularization is associated with less in-hospital mortality as compared with culprit-only revascularization.

### 000335

#### Postoperative complications in survivors of early cardiac arrest after cardiac surgery

F. Ampatzidou[1], P. Ntouma[2], A. Stratou[3], O. Ananiadou[3], R. Ioannidis[2], A. Vlahou[3], T. Asteri[2], G. Drossos[3]
[1]Cardiothoracic icu, G.Papanikolaou Hospital, Thessaloniki, Greece; [2]Cardiothoracic anesthesia, G.Papanikolaou Hospital, Thessaloniki, Greece; [3]Cardiothoracic surgery, G.Papanikolaou Hospital, Thessaloniki, Greece
**Correspondence:** F. Ampatzidou
*Intensive Care Medicine Experimental* 2019, **7(Suppl 3):**000335

**INTRODUCTION.** The incidence of cardiac arrest after cardiac surgery is around 1,4-2,7%, while reported survival rate is 30-56%.

**OBJECTIVES.** Aim of our study is to investigate whether cardiac surgical patients who survived after cardiac arrest during the first 48 hours, are more likely to experience major postoperative complications

**METHODS.** We retrospectively studied patients who suffered cardiac arrest during the first 48 h after cardiac surgery , from June 2012 to March 2019.The following complications were investigated: Post op atrial fibrillation(AF), Acute Kidney Injury based on KDIGO criteria(AKI), need for renal replacement therapy(RRT), postop stroke, sternal wound infection and low cardiac output syndrome(LCOS).We compared cardiac arrest survivors with the rest of the cohort excluding patients who died during hospital stay. Cardiopulmonary resuscitation was conducted according to the Cardiac Advanced Life Support(CALS) protocol. Chi square test was used for the statistical analysis.

**RESULTS.** From a total of 86 pts who suffered cardiac arrest, 42 survived (group A, mean age 67,2± 10,8 and mean Euro Score II 3,5± 3,1). Group B consisted of 3217 patients (mean age 65,3± 10,6 and mean Euro Score II 2,2±3,1) who underwent cardiac surgery and discharged from hospital. Results are shown in table 1.

**CONCLUSION.** Cardiac arrest survivors after cardiac surgery have higher incidence of postoperative stroke, atrial fibrillation, AKI, AKI requiring RRT, and Low Cardiac Output Syndrome compared with the rest of patients who discharged from hospital. No statistical significant difference was found regarding sternal wound infections.

**REFERENCE(S)**

1. Ngaage, Dumbor L. et al. Survival of Cardiorespiratory Arrest After Coronary Artery Bypass Grafting or Aortic Valve Surgery. Ann Thorac Surg 2009; 88(1):64 – 68
2. None

**Table 1 (abstract 000335).** See text for description

| | Group A n=42 | Group B n=3217 | p |
|---|---|---|---|
| Stroke(n,%) | 4 (9,5%) | 46 (1,4%) | <0,01 |
| AF(n,%) | 25 (59,5%) | 985 (30,6%) | <0,01 |
| AKI(n,%) | 16 (38,1%) | 414 (12,9%) | <0,01 |
| AKI-RRT(n,%) | 7 (16,7%) | 44 (1,4%) | <0,01 |
| LCOS(n,%) | 10 (23,8%) | 157 (4,9%) | <0,01 |
| Sternal infections(n,%) | 2 (4,8%) | 115 (3,6%) | 0,68 |

### 000350

#### Ischemic injury of the upper digestive tract after cardiac arrest: results of the prospective, multicentric ENTRACT study

D. grimaldi[1], S. Legriel[2], N. Pichon[3], P. Colardelle[4], S. Leblanc[5], F. Canoui-Poitrine[6], O. Ben Hadj Salem[7], G. Muller[8], N. De Prost[9], S. Herrmann[10], S. Marque[11], A. Baron[12], B. Sauneuf[13], J. Messika[14], J. Creteur[15], JP. Bedos[16], FS. Taccone[15], E. Boutin[6], A. Cariou[17]
[1]Soins intensifs, ULB Erasme, Anderlecht, Belgium; [2]Medical surgical intensive care, General Hospital Center, Versailles, France; [3]Medical surgical intensive care unit, General Hospital Center, Brive-la-Gaillarde, France; [4]Gastroenterology, C.H. de Versailles, Le Chesnay, France; [5]Gastroenterology, Hospital Cochin, Paris, France; [6]Statistic and epidemiology, Hôpital Henri-Mondor Ap-Hp, Créteil, France; [7]Icu, Hospital Center Intercommunal Poissy/Saint-Germain-En-Laye, Poissy, France; [8]Icu, The Regional Hospital of Orleans, Orléans, France; [9]Icu, Hôpital Henri-Mondor Ap-Hp, Créteil, France; [10]Gastroenterology, The Regional Hospital of Orleans, Orléans, France; [11]Icu, Hospital Center Sud Francilien, Corbeil-Essonnes, France; [12]Gastroenterology, Hospital Center Sud Francilien, Corbeil-Essonnes, France; [13]Icu, Chpc - Hospital Center Public Du Cotentin : Hospital Louis Pasteur, Cherbourg-en-Cotentin, France; [14]Icu, Louis-Mourier Hospital (AP-HP), Colombes, France; [15]Soins intensif, ULB Erasme, Anderlecht, Belgium; [16]Icu, C.H. de Versailles, Le Chesnay, France; [17]Medecine intensive reanimation, Hospital Cochin, Paris, France
**Correspondence:** D. grimaldi
*Intensive Care Medicine Experimental* 2019, **7(Suppl 3):**000350

**INTRODUCTION.** Post-CA ischemia/reperfusionn of the gastrointestinal tract is suspected to provoke endotoxin translocation, contributing to shock and organ failure (1, 2). However, incidence, characteristics and consequences of these GI ischemic injuries are unknown

**OBJECTIVES.** Main objective: to assess the incidence of upper digestive tract ischemia in a prospective cohort of patients resuscitated from an out-of-hospital cardiac arrest (OHCA).

Secondary objectives: to determine the incidence of severe digestive ischemia; to determine if upper digestive digestive tract ischemia (severe or not) is associated with occurrence and duration of post-CA resuscitation shock, and with favorable neurological outcome.

**METHODS.** The ENTRACT study was a prospective, interventional, non-controlled, multicentric study. Included patients underwent a gastroscopy 2 to 4 d after OHCA if still intubated. Digestive ischemia was determined by the gastroenterologist who performed the gastroscopy. Severe lesion were defined as ulceration or necrosis. Post-CA shock was defined by the needs for vasopressors despite appropriate fluid management and vasopressors-free days over the first 10 days. CPC 1/2 at hospital discharge defined a favorable outcome (FO). Patients with and without digestive ischemia were compared. Multivariate regression analysis were performed to identify variables associated with digestive ischemia.

**RESULTS.** 221 patients were included, 214 were suitable for complete analysis. Mean age was 62, 74% were male. CA was witnessed in 90%, initial rhythm was VT/VF in 53%, median no-flow was 5 (0-9) min and low flow 21 (15-30) min. 121 (57%, CI95% 50-63%) patients had at least one ischemic lesion lesion of upper digestive tract (103 (85%) didn't present any digestive symptoms before endoscopy). Fundus was the most frequent localization of ischemia followed by antrum, duodenum and esophagus. Ischemic lesions were severe in 55/121 (45%) patients and mild in 66 (mucosal oedema/erythema). Patients with digestive ischemia, compared to patients without, had similar demographic characteristics but were less frequently treated with proton pump inhibitor (PPI) before CA (16% vs 31%). CA characteristics were similar except that patients with digestive ischemia received more epinephrine (2(1-4) vs 1(0-3) mg) despite similar lowflow. Multivariate regression identified peripheral artery disease (OR 0.3 (0.09-0.94)) previous PPI (OR 0.49 (0.23-1.03)) and hypochloremia (OR 0.9 (0.84-0.97)) as protective factors whereas hematocrit (OR 1.04 (1.00-1.09)) and epinephrine dose during CPR (OR 1.17 (1.03-1.32) were associated with upper digestive ischemia. 138 (64%) patients presented post-resuscitation shock and 82 (38%) had a FO. These proportions were similar between the 2 groups as well as vasopressors free days. In pre-specified sub-group analysis, patients with severe digestive ischemia had a lower rate of FO (26% vs 41% and 36% for patients with moderate or no digestive ischemia, p=0.04). Analyses on post-CA shock and on organ failure are ongoing.
**CONCLUSION.** In this multicentric study, upper digestive tract ischemia was frequent after CA (57% of the patients) and associated with epinephrine dose during CPR. Digestive ischemia involved mainly the stomach, necrosis was rare. Severe ischemia seems to be associated with outcome. Main limitation is that patients extubated or dead before d2 were not included.

**REFERENCE(S)**

1) Grimaldi et al. Resuscitation. 2013 Jan;84(1):60-5
2) Grimaldi et al. Crit Care Med. 2015 Dec;43(12):2597-604
3) This study was granted by the Direction de la Recherche Clinique et de l'Innovation, CH Versailles, France

### 000382

#### Low-flow time as a predictor of weaning from extracorporeal membrane oxygenation after extracorporeal cardiopulmonary resuscitation

Y. Kwon[1], Y. Kim[2]
[1]Department of thoracic and cardiovascular surgery, Asan Medical Center, University of Ulsan College of Medicine, Seoul, Republic of Korea; [2]Department of trauma surgery, Pusan National University Hospital, Pusan National University College of Medicine, Busan, Republic of Korea
**Correspondence:** Y. Kim

*Intensive Care Medicine Experimental* 2019, **7(Suppl 3):**000382

**INTRODUCTION.** The aim of this study was to identify predictive factors for successful weaning in patients who received extracorporeal cardiopulmonary resuscitation (ECPR) after cardiac arrest.
**METHODS.** Between December 2015 and November 2018, we retrospectively analyzed all patients who had in-hospital cardiac arrest (IHCA) and out-of-hospital cardiac arrest (OHCA) and were treated with venoarterial extracorporeal membrane oxygenation (VA-ECMO) at our institution. We divided the patients into two groups (weaned and unweaned) and compared the patients' baseline characteristics, low-flow time, and laboratory findings between the two groups.
**RESULTS.** Of the 151 patients included in the study, 30 (19.9%) had OHCA and 121 (80.1%) had IHCA. Seventy-three patients (48.3%) were successfully weaned from VA-ECMO, while 78 (51.7%) were not. The mean low-flow time was 24.9 ± 16.9 minutes in all the patients, 20.9 ± 15.9 minutes in the weaned group, and 28.6 ± 17.1 minutes in the unweaned group ($p$ = 0.005). Multivariate analysis revealed shorter low-flow time as an independent predictor of successful weaning from VA-ECMO, whereas IHCA and age did not predict successful weaning from VA-ECMO.
**CONCLUSION.** Low-flow time was a significant predictor of successful weaning from VA-ECMO in the patients who received ECPR in this study.

**REFERENCE(S)**

1. Yang JH (2018) Clinical Significance of Low-flow Time in Patients Treated with Extracorporeal Cardiopulmonary Resuscitation. Korean Circ J 48:716-718.
2. Otani T, Sawano H, Natsukawa T, Nakashima T, Oku H, Gon C, Takahagi M, Hayashi Y (2018) Low-flow time is associated with a favorable neurological outcome in out-of-hospital cardiac arrest patients resuscitated with extracorporeal cardiopulmonary resuscitation. J Crit Care 48:15-20.
3. Wengenmayer T, Rombach S, Ramshorn F, Biever P, Bode C, Duerschmied D, Staudacher DL (2017) Influence of low-flow time on survival after extracorporeal cardiopulmonary resuscitation (eCPR). Crit Care 21:157.
4. D'Arrigo S, Cacciola S, Dennis M, Jung C, Kagawa E, Antonelli M, Sandroni C (2017) Predictors of favourable outcome after in-hospital cardiac arrest treated with extracorporeal cardiopulmonary resuscitation: A systematic review and meta-analysis. Resuscitation 121:62-70.
5. Jung C, Janssen K, Kaluza M, Fuernau G, Poerner TC, Fritzenwanger M, Pfeifer R, Thiele H, Figulla HR (2016) Outcome predictors in cardiopulmonary resuscitation facilitated by extracorporeal membrane oxygenation. Clin Res Cardiol 105:196-205.
6. Yu HY, Wang CH, Chi NH, Huang SC, Chou HW, Chou NK, Chen YS (2019) Effect of interplay between age and low-flow duration on neurologic outcomes of extracorporeal cardiopulmonary resuscitation. Intensive Care Med 45:44-54.

### 000428

#### Prognostic value of initial blood ammonia and lactate for hypothermia-treated cardiac arrest patients

DH. Lee[1], KN. Park[2]
[1]Department of emergency medicine, Uijeongbu St. Mary's Hospital, The Catholic University of Korea, Euijeongbu, Republic of Korea; [2]Department of emergency medicine, Seoul St. Mary's Hospital, The Catholic University of Korea, Seoul, Republic of Korea
**Correspondence:** D.H. Lee

*Intensive Care Medicine Experimental* 2019, **7(Suppl 3):**000428

**INTRODUCTION.** Current treatments for out-of-hospital comatose cardiac arrest patients have improved the survival rate and neurologic prognosis. However, early neurologic prognostication still remains a challenge. We assumed that initial serum ammonia and lactate levels may be associated with the patients neurologic outcome.
**OBJECTIVES.** This is a single-centered prospective study comprising 213 patients with 14 exclusions. Blood samples were taken after the return of spontaneous circulation (ROSC). Neurologic prognosis was evaluated using cerebral performance scale (CPC). Patients were divided into two groups: Good neurologic outcome (CPC 1, 2 = 80) and Poor neurologic outcome (CPC 3, 4, 5 = 133).
**METHODS.** Variables were compared using Chi-square or T-Test. All variables found to be significant by univariate analysis then underwent multivariate logistic regression analysis for determine whether each variable has an independent relationship with poor neurological prognosis. The primary model was constructed by extracting statistically significant variables and performing multiple logistic regression analysis. The secondary model consisted of the discriminative power of the primary model for the prediction of neurological prognosis, and the concentration of serum ammonia and blood lactate, respectively.
**RESULTS.** Serum ammonia and lactate levels in the poor outcome group were significantly higher than those of the good outcome group. On multivariate logistic regression analysis, age (OR, 1.029; 95% CI, 1.003-1.055; p =0.027), non-shockable rhythm (OR, 12.682; 95% CI, 5.855-27.471; p < 0.001), and time from collapse to ROSC (OR, 1.062; 95%CI, 1.037-1.088; p < 0.001) were the most significant predictive variables for poor neurologic outcomes, and comprised our primary prediction model for poor neurologic outcomes. Secondary model consisted of either or both of ammonia and lactic acid in the primary model. These two models were compared and no significant differences were found.
**CONCLUSION.** In conclusion, serum ammonia and lactate levels were associated with prognosis of the out-of-hospital comatose cardiac arrest patients but did not provide incremental prognostic value.

**REFERENCE(S)**

1. Yanagawa Y, Sakamoto T, Sato H. Relationship between laboratory findings and the outcome of cardiopulmonary arrest. Am J Emerg Med 2009;27:308-12.
2. Cho YM, Lim YS, Yang HJ, Park WB, Cho JS, Kim JJ, et al. Blood ammonia is a predictive biomarker of neurologic outcome in cardiac arrest patients treated with therapeutic hypothermia. Am J Emerg Med 2012;30:1395-401.
3. Shinozaki K, Oda S, Sadahiro T, Nakamura M, Hirayama Y, Watanabe E, et al. Blood ammonia and lactate levels on hospital arrival as a predictive biomarker in patients with out-of-hospital cardiac arrest. Resuscitation 2011;82:404-9.
4. Prause G, Ratzenhofer-Comenda B, Smolle-Juttner F, Heydar-Fadai J, Wildner G, Spernbauer P, et al. Comparison of lactate or BE during out-of-hospital cardiac arrest to determine metabolic acidosis. Resuscitation 2001;51:297-300.
5. Deakin CD, Morrison LJ, Morley PT, Callaway CW, Kerber RE, Kronick SL, et al. Part 8: Advanced life support: 2010 International Consensus on Cardiopulmonary Resuscitation and Emergency Cardiovascular Care Science with Treatment Recommendations. Resuscitation 2010;81 Suppl 1:e93-e174.
6. Mullner M, Sterz F, Domanovits H, Behringer W, Binder M, Laggner AN. The association between blood lactate concentration on admission, duration of cardiac arrest, and functional neurological recovery in patients resuscitated from ventricular fibrillation. Intensive Care Med 1997;23:1138-43.

#### 000490

#### Rotation compressor during CPR: is one minute better than two minutes?

N. Pechaksorn[1], V. Vattanavanit[2]
[1]Internal medicine department, Prince of Songkla university, Hat Yai, Hat Yai District, Songkhla, Thailand, Thailand; [2]Critical care medicine unit, internal medicine department, Prince of Songkla University, Karnjanavanich Road Kho Hong, Hat Yai District, Songkhla, Thailand, Kho Hong, Thailand
**Correspondence:** N. Pechaksorn
*Intensive Care Medicine Experimental* 2019, **7(Suppl 3):**000490

**INTRODUCTION.** Current basic life support guidelines recommend two minute shifts for compression between two rescuers (1). However, rescuer fatigue coupled with a decay in the quality of chest compressions occurs within one minute.

**OBJECTIVES.** To compare alternating rescuers in one-minute and two-minute chest compressions, in terms of: chest compression quality metrics and rescuer fatigue.

**METHODS.** This prospective randomized cross-over study was conducted at Songklanagarind hospital, Hat Yai, Songkhla, Thailand. The study enrolled sixth year medical students and residents. All participants were randomly grouped into pairs to perform 8 minutes of chest compression, utilizing both the one-minute and two-minute scenarios on a mannequin model. The primary end-points were: chest compression depth and rate. The secondary end-points included rescuer's fatigue, respiratory rate and heart rate.

**RESULTS.** One-hundred and four medical students and residents participated in this study. In the one-minute group, compared with the two-minute group, there was significant, higher mean (SD) compression depth [mm] (45.8 (7.2) vs 44.5 (7.1), P=0.01), but no difference in the mean (SD) compression rate [per min] (116.1 (12.5) vs 117.8 (12.4), p = 0.08). The rescuers in the one-minute group were lower in the fatigue visual analog scale (P<0.001), change in respiratory rate (P<0.001), however, there was no difference in the change of heart rate (P=0.59), compared with the two-minute group.

**CONCLUSION.** There was statistically significant, higher compression depth and lower rescuer fatigue in 1-minute chest compression groups, compared with 2-minutes. (Thai Clinical Trials Registry TCTR20170823001)

##### REFERENCE(S)

1. Kleinman ME, Brennan EE, Goldberger ZD, Swor RA, Terry M, Bobrow BJ, et al. Part 5: Adult Basic Life Support and Cardiopulmonary Resuscitation Quality: 2015 American Heart Association Guidelines Update for Cardiopulmonary Resuscitation and Emergency Cardiovascular Care. Circulation. 2015;132:S414-35.
2. This study was funded by a research grant from the Faculty of Medicine, Prince of Songkla University

#### 000549

#### Hospital survival following out of hospital cardiac arrest - is it all due to pre-hospital patient selection?

P. May[1], W. A.[1], M. J.[1], B. G.[2], P. M.[1]
[1]Intensive care, Frimley Park Hospital, Frimley, United Kingdom; [2]Accident and emergency, Frimley Park Hospital, Frimley, United Kingdom
**Correspondence:** P. May
*Intensive Care Medicine Experimental* 2019, **7(Suppl 3):**000549

**INTRODUCTION.** Out of hospital cardiac arrest (OHCA) affects 60,000 people in the UK annually and good outcomes rely on a 'chain of survival', which has pre-hospital and in-hospital components. Our 700-bedded UK DGH has a 24-7 Cardiac Cath Lab service, receives 100 OHCA patients each year, and is served by two ambulance services – South East Coast (SECAMB) and South Central (SCAS).

**OBJECTIVES.** To quantify our hospital survival after OHCA and contrast this with local ambulance service data.

**METHODS.** We analysed prospectively collected data from all adult post-OHCA patients admitted via ED over a 5-year period (01/01/12-31/12/16) using Trust electronic databases, and compared this with nationally published ambulance service data.

**RESULTS.** [Presented as median (range) or percentages] Of 499 patients identified: age 71 (20-96); primary rhythm VF 40%, VT 3%, PEA 29%, asystole 19%, none recorded 9%; died in ED 45%, admitted to ICU 43%; admitted to a ward 12%; survived 25%. Of VF patients: age 68 (20-93), admitted to ICU 59%, survived 44%. Of VT patients: age 76 (57-91), to ICU 38%, survived 38%. Of PEA patients: age 72 (23-96), to ICU 28%, survived 7%. Of asystole patients: age 74 (25-96), to ICU 26%, survived 3%. Monthly OHCA ambulance service survival data during study period: SCAS 15.4% (5.5-26.9), SECAMB 7.6% (0.7-12.3), national 8.5% (6.3-10.6). Of the 31192 ambulance arrivals in 2016: conveyed by SECAMB 77%, conveyed by SCAS 23%.

**CONCLUSION.** 1) Our data show that patients presenting with VF are younger, more likely to be selected for ICU support and survive to hospital discharge: a relationship that is well known. 2) The national incidence of cardiac arrests presenting with shockable rhythms is 20%; in our population this was 43%. 3) Our overall survival rate is well above average (observed 25%; expected 8.5%), which could be be explained, in part, by selection bias by pre-hospital teams, who consciously select in patients with favourable outcomes (those in VF and most likely to benefit from a heart attack centre) and unconsciously select out those with unfavourable outcomes via unsuccessful resuscitation. 4) Both SECAMB and SCAS follow national guidelines and are able to pronounce life extinct (PLE) at the scene. However, they have dramatically different survival data; reasons for which we will continue to investigate. We hypothesise that, among others, differences in data collection and/or PLE thresholds may be at play here. 5) SCAS conveys <¼ of our patients, so excess survival directly attributable to SCAS should have minimal impact on our outcomes. 6) Increased VF prevalence does not completely explain the extent of survival benefit that we observed.

##### REFERENCE(S)

1. Nil
2. Nil

## HSRO - Concomitants condition and their influence on outcome

#### 000773

#### Critical illness in the elderly: experience in an Intensive Care Unit of a second level Hospital

F. Dos Santos Alvernia[1], L. Castro Bournissen[1], A. Del Caño Garcia[1], A. ÁLvarez Méndez[1], C. Muñoz Leal[1], M. Mas Lodo[1], R. González González[2], N. Franco Garrobo[1]
[1]Intensive care unit, Hospital Universitario de Móstoles, Móstoles, Spain; [2]Unidad de apoyo a la investigación, Francisco de Vitoria University - Madrid, Pozuelo de Alarcón, Spain
**Correspondence:** L. Castro-Bournissen
*Intensive Care Medicine Experimental* 2019, **7(Suppl 3):**000773

**INTRODUCTION.** There is controversy on admission of critically ill elderly patients in ICU as older age has been associated with poor clinical outcome.

**OBJECTIVES.** We analyzed patients above the age of 80 admitted to our ICU during the past four years and evaluated clinical outcomes.

**METHODS.** Retrospective cohort study of patients aged 80 and above admitted to the ICU of Hospital de Mostoles in Madrid, Spain, from January 2014 to December 2018. We collected data from electronic medical records on demographics, functional status prior to admission, Simplified Acute Physiology Score III (SAPS III), admission cause, need of mechanical ventilation, vasopressor support, continuous renal replacement therapy (CRRT), mortality in ICU, hospital mortality and mortality up to one year after ICU discharge.

**RESULTS.** 347 patients aged 80 and over were admitted to our Unit during the study period. Mean age was 83 years, 47% women and 68,7% totally independent for activities of daily living according to the Barthel index. The most common cause of admission was cardiovascular, 48.1%, including cardiac arrest, acute coronary syndrome, pulmonary embolism and arrhythmias. Mean SAPS III score was 57,70. Vasopressor support was administered in 37% of patients, invasive mechanical ventilation 25% and non-invasive mechanical ventilation or high flow oxygen cannula in 19%. Only 2% of patients required CRRT. Median ICU stay 3 days. ICU mortality was 19.6% and overall hospital mortality 28,53%; 10% died within the first year after ICU discharge.

In the univariate analysis, a statistical association with mortality was found with vasopressor support, OR 4.86, 95% CI (2.75-8.61) and both invasive and non-invasive mechanical ventilation with OR 78, 95% CI (26,5-230) and OR 7.29, 95% CI ( 2.1-24.5) respectively. For each unit increase in SAPS III, there was an increased odds of death of 1.1 (95% CI 1.07–1.13). Similarly, the odds of dying in patients partially dependant for activities of daily living was 3,09 (95% CI :1-17-8.15) whereas totally independent subjects showed OR 1.07 (95% CI: 1.01-1.13). Finally, age revealed no association with increased risk of mortality (OR 0.96 with a 95% CI 0.88-1.05) and male sex had a lower risk of dead (OR 0.45 with a 95% CI 0.26-0.77). The multivariate logistic regression analysis determined that only functional status (partially dependent OR 3.09, 95% CI 1.17-8.15) and length of hospital stay (for each day increase OR 1.07, 95% CI 1.01-1.13) were associated with mortality at one year.

**CONCLUSION.** Admission of elderly patients to ICU should be considered after a careful evaluation of their functional status and comorbidities. As expected, patients requiring more ICU support (invasive mechanical ventilation, vasopresssors) have higher mortality. Age by itself does not seem to predict poor clinical outcome.

### 000775

#### Catheter related venous thrombosis in critically ill patients and ultrasound diagnosis. Prospective cohort comparing PICC vs CVC

E. SANCHO[1], P. Ramirez Galleymore[2], M. Martín Cerezuela[3], C. Vicent Perales[3], MÁ. Padrós Olmedo[3], MDC. Carrasco Jaureguízar[3], A. Martínez Yañez[3], A. Viviani[3], I. Guillén Bernardo[3]

[1]Intensive care unit, Hospital Universitario y Politécnico La Fe, València, Spain; [2]Intensive care unit, Hospital Universitario y Politécnico de La Fe, València, Spain; [3]Intensive care unit, Hospital Universitario y Politécnico La Fe, Valencia, Spain, Spain

**Correspondence:** E. SANCHO

*Intensive Care Medicine Experimental* 2019, **7(Suppl 3):**000775

**INTRODUCTION.** The use of peripherally inserted central venous catheters (PICC) is increasing in the ICU due to the easy peripheral access and the lower number of complications related to insertion. Some studies indicate a higher frequency of venous thrombosis related to PICC than to central venous catheters (CVC). However, there are no conclusive data on the incidence of catheter-related venous thrombosis (CRVT) in critically ill patients. Despite there are different diagnosis possibilities, the gold standard technique is the ecography.

**OBJECTIVES.** The aim of the study is to investigate the incidence of catheter related venous thrombosis (CRVT) in ICU and compare this incidence between patients with PICC and CVC (jugular or subclavian). A secondary objective is to locate the role of ultrasound screening for the early diagnosis of the CRVT. Additionally, the study describes the characteristics of the patient, the canalized vein, the clinical expression, and the relationship of these variables with the appearance of CRVT.

**METHODS.** The study was carried out as a prospective, open, non-randomized cohort study of consecutive patients between March and November 2018. PICC and CVC used were polyurethane 7F catheters. All PICC were placed by trained nurses. Resident or senior doctors placed CVC. Clinical (daily) and ultrasound (3 established exams) follow-up was applied to each patient during the first 20 catheter days (or until the catheter was removed). All data concerning the patient, the anticoagulation treatment and the diagnosis of other non-thrombotic complications related to the catheters was collected.

**RESULTS.** A total of 150 patients have been analyzed; 83 patients underwent PICC placement, and CVC was used in 67 patients. Both groups were comparable in terms of baseline characteristics. We found a significantly higher incidence of CRVT in the PICC group: 25% (n=21) vs 8,9% (n=6) in the CVC group (p = 0,001). PICC patients had an increased risk of thrombosis OR=3,20 (IC95% 1,18-8,69). Moreover, thrombosis appeared at day 4,81 in average in PICC patients, while appeared at day 7,83 in CVC patients (p=0,0029).

Just 8 of the 27 patients with thrombosis had clinical signs of phlebitis, while 19 (70,3%) of patients had subclinical thrombosis, only diagnosed by ecography. Then, the PPV of phlebitis is just 29,6%.

**CONCLUSION.** In our cohort, PICC catheters have an increased risk of thrombosis and this thrombosis appeared before than thrombosis related with CVC. Phlebitis was not a reliable sign of thrombosis, most patients with thrombosis had no clinical features. Ultrasound screening should be performed regularly in critically ill patients wearing central catheters.

**REFERENCE(S)**

1. Nolan ME, Yadav H, Cawcutt KA, Cartin-Ceba R. Complication rates among peripherally inserted central venous catheters and centrally inserted central catheters in the medical intensive care unit. J Crit Care. 2016 Feb;31(1):238-42
2. Zochios V, Umar I, Simpson N, Jones N Peripherally inserted central catheter (PICC)-related thrombosis in critically ill patients. J Vasc Access. 2014 Sep-Oct;15(5):329-37
3. Chopra V, Anand S, Hickner A, Buist M, Rogers MA, Saint S, Flanders SA. Risk of venous thromboembolism associated with peripherally inserted central catheters: a systematic review and meta-analysis.Lancet. 2013 Jul 27;382(9889):311-25
4. Luo L1, Jing XM2, Wang GR3, Qin Y1, Liang HX1, Liu SS4. Peripherally Inserted Central Catheter-Related Upper Extremity Venous Thrombosis in Oncology Patients: A Prospective Study Based on Doppler Sonography.J Ultrasound Med. 2016 Aug;35(8):1759-63
5. Wilson TJ, Stetler WR Jr, Fletcher JJ. Comparison of catheter-related large vein thrombosis in centrally inserted versus peripherally inserted central venous lines in the neurological intensive care unit.Clin Neurol Neurosurg. 2013 Jul;115(7):879-82
6. Chopra V, Kuhn L, Ratz D, Lee A, Krein S.Peripherally inserted central catheter-related deep vein thrombosis: contemporary patterns and predictors. J Thromb Haemost. 2014 Nov;12(11)
7. Malinoski D1, Ewing T, Bhakta A, Schutz R, Imayanagita B, Casas T, Woo N, Margulies D, Barrios C, Lekawa M, Chung R, Bukur M, Kong A Which central venous catheters have the highest rate of catheter-associated deepvenous thrombosis: a prospective analysis of 2,128 catheter days in the surgical intensive care unit. J Trauma Acute Care Surg. 2013 Feb;74(2):454-60; discussion 461-2
8. Hammes M, Desai A, Pasupneti S, Kress J, Funaki B, Watson S, Herlitz J, Hines J.Central venous catheters: incidence and predictive factors of venous thrombosis. Clin Nephrol. 2015 Jul;84(1):21-8

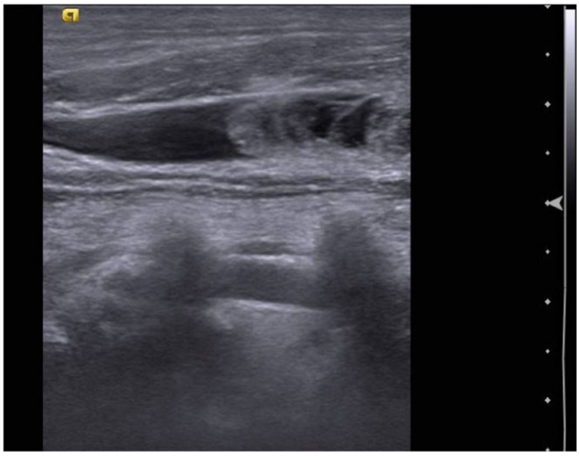

**Fig. 1 (abstract 000775).** See text for description

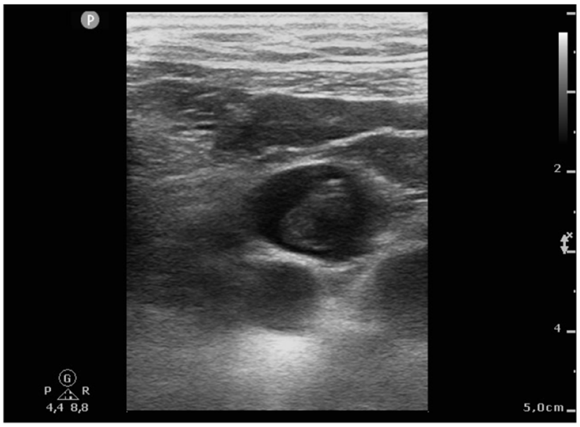

**Fig. 2 (abstract 000775).** See text for description

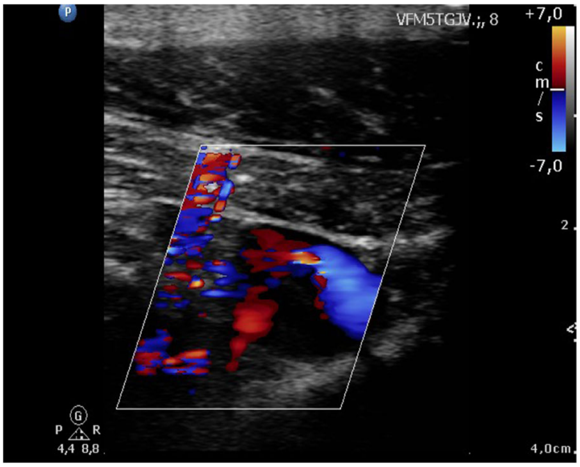

**Fig. 3 (abstract 000775).** See text for description

### 000777

#### Frailty assessment in cardiopulmonary arrest, is it necessary?

N. Arriero Fernández[1], A. Estrella Alonso[1], JA. Silva Obregón[1], R. Torres Sánchez Del Arco[1], S. Arriero Fernández[2], Z. Eguileor Marín[1], MA. Tirado Fernández[1], JE. Romo Gonzales[1], R. Viejo Moreno[1], C. Benito Punzel[1], A. Albaya Moreno[1], E. Quiros Oyaguez[1], P. Revuelta[1], E. Yañez Parareda[1], A. Moya López[1], MI. Jiménez López-Peláez[1], V. Larios Reyes[1], L. Torres Fernández[1], P. Rojo Villar[1], C. Marian Crespo[1]
[1]Intensive care, Hospital Universitario de Guadalajara, Calle Donante de Sangre, Guadalajara, Spain, Guadalajara, Spain; [2]Psychiathy, Sanatorio Privado Neuropsiquiatrico Doctor León, Plaza Mariano de Cavia, Madrid, Spain, Madrid, Spain
**Correspondence:** N. Arriero Fernández
*Intensive Care Medicine Experimental* 2019, **7(Suppl 3):**000777

**INTRODUCTION.** Frailty is characterized by age-associated declines in physiologic reserve and function across multiorgan systems, leading to increased vulnerability for adverse health outcomes.

**OBJECTIVES.** To asses the impact of frailty in the outcomes of patients suffering from a cardiac arrest during their hospital stay.

**METHODS.** A descriptive, observational and retrospective study was conducted from January 2015 to June 2018. We analyzed all calls to the ICU resuscitation team in Guadalajara Hospital in this period. Demographic variables (age and sex), frailty, causes of CPA, limitation of CPR and outcomes were determined. Frailty assessment was performed using the Clinical Frailty Scale (CFS). Frailty divide patients in 2 categories: prefrail and frail (PF-F) (CFS > 3) and non-frail (NF) (CFS ≤ 3).

For statistical analysis of the data, quantitative variables are expressed as means ± standard deviation and qualitative variables as percentages. Categorical variables were assessed using the chi-square test and continuous variables were assessed using t-Student or U-Mann-Withney tests. Multivariate analysis was performed using a logistic regression model. A p-value <0.05 was considered statistical significance

**RESULTS.** 105 calls for CPA were recruited. 17 patients were excluded because CFS could not be determined. We studied 88 patients with a main age of 74.9 ± 10,9 years, with a higher proportion of men (68.2%). In our sample, 69.3 percent of patients were considered PF-F (61/88) (Image 1). In-hospital mortality was 94,3% (83/88), with no statistically significant difference in age, sex, limitation of CPR and causes of CPA. People with frailty are significantly more likely to die. Multivariant analysis, adjusted by age, sex and CPR cause showed that only CFS>3 (PF-F patients) was a significant independent prognosis factor of mortality (OR 10,43; IC 95% 1,11 - 98,35; p=0,04).

**CONCLUSION.** Frailty is a significant independent prognosis factor of mortality in patients suffering from a CPA. Assessing frailty may help clinicians in CPR or non CPR decision-making.

#### REFERENCE(S)

1. To my mentors, Alberto Silva and Alfonso Estrella

**Table 1 (abstract 000777).** See text for description

| VARIABLE | TOTAL (N=88) | SURVIVORS (n=5) | DECEASED (n=83) | OR (IC 95%) | P value |
|---|---|---|---|---|---|
| **Age** | 74,88 ± 10,86 | 69,80 ± 11,69 | 75,18 ± 10,81 | 1,04 (0,97 - 1,12) | 0,289 |
| **Sex** | | | | | |
| Men | 60 (68,2%) | 4 (80%) | 56 (67,5%) | 1,93 (0,21 - 18,10) | 0,565 |
| Women | 28 (31,8%) | 1 (20%) | 27 (32,5%) | | |
| **Resuscitate orders** | 28 (31,8%) | 0 (0%) | 28 (33,7%) | - | - |
| **CFS** | 4,5 (3,0 - 6,75) | 3,0 (2,0 - 3,5) | 5,0 (3,0 - 7,0) | 3,64 (1,14 - 11,57) | **0,029** |
| **Frailty** | | | | | |
| Non frailty | 27 (30,7%) | 4 (80%) | 23 (27,7%) | 10,44 (1,11 - 98,35) | **0,040** |
| Pre-frailty and frailty | 61 (69,3%) | 1 (20%) | 60 (72,3%) | | |
| **PCR causes** | | | | 2,16 (0,59 - 7,87) | 0,242 |
| Cardiac | 16 (18,2%) | 2 (40%) | 14 (16,9%) | Referencia | |
| Respiratory | 41 (46,6%) | 2 (40%) | 39 (47%) | 2,78 (0,36 - 21,70) | 0,328 |
| Others | 31 (35,2%) | 1 (20%) | 30 (36,1%) | 4,29 (0,36 - 51,32) | 0,251 |
| **STOP PCR** | 17 (19,3%) | 0 (0%) | 17 (20,5%) | - | 0,578 |

### 000811

#### 'Development of a Quality Assurance Framework for Focused Echocardiography in Intensive Care

S. Curry, N. Jones, A. Roscoe, K. Salunkey, A. Rubino
Intensive care, Royal Papworth Hospital NHS Foundation Trust (new Cambridge site), Cambridge, United Kingdom
**Correspondence:** S. Curry
*Intensive Care Medicine Experimental* 2019, **7(Suppl 3):**000811

**INTRODUCTION.** The use and availability of point-of-care care ultrasound is increasingly being recognised as a valuable diagnostic and monitoring tool in the Intensive Care Unit (ICU). When used at the bedside, ultrasound-guided vascular access, drain insertion and ultrasound-guided regional anaesthesia have been shown to enhance the safety and are now considered standard practice. Equally, the use of focused transthoracic echocardiography (TTE) has become readily available for real-time haemodynamic assessment and monitoring of patients with sepsis, congestive heart failure, shock and traumatic injuries. Separate from the scope of a comprehensive TTE study, a focused TTE study is intended to answer specific questions related to the ICU patient. Limited to acquisition of four basic cardiac views and one view of each lung base, a focused scan can serve as a rapid assessment of the cardiac structure and function in acutely unwell patients. However, there are currently no robust quality assurance systems in place for ensuring responsible practice in the use of focused echocardiography by intensive care clinicians after the initial training period. To assess the quality of focused TTE studies completed on our ICU we conducted a retrospective analysis of our current practice to establish the level of consistency and reproducibility of focused TTE studies.

**METHODS.** A retrospective analysis of focused TTE studies conducted on Royal Papworth Hospital ICU. Data collection included repeated audit cycles of 3-months duration between April 2016 – Feb. 2018. All focused TTE studies conducted on Royal Papworth ICU during the audit periods were included. Focused TTE studies were analysed using a 22-point audit tool to assess technical quality, reporting, documentation and upload to the local PAC system. Data were analysed using Chi-squared tests, with a p-value <0.05 regarded as significant.

**RESULTS.** Data were collected from 409 focused TTE studies during four audit cycles. Inclusion of patient details and attachment of ECG was similar between audit cycles (64.4%). Studies with four recorded cardiac views improved from the first audit cycle (31%), with the highest percentage of complete images obtained during April-June 2017 (53%). The subcostal view was the least obtained view across all cohorts, with more than 50% of all studies missing a subcostal image. Chamber function, pericardial collections and hypovolaemia were assessed on average the same across all cohorts (>90%). Assessment of pleural collections declined from 2016 (69%) to 2018 (48%). A decrease in the number of studies uploaded to the PAC system was also observed, with only 4% of studies uploaded in June-Aug 2017.

**CONCLUSION.** Basic echocardiography training for intensive care clinicians is regarded as valuable screening tool, which can be used quickly and effectively in the management of patients in acute settings. However, variation in practice related to incomplete studies, reduced number of standard images obtained and poor compliance with clinical documentation were highlighted in this retrospective analysis. In response to these findings, we have implemented a structured system of senior review and on-going audit during monthly quality assurance meetings. BSE accredited Cardiologists and Cardiac Physiologist contribute to a quality assurance scoring system and provide direct feedback on quality of echocardiography images and on the accuracy of reporting, enabling qualitative review of focused TTE studies that was not possible in the previous quantitative audit parameters.

**REFERENCE(S)**

1. Yanick Beaulieu, Paul E Marik Bedside Ultrasonography in the ICU Elsevier Chest Journal August 2005; 128: 881-895 DOI:10.1378/chest.128.3.1766
2. S. Price, E. Nicol, D.G . Gibson, T.W Evans Echocardiography in the critically ill: current and potential roles Intensive Care Medicine January 2006; 32:48-59.
3. International Expert Statement on training standards for critical care ultrasonography- Expert Round Table on Ultrasound in ICU. Intensive Care Med 2011;37:1077-83.
4. Fox K: On behalf of a Collaborative Working Group of the British Society of Echocardiography (BSE). A position statement: echocardiography in the critically ill. JICS 2008;9:197-98.
5. none

### 000824

#### Evaluation of the dependence degree for basic activities of daily living in ICU survivors

PA. Duarte[1], TC. Lievore[2], L. Rossi[2], W. Batista[2], TV. Lordani[2], CR. Lordani[3], JB. Costa[4], AC. Jorge[1], KC. Luzzi[1]
[1]General icu, Hospital Universitario do Oeste do Parana, Cascavel, Brazil; [2]Nursing department, Hospital Universitario do Oeste do Parana, Cascavel, Brazil; [3]Department of clinical nutrition, Hospital Universitario do Oeste do Parana, Cascavel, Brazil; [4]Deparment of psychology, Hospital Universitario do Oeste do Parana, Cascavel, Brazil
**Correspondence:** P.A. Duarte
*Intensive Care Medicine Experimental* 2019, **7(Suppl 3):**000824

**INTRODUCTION.** Among the several physical, cognitive and psychological consequences of ICU stay, the activities ot daily living (ADL) are indicative tasks of the ability to lead an independent life, such as housework, shopping, self-medication.

**OBJECTIVES.** To assess the capacity of the dependence degree for basic daily activities in adult ICU survivors.

**METHODS.** Cross-sectional descriptive study by evaluating adult ICU survivors in a University Hospital, for 2 years. The sample was composed by patients who returned for the multiprofessional follow-up clinic 3 months after ICU discharge. The data were collected through consultation and application of the Katz index.

**RESULTS.** A total of 287 patients were evaluated (mean age 46.3 y; 56.8% male; 36% trauma as ICU admission cause). The pre-ICU dependence status was: 95.8% independent; 0.8% partial dependence; 3.5% total dependence. Table 1 shows the post-ICU dependence degree and some variables.

**CONCLUSION.** Although most post-ICU patients were independent, the incidence of dependence was relatively high, particularly in older and non-trauma patients. The evaluation of ADL may be an important marker of outcome in this group, as well as the quality of care performed during hospitalization.

**Table 1 (abstract 000824).** Dependence degree for basic activities of daily living in ICU survivors, n=287

| | Independent | Dependent, partial | Dependent, total |
|---|---|---|---|
| n | 213 | 25 | 49 |
| Male gender, % | 57.7% | 64.0% | 67.3% |
| Age, years, % | | | |
| <40 | 41.8% | 24.0% | 38.8% |
| 41-60 | 37.6% | 32.0% | 28.6% |
| >60 | 20.6% | 44.0% | 32.6% |
| ICU admission cause, % | | | |
| Trauma | 31.9% | 64.0% | 40.8% |
| Non-trauma | 68.1% | 36.0% | 59.2% |
| ICU length of time, mean (days) | 9.5 ± 7.8 | 14.8 ± 13.0 | 13.4 ± 8.18 |

### 000825

#### Does early lactate serum levels predicts mortality in very old critically ill patients?

R. Carvalho De Menezes[1], I. Bonifácio Brige Ferreira[2], M. Lisboa Otero[1], G. Andrade Agareno[3], AMDS. Cerqueira Junior[1], L. Pamplona Neto[1], S. Agareno De Souza Filho[1], NM. Filgueiras Filho[3]
[1]Critical care, Hospital da Cidade, Salvador, State of Bahia, Brazil, Brazil; [2]Medicine, Bahia State University, Salvador, Brazil; [3]Medicine, Salvador University, Campus Teacher Barros, Salvador, Brazil
**Correspondence:** N.M. Filgueiras Filho
*Intensive Care Medicine Experimental* 2019, **7(Suppl 3):**000825

**INTRODUCTION.** Lactate is considered to be a useful prognostic biomarker in critically ill patients. However, few studies address the association of lactate with mortality specifically in octogenarian patients, a population that deserves to be individualized given changes in clinical presentations of diseases, functional reserve, social demands and, among others that occur over the years.

**OBJECTIVES.** To assess the accuracy of lactate in predicting mortality in very old patients.

**METHODS.** A retrospective cohort study with very old patients (≥ 80 years) with a length of stay > 24 hours in a general ICU during the period August 2015 to August 2018. Lactate serum level was obtained within the first 6 hours of admission. The D'agostino test was used to determine normality in lactate values. In order to evaluate the median levels of lactate between the populations that were discharged and those who died and its accuracy, the Mann-Whitney U Test and the Area Under Receiver Operating Characteristic Curve (AUROC) were respectively used.

**RESULTS.** A total of 691 patients were analyzed, 65% (451) of whom were women, the mean ± SD and the maximum age was 87 ± 4.9 and 103 years. The mean Simplified Acute Physiology Score 3 (SAPS3) was 55.7 ± 9.4 and mean Charlson Comorbidity Index was 1.78 ± 1.73, without the assignment of points for age. Overall mortality was 28% (194), 7-day mortality was 13% (92), and there was 20% (144) of patients with an elevated lactate (≥ 2 mmol/dL). In comparison to those who survived (1.1; IQR: 0.8 – 1.8), the median lactate was higher in patients who died in all period (1.5; IQR: 1.1 – 3.05) [p < 0.0001] and in those who died in 7-day (2.0; IQR: 1.3-4.4) [p < 0.0001]. However, lactate did not demonstrate a good accuracy in determining the outcome of the patients, either to determine death during ICU stay (AUROC: 0.63) or even early death (AUROC: 0.73).

**CONCLUSION.** In the very old critically ill patients of our cohort, lactate had a poor correlation with mortality.

### 000839

#### Mortality and readmission rates of patients discharged in-hours and out-of-hours from a British ICU over a three year period

J. Cumberworth, M. Chequers, O. Boyd, B. Philips
Department of intensive care medicine, Royal Sussex County Hospital, Brighton, United Kingdom
**Correspondence:** J. Cumberworth
*Intensive Care Medicine Experimental* 2019, **7(Suppl 3):**000839

**INTRODUCTION.** We have previously reported higher in-hospital mortality in patients discharged from the ICU out-of-hours compared to in-hours.1 When patients discharged for end-of-life care or organ donation were excluded, the discrepancy in mortality rates was much smaller. This could be explained because organ donation procedures are frequently undertaken at night and the mortality rate is 100%. We hypothesised that later discharge of patients undergoing organ donation or other end of life care might be a major influence on the frequently reported finding of apparent excess mortality in out-of-hours discharges.2 This new study seeks to establish in-hospital mortality rates for those discharged in-hours and out-of-hours over a 3 year period, with those discharged for organ donation or end-of-life care excluded.

**METHODS.** This is a single centre retrospective cohort study. Discharge data were collected for all patients admitted to our unit from 01/07/2015 to 31/07/2018. Patients were excluded if they did not survive to ICU discharge or if they had an APACHE II score of 0. Readmissions were defined as patients readmitted to ICU within the same hospital stay; the first admission for these patients was included. Patients discharged for end of life care or organ donation were identified and excluded. Data collected included age, sex, APACHE II score, nature of admission, length of stay, hospital outcome and readmissions to ICU.

**RESULTS.** 3943 patients were included. The patients discharged out-of-hours were significantly older and had higher APACHE II scores, but there was no significant difference in mortality rate or readmission rate (table 1). Out-of-hours was defined as 22:00 to 07:00.

**CONCLUSION.** There was no excess mortality in patients discharged out-of-hours once the effect of end-of-life or organ donation patients had been excluded. These groups should be considered separately to other patients surviving to ICU discharge, as their prognosis is equivalent to those who have not survived. This cohort of patients may be disproportionately discharged out of hours. The inclusion of patients discharged from ICU but for end-of-life on the ward may explain the previously reported, but unexplained, finding of higher mortality rates in patients discharged from ICU out of hours.

**REFERENCE(S)**

1. Cumberworth J, Francis I, Chequers M, Gray R, Boyd O. Mortality of patients discharged in-hours and out-of-hours from a British ICU: are functional ICU practices to blame? Intensive Care Medicine Experimental 2018, 6(Suppl 2):0830
2. Vollam S, Dutton S, Lamb S, Petrinic T, Young JD, Watkinson P. Out-of-hours discharge from intensive care, in-hospital mortality and intensive care readmission rates: a systematic review and meta-analysis. Intensive Care Med 2018 Jul;44(7):1115-29

**Table 1 (abstract 000839).** Patients discharged alive from a general adult ICU over 3 years

| Characteristic | In-hours discharge | Out-of-hours discharge | Total | P value |
|---|---|---|---|---|
| n | 3943 | 735 | 4678 | n/a |
| Overall % of sample | 84.29 | 15.70 | 100 | n/a |
| Male sex (%) | 57.32 | 59.32 | 57.63 | 0.313 |
| Mean age, SD (yr) | 59.16,17.44 | 62.00,16.87 | 59.61,17.38 | < 0.001 |
| Mean APACHE II score, SD | 14.40,5.79 | 15.74,5.88 | 14.61,5.82 | < 0.001 |
| Median LOS, IQR (days) | 3.00,4.37 | 3.25,4.20 | 3.04,4.35 | 0.009 |
| Medical admission (%) | 47.05 | 51.02 | 47.67 | 0.048 |
| Post-ICU mortality (%) | 3.73 | 4.63 | 3.87 | 0.247 |
| ICU readmission rate (%) | 4.24 | 4.08 | 4.21 | 0.849 |

### 000849

#### Dissociation of glucocorticoid receptor alpha/beta expression and adrenocortical function in critically ill steroid-free patients

AG. Vassiliou[1], G. Stamogiannos[2], E. Jahaj[2], G. Floros[2], E. Botoula[3], D. Vassiliadi[3], I. Ilias[4], S. Tsagarakis[3], M. Tzanela[3], S. Orfanos[2], A. Kotanidou[2], I. Dimopoulou[2]
[1]1st department of critical care medicine & pulmonary services, gp livanos & m simou labs, Evangelismos Hospital, Medical School, National and Kapodistrian University of Athens, Athens, Greece; [2]1st department of critical care medicine & pulmonary services, Evangelismos Hospital, Medical School, National and Kapodistrian University of Athens, Athens, Greece; [3]Department of endocrinology, diabetes and metabolism, Evaggelismos General Hospital, Athina, Greece; [4]Endocrine unit, Elena Venizelou Hospital, Athens, Greece
**Correspondence:** A.G. Vassiliou
*Intensive Care Medicine Experimental* 2019, **7(Suppl 3):**000849

**INTRODUCTION.** Cortisol levels and the responsiveness of cells to steroids are important in regulating the glucocorticoid (GC) activity. GC actions are mediated by the glucocorticoid receptor (GCR) and its dysfunction leads to tissue resistance to GCs.

**OBJECTIVES.** To evaluate the expression of *GCR-α* and *GCR-β* in peripheral polymorphonuclear cells in relation to cortisol and adrenocorticotropin (ACTH) in critical illness for a 13-day period.

**METHODS.** Forty-two mechanically ventilated, steroid-free patients were studied. Blood samples were collected on admission in the ICU and on days 4, 8, and 13, to measure *GCR* expression, cortisol and ACTH. Twenty-five age- and sex-matched subjects were used as controls.

**RESULTS.** Acutely, in critically ill patients *GCR-α* mRNA expression was 10-fold that of controls (p< 0.0001), while *GCR-β* mRNA levels were 3-fold the expression of controls (p< 0.0001). Cortisol was elevated and ACTH was within normal limits. During the sub-acute phase, the expression of the isoforms was lower compared to controls, cortisol remained high and ACTH increased.

**CONCLUSION.** *GCR* expression and hypothalamic-pituitary-adrenal axis function undergo a biphasic response during critical illness; acutely, the expression of both GCRs is increased, however, *GCR-α* expression dominates and there is probably no GC resistance. During the sub-acute phase, the expression of both isoforms decreases and there is dissociation between low *GCR* expression and high cortisol, implying an abnormal stress response. Elevated cortisol is preserved through both ACTH and non-ACTH pathways.

### 000866

#### The role of communication by the ICU's healthcare personnel in the satisfaction of patients and relatives

P. Vega Ocaña[1], L. González Bautista[1], JD. Martin Santana[2], C. García Del Rosario[3], JL. Santana Cabrera[1]
[1]Intensive care unit, Hospital Universitario Insular de Gran Canaria, Las Palmas de Gran Canaria, Spain; [2]Economics, Universidad de las Palmas de Gran Canaria, Las Palmas de Gran Canaria, Spain; [3]Quality department, Hospital Universitario Insular de Gran Canaria, Las Palmas de Gran Canaria, Spain
**Correspondence:** P. Vega Ocaña
*Intensive Care Medicine Experimental* 2019, **7(Suppl 3):**000866

**INTRODUCTION.** To improve the quality of care provided to patients and their relatives in the ICU, assessing their satisfaction with the care they receive is important, being communication a crucial item to take into account.

**OBJECTIVES.** To evaluate whether the quality of communitation by the healthcare personnel in the Intensive Care Unit perceived by patients and relatives influences their level of satisfaction.

**METHODS.** Information was collected from June to September 2018 through a survey adapted to patients and family members. The questionnaire included a 7-score Likert scale conformed by different items aimed at assessing the quality of communication between patients and relatives and the healthcare personnel in the ICU. The dimensions are based on the work of Mora Lourido (2015). We conducted a difference analysis of means using the student's T, to check the differences in patients and relatives' satisfaction.

Subsequently, a multivariate linear regression analysis was performed to analyze the influence of perception of the quality of communication on satisfaction with the quality of care in the ICU.

**RESULTS.** In the scale of quality of the communication there are six variables shown in the previous table. In the regression analysis of the quality of the communication on the patients and relatives' satisfaction, we find that "Sincerity" is the variable that has the greatest influence on satisfaction (β=0.316, t=4.220, *p*=0.000), followed by the "Satisfactory communication" (β=0.247, t=2.787, *p*=0.006), being the rest of the variables not relevant to explain the satisfaction of patients and relatives.

The value of the F statistic indicates that, globally, the model is acceptable (F=52.905, *p*=0.000) and that it explains 59.1% of the total variance.

**CONCLUSION.** A high perception of the quality of the communication positively influences the satisfaction with the quality of the service on the part of patients and relatives. The most important factor in satisfaction is the sincerity in communication, followed by satisfactory communication. The communication by the healthcare personnel in the ICU is so important that it is able to explain 59.1% of the variability of satisfaction with the quality of the service, which is why it is a fundamental aspect to which health professionals should give importance.

**REFERENCE(S)**

1. Mora Lourido CJ. Análisis del impacto de la dirección de recursos humanos orientada a la gestión de la calidad y la excelencia organizativa: una aplicación en los servicios de Medicina Intensiva del Complejo Hospitalario Universitario Insular-Materno Infantil. Tesis doctoral Universidad de Las Palmas de Gran Canaria (2015).

**Table 1 (abstract 000866).** Differences in quality of communication in the ICU perceived by patients and relatives

| Variables | Mean (T.D.) | | ***t*** (***p***) |
|---|---|---|---|
| | Patients | Relatives | |
| Frequency of communication | 6.88 (0.56) | 6.60 (0.73) | 3.262 (0.001) |
| Sincerity in communication | 6.88 (0.63) | 6.69 (0.63) | 2.328 (0.021) |
| Communication time | 6.89 (0.46) | 6.65 (0.64) | 3.192 (0.002) |
| Satisfactory communication | 6.94 (0.30) | 6.64 (0.67) | 4.313 (0.000) |
| Communication form | 6.92 (0.44) | 6.66 (0.64) | 3.465 (0.001) |
| Understandable communication | 6.93 (0.25) | 6.88 (0.36) | 1.424 (0.156) |

### 000877

#### Identification of predictors for postoperative deterioration

E. Mestrom[1], T. Bakkes[2], N. Ourahou[1], P. De Andrade Serra[2], S. Turco[2], E. Korsten[1], RA. Bouwman[1]
[1]Intensive care, Catharina Ziekenhuis, Eindhoven, Netherlands; [2]Signal processing systems, Technical University, Eindhoven, Netherlands, Netherlands
**Correspondence:** E. Mestrom
*Intensive Care Medicine Experimental* 2019, **7(Suppl 3):**000877

**INTRODUCTION.** An unplanned Intensive Care Unit (ICU) admission is a serious complication in postoperative patients. Identification of patients at risk of postoperative deterioration is crucial to support the anesthesiologist's decision on patient direct admission to a higher-acuity facility, such as the ICU or the surgical ward. Currently, no evidence-based criteria exist.

**OBJECTIVES.** To identify predictors for postoperative deterioration, which could aid allocation of postoperative patients to the appropriate level of care.

**METHODS.** Retrospective data collection of surgeries was performed between January 2013 and December 2017. Only first surgery per patient per hospital admission was included for analysis. All possibly relevant preoperative, intraoperative and early postoperative factors were collected. An unplanned ICU admission was used as criterion for postoperative deterioration.

**RESULTS.** A total of 27369 postoperative patients were selected. An unplanned ICU admission complicated recovery of 187 (0.7%) patients. These patients were significantly older (65 versus 54 years), more often male (52.4 % versus 37.5%), known to have higher ASA scores (38.4% ASA III versus 16.6%) and underwent longer surgeries (273 versus 159 minutes). Multivariate analysis resulted in 9 variables as best predictors of unplanned ICU admissions, consisting of age (OR 1.02; 95% CI 1.00-1.03), general in combination with epidural anesthesia (OR 3.31; 95% CI 2.26-4.84), surgical specialism (OR 2.76; 95% CI 1.89-4.03), ASA score 3 (OR 2.02; 95% CI 1.43-2.87), administration of phenylephrine (OR 1.71; 95% CI 1.21-2.42), or erythrocytes

(OR 2.45; 95% CI1.23-4.89), or plasma (OR 5.11; 95% CI 1.20-21.9), tachycardia (OR 2.15; 95% CI 1.53-3.03) and duration of surgery in minutes (OR 1.005; 95% CI 1.004-1.006). The ROC curve of this model resulted in AUC of 0,865.

**CONCLUSION.** Combining preoperative, intraoperative and early postoperative factors results in good prediction of unplanned ICU admissions. The model could improve decision support for the patients' allocation after surgery.

**REFERENCE(S)**

1. No grant acknowledgements to declare.
2. Petersen Tym MK, Ludbrook GL, Flabouris A, Seglenieks R, Painter TW. Developing models to predict early postoperative patient deterioration and adverse events. ANZ J Surg. 2017;87(6):457-61. Epub 2017/02/02. doi: 10.1111/ans.13874. PubMed PMID: 28147435.

### 000878

#### Feasibility of renal ultrasound in critically ill patients: a SICS-II substudy

J. Wolters[1], RJ. De Haas[2], R. Wiersema[1], RJ. Eck[3], HN. Van Der Veen[1], J. Koeze[1], A. Wong[4], CFM. Franssen[3], F. Keus[1], ICC. Van Der Horst[1], Sics Study Group[1]
[1]Critical care, University Medical Center Groningen, Groningen, Netherlands; [2]Radiology, University Medical Center Groningen, Groningen, Netherlands; [3]Internal medicine, University Medical Center Groningen, Groningen, Netherlands; [4]Critical care, Royal Surrey County Hospital, Guildford, United Kingdom
**Correspondence:** R. Wiersema
*Intensive Care Medicine Experimental* 2019, **7(Suppl 3):**000878

**INTRODUCTION.** Renal ultrasound is increasingly used in critically ill patients, although studies show different levels of performance for predicting various conditions, such as acute kidney injury (1, 2). Performing renal ultrasound may be difficult in critically ill patients due to suboptimal positioning, pain or wounds. Previous studies however did not report data on feasibility, which could provide an overly optimistic impression.

**OBJECTIVES.** To explore the feasibility and image quality of renal ultrasound in a population of critically ill patients.

**METHODS.** This is a predefined substudy of SICS-II (3), a large prospective observational cohort in patients acutely admitted to the Intensive Care Unit (ICU). Patients underwent a protocolized ultrasonography exam within 24 hours of ICU admission by researchers who were not involved in patient care. All researchers were trained by experts before the start of the study and the first 20 study exams were supervised. We primarily assessed the right kidney and attempted to visualize the left kidney if image quality of the right kidney was considered insufficient. If necessary, the radiologist, who was not involved in patient care, was asked to perform the ultrasound. We recorded renal length, and arterial and venous doppler measurements. Images were validated by an expert radiologist who was blinded for patient characteristics.

**RESULTS.** So far, 403 patients were included in the SICS-II. In 81 out of 403 patients, no images were available, as ultrasound was not attempted due to severe abdominal pain, bandages or large wounds in 47 patients, and image quality was insufficient in 34 patients. In the remaining 322 patients, the right kidney was imaged in 90% of exams and the left kidney in the remaining 10%. Renal length could be obtained in 315, arterial doppler in 270 and venous doppler in 244 of the 322 patients, respectively. In 201 of the 322 patients (62%), all indices were obtained. So far, validation was completed for the first 125 exams (39%, validation is ongoing). The radiologist approved 84% of renal length measurements, 94% of arterial doppler measurements and 90% of venous doppler measurements (figure 1).

**CONCLUSION.** Renal ultrasound could be performed in 322 out of 403 patients. Kidney length could be measured in almost all patients, while doppler seems successful in around 80% of patients. When obtained, at least 84% of measurements were of sufficient quality as judged by an independent expert.

**REFERENCE(S)**

1) Darmon, M., Bourmaud, A., Reynaud, M. et al. Intensive Care Med (2018) 44: 1904. https://doi.org/10.1007/s00134-018-5386-3
2) Oliveira R, Mendes P, Park M et al. Annals of Intensive Care. 2019, 9:23. https://doi.org/10.1186/s13613-019-0500-4
3) Wiersema, R., et al. Observational Study Protocol for Repeated Clinical Examination and Critical Care Ultrasonography Within the Simple Intensive Care Studies. J. Vis. Exp. (2019) (143), e58802, https://doi:10.3791/58802
4) This research received no specific grant from any funding agency in any sector. Currently inclusion is ongoing until study completion in June 2019. In case of admission to the ESICM congress, updated results will be presented.

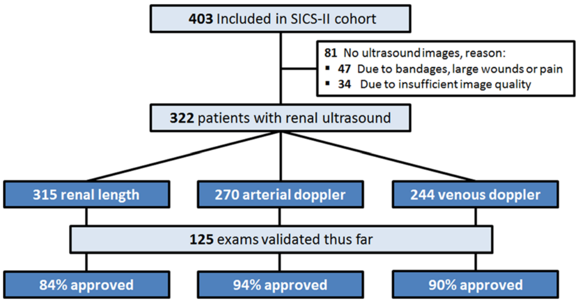

**Fig. 1 (abstract 000878).** See text for description

### 000894

#### Should a probe replace your stethoscope? Auscultation and ultrasound for interstitial syndrome

C. Dankfort[1], R. Wiersema[2], T. Kaufmann[1], B. Hiemstra[1], G. Koster[2], A. Baron[3], A. Wong[4], J. Wilkinson[5], S. Hayward[6], P. Parulekar[7], C. Veenstra[2], F. Keus[2], ICC. Van Der Horst[2], Sics Study Group[2]
[1]Anaesthesiology, University Medical Center Groningen, Groningen, Netherlands; [2]Critical care, University Medical Center Groningen, Groningen, Netherlands; [3]Emergency cardiovascular and critical care research group, St George's, University of London, London, United Kingdom; [4]Critical care, Royal Surrey County Hospital, Guildford, United Kingdom; [5]Critical care, Northampton General Hospital, Northampton, United Kingdom; [6]Physiotherapy, Blackpool Teaching Hospitals, Blackpool, United Kingdom; [7]Critical care, East Kemt hospital, Canterbury, United Kingdom
**Correspondence:** R. Wiersema
*Intensive Care Medicine Experimental* 2019, **7(Suppl 3):**000894

**INTRODUCTION.** Auscultation has historically been an essential component of physical examination in critically ill patients but may be challenging. A more advanced modality like lung ultrasound (LUS) seems easy to use and has shown to be superior to chest x-ray and comparable to CT scan in detecting interstitial syndrome (1).

**OBJECTIVES.** In this sub-study of a large observational study, we aimed to explore the level of agreement between auscultation and LUS for interstitial syndrome.

**METHODS.** This post-hoc analysis included all patients with LUS out of the SICS-I study (2). In SICS-I, patients acutely admitted to Intensive Care Unit (ICU) within 24 hours after admission were eligible for inclusion, consisting of physical examination and LUS by trained researchers not involved in patient care. Physical examination was performed first and included auscultation of six regions bilaterally for crepitation and rhonchi, if either was present, auscultation was considered abnormal. LUS consisted of the BLUE protocol (3) using a phased array probe targeting six scan-sites on the patient´s chest. Interstitial syndrome was defined as three or more B-lines in at least two scan-sites bilaterally. We evaluated sensitivity, specificity, negative predictive value and positive predictive value of the results of auscultation. We performed a sensitivity analysis to assess whether the diagnostic accuracy differs in patients with or without mechanical ventilation.

**RESULTS.** From the 1075 SICS-I patients, 926 (86%) had LUS performed in at least 2 scan sites and 535 of these patients (57%) were mechanically ventilated during the examination. A total of 302 patients (32%) had abnormal auscultation, and 307 patients (33%) had interstitial syndrome on LUS. Specificity of auscultation to diagnose interstitial syndrome on LUS was 76% (95%CI 72-79), sensitivity was 49% (95%CI 44-55), positive predictive value 50% (95%CI 45-54) and the negative predictive value was 75% (95%CI 72-77). Overall diagnostic accuracy was 67% (95%CI 64-70). Amongst those mechanically ventilated, the specificity was 73% (95%CI 68-78), sensitivity was 47% (95%CI 39-55) and accuracy was 65% (95%CI 61-69). Among those not mechanically ventilated, the specificity was 79% (95%CI 73-84), sensitivity was 52% (95%CI 43-61) and accuracy improved to 70% (95%CI 65-74).

**CONCLUSION.** The diagnostic accuracy of auscultation for interstitial syndrome on LUS was poor. Even though diagnostic accuracy and specificity of auscultation was better in patients who were not mechanically ventilated, a wide variation existed between auscultation and LUS. This leads on to whether routine use of LUS, as opposed to auscultation, should become a necessity rather than an optional skill set in the assessment of critically ill patients.

**REFERENCE(S)**

1. G. S. Shrestha*, D. Weeratunga, K. Baker. Point-of-Care Lung Ultrasound in Critically ill Patients. Reviews on Recent Clinical Trials (2018) https://doi.org/10.2174/1574887112666170911125750
2. Hiemstra, B., Koster, G., Wiersema, R. et al. Intensive Care Med (2019) 45: 190. https://doi.org/10.1007/s00134-019-05527-y
3. Lichtenstein, D. A. BLUE-Protocol and FALLS-Protocol Two Applications of Lung Ultrasound in the Critically Ill. Chest 147, 1659–1670 (2015).
4. This research received no specific grant from any funding agency in any sector. Currently more data is being analyzed to elucidate and refine the outcomes of the SICS-I. In case of admission to the ESICM congress, revised results will be presented.

### 000898

#### Change in Health-Related Quality of Life in survivors of acute exacerbation of COPD (AE/COPD) patients 3 months after discharge from ICU

A. Khedher[1], W. Zarrougui[2], N. Fraj[2], E. Ennouri[1], M. Zghidi[1], I. Elmeknassi[1], I. Ben Saida[2], A. Azouzi[1], K. Meddeb[2], M. Boussarsar[2]
[1]Medical intensive care unit, Farhat hached university hospital, Sousse, Tunisia; [2]Medical intensive care unit, farhat hached university hospital, Université de Sousse, Faculté de Médecine de Sousse, LR N° LR12SP09.Heart Failure, Sousse, Tunisia
**Correspondence:** W. Zarrougui
*Intensive Care Medicine Experimental* 2019, **7(Suppl 3):**000898

**INTRODUCTION.** It is widely known that COPD exacerbations lead to lung function Decline. Short-term Health-related quality of life (HRQL) after intensive care is poorly reported and a relevant concern for COPD patients, their relatives and providers of health care.

**OBJECTIVES.** To describe HRQL change in acute exacerbation of COPD ICU-admitted patients 3 months after discharge from a medical Tunisian ICU.

**METHODS.** We analyzed a prospective collected database including consecutive patients admitted for AE/COPD in a 9-bed medical ICU between February 1, 2017 and March 30, 2018. Patients' quality of life was assessed using Web-based version of the St George's Respiratory Questionnaire score (SGRQ) recorded at admission and within three months of ICU discharge via phone calls. The SGRQ variables (symptoms, activity, impacts) were compared before and after 3 months of ICU discharge using Wilcoxon test.

**RESULTS.** Among 102 patients admitted for AE/COPD during the study period, 75(73.5%) were included in the second evaluation. General characteristics were: age, 66.4±9.5 years old; sex ratio, 7/1; Charlson index>3, 40(53.3%); COPD GOLD D, 65(86.7%); median SAPSII score, 27 [22-34]; initial invasive mechanical ventilation, 20 (26.7%); median length of stay, 10 [6-16] days. Median SGRQ total score recorded at admission for the included patients was 33.8 [25.4-44]. The phone evaluation contact was performed in 71 (94.6%) patients. There was statistically significant increase in the total SGRQ score at admission compared to the total SGRQ score recorded after 3 months of ICU discharge 33.8 [25.4–44.] vs 55.8 [41.4-78], p<0.0001. Autonomy impairment increase was significant for all items of the SGRQ score (Symptoms 43 [41.4-78] vs 56.05 [45-74], p<0.001; Activity 47.6 [46-61.5] vs 66.1 [59.4-86], p<0.001 and impacts 21.4 [13.2-35] vs 47.7 [33.6-75], p<0.001).

**CONCLUSION.** In this study, ICU stay was associated with a significant decline in the short-term HRQL in this cohort of Tunisian COPD ICU survivors.

### 000909

#### Airway pressures and difficult weaning prediction in mechanically ventilated non-COPD patients

W. Zarrougui[1], N. Fraj[1], MA. Boujelbèn[2], H. Zorgati[2], S. Rouis[2], D. Ben Braiek[2], A. Azouzi[2], I. Ben Saida[1], K. Meddeb[1], M. Boussarsar[1]
[1]Medical intensive care unit, farhat hached university hospital, Université de Sousse, Faculté de Médecine de Sousse, LR N° LR12SP09.Heart Failure, Sousse, Tunisia; [2]Medical intensive care unit, Farhat hached university hospital, Sousse, Tunisia
**Correspondence:** W. Zarrougui
*Intensive Care Medicine Experimental* 2019, **7(Suppl 3):**000909

**INTRODUCTION.** Mechanical ventilation (MV) is used during respiratory failure to reduce work of breathing and improve gas exchange. However, its efficacy depends on the visco-elastic properties of the respiratory system(1).

**OBJECTIVES.** To identify discriminative properties of respective airway pressures to predict difficult weaning process.

**METHODS.** A retrospective charts' review of MV patients admitted to a medical ICU of Farhat Hached hospital from November 2015 to February 2018. Difficult weaning process was indirectly express d by prolonged MV (≥ 7 days), Ventilator freedays (VFD), and a composite outcome defined as death and/or length of stay ≥14 days. Were collected patients' characteristics at admission and respective airway pressures (Peak, plateau, driving and intrinsic PEEP) at admission and at day 4. High airway pressure (NDHP) is defined as the number of days spent with high pressures : Peak ≥40 and/or plateau ≥30; and/or driving pressure ≥15 and/or; intrinsic PEEP ≥6) were extracted from medical records. Univariate and multivariate regression analyses were performed to identify factors independently associated with mortality.

**RESULTS.** 304 mechanically ventilated patients were collected within the study period. 199(65%) were non-COPD patients. They were 50± 18 years aged; ARDS, 25(12.6%); pneumonia, 14(7%) Pulmonary edema, 11(5.52%); SAPS II, 35.1±15.7; pH, 7.31±0.14; PCO2, 42.4±19.9; PaO2/FiO2, 210.6±109.2mmHg; MV duration, 8.9±9.4days; tracheostomy, 18(9%); length of stay, 10±10 days. Mortality, 114(57%). Mean airway pressures were respectively for peak, plateau, driving and intrinsic PEEP at admission : 30.2±8.9, 20.2±6.22, 13.5±4.9, 2.7±4.2 cmH2O and at day 4 : 30.9±8.9, 20.8±7, 13.7±5.8, 2.4±3.8 cmH2O. Univariate analysis and multivariate logistic regression showed the following factors associated to : 1) 28VFDs, peak at day 4 (OR, 1.06; 95%CI, [1.01- 1.13]; p=0.03), 2) prolonged MV, NDHP (OR, 1.35; 95%CI,

[1.13- 1.60]; p=0.001) 3) composite outcome, plateau at day 4 (OR, 1.52; 95%CI, [1.21- 1.91]; p=0.000).

**CONCLUSION.** Peak pressure, Plateau pressure and number of days spent with high airway pressures may alter significantly the weaning process in MV non-COPD patients.

### 000910

**Post-traumatic stress disorder and the presence of memories (delusional or real) three months after ICU discharge**

PA. Duarte[1], JB. Costa[2], AC. Jorge,[1], TV. Lordani[3], CR. Lordani[4], KC. Luzzi[1], EF. Osaku[1], DP. Gund[5], S. Taba[6], LJ. Guerra[6]
[1]General icu, Hospital Universitario do Oeste do Parana, Cascavel, Brazil; [2]Deparment of psychology, Hospital Universitario do Oeste do Parana, Cascavel, Brazil; [3]Nursing department, Hospital Universitario do Oeste do Parana, Cascavel, Brazil; [4]Department of clinical nutrition, Hospital Universitario do Oeste do Parana, Cascavel, Brazil; [5]Dept of social service, Hospital Universitario do Oeste do Parana, Cascavel, Brazil; [6]Department of psychology, Hospital Universitario do Oeste do Parana, Cascavel, Brazil
**Correspondence:** P.A. Duarte
*Intensive Care Medicine Experimental* 2019, **7(Suppl 3):**000910

**INTRODUCTION.** Objective: To evaluate the relationship between psychological symptoms (particularly Posttraumattic stress disorder [PTSD]) and the memories (real or illusory) reported by ICU survivors.

**METHODS.** Prospective cohort study for 7-year period in the Post-ICU multiprofessional outpatient office of a University Hospital, attending adult patients 3 months after ICU discharge. In order to evaluate the ICU memories, a cheklist was used, consisting of 9 items grouped in 4 domains of memories: treatment-related; environmental; related to emotional experiences, and illusory memories (dreams, nightmares and hallucinations). PTSD symptoms were evaluated using the IES-R. Levels of anxiety and depression were measured using HADS.

**RESULTS.** During the study period, 834 patients were evaluated by outpatient clinic/ psychology team: 61.8% male, mean age 45.4 y, mean APACHE II 19.8, 81% used MV, 39% used continuous sedation, mean ICU length of time 10.5 days. Most patients (54.6%) described some type of ICU memory (40.0% memories of real events; 2.6% illusion memories, such as dreams, nightmares and hallucinations; 13.0% a combination of memories of real events and illusions). The most frequent memories of real events were: discharge to the ward, family visit, see other patients and feel thirsty. Remarks related to the endotracheal tube, such as aspiration and extubation were reported by only 24.7% of the patients. Among patients with post-ICU memories, the incidence of psychological disorders was high, particularly those with PTSD symptoms (Table 1).

**CONCLUSION.** The presence of ICU memories may be an additional factor for the onset of PTSD symptoms, three months after discharge from the ICU. Early identification of patients at risk for PTSD after an ICU stay may improve the patients' long-term psychological outcomes.

**Table 1 (abstract 000910).** See text for description

| | Total Outpatient Patients | No ICU Memory | With ICU Memories | p-value - ICU Memory x Psychological Disorder |
|---|---|---|---|---|
| n | 843 | 383 | 460 | |
| Any kind of psychological disorder | 30.6% | 36.0% | 63.9% | p<0.001 |
| Anxiety symptoms | 24.4% | 40.3% | 59.7% | 0.8819 |
| Depression symptoms | 19.0% | 49.1% | 50.9% | 0.3030 |
| PTSD symptoms | 18.1% | 11.1% | 88.9% | p<0.001 |

### 000915

**Prediction of mortality and multi-organ dysfunction in the ICU through MR-proADM: A prospective single-center experience**

PD. Wendel Garcia[1], P. Bardelli[2], EM. Kleinert[1], PR. Bader[1], R. Freiburghaus[3], U. Schmid[4], S. Heim[5], MP. Hilty[1], K. Spanaus[6], A. Von Eckardstein[6], M. Maggiorini[7]
[1]Institute of intensive care medicine, University Hospital of Zurich, Zürich, Switzerland, Switzerland; [2]Institute of anesthesiology, University Hospital of Zürich, Zürich, Switzerland; [3]Emergency center, Klinik Hirslanden, Zürich, Switzerland; [4]Clinic for children and teenage psychiatry, Psychiatric University Hospital Zurich (PUK), Zürich, Switzerland; [5]Center for intensive care medicine, Kantonsspital Winterthur, Winterthur, Switzerland; [6]Institute of clinical chemistry, University Hospital of Zürich, Zürich, Switzerland; [7]Institute of intensive care medicine, University Hospital of Zurich, Zürich, Switzerland
*Intensive Care Medicine Experimental* 2019, **7(Suppl 3):**000915

**INTRODUCTION.** Early mortality and severity prognostication in the critically ill is a challenge in intensive care medicine. The conception of large, trial validated Scores, as the SAPS II or the SOFA Score, offered a tool to more efficiently tackle this task. Nevertheless, these Scores were not intended for individual patient assessment. It has been shown that MR-proADM on admission is a marker of poor outcome prediction in septic ICU patients [1]. However, there is no data regarding the use of MR-proADM in a general medical ICU population.

**OBJECTIVES.** To propose MR-proADM as an admission prognostic biomarker for ICU and 28-day mortality, as well as for therapy refractory multi-organ dysfunction during the course of the ICU stay.

**METHODS.** Between January 2018 and February 2019 all patients, aged ≥18 and with given consent, admitted to the Medical ICU of the University Hospital Zurich were prospectively included. Blood samples for MR-proADM testing were obtained and the standard monitoring and laboratory analytics recorded on the moment of admission, the next 7 consecutive days and on the day of ICU-discharge.

**RESULTS.** The studied population, comprising 475 patients, presented with an age of 63 [52-72] years, a SOFA Score of 7 [4–10], a SAPS II of 43 [30–58] and a Charlson Comorbidity Score of 3 [1–5] at admission. In ICU mortality was 17% and 28-day mortality 24%. Admission MR-proADM was 2.38 [1.34–5.17] nmol/l. MR-proADM was highest on ICU admission and decreased on average by 31% until discharge. Kinetics of MR-proADM were independent of age and sex. Patients with KDIGO stage ≥ 2 presented overall higher MR-proADM levels.

MR-proADM values at admission correlated with a higher ICU and 28-day mortality ($p < 0.0001$). The AUROC of admission MR-proADM (0.73) in its prediction of ICU mortality was comparable to those of the SAPS II (0.71) and SOFA Score (0.78) (both calculated for the initial 24h in ICU). Admission MR-proADM was more specific (Cut-Off: 5 nmol/l, LR+: 4.1) than SAPS II and SOFA regarding the prediction of persistence of multi-organ impairment (organ specific SOFA subscores > 2) after 7 days. The AUROCs for admission MR-proADM showed a good predictive capacity for death or continued ICU-stay (AUC: 0.72, Cut-off: 6 nmol/l, LR+: 3.2) and for status at home (AUC: 0.7, Cut-off: 1.3 nmol/l, LR+: 3.0) after 28 days. Daily measurement of MR-proADM during the subsequent ICU days did not improve the predictive power of admission MR-proADM, except MR-proADM levels at discharge, which presented an AUROC of 0.85 (Cut-Off: 4.9 nmol/l, LR+: 10.7) for the prediction of 28-day mortality.

**CONCLUSION.** We, for the first time, show that in a general medical ICU population, admission MR-proADM is a sensitive and specific prognostic marker for sustained ICU dependency by day 7 and 28-day mortality and hence a triage tool to identify those patients with prolonged ICU dependency and poor outcome.

**REFERENCE(S)**

1. Charles, P. E., Péju, E., Dantec, A., Bruyère, R., Meunier-Beillard, N., Dargent, A., ... & Quenot, J. P. (2017). MR-ProADM elevation upon ICU admission predicts the outcome of septic patients and is correlated with upcoming fluid overload. Shock: Injury, Inflammation, and Sepsis: Laboratory and Clinical Approaches, 48(4), 418-426.

#### 000916

**Mortality review process in the intensive care unit (ICU): Are we learning from the past?**

J. Macallan, A. Cavalier, M. Alice, T. Samuels, F. Lamb
[1]Critical care, East Surrey Hospital, Redhill, UK
**Correspondence:** J. Macallan
*Intensive Care Medicine Experimental* 2019, **7(Suppl 3):**000916

**INTRODUCTION.** Mortality review meetings are used in healthcare to review patient deaths and to allow learning from these cases. National guidance recommends that these meetings are multi-disciplinary, held at least quarterly and provide non-judgemental discussion of events to allow improvements in care to be identified (1). Local guidance suggests that unexpected deaths should all be discussed in detail (2). Our ICU uses a dedicated, bespoke mortality database for review and presentation of these cases.

**OBJECTIVES.** To examine our current mortality review process using our database and to assess the recommendations produced by these cases.

**METHODS.** A retrospective search of cases entered into the mortality database of our ICU in a district general hospital in the UK. Cases were entered between January 2017 and March 2019. Recommendations from cases that have been presented were reviewed.

**RESULTS.** A total of 326 deaths were entered on to our mortality database. Cases were classified into five different categories based on whether the death was expected or not. This ranged from category 1 (anticipated due to terminal illness/ death pre-hospital arrival) to category 5 (unexpected resulting from medical intervention). 244 cases (74.8%) have been categorised, 25.2% cases are currently unclassified. Of the 326 cases entered, 202 (61.9%) have been presented at a mortality review meeting. The median number of days between death and presentation was 117 (IQR 77- 203). 77 out of 202 cases (38.1%) had recommendations for further action or suggestions for future management. 14 cases had more than one recommendation with a total of 94 recommendations made. These have been reviewed and grouped into themes (Table 1).

**CONCLUSION.** The largest category of recommendations is "feedback to another team". Our tailored database facilitates this through recording documentation and providing a presentation that can be delivered independently. The database approach also enables us to easily identify which deaths are classified as unexpected so that these can be selectively reviewed in-depth and presented as per recommendations. Reviewing every death would represent a large additional workload. Currently a quarter of deaths have not been categorised and there is a considerable time lag in presenting several of the cases. Even with our database approach, we may still be missing important leaning points. Future work should focus on reducing the interval from death to presentation through regular meetings and finding out how other ICUs manage the workload of preparing cases for mortality review.

**REFERENCE(S)**

1. Intensive Care Society. 2018. Guidelines for the provision of intensive care services Version 2 (draft) [Online]. [Accessed 8 April 2019]. Available from: http://www.ics.ac.uk/ICS/guidelines-and-standards.aspx
2. Surrey and Sussex Healthcare Trust. 2017. Learning from deaths (Mortality Review) Policy [Online]. [Accessed 8 April 2019]. Available from: https://www.surreyandsussex.nhs.uk/wp-content/uploads/2017/09/Learning-from-Deaths-Mortality-Review-Policy-1.pdf

**Table 1 (abstract 000916).** See text for description

| Recommendation | Cases |
|---|---|
| Feedback to another team involved | 29(30.9%) |
| Liaison with coroner | 17(18.1%) |
| Improvements in documentation including MCCD | 15(15.9%) |
| Suggestions for changes in management | 13(13.8%) |
| Debrief with team or family | 12(12.8%) |
| Potential earlier escalation of care | 8(8.5%) |

### SIS - Sepsis bundles and treatment

#### 001176

**Effect of obesity on critically septic patients mortality**

L. Fernandez Ruiz[1], B. Valenzuela-Mendez[2], F. Valenzuela-Sánchez[1], JF. Rodriguez-Gutierrez[3], A. Estella[1], MJ. López García[4], J. Rello[5]
[1]Servicio de medicina intensiva, Hospital Universitario de Jerez, Jerez de la Frontera, Spain; [2]Ginecologia, Hospital Universitari Germans Trias i Pujol, Badalona, Spain; [3]Hematología, Hospital Universitario de Jerez, Jerez de la Frontera, Spain; [4]Analisis clinicos. bioquimica, Hospital Universitario de Jerez, Jerez de la Frontera, Spain; [5]Ciberes barcelona. vall d'hebron research institute, Vall d'Hebron Barcelona Hospital Campus, Barcelona, Spain
**Correspondence:** F. Valenzuela-Sánchez
*Intensive Care Medicine Experimental* 2019, **7(Suppl 3):**001176

**INTRODUCTION.** Obesity and overweight are associated with an increased risk of mortality in the general population, however, in some pathological processes such as respiratory distress syndrome (ARDS) have an unexpected behavior known as "obesity paradox".

**OBJECTIVES.** The objective of this study is to assess the relation between obesity and mortality in patients admitted to the ICU with diagnosis of sepsis.

**METHODS.** Prospective observational multicenter study. The incidence of obesity in septic patients admitted to the ICU during a period of five years and it relation to mortality was studied. Obesity was defined as an IBM ≥ 30 kg / m2. Multivariable logistic regression was used to evaluate the adjusted relationship between obesity and mortality. The levels are expressed in median and interquartile range (IQR)

**RESULTS.** Among 621 patients admitted in five different hospitals in Spain with the diagnosis of sepsis, 221 (35,6%) were considered obese. Mean age was 63,7±14,4 (61,78-65,6); 41,6% were men. The global mortality was 33% and 34,1% in the obesity group (ns). Mortality subgroup analysis by origin did not show significance differences between non-obese and obese patients respectively: surgical patients: 24% vs 26,3%; abdominal focus septic patients 24,2% vs 24,6% ; bacterial pneumonia 41,3% vs 45,3% severe pneumonia caused by H1N1 virus 32,3% vs 37,5%. Kaplan-Meier survival analysis showed no significant survival variations in septic patients with and without obesity . Also the ICU and hospital stay did not differ significantly. Severity scores (APACHE II, SAPS II, SOFA), biomarkers (MR-proADM, PCR, PCT and lactate), iron metabolism and immunity (lymphocytes subsets CD14+ and HLA-DR) did not differ significantly neither among septic group vs obesity group. In the survival subgroups in obese patients, the SOFA score was higher in the non survivor than in the survivors: 12 (5-11) vs 8 (5-11) (p <0.001) and the Apache II score 27 (21- 35) vs 19 (12-25) (p = 0.0001). In the multivariate analysis (Cox proportional-hazards regression) the Apache II score at admission was statistically significant predictors for mortality in obese patients. Multivariate analysis showed that obesity was not significantly associated with an increase in mortality in septic patients.

**CONCLUSION.** Despite obesity is a common find in septic patients admitted to the ICU it does not related to an increase in mortality.

#### 001177

**Adjunctive therapy with VIT C in patients with septic shock: pre-post study**

I. Coloretti, M. Tosi, E. Munari, S. Venturelli, M. Sarti, M. Girardis
[1]Intensive care unit, Policlinico of Modena University Hospital of Modena, Modena, Italy
**Correspondence:** I. Coloretti
*Intensive Care Medicine Experimental* 2019, **7(Suppl 3):**001177

**INTRODUCTION.** Due to its pleiotropic activities vitamin C combined to steroids has been recently proposed as a potential beneficial strategy in patients with septic shock. In 2017 we decided to introduce in our institutional protocol for management of septic shock the use of vitamin C in patients receiving low-dose steroids because poorly responsive to vasopressors. In this pre-post analysis, we compared clinical outcome of septic shock patients treated and non-treated with vitamin C.

**METHODS.** Forty-one patients admitted to our ICU in 2017 and 2018 with septic shock and treated by low dose steroids (i.e. hydrocortisone, 240 mg/day) and vitamin C (1,5 g every 6 hours) were matched by a propensity score including age, SAPS II score and comorbidities with 41 control patients admitted to our ICU in 2015-2016 with septic shock and treated by low-dose steroids without vitamin C. 30-day and in-hospital mortality, length of vasoactive drug therapy and mechanical ventilation, and occurrence of renal failure requiring replacement therapy were used as clinical outcome for the comparison. P< 0,05 was used for clinical significance.

**RESULTS.** SAPS II score, age, comorbidities, source of infection and incidence of infection by multidrug resistant bacteria were similar in vitamin C treated (T) and in controls (NT). 30-day (46% T vs 49% NT) and in-Hospital (54% T and 61% NT) mortalities, duration of vasopressors (5±5 days T vs 5±4 days NT) and mechanical ventilation (6± 10 days T vs 8±9 days NT) were similar (p> 0,05) in the treated and in controls. A trend for reduction of renal replacement therapy were observed in treated patients (34%) compared to controls (46%).

**CONCLUSION.** In patients with septic shock treated with low-dose steroids, the addition of vitamin C seems to reduce the occurrence of renal failure without any significant improvement in survival rate and vasopressor time.

**REFERENCE(S)**

1. Shin TG, Kim YJ, Ryoo SM et al. Early Vitamin C and Thiamine Administration to Patients with Septic Shock in Emergency Departments: Propensity Score-Based Analysis of a Before-and-After Cohort Study. J Clin Med. 2019;8(1):102
2. Marik PE, Khangoora V, Rivera R et al. Hydrocortisone, Vitamin C, and Thiamine for the Treatment of Severe Sepsis and Septic Shock: A Retrospective Before-After Study. Chest 2017, 151, 1229–1238.
3. Fowler AA, Syed AA, Knowlson S et al. Phase I safety trial of intravenous ascorbic acid in patients with severe sepsis. J. Transl. Med. 2014, 12, 32. 10.
4. Zabet MH, Mohammadi M, Ramezani M et al. Effect of high-dose Ascorbic acid on vasopressor's requirement in septic shock. J. Res. Pharm. Pract. 2016, 5, 94–100. 9.
5. Donnino, MW, Andersen LW, Chase M et al. Randomized, Double-Blind, Placebo-Controlled Trial of Thiamine as a Metabolic Resuscitator in Septic Shock. Crit. Care Med. 2016, 44, 360–367. 8.
6. Leite HP, de Lima LF. Metabolic resuscitation in sepsis: A necessary step beyond the hemodynamic? J. Thorac. Dis. 2016, 8, E552–E557.
7. May JM, Harrison FE. Role of vitamin C in the function of the vascular endothelium. Antioxid. Redox Signal 2013, 19, 2068–2083.
8. Berger MM, Oudemans-van Straaten HM. Vitamin C supplementation in the critically ill patient. Current Curr. Opin. Clin. Nutr. Metab. Care 2015, 18, 193–201.
9. Oudemans-van Straaten H.M, Spoelstra-de Man A.M, de Waard M.C. Vitamin C revisited. Crit. Care 2014, 18, 460.
10. Rhodes A, Evans LE, Alhazzani W, Levy MM, Antonelli M, Ferrer R et al (2017) Surviving sepsis campaign: International guidelines for management of sepsis and septic shock: 2016.Crit CareMed45(3): 486–552.

### 001181

#### Ferritin as a marker of poor evolution in patients with pneumonia due to influenza A N1H1 virus

B. Valenzuela-Mendez[1], F. Valenzuela-Sánchez[2], L. Fernandez Ruiz[2], JF. Rodriguez-Gutierrez[3], I. Valiente Aleman[4], R. Bohollo De Austria[2], J. Adell Ruiz De León[5], J. Rello[6]

[1]Ginecologia, Hospital Universitari Germans Trias i Pujol, Badalona, Spain; [2]Servicio de medicina intensiva, Hospital Universitario de Jerez, Jerez de la Frontera, Spain; [3]Hematología, Hospital Universitario de Jerez, Jerez de la Frontera, Spain; [4]Unidad de medicina intensiva, University Hospital of Puerto Real, Puerto Real, Spain; [5]Analisis clinicos. bioquimica, Hospital Universitario de Jerez, Jerez de la Frontera, Spain; [6]Ciberes barcelona. vall d'hebron research institute, Vall d'Hebron Barcelona Hospital Campus, Barcelona, Spain

**Correspondence:** F. Valenzuela-Sánchez

*Intensive Care Medicine Experimental* 2019, **7(Suppl 3):**001181

**INTRODUCTION.** Different features of iron metabolism can affect bacterial growth and host response to infection. Important alterations have been described in severe viral infections.

**OBJECTIVES.** We studied the metabolism of iron in patients with severe sepsis from pulmonary origin due to influenza virus pneumonia admitted to the ICU.

**METHODS.** Prospective observational multicentre study. We recruited patients admitted consecutively to the ICU from five different hospitals in Spain with a diagnosis of severe sepsis due to influenza A and B virus pneumonia. Epidemiological data, immunological parameters, biomarkers levels (RCP, PCT, MR-proAdrenomedullin) and iron metabolism parameters were collected at admission. Data were compared with a control group (CG) of patients with pneumonia due to the different influenza viruses with less severity and who were not admitted to the ICU.

**RESULTS.** 156 patients were included: 95 patients suffered severe pneumonia caused by influenza virus (N1H1, N2H3 and B) and 61 patients were included in the control group (CG). The mortality of the group admitted to the ICU was 30.52% (29/95). Iron levels at ICU admission were 68 mcg / dl; similar values in both groups. Ferritin levels at admission were 194.8 ng / ml *versus* 95.4 ng / ml in the CG (p = 0.0047).

Regarding the different types of influenza, Ferritin levels were significantly higher in patients with pneumonia due to influenza A N1H1, both in the severe and those not admitted to the ICU: N1H1 648 ng / ml; N2H3 74.9 ng / ml; influenza B 64.25 ng / ml. In the survival subgroups Ferritin levels were higher in the non survivors than in the survivors in the total group and in the subtypes of influenza N1 H1 (2225 ng / ml vs 421 ng / ml) and influenza B (274 ng / ml vs 60 ng / ml).

The Area Under the ROC Curve (AUC) for severity prognostic (ICU admission) was 0.732 (p <0.0001) for Ferritin levels in patients with influenza A N1H1, 0.561 for type A N2H3 and 0.509 for type B. The Area Under the ROC Curve (AUC) for mortality prognostic at admission was 0.786 (p = 0.0001) for Ferritin levels in patients with influenza A N1H1.

For ferritin levels of 830 ng / ml and above, there was an increase in mortality (p <0.0001) in Kaplan-Meier survival curve. In multivariate analysis (Cox proportional-hazards regression), ferritin levels at admission were statistically significant predictors for mortalityin patients with influenza A N1H1v .

**CONCLUSION.** Ferritin levels at ICU admission help us to determine the unfavorable evolution in the Emergency Department and the risk of mortality in patients with pneumonia due to influenza A N1H1 virus but not for pneumonia due to other types of influenza viruses

### 001184

#### The immunophenotype in early bronchoalveolar Lavage has prognostic value of mortality for Intubated septic patients in ICU

F. Valenzuela-Sánchez[1], L. Fernandez Ruiz[1], B. Valenzuela-Mendez[2], JF. Rodriguez-Gutierrez[3], F. Valenzuela Mateos[4], J. Rello[5], A. Estella[1]

[1]Servicio de medicina intensiva, Hospital Universitario de Jerez, Jerez de la Frontera, Spain; [2]Ginecologia, Hospital Universitari Germans Trias i Pujol, Badalona, Spain; [3]Servicio de hematología. inmunologia, Hospital Universitario de Jerez, Jerez de la Frontera, Spain; [4]Servicio de neumología, Hospital Universitario de Jerez, Jerez de la Frontera, Spain; [5]Ciberes barcelona. vall d'hebron research institute, Vall d'Hebron Barcelona Hospital Campus, Barcelona, Spain

**Correspondence:** F. Valenzuela-Sánchez

*Intensive Care Medicine Experimental* 2019, **7(Suppl 3):**001184

**INTRODUCTION.** The analysis of lymphocyte subpopulations in BAL by flow cytometry has represented an important advance in the understanding of the physiopathology and in the diagnosis of certain pulmonary diseases. However, its usefulness for diagnosis and prognosis in mechanical ventilation (MV)critical septic patients has not been described.

**OBJECTIVES.** To study the lymphocyte populations in BAL in different types of critical septic patients admitted to the ICU with pulmonary infiltrate and to evaluate their diagnostic and prognostic usefulness.

**METHODS.** Observational and prospective study. We recruited mechanically ventilated patients admitted to the ICU with the diagnosis of sepsis with pulmonary infiltrate. Epidemiological data, APACHE II, SAPS II, SOFA, immunological parameters, biomarker levels in blood were collected at ICU admission. BAL bronchoscopy was performed for microbiologic and cellular study. Lymphocyte subpopulations in blood and BAL were studied by flow cytometry. The results were compared with a control group of patients with normal BAL. Multivariate logistic regression was used to evaluate the relationship between the immunophenotype and mortality.

**RESULTS.** 57 patients were analyzed: 43 mechanically ventilated patients and a control group of 14 without infectious disease (control group (CG)) . ICU mortality was 41,8%; mean age of 59.9 ± 13.24, with 55.8% of male patients, Apache II 18 ±7. Mechanically ventilated patients were classified according etiology: 21 bacterial pneumonia, 9 Influenza A N1H1, 3 fungal pneumonia and 9 Acute respiratory distress syndrome (ARDS) without pulmonary infection. The BAL immunophenotype of MV patients, showed a significant increase in B and NK lymphocytes and a decrease in double negative T lymphocytes (CD3+/CD4-/ CD8-) compared to the CG. In the analysis of mortality in the total group of patients in VM showed no significant differences in the CD4+T cells percentage and the CD4 / CD8 ratio. Nevertheless, In the bacterial pneumonia group, CD4+T cells percentage was significantly higher in the non-survivors (46% vs 18% p = 0.0284) and also the CD4 / CD8 ratio (1.2 vs 0.2 p = 0.0218). The Area Under the ROC Curve (AUC-ROC) for mortality prognostic was 0.778 (p = 0.0114) for CD4+ T cells percentage and 0.790 for the quotient CD4 / CD8. For CD4+ T cells levels equal to or greater than 36% and CD4 / CD8 ratios greater than 7 in BAL of patients with bacterial pneumonia, an increase in mortality (p <0.0001) was found in the Kaplan-Meier survival curve. The multivariate analysis (Cox proportional hazards regression), the percentage of CD4+ T cells in BAL was a statistically significant predictor for mortality similar to the Apache II score at admission in bacterial pneumonias in MV admitted to the ICU.

**CONCLUSION.** The appearance of NK, B lymphocytes and the disappearance of CD3+/CD4/CD8- (double negative) T cells in BAL of patients intubated with pulmonary infiltrate is suggestive of pulmonary infectious disease. The increase of CD4+ T cells and consequent increase of the CD4 / CD8 ratio in BAL in patients with bacterial pneumonia has a predictive value of poor prognosis.

### 001190

#### Antibiotics use in patients at risk of sepsis on general wards

M. Kopczynska, B. Sharif, H. Unwin' J. Lynch, A. Forrester, C. Zeicu, T. Chandy, E. Ang, E. Murphy, U. Asim, B. Payne, J. Nicholas, A. Waller, A. Owen, ZX. Tan, R. Ross, J. Wellington, Y. Amjad, V. Unadkat, T. Szakmany
[1]Department of anaesthesia, intensive care and pain medicine, division of population medicine, Cardiff University, Cardiff, UK
**Correspondence:** M. Kopczynska
*Intensive Care Medicine Experimental* 2019, **7(Suppl 3):**001190

**INTRODUCTION.** The early administration of sepsis bundles including appropriate antibiotics has been demonstrated to improve patient outcomes. However, there is a growing concern of the indiscriminate use of antibiotics and omission of microbiology investigations in patient management (1). Additionally, there is no recent description of the microbiology of sepsis on the wards or the real-life antibiotic choices used in sepsis in Wales.

**OBJECTIVES.** The primary objective of the study was to investigate antibiotic prescribing practices on general wards and emergency departments (ED) in at-risk population of patients in acute hospitals in Wales. Secondary objective was the evaluation of microbiology investigations of sepsis patients.

**METHODS.** Secondary analysis of patient episodes was performed on patient population recruited into three annual 24-hour point-prevalence studies on the general wards and ED across all Welsh acute hospitals in years 2016-2018. We recruited patients after written informed consent with a NEWS $^{3}$ 3 and proven or suspected infection documented in the clinical notes; methodology described in detail in our previous studies (2, 3). Data were collected on patient demographics; as well as radiological, laboratory and microbiology data within 48 hours of the study. For statistical analysis Mann-Whitney U and Chi-square tests were used as appropriate.

**RESULTS.** We screened 19,453 patients over the three 24 hours study periods and recruited 1252 patients who fulfilled the entry criteria. We had information about the antibiotics use for 1195 patients. 775 (64.9%) patients were treated with intravenous antibiotics. The antibiotics used were broad spectrum, with Piperacillin/Tazobactam being the most commonly used (40.5% of patients treated with Abx, 314/775). The vast majority of patients did not undergo microbiology investigations. Only in 33.65% (421/1252) of all recruited patients healthcare providers obtained blood cultures, in 25.64% (321/1252) urine cultures, in 8.63% (108/1252) sputum cultures, in 6.79% (85/1252) wound cultures and in 15.25% (191/1252) other cultures. Analysing only patients who received Abx we found that only 49.75% (300/603) had blood cultures, 13.81% (70/507) sputum cultures, 35.47% (210/592) urine cultures, 9.01% (53/588) wound cultures and 13.42% (104/775) other microbiology investigations. Out of the recruited patients 59.1% (740/1252) fulfilled SEPSIS-3 criteria. Patients with SEPSIS-3 criteria were significantly more likely to receive Abx than the non-septic cohort (p<0.0001). Nevertheless, 33.78% (250/740) of patients fulfilling SEPSIS-3 criteria did not receive any Abx. Patients who were screened for sepsis using an official tool were also significantly more likely to receive Abx in comparison to non-screened patients (p<0.0001) and to obtain blood cultures (p= 0.001) but no other microbiology investigations.

**CONCLUSION.** Our study shows that antibiotics prescription practice is not guided by microbiology investigations. Significant proportion of sepsis patients are still at risk of not receiving appropriate Abx treatment and microbiology investigations, which may be improved by more thorough implementation of sepsis screening tools.

**REFERENCE(S)**

1. Burke J, Wood S, Hermon A, Szakmany T. Improving outcome of sepsis on the ward: introducing the 'Sepsis Six' bundle. Nursing in Critical Care. 2018;(1):33-39.
2. Sharif B, Lundin RM, Morgan P, Hall JE, Dhadda A, Mann C, et al. Developing a digital data collection platform to measure the prevalence of sepsis in Wales. Journal of the American Medical Informatics Association. 2016;23(6):1185.
3. Szakmany T, Lundin RM, Sharif B, Ellis G, Morgan P, Kopczynska M, et al. Sepsis Prevalence and Outcome on the General Wards and Emergency Departments in Wales: Results of a Multi-Centre, Observational, Point Prevalence Study. PLOS ONE. 2016;11(12):e0167230.
4. Welsh Digital Data Collection Platform Collaborators
5. Fiona Elizabeth Agnew Trust

### 001193

#### A comparison between predictive sepsis models: an automated algorithm in Electronic Health Report versus Artificial Intelligence (AI) and Machine Learning (ML) techniques

M. Borges[1], A. Socias[1], A. Castillo[2], M. Aranda[1], C. Pruenza[3], J. Mena[1], V. Estrada[4], J. Diaz[3]
[1]Multidisciplinary sepsis unit, ICU. Hospital Son Llatzer, Palma, Spain; [2]Icu. hospital son llatzer, Multidisciplinary Sepsis Unit, Palma, Spain; [3]Universidad autonoma madrid, Knowledge Engineering Institute - IIC, Madrid, Spain; [4]Hospital son llatzer, Serviico de Informática, Palma de Mallorca, Spain
**Correspondence:** M. Borges
*Intensive Care Medicine Experimental* 2019, **7(Suppl 3):**001193

**INTRODUCTION.** The early detection of sepsis (SE) and septic shock (SS) with automated electronic models (AEM) is a problem due to the high percentage of false positives (FP), which generates fatigability to clinicians (1).

**OBJECTIVES.** To compare an AEM published and commonly used in our Electronic Health Record (EHR) (2) with those generated from AI and ML techniques for the detection of SE/SS.

**METHODS.** Retrospective observational study comparing predictive models for the detection of SE/SS in patients of 14 years of age or more in all hospital areas (ED, wards and ICU). We value according to SEPSIS.2, because it was the definition used during the study period. The AEM is based on 15 clinical-analytical variables with different weights and a discrimination score that we usually use (2). In contrast to others based on ML techniques that used different structured and unstructured databases (free text) of the EHR. All cases were evaluated and validated prospectively by the Multidisciplinary Sepsis Unit. The Mann-Whitney-Wilcoxon test was used to identify statistically significant clinical and analytical variables, as well as wrapper techniques, with a significance level of 0.01. And to obtain relevant unstructured data Natural Language Processing (NLP) techniques such as the Dunning test were applied. The total sample was divided into 2 groups: the 5/7 proportion of the total of randomly selected records constituted the training set and the rest of the records (2/7) formed the test set.

**RESULTS.** From January 2014 to October 2018, we included 218,562 patients, mean age of 67.5 years and 57% males, where 9301 (4.6%) patients had SE/SS. The ML models included 244 structured and unstructured variables associated with SE/SS. We have identified 75 clinical and analytical variables in our ML models compared to 15 in the EAM (p<0.0,01). Interestingly, three variables normally associated with sepsis such as Glascow Coma Score (GCS), mean arterial blood pressure (MAP) and platelet count were not significantly related to SE / SS in ML predictive models. And neither the MAP nor the platelet numbers were significantly associated in the predictive model of the AEM, which does not include the GCS in its score. There were 28,294 patients with AEM alerts, where there were 62% of false positive (FP) cases and 12% of false negatives (FN). We have obtained 3 models with ML, being that the best (named BISEPRO) identified 11,2% FP and 0,9% FN cases compared to AEM (p=0,001 in both analyzes). The AUC-ROC, sensitivity and specificity to detect SE/SS of the best ML model was 0.95 (95% CI 0,94-0,96), 0,94, 083 compared to the AEM with 0.86 (95% CI 0,83-0,88), 0,78 and 0,68, respectively. But the three models of ML were significantly superior to that of the AEM both to detect SE / SS and with lower cases of FP and FN.

**CONCLUSION.** ML predictive models were significantly higher for SE/SS detection than traditional automated ones, lowering FP by more than 50%.

**REFERENCE(S)**

1. F. J. Candel, Borges M, et al., "Current aspects in sepsis approach. Turning things around," Rev Esp Quimioter, vol. 31, n.o 4, pp. 298-315, Aug 2018.
2. B. de Dios, Borges M, et al., "Computerised sepsis protocol management. Description of an early warning system," Enferm Infecc Microbiol Clin, vol. 36, issue 2, pp. 84-90, Feb 2018.

3. Co-funded by MSD and IDISBA

### 001201

#### The Impact of Mean Arterial Pressure Changes on Cerebral Autoregulation during Sepsis

AA. Quispe-Cornejo, P. Bakos, IA. Crippa, J. Creteur, JL. Vincent, FS. Taccone

[1]Department of intensive care, Erasme University Hospital, Université libre de Bruxelles, Brussels, Belgium

**Correspondence:** A.A. Quispe-Cornejo

*Intensive Care Medicine Experimental* 2019, **7(Suppl 3):**001201

**INTRODUCTION.** The Surviving Sepsis Campaign recommends targeting a mean arterial pressure (MAP) of at least 65 mm Hg during initial resuscitation of patients with septic shock. However, the impact of such MAP target on cerebral perfusion remains unknown.

**OBJECTIVES.** To assess the effects of changes in MAP on cerebral autoregulation (CAR) in critically ill septic patients.

**METHODS.** Prospective study of 32 patients with septic shock without intracranial disease; severe arrhythmias; treatment with extracorporeal membrane oxygenation; or supra-aortic arteriopathy. MAP was progressively increased at three levels: 70±5 mmHg (low-target group), 80 ±5 mmHg (middle-target group) and 90 ±5 mmHg (high-target group). Transcranial Doppler (DWL, Germany) was performed at each target group, insonating the left middle cerebral artery (LMCA) with a 2MHz probe. LMCA blood flow velocity (FV) and arterial blood pressure (BP) signals were simultaneously recorded for at least 6 minutes; Pearson´s correlation coefficient between BP and FV (MXa) was calculated using MATLAB (MathWorks, USA). Impaired CAR was defined as MXa > 0.3.

**RESULTS.** In the 32 patients (median age: 69 [60-78] years), actual MAP were 70 (range: 55-80), 78 (63-88) and 90 (80-103) mmHg in the low-, middle- and high-target group, respectively. At different time-points, mean MXa was 0.29 (±0.36), 0.27 (±0.34) and 0.20 (±0.32), respectively (p=0.55). In patients with intact CAR (n=17), increasing MAP resulted in a significant increase of MXa over time (ANOVA; p=0.03), in particular from low- to middle-target levels (p=0.02). In patients with altered CAR (n=15), increasing MAP resulted in a significant decrease of MXa over time (p=0.003), in particular from low- to high-target levels (p=0.006).

**CONCLUSION.** Targeting higher MAP in septic shock patients can improve cerebral autoregulation when it is altered at baseline.

### 001211

#### Characteristics and outcomes of patients with severe sepsis and septic shock according to admission sources in Japan

M. Uchida[1], K. Ono[1], A. Toshikazu[2], O. Hiroshi[3], A. Shiraishi[4], K. Shigeki[5], S. Daizoh[6], F. Seitaro[7], M. Toshihiko[8], S. Yasukazu[9], N. Taka-Aki[10], T. Takehiko[11], T. Hifumi[12], Y. Otomo[13], S. Junichi[14], G. Satoshi[15]

[1]Department of emergency and critical care medicine, Dokkyoika University, Mibu, Japan; [2]Department of general medicine, Juntendo University, 2 Chome-1-1 Hongo, Bunkyo City, Tokyo, Japan, Bunkyo City, Japan; [3]Department of traumatology and acute critical medicine, Osaka University Graduate School of medicine, Osaka, Japan; [4]Emergency and trauma center, Kameda Medical Center, Kamogawa, Japan; [5]Division of emergency and critical care medicine, Tohoku university graduate school of medicine, Sendai, Japan; [6]Division of traumatology, Research Institute, National Defense Medical College, Tokorozawa, Japan; [7]Center for general medicine education, Keio University School of Medicine, Tokyo, Japan; [8]Department of emergency medicine, School of Medicine, University of Occupational and Environmental Health, Kitakyushu, Japan; [9]Department of acute medicine, Kawasaki Medical School, Kitakyushu, Japan; [10]Department of emergency and critical care medicine, Chiba University Graduate School of Medicine, Chiba, Japan; [11]Department of trauma and critical care medicine, Kyorin University School of Medicine, Tokyo, Japan; [12]Department of emergency and critical care medicine, St. Luke's International Hospital, Chuo City, Japan; [13]Trauma and acute critical care medical center, Tokyo Medical and Dental University, Tokyo, Japan; [14]Department of emergency and critical care medicine, Keio University School of Medicine, Tokyo, Japan; [15]Division of acute and critical care medicine, Hokkaido university graduate school of medicine, Sapporo, Japan

**Correspondence:** M. Uchida

*Intensive Care Medicine Experimental* 2019, **7(Suppl 3):**001211

**INTRODUCTION.** Healthcare-acquired sepsis is known to be associated with higher mortality than community-acquired sepsis. More specifically, mortality associated with intensive care unit (ICU)-acquired sepsis is extremely high. However, little is known about patient characteristics, pathogens, treatments, and outcomes according to admission sources among patients with severe sepsis and septic shock in Japan.

**OBJECTIVES.** To describe characteristics and outcomes according to admission sources in patients with severe sepsis and septic shock in Japan.

**METHODS.** We included adult patients (≥16 years old) with severe sepsis and septic shock, based on the sepsis-2 criteria published in 2003,[1] who were admitted to the ICU in the Focused Outcomes Research in Emergency Care in Acute Respiratory Distress Syndrome, Sepsis, and Trauma (FORECAST) study [2]. It conducted at 59 ICUs in Japan from January 1, 2016 to March 31, 2017. Patients whose admission sources data was missing were excluded. We compared baseline and infection characteristics, treatments such as initial antibiotics, and outcomes among patients from different admission sources which were emergency departments (ED), inside ICUs, and wards.

**RESULTS.** Overall, 1182 patients were included in this study. There were 676 (57.2%), 49 (4.1%), and 457 (38.7%) patients admitted to the ICU from EDs, inside ICUs, and wards, respectively. The median [interquartile range (IQR)] age of patients in the EDs, inside ICUs, and wards was 75 [66–83], 69 [61–81], and 71 [61–79] years old, respectively ($p < 0.01$). There was no significant difference in gender, Charlson comorbidity index, having shock, APACHE II and SOFA scores according to admission sources. The most common infected sites were as follows: the lungs (33.4%), abdomen (23.4%), and urinary tract (21.7%) in patients from the ED; the abdomen (34.7%), lungs (20.4%), and skin and soft tissue (10.2%) in patients inside the ICUs; the abdomen (29.3%), lungs (28.7%), and urinary tract (14.9%) in patients from wards. Common pathogens obtained from the blood culture were: *E. coli* (30.8%), *Staphylococcus* species (MSSA or CNS) (17.2%), and *Klebsiella* (9.7%) in patients from EDs; *Klebsiella* (20.0%), *E.coli* (16.0%), and *Pseudomonas* (12.0%) in patients inside ICUs; and *E.coli* (18.4%), *Staphylococcus* species (12.4%), and *Klebsiella* (9.8%) in patients from wards. In opportunistic pathogen positivity of blood cultures, MRSA was 1.4%, 4.0% and 3.8% and *Pseudomonas* was 1.4%, 12.0% and 2.6% in patients from the ED, inside ICUs, and wards, respectively. In regard to initial antibiotic's selection, more than 70% patients were administered carbapenem or PIPC/TAZ and approximately 20% of patients were administered vancomycin as initial antibiotic regardless of admission sources.There was no significant difference in in-hospital mortality rates according to admission sources (22.1% from EDs, 35.6% inside ICUs, and 24.2% from wards, $p = 0.108$).

**CONCLUSION.** In this multicenter study in Japan, in-hospital mortality was not different according to admission sources. The initial antibiotic selection was similar irrespective of admission location although infection characteristics such as common pathogens were different according to admission locations.

**REFERENCE(S)**

1. Levy MM et al, Crit Care Med 31: 1250-6, 2003
2. Abe et al, Crit Care 22: 322, 2018

### 001213

#### Fulfillment of the Sepsis Resuscitation Bundle is associated with lower then expected mortality: experience of a chilean University Hospital

A. Hernández, D. Pérez, A. Gajardo, F. Cayupi, N. Medel, C. Luengo
[1]Critical care unit, department of medicine, University of Chile Clinical Hospital, Santiago, Chile
**Correspondence:** C. Luengo
*Intensive Care Medicine Experimental* 2019, **7(Suppl 3):**001213

**INTRODUCTION.** Sepsis and septic shock are related to high mortality. The Hospital Clínico Universidad de Chile applies the recommendations of the Chilean Society of Intensive Care Medicine which include the sepsis bundle proposed by the Survival Sepsis Campaign (SSC) in order to reduce morbidity and mortality in septic patients. However, the adherence to these recommendations including the SSC-bundle fulfillment and it's possible impact t is unknown.

**OBJECTIVES.** To assess the adherence to fulfill the SSC bundle and its impact on in-hospital mortality and length of stay (LOS) in Intensive Care Unit (ICU) in a cohort of ICU patients with severe sepsis or septic shock.

**METHODS.** Retrospective cohort study based on patients' records. All patients diagnosed with severe sepsis or septic shock, according to the 2001 International Sepsis Definitions Conference, and who entered the ICU at Hospital Clínico Universidad de Chile between January and December 2017 were included (n = 91). We assessed the fulfillment of the SSC bundle, compared expected versus (*vs)* actual mortality, according to the Acute Physiology And Chronic Health Evaluation II (APACHE II) and Sequential Organ Failure Assessment (SOFA) (at day 1) scores, and ICU LOS in patients who fulfilled it *vs* those who did not. Comparisons were performed using Fisher's exact test and Mann Whitney's U test. In addition, logistic and linear regression models were performed to evaluate the effect of bundle compliance on mortality and LOS adjusting by age, sex, SOFA score, and lactate levels.

**RESULTS.** Patients were 61.4 ± 16.4 years old (yo) (49.5% older than 65 yo), 57.1% were men; 40.7% sepsis were diagnosed at the emergency room, and abdominal and respiratory foci were the most frequent sepsis sources (41% and 32 %, respectively). APACHE II and SOFA scores were 19 [IQR 12-23], and 7 [IQR 5-10], respectively. Patients had at least 3 dysfunctions at admission, the most frequent being the cardiovascular one with vasopressors requirement in 82% of cases. The resuscitation bundle was fulfilled in 76% of cases. The least fulfilled step during the first 3 hours was the administration of 30ml/kg of crystalloids (63%). Assessment of potential further response to volume using pulse pressure variation or echocardiography was registered in 71% of cases during the 6 hours. In-hospital mortality was 23% and was higher in cases which did not fulfill the bundle (32% *vs* 15%, p-value= 0.047). Fulfillment of the sepsis bundle was significantly and independently associated with less mortality (OR= 0.25, p-value= 0.026). On the other hand, ICU LOS in survivors who fulfilled the bundle was 11 [IQR 5-16] days, while it was 19 [IQR 10-37] days in those who did not (p-value= 0.004), and it was independent of age, sex, SOFA and lactate levels (Beta= -16.8, p-value= 0.017).

**CONCLUSION.** Fulfillment of the sepsis bundle is high but still needs improvement. SSC-bundle fulfillment is associated with lower mortality and less ICU LOS independent of age, sex and severity scores.

**REFERENCE(S)**

1. Knaus WA, Draper EA, Wagner DP, Zimmerman JE. APACHE II: a severity of disease classification system. Crit Care Med 1985 Oct;13(10):818-29
2. Rhodes A, Evans LE, Alhazzani W, et al. Surviving Sepsis Campaign: International Guidelines for Management of Sepsis and Septic Shock: 2016. Intensive Care Med 2017; 43: 304-77.
3. Levy MM, Fink MP, Marshall JC, et al. 2001 SCCM/ESICM/ACCP/ATS/SIS International Sepsis Definitions Conference. Intensive Care Med. 2003 Apr;29(4):530-8.

### 001220

#### MR-proADM levels are useful for the diagnosis and mortality risk prediction of patients with sepsis admitted to the ICU depending on the focus, type of germ and organ failure

F. Valenzuela-Sánchez[1], B. Valenzuela-Mendez[2], L. Fernandez Ruiz[1], JF. Rodriguez-Gutierrez[3], R. Bohollo De Austria[1], MA. Gonzalez-Garcia[4], J. Rello[5]
[1]Servicio de medicina intensiva, Hospital Universitario de Jerez, Jerez de la Frontera, Spain; [2]Ginecologia, Hospital Universitari Germans Trias i Pujol, Badalona, Spain; [3]Servicio de hematología. inmunologia, Hospital Universitario de Jerez, Jerez de la Frontera, Spain; [4]Analisis clinicos. bioquimica, Hospital Universitario de Jerez, Jerez de la Frontera, Spain; [5]Ciberes barcelona. vall d'hebron research institute, Vall d'Hebron Barcelona Hospital Campus, Barcelona, Spain
**Correspondence:** F. Valenzuela-Sánchez
*Intensive Care Medicine Experimental* 2019, **7(Suppl 3):**001220

**INTRODUCTION.** Levels of MR-proAdrenomedullin (MR-proADM) have been related to the diagnosis and mortality of septic patients associated with organ failure even at low SOFA scores, being more effective than other biomarkers

**OBJECTIVES.** To evaluate the usefulness of MR-proADM levels in the diagnosis and prognosis of sepsis in patients admitted to the ICU according to its focus of origin, etiology and severity.

**METHODS.** Prospective observational multicenter study. Patients with suspected sepsis were recruited to the ICU of six spanish hospitals. Epidemiological, clinical, laboratory data and MR-proADM, procalcitonin , and C reactive protein levels were collected at the time of admission, at 48 hours, at the 5th day and on the day of discharge from the ICU. Also the MR pro ADM clerance at 48 hours and at the 5th day were calculated. The results were compared by different foci of infection and germs. The results were compared with groups of patients with non-septic SIRS, critical cardiac patients and healthy volunteers. The levels are expressed in median and interquartile range (IQR)

**RESULTS.** A total of 606 patients were enrolled: 484 septic patients, 36 patients with SIRS without sepsis, 61 patients with infectious diseases not admitted to the ICU, 9 critical cardiac patients and 10 healthy volunteers. MR-proADM levels were in patients with sepsis 2,567 nmol/l, compared to patients with SIRS (1,373 nmol/l, p= 0.0061), to infectious patients not admitted to the ICU (0,034 p< 0,001), to critical cardiac patients (0.034 nmol/l p<0.001) and healthy volunteers (0.020 nmol/l;p <0.001). Regarding the distribution by source of focus: urological origin had higher levels of MR-proADM (5,147 nmol / l) compared to abdominal sepsis (3,307 nmol / l, p = 0.0195), and pulmonary sepsis ( 1.266 nmol / l, p <0.0001), even to bacterial pneumonias (2.3475 nmol / l; p <0.0001). According to the degree of SOFA the differences were significant: SOFA> 13 points were of 6.44 nmol / l for SOFA 7-12 points: 3.01 nmol / l and for SOFA < 6 points 1.84 nmol/l (p<0.0001). Depending on the type of germs, we found no significant differences except for viral sepsis (1.05 nmol / l), which were lower than gram + bacteria (2,568;p<0.05) gram- (3.13 nmol/l;p<0.05) fungi (2.95 nmol/l;p<0.05) and polymicrobial (3.01 nmol / l; p <0.05). The ROC-AUC to predict the mortality increase with the time of evolution 0.608 to the income, 0.724 to the 48 hours of income and 0.767 to the 5th day; similar improves for the biomarker clearance.The ROC-AUC comparison for germs are similar to both at admission and at 24 hours and in the 5th day except for influenza A N1H1 viruses (0.756 at admission and 0.782 at 48 hours). The AUC-ROC of the SOFA group <6 point was 0.654 vs 0.579 of the SOFA group>13 point. In multivariable model, MR-proADM levels at 48 hours and on day 5 and clearance on day 5 following admission were statistically significant predictive factors of mortality. The subgroup of severe pneumonias due to influenza A N1H1v since admission was statistically significant predictive mortality factor.

**CONCLUSION.** In clinical practice, high levels of MR-proADM help to diagnose organic failure secondary to sepsis and, after 48 hours of evolution, the prognosis of the risk of death with some differences depending on the foci of origin, germ and organic dysfunction severity.

### 001247

#### Is high sugar really bad for Septic patients? A reality check

J. Nikhilesh[1], A. Thakur[2], V. Joshi[3]
[1]Dept of critical care medicine, CHL Hospital, Indore, India; [2]Dept of critical care, CHL hospital, Indore, Madhya Pradesh, India, India; [3]Dept of critical care services, Shalby Hospitals, Indore, Madhya Pradesh, India, India
**Correspondence:** J. Nikhilesh
*Intensive Care Medicine Experimental* 2019et#//ArticleContext/IssueIDStart)>):001247

**INTRODUCTION.** There has been adequate evidence based literature to suggest and conclude that high sugars portend a worse prognosis in subsets of sepsis and there have been a gamut of trials effectively backing up on the above statement. This communication deals in diabetic patients with sepsis with variable Glycosylated hemoglobin (Hba1c) levels and a focus on correlating their outcomes with the same.

**OBJECTIVES. Objective:**
To analyze outcomes of diabetic patients with elevated HBa1C levels with sepsis and to look for an association between the same.

**Setting**: Multidiscliplinary tertiary care medical surgical ICUs of two hospitals.

**METHODS. Study Module:**
Consecutive patients with diabetes and sepsis were admitted and analyzed with reference to demographics, Hba1C levels at baseline(< 7 versus>7), SOFA scores (<10 versus>10), Length of stay in days (LOS) and type of infections. Discharges from ICU/mortality were considered as end points. SPSS version 24 was used for statistical analysis.

**RESULTS. Results:**
Sixty eight patients were included (n=68, M:F- 44:24/Range-32 -80/ Mean-59.1±10.6).HBA1C levels were 8.5±1.24(Range-6.9-12).LOS was 6.78±4 days(Range-2-27).SOFA scores were 13.04±2.64(Range-8-18).In hospital mortality was n=26 (38.24%).Infections demonstrated were urinary tract infections-36.8% (n=25), Cutaneous infections- 26.5%(n= 18) ,Pneumonias 20.6%(n=14), Intrabdominal infections-7.4% (n=5) and Mucormycosis 8.8%(n=6).Chi square test was applied. The subset with a higher HBA1C value (>7) had a higher percentage mortality vis-à-vis the subset with lower HBA1C value (<7) [38% v/s30% respectively] the test did not achieve a statistical significance when subjected to analysis (p -0.85NS).A skewing of data with reference to higher clustering of HBA1C patients in higher cut off group was noted. SOFA scores were tested [>10 versus <10] for association with mortality across the subsets and there was no demonstrable statistical significance for the same (p-0.65NS).ROC curves were drawn and AUC for HBA1C levels and SOFA scores was 0.30 and 0.47 respectively for association with mortality.

**CONCLUSION. Conclusion:**
Our study demonstrates no significant statistical differences in patient outcomes for sepsis in diabetic cohorts based on poor remote glycemic control. This would probably translate into mortality in diabetic sepsis cohorts independent of remote glycemic control. However, we will require bigger numbers with HBA1C levels <7 for data to be more equitably comparable.

### 001248

#### Effect of preconditioning on red blood cell deformability in septic patients: a preliminary study

A. Moreau[1], P. Biston[1], J. Mucha[1], BK. Zouaoui[2], M. Piagnerelli[1]
[1]Intensive care, Hopital Civil Marie Curie, Charleroi, Belgium; [2]Experimental medicine laboratory, hopital André Vésale, Charleroi, Belgium
**Correspondence:** A. Moreau
*Intensive Care Medicine Experimental* 2019, **7(Suppl 3):**001248

**INTRODUCTION.** The red blood cell (RBC), an essential actor of the microcirculation, is exposed to various levels of shear stress (SS) in the blood flow and has the capacity to modify its shape to pass through capillaries. Two recent studies (1,2) performed on RBCs from healthy volunteers, showed that after prolonged physiological SS exposure (< 30 Pa during 300s) like in blood flow, RBC deformability was improved.

**OBJECTIVES.** We investigated the effect of preconditioning on the RBC deformability from septic patients.

**METHODS.** Blood samples from 20 healthy volunteers and 20 critically ill patients with sepsis were compared. RBC deformability was assessed by the elongation index (EI) via an ektacytometry (Laser-assisted Optical Rotational Red Cell Analyzer – LORRCA) for SS from 0.3 to 50 Pa. EI was defined as (A-B)/(A+B), where A is the length and B is the width of the RBC. A higher EI indicates greater RBC deformation.

To evaluate the effect of preconditioning on RBC deformability, we studied EI just after a constant physiologic SS of 5 Pa during 300 s. Data were reported in mean ± SD or median values with 25-

75thpercentiles and compared by Kruskall-Wallis test. The effect of preconditioning on EI was assessed by Wilcoxon test. Correlations were tested by Spearman test.

**RESULTS.** Compared to volunteers, septic patients were significantly older and had a high APACHE II score: 22 (15-24). As expected, they were also significantly more anemic and had a higher inflammatory syndrome than volunteers.

RBCs deformability was significantly altered in septic patients compared to healthy volunteers for all SS > 4.89 Pa. After preconditioning, only RBCs from healthy volunteers improved EI for low SS (0.3 to 3.07 Pa) as demonstrated in previous studies (1,2). Despite a large variability in the response, no significant effect of preconditioning was observed on EI for RBCs from septic patients for all SS. Only RBCs from septic patients showed an inverse significant correlation between EI before preconditioning and delta EI (EI after – EI before preconditioning); r = -0.65; p = 0.001 for SS 0.3, 0.48, 1.21 and 1.93 Pa.

**CONCLUSION.** The RBC deformability is significantly altered during sepsis. In contrast of RBCs from healthy volunteers, preconditioning with low SS, as observed in blood flow, did not improve the deformability. Nevertheless, the response was variable and was inversely correlated with the EI at baseline. Mechanisms of these alterations should be investigated.

**REFERENCE(S)**

1. Meram E, Yilmaz BD, Bas C, Atac N, Yalcin O, Meiselman HJ, Baskurt OK. Shear stress-induced improvement of red blood cell deformability. Biorheology. 2013;50(3-4):165–176.
2. Simmonds MJ, Atac N, Baskurt OK, Meiselman HJ, Yalcin O. Erythrocyte deformability responses to intermittent and continuous subhemolytic shear stress. Biorheology. 2014;51(2-3):171-185.

### 001287

#### SEPSIS: a pre-ICU clinical emergency

E. Chrysanthopoulou, I. Karampela, P. Simitsis, C. Diakaki, F. Frantzeskaki, G. Konstantopoulou, M. Lignos, M. Theodorakopoulou, I. Tsagkaris, A. Armaganidis

2nd department of critical care, Attikon General University Hospital, Haidari, Greece

**Correspondence:** E. Chrysanthopoulou

*Intensive Care Medicine Experimental* 2019, **7(Suppl 3):**001287

**INTRODUCTION.** Sepsis is the main cause of death due to infection

**OBJECTIVES.** Aim of the study was to evaluate the impact of sepsis in critically ill patients' outcome in relation to site of sepsis acquisition ( ICU vs non-ICU acquired)

**METHODS.** We retrospectively reviewed ICU admissions during 2018. We analyzed data from patients with sepsis either on admission or during ICU stay. Demographic data, site of infection, pathogen and ICU mortality were recorded. Data of 183 patients (64% men, of mean age 60± 18 years, with APACHE II score 20± 8) were available and were analyzed.

**RESULTS.** Fifty six patients (31%) had sepsis on admission. Twenty four of them had subsequent septic episode during ICU hospitalization and were excluded from analysis, and 32 patients comprised group A. Seventeen patients (9%) had ICU acquired infection (group B). Mortality of septic-patients was more than 2fold compared to patients never presenting sepsis. Nevertheless, ICU acquired sepsis presented a significant lower mortality compared to non-ICU acquired sepsis (18% vs 63%, p<0.001).

The respiratory system was the most frequent site of infection in group A (30%) while bacteremia was in group B. Sepsis was most frequently attributed to Gam negative microbes in both groups although at different rates (22% and 59%). *Klebsiella sp. and Acinetobacter sp.* were the main causative pathogen (29% and 24%) in group B.

Among the mortality-correlated risk factors examined (sex, comorbidity, medical history, APACHE II score) only age was statistically different between the groups (66±13 vs 56±21, P=0.04). Group's A mortality risk, compared to group-B remained much higher after age-adjustment (OR: 6.7 CI 95% 1.5-30, p:0.014)

Delayed admission of septic patients was recorded to be very frequent (more than 70% of non-ICU acquired sepsis patients were admitted more than 12 hours post sepsis onset). Since immediate treatment of these patients has a significant impact on outcome, the delayed admission could explain the different mortality. Nevertheless, further studies are needed to make concrete conclusions.

**CONCLUSION.** Forty percent of ICU patients presented at least one septic episode. Non-ICU acquired sepsis had a worse outcome compared to ICU acquired sepsis, possibly due to delayed ICU admission

**REFERENCE(S)**

1. Surviving Sepsis Campaign: International Guidelines for Management of Sepsis and Septic Shock 2016 Critical Care Medicine.2017;45(3):486–552
2. Yealy DM et al. Recognizing and managing sepsis: what needs to be done? BMC Med. 2015;13:98

3. The authors received no financial support

### 001292

#### Evaluation of monocyte counts and its role in septic shock

NI. Medveczky-Ordoñez[1], MA. Amezcua-Gutiérrez[2], LA. Gorordo-Delsol[1], SE. Zamora Gómez[1], ML. Pacheco-Rivera[1], KJ. Castillo-Medrano[1], AH. Morales-Morales[1], JC. Gasca-Aldama[1], S. Sosa-Santos[1], JA. Zepeda-Pérez[1], LE. Gaytán-Medina[1], D. Sanabria-Cordero[1], A. Rodríguez-Peredo[1], I. Maldonado-Beltrán[1], GD. Hernández-López[1], JA. Castañon-Gonzalez[1], CJ. Barragán-Guadalupe[1]

[1]Adult intensive care unit, Hospital Juárez de México, Ciudad de México, Mexico; [2]Intensive care unit, Star Medica Hospital, Ciudad de México, Mexico

**Correspondence:** L.A. Gorordo-Delsol

*Intensive Care Medicine Experimental* 2019, **7(Suppl 3):**001292

**INTRODUCTION.** Sepsis, a syndrome of physiologic, pathologic, and biochemical abnormalities induced by infection, remains to be a significant health care issue associated with high mortality. Our understanding of the pathogenesis of sepsis is broader, however the function of some cellular elements that are an essential part of the innate immune line is still unknown. Unlike the alterations documented in neutrophils and lymphocytes during sepsis, the role played by monocytes is still unclear, especially if the absolute count is directly or inversely associated with the prognosis of these patients.

**OBJECTIVES.** To evaluate the association of monocytic counts with the severity and mortality in patients with septic shock at the intensive care unit (ICU) of a tertiary hospital Mexico City.

**METHODS.** Retrospective analysis during 6 months in 2018, collected patients with septic shock (Sepsis-3). Monocyte counts were categorized into <300 and >1000 cell/UL; 28-day mortality and organ dysfunction were defined as the primary endpoint and compared between the groups. Pearson´s correlation coefficient was used for statiscal test to evaluate the association of initial monocytes counts with severety. To determiny 28-day mortality risk, the odds ratio (OR) was calculated with 95% confidence intervals, and p < 0.05 was considered significant. The data are presented as absolute values and percentages. Treatment standardized protocols were followed for each patient.

**RESULTS.** We collected 48 patients (54.2% female), mean age was 48 ±17.3 years. The causes of septic shock were: medical 54.2%, surgical 43.8% and trauma 2%. Mean APACHE II and SOFA score were 17.8 ± 7.9 and 9.7 ± 4.9 respectively. The length of stay in ICU was 6.4 ± 5.8 days. Eleven patients (22.9%) died during the follow-up. Pearson´s correlation coefficient between initial monocytes counts and organ dysfunction was -0.094 (p=0.52), with determination coefficient of 0.9. Initial monocyte counts < 300 cells/uL showed highest risk of mortality at 28 days with OR 2.375 (95% IC 0.553 – 10.196, p=0.244), whereas patients group > 1000 cells/uL showed lower risk of mortality with OR 0.950 (95% IC 0.086 –10.499, p=0.966).

**CONCLUSION.** In our study monocyte count <300 cells/uL was associated with highest mortality and severity in patients with septic shock but without statistically significance contrary to international literature. Additional studies are be needed to elucidate the potentially role of monocytes on prognosis septic shock.

**REFERENCE(S)**

1. Cavaillon Jean-Marc, Minou Adib-Conquy. Monocytes/macrophages and sepsis. Crit Care Med. 2005:33(12): 506-509.
2. Chung H, Lee JH, Jo YH, Hwang JE, Kim J. Circulating Monocyte Counts and its Impact on Outcomes in Patients With Severe Sepsis Including Septic Shock. Shock. 2019;51(4):423-429.
3. Napolitano L. Sepsis 2018: Definitions and Guideline Changes. Surgical Infections. 2018;19 (2):117-125.
4. There was no financing or grants for this abstract

### 001303

#### The prognostic value of one hour bundle completion in septic shock patients in KOREA

WI. JEONG, SM. Ryoo, JS. Kim, WY. Kim
[1]Emergency medicine, University of Ulsan College of Medicine, Asan Medical Center, Seoul, Republic of Korea
**Correspondence:** W.I. JEONG
*Intensive Care Medicine Experimental* 2019, **7(Suppl 3):**001303

**INTRODUCTION.** Since 2018, Surviving sepsis campaign recommended 1 hour bundle therapy in septic shock patients. However the evidence of bundle therapy has not been established. The object of this study was to determine a prognostic value of 1 hour bundle completion in septic shock patients.

**METHODS.** This prospective, registry based, observational study was performed between January 2016 and December 2018. 1 hour bundle in septic shock was defined as a serum lactate measurement; obtain blood cultures; administration antibiotics and adequate fluid within 1 hour from emergency department admission. Eligible septic shock patients were included for analysis and we analyzed prognostic ability of all completion of 1 hour bundle and each of them. The primary outcome was 28-day mortality.

**RESULTS.** The study included 381 patients and overall 28-day mortality was 24.7%. Overall 1 hour bundle completion rate was 12.2% and each completion rate of serum lactate measurement; obtain blood cultures; administration antibiotics and adequate fluid were 85.0%, 72.5%, 18.8%, and 52.3% respectively. However, overall bundle completion as well as each bundle were not associated with 28-day mortality except adequate fluid administration (Odd ratio (OR) 0.67 [95% Confidential interval (CI) 0.30-1.50], OR 1.33 [95% CI 0.66-2.70], OR 1.50 [95% CI 0.85-2.64], OR 1.17 [95% CI 0.66-2.07], and OR 0.54 [95% CI 0.34-0.87], respectively). Multivariate logistic regression analysis shows that adequate fluid administration was an independent predictable factor in 28-day mortality (OR 0.50 [95% CI 0.29–0.84], p=0.009).

**CONCLUSION.** In this study, we found since septic shock is very complicated, most of 1 hour bundle completion were not associated with mortality. Although adequate fluid administration was associated with 28-day mortality, to generalize this result, we need multicenter interventional study with improving physician's compliance.

**REFERENCE(S)**

1. Singer M, Deutschman CS, Seymour CW, et al. The Third International Consensus Definitions for Sepsis and Septic Shock (Sepsis-3). JAMA 2016;315:801-10.
2. Rhodes A, Evans LE, Alhazzani W, et al. Surviving Sepsis Campaign: International Guidelines for Management of Sepsis and Septic Shock: 2016. Crit Care Med 2017;45:486-552.
3. Levy MM, Evans LE, Rhodes A. The Surviving Sepsis Campaign Bundle: 2018 Update. Crit Care Med 2018;46:997-1000.

### 001320

#### Addition of aerosolized colistin to the treatment of respiratory infection by multiresistant microorganisms

MT. Janer Maeso, L. Soliva Martínez, C. March Cladera, M. Garcias Sastre, M. Teruel Gimenez, TJ. Leal Rullan, M. Ferreruela Serlavós, MA. Colomar Ferrà, JI. Ayestarán Rota, JM. Raurich Puigdevall
ICU, Hospital Universitario Son Espases, Palma, Spain
**Correspondence:** M.T. Janer Maeso
*Intensive Care Medicine Experimental* 2019, **7(Suppl 3):**001320

**INTRODUCTION.** Pneumonia due to gram negative multiresistant bacteria (GNMB) is an important problem in the intensive care unit. There are few studies evaluating the efficacy of aerosolized antibiotics.

**OBJECTIVES.** To compare whether combined intravenous and inhaled colistin administration is better than intravenous colistin alone in the treatment of respiratory infection by multiresistant microorganisms in critically ill patients.

**METHODS.** This study was observational and retrospective. Patients admitted to the ICU who received intravenous colistin, inhaled colistin or both, as a treatment for a respiratory infection due to multiresistant pathogens were analyzed in the period between January 2014 and January 2019. The primary outcome was the clinical response. Secondary outcomes included microbiological response, ICU and hospital mortality and side effects such as neurologic or renal failure. Chi-square, t-Student, Mann-Whitney and Kaplan-Meyer tests were used for the statistic analysis.

**RESULTS.** Total 107 patients were studied but only 90 with lung infection were included: 43 pneumonia, 47 tracheobronchitis). 53 patients received endovenous colistin, 24 received both endovenous and aerosolized colistin and 13 received aerosolized colistin and other endovenous antibiotic. Pseudomonas aeruginosa caused lung infection in 93% of patients. No differences between groups in age, sex, gravity, previous colonization, shock development or type of infection were found. Treatment related adverse events were reported in 21 patients. Main results are shown in the table.

**CONCLUSION.** In conclusion, additional aerosolized colistin did not show better outcomes in critically ill patients with lung infection caused by multiresistant microrganisms.

**REFERENCE(S)**

1. Landersdorfer CB, Nation RL. Colistin: How should it be dosed for the critically ill? Semin Respir Crit Care Med 2015; 36: 126-135.
2. Valachis A, Samonis G, Kofteridis DP. The Role of Aerosolized Colistin in the Tratment of Ventilator-Associated Pneumonia: A Systematic Review and Metaanalysis. Crit Care Med 2015; 43: 527-533.
3. Tumbarello M, De Pascale G, Trecarichi EM, De Martino S, Bello G, Maviglia R, Spanu T, Antonelli M. Effect of Aerosolized Colistin as Adjuntive Tratment on the Outcomes of Microbiologically Documented Ventilator-Associated Pneumonia Caused by Colistin-Only Susceptible Gram-Negative Bacteria. Chest 2013; 144 (6): 1768-1775.
4. Liu D, Zhang J, Liu H-X, Zhu Y-G, Qu J-M. Intravenous combined with aerosolised polymixin versus intravenous polymyxin alone in the treatment of pneumonia caused by multidrug-resistant pathogens: a systematic review and meta-analysis. International Journal of Antimicrobial Agents 46 (2015); 603-609.
5. Boisson M, Jacobs M, Gregoire N, Gobin P, Marchand S, Couet W, Mimoz O. Comparison of Intrapulmonary and Systemic Pharmacokinetics of Colistin Methanesulfonate (CMS) and Colistin after Aerosol Delivery and

Intravenous Administration of CMS in Critically Ill Patients. Antimicrobial Agents and Chemotherapy 2014; 7331-7339.
6. Boisson M, Greogoire N, Couet W, Mimoz O. Colistin in critically ill patents. Minerva Anestesiol 2013; 79: 200-208.

**Table 1 (abstract 001320).** See text for description

| | EV colistin N:53 | INH colistin N: 37 | p value |
|---|---|---|---|
| Age, years | 56 ± 16 | 54 ± 17 | 0.50 |
| Female sex, n (%) | 10 (18.9) | 9 (24.3) | 0.60 |
| Charlson comorbidity index | 1 (0 – 1.5) | 1 (0 – 2) | 0.56 |
| SAPS II | 48 ± 16 | 48 ± 16 | 0.95 |
| APACHE II | 26 ± 11 | 26 ± 8 | 0.89 |
| Lower respiratory tract infection, n (%) | | | |
| Pneumonia | 24 (45.3) | 19 (51.4) | |
| Tracheobronchitis | 29 (54.7) | 18 (48.6) | |
| Median length of stay (interquartile range: IQR), days | | | |
| In the ICU | 29 (20 – 44) | 41 (26 – 57) | 0.02 |
| In the hospital | 46 (30 – 76) | 85 (50 – 120) | 0.004 |
| Median duration of mechanical ventilation (IQR), days | 22 (13 – 31) | 30 (16 – 39) | 0.06 |
| Mortality at 30 days, n (%) | 18 (34.0) | 6 (16.2) | 0.09 |

T1

### 001758

#### Evaluation of soluble Endoglin in septic shock patients

M. Helan[1], M. Hortová-Kohoutková[2], A. Mýtniková[3], V. Tomášková[1], P. Suk[1], J. Hruda[1], J. Frič[2], V. Šrámek[1], J. Pařenica[4]
[1]Department of anaesthesiology and intensive care, St. Anne's University Hospital, Brno, Czech Republic; [2]International clinical research centre, (ICRC), Brno, Czech Republic; [3]Faculty of medicine, Masaryk University, Brno, Czech Republic; [4]Department of internal medicine and cardiology, University Hospital Brno, Brno, Czech Republic
**Correspondence:** M. Helan
*Intensive Care Medicine Experimental* 2019, **7(Suppl 3):**001758

**INTRODUCTION.** Dysregulated endothelial activation is the key mechanism in development of septic shock, resulting in an increased permeability and dysfunction of the endothelial barrier. Endoglin as a part of the transmembrane TGF-β receptor complex is located on the surface of endothelial cells. Its extracellular domain can be cleaved, leading to the production of soluble endoglin (sEng). sEng production seems to be forced by hypoxia/oxidative stress, and can be considered to be a marker of endothelial activation/dysfunction (1,2). Endoglin is also expressed on monocytes, where it has regulatory functions during the immune response (3).

**OBJECTIVES.** The aim of the study was to evaluate a kinetics of sEng plasma concentration in septic shock patients and determine whether sEng could be used as biomarker in prediction of mortality of sepsis and whether it correlates with other biomarkers and severity of septic shock.

**METHODS.** Patients admitted to the ICU with early septic shock were prospectively enrolled into the study. Plasma samples and clinical data were collected at admission (T1) and in subsequent six days (T2-T7). Soluble endoglin concentration was measured using Human Endoglin/CD105 Quantikine ELISA Kit (R&D systems). Obtained data were compared to 3-months mortality and Pearson coefficient was used for estimation statistical correlation of sEng with patient's age, sex, CRP and procalcitonin.

**RESULTS.** Recruited patients (n=24) were predominantly male (71.4 %) with median age 68.5 (53.5-73.0) years. All patients were mechanically ventilated and the average SOFA and APACHEII scores at admission were 11.5 (9.5-13.5) and 28.0 (26.5-35.5) respectively. 3-months mortality rate was 43.8 %. There were no statistical differences in the patients' past medical history, chronic medication or sepsis severity at the time of admission between survivors and non-survivors. Median sEng concentration at admission was significantly higher (p=0.004) in deceased patients 5.18 (3.78-10.21) μg/l compared to survivors 3.24 (2.92-3.66) μg/l. There was no statistical correlation between sEng and age, APACHEII, CRP or PCT. Regarding 7-day trend, there were no changes in sEng levels in the group of surviving patients, but sEng levels were consistently elevated in the group of deceased patients with a slight drop observed on the 6-7th day. Data are provided as median (IQR).

**CONCLUSION.** Our pilot data suggests that soluble endoglin has a good predictive power in prediction of mortality in septic shock patients. Markedly higher levels of sEng in the group of deceased patients over the entire seven-day follow-up period might reflect a high degree of sEng production and therefore a high degree of endothelial dysfunction. Results needs to be verified in the bigger cohort of septic patients. Whether sEng could also differentiate between infectious (sepsis) and non-infectious etiology (SIRS) of shock or what is its specific function in endothelial dysfunction or host-response reaction in sepsis remains to be elucidated.

**REFERENCE(S)**

1. Rossi E, Bernabeu C, Smadja DM. Endoglin as an Adhesion Molecule in Mature and Progenitor Endothelial Cells: A Function Beyond TGF-β. Front Med (Lausanne). 2019.
2. Vitverova B, Blazickova K, Najmanova I, Vicen M, Hyšpler R, Dolezelova E, et al. Soluble endoglin and hypercholesterolemia aggravate endothelial and vessel wall dysfunction in mouse aorta. Atherosclerosis. 2018.
3. Ojeda-Fernández L, Recio-Poveda L, Aristorena M, Lastres P, Blanco FJ, Sanz-Rodríguez F, et al. Mice Lacking Endoglin in Macrophages Show an Impaired Immune Response. PLoS Genet. 2016.
4. This work has been supported by the Ministry of Education, Youth and Sports of the Czech Republic (LQ1605, NPU II).

## ETH - Ethics of end of life care

### 000072

#### The Role of the Spirituality of Intensive Care Unit Patient's Relatives and their Satisfaction with ICU Professionals' Communication

P. Stamou[1], M. Gouva[2], M. Christaki[1], V. Koulouras[1], G. Papathanakos[1]
[1]Intensive care unit, University Hospital of Ioannina, Ioannina, Greece; [2]Department of nursing, research laboratory psychology of patients families and health profession, University of Ioannina, Ioannina, Greece
**Correspondence:** G. Papathanakos
*Intensive Care Medicine Experimental* 2019, **7(Suppl 3):**000072

**INTRODUCTION.** Spirituality substantially influences coping strategies of family members of patients hospitalized in an Intensive Care Unit (ICU). Spirituality might also influence the communication between family members and ICU physicians and nurses.

**OBJECTIVES.** To explore the effect of spirituality of critically ill patient's family members on their satisfaction during communication with ICU health professionals.

**METHODS.** The research was conducted from March 2018 to March 2019. Family members (first degree relatives, close relatives and intimate friends) of patients hospitalized in a University ICU constituted our research material. A questionnaire for sociodemographic data and the Spiritual and Religious Attitudes in Dealing with Illness (SpREUK) Scale was completed by family members during the first week after their beloved one's ICU admission. One way ANOVA and MANCOVA was used for statistical analysis.

**RESULTS.** One hundred and three family members mean-aged 45.92±11.43 years corresponded to 86 ICU patients responded to the Study. Forty-four of them were men (42.7%) and 59 women (57.3%), Most of them were children (48.5%) and the remaining family members were patients' companions (23.3%), siblings (7.8%), parents (7.7%) or other relatives (12.7%). Spirituality was found to positively increase the satisfaction of family members (F=3.632, p=.009). General linear model analysis found strong association between "Trust in

Higher Guidance/Source" (SpREUK) and feelings of information completeness (p=.034), openness and honesty of information (p=.020), understanding of the information (p=.010) and ease of accessing information (p=.010) among family members during their communication with ICU physicians and nurses. Data analysis did not show any significant difference in spirituality of family members based on sex, marital status and place of residence except for the educational level of relatives with the SpREUK subscale "reflection" (p=.001); the higher the educational level the higher was the score in "reflection" subscale while the most prominent differences were found between 'high school' and 'postgraduate' family members (p=.041).

**CONCLUSION.** Critically ill patients' relatives are more satisfied with their communication with ICU health professionals when they have higher levels of spirituality and they believe in something "higher" to overcome the difficulties.

**REFERENCE(S)**

1. Büssing A, Ostermann T, Matthiessen PF, et al. Role of religion and spirituality in medical patients: confirmatory results with the SpREUK questionnaire. Health Qual Life Outcomes. 2005 Feb 10;3:10.
2. Koukouli S, Lambraki M, Sigala E, Alevizaki A, Stavropoulou A. The experience of Greek families of critically ill patients: Exploring their needs and coping strategies. Intensive Crit Care Nurs. 2018 Apr;45:44-51
3. Sulmasy DP. Spirituality, religion, and clinical care. Chest. 2009 Jun;135(6):1634-1642.
4. Wall RJ, Engelberg RA, Gries CJ, Glavan B, Curtis JR. Spiritual care of families in the intensive care unit. Crit Care Med. 2007 Apr;35(4):1084-90.
5. None

### 000131

#### Can fluid management influence Simultaneous Pancreas and Kidney Transplantation outcomes?

T. Isidoro Duarte[1], N. Germano[1], F. Sousa Cardoso[1], A. Martins[2], J. Paulino[2], F. Caeiro[3], I. Aires[3], P. Cotovio[3], F. Remedio[3], A. Ferreira[3], F. Nolasco[3]

[1]Intensive Care Medicine Department, Curry Cabral Hospital, Central Lisbon Hospital Center, Lisbon, Portugal; [2]Surgery department, Curry Cabral Hospital, Central Lisbon Hospital Center, Lisbon, Portugal; [3]Nephrology department, Curry Cabral Hospital, Central Lisbon Hospital Center, Lisbon, Portugal

**Correspondence:** T. Isidoro Duarte

*Intensive Care Medicine Experimental* 2019, **7(Suppl 3):**000131

**INTRODUCTION.** Simultaneous pancreas and kidney transplantation (SPK) has been established as the treatment of choice for type 1 diabetic patients with end-stage nephropathy, leading to marked improvement in cardiovascular function and long-term survival. Adequate fluid therapy is essential to maintain intravascular volume and to obtain appropriate graft perfusion. Hypovolemia can result in further organ injury or thrombosis. Furthermore, excessive volume infusion with hemodilution and tissue edema, increases oxygen diffusion distance and impairs graft perfusion.

**METHODS.** Retrospective study of patients admitted for SPK during 2018 in an Intensive Care Unit (ICU) in a Transplant Reference Center. Fluid management was implemented according to protocol (urine-output per hour plus 200mL for two days). Doppler ultrasound was done on the first post-operative day and when clinically relevant. All patients received 100mg aspirin pre and post-transplant associated with low-molecular weight heparin. Immunosuppression induction and maintenance included thymoglobulin, methylprednisolone, tacrolimus and mycophelonate mofetil. Daily fluid balance, serum urea, creatinine and C peptide were recorded every day during the first 4 days post-SPK. Fluid balance was defined as primary exposition and pancreatic thrombosis as primary outcome.

**RESULTS.** Nineteen caucasian patients (63% male) were admitted. Fourteen were under pre-transplant dialysis for more than 1 year. One needed plasmapheresis before SPK. Pancreatic graft thrombosis happened in 5 patients (26%) between the 2-6 days of ICU. No positive differences were found in both groups according to donor's age or past history of thrombosis. No relevant differences were found in fluid balance on the first three days pos-SPK transplant in both groups. Fourth day fluid balance was significantly lower in the group of pancreatic thrombosis (-1,20L *vs* +0,03L, $p < 0,05$). The percentage of patients who needed vasopressor support during surgery was higher in the thrombotic group (80% *vs* 35,7%,$p = 0,14$). Time-to-event analysis (Cox regression) with Kaplan-Meyer curves adapted for vasopressor support revealed a 30 day pancreatic graft survival worse for those who needed perioperative vasopressor (56% *vs* 90%, $p = 0,11$). Hospital mortality rate of 10,5%.

**CONCLUSION.** SPK survival can be influenced by multifactorial variables where fluid balance is one of them. Nevertheless, optimal fluid management remains a challenge for both peri and post-operative periods. The use of minimal invasive hemodynamic devices with patient-centered targets may play an important role in best understanding flow parameters in this specific group of patients with impaired cardiovascular physiology and reduced hemodynamic autoregulation. Avoiding excessive fluid resuscitation and its consequences as well as hypovolemia may be helpful in increasing graft survival and patient outcomes.

**REFERENCE(S)**

1. Wiseman A, Gralla J. Simultaneous pancreas kidney transplant versus other kidney transplant options in patients with type 2 diabetes. Clin J Am Soc Nephrol. 2012;7:656-664.
2. Stadler M, Theuert E, et al. Persistent arterial stiffness and endothelial dysfuncion following successful pancreas-kidney transplantation in Type 1 diabetes. Diabetic Medicine.2009;26:1010-1018.
3. Schnuelle P, van der Woude FJ. Perioperative fluid management in renal transplantation: a narrative review of the literature. Transplant International.2006;19:947-959.
4. Calixto Fernandes MH, Schricker T, et al. Perioperative fluid management in kidney transplantation: a black box. Critical Care.2018;22:14.

### 000187

#### Critically ill elderly patients in the emergency department: Critical information for clinical decisions

J. Wagner[1], A. Krohn[1], T. Schilling[1], D. Räpple[2], J. Heymer[1]

[1]Interdisciplinary emergency department, Klinikum Stuttgart - Katharinenhospital, Stuttgart, Germany; [2]Interdisciplinary intensive care unit, Klinikum Stuttgart - Katharinenhospital, Stuttgart, Germany

**Correspondence:** J. Heymer

*Intensive Care Medicine Experimental* 2019, **7(Suppl 3):**000187

**INTRODUCTION.** Elderly patients have a high in hospital mortality1. Physicians may support patients and families in the decision making process by providing information2.

**OBJECTIVES.** We aimed to characterize the patients admitted via the emergency department to medical and neurologic intensive care units and analyze in hospital mortality and transition to palliative care

**METHODS.** We conducted a retrospective study of patients ≥80 years who were admitted in 2016 and 2017 via the emergency department to a medical or neurointensive care unit. The electronic medical record of each patient was studied to extract data regarding the therapies, outcomes and the decision making process.

**RESULTS.** During the two- year period 248 (114 male, 134 female) patients ≥80 years were admitted from the emergency department to our ICUs. Mean age was 85,27 (±3,61; range 80,1-96,78) years, mean length of stay was 11,9 (±18,5; range 0-202) days.

In 66 (26,6%) patients, therapy was transitioned to palliative care, 68 (27,4%) patients died in hospital and 83 (33,5%) patients either died or therapy was changed to palliative care.

Frequent primary diagnosis include stroke/ICB, infection, cardiac disease, trauma, renal disease/electrolyte disorders and GI-Bleed.

113 patients required mechanical ventilation (92 patients invasive ventilation, 21 patients non invasive ventilation), 73 patients received catecholamines.

Patients requiring mechanical ventilation had a higher rate of the combined result of in hospital death or transition to palliative care

(50,4%) than patients without mechanical ventilation (19,3%) (OR 4,27, 95%CI 2,43-7,51; p < 0,0001).
In the subgroup of patients treated with invasive ventilation the rate of in hospital death was 53,3%.
In 51 of the 68 patients (75%) that eventually died therapy was changed to palliative care.
The differences in age between survivors (mean 85,3 ±3,4 years, median 84,5 years) and non survivors (mean 85,2 ±3,2 years, median 84,8 years) were not significant (p=0,7642).
**CONCLUSION.** Patients ≥ 80 years admitted to the ICU had a high rate of death or transition to palliative care. The high rate of transition to palliative care in the non- survivors underlines the importance of support in the decision making process in order to prevent futile treatments. In this group of patients ≥80 years we did not find a significant age difference between survivors and non survivors.

**REFERENCE(S)**

1. Ferrante LE et al. Functional Trajectories Among Older Persons Before and After Critical Illness. JAMA Intern Med. 2015;175(4):523-529
2. White DB et al. Prevalence of and Factors Related to Discordance About Prognosis Between Physicians and Surrogate Decision Makers of Critically Ill Patients. JAMA. 2016;315:2086-2094.
3. None

### 000370

#### Critical care in the elderly: A survey of attitudes and knowledge

B. Narayan[1], T. Samuels[2], M. Alice[2]
[1]ICU, Salford Royal NHS Foundation Trust, Manchester, UK; [2]Critical care, East Surrey Hospital, Redhill , UK
**Correspondence:** B. Narayan

*Intensive Care Medicine Experimental* 2019, **7(Suppl 3):**000370

**INTRODUCTION.** A 2009 review conducted by the UK Centre for Policy on Ageing found evidence of ageist attitudes among hospital staff and suggested that doctors may be more ageist than other healthcare professionals. The authors emphasised the need to study this in more detail.
The incidence of frailty and chronic illness increase with increasing age yet are not always present so should not be assumed to co-exist. In 2017, 18.2% of the population were aged 65 or older, compared to 10.6 % in 1948, when the NHS was created.
**OBJECTIVES.** To explore attitudes regarding age within our own multi-disciplinary team.
**METHODS.** A survey was distributed to healthcare professionals: acute internal medicine (AIM) physicians (n=14); intensive care doctors (n=8), and ICU nurses (n=9). Responses were anonymous and without conferring or using additional information. Of the 8 multiple-choice questions, three tested knowledge and two specifically asked whether the respondent felt doctors (physicians and intensivists) had an ageist attitude when making decisions about critical care referral/admission. Options were "Never", "Occasionally", "Sometimes", "Often" and "Always".
**RESULTS.** Regarding escalation and/or setting ceilings of treatment in elderly patients, **only half of doctors** (43% of AIM, 50% of intensivists), compared with a **majority of ICU nurses** (89%) **were happy with decision-making** in their workplace. A minority of AIM (36%) and intensivists (38%) but a **majority of nurses** (78%) felt it was **sometimes acceptable to use age as the main factor** in deciding whether or not a patient's treatment should be escalated. Regarding knowledge-based questions, **intensivists gave reasonably accurate answers regarding ICU mortality in the elderly and the average age of patients admitted to ICU**. The **majority of AIM over-estimated both mortality and average age**. All groups significantly **underestimated average life expectancy** in the elderly population. Both physician groups viewed both AIM and intensivists as having an ageist attitude at least some of the time, but using bootstrapping methods (10,000 replications), the difference between the opinions of the ICU and AIM doctors was not statistically significant (p = 0.1969).
**CONCLUSION.** There is significant dissatisfaction amongst both acute physicians and intensivists regarding escalation of treatment decision-making in elderly patients. ICU nurses are more satisfied, and are much more likely to accept the use of age as the main factor when decisions are made. Both doctor groups viewed both AIM and intensivists as having an ageist attitude at least some of the time, but this difference in opinion was not statistically significant. Intensivists had better awareness about ICU mortality and average patient age, but all groups significantly underestimated life expectancy in the elderly. A larger multicentre study would be useful to further explore these findings, and more training and education is needed to address the issues raised.

**REFERENCE(S)**

1. UK Office for National Statistics
2. UK Centre for Policy on Ageing and Department of Health - Ageism and age discrimination in secondary health care in the UK: A review from the literature, December 2009. Accessible at http://www.cpa.org.uk/information/reviews/CPA-ageism_and_age_discrimination_in_secondary_health_care-report.pdf

### 000405

#### Outcomes in patients perceived and not perceived as receiving excessive care by ICU clinicians : differences between patients with uncontrolled and controlled cancer versus patients without cancer

D. Benoit[1], M. Darmon[2], A. Reyners[3], V. Metaxa[4], D. Mokart[5], A. Wilmer[6], A. Hvarfner[7], K. Rusinova[8], J. Zijlstra[9], F. Vincent[10], D. Lathyris[11], F. Faria[12], AP. Meert[13], J. Devriendt[14], E. Uyttersprot[15], E. Kompanje[16], R. Piers[17], E. Azoulay[18]
[1]{street_address}, Ghent, Belgium; [2]Medical icu, Hôpital Saint-Louis, Paris, France; [3]Oncology and palliative care, University Medical Center Groningen, Groningen, Netherlands; [4]Intensive care, King's College Hospital, London, UK; [5]Medical icu, Institute Paoli-Calmettes, Marseille, France; [6]Medical intensive care, Katholieke Universiteit Leuven, Leuven, Belgium; [7]Department of anesthesiology and intensive care, Karolinska University Hospital, Stockholm, Sweden; [8]Anesthesiology and intensive care, General University Hospital in Prague, Prague, Czech Republic; [9]Intensive care, University Medical Center Groningen, Groningen, Netherlands; [10]Icu, Intercommunal Hospital Group Le Raincy Montfermeil, Montfermeil, France; [11]Intensive care, G. Gennimat Hospital, Thessaloniki, Greece; [12]Intenisve care, Instituto Português de Oncologia do Porto Francisco Gentil, Porto, Portugal; [13]Icu, Institut Jules Bordet, Bruxelles, Belgium; [14]Intensive care, CHU Brugmann, Bruxelles, Belgium; [15]Applied mathematics and computer sciences, Ghent University, Gent, Belgium; [16]Intensive care, Erasmus University Rotterdam, Rotterdam, Netherlands; [17]Geriatrics, Ghent University Hospital, Ghent, Belgium; [18]Réanimation médicale, Hôpital Saint-Louis, Paris, France

*Intensive Care Medicine Experimental* 2019, **7(Suppl 3):**000405

**INTRODUCTION.** For many years there has been a reluctance to admit cancer patients to the ICU. However, whether ICU clinicians tend to discriminate cancer patients is unknown.
**OBJECTIVES.** The aim of this study was to compare the time until treatment limitation decisions (TLDs) and death, and the one year outcomes in patients with and without concordant perceptions of excessive care (PECs) by two or more ICU clinicians in the following subgroups : uncontrolled and controlled cancer vs. patients without cancer.
**METHODS.** Subanalysis of the DISPROPRICUS study performed in 68 ICUs in 12 European countries and the USA between 2014 and 2015 [1]. Clinicians (nurses and physicians) completed daily a questionnaire about the appropriateness of care for each of their patients during a 28 day period. We compared the cumulative incidence of patients with concordant PECs, TLDs and death, and the proportion of patients attaining the combined endpoint (death, poor quality of life or not being at home) at one year across subgroups via Cox-regression (accounting for competing risk) and Fisher-exact tests, respectively. To adjust for country, ICU and patients characteristics we used inverse probability weighting based on propensity scores. Results are presented as weighed % and hazard ratios (HR)

with their 95% confidence intervals (CI). The subgroup of patients without cancer was used as reference category.

**RESULTS.** Of the 1641 patients not admitted for monitoring only during the study period, 117 (7.1%) had uncontrolled cancer and 270 (16.4%) had controlled cancer. Of the 2690 participating clinicians, 2293 (85.2%) provided 25025 perceptions of appropriateness of care, of which 2279 (9.1%) PECs by 728 (27.0%) clinicians in 334 (20.3%) patients. The cumulative incidence of concordant PECs in patients with uncontrolled and controlled cancer vs. patients without cancer was 20.4%, 9.2% and 8.9% (p=0.005 and 0.85, respectively). In patients with concordant PECs, we found no evidence for a difference in time from concordant PECs until death (HR 0.99, 95%CI 0.59-1.65 and 0.90, 95%CI 0.50-1.62) and TLDs (HR 0.93, 95%CI 0.36-2.40 and 0.68, 95%CI 0.24-1.89) across subgroups. In this group, the risk of reaching the combined endpoint at one year was 93.7% and 82.5% vs. 93.4% (p=0.99 and p<0.001). In patients without concordant PECs, we found evidence for a difference in time from admission until death (HR 2.23, 95%CI 1.58-3.15 and 1.66, 95%CI 1.28-2.15) whereas the time from admission until TLDs was statistically non-significant (NA, p=0.3 and 0.7) across subgroups. In this group, the risk of reaching the combined endpoint at one year in patients with uncontrolled and controlled cancer vs. patients without cancer was 70.8% and 63.0% vs. 54.5%,respectively (p=0.003 and p=0.02).

**CONCLUSION.** The absence of evidence of a higher risk of TLDs and death in patients with concordant PECs makes discrimination of cancer patients by ICU clinicians unlikely. However, relative to patients without cancer, our results suggest prognostic pessimism in ICU clinicians who provided PECs in patients with controlled cancer and prognostic optimism or ignorance in ICU clinicians who did not provide PECs in patients with uncontrolled cancer, respectively. This study highlights the need to improve intra-and interdisciplinary ethical reflection and subsequent decision-making in the ICU [1].

**REFERENCE(S)**

1. D.D. Benoit et al Outcome in patients perceived as receiving excessive care across different ethical climates : a prospective study in 68 intensive care units in Europe and the USA. Intensive Care Med (2018) 44:1039-1049

2. This study was supported by an European Society of Intensive Care Medicine/European Critical Care Research Network clinical research award and a Fonds voor Wetenschappelijk Onderzoek senior clinical investigators grant (1800513N) obtained in 2012 by DB.

### 000460

#### Outcomes in patients perceived and not perceived as receiving excessive care by ICU clinicians : differences between patients < 75 and ≥ 75 years old

D. Benoit[1], E. Åkerman[2], G. Meyfroidt[3],R. Gerritsen[4], M. Oikonomou[5], H. Spapen[6], A. Schouten[7], I. De Laet[8], V. Banner-Goodspeed[9], P. Deschamps[10], R. Monte[11], F. Marinangeli[12], E. Kompanje[13], E. Uyttersprot[14], R. Piers[15]

[1]{street_address}, Ghent, Belgium; [2]Anesthesiology and intensive care, Karolinska University Hospital, Stockholm, Sweden; [3]Intensive care, UZ Leuven, Leuven, Belgium; [4]Intensive care, Medical Center Leeuwarden, Leeuwarden, Netherlands; [5]Intensive care, Papageorgiou General Hospital, Thessaloniki, Greece; [6]Intensive care, UZ Brussel, Jette, Belgium; [7]Intensive care, Canisius Wilhelmina Hospital, Nijmegen, Netherlands; [8]Intensive care, ZNA Stuivenberg, Antwerpen, Belgium; [9]Anesthesia, Beth Israel Deaconess Medical Center (BIDMC), Boston, USA; [10]Intensive care, CHU Saint-Pierre, Bruxelles, Belgium; [11]Intensive care, Jose Antonio Pinho Hospital, Pedro Pimenta, Portugal; [12]Intensive care, San salvatore hospital, L'Aquila, Italy; [13]Intensive care, Erasmus University Rotterdam, Rotterdam, Netherlands; [14]Applied mathematics and computer sciences, Ghent University, Gent, Belgium; [15]Geriatrics, Ghent University Hospital, Ghent, Belgium

*Intensive Care Medicine Experimental* 2019, **7(Suppl 3):**000460

**INTRODUCTION.** The increasing number of older patients admitted to the ICU with multiple comorbidities and frailty is often at the center of debate in daily practice. However, whether ICU clinicians tend to discriminate elderly patients is unknown.

**OBJECTIVES.** The aim of this study was to compare the time until treatment limitation decisions (TLDs) and death, and one year outcomes in patients < and ≥75 year with and without concordant perceptions of excessive care (PECs) by two or more ICU clinicians.

**METHODS.** Subanalysis of the DISPROPRICUS study performed in 68 ICUs in 12 European countries and the USA between 2014 and 2015 [1]. Clinicians (nurses and physicians) completed daily a questionnaire about the appropriateness of care for each of their patients during a 28 day period. We compared the cumulative incidence of patients with concordant PECs, TLDs and death, and the proportion of patients attaining the combined endpoint (death, poor quality of life or not being at home) at one year across subgroups via Cox-regression (accounting for competing risk) and Fisher-exact tests, respectively. To adjust for differences in country, ICU and patient characteristics, we used inverse probability weighting based on propensity scores. Results are presented as weighed % and hazard ratios (HR) with their 95% confidence intervals (CI).

**RESULTS.** Of 1641 patients not admitted for monitoring only during the study period, 405 (25%) were ≥75 years. Of the 2690 participating clinicians, 2293 (85.2%) provided 25025 perceptions of appropriateness of care, of which 2279 (9.1%) PECs by 728 (27.0%) clinicians in 334 (20.3%) patients. The cumulative incidence of patients with concordant PECs was significantly higher in patients ≥75 years than in patients < 75 years (13.6 % vs. 8.5 %, p-value = 0.01). In patients with concordant PECs, we found no evidence for a difference in time from concordant PECs until death (HR 1.08, 95%CI 0.73-1.61) and TLDs (HR 0.98, 95%CI 0.51-1.91) between older and younger patients. We neither found evidence for a difference in risk of reaching the combined endpoint at one year in this group between older and younger patients (90.4% vs. 93%, p= 0.54). In patients without concordant PECs, we found evidence for a difference in risk of death (HR 1.38, 95%CI 1.11-1.73) and TLDs (HR 2.11, 95%CI 1.37-3.27), though half of the TLDs were installed already before ICU admission. The risk of reaching the combined endpoint was also significantly higher in older compared to younger patients (62.1% vs. 52.8%, p<0.001).

**CONCLUSION.** The absence of a higher risk of TLDs, death and one year outcomes in patients with concordant PECs makes discrimination of older patients by ICU clinicians unlikely. The poorer one year outcomes in older patients without concordant PECs after weighing for confounders confirms that age need to be taken into account during ethical decision-making.

**REFERENCE(S)**

1. D.D. Benoit et al. Outcomes in patients perceived as receiving excessive care across different ethical climates: a prospective study in 68 intensive care units in Europe and the USA. Intensive Care Med 2018;44:1039-1049

2. This study was supported by an European Society of Intensive Care Medicine/European Critical Care Research Network clinical research award and Fonds voor Wetenschappelijk Onderzoek senior clinical investigators grant (1800513N) obtained in 2013 and prolonged (1800518) in 2017 by DB.

### 000558

#### How is end-of-life and palliative care being performed in dying patients in intensive care units? A Brazilian multicenter observational prospective cohort study

PA. Duarte[1], AC. Jorge[1], M. Zedu Alliprandini[2], A. Ferrandin[2], A. Fernandes[2], M. Comiran Belim[2], M. Marinho Jorge[2], B. Beck[2], J. Mayumi Yaguchi[2]

[1]General icu, Hospital Universitario do Oeste do Parana, Cascavel, Brazil; [2]Medicine, Hospital Universitario do Oeste do Parana, Cascavel, Brazil

**Correspondence:** P.A. Duarte

*Intensive Care Medicine Experimental* 2019, **7(Suppl 3):**000558

**INTRODUCTION.** Objectives: To evaluate among ICU patients that evolved to death those with an indication of performing and applying end-of-life decisions and palliative care (EL-PC); and to analyse the application of items of PC, either 'unperformed items' or items of 'futile/unnecessary treatment'.

**METHODS.** Observational prospective cohort in five ICUs. Patients who evolved to death were evaluated searching for indication of EL-PC. The correct application of nine preselected items was studied.

**RESULTS.** Among 253 admissions, 52 patients died; and among these, 38.5% met criteria for EL-PC. Among PC candidates (n = 20), the PC was started later (after day 3) in 60%, and only 3 patients received adequate palliative care. 'Analgesia' and 'Daily family interviews' were the most correctly applied PC items. 'Terminal extubation/weaning' was not performed in any of the patients. A reduction in the interval from the onset of PC to death was observed in patients who underwent 'correct' interventions—66.6% died on the first day of PC.

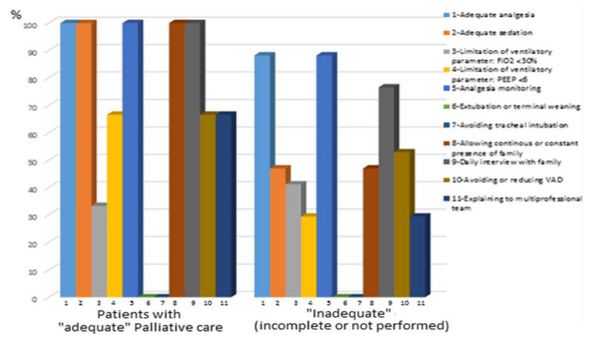

**Fig. 1 (abstract 000558).** See text for description

**CONCLUSION.** In a low/medium-income country, one-third of patients who died in the ICU had indications for EL-PC; however, the PC was adequately performed for only 15% of the candidates, with great heterogeneity and delays in regards to its initiation.

F2

### 000578

#### Swedish intensivists' experiences, conceptions and attitudes on decision- making regarding withdrawal or withholding life sustaining treatments

A. Syrous Nordenskjöld[1], A. Agard[2], L. Block[1]
[1]Dpt of anaestsia and intensive care, inst of clinical sciences, Sahlgrenska University Hospital, Gothenburg university, Sahlgrenska Academy, Gothenburg, Sweden; [2]Dpt of medicine, inst of clinical sciences, Sahlgrenska University Hospital, Gothenburg university, Sahlgrenska Academy, Gothenburg, Sweden
**Correspondence:** L. Block
*Intensive Care Medicine Experimental* 2019, **7(Suppl 3):**000578

**INTRODUCTION.** The decision-making process regarding end of life treatment is complex and challenging. Several authors report a substantial variability in this process among intensivists 1-3.

**OBJECTIVES.** The primary aim of this qualitative study was to investigate Swedish intensivists' perceptions and attitudes towards withdrawing or withholding life sustaining treatments. The secondary aim was to identify underlying factors that may contribute to inherent biases in the decision-making process.

**METHODS.** This is a descriptive study with qualitative approach. Semi-structured interviews was chosen as method for the study. The interviews were recorded and transcribed verbatim. All the data was categorized into thematic patterns as the primary basis for organizing findings and data coding was used. A two-step process, beginning with basic coding in order to distinguish overall themes, which was followed by a more in depth, interpretive code in which more specific trends and patterns were interpreted.

**RESULTS.** Nineteen semi-structured interviews with intensivists with different experience, education, age and gender, from five different hospitals were performed. The main finding of this study is that there is an unchallenged, accepted variability in end of life decision making among Swedish intensivists. The contributory factors to this variability are personality of the intensivist, lack of continuity among senior intensivists, disagreements with physicians from other specialties, lack of competence during on call hours, and concerns of criticisms for making questionable decisions. Few of the respondents had any formal education on how to approach end of life decisions and it was generally not demanded. All of the respondents knew about existing on-line guidelines, but few used them in daily clinical practice.

**CONCLUSION.** There are significant variabilities in the decision-making on withdrawing or withholding life sustaining treatments on the intensive care units. We propose a more structured approach, formal education and routine adherence to clinical guidelines in order to improve the management of this group of complex patients.

##### REFERENCE(S)

1. Mark NM, Rayner SG, Lee NJ, Curtis JR. Global variability in withholding and withdrawal of life-sustaining treatment in the intensive care unit: a systematic review. Intensive Care Med. 2015;41(9):1572-85.
2. Sprung CL, Woodcock T, Sjokvist P, Ricou B, Bulow HH, Lippert A, et al. Reasons, considerations, difficulties and documentation of end-of-life decisions in European intensive care units: the ETHICUS Study. Intensive Care Med. 2008;34(2):271-7.
3. Wunsch H, Harrison DA, Harvey S, Rowan K. End-of-life decisions: a cohort study of the withdrawal of all active treatment in intensive care units in the UK. Intensive Care Med. 2005;31(6):823-31.
4. This study was supported by The ALF agreement ALFGBG-772521, ALFGBG-722301, The Region Västra Götaland VGFOUREG-753851 and VGFOUREG-650891.

### 000610

#### Learning from stakeholders to inform good practice guidance on consent to research in intensive care units: mixed methods study

K. Paddock[1], L. Frith[1], C. Gamble[2], I. Welters[3], K. Woolfall[1], B. Young[1]
[1]Health services research, University of Liverpool, Liverpool, UK; [2]Biostatistics, University of Liverpool, Liverpool, UK; [3]Critical care, Royal Liverpool and Broadgreen University Hospitals NHS Trust, Liverpool, UK
**Correspondence:** K. Paddock
*Intensive Care Medicine Experimental* 2019, **7(Suppl 3):**000610

**INTRODUCTION.** Alternatives to prospective consent by patients are necessary for conducting clinical research with critically ill patients. It is important that such consent practices align with the perspectives of key stakeholder groups - patients, relatives and clinical practitioners.

**OBJECTIVES.** To determine the perspectives of different stakeholder groups towards current consent practices for research with critically ill patients, with the overall objective of informing good practice guidance on consent to intensive care unit (ICU) studies.

**METHODS.** Mixed methods study comprising surveys and in-depth interviews in 14 English ICUs.

**RESULTS.** 1409 surveys were included (ICU patients n=333, relatives n=488, practitioners n=588) and 58 stakeholders were interviewed. Survey responses indicated that most stakeholders agreed it was acceptable for relatives to decide about research participation on behalf of incapacitated patients, although the proportion of patients who agreed (68%) was lower than relatives (83%) or practitioners (76%). The practice of doctors consenting to research on behalf of incapacitated patients was supported by most stakeholders in situations where there were no known relatives (patients 60%, relatives, 63% practitioners 63%) or if time was too short to contact relatives (patients 57%, relatives, 51%, practitioners 53%). Most patients (55%) and relatives (52%) also supported consent by doctors when relatives were unavailable, but only 45% of practitioners supported this. Practitioner experience of research influenced their views on consent by doctors – those who had a research role were more accepting of this practice than practitioners with no research role. In all three stakeholder groups approximately 25% of members disagreed with doctor consent for ICU studies whatever the situation. However, interviews indicated that stakeholders were more flexible towards doctors consenting on behalf of incapacitated patients than their survey responses suggested, with the views of most patients and relatives, and some practitioners shifting markedly depending on particularities of the study and situation.

**CONCLUSION.** Stakeholder support for current ICU research consent practices that involve relatives in decisions was generally high. In situations where involving relatives was not possible, most stakeholders also supported consent by doctors, although a sizable minority disagreed with this consent practice. When interviewed, stakeholders were rarely fixed in their views, with many willing to countenance a range of consent practices depending on their perceptions of what the research involved and provided safeguards were in place. Findings point to the importance of explaining clearly why alternative consent practices are used in ICU studies and what safeguards are in place.

### 000619

#### Intensive Care To Facilitate Organ Donation: A report on the experience of a Spanish Center with a multidisciplinary cooperation model using a communication app and redefining referral criteria

CA. Mazo Torre[1], A. Gomez[1], A. Sandiumenge[1], JC. Ruiz[2], RA. Garcia[3], L. Abraira[4], M. Rubiera[4], S. Boned[4], J. Baena[5], M. Baguena[2], RM. Gracia[2], T. Pont[1]
[1]Transplant Coordination, Vall d'Hebron University Hospital, Barcelona, Spain; [2]Intensive care, Vall d'Hebron University Hospital, Barcelona, Spain; [3]Intenisve care, Vall d'Hebron University Hospital, Barcelona, Spain; [4]Neurology, Vall d'Hebron University Hospital, Barcelona, Spain; [5]Trauma and burns critical care, Vall D Hebron, Barcelona, Spain
**Correspondence:** C.A. Mazo Torre
*Intensive Care Medicine Experimental* 2019, **7(Suppl 3):**000619

**INTRODUCTION.** Stagnation in the number of donors after brain death (BD) requires strategies to increase the pool of donors to meet expanding waiting lists. Intensive care to facilitate organ donation (ICOD) has been defined as the initiation/continuation of intensive care measures in patients with a devastating brain injury (DBI) in whom treatment for curative purposes is deemed futile, and who are considered possible organ donors, with the aim of offering donation after brain death (DBD) inside their end-of-life care plans [1].

**OBJECTIVES.** We describe the impact on the donation activity in Vall d'Hebron University Hospital, Barcelona, following introduction of a new strategy promoting the referral of possible donors from outside the ICU to consider ICOD over a 4-year period.

**METHODS.** Retrospective analysis (2015-2018) of all patients located outside the ICU with a DBI referred to the donor coordination (DC) to be considered for ICOD. Once the DC evaluated the medical suitability and likelihood of progression to brain death (BD), families were approached to discuss the option of donation. If favourable, further evaluation and maintanance was performed in the ICU until BD ensued, leading to donation in elegible cases. In those cases where BD did not occur, life-sustaining therapies were withdrawn and the option of controlled donation after circulatory death (cDCD) was offered to the family.

**RESULTS.** Of the 983 possible donors evaluated during the study period, ICOD was considered in 206 (21%), of whom 115 (55.8%) were medically unsuitable for donation.

Family consent was obtained for 69 (76%) of the remaining patients. Refusal rate was twice as high when non-therapeutic ventilation was required for organ donation (34%) compared to those patients previously ventilated (13.6%) (p =0.02). Patients subject to ICOD died in a median of 2 days [1 – 3] and 88.4% became actual donors (AD) (39 after BD/22 after circulatory death). Nine (17.6%) donors were finally not utilized. ICOD contributed to 27% of the 195 utilized donors and 26% of the 603 organs transplanted. Over the 4 years analyzed, ICOD was responsible for a progressively greater percecentage of AD ranging from 27.7% in 2015 to 31.6% in 2018.

**CONCLUSION.** ICOD has proved to be a procedure well-accepted by families, which facilitated the offer of donation in end-of-life care to an increasing number of patients at our hospital. It also provided, with a short ICU stay, an important and sustained increment of the organ pool for transplantation.

### 000734

#### End-of-life decisions may influence ICU mortality and hospital lengths of stay

S. von Düring[1], PD. Wendel Garcia[2], JC. Schefold[3], K. Tisljar[4], S. Doll[5], H. Pargger[6], B. Ricou[1]
[1]Medical surgical intensive care unit, University Hospitals of Geneva and University of Geneva, Geneva, Switzerland; [2]Medical intensive care unit, University Hospital of Zurich, Zürich, Switzerland; [3]Department of intensive care, Inselspital, Bern University Hospital and University of Bern, Bern, Switzerland; [4]Medical intensive care unit, University Hospital of Basel, Basel, Switzerland; [5]Medical surgical intensive care unit, Cantonal Hospital of Fribourg, Fribourg, Switzerland; [6]Surgical intensive care unit, University Hospital of Basel, Basel, Switzerland
**Correspondence:** S. von Düring
*Intensive Care Medicine Experimental* 2019, **7(Suppl 3):**000734

**INTRODUCTION.** More than 70 % of ICU deaths occur after an End-Of-Life (EOL) decision consisting in withholding or withdrawing of life-sustaining therapy (LST). EOL decision-making varies greatly within Europe. Switzerland is separated into distinct cultural regions defined by languages related to the neighboring countries. The three linguistic regions are the French (FR), the German (GR) and the Italian speaking region.

**OBJECTIVES.** To determine the differences in EOL decisions between the FR and GR, and their influence on ICU and hospital length-of-stay (LOS) and mortality. As secondary objectives, we evaluated whether the type and timing of EOL decision had an influence on outcome issues.

**METHODS.** Patients admitted to ICU and who had treatment limitations over a 6 month period were included as part of the ETHICUS 2 study. Seven Swiss ICUs (3 in FR, 4 in GR) allowed us to compare two cultural regions. Patients were followed from admission until discharge from the ICU, death, or 2 months after the decision to limit the therapy.

**RESULTS.** Results of 1'115 patients are summarized in Table 1. ICU and hospital mortality differed significantly between FR and GR (33 vs 63 %, $p < 0.0001$, 48 vs 75 %, $p < 0.0001$ respectively), whereas mortality after ICU was similar in both groups. ICU LOS was similar in both groups. Post-ICU LOS was longer in FR and EOL decisions were made earlier in FR. Time from decision to death was considerably longer in FR than in GR. FR took decisions in multiple steps compared to GR (40 vs 24 %, p < 0.013), and GR withdrew LST as a single decision that impacted shortly on survival.

**CONCLUSION.** Mortality was significantly higher in GR. EOL decisions were made later in GR ICUs but with more withdrawals that were associated with more deaths. ICU LOS was similar in both regions but hospital LOS after ICU and total hospital LOS was significantly shorter in GR. Depending on the way EOL decision is made, the hospital LOS and the outcome may be impacted.

**REFERENCE(S)**

1. The authors received no financial support for the research, authorship, and/or publication of this article.
2. The authors acknowledge the assistance of Alexander Avidan and Charlie Sprung, International coordinator of Ethicus II study / Ethics Section ESICM

**Table 1 (abstract 000734).** Comparison between FR and GR

| | FR n=550 (49.3) | GR n=565 (50.7) | p |
|---|---|---|---|
| Total Hospital Length of stay (LOS) [days] | 22.27 ± 1.08 | 11.59 ± 0.77 | < 0.0001 |
| - ICU LOS [days] | 4.99 ± 0.32 | 4.15 ± 0.26 | 0.04 |
| - Hospital LOS after ICU [days] | 12.16 ± 0.76 | 3.2 ± 0.34 | < 0.0001 |
| Time to 1st EOL limitation after ICU admission [days] | 0.79 ± 0.24 | 1.92 ± 0.28 | < 0.01 |
| Time to ICU discharge after first EOL limitation [days] | 3.97 ± 0.31 | 4.18 ± 0.39 | 0.67 |
| Time to death after first EOL limitation [days] | 10.6 ± 1.01 | 2.27 ± 0.23 | < 0.0001 |

T1

### 000814

#### End-of-life Practices in German in Intensive Care Units (ICUs) – Results from the ETHICUS-2 Study

C. Denke[1], U. Jaschinski[2], R. Riessen[3], S. Bercker[4], C. Spies[1], M. Ragaller[5], M. Weiss[6], K. Dey[7], A. Michalsen[8], J. Briegel[9], A. Pohrt[10], CL. Sprung[11], A. Avidan[12], C. Hartog[13]
[1]Klinik für anästhesie m.s. operative intensivmedizin, Charité – Universitätsmedizin Berlin, Berlin, Germany; [2]Klinik für anästhesiologie und operative intensivmedizin, Universitätsklinik Augsburg, Augsburg, Germany; [3]Medizinische klinik, universitätsklinik Tübingen, Tübingen, Germany; [4]Klinik und poliklinik für anästhesiologie und intensivtherapie, Universitätsklinik Leipzig, Leipzig, Germany; [5]Klinik und poliklinik für anästhesiologie und intensivtherapie, Universitätsklinikum Carl Gustav Carus Dresden, Dresden, Germany; [6]Klinik für anästhesiologie, Universitätsklinikum Ulm, Ulm, Germany; [7]Anästhesiologie, intensivmedizin, schmerztherapie und notfallmedizin, Bundeswehrkrankenhaus Berlin, Berlin, Germany; [8]Klinik für anästhesiologie, intensivmedizin, notfallmedizin und schmerztherapie, Medizincampus Bodensee - Klinik Tettnang, Tettnang, Germany; [9]Klinik für anästhesiologie, Klinikum der Universität, LMU München, München, Germany; [10]Institut für biometrie und klinische epidemiologie, Charité – Universitätsmedizin Berlin, Berlin, Germany; [11]Department of anesthesiology, critical care and pain medicine, hadassah medical center, Hebrew University of Jerusalem, Faculty of Medicine, Jerusalem, Israel; [12]Hebrew university of jerusalem, faculty of medicine, Department of anesthesiology, critical care and pain medicine, hadassah medical center, Jerusalem, Israel; [13]Anesthesiology and operative intensive care, Charité – Universitätsmedizin Berlin, Berlin, Germany
**Correspondence:** C. Hartog
*Intensive Care Medicine Experimental* 2019, **7(Suppl 3):**000814

**INTRODUCTION.** Many ICU patients receive end-of-life care. There is a lack of knowledge about current end-of-life practices in Germany.

**OBJECTIVES.** This study presents data from the subset of German ICUs which participated in the worldwide Ethicus-2 study on ICU end-of-life care practices (EOLP) in ICUs.

**METHODS.** Observational study of consecutive ICU patients with end-of-life care, defined as patients who died and/or had end-of-life decisions (EOLD) during a 6-months period.

**RESULTS.** Eleven German ICUs participated, 9 were mixed medical/ surgical, 1 medical and 1 neurosurgical, 9 centers were in academic hospitals. A total of 1092 patients were included, representing 13,7 % of all admitted ICU patients. Median age was 72 (IQR 60-80) years, 647 (59.2%) patients were male. Patients had a median ICU and hospital length of stay of 4 (IQR 1-11) days and 10 (IQR 4-22) days, respectively. Patients with EOLD had longer median ICU and hospital stays (4 days (IQR 1-12) and 11 days (IQR 4-23), respectively) than patients without EOLD 2 (IQR 0-6) days and 5 (IQR 1-11) days respectively, both p≤ .0001). Most frequent admission diagnoses were respiratory disease (37%), surgery (35.6%) and neurological disease (23.6%). Among all patients, 270 (26.9% of all) had an advance directive, 440 (43,7%) had legal patient representatives and 126 (11.7%) of patients were mentally competent at the time of EOLD decision-making.

Among all patients, 967 (89%) had limitations of life-sustaining therapy: 555 (57%) decisions to withdraw (WD), 411 (43%) decisions to withhold (WH) and one patient with active shortening of the dying process (SDP) (0.1%). Among 125 patients without therapy limitations, 33 (26%) experienced brain death (BD) and 92 (73%) received full cardiopulmonary resuscitation (CPR). Overall, 941 (86%) of patients died in the hospital, among these 125 (13%) without and 816 (87%) with therapy limitations. In median, the time interval between ICU admission and first EOLD was 2 (IQR 0-8) days and between the first EOLD and death 1 (IQR 0-3) days.

EOLD were commonly initiated by physicians (80.4%) but rarely by nurses (0.1%). The primary reason for EOLD were unresponsiveness to maximal therapy (35%), neurologic reasons (18%) or patient preference (15%). Old age (0.4%) or poor quality of life (4%) were rarely primary reasons.

**CONCLUSION.** End-of-life decisions occurred for nine out of ten patients with end-of-life care. The final EOLD was more often to withdraw than to withhold life sustaining therapy. The fact that nurses almost never initiate EOL decisions suggests that nurses lack opportunity or motivation to make meaningful and specific contributions in the EOLD process. In a third of patients with EOLD, the primary reason was unresponsiveness to maximal therapy. Age was almost never the main reason for EOLD.

### 000836

#### Unexpected needs for follow-up bereavement care in relatives

S. Wagener[1], C. Den Uil[1], J. Rietjens[2], M. Van Mol[1]
[1]Departement of intensive care, Erasmus University Medical Center, Rotterdam, Netherlands; [2]Department of public health, Erasmus University Medical Center, Rotterdam, Netherlands
**Correspondence:** S. Wagener
*Intensive Care Medicine Experimental* 2019, **7(Suppl 3):**000836

**INTRODUCTION.** Integration of bereavement care into the management of relatives of intensive care (ICU) patients is endorsed by international ICU societies. When a patient dies at the ICU, the relatives need to make a transition from aiming at recovery of their beloved one, to preparation for an unavoidable death. Relativesmay develop complicated griefas a consequence of this unpredictable situation. Complicated grief is defined as serious and persistent grief with adjustment problems in the long run [1]. To support the relatives during ICU admission, a multidisciplinaryICU team has developed strategies to guide the bereavement process [2], e.g., an evidence-based checklist. However, little is known about needs for follow-up bereavement care.

**OBJECTIVES.** To explore relatives' needs for follow-up bereavement support.

**METHODS.** A qualitative study design in a 48-bed ICU in a university hospital with semi-structured conversations by telephone. All reported first and second contact persons of patients who died in the ICU in 2018 (n=305) have been approached by medical students. They invited the bereaved relatives to participate in a large study exploring complicated grief and gathered contact details to send additional written information and an Informed Consent Form.

**RESULTS.** In total 180 persons have been called of which 123 agreed to participate, 12 refused further contact, and 45 were unavailable. The pre- scheduled time was 5 minutes per call, although; 55 "call-hours" with an average of 20 minutes were measured.

The respondents spontaneously started to explain the patient's life and their experiences during and after the death of their loved one in the ICU. They felt acknowledged and subsequently shared their grief with the medical students. The widespread and profound need evoking from the conversations, was an unknown and unexpected result showing the need for supportive follow-up bereavement care by ICU professionals.

**CONCLUSION.** Relatives of a patient who died in the ICU showed needs to support them in their bereavement. ICU professionals should meet those needs by listening, recognizing and providing information in structured follow-up bereavement care.

**REFERENCE(S)**

[1] Holland, J.M., et al., Prolonged grief symptoms related to loss of physical functioning: examining unique associations with medical service utilization. Disability and rehabilitation, 2016. 38(3): p. 205-210.

[2] Kentish-Barnes, N., et al., CAESAR: a new tool to assess relatives' experience of dying and death in the ICU. Intensive care medicine, 2016. 42(6): p. 995-1002.

[3] This work has been supported by a grant from Erasmus MC, "Evidence Based Care by Nurses", project 2017-17407.

### 001011

#### Inconsistency in assessments of appropriateness of treatment

S. Playfor[1], B. Hunter[2]
[1]PICU, Royal Manchester Children's Hospital, Manchester, UK; [2]Medical school, The University of Manchester, Manchester, UK
**Correspondence:** S. Playfor
*Intensive Care Medicine Experimental* 2019, **7(Suppl 3):**001011

**INTRODUCTION.** Over the last 10-15 years Paediatric Critical Care has evolved from a service that provides specialist care to mostly previously-well, acutely ill children to being one part of a multidisciplinary system delivering care to children with complex chronic conditions, many of whom regularly rely on life-sustaining interventions. With this evolution there has been an inevitable increase in the frequency with which judgements are made by clinical staff of the appropriateness of therapeutic interventions being delivered. There is however little data available evaluating the consistency of such assessments made by individual team members.

**OBJECTIVES.** We undertook a prospective, interview-based project in a tertiary, general Paediatric Intensive Care Unit (PICU), to determine the consistency between assessments of appropriateness of treatment made by multidisciplinary team members for individual patients. The data was analysed according to professional group (medical staff Vs nursing staff) and according to seniority (Consultant medical staff Vs doctors in training/Advanced Practitioners (junior medics), and senior nurses Vs bedside nurses).

**METHODS.** A convenience sample of individual PICU staff were approached and asked to make an assessment, for any patient whose circumstances they felt they had sufficient understanding of, on whether the overall treatment being delivered, in their opinion, was appropriate, inappropriate or futile according to standard definitions.

**RESULTS.** In total, 534 assessments were made regarding 25 patients, by 66 PICU staff members (7 Consultants, 12 junior medics, 9 senior nurses and 33 bedside nurses). There was a significant lack of consistency in assessments of appropriateness of treatment. In only 9 patients (36%) was there complete consensus, and these patients were all considered to be receiving appropriate treatment. In 11 cases (44%) treatment for the same patient was assessed as appropriate, inappropriate and futile by different PICU staff members.

Overall, 22.1% of assessments were deemed inappropriate or futile with more senior staff, both medical and nursing staff, much more likely to draw these conclusions. Consultants considered 34.4% of assessments inappropriate or futile, compared to only 9% of junior medic assessments (p=0.00004). Senior nurses considered 26.8% of assessments inappropriate or futile, compared to 20.5% of bedside nurse assessments (p=0.11).

Even amongst the 7 Consultants involved in the project there was a considerable lack of consistency in assessments with the treatment of 4 patients being deemed appropriate, inappropriate and futile by different Consultants.

**CONCLUSION.** We have demonstrated a significant lack of consistency in assessments of appropriateness of treatment amongst PICU staff members. Junior members of medical and nursing staff are far more likely to consider treatment to be appropriate, and even within distinct professional groups there is a striking lack of consistency. These factors must be taken into account when discussing the appropriateness of treatment with critically ill children and their care-givers.

### 001031

#### End-of- Life Care in the Most Southwestern Intensive Care Unit of Europe

A. ÁVila[1], N. Pires Pereira[2], T. Cardoso[3], G. Campello[2]
[1]Serviço de medicina interna, Centro Hospitalar Universitário do Algarve - Hospital de Portimão, Portimão, Portugal; [2]Serviço de medicina intensiva 2, Centro Hospitalar Universitário do Algarve - Hospital de Portimão, Portimão, Portugal; [3]Unidade de cuidados intensivos polivalente, Centro Hospitalar Universitário do Porto, Porto, Portugal
**Correspondence:** G. Campello
*Intensive Care Medicine Experimental* 2019, **7(Suppl 3):**001031

**INTRODUCTION.** Despite technological advances death in the intensive care unit (ICU) remains common. When life support is likely to result in an unacceptable outcomes, ICU clinicians must ensure that patients die with dignity. The concept of dignity in death implies that patients remain in the center of care until the very end. Enrollment the family in the process is part of "Dying with Dignity".

**OBJECTIVES.** To determine the practice of end-of-life care in a general ICU located in South of Portugal, and understand the role of the family in the process of dying.

**METHODS.** Retrospective analysis of patients who died in the ICU from July 1, 2016, to May 30, 2018.

End-of-life care was defined as the clinical care for patients in whom a decision to withhold or withdraw therapy was made. Data were analyzed using the SPSS statistical program v. 25.0. The significance level used was 0,05.

**RESULTS.** During the period of study 602 patients were admitted to the ICU, the mean APACHE II and SAPS II for these patients was 19 and 44, respectively. Of these 164 (27%) died, 114 (70%) were males, the median (IQR) age was 72 (61-80) years and ICU length of stay (LOS) was 3 (1-8) days. The mean± SD APACHE II was 29 ± 8, SAPS II 63± 18 and SOFA score on admission 10±4. Type of admission was medical in 130 (79%), urgent surgery in 25 (15%), elective surgery in 7 (4%) and trauma in 2 (1%) patients. Cause of death was refractory septic shock in 99 (60%) patients, cardiogenic shock in 18 (11%), associated with anoxic encephalopathy in 16 (10%), massive stroke in 9 (5%), ARDS in 8(5%), hypovolemic shock in 7 (4%) and 9 (5%) from other causes.

In 150 patients (91%) death was associated with an end-of-life decision either withholding or withdrawing; always after team discussion and the decisions based on predicted outcome of the critical illness. All this patients had comfort therapy. End-of-life decision regarding ventilator support were: weaning from mechanical ventilation in 80 (53%) patients, extubation in 29 (19%) and a Do Not Intubated decision in 41 (27%).

Only in 14 patients (8.5%) dead occurred unexpectedly. These patients were older [74 (61-78) vs. 72 (61-81) years, p=0.994], with similar APACHE II 31±7 vs. 29±8 (p=0.671), SAPS II 70±19 vs. 64±18 (p=0.964) and SOFA score 10±3 vs. 10±4 (p=0.308) but with a shorter LOS [1 (0-7) vs. 3 (1-10), p=0.019] than those in whom an end-of-life decision was taken.

In 152 (93%) of the deaths, the family was informed of the prognosis in the first 24 hours and the medical decisions were reported daily. Excepted in 12 patients (7.3%) who had no any close family, in remain cases the families accompanied the patient along their end of life.

**CONCLUSION.** End-of-life care is frequent in an ICU in South of Portugal. The different end-of-life categories were withholding and/ or withdrawing life support. In 14 patients (8.5%) dead occurred unexpectedly. These patients had similar severity scores them those with decision of end-of-life, but the LOS was shorter (p=0.019). The family were involved in the dying process in 93% of death.

**REFERENCE(S)**

1. Marc Romain, M.B.B.Ch and Charles L. Sprung. End-of-life Practices in Intensive Care Unit: The Importance of Geography, Religion, Religious Affiliation and Culture. Rambam Maimonides Med J. 2014;5(1):e0003
2. deborah Cook, Graeme Rocker. Dying with Dignity in the Intensive Care Unit. N Engl J Med 2014;370:2506-14
3. ethicus study group. End-of-life practices in European Intensive Care units. the Ethicus study. JAMA.2003;290:790-797

### 001036

#### Mobile ECMO team and Donor Cardiac Death. A pilot ECMO-TT study

R. Badenes[1], JM. Segura[2], J. Carbonell[1], A. TELLEZ[2], L. Hurtado[1], B. Monleon[1], J. Galan[3], R. Zaragoza[4]

[1]Anesthesiology and surgical-trauma intensive care, Hospital Clinic Universitari de Valencia, València, Spain; [2]Intensive care, Hospital Clinic Universitari de Valencia, València, Spain; [3]Transplant coordination unit, Hospital La Fe Valenciana, València, Spain; [4]Intensive care Unit, Hospital Universitario Doctor Peset, Valencia, Spain

**Correspondence:** R. Badenes

*Intensive Care Medicine Experimental* 2019, **7(Suppl 3):**001036

**INTRODUCTION.** Scarcity of potential dead brain donors and the persistent mismatch between supply and demand of organs for transplantation has led the transplant community to reconsider donation after circulatory death (DCD) as a strategy to increase the donor pool. Normothermic regional perfusion (nRP) by extracorporeal membrane oxygenation (ECMO) may be the most effective method for preserving abdominal organs in DCD, especially in liver transplantation. A pitfall of this method is its complexity and the unavailability of this resource in some hospitals, especially in regional hospitals, where potential DCD donors may exist.

**OBJECTIVES.** Aim of this study is to report the use of Mobile ECMO team in controlled DCD.

**METHODS.** From June 2018 to Abril 2019 our group has worked as a mobile ECMO team for cDCD outside our center. The portable equipment included cannulation material and the ECMO device. The transplant team consisted of 1 transplant coordinator (anesthesiologist-intensivist, ECMO operator and organ extraction supervisor), 1 cardiac surgeon (cannulation), 1 interventional radiologist (cannulation) and one cardiovascular perfusionist (ECMO operator).

**RESULTS.** Sixteen cDCD donations were performed. Characteristics of donors and organs retrieved are summarized in Table 1. From 16 cDCD, 10 livers, 4 lungs, 27 kidneys were obtained. The evolution of grafts and receptors was favorable at day 30 post-transplant.

**Table 1 (abstract 001036).** WLST, withdrawal of life-sustaining therapy; ICU, intensive care unit; M, Male; F, Female; TBI, traumatic brain injury; L, liver; RL, Right Lung; LL, Left Lung; RK, Right Kidney; LK, Left Kidney

| | Age | Sex | Cause of WLST | ICU stay | Donation/Transplantation |
|---|---|---|---|---|---|
| Patient 1 | 71 | M | Hemorrhagic stroke | 8 | L, RK, LK |
| Patient 2 | 74 | M | Hypoxic–ischemic encephalopathy | 9 | L, RK |
| Patient 3 | 57 | F | Hemorrhagic stroke | 9 | L, RL, LL, RK, LK |
| Patient 4 | 59 | M | Hypoxic–ischemic encephalopathy | 5 | RK, LK |
| Patient 5 | 65 | F | Ischemic stroke | 6 | LK |
| Patient 6 | 62 | M | Hypoxic–ischemic encephalopathy | 10 | RK, LK |
| Patient 7 | 66 | M | Hemorrhagic stroke | 15 | L |
| Patient 8 | 64 | M | TBI | 10 | L, RK, LK |
| Patient 9 | 57 | F | Poisoning | 10 | L, RK, LK |
| Patient 10 | 70 | F | Hemorrhagic stroke | 8 | L, RK |
| Patient 11 | 52 | M | Hypoxic–ischemic encephalopathy | 17 | RK, LK |
| Patient 12 | 76 | F | Hemorrhagic stroke | 4 | RK, LK |
| Patient 13 | 68 | M | Hemorrhagic stroke | 8 | L, RK, LK |
| Patient 14 | 69 | M | Respiratory failure | 8 | L, RK, LK |
| Patient 15 | 52 | F | Hemorrhagic stroke | 14 | L, RL, LL, RK, LK |
| Patient 16 | 19 | F | Hemorrhagic stroke | 16 | RK, LK |

**CONCLUSION.** Mobile ECMO teams may enable cDCD in hospitals without these resources, thereby increasing the pool of donors and optimizing graft outcomes.

**REFERENCE(S)**

1. Oniscu GC, Randle LV, Muiesan P, et al. In situ normothermic regional perfusion for controlled donation after circulatory death – the United Kingdom experience. Am J Transplant. 2014;14:2846-2854.
2. Eren EA, Latchana N, Beal E, et al. Donations after circulatory death in liver transplant. Exp Clin Transplant. 2016;5:463-470.

### 001195

#### Nurses involvement in End-of-Life decision-making discussions in French ICUs

AS. Debue[1], JR. Curtis[2], S. Calvino Günther[3], PY. Olivier[4], N. Kentish-Barnes[5], E. Dzeng[6], L. Pourtau[7], JD. Chiche[8]

[1]Médecine intensive réanimation, Hospital Cochin, Paris, France; [2]Pulmonary & critical care medicine, Cambia Palliative Care Center of Excellence, Seattle, USA; [3]Médecine intensive réanimation, Chu Grenoble Alpes, La Tronche, France; [4]Médecine intensive réanimation, CHU Le Mans, Le Mans, France; [5]Famiréa, Saint-Louis Hospital, Paris, France; [6]Palliative medecine, UCSF, San Francisco, USA; [7]Santé publique, University of Paris-Sud, Orsay, France; [8]Réanimation médicale, Hôpital Cochin, Paris, France

**Correspondence:** A.S. Debue

*Intensive Care Medicine Experimental* 2019, **7(Suppl 3):**001195

**INTRODUCTION.** Decision-making about end-of-Life (EoL) care is frequent in Intensive Care Units (ICUs). EoL situations can be traumatic for physicians, nurses and families. Conflicts - often triggered by lack of communication - can arise from EoL situations. Since 2005, the

French law prescribes that EoL decisions should be taken after a "collegial procedure" that involves the whole ICU care team and a physician not involved in patient care. However, guidelines regarding the practical organization of the "collegial procedure" are vague. Nurses often feel that they aren't involved enough in these decisions. This may have a detrimental impact on the clinician well-being and quality of care.

**OBJECTIVES.** We hypothesized that there is significant variation in the conduct of EoL decisions in French ICUs. We conducted this study to i) describe EoL decision-making processes, ii) explore differences between ICUs in terms of organization of collegial procedures, iii) and define the place and roles granted to nurses in those EoL decision-making processes.

**METHODS.** We selected a sample of 5 university affiliated ICUs (4 adult / 1 pediatric units) representative in terms of medical case mix and geography. In each ICU, we conducted structured interviews of physicians (residents, junior and senior consultants) and nurses (young and experienced) for a total of 57 interviews in the goal of reaching theoretical saturation. Interviews wer transcribed verbatim and analyzed using the grounded theory methodology (Glaser & Strauss 1967). We also attended ICU team meetings regarding EoL decisions in those ICUs whenever possible.

**RESULTS.** We interviewed a total of 27 physicians (20 M/7F, age 33 y.o. [IQR 28.7-52]; median ICU experience 6 years [2-22]) and 27 nurses (5M/22F, age 29 y.o. [26.2-32.7]; median ICU experience 5 years [2.3-8]). Data analysis revealed a common 5-steps chronology in EoL decision-making processes. First: informal discussions beginning either inside the medical or the nursing team, or both at the same time. Second, disclosure: open questions related to appropriateness of care raised by a clinician (physician or nurse) during a moment of interprofessional communication (staff meetings, rounds, etc). Third, formalization of the EoL decision in interprofessional meeting. Fourth, information of the patient and his/her relatives when applicable. Fifth, implementation of the EoL decision, if consensus achieved. Step 3 can occur in 2 distinct models. In the vertical model, the goal of the meeting is to advise nurses and allied healthcare professionals (NAHP) of the medical decision. Those meetings aim to improve the NAHP's understanding of the overall project and promote homogeneous communication with patient and family. In the horizontal model, NAHP & physicians share information & opinions & discuss the best possible care for the patient; the meeting ends with a written summary of the decision signed by every caregiver present at the meeting.

**CONCLUSION.** The "collegial procedure" for EoL decision-making as described in the French law gives way to very different interpretations. Although the decision-making chronology includes 5 steps in all ICUs studied, processes can be very different between ICUs. The role of the ICU nurses varies a lot depending on the model of interprofessional collaboration. Further investigation is needed to understand the reasons of this variability, the factors that drive the approach in each unit, and the outcomes associated with each model of interprofessional decision-making.

**REFERENCE(S)**

1. Visser M, Deliens L, Houttekier D: Physician-related barriers to communication and patient- and family-centred decision-making towards the end of life in intensive care: A systematic review. Crit Care 2014; 18:604
2. Meltzer LS, Huckabay LM. Critical care nurses' perceptions of futile care and its effect on burnout. Am J Crit Care. 2004;13(3):202-208
3. Hamric AB, Blackhall LJ. Nurse-physician perspectives on the care of dying patients in intensive care units: collaboration, moral distress, and ethical climate. Crit Care Med. 2007;35(2):422-429
4. Ferrand E, Lemaire F, Regnier B, et al: Discrepancies between perceptions by physicians and nursing staff of intensive care unit end-of-life decisions. Am J Respir Crit Care Med 2003; 167:1310 –1315
5. EmbriacoN,AzoulayE,BarrauK,etal.High level of burnout in intensivists: prevalence and associated factors. Am J Respir Crit Care Med. 2007; 175(7):686-692.doi:10.1164/rccm.200608-1184OC
6. Flannery L, Ramjan LM, Peters K: End-of-life decisions in the Intensive Care Unit (ICU) - Exploring the experiences of ICU nurses and doctors - A critical literature review. Aust Crit Care 2016; 29:97–103
7. Eight things we would never do regarding end-of-life care in the ICU E. Wesley Ely1,2* , Elie Azoulay3 and Charles L. Sprung4 Intensive Care Med https://doi.org/10.1007/s00134-019-05562-9 © 2019 Springer-Verlag GmbH Germany, part of Springer Nature
8. Downar J, Delaney JW, Hawryluck L, et al: Guidelines for the with- drawal of life-sustaining measures. Intensive Care Med 2016; 42:1003–1017

9. Assistance Publique des Hôpitaux de Paris - Nursing PhD scholarship
10. Société de Réanimation de Langue Française - Bourse de recherche paramédicale

## NAHP - Improving outcomes in ICU populations 2

### 000430

#### Time trend analysis of the incidence of ventilator-associated pneumonia versus ventilator-associated tracheobronchitis in a Pediatric Intensive Care Unit from 2010 to 2018

Y. Peña-López[1], M. Pujol[1], M. Campins[2], D. Roca[1], J. Rello[3], J. Balcells[1]
[1]Pediatric Critical Care Department, Vall d'Hebron Barcelona Hospital Campus, Barcelona, Spain; [2]Department of preventive medicine and epidemiology, Vall d'Hebron Barcelona Hospital Campus, Barcelona, Spain, Spain; [3]Vall d'hebron research institute, Vall d'Hebron Barcelona Hospital Campus, Barcelona, Spain
**Correspondence:** Y. Peña-López
*Intensive Care Medicine Experimental* 2019, **7(Suppl 3):**000430

**INTRODUCTION.** Ventilator-associated tracheobronchitis (VAT) has not been tracked for surveillance purpose despite its increasingly recognized impact on antibiotic use and outcomes in critically ill patients. Moreover, there are no data comparing long-term effect of the ventilator care bundle on ventilator-associated pneumonia (VAP) and VAT.

**OBJECTIVES.** To compare the annual rates of VAP versus VAT among critically ill children before and after the application of a ventilator care bundle over a 9 years period.

**METHODS.** A cohort study of a prospectively collected quality improvement database from 2010 to 2018 in a 16-bed-medical-surgical Pediatric Intensive Care Unit. Basal data were recorded in 2010. A ventilator-bundle was implemented in 2011. Cases of VAP and VAT were recorded according to the United States Centers for Disease Control and Prevention criteria (1).

**RESULTS.** 5,414 patients (20,357 ventilatory-days) were analyzed over 9 years. We documented a total of 37 VAP and 115 VAT during the study period. VAP incidence decreased from 4.13 per 1,000 ventilator-days to 1.2 per 1,000 ventilator-days (p= 0.230). The decrease was greatest until 2014, three years after the implementation of the ventilator-care bundle (0.44 per 1,000 ventilator-days; p=0.010) followed by a steady rise over the subsequent years and a new decrease in 2018 after optimizing adhesion to the ventilator-bundle. VAT rates always were higher than VAP rates. As in case of VAP, VAT incidence experienced the lowest rate in 2014, decreasing from 6.88 to 3.96 per 1,000 but this was not statistically significant (p=0.357). In the following years, VAT rates remained stables between 5.81-5.98 per 1,000 ventilator-days without parallelism with the ventilator-bundle effect on VAP. Even more, in the last year there was a spike on VAT incidence (from 5.98 to 10.2 per 1,000 ventilator-days) whereas VAP incidence experienced a fall (from 2.39 to 1.2 per 1,000 ventilator-days).

**CONCLUSION.** VAT incidence were three times higher than VAP incidence. VAT rates showed a different behavior than VAP rates to the ventilator-bundle. Clinical impact of VAT must be considered when assessing emerging surveillance definitions, quality indicators and bundles.

REFERENCE(S)
1. Beardsley AL, Nitu ME, Cox EG et al. An evaluation of various ventilator-associated infection criteria in a PICU. Pediatric Critical Care Med 2016; 17 (1):73-80
2. Nseir S, Martin-Loeches I, Makris D et al. Impact of apropiase antimicrobial treatment on transition from ventilator associated tracheobronchitis to ventilator-associated pneumonia. Crit Care 2014. 23; 18 (3):R129
3. Horan TC, Andrus M, Dudeck MA. CDC/NHSN surveillance definition of health care-associated infection and criteria for specific types of infections in the acute care setting. Am J Infect Control 2008;36:309-32.

### 000444

#### Age of Child Influences Parental Consent for Research

LA. Sealey[1], J. Raddatz[1], KW. Cunningham[1], A. Zykova[1], ME. Waddell[2], NR. Shah[1], MR. Nahouraii[1], CA. Becker[3], SL. Davis[3], SL. Evans[1], T. Huynh[1]
[1]Department of acute care surgery, F H "Sammy" Ross Jr Trauma Center, Carolinas Medical Center, Charlotte, USA; [2]Department of acute care surgery, Hemby Pediatric Trauma Institute, Carolinas Medica Center, Charlotte, USA; [3]Office of clinical and translational research, Atrium Health, Charlotte, USA
**Correspondence:** L.A. Sealey
*Intensive Care Medicine Experimental* 2019, **7(Suppl 3):**000444

**INTRODUCTION.** Subject recruitment in the critically ill and injured pediatric population is challenging due to the complexities of the consent process, limited population, and vulnerabilities of the child research subject. Recruitment can be further complicated by differing perceptions of risk with respect to patient age. A critically ill or injured child may meet enrollment criteria for a clinical trial, yet parental opinions toward research may hinder research participation. Further, few studies have focused on co-enrollment (participation in multiple research studies) in this population.

**OBJECTIVES.** To examine the opinions of parents of critically ill or injured pediatric patients toward research based on age.

**METHODS.** Study participants who required a pediatric intensive care unit (PICU) stay >24 hours were identified at an urban hospital. Parents were approached to complete an anonymous electronic survey between 3-7 days following PICU admission. Surveys consisted of 20 closed-ended questions aimed at assessing parental opinion toward research participation for their ill or injured child. Subject responses were stratified based on their respective child's age and divided into three age groups: infancy (<12 months), childhood (1-9 years), adolescence (10-17 years). Data was analyzed using Chi-squared test for categorical variables and Student's t-test for continuous variables.

**RESULTS.** A total of 201 parents of critically ill or injured children were surveyed from April 2018 to April 2019. Among them, 41.8% (84) were parents of infants (I), 32.3% (65) were parents of children (C), and 25.9% (52) were parents of adolescents (A). There were no differences in mean age, race, gender or length of hospital stay among the age groups. Most parents would consider research participation for their child. Significantly fewer parents would consider enrollment in multiple studies (62.7% vs. 43.3%, p<.001). Parents of adolescents were significantly more likely to consider research participation than parents of children or infants (A=80.8% vs. C=58.5% vs. I=54.8% respectively, p=0.007). There was no significant difference in attitudes toward participation in multiple research studies across the age groups (A=50.0% vs. C=40.0% vs. I=41.7%, p=0.514). Parents were significantly more likely to consider participation in research if there was perceived direct benefit to their child as opposed to benefitting other children (81.6% vs 45.3%, p<.001).

**CONCLUSION.** Parental attitudes toward research participation in the critically ill or injured pediatric population are generally favorable. However, parents of adolescents are more inclined to participate in research compared to those with younger children. Parental enthusiasm toward research participation appears to be greatest when there is a perception of direct benefit toward their child. Many parents appear receptive to co-enrollment and this may be an acceptable recruitment practice.

### 000506

#### Characteristics of patients with Diabetic ketoacidosis treated in two Pediatric Intensive Care Units in Croatia between 2013 and 2017

J. Markic[1], I. Burcul[2], N. Arambasic[3], B. Polic[1], I. Bartulovic[3], T. Kovacevic[1], T. Catipovic Ardalic[1]
[1]Pediatrics, KBC Split, Split, Croatia; [2]-, Merkur, Zagreb, Croatia; [3]Pediatrics, Clinical Medical Center Osijek, Osijek, Croatia
**Correspondence:** J. Markic
*Intensive Care Medicine Experimental* 2019, **7(Suppl 3):**000506

**INTRODUCTION.** There is an increasing incidence of type 1 diabetes mellitus (T1D) among children in Croatia. A study published in 2014 showed annual increase of 5.87% and that is higher than the European average, which is 3.9%. Diabetic ketoacidosis (DKA) is the leading cause of morbidity and mortality in children with T1D, with cerebral edema as most severe complication. Development of brain edema is likely a consequence of abnormalities in cerebral perfusion and inflammation that occur during DKA. Since early recognition of cerebral edema leads to better outcome, it is important that patients with moderate or severe DKA are closely monitored and treated in Pediatric Intensive Care Units (PICU).

**OBJECTIVES.** The aim of this study is to investigate clinical and laboratory parameters of children treated in PICU because of DKA.

**METHODS.** Patients treated due to DKA in PICU of the University Hospitals of Split and Osijek from 2013 to 2017 were included in this study. Retrospectively collected data included age, gender, clinical signs and symptoms, and various laboratory parameters. After dividing subjects into two groups: newly diagnosed with T1D (NT1D) and previously diagnosed with T1D (PT1D), collected data was compared between the two groups.

**RESULTS.** Total of 82 patients were enrolled. Those with NT1D were more often treated in PICU. Decreased consciousness level was found in 41.5% of patients, with majority of them being somnolent. No difference was found between the groups. Of the total number of DKA patients, the rate of cerebral edema was 2.4% and both patients with cerebral edema belonged to the NT1D group. Dehydration was the most frequent clinical sign, found in 95% patients at admission. There was no significant difference regarding laboratory data at admission.

**CONCLUSION.** More children with NT1D required treatment in PICU due to DKA with two of them developing cerebral edema. Since cerebral edema is a life threatening condition, treatment of patients with moderate or severe DKA in PICU will provide necessary monitoring enabling early recognition and treatment of cerebral edema and better treatment outcome. As the dehydration is the leading sign at admission, a good estimation of dehydration severity is important during the initial management as well. To minimize the incidence of DKA among NT1D it is important to continuously carry out public health education programs aimed to early identification of signs and symptoms of T1D. For patients with PT1D it is essential to well educate and support both children and their families aiming for good control of diabetes and prevention of complications.

REFERENCE(S)
1. Kuppermann N, Ghetti S, Schunk JE, Stoner MJ, Rewers A, McManemy JK, et al. Clinical Trial of Fluid Infusion Rates for Pediatric Diabetic Ketoacidosis. N Engl J Med. 2018;378(24):2275-2287.
2. Bialo SR, Agrawai S, Boney CM, Quintos JB. Rare complications of pediatric diabetic ketoacidosis. World J Diabetes. 2015;6(1):167-74.
3. Dunger DB, Sperling MA, Acerini CL, Bohn DJ, Daneman D, Danne TP et al. ESPE. LWPES ESPE/LWPES consensus statement on diabetic ketoacidosis in children and adolescents. Arch Dis Child. 2004;89(2):188-94.

4. Rojnic Putarek N, Ille J, Spehar Uroic A, Skrabic V, Stipancic G, Krnic N et al. Incidence of type 1 diabetes mellitus in 0 to 14-yr-old children in Croatia - 2004 to 2012 study. Pediatr Diabetes. 2015;16(6):448-53.

### 000509

#### Acute Kidney Injury, Fluid Overload and ARDS - "The lethal triumvirate"

R. Iyer[1], M. Jayashree[2], A. Bansal[2], S. Bharti[3]
[1]Pediatric emergency and intensive care units, advanced pediatrics centre, Post Graduate Institute of Medical Education and Research, Chandigarh, Chandigarh, France; [2]Pediatric emergency and intensive care units, advanced pediatrics centre, Post Graduate Institute of Medical Education & Research, Chandigarh, Chandigarh, India; [3]Director, Build Healthy India Movement (Research based NGO), Chandigarh, India
**Correspondence:** R. Iyer
*Intensive Care Medicine Experimental* 2019, **7(Suppl 3):**000509

**INTRODUCTION.** Acute Kidney Injury (AKI), Fluid Overload (FO) and MODS increase mortality in Pediatric Acute Respiratory Distress Syndrome (PARDS). AKI in PARDS may be multi-factorial due to hypovolemia, SIRS and cytokine storm and exposure to nephrotoxic drugs. It has been shown in critically ill children that FO is associated with AKI and increased mortality. At our center, retrospective analysis has shown a high PARDS mortality and that FO>10% and AKI are not uncommon. Hence the need for this prospective study.

**OBJECTIVES.** To study the effects of AKI and FO on severity of PARDS and its mortality

**METHODS.** Prospective observational study from August 2017 to August 2018, in a tertiary care PICU in North India, including 48 children aged 31 days to 12 years, mechanically ventilated for PARDS (PALICC definition)

**RESULTS.** Thirty-five children (72.9%) had severe, while 9(18.8%) had moderate and 4(8.3%) had mild PARDS. The median (IQR) P/F ratio and oxygenation index(OI) were 164 (122,213) and 7.21(4.7,13.9) respectively. Thirty two (66.6%) children had FO%>10; more so in severe as compared to mild-moderate ARDS (80% vs. 30.8%;P<0.001). Odds of severe ARDS were 9 times higher in children with FO (OR 9;95%CI:6.125-13.224;P<0.0001). Linear mixed modeling regression analysis of peak FO%(PFO) on serial OI showed that PFO was a significant predictor of worst OI (F-value 23.47; P<0.001); for every 1% increase in PFO, worst OI increased by 0.67 (95% CI: 0.40-0.94;P< 0.001). Also, for every 1% increase in daily FO%(DFO), mean OI increased by 0.9 [95% CI:0.82-0.96; P<0.001].

Children with AKI had higher baseline vasoactive-inotrope score (VIS) (mean+SD=38.5±38.0;P-0.038), severe ARDS(n=26/30;P-0.005) and higher mortality (n=24/30;P-0.001), than those without AKI. Logistic regression failed to show a relationship between AKI and PFO (OR 1.02;95%CI: 0.97-1.07, P-0.40). However, on linear mixed modeling, for every 1 mg/dL rise in creatinine, there was a rise in FO by 3%(P< 0.001).

The odds of undergoing RRT increased by 6% for every 1% increase in FO (OR-1.06;95%CI:1.01-1.11,P-0.03). The area under curve (AUC) for PFO, as a predictor of RRT was 0.69 (95%CI:0.54-0.85;P-0.024). Higher proportion of non-survivors (25/30;83.3%) as compared to survivors (7/18;38.8%) had FO(P-0.002). Although higher proportion of children with progressive MODS (23/31;74.2%) had FO, this was not statistically significant.

**CONCLUSION.** AKI and FO together contribute to worsening lung injury and increased mortality in PARDS. Children with shock are at high risk for developing AKI. Early detection of AKI using non creatinine based markers and initiation of RRT based on FO% could prevent worsening ARDS.

##### REFERENCE(S)

1. Hu X, Qian S, Xu F, Huang B, Zhou D, Wang Y, et al. Incidence, management and mortality of acute hypoxemic respiratory failure and acute respiratory distress syndrome from a prospective study of Chinese paediatric intensive care network. Acta Paediatr Oslo Nor 1992. 2010 May;99(5):715–21
2. Flori HR, Church G, Liu KD, Gildengorin G, Matthay MA. Positive fluid balance is associated with higher mortality and prolonged mechanical ventilation in pediatric patients with acute lung injury. Crit Care Res Pract. 2011;2011:854142
3. Valentine SL, Sapru A, Higgerson RA, Spinella PC, Flori HR, Graham DA, et al. Fluid balance in critically ill children with acute lung injury. Crit Care Med. 2012 Oct;40(10):2883–9
4. Liu KD, Matthay MA. Advances in critical care for the nephrologist: acute lung injury/ARDS. Clin J Am Soc Nephrol CJASN. 2008 Mar;3(2):578–86.
5. Liu KD, Thompson BT, Ancukiewicz M, Steingrub JS, Douglas IS, Matthay MA, et al. Acute kidney injury in patients with acute lung injury: impact of fluid accumulation on classification of acute kidney injury and associated outcomes. Crit Care Med. 2011 Dec;39(12):2665–71.
6. Darmon M, Clec'h C, Adrie C, Argaud L, Allaouchiche B, Azoulay E, et al. Acute Respiratory Distress Syndrome and Risk of AKI among Critically Ill Patients. Clin J Am Soc Nephrol. 2014 Aug 7;9(8):1347–53.
7. None

### 000529

#### Anxiety, depression and stress in relatives of Intensive Care Unit patients - baseline data of a randomized controlled trial

M. Hoffmann[1], MM. Jeitziner[2], JC. Schefold[3], P. Eller[4], DV. Lewinski[4], P. Heindl[5], A. Bachlechner[6], R. Riedl[7], AK. Holl[8], H. Burgsteiner[9], TR. Pieber[10], K. Amrein[11]
[1]Division of endocrinology and diabetology/research unit for safety in health/ qmrm, Medical University of Graz, University Hospital Graz, Graz, Austria; [2]Department of intensive care medicine, University Hospital Bern, Bern, Switzerland; [3]Department of intensive care, Inselspital, Bern University Hospital and University of Bern, Bern, Switzerland; [4]Division of intensive care, department of internal medicine, Medical University of Graz, Graz, Austria; [5]Emergency medicine and intensive care treatment for burns, City General Hospital, Vienna - Medical University Campus of Vienna, Vienna, Austria; [6]Department of intensive care, City General Hospital, Vienna, Vienna, Austria; [7]Institute for medical informatics, statistics and documentation, Medical University of Graz, Graz, Austria; [8]Department of psychiatry and psychotherapeutic medicine, University Hospital Graz, Graz, Austria; [9]Institute for digital media education, University College of Teacher Education Styria, Graz, Austria; [10]Division of endocrinology and diabetology, department of internal medicine, Medical University of Graz, Graz, Austria; [11]Division of endocrinology and diabetology, Medical University of Graz, Graz, Austria
**Correspondence:** M. Hoffmann
*Intensive Care Medicine Experimental* 2019, **7(Suppl 3):**000529

**INTRODUCTION.** Admission to an intensive care unit (ICU) is an extraordinary experience for the patient's relatives. Critical illness paired with an uncertain future triggers substantial psychological symptoms.

**OBJECTIVES.** This study analyses the baseline data of the ongoing randomised controlled trial (RCT, ClinicalTrials.gov Identifier: NCT02931851) and examines the degree to which relatives of ICU patients are affected by symptoms of anxiety, depression and stress at the time of a relative's admission to an ICU.

**METHODS.** This RCT study was performed in Austria and Switzerland. The Hospital Anxiety and Depression Scale (HADS) and the Impact of Event Scale (IES) were assessed for relatives upon admission of the patient to the ICU (0-2 days after admission, T0) using face-to-face interviews. Clinically relevant values on the HADS score are those ≥11, while values over 15 indicate severe symptoms. For the IES, scores ≥27 points are deemed to be clinically relevant, while values ≥35 can support a diagnosis of PTSD in the right context. Data collection took place between 2017 and 2019.

**RESULTS.** 60 relatives (12 male, 48 female) were included in the study, with an average age of 46.9 13.6 years. Relatives were recruited in Graz (n=11), Vienna (n=8) and Bern (n=41).

At T0, a mean HADS score of 16.7 6.6 (min. 3.0 max. 31.0) points was observed. On average, women scored higher than men at T0 (18.1 6.3 vs. 11.1 4.9).
At T0, a mean IES score of 28.3 12.3 (min. 1.0 max. 60.0) points was observed. On average, women scored higher than men at T0 (30.5 11.3 vs. 19.4 12.5).
There is a positive correlation between HADS and IES (p <.0001, Pearson correlation coefficient 0.64). The need for nursing care of a relative before admission to ICU did not correlate with HADS score or IES score (Spearman correlation coefficients: -0.13, -0.05).
**CONCLUSION.** The relatives reported clinically relevant symptoms of anxiety, depression and stress at admission. Women reported higher scores than men, and psychological symptoms occurred regardless of whether or not the patient was urgently in need of care before admission.

1. The authors wish to express their gratitude to all participating individuals for their willingness to participate in the project. The study was funded by ÖGIAIN and the Depart-ment of Intensive Care Medicine, University Hospital Bern (Inselspital).

### 000587

#### Microcirculatory disturbances in pediatric congenital heart disease before and after cardiac surgery on cardiopulmonary bypass

Ö. Erdem[1], JW. Kuiper[1], U. Krämer[1], J. Van Rosmalen[2], J. De Graaff[3], I. De Liefde[4], A. Bogers[5], C. Ince[6], D. Tibboel[1]
[1]Intensive care and department of pediatric surgery, Erasmus University Medical Center - Sophia Children's Hospital, Rotterdam, Netherlands; [2]Department of biostatistics, Erasmus University Medical Center, Rotterdam, Netherlands; [3]Department of anesthesiology, Erasmus University Medical Center - Sophia Children's Hospital, Rotterdam, Netherlands; [4]Department of anesthesiology, Erasmus University Medical Center, Rotterdam, Netherlands; [5]Department of cardiothoracic surgery, Erasmus University Medical Center, Rotterdam, Netherlands; [6]Department of intensive care, Erasmus University Medical Center, Rotterdam, Netherlands
**Correspondence:** Ö. Erdem
*Intensive Care Medicine Experimental* 2019, **7(Suppl 3):**000587

**INTRODUCTION.** Monitoring of the sublingual microcirculation (SMC) offers insight into oxygen transport. In contrast to findings in adults, it remains unclear whether children with congenital heart disease (CHD) undergoing cardiac surgery on cardiopulmonary bypass (CPB) also show microcirculatory alterations and thus altered tissue oxygenation.
**OBJECTIVES.** 1. To assess whether the SMC differs between CHD patients and healthy controls before surgery;
2. To assess whether the SMC is altered during the first 6h after cardiac surgery on CPB.
**METHODS.** A prospective observational study was performed in a tertiary children's hospital, comparing CHD patients aged 0 – 17 years undergoing cardiac surgery on CPB and healthy controls aged 0 – 17 years undergoing minor non-cardiac surgery. The SMC was monitored with handheld vital microscopy, after induction of anesthesia (baseline) in both groups and intermittently until 6h after cardiac surgery. Parameters of vessel density and perfusion were assessed for all vessels (diameter <100μm) and small vessels (diameter <20μm). Linear mixed models were built to assess differences between groups and change over time.
**RESULTS.** Thirty-eight CDH patients (median age 0.62 years (IQR: 3.06), 16 females, 20 cyanotic heart defects, frequency RACHS-1 categories 1-6: 6, 21, 9, 1, 0 and 1 respectively) were included. In addition, 35 healthy controls (median age 1.29 years (IQR: 2.99), 15 females) were included. Survival was 100%. CHD patients showed lower median microcirculatory flow index (MFI) (MFIall 2.86 vs. 3.00, p<0.001; MFIsmall 2.83 vs. 3.00, p<0.001) and lower median proportion of perfused vessels (PPV) (PPVall 98.8% vs. 100%, p<0.001; PPVsmall 98.7% vs. 100%, p<0.001) than controls at baseline. After cardiac surgery, MFIall and MFIsmall decreased compared to baseline and did not improve during the first 6h after surgery. Other SMC parameters were unaltered after cardiac surgery and did not change over time. Also, cyanotic heart disease did not affect SMC parameters.
**CONCLUSION.** Children with CHD presented with altered microcirculatory perfusion, with lower blood flow quality and less perfused vessels than healthy controls. Blood flow quality decreased even further during the first hours after cardiac surgery.

### 000681

#### The four weaning index as predictors of mechanical ventilated patients:a systematic review and diagnostic meta-analysis

N. Ding[1], Z. Zhigang[2], Z. Caiyun[2], Y. Li[1], W. Yuchen[2], J. Biantong[3], J. Lingjie[4]
[1]Lanzhou university, Lanzhou University, Lanzhou, China; [2]Department of icu, The First Hospital of Lanzhou University, Lanzhou, China; [3]School of nursing, lanzhou university, Lanzhou University, Lanzhou, China; [4]The first hospital of lanzhou university, Lanzhou University, Lanzhou, China
**Correspondence:** N. Ding
*Intensive Care Medicine Experimental* 2019, **7(Suppl 3):**000681

**INTRODUCTION.** Mechanical ventilation(MV) is the most widely used of life support in Intensive care unit(ICU).But accurately and timely identify the "time window" to weaning successfully from mechanical ventilation is challenging.Both premature and delayed weaning can increase mortality,ICU duration and costs,So an objective and convenient predicting parameter is very important to mechanical ventilated patients.
**OBJECTIVES.** To assess the diagnostic accuracy of Tidal volume(VT),Rapid shallow breathing index(RSBI),Max inspiratory pressure(MIP) and P0.1 for weaning success by diagnostic meta-analysis
**METHODS.** PubMed, Web of Science, Embase, Cochrane Library and gray literature were searched from inception to December,2018 to collect retrospective and prospective studies about weaning index of mechanical ventilated patients in Intensive care unit.Two authors independently extracted data and evaluated study quality using the Quality Assessment of Diagnostic Accuracy Studies-2 tool and Standards for Reporting of Diagnostic Accuracy Studies Checklist. Stata 13.0 and Review Manager 5 software were used for data analysis.
**RESULTS.** A total of fourteen studies,1 720 patients were included.the pooled sensitivity and 95%*CI* of VT,RSBI,MIP and P0.1 respectively was 0.87(0.74,0.94), 0.82(0.68,0.91), 0.84(0.71,0.92) and 0.86(0.78,0.91), the pooled specificity and 95%*CI* was 0.54(0.46,0.63), 0.66(0.56,0.74), 0.41(0.21,0.65) and 0.62(0.43,0.78).the DOR respectively was 8.08, 8.94, 3.68 and 9.76,SROC and sensitivity analysis showed that all no threshold phenomenon and were stability
**CONCLUSION.** The sensitivity and specificity of the four weaning index were similar,but P0.1 showed a better diagnostic value on the weaning success from mechanical ventilation.then was the RSBI,the VT and MIP

### 000684

#### Non-pharmacological interventions to improve sleep quality in ICU patients:a network meta-analysis

LJ. JIANG
College of Nursing, Lanzhou University Lanzhou, Lanzhou, China
*Intensive Care Medicine Experimental* 2019, **7(Suppl 3):**000684

**INTRODUCTION.** Sleep is one of the most basic physiological needs of human beings. One third of life is spent in sleep. Sufficient sleep can make people relieve fatigue, better restore their mental and physical strength, maintain a good awakening state after sleep, improve work efficiency, and is also a necessary condition for the rehabilitation of patients in intensive care unit. However, a large number of studies have shown that ICU patients suffer from sleep disturbances due to various factors, and more than 80% of ICU patients have sleep disorders. Sleep disorders or sleep cycle disturbances cause great damage to the body, especially for ICU critically ill patients. Therefore, it is very important to find interventions to improve sleep disorders in ICU

patients. At present, the commonly used interventions in clinical practice are divided into drug therapy and non-drug intervention. Drug therapy includes sedative drugs, analgesic drugs and hypnotic drugs. Although the use of sedative analgesic hypnotics is quickly and effective, it is easy to cause different degrees adverse reactions and drug dependence, so non-pharmacological interventions are receiving more and more attention. At present, the study on the effect of non-drug intervention on improving sleep quality in ICU patients has been started in clinical practice, but the results are not the same, and there is no reliable clinical research literature support. Network meta-analysis is a method developed from traditional meta-analysis. Its greatest advantage is that it can summarize the different interventions for treating similar diseases and perform quantitative statistical analysis according to a certain result.

**OBJECTIVES.** To study the effect of non-drug intervention on improving sleep quality in ICU patients by network meta-analysis.

**METHODS.** Computer search database The Cochrane Library, PubMed, Embase, Web of Science, China Journal Full-text Database (CNKI), Wanfang Database (WanFang Data), VIP Database (VIP) and China Biomedical Literature Database (CBM), search and construction The library reviewed the RCT literature on non-pharmaceutical interventions to improve the sleep quality of ICU patients in December 2018, using the Stata13.0 software for network meta-analysis.

**RESULTS.** This study included 18 RCTs with a total of 1720 patients and 11 non-pharmacological interventions. The Pittsburgh Sleep Quality Index (PSQI) network meta-analysis showed that non-drug interventions improved sleep quality in ICU patients, and comprehensive nursing interventions, lavender essential oil aromatherapy, eye masks, eye masks and earplugs were statistically different from conventional care. There was no significant difference between the Richards-Campbell Sleep Scale and the VSH sleep scale. The Pittsburgh Sleep Quality Index (PSQI) ranked as follows: eye mask and earplugs > eye mask > comprehensive nursing intervention > lavender essential oil aromatherapy > routine care.

**CONCLUSION.** Based on the results of the Pittsburgh Sleep Quality Index (PSQI) network meta-analysis and ranking results, non-drug intervention eye mask and earplugs were the best in improving sleep quality in ICU patients compared with other interventions.

**REFERENCE(S)**

1. Auger R R.Clinical Sleep Disorders[M]. LWW Journals, 2012: 1010-1011.
2. Cope S, Jansen JP. Quantitative summaries of treatment effect estimates obtained with network meta-analysis of survival curves to inform decision-making. BMC Med Res Methodol, 2013, 13: 147.
3. Salanti G, Dias S, Welton NJ, et al. Evaluating novel agent effects in multiple-treatments meta-regression. Stat Med, 2010, 29(23): 2369-2383.
4. Hansen PI Langhorn L,Dreyer P.Effects of music during daytime rest in the intensive care unit[J].BACCN,2017.
5. Daneshmandi M, Neiseh F, SadeghiShermeh M, et al.Effect of Eye Mask on Sleep Quality in Patients with Acute Coronary Syndrome[J].J Caring Sci, 2012, 1(3), 135-143.
6. Babaii A, Adib-Hajbaghery M, Hajibagheri A. Effect of Using Eye Mask on Sleep Quality in Cardiac Patients: A Randomized Controlled Trial[J].Nurs Midwifery Stud, 2015, 4(4): 1-6.
7. Ryu MJ, Park JS, Park H. Effect of sleep-inducing music on sleep in persons with percutaneous transluminal coronary angiography in the cardiac care unit[J]. J Clin Nurs,2011,21,728–735.
8. Hu RF, Jiang XY, Zeng YM, et al.Effects of earplugs and eye masks combined with relaxing music on sleep, melatonin and cortisol levels in ICU patients: a randomized controlled trial [J]. Critical Care,2015,19:115.
9. Yazdannik RA,Zareie A,Hasanpour M,et al.The effect of earplugs and eye mask on patients'perceived sleep quality in intensive care unit[J].Iran J Nurs Midwifery Res,2014,19(6):673-678.
10. Hu RF, Jiang XY, Zeng YM, et al. Effects of earplugs and eye masks on nocturnal sleep, melatonin and cortisol in a simulated intensive care unit environment [J]. Crit Care, 2010, 14 ( 2 ):R66.

### 000689

#### Efficacy of urinary catheter securement in the reduction of urinary tract infection, urinary meatus injuries and discomfort, in the critically ill patient: preliminary results

N. Calpe[1], L. Wennberg[2], M. Llauradó[2]

[1]Intensive Care Unit, Hospital Universitari General de Catalunya, Barcelona, Spain; [2]Nursing department, UIC Barcelona -Universitat Internacional de Catalunya, Barcelona, Spain

**Correspondence:** N. Calpe

*Intensive Care Medicine Experimental* 2019, **7(Suppl 3):**000689

**INTRODUCTION.** Urinary catheter securement is recommended as part of care bundles for general Urinary Catheter (UC) care and for Catheter Associated Urinary Tract Infection (CAUTI) prevention (1–4). However, prevalence studies show a low prevalence of catheter securement(5). Moreover, the relationship between this care and CAUTI, discomfort and urinary meatus injuries have not been studied individually.

**OBJECTIVES.** The purpose of this study was to determinate the impact of securing urinary catheters on the rate of CAUTI, urinary meatus injuries and discomfort in critically ill patients

**METHODS.** Randomized, non-blinded multicentre clinical trial conducted in two general Intensive Care Units of two tertiary hospitals. *Inclusion criteria*: Age> 18, UC inserted in Intensive Care Unit or in the Operating room, informed consent. *Exclusion criteria*: Securement not possible, urinary tract infection at the admission, UC plan to be used and anticipated ICU stay for 48 hours or more. Participants were randomized into the control group (CG), to which the UC received traditional care, and the experimental group (EG), to which the UC was secured to the upper half thigh. Before securing, a barrier film was applied to protect the thigh skin from adhesive lesions. Protocols from both sites were equivalent. Data were obtained twice a day until study ends, including: properly done securement, sings of CAUTI, UC related injuries and discomfort. Study ends was defined as: If CAUTI was diagnosed, 48 hours after catheter removal, 48 hours after ICU discharge or after 30 days of catheter use. If CAUTI was suspected an urine sample was collected. A descriptive and bivariate analysis performed with SPSS 21 program. IRB approval from both sites was obtained.

**RESULTS.** Sixty-seven patients have been analysed at the moment (47,8% male, 52,2% female. Mean age 59 ± 14 years). Allocation: 55,2% IG (n=37), 44,8% CG (n=30).[U1] Six CAUTI have been diagnosed, all of them from CG (p<0,05). Twenty five percent of communicative patients from CG experimented UC related discomfort, in front of 5% from EG (p<0,05). The Incidence of urinary meatus injuries was found to be 10,4%, without statistically differences between two groups

**CONCLUSION.** UC securement seems to decrease CAUTI and UC-related discomfort, according to the preliminary analysis

**REFERENCE(S)**

1. European Association of Urology Nurses. Evidence-based Guidelines for Best Practice in Urological Health Care. Catheterisation. Indwelling catheters in adults. Urethral and suprapubic. 2012. 2. Wound Ostomy and Continece Nurses Society. Indwelling Urinary Catheter Securement: Best Practice for Clinicians. 2012. 3. Ansell T, Network HI. Indwelling urinary catheters: should we secure them? Br J Nurs. 2016;25(18):22–5. 4. Sampathkumar P, Barth JW, Johnson M, Marosek N, Johnson M, Worden W, et al. Mayo Clinic reduces catheter-associated urinary tract infections through a bundled 6-C approach. Jt Comm J Qual Patient Saf. 2016;42(6):254–61. 5. Appah Y, Hunter KF, Moore KN. Securement of the

Indwelling Urinary Catheter: A Prevalence Study. J Wound Ostomy Continence Nurs. 2015;43(2):173–7.
2. This project has been granted by the Societat Catalana de Medicina Intensiva i Crítica as the best research project in nursing. 2017.

### 000817

#### End-expiratory volume and lung stress and strain longitudinal changes evoked by PEEP escalation in ARDS and non-ARDS children

S. Ilia, E. Geromarkaki, P. Briassoulis, P. Bourbaki, T. Tavladaki, M. Miliaraki, G. Briassoulis
[1]Picu, university hospital, medical school, University of Crete, Heraklion, Greece
**Correspondence:** S. Ilia
*Intensive Care Medicine Experimental* 2019, **7(Suppl 3):**000817

**INTRODUCTION.** End-expiratory lung volume (EELV) is functional residual capacity (FRC) plus lung volume increased by the applied positive end expiratory pressure (PEEP). Monitoring EELV has been proposed as a valuable tool to optimize ventilator settings and improve lung protective ventilation in clinical practice

**OBJECTIVES.** To detect longitudinal changes of EELV, compliance (Crs), driving pressure (ΔPrs), stress and strain when applying incremental PEEP levels in mechanically ventilated (MV) children with or without acute respiratory disease syndrome (ARDS).

**METHODS.** Twenty-five critically-ill children were enrolled in this cohort study. Patients with ARDS (oxygenation index ≥4) or non-ARDS (at risk of ARDS or without lung injury) were subjected to PEEP trials of 4-6-8-10cmH2O. At each PEEP level, EELV (nitrogen washin/washout technique), Crs, ΔPrs, strain and stress were calculated at 6-12-24-48-72 hours.

**RESULTS.** Seven-hundred measurements were carried out. Age, height, ideal-weight, and body mass index (BMI) influenced EELV and Crs in all groups, better in ARDS ($p<0.001$). EELV was related with Crs ($p<0.001$); ΔPrs with EELV/kg and Crs/kg ($p<0.001$). Following PEEP increases, EELV increased at all time-points. EELV remained lower in the ARDS compared to at-risk group only the first 12 hours ($p<0.05$). Stepwise incremental PEEP from 4 to10 cmH2O raised static and global stress and strain earlier in non-ARDS groups compared to ARDS group ($p<0.05$). Changes in strain, stress, EELV, and Crs were evident by 72 hours of MV ($p<0.001$). Longitudinally, at all studied PEEP levels, stress and strain remained within safe limits and did not interfere with hemodynamics.

**CONCLUSION.** EELV and Crs are weight-height and ΔPrs dependent, differently in ARDS compared to non-ARDS patients. Repeated, non-invasive calculations of EELV and lung strain/stress in children with and without ARDS using the nitrogen washin/washout technique might represent a promising tool for optimizing PEEP in mechanically ventilated children.

**REFERENCE(S)**

1. Bikker IG, Scohy TV, Ad J J C Bogers, Bakker J, Gommers D. Measurement of end-expiratory lung volume in intubated children without interruption of mechanical ventilation. Intensive Care Med. 2009;35(10):1749-53.
2. Dellamonica J, Lerolle N, Sargentini C, et al. PEEP-induced changes in lung volume in acute respiratory distress syndrome. Two methods to estimate alveolar recruitment. Intensive Care Med 2011;37:1595–1604.
3. Chiumello D, Chidini G, Calderini E, et al. Respiratory mechanics and lung stress/strain in children with acute respiratory distress syndrome. Ann Intensive Care 2016;6:11.
4. Gattinoni L, Carlesso E, Caironi P. Stress and strain within the lung. Curr Opin Crit Care 2012;18:42–47
5. Chiumello D, Carlesso E, Cadringher P, et al. Lung stress and strain during mechanical ventilation for acute respiratory distress syndrome. Am J Respir Crit Care Med 2008;178:346–355
6. None

### 000855

#### Impact of implementing a cardiac surgery post-operative screening tool

R. Lefebvre[1], F. Sehmbi[1], J. Weblin[2], A. Tarrant[3], N. Thompson[4], D. McWilliams[5]
[1]Physiotherapy, Queen Elizabeth Hospital Birmingham, Birmingham, UK; [2]Specialist surgery critical care, Queen Elizabeth Hospital Birmingham, Birmingham, UK; [3]Cardio-thoracic surgery critical care, Queen Elizabeth Hospital Birmingham, Birmingham, UK; [4]Cardiac critical care, Queen Elizabeth Hospital Birmingham, Birmingham, UK; [5]Critical care rehab team, Queen Elizabeth Hospital Birmingham, Birmingham, UK
**Correspondence:** R. Lefebvre
*Intensive Care Medicine Experimental* 2019, **7(Suppl 3):**000855

**INTRODUCTION.** The benefit and necessity for prophylactic physiotherapy post-operatively remains unclear [1]. Combined with an increased demand on resources, scores to identify those patients who would most benefit are being increasingly used [2]. At present no validated tool exists for those following cardiac surgery. The Birmingham cardiac surgery screening tool (BCSST) was developed to identify patients at high risk of developing post-operative pulmonary complications (PPC's).

**OBJECTIVES.** To evaluate the effectiveness of introducing a post-operative screening tool to identify patients at risk of PPC's following cardiac surgery

**METHODS.** All patients undergoing cardiac surgery between 25th November and 24th December 2018 were included in the analysis. BCSST scores were calculated on day 1 post-operatively. For patients identified as low risk, post-operative care was led by nursing staff with no physiotherapy involvement. High risk patients received standard physiotherapy input, including respiratory interventions and mobilisation. Primary outcome was development of PPC's, assessed using the Melbourne risk prediction tool.

**RESULTS.** During the trial period 21/47 (42%) of patients were classified as low risk. None of the patients in the low risk group developed a PPC, although two patients were re-referred to physiotherapy (1 for mobility assessment and 1 for respiratory deterioration). Patients in
the low risk group mobilised 30m earlier and spent less time in hospital than the high risk group.

**CONCLUSION.** The BCSST was effective at identifying low risk patients, with no patients in this group developing a PPC. For these low risk patients care was safely led by nursing staff with no adverse events or falls reported.References

**Table 1 (abstract 000855).** See text for description

| | High Risk | Low Risk | Re-referral |
|---|---|---|---|
| Screening Outcome | 26 patients (53%) | 21 patients (42%) | 2 patients (4%) |
| Developed PPC | 8 patients (31%) | 0 patient | 0 patient |
| Time to Mob > 30 m | 5.58d | 4.65d | 5d |
| Physio complete | 6.65d | 0d | 10.5d |
| Length of stay | 9.31d | 6.60d | 11d |
| Treatment duration | 317min | 15min | 345min |

**REFERENCE(S)**

1. Ostler CM, van Willigen ZE, Gibson D, Devlin R, Bruton A: Prioritising physiotherapy services The development and implementation of a post operative screening tool. ACPRC Journal. 2008, 40: 23-30.
2. Pasquina P, Tramer MR, Granier JM, Walder B: Respiratory physiotherapy to prevent pulmonary complications after abdominal surgery. Chest.
T1 2006, 130 (6): 1887-1889.

### 000859

#### Acute Coronary Syndrome patient perceptions of their illness while hospitalized

F. DeKeyser Ganz[1], O. Raanan[2]
[1]School of nursing, Hadassah Hebrew University, Jerusalem, Israel; [2]School of nursing, Sheba Medical Center, Ramat Gan, Israel
**Correspondence:** F. DeKeyser Ganz
*Intensive Care Medicine Experimental* 2019, **7(Suppl 3):**000859

**INTRODUCTION.** Previous studies have shown that patients' perceptions of their illness can influence their health behaviours. While Acute Coronary Syndrome (ACS) is an acute event, it is indicative of a chronic illness. Few studies have described the perceptions of those with ACS in the acute phase of their illness.

**OBJECTIVES.** To describe illness perceptions of patients with ACS while still hospitalized for the event.

**METHODS.** Every Israeli patient admitted to a hospital over a 2 month period with ACS (n=1958) was included in a bi-annual, national, two month prevalence study, Acute Coronary Syndrome Israel Study (ACSIS). Clinical medical data were collected for the entire sample. A convenience sub-sample of these patients (n=990) completed the Brief Illness Perception Questionnaire. Patient demographic and clinical data were collected from the patient's medical record.

**RESULTS.** ACS patients reported mean levels of 4.8/10- 7.7/10 of their perceptions of their illness, with highest scores for medical treatment control and coherence and lower scores for identity and emotional representations. Some respondents reported that their illness would have little or no impact on their life (n=230, 30%) and its effects were for a short time period (n=314, 31%)

**CONCLUSION.** While most ACS patients perceived that they understood their illness and its impact on their lives, many did not. Increased efforts should therefore be made by nurses in the acute phase to educate patients about the full implications of coronary disease.

1. Acute Coronary Syndrome Israel Study

### 000884

#### Communication tools used with the intubated patient in the Intensive Care Units of Catalonia

N. Grané, R. Gonell, C. Cobo, A. Parera
Intensive care unit, Mútua Terrassa University Hospital, Terrassa, Spain
**Correspondence:** A.M. Parera
*Intensive Care Medicine Experimental* 2019, **7(Suppl 3):**000884

**INTRODUCTION.** Over the last years has been stablished sedation guides recommending that the mechanical ventilation (MV) should be applied with low doses of sedation, to avoid complications and prolonged admissions (6,3,2). Therefore, more often we can find ICU patients awake with MV. Not being able to communicate leads to negative emotions and frustrating levels for the health team, the patient and the family (3,5). Some needs within the context of ICU are easy to unsdesrtandand,but there are some other needs, social and emotional, where the use of a communication tool can be useful (5,10,4).

**OBJECTIVES.**

- Identify which methods and/or communication tools are used in Catalonia ICUs with intubated or tracheostomized patients.
- To know the degree of acceptance perceived by nursing professionals in the use of pictograms/ electronic devices.

**METHODS.** Multicentre, observacional , descriptive and transversal study carried out from November 2018 to March 2019. Charge nursers and nurses of all adult ICUs of Catalonia with more than 2 years of experience in ICU were included. Those nurses that are not regular staff of ICU were excluded. The nurse manager was informed by phone about the purpose of the study and her collaboration and the collaboration of two nurses per shift was requested. We collected demographic variables, professional category, work shift, years of experience at ICU, hospital name and type of ICU. An ad-hoc questionnaire was developed to know the communication tools used and the degree of acceptance perceived by nursing (Likert scale from 0 to 10) in the use of pictograms and electronic devices by their own group, the patients and the relatives. A pilot test with nursing at the ICU Universitiy Hospital Mutua Terrassa was carried out to assess the suitability of the questionnaire. Intentional non-probabilistic sampling. Descriptive statistics with averages (standard deviation) and percentages.

**RESULTS.** An 89.2% of UCIs responded (33 out of 37) and a total of 140 questionnaires were received. Low-tech tools (pen/paper, alphabet, blackboard) are used by 91% of ICUs, and 30% of them use pictograms. High-tech tools (tablets) are used by 9% of ICUs. The 73% of respondents value the use of pictograms as a tool that improves communication and 82% have the same opinion about electronic devices. The 61% of respondents did not have pictograms neither electronic devices, but 99% indicate the need to have them.

The degree of acceptance perceived by nursing in their own group, patients and relatives scored an average of 6.2 (DE=1.7) points in the use of pictograms and 6.7 (DE=1.9) in the use of electronic devices.

**CONCLUSION.** Most ICUs use basic methods and low-tech tools. The use of more technological devices is recent at ICUs and they are well accepted. The degree of acceptance perceived by nursing in the use of these devices is good. It will be necessary to revalue it when its use is more extensive. We propose a new study to know at first hand the acceptance of these tools by relatives and patients. The findings of this study are a motivation to continue working on communication and thus improve an element that will give high quality to our care.

**REFERENCE(S)**

1. Perelló, C. La comunicación con el paciente crítico intubado. Universitat de les Illes Balears. 2015.
2. Hoorn S, Elbers P, Girbes A, Tuinman P. Communicating with conscious and mechanically ventilated critically ill patients: a systematic review. Critical Care. 2016;20(1).
3. Martinho C, Rodrigues I. Communication of mechanically ventilated patients in intensive care units. Revista Brasileira de Terapia Intensiva. 2016;28(2): 132-140.
4. Duffy E, Garry J, Talbot L, Pasternak D, Flinn A, Minardi C et al. A pilot study assessing the spiritual, emotional, physical/environmental, and physiological needs of mechanically ventilated surgical intensive care unit patients via eye tracking devices, head nodding, and communication boards. Trauma Surgery & Acute Care Open. 2018;3(1).
5. Radtke J, Baumann B, Garrett K, Happ M. Listening to the Voiceless Patient: Case Reports in Assisted Communication in the Intensive Care Unit. Journal of Palliative Medicine. 2011;14(6):791-795.
6. Puiggròs Font.M, Comunicar quan no pots parlar. Projecte de recerca: metodologia quantitativa. la comunicació amb els pacients intubats a la unitat de cures intensives. Treball Fi de Grau UManresa, curs 2016/2017.

7. Sistemas de comunicación para pacientes sin habla - El blog de BJ Adaptaciones [Internet]. El blog de BJ Adaptaciones. 2019. https://blog.bjadaptaciones.com/sistemas-de-comunicacion-para-pacientes-sin-habla/
9. Rojas N, Bustamante-Troncoso CR, Dois-Castellón A. Comunicación entre equipo de enfermería y pacientes con ventilación mecánica invasiva en una unidad de paciente crítico. Aquichan. 2014;14(2):184-195
10. La veu del silenci. Humanitzant la UCI amb app,s [Internet]. Althaia.cat. 2019 http://www.althaia.cat/blogosfera/infermeria/2018/10/15/la-veu-del-silenci-humanitzant-la-uci-amb-apps/
10. To our senior staff

### 000904

#### Should time be abandoned as a definition of early mobilisation in critical care?

J. Grant, R. Haylett, E. King, P. Piper, O. Gustafson
[1]Adult intensive care unit, John Radcliffe Hospital, Headington, UK
**Correspondence:** J. Grant
*Intensive Care Medicine Experimental* 2019, **7(Suppl 3):**000904

**INTRODUCTION.** Early mobilisation has been demonstrated to be safe and feasible for patients admitted to intensive care units (ICU),[1] however there is significant heterogeneity in its definition. Mobilisation has often been defined as early when it occurs within the first 2 to 5 days of admission to ICU or alternatively, within 72 hours of admission[2]. However the course of patients' critical illness varies greatly with decisions to initiate mobilisation based on a patients' clinical condition as opposed to time. Recent interventions to facilitate earlier rehabilitation have resulted in mobilisation during a more acute phase of illness as measured by the SOFA score, despite not meeting the traditional definitions of early mobilisation[3].
**OBJECTIVES.** To compare time to first mobilisation and SOFA score on first mobilisation for critically ill adults.
**METHODS.** A retrospective review was undertaken in a general adult ICU of a UK university teaching hospital. Patients admitted to the ICU receiving invasive ventilation for more than 48 hours between August 2017 and March 2019 were included. Patients who active mobilisation was deemed inappropriate were not included e.g. palliative treatment pathway. Mobilisation was defined as sitting on the edge of the bed/dangling or any out of bed activity, and termed early when occurring within 72 hours of admission.
**RESULTS.** The analysis included 303 patients with a median duration of ventilation of 5 days (IQR 3-9) and time to first mobilisation of 4 days (IQR 3-8). For those receiving early mobilisation (n= 114), median SOFA score was 4 (IQR 2-6) and day of mobilisation was 2 (IQR 2-3). In the group receiving late mobilisation (n= 187) the median SOFA score was also 4 (IQR 2-5), however the day of mobilisation was 6 (IQR 4-9).
**CONCLUSION.** In this study of patients receiving invasive ventilation for more than 48 hours, time to first mobilisation was comparable to previous published ICU rehabilitation studies. Patients who did not meet traditional definitions of early mobilisation were mobilised with the same level of acute illness as those receiving early rehabilitation. Early mobilisation is better defined through acuity of illness as opposed to time based metrics, which would allow better evaluation of future mobilisation interventions in ICU.

##### REFERENCE(S)

1. Nydahl P, Sricharoenchai T, Chandra S, Kundt FS, Huang M, Fischill M, Needham DM. Safety of patient mobilization and rehabilitation in the intensive care unit. Systematic review with meta-analysis. Annals of the American Thoracic Society. 2017 May;14(5):766-77.
2. Hodgson CL, Berney S, Harrold M, Saxena M, Bellomo R. Clinical review: early patient mobilization in the ICU. Critical Care. 2013 Feb;17(1):207.
3. McWilliams D, Jones C, Atkins G, Hodson J, Whitehouse T, Veenith T, Reeves E, Cooper L, Snelson C. Earlier and enhanced rehabilitation of mechanically ventilated patients in critical care: a feasibility randomised controlled trial. Journal of critical care. 2018 Apr 1;44:407-12.

### 000943

#### Radiography-based severity score assessing acute respiratory failure in critically ill children: a bed-side reliable and reproducible diagnostic tool in PICU

S. Ilia[1], M. Raissaki[2], V. Katsoula[1], D. Theotokatou[1], A. Kotziamanis[2], G. Briassoulis[1]
[1]Picu, university hospital, medical school, University of Crete, Heraklion, Greece; [2]Radiology department, university hospital, medical school, University of Crete, Heraklion, Greece
**Correspondence:** S. Ilia
*Intensive Care Medicine Experimental* 2019, **7(Suppl 3):**000943

**INTRODUCTION.** Respiratory failure (RF) in children is one of the most common causes of admission in pediatric intensive care units (PICU). Chest x-ray has an important role in diagnosis and assessment upon presentation and during clinical course.
**OBJECTIVES.** We aim to propose a radiography-based severity scoring system for the assessment of children with RF in PICU and investigate possible correlations with clinical and laboratory indicators of clinical severity and with predictors of outcome.
**METHODS.** 104 children under 18 years old hospitalized with acute RF in PICU between 2014-2018 were enrolled. Disease severity (PRISM and PELOD scores), PaO2/FiO2 (PF) ratios, duration of ventilator support, length of PICU stay (LOS), and outcome were recorded. A five-point radiographic severity score was introduced by enhancing Taylor et al1 score, considering all possible radiographic abnormalities. Chest x-rays upon admission and on worst PICU day (defined by maximum respiratory support and worst oxygenation/ventilation parameters) were blindly reviewed and independently scored by four doctors, 2 radiologists and 2 intensivists, following training.
**RESULTS.** 104 children (68 males) aged 5.44±0.6 years were evaluated. 62 (59.6%) patients were mechanically ventilated for 10.8±2.1 days; PICU LOS was 15.1±2.1 days. 162/283 radiographs (85 upon admission, 77 on worst PICU day) were assessed. PRISM and PELOD scores, duration of mechanical ventilation and PICU LOS were positively related with radiographic scores, and negatively related with PF ratio (all, $p<0.001$). Higher imaging score from previous radiography, indicating deterioration, was associated with lower PF ratio, prolonged mechanical ventilation and PICU LOS (all, $p<0.001$). Mortality (5.8%) was related to increased number of radiographs ($p<0.001$) and higher radiography severity score ($p<0.001$). There was a strong statistical correlation of radiographic scores between radiologists and intensivists (Spearman's $p<0.001$).
**CONCLUSION.** We introduce a reliable radiography-based severity score for assessing respiratory failure in critical ill children. This valuable bed side tool, associated with outcome, can be reproducibly utilized by both radiologists and clinicians following training.

##### REFERENCE(S)

1. Zompatori M., Ciccarese F., Fasano L. Overview of current lung imaging in acute respiratory distress syndrome. European Respiratory Review . 2014;23(134):519–30
2. Taylor E., Haven K., Reed P., et al. A chest radiograph scoring system in patients w ith severe acute respiratory infection: a validation study. BMC Medical Imaging. 2015;15(1): 61-65.

3. Smith LS., Zimmerman JJ., Martin TR. Mechanisms of acute respiratory distress syndrome in children and adults: a review and suggestions for future research. Pediatric Critical Care Med. 2013;14(6): 641-643.

#### 000948

**Introduction of a computed tomography (CT) severity score related to intracranial pressure and cerebral perfusion pressure measurements in children with coma**

S. Ilia[1], M. Raissaki[2], D. Theotokatou[1], V. Katsoula[1], A. Kotziamanis[2], G. Briassoulis[1]
[1]Picu, university hospital, medical school, University of Crete, Heraklion, Greece; [2]Radiology department, university hospital, medical school, University of Crete, Heraklion, Greece
**Correspondence:** S. Ilia
*Intensive Care Medicine Experimental* 2019, **7(Suppl 3):**000948

**INTRODUCTION.** Coma is considered an emergency and life-threatening condition in children. According to the 2019 edition guidelines for management of pediatric brain Injury, monitoring of intracranial pressure (ICP) and cerebral perfusion pressure (CPP) is recommended. Marshall Computed tomography (CT) scores of the brain correlate with outcome in adult patients with traumatic brain injury. Similar evidence for children with traumatic and non-traumatic coma is lacking.

**OBJECTIVES.** We sought to determine whether a newly introduced imaging severity scoring system correlates with intracranial monitoring, therapeutic strategies, and outcome in children undergone head CT for coma, regardless of its aetiology.

**METHODS.** Data of all children under 18 years old with traumatic and non-traumatic coma admitted to pediatric intensive care unit (PICU) between 2014 and 2018 were retrospectively reviewed. Demographics, ICP, CPP, PICU course, and outcome were recorded. A modified Marshall CT score was evaluated for all CT scans upon admission, and on worst clinical PICU-day, when available.

**RESULTS.** 85 children (47 males) aged 7.5±5.3 years were classified in three groups, traumatic coma (n=21), non-traumatic non-surgical coma (n=62), and non-traumatic coma requiring neurosurgery (n=2). Mortality was 2.3%. 14 children (16.5%) had at least 2 CT scans. CT score was positively associated with increased PRISM (p<0.001), prolonged mechanical ventilation (p<0.01), and length of PICU stay (p< 0.01). CT score was higher in patients with ICP monitoring (p<0.01) and, particularly, when ICP>15mmHg (p<0.04) in all groups. CPP< 50mmHg was related to increased number of CT scans, higher CT score, need for intracranial hypertension treatment, and mortality in all groups (all, p<0.001). Repeated CT scan due to clinical deterioration or increased intracranial pressure exhibited higher imaging scores (p<0.02) and contributed to new information, change of diagnosis and/or treatment in 63% of re-scanned children (p<0.001).

**CONCLUSION.** The introduced modified Marshall CT score is associated with ICP/CPP measurements and outcome, contributing to therapeutic adjustments in children with coma.

**REFERENCE(S)**

1. Kochanek PM, Tasker RC, Carney N, Totten AM, Adelson PD, Selden NR, Davis-O'Reilly C, Hart EL, Bell MJ, Bratton SL10, Grant GA, Kissoon N, Reuter-Rice KE, Vavilala MS, Wainwright MS. Guidelines for the Management of Pediatric Severe Traumatic Brain Injury, ThirdEdition: Update of the Brain Trauma Foundation Guidelines, Executive Summary. Pediatr Crit Care Med. 2019 Mar;20(3):280-289.
2. Brown AW, Pretz CR, Bell KR, Hammond FM, Arciniegas DB, Bodien YG, Dams-O'Connor K, Giacino JT, Hart T, Johnson-Greene D, Kowalski RG, Walker WC, Weintraub A, Zafonte R. Predictive utility of an adapted Marshall head CT classification scheme after traumatic brain injury. Brain Inj. 2019;33(5):610-617.
3. Gerber N, Sookraj K, Munnangi S, Angus LDG, Lamba V, Kumar K, Doraiswamy B. Impact of the Pediatric Emergency Care Applied Research Network (PECARN) guidelines on emergency department use of head computed tomography at a level I safety-net trauma center. Emerg Radiol. 2019 Feb;26(1):45-52.
4. None

## CD - Fluid management

#### 000531

**Patients with Acute Coronary Syndrome and high-bleeding risk: analysis of therapeutic management and 5-years outcmomes**

AM. González González, AM. García-Bellón, C. Lara-García, M. De Mora-Martin
[1]Cardiology, Regional Hospital of Malaga, Málaga, Spain
**Correspondence:** A.M. González González
*Intensive Care Medicine Experimental* 2019, **7(Suppl 3):**000531

**INTRODUCTION.** Improvements in antithrombotic therapy have led to a significant reduction in ischemic events in patients with acute coronary syndrome (ACS), although at the expense of an increase in hemorrhagic complications.

**OBJECTIVES.** The objective of this study was to evaluate the impact of the high risk of high bleeding risk in the therapeutic treatment of patients diagnosed with ACS and its influence on the very long-term prognosis.

**METHODS.** An observational, analytical, retrospective study of patients admitted in Cardiology department with the diagnosis of ACS between May 2012 to September 2013. We included 578 patients (p).They were divided in 2 groups:
- Group A: patients with high bleeding risk (n: 126; 21,8%; 43,2% female)
- Group B:patients without high bleeding risk (n: 452; 78,2%; 27,8% female)

High bleeding risk was defined as a CRUSADES Score > 40. They were compared according to clincial and invasive management, mortality and composite primary endpoint (CPE) : reinfarction, stroke and cardiovascular death; at 5 yeras follow-up.

**RESULTS.** Patients in group A were older (A= 72,3 ± 9,3 vs B= 63,3± 13,9; p = 0,08), had more chronic kidney disease, arterial hypertension, diabetes mellitus, and chronic heart failure. (Table 1)At admission, 63,7% of group A presented with non-ST elevation ACS and Killip> 1 was presented in 54,1% of patients. Patients in group A had less invasive risk stratification (A= 54,8% vs B= 67,0%; p< 0,01). There were no differences regarding dual antiplatelet therapy (DAPT) or anticoagulantion during hospitazation or at discharge.

During hospitalization, group A presented with more bleeding complications. At 5 years follow-up, group A had higher rates of CPE. (Table 1)

**CONCLUSION.** Patients with a high risk of bleeding were associated with a worse cardiovascular profile. Although there were no statistical differences between the groups with respect to invasive stratification or antithrombotic therapies, in the very long term follow-up, these patients presented a worse prognosis.

**Table 1 (abstract 000531).** See text for description

| | Group A | Group B | P value |
|---|---|---|---|
| Chronic Kidney disease | 77% | 11,8% | P<0,01 |
| Hypertension | 82,2% | 65,5% | P<0,01 |
| Diabetes mellitus | 56,8% | 21,9% | P<0,01 |
| Chronic heart failure | 14,4% | 3% | P<0,01 |
| Bleeding complicacions during hospitalization | 12,3% | 4,8% | P=0,01 |
| CPE | 73% | 32,4% | P<0,01 |
| Mortality | 67,8% | 24,8% | P<0,01 |

### 001550

#### Impact of Mobil Intensive Care Unit (MIC) in treatment of ST elevation myocardial infarction in Guadalajara

R. Viejo Moreno[1], N. Arriero Fernández[2], A. Cabrejas Aparicio[1], Jl. Garrote Moreno[1], P. Vicente Esteban[2], Z. Eguileor Marín[2], MA. Tirado Fernández[2], P. Rojo Villar[2], E. Novo García[3], C. Benito Punzel[2], J. Balaguer Recena[3], C. Carriedo Scher[1], C. Marian Crespo[2]
[1]Emergency medical system (ems), Emergency Medical System (EMS) - GUETS - SESCAM, Guadalajara, Spain; [2]Intensive care, Hospital Universitario de Guadalajara, Calle Donante de Sangre, Guadalajara, Spain, Guadalajara, Spain; [3]Cardiology, Hospital Universitario de Guadalajara, Calle Donante de Sangre, Guadalajara, Spain, Guadalajara, Spain
**Correspondence:** N. Arriero Fernández
*Intensive Care Medicine Experimental* 2019, **7(Suppl 3):**001550

**INTRODUCTION.** Ischemic heart disease (IHD) represents the main cause of mortality worldwide. Among the ischemic heart diseases, ST-segment elevation myocardial infarction (STEMI) is noteworthy, as not only it occurs more frequently with severe complications, such as cardiogenic shock and heart failure, but also the simple presence of the ST-segment elevation is an independent risk factor for mortality prediction. Emergency medical system (EMS) -based STEMI networks allows not only STEMI diagnosis in the pre-hospital phase but also reduces treatment delays; treat your fatal complications and the immediate activation of the catheterization laboratory.
**OBJECTIVES.** The aim of study was investigate the effect of Pre-hospital versus Hospital treatment in the length stay and survival of patients with STEMI diagnosis.
**METHODS.** We performed an observational retrospective through single centre registry of consecutive patients (n = 360) admitted with STEMI to the intensive care unit of the Guadalajara Hospital, Spain, between 2015 and 2018. Two groups were established; on the one hand the intervention group or prehospital care by MICU (Group I) and the control group of patients who came to the Hospital by their own means (group II). We compared demographic variables, cardiovascular risk factors, treatment in prehospital phase or in ED, time to firs medical contact to percutaneous coronary intervention (PCI), location of coronary lesions, GRACE score, length in ICU and hospital stay as well as survival to discharge and about 30 days.
**RESULTS.** 360 were evaluated by STEMI, 219 (60.8%) by MICU and 141 (39,2%) arrived themselves to hospital. 280 (77,8%) male and mean age was 61.0 (RIC: 53.0-71.0) years old.
Patients treated for MICU had a GRACE score greater than the control group 119.85 (112.5 – 127.1) Vs 97.4 (87.2-107.5) (p= 0.002). There were differences in P2Y12 inhibitor administered. Ticagrelor was more frequent in patients admitted directly in hospital 94 (66.7%) Vs 82 (37.4%) (p=0.00) and Prasugrel was more frequent by MICU physicians 13 (9.2%) Vs 112 (51.2%) (p=0.00). Morphyne was more administered in MICU 49 (34.8%) VS 121 (55.3%) patients (p= 0.00).
The initial attention of patients with STEMI by MICU reduced time to PCI, stay length in ICU: 76.8 (37.4-191.0) Vs 44.8 (36.6-53.1) hours (p= 0.02), and was associated with lower mortality at hospital discharge 13 (9.2%)Vs 8 (3.7%) (p=0.02) and at 30 days compared to those who went to the hospital by themselves 15 (10.6%) VS 10 (4.6%) patients (p=0.02).
**CONCLUSION.** Around of 40% of patients with STEMI still come to hospital by themselves. An EMS based STEMI networks and MICU with presence of physicians on board allow to reduce the time to PCI and decrease the mortality of patients with STEMI in Guadalajara.

### 001708

#### Agreement between different non-invasive methods of ventricular elastance for the measurement and monitoring of ventriculo-arterial coupling

M. Nguyen, V. Berthoud, L. Bartamian, B. Bouhemad, PG. Guinot
[1]Department of anaesthesiology and intensive care, Hospital Center University François Mitterand, Dijon, France
**Correspondence:** M. Nguyen
*Intensive Care Medicine Experimental* 2019, **7(Suppl 3):**001708

**INTRODUCTION.** Ventriculo-arterial coupling (Ees) optimisation allows to increase stroke work efficiency and is a parameter of growing interest in intensive care unit. Chen method is the most validated non-invasive method to estimate ventriculo-arterial coupling, yet, several simplified methods are often used at bedside. Those method assume that the end systolic volume pressure relationship (ESPVR) passes through the origin and calculate Ees as a derivate from ESP/ESV. However, neither of those simplification have been tested against Chen method in the intensive care unit.
**OBJECTIVES.** The objective of the present study was to determine whether the simplifications proposed by Tanoue and Robotham might substitute to Chen formula for the measurement of ventriculo-arterial coupling at baseline and to track ventriculo-arterial coupling change with therapeutic intervention.
**METHODS.** We perfomed a monocentric retrospective study in Dijon teaching hospital. Ventriculo-arterial coupling was calculated as Ea/Ees. The 3 non-invasive Ea/Ees calculation methods were applied on 87 patients, cardiac surgical ICU patients. The reference method for measuring Ees was this of Chen (1). Ees was also calculated according to Tanoue *et al*. (2): 0.9 * SAP/ ESV and Robotham *et al.* (3)*:* Ea/(1/LVEF)-1. All patients received either fluid expansion (n=35), norepinephrine (n=32) or dobutamine (n= 20) (prescribed by the physician in charge). Measurement were performed before and after intervention.
**RESULTS.** Eighty seven patients were analysed. Median age was 67 [60-75] years, 76% were male. To measure baseline Ea/Ees, the concordance correlation coefficient (CCC) was of 0.13[-0.05;0.30], the bias was of -0.19, limit of agreement ranged from -4.1 to 3.8 and mean absolute percentage error (MAPE) was of 57% for Tanoue. Regarding Robotham, the CCC was of 0.32 [0.14;0.49] bias was of 0.26, limit of agreement ranged from -2.4 to 2.9 and MAPE was of 42%. When used to follow Ea/Ees evolution with interventions, only 65 and 70% of measures evolved in the same direction (four-quadarant plot analysis with a 15% exclusion zone) respectively for Tanoue and Robotham methods when compared to Chen.
**CONCLUSION.** Our results do not support interchangeability between Tanoue or Robotham and Chen method. These methods neither allowed a precise estimation of ventriculo-arterial coupling nor to follow its change with hemodynamic treatment.

##### REFERENCE(S)

1. Chen C-H, Fetics B, Nevo E, Rochitte CE, Chiou K-R, Ding P-A, Kawaguchi M, Kass DA. Noninvasive single-beat determination of left ventricular end-systolic elastance in humans. J Am Coll Cardiol 2001;38:2028–2034.
2. Tanoue Y, Sese A, Ueno Y, Joh K, Circulation TH-, 2001 undefined. Bidirectional Glenn procedure improves the mechanical efficiency of a total cavopulmonary connection in high-risk Fontan candidates. Am Hear Assoc
3. Robotham JL, Takata M, Berman M, Harasawa Y. Ejection fraction revisited. Anesthesiology 1991;74:172–83.

### 001756

#### Optimal hemodynamics after cardiac surgery: a cohort study on pressures and cardiac output

B. Zapletal[1], A. Schiferer[1], M. Mouhieddine[1], MH. Bernardi[1], C. Veraar[1], C. Lacom[1], G. Kargl[1], M. Hiesmayr[1]
[1]Division of cardiac thoracic vascular anaesthesia and intensive care, Medical University of Vienna, Wien, Austria
**Correspondence:** M. Hiesmayr
*Intensive Care Medicine Experimental* 2019, **7(Suppl 3):**001756

**INTRODUCTION.** Management after major cardiac surgery is typically done by the integration of data from monitoring, echocardiography, lab values, imaging techniques and patient observation. Monitoring of pressures and flow is often used to titrate vasoactive and inotropic agents postoperatively. Optimal targets for therapy are often driven by institutional habits but are not universally accepted.
**OBJECTIVES.** We aimed to determine whether an optimal level of common hemodynamic parameters can be identified that are associated with an improved outcome.

**METHODS.** We included all patients admitted with a pulmonary artery catheter after cardiac surgery to one ICU from 2012-2015. All data were extracted from the patient data management system Critical Care Manager (PICIS) that stored values every 10 minutes. We calculated first median values for a 1 hour period and excluded thereafter extreme values that are most likely artefacts (e.g. pressures below zero, heart rate below 40 and above 200). For each patient a minimum, maximum and median value for the whole stay was determined. These values were coded as categorical variables based on quintiles, the middle category serving as reference for all logistic regression analysis (STATA 15.1). Results are given as odds ratios with 95% confidence intervals from models including all 3 selected values. We analysed heart rate, systolic blood pressure, diastolic blood pressure, central venous pressure, continuous cardiac output and mixed venous saturation in 645 patients that stayed in the ICU for 219 242 hours. 59 patients died during the hospital stay.

**RESULTS.** We found a U-shaped association in the combination of minimum and maximum values with the exeption of cvp where high minimum and maximum appear detrimental.

**CONCLUSION.** We found that simple hemodynamic variables can be analysed to identify an optimal zone where both low minima and high maxima are associated with poor outcome whereas for cvp the lowest values for minimum and maximum are associted with better outcome.

A multivariable model including duration of states may allow safe margins of treatment in the future.

**Table 1 (abstract 001756).** Odds ratio for hospital death after cardiac surgery

| Variable | Q1 | Q2 | Q3 | Q4 | Q5 |
|---|---|---|---|---|---|
| heart rate minimum | 6.5(2.4-17.3) | 2.9 (1.0-7.8) | 1 | 0.4(0.1-1.6) | 0.07(0.01-0.72) |
| heart rate maximum | 0.04(0.005-0.36) | 0.11(0.03-0.43) | 1 | 0.9(0.4-2.2) | 1.4(0.6-2.2) |
| systolic minimum | 35.5(7.9-158) | 4.8(1.0-23.2) | 1 | 0.5(0.05-5.7) | - |
| systolic maximum | 0.3(0.1-0.99) | 0.6(0.2-1.8) | 1 | 1.4(0.5-4.1) | 1.1(0.4-3.3) |
| diastolic minimum | 4.7(1.9-11.6) | 0.9( 0.3-2.3) | 1 | 0.2(0.05-0.7) | 0.09(0.02-0.37) |
| diastolic maximum | 0.18(0.03-0.95) | 1.3(0.4-3.7) | 1 | 1.9(0.7-5.1) | 2.2(0.8-5.8) |
| cvp minimum | 0.9(0.3-2.4) | 0.5(0.2-1.4) | 1 | 1.7(0.7-4.5) | 1.4(0.5-3.8) |
| cvp maximum | 0.5(0.1-2.0) | 0.6(0.2-2.2) | 1 | 1.4(0.5-3.8) | 7.2(2.8-18.0) |
| cardiac output minimum | 8.4(2.8-25.4) | 1.7(0.6-6.0) | 1 | 0.5(0.2-1.7) | 0.2(0.04-0.7) |
| cardiac output maximum | 0.4(0.1-1.2) | 0.4(0.1-1.3) | 1 | 1.1(0.4-3.1) | 2.4(0.8-7.2) |
| svO2 minimum | 2.9(1.0-8.5) | 2.6(0.9-7.3) | 1 | 1.4(0.5-4.2) | 1.6(0.5-5.2) |
| svO2 maximum | 0.14(0.03-0.71) | 0.8(0.31-2.0) | 1 | 0.76(0.3-1.9) | 2.9(1.3-6.5) |

### 001350

#### Accuracy of arterial dP/dtmax for continual monitoring of left ventricular contractility depends on systemic vascular resistance, heart rate and cardiac output

P. Ostadal, D. Vondrakova, A. Krüger, M. Janotka, J. Naar, P. Neužil
[1]Cardiovascular center, Na Homolce Hospital, Prague, Czech Republic
**Correspondence:** P. Ostadal
*Intensive Care Medicine Experimental* 2019, **7(Suppl 3):**001350

**INTRODUCTION.** Continuous reliable evaluating of left ventricular (LV) contractile function in patients with advanced heart failure requiring intensive care remains challenging. Recently, continual monitoring of dP/dtmax from arterial line became available for hemodynamic monitoring. However, factors influencing the accuracy of arterial dP/dtmax for the assessment of LV dP/dtmax remain not fully understood.

**OBJECTIVES.** The aim of our study was to determine hemodynamic factors that affect the relationship between arterial dP/dtmax and LV dP/dtmax in patients with acute heart failure.

**METHODS.** Fifty patients with acute heart failure requiring intensive care, inotropes/vasopressors and hemodynamic monitoring were recruited into the study (mean age 70.2 years, 64% were males). Hemodynamic variables including arterial dP/dtmax were continually monitored using arterial line pressure waveform analysis. LV dP/dtmax was assessed using continuous-wave Doppler analysis of mitral regurgitation flow.

**RESULTS.** The values from continual arterial dP/dtmax monitoring significantly correlated with the LV dP/dtmax assessed by echocardiography (r=0.70, 95% confidence interval [CI] 0.51-0.82, P˂0.0001). Linear regression revealed that (LV dP/dtmax) = 1.25×(arterial dP/dtmax), P˂0.0001. We analyzed the relation between arterial dP/dtmax and LV dP/dtmax in the subgroups above or bellow the mean value of individual hemodynamic variables. We have found markedly stronger correlation in subjects with higher systemic vascular resistance (>900 dyn.s/cm5, N=20, Spearman r= 0.91, 95%CI 0.78 to 0.97, P<0.0001), in the subgroup with cardiac output <6 L/min, (N=26, Spearman r=0.81, 95%CI 0.60 to 0.91, P< 0.0001) and in patients with heart rate ˂92 beats/min (N=26, Spearman r=0.90, 95%CI 0.79 to 0.96, P˂0.0001).

**CONCLUSION.** Our results indicate that arterial dP/dtmax values correlate with LV dP/dtmax, particularly in patients with higher systemic vascular resistance, lower heart rate and lower cardiac output. Arterial dP/dtmax could be, therefore, used for continual monitoring of LV contractility especially in hemodynamic conditions corresponding to cardiogenic shock.

1. The study was supported by an Institutional grant MH CZ - DRO (Nemocnice Na Homolce - NNH, 00023884), IG150501.

### 001362

#### Thermal heat signal transduction by the water circuit of a VV-ECMO system

M. Nijsten[1], A. Herner[2], W. Huber[3], M. Konrad[4]
[1]Department of critical care, University Medical Center Groningen, Groningen, Netherlands; [2]Medizinische klinik und poliklinik ii, Klinikum rechts der Isar; Technische Universität München, Munich, Germany; [3]Ii medizinische klinik und poliklinik, Rechts der Isar Hospital, München, Germany; [4]Getinge, PULSION Medical Systems SE, Feldkirchen, Germany
**Correspondence:** M. Nijsten
*Intensive Care Medicine Experimental* 2019, **7(Suppl 3):**001362

**INTRODUCTION.** Treatment with VV-ECMO or VA-ECMO interferes with thermodilution methods for measuring cardiac output (CO) [1]. The thermal pulse generated in a central vein or the right atrium can be considerably disturbed by or even largely diverted into the ECMO system.

**OBJECTIVES.** Explore the feasibility of using the water heater unit (HU) circuit of the ECMO system itself as a transducer of defined thermal pulses.

**METHODS.** A pig with a body weight of 49 kg was anesthetized, ventilated and connected to a Cardiohelp (Maquet Cardiopulmonary AG, Rastatt, Germany) VV-ECMO system. Thermistor-tipped catheters were used to measure the temperature as a function of time T(t) at 3 places: at the venous inlet of the ECMO: T-VE(t), at the arterial outlet of the ECMO: T-AE(t), and in the femoral artery: T-FA(t). The membrane oxygenator was connected with a heater unit (HU) (HU-35, Maquet Cardiopulmonary AG, Rastatt, Germany) that provided a continuous water flow temporarily set at a temperature 1 to 1.5 oC above body temperature. The ability of the ECMO-HU to generate thermal pulses was assessed by interrupting the water flow from the

HU to the ECMO for a few seconds and monitoring T-AE(t), T-VE (t) and T-FA(t). The interaction of the ECMO-HU with incoming thermal pulses was assessed by comparing T-AE(t) and T-VE (t) after central venous injection of 20 ml cold boluses. High time-resolution experiments were performed in triplicate.

**RESULTS.** Interruption of HU water flow generated a rapid decrease of ≥0.5 oC in T-AE, followed after a median time of 7 seconds by a drop T-VE of 0.1 oC and after 14 seconds by a drop of T-FA by 0.3 oC. Systemic cold bolus injections generated distinct thermal spikes in T-VE (t) and T-AE(t). The nadir value for T-VE was followed 11 seconds later by a nadir value for T-AE. This interval is largely explained by the transit time resulting from the blood flow and dead volume. When the area under the curve of the thermal pulse entering the ECMO system was compared with the pulse leaving the ECMO system, we saw a 66% reduction, indicating considerable thermal transfer to the HU- water circuit.

**CONCLUSION.** Modifying heater unit water flow or temperature can be used to generate thermal signals in the arterial output of the ECMO system, which are propagated to the venous input of the ECMO system and the femoral artery. Thermal signals originating in the patient are also propagated to the ECMO system and may even be transduced to the water circuit.

**REFERENCE(S)**

[1] Haller et al. Thermodilution cardiac output may be incorrect in patients on venovenous extracorporeal lung assist. Am J Respir Crit Care Med 1995;152:1812-7.

[2] MK works for Getinge and MN and WH have received financial or material support from Getinge.

### 001367

#### Use of peep to detect fluid responsiveness during operative period in patients undergoing digestive surgery

M. Benlabed[1], S. Nedjari[2], R. Gaudy[1], A. Ladjouze[3], S. Aissaoui[4], F. Ouanes[5]

[1]Anesthesiology and Intensivecare, Lille Catholic University, Lille, France; [2]Anesthesiology, Algiers university, Alger Centre,, Algeria; [3]Anesthesiology, Algiers University, Algiers, Algeria; [4]Anesthesiology, Algiers university, Algiers, Algeria, Algeria; [5]Anesthesiology, Algiers unversity, Algiers, Algeria

**Correspondence:** M. Benlabed

*Intensive Care Medicine Experimental* 2019, **7(Suppl 3):**001367

**INTRODUCTION.** The setting of peep during operative period is recommended to avoid derecruitement and atelectasis. High peep decrease venous return and cardiac output and can be deleterious in hypovolemic patients. it is widely accepted that the dynamic indices, pulse pressure variation (PPV) , stroke volume variation (SVV) , systolic pressure variation(SPV) accurately predict fluid responsiveness in mechanically ventilated patients passively adapted to the ventilator.

**OBJECTIVES.** The **objective** of this study was to evaluate the fluid responsiveness using peep challenge in patients undergoing digestive surgery

**METHODS.** 30 patients, Asa 1, 55 +-10 years old, without cardiac failure or pulmonary pathology were enrolled for programmed digestive surgery. Anesthesia was performed using propofol, sufentanil and cisatracurium and all the patients were intubated. We evaluated the increase of peep on hemodynamics in patients Asa 1 selected for digestive surgery. This group of 30 patients was submitted to a peep challenge 60 minutes after induction, increasing peep progressively from 4cmh2o to 10 cmh2o with a time of 5 minutes to reach 10cmh2o. We monitored arterial pressure with a catheter inserted in radial left artery and central venous pressure with a three lumen catheter inserted in right jugular vein. We recorded continuously, systolic pressure variation, mean arterial pressure and pulse pressure variation from the arterial wave line. Central venous pressure(CVP) , pulse pressure variation(PPV), systolic pressure variation(SPV) and mean arterial pressure (PAM) were observed and recorded before and after the peep challenge which consisted in increasing peep from 4to 10cmh2o. So, recordings of these hemodynamic parameters were performed at peep 4cmh20 (control value) and at peep 10(peep challenge). Fluid responsiveness was defined as a more than 10% decrease of arterial pressure and pulse pressure variation more than 13% after peep challenge

**RESULTS.** Statistical analysis was performed using student's t test. We observed finally that peep challenge from control values at peep 4, increase PPV, SPV, CVP and decreases PAM. Results were expressed as mean +- std deviation. PAM(mmHg) decreases from 77,258 +- 4,016 to 69,581+-5,714.(p<0.001). PPV(%) increases from 10,097 +-1,904 to 16,677 +-1,641 (p<0.001). SPV(%)increases from 7,839+-1,393 to 14,968 +-1,835 (p<0.001). CVP(cmh20) increases from 8,516+- 2,143 to 10,129 +-2,061 (p<0,007).After fluid challenge , we came back to the control values.

**CONCLUSION.** During operative period, the setting of peep 10 (from peep 4) can detect fluid responsiveness in patients undergoing digestive surgery.

**REFERENCE(S)**

1. P .Marik et al. dynamic changes in arterial waveform derived variables and fluid responsiveness in mechanically ventilated patients: a systematic review of the literature. crit care med 2009
2. X.Monnet et al. prediction of fluid responsiveness: an update. Annals of intensive care 2016

**Table 1 (abstract 001367).** See text for description

| | Control | | Peep Challenge | | |
|---|---|---|---|---|---|
| | Mean | Std. deviation | Mean | Std. deviation | p |
| PAM mmHG | 77,258 | 4,016 | 69,581 | 5,714 | P < 0,001 |
| CVP cmH20 | 8,516 | 2,143 | 10,129 | 2,061 | P<0,007 |
| PPV % | 10,097 | 1,904 | 16,677 | 1,641 | P < 0,001 |
| SPV % | 7,839 | 1,393 | 14,968 | 1,835 | P < 0,001 |

### 001703

#### Context-sensitive indexation of global end-diastolic volume: A validation study

W. Huber, T. Lahmer, A. Herner, U. Mayr, G. Batres-Baires, S. Rasch, R. Schmid, A. Hoyos

[1]Medizinische klinik und poliklinik ii, Klinikum rechts der Isar; Technische Universität München, Munich, Germany

**Correspondence:** W. Huber

*Intensive Care Medicine Experimental* 2019, **7(Suppl 3):**001703

**INTRODUCTION.** Global end-diastolic volume is a static marker of preload. Indexation to body surface area BSA results in GEDVI. Several studies demonstrated better prediction of fluid responsiveness by GEDVI compared to CVP. However, the results are inconsistent and at least two studies suggest inappropriate indexation by BSA (1;2). Furthermore, the two commercially available devices measuring GEDVI use different indexations: The PiCCO uses predicted bodyweight (BW_pred) to calculate BSA_pred, while the EV-1000 uses actual BW to calcualte BSA_act.

Recently, the concept of context-sensitive indexation (CSI) has been introduced to optimize indexation. Based on analyses of a large haemodynamic database including about 20,000 haemodynamic measurements, CSI individually adjusts each haemodynamic parameter according to its specific association to biometric data including age and gender in addition to weight, height and BSA. Furthermore, CSI also adjusts for „contexts“ such as atrial fibrillation (AF) , mechanical ventilation and catheter position, which might have a stronger impact on the haemodynamic raw value than biometric data.

**OBJECTIVES.** This study tried to validate CSI of GEDV (GEDVI_CSI) in a group of 33 ICU-patients with shock undergoing a volume challenge (VC) with 7mL/kg saline 0.9% over 30 minutes.

**METHODS.** Methods: All patients were equipped with PiCCO-monitoring (Pulsion; Germany) and under assisted mechanical ventilation. Transpulmonary thermodilution was performed immediately before and after the VC. GEDVI_CSI was calculated as described previously using a formula adjusting GEDV for age, gender, weight, height, CVC-position (femoral or jugular) and heart rhythm (sinus rhythm or AF). Statistics: Primary endpoint: increase in Cardiac Index CI ≥10%. Second endpoint: Change in cardiac power index CPI ≥10%. ROC-analysis. SPSS 25.

**RESULTS.** 12 (36%) female, 21 (64%) male patients. SOFA 13±4. AF in 7 of 33 patients (21%). 31 of 33 patients were under vasopressor therapy (noradrenaline dosage 1476±3277µg/h).

The VC resulted in significant increases in CI (4.8±2.1 vs. 4.6±2.2L/min/m², p=0.002), CVP (16.5±4.8 vs. 13.9±6.3mmHg, p=0.002), GEDVI (831±203 vs. 790±175mL/m², p=0.020), MAP (82±10 vs. 76±8mmHg, p=0.001), cardiac power index (0.86±0.36 vs. 0.78±0.42W/m², p< 0.001) and stroke volume index (±, p<0.001), while heart rate decreased (100±17 vs. 102±18/min, p=0.006). Stroke volume variation SVV (p=0.287) and pulse pressure variation PPV (p=0.596) did not change significantly.

As expected due to the lack of controlled mechanical ventilation, baseline PPV (AUC=0.566; p=0.566) and SVV (AUC=0.664; p=0.145) did not predict an increase in CI ≥10%. While baseline CVP (AUC=0.423; p= 489), unindexed GEDV (AUC=0.510; p=0.933), GEDVI indexed to BSA_pred (AUC=0.433p=0.554) and GEDVI indexed to BSA_act (AUC= 0.400; p=0.375) did not predict the primary endpoint, GEDVI_CSI was the only significant predictor of an increase in CI ≥10% (AUC=0.745; p= 0.028). Similarly, GEDVI_CSI was the only baseline parameter to predict an increase in CPI ≥10% (AUC=0.733; p=0.025).

**CONCLUSION.** GEDVI_CSI adjusted according to CSI was the only significant predictor of fluid responsiveness, whereas CVP, SVV, PPV and GEDVI according to all other indexations were not predictive. These data support the concept of context-sensitive indexation.

**REFERENCE(S)**

(1) Huber et al., J Clin Monit Comput. 2017 Feb;31(1):143-151
(2) Wolf et al., Crit Care. 2009;13(6):R202
(3) Saugel, Huber et al. Crit Care. 2010;14(3):R95

### 001676

#### CVP reduction associated with higher CO favors good prognosis on circulatory shock: a single center, retrospective cohort study

L. Su, X. Wang, Y. Long, X. Zhou, D. Liu
ICU, Peking Union Medical College Hospital, Beijing, China
**Correspondence:** L. Su
*Intensive Care Medicine Experimental* 2019, **7(Suppl 3):**001676

**INTRODUCTION.** Changes in cardiac output (CO) caused by central venous pressure (CVP) are the most important concerns in the treatment of shock. It is a pathophysiological axiom that the lower the CVP, the better.

**OBJECTIVES.** This study was performed to explore how to use CVP and what its relevant mechanisms with respect to CO in the critically ill patients.

**METHODS.** A total of 134 patients with circulatory shock were retrospectively included and analyzed. Hemodynamic data were recorded and analyzed at PICCO initiation and 24 hr after PICCO. Data regarding 28-day mortality and renal function were also collected.

**RESULTS.** The patients were divided into a CVP↑+ CO↑ group (n=23), a CVP↑+ CO↓ group (n=29), a CVP↓+ CO↑ group (n=44), and a CVP↓+ CO↓ group (n=38) based on PICCO initiation and 24 hr after PICCO. Post hoc tests showed the CVP↓+ CO↑ group had higher 28-day survival than the other groups [log rank (Mantel-Cox)=8.758, *P=0.033*]. In terms of hemodynamic characteristics, the CVP↓+ CO↑ group had a lower cardiac function index (CFI) (4.1±1.4 /min) and higher extravascular lung water index (EVLWI) (11.0±4.7 ml/kg) at PICCO initiation. This group used more cardiotonic drugs (77.3%, *P<0.001*) and had a negative fluid balance (-780.4±1720.6 ml/24 hr, *P=0.018*) 24 hr after PICCO than the other three groups. Cardiotonic drug use and dehydration treatment was associated with increased CFI (from 4.1±1.4 /min to 4.5±1.3/min, *P=0.07*) and reduced ELVWI (from 11.0±4.7 ml/kg to 9.0±3.5 ml/kg, *P=0.029*). Renal function tests showed that SCr and BUN levels in the CVP↓+ CO↑ group were significantly improved (SCr from 197.1±128.9 mmol/L to 154.4±90.8 mmol/L; BUN from 14.3 umol/L±7.3 to 11.6±7.0 umol/L, *P<0.05*).

**CONCLUSION.** Lower CVP associated with CO increased, which may improve 28-day prognosis in patients with circulatory shock. Higher CO derived from lower CVP may also contribute to renal function improvement.

1. Supported by Capital Characteristic Clinic Project of Beijing (No. Z181100001718209).

### 001657

#### The prognostic value of the platelet-to-lymphocyte ratio in acute coronary syndrome

K. B.ismail, D. Nsib, Y. Yahia, S. Mechrgui, K. Zaouch, R. Baccouche, R. Boubaker, H. Maghraoui, K. Mejd
Urgences, Hospital Rabta, Tunis, Tunisia
**Correspondence:** I.K. Ben
*Intensive Care Medicine Experimental* 2019, **7(Suppl 3):**001657

**INTRODUCTION.** Acute Coronary Syndrome (ACS) is one of the leading causes of cardiovascular morbidity and mortality. Therefore, stratification of risk is a very important issue for the prevention and management of ACS.The platelet/lymphocyte ratio, a marker of the systemic inflammatory response, was significantly correlated with mortality during ACS according to several studies.

**OBJECTIVES.** To study the prognostic value of platelet/lymphocyte ratio in patients admitted to emergency departments for acute coronary syndrome .

**METHODS.** This was a prospective study, conducted over a period of 3 months from January to March 2019 at the emergency department of CHU La Rabta. We included all patients over the age of 18, admitted for ACS and analyzed the correlation of platelet/lymphoctic ratio (DPR) to intrahospital mortality using SPSS 22 software. The significance threshold was set at 0.05.

**RESULTS.** We included 100 patients. The average age was 65.74 ± 11.91 and the sex ratio was 1.34. The platelet/lymphoctic ratio (RPL) ranged from 10.35 to 3093.3 with an average of 199.7 ±333.99. We found STEMI in 46% of patients. The percentage of complications was 34% and the mortality rate was 15%. RPL was 150 in 48.2% of patients. We found a significant correlation between an RPL 150 and the mortality rate (p=0.000015, OR=2.6, IC=[0.8318674 -9.099468]). The area under the ROC curve was 0.61.

**CONCLUSION.** High platelet/lymphocyte ratio could be a predictor of mortality in patients with ACS.

### 001376

#### Assessment of mean systemic filling pressure using inspiration hold maneuvers is statistically significant but clinically irrelevant

L. van Loon[1], VDH. Hans[2], P. Veltink[3], J. Lemson[4]
[1]Cardiovascular and respiratory physiology group, University of Twente, Enschede, Netherlands; [2]Intensive care, Radboud University Medical Center, Nijmegen, Netherlands; [3]Biomedical signals and systems, University of Twente, Enschede, Netherlands; [4]Intensive care, Radboud University Medical Center, Nijmefgen, Netherlands
**Correspondence:** L. van Loon
*Intensive Care Medicine Experimental* 2019, **7(Suppl 3):**001376

**INTRODUCTION.** Theupstreampressureforvenous return (VR) is considered to be acombinedconceptualblood pressurewithin the systemic vessels - the mean systemic filling pressure (MSFP). The relevance of the bedside estimation of the MSFP during dynamic changes of the circulation is controversial.

**OBJECTIVES.** To studie the effect of blood volume on the relationship between VR and central venous pressure (CVP) near zero blood flow.

**METHODS.** In 9 healthy mechanically ventilatedpigs under anaesthesia, MSFP was estimated from the extrapolated CVP versus VR relationships during inspiratory hold maneuvers (IHMs) with different levels of ventilatory pressure (Pvent), see Maas et al. for a detailed description of the used method (1). VR was recorded continuously using an ultrasonic flow probe, placed around the pulmonary artery. MSFP was measured 3 times during different volumetric states, i.e. euvolemia or hypovolemia. Hypovolemia was induced by bleeding with 10 ml/kg or until CO was halved compared to baseline. The estimated MSFP values were compared to the arterial blood pressure recording after induced ventricle fibrillation.

**RESULTS.** Our results revealed a strong linear correlation between cardiac output and CVP (R^2 of 0.92 [range: 0.67-0.99]), during IHMs with different levels of Pvent. Volume status significantly alters the resulting MSFP, 20 ± 1 mmHg and 16 ± 2 mmHg for euvolemia and hypovolemia respectively (Figure 1). This estimation of the MSFP was strongly correlated - but not interchangeable- to the venous blood pressure recording after induced ventricle fibrillation (R^2=0.8 and p=0.045).

**CONCLUSION.** Absolute values of the VR curve - acquired using IHMs at the bedside – are statistically significant but clinically irrelevant and should therefore not be used to guide clinical volume therapy in its current form.

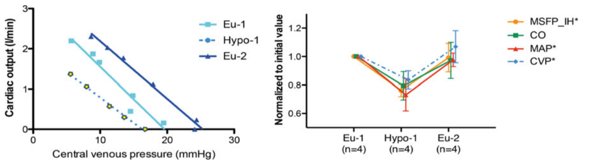

**Fig. 1 (abstract 001376).** - Left; Three constructed venous return curves during different volumetric states in order to estimate the mean systemic filling pressure. Right; Hemodynamic parameters (normalized to baseline) during the different volumetric states: Euvolemia (Eu) and Hypovolemia (Hypo). Estimation of the mean systemic filling pressure using inspiratory holds (MSFP_IH), Cardiac output (CO), Mean arterial pressure (MAP), and central venous pressure (CVP). Data are expressed as mean and SEM. *: p

### 001387

#### Diagnostic accuracy of non-invasive assessement of radial-to-central arterial pressure gradient in patients in intensive care

M. Jacquet-Lagrèze[1], D. Claveau[2], J. Cousineau[2], KP. Liu[2], JG. Guimond[2], P. Aslanian[3], Y. Lamarche[4], M. Albert[5], E. Charbonnay[2], A. Hammoud[2], L. KONTAR[6], A. Denault[2]

[1]Departement of anaesthesiology and intensive care., Hospices Civils de Lyon, Hôpital Louis Pradel, Lyon, France; [2]Intensive care unit, Institut Cardiologique de Montréal, Montréal, Canada; [3]Service de soins intensifs et centre de recherche, CHUM, Montréal, Canada; [4]Cardiac surgery, Montreal Heart Institute, Montréal, Canada; [5]Critical care, Hôpital du Sacré-Coeur de Montreal, Montréal, Canada; [6]intensive care , Montreal Heart Institute, Montréal, Canada

**Correspondence:** M. Jacquet-Lagrèze

*Intensive Care Medicine Experimental* 2019, **7(Suppl 3):**001387

**INTRODUCTION.** A radial to central pressure gradient (RCG) is a common finding during a cardiopulmonary bypass or during sepsis. This gradient leads the clinician to under evaluate the arterial pressure that can lead to fluid or vasopressor therapy overuse.

**OBJECTIVES.** We assessed in the present study the prevalence of RCG in patients in intensive care unit and the accuracy of non-invasive assessment of the radial to humeral pressure gradient (RHG) to detect the RCG.

**METHODS.** Adults patient in an intensive care unit treated with a vasopressor and equipped with a femoral and a radial artery were selected. We recorded arterial pressure at the radial and the femoral artery invasively, and non-invasively at the four limbs: on the arm at the humeral level and on the thigh and calf at the femoral and at the tibial level. This was repeated each hour for 2 hours. A significant RCG was assessed with the invasive radial and femoral arterial line, defined by either a mean arterial pressure difference of more than 10 mmHg or a systolic pressure difference of more than 25 mmHg. We assessed the prevalence of a RCG, and evaluate the correlation between RCG and RHG. We assessed the diagnostic accuracy of the RHG to detect a significant RCG.

**RESULTS.** Eighty-one patients were included. A significant gradient occurred in 15 patients with a prevalence of 18.5 % [95% CI: 10.8 - 28.7 %]. Considering the 229 measurements, 41 have shown a significant RCG 17.9% [95% CI: 13.2 - 23.5]. No demographic data were significantly associated with a significant gradient. Systolic RCG correlated with systolic RHG ($r^2$=0.36; p<0.001) and mean RCG also correlated with mean RHG ($r^2$=0.34; p<0.001).

The area under the curve of the receiving operative curve (ROC AUC) of the mean RHG to predict the RCG was 0.84 [95% CI: 0.68- 0.97]. The best threshold was 14 mmHg with a specificity of 89% [95% CI: 81- 95] and a sensitivity of 80% [95% CI: 60- 100].

**CONCLUSION.** A significant RCG occurs in almost one in every five patients Non-invasive RHG assessment can be used to detect a significant HRG.

##### REFERENCE(S)

1. Stern DH, Gerson JI, Allen FB, Parker FB. Can we trust the direct radial artery pressure immediately following cardiopulmonary bypass? Anesthesiology 1985; 62: 557–61
2. Kim WY, Jun JH, Huh JW, Hong SB, Lim C-M, Koh Y. Radial to Femoral Arterial Blood Pressure Differences in Septic Shock Patients Receiving High-Dose Norepinephrine Therapy: Shock 2013; 40: 527–31

### 001414

#### Fluid Management: Still a cause for concern but possible 'greenshoots' of improvement

R. Leach[1], N. Morton[2], M. Leach[3], M. Ostermann[1]

[1]Intensive Care, Guys & St Thomas Nhs Foundation Trust, London, UK; [2]Medicine, Guys & St Thomas Nhs Foundation Trust, London, UK; [3]Medical school, Keele Medical School, Keele, UK

**Correspondence:** R. Leach

*Intensive Care Medicine Experimental* 2019, **7(Suppl 3):**001414

**INTRODUCTION.** Daily fluid assessment, prescription and administration are essential daily tasks on intensive care, medical and surgical units. Published data suggests that intravenous (IV) fluid management is poor and patient outcomes are adversely impacted by both under and over hydration. Surgical audit data suggests that fluid related complications occurs in 17-54% of post-operative patients contributing to mortality and increased length of hospital stay.

**OBJECTIVES.** As clinician knowledge has been identified as a major factor in previous reviews of poor fluid management we examined current knowledge in 348 junior and middle-grade medical doctors in Greater London and compared this to that in consultant intensivists to determine if progress had been made to address this issue in recent years.

**METHODS.** Pre-registration (PRD; n=146) and junior medical doctors (JMD; n=70), specialty medical trainees (SpR; n=133) from 'acute' (e.g. ICU) and non-acute (e.g. rheumatology) medical specialties and consultant intensivists (CIs; n=18) were asked to complete a structured questionnaire which included: 1. Assessment of their previous training in fluid management; 2. How often they had managed fluid related complications (and whether these were reported as significant incidents); 3. Twenty 'best of 5' multiple choice knowledge questions (10 of these questions were similar to those used in previous fluid knowledge assessments (2001-2018) to enable change over time to be assessed) 4. Self-reported confidence in relation to fluid prescription. For comparison 18 CIs completed the questionnaire. Aspects of knowledge addressed included electrolyte composition of commonly administered IV fluids, normal daily fluid and electrolyte requirements and excretory mechanisms, fluid distribution, fluid and electrolyte physiology in health and disease and 4 clinical scenarios relating to the UK NICE guideline (CG 174) 'IV fluid therapy in adults in hospital'.

**RESULTS.** As in previous reports, PRD and JMD responsible for most IV fluid prescriptions, reported limited training (<10hrs) during medical school and early clinical training. Fluid related complication were common and had been managed by 25% PRD (mainly <5 episodes) and 90% of JMD and SpR (<5 to >20 episodes). The majority of these were 'never' or 'rarely' reported. On the multichoice questions CIs correctly answered 15.1±2.1 (mean±SD) questions. PRD achieved 6.66±2.4; JMD 8.37±2.6 and SpR 8.14±2.84 correct answers (with 'acute' specialty SpRs performing significantly better than non-acute specialities). Compared to previous knowledge assessments there were improvements in knowledge related to several areas including sodium content of commonly prescribed fluids, daily water and electrolyte requirements and NICE guidelines. Self reported confidence in fluid management was higher than might be expected based on multiple choice question results in all groups except CIs.

**CONCLUSION.** This study suggests that although fluid management remains a cause for concern in junior clinicians, with evidence of relatively poor training, failure to report complications, poor overall knowledge scores and over-confidence, there is evidence of improvement in a number of key areas previously highlighted in the UK NICE guideline CG174.

**REFERENCE(S)**

1. Powell-Tuck J, Gosling P, Lobo DN, Allison SP, Carlson GL, Gore M, Lewington AJ, Pearse RM and Mythen MG. British consensus guidelines on intravenous fluid therapy for adult surgical patients. 2011. www.bapen.org.uk/pdfs/bapen_pubs/giftasup.pdf
2. Powell AGMT, Paterson-Brown S. FY1 doctors still poor in prescribing intravenous fluids. BMJ 2011;342:10
3. Walsh SR, Cook EJ, Bentley R, et al. Peri-operative fluid management: prospective audit. International Journal of Clinical Practice 2008;62:492-497.
4. NICE Clinical Guideline: 'Intravenous fluid therapy in adults in hospital' CG 174. Stroud M (Chair), Al-Jayuous R, Cook P, Leach RM, Lobo D et al. December 2013
5. Walsh SR, Walsh CJ. Intravenous fluid associated morbidity in postoperative patients. Ann R Coll Surg Engl 2005;87:126-130
6. None

### 001415

#### Preload responsiveness could affect ventriculoarterial coupling in septic shock patients

S. Carelli[1], R. Shi[1], JL. Teboul[1], D. Chemla[2], N. De Vita[1], F. Gavelli[1], A. Pavot[1], W. Mongkolpun[1], C. Richard[1], X. Monnet[1]

[1]Service de médecine intensive-réanimation, Hôpital de Bicêtre, Hôpitaux universitaires Paris-Sud, AP-HP, Le Kremlin-Bicêtre, France; [2]Service de physiologie, Hôpital de Bicêtre, Hôpitaux universitaires Paris-Sud, AP-HP, Le Kremlin-Bicêtre, France

**Correspondence:** S. Carelli

*Intensive Care Medicine Experimental* 2019, **7(Suppl 3):**001415

**INTRODUCTION.** Ventriculoarterial coupling (VAC) is often impaired in septic shock patients. Fluid administration is one of the most used treatments in these patients, but its effects on VAC have been scarcely described until now. We made the hypothesis that VAC improves with a preload increase only in case of preload responsiveness because, in this condition, the fluid-induced increase in stroke volume may decrease the arterial elastance (Ea) with lesser effects on the ventricular end-systolic elastance (Ees).

**METHODS.** We included septic shock patients in whom fluid administration was mimicked by a passive leg raising (PLR) test. Hemodynamic variables and transthoracic echocardiography data before and during the PLR test were compared. Preload responsiveness was defined by a PLR-induced increase in cardiac output ≥10%. VAC was calculated as the ratio of Ea over Ees. Ea was calculated as 0.9 x systolic arterial pressure/stroke volume. Ees was calculated according to the method validated by Chen et al [1].

**RESULTS.** We performed 31 measurements among 20 patients (SAPSII on admission = 50±13, SOFA = 10±3, 28-day mortality = 30%, pneumonia in 55% of cases, norepinephrine administered in all cases). The PLR test revealed 8 preload responder cases (R) and 23 non-responder cases (NR). The left ventricular end-diastolic volume increased to a similar extent in R and NR (from 67 [57-85] to 75 [67-92] mL in R, from 75 [58-92] to 83 [68-101] mL in NR). Stroke volume increased by 19 [14-23]% in R and by 4 [0-4]% in NR. In R, Ea significantly decreased from 2.46 [1.59-2.65] to 2.01 [1.49-2.61] mmHg/mL and Ees did not change significatively (from 2.05 [1.6-2.78] to 2.09 [1.56-2.64] mmHg/mL). As a result, VAC significantly decreased from 1.14 [0.83-1.44] to 1.02 [0.74-1.3] in R cases. Among NR cases, neither Ea nor Ees showed a significant change during PLR (p=0.80 and p=0.20, respectively). Similarly, VAC did not change in these cases (p=0.18).

**CONCLUSION.** These results suggest that, in septic shock patients, increasing cardiac preload improves VAC only in cases of preload responsiveness. This suggests an improvement in cardiac efficiency with fluid under this condition. The inclusions are ongoing.

### 001424

#### Can dynamic changes of pulse pressure variation during passive leg raising or a tidal volume challenge help predicting preload responsiveness in mechanically ventilated patients with spontaneous breathing activity

O. Hamzaoui[1], R. Shi[2], S. Carelli[2], C. Gouëzel[1], B. Sztrymf[1], D. Prat[1], F. Jacobs[1], JL. Teboul[2]

[1]Service de réanimation polyvalente, Hôpital Antoine Béclère, Hôpitaux universitaires Paris-Sud, AP-HP, Clamart, France; [2]Service de médecine intensive-réanimation, Hôpital de Bicêtre, Hôpitaux universitaires Paris-Sud, AP-HP, Le Kremlin Bicêtre, France

**Correspondence:** O. Hamzaoui

*Intensive Care Medicine Experimental* 2019, **7(Suppl 3):**001424

**INTRODUCTION.** Predicting preload responsiveness by using dynamic indicators before administering fluids to critically ill patients is nowadays routinely performed at the bedside (1). Unlike other dynamic indicators of preload responsiveness that require cardiac output monitoring, pulse pressure variation (PPV) can be simply obtained via an arterial catheter (2). However, PPV is not reliable in mechanically ventilated patients with spontaneous breathing activity (2). We hypothesized that an increase in PPV after a tidal volume (TV) challenge (TVC) or a decrease in PPV during passive leg raising (PLR) will predict preload responsiveness in such cases.

**OBJECTIVES.** to examine if the change in PPV during PLR and after a TVC can predict preload responsiveness in patients with mechanical ventilation and persistent spontaneous breathing

**METHODS.** Prospective non interventional study conducted in two intensive care units. Patients under mechanical ventilation with spontaneous cycles, for whom the physician in charge decided to test preload responsiveness were included. Firstly, transthoracic echocardiography was performed to measure the velocity time integral (VTI) of the left ventricular outflow tract and to calculate delta VTI before and during PLR. Patients were considered as preload responsive when delta VTI was ≥10% (3). Secondly, a TVC was performed by increasing the TV by 2mL/kg predicted body weight from its baseline value. PPV was recorded before and after the TVC.

**RESULTS.** Thirty patients were included, their mean age was 61 ± 16 and their SAPSII was 57 ± 17. At baseline the TV was of 6.2±0.7 mL/kg predicted body weight, their PEEP was of 11±4 cmH2O and their plateau pressure was of 20±5 cmH2O. Norepinephrine was administered in 29 patients, its mean dose was of 0.37±0.44 μg/kg/min. The blood lactate was of 1.3 [1.1-1.9] and the MAP was 83 ± 12 mmHg. According to the PLR test, 14 were preload-responders and 16 non responders. PPV at baseline predicted preload responsiveness with an area under the receiver operating characteristic curve (AUROC) (with 95% CI) of 0.53 (0.33-0.71). During PLR, the absolute decrease in PPV predicted preload responsiveness with a sensitivity of 86%, a specificity of 63%, an AUROC (95% CI) of 0.72 (0.53–0.87) and a cutoff value of -1%. The increase in PPV during the TVC predicted preload responsiveness with a sensitivity of 71%, a specificity of 88%, an AUROC of 0.74 (0.55–0.89) with a cutoff value of 2. The

AUROC values for decrease in PPV during PLR and for increase in PPV during TVC were similar but were significantly different from 0.5 and from that for baseline PPV.

**CONCLUSION.** In mechanically ventilated patient with spontaneous breathing activity, PPV does not predict fluid responsiveness, however either the decrease in PPV during PLR or the increase in PPV during a TVC may help physicians to distinguish preload responders from non-responders with an acceptable accuracy.

**REFERENCE(S)**

1. Monnet X, Marik PE, Teboul JL. Prediction of fluid responsiveness: an update. Ann Intensive Care. 2016;6:111.
2. Teboul JL, Monnet X, Chemla D, Michard F. Arterial Pulse Pressure Variation with Mechanical Ventilation. Am J Respir Crit Care Med. 2019;199:22-31
3. Vignon P, Begot E, Mari A, et al. Hemodynamic Assessment of Patients With Septic Shock Using Transpulmonary Thermodilution and Critical Care Echocardiography: A Comparative Study. Chest. 2018; 153:55-64.

### 001429

#### Opioid regimensin in the resection of abdominal aorta aneurysms

D. Loncar Stojiljkovic[1]; M. Stojiljkovic[2]

[1]{street_address}, Belgrade, Serbia; [2]Department of clinical pharmacology, Institute for Cardiovascular, Belgrade, Serbia, Serbia

**Correspondence:** D. Loncar Stojiljkovic

*Intensive Care Medicine Experimental* 2019, **7(Suppl 3):**001429

**INTRODUCTION.** Surgical procedures cause pain – neuroendocrine (1), inflammatory and immune response that can be modified by anaesthesia (2, 3).

There are several opioids that are used for perioperative and postoperative analgesia: sufentanil (S), alfentanil (AL), remifentanil (R) and fentanyl (F).

**OBJECTIVES.** Objectiveof this study wasto compare the effects of these opioid analgesic regimens on cardiovascular and hormonal reaction of patientsundergoing elective aortal aneurism surgery under general endotracheal anaesthesia.

**METHODS.** A total of 60 elderly patients, ASA 3, scheduled for elective surgery of aneurysms of abdominal aorta were randomized in four equal groups to receive either *F*(0.1 μg/kg *iv*bolus +0.1 mg maintenance dose) or a equivalent doses of *AL*, *R*and *S*.

Haemodynamic parameters and concentrations of prolactine (PRO), cortisol (COR) and growth hormone (GH) were determined at critical points: baseline (BL), intubation (INT), first incision (FI), surgical manipulation (SM), extubation EXT and 24 h postop (24hPO). Analgesics were repeated hourly or if cardiovascular parameters changed by more than 20% of their BL values.

A blood sample of 10 ml was taken 10 min after insertion of *iv*catheter (BL) and sampling and was repeated at critical points of operations and 24 h postoperatively.

**RESULTS.** In the *F* group there was a significant increase in systolic and diastolic BP after INT and SM as well as pulse pressure after SM. Diastolic BP was significantly increased after EXT.

A decrease in systolic pressure was found after INT in the *R, S* and*AL*group (by 32±5%, 25±4% and 18±2%, respectively). Pulse pressure was significantly increased at SM and 24 h postop in the *R*group.

Emergence from anaesthesia was best after use of R (4.9±5 vs. 9.5±2, 12 .9±8 ;12.6±2min in F, Al and S group, respectively). Postoperative analgesia was significantly shorter after use of R (27±6 min) than of *F, S*and AL (50±6, 55±8and 42±7 min, respectively).

Only COR positively correlated with mean arterial pressure (MAP) in all opioid groups, while there were no correlations between PRO or GH and cardiovascular parameters.

During the maintenance of anaesthesia, prolactin values were significantly increased in all groups in Iphases of NT, M and EXT.

**CONCLUSION.** Cortisol plasma concentration correlates positivelywith cardiovascular parameters in patients undergoing elective surgery of abdominal aorta aneurysm during the operation and thereafter under all used opioid regimens. Its suppression is better marker of analgesia than the changes of prolactin and GH levels.

**REFERENCE(S)**

1. Desborough JP. The stress response to trauma and surgery. Br J Anaesth 2000;85(1):109-17.
2. Málek J et al. Postoperative pain menagement. Prague: Mlada fronta 2017.
3. Salartash K, Sternbergh WC 3rd, York JW, Money SR. Comparison of open transabdominal AAA repair with endovascular AAA repair in reduction of postoperative stress response. Ann Vasc Surg 2001;15:53-593.

### 001651

#### Extracorporal heart and lung support (ECLS/ECMO) for patients with refractory cardiogenic shock - boon or bane?

JKM. Fichte[1], HB. Hopf[2]

[1]Asklepios Klinik Langen, Langen (Hessen), Germany; [2]Anestesia and perioperative medicine, Asklepios Klinik Langen, Langen (Hessen), Germany

**Correspondence:** J.K.M. Fichte

*Intensive Care Medicine Experimental* 2019, **7(Suppl 3):**001651

**INTRODUCTION.** Patients with cardiogenic shock refractory to treatment still have a very poor prognosis with a hospital mortality above 50%(Thiele, EurHeartJ 2015).

Therefore in patients with refractory cardiogenic shock extracorporal support (ECLS) is increasingly used to treat such patients in order to decrease mortality. However only limited data about the results are availaible. Accordingly we evaluated all patients with refractory cardiogenic shock treated with an ECLS between 2015 and 2018 in our department.

**OBJECTIVES.** Comparing the predicted mortality (SAVE score) with the actual hospital mortality in patients with refractory cardiogenic shock and treatment with an extracorporal support (ECLS).

**METHODS.** We compared the predicted hospital mortality with the actual hospital mortality of all patients with refractory cardiogenic shock treated with extracorporal support in the Asklepios Klinik in Langen, Germany (Department of Anestesia and perioperative medicine) between January 2015 and December 2018. To predict mortality we used the SAVE-score, which has been developed by Schmidt. et al. (2).

We devided all patients treated in a palliative care group (patients with unfavorable prognosis and short survival times due to poor neurological outcome/ strict living will) and a non palliative care group.

**RESULTS.**

**CONCLUSION.** The actual in hospital mortality of our non palliative care patients was less than half oft that predicted by the SAVE-score. In addition in the non palliative care patients with refractory cardiogenic shock treated with ECLS the hospital mortality was about one third lower than the mortality published in current literature.

Thus, the use of an extracorporal support system like ECLS might improve mortality in patients with refractory cardiogenic shock.

A randomized prospective evaluation of ECLS/ECMO application in patients with refractory cardiogenic shock (ECLS-SHOCK) is under way to answer this question.

**Table 1 (abstract 001651).** RESULTS.

| | Non palliative care | Palliative care |
|---|---|---|
| | n=41 | n=33 |
| CPR | | |
| yes | n=24 | n=27 |
| no | n=17 | n=6 |
| SAVE score/pred. mortality | | |
| mean value/% | -5,8/70% | -9,4/95% |
| standard deviation | 4,4 | |
| survived | n=31 | n=0 |
| deceased | n=10 | n=33 |

## MEN - Metabolic risk and GI function

### 3000698

#### Plasma I-FABP dynamics in intensive care patients

M. Padar[1], J. Starkopf[1], A. Forbes[2], J. Wernerman[3], O. Rooyackers[3], SM. Jakob[4], M. Hiesmayr[5], T. Gold[5], M. Poeze[6], D. Meesters[6], A. Reintam Blaser[7]
[1]Department of Anaesthesiology and Intensive Care, Tartu University Hospital, Tartu, Estonia; [2]Norwich medical school, University of East Anglia, Norwich, UK; [3]Department of clinical science, intervention and technology, Karolinska Institute, Stockholm, Sweden; [4]University clinic for intensive care medicine, Bern University Hospital, Bern, Switzerland; [5]Division of cardiac thoracic vascular anaesthesia and intensive care, Medical University of Vienna, Wien, Austria; [6]Department of trauma surgery, Maastricht University Medical Centre, Maastricht, Netherlands; [7]Department of anaesthesiology and intensive care, University of Tartu, Tartu, Estonia
**Correspondence:** M. Padar
*Intensive Care Medicine Experimental* 2019, **7(Suppl 3):**3000698

**INTRODUCTION.** Intestinal fatty acid binding protein (I-FABP) is a cytosolic protein in the (mainly) small intestine that is released into circulation upon enterocyte necrosis and rapidly cleared renally (Treskes 2017). As such it parallels the onset and cessation of enterocyte damage, e.g. due to ischaemia. Little is known about I-FABP dynamics in a general ICU population.
**OBJECTIVES.** To describe I-FABP in ICU patients during their first week of stay.
**METHODS.** Consecutive adult patients in 5 ICUs were studied. Plasma was collected immediately after ICU admission and thereafter daily. Following storage at -80 °C, samples were analyzed for I-FABP using ELISA (R&D Systems Inc., US). I-FABP values between groups were compared using the SPSS Median Test for each day separately.
**RESULTS.** I-FABP was measured in 224 patients (median age 66.5, range 29-94 years; median APACHE II 18, IQR 11-23; 28 day mortality 10.3%). Median I-FABP level of all measurements was 903 (425-1862), being higher in 28 d survivors [963 (IQR 461-1882)] *vs* nonsurvivors [502 (IQR 284-1197) pg/mL]. Median of individual highest I-FABP values was 2596 (IQR 1286-4691) pg/mL and occurred on admission day in 69% of patients. I-FABP levels were lower in patients after GI surgery (days 3-4) and in patients with sepsis (days 1-3) compared to patients without these conditions respectively. Figures present I-FABP dynamics in all patients, 28 d survivors *vs* nonsurvivors, sepsis *vs* no sepsis and GI surgery *vs* no GI surgery.
**CONCLUSION.** I-FABP levels were elevated at admission, but rapidly decreased to the reference range by day 2. Interestingly, and different from previous observations, rather lower I-FABP levels were observed in nonsurvivors *vs* survivors and in sepsis *vs* no sepsis.

**REFERENCE(S)**

1. Treskes N, Persoon AM, van Zanten ARH. Diagnostic accuracy of novel serological biomarkers to detect acute mesenteric ischemia: a systematic review and meta-analysis. Intern Emerg Med. 2017; 12(6): 821–836.
2. This study is a part of the iSOFA study (NCT02613000) endorsed by ESICM and funded by Fresenius Kabi and institutional grant from the Ministry of Education and Research of Estonia (IUT34-24).

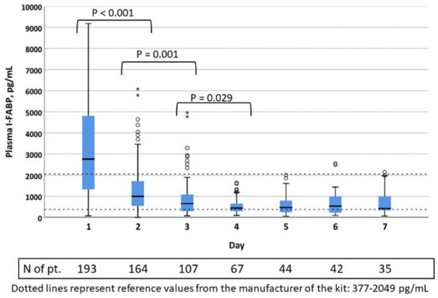

**Fig. 1 (abstract 000698).** I-FABP dynamics in all patients

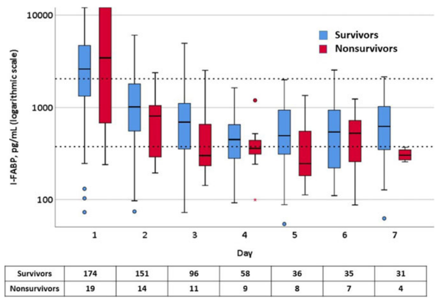

**Fig. 2 (abstract 000698).** Plasma I-FABP dynamics in 28 days survivors vs nonsurvivors

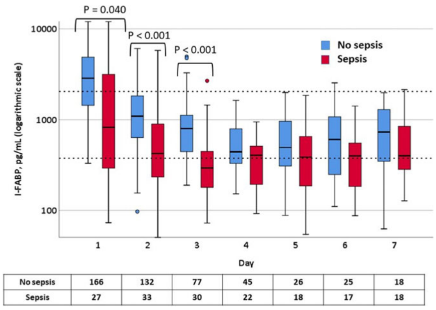

**Fig. 3 (abstract 000698).** Plasma I-FABP dynamics in sepsis vs no sepsis

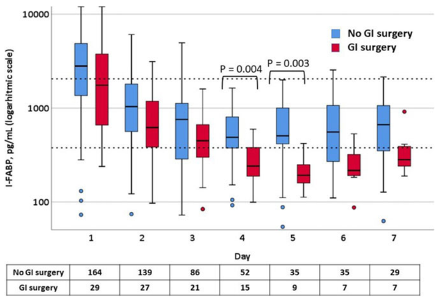

**Fig. 4 (abstract 000698).** Plasma I-FABP dynamics in GI vs no GI

### 000728

#### Factors associated with blood transfusions and early extubation in liver transplantation recipients. Prospective observational preliminary data

C. Spina[1], G. Burgio[2], G. Martucci[2], M. Barbara[3], D. Pagano[3], M. Farbo[2], F. Lullo[2], G. Chiaramonte[2], R. Fumagalli[4], S. Gruttadauria[3], A. Arcadipane[2]
[1]School of anesthesia and intensive care, University of Milano-Bicocca, Milano, Italy; [2]Anesthesia and intensive care unit, IRCCS-ISMETT, Palermo, Italy; [3]Department for the treatment and study of abdominal diseases and abdominal transplantation, IRCCS-ISMETT, Palermo, Italy; [4]Department of anesthesia and critical care, ASST Grande Ospedale Metropolitano Niguarda, Milano, Italy
**Correspondence:** C. Spina
*Intensive Care Medicine Experimental* 2019, **7(Suppl 3):**000728

**INTRODUCTION.** Fluid restriction and early extubation in operating room are recent procedures introduced in the perioperative management of liver transplantation (LT) recipients in order to avoid hemodilution, blood transfusion and to minimize ICU stay and complications.

**METHODS.** This prospective cohort study included 18 consecutive patients who underwent a deceased-donor LT at ISMETT from September 2018 to March 2019. Packed red blood cells (PRBC) transfused and early extubation were analyzed in relation to pre and intraoperative variables. LT were performed with the standard technique with or without veno-venous bypass.

**RESULTS.** Patient characteristics, intra and postoperative variables are described in table 1.

Major indications for liver transplantation were cirrhosis caused by viral diseases n= 7 (38.9%) followed by NASH syndrome n=6 (33.3%). The overall intraoperative PRBC transfusion was 1.7 ± 2.5 units on average. Patients who received blood transfusions (n=8, 44%) needed in median 3.5 PRBC (2.5-5.5).

Patients were divided between those no required blood transfusion versus those received more than one unit. The factors associated with PRBC transfusions are listed in table 2.

Patients extubated in operating room (n=13, 72%) had a significant lower dose of norepinephrine used during transplantation [0.1 vs 0.3, p 0.019] and were all treated with neuromuscular blockade reversion by Sugammadex (p<0.001).

**CONCLUSION.** These preliminary results show that low preoperative Hb, high INR value, MELD score, CP, high dose of norepinephrine and long time of surgery and anaesthesia were associated to the need of intraoperative blood transfusions during LT. These data provide also an external validation of the Mc Cluskey index as predictor of intraoperative blood loos. Hemodynamic stability and Sugammadex use were associated with extubation in OR during LT. Further increase of the sample size should confirm these preliminary results.

**REFERENCE(S)**

1. McCluskey SA, Karkouti K, Wijeysundera DN, et al. Derivation of a risk index for the prediction of massive blood transfusion in liver transplantation. Liver Transpl 2006; 12:1584–1593.
2. Aniskevich S, Pai SL. Fast track anesthesia for liver transplantation: review of the current practice. World J Hepatol 2015;7:2303–8.
3. Carrier FM , et al. Effects of perioperative fluid management on postoperative outcomes in liver transplantation: a systematic review protocol. Syst Rev. 2018 Oct 31;7(1):180. doi:10.1186/s13643-018-0841-3.

**Table 1 (abstract 000728).** Population characteristics

| | LT n=18 | | |
|---|---|---|---|
| Donor age (median [IQR]) | 62 [48.5, 66.5] | Creat (median [IQR]) | 0.8 [0.6, 1.21] |
| Recipient age (median [IQR]) | 58[50.5, 62] | INR (median [IQR]) | 1.35 [1.27, 1.43] |
| Gender = 1 (%) | 13 (72) | Hb (median [IQR]) | 10.8 [10, 11.5] |
| BMI (median [IQR]) | 24.75 [23.5, 26.8] | PLT (median [IQR]) | 67.5 [38.5, 97.75] |
| MELD (median [IQR]) | 13 [11, 16] | DoS_min (median [IQR]) | 405 [381, 472.5] |
| CP (median [IQR]) | 7.0 [6.0, 9.75] | DoA_min (median [IQR]) | 525 [480, 540] |
| HCC = 1 (%) | 11 (61) | BloodLoss (median [IQR]) | 500 [325, 1000] |
| McCluskey (%) | | Balance (median [IQR]) | 1070 [241, 2195] |
| 1 | 5 (28) | NorepMax (median [IQR]) | 0.2 [0.1, 0.3] |
| 2 | 7 (39) | LoS (median [IQR]) | 18.5 [13.0, 22.5] |
| 3 | 4 (22) | ICU.stay (median [IQR]) | 3.0 [2.0, 5.7] |
| 4 | 2 (11) | By Pass = 1 (%) | 12 (67) |

**Table 2 (abstract 000728).** Factors associated with PRBC transfusions

| | No PRBC | PRBC | p | | No PRBC | PRBC | p |
|---|---|---|---|---|---|---|---|
| N | 10 | 8 | | N | 10 | 8 | |
| MELD | 11.0 [9.2, 12] | 16 [15.5, 19] | 0.005 | McCluskey (%) | | | 0.021 |
| CP | 6 [5, 7] | 9.5 [8.8, 11] | 0.005 | 1 | 4 (40) | 1 (12) | |
| Cpclass (%) | | | 0.023 | 2 | 5 (50) | 2 (25) | |
| A | 6 (60) | 1 (12) | | 3 | 1 (10) | 3 (38) | |
| B | 3 (30) | 3 (38) | | 4 | 0 ( 0) | 2 (25) | |
| C | 1 (10) | 4 (50) | | DoS_h | 6.2 [5.6, 6.4] | 7.6 [6.9, 8.2] | 0.013 |
| INR | 1.3 [1.2, 1.4] | 1.5 [1.4, 1.6] | 0.005 | DoA_h | 8.0 [7.6,8.9] | 9.0 [8.9, 9.2] | 0.033 |
| Hb | 11.5 [11, 13] | 9.3 [7.9, 10.3] | 0.001 | NorepMax | 0.1 [0.0,0.2] | 0.3 [0.2, 0.4] | 0.014 |
| | | | | BloodLoss | 350 [300, 475] | 1000 [950, 1125] | 0.002 |

### 000788

### Comparison of Two Different Nasogastric Feeding Strategies in terms of Gastrointestinal System Complications

E. Ounde, O. Ekinci, A. Ozgultekin

[1]Anaesthesiology and intensive care, University of Health Sciences, Haydarpasa Numune Training and Research Hospital, Istanbul, Turkey

**Correspondence:** E. Ounde

*Intensive Care Medicine Experimental* 2019, **7(Suppl 3):**000788

**INTRODUCTION.** Gastrointestinal system(GIS) complications are frequent within first 7 days in patients started to feed by enteric route in Intensive Care Unit(ICU).Researches have been published reporting that these complications are due to the product, the patient or the feding method.(1)In this study, we aimed to investigate and compare the rates of complicaitions related to feeding method. Which today frequently used methods are continuous feeding(CF) and intermitent feeding(IF) via nasogastric tube.

**METHODS.** After obtaining approval of Institutional Ethics Committee, the patients, who admitted to ICU for different reasons within 4 months, aged 18-95, who had no GIS pathology, no contraindication for enteral nutrition, were included the study and randomly assigned to CF and IF group.Informed consets of subjects were obtained.After reaching the aimed calories within 72 h, diarrhea, constipation, aspiraiton, distantion, residue, nausea and reflux recorded for 7 days.Significance was defined as p<0.005.

**RESULTS.** The study was performed with a total of 62 cases (31(50%) male and 31(50%) female).The mean age was 74.45 ± 13.43 years. 37(59.7%) patients were followed-up with CF and 25 patients(40.3%) with IF. Results showed that there was no statistically significant difference between these two groups. Complications rates are summerized at Table 1.

**CONCLUSION.** In the studies comparing CF and IF, stool frequency is higher and access to aimed calories is delayed in the IF compared to CF.(2) Howerver, some other studies declared that there was no significant difference in the effects of both methods related to GIS functions. (3) In our study, although there was no statistically significant difference between the two groups, the rates of two complicitons showed clinically significance. The rate of distension was higher in patients IF (CF-3(%8,1),IF-6(%24)),while the rate of diarrhea was higher in patients CF(CF-10(%27),IF-5(%20)).As a result,CF did not show any superiorty to IF in terms of GIS complications. We suggest that IF can be considered as an option in selected critically ill patients.

**REFERENCE(S)**

1. Hiebert JM et al., Comparison of continuous vs intermittent tube feedings in adult burn patients., JPEN J Parenter Enteral Nutr. 1981 Jan-Feb;5(1):73-5
2. Patel JJ et al., Intermittent versus continuous feeding in critically ill adults., Curr Opin Clin Nutr Metab Care. 2018 Mar;21(2):116-120
3. Blumenstein I, Gastroenteric tube feeding: techniques, problems and solutions.,World J Gastroenterol. 2014 Jul 14;20(26):8505-24

**Table 1 (abstract 000788).** Evaluation of complications

| | | CF n (%) | IF n (%) | *Total n (%)* | P |
|---|---|---|---|---|---|
| Diarrhea | Absent | 27 (%73) | 20 (%80) | *47 (%75,8)* | **0,740** |
| | Present | 10 (%27) | 5 (%20) | *15 (%24,2)* | |
| Constipation | Absent | 34 (%91,9) | 24 (%96) | *58 (%93,5)* | **0,642** |
| | Present | 3 (%8,1) | 1 (%4) | *4 (%6,5)* | |
| Aspiration | Absent | 37 (%100) | 25 (%100) | *62 (%100)* | **-** |
| Distension | Absent | 34 (%91,9) | 19 (%76) | *53 (%85,5)* | **0,139** |
| | Present | 3 (%8,1) | 6 (%24) | *9 (%14,5)* | |
| Residue | Absent | 22 (%59,5) | 14 (%56) | *36 (%58,1)* | **0,993** |
| | Present | 15 (%40,5) | 11 (%44) | *26 (%41,9)* | |
| Nausea | Absent | 35 (%94,6) | 22 (%88) | *57 (%91,9)* | **0,385** |
| | Present | 2 (%5,4) | 3 (%12) | *5 (%8,1)* | |
| Reflux | Absent | 37 (%100) | 25 (%100) | *62 (%100)* | **-** |

### 000794

#### Effects of Omega-3 Fatty Acids on Proinflammatory Cytokines in an Experimental Model of Sepsis

M. Arslan, A. Kuntman, K. Demirag, M. Uyar
Anesthesiology and intensive care, Ege University Hospital, Izmir, Turkey
**Correspondence:** M. Arslan
*Intensive Care Medicine Experimental* 2019, **7(Suppl 3):**000794

**INTRODUCTION.** Cytokines play a role in the pathogenesis of sepsis by mediating the dysregulation of immune response. Omega-3 fatty acids may exert anti-inflammatory effects, affecting various elements of inflammation together with proinflammatory cytokines.

**OBJECTIVES.** The aim of the present study was to investigate the effects of omega-3 fatty acids on the level of proinflammatory cytokines and survival in a sepsis rat model.

**METHODS.** In the study, 40 adult Wistar albino rats weighing 300-350 g were divided randomly into three groups: the Sham Group (n=10): Tactile stimulation of the intestine and cecum after laparotomy, the Control Group (n=15): Saline solution (1 mL/kg/day) administered intraperitoneally for one week before the induction of an abdominal sepsis model through cecal perforation, and the Omega-3 Group (n=15): Omega-3 fatty acid lipid emulsion (1 mL/kg/day) administered intraperitoneally for one week prior to the induction of an abdominal sepsis model through cecal perforation. After one week period, serum interleukin (IL)-1, IL-6 and tumor necrosis factor-alpha (TNF-α) levels were measured prior to surgery (basal value) and 3 hours after surgery in all groups.

**RESULTS.** The 24-hour survival rates were 60% in the omega-3 group and 13.3% in the control group (p=0.008). Serum IL-1, IL-6, and TNF-α levels were significantly lower in the omega-3 group than in the control group at hour 3 (p<0.001).

**CONCLUSION.** We determined the positive effects of omega-3 fatty acids on proinflammatory cytokine levels, immune response development, and survival in experimental sepsis rat model that is considered to be related to the anti-inflammatory and immunomodulator properties of omega-3 fatty acids.

##### REFERENCE(S)

1. Schulte W, Bernhagen J, Bucala R. Cytokines in sepsis: potent immunoregulators and potential therapeutic targets–an updated view. Mediators Inflamm 2013;2013:165974.
2. Calder PC. Marine omega-3 fatty acids and inflammatory processes: Effects, mechanisms and clinical relevance. Biochim Biophys Acta 2015;1851:469-84.
3. This work was supported by a grant from the Unit of Scientific Research Projects (BAP 2016/TIP-038) at Ege University.

### 000801

#### Metabolomic alterations in ICU patients with worsening of multiorgan failure

J. Helleberg[1], J. Wernerman[2], O. Rooyackers[2]
[1]Perioperative Medicine and Intensive Care, Karolinska University Hospital, Stockholm, Sweden; [2]Department of clinical science, intervention and technology, Karolinska Institute, Stockholm, Sweden
**Correspondence:** J. Helleberg
*Intensive Care Medicine Experimental* 2019, **7(Suppl 3):**000801

**INTRODUCTION.** Multiorgan failure is the leading cause of death in the ICU. The severity of multiorgan failure, as defined by the Sequential Organ Failure Score (SOFA), as well as increases in SOFA score are known predictors of death during and after intensive care.

**OBJECTIVES.** To identify the metabolomic profile at ICU admission that is related to worsening of multiorgan failure versus improving of multiorgan failure during early ICU stay.

**METHODS.** Consecutive patients over 18 years old admitted to the ICU at the Karolinska University Hospital between September and December 2017 were included if they gave written consent and had no prior decision of withholding treatment. Plasma was sampled on ICU admission and analyzed for metabolomics. Liquid-Chromatography Mass Spectrometry (LCMS) and Gas-Chromatography Mass Spectrometry (GCMS) were used for targeted metabolomics analysis. SOFA scores were calculated automatically using data from the electronic medical record. DeltaSOFA was calculated as the difference between the highest SOFA score on ICU day 2 or 3, and the initial SOFA score. Fisher's exact test was used for non-parametric data. T-tests with False Discovery Rate adjusted P-values and Fold Change Analysis were used to identify compounds that varied significantly between the two groups. R version 3.5.3 and MetaboAnalyst 4.0 were used for all statistical analyses.

**RESULTS.** From 100 patients, data to calculate DeltaSOFA were available in 89 patients. Metabolomics data were available for 87 patients, out of which 19 patients had DeltaSOFA≥2. Mortality at 30 days was 5/19 in patients with DeltaSOFA≥2, compared to 2/68 in patients with DeltaSOFA<2 (p = 0.0049).

A total of 369 metabolites were identified. 17 metabolites were significantly different at admission between patients with DeltaSOFA<2 and patients with DeltaSOFA≥2, using a P-value cutoff of 0.1 and a fold change threshold of 2.

The 17 significant metabolites were classified according to the Human Metabolome Database (HMDB) (Table 1).

**CONCLUSION.** Early increase in SOFA score is associated with 30-day mortality. In this pilot study changes in levels of metabolites of different classes at admission are seen in patients with increase of SOFA score during their early ICU stay, suggesting alterations in multiple metabolic pathways in parallel. These findings need to be validated in cohorts with different case-mix and ethnicity.

**Table 1 (abstract 000801).** See text for description

| HMBD Class | Number of significant metabolites |
|---|---|
| Carboxylic acids and derivatives | 4 |
| Steroids and Steroid Derivatives | 6 |
| Fatty Acyls | 3 |
| Organooxygen compounds | 1 |
| Purine Nucleotides | 1 |
| Other | 2 |

### 000961

#### Usefulness of modified Nutrition Risk in Criticallly ill score to determine the risk of malnutrition in ICU patients

S. Canelles[1], MC. Gilavert[1], M. Cartanyà[1], J. Gómez[1], F. Esteban[1], M. Bodí[1]
[1]Intensive care unit, Hospital Universitari de Tarragona Joan XXIII, Tarragona, Spain
**Correspondence:** S. Canelles
*Intensive Care Medicine Experimental* 2019, **7(Suppl 3):**000961

**INTRODUCTION.** The identification of reliable parameters which can help ICU professionals to identify patients at high risk of malnutrition could be important in terms of reducing length of stay, mechanical ventilator days or mortality in ICU. According to the different societies (European Society for Clinical Nutrition and Metabolism, American Society for Enteral and Parenteral Nutrition, Society of Crtical Care Medicine, Canadian Critical Care Nutrition), there is no consensus in which might be the best values to be included in this evaluation.

**OBJECTIVES.** Determine whether there are differences between ICU patients at low against high risk of malnutrition according to modified Nutrition Risk in Critically ill (NUTRIC) score values.

**METHODS.** Retrospective study of cohorts carried out in a 30-bed polyvalent ICU. Inclusion criteria: patients admitted to ICU (May 2018 - March 2019) with nutritional risk calculated. Nutritional risk was categorized by the modified NUTRIC score (0-4 Low risk; 5-9 High risk). All data were acquired from our Clinical Information System (Centricity Critical Care from General Electric) database through Extract, Transform and Load processes using Qlik Sense scripts. Statistical analysis was performed using R (https://cran.r-project.org/), applying $chi^2$ test for categorical variables and the Mann-Whitney U test for continuous variables. P values < 0,05 were considered significant.

**RESULTS.** 506 patients out of 865 incomes were included. Median age 63 (50-73), 64% male, median APACHE II 20 (15-26), median SOFA 4 (2-6), median length of stay 4,8 (2,6-10,75) days, median days of mechanical ventilation 5 (2-13), median days with vasopressors 4 (2-7) and 13.24% of mortality.
High risk of malnutrition was determined in 124 patients. Comparing the variables obtained between both groups, there were significant differences in terms of gender, age, days of mechanical ventilation and days with vasopressors, administered KcaL, length of stay in ICU and mortality. No differences in days of renal replacement therapy or administered proteins were found.
**CONCLUSION.** Modified NUTRIC score could be a good parameter to identify patients at high risk of malnutrition in the ICU. The detection of these patients has changed the professionals' attitude towards the administered KcaL according to the required needs.

**REFERENCE(S)**

1. Singer P, Blaser AR, Berger MM, et al. ESPEN guideline on clinical nutrition in the intensive care unit. Clin Nutrit 2018. pii: S0261-614(18)32432-4.
2. Reintam Blaser A, Starkopf J, Alhazzani W, Berger MM, Casaer MP, Deane AM, et al. Early enteral nutrition in critically ill patients: ESCIM clinical practice guidelines. Intensive Care Med 2017;43:380e98.
3. McClave SA, Taylor BE, Martindale RG, et al. Guidelines for the provision and assessment of nutrition support therapy in the adult critically ill patient: Society of Critical Care Medicine (SCCM) and American Society for Parenteral and Enteral Nutrition (A.S.P.E.N.). JPEN J Parenter Enteral Nutr 2016; 40(2):159–211.
4. Dhaliwal R, Cahill N, Lemieux M, Heyland DK. The Canadian critical care nutrition guidelines in 2013: an update on corrent recommendations and implementation strategies. Nutrit Clin Pract 2014; 29:29–43.
5. Carlos III Health Institute FIS PI16/00491

### 000970

#### Estimating vitamin C status in critically ill patients with a novel point-of-care oxidation-reduction potential measurement

S. Rozemeijer, A. Spoelstra-de Man, S. Coenen, B. Smit, PWG. Elbers, HJ. de Grooth, A. Girbes, H. Oudemans-van Straaten
[1]Intensive care medicine, Amsterdam UMC, locatie VUmc, Amsterdam, Netherlands
**Correspondence:** S. Rozemeijer

*Intensive Care Medicine Experimental* 2019, **7(Suppl 3):**000970

**INTRODUCTION.** Vitamin C deficiency is common in critically ill patients. Vitamin C, the most important antioxidant, is likely consumed during oxidative stress and deficiency is associated with organ dysfunction and mortality [1-4]. Assessment of vitamin C status may be important to identify patients who might benefit from vitamin C administration. Up to now, vitamin C concentrations are not available in daily clinical practice. Recently, a point-of-care device has been developed that measures the static oxidation-reduction potential (sORP), reflecting oxidative stress, and antioxidant capacity (AOC).
**OBJECTIVES.** Aim of this study was to determine whether plasma vitamin C concentrations were associated with plasma sORP and AOC.
**METHODS.** Plasma vitamin C concentration, sORP and AOC were measured in three groups: healthy volunteers, critically ill patients, and critically ill patients receiving 2- or 10-grams vitamin C infusion. Its association was analysed using regression models and by assessment of concordance.
**RESULTS.** We measured 211 samples obtained from 103 subjects. Vitamin C concentrations were negatively associated with sORP ($R2=0.816$) and positively associated with AOC ($R2=0.842$). A high concordance of 94-100% was found between vitamin C concentration and sORP/AOC.
**CONCLUSION.** Thus, plasma vitamin C concentrations are strongly associated with plasma sORP and AOC, as measured with a novel point-of-care device. Therefore, measuring sORP and AOC at the bedside has the potential to identify and monitor patients with oxidative stress and vitamin C deficiency.

**REFERENCE(S)**

1. Borrelli, E.; Roux-Lombard, P.; Grau, G.E.; Girardin, E.; Ricou, B.; Dayer, J.; Suter, P.M. Plasma concentrations of cytokines, their soluble receptors, and antioxidant vitamins can predict the development of multiple organ failure in patients at risk. Crit Care Med 1996, 24, 392-397.
2. Grooth, H.J.; Spoelstra-de Man, A.M.E.; Oudemans-van Straaten, H.M. Early plasma vitamin c concentration, organ dysfunction and icu mortality. Intensive Care Medicine 2014, 40 (10 (Suppl 1)), S199.
3. Frei, B.; Stocker, R.; England, L.; Ames, B.N. Ascorbate: The most effective antioxidant in human plasma. Adv Exp Med Biol. 1990, 264, 155-163.
4. Carr, A.C.; Rosengrave, P.C.; Bayer, S.; Chambers, S.; Mehrtens, J.; Shaw, G.M. Hypovitaminosis c and vitamin c deficiency in critically ill patients despite recommended enteral and parenteral intakes. Crit Care 2017, 21, 300.

### 000986

#### Prognostic impact of elevated lactate levels in critically ill patients with or without preadmission metformin treatment: a Danish registry-based cohort study

RA. Posma[1], T. Froslev[2], B. Jespersen[3], I. Van der Horst[4], DJ. Touw[5], RW. Thomsen[2], M. Nijsten[1], CF. Christiansen[2]
[1]Department of critical care, University of Groningen, University Medical Center Groningen, Groningen, Netherlands; [2]Department of clinical epidemiology, Aarhus University Hospital, Aarhus, Denmark; [3]Department of renal medicine, Aarhus University Hospital, Aarhus, Denmark; [4]{street_address}, Maastricht, Netherlands; [5]Department of clinical pharmacy and pharmacology, University of Groningen, University Medical Center Groningen, Groningen, Netherlands
**Correspondence:** R.A. Posma

*Intensive Care Medicine Experimental* 2019, **7(Suppl 3):**000986

**INTRODUCTION.** Lactate is a strong prognostic marker for outcome of critically ill patients.[1] Metformin-associated lactic acidosis is a rare but severe complication of metformin therapy. It is unknown whether the prognostic value of lactate is different among critically ill metformin users, because metformin may cause elevated lactate levels in itself.[2]
**OBJECTIVES.** To determine whether the association between early lactate level and mortality during critical illness is different in metformin users compared to metformin nonusers, we conducted a registry-based cohort study of patients admitted to an intensive care unit (ICU) with available arterial or venous lactate levels and extensive data on preadmission drug prescriptions.
**METHODS.** The cohort study included patients admitted to ICUs in northern Denmark between 2010 and 2017 with any lactate measured during the period from 12 hours before until 6 hours after ICU admission. The association between mean lactate in this period and 30-day mortality was determined for metformin users and nonusers by estimating hazard ratios (HR) with 95% confidence interval (95%CI) using Cox regression analysis. The magnitude of effect measure modification was assessed by calculating the relative excess risk due to interaction (RERI).[3]
**RESULTS.** Of 37,293 included patients, 3831 (9%) used metformin at the time of ICU admission. Median (interquartile range) lactate level was 1.6 (1.0-2.7) mmol/L in metformin nonusers and 1.8 (1.2-3.2) mmol/L in metformin users, respectively. Compared to metformin nonusers with a lactate level of <1.3 mmol/L, metformin users within the same lactate category had no increased risk to die (HR 0.94, 95%CI 0.78-1.14). Compared to nonusers with a lactate level of <1.3 mmol/L, metformin nonusers with lactate level between 1.3 and 2.0 mmol/L had increased mortality (HR 1.46, 95%CI 1.36-1.57), while this association was less pronounced in metformin users (HR 1.16, 95%CI 0.97-1.39, RERI -0.24, 95%CI -0.52 to 0.03). Higher lactate categories were strongly associated with mortality, and the magnitude of effect measure modification by metformin use increased accordingly.
**CONCLUSION.** In this large observational cohort of critically ill patients, increasing lactate levels were strongly associated with mortality. However, this association was only present at higher lactate levels for metformin users compared to nonusers, indicating that the prognostic value of lactate was modified by metformin use. Therefore, elevated lactate levels should be interpreted differently in these patients.

**REFERENCE(S)**

1. Alkozai et al. Systematic comparison of routine laboratory measurements with in-hospital mortality: ICU-Labome, a large cohort study of critically ill patients. Clin Chem Lab Med, 2018.
2. Lalau et al. Metformin and other antidiabetic agents in renal failure patients. Kidney Int., 2015.
3. Knol and VanderWeele, Recommendations for presenting analyses of effect modification and interaction. Int J Epidemiol, 2012.
4. Aarhus University Research Fund

### 001038

#### The Partitioned pH Model: the new mathematical model to evaluate acid-base status

B. Gucyetmez[1], U. Sezerman,[2], K. Gucyetmez,[3], L. Telci[4]
[1]Department of anesthesiology and reanimation, Acıbadem Mehmet Ali Aydınlar University, School of Medicine, Istanbul, Turkey; [2]Department of biostatistics, Acıbadem Mehmet Ali Aydınlar University, Istanbul, Turkey; [3]-, American Robert College of Istanbul, Istanbul, Turkey; [4]General intensive care, Acıbadem International Hospital, Istanbul, Turkey
**Correspondence:** B. Gucyetmez

*Intensive Care Medicine Experimental* 2019, **7(Suppl 3):**001038

**INTRODUCTION.** Acid-base status is related to the changes in hydrogen ion concentration ([H+]), and it is affected by three independent variables: carbon dioxide, strong ion difference and weak acids (1,2). Recently, unmeasured anions (UAs), which are determined by calculating strong ion gap (SIG), have also been added as the 4th independent variable (3). Additionally, they have been mathematically linked to the total pH (pHtot) (4). However, the effects of independent variables on pHtot changes have not been made a current issue until now.
**OBJECTIVES.** We hypothesize that there are nine independent variables which directly effect pHtot and the sum of the effects of each of them on [H+] is equal to the pHtot's deviation from 7.40.
**METHODS.** The pHtot formula was revised by using 8 independent variables instead of HCO3;

$$\mathrm{pHtot} \;=\; 6.1+\frac{(\mathrm{Na\text{-}Cl})+\mathrm{K\,Ca}+\mathrm{Mg\text{-}Lactate\text{-}[Alb]\text{-}[Pi]\text{-}UA}}{0.03\times \mathrm{CO2}}$$

Delta-pHtot (ΔpHtot) was defined as the pHtot's deviation from 7.40. The normal values for Na-Cl difference and albumin were accepted as 32mmol/L and 42g/L (5). The midpoints of current normal limits were accepted as normal for K, Ca, Mg, lactate and inorganic phosphorus (Pi) (4, 1.22, 0.86, 1.1, 1.2 mmol/L respectively) whereas UAs' normal value was accepted as zero. According to the pHtot formula, the deviations from 7.40 caused by every unit change in an independent variable were named as its ΔpH while other independent variables were normal. Every ΔpH formula was obtained from correlation graphs between independent variables' deviations and ΔpHs by using Pearson correlation test. This newly created mathematical model was named as 'The Partitioned pH Model' and used in this prospective observational study.

$$\begin{aligned}\Delta\mathrm{pHtot} &= \Delta\mathrm{pHCO2}+\Delta\mathrm{pH(Na\text{-}Cl)}+\Delta\mathrm{pHK}+\Delta\mathrm{pHCa}\\ &\quad+\Delta\mathrm{pHMg}+\Delta\mathrm{pHLactate}+\Delta\mathrm{pH[Alb]}+\Delta\mathrm{pH[Pi]}\\ &\quad+\Delta\mathrm{pHUA}\end{aligned}$$

All patients over 18 years old who were admitted to ICU were included. Demographic data, blood gas parameters, and calculated ΔpHs were recorded. The multivariate linear regression model was used to detect pHtotal's independent variables. UAs values of patients were calculated with two different ways: SIG and ΔpHUA. To calculate ΔpHUA, the sum of all independent variables' ΔpHs was subtracted from ΔpHtot. Pearson correlation test was used for the correlation between ΔpHUA and SIG.
**RESULTS.** One hundred and twenty blood gas samples of thirty-one patients were evaluated. In the multivariate linear regression model, 1mmol/L increase in Na-Cl difference, K, Ca and Mg caused 0.015, 0.018, 0.023, 0.025 increase in pHtot, whereas 1mmHg increase in carbon dioxide and 1g/L increase in albumin and 1mmol/L increase in lactate, Pi and SIG caused 0.01, 0.004, 0.017, 0.032, 0.015 decrease in pHtot respectively ($p<0.05$ for all). ΔpHUA was significantly correlated with SIG ($p<0.001$ $R2=0.95$).
**CONCLUSION.** The partitioned pH model allows us to discuss the acid-base status through [H+] changes by separately determining effects of nine independent variables on ΔpHtot. Hence, the usage of this model leads us to clearly identify all metabolic components together with carbon dioxide in critically ill patients.

**REFERENCE(S)**

1. Andersen OS. The Acid-Base Status of the Blood. 4th Edition, 1976, Copenhagen, Denmark.
2. Stewart PA. Modern quantitative acid-base chemistry. Can J Physiol Pharmacol. 1983; 61:1444-63.
3. Kellum JA. Stewart's Textbook of Acid-Base.
4. Schück O. Relation between pH and the strong ion difference (SID) in body fluids. Biomed Papers. 2005; 149:69-73.
5. O'Dell E. Validation of a method to partition the base deficit in meningococcal sepsis: a retrospective study. Crit Care. 2005; 9:R464-470.

### 001046

#### Liver protein synthesis in severe liver failure

M. Amouzandeh[1], W. S.[2], J. A.[1], W. J.[3], R. O.[3], Å. Norberg[1]
[1]Department of perioperative medicine and intensive care, Karolinska Universitetssjukhuset Huddinge, Stockholm, Sweden; [2]Department of upper gastroenterology, division of hepatology,, Karolinska Universitetssjukhuset Huddinge, Stockholm, Sweden; [3]Department of clinical science intervention and technology (clintec), Karolinska Institute, Stockholm, Sweden
**Correspondence:** M. Amouzandeh

*Intensive Care Medicine Experimental* 2019, **7(Suppl 3):**001046

**INTRODUCTION.** Severe liver failure is often accompanied by low plasma albumin (P-alb). This might in part be caused by poor synthesis capacity of the liver, but the relation to other indices of compromised liver function is sparsely investigated in literature.1,2
**OBJECTIVES.** To study the absolute synthesis rates of albumin and fibrinogen in patients with advanced liver cirrhosis, Child-Pugh scores B and C. To correlate these synthesis rates to Child-Pugh and MELD scores, and to describe albumin pharmacokinetic parameters of distribution.
**METHODS.** Patients (n=28) were studied while under evaluation for liver transplantation. Synthesis rates of albumin and fibrinogen, were estimated by the flooding technique using deuterium labeled phenylalanine. Plasma volume and transcapillary escape rate of albumin was assessed by radio iodinated (125I) human serum albumin.
**RESULTS.** Albumin absolute synthesis rate was 65 (30-203) mg/kg/day and was negatively correlated to Child-Pugh (rs = -0.60, p = 0.0008) and MELD scores (rs = -0.62, p = 0.0005). Fibrinogen absolute synthesis rate was 12.8 (2.4-52.9) mg/kg/day and correlated negatively to both Child-Pugh score (rs = -0.47, p = 0.013) and MELD (rs = -0.48, p = 0.010). Spearman rank correlation (rs) between these rates was 0.717 ($p<0.0001$). Transcapillary escape rate was 6.2 ± 2.1 % per hour, and plasma volume was 4.3 ± 0.7 L. With a P-alb of 26.5 ± 4.5 g/L, the corresponding intravascular albumin mass amounted to 113 ± 29 g.
**CONCLUSION.** Worsened liver function was related to lower synthesis of both albumin and fibrinogen. P-alb was low, but, because plasma volumes were increased compared to anthropometric values i.e. an increased volume of distribution, the total intravascular albumin mass was only slightly lower than normal values.

**REFERENCE(S)**

1) Ballmer PE et al. Hepatology 1993; 18: 292-7.
2) Ballmer PE et al. Hepatology 1996; 24: 53-9.
3) County Council of Stockholm

### 001104

#### Factors associated with discrepancy between prescribed and administered enteral nutrition in general ICU

J. Nurkkala
University of Oulu, Oulu, Finland
*Intensive Care Medicine Experimental* 2019, **7(Suppl 3):**001104

**INTRODUCTION.** Enteral nutrition (EN) is reported to have several advantages over parenteral nutrition (PN) in critically ill patients[1,2]. Benefits of EN compared to PN include shorter length of stay (LOS), improved bowel function and lower incidence of infectious complications[1,2]. However, the results of previous studies suggest that there is a gap between prescribed and administered EN [3-5].

**OBJECTIVES.** The aim of this study was to find out the success rate and factors associated with inadequacy of EN delivery in mixed general intensive care unit (ICU).

**METHODS.** A retrospective single center study of 892 patients with ICU LOS ≥4 days. The factors associated with adequacy of enteral nutrition on day 4 were analyzed. These included disease specific factors, patient related factors, severity of illness and procedural factors.

**RESULTS.** Of the 892 patients, 349 (39.1%) had EN success rate of ≥70%, which was associated with lower amount of prescribed enteral energy (500 kcal [500-800] vs 800 kcal [500-1200], P<0.001), bolus administration of enteral nutrition (41 of 349 vs 27 of 543, P<0.001), GI (gastro intestinal) diagnosis OR (1.83 (1.15-2.91), P=0.01), severe inflammation (CRP≥100) OR (1.42 (1.08-1.88), P=0.01) and surgery on d4 OR (2.31 (1.12-4.78), P=0.02).

**CONCLUSION.** In this study we found that in fourth day during ICU stay discrepancy between prescribed and administered enteral nutrition was associated to severe inflammation, GI-related diagnosis, surgical procedures on day 4 and prescription policy. In our study the patients receiving bolus EN had higher caloric intake and it was closer to prescribed amount of administered enteral calories compared to those receiving continuous EN infusion.

**REFERENCE(S)**

1. [1] McClave SA, Taylor BE, Martindale RG, Warren MM, Johnson DR, Braunschweig C, et al. Guidelines for the Provision and Assessment of Nutrition Support Therapy in the Adult Critically Ill Patient: Society of Critical Care Medicine (SCCM) and American Society for Parenteral and Enteral Nutrition (A.S.P.E.N.). JPEN J.Parenter.Enteral Nutr. 2016 Feb;40(2):159-211.
2. [2] Singer P, Blaser AR, Berger MM, Alhazzani W, Calder PC, Casaer MP, et al. ESPEN guideline on clinical nutrition in the intensive care unit. Clin.Nutr. 2019 Feb;38(1):48-79.
3. [3] Cahill NE, Dhaliwal R, Day AG, Jiang X, Heyland DK. Nutrition therapy in the critical care setting: what is "best achievable" practice? An international multicenter observational study. Crit.Care Med. 2010 Feb;38(2):395-401.
4. [4] Heyland DK, Cahill NE, Dhaliwal R, Sun X, Day AG, McClave SA. Impact of enteral feeding protocols on enteral nutrition delivery: results of a multicenter observational study. JPEN J.Parenter.Enteral Nutr. 2010 Nov-Dec;34(6):675-84.
5. [5] Ramakrishnan N, Daphnee DK, Ranganathan L, Bhuvaneshwari S. Critical care 24 x 7: But, why is critical nutrition interrupted? Indian.J.Crit.Care.Med. 2014 Mar;18(3):144-8.
6. Application for travel and participating costs for ESICM LIVES 2019 congress has been send to the Finnish medical association (FMA).

### 001164

#### Effect of malnutrition scoring systems on intensive care unit outcomes

K. Inci[1], BS. Kalın[1], E. Macit Aydın[2], HN. Karaaslan[2], L. Karabıyık[2], B. Nazlıel[3], G. Aygencel[1], M. Ibiş[4], O. Yüksel[5], H. Altun[6], S. ÜNal[6], M. Türkoğlu[1]
[1]Department of internal medicine, division of intensive care medicine, Gazi University Faculty of Medicine, Ankara, Turkey; [2]Department of anesthesiology and reanimation, division of intensive care medicine, Gazi University Faculty of Medicine, Ankara, Turkey; [3]Department of neurology, Gazi University Faculty of Medicine, Ankara, Turkey; [4]Department of internal medicine, division of gastroenterology, Gazi University Faculty of Medicine, Ankara, Turkey; [5]Department of general surgery, Gazi University Faculty of Medicine, Ankara, Turkey; [6]Nutritional support team, Gazi University Faculty of Medicine, Ankara, Turkey
**Correspondence:** K. Inci
*Intensive Care Medicine Experimental* 2019, **7(Suppl 3):**001164

**INTRODUCTION.** Malnutrition is frequent in intensive care unit (ICU) patients and it is related with higher morbidity and mortality rates, more risk of infectious diseases and higher costs of illness. In this study, we aimed to evaluate the effect of frequently used malnutrition scoring systems on ICU outcome in patients who were consulted to nutritional support unit (NSU) in tertiary ICU's of a university hospital.

**METHODS.** In this study, ICU patients who were consulted to NSU between 2014-2018 in Gazi University Hospital were enrolled. Relationship between rates of reaching nutrition targets and clinical outcomes were evaluated. Nutritional Risk Screening-2002 (NRS-2002) score, Subjective Global Assesment (SGA) score and Mini Nutritional Assessment (MNA) score were used to evaluate the nutritional status. Patients with NRS-2002 score ≥ 3, SGA score < 6, MNA score <11 were accepted as risky for malnutrition. Data about reaching nutrition targets and ICU outcome were evaluated by using univariate and multivariate analysis.

**RESULTS.** 757 ICU patients were evaluated for the study. Median age was 68 [56-80] and 53% of the study patients were male. Detailed baseline characteristics of the patients were shown in Table-1. The most common causes of hospitalization were central nervous system diseases (25%), respiratory system diseases (20%) and malignancies (17%). Frequency of malnutrition risk in study patients was 90% according to NRS-2002 score, 89% according to SGA and 87% according to MNA score. Targeted calorie levels were reached only in 54% of the patients and targeted protein levels were reached only in 27% of the patients. Target calorie level was 1700 kcal/day [1500-1851], maximum delivered calorie was 1470 kcal/day [1240-1729], median delivered calorie was 1002 kcal/day [800-1100] in study patients. Target protein level was 98 gram/day [90-109], maximum delivered protein was 70 gram/day [59-90] median delivered protein was 55 gram/day [32-66]. ICU mortality was 38% in the study group. There was no significant difference in mortality between patients who reached to targeted calorie or protein levels and who did not (p<0.05). When we evaluated the independent risk factors for mortality with the aspect of nutritional parameters, the strongest independent risk factor was being under malnutrition risk according to SGA (OR (95% CI): 3.12 (1.34-7.29), p<0.01) score and others were being enterally fed with a feeding tube (OR (95% CI): 2.57 (1.87-3.51), p<0.01), being under malnutrition risk according to NRS-2002 (OR (95% CI): 1.97 (1.02-3.79), p=0,042), lover admission albumin level (OR (95% CI): 1.88 (1.42-2.48),p<0.05), being followed in medical ICU (OR (95% CI): 1.40 (1.01-1.94), p=0.042), higher admission creatinine level (OR (95% CI): 1.31 (1.16-1.48), p<0.01) and advanced age (OR (95% CI): 1.01 (1.00-1.02), p<0.01).

**CONCLUSION.** Malnutrition risk according to SGA score was the strongest independent risk factor for mortality. However, depending on complexity of ICU patients, there are many factors effecting their outcome. Thus, there may be different results about the effect of reaching nutritional targets alone on outcome and it should be evaluated along with additional parameters like ICU prognostic scoring systems.

### 001194

#### Postoperative euglycemic diabetic ketoacidosis in patients who take sodium-glucose co-uptake receptor2 inhibitors: case series

Y. Iwasaki, J. Shiotsuka, T. Masuyama, Y. Kondo, S. Masamitsu
Intensive care unit, Jichi University Saitama Medical Center, Saitama, Japan
**Correspondence:** Y. Iwasaki
*Intensive Care Medicine Experimental* 2019, **7(Suppl 3):**001194

**INTRODUCTION.** Euglycemic diabetic ketoacidosis (euglycemic DKA) is among side effects of sodium-glucose co-uptake receptor 2 inhibitors (SGLT2 inhibitor). While the incidence of euglycemic DKA associated with SGLT2 inhibitors is reported as approximately 0.1%,[1] the exact incidence of euglycemic DKA is unknown during perioperative periods.

**OBJECTIVES.** The objective of this study is to describe the incidence of diabetic ketoacidosis caused by SGLT2 inhibitors during perioperative periods.

**METHODS.** Patients who received SGLT2 inhibitors within two days prior to elective surgeries between April, 2014 and March, 2019 were included. Patients who underwent cardiovascular surgeries using cardiopulmonary bypass, emergency operations, and those who were on hemodialysis were excluded. All arterial blood gas samples during the postoperative ICU stay were analyzed. Blood ketone bodies were not measured for any of the patients. The primary outcome was the incidence of euglycemic DKA, which was defined with high anion gap metabolic acidosis (arterial blood pH< 7.3, anion gap> 12 mmol/L, and PaCO2< 45 mmHg), without significant hyperglycemia or hyperlactemia (lactate levels < 2.0mmol/L and glucose <14mmol/L).[2]

**RESULTS.** Two hundred and forty arterial blood gas samples from thirty-two patients who received preoperative SGLT2 inhibitors were analyzed. Six patients (18.5%) had at least one blood gas sample suggesting euglycemic DKA. Off-pump coronary artery bypass grafting (n=3), lung resection (n=2), and nephrectomy (n=1) were performed for those six patients, respectively. The minimum arterial blood pH was 7.252 and the maximum anion gap was 24.0 mmol/L.

**CONCLUSION.** Postoperative euglycemic ketoacidosis may be common in patients who take SGLT2 inhibitors preoperatively. Larger studies are needed to investigate the exact incidence of perioperative euglycemic ketoacidosis associated with SGLT2 inhibitors.

##### REFERENCE(S)

1. Effect of Sodium–GlucoseCotransporter2 Inhibitors on Diabetic Ketoacidosis Among Patients With Type 2 Diabetes: A Meta-analysis of Randomized Controlled Trials. Clinical Therapeutics. Clin Ther. 2016 Dec;38(12):2654-2664
2. Joint British Diabetes Societies guideline for the management of diabetic ketoacidosis. Diabet Med 2011; 28: 508–515.
3. Grant acknowledgment: None

### 001204

#### The evaluation of nutritional status in critically ill patients by Bioimpedance vector analysis (BIVA)

Y. Apichatbutr, R. Ratanarat, T. Viarasilpa
[1]Dept of medicine, siriraj hospital, mahidol university, Division of Critical Care, Bangkok, Thailand
**Correspondence:** R. Ratanarat
*Intensive Care Medicine Experimental* 2019, **7(Suppl 3):**001204

**INTRODUCTION.** Nutritional risk and malnutrition are highly prevalent among critically ill patients. Screening and assessment tools used to evaluate nutrition status, composed of subjective data. Low phase angle (PhA) derived by BIVA associated with higher severity of malnutrition defined by SGA and NRS2002 in difference populations, but there was no data in the critical ills.

**OBJECTIVES.** To assess the relationship between nutritional risk tools and PhA derived by BIVA in ICU patients, and the association of clinical outcomes and PhA compared to conventional nutritional risk tools.

**METHODS.** Nutritional risk screenings and PhA (determined by BIVA ) were performed in 64 medical ICU patients (44 male, and 22 female) within the first 24-hrs of admission. Sensitivity and specificity were calculated for PhA compared to degrees of malnutrition stratified by SGA, NRS2002, and NAF. The cut-off values for severe malnutrition risk were assessed by receiver operator characteristics area under the curve (AUC).

**RESULTS.** PhA was lower in more severe malnutrition risk classified by SGA, NAF, and NRS 2002 (p<0.001). PhA of less than 5.8 correlated with severe malnutrition risk defined by SGA [AUC 0.90, sensitivity 92% (95%CI:77-98) and specificity 78% (95%CI: 58-92)]; NAF [AUC 0.90, sensitivity 78% (95%CI:87-100) and specificity 92% (95%CI: 79-97)]; NRS2002 [AUC 0.93, sensitivity 100% (95%CI:16-100) and specificity 66% (95%CI: 53-78)]. The patients with PhA of less than 5.8 have higher mortality, prolonged ICU and hospital length of stay but not statistical significance.

**CONCLUSION.** PhA has excellent correlation with SGA, NAF, and NRS2002. It is a useful measure for assessment the patients at risk for impaired nutritional status in critically ill patients. PhA below 5.8 is cut - off point for severe malnutrition in our ICU patients.

### 001262

#### Time-course of Glucose and Insuline Resistance in 382 patients after Minor, Intermediate and Major Liver Resection

Y. van de Riet[1], R. Steenstra[2], M. Nijsten[1], M. Zeillemaker-Hoekstra[3]
[1]Department of critical care, University Medical Center Groningen, Groningen, Netherlands; [2]Department of anesthesiology, Medisch Centrum Leeuwarden , Leeuwarden, Netherlands; [3]Department of anesthesiology, University Medical Center Groningen, Groningen, Netherlands
**Correspondence:** Y. van de Riet
*Intensive Care Medicine Experimental* 2019, **7(Suppl 3):**001262

**INTRODUCTION.** Post-operative liver failure (PLF) remains a feared complication after liver resection. Related to its central role in glucose metabolism, changes in glucose metabolism can be an early sign of liver failure. The effect of the size of the functional liver remnant on the early time courses of glucose and insulin requirements are unknown.

**OBJECTIVES.** To determine the early changes in glucose levels (Glu(t)), and insulin requirements (Ins(t)) after minor, intermediate and major liver resection. A secondary objective was to compare those early changes between patients with and without PLF.

**METHODS.** This is a single centre retrospective cohort (2008-2014) of all consecutive patients admitted to the intensive care unit (ICU) after liver resections. In total 382 patients were included. Patients were classified as “minor” (n=72), “intermediate” (n=183) or “major” (n=127) liver resection based on a functional liver remnant (FLR) of >60%, 35 - 60% and <35% respectively. Patients with initial portal vein embolization were excluded. Glucose was targeted at 4 to 8 mmol/L by a computerized nurse-centered algorithm. Insulin was delivered by continuous infusion. Insulin resistance, IR(t) was estimated by Glu(t)·(Ins(t)+1) mmol·IU·L-1·h-1. PLF was defined by the ‘50/50 criteria’ [1].

**RESULTS.** Glucose levels were significantly higher in the patient group with minor liver resections (P<0.05) from ICU admission up to 5h later. After 15h the opposite occurred when the patients with the largest resection had the highest glucose levels (P<0.05). Insuline resistance (IR) was initially lowest in patients with the largest resection (P<0.05) but 18h after ICU admission IR became lowest in patients with the smallest resection (P<0.05). Post-operative liver failure was present in N=27 (7%). For the first 12h the glucose values (P<0.0001) and IR (P<0.0001) both correlated with PLF.

**CONCLUSION.** Glycometabolic changes as measured by glucose levels and insulin requirements are significantly different depending on the size of liver resection and the post-operative time. Persistent normal-low glucose levels and low insulin requirements during the first day after major liver resection could be an early indication of PLF. With regard to these findings there might be a role for protective strategies to support the liver in its early postoperative state of energy crisis and thereby improving its capacity to regenerate and restore its functions.

### 001266

#### Variable Rate Insulin therapy in Critically Ill Patients. Too Much or Too Little?

MK. Lim[1], L. Malanjum[2]
[1]Northampton General Hospital, Northampton, UK; [2]Itu, Northampton General Hospital, Northampton, UK
**Correspondence:** M.K. Lim
*Intensive Care Medicine Experimental* 2019, **7(Suppl 3):**001266

**INTRODUCTION.** Hyperglycaemia in the critically unwell is oftentimes due to a stress response and can be compounded by their existing insulin resistance. Hyperglycaemia is associated with poorer clinical outcomes but not necessarily causes it. Furthermore strict control may be more detrimental and put patients at risk of hypoglycaemia causing seizures, arrhythmias or death. For example, the COITTSS trial showed no difference between intensive and liberal insulin therapy. In addition, the NICE SUGAR trial showed a higher mortality rate and evidence of hypoglycaemia with intensive insulin therapy. A specific variable rate insulin infusion (VRII) guideline for our ITU is needed as the patients differ from the other ward patients.

**OBJECTIVES.** The primary objective was to compare our current clinical practice against the local unit guidance. Secondly we will use the results as a baseline for comparison after implementing a new critical care specific VRII guideline to determine if a quality improvement has been made.

**METHODS.** This was an observational study with a retrospective review of ITU charts.We studied adult patients >16 years old who had VRII started whilst being an ITU inpatient from Nov 2018-Jan 2019. We excluded patients on the DKA/HHS protocol, peri-op patients on 'sliding scales' or those with overly complicated glucose control eg necrotising pancreatitis.

**RESULTS.** 20 patients were included with a median age of 68 (38-78yo) and average of 4 days of VRII (1-10days). These patients had VRII when their CBG was >10mmol/L although there was a wide inconsistency of number of insulin units started; with only 7 patients (35%) having the correct units/hr as per guidelines. Only 15% of patients had their hourly CBG check for 4 hours after VRII was commenced. 60% of patients did not have the recommended CBG check an hour after a rate change. There was a 15% incidence rate (3 patients) of hypoglycaemia. Overall, we made a rough estimate of to check patients CBGs were in range of 6-10mmol/L by dividing the number of times their CBGs were 6-10 over total number of CBG taken whilst on VRII. The result was a mean of 68.6% and median of 72.1%. In addition, there was a T1DM who did not receive their regular long acting insulin and another T2DM who received it after 3 days of VRII.

**CONCLUSION.** The lack of CBG checks and absence of a clear guideline could be a strong contributing factor to poor control. We also expressed strong interest in wanting to know the events leading to their hypoglycaemia. How low were their CBGs? Were they eating, on NG feed or TPN? If so were they stopped at any point? Were they having intravenous fluids or glucose? How was the hypoglycaemia managed and how quickly did it resolve? These points will be more closely looked at during the subsequent audit after a few months. We also recommended HbA1C and ketone testing as well as diabetic team referral when appropriate. New hyperglycaemia guidelines will be disseminated to medical and nursing staff via various methods and reaudited soon. Similar VRII audits of other ITUs can also shed more light on how we treat hyperglycaemia around the country.

**REFERENCE(S)**

1. Corticosteroid Treatment and Intensive Insulin Therapy for Septic Shock in Adults. (2010). JAMA, 303(4), p.341.
2. Tanner, O. (2009). Intensive versus Conventional Glucose Control in Critically Ill Patients. Journal of the Intensive Care Society, 10(3), pp.216-217
3. Nil

### 001338

#### Early Hyperbilirubinemia in Critically-Ill Patients

J. Juschten[1], PR. Tuinman[1], LDJ. Bos[2], NP. Juffermans[3], A. Girbes, MJM. Bonten[4], OL. Cremer[5], T. Van Der Poll[6], MJ. Schultz[7]
[1]Intensive care, Vrije Universiteit Amsterdam, Amsterdam, Netherlands; [2]Pulmonology, Academic Medical Centre, Amsterdam, Netherlands; [3]Intensive care, Academic Medical Centre, Amsterdam, Netherlands; [4]Medical microbiology, University Medical Center Utrecht, Heidelberglaan, Utrecht, Netherlands, Utrecht, Netherlands; [5]Intensive care, University Medical Center Utrecht, Heidelberglaan, Utrecht, Netherlands, Utrecht, Netherlands; [6]Center for experimental and molecular medicine, Academic Medical Centre, Amsterdam, Netherlands; [7]Intensive care, Mahidol Oxford Tropical Medicine Research Unit, Bangkok, Thailand
**Correspondence:** J. Juschten
*Intensive Care Medicine Experimental* 2019, **7(Suppl 3):**001338

**INTRODUCTION.** Hyperbilirubinemia, defined as bilirubin levels ≥ 33 mmol/L, is present in 11–33% of all critically-ill patients and associated with increased mortality and a greater length of stay in the Intensive Care Unit (ICU). (1-3) However, studies investigating hyperbilirubinemia as an independent predictor of outcome have yielded conflicting results. (2-4)

**OBJECTIVES.** This study aims to investigate the association between early hyperbilirubinemia and 30-day-mortality in a large prospectively collected cohort in two tertiary ICU's in the Netherlands.

**METHODS.** This is an unplanned secondary analysis of the 'Molecular Diagnosis and Risk Stratification for Sepsis' (MARS) biorepository. Patients with measured bilirubin levels within two days after admission were included, except for patients with liver cirrhosis. Multivariable logistic regression modeling was used to determine important characteristics associated with 30-day-mortality and to estimate the impact of early hyperbilirubinemia.

**RESULTS.** Early hyperbilirubinemia was present in 559 (11.9%) of 4836 patients. These patients demonstrated higher rates of organ failure and mortality compared to patients without hyperbilirubinemia ($p < 0.001$). Multivariable logistic regression modelling revealed thrombocytopenia to be an effect modifier for the association between early hyperbilirubinemia and 30-day-mortality ($p = 0.005$). An independent association between early hyperbilirubinemia and 30-day-mortality was found in patients with a low platelet count ($p < 0.001$; Table 1), but not in patients with a normal platelet count ($p = 0.655$; Table 2).

**CONCLUSION.** In this large prospective cohort study, 11.9% of all ICU patients presented with early hyperbilirubinemia. We found an independent association between early hyperbilirubinemia and 30-day-mortality in patients with thrombocytopenia suggesting that this may possibly be feature of intravascular hemolysis, dyserythropoiesis or thrombosis in critically ill patients.

**REFERENCE(S)**

(1) Intensive care medicine. 2006;32(2):267-74.
(2) Critical care medicine. 2007;35(4):1099-104.
(3) Injury. 2017;48(1):127-32.
(4) Intensive care medicine. 2009;35(8):1406-11.

**Table 1 (abstract 001338).** Association between early hyperbilirubinemia and 30-day-mortality in patients with a low platelet count (< 150 x 109/l) (N = 1780)

| Effect | Estimate | OR (95% CI) | p-value |
|---|---|---|---|
| Crude analysis | 0.708 | 2.03 (1.60–2.58) | < 0.001 |
| Corrected for sepsis | 0.622 | 1.86 (1.45–2.38) | < 0.001 |
| Corrected for sepsis, acute renal failure and use of vasoactive medication | 0.441 | 1.56 (1.21–2.00) | < 0.001 |

**Table 2 (abstract 00138).** Association between early hyperbilirubinemia and 30-day-mortality in patients with a normal platelet count (≥ 150 x 109 / L) (N = 3014)

| Effect | Estimate | OR (95% CI) | p-value |
|---|---|---|---|
| Crude analysis | 0.084 | 1.09 (0.75–1.55) | 0.655 |

## Milan Datathon winning abstract presentations

### 000055

#### Prediction of the need for blood trasfusion in patients with GI bleeding

F. Carli[1], MM. Naldini[2], R. Levi[3], F. Grassi[4], F. Betti[3], A. Zanoni[5], N. Gozzi[3], M. Salvador[5], A. Faglia[3], D. Stein[6], Y. Altinel[7], A. Robles Arévalo[8], A. Kras[9], M. Garbulinska[10], R. Barbieri,[3], LA. Celi[11]

[1]Esomas, Università degli Studi di Torino, Torino, Italy; [2]UNISR, San Raffaele Tiget, Milano, Italy; [3]Dipartimento di elettronica, informazione e bioingegneria, Politecnico di Milano, Milan, Italy; [4]Anestesia, rianimazione e terapia del dolore, Universitá degli Studi Milano Bicocca, Mantova, Italy; [5]Dipartimento di Matematica, Politecnico di Milano, Milano, Italy; [6]Division of gastroenterology, Beth Israel Deaconess Medical Center, Boston, USA; [7]Gastrointestinal surgery, Harvard Medical School, Boston, USA; [8]Laboratory of Computational Physiology, Massachusetts Institute of Technology, Cambridge, USA; [9]Biomedical informatics, Harvard Medical School, Boston, USA; [10]Biostatistics, Harvard T.H. Chan School of Public Healt, Boston, USA; [11]Pulmonary, critical care and sleep medicine, Beth Israel Deaconess Medical Center (BIDMC), Boston, USA

**Correspondence:** R. Levi

*Intensive Care Medicine Experimental* 2019, **7(Suppl 3):**000055

**INTRODUCTION.** Gastro-intestinal bleeding is a common event which may be so severe to require hospitalization within an intensive care unit (ICU). Management of patients with an active bleeding depends on clinical examination and laboratory tests to predict the likelihood of self resolution, persisting or new episodes of bleeding. Identification of high risk patients is often challenging because changes in clinical signs and tests are delayed. Often the attending physician's own experience and practice within the institute contribute more to the final decision than evidence based data.

**OBJECTIVES.** We sought to develop an artificial intelligence algorithm aimed at predicting high risk patients for active GI bleeding or successive re-bleeding. Because the outcome we try to predict (GI bleeding) was not recorded, blood transfusion was used as a surrogate. We propose a clinically translatable model trained on a fixed time window containing the data gathered during the first hours of a patient's ICU stay able to predict the likelihood of persisting or progressing GI bleeding within the subsequent 24 hours.

**METHODS.** We exploited the publicly available MIMICIII database to extract parameters and laboratory values of interest for the first ICU stay per hospital admission of adult non-pregnant patients with diagnosed GI bleeding. Extracted parameters were filtered first by clinically driven hypothesis of relevance and subsequently by data availability. We attempted to include in our analysis the maximum number of observations (patients) available with the lowest missing features, resulting in 48 clinical, anamnestic and laboratory parameters across 2925 patient ICU stays. Patient observations gathered within the first 3 to 6 hours of ICU admission were fed into two machine learning models (Bagged logistic regression and Random Forest) in order to predict if the patient would subsequently receive a blood transfusion. The insights of the two models are then combined together through a weighted average of the predicted probabilities. All the hyperpameters of our pipeline are fixed through a model based bayesian optimization procedure and cross-validation.

**RESULTS.** Results obtained were evaluated based on model accuracy (number of correct assessments over number of all assessments), AUC ROC, recall (sensitivity) and precision (true positive rate) across different time windows of data gathering and prediction time interval as detailed in table 2. Our best performing model could successfully forecast 76.09% of patients who would then receive or not a blood transfusion within 24 hours from data gathered within the first 3 hours of ICU stay.

**CONCLUSION.** While our model could be refined and optimized to incorporate also temporal patterns within the dataset, we demonstrate a comprehensive application of machine learning models to address clinically relevant open questions from a publicly available database.

**REFERENCE(S)**

1. Johnson, Alistair EW, Tom J Pollard, Lu Shen, H Lehman Li-wei, Mengling Feng, Mohammad Ghassemi, Benjamin Moody, Peter Szolovits, Leo Anthony Celi, and Roger G Mark. 2016. "MIMIC- Iii, a Freely Accessible Critical Care Database." Scientific Data 3. Nature Publishing Group: 160035
2. Das, Ananya, Tamir Ben–Menachem, Farees T Farooq, Gregory S Cooper, Amitabh Chak, Michael V Sivak Jr, and Richard CK Wong. 2008. "Artificial Neural Network as a Predictive Instrument in Patients with Acute Nonvariceal Upper Gastrointestinal Hemorrhage." Gastroenterology 134 (1). Elsevier: 65–74
3. Cismondi, Federico, Leo A Celi, André S Fialho, Susana M Vieira, Shane R Reti, Joao MC Sousa, and Stan N Finkelstein. 2013. "Reducing Unnecessary Lab Testing in the Icu with Artificial Intelligence." International Journal of Medical Informatics 82 (5). Elsevier: 345–58
4. Chu, Adrienne, Hongshik Ahn, Bhawna Halwan, Bruce Kalmin, Everson LA Artifon, Alan Barkun, Michail G Lagoudakis, and Atul Kumar. 2008. "A Decision Support System to Facilitate Manage- ment of Patients with Acute Gastrointestinal Bleeding." Artificial Intelligence in Medicine 42 (3). Elsevier: 247–59

**Table 1 (abstract 000055).** Voting classifier: integration of logistic regression & random forest

| Time interval for data collection : | Time interval of prediction of blood transfusion : | Accuracy | AUC ROC | Recall | Precision |
|---|---|---|---|---|---|
| 0-3 hours | 4-24 hours | 0,7609 | 0,8212 | 0,7821 | 0,7688 |
| 0-3 hours | 8-24 hours | 0,7070 | 0,7659 | 0,6493 | 0,6695 |
| 0-6 hours | 8-24 hours | 0,7076 | 0,7787 | 0,6224 | 0,6466 |
| 0-8 hours | 8-24 hours | 0,7117 | 0,7898 | 0,6352 | 0,6448 |

## NIC - The brain facing acute injury

### 000204

#### Initial blood pressure as risk factor and prognostic predictor in aneurysmal subarachnoid haemorrhage

L. Galarza Barrachina, I. Catalán Monzón, L. Mateu Campos, B. Vidal Tegedor, E. Rodríguez Martínez, MD. Ferrándiz Sellés

[1]Intensive care unit, Hospital General Universitario de Castellón, Castellón de la Plana, Spain

**Correspondence:** L. Galarza Barrachina

*Intensive Care Medicine Experimental* 2019, **7(Suppl 3):**000204

**INTRODUCTION.** Hypertension has long been considered an important risk factor for aneurysm rupture. Some studies had assessed the impact of premorbid hypertension treatment on clinical course, but the data in the literature assessing the effect of blood pressure on admission is scarce.

**OBJECTIVES.** Investigate the impact of the blood pressure on the first medical contact on clinical course and outcome of patients with aneurysmal subarachnoid haemorrhage (aSAH).

**METHODS.** We retrospectively reviewed clinical data of patients with aSAH admitted to our institution from January 2014 to July 2018. Based on the blood pressure, all patients were divided into hypertensive group (BP ≥ 140/90mmHg) and non-hypertensive group (BP <140/90mmHg). Patient characteristics, imaging features, clinical complication, and outcome were analysed between the two groups using STATA 14.0.

**RESULTS.** A total of 79 patients were included in this study. 67% were women with a mean age 56.84 (SD 1.58), 53.6% were premorbid hypertension. 64.6% of aSAH patients were on the hypertensive group. There was no difference between age, sex or premorbid hypertension between both groups. Fisher grade 4 was 64.29% vs 46.43%, p 0.25 and Hunt Hess 4 or 5 was 46.43% vs 37.25%, p 0.43, for non-hypertensive vs hypertensive group. Neurological complications were vasospasm (21.34% vs 23.53%, p 0.83), hydrocephalus (21.34% vs 23.53%, p 0.83) and rebleeding (7.14% vs 9.8%, p 0.69). Medical complications as cardiac abnormalities (14.29% vs 15.69%, p 0.87) or electrolyte abnormalities (10.73% vs 11.76%, p 0.88). In-hospital mortality was 32.14 vs 31.37% (p 0.94).

Outcome evaluated at 3 month using the Glasgow Outcome Scale (GOS) is described in table 1. Difference was not significant, p 0.30.

Ordinal regression analyses for GOS at 3 months, adjusted by age and Hunt Hess grade 4 or 5, found no association with the exception of Hunt Hess grade 4 or 5 itself (OR 0.06, 0.01- 0.28, p 0.00).

**CONCLUSION.** Non-hypertensive patients had worse grade in the prognostic scales and higher mortality, but no diferences in outcome at 3 months. There was no difference on neurological and medical complications.

**Table 1 (abstract 000204).** See text for description

| | GOS at 3 months | |
|---|---|---|
| | Non hypertensive group | Hypertensive group |
| 1 | 32.14% | 31.37% |
| 2 | - | 7.84% |
| 3 | 7.14% | 7.84% |
| 4 | 32.14% | 15.69% |
| 5 | 28.57% | 37.25% |

### 000267

#### Mechanical ventilation and risk factors associated with neurocognitive disorders in critical care patients

S. Victor[1], J. Gomez[2], G. Paola[2], G. Ernesto[2], R. Tania[3]
[1]Intern medicine faculty, Dr. Jose Matias Delgado University Campus 1, Santa Tecla, El Salvador; [2]General medicine, Dr. Jose Matias Delgado University Campus 1, Antiguo Cuscatlan, El Salvador; [3]Health science faculty, Dr. Jose Matias Delgado University Campus 1, Antiguo Cuscatlan, El Salvador
**Correspondence:** J. Gomez
*Intensive Care Medicine Experimental* 2019, **7(Suppl 3):**000267

**INTRODUCTION.** In patients that need of Mechanical Ventilation (MV) have shown in multiple studies until 80% of neurocognitive disorders as part of the Post Intensive Care Syndrome (PICS).

**OBJECTIVES.** Evaluate the association between the changes of PEEP during MV, cerebral hemodynamic variables and PICS.

**METHODS.** This is a descriptive observational study, conducted in patients with MV, evaluated by TCD and TTE, who are assessed for PICS one month after the discharged from ICU between August 2018 to January 2019. The inclusion criteria involve all patients who underwent MV support and complete PICS battery evaluation. Exclusion criteria were met by any patient who had been previously diagnosed by neurocognitive disorders or was in treatment with psychoactive drugs. Head trauma, evidence of neuroinfectious disease, cardiac arrest, drug dependence or abuse and history of chronic alcoholism were excluded.

**RESULTS.** During the study period, 4 patients met the inclusion criteria of 89 patients admitted to the ICU. They received mechanical ventilation for 7 days on average. All the patients had an increase in the CaO2. However, the DO2 Index remained below normal parameters. In the cerebral hemodynamic variables, it was observed that the ICP calculated was higher than 14.3 mm Hg, while CPP was between 52.5 ml and 60.49 mm Hg respectively. The average SV at the beginning of MV support was 52.5 ml, that later with PEEP change improved. It was also observed that, with the change of PEEP, the patients presented an increase above the normal ranges in the average speeds both MCA (101.3 mm/sec) and the OA (45.23 mm/sec) which could be due to the increase in MAP and CPP. The mean PI of the MCA at the start of ventilator support was high (1.40 mm Hg), which correlates with the transient elevated PIC. All the 4 patients presented at least one neuropsychological disorder. Only one patient presented CAM-ICU. The MoCa test was positive in 3 out of 4 patients. In respect to sleep disorders, 3 of the 4 patients presented alterations in the subscales of insomnia and hyper insomnia.

**CONCLUSION.** These patients had prolonged mechanical ventilation with an average of 7 days, in which they maintained a PEEP under 10. They had low levels of DO2 Index and CaO2. In the 30 days after the ICU discharge, all had at least one neuropsychological disorder. Even though there are few cases studied on this research, the context of low O2 contribution to tissues regardless of the MV support with an increased ACM and AO velocities, it is possible to infer as a possible cause of brain damage, causing PICS because of the increase in the PEEP.

**REFERENCE(S)**

1. Schramm P, Closhen D, Felkel M, Berres M, Klein KU, David M, et al. Influence of PEEP on cerebral blood flow and cerebrovascular autoregulation in patients with acute respiratory distress syndrome. J Neurosurg Anesthesiol. April 2013 ;25(2): 162-7. Available: https://www.ncbi.nlm.nih.gov/pubmed/23211642
2. Hopkins RO, Weaver LK, Collingridge D, Parkinson RB, Chan KJ, Orme JF. Two-year cognitive, emotional, and quality-of-life outcomes in acute respiratory distress syndrome. Am J Respir Crit Care Med. February 15, 2005 ;171(4):340-7. Available: https://www.atsjournals.org/doi/full/10.1164/rccm.200406-763OC?url_ver=Z39.88-2003&rfr_id=ori%3Arid%3Acrossref.org&rfr_dat=cr_pub%3Dpubmed
3. Selldén H, Sjövall H, Ricksten SE. Sympathetic nerve activity and central haemodynamics during mechanical ventilation with positive end-expiratory pressure in rats. Acta Physiol Scand. May 1986;127(1):51-60. Available: https://www.ncbi.nlm.nih.gov/pubmed/3524116
4. We would like to give special thanks to HNSR and the Chief of staff of the ICU department, Dr. Manuel Bello.

**Table 1 (abstract 000267).** See text for description

| Parameter | Initial PEEP (3 Cm H2O) | Final PEEP AV (6Cm H2O) |
|---|---|---|
| MAP (mm Hg) | 74.5 | 97.8 |
| ICP calculated (mm Hg) | 14.09 | 9.41 |
| CPP (mm Hg) | 60.41 | 88.39 |
| CI (lt/min/m2) | 2.63 | 2.83 |
| CaO2 (ml/dl) | 14.75 | 15 |
| DO2 Index (ml/min/m2) | 387.25 | 412.5 |
| MCA Mean (cm/s) | 79.98 | 101.3 |
| OA Mean (cm/s) | 30.85 | 45.23 |

### 000414

#### Diagnostic and prognostic importance of purine metabolic disoders in acute brain ischemia

E. Oreshnikov, S. Oreshnikova, A. Oreshnikov
[1]Internal medicine, Chuvash State University named I.N.Ulyanova, Cheboksary, Russia
**Correspondence:** E. Oreshnikov
*Intensive Care Medicine Experimental* 2019, **7(Suppl 3):**000414

**INTRODUCTION.** It has been established that hypoxanthine, xanthine and uric acid (UA) are present in the brain, their content changes after ischemia, UA is the final product of degradation of purines in the brain, xanthine oxidase (XO) is also present in the brain, catalyzes the oxidation of hypoxanthine to xanthine, and then to UA, and can be a source of free radicals, inhibition of XO and exogenous administration of UA are accompanied by explicit anti-ischemicand neuroprotective effects in the experiment, whereas endogenous increased production, with "by-product" synthesis of XO free radicals oxygen reflects the severity of ischemic and reperfusion injury.

**OBJECTIVES.** Our attention was drawn to the comparative evaluation of the prognostic value ofimpairments of purine metabolism.

**METHODS.** We examined 402 patients in the acutest period (1st 7-10 days) of cerebral ischemic stroke at the age of 30-87 years. Patients, in addition to conventional laboratory parameters, determined the blood levels of guanine, hypoxanthine, adenine, xanthine and uricacid by direct spectrophotometry. Evaluated the effects of the conservation of coma and death following the onset of which drugs are widely used in intensive neurology.

**RESULTS.** The most adverse prognostic factors associated with prolongation sopor or coma are: laboratory - hyperuricemia and hyperHYPOXANTHINemia. The most favorable prognostic factor, "countering" the preservation of a coma and the coming of death in cerebral ischemic stroke is the use of antiplatelet agents. Hyperuricemia most researchers viewed as an unfavorable prognostic factor, although intravenous injection of uric acid in acute cerebral ischemia can improve the results of thrombolytic therapy. Prognostic value of supranormal blood hypoxanthine level in contrast to the hyperuricemia has been insufficiently studied.

**CONCLUSION.** Launched from the development of acute cerebral ischemia, dynamic control of the blood levels of uric acid and hypoxanthine to predict its course and outcome.

### 000417

#### Importance of purine metabolites in preeclampsia and acute brain stroke

E. Oreshnikov[1], S. Oreshnikova[1], A. Oreshnikov[1], E. Vasiljeva[2], T. Denisova[2]
[1]Internal medicine, Chuvash State University named I.N.Ulyanova, Cheboksary, Russia; [2]Obstetrics and gynecology, Chuvash State University named I.N.Ulyanova, Cheboksary, Russia
**Correspondence:** E. Oreshnikov
*Intensive Care Medicine Experimental* 2019, **7(Suppl 3):**000417

**INTRODUCTION.** Along with the classic triad: edema, proteinuria, hypertension, many clinicians as an indicator of preeclampsia using the high content of uric acid in blood serum hyperuricemia. It was also found that the hypoxanthine, xanthine and uric acid (UA) are present in the brain, and their content is changed after ischemia, UA is the endproduct of purine degradation in the brain,xanthine oxidase is also present in the brain, it catalyzes the oxidation of hypoxanthine to xanthine, and then in UA and can be a source of free radicals, inhibition of xanthine oxidase and exogenous administration of UA accompanied by explicit antiischemic and neuroprotective effects in the experiment and clinic, while the endogenous increased its production, with the"side" synthesis of xanthine oxidase oxygen free radicals, reflects the severity of ischemic andreperfusion injury. We also know that most fatal path of pathogenesis (and tanatogenesis) in preeclampsia is the development of brain stroke.

**OBJECTIVES.** Our attention was attracted by a comparative assessment of the features of purine metabolism in women with preeclampsia and acute brain stroke

**METHODS.** The study involved 33 women with preeclampsia and 350 women in acute period of cerebral stroke, in which, in addition to conventional laboratory parameters were determined in the blood and cerebrospinal fluid - guanine, hypoxanthine, adenine, xanthine and uric acid by direct spectrophotometry.

**RESULTS.** It was established that between preeclampsia and cerebral stroke there are clinical and pathobiochemical parallels, including according to the characteristics of purine metabolism. Hyperuricemia the most famous and at the same time the most pronounced adverse metabolic factor (marker or predictor) for preeclampsia, and for cerebral stroke. High value level of oxypurines (hypoxanthine, xanthine and uric acid) in the cerebrospinal fluid is good sign for a stroke, and low value level of oxypurines is good sign for preeclampsia.

**CONCLUSION.** Cerebrospinal liquor can be seen not only as a medium of administration of drugs for spinal anesthesia, but also and a source of valuable diagnostic (and predictive) information.

### 000566

#### Analysis of refractory hyponatremia in patients with neurologic critical disease

J. Zhang, C. Qi
[1]Neurology, The Chinese Peoples' Liberation Army General Hospital, Beijing, China
**Correspondence:** J. Zhang
*Intensive Care Medicine Experimental* 2019, **7(Suppl 3):**000566

**INTRODUCTION.** Refractory hyponatremia is intimately related to condition changes of patients in NICU.

**OBJECTIVES.** This study was conducted to explore the causes of refractory hyponatremia in patients with neurologic critical disease. In order to further improve the level of diagnosis and treatment of hyponatremia.

**METHODS.** A retrospective analysis was including 22 patients with refractory hyponatremia in the Chinese Peoples' Liberation Army General Hospital from June 2010 to June 2018. Hyponatremia was divided into mild, moderate and severe types according to serum sodium level (mild hyponatremia: 130mmol/L$\leq$ Na+<135mmol/L; moderate hyponatremia: 125mmol/L$\leq$ Na+<130mmol/L; and severe hyponatremia: Na+<125mmol/L).

**RESULTS.** From June 2010 to June 2018, 22 patients in the Chinese Peoples' Liberation Army General Hospital were eligible for inclusion in accordance with the diagnostic criteria of refractory hyponatremia, including 15 males (68.2%) and 7 females (31.8%). The age ranged from 20 to 88 years old, with an average age of 55.0±22.0 years old. The prevalence of young, middle-aged and elderly patients was 40.9%, 18.2% and 40.9%, respectively. Among the enrolled 22 patients with refractory hyponatremia, 5 were cerebrovascular diseases (22.7%), 8 were infectious and immunological diseases (36.4%), 3 were peripheral neuropathy (13.6%), 2 were metabolic encephalopathy (9.1%), 1 was meningeal cancer (4.5%), 1 was leukoencephalopathy (4.5%) and 1 was neurodegenerative disease (4.5%). Furthermore, mild hyponatremia occurred in 1 case (4.5%) with cerebral infarction, moderate hyponatremia in 11 cases (50%) with most of which are cerebrovascular disease, and severe hyponatremia in 10 cases (45.5%) with most of which were tuberculous meningitis, meningeal carcinomatosis or central pontine myelinolysis. The primary causes of refractory hyponatremia were infection and neuroimmune diseases (53.8%) in young and middle-aged patients, while cerebrovascular diseases (44.4%) were the main causes of refractory hyponatremia in elderly patients. Meanwhile, 0 (0%), 6 cases (46.2%) and 7 cases (53.8%) were found to have mild, moderate and severe hyponatremia in young and middle-aged patients, and 1 case (11.1%), 5 cases (55.6%) and 3 cases (33.3%) in elderly patients, respectively. The average hospitalization time of patients with moderate hyponatremia in the NICU was 40.33±46.38 days, and that was 67.75±111.57 days in patients with severe hyponatremia.

**CONCLUSION.** The prevalence rate of refractory hyponatremia is higher in patients of NICU. Cerebrovascular disease is the first cause of hyponatremia in elderly patients, while intracranial infection, especially tuberculous meningitis, is the most common pathogenic factors in young and middle-aged patients. Furthermore, in patients with high intracranial pressure, high doses of dehydrating drugs for sodium excretion may aggravate the degree of refractory hyponatremia and prolong the treatment duration of hyponatremia. In clinical practice, early detection of hyponatremia and active formulation of reasonable sodium supplementation therapy can reduce the length of stay in hospital for patients.

**REFERENCE(S)**

1. Lewis S. Blevins, External Editor.Effects of Hyponatremia on the Brain.J Clin Med. 2014 Dec; 3(4): 1163–1177.
2. Kim DK, Joo KW. Hyponatremia in patients with neurologic disorders. Electrolyte Blood Press. 2009;7(2):51–57.
3. Rabinstein AA, Wijdicks EF.Hyponatremia in critically ill neurological patients.Neurologist. 2003 Nov;9(6):290-300.

### 000590

#### Neurocritical patients – can neuromonitoring improve outcome?

R. Ferreira de Almeida[1], N. Mourão[2], I. Martins,[1], S. Vicente[1], P. Perez[1], J. Moreno[1], C. Granja[1]

[1]Intensive care department, Centro Hospitalar e Universitário do Algarve, Faro, Portugal; [2]Internal medicine, Centro Hospitalar e Universitário do Algarve, Faro, Portugal

**Correspondence:** R. Ferreira de Almeida

*Intensive Care Medicine Experimental* 2019, **7(Suppl 3):**000590

**INTRODUCTION.** Neurocritical care is a subspecialty of critical care medicine that focusses on the evaluation, diagnosis, and management of patients with acute life-threatening central nervous system diseases.(1,2) Because of their complexity, neurocritical patients needs specific continuous monitorization, to allow their recovery.

**OBJECTIVES.** To evaluate the influence of neuromonitoring in the outcome of neurocritical patients

**METHODS.** An observational retrospective study included 90 neurocritical patients in our intensive care unit (ICU) during the year of 2018. Collected variables included age, gender, diagnoses, severity scores (APACHE, SAPS, initial SOFA), in local Glasgow coma score (GCS), hospital admission GCS, Marshall, Hunt & Hess scale, Fisher scale, National institute of Health stroke scale (NIHSS), intracranial pressure (ICP) at ICU admission, time with ICP>24 in the first 72h after ICU admission, mean ICP during ICU stay, doppler variables in the first 24h of ICU stay (middle cerebral artery (MCA) blood velocity and pulsatility index) and Glasgow Outcome Scale (GOS) at ICU discharge and after 1 year from discharge.

**RESULTS.** Ninety neurocritical patients were included: traumatic brain injury (TBI) (49), aneurism (28) of which 27 had subarachnoid haemorrhage, stroke (10) and arteriovenous malformation (3).45,5% of the TBI population present Marshall scores of 4 and above. We found no statistical significant association between in local and hospital GCS, Marshal scale, mean ICP at ICU admission, time with ICP>24 in the first 72h after ICU admission, mean ICP during ICU stay and GOS at the moment of ICU discharge and GOS 1 year after discharge. In patients with aneurism, in local and hospital GCS, Hunt Hess scale, Fisher scale, time with ICP>24 in the first 72h after ICU admission, mean ICP during ICU stay and doppler variables in the first 24h of ICU stay were not statistically significantly associated with GOS at ICU discharge and GOS 1 year after discharged.

**CONCLUSION.** Neuromonitoring was not statistical significantly associated with outcome in the group of patients included in the present study. Several limitations must be acknowledged: the retrospective character of the study which implies that important data may have not be included, namely doppler records. Our sample needs to improve in order to make more robust conclusions.

**REFERENCE(S)**

1. Sekhon MS, Gooderham P, Toyota B, Kherzi N, Hu V, Dhingra VK, et al. Implementation of Neurocritical Care Is Associated with Improved Outcomes in Traumatic Brain Injury. Can J Neurol Sci. 2017;44(4):350–7.
2. Korbakis G, Bleck T. The Evolution of Neurocritical Care. Crit Care Clin. 2014;30(4):657–71.

### 000699

#### Functional situation of patients who had undergone surgery for intraventricular hemorraghe after 5 years of their admission into ICU

MC. Molina De la torre[1], MM. Gordillo-Resina[1], D. Arias-Verdú[2], JM. Mora Ordóñez[2], E. Castillo-Lorente[3], E. Aguilar-Alonso[4], MA. Arraez-Sanchez[2], LF. Guerrero[5]

[1]Intensive care, Hospital of Jaen, Jaén, Spain; [2]Intensive care, Hospital Carlos Haya, Málaga, Spain; [3]Intensive care, Hospital Universitario Virgen de las Nieves , Granada, Spain; [4]Hospital Cabra, Cabra, Spain; [5]Intensive care, Hospital Universitario Virgen de las Nieves, Granada, Spain

**Correspondence:** E. Aguilar-Alonso

*Intensive Care Medicine Experimental* 2019, **7(Suppl 3):**000699

**INTRODUCTION.** To evaluate patients admitted to ICU diagnosed with spontaneus supratentorial intracerebral hemorraghe and intraventricular hemorraghe,in terms of functional situation and it's relation to surgical intervention.

**METHODS.** Patients with spontaneus supratentorial intracerebral hemorraghe who were admitted to ICU during 2009-2012 in three different spanish hospitals with Neurosurgery Services, had been studied. Effect of surgical intervention in patients with intraventricular hemorraghe had been studied. Using a propensity index, 26 patients who had undergone surgery were paired with 26 who had not.

**RESULTS.** 163 patients were admitted with spontaneus supratentorial intracerebral hemorraghe and intraventricular hemorraghe. This patients presented at admisssion a 8+4 in Glasgow, 21.42+7.55 points in APACHE-II, and 2.75+0.9 score in ICH with a 65% predicting mortality(30 days) and a 59.5% mortality( 30 days). 13.5% presented non-reactive bilateral midriasis. 23.3% of patients (N=38) were surgically intervened. Mortality of patients intervened was 34.2% and 67.2% (p<0.001) for patients who were not intervened. In the multifactorial analysis OR for surgical intervention was 0.14(0.05-0.41).Afterwards a pairment of 26 surgically intervened patients with other 26 non-intervened in relation to a propensisty index had been done. This index was calculated in relation to age, presence of pupilar abnormalities, hemorraghe size and location, and Glasgow at admission. Patients intervened and not intervened presented similar carachteristics (age, Glasgow, ICH score, APACHE II, hemorraghe volumen and location) and non of them presented non reactive bilateral midriasis at admission. Hospital mortality of patients surgically intervened was 30.8% and 65.4% for patients not intervened (p=0.001) and OR 0.23; CI:95%: 0.07–0.75.After 5 years of admission from the 52 paired patients, 20 of them were still alive, 29 no survived and 3 were missing (1 was surgically intervened and 2 of them were not). From the 25 who were intervened, 14 were still alive (56%) and from the 24 not intervened 6 were still alive (25%) (p=0.027) OR =0.26 (0.08-0.89).For the 14 patients intervened who were still alive, the functional situation evaluated with Barthel index was 38+38 points; and for the 6 patients who were not intervened presented a 54+39 puntos, (p=0.403) in the Barthel index. For he 25 patients surgically intervened, 5 (20%) were still alive and presented a slight dependence (Barthel >60) and for the 24 non intervned, only 2 (8.3%) were still alive and presented a slight disfunction (p=0.245)

**CONCLUSION.** Patients admitted into ICU with spontaneus intracerebral hemorraghe with ventricular involucration who were surgically intervened presented less mortality after five years in comparision to the ones who were not intervned. In the other hand, survivors presented a poor functional situación with a high dependence degree.

#### 001095

#### Hyperglycemia as a Prognostic Factor In Acute Ischemic Stroke Patients

AE. Abd-Elhamid[1], HA. El Gendy[1], MA. Nosseir[1], MA. Mohamed[2]
[1]Anesthesia and intensive care, Ain Shams University Faculty of Medicine, Cairo, Egypt; [2]Critical care, Faculty of Medicine, Tanta University, Tanta, Tanta Qism 2, Tanta, Egypt, Egypt
**Correspondence:** H. El Gendy
*Intensive Care Medicine Experimental* 2019, **7(Suppl 3):**001095

**INTRODUCTION.** Whereas diabetes mellitus is a clearly risk factor for the occurrence of stroke and for its poor prognosis, hyperglycemia without pre-existing diabetes mellitus is also linked to increased mortality and morbidity in stroke patients.
**OBJECTIVES.** To assess the role of glyemic control in influencing stroke outcome regarding duration of hospital stay, motor deficit, hemorrhagic transformation and mortality.
**METHODS.** This retrospective study was conducted in Elzaiton Specialized Hospital from June 2016 to June 2017. A sample size of 80 subjects achieves nearly 90% power to detect this difference.
**Inclusion criteria**

- Age: 40-70years
- Sex: Any sex
- Patients with acute ischemic stroke without other major comorbidities within 24 hours of onset of symptoms.++

On ICU admission the random blood sugar was recorded and categorized to less than 150mg/dl (accepted) and more than 150mg/dl (not controlled). Also serial Random blood sugar daily was recorded and categorized to good control if less than 150mg/dl and poor control if more than 150mg/dl.
Patients were divided into 2 groups ( 40 patients each group)
(A) Patients with accepted random blood sugar on admission and controlled blood sugar during hospital stay
(B) Patients with increased random blood sugar on admission and poor blood sugar control during hospital stay
**RESULTS.** A total of 80 patients were enrolled in the study. There were no significant differences regarding gender, HbA1C%, body mass index (p=0.654), (p=0.114),( p=0.381) respectively between both groups, but poor control group showed significantly higher age, random blood sugar on ICU admission and during hospital stay and longer duration of hospital stay (p <0.001), (p<0.001), (p< 0.001), (p=0.022) respectively. Comorbidities (p =0.007), hemorrhagic transformation (p = 0.048) and 30 days mortality (p < 0.001) were significantly higher in poor control group. Glascow coma score on ICU admission and on discharge, motor power on ICU admission and on discharge (p < 0.001), (p= 0.044), (p < 0.001), (p=0.015) respectively showed significantly higher values in good control group.
**CONCLUSION.** Hyperglycemia is common among acute stroke patients and is associated with less favorable outcomes regarding mortality, hospital stay and functional outcome, euglycemic control is recommended for those patients.

##### REFERENCE(S)

1. Bruno A, Biller J, Adams Jr HP, et al. Acute blood glucose level and outcome from ischemic stroke. Neurology 1999; 52: 280-4. 2. Heuschmann PU1, Kolominsky-Rabas PL, Misselwitz B, et al. Pre-dictors of in-hospital mortality and attributable risks of death after ischemic stroke: the German Stroke Registers Study Group.Arch Intern Med 2004; 164: 1761–1768. 3. Kavanagh BP , McCowen KC. Clinical practice. Glycemic control in the ICU. The New England Journal of Medicine. 2010; 363(26):2540–2546. 4. Nedeltcheva K, Renza N, Karamesheva A, et al. Predictors of early mortality after acute ischemic stroke. Swiss Med Wkly. 2010; 140:254–259.
2. Authors would like to thank Elzaiton Specialized Hospital ICU team for their assistance throughout the whole work.

### ARF - Acute respiratory failure 8

#### 001029

#### Can we titrate PEEP more accurately according to transpulmonary pressures in overweight and obese patients ?

B. Acar Çinleti, Ö. Ediboğlu, C. Kıraklı
[1]Intensive care unit, Dr. Suat Seren Chest Diseases and Surgery Training Hospital, Izmir, Turkey
**Correspondence:** B. Acar Çinleti
*Intensive Care Medicine Experimental* 2019, **7(Suppl 3):**001029

**INTRODUCTION.** Determining and setting the optimal and safest PEEP levels in overweight and obese patients (body mass index>25) can be challenging for the ICU physicians. Real time transpulmonary pressure (Ptp) measurement by an esophageal balloon catheter and titrating PEEP levels according to the end expiratory Ptp shows promising results in these patients (1,2,3).
**OBJECTIVES.** To assess the feasibility and effect of Ptp guided PEEP titration in overweight and obese patients.
**METHODS.** An esophageal balloon catheter (Cooper Surgical Inc, Trumbull, CT, USA) was inserted to monitor the esophageal pressure in overweight and obese patients under invasive mechanical ventilation. Patients were connected to a ventilator capable of monitoring airway, esophageal and transpulmonary pressure waveforms real time breath-by-breath (Hamilton G5, Hamilton Medical AG, Bonaduz, Switzerland). PEEP levels were adjusted to achieve a Ptp 0 to 10 cmH2O at end expiration.
**RESULTS.** Eleven patients were enrolled. Data are expressed as mean±SD and comparisons were done using paired sample t-test. Mean age was 49± 21 years and body mass index was 29±4 . PEEP was increased to 20 ± 5 from 10±4 cmH2O after titrating PEEP according to the Ptp at end expiration (p= 0.001). This also led to a significant improvement in PaO2/FiO2 ratio (from 86±25 to 220±86 , p= 0.001) and allowed a significant decrease in FiO2 levels (from 0.9±18 to 0.67±25, p=0.008). Arterial pH increased from 7.33±0.16 to 7.40±0.10 (p =0.042) and PaCO2 decreased from 54±18 to 44±9 mmHg (p= 0.08).
**CONCLUSION.** Ptp guided PEEP titration can be effective and safe especially in overweight and obese patients who are in need of high PEEP levels.

##### REFERENCE(S)

1. Baedorf Kassis E, Loring SH, Talmor D. Should we titrate peep based on end-expiratory transpulmonary pressure?-yes. Ann Transl Med. 2018 Oct;6(19):390.
2. Talmor D, Sarge T, Malhotra A, O'Donnell CR, Ritz R, Lisbon A, Novack V, Loring SH. Mechanical ventilation guided by esophageal pressure in acute lung injury. N Engl J Med. 2008 Nov 13; 359(20):2095-104.
3. Wu X, Zheng R, Lin H, Zhuang Z, Zhang M, Yan P. [Effect of transpulmonary pressure-directed mechanical ventilation on respiration in severe acute pancreatitis patient with intraabdominal hypertension]. Zhonghua Yi Xue Za Zhi. 2015 Oct 20; 95(39):3168-72.

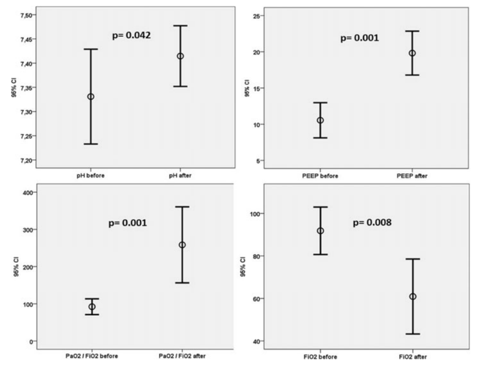

**Fig. 1 (abstract 001029).** Respiratory parameters before and after esophageal catheter replacement and titrating PEEP according to transpulmonary pressure measurement. Circles represent mean values and lines represent 95% confidence intervals

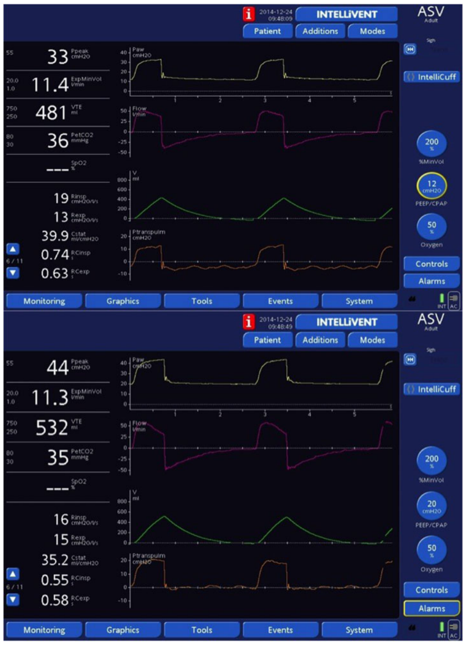

**Fig. 2 (abstract 001029).** Representative waveforms of a patient before and after PEEP titration according to transpulmonary pressure measurement.

### 001051

#### Impact of fragility in mortality of patients with tracheostomy in ICU

JA. Silva Obregón[1], A. Estrella Alonso[1], S. Saboya Sanchez[2], G. Jimenez Puente[1], C. Marian Crespo[1], N. Arriero Fernández[1], Z. Eguileor Marín[1], A. Albaya Moreno[1], C. Benito Punzel[1], MA. Tirado Fernández[1]
[1]Intensive care, Hospital Universitario de Guadalajara, Calle Donante de Sangre, Guadalajara, Spain, Guadalajara, Spain; [2]Intensive care, Hospital Puerta de Hierro-Majadahonda, Majadahonda, Spain
**Correspondence:** Z. Eguileor Marín
*Intensive Care Medicine Experimental* 2019, **7(Suppl 3):**001051

**INTRODUCTION.** Frailty is an independent predictor of short and long term mortality in patients admitted in intensive care unit (ICU).
**OBJECTIVES.** Describe the impact of frailty at 30 days (30-d) mortality from ICU admission in patients who underwent tracheostomy.
**METHODS.** Observational retrospective study from May 2014 to October 2018; patients who underwent tracheostomy in ICU were included. Exclusion criteria: patients with tracheostomy before ICU admission and re-admission patients. Demographic (age, gender) clinical (co-morbidity), IMV, tracheostomy, ICU and hospital length data, were collected. To assess de frailty we used Clinical Frailty Scale (CFS) (fragile: CFS ≥ 5). We describe qualitative variables as frequency and percentage; and continuous variables as mean ± SD or median, and interquartile range. We compared percentages using chi-square or Fisher test, and continuous variables with t-Student or U Mann-Whitney tests. Survival was described with the Kaplan-Meier technique (long-rank) and with multivariate Cox proportional-hazard models, adjusted by age, gender, co-morbidities, APACHE II (without age), re-intubation, % of time in IMV, day of tracheostomy, disconnection from the IMV and discharge from ICU without tracheostomy.
**RESULTS.** Of the 2630 patients admitted to ICU, 105 were analyzed; 16.2% were fragile (Figure 1). Median age 69.9 years (IR: 62.0-77.3) and 71.4% males; median APACHE II 23.0 (17.0-28.0). There were no significant differences in age, gender, co-morbidities, severity scores, variables related to IMV or tracheostomy. Frailty patients had significantly shorter time in IMV (p=0.038). Survival analysis showed a higher probability of 30-d mortality in frailty patients (long-rank=0.018) (Figure 2). Multivariate analysis showed fragility as independent predictor factor of 30-d mortality (OR 3.80; IC 95% 1.59 – 9.12; p= 0.003)
**CONCLUSION.** There were no differences in characteristics and management between frailty and non-frailty patients whom tracheotomy were made during ICU stay. Time of IMV in frailty patients, was probably shorter because they died earlier. Frailty is an independent predictive factor of 30-d mortality in patients who need tracheostomy during ICU admission.

**Table 1 (abstract 001051).** See text for description

| Variable | Total (N=105) | No-Fragile* (n=88) | Fragile* (n=17) | OR (IC 95%) | p valor |
|---|---|---|---|---|---|
| AGE ᵇ | 69,6 (62,0 – 77,3) | 69,0 (58,8 – 76,4) | 74,3 (66,1 – 81,8) | 1,05 (0,99 – 1,10) | 0,113 |
| SEX | | | | | |
| Men | 75 (71,4) | 65 (73,9) | 10 (58,8) | 0,51 (0,17 – 1,48) | 0,214 |
| Women | 30 (28,6) | 23 (26,1) | 7 (41,2) | | |
| Co-morbidities | | | | | |
| COPD | 18 (17,1) | 15 (17,0) | 3 (17,6) | 1,04 (0,27 – 4,1) | 0,952 |
| Cardiomiophaty (NYHA III-IV) | 5 (4,8) | 5 (5,7) | 0 (0,0) | - | - |
| Co-morbidities number | 3,0 (2,0 – 4,0) | 3,0 (2,0 – 4,0) | 3,0 (2,0 – 4,0) | 1,07 (0,77 – 1,50) | 0,683 |
| CLÍNICAS | | | | | |
| APACHE II ᵇ | 23,0 (17,0 – 28,0) | 22,0 (17,0 – 27,75) | 25,0 (16,0 – 30,5) | 1,02 (0,95 – 1,08) | 0,661 |
| APACHE II (Age) ᵃ | 18,7 ± 7,8 | 18,6 ± 7,9 | 19,1 ± 7,6 | 1,01 (0,94 – 1,08) | 0,828 |
| Reintubation | 28 (26,7) | 23 (26,1) | 5 (29,4) | 1,18 (0,37 – 3,71) | 0,780 |
| ARDS | 37 (35,2) | 34 (38,6) | 3 (17,6) | 0,340 (0,09 – 1,27) | 0,340 |
| MECHANICAL VENTILATION | | | | | |
| Days in MV ᵇ | 15,0 (8,6 – 20,1) | 15,8 (9,3 – 21,6) | 9,5 (6,8 – 14,8) | 0,92 (0,86 – 0,99) | **0,038** |
| % t in IMV ᵇ | 68,3 (52,8 – 83,8) | 69,2 (54,3 – 83,4) | 63,4 (37,7 – 99,9) | 0,99 (0,96 – 1,01) | 0,282 |
| TRACHEOTOMY | | | | | |
| Day of tracheotomy ᵃ | 10,4 ± 5,1 | 10,6 ± 4,6 | 9,4 ± 6,9 | 0,95 (0,85 – 1,06) | 0,336 |
| Days with tracheotomy ᵇ | 10,0 (6,6 – 17,0) | 10,1 (6,4 – 18,0) | 8,2 (5,5 – 15,6) | 0,97 (0,92 – 1,03) | 0,363 |
| Decanulattion | 43 (41,0) | 39 (44,3) | 4 (23,5) | 0,39 (0,12 – 1,28) | 0,120 |
| Weaning onset ᵇ | 3,0 (2,0 – 4,75) | 3,0 (2,0 – 5,0) | 2,5 (2,0 – 3,8) | 0,91 (0,74 – 1,12) | 0,371 |
| Length Disconnection (days) ᵇ | 4,3 (1,3 – 7,4) | 4,2 (1,7 – 6,9) | 4,3 (0,0 – 10,4) | 1,03 (0,93 – 1,15) | 0,532 |
| % t disconnected ᵇ | 44,2 (9,1 – 67,9) | 44,9 (11,2 – 68,1) | 38,8 (0,0 – 67,6) | 0,99 (0,98 – 1,02) | 0,841 |
| STAY | | | | | |
| ICU stay ᵇ | 21,8 (15,0 – 32,4) | 21,9 (16,2 – 32,6) | 15,2 (9,4 – 31,5) | 0,97 (0,93 – 1,02) | 0,227 |
| Hospital stay ᵇ | 37,7 (23,9 – 59,1) | 38,1 (27,1 – 62,8) | 31,2 (13,8 – 48,8) | 0,98 (0,96 – 1,01) | 0,125 |
| MORTALITY | | | | | |
| LLST | 35 (33,3) | 28 (31,8) | 7 (41,2) | 1,50 (0,52 – 4,35) | 0,456 |
| ICU mortality | 35 (33,3) | 26 (29,5) | 9 (52,9) | 2,68 (0,93 – 7,72) | 0,067 |
| Hospital mortality | 52 (49,5) | 40 (45,5) | 12 (70,6) | 2,88 (0,94 – 8,87) | 0,065 |
| 30-d mortality | 34 (32,4) | 25 (28,4) | 9 (52,9) | 2,84 (0,98 – 8,18) | 0,054 |

* *Fragile (CFS ≥ 5); No fragile (CFS < 5);*
Qualitative variables as n (%); Quantitative variables as mean ± SD ᵃ or as Median (IR) ᵇ
*Abbreviation: OR, Odds Ratio; CI, Confidence Interval; NYHA, New York Heart Association; COPD, chronic obstructive pulmonary disease; APACHE, Acute Physiology and Chronic Health Evaluation; ARDS, Acute Respiratory Distress Syndrome; IMV, invasive mechanical ventilation; LLST, limitations of life support techniques; ICU, Intensive Care Unit; t, Time*

**Table 2 (abstract 001051).** See text for description

| Variable | HR (IC 95%) | p valor |
|---|---|---|
| Age | 1,036 (1,005 – 1,067) | 0,023 |
| Frailty (CFS ≥ 5) | 3,802 (1,585 – 9,122) | 0,003 |
| % T. in IMV | 1,033 (1,004 – 1,062) | 0,025 |
| Day of tracheotomy | 0,915 (0,849 – 0,986) | 0,020 |
| Weaning | 0,164 (0,049 – 0,546) | 0,003 |

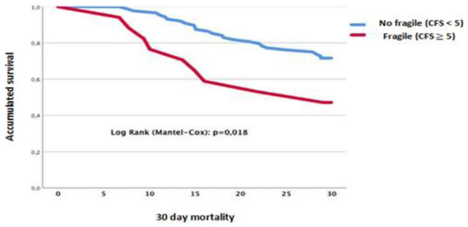

**Fig. 3 (abstract 001051).** See text for description

### 001054

#### Effect of Anti-IL-33 treatment in a murine model of Acute Respiratory Distress Syndrome

R. Muñoz-Bermúdez[1], J. Marin-Corral[1], I. Dot Jordana[1], A. Salazar-Degracia[1], O. Roca[2], L. Pijuan[3], G. Tagmouti[3], JR. Masclans[1]
[1]Critical care department, Hospital del Mar, IMIM-GREPAC, Barcelona, Spain; [2]Critical care department, Vall d´Hebron University Hospital, Vall d´Hebron Research Institute, Barcelona, Spain; [3]Anatomic pathology department, Hospital del Mar, Barcelona, Spain
**Correspondence:** R. Muñoz-Bermúdez
*Intensive Care Medicine Experimental* 2019, **7(Suppl 3):**001054

**INTRODUCTION.** Acute respiratory distress syndrome (ARDS) is characterized by pulmonary edema due to alveolar epithelial-interstitialendothelial injury, associated with profound inflammation and respiratory dysfunction. The IL-33/ST2 axis has a key role in the development of immune-inflammatory responses in the lung.

**OBJECTIVES.** To assess the anti-inflammatory and restorative capabilities of using Anti-IL33 antibody to alleviate the pathological events occurring in a murine lipopolysaccharide (LPS)-induced lung injury model.

**METHODS.** Eight to tenweeks-old male BALB/c mice (Harlan, Horst, The Netherlands) were used for the experiments (IRB: JME-16-0025). All mice were anesthetized with 2% isoflurane and challenged with a single intranasal instillation of 8 mg/kg of LPS (E. coli 055:B5:Sigma-Aldrich) dissolved in 50 μl of PBS (ARDS group), or administered 50 μl of PBS alone (control group) on day 0. Day 1, 2 and 3, mice received 3,6μg/day intraperitoneally Anti-IL-33 (treatment group), orintraperitoneally saline (placebo group). The following groups of mice were studied: 1) Control group (n=12) with non-ARDS controls+placebo (n=6) and non-ARDS controls + Anti-IL33 (n=6), 2) ARDS + placebo (n=12) and 3) ARDS + Anti-IL33 (n=12).Body weight gain was registered and bronchoalveolar lavage and blood samples were recollected. Cell count, total protein levels, IL-33 (Abnova, Walnut, CA, USA) and IL-6 (Cloud-Clone Corp, Katy, TX, USA)levels were measured in BAL fluid.IL-33 levels were also measured in blood samples. Histological evidence of lung injury was assessedthrough previous methodology (histological parameter scoring)1 and wet-to-dry lung weight ratio was also evaluated in some animals.The data was expressed as mean (standard deviation) or as median (interquartile range). Significance was set to p <0.05. Statistical analyses were performed using Portable SPSS, PASW Statistics v18.0; SPSS Inc, Chicago, IL.

**RESULTS.** There were no differences in any studied variables between non-ARDS controls + placebo and non-ARDS controls + Anti-IL33 and therefore they were analysed together as a control group. Both ARDS groups presented a decrease in the body weight gain and an increase in the wet-to-dry lung weight, neutrophil percentage in BALF, protein levels in BALFand alveolar space polymorphonuclear cells (PMN) in the histologic evaluation compared with control group. ARDS + placebo group presented higher levels of IL-33 and IL-6 in BALF, and histologic interstitial space PMN and proteinaceous debris compared with control group. ARDS + Anti-IL-33 mice presented a decrease in the wet-to-dry lung weight [0.13(0.02) vs 0.16(0.02);p=0.024], in the BALF neutrophil percentage [63.1(52.6-68.1) vs 69.4(67.4-71.1);p=0.038], interstitial space PMN [0.4(0.5) vs 1.1(0.4);p=0.015] and proteinaceous debris [0.3(0.4) vs 1.1(0.7);p=0.022] in the histologic evaluation compared to ARDS + placebo group.

**CONCLUSION.** Anti-IL-33 treatment could partially restore the acute histopathological and biochemical changes occurring after endotoxin challenge. Anti-IL-33 antibody could have a therapeutic potential for ARDS but further studies will be necessaries in the advance toward clinical applications

**REFERENCE(S)**

1. Matute-Bello G, et al. Am J Respir Cell MolBiol 2011;44:725–738
2. Martínez-González I, et al. Am J Respir Cell MolBiol 2013 Oct; 49(4):552-62
3. Grant from Instituto de Salud Carlos III-Fondo Europeo de Desarrollo Regional (FEDER) (PI14/01420)
4. Grant from Societat Catalana de Medicina Intensiva i Crítica (SOCMIC) 2017
5. Grant from Federación Española del Enfermo Crítico (FEEC) 2015

### 001055

#### Prone position enhances regional homogeneity at high and low PEEP assessed by direct pleural pressure measurement

K. Osada,D. Engelberts, L. Bastia, F. Damiani, BH. Katira, H. Chen, X. Li, T. Tetsuya, T. Yoshida, G. Otulakowski, B. Kavanagh
Translational medicine, The Hospital for Sick Children, Toronto, Canada
**Correspondence:** L. Bastia
*Intensive Care Medicine Experimental* 2019, **7(Suppl 3):**001055

**INTRODUCTION.** Prone position (PP) is a cornerstone in the treatment of acute respiratory distress syndrome (ARDS) increasing oxygenation and protecting the lung against ventilator induced lung injury (VILI). Its precise mechanism of lung protection, interaction with PEEP and the regional mechanical response of the respiratory system care still lacking.

**OBJECTIVES.** To assess the changes in pleural pressure gradient, ventilation homogeneity and regional respiratory mechanics at different PEEP levels, during supine and prone positioning.

**METHODS.** Twelve pigs (weight 36.1 ± 1.9 Kg) were anesthetized and mechanical ventilated. Catheters were inserted into dorsal and ventral pleural space to obtain a direct measurement of regional pleural pressure (Ppl), PA catheter, an esophageal balloon and an EIT belt were added. Lung injury was established with saline lavages to obtain a PaO2 < 100mmHg followed by high-stress ventilation until a decrease in respiratory system compliance (Crs) by 20%. After a recruitment manoeuvre (RM) each animal underwent a decremental PEEP protocol (PEEP from 20 to 3 cmH2O, steps of 1cmH2O every 5-6 breaths, volume control, 6 ml/kg) in both SP and PP. At every level of PEEP, Plateau pressure, Pes, regional Ppl (dorsal & ventral), and EIT were measured. Vertical gradient of Ppl, distribution of ventilation, compliance (respiratory system, lung and chest wall; total & regional) were calculated.

**RESULTS.** PP induces a uniform vertical Ppl gradient (Ppl ventral – Ppl dorsal) during expiration and inspiration (i.e. maintains lung homogeneity) and is PEEP independent. Whereas, in SP vertical gradient of Ppl is PEEP dependent (*Fig.1*). Lower PEEP distributes VT toward non-dependent regions; in SP more than PP. At lower PEEP levels, vertical gradient of ventilation (VT dependent – VT non-dependent) is lower in PP. Levels of PEEP required to achieve the best regional lung compliance (CL) in dependent and non-dependent lung was congruent in PP (13 and 12cmH2O respectively) compared to SP (15 and 10 cmH2O respectively).

**CONCLUSION.** The vertical Ppl gradient across the lung is stable during PP i.e. not affected by both high (overdistension) or low (collapse) PEEP. Thus, lung homogeneity is maintained as reflected by homogenous regional ventilation, stable gradient of ventilation and more uniform best PEEP for regionals CL.

**REFERENCE(S)**

1. Kallet RH, A Comprehensive Review of Prone Position in ARDS. Respiratory Care November 2015, 60 (11) 1660-1687
2. Gattinoni L, Taccone P, Carlesso E, Marini JJ. Prone position in acute respiratory distress syndrome. Rationale, indications, and limits. Am J Respir Crit Care Med. 2013;188:1286–93

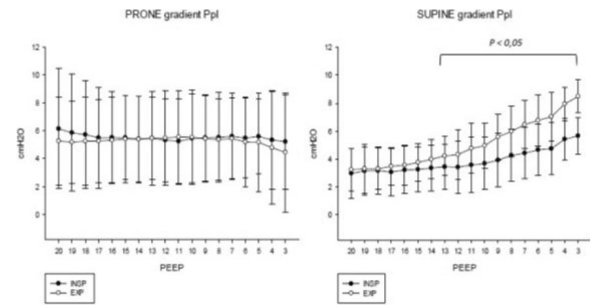

**Fig. 1 (abstract 001055).** Note: In SP interaction between PEEP and delta Ppl in both INSP and EXP (p < 0001), in PP no statistical interaction. Gradient Ppl =Ppl ventral -Ppl dorsal (in absolute value).

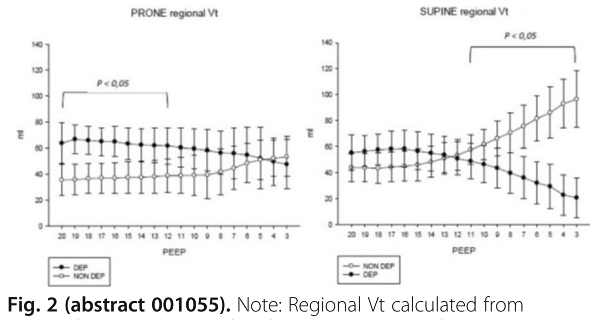

**Fig. 2 (abstract 001055).** Note: Regional Vt calculated from EIT(quadrant). Both SP and PP have interaction with PEEP (p < 0001).

### 001058

#### Reverse triggering with high respiratory effort is associated with decreased diaphragm function in an experimental model of acute lung injury

F. Damiani[1], D. Engelberts[2], L. Bastia[2], K. Osada[2], BH. Katira[2], T. Tetsuya[2], G. Otulakowski[2], EC. Goligher[3], D. Reid[4], A. Bruhn[5], L. Brochard[6], B. Kavanagh[2]
[1]Critical care medicine, Pontificia Universidad Catolica de Chile, Santiago, Chile; [2]Translational medicine, The Hospital for Sick Children, Toronto, Canada; [3]Interdepartmental division of critical care medicine, university of toronto, toronto, canada, Toronto General Hospital Research Institute, Toronto, Canada; [4]Department of physical therapy, University of Toronto, Toronto, Canada; [5]Departamento de medicina intensiva, Pontificia Universidad Católica de Chile, Santiago, Chile; [6]Interdepartmental division of critical care- university of toronto, Hospital St. Michael and Keenan research center, Toronto, Canada
**Correspondence:** F. Damiani
*Intensive Care Medicine Experimental* 2019, **7(Suppl 3):**001058

**INTRODUCTION.** Reverse triggering (RT) is a type of patient-ventilator interaction where respiratory muscle contractions are triggered by the ventilator under different patterns of entrainment. Because it occurs during expiration, RT may potentially cause eccentric myotrauma while on the other hand it could protect the diaphragm from atrophy. The impact of RT on diaphragm function remains uncertain.

**OBJECTIVES.** To establish the effect of RT in comparison to passive ventilation on diaphragm function in an experimental model of acute lung injury.

**METHODS.** Sixteen pigs (35±5kg) were anesthetized, mechanically ventilated, and monitored. Lung injury was induced by surfactant depletion and high-stress ventilation to reach $PaO_2$ <150 mmHg and 10% decrease in respiratory system compliance. After lung injury, recruiting maneuver and baseline measurements, pigs were randomized to receive either passive ventilation (n=8; VCV; RR: 35 bpm; VT: 10ml/kg), or ventilation adjusted to induce RT (n=8; VCV; variable RR and VT), for 3 hours. Based on preliminary experiments, we induced RT by stepwise changes in the following settings: (1) decreasing VT and increasing RR; (2) decreasing dose of sedatives; (3) increasing PEEP. Diaphragm function was assessed by measuring transdiaphragmatic pressure (Pdi) during phrenic nerve stimulation by single electrical twitches (PdiTw) and at different stimulation frequencies (10, 20, 30, 40 and 60 Hz) to construct force/frequency curves. PdiTw and Pdi,max after 1, 2, and 3 hours of ventilation was compared to baseline PdiTw and Pdi,max, respectively. Finally, we conducted a post-hoc analysis in the RT group based on the level of respiratory effort [average Pes swing > 10 $cmH_2O$ and 1:1 entrainment (n=4) vs. Pes swing <10 $cmH_2O$ and 1:2 entrainment (n=4)].

**RESULTS.** The RT group had lower VT, higher RR, and higher PEEP level as compared with the passive group. There was no difference in $PaCO_2$ but a higher $PaO_2$ with RT. PdiTw ratio and Pdi ratio were not different between groups but a post- hoc analysis showed that in the RT animals with high respiratory effort (n=4), PdiTw and Pdi,max was reduced by 25%. Decrease in diaphragm force was significantly different than both passive and RT with low respiratory effort (Figure 1)

**CONCLUSION.** Reverse triggering might acutely impair diaphragm strength, and this effect seems to be modulated by the level of respiratory effort.

##### REFERENCE(S)

1. Goligher EC, Brochard LJ, Reid WD et al. (2018) Diaphragmatic myotrauma: a mediator of prolonged ventilation and poor patient outcomes in acute respiratory failure. Lancet Respir Med, November 2018.
2. Akoumianaki E, Lyazidi A, Rey N, Matamis D, Perez-Martinez N, Giraud R, et al. Mechanical ventilation-induced reverse-triggered breaths: A frequently unrecognized form of neuromechanical coupling. Chest. 2013;143(4):927–38
3. FD acknowledges partial support from CONICYT-PFCHA/Doctorado Nacional/2017-folio 21171551

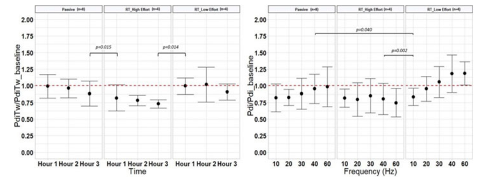

**Fig. 2 (abstract 001058).** See text for description

### 001060

#### Minimal duration of expiratory holds to verify esophageal pressure measurements

EC. de Boer[1], HJ. De Vries[2], Y. Zhang[1], AH. Jonkman[1], L. Heunks[2]
[1]Intensive care, Amsterdam University Medical Center, location VUmc, Amsterdam, Netherlands; [2]Intensive care medicine, Amsterdam UMC, locatie VUmc, Amsterdam, Netherlands
**Correspondence:** E.C. de Boer
*Intensive Care Medicine Experimental* 2019, **7(Suppl 3):**001060

**INTRODUCTION.** Esophageal pressure (Pes) measurements are increasingly used as state-of-the-art monitoring technique in ventilated ICU patients. To validate the position and filling volume of the esophageal balloon, the Baydur maneuver is performed. An expiratory hold is applied for at least one breath, during which the ratio of airway pressure (Paw) and Pes is measured. Pes measurements are considered reliable when the Paw/Pes ratio has a value between 0.8-1.2.[1] However, it is unknown if a shorter expiratory hold could be sufficient for validation of the Pes measurements. This would be beneficial as it is less uncomfortable for patients and might allow near continuous calculation of this ratio by the ventilator in the future.

**OBJECTIVES.** To determine the minimal duration of an expiratory hold in patients on partially-supported modes of ventilation in order to obtain a reliable Paw/Pes ratio.

**METHODS.** Expiratory hold maneuvers were performed in ICU patients with an esophageal balloon catheter (Nutrivent®, Sidam Italy), ventilated in a partially supported mode. A continuous ratio between Paw and Pes was calculated offline. Subsequently, it was determined how many milliseconds (ms) after the start of patient effort the Paw/Pes ratio reached the reliable range of 0.8-1.2 and stayed stable for 40ms thereafter (Fig. 1).

**RESULTS.** We analyzed 86 Baydur maneuvers obtained from 10 different patients. The Paw/Pes ratio reached the reliable range of 0.8-1.2 after a median time of 200ms [IQR 94-384ms] after the onset of patient effort.

**CONCLUSION.** In 75% of the maneuvers, a reliable and stable Paw/Pes ratio can be obtained 384ms after onset of patient effort. Therefore, we advise that an expiratory hold should have a minimal duration of 430ms after onset of patient effort. Implementation of automatic expiratory occlusions within the ventilator software enables a continuous verification of esophageal balloon measurements, increasing its clinical value.

##### REFERENCE(S)

[1] Mauri et al., "Esophageal and transpulmonary pressure in the clinical setting: meaning, usefulness and perspectives," Intensive Care Med, vol. 42, no.9, pp. 1360-73, Sep 2016
[2] HdV is supported by a research grant of Amsterdam Cardiovascular Sciences.

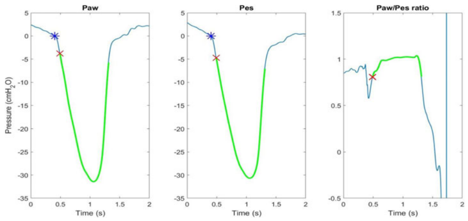

**Fig. 1 (abstract 001060).** Airway pressure (Paw), esophageal pressure (Pes) and the Paw/Pes ratio during one expiratory hold. The blue asterisks show the onset of patient effort. The green lines show the period of time that the Paw/Pes ratio is in the reliable range of 0.8-1.2. The red cross indicates the first moment when the ratio was stable in the reliable range for at least 40ms. Per expiratory hold maneuver the time between the blue asterisk and red cross is calculated.

### 001084

#### Bleeding in lung transplantation with intraoperative support by ECMO

M. López Sánchez, MI. Rubio Lopez
[1]Critical care department, University Hospital Marqués de Valdecilla, Santander, Spain
**Correspondence:** M. López Sánchez
*Intensive Care Medicine Experimental* 2019, **7(Suppl 3):**001084

**INTRODUCTION.** Intraoperative support by extracorporeal membrane oxygenation (ECMO) is widely used during lung trasplantation (LT) for cardiac, respiratory or both type of failure. Compared to cardiopulmonary bypass (CPB) ECMO is simplest, lower priming volumen, lesser anticoagulation requirement, lower coagulopathy and systemic inflammatory response. At our institution, CPB has been replaced by ECMO in this setting. Nevertheless, bleeding is an important complication in these patients.

**OBJECTIVES.** To describe demographic characteristics, ECMO indications, technical aspects, type of membrane oxygenation, ECMO weaning and mortality in LT patients with intraoperative support by ECMO and perioperative bleeding.

**METHODS.** Descriptive study between Juin 2009 to March 2019 in 12 beds ICU. Inclusion criteria for ECMO entry were cardiac failure (left, righ or both), severe pulmonay hypertension and/or respiratory failure before or during LT proceeding. ECMO systems have centrifugal pumps and polimethilpentene or polypropylene membrane oxygenation. Demographic data, APACHE II, type of ECMO support, thoracic bleeding, anticoagulation practice, ECMO weaning and ICU survival were collected.

**RESULTS.** ECMO support was used in 49 patients (p). In 35p (71.4%) ECMO was used for intraoperative support. In this group, APACHE II was 21.3 (12-29); 51.4% (18p) were woman with a median age of 49.7 years (rank 16-63). Pulmonary fibrosis was the most common disease for LT in this entity in 13 p (37.1%), COPD/emphysema in 5 p (14.2%), primary pulmonary hypertension in 4 p (11.4%), QF in 3 p (8.5%). Type of ECMO support was VA in 28 p (central 9p, peripheral 19p) and VV in 7p. Cardiac support was needed in 22p, respiratory in 7p and both in 7p. ECMO was weaned at the operating room (OR) in 51.4% (18p). Massive bleeding was present in 17p (48.5%), needing toracotomy in 10p (58.8%). Polypropylene membrane was used in 12 of them (70.5%). Only 6p (35.29%) in this group was weaned at the OR. Anticoagulation during lung transplanation was used in all patients during LT, but 5p (29.41%) didn´t receive anticoagulation at the ICU. Thrombotic complications were not seen. ICU mortality in patients with massive bleeding was 35.2% (6 p).

**CONCLUSION.** In patients with ECMO as intraoperative support, massive bleeding was present in 48.5% of patients with a ICU mortality of 35.2%. Anticoagulation regimen and type of oxygenator must be reconsidered.

**REFERENCE(S)**

1. Bharat A, DeCamp MM. Veno-arterial extracorporeal membrane oxygenation without therapeutic anticoagulation for intra-operative crdiopulmonary support during lung transplantation. J Thorac Dis 2017; 9: E629-31.
2. Moreno J, Cypel MD, McRae MD, Machuca MD, Cunningham CCP, Singer MD. The evolving role of extracorporeal membrane oxygenation in lung transplantation: implications for anesthetic management. J Cardiothor Vasc An 2018, doi: https//doi.org/10.1053/j.jvca.2018.10.007

### 001085

#### Spontaneous breathing decreases diaphragmatic fiber size in a severe ARDS experimental model assisted by ECMO

S. Dubo[1], V. Oviedo[2], A. Garcia[2], L. Alegria[2], P. García[3], ED. Valenzuela Espinoza[2], F. Damiani[2], J. Araos[4], T. Medina[5], MC. Bachmann[2], D. Soto[2], P. Cruces[5], P. Guzman[6], P. Brebi[7], E. Brandan[8], D. Rebolledo[8], J. Retamal[2], R. Cornejo[9], G. Bugedo[2], A. Bruhn[2]
[1]Departamento de kinesiología, Univerisidad de Concepción, Concepción, Chile; [2]Departamento de medicina intensiva, Pontificia Universidad Católica de Chile, Santiago, Chile; [3]Departamento de ciencias de la salud, carrera de kinesiología, Pontificia Universidad Católica de Chile, Santiago, Chile; [4]College of veterinary medicine, department of clinical sciences, Cornell University, New York, USA; [5]Unidad de pacientes críticos, Hospital El Carmen de Maipú, Santiago, Chile; [6]Departamento de anatomia patologica, Universidad de La Frontera, Temuco, Chile; [7]Bioren, laboratorio de patología molecular, Universidad de La Frontera, Temuco, Chile; [8]Centro de envejecimiento y regeneración, care; departamento de biología celular, Pontificia Universidad Católica de Chile, Santiago, Chile; [9]Hospital clínico, unidad de pacientes críticos, Universidad de Chile, Santiago, Chile
**Correspondence:** S. Dubo
*Intensive Care Medicine Experimental* 2019, **7(Suppl 3):**001085

**INTRODUCTION.** Spontaneous breathing has been suggested in ARDS patients on ECMO to prevent diaphragm dysfunction. However, the use of spontaneous breathing is controversial in severe ARDS as it may promote further lung injury.

**OBJECTIVES.** To compare the effects of spontaneous breathing versus controlled near-apneic ventilation, on lung injury and diaphragm structure, in a porcine severe ARDS model assisted by ECMO.

**METHODS.** Twelve pigs (30±5kg) were anesthetized, mechanically ventilated and monitored. Lung Injury was induced by repeated lavages (30 ml/kg of warm saline) until PaO2/FiO2 dropped below 200, followed by 2-h injurious ventilation (PEEP 0 cmH2O, PInsp 40 cmH2O, RR 10/min). After completing lung injury (T0) animals were connected to ECMO. During the first 3 hours, animals were ventilated with near-apneic ventilation (PEEP 10, driving pressure 10, RR 5/min) and NMB. After T3, animals were randomized into 2 groups: i) Near- apneic ventilation, which continued with the same settings, and ii) SB: in this group, NMB was stopped and sweep gas flow decreased until regaining respiratory efforts. Thereafter, ventilation was switched to PS mode (PS 10 cmH2O, PEEP 10 cmH2O) and sweep gas flow adjusted preserving SB at a respiratory rate of 30 to 70 bpm. ECMO weaning started in both groups at T18, sweep gas flow was stopped and animals ventilated in PS mode (adjusted to Vt=6 ml/kg) until T24. Respiratory and hemodynamic variables, as well as regional lung impedance, were collected. After 24 hours, animals were euthanized and tissues extracted (lung and diaphragm). Lung edema was estimated by the wet-dry weight ratio. Lung injury and muscle morphometrics were assessed by histological analysis.

**RESULTS.** Although the SB group developed higher respiratory rates and minute ventilation, at T18 both groups exhibited similar oxygenation and systemic hemodynamics. At T18, all animals regained spontaneous efforts and could be weaned from ECMO without hemodynamic or respiratory deterioration, and with no differences in the breathing pattern (Table 1). No differences were observed in lung wet/dry weight ratio, cytokines concentration in lung tissues or in the histological lung injury score (Fig 1). Quantification of cross-sectional area showed that slow- and fasttwitch diaphragm muscle fibers in SB group were smaller (near-apneic ventilation vs SB, fast: 662.9 ± 9.2 vs 460.7 ± 7.4 µm2; slow: 583.1 ± 10.7 vs 356.7 ± 5.6 µm2) (Fig 2). The fraction of each type of fibers did not differ between groups.

**CONCLUSION.** We found no differences between SB and Near-apneic ventilation in lung injury and lung function, but the SB group exhibited smaller slow- and fast- twitch diaphragmatic fibers.

**Table 1 (abstract 001085).** Respiratory variables

| Variable | Time | | | | | | | |
|---|---|---|---|---|---|---|---|---|
| | Baseline | $T_0$ | $T_3$ | $T_6$ | $T_{12}$ | $T_{18}$ | $T_{21}$ | $T_{24}$ |
| PaO2/FiO2 (mmHg) | | | | | | | | |
| Near-apneic ventilation | 372 ± 20 | 154 ± 27§ | 261 ± 31 | 251 ± 28 | 258 ± 25 | 251 ± 33 | - | 327 ± 37† |
| Spontaneous breathing | 423 ± 18 | 104 ± 28§ | 200 ± 53 | 197 ± 21 | 260 ± 26 | 247 ± 38 | - | 270 ± 48† |
| Respiratory rate (bpm) | | | | | | | | |
| Near-apneic ventilation | 18 ± 0 | 18 ± 1 | 5 ± 0† | 5 ± 0†,* | 5 ± 0†,* | 5 ± 0†,* | 40 ± 4 | 43 ± 5 |
| Spontaneous breathing | 17 ± 2 | 17 ± 1 | 5 ± 0† | 41 ± 2 | 41 ± 2 | 36 ± 4 | 39 ± 5 | 40 ± 5 |
| Tidal volume (ml/kg) | | | | | | | | |
| Near-apneic ventilation | 10.2 ± 0.3 | 10.3 ± 0.3 | 5.9 ± 0.7† | 6.4 ± 0.7† | 6.0 ± 0.7† | 5.6 ± 0.3† | 6.1 ± 0.5† | 6.0 ± 0.5† |
| Spontaneous breathing | 10.1 ± 0.1 | 10.1 ± 0.4 | 4.1 ± 0.5† | 4.5 ± 0.6† | 4.6 ± 0.7† | 4.1 ± 0.5† | 6.1 ± 0.1† | 6.7 ± 0.5† |
| Minute volume (L/min) | | | | | | | | |
| Near-apneic ventilation | 6.52 ± 0.46 | 6.38 ± 0.43 | 1.38 ± 0.34† | 1.92 ± 0.33†,* | 1.82 ± 0.22†,* | 1.92 ± 0.39†,* | 8.03 ± 0.71 | 8.07 ± 0.69 |
| Spontaneous breathing | 6.8 ± 0.49 | 6.63 ± 0.87 | 1.85 ± 0.44† | 5.52 ± 0.22 | 6.03 ± 0.82 | 5.48 ± 0.82 | 8.88 ± 0.82 | 8.5 ± 0.62 |
| Change in esophageal pressure ($cmH_2O$) | | | | | | | | |
| Near-apneic ventilation | 2.9 ± 0.3 | 3.5 ± 0.6 | 1.8 ± 0.4 | 2.1 ± 0.1 | - | 2.2 ± 0.2 | - | -4.7 ± 0.9† |
| Spontaneous breathing | 3.4 ± 0.5 | 2.8 ± 0.5 | 2.7 ± 0.3 | -3.6 ± 0.2†,* | - | -3.2 ± 0.3†* | - | -5.8 ± 1.2† |

Values are expressed as mean ± standard error of the mean. * p<0.05 comparing groups. All time points were compared to T0. § p < 0.05 for T0 compared to baseline, †p < 0.05 compared to T0.

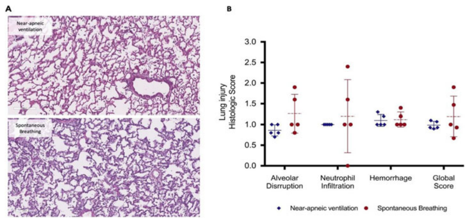

**Fig. 1 (abstract 001085).** Histological assesment of lung injury.(**A**) Representative images of lung histology for each group (original magnification: 10x hematoxilin and eosin). Images of both, near-apneic ventatilation and spontaneous ventilation groups shown similar diffuse alveolar; (**B**) Quantative score for lung injury (from 0 = normal, to 3 = maximal alteration), calculated by averaging the scores for alveolar disruption, neutrophil infiltration, and hemorrhage of the right lung, and the global score (mean of scores).

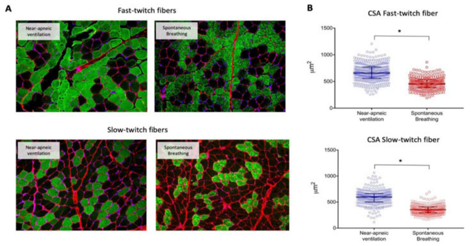

**Fig. 2 (abstract 001085).** (**A**) Immunohistochemically stained cross-sections of diaphragm muscle fiber biopsies in experimental groups by fiber type. Original magnificatio: 20x. Red: wheat germ agglutinin indication membranes. Green: Myosin heavy chain antibody specific for fast - and slow-twitch fibers.(**B**) Diaphragm fiber cross-sectional area ($µm^2$) in slow- and fast-twitch fibers by group, CSA: cross-sectional are. *p < 0.05

### 001086

#### Propofol, Ketamine and Etomidate as Induction Agents for Intubation and Outcomes in Critically Ill Patients

C. Wan[1], T. Yang[1], A. Hanson[2], P. Schulte[2], Y. Dong[3], P. Bauer[4]
[1]Department of respiratory and critical care medicine, West China Hospital, Chengdu, China; [2]Division of biomedical statistics and informatics, Mayo Clinic, Rochester, MN, USA; [3]Division of anesthesiology and perioperative medicine, Mayo Clinic, Rochester, MN, USA; [4]Division of pulmonary and critical care medicine, Mayo Clinic, Rochester, MN, USA
**Correspondence:** P. Bauer
*Intensive Care Medicine Experimental* 2019, **7(Suppl 3):**001086

**INTRODUCTION.** The intravenous agents used for induction of general anesthesia prior to intubation in ICU are either propofol, ketamine or etomidate. Their choice depends on indications, risks, benefits and preferences without clear superiority among them.

**OBJECTIVES.** To evaluate the association between agents used for endotracheal intubation and ICU and hospital outcomes in critically ill patients.

**METHODS.** Retrospective single-center cohort study of consecutive adult critically ill patients who were given a single anesthetic agent (propofol, ketamine or etomidate) for endotracheal intubation in all ICUs of a tertiary center between January 01, 2012 and December 31, 2017. Cardiac arrests and lack of research authorization were excluded. Primary outcomes were ICU and hospital mortality. Second outcomes were ICU- and hospital-free days through 28 days. An inverse probability of treatment weighed approach was used to compare treatments; multiple imputations were used for missing data. The propensity score was estimated using a generalized logit model as a function of patient characteristics: age, sex, BMI, admission source, ICU location, readmission status, length of ICU stay prior to intubation, and acute physiology score. Mortality outcomes were assessed with weighted logistic regression and –free days assessed by weighted linear regression.

**RESULTS.** The analysis included 2,673 patients, 962 (36%) received propofol, 792 (30%) received ketamine and 919 (34%) received etomidate. Overall ICU and hospital mortality were 19% and 29% respectively and lower with propofol than with ketamine or etomidate, 13, 22, 23% and 23, 32, 32% for each drug respectively. Compared with propofol, patients inducted with ketamine had higher odds of ICU mortality (Odds Ratio; 95% CI; p-value) (1.45; 1.07, 1.94; p=0.015) and higher odds of hospital mortality (1.34; 0.98, 1.84; p=0.07), and those with etomidate had higher odds of both ICU mortality (1.87; 1.40, 2.49; p<0.001) and hospital mortality (1.43; 1.09, 1.86; p=0.009). Ketamine had fewer ICU-free days (-1.24; -2.41, -0.06; p=0.039) and fewer hospital-free days (-1.21; -2.37, -0.05; p=0.041) compared to propofol. Etomidate was also associated with fewer ICU-free days (-2.10; -3.21, -1.00; p<0.001) but not hospital-free days (-0.92; -1.97, 0.13; p=0.087). When compared between them, etomidate and ketamine groups had similar outcomes except for ICU mortality which was slightly higher in the etomidate than in the ketamine group (OR, 1.29; 0.99, 1.68; p=0.057).

**CONCLUSION.** Compared to ketamine and etomidate, propofol is associated with better outcome in critically ill patients undergoing anesthesia for intubation. The hemodynamic effects of propofol might be more predictable than those with ketamine and the metabolic effects of etomidate might exert their own influence on outcome.

**REFERENCE(S)**

1. Jabre P, Combes X, Lapostolle F, Dhaouadi M, Ricard-Hibon A, Vivien B, Bertrand L, Beltramini A, Gamand P, Albizzati S, Perdrizet D, Lebail G,

Chollet-Xemard C, Maxime V, Brun-Buisson C, Lefrant JY, Bollaert PE, Megarbane B, Ricard JD, Anguel N, Vicaut E, Adnet F; KETASED Collaborative Study Group. Etomidate versus ketamine for rapid sequence intubation in acutely ill patients: a multicentre randomised controlled trial. Lancet. 2009; 374(9686):293-300.
2. Baird CR, Hay AW, McKeown DW, Ray DC. Rapid sequence induction in the emergency department: induction drug and outcome of patients admitted to the intensive care unit. Emerg Med J. 2009; 26(8):576-9.
3. Stollings JL, Diedrich DA, Oyen LJ, Brown DR. Rapid-sequence intubation: a review of the process and considerations when choosing medications. Ann Pharmacother. 2014; 48(1):62-76.
4. Koenig SJ, Lakticova V, Narasimhan M, Doelken P, Mayo PH. Safety of Propofol as an Induction Agent for Urgent Endotracheal Intubation in the Medical Intensive Care Unit. J Intensive Care Med. 2015; 30(8):499-504.
5. Upchurch CP, Grijalva CG, Russ S, Collins SP, Semler MW, Rice TW, Liu D, Ehrenfeld JM, High K, Barrett TW, McNaughton CD, Self WH. Comparison of Etomidate and Ketamine for Induction During Rapid Sequence Intubation of Adult Trauma Patients. Ann Emerg Med. 2017; 69(1):24-33.e2.
6. This project was supported by a small grant from the Research Subcommittee of the Critical Care Independent Multidisciplinary Program at Mayo Clinic, Rochester.

### 001091

#### Delphi method to clinical consensus for Bronchoscopy-guided percutaneous dilatational tracheostomy

R. de la Fuente[1], E. Kattan[2], I. Puente[3], M. Navarrete[4], J. Munoz-Gama[4],R. Fuentes[1], M. Sepúlveda[4]
[1]División anestesiología, Pontificia Universidad Catolica de Chile, Santiago, Chile; [2]Departamento de Medicina Intensiva, Pontificia Universidad Catolica de Chile, Santiago, Chile; [3]Anestesiología, Universidad de los Andes, Santiago, Chile; [4]Departamento ciencia de la computación, Pontificia Universidad Catolica de Chile, Santiago, Chile
**Correspondence:** R. de la Fuente
*Intensive Care Medicine Experimental* 2019, **7(Suppl 3):**001091

**INTRODUCTION.** Even though it's widespread implementation, the benefits of bronchoscopy-guided percutaneous dilatational tracheostomy (BG-PDT) have not been consistently demonstrated. One possible cause is the heterogeneity of techniques described(1).

**OBJECTIVES.** To develop a model for BG-PDT that includes a step by step design for successful execution, based on the literature review of published descriptions and posterior experts' consensus.

**METHODS.** Major medical databases were searched with the terms: tracheostomy, percutaneous, cricothyroidotomy, cognitive task analysis, toolkit, and evaluation. Relevant descriptions and checklists of BG-PDT and similar procedures were retrieved. Activities were extracted from each report and the adherence to McKinley's dimensions of procedural competence were analyzed: preparation, infection control, communication and work with the patient, teamwork, safety, procedural competence, and post-procedure care(2).-With this information, we developed a model represented in Business Process Model Notation (BPMN). To further validate the model, we used the Delphi method by conducting an online survey in which experts from Latin America were asked to score with a five-point Likert scale the suitability of the proposed activities. Also, experts were allowed to propose changes to the order of activities or propose new ones. Predefined criteria were used to define the "expert" group, to finalize the Delphi panel: more than 80% correlation with Pearson's R test or 3 rounds, and more than 80% of median consensus for inclusion activity in the model. Non-parametric descriptive statistics were used, dates present as median and range.

**RESULTS.** Twelve descriptions found in the literature and the local protocol were included for analysis. The descriptions presented 19 [6-33] steps and had 3 [1-5] of McKinley's dimensions. No description had all the dimensions. The most frequently represented (85%) were "preparation" and "procedural competence", while no description included "patient communication". The first model used for the Delphi panel includes all McKinley's dimensions, with two operators (bronchoscopy and tracheostomy), with a total of 51 steps divided into 6 stages. The Delphi panel was answered by 25 experts from 9 countries and was ended in the second round with a correlation of 0.83. The final model (Figure 1)included 45 activities proposed in the initial model and 6 new activities proposed by the experts; 6 activities from the initial model were excluded.

**CONCLUSION.** We have presented a BG-PDT model that includes necessary competence dimensions to be considered complete. The model has been validated by an experts' consensus, allowing to capture the inter-center variability. This model can be used to define local standards or for the training of physicians.

**REFERENCE(S)**

1. Kidane, B., & Pierre, A. F. (2018). From Open to Bedside Percutaneous Tracheostomy. Thoracic Surgery Clinics, 28(3), 263–276. http://doi.org/10.1016/j.thorsurg.2018.03.001
2. McKinley, R. K., Strand, J., Ward, L., Gray, T., Alun-Jones, T., & Miller, H. (2008). Checklists for assessment and certification of clinical procedural skills omit essential competencies: a systematic review. Medical Education, 42(4), 338–349. https://doi.org/10.1111/j.1365-2923.2007.02970.x

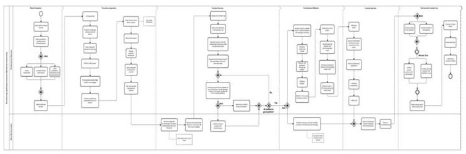

**Fig. 1 (abstract 001091).** Final Model After Delphi Panel of BG-PDT

### 001093

#### Driving pressure is a predictor of postoperative mechanical ventilation in patients undergoing emergency surgery for peritonitis: Interim analysis from a prospective observational study

R. Thakuria, A. Roy Chowdhury, CA. Kayina, N. Pangasa, S. Maitra, DK. Baidya, G. Prasad
[1]Anaesthesia,pain medicine and critical care, All India Institute Of Medical Sciences, New Delhi, NEW DELHI, India
**Correspondence:** R. Thakuria
*Intensive Care Medicine Experimental* 2019, **7(Suppl 3):**001093

**INTRODUCTION.** Patients who are undergoing emergency laparotomy are at risk of both intraoperative hypoxemia and postoperative pulmonary complications (POPC). POPCs are frequent complications following open abdominal surgery and may be associated with hospital mortality. The most important postoperative pulmonary complications are atelectasis, pneumonia, respiratory failure, and exacerbation of underlying chronic lung disease requiring postoperative mechanical ventilation.Driving pressure, a simple surrogate marker of lung strain has been found to be strongly correlated with mortality in acute respiratory distress syndrome patients and also correlated with POPC in patients undergoing surgery under general anaesthesia.

**OBJECTIVES.** We hypothesize that Driving pressure is a prognostic marker in patients undergoing emergency laparotomy and may be useful in detecting patients who might require postoperative ventilatory support.

**METHODS.** The parent study is a prospective observational single centric study, where adult patients of American Society of Anesthesiologists physical status I or II either sex aged between 18- and 65-years undergoing emergency laparotomy for clinically proven or suspected peritonitis are being recruited.The study was aimed to identify correlation between oxygen index (OI= mean airway pressure x FiO2/PaO2) and oxygen saturation index (OSI)and whether OSI is a predictor of postoperative mechanical ventilation. All patients were followed till hospital discharge.

**RESULTS.** Data of n=38 patients were analyzed in this interim analysis. Median [IQR] age of the patients was 32.5 [23- 45]y and 12 of them were female. Fifteen patients (41.7%) required postoperative mechanical ventilation and 16.7% patients died during hospital stay. Patients who required postoperative mechanical ventilation, had lower PaO2 (p=0.01), lower oxygenation index (p=0.0004) and higher driving pressure (p=0.0001) after induction of general anaesthesia. However, pCO2, pH and arterial lactate were statistically similar. In a multivariable logistic regression model which included age, baseline INR, baseline arterial lactate, OI and DP, only DP was found to be a significant predictor of postoperative mechanical ventilation [adjusted odds ratio (95% CI) 1.75 (1.14, 2.68), p=0.01; goodness-of-fit p=0.95].

**CONCLUSION.** We conclude that in patients who are undergoing surgery and having risk of POPC, intraoperative DP is a predictor postoperative mechanical ventilation irrespective of oxygenation status.

**REFERENCE(S)**

1. Neto AS, Hemmes SN, Barbas CS, Beiderlinden M, Fernandez-Bustamante A, Futier E, Gajic O, El-Tahan MR, Ghamdi AA, Günay E, Jaber S, Kokulu S, Kozian A, Licker M, Lin WQ, Maslow AD, Memtsoudis SG, Reis Miranda D, Moine P, Ng T, Paparella D, Ranieri VM, Scavonetto F, Schilling T, Selmo G, Severgnini P, Sprung J, Sundar S, Talmor D, Treschan T, Unzueta C, Weingarten TN, Wolthuis EK, Wrigge H, Amato MB, Costa EL, de Abreu MG, Pelosi P, Schultz MJ; PROVE Network Investigators. Association between driving pressure and development of postoperative pulmonary complications in patients undergoing mechanical ventilation for general anaesthesia: a meta-analysis of individual patient data. Lancet Respir Med. 2016;4:272-80.
2. Amato MB, Meade MO, Slutsky AS, Brochard L, Costa EL, Schoenfeld DA, Stewart TE, Briel M, Talmor D, Mercat A, Richard JC, Carvalho CR, Brower RG. Driving pressure and survival in the acute respiratory distress syndrome. N Engl J Med. 2015;372:747-55.
3. Balzer F, Menk M, Ziegler J, Pille C, Wernecke KD, Spies C, Schmidt M, Weber-Carstens S, Deja M. Predictors of survival in critically ill patients with acute respiratory distress syndrome (ARDS): an observational study. BMC Anesthesiol. 2016;16:108.
4. Trachsel D, McCrindle BW, Nakagawa S, Bohn D. Oxygenation index predicts outcome in children with acute hypoxemic respiratory failure. Am J Respir Crit Care Med. 2005;172:206-11.
5. None received

### 001099

#### Bilateral lung injury induced by unilateral pulmonary perfusion block

YM. Wang[1], E. Spinelli[2], F. Roma[1], E. Scotti[3], A. Mazzucco[3], I. Marongiu[3], L. Manesso[3], T. Langer[1], A. Zanella[1], G. Lopez[4], M. Battistin[3], O. Biancolilli[3], S. Ferrero[4], L. Rosso[3], T. Mauri[1], A. Pesenti[1]

[1]Department of anesthesiology,critical care and emergency,, Fondazione IRCCS Ca'Granda Ospedale Maggiore Policlinico, Milano, Italy; [2]Department of anesthesiology,critical care and emergency,, Fondazione IRCCS Ca'Granda Ospedale Maggiore Policlinico, Milan, Italy; [3]Department of pathophysiology and transplantation, University of Milan, Milano, Italy; [4]Pathology unit, Fondazione IRCCS Ca'Granda Ospedale Maggiore Policlinico, Milano, Italy

**Correspondence:** Y.M. Wang

*Intensive Care Medicine Experimental* 2019, **7(Suppl 3):**001099

**INTRODUCTION.** Pulmonary perfusion block might be associated with a combination of deleterious mechanisms, including alveolar hypocapnia and heterogeneous distribution of ventilation, which, in turn, could induce lung injury.

**OBJECTIVES.** To describe the effects of unilateral complete pulmonary perfusion block in healthy pigs undergoing protective controlled mechanical ventilation for 48 hours.

**METHODS.** Ten anesthetized pigs were randomly allocated to surgical left pulmonary artery ligation (Ligation group, n = 7) or no intervention (Control group, n = 3). All animals were ventilated on volume controlled mode with 10 ml/kg tidal volume, respiratory rate 25 bpm, FiO2 0.5 and PEEP 5 cmH2O for 48 hours or until PaO2/FiO2 fell below 100 mmHg (Tend). Gas exchange, hemodynamic parameters, and respiratory mechanics data were collected at Tend before sacrifice. Quantitative CT scan was performed to assess lung weight variation at Tend compared to initial. At autopsy, portions of left and right lung were fixed in formalin for histologic examination. Plasma samples were stored every 12 hours to measure cytokine levels (IL-6).

**RESULTS.** Two animals from the Ligation group were sacrificed at 24 and 36 hours, while all animals from the Control group safely arrived at 48 hours. Total histologic score was significantly higher in the Ligation group compared to the Control group, both for the left and the right lung (p<0,001, Figure 1). Most represented histologic alterations were alveolar neutrophils infiltration, alveolar macrophages proliferation and interstitial lymphocytes proliferation and affected both lungs of the Ligation group. In parallel, IL-6 level in the Ligation group reached a higher peak value than Controls during the study period (35.75±25.61 pg/mL vs 8.90±5.63 pg/mL, p= 0.03). At Tend, Ligation group showed lower respiratory system compliance, higher driving pressure, lower oxygenation and higher increase of the lung weight (Table 1).

**CONCLUSION.** Unilateral ligation of pulmonary artery induces bilateral lung injury in animals undergoing otherwise protective mechanical ventilation.

**REFERENCE(S)**

1. Tsuno K, Miura K, Takeya M et al. Histopathologic pulmonary changes from mechanical ventilation at high peak airway pressures. Am Rev Respir Dis. 1991 May;143(5 Pt 1):1115-20.
2. Protti A, Cressoni M, Santini A, et al. Lung stress and strain during mechanical ventilation: any safe threshold? Am J Respir Crit Care Med 2011;183(10):1354-1362.
3. Kiefmann M, Tank, Tritt MO et al. Dead space ventilation promotes alveolar hypocapnia reducing surfactant secretion by altering mitochondrial function. Thorax. 2019 Mar;74(3):219-228.
4. NONE

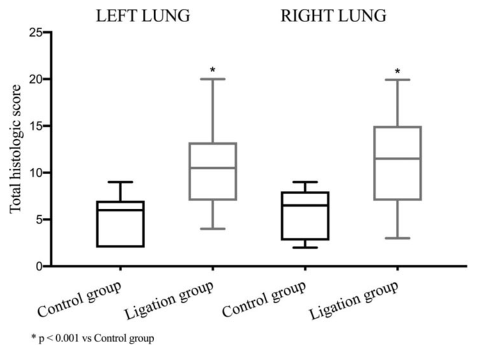

**Fig. 1 (abstract 001099).** Total histologic score between groups comparison

**Table 1 (abstract 001099).** Evaluation of respiratory and hemodynamic characateristic at Tend

| | Control group N=3 | Ligation group N=7 | P value |
|---|---|---|---|
| $PaO_2/FiO_2$ ratio | 433.33 ± 122.41 | 302.71 ± 141.73 | 0.205 |
| Peak Pressure ($cmH_2O$) | 18.00 ± 1.00 | 37.57 ± 14.50 | 0.054 |
| Driving Pressure ($cmH_2O$) | 8.33 ± 0.58 | 22.00 ± 10.65 | 0.064 |
| Static Respiratory System Compliance ($mL/cmH_2O$) | 40.05 ± 3.28 | 18.86 ± 12.28 | 0.021 |
| Left Lung Weight Variation (g) | 4.63 ± 5.69 | 44.47 ± 26.84 | 0.039 |
| Right Lung Weight Variation (g) | -72.72 ± 103.80 | 83.15 ± 95.10 | 0.049 |
| Mean Pulmonary Artery Pressure (mmHg) | 19.00 ± 2.00 | 28.14 ± 8.25 | 0.017 |
| Mean Arterial Pressure (mmHg) | 99.33 ± 15.89 | 102.17 ± 16.99 | 0.817 |

### 001106

#### Cellular profiles in different types of bronchial aspirates in mechanically ventilated patients. Is the cellular population affected?

V. Tsolaki, R. Stamatiou, E. Koutsioumpa, G. Zakynthinos, M. Xintara, E. Gerovasileiou, D. Makris
[1]Intensive care unit, University Hospital of Larissa, Larissa, Greece
**Correspondence:** V. Tsolaki
*Intensive Care Medicine Experimental* 2019, **7(Suppl 3):**001106

**INTRODUCTION.** Cellular profiles in bronchial secretions of mechanically ventilated patients are largely unknown. Different sampling procedures might result in different cellular profiles in material aspirated from the airways.[1]

**OBJECTIVES.** We aimed to evaluate bronchial secretions in terms of differential cell count and percentages obtained by mechanical ventilated critical care patients using different methods of sampling.

**METHODS.** This prospective study took place in the Intensive Care Unit of a tertiary Greek hospital during a six-month period. Patients were included if they were under MV for less than 24 hours and required bronchoscopy. Furthermore, non-mechanically ventilated subjects who underwent a single bronchoscopy, were assessed as controls. In all patients, aspiration of bronchial lining fluid (BLF) was performed, Bonchoalveolar lavage (BAL)and two types of Bronchial Washings (BW40 and BW5) were performed with the instillation of normal saline (ml) 150, 40, 5, while visible bronchial secretions were obtained via bronchoscopy (VBS) and blinded, via a common catheter for tracheobronchial aspiration (AC). Samples were homogenized in a Heidolph Silent Crusher S and total cell counting was performed after Trypan Blue staining, while cell types were assessed after May Gruenwald-Giemsa staining.

**RESULTS.** Twenty-five patients and six controls were evaluated. Mean (SE) age was 65.9(3.0) and APACHE II 18.5(1.5). Ten patients were admitted due to surgical problems. The mean total cell number (TCn) in BLF, BAL, BW40, BW5, VBS and AC were 54.2x104 (82.1x103), 11.6x105(28.8x104), 11.3x105(3.0x105), 80.7x104(13.3x104), 15.2x105(29.9x104), 10.2x105 (12.7x104) in patients and 11.2x104(17.01x104), 10.9x104(15.5x103), 12.2x104(16.8x103), 6.6x104(9.9x103), 10.9x104 (21.5x103) in controls respectively. The percentage of neutrophils (%Net) were 37.31(2.1), 22.4(2.1), 19.5(2.0), 25.8(2.1), 21.9(2.6), 24.5(1.9) vs 21.2(4.5), 23.7(5.0), 20.1(3.9), 16.2(3.9), 22.8(5.3), 11.7(5.0), in patients and controls, respectively. There were significant associations between BAL and BW40 in terms of TCn and %Net (p=0.006, p=0.03) and between VBS and AC in terms of TCn and %Net (p=0.001 and p=0.05).

**CONCLUSION.** BAL and BW40 may present similar TCn and %Net in mechanical ventilated critical care patients. Samples obtained by blind catheter tracheobronchial aspiration, as in common clinical practice, may present similar TCn and %Net with visible secretions obtained by bronchoscopy.

### 001111

#### Multidisciplinary airway training for Intensive Care staff in the workplace using the 'Bath Tea Trolley' approach

A. Serrano, G. Ball,S. Spencer, J. Chai, M. Charlton, T. Cook, F. Kelly
[1]Intensive Care, Royal United Hospital, Bath, UK
**Correspondence:** A. Serrano
*Intensive Care Medicine Experimental* 2019, **7(Suppl 3):**001111

**INTRODUCTION.** Intubation in Intensive Care Unit (ICU) is high risk ( 1,2 ) with serious airway complications 55 more likely to occur in ICU than in operating theatres (3). Airway training for ICU nurses is challenging: ICU nurses often lack anaesthetic airway experience (1,2); there are limited low risk cases for training (2);nurses receive infrequent exposure to airway management especially advanced and rescue techniques; and skill decay is an issue which affects senior and junior staff alike (4).

**OBJECTIVES.** Our aim was to design and deliver an airway training programme using the "Bath Tea Trolley" method , (5) teaching ICU staff to use a bougie and apply cricoid pressure during a rapid sequence induction.

**METHODS.** We ran a month of concentrated airway training named "Janu-Airway" using a trolley with airway training equipment on the top and pot of tea on the bottom. A trainer and the trolley travelled around the ICU providing 10-15 minutes teaching sessions for ICU staff at the bedisede during their normal working shifts. Cricoid pressure application was practiced and its effec on the view at laryngoscopy was demonstrated using a manikin and C-MAC videolaryngoscope (Karl Storz GmbH) and assisting intubation using a bougie was practised. Posters were distributed via email/closed social media groups, and handouts were given to participants to facilitate reflective learning. Feedback was collected with staff self-rating confidence before/after training using a five point Likert scale, followed by a cup of tea.

**RESULTS.** 81 members of staff received training; 18 senior nurses, 43 staff nurses, 11 junior doctors, 3 physiotherapists, 3 medical students, 1 outreach sister, 2 advanced critical care practitioners (ACCP). This included 100% of ICU nurses and junior doctors. Feedback forms were completed after 57/81 (72%) of training sessions. Resulsts as follows: increase in confidence by one point or more on the Likert scale reported for both cricoid pressure (45/58, 83%) and the use of a bougie (49/58, 84%); improvement in the abilityto assist with intubation reported by all participants - 56/58 (97%) strongly agreed, 2/58 (3%) agreed; similar training in the future was requested by all participants - 47/58 (81% strongly agreed, 11/58 (19%) agreed.

**CONCLUSION.** Our "Bath tea trolley" training programme provided effective, practical and non-threatening multidisciplinary airway training in the workplace. Training was provided to 100% of our ICU staff -during their usual shifts and for minimal cost. It was quick and easy to set up, flexible and fun. This training method could be transferable and reproducible in other ICUs

**REFERENCE(S)**

1. O ́Farrell G, McDonald M Kelly FE. Anaesthesia 2015;70(1):104
2. Lindkær Jensen NH, Cook TM, Kelly FE. Anaesthesia 2016,71,1273–1279
3. Cook TM et al. BJA 2011;1
4. Higgs A et al. BJA 2018; 120(2): 323-352
5. Nolan JP, Kelly FE. Anaesthesia 2011;66(suppl2):81–92

### 001124

#### Quantitative Lung Ultrasonography: a new algorithm for automatic detection and quantification of B-lines

C. Brusasco[1], R. Trò[2], E. Bruzzo[2], P. Boccacci[2], G. Santori[3], C. Robba[4], G. Tavazzi[5], F. Guarracino[6], F. Forfori[7], F. Corradi[7]
[1]Anaesthesia and intensive care unit, Ente Ospedaliero Ospedali Galliera, Genova, Italy; [2]Department of informatics, bioengineering, robotics and system engineering (dibris), University of Genoa, Genova, Italy; [3]Department of surgical sciences and integrated diagnostics, University of Genoa, Genova, Italy; [4]Department of anesthesia and intensive care, University of Genoa, Genova, UK; [5]Department of anesthesia and intensive care, Fondazione IRCCS Policlinico S Matteo, Pavie, Italy; [6]Cardiothoracic and vascular anesthesia and intensive care, Azienda Ospedaliero Universitaria Pisana, Pisa, Italy; [7]Department of surgical, medical and molecular pathology and critical care medicine, University of Pisa, Pisa, Italy
**Correspondence:** F. Corradi
*Intensive Care Medicine Experimental* 2019, **7(Suppl 3):**001124

**INTRODUCTION.** Lung ultrasound (LUS) is gaining recognition as a useful tool for assessing lung pathophysiology.Lung Ultrasound Scores (LUSS) have been proposed for the assessment of pulmonary edema without having been validated with a reference gold standard, i.e. invasively determined EVLW

**OBJECTIVES.** This study has two main goals. First, to test the feasibility of a modified automatic and quantitative scoring system based on the percentage of pleura involved by B-lines in an attempt to provide an objective, operator-independent and quantitative classification of the severity of pulmonary edema in comparison with the previously described semi-quantitative scores. Second, to compare the ability of this new scoring system and those previously proposed in the assessment of extra vascular lung water (EVLW) as determined by standard thermo-dilution method.

**METHODS.** Prospective observational study. Single-center, mixed medical-surgical ICU in Europe. Patients over 18 years old admitted to the Intensive Care Unit with acute respiratory distress syndrome (ARDS). Images were collected by a system (Esaote MyLab alpha or Myndray DC-N3) with high-frequency (10 MHz), linear-array probe, with the patients in a supine position. Transverse scanning was used to visualize the pleural line avoiding acoustic interference from the ribs. Six standard areas of each hemi-thorax were identified relative to sternum and axillary lines: anterior, lateral, and posterior, each ones divided into upper and lower halves. On each scan the following data were recorded: presence of A lines, maximum number of B-lines, visual percentage of lung area occupied by confluent B-lines, visual pleural involvement >50% or ≤50% and tissue-like patterns (consolidations). Five scores were defined as follows 1) maximum number of B-lines detected (nLUSS), 2) visual percentage of lung area occupied by B-lines (%LUSS)[5], 3) B-line coalescence (cLUSS)[6], 4) modified B-line coalescence score (qLUSS)[7], 5) computer-aided score (QLUSS).

**RESULTS.** 144 thoracic areas in 12 ICU patients were examined. Five scores were defined as follows 1) maximum number of B-lines detected (nLUSS), 2) visual percentage of lung area occupied by B-lines (%LUSS), 3) B-line coalescence (cLUSS), 4) modified B-line coalescence score (qLUSS), 5) computer-aided score (QLUSS). EVLW index was significantly correlated with QLUSS mean value (r=0.898; p<0.0001), qLUSS (r=0.822; p=0.001), %LUSS (r=0.743; p=0.006) and cLUSS (r=0.619; p=0.032), but not nLUSS (r=0.055; p=0.865). Absolute agreement between raters was strong for cLUSS [ICC: 0.703 (95 % CI 0.303 to 0.858)], %LUSS [ICC: 0.745 (95 % CI 0.631 to 0.822)], qLUSS [ICC: 0.825 (95 % CI 0.705 to 0.896)] and nLUSS [ICC: 0.895 (95 % CI 0.835 to 0.931)], with the best correlation for QLUSS [ICC: 0.998 (95 % CI 0.996 to 0.999)].

**CONCLUSION.** This pilot study shows that only computer-aided methods measuring the percentage of pleural line affected by B-lines can give a reliable assessment of extra vascular lung water and performs better than scores. This approach to B-lines quantification can turn images into numbers, thus providing an operator-independent analysis of large amounts of data and improving inter-observer agreement.

##### REFERENCE(S)

1. Mongodi S, Bouhemad B, Orlando A, Stella A, Tavazzi G, Via G, Iotti GA, Braschi A, Mojoli F: Modified Lung Ultrasound Score for Assessing and Monitoring Pulmonary Aeration. Ultraschall in der Medizin 2017, 38(5):530-537.
2. Corradi F, Brusasco C, Vezzani A, Santori G, Manca T, Ball L, Nicolini F, Gherli T, Brusasco V: Computer-Aided Quantitative Ultrasonography for Detection of Pulmonary Edema in Mechanically Ventilated Cardiac Surgery Patients. Chest 2016, 150(3):640-651.
3. Corradi F, Ball L, Brusasco C, Riccio AM, Baroffio M, Bovio G, Pelosi P, Brusasco V: Assessment of extravascular lung w ater by quantitative ultrasound and CT in isolated bovine lung. Respiratory physiology & neurobiology 2013, 187(3):244-249.

### 001141

#### Predictors of mortality at 48 hours in hypoxic patients initially supported by nasal high flow

L. Manesso[1], T. Mauri[1], E. Spinelli[1], E. Carlesso[2], I. Marongiu[1], A. Pacheco[3], M. García de Acilu[4], A. Pesenti[1], G. Grasselli[1], O. Roca[4]

[1]Department of anesthesiology, Fondazione IRCCS Ca'Granda Ospedale Maggiore Policlinico, Milano, Italy; [2]Department of pathophysiology and transplantation, University of Milan, Milano, Italy; [3]Oncology department, Vall d'Hebron University Hospital. VHIO, Barcelona, Spain; [4]Critical care department, Vall d´Hebron University Hospital, Barcelona, Spain

**Correspondence:** L. Manesso

*Intensive Care Medicine Experimental* 2019, **7(Suppl 3):**001141

**INTRODUCTION.** Nasal High Flow (NHF) is a non-invasive respiratory support for patients with hypoxic respiratory failure. Currently, intermediate physiologic outcomes that accurately predict mortality lack, but could be useful to test new treatments and to stratify severity.

**OBJECTIVES.** We aimed to identify physiological parameters that independently predict mortality at 48 hours in hypoxic patients initially supported by NHF.

**METHODS.** We enrolled eighty-three hypoxic patients with pneumonia and initially supported by NHF. We recorded baseline demographics, disease severity (SOFA, APACHE II, PSI scores) and quadrants involved at chest radiograph. Then, intubation rate and physiological parameters were collected at 48 hours and, finally, in-hospital mortality was recorded. By use of relevant parameters at 48 hours, we calculated a new Saturation Index as (FiO2 × Mean Airway Pressure (MAP))/SpO2; where MAP was 0 for non-intubated and not on NHF patients, flow/10 cmH2O for patients on NHF and the one measured by the ventilator in intubated patients. Finally, we conducted univariate and multivariate analysis for parameters at 48 hours to disclose independent predictors of mortality.

**RESULTS.** 66% patients were female and mean age was 59 ± 14.7 years old. APACHE II and PSI scores were 17 ± 7 and 104 ± 40. NHF was initially started with flow 42 ± 7 L/min and FiO2 0.68 ± 0.23. At 48 hours, sixteen patients were intubated (19.3%), while thirteen (15.7%) were weaned from NHF support. In-hospital mortality was 30.2% (25 patients). Univariate analysis showed that, at 48 hours, FiO2, SpO2/FiO2, Saturation Index and number of intubations were significantly different between survivors and non-survivors (Table 1). At logistic multivariate analysis performed with these four parameters, only SpO2/FiO2 ratio emerged as independent predictor of hospital mortality (OR 0.97, 95% CI 0.95 to 0.99, p=0.011). ROC curve analysis confirmed this result with AUC-ROC of 0.807 (95% CI 0.69 to 0.92, p<0.001) and cut-off of 138 (sensitivity 65% and specificity 87%).

**CONCLUSION.** In hypoxic patients initially supported by NHF, lower SpO2/FiO2 ratio measured at 48 hours independently predicts hospital mortality.

**Table 1 (abstract 001141).** Univariate statistics

| | Survivors *(n=58)* | Non-survivors *(n=25)* | |
|---|---|---|---|
| Parameters at 48 hours | | | |
| Respiratory rate | 20 (17.25; 24) | 22 (20; 26) | p=0.127 |
| FiO2 | 60 (45; 60) | 72.5 (60; 85) | **p<0.001** |
| SpO2 | 96 (95; 98) | 96 (95; 97) | p=0.843 |
| SpO2/FiO2 | 163 (156; 216.3) | 123 (112.5; 159.5) | **p<0.001** |
| Saturation Index | 2.46 (1.25; 3.16) | 3.51 (2.52; 7.94) | **p=0.002** |
| Intubation rate at 48 hours | 8 (13.8%) | 8 (32%) | **p=0.054** |
| Patients still on NHF | 40 (69%) | 14 (56%) | p=0.254 |

Data are expressed as median (interquantile range)

### 001151

#### Influence of body mass index on respiratory mechanics in acute respiratory distress syndrome: a multicenter cohort study

R. Coudroy[1], D. Vimpere[2], N. Aissaoui[2], R. Younan[2], C. Bailleul[2], A. Couteau-Chardon[2], A. Lancelot[2], L. Chen[3], JL. Diehl[4], L. Brochard[5], E. Guerot[2]

[1]Médecine intensive et réanimation, CHU de Poitiers, Poitiers, France; [2]Medical intensive care unit, Hôpital Européen Georges Pompidou APHP, Paris, France; [3]Interdepartmental division of critical care - university of toronto, Hospital St. Michae and Keenan Research Center, Toronto, Canada; [4]Intensive care, Assistance Publique - Hôpitaux de Paris, Hôpital Européen Georges Pompidou, Paris, France; [5]Interdepartmental division of critical care - university of toronto, Hospital St. Michael and Keenan Research Center, Toronto, Canada

**Correspondence:** R. Coudroy

*Intensive Care Medicine Experimental* 2019, **7(Suppl 3):**001151

**INTRODUCTION.** Overweight and obesity are increasingly prevalent worldwide and account for about 30-40% of patients with acute respiratory distress syndrome (ARDS). How body mass index (BMI) influences respiratory mechanics in ARDS is unclear.

**OBJECTIVES.** To compare the respiratory mechanics of obese and non-obese ARDS and assess the influence of BMI on respiratory mechanics and lung volumes.

**METHODS.** This study is a secondary analysis of 2 cohorts of ARDS according to the Berlin definition: a bicenter Canadian study of 45 ARDS of any BMI enrolled in a prospective study (NCT02457741), and a French monocenter cohort of selected ARDS with a BMI > 40 kg/m2. Airway pressure, flow and esophageal pressure were collected in all patients and we report data at a set positive end-expiratory pressure (PEEP) of 5 cmH2O. Presence of complete airway closure (1) and airway opening pressure were assessed using a low-flow inflation pressure-volume curve. End expiratory lung volume (EELV) was measured using the nitrogen washout/washin technique. The ratio EELV to predicted functional residual capacity (2) was calculated.Patients were sorted in 3 groups according to the World Health Organization overweight classification (BMI < 30, from 30 to < 40, and ≥40 kg/m2).

**RESULTS.** Among the 54 patients included, 18 patients (34%) had BMI < 30 kg/m2, 16 (30%) between 30 and 40 kg/m2, and 19 (36%) ≥40 kg/m2. The median PaO2/FiO2 was 138 mmHg with a PEEP of 15 cmH2O, and mortality was 32% without difference across groups.

Airway closure occurrence increased with BMI (22%, 38% and 58%, p= 0.04). When present, airway opening pressure was 9.6 cmH2O (8.5-13.2) and similar between the 3 groups. With increasing BMI, total PEEP increased from 6.0 to 9.0 cmH2O between groups (p= 0.02). All values of esophageal pressure increased with BMI. End-expiratory esophageal pressure was strongly correlated with BMI (rho= 0.71, p<0.001), as illustrated in Figure 1. Consequently end-expiratory transpulmonary pressure decreased from -2.7 to -9.3 cm H2O with increasing BMI (p= 0.008). The ratio of EELV to predicted functional residual capacity was negatively correlated with end-expiratory pressure (Rho= -0.39, p= 0.01), but not with BMI. Driving pressure and elastance of the respiratory system, chest wall and lung were similar across all ranges of BMI. Likewise, EELV was similar between groups.

**CONCLUSION.** In ARDS, increasing BMI is associated with increased occurrence of airway closure and increased values of esophageal pressure. Conversely, chest wall elastance is not influenced by BMI, as well as lung elastance. Including BMI in interpreting respiratory mechanics in ARDS patients can provide additional information for the clinical management.

**REFERENCE(S)**

1. Chen L, Del Sorbo L, Grieco DL, Shklar O, Junhasavasdikul D, Telias I, et al. Airway Closure in Acute Respiratory Distress Syndrome: An Underestimated and Misinterpreted Phenomenon. Am J Respir Crit Care Med. 2018 Jan 1;197(1):132–6.
2. Ibañez J, Raurich JM. Normal values of functional residual capacity in the sitting and supine positions. Intensive Care Med. 1982;8(4):173–7.

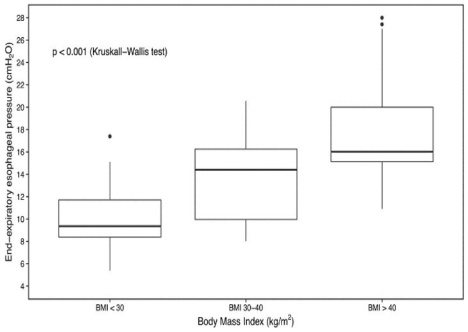

**Fig. 1 (abstract 001151).** See text for description

## DS - From data to care: Applying data science to critically ill patients

### 000199

#### Incidence of Lower Limb Mottling in Critical Care Patients and Associated Mortality Risk

M. Harford[1], J. Bedford[1], OC. Redfern[1], J. Jorge[2], M. Villarroel[2], L. Tarassenko[2], JD. Young[1], P. Watkinson[1]
[1]Critical care research group, University of Oxford, Oxford, UK;
[2]Department of engineering science, University of Oxford, Oxford, UK
**Correspondence:** M. Harford

*Intensive Care Medicine Experimental* 2019, **7(Suppl 3):**000199

**INTRODUCTION.** Mottling is a patchy discolouration of the skin, thought to be secondary to a reduction of blood flow within the superficial skin vasculature [1]. Although it may occur in the absence of acute illness, in intensive care patients it is associated with haemodynamic collapse and failing circulation. Mottling was first described as a sign of poor perfusion as early as 1894 [2], but in the last decade there has been a renewed interest in assessing mottling in critical care [3-7]. Previous studies, where attempts have been made to quantify the extent of mottling, have shown that increased mottling of the lower limb is associated with higher mortality rates [3]. The reported incidence of mottling varies considerably. Estimates range from 2.5% [8] to 70% [3], which may be due to relatively small cohorts in these studies (ranging from 14 [4] to 791 [5] patients). It has been suggested that persistent mottling of the skin is an independent risk factor for ICU mortality, after adjusting for the use of vasopressors, mechanical ventilation and hyperlactaemia [5]. However, these factors might not adequately adjust for illness severity, leading to a lack of clarity on whether mottling is truly an independent risk factor of poor prognosis.

**OBJECTIVES.** The aim of this retrospective cohort study was to assess the incidence of mottling in the intensive care unit (ICU) and its relationship with mortality and length of stay.

**METHODS.** We used data collected as part of the Post-Intensive-Care Risk-adjusted Alerting and Monitoring (PICRAM) study of patients admitted to ICU at a large UK hospital between June 2008 and December 2015. The presence of mottling was extracted from nursing lower limb skin assessment which were documented from a pre-defined list of terms including 'mottled'. Admissions were classified as either 'mottled for any duration during ICU stay' or 'never mottled'. For each admission, we also calculated the Oxford Acute Severity of Illness Score (OASIS), and outcome data comprising ICU mortality, ICU length of stay and hospital mortality were extracted. Logistic regression analysis was used to assess the effect of mottling status on outcomes.

**RESULTS.** 8,556 unique admissions to ICU were identified, of which 1,136 (13.3%) had documented mottling of the lower limb at some point during their ICU admission. Unadjusted mortality rate in the mottled group was 33.9% compared to 7.1% in the never-mottled group. Unadjusted within-ICU mortality was significantly higher in the mottled group (OR 6.71 (95% CI 5.76-7.80)). When adjusted for severity of illness using OASIS, ICU mortality risk in mottled patients remained significantly elevated at OR 3.07 (95% CI 2.59-3.64). ICU length of stay was increased by a factor of 1.54 or 4.2 days.

**CONCLUSION.** This is the largest study of mottling in critical care. We calculated the incidence of mottling to be 13.3% of ICU admissions, which is at the lower end of previous estimates. The lower estimates of our cohort may be due to under-reporting of mottling when a minor degree of mottling over the knee was present (equating to mottling score of 1 out of 5). Mottling is a clinical sign of poor skin perfusion and is associated with significantly increased mortality risk with OR 6.71. It is an independent predictor of ICU mortality.

**REFERENCE(S)**

[1] Duval, A., & Pouchot, J. (2008). Livedo: from pathophysiology to diagnosis. La Revue de medecine interne, 29(5), 380-392.
[2] Unna PG. (1984). Die Histopathoogie der Hautkrankheiten.
[3] Ait-Oufella, H., Lemoinne, S., Boelle, P. Y., Galbois, A., Baudel, J. L., Lemant, J., Joffre, J., Margetis, D., Guidet, B., Maury, E., & Offenstadt, G. (2011).

Mottling score predicts survival in septic shock. Intensive care medicine, 37(5), 801-807.
[4] Ait-Oufella, H., Bourcier, S., Alves, M., Galbois, A., Baudel, J. L., Margetis, D., Bige, N., Offenstadt, G., Maury,E., & Guidet, B. (2013). Alteration of skin perfusion in mottling area during septic shock. Annals of intensive care, 3(1), 31.
[5] Coudroy, R., Jamet, A., Frat, J. P., Veinstein, A., Chatellier, D., Goudet, V., Cabasson, S., Thille, A. W., & Robert, R. (2015). Incidence and impact of skin mottling over the knee and its duration on outcome in critically ill patients. Intensive care medicine, 41(3), 452-459.
[6] de Moura, E. B., Amorim, F. F., da Cruz Santana, A. N., Kanhouche, G., de Souza Godoy, L. G., de Jesus Almeida, L., Rodrigues, T.A., da Silveira, C. D. G., & de Oliveira Maia, M. (2016). Skin mottling score as a predictor of 28-day mortality in patients with septic shock. Intensive care medicine, 42(3), 479-480.
[7] Ferraris, A., Bouisse, C., Mottard, N., Thiollière, F., Anselin, S., Piriou, V., & Allaouchiche, B. (2018). Mottling score and skin temperature in septic shock: Relation and impact on prognosis in ICU. PloS one, 13(8), e0202329.
[8] Grissom, C. K., Morris, A. H., Lanken, P. N., Ancukiewicz, M., Orme Jr, J. F., Schoenfeld, D. A., & Thompson, B. T. (2009). Association of physical examination with pulmonary artery catheter parameters in acute lung injury. Critical care medicine, 37(10), 2720.
[9] National Institute for Health Research
[10] Oxford Biomedical Research Centre

### 000290

#### Oxygen saturation measurements in patients admitted to English intensive care units

B. Post[1], E. Palmer[2], S. Harris[3], N. MacCallum[4], D. Brealey[4], M. Singer[3], D. Martin[5]
[1]Adult critical care unit, Barts Health NHS Trust, London, UK; [2]{street_address}, London, UK; [3]Bloomsbury institute of intensive care medicine, University College London, London, UK; [4]Bloomsbury institute of intensive care medicine, University College London, Gower Street, London, UK, London, UK; [5]Division of surgery and interventional science, Royal Free Hospital, London, UK
**Correspondence:** B. Post
*Intensive Care Medicine Experimental* 2019, **7(Suppl 3):**000290

**INTRODUCTION.** Exposure to excessive oxygen concentrations may be harmful to acutely unwell patients (Girardis 2016; Helmerhorst 2016) and new guidelines recommend avoiding an oxygen saturation (SpO2) >96% (Siemieniuk 2018). Little emerging literature and guidance is specific to patients on the intensive care unit (ICU) and robust data, describing normal practice, in this cohort is absent. We aimed to determine the standard of care for oxygenation in patients admitted to ICUs in England.

**METHODS.** Data was interrogated from the Critical Care Health Informatics Collaborative (CC-HIC) data infrastructure (Harris 2018; Shi 2017) - a collaboration between five National Health Service Trusts in England, aggregating high fidelity time series data on patients from 12 ICUs. SpO2 values were queried for patient episodes from January 2014 to December 2018. Raw data were presented as time-weighted mean values for SpO2 (SpO2-tw) for a number of predetermined subgroups. Statistical analysis was not performed as the sample size would be powered to detect clinically meaningless signal.

**RESULTS.** After applying centrally determined validation procedures, we identified 6,524,918 SpO2 values from 44,150 individual patient episodes. The median [IQR] SpO2-tw for the whole cohort was 96.7% [95.5-97.8]. When patients receiving a fractional inspired oxygen concentration (FIO2) of 0.21 were excluded, the median SpO2-tw was 96.9% [95.5-98.1]. For apriori defined subgroups the median [IQR] SpO2-tw were: invasively ventilated (97.1% [95.5-98.3]) versus patients not invasively ventilated (96.5% [95.3-97.6]) (Fig. 1); primary medical (96.4% [95.0-97.6]) versus surgical diagnosis (97.0% [96.0-98.0]); prior or current diagnosis of chronic obstructive pulmonary disease (COPD) (94.5% [92.9-96.1]) versus no such diagnosis (96.8% [95.6-97.8]) (Fig 2).

**CONCLUSION.** In an unselected ICU cohort, the median SpO2-tw was higher than recommended for acutely unwell patients outside of the ICU. Statistical analysis was not performed, but visual inspection of the distributions shows clear differences between subgroups. The median SpO2-tw, for patients with COPD, is higher than current guideline recommendations (88-92%)(O'Driscoll 2017). Future study design should be mindful of this standard of care.

**REFERENCE(S)**

1. This research was conducted using NIHR Health Informatics Collaborative (NIHR HIC) data resources
2. Siemieniuk et al. 2018. "Oxygen Therapy for Acutely Ill Medical Patients: A Clinical Practice Guideline." BMJ, October, k4169.
3. Shi et al. 2017. "Critical Care Data Processing Tools." The Journal of Open Source Software 2 (20): 513.
4. O'Driscoll et al. 2017. "BTS Guideline for Oxygen Use in Adults in Healthcare and Emergency Settings." Thorax 72 (Suppl 1): ii1–90.
5. Helmerhorst et al. 2016. "Effectiveness and Clinical Outcomes of a Two-Step Implementation of Conservative Oxygenation Targets in Critically Ill Patients: A Before and After Trial." Critical Care Medicine 44 (3): 554–63.
6. Harris et al. 2018. "Critical Care Health Informatics Collaborative (CCHIC): Data, Tools and Methods for Reproducible Research: A Multi-Centre UK Intensive Care Database." International Journal of Medical Informatics 112: 82–89.
7. Massimo, et al. 2016. "Effect of Conservative vs Conventional Oxygen Therapy on Mortality Among Patients in an Intensive Care Unit: The Oxygen-ICU Randomized Clinical Trial." JAMA 316 (15): 1583–89.

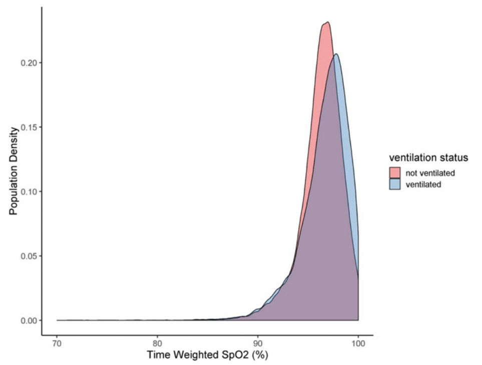

**Fig. 1 (abstract 000290).** Mechanically ventilated versus non-mechanically ventilated patients

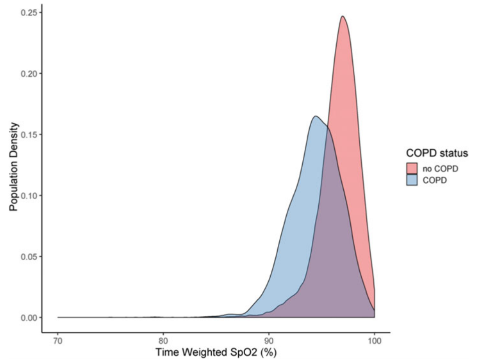

**Fig. 2 (abstract 000290).** Patients with COPD versus patients with no COPD

### 000344

#### Using machine learning in rapid response teams activation system

A. Garza de la Maza[1], GE. Carmona[2], LN. Signoret[3], ZE. Monares[4], MCA. Galindo[5], R. Lozano Zúñiga[6], EA. Ojeda Izquierdo[7], JO. Guamán Crespo[8]
[1]Intensive critical care unit, Hospital San Angel Inn Universidad, Mexico City, Mexico; [2]Data & analytics manager and econometric student., Independent, Mexico City, Mexico; [3]Medical student, Hospital San Angel Inn Universidad, Mexico City, Mexico; [4]Intensive critical care unit., Hospital San Angel Inn Universidad, Mexico City, Mexico; [5]Chief nutrition department, Hospital San Angel Inn Universidad, Mexico City, Mexico; [6]Critical Care, Hospital San Angel Inn Universidad, Mexico City, France; [7]Critical care, Hospital San Angel Inn Universidad, Mexico City, Mexico; [8]Intensive Care Unit, Hospital San Ángel Inn Universidad, Ciudad de México, France
**Correspondence:** A. Garza de la Maza
*Intensive Care Medicine Experimental* 2019, **7(Suppl 3):**000344

**INTRODUCTION.** One of the main goals of a rapid response team (RRT) is to prevent a "failure to rescue" which is the lack of a reaction for a patient which has abnormal vital signs and could increase the probability of a serious adverse event.. The ideal trigger method should increase the accuracy for detection preventing false positives, which could result in fatigue of the team, and decreasing false negatives resulting in a "failure to rescue" and its consequences. (1–3) One of the main problems using a method developed in other hospital, country or population is the implicit heterogeneity and variance, highlighting the importance of personalized tools internally developed using a self-learning machine. We developed a personalized cell phone app based on the data collected from our RRT, showing that every hospital could develop their own tool increasing its applicability and accuracy.

**METHODS.** Prospective observational study including adult hospitalized non-critically ill patients, excluding obstetric patients and "do not resuscitate" order patients. Every vital sign reported by the nursing staff, age, gender and admission diagnosis was collected in a data base with the final outcome of transfer to the Intensive Care Unit (ICU) during a 3 months period. R version 3.4.0 (20017-04-21) was used for the data analysis and shinyapps.io for the cell phone app.

**RESULTS.** 6040 cases (set of variables) were included in the final analysis. In order to program the self-learning machine the "caret" package was used in R, using the neural network method or "nnet" with the following parameters: decay of 0.0001, 100 iterations, scaling and centering pre-processing. The final formula used for the self-learning machine was "TRANSFER (to the ICU)~ Age+ SBP (systolic blood pressure)+ DBP (diastolic blood pressure)+ HR (Heart rate)+ RR (Respiratory rate)+ SAT_O2 (Oxygen saturation)". The data frame for training contained 75% of the data and the 25% left for the testing stage.

**CONCLUSION.** The use of learning machine methods to develop a personalized tool for RRT activation is a promising field. Every hospital has the potential to develop their own tools based on their unique characteristics and keep enhancing it when considered necessary.

##### REFERENCE(S)

1. Jones DA, DeVita MA, Bellomo R. Rapid-Response Teams. N Engl J Med [Internet]. 2011;365(2):139–46. Available from: http://www.ncbi.nlm.nih.gov/pubmed/21751906%0Ahttp://www.nejm.org/doi/abs/10.1056/NEJMra0910926
2. Chen J, Bellomo R, Flabouris A, Hillman K, Assareh H, Ou L. Delayed emergency team calls and associated hospital mortality: A multicenter study. Crit Care Med. 2015;43(10):2059–65.
3. Churpek MM, Yuen TC, Edelson DP. Risk stratification of hospitalized patients on the wards. Chest. 2013;143(6):1758–65.
4. The authores declare not having conflict of interest related to the present study.

F2

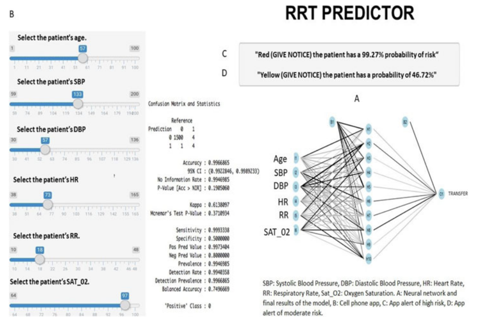

**Fig. 1 (abstract 000344).** See text for description

### 000354

#### Multimorbidity in Intensive Care: prevalence and its effects on Mortality prediction modelling

M. Blayney[1], L. Donaldson[2], P. Smith[2], S. Cole[3], D. Mcallister[4], N. Lone[5]
[1]University of edinburgh medical school, University of Edinburgh, Edinburgh, UK; [2]Scottish intensive care society audit group, NHS National Services Scotland, Glasgow, UK; [3]Ninewell's hospital, NHS Tayside, Dundee, UK; [4]Institute of health and wellbeing, University of Glasgow, Glasgow, UK; [5]Usher institute for population health sciences and informatics, The University of Edinburgh, South Bridge, Edinburgh, UK, Edinburgh, UK
**Correspondence:** M. Blayney
*Intensive Care Medicine Experimental* 2019, **7(Suppl 3):**000354

**INTRODUCTION.** ICU populations have increasing levels of multimorbidity. The APACHE-II model is currently used in Scotland to publicly report risk-adjusted mortality in ICUs and benchmark care quality(1,2). However, the model only accounts for severe comorbidity. In Scotland, two national population-wide datasets exist from which milder comorbidities can be derived. These are the Scottish Morbidity Record (SMR01) comprising hospital discharge records for all Scottish hospitals(3), and the Prescribing Information System (PIS), a dataset comprising all community dispensed medical prescriptions(4).

**OBJECTIVES.**

- To derive comorbidity measures from SMR01 and PIS, report prevalence of comorbidities and their association with mortality in a Scottish ICU population
- To ascertain whether adding comorbidities derived from the two data sources to the APACHE-II-derived risk prediction model improves mortality prediction

**METHODS.** Cohort study of all patients admitted to Scottish ICUs from 2010-2017 (excluding readmissions) derived from the Scottish Intensive Care Society Audit Group (SICSAG) database, comprising all admissions to Scottish ICUs(5). Comorbidities were derived from two datasets: SMR01-derived comorbidities defined by Charlson(6); PIS-derived comorbidities using British National Formulary codes, adapted from a study using Anatomical Therapeutic Chemical codes(7). Prevalence of derived comorbidities was reported. Logistic regression was used to report the univariable association of each comorbidity with ultimate hospital mortality. After excluding patients ineligible for APACHE-II risk scoring, three risk prediction models for ultimate hospital mortality were developed using multivariable logistic regression: current APACHE-II model, APACHE-II plus PIS comorbidities, and APACHE-II plus SMR01 comorbidities. ROC graphs, area under the receiver operating characteristic curve (AUROC), AIC, BIC and Brier's score were compared.

**RESULTS.** 99773 patients were included during the study period. 19108 (19.2%) died before ultimate hospital discharge. 17 comorbidity categories were derived from SMR01, and 20 from PIS. The most prevalent SMR01 comorbidity was cancer (15521 (15.6%)), the least was HIV (162 (0.2%)). The most prevalent PIS comorbidity was pain (42354 (42.5%)), the least was dementia (266 (0.3%)). The median number of comorbidities by SMR01 was 1 (IQR 0,1); PIS was 2 (IQR 0,4). On univariable analysis of SMR01, "moderate-severe liver disease" held the highest OR for mortality (OR 3.76 (CI 3.46,4.08), p<0.001); from PIS, the highest was dementia (OR 1.68 (CI 1.44,2.49), p<0.001). Adding comorbidity increased the predictive ability of both models compared to the APACHE-II model, as measured by AUROC, AIC, BIC and Brier's score. The best model measured by AUROC was "APACHE plus SMR01 comorbidities" (AUROC=0.871 vs 0.865, p<0.001).

**CONCLUSION.** Prescribing data yielded greater prevalence of comorbidities than hospital discharge records. Adding comorbidities to risk prediction models for patients admitted to ICU leads to a small improvement in prediction of mortality compared with the current APACHE II model. Current ICU risk prediction models may need to be revised to include wider comorbidity measures as increasingly multimorbid patients are admitted to ICUs.

**REFERENCE(S)**

1. Knaus W, Draper E, Wagner D, Zimmerman J. APACHE II: a severity of disease classification system. Crit Care Med. 1985;13(10):818–29.
2. Scottish Intensive Care Society Audit Group. Audit of Critical Care in Scotland 2018 report - reporting on 2017 [Internet]. 2018. Available from: http://www.sicsag.scot.nhs.uk/publications/_docs/2018/2018-08-14-SICSAG-report.pdf?55
3. Information Services Division Scotland. General Acute Inpatient and Day Case - Scottish Morbidity Record (SMR01) [Internet]. National Data Catalogue. 2016 [cited 2019 Apr 8]. Available from: https://www.ndc.scot.nhs.uk/National-Datasets/data.asp?SubID=5
4. Information Services Division Scotland, National Services Scotland. Prescribing and Medicines [Internet]. 2018 [cited 2019 Apr 8]. Available from: https://www.isdscotland.org/Health-Topics/Prescribing-and-Medicines/Prescribing-Datamarts/#pis
5. Scottish Intensive Care Society Audit Group. Data Quality [Internet]. 2014 [cited 2018 Aug 22]. p. 1. Available from: http://www.sicsag.scot.nhs.uk/quality/data.html
6. Charlson ME, Pompei P, Ales KL, MacKenzie RC. a New Method of Classifying Prognostic in Longitudinal Studies : Development and validation. J Chron Dis. 1987;40(5):373–83.
7. Huber CA, Szucs TD, Rapold R, Reich O. Identifying patients with chronic conditions using pharmacy data in Switzerland: An updated mapping approach to the classification of medications. BMC Public Health. 2013;13(1):1–10.
8. No grants were used for this project.

### 000416

#### Artificial Intelligence Assists Junior Clinicians in Assessing Risk of Severe Cardio-respiratory Instability in Monitored Patients

L. Chen[1], M. Hravnak[2], T. Pellathy[2], J. Yoon[3], G. Clermont[3], M. Pinsky[3], A. Dubrawski[1]

[1]Robotics institute, auton lab, Carnegie Mellon University, Pittsburgh, USA; [2]School of nursing, University of Pittsburgh, Pittsburgh, USA; [3]School of medicine, University of Pittsburgh, Pittsburgh, USA

**Correspondence:** M. Hravnak

*Intensive Care Medicine Experimental* 2019, **7(Suppl 3):**000416

**INTRODUCTION.** To decide whether or not to escalate care, clinicians must accurately assess risk of patients' cardio-respiratory instability (CRI) based on continuously monitored multi-parameter Vital Signs (VS). Assessment accuracy depends on experience, and is a challenge for clinicians.

**OBJECTIVES.** We propose that an Artificial Intelligence (AI) model built on expert clinicians' adjudication could assist junior nurses (RN) and doctors (MD) to improve their assessments.

**METHODS.** VS data (heart rate, respiratory rate, blood pressure, oxygen saturation by pulse oximetry) were collected from 1087 continuously monitored step-down unit patients. We fit a multi-class random forest AI model using four-level annotation labels provided by expert senior clinicians (1 RN, 1 MD) suggestive of patients' CRI severity levels and need for care escalation. We also asked two junior clinicians (1 RN and 1 MD) to provide independent assessment of patients' severity levels, blinded to experts' annotations. To emulate scenarios where AI models may be used in practice to assist clinicians' subjective assessment, we applied a conservative adjustment rule on junior clinicians' assessment by overriding their decisions only if the severity level determined by the model was higher than theirs.

**RESULTS.** We computed pairwise disagreement (1-Cohen's kappa) between expert clinicians, junior clinicians, and the AI model, visualized as distances in the multidimensional scaling (MDS) plot in Fig. 1. Before adjustment, the Junior RN and Junior MD widely disagreed with expert assessment, but converged toward experts when supplemented by the model's suggestions (arrows). Changes of Cohen's kappa with respect to experts' consensus are from .28 to .56 for Junior RN, and .36 to .49 for Junior MD. Also, disagreement between the Junior RN and Junior MD decreased, with Cohen's kappa increased from .27 to .62. After incorporating the AI model's suggestions, the true positive rate (proportion of high CRI risk patients correctly identified, computed using experts' consensus as ground truth) increased (.376 to .836 for Junior RN; .633 to .907 for Junior MD), both converging towards the model's and experts' rating, at a low false positive rate (.03 for junior RN, .22 for junior MD).

**CONCLUSION.** Incorporating AI encoding expert opinion into the decisional process of junior clinicians' assessment of patient's propensity for severe CRI could improve their clinical decisions.

1. NIH R01NR013912

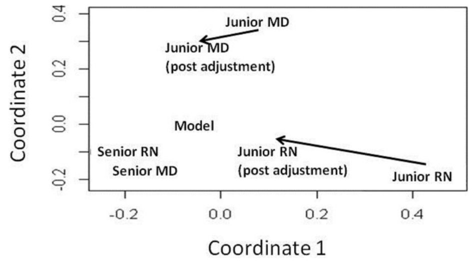

**Fig. 1 (abstract 000416).** MDS of inter-rater agreements measured with Cohen's kappa. Distance between points represent the extent of disagreement between ratings by different individuals. Arrows show changes of Junior MD and RN assesment adjusted with the AI model trained on data from senior clinicians.

### 000487

#### Development of a Prediction Model for Correction of Hypernatremia in the ICU Using Machine Learning Methods

E. Mlodzinski[1], G. Chua[2], R. Sherak[3], D. Chiu[4], N. Qiao[5], LA. Celi[6]

[1]Medicine, Beth Israel Deaconess Medical Center (BIDMC), Boston, USA; [2]Operations research center, MIT Sloan School of Management, Cambridge, USA; [3]Medicine, Albert Einstein College of Medicine, New York, USA; [4]Medicine, Boston University School of Medicine, Boston, USA; [5]Neurosurgery, Huashan Hospital, Shanghai, China; [6]Pulmonary, critical care and sleep medicine, Beth Israel Deaconess Medical Center (BIDMC), Boston, USA

**Correspondence:** E. Mlodzinski

*Intensive Care Medicine Experimental* 2019, **7(Suppl 3):**000487

**INTRODUCTION.** Hypernatremia is a known independent risk factor for mortality and increases length of stay in the ICU, and slow rates of correction have also been shown to increase mortality. Most patients are undercorrected based on previous studies. Machine learning methods have the potential to improve the management of this condition.

**OBJECTIVES.** The study is aimed to use machine learning methods to develop a model which can predict appropriate serum sodium correction in patients with hypernatremia in 24 hours from ICU admission. The secondary aim is to identify which factors are most influential in recovery from hypernatremia.

**METHODS.** Using the Medical Information Mart for Intensive Care Database (MIMIC-III), we performed a retrospective analysis of patients with hypernatremia (defined as sodium >145 mmol/L) on admission to the ICU. Appropriate correction was defined as either a decrease in serum sodium by 6 to 10 mmol/L or to less than 145 mmol/L without correcting >10 mmol/L in the first 24 hours. The primary exposure was fluid volume controlled for sodium content received in the first 24 hours of admission. We calculated the total sodium content of fluids received and created categories of total free water intake and total crystalloid intake. We queried the database for clinically relevant covariates, including lab data and admission diagnoses. We trained our model in 75% of the patients and tested the model in 25% of the patients. We used both a regularized logistic regression and a random forest classifier to build predictive models. We examined the importance of the covariates in the random forest model.

**RESULTS.** 1902 patients met criteria for inclusion. 54.6% of patients achieved appropriate correction. Factors associated with lack of correction included older age, sepsis, higher initial sodium value, and elevated BUN. Those who did not correct had a higher mortality rate (24.6% vs. 20.2%). The volume of free water and total fluid volume were not significantly different between the groups. The logistic regression model produced an AUC of 0.74, and the random forest model produced an AUC of 0.76. By examining the variable importance of the random forest model, we identified baseline sodium and BUN as influential predictors for hypernatremia correction.

**CONCLUSION.** The management of hypernatremia is challenging, and inappropriate correction rates lead to worse outcomes in both the ICU and general hospital population. We were able to create a predictive model for hypernatremia correction using a random forest classifier with a 76% success rate, suggesting that machine learning can be a valuable tool in managing this condition. We also found that baseline sodium and BUN level were influential in predicting correction. Our next step will be to utilize the larger eICU Collaborative Research Database and apply similar methods to create a more reliable model. We hope to eventually develop more effective and efficient methods of hypernatremia correction.

#### REFERENCE(S)

1. Goldberger AL, Amaral LAN, Glass L, Hausdorff JM, Ivanov PCh, Mark RG, Mietus JE, Moody GB, Peng C-K, Stanley HE. PhysioBank, PhysioToolkit, and PhysioNet: Components of a New Research Resource for Complex Physiologic Signals. Circulation 101(23):e215-e220 [Circulation Electronic Pages; http://circ.ahajournals.org/content/101/23/e215]; 2000 (June 13)
2. Pollard TJ, Johnson AEW, Raffa JD, Celi LA, Mark RG, Badawi O.The eICU Collaborative Research Database, a freely available multi-center database for critical care research. Scientific Data, 5:180178, Sept. 2018
3. Johnson, A. E., Pollard, T. J., Shen, L., Li-wei, H. L., Feng, M., Ghassemi, M., ... Mark, R. G. (2016). MIMIC-III, a freely accessible critical care database. Scientific data, 3, 160035.
4. Alshayeb, H. M., Showkat, A., Babar, F., Mangold, T., Wall, B. M. (2011). Severe Hy- pernatremia Correction RateMortality in Hospitalized Patients. The American journal of the medical sciences, 341(5), 356-360.
5. Bataille, S., Baralla, C., Torro, D., Buffat, C., Berland, Y., Alazia, M., ... Vacher-Coponat, H. (2014). Undercorrection of hypernatremia is frequent and associated with mortality. BMC nephrology, 15(1), 37
6. Sun, T., et al. "The influence of hypernatremia on mortality in intensive care unit patients: a meta-analysis." (2014): 228-232
7. Lindner, G., Funk, G. C. (2013). Hypernatremia in critically ill patients. Journal of critical care, 28(2), 216-e11

### 000511

### The impact of using historical data on the performance of Bayesian forecasting of vancomycin plasma concentration in intensive care patients

T. Guo[1], RM. Van Hest[2], LM. Fleuren[1], LF. Roggeveen[1], PJ. Thoral[1], RJ. Bosman[3], PHJ. Van Der Voort[3], A. Girbes, RAA. Mathot[2], JGC. Van Hasselt[4], PWG. Elbers[1]

[1]Intensive care medicine, Amsterdam UMC, locatie VUmc, Amsterdam, Netherlands; [2]Department of pharmacy, Amsterdam UMC, locatie AMC, Amsterdam, Netherlands; [3]Intensive care medicine, OLVG location East, Amsterdam, Netherlands; [4]Leiden academic centre for drug research (lacdr), Leiden University, Leiden, Netherlands

**Correspondence:** T. Guo

*Intensive Care Medicine Experimental* 2019, **7(Suppl 3):**000511

**INTRODUCTION.** Population pharmacokinetic (PK) model-based Bayesian analysis is playing an important role in optimizing vancomycin treatment for intensive care (ICU) patients. A strength of Bayesian analysis is the ability to include historical PK data to improve the estimation of PK parameters for the betterment of concentration forecasting. However, this strength may turn into a potential weakness due to the high PK variabilities in ICU patients which may affect Bayesian forecasting. In this study, we investigated the impact of using historical data of ICU patients on the performance of Bayesian forecasting of vancomycin plasma concentration.

**METHODS.** A published one-compartmental population PK model of vancomycin in ICU patients was used in this study [1]. The model has been validated in a large cohort of 839 ICU patients of our own [2]. To assess the impact of historical data in Bayesian forecasting of the vancomycin concentrations in ICU patients, we performed Bayesian analysis in a real dataset of 490 ICU patients including multiple days where PK data was available. By reorganizing the data set, the future 1-day concentrations were forecasted by using the Bayesian estimates of the last 1, 3, 5, 7, 10, and 14 days. Thereafter, we calculated the bias of forecasted versus actual plasma concentrations. Nonlinear mixed-effects modeling software (NONMEM, version 7.4.3; ICON Development Solutions, MD, USA) was used to perform the analysis. Data organization and visualization were carried out with R (version 3.5.2; R-project.org).

**RESULTS.** The forecasted plasma vancomycin concentration was not significantly biased (-3.8%) when using only the last 1-day samples for Bayesian analysis. However, when including further historical data (last 3 to 14 days), the concentration was increasingly under-forecasted (-6.1% to -18.6%). The Bayesian estimates of every day for clearance decreased over time indicating the potential deterioration in the renal function of patients staying in ICU for long, which was in line with our clinical experience.

**CONCLUSION.** Through Bayesian analysis, vancomycin plasma concentration can be reasonably forecasted for ICU patients when the PK data from last 1-day was used. Inclusion of further historical data could be detrimental to Bayesian forecasting, which may mislead clinicians into treating patients inadequately.

#### REFERENCE(S)

[1] Roberts, Jason A., Fabio Silvio Taccone, Andrew A. Udy, Jean-Louis Vincent, Frédérique Jacobs, and Jeffrey Lipman. "Vancomycin dosing in critically ill patients: robust methods for improved continuous-infusion regimens." Antimicrobial agents and chemotherapy 55, no. 6 (2011): 2704-2709.

[2] Guo, Tingjie, Reinier M. van Hest, Luca F. Roggeveen, Lucas M. Fleuren, Patrick J. Thoral, Rob J. Bosman, Peter HJ van der Voort, Armand RJ Girbes, Ron AA Mathot, and Paul WG Elbers. "External evaluation of population pharmacokinetic models of vancomycin in large cohorts of intensive care patients." Antimicrobial agents and chemotherapy (2019): AAC-02543.

[3] This work is part of the Right Dose, Right Now project by Amsterdam UMC, location VUmc and OLVG Oost Amsterdam and was partially funded by the ZonMw Rational Pharmacotherapy program.

### 000541

#### Inform-DB: a clinical data warehouse design to enable forecasting of intensive care unit bed occupancy

T. Keen[1], R. Klapaukh[2], J. Stein[2], M. Gillman[2], J. Cooper[2], A. Shah[3], T. Bonnici[1], S. Harris[4], N. MacCallum[4], D. Brealey[5], M. Grocott[6], M. Singer[7], M. Mythen[8]
[1]Inform lab, University College London, London, UK; [2]Research software development, University College London, London, UK; [3]Institute of health informatics, University College London, London, UK; [4]Inform lab, University College London, Gower Street, London, UK, London, UK; [5]Inform lab, UCL Hospitals NHS Foundation Trust, London, UK; [6]Faculty of medicine, University of Southampton, Southampton, UK; [7]University College London, Bloomsbury Institute of Intensive Care Medicine, London, UK; [8]Department of anaesthesia and perioperative medicine, University College Hospital, London, UK
**Correspondence:** T. Keen
*Intensive Care Medicine Experimental* 2019, **7(Suppl 3):**000541

**INTRODUCTION.** A recent UK study of elective surgery found that one in ten patients had experienced a prior cancellation with lack of Intensive Care Unit (ICU) beds being a major contributory factor [1]. Forecasting ICU bed occupancy to prevent on-the-day cancellation could potentially curb the physical, emotional and economic costs of postponement [2]. Occupancy forecasting requires an optimal database schema to house clinical data – "Clinical Data Warehouse (CDW) design". If forecasts are to be based on real-time Electronic Health Record (EHR) data, the database powering them must integrate directly into the hospital's outward data flows. Then, once the data is stored in the CDW, the needs of data scientists creating new models differ from those of software developers looking to deploy transferable forecasting systems. Good CDW design should support both use cases.

**OBJECTIVES.** The core objective was to design and build a CDW to enable research into EHR-driven occupancy forecasting. As a first milestone, we focused on ingesting ADT (Admission Discharge Transfer) data as important indicators of short term occupancy. A key outcome was to demonstrate the CDW's robustness to the high data volumes leaving the hospital. To be viable, any design also has to support two major output layers for our data science users. The first was the Observational Medical Outcomes Partnership (OMOP) Common Data Model, a well-documented patient-centric data model with uptake in the ICU community for retrospective research [3,4]. The second was the Fast Health Interoperability Resource (FHIR), an emerging interoperability standard, with a clear API specification for app development [6].

**METHODS.** The design process began with a literature review. This informed a prototype transformation of our ICU EHR to the OMOP Common Data Model. We then formalised the CDW design, named 'Inform-DB'. This schema was load-tested in PostgreSQL 11.2 with a million fabricated patients, twice the number of yearly admission messages to our hospital.

**RESULTS.** The design process for Inform-DB resulted in a schema specification for ADT messages [6]. A preliminary translation to the OMOP data model has been built. This design withstood load testing across two years' worth of admission data in less than 36 hours. A standard query of patients admitted across one week was returned in less than 3 seconds, and from the third repetition onwards in less than 450 milliseconds (Fig. 1).

**CONCLUSION.** This early work shows that Inform-DB has the necessary speed to handle real-time data flows from a large tertiary hospital. Work is underway to integrate the schema with our hospital's live outbound messages. This will enable incorporation of real-time data into EHR-driven forecasting systems.

**REFERENCE(S)**

[1] Wong DJN, Harris SK, Moonesinghe SR on behalf of the SNAP-2: EPICCS collaborators. Cancelled operations: a 7-day cohort study of planned adult inpatient surgery in 245 UK National Health Service hospitals. Br J Anaesth 2018; 121:730-8
[2] Pagel C et al. Development, implementation and evaluation of a tool for forecasting short term demand for beds in an intensive care unit. Oper Res Heal Care 2017; 15:19–31
[3] Voss EA et al. Feasibility and utility of applications of the common data model to multiple, disparate observational health databases. JAMIA 2015; 22: 553–64
[4] Health Level 7 (2018) FHIR Specification Home Page. http://hl7.org/fhir/ Accessed April 11, 2019
[5] Inform-DB ADT Schema. https://github.com/ThomasKeen/Inform-DB/blob/master/ADT_Schema.png Accessed April 12, 2019
[6] University College London Hospital Charity
[7] University College London Hospital NIHR Biomedical Research Centre
[8] Health Foundation
[9] Paris N et al. MIMIC-III into OMOP : 48h hackathon evaluation. OHDSI Europe Symposium, Rotterdam 2018

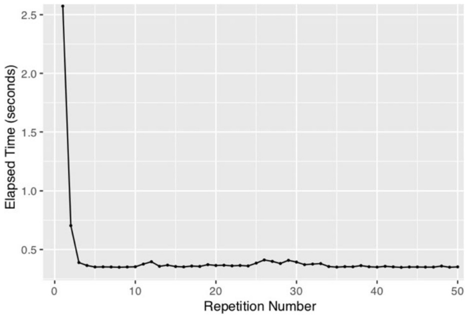

**Fig. 1 (abstract 000290).** Elapsed time for our example Inform-DB query to run. The query, which runs to return a single week's admissions, quickly falls below 450ms with Postgres caching.

### 000551

#### Comparing an artificial neural network against a generalised multivariate regression model prediction of hospital mortality using physiological parameters from the Medical Information Mart for Intensive Care III (MIMIC III) publicly available dataset

T. Sanderson[1], M. Alice[2], T. Samuels[2]
[1]Critical care, East Surrey Hospital, London, UK; [2]Critical care, East Surrey Hospital, Redhill , UK
**Correspondence:** T. Sanderson
*Intensive Care Medicine Experimental* 2019, **7(Suppl 3):**000551

**INTRODUCTION.** The MIMIC-III dataset is a large, freely-available database containing anonymised healthcare data associated with over forty thousand patients who stayed in critical care units of the Beth Israel Deaconess Medical Centre between 2001 and 2012 (1). It can be a challenging process to manipulate the data in such a way as to make it useful. Artificial neural networks (ANNs) are constructed by a set of computing units that simulate neurons linked together. An architecture for the network is established and then an algorithm is used to find the weights of the connections between the neurons. We sought to determine whether hospital outcome (e.g. alive or dead) could be predicted more accurately using an ANN or by more familiar methods (e.g. generalised linear modelling) using the MIMIC-III dataset.

**METHODS.** Following data cleansing (e.g. removal of all multiple admissions, selection of data recorded within first 24 hours of admission) our dataset comprised of 11,759 patients (18 to 89 years old). We chose similar physiological variables as used by the Intensive Care National Audit & Research Centre (ICNARC) Case Mix Programme ICNARCH-2018 regression model (2): age, lowest pH, highest lactate, highest urea, highest creatinine, highest sodium, lowest white blood cell count and lowest platelet count. We chose to use one hidden layer with 5 neurons for our ANN. Prior to training the neural network the dataset was normalised using a min-max scale method. The dataset was randomly divided into a training and test subset using a 70:30 ratio respectively. To evaluate the accuracy of our predictions we used the mean squared error (MSE), confusion matrices and ROC curves for both the ANN and multivariate regression model. All data mining and statistical analyses was performed using R version 3.5.3 (packages *dplyr, lubridate, ggplot2, neuralnet, plotROC*); computation was performed using an Intel Core i7-8750H hexacore processor.

**RESULTS.** The median age of our dataset was 64 [IQR 52 – 76] years, with the training and testing dataset comprising of 8231 and 3528 patients respectively. The ANN training time was approximately 4 minutes and produced an MSE of 0.118 (figure 1) and ROC AUC of 0.81 (figure 2). The multivariate regression model required approximately 0.04 seconds of computational time and produced an MSE of 0.127 and ROC AUC of 0.77 (figure 2); all variables were considered statistically significant at an alpha level of 0.05. The confusion matrices for the ANN and regression model demonstrated an accuracy of 84% and 83% respectively.

**CONCLUSION.** The ANN performed only marginally better when compared to the multivariate regression model, suggesting little difference between the two methods for predicting hospital mortality. However, neural networks remain flexible and once trained can compute predictions as quickly as other well-known methods. In addition, given that datasets such as MIMIC-III continue to grow and encompass more and more data, being able to feed this new data into an ANN as it accumulates over time will inevitably be less resource intensive due to its nature of 'online' learning as opposed to 'batch' learning.

**REFERENCE(S)**

1. MIMIC-III, a freely accessible critical care database. Johnson AEW, Pollard TJ, Shen L, Lehman L, Feng M, Ghassemi M, Moody B, Szolovits P, Celi LA, and Mark RG. Scientific Data (2016)
2. INARC.org. (2019). ICNARC. [online] Available at: https://www.icnarc.org/Our-Audit/Audits/Cmp/About [Accessed 17 Mar. 2019].

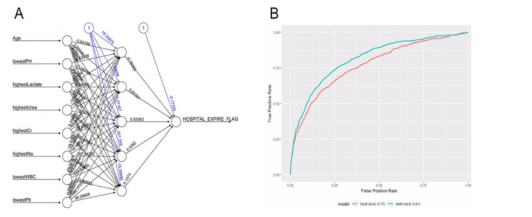

**Fig. 2 (abstract 000551).** See text for description

### 000555

#### Trajectory of illness predicts outcome independently of illness severity at time of initial assessment of patients admitted to ICU, and is significantly different in sepsis

J. Hunter[1], S. Harris,[2]

[1]Anaesthetic department, University College Hospital, Euston Road, London, UK; [2]Critical care department, University College Hospital, Euston Road, London, UK

**Correspondence:** J. Hunter

*Intensive Care Medicine Experimental* 2019, **7(Suppl 3):**000555

**INTRODUCTION.** Acute physiology scores (APS) are used to risk stratify patients on admission to Intensive Care (ICU), and intuition would suggest determining illness trajectories could improve prognostication. Underlying pathology is likely to be a significant factor in the trajectory of critical illness, but there is little data supporting this. The trajectory of sepsis is of particular interest as the SEPSIS-3 definition requires a change in SOFA score (1).

**METHODS.** This was a planned analysis of data from a prospective cohort study of consecutive deteriorating ward patients assessed for ICU admission in 49 NHS hospitals (1 November 2010 to 31 December 2011). Trajectory was defined as the change per hour in severity score between the ward assessment and first 24 hours within ICU, calculated based on the ICNARC APS score (2). A negative trajectory represents improvement. Multiple imputation via chained equations was used to manage missing data. The primary admission diagnosis was extracted from the clinical coding on admission.

**RESULTS.** Imputed data for 5,429 patients were analysed. 1,791 patients had a confirmed ICNARC sepsis diagnosis occurring with a frequency of greater than 1 per cent (pneumonia, septicaemia, septic shock, acute kidney injury (AKI) related to infection, or urosepsis). Patients with a confirmed ICNARC sepsis diagnosis are on an improving median trajectory of -0.5 (IQR -3.00 – 0.08) at time of admission to ICU, but show less improvement than patients without a sepsis diagnosis, with a median trajectory of -0.75 (IQR -4.00 – 0.08). The difference is significant (Kruskal-Wallis test, H = 9.49, 1 d.f., p = 0.002)

Using a binomial generalised linear model including age, lactate, presence of ICNARC sepsis diagnosis, and ward severity score, trajectory was an independent predictor of death at 28 days (odds ratio 1.084, 95% confidence interval 1.079 – 1.092, p = <2 x 10-16). The interaction between severity score trajectory and ICNARC diagnosis of sepsis, modeled in a separate binomial generalised linear model, was not significantly predictive of outcome.

**CONCLUSION.** This dataset reveals that patients with a sepsis diagnosis are improving on admission to ICU, which is of interest given the new SEPSIS-3 definition, and there is significant variation in trajectory between patients with sepsis and non-sepsis diagnoses. Further work is needed on the potential for trajectory before and during ICU admission as a predictor of outcome.

**REFERENCE(S)**

1. Singer M, Deutschman CS, Seymour CW, et al. The Third International Consensus Definitions for Sepsis and Septic Shock (Sepsis-3). JAMA. 2016;315(8):801–810. doi:10.1001/jama.2016.0287
2. Harrison DA, Parry GJ, Carpenter JR, Short A, Rowan K. A new risk prediction model for critical care: the Intensive Care National Audit & Research Centre (ICNARC) model. Crit Care Med 2007; 35:1091–8.

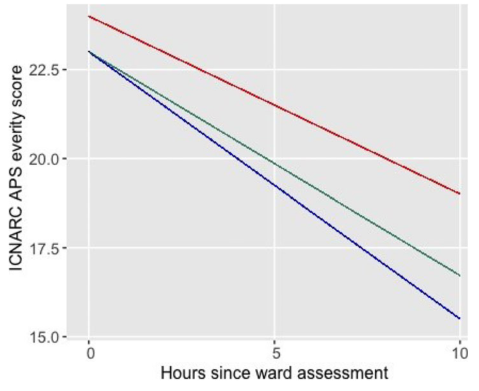

**Fig. 1 (abstract 000555).** See text for description

### 000584

#### Deep learning for predicting in-hospital mortality for cardiac arrest patients with national-wide healthcare data

CH. Huang[1], YB. Chen[1], CL. Tsai[1], J. Xu[2], R. Soltani[2], A. Tomberg[2], T. Min-Shan[1], W. Shih-Ni[1], W. Chien-Kai[1], WJ. Chen[1]

[1]Emergency medicine, National Taiwan University Hospital, Taipei, Taiwan; [2]Knowtions, Knowtions, Toronto, Canada

**Correspondence:** C.H. Huang

*Intensive Care Medicine Experimental* 2019, **7(Suppl 3):**000584

**INTRODUCTION.** Cardiac arrest carries a significant rate of in-hospital mortality, which is a major catastrophic event and hard to predict. Various early warning scores have been developed, but they mostly rely on in-hospital data such as vital signs from the current events, and suffer from low accuracy and high false positive rate. Here we tested if claims data can be used to predict in-hospital mortality, because we hypothesized that historical events play a role in predicting patient outcomes. We employed a deep learning approach, which has been shown to be useful in learning hidden patterns.

**OBJECTIVES.** To develop models capable of predicting in-hospital mortality based on historical claim data from Taiwan National Health Insurance Research Database (NHIRD).

**METHODS.** Taiwan NHIRD is derived from its single payer health insurance program that covers 99% of the population. It provides nationwide longitudinal claim records from all healthcare providers. We selected 8 years of de-identified claims (2003–2010) corresponding to persons with cardiac arrest and resuscitation with at least one emergency department (ED) visit or one hospitalization event. Primary outcome was all-cause in-hospital mortality. We constructed a deep learning system involving two steps. First, we used a taxonomy mapping system Text2Node, to generate a distributed representation for each concept. Second, we applied a multilevel hierarchical deep learning model based on the long short-term memory (LSTM) architecture. The resulting model was trained using the multi-task learning approach,

**RESULTS.** We included a total of 1,227,739 hospitalizations and 1,346,782 ED visits involving 326,180 patients. In in-patient events, in-hospital mortality is 7% (109,131 cases).The dataset is split randomly into training (70% of the patients), test (15%) and validation (15%) sets (figure 1). Hyper-parameters for each model was optimized by maximizing the area under the receiver operating characteristic curve (AUROC) for the validation set. The optimized set of hyper-parameters was then used to train the model on all events excluding the test set. For model evaluation, the AUROC, test accuracy, and F1 score were calculated on the held-out test set. Our model achieved high accuracy, with an AUROC value of 0.96, 95% of accuracy, and an F1-score of 0.63.

**CONCLUSION.** Longitudinal claim databases such as NHIRD provide valuable information in predicting rare catastrophic events when employing a deep learning approach. To implement this system in a real-world ED setting, a study on interpretability is underway.

**REFERENCE(S)**

1. Huang CH, Yu PH, Tsai MS, et al .Relationship Between Statin Use and Outcomes in Patients Having Cardiac Arrest (from a Nationwide Cohort Study in Taiwan). Am J Cardiol. 2019 Feb 22. pii: S0002-9149(19)30223-1
2. Huang CH, Yu PH, Tsai MS, et al. Acute hospital administration of amiodarone and/or lidocaine in shockable patients presenting with out-of-hospital cardiac arrest: A nationwide cohort study. Int J Cardiol. 2017 Jan 15;227:292-298
3. MOST-108-2634-F-002 -020

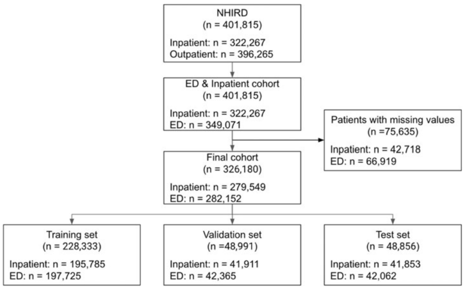

**Fig. 1 (abstract 000584).** See text for description

### 000675

#### Supervised classification techniques and deep learning for mortality prediction in sepsis patients

A. Rodriguez[1], D. Mendoza[2], J. Ascuntar[2], F. Jaimes[2]

[1]System engineering, Universidad Nacional, Medellín, Colombia; [2]Internal medicine, Universidad de Antioquia, Medellin, Colombia

**Correspondence:** F. Jaimes

*Intensive Care Medicine Experimental* 2019, **7(Suppl 3):**000675

**INTRODUCTION.** Sepsis mortality is still unacceptably high and an appropriate prognostic tool may increase the accuracy for clinical decisions. Several techniques of artificial intelligence (AI) are currently providing efficient ways for data mining and analysis. Thus, sepsis is a very attractive area for implementation and evaluation of several models of AI

**OBJECTIVES.** To develop and to evaluate several supervised techniques of data mining and AI for classification and prediction of mortality in adult patients hospitalized by emergency services with sepsis diagnosis

**METHODS.** Secondary data analysis of a prospective cohort conducted between June 2014 and February 2016 in three emergency services of university hospitals in Medellin, Colombia. Patients 18 years older were included, provided they were hospitalized because of suspected or confirmed infection and any organ dysfunction according to SOFA score. The outcome variable was hospital mortality and the prediction variables were grouped in either those related with treatment and initial clinical attention or those that measure directly physiological derangements. Five supervised classification techniques were analyzed: C4.5 Decision Tree (C4.5), Random Forest (RF), Artificial Neural Network (ANN) and Support Vector Machine (SVM) by either *dot* or ANOVA. Their performance was evaluated by the concordance between observed and predicted mortality (accuracy) and by the discrimination according to AUC-ROC

**RESULTS.** The study cohort was composed of 2510 patients with a median age of 62 years (IQR=46-74) and an overall hospital mortality rate of 11.5 % (n=289). The best discrimination was provided by the ANN using physiological variables (Table).

**CONCLUSION.** Deep learning and artificial intelligence are increasingly used as supporting tools in clinical medicine. Their performance in such a complex and heterogeneous syndrome as sepsis may be a new a horizon in clinical research. Artificial Neural Networks seem promising for improving sepsis classification and prognosis

**REFERENCE(S)**

1. Jaimes F, Farbiarz J, Alvarez D, Martínez C. Comparison between logistic regression and neural networks to predict death in patients with suspected sepsis in the emergency room. Crit Care. 2005 Apr;9(2):R150-6. Epub 2005 Feb 17. PubMed PMID: 15774048; PubMed Central PMCID: PMC1175932.
2. Grant COLCIENCIAS (Departamento Administrativo de Ciencia, Tecnología e Innovación) - 1115-5693-3362 and Universidad de Antioquia - CODI 2582-2014

**Table 1 (abstract 000675).** See text for description

| Model | Physiological | | Treatment | |
|---|---|---|---|---|
| | Accuracy | AUC-ROC | Accuracy | AUC-ROC |
| C4.5 | 56,7 | 0,64 | 54,9 | 0,55 |
| RN | 60,6 | 0,66 | 59,3 | 0,55 |
| **ANN** | 64,7 | **0,88** | 60 | 0,58 |
| SVM *dot* | 68,9 | 0,71 | 60,3 | 0,61 |
| SVM Anova | 70,5 | 0,80 | 61 | 0,61 |

### 000716

#### Utilizing a critical care database to evaluate glucose control and optimal glucose targets for critically ill patients

F. DeMichele[1], A. Robles Arévalo[2], L. Baker[3], LA. Celi[4]
[1]Health policy and management, Harvard T.H. Chan School of Public Health, Boston, USA; [2]Laboratory of Computational Physiology, Massachusetts Institute of Technology, Cambridge, USA; [3]Institute for data, systems and society, Massachusetts Institute of Technology, Cambridge, USA; [4]Pulmonary, critical care and sleep medicine, Beth Israel Deaconess Medical Center (BIDMC), Boston, USA
**Correspondence:** F. DeMichele
*Intensive Care Medicine Experimental* 2019, **7(Suppl 3):**000716

**INTRODUCTION.** The target for optimal glycemic control in the intensive care setting remains uncertain, despite more than a decade of debate. Randomized clinical trials suggest that different patient populations may benefit from different targetss. Van den Berghe et al. showed that glucose targets of 80 – 110 mg/dL resulted in a decrease in morbidity and mortality for surgical ICU patients, without the same effect observed in medical ICU patients. Meanwhile Finfer et al. showed that an intensive blood glucose target of 81 – 108 mg/dL compared to the conventional target of ≤ 180 mg/dL resulted in significantly higher 90 day mortality in both medical and surgical patients. Current research is limited with regards to evidence supporting specific glucose targets for heterogeneous patient populations.

**OBJECTIVES.** We hypothesize that dynamic glucose targets (based on variables such as patient physiologic parameters, duration of ICU stay and disease trajectory) may improve outcomes in the ICU patients. Since randomized controlled trials for different glucose targets and sub-populations of patients are impractical, we have utilized observational data in large publicly available datasets. To begin to analyze outcomes, we have first curated the database and performed descriptive analysis of current practices.

**METHODS.** This study utilized the publicly available MIMIC-III database which contains over 58,000 ICU admissions between 2008-2012 for a cohort of approximately 38,600 adults. Retrospective data were extracted in the following categories: blood glucose measurements (finger-stick glucometer and serum values), dextrose inputs, and insulin inputs (short, intermediate and long acting). Glucose and insulin values were paired to match them according to timestamp criteria. Data extraction and alignment was performed with SQL queries executed in Python.

**RESULTS.** A combined 373,239 glucose values were extracted between 155,168 laboratory chemistry readings and 218,071 finger-stick readings. 151,184 total insulin values were extracted; 138,561(91%) short-acting, 3,982(3%) intermediate-acting and 8,641(6%) long-acting. When frequency of insulin dosing was plotted it was shown that the majority of short acting insulin administered was in the range of 2 to less than 4 units (nearly 35,000 occurrences). After applying the algorithm for alignment, 41,387(95.9%) of short-acting intravenous infusions and 84,070(94.6%) of short-acting subcutaneous boluses were matched to corresponding glucose values. Dashboards were created for each ICU admission (plotting glucose and insulin values over time) to clinically validate the alignments. To evaluate the quality of glucose control, glucose values after short-acting subcutaneous insulin administration were plotted for the following intervals, <60 min, 60-120 min and 2-6 hours.

**CONCLUSION.** Blood glucose control in the ICU does not meet the current recommended guidelines. Despite suggested targets, glucose often falls outside of this range, and patients often experience hyperglycemia. For research on targets to be effective, we need to be able to control glucose well. Strategies for management need to be developed in tandem with accurate personalised targets if we are to improve outcomes. Future analysis with this dataset will seek to find trends between glucose control and clinically meaningful intermediary outcomes, such as indices of illness severity such as SOFA scores.

**REFERENCE(S)**

1. Pollard, T. J., & Johnson, A. E. W. The MIMIC-III Clinical Database http://dx.doi.org/10.13026/C2XW26 (2016)
2. Finfer, S. et al. Intensive versus Conventional Glucose Control in Critically Ill Patients. New England Journal of Medicine 360, 1283–97 (2009).
3. Van den Berghe, G. et al. Intensive Insulin Therapy in the Medical ICU. New England Journal of Medicine 354, 449–461 (2006).
4. Van den Berghe, G. et al. Intensive Insulin Therapy in Critically Ill Patients. New England Journal of Medicine 345, 1359–1367 (2001).
5. The work of Aldo Arévalo was supported by the PhD scholarship PD/BD/114107/2015 from FCT under MIT-Portugal Program - Bioengineering Systems focus area.

### 000956

#### Deep-learning neural networks for accurate diagnosis of sepsis using microarray gene expression data

D. Schaack, T. Brenner, M. Weigand, F. Uhle
[1]Department of anesthesiology, University Hospital Heidelberg, Heidelberg, Germany
**Correspondence:** D. Schaack
*Intensive Care Medicine Experimental* 2019, **7(Suppl 3):**000956

**INTRODUCTION.** Sepsis is a life-threatening condition, arising from a dysregulated and harmful host response to infection [1]. Due to its syndromic nature, no solitary biomarker with sufficient clinical performance is available and reliable diagnosis of sepsis remains challenging. Apart from rapid identification of the causative pathogen, the bloods' immune system is proposed to contain important information, especially on gene expression level. In recent years, machine learning approaches revolutionized the way of using high-dimensional data and also proved its diagnostic value when integrating electronic health record data of ICU patients [2].

**OBJECTIVES.** We performed a pilot study utilizing deep-learning artificial neural networks based on a large set of public microarray data and aiming to evaluate its performance for the accurate diagnosis of sepsis.

**METHODS.** Public repositories (NCBI GEO, EMBL-EBI Array Express) were searched for microarray data series containing septic patients (n=1,354), trauma patients (n=478), and healthy controls (n=383). Those were integrated into a comprehensive meta-dataset, containing gene expression values of a consensus of 5,932 genes for further analysis. After data preprocessing, samples were randomly divided into subsets for training (65%), validation (20%), and testing (15%). Training and validation samples were used as input for the step-wise training process of neural networks within the Google TensorFlow framework to learn binary discrimination of the entity of patients with sepsis and "controls" (trauma patients and healthy controls). Classification performance of trained neural networks was subsequently tested with the unknown samples. The full process of random data splitting, network training and prediction was repeated 250 times to test the robustness. Furthermore, stratified ShuffleSplit cross-validation of the whole approach was used to verify the reproducibility.

**RESULTS.** A total of 250 iterations resulted in an area under the curve (AUC) of 0.999 (Min=0.998, Max=1.000), with a corresponding sensitivity of 0.98 (Min=0.94, Max=1.00), specificity of 0.99 (Min=0.95, Max=1.00), and diagnostic accuracy of 0.96 (Min=0.96, Max=1.00). Random limitation of the provided genes to a fraction of 25% still enables reliable classification with nearly unaltered performance (n=250; AUC=0.99 (Min=0.99, Max=1.00), sensitivity=0.98 (Min=0.91, Max=1.00), specificity=0.97 (Min=0.90, Max=1.00), accuracy=0.97 (Min=0.94, Max=0.99)).

**CONCLUSION.** The presented approach surpasses the most advanced solutions for sepsis classification and closes the gap to comparable studies on cancer diagnosis. By limiting the number of available genes for prediction, we can prove that, instead of learning idiosyncratic features tailored to specific data series, generalized strategies for sample discrimination have developed in the trained artificial neural networks. The combination of artificial neural networks and microarray gene expression data is therefore capable of achieving sepsis diagnosis with superior accuracy and thus augments the current diagnostic scope.

**REFERENCE(S)**

1. Singer M et al. The Third International Consensus Definitions for Sepsis and Septic Shock (Sepsis-3). JAMA. 2016; 315:801-810. 2. Kam HJ, Kim HY. Learning representations for the early detection of sepsis with deep neural networks. Comput Biol Med. 2017; 89:248-55.

2. No financial support was received.

### 001065

### The Influence of Organ Dysfunction Trajectories on Death in Sepsis: A Joint Modelling Approach

E. Palmer[1], R. Klapaukh[2], M. Singer[3], N. MacCallum[4], D. Brealey[5], S. Harris[4]
[1]{street_address}, London, UK; [2]Research software development, University College London, London, UK; [3]University College London, Bloomsbury Institute of Intensive Care Medicine, London, UK; [4]Bloomsbury institute of intensive care medicine, University College London, Gower Street, London, UK, London, UK; [5]Critical care, UCL Hospitals NHS Foundation Trust , London, UK
**Correspondence:** E. Palmer

*Intensive Care Medicine Experimental* 2019, **7(Suppl 3):**001065

**INTRODUCTION.** With the rise of the electronic health record (EHR), new data resources have become available to explore the dynamics of sepsis through longitudinal physiology. The Critical Care Health Informatics Collaboration (CC-HIC) is a multicentre research project, aggregating high-fidelity, time-varying data on critical care patients from 12 intensive care units across five Biomedical Research Centres in the UK (1). 263 fields are available including demographics, bed-side monitoring, drug infusions, microbiology and organ support. We set out to explore the impact of organ dysfunction trajectories, measured as the Sequential Organ Failure Assessment (SOFA) score, on outcomes in sepsis.

**OBJECTIVES.** To determine how the rate of change of organ dysfunction (organ dysfunction trajectory) impacts upon prediction of death in sepsis from a respiratory source. Particular focus is given to addressing the methodological aspects of informative censoring and measurement error that characteristically bias analysis of this type.

**METHODS.** Joint models allow simultaneous modelling of time-to-event and mixed effects models through shared latent parameters (2). Joint models are a class of model that account for informative censoring, and error-prone repeated measures biomarkers. We applied joint models to a sample of patients with a diagnostic label of pneumonic sepsis between 1st January 2014 and 31st December 2018.

**RESULTS.** A pilot cohort of 611 patients met the entry criteria. There was a 1.17 fold increase (p = 1.32 x 10-6) in the hazard for death for every 1 unit increase in organ dysfunction severity (absolute value of SOFA). The same model describes a 4.93 fold (p = 5.67 x 10-4) increase in the hazard for death for every 1 unit increase in the trajectory of SOFA. The likelihood ratio comparing this model to a model omitting the trajectory term was 13.57 (p = 0.0002, chi-square, 1 d.f), suggesting a significant improvement in model fit from the addition of the trajectory parameter.

**CONCLUSION.** In pneumonic sepsis, the trajectory of organ dysfunction (measured as the rate of change of SOFA) not only adds important information for predicting outcomes, but exerts a substantially greater effect size over death than organ dysfunction severity alone.

**REFERENCE(S)**

1) Harris S et al. Critical Care Health Informatics Collaborative (CCHIC): Data, Tools and Methods for Reproducible Research: A Multi-Centre UK Intensive Care Database." Int J Med Inform 2018; 112:82–89.
2) Rizopoulos D. Joint Models for Longitudinal and Time-to-Event Data: With Applications in R. Chapman & Hall/CRC Biostatistics Series 6. Boca Raton: CRC Press, 2012.

3) This research was conducted using NIHR Health Informatics Collaborative (NIHR HIC) data resources.
4) Dr. Palmer is funded by the Medical Research Council.

### 001080

### Machine learning can accurately predict pre-admission baseline hemoglobin and creatinine in intensive care patients, bringing context to abnormal admission lab values

A. Dauvin[1], D. Carolina[2], B. Patrik[3], H. Ke-Chun[4], MS. Christopher[5], R. Daniele[6], B. Matteo[7], C. Leo Anthony[4], D. Molly[8]
[1]Operations research center and sloan school of management, Massachusetts Institute of Technology, Cambridge, USA; [2]Anesthesiology, perioperative, and pain medicine, Harvard Medical School, Boston, USA; [3]Health policy and management, Harvard T.H. Chan School of Public Health, Boston, USA; [4]Laboratory for computational physiology, Massachusetts Institute of Technology, Cambridge, USA; [5]Public health, Harvard T.H. Chan School of Public Health, Boston, USA; [6]Department of pathology, Stanford University, Serra Mall, Stanford, CA, USA, Stanford, USA; [7]Department of statistics & data science, Carnegie Mellon University, Pittsburgh, USA; [8]Acute care, trauma & critical care, Beth Israel Deaconess Medical Center (BIDMC), Boston, USA
**Correspondence:** A. Dauvin

*Intensive Care Medicine Experimental* 2019, **7(Suppl 3):**001080

**INTRODUCTION.** Patients are commonly admitted to the intensive care unit (ICU) in the absence of historical baseline laboratory blood results, with derangements in hemoglobin and creatinine levels being particularly common. However, the chronicity and therefore clinical significance of the derangement is often unknown (Pivovarov et al., 2014). The richness of data collected in the electronic health record presents an opportunity to deploy machine learning techniques to impute baseline hemoglobin and creatinine values. This lends context to abnormal laboratory results to distinguish between acute and chronic conditions and can aid optimal medical decision making.

**METHODS.** ***Design & Participants:*** Data from the Medical Information Mart for Intensive Care (MIMIC-III) was used. The MIMIC-III database is compiled from adult and neonatal ICU stays at an urban tertiary care center between 2001 and 2012. Adult patients with pre-admission ("baseline") hemoglobin and creatinine values available were selected (6,435 and 5,470 patients respectively). Demographics, vital signs, and admission laboratory results obtained within two hours of ICU admission were extracted as model inputs. Patient cohorts were split into training (75%) and testing datasets (25%). Prediction was done as both a classification task, using a threshold of 10 g/dL for hemoglobin and 1.2 mg/dL for creatinine, and as a regression task, for which the actual baseline value was predicted. We trained and compared several different types of model: linear regression, logistic regression, gradient boosting, random forest, and optimal classification trees.

***Outcome measures:*** Model performance on the classification task was evaluated with the accuracy and AUC. Performance on the regression tasks was evaluated using different metrics: R2, MAE, and MSE. Models were also qualitatively assessed for interpretability. Strong predictors of baseline hemoglobin and creatinine were identified via the machine learning techniques.

**RESULTS.** For the classification task, the AUC across the models developed was 0.87-0.88 for hemoglobin and 0.88-0.92 for creatinine. For the regression task, the best-performing model yielded a mean absolute error (mean difference between predicted and observed value) of 1.0 g/dL for hemoglobin and 0.30 mg/dL for creatinine, and with a mean squared error of 1.68 g/dL for hemoglobin and 0.54 mg/dL for creatinine. R2 values ranged from 0.55-0.58 for hemoglobin and 0.78-0.81 for creatinine. Given that inter-analyzer laboratory variability for hemoglobin measurement has been reported at 0.3-1.5 g/dL,36 and for creatinine at 0.1-0.2 mg/dL,37 these results are quite significant. We provided an optimal trees diagram (Decision Tree) and ready-to-use application that shows how the predictions are made (with an AUC of 88-89%).

**CONCLUSION.** This study is proof-of-concept that machine learning methods can predict pre-admission laboratory values when historical data are lacking. It also proposes a workflow that could be applied to predict other types of laboratory results. Expansion of these tools promises to add a new dimension of clinical parameters, help to differentiate acute from chronic conditions using limited data, and aid in real-time clinical decision making.

**REFERENCE(S)**

1. 48. Pivovarov R, Albers DJ, Sepulveda JL, Elhadad N: Identifying and mitigating biases in EHR laboratory tests. J Biomed Inform. 2014. 49. Jean-Louis Vincent and Rui Moreno: Clinical review: Scoring systems in the critically ill. Critical Care 2014.
2. References 1. Deyo, D., Khaliq, A., Mitchell, D. & Hughes, D. R. Electronic sharing of diagnostic information and patient outcomes. Am J Manag Care 24, 32–37 (2018). 2. Rudin, R. S., Motala, A., Goldzweig, C. L. & Shekelle, P. G. Usage and effect of health information exchange: a systematic review. Ann. Intern. Med. 161, 803–811 (2014). 3. World Health Organization. Haemoglobin concentrations for the diagnosis of anaemia and assessment of severity. (2011). 4. Tyler, P. D. et al. Assessment of Intensive Care Unit Laboratory Values That Differ From Reference Ranges and Association With Patient Mortality and Length of Stay. JAMA Netw Open 1, e184521–e184521 (2018). 5. Hébert, P. C. et al. A multicenter, randomized, controlled clinical trial of transfusion requirements in critical care. Transfusion Requirements in Critical Care Investigators, Canadian Critical Care Trials Group. N. Engl. J. Med. 340, 409–417 (1999). 6. Carson, J. L. et al. Red blood cell transfusion: a clinical practice guideline from the AABB*. Ann. Intern. Med. 157, 49–58 (2012). 7. Khwaja, A. KDIGO clinical practice guidelines for acute kidney injury. Nephron Clin Pract 120, c179-184 (2012). 8. Levey, A. S. et al. Definition and classification of chronic kidney disease: A position statement from Kidney Disease: Improving Global Outcomes (KDIGO). Kidney International 67, 2089–2100 (2005). 9. Esteva, A. et al. A guide to deep learning in healthcare. Nat. Med. 25, 24–29 (2019). 10. Celi, L. A. G. et al. A Clinical Database-Driven Approach to Decision Support: Predicting Mortality Among Patients with Acute Kidney Injury. Journal of Healthcare Engineering (2011). doi:10.1260/2040-2295.2.1.97 11. Moreno, R. P. et al. Sepsis mortality prediction based on predisposition, infection and response. Intensive Care Med 34, 496–504 (2008). 12. Somanchi, S., Adhikari, S., Lin, A., Eneva, E. & Ghani, R. Early Prediction of Cardiac Arrest (Code Blue) using Electronic Medical Records. in Proceedings of the 21th ACM SIGKDD International Conference on Knowledge Discovery and Data Mining - KDD '15 2119–2126 (ACM Press, 2015). doi:10.1145/2783258.2788588 13. Frizzell, J. D. et al. Prediction of 30-Day All-Cause Readmissions in Patients Hospitalized for Heart Failure: Comparison of Machine Learning and Other Statistical Approaches. JAMA Cardiology 2, 204 (2017). 14. Kourou, K., Exarchos, T. P., Exarchos, K. P., Karamouzis, M. V. & Fotiadis, D. I. Machine learning applications in cancer prognosis and prediction. Computational and Structural Biotechnology Journal 13, 8–17 (2015). 15. Celi, L. A., Hinske Christian, L., Alterovitz, G. & Szolovits, P. An artificial intelligence tool to predict fluid requirement in the intensive care unit: a proof-of-concept study. Critical Care 12, R151 (2008). 16. Cismondi, F. et al. Reducing unnecessary lab testing in the ICU with artificial intelligence. Int J Med Inform 82, 345–358 (2013). 17. Hannun, A. Y. et al. Cardiologist-level arrhythmia detection and classification in ambulatory electrocardiograms using a deep neural network. Nature Medicine 25, 65 (2019). 18. Lee, J.-G. et al. Deep Learning in Medical Imaging: General Overview. Korean Journal of Radiology 18, 570–584 (2017). 19. Gulshan, V. et al. Development and Validation of a Deep Learning Algorithm for Detection of Diabetic Retinopathy in Retinal Fundus Photographs. JAMA 316, 2402 (2016). 20. Wang, S. & Summers, R. M. Machine learning and radiology. Medical Image Analysis 16, 933–951 (2012). 21. Caruana, R. et al. Intelligible Models for HealthCare: Predicting Pneumonia Risk and Hospital 30-day Readmission. in Proceedings of the 21th ACM SIGKDD International Conference on Knowledge Discovery and Data Mining 1721–1730 (ACM, 2015). doi:10.1145/2783258.2788613 22. Vandenbroucke, J. P. et al. Strengthening the Reporting of Observational Studies in Epidemiology (STROBE): explanation and elaboration. Ann. Intern. Med. 147, W163-194 (2007). 23. Johnson, A. E. W. et al. MIMIC-III, a freely accessible critical care database. Scientific Data 3, 160035 (2016). 24. R Core Team, R. F. for S. C. R: A Language and Environment for Statistical Computing. (2018). 25. Bertsimas, D., Pawlowski, C. & Zhuo, Y. D. From Predictive Methods to Missing Data Imputation: An Optimization Approach. Journal of Machine Learning Research 18, 1–39 (2018). 26. Chen, T. & Guestrin, C. XGBoost: A Scalable Tree Boosting System. in Proceedings of the 22Nd ACM SIGKDD International Conference on Knowledge Discovery and Data Mining 785–794 (ACM, 2016). doi:10.1145/2939672.2939785 27. Breiman, L. Random Forests. Mach. Learn. 45, 5–32 (2001). 28. Bertsimas, D. & Dunn, J. Optimal Classification Trees. Mach. Learn. 106, 1039–1082 (2017). 29. Bertsimas, D. & King, A. OR Forum—An Algorithmic Approach to Linear Regression. Operations Research 64, 2–16 (2015). 30. Perez, F. & Granger, B. E. IPython: A System for Interactive Scientific Computing. Computing in Science Engineering 9, 21–29 (2007). 31. Hunter, J. D. Matplotlib: A 2D Graphics Environment. Computing in Science Engineering 9, 90–95 (2007). 32. Pedregosa, F. et al. Scikit-learn: Machine Learning in Python. Journal of Machine Learning Research 12, 2825–2830 (2011). 33. Jones, E., Oliphant, T. & Peterson, P. SciPy: Open source scientific tools for Python. http://www. scipy. org/ (2001). 34. McKinney, W. Data Structures for Statistical Computing in Python. 6 (2010). 35. Breiman, L., Friedman, J., Stone, C. J. & Olshen, R. A. Classification and Regression Trees. (Wadsworth. Republished by CRC Press, 1984). 36. Shah, N., Osea, E. A. & Martinez, G. J. Accuracy of noninvasive hemoglobin and invasive point-of-care hemoglobin testing compared with a laboratory analyzer. International Journal of Laboratory Hematology 36, 56–61 (2014). 37. Lee, E., Collier, C. P. & White, C. A. Interlaboratory Variability in Plasma Creatinine Measurement and the Relation with Estimated Glomerular Filtration Rate and Chronic Kidney Disease Diagnosis. Clin J Am Soc Nephrol 12, 29–37 (2017). 38. Vayena, E., Blasimme, A. & Cohen, I. G. Machine learning in medicine: Addressing ethical challenges. PLOS Medicine 15, e1002689 (2018). 39. Bertsimas, D., Dunn, J., Velmahos, G. C. & Kaafarani, H. M. A. Surgical Risk Is Not Linear: Derivation and Validation of a Novel, User-friendly, and Machine-learning-based Predictive OpTimal Trees in Emergency Surgery Risk (POTTER) Calculator. Ann. Surg. 268, 574–583 (2018). 40. Sarma, P. R. Red Cell Indices. in Clinical Methods: The History, Physical, and Laboratory Examinations (eds. Walker, H. K., Hall, W. D. & Hurst, J. W.) (Butterworths, 1990). 41. Zhang, Z., Xu, X., Fan, H., Li, D. & Deng, H. Higher serum chloride concentrations are associated with acute kidney injury in unselected critically ill patients. BMC Nephrology 14, 235 (2013). 42. Oh, T. K. et al. Hyperchloremia and postoperative acute kidney injury: a retrospective analysis of data from the surgical intensive care unit. Critical Care 22, 277 (2018). 43. Complete Blood Count (CBC): MedlinePlus Lab Test Information. Available at: https://medlineplus.gov/lab-tests/complete-blood-count-cbc/. (Accessed: 11th March 2019) 44. Jiang, F. et al. Artificial intelligence in healthcare: past, present and future. Stroke Vasc Neurol 2, 230–243 (2017). 45. Chouldechova, A. & G'Sell, M. Fairer and more accurate, but for whom? preprint arXiv:1707.00046 [cs, stat] (2017). 46. Hall, W. J. et al. Implicit Racial/Ethnic Bias Among Health Care Professionals and Its Influence on Health Care Outcomes: A Systematic Review. Am J Public Health 105, e60–e76 (2015). 47. Forhan, M. & Salas, X. R. Inequities in Healthcare: A Review of Bias and Discrimination in Obesity Treatment. Canadian Journal of Diabetes 37, 205–209 (2013).
3. This paper was composed by participants in the HST.953 course at the Massachusetts Institute of Technology, Fall 2018. For more information about the course, please visit: https://criticaldata.mit.edu/course/
4. Software: This research made use of the community-developed statistical software R,23 community-developed core Python and Julia packages, and packages developed by the Operations Research Center of MIT including: IPython30 Matplotlib,31 Pandas34 Scikit-learn,32 SciPy33, OptImpute, and OptimalTrees28

## HSRO - Organisation in the ICU

### 000921

#### Contact isolation precautions in the ICU: can audit and feedback effectively change practice?

D. Mano[1], R. Pereira[1], S. Faria[2], MJ. Susano[1], M. Silva[1], T. Tinoco[1], D. Fernandes[3], M. Alves[1], J. Pinho[1], P. Maia[1]

[1]Department of anesthesiology, emergency and intensive care, Hospital Geral de Santo António, Porto, Portugal; [2]Internal medicine, Hospital Distrital Figueira da Foz, Figueira da Foz, Portugal; [3]Internal medicine, Unidade Local de Saúde do Nordeste, Bragança, Portugal

**Correspondence:** D. Mano

*Intensive Care Medicine Experimental* 2019, **7(Suppl 3):**000921

**INTRODUCTION.** Healthcare-associated infections (HAIs) remain a major issue among critical ill patients in the intensive care unit (ICU), associated with substantial morbidity and mortality. Even if the use of contact precautions (CPs) is highly recommended for patients who have Multi-Drug Resistant Organisms (MDROs) (1), and considered a Quality Indicator in Intensive Care Medicine (2), Health Care Workers (HCWs) effective compliance with its practice seems to be low. Outbreaks of MDROs strengthened the role of improving adherence to CPs as part of a multifaceted infection prevention strategy, which may be a controversial recommendation for some (3). Auditing and giving feedback may be a strategy to improve professional practice (4).

**OBJECTIVES.** Auditing the HCWs (senior physicians, residents, nurses and healthcare assistants) compliance with indications for isolation (either preventive or documented) and with supplemental measures; to evaluate the effects on HCWs practice of giving them feedback and performing an educational intervention.

**METHODS.** This is a single ICU study. The ICU includes: 10 level 3 beds and 12 level 2 beds; protocols for preventive isolation and active screening for carbapenem-resistant *Enterobacteriaceae* (CRE) on ICU admission, hand hygiene (HH) and documented isolation. The audit tool was developed and approved institutionally. The observation methodology was explained to the auditing team and had 2 periods of 2 weeks to collect data (November/December 2018 and March/April 2019), once on each nurse shift, 1 to 3 shifts a day, for 30 minutes, either in level 2 and level 3 ICU beds. In between the two periods, results of the first audit were disclosed to ICU HCWs, followed by an intervention (educational sessions with targets and action plan included).

**RESULTS.** On the pre-intervention audit, data were collected in 57 shifts (228 observations of patients assigned to contact isolation precautions); after the educational intervention, data were collected in 37 shifts (162 observations). Main results are presented (pre-intervention %; post-intervention %). Documented isolation was mostly related to CRE (64%; 72%) and *Methicillin-resistant Staphylococcus aureus (*18%; 12%). Personal protective equipment (gown, gloves, mask) was available at the isolation unit (48%; 57%); was adequately wore (77%; 87%) and removed (73%; 90%). Glove use was appropriate (84%; 91%) more often than mask (46%; 92%).

Supplemental measures included barriers (69%; 61%) and isolated rooms (32%; 39%), waste sorting bags next to the bed (98%, 90%). Concerning HCWs groups, nurses were the most audited (46%; 43%) and the most compliant with CPs and senior doctors the least audited (6%; 6%).

**CONCLUSION.** The adherence of HCWs to CPs was low. Audit and feedback resulted in practice improvement, however we still seem to be far from the desirable target of 100%.

**REFERENCE(S)**

1. CDC. Guideline for Isolation Precautions: Preventing Transmission of Infectious Agents in healthcare Settings. 2007
2. Quality Indicators in Critically Ill Patients. SEMICYUC 2011.
3. Cohen CC et al. Effectiveness of contact precautions against multidrug resistant organism transmission in acute care: a systematic review of the literature. Journal of Hospital Infection 2015;90:275-284.
4. Ivers N, et al. Audit and feedback: effects on professional practice and healthcare outcomes (Review). 2012 The Cochrane Collaboration

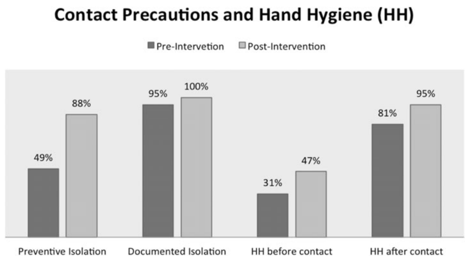

**Fig. 1 (abstract 000921).** See text for description

### 000926

#### Severe ICU -immobility and 28- days mortality

P. Vargas, M. Mellado, L. Ilaja, M. Bozinovic, F. Valle, M. Cantillano, C. Caceres, C. Herrera, A. Cid, D. Ramos, D. Navarrete, J. Dahdal[1]

Upc, Hospital del Salvador, Providencia, Chile

**Correspondence:** P. Vargas

*Intensive Care Medicine Experimental* 2019, **7(Suppl 3):**000926

**INTRODUCTION.** Immobilization during hospitalization stimulates a complex adaptative response that results in muscle atrophy and loss of functional performance. Immobility is a frequently understimated phenomenon during ICU stay and it could be associated with worse outcomes. Its prevalence and association with mortality is unknown.

**OBJECTIVES.** To estimate the prevalence of severe ICU-immobility and its association with 28-day mortality

**METHODS.** Retrospective study. Over a 6-month period we included all adults who were admitted to ICU and stayed for more than 48 hours. Patient mobilization was carried out according to local protocol. Mobility status was assesed daily using the Intensive Care Unit Mobility Scale (IMS). We considered severe ICU immobility as the inability to sit with active trunk control (over edge of bed) or IMS <3 during all over ICU stay (may be assisted by staff). Observed outcomes were 28-day mortality and ICU lenght of stay (LOS). We performed a multivariate analysis and Kaplan Meier plots with log-rank test.

**RESULTS.** 178 patients were included, mean age 60 years old (±18), APACHE II 16 (±9). The main diagnosis was sepsis (n=60 % 33,7). 96 patients (53,9%) required mechanical ventilation. ICU LOS was 7,2 days (±6), 28-days mortality was 24% (n=42). Severe ICU-immobility prevalence was 68,5% (n= 122) and was independently associated with both 28-days mortality OR 6,0 CI 95% 1,8 -19,8 (Figure 1) and longer stay in ICU (6,2±5,4 vs 9,1±6,8). Requirements of mechanical ventilation and emergency surgery were independently associated with severe ICU-immobility (Table 1).

**CONCLUSION.** Severe ICU- immobility defined as the inability to sit actively during hospitalization has a high prevalence and is independently associated with a higher 28-days mortality.

**REFERENCE(S)**

1. Doiron KA, Hoffmann TC, Beller EM. Early intervention (mobilization or active exercise) for critically ill adults in the intensive care unit. Cochrane Database of Systematic Reviews 2018, Issue 3. Art. No.: CD010754.
2. Morris, Peter E.. "Moving our critically ill patients: mobility barriers and benefits." Critical care clinics 23 1 (2007): 1-20 .
3. Brown, C. J. et al. "Prevalence and outcomes of low mobility in hospitalized older patients." Journal of the American Geriatrics Society 52 8 (2004): 1263-70 .
4. Winkelman, Chris. "Inactivity and inflammation in the critically ill patient." Critical care clinics 23 1 (2007): 21-34 .
5. Tipping, Claire J. et al. "The ICU Mobility Scale Has Construct and Predictive Validity and Is Responsive. A Multicenter Observational Study." Annals of the American Thoracic Society 13 6 (2016): 887-93 .
6. Hodgson, C. et al. Feasibility and inter-rater reliability of the ICU Mobility Scale. Heart and Lung: Journal of Critical Care, 43(1), 19-24.

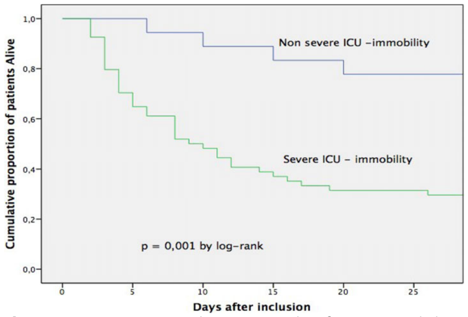

**Fig. 1 (abstract 000926).** Kaplan Meier plot for ICU mobility status and 28-days survival

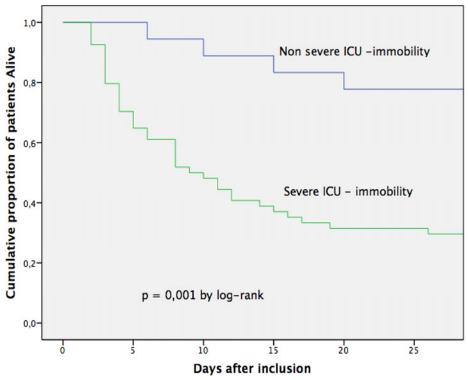

**Fig. 2 (abstract 000926).** See text for description

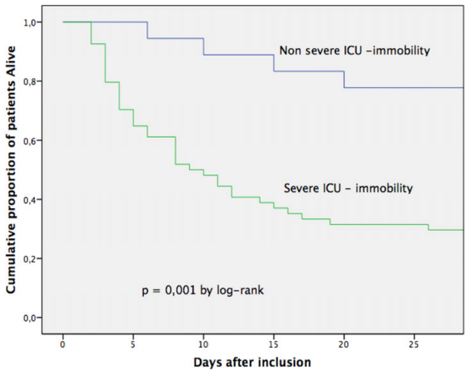

**Fig. 3 (abstract 000926).** See text for description

**Table 1 (abstract 000926).** See text for description

| Variable | Non Severe immobility (n =56) | Severe immobility (n=122) | p |
|---|---|---|---|
| Age - yr (SD) | 60,5 (±18,7) | 59,7 (±16,7) | NS |
| APACHE II - (SD) | 14,1 (±8,5) | 16,3 (±8,8) | NS |
| ICU stay- days (SD) | 6,2 (±5,4) | 9,1 (±6,8) | 0,003 |
| Mechanical Ventilation- n (%) | 21 (37,5) | 75 (61,4) | 0,04 |
| 28 days Mortality- n (%) | 4 (7,1) | 38 (31,1) | < 0,001 |
| Sepsis- n (%) | 16 (28,5) | 44 (36) | NS |
| Emergency Surgery- n (%) | 2 (3,5) | 18 (14,7) | 0,038 |
| Immunocompromised- n (%) | 12 (21,4) | 29 (23,7) | NS |
| Acute Kidney Injury- n (%) | 14 (25) | 27 (22,1) | NS |

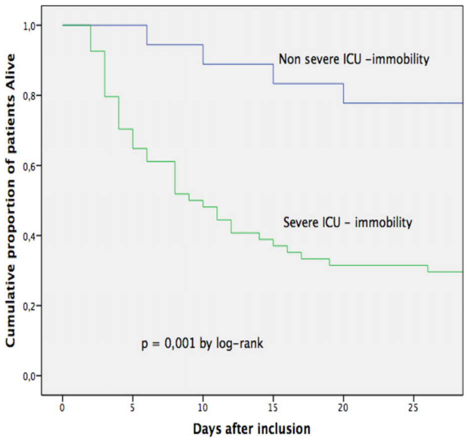

**Fig. 4 (abstract 000926).** See text for description

### 000936

#### Airway pressures' trends impact on weaning in mechanically ventilated patients

MA. Boujelbèn[1], W. Zarrougui[2], N. Fraj[2], I. Ben Saida[2], M. Zghidi[1], S. Rouis[1], A. Azouzi[1], K. Meddeb[2], M. Boussarsar[2]
[1]Medical intensive care unit, Farhat hached university hospital, Sousse, Tunisia; [2]Medical intensive care unit, farhat hached university hospital, Université de Sousse, Faculté de Médecine de Sousse, LR N° LR12SP09.Heart Failure, Sousse, Tunisia
**Correspondence:** W. Zarrougui
*Intensive Care Medicine Experimental* 2019, **7(Suppl 3):**000936

**INTRODUCTION.** The evolvement of airway pressures (Paw) within the mechanical ventilation may reflect the modifications of the viscoelastic properties of the respiratory system and may predict difficult weaning.

**OBJECTIVES.** To investigate the impact of airway pressures trends on weaning in mechanically ventilated (MV) patients.

**METHODS.** Medical records were abstracted for all consecutive MV patients who were admitted from November, 2015 to February, 2018 in the MICU of Farhat Hached teaching hospital, Sousse,Tunisia. Data regarding demographics, clinical variables, trends of Paw (Paw at day 4 of hospitalization - Paw upon admission) and outcomes were recorded from chosen eligible charts. Poor outcomes were defined as : a ventilator-free days at day 28 (VFDs) = 0 and a composite outcome : death or length of stay ≥ 14 days. Univariate and multivariate regression analyses were used to identify factors independently associated to difficult weaning.

**RESULTS.** A total of 304 MV patients were included. Their main characteristics were : mean age, 56±18 years; male sex ratio, 64.8%(n=197); mean SAPS II, 34.9±14.3; pH, 7,3±0,1; pCO2, 50± 23mmHg; P/F ratio, 204±101; AE/COPD, 105(34.5%); ARDS, 25(8.2%); restrictive lung disease, 20(6.6%); pneumonia, 14(4.6%); pulmonary edema, 11(3.6%); median mechanical ventilation duration, 3[6-14] days; tracheostomy, 44(14.5%); median length of stay, 13[6-21] days; median VFDs, 0[0-2] days; mortality, 173(56.9%). Median differential airway pressures for delta peak, delta plateau, delta driving and delta auto-PEEP were respectively : 0[-5;5], 1[-2;3], 0[-2;3] and 0[-2;0] cmH2O. Univariate analysis than multivariate logistic regression showed that an elevated delta plateau is associated with a zero VFDs

(OR, 1.05; 95%CI, [1.002-1.11]; p=0.041) and an elevated delta peak is associated with poor composite outcome : (OR, 1.05; 95%CI, [1.009-1.09]; p=0.017). In COPD patients : an elevated delta plateau was associated with zero VFDs (OR, 1.1; 95%CI,[1.01-1.21]; p=0.028) and poor composite endpoint (OR, 1.13; 95%CI, [1.01-1.27]; p=0.026). While in non-COPD patients it was the elevated delta peak that was associated with poor outcomes : zero VFDs (OR, 1.06; 95%CI, [1.01-1.12]; p=0.016) and composite outcome (OR,1.1; 95%CI, [1.03-1.18]; p=0.002).

**CONCLUSION.** The trends of airway pressures (delta peak and delta plateau) in mechanically ventilated patients could predict difficult weaning and so poor outcomes.

### 000945

#### Comparison of two ultrasound guided approaches, distal internal jugular vein and subclavian vein for central venous catheterization: a randomized controlled open-label pilot trial

C. Fournil[1], S. Bastide[2], JY. Lefrant[1], L. Muller[1], C. Roger[1]
[1]Department of intensive care medicine, Nimes University Hospital, Nîmes, France; [2]Department of biostatistics, Nimes University Hospital, Nîmes, France
**Correspondence:** C. Roger
*Intensive Care Medicine Experimental* 2019, **7(Suppl 3):**000945

**INTRODUCTION.** Internal jugular vein (IJV) and subclavian vein (SV) approaches are commonly used for central venous catheterization (CVC) under ultrasound (US) guidance. However, the lateral short axis approach of distal IJV has been poorly described and never compared to SV approach.

**OBJECTIVES.** The aim was to describe the lateral short axis in plane technique of distal internal jugular vein catheterization and to compare this approach to the SV approach.

**METHODS.** Single centre randomized controlled open-label pilot study. All patients requiring CVC in intensive care unit (ICU) were randomly assigned to US-guided IJV or to SV approach (Figure 1). After two unsuccessful punctures, an alternative CVC site was considered. The primary outcome was the success rate at the second puncture. The secondary outcomes were procedure characteristics and complications.

**RESULTS.** A total of 210 patients were included over 18 months. Nine patients were excluded leading to 201 patients analyzed: 100 in the IJV group, 101 in the SV group. 33% were female, mean (SD) age was 63 ± 15 years, median (IQR) BMI was 26 (23-30) and median SAPS II was 44 (35-56). The success rate at the second puncture was 96% (IC 95% 90-99) for IJV and 89% (IC 95% 81-94) for SV, p=0.06. In the IJV group, 7 complications occurred: 2 hematoma, 1 arrhythmia and 4 arterial punctures whereas 14 complications were observed in the SV group: 1 pneumothorax, 2 hematoma, 3 arterial punctures and 8 catheter misplacements. The first puncture success rate was 90% for IJV and 81% for SV, p=0.05. The duration until guidewire insertion was 60 sec in IJV group and 96 sec in SV group.

**CONCLUSION.** US-guided IJV and SV approaches are safe and efficient techniques for CVC insertion. The lateral short axis in-plane IJV approach deserves first consideration when CVC is performed in ICU patients given its high success rate, low complication rate and its facility to perform. US-guided SV approach is a good alternative when no IJV vascular access is possible.

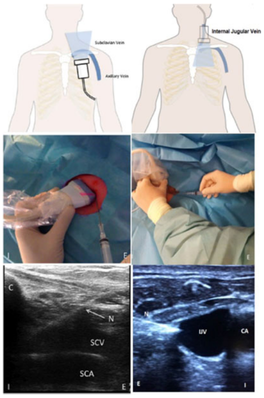

**Fig. 1 (abstract 000945).** See text for description

### 000957

#### Neuroradiological Investigation and Outcomes in Post Cardiac Arrest ICU Patients: a Clinical Audit

J. Kirk, S. White, N. Robin
[1]Intensive care, Countess of Chester Hospital NHS Foundation Trust, Chester, UK
**Correspondence:** J. Kirk
*Intensive Care Medicine Experimental* 2019, **7(Suppl 3):**000957

**INTRODUCTION.** Following cardiac arrest and return of spontaneous circulation patients are frequently admitted to the intensive care unit (ICU) for cardiovascular support and neuroprotection followed by neuroprognos itication1. This process involves neuroradiological imaging demonstrating hypoxic brain injury along with other indicators of poor neurological outcome. Anecdotally, lack of concordance of local reporting of neurorad iological imaging with clinical and other neurophysiological findings causes delay in clinical decision making, pending specialist radiological reporting.

Any improvement in this process is likely to improve timely decision making and lighten the burden of delay and uncertainty for relatives.

**OBJECTIVES.** A clinical audit to assess for the incidence of discrepancy between neuroradiological reporting, clinical assessment and specialist neurological opinion and its impact on definitive management of post cardiac arrest ICU patients.

**METHODS.** A retrospective clinical audit was performed on post cardiac arrest patients admitted to a 15 bed district general ICU over a 3 year period. Data was collected to evaluate for neuroradiology reporting by general radiologist, neurologist opinion, neuroradiologist reporting; whether there was any discrepancy between the opinions and with clinical findings or other prognostic tests; whether such discrepancies incurred a delay in definitive management.

**RESULTS.** 68 applicable patients were identified of which 7 (10.3%) were found to have a discrepancy between neuroradiology reporting by general radiologist, neurologist opinion or neuroradiologist reporting. 3 patients experienced a delay in definitive management as a direct result of this (mean delay 3.3 days).

Discrepancies between neuroradiology reports by general radiologists and other modalities of assessment were not fed back to the radiology department and the quality of clinical information supplied in scan requests was variable. There was a lack of consistency in the approach to investigating patients post cardiac arrest which may also have contributed to delays in definitive care.

**CONCLUSION.** Although only a small population stands to benefit from improved management of post cardiac arrest hypoxic brain injury on ICU, the individual burden is high and potential benefits are to the patient, their relatives and the efficient use of ICU resources.

In order to improve this management a local guideline has been designed to streamline the investigation and treatment of post cardiac arrest hypoxic brain injury and a referral pathway for neuroradiology MDT for general radiologists with an interest in neuroradiology has been created.

REFERENCE(S)

1. Nolan J et al. Post-resuscitation care: 8. Prognostication. Resuscitation Council (UK). 2015
2. Nil

**Table 1 (abstract 000957).** Results of patients with image reporting discrepancies

| | |
|---|---|
| % of patients affected by discrepancies in reporting/specialist opinion | 10.3% (7) |
| Mean length of time between MRI head and withdrawal of care | 1.7 days (0-7) |
| Percentage of patients affected by delay in definitive management | 28.6% (3) |
| Mean length of delay | 3.3 days (1-7) |

### 000971

#### Stress Ulcer prophylaxis audit in the Intensive Care Unit

S. Millington[1], A. Wilkinson[2]

[1]William Harvey Hospital, Kennington Road, Willesborough, Ashford, UK, Sevenoaks, UK; [2]Intensive care, William Harvey Hospital, Willesborough, UK

**Correspondence:** S. Millington

*Intensive Care Medicine Experimental* 2019, **7(Suppl 3):**000971

**INTRODUCTION.** Previously, stress ulcer prophylaxis was prescribed routinely for all patients admitted to the William Harvey Hospital (WHH) intensive care unit (ICU). Recent RCT and meta-analyses question the rationale behind the recommendation of stress ulcer prophylaxis in ICU as the incidence of gastrointestinal (GI) bleeding in these patients is low(1). Furthermore, evidence suggests this approach may not reduce incidence of upper GI bleeds, and may contribute to increased risk of hospital acquired pneumonia and mortality (2). Many factors put patients at higher risk of GI bleeding; including mechanical ventilation, coagulopathy, and recent GI bleed. For those patients, evidence suggests stress ulcer prophylaxis outweighs the negatives.

**OBJECTIVES.** An audit in 2017 aimed to compare the practice at WHH ICU against published best practice. The findings showed that many patients admitted to the ICU were prescribed ulcer prophylaxis unnecessarily. As a result, local guidelines were updated and rolled out. After 3 months, a re-audit was conducted to assess whether these guidelines were being followed.

**METHODS.** Data was collected from patient notes and prescription charts over two cycles, 1 year apart. Each assessed whether patients met the accepted criteria for ulcer prophylaxis. Also, if prophylaxis was prescribed, the drug chosen and route were audited. Finally, we assessed whether the prescription overall adhered to the guidelines. In total 97 adult ICU patients were included, 47 in the first cycle, and 50 in the second. Standards are based on Guidelines for the Provision of Intensive Care Services, edition 1, 2015. The guidelines state that stress ulcer prophylaxis should be used judiciously, and only in patients considered to be at high risk of upper GI bleeding. Risk Factors are: Coagulopathy, (platelet count <50,000, INR >1.5, or PTT >2 times control value); Mechanical ventilation for >48 hours; History of GI ulceration or bleeding within the past year; Traumatic brain injury, traumatic spinal cord injury, or burn injury; Two or more of the following minor criteria: sepsis, ICU stay >1 week, occult GI bleeding, or glucocorticoid therapy (>250 mg hydrocortisone).

**RESULTS.** In the first cycle ulcer prophylaxis was appropriately prescribed in 15 patients (35%), and prescribed inappropriately in 28 (65%).

This improved in the second cycle as ulcer prophylaxis was appropriately prescribed in 40 patients (80%), and inappropriately in 10 (20%)

**CONCLUSION.** These findings show a 45% increase in patients that have ulcer prophylaxis prescribed appropriately between the initial audit and this repeat.

REFERENCE(S)

1. Marik PE Vasu T Hirani A Pachinburavan M 'Stress ulcer prophylaxis in the new millennium: a systematic review and meta-analysis'. Crit Care Med 2010; 38(11):2222
2. Krag M Perner A Wetterslev J Moller MH 'Stress ulcer prophylaxis in the intensive care unit: is it indicated? A topical systematic review' Acta Anaesthesiol Scand 2013; Aug:57(7):835-47
3. n/a

### 000979

#### Implementation of an ICU without walls team can really be helpful?

J. Monclou, N. Montserrat, S. Iglesias, S. Carvalho Brugger, M. Miralbés Torner, J. Prados, M. Vallverdú, JJ. Trujillano, J. Caballero-López

Intensive care department, University Hospital Arnau de Vilanova, Lleida, Spain

**Correspondence:** J. Monclou

*Intensive Care Medicine Experimental* 2019, **7(Suppl 3):**000979

**INTRODUCTION.** To describe the results of the early attention of critical patients outside the ICU, through a team of intensivists intended for the "ICU without walls (ICUWW)" from 8 a.m. to 8 p.m. in a hospital of 400 beds in Lleida, Spain.

**METHODS.** A descriptive study from January to December of 2018. Developed in 4 phases: 1: elaboration of the project of ICUWW. 2: establish activation criteria. 3: dissemination to the services involved 4: implementation and evaluation of the results. The IWW is formed by a team that is activated with calls from the ER and other services of the hospital; for the assessment, assistance and follow-up of patients with clinical impairment and/or risk patients previously assessed by the ICU night shift or after the discharge of the ICU. The variables are described as mean± standard deviation or percentage. For the comparison of the variables the statistical test of Chi-squared ($\chi2$) was used for categorical variables, and the Mann- Whitney test for the continuous variables.

**RESULTS.** The ICUWW attended 932 patients, with a total of 1496 interventions. 63% were men and 37% women, with an average age of 61±17 years. The average time of attention was 81±60 minutes/patient with one average follow-up of 1.36 days. 52% had medical pathologies, 38.2% surgical and 9.8% traumatic. The 29.7% were assessed at the ER, 13.2% at the general surgery unit and the rest in other areas. 49.3% of the attention was made by a direct call; 5.6% was a follow-up of patients assessed in the previous ICU night shift, 11,4% required central venous catheter placement, 14.2% any other procedure, 5.7% were the follow-up of fragile patients after the ICU discharge and the rest 19,5% by other causes. The 17.2% required admission to ICU, 13.5% did not require admission because of the absence of criteria or for having completed adequate management outside of the ICU. A limitation of therapeutic effort was decided in 8.5%. Of the patients that required follow-up (N=193), 20.7% needed more than 2 days; 14% admission to ICU and the rest avoided the admission. Only (N=53) 24.5% of the patients with a follow-up after the discharge from ICU required re-entry out the global of our unit (N=1053) that is 6,12%.

**CONCLUSION.** The number of patients treated outside of the ICU justifies the creation of the ICUWW. We also describe different groups of patients attended by the ICUWW. During the study period, the ICUWW avoided admission to ICU in 26.3% of cases thanks to the early care of patients with clinical deterioration and the management of these by an intensivist regardless of the location and pathology.

REFERENCE
1. ICU without walls project. Effect of the early detection of patients at risk. Med Intensiva. 2013 Jan-Feb;37(1):12-8.

### 000992

#### The impact of ECMO on septic shock morbidity, mortality and resource utilization

S. Sangli[1], N. Smischney[2], T. Seelhammer[2]
[1]Critical Care, Mayo Clinic Hospital, Saint Marys Campus, Rochester, USA; [2]Anesthesiology and perioperative medicine, critical care, Mayo Clinic Hospital, Saint Marys Campus, Rochester, USA
**Correspondence:** S. Sangli
*Intensive Care Medicine Experimental* 2019, **7(Suppl 3):**000992

**INTRODUCTION.** Practitioners have limited evidence to guide them in the use of extracorporeal membrane oxygenation (ECMO) for adult patients with septic shock. Studies suggest the mortality of septic shock not on ECMO can be greater than 40% while the mortality of septic shock on ECMO may be as high as 70%. Given the high incidence of sepsis in intensive care units (ICUs), it's important to understand the impact of ECMO on resource utilization and survival.

**OBJECTIVES.** Our primary objective was to determine the mortality difference in septic shock patients in those requiring ECMO and those who did not. Secondary outcomes included the rates of blood product transfusions, subsequent organ failure, and ICU. We then sought to determine the risk factors for death in the ECMO group using multivariate analysis.

**METHODS.** A single center retrospective study was performed on adult patients (≥ 18 years) at Mayo Clinic Rochester with septic shock between 01/01/2009 and 06/30/2018. Post-cardiotomy patients were excluded. Data extraction was accomplished using digital queries from the institutional data repository and included demographic, laboratory, blood product transfusion, and outcome data. Patients were categorized into those with septic shock and those with septic shock subsequently requiring ECMO and assessed for differences in the primary and secondary outcomes. Multivariate analysis was performed on the ECMO patients exploring risk factors for death.

**RESULTS.** Of the 3335 patients with septic shock, 52 of them underwent ECMO. Significant differences existed between the two groups with those requiring ECMO being younger and having an elevated SOFA score as well as increased lab derangements at baseline. In-hospital mortality in the ECMO group was 50% as compared to those not on ECMO with 32% (P=<0.0074). Secondary outcomes in the ECMO and non-ECMO groups respectively included: i) packed red blood cell use of 10,567.25 ± 9572.46 ml vs 1136.60 ± 4094.56 ml (P=<0.0001) ii) acute kidney injury incidence during the hospital stay was 69% vs 29% (P=<0.0001) with 77% patients requiring hemodialysis vs 26% iii) the median ICU length of stay of 20.56 ± 33.8 vs 6.01 ± 8.34 days (P=<0.0001) and hospital length of stay of 37.74 ± 38.35 vs 16.32 ± 23.99 days (P=<0.0001). Multivariate analysis of risk factors for death on ECMO included association of diabetes (P=0.0575), maximum total serum bilirubin concentrations > 10.9 mg/dl (P=0.0146), maximum lactate elevation > 11.9 mmol/L (P=0.0319) and charlson score >2.8 (P=0.0250). Area under the curve with these four variables was 0.89.

**CONCLUSION.** Mortality is significantly increased in patients with septic shock requiring ECMO compared to those not requiring ECMO with mortality of 50% while also suffering from increased rates of blood product transfusion, multiorgan failure and overall length of stay. Risk factors for mortality in septic shock patients supported on ECMO included lactic acidosis, hyperbilirubinemia, diabetes and high charlson score.

REFERENCE(S)
1. Park, T.K., et al., Extracorporeal membrane oxygenation for refractory septic shock in adults. Eur J Cardiothorac Surg, 2015. 47(2): p. e68-74.
2. Singer, M., et al., The Third International Consensus Definitions for Sepsis and Septic Shock (Sepsis-3). JAMA, 2016. 315(8): p. 801-10.

### 000993

#### Factors influencing long term-mortality of ICU patients

C. Mendes Silva, JP. Baptista, P. Martins
[1]Intensive care medicine service, Hospitalar and University Center of Coimbra, Coimbra, Portugal, Portugal
**Correspondence:** C. Mendes Silva
*Intensive Care Medicine Experimental* 2019, **7(Suppl 3):**000993

**INTRODUCTION.** Advances in intensive care medicine is increasing patient survival in Intensive Care Units (ICU) and outcome assessment should shift from short-term mortality to long-term outcomes of ICU survivors (1). Long-term mortality after acute critical illness can be considered a patient-centered outcome (2).

**OBJECTIVES.** This study aimed to evaluate factors influencing survival of ICU patients 5 years after hospital discharge.

**METHODS.** A retrospective study was conducted in adult patients admitted to a 20-bed multipurpose ICU of a tertiary center during 6 months.

**RESULTS.** Of a total of 209 patients, 21.5% died during ICU stay. Factors statistically associated with death in ICU were age (p= 0.005), Charlson Score (p<0.001), SAPS II (p<0.001), APACHE II (p< 0.001), SOFA at admission (p<0.001), need for vasoactive drugs (p=0.015), invasive mechanical ventilation (<0.001) and need for renal replacement therapy (p=0.024). All these factors were also associated with in-hospital mortality in ICU survivors (cumulative mortality of 36.3%). In addition, late readmission (>72h) in ICU (p=0.008), but not early readmission, was associated with in-hospital death. Overall mortality of ICU patients was 46.9%, 53.6% and 56.9%, 1, 3 and 5 years after hospital discharge, respectively. Mortality in patients surviving hospital admission (N=133) at 3 and 5 years after discharge was associated with age (p<0.001) and Charlson Score (p<0.001).

**CONCLUSION.** Long-term mortality of critically ill patients was associated with age and comorbidities, but not with factors of acute illness that were associated with ICU and in-hospital mortality.

### 000994

#### Admission lactate is a marker of severity, not of infection, on ICU admission

C. Mendes Silva[1], JP. Baptista[1], P. Mergulhão[2], F. Froes[3], J. Gonçalves-Pereira[4], JM. Pereira[2], C. Dias[5], JA. Paiva[2]
[1]Intensive care medicine service, Hospitalar and University Center of Coimbra, Coimbra, Portugal, Portugal; [2]Emergency and intensive care department, Centro Hospitalar de São João, Porto, Portugal; [3]Intensive care unit, Hospital Pulido Valente, Centro Hospitalar Lisboa Norte, Lisboa, Portugal; [4]Intensive care unit, Hospital de Vila Franca de Xira, Vila Franca de Xira, Portugal; [5]Department of community medical information and health decision sciences, Faculty of Medicine, University of Porto, Porto, Portugal
**Correspondence:** C. Mendes Silva
*Intensive Care Medicine Experimental* 2019, **7(Suppl 3):**000994

**INTRODUCTION.** Since its first description in humans, increased blood lactate levels have been related to morbidity and mortality (1). Although lactate is mistakenly considered a biomarker for sepsis, there are many other causes for hyperlactatemia in Intensive Care Unit (ICU) general population (2).

**OBJECTIVES.** To evaluate the influence of patient and clinical characteristics on blood lactate values in a non-infected versus an infected population at ICU admission and define differences in outcomes.

**METHODS.** Post-hoc analysis on hyperlactatemia in the INFAUCI study, a prospective, observational, cohort, multicenter study, conducted in 14 Portuguese ICUs with data collected between 1

May 2009 and 31 December 2010. Continuous variables were dichotomized around the median values for all the patients.
**RESULTS.** A total of 3766 patients were admitted to INFAUCI study, with 1619 patients being included in the non-infected group and 1640 patients in the group infected at admission for the purpose of this post-hoc analysis, having a median lactate of 2.0 and 2.15 mmol/L (p<0.001), respectively. In both non-infected and infected groups, lactate significantly increased with SAPS II score, SOFA score and Charlson score. Only in infected group lactate was also higher with age. Longer ICU length of stay was not associated with differing lactate at admission in the non-infected group, but a significant decrease in lactate was observed in the infected group. ICU survivors had a median lactate at admission of 1.9 mmol/L [1.1-3.0] in the non-infected group and 2.0 mmol/L [1.2-3-1] in the infected group, while non-survivors had significantly higher values of 3.3 mmol/L [1.8-7.0] and 3.4 mmol/L [1.9-7.0], respectively. This difference between survivors and non-survivors was higher in both groups when ICU length of stay was less than 6 days. The ROC curve for ICU mortality in the non-infected group had an AUC of 0.679 [0.637-0.722] compared with 0.865 [0.838-0.892] for SAPS II. In the infected group, the AUC for lactate was 0.667 [0.63-0.70] compared with 0.734 [0.710-0.767] for SAPS II.
**CONCLUSION.** Lactate is a predictor of mortality for both non-infected and infected patients, showing similar variations in both groups. Lactate levels were similar in both groups meaning that is not a good biomarker to differentiate between infection and other causes of admission.

**REFERENCE(S)**

(1) Bakker J et al. Clinical use of lactate monitoring in critically ill patients. Ann Intensive Care. 2013;3(1):12.
(2) Ferreruela M et al. Hyperlactatemia in ICU patients: Incidence, causes and associated mortality. J Crit Care. 2017;42:200-5.

### 001015

#### The impact of Novel Interactive Shared Decision Making on Early Tracheostomyin Prolonged Intubated Critical Patients

SH. Kuo[1], KC. Lin[2], CP. Yang[1], TH. Yang[1], HC. Chen[1], MC. Wu[1], HN. Chen[1], SC. Chang[1], SY. Chang[2], HL. Liang[1], WC. Huang[3], CP. Liu[1]
[1]Critical care medicine and cardiovascular center, Kaohsiung Veterans General Hospital, Kaohsiung, Taiwan; [2]Department of critical care medicine and cardiovascular center, Kaohsiung Veterans General Hospital, Kaohsiung, Taiwan; [3]Critical care medicine and cardiovascular center, Kaohsiung Veterans General Hospital, Taipei, Taiwan
**Correspondence:** W.C. Huang
*Intensive Care Medicine Experimental* 2019, **7(Suppl 3):**001015

**INTRODUCTION.** The shared decision making(SDM)has became the major care model enbodying patient centered care and physician-patient equality interaction. Tracheostomy creation was not well accepted in critical patients due to mis-understanding of patients or their family, espcially in Taiwan. The aim of this study is to investigate the impact of novel interactiveSDM on early tracheostomyin prolonged intubated critical patients.
**METHODS.** Medical record ofconsecutive patients with respiratory failure in adult intensive care unitof tertiary medical centerfrom 2016 to 2017 was retrospectively reviewed. These patients were further divided into pre-SDM (2016) and post-SDM(2017) intervention period for analysis. Prolonged intubation was defined as duration of mechanical ventilation more than 14 days and sub-acute respiratory care center transferal needed. Early tracheostomywas defined as tracheostomy created within 14 days since being intubated and ventilated. Conversely, late tracheostomy was defined as tracheostomy created beyond the 14th day after intubation and mechanical ventilation. Patient who was successful weaning, chronic ventilator dependent before admission, post tracheostomy creation surgery, or expired were excluded. Data including total tracheostomy rate, early and late tracheostomy rate, ventilator weaning rate and ventilator days, in-hospital mortality, and length of hospital stay of prolonged intubated patient were collected. SPSS was applied for statistically analysis, and a p value less than 0.05 was considered significant difference.
**RESULTS.** Total 1253 patients with 7137 patient ventilator days were enrolled. Total tracheostomy rate improved from 8.8% in pre-SDM(2016) phase to 9.6% in post-SDM(2017) phase. Among 1253 patients, there were 128 patients with prolonged intubation in2016 and 112 patients in 2018for further analysis.After introducing SDM, late tracheostomy rate reduced from 43.8% to 40% and ventilator weaning rate improved from 69.5% to 74.1%in prolonged intubated patients. However there was no significant difference in in-hospital mortality(4% v.s 5%), and length of hospital stay(65.2 v.s 64.5) in prolonged intubated patients. In comparison late tracheostomy, the early tracheostomy patients had lower ventilator days(34.6+/-17.6 v.s 47.5+/-28.4, p: 0.004) and the length of hospital stay(57.2+/-21.8 v.s 68.4+/-26.8, p: 0.028). However, there was no difference in the ventilator weaning rate(69.2% v.s 76.7%, p: 0.389) and in-hospital mortality(10.3% v.s 8.2%, p: 0.737) between early tracheostomy and late tracheostomy of prolonged intubated patient.The tracheostomy decision making time delay in post-SDM phage was shown 5 days less than pre-SDM phase.
**CONCLUSION.** This studydemonstrated that introducing novel interactive shared decision making can shorten the tracheostomy decision time delay, further improve total tracheostomy rate,late tracheostomy rate and ventilator weaning rate.The latetracheostomy patients had higher ventilator daysand length of hospital stay than early tracehostomypatients.

### 001022

#### Relationship between diaphragm measurements and nutritional status in medical intensive care unit patients

K. Inci[1], E. Macit Aydın[2], M. Türkoğlu[1], G. Aygencel[1]
[1]Department of internal medicine, division of intensive care medicine, Gazi University Faculty of Medicine, Ankara, Turkey; [2]Department of anesthesiology and reanimation, division of intensive care medicine, Gazi University Faculty of Medicine, Ankara, Turkey
**Correspondence:** K. Inci
*Intensive Care Medicine Experimental* 2019, **7(Suppl 3):**001022

**INTRODUCTION.** Critical illness is strongly linked to malnutrition and diaphragm dysfunction (DD) in intensive care unit (ICU) patients. Bedside ultrasonography is a useful tool to detect DD with high specifity. In this study, we aimed to adress the relationship between malnutrition risk and diaphragmatic measurements in medical ICU patients.
**METHODS.** We measured the diaphragmatic excursion, thickness and thickening fraction (TF) in all patients with B-Mode ultrasonography. Nutritional risk screening-2002 (NRS-2002), Subjective global assesment (SGA), Modified Nutrition Risk in Critically ill (MNUTRIC) scores and measurement of Mid-upper arm circumference (MUAC) were used to differentiate the nutritional status. Patients with NRS-2002≥3, SGA<6, mNUTRIC score>4, MUAC<22 cm for female and<23 cm for male were accepted under malnutrition risk. Definition of DD was accepted TF≤20% and/or tidal DE<10 mm, diaphragm atropy (DA) was accepted diaphragm thickness of <0.2 cm measured at the end of expiration. Comparisons between DD, DA and nutritional status were analyzed by using Fisher's exact and Chi square test.
**RESULTS.** Median age was 71 [60-72], 52% were female, 62% were mechanically ventilated and ICU mortality was 32% in 50 study patients. APACHE-II score was 20±6, SOFA score was 6±2. The most common cause of ICU admission was pulmonary diseases (68%) and the most common comorbidity was heart failure (40%). Malnutrition risk rate was varied between 54% and 78%. 28% of the patients had DA and 24% had DD. Malnutrition risk diagnosed by all scoring systems were significantly correlated with DD ($p<0.05$) but was not correlated with DA ($p>0.05$). Inversely, malnutrition risk diagnosed by MUAC was not correlated with DD but it was correlated with DA. Malnutrition risk according to mNUTRIC score was an independent risk factor for DD (OR:6.6, 95%CI, 1.3-34; p=0.026). DD, DA and malnutrition risk as mNUTRIC score were more frequent in non-survivors ($p<0.05$) but none of them were independent risk factors for mortality ($p>0.05$)

**CONCLUSION.** Diaphragmatic measurements can be a good indicator for malnutrition risk or nutritional status in medical ICU patients.

**Table 1 (abstract 001022).** Relationship between diaphragmatic measurements and malnutrition risk in medical ICU patients

| According to diaphragmatic dysfunction (DD) | | | |
|---|---|---|---|
| | Patients with DD, n% n=12 | Patients without DD, n% n=38 | p value |
| Malnutrition risk | | | |
| NRS-2002 | 12 (100%) | 27 (71%) | **0.04** |
| mNUTRICScore | 10 (83%) | 16 (42%) | **0.02** |
| SGA, n (%) | 10 (83%) | 18 (47%) | **0.04** |
| Mid-Upper Arm Circumference, n (%) | 8 (67%) | 18 (47%) | >0.05 |
| According to diaphragm atrophy (DA) | | | |
| | Patients with DA, n% n=14 | Patients without DA, n% n=36 | p value |
| Malnutrition | | | |
| NRS-2002 | 14 (100%) | 28 (78%) | >0.05 |
| mNutric Score | 9 (64%) | 17 (47%) | >0.05 |
| SGA, n (%) | 10 (71%) | 18 (50%) | >0.05 |
| Mid-Upper Arm Circumference, n (%) | 11 (79%) | 15 (42%) | **0.02** |

n:number, NRS-2002: Nutritional risk screening-2002, mNUTRIC: Modified Nutrition Risk in Critically ill, SGA: Subjective global assesment

### 001028

#### The Rate and Cost of Chronic Critical Illness in a Single Intensive Care Unit in Turkey

S. Yıldırım[1], M. Mercan Kaya[1], İ. Nesil[1], O. Yeniay[1], Y. Durmaz[1], Y. ŞAn[1], C. Kıraklı[2]

[1]Intensive Care, Dr. Suat Seren Göğüs Hastalıkları Ve Cerrahisi Eğitim Ve Araştırma Hastanesi Acil Servisi, Izmir, Turkey; [2]Intensive care, Dr. Suat Seren Chest Diseases Hospital, Izmir, Turkey

**Correspondence:** S. Yıldırım

*Intensive Care Medicine Experimental* 2019, **7(Suppl 3):**001028

**INTRODUCTION.** Chronic critical illness (CCI) is defined as prolonged length of stay (LOS) in the intensive care unit (ICU) (longer than 21 days) due to mechanical ventilation dependence or other intensive care treatments[1].

**OBJECTIVES.** We aimed to determine CCI rates and its cost for our hospital.

**METHODS.** A retrospective cohort study was designed in patients admitted between 1 January 2017 and 31 December 2018 in Dr. Suat Seren Chest Disease and Surgery Teaching and Research Hospital ICU, İzmir, Turkey. Patients with a LOS less than 24 hours in ICU were excluded. We collected patient data from the hospital electronic system.

**RESULTS.** Data are expressed as median (25th-75th percentiles). 1015 patients were included to the study and 152 of them (15%) were chronic critically ill. Age, APACHE-2 score, LOS and daily cost were higher in patients with CCI (Table 1). LOS of patients with CCI was 31 (25-48) days. Daily cost for patients with CCI was 1020 (212$) Turkish Liras (TL) and 956TL (199$) patients without CCI ($p<0.001$). The need for vasopressor drugs and renal replacement therapy (RRT) in patients with CCI was 73% and 22%, respectively ($p<0.001$).

**CONCLUSION.** The rate of CCI was 15% in our ICU. More use of vasopressor drugs and RRT in patients with CCI may result in a slightly higher daily cost.

.

**Table 1 (abstract 001028).** Characteristics of Patients With and Without CCI

| | Patients without CCI | Patients with CCI | p |
|---|---|---|---|
| Age (yr), median (IQR) | 68 (59-76) | 71 (62-79) | 0,019 |
| APACHE-2, median (IQR) | 18 (14-23) | 20 (16-25) | 0,001 |
| LOS (Days) median, (IQR) | 6 (4-9) | 31 (25-48) | < 0,001 |
| Tracheostomized patients n, (%) | 32 (4%) | 77 (51%) | < 0,001 |
| Day of Tracheostomy, median (IQR) | 7 (6-11) | 16 (9-22) | 0,001 |
| Daily Cost (TL)*, median (IQR) | 956 (888-1126) | 1020 (956-1099) | < 0,001 |

*CCI* Chronical critical illness, *LOS* Length of stay, *TL* Turkish Lira
*1TL≅0.20$

### 001034

#### Altered risk of malignant disease after sepsis, a register-based study

J. Hästbacka[1], A. But[2], G. Strandberg[3], M. Lipcsey[4]

[1]Intensive care units, department of anaesthesiology, intensive care and pain medicine, University of Helsinki and Helsinki University Hospital, Helsinki, Finland; [2]Depatrment of public health, University of Helsinki, Helsinki, Finland; [3]Intensive care unit, department of surgical sciences, Uppsala University Hospital, Uppsala, Sweden; [4]Intensive care units, department of surgical sciences, Uppsala University Hospital, Uppsala, Sweden

**Correspondence:** J. Hästbacka

*Intensive Care Medicine Experimental* 2019, **7(Suppl 3):**001034

**INTRODUCTION.** Sepsis survivors are at increased risk of death up to 10 years after their ICU treatment.(1)The reasons are not fully known, but post-sepsis abnormal immune function may play a role. Acute inflammation may be followed by a chronic anti-inflammatory state with a severely impaired immune function which could associate with increased morbidity.(2) The immune suppressed state may last long and could lead to an increased risk for malignant disease as has been reported in other immune suppressed states, such as organ transplantation.(3, 4) Sepsis and cancer may also share common risk factors.

**OBJECTIVES.** To study the incidence of cancer in patients admitted to intensive care units for the treatment of sepsis and compare it with that of the general population.

**METHODS.** The study cohort was formed of adult patients admitted to an intensive care unit (Swedish Intensive Care Registry) in 2005-2015 and who had a diagnosis of sepsis (ICD-10 codes A41.9, R57.2, R65.1). Patients with no record in the population register or aged <20 years at the index admission were excluded. The data on cancer diagnoses were obtained by linking the cohort to the Swedish Cancer database of The National Board of Health and Welfare. The individuals were followed from the index admission until death, emigration or end of 2015, whichever occurred first. We calculated the standardized incidence ratio (SIR) of any cancer and ten most frequent cancer types as the ratio between the observed and the expected number of cancers. The Swedish population served as the reference and cancer statistics on this population was retrieved from the NORDCAN database.(5, 6) To account for the potential detection bias and reverse causation, we assessed the SIRs by follow-up time divided into smaller intervals and focused the SIR calculated among 6-month survivors.

**RESULTS.** After applying the exclusion criteria, the study population comprised 30051 individuals, of whom 841 patients died on admission day. The median follow-up time was 2.2 years (IQR 0.25-4.86). A total of 6928 patients died within six months and 10091 during the whole follow-up period. Six-month survivors contributed to the follow-up with 85321.3 person years. The median age at the beginning of the follow-up was 69 (IQR 60-77). New cancer diagnoses were found on 1801 patients. Focusing on new diagnoses six months after sepsis and later, we found an increased SIR in non-melanoma cancer of skin and cancer of liver and gallbladder. Decreased SIRs were observed in colorectal, prostate, lung, bladder, breast and ovarian cancers. Forest plots demonstrating the standardized incidence ratios for each gender are shown in Figure 1.

**CONCLUSION.** Long-term incidence of malignant disease in patients treated in intensive care units for sepsis is altered, as compared with age and gender-matched general population.

**REFERENCE(S)**

1) Linder, A., D. Guh, J. H. Boyd, K. R. Walley, A. H. Anis and J. A. Russell (2014). "Long-term (10-year) mortality of younger previously healthy patients with severe sepsis/septic shock is worse than that of patients with nonseptic critical illness and of the general population." Crit Care Med 42(10): 2211-2218
2) Delano, M. J. and P. A. Ward (2016). "Sepsis-induced immune dysfunction: can immune therapies reduce mortality?" J Clin Invest 126 (1): 23-31.
3) Garrett, G. L., S. E. Lowenstein, J. P. Singer, S. Y. He and S. T. Arron (2016). "Trends of skin cancer mortality after transplantation in the United States: 1987 to 2013." J Am Acad Dermatol 75(1): 106-112.
4) Mukthinuthalapati, P. K., R. Gotur and M. Ghabril (2016). "Incidence, risk factors and outcomes of de novo malignancies post liver transplantation." World J Hepatol 8(12): 533-544.
5) Danckert B, Ferlay J, Engholm G , Hansen HL, Johannesen TB, Khan S, Køtlum JE, Ólafsdóttir E, Schmidt LKH, Virtanen A and Storm HH. NORDCAN: Cancer Incidence, Mortality, Prevalence and Survival in the Nordic Countries, Version 8.2 (26.03.2019). Association of the Nordic Cancer Registries. Danish Cancer Society. Available from http://www.ancr.nu, accessed on day/month/year.
6) Engholm G, Ferlay J, Christensen N, Bray F, Gjerstorff ML, Klint A, Køtlum JE, Olafsdóttir E, Pukkala E, Storm HH (2010). NORDCAN-a Nordic tool for cancer information, planning, quality control and research. Acta Oncol. 2010 Jun;49(5):725-36.

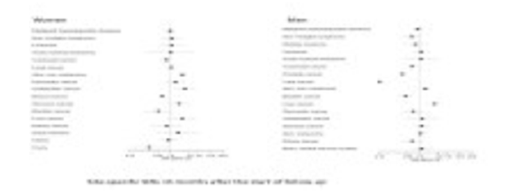

**Fig. 2 (abstract 001034).** See text for description

### 001044

#### Fluid overload is associated with an increased risk mortality in the critically ill and post-operative patient in Bogotá, Colombia

EL. Rojas Diaz, JA. Carrizosa, E. Celis

[1]Critical Care, Fundación Santa Fe de Bogotá, Bogotá, Colombia

**Correspondence:** E.L. Rojas Diaz

*Intensive Care Medicine Experimental* 2019, **7(Suppl 3):**001044

**INTRODUCTION.** The most frequent management strategy in critically ill patients is intravenous fluids. There is still debate about the amount administered, becoming a problematic intervention. For this reason, it is recommended to perform a liquid therapy guided by goals, given that the fluid overload, defined as an increase of 15% of the body weight of the income (equivalent to approximately 2.5 liters of absolute balance), is associated with increased morbidity, mortality, over-cost and prolonged hospital stay.

**OBJECTIVES.** To determine if a positive balance of fluids is an independent factor of mortality in the postoperative critically ill patient in the Intensive Care Unit at the University Hospital Fundacion Santa Fe de Bogotá in Colombia, between January and December 2017.

**METHODS.** We conducted a retrospective, cross-sectional, observational study in an Intensive Care Unit in Colombia for one year. We collect data on patient characteristics. The primary outcome was hospital mortality. Bivariate and logistic models were done to explore the association between Fluid overload and mortality in this cohort

**RESULTS.** Three hundred sixty-four patients were meeting the inclusion criteria. Three hundred thirty-five patients with complete data on fluid balance. Mean age was 60 years; 51,9% were women. The acute physiologic and chronic health evaluation (APACHE) II score was nine at ICU admission, and median ICU stay duration was 6,4 days. The fluid balance median in 24 hours is 1200 ml, and 60 % of patients had a fluid overload at ICU discharge. Overall mortality was 6%, and statistically association was found between the positive balance of fluids and mortality at ICU admission, at 24 hours, at seven days after admission, and a total balance of 3.5 liters or more with inhospital mortality.

**CONCLUSION.** Positive fluid balances more than 3.5 liters lead to negative outcomes for patients, in this case in the critically ill and post-operative should be goal directed therapy, maintaining a neutral fluid balance. In case that not check this therapy in your bundles it could be negatively impacted in mortality, especially at the 7th day ICU stay.

**REFERENCE(S)**

(1) Myburgh J a., Finfer S, Bellomo R, Billot L, Cass A, Gattas D, et al. Hydroxyethyl Starch or Saline for Fluid Resuscitation in Intensive Care. N Engl J Med. 2012 Nov 15;367(20):1901-11. doi: 10.1056/NEJMoa1209759.
(2) Subramanian S, Ziedalski TM. Oliguria, volume overload, Na+ balance, and diuretics. Crit Care Clin. 2005;21(2):291–303. doi: 10.1016/j.ccc.2005.01.009
(3) Bartels K, Thiele R, Gan T. Rational fluid management in today's ICU practice. Crit Care [Internet]. 2013;17(Suppl 1):S10. Available from: http://ccforum.com/content/17/S1/S9\npapers3://publication/doi/10.1186/cc12771
(4) Cecconi M, Corredor C, Arulkumaran N, Abuella G, Ball J, Grounds RM, et al. Clinical review: Goal-directed therapy - what is the evidence in surgical patients ? The effect on different risk groups. Crit Care. 2013 Mar 5;17(2):209. doi: 10.1186/cc11823.
(5) Basso F, Berdin G, Virzì GM, Mason G, Piccinni P, Day S, et al. Fluid management in the intensive care unit: bioelectrical impedance vector analysis as a tool to assess hydration status and optimal fluid balance in critically ill patients. Blood Purif. 2013;36(3-4):192-9. doi: 10.1159/000356366.
(6) Solomon AW, Kirwan CJ, Alexander NDE, Nimako K, Jurukov A, Forth RJ, et al. Urine output on an intensive care unit: case-control study. BMJ. 2010 Dec 14;341:c6761. doi: 10.1136/bmj.c6761.
(7) Brown H, Greenberg J, Swanson A, Sewall MR, Robinson SB. The costs of fluid overload in the adult intensive care unit : is a small-volume infusion model a proactive solution ?. Clinicoecon Outcomes Res. 2014 Dec 15;7:1-8. doi: 10.2147/CEOR.S72776

### 001059

#### Organisational Burden of Palliative Care in the Intensive Care Unit (ICU)

U. Jaschinski[1]; B. Rupprecht[1]; C. Hartog[2]

[1]Anesthesiology and intensive care medicine, University Hospital, Augbsurg, Germany; [2]Anesthesiology and operative intensive care, Charité – Universitätsmedizin Berlin, Berlin, Germany

**Correspondence:** U. Jaschinski

*Intensive Care Medicine Experimental* 2019, **7(Suppl 3):**001059

**INTRODUCTION.** End of life practice (EOLP) – i.e. care of ICU patients with an end-of-life decision (EOLD) to limit life-sustaining therapies – is common practice nowadays. Critical Care Physicians face these decisions nearly every day However, data on the organizational burden accompanying this procedure is sparse.

**OBJECTIVES.** We evaluated ICU days spent on patients with an EOL decision order (EOLD).

**METHODS.** This study presents data from our ICU which participated in the worldwide Ethicus-2 study on ICU end-of-life care practices (EOLP) in ICUs.

**RESULTS.** During a 6 month period 1958 adult patients were admitted to a 42 bed surgical ICU of a tertiary hospital. In 183 (9,34%) - 115 male; mean age 71,7 ± 11,7 SD; 68 female; mean age 74,3 ± 11,2 SD - EOLP was applied. 26 left the ICU alive and 16 survived the hospital stay which corresponds to an ICU mortality of 85,8% and a hospital mortality of 91,3% respectively. The ICU stay (LOS) until the EOLD was 104,36 hours [mean, 95% CI 128,57-80,15] and 47,86 hours [mean, 95% CI 61,65-34,07] elapsed between the decision and death or discharge. The mean LOS in the EOLD patients was 6,7 ± 9,3 days (mean, SD). The LOS from EOLD until ICU demission corresponded to 364,17 patients days.

**CONCLUSION.** In times of shortage of ICU beds in level 3 hospitals this may be an important finding. Since the mean LOS in this particular ICU is 3,3 days this means that during the 6 month observation period about 110 additional patients did not get access to the ICU and thus possibly received suboptimal care. This capacity strain (1) may further impede the process of triage recently recommended by a Task Force of the World Federation of Societies of Intensive and Critical Care Medicine (2). The considerable organizational burden of palliative care in the ICU calls for alternative resource allocation and end-of-life care pathways.

**REFERENCE(S)**

1. Blanch L et al. J Crit Care (2016) 36:301-305
2. Bagshaw SM et al. Crit Care Med (2017) 45:e347-e356

#### 001073

**MET implementation evaluation - education as a cornerstone for success**

I. Jesus Pereira, P. Barbosa, I. Hubert, C. Granja
[1]Serviço de medicina intensiva 1, Centro Hospitalar Universitário do Algarve, Faro, Portugal
**Correspondence:** I. Jesus Pereira

*Intensive Care Medicine Experimental* 2019, **7(Suppl 3):**001073

**INTRODUCTION.** Medical Emergency Teams (METs) provide assessment and early intervention for patients outside critical care areas. The implementation of METs led to the decrease of resuscitations, critical care transfers, overall mortality and cost.

In 2015 the authors implemented a MET in their hospital. Activation criteria were adapted to the local reality and were grouped under: airway, ventilation, circulation, neurological status and clinical criteria. After 3 years of implementation, the results obtained from the present and previous studies (1,2) are worrying. The future interventions are now set to improve education and early recognition of signs and symptoms of patient clinical deterioration.

**OBJECTIVES.** To evaluate the MET implementation adequacy.

**METHODS.** Observational prospective study of the MET activity, from October 2015 to December 2018. Data was obtained from the MET registry and from the patient clinical charts. Categorical variables were described as absolute frequencies (n) and relative frequencies (%). Median and interquartile range were used for continuous variables. Statistical analysis was performed using the software Statistical Package for the Social Sciences v. 25.0.

**RESULTS.** During this period, 1672 primary activations were registered, 56.1% of which for male patients. The median age was 76 years (IQR 65, 84); the youngest patient was 1 year old and the oldest was 99 years old.

The surgical department was responsible for the majority of activations, representing 47.2% (n= 789) of the total events.

Going through the evolution of the number of activations there was a progressive decrease since the first quarter of activity (2015: n=185; 2016: n=628; 2017: n=410: 2018: n=449).

Mortality was evaluated at 30 days, with 773 deaths (46.2% of total activations), of which 43.9% were under the event Circulation and 38.6% under Airway/Ventilation. Of the total deaths, 66.8% occurred within 72 hours of the MET intervention. The MET was activated 197 times for the event Cardiac Arrest, with an increase in the relative frequency from 2015 until 2017 and a slight drop in 2018 (2015: 8.4%, 2016: 11.3%, 2017: 13.6%, 2018: 12.0%). This subgroup of events presents a 30-day mortality of 94%. Available hospital mortality rate data are 7.46 in 2015; 8.35 in 2016 and 8.64 in the first quarter of 2017.

**CONCLUSION.** Hospital mortality is high and consistent with MET data. However, it should be emphasized that it was not possible to exclude end-of-life decisions from both the hospital and the MET mortality.

The activation decline over time reinforces the importance of continuous education.

The mortality results, even after the MET intervention, point towards a delay in activation, with a small benefit to the intervened population.

A multidisciplinary intervention is necessary. A certified training center has already been set in 2019 and the official training program has started. Besides basic and advanced life support formation, the team activation criteria will be recalled, and emphasis will be given on the need for early recognition of signs of clinical instability.

**REFERENCE(S)**

1. A. Correia, I.J. Pereira, P. Barbosa, C. Granja “Medical Emergency Team - is afferent limb (METal) working?“ Intensive Care Medicine Experimental 2017, 5 (Suppl 2):0719. DOI 10.1186/s40635-017-0151-4.
2. A.Correia, I. Jesus Pereira, P. Barbosa, C. Granja. “Crew Resource Management: is it lacking in Basic Life Support training? – CRM study”. Intensive Care Medicine Experimental 2018, 6(Suppl 2): 0677. DOI 10.1186/s40635-018-0201-6

### SIS - Sepsis evaluation

#### 001323

**Pentraxin-3 and sepsis: diagnosis may not be the right search!**

G. Cogo, K. Donadello, V. Schweiger, M. Citino, L. Cangemi, E. Polati
[1]Anesthesia and intensive care b unit, University of Verona, AOUI-University Hospital Integrated Trust of Verona, Verona, Italy
**Correspondence:** K. Donadello

*Intensive Care Medicine Experimental* 2019, **7(Suppl 3):**001323

**INTRODUCTION.** Sepsis still represent a major health issue. Many sepsis biomarkers have been tested in the past years. Pentraxin-3 (PTX-3) has been proposed as a valuable diagnostic tool.

**OBJECTIVES.** To compare PTX3 and Procalcitonin (PCT) in the diagnosis and prognostication of sepsis in medical critically ill patients in a medico-surgical intensive care unit.

**METHODS.** PTX3 and PCT plasma concentrations were measured in prospectively adult medical intensive care unit (ICU) patients within 24 hours from admission and thereafter daily. Their levels were compared in infectious and non-infectious conditions. The ability of PTX3 and PCT to discriminate sepsis was evaluated using receiver operating characteristic (ROC) curves and the respective areas under curves (AUCs). The best cut-off values of both sepsis markers were calculated using diagnostic odds ratio (DOR). To assess the prognostic value of the day-by-day changes of PCT and PTX3 plasma levels wecomparedthe incidence of ICU- and in-hospital mortality in patients with and without two consecutively increases of these markers above the value exceeding the cut-off level.

**RESULTS.** We included 37 consecutive patients who were admitted to our ICU for an expected time longer than 48 hrs (186 patient-days-pd). The mortality rate was 21.6%. Patients with sepsis (66 pd) and septic shock (29 pd) had higher PTX3 (3.69± 2.83 µg/L and 5.35 ± 3.13 µg/L) and PCT plasma concentrations (7.82 ± 13.6 µg/L and 8.45 ± 11.5 µg/L), compared to those without infection (91 pd, 3.18 ± 2.9 and 0.88 ± 0.93 µg/L, respectively, p < 0.01). The area under the ROC curve for PCT was greater than the one for PTX3: 0.89 (CI 95%: 0.83-0.93) vs 0.65 (CI 95%: 0.57-0.72) (p=0.037). The cut-off values of 2.4 µg/Lfor PTX-3, and of 1.4 µg/Lfor PTX3 yielded the highest DOR for sepsis diagnosis. Patients who survived had lower values of PCT and PTX3 compared to the group of patients that died, both at admission time (7.14 ± 15.08 µg/L vs 12.3 ± 14.3 µg/L p=0.4 for PCT

and 5.08 ± 3.42 μg/L vs 9.03 ± 1.0 μg/L p<0.009 for PTX3), anddaily (PCT: 4 ± 9.6 μg/L vs 7.1 ± 10.7 μg/L p=0.09 and PTX3: 3.1 ± 2.7 μg/L vs 6.2 ± 3.2 μg/L p<0.001). Patients with two consecutive daily increases of PCT and PTX3 had in-hospital mortality rate of 66.7% and 61.5% respectively, compared to patients without two consecutive daily increases of PCT and PTX3 that had an in-hospital mortality rate of 8.0% and 8.3% (p < 0.01) respectively.

**CONCLUSION.** In our small cohort of medical ICU patients, both PCT and PTX3 could discriminate sepsis, PCT being a highly better diagnostic tool. Both marker time course revealed to have some prognostic power to be further investigated.

### 001330

#### Muscle wasting in septic shock: qualitative and quantitative echographic assessment of sarcopenia during one week of admission to the ICU

LE. López, MP. Benitez, JF. Martínez Carmona, MFA. Hijano, AMJ. Delgado
[1]Intensive care unit, Hospital Carlos Haya, Málaga, Spain
**Correspondence:** J.F. Martínez Carmona
*Intensive Care Medicine Experimental* 2019, **7(Suppl 3):**001330

**INTRODUCTION.** Patients with septic shock have a catabolic state that leads to a depletion of the skeletal muscle compartment early. The loss of muscle mass is associated with an increased risk of nosocomial infections, prolonged mechanical ventilation, longer ICU and hospital stays, and increased mortality.

**OBJECTIVES.** To determine the loss of muscle mass in patients with septic shock by ultrasound evaluation of the right rectus femoris muscle, and its correlation with morbidity and mortality.

**METHODS.** Prospective study that includes 11 patients with septic shock of different focus. Ultrasound measurement of the area of the rectus femoris muscle (CSA RF) was performed on admission and after 7 days, as well as a qualitative assessment using Heckmatt Score. Demographic variables were collected, time MV, ventilator-associated pneumonia, weaning failure, tracheostomy, ICU and hospital stay, mortality.

**RESULTS.** The average age was 60.6 years +/- 11.9. 72.7% were male. Reason for admission: Respiratory (72.7%), abdominal (18.2%) and urological (9.1%) sepsis. Apache II mean at admission was 23.73 +/- 9.27; the mean SOFA upon admission was 10.64 +/- 2.76. The median time on mechanical ventilation was 18 days. 45.5% had VAP and 72.7% required a tracheostomy. The median stay in the ICU was 24 days. The mortality in the ICU was 27.3%, the survival at 6 months was 54.55%.

We observed significant differences between the loss of muscle mass during the first 7 days and mortality (Test Chi2 p 0.027), however, when performing the ANOVA analysis we found no significant relationship between the variables (F 3.4, p 0.102). We did not find significant differences with the duration of mechanical ventilation, VAP or stay. It is a small sample, so it is not yet possible to draw conclusions.

**CONCLUSION.** - Muscle ultrasound is a useful tool at bedside and easy to perform, specifying a small learning curve

- Wasting muscle plays a fundamental role in the evolution of the critical patient, so its monitoring and minimization is key to modify the prognosis of our patients.

##### REFERENCE(S)

1. - Mueller et al. Can Sarcopenia Quantified by Ultrasound of the Rectus Femoris Muscle Predict Adverse Outcome of Surgical Intensive Care Unit Patients as well as Frailty? A Prospective, Observational Cohort Study. Ann Surg 2016; Dec 264 (6): 1116-1124.
2. - Turton P, Hay R, Taylor J, McPhee J, Welters I. Human limb skeletal muscle wasting and architectural remodeling during five to ten days intubation and ventilation in critical care - an observational study using ultrasound. BMC Anesthesiology 2016; 29: 16(1): 119.
3. - Mourtzakis M, Parry S, Connolly B, Puthucheary Z. Skeletal Muscle Ultrasound in Critical Care: A Tool in Need of Translation. Ann Am Thorac Soc 2017; Oct 14 (10): 1495-1503.

### 001332

#### Interleukin-8 is a major factor in inducing Neutrophil Extracellular Traps (NETs) in sepsis

M. Alsabani[1], S. Abrams[1], Z. Cheng[1], Y. Alhamdi[1], B. Morton[2], G. Wang[1], CH. Toh[1]
[1]Clinical infection, microbiology and immunology, Institute of Infection and Global Health, University of Liverpool, Liverpool, UK; [2]Clinical sciences, Liverpool School of Tropical Medicine, Liverpool, UK
**Correspondence:** M. Alsabani
*Intensive Care Medicine Experimental* 2019, **7(Suppl 3):**001332

**INTRODUCTION.** Neutrophil extracellular traps (NETs) formation is a novel mechanism by which neutrophils immobilise and kill pathogens and are increase in critical illnesses such as sepsis (1, 2). Recent studies highlight the harmful effects of NETs by providing a scaffold for thrombosis, resulting in microvascular occlusion and multiple organ failure (MOF) (3). Interleukin-8 (IL-8) is a potent factor for neutrophil recruitment and activation during sepsis, and has recently been demonstrated to induce NETs *in vitro*(4, 5).

**OBJECTIVES.** To establish the pathophysiological role of IL-8 induced NETs formation in a murine model of sepsis.

**METHODS.** Sepsis was induced in mice by caecal ligation and puncture (CLP) or intraperitoneal (i.p) injection of *Escherichia coli*without and with anti-IL-8 therapy. Histopathological analysis was used to measure NETs formation and Acute Lung Injury (ALI). Blood Urea Nitrogen (BUN) and Alanine aminotransferase (ALT) were measured to investigate the levels of organ injury. Survival analysis was observed in CLP septic mice.

**RESULTS.** We specifically observed NETs positive staining (cit-H3) in the lung tissue of mice following sepsis, which was associated with increases in lung injury scores compared to control mice. Ten hours following induction of both sepsis models, lung injury was significantly attenuated by anti-IL-8 therapy (CLP: P<0.001, E.coli: P<0.001), along with circulating markers of both liver (BUN [CLP: P=0.005, E.coli: P<0.001]) and kidney (ALT [CLP: P= 0.01, E.coli: P=0.002]) injury. Finally, we observed significantly improved survival times in septic mice treated with anti-IL-8 therapy compared to septic mice without therapeutic intervention (P=0.004).

**CONCLUSION.** Targeting IL-8 reduced NETs-induced organ injury and increases survival rates in mice with sepsis. This could provide a novel therapy in improving clinical outcome in critically ill patients.

##### REFERENCE(S)

1. Brinkmann V, Reichard U, Goosmann C, Fauler B, Uhlemann Y, Weiss DS, et al. Neutrophil extracellular traps kill bacteria. science. 2004;303(5663):1532-5.
2. Czaikoski PG, Mota JMSC, Nascimento DC, Sônego F, Melo PH, Scortegagna GT, et al. Neutrophil extracellular traps induce organ damage during experimental and clinical sepsis. PloS one. 2016;11(2):e0148142.
3. McDonald B, Davis RP, Kim S-J, Tse M, Esmon CT, Kolaczkowska E, et al. Platelets and neutrophil extracellular traps collaborate to promote intravascular coagulation during sepsis in mice. Blood. 2017;129(10):1357-67.
4. Ness TL, Hogaboam CM, Strieter RM, Kunkel SL. Immunomodulatory role of CXCR2 during experimental septic peritonitis. The Journal of Immunology. 2003;171(7):3775-84.
5. Hazeldine J, Harris P, Chapple IL, Grant M, Greenwood H, Livesey A, et al. Impaired neutrophil extracellular trap formation: a novel defect in the innate immune system of aged individuals. Aging cell. 2014;13(4):690-8.

### 001366

#### Alfatorquetenovirus: a new sepsis biomarker?

N. Segura-Marín[1], E. Albert[2], S. Martinez[1], P. Pardo[1], D. Navarro[2], G. Aguilar[1]
[1]Anaesthesia and critical care, University Hospital, València, Spain; [2]Microbiology, University Hospital, València, Spain
**Correspondence:** N. Segura
*Intensive Care Medicine Experimental* 2019, **7(Suppl 3):**001366

**INTRODUCTION.** The use of biomarkers in sepsis is very useful for the early diagnosis of and to establishing a prognosis. The desired marker should be sensitive, specific, fast and accurate. Procalcitonin (PCT) is one of the measurements approved by the FDA, although its efficacy is still questioned1. The determination of alfatorquetenovirus (TTV) could be a useful marker2.

**OBJECTIVES.** 1. Establish a relationship between the viral TTV load and the prognosis of sepsis

2. Relate the viral TTV load with inflammatory parameters

**METHODS.** We analyzed 55 samples from 23 patients admitted to intensive care with clinical suspicion of sepsis. Analytical data of C-reactive protein (CRP), neutrophils and procalcitonin were collected. The SOFA and APACHE II scores were calculated, and the patients were stratified according to these five values in good and poor prognosis.

A quantitative determination of TTV was carried out from each sample using a quantitative PCR3. We calculate the area under the curve of the TTV plasma values as a function of time.

The differences between medians were compared using the U-Mann-Whitney test. The correlation between variables was measured using the Spearman correlation. The relationship between qualitative variables was studied through Chi2 analysis. The value of P <0.05 was considered statistically significant.

**RESULTS.** We have found an inverse relationship, not significant, between the TTV AUC and the patient´s proinflammatory level and therefore with the disease´s prognosis.

A tendency (not significant) was found between poor prognosis and the procalcitonin´s median values and CRP being higher in the group with poor prognosis.

A trend was observed indicating that at a lower TTV DNAnemia is related to worse prognosis.

An inverse relationship was found between procalcitonin and CRP values and the TTV copies / ml in plasma, (rho = -0.132 and -0.349 respectively) nonsignificant correlation in the case of procalcitonin.

There was a clear trend between the neutrophils´ expansion and the regression line slope, obtained between the TTV loads in the first two study times.

The results obtained indicate a possible relationship between immune exacerbation degree and TTV DNAemia.

**CONCLUSION.** The TTV quantitative determination could be useful as a proinflammatory marker in sepsis, both for its low cost and its ease in the determination as for its relationship with the functionality of the patient's immune system. It is necessary to carry out a study with a larger sample size to corroborate the validity of our hypothesis and in turn to establish a TTV threshold that allows us to anticipate the disease clinical outcome.

**REFERENCE(S)**

1. Overview of procalcitonin assays and procalcitonin-guided protocols for the management of patients with infections and sepsis. Schuetz. P, Bretscher. C, Bernasconi. L, Mueller. B. Expert Rev Mol Diagn. 2017 Jun
2. Torquetenovirus (TTV): The Human Virome From Bench to Bedside. Daniele Focosi, Guido Antonelli, Mauro Pistello, Dr. Fabrizio Maggi, 2016, Clinical Microbiology and Infection
3. Dynamics of Torque Teno plasma virus DNAemia in allogeneic stem cell transplant recipients. Albert E. J Clin Virol 94 (2017) 22-28)
4. Dr, Gerardo Aguilar. Head of Critical Care Unit. Clinic Universitary Hospital, Valencia, Spain
5. Dr. David Navarro. Head of Microbiology Unit. Clinic Universitary Hospital Valencia, Spain

### 001370

#### Proteomic profiling of lysine acetylation reveals SIRT3 deficiency exacerbates sepsis-induced myocardial dysfunction via inducing mitochondrial dysfunction

S. Zhang[1], J. Rong[2], Y. Xu[2], Y. Wang[1], Z. Zhang[3]
[1]College of pharmaceutical sciences, Zhejiang University, Hangzhou, China; [2]Department of cardiology, cardiovascular key laboratory of zhejiang province, Second Affiliated Hospital, Zhejiang University School of Medicine, Hangzhou, China; [3]Department of intensive care unit, Second Affiliated Hospital, Zhejiang University School of Medicine, Hangzhou, China
**Correspondence:** Z. Zhang
*Intensive Care Medicine Experimental* 2019, **7(Suppl 3):**001370

**INTRODUCTION.** Sepsis-induced myocardial dysfunction (SIMD) leads to increased mortality in septic patients. Mitochondrial dysfunction is confirmed to be involved in the development of SIMD. Sirt3 is identified as one of the major mitochondrial deacetylases, whose activity is necessary to prevent mitochondrial dysfunction. However, the role of Sirt3 in the development of mitochondrial dysfunction associated with SIMD have not been determined.

**OBJECTIVES.** To investigate the potential role of Sirt3 in SIMD and identify the key protein post-translational modifications related to mitochondrial dysfunction.

**METHODS.** In a lipopolysaccharide (LPS)-induced sepsis mouse model, animals were divided into 4 groups: WT-PBS, WT-LPS, Sirt3(-/-)-PBS, and Sirt3(-/-)-LPS. Cardiac function of animals was evaluated by echocardiography, and the indicators of cardiac hypertrophy and inflammatory factors were detected in heart tissues. The level of mitophagy in the hearts by examining the expression of autophagy-related proteins and autophagosome, as regulated by LPS and/or SIRT3 was also detected. Furthermore, label-free quantitative proteomic analysis by using mass spectrometry was performed to characterize the acetyl-proteome of hearts and the role of SIRT3-dependent regulation of the acetylation under LPS stimulation.

**RESULTS.** LPS-induced cardiac dysfunction was more severe in Sirt3(-/-) mice compared with WT mice. The indicators of cardiac hypertrophy as well as the inflammatory mediators including IL-1β, IL-6, and TNF-α in LPS groups increased significantly compared with those in PBS groups. The expression of autophagy-related protein LC3 and the number of autophagosomes increased in Sirt3(-/-) mice and aggravated under LPS stimulation. A total of 1600 unique sites were identified as acetylated, most belonged to mitochondrial proteins. Several metabolic pathways including fatty acid catabolic process were significantly hyperacetylated in Sirt3(-/-) mice (WT-LPS vs WT-PBS, Sirt3(-/-)-LPS vs Sirt3(-/-)-PBS). Finally, we identified that key enzymes in the tricarboxylic acid (TCA) cycle could be commonly regulated by acylation and exacerbated by Sirt3 deficiency in sepsis, among which malate dehydrogenase (MDH2) and isocitrate dehydrogenase (IDH2) might be the key targets for further intervention.

**CONCLUSION.** These findings established Sirt3 as a global regulator of lysine acetylation in mitochondria. Sirt3 deficiency exacerbates SIMD via changing the level of mitochondrial proteins acetylation, which enriched in the metabolic pathways including fatty acid degradation and TCA cycle.

**REFERENCE(S)**

1) Martin L, Derwall M, Zoubi SA, et al. The septic heart: Current understanding of molecular mechanisms and clinical implications. Chest 2019; 155 (2): 427-437

2) Durand A, Duburcq T, Dekeyser T, et al. Involvement of mitochondrial disorders in septic cardiomyopathy. Oxid Med Cell Longev 2017; 2017:4076348
3) Sack MN. Emerging characterization of the role of SIRT3-mediated mitochondrial protein deacetylation in the heart. Am J Physiol Heart Circ Physiol 2011; 301: H2191–H2197
4) Sheeran FL, Pepe S. Posttranslational modifications and dysfunction of mitochondrial enzymes in human heart failure. AM J Physiol Endocrinol metab 2016; 311: E449-E460
5) This study was supported by national natural science foundation of China (No.81471889 and No. 81772110 to ZZC, No. 81500176 to XYC).

### 001391

#### Renal and splanchnic sympathetic overactivation in sepsis survivors. Experimental study

IM. Milanez[1], AMA. Liberatore[2], EE. Nishi[1], TC. Bergamaschi[1], R. Campos[1], IHJ. Koh[2]
[1]Physiology, cardiovascular division, Universidade Federal de São Puo, Sao Paulo, Brazil; [2]Surgery, Universidade Federal de São Puo, Sao Paulo, Brazil
**Correspondence:** I.H.J. Koh
*Intensive Care Medicine Experimental* 2019, **7(Suppl 3):**001391

**INTRODUCTION.** Post-sepsis mortality has been related to a syndrome with persistent inflammation, immunosuppression and catabolism (PICS) [1], Among varying alterations, septic associated cognitive dysfunction is a common condition [2] and a change in sympathetic nerve activity (SNA) during the acute sepsis, such as an increase in cardiac SNA and the display of a biphasic pattern of renal SNA (rSNA). However, little is known about the role of the sympathetic nervous system over abdominal organs commonly compromised in sepsis and after sepsis recovery.

**OBJECTIVES.** Evaluate the renal (rSNA) and splanchnic (sSNC) baseline sympathetic nerve activity kinetics in the acute sepsis phase and in post-sepsis survivals

**METHODS.** Female Wistar rats (250-300g) underwent DL50 sepsis model induction (2mL *E. coli* 108 CFU/mL, iv.) After 6 hours of sepsis induction (S8-6h group), 1 month (S8-1mo group) and 3 months (S8-3mo group) surviving rats had their femoral vein and artery cannulated. After 24h of cannulation, BP and HR were recorded in conscious rats and then they were anesthetized with urethane (1,2 g/Kg, iv) and underwent rSNA and sSNA monitoring.

**RESULTS.** All animals showed no clinical signs of illness in 1- and 3-months post-sepsis, and their BP and HR were within the normal range. However, there was a clear trend to the increase in SNA in post-sepsis periods, the sSNA was augmented in the S8-1mo and both rSNA and sSNA were significantly increased in the S8-3mo.

**CONCLUSION.** These preliminary findings suggest that there is a sympathetic overactivation state after sepsis which may contribute to a handicapped cardiovascular physiological response to overcome the new pathological challenge events in post-sepsis recovery periods. Ongoing studies are in course to check other neurological mechanisms related to PICS.

##### REFERENCE(S)

1. Allan J. C. Calsavara, Priscila A. Costa, Vandack Nobre & Antonio L. Teixeira. Factors Associated with Short- and Long-Term Cognitive Changes in Patients with Sepsis. SCIENTIFIC REPORTS | (2018) 8:4509
2. Lori F. Gentile, MD, Alex G. Cuenca, MD, et col. Persistent inflammation and immunosuppression: A common syndrome and new horizon for surgical intensive care. Journal of Trauma and Acute Care Surgery,2012, 72(6), 1491–1501.
3. Grant 2017/21052-0, São Paulo Research Foundation (FAPESP).

### 001445

#### Role of Presepsin in Diagnosis and prediction of mortality in ICU patients with infection

A. Al Tayar, E. Abdelshafey, M. Rashwan, M. Fawzy, A. Gohary
[1]Icu, SFHD, Dammam, Saudi Arabia
**Correspondence:** A. Al Tayar
*Intensive Care Medicine Experimental* 2019, **7(Suppl 3):**001445

**INTRODUCTION.** sepsis is defined as life-threatening organ dysfunction caused by dysregulated host response to infection. The systemic inflammatory response syndrome (SIRS) criteria are replaced now by SOFA score inside intensive care unit (ICU) and quick SOFA (qSOFA) for patients outside ICU to identify patients who are potentially at risk of dying from sepsis. Soluble CD14 subtype (sCD14-ST), known as presepsin, is an immunological biomarker which can be considered as an indicator of activated innate immune effector cells in response to invasive pathogens.

**OBJECTIVES.** The aim of this study is to evaluate significance of presepsin for early identification of sepsis and prediction of mortality in ICU patient in comparison to SIRS criteria and qSOFA scores.

**METHODS.** 40 patients were selected randomly after admission to adult ICU department from ER in Security Forces Hospital Dammam in the period from June to December 2018 without respect of admission diagnosis. Data from ER triaging and initial laboratory results were gathered to calculate qSOFA score, SIRS criteria and SOFA score. Presepsin measurement in full blood sample was performed within 6 hours from ER triaging.

After complete course in ICU patients were divided retrospectively into 2 groups; group A (patients with sepsis) and group B (patients with no sepsis) depending on clinical and microbiological criteria and SOFA score changes

**RESULTS.** 26 patients were diagnosed as sepsis 16 males and 10 females with average age of 68.04 ± 18.60 years while 14 patients were remarked as non-septic 8 males and 6 females and average age was 51.71 ± 24.88 years with significant difference regarding Age and APACHE 2 score being higher in septic group while no significant difference was found regarding sex.

With significant AUC of 0.848 ($p<0.001$), prespsin with cutoff value >640 pg/ml identified septic cases with sensitivity of 73.08% and specificity of 92.86% compared to non-significant SIRS criteria (AUC 0.670, sensitivity 69.23% and specificity of 57.14%) or qSOFA (AUC 0.652, sensitivity 38.46% and specificity of 78.57%).

Also, with significant AUC of 0.920 ($p<0.001$) prespsin with cutoff value >775 pg/ml predicted mortality with sensitivity of 100.0% and specificity of 76.67 % compared to non-significant SIRS criteria (AUC 0.540, sensitivity 70.0% and specificity 43.33%) or qSOFA (AUC 0.670, sensitivity of 60% and specificity of 76.67%)

**CONCLUSION.** Early presepsin measurement in ICU patients has significant role in diagnosis of sepsis and prediction of mortality compared to SIRS criteria or qSOFA score

##### REFERENCE(S)

1. Singer M, Deutschman CS, Seymour CW, et al. The Third International Consensus Definitions for Sepsis and Septic Shock (Sepsis-3). JAMA 2016; 315:801.

2. M. Sandquist, H.R. Wong, Biomarkers of sepsis and their potential value in diagnosis,prognosis and treatment, Expert Rev. Clin. Immunol. 10 (10) (2014) 1349–1356.
3. no grant was given , unfunded research

### 001448

#### Prognostic value of Reactive C Protein (RCP) and Procalcitonin (PCT) in critically ill patients with community-acquired respiratory sepsis

R. Carbonell[1], G. Moreno[1], J. Gómez[2], M. Bodí[3], A. Rodríguez[3]
[1]Intensive care, Hospital Universitari de Tarragona Joan XXIII, Tarragona, Spain; [2]Intensive care unit, Hospital Universitari de Tarragona Joan XXIII, URV, Tarragona, Spain; [3]Intensive care unit, Hospital Universitari de Tarragona Joan XXIII, URV, IISPV/CIBERES, Tarragona, Spain
**Correspondence:** R. Carbonell
*Intensive Care Medicine Experimental* 2019, **7(Suppl 3):**001448

**INTRODUCTION.** Severe sepsis and septic shock are frequent and serious complications in critically ill patients. Timely diagnosis and treatment are highly important in reducing the morbidity and mortality associated with sepsis. Serum biomarkers are very helpful tools in the early diagnosis and management of sepsis. PCT has been reported as a useful biochemical marker to differentiate early sepsis from other non-infectious causes of systemic inflammatory response syndrome, and its clearance is useful to check the response to therapy (1). However, the use of PCT as a prognostic marker is still uncertain.

**OBJECTIVES.** To evaluate the association between serum PCT and RCP levels on ICU admission and ICU-mortality in patients with community-acquired respiratory sepsis.

**METHODS.** Prospective cohort study of critically ill patients with confirmed Influenza pneumonia admitted to 184 ICUs in Spain, between June 2009 and April 2018. Only patients with determination of PCT and CRP levels on ICU admission were included. We analyzed global population, and differentiating those with primary viral pneumonia (PVP) and those with bacterial co-infection (BC). We recorded demographic characteristics, comorbidities, outcomes, as well as clinical variables and complications during admission. The statistical analysis was performed using chi-squared test (categorical variables) and Student *t* test (continuous variables). The multivariate analysis (binary logistic regression) was performed to determine variables independently associated with ICU-mortality.

**RESULTS.** We included 1610 patients, 1186 (73.6%) with PVP and 424 (26.3%) with BC. The median age was 56 years old (IQR 46-67), with median APACHE II and SOFA scores of 17 (IQR 12-22) and 6 (IQR 4-9) respectively. The most frequent comorbidity was chronic obstructive pulmonary disease (20.4%); 32.9% (530 patients) developed acute renal failure, 57.1% had shock on admission and 81.9% required mechanical ventilation. Overall mortality was 23.7% (n = 382). Patients with BC had higher PCT levels (4.25 [0.6-19.5]) and CRP (36.7 [20.23-118]) compared with PVP group (PCT 0.6 [0.2-2.3, p=0.001], and CRP (28.05 [13.3-109, p=0.001]). PCT levels were lower in survivors compared with non survivors, in both groups: PVP (0.53 vs 0.82, p <0.001), BC (3.88 vs 6.94, p=0.03). However, no differences were found in RCP levels between both groups. The multivariable analysis showed that PCT and RCP were not variables independently associated with mortality in global population neither at differentiating PVP from BC.

**CONCLUSION.** Serum PCT and RCP levels on ICU-admission in patients with viral pneumonia and/or bacterial co-infection are not factors independently associated with the prognosis.

##### REFERENCE(S)

(1) Sudhir Agarwal, MD; Arvind Mishra, MBBS. Role of Serum Procalcitonin as a Prognostic Biomarker in Community Acquired Pneumonia. Chest. 2013;144: 388A.
(2) GETGAG working group

### 001494

#### Prognostic value of initial high levels of serum interleukin -6 (IL-6) in patients with septic shock

A. Cortes Herrera[1], M. Perez[1], A. Ruiz[1], L. Chiscano[1], V. Casares[2], A. Fabrega[3], JJ. González[3], R. Ferrer Roca[1], JC. Ruiz-Rodríguez[1]
[1]Intensive care department, Vall d'Hebron University Hospital, Barcelona, Spain; [2]Intensive care department, Vall d'Hebron Research Institute, Barcelona, Spain; [3]Microbiology department, Hospital Vall d'Hebron, Barcelona, Spain
**Correspondence:** A. Cortes Herrera
*Intensive Care Medicine Experimental* 2019, **7(Suppl 3):**001494

**INTRODUCTION.** We analyse whether there is an association between high levels of initial IL-6 and the development of multiorgan dysfunction (MODS) and higher mortality in septic shock patients admitted to ICU.

**METHODS.** Retrospective analysis of prospectively collected data of patients with abdominal, respiratory and urinary septic shock that were activated by Sepsis Code (SC) in the hospital and admitted to ICU, whose samples were stored in the Sepsis Bank during the period 2015-2017.

The cohort was divided into two groups depending on IL-6 levels > 1000 pg/mL (1, 2). We analyse demographic variables, severity (APACHE II), the development of MODS defined through the Sequential Organ Failure Assessment score (SOFA) >9, at ICU admission (ICU-SOFA), and its evolution through the highest levels (H-SOFA) during the first 96 hours, the mortality, the levels of Procalcitonin (PCT) and Lactate.

The data has been expressed in the form of "n" (%) if they are categorical, median and interquartile range if they are quantitative. The comparison of qualitative data was done with the Chi-square test. The comparison of quantitative data has been made with the Mann Whitney U test. The study was authorized by CEIC (PR (AG) 11/2016, PR (AG) 336/2016) and the patients or their representatives have signed the informed consent

**RESULTS.** During the study period, 72 activated patients by SC presented the inclusion criteria. Characteristics of the study population: 66.7% men, age= 61.9 (54.2-73) years, APACHE II= 23.1 (18.2-28), ICU- SOFA= 8.9 (7-10), H-SOFA=10.5 (7-13).

63.9% of patients had IL-6 > 1000 pg/ml, their most frequent focus was the respiratory (38.9%), the mortality rate was 27.8% and in the ICU (16.7%). High IL-6 levels was associated with the development of MODS at admission (58.7% vs. 41.3% p = 0.09), ICU-SOFA= 9.7 (7-10) vs. 7.5 (6-7) p=0.01, and tend to be associated with a worsening evolution H-SOFA= 11 (8-10) vs 9.5 (6.7-10) p = 0.06.

We found no association between high IL-6 levels and hospital Mortality (32.6 % vs 19.2 % p = 0.24), and ICU Mortality (21.7 % vs.7.6 % p = 0.11).

The highest levels of IL6 were observed in patients with abdominal focus (91.3% vs 53.6 and 47.6 % p = 0.02). There was association between IL 6 and PCT =43.5 mg/dl (7.1-18) vs 28.9 mmol/L (0.3-3) p = 0.00 and Lactate= 5.1 mmol/L (2.6-4.2) vs 2.2 mmol/L (1.3-1.9) p= 0.00.

In multivariate analysis, high levels of IL 6 are associated independently with high levels of lactate OR=2.4 (1.4 – 4,1) p= 0,01.

**CONCLUSION.** Elevated initial levels of IL 6 in septic patients are not associated with the development of MODS and is not associated with an increased in the mortality. Only the high levels of lactate are associated independently with high levels of IL 6.

##### REFERENCE(S)

1. Tieying Hou, Dehong Huang, Rong Zeng, Zhiming Ye, Yu Zhang. Accuracy of serum interleukin (IL)-6 in sepsis diagnosis:a systematic review and meta-analysis. Int J Clin Exp Med 2015;8(9):15238-15245
2. Oberhoffer M, Karzai W, Meier-Hellmann A, Bogel D, Fassbinder J and Reinhart K. Sensitivity and specificity of various markers of inflammation for the prediction of tumor necrosis factor-alpha and interleukin-6 in patients with sepsis. Crit Care Med 1999; 27: 18141818

### 001495

#### PILOT STUDY: Alert system with serum lactate

MT. Cruces Moreno[1], J. Tejero Aranguren[1], A. Carranza Pinel[2], I. Cruz Valero[1], PB. Ana María[1], M. Colmenero Ruíz[1]
[1]Intensive care unit, Hospital Universitario San Cecilio, Granada, Spain; [2]Intensive care unit, Hospital Universitario Clínico., Granada, Spain
**Correspondence:** J. Tejero Aranguren
*Intensive Care Medicine Experimental* 2019, **7(Suppl 3):**001495

**INTRODUCTION.** Serum lactate is an interesting biomarker that allows the clinician a diagnostic, prognostic and therapeutic approach. Therefore, at present, it is included in the most commonly used prognostic scales in the Intensive Care Units.[1 2]

We have created an alert system in which the LAB contacts us when they detect a serum lactate >4 mmol/L in a blood test. After that, we review individualy each case in order to know if there is a change in the approach of the patient that could be done.

**OBJECTIVES.** To determine the adequacy of the therapeutic rescue measures performed in the first hour of patients whose serum lactate is greater than 4 mmol/L in a blood test.

**METHODS.** Emergency patients and inpatients with AL levels> 4 mmol / L were included. The period of analysis was from August 10th, 2018 to February 4 th, 2019. Post anesthesia Unit Care, obstetric, pediatric and palliative areas were excluded. The measures evaluated in case of septic patients are those promoted by the guidelines of the Surviving Sepsis Campaign. We determine descriptive parameters by means, medians and frequencies. The computer program used was IBM SPSS v.20

**RESULTS.** 114 patients were included with a mean age of 68.25 years (18-95), 37.7% were women and 62.3% were men. 93% of the cases obtained the sample by venipuncture with mean values of 6.58 mmol / L (4-17).

After all cases were evaluated by two physicians, 69 of them would met criteria for admission to Intensive Care in case of requiring it. Thirty-three of them were admitted to ICU.

The diagnoses were sepsis (33%), cardiological pathology (15.2%), neurological (15.2%), renal (15.2%), digestive (12.1%) and bleeding (9.1%). 81.8% of cases of sepsis received all the required measures of fluid therapy with crystalloids, antibiotherapy and taking cultures prior to admission; 90.9% of patients with other pathologies also received appropriate treatment. A total of 3 patients who were candidates for invasive therapy were admitted to intensive care days after admission to the hospital ward.

**CONCLUSION.** We have been able to observe a possible room of improvement in 18.2% of the cases of sepsis and 9.1% for other pathologies, about the measures that could be adopted in the first hour.

##### REFERENCE(S)

1. Rhodes A. et Al. Surviving Sepsis Campaign: International Guidelines for Management of Sepsis and Septic Shock: 2016. Intensive Care Med. 2017 Mar;43(3):304-377
2. Song YH, Shin TG, Kang MJ et al (2012) Predicting factors associated with clinical deterioration of sepsis patients with intermediate levels of serum lactate. Shock 38(3):249–254
3. Dellinger P, Okorie ON. Lactate: biomarker and potential therapeutic target. Crit Care Clin 2011; 27: 299-326
4. The authors declare that they have not received any funding.

### 001521

#### Presepsin usefulness in intensive care unit

K. Donadello, G. Cogo, B. Cazzetta, V. Schweiger, S. Miori, S. Simari, E. Polati
[1]Anesthesia and intensive care b unit, University of Verona, AOUI-University Hospital Integrated Trust of Verona, Verona, Italy
**Correspondence:** K. Donadello
*Intensive Care Medicine Experimental* 2019, **7(Suppl 3):**001521

**INTRODUCTION.** Sepsis still represent a major health issue, mostly in intensive care medicine. Among the available diagnostic tools, Presepsin (P-SEP) has been proposed as a valuable point-of-care biomarker.

**OBJECTIVES.** We investigated the potential role of P-SEP during the diagnosis of sepsis and as an early prognostic tool in an acute care setting within our medico-surgical intensive care unit (ICU).

**METHODS.** From May 2016 to February 2018 we evaluated all consecutive patients admitted to our ICU with an expected length of stay of more than 48 hours. We reported demographic characteristics, daily clinical and laboratory data. For each patients we recorded procalcitonin (PCT) and P-SEP levels. P-SEP assay was obtained with luminescent monoclonal antibodies (the so called "pathfast method). At admission and every day during the ICU stay we assigned every patient according to the last published sepsis criteria (Sepsis 3). Daily SOFA score was used to categorize patient-days in three different quartiles (SOFA <6, SOFA 7-12, SOFA >=13). At the fifth day we used SOFA score variation (delta SOFA score) to divide patients in good or unfavourable clinical performance.

Primary and secondary aims were to evaluate diagnostic and prognostic ability of P-SEP, compared to PCT.

**RESULTS.** 284 patients (1912 patient-days) were included. Mean P-SEP levels in septic shock patients (343 patient-days) were significantly higher than those classified as septic (955 patient-days) or not-septic (614 patient-days)(mean and 95% CI): 3763 (3290,778 - 4235,810) vs 2081 (1911,970 - 2251,574) vs 767 (673,40 - 860,61) pg/ml, respectively. PCT showed the same trend and indeed PCT and PSEP had similar accuracy (AUC ROC curves for sepsis diagnosis were 0.784 for P-SEP and 0.787 for PCT). The best diagnostic cut-off for P-SEP was 1039 pg/ml.

We did not find a correlation (r=0,29) with APACHE score at admission. Both markers presented significantly different values when patient-days were divided in the three SOFA quartiles. During the first five days P-SEP trend was significantly different (two way ANOVA) between survivors and non-survivors: 1187 (1058-1315) vs 2726 (1816-3636) pg/ml respectively. PCT trend was conversely not statistically different between the two groups. Patients with unfavourable outcome presented a continuous and progressive P-SEP increase, meanwhile this was not found for PCT.

**CONCLUSION.** This study confirms that P-SEP has similar diagnostic potential as PCT, but is far more rapid in its execution and point of care. P-SEP trend may represent a promising early prognostic marker of recovery and mortality.

### 001522

#### Bacterial translocation and epithelial permeability in patients with major trauma

J. Schäper[1], JR. Herrmann[1], R. Lugert[2], AR. Asif[3], M. Quintel[1], O. Mörer[1]
[1]Department of anesthesiology, emergency and intensive care medicine, University Hospital Göttingen - University Medical Center Göttingen, Göttingen, Germany; [2]Department of microbiology, University Hospital Göttingen - University Medical Center Göttingen, Göttingen, Germany; [3]Department of clinical chemistry, University Hospital Göttingen - University Medical Center Göttingen, Göttingen, Germany
**Correspondence:** J. Schäper
*Intensive Care Medicine Experimental* 2019, **7(Suppl 3):**001522

**INTRODUCTION.** The gut as a motor of sepsis hypothesis was first investigated in trauma patients 30 years ago. Although the hypothesis is well accepted among physicians, there has been a lack of evidence so far that bacterial translocation directly causes the development and perpetuation of systemic inflammation.

**OBJECTIVES.** Quantification of bacterial translocation and correlation with markers of gut barrier failure, systemic inflammation and severity of illness in an ICU patient cohort with major trauma.

**METHODS.** Prospective monocentric observational cohort study (ethical approval Göttingen University 22/1/14) in 14 subsequent ICU patients with major trauma. Inclusion into the study was within eight hours after hospital admission. Quantification of Bacteroides and Enterococcus species DNA und surrogate parameters for altered epithelial permeability (iFABP, Claudin 3, sCD14) in patient blood and urine was performed on admission (d0), d1, d2, d3, d5, d7, d10, and d14 of ICU stay. Severity of illness scores were calculated on admission, organ failure scoring was used in the further course of ICU stay.

**RESULTS.** Enterococcus or Bacteroides DNA was detected in 54% of patients with major trauma and in 45% of those patients, who developed sepsis. Patients with bacterial translocation developed sepsis in 63 % of cases, whereas all patients without bacterial translocation developed sepsis. Neither disease severity scores nor organ failure scores differed between patients with or without bacterial translocation. iFABP concentration in urine was comparable in patients with and without bacterial translocation (466 [121; 3435] vs. 674 [208; 3758] pg/mL) as well as in patients with and without sepsis (505 [128; 3520] vs. 514 [263; 6513] pg/mL). This also applied for Claudin 3 and sCD14.

**CONCLUSION.** Bacterial translocation of two gut-specific bacteria into the blood was present in approximately half of patients with major trauma. There was no association between bacterial translocation and the development of sepsis in this cohort of patients. Neither iFABP nor other surrogate markers for gut barrier failure were associated with bacterial translocation or sepsis.

### 001523

#### Prior statin therapy and influence on survival and levels of cholesterol and triglycerides in critically ill patients

G. Cavrić[1], I. Prkačin[1], HD. Bartolek[2], B. Bedenić[3], M. Zeljko[1], D. Kurbegović[1], M. Vučković[1]

[1]Department of internal medicine, University Hospital Merkur, Zagreb, Croatia; [2]Anesthesiology, reanimation and intensive medicine, University Hospital Centre Zagreb, Zagreb, Croatia; [3]Department of microbiology, University Hospital Centre Zagreb, Zagreb, Croatia

**Correspondence:** G. Cavrić

*Intensive Care Medicine Experimental* 2019, **7(Suppl 3):**001523

**INTRODUCTION.** Statins have anti-inflammatory effects that are independent of their lipid-lowering abilities, but their role in critically ill patients is still a matter of debate. On the other hand lower levels of cholesterol and a higher level of triglycerides are usual findings in that population oh patients especially in patients with sepsis.

**OBJECTIVES.** Aim of this study is to examine the survival of critically ill patients with or without previous taking statins in regular therapy and also compare levels of cholesterol and triglycerides in these patients.

**METHODS.** We provided a retrospective analysis of 311 patients who were hospitalized in the medical intensive care unit.

**RESULTS.** We analysed data of 311 patients. Forty-two (13.5%) of them were taking statins (statin group- SG) as regular therapy before their hospitalization in MICU. Two hundred and eight patients (73.31%) have infection and/or sepsis. Survival rate of SG was 80.95%, whilst survival rate of the rest of the patients (non-statins group- NSG) was 68.4%. Mean APACHE II score in SG was 19.79 (SD 9.814) and in NSG group 18.66 (SD 9.945). Mean GCS was 13.45 (SD 3.964) in SG, and 12.97 (SD 3.464) in NSG. The average level of the lowest values of cholesterol was 3.878 mmol/l in SG, and 3.853 in NSG. Mean level of the lowest values of triglycerides was 1.510 mmol/l in SG, and 1.709 mmol/l in NSG. We didn't find statistically significant difference in levels of cholesterol and triglycerides, mean APACHE II score or GCS. When we compare APACHE II score in the group of patients with infection and/or sepsis according to their taking or not taking statins we also didn't find statistical significance. But in the rest of patients (critically ill without infection and/or sepsis (83 patient, 26.68%) – patients with statins had mean APACHE II core 17.85, and without statins 12.51 which was statistically significant (p= 0.009). The mean age of patients in SG was 70.74 years (SD 12.445) and in NSG 66.17 years (SD 16.323) which wasn't statistically significant but there was a trend to higher age in SG (p= 0.084).

**CONCLUSION.** Although SG in the population of analysed patients was small, our findings could show some trends towards the benefits of statins. Survival of SG was better despite they were older and despite the fact that some of them had statistical higher APACHE II score than NSG. Also, it is very interesting that the levels of cholesterol and triglycerides weren't statistical significant between SG and NSG. That fact could speak towards the protective effect of statin. During inflammation we usually can see the trend to lower cholesterol and higher triglycerides. We couldn't prove reverse effect in our SG but can maybe see some nonsignificant trends towards that. It would be necessary to provide further research with more patients.

**REFERENCE(S)**

1. Almog Y, Shefer A, Novack V etal. Prior statin therapy is associated with a decreased rate of severe sepsis. Circulation 2004; 110: 880- 885
2. Lee MG, Lee CC, Lai CC et al.Preadmission statin use improve the outcome of less severe sepsis patients- a population -based propensity score matched cohort study. Br J Anaesth 2017; 119:645- 654.

### 001528

#### The neutrophil activation product heparin-binding protein, suggested biomarker of sepsis, is markedly increased during cardiopulmonary bypass

A. Olsson[1], S. Berg[2]

[1]Cardiothoracic surgery, Blekinge Hospital, Karlskrona, Sweden; [2]Cardiothoracic Intensive Care, University Hospital, Linköping, Sweden

**Correspondence:** S. Berg

*Intensive Care Medicine Experimental* 2019, **7(Suppl 3):**001528

**INTRODUCTION.** Heparin-binding protein (HBP) is a bactericidal protein stored in granule of neutrophils and released upon neutrophil activation. It alters endothelial barrier function and induces plasma leakage. HBP has been recognized as a possible marker for sepsis and high circulating levels of HBP have been demonstrated early in septic patients.

Cardiopulmonary bypass (CPB) during heart surgery is associated with a pronounced inflammatory reaction with activation of leukocytes. This reaction is thought to be induced by contact activation of the CPB circuit, and is elicited without any exposure to bacterial agents.

**OBJECTIVES.** To study how non-infectious activation of leukocytes during CPB affects plasma levels of HBP and to correlate this to changes in plasma myeloperoxidase (MPO), a neutrophil granule product known to be released during CPB.

**METHODS.** Forty adult patients undergoing coronary artery bypass surgery with CPB were studied. Plasma samples were obtained at baseline, before induction of anesthesia, at the end of CPB before protaminisation and at 4 hours after surgery. HBP was measured by an ELISA assay and MPO by a multiplex bead assay. Friedmans ANOVA on ranks followed by Wilcoxon paired test with Bonferroni correction and Spearmans rank correlation was used for statistical analysis, results are presented as median and interquartile range.

**RESULTS.** HBP increased from normal low levels at baseline 5.9 (5.9-5.9) µg/L to very high levels at end of CPB 513 (436-688) µg/L and decreased to 44.9 (31-62) µg/L at 4 h after surgery (all $p<0.001$). MPO increased in parallel from 30 (0.2-44) µg/L at baseline to 3034 (2292-3763) at end of bypass and 457 (290-602) µg/L at 4 h after surgery (all $p<0.001$). Plasma HBP correlated with MPO levels at end of CPB R=0.64 and at 4 h after surgery R=0.84 (both $p<0.001$).

**CONCLUSION.** CPB is accompanied by a marked, up to 100-fold, increase in plasma levels of HBP. These concentrations are approximately 10 times higher than those seen in sepsis and septic shock. The increase in plasma HBP is parallel to increased plasma MPO and presumably reflects activation of neutrophils by the extracorporeal circle. Increased plasma HBP seems therefore not to be a marker of neutrophil activation exclusively by bacteria or infection, but rather a non-specific marker of neutrophil activation. Whether heparin present in the blood during CPB influences the high HBP levels seen requires further studies.

### 001533

#### Impact of the 1-hour bundle in patients with sepsis and septic shock admitted in the emergency department of a tertiary Hospital in Spain

VD. Gumucio Sanguino[1], L. Anguela-Calvet[1], MD. Belda-Ley[1], V. Corral-Velez[1], P. Malchair[2], J. Sabater Riera[1], F. Llopis[2], HB. Alanez-Saavedra[1], JL. Pérez Fernández[1]

[1]Intensive care medicine, Bellvitge Hospital, Hospitalet de Llobregat, Spain; [2]Emergency medicine, Bellvitge Hospital, Hospitalet de Llobregat, Spain

**Correspondence:** V.D. Gumucio Sanguino

*Intensive Care Medicine Experimental* 2019, **7(Suppl 3):**001533

**INTRODUCTION.** Recently was proposed for the Surviving Sepsis Campaign a 1-hour bundle for the treatment initial of patients with sepsis and sepsis shock. The Impact in-hospital mortality in comparison with the 3-hour bundle stills unclear.

**OBJECTIVES.** To evaluate the accomplished of the 3- hour bundle vs. 1-hour bundle in patients admitted in the emergency department (ED).

To assess the impact on the mortality between 1-hour vs. 3 -hour bundle.

**METHODS.** We collected prospective data from patients admitted in the emergency department from January 2016 to December 2018 with the diagnosis of sepsis and septic shock activated with the informatics system. General characteristics of patients and the time in minutes from admission until the completion of the 1-hour and 3-hour bundle was analyzed.

**RESULTS.** 497 patients were collected from the informatic activation system, were men 58,4%, mean age was 68 years +/- 15,5 (SD). From advanced medical ED were 62%, basic medical ED 26,6% and from advanced surgical ED 8%. Mainly patients were medical in 64%, surgical 11,9%, hematological 7,5% and neoplastic 16,5%. Respiratory sepsis was the principal diagnosis in 39% of patients, urinary sepsis 27,5%, abdominal sepsis 16,2% and medical sepsis (soft-tissue, endocarditis, etc.) 12%, blood cultures were positive in 34%. The mean SOFA was 7,87 +/- 2,27SD. Patients with hypotension and lactate > 4mmol/l were 65% and had septic shock in 19%. Received hydrocortisone in 33% and fluid bolus 30ml/kg in 51% of patients with hypotension. Mechanical ventilation was required in 4,5% of patients, non-invasive ventilation in 10,9%, renal replacement therapy 5,1% and was admitted to the ICU in 21%. In-hospital mortality was 13,3%. Time from admission to complete the bundle was 350min +/- 822 SD. For each item of the bundle, from admission to antibiotic administration 211min +/- 353 SD, to lactate obtention 192min +/- 567 SD and blood cultures obtained in 276min +/-1474 SD. The initial bundle was completed in 82,9% of patients, in less of 3-hour in 76,9% and less of 1- hour in 38,1%. In-hospital mortality was 13,8% in less 1- hour bundle vs. 14,4 % in less than 3-hour group without statistical significative differences between groups.

**CONCLUSION.** In this series of septic patients seems there is no impact in mortality between 1-hour bundle vs. 3-hour bundle, but the use of 1- hour bundle is still low, and patients probably were sicker.

### 001534

#### Effects of very early start of norepinephrine in patients with septic shock and sepsis-related cardiovascular dysfunction

GA. Ospina-Tascón[1], G. Hernandez[2], I. Alvarez[3], A. Sanchez-Ortiz[3], L. Calderón-Tapia[3], R. Manzano-Nuñez[3], E. Quiñones[3], H. Madriñan-Navia[3], J. Ruiz[3], J. Aldana[3], J. Bakker[4]

[1]Department of intensive care, Fundacion Valle del Lili - Universidad ICESI, Cali, Colombia; [2]Departamento de medicina intensiva, Pontificia Universidad Catolica de Chile, Santiago, Chile; [3]Department of intensive care, Fundacion Valle del Lili, Cali, Colombia; [4]Department of intensive care medicine, Erasmus University Medical Center, Rotterdam, Netherlands

**Correspondence:** G.A. Ospina-Tascón

*Intensive Care Medicine Experimental* 2019, **7(Suppl 3):**001534

**INTRODUCTION.** Optimal timing for starting vasopressors (VP) in septic shock has not been widely studied since it is assumed that fluids must be administered in advance

**OBJECTIVES.** To evaluate whether a very early start of VP (preceding or simultaneously to the first resuscitation fluid load) could impact clinical outcomes in patients with septic shock

**METHODS.** Retrospective analysis of 337 patients with sepsis requiring vasopressor support. The attending physician decided in each case when start VP support according his clinical judgment. Very early start of VP (VE-VPs) was defined as VP support initiatedbefore or within the next hour of the first fluid load with resuscitative intention (FRLoad). A VP start >1hour after the FRLoad was considered as delayed (D-VPs). A propensity score was fitted using the age, previous chronic hypertension; lactate, heart rate, systolic and diastolic pressure at VPs; source of admission (emergency room, general wards, intensive care unit); andthe heart rate/diastolic blood pressure ratio at the first fluid load. Then, a nearest neighbor-matching algorithm extracted 1:1 matched pairs of VE-VPs (n=93) and D-VPs (n=93) individuals. A risk-adjusted Cox proportional hazard model was fitted to assess the association between VE-VPs and day-28 mortality. A p<0.05 was considered as significant

**RESULTS.** Patients subjected to VE-VPs received significantly less fluid at the VP start (0[0-510] vs. 1,500 [650-2,300] mL, p<0.001) and at 6-hours (1,000[500-1,680 vs. 1,965[1,200-3,050] mL, p<0.001). VE-VPs strategy was related with significant lower net fluid balances at VP start, 6-hours and 24-hours after. The Cox-proportional hazard model revealed a significant lower risk of death for VE-VPs at day-28 (HR:0.44, CI95% 0.24–0.79, p=0.006). There were no significant differences for renal replacement therapy requirements between VE-VPs and D-VPs groups

**CONCLUSION.** Very early start of VP, even preceding initial resuscitation fluid load, seems to be safe and it could be related with better clinical outcomes in sepsis-related cardiovascular dysfunction

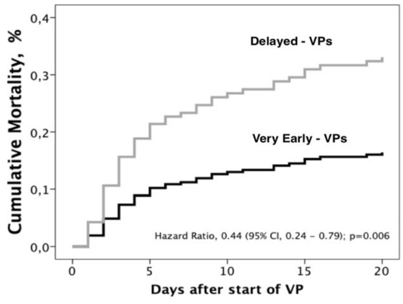

| | | | | | |
|---|---|---|---|---|---|
| C-VPs | 93 | 80 | 78 | 78 | 76 |
| NC-VPs | 93 | 70 | 63 | 57 | 56 |

**Fig. 4 (abstract 001534).** See text for description

### 001542

#### Patterns of modified shock indices between survivors and nonsurvivors across cohorts of similar vasopressor equivalents

S. Falini[1], G. Angelotti[1], M. Greco[1], P. Morandini[2], A. Chiti[3], R. Barbieri[2], M. Cecconi[1]

[1]Anesthesia and intensive care, Humanitas Research Hospital, Milano, Italy; [2]Electronics, information and bioengineering, Politecnico di Milano, Milano, Italy; [3]Nuclear medicine, Humanitas Research Hospital & Humanitas University, Milano, Italy

**Correspondence:** S. Falini

*Intensive Care Medicine Experimental* 2019, **7(Suppl 3):**001542

**INTRODUCTION.** Sepsis is a severe condition in which a patient's hemodynamics are compromised in response to a generalized infectious insult(1). Its prognosis is often ill-fated, and various indices have been investigated to predict response to therapy, including the shock index (SI), a ratio of systolic pressure (SP) and heart rate (HR)(2). We hypothesize that modified shock indices based on the diastolic (DP), mean (MP), or pulse pressure (PP) could be as good in predicting survival in sepsis.

**OBJECTIVES.** The purpose of the analysis was to compare the discriminative ability of modified shock indices with regard to response to therapy of septic patients.

**METHODS.** The Medical Information Mart for Intensive Care version III (MIMIC-III)(3) database was used for the analysis. Patients in the 18-89 age range, admitted for at least 48 hours and fulfilling the international consensus sepsis-3 criteria(4) were included. In-ICU mortality was chosen as a surrogate outcome for response to therapy, and patients dying within 48 hours from admission were excluded. Clinical data relative to the first 6 hours of ICU stay were used to formulate predictions. Patients were stratified in 5 subcohorts of increasing severity using mean dosage of vasopressor equivalents (VPE), and divided in survivors (S) and non-survivors (D). The discriminative ability of SI, diastolic shock index (DSI = DP/HR), mean shock index (MSI = MP/HR) and pulse pressure shock index (PPSI = PP/HR) was compared. In addition, machine learning models including routinely measured values were built and validated for outcome prediction.

**RESULTS.** A common pattern was observed when plotting the mean of the selected indices across the 5 subcohorts of patients (figures 1-4): none was able to distinguish between survivors and nonsurvivors in the 'no vasopressor' subcohort, while a significant difference appeared in the 'low VPE' subcohorts (with survivors showing higher values than nonsurvivors), becoming again non significant for higher VPEs. For MP and DP, a significant difference was found in the 'very high VPE' sub-cohort, although reversed for survival classes (i.e. with survivors showing lower index values than nonsurvivors). Machine learning models considering these indices and other routinely measured values showed poor discrimination.

**CONCLUSION.** All modified shock indices significantly stratify survivors and nonsurvivors of sepsis on low vasopressor dose, suggesting that they could be used to predict the response to or guide the treatment with vasopressors in the early phases of septic shock. Indices based on MP and DP also significantly stratify septic patients receiving very high VPE.

**REFERENCE(S)**

1. Johnson AEW, Aboab J, Raffa JD, Pollard TJ, Deliberato RO, Celi LA, et al. A Comparative Analysis of Sepsis Identification Methods in an Electronic Database. Crit Care Med. 2018 Apr;46(4):494–9.
2. Johnson AEW, Pollard TJ, Shen L, Lehman L-WH, Feng M, Ghassemi M, et al. MIMIC-III, a freely accessible critical care database. Sci Data. 2016 May 24;3:160035.
3. Tseng J, Nugent K. Utility of the shock index in patients with sepsis. Am J Med Sci. 2015 Jun;349(6):531–5.
4. Singer M, Deutschman CS, Seymour CW, Shankar-Hari M, Annane D, Bauer M, et al. The Third International Consensus Definitions for Sepsis and Septic Shock (Sepsis-3). JAMA. 2016 Feb 23;315(8):801–10.

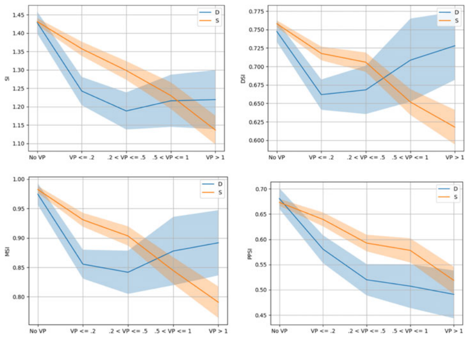

**Fig. 1 (abstract 001542).** See text for description

## POIC - Between the theatre and the ICU

### 000959

#### Consumption profile of analgosedative drugs and level of sedation in critical care after an educational intervention: a before-after study

Cl. Loudet[1], M. Sarubbio[1], MJ. Meschini[2], MC. Marchena[1], S. Bottaia[1], JF. Caminos[1], R. Reina[1], V. Pazos[1], E. Estenssoro[1]
[1]Intensive care unit, Hospital General San Martín de LA PLATA, La Plata, Argentina; [2]Pharmacy department, Hospital General San Martín de LA PLATA, La Plata, Argentina
**Correspondence:** C.I. Loudet

*Intensive Care Medicine Experimental* 2019, **7(Suppl 3):**000959

**INTRODUCTION.** Sedation guidelines are widely available, but international surveys have shown low compliance with them. In our ICU, we implemented a quality improvement strategy to achieve appropriate sedation levels.

**OBJECTIVES.** To evaluate the effect of an educational program to improve consumption profile and dosing of analgosedative (AS) drugs, and sedation level in patients on mechanical ventilation (MV)

**METHODS.** Before-after study in a 14-bed medical-surgical ICU in Argentina. We included consecutive patients on MV>2 days, and recorded characteristics and outcomes, prescribed doses of midazolam and fentanyl, level of sedation (RASS), midazolam and fentanyl consumption -as equivalents of benzodiazepines (BZD) and opioids-, propofol and dexmedetomidine. A special team (pharmacists/intensivists) designed nomogram templates for each drug, as a tool for proper prescription (maximum doses, adequate ranges/dilution, encouragement for not using BZD). After an educational session, nomogram templates were implemented. We compared prescribed doses of AS drugs, level of sedation, and consumption (mg of AS drug/100 occupied bed days) among Pre-I/Post-I. P value<0.05 significant for comparisons.

**RESULTS.** We included 231 and 186 patients in Pre-I/Post-I periods respectively; age was 44±20, 44±18; APACHEII 16±7,16±8; MV-days 10[3-24], 7[2-27] respectively; ICU mortality was 74(32%) and 58 (31%). RASS scale - 4[- 2 to -5], - 2[-0.5 to -4](p<0.01); midazolam prescribed dose (mg/kg/h): 0.23 [0.1-0.4], 0.12[0.04-0.2] and fentanyl prescribed dose(mg/kg/h): 1.04[0.7-2.4], 3[2-5.6] (p<0.01) respectively.The evolution of consumption drugs for AS is shown in Table 1. In Post-I, consumption profile was significantly higher, for propofol, dexmedetomidine, and opioid equivalents, and lower, for BZD equivalents.

**CONCLUSION.** After an educational intervention with nomogram templates as main tools, it was possible to decrease the level of sedation, to better adjust the prescription doses of AS drugs, and to modify their consumption profile.

**Table 1 (abstract 000959).** Consumption profile of AS drugs (expressed as mg/100 occupied bed day)

| AS drug | Year 2014 | Year 2015 | Year 2016 | Year 2017 | p value |
|---|---|---|---|---|---|
| Opioid eq. (mg x $10^3$) | 0.4 [0.3-0.6] | 0.5 [0.4-0.9] | 1.7 [1.1-1.8] | 2.3[1.4-2.6] | <0.01 |
| BZD eq. (mg x $10^3$) | 26 [16-30] | 25 [22-29] | 20 [16-27] | 12 [8-13] | <0.01 |
| Propofol (mg x $10^3$) | 0.5 [0-15] | 33 [31-41] | 71 [39-89] | 89 [70-99] | <0.01 |
| Dexmedetomidin(mg) | 0 [0-0] | 0 [0-0.02] | 2 [0.5-8.5] | 28 [18-43] | <0.01 |

### 001007

#### Agitation during noninvasive ventilation: an observational prospective study in a high dependency unit

E. Segre[1], E. Greco[1], G. Labarile[1], A. Bartalucci[1], M. Melchio[1], E. Lupia[1]
[1]Emergency medicine department, Azienda Ospedaliero-Universitaria Città della Salute e della Scienza di Torino, Torino, Italy
**Correspondence:** E. Segre

*Intensive Care Medicine Experimental* 2019, **7(Suppl 3):**001007

**INTRODUCTION.** Patient tolerance is critical for non-invasive ventilation (NIV) success. However, sedation is not routinely used in agitated patients during NIV, due to lack of specific protocols and safety concerns (1,2). Literature on the topic is scarce and no studies are available on sedation in patients treated with NIV outside intensive care units.

**OBJECTIVES.** To assess characteristics of patients treated with NIV in our high dependency units (HDU) and outcome of agitated patients requiring sedation.

**METHODS.** A single centre observational prospective study was performed in adult patients receiving NIV, in a medical HDU in Città della Salute e della Scienza Hospital (Turin, Italy) from April 2018 to March 2019. Patient and ventilation characteristics were collected. Richmond Agitation Sedation Scale was used to classify agitation during observation time (48 hours since start of NIV) and pharmacological/behavioural interventions were recorded. The escalation plan to intubation was collected from clinicians on admission (not routinely or formally done in Italy): patients were classified accordingly as "for full escalation" or "do not intubate" (DNI). NIV and sedation were started/continued in some patients as part of palliative care ("palliation" group). Primary outcomes were rate of intubation, discontinuation of NIV and mortality.

**RESULTS.** 104 patients were screened and 99 included. Mean age was 75 and mean Apache II 20. Indications for NIV were chronic obstructive pulmonary disease exacerbation (21%), acute pulmonary oedema (26%), pneumonia (25%), mixed diagnosis or neuromuscular diseases (28%). 45% of patients were considered "for full escalation", 42% were classified as DNI (remaining 13% unknown). 40% of patients had at least one episode of agitation during the first 48 hours, 71.6% of which received pharmacological sedation. Only 17% of agitated patients required discontinuation of NIV (3 patients were not sedated, 7 after sedation). 11% of patient received NIV and sedation for palliation and were excluded from mortality analyses. Overall 4 patients required intubation, two of which were agitated. Agitation was not correlated with higher intubation rate, mortality during HDU admission and at 30 days, both in DNI and full escalation group. Mortality was higher in patient with pneumonia.

**CONCLUSION.** In our study, agitated patients receiving sedation during NIV did not present a greater rate of intubation or mortality during HDU-stay and at 30 days.

**REFERENCE(S)**

1. Devlin JW, Nava S, Fong JJ, Bahhady I, Hill NS. Survey of sedation practices during noninvasive positive pressure ventilation to treat acute respiratory failure. Crit Care Med 2007;35:2298-302.
2. Carlucci A, Richard JC, Wysocki M, Lepage E, Brochard L. Noninvasive versus conventional mechanical ventilation: an epidemiologic survey. Am J Respir Crit Care Med 2001;163:874-80.

### 001014

#### Innovative Artificial Intelligence System can Improve quality care of pain, agitation and delirium in critical patients

MS. Huang[1], HC. Chung[1], HL. Lin[2], SH. Huang[1], YP. Yin[1], PL. Wu[1], TX. Guo[1], HY. Hsu[1], YM. Lue[1], HF. Yang[1], ML. Yeh[1], YH. Huang[1], KP. Chen[1], SH. Kuo[2], HL. Liang[2], WC. Huang[2], CP. Liu[2]
[1]Department of nursing, Kaohsiung Veterans General Hospital, Kaohsiung, Taiwan; [2]Critical care medicine and cardiovascular center, Kaohsiung Veterans General Hospital, Kaohsiung, Taiwan
**Correspondence:** W.C. Huang
*Intensive Care Medicine Experimental* 2019, **7(Suppl 3):**001014

**INTRODUCTION.** In the environment of intensive care unit, the incidence rate of delirium is 16-85%, 63~75% of patients have pain experience and 71% have had agitation, which might be related todisease, treatment, noise, lighting or other multiple factors. Therefore, the assessment and management of pain, agitation, and delirium are critical for the quality of care in critical patients.The aim of this study is to investigate the impact of Innovative*artificial intelligence system on quality care of pain, agitation and delirium (PAD) in critical patients*

**METHODS.** A multidisciplinary team among intensivists, cardiologists, surgeon and nursing staffs in a tertiary medical center was organized since May 2017. The key interventions include analgesic and sedative drug dosage *artificial intelligence*information system, smart healthcare digital communication platform among patients, family, nursing and intensivists, wisdom situation lighting system, PAD information system, and PADIS integrated information monitoring dashboard.All patients admitted between January 2017 and December 2018 were enrolled. The patientswere divided into three groups: pre-interventional group from Jan to July 2017, Interventional group from August to September 2017 and post-interventional group from October 2013 to December 2018.

**RESULTS.** The pain assessment rate improved from 75.7%in pre-interventional group, to 98‰ in interventional group and to 102% in post-interventional group (p<0.05). The agitation assessment rate increased from 0%, 96% to 102% in post-interventional group (p< 0.05).The delirium assessment rate increased from 0%, 77% to 103% in post-interventional group (p<0.05). The pain control rate improved from 55.8%, 66.6% to 84.3% in post-interventional group (p<0.05). The agitation control rate improved from 91.5% to 95.1% in post-interventional group (p<0.05). The delirium control rate also improved from 70.8% to 85.3% in post-interventional group (p<0.05).

**CONCLUSION.** The study demonstrated that implementation of *artificial intelligence system* could increase pain, agitation and delirium assessment rate. Furthermore, pain, agitation anddelirium control rate also significant improved.

### 001039

#### New Oral Anticoagulants (NOACs) and neuraxial anesthesia in hip fracture: a retrospective analysis

M. Teggia Droghi[1], M. Giani[2], V. Coccini[3], V. Vitullo[2], L. Bosa[1], F. Rossi[1], M. Corsi[4], M. Turati[5], E. Martinez[2], R. Rona[2], F. Rossi[3], G. Foti[2]
[1]Department of emergency and intensive care, University Of Milano-Bicocca, Monza, Italy; [2]Department of emergency and intensive care, ASST Monza, University Of Milano-Bicocca, Monza, Italy; [3]Immunotransfusional unit, ASST Monza, Monza, Italy; [4]Acute geriatric unit, ASST Monza, Monza, Italy; [5]Orthopedic department, ASST Monza, Monza, Italy
**Correspondence:** M. Giani
*Intensive Care Medicine Experimental* 2019, **7(Suppl 3):**001039

**INTRODUCTION.** Neuraxial anesthesia represents the clinical standard for hip fracture surgery. However, lumbar puncture is contraindicated in coagulation disorders. The most common alteration of coagulation in the elderly patient is the prophylactic or therapeutic use of anticoagulant drugs. Indications for New Oral Anticoagulants (NOACs) have increased: NOACs has become the first choice in many diseases such as non-valvular atrial fibrillation and deep vein thrombosis.

Common laboratory test cannot detect the anticoagulation activity of these medications. However, monitoring of NOAC plasma concentration has become widely available, and was recently implemented in our clinical practice together with standard laboratory tests and tromboelastography (TEG) for urgent orthopedic surgery. Concentration cutoff depends on laboratory technique and at our institution it was fixed at 30 ng/mL as a safe threshold for surgery[1]. Contrarily, anesthesiology societies only suggest a temporal criterion to define the security of neuraxial blocks after NOAC discontinuation[2,3]. NOACs, like all drugs, have an inter-individual variable metabolism time, and a great variability in drug clearance has been reported. The aim of the study is to verify if the monitoring of NOAC plasma concentration could guide the clinical decision more precisely than the timing criteria alone.

**METHODS.** We included in our retrospective single center study patients on NOACs admitted to Emergency Department (ED) of ASST Monza for hip fracture. NOAC concentration, Kaolin TEG, aPTT, PT, platelets and serum creatinine were collected at the admission. NOAC levels and TEG tracings were recorded up to 96 hours after the last anticoagulant administration.

**RESULTS.** 38 patients on NOACs (4 on Dabigatran, 23 on Apixaban and 11 on Rivaroxaban) were enrolled. Average age was 80 ± 10 years, serum creatinine at ED admission was 1.0 ± 0.3 and calculated creatinine clearance 57 ± 26. Decrease of NOAC levels over time is shown in Figure 1. Interestingly, DOACs concentration was already less than 30 ng/mL at 24 h in 4 patients, whereas in 7 patients the concentration was higher than the threshold at 72 hours. Kaolin TEG R time did not correlate significantly with DOAC levels, except for dabigatran (p=0.19, R2 0.9).

**CONCLUSION.** Interindividual difference in metabolism of the drugs affects the NOACs half-life. Measuring NOAC concentration may support the clinical decision of the anesthesia strategy, regardless the time from the last administration.

**REFERENCE(S)**

1. Steffel J, Verhamme P, Potpara TS, Albaladejo P, Antz M, Desteghe L, Haeusler KG, Oldgren J, Reinecke H, Roldan-Schilling V, Rowell N, Sinnaeve P, Collins R, Camm AJ, Heidbüchel H; ESC Scientific Document Group . The 2018 European Heart Rhythm Association Practical Guide on the use of non-vitamin K antagonist oral anticoagulants in patients with atrial fibrillation. Eur Heart J. 2018 Apr 21;39(16):1330-1393.
2. Horlocker TT, Vandermeulen E, Kopp SL, Gogarten W, Leffert LR, Benzon HT. Regional Anesthesia in the Patient Receiving Antithrombotic or Thrombolytic Therapy: American Society of Regional Anesthesia and Pain Medicine Evidence-Based Guidelines (Fourth Edition). Reg Anesth Pain Med. 2018 Apr;43(3):263-309.
3. Ebner M, Birschmann I, Peter A, Spencer C, Härtig F, Kuhn J, Blumenstock G, Zuern CS, Ziemann U, Poli S. Point-of-care testing for emergency assessment of coagulation in patients treated with direct oral anticoagulants. Crit Care. 2017 Feb 15;21(1):32.

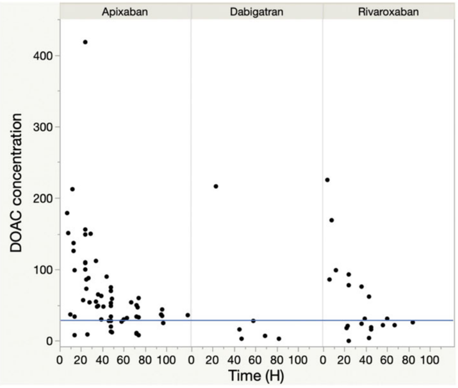

**Fig. 1 (abstract 001039).** See text for description

### 001064

#### Retrospective Analysis of Fluid Balance and Patient Outcome in Acute Pancreatitis

L. Bonito Moreira[1], T. Isidoro Duarte[2], M. Ilharco[1], A. Monteiro[2], N. Germano[2]

[1]Internal medicine department, São José Hospital, Central Lisbon Hospital Center, Lisbon, Portugal, Portugal; [2]Intensive care medicine department, Curry Cabral Hospital, Central Lisbon Hospital Center, Lisbon, Portugal

**Correspondence:** L. Bonito Moreira

*Intensive Care Medicine Experimental* 2019, **7(Suppl 3):**001064

**INTRODUCTION.** Acute Pancreatitis (AP) is a low incidence disease with high mortality rates. It is characterized by a systemic proinflammatory state many times associated with distributive and hypovolemic shock. Several studies report a better outcome when high volume of fluids is given in the first hours of disease.1

**OBJECTIVES.** We conducted this study to assess if the amount of fluids given in the first 24h of admission in the Intensive Care Unit (ICU) had impact in AP's outcome.

**METHODS.** We included all patients admitted between 2017 and 2018, with AP as main diagnosis and made a retrospective analysis using IBM SPSS V20 (2011). Comparison between groups was made using the non-parametric test Mann-Whitney.

**RESULTS.** There were 19 patients with diagnosis of AP (11 male), 5 of whom died. The average age was 56.4±21.2. The majority of cases occurred in Portuguese people (16). There were 3 cases in foreigners from Argentina, Finland and Honduras. The most common cause of AP was lithiasis (10 cases) followed by alcohol (4 cases); 2 cases occurred in context of hypertriglyceridemia and 1 after endoscopic retrograde cholangiopancreatography; in 2 cases no etiology was found. The APACHE score at admission ranged from 7 to 34 (15.7± 6.3). We found that the APACHE score of patients who died was higher and had statistic significance (U=13, p<0.05). Nine of the 19 patients were directly admitted from the Emergency Room (ER) to the ICU. The mean APACHE score in those was 18.2 ± 7.14 (vs 13± 4.37 in those who were admitted later in the course of disease) but this difference had only borderline statistic value (U=23, p=0.07). Those who died had a greater fluid balance (FB) in the first day after admission than those who survived (4302±5020 vs 783±1666mL), but that difference was not statistically significant (U=15.5 p=0.71). Patients with local AP complications had a FB in the first 24h superior to those who did not (1791±3848 vs 1531±960mL), whereas in patients with systemic complications, FB was smaller than in those without such complications (1495±2960 vs 2076±3757mL). In both comparisons, no statistical difference was found (U=38, p=0.93; U= 38.5, p=0.767).

**CONCLUSION.** In sates of shock, FB is an important tool in aiding monitoring and adjustment of tissue O2 delivery. However, it is known that excess intake of fluids can negatively affect the outcome of such patients. We report a higher FB in patients who died, fact that can be justified by a greater complexity and severity of disease in those patients. In such cases, hemodynamic monitoring can be challenging. Invasive tools can be helpful and may be useful in guiding clinical decision. International guidelines recommend aggressive fluid resuscitation in the first hours of disease. Given the timming of admission, we weren't able to access this point, and cannot draw conclusions related to the outcome.

### 001074

#### Oxygen delivery versus lactate measurement during cardiac surgery with cardiopulmonary bypass

J. Tieman[1], R. Haumann[2], D. Ikink[1], A. Vonk[2], C. Bulte[1], C. Boer[1], C. Van Den Brom[1]

[1]Anesthesiology, Amsterdam UMC, locatie VUmc, Amsterdam, Netherlands; [2]Cardiothoracic surgery, Amsterdam UMC, locatie VUmc, Amsterdam, Netherlands

**Correspondence:** J. Tieman

*Intensive Care Medicine Experimental* 2019, **7(Suppl 3):**001074

**INTRODUCTION.** Inadequate microcirculatory perfusion during cardiac surgery with cardiopulmonary bypass (CPB) contributes to postoperative organ failure. Several techniques have been proposed to estimate microcirculatory perfusion during CPB, of which intraoperative lactate measurement is widely used. Lately, oxygen derived parameters are suggested as even better predictors of anaerobic metabolism. As a first step, we compared the course of oxygen delivery (DO2) with lactate levels during cardiac surgery with CPB.

**METHODS.** This single-center, prospective cohort study included 109 adult patients undergoing elective cardiac surgery with CPB. Intraoperative DO2 was calculated via the heart-lung machine (System M - M4) at the start, during and the end of aortic cross-clamping. Lactate measurement was performed at the same time-points by arterial blood gas analysis.
**RESULTS.** The study population consisted of 83 male and 26 female patients, aging 69 [63-74] years and had a mean body mass index of 26±4 kg/m2. Hypertension (n=65) and hypercholesterolemia (n=59) were the most common comorbidities. Mean duration of CPB and aortic cross-clamping was 117 [91-144] and 84 [62-102] minutes, respectively. When compared to the start of aortic cross-clamping, DO2 increased during (315±44 mL/min/m2 vs. 310±49 mL/min/m2; *P*=0.001) and up to the end of aortic cross-clamping (325±52 mL/min/m2 vs. 310±49 mL/min/m2; *P*=0.026). Lactate levels showed an initial decrease mid cross-clamping compared to starting levels (1.06±0.49 mmol/L vs. 1.25±0.49 mmol/L; *P*<0.001). At the end of cross-clamping, the lactate level returned to baseline level (1.24±0.56 mmol/L vs. 1.25±0.49 mmol/L). Only at the start of aortic cross-clamping a weak correlation between DO2 and lactate level was present (r=-0.25; *P*=0.017). No correlation was found between DO2 and lactate levels at any other time-point.
**CONCLUSION.** DO2 and lactate levels have a divergent course and a merely weak correlation during cardiac surgery with CPB. This difference suggests that one parameter could be a superior predictor of postoperative complications when compared to the other.

### 001107

#### Blood transfusion improves skin blood flow when initially impaired

E. Cavalcante dos Santos, W. Mongkolpun, P. Bakos, FS. Taccone, JL. Vincent, J. Creteur
[1]Department of intensive care, Erasme Hospital, Brussels, Belgium
**Correspondence:** E. Cavalcante dos Santos
*Intensive Care Medicine Experimental* 2019, **7(Suppl 3):**001107

**INTRODUCTION.** Red blood cell transfusion (RBCT) increases tissue oxygen delivery (DO2) and may improve microcirculation. However, the effects on blood flow are inconsistent.
**OBJECTIVES.** To assess the effects of RBCT on tissue perfusion and identify the hemodynamic variables predicting such response.
**METHODS.** We studied 36 ICU patients with stable hemodynamic status (mean arterial pressure (MAP) ≥ 65 mmHg for at least 6 hours) and without active bleeding, who required RBCT. Skin blood flow (SBF) was determined (Periflux System 5000, Perimed, index finger; perfusion unit, PU) together with MAP, heart rate (HR), hemoglobin (Hb), lactate levels and ScvO2 before and after RBCT. In each case, SBF was measured at basal temperature (BT) for 3 min. According to previous data indicating an SBF of 151 PU in non-infected patients, all patients were analyzed according to the baseline SBF (i.e. <151 PU - low SBF vs. ≥151 PU – high SBF). A positive RBCT responder was defined as a > 10% increase in SBF. Data were expressed by median (25th-75th percentile).
**RESULTS.** In all patients, RBCT was associated with increases in Hb, MAP, and ScvO2 but no change in SBF (Table 1). At baseline, there were no difference in Hb (p=0.6), MAP (p=0.5), lactate (p=0.2), and ScvO2 (p=0.3), between the patients with low and high SBF (Table 1). During the transfusion, MAP and Hb increased in both groups (Table 1) but SBF rose only in patients with an initially low SBF (Table 1). Baseline ScvO2 was somewhat lower in the responders (n=19) than in the non-responders to RBCT (p=0.07).
**CONCLUSION.** RBCT increases skin blood flow when it is low at baseline.

**Table 1 (abstract 001107).** Hemodynamic and oxygenation variables before and after RBCT in patients with lower and higher baseline skin blood flow

| | All patients (N=36) | | | Low SBF (N=19) | | | High SBF (N=17) | | |
|---|---|---|---|---|---|---|---|---|---|
| | Before | After | p | Before | After | p | Before | After | p |
| Hb (g/dL) | 7.4(7.1–7.7) | 8.5(8.2–9.2) | <0.01 | 7.4(7.1–7.7) | 8.5(8.2–9.2) | <0.01 | 7.4(7.1–7.7) | 8.9(8.2–9.5) | <0.01 |
| MAP (mmHg) | 74(68.0–79) | 82(76–91) | <0.01 | 74(70–79) | 82(76–87) | 0.03 | 74(66–84) | 83(75–95) | 0.02 |
| HR (bpm) | 83(71–96) | 83(69–96) | 0.6 | 84(70–90) | 84(69–92) | 0.8 | 79(73–99) | 81(70–100) | 0.4 |
| CVP (mmHg) | 7(5–12) | 8(5–14) | 0.9 | 7(4–10) | 8(3–11) | 0.5 | 6(5–21) | 8(5–18) | 0.50 |
| ScvO2 (%) | 67(59–74) | 69 (62–76 | <0.01 | 65(59–70) | 68 (63–76) | <0.01 | 70(59–74) | 69(62–77) | 0.2 |
| SBF(PU) | 130.1 (24.2–241.3) | 120.5(63.0–218.8) | 0.5 | 26.7 (15.4–68.9) | 72.0 (39.0–169.4) | 0.001 | 242.0(190.6–308.0) | 183.1(107.4–251.6) | 0.7 |

### 001122

#### Music interventions for preventing ICU delirium: a randomized controlled trial

K. Wakabayashi[1], S. Sato[1], N. Nawa[2], E. Takezawa[1], T. Fujiwara[2], H. Shigemitsu[1]
[1]Department of intensive care medicine, Tokyo Medical and Dental University, Bunkyo City, Japan; [2]Department of global health promotion, Tokyo Medical and Dental University, Bunkyo City, Japan
**Correspondence:** K. Wakabayashi
*Intensive Care Medicine Experimental* 2019, **7(Suppl 3):**001122

**INTRODUCTION.** Critically ill patients are subject to a multitude of environmental changes in the Intensive Care Unit (ICU) which contribute to the development of delirium, ultimately affecting their long-term morbidity and mortality in the ICU. ICU patients may register these environmental changes through their five senses including their auditory sensation. One way of enhancing auditory sensation is through music either with or without self-controlled patient-directed music (PDM).
**OBJECTIVES.** To investigate whether a novel music delivery system produced by Yamaha with capabilities of self-controlled PDM can prevent ICU delirium.
**METHODS.** We performed a randomized controlled open-label trial with intubated and non-intubated patients in the ICU at a tertiary university hospital between December 2017 and June 2018. Patients in the intervention group were allowed to choose their preferred genre of music at any time and listen to it as much as they like through Yamaha's sound system under development for medical use until ICU discharge. The control group was not able to listen to music through the Yamaha sound system. For patients who had altered level of consciousness or who were unable to operate the system by themselves, ICU nurses instead chose the music genre and the duration of music delivery for their patients. Delirium was assessed every 3 hours by ICU nurses using the Confusion Assessment Method for the ICU. A competing risk survival analysis was used to examine the relative risk of delirium in the intervention group vs the control group. We performed additional analyses with data stratified by the length of ICU stay and the age of the patients.
**RESULTS.** A competing risk survival analysis indicated that the protective effect of the intervention on delirium incidence was not statistically significant (subdistribution hazard ratio: 0.79, 95% CI 0.42-1.51) in the whole sample with intubation. Stratified analysis by the length of ICU stay and the age of the patients suggested that the intervention may reduce the risk of delirium among intubated elderly (> 70 years old) patients who stayed in the ICU ≤ 7 days (Figure; subdistribution hazard ratio: 0.11, 95% CI 0.02-0.63). Among patients without intubation no effect of the intervention was detected.

**CONCLUSION.** PDM did not reduce delirium progression in the ICU, however subgroup analysis showed that PDM may reduce the onset of ICU delirium in intubated elderly patients.

1. This work was supported by Yamaha Co.

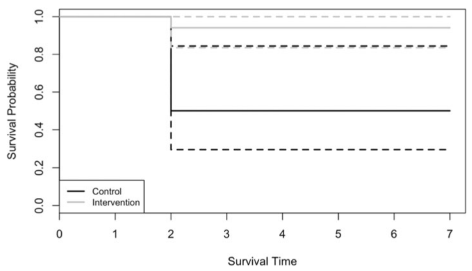

**Fig. 1 (abstract 001122).** See text for description

### 001126

#### Association of post-operative fluid balance with mortality and morbidity in critically ill patients with complicated intra-abdominal infection

SS. Hong, J. Shim, HJ. Shin, YT. Jung
[1]Surgery, Ajou University Hospital, Suwon, Republic of Korea
**Correspondence:** Y.T. Jung
*Intensive Care Medicine Experimental* 2019, **7(Suppl 3):**001126

**INTRODUCTION.** Fluid resuscitation is one the most influential aspects in perioperative management for critically ill patients with complicated intra-abdominal infection (cIAI). However, fluid overload may increase risk of developing pulmonary complications and result in adverse outcomes. Therefore, we evaluated the impact of excessive fluid administration on mortality and morbidity of the post-operative patients with cIAI in surgical ICU (SICU).

**METHODS.** We reviewed medical records of 320 patients administered to SICU after emergency abdominal surgery for cIAI between January 2013 and December 2018. Firstly, fluid balance of the patients was reviewed for maximum of seven days including the day of operation. Next, average fluid balance over body weight per day was calculated. Then, the patients were divided into two groups with a cut-off value of 20mL/kg/day. Baseline differences of patients in each group are matched using propensity score matching method.

**RESULTS.** Fluid-overloaded patients (≥20mL/kg/day) were associated with higher rates of 30-day mortality (11.8% vs 2.4%; p = 0.036) than those who received less fluid (<20mL/kg/day). Kaplan-Meier survival curves for 30-day mortality in these groups also showed a statistical significance (p = 0.020). Pneumonia occurrence (47.1% vs 24.7%; p = 0.004) were also higher in fluid-overloaded group. Rate of pleural effusion development (61.2% vs 57.7%; p = 0.755), reintubation (18.8% vs 10.6%; p = 0.194), and infectious complication (55.3% vs 49.4%; p = 0.539) showed no significant differences between two groups.

**CONCLUSION.** Fluid overload in patients after emergency surgery for cIAI was associated with higher rates of 30-day mortality, and pneumonia. Postoperative fluid balance in these patients should be carefully adjusted to avoid adverse clinical outcomes.

### 001130

#### Case series of prolonged anticoagulation-free veno-venous Extra-Corporeal Membrane Oxygenation (ECMO)

E. Cipriani[1], M. Panigada[2], M. Rossetti[1], E. Spinelli[2], P. Properzi[2], D. Tubiolo[2], A. Pesenti[1], G. Grasselli[1]
[1]Department of pathophysiology and transplantation, University of Milan, Milano, Italy; [2]Department of anesthesiology, Fondazione IRCCS Ca'Granda Ospedale Maggiore Policlinico, Milano, Italy
**Correspondence:** E. Cipriani
*Intensive Care Medicine Experimental* 2019, **7(Suppl 3):**001130

**INTRODUCTION.** Recent evidence suggests that a lower than recommended range for anticoagulation during ECMO may be safely used without major side effects [1]. There are also a few case reports of heparin-free ECMO runs in polytrauma patients indicating that ECMO could be performed without systemic anticoagulation even for prolonged periods of time in the presence of an high bleeding risk [2]. The safety of prolonged (> 24hours) periods of anticoagulation-free veno-venous (vv) ECMO in non-trauma patients has not been studied yet. The aim of this study was to evaluate the complications occurred during anticoagulation-free vvECMO in our cohort of patients with respiratory failure.

**METHODS.** We retrospectively reviewed our digital database on patients supported with vvECMO for severe respiratory failure from January 2012 to February 2019 at Fondazione IRCCS Ca' Granda Ospedale Maggiore Policlinico di Milano and selected vvECMO patients not receiving heparin or any other anticoagulant drug for at least 24 hours (and a concomitant aPTT ratio in the normal ranges). Thrombotic adverse events defined as cannula related thrombosis, deep vein thrombosis (DVT) checked at ultrasonography within 24 hours after ECMO removal and exchange of the ECMO circuit due to induced coagulopathy (defined as hypofibrinogenemia, elevation of DDimers and decrease of platelet count, once ruled out other causes) during the anticoagulation-free period are reported.

**RESULTS.** Ten out of 148 (6.8%) patients supported with vvECMO from 2012 to 2019 were identified and reviewed. Heparin coated circuits were used in all patients. Median time of vvECMO without anticoagulation was 42.25 (range 28.5-617.5) hours and median total ECMO time was 17 days. Main reasons for withholding anticoagulation are reported in table 1. Six (60%) patients died on ECMO or after removal. No cannula related thrombosis nor DVT were observed. The ECMO circuit was exchanged in 3/10 (30%) of patients during the anticoagulation-free period.

**CONCLUSION.** Prolonged periods of vvECMO without anticoagulation occurred rarely in our cohort and mostly because of uncontrolled bleeding. Although it was not possible to assess thrombosis in all patients, its incidence was low.

**REFERENCE(S)**

1. Panigada et al. Thromboelastography-based anticoagulation management during extracorporeal membrane oxygenation: a safety and feasibility pilot study. Ann Intensive Care. 2018 Jan 16;8(1):7

2. Wen et al. Non-heparinized ECMO serves a rescue method in a multi-trauma patient combining pulmonary contusion and nonoperative internal bleeding: a case report and literature review. World J Emerg Surg. 2015 Mar 12;10:15

**Table 1 (abstract 001130).** LTX (lung transplant), ARDS (Acute Respiratory Distress Syndrome). *Two patients had more one anticoagulation-free vvECMO period (total anticoagulation-free time is considered). §ECMO circuit exchange during the anticoagulation-free period. °Evaluated through Compression Ultrasound (CUS) 24-48 hours after ECMO removal. ^Not evaluated because patient died during vvECMO support or soon after removal

| | Age (years) | Indication for vvECMO | Cause of no anticoagulation | Total anticoagulation-free time (hours) | Total ECMO time (days) | ECMO circuit exchange§ | Outcome (ICU) | Deep Vein Thrombosis° |
|---|---|---|---|---|---|---|---|---|
| **Patient n°1** | 30 | Perioperative to LTX | Bleeding | 38 | 10 | NO | Dead | No |
| **Patient n°2** | 58 | ARDS (Unknown aetiology) | Haemorrhagic shock | 94.5 | 31 | NO | Dead | Not evaluated^ |
| **Patient n°3** | 34 | Perioperative to LTX | Haemorrhagic shock | 29 | 9 | NO | Alive | No |
| **Patient n°4** | 54 | ARDS (Pneumocystis) | Bleeding | 66.5* | 46 | YES | Dead | Not evaluated^ |
| **Patient n°5** | 48 | ARDS (Unknown aetiology) | Gluteal hematoma | 44 | 49 | YES | Dead | Not evaluated^ |
| **Patient n°6** | 57 | Postoperative to LTX | Haemorrhagic shock | 40.5 | 3 | NO | Alive | No |
| **Patient n°7** | 50 | Postoperative to LTX | Recent surgery | 31 | 2 | NO | Alive | No |
| **Patient n°8** | 28 | Postoperative to LTX | Recent surgery and bleeding | 617.5* | 38 | YES | Dead | Not evaluated^ |
| **Patient n°9** | 57 | ARDS (Legionella) | Intracranial hematoma | 222 | 13 | NO | Dead | Not evaluated^ |
| **Patient n°10** | 38 | ARDS (Influenza A H3 and S.Pyogenes) | Bleeding | 28.5 | 21 | NO | Alive | No |

### 001158

#### Midodrine for persistent vasoplegia after cardiac surgery – an exploratory cohort study

JA. Tremblay[1], P. Larammée[2], Y. Lamarche[3], K. Serri[4], AJ. Frenette[4], A. Denault[5], E. Charbonney[4]

[1]Critical care, Université de Montréal, Montréal, Canada; [2]Faculté de médecine, Université de Montréal, Montréal, Canada; [3]Cardiac surgery, Montreal Heart Institute, Montréal, Canada; [4]Critical care, Hôpital du Sacré-Coeur de Montreal, Montréal, Canada; [5]Critical care, Montreal Heart Institute, Montréal, Canada

**Correspondence:** J.A. Tremblay

*Intensive Care Medicine Experimental* 2019, **7(Suppl 3):**001158

**INTRODUCTION.** Vasoplegia is a frequent complication after cardiac surgery with cardiopulmonary bypass (CPB) and is associated with increased complications. As most healthcare structures do not allow for intravenous vasopressors outside of the intensive care unit (ICU), midodrine, an orally administered alpha agonist, could potentially reduce intravenous vasopressor use and accelerate ICU discharge of otherwise stable patients.

**OBJECTIVES.** We aimed to explore the clinical contexts in which midodrine is used in a high-volume cardiac surgery center as well as the prescription patterns and safety of this strategy.

**METHODS.** This is a retrospective cohort study that included all consecutive patients having received midodrine while being on vasopressor support in the ICU within 72 hours after cardiac surgery with CPB, between January 2013 and January 2017 at the Montreal Heart Institute. Outcomes included time on intravenous vasopressors, ICU length of stay, ICU readmission rate, occurrence of kidney injury and in-hospital mortality.

**RESULTS.** A total of 72 patients fulfilled inclusion criteria during the study period. Surgical interventions consisted of revascularization (N=39, 54.4%), valve surgery (N=13, 18.1%), combined surgery (N=15, 20.8%) and other (N=5, 6.9%). Median Euroscore II [IQR] was 1.94 [0.98-2.78], and median CPB duration was 77 [61-111] min. The median norepinephrine dose at midodrine initiation was 0.04 [0.03-0.08] mcg/kg/min with a cumulative vasopressor index1 at that time of 2.5 [2-4]. The first dose of midodrine was administered at a post-operative median time of 24 [20-44] hours at an initial dose of 10 [8.75-10] mg and treatment duration was 37 [22-73] hours. Median increase in creatinine after midodrine initiation was 5 [0-26.5] μmol/L and acute kidney injury (AKIN stage 1 or more) occurred in 18 patients (25%). Intravenous vasopressors were stopped 17 [4-40] hours after midodrine initiation, for a total of 52 [36-72] hours post-op. In 16 patients (22.2%), intravenous vasopressors had to be reinstituted after more than 2 hours of cessation. ICU length of stay was 93 [53-120] hours and 17 patients (23.6%) were still receiving midodrine on the surgical ward after ICU discharge. A total of 7 patients (9.7%) had to be readmitted to the ICU, of which one (1) was initially discharged while still receiving midodrine. Overall in-hospital mortality was 8.3% (N=6).

**CONCLUSION.** We report the practice of midodrine administration as an adjunct to intravenous vasopressors for vasoplegia syndrome after cardiac surgery. In a selected cohort of patients, this therapeutic approach appears to be associated with higher than expected ICU readmission rate and mortality. Even though these results may represent confounding by indication, routine prescription of midodrine to hasten ICU discharge after cardiac surgery should be done very cautiously until further prospective study is conducted.

### 001162

#### PRE-DELIRIC model as predictor of delirium development in patients in Intensive Care Unit (ICU)

R. Carbajal Serrano, OE. Palacios Calderon, CA. Rojas Gomez, J. Franco Granillo, AG. Camarena, J. Aguirre Sánchez

[1]Intensive care unit, The American British Cowdray Medical Center, Mexico City, Mexico

**Correspondence:** R. Carbajal Serrano

*Intensive Care Medicine Experimental* 2019, **7(Suppl 3):**001162

**INTRODUCTION.** Delirium is a neuropsychiatric disorder that affects patients in the ICU, is associated with increased mortality, prolonged hospitalization and long-term cognitive impairment. There are few tools that evaluate the risk of presenting it. The prediction model PRE-DELIRIC contains 10 risk factors, age, APACHE-II score, type of admission, coma, infection, metabolic acidosis, use of sedatives and morphine, urea, and urgent admission. The model had an area under the receiver operating characteristics curve of 0.87 (95% confidence interval 0.85 to 0.89) and 0.86 after bootstrapping. It is divided into 4 groups of low, moderate, high and very high risk.

**OBJECTIVES.** To determine whether the PRE-DELIRIC model is a good predictor of delirium in patients admitted to the ICU.

**METHODS.** Observational, analytical, prospective longitudinal study in 400 patients admitted to the ICU to whom PRE-DELIRIC was applied at 24 hours of admission, during the period from June 2018 to January 2019, sociodemographic and clinical data were obtained, in addition to risk factors of the predictive scale. To identify the presence of delirium, patients were evaluated with the CAM-ICU scale. The information was analyzed by descriptive statistics. The variables were evaluated on a numerical scale and reported in terms of percentages and standard deviation.

**RESULTS.** Low risk 136 (34%), moderate 96 (24%), high 48 (12%), very high 120 (30%), of the latter group 73% male, age: 63.8 ± 16.82 years, with diabetes 7%, hypertension 33%, 70% developed delirium, 50% with sepsis, APACHE-II 18 ±7.5 points, SOFA 8.3 ± 3.8, use of sedatives 83%, use opioids 77%, 75% with metabolic acidosis, 50% with neoplasia, emergency admission 77%, medical 43%, surgical 20%, neurological or neurosurgical 30%, mortality 37% in this group.

**CONCLUSION.** PRE-DELIRIC is a static model that shows calculated probability for delirium 24 hours after admission, it is a useful tool to detect risk of delirium in ICU. However, it was found that it is not a good predictor of delirium for cancer patients. A predictive scale is needed for this group of patients. By using this predictive scale routinely we could establish non-pharmacological preventive measures to reduce mortality and days of hospital stay due to delirium.

**REFERENCE(S)**

1. Jiyeon Kang. Effect of nonpharmacological interventions for the prevention of delirium in the intensive care unit: Asystematic review and metaanalysis. Journal of Critical Care 2018.
2. M van den Boogaard. Development and validation of PRE-DELIRIC (PREdiction of DELIRium in ICu patients) delirium prediction model for intensive care patients: observational multicentre study. BMJ 2012 Feb;344:e420

### 001180

#### Predictors of in-hospital mortality in adult patients undergoing emergency laparotomy for peritonitis: Analysis from a randomized controlled trial

S. Maitra[1], DK. Baidya[2], A. Roy Chowdhury[3], S. Rajeshwari[4], MK. Arora[5], R. Subramanian[6]

[1]Anaesthesia,critical care and pain medicine, All India Institute Of Medical Sciences, New Delhi, India; [2]Anaesthesia,pain medicine and critical care, All India Institute Of Medical Sciences, New Delhi, NEW DELHI, India; [3]Anaesthesia,pain medicine andcrotocal care, All India Institute Of Medical Sciences, New Delhi, NEW DELHI, India; [4]Anaesthesiology, pain medicine & critical care, All India Institute Of Medical Sciences, New Delhi, India; [5]Anaesthesiology & critical care, Institute of Liver and Biliary Sciences, New Delhi, India; [6]Liver transplant anaesthesiology, Max Super Speciality Hospital, Saket (Max Saket), New Delhi, India

**Correspondence:** S. Maitra

*Intensive Care Medicine Experimental* 2019, **7(Suppl 3):**001180

**INTRODUCTION.** Patients undergoing emergency laparotomy for intra-abdominal infection experience high peri-operative risk. Mortality rate remains high despite of advancement of surgical technique, antibiotic therapy and intensive care support. In the UK, 30-day mortality is one in seven overall, and for the elderly (age ≥ 80 years), almost one in four (1). Perforative peritonitis is linked with Multiple Organ Dysfunction Syndrome (MODS) in up to 73% patients and mortality reaches 30% in such cases (2, 3). Early identification of patients of severe peritonitis with higher risk of mortality will helpful earlier institution of intensive care management (4)

**OBJECTIVES.** In this study we have evaluated the risk factors for in-hospital mortality in peritonitis patients who are undergoing emergency laparotomy.

**METHODS.** This study is a secondary analysis of a previously conducted randomized controlled trial (5). Briefly, in that trial n=100 adult patients of either sex undergoing emergency laparotomy for clinically proven or suspected peritonitis were included. Patients were randomized in two arms; in group A mechanical ventilation was achieved with 6-8 ml/kg of tidal volume, 6- 8 cm H2O of PEEP with additional recruitment maneuver at thirty minutes. In group B, mechanical ventilation was delivered with 10 ml/kg of tidal volume, PEEP of 5 cm H2O and maximum allowable plateau pressure of 30 cm H2O. All patients were followed till hospital discharge and baseline preoperative demographic and laboratory parameters were noted.

**RESULTS.** During hospital stay, 25 patients died, and 69 patients were discharged from the hospital. Rate of in- hospital mortality (95% CI) was 26.6 (18, 36.7) %. Median [IQR] duration of ICU/ HDU stay was 6 [3- 11] days and duration of total hospital stay was 13 [9-21] days. Non survivors had similar length of ICU/ HDU stay (p=0.16) but shorter length of hospital stay (p=0.001). Non- survivors had longer duration of symptoms before surgery (p=0.003), lower platelet count (p=0.02), higher international normalized ratio (p=0.009) and higher arterial lactate (p=0.001). We have found that only duration of symptoms before surgery [adjusted odds ratio (95% CI) 1.61 (1.14, 2.27); p=0.007] and arterial lactate [adjusted odds ratio (95% CI) 2.25 (1.08, 4.70); p=0.031] were significant predictor of mortality (p=0.22, goodness-of-fit for the model) in a binary logistic regression model.

**CONCLUSION.** Perforation peritonitis still carries a high hospital mortality and duration of symptoms before surgery and baseline arterial lactate was found to be a significant predictor of mortality in these patients.

**REFERENCE(S)**

1. Saunders DI, Murray D, Pichel AC, Varley S, Peden CJ, UK Emergency Laparotomy Network. Variations in mortality after emergency laparotomy: the first report of the UK Emergency Laparotomy Network. Br J Anaesth . 2012 ;109:368–75.
2. Barie PS, Hydo LJ, Fischer E. Development of multiple organ dysfunction syndrome in critically ill patients with perforated viscus. Predictive value of APACHE severity scoring. Arch Surg . 1996 ;131:37–43.
3. Bohnen JM, Mustard RA, Oxholm SE, Schouten BD. APACHE II score and abdominal sepsis. A prospective study. Arch Surg . 1988 ;123:225–9.
4. V A M, C P M, S S, Srinivasarangan M. Efficacy of Mannheim Peritonitis Index (MPI) Score in Patients with Secondary Peritonitis. J Clin Diagn Res. 2014 ;8:NC01-3.
5. National Clinical Trial Registry of India (http://ctri.nic.in; Ref No: CTRI/2017/12/010732)
6. Dr. Dalim K. Baidya received research grant from Research Section, All India Institute of Medical Sciences, New Delhi, India

### 001189

#### Hospital-induced delirium associated with post-intensive care syndrome one year after discharge in severe trauma patients

N. Saito, Y. Takanori, K. Okada, M. Kujo, M. Hisashi

[1]Shock and Trauma Center, Nippon Medical School Chiba Hokusoh Hospital, 1715 Kamagari, Inzai, Chiba, Japan, Inzai, Japan

**Correspondence:** N. Saito

*Intensive Care Medicine Experimental* 2019, **7(Suppl 3):**001189

**INTRODUCTION.** Patients with severe trauma often experience delirium due to head injury, pain, and surgery. Hospital-induced delirium is a risk factor for increased long-term mortality. Additionally , post-intensive care syndrome (PICS) in a critically ill patient is a long-term health problem, but the hospitalization-related factors are unknown.

**OBJECTIVES.** We aimed to clarify the association between hospital-induced delirium and PICS, one year after discharge, in severe trauma patients.

**METHODS.** This single-institutional prospective study included 213 injured patients who were admitted to the trauma surgical intensive care unit (TSICU) and survived until discharge to home between 2016 and 2017.We evaluated the activities of daily living one year after the discharge using physical and mental component scores of SF-36®and defined physical dysfunction and mental disorder as a score of less than the 25thpercentile of the national standard value for the same age. Further, cognitive impairment was determined by self-administered screening. PICS was diagnosed when one of the following three components was recognized: physical dysfunction, mental disorder, and cognitive impairment. We divided the patients in the PICS and control (without PICS) groups and compared the groups.

**RESULTS.** The patients had experienced blunt injuries, including pedestrian injuries (19.7%) and falls (34.3%). The mean age was 61.6 years (men: 70%); the median injury severity score (ISS) was 21 (interquartile range: 16–29); and the mean length of TSICU stay was 4.3 days. The average period from the injury until the investigation was 17.9 months. The PICS group included 121 patients (56.8%), of which 99 (46.5%), 46 (21.6%), and 28 (13.1%) had physical dysfunction, mental disorder, and cognitive impairment, respectively. More patients in the PICS group were transferred to a rehabilitation facility and were receiving outpatient treatment at the time of the study compared to the control group. Furthermore, they had more health-related and financial problems ($P < 0.01$). The frequency of emergency surgery (50.4% vs. 33.7%) and mechanical ventilation (37.2% vs. 23.9%) in the PICS group were significantly higher compared to the control group. The incidence of delirium in the PICS group was significantly higher compared to the control group (33.1

% vs. 12.0%). A multivariate analysis, which was adjusted for age, severity, and gender, revealed the factors affecting PICS: delirium (odds ratio [OR]: 3.54; 95% confidence interval [CI]: 1.54–8.16; *P*=0.003), emergency surgery (OR: 2.36; 95% CI: 1.24–4.46; *P*=0.08), and spinal cord injury (OR: 4.42; 95% CI: 1.48–13.12; *P*=0.07).

**CONCLUSION.** In severe trauma patients, hospital-induced delirium was independently associated with PICS, lasting up to one year after discharge.

### 001197

#### Protective effect and mechanism of mesenchymal stem cells on intestinal injury induced by heat stroke

L. Wang[1], R. Yuan[1], Y. Zhang[2], M. Yang[3], Y. Zhao[4], H. Kang[4]
[1]Departmentof critical care medicine, Chinese PLA General Hospital, Beijing, China; [2]Departmentof critical care medicine, Chinese PLA General Hospital, Beijing, China; [3]Departmentof critical care medicine, Departmentof Critical Care Medicine,Chinese PLA General Hospital,Beijing,China, Beijing, China; [4]Department of critical care medicine, Chinese PLA General Hospital, Beijing, China
**Correspondence:** L. Wang
*Intensive Care Medicine Experimental* 2019, **7(Suppl 3):**001197

**INTRODUCTION.** Heat stroke is a high fever associated with systemic inflammatory response, which can lead to multiple organ dysfunction syndrome, mainly encephalopathy[1]. High heat stimulation causes intestinal ischemia, mucosal barrier dysfunction and release of inflammatory factors[2], eventually leading to systemic inflammatory response. Mesenchymal stem cells have many mechanisms, such as immune regulation and repair of tissue damage. Many studies have found that MSCs can regulate the level of inflammatory factors through paracrine effect, and also affect regulatory T cells[3] and macrophages[4], thus playing an anti-inflammatory role. MSCs can also repair tight junctions[5] and alleviate ischemia-reperfusion injury, thereby improving intestinal mucosal barrier function.

**OBJECTIVES.** To explore whether MSCs can reduce intestinal damage and systemic inflammation caused by heat stroke, and ultimately improve survival rate.

**METHODS.** Sprague-Dawley (SD) male rats were used to establish heat stroke injury model, and MSCs were infused into HS rats treated with MSC. Rats in model group and treatment group were divided into early, middle and late stages. The levels of biochemical markers (ALT, AST, etc.), inflammatory factors (IL-1beta, IL-6, TNF-alpha) and chemokines (Eotaxin, Rantes, MIP-2) in intestine and blood were detected respectively. The intestinal injury was compared by intestinal histopathology. The intestinal injury was compared by intestinal histopathology. Survival rate was estimated by 28-day observation.

**RESULTS.** The 28-day survival rate of rats in the treatment group was significantly higher than that in the model group, and the level of biochemical markers was significantly better than that in the model group. According to Chiu's score of intestinal mucosa, the treatment group was significantly lower than the model group. In intestinal tissue and blood, the level of anti-inflammatory factors in the treatment group was higher than that in the model group, and the level of pro-inflammatory factors was lower than that in the model group, with statistical difference. The change trend of chemokine levels in the two groups was the same, but the overall level in the treatment group was lower than that in the model group.

**CONCLUSION.** In animal models of heat stroke, MSCs can play an anti-inflammatory role, reduce organ damage and improve survival rate. MSCs can also reduce stress injury of intestinal mucosa and promote tissue repair.

**REFERENCE(S)**

[1]. Abderrezak Bouchama JPK.Heat stroke[J].The New England Journal of Medicine.2002,346(25):1978-88.
[2]. Yanwei Du YM, Xuejiao Lv, Lirong Guo, Xiaoqin Wang, Zhenzhong Su, Lu Li, Na Li, Shuhua Zhao, Lijing Zhao, Xuejian Zhao.Dexamethasone attenuates LPS-induced changes in expression of urea transporter and aquaporin proteins, ameliorating brain endotoxemia in mice.[J].Int J Clin Exp Pathol.2014,7(12):8443-52.
[3]. Zappia E, Casazza S, Pedemonte E, Benvenuto F, Bonanni I, Gerdoni E, et al.Mesenchymal stem cells ameliorate experimental autoimmune encephalomyelitis inducing T-cell anergy[J].Blood.2005,106(5):1755-61.
[4]. Park HJ, Kim J, Saima FT, Rhee KJ, Hwang S, Kim MY, et al.Adipose-derived stem cells ameliorate colitis by suppression of inflammasome formation and regulation of M1-macrophage population through prostaglandin E2[J].Biochem Biophys Res Commun.2018,498(4):988-95.
[5]. Wen Zhang Z-YS, Hong-Li Song, Yang Yang, Ben-Juan Wu, Nan-Nan Fu, Tao Liu.Protective effect of bone marrow mesenchymal stem cells in intestinal barrier permeability after heterotopic intestinal transplantation[J].-World Journal of Gastroenterology.2014,20(23):7442-51.

### 001241

#### Albumin mass balance in pancreatic surgery

A. Komáromi[1], J. Wernerman[1], O. Rooyackers[2], Å. Norberg[1]
[1]Clintec, Karolinska Institutet, Stockholm, Sweden; [2]Perioperative medicine and intensive care, Karolinska University Hospital Huddinge, Stockholm, Sweden
**Correspondence:** A. Komáromi
*Intensive Care Medicine Experimental* 2019, **7(Suppl 3):**001241

**INTRODUCTION.** In major abdominal surgery plasma albumin concentration decreases more than explained by bleeding, presumably due to increased transcapillary leakage into the interstitial space and alterations in plasma volume. The contribution of compromised lymphatic return to net albumin leakage has not been investigated. Here we study this contribution by using pancreatic Whipple surgery with aortocaval lymphadenectomy as a model for compromised lymphatic return in the postoperative period.

**METHODS.** Patients scheduled for pancreatic resection were studied in two groups, A (n=12, Whipple procedure) and B (n=13, Whipple + aortocaval lymphadenectomy), Albumin mass balance was assessed from start of anesthesia until postoperative day 3 (POD 3). Endpoints were albumin net leakage at end of surgery (EOS) and on POD 3.

**RESULTS.** Plasma albumin decreased from 35.0 ± 3.6 g/L and 32.7 ± 3.6 g/L at start of anesthesia to 28.6 ± 4.4 g/L (p<0.0001) and 27.9 ± 4.8 g/L (p=0.0002) at EOS for groups A and B respectively, corresponding to decreases of 18 ± 8 and 15 ± 10 %, respectively. At EOS this corresponds to an albumin shift from plasma of 22 ± 10 g and 23 ± 12 g in group A and B, respectively. At POD 3 the shifted amount in group A was 9 ± 29 g (p = 0.34 compared to EOS) and 23 ± 29 g (p = 0.99) in group B.

**CONCLUSION.** Plasma albumin decreased by 16 % until EOS. The decrease in albumin leakage in group A postoperatively failed to reach statistical significance, indicating that lymphatic return might not be the main contributor. The large variability suggests a multifactorial explanation behind the postoperative shift of albumin.

**REFERENCE(S)**

1. TRIAL REGISTRATION: ACTRN12616001381471. Registered on 5 Oct 2016.
2. Grant acknowledgement: County Council of Stockholm

### 001298

#### Impact of noninvasive hemoglobin-level measurement by Radical 7 Pulse Co-Oximetry on clinical decision: a prospective observational study

A. Herner, I. Hartter, R. Schmid, W. Huber
[1]Medizinische klinik und poliklinik ii, Klinikum rechts der Isar; Technische Universität München, Munich, Germany
**Correspondence:** A. Herner
*Intensive Care Medicine Experimental* 2019, **7(Suppl 3):**001298

**INTRODUCTION.** Accurate measurement of hemoglobin (Hb) concentration is a major target of monitoring in critically ill. Furthermore, it guides decision making regarding blood transfusion. Standard laboratory hemoglobin measurement requires time and resources. Therefore, noninvasive real-time Hb measurement devices as Radical 7 Pulse Co-Oximeter (MASIMO, USA) are of high interest. Despite a number of attempts to validate this device, its accuracy especially at low Hb values remains unclear.

**OBJECTIVES.** Therefore, we investigated, if noninvasive pulse-oximetry Hb (SpHb) appropriately indicates the need for blood transfusion according to invasive hemoglobin measurements (primary endpoint) in critical ill patients.

**METHODS.** This prospective observational study was performed on a general ICU of a University Hospital in Munich. Only patients with a need to blood transfusion (Laboratory Hb <7mg/dl; in case of pre-existing cardiovascular disease: Hb <8 mg/dl) were included in this study. All patients (n=44) were equipped with Radical 7. Reference laboratory Hb (Hb_BGA) values were determined by RAPIDPoint 500 Blood Gas analyzer (Siemens, Germany). 35 out of 44 patients were equipped with transpulmonary thermodilution monitoring (PiCCO, Germany). All measurements were performed prior and after the transfusion of two units of red blood cells. Statistics: Fisher´s exact test. Wilcoxon-test for paired samples. IBM SPSS 25.

**RESULTS.** All 44 patients fulfilled the pre-defined criteria for blood transfusion according to Hb_BGA. Pre-transfusion Hb_BGA was 6.7± 0.6 mg/dl. In 8 out of these 44 patients (18%) Radical 7 was not able to generate a hemoglobin value. In none of the remaining 36 patients SpHb would detected the need for blood transfusion. Consequently, the need for transfusion was detected in 0/44 (0%) by SpHb compared to Hb_BGA (44/44 (100%); p<0.001).

SpHb prior to transfusion significantly overestimated Hb according to the gold standard laboratory measurement (9.3±1.3 vs. 6.7±0.6 mg/dl, p<0.001) with a bias of 2.6±1.2 mg/dl, lower and upper limits of agreement f of 0.02 and 4.97 mg/dl, and a percentage error of 30%.

Similarly SpHb overestimated Hb_BGA after transfusion of 2 units of red cells (10.2±1.5 vs. 8.8±0.8mg/dl, p<0.001), with a bias of 1.4±1.5 mg/dl and limits of agreement of -1.4 and 4.2 mg/dl, and a percentage error of 29%.

Also trending capacities of SpHb were poor: Changes in SpHb after transfusion did not correlate with changes in Hb_BGA (r=0.206; p= 0.220).

While Hb_BGA increased in 44 of 44 patients (100%), SpHb increased in only 32 of 37 displayed mesaurements (p=0.017).

Increases in Hb_BGA after transfusion compared to baseline were significantly higher than changes in SpHb (2.1±0.6 vs. 1.0±0.9g/dl; p< 0.001).

**CONCLUSION.** 1.) Radical 7 Pulse CO-Oximeter does not accurately measure decreased Hb-values in critically ill patients.

2.) SpHb is not appropriate to trigger transfusion and and to track changes in Hb critically ill patients with a low hemoglobin values.

## TEM - New aspects in cardiac arrest management

### 000070

#### Patient Survival Rates and their Correlated Factors in a Dust Explosion Incident

CC. Chao
[1]{street_address}, Taipei, Taiwan
**Correspondence:** C.C. Chao
*Intensive Care Medicine Experimental* 2019, **7(Suppl 3):**000070

**INTRODUCTION.** Triage plays a critical role in mass casualty incidents by optimizing the use of medical resources. The Formosa Fun Coast (Baxian Water Park) dust explosion incident in 2015 revealed the lack of resources for mass burn and scald casualties in the Taiwanese medical system, however mortalities in this incident were only 3% (15/499) by the end of 2015.

**OBJECTIVES.** This study examined the key feature of the prehospital settings of the 15 mortalities.

**METHODS.** This was a retrospective cohort study, and all the patients from the Formosa Fun Coast incident (N = 499) were enrolled. The follow-up period was from June 27, 2015 to December 31, 2015. We first examined the correlation between patient survival and various variables and then tested the correlation between the survival-correlated variables and the level of the hospitals that provided treatment.

**RESULTS.** The survivor and nonsurvivor groups shared similar distributions of all the study variables.The patients were divided into two groups: survivors (N = 484) and nonsurvivors (N = 15). The two groups shared a similar average age and sex division. Most of the patients were sorted to more severe levels; this is evident from both the emergency severity index and EMT-performed triage assessment. Regarding the emergency severity index, the majority of the patients were Level 1 or Level 2 (100% of nonsurvivors; 74.7% of survivors). For EMT-performed triage assessment, most of the patients' conditions were of high or medium severity (100% of nonsurvivors; 79.6% of survivors). The burn and scald classification and Baux score were higher in the nonsurvivor group; the majority of nonsurvivors had a third-degree burn (86.7%) and a Baux score of more than 50 (100%).

**CONCLUSION.** The lack of effect of hospital level on patient mortality indicated that in addition to a satisfactory EMT-performed triage system, all hospitals, regardless of their level, are equipped with adequate medical capacity for handling patients with burns and scalds.

**Table 1 (abstract 000070).** Correlation between patient survival and study variables

| Variables | Value | p value |
|---|---|---|
| Age | 2.8883 | 0.4092 |
| Sex | 0.5582 | 0.4550 |
| Emergency severity index | 1.4323 | 0.4886 |
| EMT-performed triage assessment | 6.0798 | **0.0478** |
| Burn classification | 5.2554 | 0.0722 |
| Baux score | 7.4863 | **0.0062** |
| Hospital level | 0.1509 | 0.6977 |
| Method of transportation to hospital | 3.7658 | 0.1522 |

### 000077

#### Increase of platelet cellular density can predict infectious complication after severe burn injury

C. Dr.Loibl[1], S. Rendeki[1], E. Ezer[1], M. Rozanovic[1], A. Pankaczi[1], P. Kovacs[1], M. Matancic[2], L. Bogar[1], L. Szelig[1], T. Nemeth[3], A. Tamas[4], A. Miseta[5], T. Molnar[1], C. Csontos[1]
[1]Department of anaesthesiology and intensive care, University of Pécs Medical School, Pecs, Hungary; [2]1st department of internal medicine, University of Pécs Medical School, Pécs, Hungary; [3]Department of languages for specific purposes, University of Pécs Medical School, Pécs, Hungary; [4]Department of anatomy, University of Pécs Medical School, Pecs, Hungary; [5]Department of department of laboratory medicine, University of Pécs Medical School, Pecs, Hungary
**Correspondence:** C. Dr.Loibl
*Intensive Care Medicine Experimental* 2019, **7(Suppl 3):**000077

**INTRODUCTION.** Major burn injury can cause massive tissue destruction and initiates cytokine-mediated inflammatory response, impaired coagulation and fibrinolysis. This condition features an initial transient thrombocytopenia followed by normalization of platelet count and eventual reactive thrombocytosis.

**OBJECTIVES.** Our aim was to investigate the kinetics of platelet antisedimentation rate (PAR), C reactive protein (CRP) and procalcitonin (PCT) levels in the early period after burn and to assess whether these parameters can predict development of septic complications and ICU outcome.

**METHODS.** In a prospective, observational study 23 consecutive patients with more than 20% body surface burn injury were followed for five days (T1-T5) after admission to ICU. PAR was measured by one-hour gravity sedimentation indicating the percentage of platelets had crossed the half line of blood sample column. Serum CRP and PCT values were measured by standard laboratory methods and sepsis diagnosis was based on American Burn Association guideline.

**RESULTS.** Ten patients developed septic complications. In the whole group platelet concentration decreased by time and became significant at T3 ($p<0.001$) in survivors (n=16) and at T2 ($p<0.05$) in non survivors comparing to T1. In survivors mean PAR continuously increased between T1 and T5. Mean PAR of survivors were significantly higher than that of non survivors at T1, T4 and T5 (all $p<0.05$). In non survivors PAR increased significantly ($p<0.05$) from T3 and reached its peak at T5. PAR had showed a decreasing tendency in septic patients during three days before clinical diagnosis of sepsis was made. Drop of mean CRP was significant ($p<0.05$) just a day before sepsis was clinically evident. Serum PCT increased right on the day of sepsis commencement ($p<0.05$).

**CONCLUSION.** In patients with a significantly higher PAR value on T2 ($p<0.05$) were more likely to survive ICU treatment. A sudden drop in the PAR levels may predict the onset of sepsis earlier than conventional clinical signs. Earlier detection of septic complications can result in earlier therapeutic interventions. Ten patients who developed sepsis as a consequence of burn injury do not provide enough data required for the complete description of sepsis kinetics. A greater number of patients will be needed in the future.

**REFERENCE(S)**

1. Cato et al. Burns 2018; 44: 288-97, Midura et al. Shock 2016; 45: 82-7.
2. None.

### 000081

#### Incidence Of Multidrug-Resistant Organisms (MDRO) And Association With Worse Outcome In Severely Injuried Patients

A. Nohl[1], U. Hamsen[2], M. Dudda[3], TA. Schildhauer[2], A. Wegner[3]
[1]Department of trauma surgery and orthopedics, BG Klinikum Duisburg, Duisburg, Germany; [2]Department of general and trauma surgery, BG University Hospital Bergmannsheil, Bochum, Germany; [3]Center for orthopedics and traumatology, University Hospital Essen, Essen, Germany
**Correspondence:** A. Nohl

*Intensive Care Medicine Experimental* 2019, **7(Suppl 3):**000081

**INTRODUCTION.** MDRO are an increasing problem in hospitals and especially in intensive care units(1). Furthermore, infections with MDRO have significant economic effects(2). Taking into account the increasing health and socioeconomic issues, recent findings show that up to 37% of healthcare facilities in a rich industrialized country do not perform any screening at admission(2). An effect of MDRO in injured patients is not yet described.

**OBJECTIVES.** This study describes for the first time the rate and impact of MDRO in severely injured patients.

**METHODS.** Retrospective, multicenter study from three trauma level-1 centers in Germany. The study was approved by local ethics committees.

All patients treated in the trauma room and admitted to an intensive care unit between 2013 and 2017 were included. Data were extracted from the german trauma registry( Traumaregister DGU ®) and completed with data of microbiological screening. Data are presented as mean +/- standard deviation.

**RESULTS.** We included 3887 trauma patients, mean ISS was 16.25 (+/- 13.18). Incidence of positive screening for MDRO was 1.4 %. Comparing MDRO positive vs MDRO negative patients , MDRO positive patients showed an extended length of stay in icu (26.80 days +/- 24.29 vs. 6.42 days +/- 12.21; p=<0.001), duration of mechanical ventilation (395.3 hours, +/- 455.9 vs 64.94 hours, +/-194.7 ; p=<0.001), sepsis (n=25=45,5% vs n=214=5,5%, p=<0.001), multi organ failure (n=9=16,4% vs n=271=7%, p=<0.007), Glasgow Outcome Scale (median 2 vs median 1, p=<0.001).

**CONCLUSION.** MDRO are rare in severely injuried patients but they are associated with a devasting worser outcome . We strongly recommend extented screening for MDRO in critically ill trauma patients.

**REFERENCE(S)**

(1) European Centre for Disease Prevention and Control (2013) Point prevalence survey of healthcare-associated infections and antimicrobial use in European acute care hospitals. ECDC https://ecdc.europa.eu/sites/portal/files/media/en/publications/Publications/healthcare-associated-infections-antimicrobial-use-PPS.pdf. Accessed 7 Jul 2018
(2) Evans HL, Lefrak SN, Lyman J, Smith RL, Chong TW, McElearney ST, Schulman AR, Hughes MG, Raymond DP, Pruett TL, Sawyer RG (2007) Cost of Gram-negative resistance. Crit Care Med 35:89–95
(3) Romain Martischang, MD,1 Niccolo Buetti, Carlo Balmelli, Mirko Saam, Andreas Widmer, Stephan Harbarth. Nation-wide survey of screening practices to detect carriers of multi-drug resistant organisms upon admission to Swiss healthcare institutions. Antimicrob Resist Infect Control. 2019; 8: 37
(4) no financial interests, no fundings

### 000137

#### Investigation of the Antioxidant Effects of Dexmedetomidine and Ascorbic Acid Against Hydrogen Peroxide-Induced DNA Damage in In-Vitro Cell Cultures by Alkali Comet Technique

M. Kotanoglu[1], E. Kadioglu[2], E. Emerce[2], C. Kaymak[1], A. Ozcan[1], H. Basar[1]
[1]Department of anesthesiology and reanimation, University of Health Sciences, Ankara Health Application and Research Center, Ankara, Turkey; [2]Department of pharmaceutical toxicology, Gazi University, Faculty of Pharmacy, Ankara, Turkey
**Correspondence:** C. Kaymak

*Intensive Care Medicine Experimental* 2019, **7(Suppl 3):**000137

**INTRODUCTION.** Oxidative stress induced by ischemia, mechanical stress or toxins is a condition that results from the imbalance between the production of oxygen species and free radicals as well as inappropriate antioxidant function. The dexmedetomidine (DEX) is a highly selective alpha-2 adrenergic receptor agonist that is commonly used in the clinic as a sedative and anesthetic. In addition, DEX protective effects against oxidative damage in both in-vitro and in vivo conditions have been demonstrated. The Alkali Comet Method is an in-vitro method that allows the determination of single strand fractures of DNA.

**OBJECTIVES.** It was aimed to evaluate the protective effects of DEX and Ascorbic Acid (AA) on DNA against alkali-induced DNA damage caused by Hydrogen Peroxide ($H_2O_2$) induced oxidative stress in human lymphocyte cell cultures in vitro by alkaline comet method.

**METHODS.** Lymphocyte cell cultures were divided into five groups as negative control; solvent control; positive control; $H_2O_2$ (150 μM) + DEX (1μM; 2.5μM; 5μM); $H_2O_2$ (150μM) + AA (1μM; 2.5μM; 5μM) and incubated at 37°C for 1 hour. Cell viability was measured by Trypan Blue test. DNA damage was measured by Alkali Comet Technique and % tail intensity was evaluated. Statistical analysis was performed by one-way analysis of variance and Tukey's multiple comparison test.

**RESULTS.** It was observed that $H_2O_2$ significantly induced DNA damage in lymphocytes and this damage decreased significantly with AA and DEX. It was observed that AA at 1μM and 2.5μM doses had a significantly stronger antioxidant effect, but there was no significant difference between the antioxidant effects of AA and DEX in 5μM doses. The concentration of 5μM DEX was found to be most effective in reducing oxidative DNA damage.

**CONCLUSION.** The anti-oxidative effects of dexmedetomidine have been reported to be associated with extracellularly signal-regulated kinase signaling pathways. It has been reported that produces protective effects by suppressing in toll-like receptor, nuclear factor-kappa B like and phosphoinositide-3 kinase signaling pathways against oxidative injury. Our results showed that dexmedetomidine is a protective against H2O2 induced in vitro oxidative DNA damage in lymphocyte cell cultures in a dose-dependent manner.

**REFERENCE(S)**

1. Dexmedetomidine inhibits the secretion of high mobility group box 1 from lipopolysaccharide-activated macrophages in vitro. J Surg Res 2013; 181:308-14. 2. Dexmedetomidine attenuates H2O2-induced cell death in human osteoblasts. J Dent Anesth Pain Med 2016;16:295-302. 3. Dexmedetomidine Protects Cardiomyocytes against Hypoxia/Reoxygenation Injury by Suppressing TLR4-MyD88-NF-κB Signaling. Biomed Res Int 2017;2017:1674613.
2. None

### 000169

#### The impact of the use of Oxandrolone in the adult burn patient

J. Goethals[1], K. Claes[2], J. Fierens[3], S. Witdouck[4], L. De Bus[5], K. Colpaert[5]
[1]Faculty of medicine and health sciences, Ghent University, Ghent, Belgium; [2]Plastic surgery, Ghent University Hospital, Gent, Belgium; [3]Intensive care, Ghent University, Ghent, Belgium; [4]Burn center, Ghent University Hospital, Gent, Belgium; [5]Intensive care, Ghent University Hospital, Gent, Belgium
**Correspondence:** K. Colpaert
*Intensive Care Medicine Experimental* 2019, **7(Suppl 3):**000169

**INTRODUCTION.** Burn patients experience a hypermetabolic response that induces several pathophysiological changes leading to catabolism. This response can be potentially countered with optimal fluid resuscitation, excision and grafting, early enteral nutrition and physical rehabilitation. Pharmacological modulation of the hypermetabolic response due to severe burns can help improve the prognosis. The anabolic steroid, Oxandrolone, possibly finds its place here.

**OBJECTIVES.** The objective of this systematic review on the use of Oxandrolone in adult burn injury patients is to provide an overview of the effectiveness on weight, nitrogen balance, metabolism, length of stay, side effects and mortality.

**METHODS.** PubMed, Google Scholar, Web of Science, Embase and Cochrane Library databases were searched up to November 2018. Forest plots were created in Review Manager.

**RESULTS.** Six randomized controlled trials (RCTs) and two retrospective studies with a total of 541 patients were included in this study. The RCTs had a total of 257 patients; 5 single centre studies from one author (total of 176 patients), and 1 multicentric study from a second author (total of 81 patients). The systematic review on the six RCTs showed that Oxandrolone had a potentially beneficial effect on the healing process of the adult burn patient in a dosing of 2 times 10mg Oxandrolone per day. No additional side effects could be observed by the use of oxandrolone on liver function (P=0.41), glycemic control or hirsutism. There was less weight loss in the acute phase (P<0.001) as well as a better increase in weight in the rehabilitation phase (P<0.001). This beneficial effect on weight remained noticeable up to six months after discontinuation of Oxandrolone. Furthermore, there was a better nitrogen balance (P<0.001). No effects on metabolism could be observed (P=0.47). There was a significant faster wound healing (P<0.001). No impact on length of stay or mortality could be demonstrated.

**CONCLUSION.** Although this systematic review clearly shows a significant benefit towards adequate weight control and wound healing, currently no clear recommendation can be made. The low quality nature of the evidence does not allow for therapeutic advices to be drawn. Further methodologically robust research including more patients as well as centres needs to be conducted.

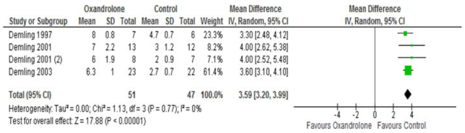

**Fig. 2 (abstract 000169).** Forest plot of the gain of weight in the rehabilitation phase.

### 000206

#### ALEC STUDY : Analysis of phenytoin use in acute seizures at Chicoutimi Hospital, Canada

A. Noël[1], A. Bouchard[2], C. Côté[2], A. Lavoie[2], G. Babin[2], F. Calon[3]
[1]Pharmacy, Hôpital de Val-d'Or, Val-d'Or, Canada; [2]Pharmacy, Hôpital De Chicoutimi, Saguenay, Canada; [3]Research, Centre recherche CHU de Québec, Quebec City, Canada
**Correspondence:** A. Noël
*Intensive Care Medicine Experimental* 2019, **7(Suppl 3):**000206

**INTRODUCTION.** Phenytoin (PHT)is frequently used in the treatment of acute seizures. Inadequate dose or wrong interpretation of the total phenytoin plasma level (TPPL) could lead to significant consequences. Five different formulas can be used to estimate the concentration of free PHT, but no clinical guidelines are available to determine which formula to use(1).

**OBJECTIVES.** The main objective was to determine the proportion of therapeutic TPPL within the first 48 hours of treatment. The secondary objectives were to describe the doses of PHT used and the TPPL interpretation, evaluate the proportion of patients to whom a TPPL is prescribed, the impact of different loading doses (LD) and interventions of a clinical pharmacist.

**METHODS.** A descriptive, longitudinal and retrospective study design was used. The health records from a single university hospital of all adult patients receiving intravenous PHT were included. The exclusion criterias were therapeutic hypothermia and pregnancy. The TPPL was analysed if the patient file contained one albumin and creatinine plasma concentration measured within 72 hours of the TPPL. The adequacy of the formula used was determined by an algorithm developed by the authors after a literature review.

**RESULTS.** 401 patients from January 3rd 2014 to April 19th 2018 were included. After excluding files with missing data, 156 patients and 383 PHT dosages were included in the analysis. 60.7% of the TPPL were within the therapeutic range at 48 hours. The mean initial LD was 12.4 ± 3.7 mg/kg. A TPPL was ordered for 44.1% of patients in the first 7 days of treatment. The TPPL were corrected with the appropriate formula in 53.4% of cases by pharmacists and in 10.9% by physicians. The initial LD of 1g resulted in 65.5% therapeutic dosages at 48 hours and those between 15-20 mg/kg in 80.6% (p=0.04).

**CONCLUSION.** The PHT levels of four out of ten patients were subtherapeutic due to insufficient initial LD. Drug monitoring of PHT was inadequate in a large proportion of patients because the wrong formula was used. Less than half of the patients had a TPPL prescribed and even less had an analysable result without missing data. This is worrisome, seeing as it could lead to therapeutic failure or adverse events with this narrow therapeutic index drug. This study demonstrates that education is still needed to insure an optimal treatment of seizures with PHT.

### 000271

#### Shock index for early detection of low plasma fibrinogen in trauma: a prospective observational trial

J. Škola[1], M. Bílská[1], J. Beneš[2], M. Peltanová[2], V. Tegl[2], R. ŠKulec[1], V. ČErný[1]
[1]Department of Anaesthesia, Perioperative Medicine and Intensive Care, Krajská zdravotní, a.s., Ústí nad Labem, Czech Republic; [2]Department of anaesthesiology, resuscitation and intensive care, University Hospital in Pilsen, Plzeň, Czechia, Czech Republic
**Correspondence:** J. Škola
*Intensive Care Medicine Experimental* 2019, **7(Suppl 3):**000271

**INTRODUCTION.** Shock index (a ratio between heart rate and systolic blood pressure, SI) has been shown to predict transfusion requirements and the need for hemostatic resuscitation in severe trauma patients. Trauma-induced coagulopathy frequently occurs in patients with traumatic shock and increases mortality by up to four times. Fibrinogen is the first clotting factor that reaches critical levels. Resuscitation strategy based on early fibrinogen supplementation has recently been shown to be superior in coagulopathy reversal compared to fixed plasma to packed red blood cells ratio approach. In the present study, we aimed to determine whether prehospital and on-admission shock index values can be used to predict low plasma fibrinogen in trauma patients.

**METHODS.** Between January 2016 and February 2017, all trauma patients whose prehospital vital signs record was available were assessed for demographics, injury characteristics, shock index and admission plasma fibrinogen level. An area under the receiver operating characteristics curve (AUROC), sensitivity, specificity, positive and negative predictive values and accuracy were calculated. Clinicaltrial.gov ID: NCT0269539 (registered 27 November 2015).

**RESULTS.** AUROC for prespecified cut-offs of plasma fibrinogen concentrations and the worst prehospital shock index was 0.7 (95% CI 0.63 - 0.76) for fibrinogen 2.3 g.l-1, 0.78 (95% CI 0.71 - 0.84) for 2.0 g.l-1 and 0.76 (95% CI 0.64 - 0.86) for 1.5 g.l-1. For the admission SI, AUROC was 0.65 (95% CI 0.59 - 0.71), 0.8 (95% CI 45 0.68 - 0.91) and 0.8 (95% CI 0.68 - 0.91) for the same levels of fibrinogen as above. For the worst prehospital shock index $\geq$ 1 and plasma fibrinogen $\leq$ 1.5 g.l-1 sensitivity was 0.54 (0.25 - 0.81), specificity 0.87 (0.83 - 0.91), positive predictive value 0.16 (0.10 - 0.25), negative predictive value 0.98 (0.96 - 48 0.99) and accuracy 0.86 (0.81 - 0.90).

**CONCLUSION.** Shock index is an easy-to-perform clinical tool, which may help identify adult trauma patients at risk of hypofibrinogenemia from the very early contact with the patient. Levels < 1 excluded severe hypofibrinogenemia with 98% predictive value. Taken to the patient's bedside, this means that whenever the systolic blood pressure remained higher than the heart rate, the risk of critical hypofibrinogenemia was low. These findings should prompt emergency physicians to consider early fibrinogen administration when fibrinogen levels are not available, and shock index is high.

**REFERENCE(S)**

1. Hagemo JS. Prehospital detection of traumatic coagulopathy. Transfusion. 2013;53(SUPPL. 1).
2. Vandromme MJ, Griffin RL, Kerby JD, McGwin G, Rue LW, Weinberg JA. Identifying risk for massive transfusion in the relatively normotensive patient: Utility of the prehospital shock index. J Trauma - Inj Infect Crit Care. 2011;70(2):384–90.
3. Allgöwer M, Burri C. Schockindex. Dtsch med Wschr. 1967;43:1947-1950.
4. Innerhofer P, Fries D, Mittermayr M, Innerhofer N, von Langen D, Hell T, et al. Reversal of trauma-induced coagulopathy using first-line coagulation factor concentrates or fresh frozen plasma (RETIC): A single-centre, parallel-group, open-label, randomised trial. Lancet Haematol. 2017;258–71.
5. Rossaint R, Bouillon B, Cerny V, Coats TJ, Duranteau J, Fernández-Mondéjar E, et al. The European guideline on management of major bleeding and coagulopathy following trauma: fourth edition. Crit Care. 2016;20(1):100.
6. Hagemo JS, Stanworth S, Juffermans NP, Brohi K, Cohen MJ, Johansson PI, et al. Prevalence, predictors and outcome of hypofibrinogenaemia in trauma: A multicentre observational study. Crit Care. 2014;18(2).
7. Chang R, Cardenas JC, Wade CE, Holcomb JB. Advances in the understanding of trauma-induced coagulopathy. Blood. 2016;128(8):1043–9.
8. Brohi K, Singh J, Heron M, Coats T. Acute Traumatic Coagulopathy. J Trauma. 2003;54:1127–271 1130.

9. The study was funded by an internal grant of Krajska zdravotni, a. s. Participation of the University Hospital Pilsen was supported by the Q39 (PROGRES) Charles University research project. The funding bodies had no role in the design of the research, collection, analysis or interpretation of data and in writing the manuscript.

### 000272

#### Septic shock as major predictor for mortality in critically ill patients admitted due to burn injuries in Catalonia

C. Vizcaino Urresta[1], M. S.[2], A. Rey-Pérez[2], J. Baena[2], L. Pérez[2], L. Lagunes[3], M. Baguena[2], J. Barret[4]
[1]Critical care, Vall d'Hebron University Hospital, Barcelona, Spain; [2]Trauma and burns critical care, Vall D Hebron, Barcelona, Spain; [3]Critical care, Vall D Hebron, Barcelona, Spain; [4]Plastic and reconstructive surgery and burn unit, Vall D Hebron, Barcelona, Spain
**Correspondence:** C. Vizcaino Urresta
*Intensive Care Medicine Experimental* 2019, **7(Suppl 3):**000272

**INTRODUCTION.** Burn injury is a complex process with high mortality with a high economic burden and social implications. Specific risk factors for mortality has been outlined in recent reports, including the total body surface area (TBSA) of burns, the TBSA of full-thickness burns, age, and presence of inhalation injury. However information on our environment is scarce.

**OBJECTIVES.** To assess mortality risk factors and to update the epidemiological distribution of patients with major burns treated in Catalonia.

**METHODS.** Retrospective monocentric cohort study of patients admitted to Burns ICU (BICU) at Vall d'Hebron university Hospital in Barcelona between February 2009 and January 2017 were included. Demographic data, presence of inhalation injury defined by the presence of facial burns, singed nasal vibrissae, soot in the mouth or sputum, history of closed space injury and patient`s history of breathed smoke. Infection related variables and resuscitation related variables as hemodilution were recorded, Multivariate stepwise logistic regression was performed to identify factors associated with mortality.

**RESULTS.** 276 patients were admitted in the BICU during the study period. All population mortality was around 18% (51) patients. Septic shock was identified as number one predictive factor of mortality in this cohort (OR 46.7; IC95% 6-363.8; p=0.001), followed by age (OR 1.12; IC95% 1.07-1.17; p=0.001) and TBSA (OR 1.07; IC95% 1.04-1.12; p=0.001). A total liquid balance near to 0 measure by hemodilution on deltaHb at first 48h and an initial management in the ICU with a shorter length of stay in this unit were identified as protective factors for mortality.

**CONCLUSION.** Septic shock was identified as major risk factor for mortality, other non modifiable factors such as age and higher TBSA

were identified. Further studies are necessary to early detect sepsis in this group of patients. Neutral liquid balance in the first 48h was associated with a better survival.

**REFERENCE(S)**

1. Brusselaers N, Monstrey S, Vogelaers D, Hoste E, Blot S. Severe burn injury in Europe: a systematic review of the incidence, etiology, morbidity, and mortality. Crit Care 2010;14:R188.
2. Jeschke MG, Patsouris D, Stanojcic M, et al. The patho- physiological response to burns in elderly. EBioMedicine 2015;2:1536–1548.
3. Greenhalgh DG, Saffle JR, Holmes JH, et al. American burn association consensus conference to define sepsis and infection in burns. J Burn Care Res. 2007;28: 776e790.

### 000280

#### S-100 B levels elevations in polytrauma patients – a multivariate analysis

M. Martin[1], J. Gerber[2], W. Hautz[3], JC. Schefold[2], A. Exdaktylos[3], C. Pfortmueller[4]
[1]Institute of health economics and clinical epidemiology, University Hospital of Cologne, Cologne, Bern, Switzerland; [2]Department of intensive care, Inselspital, Bern University Hospital and University of Bern, Bern, Switzerland; [3]Department of emergency medicine, Inselspital, Bern University Hospital and University of Bern, Bern, Switzerland; [4]Department of Intensive Care, Inselspital, Bern University Hospital and University of Bern, Bern, Switzerland
**Correspondence:** C. Pfortmueller

*Intensive Care Medicine Experimental* 2019, **7(Suppl 3):**000280

**INTRODUCTION.** S-100 B Protein has been identified as a biomarker for traumatic brain injury, but studies suggest that extracranial injuries lead to elevated S-100 B levels as well. Hence, the impact of different concomitant injuries on S-100 B levels in trauma and its role in multiple trauma patients remain unclear.
**OBJECTIVES.** . In this study, we therefore aimed to quantify the impact of concomitant injuries on S-100 B levels in trauma patients and further investigate the role of S100-B levels to predict mortality and injury severity.
**METHODS.** All patients with suspected multiple trauma treated at a Level 1 Trauma centre in Switzerland over a three-year period were included in this retrospective cohort analysis. The extent of injuries and their severity, reflected by the abbreviated injury scale (AIS) and injury severity score (ISS), was assessed and S-100 B levels on admission were obtained. Potential predictors of pathological S-100 B levels (>2.0 µg/L) were identified through uni- and multivariable analysis.
**RESULTS.** In total, 1,338 patients with suspected multiple trauma, 76.5% of them with pathological S-100 B levels, were included in the analysis. In multivariable logistic regression adjusting for sociodemographic characteristics, concomitant injuries and trauma severity, the following predictors showed a significant association (p<0.001) with pathological S-100 B levels: Longbone fracture (OR 3.0, 95% CI: 2.2-4.3, p<0.001), non-longbone fracture (OR 2.3, 95% CI: 1.3-4.1, p= 0.004), thoracic injury (OR 2.6, 95% CI 1.6-4.2, p<0.001), flesh wounds (OR 1.9, 1.4-2.6, p<0.001). Head trauma with intracerebral bleeding was only weakly associated (OR 2.0 (1.19 - 3.45) p< 0.010) and head trauma without intracranial bleeding was not associated with increased S-100 B protein levels at all (p< 0.706). Trauma severity was also related to S-100 B protein increase (OR per ISS: 1.1, 95% CI 1.0-1.1, p<0.001). In-hospital death was strongly associated with pathological S-100 B levels (OR 24.8, 95% CI: 3.4-179.4, p<0.001).S-100 B levels below 0.575 µg/L had a high diagnostical value to rule out in-hospital mortality (negative predictive value: 0.99, 95% CI: 0.98-1.00).
**CONCLUSION.** Fractures and thoracic injuries are the main predictors for elevated S-100 B levels. Despite common perception, head injuries play a minor role in S100-protein elevation in multiple trauma patients S-100 B has a good negative predictive value for in-hospital mortality and is associated with trauma severity and hence might be of use as a prognostic marker in trauma patients.

### 000286

#### Is Haloperidol Safe in the Presence of Other QT Prolonging Drugs in the Intensive Care Unit?

J. McLuckie, K. Puxty, L. Jarvie, M. Shaw
[1]Intensive care unit, Glasgow Royal Infirmary, Glasgow, UK
**Correspondence:** J. McLuckie

*Intensive Care Medicine Experimental* 2019, **7(Suppl 3):**000286

**INTRODUCTION.** ICU delirium is a neuropsychiatric disorder, characterised by an acute fluctuation in consciousness, which affects between 30-50% of the critically ill [1]. Haloperidol is the most commonly used pharmacological agent in the management [2]. However, the European Medicines Agency recently suggested that Haloperidol is now contraindicated for utilisation in combination with other QT prolonging drugs due to the risk of ventricular tachyarrhythmia [3].
**OBJECTIVES.** However, before practice changes to another potentially harmful alternative, it is essential to understand the influence of Haloperidol in combination with other QT prolonging drugs on VT in comparison to Haloperidol alone. It is also necessary to correct for the influence of other risk factors including age, gender, electrolyte disturbances such as mild hypokalaemia, severe hypokalaemia, hypomagnesaemia or hypocalcaemia and a past medical history of Ischaemic Heart Disease or previous arrhythmia on VT. To ascertain if episodes of VT are clinically significant, it is important to determine the odds of VT requiring intervention in relation to each factor.
**METHODS.** A case control study involving 4,189 admissions was performed. Electronic records for each were interrogated to provide information pertaining to the aforementioned factors and receipt use of Haloperidol or other QT prolonging drugs. It was also documented as to whether the patient experienced VT during their stay. Multivariate logistic regression analysis calculated odds ratios to ascertain factors associated with VT and to determine if these episodes were clinically significant, odds ratios were calculated for VT requiring treatment.
**RESULTS.** Our results identified that 2.1% of patients in ICU developed VT and that hypocalcaemia, IHD, previous arrhythmia and the administration of QT prolonging drugs were all associated with VT. The effect was strongest for Haloperidol administration without another QT prolonging drug (OR 12.309, 95% CI 3.396 – 44.618). The administration of a QT prolonging agent in combination with Haloperidol did not further increase this (OR 8.599, 95% CI 3.175 – 23.291). Intervention was required for 48.3% of patients that developed VT and included electrolyte replacement, antiarrhythmic drugs and DCCV. The administration of any QT prolonging drug resulted in the greatest odds of VT requiring treatment (OR 4.373, 95% CI: 1.337 – 14.299).
**CONCLUSION.** Despite concerns regarding its safety, Haloperidol should remain the first line treatment option in delirium since the chance of developing VT remains low, even though the odds are significantly increased. Additional QT prolonging drugs do not increase this further and this combination in patients whom it is clinically appropriate appears to be safe. However, alternatives including atypical antipsychotics should be considered in patients with concomitant risk factors such as IHD and previous arrhythmias.

**REFERENCE(S)**

1. Yoon H, Park K, Choi W et al. Efficacy and safety of haloperidol versus atypical antipsychotic medications in the treatment of delirium. BMC Psychiatry. 2013;13(1).
2. Meagher D. Regular review: Delirium: Optimising management. BMJ. 2001;322(7279):144-149
3. [Internet]. Ema.europa.eu. 2017 [cited 8 February 2019]. Available from: https://www.ema.europa.eu/documents/referral/haldol-decanoate-article-30-assessment-report_en.pdf

### 000334

#### Effects of hypothermia on microcirculation in conditions of hemodynamic stability and hemorrhagic shock

JF. Caminos Eguillor, G. Ferrara, VS. Kanoore Edul, MG. Buscetti, HS. Canales, B. Lattanzio, L. Gatti, FJ. Gutierrez, A. Dubin
[1]Cátedra de farmacología aplicada, Universidad Nacional de La Plata, Facultad de Ciencias Médicas, La Plata, Argentina
**Correspondence:** A. Dubin
*Intensive Care Medicine Experimental* 2019, **7(Suppl 3):**000334

**INTRODUCTION.** The presence of hypothermia is an independent predictor of outcome in traumatic shock. Nevertheless, hypothermia may be an effective therapeutic approach for otherwise lethal traumatic hemorrhage.

**OBJECTIVES.** To characterize the effects of hypothermia on microcirculation in normal conditions and in hemorrhagic shock.

**METHODS.** We studied 24 anesthetized and mechanically ventilated sheep. We measured cardiac output (CO, mL/min/kg) by thermodilution, renal (RBF, mL/min/100g) and intestinal (IBF, mL/min/100g) blood flow by ultrasonic probes, and systemic $O_2$ consumption ($VO_2$, mL/min/kg) by a metabolic cart. Renal ($rVO_2$, mL/min/100g) and intestinal ($rVO_2$, mL/min/100g) $O_2$ consumption were calculated from RBF and IBF and the respective arteriovenous $O_2$ content difference. Cortical renal, intestinal villi and sublingual microcirculation were assessed by IDF-videomicroscopy. After basal measurement, sheep were assigned to normothermia (N, n=12) and hypothermia (H, n=12) groups. In the former, central temperature (Temp, °C) was maintained at basal values and in the latter, it was reduced to ~34°C. Measurements were repeated after 1 h of hemodynamic stable conditions and 1 h of hemorrhagic shock.

**RESULTS.** During hemodynamic stability, hypothermia decreased CO, RBF, and systemic and regional $VO_2$. Renal, villi and sublingual red blood cell velocity (RBCV, µm/s) decreased while perfused vascular density (PVD, mm/mm²) remained unchanged. In normothermia group, all these variables were unchanged.

During hemorrhagic shock, systemic and regional flow and $VO_2$, and RBCV and PVD were similarly reduced in normo- and hypothermia groups. The microcirculatory derangements were higher in the renal cortex than in the villi and the sublingual mucosa: PVD was reduced to 25±28, 67±29, 82±21% from basal values, in renal, villi and sublingual microcirculation; and RBCV to 37±28, 56±15, and 54±9% ($P<0.001$ for both, renal vs. villi and sublingual microcirculation).

**CONCLUSION.** 1) In stable hemodynamic conditions, hypothermia decreased peritubular, villi and sublingual RBCV but PVD was unaffected. 2) In hemorrhagic shock, hypothermia showed neither beneficial nor detrimental effects on microcirculation. 3) Renal microcirculation was more sensitive than villi and sublingual microcirculation to hemorrhagic shock.

**Table 1 (abstract 000334).** Behavior of hemodynamic, metabolic and microcirculatory variables

| Period | Group | Temp | CO | RBF | IBF | $VO_2$ | $rVO_2$ | $iVO_2$ | RBCV | PVD |
|---|---|---|---|---|---|---|---|---|---|---|
| Basal | H | 38.7± 0.6 | 104± 32 | 309± 75 | 63± 45 | 6.4± 1.6 | 7.0± 3.2 | 2.5± 1.1 | 1094± 101 | 19.0± 1.41 |
| | N | 39.1± 0.7 | 108± 14 | 324± 117 | 52± 24 | 6.8± 1.5 | 6.6± 2.5 | 2.2± 0.9 | 1159± 94 | 18.4± 2.7 |
| Stability | H | 33.7± 0.5*§ | 88± 19*§ | 199± 84*§ | 58± 25 | 4.6± 1.3*§ | 4.3± 1.7*§ | 1.8± 0.9* | 836± 195*§ | 16.1± 4.1* |
| | N | 38.9± 0.7 | 104± 19 | 306± 121 | 50± 23 | 6.3± 1.3 | 7.3± 2.7 | 2.2± 0.9 | 1066± 162 | 17.8± 3.4 |
| Shock | H | 33.8± 0.4*§ | 34± 10* | 44± 32* | 24± 9* | 3.2± 0.7* | 2.6± 1.2* | 1.6± 0.6* | 403± 298* | 5.6± 5.7 |
| | N | 39.0± 1.1 | 45± 10* | 50± 34* | 23± 14* | 3.9± 1.1* | 2.5± 1.2* | 1.6± 0.9* | 420± 337* | 4.3± 5.6* |

*$P<0.05$ vs. baseline. §$P<0.05$ vs. normothermia group

### 000374

#### Study of the clinical profile of patients admitted to UCI due to acute intoxication

MP. Benitez Moreno[1], MC. Martínez González[1], A. Barroso González[2]
[1]Intensive care, HOSPITAL CARLOS HAYA, MALAGA, Spain; [2]Anesthesia, HOSPITAL CARLOS HAYA, MALAGA, Spain
**Correspondence:** M.C. Martínez González
*Intensive Care Medicine Experimental* 2019, **7(Suppl 3):**000374

**INTRODUCTION.** Patients suffering from acute intoxication, whether voluntarily for autolytic or accidental purposes, often require life support in intensive care units.

**METHODS.** Retrospective observational study of all patients admitted for acute intoxication who required admission to the ICU of the Regional Hospital of Malaga between January 2012 and August 2016, older than 14 years with admission to the ICU for intoxication of any kind. We study patient characteristics in terms of age, sex and medical history, type of toxicity, severity and evolution in our unit.

**RESULTS.** We found 70 cases of patients who required admission to the ICU due to acute intoxication, of which 55.6% were women. The average age was 47.36 (standard deviation 18.22). The average stay in ICU was 5.04 (standard deviation 8.09). 54.2% of patients had a psychiatric history. As other background highlights, 19.4% were addicted to illegal drugs and 25% were hipertensive. Most patients took more than one toxic 83.3% and intoxication was voluntary in 84.7% versus accidental in 12.5% of cases. The toxic was known in 68%. The most used benzodiazepines in 26.4% of the total. The main cause of admission to the ICU was due to neurological deterioration in 49 of the cases registered and mechanical ventilation was necessary in 44 patients. The maximum time in mechanical ventilation was 34 days. The infection occurred in 24.3%, with the majority being respiratory infection. The 4.7% died in ICU. The hospital stay presented an average of 9.3 days.

**CONCLUSION.** The profile of patient admitted to the ICU due to acute intoxication is a woman of middle age and psychiatric history, with voluntary intoxication of several toxic substances and requiring mechanical ventilation for a low level of consciousness for an average of 3 days. The survival is very high it would be necesary to analyze the possible relapses of these patients

### 000376

#### Acute drug intoxication in adult patients admitted to ICU: Analysis of the complications and prognostic value

MC. Martínez González[1], MP. Benitez Moreno[1], S. Jimenez Jimenez[1], A. Barroso González[2]
[1]INTENSIVE CARE, HOSPITAL CARLOS HAYA, MALAGA, Spain; [2]Anesthesia, HOSPITAL CARLOS HAYA, MALAGA, Spain
**Correspondence:** M.C. Martínez González
*Intensive Care Medicine Experimental* 2019, **7(Suppl 3):**000376

**INTRODUCTION.** Only some of the cases of exposure to toxic substances entails a serious poisoning that requires admission to the ICU. The initial instability in need of supportive treatment, alteration of the internal environment, or the appearance of sequelae differs widely depending on the degree, etiology and characteristics of the intoxication.

**OBJECTIVES.** To analyze the possible complications of the poisoning admitted in the ICU, as well as make a prognostic assessment of cases through the use of some analytical markers such as pH, lactate levels and the clearance of this one (obtained by the difference between the level of the first and the second day)

**METHODS.** We performed a retrospective observational study of all patients admitted for acute intoxication that required admission to the intensive care unit of the Regional Hospital of Malaga between January 2012 and August 2016. The inclusion criteria were age above14 years admitted to the ICU due to poisoning of any kind. We used the t-test or chi 2 for the univariate analysis according to need with an alpha error of 5%.

**RESULTS.** We recorded 70 patients, in which a 68% the poison was known, being 83.3% of the total caused by multiple toxics. The 21.7% of the patients underwent early gastric lavage or activated charcoal was administered, not finding significant differences with the group that did not receive treatment when compared to alterations in the internal environment (pH, lactate in the blood). The mortality of patients admitted for poisoning was 4.7%. There was a tendency, although not statistically significant between patients with worse pH and less of lactate clearance in the first 6 hours with mortality (p=0.09). No significant relationship between the patients who have neurological sequelae at discharge with those blood levels studied.

A 7.1% of the patients required dialysis during the stay in the ICU. There is a association of this fact with the finding of an average value of lactate in the early hours (p=0.038).

**CONCLUSION.** The mortality of the intoxicated patient is not negligible in our environment. The profile is of a middle-aged male patient, with voluntary intoxication with several medications that requires VMI or dialysis in a variable percentage. The highest levels of lactate to income or in the early hours were associated with greater need of CRRT. Perhaps the concordance of these values with other complications can be demonstrated continuing the study and increasing the sample.

### 000432

#### Low Meropenem Concentration in Brain-Dead Organ Donors: A Single-Center Pharmacokinetic Study and Simulation

JM. Lee
[1]Acute care surgery, Korea University Anam Hospital, Seoul, Republic of Korea
**Correspondence:** J.M. Lee
*Intensive Care Medicine Experimental* 2019, **7(Suppl 3):**000432

**INTRODUCTION.** Meropenem is an ultrabroad-spectrum antibiotic of carbapenem family. In brain-dead organ donors, administration of standard meropenem dosages does not reach therapeutic levels.

**OBJECTIVES.** Our objectives were to determine the plasma concentration of meropenem after administration of standard meropenem dose and to estimate an improved dosage regimen for these patients.

**METHODS.** One gram of meropenem was administered as 1-h infusion every 8 h for 1–3 days, and blood samples were collected. The plasma concentration of meropenem was measured and subjected to pharmacokinetic analysis. Simcyp simulation was performed to predict the optimum plasma levels and dosage based on the patients' individual pharmacokinetic parameters.

**RESULTS.** The maximum plasma concentration of meropenem was 3.29 μg/ml, which was lower than four times the minimum inhibitory concentration of 8 μg/ml. Although mean creatinine clearance of patients was moderately low (67.5 ml/min), apparent volume of distribution at steady state (*V*ss) and time-averaged total body clearance (CL) of meropenem were markedly elevated (4.97 l/kg and 2.06 l/h/kg, respectively) owing to massive fluid loading to decrease the high sodium levels and to treat shock or dehydration. The simulation revealed that dose and infusion time of meropenem should be increased based on patients' *V*ss and CL and loading dose is recommended to reach rapidly the target concentration.

**CONCLUSION.** In conclusion, standard meropenem regimen is insufficient to achieve optimal drug levels in brain-dead patients and increase in dose and extended or continuous infusion with intravenous bolus administration of loading dose are recommended for these patients.

### 000458

#### Point-of-care 24/7 "load & go" flow cytometry to assess the innate immune responses in trauma patients: the start of a new era

R. Spijkerman[1], L. Hesselink[1], F. Hietbrink[1], L. Koenderman[2], L. Leenen[1]
[1]Traumasurgery, University Medical Center Utrecht, Utrecht, Netherlands; [2]Laboratory of translational immunology, University Medical Center Utrecht, Utrecht, Netherlands
**Correspondence:** R. Spijkerman
*Intensive Care Medicine Experimental* 2019, **7(Suppl 3):**000458

**INTRODUCTION.** Trauma patients are at risk for severe infections after trauma. The risk of developing these infections is associated with the severity of tissue damage and the amplitude of the following immune response. Neutrophils act as important effector cells in the immune response to trauma. Previous studies showed that shifts in neutrophil phenotypes directly after trauma are a promising predictor for inflammatory complications. Unfortunately, technical and logistical difficulties preclude application of such a test in the clinical setting. However, recently a fully automated point-of-care 24/7 "load & go" flow cytometry system became available, that can possibly overcome these challenges.

**OBJECTIVES.** The aim of this study was to assess the feasibility of implementing a point-of-care 24/7 "load & go" flow cytometry test in the shock room.

**METHODS.** A prospective mono-center cohort study was performed in our level one trauma center from November 2018 until February 2019. All trauma patients >18y initially presented at the shock room were included by the trauma team. An extra tube of blood was obtained during standard diagnostic workup and was placed in the 24/7 available "load & go" flowcytometer, AQUIOS CL©, co-located in the shock room by the trauma team. The markers CD35, CD16, CD62L, CD11b, active CD11b, CD10, CD66b and CD11c were immediately measured with and without the bacterial stimulus fMLF. All patient characteristics and follow-up data were collected from the electronic medical record.

**RESULTS.** A total of 235 patients were presented in the shock room during the inclusion period, of whom 166 patients were eligible for inclusion. Only in 3 patients the trauma team failed to start the analysis. This resulted in a total of 163 successfully placed tubes in the machine. Technical difficulties resulted in the exclusion of 4 samples, resulting in a total of 159 successfully analyzed patients. A total of 38 patients were marked as multi trauma (ISS ≥16) patients. Patients without any significant tissue damage had one clear population of mature (CD16high/CD62Lhigh) neutrophils. Multi trauma patients with significant tissue damage always developed two new neutrophil subsets, CD16dim/CD62Lhigh and CD16high/CD62Ldim neutrophils. The amount of CD16dim neutrophils (5-50%) was related to the extent of tissue damage after trauma. All trauma patients that are admitted to the ICU showed CD16dim cells, however the amount of CD16dim cell differed between patients. Moreover some patients showed about 50% progenitor cells and all of them died <48h after admission.

**CONCLUSION.** Implementation of routine neutrophil analysis by point-of-care 24/7 "load & go" flow cytometry in the shock room is applicable. The percentage of CD16dim neutrophils is associated with the amount of tissue damage that can result in immune dysregulation and immune related complications after trauma. This quick analysis of CD16dim neutrophils as read out for tissue damage might proof a valuable tool to predict immune related complications in the ICU. A large prognostic study should be deployed.

### 000522

#### Persistent inflammation, immunosuppression and catabolism syndrome after polytrauma: a rare syndrome with major consequences

L. Hesselink[1], R. Hoepelman[1], R. Spijkerman[1], A. Huisman[2], M. Ten Berg[2], K. Van Wessem[1], L. Koenderman[3], L. Leenen[1], F. Hietbrink[1]
[1]Trauma surgery, University Medical Center Utrecht, Utrecht, Netherlands; [2]Laboratory of clinical chemistry and hematology, University Medical Center Utrecht, Utrecht, Netherlands; [3]Laboratory of translational immunology, University Medical Center Utrecht, Utrecht, Netherlands
*Intensive Care Medicine Experimental* 2019, **7(Suppl 3):**000522

Correspondence: L. Hesselink
*Intensive Care Medicine Experimental* 2019, **7(Suppl 3):**000522

**INTRODUCTION.** More and more severely injured patients survive the critical first phase after trauma. A substantial portion of these patients require long-term critical care support and suffer from recurrent infections. This clinical condition fits in a syndrome also referred to as "Persistent inflammation, immunosuppression and catabolism syndrome" (PICS).

**OBJECTIVES.** The aim of this study was to investigate the incidence of PICS and clinical outcomes of these trauma patients with PICS in a level one trauma center.

**METHODS.** All trauma patients ≥ 16 years who were admitted to the intensive care unit (ICU) for ≥ 14 days between 2007 and 2017, were included. Patients with isolated neurological injuries were excluded. PICS patients were identified by ICU stay ≥ 14 days, ≥ 3 infectious complications and increased catabolism. Infectious complications included infections during hospitalization and readmissions due to an infection. Increased catabolism was defined as weight loss >10%, a body mass index <18 or persistent albumin levels <30g/L. Long term clinical outcomes of PICS patients were analyzed.

**RESULTS.** Of the 3859 polytrauma patients, only 194 patients had an ICU stay ≥ 14 days. After exclusion of patients with isolated neurological injuries, 78 patients were included. Of these patients, 18 developed PICS. PICS patients sustained 5 infectious complications on average (compared to 1 in the non-PICS group, p<0.001) and 72.2% of the PICS patients developed sepsis. There were no significant differences in Injury Severity Score and in-hospital mortality between PICS patients and the other patients with an ICU stay ≥ 14 days. However, these PICS patients had a longer hospital stay (mean of 90 days versus 50 days, p<0.001) and sustained more surgical procedures (mean of 13 versus 4 per patient, p<0.001). Infectious readmissions occurred until 5 years after the initial trauma.

**CONCLUSION.** Patients who develop PICS experience long-term inflammatory complications that lead to frequent readmissions and surgical procedures. Therefore, this clinical condition forms a burden on patients and a substantial financial burden on society, despite its low incidence.

**REFERENCE(S)**

1. Mira JC, Brakenridge SC, Moldawer LL, Moore A. Persistent inflammation, immunosuppression and Catabolism syndrome (PICS). Crit Care Clin. 2017;33(2):245–58.
2. Gentile LF, Cuenca AG, Vanzant EL, Efron P a, Mckinley B, Moore F, et al. Persistent inflammation and immunosuppression: A common sydrome and new horizon for surgical intensive care. j Trauma Acute Care Surg. 2012;72(6):1491–501.

### 000524

#### Neutrophil acidification in critically ill trauma patients detected by point of care flow cytometry

L. Hesselink[1], S. Bongers[2], R. Spijkerman[2], L. Koenderman[3], L. Leenen[2], F. Hietbrink[2]
[1]Traumasurgery, University Medical Center Utrecht, Utrecht, Netherlands; [2]Trauma surgery, University Medical Center Utrecht, Utrecht, Netherlands; [3]Laboratory of translational immunology, University Medical Center Utrecht, Utrecht, Netherlands
**Correspondence:** L. Hesselink
*Intensive Care Medicine Experimental* 2019, **7(Suppl 3):**000524

**INTRODUCTION.** Patients often develop life-threatening infections after severe trauma. Neutrophils are the main immune cells to combat these infections by phagocytosis and killing of pathogens. However, severe injuries can induce malfunction of neutrophil killing. Analysis of this dysfunction is laborious and can therefore not be applied in routine diagnostics. Neutrophil phagosomal acidification, however, one of the last steps in the killing process, can nowadays be measured within an hour. Hence, we developed a quick and automated method to assess phagosomal acidification.

**OBJECTIVES.** The aim of this study was to investigate the relation between neutrophil phagosomal acidification and infectious complications in critically ill trauma patients.

**METHODS.** Multitrauma patients aged 18-80 years with an expected stay in the intensive care unit of ≥ 2 days, were prospectively included. Blood samples were obtained <12 hours after trauma, after 3 days, 6 days, 10 days and 15 days. Phagosomal acidification was analyzed using the fully automated "Load & Go" AQUIOS CL© flow cytometer after incubation of whole blood with double labeled (promofluor[PF]/pH insensitive and pHrodo/pH sensitive) Staphylococcus Aureus bioparticles for 60 minutes. Data are presented as mean ratio pHrodo/PF after 60 minutes ± standard error (SE).

**RESULTS.** Ten patients were included. Of these patients, 5 developed an infection between day 6 and 10 after trauma. Neutrophil phagosomal acidification remained stable and within reference values (0.83 – 1.05) in patients who did not develop an infection. In patients with infectious complications however, phagosomal acidificiation was slightly elevated on day 0 and day 3 (1.10±0.11 and 1.16±0.12 respectively), after which phagosomal acification decreased to below reference values (day 10: 0.78±0.11 and day 14: 0.74±0.07).

**CONCLUSION.** Flow cytometry analysis of neutrophil phagosomal acidification is a fast and clinical applicable method to assess neutrophil function. This method can be used to detect changes in neutrophil function in critically ill trauma patients and could therefore potentially be a useful method to identify patients at risk for infectious complications.

## ARF - Acute respiratory failure 9

### 000385

#### New Targets for Veno-Venous Extracorporeal Membrane Oxygenation Support in Acute Respiratory Distress Syndrome: Insights from the Inflammatory/Coagulation miRNAs patterns

G. Martucci[1], V. Miceli[2], G. Occhipinti[1], A. Papeo[1], G. Panarello[1], C. Spina[3], C. Carcione[4], V. Agnese[5], F. Tuzzolino[6], PG. Conaldi[5], A. Arcadipane[1]
[1]Department of anesthesia and intensive care, IRCCS-ISMETT, Palermo, Italy; [2]Department of laboratory medicine and advanced biotechnologies, IRCCS-ISMETT, Palermo, Italy; [3]School of anesthesia and intensive care, University of Milano-Bicocca, Milano, Italy; [4]Laboratory medicine and advanced biotechnologies, Ri.MED Foundation, Palermo, Italy; [5]Department of research, IRCCS-ISMETT, Palermo, Italy; [6]Research office, IRCCS-ISMETT, Palermo, Italy
**Correspondence:** G. Martucci
*Intensive Care Medicine Experimental* 2019, **7(Suppl 3):**000385

**INTRODUCTION.** Implementing the biological knowledge on the pathophysiology of ARDS and on different coagulative and inflammatory pathways activated by ECMO may improve patient's survival.

**OBJECTIVES.** Explore miRNAs expression changes in ARDS patients during ECMO treatment, as predictive markers of survival and monitoring of the disease.

**METHODS.** 754 human miRNAs were screened in whole blood before cannulation and on day 7 and 14 of ECMO support in ARDS patients weaned from ECMO. The miRNAs were analyzed by PCR using TaqMan™ Array Human MicroRNA A+B Cards Set v3.0 (Applied Biosystems).

**RESULTS.** From September 2018 to March 2019, 9 patients were supported with VV-ECMO for ARDS and n=7 survived (77.8%): age 53 (44-57) years, male gender n=6 (86%), BMI 26.5 (24.7-27.8), SAPS II at admission 37 (34.5-43.5), PaO2/FiO2 preECMO 60.5 (56.3-64.8), Pre-serve score 3.5 (2.3-5), Respscore 3 (-2.5-4.7), duration of ECMO support 19 days (9-31).

After quality control screening (amplification score > 1.1, Cq confidence > 0.8, high expression (Ct < 33), and detectable expression in more than half of the samples), 318 miRNAs were selected. Volcano plot analysis (p < 0,05 and fold change > 2) identified 12 miRNAs deregulated on day 7 and 27 miRNAs on day 14. In particular, we identified 6 upregulated miRNAs (figure 1A) and 6 downregulated miRNAs (figure 1B) on day 7, whereas 27 upregulated miRNAs on day 14

(figure 1C). Hierarchical clustering analysis showed systematic variations in the miRNAs expression among the different groups.

To correlate deregulated miRNAs and ARDS, we considered, through DIANA-miRPath v3.0, representative pathways for ARDS in which deregulated miRNAs are involved, reducing to 7 the miRNA deregulated on day 7, and to 20 the miRNA on day 14. The clustering of this new list of miRNAs leads to a complete cluster separation after 7 days of treatment, and an almost complete separation after 14 days of treatment.

These miRNAs are correlated to the pathways tissue remodeling cellular stress and apoptosis, regulation of immune system and regulation of coagulation process.

**CONCLUSION.** Up- and down-regulated miRNAs, tend to cluster in ARDS and ECMO support. MiRNAs from the whole blood are promising biomolecular markers to monitor ARDS evolution and the impact of ECMO on inflammation/coagulation system.

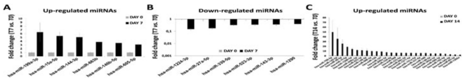

**Fig. 1 (abstract 000385).** See text for description

### 000388

#### Prone positioning during veno-venous ECMO support for severe ARDS: a multicenter retrospective trial

M. Giani[1], F. Fossi[1], G. Grasselli[2], E. Garofalo[3], G. Martucci[4], M. Belliato[5], V. Fanelli[6], U. Simonetti[6], N. Peroni[7], A. Lucchini[1]; P. Navalesi[3], A. Arcadipane[4], GA. Iotti[7], A. Pesenti[2], G. Foti[1]
[1]Department of emergency and intensive care, ASST Monza, University Of Milano-Bicocca, Monza, Italy; [2]Department of anesthesia, critical care, and emergency, Fondazione IRCCS Ca' Granda Ospedale Maggiore Policlinico, Università di Milano, Milano, Italy; [3]Department of medical and surgical sciences, University Hospital Mater Domini, Magna Grecia University, Catanzaro, Italy; [4]Anesthesia and intensive care unit, IRCCS-ISMETT, Palermo, Italy; [5]U.o.c. anestesia e rianimazione 1, Fondazione I.R.C.C.S. Policlinico San Matteo, Pavia, Italy; [6]Surgical sciences department, Città della Salute e della Scienza, University of Torino, Turin, Italy; [7]Department of anesthesia and intensive care, Fondazione IRCCS Policlinico S Matteo, Università degli studi di Pavia, Pavia, Italy
**Correspondence:** M. Giani
*Intensive Care Medicine Experimental* 2019, **7(Suppl 3):**000388

**INTRODUCTION.** Prone positioning in severe ARDS patients improves oxygenation, reduces ventilator-induced lung injury and is associated with mortality reduction[1]. A strong physiologic rationale supports prone positioning (PP) in ARDS patients receiving ECMO therapy. However, the fear of complications historically limited its use during ECMO. To date, despite an increase in utilization of this procedure in ECMO patients[1], only few monocentric retrospective studies have been published on this topic.

**OBJECTIVES.** We conducted a multicentric retrospective study to evaluate safety, efficacy of PP during ECMO support. Secondly, we aim to compare outcomes between patients who underwent PP during ECMO (PP-ECMO group) with a matched population of patients treated with extracorporeal support (supine-ECMO group).

**METHODS.** A multicenter, retrospective trial including patients admitted to 6 ECMO referral centers between 2014 and 2018 and treated with veno-venous ECMO for respiratory failure: patients in the PP-ECMO group are enrolled in 4 ECMO centers where PP is a standard procedure during extracorporeal support, whereas patients in the supine-ECMO group are enrolled in 2 ECMO centers where PP is not performed during ECMO. In the PP-ECMO group, respiratory and hemodynamic parameters were recorded at four different steps: 1 - Supine; 2 - Start PP; 3 - End PP; 4 - Return to supine position We plan to include about 130 patients in the PP-ECMO group and 100 patients in the supine-ECMO group. Outcomes (survival, icu LOS, duration of ECMO support) will be compared between groups after individual and propensity-score matching.

**RESULTS.** We report preliminary data from 2 of 6 centers. During the study period 37 patient were included in the PP-ECMO group and 69 patients in the supine-ECMO group. In the PP-ECMO group the number of PP cycles was 3 [2-4] per patient and the duration was 12 [8-17] hours per cycle.

No major complication related to the procedure (extubation, cannula displacement, uncontrolled bleeding) was recorded.

Prone positioning was associated with an improvement of oxygenation and respiratory mechanics (see Figure 1 below). Lung Injury Score, SOFA and SAPS II did not differ between PP-ECMO and supine-ECMO group. ICU mortality did not differ (35% and 41% respectively, p=0.58).

**CONCLUSION.** Prone positioning during ECMO was safe and effective. The impact of this procedure on outcome during ECMO has still to be assessed.

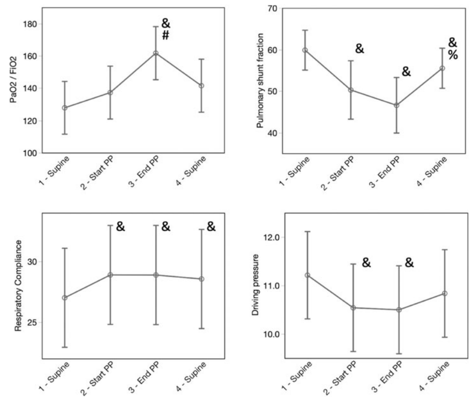

**Fig. 1 (abstract 000388).** See text for description

### 000391

#### Diaphragmatic atrophy is less when patients are ventilated using SIMV+PS than with ACMV

RN. Thiriveedhi[1], LN. Yaddanapudi[2], V. saini[3]
[1]Anaesthesia and critical care, Post Graduate Institute of Medical Education & Research, Chandigarh, Chandigarh, India; [2]Anaesthesia and critical care, Post Graduate Institute of Medical Education & Research, Chandigarh, USA; [3]Anaesthesia and critical care, post graduate institute of medical education and research, Chandigarh, India
**Correspondence:** R.N. Thiriveedhi
*Intensive Care Medicine Experimental* 2019, **7(Suppl 3):**000391

**INTRODUCTION.** Diaphragmatic atrophy starts within 12-18 hours after initiation of mechanical ventilation[1] and reaches a peak by 69-72 hours[2]. Both the duration and mode of ventilation are significant factors in determining the magnitude of atrophy[3, 4] Assist Control Mechanical Ventilation (ACMV) and Synchronized Intermittent Mandatory Ventilation with Pressure Support (SIMV+PS) are commonly used in ICUs. In ACMV the ventilator delivers the set tidal volume in both mandatory and spontaneous breaths. In SIMV+PS the inspiratory pressure support is tailored to the patient's effort and the mechanical properties of the lungs.

**OBJECTIVES.** We compared the decrease in diaphragmatic thickness (DT) measured ultrasonically between patients on ACMV and SIMV+PS.

**METHODS.** Ethical clearance and informed consent from the next of kin were obtained. 42 patients were randomly allocated to two equal groups (Group A: ACMV, Group B: SIMV+PS) within 6 hours of ICU admission. Tidal volume was set to 6-7 ml/kg. PS level was adjusted to deliver ~80% of the set tidal volume. DT was measured using a straight probe at the zone of transition on the mid-axillary line at the liver and spleen windows on the right and left sides (Sonosite Fuzifilm M-Turbo) within 6 hrs of initiation of mechanical ventilation and after 72 hours. Three repeat measurements were done separately for each hemidiaphragm and averaged. The quadriceps muscle thickness was also measured at each time point. Enteral feeding (1500-1600 Kcal/day) was started within 24 hours of ICU admission and serum procalcitonin was measured at 72 hours in all patients.

**RESULTS.** The decrease in DT was significantly greater in ACMV compared to SIMV+PS (7.2 vs 5.1 mm; Difference between groups: mean 1.9 mm, 95% CI: 0.9-3.8 mm, p=0.002, Welch's T-test). Quadriceps thickness and serum procalcitonin did not significantly change after 72 hours in either group. (Figure 1)

**CONCLUSION.** Using SIMV+PS reduces the magnitude of diaphragmatic atrophy compared to ACMV in critically ill patients on mchanical ventilation.

**REFERENCE(S)**

1. Powers SK, Kavazis AN, Levine S. Prolonged Mechanical ventilation alters diaphragmatic structure and function. Crit Care Med. 2009; 37(10): S347-53.
2. Schepens T, Verbrugghe W, Dams K, Corthouts B, Parizel PM, Philippe JG, et al. The course of diaphragm atrophy in ventilated patients assessed with ultrasound: a longitudinal cohort study. Crit Care. 2015; 19: 422.
3. Levine S, Nguyen T, Taylor N, Friscia ME, Budak MT, Rothenberg P, et al. Rapid disuse atrophy of diaphragm fibers in mechanically ventilated humans. N Engl J 3. Med. 2008; 358(13): 1327–35.
4. Futier E, Constantin JM, Combaret L, Mosoni L, Roszyk L, Sapin V, et al. Pressure support ventilation attenuates ventilator-induced protein modifications in the diaphragm. Crit Care. 2008;12(5):R116.

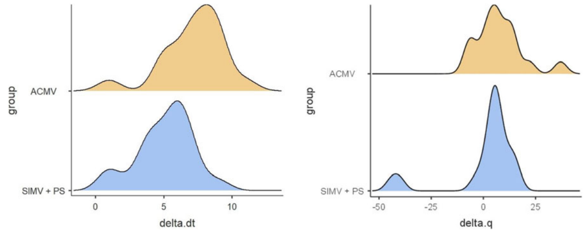

**Fig. 1 (abstract 000391).** See text for description

### 000412

#### Is Chronic Obstructive Pulmonary Disease a risk factor for microaspiration in intubated critically-ill patients?

T. Degroote[1], E. Jaillette[2], J. Reignier[3], JB. Lascarrou[3], F. Zerimech[4], S. Nseir[2]
[1]Réanimation polyvalente, Hôpital Saint Joseph, Paris, France; [2]Réanimation médicale, Hôpital Roger Salengro, Lille, France; [3]Médecine intensive réanimation, Nantes University Hospital Hotel-Dieu, Nantes, France; [4]Centre de biologie pathologie, Chu, Lille, France
**Correspondence:** T. Degroote
*Intensive Care Medicine Experimental* 2019, **7(Suppl 3):**000412

**INTRODUCTION.** Microaspiration of gastric and oropharyngeal contaminated secretions occurs frequently in intubated critically-ill patients, and plays a major role in the pathogenesis of ventilator-associated pneumonia (VAP). At steady state, patients with chronic obstructive pulmonary disease (COPD) have an increased risk of microaspiration (due to gastro-esophageal reflux disease, pharyngo-laryngeal dysfunction…), this risk may even be more important under mechanical ventilation.

**OBJECTIVES.** The purpose of this study is to determine if COPD is a risk factor for global abundant microaspiration (GAM) and its complications (ventilator-associated tracheobronchitis (VAT), VAP, death) in intubated critically-ill patients.

**METHODS.** We gathered data from three prospective randomized trials (1–3) focused on enteral nutrition or microaspiration in intubated patients. In all included patients, pepsin and salivary amylase were prospectively measured in all tracheal aspirates for 48h. Data on COPD were retrospectively collected. GAM was defined as the presence at significant level of pepsin (>200 ng/ml) and/or salivary amylase (>1685UI/L) in at least 30 % of the tracheal aspirates during the first 48 hours after intubation.

**RESULTS.** 540 patients were included among which 121 had COPD (70 with spirometric-proven COPD). GAM was present in 102 COPD patients and in 349 non-COPD patients (84.3% vs 83.3%, p=0,79). Abundant gastric microaspiration was found in 59 COPD patients and in 194 non-COPD patients (48.7% vs 46.3%, p=0.63). Abundant oropharyngeal microaspiration was found in 86 COPD patients and in 304 non-COPD patients (71% vs 75.5%, p=0.83). Neither spirometric-proven COPD nor COPD severity were found as risk factors for GAM. There was no statistical difference between COPD and no-COPD groups for the rates of VAT (2.4% vs 2.1%, p=0.82), VAP (18% vs 22%, p=0.34), or ICU mortality (27% vs 27%, p=0.97). Similar outcomes were found in no-COPD and spirometric-proven COPD groups.

**CONCLUSION.** COPD is not associated with increased risk for GAM or VAP.

**REFERENCE(S)**

1. Nseir S, Zerimech F, Fournier C, et al. Continuous control of tracheal cuff pressure and microaspiration of gastric contents in critically ill patients. Am J Respir Crit Care Med. 2011;184:1041-7.
2. Jaillette E, Girault C, Brunin G, et al. Impact of tapered-cuff tracheal tube on microaspiration of gastric contents in intubated critically ill patients: a multicenter cluster-randomized cross-over controlled trial. Intensive Care Med. 2017;43:1562-71.
3. Nseir S, Le Gouge A, Lascarrou J-B, et al. Impact of nutrition route on microaspiration in critically ill patients with shock: a planned ancillary study of the NUTRIREA-2 trial. Crit Care 2019;23:111.

### 000425

#### High-flow nasal cannula versus non-invasive ventilation on re-intubaion in patients with established post-extubation respiratory failure

M. Park[1], J. Kim[1], K. M.[1], HL. Lee[2], SM. Kwak[2], JS. Ryu[2], HS. Nam[2]
[1]Division of pulmonary medicine, Inha University Hospital, Incheon, South Korea, Republic of Korea; [2]Pulmonary medicine, Inha University Hospital, Incheon, Republic of Korea
**Correspondence:** M. Park

*Intensive Care Medicine Experimental* 2019, **7(Suppl 3):**000425

**INTRODUCTION.** Post-extubation respiratory failure (PERF) is a common complication in electively extubated patients, it is unclear whether high-flow nasal cannula (HFNC) and non-invasive ventilation (NIV) decrease rates of re-intubation in patients with established post-extubation respiratory failure. As high-flow oxygen therapy delivered through nasal cannula could improve oxygenation, secretions management and reduce work of breathing, we assessed whether HFNC could reduce the rate of re-intubation in patients with established PERF compared with NIV by helmet or oronasal mask.

**METHODS.** Between March 1, 2017 and December 31, 2018, eighty-nine patients had established respiratory failure after elective extubation and were applied HFNC or NIV. The data of these 89 patients were collected prospectively in a medical intensive care unit (ICU), academic, tertiary care hospital and retrospectively analyzed.

**RESULTS.** The data of 89 patients were collected. HFNC was administered to 46 and NIV by helmet or oronasal mask was administered to 43. There were no differences between baseline characteristics in the two groups. A comparison of the HFNC and NIV groups showed no differences between rates of re-intubation, ICU or hospital mortalities in patients with established post-extubation respiratory failure. pH, PaO2/FiO2 in the NIV group were significantly lower and PaCO2 was higher than in the HFNC group before HFNC or NIV application.

**CONCLUSION.** In previous study, among high-risk patients who have undergone extubation, there was no difference in preventing re-intubation between HFNC and NIV. Similar to previous study, in patients with established pos-extubation respiratory failure, the effectiveness of HFNC was not significantly inferior to NIV in our study. A prospective study that takes into account patient selection and HFNC or ventilator settings is required to determine the clinical efficacy of HFNC or NIV.

**REFERENCE(S)**

1. JAMA 2018;320(20):2099-2107
2. CHEST 2017; 151(4):764-775
3. N Engl J Med 2015;372:2185-96
4. JAMA 2016;316(15):1565-1574

5. The authors received no financial support for the research, authorship, and/or publication of this article.

### 000431

#### Acute severe asthma requiring invasive mechanical ventilation: a 10-year exhaustive analysis in a French department

A. Binachon[1], J. Jabot[1], N. Allou[1], J. Allyn[1], O. Martinet[1], V. Boisson[2], A. Gauthier[2], R. Persichini[1]
[1]Medical-surgical intensive care unit, CHU Felix-Guyon, Saint-Denis, La Réunion, France; [2]Medical-surgical intensive care unit, CHU Sud-Réunion, Saint-Pierre, La Réunion, France
**Correspondence:** R. Persichini

*Intensive Care Medicine Experimental* 2019, **7(Suppl 3):**000431

**INTRODUCTION.** Acute severe asthma (ASA) is a common cause of admission in intensive care units (ICU). In some rare cases, it can lead to invasive mechanical ventilation. There is currently few recent data in the literature about this specific subtype of patient.

**OBJECTIVES.** The aim of this retrospective and bicentric study was to investigate the factors associated with mortality of ASA requiring invasive mechanical ventilation.

**METHODS.** All ASA requiring invasive mechanical ventilation between 2008 and 2017 were analyzed.

Patients' characteristics and laboratory tests were tested in bivariate analysis for association with 28-day mortality. Factors achieving $p < 0.20$ were introduced into a multivariable model.

**RESULTS.** The main strength of this study is its completeness: it concerns the Reunion Island, a French overseas territory with only 2 ICU, both having participated in this study. Therefore, all ASA requiring invasive mechanical ventilation during the study period were analyzed without exception.

Eighty-one patients were evaluated in this study, among whom 52 (64%) were women. All patients were known to have asthma. The median age was 49 years old [interquartile range, 42-60] with a median Simplified Acute Physiology Score II (SAPS II) of 40 [30–54]. About a quarter of the patients (n=20) had experienced a previous ASA that required invasive mechanical ventilation. No decompensation risk factor was found in almost half of the cases (49% - n=40). The other decompensation risk factors found were cessation of asthma treatment (12% - n=10), allergenic exposure (10% - n=8), extra-respiratory sepsis (11% - n=9), bacterial or viral pneumonia (15% - n=12) and acute bronchitis (14% - n=11). Seven patients underwent salvage therapy, sometime in combinaison: ECMO for 6 patients, hallogenated gaz for 1 patient and anti-IL5 antibody (mepolizumab) for 2 patients.

The mortality rate was 14% (n = 11). In bivariate analysis, the occurrence of a cardiac arrest prior to admission, a cardiac arrest as the reason for intubation, the absence of a decompensating risk factor found and an intubation in prehospital setting were significantly associated with a poorer outcome. In non-survivor, SAPS II, creatinine level and lactatemia at admission were significantly higher whereas pH and HCO3 at admission were significantly lower. Multivariable analyses retained a positive fluid balance at day 1 (OR 6 [1-11] for 1L, $p = 0.02$) and the occurrence of a cardiac arrest prior to admission (OR 36.6 [3.6-374.9], $p < 0.01$) as being independently associated with 28-day mortality.

**CONCLUSION.** Nowadays, ASA requiring invasive mechanical ventilation is still responsible of high 28-day mortality. The prognostis is mainly related to multiorgan failure with the occurrence of a cardiac arrest prior to admission as the worst risk factor. A positive fluid balance at day 1 is also associated with poor outcome, challenging the usual dogma that recommends a liberal hydratation in such patients.

### 000435

#### Risk modifiers for acute respiratory distress syndrome in patients with non-pulmonary sepsis

H. Iriyama[1], T. Abe, T. Naito[1], H. Ogura[2], A. Shiraishi[3], S. Kushimoto[4], D. Saitoh[5], S. Fujishima[6], T. Mayumi[7], A. Komori[1], T. Kainoh[1], Y. Shiino[8], TA. Nakada[9], T. Tarui[10], T. Hifumi[11], Y. Otomo[12], Y. Sakamoto[13], J. Sasaki[14], S. Junichi[14], S. Gando[15]
[1]Department of general medicine, Juntendo University, 2 Chome-1-1 Hongo, Bunkyo City, Tokyo, Japan, Bunkyo City, Japan; [2]Department of traumatology and acute critical medicine, Osaka University Graduate School of Medicine, Suita, Japan; [3]Emergency and trauma center, Kameda Medical Center, Kamogawa, Chiba, Japan, Japan; [4]S of emergency and critical care medicine, Tohoku University Graduate School of Medicine, Sendai, Japan; [5]Division of traumatology, research institute, National Defense Medical College, Tokorozawa, Japan; [6]Center for general medicine education, Keio University School of Medicine, Tokyo, Japan; [7]Department of emergency medicine, University of Occupational and Environmental Health, Kitakyushu, Fukuoka, Japan, Japan; [8]Department of acute medicine, Kawasaki Medical School Hospital, 577 Matsushima, Kurashiki, Okayama, Japan, Kurashiki, Japan; [9]Department of emergency and critical care medicine, Chiba University Graduate School of Medicine, Chiba, Japan, Japan; [10]Department of trauma and critical care medicine, Kyorin University, 5 Chome-4 Shimorenjaku, Mitaka, Tokyo, Japan, Mitaka, Japan; [11]Department of emergency and critical care medicine, St. Luke's International Hospital, Chuo City, Japan; [12]Trauma and acute critical care center, Tokyo Medical and Dental University, Bunkyo City, Japan; [13]Emergency and critical care medicine, Saga University Hospital, Saga, Japan; [14]Department of emergency and critical care medicine, Keio University School of Medicine, Tokyo, Japan; [15]Division of acute and critical care medicine, Hokkaido University Graduate School of Medicine, Sapporo, USA
**Correspondence:** H. Iriyama
*Intensive Care Medicine Experimental* 2019, **7(Suppl 3):**000435

**INTRODUCTION.** Predisposing conditions and risk modifiers have been recently used to identify patients at risk for acute respiratory distress syndrome (ARDS) instead of causes and risk factors. Risk modifiers of ARDS caused by sepsis due to pneumonia which is one of the most common predisposing conditions are well known. However, little is known about risk modifiers of ARDS especially among patients with non-pulmonary sepsis.

**OBJECTIVES.** Our aim was to evaluate risk modifiers associated with ARDS among patients with non-pulmonary sepsis.

**METHODS.** This is a secondary analysis of the sepsis cohort in the Focused Outcomes Research in Emergency Care in Acute Respiratory Distress Syndrome, Sepsis and Trauma (FORECAST) study. [1] This was a multicenter, prospective cohort study conducted in 59 intensive care units (ICUs) in Japan from January 2016 to March 2017.We included adult patients with severe sepsis and septic shock caused by non-pulmonary infection, who admitted to ICUs. The primary outcome was having ARDS defined as meeting the Berlin definition [2] at the first or the fourth day of ARDS screening. The secondary outcome was developing ARDS defined as both of (1) absence of ARDS at the first day of ARDS screening and (2) presence of ARDS at the fourth day of ARDS screening. The explanatory variables were prespecified a priori based on clinical experience and prior studies, such as age, gender, admission source, body mass index, smoking status, congestive heart failure, chronic obstructive pulmonary disease, diabetes mellitus, steroids, statin, site of infection, septic shock, and APACHE II score. After carefully checking collinearity, to identify risk modifiers associated with having ARDS and developing ARDS, we developed multivariate logistic regression models.

**RESULTS.** 594 patients with non-pulmonary sepsis met the inclusion and exclusion criteria of the study. 85 patients had ARDS at the first or the fourth day of ARDS screening, and 16 patients developed ARDS at the fourth day of ARDS screening.
There was no significant difference between patients with and without ARDS regarding baseline characteristics such as age, body mass index, coexisting conditions, smoking, and sites of infection. 80% of patients with ARDS and 66% of patients without ARDS had septic shock (P = 0.01). APACHE II scores were higher in patients with ARDS than those without ARDS (26.0 vs 21.0, P < 0.01). In the multivariate logistic regression model, ICU admission from other wards compared with emergency departments (odds ratio (OR) [95% confidence interval (CI)], 0.53 [0.294–0.947]), smoking status (current smoking compared with never having smoked: OR [95%CI], 0.18 [0.056–0.587]), soft tissue infection compared with abdominal infection (OR [95% CI], 2.39 [1.040–5.400]), and higher APACHE II score (OR [95% CI], 1.08 [1.050–1.120]) were independently associated with having ARDS. No risk modifiers were independently associated with developing ARDS in our cohort.

**CONCLUSION.** Soft tissue infection, ICU admission from hospital wards, and higher APACHE II score may be risk modifiers of ARDS in patients with non-pulmonary sepsis.

**REFERENCE(S)**

1. JAMA. 2012;307(23):2526-33.
2. Crit Care. 2018;22(1):322.

### 000443

#### Retrospective Evaluation of Factors Affecting Development of Tracheomalacia in Critically ill Patients with Prolonged Intubation

ZO. Simsek[1], K. Gundogan[2], M. Sungur[1]
[1]Department of internal medicine, division of intensive care, Erciyes University, School of Medicine, Kayseri, Turkey; [2]Department of internal medicine, division of intensive care, Erciyes University, School of medicine,, Kayseri, Turkey
**Correspondence:** K. Gundogan
*Intensive Care Medicine Experimental* 2019, **7(Suppl 3):**000443

**INTRODUCTION.** Tracheomalacia (TM) is defined as diffuse or segmental weakness of the trachea. Acquired TM, is more common than the congenital form and more frequent in male gender. The most important diagnostic method is observation of airway collapse via bronchoscopy.

**OBJECTIVES.** Aim of this study is to identify development frequency of and factors affecting TM in critically ill patients with prolonged intubation.

**METHODS.** This study was performed in Intensive Care Unit. Intubation time longer than 7 day patients were included into the study. A chest disease specialist examined the bronchoscopy video records and the study included patients who had a bronchoscopy video record on the first day of intubation and at least one bronchoscopy video record in the period between seventh day of intubation and extubation.

**RESULTS.** The study included 40 patients. Mean age of the patients was 59.9±13.2 years. Mean body mass index (BMI) of the patients was 26.4±4.9 kg/m2. Patients' median APACHE II and SOFA scores were 18 (range: 9-35) and 6 (range: 3-13), respectively. TM was identified in 4 patients (%10) among all the patients included in the study. When we checked risk factors for TM development, two patients had history of COPD. One patient had history of intubation. No risk factor was identified in the other patient. During follow-up weaning failure, requirement of re-intubation and fatal progress were identified in patients with TM. Gender distribution was equal in patients with TM.

**CONCLUSION.** We identified weaning failures, re-intubation requirements and fatal progress in all patients with tracheomalacia. TM is an important condition that increases morbidity and mortality in the settings of intensive care units and suspected patients in terms of TM should undergo bronchoscopy.

5. Yusuff HO, Zochios V, Vuylsteke A. Extracorporeal membrane oxygenation in acute massive pulmonary embolism: a systematic review. Perfusion 2015;30:611-6.

### 000449

#### Use of Extracorporeal Membrane Oxygenation in Patients with Acute High-risk Pulmonary Embolism: A Case Series with Literature Review

O. You Na[1], O. Dong Kyu[1], K. Younsuck[1], L. Chae-Man[1], H. Jin-Won[1]; L. Jae Seung[1], J. Sung-Ho[2], K. Pil-Je[2], H. Sang-Bum[1]
[1]Department of pulmonary and critical care medicine, Asan Medical Center, Seoul, Republic of Korea; [2]Department of thoracic and cardiovascular surgery, Asan Medical Center, Seoul, Republic of Korea
**Correspondence:** Y.N. OH
*Intensive Care Medicine Experimental* 2019, **7(Suppl 3):**000449

**INTRODUCTION.** Acute pulmonary embolism (PE) is the most serious clinical presentation of venous thromboembolism and causes obstructive shock and hemodynamic instability [1-3]. It is stratified on the basis of the early PE-related mortality risk, and the high-risk group is defined as patients who have shock or systemic hypotension [2,3]. Unfortunately, despite the expedited lifesaving treatments such as systemic thrombolysis and surgical embolectomy, acute high-risk PE is associated with significant morbidity and mortality [2-5].

**OBJECTIVES.** Although extracorporeal membrane oxygenation (ECMO) has been used for the treatment of acute high-risk pulmonary embolism (PE), reports focusing on this approach are limited. We describe our experience with ECMO for acute high-risk PE.

**METHODS.** We retrospectively reviewed the medical records of patients treated for acute high-risk PE with ECMO between January 2014 and December 2018.

**RESULTS.** Among the 16 patients included, 6 (37.5%) were men. The median age was 51 years (interquartile range [IQR], 38–71 years). Cardiac arrest occurred in 12 patients (75.0%), including 2 with out-of-hospital arrest. All the patients underwent venoarterial ECMO for a median duration of 1.5 days (IQR, 0.0–4.5 days). Seven (43.8%) and 9 patients (56.3%) underwent systemic thrombolysis and surgical embolectomy, respectively, including 3 (18.8%) who received both treatments. The overall 30-day mortality rate was 43.8% (95% confidence interval, 23.1%–66.8%) and showed no significant difference among the treatment groups (thrombolysis vs. non-thrombolysis: 42.9% [n=3/7] vs. 44.4% [n=4/9], $p$=0.870 and embolectomy vs. non-embolectomy: 44.4% [n=4/9] vs. 42.9% [n=3/7], $p$=0.870).

**CONCLUSION.** Despite treatment efforts, the patients with acute high-risk PE had substantial morbidity and mortality. ECMO may be considered in these patients in conjunction with either systemic thrombolysis or surgical embolectomy.

##### REFERENCE(S)

1. Aissaoui N, Konstantinides S, Meyer G. What's new in severe pulmonary embolism? Intensive Care Med 2019;45:75-7.
2. Konstantinides SV, Torbicki A, Agnelli G, Danchin N, Fitzmaurice D, Galie N, et al. 2014 ESC guidelines on the diagnosis and management of acute pulmonary embolism. Eur Heart J 2014;35:3033-69, 69a-69k.
3. Jaff MR, McMurtry MS, Archer SL, Cushman M, Goldenberg N, Goldhaber SZ, et al. Management of massive and submassive pulmonary embolism, iliofemoral deep vein thrombosis, and chronic thromboembolic pulmonary hypertension: a scientific statement from the American Heart Association. Circulation 2011;123:1788-830.
4. Logan JK, Pantle H, Huiras P, Bessman E, Bright L. Evidence-based diagnosis and thrombolytic treatment of cardiac arrest or periarrest due to suspected pulmonary embolism. Am J Emerg Med 2014;32:789-96.

### 000456

#### The use of ward based high flow nasal oxygenation in patients with single organ respiratory failure reduces critical care admission

E. Hubbard[1], J. walton[2]
[1]Outreach nursing service, Newcastle Freeman Hospital, High Heaton, UK; [2]Intensive care medicine, Freeman Hospital, Newcastle upon Tyne, UK
**Correspondence:** J. walton
*Intensive Care Medicine Experimental* 2019, **7(Suppl 3):**000456

**INTRODUCTION.** 25% of critical care admissions in our hospital are for respiratory failure. High flow nasal oxygenation (HFNO) in respiratory failure has been shown to be safe and effective (1) and has been used at the Freeman Hospital since 2009. By introducing a formalized outreach nursing service led, ward based HFNO service for patients with single organ respiratory failure we aimed to reduce unnecessary critical care admissions without compromising patient safety.

**OBJECTIVES.** 2 prospective audits were conducted assessing the outcome of patients requiring critical care ventilatory support for single organ respiratory failure but managed with HFNO in a ward setting under outreach nursing team care.

**METHODS.** In a pilot audit 53 patients referred by the ward team to critical care for single organ respiratory failure via an early warning scoring system (NEWS) were reviewed by the critical care outreach team over a 12 month period.

If assessed as suitable for ward management, the patients were commenced on HFNO (Airvo 2 Fisher & Paykel) on the ward. The prescription was determined clinically to maintain patient comfort with targeted SpO2 and arterial blood gas monitoring as necessary. A medical decision was taken daily to either: escalate to critical care, continue HFNO or move to palliative care.

Triggering of the NEWS score or an admitting team request led to an urgent medical critical care review in accordance with hospital policy. Patient demographics and ultimate outcomes were recorded on every patient.

The second audit was of 200 patients using the same referral and assessment system over another 12 month period in 2016-17.

**RESULTS.** In the pilot audit of 53 patients, 43% remained on the ward and survived to discontinuation of HFNO. 42% were admitted to critical care and 15% received palliative care. In the follow up audit of 200 patients, 57.5% remained on the ward, 23.5% were admitted to critical care with 19% receiving palliative care.

No difference was seen in mortality rates between those managed on the ward and those admitted directly to critical care.

**CONCLUSION.** In our 2 prospective audits, ward based HFNO for respiratory failure significantly reduced critical care admissions without affecting patient outcomes, saving 582 bed days and potentially a net cost saving of £459,174(2).

##### REFERENCE(S)

1. Yigal Helviz and Sharon Einav. 29558988A Systematic Review of the High-flow Nasal Cannula for Adult Patients Crit Care. 2018; 22:71. Published online 2018 Mar 20.
2. Welsh Government: Together for Health – a delivery plan for the critically ill. 2016. http://www.wales.nhs.uk/documents/delivery-plan-for-the-critically-ill.pdf
3. No grant was made for this study. The results have been used by Fisher and Paykel in their advertising literature.

### 000462

#### Outcome of Percutaneous Dilatational Tracheostomy without Endotracheal Guidance

D. Lee[1], T. Kim[2], S. Kim[3]
[1]Intensive care medicine, Dong-a University Hospital, Busan, Republic of Korea; [2]Internal medicine, Gyeongsang National University, College of Medicine, Changwon, Republic of Korea; [3]Pulmonology, Inje University Busan Paik Hospital, Busan, Republic of Korea
**Correspondence:** D. Lee
*Intensive Care Medicine Experimental* 2019, **7(Suppl 3):**000462

**INTRODUCTION.** Percutaneous dilatational tracheostomy (PDT) is commonly performed in intensive care unit (ICU). Although it is safe and easily performed at bedside, it always needs endotracheal guidance such as bronchoscopy or light wand.

**OBJECTIVES.** We sought to the feasibility of PDT without endotracheal guidance.

**METHODS.** In the presenting ICU, PDT has been routinely performed without endotracheal guidance by single medical intensivist using Griggs technique PDT kit (Portex percutaneous tracheostomy kit, Smith Medical, England). We retrospectively reviewed the outcome of PDT without endotracheal guidance.

**RESULTS.** From January 2018 to December 2018, 78 cases of PDT were performed in medical ICU and coronary care unit. Mean age was 71.9 ± 11.5 years and 29 patients (37.2%) were female. Most patients were admitted to pulmonology (46 patients, 59.0%) and followed by cardiology (7 patients, 9.0%). PDT was successful in 76 patients (97.4%). Two patients were converted to surgical tracheostomy because of failure of guidewire passage. One patient were re-intubated for the surgical tracheostomy and in the other patient, supraglottic airway device (i-gel, Intersurgical, Wokingham, UK) were inserted during tracheostomy. No significant procedure related complication was observed in both patients. Another patient was re-intubated due to guidewire passage failure, however PDT was successfully performed.

Procedure related complication was observed in 5 patients (6.4%) and all of them were observed with 24 hours after PDT and controlled with simple local compression. In-hospital mortality was 33.3% (26 of 78 patients) and mean time from PDT to death was 41.7 ± 43.2 days.

**CONCLUSION.** PDT without endotracheal guidance is safe and feasible.

##### REFERENCE(S)

1. Feasibility of Percutaneous Dilatational Tracheostomy with a Light Source in the Surgical Intensive Care Unit Acute and Critical Care. 2018 May 33(2):89-94
2. No grant acknowledgment

### 000464

#### Utility of success predictors for mechanical ventilation removal

JO. Guamán Crespo[1], ZE. Monares[2], MCA. Galindo[3], EA. Ojeda Izquierdo[4], R. Lozano Zúñiga[5], A. Garza de la Maza[4]
[1]Critical care unit, Hospital San Angel Inn Universidad, Mexico City, Mexico; [2]Intensive critical care unit., Hospital San Angel Inn Universidad, Mexico City, Mexico; [3]Chief nutrition department, Hospital San Angel Inn Universidad, Mexico City, Mexico; [4]Critical care, Hospital San Angel Inn Universidad, Mexico City, Mexico; [5]Critical Care, Hospital San Angel Inn Universidad, Mexico City, France
**Correspondence:** J.O. Guamán Crespo
*Intensive Care Medicine Experimental* 2019, **7(Suppl 3):**000464

**INTRODUCTION.** The invasive mechanical ventilation weaning protocol aims at the progressive removal of ventilatory support, after presenting acute respiratory insufficiency or not, and having overcome it totally or partially, this process is relayed after a standardized evaluation with international guidelines. Several authors have explored risk factors and prediction rates that are associated with failure in the release of ventilation.

**OBJECTIVES.** Evaluate if there is an association between the use of release failure rates in our population and the initial evaluation with Tobin's mandatory criteria.

**METHODS.** A retrospective study was conducted, which included 65 patients, in the intensive therapy unit of the "Hospital San Ángel Inn Universidad", in the period from May 2015 to July 2018, the included patients, were in mechanical ventilation for any cause with more than 24 hours of intubation, the included patients were over 18 years old; patients with incomplete records of the ventilation withdrawal protocol, diagnosis of failed extubation in the current hospitalization, pregnant or puerperal patients were excluded; criteria for elimination were voluntary discharge and signature of advance will. The patients were analyzed in two groups: success, 58 patients, or failure to withdraw, 7 patients. The data were analyzed by Student's t and sum ranges of Wilcoxón or Man-Whitney U as the case may be. A ROC curve was performed to describe the effect on the release of the different ventilation withdrawal rates.

**RESULTS.** There was no significant difference in the variables collected between the groups. Later in the analysis of ROC curves the variables Tobin 1, Tobin 2, p.01 and IWI did not show significant performance in the detection of failure in the extubation of this sample (p> 0.05 against Area under the Curve of 0.5), so that is not continued with subsequent corresponding analyzes.

**CONCLUSION.** In patients who undergo removal of invasive mechanical ventilation, using a protocol that evaluates the mandatory criteria cited by Tobin, it is not relevant to measure predictors of success as index of superficial respiration, at 1and 30 minutes, as well as the index of IWI.

##### REFERENCE(S)

1. Nemer SN, Barbas CS, Caldeira JB, et al. A new integrative weaning index of discontinuation from mechanical ventilation. Crit Care. 2009;13(5):R152
2. Meade M, Guyatt G, Cook D, et al. Predicting success in weaning from mechanical ventilation. Chest 2001; 120:400S
3. Ladeira MT, Vital FMR, Andriolo RB, Andriolo BNG, Atallah ÁN, Peccin MS. Pressure support versus T-tube for weaning from mechanical ventilation in adults. Cochrane Database of Systematic Reviews 2014, Issue 5. Art. No.: CD006056
4. Epstein SK, Nevins ML, Chung J. Effect of unplanned extubation on outcome of mechanical ventilation. Am J Respir Crit Care Med
5. Tobin MJ. Physiologic Basis of Mechanical Ventilation. Annals ATS 2018. Issue:1; S49–S52. DOI: 10.1513/AnnalsATS.201705-417KV
6. Tobin MJ, Variable performance of weaning-predictor tests: role of Bayes' theorem and spectrum and test-referral bias. Intensive Care Med (2006) 32:2002–2012. DOI 10.1007/s00134-006-0439-4
7. Tobin MJ. et al. Role and interpretation of weaning predictors. As presented at the 5th International Consensus Conference in Intensive Care Medicine: Weaning from Mechanical VentilationBudapest, April 28–29, 2005
8. Brochard L. Pressure support is the preferred weaning method. As presented at the 5th International Consensus Conference in Intensive Care Medicine: Weaning from Mechanical Ventilation. Budapest, April 28–29, 2005
9. Coplin WM, Pierson DJ, Cooley KD, et al. Implications of extubation delay in brain-injured patients meeting standard weaning criteria. Am J Respir Crit Care Med 2000; 161: 1530–1536
10. Esteban A, Anzueto A, Frutos F, et al. Mechanical Ventilation International Study Group. Characteristics and outcomes in adult patients receiving mechanical ventilation: a 28-day international study. JAMA 2002; 287: 345–355

### 000476

#### Impact of tidal volume on outcome of patients under Pressure Support Ventilation during moderate and severe Acute Respiratory Distress Syndrome: a monocentric retrospective study

G. David, D. Castanares, X. Wittebole, J. Roeseler, J. Dugernier, C. Hickmann, C. Collienne, PF. Laterre, L. Gerard
[1]Intensive care unit, Cliniques Universitaires Saint-Luc, Brussels, Belgium
**Correspondence:** G. David
*Intensive Care Medicine Experimental* 2019, **7(Suppl 3):**000476

**INTRODUCTION.** While controlled mechanical ventilation (MV) still represents the standard of care in acute respiratory distress syndrome (ARDS), assisted MV has gained interest in recent years because of its recognized beneficial effects (e.g. more homogeneous ventilation, prevention of diaphragmatic atrophy, reduction in sedative requirements). Nevertheless, maintaining some degree of spontaneous ventilation also has a potential for harm (e.g. occult increase in transpulmonary pressure, Pendelluft phenomenon, loss of control of tidal volume). No clinical studies have compared spontaneous breathing to controlled MV during acute respiratory failure, and none has been conducted in the context of moderate to severe ARDS. Moreover, guidelines recommend limiting tidal volume (TV) to 4-8 ml/kg of predicted body weight (PBW), but the usefulness of TV limitation during *assisted* MV remains unproven.

**OBJECTIVES.** We aimed to evaluate the impact of spontaneous breathing on TV during the initial phase of moderate and severe ARDS, and to assess the association between high tidal volume (HTV) and outcome in spontaneously breathing patients.

**METHODS.** We conducted a retrospective analysis including all moderate to severe ARDS patients mechanically ventilated in pressure support mode for at least 72 hours between 2012 and 2018, within a 22-bed mixed ICU of a tertiary medical center. All data were retrieved from electronic medical recordings. Patients were divided into a HTV group and a low tidal volume (LTV) group based on the mean TV relative to PBW, patients with a mean TV > 8 ml/kg PBW being classified in the HTV group. LTV and HTV groups were compared in terms of demographics, comorbidities, MV and respiratory parameters, hemodynamics, adjuvant therapies and outcomes. In a subgroup analysis, we compared patients' characteristics and outcomes of the HTV group with the LTV group, stratified by ARDS severity.

**RESULTS.** Among 100 patients who fulfilled inclusion criteria, forty-six (46%) had a mean TV > 8 ml/kg PBW and were classified in the HTV group. The 90-day mortality rate was not significantly different between the LTV and the HTV group (44.4 vs 56.5%; p=0.25), despite higher severity in the HTV group. Median number of Ventilator-Free days at day 28 (9.5 days vs 11 days, p=0.89) and length of stay in the ICU (15.5 days vs 14 days, p=0.48) did not differ between LTV and HTV group. In the most severe patients (PaO2/FiO2 < 100, n=31), HTV was associated with a trend towards higher 90-day mortality (43 vs 73%, p=0.09), whereas in moderate ARDS patients (n=69), no difference between groups was shown, despite patients in the HTV group being significantly older.

**CONCLUSION.** In this retrospective analysis of patients spontaneously breathing during the first 72 hours of moderate-to-severe ARDS, TV > 8 ml/kg PBW was frequent and was not associated with adverse outcomes.

**REFERENCE(S)**

1. Yoshida T, Fujino Y, Amato M, et al: Fifty Years of Research in ARDS. Spontaneous Breathing during Mechanical Ventilation. Risks, Mechanisms, and Management. Am J RespirCrit Care Med 2017; 195(8):985-92.
2. Yoshida T, Uchiyama A, Matsuura N et al: The comparison of spontaneous breathing and muscle paralysis in two different severities of experimental lung injury. Crit Care Med 2013; 41:536-45.
3. Van Haren F, Pham T, Brochard L, et al: Spontaneous Breathing in Early Acute Respiratory Distress Syndrome: Insights From the Large Observational Study to UNderstand the Global Impact of Severe Acute Respiratory FailurE Study. Crit Care Med 2019; 47(2):229-238.

### 000479

#### Characteristics of drowning patients admitted to an Intensive Care Unit (ICU) in a second-level Hospital in a six-year period (2013-2018)

JL. Martinez Melgar[1], E. Moreno Lopez[2], I. Gallego Barbachano[1], E. Sanmartin Mantiñan[1], A. Ortega Montes[1], JI. Cenoz Osinaga[1], JV. Bravo Doviso[1], T. Sanchez De Dios[1], A. Pais Almozara[1], P. Posada Gonzalez[1]
[1]Intensive care unit, Complexo Hospitalario De Pontevedra, Pontevedra, Spain; [2]Anesthesia and perioperative care, Complexo Hospitalario Universitario de Ferrol, Ferrol, Spain
**Correspondence:** E. Moreno Lopez
*Intensive Care Medicine Experimental* 2019, **7(Suppl 3):**000479

**INTRODUCTION.** The WHO defines drowning as " the process of experiencing respiratory impairment from submersion/immersion in liquid". It is a mayor public health problem that causes 7% of deaths from unintentional injury worldwide

**OBJECTIVES.** To know the epidemiologic and clinical characteristics (by organs), as well as therapeutic measures and evolution of drowning patients admitted to a medical ICU in a second-level hospital in a six-year period ( from January 2013 to December 2018).

**METHODS.** Retrospective and descriptive study of drowning patients admitted to a medical ICU. The following parameters were analyzed: gender, mean age by group, location of drowning, causes of drowning, period of the year, source of admission, Simcock classification on hospital admission, alterations: respiratory, neurological, cardiac, renal and temperature, as well as therapeutic measures in terms of ventilatory support, inotropic support and rewarming. Time spent under mechanical ventilation and mortality were also analyzed. Statistical analysis: quantitative variables are expressed as mean and standard deviation (SD) and qualitative variables as percentages (%). The difference between qualitative variables are expressed using Chi-square test and quantitative using an ANOVA analysis.

**RESULTS.** 19 drowning patients admitted to our ICU were analyzed: 11 female (8 male), mean age by group: <30 years old: 1, 30-50 years old: 3, 51-70 years old: 8 (42%) and >70 years old: 7 ( 37%). Location of drowning: ocean 14 (73,5%), river 3 and swimming pool 2. Period of the year: between May - September: 12 ( 63%), between October - April: 7. Causes of drowning: unintentional l 8 (42%) , trauma 4 (21%), syncope 4 (21%), suicide attempt 2, seizures 1. Source of admission: paramedics 12 (63%), hospital A&E 4 , other hospital 3. Simcock classification: group II 1, group III 11 (58%), group IV (cardiac arrest) 7 (37%), shockable rhythm 5 (71%) non-shockable rhythm 2. Respiratory alterations: noncardiogenic pulmonary edema 12 (63%), single alveolar pulmonary infiltrate 4, barotrauma 1 and no alterations 2. Neurological alterations: category A (awake) 9 (47%), category B ( blunted) 4 and category C ( comatose) 6 (31,5%): C1 ( decorticate) 1, C2 ( decerebrate) 3 and C3 (flaccid) 2. Renal alterations: acute kidney injury ( AKI) 2, rhabdomyolysis 1. Hypothernia: >32ºC 12 (67%) and between 32-28ºC 7 (33%), Ventilatory support: NIV 6 ( 31,5%): BiPAP 4 and CPAP 2, High Flow nasal cannula (HFNC) 1, low flow oxygen therapy ( Venturi face mask) 2, intubation + mechanical ventilation 10 (52,6%), lung protective mechanical ventilaton (ARDS) 3; inotropic support 6 (31,5%) and rewarming 7 (37%) (4 active external and 3 active internal). In patients who required intubation (10), 8 (80%) were extubated, 5 within 72 hours (50%) and >72 hours 3 (20%). 2 patients (10,5%) died.

**CONCLUSION.** In our series, the drowning patient is a female between 51-70 years old, who suffered an unintentional drowning in the ocean, Simcock type III who required intubation and mechanical ventilation ( 52,6%), was extubated within 72 hours (50%) and required inotropic support (31,5%) and rewarming (37%) with a mortality rate of 10,5%.

### 000492

#### Effect of early versus late or no tracheostomy on clinical outcomes in patients with prolonged acute mechanical ventilation: A single-center experience in Korea

Y. Kang, JS. Eom, EJ. Jo, J. Mok, MH. Kim, KU. Kim, HK. Park, MK. Lee, K. Lee
[1]Department of internal medicine, Pusan National University Hospital, Busan, South Korea, Republic of Korea
**Correspondence:** Y. Kang
*Intensive Care Medicine Experimental* 2019, **7(Suppl 3):**000492

**INTRODUCTION.** In Asian countries, critical care delivery systems are underdeveloped compared to Western countries: there would be distinct characteristics regarding tracheostomy in patients with translaryngeal intubation

**OBJECTIVES.** Our study aimed to compare effect of early versus late or no tracheostomy on clinical outcomes in patients with prolonged acute mechanical ventilation (ventilator care ≥ 96 hrs).

**METHODS.** Data were obtained from 592 patients [median age 68 yrs (range, 17-93 yrs), 69.4% were male] at a medical intensive care unit (ICU) of a university-affiliated tertiary care hospital during 9 years. Early tracheostomy was defined as ≤ 10 days after translaryngeal intubation.

**RESULTS.** Of total patients, 271 (45.8%) patients underwent early tracheostomy. Early tracheostomy group showed significantly lower Acute Physiology and Chronic Health Evaluation II [median 18, (range 3-41) vs 22 (6-35), p<0.001] and Sequential Organ Failure Assessment [5 (1-18) vs 8 (2-17), p<0.001] scores, and Charlson's Comorbidity Index [2 (0-14) vs 4 (0-15), p<0.001)] as well as shorter ICU and hospital length of stay [15 (1-776) vs 22 (4-219) and 24 (6-745) vs 36 (6-374) days, (all p<0.001, respectively)] than late or no tracheostomy group. Also, they had lower healthcare-associated costs [19155.8 (2482.4–240960.5) vs 26677.9 (5711.4-206636.3) USD, p<0.001] and higher successful decannulation (41.0 vs 24.5%, p=0.004) during hospital stay. However, there were no significant differences between the both groups for in-hospital, 6-months, and 1-year mortality (38.4 vs 42.1%, 55.0 vs 55.5%, and 62 vs 62%, respectively).

**CONCLUSION.** The patients who underwent early tracheostomy were associated with lower severity-of-illness, comorbidities on ICU admission, lower total medical cost, and more success of decannulation. However, there was no significant difference in mortality.

**REFERENCE(S)**

1. Siempos, II, Ntaidou TK, Filippidis FT, Choi AMK (2015) Effect of early versus late or no tracheostomy on mortality and pneumonia of critically ill patients receiving mechanical ventilation: a systematic review and meta-analysis. Lancet Respir Med 3:150-158. https://doi.org/10.1016/S2213-2600(15)00007-7
2. Gomes Silva BN, Andriolo RB, Saconato H, Atallah AN, Valente O (2012) Early versus late tracheostomy for critically ill patients. Cochrane Database Syst Rev:CD007271. https://doi.org/10.1002/14651858.CD007271.pub2

### 000496

#### Pleth variability index predicts fluid responsiveness in acute respiratory failure patients supported by nasal high-flow

M. García de Acilu, A. Pacheco, M. Santafé, F. Ramos, J. Ruíz-Rodríguez, R. Ferrer Roca, O. Roca
[1]Critical care department, Vall d´Hebron University Hospital, Vall d´ Hebron Research Institute, Barcelona, Spain
**Correspondence:** M. García de Acilu
*Intensive Care Medicine Experimental* 2019, **7(Suppl 3):**000496

**INTRODUCTION.** The pleth variability index (PVi) has been shown to be a predictor of fluid responsiveness (FR) in mechanically ventilated (MV) patients(1). Patients treated with nasal high-flow (NHF) show similar haemodynamic changes to those observed in MV patients(2). However, the usefulness of PVi for predicting FR in NHF patients has not been evaluated.

**OBJECTIVES.** To analyze whether PVi can predict FR in acute respiratory failure (ARF) patients supported by NHF.

**METHODS.** Proof of concept study that included ARF patients ≥18 y treated with NHF (≥40lpm) showing signs of inadequate tissue perfusion. At baseline, they were all in Fowler position (head of the bed at 45º angle). Stroke volume (SV) was measured by transthoracic echocardiography and PVi was monitored using a pulse oximeter (Masimo Radical-7® Pulse CO-Oximeter®, Irvine, CA). Fluid-response was defined as an increase ≥10% in SV after passive leg raising (PLR). Responders then received a 250ml fluid challenge for 10 minutes. After PLR and fluid challenge, SV and PVi were reassessed. Results are expressed as mean (95% CI) or frequency (%).

**RESULTS.** Twenty patients were included, with a mean age of 63 years (56-59). They were treated with a mean flow of 48 (40-57) lpm and FIO2 of 0.6 (0.5-0.7). Mean SOFA at the moment of inclusion was 9 (7-10) and SAPS-II 48 (40-51), with a PaO2/FIO2 of 141 (112-171). Eleven (55%) patients required noradrenaline, with a mean lactate level of 3.7 (2.7-4.7) mmol/L. Twelve patients (60%) responded to fluid administration. Responders showed higher baseline values of PVi (24 [20-28]% vs 13 [8-18]%;p=0.001) and higher PVi variation (ΔPVi) after PLR (6.8 [5.0-8.5]% vs -1.7 [-4.8-1.4]%;p<0.001). Moreover, ΔPVi after PLR was similar to ΔPVi after fluid challenge (6.8 [5.0 – 8.5] vs 7.4 [5.3 – 9.4];p=0.237); both values were strongly correlated (r= 0.84;p<0.001). PVi and ΔPVi after PLR showed excellent diagnostic accuracy (AUROC 0.92 [0.77-1.00];p< 0.001 and AUROC 1.00 [1.00-1.00];p<0.001 respectively). The best cut-off points using the Youden index were baseline PVi ≥16 and ΔPVi after PLR ≥2 (Sensitivity, Specificity, PPV and NPV are presented in Table 1).

**CONCLUSION.** PVi may predict fluid responsiveness in ARF patients treated with NHF.

**REFERENCE(S)**

(1) Loupec T et al. Pleth variability index predicts fluid responsiveness in critically ill patients. Crit Care Med. 2011;39:294-9.
(2) Roca O et al. Patients with New York Heart Association class III heart failure may benefit with high flow nasal cannula supportive therapy: high flow nasal cannula in heart failure. J Crit Care. 2013;28:741-6.
(3) Equipment supplied by Masimo.
(4) This study was supported by grants from "Sociedad Española de Neumología y Cirugía Torácica – SEPAR", (169/2016).

**Table 1 (abstract 000496).** See text for description

| | Sensitivity | Specificity | PPV | NPV | LR+ | LR- |
|---|---|---|---|---|---|---|
| Baseline PVi ≥16 | 91.7% | 87.5% | 91.7% | 87.5% | 7.33 | 0.10 |
| ΔPVi after PLR ≥2 | 100% | 100% | 100% | 100% | NA | NA |

*PPV* Positive predictive value, *NPV* Negative predictive value, *LR* Likelihood ratio

### 001289

#### Respiratory rate is associated with mortality in ARDS patients: An ancillary analysis of the Calgary ARDS observational cohort

K. Parhar[1], D. Niven[1], HT. Stelfox[1], G. Rubenfeld[2], C. Doig[1], K. Fiest[1], A. Soo[1]
[1]Department of Critical Care Medicine, University of Calgary, Calgary, Canada; [2]Interdepartmental division of critical care, Sunnybrook Health Sciences Center, Toronto, Canada
**Correspondence:** K. Parhar
*Intensive Care Medicine Experimental* 2019, **7(Suppl 3):**001289

**INTRODUCTION.** Acute respiratory distress syndrome (ARDS) is an inflammatory syndrome of the lungs and associated with significant morbidity and mortality. Mechanical power is an estimation of the energy delivered to a patient during mechanical ventilation and is associated with both ventilator-induced lung injury and survival outcomes. Respiratory rate is one of the determinants of mechanical power; however, its influence on outcomes remains unclear.

**OBJECTIVES.**

1. Determine the association between respiratory rate and 28-day ventilator free days (VFDs), 28-day hospital survival and 3-year survival in patients with ARDS
2. Determine patient factors associated with an increased respiratory rate
3. Determine the optimal respiratory rate threshold for mortality

**METHODS.** A prospective standardized screening program for ARDS (4 intensive care units (ICUs) within Calgary Alberta from 2010 to 2012) was conducted. Demographics and outcomes of this cohort have previously been reported. An ancillary analysis of this database was performed. Outcomes included 28-day hospital survival, 3-year survival as well as 28-day VFDs. Adjusted logistic and linear regression were used to model the association between respiratory rate and these outcomes; models were adjusted for sex, age, height, weight, admission APACHE, pH, PF ratio, and PaCO2. Multivariable linear regression was also used to determine patient factors associated with increased respiratory rate. Youden's J statistic and area under the receiver operating curve analysis was used to determine the optimal respiratory rate threshold for 28-day and 3-year mortality.

**RESULTS.** During the study period 7944 patients were screened and 633 patients (7.9%) met criteria for sustained ARDS. The median respiratory rate was higher in hospital non-survivors vs survivors (24, interquartile range [IQR] 20-28 vs 21, IQR 18-24 respectively, p< 0.001). Adjusted models showed that respiratory rate was associated with an increase in 28-day hospital mortality (odds ratio [OR]1.11, 95% confidence interval 1.06-1.17, p<0.001), 3-year mortality (OR 1.11, 95% CI 1.06-1.16, p<0.001), and reduced VFDs (mean difference -0.75, 95% CI -0.91 to -0.58, p<0.001). Patient factors associated with increased respiratory rate in a multivariable model included age, pneumonia or sepsis as ARDS etiology, admission pH, and initial mechanical ventilation on spontaneous mode. The respiratory rate threshold associated with an increase in 28-day hospital mortality and 3-year mortality was 22 for both.

**CONCLUSION.** Increased respiratory rate is associated with increase mortality and reduced 28-day VFDs. Further study will be required to determine if interventions targeted at reducing the respiratory rate can modify outcomes.

**REFERENCE(S)**

1. Quality improvement grant from the Chief Medical Office/Calgary Zone Medical Affairs – Alberta Health Services (KP)
2. Seed grant from the Critical Care Strategic Clinical Network (KP)

## ARF - Acute respiratory failure 10

### 001167

#### Individualized selection of PEEP in patients with Acute Respiratory Distress Syndrome: Electrical Impedance Tomography vs. Transpulmonary Pressure

G. Scaramuzzo[1], T. Mauri[2], F. Dalla Corte[1], AD. Waldmann[3], SH. Böhm[3], E. Spinelli[2], E. Marangoni[1], S. Spadaro[1], CA. Volta[1]
[1]Department of morphology, surgery and experimental medicine, University of Ferrara, Ferrara, Italy; [2]Department of anesthesiology, Fondazione IRCCS Ca'Granda Ospedale Maggiore Policlinico, Milano, Italy; [3]Klinik und poliklinik für anästhesiologie und intensivtherapie, University Hospital of Rostock, Rostock, Germany
**Correspondence:** G. Scaramuzzo
*Intensive Care Medicine Experimental* 2019, **7(Suppl 3):**001167

**INTRODUCTION.** Personalized positive end-expiratory pressure (PEEP) might attenuate lung injury and improve patient's outcome in acute respiratory distress syndrome (ARDS). However, how to set the optimal PEEP level remains controversial. Electrical impedance tomography (EIT), which monitors ventilation distribution, and esophageal pressure, which allows measure of transpulmonary pressure (PL), have been proposed as guide for personalized PEEP setting.

**OBJECTIVES.** To test if PEEP-EIT and PEEP-PL differs in terms of values,gas exchange and lung mechanics.

**METHODS.** After obtaining consent, an esophageal catheter (Nutrivent, SIDAM, Mirandola, Italy) and a 32-electrodes EIT monitor (Swisstom BB2, Swisstom, Landquart, Switzerland) were positioned in 20 ARDS patients . Each patient underwent, in random order, two PEEP titration trials to find the levels of PEEP associated with 1) positive end-expiratory PL based on a PL/FiO2 table (PEEP-PL) [1] and 2) the lowest PEEP providing silent spaces ≤15% (PEEP-EIT). FiO2 was set to obtain SpO2=88-95%, Vt was 6 ml/kg/PBW and RR to maintain normocapnia. After identifying the 2 PEEP levels, each was maintained for 20 minutes in a random order. Lung mechanics, gas exchange, hemodynamics and EIT data were recorded at the end of each step. Regional respiratory system compliance was calculated in 4 cranio-caudal regions of interest (ROI1=ventral;ROI4=dorsal). Wilcoxon signed-rank test was used to evaluate if the difference between variables during the two steps was significant.

**RESULTS.** Baseline P/F was 149 [108-210], RR 19 [16-24] bpm and Vt 380[358-440] ml. Median values of PEEP-EIT and PEEP-PL were 14 [8-17] cmH2O and 13 [11-14] cmH2O, with a median difference within patient of 1.5 [(-)7-5.8] cmH2O (p=0.95). No patient was assigned the same PEEP and correlation between the levels of PEEP was not significant (Rs=0.25; p=0.29). PEEP-EIT and PEEP-PL resulted in no statistically significant differences in gas exchange, but airway driving pressure (DP) was significantly higher and regional ventral lung compliance was lower at PEEP-EIT. On the other hand, PEEP-PL was associated with higher total and dependent hypoventilated regions (table 1).

**CONCLUSION.** Personalized PEEP levels assigned by EIT and PL were not correlated at the individual patient-level. PEEP-PL might be associated with lower risk of dynamic lung stress and overdistension while PEEP-EIT might be more protective from excessive lung inhomogeneity.

**REFERENCE(S)**

[1] Talmor D, Sarge T, Malhotra A, et al (2008) Mechanical ventilation guided by esophageal pressure in acute lung injury. N Engl J Med 359:2095–2104. https://doi.org/10.1056/NEJMoa0708638
[2] The study was supported by SIAARTI (Società Italiana di Anestesia Analgesia Rianimazione E Terapia Intensiva).

**Table 1 (abstract 001167).** See text for description

| | Difference ($PEEP_{EIT} - PEEP_{PL}$) | P value |
|---|---|---|
| $PaCO_2$ | 0,5[-0,9;1,9] | 0,53 |
| P/F | -7[-19;3,5] | 0,14 |
| Respiratory system DP ($cmH_2O$) | 1,2[-0,32;2,1] | **0,03** |
| Lung DP ($cmH_2O$) | 1[-0,83;2,6] | 0,11 |
| Total Silent Spaces | -1,9[-6,4;0,72] | **0,02** |
| Dependent Silent Spaces | -2,3[-7;0,72] | **0,02** |
| Non-Dependent Silent Spaces | 0[-0,82;0,73] | 0,71 |
| ROI1 regional compliance (RS) | -0,37[-3,1;0,54] | 0,10 |
| ROI2 regional compliance (RS) | -2,6[-3,5;0,17] | 0,06 |
| ROI3 regional compliance (RS) | -0,19[-3,4;0,87] | 0,23 |
| ROI4 regional compliance (RS) | -0,013[-1,1;0,72] | 0,67 |
| ROI1 regional compliance (Lung) | -2,2[-6,2;-0,016] | **0,03** |
| ROI2 regional compliance (Lung) | -4[-9,2;0,16] | **0,04** |
| ROI3 regional compliance (Lung) | -1,9[-3,7;0,86] | 0,10 |
| ROI4 regional compliance (Lung) | 0,17[-1,4;0,94] | 0,68 |

### 001175

#### Effect of Use Preemptive APRV According to Lung Injury Prediction Score (LIPS) on Preventing the Development of ARDS in the Intensive Care Unit

M. Pehlivanlar[1], ÇE. ÖZtürk[2], IN. Köylü[3], AO. Küçük[4], DF. Ergül[5], F. ÜLger[6]
[1]Departmen of chest disease, division of intensive care medicine, KTU Faculty of Medicine, Trabzon, Turkey; [2]Division of intensive care medicine, Samsun Training and Research Hospital, Samsun, Turkey; [3]Division of intensive care medicine, Samsun Gazi State Hospital, Samsun, Turkey; [4]Department of anesthesiology and reanimation, division of intensive care medicine, KTU Faculty of Medicine, Trabzon, Turkey; [5]Division of intensive care medicine, Hittite University Faculty of Medicine, Çorum, Turkey; [6]Department of anesthesiology and reanimation, division of intensive care medicine, Ondokuz Mayıs Üniversitesi Tıp Fakültesi , Samsun, Turkey
**Correspondence:** M. Pehlivanlar
*Intensive Care Medicine Experimental* 2019, **7(Suppl 3):**001175

**INTRODUCTION.** Airway pressure release ventilation(APRV) is a type of inverse-ratio, pressure-controlled,
mode.In addition to being used in patients with acute respiratory distress syndrome(ARDS), lung protective effects have recently become prominent (1).The aim of this study is to investigate whether the use of early airway pressure release ventilation(APRV) mode as a lung protective strategy is superior to conventional methods in ARDS development.

**METHODS.** The study was planned as, randomized controlled study between 2016-2018 in an 18-bed general intensive care unit(ICU).Patients with invasive mechanical ventilation but who were not diagnosed with ARDS at baseline, had LIPS(Lung Injury Prediction Score) >7 and intensive care hospitalization for more than 24 hours were included in the study.The patients were evaluated as APRV and P-SIMV groups.

**RESULTS.** 65 patients who met the eligibility criteria were included in the study.Thirty-three patients (50.8%) underwent P-SIMV mode and 32(49.2%) patients underwent APRV mode.P/F ratio values were higher in APRV group.This difference was statistically significant on day 3(p=0.032).The FiO2 value was lower in the APRV group.There was a statistically significant difference between the groups at day 7(p=0.011).Patients who needed sedation in the groups were statistically lower in the APRV group(p=0.048).The duration of mechanical ventilation was 9(3-65) days in the P-SIMV group and 7.5(2-29) days in the APRV group.No difference was found between the groups(p = 0.171).Duration of ICU stay was 8(2-11) days in the APRV group and 13(8-81) days in the P-SIMV group(p=0.019). (Table 1)

**CONCLUSION.** The goal of ventilation with APRV is to remain on the steep portion of the compliance curve between the lower and upper inflection points to prevent atelectrauma/barotraumas.Our study contributed to the results of a few preemptive studies in the literature.The same positive effects were also demonstrated in healthy lungs.Follow-up period, duration of ICU stay, sedation requirement and oxygenation parameters improved significantly.

**REFERENCE(S)**

1. Preemptive Application of Airway Pressure Release Ventilation (APRV) Prevents Development of Acute Respiratory Distress Syndrome (ARDS) in a Rat Traumatic Hemorrhagic Shock Model Shreyas K. Roy, M.D., C.M.,1 Bryanna Emr, M.D.,1 Benjamin Sadowitz, M.D.,1 Louis A. Gatto, Ph.D.,1,2 Auyon Ghosh, B.Sc.,1 Joshua M. Satalin, B.S.,1 Kathy P. Snyder, B.S.,1 Lin Ge, Ph.D.,1 Guirong Wang, Ph.D.,1 William Marx, D.O.,3 David Dean, Ph.D.,4 Penny Andrews, R.N.,5 Anil Singh, M.D.,1 Thomas Scalea, M.D.,5 Nader Habashi, M.D.,5 and Gary F. Nieman, B.A.1

**Table 1 (abstract 001175).** Comparison of end points without mortal patients

| | Total | P-SIMV+PS | APRV | p |
|---|---|---|---|---|
| APACHE II | 17 (7 - 35) | 16 (7 - 24) | 17 (7 - 35) | 0.164 |
| MV / Extubation duration (total day) | 9 (2 - 65) | 10 (3 - 65) | 8 (2 - 26) | 0.211 |
| ICU stay (day) | 16.5 (2 - 81) | 23.5 (10 - 81) | 11 (2 - 58) | **0.027** |
| Hospital stay (day) | 27.5 (8 - 85) | 33 (17 - 85) | 25.5 (8 - 79) | 0.297 |

All values are shown as med (min – max), p < 0.05 is istatistically significant. Bold P values are < 0.05 APACHE II, Acute Physiology, Age, Chronic Health Evaluation II; MV, mechanical ventilation; ICU, intensive care unit

### 001178

#### Safety and Tolerability of Mechanical Insufflation-Exsufflation (MIE) and Hypertonic Saline with Hyaluronic Acid (HS-HA) for Respiratory Secretion Suctioning (RSS) in Intubated Patients

M. Sanchez Garcia, M. Alvarez, S. Domingo, A. Del Pino, F. Martínez, P. González, C. Cardenal, E. Velasco, MJ. Jimenez, A. Núñez
[1]Critical care, Hospital Clínico San Carlos, Madrid, Spain
**Correspondence:** M. Sanchez Garcia
*Intensive Care Medicine Experimental* 2019, **7(Suppl 3):**001178

**INTRODUCTION.** Conventional catheter RSS causes pain and injuries to the tracheobronchial mucosa, and other complications. MIE generates high expiratory air flow, simulating spontaneous cough, and is used extensively in debilitated non-intubated patients for airway clearance. HS-HA is nebulized to fluidify secretions and facilitate RSS.

**OBJECTIVES.** Safety and tolerability of both interventions in intubated critically ill patients.

**METHODS.** Randomised controlled trial of the effect of a single RSS manoeuvre on respiratory and cardiovascular (CV) parameters and pain, sedation-agitation scores in 120 consecutive patients, assigned to 1) catheter RSS, 2) catheter RSS after HS-HA, 3) MIE, and 4) MIE with HS-HA. Patients with an artificial airway (AAW) with cuff, on mechanical ventilation (MV) or spontaneous breathing (SB), without bronchospasm or haemoptysis were included after obtaining informed consent. Catheter RSS was done according to local protocol (-120 to -150 mBar), MIE was programmed at +50/-50 cmH2O and a jet nebulizer administered 5 ml of HS-HA placed at the AAW (group 2) or MIE tubing (group 4). Data were collected before, during and 5 and 60 minutes after the intervention, and occurrence of adverse events (predefined respiratory and CV and other) was monitored from inclusion to ICU discharge. We used chi2, one-way Anova and Bonferroni post hoc test.

**RESULTS.** 19 eligible subjects met exclusion criteria and 30 per group were enrolled and completed the study protocol. There were no differences in baseline characteristics and total, respiratory and CV adverse events (Table). Pain Scale (Behavior Indicators of Pain, "ESCID") rose significantly during RSS in groups 1 and 2 and were significantly higher during RSS in groups 1 and 2 for all comparisons. No difference in ESCID was observed at 5 and 60 minutes after RSS. Richmond Agitation-Sedation Scale did not differ between time-points or study groups. Chest x-rays within 24 hours after RSS did not disclose AEs.

**CONCLUSION.** MIE and HS-HA are save and better tolerated than conventional catheter RSS in critically ill patients with AAW. Future studies should address the efficacy of both measures.

**Table 1 (abstract 001178).** See text for description

| | Catheter RSS | Catheter RSS + HS-HA | MIE | MIE + HS-HA |
|---|---|---|---|---|
| Age | 64.7±16.2 | 69.7±11.1 | 64.5±15.9 | 62.5±14.2 |
| Sex, Male/Female | 24/6 | 23/7 | 21/9 | 22/8 |
| Diagnostic group (M/S/T) | 15/14/1 | 12/16/2 | 13/12/5 | 12/14/4 |
| Apache II score, mean±SD | 22.8±8.1 | 22.1±7.0 | 22.3±8.1 | 21.2±6.7 |
| Endotrachel Tube/Tracheal cannula Size, mean±SD (mm) | 25/5; 7.9± 0.5 | 24/6; 7.9±0.4 | 25/5; 7.8±0.6 | 26/4; 7.9±0.4 |
| PaO2/FiO2 | 281±101 | 254±98 | 239±85 | 299±86 |
| Noradrenaline, n (%) | 17 (56.7) | 18 (60) | 15 (50) | 14 (46.7) |
| Adverse events, total, n (%) | 9 (30) | 8 (26.7) | 4 (13.3) | 9 (30) |
| Respiratory, n (%) | 3 (10) | 4 (13.3) | 1 (3.3) | 2 (6.7) |
| CV, n (%) | 8 (26.7) | 4 (13.3) | 6 (20) | 8 (26.7) |

### 001179

#### Esophageal balloon calibration versus conventional technique to assess esophageal pressure during volume control, pressure support, and pressure support – Sigh ventilation

G. Cammarota[1], E. Santangelo[2], G. Lauro[2], N. Devita[2], E. Boniolo[2], R. Tarquini[2], F. Verdina[2], R. Perucca[1], I. Sguazzotti[1], E. Spinelli[3], A. Bruni[4], E. Garofalo[4], F. Longhini[4], A. Messina[5], F. Della Corte[2], E. Bignami[6], P. Navalesi[7], T. Mauri[8], R. Vaschetto[2]
[1]Anesthesia and intensive care, Azienda Ospedaliero Universitaria Maggiore della Carità di Novara, Novara, Italy; [2]Department of translational medicine, Università Degli Studi Del Piemonte Orientale, Novara, Italy; [3]Department of anesthesiology, Fondazione IRCCS Ca'Granda Ospedale Maggiore Policlinico, Milano, Italy; [4]Anesthesia and intensive care unit, Magna Græcia University, Catanzaro, Italy; [5]Anesthesia and intensive care, Humanitas Research Hospital, Rozzano, Italy; [6]Anesthesia and intensive care, University of Parma, Parma, Italy; [7]Department of medical and surgical sciences, University Hospital Mater Domini, Magna Grecia University, Catanzaro, Italy; [8]Department of anesthesiology, Fondazione IRCCS Ca'Granda Ospedale Maggiore Policlinico - Servizio Beni Culturali, Milano, Italy
**Correspondence:** G. Cammarota
*Intensive Care Medicine Experimental* 2019, **7(Suppl 3):**001179

**INTRODUCTION.** Esophageal balloon pressure calibration (Ebc) is a 2-steps procedure that was proven to increase accuracy of esophageal pressure (Pes) measure in patients undergoing invasive volume-controlled mechanical ventilation (VCV) in the intensive care unit 1 (ICU) and the operating room 2.

**OBJECTIVES.** Aim of present study was to compare Pes measures after Ebc during VCV, pressure support (PSV), and PSV+Sigh with those obtained with conventional inflating at volume of 4 ml as manufacturer-recommended (V4).

**METHODS.** In 11 adult patients admitted in ICU, after esophageal balloon catheter was inserted, Ebc was performed to obtain optimal filling volume (Vbest) and to correct for pressure generated by esophageal wall and balloon during VCV, PSV and PSV+Sigh, applied in random order. The expiratory and inspiratory calibrated Pes values (Pescal), those obtained at Vbest (PesVbest) and at V4 (PesV4) were recorded.

**RESULTS.** PesV4 and PesVbest were higher than Pescal at end-expiration (13.1 ± 3.2 cmH2O vs 8.4 ± 3.1 cmH2O, *P*<0.0001; 11.1 ± 4.6 cmH2O vs 8.4 ± 3.1 cmH2O, *P*=0.0427) and end-inspiration (15.6 ± 3.1 cmH2O vs 11.7 ± 3.3 cmH2O, *P*<0.0001; 14.3 ± 5.1 cmH2O vs 11.7 ± 3.3 cmH2O, *P*=0.0427), respectively, during VCV. During PSV, expiratory and inspiratory PesV4 was greater than Pescal (14.1 ± 4 cmH2O vs 9.6 ± 3.9 cmH2O, *P*=0.0020; 17.2 ± 4 cmH2O vs 13.8 ± 4.4 cmH2O, *P*=0.0020). During Sigh, expiratory and inspiratory Pescal was lower compared to PesVbest (9.3 ± 3.9 cmH2O vs 14.9 ± 5 cmH2O, *P*=0.0002; 16.9 ± 4 cmH2O vs 22.4 ± 5.2 cmH2O, *P*=0.0002) and PesV4 (9.3 ± 3.9 cmH2O vs 13.4 ± 3.7 cmH2O, *P*=0.0060; 16.9 ± 4 cmH2O vs 20.1 ± 3.5 cmH2O, *P*=0.0232), respectively.

Vbest for Sigh was greater compared to that computed at VCV (5 ± 1.6 cmH2O vs 2.4 ± 1.9 cmH2O, *P*=0.0086) and PSV (5 ± 1.6 cmH2O vs 2.5 ± 1.8 cmH2O, *P*=0.0168), respectively. Esophageal wall pressure at Vbest increased moving from VCV and PSV to Sigh (2.6 ± 2.9 cmH2O vs 5.6 ± 2.7 cmH2O, *P*= 0.0086; 2.6 ± 2.6 cmH2O vs vs 5.6 ± 2.7 cmH2O, *P*= 0.0168), whereas no modifications were noted at V4 over the trials. Esophageal wall pressure was higher at V4 in VCV (5 ± 1.2 cmH2O vs 2.6 ± 2.9 cmH2O, *P*=0.0371) and PSV (5.1 ± 1 cmH2O vs 2.6 ± 2.6 cmH2O, *P*=0.0195) compared to Vbest.

**CONCLUSION.** Ebc improved Pes assessment during VCV, PSV and sigh. Average Vbest at VCV and PSV never achieved V4 and Vbest at Sigh. Balloon filling volume and esophageal wall artifacts were the only factors that negatively affected conventional Pes assessment in our series.

**REFERENCE(S)**

1. Mojoli et al, Critical Care 2016
2. Cammarota et al, Intensive Care Medicine Experimental 2018 (Suppl 2).

### 001185

#### Initial assessment of the Percutaneous Electrical Phrenic Nerve Stimulation (PEPNS) System in patients on mechanical ventilation

J. O'rourke[1], M. Soták[2], G. Curley[1], A. Doolan[1], T. Henlín[2], W. Omlie[3], T. Tyll[2]

[1]Department of anaesthesia and critical care medicine, Royal College of Surgeons in Ireland, Beaumont Hospital, Dublin, Ireland; [2]Department of anesthesia and intensive medicine, 1st Medical Faculty and University Military Hospital, Prague, Czech Republic; [3]Vascular and general surgery, University of Minnesota and Fairview Health System, Minneapolis, MN, USA

**Correspondence:** L. Yost

*Intensive Care Medicine Experimental* 2019, **7(Suppl 3):**001185

**INTRODUCTION.** Ventilator-Induced Diaphragm Dysfunction (VIDD) is considered a major determinant of the ability to successfully wean patients from mechanical ventilation (1). Maintaining diaphragm work using electrical stimulation during mechanical ventilation (MV) has been proposed to attenuate VIDD (2).

**OBJECTIVES.** This first-in-human study assessed the safety and feasibility of temporary Percutaneous Electrical Phrenic Nerve Stimulation (PEPNS) on user-specified inspiratory breaths while on MV. Primary endpoints included the ability to synchronize electrical stimulation with inspiration to mobilize the diaphragm and to maintain work of breathing (WOB) within defined limits. Secondary endpoints included the percentage of patients with successful pdSTIM multipolar lead placement via ultrasound guidance and serious device/procedure-related adverse events.

**METHODS.** This prospective, multi-center, single-arm trial enrolled ICU patients on MV. PEPNS was used for 6 two-hour sessions at eight-hour intervals over 48 hours. Electrical stimulation was used to activate the diaphragm in synchrony with inspiration while on MV. Data collected included lead deployment success, nerve integrity, ventilation parameters, blood gasses, vital signs, WOB, electrical stimulation parameters, stimulation-breath synchrony and diaphragm thickness measured by ultrasound at 0, 24 and 48 hours.

**RESULTS.** Twelve patients were enrolled with 2 initial pilot patients having leads inserted on the left side only. Lead insertion was successful in 21 of 22 attempts (95.5%). An analysis of 36,059 stimulated breaths from the 10 non-pilot patients in whom the pdSTIM leads were placed bilaterally demonstrated phrenic nerve stimulation had a mean inspiratory lag of 23.66 msec (p<0.001 vs. null hypothesis of <88msec). WOB was maintained between 0.2 and 2.0 joules/L for 96.77% of the time, exceeding the 80% target. No serious device/procedure-related adverse events were reported. Diaphragm thickness increased for both stimulated and unstimulated diaphragm hemispheres.

**CONCLUSION.** Our results demonstrate the ability to safely and successfully place PEPNS leads in MV patients and the feasibility of using this approach to synchronize electrical stimulation with inspiration while also maintaining WOB between defined limits.

**REFERENCE(S)**

1. Jaber S, Petrof BJ, Jung B, et al. Rapidly Progressive Diaphragmatic Weakness and Injury during Mechanical Ventilation in Humans. Am J Respir Crit Care Med 2011; 183:364-371.
2. Pavlovic D, Wendt M. Diaphragm pacing during prolonged mechanical ventilation of the lungs could prevent from respiratory muscle fatigue. Med Hypotheses 2003; 60:398-403.
3. This study was funded by a research grant from Stimdia Medical, Inc. Edina, Minnesota, USA.

### 001196

#### Predictors of successful weaning from high-flow nasal oxygen therapy in patients with acute respiratory failure: a retrospective monocenter study

M. Rodriguez, AW. Thille, F. Boissier, A. Veinstein, D. Chatellier, R. Robert, S. Le Pape, JP. Frat, R. Coudroy

Médecine intensive et réanimation, CHU de Poitiers, Poitiers, France

**Correspondence:** R. Coudroy

*Intensive Care Medicine Experimental* 2019, **7(Suppl 3):**001196

**INTRODUCTION.** Several trials have reported promising clinical effects of high flow nasal oxygen therapy (HFOT) as compared to standard oxygen therapy or noninvasive ventilation in various settings. However, factors associated with successful HFOT weaning have never been assessed. As HFOT continuation might lead to unnecessarily prolonged ICU stay, we aimed at identifying predictors of successful HFOT weaning.

**METHODS.** This is a retrospective monocenter study over a 2-year period including all patients treated with HFOT for acute respiratory failure. Patients who died or were intubated without prior HFOT weaning attempt, those who were treated with noninvasive ventilation at the time of HFOT weaning, and those who received HFOT as a preventive treatment during the post-extubation period were excluded. HFOT weaning was driven by the attending physician. HFOT weaning failure was defined as a respiratory failure requiring HFOT resumption, noninvasive ventilation initiation, intubation, or death within the first 48 hours after HFOT weaning. Demographic data, ventilatory settings, and vital parameters under HFOT were collected before each weaning attempt. The pulse oximetry to fraction of inspired oxygen ratio (SpO2/FiO2) and the ROX index (SpO2/FiO2 to respiratory rate) were calculated under HFOT before each weaning attempt (1).

**RESULTS.** Among the 190 patients retained in the analysis, maximal gas flow and FiO2 set under HFOT were 50 ± 3 L/min and 75 ± 21 %, respectively. The main reason for HFOT initiation was pneumonia in 46% of cases. The first HFOT weaning attempt was successful in 168 patients (88%). Their baseline characteristics were not different from those who failed. They required lower FiO2 under HFOT than those who failed (39 ± 7% vs. 48 ± 16%, respectively, p= 0.02). A FiO2 ≤ 40% was a predictor of successful HFOT weaning with a sensitivity of 85%, a specificity of 41%, a positive predictive value of 92%, a negative predictive value of 26% and an accuracy of 80%. Likewise, the ROX index was higher in patients who were successfully weaned from HFOT than in those who failed (12.7 ± 1.2 vs. 10.2 ± 3.0, p = 0.002). A ROX index > 9.2 was a predictor of successful HFOT weaning with a sensitivity of 84%, a specificity of 50%, a positive predictive value of 93%, a negative predictive value of 30% and an accuracy of 80%.

ICU length of stay was longer in patients who failed the first HFOT weaning attempt than in those who succeeded: 10 (7-12) vs. 5 days (4-8) respectively (p < 0.0001). The main reason for failure was hypoxemia in 95% of cases (21 out of 22) and required HFOT resumption after 13 (5-26) hours under standard oxygen. After the first HFOT weaning failure, 86% of patients (19 out of 22) were eventually weaned successfully from HFOT. Their ROX index before the weaning success was higher than before their prior weaning failure (12.4 ± 3.8 vs. 10.1 ± 3.1, respectively, p = 0.04).

**CONCLUSION.** In this retrospective monocenter study, a FiO2 ≤ 40% and a ROX index > 9.2 were the best predictors of successful HFOT weaning at the bedside. A multicenter prospective study is mandatory to confirm the usefulness of these two easy-to-assess parameters to predict the successful HFOT weaning.

### 001208

#### Non-invasive ventilation and energy expenditure in healthy subjects. A pilot

J. Jonckheer[1], A. Sablon[1], S. Du Four[2], M. Borremans[3], J. Demol[4], MLNG. Malbrain[5], E. De Waele[1]

[1]Intensive care, UZ Brussel, Jette, Belgium; [2]Neurosurgery, UZ Brussel, Jette, Belgium; [3]Physiotherapy, UZ Brussel, Jette, Belgium; [4]Nutrition, UZ Brussel, Jette, Belgium; [5]Intensive care unit, University Hospital Brussels (UZB), Jette, Belgium

**Correspondence:** J. Jonckheer

*Intensive Care Medicine Experimental* 2019, **7(Suppl 3):**001208

**INTRODUCTION.** Acute respiratory failure with respiratory acidosis is characterized by hyperinflation and insufficient, inappropriate muscle function with usually high muscle energy expenditure. Non-invasive assisted ventilation (NIV) via face mask can be used to facilitate gas exchange in the lungs but also to assist the distressed respiratory muscle function and diminish dyspnea levels. Indirect calorimetry uses gas exchange at the level of the lungs to calculate energy expenditure (EE) by incorporating O2consumption and CO2production in the Weir equation.

**OBJECTIVES.** The aim was to investigate the influence of NIV on indirect calorimetry and its correlation with respiratory effort.

**METHODS.** In 5 healthy subjects NIV (V60, Philips, Eindhoven, The Netherlands) was administered at a level of positive end expiratory pressure (PEEP) around 4 cmH2O and without additional inspiratory support. Inspiratory support was then increased by 2 cmH2O every 2 minutes until 8 cmH2O was achieved for 2 minutes. The EE was measured using indirect calorimetry (Q-NRG, Cosmed, Italy). To measure respiratory effort, the combined reversed RPE-scale was created for the purpose of the study. It uses the validated "rate of perceived exertion" scale (RPE-scale) which was altered by adding a reversed part. This could result in a score of -10 (no respiratory effort) until +10 ( maximal respiratory effort) where 0 represents the basal respiratory condition.

**RESULTS.** The application of NIV without inspiratory support resulted in a mean EE of 1080 ±267 Kcal/day and a combined reversed RPE-score of 1.8 ±1.1. After incremental increase of inspiratory support to 8 cmH2O, mean EE changed to 1138 ±397 Kcal/day (p=0.46)and the combined reversed RPE-score to -4.6 ±2.1 (p=0.004). From the inspiratory support, EE could be predicted as 16.91* X + 1037 (R2= 0.74) and combined reversed RPE-score as -0.845*X + 1.12 (R2= 0.91) using linear regression. Pearson r test showed a correlation coefficient of -0,828 (p=0.083).

**CONCLUSION.** NIV seems to induce a minimal metabolic burden in healthy subjects. Surprisingly a reversed relationship was observed between respiratory relieve by NIV and energy expenditure in healthy subjects. This suggests that in healthy subjects, respiratory relieve is not due to altered muscle energy expenditure.

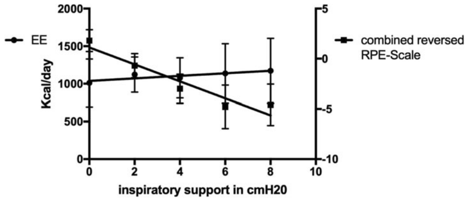

**Fig. 1 (abstract 001208).** See text for description

### 001215

#### Haemodynamic and ventilatory impact of tracheotomy: An observational studyy

W. Huber, F. Sehl, A. Herner, U. Mayr, G. Batres-Baires, S. Rasch, S. Schreiber, R. Schmid, T. Lahmer

[1]Medizinische klinik und poliklinik ii, Klinikum rechts der Isar; Technische Universität München, Munich, Germany

**Correspondence:** W. Huber

*Intensive Care Medicine Experimental* 2019, **7(Suppl 3):**001215

**INTRODUCTION.** Tracheotomy (TT) is a frequent procedure in critically ill patients. Its risks and benefits are matter of an ongoing debate. At least in theory there are some advantages including lower airway resistance due to the shorter tube length and less dead space. However, data on improved outcome are conflicting, and there is consensus about early and late complications as well as a substantial procedural mortality of up to 0.6%.

Little is known about short term haemodynamic effects.

**OBJECTIVES.** Therefore, we compared pre- and post TT hamodynamics and respiratory effects in 30 ICU-patients with transpulmonary thermodilution (TPTD) and pulse contour analysis (PCA) monitoring with the PiCCO-device (Pulsion; Germany).

**METHODS.** Analysis of a prospectively maintained database on haemodynamic monitoring with the PiCCO device. Comparison of the last values before and the first values after TT (primary endpoint).

**RESULTS.** 30 patients undergoing surgical tracheotomy for prolonged mechanical ventilation, 17 male; 13 female. APACHE-II 26± 8; age 72±10 years; height 170±10cm; weight 75±15kg.

Haemodynamics: SVRI was higher before compared to after TT: 1552±524 vs. 1361±437dyn*s/cm5/m$^2$; p=0.020.

All other haemodynamic parameters were comparable before and after TT: Heart rate 89±15 vs. 93±18/min; p=0.156. MAP 87±18 vs. 83±13mmHg; p=0.304; CVP 13±5 vs. 13±6mmHg; p=0.775. dPmax 1447±522 vs. 1469±553 mmHg/s; p=0.516; global enddiastolic volume index GEDVI 799±244 vs. 830±231mL/m$^2$; p=0.897; extravascular lung water index EVLWI 9.6±3.0 vs. 9.8±3.1mL/kg; p= 0.894; stroke volume index SVI 46±14 vs. 46±14mL/min; p=0.869;

Cardiac index CI 4.11±1.44 vs. 4.13±1.11L/min/m²; p=0.674; global ejection fraction GEF 24±7% vs. 24±8%; p=0.418.
Respiratory parameters: Tracheotomy resulted in an increase in the number of patients with controlled compared to assisted or spontaneous ventilation (8/30 vs. 2/30; p=0.038). All other respiratory parameters were comparable before and after TT: Respiratory rate 22±8 vs. 22±8/min; p=0.920; tidal volume 488±167 vs. 493±158mL; p=0.728; PEEP 7.6±1.9 vs. 7.9±1.9cmH2O; p=0.382; P_peak 24±6 vs. 23±6cmH2O; p=0.305; P_mean 12±4 vs. 12±3cmH2O; p=0.972; FiO2 0.42±0.11 vs. 0.42±0.10; p=0.859. P/F-ratio 240±77 vs. 234±84mmHg; p=0.349; Oxygenation index 6.2±3.9 vs. 6.6±4.0cmH2O/mmHg; p= 0.313; compliance 35±15 vs. 42±29mL/cmH2O; p=0.241.
**CONCLUSION.** 1.) TT did not result in haemodynamic impairment.
2.) TT induced a slight short-term increase in the rate of patients under controlled mechanical ventilation, but did not improve any respiratory parameter.

### 001230

#### Esophageal balloon calibration in patients undergoing invasive pressure support ventilation

G. Cammarota[1], G. Lauro[2], E. Santangelo[2], E. Boniolo[2], R. Tarquini[2], F. Verdina[2], R. Perucca[1], E. Spinelli[3], N. Devita[2], I. Sguazzotti[1], A. Bruni[4], E. Garofalo[4], F. Longhini[4], F. Della Corte[2], E. Bignami[5], A. Messina[6], P. Navalesi[7], T. Mauri[8], R. Vaschetto[2]
[1]Anesthesia and intensive care, Azienda Ospedaliero Universitaria Maggiore della Carità di Novara, Novara, Italy; [2]Department of translational medicine, Università Degli Studi Del Piemonte Orientale, Novara, Italy; [3]Department of anesthesiology, Fondazione IRCCS Ca'Granda Ospedale Maggiore Policlinico, Milano, Italy; [4]Anesthesia and intensive care unit, Magna Græcia University, Catanzaro, Italy; [5]Anesthesia and intensive care, University of Parma, Parma, Italy; [6]Anesthesia and intensive care, Humanitas Research Hospital, Rozzano, Italy; [7]Department of medical and surgical sciences, University Hospital Mater Domini, Magna Grecia University, Catanzaro, Italy; [8]Department of anesthesiology, Fondazione IRCCS Ca'Granda Ospedale Maggiore Policlinico - Servizio Beni Culturali, Milano, Italy
**Correspondence:** G. Cammarota
*Intensive Care Medicine Experimental* 2019, **7(Suppl 3):**001230

**INTRODUCTION.** Esophageal balloon calibration (Ebc) has been proposed in intensive care unit 1 (ICU) and the operating room 2 to improve accuracy of esophageal pressure (Pes) measure in patients undergoing invasive mechanical ventilation. Ebc is a 2-step procedure and consists in 1) identifying optimal balloon filling volume (Vbest) and 2) subtracting esophageal wall recoil pressure to Pes measured at Vbest (PesVbest) to obtain calibrated Pes (Pescal).
**OBJECTIVES.** The present study aimed to evaluate Ebc and Pescal in intubated patients undergoing pressure support ventilation (PSV) compared to the technique recommended by the manufacturer (i.e., standard filling volume of 4 ml (V4)).
**METHODS.** In intubated and mechanically ventilated adult patients, admitted to the ICU for acute hypoxemic respiratory failure, a naso-gastric tube equipped with esophageal balloon was inserted (Nutrivent, Sidam, Mirandola, Italy). After correct insertion depth was assured, Ebc was carried out while patients were ventilated on PSV. Briefly, the balloon was progressively inflated with volume ranging between 0 and 8 ml. Aiming to construct the end-expiratory and inspiratory balloon pressure-volume curve, a series of short end-inspiratory and end-expiratory holds were applied at each filling volume, assuring patient's relaxation during occlusions and interval of 15-30 breaths between each occlusion 3 - 5. Vbest, was identified as the filling volume associated with the maximal tidal Pes swing (Pesswing) on tidal volume (Vt) ratio (Pesswing/Vt). Then, on the end-expiratory pressure-volume curve, esophageal wall elastance was quantified to quantify esophageal recoil pressure at Vbest and, finally, Pes measured at Vbest (PesVbest) was corrected for esophageal wall pressure to obtain Pescal. Raw Pes at V4 values (PesV4) were recorded, too. Finally, the Baydur occlusion calibration test was performed to compare accuracy of the two methods. Finally, pressure generated by esophageal balloon pressure was computed.
**RESULTS.** Ebc was performed in 11 patients. Occlusion test was accurate (0.8-1.2 ratio) in 11/11 (100%) at Vbest and 5/11 (45.5%) patients at V4 ($P$ = 0.0124), being 0.87 ± 0.07 with Vbest and 0.79 ± 0.1 with V4 ($P$ = 0.0098), respectively. Vbest and Vmin was lower than Vmax (2.5 ± 1.8 vs 6.3 ± 1.3 ml, $P$ = 0.0316; 0.6 ± 0.2 vs 6.3 ± 1.3 ml, $P$ < 0.0001). Pesswing/Vt at Vbest was higher than that computed at V4 (8.5 ± 3.1 vs 5.9 ± 3.5 cmH2O/l, $P$ = 0.0010). Esophageal wall pressure computed at Vbest was lower than at V4 (2.6 ± 2.6 vs 5.1 ± 1 cmH2O, $P$ = 0.0195). Esophageal balloon pressure was negligible at Vbest and V4. Expiratory and inspiratory Pescal was lower than PesV4 (9.6 ± 3.9 vs 14.1 ± 4 cmH2O, $P$ = 0.0020; 13.8 ± 4.4 cmH2O, $P$ 0.0020).
**CONCLUSION.** Ebc is feasible in patients undergoing PSV and it might be associate with more accurate assessment of the esophageal pressure swings, which are a critical component of the risk of lung and diaphragm injury during assisted ventilation.

**REFERENCE(S)**

1. Mojoli et al, Critical Care 2016
2. Cammarota et al, Intensive Care Medicine Experimental 2018 (Suppl 2).
3. Bellani et al, Critical Care 2016
4. Bellani et al, Intensive Care Medicine 2018
5. Younes et al, Am J Resp Crit Care Med 2001

### 001235

#### Calibrated esophageal pressure versus conventional technique to monitor transpulmonary pressure during Sigh

G. Cammarota[1], E. Santangelo[2], G. Lauro[2], F. Verdina[2], E. Boniolo[2], N. Devita[2], R. Tarquini[2], R. Perucca[1], I. Sguazzotti[1], E. Spinelli[3], F. Longhini[4], E. Garofalo[4], A. Bruni[4], F. Della Corte[2], E. Bignami[5], A. Messina[6], P. Navalesi[7], T. Mauri[8], R. Vaschetto[2]
[1]Anesthesia and intensive care, Azienda Ospedaliero Universitaria Maggiore della Carità di Novara, Novara, Italy; [2]Department of translational medicine, Università Degli Studi Del Piemonte Orientale, Novara, Italy; [3]Department of anesthesiology, Fondazione IRCCS Ca'Granda Ospedale Maggiore Policlinico, Milano, Italy; [4]Anesthesia and intensive care unit, Magna Græcia University, Catanzaro, Italy; [5]Anesthesia and intensive care, University of Parma, Parma, Italy; [6]Anesthesia and intensive care, Humanitas Research Hospital, Rozzano, Italy; [7]Department of medical and surgical sciences, University Hospital Mater Domini, Magna Grecia University, Catanzaro, Italy; [8]Department of anesthesiology, Fondazione IRCCS Ca'Granda Ospedale Maggiore Policlinico - Servizio Beni Culturali, Milano, Italy
**Correspondence:** G. Cammarota
*Intensive Care Medicine Experimental* 2019, **7(Suppl 3):**001235

**INTRODUCTION.** Application of Sigh in patients with the acute respiratory distress syndrome undergoing invasive assisted mechanical ventilation has been shown to improve oxygenation and lung recruitment 1.
**OBJECTIVES.** Aim of present study was to compare, in patients undergoing pressure support ventilation (PSV) + Sigh, the pressure applied to the lung (Pl) during Sigh breaths, measured at optimal esophageal balloon filling volume (Vbest) versus at recommended filling volume of 4 ml (V4).
**METHODS.** In intubated ICU adult patients with hypoxic acute respiratory failure of various etiologies, mechanically ventilated in PSV, a naso-gastric catheter equipped with esophageal balloon (Nutrivent, Sidam, Mirandola, Italy) was inserted. During PSV, inspiratory 4 sec-long PCV mode Sigh at a pressure of 35 cmH2O was set at a rate of 1 per minute. After the correct position of esophageal balloon was reached, esophageal balloon calibration was performed to obtain calibrated esophageal pressure at Vbest during Sigh. Hence, expiratory and inspiratory Pl for dependent (Pld) and non-dependent (Plnd) lung regions was calculated at Vbest and V4 2.
**RESULTS.** In 11 patients we performed esophageal balloon calibration during Sigh. Vbest was similar to V4 (5 ± 1.6 vs 4 ± 0 ml, $P$ = 0.0859). Expiratory Pld at Vbest was 0.1 ± 4.2 cmH2O vs -4 ± 4.2 cmH2O at V4 ($P$ = 0.0010). Expiratory Plnd was slightly lower at Vbest compared to V4 (6.4 ± 2.5 vs 6.8 ± 2.5 cmH2O, $P$ = 0.0020).

Inspiratory Pld was 18.2 ± 4 cmH2O at Vbest vs 14.9 ± 3.5 at V4 ($P$ = 0.0049) whereas Inspiratory Plnd at Vbest was 24.5 ± 4.5 cmH2O vs 25.9 ± 4.5 cmH2O at V4 ($P$ = 0.0020). Lung, chest wall, and respiratory system elastances at sigh pressure were 23.8 ± 25.2 cmH2O/l, 7.5 ± 6.6 cmH2O/l, and 31.35 ± 28.5 cmH2O/l, respectively. Tidal volume during sigh was 1,371 ± 0.841 l (tidal volume - predicted body weight ratio 21.3 ± 11. 04 ml/Kg). Lung and respiratory system driving pressure was 18.1 ± 4.9 cmH2O and 25.6 ± 3.8 cmH2O, respectively. Inspiratory Plnd was directly correlated with lung driving pressure (Spearman r = 0.8091, confidence interval 0.3891 – 0.9506, $P$ = 0.0039).

**CONCLUSION.** In patients undergoing mechanical ventilation under PSV + Sigh, Pl assessment was more accurate applying Vbest compared to V4. Noteworthy, when Sigh is delivered at 35 cmH2O, inspiratory Plnd exceeded the safety threshold of 20-22 cmH2O, suggesting that setting sigh at lower pressure might be safer.

**REFERENCE(S)**

1. Pelosi et al, Am J Respir Crit Care Med 1999
2. Yoshida et al, Am J Respir Crit Care Med 2018

### 001680

#### Validation of lung injury prediction score in high risk trauma and surgical patients

M. Ahmed[1], K. Taema[2], S. Fawzi[2], G. Hamed[2]
[1]Intensive care medicine, Al Haram hospital, Cairo, Egypt; [2]Critical care medicine, Cairo University, Faculty Of Medicine, Kasr Al Ainy, Cairo, Egypt
*Intensive Care Medicine Experimental* 2019, **7(Suppl 3):**001680

Correspondence: K. Taema
*Intensive Care Medicine Experimental* 2019, **7(Suppl 3):**001680

**INTRODUCTION.** Major suegery and taruma patients are important subset of patients who are liable to acute respiratory distress syndrome (ARDS).

**OBJECTIVES.** We intended to validate a previously derived lung injury prediction scores (LIPS) in predicting ARDS and in-hospital mortality in high risk major trauma and surgical ICU patients.

**METHODS.** 79 patients with APACHE-II score≥15 admitted to the ICU by major trauma and/or major surgery were included in our prospective observational study. LIPS was calculated within six hours of admission using two previously derived formulas by Cartin-Ceba et al, 2009 and Trillo-Alvarez et al, 2011. We measured C-reactive protein on admission (CRP-0) and 48-hours later (CRP-48). Our endpoints included ARDS development and in-hospital mortality.

**RESULTS.** 33 patients (41.8%) developed ARDS after median(Q1-Q3) of 4(2-7) days. The LIPS of Cartin-Ceba et al and Trillo-Alvarez et al were 5(4-6) and 4.5(3-6.3) in ARDS patients compared to 3(1.8-4.3) and 3(1.5-4.6) in non-ARDS patients ($P$=0.000 & 0.001 for them respectively). CRP-48 was 96(57-192)mg/L and 48(24-96)mg/L in both groups respectively ($P$=0.000). The AUC was 0.741 and 0.712 for Cartin-Ceba et al and Trillo-Alvarez et al respectively. LIPS of Cartin-Ceba et al of 3.5 was 79% sensitive and 59% specific while LIPS of Trillo-Alvarez et al of 2.25 was 91% sensitive and 41% specific for ARDS prediction. CRP-48 was the only variable which was significantly higher in non-survivors ($P$=0.000). CRP-48 of 94 mg/L was 73% sensitive and 69% specific in predicting in-hospital mortality.

**CONCLUSION.** We concluded that despite both LIPS scores are equally effective in predicting ARDS in ICU trauma and major surgery patients, they were less accurate than in general ICU population.

### 001689

#### Acute Respiratory Distress Syndrome in patients with severe P. falciparum Malaria admitted to an Intensive Care Unit of a Tertiary Teaching Hospital from Portugal

L. Graça[1], I. Abreu[1], L. Graça[2], A. Ferreira[1], S. Xerinda[1], AS. Faustino[1], P. Figueiredo[1], L. Santos[1]
[1]Infectious Diseases, Saint John Hospital, Porto, Portugal; [2]Nursing, School of Health Sciences of Viana do Castelo, Viana do Castelo, Portugal
**Correspondence:** L. Graça
*Intensive Care Medicine Experimental* 2019, **7(Suppl 3):**001689

**INTRODUCTION.** ARDS (Acute Respiratory Distress Syndrome) is a common (up to 25%) and serious complication of malaria. Factors associated with its development and prognosis are poorly understood.

**OBJECTIVES.** This study aims to identify risk factors for ARDS and related prognosis.

**METHODS.** We retrospectively evaluated the clinical records of 99 patients with severe malaria due to P. falciparum who were admitted to an Intensive Care Unit (ICU) of a Tertiary Care Hospital from January 2009 to December 2018. We defined severe malaria according to World Health Organization (WHO) 2015 guidelines and used the Berlin criteria for ARDS. Continuous variables were compared among groups using the t test or Mann-Whitney test and categorical variables using the chi-square test.

**RESULTS.** We identified 33 ARDS cases (33%). The mean age was 44.7 ± 11.3 years and 87.9% were males. Patients developed ARDS 7.4 ± 3.5 days after the onset of symptoms. At the time of the admission to the ICU, 36.4% of patients already had ARDS. Three patients were managed with non-invasive ventilation (NIV) only, 30 patients required invasive ventilation (IMV), 10 of which had failed NIV trial. The ventilator parameters were available for 30 patients: none had mild ARDS, 43.3% had moderate ARDS and 56.7% had severe ARDS. The mean time between ICU admission and intubation was 1.9 days (minimum 0, maximum 5 days) and the mean duration of IMV was 8.95 days (min 1, max 52 days). Thirteen patients required curarization, 3 prone positioning and 3 Extra Corporeal Membrane Oxygenation (ECMO), for 7, 15 and 45 days, respectively. Cough was the only factor significantly associated with ARDS (OR 2.7; 95% CI 1.1 - 6.7). Time to endotracheal intubation was not associated with duration of IMV, need for curarization, prone positioning or ECMO. The overall fatality rate was 4% and the fatality rate in those with ARDS was 6.4%, with no deaths directly attributed to ARDS. Patients with ARDS had longer ICU stay (16.6 ± 11.3 vs 4.4 ± 4.4), longer hospital stay (24.7 ± 14.9 vs 9.5 ± 7.7) and a higher risk of nosocomial infection (OR 2.1; 95% CI 1.4-3.2), namely ventilator-associated pneumonia (OR 1.4; 95% CI 1.1 – 1.8).

**CONCLUSION.** Cough, a clinical parameter, was the best predictor of development of ARDS in our study which highlights the need to evaluate these patients thoroughly and monitor for the development of respiratory failure. NIV trial, although with a high failure rate, did not worsen the prognosis of these patients and might be an adequate therapy for some patients. The treatment of malaria ARDS by specialized teams with access to advanced techniques dramatically improves the chance of survival.

**REFERENCE(S)**

1. World Health Organization. (2015). Guidelines for the treatment of malaria. World Health Organization.

2. Definition Task Force, A. R. D. S., Ranieri, V. M., Rubenfeld, G. D., Thompson, B. T., Ferguson, N. D., Caldwell, E., ... & Slutsky, A. S. (2012). Acute respiratory distress syndrome: the Berlin Definition. JaMa, 307(23), 2526-2533.
3. Taylor, W. R., Hanson, J., Turner, G. D., White, N. J., & Dondorp, A. M. (2012). Respiratory manifestations of malaria. Chest, 142(2), 492-505.

### 001702

#### Right and Left Ventricular Functions during Proportional Assist Ventilation and During Weaning from Mechanical Ventilation

M. Elansary, YS. Nassar, R. Soliman, H. Mowafy
[1]Critical care medicine, Kasr El Aini Teaching Hospital, Cairo, Egypt
**Correspondence:** M. Elansary
*Intensive Care Medicine Experimental* 2019, **7(Suppl 3):**001702

**INTRODUCTION.** Mechanical ventilation (MV) induces changes in lung volumes and intrathoracic pressures which are transmitted to the heart, great arteries and veins and therefore independently affect the key determinants of cardiovascular performance and consequently Heart rate (HR) and myocardial contractility. New ventilatory modes as Proportional assist Ventilation (PAV+) with more patient synchrony may have better hemodynamic effects.

**OBJECTIVES. T**o evaluate the changes in hemodynamics , left & right ventricular functions during weaning from MV & to document the hemodynamic impact of MV in patients with normal & impaired left ventricular systolic function.

**METHODS.** 100 patients who received MV using pressure control mode were recruited in the study after fulfilling all the criteria for a spontaneous breathing trial. Every patient underwent 4 phases of ventilation with 2 hours apart and each phase lasted for 3 hours which were Pressure control – Assisted control ventilation (PC-ACV) with a preset pressure sufficient to maintain tidal volume of 4-6ml/kg & preset respiratory rate (RR) which is sufficient to eliminate CO2 in arterial blood gases, spontaneous breathing trial (SBT) with Pressure Support (PS) 10 , Positive end Expiratory pressure (PEEP) 5 , PAV+ mode with 20-30% assist and finally after discontinuation of MV. During each phase, the changes in change in HR, RR, systolic and diastolic blood pressures (SBP & DBP) were followed. Thoracic electrical bioempedance was used to measure stroke index (SI), cardiac index (CI) and systemic vascular resistance (SVR) in each phase of ventilation. Echocardiography and tissue Doppler imaging (TDI) were used to assess the changes in Left and right ventricular systolic and diastolic functions during each phase of ventilation. Patients with atrial fibrillation (AF), receiving vasopressors and inotropes and patients who failed weaning trial within less than 24 hours post extubation were excluded.

**RESULTS. SBP, DBP, SI, CI & SVR** did not show any significant changes between all phases of ventilation in all groups **(P: Non significant). HR & E/A** were higher during spontanous breathing in all patient groups **(P<0.001 & P<0.012 respectively). EF** was highest during PAV+ in all patient groups **(P<0.018). Left ventricular "S" wave** was higher during PAV+ & PC-ACV in Normal & Impaired systolic function groups **(P 0.024). E/E'** was lower during PC-ACV in Normal systolic function group **(P 0.037).**

**TAPSE** was higher during PAV+ & PC-ACV in Normal systolic function group **(P<0.008). Right ventricular "S" wave** was higher in PAV+ in impaired systolic function group **(P 0.024). Tricuspid E/E'** was higher during Spontanous breathing in all patient groups **(P 0.002). Pulmonary artery systolic pressure (PASP)** was lower during PC-ACV in impaired systolic function group **(P 0.04).**

**CONCLUSION.** PAV+ may have favourable effects on hemodynamics, left & right ventricular functions in patients ready for weaning.

### 001704

#### Association between extravascular lung water and computed tomography lung attenuation pattern in acute respiratory failure

J. Graf, P. Vargas, A. Salazar, P. Mercado, R. Perez
[1]Departamento de Paciente Crítico, Clinica Alemana, Vitacura, Chile
**Correspondence:** J. Graf
*Intensive Care Medicine Experimental* 2019, **7(Suppl 3):**001704

**INTRODUCTION.** Extravascular lung water (EVLW) and pulmonary vascular permeability index (PVPI) measured by transpulmonary thermodilution (TPTD) add objectivity to ARDS diagnosis. Paradoxically, up to a third of patients who meet ARDS criteria have near-normal EVLW. In addition, ARDS can be classified as focal or non-focal according to computed tomography (CT) lung attenuation pattern.

**OBJECTIVES.** To assess the association between EVLW or PVPI and CT lung attenuation pattern in patients with acute respiratory failure (ARF).

**METHODS.** Retrospective observational study. Inclusion criteria were invasive mechanical ventilation, $PaO_2$:$FiO_2$ ratio <300, hemodynamic monitoring with TPTD (PiCCO®, Pulsion Medical System) and CT of the chest performed <8 hours apart form a TPTD measurement. Patients with cardiogenic pulmonary edema or chronic lung diseases were excluded. Measurement of EVLW indexed by predicted body weight (EVLWi), PVPI (EVLW/pulmonary blood volume), $PaO_2$:$FiO_2$ and oxygenation index (mean airway pressure/$PaO_2$:$FiO_2$*100) closest to the CT acquisition were recorded. Patients were classified according to CT attenuation distribution as non-focal ARDS (bilateral diffuse), focal ARDS (bilateral dependent) and non-ARDS (unilateral). Data are expressed as median [IQR]; variables were compared according to morphologic categories using the Kruskal-Wallis test and p<0.05 was considered significant.

**RESULTS.** 36 patients were included, 29 fulfilled the Berlin definition for ARDS. Age 65 [51-71] years, APACHE II 18 [13-26], $PaO_2$:$FiO_2$ 176 [107-252], oxygenation index 7.6 [3.7-13.2]. Oxygenation and TDTP variables according to morphologic categories are presented in table 1. There were no differences in oxygenation between groups. EVLWi was significantly higher in patients with non-focal ARDS (n=20) than in those with focal ARDS (n=9) or non-ARDS (n=7). PVPI was also higher in non-focal than in focal ARDS. EVLWi and PVPI were not different between focal ARDS and non-ARDS patients. Among the 29 patients with ARDS, 10 (34%) had EVLWi <10 ml/Kg; 7 of these (70%) were focal.

**CONCLUSION.** Patients who meet conventional ARDS criteria with EVLWi <10 ml/Kg are likely to have focal morphology. Conversely, ARDS with EVLWi >10 ml/Kg is likely non-focal. Focal ARDS has an oxygenation and TPTD profile similar to ARF with unilateral lung opacities. Non focal or difuse ARDS may represent true permeability pulmonary edema.

**REFERENCE(S)**

1. Kushimoto S, Taira Y, Kitazawa Y, et al (2012) The clinical usefulness of EVLW and pulmonary vascular permeability index to diagnose and characterize pulmonary edema. Critical Care 16:R232
2. Michard F, Zarka V, Alaya S (2004) Better characterization of ALI/ARDS using lung water. Chest 125:1166
3. Constantin JM, Grasso S, Chanques G, et al (2010) Lung morphology predicts response to recruitment maneuver in patients with ARDS. Crit Care Med 38:1108-1117

**Table 1 (abstract 001704).** See text for description

| | Non-focal ARDS | Focal ARDS | Non-ARDS |
|---|---|---|---|
| $PaO_2/FiO_2$ | 133 (98-216) | 117 (86-222) | 206 (138-222) |
| Oxygenation index | 10.3 (6.6-16.7) | 7.9 (4.0-14.2) | 7.6 (3.9-8.7) |
| EVLWi (ml/Kg) | 12.9 (10.9-18.6)*'** | 8.1 (7.1-10.7) | 6.6 (5.8-9.4) |
| PVPI | 2.50 (1.85-2.75)** | 1.60 (1.05-1.60) | 1.45 (0.85-2.35) |

### 001734

#### An Initial Study of Delivery of Nebulised Drugs During High Flow Nasal Cannula Oxygenation

M. McCullagh[1], T. Young[2], J. Martin-Lazaro[2]
[1]Newham University Hospital, London, France; [2]Icu, Newham University Hospital, London, France
**Correspondence:** M. McCullagh
*Intensive Care Medicine Experimental* 2019, **7(Suppl 3):**001734

**INTRODUCTION.** High flow nasal cannula (HFNC) oxygenation is an emerging therapy for hypoxic respiratory failure(1,2,3). Frequently patients receiving HFNC therapy also require nebulised drug delivery; in particular, bronchodilators such as salbutamol. Some of these patients are unable to tolerate even brief periods of time without HFNC oxygen and desaturate if the HFNC is swapped to a face mask with a nebuliser attached. Therefore, nebulised drugs should ideally be delivered simultaneously to HFNC therapy. The optimal method to achieve this simultaneous delivery is not yet established. Studies exist on HFNC therapy combined with nebuliser therapy - showing optimal aerosol particle sizes in vitro in nebuliser delivered through HFNC compared to via face mask with jet nebulisation(4); and looking at identification of optimal settings for the implementation of nebulisation within HFNC circuits(5) - but comparison of nebuliser delivery method with ongoing HFNC has not been widely studied.

**OBJECTIVES.** To compare effects of nebulisation therapy in conjunction with HFNC therapy between delivery with face mask plus nebuliser attachment, and delivery with nebuliser attachment in-line with HFNC tubing.

**METHODS.** A retrospective observational study on critical care patients with respiratory failure requiring HFNC therapy and bronchodilators (n=20). Group 1 (n=9) received nebuliser therapy via a face mask fitted with a jet nebulisation chamber which was placed over the nose and mouth, with the HFNC still in place.Group 2 (n= 11) received nebuliser therapy via a nebulisation chamber which was attached upstream of the HFNC in-line with the HFNC tubing. Clinical and biochemical parameters were compared in each patient within 1 hour before and within 1 hour after nebuliser therapy with 2.5 mg salbutamol. Heart rate (HR), respiratory rate (RR) and peripheral capillary oxygen saturation (SpO2) were measured as the clinical parameters. Arterial blood gas sampling was used to assess biochemical parameters: pH, PaCO2, PaO2 and HCO3-.

Data collection and analysis was carried out using Excel.

**RESULTS.** Twenty patients were included in the study with an average age of 59 +/- 15 years.

An initial direct comparison of means showed a generally favourable outcome in parameters post nebuliser therapy delivered via tubing compared with delivery via face mask. Using t-test analysis (significance set a p value of <0.05), we observed a statistically significant reduction (p=0.037) in heart rate with nebuliser therapy delivered through tubing compared to delivery via face mask. Changes in other parameters were statistically comparable between the two groups (RR: p = 0.107; SpO2: p = 0.316; pH p = 0.238; PaCO2 p = 0.313; PaO2: p = 0.298, HCO3-: p = 0.298).

**CONCLUSION.** Current statistical analysis suggests that nebuliser therapy delivered in conjunction with HFNC via tubing is at least as effective as nebuliser therapy delivered in conjunction with HFNC via face mask, with a statistically significant improvement in heart rate in the tubing group. The improvement in mean parameters for nebuliser delivered via tubing compared to face mask is promising, although not statistically significant. However, it is possible that the small population size could lead to a Type II error (failure to reject a false null hypothesis), and it would certainly be of value to extend this initial study to a prospective study over a larger timescale with a greater sample population.

**REFERENCE(S)**

[1] Song, H. Z., Gu, J. X., Xiu, H. Q., Cui, W., & Zhang, G. S. (2017). The value of high-flow nasal cannula oxygen therapy after extubation in patients with acute respiratory failure. Clinics (Sao Paulo, Brazil), 72(9), 562–567. doi:10.6061/clinics/2017(09)07

[2] Xu, Z., Li, Y., Zhou, J., Li, X., Huang, Y., Liu, X., … Zhang, H. (2018). High-flow nasal cannula in adults with acute respiratory failure and after extubation: a systematic review and meta-analysis. Respiratory research, 19(1), 202. doi:10.1186/s12931-018-0908-7

[3] Drake MG (2018). High-flow nasal cannula oxygen in adults: an evidence-based assessment. Ann Am Thorac Soc. 2018; 15(2): 145–155.

[4] Juan Valencia-Ramos, Juan Arnaez, José Manuel Benito, Alicia Mirás, Carlos Ochoa & Sagrario Beltrán (2019) A comparative in vitro study of standard facemask jet nebulization and high-flow nebulization in bronchiolitis, Experimental Lung Research, DOI: 10.1080/01902148.2019.1599084

[5] François Réminiac, Laurent Vecellio, Nathalie Heuzé-Vourc'h, Antoine Petitcollin, Renaud Respaud, Maria Cabrera, Deborah Le Pennec, Patrice Diot, and Stephan Ehrmann (2016). Aerosol Therapy in Adults Receiving High Flow Nasal Cannula Oxygen Therapy, Journal of Aerosol Medicine and Pulmonary Drug Delivery 29:2, 134-141

### 001736

### Possible Complications of Enzymatic Debridement In Critically Burn Patients with Inhalation Injury

C. Gutiérrez Mavarez, A. Garcia Muñoz, C. Arevalo Martin, M. Sanchez, L. Cachafeiro, A. Agrifoglio, E. Flores, B. Civantos Martin, A. García De Lorenzo

[1]Intensive care unit, Hospital La Paz, Madrid, Spain, Spain

**Correspondence:** C. Gutierrez

*Intensive Care Medicine Experimental* 2019, **7(Suppl 3):**001736

**INTRODUCTION.** Enzymatic debridement has supposed a great advance in the treatment of critically burned patients, reducing time until debridement and therefore ICU stay. There is a great amount of systemic implications that hasn't been fully studied about these topic treatments, including those in patients with Inhalation injury.

**OBJECTIVES.** To describe our experience using enzymatic debridement in critically burn patients with inhalation injury and determine possible complications of its use in this set of patients.

**METHODS.** We performed a Retrospective observational study in the Critical Burn Unit of the La Paz Hospital in Madrid Spain from January 2017 until February 2019. All critically burned patients over 18 years old with a high suspicion or confirmed Inhalation Injury were included. Mean and standard deviation were used for normal quantitative variables and median and interquartile range in the opposite case. Qualitative variables were presented by absolute and relative frequencies.

**RESULTS.** We studied 152 patients admitted in our Critical Burn Unit. 16 patients (10.5%) had high suspicion or compatible criteria with Inhalation Injury, with initial carboxyhemoglobin over 4% at admission. Mean age was 59 (+- 24.5), Median TBSA was 17% (RI 6-42%), APACHE II was 15 (RI 10-19), ABSI score was 7 (RI 5-9). The median initial levels of Carboxyhemoglobin at admission in the ICU was 13 (RI 5-22), mean Lactate at admission was 4.2. Out of the 16 patients studied, 5 patients (31%) underwent enzymatic debridement in the first 12 hours of admission. 3 out of 5 patients (60%) in whom enzymatic debridement was performed presented a prolongation of corrected QT interval, two of them developed Ventricular Tachycardia followed by cardiac arrest in the first 24 hours.

**CONCLUSION.** Inhalation of toxic gas in critically burn patients might worsen the systemic response typical of these patients. 60% of the patients that received enzymatic debridement presented abnormalities in the electrocardiogram, 2 of them resulting in fatal Ventricular Tachycardia. Although more studies are needed, we suggest close monitoring and follow-up of patients with inhalation injury who undergo early enzymatic debridement because it can increase the inflammatory response or potentiate the effects of inhaled toxins.

**REFERENCE(S)**

1. Kaita, Yasuhiko et al. Cyanide poisoning is a possible cause of cardiac arrest among fire victims, and empiric antidote treatment may improve outcomes. The American Journal of Emergency Medicine , Volume 36 , Issue 5 , 851 - 853
2. Peers C, Steele DS. Carbon monoxide: a vital signalling molecule and potent toxin in the myocardium. J Mol Cell Cardiol 2012;52:359–365
3. Bajraktarova-Valjakova E, Korunoska-Stevkovska V, Georgieva S, et al. Hydrofluoric Acid: Burns and Systemic Toxicity, Protective Measures, Immediate and Hospital Medical Treatment. Open Access Maced J Med Sci. 2018;6(11):2257–2269. Published 2018 Nov 20. doi:10.3889/oamjms.2018.429

4. Walker PF, Buehner MF, Wood LA, et al. Diagnosis and management of inhalation injury: an updated review. Crit Care. 2015;19:351. Published 2015 Oct 28. doi:10.1186/s13054-015-1077-4

#### 001753

#### High Flow Oxygen Therapy application outside the ICU in Do Not Intubate patients with hypoxemic respiratory failure

N. Corcione[1]; A. Guzzardella[2]; SM. Colombo[2]; R. Russo[1]; A. Galazzi[1]; MC. Paleari[2]; P. Tagliabue[1]; T. Mauri[2]; A. Pesenti[2]; G. Grasselli[2]
[1]Department of anesthesia, critical care and emergency, Ospedale Maggiore Policlinico, Milan, Italy; [2]Department of pathophysiology and transplantation, University of Milan, Milan, Metropolitan City of Milan, Italy, Italy
**Correspondence:** N. Corcione
*Intensive Care Medicine Experimental* 2019, **7(Suppl 3):**001753

**INTRODUCTION.** Since the definition of "subjective experience of breathing discomfort" provided in 1999 by the ATS consensus statement, the concept of dyspnea has been widened covering not only a sensory-perceptual domain (i.e., dyspnea due to increased work of breathing) but also a behavioral-emotional area. Hence, the relief of dyspnea may not be strictly linked to the improvement of gas exchange, but also to the reduction of psychological suffering. Do-not-intubate (DNI) patients with acute hypoxemic respiratory failure (AHRF) are usually treated with noninvasive respiratory supports, mainly aimed at improving patients' comfort. High flow oxygen therapy (HFOT) seems to have a significant impact on dyspnea Limited literature data are available about the treatment of AHRF in DNI patients, included the relief of dyspnea

**OBJECTIVES.** To assess the effects of HFOT in DNI patients affected by AHRF, hospitalized in general wards

**METHODS.** We selected a subset of patients included in a larger trial evaluating the use of HFOT outside the ICU under the constant supervision of an Intensivist. 9 wards of Ospedale Policlinico (Milan, Italy) were equipped with HFOT devices; in each ward, educational meetings were given by an ICU physician and an ICU nurse. HFOT was started in patients with AHRF (PaO2/FiO2 ≥150 and <300). The ICU Outreach team followed the patients daily. Arterial blood gases, respiratory rate (RR), dyspnea (assessed by Borg scale) and comfort (assessed through a visual analogic scale) were collected just before the beginning of HFOT, after 2 hours and then every 24. Data are presented as median and interquartile range.

**RESULTS.** From November 2017 to April 2019, the ICU Outreach Team followed 118 patients. 50 patients (42%) were classified as DNI; 18 (36%) were terminally ill (End-Of-Life). Median age was 76 [66.75-85] years. Hospital mortality was 68%. Twenty-four patients (48%) had oncologic comorbidities and 16 (32%) had COPD. Median PaO2/FiO2 was 156 [116-191]. The duration of HFOT was 10 [4-15] days. After 2 hours from the beginning of HFOT, we observed a significant reduction of RR from 28 [24-31] to 23 [20-28] breaths/min (p <0.05), a reduction of Borg scale value from 3 [2-5] to 2 [1-3] points (p < 0.05) and an increase in comfort scale from 3 [2-3] to 3.5 [3-4] points (p <0.05). Gas exchange did not improve

**CONCLUSION.** HFOT is effective inalleviating dyspnea and improving comfort in DNI patients with AHRF hospitalized in general wards. The effect on dyspnea is not explained by an improvement of PaO2/FiO2 ratio

##### REFERENCE(S)

1. Peters SG, Respir. Care 2012
2. Mauri T, Crit. Care 2018
3. Laviolette L, Eur Respir J 2014
4. Parshall MB, Am J Respir Crit Care 2012
5. Kato S, Support Care Cancer 2008

**Table 1 (abstract 001753).** See text for description

| | Before HFOT | After 2 h HFOT | After 24 h HFOT | p-value |
|---|---|---|---|---|
| pH | 7.45 [7.41-7.51] | 7.48 [7.42-7.52]* | 7.47 [7.41-7.50] | **<0.01** |
| pO2 (mmHg) | 69 [59-89] | 76.5 [63-99.5] | 75 [62.3-85.5] | 0.39 |
| pCO2 (mmHg) | 41 [35-52] | 41.5 [37-54.2] | 43 [37.2-57.5] | 0.26 |
| PaO2/FiO2 | 156 [116-191] | 147 [123-208] | 156 [120-195] | 0.59 |
| Respiratory Rate (breaths/minute) | 28 [24-31] | 23 [20-28]* | 24 [20-30]* | **< 0.0001** |
| Borg | 3 [2-5] | 2 [1-3]* | 2 [1-3.7]* | **< 0.0001** |
| Comfort | 3 [2-3] | 3.5 [3-4]* | 3 [3-4]* | **< 0.0001** |
| FiO2 (%) | 50 [39-54] | 50 [40-60]* | 50 [35-60]* | **<0.01** |
| HFOT Flow (L/min) | - | 60 [50-60] | 60 [50-60] | 0.23 |
| HFOT Temperature (°C) | - | 34 [31-36.5] | 34 [31-36] | 0.78 |

* p<0.05 vs Before HFNC

### SIS - Sepsis treatment and biomarkers

#### 000347

#### Quantification of plasma exosome as potential diagnostic marker for septic shock

Y. Im, JY. Lee, H. Yoo, GY. Suh, K. Jeon
[1]Division of pulmonary and critical care medicine, department of medicine, Samsung Medical Center, Irwon-ro, Irwon-dong, Gangnam-gu, Seoul, South Korea, Seoul, Republic of Korea
**Correspondence:** Y. Im
*Intensive Care Medicine Experimental* 2019, **7(Suppl 3):**000347

**INTRODUCTION.** Exosomes have been studied extensively in several diseases including cancers and cardiovascular diseases as an inflammatory marker, however, little is known about their role and behavior in sepsis. Sepsis is a dysregulated inflammatory response syndrome that leads to multiple organ failure. Current sepsis biomarkers may be helpful in determining organ failure and evaluating the patients' clinical course, but there are no direct molecular biomarkers to predict subsequent organ failure. Exosome play an important role in the inflammatory response, coagulation process, and cardiac dysfunction in sepsis.

**OBJECTIVES.** The objective of this study was to evaluate the association of plasma exosome with severity of sepsis and mortality in critically ill patients with sepsis.

**METHODS.** Plasma exosome levels were measured in 80 patients prospectively enrolled in ongoing ICU cohort in Korea between April 2014 and January 2019, in comparison to healthy controls (n = 4). The levels of plasma exosomes expressing CD9 among groups of healthy donor, sepsis and septic shock were compared. To detect and quantify exosomes in plasma, we used a CD9 expression based enzyme-linked immunosorbent assay (ELISA) kit according to the manufacturer's protocol (Novusbio, Littleton, CO, USA).

**RESULTS.** A total of 80 patients admitted to the medical ICU and diagnosed sepsis or septic shock at our institution were recruited, among which 36.5% presented with septic shock. The median age of patients was 68 (IQR; 61–75) years and 73% were male. Patient with septic shock had higher exosome levels in the plasma compared with healthy controls and patients without shock (median(IQR);

200(158–242) μg vs. 490(468–473) μg vs. 928(899–946) μg, $p < 0.001$). Serum levels of lactic acid and procalcitonin were also higher among patients with septic shock ($p < 0.001$) compared to sepsis patients. We also performed ROC analysis and Delong's test to demonstrate and compare the predictive value of exosomes as a prognostic predictor of 28-day all-cause mortality. The area under the ROC curve (AUC) was 0.68 (95% CI; 0.44–0.92) for plasma exosomes, while the AUC of serum lactic acid and procalcitonin were 0.50 (95% CI; 0.30–0.69) and 0.65 (95% CI; 0.38–0.92), respectively. Pairwise comparisons of ROC curves showed that there was no difference to predict 28-day mortality between exosome and either lactic acid ($p = 0.11$) or procalcitonin ($p = 0.86$) in this study.

**CONCLUSION.** Our findings suggest that the elevated levels of plasma exosomes in patients admitted to the ICU are associated with septic shock and organ failure. Further studies involving molecular characterization of exosomes are required to fully understand their role in the pathophysiology of sepsis.

### 000381

#### A risk scoring model predicting ICU mortality in severe septic shock patients treated with combined vitamin C, hydrocortisone, and thiamine therapy

H. Kim, WY. Kim

[1]Internal medicine, Chung ang University Hospital, Seoul, Republic of Korea

**Correspondence:** W.Y. Kim

*Intensive Care Medicine Experimental* 2019, **7(Suppl 3):**000381

**INTRODUCTION.** Recent observational studies demonstrated that septic patients who received the combination of vitamin C, hydrocortisone, and thiamine had a substantial mortality benefit [1, 2]. Several randomized controlled trials are ongoing to assess the effect of this combination therapy on clinically important outcomes in sepsis. Better prediction of mortality in these patients may help clinicians to target patients most likely to benefit from vitamin C protocol.

**OBJECTIVES.** To develop a model for predicting ICU mortality at initiation of vitamin C protocol for septic shock.

**METHODS.** Data were obtained from severe septic shock patients (requiring high-dose vasopressors such as norepinephrine ≥0.2 ug/kg/min ± vasopressin) treated with the vitamin C protocol between September 2018 and February 2019 at a university-affiliated tertiary care hospital in Korea. Binary logistic regression was used to identify pre-treatment variables predicting ICU mortality. The Vitamin for Sepsis (VITASEP) score was calculated as the sum of simplified regression weights (SRW).

**RESULTS.** Data from 55 patients were reviewed. A total of 22 patients (40%) died during ICU admission. On the basis of multivariate analysis, the following factors were included in the VITASEP score: non-urosepsis (SRW 1), body temperature <37.1°C (SRW 1), white blood cell <14,000/mm3 (SRW 1), and C-reactive protein <200 mg/L (SRW 2). The area under the receiver operating characteristic curve of the VITASEP score predicting ICU mortality was 0.77 (95% confidence interval, 0.64–0.89). For VITASEP scores 0–1, 2, 3, and 4–5, the 28-day mortality rates were 17%, 27%, 35%, and 71%, respectively (log-rank test, $P = 0.02$). For VITASEP scores 0–1, 2, 3, and 4–5, the mean number of vasopressor-free days at day 28 were 24.7, 17.1, 13.6, and 9.0, respectively (Kruskal-Wallis test, $P = 0.02$).

**CONCLUSION.** Our results suggest that VITASEP score may predict ICU mortality among septic patients treated with combined vitamin C, hydrocortisone, and thiamine therapy. Vitamin C protocol may benefit septic patients with a "hyperinflammatory" subphenotype. Further validation in a larger sample is required.

**REFERENCE(S)**

1. Marik PE, Khangoora V, Rivera R, Hooper MH, Catravas J (2017) Hydrocortisone, Vitamin C, and Thiamine for the Treatment of Severe Sepsis and Septic Shock: A Retrospective Before-After Study. Chest 151:1229-1238
2. Kim WY, Jo EJ, Eom JS, Mok J, Kim MH, Kim KU et al (2018) Combined vitamin C, hydrocortisone, and thiamine therapy for patients with severe pneumonia who were admitted to the intensive care unit: Propensity score-based analysis of a before-after cohort study. J Crit Care 47:211-218
3. This research was supported by the Chung-Ang University Research Grants in 2019.

### 000393

#### Hyperphosphatemia as an independent prognostic factor in patients with sepsis

CU. Lee, YH. Jo, DH. Jang, JH. Lee, J. Kim, SM. Park, JE. Hwang, DK. Lee, I. Park

[1]Department of emergency medicine, Seoul National University Bundang Hospital, Seongnam-si, Republic of Korea

**Correspondence:** C.U. Lee

*Intensive Care Medicine Experimental* 2019, **7(Suppl 3):**000393

**INTRODUCTION.** Sepsis may result in life-threatening organ dysfunction, which is caused by various infections and dysregulated immune responses of host. Phosphorus is an important substance that plays various roles in the body such as intracellular energy exchange and mineral metabolism. Several studies reported that the phosphate concentration is associated with prognosis in diseases which cause ischemic injury of tissues such as intestinal ischemia, coronary heart disease, critical limb ischemia, and cardiac arrest. In sepsis, tissue hypoperfusion induces cytopathic hypoxia at the cellular level and it results in ischemic tissue injury and organ dysfunction. Therefore, we hypothesized that the phosphate concentration, which was a marker of ischemic tissue injury, could be used to predict prognosis in patients with sepsis.

**OBJECTIVES.** Ischemic injury caused by tissue hypoperfusion is one of the major mechanisms of sepsis, and phosphate concentration is elevated in ischemic tissue injury. This study was performed to investigate the association of the phosphate concentration with the mortality of patients with sepsis.

**METHODS.** This was a retrospective cohort study of patients with sepsis. The patients were divided into three groups according to the phosphate concentration: hypophosphatemia ($P < 2.5$ mg/dl), normophosphatemia ($2.5 \leq P \leq 4.5$ mg/dl), and hyperphosphatemia ($P > 4.5$ mg/dl). Multivariable Cox proportional hazard regression analysis was performed to evaluate the independent association of phosphate concentration with 28-day mortality.

**RESULTS.** A total of 3,034 patients were included in the study and the overall mortality rate was 21.9%. The 28-day mortality rates of the hypophosphatemia, normophosphatemia, and hyperphosphatemia group were 14.8%, 19.8%, and 38.1%, respectively ($p <0.001$). In the multivariable Cox proportional hazards regression analysis, hyperphosphatemia was independently associated with 28-day mortality compared with normophosphatemia (hazard ratio, 1.27; 95% confidential interval, 1.05-1.54).

**CONCLUSION.** Hyperphosphatemia was associated with 28-day mortality in patients with sepsis and could be used as a prognostic factor in sepsis.

### 000397

#### Implementation of aggressive antibiotic administration for patients with severe sepsis and septic shock in Japan

A. Toshikazu[1], S. Kushimoto[2], Y. Tokuda[3], G. Phillips[4], M. Levy[5], A. Rhodes[6], T. Sugiyama[7], H. Ogura[8], S. Fujishima[9], A. Shiraishi[10], S. Saitoh[11], T. Mayumi[12], K. Takuma[13], A. Komori[1], H. Iriyama[1], T. Kainoh[1], T. Naito[1], Y. Otomo[14], T. Tarui[15], S. Gando[16]
[1]Department of general medicine, Juntendo University, 2 Chome-1-1 Hongo, Bunkyo City, Tokyo, Japan, Bunkyo City, Japan; [2]Division of emergency and critical care medicine, Tohoku University, 2 Chome-1-1 Katahira, Aoba Ward, Sendai, Miyagi, Japan, Sendai, Japan; [3]Department of medicine, Muribushi Project for Okinawa Residency Programs, Naha, Okinawa, Japan, Japan; [4]Retired from the center for biostatistics, department of biomedical informatics, Ohio State University, Columbus, USA; [5]Division of pulmonary, critical care and sleep medicine, Warren Alpert School of Medicine at Brown University, Providence, USA; [6]Anaesthesia and intensive care medicine, St George's University Hospitals NHS Foundation Trust, Londres, UK; [7]Department of health services research, University of Tsukuba, Kasuga area, Tsukuba, Japan; [8]Department of traumatology and acute critical medicine, Osaka University Graduate School of Medicine, Suita, Japan; [9]Center for general medicine education, Keio University School of Medicine, Tokyo, Japan; [10]Emergency and trauma center, Kameda Medical Center, Kamogawa, Chiba, Japan, Japan; [11]Division of traumatology, research institute, National Defense Medical College, Tokorozawa, Japan; [12]Department of emergency medicine, University of Occupational and Environmental Health, Kitakyushu, Fukuoka, Japan, Japan; [13]Emergency & critical care center, Kawasaki Municipal Kawasaki Hospital, Kawasaki, Kanagawa, Japan, Japan; [14]Trauma and acute critical care center, Tokyo Medical and Dental University, Bunkyo City, Japan; [15]Department of trauma and critical care medicine, Kyorin University, 5 Chome-4 Shimorenjaku, Mitaka, Tokyo, Japan, Mitaka, Japan; [16]Division of acute and critical care medicine, Hokkaido University Graduate School of Medicine, Sapporo, USA
*Intensive Care Medicine Experimental* 2019, **7(Suppl 3):**000397

**INTRODUCTION.** The surviving sepsis campaign guidelines recommend empirical broad-spectrum therapy within earlier time from triage such as one hour for both sepsis and septic shock.

**OBJECTIVES.** To describethe association timing of antibiotic administration and outcomes among patients with severe sepsis and septic shock in real-world clinical setting in Japan.

**METHODS.** We included adult patients (≥16 years) with severe sepsis based on the sepsis-2 criteria published in 2003 [1], who were admitted to the ICU in the Focused Outcomes Research in Emergency Care in Acute Respiratory Distress Syndrome, Sepsis and Trauma (FORECAST) study [2]. It used a sample of 59 ICUs in Japan from January 1, 2016 to March 31, 2017. The primary outcome was in-hospital mortality. We compared characteristics and outcomes among patients initiating antibiotics at different timing (0-60, 61-120, 121-180, 181-240, 241-360, and 361-1440 minutes after the time of sepsis recognition), with analysis of variance, Kruskal-Wallis test, or chi-square tests. We estimated the impact of antibiotic timing on risk-adjusted hospital mortality using the generalized estimating equation (GEE) model with an exchangeable within-group correlation matrix where hospital was the panel or grouping variable. The covariates were prespecified a priori based on clinical experience and prior studies, such as patient age; gender; admission source (emergency department (ED), ward, or in intensive care unit (ICU)); CCI; antibiotic use before arrival; site of infection (e.g., lung, abdomen, urinary tract, soft tissue, central nerve system, blood stream-related, or others); and SOFA score.

**RESULTS.** 1124 patients met criteria in 54 hospitals. Of these patients,30.5% of patients received antibiotics within one hour and 73.9% of patients received antibiotics within three hours. The median time to antibiotic administration was 102 min (IQR, 55–189 min). Patients in ED or those diagnosed sepsis in ICU received antibiotics earlier than those in ward. The earlier antibiotic use group was likely to achieve the 3-hour bundle. Overall crude mortality was 23.4% in the study population. Patients who received antibiotic within 60 minutes had highest mortality (28.0%). Patients received antibiotics within the first 60 min had also highest adjusted mortality rate (28.5% [23.1–33.9]), whereas that of patients administered antibiotics between 61 and 120 min was the lowest (21.6% [16.5–26.6]). Differences in mortality were noted only between the 0–60 min and the 61–120 min groups. There was not significant difference among patients initiating antibiotics at different timing in ICU-free days, ventilator free days, and length of hospital stay. Similar results were observed even if patients from ED were stratified.

**CONCLUSION.** This prospective observational study failed to show the association between earlier antibiotic administration and reduction of in-hospital mortality among patients with severe sepsis.

##### REFERENCE(S)

1. Levy MM et al, Crit Care Med 31: 1250-6, 2003
2. Abe et al, Crit Care 22: 322, 2018
3. Japanese Association for Acute Medicine

### 000400

#### Extra-corporeal blood purification in sepsis A meta-analysis and trial sequential analysis

T. Snow[1], S. Littlewood[2], C. Corredor[3], M. Singer[1], N. Arulkumaran[1]
[1]Bloomsbury Institute of Intensive Care Medicine, University College London, London, UK; [2]Gicu, St George's Hospital Atkinson Morley Wing, London, UK; [3]Department of perioperative medicine, St Bartholomew's Hospital, London, UK
**Correspondence:** T. Snow
*Intensive Care Medicine Experimental* 2019, **7(Suppl 3):**000400

**INTRODUCTION.** Extracorporeal blood purification techniques aim to ameliorate the excessive host response to sepsis and/or remove circulating endotoxin. Various devices have been evaluated in randomised clinical trials (RCTs) (1-3) although not in routine use.

**OBJECTIVES.** We conducted a meta-analysis and trial sequential analysis (4) of RCTs to determine the potential mortality benefit of extracorporeal blood purification techniques in sepsis.

**METHODS.** A systematic search of MEDLINE, Embase, clinicaltrials.gov, and the Cochrane Central Register of Controlled Trials for RCTs was conducted. Mortality was assessed using Mantel-Haenszel models and *I2* used for heterogeneity. Data are presented ad odds ratios (confidence intervals); p-values; *I2*).

**RESULTS.** We identified 28 RCTs including 2,194 patients, with 1,115 (51%) in the treatment arm. Eight studies used haemofiltration, 15 endotoxin absorption devices, four non-specific cytokine removal devices, and one used plasma exchange. An improvement in mortality was associated with the use of endotoxin removal devices (0.47 (0.28-0.79); p=0.004; *I2*=68%), but not haemofiltration (0.74 (0.49-1.13); p=0.17; *I2*=0%) nor non-specific cytokine removal (0.81 (0.33-2.00); p=0.65; *I2*=72%). Subgroup analyses revealed that mortality benefit was limited to the use of endotoxin removal devices in Asian (0.23 (0.12-0.47); p<0.001; *I2*=47%) but not Western countries (1.10 (0.82-1.48); p=0.54; *I2*=5%). The use of endotoxin removal devices (0.21 (0.12-0.34); p<0.001; *I2*=0%) and cytokine removal devices (0.43(0.20-0.94); p=0.02; *I2*=51%) was associated with an improvement in mortality only among patients with a high risk of death (control arm mortality >50%). Patients with documented Gram-negative sepsis were most likely to benefit from endotoxin removal devices (0.22 (0.12-0.42); p<0.001; *I2*=0%).

**CONCLUSION.** The use of endotoxin removal devices in sepsis is associated with mortality benefit, particularly in patients with high mortality risk and with documented Gram-negative sepsis.

##### REFERENCE(S)

1. Dellinger RP et al. JAMA. 2018;320:1455-63.
2. Livigni S, et al. BMJ Open. 2014;4:e003536.
3. None

### 000401

#### Characterization of fecal peritonitis-induced sepsis in a porcine model

I. Park[1], JH. Lee[1], DH. Jang[1], D. Kim[1], H. Chang[1], H. Kwon[1], S. Kim[1], TS. Kim[2]
[1]Department of emergency medicine, Seoul National University Bundang Hospital, Seongnam-si, Republic of Korea; [2]Department of laboratory medicine, Seoul National University Hospital, Seoul, Republic of Korea
**Correspondence:** I. Park
*Intensive Care Medicine Experimental* 2019, **7(Suppl 3):**000401

**INTRODUCTION.** Although small animal sepsis model is well established, longitudinal assessment of hemodynamic variables, laboratory values, and blood culture in a single living sepsis model is limited.

**OBJECTIVES.** Therefore, we aimed to comprehensively characterize the fecal peritonitis-induced sepsis in the porcine model.

**METHODS.** Autologous feces (1g/kg) were administered into the peritoneum of eleven male pigs (49 ± 8 kg). The pigs were monitored up to 12 hours with the full support of fluid and vasopressors to maintain over 65mmHg of the MAP. Longitudinal blood culture and laboratory values were obtained every one or two hours. The cytokine levels in plasma were analyzed. Moreover, clinical registry of sepsis patients in single emergency department was utilized to compare the SOFA score with those of the porcine model.

**RESULTS.** The hyperdynamic phase of increasing cardiac output with decreasing systemic vascular resistance was maintained until 2 hours, followed by the reverse hypodynamic phase. With the escalating requirement of fluid and vasopressor, the lactate level was progressively increased while platelet counts, urine output, and serum albumin level were consistently decreased. Bacteremia was developed at 7 hours after administration of feces and the *Escherichia coli* was the most common pathogen. The pattern of SOFA score with prominent cardiovascular failure was comparable to clinical data.

**CONCLUSION.** We implemented the porcine fecal peritonitis-induced sepsis model which demonstrates culture-proven bacteremia and multiple organ failure, particularly the cardiovascular system. This model could be utilized in the development of early diagnostic technology of bacterial pathogens in the blood.

1. Bio & Medical Technology Development Program of the National Research Foundation (NRF) funded by the Ministry of Science & ICT (NRF-2017M3A9E2062210)

### 000402

#### Effect of recombinant human soluble thrombomodulin on coagulation and inflammatory markers in patients with sepsis-induced disseminated intravascular coagulation: a retrospective observational study

C. Mitaka, I. Kawagoe, D. Satoh, E. Inada
[1]Anesthesiology and pain medicine, Juntendo University, Hongo Tokyo, Japan
**Correspondence:** C. Mitaka
*Intensive Care Medicine Experimental* 2019, **7(Suppl 3):**000402

**INTRODUCTION.** Thrombomodulin (TM) on vascular endothelium binds to thrombin and acts as anticoagulant. In addition, the thrombin-TM complex activates protein C to produce activated protein C, which inactivates factors Va and VIIIa, thereby inhibiting thrombin formation. Furthermore, TM has anti-inflammatory and cytoprotective activities [1]. In Japan, recombinant human soluble TM (rhTM) has been used for treatment of disseminated intravascular coagulation (DIC) since 2008.

**OBJECTIVES.** The objectives of this study are to evaluate the effectiveness of rhTM on platelet count, fibrin/fibrinogen degradation products (FDP), prothrombin time (PT) ratio, and C-reactive protein (CRP) levels in patients with sepsis-induced DIC.

**METHODS.** We retrospectively searched patients with sepsis who were admitted to ICU between April 2015 and January 2019, and investigated patients who are diagnosed as sepsis-induced DIC and received rhTM. Sepsis was defined according to Sepsis-3. DIC was diagnosed based on DIC score ≥ 4 on the Japanese Association for Acute Medicine (JAAM) DIC scoring system [2]. Platelet count, FDP, PT ratio, DIC score, and CRP levels were collected. Dose and duration of rhTM was also collected. DIC resolution was defined as DIC score ≤ 3 by the day after the last rhTM.

**RESULTS.** Total 137 patients with sepsis were admitted to ICU and 34 patients (25%, 66 ± 15 years) out of them were diagnosed as sepsis-induced DIC and received rhTM. Sources of sepsis are abdomen (n =19), urine (n = 5), lungs (n = 3), skin (n = 2), blood (n = 2), and unknown (n = 3). The mean ± SD values of dose and the duration of rhTM were 292 ± 121 U/kg/day and 5 ± 2 days, respectively. The platelet count (median, IQR) increased significantly ($p < 0.001$) from 99 [41-99] to 147 [87-379] (×1000/μL) after rhTM administration. The PT ratio and FDP level (median, IQR) significantly ($p < 0.001$) decreased from 1.49 [1.27-1.66] to 1.13 [1.06-1.27] and from 30.75 [21.15-51.25] to 14.05 [6.7-17.8] (μg/mL), respectively. The DIC score (median, IQR) significantly ($p < 0.001$) decreased from 5[4.25-7] to 2[1-4] and 23 patients (68 %) were recovered from DIC after rhTM administration. The CRP levels (median, IQR) significantly ($p < 0.001$) decreased from 21.1 [14.4-28.0] to 8.2 [5.3-11.5] (mg/dL). Twenty eight-day mortality was 20.6%.

**CONCLUSION.** This study showed that rhTM significantly increased platelets counts and significantly reduced PT ratio, FDP level, DIC score and CRP levels, resulting in improvement of DIC in patients with sepsis-induced DIC. Further studies are needed to verify the effectiveness of TM-α on these patients.

##### REFERENCE(S)

1. Ikezoe T. Thrombomodulin/activated protein C system in septic disseminated intravascular coagulation. J Intensive Care 2015:3:1 DOI 10.1186/s40560-014-0050-7
2. Gando S, et al. A multicenter, prospective validation of disseminated intravascular coagulation diagnostic criteria for critically ill patients: comparing current criteria. Crit Care Med 2006;34:625-631

### 000404

#### Description of hemodynamic patterns of pediatric patients with septic shock during admission and after 48 hours in the ICU of IPPMG-UFRJ

C. cury, E. Gomes, R. Belmino
[1]PICU, Instituto de Puericultura e Pediatria Martagão Gesteira - IPPMG, Rio de Janeiro, Brazil
**Correspondence:** C. cury
*Intensive Care Medicine Experimental* 2019, **7(Suppl 3):**000404

**INTRODUCTION.** Severe sepsis and septic shock are important issues of public health worldwide and persist as the leading causes of death in children under 5 years-old (with mortality rates around 25%).

We classified shock under two different patterns during clinical evaluation: hypodynamic shock, most frequent in patients who present with low cardiac output, and hyperdynamic shock, most frequent in patients who present with high cardiac output. Hypodynamic shock has been reported as the predominant type of shock in children. However, diagnosis made by clinical findings alone has its limitations and we propose that, with adequate hemodynamic monitoring, we can find a change in the initial patterns of shock, often changing from the most prevalent hypodynamic shock to a hyperdynamic shock pattern 48 hours after admission.
It is of utmost importance identifying the hemodynamic type of shock to improve our clinical approach and treatment plans, both relevant to the outcome of patients

**METHODS.** This was a retrospective observational study aimed to classify and identify the hemodynamic profiles of patients admitted in the Pediatric Intensive Care Unit of Martagão Gesteira Pediatric Childcare Institute – Rio de Janeiro Federal University (IPPMG-UFRJ), between November 2016 and December 2018, as well as assess if there was any change, 48 hours after PICU admission, in the pattern initially identified.

**RESULTS.** 44 patients were included in the study, with median age 13.5 months, 75% presenting comorbidities, 40.9% with primary respiratory disease, and death occurring in 20.5% of patients. The shock pattern was observed at admission and 48 hours later for us to classify patients with hypodynamic or hyperdynamic shock. At admission, 9 patients (20.5%) had hyperdynamic shock and 35 patients (79.5%) presented with hypodynamic shock, with those numbers changing after 48 hours post admission, with 19 patients (43.2%) presenting with hyperdynamic and 25 patients (56.8%) presenting with hypodynamic shock. Within the patients who modified the shock pattern, 85.7% were from the hypodynamic group at admission, with statistical significance in the associations observed ($p < 0.05$ – Fisher's test). Clinical/Non-invasive criteria (97%) were predominant for the definition and initial approach of shock in the first evaluation at admission, followed by lactate (75%), central venous saturation and oxygen extraction rate (40.9%). Monitoring with invasive blood pressure (45%) and central venous pressure (34%) were less utilized as a primary tool.

**CONCLUSION.** Our results were compatible with recent researches, meaning that we observed a considerable percentual change in the hemodynamic profiles, mostly from a hypodynamic to a hyperdynamic type of shock, between ICU admission and the following 48 hours. Patients' evaluation was mostly through clinical evaluation, with only 40-45% of patients receiving invasive monitoring, which may have contributed to our findings. However, even within our limitations, it was indeed observed a clear pattern that seems to occur somewhat frequently, as described in similar studies on the subject.

**REFERENCE(S)**

1. BRIERLEY, J.; PETERS, M. Distinct hemodynamic patterns of septic shock at presentation to pediatric intensive care. Pediatrics, n. 122, p. 752 – 759, oct 2008
2. CECCONI, M.; BACKER, D. D.; ANTONELLI, M. Consensus on circulatory shock and hemodynamic monitoring. Task force of the European Society of Intensive Care Medicine. Intensive Care Med, v. 12, n. 40, p. 1795 – 1815, dec 2014.
3. CAVAZZONI, S. L. Z.; R, P. D. Hemodynamic optimization of sepsis-induced tissue hypoperfusion. Critical Care, BioMed Central, v. 10, n. Suppl 3, p. S2 –, nov 2006. ISSN 1364-8535.
4. RHODES, A. Surviving Sepsis Campaign Guidelines Committee. Surviving Sepsis Campaign: International Guidelines for Management of Sepsis and Septic Shock: 2016. Intensive Care Med, v. 3, n. 45, march 2017
5. ABRAHAM, E. New Definitions for Sepsis and Septic Shock. Continuing Evolution but With Much Still to Be Done. JAMA, v. 8, n. 315, p. 757 – 759, february 2016
6. RANJIT, S. et al. Multimodal monitoring for hemodynamic categorization and management of pediatric septic shock: a pilot observational study. Pediatr Crit Care Med, v. 26, n. 15, p. – 17, jan 2015.

7. This work was supported by Plataforma Brasil and approved by the Ethics in Research Committee of IPPMG under grant number 94230718.0.0000.5264

### 000437

#### Characteristics and outcomes of bacteremia among patients with severe sepsis

A. Komori[1], A. Toshikazu[1], T. Naito[1], H. Ogura[2], A. Shiraishi[3], S. Kushimoto[4], D. Saitoh[5], S. Fujishima[6], T. Mayumi[7], H. Iriyama[1], T. Kainoh[1], Y. Shiino[8], TA. Nakada[9], T. Tarui[10], T. Hifumi[11], Y. Otomo[12], Y. Sakamoto[13], J. Sasaki[14], K. Yamakawa[15], S. Gando[16]
[1]Department of general medicine, Juntendo University, 2 Chome-1-1 Hongo, Bunkyo City, Tokyo, Japan, Bunkyo City, Japan; [2]Department of traumatology and acute critical medicine, Osaka University Graduate School of Medicine, Suita, Japan; [3]Emergency and trauma center, Kameda Medical Center, Kamogawa, Japan; [4]Division of emergency and critical care medicine, Tohoku University Graduate School of Medicine, Sendai, Japan; [5]Division of traumatology, research institute, National Defense Medical College, Tokorozawa, Japan; [6]Center for general medicine education, Keio University School of Medicine, Tokyo, Japan; [7]Department of emergency medicine, University of Occupational and Environmental Health, Kitakyushu, Fukuoka, Japan, Japan; [8]Department of acute medicine, Kawasaki Medical School Hospital, 577 Matsushima, Kurashiki, Okayama, Japan, Kurashiki, Japan; [9]Department of emergency and critical care medicine, Chiba University Graduate School of Medicine, Chiba, Japan, Japan; [10]Department of trauma and critical care medicine, Kyorin University, 5 Chome-4 Shimorenjaku, Mitaka, Tokyo, Japan, Mitaka, Japan; [11]Department of emergency and critical care medicine, St. Luke's International Hospital, Chuo City, Japan; [12]Trauma and acute critical care center, Tokyo Medical and Dental University, Bunkyo City, Japan; [13]Emergency and critical care medicine, University Hospital attached to the Faculty of Medicine, Saga University, Saga, Japan; [14]Department of emergency and critical care medicine, Keio University School of Medicine, Tokyo, Japan; [15]Division of trauma and surgical critical care, Osaka General Medical Center, Osaka, Japan; [16]Division of acute and critical care medicine, Hokkaido University Graduate School of Medicine, Sapporo, USA
**Correspondence:** A. Komori

*Intensive Care Medicine Experimental* 2019, **7(Suppl 3):**000437

**INTRODUCTION.** Bacteremia is common in severely ill patients. However, little research has been done on clinical characteristics and outcomes of bacteremia among patients with sepsis.

**OBJECTIVES.** We aimed 1) to compare the clinical characteristics and outcomes of severe sepsis patients with and without bacteremia, and 2) to assess the relationship between procalcitonin (PCT) and bacteremia, including different pathogen species.

**METHODS.** This is a secondary analysis of the sepsis cohort of the Focused Outcomes Research in Emergency Care in Acute Respiratory Distress Syndrome, Sepsis and Trauma (FORECAST) study, a multicenter, prospective cohort study of patients with severe sepsis which was conducted in 59 ICUs in Japan over 15 months [1]. Adult patients (≥16 years) with severe sepsis or septic shock admitted to a participating ICU were included. We compared characteristics and outcomes of patients with and without bacteremia. To know a characteristic of bacteremia of each pathogen in details, we also assessed the prognostic utility of PCT for bacteremia of each pathogen by drawing a receiver operating characteristics (ROC) curve.

**RESULTS.** One thousand one hundred sixty-seven patients from the FORECAST database met the study criteria. Among those, 636 patients (54.5%) with bacteremia were identified. Patients with bacteremia had significantly higher rates of septic shock than those without (422 [66.4%] vs. 313 [58.9%], $p<0.01$). There was no significant difference in the comorbidities of the groups. Sepsis severity scores such as APACHE II and SOFA were higher for patients with bacteremia than patients without bacteremia (median [interquartile (IQR)] 23 [18–30] vs. 22 [16–29], p=0.02 and 9 [6–12] vs. 8 [5–11], $p< 0.01$, respectively). Regarding the laboratory data, white blood cell count and platelet count were lower in patients with bacteremia than in patients without bacteremia (median [IQR] 10,600 [4,760–17,300]/μL vs. 12,440 [6,620–18,430]/μL, p=0.02 and 12.4 [7.5–18.6] ×104/μL vs. 17.5 [11.1–24.5] ×104/μL, p<0.01, respectively). PCT concentration was significantly higher in patients with bacteremia than in patients without bacteremia (median [IQR] 21.3 [3.5–75.6] ng/mL vs. 5.2 [1.1–34.4] ng/mL, p<0.01). In-hospital mortality was 158/614 (25.6%) for patients with bacteremia and 108/515 (21.0%) for those without (p=0.08). Length of hospital stay was 23 (IQR 12–44) days for patients with bacteremia and 24 (IQR 13–50) days for those without (p=0.10). The area under the ROC of PCT for predicting bacteremia was 0.631. The optimal cutoff value of PCT for predicting bacteremia was 3.84 ng/mL. The highest PCT level was found in *Klebsiella* (median [IQR] 60.8 [20.8–130.1] ng/mL), followed by *E. coli* (30.0 [5.5–100] ng/mL) and *Pseudomonas* (24.7 [5.6–49.1] ng/mL). The lowest PCT level was found in *Staphylococcus* (7.7 [2.0–25.4] ng/mL).

**CONCLUSION.** Severe sepsis patients with bacteremia had more shock and higher severity scores than those without. However, there was not much difference between the two groups in outcomes such as in-hospital mortality. PCT levels were different among patients with bacteremia of different pathogen species.

### 000445

#### Sepsis induces early structural and mitochondrial muscle dysfunction

A. Bouglé[1], L. Duarte[1], B. Duceau[1], D. Briand[1], J. Bardon[1], L. Boccara[1], Q. Giai Gianetto[2], T. Chaze[2], L. Chatre[3], C. Crochemore[3], C. Tresallet[4], M. Raux[5], B. Riou[6], T. Lescot[7], O. Langeron[8], F. Cook[9], T. Sharshar[1], F. Chrétien[1]

[1]Neuropathologie expérimentale, Institut Pasteur, Paris, France; [2]Spectrométrie de masse, Institut Pasteur, Paris, France; [3]Cellules souches et développement, Institut Pasteur, Paris, France; [4]Chirurgie générale et endocrinienne, Groupe Hospitalier Pitié-Salpêtrière, Paris, France; [5]Sspi et accueil polytraumatisés, Groupe Hospitalier Pitié-Salpêtrière, Paris, France; [6]Department of emergency medicine and surgery, Groupe Hospitalier Pitié-Salpêtrière, Paris, France; [7]Réanimation chirurgicale, Hospital Saint-Antoine, Paris, France; [8]Réanimation chirurgicale polyvalente, Groupe Hospitalier Pitié-Salpêtrière, Paris, France; [9]Polyvalent and surgical icu, Hôpital Henri-Mondor Ap-Hp, Créteil, France

**Correspondence:** A. Bouglé

*Intensive Care Medicine Experimental* 2019, **7(Suppl 3):**000445

**INTRODUCTION.** Despite a decrease in the mortality rate of septic shock, morbidity remains high due to the sequelae of sepsis and prolonged stay in intensive care unit. Among the different sequelae included in the post-Intensive Care syndrome, Intensive Care Unit - associated weakness (ICU-AW) plays a particular role. This generalized muscle weakness affects 30 to 50% of septic shock patients and is associated with an increased duration of both mechanical ventilation and hospitalization. In addition, one-third of patients will suffer severe sequelae such as the inability to walk or breathe on their own. The pathophysiology of this syndrome appears to be multifactorial but is largely based on pre-clinical data: sodium channel inactivation, oxidative stress, proteolysis, mitochondrial dysfunction and muscle stem cell dysfunction.

**OBJECTIVES.** The objective of this study was to characterize in a multimodal way the early muscle dysfunction in patients with septic shock or severe sepsis compared to control patients.

**METHODS.** Three patient populations were included in the DISCUSS study: a septic patient population requiring surgery; an inflammatory non-septic patient population in brain death or cardiogenic shock; and a non-inflammatory non-septic patient population operated on scheduled visceral surgery. After obtaining the consent or non-opposition of the relatives (brain-dead state), patients were sampled when they went to the operating room or when they were placed on the ECMO with 2 cm3 of muscle and 5 ml of blood. This research has been approved by the Comité de Protection des Personnes Ile de France V (06/10/2015, N°15051). The samples were processed at the Institut Pasteur for mitochondrial morphological analysis by Transmission Electron Microscopy (TEM), proteomic analysis and analysis of mitochondrial parameters of muscle stem cells and serum.

**RESULTS.** Sixty-seven patients were included between June 2016 and April 2018. Eighteen septic patients, eighteen brain-dead patients, twelve cardiogenic shock patients and nine controls. The TEM analysis showed a difference in mitochondrial ultrastructure and a significantly different morphotype distribution: more type 2 morphotypes were found in the control group while there were more type 3 morphotypes (mitochondrial suffering with swelling and loss of peaks) in septic patients. Compared to the other conditions, proteomic analysis of septic patients showed significant differences in protein expression related to acetyl-CoA metabolism pathways, Krebs cycle, propanoate metabolism, amino acid biosynthesis and pyruvate metabolism. Serum analysis showed a significant difference between groups regarding the amounts of reactive oxygen species, of acetyl-CoA and of ATP but no differences were found regarding reactive nitrogen species and SOD activity.

**CONCLUSION.** In the acute phase of sepsis in humans, we therefore show an early muscle dysfunction secondary to mitochondrial damage and to a structural impairment of the proteins involved in muscle synthesis and proteolysis. Yet, we did not find any evidence for specific damage to satellite cells.

**REFERENCE(S)**

1. Rocheteau P et al. Sepsis induces long-term metabolic and mitochondrial muscle stem cell dysfunction amenable by mesenchymal stem cell therapy. Nat Commun. 2015 Dec 15;6:10145
2. Brealey D et al. Association between mitochondrial dysfunction and severity and outcome of septic shock. Lancet. 2002 Jul 20;360(9328):219-23

### 000447

#### Long-term immune dysfunction induced by sepsis is dependent of age

DF. Colon[1], C. Wanderley[2], AL. Souza[2], F. Castanheira[3], AP. Carlotti[4], F. Carmona[4], F. Ramalho[5], JC. Alves-Filho[1], F. Liew[6], FQ. Cunha[2]

[1]Department of biochemistry and immunology, University of São Paulo, Ribeirao Preto, Brazil; [2]Department of pharmacology, University of São Paulo, Ribeirao Preto, Brazil; [3]Department of pharmacology, University of Calgary, Calgary, Canada; [4]Department of pediatrics, University of São Paulo, São Paulo, Brazil; [5]Department of pathology, University of São Paulo, Ribeirao Preto, Brazil; [6]Division of infection, immunity and inflammation, University of Glasgow, Glasgow, UK

**Correspondence:** D.F. Colon

*Intensive Care Medicine Experimental* 2019, **7(Suppl 3):**000447

**INTRODUCTION.** Sepsis is a life-threatening multi-organ dysfunction caused by dysregulated host response to infection with unmet clinical needs. In the context of adults, patients who survive sepsis can develop long-term immune dysfunction, with an expansion of the IL-33/M2 macrophages/regulatory T (Treg) cell axis (1). However, these findings were reported in humans and adult mice; however, there is no evidence of these alterations in the pediatric sepsis.

**OBJECTIVES.** To investigate the role of age in the genesis of immunosuppression following sepsis

**METHODS.** C57BL/6 mice (infant and adults) were submitted to sepsis and treated with antibiotic for three days. On day 15 after infection, Treg cells frequency and the activation of IL-33/Th2 profile cytokines/ILC2 cells/M2 macrophages axis were evaluated. To assess the immunosuppression, surviving sepsis mice were inoculated intranasally with *Pseudomonas aeruginosa* or by subcutaneous inoculation of B16LucF10 Melanoma cell line. Finally, blood samples from pediatric and adult surviving sepsis patients were collected and the Treg cells and Th2 profile cytokines were evaluated

**RESULTS.** Here we showed that sepsis surviving-infant mice, in contrast with the adult mice, were resistant to secondary infection and controlled the tumoral growth suggesting the non-development of immunosuppression. Mechanistically, the "resistance" was related to the decrease in *Foxp3* expression, lower phosphorylation of SMAD2/3

on TCD4+ cells and reduction in Tregs cell expansion and FOXP3 stability. Furthermore, infant mice presented lower production of IL-33 as well as a lower expansion of ILC2 cells and production of Th2 profile cytokines (IL-4 and IL10), leading to a progressive decrease in the M2 macrophages population and Tregs cell expansion. Importantly, in a translational manner, we demonstrated that sepsis-surviving pediatric patients, in contrast to adult patients, did not exhibit an increase in Treg cell, IL-33 and IL-10 in their peripheral blood, indicating that "resistance" to the development of immunosuppression observed in infants could be associated with the impairment of the IL-33/cytokines of the Th2 profile/ILC2 cells/M2 macrophages/Tregs cells axis

**CONCLUSION.** These findings demonstrate for the first time that the immunosuppression post-sepsis is related to the age of host. Thus, a better understanding of the process could lead to differential therapeutic treatments of adult and pediatric sepsis.

### 000463

#### Preload Functional Status and Patient Outcome in Sepsis

I. Douglas[1], P. Alapat[2], K. Corl[3], M. Exline[4], L. Forni[5], D. Kaufman[6], A. Khan[7], A. Holder[8], M. Levy[9], G. Martin[8], J. Sahatjian[10], E. Seeley[11], W. Self[12], J. Weingarten[13], M. Williams[14], D. Hansell[15]

[1]Icu, Denver Health, Denver, USA; [2]Icu, Ben Taub General Hospital , Houston, USA; [3]Icu, Rhode Island Hospital, Providence, USA; [4]Icu, Ohio State University Hospital, Columbus, USA; [5]Icu, Royal Surrey County Hospital, Guildford, UK; [6]Icu, NYU School of Medicine, New York, USA; [7]Icu, Oregon Health & Science University, Portland, USA; [8]Icu, Emory University Hospital Midtown, Atlanta, USA; [9]Division of pulmonary, critical care and sleep medicine, Warren Alpert School of Medicine at Brown University, Providence, USA; [10]Clinical affairs, Cheetah Medical, Newton Center, USA; [11]Icu, University of California San Francisco Parnassus Campus, San Francisco, USA; [12]Icu, Vanderbilt University, Nashville, USA; [13]Icu, NewYork-Presbyterian Brooklyn Methodist Hospital, New York, USA; [14]Icu, Methodist Hospital , Indianapolis, USA; [15]Anesethesiology, Massachusetts general hospital, Boston, USA

**Correspondence:** J. Sahatjian

*Intensive Care Medicine Experimental* 2019, **7(Suppl 3):**000463

**INTRODUCTION.** Cardiac function is known to be negatively impacted by sepsis. Stroke volume (SV) change in response to Passive Leg Raise (PLR) is an effective method to predict fluid responsiveness (FR) or cardiac response to preload expansion. Preload functional status and trends in cardiac output may identify patient phenotypes with varying cardiac reserve, dysfunction and outcome.

**OBJECTIVES.** The goal of this study is to identify resuscitation phenotypes based on fluid responsiveness and evidence of cardiac dysfunction, and assess outcome differences between them

**METHODS.** FRESH is randomized controlled study, evaluating the incidence of FR in critically ill patients with sepsis or septic shock (NCT02837731). Patients randomized to PLR guided resuscitation were classified as FR if SV increased ≥ 10% when measured with non-invasive bioreactance (Starling SV, Cheetah Medical). Patients were characterized asNon FR if SV increased <10%. Patients were grouped into 6 different sextets based on the percentage of FR PLRs. Patients in Group 1 (N=11) exhibited 100% FR PLRs, where Group 2 (N=5) exhibited between 80-90% FR PLRs. Groups 3-5 (N= 19, N=26, and N=20) exhibited FR rates between 5-75%, with Group 6 (N=15) exhibiting 0% incidence of FR PLRs.

**RESULTS.** A total of 670 PLR assessments were performed in 96 patients over a 72 hour monitoring period. 60 % were female, and the average age was 61 years. Overall, 40% of assessments demonstrated a FR positive response after receiving initial resuscitation fluid of 2.3 (+/- 0.6) L. There were no differences among the 6 groups with respect to age, gender, or QSOFA score. Fluid responsiveness was dynamic changing over the 72 hours of the study. When Groups 1-3 (54%-100% incidence FR PLRs) were compared to Groups 4-6 (50%-0% incidence FR PLRS) there were differences in patient outcome. Patients in Groups 1-3 exhibited a significant reduction in need for new onset dialysis (0%) compared to groups 4-6 (18%), p=0.012, and a decreased incidence of MACE (0% compared to 11.7%, p=0.04). Patients in Groups 1-3 also exhibited a notable trend toward decreased mortality (8.6%) compared to Groups 3-6 (25%) p=0.059. Patients in Group 6 exhibited the highest incidence of mortality during the 30 day follow up period (40%).

**CONCLUSION.** Short-term sepsis resuscitation phenotypes based on SV and responses to PLR identify discrete and significantly different patient sub-groups. Patients who were FR, and who augmented CO in response to the resuscitation experience decreased need for dialysis and a decreased incidence of MACE, as well as a trend towards decreased mortality. Periodic dynamic monitoring may predict patient outcome and guide treatment for sepsis-associated cardiac dysfunction.

### 000471

#### Splanchnic blood flow dynamics in sepsis and in sepsis survivors. Experimental study

GC. Obara[1], YR. Kang[2], MN. Nakamae[3], AMA. Liberatore[4], RB. Souza[5], SA. De Moura[1], IHJ. Koh[6]

[1]Cirurgia, Universidade Federal de São Paulo, São Paulo, Brazil; [2]Cirurgia, Unversidade Federal de São Paulo, São Paulo, Brazil; [3]Cirugia, Universidade Federal de São Paulo, São Paulo, Brazil; [4]Cirurgia, Universidade Federal de São Puo, Sao Paulo, Brazil; [5]Genetics and evolutionary biology, University of São Paulo, São Paulo, Brazil; [6]Surgery, Universidade Federal de São Puo, Sao Paulo, Brazil

**Correspondence:** I.H.J. Koh

*Intensive Care Medicine Experimental* 2019, **7(Suppl 3):**000471

**INTRODUCTION.** In sepsis, blood hypo-flow into the splanchnic territory has been related to cytopathic hypoxia, microcirculatory dysfunction, gut bacterial overgrowth, gut barrier dysfunction, bacterial translocation, activation of lymphoid tissue associated with the intestine and exacerbation of systemic inflammation in the host, culminating with organic dysfunction and clinical worsening [1-3]. The main hypothesis for these events has been attributed to the selective reduction of physiological blood flow to the gut in order to preserve the nobler organs for the preservation of life. However, the dynamics of splanchnic blood flow distribution during sepsis and survivors remains relatively unknown.

**OBJECTIVES.** Evaluate the kinetics of blood flow distribution to splanchnic territory in the acute phase of sepsis and after recovery from sepsis.

**METHODS.** Following DL50 sepsis model induction (2mL E.coli 108 CFU/mL, iv) in adult Wistar female rats, the animals were monitored to macro (MAP, HR and abdominal aorta blood flow by Transonic, TS420 transit-time flowmeter), regional (mesenteric artery, celiac trunk artery and portal vein by Transonic, TS420 transit-time flowmeter), and micro hemodynamics (kidney cortical microcirculation by SDF videomicroscopy). Animals were monitored at before sepsis (T-0) and at 3 hours (T-3h), 6 hours (T-6h) and 1month (T-1m) post-sepsis periods. Naïve animals (N) were used as control. (n=4-5/period). All rats were euthanized at the end of experiments.

**RESULTS.** The results showed a significant fast fall of the aorta's blood flow until 6h after sepsis induction and partial recovery at 1 month, but not reaching T-0 value. The celiac trunk artery, mesenteric artery and portal vein showed a significant blood flow decrease up to 6h after sepsis induction and remained significantly low up to 1 month after sepsis (fig1). These observations were even more evident when verifying the delta-percent of their blood flow decay in relation to the naive group without sepsis (T-0h). (Fig.2). Following, when examined the proportion of blood flow of the same vessels in relation to of the aorta flux to evaluate the blood fraction distribution, we observed a similar pattern in all splanchnic vessels (fig.3). The percentage of flow from the aortic artery to the celiac trunk artery increased in the acute phase of sepsis (T-3h and T-6h)

suggestive of the hyperdynamic phase in this DL50 model of sepsis, and after 1-month survival presented a significant reduction of their distributive percentage compared to the other periods. The blood flow to the mesenteric artery and portal vein showed a similar pattern between T-0 and T-3h periods but was significantly decreasing in other periods. These data suggest that the blood flow of splanchnic vessels and of the abdominal aorta do not return to normal levels until 1 month after sepsis, and this hypovolemic feature may be associated with the chronic oxygen deficiency stress in splanchnic organs.

**CONCLUSION.** Splanchnic hemodynamics decays substantially since the early acute phase of sepsis and remains in a state of hypovolemic stress up to one month after recovery. Studies are evaluating if this hemodynamic dysfunction in post-sepsis is transitory or permanent.

**REFERENCE(S)**

1. Konig J, Wells J, Cani P.D, Garcia-Rodenas C.L, Mac-Donald T, Mercenier A, Whyte J, et al. Human intestinal barrier function in health and disease. Clin Transl Gastroenterol. 2016;7(10):e196.
2. Stelios F et al.The Role of the Gut Barrier Function in Health and Disease. Gastroenterol Res. 2018;11(4):261-263.
3. Clark JA & Coopersmith CM. Intestinal crosstalk: a new paradigm for understanding the gut as the "motor" of critical illness. Shock. 2007;28:384–393.

4. Grant 2017/21052-0, São Paulo Research Foundation (FAPESP).

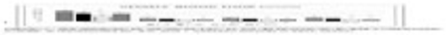

**Fig. 1 (abstract 000471).** See text for description

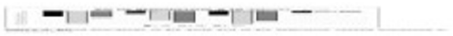

**Fig. 2 (abstract 000471).** See text for description

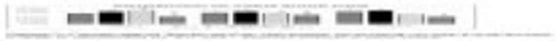

**Fig. 3 (abstract 000471).** See text for description

### 000472

#### Procalcitonin (PCT) and lactate utility as biomarkers of bacterial infection in the central nervous system (CNS)

M. Morales Moli, PJM. Morales, S. Foradada Ubach, A. Tache Sala, À. Castillo Niell, P. Pujol Valverde, J. Gonzalez Londoño, JM. Sirvent Calvera
[1]Intensive care medicine, Hospital Universitari de Girona Dr Josep Trueta, Girona, Spain
**Correspondence:** M. Morales Moli

*Intensive Care Medicine Experimental* 2019, **7(Suppl 3):**000472

**INTRODUCTION.** Bacterial CNS infections lead to high morbidity and mortality requiring early diagnosis and treatment. The aim of the study is to assess the utility of PCT and lactate levels in the cerebralspinal fluid (CSF) as biomarkers of bacterial infection in neurocritical patients.

**METHODS.** An observational study of a group of 65 patients admitted to an ICU of 18 beds between the period of July 2017 and December 2018. Patients who, due to admission to ICU, were included in the analysis of the CSF. Demographic variables, lactate and PCT in blood and lactate, PCT, Gram staining and CSF culture were collected. The CSF PCT/serum PCT and CSF lactate/serum lactate quotients were also analyzed. The patients were put into 2 groups and were classified according to the final diagnosis: bacterial infections of the CNS and other CNS affections.

**RESULTS.** In the sample analyzed (n = 65), 61.5% were men. The mean age was 54 ± 16. Mortality was 21.5%. The average APACHE II was 25.3 ± 6.4 and the average SOFA of 6.1 ± 3.2. With an average PCT in CSF of 0,5 ± 1,6 ng/mL and an average lactate in CSF of 36,4 ± 30,4 mg/dL. In the group of bacterial infections of the CNS, Gram staining was positive in 54.55% of the cases. The culture was positive in all of them. The isolated germs were: *S. Pneumoniae* (45.4%), *E. Coli* (18.2%), *L. Monocytogenes* (18.2%) and *M. Tuberculosis* (18.2%).

**CONCLUSION.** PCT in CSF is not a useful biomarker for the diagnosis of bacterial infections of the CNS. Lactate in CSF is elevated in different pathologies of the CNS, being significantly higher in bacterial infectious pathology. In addition, lactate index CFS/serum could be a good early marker of bacterial infection of the CNS.

**Table 1 (abstract 000472).** See text for description

| | Bacterial infections CNS (n = 11) | Other affectations CNS (n = 54) | p |
|---|---|---|---|
| Serum PCT (ng/mL) | 9.3 ± 13.6 | 7.1 ± 28.9 | NS |
| Serum lactate (mg/dL) | 15 ± 6.1 | 22.1 ± 25.1 | NS |
| CSF PCT (ng/mL) | 0.7 ± 1.5 | 0.4 ± 1.6 | NS |
| CSF Lactate (mg/dL) | 90.4 ± 38.2 | 25.4 ± 10.6 | < 0,01 |
| CSF PCT/serum PCT | 9.2 ± 13.6 | 11.2 ± 29.1 | NS |
| CSF lactate/Serum lactate | 6.4 ± 2.5 | 1.9 ± 1.6 | < 0,01 |

### 000498

#### Hypochloremia is associated with 28-day mortality in patients with septic shock: A retrospective analysis of a multicenter prospective registry

H. Kwak[1], M. Lee[2], TG. Shin[3], WY. Kim[4], YH. Jo[5], YJ. Hwang[6], SH. Choi[7], TH. Lim[8], KS. Han[9], J. Shin[10], GJ. Suh[11], KS. Kim[11], GH. Kang[12]
[1]seoul national university hospital, Seoul, Republic of Korea; [2]Emergency medicine, critical care medicine, Seoul National University Hospital, Seoul, Republic of Korea; [3]Emergency medicine, Sungkyunkwan University School of Medicine, Seoul, Republic of Korea; [4]Emergency medicine, Asan medical center, Seoul, Republic of Korea; [5]Department of emergency medicine, Seoul National University Bundang Hospital, Seongnam-si, Republic of Korea; [6]Emergency medicine, Yonsei University College of Medicine, Seoul, Republic of Korea; [7]Emergency medicine, Korea University Medical Center, Seoul, Republic of Korea; [8]Emergency medicine, Hanyang University College of Medicine, Seoul, Republic of Korea; [9]Emergency medicine, Korea University College of Medicine and School of Medicine, Seoul, Republic of Korea; [10]Emergency medicine, Boramae Medical Center, Seoul National University College of Medicine, Seoul, Republic of Korea; [11]Emergency medicine, Seoul National University Hospital, Seoul, Republic of Korea; [12]Emergency medicine, Hallym University College of Medicine, Seoul, Republic of Korea
**Correspondence:** H. Kwak

*Intensive Care Medicine Experimental* 2019, **7(Suppl 3):**000498

**INTRODUCTION.** Hyperchloremia is known to be associated with poor clinical outcomes in sepsis patients. However, the clinical significance of hypochloremia is not well studied.

**OBJECTIVES.** We investigated the association between hypochloremia and mortality in patients with septic shock.

**METHODS.** This is a retrospective analysis of prospectively collected multicenter registry including 11 emergency departments. The Outcome was defined as 28-day mortality. Patients were divided into three groups according to initial serum chloride level; hypochloremia (<98 mEq/L, 760/2037 [37.3%]), normochloremia (between 98 mEq/L and 110 mEq/L, 1182/2037 [58.0%]), and hyperchloremia (>110 mEq/L, 95/2037 [4.6%]). Multivariate logistic regression analysis was used to test the independent association between chloride categories and the 28-day mortality.

**RESULTS.** The 28-day mortality rates were 34.7% (33/95), 23.8% (181/760), and 19.2% (227/1182) (Chi square test, p <0.001) in hyperchloremia, hypochloremia, and normochloremia groups, respectively. Multivariate logistic regression analysis showed that both hyperchloremia (adjusted odds ratio [OR], 1.65, 95% confidence intervals [CI], 1.00–2.70; p value, 0.048) and hypochloremia (adjusted OR, 1.30, 95% CI, 1.02–1.67; p value, 0.035) were independently associated with 28-day mortality.

**CONCLUSION.** Hypochloremia (37.3%) was more frequently observed than hyperchloremia (4.6%) and it was independently associated with 28-day mortality in patients with septic shock in the emergency departments.

**REFERENCE(S)**

[1] Neyra JA, Canepa-Escaro F, Li X, Manllo J, Adams-Huet B, Yee J, et al. Acute kidney injury in critical illness study group: association of hyperchloremia with hospital mortality in critically ill septic patients. Crit Care Med 2015;43:1938-44.
[2] McCluskey SA, Karkouti K, Wijeysundera D, Minkovich L, Tait G, Beattie WS. Hyperchloremia after noncardiac surgery is independently associated with increased morbidity and mortality: a propensity-matched cohort study. Anesth Analg 2013;117:412-21.
[3] Boniatti MM, Cardoso PR, Castilho RK, Vieira SR. Is hyperchloremia associated with mortality in critically ill patients? A prospective cohort study. J Crit Care 2011;26:175-9.
[4] Rochwerg B, Alhazzani W, Sindi A, Heels-Ansdell D, Thabane L, Fox-Robichaud A, et al. Fluid resuscitation in sepsis: a systematic review and network meta-analysis. Ann Intern Med 2014;161:347-55.
[5] Yunos NM, Bellomo R, Hegarty C, Story D, Ho L, Bailey M: Association between a chloride-liberal vs chloride-restrictive intravenous fluid administration strategy and kidney injury in critically ill adults. JAMA 2012;308:1566-72.
[6] Raghunathan K, Shaw A, Nathanson B, Sturmer T, Brookhart A, Stefan MS, et al. Association between the choice of IV crystalloid and in-hospital mortality among critically ill adults with sepsis. Crit Care Med 2014;42:1585-91.
[7] Semler MW, Self WH, Wanderer JP, Ehrenfeld JM, Wang L, Byrne DW, et al. Balanced crystalloids versus saline in critically ill adults. N Engl J Med 2018;378:829-839.
[8] Funk GC, Zauner C, Bauer E, Oschatz E, Schneeweiss B. Compensatory hypochloraemic alkalosis in diabetic ketoacidosis. Diabetologia 2003;46:871-3.
[9] Yasuda K, Hayashi M, Murayama M, Yamakita N. Acidosis-induced hypochloremic alkalosis in diabetic ketoacidosis confirmed by the modified base excess method. J Clin Endocrinol Metab 2016;101:2390-5.
[10] Shin TG, Hwang SY, Kang GH, Kim WY, Ryoo SM, Kim K, et al. Korean Shock Society septic shock registry: a preliminary report. Clin Exp Emerg Med 2017;4:146-53.
[11] Angus DC, Barnato AE, Bell D, Bellomo R, Chong CR, Coats TJ, et al. A systematic review and meta-analysis of early goal-directed therapy for septic shock: the ARISE, ProCESS and ProMISe Investigators. Intensive Care Med 2015;41:1549-60.
[12] ProCESS Investigators. A randomized trial of protocol-based care for early septic shock. N Engl J Med 2014;370(18):1683-93.
[13] Mouncey PR, Osborn TM, Power GS, Harrison DA, Sadique MZ, Grieve RD, et al. Trial of early, goal-directed resuscitation for septic shock. N Engl J Med 372(14):1301-11, 2015.
[14] Dellinger RP, Levy MM, Rhodes A, Annane D, Gerlach H, Opal SM, et al. Surviving Sepsis Campaign: international guidelines for management of severe sepsis and septic shock, 2012. Intensive Care Med 2013;39:165-228.
[15] Kaukonen KM, Bailey M, Pilcher D, Cooper DJ, Bellomo R. Systemic inflammatory response syndrome criteria in defining severe sepsis. N Engl J Med 2015;372:1629-38.
[16] Shao M, Li G, Sarvottam K, Wang S, Thongprayoon C, Dong Y, et al. Dyschloremia is a risk factor for the development of acute kidney injury in critically ill patients. PLoS One 2016;11:e0160322.
[17] Thongprayoon C, Cheungpasitporn W, Cheng Z, Qian Q. Chloride alterations in hospitalized patients: Prevalence and outcome significance. PLoS One 2017;12:e0174430.
[18] Ryoo SM, Kang GH, Shin TG, Hwang SY, Kim K, Jo YH, et al. Clinical outcome comparison of patients with septic shock defined by the new sepsis-3 criteria and by previous criteria. J Thorac Dis 2018;10:845-53.
[19] Bone RC, Balk RA, Cerra FB, Dellinger RP, Fein AM, Knaus WA, et al. Definitions for sepsis and organ failure and guidelines for the use of innovative therapies in sepsis. The ACCP/SCCM Consensus Conference Committee. American College of Chest Physicians/Society of Critical Care Medicine. Chest 1992;101:1644-55.
[20] Yessayan L, Neyra JA, Canepa-Escaro F, Vasquez-Rios G, Heung M, Yee J, et al. Effect of hyperchloremia on acute kidney injury in critically ill septic patients: a retrospective cohort study. BMC Nephrol 2017;18:346.
[21] Suetrong B, Pisitsak C, Boyd JH, Russell JA, Walley KR. Hyperchloremia and moderate increase in serum chloride are associated with acute kidney injury in severe sepsis and septic shock patients. Crit Care 2016;20:315.
[22] Oh HJ, Kim SJ, Kim YC, Kim EJ, Jung IY, Oh DH, et al. An increased chloride level in hypochloremia is associated with decreased mortality in patients with severe sepsis or septic shock. Sci Rep 2017;7:15883.
[23] Stewart PA. Modern quantitative acid-base chemistry. Can J Physiol Pharmacol 1983;61:1444-61.
[24] Palmer BF, Alpern RJ. Metabolic alkalosis. J Am Soc Nephrol 1997;8:1462-9.
[25] Edwards SL. Pathophysiology of acid base balance: the theory practice relationship. Intensive Crit Care Nurs 2008;24:28-38.
[26] Kreu S, Jazrawi A, Miller J, Baigi A, Chew M. Alkalosis in critically ill patients with severe sepsis and septic shock. PLoS One 2017;12:e0168563.
[27] Tani M, Morimatsu H, Takatsu F, Morita K. The incidence and prognostic value of hypochloremia in critically ill patients. ScientificWorldJournal. 2012;2012:474185.

### 000520

#### Candidemia in intensive care unit patients, study from a tertiary care hospital in Dubai

A. Alsabbah[1], M. Salam[1], H. Celiloglu[2], S. Tausif[2], S. Al-Sabbah[3]
[1]Intensive care unit, Mediclinic City Hospital, Dubai, UAE; [2]Microbiology department, Mediclinic City Hospital, Dubai, UAE; [3]Department of statistics, Administration and Economics college, Kerbala University, Karbala, Iraq
**Correspondence:** A. Alsabbah

*Intensive Care Medicine Experimental* 2019, **7(Suppl 3):**000520

**INTRODUCTION.** · Candidemia remains a major cause of morbidity and mortality in the health care setting, and the epidemiology of Candida infection is changing.

· . Non-albicans Candida species have emerged as major causes of candidemia in many countries. Added to it is the problem of antifungal resistance in Candida isolates.

**OBJECTIVES.** · To find out the prevalence of candidemia in our intensive care unit (ICU), the ratio of candida albicans to non albicans and what are the difference in the outcome of each type along with the antifungal susceptibility pattern of Candida isolates and various risk factors associated with candidemia.

**METHODS.** · A single-center, retrospective analysis over 5 years covering the period from January 1, 2012 to December 31, 2017 was performed.

· The epidemiology, risk factors, demographic features, species distribution, and clinical outcome associated with candidemia in patients admitted to a single tertiary-care hospital in Dubai, were analyzed.

**RESULTS.** A total of 30 Candida isolates were isolated during the study period (overall incidence of 13.5 cases/1,000 ICU admission). Candida Non albicans were isolated more than Albicans in 63% (n= 19) vs 37% (n=11). Among Non albicans Candida parapsilosis was the most common isolate 58.8% (n=10) followed by Candida tropicalis 26.3% (n=5), Candida glabrata 11.8% (n=2) while only one case isolated of Candida kefyr and Candida auris. 26 Candida isolates (86.7%) were pansensitive while 4 isolates (13.3%) were resistant to fluconazole & amphotericine B.

· The most common medical problem associated with candidemia was cancer patient followed by Diabetes Mellitus, CVA and renal failure. The risk factors commonly associated with candidemia patients were use of central venous catheters, mechanical ventilation, hemodialysis or CRRT, abdominal or pleural drain, Trans parenteral nutrition and post abdominal surgery.
· There was no significant difference in mortality between male and female 60% vs 55% while the mortality was significantly higher in old age group above 60 year old comparing to younger patient than 60 ( 58% vs 33%). The species distribution and outcome of candidemia showed significant difference in the crude mortality between patients infected with C. albicans and non-albicans 45% vs 63% while among non albicans candidemia both candida Tropicalis & candida Parapsilosis had the highest mortality rate around 60%.
**CONCLUSION.** Candidemia is emerging as a significant problem in hospitalized patients. Non-albicans Candida species are the major cause of candidemia as found in our study. The epidemiology and choice of therapy for candidemia are rapidly changing. Additional multicentric studies involving many hospitals are required to know the true prevalence of candidemia, differences in virulence among Candida species and to determine the best therapeutic regimen.

### 000546

#### Time for antibiotics on sepsis: ¿How much earlier is the earliest? A propensity score analysis

J. Ascuntar[1], D. Mendoza[1], F. Jaimes[2]
[1]Internal medicine, Universidad de Antioquia, Medellin, Colombia; [2]Direccion de investigaciones, Hospital Universitario San Vicente Fundacion, Medellín, Colombia
**Correspondence:** F. Jaimes
*Intensive Care Medicine Experimental* 2019, **7(Suppl 3):**000546

**INTRODUCTION.** Early use of antibiotics is a critical point in the treatment of patients with sepsis. The exact time of initiation is controversial and may be a difficult intervention in crowded emergency services
**OBJECTIVES.** To estimate, using a matched propensity score, the effectiveness on hospital mortality of using antibiotics within one or three hours of diagnosis of sepsis, on patients hospitalized by emergency services (ES)
**METHODS.** Secondary data analysis of a prospective cohort conducted between 2014 and 2016 in three emergency services of university hospitals in Medellín, Colombia. A propensity score analysis for use of antibiotic, both within one and three hours of admission by the ES, was fitted with 28 variables related with clinical attention and physiological changes. As a sensitivity analysis, a logistic regression model was fitted for antibiotic use adjusted both by propensity score and confounding variables.
**RESULTS.** The study cohort was composed of 2,454 patients with a median age of 62 years (IQR=46-74). Among them, 32% (n=781) received antibiotics within three hours and 14% (n=340) within the first hour. The main diagnosis were urinary tract infection (28%, n=682) and pneumonia (27%, n=671). Blood cultures were requested in 87% (n=2140) and yielded positive in 29% (n=629), mainly with *Escherichia coli* (37%, n=230), *Staphylococcus aureus* (21%, n=132) and *Klebsiella pneumoniae* (102%, n=64). Hospital mortality rate was 11.5% (n=283). There were no significant differences in mortality using antibiotics either in the first hour (OR 1.09; 95% CI = 0.69; 1.72) or three hours (OR 0.98; 95% CI = 0.74; 1.32). There were no changes with different models for sensitivity analysis (Table).
**CONCLUSION.** Despite the obvious constraints given for sample size and residual confounding, our results suggest that we need a more comprehensive approach to sepsis treatment, considering multiple interventions and goals beyond the simple time-to-antibiotics

##### REFERENCE(S)

1. Grant COLCIENCIAS (Agencia de Ciencia, Tecnología e Innovación de Colombia) - 111556933362
2. Grant CODI - Universidad de Antioquia – 2582

**Table 1 (abstract 000546).** Effectiveness of antibiotic prescription within one or three hours of admission by emergency services in patients with sepsis

| Model | ≤ 1 hour | ≤ 3 hours |
|---|---|---|
| Matched by propensity score | OR (95%CI), n=680 | OR (95%CI), n=1562 |
| Without adjustment | 1,09 (0,69-1.72) | 0,98 (0,74-1,32) |
| Adjusted by propensity score | 1,05 (0,66-1,77) | 0,86 (0,63-1,17) |
| Adjusted by covariates* | 1,07 (0,65-1,75) | 0,93 (0,67-1,28) |
| Adjusted by PS and covariates* | 1,03 (0,63-1,7) | 0,85 (0,61-1,20) |
| Total Cohort (n=2454) | | |
| Without adjustment | 1,13 (0,8-1,6) | 1,28 (0,99-1,66) |
| Adjusted by propensity score | 0,94 (0,63-1,40) | 0,89 (0,67-1,2) |
| Adjusted by covariates* | 0,98 (0,66-1,45) | 1,04 (0,77-1,4) |
| Adjusted by PS and covariates* | 0,89 (0,58-1,37) | 0,87 (0,63-1,22) |

*Age, Charlson, IVF ≥ 1500 first hour, blood culture within three hours, lactate, SOFA, APACHE II, confirmed infection, appropriate antibiotics

## POIC - ICU treatment within treatment pathways

### 000095

#### Associated factors to the presence of agitation in maxillofacial surgery postoperative

R. Padilla[1], M. Dalorzo[1], F. Moran[1], M. Sanchez Casado[1], R. Martin[2]
[1]Icu, Virgin Health Hospital, Toledo, Spain; [2]Maxillofacial surgery, Virgin Health Hospital, Toledo, Spain
**Correspondence:** R. Padilla
*Intensive Care Medicine Experimental* 2019, **7(Suppl 3):**000095

**INTRODUCTION.** Patients undergoing elective and major maxillofacial surgery are one of the groups of greater risk of psychomotor agitation. It is associated with a greater frequency of complications, longer stay in ICU and hospital stay.
**OBJECTIVES.** Evaluate the factor associated to the presence of agitation in maxillofacial surgery postoperative.
**METHODS.** Retrospective revission in the clinical histories of the patients entered into ICU in the prospector of maxillofacial surgery higher in the last 6 years.
It was recorded basal variables, records, clinics and evolutive. To determinate the agitation it was used Riker scale and AgitatedBehaviorScales (ABS) scale. And we define psychomotor agitation as a ABC value bigger than or equal to 21. The cuantitative data are expressed like medium (interquartile range) and cathegorics like counting (percentage).
**RESULTS.** We get 60 patients, of wich 28 (46,7%) were agitated patients. We compare patients without agitation regarding patients with agitation. There are differences in males (78,1% vs 96,4%; p=0,037); the most important history antecedent was to be an active drinker (43,8% vs 64,3%; p=0,112); SOFA (1(1-1) vs 1(1-2);p=0,022). Tracheostomy was present (78,1% vs 96,4%;p=0,037).
There are evolutionary differences in re-surgery (21,9% vs 50%;p=0,023), infectious complications in ICU (12,5% vs 42,9%;p=0,008), rebleeding (9,4% vs 28,6%; p=0,055) and graft thrombosis (6,3% vs 21,4%;p=0,084); longer sedation time, (1 (1-1,5) vs 4 (2-7) days; p=0,001); start weaning (1 (1-1) vs 2 (1-3) days; p<0,001); mechanical ventilation duration (1 (1-2) vs 4 (2,5-9,5) days; p<0,001); days in ICU (3 (2-5,5) vs 4 (1,5-6,5) days; p<0,001).
**CONCLUSION.** Know the factors asociated to psychomotor agitation in the maxillofacial surgery postoperative will help us to get better in knowledge and use prophylaxis of it in certain treatment groups

(male patients, tracheostomized, higher SOFA punctuation), as it is associated to longer stays and postoperative complications.

**REFERENCE(S)**

1. Fields A, Huang J, Schroeder D, Sprung J, Weingarten T. Agitation in adults in the postanaesthesia care unit after general anaesthesia. Br J Anaesth. 2018;121(5):1052-1058.
2. Chen ZF1, Chen YK, Guo Y, Jiang H. [Effect of dexmedetomidine on emergence agitation after oral and maxillofacial surgery]. Shanghai Kou Qiang Yi Xue. 2013;22(6):698-701.

3.Chen
JW, Lv X, Zhang L, Chen ZF. [Effects of remifentanil and dexmedetomidine on recovery profiles after oral and maxillofacial surgery]. Shanghai Kou Qiang Yi Xue. 2016;25(1):101-4.

4. Curiel Balsera E, Prieto Palomino MA, Muñoz Bono J, Arias Verdú MD, Mora Ordóñez J, Quesada García G. [Decision on the time for post-operative extubation of maxillofacial surgery patient in the intensive care unit]. Med Intensiva. 2009 Mar;33(2):63-7.
5. Curiel Balsera E, Macias Guarasa I, Prieto Palomino MA, Muñoz Muñoz JL, Salguero Piedras M, Chaparro Sánchez MJ. Eficacia y seguridad de la analgesia con remifentanilo en el postoperatorio inmediato de cirugía maxillofacial. Med Intensiva. 2012;36(2):154-5.
6. Terada Y, Inoue S, Konda M, Egawa J, Ueda J, Kirita T et al. Effects of deep sedation under mechanical ventilation on cognitive outcome in patients undergoing surgery for oral and maxillofacial cancer and microvascular reconstruction. Med Intensiva. 2019;43(1):3-9.

7Devlin
JW, Skrobik Y, Gélinas C, Needham DM, Slooter AJC, Pandharipande PP et al. Clinical Practice Guidelines for the Prevention and Management of Pain, Agitation/Sedation, Delirium, Immobility, and Sleep Disruption in Adult Patients in the ICU. Crit Care Med. 2018;46(9):e825- e873.

8. Corrigan JD. Development of a scale for assessment of agitation following traumatic brain injury. J Clin Exp Neuropsychol. 1989;11:262-77.
9. Riker RR, Fraser GL, Simmons LE, Wilkins ML. Validating the sedation-agitation scale with the bispectral index and visual analog scale in adult ICU patients after cardiac surgery. Intensive Care med. 2001; 27(4):853-8.
10. Zhu Y, Wang G, Liu S, Zhou S, Lian Y, Zhang C et al. Risk factors for postoperative delirium in patients undergoing major head and neck cancer surgery: a meta-analysis. Jpn J Clin Oncol. 2017;47(6):505-511.

### 000266

#### Another New Attempt of Combination of Sedative Agents in Repeated Treatment for Pediatric Patients with Hemato-Oncology Disease

H. Koh, J. Huh, L. Min Soo
[1]Anesthesiology and pain medicine, The Catholic University of Korea Seoul St. Mary's Hospital, Seoul, Republic of Korea
**Correspondence:** H. Koh
*Intensive Care Medicine Experimental* 2019, **7(Suppl 3):**000266

**INTRODUCTION.** Many procedures and tests have been performed for the diagnosis and treatment of children. Young patients feel anxious and fear for repeated tests and procedures irrespective of the occurrence of pain. Many hours and manpower are consumed throughout all these process.

**OBJECTIVES.** These were two cases about the deep sedation which was undergone daily radiation therapy (RT) due to malignant solid tumor. They were failed in previous sedation with other agents (chlroral hydrate and midazolam), so referred to our department to solve the problem.

**METHODS.** One is the case for a 5 year-old-child (weight 20.8~22.9 kg) who was diagnosed into anaplastic ependymoma. After suboccipital craniotomy with tumor removal, they were scheduled to 28 fractions tomotherapy 1.8 Gy/d (total of 50.4 Gy/28d) at two target sites; posterior cranial fossa and spine. The other is for a 34-month-old infant (weight: 12.7~12.9 kg) was diagnosed to neuroblastma in adrenal gland with metastasis. After liver wedge resection and Lt. adrealectomy, they were scheduled to 12 fractions tomotherapy 1.73 Gy/d (total of 20.76Cy/12d) at two target sites; Lt. adrenal area and Rt. Fibular. Before RT, the former, midazolam 0.05mg/kg was injected and the latter, dexmedetomidine (Dex) 1mcg/kg for 10minutes as premedication. In both cases, after premedications were applied, three times of 1mg/kg of 1% propofol at a time were administered and then 150mcg/kg/min were continuously infused during RT. Blood tests were performed the next days after radiotherapy.

**RESULTS.** In both of these cases, there were no abnormal findings other than mild decreased hemoglobin, platelet and ANC (absolute neutrophil count) by bone marrow suppression. They were done all RT cycles well and discharged without complications. there were no gradual increases in dose of sedatives and drug dependence which were resulted from repeated sedation. In addition, hemodynamic adverse reactions; severe hypo or hypertension and bradycardia except for slight hypotension (systolic blood pressure 85~95mmHg) during sedation were not observed and other vital signs; pulse rate, SpO2 (saturation of percutaneous oxygen), respiration rate were kept constant within normal range. In sedation status, the RSS (Ramsay sedation scale) was maintained satisfactorily at level 4~5 and the Bispectral Index for measuring anesthetic depth was also showed at 40~50.

**CONCLUSION.** Generally, midazolam and propofol are widely used in procedural sedation. Recently, various trials through Dex are applied. The use of Dex is increasing as the way to maintain a safe sedation in cases of respiratory problems or in ICU. However, Dex alone is not enough to maintain an effective anesthetic depth during procedures or clinical tests, and does not show the same effect as propofol with shorter duration and recovery. However, due to the side effects of benzodiazepine as a sedative, it has gradually been replaced by other stable sedative agents, one of which is Dex. Therefore, Dex instead of midazolam was used as an adjuvant agent based on propofol sedation. Through this case, we suggest that Dex was able to maintain the sedation reliably when it is used as only pretreatment loading and also strengthen the sedation state even though not a the continuous infusion of Dex.

**REFERENCE(S)**

1. Management of Pain and Agitation for Patients in the Intensive Care Unit J Neurocrit Care 2015;8(2):53-65
2. Park SW, Choi JH, Kim HT, Cho YH. Replacement of dexmedetomidine loading with midazolam for sedation in elderly patients with spinal anesthesia. Medicine
3. Boriosi JP, Eickhoff JC, Klein KB, Hollman GA. A retrospective comparison of propofol alone to propofol in combination with dexmedetomidine for pediatric 3T MRI sedation. Paediatr Anaesth 2017;27:52-9. doi: 10.1111/pan.13041.
4. Kim SK, Song MH, Lee IJ, Lee JH, Choi IC. Dexmedetomidine for sedation in pediatric patients who received more than 20 sessions of radiation therapy: two cases report. Korean J Anesthesiol 2016;69:627-31. doi: 10.4097/kjae.2016.69.6.627.

### 000273

#### The effect of polyolefin bag preservation with shaking on human platelet functions in hemodilutional autologous blood transfusion

Y. Murata, S. Kawamoto, K. Fukuda
[1]Department of Anesthesia, Kyoto University Hospital, Kyoto, Japan
**Correspondence:** Y. Murata
*Intensive Care Medicine Experimental* 2019, **7(Suppl 3):**000273

**INTRODUCTION.** In recent years, hemodilutional autologous blood transfusion (HAT) has attracted attention for its effects in reducing hospital stay days and postoperative complications in cardiovascular surgery. HAT is a blood conservation technique using polyvinyl chloride bag with CPDA fluid including citrate sodium, glucose, adenine and sodium dihydrogenphosphate at room temperature without shaking. But this is not suitable for maintaining platelet functions because of low pH of CPDA fluid, and low gas permeability of polyvinyl chloride bag. At present, HAT has not been implemented mainly for the purpose of hemostasis. Therefore, there is a need to develop new HAT that can preserve platelet functions and be used as an optimal hemostatic products in cardiovascular surgery.

**OBJECTIVES.** The aim of this study is to find storage conditions that can best maintain platelet functions. We used polyolefin bag with shaking to maintain platelet functions. Since polyolefin have high gas permeability, shaking storage can release carbon dioxide generated by the equilibrium reaction of lactic acid and bicarbonate, and maintain an appropriate pH.

**METHODS.** Informed consent was given and the protocol was approved by the ethical committee. Venous blood was taken from 3 healthy volunteers who had not taken any medication at least for two weeks before blood collection. 50 ml of whole blood were preserved in polyolefin bag with shaking at 50/min or polyvinyl chloride bag without shaking for 8 hours at 22 degrees Celsius. CPDA fluid was used as preservative solution. We performed blood gas analysis (PaO2, PaCO2, pH, BE, lactate, HCO3-), complete blood count (hematocrit, platelet count, mean platelet volume (MPV)) and platelet function tests including ADP-induced aggregability using light transmission method and P-selectin expression with or without ADP stimulation using flow cytometry at 0 and 8 hours after blood collection. Wilcoxon signed rank test was used to compare the two groups. P values less than 0.05 were considered to indicate statistical significance.

**RESULTS.** No statistically significant difference was found between the two groups in any of the items, but in the polyvinyl chloride group and the polyolefin group, the aggregability was reduced by 61.0% and 25.3%, non-ADP stimulated P-selectin expression was increased by 193.7% and 104.5%, after 8 hours, respectively. MPV and ADP-stimulated P-selectin expression were almost similar. The polyolefin group had tendency to keep PaO2 high, PaCO2 low, and pH high. P-selectin expressions with or without ADP stimulation represent potential platelet adhesivity and platelet activation level by HAT procedure itself, respectively. MPV indicates platelet activation. Further cases are needed to determine whether platelet functions are maintained in the polyolefin group due to the high oxygen permeability with shaking. 8 hours might be too short to detect the superiority of polyolefin storage in platelet aggregability and adhesivity. Other platelet function evaluations, including release ability, hypotonic shock recovery rate, intracellular Ca concentration, clot dynamics, and morphology are needed to clarify the effectiveness of polyolefin preservation with shaking.

**CONCLUSION.** Further evaluations are needed to confirm the usefulness of polyolefin preservation with shaking on maintaining platelet functions.

1. Grant-in-Aid for Scientific Research (KAKENHI) :19K18293

### 000624

#### Use of parenteral iron in the perioperative resuscitation of hip fracture patients – a quality improvement project

JM. Dudziak, A. Ahsan, D. Factor
Anaesthetics, Queen Elizabeth Hospital, London, UK, UK
**Correspondence:** J.M. Dudziak
*Intensive Care Medicine Experimental* 2019, **7(Suppl 3):**000624

**INTRODUCTION.** Blood loss in hip fracture patients is common, resulting both from initial trauma and subsequent operative intervention. As UK national standards mandate operative fixation within 36h of presentation to hospital, rapid correction of anaemia is paramount to optimise postoperative mobility (*Lawrence 2003*). However, multi-centre trials have found no benefit from using a liberal (transfusion if Hb <100 g/L) over a restrictive strategy (Hb <80 g/L or patient symptomatic) (*Carson 2011*), indicating that adverse effects of red cell transfusion could offset the potential benefits of higher Hb levels. Parenteral iron replacement promises to increase Hb without transfusion-related morbidity (*Bernabeu-Wittel 2016*).

**OBJECTIVES.** To assess and optimise our use of parenteral iron to treat iron deficiency in anaemic hip fracture patients.

**METHODS.** Initial cohort of 43 consecutive hip fracture patients, median age 81, 63% female, admitted to our UK district general hospital over six weeks from November 2018 to January 2019. Forty patients survived to operative fixation and were included in the primary analysis. We deployed a care bundle to improve testing for and correction of perioperative iron deficiency in April 2019, will assess its impact in June and present our findings at the congress.

**RESULTS.** All but one (97%) patient were anaemic (Hb <130 g/L, from *Muñoz 2017*) during their inpatient episode. Thirty-seven (93%) patients had their serum iron levels measured, which was low in 29 (78%) cases. Only four patients (10%) had transferrin saturation (TSAT) measured, which were low in all cases (100%). Ferritin was measured in 21 (53%) patients, but only in three (13%) cases was less than 100 mcg/L (threshold in presence of inflammation, from *Muñoz 2017*). Twelve patients (30%) received ferric carboxymaltose (FCM), all with low serum iron levels. However, only two (50%) of the patients with low TSAT, and only one (33%) of the patients with low ferritin received FCM. Seventeen patients (59%) with low serum iron did not receive FCM, although four of those patients additionally had either low TSAT or low ferritin, see above.

**CONCLUSION.** Both anaemia and iron deficiency were extremely common. Serum iron was the most commonly used biomarker of iron deficiency and showed overwhelmingly low iron stores in our cohort. TSAT was underused, despite being the most sensitive marker for insufficient iron supply in the presence of trauma. Ferritin, despite still being the diagnostic mainstay (*Muñoz 2017*) was poorly sensitive in our cohort, likely due to false elevation as part of the acute phase response. Although most patients would likely have benefited from parenteral iron replacement, only a minority received it. We will intensify staff education, deploy a care bundle containing prompts for blood sampling as well as indications for parenteral iron replacement and will report the result of this intervention at the congress.

**REFERENCE(S)**

1. Muñoz M, Acheson AG, Auerbach M, et al. International consensus statement on the peri-operative management of anaemia and iron deficiency. Anaesthesia 2017; 72: 233-247.
2. Bernabeu-Wittel M, Romero M; PAHFRAC-01 Investigators. Ferric carboxymaltose with or without erythropoietin in anemic patients with hip fracture: a randomized clinical trial. Transfusion 2016; 56(9): 2199-211.
3. Carson JL, Terrin ML et al; FOCUS Investigators. Liberal or restrictive transfusion in high-risk patients after hip surgery. New England Journal of Medicine 2011; 365(26): 2453-62.
4. Lawrence VA, Silverstein JH, Cornell JE, Pederson T, Noveck H, Carson JL. Higher Hb level is associated with better early functional recovery after hip fracture repair. Transfusion 2003; 43: 1717-1722.

### 000852

#### New recombinant antithrombin gamma may improve its activity and coagulation abnormality

H. Miyazaki, R. Furuya, K. Takada, R. Matsumura
Emergency and critical care medicine, National Health Organization Yokohama Medical Center, Yokohama, Japan
**Correspondence:** H. Miyazaki
*Intensive Care Medicine Experimental* 2019, **7(Suppl 3):**000852

**INTRODUCTION.** The coagulation system in human is extraordinarily fragile. Various stimuli may deteriorate the system, cause life-threatening events such as Disseminated intravascular coagulation (DIC). Replacement of decreased antithrombin may improve the coagulopathy caused by noxious stimuli such as sepsis or severe trauma.

Recently, recombinant antithrombin gamma (Kyouwa Hakkou Kirin Co.Ltd. Tokyo) has been introduced into Japan. This novel recombinant protein will reduce adverse effects related to the blood extracts, but also may diminish the problem of blood supply.

We investigated the efficacy of the newly introduced recombinant antithrombin. This study is a brief report of the administration of the antithrombin gamma for 54 patients who were diagnosed as DIC or decreased antithrombin activity.

**METHODS.** From December 2017 to November 2018, 54 patients who were diagnosed DIC and were administered the antithrombin gamma were surveyed by retrospectively using medical records. The collected data were as follows, patients age, sex, body weight, the illness of patients, acute DIC scores (defined by the Japanese Society of Emergency and Critical Medicine), dose of study drug, other combination therapies and prognosis of the patient.

**RESULTS.** In 54 studied patients, 34 cases were diagnosed DIC; however, all patients showed antithrombin activities below 70% of normal. Twenty-eight in 54 patients had sepsis. The average dose of the antithrombin gamma was 1877±450U. The administration days were 1 day (1 to 14 days) in 65% of cases. Administration of the antithrombin gamma had increased antithrombin activities linearly. Average activity before administration and after were 47±12% and 72±12%, respectively. Eleven in 34 patients recovered from DIC after administration. Also, 36 in 54 patients were survived at the time of discharge.

**CONCLUSION.** Various stimuli may cause abnormality of coagulation systems. Despite a long history of investigations, there have been no well-established therapies. However, antithrombin administration for the patients who were suffered by coagulopathy is a controversial issue; some guidelines mention about this therapy. Recently, recombinant antithrombin has been developed and introduced into clinical practice. Our result showed the recombinant antithrombin gamma restored the activity of antithrombin, and recover from DIC in one-third of the study group. This finding is compatible with the former studies of antithrombin administration which was extracted from the human blood. In the study group, we have not experienced any adverse events related to the antithrombin gamma. A phase I trial of the antithrombin gamma has been conducted in Europe from April 2018. Still, little literature revealed its feasibility and equivalency of AT gamma over conventional antithrombin. We will need to further investigations, especially a RCT, to assess the efficacy and safety of the recombinant antithrombin gamma.

1. Shigeatsu Endo, Ryutaro Shimazaki and the Antithrombin Gamma Study Group. An open-label, randomized, phase 3 study of the efficacy and safety of antithrombin gamma in patients with sepsis-induced disseminated intravascular coagulation syndrome. Journal of Intensive Care.201; 86:75

### 000962

#### A Descriptive Study of postoperative Radical Cystectomy in ICU

C. Joya Montosa[1], MP. Benítez Moreno[1], FA. Hijano Muñoz[1], MJ. Delgado Amaya[1], MA. Barbancho Fernández[2]
[1]Intensive care unit, Hospital Regional Universitario de Málaga, Málaga, Spain; [2]Fisiology department, University of Malaga, Málaga, Spain
**Correspondence:** C. Joya Montosa
*Intensive Care Medicine Experimental* 2019, **7(Suppl 3):**000962

**INTRODUCTION. Objective**: Presenting clinical and epidemiological data of patients with a radical cystectomy with urinary diversion who required ICU admission.

**METHODS.** Retrospective, descriptive study of patients admitted in ICU from May 2018 to December 2018. Clinical, epidemiological and result-related variables were analyzed. Quantitative variables are expressed as mean and standard deviation, while qualitative variables are expressed as ratios and absolute value.

**RESULTS.** A total number of 15 patients took part in this study; mean age was 60.73 ± 9.49 years (73.3% were males). APACHE II at admission was 10.6 ± 1.35. SAPS3 52.6 ± 10.03. Between the comorbidities 40% of the patients presented high blood pressure, 26.7% had dyslipidemia, 20% were diabetics, 46.7% were smokers, 13.3% took alcohol, and 26.7% had others as obesity or COPD. 6.7% had no comorbidities. All of the patients had history of urological disease; the most frequent were prostatic hyperplasia (13.3%) and urinary tract infections (UTI) (13.3%). Previous abdominal surgery was performed in 26.7% of the patients. In 12 of the cases the radical cystectomy was necessary because of muscle-infiltrating urothelial bladder carcinoma. Two patients required the surgery because of obstructive problems, and one of them because of neurogenic bladder (multiple sclerosis). 66.7% patients received at first transurethral resection (TUR). 6.7% required chemotherapy before the procedure; braquitherapy was administered also in 6.7%. None of them had neoadjuvant radiotherapy. The most common urinary diversion was Bricker (only one was Nesbitt). At the ICU admission 53.5% of the patients presented creatinine values between 1 and 1.5mg/dl. 33.3% had light metabolic acidosis and none of them hydroelectrolyte imbalance.Peri and postoperative antimicrobial prophylaxis with Amoxicillin-Clavulanic was done in 80% of the patients; 13.3% were treated with Quinolones and only in 6.7% Piperacillin-Tazobactan was used. 26.7% (4) of our patients had complications, the most frequent was paralytic ileus (50%); 25% had acute renal failure, and only one (25%) had minor bleeding which didn't need surgery. A patient need re-entry en UCI due to Sepsis of unknown origin, dying after 7 days of intensive therapy. Average ICU stay was 1.26 ± 0.59 days. ICU mortality rate was 6.7% and one-year mortality after the cystectomy was 20%.

**CONCLUSION.** The profile of the patient admitted to the ICU after cystectomy is a 60 year old patient with comorbidities, muscle-infiltrating urothelial carcinoma and normal renal function, to those who underwent prophylactic treatment with Amoxicillyn-Calvulanic. A good postoperative progress and no re-entry en ICU was observed.

### 000966

#### Preeclamsia patients who need ICU: A descriptive study

MP. Benítez Moreno[1], C. Joya Montosa[1], JF. Martínez Carmona[1], MJ. Delgado Amaya[1], MA. Barbancho Fernández[2]
[1]Intensive care unit, Regional Hospital of Malaga, Málaga, Spain;
[2]Fisiology department, University of Malaga, Málaga, Spain
*Intensive Care Medicine Experimental* 2019, **7(Suppl 3):**000966

**INTRODUCTION.** We consider severe preeclampsia when the increase in blood pressure is associated with certain symptoms, analytical alterations, oligohydramnios or Intrauterine *growth restriction* (IUGR). Treatment sometimes requires admission to the ICU, depending on the severity of the episode, aimed at controlling blood pressure levels with maternal and fetal monitoring, maturing the fetus, if necessary, and avoiding possible complications.

**METHODS.** We conducted a descriptive retrospective study in which the patients who required admission to the gynecological ICU in our hospital were collected during 2018 with a diagnosis of gestosis to evaluate the type of preeclampsia, the treatment performed and the results obtained.

**RESULTS.** A total of 14 patients diagnosed with preeclampsia, who required admission to the ICU during 2018, were collected. The gestational age was between 22 and 39 weeks. Fetal maturation with betamethasone was performed in 64.3% of the cases, only 14.7% if the pregnancies were multiple. All patients who required admission to the ICU were diagnosed with severe preeclampsia. The most frequent symptoms that they presented were visual anomalies (85.7%), followed by edema and abdominal pain (71.4% each), headache (57.1%) and proteinuria (42.9%). No cases of acute pulmonary edema (APE) or oliguria were observed. Upon admission to the ICU, 71.4% had SBP ≥ 160 mmHg with a medium of 120.7 and a typical deviation of 14.16. 100% required more than one antihypertensive to control blood pressure and in all cases labetalol was used, followed by hydralazine (71.4%), alpha methyl dopa (42.9%), and nifedipine (21.4%) . Magnesium sulfate was used in

71.4% of the cases, with a mean dose of 6.28 g /day and a standard deviation of 4.87. End of the pregnancy for maternal or fetal benefit was needed in most of the cases, been early caesarean section performed in 85.7% of patients; only 1 case was delayed 9 days due to low gestational age. Maternal complications after ICU admission only appeared in 3 of the 14 cases and were: 1 post-cesarean bleeding, 1 urinary tract infection and 1 case of paralytic ileus. As far as the fetus IUGR was observed in 14.3% of the cases and only one presented oligohydramnios. Maternal survival was 100% meanwhile 87.5% was observe in newborns.

**CONCLUSION.** All patients admitted to the ICU due to gestosis present criteria of severe preeclampsia. The most frequent symptoms were visual alterations, abdominal pain and edema. A 71.4% on admission showed arterial hypertension. 100% of the cases required more than 1 antihypertensive for the control of blood pressure in the ICU, being labetalol the antihypertensive that was administered in all cases. It is a small sample but with full survival despite the severity of the case we continue with the collection of data to confirm that patients diagnosed with severe preeclampsia benefit from admission in ICU.

### 000968

#### Andalusian Women preeclampsia: ICU epidemiology

MP. Benítez Moreno[1], C. Joya Montosa[2], E. Trujillo García[2], MJ. Delgado Amaya[2], MA. Barbancho Fernández[3]
[1]Intensive care, HOSPITAL CARLOS HAYA, MALAGA, Spain; [2]Intensive care unit, Regional Hospital of Malaga, Málaga, Spain; [3]Fisiology department, University of Malaga, Málaga, Spain
*Intensive Care Medicine Experimental* 2019, **7(Suppl 3):**000968

**INTRODUCTION.** The incidence of hypertensive disorders associated with pregnancy is increasing due to maternal age, obesity, assisted reproductive techniques, etc. Preeclampsia refers to a blood pressure ≥140/90 mmHg, with proteinuria in pregnant women over 20 weeks or up to 2 weeks after delivery. Risk factors are: maternal age, (<20 or > 35), personal or family history of preeclampsia, hypertension, diabetes, obesity, renal failure, primiparous or multiparity, multiple pregnancy, and coagulation disorders.

**OBJECTIVES.** We wanted to know the epidemiology of the patients who needs ICU admission due to gestosis, risk factors, mortality scores and survival of pregnant women and newborns.

**METHODS.** Retrospective, descriptive study of patients admitted in ICU from January 2018 to December 2018 . Clinical, epidemiological and result-related variables were analyzed. Quantitative variables are expressed as mean and standard deviation, while qualitative variables are expressed as ratios and absolute value.

**RESULTS.** During 2018, 14 patients with a diagnosis of gestosis required ICU admission. The patients had between 23 and 50 years, medium age of 35.14 years and standard deviation of 7.8. Gestational age at admission was between 22 and 39 weeks with a medium of 31.5. 100% of pregnant women had at least a risk factor (nuliparity was the most frequent (57.14%), 42.9% were older than 35 years, diabetes and coagulation disorders were observe in 28.6% of the patients, and hypertension, preeclampsia history and multiple pregnancy in 21.4%. Only 2 patients were in vitro fertilisation, in both cases were their first pregnancy. ICU stay was between 1 and 6 days with a medium of 2.6 days and standard deviation of 1.5. APACHE II at admission was 6.3 with a standard deviation of 3.6. All of our patients were alive when the results were taken. Two of the sixteen babies died: one of them was a twin who died in uterus a month before the admission; the second one had a gestational age of 22 weeks.

**CONCLUSION.** Our low incidence of preeclamsia, which need ICU admission, could be explained by primary prevention and treatment in healthcare centres. Patient admitted in our ICU with preeclamsia, is a 35 years old woman who at least has one risk factor, been nuliparity the most frequent, as well as age over 35 year, diabetes and coagulation disorders; and with a low mortality predicted by APACHE II at admission.

## HSRO / TEM - Predicting outcome: Influenceable variables

### 001079

#### Scientific evidences as a policy for the optimization of beds in public ICUs in Brazil

G. Rossone Reis[1], W. Rodrigues[2], GJ. Von Glehn Dos Santos[3], E. Barbosa[4], SE. Bazzo Júnior[5]
[1]Physiotherapy, UnirG - Universidade de Gurupi, Gurupi, Brazil; [2]Economics, UFT - Universidade Federal do Tocantins, Palmas, Brazil; [3]Medicine, UnirG - Universidade de Gurupi, Gurupi, Brazil; [4]Critical care, HGP - Hospital Geral de Palmas, Palmas, Brazil; [5]Physiotherapy, HDT - Hospital de Doenças Tropicais da Universidade Federal do Tocantins, Araguaína, Brazil
**Correspondence:** G. Rossone Reis
*Intensive Care Medicine Experimental* 2019, **7(Suppl 3):**001079

**INTRODUCTION.** The availability of beds in intensive care units in Brazil has become a serious public health problem. Through the identification of studies and data collection that resulted from the analysis of variables that generated better indicators of quality in intensive care, such as the reduction of length of stay, costs of care and mortality rate.

**OBJECTIVES.** To analyze whether the implementation of public policies based on scientific evidence focused on ICU patient admission criteria and infection control can contribute to increase the availability of beds and reduce hospital costs in public ICUs in Brazil.

**METHODS.** The retrospective, quantitative and statistical documentary research through collection, correlation and comparison of data, aiming to analyze if the admission criteria and the incidence of nosocomial infection is related to length of stay, costs and mortality rate in patients hospitalized in Brazilian public ICUs. Data were analyzed of all hospitalizations of patients admitted to the three largest public ICUs in the State of Tocantins, in the north of Brazil, from January to December, 2018. The clinical data of the 1013 patients admitted to the ICUs in the year of 2018 were collected, based on the ICU vacancy request form sent to the Regulation Center, where they were allocated to the priority admission groups (Groups with priority 1, 2 and 3, and Group with priority 4). After the analysis of the patients' records at the respective hospitals, the length of stay, the infection index, the mortality rate and the cost estimate were collected. Regarding the statistical analysis, the correlation data between the quality indicators (length of stay, costs and mortality rate) on the presence or absence of hospital infection and the admission criteria, were analyzed by the coefficient of Pearson and Staverman in order to evaluate the degree of linear association.

**RESULTS.** A total of 1013 patients were collected, however, some patients did not present the necessary information to perform the study, and only 634 patients were considered to compose the sample. Through analysis of the pearson correlation, it can be observed that high infection rates (n=260 or 41%) and lack of admission criterion (criteria 4 with n=134 or 21%) corroborate for higher mortality ($p<0.001$), longer stay time ($p=0.0019$) and higher costs ($p<0.001$), reducing the quality indicators of public ICUs in Brazil.

**CONCLUSION.** Evidence shows that the adoption of continuous assessment measures based on technical knowledge and the application of evidence-based medicine in the management of public health policies, especially in the intensive care unit, can corroborate better quality indicators, with a greater supply of beds and, consequently lower waiting time for vacancy in ICU, lower mortality rate and lower costs, without investments in new units and/or opening of new beds.

**REFERENCE(S)**

1. REIS, Geovane Rossone et al. Correlação entre níveis de sedação e tempo de ventilação mecânica. AMAZÔNIA: SCIENCE & HEALTH, v. 2, n. 2, p. 15-20, 2014.

2. MOGYORÓDI, Bence et al. Ventilator-associated pneumonia and the importance of education of ICU nurses on prevention–Preliminary results. Interventional Medicine and Applied Science, v. 8, n. 4, p. 147-151, 2016.
3. GARCÍA-GIGORRO, R. et al. Impact on patient outcome of emergency department length of stay prior to ICU admission. Medicina Intensiva, 2016.
4. ELY, E. et al. The impact of delirium in the intensive care unit on hospital length of stay. Intensive care medicine, v. 27, n. 12, p. 1892-1900, 2001.
5. DE SOUZA MIQUELIN, Priscila Rodrigues; REIS, Geovane Rossone. Comparação entre as taxas de morbimortalidade de pacientes com septicemia em todos os estados da federação e o Distrito Federal. AMAZÔNIA: SCIENCE & HEALTH, v. 4, n. 4, p. 20-24, 2016.
6. DAL FORNO, Claudia Balbuena et al. Bloodstream infection in the intensive care unit: Preventable adverse events and cost savings. Value In Health Regional Issues, v. 1, n. 2, p. 136-141, 2012.
7. CHACKO, Binila et al. Attributable cost of a nosocomial infection in the intensive care unit: A prospective cohort study. World Journal of Critical Care Medicine, v. 6, n. 1, p. 79, 2017.
8. CARDOSO, Lucienne TQ et al. Impact of delayed admission to intensive care units on mortality of critically ill patients: a cohort study. Critical Care, v. 15, n. 1, p. R28, 2011.
9. CALDEIRA, Vanessa Maria Horta et al. Criteria for patient admission in the intensive care unit and mortality rate. Revista da Associação Médica Brasileira, v. 56, n. 5, p. 528-534, 2010.
10. ASSIS, Michelli Cristina Silva de et al. Is duration of postoperative fasting associated with infection and prolonged length of stay in surgical patients?. Nutricion hospitalaria. Madrid. Vol. 30, no. 4 (2014), p. 919-926, 2014.

### 001082

#### Medical Emergency Team in Italy: a national survey

A. Galazzi[1], NM. Bonasera Vincenti[2], I. Adamini[1], GD. Giusti[3], M. Brioni[1], A. Zampetti[2], D. Laquintana[4], G. Grasselli[1]
[1]Intensive care unit, Foundation IRCCS Ca' Granda Ospedale Maggiore Policlinico, Milano, Italy; [2]Nursing, University of Milan, Milano, Italy; [3]Intensive care unit, A.O.U. Ospedale di Perugia, Perugia, Italy; [4]Direction of healthcare professions, Foundation IRCCS Ca' Granda Ospedale Maggiore Policlinico, Milano, Italy
**Correspondence:** A. Galazzi
*Intensive Care Medicine Experimental* 2019, **7(Suppl 3):**001082

**INTRODUCTION.** Medical Emergency Team (MET) is implemented in many hospitals all over the world and it seems associated with the prevention of cardiac arrests and unexpected deaths in wards, reduced rates of admissions to intensive care units and hospital mortality (1). Therefore, international bodies of quality certification, such as the Joint Commission and the Institute for Healthcare Improvement, support creation and implementation of MET in every hospital (2). Although its implementation is recommended, little is known about the presence of MET and its organization in Italian National Health Service hospitals (3).
**OBJECTIVES.** To investigate the presence of MET and its organization in Italian National Health Service hospitals.
**METHODS.** A national survey was conducted for two months in 2018. All public and private National Health Service associated hospitals with a service of intensive care were invited to participate. An online questionnaire was sent via e-mail to nursing coordinators or MET referents.
**RESULTS.** Among the 544 Italian hospitals identified, 197 (36.2%) answered the questionnaire. In every hospital there was a physician who answered the emergency calls, but a real MET (intensivist physician and critical care nurse) was present only in 59.9% of the hospitals, particularly in the north of Italy and in the community hospitals (61.5%). MET activity started about 10 years ago (51.7%) and in most hospitals the service is available 24/24 hours, 7/7 days (84.8%). The team members' minimal education was a specialization on intensive care medicine (100%) for physicians, while the most usual criteria for nurses were working experience in critical care (70.3%), besides specific internal courses were also performed (21.2%). The MET nurses belonged to the intensive care unit (83.1%) or to the emergency room (21.2%). Unfortunately, the intensivist physician and the critical care nurse weren't dedicated to MET (67.8% and 69.5% respectively), in particular in the community hospitals. The Modified Early Warning Score was used in 22.9%, while in 34.8% of hospitals no warning score was used.
**CONCLUSION.** This survey has shown that in Italian National Health Service hospitals MET appears structured and consolidated in some realities, while in others it is totally absent or lacking in some fundamental peculiarities of an intra-hospital emergency system.

**REFERENCE(S)**

1) Hamsen U, et al. Unfallchirurg 2018
2) Silva R, et al. SJTREM 2016
3) Savoia G, et al. Minerva Anestesiol 2007
4) None

### 001083

#### Burnout experience in the ICU staff of the Regional University Hospital in Ukraine

M. Grynovska[1], A. Wong[2]
[1]Anesthesia and intensive care, Ivano-Frankivsk National Medical University and Regional University Teaching Hospital, Ivano-Frankivsk, Ukraine; [2]{street_address}, Guildford, UK
**Correspondence:** M. Grynovska
*Intensive Care Medicine Experimental* 2019, **7(Suppl 3):**001083

**INTRODUCTION.** Burnout (BO) is reaching epidemic levels in the medical population with prevalence reported around or over 50%.[1] Its presence is associated with poor patient safety outcomes.[2] Those working in ICM are disproportionately affected, rating higher on stress, BO and compassion fatigue indices.[3] In Ukraine, specifically, the concept of BO in ICM remains poorly understood and is largely under recognized, or dismissed. With paucity of literature addressing the issue, we decided to investigate the reality of BO experience in our country.
**OBJECTIVES.** To assess the BO experience and identify associated risk factors in the ICU of a university teaching hospital.
**METHODS.** The focus of the study was the frontline ICU staff nurses (n=40) and doctors (n=49). Data collection was performed with the help of the Maslach Burnout Inventory (MBI) survey. The BO rate was assessed across various ICU types, seniority, gender and age groups.
**RESULTS.** The overall prevalence of BO in ICU staff was 60.7 % (nurses 62.5% doctors 59.2%). There was no difference on BO subscales between doctors and nurses (95%CI,P>0,05).We also found no statistically significant difference in the incidence of BO between doctors and nurses (P>0,05). Our study showed no difference (P>0,05) in the incidence of BO in doctors across ICU type, seniority level, gender or age group. In contrast, there was a difference in the incidence of BO in nurses across the ICU type (P < 0,05).
**CONCLUSION.** The response rate was 92%. BO prevalence was notably high in both ICU nurses and doctors. Our findings indicate that doctors and nurses are equally at risk of a BOexperience. The study showed no clear association between any of the risks and the incidence of BO in doctors. With regard to nurses,the ICU type proved to be a factor in the incidence of burnout. The nurses of general and neurosurgery ICUs were most affected by BO possibly due to demanding workload and severity of the patients in these units. Limitations of our study include a relatively small number of respondents that prevents from drawing conclusions in the context of a country/healthcare system. Further research across more hospitals in Ukraine is warranted to determine the BO rates and risk factors. In conclusion, we expect to raise awareness of the ICU staff BO in Ukraine and advocate for more research in this field. In a wider perspective, we hope that our study contributes to a better understanding of BO experience and promotes a broader discussion on the welfare and sustainability of the ICM workforce.

**REFERENCE(S)**

1. Shanafelt TD et al., Mayo Clinic Proceedings. 2015; 90(12):1600–1613.
2. Hall LH et al., PLoS One.2016;11(7):e0159015.

3. Burki TK. How do you deal with burnout in the clinical workplace? Lancet Respiratory 2015; 3: 610-611.
4. I would like to thank ESICM NEXT Mentoring and my mentor Adrian Wong for supervision and guidance.

### 001089

#### Uric Acid Correlates with Circulating Mitochondrial DNA and Predicts Mortality in Critical Ill Patients

K. Krychtiuk, M. Lenz, P. Hohensinner, S. Ruhittel, C. Kaun, J. Wojta, G. Heinz, WS. Speidl

[1]Department of internal medicine ii, Medical University of Vienna, Vienna, Austria

**Correspondence:** K. Krychtiuk

*Intensive Care Medicine Experimental* 2019, **7(Suppl 3):**001089

**INTRODUCTION.** It has recently been shown that mitochondrial DNA (mtDNA) is associated with outcome in critically ill patients, however measurement of mtDNA is time consuming and cumbersome. Uric acid is a product of the metabolic breakdown of purine nucleotides and can be measured routinely.

**OBJECTIVES.** To analyze whether uric acid is associated with levels of mtDNA and predicts 30-day survival in ICU.

**METHODS.** In this prospective, observational cohort study, 222 consecutive patients admitted to a cardiac ICU at a tertiary care center were enrolled. Blood was taken at admission to the ICU and levels of circulating mtDNA were quantified by real-time.

**RESULTS.** Mean Apache II score was 19.6±8.3 and 30-day mortality was 26.1%. Uric acid correlated with circulating mtDNA plasma levels (R=0.21; p<0.002). Non-survivors showed significantly higher uric acid levels as compared to survivors (7.8±3.9 vs 6.2±2.5mg/dL). Patients with uric acid levels in the highest quartile (≥8.5mg/dL) showed a 2.8 (1.7 - 4.8)-fold risk of death as compared to patients in the lower quartiles (p<0.001). This increased risk of death was independent of age, gender, serum creatinine and APACHE II score.

**CONCLUSION.** Uric acid levels correlate with circulating mtDNA and predict mortality in critically ill patients.

### 001090

#### Comparison of the functionality and the peripheral muscular strength of patients hospitalized in the ICU and evaluated at the outpatient clinic

G. Antonelli[1], EF. Osaku[2], CRLDM. Costa[2], JBD. Anjos[1], A. Tomazelli[1], JRGD. Costa[1], LSDP. Ferreira[1], MCLD. Jesus[1], M. Gossler[1], PN. Piñeiro[1], TC. Corsi[1], SM. Ogasawara[2], MA. Leite[2], AC. Jorge[3], PA. Duarte[3]

[1]Physiotherapy resident, Hospital Universitario do Oeste do Parana, Cascavel, Brazil; [2]Physiotherapy department, Hospital Universitario do Oeste do Parana, Cascavel, Brazil; [3]General icu, Hospital Universitario do Oeste do Parana, Cascavel, Brazil

**Correspondence:** G. Antonelli

*Intensive Care Medicine Experimental* 2019, **7(Suppl 3):**001090

**INTRODUCTION.** Critically ill patients have a decline in their muscle strength and in their functional capacity, which may occur after their hospital discharge.

**OBJECTIVES.** To evaluate the functionality and peripheral muscular strength of patients who were hospitalized in the ICU after three months of their discharge

**METHODS.** Retrospective study with data collected from January to December 2018, performed through the analysis of medical records of patients who were admitted to the ICU in the Hospital Universitário do Oeste do Paraná - Brazil, who returned for evaluation in the outpatient clinic three months after their discharge from the ICU. This study was approved by the Ethics Research Committee from the Western Parana State University, protocol number 436.770/2013. During ICU the patients were allocated into three groups: passive group (PG), Active-Assisted Group (AAG) Active Group (AG), according to their condition and clinical evolution. Peripheral muscle strength was assessed by the Medical Research Council (MRC), and functionality by the Functional Independence Measure scale (FIM) on discharge from the ICU and outpatient clinic. Variables with normal distribution were compared using the *Student's T-Test,* and the non-normally distributed variables were compared using the *Man Whitnney* tests. All analyzes were performed at 5% significance.

**RESULTS.** The sample had 54 patients, 18 in the passive group, 17 in the active-assisted, and 19 in the active, predominating the masculine gender, with age of 46.83 ± 21.37 vs 45.05 ± 19.24 vs 48.73 ± 15.56; p=0.91, with neurological (44%), non-neurological (35%) and neurological (31%) admission causes in PG vs AAG vs AG, respectively. The variables APACHE II (27.05 ± 5.47 vs 24.35 ± 8.50 vs 23.57 ± 8.19, p = 0.40), SOFA (11.16 ± 2.92 vs 7.23 ± 3.71 vs 8.11 ± 2.28, p = 0.002), sedation time in hours (93.17±76.80 *vs* 80.71±84.52 *vs* 20.17±31.27; p=0.008), mechanical ventilation in hours (224.16± 130.56 *vs* 162.31±154.86 *vs* 50.47±56.95; p=0.0009), ICU length of stay in days (14.72 ± 8.52 vs. 13.11 ± 11.27 vs 5.63 ± 4.33, p = 0.0004), Glasgow coma scale at discharge from the ICU (9.5 ± 3.11 vs 13.23 ± 2.01 vs. 14.38 ± 1.41; p <0.0001) in PG vs AAG vs AG respectively. Table 1 presents a comparison of the variables of the peripheral muscle strength and functionality in the ICU and in the outpatient clinic. In all three groups there were an increase in MIF and MRC, showing a recovery of the patient.

**CONCLUSION.** Functional and peripheral muscle strength improved in all groups three months after their discharge from the ICU.

**Table 1 (abstract 001090).** See text for description

| | | ICU | Clinics | p-value |
|---|---|---|---|---|
| Passive Group (PG) | FIM | 33.44 ± 21.05 | 109 ± 26.76 | <0.001 |
| | MRC | 12 ± 14.49 | 44.33 ± 16.92 | 0.0004 |
| Active-Assisted Group (AAG) | FIM | 72.35 ± 35.53 | 118.11 ± 17.57 | <0.001 |
| | MRC | 38.35 ± 8,81 | 52.70 ± 5 | 0.0005 |
| Active Group (AG) | FIM | 69.15 ± 32,12 | 115.15 ± 24.05 | <0.001 |
| | MRC | 52.31 ± 5,55 | 54.05 ± 6.60 | 0.46 |

### 001131

#### Prevalence of alcohol use disorders in intensive care patients: a multi-centre registry based study

E. Uljas[1], R. Suojaranta[2], AM. Korhonen[1], M. Hynninen[1], P. Loisa[3], T. Kaminski[4], I. Parviainen[5], A. Mäkelä[6], H. Laine[7], T. Ahtiainen[8], M. Reinikainen[5], J. Hästbacka[1]

[1]Intensive care units, department of anaesthesiology, intensive care and pain medicine, University of Helsinki and Helsinki University Hospital, Helsinki, Finland; [2]Department of cardiology, heart and lung center, University of Helsinki and Helsinki University Hospital, Helsinki, Finland; [3]Department of intensive care, Päijät Häme Central Hospital, Lahti, Finland; [4]Department of intensive care, Central Ostrobotnia Central Hospital, Kokkola, Finland; [5]Department of intensive care, Kuopio University Hospital, Kuopio, Finland; [6]Department of intensive care, South Carelia Central Hospital, Lappeenranta, Finland; [7]Department of intensive care, Mikkeli Central Hospital, Mikkeli, Finland; [8]Department of intensive care, Satakunta Central Hospital, Pori, Finland

**Correspondence:** J. Hästbacka

*Intensive Care Medicine Experimental* 2019, **7(Suppl 3):**001131

**INTRODUCTION.** Depending on the definition and the method used for detecting, up to 34% of intensive care unit (ICU) patients have a history of an alcohol use disorder. (1-3) One study has reported high prevalence of risky alcohol use and its association with increased incidence of bacterial infection, and a recent systematic review found an increased risk for ARDS among patients with harmful alcohol use. (3,4) However, reports on its association with mortality are contradictory, partly because of the diagnostic methods used are variable (1-3).

**OBJECTIVES.** To study the prevalence of alcohol use disorders and their association with the length of stay (LOS), treatment intensity and outcome in ICU treated patients.

**METHODS.** Since the beginning of 2017, ICUs in Finland have collected AUDIT-C (Alcohol use disorder identification testconsumption) data from ICU patients into the Finnish Intensive Care Consortium Database. AUDIT-C is based on the interview of the patient or a close relative. Risky drinking is defined as ≥5 points (of 12) for females and ≥6 for males. We used the registry data from January 1 to March 31 2018 to study the prevalence of risky alcohol use. Data from centres with an AUDIT-C recording rate less than 70% of admissions were excluded. We compared the disease severity scores, ICU LOS, Therapeutic Intervention Scoring Systems (TISS) and ICU-, hospital-, and one-year mortality in patients with and without risky drinking. Here, we report the results of the preliminary analyses.

**RESULTS.** Ten centres had a 70% or higher availability of AUDIT-C score. Of the 2186 admissions in these centres, AUDIT-C was available in 1660. AUDIT-C scores suggesting risky drinking were recorded in 22% of the patients. Patients with risky drinking were younger [median age 55 (IQR 42-65) vs 67 (IQR 57-74), (p>0.001)] and more often male, 78.1% vs 60.9% (p<0.001) than other patients. In unadjusted analyses we found no difference in disease severity scores, LOS, TISS, ICU or hospital mortality. According to preliminary analyses (data of 299 patients pending), the 1-year mortality curve according to AUDIT-C divided in subclasses is U-shaped with the highest mortality in the group of AUDIT-C 11 to 12 points (Figure). Final analyses will be performed after completing the mortality data.

**CONCLUSION.** Based on results obtained by using a validated self-report tool AUDIT-C, the prevalence of AUDs in Finnish ICU:s was 22%. We did not find an association of AUDs with patient outcomes in preliminary unadjusted analyses. However, one-year mortality appears highest in patients with very high AUDIT-C score as well as abstainers.

### REFERENCE(S)

(1) Uusaro A, Parviainen I, Tenhunen JJ, Ruokonen E. The proportion of intensive care unit admissions related to alcohol use: a prospective cohort study. Acta Anaesthesiol Scand 2005;49(9):1236-1240.

(2) McPeake JM, Shaw M, O'Neill A, Forrest E, Puxty A, Quasim T, et al. Do alcohol use disorders impact on long term outcomes from intensive care? Crit Care 2015;19:6.

(3) Gacouin A, Tadie JM, Uhel F, Sauvadet E, Fillatre P, Letheulle J, et al. At-risk drinking is independently associated with ICU and one-year mortality in critically ill nontrauma patients. Crit Care Med 2014;42(4):860-867.

(4) Simou E, Leonardi-Bee J, Britton J. The Effect of Alcohol Consumption on the Risk of ARDS: A Systematic Review and Meta-Analysis. Chest. 2018 154(1):58-68.

(5) Supported by government research funding for Helsinki hospital district TYH 2017105

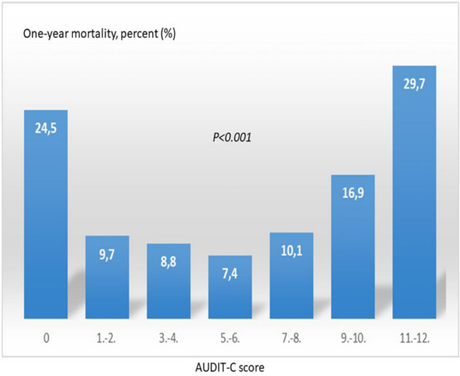

**Fig. 1 (abstract 001131).** See text for description

### 001183

#### Caregiver predictions of in-hospital mortality of the critically ill – a multidisciplinary study

R. Wiersema[1], RJ. Eck[2], T. Kaufmann[3], J. Castela Forte[1], EGM. Cox[1], M. Onrust[1], W. Dieperink[1], F. Keus[1], ICC. Van Der Horst[1], .. Sics Study Group[1]
[1]Critical care, University Medical Center Groningen, Groningen, Netherlands; [2]Internal medicine, University Medical Center Groningen, Groningen, Netherlands; [3]Anaesthesiology, University Medical Center Groningen, Groningen, Netherlands
**Correspondence:** R. Wiersema

*Intensive Care Medicine Experimental* 2019, **7(Suppl 3):**001183

**INTRODUCTION.** Predictions of survival or functional outcome may influence clinical decision making in critically ill patients. One study investigating the discriminative accuracy of mortality estimations by physicians and nurses found that nurse's estimations of in-hospital mortality were most accurate. (1)

**OBJECTIVES.** To validate previous observations on forecasting in-hospital mortality. To study the accuracy of students' estimations and the association of combined estimations by physicians, nurses and students with in-hospital mortality.

**METHODS.** This is a substudy of the Simple Observational Critical Care Study (SOCCS, NCT0355306), by the SICS study group. (2) All patients acutely admitted to a tertiary centre Intensive Care Unit (ICU) were included. Within three hours of ICU admission a medical student, an ICU nurse and a physician independently predicted in-hospital mortality. Medical students had at least completed their first year of medical school and were trained to perform a structured one-time physical examination. Physicians and nurses received no additional training.

**RESULTS.** So far, 702 patients were included between May 12, 2018 and March 28, 2019. In 151 patients no estimations were made, due to absence of the researcher or interference with clinical care, leaving 551 patients for analysis. Estimations concerning in-hospital mortality were obtained from medical students in all cases, from the nurses in 486 cases (88%) and from the physicians in 331 cases (60%). Medical students predicted an in hospital-mortality of 11.4%, nurses 15.2%, and physicians 18.1%. Observed in-hospital mortality was 19.2%. Estimations by medical students, nurses and physicians all were correct in 81% with no differences between groups (p=0.6). The estimations of students, nurses and physicians were all associated with mortality (OR 5.8, CI 3.3-10.1, OR 6.8, CI 4.1-11.5 and OR 8.3, CI 4.5-15.2 respectively), and when more estimators forecasted non-survival, odds of non-survival increased (OR 2.7, CI 2.1-3.5 per additional estimate).

**CONCLUSION.** Physicians, nurses and students accurately estimate in-hospital mortality in 81% of cases. When more caregivers estimated non-survival, the risk of death increased. Future research should focus on investigating the conditional dependencies between the estimates and the variables obtained from clinical examination, ie using Bayesian networks, to assess how caregivers come to their predictions.

### REFERENCE(S)

1. Detsky ME, Harhay MO, Bayard DF, et al. Discriminative Accuracy of Physician and Nurse Predictions for Survival and Functional Outcomes 6 Months After an ICU Admission. JAMA. 2017;317(21):2187–2195. doi:10.1001/jama.2017.4078
2. Hiemstra, B., Koster, G., Wiersema, R. et al. Intensive Care Med (2019) 45: 190. https://doi.org/10.1007/s00134-019-05527-y

3. This research received no specific grant from any funding agency in any sector. Currently inclusion is on-going. In case of acceptance to the ESICM congress, updated results will be presented.

### 001192

#### Mortality predicting model for the patients in surgical intensive care units and importance of increased respiratory rate

HJ. Shin[1], SH. Sung[1], HS. Joo[2], TJ. Yun[1]
[1]Department of transplantation and vascular surgery, Ajou University Hospital, Suwon, Republic of Korea; [2]Department of pediatric surgery, Ajou University Hospital, Suwon, Republic of Korea
**Correspondence:** H.J. Shin
*Intensive Care Medicine Experimental* 2019, **7(Suppl 3):**001192

**INTRODUCTION.** Early and proper management of multimorbid hospital patients have an important role in improving the quality of care. The aim of this study is to determine which pre-operative variables were significantly associated with mortality of patients before admission to the surgical intensive care units(SICU).

**METHODS.** 307 patients who admitted to SICU after emergency operation from 2013 to 2018 were recruited at the Department of General Surgery at Ajou university hospital. A total of 35 variables from database were extracted. Cox regression model and restricted cubic spline (RCS) were used to evaluated the association between preoperative variables and mortality.

**RESULTS.** In the final model, serum albumin level was the most important predictor of mortality ($\chi2$-df=23.7, p<0.001). The respiratory rate(RR) was the 2nd most important predictor. ($\chi2$-df= 17.7, p<0.001). The likelihood ratio $\chi2$ test showed significant added value of RR (p=0.008). The bias corrected C-statistic was 0.788 with RR and 0.768 without RR.

**CONCLUSION.** Compared with a set of available clinical risk factors, increase of RR was a stronger predictor of preoperative mortality. In critically-ill patients who need emergency operation, increased RR could be a good surrogate marker for predicting a need for prompt management including surgery and careful intensive care.

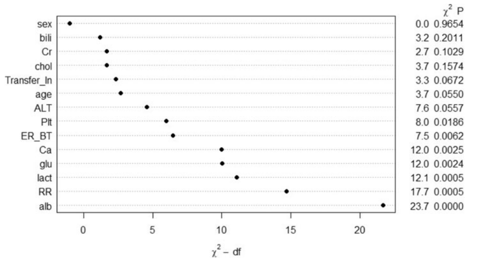

**Fig. 1 (abstract 001192).** See text for description

### 001199

#### Critical care consultants versus parent team: Predicting mortality on a Critical Care Unit in a tertiary oncology centre

BU. Adam, UU. Adam, P. Haji-Michael
Critical care, Christie Hospital, Manchester, UK
**Correspondence:** B.U. Adam
*Intensive Care Medicine Experimental* 2019, **7(Suppl 3):**001199

**INTRODUCTION.** The Christie Hospital is a tertiary cancer centre in northwest England. The Critical Care Unit (CCU) provides level 2 and 3 support to patients with cancer. Patients are referred by a patient's oncologist, haematologist or surgeon.

**OBJECTIVES.** To compare mortality of non-elective patients admitted to CCU at The Christie based on the opinion of the patient's oncologist and the admitting critical care consultant (CCC).

**METHODS.** Non-elective patients referred to CCU at The Christie are always reviewed by a CCC. Following initial review, the on-call CCC was asked what their opinion of the ceiling of care should be. This was either level 1, 2 or 3 care. The patient's parent consultant was also asked the same question. Where possible, the opinion of a second CCC was obtained and recorded. All consultants involved were blinded to the study.

The responses were recorded and compared against the maximum level of treatment the patient went onto receive during that episode and the subsequent outcome. Primary outcome was mortality on CCU.

**RESULTS.** 91 referrals to critical care led to 74 (81%) non-elective admissions. Medical oncology made up over half of referrals (56%), and others were from haematology (20.8%), clinical oncology (12.1%) and surgery (11%).

There was agreement between the CCC and the parent oncologist/ surgeon about the ceiling of care in 49.5% of cases. In 47.2% of cases the CCC recommended a lower level of care and in 3 patients (3.2%) the CCC recommended a higher level of care than the admitting oncologist.

When a second CCC reviewed the patient, there was agreement between CCC about the ceiling of care in 46/91 cases, disagreement in 28/91 cases and no data in 17 cases.

13 patients (14.3%) went on to receive a higher level of treatment than that recommended by a CCC. These patients had the highest mortality on the unit (table 1).

**CONCLUSION.** This study shows that while the parent team may know a patient well, the opinion of the CCC is more accurate in predicting mortality on a critical care environment. Mortality was 100% if a patient received level 3 support against the advice of the CCC.

**Table 1 (abstract 001199).** Mortality on CCU based on the opinion of the admitting CCU consultant and the levels of care given

| CCU Consultant recommended ceiling of care | Actual maximal level of care received | Number of patients | Number of patients who died on CCU (mortality rate %) |
|---|---|---|---|
| Received care at a or below the level recommended by a CCC | | 61 | 16 (26.2%) |
| Level 1 | Level 2 | 3 | 1 (33.3%) |
| Level 1 | Level 3 | 3 | 3 (100%) |
| Level 2 | Level 3 | 7 | 5 (71.4%) |

### 001203

#### A study on the effect of regionalization strategy for the reduction of reperfusion time in the patient with ST-elevation myocardial infarction transferred from non-PCI possible hospital

M. JUNG[1], D. Hanho[2], L. Jeonghun[2], L. Seungchul[2], SS. Jun[2], WK. Yong[2], L. Sanghun[2]
[1]Dongguk University Ilsan Hospital, Goyang-si, Republic of Korea; [2]Emergency medicine, Dongguk University Ilsan Hospital, Goyang-si, Republic of Korea
**Correspondence:** M. JUNG
*Intensive Care Medicine Experimental* 2019, **7(Suppl 3):**001203

**INTRODUCTION.** Prompt reperfusion treatment is important for the patients with ST elevation myocardial infarction (STEMI). However, patients often need interhospital transfer for percutaneous coronary intervention (PCI) because not all facilities are available for this procedure. The purpose of this study is to reduce the PCI delay through the regionalization protocol, in the patients with STEMI when they are transferred from the hospital where PCI not available.

**METHODS.** We established revascularization protocol named PREPARE (Preparing Revascularization Equipment before Patients Arrival as Regionalization Engagement) for the STEMI patients transferred from an outside regional hospital. The protocol included immediate referral acceptance by emergency physician, real-time electrocardiogram sharing via messenger service and early activation of the PCI team. We analyzed the differences between PREPARE group with non-PREPARE group about time consumption for PCI, length of hospital stay and major adverse cardiac events within 4 weeks.

**RESULTS.** In PREPARE group, the median time from the visit of first hospital to the PCI in receiving facility (D1toB time) was 111 minutes, and it was significantly shorter than non-PREPARE group (147 minutes). Rate of D1toB time achieved within 120 minutes was 26.0% (13/50%) in Non-PREPARE and 60.0% (30/50) in PREPARE and showed meaningful differences between the two groups(p=0.000). There were no statistically significant differences in hospital length of stay and major adverse cardiac events within 4 weeks.

**CONCLUSION.** PREPARE protocol as a regionalization strategy was effective to reduce revascularization time in transferred STEMI patients.

### 001217

#### Investigating the Association between shift level Nurse Staffing and Adverse Events in a Swiss Adult Intensive Care Unit. A Retrospective Analysis

MT. Exl[1], MM. Jeitziner[2], S. Musy[3], K. Thormann[4], M. Simon[5]
[1]Department of intensive care medicine, University Hospital Bern (Inselspital), University of Bern, Bern, Switzerland; [2]Department of intensive care medicine, University Hospital Bern (Inselspital), University of Bern, Bern, Switzerland; [3]Nursing & midwifery research unit, University Hospital Bern (Inselspital), Bern, Switzerland; [4]Department of dermatology, University Hospital Bern (Inselspital), University of Bern, Bern, Switzerland; [5]Institute of nursing science, University of Basel, Basel, Switzerland
**Correspondence:** M.T. Exl
*Intensive Care Medicine Experimental* 2019, **7(Suppl 3):**001217

**INTRODUCTION.** Patients on the Intensive Care Unit (ICU) require high levels of nurse staffing. Critically ill patients have complex multiple diseases and are exposed to many diagnostic and therapeutic interventions. Therefore, they are at risk for Adverse Events (AEs). Many studies have looked at the association between nurse staffing and AEs with contradictory results. The omission of variables such as units, days of the week, shifts and nurse education are potential sources of bias.

**OBJECTIVES.** The aims of this study were, 1) to describe the shift variation of the patient-to-nurse ratio (PNR) of an ICU in the Swiss setting; 2) to describe, whether AEs vary by shifts and days of the week and 3) to investigate nurse staffing per shift and their association with AEs.

**METHODS.** Retrospective analysis of nurse staffing, routine patients and AE data was performed on one interdisciplinary ICU with eight units. Data were from January 1st, 2014 to January 31st, 2016. After merging the three data sources, PNR were calculated for each nurse educations (critical care nurse, critical care nurse in training, and registered nurse (RN)) and for every unit, day of the week and shift separately. To assess the association between nurse staffing per shift and AEs, two regression models were fitted: 1) model to predict nurse staffing with patient, units, days of the week, shifts and nurse educations variables; and 2) model to predict AEs with shifts and observed over expected (OE) estimator. The OE was obtained by dividing the observed (actual data) PNR through the expected (predicted data from staffing model) PNR. Three values were possible: 1) OE = 1 meant predicted staffing equals the actual staffing; 2) OE < 1 meant actual staffing higher than expected (unusually high staffing); and 3) OE > 1 meant actual staffing lower than expected (unusually low staffing).

**RESULTS.** A total of 6'938 shifts with 11'979 patients and 235 nurses (115 critical care nurses, 45 critical care nurses and 75 RN) were analysed. The PNR ranged from 0.93 to 1.01 for morning, from 0.98 to 1.10 for afternoon, and from 1.25 to 1.37 for the night shift. There was little to no PNR variation between units, days of the week, shifts and nurse education. No pattern could be seen between the occurrence of AEs and days of the week, but an increase of AEs from morning to afternoon to night shifts was observed. For the OE, the percentage of shifts with unusually low and high staffing were from 10.4% to 14.4% and from 13.0% to 17.1%, respectively. No association between the occurrence of AEs and shifts with unusually low or high nurse staffing could be established.

**CONCLUSION.** Although no association between PNR and AEs was identified with the current statistical approach and variables, the OE estimator allowed to describe the low and high staffing variability in the given ICU units on a shift basis. The OE estimator provided a new perspective to describe nurse staffing, which opens new possibilities for the future.

### 001232

#### Evaluation of inspiratory muscle strength in patients who were tracheostomy versus extubated

G. Antonelli[1]; EF. Osaku[2]; CRLDM. Costa[2]; JBD. Anjos[1]; A. Tomazelli[1]; TC. Corsi[1]; JRGD. Costa[1]; LSDP. Ferreira[1]; MCLD. Jesus[1]; M. Gossler[1]; PN. Piñeiro[1]; SM. Ogasawara[2]; MA. Leite[2]; AC. Jorge,[3]; PA. Duarte[3]
[1]Physiotherapy resident, Hospital Universitario do Oeste do Parana, Cascavel, Brazil; [2]Physiotherapy department, Hospital Universitario do Oeste do Parana, Cascavel, Brazil; [3]General icu, Hospital Universitario do Oeste do Parana, Cascavel, Brazil
**Correspondence:** G. Antonelli
*Intensive Care Medicine Experimental* 2019, **7(Suppl 3):**001232

**INTRODUCTION.** The prolonged mechanical ventilation (MV) causes deleterious effects to the patients, such as the decrease of muscle strength, interfering negatively in the weaning of the MV.

**OBJECTIVES.** To compare Maximal Inspiratory Pressure (MIP) and Glasgow Coma Scale (GCS) in extubated and tracheostomy patients for weaning from MV, evaluated in the ICU and in the outpatient clinic.

**METHODS.** Retrospective study with data collected from January to December 2018 of patients who were admitted to the ICU in the Hospital Universitário do Oeste do Paraná- in south of Brazil, and who returned for evaluation in the outpatient clinic three months after their discharge from the ICU. Variables with normal distribution were compared using the Student's T-Test, and the non-normally distributed variables were compared using the Man Whitnney and Kruskal-Wallis test. All analyzes were performed at 5% significance.

**RESULTS.** The sample consisted of 57 patients, 36 extubated and 21 requiring tracheostomy. The main causes of admission were, extubated group (EG): 33% clinical neurological condition, 19% clinical non-neurological condition, and 19% postoperative elective; tracheostomy group (TG): 66% clinical neurological and trauma with TBI and 29% clinical non-neurological. The variables comparing EG vs TG: age in years (47.33±16.81 vs 46.90±19.06; p=0.88), male gender (61% vs 52%); APACHE II (24.8±6.80 vs 26.5±6.38, p=0.39), SOFA (8.25± 3.07 vs 10.95±2.87, p=0.002), sedation time in hours (26.64± 44.73 vs 104.08±83.56, p=0.0007), time of MV (64.97±75.82 vs 256± 136, p=<0.0001), ICU *length of stay in days* (6.34±4.68 vs 17.14±10.15, p=<0.0001). GCS of EG on the day of extubating, on the day of discharge, and in the clinics (10.58±0.64 vs 14.05±1.09 vs 14.06±1.04, p=<0.001). GCS of TG on the day of tracheostomy, on day of discharge, and in the clinics (5.23±1.26 vs 9±1.92 vs 14.71±0.88, p=< 0.001). Both groups had gain MIP in the discharge vs clinics, EG (-38.05 vs -68.47, p=<0.0001) and TG (-28.23 vs -54.76; p=0.0003). Table 1 shows the EG and TG variables. TG had a lower level of consciousness on the day of the procedure and on discharge. In the outpatient clinic, there was no difference in GCS between the groups. The MIP of the TG on discharge from the ICU was low when compared to the EG.

**CONCLUSION.** Patients submitted to the TG, presented worse SOFA, and even with tracheostomy, had a longer time of sedation and MV. This may be related to the cause of admission, with a prevalence of neurological patients. The TG also demonstrated lower MIP, and lower level of consciousness compared to extubated patients.

**Table 1 (abstract 001232).** See text for description

| Variables | Tracheostomy Group | Extubation Group | p-value |
|---|---|---|---|
| GCS on the day of the procedure | 5.23 ± 1.26 | 10.58 ± 0.64 | p< 0.0001 |
| GCS discharge ICU | 9 ± 1.92 | 14.5 ±1.09 | p< 0.0001 |
| GCS clinics | 14.71 ± 0.88 | 14.6 ± 1.04 | 0.86 |
| MIP discharge ICU | -28.23 ± 12.37 | -38.05 ± 19.23 | 0.06 |
| MIP clinics | -54.76 ± 23.01 | -68.47 ± 25.97 | 0.04 |

### 001234

#### Value of the combination of renal resistive index and the venous-arterial PCO2 to arterial-venous O2 content difference index in the presence of shock and in the prediction of clinical outcome

G. Fotopoulou[1]; I. Poularas[1]; S. Kokkoris[1]; E. Broutzos[2]; S. Zakynthinos[1]; C. Routsi[1]
[1]Department of intensive care, medical school, national and kapodistrian university of athens, Evaggelismos General Hospital, Athens, Greece, Greece; [2]Department of radiology, medical school, national and kapodistrian university of athens, Attikon , Athens, Greece, Greece
**Correspondence:** G. Fotopoulou
*Intensive Care Medicine Experimental* 2019, **7(Suppl 3):**001234

**INTRODUCTION.** The Doppler-based renal resistive index (RRI) [(peak systolic velocity – end-diastolic velocity)/peak systolic velocity] is a newly recognized non-invasive technique to detect earlier patients with renal tissue hypoxia and imminent acute kidney injury [1]. Moreover, the ratio of veno-arterial PCO2 difference to arterio-venous O2 content difference (ΔPCO2/ C(a-v)O2), has been recognized as a reliable marker of global anaerobic metabolism [2].

**OBJECTIVES.** The objective of our study was to evaluate whether the combination of both RRI and ΔPCO2/ C(a-v) O2 ratio is better associated with the presence of shock and clinical outcome in intensive care unit (ICU) patients than either of them.

**METHODS.** A prospective observational study was conducted in our multidisciplinary university intensive care unit (ICU). Patients requiring mechanical ventilation, without pre-existing chronic kidney disease were enrolled. Illness severity scoring systems (APACHE II and SOFA) were calculated on ICU admission. RRI measurements were obtained within the first 24 hours post-ICU admission, after the initial hemodynamic resuscitation. At the same time the ΔPCO2/ C(a-v) O2 ratio was calculated. A cutoff value ≥ 1.4 was used to characterize a high ΔPCO2/C(a-v) O2 ratio, whereas a cutoff ≥ 0.7 was used to characterize a high RRI value.

**RESULTS.** A total of one hundred and twenty-six critically ill patients (median age 61 years, 58.7% males) were included. Median values (IQR) for APACHE and SOFA scores, RRI and ΔPCO2/ C(a-v)O2 ratio were 18 (15-25), 9 (7-10), 0.7 (0.7-0.8) and 1.7 (0.6-3.4) respectively. Shock was present in 60 patients (47.6%) and sepsis in 57 patients (45.2%). The ICU mortality rate was 27.8% (35/126 patients). Patients with high values of both RRI and ΔPCO2/ C(a-v)O2 had an increased likelihood of shock presence (77.2% ), compared to those who had only one of these markers high (30.4%) or those with both values low (12.5%), p=0.001. Similarly, patients with high values of both RRI and ΔPCO2/ C(a-v)O2 were more likely to have a fatal ICU outcome (49.1%), than those having one of them high (13%) or those with both values low (2.5%), p=0.001.

**CONCLUSION.** The combination of RRI and ΔPCO2/ C(a-v) O2 measurements was better associated with the presence of shock and the prediction of clinical outcome, than either of them.

**REFERENCE(S)**

1. Shock. 2012; 37:360-5.
2. Intensive Care Med 2002; 28:272–77

### 001258

#### VAP due to XDR acinetobacter baumanii: characteristics and outcomes

E. Koutsioumpa[1], E. Gerovasileiou[2], A. Rentzoulas[1], A. Gavanou[1], G. Zakynthinos[1], D. Makris[1]
[1]Intensive care unit, University Hospital of Larissa, Larissa, Greece; [2]Intensive care unit, University Hospital of Larissa, Larisa, Greece
**Correspondence:** E. Koutsioumpa
*Intensive Care Medicine Experimental* 2019, **7(Suppl 3):**001258

**INTRODUCTION.** Infections due to extensively drug-resistant (XDR) Acinetobacter baumannii is a therapeutic challenge in critical care patients.

**OBJECTIVES.** We aimed to study prospectively clinical characteristics and outcomes of patients with ventilator associated pneumonia (VAP) due to XDR Acinetobacter baumannii (VAPxdrAB) and to compare them with similar characteristics and outcomes of patients with non-XDR VAP or no-VAP

**METHODS.** The present is an one-centre prospective study. Consecutive patients were recruited from the ICU of a tertiary hospital of central Greece in a three month period in 2018. Inclusion criteria included mechanical ventilation and ICU stay > 48 hours while patients with pneumonia at admission or COPD, Asthma were excluded. Diagnosis of VAP required microbiologic confirmation of the bacterial agent.

**RESULTS.** Sixty-one patients were assessed. Mean(SE) age was 62.6(1.9), APACHE II score 18.5(1.9), SOFA score 7(0.4). There were 16(26.2%) patients with VAP, 8(13.1%) of them had VAPxdrAB. No patient presented a second VAP episode. Mean(SE) age and APACHE II scores in VAPxdrAB , VAP-non-XDR and no-VAP patients were 60.2(6), 57.0(4), 61.9(2) and 16.5(1.4), 17(2.5), 18.2(1.2) respectively, (p=ns). Mean ICU day of VAP diagnosis in VAPxdrAB and VAP non XDR were 6.4(0.9) and 7.8(1.1)(p=ns). VAP treatment duration and overall antibiotic treatment duration were not significantly different; ICU duration (days) were 31.4(3), 24.1(3), 11.3(1) (p=0.001 between VAPxdrAB and non VAP and p=0.15 between VAPxdrAB and VAP non-XDR). ICU mortality in VAPxdrAB , VAP-non-XDR and non VAP patients was 0(0%), 2(25%) and 10(22.2%).

**CONCLUSION.** VAP due to XDR Acinetobacter baumannii was associated with significant prolongation of ICU stay in our population, underlying that the necessity of the implementation of strict infection control measures.

### 001471

#### DdAVP improves coagulation in a trauma-transfusion rat model

MR. Wirtz[1], V. Jan[2], J. Roelofs[3], J. Goslings[4], N. Juffermans[5]
[1]Department of intensive care medicine, Amsterdam University Medical Centers, location AMC, Amsterdam, Netherlands; [2]Department of plasma proteins, Sanquin, Amsterdam, Netherlands; [3]Department of pathology, Amsterdam University Medical Centers, location AMC, Amsterdam, Netherlands; [4]Department of surgery, Onze Lieve Vrouwe Gasthuis, Amsterdam, Netherlands; [5]Intensive care, Academic Medical Centre, Amsterdam, Netherlands
**Correspondence:** M.R. Wirtz
*Intensive Care Medicine Experimental* 2019, **7(Suppl 3):**001471

**INTRODUCTION.** Trauma is characterized by endothelial dysfunction and is especially apparent in patients in hemorrhagic shock. A disruption of intercellular connections may be an underlying mechanism(1). Vasopressin analog ddAVP (desmopressin) acts through a cAMP-dependent pathway, resulting in reorganization and redistribution of adhesive and tight junction molecules, enhancing endothelial barrier function. Furthermore, ddAVP increases von Willebrand Factor plasma levels and thereby potentially enhances platelet-based coagulation

**OBJECTIVES.** We aimed to assess whether the use of ddAVP will result in a restoration of the endothelial barrier function and an improvement in platelet-based coagulation without aggravating organ failure in a rat model of trauma and transfusion.

**METHODS.** Blood products were prepared from syngeneic rat blood according to blood bank standards. Polytrauma was induced in

Sprague Dawley rats by crush injury to the intestines and liver and by fracture of the femur. The rats were hemorrhaged by phlebotomy until a mean arterial pressure (MAP) of 40mmHg was reached. Rats were randomized (n=8 per group) to receive transfusion of RBCs, FFPs and platelets in a 1:1:1 ratio to achieve a MAP of 60 mmHg, with or without a single dose of ddAVP. Blood samples were taken up to 6h after trauma to assess biochemistry and coagulation status by rotational thromboelastometry (ROTEM). Organ damage was assessed by histopathology and biochemical parameters. Animals were continuously monitored with an arterial and urinary catheter.

**RESULTS.** Rats receiving ddAVP had significantly higher MAP values after the bolus was given. As reflected by their pH levels, a trend towards a more effective correction of shock was found in rats treated with ddAVP (7.36 vs 7.25, p=0.10). EXTEM CT and MCF remained stable in the ddAVP group, while rats in the control group showed a prolongation of their CT and a decrease in their MCF. This difference between the groups increased for both parameters over time. Platelet function relative to baseline values (reflected by EXTEM minus FIBTEM) also remained stable in the ddAVP group (around 100%), while it dropped markedly in the control rats (90%, 88% and 83% after 45, 75 and 120 minutes respectively). A trend towards lower soluble VE-cadherin levels was found in rats having received ddAVP compared to controls (133 vs 311%, p=0.17). No difference in organ injury was found between groups.

**CONCLUSION.** The use of ddAVP in a rat trauma-transfusion model increased blood pressure and improved ROTEM parameters of clot formation, with a trend towards an increased endothelial barrier function. However, this did not abrogate the amount of organ injury. The use of ddAVP should be further studied as an adjunctive therapy in trauma hemorrhage.

#### 001480

#### The impact of blood product ratio and pro-coagulant therapy on the development of thromboembolic events in severely injured hemorrhaging trauma patients

MR. Wirtz[1], D. Schalkers[1], J. Goslings[2], N. Juffermans[3]
[1]Department of intensive care medicine, Amsterdam University Medical Centers, location AMC, Amsterdam, Netherlands; [2]Department of surgery, Onze Lieve Vrouwe Gasthuis, Amsterdam, Netherlands; [3]Intensive care, Academic Medical Centre, Amsterdam, Netherlands
**Correspondence:** M.R. Wirtz
*Intensive Care Medicine Experimental* 2019, **7(Suppl 3):**001480

**INTRODUCTION.** Current treatment of trauma-induced coagulopathy consists of large volumes of plasma and early administration of anti-fibrinolytic and pro-coagulant medication. While half of trauma-related deaths occur in the first hours post-injury due to bleeding, the other half occurs during or after hospital admission(1). The development of thromboembolic events is an important contributor to morbidity and late mortality(2). Currently it is unknown whether the changes in resuscitation strategies increases the risk of developing thromboembolic complications.

**OBJECTIVES.** The aim of this systematic review is to identify risk factors of current resuscitation strategies for the development of thromboembolic complications.

**METHODS.** An electronic search was conducted in MEDLINE, PubMed and Embase. Relevant studies were screened by two independent reviewers and were included if they reported on thromboembolic complications in patients with severe trauma (ISS≥16) who received transfusion. Thromboembolic events was the primary outcome measure, either diagnosed when patients showed clinical symptoms or by routine screening using ultrasound or CT scan.

**RESULTS.** A total of 11.074 bleeding trauma patients were included, in which a total of 1.145 thromboembolic events were reported (incidence 10%). In studies performing routine screening for thromboembolic complications, the incidence was 12 to 23%. The risk of thromboembolic events in trauma patients was increased after administration of tranexamic acid (OR 2.6, 95%CI 1.7-4.1, p< 0.001) and after fibrinogen concentrate (OR 2.1, 95%CI 1.0-4.2, p= 0.04). Blood product ratio, the use of prothrombin complex concentrate or the use of recombinant factor VIIa showed no association with thromboembolic complications.

**CONCLUSION.** This systematic review of the literature identified an incidence of thromboembolic events of 10% in severely injured hemorrhaging trauma patients. The use of tranexamic acid and fibrinogen concentrate showed an association with the development of a thromboembolic complication. These results may beg the question whether routine screening in these patient populations should be implemented, in particular in patients treated with tranexamic acid and/or fibrinogen concentrate.

**REFERENCE(S)**

1. Dutton RP, Stansbury LG, Leone S, Kramer E, Hess JR, Scalea TM. Trauma mortality in mature trauma systems: are we doing better? An analysis of trauma mortality patterns, 1997-2008. J Trauma. 2010;69(3):620-6.
2. Ruskin KJ. Deep vein thrombosis and venous thromboembolism in trauma. Curr Opin Anaesthesiol. 2018;31(2):215-8.

## MEN / DS - Evaluation of metabolic therapeutic interventions

#### 001351

#### Prevalence, Determinants And Outcomes Of Hypoxic Hepatitis In Critically Ill Cirrhotics

R. Maiwall, P. Jain, S. Sarin
[1]Hepatology, Institute of Liver and Biliary Sciences, New Delhi, India
**Correspondence:** R. Maiwall
*Intensive Care Medicine Experimental* 2019, **7(Suppl 3):**001351

**INTRODUCTION.** Hypoxic hepatitis or "shock liver" is an acute liver injury characterised by a massive and transient rise in serum aminotransferases secondary to the anoxic necrosis of centrilobular liver cells. It is frequently observed in the intensive care units varying from 10-15%, however currently there is no study evaluating the prognostic relevance of this entity in critically ill cirrhotics. We evaluated the prevalence, predictors and outcome of hypoxic hepatitis in a prospective cohort of cirrhotics admitted to a dedicated liver intensive care.

**METHODS.** Consecutive patients with cirrhosis admitted to the liver intensive care were screened for presence of hypoxic hepatitis. Hypoxic hepatitis was defined as an increase in AST more than 5 fold from the baseline in the absence of viral or drug induced liver injury in an appropriate clinical setting of cardiac, respiratory or circulatory failure.

**RESULTS.** Five hundred and fifty-three patients with mean age 44(± 12) years, 89% males with mean MELD 32 (±9) and SOFA score of 11 (±3.5) were evaluated. Alcohol was the predominant etiology in 310 (56%). Hypoxic hepatitis was seen in 119 (21%) patients. On multivariate logistic regression analysis it was significantly associated to the MELD score (P,OR,95%CI) (<0.001,1.06,1.03-1.09), presence of hypoxia (<0.001,3.77, 1.78-7.98), variceal bleed (<0.001,3.19,1.72-5.93) and female gender (p<0.001,4.77,2.23-10.2). Patients with hypoxic hepatitis had significantly higher worsening of serum bilirubin (p= 0.002), Lactate (p=0.04), MELD (p=0.014) and SOFA (p<0.001) scores from baseline till day 15 as compared to patients without hypoxic hepatitis. On multivariate analysis, using Cox-Proportional hazard model, presence of hypoxic hepatitis (p<0.001, HR 2.06, 95%CI1.58-2.6), hepatic encephalopathy (p=0.018,HR 1.36,95%CI1.05-1.74), high MELD (≥32) (P<0.001, HR1.96, 95%CI1.52-2.52), Leucocyte counts(≥16,000) (p=0.005, HR1.38,95%CI1.10-1.74), serum sodium(≥130) (p=0.03,HR1.27,95%CI 1.01-1.60) and serum lactate(≥2) (p=0.003, HR1.42, 95%CI1.13-1.8) were independent predictors of mortality.

**CONCLUSION.** Hypoxic hepatitis is frequently seen in critically ill cirrhotics which correlates with the severity of liver disease. Development of hypoxic hepatitis is associated with worsening liver functions and is an independent predictor of mortality in critically ill cirrhotics.

### 001354

#### Cystatin C And Urinary Neutrophil-Gelatinase-Associated Lipocalin Can Predict Need Of Renal Replacement Therapy And Poor Outcomes In Critically Ill Cirrhotics

R. Maiwall, P. Jain, S. Sarin
Hepatology, Institute of Liver and Biliary Sciences, New Delhi, India
**Correspondence:** R. Maiwall
*Intensive Care Medicine Experimental* 2019, **7(Suppl 3):**001354

**INTRODUCTION.** Bacterial infections and systemic inflammation(SIRS) are the major precipitants of organ failure including acute kidney injury (AKI) in critically ill(CIC) and predispose to increased progression requiring dialysis. Timely identification of AKI is therefore an unmet need. Biomarkers can help in differential diagnosis and prediction of the trajectory of AKI course. We evaluated Cystatin C(CysC) and urinary neutrophil-gelatinase-associated lipocalin (NGAL) as biomarkers of glomerular and tubular injury respectively in predicting a progressive AKI requiring dialysis in CIC.

**METHODS.** CIC admitted to the Liver ICU were prospectively followed up. Dialysis was done for standard indications.

**RESULTS.** A total of 688 CIC, mean age 48.4±11.5 years, 87% males were followed until death or recovery. The median serum NGAL and CysC was 1004(803-1386) ng/ml and 2.1(1.7-2.6) mg/l respectively. SIRS was seen in 52% and sepsis in 60%. AKI at admission was present in 82% (Stage 1:2:3 18%vs 38%vs 44%) of which 65(11%) had progressive AKI requiring dialysis by day 7. On univariate analysis, lower MAP, higher arterial lactate, presence of leucocytosis and higher urine NGAL and serum CysC predicted requirement of dialysis at day 7. However, on multivariate analysis (OR,95% CI), u-NGAL (OR 6.3, 2.6-14.9), Cyst C (OR 6.4, 1.9-21.3) significantly predicted need of renal replacement therapy at day 7. The levels of NGAL (HR 2.3, 1.7-3.1) and Cyst C( HR 5.1,3.5-7.5) could also predict 30-day mortality in CIC.

**CONCLUSION.** Almost 80% of CIC have AKI at presentation, which in majority is a result of sepsis, is progressive and is associated with worse outcome. u-NGAL and CysC can accurately predict progression of AKI to dialysis and adverse outcomes which can help in stratifying patients for early therapeutic intervention.

### 001358

#### Validation of ultrasound subcutaneous fat thickness in comparison to L3 subcutaneous and visceral fat area in computed tomography in 119 non-ICU patients: the prospective USVALID study

A. Fischer[1], M. Pesta[1], I. Timmermann[1], T. Siebenrock[1], K. Liebau[1], R. Hahn[1], M. Anwar[1], A. Hertwig[1], D. Tamandl[2], H. Ringl[3], M. Hiesmayr[1]
[1]Anesthesia and intensive care medicine, Medical University of Vienna, Wien, Austria; [2]Radiology, Medical University of Vienna, Wien, Austria; [3]Radiology, Social Medical Center East - Donauspital, Wien, Austria
**Correspondence:** A. Fischer
*Intensive Care Medicine Experimental* 2019, **7(Suppl 3):**001358

**INTRODUCTION.** CT fat area at the level of the third lumbar vertebra is a gold standard of fat mass measurement, because it correlates very well with whole body fat mass. So far only abdominal but not appendicular ultrasound fat thickness has been validated to CT fat area (1). Moreover a large-scale study comparing US fat thickness to CT fat area in patients does not exist so far.

**OBJECTIVES.** The objective of the study was to validate appendicular ultrasound fat thickness in comparison to subcutaneous and visceral CT area in non-ICU patients.

**METHODS.** 119 non critically ill patients were recruited after routine abdominal CT scanning. Ultrasound fat thickness was measured at 2 measuring points on each upper arm and 3 measuring points on each thigh. Mean of upper arm fat thickness and mean of thigh fat thickness was calculated. Limb length was measured. Minimal compression was applied during ultrasound measurement. The ultrasound fat thickness was compared to CT L3 subcutaneous and visceral fat area by regression model. The covariables thigh fat thickness, upper arm fat thickness, thigh length, upper arm length, height, weight, age and sex were taken into account in multivariable regression models. Only statistically significant covariables were retained in the final model.

**RESULTS.** 79 men and 40 women were recruited. Median (IQR) age was 61 (47-69) years. Mean upper arm fat thickness better predicted CT L3 subcutaneous fat area (r2=0,69) than mean thigh fat thickness did (r2=0,54). In a multivariable regression model for prediction of CT L3 subcutaneous fat area only upper arm fat thickness and weight remained statistically significant (r2=0,73) (see Table 1). For prediction of CT L3 visceral fat area only age and weight were statistically significant covariables (r2=0,57).

**CONCLUSION.** Upper arm fat thickness but not thigh fat thickness correlated with subcutaneous L3 fat area in a multivariable model. Neither upper arm fat thickness nor thigh fat thickness correlated with visceral L3 fat area. Therefore upper arm fat thickness may be used as a surrogate for subcutaneous L3 fat mass but not for visceral L3 fat mass.

**Table 1 (abstract 001358).** Multivariable linear regression model for CT L3 subcutaneous fat area (cm2)

| | Estimate of CT L3 subcutaneous fat area (cm2) | 95% CI of estimate | P |
|---|---|---|---|
| Constant | -138,0 | -185,0 to -91,1 | < 0,001 |
| Mean upper arm fat thickness (cm) | 170,4 | 142,2 to 198,6 | < 0,001 |
| Weight (kg) | 1,9 | 1,2 to 2,6 | < 0,001 |

### 001388

#### Potential impact of fructose-containing pre-operative drink on lactate levels during surgery

W. Bosma[1], G. Nieuwenhuijs-Moeke[2], M. Nijsten[3]
[1]Department of cardiac anesthesiology, Medisch Spectrum Twente, Enschede, Netherlands, Netherlands; [2]Department of anesthesiology, University Medical Center Groningen, Groningen, Netherlands; [3]Department of critical care, University Medical Center Groningen, Groningen, Netherlands
**Correspondence:** M. Nijsten
*Intensive Care Medicine Experimental* 2019, **7(Suppl 3):**001388

**INTRODUCTION.** Intravenous administration of fructose leads to increased lactate levels [1]. Oral co-ingestion of fructose together with glucose during sustained exercise has been associated with both a better performance and higher lactate levels as part of the so called “reverse Cori cycle” [2,3].

**OBJECTIVES.** We examined whether a pre-operative drink containing fructose might affect intra-operative lactate levels.

**METHODS.** As part of the VAPOR-1 trial, renal transplant donors received 800ml of Preop® (Nutricia) orally the night before and 400ml at least 2h before surgery. This latter volume contained 0.8 g glucose, 5.2 g fructose, 2.4 g maltose and 40 g of polysaccharides. The transplant recipients did not receive Preop®. Mean ±SD lactate levels (reference value <1.5 mmol/L; <14 mg/dL) from preoperative arterial blood gas measurements were compared between renal transplant donors and recipients at four subsequent intra-operative time points (T1 through T4). Patients were carefully monitored for complications that might lead to increased lactate levels.

**RESULTS.** Significantly elevated lactate levels were found in the donor (Preop®) group compared to both the reference range (T1: 1.8 ±0.8 mmol/L, P<0.005), (T2: 2.2 ±0.7 mmol/L,P<0.0001) and also to the recipients values at T1: 1.1 ±0.5 mmol/L (P<0.0001) and T2: 1.6 ± 0.8 mmol/L, (P<0.0001). Lactate levels normalized and were similar for the two groups at T3 (1.6 ±0.5 mmol/L) and T4 (1.5 ±0.5 mmol/L).
**CONCLUSION.** The early mild intra-operative lactate increase observed in the donor group may be related to the preoperative carbohydrate drink. This lactate may originate from hepatic conversion of fructose to lactate as part of the reverse Cori cycle [3]. If analogous to exercise physiology, this hepatogenic lactate may actually be a useful fuel for stressed tissues and should thus not be considered an adverse but a benign effect. Planned trials where oral preoperative feeding is randomized, can verify this phenomenon.

**REFERENCE(S)**

1. Mendeloff AI, Weichselbaum TE. Role of the human liver in the assimilation of intravenously administered fructose. Metabolism 1953;2:450-8.
2. Lecoultre V, et al. Fructose and glucose co-ingestion during prolonged exercise increases lactate and glucose fluxes and oxidation compared with an equimolar intake of glucose. Am J Clin Nutr 2010;92:1071-9.
3. Tappy L, Rosset R. Fructose metabolism from a functional perspective: Implications for athletes. Sports Med 2017;47:23-32.

4. The research was supported by an efficacy grant from the UMC Groningen.

### 001431

#### Treatment of hypoxic liver failure using molecular adsorbent recirculating system (MARS®) – a randomized controlled study

V. Fuhrmann[1], H. Herkner[2], K. Roedl[1], A. Drolz[1], T. Horvatits[1], K. Horvatits[1], D. Jarczak[1], C. Zauner[3], P. Schellongowski[4], G. Heinz[5], M. Trauner[3], S. Kluge[1]

[1]Department of intensive care medicine, University Medical Center Hamburg-Eppendorf, Hamburg, Germany; [2]Department of emergency medicine, Medical University of Vienna, Wien, Austria; [3]Department of internal medicine iii, Medical University of Vienna, Wien, Austria; [4]Department of medicine i, Medical University of Vienna, Wien, Austria; [5]Department of internal medicine ii, Medical University of Vienna, Vienna, Austria

*Intensive Care Medicine Experimental* 2019, **7(Suppl 3):**001431

**INTRODUCTION.** Secondary acute liver failure, also known as hypoxic liver injury (HLI) or shock liver, is the most frequent cause of liver failure in hospital. Up to ten percent of critically ill patients develop HLI during their stay at the intensive care unit and have dramatically increased mortality rates. The only established procedures in these patients are symptomatic therapy and treatment of the underlying disease. Liver transplantation is no therapeutic option. Albumin dialysis by molecular adsorbent recirculating system (MARS®) can be used in patients with acute liver failure as symptomatic therapy for bridging to regeneration or liver transplantation.
**OBJECTIVES.** The aim of this study is to investigate the clinical impact of MARS® therapy in critically ill patients with HLI.
**METHODS.** We performed a binational randomized controlled study in two tertiary care centers (Medical University Vienna, Austria and University Medical Center Hamburg-Eppendorf, Germany). Forty patients with severy hypoxic liver injury (defined as aminotransferase levels 40 times upper the limit of normal) were included in this study and randomized to early initiation of MARS therapy (MARS, 4 sessions) (n=20) or standard therapy (SMT) (n=20). One individual in the SMT group was not included in the subsequent analyses due to transfer to another hospital immediately after randomisation without receiving any study related treatment.
Primary end point was the difference of the indocyanine plasma disappearance rate (ICG-PDR) at day 7. ICD-PDR is an independent predictor of mortality and represents liver function idependently of established liver function parameters in patients with HLI. (1) Secondary end points included among others mortality, days free of vasopressors and mechanical ventilation within the first 28 days after randomisation.
**RESULTS.** There were no significant differences in the MARS and SMT group at baseline.
Patients in the MARS group had significantly higher median ICG-PDR at day 7 compared to SMT (17.75%/min, IQR 5.8-9.8 versus 6.3%/min, IQR 4-11.9, p < 0.05).
We observed a non-significant higher 90-day mortality rate in the SMT group (63%) compared to the MARS-group (40%). Median days free of vasopressors within the first 28 days after randomisation were significantly higher in the MARS group (24 days) compared to SMT, median days free of mechanical ventilation within the first 28 days after randomisation did not differ significantly in the MARS group (20.5 days) compared to SMT.
**CONCLUSION.** Early initiation of albumin dialysis using the MARS-system lead to significant improvement of the liver function represented by ICD-PDR in critically ill patients with severe HLI.

**REFERENCE(S)**

1. Horvatits T, Kneidinger N, Drolz A et al. Prognostic impact of ICG-PDR in patients with hypoxic hepatitis. Ann Intensive Care 2015;5:47

2. This investigator initiated study was supported in part by Gambro, Lund, Sweden

### 001562

#### A comparison of Beacon Caresystem and Deltatrac II for indirect calorimetry in mechanically ventilated patients

M. Sundström Rehal[1], P. Promsin[2], I. Tjäder[1], O. Rooyackers[1], J. Wernerman[3]

[1]Department of perioperative medicine and intensive care, Karolinska University Hospital, Stockholm, Sweden; [2]Department of medicine, Siriraj Hospital, Bangkok, Thailand, Thailand; [3]Department of clinical science, intervention and technology, Karolinska Institutet, Stockholm, Sweden

**Correspondence:** M. Sundström Rehal

*Intensive Care Medicine Experimental* 2019, **7(Suppl 3):**001562

**INTRODUCTION.** Indirect calorimetry is a reliable method for assessing resting energy expenditure (REE) in mechanically ventilated patients. The Deltatrac Metabolic Monitor (Datex-Ohmeda, Helsinki, Finland) has been considered the gold standard in the ICU setting, using a mixing chamber technology. The Beacon Caresystem (Mermaid Care, Nørresundby, Denmark) is a modern device allowing calorimetry with breath-by-breath analysis; however, it needs to be validated against the reference device.
**OBJECTIVES.** To determine the level of agreement between Beacon Caresystem and Deltatrac II for gas exchange and REE in mechanically ventilated ICU patients.
**METHODS.** Mechanically ventilated adult patients admitted to the ICU of a university hospital during June to October 2018 were screened for recruitment. Patients with respiratory rates >35, an air leak in the ventilator circuit of >10% of minute volume or requiring FiO2 ≥ 0.6 were excluded. Repeated measurements were allowed in the same patient on different days. Measurements of oxygen consumption (VO2) and carbon dioxide production (VCO2) with both devices were performed concurrently for at least 20 minutes. REE was calculated with the modified Weir equation using average values of VO2 and VCO2 for each measurement. Bland-Altman plots were used to depict bias and limits of agreement (bias ± 2 standard deviations).
**RESULTS.** 60 measurements were performed. 11 measurements were excluded (n=9 RQ <0.6 from Deltatrac, n=1 lack of informed consent, n=1 missing VO2 values from Beacon), with 49 valid measurements in 18 patients analyzed. Patients had a mean age of 54±17 and body mass index of 28±6 kg/m2. Mean VO2 between Beacon and Deltatrac was not different (289±90 vs 280±87 ml/min, p=0.068), while there were differences in terms of mean VCO2, RQ and REE (265±83 vs 201±62 ml/min; 0.92±0.06 vs 0.72±0.08;2063±643 vs 1909±586 kcal/day, respectively, p <0.001 for all). Compared to Deltatrac, Beacon had a bias for VO2 of +9 ml/min with limits of agreement (LoA) of ±68 ml/min and a percentage error (PE) of ±24%, whereas bias and LoA for VCO2 were +64 ml/min and ±58 ml/min (PE±25%). For REE there was a bias of +154 kcal/day (about 7% of REE measured by Beacon) with LoA of ±433 kcal/day (PE±22%). Linear regression did not show a proportional bias for VO2 and REE (R2=0.01 and 0.07; p=0.45 and 0.06, respectively).

**CONCLUSION.** In this validation study of Beacon Caresystem compared to Deltatrac, we found a good agreement between mean VO2 of both instruments. Despite a significant bias for VCO2, REE measured by Beacon was overestimated by only 7%, with similar limits of agreement compared to published data from other breath-by-breath devices in relation to Deltatrac [1].

### 001568

#### Is it safe to use protocols for glycemic control in ICU?

R. Francisco, J. Silvestre, R. Dias, R. Inacio, C. Ponte, N. Candeias, R. Marques, A. Ricardo, J. Nunes
Intensive care unit, Hospiltal Lusiadas Lisboa, Lisbon, Portugal
**Correspondence:** J. Silvestre
*Intensive Care Medicine Experimental* 2019, **7(Suppl 3):**001568

**INTRODUCTION.** Stress-induced hyperglycemia is a common feature of intensive care unit (ICU) patients. Hyperglycemia and hypoglycemia have been highlighted as independent predictors of ICU and hospital mortality. Recent ICU recommendations suggest using insulin infusion protocols that can minimize glucose variability and reduce hypoglycemic risk.
**OBJECTIVES.** Our aim was to assess the efficacy, safety, and acceptance by nurses of a paper-based dynamic insulin protocol.
**METHODS.** This was a prospective study 7-month period, which included all adult patients admitted in the intensive/intermediate care unit of a private Hospital of Lisbon. Patients with diabetic ketoacidosis and hyperglycemic hyperosmolar state were excluded. Patients were divided in 2 groups: A, non-critical ill patients and B, critical ill patients. Critical ill patients were classified if they had one or more organ dysfunction, if they were submitted to cardiac surgery or to a major postoperative surgery. In A group, subcutaneous insulin protocol was applied. In B group protocol insulin was administrated according to modified Yale protocol. The target glucose level was between 140-180 mg/dl.
**RESULTS.** We included 999 patients, 75 (7,5 %) in B group and 924 (92,5%) in A group. In B group: 35 patients needed insulin perfusion; the mean time to achieve the target was 3,81 h; 9 patients needed more than 4 hours to achieve the target glucose level and only 1 episode of hypoglycemia was registered. In A protocol no hypoglycemia were observed; 3 patients transited to B protocol due to failure to glycemic control or presence of a critical event.
**CONCLUSION.** Our insulin protocols demonstrated to be very safe. Individualized protocols in Intensive/intermediate care units should be individualized according to patients severity. The great majority of our patients achieved the target glucose level in less than four hours, minimizing the potential risks of hyperglycemia.

### 001616

#### Increased levels of reverse triiodothyronine and mortality in clinical critical ill patients

FF. Amorim[1], CDG. Da Silveira[1], RB. De Santana[2], EBD. Moura[1], BTG. Da Silveira[3], SF. Da Silva[1], FFP. Amorim[4], DJVF. Palhano[1], MDO. Maia[1]
[1]Intensive Care Unit, Hospital Santa Luzia Rede D´or São Luis, Brasília, Brazil; [2]Curso de graduação em medicina, Escola Superior de Ciências da Saúde, Brasília, Brazil; [3]Coordenação de pós-graduação e extensão, Escola Superior de Ciências da Saúde, Brasília, Brazil; [4]Curso de graduação em medicina, Universidade Católica de Brasília, Brasília, Brazil
**Correspondence:** F.F. Amorim
*Intensive Care Medicine Experimental* 2019, **7(Suppl 3):**001616

**INTRODUCTION.** Disturbances of the endocrine function are a frequent finding in critically ill patients. Among thyroid hormone system, the downregulated of the thyroid function may be an adaptative response to conserve energy. However, this condition can limit response to additional stress factors such as a onset of circulatory shock. In later stage, this adaptive response may result in the condition known as euthyroid sick syndrome. In the early stages, there is commonly observed a drop of the peripheral conversion of thyroxine (T4) to triiodothyronine (T3) with a consequent increase in reverse triiodothyronine level (rT3).
**OBJECTIVES.** To evaluate the association of increased level of rT3 and mortality in clinical critical ill patients.
**METHODS.** This was a retrospective analysis of observational data prospectively collected during a 7-month period (2018) in a mixed medical/surgical adult ICU in a tertiary hospital in Brasilia, Federal District, Brazil. All consecutive clinical patients, older than 18 years, admitted to the ICU were included. Free T4 (fT4), total T4 (tT4), thyroid-stimulating homone (TSH), free T3 (fT3) and rT3 was collected in the first 48 hours after admission. Patients with a prior diagnosis of hyperthyroidism or hypothyroidism and surgical patients were excluded. According to rT3 levels, patients were divided into 2 groups: increased rT3 group and non-increased rT3 group.
**RESULTS.** During the study period, 304 patients were included. Age was 68.3±18.2 years, APACHE II score 16.9±8.1, SOFA 3.2±2.9, and ICU mortality was 17.1% (n=52). Increased rT3 level was observed in 216 patients (71.1%). Patients with incresead rT3 level had higher mortality (19.9% vs 10.2%, p=0.042). Non-survivors patients also had higher age (75.2±16.3 years versus 66.5±19.4 years, p=0.001), APACHE II (23.5±7.5 versus 15.3±7.4, p<0.001), and SOFA (5.7±3.0 versus 2.6±2.5, p<0.001). In the multivariate analysis, increased rT3 (p=0.039), APACHE II (p=0.024), and SOFA (p=0.041) were independently associated with ICU mortality.
**CONCLUSION.** Increased rT3 was independently associated with ICU mortality. Determination of reverse T3 levels may be a valuable aid to improve identification of clinical critical ill patients with higher risk of mortality.

**REFERENCE(S)**

1. Friberg L et al. Association between increased levels of reverse triiodothyronine and mortality after acute myocardial infarction. Am J Med 2001;111(9):699-703.
2. Sakharova OV, Inzucchi SE. Endocrine assessments during critical illness. Crit Care Clin. 2007;23(3):467-90.
3. Sharshar T et al. Hormonal status in protracted critical illness and in-hospital mortality. Crit Care.2011;15(1):R47.

### 001716

#### Effects of Single Dose and Maintenance Cholecalsiferol Treatment on Infection and Mortality in Critical Patients with Vitamin D Deficiency

C. Balci Altin[1], S. Izdes[2], KD. Calili[1], T. Bozkurt[1], L. Ozturk[2], B. Kayaaslan[3], A. But[2]
[1]Anesthesiology and intensive care unit, Ankara City Hospital, Ankara, Turkey; [2]Anesthesiology and intensive care unit, Ankara City Hospital, University of Yildirim Beyazit Medical Faculty, Ankara, Turkey; [3]Infection and clinical microbiology, Ankara City Hospital, University of Yildirim Beyazit Medical Faculty, Ankara, Turkey
**Correspondence:** C. Balci Altin
*Intensive Care Medicine Experimental* 2019, **7(Suppl 3):**001716

**INTRODUCTION.** Sepsis, owing to its wide spectrum of pathophysiological mechanisms but limited treatment options, is a condition too difficult to manage and is the most important causes of increased mortality among critical patients admitted to intensive care units (ICUs). It is still hypothetical that vitamin D, with its immunomodulator function along with many other functions, has a therapeutic effect in sepsis when supplemented in critical patients.

**OBJECTIVES.** We aimed to determine if cholecalciferol treatment, when enterally administered in two different doses, had any relationship with infectious agent, sepsis, and mortality among critical patients diagnosed with vitamin D deficiency at the time of ICU admission.

**METHODS.** This study included all patients older than 18 years of age not meeting the exclusion criteria, who were admitted to and expected to stay for more than 24 hours at a tertiary, mixed type, 20-bed ICU for a period of 1 year between 2016 and 2017. Based on vitamin D level checked in the first 24 hours of admission, the patients were grouped into four groups as those with a normal vitamin D level; those with a low vitamin D level but not receiving therapy; those with a low vitamin D level receiving a single 300.000 IU bolus dose enteral cholecalciferol; and those with a low vitamin D level and receiving a bolus dose of 300.000 IU flowed by 2000 IU daily maintenance dose of cholecalciferol. Demographic data; APACHE-II, SOFA, and GCS scores as predictors of clinical prognosis; diagnoses of sepsis, septic shock, and MODs at the time of admission and during follow-up; the highest among SOFA scores daily calculated during follow-up; duration of ICU and hospital stay; clinical outcomes; infectious markers; liver and kidney function tests and parathormone (PTH) levels were analyzed and compared across the groups.

**RESULTS.** The study included a total of 210 patients of whom 185 had vitamin D deficiency (61.08% with severe deficiency, 24.32% with deficiency, and 14.59 with insufficiency). Patients with vitamin D deficiency had a significantly higher rate of sepsis ($p < 0.05$). The group with severe vitamin D deficiency had significantly higher rates of sepsis, septic shock, MODs compared to those with a normal vitamin D level ($p < 0.05$). The patients with higher PTH levels had significantly greater age, BMI, rate of immobility, and rate of tight dressing compared to those with a normal level ($p < 0.05$). Moreover, as compared to those with a normal PTH level, patients with a higher PTH level had a significantly higher admission APACHE-II and SOFA scores; higher rates of sepsis, septic shock, and MODs at admission; higher rates of sepsis, septic shock, and MODs during follow-up; higher rates of acute renal failure (ARF) and mortality ($p < 0.05$). However, vitamin D levels of both groups were similar (p=0,691). The group that did not receive vitamin D supplementation had a higher rate of MODs at admission ($p < 0.05$). Nevertheless, the groups without vitamin D supplementation had a significantly higher rates of sepsis, septic shock, MODs, ARF, and mortality than the group that received vitamin D supplementation. The rates of sepsis, septic shock, or MODs were higher, albeit statistically non-significant, in the group that received a single dose of cholecalciferol compared to those receiving a maintenance dose.

**CONCLUSION.** This study showed that critical patients with vitamin D deficiency and PTH elevation with and without vitamin D deficiency were prone to sepsis, septic shock, and MODs development. PTH elevation was also shown to increase mortality by exacerbating the severity and course of critical illness. Even though serum vitamin D levels similarly rose, patients put on maintenance therapy with cholecalciferol, as compared to those that were administered a single dose of the same drug, developed less sepsis, septic shock, and MODs and hence maintenance therapy was deemed superior to single dose treatment.

### 001732

**Vitamin D in adult critically ill patients. A systematic review and meta-analysis of randomized trials**

A. Putzu[1]; G. Landoni,[2]

[1]Department of anesthesiology, pharmacology, intensive care and emergency medicine, Hôpitaux Universitaires de Genève (HUG), Genève, Switzerland; [2]Department of anesthesia and intensive care, San Raffaele Hospital, Milan, Italy

**Correspondence:** A. Putzu

*Intensive Care Medicine Experimental* 2019, **7(Suppl 3):**001732

**INTRODUCTION.** Vitamin D, beside its involvement in bone and electrolyte metabolism homeostasis, has pleiotropic effects, including immunomodulatory, cardiovascular, and muscular effects. Low vitamin D blood levels are associated with high morbidity and mortality in critically ill patients. However, there is controversy about vitamin D supplementation in this population.

**OBJECTIVES.** The objective of this systematic review and meta-analysis was to evaluate if vitamin D administration reduces mortality in critically ill patients.

**METHODS.** PubMed, Embase, and the Cochrane Library were searched up to April 1st, 2019 for randomized placebo-controlled trials on the use of vitamin D in adult patients with critical illness in comparison to placebo. The primary endpoint was mortality at longest follow-up available. We performed a random-effects meta-analysis with results expressed as risk ratio (RR) and 95% confidence interval (CI). Cochrane methodology was employed [1].

**RESULTS.** Ten studies published between 2011 and 2019, for a total of 926 patients, were included in the present meta-analysis. Nine trials administered enteral or intramuscular vitamin D3, also known as cholecalciferol, while 1 trial administered intravenous calcitriol. Vitamin D administration was associated with a significantly lower mortality compared to placebo (119/474 [25%] in the Vitamin D group versus 143/452 [32%] in the placebo group, RR = 0.80 [95% CI, 0.66 to 0.97], p = 0.03, I2 = 0%) (Figure 1).

At subgroup analysis according to clinical setting, the positive effects were mostly evident in a subgroup of trials including a heterogeneous mixed intensive care unit population (Figure 1). The risk of mortality was similar in vitamin D-deficient patients randomized to receive vitamin D supplementation (3 trials and 551 patients, RR = 0.64 [95% CI, 0.37 to 1.11]) or in trials enrolling patients with low or normal vitamin D levels (RR = 0.78 [95% CI, 0.39 to 1.57]). Trial sequential analysis was inconclusive for a relative risk reduction in mortality of 15% (RR = 0.80 [adjusted 95% CI, 0.54 to 1.17]), since only 32.61% of the information size was accrued and no firm conclusions on benefit, harm, or futility of vitamin D could be supported at the current stage.

**CONCLUSION.** In critically ill patients, vitamin D administration may be associated with a reduction in mortality. However, the quantity of evidence is still insufficient to draw a firm conclusion. Large multicenter randomized trials are warranted before systematic implementation of this therapy in clinical practice.

**REFERENCE(S)**

1. Higgins JP, Green S. Cochrane handbook for systematic reviews of interventions. Version 5. The Cochrane Collaboration; 2011.

2. No funding or conflict of interests to report.

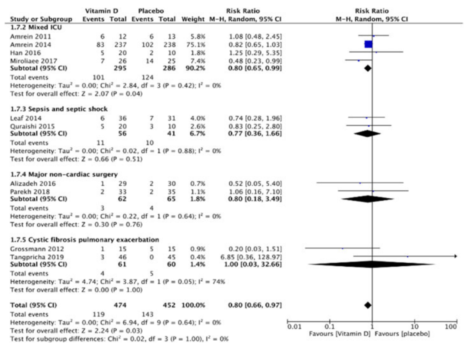

**Fig. 1 (abstract 000471).** See text for description

### 001744

#### Adecuate use of parenteral nutrition as a clinical quality indicator in an intensive care unit

R. Morón Romero[1], P. Nieto Gómez[1], I. Cruz Valero[2], A. Carranza Pinel[2], M. Colmenero Ruíz[2]
[1]Service hospital pharmacy, Hospital Universitario San Cecilio, Granada, Spain; [2]Intensive care unit, Hospital Universitario San Cecilio, Granada, Spain
**Correspondence:** R. Morón Romero
*Intensive Care Medicine Experimental* 2019, **7(Suppl 3):**001744

**INTRODUCTION.** Parenteral Nutrition (PN) is an alternative route to feed the critical patient, completely or partially, when the digestive tract can not be used for any reason.
PN monitoring has been established by the Spanish Society of Intensive Medicine and Coronary Care Unit (SEMICYUC) as a clinical quality indicator for intensive care units (ICU).
**OBJECTIVES.**
To evaluate the fulfillment of the Clinical Indicator: "adequate use of parenteral nutrition in an Intensive Medicine Service".
**METHODS.** Our investigation is a retrospective descriptive study. Data were collected from every patient admitted to our intensive care unit who received artificial nutrition (Enteral or Parenteral Nutrition) in the last four months. The variables collected were age, sex, PN indication and number of days from admission until PN was prescribed. The quality indicator was evaluated using SEMICYUC criteria: number of patients with indication of PN \ total number of patients needing artificial nutrition x 100 (standard = 16% with PN, and 25% with complementary PN). To assess whether the indication of PN is correct or not, we followed the criteria included in the guide of quality indicators in critical patient of SEMICYUC 2017.
**RESULTS.** During the study period 75 patients were analyzed. 66.6% were men, the mean age was 64.77 years and the average number of days from admission to the prescription of NP was 5.3 days. Only 24 were indicated to receive PN, 8 with complementary parenteral nutrition (CPN) and only 2 patients received PN without indication. 6 patients received PN as they were not expected to be fed in 5-7 days by oral or enteral route, 4 had intestinal insufficiency, 1 mesenteric ischemia and 5 intestinal obstruction. Using the formula described, the proportion of patients with NP indication was 32% and 10.6% with CPN.
**CONCLUSION.** The quality standard of this indicator is not reached. The exclusive administration of EN is impossible in certain scenarios like gastrointestinal dysfunction associated with the critical process. Depending on the clinical diagnosis at admission, the value of this standard will fluctuate, but monitoring this indicator is important to assess both, under and overprescription.

### 001757

#### Safety of parenteral nutrition in patients with severe combined traumatic brain injury

A. Shakotko[1], S. Petrikov[2], V. Movsisyan[1], E. Klychnikova[1], A. Ryk[1], A. Evdokimov[1]
[1]intensive therapy, N.V. Sklifosovsky Research Institute of Emergency Medicine, Moscow, Russia; [2]Neurosurgical icu, Sklifosovsky Research Institute for Emergency Medicine, Moscow, Russia
**Correspondence:** A. Shakotko
*Intensive Care Medicine Experimental* 2019, **7(Suppl 3):**001757

**INTRODUCTION.** Parenteral nutrition (PN) is indicated when it is impossible or insufficient to cover the estimated energy requirements and substrates by enteral route but PN can be accompanied by a number of complications.
**OBJECTIVES. The Aim of work** is to determine the safety of PN in patients with severe combined traumatic brain injury (SCMT).
**METHODS.** 20 pts enrolled in the study in all patients started the EN from the 2nd day after the injury. From 8.8 ± 1.3 days after admission, PN is added. The average duration of a PN is 3-7 days. For PN - three-component blend Nutriflex Lipid 70/180 (B. Braun, Germany), volume 625 ml (amino acids 35.9 g, carbohydrates 90 g, fats 25 g, total nitrogen 5 g, total energy value 740 kcal). Mixture was infused during 12 hours (52 ml / hour). The concentration of triglycerides (TG) and glucose level in venous blood was determined and (PaO2 / FiO2) in arterial blood, were investigated according to the following scheme:
• Prior to the start of infusion of PN (11: 30-12: 00)
• 2 hours after the start of PN infusion (14:00)
• Immediately after the end of the infusion of PN (24:00)
• 6 hours after the end of the infusion of PN (06:00)
**RESULTS.** The introduction of PN was accompanied by the development of hyperglycemia and a slight increase in the concentration of TG. Glucose level increase was noted after 2 hours and 12 hours after the start of PN, 6 hours after the end of infusion the glucose concentration tended to decrease. An increase in TG 2 hours after the start of the infusion did not affect the pulmonary gas exchange.
The glucose concentration before the introduction of PN was 7.8 ± 1.9 mmol / l (n = 90), two hours after the start of the PN 9.6 ± 2.8 mmol / l (n = 90) ($p < 0.05$ ), immediately after the end of the administration of PN 10.2 ± 3.9 mmol / l (n = 90) ($p < 0.05$), six hours after the end of the administration of PN 8.9 ± 3 mmol / l (n = 90) ($p < 0.05$). The concentration of TG before the introduction of PN was 1.48 ± 0.64 mmol / l (reference values <1.71 mmol / l) PaO2 / FiO2 333 ± 81 (n = 90), two hours after the start of PN TG 1.67 ± 0.9 mmol / l, PaO2 / FiO2 363 ± 72 (n = 90), immediately after the introduction of PN TG 1.53 ± 0.82 mmol / l, PaO2 / FiO2 357 ± 63 (n = 90), after the end of the introduction of PN TG 1.51 ± 0.8 mmol / l, PaO2 / FiO2 352 ± 64 (n = 90).
**CONCLUSION.** Introduction of parenteral nutrition as part of mixed artificial nutrition in patients with severe combined traumatic brain injury is not accompanied by hypertriglyceridemia and pulmonary dysfunction, but lead to slight glucose plasma level increase.

### 001221

#### Daily temperature variability of patients is associated with changes in ICU mortality

T. Hinoshita[1], J. Young Seok[2], T. Hensman[3], D. Garner[4], S. Sturland[5], T. Sugawara[6], R. Hayes[7], N. Nonaka[8], R. Kameya[9], M. Izumi[10], M. Adomi[11], M. Feng[2], D. Pilcher[12], LA. Celi[13], S. Hashimoto[14], H. Shigemitsu[1]
[1]Intensive care medicine, Tokyo Medical and Dental University, 1-5-45 Yushima, Bunkyo, Tokyo, Japan; [2]Saw swee hock school of public health, National University of Singapore, Singapore, Singapore; [3]Intensive care unit, Austin Health, Melbourne, Australia; [4]Respiratory and sleep medicine, Hawkes Bay DHB, Hastings, Australia; [5]Intensive care medicine, Wellington Hospital, Wellington, New Zealand; [6]Anesthesiology, Kagawa University, Kagawa, Japan; [7]Software engineering, Google LLC, Waterloo, Canada; [8]Medical sciences innovation hub program, Riken, Tokkyo, Japan; [9]Data service, Perspective Co., Ltd, Tokyo, Japan; [10]Data service, Dank NET Inc., Tokyo, Japan; [11]School of medicine, University of Tsukuba, Tsukuba, Japan; [12]Core chair, ANZICS Centre for Outcome and Resource Evaluation (CORE), Melbourne, Australia; [13]Pulmonary, critical care and sleep medicine, Beth Israel Deaconess Medical Center (BIDMC), Boston, USA; [14]Intensive care medicine, Kyoto Prefectural University of Medicine, Kyoto, Japan
**Correspondence:** T. Hinoshita
*Intensive Care Medicine Experimental* 2019, **7(Suppl 3):**001221

**INTRODUCTION.** Instability of daily body temperature rhythm is known to correlate with worse prognosis. Yet, the management of body temperature rhythm in critically ill patients remains elusive and is still left up to facilities. We aimed to assess whether body temperature variation beyond expected with circadian rhythm affect intensive care unit (ICU) mortality by machine learning. We also hypothesized if other physiological variables affect ICU mortality.
**METHODS.** We extracted 15,365 from 61,132 patients in the single center ICU database, MIMIC-III®, whose average Oxford Acute Severity of Illness Score (OASIS) was 37 points. They had at least two measurements of body temperature in a day and were compared the difference of body temperature between morning and afternoon for 28 days survival (OASIS=36) vs. non-survival (OASIS=40) patients in ICU.

Using a machine learning approach, we created a predictive linear regression model to investigate the effect of physiological variables of all extracted patients in the same database against ICU mortality.

**RESULTS.** Variations of nycterohemeral body temperature change in ICU were distinct between survival and non-survival groups (adjusted P=0.03). Hyperthermia (standardized weight (SW)=0.138), hypothermia (SW=0.514), and the chronological phase shift of body temperature circadian rhythm (SW=0.499) worsened ICU mortality.

**CONCLUSION.** Our study shows that permissive body temperature management for ICU patients is harmful by itself. Not only magnitudal but chronological deviation of body temperature directly increases ICU mortality. Further studies utilizing multicenter data are needed to evaluate this effect.

### 001228

#### Developing a prediction model for quality of life 1 year after ICU admission for shared decision-making in the ICU: preliminary results

N. Wubben[1], M. Van Den Boogaard[1], J. Ramjith[2], LLA. Bisschops[1], T. Frenzel[1], JG. Van Der Hoeven[1], M. Zegers[1]
[1]Intensive care medicine, Radboud University Medical Center, Nijmegen, Netherlands; [2]Health evidence, Radboud University Medical Center, Nijmegen, Netherlands
**Correspondence:** N. Wubben
*Intensive Care Medicine Experimental* 2019, **7(Suppl 3):**001228

**INTRODUCTION.** Decision-making in the ICU is largely based on doctors' experience and intuition, due to a lack of patient-reported outcome information regarding long-term physical and mental functioning. Moreover, patients and relatives are often not involved in the decision-making process, which can lead to a decline in patient satisfaction and lower patient quality of life (QoL).

**OBJECTIVES.** To develop a prediction model for QoL one year after ICU admission.

**METHODS.** Adult patients admitted ≥12 hours to a university hospital ICU between July 2016 and December 2017 were included (MONITOR-IC study, clinicaltrials.gov NCT03246334). Moribund patients were excluded. Patients (or relatives) rated their QoL before and one year after ICU admission by completing the Short-Form (SF) 36, that consists of the following outcome domains: physical functioning, role limitations due to either physical or emotional problems, social functioning, pain, vitality, mental health, general health and change in health (ranging 0-100; higher scores indicate better functioning or health). Demographic and medical data were extracted from the patients' electronic health record (EHR). Multivariate linear regression analysis was utilized using a subset of selected variables based on expert opinion as predictors.

**RESULTS.** Modelling with candidate predictors in 2603 ICU patients showed that if there was an association, higher age, female gender, medical admission, emergency surgery, lowest haemoglobin level, APACHE-IV, COPD and immunodeficiency were generally negatively associated with the QoL domains, whereas QoL at baseline was positively associated with the domains. The explained variance of the prediction model was 0.17. Women scored worse for nearly all SF-36 domains, most notably physical functioning and role limitations due to physical problems (β= -10.24, p<.01 and β= -9.65, p<.01, respectively). Especially medical admission (compared to planned admission) was strongly negatively associated with these two domains as well (β= -10.38, p<.01 and β= -12.21, p<.01, respectively). None of the predictors except QoL at baseline (β= 0.13, p<.01) were associated with mental health.

**CONCLUSION.** From a subset of predictors, gender and admission type emerged as strong predictors for various SF-36 domains one year after ICU admission. The explained variance of this preliminary model needs to be improved. The final prediction model can be used before or during admission to facilitate healthcare professionals, patients and their relatives in the shared decision-making process, by providing them information regarding prognosis and by management of health expectations, thereby possibly enhancing long-term patient care satisfaction and QoL.

### 001280

#### Online Personalized Prediction of Acute Hypotensive Episodes using the Online Super Learner

I. Malenica[1], C. Menyssa[2], M. Resche Rigon[2], A. Hubbard[1], M. Van Der Laan[1], R. Pirracchio[3]
[1]Biostatistics, UC Berkeley, Berkeley, USA; [2]Biostatistics, INSERM , Paris, France; [3]Anesthesia and perioperative care, UCSF, San Francisco, France
**Correspondence:** R. Pirracchio
*Intensive Care Medicine Experimental* 2019, **7(Suppl 3):**001280

**INTRODUCTION.** Prediction analytics using machine learning and big data generated by the electronic health records and patients' monitoring have recently gained a lot of traction, especially in critical care. However, most algorithms are trained and evaluated at the population level, meaning that good overall performance may not translate into accurate individual predictions. We argue that online machine learning algorithms that can leverage time-series obtained from individual patients may generate better personalized predictions.

**OBJECTIVES.** The goal was to use innovative machine approaches to predict acute hypotensive episodes, defined as a mean arterial pressure (MAP) < 65 mmHg for more than 5 minutes.

**METHODS.** We used the data from the MIMIC II, a publicly and freely available database associating medico-administrative data, physiologic measurements and treatment administration prospectively and consecutively collected at the bedside over a seven-year period (2001-2008) from any of the five ICUs of Boston's Beth Israel Deaconess Medical Center (BIDMC).1 Our methodology extends the supervised ensemble machine learning algorithm called the SuperLearner, allowing for online, fast prediction tailored for each subject.2 In particular, we develop an online Super-Learner for a collection of unit-specific time-series that leverages information learned from the population of time-series, stratified population of time-series taking into account their baseline covariates as well as treatment, and individually trained single-unit fits. As such, we are able to utilize the wealth of subject trajectories in order to learn global patterns in data, while tailoring the prediction for the single-unit of interest. We demonstrate the benefits of using the Online Super-Learner for a collection of unit-specific time-series, and clear advantages over population-based on one-sample prediction settings in a clinical setting using MIMIC II data.

**RESULTS.** 200 patients were included in this pilot study, with 50 samples having at least one hypotensive episode. The performance of the Combined Online SuperLearner to predict acute hypotension are illustrated in figure 1. The area under the receiver operating curve for the Combined Online SuperLearner was 98%.

**CONCLUSION.** Most algorithms are trained and evaluated at the population level, while the clinicians are interested in taking accurate clinical decisions at the individual levels. We show for the first time that online machine learning algorithms that have the ability to leverage the information encompassed in individual time-series may be extremely useful in predicting short-term outcomes such as acute hypotension episodes in critically ill patients.

**REFERENCE(S)**

1. Saeed M, Villarroel M, Reisner AT, et al. Multiparameter Intelligent Monitoring in Intensive Care II: A public-access intensive care unit database*. Crit Care Med. 2011;39(5):952-960. doi:10.1097/CCM.0b013e31820a92c6
2. van der Laan MJ, Polley EC, Hubbard AE. Super Learner. Stat Appl Genet Mol Biol. 2007. doi:10.2202/1544-6115.1309
3. None

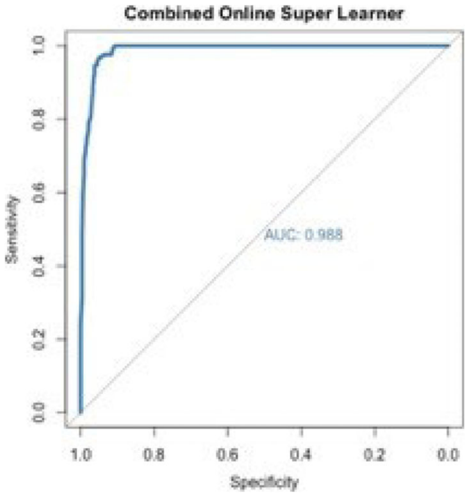

**Fig. 2 (abstract 001280).** See text for description

### 001423

#### Right Data, Right Now - Robust external validation of a deep reinforcement learning model to optimize hemodynamic treatment of critically ill patients with sepsis

L. Roggeveen[1], T. Guo[1], L. Fleuren[1], PJ. Thoral[1], A. Girbes, AE. Eiben[2], M. Hoogendoorn[2], PWG. Elbers[1]
[1]Intensive care medicine, Amsterdam UMC, locatie VUmc, Amsterdam, Netherlands; [2]Computer science, VU, Amsterdam, Netherlands
**Correspondence:** L. Roggeveen
*Intensive Care Medicine Experimental* 2019, **7(Suppl 3):**001423

**INTRODUCTION.** Sepsis is defined as life-threatening organ dysfunction caused by a dysregulated host response to infection [1]. Sepsis leads to haemodynamic changes, low blood pressure and insufficient tissue oxygenation. To determine whether a patient has received 'adequate fluid resuscitation' or needs vasopressor therapy, no criteria can be explicitly specified. Furthermore, it is recommended to individualize the target blood pressure during shock resuscitation [2]. Truly, there is a need for a more personalised treatment protocol.
In 2017, Raghu et al. developed a deep reinforcement learning algorithm to determine optimal treatment policies for septic patients by using a continuous state space reinforcement learning model [3]. Their model was developed on data 24hours prior to 48hours after the onset of sepsis. They found that their model could reduce patient mortality in the hospital. To the best of our knowledge, no external validation of this model design has been performed.
**OBJECTIVES.** To perform a clinical external validation of reinforcement learning models for the haemodynamic treatment of sepsis. The goal is to evaluate whether a reinforcement learning model could improve sepsis treatment if incorporated in an intensive care clinical workflow.
**METHODS.** We perform a quantitative and qualitative analysis of reinforcement learning for sepsis treatment. Due to data limitations, we redeveloped a double deep duelling Q-learning model on the US based on Raghu's design on the MIMIC dataset. We applied the model to a new Dutch dataset of over 4000 critically ill patients treated in the ICU between 2003 and 2016. We perform robust external validation by assessing model performance and transferability under clinically applicable inclusion criteria on two time periods with different data granularity. Furthermore, we develop a sparse model based on physiological parameters only to investigate the importance of severity scores in relation to transferability. We quantify the performance using the weighted doubly robust (WDR) estimator and the weighted importance sampling (WIS) methods.
**RESULTS.** Physician performance on the MIMIC dataset was 9.2 (WDR) and 7.8 (WDR) on the Dutch dataset consistent with the higher observed mortality in the Dutch dataset. Further results of the model performance are pending.
**CONCLUSION.** Reinforcement learning based model performance varies across different patient populations. It is possible to develop a model based on sparse physiological parameters only. It is vital to evaluate model performance under clinical settings that simulate how a model would be incorporated into a clinical workflow. Model performance should be robust against conceptual drift; differences in data granularity, treatment plans and patient populations. More research is needed to evaluate model performance before such models can be applied in a clinical setting and potentially help reduce ICU mortality.

**REFERENCE(S)**

1. Singer M, Deutschman CS, Seymour CW, et al. The Third International Consensus Definitions for Sepsis and Septic Shock (Sepsis-3). JAMA. 2016;315(8):801. doi:10.1001/jama.2016.0287
2. Cecconi M, De Backer D, Antonelli M, et al. Consensus on circulatory shock and hemodynamic monitoring. Task force of the European Society of Intensive Care Medicine. Intensive Care Med. 2014;40(12):1795-1815. doi:10.1007/s00134-014-3525-z
3. Raghu A, Komorowski M, Ahmed I, Celi L, Szolovits P, Ghassemi M. Deep Reinforcement Learning for Sepsis Treatment. November 2017. http://arxiv.org/abs/1711.09602. Accessed April 22, 2019.
4. This work has not received grant funding.

## NIC - Pearls in neurocritical care

### 000900

#### Clinical scores may not predict Delayed Cerebral Ischemia in aneurismatic subarachnoid hemorrhage: Two-year observational prospective study

M. Santafe[1], AE. Mera[1], A. Sánchez[1], RM. Gràcia[1], F. Arikan[2], E. Santamarina[3], O. Maisterra[3], M. García-De-Acilu[4], R. Ferrer Roca[1]
[1]Intensive care department, Vall d'Hebron University Hospital, Barcelona, Spain; [2]Neurosurgical department, Vall d'Hebron University Hospital, Barcelona, Spain; [3]Neurology department, Vall d'Hebron University Hospital, Barcelona, Spain; [4]Critical care department, Vall d´Hebron University Hospita, Barcelona, Spain
**Correspondence:** M. Santafe
*Intensive Care Medicine Experimental* 2019, **7(Suppl 3):**000900

**INTRODUCTION.** Delayed Cerebral Ischemia (DCI), manifests in approximately 30% of patients with Aneurismal subarachnoid hemorrhage (aSAH), and is one of the most important causes of morbidity (1). Several clinical and radiological scales have been proposed to predict DCI. We hypothesized that easy-to use clinical scales, such as Hunt and Hess (HH), World Federation of Neurosurgical Societies (WFNS), Glasgow Coma Scale (GCS), Fisher Scale and the recently proposed VASOGRADE scale may be associated with DCI development (2,3).
**OBJECTIVES.** To analyze if higher values in the above mentioned clinical scales may be associated with DCI development in patients with aSAH.
**METHODS.** Single-centre observational prospective cohort study including adult (+18 years) patients with aSAH diagnosis who were admitted to a tertiary ICU between June 1st 2017 and April 1st 2019. Patients with do-not-intubate order were excluded. DCI was defined as the occurrence of focal neurological impairment or a decrease of $\geq$ 2 points on the GCS for at least 1 hour (4). Cerebral ischemia was defined as hypodensities on CT or MR imaging. Vasospasm is defined as a radiological finding, either CT angiography, MR angiography, or digital subtraction angiography. GCS, HH, WFNS, Fisher and VASOGRADE were assessed at ICU admission. Quantitative variables are presented as mean (95%CI) and categorical as frequency (percentage). Differences between DCI and non-DCI patients were assessed using Chi square or Fisher's *exact test*, as appropriate.
**RESULTS.** Sixty-six patients were included, with a mean age of 58.77(55.56-31.98) years, 49(74.2%) were female. Mean SOFA at ICU

admission was 3.36(2.52-4.21) and APACHE-II score was 18.26(16.30-20.21). Twenty-one(31.8%) aneurisms were located at *anterior* communicating artery, 17(25.8%) at *posterior* communicating artery and 12(18.2%) at *middle cerebral* artery. Twenty-three (34.8%) patients developed DCI. Nine(39.1%) of them showed radiological signs of cerebral ischemia, and 10(43.5%) had radiologic signs of vasospasm. Mean GCS and GOS at ICU discharge of all patients were 13.60(13.06-14.11) and 3.39(2.99-3.78) respectively. The scales: GCS≤8 [5(22.7%) vs. 18(41.9%);p=0.173], WFNS≥4 [6(24.0%) vs 17(42.5%); p=0.18)], HH≥4 [5(21.7%) vs 18(42.9%);p=0.11], Fisher=4 [18(36.7%)vs 5(31.3%);p=0.77] and Vasograde=3 [4(20%)vs19(42.2%);p=0.10] were not associated with a higher incidence of DCI.

**CONCLUSION.** worse values of clinical-radiological scales were not statistically associated with DCI development in patients with aSAH, probably by the small sample size. However, more studies are required to identify predictors of DCI in patients with aSAH.

**REFERENCE(S)**

1. Van der Schaaf et al Stroke. 2002. Aug;33(8):2043-6.
2. de Oliveira Manoel et al. Stroke 2015 Jul;46(7):1826-31.
3. Johnston et al. Neurology. 1998 May;50(5):1413-8.
4. Vergouwen et al. Stroke 2010 Aug 41:2391–23955

### 000911

#### Thromboembolism in stroke patients in an ICU setting

I. Andrianopoulos, G. Papathanakos, X. Zikou, A. Papathanasiou, M. Saranti, E. Kostanti, V. Koulouras
[1]Intensive care unit, University Hospital of Ioannina, Ioannina, Greece
**Correspondence:** G. Papathanakos
*Intensive Care Medicine Experimental* 2019, **7(Suppl 3):**000911

**INTRODUCTION.** Patients with acute stroke may develop venous thromboembolism (VTE) during their hospitalization. The incidence of clinically evident deep vein thrombosis (DVT) and pulmonary embolism (PE) is 1-5% and 1% respectively. When stroke patients are routinely assessed using imaging techniques VTE incidence rises to 40%. Patients admitted in Intensive Care Unit (ICU) have high VTE risk and the incidence of DVT and PE in the general ICU population is 5.4%-23.6% and 7-27% respectively. Although ICU stroke patients have prolonged immobilization, undergo interventions that increase their VTE risk (i.e. central venous catherization (CVC), surgical operations etc.) and have high mortality, their incidence of clinically evident thromboembolism is not well known.

**OBJECTIVES.** Our objective was to identify the incidence of clinically evident VTE in our ICU stroke population and to assess whether the outcome of these patients differed from non VTE stroke patients.

**METHODS.** We retrospectively identified all stroke patients admitted in our academic ICU over a three-year period (2016-2018) and investigated which of them developed clinically suspected VTE confirmed by imaging techniques during their ICU stay. It must be emphasized that not all patients underwent a diagnostic test for VTE but only those with a clinical suspicion of a possible VTE. A descriptive statistical analysis followed comparing survival, disability and length of ICU hospitalization between VTE- and non VTE- ICU stroke patients.

**RESULTS.** Seventy-five patients, 47 (62.7%) men and 28 (37.3%) women, mean-aged 66.5±12 years with stroke were admitted in our ICU over a three-year period. Thirty-six (48%) patients had an ischemic stroke and 39 (52%) a hemorrhagic stroke. Eleven out of 75 (14.6%) patients (4 ischemic/7 hemorrhagic stroke) developed clinically evident VTE (5 DVT and 6 PE cases). All DVT cases were related to a central venous catheter placement. There was no difference in age or gender among VTE and non-VTE group. Stroke patients with a VTE episode had higher mortality (45% vs 36%), longer ICU hospitalization (30.7 days vs 16.9 days), required more often a tracheostomy (72% vs 50%) and had more severe disability on discharge (modified Rankin scale 4.2 vs 3.7). Adverse events related to anticoagulation were noted in 3/11 stroke VTE patients (2 patients developed subcutaneous hematoma requiring blood transfusion and a patient suffered hemorrhagic transformation of his ischemic stroke.

**CONCLUSION.** Clinically evident thromboembolism is more common in ICU stroke patients than in the general stroke population. Patients suffering a thromboembolic event have a longer ICU hospitalization, higher mortality and more disability than non-VTE stroke patients and maybe these patients should be routinely assessed for VTE using imaging techniques. Larger multicenter studies are required to confirm the above findings.

**REFERENCE(S)**

1. Sonneville R, Gimenez L, Labreuche J, Smonig R, Magalhaes E, Bouadma L, Timsit JF, Mazighi M. What is the prognosis of acute stroke patients requiring ICU admission? Intensive Care Med. 2017 Feb;43(2):271-272
2. Pongmoragot J, Rabinstein AA, Nilanont Y, Swartz RH, Zhou L, Saposnik G; Investigators of Registry of Canadian Stroke Network (RCSN) and University of Toronto Stroke Program for Stroke Outcomes Research Canada (SORCan [www.sorcan.ca]) Working Group. Pulmonary embolism in ischemic stroke: clinical presentation, risk factors, and outcome. J Am Heart Assoc. 2013 Nov 25;2(6):e000372.
3. CLOTS (Clots in Legs Or sTockings after Stroke) Trials Collaboration, Dennis M, Sandercock P, Reid J, Graham C, Forbes J, Murray G. Effectiveness of intermittent pneumatic compression in reduction of risk of deep vein thrombosis in patients who have had a stroke (CLOTS 3): a multicentre randomised controlled trial. Lancet. 2013 Aug 10;382(9891):516-24.
4. Kappelle LJ. Preventing deep vein thrombosis after stroke: strategies and recommendations. Curr Treat Options Neurol. 2011 Dec;13(6):629-35.
5. None

### 000913

#### Non-atherosclerotic ischemic stroke vasculopathies in ICU

I. Andrianopoulos, X. Zikou, G. Papathanakos, D. Kantas, A. Kittas, V. Koulouras
[1]Intensive care unit, University Hospital of Ioannina, Ioannina, Greece
**Correspondence:** G. Papathanakos
*Intensive Care Medicine Experimental* 2019, **7(Suppl 3):**000913

**INTRODUCTION.** The main causes of an ischemic stroke are embolism or vascular atherosclerosis. Stroke can also occur because of inflammatory, infectious, congenital, vasospastic and other non-atherosclerotic vascular causes. This heterogeneous group of disorders is called non-atherosclerotic vasculopathies and its true incidence and outcome in an Intensive Care Unit (ICU) setting is unknown.

**OBJECTIVES.** The purpose of this study was to identify the patients with a non-atherosclerotic vasculopathy admitted in our academic ICU with an ischemic stroke over the last three years and compare their outcome with that of patients with an atherosclerotic/embolic ischemic stroke.

**METHODS.** All cases of ischemic stroke admitted in our ICU over the last three years were retrospectively identified. They were divided in two groups according to their cause: either atherosclerotic vascular/embolic or non-atherosclerotic vascular. Clinical outcome (ICU survival, ICU length of stay and the modified Rankin scale for measuring the degree of disability or dependence in the daily activities) was assessed and compared using descriptive statistical analysis.

**RESULTS.** Overall, the last three years 36 patients, 22 (61%) males, 14 (38.8%) females, mean-aged 66.5±12.6 years, with ischemic stroke were admitted to our ICU. Thirty-one patients suffered an atherosclerotic/embolic ischemic stroke while the remaining 5/36 patients suffered a stroke of non-atherosclerotic vascular origin: one case of thrombotic thrombocytopenic purpura, one case of HELLP syndrome, one case of Listeria-associated stroke, one tuberculous meningitis-associated stroke case and one case of reversible vasoconstriction syndrome. The two groups were of similar average age and sex distribution. The non-atherosclerotic vasculopathy group had worse ICU survival (60% vs 67.7%), longer ICU length of stay (41.6±28.6 vs 11.42±11.6 days) and more disability (modified Rankin scale 5±0.89 vs 3.77±1.9). All survivals of the non-atherosclerotic vasculopathy group had a prolonged rehabilitation period for several months after their initial admission, requiring assistance for most of their daily activities.

**CONCLUSION.** Although non-atherosclerotic vasculopathies represent only a minor proportion of patients suffering an ischemic stroke, they are an existent entity in ICU, with a high impact on mortality, ICU length of stay and disability.

**REFERENCE(S)**

1. Bang OY, Toyoda K, Arenillas JF, Liu L, Kim JS. Intracranial Large Artery Disease of Non-Atherosclerotic Origin: Recent Progress and Clinical Implications. J Stroke. 2018 May;20(2):208-217.
2. Kim JS, Caplan LR. Non-Atherosclerotic Intracranial Arterial Diseases. Front Neurol Neurosci. 2016;40:179-203.
3. Chen PH, Gao S, Wang YJ, Xu AD, Li YS, Wang D. Classifying Ischemic Stroke, from TOAST to CISS. CNS Neurosci Ther. 2012 Jun;18(6):452-6.
4. Kalaria RN, Kalimo H. Introduction: Non-atherosclerotic cerebrovascular disorders. Brain Pathol. 2002 Jul;12(3):337-42.
5. None

### 000937

#### EEG abnormalities in patients undergoing ECMO

L. Peluso[1], S. Rechichi[2], F. Franchi[2], N. Gaspard[3], JL. Vincent[1], J. Creteur[1], FS. Taccone[1]

[1]Department of intensive care, Erasme Hospital, Université Libre de Bruxelles, Brussels, Belgium; [2]Department of biotechnologies, uoc rianimazione e medicina critica, Azienda Ospedaliera Universitaria Senese, Siena, Italy; [3]Department of neurology, Erasme Hospital, Université Libre de Bruxelles, Brussels, Belgium

**Correspondence:** L. Peluso

*Intensive Care Medicine Experimental* 2019, **7(Suppl 3):**000937

**INTRODUCTION.** Neurologic injury is one of the most frequent causes of death in patients undergoing extracorporeal membrane oxygenation (ECMO). As neurological examination is often unreliable in sedated patients, additional neuromonitoring is needed. However, the value of electroencephalogram (EEG) in adult ECMO patients has not been well assessed.

**OBJECTIVES.** To assess the occurrence of EEG abnormalities and their relationship to outcome in patients treated by ECMO.

**METHODS.** Single-center analysis of all patients undergoing veno-venous (VV) or veno-arterial (VA) ECMO with a contemporary (either intermittently or continuous) EEG monitoring (April 2009 - December 2018). EEG findings of interest were: a) "mild-moderate encephalopathy" (i.e. diffuse slowing with reactivity/variability) vs. "severe encephalopathy" (i.e. diffuse slowing without reactivity/variability); b) "burst suppression" or flat; c) epileptiform activity (i.e. ictal EEG pattern, sporadic epileptiform discharges or periodic discharges); d) EEG reactivity. EEG findings were analyzed according to the primary diagnosis (presence of cardiac arrest (CA) or not) and the use of VA vs. VV ECMO.

**RESULTS.** We studied 139 sedated patients (54 [41-62] years; 60 [43%] male gender) out of 596 treated with ECMO. ICU mortality was 65% (n=90). There were more unreactive EEGs among VA-ECMO (n=98) patients than in VV-ECMO (n=41; 58% vs. 37%; p=0.03). As expected, patients after cardiac arrest (n=86) more frequently had burst suppression or flat EEG than the others (n=53; 28% vs. 13%; p=0.05) but less epileptiform activity (6% vs. 21%, p=0.01). Severe encephalopathy (28/42, 67%) or burst suppression/flat EEG (30/31, 97%; p< 0.001) had a higher mortality than mild/moderate encephalopathy (32/66, 48%); the frequency of epileptiform activity was similar in survivors and non-survivors. Burst suppression/flat EEG was strongly associated with ICU mortality (OR 24.02 [4.16-251.25]; p<0.001), even after adjustment for confounders.

**CONCLUSION.** EEG monitoring can identify sedated patients with severe neurological injury during ECMO.

### 000951

#### Physiological parameters to predict successful ventilator liberation in brain injured patients

Z. Shi[1], AH. Jonkman[1], YL. Yang[2], GQ. Chen[2], M. Xu[2], JX. Zhou[2], L. Heunks[1]

[1]Intensive care medicine, Amsterdam UMC, locatie VUmc, Amsterdam, Netherlands; [2]Critical care medicine, Beijing Tiantan Hospital, Capital Medical University, Beijing, China

**Correspondence:** Z. Shi

*Intensive Care Medicine Experimental* 2019, **7(Suppl 3):**000951

**INTRODUCTION.** Nearly 50% of the brain-injured patients require acute invasive mechanical ventilation. Very few studies have investigated predictors for successful ventilator liberation in these patients.

**OBJECTIVES.** To identify physiological parameters obtained during a successful spontaneous breathing trial (SBT) to predict successful ventilator liberation in brain-injured patients.

**METHODS.** A prospective study was conducted in a general ICU. Patients meeting criteria for a SBT [1] and who successfully passed this 30-min. SBT (CPAP 5 cmH2O) were enrolled. Respiratory rate (RR), tidal volume (Vt), esophageal pressure (Pes) and diaphragm electrical activity (EAdi) were continuously measured during the SBT. From these data, RR/Vt, neuromechanical efficiency (Pes/EAdi) and neuroventilatory efficiency (Vt/EAdi) were calculated breath-by-breath. Successful liberation from the ventilator was defined as breathing without ventilator support for more than 48 hours after the SBT.

**RESULTS.** Forty-five patients (51.6±14.6 years; 60.0% male) were enrolled. The proportion of patients without requirement for ventilator support is shown in Fig.1. 68.9% patients were successfully liberated. There were no differences in baseline characteristics or physiological parameters (see Table) between patients in successful and failed ventilator liberation groups.

**CONCLUSION.** These data provide new and clinically relevant insights in ventilator liberation in brain-injured patients. First, 30% of patients successfully completed a SBT failed liberation < 48h. Second, in depth respiratory physiological parameters obtained during a SBT do not predict liberation success.

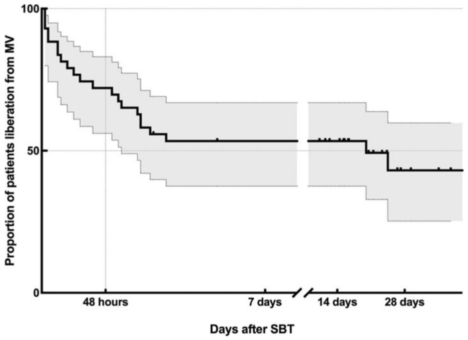

**Fig. 3 (abstract 000951).** See text for description

**Table 1 (abstract 000951).** Physiological parameters at start and end of SBT in both groups

| | Success (n=31) | Failure (n=14) | P-value |
|---|---|---|---|
| RR/Vt(breaths·min-1·L-1) | 50.0±37.4 | 61.2±37.3 | 0.419 |
| | 41.7±29.8 | 52.3±31.2* | 0.172 |
| Pes (cmH2O) | 6.5±4.2 | 8.8±4.3 | 0.113 |
| | 7.9±3.3* | 10.3±5.2* | 0.072 |
| EAdi (mV) | 7.6±5.8 | 7.4±4.4 | 0.842 |
| | 8.6±4.7 | 9.5±4.8 | 0.548 |
| NME (cmH2O/mV) | 1.2±0.8 | 1.5±1.1 | 0.275 |
| | 1.2±0.7 | 1.3±0.9 | 0.635 |
| NVE (ml/mV) | 118±127 | 94.8±72.3 | 0.539 |
| | 95.8±97.1 | 62.4±31.3 | 0.219 |

* compared within groups, p < 0.05

### 001016

#### Risk factors associated with disability and mortality in patients in a neurotraumatic ICU with decompressive craniectomy six months after discharge. A six year study

C. Sánchez Ramírez, CF. Lübbe Vázquez, LDM. Díaz Suárez, C. Agüero Senovilla, C. Vázquez Pineda, S. Ruiz-Santana
[1]Intensive care medicine, University Hospital of Gran Canaria Dr. Negrin, Las Palmas de Gran Canaria, Spain
**Correspondence:** C. Sánchez Ramírez
*Intensive Care Medicine Experimental* 2019, **7(Suppl 3):**001016

**INTRODUCTION.** Second level therapeutic maneuvres for controlling intracranial hypertension (ICH) proposed by the European Brain Injury include barbiturates, moderate hypothermia and decompressive craniectomy (DC) but outcome is controversial. Our aim was to evaluate the factors associated with disability and mortality after ICU discharge in patients undergoing DC.

**OBJECTIVES.**

**METHODS.** Prospective study of patients admitted from January 1, 2013 to March 2019 who required DC. DC was performed due ICH refractory to medical treatment. We analyzed: main admission diagnosis; demographic data; neurological data (clinical examination and Glasgow Coma Score: GCS); hypotension type of craniectomy and DC complications; Rankin scale, and Glasgow outcome scale (GOS) at 30, 60 days after ICU admission, at ICU discharge and 6 months after ICU discharge; preoperative serum lactate levels ; hypo and hyperglycemia; application of mannitol or hypertonic saline solution before and after DC; leukocytes and platelets previous and after DC and other factors related to prognosis. Univariate analysis of disability (Rankin > 3) and mortality in ICU, 60 days after DC and 6 months after ICU discharge were performed. Statistical significance was set at p ≤ 0.05.

**RESULTS.** Twenty eight DC patients were collected. Demographic data and types of admission are shown in Table 1.Most DC were subarachnoid haemorrhages (SAH) 13 (46%) and 75% of then were hemicranial. The most frequent complications were reoperation due to complications (50%) and hydrocephalus (46%). Six patients died at ICU discharge (39.3%), 4 (66,6%) of them were SAH. Rankin score at ICU discharge was 4,5 and GOS was 3..Rankin score 6 months after ICU discharge was 2 and GOS was 4. Reoperation due to complications and the highest ICU GCS was associated with disability, 60 days after DC (Table 2).Mortality 6 months after discharge was significantly associated with bilateral pupillary reactivity prior DC (less mortality), female sex, the presence of focal contusion with edema and expansivity, Rankin 30 days after admission and at ICU discharged (Table 3). We found no significant risk factors associated with disability in the ICU and six months after discharge.

**CONCLUSION.** DC patients showed low ICU mortality (21,4%) Rankin and GOS reflected moderate / severe disability of these patients, at ICU discharge. Rankin and GOS also showed disability deterioration, sixty days and 6 months after ICU discharge. Reoperation due to complications and the highest ICU GCS was associated with disability and 60 days after DC. Mortality at 6 months after discharge was significantly associated with bilateral pupillary reactivity prior DC, female sex, presence of focal contusion with edema and expansivity, Rankin 30 days after admission and at ICU discharge.

**Table 1 (abstract 001016).** See text for description

| Table 1. | N 28 |
|---|---|
| Age years, n (IQR) | 62 (52 ; 83) |
| Male/Female, n (%) | 10 /18 |
| Diabetes, n (%) | 2 (7.1) |
| Hypertension, n (%) | 4 (14) |
| APACHE-II, n ,(IQR) | 23 (20 ; 26) |
| TBI on admission, n (%) | 11 (39.3) |
| **Diagnosis on admission** | |
| Subarachnoid hemorrhage, n (%) | 13 (46) |
| Stroke Malignant middle cerebral artery, n (%) | 7 (25) |
| Tumor, n (%) | 3 (10.7) |
| Acute Subdural | 13 (46) |
| **Type of craniectomy** | |
| Primary, n (%) | 16 (57.1) |
| Secondary, n (%) | 12 (42.9) |
| Bifrontal, n (%) | 3 (10.7) |
| Hemicraneal, n (%) | 21 (75) |
| Other, n (%) | 2 (7.1) |
| **Mortality** | 11 (39.3) |
| ICU, n (%) | 6 (21.4) |
| Hospital, n (%) | 1 (3.6) |
| Upon discharge from hospital, n (%) | 4(15,3) |
| ICU admission days, days | 25,5 (18.7;36) |
| Days with craniectomy, days | 46 (40;65) |
| ICU admission days until EXITUS, days | 57 (16.2; 231.2) |
| Highest GCS, in ICU | 14 (9;15) |
| GOS at ICU discharge | 3 (2; 3,75) |
| GOS 30 days after ICU admission | 3 (2;4) |
| GOS 60 days after ICU admission / 6 months after ICU discharge | 2 (1;3.25) |
| Rankin at CU discharge | 4,5 (3;5) |
| Rankin 30 days after ICU admission | 4,5 (3,5:5) |
| Rankin 60 days after ICU admissio / 6 months after ICU discharge | 5 (5;6) |

GCS Glasgow Coma Score, GOS Glasgow outcome score, TBI trauma brain injury

**Table 2 (abstract 001016).** See text for description

| Table 2. | Disablility: 60 days after ICU admission | | |
|---|---|---|---|
| | Yes N=22 | No N=6 | P |
| Female/Male | 7/15 | 3/3 | 0.634 |
| Diabetes mellitus, n (%) | 2 (9.1) | 0 (0) | 1 |
| Hypertension | 3 (13.6) | 1 (16.7) | 1 |
| Dyslipidemia | 2 (10.1) | 2 (33.3) | 0.191 |
| **Reason for admission** | | | |
| Tumor | 2 (10.1) | 1 (16.7) | 0.545 |
| SAH | 9 (40.9) | 4 (66.6) | 0.372 |
| Stroke Malignant middle cerebral artery | 7 (31.8) | 0 (0) | 0.288 |
| Acute subdural hematoma | 11 (55) | 2 (33.3) | 0.648 |
| Obliteration of basal cisterns | 8 (38.1) | 1 (16.7) | 0.628 |
| Focal contusion with edema and expansivity | 8 (38.1) | 4 (66.6) | 0.357 |
| Evacuated Injury | 5 (23.8) | 0 (0) | 0.555 |
| TBI | 8 (38.1) | 3 (50) | 0.662 |
| **Pre Pupillary reactivity** | | | |
| Both | 2 (10.1) | 0 (0) | 1 |
| None | 14 (66.7) | 5 (83.3) | 0.633 |
| **Endotracheal intubation** | | | |
| Pre Hospital | 4 (18.2) | 2 (33.3) | 0.581 |
| Emergency | 9 (40.9) | 2 (33.3) | 1 |
| ICU | 3 (27.3) | 4 (23.6) | 1 |
| Abscess, n (%) | 4 (18.2) | 0 (0) | 0.545 |
| Seazures before CD, n (%) | 4 (18.2) | 1 (16.7) | 1 |
| Previous transfusion, n (%) | 4 (36.4) | 2 (11.8) | .163 |
| Midline shift, on CT at admission, n (RIQ) | 3 (0;9) | 2 (0;8) | 0.383 |
| GCS at admission, n (RIQ) | 11 (5;15) | 10 (5;15) | 0.436 |
| Highest GCS | 11 (8;15) | 15 (14;15) | 0.024 |
| Number of platelets, after DC: n (IQR) x $10^3$ | 210 (134;289) | 243 (126;452) | 0.341 |
| APACHE II, n (IQR) | 22 (19;25) | 25 (22;27) | 0.191 |

ICU: Intensive Care unit;GCS: Glasgow Coma Score; TBI trauma brain injury;; SAH subarachnoid hemorrhage

**Table 3 (abstract 001016).** See text for description

| Table 3 | Mortality six months after ICU discharge | | |
|---|---|---|---|
| Variables | Yes | No | P |
| | **N=11** | **N=17** | |
| Female/Male | 7/4 | 3/14 | .020 |
| Diabetes mellitus, n (%) | 1 (9.1) | 1 (5.9) | 1 |
| Hypertension | 1 (9.1) | 3 (17.6) | 1 |
| Dyslipemia | 2 (18.2) | 2 (11.8) | 1 |
| **Reason for admission** | | | |
| Tumor | 2 (18.2) | 1 (5.9) | .549 |
| SAH | 6 (54.6) | 7 (41.3) | .700 |
| Stroke Malignant middle cerebral artery | 4 (36.4) | 3 (17.6) | .381 |
| Acute subdural hematoma | 4 (36.4) | 9 (53.1) | .440 |
| Obliteration of basal cisterns | 3 (27.3) | 6 (42.9) | .649 |
| Focal contusion with edema and expansivity | 2 (18.2) | 10 (58.8) | .047 |
| Evacuated Injury | 0 (0) | 6 (42.9) | .054 |
| TBI | 3 (27.3) | 8 (47.2) | .427 |
| **Pre Pupillary reactivity** | | | |
| Both | 4 (36.4) | 15 (88.5) | .002 |
| None | 1 (9.1) | 1 (5.9) | 1 |
| **Endotracheal intubation** | | | |
| Pre Hospital | 2 (18.2) | 4(23.6) | 1 |
| Emergency | 4 (36.4) | 4(23.6) | 1 |
| ICU | 3 (27.3) | 4 (23.6) | 1 |
| Hemicranial DC n (%) | 6 (54.6) | 15 (88.5) | .076 |
| Seazures before CD, n (%) | 3 (27.3) | 1 (5.9) | .264 |
| Previous transfusion, n (%) | 4 (36.4) | 2 (11.8) | .163 |
| Midline shift on CT at admission, n (IQR) | 5.5 (0 ; 9.25) | 2.5 (0 ; 7.5) | .383 |
| GCS at admission, n (IQR) | 11 (6 ; 14) | 10 (3 ; 15) | .109 |
| Number of platelets, after DC: n (IQR) x $10^3$ | 182 (112 ; 278) | 222 (158 ; 425) | .341 |
| APACHE II, n (IQR) | 25 (22; 27) | 22 (17 ; 25) | .191 |
| | | | |

ICU: Intensive Care unit;GCS: Glasgow Coma Score; TBI trauma brain injury;CT: computer tomography; SAH subarachnoid hemorrhage .

### 001017

#### Acute neurocardiogenic injury in patients with subarachnoid haemorrhage. Pilot study in Italian Piedmont Area

G. Catozzi[1], S. Cappio Borlino[1], G. Montrucchio[1], V. Bonicalzi[1], V. Tardivo[1], S. Malerba[1], S. Bosso[1], M. Di Cuia[2], S. Palacio[2], S. Hammad[2], F. Arruga[3], S. Deaglio[3], A. Amoroso[3], W. Grosso Marra[2], AT. Mazzeo[1]

[1]Department of surgical sciences, anaesthesia and intensive care, University of Turin, Turin, Italy; [2]Department of medical sciences, cardiology, University of Turin, Turin, Italy; [3]Department of medical sciences, medical genetics, University of Turin, Turin, Italy

**Correspondence:** A.T. Mazzeo

*Intensive Care Medicine Experimental* 2019, **7(Suppl 3):**001017

**INTRODUCTION.** In the acute phase of subarachnoid haemorrhage (SAH), patients may develop neurogenic stress cardiomyopathy (NSC), expressed as cardiac necrosis markers elevation, ECG alterations and left ventricular wall motion abnormalities (WMA). A catecholamine storm induced by SAH is central in its pathogenesis. NSC may worsen the patient outcome. Several predictors have been proposed in literature for the development of NSC, without definitive evidences.

**OBJECTIVES.** Primary aim of this study was to evaluate the occurrence of cardiac dysfunction measured as cardiac necrosis markers, ECG and echocardiographic abnormalities in SAH patients, during the first 5 days after admission. Secondary aim was to evaluate whether polymorphisms of adrenergic receptors and related proteins may be associated with an increased risk of cardiac injury after SAH.

**METHODS.** Adult SAH patients (World Federation of Neurosurgical Societies score IV-V) admitted at the university ICU in Torino were enrolled. Exclusion criteria: admission >48hrs, previous myocardial infarction, prior left ventricle ejection fraction <40%, pregnancy. Myocardial necrosis markers, ECG and WMA were evaluated daily during the first 5 days. Severity indices and physiological variables were collected at the same time points. For the genetic analysis, we evaluated polymorphisms of Catecol-OssiMethyl Transferase (COMT) rs4680, endothelial Nitric Oxide Synthase (eNOS) rs20707144, β2 adrenergic receptor (ADRB2) rs1042713 and ADRB2 rs1042714 and their association with cardiac dysfunction.

**RESULTS.** 49 patients were enrolled in this pilot study: 82% female, 63±12 yrs. Median (IQR) for APACHE II (Acute Physiology and Chronic Health Disease Classification System II) and SAPS II (Simplified Acute Physiology Score II) were 19 (16-23) and 48 (39-53) respectively. Aneurysm was secured by coiling in 67% of patients, clipping in 25%. The prevalent location of aneurysms was the mean cerebral artery (24.5%), followed by anterior communicating (2.4%) and intracranial carotid arteries (8.2%). In-hospital mortality was 24.5%. Troponin T was elevated in 53% of patients, with median (IQR) decreasing from 56 (16-261) ng/L at day0 to 23 (10-75) at day4. NT proBNP increased in 59% of patients and its value remained stable during the first 5 days. Copeptin was elevated in all patients and remained elevated during the study period. ECG abnormalities were recorded in 76% of patients and WMA in 25%. Genetic analysis was performed in the first 25 patients and the AA genotype of COMT rs4680 polymorphism was more common in patients with WMA (p= 0.042). This genetic variant is associated with a reduction in catecholamines degradation, suggesting a possible catecholamine toxic injury on myocardium.

**CONCLUSION.** We demonstrated a high incidence of cardiac abnormalities in the early period of ICU admission after SAH. A possible association between NSC and specific genetic polymorphisms has been observed. Larger studies are needed to confirm these results and to evaluate the role of other possible predictors.

1. Sanpaolo Grant S1618_L2_MAZA_01

### 001069

#### The value of brain tissue pO2 monitoring for the prevention of secondary brain injury

T. Schizodimos, E. Siomos, C. Iasonidou, E. Lazoudi, E. Setsidou, N. Kapravelos

B icu, General Hospital "G. Papanikolaou", Thessaloniki, Greece

**Correspondence:** T. Schizodimos

*Intensive Care Medicine Experimental* 2019, **7(Suppl 3):**001069

**INTRODUCTION.** Neurocritical care patients are at risk of developing secondary brain injury (SBI) following primary lesion. Prevention, detection and treatment of SBI are of paramount importance for the clinical outcomes of patients with acute brain injury (ABI). Taking into account the complex pathophysiology of ABI, the use of multimodal neuromonitoring, including brain tissue pO2 (PbtO2), may have a major role in this purpose.

**OBJECTIVES.** To investigate the association between PbtO2 monitoring and prevention of SBI in neurocritical care patients.

**METHODS.** An observational (preliminary) study was performed in a Greek ICU from July 2018 to March 2019, including 23 patients with ABI (11 Traumatic brain injury, 9 Subarachnoid haemorrhage, 2 Intracerebral haemorrhage, 1 Ischemic stroke) and intracranial pressure (ICP) / PbtO2 monitoring. We selected 19 of them who survived and we divided them in two groups. The first group included all survivors who developed SBI and the second group all survivors without SBI. Monitoring of ICP and PbtO2 was performed, considering as critical thresholds of ICP > 22 mmHg and PbtO2 < 15 mmHg. We collected data from ICP and PbtO2 measurements in the first 24 h and > 24 h.

**RESULTS.** Included 23 patients had a mean age of 48.7 (± 16.8) years, ICU admission GCS 7.8 (± 3.9) and APACHE II score 17 (± 6.3). An external ventricular drain was inserted in 11 patients. 19 patients (82.6%) survived and 4 (17.4%) died. Among survivors, 7 patients (36.8%) developed SBI. In this group during the first 24 h PbtO2 < 15 mmHg was detected in all patients (100%) and remained low for time interval > 24 h in 5 patients (71.4%). It was remarkable that ICP > 22 mmHg was detected in only 2 patients (28.6%). We observed that 4 patients with SBI (57.1%) had a low PbtO2 and a normal ICP. From the group of 12 patients who did not develop SBI 7 of them (58.3%) had brain hypoxia in the first 24 h, but after 24 h of treatment none had.

**CONCLUSION.** Brain hypoxia may occur despite normal ICP. Therefore, monitoring of PbtO2 can provide additional information on early detection and prevention of SBI. Measurement of brain tissue oxygenation may be part of multimodality monitoring in order to achieve appropriate guidance of treatment and consequently improve outcomes. However, there remains a need for more, high quality trials in patients with ABI.

**REFERENCE(S)**

1. Roh D, Park S. Brain Multimodality Monitoring: Updated Perspectives. Curr Neurol Neurosci Rep 2016;16(6):56
2. Le Roux P, et al. Consensus summary statement of the International Multidisciplinary Consensus Conference on Multimodality Monitoring in Neurocritical Care: a statement for healthcare professionals from the Neurocritical Care Society and the European Society of Intensive Care Medicine. Intensive Care Med 2014;40(9):1189-209
3. None

### 001075

#### Cerebral consequences of prone positioning in patients with brain injury and moderate to severe ARDS, a retrospective multicentric analysis

P. BERNON[1], B. Balança[2], S. Mrozek[3], G. Dupont[4], F. Dailler[5], AC. Lukaszewicz[2]

[1]Reanimation Medicale Hôpital de la Croix Rousse , Hospices civils de Lyon , LYON, France; [2]Reanimation neurologique, Hospices Civils de Lyon, Lyon, France; [3]Anesthesie en neurochirurgie, Centre Hospitalier Universitaire de Toulouse, Toulouse, France; [4]Reanimation polyvalente, Centre Hospitalier Universitaire de Saint Etienne, Saint Etienne, France; [5]Reanimation neurologique, Hospices Civils de Lyon , Lyon, France

**Correspondence:** P. BERNON

*Intensive Care Medicine Experimental* 2019, **7(Suppl 3):**001075

**INTRODUCTION.** Acute respiratory distress syndrome (ARDS) is associated with a high mortality rate (1). In patients with ARDS without intracranial pathology, prone positioning (PP) sessions significantly decrease mortality and improve oxygenation (2). We can presume similar benefits on ARDS in patients with acute brain injury; however the effect of PP on cerebral hemodynamics remains controversial.

**OBJECTIVES.** Our objective was to evaluate the tolerance of PP on intracranial pressure (ICP), in patients with acute brain injury and ARDS.

**METHODS.** This study was conducted in three intensive care units (ICU) in France between January 2013 and January 2018. We retrospectively analyzed data from patients with moderate and severe ARDS (PaO2/FiO2 ratio < 150, according to Berlin definition(3)) and a continuous monitoring of intracranial pressure. Cerebral, respiratory and hemodynamic parameters were collected hourly before, during and after PP. The primary endpoints were PP efficacy on PaO2/FiO2, and tolerance through the number of patients who had at least one ICP measurement over 25 mmHg during PP. Secondary endpoints were the effect of prone positioning on cerebral oxygenation via the measuring of brain oxygen partial pressure (PbtO2). This study was conducted in accordance with French and European ethical guidelines.

**RESULTS.** Twenty-seven patients (46 years old [36.5-55], 5 female) were included: 10 traumatic brain injury, 11 subarachnoid hemorrhage and 6 stroke. During PP, PaO2/FiO2 rose from 100 [89.5-126] to 216 [171-257] and was at 146 [122-186] after PP. During PP, ICP increase of +10.5 mm Hg [5.25-17.75] and rose above 25 mmHg in 14 (52%) patients. The ICP increase was greater in patient who had intracranial hypertension (ICH) than those without ICH during PP (+19mmHg [13.5-20] *vs* +6mmHg [3.5-8.5] respectively, p=0.025). Before PP, patients who developed ICH had a significantly higher ICP compared to those without ICH (20mmHg [13-26] *vs* 11mmHg [7-12], p = 0.005) and the cerebral perfusion pressure was also significantly lower (67 [64.25-74.75] *vs* 79 [77-87] respectively, p = 0.04). Other variable before prone position onset were not significant different. In this population, all patients with an ICP > 17.5 mmHg before PP had ICH during PP.

There were no significant differences in the duration of mechanical ventilation, mortality and Rankin Scale at ICU discharge between patients with or without ICH during PP. PbtO2 was monitored in 4 patients and rose from 20.5 mmHg [18.75-23.5] before PP to 28mmHg [22-31] during PP. Two patients with initial PIC at 19mm Hg and 9mm Hg died of brain death following complicated refractory ICH after PP. In total, the mechanical ventilation time was 23 days[11.5-36], mortality rate was 25.93% and Rankin's score at ICU discharge was 4[4-5.5].

**CONCLUSION.** These data suggest that PP could be beneficial safe in brain injured patient with ARDS. However, patients with ICH before are at risk of worsening ICH during PP, and require precautions. Special handling would have to be defined for PP in patients with ICH for securing such maneuver.

**REFERENCE(S)**

1. Bellani G, Laffey JG, Pham T, Fan E, Brochard L, Esteban A, et al. Epidemiology, Patterns of Care, and Mortality for Patients With Acute Respiratory Distress Syndrome in Intensive Care Units in 50 Countries. JAMA. 23 févr 2016;315(8):788-800.
2. Guérin C, Reignier J, Richard J-C, Beuret P, Gacouin A, Boulain T, et al. Prone positioning in severe acute respiratory distress syndrome. N Engl J Med. 6 juin 2013;368(23):2159-68.
3. ARDS Definition Task Force, Ranieri VM, Rubenfeld GD, Thompson BT, Ferguson ND, Caldwell E, et al. Acute respiratory distress syndrome: the Berlin Definition. JAMA. 20 juin 2012;307(23):2526-33.

### 001097

#### Is it peak or trough meropenem concentration the main determinants of EEG abnormalities in septic patients?

F. Montanaro[1], B. Marjorie[2], MS. Bedrana[2], G. Nicolas[2], H. Maya[2], W. Fleur[2], V. Jean-Louis[2], J. Frederique[2], C. Jacques[2], FS. Taccone

[1]{street_address}, Bruxelles, Belgium; [2]Intensive care, Hospital Erasme, Bruxelles, Belgium

**Correspondence:** F. Montanaro

*Intensive Care Medicine Experimental* 2019, **7(Suppl 3):**001097

**INTRODUCTION.** Although high meropenem concentrations have been associated with clinical neurological deterioration in septic patients, no data have been reported

**OBJECTIVES.** To assess the association between meropenem concentrations and EEG abnormalities in ICU patients with sepsis.

**METHODS.** We reviewed all ICU patients over a 5-year period (2012-2017), who were treated with meropenem and in whom at least two β-lactam concentrations (trough, Cmin; concentration 2 hours after the bolus injection, C2H) and a concomitant EEG monitoring were available. Drug levels were measured using high-performance liquid chromatography. An "altered" EEG was defined when at least one between ictal EEG pattern, generalized periodic discharges (GPDs) or burst suppression was identified.

**RESULTS.** We collected 526 meropenem concentrations (263 at Cmin and 263 at C2H) from 193 patients over the study period. Altered EEG was found in 52 (20%) of cases, with ictal EEG pattern in 32 of them. Meropenem Cmin and C2H were higher in patients with an altered EEG than others (7.2 [5.1-11.8] vs. 4.8 [2.2-8.1] mg/L; p<0.001 – 25.1 [17.5-33.9] vs. 19.6 [12.9-29.1] mg/L, p=0.01). A multivariate analysis (adjusted for patients) identified the following as independent predictive factors for altered EEG: mechanical ventilation (OR 1.225 [1.072-1.978], p=0.04), high Cmin (OR 1.128 [1.072-1.188], p<0.001) and high pH (OR 1.072 [1.019-1.128], p=0.007).

**CONCLUSION.** There was a significant association between both trough and maximal meropenem concentrations and an increased occurrence of EEG abnormalities in ICU patients with sepsis. However, only high Cmin were the main determinant of potential drug toxicity.

### 001113

#### Delayed cerebral ischemia associated risk factors in patients with subarachnoid hemorrhage in a neurotraumatic ICU

C. Sánchez Ramírez[1], CF. Lübbe Vázquez[1], JL. Vicente Arranz[1], J. López Pérez[1], C. Vázquez Pineda[1], J. Garriga Segarra[1], JM. Ríos Bort[1], A. Sánchez Del Rio[1], P. Saavedra-Santana[2], S. Ruiz-Santana[1]
[1]Intensive care medicine, University Hospital of Gran Canaria Dr. Negrin, Las Palmas de Gran Canaria, Spain; [2]Mathematics and informatics deparment, University of Las Palmas, Las Palmas de Gran Canaria, Spain
**Correspondence:** C. Sánchez Ramírez
*Intensive Care Medicine Experimental* 2019, **7(Suppl 3):**001113

**INTRODUCTION.** Delayed cerebral ischemia (DCI) is a major contributor to the high case fatality rate and morbidity of aneurysmal subarachnoid hemorrhage. About 30% of the subarachnoid hemorrhage (SAH) patients develop DCI but it is difficult to predict which patients will develop it

**OBJECTIVES.** To assess the development of DCI risk factors in patients with SAH, admitted in a neurotraumatic ICU.

**METHODS.** Prospectively collected data of patients admitted from October, 2013 to December 2017 to a 10-bed Neurotraumatic ICU. We analyzed: main diagnosis at admission; demographic data including sex and race; neurological data (clinical examination, pupils reactivity and size, and Glasgow Coma Score: GCS; localization and aneurysm size; presence of intracranial hematoma (ICH); presence and volume of intraventricular bleeding; days to develop vasoespam; development of DCI; Fisher scale, Modified Fisher scale, Hunt and Hess scale, Word Federation of Neurosurgeons (WFNS) scale; presence of vasoespasm in doppler or arteriography; delayed of admission in ICU; treatment of the aneurysm; complications, including infections; Glasgow Outcome Scale (GOS) at ICU discharge and 6 months after ICU discharge and several other risk factors. A univariate and a multivariate logistic regression analysis of DCI were performed. It was considered significant if p $\leq$.05.

**RESULTS.** Eighty three SAH patients were collected, 24 (28,9%) of them developed DCI. Demographic data and types of admission are shown in Table 1a and 1b. Anterior /Aco) and posterior (Pco) communicating artery aneurysms were the most frequently found and most of them were localized in the left side. The most frequent complications were hydrocephalus 36 (43.9%) and ventriculitis 9 (11.4%).The Fisher scale was 3 in SAH and in DCI patients, modified Fisher scale was 4 for the SAH and DCI patients. Hunt and Hess scales was 2 for SAH patients and DCI patients. WFNS was 2 in SAH and DCI patients. GOS at ICU discharge was 4 and, six months after ICU discharged, was 5 in patients with SAH. Patients who developed DCI had GOS at ICU discharged of 4 and it was also 4 six months after ICU discharge. Eleven SHA patients died (13.4%) Three DCI patients died.To be orientated OR, IC 95% (IQR): 0.184 (0.058 ; 0.579) and PCo aneurysms OR, IC 95% (IQR): 4.559 (1.314 ; 15.817) were independently associated with DCI.

**CONCLUSION.** Our data showed 28,9 % of DCI patients. GOS reflect low disability of DCI patients both at ICU discharge and six months after it. To be orientated and PCo aneurysm were independently associated with the development of DCI.

**Table 1a (abstract 001113).** Characteristics of the patients

| | Overall | *Delayed cerebral ischemia* | | P |
|---|---|---|---|---|
| | N = 83 | No N = 59 | Yes N = 24 | |
| Age, years | 57.7 ± 13.0 | 59.1 ± 13.3 | 54.5 ± 12.0 | 0.147 |
| Age > 55 | 45 (54.2) | 35 (59.3) | 10 (41.7) | 0.143 |
| Sex female | 55 (66.3) | 38 (64.4) | 17 (70.8) | 0.575 |
| Apache II at admission | 14.1 ± 7.5 | 12.9 ± 7.1 | 17.1 ± 8.0 | 0.021 |
| SOFA at admission | 2 (0 - 4) | 2 (0 - 4) | 2 (1 - 6) | 0.143 |
| GCS on site | 15 (13 - 15) | 15 (13 - 15) | 15 (13 - 15) | 0.537 |
| GCS in emergency room | 14 (10 - 15) | 15 (12 - 15) | 13 (9 - 15) | 0.140 |
| GCS at ICU admission | 13 (4 - 15) | 14 (4 - 15) | 7 (3 - 14) | 0.150 |
| ICU re-admission by vasospasm | 7 (8.6) | 2 (3.5) | 5 (20.8) | 0.022 |
| Death at ICU discharge | 11 (13.4) | 8 (13.8) | 3 (12.5) | 1 |
| Death in hospital | 6 (7.4) | 3 (5.3) | 3 (12.5) | 0.354 |
| Anti-Vitamin K drugs | 3 (3.7) | 2 (3.5) | 1 (4.2) | 1 |
| Diabetes | 13 (15.8) | 8 (13.8) | 5 (20.8) | 0.510 |
| Dyslipemia | 29 (35.4) | 19 (32.8) | 10 (41.7) | 0.443 |
| Chronic renal failure | 4 (4.9) | 3 (5.2) | 1 (4.2) | 1 |
| Neoplasm | 2 (2.4) | 2 (3.5) | 0 | 1 |
| Malnutrition | 1 (1.2) | 1 (1.7) | 0 | 1 |
| Smoker | 31 (37.8) | 19 (32.8) | 12 (50.0) | 0.143 |
| Alcoholic | 8 (9.8) | 6 (10.3) | 2 (8.3) | 1 |
| Cocaine use | 4 (4.9) | 2 (3.5) | 2 (8.3) | 0.577 |
| Stroke family history | 1 (1.4) | 1 (2.1) | 0 | 1 |
| Caucasian race | 71 (96.0) | 51 (98.1) | 20 (90.9) | 0.209 |
| Platelet inhibitor | 9 (11.1) | 6 (10.5) | 3 (12.5) | 1 |
| Emergency surgery at admission | 7 (8.4) | 3 (5.1) | 4 (16.7) | 0.185 |
| Previous surgery | 2 (2.4) | 1 (1.7) | 1 (4.2) | 0.497 |
| SDD | 21 (28.0) | 14 (25.9) | 7 (33.3) | 0.521 |
| Oriented | 46 (55.4) | 38 (64.4) | 8 (33.3) | 0.010 |
| Alert | 54 (65.1) | 43 (72.9) | 11 (45.8) | 0.019 |
| Confused | 13 (15.7) | 8 (13.6) | 5 (20.8) | 0.507 |
| Stuporous | 8 (9.9) | 3 (5.2) | 5 (21.7) | 0.038 |
| Bilateral mydriasis | 3 (3.8) | 2 (3.6) | 1 (4.2) | 1 |
| Anisochoric pupils | 8 (10.0) | 5 (8.9) | 3 (12.5) | 0.691 |
| Isochoric pupils | 70 (87.5) | 49 (87.5) | 21 (87.5) | 1 |
| One reactive pupil | 5 (6.2) | 2 (3.6) | 3 (12.5) | 0.156 |
| Boths reactive pupils | 66 (82.5) | 47 (83.9) | 19 (79.2) | 0.749 |
| None-reactive pupils | 6 (10.0) | 5 (11.6) | 1 (5.9) | 0.665 |
| Right aneurysm | 19 (24.1) | 14 (25.0) | 5 (21.7) | 0.758 |
| Left aneurysm | 28 (35.4) | 18 (32.1) | 10 (43.5) | 0.339 |
| Bilateral aneurysm | 5 (6.2) | 4 (7.0) | 1 (4.2) | 1 |
| Aneurysm in the midline | 16 (20.2) | 10 (17.9) | 6 (26.1) | 0.538 |
| Anterior Cerebral Artery aneurysm | 6 (7.7) | 4 (7.1) | 2 (9.1) | 1 |
| Anterior Communicating Artery aneurysm | 26 (32.1) | 18 (31.6) | 8 (33.3) | 0.877 |
| Posterior Communicating Artery aneurysm | 19 (24.1) | 10 (17.9) | 9 (39.1) | 0.044 |
| Anterior Cerebral Artery aneurysm | 6 (7.6) | 4 (7.1) | 2 (8.7) | 1 |
| Ophthalmic Artery aneurysm | 1 (1.3) | 0 | 1 (4.3) | 0.291 |
| Middle Cerebral Artery aneurysm | 7 (8.9) | 6 (10.7) | 1 (4.3) | 0.667 |

**Table 1b (abstract 001113).** Characteristics of the Patients

| Characteristics of the Patients | Overall | *Delayed cerebral ischemia* | | P |
|---|---|---|---|---|
| | N = 83 | No N = 59 | Yes N = 24 | |
| Posterior Inferior Cerebellar Artery aneurysm | 4 (5.1) | 4 (7.1) | 0 | 0.316 |
| Carotid aneurysm | 2 (2.5) | 2 (3.6) | 0 | 1 |
| Multiple aneurysm | 6 (7.6) | 3 (5.4) | 3 (13.0) | 0.350 |
| Aneurysm clipping | 15 (19.0) | 10 (17.9) | 5 (21.7) | 0.755 |
| Lumbar drainage | 18 (21.7) | 11 (18.6) | 7 (29.2) | 0.292 |
| Embolization of the aneurysm | 1 (1.2) | 1 (1.7) | 0 | 1 |
| Embolization and surgery treatment | 50 (60.2) | 33 (55.9) | 17 (70.8) | 0.209 |
| Conservative treatment | 2 (2.4) | 1 (1.7) | 1 (4.2) | 0.497 |
| Decompressive craniectomy | 12 (14.5) | 11 (18.6) | 1 (4.2) | 0.165 |
| Intraoperative aneurysm rupture | 3 (3.7) | 2 (3.5) | 1 (4.2) | 1 |
| Died after treatment | 7 (8.4) | 4 (6.8) | 3 (12.5) | 0.407 |
| External ventricular device | 2 (2.4) | 1 (1.7) | 1 (4.2) | 0.497 |
| Cerebrospinal fluid fistula | 38 (47.5) | 23 (41.1) | 15 (62.5) | 0.079 |
| Hidrocephalus | 1 (1.2) | 0 | 1 (4.2) | 0.289 |
| MV > 7 days | 36 (43.9) | 24 (41.4) | 12 (50.0) | 0.474 |
| ICH | 25 (30.1) | 18 (30.5) | 7 (29.2) | 0.904 |
| Frontal ICH | 16 (19.5) | 14 (24.1) | 2 (8.3) | 0.131 |
| Perisylvian ICH | 13 (16.2) | 11 (19.6) | 2 (8.3) | 0.324 |
| Temporal ICH | 14 (17.5) | 11 (19.6) | 3 (12.5) | 0.536 |
| Subdural hematoma | 8 (10.0) | 8 (14.3) | 0 | 0.097 |
| Vasoespasm_doppler | 6 (7.5) | 4 (7.1) | 2 (8.3) | 1 |
| Vasoespasm_arteriography | 28 (36.4) | 10 (18.5) | 18 (78.3) | < .001 |
| Internal carotid artery aneurysm | 17 (23.0) | 5 (9.6) | 12 (54.5) | < .001 |
| Rebleeding 72 hours | 4 (4.9) | 2 (3.5) | 2 (8.3) | 0.577 |
| Ventriculitis | 9 (11.4) | 6 (10.9) | 3 (12.5) | 1 |
| McCabe | | | | 0.480 |
| 1 | 17 (22.4) | 10 (18.9) | 7 (30.4) | |
| 2 | 45 (59.2) | 32 (60.4) | 13 (56.5) | |
| 3 | 14 (18.4) | 11 (20.8) | 3 (13.0) | |
| Delayed admission after bleeding | 10 (4 - 24) | 8 (3 - 24) | 12 (8 - 24) | 0.155 |
| GOS ICU discharge | 4 (3 - 5) | 5 (3 - 5) | 4 (3 - 5) | 0.172 |
| GOS 6 months after discharge | 5 (2 - 5) | 5 (2 - 5) | 4 (2 - 5) | 0.231 |
| Fisher scale | 3 (3 - 4) | 3 (3 - 4) | 3 (3 - 4) | 0.809 |
| Fisher modified scale | 4 (3 - 4) | 3 (3 - 4) | 4 (3 - 4) | 0.060 |
| Hunt and Hess scale | 2 (1 - 3) | 1 (1 - 3) | 2 (1 - 4) | 0.076 |
| WFNS scale | 2 (1 - 4) | 2 (1 - 4) | 2 (1 - 4) | 0.063 |

Data are means SD (standard deviation), frequencies (%) and medians (IQR: Interquartile range)., MV: mechanical ventilation, ICH: intracerebral hematoma, GOS: Glasgow outcome score, WFNS: World Federation Neurosurgical Societies

**Table 2 (abstract 001113).** Multivariate logistic regression for the DCI. Variables were selected using the best subset regression and BIC criteria

| | P | OR (95% CI) |
|---|---|---|
| Oriented | 0.004 | 0.184 (0.058 ; 0.579) |
| Posterior communicating artery aneurysm | 0.017 | 4.559 (1.314 ; 15.817) |

### 001144

#### Cerebral haemorrhage secondary to aneurysms and arteriovenous malformations in pregnant women. follow-up and prognosis

D. Arias-Verdú[1], MP. Benitez Moreno[1], J. Barrueco-Francioni[1], A. Muñoz-López[1], G. Quesada-García[1]; R. Rivera-Fernández[2]
[1]Intensive care, Regional Hospital of Malaga, Málaga, Spain; [2]Intensive care, Hospital Neurotraumatologico Universitario , Jaén, Spain, Spain
*Intensive Care Medicine Experimental* 2019, **7(Suppl 3):**001144

**INTRODUCTION.** Stroke in women of child-bearing age could be more common during pregnancy. Subarachnoid haemorrhage (SAH) is the third leading cause of non-obstetrical death in pregnant women
**OBJECTIVES.** We aimed to study the clinical and epidemiological characteristics of pregnant patients with spontaneous brain haemorrhage or SAH secondary to aneurysms or arteriovenous malformations (AVM).
**METHODS.** This descriptive retrospective study was undertaken in 2008-2013 in a third-level ICU attending pregnant patients. All pregnant women with brain haemorrhage were included. Analyses were made of the scores of severity, Glasgow on admission and discharge from the ICU, clinical and epidemiological variables, CT, cause of the haemorrhage, treatment if given, term, pregnancy outcome and delivery, and prognosis according to the GOSE scale and Barthell index, among others.
**RESULTS.** During the 6-year study period 6 patients experienced a brain haemorrhage. The mean age was 37.16±55 years. The haemorrhages presented in the third term, except for one in the second term. The mean Glasgow score on admission was 12.33±2.8 and on discharge 14.33±0.63. All had SAH, and 4 patients also had associated parenchymal haemorrhages. The mean Hunt and Hess on admission was 2.5±0.84 and the Fisher 3.67±0.82. The cause detected was: 3 with anterior communicating artery aneurysms, 1 with AVM, 1 with vasculitis and haemorrhage at the time of delivery, and another with gestosis and haemorrhage at the time of delivery. The aneurysms (3 patients) were treated with embolization at a mean of 2.66 days. Only one of the hematomas required neurosurgical evacuation. Caesarean section was required in all 6 pregnancies, 4 between 3 months and 4 days posthaemorrhage, and in 2 (gestosis and vasculitis) the haemorrhage occurred at the time of delivery. The GOS at 6 months and one year was 4 (moderate disability) in three patients and 5 (good recovery) in the other three. Sequelae included headache, epilepsy without aura and depression. No mother and no newborn died.
**CONCLUSION.** SAH in pregnant women is a severe condition, requiring diagnosis and interdisciplinary treatment. In our series, as in others, the attitude was to treat the aneurysm and/or its cause as soon as possible, and to use a caesarean delivery.

### 001148

#### Early lymphopenia and infections in non-traumatic subarachnoid hemorrhage

L. Attanasio[1], D. grimaldi[2], R. Akhtar Ramiz[2], S. Schuind[3], J. Creteur[2], S. Spadaro[4], FS. Taccone[2]
[1]Dipartimento di medicina interna, Università degli studi della Campania Luigi Vanvitelli, Naples, Italy; [2]ICU, Hospital Erasme, Bruxelles, Belgium; [3]Neurosurgery department, Hospital Erasme, Bruxelles, Belgium; [4]Ferrara, Ospedale Sant'Anna, Ferrara, Italy
**Correspondence:** L. Attanasio
*Intensive Care Medicine Experimental* 2019, **7(Suppl 3):**001148

**INTRODUCTION.** Subarachnoid hemorrhage (SAH) is an acute cerebrovascular event associated with high morbidity and mortality. Several studies showed the independent impact of a lymphopenia with poor outcome during critically illness but no data are available for SAH.
**OBJECTIVES.** To evaluate the prevalence of lymphopenia among SAH patients and its association with infections occurrence
**METHODS.** Retrospective analysis of an institutional database of adult (>18y) patients admitted to the Department of Intensive Care after non-traumatic SAH between January 2011 and May 2016. Lymphocyte count was obtained daily for a maximum of 5 days; lymphopenia was defined as lymphocyte count < 1000/mm3. We collected the occurrence of infection during the hospital stay as well as ICU mortality and unfavorable neurological outcome (UO; i.e. Glasgow Outcome Scale of 1-3 at 3 months).
**RESULTS.** Data from 270 patients were analyzed (median age 54 years; male 45%); 62 (23%) patients developed infections during hospital stay. Mortality was 29% and UO 40%. The median lymphocyte count on admission was 1280 (890-1977)/mm3 and 45% (n= 121) had lymphopenia. Lymphopenic patients had more frequently infections (38/121, 31% vs. 24/139, 17% - p= 0.003), while mortality and UO were similar when compared to non-lymphopenic patients. In a multivariate analysis, seizures on admission and the use of mechanical ventilation were independent predictors of infection, while lymphopenia was not. Independent predictors of lymphopenia was the Fisher score on admission and the development of fever
**CONCLUSION.** Early lymphopenia is common after SAH; however, it is not significantly associated with the development of infections.

### 001206

#### Risk factors for the development of fever in traumatic brain injury patients admitted to the intensive care unit

M. Kofler[1], BA. Ianosi[1], V. Rass[1], F. Ortolano[2], S. Rossi[3], AJ. Schiefecker[1], R. Beer[1], B. Pfausler[1], E. Picetti[3], N. Stocchetti[2], R. Helbok[1]
[1]Department of neurology, Medizinische Universität Innsbruck, Innsbruck, Austria; [2]Department of anaesthesia and critical care, Fondazione IRCCS Cà Granda Ospedale Maggiore Policlinico, Milano, Italy; [3]Department of anesthesia and intensive care, Parma University Hospital, Parma, Italy
**Correspondence:** M. Kofler
*Intensive Care Medicine Experimental* 2019, **7(Suppl 3):**001206

**INTRODUCTION.** Fever is a modifiable complication contributing to poor functional outcome and increased mortality in traumatic brain injury (TBI) patients.
**OBJECTIVES.** We sought to identify risk factors for the development of fever in TBI patients admitted to the intensive care unit (ICU).
**METHODS.** This is a retrospective analysis of data acquired in a European prospective, multicenter, observational cohort study (CENTER-TBI). Patients were included if they were admitted to an ICU as a direct consequence of TBI and data on highest body temperature were available on at least 2 different days. Fever was defined as body temperature above 38.3°C. Statistical analysis was performed using binary logistic regression models.
**RESULTS.** Two-thousand and seventy-four patients were included. Median age was 49 (29-65) years. Pre-injury variables associated with the development of fever were younger age (p=0.035), male sex (p< 0.001) and a medical history of a neurologic disease (p=0.036). Fever was most common in patients with diffuse brain injury as primary intracranial pathology (59%), followed by subdural hematoma (49.6%), contusions (49.1%), traumatic subarachnoid hemorrhage (43.3%) and epidural hematoma (34.7%). Regarding injury details and pre-ICU management, a Glagow Coma Scale score below 14 (p< 0.001), injury severity score above 30 (p<0.001), episodes of hypoxia (p<0.001), hypotension (p=0.004), or hypothermia (p=0.009), neuroworsening (p<0.001), clinical symptoms of skull base fracture (p< 0.001), a focal neurological deficit (p<0.001), any airway treatment (p<0.001), and any circulatory support (p<0.001) were associated with a more common occurrence of fever. During the ICU stay, any surgery (p<0.001), any infectious complication (p<0.001), seizures (p<

0.001), and intracranial lesion progression (p<0.001) were risk factors for the development of fever.

**CONCLUSION.** In this study we identified risk factors for the development of fever in a European cohort of TBI patients admitted to the ICU. Demographic variables, markers of injury severity, pre-ICU course and complications during the ICU stay were associated with the development of fever. Multivariable models and a differentiation between infectious and non-infectious fever will be presented at the meeting.

### 001210

#### Reliability of the pulsatility index to assess cerebral autoregulation in septic patients

AA. Quispe-Cornejo, S. Mcduff, IA. Crippa, J. Creteur, JL. Vincent, FS. Taccone

Department of intensive care, Erasme University Hospital, Université libre de Bruxelles, Brussels, Belgium

**Correspondence:** A.A. Quispe-Cornejo

*Intensive Care Medicine Experimental* 2019, **7(Suppl 3):**001210

**INTRODUCTION.** The pulsatility index (PI), measured by transcranial Doppler (TCD), is useful to assess cerebrovascular resistances. Although it has been associated with septic encephalopathy, the relationship between PI and cerebral autoregulation (CAR) in septic patients has not been well defined.

**OBJECTIVES.**

- To evaluate if pulsatility index (PI) is a surrogate of CAR in critically ill septic patients.

**METHODS.** Observational study of 50 adult septic patients (with or without shock). Exclusion criteria were: any intracranial disease; major arrhythmias; treatment with extracorporeal membrane oxygenation; any supra-aortic arteriopathy. Transcranial Doppler (DWL, Germany) was performed by insonating the left middle cerebral artery (LMCA) with a 2MHz probe. LMCA blood flow velocity (FV) and arterial blood pressure (BP) signals were simultaneously recorded for at least 6 minutes; Pearson´s correlation coefficient between BP and FV (MXa) was calculated using MATLAB (MathWorks, USA). Impaired CAR was defined as MXa > 0.3. High PI was defined as > 1.2. The first PI available was registered from the same TCD recording.

**RESULTS.** The 50 patients had a median age of 64 [54-74] years. Median MXa was 0.25 (-0.07 - 0.43) and median PI was 0.97 (0.76 - 1.18). There was no correlation between MXa and PI (p=0.83). Both in the 24 patients with altered and in the 26 patients with intact CAR, there was no significant correlation between MXa and PI (p=0.58 and 0.93, respectively). Mean PI were similar in patients with altered and intact CAR (1.04 and 1.06, respectively). Also, the number of patients with high PI was similar in patients with altered (7/24) or preserved CAR (6/26).

**CONCLUSION.** Pulsatility index cannot reliably assess cerebral autoregulation in septic patients.

### 001261

#### Level SVI and SVRI in anesthesia, including alpha2-adrenoagonisty

M. Rumiantceva[1], L. Tsentsiper[1], N. Lesteva[1], R. Nazarov[2], A. Kondratyev[1]

[1]Anesthesiology and intensive care, RNSI n.a.A.L. Polenov at V.A. Almazov National Medical Research Center, Saint Petersburg, Russia, Russia; [2]Anesthesiology and intensive care, FMBC-FMBA n.a. A.I.Burnazyan, Moscow, Russia

**Correspondence:** M. Rumiantceva

*Intensive Care Medicine Experimental* 2019, **7(Suppl 3):**001261

**INTRODUCTION.** An integral part of neuroregulatory systems of the brain stem are opioid and adrenergic antinociceptive systems. The use of anesthesia, including a combined effect on opioid and adrenergic antinociceptive systems, creates favorable conditions for performing operations on brain tumors.

**OBJECTIVES.** To assess changes in SVI and SVRI during anesthesia, including alpha2-adrenoagonist.

**METHODS.** The study included 118 patients (mean age 50.5±13.6 (Mean ± Std)) who underwent planned surgical treatment about brain tumors.

In all patients induction of anesthesia included: muscle relaxants, hypnotics (propofol), opioid analgetic (fentanyl 4.8±0.6mcg/kg) + alpha2-adrenoagonist (clonidine or dexmedetomidine). Maintenance of anesthesia: hypnotic (propofol), opioid analgetic (fentanyl 1.3±0.4mcg/kg/h) + alpha2-adrenoagonist (clonidine or dexmedetomidine).

All patients were divided into three groups, depending on the alpha2-adrenoagonist used and its dosage.

In group I (26 patients) induction of anesthesia: clonidine 1.5±0.4mcg/kg; maintenance of anesthesia: clonidine 0.4±0.15mcg/kg/h. In group II (58 patients) induction of anesthesia: dexmedetomidine 1.5±0.4mcg/kg; maintenance of anesthesia: dexmedetomidine 0.4±0.2mcg/kg/h. In group III (34 patients) induction of anesthesia: dexmedetomidine 0.7±0.1mcg/kg; maintenance of anesthesia: dexmedetomidine 0.2±0.1 mcg/kg/h. All three groups are statistically comparable by sex, age, initial blood pressure, initial heart rate, position on the operating table and localization of the brain tumor.

**RESULTS.** In group I, after induction anesthesia, SVI 37,4 ± 6 ml/m2, SVRI 2487 ± 620 dyn·sec/cm5/m2. After positioning the patient on the operating table, SVI 34,4 ± 11 ml/m2, SVRI 3078 ± 1490 dyn·sec/cm5/m2. At the stage of tumor removal, SVI 34,2 ±9 ml/m2, SVRI 3176 ± 921 dyn·sec/cm5/m2. At the stage of wound closure, SVI 34,1±8 ml/m2, SVRI 3265 ± 1201 dyn·sec/cm5/m2.

In group II, after induction anesthesia, SVI 36,2± 8 ml/m2, SVRI 4006±1692 dyn·sec/cm5/m2. After positioning the patient on the operating table, SVI 34,2±7 ml/m2, SVRI 3971±1179 dyn·sec/cm5/m2. At the stage of tumor removal, SVI 33,6±6 ml/m2, SVRI 3894±1042 dyn·sec/cm5/m2. At the stage of wound closure, SVI 34,5±7 ml/m2, SVRI 3842±1123 dyn·sec/cm5/m2.

In group III, after induction anesthesia, SVI 38,9±8 ml/m2, SVRI 3294±1198 dyn·sec/cm5/m2. After positioning the patient on the operating table, SVI 35,4±9 ml/m2, SVRI 3170±808 dyn·sec/cm5/m2. At the stage of tumor removal, SVI 36,1±7 ml/m2, SVRI 3400±795 dyn·sec/cm5/m2. At the stage of wound closure, SVI 36,9±6 ml/m2, SVRI 3747±1001 dyn·sec/cm5/m2.

**CONCLUSION.** SVI lower and SVRI higher, than the reference value in the during anesthesia, including alpha2-adrenoagonist. These values are stored at all stages of the operation.

### 000114

#### Disturbance of platelet aggregation in Spontaneous Cerebral Hemorrhage

A. TELLEZ[1], A. Serrano Lazaro[1], B. Ruiz Orenga[2], ML. Blasco Cortes[1], R. Huerta Bravo[1], F. Rosa Rubio[1], E. Martí[3], EM. Andrés Esteban[4], M. Quintana Diaz[4]

[1]Intensive care unit, Hospital Clínic Universitari de València, València, Spain; [2]Intensive care unit, Hospital Universitario y Politécnico de La Fe, València, Spain; [3]Department of hematology, Hospital Clínic Universitari de València, València, Spain; [4]Intensive care unit, Hospital La Paz-madrid, Madrid, Spain

**Correspondence:** A. TELLEZ

*Intensive Care Medicine Experimental* 2019, **7(Suppl 3):**000114

**INTRODUCTION.** The functionality of platelets during coagulation is essential for stopping bleeding. Our study aims to assess the disturbance of platelet aggregation capacity in spontaneous cerebral hemorrhages (SCH), both intraparenchymal hemorrhage (IPH) and subarachnoid hemorrhage (SAH), and its correlation with severity and mortality.

**METHODS.** Patients with SCH admitted in ICU were included. A blood sample is analyzed with the Multiplateâ platelet function test at admission (day 0) and at 24h (day 1). This system measures platelet aggregability to arachidonic acid (AA), adenosine diphosphate (ADP), collagen (Col), Ristocetin (Ris) and thrombin (Trom). Hematoma volume was determined as a marker of severity in HIP and Hunt and Hess scale for HSA. Patients with oral antiplatelet therapy were excluded. The prevalence of the platelet alteration was estimated, and it was related to severity and mortality using the chi-square test or Fisher's exact test.

**RESULTS.** 69 patients included. IPH 37 (53.62%), SHA 32 (46.38%); Mean age 50-60 years. Mean hematoma volume 48.28 cc. In SHA Hunt & Hess scale were IV-V in 35.48% of patients. Mortality rate at 6 months 33.33%.

A disturbance in platelet aggregability (at least one of the tests) was observed in 100% of patients, both on day 0 and day 1. Platelet dysfunction is associated with higher mortality in all tests, although it was only significant with Collagen test (p 0.048). It´s also associated with a greater hematoma volume in IPH, only significant with Ristocetin test (p 0.015) (Figure 1).

**CONCLUSION.** There is a disturbance in platelet aggregability at the global of SCH, also correlated with a higher mortality and greater hematoma volume in the group of IPH.

## NAHP / DS - From basics to advances in care delivery

### 000980

#### Barriers to achieving a higher level of activity during early mobilization of mechanically ventilated patients

K. Tavares Timenetsky[1], CF. Lopes[2], M. Buttignol[3], RH. Moura[4], KSDS. Pereira[5], AP. Paiva[5], LHR. Gonçalves[3], RAC. Eid[1]
[1]Critically ill, Hospital Israelita Albert Einstein, São Paulo, Brazil; [2]Crtiically ill, Hospital Israelita Albert Einstein, São Paulo, Brazil; [3]Critically ill, Hospital Municipal Vila Santa Catarina, São Paulo, Brazil; [4]Critically ill, Hospital Municial Vila Santa Catarina, São Paulo, Brazil; [5]Critically ill, Hospital Municipal Dr Moysés Deutsch, São Paulo, Brazil
**Correspondence:** K. Tavares Timenetsky
*Intensive Care Medicine Experimental* 2019, **7(Suppl 3):**000980

**INTRODUCTION.** Recent studies have shown that in-bed exercises are the most prevalent activity performed in mechanically ventilated patients receiving early mobilization. However, the barriers to achieving a higher activity level may vary in different cultures, and the reasons for this may be both clinical and structural. The objective of this study was to evaluate the clinical and structural barriers to achieving a higher activity level in mechanically ventilated patients. Recent studies have shown that in-bed exercises are the most prevalent activity performed in mechanically ventilated patients receiving early mobilization. However, the barriers to achieving a higher activity level may vary in different cultures, and the reasons for this may be both clinical and structural.

**OBJECTIVES.** The objective of this study was to evaluate the clinical and structural barriers to achieving a higher activity level in mechanically ventilated patients.

**METHODS.** A multicenter observational study performed in 3 ICUs in Sao Paulo, Brazil during a 3-month period (from January to March 2019). Demographic data, prevalence of early mobilization, types of mobility activities, clinical and structural barriers to achieving a higher activity level were collected daily from all adult patients in mechanical ventilation longer than 24 hours.

**RESULTS.** In total, 85 patients were included in this study, with a total of 327 observations. The mean (SD) age was 72(4.5) years and 58% of patients were male. Early mobilization during MV was performed in 92% [301/327] of the observations. Early mobilization activities encompassed in-bed exercises (n=301; 92%), a sit over the edge of bed (n=21; 6.4%), and actively transferring from bed to chair (n=5; 1.5%); no patients were engaged in standing or walking activities. Sedation represented the most common clinical barrier to achieving a higher activity level (n=125; 38.2%), followed by use of vasoactive drug (n=107; 32.7%), consciousness impairment (n=107; 32.7%), hemodynamic instability (n=91; 27.8%), respiratory instability (n=54; 16.5%), continuous hemodialysis (n= 46; 14%), and delirium (n=16; 4.9%). Limited resources were identified as the most common structural barrier (n= 17; 5.2%), followed by time for therapy (n= 3; 0.9%) and limited staff (n=2; 0.6%).

**CONCLUSION.** In order to achieve a higher activity level during mobilization of mechanically ventilated patients, clinical barriers were more prevalent than structural barriers. The most prevalent clinical barriers were sedation, use of vasoactive drug, and consciousness impairment. Structural barriers were more related to limited resources.

**REFERENCE(S)**

1. Dubb R, Nydahl P, Hermes C, Schwabbauer N, Toonstra A, Parker A, Kaltwasser A, Needham D (2016) Barriers and strategies for early mobilization in intensive care units. Ann ATS 13(5):724–730
2. Berney SC, Harrold M, Webb SA, Seppelt I, Patman S, Thomas PJ et al. Intensive care unit mobility practices in Australia and New Zealand: a point prevalence study. Crit Care Ressusc 2013;15(4): 260-5.
3. Nydahl P, Ruhl AP, Bartoszek G, Dubb R, Filipovic S, Flohr HJ, et al. Early mobilization of mechanically ventilated patients: a 1-day point-prevalence study in Germany. Crit Care Med 2014; 42(5): 1178-86.
4. Jolley SE, Moss M, Needham DM, Caldwell E, Morris PE, Miller RR et al. Point prevalence study of mobilization practices for acute respiratory failure patients in the United States. Crit Care Med 2017; 45(2): 205-215.

### 001000

#### Family involvement at the ICU, needs are changing

T. van Galen, EE. Rouw
[1]Intensive care, Amsterdam UMC - locatie VUmc, Amsterdam, Netherlands
**Correspondence:** T. van Galen
*Intensive Care Medicine Experimental* 2019, **7(Suppl 3):**001000

**INTRODUCTION.** There is growing interest in involving family members at the ICU. Family Centered Care (FCC) and Shared Decision Making (SDM) are becoming more common in hospitals (Burns, Gerritsen). However, introducing FCC and SDM at the ICU is difficult due to limited evidence and despite a widely supported 23 recommendations guideline as provided by Davidson. At our 20 bed referral teaching ICU we developed a small size questionnaire to assess families needs, based on the five components of family involvement as described by Olding.

**OBJECTIVES.** To perform a small volume qualitative study to assess new or growing needs of family members of ICU patients to improve our medical and/or nursing services as provided on a daily basis. The questionnaire was aimed at five areas: ICU presence; having needs met/families being supported; communication and information; shared decision making (related to family expectancy) and families contributing to ICU care.

**METHODS.** The study was performed using a semi structured questionnaire (20 questions) and questions to determine patient and respondent characteristics (3 questions). During a six month period 34 families, usually one or two representative(s), were interviewed. Only families from patients with a ICU length of stay over 48 hour were included.

**RESULTS.** Family members of ICU patients endure a lot of stress and hardship during ICU admittance of their loved one. They experience disturbed sleep (76%), mental exhaustion (86%) and struggle balancing work (36%) and family (13%) as key issues. Despite their problems, 85% of families are coping sufficiently to still be able to support the ICU patient during the recovery period. 25% of families stated needing an (undefined) form of support from the hospital. Also a surprising need is found in a high number of families wanting to attend medical and nursing meetings and/or handovers and access to specific information as quality of life, incidents and risks of treatment and procedures. These needs are supported by almost 80% of the interviewed respondents, however the current offering of information and accessibility at our ICU is valued very positively. With concerns to SDM expectations, 18% of families stated needing no influence at all and 76% wanting only a consulting role. Only 3% stated that all (medical) decisions must be made by the family or only with full family consent. Despite insecure positive effects, families are willing to participate in daily ICU care. This is applicable on non-physical and physical (nursing) tasks. In general no statistical significant differences were found between patient or respondent characteristics.

**CONCLUSION.** Not only ICU or critical care is developing, needs of patient's' family members are developing as well. This provides a growing challenge for hospitals and ICU nurses and doctors to meet these needs of those family members and balance it with the daily care for the ICU patient. It is time to define new frontiers related to the role and participation of family members at the ICU. Maybe it is also time to shape the ICU workflow more to patient and families needs instead of being based on the doctors and/or nurses workflow. This will not be an easy transition. One simple question can be asked to family members today and immediately: what are your current needs and how can we support this?

**REFERENCE(S)**

1. None
2. Burns, K.E.A. et al. Patient and Family Engagement in the ICU. American Journal of Respiratory and Critical Care Medicine. Vol. 198, No. 3 Aug 01, 2018.
3. Gerritsen, R.T. Hartog, C.S. Curtis, J.R. New developments in the provision of family-centered care in the intensive care unit. Intensive Care Medicine. April 2017, Volume 43, Issue 4, pp 550–553.
4. Davidson, J.E. et al. Guidelines for Family-Centered Care in the Neonatal, Pediatric, and Adult ICU. Crit Care Med. 2017 Jan;45(1):103-128.
5. Olding, M. et al. Patient and family involvement in adult critical and intensive care settings: a scoping review. 2015.

### 001053

#### Impact of Family Care Journals in Understanding the Family Experience in Pediatric Intensive Care Unit

J. Tcharmtchi[1], K. Abela[1], D. Acorda[1], J. Coss-Bu[2], H. Tcharmtchi[2]
[1]Pediatrics, Pediatric Intensive Care Unit, Texas Children's Hospital, Houston, USA; [2]Pediatric critical care medicine, Baylor College of Medicine, Texas Children's Hospital, Houston, USA
**Correspondence:** H. Tcharmtchi

*Intensive Care Medicine Experimental* 2019, **7(Suppl 3):**001053

**INTRODUCTION.** The significance of multidisciplinary rounds and patient care where family members and parents of critically ill children are actively involved in their child's care is receiving increased recognition as a vital complement to medical treatment. There are several studies in adults showing that the use of ICU diaries has decreased anxiety and depression in both patients and families. Despite the potential benefits, there is a paucity of information regarding the use of family care journals (FCJ) in the Pediatric Intensive Care Unit (PICU).

**OBJECTIVES.** The objectives of this quality improvement study are: 1) to develop a reliable process to learn about the experiences of families in the PICU, 2) to give families a resource tool to enhance their critical care stay, and 3) to develop improvement strategies for communication with families and enrich their PICU experience.

**METHODS.** An electronic point of care (POC) 10-item questionnaire was designed to obtain a baseline assessment during the first phase of the project. The survey was administered upon transfer from the PICU. Gaps were identified and the FCJ, developed by the Family-Centered Care Program, was distributed in the second phase. Throughout the second phase of the project, the survey administration continued to evaluate the impact of the FCJ on the identified gaps.

**RESULTS.** A total of 500 FCJ were distributed and 188 completed surveys were collected. A significant improvement in all of the questions between the two phases of the project was seen: Encouraging families to write down questions (+35%), inviting families to join rounds (+16%), and understanding the patient's plan of care (+9.7%). Of all the respondents, 90% found the FCJ to be a useful tool during their PICU stay. Open-ended questions revealed more improvement opportunities including: communication with non-ICU consultants, comfort level at the time of transfer to a lower level of care environment. Additionally, the majority of respondents gave positive feedback about the excellent nursing care provided while in PICU.

**CONCLUSION.** Using a readily-accessible electronic POC survey in combination with the FCJ can provide teams with real-time meaningful and actionable information to improve the partnership between clinical providers and families. The clinical team can use this information to drive improvement strategies focused on the partnership between families and ICU teams.

**REFERENCE(S)**

1. Garrouste-Orgeas, M., Périer, A., Mouricou, P., Grégoire, C., Bruel, C., Brochon, S., Philippart, F., Max, A., and Misset, B. (2014). Writing In and Reading ICU Diaries: Qualitative Study of Families' Experience in the ICU. PLoS ONE, 9(10).
2. Herrup A., Wieczorek B., Kudchadkar S. (2019) Feasibility and Perception of PICU Diaries. Pediatric Critical Care Medicine; (2) e83-e-90

### 001174

#### Effect of health insurance status on outcome of critical care in pediatric patients

E. Park[1], C. Joongbum[2]
[1]Critical care medicine, Samsung Medical Center, Seoul, Republic of Korea; [2]Department of critical care medicine, Samsung Medical Center, Seoul, Republic of Korea
**Correspondence:** E. Park

*Intensive Care Medicine Experimental* 2019, **7(Suppl 3):**001174

**INTRODUCTION.** Health care disparity in critically ill children according to health insurance status is not well known.

**OBJECTIVES.** We aim to study whether there are differences in management and outcome between health insurance and Medical aid patients.

**METHODS.** We conducted a retrospective cohort analysis of the Health Insurance Review and Assessment (HIRA) database. All pediatric admission (<18 years of age) to intensive care units in Korea from August 1, 2009, through September 30, 2014, were enrolled, and we excluded i) admissions to neonatal intensive care units, ii) patients under 28 days of age, iii) primary diagnosis Z of International Classification of Disease, and iv) other than the first admission during study periods. We compared the management procedure and hospital death, Intensive care unit (ICU) length of stay between health insurance group and Medical aid group.

**RESULTS.** A total of 18,291 patients were enrolled, and 17,147 (93.7%) were health insurance status and 1,144 (6.3%) were Medical aid status. Age was higher in Medical aid patients (11 vs. 7 years, P< 0.001), the proportion of tertiary hospital admission was lower in Medical aid patients (67.5 vs. 39.5%, P<0.01). The proportion of mechanical ventilation (43.2 vs. 35.1%, < 0.01) and vasopressor drugs (15.8 vs. 12.0%, P<0.01) were lower in Medical aid patients. Crude mortality rate was not different between 2 groups (5.0 vs. 5.9%, P= 0.223). In adjusted analyses, medical aid state was not significant risk factor of mortality (Odds ratio 1.09, Confidence interval 0.79-1.51). However medical aid state was a risk factor of re-admission (Odds ratio 1.25, confidence interval 1.11-1.43) and visit to emergency room (Odds ratio 1.32, confidence interval 1.07-1.62).

**CONCLUSION.** The mortality rate of critically ill children who admitted to intensive care unit was not different between health insurance and medical aid patients, but the incidence of hospital readmission and emergency room visits within 3 months after discharge was significantly higher in medical aid patients. Further studies about health care disparities outside ICU are neede according to health insurance status.

**REFERENCE(S)**
1. Effects of public and private health insurance on medical service utilization in the National Health Insurance System: National panel study in the Republic of Korea
2. Samsung medical center

### 001200

#### Use of high frequency oscillatory ventilator in neonates with respiratory failure: the clinical practice in Taiwan and risk factors of treatment failure

MH. Tsai[1], FH. Jen[2], CY. Mei[3]
[1]Pediatrics, Chang Gung Memorial Hospital, Yunlin, Taiwan; [2]Pediatrics, Linkou Chang Gung Memorial Hospital, Linkou, Taiwan; [3]Pediatrics, Taipei Chang Gung Memorial Hospital, Linkou district New Taipei City, Taiwan
**Correspondence:** M.H. Tsai
*Intensive Care Medicine Experimental* 2019, **7(Suppl 3):**001200

**INTRODUCTION.** High-frequency oscillatory ventilation (HFOV) is often considered as the final rescue therapy for patients with refractory respiratory failure. However, there is limited data regarding the treatment outcomes and the risk factors of mortality after neonates on HFOV.

**OBJECTIVES.** To describe current clinical practice and predict the mortality risk for neonates receiving HFOV.

**METHODS.** A single retrospective, observational study was conducted and all neonates treated with HFOV during the study period from January 2010 to December 2017 were enrolled. Patients were classified into five cohorts based on underlying diagnosis. We used univariate analysis to identify factors associated with 30-day mortality following HFOV use and multivariate logistic regression to identify independent predictors of mortality risk.

**RESULTS.** A total of 1036 neonates ever supported on HFOV were enrolled, of which 64.1% had HFOV as the rescue therapy, 27.2% as elective use, and 8.7% for air leak. 755 (67.1%) had good response to HFOV and can be weaned within 3 days, whereas 87 (7.7%) patients died within 3 days after initiation of HFOV. The overall in-hospital mortality rate was 31.7%. An average oxygenation index (OI) greater than 25 within the first 24 hours after initiation of HFOV and patients with secondary pulmonary hypertension exhibited the greatest predictive power ($p < 0.001$) for increased mortality risk. After decision tree regression analysis, a pre-HFOV OI of more than 20.5 was identified as the cut-off point to be highly associated with final in-hospital mortality.

**CONCLUSION.** We identified the predictive factor and cut-off point that is independently associated with treatment failure in neonates on HFOV. Further efforts to optimize the outcomes are still needed.

### 001226

#### Current physiotherapy practice in Albanian Intensive Care Units

V. SHPATA, M. Kreka, X. Prendushi, T. Çlna
Faculty of Medical Technical Sciences, University of Medicine, Tirana, Albania
**Correspondence:** V. SHPATA
*Intensive Care Medicine Experimental* 2019, **7(Suppl 3):**001226

**INTRODUCTION.** Although the physiotherapists are routinely involved in the critical patients care, the albanian intensive care units (ICU) have no exclusive ICU Physiotherapists.

**OBJECTIVES.** To give a view of physiotherapy practice in the ICU, to determine the role of intensive care nurses and their involvement in physiotherapeutic techniques.

**METHODS.** ICU nurses of 6 Intensive Care Units in the university hospitals of Tirana were requested to fill a questionnaire. The questionnare consisted of 25 short-form questions, which examined the nurses' involvement in respiratory care, early mobilisation and other physiotherapeutic techniques.

**RESULTS.** Physiotherapists do not work exclusively in the ICU-s, they perform therapeutic procedures only in some patients, they work in the ICU for 2-3 hours during the morning shifts. They are absent during the afternoon and night shifts, and also on public holidays and at the weekends.
100 completed questionnaries were analyzed. Nurses are routinely involved in ICU patients care. Treatment techniques performed "very often" included out-of-bed mobilisation (60%), passive and active limb mobilization (23%), in-bed mobilisation and positioning (81%) and airway suctioning (100%).
The nurses were no involved in the adjustment of mechanical ventilation, in weanings from mechanical ventilation, in extubation and in the implementation of invasive mechanical ventilation, considering these procedures the doctors' responsibility.
ICU nurses had graduated as general nurses, and had not post-graduate specialisation in ICU therapy or respiratory therapy. They also state that their knowledge in ICU physiotherapy is insuficient (73%), and absolutely absent (27%).

**CONCLUSION.** There is a lack of regular physiotherapy service in Albanian ICU-s. The low availability shows that the need and importance of regular physiotherapists in ICU is still not clearly recognized in Albania. Physiotherapists should be part of multidisiplinary intensive care team, in order to offer high-quality physiotherapy servises and improving the outcome of patients. Besides, systematic educational programs are necessary for improving nurses knowledge on physiotherapeutic procedures, especially in early mobilisation and respiratory physiotherapy.

### 001227

#### Musculoskeletal complications following critical illness: an under recognised source of disability

O. Gustafson[1], S. Mckechnie[1], PJ. Watkinson[2], M. Williams[3], MJ. Rowland[2]
[1]Adult intensive care unit, John Radcliffe Hospital, Headington, UK;
[2]Critical care research group, University of Oxford, Oxford, UK;
[3]Department of sport and health science, Oxford Brookes University, Oxford, UK, UK
**Correspondence:** O. Gustafson
*Intensive Care Medicine Experimental* 2019, **7(Suppl 3):**001227

**INTRODUCTION.** Patients surviving an admission to an Intensive Care Unit (ICU) frequently experience long-term physical impairment, decreased health related quality of life (QoL) and low rates of return to employment.[1] Despite this there has been limited investigation of the reasons for poor physical function. Musculoskeletal (MSK) conditions are wide raging, affecting approximately 25% of the UK population and are one of the main causes of sickness absence at work.[2] Patients in ICU experience rates of muscle mass loss of 20% in the first week of admission,[3] which may put them at risk of developing further MSK complications. However, the MSK complications experienced by ICU survivors are unknown.

**OBJECTIVES.** To undertake a scoping review to gain an understanding of the evidence base surrounding the long term MSK complications experienced by ICU survivors.

**METHODS.** A systematic search was conducted of the following databases: Cochrane, CINAHL, AMED and EMBASE. Studies were included if they evaluated at least on aspect of MSK health in ICU survivors following hospital discharge. Abstracts and case reports were excluded.

**RESULTS.** Of the 22 included papers: 8 reported decreased muscle mass or muscle weakness; 7 reported MSK related chronic pain; 3 reported abnormal neuromuscular function; 3 reported joint related complications; and 1 paper reported increased fracture risk. Most papers (n=21) reported on a single aspect of MSK health.

**CONCLUSION.** The nature of critical illness and its long term consequences, along with the prevalence and detrimental effect of MSK conditions in the general population, means that it is highly likely that MSK conditions are having a negative impact on patient's physical function and QoL. The small number of studies investigating long-term MSK complications to date has identified a variety of problems extending beyond weakness. Further investigation of MSK complications following critical illness may guide future post-ICU rehabilitation interventions.

REFERENCE(S)

1. Kamdar, B.B. et al., 2018. Return to work and lost earnings after acute respiratory distress syndrome: a 5-year prospective, longitudinal study of long-term survivors. Thorax, 73(2)
2. NIHR Dissemination Centre, 2018. Moving Forward: Physiotherapy for Musculoskeletal Health and Wellbeing https://www.dc.nihr.ac.uk/themed-reviews/Moving-Forward-Final.pdf
3. Puthucheary, Z.A. et al., 2013. Acute skeletal muscle wasting in critical illness. Jama, 310(15)

### 001316

#### Pressure ulcers following prone positioning in ARDS patients undergoing ECMO treatment

F. Binda[1], A. Galazzi[1], F. Marelli[1], M. Bruno[1], E. Vinci[1], T. Mauri[2], I. Adamini[1], D. Laquintana[3]
[1]Intensive care unit, Fondazione IRCCS Ca' Granda Ospedale Maggiore Policlinico, Milano, Italy; [2]Department of anesthesiology, University of Milan, Milano, Italy; [3]Direction of healthcare professions, Fondazione IRCCS Ca' Granda Ospedale Maggiore Policlinico, Milano, Italy
**Correspondence:** F. Binda
*Intensive Care Medicine Experimental* 2019, **7(Suppl 3):**001316

**INTRODUCTION.** The application of prone positioning (PP) during veno-venous extracorporeal membrane oxygenation (VV-ECMO) has shown to be a safe and reliable technique when performed in a recognized ECMO center with the appropriately trained staff and standard procedures. [1] Several clinical studies evaluated the safety and efficacy of PP in mechanically ventilated patients, however a higher frequency of pressure ulcers has been reported. [2]
**OBJECTIVES.** To detect the incidence and the characteristics of pressure ulcers in PP patients with severe acute respiratory distress syndrome undergoing ECMO treatment.
**METHODS.** Observational retrospective analysis of all the patients admitted to our intensive care unit (ICU) of a tertiary level hospital from January 2013 to December 2017. Only the patients undergoing PP for at least 12 hours were included. The Braden scale was used to assess the patients' risk of developing a pressure ulcer at ICU admission while the pressure ulcers were staged according to the NPUAP staging system (National Pressure Ulcer Advisory Panel). Age, sex, BMI, ICU length of stay, SAPS and SOFA score and mortality were recorded.
**RESULTS.** A total of 50 PP patients undergoing ECMO were identified in the medical records. The pressure ulcers incidence was 46% and the main part of the body with skin lesions were: face (51.3%), rib cage (15.4%), hip bones (7.7%) knees (5.1%) and others (20.5%). Fourteen patients (28%) report 3 pressure ulcers present simultaneously in different anatomical sites. During all PP maneuvers, no adverse events, like ECMO cannula dislocation, were recorded.
The other results are summarized in table 1.
**CONCLUSION.** In this sample, PP is a safe procedure but it is associated with a high risk of pressure ulcers on the face. For this reason, further preventive measures to protect the skin should be implemented.

REFERENCE(S)

1. Lucchini et al. Intensive Crit Care Nurs. 2018;48:61-68.
2. Kim et al. J Wound Ostomy Continence Nurs. 2016;43(4):427-9.

**Table 1 (abstract 001316).** See text for description

| Characteristics | | Clinical score | |
|---|---|---|---|
| Age (mean, SD) | 55 ± 16 | BMI (median, IQR) | 25 (21 - 29) |
| Sex (male) | 31 (62%) | Braden score (median, IQR) | 10 (9 - 12) |
| ICU length of stay (days) | 28 (19 - 39) | SAPS II (median, IQR) | 42 (29 - 54) |
| Mortality (%) | 14 (28%) | SOFA (median, IQR) | 9 (7 - 12) |

### 001403

#### Effect of serum from critically ill children on autophagy in human skeletal muscle cells

P. Promsin[1], U. Fläring[2], O. Rooyackers[3], N. Tardif[3]
[1]Division of critical care, Department of Medicine, Faculty of Medicine Siriraj Hospital, Mahidol University, Bangkok, Thailand; [2]Department of pediatric perioperative medicine and intensive care, Astrid Lindgren Children's Hospital, Karolinska University Hospital, Stockholm, Sweden; [3]Division of anaesthesia and intensive care, Department of Clinical Science Intervention and Technology (CLINTEC), Karolinska Institutet, Stockholm, Sweden
**Correspondence:** P. Promsin
*Intensive Care Medicine Experimental* 2019, **7(Suppl 3):**001403

**INTRODUCTION.** Autophagy executes an intracellular recycling process through the lysosomal-mediated pathway. Previous studies have revealed a possible association between insufficient autophagy and a detrimental outcome in critically ill patients. However, the autophagy flux and its dysregulation by critical illness is not well described and cannot be measured in critically ill pediatric patients in vivo.
**OBJECTIVES.** To examine how serum from critically ill children influence the autophagy flux in human skeletal muscle cells in vitro, compared with serum from healthy children.
**METHODS.** Three independent experiments were conducted. Serum from 82 pediatric ICU (PICU) patients (age < 18 years) and 50 age-matched healthy subjects were incubated with cultured primary human skeletal muscle cells in 96-well plates for 24 hours. The expression of p62 protein in the muscle cells was analysed with and without an autophagy blocker (chloroquine or bafilomycin A1) using In-Cell Western assay. The autophagy flux was determined by the difference in p62 levels between the conditions with and without the autophagy blocker. Serum-free media was used as a control condition and the autophagy flux was reported as the percentage of this control. Clinical parameters of the PICU patients were also recorded. Mann-Whitney U test and Kruskal-Wallis H test were used for statistical analysis because of non-parametric distribution of the data. Data is presented as median and interquartile range[IQR].
**RESULTS.** The levels of basal p62 expression were comparable between PICU and control group (98.7 [88.0-111.1] vs 91.9 [86.4-104.5] A.U., p=0.13). No difference in autophagy flux was observed between the PICU and control group (44.4 [19.3-76.6] vs 54.7 [25.9-78.0] %control, p=0.21). However, the PICU group had more outliers of autophagy flux, with 13% (n=11) of values lower than the mean minus SD (blockers) and 15% (n=12) of values higher than the mean plus SD (inducers). The blockers group had significantly higher p62 levels than the non-responder group (autophagy flux within mean ± SD, n=59) and the inducers group (121.5 [100.7-164.2] vs 98.3 [90.6-110.3] vs 87.8 [82.2-99.6] A.U. respectively, p=0.015). There was no difference in terms of number of organ failure among these groups (p=0.675); however, days of mechanical ventilation tended to be higher in the blockers group (7 [4-18.5] vs 3 [0.3-7] vs 2.5 [0-4] days respectively, p=0.07).
**CONCLUSION.** We observed a variation of autophagy in muscle cells modulated by serum from critically ill children with 13% of serum initiating a block in autophagy.

1. This work was supported by the Swedish Research Council.

### 001538

#### Right Data, Right Now: paving the way for implementation of a machine learning ICU readmission model by validating on external datasets and adjusting for concept shift

PJ. Thoral[1], M. Fornasa[2], AM. Curth[2], DP. De Bruin[2], H. Hovenkamp[2], RH. Driessen[1], A. Girbes, PWG. Elbers[1]
[1]Department of intensive care medicine, Amsterdam UMC, Vrije Universiteit Amsterdam, Amsterdam, Netherlands; [2]Pacmed.ai, Pacmed B.V., Amsterdam, Netherlands
**Correspondence:** P.J. Thoral
*Intensive Care Medicine Experimental* 2019, **7(Suppl 3):**001538

**INTRODUCTION.** Transfer of care from the ICU to the ward may lead to preventable errors and adverse events. In particular, unexpected ICU readmission is associated with longer length of stay and an increase in mortality.[1] Many prediction models have been developed but none are currently widely implemented to prevent ICU readmission. Our recently developed model employed extensive feature engineering and start-of-the-art machine learning algorithms and achieved an ROC AUC of 0.82. (P.J. Thoral, et al, ESICM LIVES 2018 Meeting Abstract. Manuscript in preparation). An important part of implementation is applying the model to external datasets, different from and independent of the one it was originally trained on, in order to both validate the model and increase its versatility and value.

**OBJECTIVES.** To increase the performance and implementability of our prediction model for ICU readmission and mortality by extending the training dataset with clinical data collected during the last two years in the electronic health records (EHR) at the ICU of Amsterdam UMC, as well as datasets from other Dutch ICUs.

**METHODS.** Our model was developed using patient data from 2004 to 2016 extracted from our Patient Data Management System (MetaVision, iMDsoft). We tested performance of our model on data extracted from our current EHR (EpicCare, Epic Systems) from 2016-2018 and are currently testing performance on data from other Dutch ICU's. Since a change in clinical practice associated with transition to another EHR (e.g. different standardization of orders using order sets) and different patient populations between ICUs will lead to datasets with features characterized by different distributions, we explore multiple methods suggested in the literature to ensure external validity.[2, 3] To address possible covariate shift caused by different patient populations, we implement importance weighting to better align feature distributions between ICUs. To address potential concept shift, due to, for example, differences in practice, we add data from multiple sources to the training set and restrict the feature set to features that are expected to generalize well. Code for model development and analysis was written in Python using the scikit-learn package.

**RESULTS.**

Applying our model without adjustments to our current EHR results in a lower ROC AUC of 0.77 with an average precision (AP) on the precision-recall curve (PRC) of 0.21. (Fig. 1 and 2). Through careful feature selection, combination of training data and domain shift mitigation techniques we improve the model performance and increase the ROC AUC to 0.83 and the AP to 0.26. Results for validation on and combination with datasets from other Dutch ICUs are pending.

**CONCLUSION.** Our validation approach overcomes the problem of concept shift due to datasets originating from different sources and leads to a model with increased performance and versatility that is ready for implementation.

**REFERENCE(S)**

1. Chen LM, Martin CM, Keenan SP, Sibbald WJ (1998) Patients readmitted to the intensive care unit during the same hospitalization: Clinical features and outcomes. Crit Care Med 26:1834–1841. https://doi.org/10.1097/00003246-199811000-00025
2. Daumé H (2009) Frustratingly Easy Domain Adaptation. http://arxiv.org/abs/0907.1815
3. Kouw WM, Loog M (2018) An introduction to domain adaptation and transfer learning. http://arxiv.org/abs/1812.11806

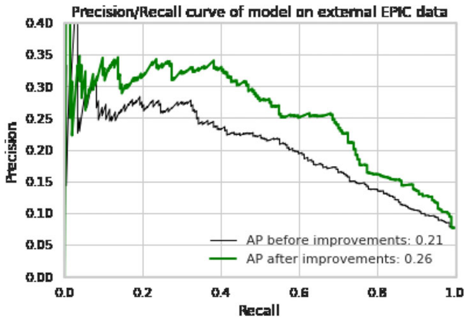

**Fig. 1 (abstract 001538).** See text for description

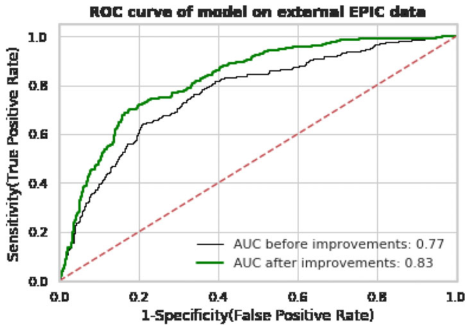

**Fig. 2 (abstract 001538).** See text for description

### 001514

#### Experience in cytokine release syndrome after CAR-T cells therapy in a pediatric intensive care unit

S. Bobillo[1], M. Balaguer[1], A. Català[2], P. Castro[3], A. Alonso[2], C. Llanos[4], M. Torrebadell[5], FJ. Cambra[1], M. Juan[6], J. Delgado[3], S. Rives[2], I. Jordan[1]
[1]Picu, H Sant Joan de Déu, Barcelona, Spain; [2]Hematology and oncology, Hospital Sant Joan de Déu Barcelona, Esplugues de Llobregat, Spain; [3]Hematology, Hospital Clinic, Barcelona, Spain, Spain; [4]Unitat de recerca, H Sant Joan de Déu, Barcelona, Spain; [5]Laboratory, H Sant Joan de Déu, Barcelona, Spain; [6]Immunology, Hospital Clinic, Barcelona, Spain, Spain
**Correspondence:** S. Bobillo

*Intensive Care Medicine Experimental* 2019, **7(Suppl 3):**001514

**INTRODUCTION.** Immunotherapy with CAR-T cells is a new approach to treat B-cell lymphoblastic leukemia in children and young adults. The most important acute adverse event is the cytokine release syndrome (CRS).

**OBJECTIVES.** To evaluate the CRS after infusion of CAR-T cells in children that required admission to the pediatric intensive care unit (PICU).

**METHODS.** Retrospective review of the patients that had received CAR-T therapy between April 2016 to February 2018 and needed PICU due to the CRS. During these period two different CAR-T cells therapies were used (Group 1 and group 2), both with 4-1BB as costimulatory signal. The CRS was graded using the Penn definitions.

**RESULTS.** Twenty-five patients were treated with CAR-T cells, 2 required a reinfusion. Seven patients required intensive care due to CRS (7/27, 25.9%). Four were girls (57.1%), and the median age was 8 years (IQR 6-19). Five patients were in the group 1 and 2 in the group 2. Six patients presented a high percentage of blasts before infusion.

In group 1 there were 3 girls (60%) and the median age was 8 years (IQR 7-21.5). The CRS began in the 3th day (IQR 2.5-4.5) and the median day since the infusion until the admission to the PICU was 6 day (IQR 3.5-6). Four presented CRS grade 3 (80%) and one presented CRS grade 4 (20%). All required inotropic support with a median inotropic score of 15 (IQR 9-92.5). None required mechanical ventilation. Three patients received one dose of tocilizumab (60%) in the day 1 of admission to the PICU. Two patients (40%) received steroids (prednisone) in the day 2.5. Three patients (60%) presented mild neurological impartment. The median days in the PICU was 5 (IQR 2-6.5) and in hospital was 31 days (IQR 15-47.5).

In group 2 there were 1 girl and 1 boy with an age of 4 and 11 years old, respectively. Both patients began the CRS symptoms in the day of infusion and were admitted to the PICU at the day 2 and day 1 after infusion. The maxim CRS were grade 4 and 5, respectively, and the second patient presented mild neurological toxicity. Both required inotropic support and mechanical ventilation. They were intubated at the second and third day after be admitted to the PICU. Both received 2 dose of tocilizumab and prednisone. The first patient was extubated at the day 5 and was transferred to the ward at the day 10th. The second patient behaved like a hemophagocytic lymphohistiocytosis and died due to the CRS at the 4th day of admission. This patient presented higher values of procalcitonin, lactate and ferritin.

The global survival of the CRS was 85.7%, considering all the infusion of CART-T cells 96.3% (26/27).

**CONCLUSION.** CRS was a frequent adverse event after infusion CAR-T therapy. In a third cases needed admission to the PICU. Tocilizumab was administered in CRS 3 and 4, always before steroids. The most severe CRS began with symptoms in the first day of infusion and needed two dose of tocilizumab. The survival of CRS was high.

**REFERENCE(S)**

1. Nat Rev Clin Oncol. 2019 Jan;16(1):45-63. doi: 10.1038/s41571-018-0075-2. Management guidelines for paediatric patients receiving chimeric antigen receptor T cell therapy. Mahadeo KM
2. N Engl J Med. 2018 Feb 1;378(5):439-448. doi: 10.1056/NEJMoa1709866. Tisagenlecleucel in Children and Young Adults with B-Cell Lymphoblastic Leukemia. Maude SL

### 001497

#### Right Data, Right Now: Improving a machine learning based ICU readmission tool by targeting model explainability and software usability with end-user testing

AA. De Beer[1], PJ. Thoral[2], H. Hovenkamp[1], WJ. Van Den Wildenberg[3], M. Platenkamp[4], A. Girbes, PWG. Elbers[2]

[1]Pacmed.ai, Pacmed B.V., Amsterdam, Netherlands; [2]Department of intensive care medicine, Amsterdam UMC, Vrije Universiteit Amsterdam, Amsterdam, Netherlands; [3]Department of intensive care medicine, Elisabeth-TweeSteden Ziekenhuis, Tilburg, Netherlands; [4]Department of intensive care medicine, University Medical Centre Utrecht, Utrecht University, Utrecht, Netherlands

**Correspondence:** P.J. Thoral

*Intensive Care Medicine Experimental* 2019, **7(Suppl 3):**001497

**INTRODUCTION.** Clinical data science, and machine learning in particular, is on the rise with new ICU related prediction models being published continuously. However, few models make it to the bedside and are able to influence clinical decision making. Recently, we developed a machine learning based ICU readmission model (P.J. Thoral, et al, ESICM LIVES 2018 Meeting Abstract. Manuscript in preparation). We hypothesize that a tool based on our model should be adapted to be explainable to the intensivist and offer an intuitive user interface (UI).

**OBJECTIVES.**

To test both model output explainability from a software prototype based on our ICU readmission model and usability of the UI.

**METHODS.** A two-phase multicentre usability study was performed at the Amsterdam University Medical Center, location VUmc, Amsterdam, the Elisabeth-TweeSteden Ziekenhuis, Tilburg and the University Medical Center Utrecht, Utrecht. The study was performed using a software prototype of our ICU readmission model showing twelve representative patients, predicting the combined 7-day risk of ICU readmission or mortality on the moment of ICU discharge (see Figures 1 and 2 ).

As part of the technical file for CE application of the software, the study was set up according to the applicable guideline[1]. In phase 1 the software was tested by scripted end-user interaction. Results of these tests were used to improve the software. In phase 2 the improved software was tested. Both phases focused on the users' understanding of the model predictions and the user

-friendliness of the interface design. All responses were scored against the requirements of the software in order to evaluate safe use of the tool.

**RESULTS.** Phase 1 was completed by 15 individual intensivists; the second phase by six new and three recurrent intensivists. In phase 1 the explainability of the predictive features shown per patient was insufficient with the average number of difficult to interpret features being 4.9 out of 10 displayed features.

Table 1 shows the identified problems.

Furthermore, the graphical indicators and their meaning for specific ICU supportive care were potentially confusing. Based on these results explainability was improved by removing some less predictive features and by simplifying feature aggregates. The UI was enhanced by changing the display of feature importance and the supportive care indicators. In phase 2, after these modifications, the participants showed better understanding of the model output. The average number of clinically difficult to interpret features dropped to 2.2/10. In both phases all safety related equirements were met, meaning that >90% of participants correctly understood and applied the intended purpose of the tool.

**CONCLUSION.** We evaluated the use of an ICU readmission model in a software prototype and improved both the explainability of the model and usability of the software towards a valuable tool intensivists can understand and safely use in their decision on discharging a patient from the ICU.

**Fig. 1 (abstract 001497).** See text for description

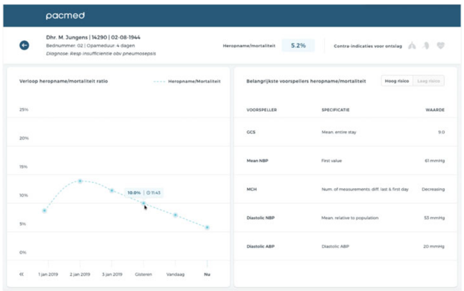

**Fig. 1 (abstract 001497).** See text for description

**Table 1 (abstract 001497).** See text for description

| Base Feature | Potential issue |
|---|---|
| Origin department | Poor generalizability when used as a substitute for admission diagnosis. |
| Serum urea | Poor generalizability when not measured frequently in all ICU's |
| Serum albumin | Unsafe use: may lead to clinicians directly targeting albumin (e.g. by albumin infusion) |
| Ionised calcium | Difficult to appreciate clinically since is often targeted directly (e.g. during renal replacement therapy) |
| **Feature aggregation** | **Potential issue** |
| Average over whole admission | Difficult to interpret since these averages are often not known by clinicians |
| Minimum first 24hrs – minimum last 24hrs<br>Maximum first 24hrs – maximum last 24hrs<br>SD first 24hrs – SD last 24hrs<br>Slope first 24hrs – slope last 24hrs | Difficult to interpret without absolute values and reference values. |
| Minimum first 24hrs<br>Maximum first 24hrs<br>Standard deviation first 24hrs<br>Slope first 24hrs | Difficult to interpret without absolute values or trend of the value. Disagreement among clinicians whether this is clinically relevant for patients with longer length of stay |
| Standard deviation last 24hrs<br>Slope last 24hrs | Difficult to interpret without absolute value or trend of the value |
| Binary: measured in first/last 24 hours?<br>Binary: at least measured once during admission? | Poor generalizability: could be process dependent, e.g. dependent on ICU routines and customs |

### 001720

#### Are we following the best practice to prevent Intensive Care Unit – acquired weakness, shoulder subluxation and ankle limitations

B. Mariano[1], G. Burgio[1], F. Rubulotta[2]
[1]Anesthesia and intensive care unit, IRCCS-ISMETT, Palermo, Italy; [2]{street_address}, London, UK
**Correspondence:** F. Rubulotta
*Intensive Care Medicine Experimental* 2019, **7(Suppl 3):**001720

**INTRODUCTION.** Intensive Care Unit –acquired weakness (ICUAW) is a clinical syndrome that develops while a patient is critically ill. In a recent multi-centre cohort study, ICUAW was present in more than 50% of patients at ICU discharge. In an official American Thoracic Society Clinical Practice Guideline, ICUAW was reported to be more common in patients with severe sepsis, prolonged mechanical ventilation, or difficulty with liberation from mechanical ventilation. There is no consensus on the approach to diagnosis of ICUAW. Physical examination of three muscle groups in each of the upper and lower limbs has been developped by the medical research council (MRC) in the UK and it results in a composite score of 60. This method has been proven reliable in a cooperative patient. In a prospective, multicentre cohort study, hand-held dynamometry was used in non cooperative patients to identify ICUAW and this method was independently associated with increased hospital mortality in patients with ICUAW. The leuven protocol is the most frequently used in ICU and it also correlates with mortanily.

**OBJECTIVES.** To identify the incidence of ICU-AW in patients admitted to a tertiary neursugical ICU in a large teaching hospital. To implement measures to prevent ICU-AW

**METHODS.** The study was conducted in a 24 beds neuro ICU in a teaching hospital. This is an adult ICU admitting over 2000 neurneurosulgical patients per year and with over 200 cases per year remaining in the ICU for over 7 days. One doctor and one physiotherapist performed daily rounds to assess the incidence of ICU AW, shoulder subluxation and ankle limitations in all neurosurgical patients. General characteristics were collected including age, gender, reason for admission, length of ICU stay, others. The Leuven protocol and planning was used to assess ICU-AW. Investigators collected data related to the position of the patient, presence or supporting devices for the shoulders, for the ankles, and the time and duration of physiotherapy provided every day. Data collected were daily compared and the diagnosis was agreed among doctors and the rehabilitation team. The nurses educators were involved in implementing new meaasures to prevent ICU-AW and to support the rehabilitation team .

**RESULTS.** Data were collected between the 13th decemebr and the 11 of November 2018. Interventions to reduce the incidence of ICU AW were implemented in January 2019. Data were anonymous and patients consented for the study. 101 measurements were performed in 25 patients. 100% of the patients had low cooperation (0-2) and low MRC score. 28% of the patients had shoulder subluxation at ICU discharge. 36% of the patients had ankle limitations (of whom 5% bilateral). Doctor was more likely to detect the shoulder subluxation (80% of relevations) and physiotherrapists the ankle limitations (65%). Nurses were not aware of this problem and they did not felt this could affect outcome.

**CONCLUSION.** ICU-AW and limitations are frequent in long standing neuro-surgical patients. Nursing staff is not aware and physiotherapy cannot prevent alone this complication. Combined complex interventions are needed to prevent ICU-AW. The nursing staff should be trained in positioning adequately the patient and support shoulders and ankles. Pillow and simple support devices are sufficient. Physiotherapy cannot provide enough resources to prevent these complications and these are frequently not diagnosed. Doctors should be trained in examining and recognise ICU AW because mortality is associated with the presence of sub-luxations or ankle limitations at ICU discharge. The Leuven protocol is ideal because it has a progression and an intervention scale which is easy to use. However, awareness and training of the staff in the ICU is needed.

1. Charing Cross Hospital nursing and physiotherapy staff

### 001731

#### Develop a "Therapeutic Nurse Relationship Program" to improve communication between nurses, patient, patient's relatives in ICU

NW. Chu, ST. Poon, SFL. Tang, HW. Cheng, BKG. Chan, WY. Ho, CM. Leung, NL. Tse, KYR. Cheung, E. Cham, KH. Chau, CK. Chan, WH. Chu, PN. Lau, SH. Siu, KM. Ho
[1]Icu, Prince of Wales Hospital, Hong Kong, Hong Kong
**Correspondence:** N.W. Chu
*Intensive Care Medicine Experimental* 2019, **7(Suppl 3):**001731

**INTRODUCTION.** ICU nurses are sometimes criticized as "high tech. & low touch" in the medical literature, which may create patient and nurse dissatisfaction patient but also to nurses. Stayt (2007) studied ICU nurse's emotional experiences and reflected ICU nurses face a fundamental conflict both between the role expectations of patient care, professional ideals and being a human which may contribute to occupational stress.

**OBJECTIVES.** To test a "Therapeutic Nurse Relationship Program" designed to improve nursing communication skill with patients and relatives in ICU

**METHODS.** From Nov.2018, a pre-survey was conducted to explore nurse communication within established therapeutic relationships with patients/families. Inclusion criteria: patient ICU stay > 72 hours, either patient or relatives were recruited by convenience sampling.

The survey questions included 6 questions used an anonymous 5-item questionnaire with a 5-point Likert response scale (1=poor to 5=excellent).
It focused on nursing communication with patient / relatives regarding: 1. Explanation before performing a procedure (such as suction/turning), 2. Care about patient's comfort, 3. Proactively updating the condition, 4. Providing psychological / mental support, 5. Offering religion support / referral, and 6. Overall satisfaction to nursing care
3 qualitative questions were also used to explore the subject's views of nursing communication. A teaching program was conducted at Dec., 2018 & focused on: 1. Different skills on communication - including verbal & non-verbal, and 2. Promotion of various referral service from hospital
79 (70%) of nurses attended the program and a post survey was conducted from Jan.- Feb.,2019 for comparison.
**RESULTS**
Data in table one shows that there was a substantial increase in positive response in three areas: 1. Providing explanation to patient / relatives before perform procedure (from 65% to 90%), 2. Providing psychological / mental support (from 55% to 70%), and 3. Offer religion support / referral (from 15% to 25%).
There was no substantial increase in the other domains, although overall baseline responses in the remaining domains were already good (>70%). Overall satisfaction baseline responses (excellent & very good) was already 80%, and increased to 90%.
**CONCLUSION.** The post-survey demonstrated a more positive feedback from patient's relatives than the pre-survey. This positive trend was confirmed in the in qualitative descriptions. It suggests the program may enhance nursing communication to improve the therapeutic relationship between nurses to patient & patient's relatives.

**Table 1 (abstract 001731).** Results of pre-intervention and post-intervention survey

| Questions: | Pre-test (% rated excellent OR very good) | Post-test (% rated on excellent OR very good) |
|---|---|---|
| 1.Explain before perform procedure (such as suction / turning) | 65% | 90% |
| 2.Care about patient's comfort | 70% | 75% |
| 3.Proactively update the condition | 75% | 80% |
| 4.Provide psychological / mental support | 55% | 75% |
| 5.Offer religion support / referral | 15% | 25% |
| 6.Overall satisfaction to nursing care | 80% | 90% |

### 001735

#### Machine learning algorithm to predict 30 day mortality of post cardiac arrest patients admitted to intensive care unit

K. MOHEE[1], A. Anastasiou[2], H. Haboubi[3], M. Protty[4], S. Pillai[5]
[1]Ed major critical care unit, Morriston Hospital, Treforys, UK; [2]Health data science, Swansea University, Swansea, UK; [3]Medical school, Swansea University, Swansea, UK; [4]Systems immunity university research institute, Cardiff University, Cardiff, UK; [5]Ed major critical care unit, Morriston Hospital, Morriston, UK
**Correspondence:** K. MOHEE
*Intensive Care Medicine Experimental* 2019, **7(Suppl 3):**001735

**INTRODUCTION.** Cardiac arrest (CA) is a leading cause of mortality in Europe and United States. The average survival to discharge rate in out-of-hospital cardiac arrest patients (OHCA) is approximately 10%, in contrast to in-hospital cardiac arrest (IHCA) where it is 17-20%. The risk of neurological disability remains high among survivors in both groups.
Machine Learning (ML) is a type of artificial intelligence that provides systems the ability to automatically learn and improve from experience without being explicitly programmed.
**OBJECTIVES.** The aim of this study was to develop a type of ML algorithm using data from the Wardwatcher Intensive Care Unit (ICU) Swansea database to predict 30-day survival of patients admitted to ICU post cardiac arrest.
**METHODS.** All cardiac arrest patients admitted to our regional intensive care unit in South West Wales, United Kingdom between January 2007 and June 2018 were included in this analysis. Data included were age, gender, comorbidities, biochemical and haematological results as well as temperature within 24h of admission, length of stay, follow-up time and survival status.
**RESULTS.** Of the 1034 patients (532 OHCA and 502 IHCA) included in the study, 600 (58%) patients died within 30 days. Random forest (RF) was used as the type of ML algorithm. The area under the receiver operating characteristic curve (AUROC) and area under the precision-recall curve (AUPRC) predicting survival within 30 days of admission was AUROC: 0.89 and AUPRC: 0.91 (.80-.90= good and .90-1= excellent). For patients admitted with OHCA alone, the same RF algorithm showed AUROC: 0.86 and AUPRC: 0.91. The ML modelling took 0.99-2.32 seconds to be built.
**CONCLUSION.** ML modelling of the data of post cardiac arrest patients admitted to ICU can predict 30-day survival with a reasonable level of accuracy. Therefore, the data obtained from ML modelling may be used as an adjunct in making treatment decisions in these group of patients, however larger studies comparing different ML modelling are required to establish this.

### 001751

#### Concordance of prospectively assessed comorbidities of the Charlson comorbidity index with routinely documented ICD-10 codes in electronic health records

AS. Poncette[1], G. Vorderwülbecke[1], PB. Opitz[2], B. Weiss[1], D. Fürstenau[2], F. Balzer[1]
[1]Department for anesthesiology and intensive care medicine, Charité–Universitätsmedizin Berlin, Berlin, Germany; [2]Department of information systems, Freie Universität Berlin, School of Business & Economics, Berlin, Germany
**Correspondence:** A.S. Poncette
*Intensive Care Medicine Experimental* 2019, **7(Suppl 3):**001751

**INTRODUCTION.** Coding of diagnoses based on the International Classification of Diseases (ICD) is a world-wide standard used for the identification of health trends and statistics. Coded data is used for multiple purposes including billing, health care allocation, and research, presenting thus an urgent need for valid routine data. However, data validation of ICD-10 routine data in relation to the completeness and correctness of comorbidity documentation is rare.
**OBJECTIVES.** Purpose of this study is to examine whether co-morbidities of the Charlson-Comorbidity Index (CCI) that are derived from routinely documented ICD-10 codes in patient records correspond to manually assessed co-morbidities by a health professional on the basis of anamnesis data.
**METHODS.** This is a secondary analysis of the FRAIL-AMB project, a prospective observational study of elderly patients undergoing surgery in a German university hospital between 2017 and 2018. Ethical approval from the local authorities was obtained (EA1/227/16). The CCI was calculated according to Quan et al. from the ICD-10 coded routine data and compared to prospectively collected data from the FRAIL-AMB project that was defined as gold standard. We drew on result pair analyses.
**RESULTS.** A total of 1,186 patients were included into analyses. CCI completeness of 52% (95% CI: 51.4-52.7) and a correctness of 60.5% (95% CI: 59.9-61.2) was calculated for all comorbidity classes in the ICD-coded routine data (Figure 1, SAP_CCI1=ICD-coded routine data). Overall, a general undercoding was found.

**CONCLUSION.** The study suggests that ICD-10 routine data cannot provide consistently reliable information on all CCI comorbidity classes and should be used with caution. In a next step, improvement potentials of critical CCI comorbidity classes should be investigated.

**REFERENCE(S)**

1. Stausberg, J. Bundesgesundheitsbl. (2007) 50: 1039. https://doi.org/10.1007/s00103-007-0296-5
2. Quan, H., Li, B., Couris, C.M., Fushimi, K., Graham, P., Hider, P., Januel, J.-M., Sundararajan, V.: Updating and Validating the Charlson Comorbidity Index and Score for Risk Adjustment in Hospital Discharge Abstracts Using Data From 6 Countries. Am. J. Epidemiol. 173, 676–682 (2011). doi:10.1093/aje/kwq433
3. Quan, H., Sundararajan, V., Halfon, P., Fong, A., Burnand, B., Luthi, J.-C., Saunders, L.D., Beck, C.A., Feasby, T.E., Ghali, W.A.: Coding algorithms for defining comorbidities in ICD-9-CM and ICD-10 administrative data. Med. Care. 43, 1130–1139 (2005)
4. https://www.who.int/classifications/icd/factsheet/en/
5. This work has been supported by an institutional grant of Charité - Universitätsmedizin Berlin and Einstein Center Digital Future.

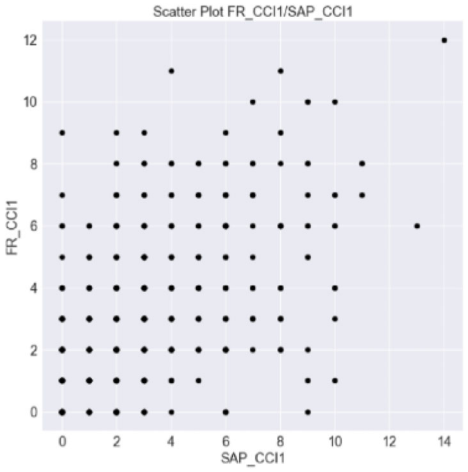

**Fig. 1 (abstract 001751).** See text for description

### 001755

### Association of mean arterial pressure and acute kidney injury after high risk surgery

M. Greco, S. Falini, G. Angelotti, M. Cecconi
[1]Department of anesthesia and intensive care, Humanitas Clinical and Research Center, Milano, Italy
**Correspondence:** M. Greco
*Intensive Care Medicine Experimental* 2019, **7(Suppl 3):**001755

**INTRODUCTION.** Acute Kidney Injury (AKI) is a frequent and severe complication after major surgery, and is a common critical care outreach call and a frequent reason for ICU admission. With millions of surgeries performed worldwide every year, AKI have a high impact in terms of morbidity, mortality and costs. According to KDIGO guidelines, AKI is amenable to prevention, treatment and resolution (1). However, early AKI detection and patients stratification in standard surgical wards remains a significant challenge.

**OBJECTIVES.** To assess if mean arterial pressure (MAP) is associated with AKI after high-risk surgery

**METHODS.** Deidentified data from Electronic Health Records (EHR) of a large teaching hospital were included in the analysis. We selected a mixed surgical population of 1700 adult patients undergoing high risk vascular and thoracic surgery from Jan 2015 to Dec 2018. AKI was defined according to KDIGO guidelines (1), applying urinary and creatinine criteria to vitals and laboratory data derived from EHR. A subset including the first 7 postoperative days (POD) was selected for the analysis. MAP was estimated from systolic and diastolic pressure. Standard statistical regressions and machine learning techniques were applied using R (R Core Team 2019) for data analysis.

**RESULTS.** 1441 patients were finally included in the analysis, with 245 patients (17%) developing AKI. Mean MAP was lower in patients with AKI compared to controls on POD 1, 2 and 3, while there was no difference on the day of surgery or after POD 4. (Fig 1). A similar pattern was detected when plotting mean MAP up to AKI development against control MAP (Fig2). The association between mean MAP and AKI was confirmed when controlling for baseline variables and type of surgery in a logistic regression model, (OR 0.96 [0.94-.98]), while statistical significance was lost when adjusting for panel data. Machine learning models resulted in lower accuracy than standard models.

**CONCLUSION.** MAP in the first days after high-risk surgery is associated with AKI development. While both AKI and lower MAP can reflect an insult occurred during surgery, models based on EHR data may permit early stratification and detection of AKI, allowing to start timely treatment.

**Fig. 1 (abstract 001755).** See text for description

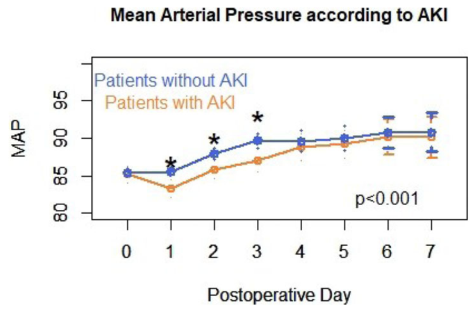

**Fig. 2 (abstract 001755).** See text for description

## CD / AKI - Haemodynamic and kidney issues in the ICU

### 001301

#### The complex effect of protamine on platelet function: an in vitro study using impedance aggregometry and flow cytometry

M. Törnudd[1], S. Ramström[2], JP. Kvitting[3], J. Alfredsson[4], S. Berg[5]
[1]Department of Cardiothoracic and Vascular Surgery and Department of Medical and Health Sciences, University hospital, Linköping, Sweden; [2]Cardiovascular research centre, school of medical sciences, Örebro University, Örebro, Sweden; [3]Department of cardiothoracic surgery, Oslo University Hospital, Oslo, Norway; [4]Department of cardiology and department of medical and health sciences, University hospital, Linköping, Sweden; [5]Department of cardiothoracic and vascular and department of medical and health sciences, Linkoping University Hospital, Linköping, Sweden
**Correspondence:** M. Törnudd
*Intensive Care Medicine Experimental* 2019, **7(Suppl 3):**001301

**INTRODUCTION.** In cardiac surgery, heparin and protamine are fundamental for management of anticoagulation, and its management is known to affect bleeding. The effect of protamine on platelet function, however, is not fully understood. In previous *in vivo*-studies(1) using impedance aggregometry, a decrease in platelet function after exposure to protamine has been seen.

**OBJECTIVES.** The aim of this study was to further examine the effect of protamine on platelet function *in vitro,* using both aggregometry and flow cytometry.

**METHODS.** Seven elective CABG patients were studied using impedance aggregometry (Multiplate). Blood was mixed with protamine to a concentration of 0, 20, 40 and 80 µg/ml. Adenosine diphosphate (ADP) was used as activator and aggregation was expressed as the area under the curve (AUC).

Blood from 7 healthy volunteers was studied using flow cytometry with protamine added in the same concentrations as above, either alone or together with ADP. Median fluorescence intensity (MFI) from the monoconal antibody PAC-1-FITC, which binds to the activated fibrinogen receptor on activated platelets, was studied. We also studied expression of P-selectin using the antibody anti-P-selectin-PE (CD62P, clone: AK-4).

Statistics used were ANOVA and paired T-test. Data are expressed as mean ± standard deviation.

**RESULTS.** With aggregometry, mean AUC for platelets activated by ADP decreased with protamine from 73.8 ± 29.4 U (no protamine) to 61.8 ± 29.4 U (20 µg/ml), 51.7 ± 26.8 U (40 µg/ml), and 46.9 ± 26.1 U (80 µg/ml); p= 0.003. With flow cytometry, when activated with ADP, protamine reduced platelet activation seen by MFI from 5.1± 2.0 (no protamine) to 4.1 ± 0.7 (20 µg/ml), and 3.1 ± 0.4 (40 µg/ml); p=0.02. When only activated with protamine, mean MFI increased from 0.4 ± 0.04 (no protamine) to 0.7± 0.2 (20 µg/ml), 1.9± 1.0 (40 µg/ml), and 3.1± 0.8 (80 µg/ml); p<0,001. At 80 µg/ml protamine, mean MFI was similar both with and without activation with ADP (3.4 ± 0.2 and 3.1 ± 0.8, respectively).

Protamine with and without any activator, produced a significant concentration-dependent increase in the proportion of platelets exposing P-selectin, which indicates release of alfa-granule. The mean proportion of P-selectin-positive platelets when no other activator was used, increased from 4.0 ± 2.7 % (no protamine) to 95.3 ± 3.0 %(80 µg/ml); p<0.001. When activated with ADP, the proportion increased from 22.8 ± 17.6 % (no protamine) to 95.8 ± 2.5 % (80 µg/ml); p<0.001.

**CONCLUSION.** Protamine *in vitro* interacts with platelets with both a direct activating effect and impairment of activation by other activators. This impairment is consistent with previous *in vivo*-data showing reduced platelet function after protamine exposure. The direct activating effect may have significance for the risk of thrombosis in cardiac surgery.

### 001336

#### Effect of albumin based resuscitation on spinal cord microcirculation in an experimental model of shock

M. Gräßler[1], S. Wipper[2], C. Behem[1], R. Kluttig[1], D. Hinck[3], C. Trepte[1]
[1]Department of anaesthesiology, University Medical Center Hamburg-Eppendorf, Hamburg, Germany; [2]Department of vascular surgery, University Heart Center Hamburg GmbH, Hamburg, Germany; [3]Department of general and vascular surgery, BWK Hamburg, Hamburg, Germany
**Correspondence:** M. Gräßler
*Intensive Care Medicine Experimental* 2019, **7(Suppl 3):**001336

**INTRODUCTION.** Alterations in microcirculation have been shown to influence patient outcome and survival in different types of shock. [1] Spinal cord is extremely sensitive to changes in microcirculation and spinal cord hypoperfusion or ischemia with consecutive paraplegia are major complications in thoraco-abdominal aortic repair [2] The positive effects of albumin administration on macro and microcirculatory state in shock have recently been described [3]. However none of this studies primarily focused on the effects on spinal cord microcirculation.

**OBJECTIVES.** To date the direct effect of albumin administration on spinal cord microcirculation has not been systematically assessed. Therefore the aim of this study was to test whether a albumin based volume resuscitation can improve spinal cord microcirculation in a experimental model of shock

**METHODS.** The study was designed as prospective trial in 10 domestic pigs. Animals were anaesthetized and mechanically ventilated. A laser – Doppler fluxmetry (LDF) Needle probe was directly placed in the lumbar spinal cord for measurement of microcirculatory blood flow (mFLUX). After baseline measurements (M0) . Shock was in induced by hypovolemia (M1) (withdrawal of blood 20ml/kg bodyweight). Thereafter volume loading was performed using 5 % human albumin until no positive macro-hemodynamic response to volume administration occurred (M2). An increase in stroke volume >10% was considered as a positive volume response.

**RESULTS.** Spinal cord mFLUX significantly decreased (47,8 at M0 to 22,7 at M1 p<0.05) during shock and was significantly increased after albumin administration ( 22,7 at M1 to 51,2 at M2 p<0.05). Furhtermore there was no significant difference between the mFLUX values at M2 and baseline.

**CONCLUSION.** In our experimental animal model albumin based volume resuscitation was able to improve spinal cord microcirculation.

##### REFERENCE(S)

1. Sakr Y, Dubois MJ, De Backer D, Creteur J, Vincent JL. Persistent microcirculatory alterations are associated with organ failure and death in patients with septic shock. Crit care med 2004;32:1825-31.
2. Augoustides JG, Stone ME, Drenger B. Novel approaches to spinal cord protection during thoracoabdominal aortic interventions. Curr Opin Anaethesiol 2014;27:98-105
3. Vincent JL, De Backer D, Wiedermann CJ Fluid management in sepsis: The potential beneficial effects of albumin J Crit Care. 2016 Oct;35:161-7.

### 001340

#### Protein kinase C expression and cardioprotective effects of remote ischemic preconditioning in cardiac surgery

D. Tashkhanov, A. Bautin, S. Datsenko, A. Marichev, A. Radovskiy
[1]Anesthesiology and intensive care medicine, National Medical Research Center. VA Almazov, Sankt-Peterburg, Russia
**Correspondence:** D. Tashkhanov
*Intensive Care Medicine Experimental* 2019, **7(Suppl 3):**001340

**INTRODUCTION.** Remote ischemic preconditioning is a perspective method of cardioprotection.

**OBJECTIVES.** To evaluate cardioprotective effects of remote ischemic preconditioning (RIPC) and myocardial protein kinase C epsilon (PKC-

ε) release in patients undergoing aortic valve replacement under different types of anesthesia.

**METHODS.** In prospective randomized study we enrolled 48 patients, aged 64(56;69) years, which were scheduled for elective aortic valve replacement using cardiopulmonary bypass (CPB). We divided them into 4 groups: 1) RIPC performed during propofol anesthesia (RIPC prop, n=12), 2) RIPC performed during sevoflurane anesthesia (RIPC sevo, n=12), 3) propofol anesthesia without RIPC (CONTROL prop, n=12), 4) sevoflurane anesthesia without RIPC (CONTROL sev, n=12). Initial data of participants was similar in all groups. RIPC protocol consisted of 3 simultaneous 5-min ischemic episodes of both lower limbs with 5-min reperfusion intervals. Right atrium myocardium incision biopsy was performed for PKC-ε expression assessment using Western immunoblot. Troponin I levels (cTnI) were measured before anesthesia induction, after 30 min, 6, 12, 24, 48 hours after CPB completion. Data were assessed using Mann-Whitney U-test and Newman-Keuls method for multigroup comparison. p<0.05 was considered significant. The data are presented as median (25th;75th percentile).

**RESULTS.** RIPC showed cardioprotective effects only after sevoflurane anesthesia: significant differences in cTnI were found between RIPC sevo and CONTROL sevo groups at 6, 12 and 24 hours: 1.68(1.28;2.09) ng/ml vs 3.66(2.07;4.49) ng/ml, respectively at 6 hours (p=0.04); 1.89(1.59;2.36) ng/ml vs 3.66(2.91;5.64) ng/ml, respectively at 12 hours (p=0.001); 1.68(1.55;2.23) ng/ml vs 3.32(2.10;5.46) ng/ml, respectively at 24 hours (p=0.01). There were no differences found in cTnI between RIPC prop and CONTROL prop groups in all timepoints. RIPC applied during sevoflurane anesthesia also significantly increased PKC-ε expression: 1221(921;1438)Units in CONTROL sev group vs 1882(1564;2131)Units in RIPC sevo group (p<0.05). Performing RIPC during propofol anesthesia was not associated with any significant difference in PKC-ε expression compared with control group: 620(436;782)Units in CONTROL prop versus 788(574;1063)Units in RIPC prop group. PKC-ε expression between control groups was significantly higher in sevoflurane anesthesia than propofol anesthesia.

**CONCLUSION.** RIPC showed cardioprotective effects only during sevoflurane anesthesia. This was confirmed by PKC-ε expression increase and lower value of cTnI.

**REFERENCE(S)**

1. Przyklenk K.,Bauer B.,Ovize M.,Kloner R.A.,Whittaker P. Regional ischemic 'preconditioning' protects remote virgin myocardium from subsequent sustained coronary occlusion//Circulation 1993,Vol.87(3):pp.893-899.
2. None

### 001344

#### Improvement in knowledge of fluid responsiveness assessment amongst healthcare workers in a district general hospital critical care unit

D. Wright[1], J. Morris[2], M. Alice[3], T. Samuels[1], P. Morgan[1]
[1]Intensive care, East Surrey Hospital, Redhill, UK; [2]Intensive care, Croydon University Hospital, Croydon, UK; [3]Icu, East Surrey Hospital, Redhill, UK
**Correspondence:** D. Wright
*Intensive Care Medicine Experimental* 2019, **7(Suppl 3):**001344

**INTRODUCTION.** The ability to assess and appropriately interpret a patient's fluid responsive status is a critical skill for all ICU staff when managing complex patients with hemodynamic compromise. There are several techniques of varying complexity available to assess preload responsiveness. Intravenous fluid administration is not risk-free and should only be used under the right circumstances.

**OBJECTIVES.** The primary outcome was to assess the overall change in knowledge about fluid responsiveness assessment amongst the ICU staff in response to interventional educational processes. Secondary outcomes looked at the changes amongst the varying groups and the change in opinion/practice following education.

**METHODS.** A 25-question survey was composed based on a landmark paper by Monnet et al and it was given out to all ICU staff, including consultants, trainees, nurses and medical students. Results from this were determined at baseline and after education by means of a fact sheet, a tutorial and it being a "hot topic of the week". Each stage of the project was administered over 2 weeks: that is, the first round of surveys was given out and collected over the first 2 weeks, then the educational processes were delivered over the following 2 weeks, and finally, the second round of surveys was given out and collected over the subsequent 2 weeks. There were 25 participants in the first round and 14 participants in the second round.

**RESULTS.**

**CONCLUSION.** There was an overall improvement in the knowledge of fluid responsiveness assessment in the critical care unit healthcare workers in response to the educational processes. There was a decrease in the number of people who used CVP, CXR and lactate in their assessment, for which there is no role in gauging fluid responsiveness. There was also a decrease in the number of people who used fluid challenges as their technique of choice with increased usage of other techniques, such as pulse pressure variation and passive leg raising. This highlighted an increase in awareness of the fact that a fluid challenge is a treatment in and of itself with associated risks and less invasive means should be used, where feasible. Review of cases of iatrogenic fluid overload is needed in the future to assess the impact of this increase in knowledge, change in opinion and change in practice of fluid responsiveness assessment on patient outcome.

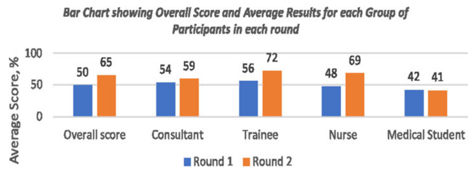

**Fig. 1 (abstract 001344).** See text for description

**Table 1 (abstract 001344).** Table showing the method of Assessment used and the opinion of their accuracy in assessing fluid responsiveness in each round

| Method of Assessment | Technique Used | | Opinion of Accuracy | |
|---|---|---|---|---|
| | Round 1 (%) | Round 2 (%) | Round 1 (%) | Round 2 (%) |
| Fluid Bolus | 100 | 93 | 32 | 50 |
| Transthoracic Echo | 12 | 14 | 24 | 29 |
| CXR | 8 | 0 | 0 | 0 |
| Lung ultrasound | 0 | 0 | 0 | 0 |
| Pulse Pressure Variation | 48 | 71 | 8 | 29 |
| Lactate | 68 | 36 | 12 | 7 |
| Central Venous Pressure | 48 | 29 | 20 | 0 |
| Passive leg raise | 44 | 71 | 8 | 14 |
| Pulse Contour Analysis | 60 | 57 | 60 | 43 |
| End-Expiratory Occlusion Test | 0 | 7 | 0 | 14 |

### 001473

#### Heart-lung interaction during whole lung lavage for alveolar proteinosis. An insight into transpulmonary pressure and early right ventricular impairment

V. Dammassa[1], G. Tavazzi[1], M. Pozzi[2], S. Bianzina[3], F. Torriglia[2], A. Orlando[1], M. Mazzocchi[1], G. Rodi[2], GA. Iotti[1], F. Mojoli[1]
[1]Department of clinical, surgical, diagnostic and pediatric sciences; intensive care unit, University of Pavia, Pavia, Italy; [2]Intensive care department, Fondazione Policlinico San Matteo IRCCS, Pavia, Italy; [3]Neonatal and Pediatric Intensive Care Unit, G. Gaslini Institute, Genoa, Italy, Genoa, Italy
**Correspondence:** V. Dammassa
*Intensive Care Medicine Experimental* 2019, **7(Suppl 3):**001473

**INTRODUCTION.** Right ventricular (RV) dysfunction has been largely investigated in ARDS in terms of acute cor pulmonale (ACP). However, ACP represents the last stage of RV failure. We aim to investigate early RV dysfunction signs with echocardiography in patients undertaking whole lung lavage (WLL) for pulmonary alveolar proteinosis (PAP).

**OBJECTIVES.** To evaluate the correlation between echocardiographic parameters of RV function and respiratory mechanics at different steps of transpulmonary pressure during WLL.

**METHODS.** Prospective observational study of patients undertaking WLL for PAP from January 2014 to March 2019. All patients were intubated with selective orotracheal tube. WLL entails the performance of alternatively single-lung ventilation while large-volume (20 L) lavages are instilled and drained on the non-ventilated lung along with chest physiotherapy and postural positioning. Calibrated values of oesophageal pressure were used as surrogate for pleural pressure (Ppl); transpulmonary pressure (PL) was calculated during end-inspiratory (insp) and end-expiratory (exp) occlusion manoeuvres (1). Paw is the airway pressure. Right ventricular longitudinal function (TAPSE) and the ratio between trans-tricuspid early diastolic flow (E wave) and tricuspid annular tissue velocity displacement (E′) were collected to evaluate respectively systolic and diastolic function of RV (2). Ratio of RV to LV end-diastolic area – RVEDA/LVEDA- is a marker of ventricular interdependence sensitive to RV afterload variations (3). Respiratory mechanics and echocardiographic parameters were collected at ZEEP (t0), at clinical PEEP right before the procedure (t1), after the first lung lavage (t2), and at end of procedure after the lavage of the second lung (t3).

**RESULTS.** Preliminary results on 13 patients (69% males). RVEDA/LVEDA ratio correlates with Paw insp (p < 0.0001, r 0.61), Paw exp (p < 0.0001, r 0.6), PL insp (p 0.0001, r 0.5) and PL exp (p 0.0006, r 0.46), but not with Ppl. RV E/E′ was related to Paw insp (p < 0.001, r 0.47), Paw exp (p< 0.001, r 0.49) and PL insp (p<0.001, r 0.5269). TAPSE was not related to respiratory mechanics parameters. **Table** Values of respiratory and echocardiographic variables tested: mean (± standard deviation)

**CONCLUSION.** In the setting of WLL, the increment in RVEDA/LVEDA and in E/E′ could be considered sensitive markers of early RV dysfunction. Absolute values of transpulmonary and alveolar pressures, and not their respiratory variations (the driving pressures), are correlated with RV function.

**REFERENCE(S)**

1. Mojoli F. et al. In vivo calibration of esophageal pressure in the mechanically ventilated patient makes measurements reliable. Critical Care (2016) 20:98
2. Rudski L.G. et al. Guidelines for the Echocardiographic Assessment of the Right Heart in Adults. J Am Soc Echocardiogr. 2010;23:685-713
3. Magder S. The left heart can only be as good as the right heart: determinants of function and dysfunction of the right ventricle. Crit Care Resusc. 2007 9:344–351

**Table 1 (abstract 001473).** See text for description

| Variables | t0 | t1 | t2 | t3 |
|---|---|---|---|---|
| Paw end-insp | 15,4 (±4) | 19,5 (±4,4) | 25,4 (±5,6) | 26,4 (±4,2) |
| Paw end-exp | 1,03 (±0,9) | 5,7 (±0,6) | 11,7 (±1) | 12,9 (±1,7) |
| PL insp | 8,5 (±3,9) | 11,3 (±5) | 16,3 (±3,9) | 16,8 (±3,6) |
| PL exp | -3 (±3,9) | -0,3 (±4,3) | 5,2 (±3) | 5,8 (±3,5) |
| RVEDA/LVEDA | 0,36 (±0,04) | 0,4 (±0,07) | 0,51 (±0,09) | 0,45 (±0,05) |
| E/E′ | 5,1 (±1,25) | 5,7 (±1,4) | 10,5 (±1,26) | 6,75 (±1,6) |
| TAPSE | 20,3 (±2,64) | 18,7 (±3,3) | 16,2 (±1,9) | 18,1 (±1,5) |

### 001475

#### The Use of Central Venous to Arterial Carbon Dioxide Tension Gap for Outcome Prediction in Critically Ill Patients. A systematic Review and Meta-analysis

Z. Al Duhailib[1], R. Lalli[2], K. Fiorini[3], F. Priestap[4], A. Hegazy[5], M. Slessarev[1]
[1]Adult critical care medicine, University of Western Ontario, London, ON, Canada; [2]Paediatric department, University of Western Ontario, London, ON, Canada; [3]Internal medicine, University of Western Ontario, London, ON, Canada; [4]Clinical epidemiology and biostatistics, University of Western Ontario, London, ON, Canada; [5]Adult critical care medicine and anaesthesia, University of Western Ontario, London, ON, Canada
**Correspondence:** Z. Al Duhailib

*Intensive Care Medicine Experimental* 2019, **7(Suppl 3):**001475

**INTRODUCTION.** Circulatory shock is common, accounting for one third of all ICU admissions. Despite current resuscitative practices aimed at restoring macro-hemodynamics, mortality remains high at over 50%. (1) This residual mortality has been attributed to microcirculatory failure, yet clinical monitoring of microcirculatory function remains elusive. Venous-to-arterial carbon dioxide difference (Pcv-aCO2 gap) has emerged as potential clinical marker of microcirculatory function, with some studies suggesting that values greater than 6 mmHg are predictive of poor outcomes. (2,3)

**OBJECTIVES.** We carried out a systematic review and quantitative meta-analysis to determine if high Pcv-aCO2 gap is associated with increased mortality in adult critically ill patients presenting with circulatory shock admitted to medical, cardiovascular and surgical ICUs.

**METHODS.** We conducted a systematic search using MEDLINE® and EMBASE® electronic databases, using OvidSP search interface, from inception through October 2017. Searches were restricted to studies published in English language. The MOOSE checklist was followed and methodological quality was assessed using Newcastle-Ottawa Scale (NOS).

**RESULTS.** We included 50 studies (n=3948 patients) from medical (n=26), cardiovascular (n=15), surgical (n=5) and mixed (n=4) ICUs. The majority of studies were observational with prospective (n=40) or retrospective (n=8) designs, and two were randomised controlled trials (RCT). Eighteen studies reported the primary outcome (either hospital or 28-day mortality). In these studies, high Pcv-aCO2 gap (> 6mmHg) was associated with higher hospital mortality with an odds ratio of death of 2.6 (95% CI [1.6, 4.4], *P*<0.001). Subgroup analysis, showed that Pcv-aCO2 gap may be a stronger predictor of mortality in the medical ICU patients in comparison to cardiac and surgical population. This occurred on the background of similar cardiac index (0 hr: *P*=0.07; 6 hrs: *P*=0.2; 24 hrs: *P*= 0.96), lactate (0 hr: *P*=0.15; 6 hr: *P*=0.06; 24 hrs: *P*=0.49), and central venous oxygen saturation (0 hr: *P*=0.5; 6 hr: *P*=0.41; 24 hrs: *P*=0.93) between high and low Pcv-aCO2 gap groups. Meta-regression showed that the high gap has a predictive power even if the differences in the established predictors of mortality (APACHE II and SOFA) are accounted for (*P*= 0.0296, $R^2$ = 0.60 and *P*= 0.0151, $R^2$ = 0.83, respectively). There was no statistical difference in terms of secondary outcomes such as ICU and hospital length of stay, mechanical ventilation days and requirements for vasopressors and inotropes between the two groups. However, a high Pcv-aCO2 gap was associated with an increased odds of requiring renal replacement therapy (odds ratio: 1.95, 95% CI [1.32, 2.9], *P*= 0.001).

**CONCLUSION.** In patients with circulatory shock, high Pcv-aCO2 adds predictive power over established predictors of hospital mortality despite similar indices of macro-circulation and tissue perfusion. Future studies should evaluate whether resuscitation aimed at closing the Pcv-aCO2 gap Improves mortality in shock.

**REFERENCE(S)**

1. Sakr Y, Reinhart K, Vincent JL, Sprung CL, Moreno R, Ranieri VM, et al. Does dopamine administration in shock influence outcome? Results of the Sepsis Occurrence in Acutely Ill Patients (SOAP) Study. Crit Care Med. 2006;34(3):589-97.
2. Mallat J, Pepy F, Lemyze M, Gasan G, Vangrunderbeeck N, Tronchon L, et al. Central venous-to-arterial carbon dioxide partial pressure difference in early resuscitation from septic shock: a prospective observational study. Eur J Anaesthesiol. 2014;31(7):371-80.
3. Vallet B, Pinsky MR, Cecconi M. Resuscitation of patients with septic shock: please "mind the gap"! Intensive Care Med. 2013;39(9):1653-5.

### 001489

#### Assessment of volumresponsivness (VR) by ultrasound parameters of carotic blood flow in patients with heart failure (HF) or cardiogenic shock (CS)

M. Janotka, P. Ostadal, A. Krüger, D. Vondrakova, J. Naar, P. Neužil
[1]Cardiovascular center, Na Homolce Hospital, Prague, Czech Republic
**Correspondence:** M. Janotka
*Intensive Care Medicine Experimental* 2019, **7(Suppl 3):**001489

**INTRODUCTION.** Fluidoverload in critical ill patients leads to worse prognosis.Therefore the assessment of VR is crucial and we should refrain from blind administering of volume in order to evaluate the VR.Tests looking at change of cardiac output (CO) using reversible increase of preload (e.g. passive leg raise PLR) are preferred instead.Continual monitoring of CO for assesment of fast changes is necessary e.g. echography or pulse contour analysis (PCA) monitors.Recent data showed promising results of assessing the VR by easy ultrasound (US) examination of carotic blood flow changes in patients on ICU.Unlike echography this method has short learning curve and does not need expensive PCA monitor.But there are no data for patients with HF or CS yet.

**OBJECTIVES.** The first aim was evaluating the relationship between changes of CO measured by echography and PCA monitor and changes of carotic flow measured by US in patiens with HF or CS on mechanical ventilation.The second aim was to find out cut-off values of these parameters for determination of VR.

**METHODS.** We examined 21 patients with HF or CS on mechanical ventilation from Jan 2018 to Mar 2019.17 patients required inotropes at the time of examination being in state of CS either alone or combined with distributive shock.All patiens underwent complex analysis of VR (increase of CO by 15% after volume challenge or after PLR were considered as VR).All patients had PLR test and stroke volume variation examined,measured by echography (velocity-time integral in outflow tract) and PCA monitor.Additional parameters were taken into account-echographic signs of left ventricle(LV) filling pressure (transmitral diastolic flow /E,A,E´/,gradients on pulmonary and mitral regurgitation jets) and colabsibility of inferior vena cava,-pulmonary capillary wedge pressure (4patients had Swan-Ganz/SG/ catheter).Patients considered as volumresponsive underwent volum challenge.Those considered nonresponsive underwent volum challenge only if it was regarded as safe.We examined following carotic parameters: respiratory variation of peak velocity(Vmax),effect of PLR test on corrected carotic flow time (CFT) and carotic blood flow (CBF).

**RESULTS.** Average LV ejection fraction was 35.6%.10 patients were deemed as volumresponsive.All of them had positive response on volume administration.11patients were deemed as nonresponsive.5 of them had volum challenge done without positive response, remained 6 patients were considered not safe for volume challenge but 4 of them had SG catheter and PCWP above 20torr.Linear regression analysis revealed relationship between relative change of CO (ΔCO) and CBF calculated by equation ΔCBF=1,21*ΔCO+8,53. We found linear relationship also between ΔCO and changes of both Vmax and CFT (ΔVmax=0,62*ΔCO+3,64 and ΔCFT=0,61*Δ+1,82)The cut-off value for volumresponsivness was found by ROC analysis 15% for ΔCBF,10.7% for ΔVmax and 6.2% for ΔCFT.

**CONCLUSION.** Although it is a small cohort of patients, our data imply possible applicability of easy carotic US examination in fluid management in patients with HF or CS.Change of ΔCBF seems to be the most promising parameter.

**REFERENCE**
1. The study was supported by an Institutional grant MH CZ - DRO (Nemocnice Na Homolce - NNH, 00023884), IG150501.

### 001499

#### Inflammation and resolution of inflammation in human acute heart failure

M. Reina-Couto[1], C. Silva-Pereira[2], JP. Bessa[2], M. Oliveira-Santos[2], P. Serrão[2], J. Afonso[2], R. Roncon-Albuquerque[3], JA. Paiva[4], A. Albino-Teixeira[2], T. Sousa[2]
[1]Intensive care department & dep. biomedicina – unid. farmacologia e terapêutica, Centro Hospitalar São João & Faculdade de Medicina da Universidade do Porto, Porto, Portugal; [2]Dep. biomedicina – unid. farmacologia e terapêutica, Faculdade de Medicina da Universidade do Porto, Porto, Portugal, Portugal; [3]Intensive care department & cirurgia e fisiologia department, Centro Hospitalar São João & Faculdade de Medicina da Universidade do Porto, Porto, Portugal; [4]Emergency and intensive care department, Centro Hospitalar de São João, Porto, Portugal
**Correspondence:** M. Reina-Couto
*Intensive Care Medicine Experimental* 2019, **7(Suppl 3):**001499

**INTRODUCTION.** Although inflammation is recognized as a major contributor for heart failure (HF) progression, clinical trials targeting proinflammatory cytokines in HF have been disappointing. A better understanding of the inflammatory pathways triggered in acute HF (AHF) is urgently needed to identify putative therapeutic targets to alter the course of this disease. A new paradigm of the inflammatory process has emerged recently, introducing endogenous specialized pro-resolving mediators (SPMs) that stimulate the resolution of inflammation and tissue regeneration. These include resolvins (Rvs) and lipoxins (LXs) that were never studied in human AHF.

**OBJECTIVES.** To evaluate SPMs and their correlation with markers of inflammation, oxidative stress, cardiovascular dysfunction and also with prognostic scores/biomarkers in human AHF.

**METHODS.** AHF (n=10) and cardiogenic shock (CS) (n=9) patients were included and blood samples were collected at days 1-2, 3-4 and 5-7. Blood donors were used as controls (n=10). LXA4, 15-epi-LXA4, RvD1, RvE1, endocan (endothelial dysfunction/proinflammatory marker), IL-10, myeloperoxidase (MPO) and nitrotyrosine were measured with ELISA kits. C-reactive protein (CRP), B-type natriuretic peptide (BNP), high-sensitivity troponin I (hsTi), lactic acid, APACHE II and SAPS II scores were also evaluated.

**RESULTS.** At admission, CS was associated with significantly lower RvD1 (CS vs AHF, p=0.024) and higher values of RvE1 (CS vs controls, p=0.004) and IL-10 (CS vs controls, p=0.044). Both AHF and CS groups exhibited higher endocan and MPO serum concentrations (p<0.05 or p<0.001 vs controls) and unaltered LXs values. During hospitalization, we only observed significant marked reductions for LXs values. Within patients, we observed that RvD1 presented inverse correlations with endocan (r=-0.38, p=0.009), lactic acid (r=-0.51, p=0.041), 15-epi-LXA4 (r=-0.32, p=0.029), IL-10 (r=-0.35, p=0.004) and SAPS II (r=-0.50, p=0.029), while positive correlations were observed for RvE1 with CRP (r=0.30, p=0.047) and MPO (r=0.29, p=0.048), LXA4 with 15-epi-LXA4 (r=0.70, p<0.001), and IL-10 with endocan (r=0.43, p=0.004), nitrotyrosine (r=0.35, p=0.018), hsTi (r=0.37, p=0.017), CRP (r=0.41, p=0.007) and APACHE II (r=0.62, p=0.006).

**CONCLUSION.** RvE1 increases with clinical severity, being positively associated with proinflammatory status. RvD1 appears to be exhausted/inactivated in worse clinical scenarios in AHF spectra, being probably a protective mediator and a potential therapeutic target as suggested by its inverse correlations with biomarkers of endothelial dysfunction, inflammatory status and prognostic scores/ biomarkers. The drastic reductions in LXs values during hospitalization may be related to poor prognosis in AHF after discharge, as higher severity of chronic HF was previously shown to be associated with lower values of these SPMs.

### 001500

#### Transcatheter aortic valve implantation (TAVI) in high risk patients for surgical aortic valve replacement: analysis of mortality in the study period

R. Vara Arlanzón, O. Badallo Arévalo, M. Arroyo Díez, M. Martínez Barrios, M. Montero Baladía, JA. Fernández Ratero, M. Del Valle Ortíz, M. Gero Escapa, ME. Perea Rodríguez, E. Martínez Barrio
Hospital universitario de burgos, Intensive care unit, Burgos, Spain
**Correspondence:** R. Vara Arlanzón
*Intensive Care Medicine Experimental* 2019, **7(Suppl 3):**001500

**INTRODUCTION.** Nowadays, the prevalence of aortic stenosis is increasing and some of these patients present comorbidities where the surgical replacement is contraindicated. As an alternative solution, there is TAVI intervention, available in our hospital since 2010 with an important rise in survival for this population.

**OBJECTIVES.** Mortality rates review during hospital stay and one year mortality, for patients who underwent TAVI in a tertiary level hospital without Cardiac Surgery Department.

**METHODS.** Between January 2011 and November 2018, all patients undergoing TAVI with transfemoral self-expandable and balloon-expandable bioprotesis at Burgos University Hospital, were included in a retrospective registry (CEIC: 2062). Demographic characteristics, periprocedural complications and comorbidities were recorded in Excel database with posterior analyses in SPSS. Survival was estimated using the Kaplan-Meier method.

**RESULTS.** During study period, 103 TAVI were implanted in our hospital. 30-day mortality was 7%, one year mortality was 18% instead. Mortality during Intensive Care Unit stay was 4%. Survival probability was calculated with mortality rates: 50% at 45 months since TAVI implantation.

Once all mortality causes were bundled, a 6,8% of patients died due to severe complications during the implantation, predominantlyvascular issue. During post-implant period, 7% of the patients died because of acute heart failure, 1,9% cardiac block, 1,9% had severe hemorrhage to death and 3,9% had systemic infection to death with organ failure.

Presenting previous malignant illness or an active one, was associated with global mortality independently (OR 1,42, 95% [IC] 0,258 – 7,896; p= 0,027).

**CONCLUSION.** TAVI implantation is being introduced as a treatment with high survival in our center with similar outcomes comparing with the available data. The careful selection of best candidates for TAVI implantation based on patient risk factors and comorbidities is the clue for success.

##### REFERENCE(S)

1. Manoharan et al. 1- Year Outcomes With the Evolut R Self Expanding Transcatheter Aortic Valve. The FORWARD study 1- Year Outcomes. JACC: Cardiovascular interventions 2018; 11 (22): 2326-34.
2. M.B. Leon et al. Transcatheter Aortic-Valve Implantation for Aortic Stenosis in Patients Who Cannot Undergo Surgery. N Engl J Med 2010; 363 (17): 1597-1607.
3. G.C.M. Siontis et al. Transcatheter aortic valve implantation vs. Surgical aortic valve replacement for treatment of severe aortic stenosis: a meta-analysis of randomized trials. European Heart Journal 2016.
4. R. Puri et al. TAVI or No TAVI: identifying patients unlikely to benefit from transcatheter aortic valve implantation. European Hearth Journal 2016; 37: 2217-2225.

### 001547

#### Circulating dipeptidyl peptidase 3 is a myocardial depressant factor quickly and promptly reversed by Procizumab

B. Deniau[1], K. Santos[2], A. Dienelt[2], L. Rehfeld[2], A. Bergman[2], A. Blet[1], A. Mebazaa[1]
[1]Anesthesiology and intensive care, Lariboisière hospital, Paris, France; [2]Hennigsdorf, sphingotec therapeutics, hennigsdorf, Germany
**Correspondence:** B. Deniau
*Intensive Care Medicine Experimental* 2019, **7(Suppl 3):**001547

**INTRODUCTION.** Acute heart failure (AHF) is a high mortality disease with a complex pathophysiology, including renin angiotensin aldosterone system disorders. Therapeutics of AHF are lacking (1). Dipeptidyl peptidase 3 (DPP3) is a protein whose main substrate are angiotensin II and enkephalins (2). Recently, high DPP3 levels were associated to a high mortality and organs failure in septic patients (3).

**OBJECTIVES.** The aim of our work is to describe consequences of high DPP3 levels and effects of its inhibition in an experimental mice model of AHF.

**METHODS.** Our study obtained approval of local ethic committee and used 3 month old C57Bl6 mice. (A) DPP3 was IV injected in mice followed during 2h after injection. (B) In an isoproterenol (ISO) induced stress protocol mice followed during 24h were blinded injected by a antibody against DPP3, the Procizumab (PCZ), or PBS. Myocardial oxidative stress staining was realized by dihydroethidium (DHE) immunofluorescence. Datas were expressed as mean ± SEM (intergroup comparison by 2 ways of ANOVA and Mann Whitney test, p<0.05 was significant).

**RESULTS.** (A) 7 mice were included in DPP3 injection protocol (n=3 sham, n=4 DPP3). Shortening fraction (SF) decreased and reached low value at 15 min after DPP3 injection (53 ± 1 vs 62 ± 1, p<0.05), renal resistive index (RRI) increased at 60 min (0.77 ± 0.05 vs 0.56 ± 0.01, p<0.05) and increased myocardial oxidative stress at 120 min (4.8±0.7 AU vs 1.4±0.3 AU, p<0.05). (B) 15 mice were included in ISO + Procizumab protocol (n=5/group). In ISO + PCZ group, SF increased from the first hour (48 ± 1% vs 60 ± 1, p< 0.0001), RRI decreased at 6h (0.62 ± 0.02 vs 0.81 ± 0.06, p<0.05), and decreased myocardial oxidative stress at 1h (2.6 AU ± 0.5 vs 9.3 AU ± 0.9 p<0.05) after Procizumab injection, compared to ISO + PBS group.

**CONCLUSION.** Excess of circulating DPP3 induced myocardial depression. DPP3 inhibition by Procizumab restore cardiac function and kidney hemodynamics and decrease oxidative stress. Studies are necessary to understand the role of DPP3 in AHF pathophysiology. Procizumab could be an interesting therapeutic in AHF.

##### REFERENCE(S)

1. Eur. Heart J. 2016
2. Biol. Chem. 2016
3. J. Appl. Lab. Med.,2018.

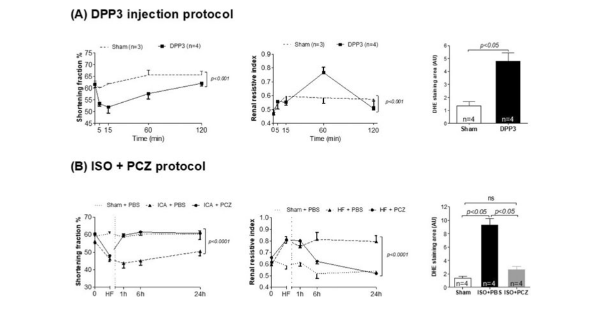

**Fig. 1 (abstract 001547).** See text for description

### 001559

#### Effects of transfusion of two units of red blood cells on haemodynamics in severely anaemic critically ill patients: an observational study in patients with MASIMO (Radical 7) and PiCCO monitoring

A. Herner, W. Huber, R. Schmid, I. Hartter
[1]Medizinische klinik und poliklinik ii, Klinikum rechts der Isar; Technische Universität München, Munich, Germany
**Correspondence:** W. Huber
*Intensive Care Medicine Experimental* 2019, **7(Suppl 3):**001559

**INTRODUCTION.** Anaemia due to acute or chronic loss of blood or due to dilution is frequent in critically ill patients. Thresholds to initiate transfusion with red blood cells (RMC) are matter of an ongoing debate. Nevertheless, there are only few studies investigating the impact of transfusion on extended haemodynamic monitoring (1).

Therefore, we investigated changes in haemodynamic parameters induced by transfusion of two units of red blood in 45 critically ill patients under haemodynamic monitoring with Radical 7 (MASIMO, USA; 45 patients) and PiCCO (35 patients; 10 patients required transfusion before initiation of PiCCO-monitoring).

**METHODS.** This prospective observational study was performed on a general ICU of a University Hospital in Munich. Only patients at predefined need of blood transfusion (laboratory haemoglobin Hb < 7mg/dL; in case of pre-existing severe cardiovascular disease: Hb <8 mg/dL; measured with RAPIDPoint 500 Blood Gas analyzer (Siemens, Germany)) were included. All measurements were performed prior and after the transfusion of two units of red blood cells. Statistics: Wilcoxon-test for paired samples. IBM SPSS 25.

**RESULTS.** 12 (27%) female and 33 (73%) male patients, height 175±8cm, weight 79±17kg. Hb measured by the blood gas analyzer increased from 6.7±0.6 to 8.8±0.8g/dL (p<0.001). Among MASIMO-parameters, perfusion index PI decreased from 2.7±2.3 to 2.3±1.7 (p=0.024) and pleth variability index (PVI; only analyzed in patients with sinus rhythm SR and controlled mechanical ventilation CMV) increased from 10.4±5.5 to 14.5±10.4; p<0.001. By contrast, PiCCO-derived stroke volume variation SVV (9.4±7.9 to 8.5±7.2%; p=0.442) and pulse pressure variation PPV (6.8±5.9 to 7.6±7.3%; p=0.719) did not change. Among the other PiCCO-derived parameters, the most pronounced change was an increase in mean arterial pressure MAP (87.6±11.6 vs. 97.2± 14.1mmHg; p<0.001). Furthermore, cardiac power index CPI increased from 0.82±0.25 to 0.91±0.26W/m$^2$ (p=0.009), and SVRI increased from 1093±483 to 1353±653dyne/cm2/m5; p<0.001. Global ejection fraction decreased from 25.3±6.6 to 23.7±6.8% (p<0.001). All other parameters did not change significantly from before to after transfusion: Heart rate 93±15 vs. 92±17/min (p= 0.154); cardiac index CI 4.9±1.4 vs. 4.7±1.3L/min/m$^2$; p=0.064; stroke volume index SVI 53.6±13.2 vs. 52.2±14.1mL/m$^2$; p=0.098; extravascular lung water index EVLWI 10.9±3.8 vs. 11.4±3.2mL/kg; p=0.099; CVP 19.9±17.8 vs. 17.7±8.1mmHg; p=0.203; global end-diastolic volume index GEDVI 878±187 vs. 894±172mL/m$^2$; p= 0453; dPmax 1328±414 vs. 1354±393mmHg/s; p=0.184. Furthermore, the noradrenaline dosage was comparable before and after transfusion (413±317 vs. 398±345µg/h; p=0.465).

**CONCLUSION.** 1.) Transfusion of two units of red blood cells in severely anaemic patients resulted in marked increases in MAP (11%) and CPI (11%). 2.) In patients with SR and CMV, changes in dynamic indices of fluid responsiveness derived from MASIMO and PiCCO were not congruent: While PVI increased despite the volume load, SVV and PPV did not change. 3.) MASIMO-measured perfusion index PI decreased despite the volume load by two units of red blood cells.

### 001570

#### Evaluating the accuracy and reliability of sublingual microcirculation measurements as an adjunct to fluid management in cardiac surgery patients

G. Guven[1], J. Montomoli[2], S. Roelen[3], P. Giaccaglia[2], BAD. Mol[3], C. Ince[2]
[1]Intensive care unit, Erasmus Medical Center, Rotterdam, Netherlands; [2]Translational physiology, Academic Medical Centre, Amsterdam, Netherlands; [3]Thoraxchirurgie, Academic Medical Centre, Amsterdam, Netherlands
**Correspondence:** G. Guven
*Intensive Care Medicine Experimental* 2019, **7(Suppl 3):**001570

**INTRODUCTION.** Fluid administration is the first therapeutic response in perioperative patients where there are signs of decreased tissue oxygenation and impaired organ perfusion [1]. Inadequate fluid therapy can cause hypovolemia. Conversely, excessive fluid administration leads to a positive cumulative fluid balance which can lead to complications for the cardiac surgery (CS) patient [2]. No real endpoint for optimal administration of fluids is currently available and global macrohemodynamic parameters may not always reflect microcirculatory dysfunction [3]. Here we introduce a non-invasive integrative hemodynamic monitoring (IHM) approach to monitor the hemodynamic impact of fluid therapy by monitoring the systemic circulation microcirculation and tissue water composition. We propose this integrative hemodynamic platform as a comprehensive physiologically based approach for fluid management of the CS patient.

**OBJECTIVES.** We aimed to determine how microcirculation, macrocirculation, and body fluid compartments are affected in post-ICU CS patients.

**METHODS.** Twenty-five patients whom underwent open CS were included in the study. Non-invasive IHM was accomplished by measurement of sublingual microcirculation (Cytocam-IDF, Braedius Medical, Huizen, the Netherland), systemic hemodynamic parameters (Nexfin, BMEYE, Amsterdam, The Netherlands) and of the body fluid status (Body Composition Monitor, Fresenius Medical Care, Bad Homburg, Germany). Measurements were performed before CS (T0), first (T1) and on day 3 (T3) after admission to the ward from the ICU.

**RESULTS.** Seventeen patients with three-time points and eight patients with two-time points were analyzed using dedicated software. The baseline body weight was 84.5 kg (79.4; 93.8) which T0, increased to 89.4 kg (82;101) at T1 and 86.5 kg (80.9; 97.3) at T3 (p< 0.001). Total vessel density showed an inverse relationship to body weight (T0: 24 mm/mm2(22.9;25.3), T1: 20.2 mm/mm2(18.5;21;6), T3: 21.6 mm/mm2(19.4;23.5), p<0.001). Total body water and extracellular water changed in parallel with the change in body weight (p= 0.002 and p<0.001, respectively). Interestingly, intracellular water (ICW) at T3 was found to decrease significantly lower than baseline although ICW at T1 was not significantly different from T0 (T0: 20.3 lt (17.5; 22.8), T1: 20.7 lt (18.8; 23.1), T3: 18.9 lt (16.4; 21.1), p=0.012). Systolic, diastolic and mean arterial pressure at T1 and T3 were found to be lower than baseline (p=0.004, p=0.024, p=0.005, respectively). Cardiac output/index and dP/dt did not differ within time points (p= 0.08 and p=0.42, respectively). Stroke volume was 96 ml (85;104) at T0, and decreased to 82 ml (74; 94) at T1, and 85 ml (67; 93) at T3 (p=0.004).

**CONCLUSION.** Our study shows that non-invasive IHM allows comprehensive identification of behaviour of fluid therapy in CS patients. Results show that CS related microcirculatory perfusion disturbances and loss of hemodynamic coherence occur as a result of fluid therapy which persists even after discharge from the ICU.

##### REFERENCE(S)

1. Kara, A., S. Akin, and C. Ince, The response of the microcirculation to cardiac surgery. Curr Opin Anaesthesiol, 2016. 29(1): p. 85-93.
2. Bellomo, R., J. Raman, and C. Ronco, Intensive care unit management of the critically ill patient with fluid overload after open heart surgery. Cardiology, 2001. 96(3-4): p. 169-76.

3. Ince, C., Hemodynamic coherence and the rationale for monitoring the microcirculation. Crit Care, 2015. 19 Suppl 3: p. S8.

### 001587

#### Acute Myocardial Infarction in Polyvalent Intensive Care Units

AT. Ferreira, D. Tiago, V. José, G. Nuno, L. Bento
Intensive care unit, Centro Hospitalar Universitário de Lisboa Central, Lisbon, Portugal, Portugal
**Correspondence:** A.T. Ferreira,
*Intensive Care Medicine Experimental* 2019, **7(Suppl 3):**001587

**INTRODUCTION.** Acute Myocardial Infarction (AMI) is a potentially life-threatening condition, frequently diagnosed in critical care environment. The presence of concomitant medical conditions such as infection or hematological disorders may reduce therapeutic options. It is therefore important to characterize this subset of patients, since the medical approach is often challenging.

**OBJECTIVES.** To analyze the population of patients admitted in Polyvalent ICUs with the diagnosis of AMI in a two-year period.

**METHODS.** We performed a retrospective characterization of patients admitted in two Polyvalent Intensive Care Units between January of 2017 and December of 2018, with the primary or secondary diagnosis of AMI.

**RESULTS.** The final diagnosis of AMI was made in 72 patients, 31,9% of which (n=23) classified as STEMI. The median age of the population was 71 years.

72,2% (n=52) of the patients had a previous diagnosis of Arterial Hypertension, and 48,6% (n=35) of Diabetes mellitus. 26,3% of the patients (n=19) had previously known coronary heart disease.

51,4% (n=37) were admitted in circulatory shock (cardiogenic or other).

43% of the patients (n=31) did not perform coronary angiography, either due to clinical instability from concomitant medical conditions or to the likely diagnosis of type 2 myocardial infarction.

Among the STEMIs, 7 cases were admitted in Polyvalent ICUs after successful cardiopulmonary resuscitation and immediately after cardiac revascularization in order to initiate hypothermia protocol.

56,9% (n=41) of the total number of patients were submitted to coronary angiography, and in 33,3% (n=24) Primary Coronary Angioplasty of one or more coronary arteries was performed. Seven patients were proposed for Coronary Artery Bypass Graft surgery. The remaining patients were considered to have no benefit from revascularization.

The intra-hospital mortality was of 37,5% (n=27).

**CONCLUSION.** The patients diagnosed with AMI in Polyvalent ICUs require different medical and interventional approaches, since there are often distinct pathophysiology processes involved and conditions preventing early revascularization. An experienced team and availability of resources are fundamental to ensure the best outcomes among these patients.

### 001617

#### How common is new onset atrial fibrillation in critical care? A single centre retrospective study

J. Macfarlane[1], B. Johnston[2], I. Welters[2], A. Waite[2]
[1]Institute of ageing and chronic disease, University of Liverpool, Liverpool, UK; [2]Critical care, Royal Liverpool and Broadgreen University Hospitals NHS Trust, Liverpool, UK
**Correspondence:** A. Waite
*Intensive Care Medicine Experimental* 2019, **7(Suppl 3):**001617

**INTRODUCTION.** Atrial fibrillation (AF) is the most common arrhythmia in critical ill patients (1). New onset AF (NOAF) is linked with severity of disease and is associated with poorer outcomes, including prolonged ICU stays and higher mortality rates (1-3). National guidelines exist in the UK for management of AF, but they are not specific to critical care (4). The most effective treatment for AF in critical care remains unclear.

**OBJECTIVES.** To retrospectively assess the incidence and management of new-onset atrial fibrillation in a single centre critical care setting.

**METHODS.** Notes of 909 patients admitted to critical care at the Royal University Liverpool Hospital between 2008 and 2014 were reviewed. Patients were identified as having pre-existing AF (PEAF, either chronic or paroxysmal), NOAF, or no AF during a period of up to 7 days. Pharmacological treatment regimes for rate or rhythm control of AF, heart rate before and after onset of AF, inotrope usage at onset of AF, presence of sepsis, age, APACHE II score and 28 day mortality rates were also recorded.

**RESULTS.** 199 patients (21.9%) were identified as having new onset AF; 53 patients had pre-existing AF (5.8%), and the remaining 657 patients (72.3%) did not have AF. The mean age of patients with new or pre-existing AF was higher than those without AF (72, 70 and 56 respectively) and APACHE II scores followed a similar pattern (21, 22 and 18). Mortality rates were higher amongst patients with NOAF than PEAF or no AF (30.7%, 24.5% and 16.2%). 481 patients had sepsis (52.9%); 142 of these patients had NOAF and 14 had PEAF. 55 patients only had electrolyte replacement ($Mg^{2+}$ and $K^{+}$) after the onset of AF, and 53% of these patients reverted to sinus rhythm; but the majority of patients were given amiodarone, in combination with electrolytes, alone, or with other anti-arrhythmics.

**CONCLUSION.** New-onset atrial fibrillation is more common in older patients with higher APACHE II scores, and these patients have a higher 28 day mortality rate than patients with pre-existing AF. Further research is needed to clarify the best treatment strategy for new onset AF in critically ill patients.

**REFERENCE(S)**

1. Bosch NA, Cimini J, Walkey AJ. Atrial Fibrillation in the ICU. Chest 2018; 154 (6):1424-1434
2. Walkey AJ, Hogarth DK, Lip GYH. Optimizing Atrial Fibrillation Management - From ICU and Beyond. Chest, 2015; 148(4); 859-864
3. Klein Klouwenberg PM, et al. Incidence, Predictors, and Outcomes of New-Onset Atrial Fibrillation in Critically Ill Patients with Sepsis. A Cohort Study. Am J Respir Crit Care Med, 2017; 195(2): 205-211
4. National Institute for Health and Care Excellence (NICE) Clinical guideline 180: Atrial Fibrillation, 2014.

### 001717

#### The impact of CCRT (continous renal replacement therapy) on survival of a specific geriatric subgroup (>80y.o.)

LF. Frade[1], P. Valério[2], M. Toscano[3], C. Pereira[4], C. Carvalho[5], I. Botelho[5], A. Ramos[5]
[1]Internal medicine, São Francisco Xavier Hospital, Lisboa, Portugal; [2]Nephrology, Hospital São Bernardo, Setúbal, Portugal; [3]Internal medicine, Hospital de Cascais, Alcabideche, Portugal; [4]Oncology, IPO Instituto Portugues De Oncologia De Lisboa, Lisboa, Portugal; [5]Intensive care unit, Hospital de Cascais, Alcabideche, Portugal
**Correspondence:** L.F. Frade
*Intensive Care Medicine Experimental* 2019, **7(Suppl 3):**001717

**INTRODUCTION.** There is an increased concern on the specifics of the geriatric population, still the role of continuous renal replacement therapies in survival is not very clear and most certainly not for a subgroup of very old patients (> 80y.o.)

**OBJECTIVES.** The primary objective of this study was to access the impact of CCRT (*continous renal replacement therapy*) on survival of a specific geriatric subgroup, the so called, very old patients (> or = 80 years old), comparing it with the younger population who was also submitted to CCRT on the same time period.

**METHODS.** 141 ICU patients were included, from March/2010 to March/2019 (84 patients were excluded due to lack of data needed for the analysis). Age group: the younger patient was 18y.o., the oldest was 92 y.o. (mean 69.4 ±14.1), from those, 80 patients were > 80 y.o. (it means 70.9% of the patients). Concerning the gender, 37.6 % (n=53) were females, 62.4% (n=88) were males and 95% (n=134) Caucasians vs. 5% (n=7) Blacks. The data was collected through a retrospective analysis of these patient´s medical reports and analyzed using SPSS version 23.

**RESULTS.** The survival curve (Kaplan-Meier) showed that patients in an age range = or > 80y.o. submitted to CCRT had lower survival rates after starting the therapy, this data is of statistical significance (Log Rank test, p<0.05). Their survival was influenced by: age > 80 (H.R 4.3, confidence interval 95%, p value <0.05) and by duration of CRRT (measured in days), the shortest the therapy the highest the mortality (HR 15.7, confidence interval 95%, p- value < 0.01). Using Cox regression, it was found that the cause of patient´s admission on ICU had no impact on survival (p-value >0.05), likewise if it was a newly diagnosed acute renal failure or an acute renal failure on a patient with stablished chronic kidney disease. Serum levels of creatinine, urea, potassium, pH and HCO3 on arterial blood gas analysis, immediately before starting the CRRT also did not have impact on survival. Oliguria, defined as urine output < or = 0.3 mL/kg/min previous to the therapy, as well as type of therapy applied (hemodialysis, hemofiltration, both), type of anticoagulant (citrate or heparin), prescribed dose of therapy, did not have impact on survival of this geriatric subgroup (very old patients).

**CONCLUSION.** The geriatric patients, specially the very old patients (> or = 80 y.o.) should be thought as a particular population and that should guide the clinician to make a different clinical approach comparing to younger patients. The highlight of this study was to find out that regardless of the etiology of renal insufficiency, regardless of lab values or presence/absence of oliguria, age prevails and that variable itself should help on making decisions on weather to start/not to start CRRT since it showed no improvement on survival of this age group

### 001718

#### Survival analysis of a group of patients submitted to CRRT (continuous renal replacement therapy) – convective vs diffusive techniques (CVVHF vs CVVHD)

P. Valério[1], LF. Frade[2], M. Toscano[3], C. Pereira[4], S. Cunha[5], I. Botelho[6], A. Ramos[6]
[1]Nephrology, Centro Hospitalar De Setúbal E.P.E., Setúbal, Portugal; [2]Internal medicine, São Francisco Xavier Hospital, Lisboa, Portugal; [3]Internal medicine, Hospital de Cascais, Alcabideche, Portugal; [4]Oncology, IPO Instituto Portugues De Oncologia De Lisboa, Lisboa, Portugal; [5]Anesthesiology, IPO, Lisbon, Portugal; [6]Intensive care unit, Hospital de Cascais, Alcabideche, Portugal
**Correspondence:** ,.P. Valério
*Intensive Care Medicine Experimental* 2019, **7(Suppl 3):**001718

**INTRODUCTION.** The prevalence of acute renal failure in intensive care units (ICU) is about 66%. From those, 13,5% meet the criteria for CRRT. The short term mortality of these patients is around 70%. The ideal CRRT technique is not yet stablished and some studies showed no difference on mortality.

**OBJECTIVES.** Retrospective survival analysis of patients submitted to CVVHF (continuous venovenous hemofiltration) vs CVVHD (continuous venovenous hemodyalisis).

**METHODS.** One hundred forty four ICU patients submitted to CRRT were selected, from March 2010 to March 2019. Seventy two patients were excluded, because they were submitted to continuous venovenous hemodiafiltration.

Those on regular program of hemodialysis were also excluded.

The demographic data were collected from patient's medical reports and it was analyzed through SPSS 23 statistics software.

**RESULTS.** Seventy two patients were included. From those 63,9% (n= 46) were male. The mean age was 69,0 ± 5,6 years old (18 to 87 years old).

Most of the patients were submitted to CVVHD (n=47; 65,3%). The mean survival was 4 days (0-33 days) and 70,8% (n=51) died during ICU admission.

The survival curve (using Kaplan-Meier method) showed lower survival for patients submitted to CVVHD, although it was not statistically significant.

Excluding the patients who died within 48 hours after hospital admission, in whom the mortality is strongly influenced by admission cause, the results weren´t any different.

The other variables analyzed (age, gender, cause of admission, previous diagnosis of chronic renal insufficiency, duration of CRRT, degree of metabolic acidosis or creatinine and urea serum levels) had no impact on survival.

**CONCLUSION.** This study showed that there is no statistically significant difference between the CVVHD vs CVVHF groups survival. Like other studies available, this analysis has a small sample and it's a retrospective and monocentric study.

A bigger scale study is needed so that we have more reliable information to approach these patients.

### 001762

#### Predicting outcome in ICU trauma patients with AKI

M. dlela[1], T. Olfa[2], T. Aziza[3], M. Bahloul[2], M. Bouaziz[2]
[1]Intensive care unit, Habib bourguiba university hospital, Sfax, France; [2]Intensive care unit, habib bourguiba university hospital, sfax, Tunisia; [3]Emergency departement, habib bourguiba university hospital, sfax, Tunisia
**Correspondence:** M. dlela
*Intensive Care Medicine Experimental* 2019, **7(Suppl 3):**001762

**INTRODUCTION.** Acute kidney injury in trauma patients is a problem that has been little studied in the ICU. Its occurrence has been shown to be associated with high morbidity and mortality.

**OBJECTIVES.** To determine the outcome in ICU trauma patients with AKI, including the incidence of death in the ICU, of non-reversible renal impairment and ICU complications. As well as to identify predictive factors of ICU mortality and poor outcome.

**METHODS.** This is a ten-month long prospective cohort-study, conducted in the department of emergencies and intensive care unit (ICU) of a university hospital, including trauma patients with a minimum ICU stay of 7 days. Renal failure was defined based on the new KDIGO classification. Predictors of mortality and poor outcome were identified using univariate and then multivariate analysis.

**RESULTS.** One hundred thirty patients were admitted during the study period for the management of post-traumatic injuries, among which 86 patients were included. The incidence of AKI in the studied population was 53% (46 cases) with 26 (56%) diagnosed with stage one, ten (22%) with stage two and ten (22%) with stage three. The overall mortality of patients with post-traumatic AKI was 34.8% (16 patients) with a mean lengh of ICU stay (LOS) at 22 ± 18 days (extremes ranging from 5 to 68 days), and of days on ventilator at 17 ± 15 days (extremes ranging from 2 to 68 days). Eight patients (17.4%) needed renal replacement therapy and thirty-four had non-reversible renal impairement (74%). During ICU stay, thirty-two (69%) patients developed nosocomial infections and eight (17%) were diagnosed with pulmonary embolism.

On univariate analysis, the following variables were associated to mortality in patients with post-tramatic AKI including; age, hemodynamic instability on the day of diagnosis, the use of vasoactive support, transfusions and bilirubin levels on the day of AKI diagnosis.

Besides, according to our analysis, the use of renal replacement therapy and the non-reversibility of renal impairment during ICU stay were also associated to ICU mortality.

Among these factors, the non-reversibility of renal impairment in the ICU was a predictor of mortality on multivariate analysis (p = 0.009, OR = 29, CI: 4-142).

Moreover, in this cohort, the following variables were predictive of non-reversible renal impairment during ICU stay; including age (with a best cut-off of 55 years old), medical history of diabetes or hypertension, high injury severity score and diuretics' administration.
The early delay to AKI from trauma was also predictive of the non-reversibility with a best cut-off at 2.5 days (AUC ROC= 0.87, Se= 83.3%, Sp=82.4%). On multivariate analysis, the age(p = 0.004, OR = 0.9, CI: 0.80-0.97) and use of diuretics (p = 0.003, OR = 33, CI: 3.1-359) were associated to non-reversible AKI in the ICU.
**CONCLUSION.** Our study confirms that post-traumatic AKI in the ICU is associated to high morbidity and mortality. The identification of outcome predictors could be valuable to guide the management AKI.

## INF / ETH - Onco-hematologic issues and approaches to improve ICU outcomes

### 000556

#### ICU mortality among oncological patients at a public cancer hospital in São Luís-Northeast Brazil: three-year evaluation

AP. Pierre de Moraes[1], G. Alves[1], JR. Lima[1], AA. Silva[2], Y. Assis[1]
Intensive Care Unit, Cancer Hospital of Maranhao Tarquinio Lopes Filho, São Luís, Brazil; [2]Public health, Federal University of Maranhão, São Luís - Vila Maranhão, São Luís - State of Maranhão, Brazil, Brazil
**Correspondence:** A.P. Pierre de Moraes
*Intensive Care Medicine Experimental* 2019, **7(Suppl 3):**000556

**INTRODUCTION.** Recent studies point out that the severity of acute complications, the number of organ disfunction, the management complexity and performance status have more impact on ICU mortality among cancer patients than the underlying neoplasm disease [1-3].
**OBJECTIVES.** To evaluate risk factors for ICU mortality among cancer patients
**METHODS.** A retrospective study conducted at a 11-bed ICU of a public cancer hospital in São Luis, one of the capitals of northeastern Brazil. We evaluated all cancer patients > 18 years old requiring ICU admission from January 2016 to December 2018, excluding those in palliative care support, readmission, and those that had ICU stay <24 hours. We evaluated demographic and clinical variables, ICU support at admission and during ICU stay by univariate and multivariate analysis. The risk factors for ICU mortality were investigated through multiple logistic regression analysis.
**RESULTS.** Out of 1021 patients, 699 (68%) had solid locoregional tumors, 182 (18%) had solid metastatical tumors and 140 (14%) were onco-hematology patients. The main solid tumor sites were gastrointestinal 254 (25%), gynecological 152 (15%), and urological 107 (11%). There were 438 (43%) admissions due to medical reasons, 553 (52%) and 50 (5%) for postoperative care after elective and emergency surgery, respectively. The overall ICU mortality was 34%. At multivariate analysis, the independent risk factors for ICU mortality were: admission due to medical reasons (OR = 7,57; 95%CI: 4,39-13,08) or due to emergency surgery (OR=2,78; 95%CI 1,27-6,08), high SAPS 3 (OR = 1,03; 95%CI: 1,01-1,04) and high SOFA scores at admission (OR = 1,17; 95%CI: 1,08-1,26), need of mechanical ventilation (OR = 2,94; 95%CI: 1,94-4,46), use of vasoactive drugs during ICU stay (OR = 2,19; 95%CI: 1,36-3,55) and nosocomial ICU infection (OR = 3,96; 95%CI: 2,26-6,93).
**CONCLUSION.** An unplanned ICU admission, higher severity scores upon ICU admission, complex support and infection during ICU stay were identified as risk factors for ICU mortality. The severity of acute illness at ICU admission may suggest that prompt recognition of organ dysfunction and the possibility of early ICU referral could offer opportunities to better management of acute critical complications. The expansion of prevention strategies to reduce hospital-acquired infection would be important for improving ICU mortality rates.

**REFERENCE(S)**
1. Soares M, Caruso P, Silva E, et al. Characteristics and outcomes of patients with cancer requiring admission to intensive care units: a prospective multicenter study. Crit Care Med 2010;38(1):9-15
2. Biskup E, Cai F, Vetter M, Marsch S. Oncological patients in the intensive care unit: prognosis, decision-making, therapies and end-of-life care. Swiss Med Wkly. 2017;147: w14481
3. Azoulay E, Schellongowski P, Darmon M, Bauer PR, Benoit D, Depuydt P, et al. The Intensive Care Medicine research agenda on critically ill oncology and hematology patients. Intensive Care Med 2017;43(9):1366-1382.

### 000559

#### Characteristics and outcomes of patients with advanced uterine cervical cancer requiring intensive care in São Luís- Northeast Brazil

AP. Pierre de Moraes[1], G. Alves[1], JR. Lima[1], AA. Silva[2], Y. Assis[1], FJ. Lobato[3]
[1]Intensive Care Unit, Cancer Hospital of Maranhao Tarquinio Lopes Filho, São Luís, Brazil; [2]Public health, Federal University of Maranhão, São Luís - Vila Maranhão, São Luís - State of Maranhão, Brazil, Brazil; [3]Medicine, University CEUMA Matrix, São Luís - Vila Maranhão, São Luís - State of Maranhão, Brazil, Brazil
**Correspondence:** A.P. Pierre de Moraes
*Intensive Care Medicine Experimental* 2019, **7(Suppl 3):**000559

**INTRODUCTION.** Cervical cancer presents large geographic variations in incidence and mortality rates. Control and prevention measures also differ markedly worldwide. It is estimated that almost 90% of deaths occur in low- and middle-income countries. [1-2]
**OBJECTIVES.** To evaluate the characteristics and outcomes of cervical cancer patients requiring ICU admission and compare them to those with other solid cancer patients also requiring ICU admission
**METHODS.** This retrospective study was conducted at a 11-bed ICU of a public cancer hospital in São Luis, one lower income capital of northeastern Brazil. All patients > 18 years old with a solid tumor requiring ICU admission from January 2016 to December 2018 were included and were classified based on their primary solid tumor site in cervical and other solid tumor patients. We evaluated demographic and clinical variables at ICU admission, ICU support. The primary outcome were ICU and in-hospital mortality. Statistical differences were tested using Pearson´s chi-square or Mann-Whitney tests as appropriate. The significance level adopted was 0,05.
**RESULTS.** Out of 1369 patients, 148 (11%) had cervical uterine cancer and 1221 (89%) had other solid tumors. The main admission reasons were elective surgery 80 (54%), medical admission due to infection/sepsis 20 (14%) and renal disfunction 17 (12%). Compared to other solid cancer patients, cervical cancer patients were younger (p< 0,001), but did not differ in the presence of metastatical disease(p= 0,59), comorbidities (p=0,73), functional status (p=0,40), SAPS 3 score (p=0,58) and SOFA score on the first ICU day (p=0,38). Regarding ICI support, cervical cancer patients had higher need for renal replacement therapy (p<0,001), but lower need for mechanical ventilation (p=0.01). Compared to other solid cancer patients, ICU mortality was lower in cervical cancer patients (29% versus 19%, p= 0,02), but there is no difference between groups in in-hospital mortality (40% versus 39%, p=0,69).
**CONCLUSION.** There was a considerable occurrence of ICU admission of young or middle-aged women with a preventable disease that had high morbidity and mortality. The results of this study reinforce the importance of providing adequate prevention programs, early diagnosis, access to medical attendance and prompt reference in order to improve cervical cancer outcomes.

**REFERENCE(S)**

1. World Heath Organization. International Agency for Research on cancer. http://gco.iarc.fr/today/data/factsheets/cancers/23-Cervix-uteri-fact-sheet.pdf
2. Pimple S, Mishra G, Shastri S. Global strategies for cervical cancer prevention. Curr Opin Obstet Gynecol 2016; 28(1):4-10

### 001112

#### Intrahospital mortality after discharge from the intensive care unit (hidden mortality) in hematopoietic stem cell transplantation recipients admitted to a tertiary Intensive Care Unit

C. Díaz[1], L. Fox[2], I. Romera[1], P. Barba[2], M. Santafe[1], A. Pacheco[1], A. García[1], M. Pérez[1], R. Ferrer Roca[1]
[1]Intensive care department, Vall d'Hebron University Hospital. SODIR. VHIR, Barcelona, Spain; [2]Hematology department, Vall d'Hebron University Hospital, Barcelona, Spain
**Correspondence:** C. Díaz
*Intensive Care Medicine Experimental* 2019, **7(Suppl 3):**001112

**INTRODUCTION.** Hidden mortality is a relevant problem in many critical conditions, including hematopoietic stem cell transplantation recipients (HSCT-R). Despite a significant number of HSCT-R die in the Hematology ward after been discharged from the Intensive Care Unit (ICU), this aspect has not been extensively studied. So, we believe that a better understanding of this population would help to improve their prognosis.

**OBJECTIVES.** Our aim was to analyze the differences between HSCT-R discharged from the ICU who died before being discharged from hospital and those who survived.

**METHODS.** Retrospective study, including adult (+18 years) patients who received an HSCT and required ICU admission between 2010 and 2018, and were discharged alive to the Hematology ward. X-Square, Fisher´s test, T test, U Mann-Whitney and logistic regression were employed as required. Quantitative variables are reported as median (IQR) and categorical as frequency (%).

**RESULTS.** During this eight year period, 79 HSCT-R were admitted to the ICU. Thirty-three (42%) of them were discharged to the Hematology ward and 20 (25%) finally survived the hospital admission. Nineteen (58%) were men, with an age of 57 (37-62) years. Twenty-five (76%) were allogeneic HSCT and 18 (55%) were neutropenic. APACHE was 20 (14-24) and SOFA score 8 (6-9). Twenty-one (64%) were treated with vasoactive drugs (VAD), 15 (47%) with high flow nasal cannula (HFNC), 9 (27%) received mechanical ventilation (MV) and 2 (6%) renal replacement therapy (RRT). Seven (6%) were readmitted to the ICU. Allogeneic HSCT (OR: 1.9, IC95%: 0.3-11.7, p= 0.476) and neutropenia (OR: 4, IC95%: 0.8-19.8, p=0.09) were not related to hidden mortality, nor were the use of HFNC (OR: 1.8, IC95%: 0.4-8.6, p=0.441), MV (OR: 1.1, IC95%: 0.2-6, p=0.890), VAD (OR: 0.75, IC95%: 0.2-3.6, p=0.75) or RRT (OR: 1.9, IC95%: 0.1-33.7, p=0.662). APACHE score (OR: 1.1, IC95%: 1-1.2, p=0.233) and SOFA score (OR: 1.2, IC95%: 0.9-1.7, p=0.268) were not different in both groups, but those who did not decrease their SOFA score at fifth day had a greater hidden mortality (OR: 18.3, IC95%: 1.5-222.9, p=0.022).

**CONCLUSION.** A significant amount of HSCT-R discharged from the ICU die before being discharged home. We did not find any relationship with the type of HSCT or the requirements of organ support, but we found a strong relationship between the persistence of organ dysfunction at day five. Those with no improvement in SOFA score in the first five days, despite being discharged to the ward are at greater risk of mortality before being discharged home.

### 001225

#### Comparison of long-term outcomes of de novo acute lymphoblastic leukemia (ALL) patients (pts) and acute myeloblastic leukemia (AML) pts who require intensive care unit admission

A. Bazhenov[1], G. Galstyan[1], E. Parovichnikova[2], V. Troitskaya[2], V. Savchenko[2]
[1]Icu, National Research Center for hematology, Moscow, Russia; [2]Hematology, National Research Center for hematology, Moscow, Russia
**Correspondence:** A. Bazhenov
*Intensive Care Medicine Experimental* 2019, **7(Suppl 3):**001225

**INTRODUCTION.** Treatment of acute leukemia pts can be associated with the life-threating complications requiring ICU admission.

**OBJECTIVES.** To compare frequency of ICU admission and short and long-term outcomes of *de novo* ALL and AML pts who required and not required ICU admission during induction chemotherapy.

**METHODS.** All *de novo* ALL (median age 28 yo, 17-60 yo) and AML (median age 28 yo, 17-60 yo), treated from 2013 to 2017, were enrolled in the study. ALL and AML pts were divided into 4 groups: pts required ICU admission (ALL and AML ICU-groups), and pts not required ICU admission (ALL and AML non-ICU groups).

**RESULTS.** ICU admission was required in 18 (20.6%) of 87 ALL pts and in 44 (27.5%) of 160, ($p$ = 0.2394) AML pts. The reasons for ICU admissions were similar for ALL and AML pts: acute respiratory failure (44% and 47.7%, $p$ = 0.637), acute neurological events (28% and 13.6%%, $p$ = 0.759), tumor lysis syndrome (17% and 4.6%%, $p$ = 0.112), septic shock (SS) (11% and 20.4%%, $p$ = 0.383) respectively. Needs of mechanical ventilation and infusions of vasopressors were independent predictors of ICU mortality ($p$<0.05) in ALL and AML pts. 28-day mortality was 47.5% for AML ICU-group and 50% for ALL-CU-group ($p$=0.196). Any pts did not die in both non-ICU groups during 28 days. In ALL pts 5-year overall survival (OS) was worse in ICU-group (44%) than in non-ICU group (44% vs. 76% $p$ <0.05) (fig. 1). There were no significant differences in 5-year OS of AML pts from ICU and non-ICU groups (42.6% and 48.6%, *p=0.946)* (fig 2).

**CONCLUSION.** The frequency of ICU admission, reasons for ICU admission and 28-day mortality rate were similar for ALL and AML pts**.** The long-term OS was lower in discharged from ICU ALL pts than in non-ICU ALL pts. In AML pts long term OS was similar in pts discharged from ICU and non-ICU pts.

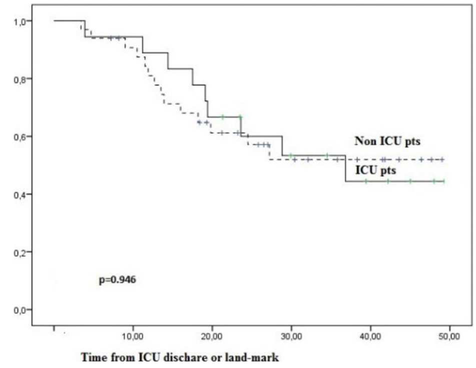

**Fig. 1 (abstract 001225).** See text for description

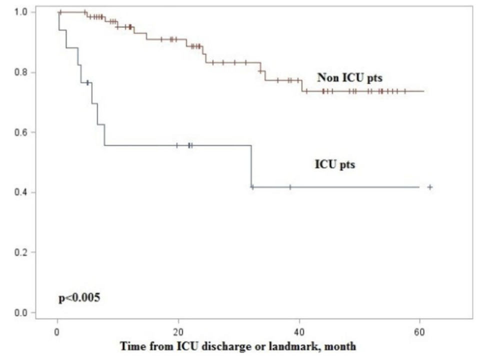

**Fig. 1 (abstract 001755).** See text for description

### 001327

#### Outcome of pediatric acute leukemia in patients admitted to the Intensive Care Unit

M. Caballero Bellon[1], A. Faura Morros[2], A. Margarit Soler[3], M. Balaguer[4], J. Marsal[2], E. G. Forster[2], A. Alonso[2], A. Català[2], A. Ruiz[5], M. Mesegue[2], N. Conde[2], R. Berrueco[5], S. Rives[2], I. Jordan[4]
[1]Paediatrics, Hospital Sant Joan de Déu Barcelona, Esplugues de Llobregat, Spain; [2]Hematology and oncology, Hospital Sant Joan de Déu Barcelona, Esplugues de Llobregat, Spain; [3]Bone marrow transplant, Great Ormond Street Hospital, London, UK; [4]Paediatric intensive care unit, Hospital Sant Joan de Déu Barcelona, Esplugues de Llobregat, Spain; [5]Hematology, Hospital Sant Joan de Déu Barcelona, Esplugues de Llobregat, Spain
**Correspondence:** A. Faura Morros
*Intensive Care Medicine Experimental* 2019, **7(Suppl 3):**001327

**INTRODUCTION.** Acute leukemia (AL) is the most common cancer of childhood. AlthoughEven great advances have been made, admission to Pediatric Intensive Care Unit (PICU) is related with an increase inof mortality rate. Data in pediatric population is scarce, which leads to a lack in information regarding outcome of patients with AL admitted to the PICU.

**OBJECTIVES.** The objective was to describe the clinic and epidemiologic characteristics of the pediatric patients with AL admitted to PICU, and the differences between emergent or planned admission. Second goal was to evaluate mortality during their PICU stay.

**METHODS.** Retrospective study conducted in a tertiary referral hospital in Barcelona, Spain (July 2010-July 2016).

All patients with acute lymphoblastic leukemia (ALL) or acute myeloid leukemia (ALM) (28 days-18 years) who required PICU admissions were included. Patients with hematopoietic stem cell transplant and CAR-T cell therapy were excluded. Mortality was differentiated between PICU and out PICU mortality. The study was approved by the ethic review board of our hospital.

**RESULTS.** Out of 156 consecutive patients diagnosed with acute leukemia, 48 patients (69 episodes), required PICU admission, 58% were female, and the median age was 10.5 years (range 0-18). There were 38 (80%) diagnosed with ALL and 10 (20%) with AML. The admission was urgent in 45 episodes (65%), the planned admission was for bronchoalveolar lavage procedure (BAL) in 12 (50%) cases. Main reasons for admission were respiratory failure (11, 16%), sepsis (11, 16%) and BAAL (12, 17%). The Pediatric Risk Score Mortality resulted in 3 points. 23 (33.3%) patients required mechanical ventilation (MV) (mean days 1;1-7) due to pneumonia in 64,3%. Inotropic support was administered in 11 (15.9%) and 2 (2.9%) required hemofiltration. 28 (40.6%) patients had multiorgan failure, and 6 (12,5%) died (2 in PICU due to sepsis and 1 due to a BAL pulmonary haemorrhage). Mean days of PICU stay were 3,8 (0-58). All these variables were statistically significantly worse in emergency admission cases compared with a planned one, p< 0.001.

Two children died during the following 60 days after PICU discharge and one during the following 6 months; all of them were patients with relapsed ALL , 2 patients were in palliative care treatment and died from progressive disease and one due to treatment toxicity (hepatic sinosuoidal obstructive syndrome). Median length of stay for patients who died in PICU was 14 days (13-15.5).

PRISM ≥5 (OR 3,12; IC95%0,9-10,86), requiring MV (OR 9,47; IC95%0,99-90) or inotropes(OR 4,97; IC95%0,6-27,87) and severe neutropenia (OR 1,26; IC95%0,36-4,44) were independent factors for death

**CONCLUSION.** Pediatric patients with AL frequently required PICU admission (almost one third) and respiratory failure and sepsis were the main cause of admission. Patient who died were the higher risk ALL cases (relapsed patients) or AML. Accurate efforts need to be conducted when these patients require MV, or inotropes and if data of multiorgan failure are present.

**REFERENCE(S)**

1. None
2. Pound CM, Johnston DL, Armstrong R, Gaboury I, Menon K (2008) The morbidity and mortality of pediatric oncology patients presenting to the intensive care unit with septic shock. Pediatr Blood Cancer 51:584–588.
3. Pollack MM, Ruttimann UE, Getson PR: Pediatric risk of mortality (PRISM) score. Crit Care Med 1988, 16: 1110–1116.
4. Kraguljac AP, Croucher D, Christian M, et al. Outcomes and predictors of mortality for patients with acute leukemia admitted to the intensive care unit. Can Respir J. 2016;2016:3027656.
5. Zinter, M.S., DuBois, S.G., Spicer, A., Matthay, K., and Sapru, A. Pediatric cancer type predicts infection rate, need for critical care intervention, and mortality in the pediatric intensive care unit. Intensive Care Med. 2014; 40: 1536–1544
6. Demaret, P., Pettersen, G., Hubert, P., Teira, P., and Emeriaud, G. The critically-ill pediatric hemato-oncology patient: epidemiology, management, and strategy of transfer to the pediatric intensive care unit. (14-5820-2-14)Ann Intensive Care. 2012; 2
7. Thakkar SG, Fu AZ, Sweetenham JW, Mciver ZA, Mohan SR, Ramsingh G, Advani AS, Sobecks R, Rybicki L, Kalaycio M, Sekeres MA. Survival and predictors of outcome in patients with acute leukemia admitted to the intensive care unit. Cancer. 2008;112(10):2233–40.
8. Maude SL, Fitzgerald JC, Fisher BT, et al. Outcome of pediatric acute myeloid leukemia patients receiving intensive care in the United States. Pediatr Crit Care Med. 2014;15:112–120.
9. Lüthi, Fabienne & Leibundgut, Kurt & Niggli, Felix & Nadal, David & Aebi, Christoph & Bodmer, Nicole & Ammann, Roland. (2012). Serious medical complications in children with cancer and fever in chemotherapy-induced neutropenia: Results of the prospective multicenter SPOG 2003 FN study. Pediatric blood & cancer. 59. 90-5.

### 000101

#### Ultrasound-guided subclavian venous access: regional survey of experience followed by tailored teaching programmes for both trainees and consultants

B. Millette[1], N. Suarez[2]
[1]Oxford University Hospitals NHS Foundation Trust, Oxford, UK;
[2]Intensive care, Oxford University Hospitals NHS Foundation Trust, Oxford, UK
**Correspondence:** B. Millette
*Intensive Care Medicine Experimental* 2019, **7(Suppl 3):**000101

**INTRODUCTION.** Subclavian central venous access (SCVA) has a lower rate of infective and thrombotic complications compared to femoral and internal jugular venous access(1). This data also demonstrates higher rates of mechanical complications such as pneumothorax, but in the context of high rates of insertion guided by landmark technique(1). The subclavian vein's anatomical position is variable(2) and the rate of mechanical complications is reduced by the use of real time ultrasound scanning (USS)(3). We hypothesised that few anaesthetic or intensive care medicine (ICM) trainees in our region had received formal training in landmark or ultrasound-guided SCVA and are not confident in it. We sought to establish if a short teaching session with live human USS and subclavian gel phantom needling practice would improve confidence.

Having developed this teaching programme, we had a great deal of interest in developing these skills from consultants in anaesthesia and ICM. We therefore refined and extended it to further promulgate these skills in our region. We aimed to increase regional use of ultrasound-guided SVCA in order to minimise central line-associated bloodstream infection (CLABSI) and mechanical complications associated with SCVA.

**METHODS.** We conducted a survey of all Anaesthetic and ICM trainees in the Thames Valley deanery in the UK to ascertain previous experience of SCVA. Having identified this need for training, we conducted teaching sessions for anaesthetic and ICM physicians. These comprised a lecture, in vivo scanning of the subclavian vein and needling a subclavian gel phantom with USS.

We also gathered data on participant grade, formal teaching experience in SCVA and number of times they had performed SCVA. We compared their confidence in performing ultrasound-guided SCVA before and after the training session using a visual analogue scale (VAS) running from 0 to 100. Continuous data were checked for normality using the Shapiro-Wilk test. When this failed to detect normality, data were compared using the Mann-Whitney test.

**RESULTS.** 49 specialty registrars in anaesthetics or ICM responded to the SCVA training survey (response rate of 49% of deanery trainees). This revealed that 33% of respondents had sited no subclavian lines and 71% had sited 5 or fewer. Only 29% had any formal training in SCVA and mean confidence score was 35 out of 100 on the VAS.

42 physicians in total were then taught over four teaching sessions. 86% had received no previous instruction in ultrasound-guided SCVA. 64% had inserted no subclavian lines under ultrasound guidance and 93% had inserted five or fewer. The median baseline confidence in SCVA was 12 and after teaching this rose to 52 on the VAS ($p < 0.001$ by Mann-Whitney U test). The sessions were highly valued by trainees and consultants alike.

**CONCLUSION.** This study demonstrates that anaesthetic/ICM physicians in our region have little experience in inserting SCVA and receive little formal training in it. Furthermore, we have shown that a short training session can improve their confidence. We have used the teaching sessions to encourage use of this technique in order to help minimise overall complications of central venous access including CLABSI and mechanical complications. Further teaching sessions are planned given the ongoing demand for these skills in our region.

**REFERENCE(S)**

1. Parienti J-J, Mongardon N, Mégarbane B, Mira J-P, Kalfon P, Gros A, et al. Intravascular Complications of Central Venous Catheterization by Insertion Site. NEJM. 2015;373(13):1220–9.
2. Lavallée C, Ayoub C, Mansour A, Lambert J, Lebon J-S, Lalu MM, et al. Subclavian and axillary vessel anatomy: a prospective observational ultrasound study. Can J Anesth. 2018;65(4):350–9.
3. Lalu MM, Fayad A, Ahmed O, Bryson GL, Fergusson DA, Barron CC, et al. Ultrasound-Guided Subclavian Vein Catheterization. Crit Care Med. 2015;43(7):1498–507.

### 000300

#### Antimicrobial effect of lysozyme in serum as a nonspecific humoral factor of immune system

V. Ziamko[1], A. Dzyadzko[2], V. Okulich[3]

[1]Viciebsk State Order of Peoples' Friendship Medical University, Viciebsk, Belarus; [2]Anestheology and intensive care unit department, Minsk Scientific and Practical Center of Surgery, Transplantation and Hematology , Minsk, Belarus; [3]Clinical microbiology department, Viciebsk State Order of Peoples' Friendship Medical University, Viciebsk, Belarus

**Correspondence:** V. Ziamko

*Intensive Care Medicine Experimental* 2019, **7(Suppl 3):**000300

**INTRODUCTION.** In modern concepts of pathogenesis of inflammatory diseases, much attention is paid to nonspecific humoral factors of immune system, including lysozyme.

**OBJECTIVES. The aim of the study** was to evaluate level of lysozyme activity in serum in patients with ventilation-associated pneumonia.

**METHODS.** A complex examination of 43 patients with ventilation-associated pneumonia and 43 practically healthy people was performed. Blood was taken from the ulnar vein before meals into sterile test tubes.

**RESULTS.** We have created a simpler and a cheaper way of evaluating lysozyme activity in biological fluids, which demands little time and has clear evaluation criteria. The invention relates to medicine, namely to laboratory diagnosis. From cell walls *of Micrococcus lysodeikticus* culture ATCC 4698 peptidoglycan substrate was prepared, and the activity of lysozyme was calculated by the formula obtained after constructing calibration curve for lysozyme, which showed the dependence of the concentration of lysozyme and the optical density of the solution of Congo red. The positive effect of the proposed method was that the substrate for the production of reaction was prepared once and could be used for a long time. The way was easy and simple in play. The sensitivity of this method to lysozyme was 0.06 mg / ml. This is enough to determine immunodeficiency states, because the content of lysozyme in biological fluids varies according to various data in a wide range from 0.2 to 28 mg / ml in normal.

It was found that the lysozyme activity in the serum was lower in patients with ventilation-associated pneumonia (246.7; 141.2-298.7 mcg/ml) than that in donors (445.5; 350.1-816.1 mcg/ml). After inactivation of the complement both in patients with ventilation-associated pneumonia (116.0; 56.5-160.1 mcg/ml) and in donors (246,0; 183,6-305,7 mcg/ml) a statistically significant decrease in lysozyme activity occured.

**CONCLUSION.** There was a statistically significant decrease of nonspecific humoral resistance in patients with bacterial infection, namely, lysozyme, which was a risk factor for the development of ventilation-associated pneumonia.

### 001699

#### Functional mechanisms of common human SP-B genetic variants-mediated susceptibility to Staphylococcus aureus pneumonia through p38 MAPK-mediated NF-κB pathway

J. Liu[1], Z. Liu[2], L. Ge[2], G. Wang[2]

[1]Department of Critical Care Medicine, Shanghai Jiaotong University, School of Medicine, Ruijin Hospital North, Shanghai, China; [2]Department of surgery, SUNY Upstate Medical University, Syracuse, USA

**Correspondence:** J. Liu

*Intensive Care Medicine Experimental* 2019, **7(Suppl 3):**001699

**INTRODUCTION.** Surfactant protein B (SP-B), a member of Saposin-like family of proteins (SAPLIP), plays roles in both lowering surface tension and antimicrobial activity in the lung. Recent studies demonstrated the patients with the C allele of SP-B gene are susceptible to bacterial pneumonia. In the study we examined the effects and molecular mechanisms of the T and C alleles of human SP-B gene with humanized transgenic mice in bacterial pneumonia model.

**METHODS.** SP-B 1580 locus polymorphisms and SP-B protein level in BALF of thirty *S. aureus*-infected pneumonia patients and thirty-five age-matched, unrelated healthy subjects were analyzed. Then, humanized transgenic (hTG) mice, which expressed the C or T allele of human SP-B genetic variants in a mouse SP-B (-/-) background, was generated and used in this study. Pneumonia model was induced using *S. aureus*(CFU: 5x108/mouse) intratracheal injection in hTG SP-B mice. Lung tissue and bronchoalveolar lavage fluid (BALF) were harvested 24h after exposure to *S. aureus*or saline (control). Histological changes were consistent with pneumonia. Cellular and molecular analysis were performed by Western blot, ELISA, CFU counts.The p38 mitogen-activated protein kinase (MAPK) and the nuclear factor-kappa B(NF-κB) signaling pathways were measured by Western blot. In vivo imaging system was also used to analyze the dynamic bacterial clearance in the lung.

**RESULTS.** Human subjects with the SP-B1580 CC genotype were more susceptible to *S. aureus*-infected pneumonia.*S. aureus*-infected pneumonia patients with SP-B-1580CC genotype exhibited significantly lower SP-B level in BALF. *S. aureus*-treated hTG SP-B-C mice exhibited higher mortality than hTG SP-B-T mice ($p<0.05$). In vivo results demonstrated that infected hTG hSP-B-C mice displayed decreased bacterial clearance 24h post-infection compared to infected hTG hSP-B-T mice. Infected hTG SP-B-C mice showed more severe lung injury (p<0.05), inflammation (p<0.01) and bacterial load in the lung compared to infected hTG SP-B-T mice 24 hrs after infection.The levels of surfactant phospholipid and surfactant proteins B, A and D in BALF, decreased significantly (p<0.01) in infected hTG SP-B-C mice, when compared with infected hTG SP-B-T mice. Moreover, infected hTG SP-B-C mice showed increased apoptotic cells and caspase-3 as well as decreased levels of Bcl-2 in the lung compared to infected hTG SP-B-T mice. Furthermore, levels of NF-κB P65, phosphorylated IkB-α and P38MAPK increased significantly in the lung of infected hTG SP-B-C mice which were related with significant increased levels of IL-6 and TNF-a in BALF than infected hTG SP-B-T mice.

**CONCLUSION.** Mice with C allele or T allele of human SP-B genetic variants exhibited different susceptibility to bacterial Staphylococcal pneumonia probably through regulating p38 MAPK-mediated NF-κB pathway related inflammation and apoptosis.

### 001373

#### Effect of a real-time automatic alerting system based on electronic medical record on rapid response system

S. Kim[1], HJ. Lee[2], E. Yang[1], SM. Lee[3], HG. Ryu[4], SB. Go[5], J. Lee[3], H. Lee[4], SY. Oh[1], EJ. Ha[1]

[1]Department of critical care medicine, Seoul National University Hospital, Seoul, Republic of Korea; [2]Department of thoracic and cardiovascular surgery and critical care medicine, Seoul National University Hospital, Seoul, Republic of Korea; [3]Department of pulmonary and critical care medicine, Seoul National University Hospital, Seoul, Republic of Korea; [4]Department of anesthesiology and critical care medicine, Seoul National University Hospital, Seoul, Republic of Korea; [5]Department of neurology and critical care medicine, Seoul National University Hospital, Seoul, Republic of Korea

**Correspondence:** H.J. Lee

*Intensive Care Medicine Experimental* 2019, **7(Suppl 3):**001373

**INTRODUCTION.** Rapid response system (RRS) has been developed for early detection of deteriorated patients. To improve the results of RRS, early detection and early intervention is warranted. However, there is no definite criteria of activation in RRS or no universal consensus for method of activations.

**OBJECTIVES.** The aim of study is to estimate the effect of a real-time automatic alerting system (AAS) based on electronic medical record (EMR) on RRS.

**METHODS.** We retrospectively reviewed the clinical data of patients who were activated by RRS in a tertiary university hospital. From August, 2015, RRS have been developed with activation by clinicians' calls and by active screening of high-risk patients in surgical wards. Activation criteria was suggested as Table. From November, 2016, we added a new real-time AAS to conventional activation systems. We compared the periods before and after the application of AAS in RRS as unplanned ICU transfer and other clinical outcomes; from January to October, 2016 (pre-AAS) vs from January to October, 2017 (post-AAS1) vs from January to October, 2018 (post-AAS2).

**RESULTS.** After application of AAS, the number of RRS activation significantly increased from 15.5 per 1,000 admissions in pre-AAS to 30.7 in post-AAS1 (p < 0.001), but decreased after adaptation (9.3 per 1,000 admission in post-AAS2). AAS activation (42.7%) was the most common cause of RRS activation in post-AAS1, followed by active screening (41.4%). Modified early warning score of activated patients in post-AAS2 was significantly higher than that of pre-AAS or post-AAS1 (p < 0.001). During study period, 264 unplanned ICU transfer occurred (2.88 per 1,000 admission in pre-AAS, 2.77 in post-AAS1, and 2.40 in post-AAS2, p = 0.475). In post-AAS1, 91.1% were activated by AAS before unplanned ICU transfer (62.5% in pre-AAS, 64.1% in post-AAS2, p < 0.01). There were no differences in ICU days and cardiopulmonary resuscitation rate among three groups (p = 0.341 and p = 0.49).

**CONCLUSION.** Use of a real-time AAS based on EMR might be helpful to detect unplanned ICU transfer. However, a learning period would be required for adaptation with increased RRS alarms.

1. HJ Lee is supported by the international fellowship program funded by Takeda Science Foundation.

**Table 1 (abstract 001373).** See text for description

| System | Pre-AAS (call activation criteria) | Post-AAS (automatic alert system) |
|---|---|---|
| Respiratory | - RR ≤ 8/min, or ≥ 28/min<br>- SpO2 ≤ 90%<br>- Sudden respiratory distressed symptom | - RR ≤ 8/min, or ≥ 28/min<br>- SpO2 ≤ 90% |
| Cardiovascular | - HR ≤ 40/min, or ≥ 130/min<br>- SBP ≤ 80mmHg, or ≥ 200mmHg<br>- SBP 80~90 mmHg with symptoms<br>- Chest pain sustained with nitroglycerin medication | - HR ≤ 40/min, or ≥ 130/min<br>- SBP ≤ 80mmHg, or ≥ 200mmHg |
| Neurologic | - Sudden deterioration of consciousness<br>- Sudden onset facial or extremities paralysis<br>- New onset epilepsy<br>- Non-specific unexplained agitation | |
| Others | - Concerns by physician<br>- Hypoperfusion signs | |

### 000429

#### Comparison of factors associated with adult and pediatric ventilator-associated events

Y. Peña-López[1], S. Ramírez-Estrada[2], A. Romero[2], L. Lagunes[3], D. Koulenti[4], J. Rello[2]

[1]Pediatric Critical Care Department, Vall d'Hebron Barcelona Hospital Campus, Barcelona, Spain; [2]Vall d'hebron research institute, Vall d'Hebron Barcelona Hospital Campus, Barcelona, Spain; [3]Critical care, Vall D Hebron, Barcelona, Spain; [4]Critical care department, Attikon University Hospital, Athens, Greece

**Correspondence:** Y. Peña-López

*Intensive Care Medicine Experimental* 2019, **7(Suppl 3):**000429

**INTRODUCTION.** Few studies have evaluated risk factors for ventilator-associated events (VAE) and there is no data comparing them between the adult and children population.

**OBJECTIVES.** To identify differences in factors associated with VAE in patients who underwent mechanical ventilation (MV) ≥48 h, between adults and children.

**METHODS.** Secondary analysis of a prospective combined multicentre cohort (adult and children). Six Intensive Care Units (ICU) in 4 European countries (France, Greece, Spain, and Turkey) and one children's ICU (Spain). Subjects who had undergone MV ≥48 hours were included. Age ≤ 16 yr was categorized as children. In subjects with multiple episodes of MV, only the first one was eligible. The adult definitions for VAEs were adjusted to the 2015 update of the 2013 United States Center for Disease Control and Prevention definition (1). The pediatric definition of VAE (PedVAE) was reported by Peña-López et al. (2). Association of risk factors with VAE and PedVAE was estimated through multivariate logistic regression analysis.

**RESULTS.** A hundred and sixty-three MV episodes in adults and 88 in children (2,178 and 731 ventilator-days respectively) were included. Median age was 60 yr in adults and 1.0 yr in children. Eighty-three adults (50.9%) and 43 children (48.9%) had high severity ICU index

(p=0.650). The median (IQR) duration of MV was 9 (6 – 20) in adults and 7 (4 - 9) days in children (p<0.005). We documented 76 VAE in adults (46.6%) and 23 PedVAE in children (26.1%) (p=0.002). The median (IQR) onset time of VAE and PedVAE was 6 (4-9) and 4 (3 - 6) days (p=0.065), with 52 (68.4%) and 19 (82.6%) presenting within the first 7 days of MV, respectively (p=0.186). MV> 7 days was associated with VAE (OR 5.03, 95%CI 1.95-13; p=0.001) and PedVAE (OR 4.55; 95%CI 1.3-15.86; p=0.017). Other independent predictors of VAE were selective digestive decontamination (SDD) absence (OR 4.45; 95%CI 1.78-11.12), tracheostomy (OR 3.55; 95%CI 1.35-9.32), midazolam use>3 days (OR 3.48; 95%CI 1.19-10.21), and surgical/trauma patients (OR 2.62; 95%CI 1.1-6.26) (p<0.05). High severity score, delirium, early mobility, and pharmacological paralysis did not show a statistically significant association with VAE or PedVAE on multivariate logistic regression analysis. In children, SDD and tracheostomy were not performed on the analysis due to the low number of cases. In contrast to adults, surgical/trauma patients and midazolam use>3 days in children did not show a statistically significant association with PedVAE and there was a trend toward increased risk of PedVAE in the pediatric population when the reason for intubation was respiratory failure (OR 3.35; 95%CI 0.89-12.57; p=0.071).

**CONCLUSION.** Future bundles should include SDD implementation and midazolam restriction as potential strategies to prevent VAEs in adults. Respiratory failure as cause of intubation may be more determinant for PedVAE than prolonged midazolam use in children.

**REFERENCE(S)**

1. CDC Device-associated Module. Ventilator-Associated Event (VAE), January 2019. https://www.cdc.gov/nhsn/PDFs/pscManual/10-VAE_FINAL.pdf
2. Peña-Lopez Y, Pujol M, Campins M et al. Clin Microbiol Infect. 2018;24(7):732-7.
3. Supported in part by CIBERES; I Salud Carlos III, Madrid, Spain.
4. Supported in part by the European Society of Clinical Microbiology and Infectious Diseases (ESCIMID) Study Group for Infections in Critically ill Patients (ESGCIP), Basel, Switzerland

### 000495

#### Reducing Unplaned extubation and 48 hours re-intubation rate by implantation of Informatics System and Care Bundle in ICU

CC. Chao

{street_address}, Taipei, Taiwan

*Intensive Care Medicine Experimental* 2019, **7(Suppl 3):**000495

**INTRODUCTION.** The ventilator protects airway in patients who are critically ill, and there are many reasons for a patient with failure to wean from ventilator. How to successfully wean patients from ventilator requires consideration of various factors.

**OBJECTIVES.** We want to detect the effect of implantation of informatics system and care bundle in ICU on unplaned extubation and re-intubation rate in the ICU.

**METHODS.** Taipei Medical University Hospital is a community based teaching hospital with 736 beds capacity (includes 58 ICU beds). We set up electronic ICU (e-ICU) to automatically upload ventilator parameters and clinical data within critical care unit for providing real time information to the care team in Feb 2017. We use e-ICU software with automatic upload clinical information of ventilator: FiO2,, PaO2/FiO2, PEEP, RSBI(rapid shallow breathing index)Tidal volumeRespiratory rateMinute ventilation, minute Ventilation. We use ventilator associated bundle (VAP) and continuously monitor all the parameters including laboratory results, image findings and vital signs. This is a before and after study aims to detect the effect of implantation of informatics system and care bundle in ICU.

**RESULTS.** There are 1857 patients who admitted to our ICU during this period (915patients before e-ICU and 942 after). There are 458 (50.1%) patients received intubation with ventilator support and 472 patients (50.1%) after. There is no significant difference in background (APACHE II, gender and age) and mortality rate (33.5% vs 32.9%; p=0.39) between the two groups. The duration of ventilator usage was not significantly different (10.3 days before and 10.1 days after; p=0.46), and 48 hours re-intubation rate reduced from 6.98% (n=32) to 3.39% (n=16) and un-expected extubation reduced from 2.0% (n=9) to 0.2 % (n=1).

**CONCLUSION.** Implantation of information system (e-ICU) and care bundle in ICU can help reduce unplaned extubation and 48 hours re-intubation rate.

**Table 1 (abstract 000495).** See text for description

| | Before (n=915) | After (n=942) | P value |
|---|---|---|---|
| Male | 546 (69.7%) | 549(58.2%) | 0.40 |
| Age | 68.0 ± 0.6 | 67.5 ± 0.7 | 0.12 |
| APACHE II | 11.9 ± 0.3 | 11.8 ± 0.3 | 0.20 |
| Mortality | 317 (33.5%) | 310(32.9%) | 0.39 |
| Patient with ventilator support | 458 (50.1%) | 472(50.1%) | 0.59 |
| Ventilator days | 10.3±12.5 | 10.1±13.6 | 0.46 |
| Re-intubation with  48 hours | 32(6.98%) | 16(3.39%) | 0.001 |
| Un-expected extubation | 9(2.0%) | 1(0.2%) | 0.007 |

### 000923

#### Assessment of the adherence to tidal volume (TV) recommendations through the information stored into the clinical information system (CIS). Creation of fully automated and more accurate quality indicators

S. MANRIQUE[1], J. Gómez[1], G. Sirgo[1], F. Estaban[1], A. Rodriguez[2], M. Bodí[2]

[1]MEDICINA INTENSIVA, Hospital Universitari de Tarragona Joan XXIII, Tarragona, Spain; [2]Uci, Hospital Universitari de Tarragona Joan XXIII, Tarragona, Spain

**Correspondence:** S. MANRIQUE

*Intensive Care Medicine Experimental* 2019, **7(Suppl 3):**000923

**INTRODUCTION.** Mechanical ventilation (MV) may produce ventilation induced lung injury (VILI). To solve it, protective mechanical ventilation (PMV) appears as TV<8ml/Kg of predicted body weight (PBW) and Plateau pressure <30 cmH2O. Spanish society of intensive critical care and coronary units (SEMYCIUC) have created an indicator to evaluate the adherence to clinical practice guidelines. MV is dynamic, so few manual data collection entails a huge loss of information. Thanks to CIS we can collect MV data every two minutes, and create fully automated and more accurate quality indicators.

**OBJECTIVES.** Evaluate clinical practice adherence to TV recommendations. Create fully automated and more accurate quality indicators through the data stored into the CIS.

**METHODS.** Retrospective study of cohorts carried out in a 30-bed polyvalent ICU. Inclusion criteria: patients admitted to ICU (2015-2018) connected to MV. Exclusion criteria: Patient whose height and/or APACHE was not registered and height <140cm. PBW and the % of time in which each patient had a TV> 8ml/Kg PBW (%_OV) was calculated automatically in the ETL (Extract, Transform and Load) process. Cohort was divided into quartiles according to % T_OV (Q1: lower % T_OV and Q4: higher % T_OV) and extreme quartiles were compared based on 24 variables: demographic, clinical, respiratory modality (volume control (VC) or pressure support (SP)) and evolution. A subanalysis of extreme quartiles according to %_OV within the Q4 of % T_OV was carried out using the same methodology. We proposed two indicators: 1) Patients with adequate TV (nº of patients>80%time with TV<8ml/Kg PBW/ total nº of patients with MV x100; proposed standard> 90%), 2) Time of inadequate TV (Total time_OV/ Total time in MV x100, proposed standard <20%). Statistical analysis was performed using R (https://cran.r-project.org/), applying the 'chi2' test for categorical variables and the 'Mann-Whitney U' for continuous ones.

**RESULTS.** 1071 patients were included. Median age 64 (51-73), 67,3% male, median APACHE 25 (20-31) and median SOFA 7 (4-9). Median TV was 0,53L (0,49-0,58). % of patients with adequate TV was 15,8% and total time of inadequate TV was 63,2% (Q1: 14,9%, Q2: 47,4%, Q3: 76% and Q4: 95,5%). Higher height (and as consequence higher

PBW), males, lower age and lower APACHE II and SOFA values were associated with lower %T_OV. There were also significant differences between respiratory modality, being higher those referred to VC. Overall mortality was 27,7%, without significant differences between Q1 and Q4, neither in MV days nor stay in ICU. In our subanalysis, patients in Q4 of % T_OV with <%OV had shorter ICU stays.

**CONCLUSION.** CIS are an opportunity to create fully automated and more accurate quality indications to evaluate PMV adherence. Patients with lower TV are taller, with higher PBW. It will be necessary future investigation to adjust the proposed standards.

**REFERENCE(S)**

1. Murias,G; Nin, N and Bruhn, A. Lesión pulmonar inducida por la ventilación mecánica invasiva en Rodríguez, A; Bodí, M; Bruhn, F; Gordo, F; Magret, M; Nin, N y Rios, F. (2017) Ventilación mecánica. Fisiopatología respiratoria aplicada. 175-180. Argentina: Ediciones journal.
2. Ramos, LA y Benito S. Complicaciones de la ventilación mecánica en Ramos, LA y 3Benito S. 2012. Fundamentos de ventilación mecánica.197-208. Barcelona: Marge Medica Books.
3. Claverias, L; De Mendoza, D y Fernández, E. Fisiopatologia de la lesión pulmonar aguda y del síndrome de distrés respiratorio agudo. en Rodriguez, A; Bodí, M, Do Pico, JL y Restrepo, M. 2013. Medicina Intensiva. Bases fisiopatológicas del tratamiento. (127-131). Argentina: Ediciones journal
4. Claverias, L; Albaiceta, GM y Fernández, E. Bases del tratamiento de la lesión pulmonar aguda y el síndrome de distrés respiratorio agudo. en Rodriguez, A; Bodí, M, Do Pico, JL y Restrepo, M. 2013. Medicina Intensiva. Bases fisiopatológicas del tratamiento. (132-135). Argentina: Ediciones journal
5. Peñuelas, O; Lorente, JA; Llompart, JA; Terceros, L; Sánchez, M; Domínguez, JM; Guerrero, F; Homar, J; Enríquez, P; Herrán, R. Indicadores de calidad en insuficiencia respiratoria aguda. en Sociedad Española de Medicina Intensiva crítica y unidades coronarias (SEMICYUC). Indicadores de calidad del enfermo crítico. (pp 64.) Actualización 2017
6. Serpa, A; Oliveira, S; Manetta, JA; Galvao, V; Crepaldi, D; De Oliveira, M; Toledo,MC and Schultz, MC. "Association between use of lung protective ventilation with lower tidal volumes and clinical outcomes among patients without acute respiratory distress syndrome. Metananalysis." JAMA Vol 308, No. 16 (October 2012):1651-1659.
7. Guidelines on the management of acute respiratory distress syndrome. Version 1. July 2018.
8. Bodí, M; Blanch, LI y Maspon, R. "Clinical information systems: An opportunity to measure value, investigate and innovate from the real world". Med Intensiva. 2017;41(5):316-318.
9. Sirgo, G; Esteban, F; Gómez, J; Moreno, G; Rodríguez, A; Blanch, L; Guardiola, JJ; Garcia R, De Haro, L y Bodí, M. Validation of the ICU-DaMa tool for automatically extracting variables for minimum dataset and quality indicators: The importance of data quality assessment. International Journal of Medical Informatics 112 (2018) 166–172.
10. Sjoding, MW;Gong, MN; Haas,CF y Iwashyna, YJ. "Evaluating Delivery of Low Tidal Volume Ventilation in Six ICUs Using Electronic Health Record Data". Society of Critical Care Medicine. 2019. Volume 47: 56-61.

11. Grant acknowledgment to FIS PI16/00491

### 001010

#### Early goal directed mobility using novel information system improve outcome in intensive care unit

YM. Lue[1], HF. Yang[1], HC. Chung[1], HL. Lin[2], SH. Huang[1], MS. Huang[1], YP. Yin[1], PL. Wu[1], TX. Guo[1], HY. Hsu[1], ML. Yeh[1], YH. Huang[1], KP. Chen[1], SH. Kuo[2], HL. Liang[2], WC. Huang[2], CP. Liu[2]
[1]Department of nursing, Kaohsiung Veterans General Hospital, Kaohsiung, Taiwan; [2]Critical care medicine and cardiovascular center, Kaohsiung Veterans General Hospital, Kaohsiung, Taiwan
**Correspondence:** W.C. Huang

*Intensive Care Medicine Experimental* 2019, **7(Suppl 3):**001010

**INTRODUCTION.** Early mobilization in intensive care unit (ICU) is a candidate intervention to reduce the incidence and severity of ICU acquired weakness and improve outcomes. Implementing early goal directed mobility (EGDM) was shown to improve duration of mechanical ventilation, ICU stay, long-term functional independence, and possibly mortality. However, it remained challenging issue in daily practice.

**OBJECTIVES.** The aim of this study is to investigate the impact of early goal directed mobility using novel information system on patients' outcome in intensive care unit

**METHODS.** All consecutive patients form 2017-2018 in adult ICU were enrolled. The key interventions include novel early rehabilitation*information system* and virtual reality rehabilitation system for critical patients.The patientswere divided into three periods: pre-EDGM system period from Jan to July 2017, EDGM system setting period from August to September 2017 and post-EDGM system period from October 2017 to December 2018.

**RESULTS.** The early rehabilitationrate improved from 17.1%in pre-EDGM system period, to 20‰ in EDGM system setting period and to 95.1% in post-EDGM system period (p<0.05).Average ICU stay decrease from 7.9 days to 6 days after intervention (p<0.05).Average ventilator days improved from 5.5 days to 4.9 days in post-EDGM system period (p<0.05). The incidence of ventilator-associated pneumonia decreased from 1.3%to 0.83‰(p<0.05).

**CONCLUSION.** The study showed that implementation ofearly goal directed mobility using novel information system could increase early rehabilitation rate, and reduceaverage ICU stay and ventilator days. Furthermore, the incidence of ventilator-associated pneumonia also improved.

### 001359

#### Intravenous immunoglobulin therapy is associated with favorable outcome in patients with severe infection having a low level of immunoglobulin G

M. Akatsuka[1], Y. Masuda[1], H. Tatsumi[1], M. Yamakage[2]
[1]Department of Intensive Care Medicine, Sapporo Medical University School of Medicine, Sapporo, Japan; [2]Department of anesthesiology, Sapporo Medical University School of Medicine, Sapporo, Japan
**Correspondence:** M. Akatsuka

*Intensive Care Medicine Experimental* 2019, **7(Suppl 3):**001359

**INTRODUCTION.** Immunoglobulins act as one of host defense systems against microorganisms such as bacteria, virus, and fungi. Severe infection causes sepsis and the related multiple organ failure, resulting in the cause of death in intensive care unit. Recent study shows that serum IgG concentration in sepsis patients is often low and the patients with hypo-IgG have high mortality (1). It is assumed that increased capillary permeability, increased catabolism, and the consuming effect of antigen-antibody reactions can reduce immunoglobulin concentrations. However, the precise mechanisms underlying low IgG in sepsis remains unclear. There have been many studies showing that low IgG level is associated with poor prognosis in sepsis patients. However, the effectiveness of supplementation of intravenous immunoglobulin (IVIG) for improving mortality has not been elucidated.

**OBJECTIVES.** We conducted this study to determine whether administration of IVIG for patients with a low IgG level can improve the prognosis.

**METHODS.** This study was conducted retrospectively. Inclusion criteria were the patients with severe infection in whom serum IgG level was determined on admission to the ICU. First, we investigated the prognosis of the patients and drew the Receiver-operating characteristic (ROC) curve. From the ROC curve, we determined the cut off of serum IgG level. Second, we identified the patients with low IgG below the cut off level. Information on patients' characteristics, severity score (APACHE II, SOFA score), IgG level, and the dose of IVIG given was obtained from medical records in the period from January 2013 to August 2018. The primary outcome was set to 28-day mortality.

**RESULTS.** There were 247 patients with infection in whom serum IgG level was determined on admission to the ICU during the study period. The mortality rate of the patients was 23.9%, and we determined the low IgG level below 663mg/dL from the ROC curve. Sixty-one of those patients had a low IgG level. We divided the patients into two groups according to administration of IVIG given: non-IVIG group (n=21) and IVIG group (n=40). The 28-day mortality rate in the IVIG group were significantly lower than that in the non-IVIG group (p = 0.024).

**CONCLUSION.** Our results demonstrated that administration of IVIG for patients with a low IgG level is associated with improvement in mortality. These findings indicate the rationale for performing a prospective study with a large number of patients to investigate the efficacy of IVIG therapy for patients with severe infection with low IgG levels.

### 001600

#### Organ Donation Trends in the UK: a Cardio-thoracic Intensive Care Unit Perspective

V. Della Torre[1], A. Rubino[2], D. Walford[3]
[1]Intensive Care, Imperial Healtha, London, UK; [2]Intensive care, Royal Papworth Hospital - Opening April 2019, Cambridge, UK; [3]Intensive care-specialist nurse-organ donation, east organ donation service team, Royal Papworth Hospital - Opening April 2019, Cambridge, UK
**Correspondence:** V. Della Torre
*Intensive Care Medicine Experimental* 2019, **7(Suppl 3):**001600

**INTRODUCTION.** The last decade has seen a remarkable change in the landscape of organ donation after brain death (DBD) and circulatory death (DCD) in the UK1. The Organ Donation Taskforce highlighted the urgent need to address the lack of a clear ethical, legal and professional practice framework2, and it has been established that higher referral rates and the presence of a specialist nurse for organ donation (SNOD) lead to higher successful donors3,4.We publish the trends in organ donation of Cardiothoracic (CT) and non-CT Intensive Care Units (ICUs) in the UK, from 1st April 2014 to 31stDecember 2018.

**OBJECTIVES.** We conducted a retrospective analysis to demonstrate the reasons behind these sustained improvements in number of referrals and organ donations. We identified the differences between CT-transplant and non CT-transplant ICUs.

**METHODS.** Data analysed were extracted from the NHSBlood&Transplant (NHSBT) potential donor audit, the organ donor register and renal registry. Data collection included all patients deceased at Royal Papworth CT-ICU from April 2014 to December 2018 and we compared these with other CT-ICUs. Variables analysed (Key metrics):

- *Referral*– If suitable patients are not referred, the patient's decision to be an organ donor is not honored.
- *SNOD*- The consent rate in the UK is higher when a SNOD is present.
- *Waiting lists* - The number of patients receiving a life-saving or life-changing organ transplant in the UK is increasing, but patients are still dying while waiting.

**RESULTS.** In 2007/08 there were 13.4 deceased donors pmp, in total 809 donors. By 2017/18 this has risen to 24.9 pmp (1574 donors). However during 2017/18 there were still 426 deaths waiting for transplant. Transplant CT-ICUs had lower referral rates that non-transplant CT-ICUs.

**CONCLUSION.**

The reasons why transplant CT-ICUs have lower referral rates have raised questions, as it would seem these units should have a vested interest in the benefits of transplant. Some have postulated that transplant CT-ICUs are more likely to make local decisions that certain patients may not be candidates. Furthermore, transplant centres offers invasive organ support, including extracorporeal life support (ECMO) and mechanical circulatory assist device; therefore withdrawal of patients happens in a later stage. In addition, the issue of confirming death on such devices is contentious5,6,7. Clear definition of key metrics (referral, SNOD and waiting list), that demonstrably change outcome, contributes to improvement in outcome towards the target donor numbers, advocated in the latest NHS Blood & Transplants document *"Taking Organ Transplantation to 2020"4*. Royal Papworth Hospital has tackled this by empowering the "embedded SNODs" in promoting teaching within the staff, and supporting a multidisciplinary team-based approach. This has contributed to increase our referral rates and SNOD involvement from 79.5% and 76% respectively, in 2017, to a 100% in both metrics in 2018. Donation is now considered to be a component of good end of life care. Furthermore, the UK is moving towards deemed consent for organ donation8. An implementing strategy has been used at Royal Papworth in the last year: referrals of all deteriorating patients, at the very early stage of the withdrawal process, identification of potential donors, embedded SNODs, incorporation of organ donation audit into the monthly Morbidity&Mortality Meetings. The results of this strategy have been: increased number of referral to the success of 100% referrals, earlier referrals, and a more efficient multidisciplinary work with Transplant Surgeons.

**REFERENCE(S)**

1. Jonson, RL., Bradbury, LL., Martin, K. Organ Donation and Transplantation in the UK - The Last Decade: A Report From the UK National Transplant Registry. Transplantation. 2014; 19: S1-S27.
2. Organ Donation Taskforce, Department of Health UK, Organs for transplant: A report from the Organ Donation Taskforce 2008.
3. NHS Blood and Transplant. NHS Blood and Transplant annual report and accounts 2016 to 2017.
4. UK Organ Donation Taskforce. Taking Organ Donation to 2020 NHS Blood and Transplant. NHS Blood and Transplant annual report and accounts 2016to2017.https://www.gov.uk/government/uploads/system/uploads/attachment_data/file/625326/NHSBT_annual_report_accounts_2016_2017.pdf
5. Meadows C, Toolan M, Slack A, et al. Diagnosis of death using neurological criteria in adult patients on extracorporeal membrane oxygenation: Development of UK guidance. Journal of the Intensive Care Soiety.2019. 0(0):1-5
6. Bein T, Muller T and Citerio G. Determination of brain death under extracorporeal life support. Intensive Care Med.2019;45:364-366
7. Bein T, Brodie D. Understanding ethical decisions for patients on extracorporeal life support. Intesive Care Med. 2017;43:1510-1511 8. Max and Keyra's Law- The Opt out Bill, English Organ Donation Law,2019.
8. Acknowledgments: With special thanks to the Embedded Specialist Nurse Team for Organ Donation, Royal Papworth Hospital)

### 001691

#### Conditions for responsible innovation of prognostication in postanoxic coma

M. Boenink[1], M. Mertens[1], M. Weernink[2], J. Van Til[2], J. Hofmeijer[3]
[1]Philosophy, University of Twente / Universiteit Twente, Enschede, Netherlands; [2]Health technology & services research, University of Twente / Universiteit Twente, Enschede, Netherlands; [3]Clinical neurophysiology, University of Twente / Universiteit Twente, Enschede, Netherlands
**Correspondence:** M. Boenink
*Intensive Care Medicine Experimental* 2019, **7(Suppl 3):**001691

**INTRODUCTION.** There is increasing evidence that continuous EEG monitoring (cEEG) can reliably predict poor, and possibly also good outcome of patients in postanoxic coma within 24 hours after cardiac arrest (1, 2). As a result, cEEG may soon be included in clinical guidelines for prognostication of these patients. The acceptability of a novel medical technology, however, also hinges on its broader impacts on practices of care and society at large. Responsible innovation implies that possible impacts of a new technology are anticipated and discussed with stakeholders and responded to during its development (3).

**OBJECTIVES.** To explore the broader impacts of cEEG on practices of care and identify under which conditions cEEG may be a responsible innovation.

**METHODS.** Ethnographic fieldwork was conducted on 2 Dutch IC's and 1 American IC, all engaged in clinical research with cEEG. Explorative, semi-structured interviews were held with 11 care professionals, 2 surviving patients and 7 relatives of patients who were or had been in postanoxic coma (all Dutch), focusing on what they valued in and were concerned about in relation to prognostic practice and the impacts of cEEG. Additionally, semi-structured interviews were held with 22 Dutch professionals who may become involved in the implementation of cEEG, focusing on enablers and constraints of implementation. Field notes and the first set of interview transcripts were qualitatively analysed, first inductively and then deductively with insights from the ethical literature. The second set of transcripts was analysed deductively, based on well-known determinants of implementation. Findings were discussed with and validated by a panel of representatives of stakeholders.

**RESULTS.** It was clear to most of our interviewees that cEEG may have added value for prognostication of patients in postanoxic coma, but they also pointed to potential impacts that may be less desirable. The concerns identified centred around: 1. the meaning of cEEG measurements (for example of 'grey' results); 2. the interpretation of measurements (like the tension between ease of use and careful interpretation); and 3. communication about measurements (for example with regard to timing).

**CONCLUSION.** The concerns identified point to several conditions for the responsible development, design as well as organizational embedding of cEEG, including: more research on long term outcomes, in particular of those with 'grey' cEEG results; an interface that reduces the risk of lay interpretations of measurements; and different communication policies for poor, good and 'grey' results.

**REFERENCE(S)**

1. Hofmeijer, J. et al. Early EEG Contributes to Multimodal Outcome Prediction of Postanoxic Coma. Neurology 2015, 85(2): p.137–143.
2. Spalletti, M. et al. Single Electroencephalographic Patterns as Specific and Time-dependent Indicators of Good and Poor Outcome After Cardiac Arrest. Clin Neurophysiol 2016, 127(7): p.2610–2617.
3. Stilgoe, J. et al. Developing a framework for responsible innovation. Res Policy 2013, 42(9): p.1568-1580.
4. Funded by NWO (Dutch Organization for Scientific Research), Responsible Innovation programme, grant nr. 313-99-309.

#### 001618

**Effects after mandatory documentation of therapeutic interventions limiting decisions in Neuro-ICU**

H. Novak[1], D. Pavlovic[2]
[1]Neuro Intensive Care Unit, Christian Doppler Klinik, Salzburg, Austria; [2]Neurosurgery intensive care unit, Christian Doppler Klinik, Salzburg, Austria
**Correspondence:** H. Novak
*Intensive Care Medicine Experimental* 2019, **7(Suppl 3):**001618

**INTRODUCTION.** Within a six-years-period (2013-2018) 3554 patients were treated ad Neuro-ICU of CDK Salzburg. Average ICU-mortality was 6.7%, average length of stay in ICU (LOS) was 5.4 days.

Average LOS of ICU-survivers was 5.2 days, of ICU-fatalities was 7.9 days.

From 2016 to 2017 we started standardized mandatory documentation of formerly only sporadic decision for limitation of therapeutic interventions (TLD) in order to prevent futile ICU-interventions.

**OBJECTIVES.** This paper aims to compare ICU-mortality and LOS during first of four years before (2013) to last of two years after (2018) implementation of TLD.

**METHODS.** ICU-patients-statistics and patients records were reviewed retrospectively for fatalities, LOS and TLD.

**RESULTS.** Within six years overall ICU-mortality sunk from 7.1% four years before to 4.8% two years after implementation of standardized documentation of TLD. Overall LOS was 6.1 days in 2013 and 5.2 days in 2018, average LOS of deceased patients was 7.9 days, 11.9 days four years before and 5.5 days two years after implementation of TLD.

**CONCLUSION.** Against opposite apprehensions implementation of TLD decreased average Neuro-ICU-mortality and LOS of deceased patients. As intended but exceeding anticipations, length of futile ICU-treatment could be cut into half.

**REFERENCE(S)**

1. Kagerl, T. (2014). Analyse von ethischen Konflikten in der Intensivmedizin bei Ärzten und Pflegenden. Unveröffentlichte Inaugural – Dissertationsarbeit, Fakultät für Medizin, Regensburg.
2. No grants to be acknowledged

### HSRO / NIC - Surgery and brain injury

#### 001441

**Real world data on cancer patients admitted to an intensive care unit after Major Head and Neck Surgery**

R. Neto[1], I. Leão[2], A. L. Rios[1], D. Adrião[1], P. Fernandes[1], P. Castelões[1]
[1]Intensive care medicine, Centro Hospitalar de Vila Nova Gaia/Espinho, Vila Nova de Gaia, Portugal; [2]Medical oncology, Centro Hospitalar de Vila Nova Gaia/Espinho, Vila Nova de Gaia, Portugal
**Correspondence:** R. Neto
*Intensive Care Medicine Experimental* 2019, **7(Suppl 3):**001441

**INTRODUCTION.** Major head and neck surgery with flap reconstruction is a frequent treatment option for cancer patients. It may be indicated for primary tumor care (head and neck cancer) or to manage treatment' complications (e.g. osteonecrosis).

This intervention presents a high risk of airway compromise(1) and most patients require post-operative vigilance in an intensive care setting due to their comorbidities, aggressiveness of the surgical procedure and to ensure adequate free flap monitoring perfusion(2).

**OBJECTIVES.** Understand critical care outcomes of cancer patients submitted to major head and neck surgery with flap reconstruction.

**METHODS.** Retrospective cohort study of cancer patients admitted to a polyvalent intensive care unit (ICU), between 2015–2018, after major head and neck surgery with flap reconstruction. Exclusion criteria were ICU stay <24h and trauma patients.

**RESULTS.** During this period, 209 patients were admitted to the ICU after this procedure and 55 fulfilled the enrollment criteria.

The majority of these patients were proposed for surgery as part of their anti-cancer treatment, but 12 were due to osteonecrosis (2 medical and 10 after radiotherapy). Median age was 60 years, most patients were male (72.7%) with an ECOG-PS of 0-1 (94.5%) and a locally advanced disease (52.7%). The median weight at admission was 68Kg (min. 57Kg - max. 76.25Kg).

Median surgery length was 10h30 (min. 7h20 – max. 14h30), 36.4% of patients required intra-operative blood transfusion and intra-operative net fluid balance was positive in the majority of patients (69.1%). Median APACHE and SAPS II scores were 13 and 24, respectively. In the post-operative setting, 67.3% of patients required mechanical ventilation (median duration of 4 days) and 29.1% vasopressor support (median 3.5 days). Flap necrosis was the most common surgical complication (10.9%) and 13 patients (23.6%) required re-intervention. One patient developed bacteremia associated with central venous line. Median ICU length of stay was 4 days (min. 24h - max. 28 days). There were no deaths in the ICU, with a 9% mortality rate at 6 months after discharge.

Weight correlated with the duration of ventilation (r: 0.370; p=0.026) and with the ICU stay (r: 0.305; p=0.025). Longer surgery duration was associated with prolonged ICU stay (>5 days) (p=0.049) on univariate analysis. On multivariate analysis, positive fluid balance was associated with prolonged ICU stay (p=0.049).

**CONCLUSION.** Patients submitted to major head and neck surgery had good outcomes during ICU stay. Positive net fluid balance during surgery was significantly associated with prolonged ICU stay in this subset of patients and should be closely monitored.

**REFERENCE(S)**

1. Arshad H, Ozer HG, Thatcher A, Old M, Ozer E, Agarwal A, et al. Intensive care unit versus non-intensive care unit postoperative management of head and neck free flaps: comparative effectiveness and cost comparisons. Head & neck. 2014;36(4):536-9.
2. Marsh M, Elliott S, Anand R, Brennan PA. Early postoperative care for free flap head & neck reconstructive surgery–a national survey of practice. The British journal of oral & maxillofacial surgery. 2009;47(3):182-5.
3. None

### 001669

#### Outcome of Diabetic patients with Cardiomyopathy in Critical Care Unit: Hospital and Short Term Outcome in a period of 6 Months to One Year

N. awadh[1], S. El-Hadidy-Samir[2], K. Hassan[2]

[1]Cairo University, Faculty Of Medicine, Kasr Al Ainy, Cairo, Egypt; [2]Critical care medicine, Cairo University, Faculty Of Medicine, Kasr Al Ainy, Cairo, Egypt

**Correspondence:** N. awadh

*Intensive Care Medicine Experimental* 2019, **7(Suppl 3):**001669

**INTRODUCTION.** : Diabetes mellitus (DM) is major risk factor for heart failure (HF) and coronary artery disease (CAD).DM causes structural changes involving the left ventricle (LV) systolic and diastolic function.

**OBJECTIVES.** To compare diabetic patients who have ischemic cardiomyopathy (ICM) to those with non ischemic cardiomyopathy (NICM) in terms of clinical course, left ventricular (LV) systolic function, diastolic function, in hospital, short term and long term mortality.

**METHODS.** Sixty diabetic patients with heart failure and left ventricular ejection fraction ( LVEF ≤ 35% ) admitted to Critical Care Medicine department Cairo University in a period of 16 months divided into two groups based on coronary angiography results group I ( ICM) n=32 patients and group II (NICM)n=28 patients.

**RESULTS. Results** Group I patients had higher mean age (63±7) years, (p=0.004). Hypertension ( p ˂0.001) and dyslipidemia ( p ˂0.008) was significant in group I compared to group II. No significant differences were found regarding LVEF, global longitudinal strain (GLS), E/A and E/É ratio in both groups. A significant wall motion score index (WMSI) in group I; (1.4±0.4) vs group II;(1.1±0.2),( p= 0.005).

In the study, 4 patients in group I had cardiogenic shock with no documented in hospital mortality. At 6 months a significant mortality difference was found between the two groups, (p= 0.006), while at one year there was no significant mortality difference between the two groups,(p=0.077).In comparison of survived and non survived patients at 6 months the LVEF,GLS were significantly higher in those who survived (41±5.3%) vs (23±6.3%),(p˂0.001) and GLS (-8±2.4%) vs (-4.67±2.7%),(p= 0.027).The E/A and E/É ratio was significantly lower at 6 months in survived patients (1.33±0.91 vs 1.8±0.61),(p 0.037) and E/É (12.12±7.8 vs 21±3.7),(p= 0.016) respectively. At one year, there was a significant difference between survived and non survived; LVEF (39±6.8%) vs (25%), (p= 0.001) and GLS ( -8±2.9%C) vs (-5 %),(p= 0.011).The E/A ratio in survived patients was(1.5±0.94 vs 3.3), p= (0.033) while E/É was not significantly different between survived and non survived patients (15±5.2 vs 15 ),(p= 0.868).

**CONCLUSION.** The combination of cardiomyopathy and diabetes affects LV systolic and diastolic function. ICM is associated with the worst prognosis.

**REFERENCE(S)**

1. Maguy Chiha, Mario Njeim and Edgar G. Chedrawy: Diabetes and Coronary Heart Disease: A Risk Factor for the Global Epidemic. International Journal of Hypertension Volume , Article ID 697240 (2012).
2. Giuseppe MC Rosano,CristianaVitale,PetarSeferovic: Heart failure in patients with diabetes mellitus. Card Fail Rev. (2017) 3(1): 52–55.
3. Réal Lebeau, Karim Serri Marie-Claude Morice et al.,: Assessment of left ventricular ejection fraction using the wall motion score index in cardiac magnetic resonance imaging. Archives of Cardiovascular Disease 105, 91—98 (2012).
4. Roberto M. Lang, Luigi P. Badano and Victor Mor-Avi: Recommendations for Cardiac Chamber Quantification by Echocardiography in Adults: An Update from the American Society of Echocardiography and the European Association of Cardiovascular Imaging.European Heart Journal - Cardiovascular Imaging, Volume 16, Issue 3, 1 March (2015).
5. Nagueh SF, Smiseth OA, Appleton CP etal.,: Recommendations for the Evaluation of Left Ventricular Diastolic Function by Echocardiography: An Update from the American Society of Echocardiography and the European Association of Cardiovascular Imaging's 29(4):277-314(2016).
6. Thomas J Ford and David Corcoran: Stable coronary syndromes: pathophysiology, Diagnostic advances and therapeutic need. Heart 104:284–292(2018).
7. Chan YH a: Biostatistics102: Quantitative Data – Parametric & Non-parametric Tests. Singapore Med J.;44(8): 391-396 (2003).
8. Chan YH b :Biostatistics 103: Qualitative Data –Tests of Independence. Singapore Med J.;44(10): 498-503(2003)
9. Abdulfatai B. Olokoba, Olusegun A, et al: Type 2 Diabetes Mellitus: A Review of Current Trends. Oman Medical Journal Vol. 27, No. 4: 269-273(2012).
10. Petar M. Seferovic and Walter J. Paulus: Clinical diabetic Cardiomyopathy: A two-faced disease with restrictive and dilated phenotypes. European Heart Journal (2015) 36, 1718–1727.
11. We acknowledge the patients who have participated in research studies cited in this article.

### 001674

#### Risk Analysis of Death in Critically Ill Patients based on China's ICU Medical Quality Survey

L. Su, Y. Long, D. Liu, X. Zhou

[1]Icu, Peking Union Medical College Hospital, Beijing, China

**Correspondence:** L. Su

*Intensive Care Medicine Experimental* 2019, **7(Suppl 3):**001674

**INTRODUCTION.** Chinese Critical Care Medicine has developed rapidly in recent years. The uneven level of economic and medical development in different regions lead to differences in treatment efficiency and clinical outcomes.

**OBJECTIVES.** In order to understand the quality of medical care in Chinese critical Care Medicine, a quality survey on the medical quality of ICUs in China in 2015 was used to explore the risk factors of critically ill patients in the intensive care unit in China.

**METHODS.** 15 indicators of medical quality of critical care medicine were recommended by the experts' panels of Chines Society of Critical Care Medicine. The database of the National Clinical Improvement System was desinged to collect this information. (https://ncisdc.medidata.cn/login.jsp). The multivariate logistic analysis was performed to identify factors related to mortality in ICU.

**RESULTS.** Data on 15 indicators of medical quality were collected from ICUs in 1,174 hospitals in China. The indicators were as follows: the proportion of the total inpatients in the ICU (1.83%), proportion of total inpatient bed occupancy accounted for by the ICU (1.44%), proportion of ICU patients with APACHEII scores ≥15 (51.08%), 3-hour Surviving Sepsis Campaign (SSC) bundle compliance (74.37%), 6-hour SSC bundle compliance (76.6%), rate of microbiology detection before the administration of antibiotics (62.93%), proportion of ICU patients administered deep vein thrombosis (DVT) prophylaxis (58.24%), proportion of endotracheal extubations that was unplanned (1.49%), proportion of extubated patients reintubated within 48 h (1.99%), proportion of patients whose transfers to the ICU were unplanned (6.38%), 48-hour ICU readmission rate (1.20%), incidence of ventilator-associated pneumonia (VAP) (1.28 per 1000 ventilator days), incidence of catheter-related bloodstream infections (CRBSI) (3.06 per 1000 catheter days), incidence of catheter-associated urinary tract infections (CAUTI) (3.65 per 1000 catheter days), and in-hospital mortality rate (10.19%). Unplanned transfer to the ICU and higher APACHE II scores are mortality risk factors in Chinese ICUs

(OR=3.798 & OR=4.588, respectively). Treatment in certain regions (Central and North China) and 3-hour SSC bundle compliance may benefit critically ill patients (OR=0.560, OR=0.581 & OR=0.667, respectively).

**CONCLUSION.** Unplanned transfer to the ICU and higher APACHE II scores are risk factors for higher mortality rates in Chinese ICU. 3-hour SSC bundle compliance and the economic level of the area where the hospital is located maybe benefits. Improving the identification of critically ill patients and the implementation of appropriate and timely treatment interventions are of very important for improving the level of critical care in China.

**REFERENCE(S)**

1. Ray B, Samaddar DP, Todi SK, Ramakrishnan N, John G, Ramasubban S. Quality indicators for ICU: ISCCM guidelines for ICUs in India. Indian J Crit Care Med. 2009 Oct;13(4):173-206.
2. Chrusch CA, Martin CM, Project TQ. Quality Improvement in Critical Care: Selection and Development of Quality Indicators. Can Respir J. 2016;2016:2516765.
3. Rhodes A, Moreno RP, Azoulay E, Capuzzo M, Chiche JD, Eddleston J, Endacott R, Ferdinande P, Flaatten H, Guidet B, Kuhlen R, León-Gil C, Martin Delgado MC, Metnitz PG, Soares M, Sprung CL, Timsit JF, Valentin A; Task Force on Safety and Quality of European Society of Intensive Care Medicine (ESICM). Prospectively defined indicators to improve the safety and quality of care for critically ill patients: a report from the Task Force on Safety and Quality of the European Society of Intensive Care Medicine (ESICM). Intensive Care Med. 2012 Apr;38(4):598-605.
4. NA

### 001681

#### Hypercapnic exacerbations of COPD carry a high risk of readmission or death independently of the severity

G. Cavalot[1], T. Piraino[2], R. Coudroy[1], F. Damiani[1], N. Philips[1], V. Dounaevskaia[3], O. Smith[1], K. White[2], L. Brochard[1]
[1]Interdepartmental division of critical care medicine - university of toronto, Hospital St. Michael and Keenan research center, Toronto, Canada; [2]Department of respiratory therapy, St Michael's Hospital, Toronto, Canada; [3]Department of medicine, St Michael's Hospital, Toronto, Canada
**Correspondence:** G. Cavalot
*Intensive Care Medicine Experimental* 2019, **7(Suppl 3):**001681

**INTRODUCTION.** Patients with chronic obstructive pulmonary disease(COPD) admitted to the hospital for acute hypercapnic respiratory failure(AHRF) have shown poor outcomes including high readmission rates and mortality at one year.(1) Studies suggested that the use of non-invasive ventilation(NIV) reduces in-hospital mortality but is followed by poorer outcomes after discharge.(2) Recent data also showed that most of these patients have multiple comorbidities often untreated. (3)This study examined 1-year outcomes in COPD patients surviving hospital admission for exacerbation and the impact of the initial severity (respiratory support, intensive care unit(ICU) admission) on readmission and death.

**OBJECTIVES.** To describe characteristics and evaluate 1-y readmission and mortality rates of COPD patients admitted for exacerbation to one academic hospital in Toronto.

**METHODS.** We conducted a retrospective study including patients admitted to the emergency department (ED) of St Michael's Hospital in 2017 with AHRF defined on arterial or venous blood gas and presence of respiratory symptoms at admission. We selected COPD patients using appropriate ICD10 codes (J44.0,J44.1). Demographic, clinical and laboratory data, admission to ICU, use of NIV and 1-y readmission rates and mortality were collected. Patients readmitted and not readmitted were compared. We conducted a survival analysis comparing patients who didn't receive any respiratory support,received NIV in the ED or received NIV and were admitted to ICU.

**RESULTS.** 350 patients met our inclusion criteria for AHRF; among those, 80 patients were labelled as COPD as per ICD10 codes. Most patients were male (59%) with mean age of 67±10years; at admission mean pH was 7.28±0.06, mean $pCO_2$ was 67±12 mmHg. 50% of patients had more than 2 comorbidities. 24 patients (30%) received respiratory support (NIV and/or invasive ventilation), 12 (15%) were admitted to ICU and 3(4%) died. 48 out of 77 patients (62%) were readmitted at least once or died in the follow-up year. No differences were seen between patients readmitted and patients not readmitted in terms of demographics, clinical data and laboratory values. We performed the survival analysis in the three groups: patient that didn't receive any NIV, patients that received NIV only and patients that received NIV and were admitted to ICU. The Kaplan-Meier curves for these three groups (figure) did not differ. The readmission and mortality rate in the following year was 64%(7/11) in patients that received NIV only and 73%(8/11) in patients that received NIV and were admitted to the ICU.

**CONCLUSION.** All patients that survive an hypercapnic episode of COPD exacerbation are at high risk of hospital readmission or death no matter if they received NIV or were admitted to the ICU during index admission. This whole group should be targeted for prevention of readmissions.

**REFERENCE(S)**

1. Chu CM, Chan VL, Lin AWN, Wong IWY, Leung WS, Lai CKW. Readmission rates and life threatening events in COPD survivors treated with non-invasive ventilation for acute hypercapnic respiratory failure. Thorax. 2004;59(12):1020–5.
2. Ankjærgaard KL, Rasmussen DB, Schwaner SH, Andreassen HF, Hansen EF, Wilcke JT. COPD: Mortality and Readmissions in Relation to Number of Admissions with Noninvasive Ventilation. COPD. 2017 Jan 2;14(1):30–6.
3. Adler D, Pepin JL, Dupuis-Lozeron E, Espa-Cervena K, Merlet-Violet R, Muller H, et al. Comorbidities and subgroups of patients surviving severe acute hypercapnic respiratory failure in the intensive care unit. Am J Respir Crit Care Med. 2017;196(2):200–7.

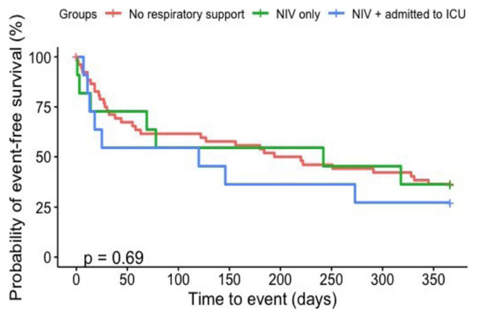

**Fig. 2 (abstract 001755).** See text for description

### 001752

#### Preliminary results of the First dedicated Dutch ICU for complex weaning from mechanical ventilation

T. Frenzel[1], L. Roesthuis[1], M. van den Boogaard[1], M. Zegers[1], J. Van Der Hoeven[2]
[1]Intensive care, Radboud University Medical Center, Nijmegen, Netherlands; [2]Department of intensive care medicine, Radboud University Medical Center, Nijmegen, Netherlands
**Correspondence:** T. Frenzel
*Intensive Care Medicine Experimental* 2019, **7(Suppl 3):**001752

**INTRODUCTION.** Weaning from mechanical ventilation (MV) is prolonged (>7 days) in 8.7% of all ICU patients (1). Previously, there was no dedicated weaning unit in the Netherlands. In January 2016, a specialized ICU for patients with complex weaning from MV was opened at Radboud University (**Radboudumc Centre of Expertise for Weaning from MV, @NExCOB**) aiming to improve outcome with a better cost efficiency for this group of patients.

**METHODS.** We use a **multidisciplinary approach** including a **dedicated team** of ICU nurses, physiotherapists, speech therapists, intensivists, a technical physician, and if indicated other consultants (2). If possible (hygiene) and indicated, patients receive **hydrotherapy** (3). Patients with **difficult or prolonged weaning** are eligible for admission (team decision). **Descriptive analysis** of the first cohort, **3-month outcome data**, derived from **MONITOR-IC** study (4), and **survival data** (population register data) are reported.

**RESULTS.** From January 2016 to November 2017, 109 patients were admitted. 28% came from other hospitals, 36% had a history of COPD and 15% of cardiac failure. Median duration of MV before admission was 19 days [IQR 12-25]. 61% suffered from delirium, 41% from anxiety, 39% from sleep disturbances. Most patients (75%) were highly motivated for treatment. Median length of stay in our unit was 11 days [IQR 6-20]. Dysphagia was observed frequently resulting in prolonged admission. Survival was 22±1 months (mean±SE, fig. 1).

**CONCLUSION.** Patients treated at a new dedicated ICU for complex weaning from MV in the Netherlands have a **high rate** of **successful weaning**. Patients were **vulnerable before admission** with increase of frailty at discharge improving modestly after 3 months, while mental health was comparable to before admission. Long-term **survival** in this population was reasonable.

**REFERENCE(S)**

1. Béduneau G. et al., Am J Respir Crit Care Med 2017;195:772-783
2. Heunks LM, van der Hoeven, Crit Care. 2010;14(6):245
3. Felten-Barentsz et al., Am J Respir Crit Care Med 2015 Feb 15;191(4):476-7
4. Geense W. et al., BMJ Open 2017 Nov 14; 7(11)
5. Betaalbaar beter, Radboudumc / VGZ

**Table 1 (abstract 001752).** Outcomes of treatment

| | |
|---|---|
| Successful weaning | 86 (79%) |
| Death during admission | 7 (6%) |
| Readmission to ICU due to deterioration | 8 (7%) |
| Back to referring ICU after diagnosis with detailed treatment plan | 9 (8%) |

**Table 2 (abstract 001752).** Long-term outcomes

| Parameter median [IQR] | Before admission | At hospital discharge | 3 months after ICU admission |
|---|---|---|---|
| Frailty (CFS) (0-9) | 3.5 [3-5.25] | 6.0 [5.5-7] | 5.0 [4-6] |
| Quality of life (SF36) (0-100) | | | |
| PCS | 30 [21-44] | n.a. | 27 [22-37] |
| MCS | 49 [32-54] | n.a. | 48 [37-58] |

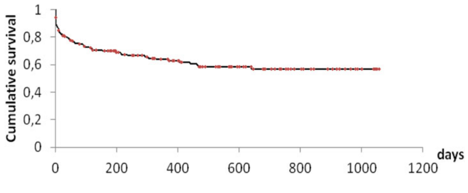

**Fig. 1 (abstract 001752).** See text for description

### 001760

#### Identification of increased microcirculatory leukocytes using Incident Darkfield imaging in liver resection patients

Z. Uz[1], C. Ince[1], L. Shen[1], B. Ergin[1], M. Heger[2], T. Van Gulik[2]
[1]Translational physiology, Amsterdam UMC, Amsterdam, Netherlands; [2]Surgery, Amsterdam UMC, Amsterdam, Netherlands
**Correspondence:** Z. Uz

*Intensive Care Medicine Experimental* 2019, **7(Suppl 3):**001760

**INTRODUCTION.** The surgical resection of the liver is the only curative treatment for primary liver cancers.

To reduce blood loss, surgeons will apply the Pringle Manoeuvre which occlude inflow of blood to the liver. However, the application of the Pringle will cause ischemia reperfusion injury (IRI) to the liver, characterized by the infiltration of leukocytes. Leukocytes can be visualized using Incident Darkfield (IDF) imaging. Monitoring the inflammation in the sublingual microcirculation during and 24 hours after liver resection, provides a potential clinical parameter (microcirculatory leukocytes) to monitor critically ill patients whom would benefit from early intervention.

**METHODS.** 19 patients undergoing major or minor hepatectomy were enrolled. Using Cytocam-IDF imaging, their sublingual microcirculation was measured before skin incision but after the induction of anaesthesia (T0), before skin closure (T1) and 24 hours after surgery (T2). The microcirculatory parameters measured were TVD, PVD, PPV, MFI and PVS. Systemic leukocytes and microcirculatory leukocytes were counted in all time points.

**RESULTS.** 8 patients of the 19 included patients were analysed. An increase in the number of rolling(r), non-rolling(nr) and total(tl) sublingual leukocytes can be seen from T0 to T1 to T2 (r= 2±1, nr= 5±3, tl= 8±7 to r= 5±6, nr=7±3, tl=4±3 to r=6±5, nr=13±3, tl=12±6, p= 0.62 p=0.309 and p=0.055 respectively). There was also an increase in systemic leukocyte count seen (8±4 to 12±6 to 20±13, p=0.097). There was a significant decrease seen in the MFI from To to T1 to T2 (2.7±0.3 to 2.8±0.0 to 2.3±0.4, p= 0.029).

**CONCLUSION.** The number of microcirculatory leukocytes increased from T0 to T2 in concordance with the increase in systemic leukocyte count. This study shows that the monitoring of the sublingual microcirculation using IDF imaging identifies non-invasively the level of inflammation and perfusion.

### 001449

#### Anemia and red blood cell transfusions after non-traumatic subarachnoid hemorrhage

A. Castella[1], L. Attanasio[2], S. Schuind[3], E. Bogossian[2], JL. Vincent[2], J. Creteur[2], FS. Taccone[2]
[1]Anestesia e rianimazione, IRCCS Ospedale San Raffaele, Milano, Italy; [2]Soins intensifs, ULB Erasme, Anderlecht, Belgium; [3]Service de neurochirurgie, Hospital Erasme, Bruxelles, Belgium
**Correspondence:** A. Castella

*Intensive Care Medicine Experimental* 2019, **7(Suppl 3):**001449

**INTRODUCTION.** Subarachnoid hemorrhage (SAH) is associated with high morbidity and mortality. Studies have shown that both anemia and red blood cell transfusions (RBCT) are independent risk factors for poor outcome.

**OBJECTIVES.** To evaluate the role of anemia and RBCT on long-term neurological outcome after SAH.

**METHODS.** We reviewed our institutional database of adult (>18y) patients admitted to the Department of Intensive Care after non-traumatic SAH over a 5-year period. We recorded hemoglobin (Hb) levels daily for a maximum of 15 days, as well as RBCT (with the pre-RBCT Hb values). We collected unfavorable neurological outcome (UO; i.e. Glasgow Outcome Scale of 1-3) at 3 months.

**RESULTS.** We collected data from 270 patients (median age 54 years; male 45%); UO was 40%. Hb on admission was 13.4 (12.2-14.3) g/dL; highest and lowest Hb values were 14.3 (12.9-15.2) and 10.3 (8.4-11.9) g/dL, respectively. The patients with UO were more likely to have Hb <11, <10, <9, <8 or <7 g/dL than the others (79 vs. 51% - 63 vs. 32% – 51 vs. 18% - 37 vs. 11% – 20 vs. 5% - respectively; p< 0.05 for all). Patients with UO had consistently lower Hb over the study period, in particular since the second day (p<0.001). More patients with UO received RBCT (15/109, 14% vs. 6/161, 4% - p<0.01); pre-RBCT Hb values were similar in UO and in the other patients (6.9 [6.6-7.1] vs. 7.3 [6.3-8.1] g/dL – p=0.21). In a multivariable analysis, older age, chronic renal failure, neurological status on admission, the use of mechanical ventilation and the development of delayed cerebral ischemia, but neither Hb nor RBCT were independent predictors of UO.

**CONCLUSION.** Although anemia is associated with unfavorable neurological outcome after SAH, it does not seem to be an independent prognostic factor.

### 001485

#### Use of automated pupillometry to assess autonomic nervous system dysfunction

G. Ponzone, AA. Quispe-Cornejo, J. Creteur, IA. Crippa, JL. Vincent, FS. Taccone
[1]Department of intensive care, Erasme University Hospital, Université libre de Bruxelles, Brussels, Belgium
**Correspondence:** G. Ponzone
*Intensive Care Medicine Experimental* 2019, **7(Suppl 3):**001485

**INTRODUCTION.** Autonomic nervous system (ANS) dysfunction is frequently observed in critically ill patients. Heart rate variability (HRV) can provide an estimation of the ANS integrity but requires specific software and expertise. Assessment of pupillary light reflex using automated pupillometry (AP) could also assess ANS dysfunction at the bedside.

**OBJECTIVES.** To explore whether changes in AP-derived variables correlate with changes in HRV in critically ill patients.

**METHODS.** We prospectively studied (Nov 2018 - March 2019) 53 adult patients (median age 62 [52-69] years – APACHE II on admission 18 [14-25]), with normal sinus rhythm and an estimated ICU stay > 3 days. Patients with pupillary abnormalities were excluded. Assessment of HRV and AP was performed within the first 24 hours in the ICU (T1) and at least 48 hours thereafter (T2). HRV was assessed for at least a 6-minute period using a specific software (Kubios HRV Premium version 3.2.0) analysing both time-domain and frequency-domain methods of RR intervals. Automated pupillometry (NPi-200 – Neuroptics) was performed with light stimulation three times during the HRV recording on each eye; and mean values were averaged. Changes in HRV and AP-variables were calculated as: (T2-T1)/T2*100.

**RESULTS.** The 53 patients had an ICU length of stay of 8 [5-13] days and a 28% ICU mortality. There was no significant correlation between changes in AP derived Neurologic Pupil Index and the dilation velocity and changes in HRV. However, changes in pupil size, constriction rate and constriction velocity were significantly correlated with changes in HRV variables, such as root mean square of differences between successive NN intervals (RMSSD), low- and high-frequency power (which captures the magnitude of underlying oscillations in the different HR patterns) and the Poincaré plot standard deviation perpendicular (SD1) or along the line of identity (SD2) parameters. Changes in pupillary latency correlated with changes in other HRV variables, such as the mean R - R interval (mRR), the normalized low-frequency power (FFT-LFnu) and the detrended fluctuation analysis (DFAa1).

**CONCLUSION.** Automated pupillometry could be used to assess changes in autonomic nervous system dysfunction in critically ill patients.

### 001505

#### Acute Stroke Patients in Intensive Care Unit – is it really worth it?

P. Campos[1], G. Videira[2], R. Antunes[1], T. Cardoso[1], R. Felgueiras[2], I. Aragão[1]
[1]Unidade de cuidados intensivos polivalente, Centro Hospitalar e Universitário do Porto, Porto, Portugal, Portugal; [2]Serviço de neurologia, Centro Hospitalar e Universitário do Porto, Porto, Portugal, Portugal
**Correspondence:** P. Campos
*Intensive Care Medicine Experimental* 2019, **7(Suppl 3):**001505

**INTRODUCTION.** Stroke is the major cause of disability and the second most common cause of death. The majority of acute strokes are managed on the ward; however, a significant proportion require more specialized care. Neurological impairment with airway or respiratory compromise, seizures, and need for close management of blood pressure and monitoring of post stroke complications are the common indications for Intensive Care Unit (ICU) admission.

**OBJECTIVES.** This study aims to determine the rate of ICU admission for acute stroke, to analyze and characterize this population, and to determining the functional status at ICU and hospital discharge and at 3 months follow up.

**METHODS.** Retrospective and observational study conducted at Centro Hospitalar e Universitário do Porto, a tertiary hospital and reference center for thrombolysis and neurosurgery, from January 2013 to December 2016. All patients with acute stroke admitted in the ICU were included. Patients under 18 years of age, stoke due to subarachnoid hemorrhage and due to traumatic etiology and admitted for organ donation were excluded. The functional score was evaluated by Modified Rankin Scale (mRS).

**RESULTS.** A total of 156 patients, 4.3% of all ICU admission, were included, 78 (50%) for ischemic stroke. The mRS at admission was similar in ischemic and hemorrhagic stroke. The Glasgow Coma Scale (GCS) was lower in hemorrhagic stroke and cardiovascular risk factors were more prevalent for ischemic. SAPS II and SOFA were similar in both groups. The most common locals of occlusion for ischemic stroke were basilar artery and segment M1 of middle cerebral artery. 51% of patients had no infarction in brain computed tomography. Thrombolysis was performed in 35 patients (49%) and 56 (74%) were submitted to thrombectomy. Complete recanalization (TICI 3) was obtained in 12 (21%) of patients and 9 (16%) had no recanalization (TICI 0). Deep hemorrhage was the most common type, being responsible for 41 (53%) of hemorrhagic strokes. Neurosurgical procedure was performed in 62 (80%) of patients with hemorrhagic stroke, being external ventricular shunt the most common procedure. Time to neurosurgical procedure was higher in ischemic stroke (1977 min vs 1089 min). The ICU length of stay was higher in hemorrhagic stroke (12 days vs 6 days). Most of the patients had an mRS score of 5 at ICU and hospital discharge in both types of stroke. Three months after discharge the mortality rate was 44% for ischemic stroke and 40% for hemorrhagic stroke.

**CONCLUSION.** Despite the incidence of ischemic stroke being higher than hemorrhagic stroke, in our population the rate of admission for both types of stroke was similar. As expected, the cardiovascular risk factors were more frequent in ischemic stroke. Time to neurosurgical procedure was higher in ischemic stroke since it is performed most often due to hemorrhagic complications. Despite the ICU management, the mortality rate and the functional status is poor in the population of patients admitted to ICU.

**REFERENCE(S)**

1. M. McDermott et al.; Critical care in acute ischemic stroke; Handb Clin Neurol. 2017;140:153-176.
2. A. Alonso et al.; Outcome Predictors of Acute Stroke Patients in Need of Intensive Care Treatment; Cerebrovasc Dis 2015;40:10–17.

### 001507

#### Defining coagulopathy by Thromboelastometry (ROTEM) in patients with traumatic brain injury

E. Lazoudi[1], C. Iasonidou[2], E. Siomos[2], A. Kosmas[3], E. Seitsidou[2], N. Kapravelos[2]
[1]{street_address}, Thessaloniki, Greece; [2]B icu, General Hospital "G. Papanikolaou", Thessaloniki, Greece; [3]Icu, GENERAL HOSPITAL "GEORGE PAPANIKOLAOU", Thessaloniki, Greece, Greece
**Correspondence:** E. Lazoudi
*Intensive Care Medicine Experimental* 2019, **7(Suppl 3):**001507

**INTRODUCTION.** Severe traumatic brain injury (TBI) is often accompanied by coagulopathy and its presence has been shown to be a poor prognostic factor. Rapid assessment of coagulation status and successful treatment of bleeding disorders are crucial. Standard coagulation tests are usually time consuming and provide information only on the initiation of clot formation. In contrast, thromboelastometry (ROTEM) assess coagulation status within minutes and offer information on clot firmness and clot stability, as well as the initiation of coagulation.

**OBJECTIVES.** This retrospective study was designed to assess the potential role of ROTEM in patients (pts) with TBI, to identify associated coagulopathy and to correlate these hemostatic disorders with outcome.

**METHODS.** This retrospective evaluation comprised ICU pts with TBI. Blood samples were obtained on admission, after 12 and after 24-48 hours. ROTEM analysis (EXTEM, INTEM, FIBTEM tests) and standard laboratory coagulation tests ( PT, INR, aPTT, platelets count, fibrinogen concentration) were compared. The hemostatic parameters were compared between survivors and non survivors. We evaluated the time course of hemostatic disorders and their relationship with the expansion of hemorrhage, as confirmed by CT-scan. Using standard laboratory tests coagulopathy was defined as one or more of following: INR>1.5, aPTT>35s, fibrinogen<150mg/dL, PLT<100000/μL. Using ROTEM coagulopathy was defined as one or more of the following results: CTextem>80s, CFTextem>159s, MCFextem<50mm, CTintem>240s, CFTintem>110s, MCFintem<50mm, A10fibtem<7mm.

**RESULTS.** 29 pts with TBI aged 48.31 years were evaluated. 23 pts survived (79.3%) and 6 (20.6%) died. Coagulopathy as defined by both tests were observed in 10 pts (34.48%). In this group 7 pts survived (70%) and 3 (30%) died, while 8 developed expansion of hemorrhage on CT-scan (80%). Out of 29 pts in 19 (65.5%) were observed abnormalities in ROTEM variables, while 9(47.36%) pts of them revealed normal standard coagulation tests. In this group 15 (78.9%) pts survived and 3 died. Remarkably, 16 pts with abnormal ROTEM analysis developed expansion of hemorrhage and 3 did not, which difference is statistically significant (p<0.05). Also we observed abnormal CTextem in 18 (62%), CTintem in 8 (27.5%) and A10fibtem in 7 (24.1%). Abnormalities in ROTEM were observed in 15pts (51.7%) on admission, in 13pts (44.8%) after 12h and in 15pts (51.7%) after 24-48h.

**CONCLUSION.** Coagulopathy is a frequently encountered and highly morbid complication of TBI that has been consistently linked to poor outcomes. ROTEM provides rapid and useful information on the coagulation status of pts with TBI. ROTEM appeared to offer an early signal of life-threatening TBI. Further studies are warranted to confirm these results and to investigate the role of ROTEM in optimizing coagulation therapy.

**REFERENCE(S)**

1. Advances in the Management of Coagulopathy in Traumatic Brain Injury. Cheng et al. J Cerebrovasc Dis And Stroke 2017: 4(5):1073
2. Tromboelastometric (ROTEM) Findingsin Patients Suffering from Isolated Severe Traumatic Brain Injury. Schochl et al. Journal of Neurotrauma 28:1-9, 2011

### 001537

#### Association of timing to aneurysm repair and mortality in subarachnoid hemorrhage – A multicentric prospective study

B. Gonçalves[1], C. Rynkowski[2], R. Turon[1], F. Miranda[1], C. Tibau[1], A. Aded[1], T. Santos[1], M. Prazeres[1], C. Righy[1], F. Bozza[3], P. Kurtz[1]

[1]Intensive care unit, Paulo Niemeyer State Brain Institute, Rio de Janeiro, Brazil; [2]Intensive care unit, Cristo Redentor Hospital, Porto Alegre, Brazil; [3]National institute of infectology, Oswaldo Cruz Foundation, Rio de Janeiro, Brazil

**Correspondence:** B. Gonçalves

*Intensive Care Medicine Experimental* 2019, **7(Suppl 3):**001537

**INTRODUCTION.** Aneurysmal subarachnoid hemorrhage (SAH) is an acute and severe cerebrovascular event, with high mortality and morbidity. The moment of aneurysm repair could have an impact on outcomes, but the optimal timing remains controversial.

**OBJECTIVES.** Our goal is to describe the association of the moment of aneurysm repair procedure with the mortality.

**METHODS.** This study was conducted in two reference centers – one in Rio de Janeiro and one in Porto Alegre. From July 2015 to March 2019, every adult patient admitted to the ICU with aneurysmal SAH was enrolled in the study. Data were collected prospectively during the hospital stay. Patients were divided into four groups according to the moment of aneurysm repair after bleeding – up to the 3rd day, 4 to 8 days, above eight days and those not repaired. The primary endpoint was mortality at hospital discharge. A multivariate model was made using the group with higher mortality as the reference group (4 to 8 days).

**RESULTS.** A total of 437 patients were enrolled in the study. Median age was 55 years, mostly female (73%). 31% of patietes had poor-grade SAH (WFNS 4-5), with 22% mortality and 50% poor functional outcome (modified Rankin of 4 to 6). In the multivariate model for mortality (table 1), hydrocephalus, post-procedure neurological worsening, DCI were associated with higher mortality, but late repair (after eight days) was associated with lower mortality (OR 0.4) against aneurysm occlusion between 4 to 8 days, and surgical treatment was associated with lower mortality than endovascular treatment (OR 0.3).

**CONCLUSION.** Our study shows higher mortality in patients submitted to aneurysm occlusion procedure from day 4 to 8 when compared to a late repair, with higher mortality on patients who underwent endovascular treatment. Still a controversial topic, the timing of aneurysm repair needs more studies to define what would yield the best outcome.

**REFERENCE(S)**

1. Yao Z, Hu X, Ma L, You C, He M. Timing of surgery for aneurysmal subarachnoid hemorrhage: A systematic review and meta-analysis. International Journal of Surgery. 2017;48:266-274.
2. Not applicable

**Table 1 (abstract 001537).** Association of studied variables and mortality – Multivariate model

| Variable | | Odds Ratio | 95% CI | P value |
|---|---|---|---|---|
| Age | | 1.05 | 1.02-1.09 | 0.002 |
| Time to occlusion | <96h | 0.6 | 0.2-1.6 | 0.2 |
| | 4 – 8 days (Ref) | - | | |
| | >8 days | 0.4 | 0.2-0.9 | 0.03 |
| Surgical treatment vs endovascular treatment | | 0.3 | 0.14-0.64 | 0.002 |
| Poor Grade (WFNS 4 or 5) | | 2.2 | 1-4.7 | 0.05 |
| Neurological deterioration post-procedure | | 3.15 | 1.5-6.8 | 0.003 |
| Hydrocephalus | | 2.9 | 1.34-6.41 | 0.007 |
| DCI | | 4.1 | 1.9-8.9 | < 0.0001 |

### 001552

#### Restrictive versus goal-directed perioperative fluid therapy in neurosurgery

K. Debricka[1], MC. Casadio[2], C. Viola[3], A. Cuoci[2], G. Gentili[2], M. Zanello[3]

[1]School of anesthesia and intensive care, University of Modena and Reggio Emilia, Modena, Italy; [2]Anesthesia and intensive care, IRCCS Istituto delle Scienze Neurologiche di BOLOGNA, Bologna, Italy; [3]School of anesthesia and intensive care, Alma Mater Studiorum - Università di Bologna, Bologna, Italy

**Correspondence:** M.C. Casadio

*Intensive Care Medicine Experimental* 2019, **7(Suppl 3):**001552

**INTRODUCTION.** Maintaining blood flow and oxygen transport are of primary importance in neurosurgical patients, avoiding overload and cerebral edema.

**OBJECTIVES.** We compared 2 fluid therapies in patients undergoing neurosurgery: a restrictive one based on literature data and a goal-directed (GD) one through the evaluation of the stroke volume variation. The primary outcome variable was the hospital length of stay (LOS).

**METHODS.** For 2 months we included all patients undergoing elective craniotomy for a supratentorial non-vascular lesion, in the IRCCS Neurological Science Institute of Bologna. We excluded all the patients with an age<18 years old, pregnancy, BMI>35, ASA≥4, Revised Cardiac Risk Index≥2, a respiratory disease requiring home oxygen therapy, renal replacement therapy or an increase of more than 50% of the normal values of creatinine. We randomly assigned these patients to the restrictive group (Z Group) or the GD one. In figure 1 we showed the 2 strategies; SVV was measured through the FloTrack/Vigileo (Edwards Lifescience©, Irvine, CA, USA). We recorded demographics, neurosurgery type and indication, intraoperative hemodynamic parameters, postoperative complications, ICU and hospital LOS. We collected neurological status and biochemical data before and after surgery.

**RESULTS.** We enrolled 20 patients; the mean age was 51.8±7.8 years with a prevalence of female (65%); 45% of the lesions were meningiomas, 20% glial lesions, 20% epileptogenic dysplasia and the remaining 15% brain metastasis or colloidal cysts. The groups didn't show any differences regarding patients basal characteristics (age, ASA, preoperative GCS, Apfel score, biochemical data), mean surgical duration (283±88.6vs273±94.7,p>0.05) and types of crystalloids. In the GD group we infused a less amount of crystalloids (1862±757mlvs2254±851.6ml,p>0.05); this data reaches the statistical significance when we translate it in terms of perioperative fluid balance (-319.2±634.7ml for the GD group vs 107±229.4ml for the Z group,p=0.04). Arterial pH was more close to the physiological value in the GD group (7.4±0.04 vs 7.36±0.05, p=0.005); other markers of perfusion and homeostasis such as arterial lactate and base excess tended to approach more physiological values in this group (lactate:1.75±1.3 vs 2.02±0.6,BE: 2.97±3.4vs -1.99±2.5,p>0.05). We didn't find any other differences in terms of neurological evaluation (GCS/RASS), postoperative complications (NRS, nausea/vomit), hydroelectrolytic status and inflammations. While the ICU-LOS is similar, the hospital one tends to be shorter in the GD group (6.4±1.3days vs 7.4±2.5days, p=0.07).

**CONCLUSION.** We observed a better preservation of the physiological status of the patient with a GD fluid therapy.

**REFERENCE(S)**

1. Navarro et al. Perioperative Medicine (2015)4:3
2. Luo et al. Ann.Intensive Care(2017)7:16

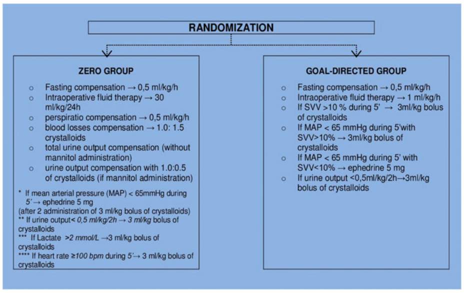

**Fig. 1 (abstract 001552).** See text for description

### 001615

### Brain injury biomarkers as prognostic factors in patients with spontaneous intracerebral hemorrhage

HB. Rotzel, A. Serrano Lazaro, ML. Blasco Cortes, TA. Tellez, S. Mulet Mascarell, C. Sanchis Piqueras, DL. Aguillón Prada, N. Carbonell Monleón, GM. Rodriguez, A. M A., MM. Juan Díaz
[1]Intensive care unit, Hospital Clínic Universitari de València, València, Spain
**Correspondence:** H.B. Rotzel

*Intensive Care Medicine Experimental* 2019, **7(Suppl 3):**001615

**INTRODUCTION.** Spontaneous intracerebral hemorrhage (sICH) are responsible for 9-27% of cerebrovascular diseases worldwide, with 37-52 % of mortality at 30 days and 54% a year. Brain injury biomarkers (BIB) have been studied to predict prognosis in this pathologies.

**OBJECTIVES.** To related BIB with mortality and functional outcome in patients with sICH.

**METHODS.** We performed a prospective study with patients admitted in ICU with sICH. BIB were determined (Enolase, S-100B, Dimer D/DD, Brain natriuretic peptide/BNP) at admission,1,2, and 3rd day. APACHE II, SOFA, GCS and GRAEB were determined at admission and the modified Rankin Scale (mRS: poor outcome >2) and Glasgow Outcome Scale (GOS: poor outcome <4) at ICU discharge and 6 months after the event. Variables were summarized using %, mean (SD) and median. We used T-Student and χ2(p <0.05) for univariable analysis.

**RESULTS.** We enrolled 120 patients, 63,3% were men, mean age 63,3 ± 12.6 ys. Global mortality was 30,8%. In the univariable analysis of mortality we obtain an stadistically significance with APACHE II 18,4 ± 5,3 vs 12,4 ± 5,4 (p<0,001), SOFA 6,7 ± 2 vs 3,5 ± 2 (p< 0,001); GCS 8 ± 3 vs 11,8 ±3 (p < 0,001), GRAEB 3,84±0,56 vs 2,46±0,39 (p 0,05); it was also significantly associated with BNP at admission, 1, 2 and 3rd day (p: 0,005, 0,027, 0,029 and 0,012, respectively) and DD at 1, 2 and 3rd day (p 0,041, 0,005, 0,004 respectively). Otherwise, the univariable analysis of poor outcome with mRS and GOS at ICU discharge were associated with DD at admission, 1, 2 and 3rd day (GOS: p 0,005, 0,05, 0,001, 0,001; mRankin p 0,001, 0,001, <0,001, <0,001; respectively) and S100B at admission, 1, 2 and 3rd day (GOS p: 0,015, 0,003, 0,003, 0,008; mRankin p: 0,016, 0,003, 003 and 0,004; respectively). We obtained a significance association between poor mRS and GOS at 6 months with S100B 2nd day(p 0,003).

**CONCLUSION.** BIB as BNP (at admission, 1, 2 and 3rd day) and DD (1, 2 and 3rd day) were related to mortality. S-100B and DD (both at admission, 1, 2 and 3rd day) in an early stage were related to worse outcome at ICU discharge. But only S100B 2 day was related to poor outcome at 6 months.

**REFERENCE(S)**

1. Senn R, Elkind M, Montaner J, Christ-Crain M, Katan M. Potential Role of Blood Biomarkers in the Management of Nontraumatic Intracerebral Hemorrhage. Cerebrovasc Dis 2014;38:395–409
2. Seelora Sahu, Deb Sanjay Nag, Amlan Swain, Devi Prasad Samaddar. Biochemical changes in the injured brain. World J Biol Chem 2017 February 26; 8(1): 21-3

**Table 1 (abstract 001615).** Univariable analysis of BIB associated with mortality. +Mean±SDE

| BIB | DEATH+ | ALIVE+ | p |
|---|---|---|---|
| BNP 0 | 199,1±37 | 84,3±10 | 0,005 |
| Day 1 | 153,2±27,7 | 86,8±8,2 | 0,027 |
| Day 2 | 154,1±28.6 | 86,4±8,3 | 0,029 |
| Day 3 | 176,5±29,9 | 92,5±10,8 | 0,012 |
| DD Day 1 | 1599,5±378 | 763,7±116 | 0,041 |
| Day 2 | 1786,9±318 | 776,2±112 | 0,005 |
| Day 3 | 2196,1±386 | 962,5±118 | 0,004 |

**Table 2 (abstract 001615).** Univariable analysis of BIB associated with mRS and GOS at ICU discharge. +Mean±SDE

| BIB | POOR | | GOOD | | p | |
|---|---|---|---|---|---|---|
| | GOS+ | mRankin+ | GOS+ | mRankin+ | GOS | mRankin |
| DD 0 | 693±106 | 693±99,7 | 358±42 | 320±42,4 | 0,005 | 0,001 |
| Day 1 | 966±156,8 | 1017±157 | 554±113 | 419±54 | 0,05 | 0,001 |
| Day 2 | 1105±171,9 | 1118±163 | 475±60 | 390±39,7 | 0,001 | <0,001 |
| Day 3 | 1415±201 | 1446±191 | 612±97,4 | 448±53,2 | 0,001 | <0,001 |
| S100B 0 | 0,4±0,09 | 0,39±0,08 | 0,16±0,02 | 0,16±0,02 | 0,015 | 0,016 |
| Day 1 | 0,34±0,06 | 0,33±0,06 | 0,12±0,02 | 0,13±0,02 | 0,003 | 0,003 |
| Day 2 | 0,19±0,02 | 0,19±0,02 | 0,10±0,01 | 0,09±0,01 | 0,003 | 0,003 |
| Day 3 | 0,12±0,02 | 0,12±0,02 | 0,06±0,01 | 0,06±0,01 | 0,008 | 0,004 |

### 001655

#### Shift Matters: CT scan Predictors of Diabetes Insipidus in Brain Death

A. Daneshmand, EF. Wijdicks
Neurology, Mayo Clinic Hospital, Saint Marys Campus, Rochester, USA
**Correspondence:** A. Daneshmand
*Intensive Care Medicine Experimental* 2019, **7(Suppl 3):**001655

**INTRODUCTION.** Diabetes insipidus (DI) is a known complication of patients with catastrophic brain injury resulting in brain death. The pathophysiology of the DI is not fully understood. The presumed mechanism is the injury of the pituitary gland and stalk from brain tissue shift. In the present study, we reviewed the relationship of brain shift and edema and the appearance of DI.

**METHODS.** We reviewed a series of patients with near complete loss of brainstem reflexes leading to brain death. Baseline Mean Arterial Pressure (MAP) and baseline demographic information were collected. Effacement of basal cisterns and midline shift were assessed using baseline CT scan and correlated to DI using standard diagnostic criteria of specific gravity, and serum and urine osmolarity.

**RESULTS.** In our series, 26% of patients developed DI before the diagnosis of brain death. The mean midline shift on baseline CT scan in patients who developed DI was 7.2 mm, comparing to 3.6 mm in patients with no DI. (p=0.005). Effacement of basal cisterns was noted in 84% of patients with DI as opposed to 44% of patients without DI (p<0.05). The MAP was not significantly different between the two groups (p=0.33). 40% of patients with subarachnoid hemorrhage, 32% of patients with intracerebral hemorrhage, 22% of patients with anoxic brain injury and 10% patient with traumatic brain injury developed DI.

**CONCLUSION.** DI is associated with much more profound brain tissue shift and effacement of the basal cisterns in patients who progress to brain death.

##### REFERENCE(S)

1. Wijdicks EF, Varelas PN, Gronseth GS, Greer DM. Evidence-based guideline update: determining brain death in adults: report of the Quality Standards Subcommittee of the American Academy of Neurology. Neurology. 2010 Jun 8;74(23):1911-8.
2. None

### 001714

#### Electronic Observation Chart and Education improved accuracy of Cerebral Perfusion Pressure measurement in patients with Severe Traumatic Brain Injury

S. Gudibande[1], M. Belal[2], D. Kelly[3], T. Owen[1], D. Cottle[1]
[1]Consultant, regional neurosciences critical care, Lancashire Teaching Hospitals NHS trust, Preston, UK; [2]Specialty doctor, regional neurosciences critical care, Lancashire Teaching Hospitals NHS trust, Preston, UK; [3]Foundation year 2 doctor,regional neurosciences critical care, Lancashire Teaching Hospitals NHS trust, Preston, UK
**Correspondence:** S. Gudibande
*Intensive Care Medicine Experimental* 2019, **7(Suppl 3):**001714

**INTRODUCTION.** Monitoring and management of intracranial pressure (ICP) and cerebral perfusion pressure (CPP) based on brain trauma foundation guidelines(1)is the standard of care in the management of patients with traumatic brain injury (TBI). However, there is a wide variation in the way CPP is measured.Councils of Neuroanaesthesia and Critical Care Society of Great Britain and Ireland (NACCS) and the Society of British Neurological Surgeons (SBNS) **recommend that in the management of TBI, when calculating CPP, the arterial transducer used to estimate mean arterial pressure (MAP) for the calculation CPP =MAP–ICP should be positioned at the level of the tragus (which is an approximation for middle cranial fossa level)(2).**In a person with 30 degrees head elevation and 30 cm distance between heart(Phlebostatic Axis)and the head,the difference in measured MAP/CPP levels will be 11 mmHg depending on the calibration level.

**METHODS.** We conducted a retrospective audit of the position of arterial transducer in patients with TBI who had continuous ICP and CPP monitoring with **an aim to ensure correct positioning of transducer at tragus level,**as per national recommendations (2).We implemented continuous teaching sessions for nursing staff and introduced a mandatory drop-down list for arterial transducer position(Figure 1)on our electronic patient observation chart. We re-audited the position of transducer after implementation of above changes. Audits were conducted for a period of 1 month where patients with TBI were observed over a period of 3 days with twice daily recording of the position of the transducer. We excluded patients with non-traumatic brain injury and patients with significant extracranial polytrauma even if they had continuous ICP monitoring.**Figure 1 showing the ''drop-down list'' of arterial transducer position in our electronic patient observation chart**.

**RESULTS.** A total of 24 observations were recorded from 4 patients with TBI and continuous ICP and CPP monitoring over their first 3 days of admission, checking the position of transducer twice daily, corresponding to nursing shift changes.**Only on 1(4%)occasion was the transducer positioned correctly (tragus) in the 1st audit. After the implementation of nursing education and change in electronic patient observation chart,on 18(75%) occasions, positioning of transducer was at correct level.**

**CONCLUSION.** We demonstrated significant improvements in adherence to national recommendations on arterial transducer position in patients with isolated TBI requiring continuous ICP and CPP monitoring, after implementation of changes to our patient observation chart as well as improved nursing education on this topic.We recognise that there is scarcity of evidence of benefit for this practice,in terms of outcomes for this cohort of patients.It is perhaps time to design high quality studies to see if there are benefits,in terms of neurological outcomes,of measuring cerebral perfusion pressure at midbrain level.

**REFERENCE(S)**

1. Carney, Nancy, et al. "Guidelines for the management of severe traumatic brain injury." Neurosurgery 80.1 (2017): 6-15.
2. Thomas, E., M. Czosnyka, and P. Hutchinson. "Calculation of cerebral perfusion pressure in the management of traumatic brain injury: joint position statement by the councils of the Neuroanaesthesia and Critical Care Society of Great Britain and Ireland (NACCS) and the Society of British Neurological Surgeons (SBNS)." (2015): 487-488.
3. No grants received

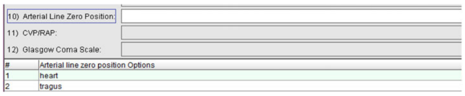

**Fig. 1 (abstract 001714).** showing the "drop-down list" of arterial transducer position in our electronic patient observation chart.

### 001726

#### Optic nerve ultrasound in patients with intracranial hemorrhages

M. Andreytseva[1], L. Khamidova[1], S. Petrikov[2], A. Solodov[2]

[1]Ultrasound, Sklifosovsky Research Institute for Emergency Medicine, Moscow, Russia, Moskva, Russia; [2]Neurosurgical icu, Sklifosovsky Research Institute for Emergency Medicine, Moscow, Russia

**Correspondence:** M. Andreytseva

*Intensive Care Medicine Experimental* 2019, **7(Suppl 3):**001726

**INTRODUCTION.** Intracranial hypertension (ICH) - the most frequent and terrible complication that occurs in patients with intracranial hemorrhage.One of the main tasks of the intensive care of patients with intracranial hemorrhage is the diagnosticsand correction of ICH.

**OBJECTIVES.** We evaluated the possibilities of the optic nerve ultrasound in the diagnosticsof ICH.

**METHODS.** This prospective observational study enrolled 51 adult patients with SAH (n=14) and TBI (n=37) with mean age of 48±14 years.All patients underwent neurological examination, performed CT scan of the brain, invasive monitoring using intracranial pressure sensors and dynamic measurement of the optic nerve sheath diameter (ONSD) and optic nerve diameter (OND) within the first 14 days following the onset of the disease. All patients were operated on. We examined 26 healthy volunteers to determine the normal values of ONSD and OND (n=26).

**RESULTS.** The normal values of the ONSD according to ultrasound in healthy volunteers were 4,7±0,2mm, OND - 2,3±0,1mm.In patients with SAH the mean ICP initially was 12,4±2,0mmHg, OND - 2,5±0,1mm, ONSD - 5,1±0,2mm. Further was found a tendency to ICH (average ICP values - 32±9mmHg, OND - 2,5±0,2mm, ONSD - 6,3±0,2mm), then ICP returned to normal values - 14±1,9mmHg, OND - 2,6±0,2mm, ONSD - 5,5±0,2mm. In patients with TBI ICP initially and during first 5 days was high - 37,6±3,1mmHg, OND - 2,6±0,2mm, ONSD - 6,4±0,1mm, then the level of ICP returned to normal values (mean ICP values 14,1±3,2mmHg, OND - 2,4±0,2mm, ONSD - 4,9±0,2mm). A strong correlation was found in patients with ICP >= 20mmHg and ONSD compared to healthy volunteers (Spearman N=803, R=0,3, p<0,001; Kruskal-Wallis p<0,001), the OND in the examined patients and in healthy volunteers did not differ (Spearman N=829, R=0,0055, p=0,9). The ROC analysis showed the ONSD of 5,9 mm was the best cutoff value with a sensitivity of 91% and a specificity of 86% for identifying ICP $^{3}$20mmHg.

**CONCLUSION.** The use of ultrasound of the optic nerve can serve as an additional screening non-invasive method for diagnostics ICH.

### 001371

#### Non-traumatic subarachnoid hemorrhage in Intensive Care Unit: prognostic factors and outcome. A long-term study

ML. Pérez Pérez[1], B. Balandín Moreno[1], J. Palamidessi Domínguez[2], R. Fernández Rivas[1], C. Martín Dal Gesso[3], C. De La Rosa Ruíz[4], S. Alcántara Carmona[1], I. Fernández Simón[1], N. Martinez Sanz[1], JJ. Rubio Muñoz[1]

[1]Intensive care, Hospital Puerta de Hierro-Majadahonda, Majadahonda, Spain; [2]Anesthesiology, Hospital Puerta de Hierro-Majadahonda, Majadahonda, Spain; [3]Intensive care, Hospital Universitario del Sureste, Arganda del Rey, Spain; [4]Radiology, Hospital Puerta de Hierro-Majadahonda, Majadahonda, Spain

**Correspondence:** M.L. Pérez Pérez

*Intensive Care Medicine Experimental* 2019, **7(Suppl 3):**001371

**INTRODUCTION.** Non-traumatic subarachnoid hemorrhage (SAH) represents a dramatic disease.

**OBJECTIVES.** To analyze prognostic factors and long-term outcomes of patients with SHA.

**METHODS.** Descriptive study (2009-2017). We collected: age, sex, Hunt & Hess (HH), Modified Fisher (MF), arteriography results and treatment: endovascular coiling (EVC) or surgical clipping (SC). We recorded the complications during ICU stay and the necessity of invasive procedures as external ventricular drain (EVD), mechanical ventilation (VM) and tracheostomy (TR). The time between diagnosis and treatment was analyzed too. Mortality and Glasgow Outcome Score (GOS) was evaluated at hospital discharge and at 6 months.

**RESULTS.** For 9 years, 184 patients with SAH were recruited, 58.7% were women, median age was 53 (46-64). HH was I-II in 120 patients (65.5%), III in 24 and IV-V in 39 (21.3%). MF was III-IV in 149 patients (81.4%). Arteriography (180) was positive for aneurysm in 134 cases (72.8%), arteriovenous malformation in 6 patients and negative in 40. The median size of aneurysm was 5 mm (4-7). EVC was performed in 117 patients (64.6%), surgical clipping in 17. The time between diagnosis and treatment was less than 24 hours in 58.8% cases. No spontaneous rebleeding was observed. Complications rates were: hydrocephalus 40.4%, vasospasm 26.9%, hyponatremia 17.1%, seizures 5.7%, myocardial injury 9%, lung injury 3% and infection 23.7% (urinary tract infection 51.1%, pneumonia 25.6% and ventriculitis 11.6%). Sixty-eight patients needed an EVD, 59 MV and 26 TR. The average hospital stay was 21.5 (13-33) days and 9 in the ICU (4-19). At hospital discharge 63.5% of the patients had GOS IV-V (moderate disability - good recovery), 22.6% GOS III (severe disability) and 13.8% GOS I-II (dead - vegetative state). At 6 moths 73.3% patients presented GOS IV-V. The overall mortality at six months was 11.4%. In the univariate analysis HH, MF, aneurysm, treatment, timing for EVC, EVD, hydrocephalus, infection, MV and age were related with worse outcome at hospital discharge (GOS I-III) (p< 0.05) (table 1). After the logistic regression analysis, the factors independently associated with worse functional status (GOS I-III) were: HH IV-V, hydrocephalus, necessity of MV and age (p<0.05) (table 2).

**CONCLUSION.** SAH had a high incidence of complications during ICU stay. HH IV-V, hydrocephalus, MV and age were independent risk factors for a worse outcome at hospital discharge. However, we observed a high rate of good-moderate recovery. In our series, no rebleeding was found, probably due to the early treatment.

**Table 1 (abstract 001371).** See text for description

| | GOS IV-V n= 115 | GOS I-III n=66 | *P* value |
|---|---|---|---|
| Age, *median (IQR)* | 50 (44-59) | 57 (51-68) | 0.01 |
| **Hunt & Hess,** I-III | 109 (95) | 34 (51) | < 0.001 |
| IV-V | 6 (5) | 32 (48) | |
| **Modified Fisher,** I-II | 29 (25) | 5 (7) | 0.003 |
| III-IV | 86 (74) | 61 (91) | |
| **Aneurysm** | 76 (67) | 56 (89) | 0.01 |
| **Treatment** | | | |
| None | 39 (33) | 11 (14) | |
| Surgical clipping | 8 (7) | 9 (14) | 0.012 |
| Endovascular coiling | 68 (60) | 46 (72) | |
| <24 77 (58.8%) | 36 (48) | 39 (73) | |
| 25-48 24 (18.3%) | 15 (20) | 8 (15) | 0.015 |
| 48-72 9 (6.8%) | 6 (8) | 3 (6) | |
| >72 21 (16 %) | 18 (24) | 3 (6) | |
| **Complications** | | | |
| Hydrocephalus | 31 (27) | 39 (64) | < 0.001 |
| Infection | 20 (17) | 20 (32) | 0.038 |
| External ventricular drain | 18 (60) | 38 (93) | 0.004 |
| Mechanical ventilation | 17 (15) | 40 (62) | < 0.001 |

**Table 2 (abstract 001371).** See text for description

| GOS I-III | Odds Ratio | P | (95% Conf. Interval) |
|---|---|---|---|
| Hunt & Hess IV-V | 5.71 | 0.01 | 1.50 - 21.74 |
| Mechanical Ventilation | 4.81 | 0.007 | 1.52 - 15.12 |
| Hydrocephalus | 2.32 | 0.05 | 1.52 - 5.40 |
| Age | 1.07 | 0.01 | 1.04 – 1.11 |

## ARF / ETH - Acute respiratory failure and ethics

### 000502

#### Surrogates of Mechanical Power:

M. Busana[1]; I. Pasticci[1]; L. Giosa[1]; F. Vassalli[1]; MM. Macrì[1]; M. Bonifazi[1]; R. D'albo[1]; F. Romitti[1]; M. Quintel[1]; L. Gattinoni[1]
[1]Department of Anesthesiology, Emergency and Intensive Care Medicine, University Hospital Göttingen - University Medical Center Göttingen, Göttingen, Germany
**Correspondence:** M. Busana
*Intensive Care Medicine Experimental* 2019, **7(Suppl 3):**000502

**INTRODUCTION.** Mechanical power has been increasingly recognized as the unifying variable that gathers the ventilator associated parameters which are responsible for VILI. Since the assessement of mechanical power is somehow difficult at the bedside, finding an easily available surrogate to estimate the energy delivered to the lung over time could be clinically interesting. Our aim is to evaluate the agreement between the measured mechanical power and two of its possible surrogates that we built up starting from the most available ventilation parameters.

**METHODS.** We collected experimental data from 78 mechanically ventilated pigs and clinical measurements obtained from 36 ICU patients for a total of 1002 observations. The so called "Peak power" was calculated as the product between peak pressure, tidal volume and respiratory rate. For the "Driving power" we used driving pressure instead of peak pressure.

**RESULTS.** Peak power overstimate actual mechanical power both in animals and human patients, with a significant positive bias of 7.68 J/min and 3.48 J/min respectively. The proportional error was similar in the two groups, with an increasing difference of 0.27 J/min (animals) and 0.25 J/min (patients) for each J/min increase of mechanical power delivered. The R2 of the linear regression was 0,97 for animals and 0,98 in patients, indicating an almost perfect linear relationship. When driving pressure was used instead of the peak pressure the goodness of fit of the model was reduced with an R2 of 0,65 in animals and 0,66 in patients. Driving power leads to an underestimation of the actual mechanical power of 7.35 J/min (animals) and 5.79 J/min (human patients) Both animals and patients showed a proportional error, with an increasingly negative difference of 0.33 J/min (animals) and 0.45 J/min (patients) for each J/min increase of mechanical power delivered.

**CONCLUSION.** Between the two surrogates we analyzed, peak power compared to driving power. Peak power overestimate the value of power, but this bias is moderate in the range of ventilation parameters commonly used in clinical practice. Moreover, an overestimation is probably safer than the underestimation provided by driving power which also does not take into account the energy needed to overcome PEEP. This might be relevant not only in the context of VILI prevention, but also in the hemodynamic management of the patients.

**REFERENCE(S)**

1. Gattinoni L, Tonetti T, Cressoni M, Cadringher P, Herrmann P, Moerer O, Protti A, Gotti M, Chiurazzi C, Carlesso E, Chiumello D, Quintel M. Ventilator-related causes of lung injury: the mechanical power. Intensive Care Med. 2016 Oct;42(10):1567-1575. doi: 10.1007/s00134-016-4505-2
2. None

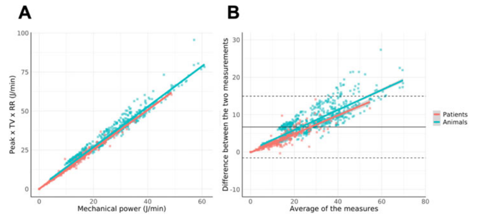

**Fig. 2 (abstract 000502).** See text for description

### 001155

#### Use of respiratory samples in the diagnosis of pneumonia associated to mechanical ventilation

M. Las Heras[1], I. Carboni Bisso[1], C. Videla[1], S. Di Stefano[1], A. Martinez[1], I. Staneloni[2], G. Almada[2], JM. Dianti[1], E. San Román[1]
[1]Unidad de terapia intensiva adultos, Hospital Italiano de Buenos Aires, Ciudad Autónoma de Buenos Aires, Argentina; [2]Infectology, Hospital Italiano de Buenos Aires, Ciudad Autónoma de Buenos Aires, Argentina
**Correspondence:** I. Carboni Bisso
*Intensive Care Medicine Experimental* 2019, **7(Suppl 3):**001155

**INTRODUCTION.** Ventilator associated pneumonia (VAP) is usually suspected when a patient develops a new or progressive infiltrate on chest radiograph, leukocytosis, purulent tracheobronchial secretions, and worsening gas exchange evidenced either by increasing oxygen requirements or ventilator demand.

Taken from respiratory samples, endotracheal aspirate (EA) and bronchoalveolar lavage (BAL) play an important role by allowing to know bacteriology, antibiotic sensitivity and epidemiology of different centers. Nevertheless respiratory samples are only one of various components in diagnosing VAP.

**OBJECTIVES.** Measurement of the impact of respiratory samples (EA and BAL) in diagnosing VAP.

**METHODS.** A retrospective, single-center observational study over a period of 1 year (2017- 2018) was carried out in 233 respiratory samples of 145 patients with suspected VAP.

**RESULTS.** Out of the 233 samples in 145 patients with suspected VAP, 52% (122) were EA and 48% (111) BAL. 64% of the patients were men and the average age was 53 years.

The most frequent pathologies were respiratory (38%) neurological (18%) and polytrauma (14%).
Clinical criteria, radiological evidence, alterations in gas exchange and respiratory samples were used to define VAP (as Criteria of centers for the control and prevention of diseases). Only in 18% (27) of the patients did the respiratory samples define an accurate diagnosis of VAP in the context of the suspected criteria.
**CONCLUSION.** The sole use of the respiratory samples (EA and BAL) for the diagnosis of VAP without taking into account clinical criteria, does not increase the accuracy of the diagnosis and it can lead to error, with an important impact on health costs and inadequate management of antibiotic therapy which generates greater bacterial resistance. Among the population studied with the implementation of international guidelines, in 18% of the patients the respiratory samples concluded a definitive diagnosis of VAP.

**REFERENCE(S)**

1. Steven M. Koenig and Jonathon D. Truwit .Ventilator-associated pneumonia: diagnosis, treatment, and prevention. Clin Microbiol Rev. 2006 Oct;19(4):637-57. DOI:10.1128/CMR.00051-05
2. Cristina Mietto, Riccardo Pinciroli, Niti Patel and Lorenzo Berra. Ventilator Associated Pneumonia: Evolving Definitions and Preventive Strategies. Respiratory Care June 2013, 58 (6) 990-1007. doi:10.4187/respcare.02380.
3. We would like to thank Fundación de Cuidados Críticos del Hospital Italiano de Buenos Aires (FCC-HIBA), Ignacio Fernández Ceballos, Ana Montserrat Rivera, Manuel Tisminetzky and Servicio de Neumonología del Hospital Italiano for their invaluable collaboration in this work.

### 001239

#### Profile of ARDS patients who were admitted to an Intensive Care Unit in a University Hospital in Southern Brazil

G. Antonelli[1], EF. Osaku[2], CRLDM. Costa[2], JBD. Anjos[1], A. Tomazelli[1], SM. Ogasawara[2], MA. Leite[2], T. Lordoni[3], AC. Jorge[4], PA. Duarte[4]
[1]Physiotherapy resident, Hospital Universitario do Oeste do Parana, Cascavel, Brazil; [2]Physiotherapy department, Hospital Universitario do Oeste do Parana, Cascavel, Brazil; [3]Nursing departament, Hospital Universitário do Oeste do Paraná, Cascavel, Brazil; [4]General icu, Hospital Universitario do Oeste do Parana, Cascavel, Brazil
**Correspondence:** G. Antonelli
*Intensive Care Medicine Experimental* 2019, **7(Suppl 3):**001239

**INTRODUCTION.** ARDS is a heterogeneous disease and its mortality rate is still high in intensive care unit (ICU). Causes of death generally are not only pulmonary pathologies, but concomitant extrapulmonary dysfunction.
**OBJECTIVES.** To characterize patients who developed ARDS.
**METHODS.** Retrospective study with data collected from January to December in 2017, performed through the analysis of the medical records of patients admitted to the ICU in the Hospital Universitário do Oeste do Paraná - Brazil. The variables were described by mean, standard deviation and percentage. Variables with normal distribution were compared using the *Student's T-Test,* and the non-normally distributed variables were compared using the *Man Whitnney* tests. All analyzes were performed at 5% significance.
**RESULTS.** The sample consisted of 36 patients who developed ARDS, 22 survivors (SG) and 14 deaths (DG). The variables comparing were SG *vs* DG: age in years (45.6 ± 21.8 *vs* 52.7 ± 26.8; p=0.55), male (63% *vs* 50%), non-neuro clinical causes of admission (29.16 *vs* 50%), severe traumatic brain injury (29.16% *vs* 21.42%), APACHE II (29.90 ± 7.13 *vs* 34.92 ± 7.42; p=0.05), SOFA (10.90 ± 3.03 *vs* 15.28 ± 3.02; p= 0.0002), higher FiO2 (57.5 ± 17.30 *vs* 53.21 ± 12.95; p=0,48), worst plateau pressure (19.13 ± 6.95 *vs* 21.5 ± 4.20; p=0.55), worst static complacency (31.40 ± 8.16 *vs* 32.78 ± 9.93; p=0.66), worst PaO2/FiO2 (155.54 ± 59.85 *vs* 142.92 ± 62.07; p=0.55), tidal volume ml/kg (6.90 ± 0.52 *vs* 6.57 ± 0.64; p=0.08), higher PEEP (12.40 ± 4.15 *vs* 12.85 ± 2.87; p=0.70), drive pressure (12.07 ± 3.02 *vs* 11.86 ± 4.45; p=0.85). The SG had an ICU length of stay of (24.04 ± 24.04) days, time of sedation in hours (253.10 ± 164.21), and time of the mechanical ventilation in hours (398 ± 338.29). The death group had an ICU length of stay of 14 ± 16 days. The alveolar recruitment maneuver was used in all patients and the prone position was used in 27% SG and 79% DG.
**CONCLUSION.** Patients with ARDS who died had the higher APACHE II and SOFA scores.

### 001253

#### Evaluating total protein in aspirates from different sampling methods in mechanically ventilated patients

R. STAMATIOU[1], V. Tsolaki[2], E. Koutsioumpa[3], G. Zakynthinos[3], M. Xintara[4], D. Makris[5]
[1]Icu university hospital of larisa, university of thessaly, greece, BIOPOLIS, Larisa, Greece; [2]Intensive care, University Hospital of Larissa, Larisa, Greece; [3]Intensive care unit, University Hospital of Larissa, Larissa, Greece; [4]Intensive care unit, University Hospital of Larissa, Larisa, Greece; [5]Department of intensive care medicine, University of Thessaly, Medical SchooL, Larissa, Greece
**Correspondence:** R. STAMATIOU
*Intensive Care Medicine Experimental* 2019, **7(Suppl 3):**001253

**INTRODUCTION.** Mechanically ventilated (MV) patients may present airway inflammation and elevated secretion production. However, it is not known whether total protein counts in bronchial samples may be useful to evaluate the clinical condition of patients and bronchial sampling for such purposes is not yet standardized.
**OBJECTIVES.** We aimed to evaluate bronchial secretions, with or without cells, in terms of differential total protein count obtained by mechanical ventilated critical care patients using different methods of sampling
**METHODS.** This study took place in the Intensive Care Unit of a tertiary Greek hospital during a six-month period. Patients were included if they were under MV for less than 24 hours and required bronchoscopy. Furthermore, non-mechanically ventilated subjects who underwent a single bronchoscopy, were assessed as controls. In all patients, aspiration of bronchial lining fluid (BLF) was performed, Bonchoalveolar lavage (BAL)and two types of Bronchial Washings (BW40 and BW5) were performed with the instillation of normal saline (ml) 150, 40, 5, while visible bronchial secretions were obtained via bronchoscopy (VBS) and blinded, via a common catheter for tracheobronchial aspiration (AC). Samples were divided in two and one of them was homogenized in a Heidolph Silent Crusher S and total protein was measured using the Bradford method after treatment with Lysis buffer (20 mM Tris-Cl pH 8.0, 150 mM NaCl, 1% Triton X-100, 1 mM dithiothreitol, 100 mg/mL-1 PMSF). Cell extracts were cleared by centrifugation (1000g, 20min, 4°C) and optical density was measured photometrically in samples with or without cells.
**RESULTS.** Twenty-five patients and six controls were evaluated. Mean (SE) age was 65.9 (3.0) and APACHE II 18.5(1.5). Ten patients were admitted due to surgical problems. Protein concentration was elevated in homogenized samples [14.98(1.45) μg/ml] compared to non-homogenized ones [13.69(2.25) μg/ml, *p<0.05]. Regarding the sampling technique, AC and VBS appear to be no different, while protein concentrations were elevated in VBS compared to BAL and BW samples (*p,0.05). There appeared to be no differences in samples with or without cells.
**CONCLUSION.** Both the sampling technique chosen and the homogenization of the sample, but not the presence of cells in the sample, can affect protein concentration in both MV patients and controls. These results show that total protein concentration in bronchial secretions could represent a potential inflammation biomarker.

**REFERENCE(S)**

1. Keatings VM, Evans DJ, BJ O'Connor, PJ Burnes. Cellular profiles in asthmatic airways: a comparison of induced sputum, bronchial washings and bronchoalveolar lavage fluid. Thorax 1997;52(4):372-374
2. The study did not receive any grant

### 001256

#### DNA fragmentation in bronchoalveolar lavage and bronchial washing of patients under mechanical ventilation

E. Golomazou[1], R. STAMATIOU[2], V. Tsolaki[3], M. Xintara[4], G. Zakynthinos[5], D. Makris[6]

[1]Department of ichthyology and aquatic environment - aquaculture laboratory, school of agricultur, University of Thessaly, Volos, Greece, Volos, Greece; [2]Icu university hospital of larisa, university of thessaly, greece, BIOPOLIS, Larisa, Greece; [3]Intensive care, University Hospital of Larissa, Larisa, Greece; [4]Intensive care unit, University Hospital of Larissa, Larisa, Greece; [5]Intensive care unit, University Hospital of Larissa, Larissa, Greece; [6]Department of intensive care medicine, University of Thessaly, Medical SchooL, Larissa, Greece

**Correspondence:** R. STAMATIOU

*Intensive Care Medicine Experimental* 2019, **7(Suppl 3):**001256

**INTRODUCTION.** The single cell gel electrophoresis (comet assay) is a sensitive technique for the detection of DNA damage which could serve as an index of cellular stress in the lung during mechanical ventilation (MV).

**OBJECTIVES.** DNA damage caused in lung parenchyma and bronchi in mini-bronchoalveolar lavage (mini-BAL) and bronchial washing (BW) samples obtained from patients under mechanical ventilation, was presently assessed.

**METHODS.** This prospective study took place in the Intensive Care Unit of a tertiary Greek hospital during a six-month period. Patients were included if they were under MV and required two bronchoscopy sessions; a. within the first 48 hours and b. at the 7th MV-day. Non-mechanically ventilated subjects who underwent a single bronchoscopy, were assessed as controls, as well. In all patients mini-BAL and BW were performed with the instillation of 40 ml and 5 ml of normal saline, respectively. Cellular stress response was assessed by the comet assay in which the fragmented DNA migrates out of the nucleus forming a tail, known as a "comet". Damage was quantified using the parameter %DNA in the comet's tail, which represents the percentage of total DNA migrated in the tail and is calculated as ratio of total intensity of the tail and total intensity of the comet.

**RESULTS.** Nine patients were assessed. Mean±SE age was 66.4±3.4 and APACHE II 18±1.8. Five patients were admitted due to medical problems (three of them with respiratory failure). Mean % DNA in the comet's tail were 9.84±3.1 and 6.3±2.7 in BW and mini-BAL, respectively; 7.6±2.0, 5.2±1.5 and 12.9±7.0, 7.6±3.3 in the first and second week, respectively, whereas % DNA damage in control samples was lower (1.10±0.3).

**CONCLUSION.** The comet assay may be useful in the evaluation of the cellular stress response in the lung due to mechanical stress of MV. A larger study will help to assess separately the role of various insults that can affect the lung during MV.

1. The study did not receive any grant.

### 001263

#### Hemoglobin Threshold for Transfusion in Critically Ill Adults with ARDS

O. Hunsicker[1], L. Materne[1], V. Bünger[1], A. Krannich[2], F. Balzer[1], C. Spies[1], R. Francis[1], S. Weber-Carstens[1], M. Menk[1], JA. Graw[3]

[1]Department of anesthesiology and operative intensive care medicine (ccm, cvk), Charité - Universitätsmedizin Berlin, Berlin, Germany; [2]Clinical trial office, Charité - Universitätsmedizin Berlin, Berlin, Germany; [3]Department of anesthesiology and operative intensive care medicine (ccm, cvk), Charité - Universitätsmedizin Berlin and Berlin Institute of Health (BIH), Berlin, Germany

**Correspondence:** O. Hunsicker

*Intensive Care Medicine Experimental* 2019, **7(Suppl 3):**001263

**INTRODUCTION.** The optimal hemoglobin threshold to trigger transfusion of packed red blood cells (PRBCs) in critically ill adults with acute respiratory distress syndrome (ARDS) is unknown. While several guidelines recommend to maintain the hemoglobin concentration within normal ranges, a restrictive transfusion strategy has been proven safe in many other clinical settings. However, the outcome of different hemoglobin thresholds that trigger blood transfusions in patients with ARDS has not been evaluated.

**OBJECTIVES.** To assess the effect of different transfusion thresholds on short-term outcome in patients with ARDS. We hypothesized that short-term outcome in patients transfused at lower hemoglobin thresholds is not inferior compared to patients transfused at higher hemoglobin thresholds.

**METHODS.** This retrospective observational study was conducted in a tertiary ARDS referral center. Patients admitted with an ARDS between January 2007 and December 2018 that received at least one PRBC transfusion were included into the study. For each patient, the hemoglobin concentration prior to each PRBC transfusion was identified and then the average over the number of PRBC transfusions was calculated. According to this individual hemoglobin threshold, patients were assigned to 5 different groups (<7 g/dl, 7-8 g/dl, 8-9 g/dl, 9-10 g/dl, 10-13 g/dl). In each group, a transfusion was not given until the hemoglobin concentration dropped into the group specific range. The primary endpoint was ICU mortality. Cox proportional hazards regression was used to estimate the risk of mortality in each group and to control for between-group differences of prognostic determinants in ARDS. (Ethical approval: Charité - Universitätsmedizin Berlin, No. EA2/018/19).

**RESULTS.** A total of 1042 ARDS patients were screened and 904 (86.8%) patients receiving overall 17.197 PRBC units were included in the analysis. The main cause for ARDS was pneumonia (64.6%). Most patients were graded with severe ARDS (84.8%) and 569 (62.9%) patients were treated with an extracorporeal lung assist device (ELAD). A total of 42 (18; 101) [median (25%; 75% quartiles)] hemoglobin values per patient were screened to calculate the individual hemoglobin threshold for each patient, resulting in 67 patients transfused at <7 g/dl, 275 patients at 7-8 g/dl, 309 patients at 8-9 g/dl, 147 patients at 9-10 g/dl, and 106 patients at 10-13 g/dl, respectively. After adjusting for confounders of mortality in ARDS, the risk for ICU mortality was not higher in patients transfused at lower hemoglobin thresholds (<7 g/dl and 7-8 g/dl) compared to patients transfused at higher hemoglobin thresholds (8-9 g/dl, 9-10 g/dl, and 10-13 g/dl). This finding was concordant with the results found in the subgroup of patients with ARDS and therapy with an ELAD.

**CONCLUSION.** In critically ill ARDS patients, the risk of death in the ICU is not higher when a restrictive transfusion strategy is applied compared to a liberal approach.

### 001274

#### Diaphragm Dysfunction from Phrenic Nerve Injuries during Cardiac Procedures: The Evolving Positive Role of Diaphragm Pacing to Improve Nerve and Diaphragm Function

R. Onders[1], H. R.[2], M. Elmo[1]

[1]Surgery, University Hospitals Cleveland Medical Center, Cleveland, USA; [2]Pulmonary, University Hospitals Cleveland Medical Center, Cleveland, USA

**Correspondence:** R. Onders

*Intensive Care Medicine Experimental* 2019, **7(Suppl 3):**001274

**INTRODUCTION.** Phrenic nerve damage from cardiac surgery occurs in 1 to 60% of cases depending on the procedure and methods of diaphragm evaluation. During atrial fibrillation ablation procedures it occurs in up to 20% of patients. Phrenic injures can results in symptoms of mild dyspnea, dependency on non-invasive ventilation (NIV) and failure to wean off mechanical ventilation (MV) with associated morbidity and mortality. Data on spontaneous recovery post injury is sparse and historically nerve recover can take up to 18 months.

**OBJECTIVES.** Report on the use of diaphragm pacing (DP) as a modality to improve diaphragm function post phrenic nerve injury.

**METHODS.** This is a retrospective review of a compassionate off label use of an FDA approved humanitarian use device under IRB approval (#02-10-18). Patients with diaphragm dysfunction (DD) from phrenic nerve injury underwent laparoscopic direct evaluation of the diaphragm. If the diaphragm was stimulateable, two intramuscular electrodes were placed in each diaphragm and electrical stimulation was begun to facilitate diaphragm strengthening and phrenic nerve recovery. Electrical stimulation therapy to strengthen the diaphragm ensued. Serial diaphragmatic electromyography (dEMG) through the implanted electrodes, chest radiographs (CXR), ventilation use and patient reported symptoms were obtained.

**RESULTS.** Twenty-three of 115 patients in the database had DD post cardiac procedure. Three developed DD post ablation and 1 after pericardial effusion procedure. Nineteen had median sternotomies: 3 left ventricular assist devices, 3 heart transplants, 2 atrial myxoma resections and 9 valve or coronary bypass isolated or combined cases. Nine were dependent on invasive MV with 8 tracheostomis. Nine used NIV with 5 continuous full-time NIV. Average age was 66.7 years (47-85). Time from insult to implant was 2 weeks to 15.5 years( median 2 months). Eight of the nine MV were weaned with five tracheostomy decannulations. Complete weaning averaged between 1 to 35 days (13.45 average). Initial dEMG data, available for 19, showed minimal to no activity on the affected side. Seventeen had significant improvement in dEMG. Overall survival is 23.6 months(2 weeks-7.5 years) with 6 deaths. Twenty patients had demonstrated diaphragm and phrenic nerve improvement (86.9%).

**CONCLUSION.** Patients requiring prolonged MV post cardiac procedure have significant morbidity with only a 30% one year survival. Reported spontaneous improvement of diaphragm paralysis is less than 45%. In this report DP showed improvement in 86.9% of patients utilizing diaphragm electrical stimulation as physical therapy to overcome the atrophied diaphragm and help nerve recovery. Early use of DP can help prevent the elevated diaphragm from paradoxical movement that can lead to lung collapse which would increase pneumonia risk. Early use may decrease the morbidity of tracheostomies. Future randomized trials will elucidate the role of early diaphragm pacing in preventing prolonged MV.

**REFERENCE(S)**

1. Onders RP, Markowitz A, Ho VP, Hardacre J, Novitsky Y, Towe C, Elmo M, Kaplan C, Schilz R. Completed FDA feasibility trial of surgically placed temporary diaphragm pacing electrodes: A promising option to prevent and treat respiratory failure.Am J Surg. 2018 Mar;215(3):518-521
2. Onders R, Elmo MJ, Kaplan C, Katirji B, Schilz R. Extended Use of Diaphragm Pacing in Patients with Unilateral of Bilateral Diaphragm Dysfunction: A New Therapeutic Option. Surgery 2014;156:772-86
3. Onders RP, Elmo MJ, Kaplan C, Schilz R, Katirji B, Tinkoff G. Long-term experience with diaphragm pacing for traumatic spinal cord injury: Early implantation should be considered. Surgery 2018. 164(4):705-711

### 001630

#### Patient-ventilator synchrony in Neurally Adjusted Ventilatory Assist (NAVA) and Pressure Support Ventilation (PSV)

M. Fakher[1], H. Kamal[2], M. Yehia[1], A. Abdelfatah[1], K. Abdelwahab[1]
[1]Critical care, Cairo University, Faculty Of Medicine, Kasr Al Ainy, cairo, Egypt; [2]Critical care, Suez Canal, Ismailia, Egypt
**Correspondence:** M. Fakher

*Intensive Care Medicine Experimental* 2019, **7(Suppl 3):**001630

**INTRODUCTION.** Neurally adjusted ventilatory assist (NAVA) is a mode that delivers proportional ventilation using esophageal probe to detect the electrical activity of the diaphragm (EAdi).

Difficulty in weaning is partly associated with the presence of asynchronies between the patient and the ventilator ,which occurs when either the initiation and/or termination of mechanical breath is not in time agreement with the neural inspiration, or if the magnitude of mechanical assist does not respond to the patient's respiratory demands.

**OBJECTIVES.** To evaluate role of NAVA mode in reducing asynchrony versus PSV mode

**METHODS.** A prospective, randomized, interventional, single-center study, which was approved by the lEthics and Research Committee in both Cairo University and Suez Canal Authority. Before inclusion, patients and/or families (next of kin) gave their consent about participation after information on the aim of this study.The study was conducted in the service of the ICU of Suez Canal Authority Hospital over a period of 14 months (from June 2017 till August 2018)

**Inclusion criteria:**

Invasively ventilated patients with predictive criteria of difficult weaning

**Exclusion criteria:**

- Contraindication to EAdi catheter placement

Patients who met the inclusion criteria and give their or their families consent about participation were successively ventilated for 24 h using PSV and then they were ventilated for another 24 h in the NAVA. dysynchronies were recorded and calculated .

**RESULTS.** Fifteen critically ill invasively ventilated patients were included in this study after fulfilling the inclusion criteria and giving their or their families consents.

**CONCLUSION.**

- Using NAVA mode was helpful in reducing patient ventilator desynchronies significantly when compared to PSV (which allow more patient comfort).
- There were significant increase in oxygenation index during NAVA than during PSV

1. The author(s) received no financial support for the research, authorship, and/or publication of this article.

**Table 1 (abstract 001630).** prevalance of dyssynchrony in both groups

| | PSV | NAVA | *P* |
|---|---|---|---|
| Asynchrony index | 2.8 | 1.1 | 0.001 |
| Ineffective efforts (median value) | 0.9% | 0.1% | 0.004 |
| Auto triggering (median value) | 0.66 % | <0.001 | 0.005 |
| Double triggering (median value) | 0.3% | 0.8% | 0.47 |
| Delayed cycling (median value) | 0.15% | 0 | 0.007 |
| Auto peep | <0.001 | 0 | 0.16 |

**Table 2 (abstract 001630).** arterial blood gases and hemodynamics during PSV and NAVA

| | PSV | NAVA | *P* |
|---|---|---|---|
| PaO2 / FiO2 | 210±37.2 | 258±31.4 | <0.001 |
| Pa CO2 (mmHg) | 38.6±4.5 | 40±2 | 0.92 |
| HCO3- (mmol/L) | 21.2±2.6 | 22.4±1.5 | 0.35 |
| MAP (mmHg) | 78±9.8 | 74±6.6 | 0.015 |
| HR (beats/min) | 108±14.5 | 88±7.7 | <0.0 |

### 001663

#### Transpulmonary pressure guided ventilation in ECMO eligible obese severe ARDS patients

S. Jog, P. Kalyani, D. Pradip, R. Prasad
[1]Intensive care medicine, Deenanath Mangeshkar Hospital and Research Center, Pune, India
**Correspondence:** S. Jog

*Intensive Care Medicine Experimental* 2019, **7(Suppl 3):**001663

**INTRODUCTION.** Transpulmonary Pressure(TPP) measurement is a promising tool used for ventilatory adjustments in ARDS patients. Its usefulness and application in the management of severe ARDS remains unclear.

**OBJECTIVES.** 1) Does TPP measurement help in ventilatory adjustments in severe ARDS patients?

2)Do TPP guided ventilator strategies improve gas exchange?

3) Does TPP guided PEEP adjustment increases incidence of barotraumas?

**METHODS.** Prospective observational study. TPP measured by an esophageal balloon catheter(Nutrivent, Sidam, Italy) and a suitable ventilator( Hamilton G5, Switzerland)

Inclusion criteria – Adult patients with Body Mass Index > 25, Severe ARDS (PO2/FiO2 ratio < 100) having set PEEP ≥ 15 cm, Airway plateau pressure > 30 cm and under deep sedation with neuromuscular blockade and at least one 16 hour prone position trial instituted.

Exclusion Criteria – 1)Patients receiving ECMO 2) Patients in whom position of esophageal balloon and measurement of TPP could not be confirmed by cardiac oscillation and abdominal pressure technique. Ventilatory parameters and blood gas parameters were documented before measuring TPP and after measuring TPP. Ventilatory setting adjustments done after TPP measurements were – i)increasing PEEP in hypoxic patients( SpO2 < 88 %) till oxygen saturation remained > 90% provided plateau TPP remained < 23 cm and end expiratory TPP remained > 0 cm ii)Increasing tidal volume( > 6 ml / KG) in hypercapnoeic acidotic patients ( pH< 7.20 and PCO2> 80mm Hg) provided plateau TPP remained < 25 cm.

**RESULTS.** Consecutive 18 patients who met inclusion criteria were screened only 10 patients included in the study. All 10 of our study patients were eligible for ECMO. However ECMO therapy could not be offered to these patients due to resource limitations. Average weigh of these patients was 88.3 ± 20.3 with BMI 30.8 ± 6.8 . Average PEEP set was 15.1 ±2.1 cm of H2O prior TPP measurement. Post intervention PEEP was 20.5 ± 3.1 cm of H2O. (p = 0.000043). The mean PO2/FiO2 ratio before intervention was 85.5 ± 24.41 and after intervention it was 103.4 ± 20.9 . The difference in PO2/FiO2 ratio before and after intervention was significant, 68.7 ± 14.34 versus 81 ± 12.28 (p = 0.0473). None of these 10 patients developed pneumothorax even at average PEEP of 20.5 ± 3.1 cm and average airway plateau pressure of 41.2 ± 4.2 cm. 6 out of 10 patients survived till hospital discharge and 4 patients died due to sepsis and MODS.

**CONCLUSION.** TPP measurement is useful in setting appropriate PEEP and tidal volume in severe ARDS patients without increasing the risk of barotrauma. TPP guided ventilation strategy in ECMO eligible severe ARDS patients , can improve outcome in resource limited settings.

**REFERENCE(S)**

1. Grasso S, Birocco A, Sorbo L Del, Gattinoni L, Ranieri VM. ECMO criteria for influenza A ( H1N1 ) - associated ARDS : role of transpulmonary pressure. Intensive Care Med. 2012;38:395–403.
2. Daniel Talmor, Todd Sarge, Atul Malhotra, Carl R. O'Donnell, Ray Ritz, Alan Lisbon VN and SHL. Mechanical Ventilation Guided by Esophageal Pressure in Acute Lung Injury. N Engl J Med. 2008;359(20):2095–104.
3. Akoumianaki E, Maggiore SM, Valenza F, Bellani G, Jubran A, Loring SH, et al. The Application of Esophageal Pressure Measurement in Patients with Respiratory Failure. Am J Respir Crit Care Med. 2014;189(5):520–31
4. NIL

### 001664

#### PEEP effect of tracheotomy high-flow oxygen therapy in patients with severe head injury

P. Xu[1], H. Ge[2]

[1]Sir Run Run Show Hospital, School of Medicine, Zhejiang University, Hangzhou, China; [2]Respiratory care, Sir Run Run Show Hospital, School of Medicine, Zhejiang University, Hangzhou, China

**Correspondence:** P. Xu

*Intensive Care Medicine Experimental* 2019, **7(Suppl 3):**001664

**INTRODUCTION.** The current research is mostly focused on nasal high-flow oxygen therapy (NHFO). Whether the high-flow oxygen therapy has the same physiological effect through tracheotomy catheter is still unknown(THFO) , and there are certain doubts in the clinic.

**OBJECTIVES.** Investigate the physiological role of tracheotomy high-flow oxygen therapy in the patients with severe craniocerebral injury after tracheotomy.

**METHODS.** From July 1st to December 31st, 2018, we entered the emergency department of our hospital with craniocerebral injury (GCS ≤ 8) and underwent tracheotomy. Patients who successfully detached from the ventilator for 24 hours were followed up by themselves. Give venturi mask gas mask (MO) and tracheotomy high flow oxygenation (THFO), high flow rate from 30L / min, 45L / min to 60L / min in turn, each method lasted for 30 minutes. The main observation was the measurement of end-tidal lung volume (△EELI) during the different oxygenation modes of patients by electrical impedance tomography (EIT). Secondary observations included different oxygenation methods for vital signs, oxygenation and the impact of gas exchange.

**RESULTS.** Total of 20 patients with severe craniocerebral injury were included in the study. The end-expiratory lung volume (EELI) decreased significantly with the increase of flow rate after high-flow oxygen therapy (p<0.05), mainly in the non-gravity lung. The decline in lung volume in the dependent area. The oxygenation index and carbon dioxide partial pressure were significantly improved compared with the oxygen absorption in the venturi, which were p <0.01 and p <0.05, respectively. However, the oxygen concentration did not increase significantly with the increase of THFO flow, p = 0.234, and the change of PaCO2 did not cause a significant change in blood pH (p = 0.298). The patient's respiratory rate was lower at high flow oxygen therapy (p < 0.05), but the increase in the THTO flow did not decrease further (p = 0.554). Other vital signs such as tidal volume, heart rate, blood pressure, and mean arterial pressure were not statistically different between high-flow oxygen therapy and venturi.

**CONCLUSION.** Tracheotomy high flow oxygen therapy can reduce the respiratory rate, end-tidal and arterial blood carbon dioxide partial pressure in patients with severe craniocerebral injury, and improve the patient's oxygen and without affecting other vital signs. THFO does not produce PEEP effect, and the end-expiratory lung volume even has a tendency to decline. The specific reasons need further study.

**REFERENCE(S)**

1. Corley A, Caruana LR, Barnett AG, Tronstad O, Fraser JF. Oxygen delivery through high-flow nasal cannulae increase end-expiratory lung volume and reduce respiratory rate in post-cardiac surgical patients. Br. J. Anaesth. 2011; 107: 998–1004.
2. Mauri T, Eronia N, Turrini C, Battistini M, Grasselli G, Rona R, Volta CA, Bellani G, Pesenti A (2016) Bedside assessment of the effects of positive end-expiratory pressure on lung inflation and recruitment by the helium dilution technique and electrical impedance tomography. Intensive Care Med 42(10): 1576–1587.
3. Van der Burg PS, Miedema M, de Jongh FH, Frerichs I, van Kaam AH(2014) Cross-sectional changes in lung volume measured by electrical impedance tomography are representative for the whole lung in ventilated preterm infants. Crit Care Med 42(6):1524–1530.
4. Kobylianskii J, Murray A, Brace D, Goligher E, Fan E. Electrical impedance tomography in adult patients undergoing mechanical ventilation: a systematic review. J Crit Care. 2016;35:33–50.
5. Mauri T, Eronia N, Abbruzzese C, Marcolin R, Coppadoro A, SpadaroS, Patroniti N, Bellani G, Pesenti A (2015) Effects of sigh on regional lung strain and ventilation heterogeneity in acute respiratory failure patients undergoing assisted mechanical ventilation. Crit Care Med43(9):1823–1831.

### 001665

#### Outcome Predictors of Prone Position Ventilation in Severe ARDS

S. Jog[1], P. Kalyani[2], M. Jai[2], G. Arpit[2], R. Prasad[2]

[1]Intensive care medicine, Pune University, Pune, India; [2]Intensive care medicine, Deenanath Mangeshkar Hospital and Research Center, Pune, India

**Correspondence:** S. Jog

*Intensive Care Medicine Experimental* 2019, **7(Suppl 3):**001665

**INTRODUCTION.** Prone ventilation (PV) is a standard of care for severe ARDS . Factors associated with outcome of PV are not well studied.

**OBJECTIVES.** This retrospective analysis aims at finding the factors associated with survival as an outcome in patients who received PV in severe ARDS.

**METHODS.** Single center retrospective observational study. Patients with severe ARDS who received lung protective ventilation as per the ARDSnet protocol and received prone ventilation(PV) were considered for the analysis. Inclusion criteria - 1)PO2/FiO2 ratio < 100 2)Plateau Pressure > 28 cm 3)PEEP > 12 cm 4) Received at least 1 session of PV for consecutive 14 hours. Exclusion criteria –i)Patients who received ECMO or ECCO2R Extended prone session was defined as a prone ventilation session continued for more than 24 hours consecutively. Multivariable regression analysis used as a statistical method.

**RESULTS.**

Data of 44 patients was analysed. Mortality was 54.44%( 24/44).Average days of mechanical ventilation were 14.84 days.ICU length of stay was 18.72 days. Average days of mechanical ventilation were 14.84 days.ICU length of stay was 18.72 days .In univariate analysis, we analysed 32 clinical, organ function, gas exchange and lung mechanics parameters of each patient. Survival as an outcome was significantly associated with Extended Prone Session ( P<0.005) and number of Extended Prone Sessions ( P < 0.05) while following factors were significantly associated with death as an outcome:

1)pH < 7.15 at the end of first prone session ( P = 0.000) 2)Presence of shock needing vasopressors ( OR 9.86 ; CI 1.07 to 90.7) 3)Need for Renal Replacement Therapy (OR 5.4;CI 1 to 28.93) 4)PCO2 > 55 mm Hg at the end of first prone session ( OR 4.46; CI 1.24 to 17.41) In multiple regression analysis, 2 factors were independently associated with outcome. pH < 7.15 at the end of first prone session( 1.25 ± 0.57;P=0.03) was associated with Mortality and number of extended prone position sessions ( 0.10 ± 0.04;P=0.02) was associated with Survival

**CONCLUSION.** Extended sessions of prone position are associated with survival and persistent respiratory acidosis at the end of first prone session is associated with death in patients of severe ARDS treated with prone position.

### 001668

#### Neurally Adjusted Ventilatory Assist versus Pressure Support Ventilation during SBT in patients with difficult weaning

M. Fakher[1], H. Kamal[2], M. Yehia[1], K. Abdelwahab[1], A. Abdelfatah[1]
[1]Critical care, Cairo University, Faculty Of Medicine, Kasr Al Ainy, cairo, Egypt; [2]Critical care, Suez Canal, Ismailia, Egypt
**Correspondence:** M. Fakher
*Intensive Care Medicine Experimental* 2019, **7(Suppl 3):**001668

**INTRODUCTION.** Neurally adjusted ventilatory assist (NAVA) is a mode that delivers proportional ventilation using esophageal probe to detect the electrical activity of the diaphragm (EAdi).Difficulty in weaning is partly associated with the presence of asynchroniesbetween the patient and the ventilator ,using Proportional modes vs traditianoal PSV in weaning appears to reduce dyssynchrony and aid weaning(1)

**OBJECTIVES.** To evaluate the role of NAVA compared to PSV in patient with difficult weaning

**METHODS.** This is a prospective randomized control study conducted on 30 critically ill, invasively ventilated patients with predictive criteria of difficult weaning(2) admitted during the period from June 2017 till August 2018 ,the patients were evaluated for readiness criteria and presence of weaning indices then they were randomly divided into two groups: group A include fifteen patients were ventilated in PSV (as a weaning mode) and evaluated for successful weaning and group B: including Fifteen patients were NAVA was used as a weaning mode for 24 hours.Research and Ethics comittee approval was taken prior to performing the study and a written consent from each patient and or his next of kin before participating in the study.

**RESULTS.** There was no difference between both groups regarding demographics ,baseline charecteristics and initial cause of venilation or initial ventilatory support.

There was no significant difference between both groups regarding length of mechanical ventilation ,susessful weaning, length of hospital stay.see ***table(1)***

The assyncrony index was lower in group B using NAVA while the oxygenation index was significantly higher in same group compared to group A using PSV during weaning .

**CONCLUSION.** Using NAVA during sponteneous breathing trial in difficult weaning patients reduced Assynchrony index and improved oxygenation index but this did not affect the sucess of weaning nor the outcome of patients(mortality LOS nor LOV ).

**REFERENCE(S)**

1. Rozé H, Lafrikh A, Perrier V, Germain A, Dewitte A, Gomez F, Janvier G, Ouattara A. Daily titration of neurally adjusted ventilatory assist using the diaphragm electrical activity. Intensive care medicine. 2011 Jul 1;37(7):1087-94.
2. Spadaro S, Mirabella L, Volta CA, Serio G, Staffieri F, Dambrosio M, Cinnella G, Bruno F, GrasSo, S. Impact of prolonged assisted ventilation on diaphragmatic efficiency: NAVA versus PSV. Critical Care. 2015 Dec;20(1):1.
3. There is no specific research grant received by the authors.

**Table 1 (abstract 001668).** Outcome of both groups

| | Group A | Group B | p |
|---|---|---|---|
| Weaninig sucess(N/%) | 6/40 | 8/53.3 | 0.71 |
| length of MV(Days) | 14.5±6.4 | 16.1±6.1 | 0.58 |
| Length of stay(Days) | 17.3 ± 7.7 | 19.7 ± 7.7 | 0.4 |
| Mortality(N/%) | 6/40 | 4/26.7 | 0.7 |
| PaO2/FiO2 index | 210±37.22 | 258±31.37 | 0.001 |

### 001677

#### Vimentin regulation on the activation of autophagy in lung fibroblast in response to LPS exposure

P. Pan[1], L. Su[2], D. Liu[2], Y. Long[2]
[1]Icu, Beijing Tiantan Hospital, Beijing, China; [2]ICU, Peking Union Medical College Hospital, Beijing, China
**Correspondence:** L. Su
*Intensive Care Medicine Experimental* 2019, **7(Suppl 3):**001677

**INTRODUCTION.** Activation and assembly of the NLRP3 inflammasome is dependent on interaction between NLRP3 and the intermediate filament protein vimentin in ARDS model.

**OBJECTIVES.** The roles of vimentin in the process of ARDS were further investigated.

**METHODS.** The human fetal lung (HFL-1) fibroblasts with vimentin transfergene or knockout were treated with LPS intervention. The oxidative stress damage, inflammation and cytokines, apoptosis, autophagy, and pyroptosis were measured and analyzed the relevant mechanism.

**RESULTS.** We demonstrated that vimentin expression less in the HFL-1 cells with vimentin knockout in response to LPS. Specifically, an increase of oxidative stress, a decrease of mitochondrial membrane potential, and an increase of calcium ion permeability resulted in an increase in the level of fibroblast apoptosis. As the same time, inflammatory response of vimentin knocked out were also upregulated, including the higher levels of TNF-a, IL-1ß, IL-6, and IL-10. Importantly, the mechanism of suppression of vimentin in lung fibroblasts was caused by a decrease in autophagy, a decrease in mitochondrial membrane protein, and a decrease in mitochondrial function, which might contribute to the augmented cellular injury in response to LPS exposure.

**CONCLUSION.** This study provided insights into that vimentin may interfere inflammatory cascade by activating the mitochondrial autophagy pathway of lung fibroblasts in the early stage of acute lung injury.

**REFERENCE(S)**

1. Su LX, Pan P, Wang XT, Long Y, Liu DW, Zhou X. Vimentin modulates apoptosis and inflammatory cytokine release by a human monocytic cell line (THP-1) in response to lipopolysaccharides in vitro. Chin Med J (Engl). 2019.
2. Su L, Pan P, Yan P, Long Y, Zhou X, Wang X, Zhou R, Wen B, Xie L, Liu D. Role of vimentin in modulating immune cell apoptosis and inflammatory responses in sepsis. Sci Rep. 2019; 9: 5747.
3. dos Santos G, Rogel MR, Baker MA, Troken JR, Urich D, Morales-Nebreda L, Sennello JA, Kutuzov MA, Sitikov A, Davis JM, Lam AP, Cheresh P, Kamp D, Shumaker DK, Budinger GR, Ridge KM. Vimentin regulates activation of the NLRP3 inflammasome. Nat Commun. 2015; 6: 6574.
4. This study was supported by the National Science Foundation for Young Scientists of China (Grant No.81501707).

### 001678

#### Quality control targets (SpO2≠100%, PaCO2≮40mmHg, Pmean ≯10cmH2O) on Outcome Among Patients in ICU

L. Su[1], P. Pan[2], D. Liu[1], X. Wang[1]

[1]ICU, Peking Union Medical College Hospital, Beijing, China; [2]Icu, Beijing Tiantan Hospital, Beijing, China

**Correspondence:** L. Su

*Intensive Care Medicine Experimental* 2019, **7(Suppl 3):**001678

**INTRODUCTION.** A series of quality control (QC) targets (SpO2≠100%, PaCO2≮40mmHg, Pmean ≯10cmH2O) was put forwarded and widely used in a single intensive care unit (ICU) setting.

**OBJECTIVES.** The aim of this study is to assess whether these QC targets could improve outcomes of the critically ill patients.

**METHODS.** The real-time clinical data of patients with mechanical ventilation at ICU admission between May 2013 to May 2017 in the Department of Critical Care Medicine of Peking Union Medical College Hospital were collected and analysed.

**RESULTS.** A total of 7,670 patients (mean age, 58 years; 3,943 [51.5%] male) were divided into QC before (n = 3,936) or QC after (n = 3,734) group. QC targets (SpO2, PaCO2, Pmean) and respiratory parameters (FiO2%, PaO2, PEEP, tidal volume, respiratory rate) within 72-hour ICU admission, primary outcome (ICU mortality, 28-day, 60-day, and 90 day mortality) and secondary outcomes (against advise discharge, ICU admission days, mechanical ventilation times, and central venous pressure) were calculated and made the comparisons between QC before and after.72-hour averaged SpO2, Pmean, FiO2%, PaO2, and VT were significantly lower and PaCO2 was higher after QC (*P<0.05*). Lower 90-day mortality, less against advise discharge, ICU admission days and mechanical ventilation times were found after QC (*P<0.05*). Interestingly, CVP significantly decreased accordingly in the QC after group (*P<0.05*).

**CONCLUSION.** The QC targets (SpO2≠100%, PaCO2≮40mmHg, Pmean ≯10cmH2O) contributed to avoid the high oxygen hazards, protect the lung injury, and improve the circulatory function, which resulted in better prognosis of critically ill patients.

1. Supported by Capital Characteristic Clinic Project of Beijing (No. Z181100001718209).

### 001171

#### Elder donors as a way of increasing the organ donor pool. Establishment of a program of intensive care to facilitate organ donation: a three-year experience in a tertiary-level hospital

J. Garcia-Sanz[1], M. Pérez Redondo[1], JJ. Rubio Muñoz[1], I. Fernández Simón[1], S. Alcántara Carmona[1], I. Lipperheide Vallhonrat[1], J. Veganzones Ramos[2], S. Tejado Bravo[1], P. Rodríguez Villamizar[1]

[1]Intensive care department, Hospital Puerta de Hierro, Majadahonda, Spain; [2]Anesthesiology department, Hospital La Paz, Madrid, Spain

**Correspondence:** J. Garcia-Sanz

*Intensive Care Medicine Experimental* 2019, **7(Suppl 3):**001171

**INTRODUCTION.** The imbalance between organ demand and organ availability has led to the development of new strategies to increase the donor pool. The ACCORD-Spain project, carried out in 68 Spanish hospitals between November 2014-April 2015, evaluated the number of patients deceased after a devastating brain injury (DBI) and not admitted to intensive care (ICU) due to their bad neurological prognosis, that could have become potential donors. This project laid the grounds for what it is known as intensive care to facilitate organ donation (ICOD).

**OBJECTIVES.** To evaluate the impact of a new protocol aimed at promoting ICOD in patients with a DBI once active treatment (medical and/or surgical) has been deemed futile.

**METHODS.** Retrospective descriptive study (January 2016-December 2018) carried out in a Spanish tertiary hospital with a transplantation program that performs all transplants except small bowel and pancreas. The new protocol for ICOD comprehended the elaboration of a banner explaining which patients could be eligible for ICOD together with informative talks in the Emergency, Neurology, Neurosurgery and Internal Medicine Departments. We reviewed all patients notified to the Transplant Coordination Team and data were recovered from their database. Evaluation and analysis of all notifications were carried out.

**RESULTS.** During the period evaluated, 27 cases were notified; 21 (77%) out of the Emergency Department, four (15%) out of the neurology ward and two (8%) were ICU patients admitted after a DBI secondary to complications of an endovascular neurointervention. Mean age: 84.5 years with 21 (77%) females. The most frequent cause of DBI was intracranial hemorrhage (n= 18; 66%) followed by severe traumatic brain injury (n=6; 22%), stroke (n=2; 8%) and acute subarachnoid hemorrhage (n=1; 4%). Eleven (40%) out of the 27 patients were admitted to ICU with five (18.5%) of them becoming effective organ donors. An average of 2 organs were retrieved from each donor, with a total of 10 organs being implanted (1 bilateral and 1 single lung, 4 livers and 4 kidneys). This accounted for 7% of our total annual donor pool. Reasons for discarding the other six (21.5%) patients as donors were: absence of progression to brain death (n=2), new diagnosis of a neoplastic process, hemodynamic instability, medical contraindication and dismissal by the National Organ & Tissue Transplantation Organization due to lack of recipient (one case each).

Sixteen patients (60%) were not eligible for ICOD because of: age above 90 years plus comorbidities (n=5); clinical and radiological criteria that oriented to a low probability of evolving towards brain death (n=3); family refusal for ICOD (n=3) and absence of free ICU beds (n=2). Hemodynamic instability, the impossibility of correctly studying a neoplastic process at the time of admission and reasons not detailed in the clinical history constituted the three remaining cases.

**CONCLUSION.** ICOD represents a good and feasible option to increase the organ donor pool. For this reason, we believe all patients with a DBI in which treatment has been deemed futile should be evaluated by the Transplant Coordination Team.

ICOD will only be possible after efforts have been made to raise awareness towards organ donation across different hospital departments.

**REFERENCE(S)**

1. Domínguez-Gil B, Coll Torres E, Luengo Calvo A, Ramos Suárez B. Extensión de la Acción Conjunta Europea Achieving Comprehensive Coordination in ORgan Donation throughout the European Union (ACCORD). ACCORD-España. Informe 1. Prácticas clínicas al final de la vida en pacientes fallecidos con daño cerebral catastrófico y donación de órganos en España. Organización Nacional de Trasplantes [internet]. November 2015. Available from: http://www.ont.es/infesp/

ProyectosEnMarcha/Proyecto%20ACCORD%20España_INFORME%20-FASE%20PRIMERA%20COMPLETO%20FINAL_20151101.pdf

2. Domínguez-Gil B et al. Expanding the Donor Pool Through Intensive Care to Facilitate Organ Donation: Results of A Spanish Multicenter Study. Transplantation.2017 Aug;101(8):e265-e272. doi : 10.1097/TP.0000000000001701.

### 001272

#### Use of DNR orders in Worldwide Intensive Care Units: The Ethicus-2 Study

P. Maia[1], A. Avidan[2], M. Weiss[3], C. Feldman[4], R. Sreedharan[5], C. Danbury[6], M. Hache-Marliere[7], S. Mullick[8], C. Sprung[2]
[1]Intensive care medicine, Hospital Geral de Santo António, Porto, Portugal; [2]Anesthesiology and critical care, Hadassah Medical Center, Hebrew University of Jerusalem,Faculty of Medicine, Jerusalem, Israel; [3]Anesthesiology, University Hospital Ulm, Ulm, Germany; [4]Internal medicine, University of the Witwatersrand, Johannesburg, South Africa; [5]Critical care, Cleveland Clinic Foundation, Cleveland, OH, USA; [6]Intensive care, Royal Berkshire Hospital, Reading, UK; [7]Intensive care medicine, CEDIMAT, Santo Domingo, Dominican Republic; [8]Department of critical care medicine, Tata Medical Center, New Town, Kolkata, India
**Correspondence:** P. Maia
*Intensive Care Medicine Experimental* 2019, **7(Suppl 3):**001272

**INTRODUCTION.** *Do-not-resuscitate* (DNR) orders apply to the decision to withhold *cardiopulmonary resuscitation* (CPR) and should not impact on other diagnostic or therapeutic measures. When there is *limitation of life-sustaining therapies* (LT), DNR is frequently the only or the first therapy to be limited and to many physicians a pre-requisite for other limitations.

**OBJECTIVES.** To evaluate the use of DNR orders worldwide and how they are related to LT.

**METHODS.** ICUs worldwide were invited to participate in Ethicus-2 through their country societies. Consecutively admitted patients who died or had LT during a 6 month period from 1.9.2015 to 30.9.2016 were prospectively studied. Regions included *North, Central and Southern Europe* (NE, CE, SE), *North and Latin America* (NA, LA), *Asia* (As), *Australia/New Zealand* (Au/NZ) and *Africa* (Af). Previous *end-of-life practices* (EOLP) definitions (1) were used.

**RESULTS.** 199 ICUs in 36 countries participated, enrolling 12850 patients. Decisions to LT, either *withholding* (WH) or *withdrawing* (WD) or to *actively shortening the dying process* (SDP) for the different regions were (WH %,WD %, SDP %): Af (20;13;0); As (41;39;0); NE (38;53;0.2); CE (47; 37; 1); SE (41;25;1); LA (60;6;0); NA (54;36;1); Au/NZ (45;46;0). The % of use of DNR orders in patients with LT for region was: Af (8); As (80); NE (92); CE (85); SE (50); LA (41); NA (95); Au/NZ (97). DNR orders are common when a decision to WH, WD or SDP is taken in Asia, NE and CE, NA and Au/NZ, but not in Af, SE and LA.

**CONCLUSION.** DNR orders are used differently with other limitations of LT around the world.

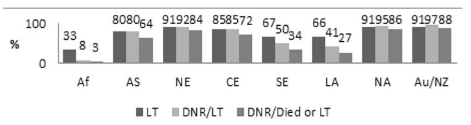

**Fig. 1 (abstract 001272).** See text for description

### 001580

#### Moral distress in organ donation decisions in ICU

U. St Ledger[1], DF. Mc Auley[2], J. Reid[3], L. Prior[4], B. Blackwood[2]
[1]{street_address}, Belfast, UK; [2]Centre for experimental medicine, Queen's University Belfast, Belfast, UK; [3]School of nursing & midwifery, Queen's University Belfast, Belfast, UK; [4]Centre of excellence for public health, Queen's University Belfast, Belfast, UK
**Correspondence:** U. St Ledger
*Intensive Care Medicine Experimental* 2019, **7(Suppl 3):**001580

**INTRODUCTION.** Moral distress, a powerful and detrimental psychological response experienced when moral action is constrained (Jameton, 2008) has been reported in end-of-life care (Hamric and Blackhall, 2007). Increasingly intensive care unit (ICU) end-of-life decisions were expected to involve approaches for organ donation and opt in opt out approaches to organ donation high on international political agendas. However, organ donation related moral distress and the impact on clinicians and patients' relatives was under-investigated.

**OBJECTIVES.** To explore the triggers for moral distress and the consequences for physicians, nurses and relatives involved in approaches for organ donation in end-of-life decisions.

**METHODS.** This qualitative narrative inquiry thematic analysis was undertaken, in a large 19-bedded ICU in Northern Ireland, from August 2012-November 2013. Twenty bereaved relatives and 45 nurses and physicians involved in 21 end-of-life patient cases were purposively recruited and interviewed using in-depth digitally recorded methods.

**RESULTS.** The timing and sensitivity of the organ donation approach was important for supporting relatives to make morally comfortable decisions. Physicians and nurses roles of carer versus broker for organs created moral conflict. Physicians perceived pressure to challenge relatives' decisions not to donate. Such challenge was morally distressing for relatives and nurses, jeopardising autonomy, integrity and relationships. Rapid therapy withdrawal in donation after circulatory death clashed with notions of 'a good death'. Lengthy delays in organ retrieval threatened relatives' fulfilment of culturally important after-death rituals and obligations and risked withdrawal of consent to donate. Moral distress impacted negatively on the grieving process, well-being and attitudes about organ donation and positively on quests to promote organ donation.

**CONCLUSION.** Organ donation decisions can generate considerable moral distress. Findings have important implications for: (a) the educational preparation and support of ICU clinicians to recognise and manage the ethical challenges and sensitivities of organ donation approaches and processes; (b) timely organ retrieval supportive of important familial and cultural obligations and beliefs; and (c) for organ donation opt in-opt out policies and legislation and organ donation consent rates.

**REFERENCE(S)**

1. Organ Donation Taskforce (ODT) (2008) The potential impact of an opt out system for organ donation in the UK: an independent report from the Organ Donation Taskforce. DH Publications: London.
2. Hamric, A. B., Blackhall, L. J. (2007). Nurse-physician perspectives on the care of dying patients in intensive care units: collaboration, moral distress, and ethical climate. Critical Care Medicine, 35(2), 422-429.
3. Jameton A. (1984) Nursing practice: the ethical issues. Englewood Cliffs, New Jersey: Prentice Hall.
4. Funded by The Research and Development Office, Public Health Agency, Northern Ireland

## POIC / AKI - ICU care after surgery

### 001558

#### Continuous non-invasive haemodynamic monitoring during caesarean section in hypertensive patients

E. Pedrazzoli, G. Frison, K. Donadello, L. Cattin, V. Schweiger, E. Polati
[1]Anesthesia and intensive care b unit, University of Verona, AOUI-University Hospital Integrated Trust of Verona, Verona, Italy
**Correspondence:** K. Donadello
*Intensive Care Medicine Experimental* 2019, **7(Suppl 3):**001558

**INTRODUCTION.** Hypertensive derangements, preeclampia included, complicate 8-10% of pregnancies worldwide and represent a major cause of maternal and foetal morbidity and mortality. Preeclamptic patients are often hypovolemic and oedematous with increased systemic vascular resistances (SVR), low cardiac output (CO) and variable degree of left ventricular hypertrophy and dysfunction. In these patients the haemodynamic variations induced by loco-regional anesthesia (LRA) during caesarean section (CS), despite common, may be unpredictable and risky.

**OBJECTIVES.** This observational study aimed to minutely evaluate the haemodynamic effects of LRA in preeclamptic and hypertensive patients, compared to healthy controls.

**METHODS.** Pregnant(18-40 y) and informed women, single fetus, gestational age> 31weeks, ASA II or ASA III, elective or urgent CS under LRA. Used monitoring system: Clear-sight finger cuff (Edwards Lifescience). Recorded hemodynamic parameters were: CO, cardiac index (CI), systolic, diastolic and mean arterial pressure (SAP,DAP,-MAP) and heart rate (HR), stroke volume (SV), stroke volume index (SVI), stroke volume variation (SVV). Precise chosen waypoints were: baseline,left lateral decubitus, LRA, skin incision, fetal extraction, afterbirth.

**RESULTS.** 10 preeclamptic patients (PE, 5 early and 5 late preeclampsia), 5 hypertensive patients (HT) and 22 controls (CN). No statistically significant differences for preoperative data (age, weight, height, comorbidities, chronic therapy) were found, except hypertension and hypertensive drugs. Hemodynamic parameters varied during surgery within all groups, being pathological from the beginning in PE and HT patients; pressure followed physiological and LRA-induced changes, PE and HT pts maintained higher PAM values than controls; CI downward variation was higher in preeclamptic patients than in hypertensive ones, with a 25% increase in CN and HT patients after LRA nadir; non compensatory HR increase was witnessed in PE patients; SVI variation pattern was group specific after LRA maximal effect. Fig.1-2.

**CONCLUSION.** Pathological pregnancy is characterized by a heterogenous response to LRA. Preeclampsia may not be treated as hypertension.

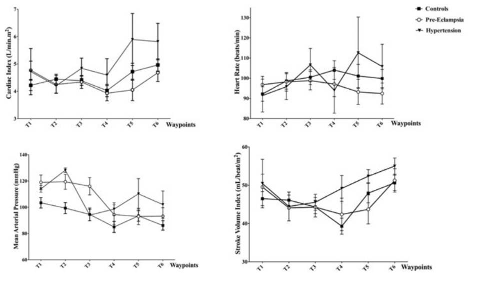

**Fig. 1 (abstract 001558).** See text for description

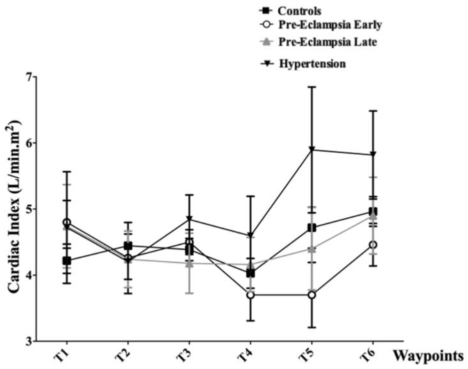

**Fig. 1 (abstract 001558).** See text for description

### 001566

#### Developement of pediatric delirium after anesthesia in maxillofacial surgery

R. Varutti[1], A. Mosca[1], C. Meldini[1], T. Iob[1], AM. Giannini[2], N. Latronico[1]
[1]Intensive care unit and anesthesia 2, Spedali civili di brescia, Brescia, Italy; [2]Intensive care unit, Ospedale dei Bambini, Brescia, Italy
**Correspondence:** R. Varutti
*Intensive Care Medicine Experimental* 2019, **7(Suppl 3):**001566

**INTRODUCTION.** Emergence delirium is defined as a cognitive disturbance during emergence from general anesthesia resulting in hallucinations, delusions and confusion manifested by agitation, restlessness, involuntary physical movement and extreme flailing in bed. Postoperative emergence delirium develops in 12% to 18% of al children undergoing general anesthesia for surgery. This postanesthetic phenomenon changes cognitive and psychomotor behavior, and puts pediatric patients and health care personnel at risk of injury.

**OBJECTIVES.** To describe a single-institution pilot study regarding prevalence and risk factors for delirium in children after maxillofacial surgery.

**METHODS.** A ptospective observational pilot study was made, from Jenuary to March 2019 in the pediatric operating room (maxillofacial surgery) at an urban academic medical center. Thirty consecutive patients, aged 28 to 185 months were enrolled. When the patients were awake (Richmond Agitation and Sedation Score > -3), two trained investigators conducted the Paediatric Assessment of Emergent Delirium (PAED) and the pediatric Confusion Assessment Method for the Intensive Care Unit (pCAM-ICU) three times: basal (at the preliminary anesthetic visit, before surgery), 30 minutes after the end of general anesthesia and 6 h later.

**RESULTS.** All children have been accopanied to operating room by their caregivers. Only 6% recived an oral premedication before surgery. 86% received opioids during anesthesia. Pediatric delirium was present on 2% patient at awake time, 12 % after 30 min and 3% 6 h late. Both pediatric delirium scales was similar in recognizing the presence of delirium. All of this children with delirium received opioids during induction and/or general anestehesia. Only 1 of this patients had a IV cannula at the operating room admission. No one of them received a premedication before surgery. The children who developed delirium were the youngest.

**CONCLUSION.** In our institution, pediatric delirium is a prevalent problem, with identifiable risk factors (opioids during anesthesia, no premedication before surgery, smal age). Some have a short-lasting course, which underlines the need for early screening. The pCAM-ICU scale, usually used in the intensive care unit to recognize pediatric delirium, could be used in other departments than in intensive care setting, as demonstrated. Our findings support the view of delirium as a continuum of acute neurocognitive disorder. Further research is needed to investigate prophylactic and treatment approaches for postoperative pediatric delirium.

**REFERENCE(S)**
1. Patterns of postoperative delirium in children. Meyburg et al. Pediatric critical care Medicine 2017; 18 (2):128-134
2. Emergence delirium in children: an update. Dahmani et al. Pediatric anesthesia 2014; 27 (3):310-315
3. None

### 001577

#### Analgesia, sedation and delirium in ICU. WHAT DO WE PERCEIVE?

J. Priego Sanz[1], A. Fernandez Ferreira[2], JL. Martinez Melgar[3], I. Perez Martin[4], D. Combarros Mendez[1], S. Gonzalez Prado[2]
[1]Intensive care unit, Complexo Hospitalario Universitario Ourense, Ourense, Spain; [2]Intensive care unit, Hospital Alvaro Cunqueiro, Vigo, Spain; [3]Intensive care unit, Complexo Hospitalario De Pontevedra, Pontevedra, Spain; [4]Intensive care unit, Clinica Mompia Cantabria, Santander, Spain
**Correspondence:** J. Priego Sanz
*Intensive Care Medicine Experimental* 2019, **7(Suppl 3):**001577

**INTRODUCTION.** Guidelines published suggested appropriate management of pain, agitation and delirium (PAD) is crucial in improving patient outcomes. However, the practice of PAD assessment and management in community hospitals is unclear and the mechanisms contributing to the potential care gap are unknown. Because of this, we want to know their perception in our ICUs

**OBJECTIVES.** To describe the perception of the management of PAD in ICU by its professionals in 4 ICUs in Northern Spain.

**METHODS.** Multicenter descriptive study on the management of PAD in 4 ICUs of 4 Medical centers: Complexo Hospitalario Universitario de Ourense (CHUO), Hospital Álvaro Cunqueiro de Vigo (HAC), Hospital Montecelo de Pontevedra (CHOP) and Clínica Mompía de Cantabria (CMC).

An anonymous survey was conducted from December 2018 to March 2019 to the ICUs staff (physicians and nurses). We ask about demographic variables and different characteristics of PAD assessment and management in the 4 ICUs.

**RESULTS.** They answered 152 surveys (CHUO 41.4%, HAC 36.8%, CHOP 15.1% and CMC 6.6%). The 74.3% were nurses with age between 31-45 years (56.6%). 45.4% were permanent staff and 36.8% had more than 10 years of experience. Only 1 center had a PAD protocol.

The pain scales used were: VAS and NRS in communicative patients (95.8%) and ESCID in non-communicative (61.8%). The most used analgesic drugs were acetaminophen (64.5%) and fentanyl (32.9%). Preemptive analgesia before nursing procedures was ocassional. The 46.7% of the staff thought that patients had pain. The 50.7% believed that the response to treatment was satisfactory.

97.4% used RASS as a sedation scale. Light sedation strategy was the most used (59.9%). The preferred sedatives were midazolam (83.6%) and propofol (72.4%). The combination of midazolam and fentanyl was the most popular (88.2%). The sedation degree was uneven: 33.3% poor, 36.2% correct and 27% over sedated. Physicians subgroup consider that patients were overstated (71.8%).

Delirium evaluation was uncertain: occasional (52.6%). Among those who used a diagnostic tool, CAM-ICU was preferred (74.3%). Our results indicated that delirium was underdiagnosed (66%) and oversedation contributed to its appearance (75.7%).

Non-pharmacological measures were used in 69.1%. The preferred by our surveyed were: light control (32.2%) and family support (47.3%). Only 1 of the ICUs had an open visits schedule more than 6 hours. Haloperidol is the most commonly drug used in treatment (78.9%)

**CONCLUSION.**

- The global use of scales is high.
- Despite of the Guidelines recommendations, benzodiazepines (midazolam) use is elevated.
- Exist a different perception in sedation degree between nurses and physicians.
- It is necessary to implement protocols to optimize and standardize the handling of PAD.

**REFERENCE(S)**
1. Maximous R, Miller F, Tan C, et al. Pain, agitation and delirium assessment and management in a community medical-surgical ICU: results from a prospective observational study and nurse survey. BMJ Open Quality 2018;7:e000413. doi:10.1136/ bmjoq-2018-000413
2. D.H. Cerasoa, C. Duenas-Castelb, N. Raimondic, E. Celisd, R. Carrilloe, S. Ugarte Ubiergof y F. Rodriguezg. Encuesta iberoamericana sobre delirium en pacientes críticos. Med Intensiva. 2010;34(8):495–505. doi:10.1016/ j.medin.2010.04.003
3. García-Sánchez M, et al. Prácticas de analgosedación y delirium en Unidades de Cuidados Intensivos espa˜nolas: Encuesta 2013-2014. Med Intensiva. 2018. doi.org/10.1016/j.medin.2018.12.003
4. We have not any grant acknowledgemnt to make this activity

### 001601

#### Delirium: impact in cognition, executive functions, and quality of life in a Colombian intensive care unit (ICU)

M. Sanchez[1], C. Restrepo[1], A. Garcia[2], J. Gutierrez[3]
[1]Critical care medicine, Universidad Tecnologica de Pereira, Pereira, Colombia; [2]Department of cellular and applied physiology, Universidad Tecnologica de Pereira, Pereira, Colombia; [3]Department of psychiatry, Universidad Tecnologica de Pereira, Pereira, Colombia
**Correspondence:** M. Sanchez
*Intensive Care Medicine Experimental* 2019, **7(Suppl 3):**001601

**INTRODUCTION.** Exist a large number of scientific publications that describe the importance of delirium in terms of outcomes (mortality, days of hospital stay and sequelae), but the association with the alterations in the quality of life and executive function is not well understood1,2.

**OBJECTIVES.** Evaluating the impact of delirium in the decline of cognitive and executive functions, quality of life and survival of the critically ill.

**METHODS.** A nested case-control study in a cohort of six months. The prevalence of delirium, mortality, cognitive and executive disorders, and the quality of life in survivors in a period greater than three months after discharge from the ICU were evaluated. The CAM - ICU for delirium screening was performed daily for all patients to find the cases, defined as a positive score, and a homogeneous sample with a negative score was considered as a control group3. For the follow-up, the scales of quality of life in health (SF-36), instrumental activities of daily life (PGC - IADL) and minimental test (MMSE) were carried out.

**RESULTS.** 457 patients were hospitalized between April and September of 2018. 81 patients were evaluated after three months of discharge from the ICU, with a mean of 61.3 ± 18 years. Of this group, 43 subjects had delirium and 38 did not. We found differences with statistical significance in the cognitive functions ($p < 0.01$), with greater affectation of the domains of the orientation, the calculation and the language / praxis ($p < 0.05$) and deterioration in the quality of life (QoL) in the subscales of social function ($p < 0.006$), emotional role ($p < 0.04$) and mental health ($p < 0.04$) in cases. Although the executive functions were not affected by the presence of delirium in the hospitalization, it was diminished in both groups.

**CONCLUSION.** Cognitive capacity, social function, emotional role, and mental health were affected with statistical significance in the group of patients who had delirium during the stay in ICU for any reason when compared with a control group not exposed to this factor, but with similar socio-demographic characteristics.

**REFERENCE(S)**

1. Cavallazzi R, Saad M, Marik PE. Delirium in the ICU: an overview. Ann Intensive Care 2012;2:49.
2. Pandharipande PP, Girard TD, Jackson JC, et al. Long-Term Cognitive Impairment after Critical Illness. N Engl J Med 2013;369:1306-16
3. Ely EW, Inouye SK, Bernard GR, et al. Delirium in Mechanically Ventilated Patients. Validity and Reliability of the Confusion Assessment Method for the Intensive Care Unit (CAM-ICU). JAMA. 2001;286:2703-2710

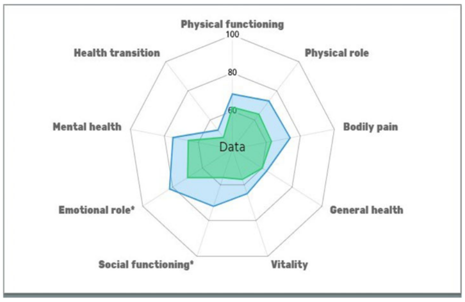

**Fig. 1 (abstract 001601).** Radar plot for SF-36 domain scores by presence/absence of delirium in critically ill patients

### 001634

#### Correlation between thromboelastometry and laboratory clotting parameters in surgical patients

A. Diaz-Martin, M. Casado-Mendez, V. Arellano-Orden, D. Cuenca-Apolo, A. Pastor, R. Dusseck, S. Leal-Noval

[1]I.C.U., H.U. Virgen del Rocio, Seville, Spain

**Correspondence:** A. Diaz-Martin

*Intensive Care Medicine Experimental* 2019, **7(Suppl 3):**001634

**INTRODUCTION.** In patients undergoing surgical procedures, the type of surgical procedure and the perioperative sampling time may influence the correlation between the standard laboratory tests (SLT) and their equivalent ROTEM parameters.

**OBJECTIVES.** Prospective study (ABCD study, NCT:026552897) funded by Carlos III Institute (PI15/00512), aimed at investigating the correlation between prothrombin time (PT) *vs.* clotting time of ex-TEM (CT-exTEM), and between plasmatic Clauss fibrinogen level (pFBN) *vs.* maximum clotting firmness (MCF-fibTEM), in patients undergoing procedures with high (glioblastoma multiforme resection: GBR) or moderate (colon cancer resection, by laparoscopy: CCR) degree of surgical injury, at different perioperative time-points.

**METHODS.** ExTEM clotting-time (CT-exTEM) and FibTEM maximum clot firmness (MCF-fibTEM), as measured by ROTEM, were correlated with prothrombin time (PT) and plasma fibrinogen (pFBN, Clauss method), as measured at SLTs, in GBR (N=60) and CCR (N=40) patients. Blood samples for SLTs and ROTEM assessments were drawn simultaneously within 24-hours before surgery (baseline) and 2, 24 and 48-hours after surgery. Correlations between ROTEM parameters and SLTs values assessing similar coagulation profile were evaluated by Spearman r rank-order. Receiver-Operator Characteristic (ROC) curves were performed in order to screening the efficiency of best CT-exTEM and MCF-fibTEM cut-offs for detecting prolonged PT (>15 s) and low pFBN values (<2 g/L), respectively.

**RESULTS.** The correlations between PT and CT-exTEM and between pFBN and MCF-fibTEM were weak ($r < 0.40$) or moderate ($r \approx 0.40 – 0.65$), respectively, and remained unchanged over the studied perioperative period in both groups. Recommended cut-offs for CT-exTEM (>75 s) and MCF-fibTEM (<10 mm) had poor sensitivity (% [95% confidence interval]) for identifying a prolonged prothrombin time (17 % [8, 31]) or a low pFBN (46 % [32, 62]).

**CONCLUSION.** As SLTs and their ROTEM equivalents parameters are poorly related, they should not be used interchangeably in patients undergoing GBR or CCR. Neither the type of surgery nor the perioperative timing significantly influenced these correlations.

1. This study has been partially supported by Fondo de Investigación Sanitaria (FIS) PI 15/00512, Carlos III Institute, Ministry of Health, Government of Spain, and European Regional Development Fund (ERDF)

### 001644

#### The factors influencing ventilator weaning time in sequential sedation patients

J. Yang[1], Y. Zhou[1], Y. Kang[1], J. Liu[2]

[1]Department of critical care medicine, West China hospital of Sichuan University, Chengdu, China; [2]Department of anesthesiology, West China hospital of Sichuan University, Chengdu, China

**Correspondence:** J. Yang

*Intensive Care Medicine Experimental* 2019, **7(Suppl 3):**001644

**INTRODUCTION.** Critically ill, mechanically ventilated patients in intensive care units generally require sedation treatment to relieve anxiety and agitation. But long-term mono-sedative use is associated with some adverse effects. Different sedatives sequentially used after meeting the sequential criteria may reduce these adverse effects, leading to patient faster recovery, earlier extubation and better outcome.

**OBJECTIVES.** This study aimed to evaluate the factors influencing ventilator weaning time in sequential sedation patients.

**METHODS.** A total of 216 ventilated patients who received sequential sedation and completed sequential sedation protocol were enrolled to this analysis. The primary endpoint in this study was patient weaning time defined as duration of mechanical ventilation after patients met the sequential criteria. Sequential criteria indicated patients passed spontaneous breathing trial (SBT) safety screen, but failed the 30-minute SBT. Univariate and multivariate Cox proportional hazards regressions were used to predict factors influencing weaning time.

**RESULTS.** In this analysis, older age (RR=0.984, 95%CL 0.974-0.993, p=0.001), longer time from meeting the sequential criteria to stopping sedation (RR=0.968, 95%CL 0.959-0.977, p<0.001), more accumulated dose of fentanyl after patients met the sequential criteria for extubation (RR=0.891, 95%CL 0.801-0.993, p=0.036), and faster rapid shallow breathing index (RSBI) when patients undergoing SBT trial (RR=0.992, 95%CL 0.985-0.998, p=0.008) were independent risk factors for prolonging patient weaning time. Non-smokers (RR=2.456, 95%CL 1.489-4.053, p<0.001), lighter average sedation depth (RR=1.221, 95%CL 1.053-1.416, p=0.008), sequential sedation with propofol (RR=2.612, 95%CL 1.796-3.800, p<0.001), and sequential sedation with dexmedetomidine (RR=2.543, 95CL% 1.727-3.744, p<0.001) after patients met the sequential criteria were associated with earlier ventilator weaning.

**CONCLUSION.** In sequential sedation patients, older age, longer time from meeting the sequential criteria to stopping sedation, more accumulated dose of fentanyl after patients met the sequential criteria for extubation and faster RSBI were independent risk factors for delaying ventilator weaning. However, non-smokers comparing with current smokers, lighter average sedation depth, sequential sedation with propofol or dexmedetomidine after patients met the sequential criteria could shorten patient weaning time.

### 001647

#### Intraoperative Lung Protective Ventilation in Peritonitis Patients Undergoing Emergency Laparotomy: A Randomized Controlled Trial

DK. Baidya[1], S. Maitra[2], S. Rajeshwari[3], MK. Arora[4], VK. Bansal[5], A. Roychowdhury[6], R. Subramanian[7]

[1]All India Institute Of Medical Sciences, New Delhi, New Delhi, India; [2]Anaesthesia,critical care and pain medicine, All India Institute Of Medical Sciences, New Delhi, India; [3]Anaesthesiology, pain medicine & critical care, All India Institute Of Medical Sciences, New Delhi, India; [4]Anaesthesiology & critical care, Institute of Liver and Biliary Sciences, New Delhi, India; [5]Surgical disciplines, all india institute of medical sciences, new delhi, Surgical Disciplines, New Delhi, India; [6]All india institute of medical sciences, Anaesthesiology Pain Medicine and Critical Care, New Delhi, India; [7]Liver transplant anaesthesiology, Max Super Speciality Hospital, Saket (Max Saket), New Delhi, India

**Correspondence:** D.K. Baidya

*Intensive Care Medicine Experimental* 2019, **7(Suppl 3):**001647

**INTRODUCTION.** Lung protective ventilation improves survival in ARDS1. It's role in elective major abdominal surgery is controversial2. Peritonitis patients undergo laparotomy and often develop ARDS.

**OBJECTIVES.** To identify whether use of lung protective ventilation during laparotomy in abdominal sepsis patients affect mortality and morbidity.

**METHODS.** After Institutional ethics committee clearance and informed written consent, 100 adult patients undergoing emergency laparotomy for peritonitis were randomized in to two groups. In Group1: Vt 6-8ml/kg, PEEP 6-8cmH2O and recruitment maneuver every 30min and in Group 2 Vt 10-12ml/kg, no PEEP and no recruitment were used. All the patients were followed up till death/discharge.

**RESULTS.** All the patients received allocated treatment. Follow up was lost for six patients and data from 94 patients (45 patients in Group1 & 49 patients in Group2) were analyzed.

Demographic & laboratory parameters were comparable in two groups. In-hospital mortality was 26.7% in Group1 versus 26.5% in Group2 (risk difference 0.14%, 95% CI -17.7- 18.0; p=0.98, Chi- square test). Kaplan Mayer survival analysis confirmed similar in-hospital mortality in two groups (p=0.79, log-rank test). Duration of hospital stay [median (IQR) 13(9-18) days in Group1 versus 13(8-21) days in Group2; p=0.82] & duration of ICU stay [median (IQR) 7(4-10) days versus 6(3-12) days; p=0.88] were also similar. Postoperative pulmonary complications (POPC) during hospital stay were also similar [median (95% CI) of POPC grade in Group1 was 2(1-4) versus 3(1-4) in Group2; p=0.45].

**CONCLUSION.** Intraoperative lung protective ventilation does not provide mortality benefit in peritonitis patients undergoing emergency laparotomy.

**REFERENCE(S)**

1. The Acute Respiratory Distress Syndrome Network. Ventilation with lower tidal volumes as compared with traditional tidal volumes for acute lung injury and the acute respiratory distress syndrome. N Engl J Med 2000;342:1301-8.
2. Futier E, Constantin JM, Paugam-Burtz C, Pascal J, Eurin M, Neuschwander A et al; IMPROVE Study Group. A trial of intraoperative low-tidal-volume ventilation in abdominal surgery. N Engl J Med 2013;369:428-37.

3. The study was funded by All India Institute of Medical Sciences, New Delhi

### 001648

#### Relationship between thromboelastography and conventional clotting tests values with the severe bleeding in critically ill patients with coagulopathy

M. Casado-Mendez, A. Diaz-Martin, V. Arellano-Orden, A. Pastor, R. Dusseck, D. Cuenca-Apolo, S. Leal-Noval

I.c.u., H.U. Virgen del Rocio, Seville, Spain

**Correspondence:** A. Diaz-Martin

*Intensive Care Medicine Experimental* 2019, **7(Suppl 3):**001648

**INTRODUCTION.** Certain groups of critically ill patients are archetypally at high risk of coagulopathy and severe bleeding. Detection and correction of coagulopathy frequently relies on standard laboratory tests (SLTs). The viscoelastic tests (VET) offers an alternative approach to assess coagulation alterations, offering faster turn-around-time and performing a more global assessment of coagulation.

**OBJECTIVES.** This study aimed to ascertain the relationship between thromboelastography (TEG®) and standard laboratory tests (SLTs) values with the presence of bleeding in critically ill patients with known coagulopathy.

**METHODS.** Consecutive coagulopathic patients with hepatic failure, postoperative period of prolonged cardiac surgery or complex abdominal surgery with sepsis, were prospectively included in this study. On intensive care unit (ICU) admission, patients were stratified into two groups according to whether yes or not they had major bleeding (MB) [whether evident overt bleeding, important bleeding apparent on image studies and/or need of moderate-massive blood transfusion and hemodynamic instability]. Blood samples were drawn for SLTs [international normalized ratio (INR), activated partial thromboplastin time (aPTT), platelets count and fibrinogen level (Clauss)] and TEG (R, reaction time; MA, maximal amplitude and Ly30, clot lysis at 30 min.). Receiver-Operator Characteristic (ROC) curves analysis were performed in order to screening the efficiency of TEG and SLTs in detecting bleeding. Correlation between SLTs and TEG parameters assessing similar coagulation profile was evaluated by Spearman rank-order.

**RESULTS.** Eighty-tree patients were included and bleeding was confirmed in 45 (54%). Fibrinogen level demonstrated the best accuracy, even low, for detecting bleeding with an area under curve and 95% confidence intervals (AUC (95% CI)): 0.74 (0.63-0.85) for a best cut-off ≤ 2 g/L. Regarding to TEG-MA, values for an optimal cut-off ≤ 51 mm were AUC: 0.68 (0.56-0.80).

**CONCLUSION.** Both conventional clotting tests and TEG values were poorly associated with bleeding in this critically ill cohort patients with coagulopathy.

**Table 1 (abstract 001648).** Accuracy of thromboelastography and laboratory tests in predicting bleeding from variables assessing initiation of coagulation or final clot firmness

| Variables | AUC (95%) | Optimal cut-off | Sensitivity | Specificity | LR+ | LR- |
|---|---|---|---|---|---|---|
| INR | 0.55 (0.44-0.66); p=0.4161 | > 1.2 | 64 (49-78) | 29 (15-46) | 0.91 | 1.23 |
| aPTT (s) | 0.54 (0.43-0.65); p=0.4940 | > 36 | 35 (22-51) | 66 (48-80) | 1.04 | 0.98 |
| TEG-R (min) | 0.62 (0.50-0.72); p=0.0585 | > 10 | 35 (22-51) | 84 (68-94) | 2.25 | 0.77 |
| Fibrinogen level (g/l) | 0.74 (0.63-0.83); p<0.0001 | ≤ 2 | 47 (32-62) | 82 (66-92) | 2.53 | 0.65 |
| Platelet count (109g/l) | 0.62 (0.50-0.72); p=0.0630 | ≤ 100 | 47 (32-62) | 74 (72-95) | 1.77 | 0.72 |
| TEG-angle (degree) | 0.65 (0.54-0.75); p=0.0126 | ≤ 52 | 35 (21-51) | 86 (71-95) | 2.70 | 0.74 |
| TEG-MA (mm) | 0.68 (0.57-0.78); p=0.0028 | ≤ 51 | 33 (20-49) | 87 (72-95) | 2.53 | 0.77 |

### 001658

#### Does CT-ProET-1 predict myocardial injury in patients undergoing major abdominal surgery?

H. Hansson[1], MS. Chew[2], W. Gääw Rolander[1], H. Didriksson[1], L. Sundin[1], HA. Andersson[1]

[1]Anesthesiology and intensive care, Linkoping University Hospital, Linköping, Sweden; [2]Department of anaesthesia and intensive care, medical and health sciences, Linköping University Hospital, Linköping, Sweden

**Correspondence:** H. Hansson

*Intensive Care Medicine Experimental* 2019, **7(Suppl 3):**001658

**INTRODUCTION.** Cardiovascular complications due to ischemic injury of the myocardium after major non-cardiac surgery are frequent and cause a considerable amount of morbidity and mortality in patients undergoing noncardiac surgery. Major Cardiovascular and Cerebrovascular Events (MACCE), are the most common causes of serious postoperative complications. Current risk stratification systems have limited sensitivity and specificity in identifying patients at risk of MACCE and postoperative symptoms and clinical signs of myocardial injury are often diffuse or lacking. Endothelin-1, a potent vasoconstrictor considered to have an important pathophysiological role in cardiovascular dysfunction, is proposed as a promising biomarker in predicting adverse cardiovascular events.

**OBJECTIVES.** The purpose of this study is to identify if ECG-changes and levels of C-terminal pro-endothelin-1, CT-proET1 can predict MACCE in patients undergoing major abdominal surgery.

**METHODS.** We conducted a multicenter prospective study including 387 surgical patients. This was a preplanned substudy of the MINSS study (NCT03436238). Perioperative blood samples and ECGs were obtained at five sample points: pre-surgery, immediately post-surgery, and on Days 1,2,3 after surgery. We analysed plasma levels of CT-proET-1 and hsTnT levels, as well as ECG-changes and tested for their association with MACCE within one month of surgery.

**RESULTS.** Perioperative plasma levels of CT-proET-1 were significantly higher in patients affected by MACCE and the prognostic value exceeded that of cTnT, the number one cardiac biomarker used today, in all analyses. The number of ECGs interpreted at the end of this study was not sufficient to run statistical analyses, however, the distribution of ECG-changes did not seem to be a predictor of MACCE.

**CONCLUSION.** Our analysis suggests that increased perioperative CT-proET-1 levels significantly identify patients at risk of MACCE in the ensuing month following major abdominal surgery and the prognostic value exceeded that of cTnT.

**REFERENCE(S)**

1. Setianto, B. Y., Hartopo, A. B., Sukmasari, I., & Puspitawati, I. (2016). On-admission high endothelin-1 level independently predicts in-hospital adverse cardiac events following ST-elevation acute myocardial infarction. International Journal of Cardiology, 220, 72–76.
2. Bhandari, S., Davies, J. E., Struck, J., & Ng, L. L. (2014) Plasma C-terminal proEndothelin-1 (CTproET-1) is affected by age, renal function, left atrial size and diastolic blood pressure in healthy subjects. Peptides, 52, 53-57.
3. Zhang, C.-L., Qiao, X., An, Y.-M., Zhang, Y., Li, L., Wu, L.-L., … Zhang, F.-C. (2017). Plasma endothelin-1-related peptides as the prognostic biomarkers for heart failure A PRISMA-compliant meta-analysis. Medicine, 96(50).
4. Haaf, P., Zellweger, C., Reichlin, T., Zbinden, A., Wildi, K., Mosimann, T., … Mueller, C. (2014). Clinical Research: Utility of C-terminal Proendothelin in the Early Diagnosis and Risk Stratification of Patients With Suspected Acute Myocardial Infarction. Canadian Journal of Cardiology, 30(2), 195-203.
5. Noordzij, P. G., van Geffen, O., Dijkstra, I. M., Boerma, D., Meinders, A. J., Rettig, T. C. D., … van Dongen, E. P. A. (2015). High-sensitive cardiac troponin T measurements in prediction of non-cardiac complications after major abdominal surgery. The British Journal of Anaesthesia, 114(6), 909–918.
6. Protopapa, K. L., Simpson, J. C., Smith, N. C. E., & Moonsinghe, S. R. (2014). Development and validation of the Surgical Outcome Risk Tool (SORT). British Journal of Surgery, 101(13), 1774–1783.
7. Sabate, S., Mases, A., Guilera, N., Canet, J., Castillo, J., Orrego, C. … Sabate, M. (2011). Incidence and predictors of major perioperative adverse cardiac and cerebrovascular events in non-cardiac surgery. British Journal of Anaesthesia, 107(6), 879-890
8. Devereaux, P. J. & Sessler, D. I. (2015). Cardiac Complications in Patients Undergoing Major Noncardiac Surgery. New England Journal of Medicine, 373(23), 2258-2269
9. Sessler, D. I. & Khanna, A. K. (2018). Perioperative myocardial injury and the contribution of hypotension. Intensive Care Medicine, 44(6), pp. 811–822.
10. Botto, F., Alonso-Coello, P., Chan, M. T. V., Carlos Villar, J., Xavier, D., Srinathan, S., … Wildes, T. (2014). Myocardial Injury after Noncardiac Surgery: A Large, International, Prospective Cohort Study Establishing Diagnostic Criteria, Characteristics, Predictors, and 30-day Outcomes. Anesthesiology, 120(3), 564–578.
11. Swedish Research Council
12. Medical Research Council of Southeast Sweden
13. Region Östergotland County Council

### 001671

#### The relationship between near infrared spectroscopy (NIRS) and carotid blood flow as an indicator of cerebral blood flow

U. Borg
[1]Medical affairs, Medtronic Surgical Solutions, Boulder, USA
**Correspondence:** U. Borg
*Intensive Care Medicine Experimental* 2019, **7(Suppl 3):**001671

**INTRODUCTION.** NIRS is frequently used in cardiac surgery to assess adequacy of cerebral oxygenation (rSO2). Current NIRS devices are categorized as oxygen saturation monitors although there are possibly additional benefits to NIRS monitoring. This study was performed to investigate the ability of NIRS monitoring to detect changes in cerebral blood flow during hypovolemia and resuscitation.

**OBJECTIVES.** To investigate the ability of the INVOS 5100C system (Medtronic, Boulder USA) to detect changes in cerebral blood flow during hypovolemic shock and resuscitation.

**METHODS.** In an animal study approved by the local animal use committee we anesthetized, intubated and ventilated 19 pigs. Hypovolemic shock was accomplished by removing 50% of the animal's blood volume over a 20-minute period in 3 steps. Resuscitation by blood returned to the animal over a 20-minute period in 3 steps. 30-minutes allowed between end of blood removal and start of resuscitation. Blood pressure, heart rate, arterial oxygen saturation, internal carotid blood flow (CBF), rSO2 and end-tidal carbon dioxide was measured continuously. Normocapnia was maintained in all animals.

**RESULTS.** CBF and rSO2 correlated during bleeding (r2 0.88). Bleeding resulted in a final CBF of 66 ml/min a decrease of 58% while rSO2 fell by 31% (Fig 1).

During resuscitation CBF and rSO2 correlated during the first infusion of 10% of the shed volume increasing the CBF from 115 ml/min to 176 ml/min and rSO2 from 46% to 55%. The second and third infusion increased CBF to 207 ml/min without a major change in rSO2 (59%) (Fig 2).

**CONCLUSION.** During blood loss rSO2 may be an indicator of cerebral blood flow since fall in rSO2 and decrease in CBF correlated. Allowing 30 minutes for stabilization after final blood removal resulted in recovery of CBF due to peripheral vasoconstriction. Increased CBF did not result in increased rSO2 probably because of increased cerebral oxygen extraction. Infusion of the first 10% of shed volume led to rSO2 correlation with increase in CBF. Further infusionsion did not result in increased rSO2 despite increase in CBF. During resuscitation there may be very limited correlation between rSO2 and cerebral blood flow due to compensatory oxygen extraction and vasoactive behavior because of prior ischemia during shock.

1. Ulf Borg and Julia Katilius are full time employees at MEDTRONIC. The study was payed for by MEDTRONIC.

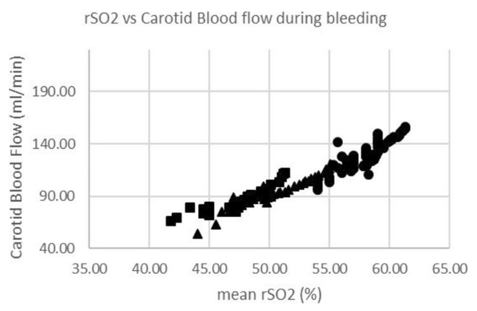

**Fig. 1 (abstract 001671).** See text for description

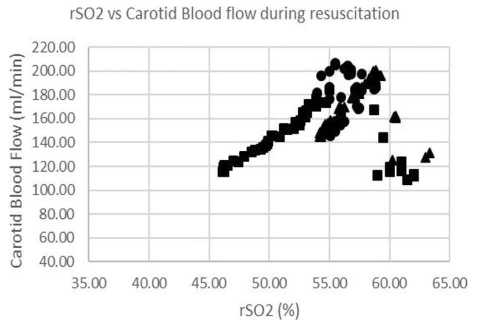

**Fig. 1 (abstract 001671).** See text for description

### 001721

#### Therapeutic plasma exchange in critical care: a 5-year review

M. Toscano[1], P. Valério[2], LF. Frade[3], FC. C.[4], I. Botelho[3], A. Ramos[3]
[1]Internal medicine, Hospital de Cascais, Alcabideche, Portugal; [2]Nephrology, Centro Hospitalar De Setúbal E.P.E., Setúbal, Portugal; [3]Intensive Care Unit, Hospital de Cascais, Alcabideche, Portugal; [4]Oncology, IPO, Lisboa, Portugal
**Correspondence:** M. Toscano,
*Intensive Care Medicine Experimental* 2019, **7(Suppl 3):**001721

**INTRODUCTION.** Therapeutic plasma exchange (TPE) is an extracorporeal treatment used in the removal of high molecular weight particles causing acute toxicity, whose applications have been undergoing constant development.

**OBJECTIVES.** Retrospective analysis of patients undergoing TPE and respective outcomes

**METHODS.** Review of the clinical files of patients who underwent TPE since 2014

**RESULTS.** Between January 2014 and April 2019, 15 patients underwent TPE. The most frequent indication was hypertriglyceridemic pancreatitis, in 7 cases. All were young male patients, most commonly with favorable response after a single session. We highlight the occurrence of 2 deaths due to necrohemorrhagic pancreatitis and multiorgan dysfunction. There were 3 cases of thrombotic microangiopathy. In 2 of these (HELLP syndrome in a 41-year-old puerperal patient and atypical hemolytic-uremic syndrome in a 31-year-old patient), there was resolution of hemolysis and complete recovery of renal function. In a case of thrombotic thrombocytopenic purpura in a 66-year-old patient, refractory shock and death was observed. There was only 1 case of dialysis-dependent ANCA+/PR3 vasculitis with diffuse alveolar hemorrhage in a 79-year-old patient, with unfavourable outcome. In 2 cases of myasthenic crisis submitted to short cycles of TPE, along with immunosuppressive therapy, significant symptomatic improvement and reduction of fatigability were observed. Finally, in 2 patients diagnosed with autoimmune encephalitis, who underwent prolonged cycles of TPE, response varied, with ad integrum recovery in a 19-year-old patient with anti-NMDAr encephalitis, and persistence of neurological deficits and development of hydrocephalus in a 51-year-old patient. Complications included pneumothorax associated with placement of central venous catheter, transient hemodynamic instability and electrolyte imbalance.

**CONCLUSION.** TPE has a proven benefit, and is sometimes a life-saving therapy. This is the case of thrombotic microangiopathy and myasthenic crisis, where we also observed the most favorable results. On the other hand, its role is not established in hypertriglyceridemic pancreatitis as well as in autoimmune encephalitis (except for anti-NMDAr), where it remains a reasonable alternative, to be considered in cases of immunosuppressive therapy failure. Complications are rare and generally related to central venous access catheterization, need for anticoagulation or associated with the replacement fluid used.

In sum, TPE is a useful treatment in a wide range of diseases observed in the critical patient.

**Table 1 (abstract 001721).** See text for description

| Gender | Age | Indication | ASFA category | Nr. of sessions | Replacement fluid | Exchange volume | Weight (kg) | Hematocrit (%) | Complications | Death |
|---|---|---|---|---|---|---|---|---|---|---|
| ♂ | 34 | hypertriglyceridemic pancreatitis | III | 1 | 5% albumin + saline | 1 | 120 | 42 | - | N |
| ♂ | 47 | hypertriglyceridemic pancreatitis | III | 1 | 5% albumin + saline | 1 | 84 | 37 | hypokalemia | Y |
| ♂ | 42 | hypertriglyceridemic pancreatitis | III | 1 | 5% albumin + saline | 1 | 67 | 33 | - | Y |
| ♂ | 41 | hypertriglyceridemic pancreatitis | III | 1 | 5% albumin + saline | 1 | 130 | 44 | - | N |
| ♂ | 42 | hypertriglyceridemic pancreatitis | III | 2 | 5% albumin + saline | 1 | 75 | 43 | hypokalemia | N |
| ♂ | 36 | hypertriglyceridemic pancreatitis | III | 2 | 5% albumin + saline | 1 | 80 | 35 | - | N |
| ♂ | 50 | hypertriglyceridemic pancreatitis | III | 2 | 5% albumin + saline | 1 | 95 | 47 | - | N |
| ♀ | 41 | post-partum HELLP syndrome | III | 6 | fresh frozen plasma | 1.5 | 60 | 20 | hypokalemia | N |
| ♂ | 31 | atypical HUS | I | 5+5* | fresh frozen plasma | 1.5 | 70 | 29 | bradycardia | N |
| ♂ | 66 | acquired TTP | I | 3 | fresh frozen plasma | 1.5 | 95 | 25 | hypocalcemia | N |
| ♀ | 81 | myasthenic crisis | I | 7 | 5% albumin + saline | 1 | 45 | 32 | - | N |
| ♂ | 73 | myasthenic crisis | I | 4 | 5% albumin + saline | 1 | 72 | 36 | - | N |
| ♀ | 79 | ANCA+ vasculitis | I | 5 | 5% albumin | 1 | 55 | 27 | pneumothorax | Y |
| ♀ | 19 | anti-NMDAr encephalitis | I | 11 | 5% albumin + saline | 1 | 56 | 35 | - | N |
| ♀ | 51 | autoimmune encephalitis | I | 10 | 5% albumin + saline | 1 | 50 | 24 | hypokalemia | Y |

* 5 consecutive sessions followed by 5 sessions in alternate days; HUS – hemolytic-uremic syndrome, TTP - thrombotic thrombocytopenic purpura

### 001728

#### Is RECOMBINANT FACTOR VIIa overused in critically ill patients?

P. Routray[1], NR. Dr[2], RV. Dr[2]
[1]critical care, care hospitals, BHUBANESWAR, India; [2]Critical care, APOLLO HOSPITALS, Chennai, Tamil Nadu, India, India
**Correspondence:** P. Routray
*Intensive Care Medicine Experimental* 2019, **7(Suppl 3):**001728

**INTRODUCTION.** Recombinant activated Factor VII (rVIIa) enhances haemostasis with action limited to areas of injury. It is approved for use only in haemophilia and presence of clotting factor inhibitors. We sought to audit its use outside these recommendations and evaluate its efficacy in improving outcome.

**OBJECTIVES.** To observe efficacy of Factor VII (rVIIa) outside its prescribed indications and monitor its outcome.

**METHODS.** We performed a retrospective observational study of all patients who received factor rVIIa in two tertiary care hospitals between January 2008-December 2012 for persistent haemorrhage refractory to conventional treatment. Efficacy of factor rVIIa was assessed by mortality and blood product requirements.

**RESULTS.** Total of 42 patients were given factor rVIIa. Indications included cardiac surgery (26%), haematological disorders (21%), intracerebral haemorrhage (14%), obstetric bleeding (9.5%), abdominal surgeries (7.1%), sepsis (7.1%), trauma (7.1%), liver diseases (4.7%) and dengue (2.3%). The median of units of packed red blood cell (PRBC), platelets, fresh frozen plasma (FFP) and cryoprecipitate requirement before and after factor rVIIa administration in medical patients were 4,3,5,2 and 1.5,0,0.5,0 respectively. In surgical patients requirements of PRBC, platelets, FFP and cryoprecipitate before and after use of rVIIa were 6,3.5,5,6 and 3,0,2,1 respectively. Mortality was 28% among medical patients and 46% among surgical patients. There was no documented thromboembolic phenomenon in the study patients.

**CONCLUSION.** Recombinant Factor rVIIa is widely used beyond conventional recommendations. It appears to be effective in reducing further need for blood product transfusions in medical patients but not in surgical patients. Higher mortality in surgical patients receiving factor rVIIa may be contributed by inadequate source control of the bleeding. Cost effectiveness of rVIIa therapy needs to be factored in prior to use in all patients.

##### REFERENCE(S)

1. Lawrence Tim Goodnough, Douglas M Lublin, Lini Zhang, George Despotis, Charles Eby. Transfusion medicine service policies for recombinant factor VIIa administration. Transfusion 2004;44:1325-31
2. Martinowitz U, Kenet G, Segal E, et al. Recombinant activated factor VII for adjunctive hemorrhage control in trauma. J Trauma 2001;51:431-8.

### 001517

#### Is citrate anticoagulation better than anticoagulation with unfractionated heparin to prevent coagulation of the circuit?

K. Maisterra Santos, ND. Toapanta Gaibor, J. Puentes Yáñez, C. Sanz Mellado, S. Gonzalez Del Hoyo, J. Castaño Camuñez, J. Pérez Sánchez, I. Romera Peregrina, M. González Romero, V. Fuentes Mila, R. Buendía Flores, L. Costa Capella, E. Puente Mora, JL. Pérez Fernández, J. Sabater Riera
[1]Intensive care, Hospital Universitari de Bellvitge, Barcelona, Spain
**Correspondence:** N.D. Toapanta Gaibor
*Intensive Care Medicine Experimental* 2019, **7(Suppl 3):**001517

**INTRODUCTION.** The continuous techniques of renal clearance in patients admitted to the intensive care units (ICU) continue to be used up to 20%, in which anticoagulation of the extracorporeal circuit is mandatory. The regional anticoagulation with citrate has been used in recent years to prevent circuit coagulation, maintain filter performance and reduce the risk of bleeding.

**OBJECTIVES.** To determine the prevalence of filter consumption according to the use of anticoagulation with unfractionated heparin in relation to the regional anticoagulation with citrate.

**METHODS.** Retrospective observational study, performed on patients admitted to the intensive care unit with renal clearance in the period from 2016 - 2018 at the University Hospital of Bellvitge.

**RESULTS.** We analyzed 186 patients admitted to the ICU which required renal clearance techniques, 41 patients of them received anticoagulation with unfractionated heparin, 15 ± patients with citrate anticoagulation and 115 of the patients did not receive anticoagulation.

The average of the days that required techniques of renal clearance with unfractionated heparin was 7.6 ± 7.9, that of regional citrate anticoagulation was 8.4 ± 7.9; the average daily use of filters was 0.8 ± 0.6, in patients with anticoagulation of unfractionated heparin, 0.7 ± 0.8 in patients with regional citrate anticoagulation and 0.8 ± 0.6 in patients who did not receive anticoagulation. The mean number of filters used with unfractionated heparin was 4.8 ± 4.6 and 4.6 ± 4.7 for citrate anticoagulation.

**CONCLUSION.** No significant differences were found between the consumption of filters of patients who received anticoagulation with fractionated heparin in relation to patients who received anticoagulation with citrate. It was difficult to find significant difference due to a small population.

**REFERENCE(S)**

1. J Nephrol. 2007 Sep-Oct; 20 (5): 602-8.
2. J Intensive Care Soc. 2017 Aug; 18 (3): 184-192.
3. Crit Care Med. 2015 Aug; 43 (8): 1622-9.

### 001560

#### Predictors of post-traumatic AKI in the ICU

M. dlela, T. Olfa, T. Amal, M. Bahloul, M. Bouaziz

[1]Intensive care unit, Habib bourguiba university hospital, Sfax, Tunisia

**Correspondence:** M. dlela

*Intensive Care Medicine Experimental* 2019, **7(Suppl 3):**001560

**INTRODUCTION.** The occurrence of acute kidney injury (AKI) in trauma patients is a problem that has been little studied to date. Its presence has been shown to be associated with an increased risk of morbidity and mortality in affected individuals.

**OBJECTIVES.** To determine the incidence of post-traumatic AKI and identify its predictive risk factors that could be eventually prevented.

**METHODS.** This is a ten-month long prospective cohort-study, conducted in the department of emergencies and intensive care unit (ICU) of a university hospital, including trauma patients with a minimum ICU stay of 7 days. Renal failure was defined based on the new KDIGO classification. Predictors of AKI were identified using univariate and then multivariate analysis.

**RESULTS.** One hundred thirty patients were admitted during the study period for the management of post-traumatic injuries, among which 86 patients were included. The incidence of AKI in the studied population was 53% (46 cases) with 26 (56%) diagnosed with stage one, ten (22%) with stage two and ten (22%) with stage three.

On univariate analysis, older age and medical history of diabetes or hypertension were predictors of AKI. Injury assessment found traumatic brain injury (AIS>3), Glasgow (GCS) on admission, and the diagnosis of fat embolism to be associated to post-traumatic AKI. Moreover, hemodynamic instability on admission and during ICU stay, shock-index on admission, the amount of fluid administered the use of vasoactive drugs, sepsis, hyperbilirubinemia, P/F ratio and acute respiratory distress syndrome (ARDS) were also associated to post-traumatic AKI.

Among these factors, ARDS (p = 0.001, OR = 9 , CI: 6-100), fat embolism (p = 0.028, OR = 2, CI: 1.6-2.5), Shock index (p = 0.02, OR = 15.2, CI: 2.2-105), and bilirubin levels (p = 0.006, OR = 1.035, CI: 1.01-1.06) were identified as independent predictors of post-traumatic AKI on multi-variate analysis.

Besides, according to our analysis, the following variables were predictive of stage3 AKI, including bilirubin levels on the day of AKI diagnosis (p = 0.026, OR = 1.032, CI: 1.004-1.061), transfusions (p = 0.02, OR = 1.5, CI: 1.1-1.9), fat embolism (p = 0.015, OR = 11.3, CI: 1.6-75), and diuretics' administration (p = 0.004, OR = 10.4, CI: 1.8-57).

**CONCLUSION.** Post-traumatic AKI could be associated with significant morbi-mortality in the ICU. The identification of predictors from the initial onset of trauma could be valuable to guide its management.

### 001636

#### Vancomycin and Teicoplanin Clearance during an in vitro Model of Continuous Venovenous Hemofiltration using PMMA hemofilter

I. Godi[1], A. Lorenzin[2], S. De Rosa[3], M. Decal[2], C. Ronco[4]

[1]Department of Anesthesia and Intensive Care Unit, University of Padua, Padova, Italy; [2]International renal research institute of vicenza, San Bortolo Hospital Of Vicenza, Vicenza, Italy; [3]Department of anesthesia and intensive care unit, San Bortolo Hospital Of Vicenza, Vicenza, Italy; [4]Department of nephrology, San Bortolo Hospital Of Vicenza, Vicenza, Italy

**Correspondence:** I. Godi

*Intensive Care Medicine Experimental* 2019, **7(Suppl 3):**001636

**INTRODUCTION.** Sepsis-associated acute kidney injury is the most common syndrome in ICU. CRRT may be useful during sepsis, but definitive recommendations are lacking. Early and appropriate antibiotic therapy is the mainstay to successfully treat sepsis, but no comprehensive guidelines exist that provide antibiotic dosing recommendations for adult patients receiving CRRT.

The factors that need to be considered when dosing patients on RRT are related to the drug being used, type of RRT, and patient status. Particularly, the type of membrane plays a major role in drug removal. New synthetic and biocompatible membranes have high adsorptive affinity to proteins, endotoxins, inflammatory mediators, but also drugs. Two antibiotics, Vancomycin (VAN) and Teicoplanin (TEC), are frequently used in critically ill adult patients receiving CRRT to treat Gram-positive infections, especially sustained by methicillin-resistant Staphylococcus aureus.

**OBJECTIVES.** The aim of this study was to evaluate, in an in vitro system simulating the in-vivo permeability drugs, the convective and adsorptive drug clearance of VAN and TEC during CVVH with polymethylmethacrylate filter.

**METHODS.** VAN (1448 Da) and TEC (1885 Da) clearance was assessed in vitro in blood from healthy donors, spiked with one antibiotic under investigation. Closed circuit simulating CVVH was performed using PMMA filter at 10 ml/min ultrafiltrate and 50 ml/min blood flow rates. The duration of the experiment was of 360 min. Samples were collected at 5, 10, 30, 60, 120, 240 and 360 minutes from in-flow, out-flow and ultrafiltrate line; antibiotic concentrations were measured with biochemistry analyzer. Convective clearance was evaluated in terms of sieving coefficient, and adsorptive clearance was calculated using mass balance analysis.

**RESULTS.** The target initial VAN and TEC plasma concentrations set in this study were respectively 100 and 130 mg/L. Although the initial VAN concentration was 99.7 mg/L, after 360 minutes of CVVH, VAN concentration slightly decreased to 90.7 mg/L, with a total estimated adsorbed mass per surface area of 10.86 mg/m2. The blood SC ranged from 0.84 to 0.87. The initial TEC concentration was of 129 mg/L. After two hours, the concentration decreased of 25% and of 44% at the end of simulation, with a total estimated adsorbed mass per surface area of 65.12 mg/m2. The blood SC ranged from 0 to 0.19.

**CONCLUSION.** Our findings suggest that VAN prescription should take into account the elevated SC with PMMA membrane and dosage should be adjusted proportionally with ultrafiltration flow. Otherwise, TEC is largely adsorbed by PMMA membrane; a loading dose should be considered at the beginning of each treatment to balance the adsorptive phenomenon.

**REFERENCE(S)**

1. Bellomo R, Kellum JA, Ronco C, et al. Acute kidney injury in sepsis. Intensive Care Med. 2017 Jun;43(6):816-828.
2. González de Molina FJ, Ferrer R. Appropriate antibiotic dosing in severe sepsis and acute renal failure: factors to consider. Crit Care. 2011 Aug 1;15(4):175.

### 001679

#### Acute Kidney Injury Requiring Renal Replacement Therapy In Patients With Septic Shock

F. Sadaka, D. Bhargavi, G. Justin, N. Organti, D. Tannehill, J. O'brien
[1]Critical Care Medicine, Mercy Hospital St. Louis, St. Louis, USA
**Correspondence:** F. Sadaka
*Intensive Care Medicine Experimental* 2019, **7(Suppl 3):**001679

**INTRODUCTION.** Acute Kidney Injury (AKI) occurs frequently among patients with septic shock (SS) and is associated with worse prognosis, particularly if renal replacement therapy (RRT) is required. International guidelines recommend rapid recognition and initiation of treatment of SS.
**OBJECTIVES.** We examined the characteristics and outcomes of patients with SS who developed AKI requiring RRT.
**METHODS.** Data on 53 SS patients (requiring vasopressors - VP) were collected from acute physiologic and chronic health evaluation (APACHE) Outcome database and medical records. Data included age, gender, APACHE III score, APS (acute physiologic score), Sequential Organ Failure Assessment (SOFA) score at time of VP initiation (T0) and at 72 hours (T72), Delta SOFA (SOFA T0 - SOFA T72), lactic acid at T0, serum creatinine (Cr) at T0, duration of vasopressors in days, Input/ Output (I/O in cc) at 6 hours and 24 hours, hospital length of stay (LOS in days), and hospital mortality. AKI was diagnosed based on Kidney Disease Improving Global Outcomes (KDIGO) criteria.
**RESULTS.** Of the 53 patients with SS, AKI occurred in 24 patients (65%) of which 16 patients (67% of AKI cases) required RRT. Overall, the prevalence of AKI requiring RRT among SS patients was 30%. The SS-RRT group (16) and the SS without RRT group (37) were comparable on age (65 ± 12 vs 66 ± 15, p = 0.8), gender (male, 63 % vs 65 %, p = 0.9), APACHE III scores (114 ± 26 vs 99 ± 36, p = 0.2), SOFA T0 (13.4± 3.6 vs 11.6 ± 3.8, p = 0.1), lactic acid (4.9 ± 1.9 vs 5.9± 4.2, p = 0.3), I/O at 6 hrs (2200 ± 1800 vs 2100 ± 2100, p = 0.9), I/O at 24 hrs (6900 ± 4000 vs 5900 ± 3300, p = 0.4), and hospital mortality (38% vs 48%, p = 0.5). The SS-RRT group had higher APS (102 ± 24 vs 84 ± 33, p = 0.05), higher SOFA T72 (11.6 ± 3.8 vs 9.2 ± 4.4, p = 0.05), less Delta SOFA (1.8 ± 5.0 vs 4.4 ± 5.9, p = 0.03), higher Cr (3.7 ± 1.5 vs 2.2 ± 1.3, p < 0.001), longer duration of VP (6.0 ± 3.5 vs 3.3 ± 2.3, p < 0.01), and longer hospital LOS (24 ± 13 vs 14 ± 12, p < 0.01).
**CONCLUSION.** AKI and AKI requiring RRT are prevalent among patients with septic shock. Patients who developed AKI requiring RRT during septic shock have a significantly higher acute physiologic derangements, worsening organ failures, higher creatinine, longer duration of vasopressors, and longer hospital LOS than those without.

##### REFERENCE(S)

1. Bagshaw SM, Uchino S, Bellomo R, et al. Timing of renal replacement therapy and clinical outcomes in critically ill patients with severe acute kidney injury. J Crit Care 2009;24:129–40.
2. Rhodes A, Evans LE, Alhazzani W, Levy MM, Antonelli M, Ferrer R, Kumar A, Sevransky JE, Sprung CL, Nunnally ME, Rochwerg B, Rubenfeld GD, Angus DC, Annane D, Beale RJ, Bellinghan GJ, Bernard GR, Chiche JD, Coopersmith C, De Backer DP, French CJ, Fujishima S, Gerlach H, Hidalgo JL, Hollenberg SM, Jones AE, Karnad DR, Kleinpell RM, Koh Y, Lisboa TC, Machado FR, Marini JJ, Marshall JC, Mazuski JE, McIntyre LA, McLean AS, Mehta S, Moreno RP, Myburgh J, Navalesi P, Nishida O, Osborn TM, Perner A, Plunkett CM, Ranieri M, Schorr CA, Seckel MA, Seymour CW, Shieh L, Shukri KA, Simpson SQ, Singer M, Thompson BT, Townsend SR, Van der Poll T, Vincent JL, Wiersinga WJ, Zimmerman JL, Dellinger RP.Surviving Sepsis Campaign: International Guidelines for Management of Sepsis and Septic Shock: 2016.Intensive Care Med. 2017 Mar;43(3):304-377.

### 001697

#### Surfactant protein D (SP-D) gene polymorphismrs721917 is an independent predictor of acute kidney injury development in sepsis patients: A prospective cohort study

J. Liu[1], L. Zhang[1], J. Yao[2], Y. Chen[1], D. Chen[1]
[1]Department of Critical Care Medicine, Shanghai Jiaotong University, School of Medicine, Ruijin Hospital North, Shanghai, China; [2]Intensive care unit, The First People,s Hopspital of Kunshan, Kunshan, China
**Correspondence:** J. Liu
*Intensive Care Medicine Experimental* 2019, **7(Suppl 3):**001697

**INTRODUCTION.** Currently, there are no reliable predictors of risk of development and severity of acute kidney injury (AKI) in septic patients. In the prospective study, we sought to determine plasma surfactant protein D (SP-D) levels and *SP-D*polymorphisms rs721917C/T and rs2243639A/G of septic patients with AKI(defined with KDIGO criterion) *versus*those without AKI. The association between *SP-D*polymorphisms and plasma SP-D level and subsequent development and severity of AKI were analyzed.
**METHODS.** The study enrolled septic and septic shock patients admitted to the Department of Critical Care Department of two tertiary care hospitals. *SP-D*rs721917C/T and rs2243639A/G polymorphisms were determined using the PCR-SSP method. Furthermore, plasma SP-D and urine NGAL contents were examined using commercially available ELISA kits.
**RESULTS.** Totally 330 septic patients including 121 patients with septic shock were included. Their mean SOFA scores were 12 ± 3 (range 8-21). Septic patients with AKI (n=156) had significantly higher plasma SP-D levels (median: 150 ng/mL, range: 138-160 ng/mL) than those without AKI (median: 101 ng/mL, range: 82-111 ng/mL;*P*= 0.01).Plasma SP-D levelsof septic AKI patients were correlated with urine NGAL contents(*r*=0.596) and duration for CRRT(*r*=0.448). Septic patients with AKI had a significantly higher rate of rs721917 CC genotype (AKI: 35% *vs.* non-AKI:20%; *P* = 0.012), but a significantly lower rate of TT genotype (AKI: 19%*vs.*non-AKI: 26%; *P*= 0.005). Our multivariate analysis showed that SP-D rs721917 CC genotype was a significant independent predictor of AKI (*P*= 0.044) and mortality (*P*= 0.014) of septic patients.
**CONCLUSION.** Our study showed that septic patients with AKI had significantly higher plasma SP-D levels *versus*those without AKISP-D rs721917CC genotype is an independent and significant predictor of AKI development and mortality of septic patients. SP-D rs721917C/T polymorphisms should be further studied as a diagnostic and prognostic biomarker to facilitate early recognition of AKI so that treatment can be instituted promptly.

### 000446

#### AKI-ECMO: Predicting acute kidney injury after cardiac surgery in patients assisted by VA ECMO

V. Lepere[1], B. Duceau[1], C. Bombled[1], O. Dujardin[1], A. Charfeddine[1], G. Lebreton[2], J. Amour[3], D. Hajage[4], A. Bouglé[5]
[1]Department of intensive care medecine, University Hospitals Pitié Salpêtrière - Charles Foix, Paris, France; [2]Cardiac surgery, University Hospitals Pitié Salpêtrière - Charles Foix, Paris, France; [3]Department of intensive care medicine, University Hospitals Pitié Salpêtrière - Charles Foix, Paris, France; [4]Department of statistics, University Hospitals Pitié Salpêtrière - Charles Foix, Paris, France; [5]Department of intensive care, University Hospitals Pitié Salpêtrière - Charles Foix, Paris, France
**Correspondence:** V. Lepere
*Intensive Care Medicine Experimental* 2019, **7(Suppl 3):**000446

**INTRODUCTION.** Cardiogenic shock occurs in 2-6% of patients undergoing cardiac surgery. One percent will require mechanical support using VA ECMO. Acute Kidney Injury (AKI) is one of the most frequent complications in patients undergoing ECMO support and is associated with mortality.
**OBJECTIVES.** The aims of our study were to 1/ describe the epidemiology and 2/ identify the risk factors of AKI in adult patients assisted by VA ECMO in perioperative of cardiac surgery.
**METHODS.** We conducted an observational, retrospective and monocentric study in the Surgical Intensive Care Unit of the Cardiology Institute of La Pitié-Salpêtrière University Hospital in Paris, France. All patients hospitalized in the department between January 1, 2013 and December 31, 2016 on a cardiac surgical peri-operative basis and assisted by VA ECMO for more than 48 hours were included in the study. A multivariate statistical analysis identified the risk factors for severe AKI in this cohort.
**RESULTS.** Of the 319 patients included, 211 (66%) developed severe AKI (KDIGO 3 stage), for an overall 30-day mortality of 48%, increased to 59% in patients undergoing renal replacement therapy (86% of patients with AKI). Risk factors for AKI werehigh preoperative LVEF

(OR 1.02 CI 95%[1.01-1.04], p=0.006); maximumperoperative adrenaline dose (OR 1.04 CI 95%[0.75-1.45], p=0.002); presence of right ventricular dysfunction (OR 2.20 CI 95%[1.14-4.23], p=0.009); arterial lactate level (OR 1.07 CI95%[0.97-1.18], p=0.009), bilirubinemia (OR 1.01 CI95%[1.004-1.020], p<0.001) and creatinine level (OR 1.01 CI 95%[1.01-1.02],p=0.001) on the day of VA ECMO implantation. The implantation of VA ECMO percardiotomy (OR 0.51 CI 95%[0.28-0.94], p=0.02) and high protidemia (OR 0.96 CI95%[0.92-0.99], p=0.04) were protective factors for AKI. Several risk factors ofday 30 mortality have been identified: chronic arteriopathy (OR 3.59 CI 95%[1.42-9.10], p=0.01); biventricular failure (OR 1.77 CI 95%[1.00-3.11], p=0.048) and high arterial lactate level (OR 1.17 CI 95%[1.07-1.28], p<0.001) at VA ECMO implantation. Heart transplantation was a protective factor of mortality compared to other types of surgery (OR 0.34 CI 95%[0.14-0.80], p=0.01). In this cohort, AKI was not associated with increased mortality.

**CONCLUSION.** AKI in patients assisted with VA ECMO undergoing cardiac surgery is frequent and associated with significant mortality. Several risk factors for AKI are identified. The implantation of VA ECMO in per cardiotomy is a protective factor.

## HSRO / NAHP - Outcomes and quality assessments in the intensive care setting

### 000136

#### Correlation among Pseudomonas Aeruginosa and nursing, severity and outcome indexes in ICU patients

A. Vakalos, M. Piperidou
[1]Icu, Xanthi General Hospital, Xanthi, Greece
**Correspondence:** A. Vakalos
*Intensive Care Medicine Experimental* 2019, **7(Suppl 3):**000136

**INTRODUCTION.** The ability of Pseudomonas Aeruginosa strains to cause infection is complex, because not only they provide strong infection ability, but because invade the tissues with discretion as well. Spreading of these strains may lead in developing severe infections in critically ill ICU patients.

**OBJECTIVES.** The aim of our observation retrospective study was to test the hypothesis that a statistical significant correlation exists among nursing, severity and outcome indexes and percentage of Pseudomonas Aeruginosa positive cultures in severely ill patients in our both medical and surgical ICU served in community hospital.

**METHODS.** From January 2006 to December 2017, 1107 patients admitted to our ICU. Mean age 66 years, mean length of stay (LOS) 12.2 days, mean APACHE II score on admission 21.5, predicted mortality 39.5 %, actual mortality 29.3 %, Standardized mortality ratio (SMR) 0.74. From our database we looked for percent of total of Pseudomonas Aeruginosa positive cultures and nursing (number of patients, number of patients ventilated, length of stay, hospitalization days, ventilation days per patient ventilated, percentage of ventilated patients, plentitude, mechanical ventilation days), severity (age, APACHE II score, predicted mortality) and outcome (actual mortality, Standardized mortality ratio) indexes per year from 2006 to 2017 (mean values). Using linear correlation method, we looked for linear slope, correlation coefficient (r), and coefficient of determination (r2), and by linear regression method using ANOVA test we looked for p value, according Pseudomonas Aeruginosa % and ICU indexes.

**RESULTS.**

**CONCLUSION.** According to our data, there was no statistical significant correlation detected among all severity, all outcome and all of the nursing indexes and the percentage of Pseudomonas Aeruginosa positive cultures. Our data suggest that in our study, the impact of the incidence of Pseudomonas Aeruginosa infection was not statistical significant or not strong enough to influence the length of stay or the outcome.

**Table 1 (abstract 000136).** See text for description

| | Slope | r | r2 | St. error | P value |
|---|---|---|---|---|---|
| Noof patients | 3.3180 | 0.4544 | 0.2065 | 2.0570 | 0.1378 |
| Hosp days | 28.2890 | 0.4230 | 0.1790 | 19.1610 | 0.1706 |
| LOS | -0.1122 | -0.2409 | 0.0585 | 0.1423 | 0.4488 |
| Age | 0.1823 | 0.2349 | 0.0551 | 0.2386 | 0.4624 |
| % ventilated | 0.1009 | 0.1361 | 0.0185 | 0.2323 | 0.6733 |
| Plentitude | 0.0539 | 0.0393 | 0.0015 | 0.4336 | 0.9035 |
| Pts ventilated | 3.1260 | 0.4627 | 0.2141 | 1.8940 | 0.1299 |
| Days M V | 20.5140 | 0.3569 | 0.1274 | 16.9760 | 0.2547 |
| VD per pt | -0.0607 | -0.1414 | 0.0199 | 0.1345 | 0.6612 |
| APACHE | 0.0569 | 0.1066 | 0.0113 | 0.1681 | 0.7415 |
| Pred Mort. | -0.0054 | -0.0034 | 1.2200 | 0.4968 | 0.9914 |
| Actual mort | 0.4760 | 0.2792 | 0.0779 | 0.5176 | 0.3794 |
| SMR | 0.0133 | 0.4244 | 0.1801 | 0.0090 | 0.1691 |

### 000843

#### ICU mortality in relation with age, co morbidities and Apache II Score, what's the correlation for the outcome?

K. Tsikritsaki, G. Koukoulitsios, C. Mandila, E. Koutrouba, D. Belesiotis, M. Anifanti, D. Toumpanakis, I. Tsoni, EM. Papadimitriou, L. Avramopoulou, N. Panagiotopoulou, S. Bakouli, I. Poularas, V. Romanou, S. Antonopoulou, M. Gianoulopoulou, V. Koutsoukou, A. Dafni, K. Tsironas, A. Kalogeromitros
icu, G.Gennimatas General Hospital, Athens, Greece
**Correspondence:** K. Tsikritsaki
*Intensive Care Medicine Experimental* 2019, **7(Suppl 3):**000843

**INTRODUCTION.** Advanced age is associated with higher mortality and adverse outcomes in ICUs

**OBJECTIVES.** The aim of this study was to evaluate the possible effect of age correlated to apache II score, co morbidities and reason for admission for the final outcome.

**METHODS.** Our study included 280 patients aged 18 and older that were admitted in the ICU from January 2016 till March 2019. Demographic parameters, apache score II, admission diagnosis, underling diseases, ICU length of stay and finally outcome were recorded.

**RESULTS.** We enrolled 280 patients, 156 males and 124 females with mean age 55±37 years. The main reasons for ICU admission were: post operative (after elective operation), trauma, acute respiratory failure and sepsis. Out of 280 patients the 171 had co morbid medical illness (e.g. ischemic heart disease, arterial hypertension, diabetes mellitus, COPD, chronic renal failure). The mean length of stay was 22 days but it ranged from 1 to 72 days. 196 patients were discharged from the ICU and 84 patients died. Apache II score ranged from 6 to 39, higher values were correlated with longer hospitalization and worse outcome. Post operative patients independently from the age and co morbidities had the best outcome. There were not differences in the outcome as far as the gender was concerned.

**CONCLUSION.** Reasons for mortality appears to be multifactorial. High age alone is not a factor to predict morbidity. Previous health state, co morbidities, high apache II score and the reason of admission in the ICU play a very important role for the final outcome.

**Table 1 (abstract 000843).** See text for descriptio

| | Patients | Males | Females | CO MORB | Age | Apache II | Length of stay (Days) | Discharged | Deaths |
|---|---|---|---|---|---|---|---|---|---|
| Post operative | 88 | 48 | 40 | 65 | 18-55 | 10±4 | 1-14 | 76 | 12 |
| Trauma | 96 | 62 | 34 | 40 | 18-62 | 21±7 | 12-72 | 71 | 25 |
| Acute Respiratory Failure | 20 | 12 | 8 | 18 | 32-92 | 25±11 | 7-31 | 15 | 5 |
| Sepsis | 65 | 32 | 33 | 40 | 50-92 | 27±12 | 1-33 | 25 | 40 |
| Miscellaneous | 11 | 2 | 9 | 8 | 22-92 | 22±11 | 1-45 | 9 | 2 |
| Total | 280 | 156 | 124 | 171 | | | mean ≈22 days | 196 | |
| | | | | | | | | | 84 |

### 000864

#### Evaluation of the quality of care in the Intensive Care Unit from the perspective of patients and relatives

P. Vega Ocaña[1], L. González Bautista[1], JD. Martin Santana[2], C. García Del Rosario[3], JL. Santana Cabrera[1]
[1]Intensive care unit, Hospital Universitario Insular de Gran Canaria, Las Palmas de Gran Canaria, Spain; [2]Economics, Universidad de las Palmas de Gran Canaria, Las Palmas de Gran Canaria, Spain; [3]Quality department, Hospital Universitario Insular de Gran Canaria, Las Palmas de Gran Canaria, Spain
**Correspondence:** P. Vega Ocaña
*Intensive Care Medicine Experimental* 2019, **7(Suppl 3):**000864

**INTRODUCTION.** In the past, quality and improvement in healthcare have focused on what proffesionals think should be valued and have been less interested in what serve userd felt was important. In the Intensive Care Unit, the patient's perspective, as well as their relatives', is central to quality improvement.

**OBJECTIVES.** To evaluate the level of technical and structural human quality of an Intensive Care Unit (ICU) from the perspective of patients and their relatives.

**METHODS.** Information was collected from June to September 2018 through a survey adapted to patients and family members. The questionnaire included a 7-score Likert scale conformed by different items aimed at assessing the quality of the service provided to patients in the ICU.

Based on the work of Mora Lourido (2015), we took the dimensions created to measure the technical and structural human quality in the ICU, and we conducted an analysis of means using the student's T, to check the differences in the perceived quality by patients and relatives.

**RESULTS.** The measurement scale was consisted of 7 dimensions, whose average values are shown in the attached table.

**CONCLUSION.** We may conclude that the relatives' group is more critical when evaluating the technical and structural human quality of the ICU. Statistically significant differences were found in the valuation of all the dimensions, excepto for "food" and "facilities, available resources and environmental atmosphere", being able to explain why there are dimensions where aspects in which the human factor does not intervene are valued. On the other hand, "waiting room" was punctuated only by relatives, since this ítem was not included in the patient's questionnaire.The human dimension and professionalism in patient care were the aspects that were best valued by both patients and relatives. On the other hand, the dimension referring to visits has been the worst evaluated by both groups, so an improvement should be considered in the future.

**Table 1 (abstract 000864).** Descriptive analysis of the factors of the ICU quality scale

| Dimensions | Mean (T.D.) | | *t* (*p*) |
|---|---|---|---|
| | Patients | Relatives | |
| Human dimension | 6.94 (0.19) | 6.79 (0.33) | 3.809 (0.000) |
| Professionality | 6.93 (0.26) | 6.81 (0.32) | 2.909 (0.004) |
| Waiting room | - | 5.69 (1.22) | - |
| Facilities, available resources and enviromental atmosphere | 6.52 (0.38) | 6.43 (0.53) | 1.439 (0.152) |
| Visits | 6.24 (0.98) | 5.54 (1.87) | 3.503 (0.001) |
| Food | 6.48 (1.61) | 6.44 (0.70) | 0.207 (0.836) |
| Cleaning | 6.94 (0.31) | 6.34 (0.72) | 7.985 (0.000) |

### 001594

#### Potassium Changes in Critical Illness in a sub-Saharan Teaching Hospital

M. Oladimeji[1], A. Fadeyi[2], G. Asiyanbi[3], S. Olanipekun[2], O. Adekola[4]
[1]Anesthesia & intensive care unit, Lagos University Teaching Hospital, LAGOS, Nigeria, Federal Republic of; [2]Intensive care unit, Onelife Hospital, LAGOS, Nigeria, Federal Republic of; [3]Anaesthesia & intensive care, Lagos University Teaching Hospital, LAGOS, Nigeria, Federal Republic of; [4]Intensive care unit, Onelife Hospital, LAGOS, France
**Correspondence:** O. Adekola
*Intensive Care Medicine Experimental* 2019, **7(Suppl 3):**001594

**INTRODUCTION.** Potassium is one of the body's major ions, 98% of the body's potassium is intracellular. It is important for generating action potentials. Disturbance in potassium homeostasis common in critically ill patients s it can induces lethal arrhythmias and result in cardiac dysfunction

**OBJECTIVES.** We determined the incidence of potassium abnormalities in critically ill patients.

**METHODS.** The stduy was conduct at a University Teaching Hospital between June 2018 and December 2018. changes in potassium concentration in adult critically ill patients was studied. The serum potassium was measured on admission and dialy for seven days.

Normal range: for potaasium described as 3.5 -5.0, Mild Hypokalemia as 3.0-3.4 and severe: as potassium <3.0. Mild Hyperkalemia defined as potassium > 5.0 -6.0 and severe potassium >6.0

**RESULTS.** A total of 120 patients were studied. The median age 36 years, the meadian duration of ICU admission was 5 days. Hypokalemia was observed n 61.6%, In patients with hypokalemia; mild hypokalemia observed in 37.3% and severe hypokalemia was observed in 24.3%. Hyperkalemia in 17.6% of patients. Mild hyperkalemia in 12.7% and severe hyperkalemia in 4.9% of patients. The median duration of onset of hypokalemia and hyperkalemia was 2 days. While the median duration of hypokalemia and hyperkalemia were 2 and 2.5 days respectively.

**CONCLUSION.** Potassium derangement is not uncommon in critically ill patients at our institution.

### 001649

#### Use of APACHE II score for predicting prognosis in elderly patients admitted to emergency department

K. B.ismail, N. Zaouek, Y. Yahia, K. Zaouch, R. Boubaker, R. Baccouche, H. Maghraoui, K. Mejd
Urgences, Hospital Rabta, Tunis, Tunisia
**Correspondence:** I.K. Ben
*Intensive Care Medicine Experimental* 2019, **7(Suppl 3):**001649

**INTRODUCTION.** Emergency departments receive an increasing number of elderly patients in often critical condition. The APACHE II score usually calculated at intensive care unit admission could be useful to determine the prognosis in elderly patients admitted to emergency department.

**OBJECTIVES.** The aim of this study is to evaluate the ability of APACHE II score to predict prognosis in elderly patients hospitalized at emergency department (ED).

**METHODS.** This is a retrospective study conducted in all old patients (≥65years) admitted to ED between September 2018 and March 2019. Data of all patients were collected and the APACHE II score was calculated at admission. The main study endpoints were the use of mechanical ventilation or vasoactive drugs and inpatient mortality.

**RESULTS.** •A total of 257 elderly patients were included.The mean age was 75±7 years.

• 134 (52%) were females and 123 (48%) were males. Patients had an history of hypertension (56%), diabete (56%) and coronary disease (29,6%).

• Diagnosis of hospitalization were: diabetic ketoacidosis (29%), acute coronary syndrom (16%), acute heart failure (9%), meningoencephalitis (9%), sepsis (8%), hypoxemic pneumonia (8%), acute exacerbation of chronic obstructive pulmonary disease (6%), hemorrhagic syndrome (6%), stroke (4%).

•ventilation was performed in 24% of patients and vasoactive drugs were used in 13%.

•35% were discharged, 45% were transferred to other services including 5% to intensive care unit.

•The mean duration of stay was 35,7±7hours.

• In hospital mortality rate was 20% and mean score of APACHE II was 18±23.

•The APACHE II score was significantly correlated with using mechanical ventilation (p=0,000) and vasoactive drugs (p=0,04) but not with inhospital mortality (p=0,99).

**CONCLUSION.** The APACHE II score wasn't useful for predicting inhospital mortality in elderly patients admitted to ED but it was significantly correlated to the use of mechanical ventilation or vasoactive drugs.

Further studies seem necessary to confirm these results.

### 001745

#### When pregnancy becomes a disease in an Intensive Care Unit - a retrospective study

S. Cunha[1], R. Reis[2], F. Pulido Adragão[3], C. Parente[4], ,P. Valério[5], Al. Pedroso[6], A. Ramos[6]
[1]Anesthesiology, IPO, Lisbon, Portugal; [2]Internal medicine, Barreiro Montijo Hospital Center, Lisbon, Portugal; [3]Internal medicine, Algarve Hospital Center, Lisbon, Portugal, Portugal; [4]Internal medicine, Barreiro Montijo Hospital Center, Lisbon, Portugal, Portugal; [5]Nephrology, Hospital São Bernardo, Setúbal, Portugal; [6]Intensive care unit, Hospital de Cascais, Cascais, Portugal
**Correspondence:** S. Cunha,
*Intensive Care Medicine Experimental* 2019, **7(Suppl 3):**001745

**INTRODUCTION.** Pregnancy involves many physiologic adaptations and sometimes physiopathological changes also ensue.

Per year, around 200-700/100.000 pregnant women are admitted into an Intensive Care Unit (ICU). The admission reasons are diverse, involving obstetric and also non obstetric causes. Thereafter, this population is frequently a challenge when in the ICU.

**OBJECTIVES.** This study's aim is to describe the demographic, clinical data and outcomes of patients admitted in the ICU of Cascais' Hospital for obstetric reasons.

**METHODS.** We conducted a retrospective descriptive analysis of patients admitted to the ICU with the following inclusion criteria: pregnant or puerpera women admitted for obstetric reasons, either in the antepartum, postpartum period or late puerperium. The statistical analysis of epidemiological and clinical data was performed with the software SPSS®. The study period comprised between June 1st 2017 to 31th December 2018.

**RESULTS.** During the study period, 31 patients were admitted to the ICU for obstetric reasons: 14.8% in the antenatal period, 11.1% on the vaginal pospartum period and 70.4% post-cesarean section. The medium age of this sample was 33 years (SD=6), the median of the gestational age at admission was 34 weeks (IQR 10) and 81,5% had a surveilled pregnancy. The main reason of admission was pre-eclampsia (18.5%), followed by hemorrhage and HELLP syndrome, both accounting 14.8% of admissions. In our sample, the mortality rate was 0%, nevertheless 29.6% required ventilatory support, 14.8% vasopressor/inotropes and 7.4% renal replacement therapy. The median duration of stay in the ICU was 3 days (IQR 3).

**CONCLUSION.** Despite the low incidence of admissions in ICU among pregnant women, the higher maternal age and increasing cesarean deliveries, are contributing to the increasing physiopathological changes and potentially fatal disorders, such as hypertensive disorders of pregnancy (pre-eclampsia, eclampsia and HELLP syndrome) and post-partum hemorrhage.

The authors highlight the importance of the prompt diagnosis and multidisciplinary approach (involving obstetricians, neonatology and ICU) of this obstetric disorders, aiming for the best maternal and neonatal outcome.

**REFERENCE(S)**

1. Guntupalli et al. Critical Illness in Pregnancy Part I: An Approach to a Pregnant Patient in the ICU and Common Obstetric Disorders. CHEST 2015; 148 (4): 1093 - 1104
2. Crozier, T. General Care of the Pregnant Patient in the Intensive Care Unit. Semin Respir Crit Care Med 2017;38:208–217

### 000011

#### Multiple boluses of intravenous tranexamic acid to reduce hidden blood loss and the inflammatory response following major surgery after severe traumatic injury: a randomised clinical trial

D. Dmytriiev
Grushevskogo 21/15, Vinnytsia, Ukraine
*Intensive Care Medicine Experimental* 2019, **7(Suppl 3):**000011

**INTRODUCTION.** The aim of this study was to examine the efficacy and safety of multiple boluses of intravenous (IV) tranexamic acid (TXA) on the hidden blood loss (HBL) and inflammatory response following **major surgery after severe traumatic injury** (STI).

**METHODS.** A total of 102 patients were allocated randomly to receive a single bolus of 20 mg/kg IV TXA before the incision (group A), a single bolus followed by a second bolus of 1 g IV-TXA three hours later (group B) or a single bolus followed by two boluses of 1 g IV-TXA three and six hours later (group C). All patients were treated using a standard perioperative enhanced recovery protocol. Primary outcomes were HBL and the level of haemoglobin (Hb) as well as the levels of C-reactive protein (CRP) and interleukin-6 (IL-6) as markers of inflammation. Secondary outcomes included the length of stay in hospital and the incidence of venous thromboembolism (VTE).

**RESULTS.** The mean HBL was significantly lower in group C (244.14 ml standard deviation (sd) 125.97) than group A (464.23 ml sd 198.23, $p < 0.001$) or B (334.12 ml sd 193.26, $p = 0.012$). The decrease in the level of Hb between the pre-operative baseline and the level on the third post-operative day was 29.32 g/L (sd 5.84 g/L) in group A, 30.16 g/L (sd 6.44) in group B and 29.94 g/L (sd 4.32) in group C. This decrease differed significantly among the three groups

($p < 0.01$). The mean level of CRP was significantly lower in group C than in the other two groups on the second ($p \leq 0.030$) and third post-operative days ($p \leq 0.010$). The levels of IL-6 were significantly lower in group C than group A on the first three post-operative days ($p = 0.020$). The mean length of stay was significantly lower in group C than group A ($p = 0.024$). No VTE or other adverse events occurred.

**CONCLUSION.** Anterior rectus sheath blocks using local anesthetics and steroids are safe and long-term successful in more than one-third of children suffering from abdominal pain due to anterior cutaneous nerve entrapment syndrome after laparoscopic oncology surgery.

#### 001147

**Perception of discomfort in the ICU by caregivers and patients: a qualitative study**

S. Ashkenazy[1], F. DeKeyser Ganz[2]
[1]Hadassah medical center, Hebrew University, Jerusalem, Israel; [2]School of nursing, Hadassah Hebrew University, Jerusalem, Israel
**Correspondence:** S. Ashkenazy
*Intensive Care Medicine Experimental* 2019, **7(Suppl 3):**001147

**INTRODUCTION.** Being hospitalized in the Intensive Care Unit (ICU) can lead to unpleasant experiences that involve pain and discomfort for most patients. Sedation and analgesia have become common treatments for alleviation of these symptoms, especially for those receiving mechanical ventilation. As a result, patient – caregivers communication is impaired, leading to caregivers' decreased ability to distinguish between pain and other sources for discomfort. Since patient behaviour might look the same whether the patient is in pain agitated or just uncomfortable. Caregivers may fail to recognize sources for discomfort as opposed to pain. This in turn lead to inappropriate treatment when the patient is uncomfortable but not in pain

**OBJECTIVES.** The present study is aimed at exploring and understanding caregivers and patients' perception of discomfort in the ICU.

**METHODS.** This is a descriptive qualitative study using content analysis. Caregivers (n=25, nurses and physicians) and patients (n= 12), treated in the ICU for more than 48h and receiving mechanical ventilation, were interviewed after ICU discharged using a semi-structed interview.

**RESULTS.** Two main categories (physical and emotional discomfort) and other sub-themes categories emerged from this analysis. Differences and similarities were found in the perception of discomfort in the ICU discomfort as described by patients and caregivers. Caregivers described both objective and subjective ways to differentiate between discomfort pain and agitation.

**CONCLUSION.** Patients experienced discomfort in the ICU. In order to improve quality of care it is important, that caregivers be aware of other sources of discomfort other than pain, while caring for the ICU patients. Strategies to identify discomfort where described.

### INF - Insights in specific infectious situations

#### 000521

**Intensive care unit: nosocomial acinetobacter spp. multi-drug-resistant strain monobacteremia**

D. Adukauskiene[1], D. Valanciene[2], G. Kasputyte[3]
[1]Intensive care unit, Lithuanian University of Health Sciences, Kaunas, Lithuania; [2]Anesthesiology and intensive care department, Klaipeda Seamen's Hospital, Klaipėda, Lithuania; [3]Department of anesthesiology, Lithuanian University of Health Sciences, Kaunas, Lithuania
**Correspondence:** D. Valanciene
*Intensive Care Medicine Experimental* 2019, **7(Suppl 3):**000521

**INTRODUCTION.** Patients in Intensive Care Units (ICU) are at higher predisposition to acquire nosocomial infection with high mortality rates. Vast majority of bacteremias in ICU is caused by Gram-negative rods. The rate and resistance of nosocomial infections is increasing in hospitals all over the world.

**OBJECTIVES.** The aim of study was to analyze sensitivity of multi-drug-resistant (MDR) Acinetobacter spp. strains of nosocomial monobacteremia and associating factors for primary bacteremia and mortality in ICU.

**METHODS.** Ongoing work: the retrospective data analysis of patients treated in Kaunas Clinic's ICU with positive blood culture of nosocomial MDR Acinetobacter spp. strain during past 10yrs period was carried out. Bacteremia was defined as nosocomial, if positive blood culture was taken after 72hrs of hospitalisation. Strain was defined as MDR, if resistant to ≥ 3 classes of antibiotics.

**RESULTS.** There were recorded 79 cases of nosocomial MDR Acinetobacter spp. strain monobacteremia with sensitivity to antimicrobial drugs: imipenem and colistin (n=79, 100%, P=0.001), meropenem (n=71, 89.9%, P=0.03), amikacin (n=36, 45.6%), ciprofloxacin (n=18, 22.8%), piperacillin/tazobactam (n=14, 17.7%), gentamicin (n=13, 16.5%), piperacillin (n=12, 15.2%), cefuroxim (n= 10, 12.7%), ampicillin and ampicillin/sulbactam (n=2, 2.5%). For patients with primary origin of nosocomial MDR Acinetobacter spp. monobacteremia (n=58, 73.4%, P=0.02, OR=2.06, CI95%=2.67-14.98) invasive procedures (n=62, 78.5%, P=0.01, OR=2.75, CI95%=1.67-15.87) and abdominal surgery were performed (n=56, 70.9%, P=0.02, OR=3.66 CI95%=4.76-11.65), previous hospitalization recorded (n=48, 60.8%, P=0.04, OR=1.48 CI95%=3.56-7.86), treatment with wide-spectrum antibiotics during previous 30 days was used (n=45, 57.0%, P=0.04, OR=1.77, CI95%=4.67-8.67). Lethal outcome (n=68, 86.1%) was found in patients with mechanical ventilation (n=68, 100%, P= 0.001, OR=1.73, CI95%=2.56-7.89), septic shock (n=62, 91.2%, P= 0.001, OR=1.15 CI95%=3.78-9.64), primary bacteremia (n=58, 85.3%, P=0.03, OR=2.08, CI95%=2.56-7.56), inadequate empiric antimicrobial treatment (n=58, 85.3%, P=0.03, OR=2.08, CI95%=2.56-7.56), SOFA score $^{3}$13 (n=55, 80.9%, P=0.02, OR=2.06 CI95%=4.78-11.67), $^{3}$3 co-morbidities (n=41, 60.3%, P=0.04, OR=1.39 CI95%=3.65-9.65), elderly (n=35, 51.5%, P=0.04, RR=16.5).

**CONCLUSION.** Acinetobacter spp. MDR strains of nosocomial bacteremia were found to be sensitive to imipenem and colistin. Primary bacteremia was associated with invasive procedures, abdominal surgery, previous treatment in hospital and use of wide spectrum antibiotics during 30 days before bacteremia. Nosocomial MDR Acinetobacter spp. monobacteremia was characterized by high mortality rate of 86% and associated with mechanical ventilation, septic shock, primary bacteremia, inadequate antibacterial treatment, high SOFA score, comorbidities and elderly.

**REFERENCE(S)**

1. Xu S et al. A case-control study: clinical characteristics of nosocomial bloodstream infections versus non-bloodstream infections of Acinetobacter spp. Clinical Infectious Diseases, 2018;67:189-95.
2. Baviskar AS et al. Nosocomial infections in surgical intensive care unit: A retrospective single-centre study. Int J Crit Illn Sci 2019;9:16-20.
3. none

#### 001727

**Carbapenem resistant bacteria infections in a general ICU**

C. Cardoso, F. Faria, R. Cavaco, A. Graça, L. Bento
Unidade de urgência médica, São José Hospital, Lisboa, Portugal
**Correspondence:** C. Cardoso
*Intensive Care Medicine Experimental* 2019, **7(Suppl 3):**001727

**INTRODUCTION.** Carbapenem resistant bacteria (CRB) infection is currently one of the most common causes of high mortality in ICU with very few antibiotic options.

No studies have been published about epidemiology, antibiotic susceptibility patterns and treatment outcome related to these infections in portuguese ICU's.

**OBJECTIVES.** Analyze epidemiology and treatment outcomes of CRB infections in a portuguese general ICU

**METHODS.** Retrospective study on patients who were admitted or developed CRB infections in a general ICU in a two year period

**RESULTS.** Forty four infections were identified : thirty nine due to *Klebsiella pneumoniae* and five due to *Pseudomonas aeruginosa*. Eleven pneumonias, thirty bloodstream infections, one urinary tract , one abdominal and one soft tissue infection. Main admission diagnosis was sepsis and thirteen patients had hematologic malignancies. Mean days of hospitalization for infection development was 22,2 days.

Half of patients presented with septic shock. All patients were initially treated with dual antibiotic therapy; Colistin and Tigecycline being the most used combination. Ceftazidime Avibactam was used in nine patients, three of them due to persistent bacteremia. Ceftolozane Tazobactam was prescribed in three patients due to multidrug resistant *Pseudomonas aeruginosa*. With these newer antibiotics all patients survived, except one. Mortality for all causes was high (52,3%), especially in hematologic patients. In a significant proportion it was not possible to determine outcome since some were transferred to other hospitals with contact loss or were under life-support interventions limitation at the time of antibiotic treatment.

**CONCLUSION.** In a portuguese general ICU, CRB infections are related to high mortality especially in groups such as hematologic patients. Susceptibility rates are high to Colistin so it still seems to be an adequate treatment combined to another antibiotic. Ceftazidime Avibactam and Ceftolozane Tazobactam achieve good results regarding outcome but more studies are needed to determine which patients might benefit from their prescription.

**REFERENCE(S)**

1. McCann Eilish, Srinivasan Arjun, DeRyke C Andrew, Ye Gang, DePestel D Daryl, Murray John and Gupta Vikas Carbapenem-Nonsusceptible Gram-Negative Pathogens in ICU and Non-ICU Settings in US Hospitals in 2017: A Multicenter Study Open Forum Infect Dis. 2018 Oct; 5(10): ofy241.
2. Xu L, Sun X, Ma X Systematic review and meta-analysis of mortality of patients infected with carbapenem-resistant Klebsiella pneumoniae Ann Clin Microbiol Antimicrob. 2017 Mar 29;16(1):18
3. Falagas M. E., Tansarli G. S., Karageorgopoulos D. E., Vardakas K. Z. Deaths attributable to Carbapenem-resistant Enterobacteriaceae infections Emerging Infectious Diseases 2014;20(7):1170–1175
4. Karlijn van Loon, a Anne F. Voor in 't holt, a Margreet C. Vos A Systematic Review and Meta-analyses of the Clinical Epidemiology of Carbapenem-Resistant Enterobacteriaceae Antimicrobial Agents and Chemotherapy Dec 2017, 62 (1) e01730-17

### 000816

#### Community acquired pneumonia in the intensive care unit

M. López De Olivencia, S. Gallego Zarzosa, D. Cabestrero, R. De Pablo, C. Soriano Cuesta, J. Higuera
[1]Intensive medicine, Hospital Ramón YCajal, Madrid, Spain
**Correspondence:** J. Higuera
*Intensive Care Medicine Experimental* 2019, **7(Suppl 3):**000816

**INTRODUCTION.** Community-acquired pneumonia is a pathology with an important impact on public health. The term community-severe pneumonia identifies the group of patients with pneumonia who require admission to the intensive care units.

**OBJECTIVES.** Our goal is to study the pneumonia acquired in our community. The incidence of each microorganism, bacteria or virus. The need of mechanical ventilation of the pathology itself and by etiological group.

**METHODS.** A retrospective, observational study was performed with all the patients who required admission to the Intensive Care Service in a Tertiary and University Hospital with the diagnosis of Community acquired pneumonia (CAP) from March 2015 to February 2019.

**RESULTS.** A total of 141 patients were collected. The characteristics of the patients were: Age 59.6 ± 13.9, SOFA 7.36 ± 3.9, APACHE II 18.5 ± 8.7, SAPS II 40.4 ± 17.5, Sex (Male) 60 , 6%, Diabetes Mellitus 26.8%, Arterial hypertension 39.4, Cardiovascular pathology 22.5%, Chronic lung disease 43.7%, Cirrhosis 4.2%, Need for vasoactive drugs 69.7%, Need for mechanical ventilation 55.6%, Our sample shows a mortality rate of 5.6%.

Viral microorganisms were isolated in 46 patients, whereas in 62 cases, the isolation was bacterial. The two microorganisms most frequently isolated were Streptococcus pneumoniae (41) and Influenza virus (32), followed by Staphilococcus Aureus (8). In the comparative analysis of CAP due to influenza virus and streptococcus pneumoniae and eliminating co-infections, we found that viral pneumonia presents a higher need of mechanical ventilation (82% vs 58%;P = 0.05). Bacterial pneumonia presents higher renal failure (61.2% vs 30%; P = 0.024). No statistically significant differences were found in terms of mortality (0% vs 6.4%; P = 0.325) or previous personal history.

**CONCLUSION.** CAP is a very common pathology in the ICU. Although the most frequent cause is bacterial, community viral pneumonia plays an important role, being the Influenza virus the second most frequently isolated microorganism. In our sample, viral pneumonia was associated in a statistically significant way with the need of mechanical ventilation.

### 000793

#### Ventilator Associated Pneumonia Rate in an Anesthesiology and Reanimation Intensive Care Unit

CA. Hatipoglu[1], C. Kaymak[2], FS. Erdinc[1], A. Ozcan[2], GT. Ertem[1], H. Basar[2], S. Kinikli[1], N. Tulek[1]
[1]Department of clinical microbiology and infectious diseases, University of Health Sciences, Ankara Health Application and Research Center, Ankara, Turkey; [2]Department of anesthesiology and reanimation, University of Health Sciences, Ankara Health Application and Research Center, Ankara, Turkey
**Correspondence:** C. Kaymak
*Intensive Care Medicine Experimental* 2019, **7(Suppl 3):**000793

**INTRODUCTION.** Ventilator associated pneumonia (VAP) is a significant cause of morbidity and mortality in critically unwell patients within the intensive care unit (ICU). Implementation of protective measures for VAP and early diagnosis can reduce mortality and reduce the development of multidrug resistant organisms.

**OBJECTIVES.** The aim of this study was to evaluate the VAP rate, ventilator use rate and causative microorganisms isolated in VAP cases in a five year study period.

**METHODS.** This study was conducted in the 25-bed anesthesiology and reanimation intensive care unit (AR-ICU) between 2014 and 2018. VAP rate per 1000 mechanical ventilator-days, device utilization ratio for mechanical ventilator and pathogens isolated in patients with VAP were evaluated retrospectively (VAP Rate = VAP Number / Ventilator Day x 1000). Hospital acquired infection definitions are made according to the US Centers for Disease Control and Prevention (*CDC*) criteria.

**RESULTS. Results:** During the study period totally 2944 patients were followed in the AR-ICU for 37402 bed days. In 2014, the VAP rate was 14.5 per 1000 mechanical ventilator days and the ventilator use rate was 55%, whereas the VAP rate was 5.7 per 1000 mechanical ventilator days and the ventilator use was 55% in 2018. Between 2015-2017, the VAP rate and the ventilator use rate were 13.6, 8.8, 9.5 and 58%, 62%, 52%, respectively. The number of VAP was 44, 44, 47, 43 and 27 in years of 2014-2018, respectively. Generally during the five-year study period, the mostly isolated pathogens of VAP were Acinetobacter baumannii (42.6 %) and Pseudomonas aeruginosa (25.5 %). The rate of Acinetobacter baumannii in VAP patients was decreased form 55.7% in 2014 to 27.3% in 2018.

**CONCLUSION.** In the AR-ICU, the VIP rate decreased gradually in the last 5 years, while the ventilator usage rate was generally similar. We think that infection control measures as well as early diagnosis and appropriate treatment of VAP can be effective in reducing the VAP rate.

### 001412

#### Klebsiela pneumoniae carbapenemase-producing: epidemiology and comparisons of mortality in a retrospective cohort between treatments and site infections from a single-center

B. Lucena[1], P. Zamith[1], SRS. Fonseca[1], L. Junior[2], MJC. Carmona[1], L. Malbouisson[1]
[1]Departamento de Anestesiologia, Hospital das Clínicas - Faculdade de Medicina da Universidade de São Paulo, Sao Paulo, Brazil;
[2]Departamento de pacientes graves, Hospital Vila Santa Catarina, São Paulo, Brazil
**Correspondence:** B. Lucena
*Intensive Care Medicine Experimental* 2019, **7(Suppl 3):**001412

**INTRODUCTION.** Infections caused by Klebsiela pneumoniae carbapenemase-producing (KPC-p)has become a worldwide emergent health problem considering the difficulty involved in the treatment of these multi resistant organisms and the highmortality rates. Despite the severity from this disease, treatments are divergent in medical literature.

**OBJECTIVES.** This study aims to analyze the characteristics of patients infected by KPC-p in a single center, in a cohort retrospective analyzing mortality, treatment options and site infections during a period from January 1st 2014 until December 31th2016.

**METHODS.** This study was conducted in a surgical intensive care unit from a tertiaryhospital in Brazil. KPC-p was observed in 41 patients, with a total of 42 positivecultures for KPC-p. The site infections were distributed between bloodstream(40,47%), respiratory tract (23,80%), abdominal (19,04%), urinary tract (9,52%)and soft tissues (7,14%). Average of SAPS 3 score at the day of admission at ICU was 63,23 (estimated mortality of 41,2%) and average of SOFA score atthe day of the culture was 9,30 (estimated mortality of 35%).The average of patients age was 59,5 years old, and sex distribution was 65,85% male, 34,35% female. All the patients received vasoactive drugs during the stay in the ICU and 56,07% of them required hemodialysis. A total of 12,19% of patients did notreceive any specific treatment against KPC-p infection, 12,19% receivedmonotherapy treatment, 36,58% double therapy and 39,02% three or moredrugs directed to the KPC-p treatment. The drugs considered as specific treatment to KPC-c were carbapenems and tigecycline combined with another drug; polymyxins and aminoglycosides combined or in monotherapy.

**RESULTS.** General mortality was 80,48% in our sample and the mortality when compared initial antibiotherapy containing a specific treatment to KPC-p or not was,respectively, 84,6% and 80,6%.The mortality when analyzed the stablished treatment after results from cultures was 100%, 82,3% and 76,4% respectively in mono, double and triple therapy. Comparing infection sites as mortality predictor, this outcome was observed in 89,4%, 85%, 81%, 75% and 33%respectively in bloodstream, abdominal, respiratory, urinary and soft tissues infections.

**CONCLUSION.** This study has several limitations. The higher mortality ratio in all groups canoverestimate the importance from some variables and our sample wasinsufficient to stablish differences statistically significance. Despite this,differences of mortality in monotherapy vs. double therapy vs. triple therapy,can indicate a better option to treat patients infected by KPC-p. Differences ratios between site of infections and mortality can be useful as a predictormortality in future models. Studies with increase in number of patients are necessary to stablish better recommendations in treatment.

**REFERENCE(S)**

1. Qureshi ZA, Paterson DL, Potoski BA, Kilayko MC, Sandovsky G, Sordillo E, et al.Treatment outcome of bacteremia due to KPC-producing Klebsiella pneumoniae: superiority of combination antimicrobial regimens. Antimicrob Agents Chemother 2012;56(4):2108–13.
2. Maltezou HC, Kontopidou F, Dedoukou X, Katerelos P, Gourgoulis G-M, Tsonou P, et al. Action plan to combat infections due to carbapenem-resistant, Gram negativenpathogens in acute-care hospitals in Greece. J Glob Antimicrob Resist 2014;2(1):11–6.
3. None

### 001519

#### Surgical treatment of infective endocarditis. Nine years of experience

R. Hernandez Estefanía[1], G. Aldamiz Echevarria Del Castillo[1], E. Rosas Carvajal[2], VA. Hortigüela Martín[2], Á. Vidal González[2], AI. Tejero Redondo[2], D. Robaglia[2], LM. Polanco Mahecha[2], M. Pérez Márquez[3], C. Pérez Calvo[2]
[1]Cardiac surgery, Hospital Universitario Fundación Jiménez Díaz, Madrid, Spain; [2]Intensive care unit, Hospital Universitario Fundación Jiménez Díaz, Madrid, Spain; [3]Intensive care unit, Hospital Universitario Rey Juan Carlos, Madrid, Spain
**Correspondence:** E. Rosas Carvajal
*Intensive Care Medicine Experimental* 2019, **7(Suppl 3):**001519

**INTRODUCTION.** The treatment of infective endocarditis (IE) has been widely discussed in order to decide which patients are going to benefit from a surgical approach.

Surgical indication should be established with caution due to its technical difficulties and potential postoperative complications.

**OBJECTIVES.** Describe the clinical and evolutionary characteristics of patients with infective endocarditis both in native or prosthetic valves undergoing cardiac surgery.

**METHODS.** Retrospective study in patients operated of IE between 2010 and 2018, at Fundación Jiménez Díaz and Rey Juan Carlos Hospitals.

Demographic variables (sex, age), affected valve (native, prosthetic), microorganism (blood cultures, valve or explanted prosthesis), type of intervention, postoperative complications, lenght of ICU and hospitalization stay were collected.

In January 2019, the status and functional status (NYHA) of the survivors was assessed by telephone call or review of the medical record.

**RESULTS.** 91 patients were operated (67% males) with a mean age of 65.1 years. 58 (63.7%) had native valve endocarditis. The average EuroSCORE II was 18.3. There was mitral involvement in 38 patients (41.8%), aortic in 33 (36.2%) and both in 18 (19.8%). In the mitral group 23 were native and 15 prosthetic; 25 bioprostheses and 12 mechanics were implanted. In the aortic group 24 were native and 9 prosthetic; 30 bioprostheses and 3 mechanics were implanted. In the mitral-aortic group 66.6% were pure native and the rest had at least one previous prosthesis.

The microorganisms isolated in blood cultures (BC) were: 39.5% Staphylococcus, 25.2% Streptococcus, 16.4% Enterococcus and 2% fungi. In 7.6% BC were negative.

The postoperative complications were: renal failure (16.4%), atrial fibrillation (9.8%), bleeding (5.4%), pneumonia (5.4%), polyneuropathy (5.4%) and stroke (4, 3%). The mechanical ventilation time (MV) was lower than 72 hours in 71.4% of the patients. 12% required tracheostomy. 9.8% required a definitive pacemaker (55% within the mitral-aortic group). 4% of patients were re-admitted to the ICU after being discharged to the ward. There was a hospital mortality of 13 patients (14.3%).

At the time of follow-up, 13 patients had died, 3 were hospitalized and the rest (62, 68.1%) were functional grade I (45.1%), II (56.4%) and III (3.2%), of NYHA respectively.

**CONCLUSION.** The surgery of the IE has a very good vital and functional prognosis, with a low mortality, although it is not free of post-operative complications. There are no major differences in relation to the native valve affected. The bioprostheses are the most used. Patients with bivalvular infection have a higher incidence of permanent pacemaker due to affectation of the auriculo-ventricular conduction zone.

#### REFERENCE(S)

1. Hoen B, Duval X. Infective endocarditis. New England Journal of Medicine. 2013 Aug 22;369(8):785.
2. Wolff M, Mourvillier B, Sonneville R, Timsit JF. My paper 10 years later: infective endocarditis in the intensive care unit. Intensive Care Medicine. 2014 Dec;40(12):1843-52.
3. The authors declare no conflict of interest.

### 001576

#### Predictors and outcome of acalculous cholecystitis in the ICU

M. dlela[1], R. Daoud[2], T. Olfa[3], M. Bahloul[1], M. Bouaziz[1]
[1]Intensive care unit, Habib bourguiba university hospital, Sfax, Tunisia; [2]Departement of general surgery, Habib bourguiba university hospital, Sfax, Tunisia; [3]Intensive care, habib bourguiba university hospital, sfax, Tunisia
**Correspondence:** M. dlela
*Intensive Care Medicine Experimental* 2019, **7(Suppl 3):**001576

**INTRODUCTION.** Acute acalculus cholecystitis (AAC) is a serious complication of critical illness.
However, up until now there are no specific criteria to diagnose AAC in intensive care units (ICU).
**OBJECTIVES.** The aim of this study was to evaluate the underlying diseases, clinical and diagnostic features, and outcome of operatively treated AAC among ICU population.
**METHODS.** We conducted a retrospective study, over a five-year period, between September 1st, 2013 and august 31st, 2018, including the patients admitted to our ICU, who undergone surgery for suspected AAC during their ICU stay.
AAC group was defined as the group of patients with confirmed AAC based on operative findings and anatomopathological examination.
**RESULTS.** Fourty-eight patients underwent open cholecystectomy for AAC during the study's period. Traumatic injury was the most common admission diagnosis (66.7%), followed by acute respiratory failure (12.5%), hemorrhagic stroke (8.3%) and cardiac surgery (4.2%). The mean Sequential Organ Failure Assessment (SOFA) score was 5.8. On admission, 12 (50%) patients presented with shock and 4 (16.7%) with acute respiratory distress syndrome (ARDS).
According to operative findings and anatomopathological exam, thirty patients had confirmed AAC (65%). The mean length of ICU stay before AAC was suspected was 16.7 days, and the mean delay of surgical treatment was 2.6 days.
Ultrasonographic (US) abnormalities included gallbladder distension (66.7%), thickened gallbladder wall (66.7%), ultrasonographic Murphy's sign (12.5%) and pericholecystic fluid (50%).
On the day of cholecystectomy, average SOFA score was 5, twenty patients presented with positive blood cultures including klebsiella pneumonia in ten cases, pseudomonas in four cases, stenotrophomonas maltophilia in two cases and candidemia in four cases.
According to our analysis risk factors associated to AAC were trauma, cardiac surgery, diabetes and blood transfusions. On the day of diagnosis, hyperglycemia, increased gastric residual volume and jaundice were predictive of AAC. Moreover the delay to AAC was also predictive of the diagnosis with a best cut-off at 12.5 days (AUC ROC =0.77, Se=86% ,Sp=73%).
The mean total length of ICU stay was 31.9 days, and overall mortality was 17%. Subjects with confirmed AAC had higher mortality rates (21%). Our study showed that compared to the survivors, nonsurvivors had higher SOFA scoring on the day of cholecystectomy (p= 0.001, OR= 1.5, CI= 1.2-2). Intraoperative bleeding was also predictive of mortality (p= 0.001, OR= 4, CI= 2.5-7.8).
**CONCLUSION.** Acute acalculous cholecystitis is associated with multiple organ failure, long ICU stay and death. The diagnosis of AAC remains challenging and should be suspected especially in critically ill patient with traumatic injuries or cardiac surgery and jaundice.

### 001645

#### Nosocomial infections in Medical ICU- A Retrospective study comparing trends from 2011 and 2018

S. Dixit[1], K. Khatib[2], K. Borawake[3], H. Dongare[4]
[1]Critical care, Sanjeevan Hospital, Karve Road, Pune, Pune, India; [2]Medicine, Smt. Kashibai Navale Medical College, Pune, India; [3]Icu, VishwaRaj Hospital, Pune, India; [4]Anaesthesia, Smt. Kashibai Navale Medical College , Pune, India
**Correspondence:** K. Khatib
*Intensive Care Medicine Experimental* 2019, **7(Suppl 3):**001645

**INTRODUCTION.** Patients admitted to ICU are prone to various nosocomial infections (NI) due to various reasons. Gram negative bacteria (GNB) are often the most important causative organisms for the same. Of these, multi-drug resistant (MDR) GNB lead to difficulty in treatment, increase length of ICU stay, cost of treatment and increase morbidity and mortality.
**OBJECTIVES.** a) To investigate the trends of NI in our ICU over the period from 2011 to 2018. b) To study the trends in NI caused by MDR-GNB in that period.
**METHODS.** A retrospective observational study design. Charts were reviewed of all patients admitted in ICU in the 12 months of 2011 and 2018. Patients developing NI (defined as new onset infection developing after 48 hours of ICU admission) were included in the study. Data was collected regarding socio-demographic factors, cause of ICU admission, type of infection (organ system involved, type of bacteria, sensitivity pattern on culture, etc.), outcomes [ICU length of stay (LOS), mortality].Variables were characterised by number and percentage and were compared by applying Pearson's Chi-square with Fisher's exact test. Trends over time were determined by the Cochran–Armitage trend test and linear regression.
**RESULTS.** The results are shown in Table no 1.Of the organisms causing NI, 20% were found to be MDR in 2011 as compared to 40% in 2018 ($p<0.05$). The average ICU LOS was 16.5 and 17.4 days (p= NS) in 2011 and 2018 respectively, and mortality was 25.3 and 28.4% (p=0.06) in 2011 and 2018 respectively.
**CONCLUSION.** There was a significant increase in occurrence of NI. Though there was a reduction in Respiratory tract infections over the period of the study, there was an increase in Urinary tract infections as well as Skin & soft tissue infections. The number of MDR organisms isolated also saw a significant increase. There was an increase in ICU LOS and mortality though not statistically significant.

#### REFERENCE(S)

1. Choudhuri AH, Chakravarty M, Uppal R. Epidemiology and characteristics of nosocomial infections in critically ill patients in a tertiary care intensive care unit of Northern India. Saudi J Anaesth 2017;11:402-7.
2. Vincent JL, Bihari DJ, Suter PM, Bruining HA, White J, Nicolas- Chanoin MH, Wolff M, Spencer RC, Hemmer M: The prevalence of nosocomial infection in intensive care units in Europe. Results of the European Prevalence of infection in Intensive Care (EPIC) Study. EPIC International Advisory Committee. JAMA 1995;274:639-644.
3. Horan TC, Andrus M, Dudeck MA. CDC/NHSN surveillance definition of health care-associated infection and criteria for specific types of infections in the acute care setting. Am J Infect Control 2008;36:309-32.
4. NIL

**Table 1 (abstract 001645).** See text for description

| | 2011 | 2018 | P value |
|---|---|---|---|
| Total ICU patients admitted/ Developed NI(% Incidence) | 32/366 (8.74%) | 350/ 1051(33.3%) | p< 0.05 |
| Male:Female (%) | 63.7/36.3 | 76/24 | |
| APACHE II score, Mean+_SD | 22+_8 | 20+_11 | p=NS |
| Types of Nosocomial infections | | | |
| Respiratory tract infections | 55.8 | 24.46 | p< 0.05 |
| Urinary tract infections | 17.1 | 36.4 | p< 0.05 |
| Skin & soft tissue infections | 10.2 | 23.3 | p< 0.05 |
| Organisms causing Nosocomial infections | | | |
| Acinetobacter | 34.5 | 18.08 | p< 0.05 |
| E. coli | 12.1 | 26.5 | p< 0.05 |
| Pseudomonas | 32 | 15.6 | p< 0.05 |
| Klebsiella | 13.9 | 11.7 | p=NS |

### 001700

#### Risk Factors and Molecular Epidemiology of Complicated Intra-Abdominal Infections with Carbapenem-Resistant Enterobacteriaceae: A Multicenter Study in China

J. Liu[1], L. Zhang[1], J. Pan[2], D. Chen[1]
[1]Department of Critical Care Medicine, Shanghai Jiaotong University, School of Medicine, Ruijin Hospital North, Shanghai, China; [2]Department of critical care medicine, The First Affiliated Hospital of Wenzhou Medical School, Zhejiang, China
**Correspondence:** J. Liu
*Intensive Care Medicine Experimental* 2019, **7(Suppl 3):**001700

**INTRODUCTION.** Carbapenem-resistant Enterobacteriaceae (CRE) infections are associated with poor patient outcomes. Data on risk factors and molecular epidemiology of CRE in complicated intra-abdominal infections (cIAI) are limited.

**METHODS.** In this retrospective analysis, we identified cIAI patients hospitalized during a period from 1 January 2013 to 31 October 2018 in 14 hospitals in China. 30 CRE isolates were genotyped.

**RESULTS.** *Escherichia coli* (34.5%)and *Klebsiella pneumoniae* (21.2%) were the leading pathogens. Patients with hospital-acquired (HA)-cIAIhad lower rate of *E. coli*(26.0% vs. 49.1%; $p$<0.001) and highercarbapenem-resistant Gram-negative bacteria (31.7% vs. 18.8%; $p$=0.002)than patients with community-acquired (CA)-cIAI. 16.0% of the *Enterobacteriaceae*isolates and 23.4% of the *K. pneumoniae* isolates were resistant to carbapenem.The 28-day mortality was 12.2% and 9.0% in patients with CRE-cIAI versus non-CRE-cIAI( $p$ <0.001). In-hospital mortality was 4.7-fold higher for carbapenemase-producing(CP)-CRE versus non-CP-CRE infection ($p$= 0.049), which was regardless of carbapenem-containing combinations.The risk factors for 28-day mortality in patients with CRE-cIAIincluded sepsis or septic shock,antibiotic exposure during the preceding 30 days,and comorbidities.

**CONCLUSION.***Klebsiella pneumoniae*is the highest prevalence in CRE-cIAI. Infection with CRE, and CP-CRE in particular, is associated with increased mortality in cIAI.

## Hot Topics Session

### 000143

#### Targeted temperature management at 33°C versus 37°C after non-shockable cardiac arrest: the HYPERION randomized clinical trial

JB. Lascarrou[1], F. Meziani[2], A. Le Gouge[3], G. Colin[4], G. Grillet[5], P. Girardie[6], E. Coupez[7], PF. Dequin[8], A. Cariou[9], I. Runge[10], JP. Frat[11], P. Asfar[12], N. Pichon[13], M. Landais[14], G. Plantefeve[15], JP. Quenot[16], JC. Chakarian[17], M. Sirodot[18], S. Legriel[19], J. Reignier[1]
[1]Médecine Intensive Réanimation, Nantes University Hospital Hotel-Dieu, Nantes, France; [2]Medical intensive care, Nouvel Hôpital Civil, Strasbourg, France; [3]Inserm cic1415, Chru Hôpitaux De Tours, Hospital Bretonneau, Tours, France; [4]Medical surgical intensive care unit, Hospital Center Departmental Vendée, La Roche-sur-Yon, France; [5]Medical-surgical intensive care unit, South Brittany General Hospital Center, Lorient, France; [6]Medicine intensive reanimation, Chu De Lille, Lille, France; [7]Medical intensive care, C.H.U, Clermont-Ferrand, France; [8]Medecine intensive reanimation, Chru Hôpitaux De Tours, Hospital Bretonneau, Tours, France; [9]Medecine intensive reanimation, Hospital Cochin, Paris, France; [10]Medicine intensive reanimation, The Regional Hospital of Orleans, Orléans, France; [11]Medecine intensive reanimation, Poitiers University Hospital, Poitiers, France; [12]Medecine intensive reanimation, centre hospitalier universitaire d'Angers, Angers, France; [13]Medical surgical intensive care unit, General Hospital Center, Brive-la-Gaillarde, France; [14]Medical surgical intensive care unit, Hospital Center Le Mans , Le Mans, France; [15]Medical surgical intensive care unit, General Hospital Center, Argenteuil, France; [16]Medical intensive care unit, Chu Dijon, Dijon, France; [17]Medical surgical intensive care unit, General Hospital Center, Roanne, France; [18]Medical surgical intensive care unit, General Hospital Center, Annecy, France; [19]Medical surgical intensive care, General Hospital Center, Versailles, France
**Correspondence:** J.B. Lascarrou
*Intensive Care Medicine Experimental* 2019, **7(Suppl 3):**000143

**INTRODUCTION.** Moderate therapeutic hypothermia (MTH) is currently recommended to improve neurological outcomes in adults with persistent coma after resuscitated out-of-hospital cardiac arrest (OHCA). However, the effectiveness of MTH in patients with non-shockable rhythms (asystole or pulseless electrical activity) is debated. We tested the effectiveness of MTH at 33°C in patients with non-shockable in- or out-of-hospital cardiac arrest (CA).

**METHODS.** HYPERION was a randomized controlled trial comparing MTH (33°C) to targeted normothermia (37°C) in comatose patients admitted to 25 intensive care units after non-shockable CA. Outcome assessment was blinded. The primary outcome was neurological recovery assessed on post-randomization day 90 using the Cerebral Performance Categories (CPC) scale, with CPC 1 and 2 defining a good outcome.

**RESULTS.** Between February 2014 and November 2017, 584 patients were randomized and 581 were included in a modified intention-to-treat analysis (3 patients withdrew consent). On day 90, 29 (10.2% [95% confidence interval, 6.7%;13.7%] of the 284 MTH patients were CPC 1 or 2, compared to 17 (5.7% [3.1;8.4%]) of the 297 normothermia patients (OR, 1.87 [1.01;3.50]; $P$=0.048). Day-90 mortality did not differ between groups (MTH: 81.3% [76.8%;85.9%]; normothermia: 83.2% [78.9;87.4%]; $P$=0.81). Pre-specified adverse events were not different between the groups.

**CONCLUSION.** Neurological outcomes after non-shockable CA are poor. Compared to normothermia, MTH at 33°C improved neurological recovery in survivors but had no effect on mortality.

**REFERENCE(S)**

1. Funded by French Ministry of Health PHRCI1369057N, La Roche Sur Yon District Hospital Center France and Laerdal Foundation
2. Lascarrou JB, Meziani F, Le Gouge A, Boulain T, Bousser J, Belliard G, Asfar P, Frat JP, Dequin PF, Gouello JP, Delahaye A, Hssain AA, Chakarian JC, Pichon N, Desachy A, Bellec F, Thevenin D, Quenot JP, Sirodot M, Labadie F, Plantefeve G, Vivier D, Girardie P, Giraudeau B, Reignier J; Clinical Research in Intensive Care and Sepsis (CRICS) Group and HYPERION Study Group. Therapeutic hypothermia after nonshockable cardiac arrest: the HYPERION multicenter, randomized, controlled, assessor-blinded, superiority trial. Scand J Trauma Resusc Emerg Med. 2015 Mar 7;23:26. doi: 10.1186/s13049-015-0103-5. PubMed PMID: 25882712; PubMed Central PMCID: PMC4353458.

### 000228

#### Increased risk of dying if discharged with inter-hospital transfer due to lack of ICU beds. A nationwide study from the Swedish Intensive Care Registry

F. Parenmark[1], S. Walther[2]

[1]Dept of anaesthesia and intensive care, Gävle Sjukhus, Gävle, Sweden; [2]Dept of cardiothoracic anaesthesia and intensive care, Linkoping University Hospital, Linköping, Sweden

**Correspondence:** F. Parenmark

*Intensive Care Medicine Experimental* 2019, **7(Suppl 3):**000228

**INTRODUCTION.** Most patients admitted to intensive care are discharged to a general ward in the same hospital, but some patients require transfer to another hospital. Indications for inter-hospital transfers (IHT) include referral for specialist treatment, lack of intensive care beds at the referring ICU and repatriation to ICU in home hospital [1].

**OBJECTIVES.** To review mortality of ICU-patients undergoing IHT and analyse whether different indications for transfer render different mortalities.

**METHODS.** Retrospective cohort register study using the Swedish Intensive Care Registry (SIR) during 2016-2018. The SIR collects data from 98.8% of Swedish ICUs including data on discharge from ICUs to other hospitals/ICUs. Transfers were divided into three categories: transfer due to medical reasons, lack of ICU beds or repatriation to ICU in home hospital. We analysed odds ratios (ORs) for dying within 30 days after discharge from ICU using risk adjusted (SAPS3 score) multi-level mixed effect logistic regression with ICUs as random effect.

**RESULTS.** We identified 12,356 patients who were discharged to another ICU and hospital, i.e. inter-hospital transfers. The unadjusted mortality 30 days after IHT was 17.2 % compared to 12.4 % if discharged to ward in the same hospital. Mortality after IHT varied with the cause of discharge (Figure).Main diagnoses for transfer due to specialist treatment were subarachnoid haemorrhage, head injury and multi-trauma whilst for lack of ICU beds post cardiac arrest, respiratory failure and pneumonia dominated. Risk adjusted analysis showed a significantly increased risk of dying after discharge due to lack of ICU-beds in comparison with other reasons for IHTs

**CONCLUSION.** The adjusted risk of dying within 30 days after inter-hospital transfer was greater among critically ill patients when the transfer was due to lack of beds in the referring ICU. The increased mortality lingered for at least 6 months underlining the importance to identify causes and intervene to avoid unnecessary loss of life.

**REFERENCE(S)**

1. A Gray, S Bush, S Whiteley. Secondary transport of the critically ill and injured adult. Emerg Med J 2004;21:281–285
2. Supported by institutional grants from Centre for Research and Development Region Gävleborg and Region Östergötland, Sweden.

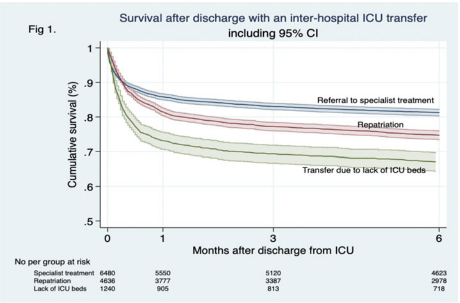

**Fig. 1 (abstract 000228).** See text for description

**Table 1 (abstract 000228).** See text for description

| | Medical reasons N=6,480 | Repatriation N=4,636 | Lack of ICU beds N=1,240 |
|---|---|---|---|
| Age yrs, mean (SD) | 49.9 (23.6) | 58.4 (20.1) | 60.0 (20.1) |
| Women % | 41.7 | 37.1 | 38.5 |
| SAPS3 risk, median (IQR) | 0.07 (0.02 – 0.23) | 0.18 (0.07 – 0.34) | 0.24 (0.10 – 0.42) |
| ICU stay hrs, median (IQR) | 14 (4 – 37) | 62 (27 – 138) | 42 (14 – 104) |
| 30-day mortality, % | 14.4 | 18.5 | 27.0 |
| 30-day mortality, adjusted OR (95% CI) | 1.0 (ref) | 0.93 (0.83-1.06) | 1.24 (1.05-1.46) p=0.011 |

### 001556

#### Early Oseltamivir therapy improves survival in critically ill patients with severe influenza infection

G. Moreno[1], R. Carbonell[1], E. Papiol[2], J. Sole-Violan[3], I. Martin-Loeches[4], E. Díaz[5], M. Bodí[6], LF. Reyes[7], J. Gómez[8], J. Guardiola[9], S. Trefler[6], E. Correig[10], M. Restrepo[11], A. Torres[12], A. Rodriguez[6]

[1]Intensive care, Hospital Universitari de Tarragona Joan XXIII, Tarragona, Spain; [2]Intensive care, Hospital Vall d'Hebrón, Barcelona, Spain; [3]Intensive care, Hospital Dr Negrin, Las Palmas de Gran Canaria, Spain, Spain; [4]School of medicine, Trinity College Dublin, Dublin, Ireland; [5]Intensive care, Hospital Parc Taulí, Sabadell, Spain; [6]Intensive care, Hospital Universitari de Tarragona Joan XXIII/URV/IISPV/CIBERES, Tarragona, Spain; [7]Critical Care Medicine - Infectious Disease Department, Universidad de la sabana, Bogotá, Colombia; [8]Intensive care unit, associate professor at urv, PhD, Tarragona, Spain; [9]Division of pulmonary critical care and sleep medicine, University of Louisville and Robley Rex VA Medical Center, Louisville, USA; [10]Catedra de bioestadística, Universitat Rovira i Virgili, Tarragona, Spain; [11]Intensive care, South Texas veterans health care system , San Antonio, USA; [12]Servei de pneumologia i al·lèrgia respiratoria, Hospital Clinic, Barcelona, Spain

**Correspondence:** G. Moreno

*Intensive Care Medicine Experimental* 2019, **7(Suppl 3):**001556

**INTRODUCTION.** Influenza virus epidemics can cause complications resulting in severe illness and are associated with significant mortality each year worldwide. Up to one third of adults hospitalized with influenza require admission to an intensive care unit (ICU) and up to 80% are mechanically ventilated (MV), due to viral pneumonia and acute distress respiratory syndrome (ARDS), with a resultant mortality rate of up to 30%.

**OBJECTIVES.** To determine if early oseltamivir treatment (within 48 hours of symptom onset) compared to no treatment and late treatment is associated with decreased ICU-mortality among patients with severe influenza infection.

**METHODS.** Prospective, observational study including subjects with confirmed influenza infection admitted to 184 ICUs in Spain. Patients with missing data on antiviral therapy or on outcome were excluded. The primary outcomes were to investigate the impact on ICU-mortality of antiviral therapy (All treated [AT], early therapy [ET], and late therapy [LT]) compared to untreated (UT) patients and the effect of ET vs LT. To evaluate endpoints, Cox proportional hazards regression models were made. Propensity Score Matching (PSM) Competing Risks analysis (CRA) were used to control bias.

**RESULTS.** Between June 2009 and April 2018, 4175 patients were enrolled. 3537 patients met the inclusion criteria. AT patients (n=3439) were compared to UT group (n=98). After adjusting for severity of illness, AT patients had higher survival compared to UT patients (HR 0·64; p=0·022). Afterwards, 149 patients were excluded for timing of oseltamivir study, remaining 3388 patients (ET, n=807 and LT, n=2581). Most of patients developed influenza pneumonia (84·7%). Overall ICU-mortality was higher in LT group (23·1% vs 19·8%; p=0·05). In the multivariable analysis, ET with oseltamivir was associated with lower ICU-mortality rate (OR 0·7; p=0·004). PSM was applied performing the new matched cohort (ET, n=790 vs LT, 2522). ET with oseltamivir was associated with lower ICU-mortality over time (HR 0·77; p=0·005) compared to LT and this result remained firm after CRA (sHR 0·80; p<0·05).

**CONCLUSION.** In patients with confirmed severe influenza infection, ET with oseltamivir is associated with better survival rates.

**Publisher's Note**

**Submit your manuscript to a SpringerOpen journal and benefit from:**

- Convenient online submission
- Rigorous peer review
- Open access: articles freely available online
- High visibility within the field
- Retaining the copyright to your article

**Submit your next manuscript at ► springeropen.com**

Publisher’s Note

Springer Nature remains neutral with regard to jurisdictional claims in published maps and institutional affiliations.

Change history

1/21/2020

After publication of this supplement [1], it was brought to our attention that the author ‘L. Vegnuti’ has been erroneously omitted from the author list of the following abstract.

