# Correction to: A case of intracoronary and endovenous levosimendan as a rescue therapy for refractory cardiac arrest

**DOI:** 10.1186/s40635-019-0289-3

**Published:** 2020-01-21

**Authors:** B. Ferro, R. Tofani, L. Vegnuti, G. Borelli, L. Gargani, P. Roncucci

**Affiliations:** 1 Anestesia e rianimazione, Spedali Riuniti Livorno ATNO ESTAR, Livorno, Italy; 2 Cardiology, CNR PISA, Pisa, Italy; 3 Cardiology, Spedali Riuniti Livorno ATNO ESTAR, Livorno, Italy

**Correction to: Intensive Care Med Exp (2019) 7**

**https://doi.org/10.1186/s40635-019-0265-y**

After publication of this supplement [1], it was brought to our attention that the author ‘L. Vegnuti’ has been erroneously omitted from the author list of the following abstract.

Please find the corrected author list detailed in this correction.

## 001693 A case of intracoronary and endovenous levosimendan as a rescue therapy for refractory cardiac arrest

**INTRODUCTION.** Reducing time of cardiac arrest is the goal to obtain better prognosis in every setting from the street to the cardiac catheterization laboratory. Epinephrine is the standard medication for acls but its effect on both recovery of spontaneous circulation and amelioration of neurological prognosis remains controversial (1). As a consequence there’s need to develop pharmacological strategies to reduce time to ROSC and to cerebral reperfusion especially when ECLS is not available.

**METHODS.** We report a case of a 60 years old male patient with refractory cardiac arrest secondary to myocardial infarction unresponsive to common therapies in whom intracoronary injection followed by endovenous infusion of calcium sensitizer levosimendan was able to gain and mantain ROSC.

**RESULTS.** The patient was admitted to cardiac catheterization laboratory with diagnosis of cardiogenic shock secondary to acute anterior myocardial infarction. Patient was intubated, an aortic counterpulsation balloon was placed and PTCA of IVA was performed. During procedure numerous episodes of shockable rhythms (ventricular fibrillation and ventricular tachycardia) were treated following ACLS guidelines. After coronary reperfusion an episode of ventricular tachycardia followed by pulseless electric activity of more ten minutes was unresponsive to therapy. A bolus of 10 mcg/kg of levosimendan was injected in the left coronary artery followed by a continuous endovenous infusion (0,2 mcg/kg/min). After 30 s ROSC was obtained, foretold by increasing etCO2 from 10 to 25 mmhg during chest compressions (figure). Hemodynamic was mantained with low dosage of norepinephrine and continuous infusion of levosimendan. In intensive care unit first Bispectral index was 38.

Patients was treated with targeted temperature management and then gradually weaned from vasoactives, aortic counterpulsation and ventilation, recovering good neurological condition and discharged with cpc score of 2.

**CONCLUSION.** To our knowledge, this is the first report of successful reversal of refractory cardiac arrest with intracoronary followed endovenous infusion of levosimendan. Only preclinical studies have analyzed the potentiality of levosimendan in cardiac arrest. Nan et al. showed that a combination of levosimendan and epinephrine was superior to epinephrine alone to reverse bupivacaine-induced cardiac arrest in a rat model (2).

There is evidence that intracoronary injection of levosimendan in cardiac surgery guarantees optimal drug spread, increasing contractility, global cardiac function, coronary perfusion pressure and reducing arrhytmic ischemic events (3). In this case intracoronary administration was possible because arrhytmic events followed coronary reperfusion during catheterization procedure. Intracoronary administration of drug is important to hypothesize the direct effect of levosimendan on arrhytmic unresponsive events and ROSC.

**Fig. 1:**
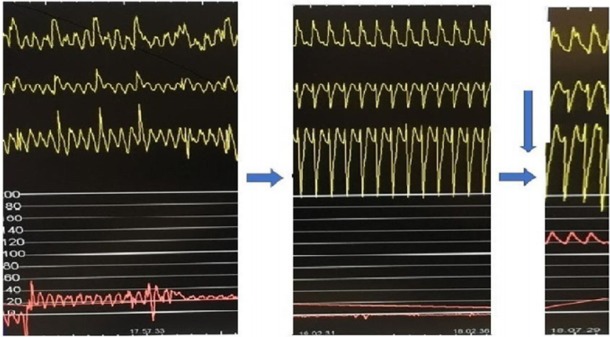
**(abstract 001693).** See text for description
